# Draft Sequences of the Radish (*Raphanus sativus* L.) Genome

**DOI:** 10.1093/dnares/dsu014

**Published:** 2014-05-16

**Authors:** Hiroyasu Kitashiba, Feng Li, Hideki Hirakawa, Takahiro Kawanabe, Zhongwei Zou, Yoichi Hasegawa, Kaoru Tonosaki, Sachiko Shirasawa, Aki Fukushima, Shuji Yokoi, Yoshihito Takahata, Tomohiro Kakizaki, Masahiko Ishida, Shunsuke Okamoto, Koji Sakamoto, Kenta Shirasawa, Satoshi Tabata, Takeshi Nishio

**Affiliations:** 1 Graduate School of Agricultural Science, Tohoku University, 1-1 Tsutsumidori-Amamiyamachi, Aoba-ku, Sendai, Miyagi 981-8555, Japan; 2 Kazusa DNA Research Institute, 2-6-7 Kazusa-Kamatari, Kisarazu, Chiba 292-0818, Japan; 3 Faculty of Agriculture, Iwate University, 3-18-8 Ueda, Morioka, Iwate 020-8550, Japan; 4 National Institute of Vegetable and Tea Science, 360, Kusawa, Ano, Tsu, Mie 514-2392, Japan; 5 Plant Breeding Experimental Station, Takii Seed Co. Ltd, Kohsei, Kohka-gun, Shiga 520-20, Japan

**Keywords:** radish, draft sequence, high-density genetic map

## Abstract

Radish (*Raphanus sativus* L., *n* = 9) is one of the major vegetables in Asia. Since the genomes of *Brassica* and related species including radish underwent genome rearrangement, it is quite difficult to perform functional analysis based on the reported genomic sequence of *Brassica rapa*. Therefore, we performed genome sequencing of radish. Short reads of genomic sequences of 191.1 Gb were obtained by next-generation sequencing (NGS) for a radish inbred line, and 76,592 scaffolds of ≥300 bp were constructed along with the bacterial artificial chromosome-end sequences. Finally, the whole draft genomic sequence of 402 Mb spanning 75.9% of the estimated genomic size and containing 61,572 predicted genes was obtained. Subsequently, 221 single nucleotide polymorphism markers and 768 PCR-RFLP markers were used together with the 746 markers produced in our previous study for the construction of a linkage map. The map was combined further with another radish linkage map constructed mainly with expressed sequence tag-simple sequence repeat markers into a high-density integrated map of 1,166 cM with 2,553 DNA markers. A total of 1,345 scaffolds were assigned to the linkage map, spanning 116.0 Mb. Bulked PCR products amplified by 2,880 primer pairs were sequenced by NGS, and SNPs in eight inbred lines were identified.

## 1. Introduction

Radish (*Raphanus sativus* L.), also called ‘Daikon’, is an important vegetable root crop especially in Asia. There is a large variation in size and shape of roots from smaller than 3 cm in diameter in the case of the European garden radish to more than 30 cm in diameter for ‘Sakurajima Daikon’ and from a round type in the case of the European garden radish and ‘Sakurajima Daikon’ to a long type such as ‘Moriguchi Daikon’ having a root more than 2 m in length. Fresh sprouts are used as a vegetable, and in tropical Asia, immature siliques are consumed as a vegetable. Radish is also produced as an oil crop, oil being extracted from mature seeds. Radish roots contain glucosinolates, which are hydrolyzed by inherent myrosinase (EC3.2.1.147) after disruption of cells, resulting in production of pungent components, i.e. isothiocyanates. Since 4-methylthio-3-butenyl isothiocyanate generated from the major glucosinolate in radish has been reported to have anti-mutagenicity^1,2^ and anti-carcinogenicity,^3^ radish may become more popular for use in salads.

Radish belongs to a genus different from that of turnip (*Brassica rapa*), but they are highly similar in morphology to each other as vegetables. Shapes of siliques and seed sizes are obviously different between them. Phylogenetic analyses of Brassicaceae species using DNA markers or nucleotide sequences of genes have revealed that *R. sativus* belongs to the *rapa*/*oleracea* lineage not to the *nigra* lineage.^4,5^ Chromosome numbers of these species are different, i.e. *n* = 8 in *Brassica nigra*, *n* = 9 in *Brassica oleracea* and *R. sativus*, and *n* = 10 in *B. rapa*. Genome syntenies between these species are complicated,^6,7^ suggesting that extensive genome rearrangements have occurred during or after speciation of these species, while overall genome syntenies are well conserved in Poaceae crops, e.g. rice, wheat, maize, barley, and sorghum,^8^ and Solanaceae crops, e.g. tomato, potato, and eggplant.^9^

The development of next-generation sequencers (NGSs) has enabled accumulation of a large amount of genomic nucleotide sequence data of many organisms at relatively low cost. *De novo* assembly of the genomic sequence data can provide whole-genome sequences, which can be assigned to chromosomes using the sequences of mapped DNA markers in a linkage map. Although the draft genome sequences of Chinese cabbage in *B. rapa* have been obtained and published,^10^ it is difficult to use these sequence data as references to determine the radish genome sequences because of highly complicated genome synteny between *B. rapa* and *R. sativus*.^7^

In the present study, *R. sativus* draft genome sequences were determined by a NGS along with bacterial artificial chromosome (BAC)-end sequences. Using the sequence information, we constructed a high-density linkage map by adding new DNA markers and combining two different linkage maps, resulting in 2,553 DNA markers including 2,351 sequence-characterized markers (954 dot-blot-SNP markers, 768 PCR restriction fragment length polymorphism (PCR-RFLP) markers, and 629 expressed sequence tag-simple sequence repeat (EST-SSR) markers), and revealed detailed synteny between *R. sativus* and *B. rapa.* Additionally, single nucleotide polymorphisms (SNPs) between several inbred lines were surveyed.

## 2. Materials and methods

### 2.1. Plant materials

A genetic linkage map has been previously constructed using an F_2_ population derived from a cross between two radish lines, which were self-pollinated for three generations from ‘Sayatori 26704’ (hereafter ‘Sayatori’) (National Institute of Vegetable and Tea Science, Japan) and ‘Aokubi *S-h*’ (hereafter ‘Aokubi’) (Takii Seed Co., Japan), respectively.^7^ ‘Sayatori’ is a seedpod vegetable with a very thin and small root like a rat tail and ‘Aokubi’ is Japanese radish with a long and thick root. Crossing these two lines yielded 189 F_2_ plants, which were used for construction of a linkage map. Total genomic DNAs were extracted from leaves with the CTAB method^11^ and subjected to genotype analysis and *de novo* sequencing analysis. For SNP identification by sequencing of bulked PCR products, three inbred lines, such as ‘Yumehomare’, ‘Sakurajima’, and ‘Nishimachi-Risou’, and an inbred line, ‘N1-3’, obtained from a cross between ‘Mino-wase’ and ‘Miyashige-Soubutori’ were used.

### 2.2. Sequencing analysis

Total genomic DNA of ‘Aokubi’ was subjected to library construction according to the standard protocol (Illumina) for paired-end (PE; insert size of 250 bp) and mate-pair (MP) libraries (insert size of 5 kb). Sequencing analysis was carried out with a HiSeq 2000 sequencer (Illumina) in the paired-end sequencing mode (101 and 38 bases each for PE and MP libraries, respectively). Massive sequencing of a PE library for a radish line, ‘Sayatori’, was also carried out with an Illumina GAIIx sequencer in the paired-end mode (101-base each). The obtained Illumina reads were trimmed with quality scores of <10 by PRINSEQ 0.19.5.^12^ The end sequences of BAC clones, which were randomly selected from a BAC library of a doubled haploid line derived from ‘Aokubi’, were determined by the Sanger method^13^ using ABI3730xl (Applied Biosystems, USA).

### 2.3. Genome assembly

The low-quality and contaminated Sanger reads were eliminated by Cross_match (-minmatch 10 -minscore 18) for masking vector sequences (NCBI's UniVec), Trim2 (-m 100 -q 20 -x 10) for trimming low-quality bases, and Blast (*E*-value cut-off of 1*E*−10) for eliminating sequences similar to bacteria (all the bacterial genome sequences of NCBI), chloroplasts (accession number: NC_000932.1), and mitochondria sequences (accession number: NC_001284.2) of *Arabidopsis thaliana*.

The Illumina PE reads of ‘Aokubi’ were assembled by the SOAPdenovo 2r223 assembler^14^ with a *k*-mer size of 81 and the default parameters. The resultant scaffolds were subjected to gap-filling with the Illumina reads by GapCloser 1.10 (p = 31) (http://soap.genomics.org.cn). Then, the scaffolds were bridged with the Illumina MP reads by SSPACE2.0.^15^ Furthermore, BAC-end sequences of ‘Aokubi’ were employed to construct super-scaffolds with SSPACE2.0.

### 2.4. Gene prediction and annotation

From the RSA_r1.0, genes were predicted by Augustus 2.7^16^ with a training set of *A. thaliana* (TAIR10). The parameters used were –species = arabidopsis –genemodel = partial –protein = on –introns = on –start = on –stop = on –cds = on –codingseq = on –alternatives-from-evidence = true –alternatives-from-sampling = true –gff3 = on –UTR = on. The predicted genes were classified into four categories, i.e. intrinsic (with start and stop codons), partial (without start and/or stop codons), pseudo (with in-frame stop codons), and short genes (encoding <50 amino acids). Transposable elements (TEs) were judged from the results of hmmscan^17^ against GyDB^18^ with an *E*-value cut-off of 1.0, BLASTP against NCBI non-redundant protein database (nr: http://blast.ncbi.nlm.nih.gov/Blast.cgi?PAGE=Proteins) with an *E*-value cut-off of 1*E*−10, and InterProScan^19^ against InterPro databases.^20^ To evaluate the accuracy of the gene prediction, radish unigene sequences available from the RadishBase (http://bioinfo.bti.cornell.edu/cgi-bin/radish/index.cgi)^21^ were used for BLAST searches (*E*-value cut-off of 1*E*−10) against the sequences of the RSA_r1.0.

Functional domains in the predicted genes, which were searched for against InterPro databases^20^ using InterProScan,^19^ were assigned to the plant GO slim categories by using the map2slim program.^22^ Subsequently, the predicted genes were classified into eukaryotic Clusters of Orthologous Groups of proteins (KOG) categories^23^ by BLAST searches with an *E*-value cut-off of 1*E*−20. In addition, the predicted genes in the radish genome together with those in the *A. thaliana* and *B. rapa* genomes and unigenes for *B. oleracea* and *Raphanus raphanistrum* were clustered by CD-hit^24^ with parameters of *c* = 0.4; and aS = 0.4.

### 2.5. Repetitive sequence analysis

Putative repetitive sequences in the RSA_r1.0 were identified by RepeatScout^25^ with default parameters. In parallel, similarity searches and repeat masking were performed by RepeatMasker (http://www.repeatmasker.org) on RSA_r1.0 against known repetitive sequences registered in the RepBase.^26^ SSR motifs were searched for the RSA_r1.0 using SciRoKo^27^ with the MISA mode. The same analyses were carried out on the *A. thaliana* and *B. rapa* genomes.

### 2.6. Discovering SNPs with other *Raphanus* lines

The Illumina reads obtained from the resequencing of ‘Sayatori’ described above were mapped onto the RSA_r1.0 for SNP discovery using the Bowtie 2 (http://bowtie-bio.sourceforge.net/index.shtml)^28^ and SAMtools (http://samtools.sourceforge.net/) with default parameters.

In our previous studies,^7,29^ 2,880 primer pairs were designed for specific amplification of coding regions of genes containing 3′-untranslated regions. Using this primer set, sample preparations for sequencing were conducted for four *R. sativus* lines independently according to Zou *et al*.^29^ Sequences were determined using the Illumina GAIIx and the obtained reads were analysed by mapping to reference sequences of ‘Aokubi’ (RSA_r1.0) to discover SNPs between each *R. sativus* line using the program Bowtie 2 and SAMtools with default parameters.

### 2.7. Development of SNP markers

Two strategies were adopted to discover SNPs between the parental lines ‘Sayatori’ and ‘Aokubi’. One was sequencing of PCR products of the parental lines by the Sanger method^13^ as described by Li *et al.*^7^ PCR primer pairs were designed for amplification of the unigenes from the RS2 library of the Radish Database (http://radish.plantbiology.msu.edu). SNPs were discovered by the comparison of determined sequences. Another strategy was the use of NGS data of both parents. SNPs were surveyed by mapping of reads of ‘Sayatori’ to ‘Aokubi’ reference sequences as described above. Polymorphic sequences for eight kinds of restriction enzymes, i.e. *Bam*HI, *Eco*RI, *Hin*dIII, *Pst*I, *Sac*I, *Sal*I, *Xba*I, and *Xho*I, were also surveyed by CLC Genomics Workbench 5.5 (CLC Bio., Denmark).

PCR primer pairs were designed to amplify 400–700 bp products spanning SNPs. The sequences having SNPs were used for designing bridge probes^30^ for MPMP dot-blot-SNP analysis.^7^ In this case, the 189 F_2_ plants from the cross between both parents were used. In PCR-RFLP analyses, PCR primer pairs were designed spanning the polymorphisms and each PCR product was digested by a proper restriction enzyme and then separated by 2% agarose gel in 1× tris-acetate-EDTA buffer. The resulting DNA bands were stained with ethidium bromide. For this analysis, 29 F_2_ plants from the 189 F_2_ were selected by selective mapping software MapPop 1.0^31^ and subjected to genotyping.

### 2.8. Linkage analysis

First, a new marker data set for SNPs was added to the original data to produce a combined data set. Linkage analysis was carried out using the JoinMap 4.0 software (Kyazma B.V., Wageningen, The Netherlands). The markers were grouped into nine linkage groups (R1–R9)^7^ at high logarithm of ODDs (LOD) threshold (≥6). Marker order was determined by a regression mapping algorithm on the basis of a minimum LOD score of 1.0 and a recombination threshold of 0.4 in each LG. Recombination frequencies were converted into map distances in centimorgan (cM) using the Kosambi mapping function.

Secondly, a new marker data set for polymorphisms by PCR-RFLP was also added to the renewed genotype data, and linkage analysis was carried out in the same manner described above. The linkage map was graphically visualized with MapChart.

### 2.9. Integration of genetic maps

To integrate a radish linkage map of EST-SNP markers with the linkage map of EST-SSR markers constructed by Shirasawa *et al.*,^32^ 116 EST-SSR markers evenly distributed along the nine linkage groups were used to analyse polymorphism between the two parental lines and the EST-SSR markers having polymorphism were used for analysis of the F_2_ population. The PCR products were separated by 2% agarose gel or 8% polyacrylamide gel in 1× tris-borate-EDTA buffer.

The sequences of the unigenes located in the newly constructed linkage map and the map of Shirasawa *et al.*^32^ were aligned to identify the same unigenes using the SEQUENCHER version 4.7 (Gene Codes Corporation, MI, USA) with the following parameters: window = 100, similarity = 90. Prior to construction of an integrated map, the orientation of each linkage group in the linkage map of Shirasawa *et al.*^32^ was adjusted in accordance with the linkage map using the consensus SSR markers. Using a software MergeMap (http://138.23.178.42/mgmap/), these two linkage maps were integrated to be a consensus map.

### 2.10. Assignment of scaffolds to a linkage map

The sequences of scaffolds were searched by BLAT with sequences of DNA markers on the linkage map. The scaffolds with identity ≥90% and score ≥120 were assigned to their corresponding DNA markers.

### 2.11. Comparison with the *B. rapa* genome sequences

For a comparison analysis between the sequences of DNA markers and genomic sequences of *B. rapa*,^10^ homology search was performed using the local BLAST software included in the CLC Genomics Workbench 5.5 (CLC Bio.). The genome sequence fragments of *B. rapa* with the lowest *E*-value of <1*E*−50 were regarded as the homologous sequences. Syntenic regions (SRs) were identified according to conserved collinearity of EST sequences in the linkage map of *R. sativus* and the *B. rapa* genome sequences.

For a dot-plot view of SRs of *R. sativus* and *B. rapa* genomes, genomic sequences of scaffolds anchored to the integrated high-density linkage map of *R. sativus* in this study were aligned to genomic sequences of *B. rapa* according to the following step. Since the linkage map for assignment of the scaffolds was an integrated high-density linkage map combining an SNP-based map, a PCR-RFLP-based map by a selective mapping method, and an SSR-based map, the accuracy of the positions of the marker types might be in the order of SNP, SSR, and PCR-RFLP markers. If a scaffold was assigned to multiple markers on a linkage group, the most accurate marker position as the unique position of the scaffold was preferentially selected. In addition, if a scaffold was assigned to multiple markers of the same type, the position of the marker whose neighboured markers' syntenic relationship with the *B. rapa* genome was consistent with the microsyteny between the scaffold and *B. rapa* genome was regarded as being the proper position of the scaffold. Thus, the ‘pseudomolecules’ representative of the genome of *R. sativus* was established and the genetic distances between the scaffolds were converted to physical distances based on the ratio of total length of linkage map and genome size of *R. sativus*. Furthermore, physical distances between the predicted genes were also estimated. All genomic sequences of predicted genes in the pseudomolecules of *R. sativus* and those in the *B. rapa* genome were compared with each other using nucleotide BLAST. The genes of *B. rapa* with the lowest *E*-value and the *E*-value of <1*E*−100 were regarded as syntenic homologues. A list of syntenic homologues between genes in *R. sativus* and *B. rapa* was compiled and the dot-plot view was constructed by EXCEL based on the position of the syntenic homologues in two genomes.

## 3. Results and discussion

### 3.1. Genome assembly

In the whole-genome shotgun sequencing of ‘Aokubi’ with an Illumina HiSeq 2000 sequencer in the paired-end mode, a total of 1,142 million (M) and 924 M reads corresponding to 103.7 Gb and 87.4 Gb DNA were obtained in the PE and MP libraries, respectively. Total depth of the obtained sequence data (191.1 Gb) was shown by calculation to be ∼246.5 times as the estimated size of the radish genome being 528.6 Mb (Supplementary Fig. S1), which is almost the same size as 530 Mb of a predicted *R. sativus* genome size.^33^ After trimming the reads with quality scores of <10 by PRINSEQ 0.19.5^11^ and the adaptor sequence used in paired-end reads by fastx_clipper in FASTX-Toolkit (http://hannonlab.cshl.edu/fastx_toolkit), the remaining paired-end reads were assembled into 1,020,003 scaffolds containing 435,331,541 bases, and the gaps in the scaffolds were subsequently filled with the Illumina reads by GapCloser 1.10 (p = 31) (http://soap.genomics.org.cn/soapdenovo.html). The total length of the gap-filled scaffolds was 438,973,418 bases consisting of 1,020,003 scaffolds. The Illumina MP reads were used for extension of the length of the scaffolds, resulting in 473,904,309 bases consisting of 992,801 scaffolds (Supplementary Table S1). Both ends of 20,736 BAC clones were sequenced by the Sanger method.^13^ After removing end sequences with low-quality values of <20 and those showing similarities to contaminated sequences of chloroplasts, mitochondria, bacteria, and a cloning vector, the remaining 27,904 high-quality BAC-end sequences (accession no. GA872392–GA901611 in DDBJ) representing the radish genome were subjected to construction of super-scaffolds (Supplementary Table S2). An assembly analysis using SSPACE 2.0 constructed 737 super-scaffolds. Finally, 76,592 scaffolds with ≥300 bp spanning 402,330,269 bases (N50: 46,262 bases; GC%: 34.9) were obtained and named RSA_r1.0; they covered 75.9% of the estimated genome size in radish (530 Mb) (Table 1). Sequences of RSA_r1.0 were registered in the DDBJ database as accession DF384214-DF396802 and are published in the ‘*Raphanus sativus* Genome DataBase’ (http://radish.kazusa.or.jp).

**Table 1.** Statistics of RSA_r1.0

Number of scaffolds (≥300 bp)	76,592
Total length (Mb)	402
Average size (bp)	5,253
Maximum size (bp)	831,256
N50 (bp)	46,262
GC content (%)	34.9

Comparative analyses of linkage maps between *B. rapa* and *A. thaliana*^34^ and between *R. sativus* and *A. thaliana*^7^ have suggested that the diploid *R. sativus* and *B. rapa* species possess triplicated genomes. Nucleotide sequencing^35,36^ and cytogenetic analysis^37,38^ in *Brassica* species have also suggested this. Therefore, a possibility of mis-assemblies of scaffolds should be considered. To evaluate validities of scaffolds, a linkage of both ends of each scaffold was tested. For this purpose, DNA markers derived from both ends of each scaffold were produced for 59 comparatively long ones (>100 kb), which were selected randomly, and used them for genotyping analyses of 48 F_2_ plants derived from a crossing between ‘Aokubi’ and an inbred line from ‘Sayatori’.^7^ Of the 59 examined scaffolds, 56 exhibited complete linkages (Supplementary Table S3), suggesting that the possibility of mis-assembly must be low, ca. 5%, in the present study.

### 3.2. Gene annotation

A total of 80,521 genes were predicted in RSA_r1.0 (Table 2 and Supplementary Table S4) through an analysis by Augustus 2.7^16^ with a training set of *A. thaliana*. Using the hmmscan module in HMMER 3.0^17^ against the Database GyDB 2.0,^18^ BLASTP search against NCBI's non-redundant protein sequence database, and InterProScan^19^ against the InterPro database,^20^ 61,572 genes were predicted as intrinsic genes, i.e. genes with start and stop codons (45,002) and partial genes (16,570) (Table 2 and Supplementary Table S5). There were 15,545 genes predicted to be transposable elements and 3,404 pseudo and short genes. Therefore, the 61,572 predicted genes (average length: 874 bases; GC contents: 46.6%) were employed for further analysis (Table 2). Among them, 1,335 genes for transfer RNAs were identified, a number similar to that in *B. rapa* and twice that in *A. thaliana* (Supplementary Table S6). Of 85,083 radish unigene sequences available from the RadishBase,^21^ 84,165 (98.9%) were found in the genome sequences of RSA_r1.0 (Supplementary Table S7), indicating that the genome coverage of RSA_r1.0 was sufficient to identify genes.

**Table 2.** Statistics of genes predicted by Augustus 2.7

	Total	Intrinsic and partial
Number of sequences	80,521	61,572
Total length (bp)	78,403,816	53,787,862
Average length (bp)	974	874
Max length (bp)	16,173	16,173
N50 length (bp)	1,470	1,266

The total length of repetitive sequences in RSA_r1.0 was 107.2 Mb. The size was not so different from that in the *B. rapa* genome (93.4 Mb), while it was much larger than that in *A. thaliana* (23.6 Mb) (Supplementary Table S8). Predominant repetitive sequences in RSA_r1.0 were novel ones occupying 14.7%, as in *B. rapa* (19.1%). In the known interspersed repeats, long terminal repeat elements of the Class I elements including *copia*- and *gypsy*-types were the most frequent repeat sequences in RSA_r1.0 (4.1%) as in *B. rapa* (4.4%) and *A. thaliana* (8.7%).

**Figure 1.**
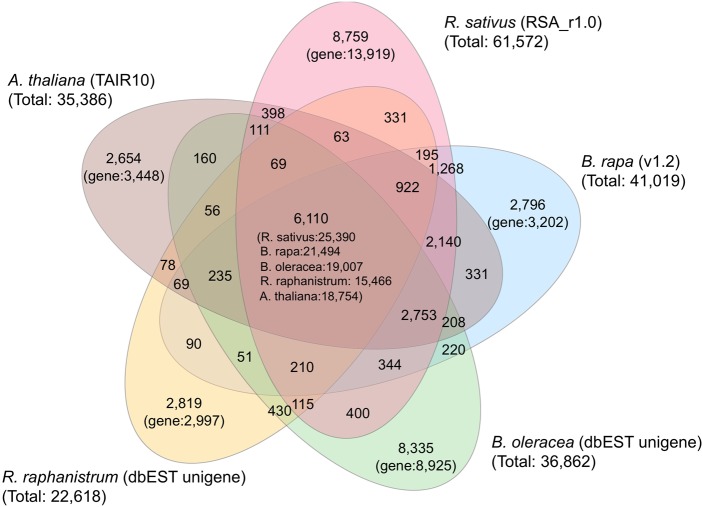
Venn diagram showing unique and shared gene families in *R. sativus*, *A. thaliana*, *Brassica rapa*, *B. oleracea*, and *R. raphanistrum*. Numbers in the individual sections represent the number of clusters. The number below the species name marks the total number of genes used as an input for the software. Genome data sets were used in *R. sativus* (RSA_r1.0), *A. thaliana* (TAIR10), and *B. rapa* (ver. 1.2), and EST-unigene data sets were used in *B. oleracea* and *R. raphanistrum*.

**Figure 2.**
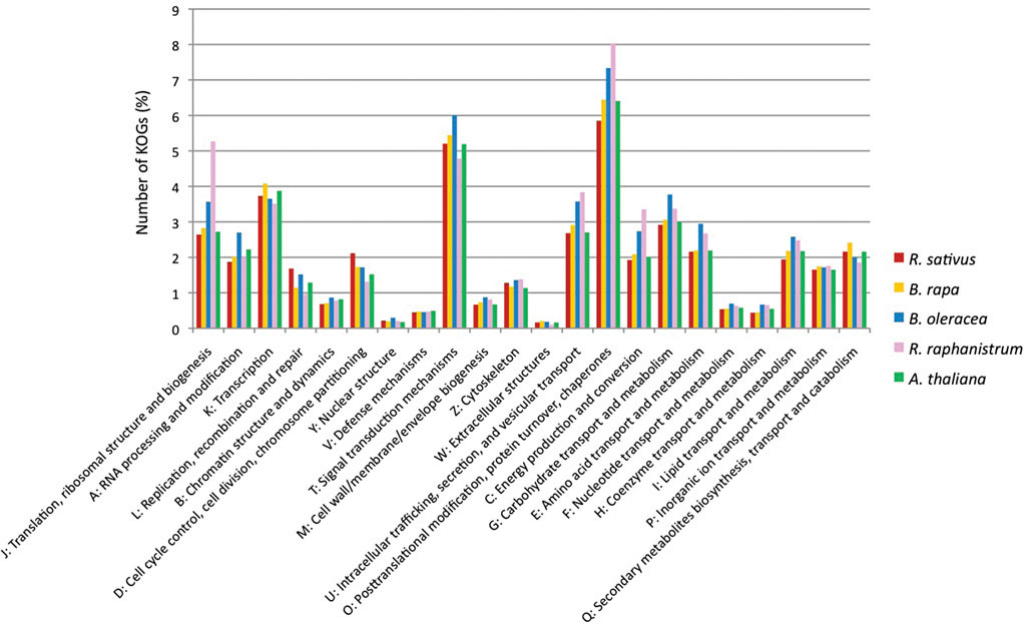
Comparison of KOG (Clusters of Orthologous Groups of proteins) classifications between *R. sativus*, *B. rapa*, *A. thaliana*, *R. raphanistrum*, and *B. oleracea*.

The 61,572 genes predicted by Augustus were annotated by the following analyses. First, the predicted genes in the radish genome of ‘Aokubi’ together with those in the *A. thaliana* and *B. rapa* genomes and EST-derived unigenes for *B. oleracea* and *R. raphanistrum* were clustered. The 61,572 genes in *R. sativus*, 41,019 in *B. rapa*, 35, 386 in *A. thaliana*, 36,862 in *B. oleracea*, and 22,618 in *R. raphanistrum* were clustered into 24,188; 17,942; 16,357; 19,807; and 11,843 families, respectively (Fig. 1). Of them, 6,110 families were common among the five species. The number of families specific to *R. sativus* was 8,759 and the distribution of the species-specific families to total families was 36.2%, which was much higher than those in *B. rapa* (15.6%) and *A. thaliana* (16.2%), suggesting that unique sequences are richer in radish than in *B. rapa* and *A. thaliana*. Functions of the predicted genes were investigated and compared with those in *R. sativus*, *A. thaliana*, *B. rapa*, *B. oleracea*, and *R. raphanistrum*. Among the radish predicted genes, 21,828 showed similarities to protein-encoding sequences in NCBI's KOG database^23^ with functional classification (Supplementary Table S9). Although their distributions are similar to those of the five species (Fig. 2), comparatively higher values in two KOGs, i.e. ‘replication, recombination, and repair’ and ‘cell cycle control, cell division, and chromosome partitioning’, than those in *B. rapa* and *A. thaliana* were displayed in *R. sativus*.

### 3.3. SNP identification by whole-genome sequencing

Genome-wide SNPs were identified by a sequencing strategy. Whole-genome resequencing of a radish line, ‘Sayatori’, was carried out using an Illimina GAIIx sequencer, and a total of 14.3 Gb data, mean depth of 28 times, were obtained. The reads were filtered with a quality score of <10 and mapped on RSA_r1.0 to discover SNP candidates (Supplementary Table S10). Consequently, a total of 1,137,732 SNP candidates were identified from 151,012,313 bases of RSA_r1.0 with a read depth of 3–18 times. Of them, 500,113 and 637,619 SNP candidates were found in the intrinsic and partial genic regions (77,327,761 bases) and in intergenic regions including TEs, pseudogenes, and short genes (73,684,552 bases), respectively. SNP densities were, therefore, calculated to be 1/155 and 1/116 bp in the genic regions and the intergenic regions, respectively. The ratios of transitions to transversions were 1.3 (376,530/261,089) and 1.4 (281,810/218,303) in the genic regions and the intergenic regions, respectively.

### 3.4. Construction of a high-density linkage map of DNA markers

Of the 670 primer pairs newly designed from the radish unigene sequences (http://radish.plantbiology.msu.edu), single DNA fragments were amplified by 528 primer pairs; of which, 351 showed nucleotide polymorphism between ‘Sayatori’ and ‘Aokubi’, which are the parents of F_2_ plants used for DNA marker mapping, by the Sanger sequencing method. According to the identified SNPs, 351 dot-blot-SNP markers were developed and named <RS2> <EST name> <s>. Additionally, SNPs were surveyed between ‘Aokubi’ and ‘Sayatori’ by mapping of ‘Sayatori’ reads to ‘Aokubi’-scaffold sequences, whose sequence data were collected by the Illumina sequencer as described in the previous paragraph. SNPs were randomly selected and 140 primer pairs were designed for amplification of the regions containing SNPs. Of these, 129 primer pairs amplified single DNA fragments of both ‘Aokubi’ and ‘Sayatori’. Dot-blot-SNP markers were designed and named <RGA> <scaffold name> <s>. The MPMP dot-blot-SNP method^7^ was employed for SNP genotyping.

Of the 351 and 129 dot-blot-SNP markers, 181 and 94, respectively, showed clear dot-blot signals with distinct differences between SNP alleles. In total, 275 DNA markers were used for analysis of 189 F_2_ plants. Taken together with the genotype data of 746 markers in the previously published map,^7^ linkage analysis was performed by the JoinMap 4.0. As a result, 954 markers including 889 RS2-SNP markers and 65 RGA-SNP markers were assigned to nine LGs, designated as R1–R9.^7^ The information of new dot-blot-SNP markers is shown in Supplementary Table S11.

To map more DNA markers onto the linkage map, selective mapping was carried out by genotyping analysis using 29 of the 189 F_2_ plants. Preliminarily, using a part of the Illumina sequence data of ‘Aokubi’ and ‘Sayatori’, we mapped reads of ‘Sayatori’ to contigs of ‘Aokubi’ by CLC Genomics Workbench 5.5 (CLC Bio.) to design PCR-RFLP markers. One hundred and sixteen PCR-RFLP markers were found to be available for genotyping of F_2_ plants and those were named <RGB> <contig name> <c> (Supplementary Table S12). Furthermore, after construction of RSA_r1.0 scaffolds, 1,028 PCR-RFLP markers were designed by the comparison of sequences between RSA_r1.0 scaffolds of ‘Aokubi’ and reads of ‘Sayatori’. Six hundred and fifty-two markers were added to the linkage map and the markers were named <RGC> <order of design of primer pair> <c> (Supplementary Table S13). Consequently, a linkage map of 1,020 cM with 1,722 markers was constructed.

Another linkage map reported by Shirasawa *et al.*^32^ has been constructed with 832 markers including mainly 630 EST-SSR markers using the different population. Among them, 12 makers were common between both linkage maps. One hundred and sixteen SSR markers were used for analysis of ‘Aokubi’ and ‘Sayatori’; of which, 41 showed polymorphism between them. Of the 41 markers, 37 were available for genotyping of the 189 F_2_ plants. Using a total of 49 markers, an integrated map was constructed by the MergeMap software. The integrated map consisted of 2,553 markers (Supplementary Fig. S2 and Table S14). Respective linkage groups for the *R. sativus* LGs were assigned from R1 to R9, according to Li *et al.*^7^ (Supplementary Table S15). The total length covered by the integrated linkage map was 1,165.8 cM with an average interval distance between neighbouring markers of 0.46 cM (Supplementary Table S15).

SNP markers showing distorted segregation were surveyed. Five regions showed segregation ratios significantly deviated from the expected ratio, i.e. 1 : 2 : 1. A region from RSCL4186s to RGA1553s in R3 had segregation ratio of 2 : 3 : 1. Segregations of a region from RS2CL1405s to RS2CL3657s in R5, a region from RS2CL7837s to RS2CL7123s in R6, and a region from RS2CL6859s to RSCL8726s in R6 were approximately 1 : 1 : 1. A region from RS2CL1468s to RS2CL1940s in R8 showed a segregation ratio of 1 : 3 : 1.

### 3.5. Assignment of scaffolds to the integrated map and comparison with the genome of *B*. *rapa*

RSA_r1.0 scaffolds were assigned to the integrated linkage map using alignments with the marker sequences. To R1, R2, R3, R4, R5, R6, R7, R8, and R9, 98, 164, 122, 212, 196, 189, 108, 120, and 136 scaffolds were assigned, respectively (Supplementary Table S16). A total of 1,345 scaffolds spanned 116.0 Mb, which covers 21.8% of the *Raphanus* genome.

Since *B. rapa* and *R. sativus* are considered to have originated from the same ancestral species after genome triplication, which was followed by extensive genome rearrangements, chromosome synteny was investigated by comparative mapping. The sequences of the DNA markers on the integrated linkage map were compared with the genome sequences of *B. rapa* by BLASTN. Under a significance *E*-value threshold of <1*E*−50, *B. rapa* homologous sequences were identified (Supplementary Table S14). According to the genome collinearity between *B. rapa* and *R. sativus,* 49 SRs were identified (Supplementary Table S14)*.* The whole linkage groups of R3 and R8 were confirmed to show almost complete synteny with the upper part of A3 and all of A8, respectively. The collinearities of R3 and R8 were also observed in C3, B3, and S3, and in C8, B7, and S8.^7^ This observation suggested that a rearrangement event has seldom occurred in these chromosomal regions, although the reason is unknown. On the other hand, the R5 and R6 linkage groups showed very complicated genome syntenies, being composed of nine and eight SRs derived from parts of five and six linkage groups of *B. rapa*, respectively (Supplementary Table S14). In the other *R. sativus* linkage groups, such as R1, R2, R4, R7, and R9, similar complicated compositions were also observed (Supplementary Table S14). In Poaceae genomes such as rice and barley and in Solanaceae genomes such as tomato and potato, highly syntenic relationships between close relative species have been reported.^8,9^ To the contrary, highly complicated relationships have been observed between *B. rapa* and *B. oleracea*.^7^ Similar complexity was also detected between *B. rapa* and *R. sativus*. Whole genome triplication (WGT) of ancestral species of these *Brassica* crop species has been estimated to have occurred between 13 and 17 million years ago.^35,36^ After WGT, chromosome rearrangements might have occurred many times by the time *R. sativus* was established.

**Figure 3.**
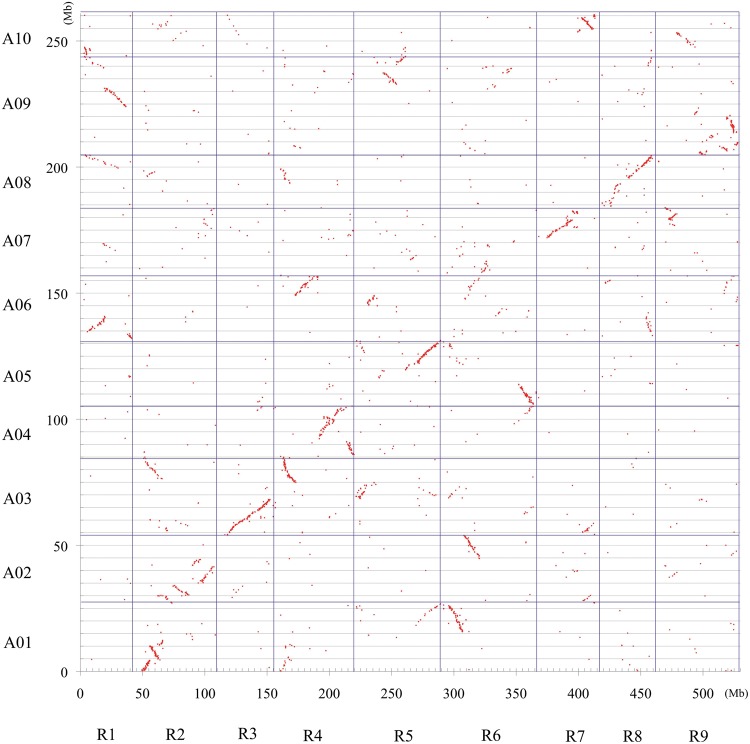
Dot-plot view of SRs of *R. sativus* (horizontal) and *B. rapa* (vertical) genomes. All genomic sequences of predicted genes in the pseudomolecules of scaffolds assigned to the *R. sativus* linkage map and those in the *B. rapa* genome were compared with each other, using nucleotide BLAST. The genes of *B. rapa* with the lowest *E*-value, which meets <1*E*−100, were regarded as the syntenic homologues, and the dots were plotted on the chart (see Materials and Methods). The genetic distances between the scaffolds were converted to physical distances based on the ratio of total length of linkage map and genome size of *R. sativus*. Axes represent the concatenation of all chromosomes for the corresponding genomes. Gridlines indicate the boundaries between chromosomes.

Based on the genomic sequences of the scaffolds that were anchored to the linkage map, collinearity with the *B. rapa* genome was surveyed. Genomic sequences of predicted genes in the anchored scaffolds were aligned with those of *B. rapa*^10^ by BLAST and those with low *E*-values (<1*E*−100) were 10,995 genes in *R. sativus* and 10,422 in *B. rapa*. The dot-plot view (Fig. 3) revealed the same large SRs between genomes of *R. sativus* and *B. rapa*, corresponding to the 49 SRs (Supplementary Table S14).

### 3.6. SNP identification by sequencing of bulked PCR products in other *Raphanus* lines

In our previous studies, 2,880 primer pairs were designed to construct an SNP-based linkage map,^7^ and Zou *et al.*^29^ developed a highly efficient method for identification of SNPs by determining nucleotide sequences of the bulked PCR products amplified by these primer pairs using an NGS. Using the same primer pairs, multiplex PCRs were carried out in four inbred lines of ‘Yumehomare’, ‘Sakurajima’, ‘N1-3’, and ‘Nishimachi-Risou’, and the nucleotide sequences were determined by an Illumina sequencer. The short reads of these lines along with those of ‘Taibyosobutori’ and ‘AZ26H’^29^ were mapped onto fragments of RSA_r1.0 scaffold sequences. Taken together with the identified fragments of ‘Sayatori’, SNPs were surveyed between all inbred lines and the results for the number of SNPs and the number of common amplicons containing SNPs between different lines are shown in Supplementary Table S17 and Table S18, respectively. A great number of SNPs were detected in the combination of ‘Sayatori’ and the other inbred lines. The number of SNPs per common amplicon was over 5.5 and was the most, i.e. 6.95, between ‘Aokubi’ and ‘Sayatori’. In the other combinations, the number of SNPs ranged from 2,066 at minimum between ‘Taibyosobutori’ and ‘Aokubi’ to 3,568 at maximum between ‘Sakurajima’ and ‘Aokubi’. Consequently, many SNPs were detected in every combination and will certainly be useful for molecular genetic studies such as QTL analyses, as described by Zou *et al*.^29^

## 4. Database

The draft genome sequences (RSA_r1.0), gene sequences, and SNP information between cultivars are available from the *Raphanus sativus* Genome DataBase (http://radish.kazusa.or.jp). The sequence data used in this study are available from the DDBJ Sequence Read Archive (DRA) under the following accession numbers: DRR014095 [Illumina Paired-end (PE) (insert size 100 bp) of ‘Aokubi’], DRR014096, and DRR014097 [Illumina Mate-pair (MP) (insert size 5 Kb) of ‘Aokubi’], DRR014098 [Illumina PE (insert size 100 bp) of ‘Sayatori’], DRR015470 [Illumina Single-end (SE) of Taibyosobutori], DRR015471 (Illumina SE of Yumehomare), DRR015472 (Illumina SE of Sakurajima), DRR015473 (Illumina SE of AZ26H), DRR015474 (Illumina SE of N1-3), and DRR015475 (Illumiina SE of Nishimachi-Risou). The BAC-end sequences are available from accession numbers GA872392–GA901611 (29,220 entries).

## Supplementary data

Supplementary data are available at www.dnaresearch.oxfordjournals.org.

## Funding

This work was supported by the Program for the Promotion of Basic and Applied Researches for Innovations in Bio-oriented Industry (BRAIN), Japan.

## Supplementary Material

Supplementary Data
