# Supplementary material for: Draft Sequences of the Radish (Raphanus sativus L.) Genome
Source: DNA Res. 2014 May 16;21(5):481–90. doi: 10.1093/dnares/dsu014 (PMC4195494; doi:10.1093/dnares/dsu014)
Supplement: Supplementary Data [file supp_21_5_481__index.html]

Draft Sequences of the Radish (Raphanus sativus L.) Genome — Supplementary Data 

# Draft Sequences of the Radish (*Raphanus sativus* L.) Genome

## Supplementary Data

Supplementary Data

**Files in this Data Supplement:**

- Supplementary Data - Pdf file
- Supplementary Table 1-4 - pdf file
- Supplementary Table 5-7 - pdf file
- Supplementary Table 8-18 - pdf file
- Supplementary Table 4-3 - xls file
- Supplementary Table 4-4 - xls file
- Supplementary Table 4-5 - xls file
- Supplementary Table 4-6 - xls file
- Supplementary Table 4-7 - xls file
- Supplementary Table 4-8 - xls file
