# Supplementary material for: Draft Sequences of the Radish (Raphanus sativus L.) Genome
Source: DNA Res. 2014 May 16;21(5):481–90. doi: 10.1093/dnares/dsu014 (PMC4195494; doi:10.1093/dnares/dsu014)
Supplement: Supplementary Data [file supp_dsu014_dsu014supp_tables1.pdf]

Table S1 Statistics of the assembled scaffolds only by short-read sequences

Number of scaffolds (>100 bp)	992,801
Total length (bp)	473,904,309
Average size (bp)	477
Maximum size (bp)	343,168
N50 (bp)	13,462

Table S2　Summary of BAC clones

BAC clones (Number of ends)	20,736 (41,476)
Number of sequenced ends	34,417
Number of nuclear sequences	27,904
Number of chloroplast	6,396
Number of mitochondria	36
Number of bacteria	80
Number of vecotor	1

Table S3 Linkages of both ends of scaffolds

ID of scaffold	Size of scaffold (bp)	Name of marker	Type of DNA marker		Primer sequence		Restriction enzyme	Probe seq.	Hybridization condition		Probe seq.	Hybridization condition		Linkage
					Forward	Reverse		Saya	temperature(°C)	SSC (x)	Aokubi	temperature(°C)	SSC (x)	
Rsa1.0_00002.1	601,661	Rsa00002_5'	dCAPS	co-dominant	ATCTCATTCAGAATTATAAGACCCG	TGTATTGTGTGTGGCATCTC	Hap II	–	–	–	–	–	–	LK
		Rsa00002_3'	CAPS	dominant	GGTGACTGCATTGCTTTCG	TCCATCCTGACGTTCTCCTC	Taq I	–	–	–	–	–	–	
Rsa1.0_00004.1	253,996	Rsa00004_5'	dCAPS	dominant	ATTTGTTTTTTTATGTTAAATTTCG	GGAAAGAGTTAAGGGCACTTGT	Taq I	–	–	–	–	–	–	LK
		Rsa00004_3'	dot-blot	dominant	CGCCATAGCTGTTCTCATCA	AGCTCGACCCAGAACATGAC	–	ACTCGGATGTATCTCTC	40	0.5	ACTCGGATATATCTCTC	40	0.5	
Rsa1.0_00019.1	301,669	Rsa00019_5'	dot-blot	co-dominant	GCTCTCCTTGTTTGCTCGTC	TCCAAGCAGATCGCCTTATC	–	ACCATCATTTTATATTA	40	0.5	ACCATCATCTTATATTA	40	0.5	LK
		Rsa00019_3'	dot-blot	dominant	TGCTTACGGTTCATCTTTGG	CCCTCATTTTCTACGAACTCTC	–	ATGTAATATAAAGAGAA	35	1	ATGTAATACAAAGAGAA	40	1	
Rsa1.0_00022.1	200,461	Rsa00022_5'	CAPS	co-dominant	GAGCTGGTCCTCCAGAGAAA	GTGGTCGTTGATGTCGTGAA	Alu I	–	–	–	–	–	–	LK
		Rsa00022_3'	dot-blot	co-dominant	ACTTCTGCCGTTGTGCTTTG	CATCTGCATGGGACAAAATG	–	CCAGTCAAGGATTTCTGG	40	0.5	CCAGTCAATAATTTCTGG	40	0.5	
Rsa1.0_00023.1	194,764	Rsa00023_5'	CAPS	dominant	GCTGCCTGCAGTTAAAGGAG	TGGGCGCTAAGGAATTGTAG	Kpn I	–	–	–	–	–	–	LK
		Rsa00023_3'	dot-blot	co-dominant	GATTTTCTCCCCAAAACCTC	GCGAAGTAGCGAGAATGATG	–	GTCTAACATGTTCAGGG	40	0.5	GTCTAACACGTTCAGGG	40	0.2	
Rsa1.0_00025.1	277,312	Rsa00025_5'	CAPS	dominant	TGGTGGGAGGTTCTTTAG	TCACAGTTCATCACTCTAGG	Mun I	–	–	–	–	–	–	LK
		Rsa00025_3'	CAPS	co-dominant	ATGGGGATTTTTGATCATGG	TGGTGAATTTGGAAGGGAAG	Ssp I	–	–	–	–	–	–	
Rsa1.0_00027.1	283,362	Rsa00027_5'	dot-blot	co-dominant	GTGAATGCTAGCAGTTGTCCA	ATACAAGTCTCCGGCACCAC	–	CATTGGAAGTTTAGACA	40	0.5	CATTGGAATTTTAGACA	40	1	LK
		Rsa00027_3'	dot-blot	co-dominant	GGAAGCGGTGATGGAGATAA	TGGAAGGGTAACGGATCTTG	–	TTTATTATGTTATTACA	35	1	TTTATTATATTATTACA	35	1	
Rsa1.0_00036.1	176,186	Rsa00036_5'	CAPS	dominant	TCGAACTTACCGTCGAAACC	AAAGATCCCACATTGCAAGC	Hae III	–	–	–	–	–	–	LK
		Rsa00036_3'	dot-blot	co-dominant	CGGTGTGAAGTTGTTTGTGG	AGTGGCACTGGGAAGACAAG	–	TTAGCTTTGACTTTGAG	50	0.5	TTAGCTTTAACTTTGAG	40	0.5	
Rsa1.0_00042.1	171,507	Rsa00042_5'	dCAPS	dominant	AGAGAATGAGAGCGAAGATG	CTATAGATAAAGTTTGACCAAACTCG	Taq I	–	–	–	–	–	–	LK
		Rsa00042_3'	CAPS	dominant	CTTGGCAGAAAAATAACCTC	TGAGAGAGTTTGCGGATGTG	Xba I	–	–	–	–	–	–	
Rsa1.0_00043.1	264,937	Rsa00043_5'	dCAPS	co-dominant	GTATTTGGATCCGTTCTCG	GTTATTACCTGACAAAAGAGTGATC	Taq I	–	–	–	–	–	–	LK
		Rsa00043_3'	CAPS	dominant	GCTCATCGTGTAGCTGCTGA	AAGCCGTTCTCATGGACTTG	Xsp I	–	–	–	–	–	–	
Rsa1.0_00048.1	167,185	Rsa00048_5'	dot-blot	co-dominant	TTGGGCTTGGGATTTGATAG	ACGAAACACAACACGACCAA	–	TAACACATTAAGAAATG	40	1	TAACACATGAAGAAATG	40	1	LK
		Rsa00048_3'	dot-blot	co-dominant	ACGTGGGCTTAAACTGAACG	TCCACAACATGCCGTATCTC	–	GCCGTCATCATTGCAGT	50	1	GCCGTCATGATTGCAGT	50	1	
Rsa1.0_00065.1	454,616	Rsa00065_5'	dot-blot	co-dominant	CGCCTTTAAAAGAGCTGGTA	TTTAGACGGCTGGGATGAAG	–	ATCAATAGGTCATATTC	35	1	ATCAATAGATCATATTC	40	0.5	LK
		Rsa00065_3'	CAPS	co-dominant	GACACATGGTTCAGTGACGTG	GCCTAAGTTGGCACGTTTAAG	Alu I	–	–	–	–	–	–	
Rsa1.0_00100.1	249,232	Rsa00100_5'	SCAR	co-dominant	AGTCACGGACAACGAAATCC	TGACACCGAGAACAGCAAAG	–	–	–	–	–	–	–	LK
		Rsa00100_3'	dot-blot	co-dominant	AATGGATAGCCAACCAAACG	ACACGCATTATCGCCTTCC	–	TTACCATAGATCATTGC	35	1	TTACCATAAATCATTGC	35	1	
Rsa1.0_00123.1	325,650	Rsa00123_5'	dot-blot	dominant	CCCTCCAAATAACGAAAGAGC	AGAGAAGATCGGTCCGAAGAG	–	GCCCTAATTCACTCGCC	50	1	GCCCTAATCCACTCGCC	50	1	LK
		Rsa00123_3'	dot-blot	co-dominant	AGAGTGCCAACATTTTATTGATACC	GAGCGCGACACATAGGAAAC	–	ATGACATGGCTTATAGT	35	1	ATGACATGACTTATAGT	35	1	
Rsa1.0_00125.1	253,043	Rsa00125_5'	dot-blot	co-dominant	AGTCTTGCCTTTTTGCTCCA	AAACTCTCAGCCGTTTGGAA	–	CAATCTTCTGACCGATT	45	0.5	CAATCTTCCGACCGATT	45	0.5	LK
		Rsa00125_3'	dot-blot	co-dominant	TTCGTCAACACCAAGCATTC	TTTTGGAGCCAAGTTTCTCA	–	AGAGAACCTTTCTCTATT	40	1	AGAGAAACTTTATCTATT	50	1	
Rsa1.0_00134.1	390,219	Rsa00134_5'	CAPS	co-dominant	GTCAAAATTCCAGGAGTTCCAC	AAGGTCACCATCTCCACCAG	Mun I	–	–	–	–	–	–	LK
		Rsa00134_3'	CAPS	co-dominant	GGCGCCAATTTGATGTTAG	TCCAATTCACTGATTGCAC	Dde I	–	–	–	–	–	–	
Rsa1.0_00152.1	257,730	Rsa00152_5'	dot-blot	co-dominant	AAACCCCTCAACCGTGAAG	CGTGCACTACAAGAAAACATCG	–	CGAGGAAACCCTATTGT	50	1	CGAGGAAATCCTATTGT	40	0.5	LK
		Rsa00152_3'	dot-blot	co-dominant	GCTCGCTGCTCAGTAAGAAAA	ACGCAGGCATAGACATCAAAC	–	AGTCACAACTCGTAAGT	50	1	AGTCACAAATCGTAAGT	40	0.5	
Rsa1.0_00173.1	288,743	Rsa00173_5'	dot-blot	co-dominant	GAATCAATGGGGAATCGAAG	TTTATCGGTTCGGTTCTGGA	–	ATCGAAGACGGGTTATT	50	1	ATCGAAGATGGGTTATT	50	1	LK
		Rsa00173_3'	dCAPS	co-dominant	AGTGTTTAGTATCGTCTCTCGAGAT	TAGAGCCTCTTGGGATTTTGAG	Mbo I	–	–	–	–	–	–	
Rsa1.0_00179.1	393,324	Rsa00179_5'	dCAPS	co-dominant	GAGACACAACATACGACAAC	CTTATGGAGTTCATGCTTTGAT	Mbo I	–	–	–	–	–	–	LK
		Rsa00179_3'	dot-blot	co-dominant	AGTAACAAACGAACGCCACA	CAACGATGTGGTGAAGAACG	–	TTAGTTAATTAGTGATC	35	1	TTAGTTAAGTAGTGATC	40	1	
Rsa1.0_00215.1	201,775	Rsa00215_5'	dot-blot	co-dominant	TCCTTCTTCCAAACCACCAC	CCTTGTGGAAAACCTCCTGA	–	GCATGCCTAGAGTAGGA	50	1	GCATGCCTCGAGTAGGA	50	0.5	LK
		Rsa00215_3'	dot-blot	co-dominant	ACTGAAATGCAACTGCGATG	TGACCTTGGGAAAGATGGAG	–	GTATTGAATACACTTTCA	40	0.5	TGTATTGAACACTTTCAA	40	0.5	
Rsa1.0_00224.1	327,530	Rsa00224_5'	dot-blot	dominant	CTGGGCTTTGTGCATTTTG	GCCACATTTGTGGTTCTCCT	–	AATATGTGAGAAACCTGT	40	0.2	AATATGTGTAAAACCTGT	40	1	LK
		Rsa00224_3'	dCAPS	dominant	GCACTCTGCAGCAGCCTGG	TCCCTAAGCAGCCGGTCAAG	Taq I	–	–	–	–	–	–	
Rsa1.0_00234.1	197,060	Rsa00234_5'	CAPS	dominant	GGCAACATCACGACTCTTCA	CTTCCGAAACAGAGCGATTC	Sac I	–	–	–	–	–	–	LK
		Rsa00234_3'	CAPS	co-dominant	CATCAACGCTCTGCTCTTTG	AGAAACAAAGCACGGTGTCC	Sph I	–	–	–	–	–	–	
Rsa1.0_00238.1	193,193	Rsa00238_5'	dCAPS	co-dominant	TTCCTTGGGGGTTTTAAGTG	GATGGTCGACTTGAATGGTAG	Bgl II	–	–	–	–	–	–	LK
		Rsa00238_3'	CAPS	co-dominant	ATGTGACGGAGGTTCCTTTG	ACCGCCATATTCTCATCGTC	Hae III	–	–	–	–	–	–	
Rsa1.0_00255.1	204,967	Rsa00255_5'	dot-blot	co-dominant	AGGTGCTGAATGGTGGTCTC	CTGCAAGTAGCACGACGAAG	–	TCTTGCAAGTTTATTGA	40	0.2	TCTTGCAAATTTATTGA	40	0.5	LK
		Rsa00255_3'	CAPS	dominant	ACACGTGTCCAAAGGAAACC	CGTGGAAGCATGAATCAAGA	Alu I	–	–	–	–	–	–	
Rsa1.0_00273.1	326,186	Rsa00273_5'	dCAPS	dominant	TTCCTAAACTTTCACTTGTCCTATC	GGACTTGAGCGGTGTTTTATAG	Taq I	–	–	–	–	–	–	LK
		Rsa00273_3'	dot-blot	dominant	TTCACCTACCAGGGAAGCAG	ACCTCCTCAAAACACGTTGG	–	AGAAGACTAACTTTAGC	40	1	AGAAGACTGACTTTAGC	40	1	
Rsa1.0_00307.1	199,003	Rsa00307_5'	CAPS	co-dominant	GTATGGGAGTTGCCCAAAA	CGGAGAATCTATTTGTGTTGAG	Dra I	–	–	–	–	–	–	LK
		Rsa00307_3'	dCAPS	co-dominant	AAAGCATTTGTTTTGATGTAC	ATATTCAAACAGCAAACCTGTA	Bsp1407 I	–	–	–	–	–	–	
Rsa1.0_00344.1	191,774	Rsa00344_5'	dCAPS	co-dominant	GAATTCAACTGCTTGGACAG	CTTACCACTACAAGTTTGCTTCG	Taq I	–	–	–	–	–	–	LK

		Rsa00344_3'	CAPS	co-dominant	GCCGCTAGAGCAAACTATGG	ACCCTAACCGTGTGAGCAAG	Xsp I	–	–	–	–	–	–	
Rsa1.0_00351.1	413,096	Rsa00351_5'	CAPS	co-dominant	GAATCCTAGGGCGATCATACTG	GGGTGTGAGTGTGATTGTATGG	EcoR V	–	–	–	–	–	–	LK
		Rsa00351_3'	dot-blot	dominant	GGACACAAATGAAACCGAGAC	CAAAGTCGGATTTGGATTGC	–	AATAATCTGCATTTTTG	40	1	AATAATCTTCATTTTTG	40	1	
Rsa1.0_00387.1	495,407	Rsa00387_5'	dot-blot	co-dominant	TGCATTGTATAGGAGGCATGA	ACAGAAGCCGTTCTTTGTGG	–	GAGAACTTCAAATAGTA	40	1	GAGAACTTTAAATAGTA	40	1	LK
		Rsa00387_3'	CAPS	dominant	GCGACAAAACGAGTTCTTCC	GCAACTCACTCGCAACTCAA	Xsp I	–	–	–	–	–	–	
Rsa1.0_00427.1	189,678	Rsa00427_5'	dot-blot	co-dominant	CTTGATGCCATTGATGTTGC	AAGTGGCATGCAAGGTTAGG	–	GAATGACTGTCGTGTTT	50	1	GAATGACTTTCGTGTTT	50	1	LK
		Rsa00427_3'	CAPS	co-dominant	GAGAGTCGGCAGAAAATCCA	CGGAGCTGTTCATTCGGTA	Taq I	–	–	–	–	–	–	
Rsa1.0_00490.1	186,713	Rsa00490_5'	CAPS	co-dominant	CCAGTCTCCATTGCAGAACA	GCGAGAATCTGACCAAGAGG	Alu I	–	–	–	–	–	–	LK
		Rsa00490_3'	dot-blot	co-dominant	ATCAAACACATGGCTTGCTG	TCCACGTCCGAGTTACATTG	–	TTAAAAGAGATCTTCAG	40	1	TTAAAAGAAATCTTCAG	40	1	
Rsa1.0_00576.1	178,949	Rsa00576_5'	dot-blot	dominant	TTGCAGTTTGCACGAAAAAG	ATCGTCGACCGTGTTCTCTC	–	TTATAGGATTGCAAACA	40	0.5	TTATAGGACTGCAAACA	40	0.5	LK
		Rsa00576_3'	dot-blot	dominant	GATCTTTGCGGGTTCATTTC	TAAGAGCCAAAACCGAATCC	–	AAAAGTAACATATCAAA	35	1	AAAAGTAAAATATCAAA	35	1	
Rsa1.0_00577.1	174,827	Rsa00577_5'	dot-blot	co-dominant	CAAATCCTCACGCCACACTA	GAGAGAGCATCGGAGACACC	–	CCGATTCTATTATATTC	40	0.5	CCGATTCTGTTATATTC	40	1	LK
		Rsa00577_3'	dot-blot	co-dominant	CTTGGGGAGACAGCAAAATC	AACGCTTCGACTCTTCCTTG	–	ACTCCTCATATTGCTTG	40	1	ACTCCTCACATTGCTTG	40	0.5	
Rsa1.0_00645.1	189,716	Rsa00645_5'	dCAPS	co-dominant	CCATGTTGTGGTGTTTGGAC	CAAACCCATTCTTAGCATTCCG	Hap II	–	–	–	–	–	–	LK
		Rsa00645_3'	CAPS	co-dominant	TTTCCATGTTTGCATCTTGC	CTCGCTTGTTGGTGTTGTTG	Hinf I	–	–	–	–	–	–	
Rsa1.0_00662.1	192,183	Rsa00662_5'	CAPS	co-dominant	CCCATGGACACACCTTCTG	GGAAGCTTTCTTTGAAGATTGG	AluI	–	–	–	–	–	–	LK
		Rsa00662_3'	CAPS	co-dominant	CGACCATTGCACAGAGAGAA	TGCTCCTGCGATTGTAGTTG	Dde I	–	–	–	–	–	–	
Rsa1.0_00743.1	177,459	Rsa00743_5'	dCAPS	dominant	CATACACATGAAAACGTATCATTA	TCTTCAGCATCACCATCACC	Vsp I	–	–	–	–	–	–	LK
		Rsa00743_3'	dot-blot	co-dominant	TTGATCTGCTTGTTCTGCAAG	GAGTACCTCCTCCGGGAAAC	–	CTTAAACAACTATAACT	35	0.5	TGCTTAAACTATAACTAT	35	1	
Rsa1.0_00750.1	190,548	Rsa00750_5'	dot-blot	dominant	CAGACCCGTGCATATACACATAA	TTGCTGCATAGCTGCATACG	–	AGAATCTACACAAATAT	40	1	AGAATCTATACAAATAT	35	1	LK
		Rsa00750_3'	dot-blot	co-dominant	CCCAGCTCTACCTGTATAATTTGA	ACCCATGGACGCTCTTAGTG	–	TATCAACCAGAATAGCA	50	1	TATCAACCATAATAGCA	50	1	
Rsa1.0_00816.1	403,350	Rsa00816_5'	dot-blot	dominant	GTCTATCGCGAGCAAGGAAC	TCTCCACCTCCGTTTCAATC	–	CGGATGAGAATGGAAAG	50	0.5	CGGATGAGGATGGAAAG	50	0.5	LK
		Rsa00816_3'	CAPS	co-dominant	GGACTAAAAGCAGCGTTCCA	CCGTGCAGTTTTGTGGTAAA	Hae III	–	–	–	–	–	–	
Rsa1.0_00835.1	278,058	Rsa00835_5'	dot-blot	co-dominant	TCATATGTAACATGGTCACG	GGGATTCAAAGTTGTAGAAGC	–	ACCTAACTATCATTACT	35	1	ACCTAACTGTCATTACT	35	1	LK
		Rsa00835_3'	CAPS	co-dominant	ATGTGTTGGAAGTTGCTTCG	CATGTGAATCGACATTGACC	Hind III	–	–	–	–	–	–	
Rsa1.0_00878.1	330,303	Rsa00878_5'	dCAPS	co-dominant	ACTATTTTAGCACAAGACCGGAGC	AGGATATGACCTGCGCCTTG	Alu I	–	–	–	–	–	–	LK
		Rsa00878_3'	dot-blot	co-dominant	CAATCACCATCCGACCTTTC	AACGTGGATTGATGACATGC	–	ACCCATCCTTCCACTAC	40	0.5	ACCCATTCTTTCACTAC	40	0.5	
Rsa1.0_00894.1	269,700	Rsa00894_3'	CAPS	co-dominant	TGACCATGCTACAGGAGAACA	CATTCTCGTGGTAGGTCTCG	Hinc II	–	–	–	–	–	–	LK
		Rsa00894_5'	CAPS	co-dominant	CAGCGGAAACTGCAACTTAAC	GGTGAAACTTATGGTGCCAGAC	Alu I	–	–	–	–	–	–	
Rsa1.0_00904.1	180,594	Rsa00904_5'	dot-blot	co-dominant	AAAACCGATCCGAAAATCAG	AGACCTGGACGTTTGTGTAT	–	CCGAATGAGTACACAAA	35	1	CCGAATGAATACACAAA	35	1	LK
		Rsa00904_3'	CAPS	co-dominant	CAAAACCATCCTCCATCTCC	TCCCAATGAGGTTCTTGAGG	Afl II	–	–	–	–	–	–	
Rsa1.0_00920.1	601,703	Rsa00920_5'	dot-blot	dominant	GCAATCAGCAAGTTGTTCCA	TCTGCTCGGTTGCATTACAG	–	GCCAGCAATTTCAGGTC	50	0.5	GCCAGCAACTTCAGGTC	50	1	LK
		Rsa00920_3'	dCAPS	dominant	AGAACATGAAGCCATTGTCTA	AAACTCCCCCTGATGAATGC	Xsp I	–	–	–	–	–	–	
Rsa1.0_00926.1	195,072	Rsa00926_5'	CAPS	co-dominant	CAGAATATCAAATCGGCGTTAC	TGTTACACTCTAGGGCCAACG	Afa I	–	–	–	–	–	–	LK
		Rsa00926_3'	CAPS	dominant	CTACTTCGCCCTACGTCTCG	GTTCCGGCCAAAAGAAGAAG	Alu I	–	–	–	–	–	–	
Rsa1.0_00934.1	193,516	Rsa00934_5'	dot-blot	dominant	ACTTCGGATACCCCCGAATA	TTTGGGTCTGTTTGGGTTTC	–	TATATAATGAATTTTAA	35	1	TATATAATAAATTTTAA	35	1	LK
		Rsa00934_3'	dot-blot	dominant	ATGTTGGCGCAGAAAAAGAG	CGACAGCTCCTCCTTGAGAC	–	TCCTTTTGAGATATCGA	40	0.5	TCCTTTCGAGCTATCGA	50	1	
Rsa1.0_00938.1	193,891	Rsa00938_5'	dot-blot	co-dominant	AAATCGTCGACCCCTCTCTC	CAGGGGCAGATCCAAGATAA	–	GGGATAAAAATGCTCTA	40	0.5	GGGATAAAGATGCTCTA	40	0.5	LK
		Rsa00938_3'	dot-blot	dominant	TGAAAGAAGCGAAGCAGAGAG	TTACCAAATCACACCGAAACC	–	ATAGCTCAAAGCTGAAA	50	0.5	ATAGCTCAGAGCTGAAA	50	0.5	
Rsa1.0_00977.1	198,001	Rsa00977_5'	dot-blot	dominant	TGGAGCAAAAAGGATCTTG	ATACGCTCTACCACCGTTGC	–	TAGTCTCTGTTCTTGCAG	50	0.5	TAGTCTCTATTCTTGCAG	50	1	LK
		Rsa00977_3'	dCAPS	dominant	GATGAGAGATCCAGATTACTGCG	ATCACTCCTATTGCGGTTCC	Hha I	–	–	–	–	–	–	
Rsa1.0_01029.1	256,254	Rsa01029_5'	CAPS	co-dominant	GGAGCAAAAGGTGGTAACGA	TTTCGGTAAGACGCAGGTTC	Stu I	–	–	–	–	–	–	LK
		Rsa01029_3'	CAPS	dominant	TTGAGGTGGTGATTGTGGAG	TTTCCCACCCATATCCAAGA	Nde I	–	–	–	–	–	–	
Rsa1.0_01112.1	310,601	Rsa01112_5'	dot-blot	dominant	CTATTAGTGATATTGCAAATTAATGC	GCAAGGTCAATTGGTTATAG	–	AATAATAACATACATGT	30	1	AATAATAATATACATGT	30	1	LK
		Rsa01112_3'	CAPS	dominant	TCCATGGAGTTCAGTTCACC	AATACGCCCAAAGACCAATG	Sac I	–	–	–	–	–	–	
Rsa1.0_01504.1	189,978	Rsa01504_5'	dot-blot	dominant	GAATAGCCGTTTTCCGTCAA	TCGTCTGATGAGTTGCAAGG	–	TTTTAGCACAAGACCGG	50	1	TTTTAGCATAAGACCGG	40	0.5	LK
		Rsa01504_3'	dot-blot	dominant	TTTTGCAACTCGGAATCATC	ATGCACCAGGGAGTAAATGC	–	TAGAGTTAGTCAAAGTG	40	0.5	TAGAGTTATTCAAAGTG	40	1	
Rsa1.0_01544.1	185,602	Rsa01544_5'	CAPS	dominant	CCAAAGACAAAACCAACTCTC	CCGGGTTAGAGTTTCTTCAAA	Alu I	–	–	–	–	–	–	LK
		Rsa01544_3'	CAPS	dominant	TAAAGCCTTTGACCCATTCG	TGGAAAACGTGAACGTACCA	EcoR V	–	–	–	–	–	–	
Rsa1.0_01608.1	170,540	Rsa01608_5'	dot-blot	dominant	GTGGACTTCACGAGGAAACC	TCGTTTATCGTCCGTCGTAAC	–	GGAACTTACGACGAAAT	40	0.5	GGAACTTATGACGAAAT	40	0.5	LK
		Rsa01608_3'	dot-blot	dominant	CTCAACTCCATGTTCCCTCTG	TGGAAGCGCTTTAAAATTGG	–	TGAATCAAGATTTTGAA	40	0.5	TGAATCAAAATTTTGAA	40	0.5	
Rsa1.0_01674.1	168,300	Rsa01674_5'	dot-blot	co-dominant	TGTTGGAGCAGGCAATGTAG	CCACAAAAGGGTCTTTTCCA	–	GATGTCTTCTCCCTTAG	40	0.5	TGATGTCTCCCTTAGA	40	0.5	LK
		Rsa01674_3'	CAPS	co-dominant	TATCCCAACTTTCCCGTCTG	GCGGAGCCAGAAAACATTTA	Spe I	–	–	–	–	–	–	
Rsa1.0_02020.1	173,211	Rsa02020_5'	CAPS	dominant	TTAACGCCGACGAAAACAAC	TGATGCGTATGCAGGAAGAG	TaqI	–	–	–	–	–	–	LK
		Rsa02020_3'	dot-blot	dominant	TCAATTAAGGGGCTGACTGG	TTTGGGTTCGGTCTGAGTTC		TAAAAATTCAAAACCAA	35	0.5	TAAAAATTTAAAACCAA	35	1	
Rsa1.0_02270.1	249,821	Rsa02270_5'	CAPS	dominant	GATCACCCAAGGTCAATTCC	AGCTCTTTTCGGATCCATGA	Nco I	–	–	–	–	–	–	LK
		Rsa02270_3'	CAPS	dominant	ACATGACCGAGATGGGAGAC	GGGGAATAAGGTGCAGTCAA	Hind III	–	–	–	–	–	–	

Rsa1.0_02589.1	300,828	Rsa02589_5'	dot-blot	co-dominant	CCCATGAGTTCTCCTTTCCA	AACCACCTACATCCGCAAAC	–	ATAGCTTAAAAGTATCA	40	1	ATAGCTTACAAGTATCA	40	0.5	LK
		Rsa02589_3'	dot-blot	co-dominant	AAGTTTGGTTCGGGTTCGTA	AACCAAACCTGATCCGACTG	–	TTAACTATCTTTATATT	35	1	TTAACTATTTTTATATT	35	1	
Rsa1.0_00021.1	263,655	Rsa00021_5'	CAPS	dominant	TGGAGTCTCTCTTGGGGATG	TCTTGGAGTTGGCCTTGTTC	Taq I	–	–	–	–	–	–	NL
		Rsa00021_3'	CAPS	dominant	CGCACCTTCACAAAATACGA	TCATTTTGATCCGTGCAAAG	Afl II	–	–	–	–	–	–	
Rsa1.0_00915.1	194,446	Rsa00915_5'	dCAPS	co-dominant	GTGATATGGATATGCCACACAGGC	GCACGAACACCTCCAACAAC	Hae III	–	–	–	–	–	–	NL
		Rsa00915_3'	dot-blot	co-dominant	GGGTAAGCAGCAGAACTTCG	GCCCTTGCCATTTTAACAAA	–	CTGCTTCTTTCAACGTC	50	1	CTGCTTCTGTCAACGTC	50	1	
Rsa1.0_00917.1	182,012	Rsa00917_5'	CAPS	co-dominant	CAGAAATTAGTAGCCGTGACA	GCCAATGCAGATTTACAAG	Dde I	–	–	–	–	–	–	NL
		Rsa00917_3'	dot-blot	dominant	CTCACCCAAAGCCCAAATAA	TTCGGAGTGCTATGTCCAAG	–	AGGTATGGGATGCTAAA	40	0.5	AGGTATGGATGCTAAA	40	0.5	

LK, Both ends of scaffolds are linked.

NL, Both ends of scaffolds are not linked

Table S4 Genes of 80,521 predicted by Augustus.

	vs NR (Non Redundant Database: http://blast.ncbi.nlm.nih.gov/Blast.cgi?PAGE=Proteins)										vs TAIR10 of A. thaliana						
Query	NR	Length (query)	Length (subject)	E-value	Length%	Identity%	Positive%	Product	Genbank	Species	Subject	Query	Subject	E-value	Length%	Identity%	Positve%
Rsa1.0_00001.1.g1.t1	ref|XP_002867679.1| hydrolase, alpha/beta fold family protein [Arabidopsis lyrata subsp. lyrata] gi|297313515|gb|EFH43938.1| hydrolase, alpha/beta fold family protein [Arabidopsis lyrata subsp. lyrata]	498	498	0	100.0	88.6	93.4	hydrolase, alpha/beta fold family protein	gbpln	Arabidopsis lyrata	AT4G24140.1 | Symbols: | alpha/beta-Hydrolases superfamily protein | chr4:12530032-12533664 REVERSE LENGTH=498	498	498	0	100.0	88.0	93.4
Rsa1.0_00001.1.g2.t1	gb|AAB63610.1| hypothetical protein [Arabidopsis thaliana]	384	430	1.00E-128	112.0	71.1	78.9	hypothetical protein	gbpln	Arabidopsis thaliana	AT4G24150.1 | Symbols: AtGRF8, GRF8 | growth-regulating factor 8 | chr4:12535972-12539387 FORWARD LENGTH=493	384	493	1.00E-126	128.4	64.1	70.8
Rsa1.0_00001.1.g3.t1	ref|XP_002869734.1| hydrolase, alpha/beta fold family protein [Arabidopsis lyrata subsp. lyrata] gi|297315570|gb|EFH45993.1| hydrolase, alpha/beta fold family protein [Arabidopsis lyrata subsp. lyrata]	407	417	0	102.5	90.4	95.8	hydrolase, alpha/beta fold family protein	gbpln	Arabidopsis lyrata	AT4G24160.1 | Symbols: | alpha/beta-Hydrolases superfamily protein | chr4:12539871-12542210 FORWARD LENGTH=418	407	418	0	102.7	88.7	94.8
Rsa1.0_00001.1.g4.t1	emb|CAB51660.1| putative protein [Arabidopsis thaliana] gi|7269267|emb|CAB79327.1| putative protein [Arabidopsis thaliana]	1134	1263	0	111.4	45.5	50.8	putative protein	gbpln	Arabidopsis thaliana	AT4G24170.1 | Symbols: | ATP binding microtubule motor family protein | chr4:12543206-12546805 FORWARD LENGTH=1004	1134	1004	0	88.5	45.5	50.8
Rsa1.0_00001.1.g5.t1	ref|XP_002869733.1| hypothetical protein ARALYDRAFT_492444 [Arabidopsis lyrata subsp. lyrata] gi|297315569|gb|EFH45992.1| hypothetical protein ARALYDRAFT_492444 [Arabidopsis lyrata subsp. lyrata]	315	305	1.00E-131	96.8	77.1	87.0	hypothetical protein ARALYDRAFT_492444	gbpln	Arabidopsis lyrata	AT4G24175.1 | Symbols: | unknown protein; CONTAINS InterPro DOMAIN/s: Uncharacterised protein family UPF0307 (InterPro:IPR006839); Has 30201 Blast hits to 17322 proteins in 780 species: Archae - 12; Bacteria - 1396; Metazoa - 17338; Fungi - 3422; Plants - 5037; Viruses - 0; Other Eukaryotes - 2996 (source: NCBI BLink). | chr4:12547018-12548810 FORWARD LENGTH=306	315	306	1.00E-116	97.1	76.8	85.7
Rsa1.0_00001.1.g6.t1	gb|EOA17059.1| hypothetical protein CARUB_v10005297mg [Capsella rubella]	255	312	1.00E-132	122.4	89.4	94.9	hypothetical protein CARUB_v10005297mg	gbpln	Capsella rubella	AT4G24180.1 | Symbols: TLP1, ATTLP1 | THAUMATIN-LIKE PROTEIN 1 | chr4:12550356-12551221 REVERSE LENGTH=260	255	260	1.00E-130	102.0	88.6	93.3
Rsa1.0_00001.1.g7.t4	ref|XP_002867675.1| hypothetical protein ARALYDRAFT_329238 [Arabidopsis lyrata subsp. lyrata] gi|297313511|gb|EFH43934.1| hypothetical protein ARALYDRAFT_329238 [Arabidopsis lyrata subsp. lyrata]	162	157	2.00E-61	96.9	73.5	82.1	hypothetical protein ARALYDRAFT_329238	gbpln	Arabidopsis lyrata	AT4G24204.2 | Symbols: | RING/U-box superfamily protein | chr4:12562069-12562646 REVERSE LENGTH=157	162	157	2.00E-61	96.9	71.0	80.9
Rsa1.0_00001.1.g8.t1	gb|ACV88719.1| SLY1 F-box protein [Brassica napus]	147	146	3.00E-69	99.3	93.2	95.2	SLY1 F-box protein	gbpln	Brassica napus	AT4G24210.1 | Symbols: SLY1 | F-box family protein | chr4:12563658-12564113 FORWARD LENGTH=151	147	151	6.00E-62	102.7	78.2	86.4
Rsa1.0_00001.1.g9.t1	gb|EOA16797.1| hypothetical protein CARUB_v10005016mg [Capsella rubella]	390	388	0	99.5	85.9	93.1	hypothetical protein CARUB_v10005016mg	gbpln	Capsella rubella	AT4G24220.1 | Symbols: VEP1, AWI31 | NAD(P)-binding Rossmann-fold superfamily protein | chr4:12565219-12566474 FORWARD LENGTH=388	390	388	0	99.5	83.6	92.3
Rsa1.0_00001.1.g10.t1	gb|AAS21130.1| At4g24230 [Arabidopsis thaliana] gi|45773894|gb|AAS76751.1| At4g24230 [Arabidopsis thaliana]	392	362	6.00E-82	92.3	56.9	66.3	At4g24230	gbpln	Arabidopsis thaliana	AT4G24230.5 | Symbols: ACBP3 | acyl-CoA-binding domain 3 | chr4:12567240-12568754 REVERSE LENGTH=362	392	362	3.00E-84	92.3	56.9	66.3
Rsa1.0_00001.1.g11.t1	gb|ACQ99198.1| WRKY7 [Brassica rapa subsp. pekinensis]	341	307	1.00E-155	90.0	81.5	84.8	WRKY7	gbpln	Brassica rapa	AT4G24240.1 | Symbols: WRKY7, ATWRKY7 | WRKY DNA-binding protein 7 | chr4:12571930-12573446 FORWARD LENGTH=353	341	353	1.00E-134	103.5	77.1	84.5
Rsa1.0_00001.1.g12.t2	ref|XP_002867673.1| hypothetical protein ARALYDRAFT_914165 [Arabidopsis lyrata subsp. lyrata] gi|297313509|gb|EFH43932.1| hypothetical protein ARALYDRAFT_914165 [Arabidopsis lyrata subsp. lyrata]	498	482	0	96.8	84.7	90.0	hypothetical protein ARALYDRAFT_914165	gbpln	Arabidopsis lyrata	AT4G24250.1 | Symbols: MLO13, ATMLO13 | Seven transmembrane MLO family protein | chr4:12575008-12577517 REVERSE LENGTH=478	498	478	0	96.0	83.7	89.2
Rsa1.0_00001.1.g13.t1	ref|NP_194157.1| endoglucanase 21 [Arabidopsis thaliana] gi|75266338|sp|Q9STW8.1|GUN21_ARATH RecName: Full=Endoglucanase 21; AltName: Full=Endo-1,4-beta glucanase 21 gi|5051768|emb|CAB45061.1| endo-1, 4-beta-glucanase like protein [Arabidopsis thaliana] gi|7269276|emb|CAB79336.1| endo-1, 4-beta-glucanase like protein [Arabidopsis thaliana] gi|332659479|gb|AEE84879.1| endoglucanase 21 [Arabidopsis thaliana]	764	620	0	81.2	58.9	67.7	endoglucanase 21	gbpln	Arabidopsis thaliana	AT4G24260.1 | Symbols: ATGH9A3, KOR3, GH9A3 | glycosyl hydrolase 9A3 | chr4:12577871-12580133 REVERSE LENGTH=620	764	620	0	81.2	58.9	67.7

Rsa1.0_00001.1.g14.t1	ref|XP_002867670.1| RNA recognition motif-containing protein [Arabidopsis lyrata subsp. lyrata] gi|297313506|gb|EFH43929.1| RNA recognition motif-containing protein [Arabidopsis lyrata subsp. lyrata]	835	820	0	98.2	82.2	88.6	RNA recognition motif-containing protein	gbpln	Arabidopsis lyrata	AT4G24270.1 | Symbols: EMB140 | EMBRYO DEFECTIVE 140 | chr4:12581765-12587293 REVERSE LENGTH=816	835	816	0	97.7	82.0	88.6
Rsa1.0_00001.1.g15.t1	gb|EOA15374.1| hypothetical protein CARUB_v10006044mg [Capsella rubella]	109	121	2.00E-25	111.0	67.9	77.1	hypothetical protein CARUB_v10006044mg	gbpln	Capsella rubella	AT4G24275.1 | Symbols: | Identified as a screen for stress-responsive genes. | chr4:12588579-12588959 FORWARD LENGTH=126	109	126	3.00E-22	115.6	63.3	74.3
Rsa1.0_00001.1.g16.t1	gb|AAD25622.1|AC005287_24 Hypothetical protein [Arabidopsis thaliana]	161	224	3.00E-24	139.1	45.3	61.5	Hypothetical protein	gbpln	Arabidopsis thaliana	AT1G14800.1 | Symbols: | Nucleic acid-binding, OB-fold-like protein | chr1:5099572-5102558 FORWARD LENGTH=384	161	384	1.00E-26	238.5	41.6	57.1
Rsa1.0_00001.1.g17.t1	ref|XP_002869727.1| cphsc70-1 [Arabidopsis lyrata subsp. lyrata] gi|297315563|gb|EFH45986.1| cphsc70-1 [Arabidopsis lyrata subsp. lyrata]	715	718	0	100.4	92.9	95.7	cphsc70-1	gbpln	Arabidopsis lyrata	AT5G49910.1 | Symbols: CPHSC70-2EAT SHOCK PROTEIN 70-2, HSC70-7, cpHsc70-2 | chloroplast heat shock protein 70-2 | chr5:20303470-20306295 FORWARD LENGTH=718	715	718	0	100.4	91.5	95.2
Rsa1.0_00001.1.g18.t1	ref|NP_194159.1| chloroplast heat shock protein 70-1 [Arabidopsis thaliana] gi|75313889|sp|Q9STW6.1|HSP7F_ARATH RecName: Full=Heat shock 70 kDa protein 6, chloroplastic; AltName: Full=Chloroplast heat shock protein 70-1; Short=cpHsc70-1; AltName: Full=Heat shock protein 70-6; Short=AtHsp70-6; Flags: Precursor gi|5051770|emb|CAB45063.1| hsp 70-like protein [Arabidopsis thaliana] gi|7269278|emb|CAB79338.1| hsp 70-like protein [Arabidopsis thaliana] gi|18175969|gb|AAL59960.1| putative hsp 70 protein [Arabidopsis thaliana] gi|25054945|gb|AAN71949.1| putative hsp 70 protein [Arabidopsis thaliana] gi|332659484|gb|AEE84884.1| chloroplast heat shock protein 70-1 [Arabidopsis thaliana]	240	718	1.00E-66	299.2	55.4	62.9	chloroplast heat shock protein 70-1	gbpln	Arabidopsis thaliana	AT4G24280.1 | Symbols: cpHsc70-1 | chloroplast heat shock protein 70-1 | chr4:12590094-12593437 FORWARD LENGTH=718	240	718	4.00E-69	299.2	55.4	62.9
Rsa1.0_00001.1.g19.t1	ref|XP_002869727.1| cphsc70-1 [Arabidopsis lyrata subsp. lyrata] gi|297315563|gb|EFH45986.1| cphsc70-1 [Arabidopsis lyrata subsp. lyrata]	935	718	0	76.8	53.8	58.8	cphsc70-1	gbpln	Arabidopsis lyrata	AT4G24280.1 | Symbols: cpHsc70-1 | chloroplast heat shock protein 70-1 | chr4:12590094-12593437 FORWARD LENGTH=718	935	718	0	76.8	53.4	58.6
Rsa1.0_00001.1.g20.t1	gb|EOA16263.1| hypothetical protein CARUB_v10004407mg [Capsella rubella]	606	606	0	100.0	88.9	92.7	hypothetical protein CARUB_v10004407mg	gbpln	Capsella rubella	AT4G24290.2 | Symbols: | MAC/Perforin domain-containing protein | chr4:12594856-12597815 FORWARD LENGTH=606	606	606	0	100.0	87.5	91.9
Rsa1.0_00001.1.g21.t1	ref|XP_002869724.1| hypothetical protein ARALYDRAFT_492419 [Arabidopsis lyrata subsp. lyrata] gi|297315560|gb|EFH45983.1| hypothetical protein ARALYDRAFT_492419 [Arabidopsis lyrata subsp. lyrata]	220	213	1.00E-103	96.8	87.7	90.5	hypothetical protein ARALYDRAFT_492419	gbpln	Arabidopsis lyrata	AT4G24310.1 | Symbols: | Protein of unknown function (DUF679) | chr4:12600900-12601541 FORWARD LENGTH=213	220	213	1.00E-98	96.8	86.4	90.0
Rsa1.0_00001.1.g22.t1	ref|NP_194163.1| ubiquitin carboxyl-terminal hydrolase-like protein [Arabidopsis thaliana] gi|5051774|emb|CAB45067.1| putative protein [Arabidopsis thaliana] gi|7269282|emb|CAB79342.1| putative protein [Arabidopsis thaliana] gi|91805609|gb|ABE65533.1| hypothetical protein At4g24320 [Arabidopsis thaliana] gi|332659489|gb|AEE84889.1| ubiquitin carboxyl-terminal hydrolase-like protein [Arabidopsis thaliana]	379	395	1.00E-170	104.2	80.5	88.7	ubiquitin carboxyl-terminal hydrolase-like protein	gbpln	Arabidopsis thaliana	AT4G24320.1 | Symbols: | Ubiquitin carboxyl-terminal hydrolase family protein | chr4:12602298-12603485 REVERSE LENGTH=395	379	395	1.00E-172	104.2	80.5	88.7
Rsa1.0_00001.1.g23.t1	ref|NP_194164.1| uncharacterized protein [Arabidopsis thaliana] gi|5051775|emb|CAB45068.1| putative protein [Arabidopsis thaliana] gi|7269283|emb|CAB79343.1| putative protein [Arabidopsis thaliana] gi|58652054|gb|AAW80852.1| At4g24330 [Arabidopsis thaliana] gi|332659490|gb|AEE84890.1| uncharacterized protein AT4G24330 [Arabidopsis thaliana]	520	478	0	91.9	76.2	81.9	uncharacterized protein	gbpln	Arabidopsis thaliana	AT4G24330.1 | Symbols: | Protein of unknown function (DUF1682) | chr4:12603848-12606229 REVERSE LENGTH=478	520	478	0	91.9	76.2	81.9

Rsa1.0_00001.1.g24.t1	gb|AAG51046.1|AC069473_8 gypsy/Ty-3 retroelement polyprotein; 69905-74404 [Arabidopsis thaliana] gi|10998138|dbj|BAB03109.1| retroelement pol polyprotein [Arabidopsis thaliana]	1513	1499	0	99.1	64.8	79.0	gypsy/Ty-3 retroelement polyprotein; 69905-74404	gbpln	Arabidopsis thaliana	ATMG00860.1 | Symbols: ORF158 | DNA/RNA polymerases superfamily protein | chrM:235916-236392 FORWARD LENGTH=158	1513	158	7.00E-31	10.4	4.4	5.5
Rsa1.0_00001.1.g25.t1	ref|NP_567699.1| phosphorylase family protein [Arabidopsis thaliana] gi|26453148|dbj|BAC43650.1| unknown protein [Arabidopsis thaliana] gi|28950909|gb|AAO63378.1| At4g24340 [Arabidopsis thaliana] gi|332659491|gb|AEE84891.1| phosphorylase family protein [Arabidopsis thaliana]	343	338	1.00E-161	98.5	84.0	88.9	phosphorylase family protein	gbpln	Arabidopsis thaliana	AT4G24340.1 | Symbols: | Phosphorylase superfamily protein | chr4:12607475-12609148 FORWARD LENGTH=338	343	338	1.00E-164	98.5	84.0	88.9
Rsa1.0_00001.1.g26.t1	gb|AAM65580.1| unknown [Arabidopsis thaliana]	350	336	1.00E-159	96.0	78.3	85.4	unknown	gbpln	Arabidopsis thaliana	AT4G24350.1 | Symbols: | Phosphorylase superfamily protein | chr4:12609637-12611328 FORWARD LENGTH=336	350	336	1.00E-160	96.0	77.7	84.9
Rsa1.0_00001.1.g27.t1	ref|NP_567700.1| uncharacterized protein [Arabidopsis thaliana] gi|5051779|emb|CAB45072.1| hypothetical protein [Arabidopsis thaliana] gi|7269287|emb|CAB79347.1| hypothetical protein [Arabidopsis thaliana] gi|17381050|gb|AAL36337.1| unknown protein [Arabidopsis thaliana] gi|21436263|gb|AAM51270.1| unknown protein [Arabidopsis thaliana] gi|21555804|gb|AAM63937.1| unknown [Arabidopsis thaliana] gi|332659494|gb|AEE84894.1| uncharacterized protein AT4G24370 [Arabidopsis thaliana]	176	164	2.00E-41	93.2	65.3	77.3	uncharacterized protein	gbpln	Arabidopsis thaliana	AT4G24370.1 | Symbols: | unknown protein; Has 30201 Blast hits to 17322 proteins in 780 species: Archae - 12; Bacteria - 1396; Metazoa - 17338; Fungi - 3422; Plants - 5037; Viruses - 0; Other Eukaryotes - 2996 (source: NCBI BLink). | chr4:12611608-12612102 REVERSE LENGTH=164	176	164	7.00E-44	93.2	65.3	77.3
Rsa1.0_00001.1.g28.t1	ref|NP_567701.1| uncharacterized protein [Arabidopsis thaliana] gi|26451917|dbj|BAC43051.1| unknown protein [Arabidopsis thaliana] gi|88196765|gb|ABD43025.1| At4g24380 [Arabidopsis thaliana] gi|332659495|gb|AEE84895.1| uncharacterized protein AT4G24380 [Arabidopsis thaliana]	234	234	1.00E-114	100.0	84.6	92.3	uncharacterized protein	gbpln	Arabidopsis thaliana	AT4G24380.1 | Symbols: | INVOLVED IN: 10-formyltetrahydrofolate biosynthetic process, folic acid and derivative biosynthetic process; EXPRESSED IN: 24 plant structures; EXPRESSED DURING: 15 growth stages; CONTAINS InterPro DOMAIN/s: Serine hydrolase (InterPro:IPR005645); BEST Arabidopsis thaliana protein match is: alpha/beta-Hydrolases superfamily protein (TAIR:AT5G65400.1); Has 30201 Blast hits to 17322 proteins in 780 species: Archae - 12; Bacteria - 1396; Metazoa - 17338; Fungi - 3422; Plants - 5037; Viruses - 0; Other Eukaryotes - 2996 (source: NCBI BLink). | chr4:12612554-12613586 FORWARD LENGTH=234	234	234	1.00E-117	100.0	84.6	92.3
Rsa1.0_00001.1.g29.t1	gb|EOA18169.1| hypothetical protein CARUB_v10006645mg [Capsella rubella]	615	622	0	101.1	85.0	90.9	hypothetical protein CARUB_v10006645mg	gbpln	Capsella rubella	AT4G24390.1 | Symbols: | RNI-like superfamily protein | chr4:12613909-12615966 REVERSE LENGTH=623	615	623	0	101.3	84.2	90.7
Rsa1.0_00001.1.g30.t1	ref|NP_194171.1| CBL-interacting serine/threonine-protein kinase 8 [Arabidopsis thaliana] gi|75337651|sp|Q9STV4.1|CIPK8_ARATH RecName: Full=CBL-interacting serine/threonine-protein kinase 8; AltName: Full=SNF1-related kinase 3.13; AltName: Full=SOS2-like protein kinase PKS11 gi|19343483|gb|AAK16683.2|AF290193_1 CBL-interacting protein kinase 8 [Arabidopsis thaliana] gi|5051782|emb|CAB45075.1| serine/threonine kinase-like protein [Arabidopsis thaliana] gi|7269290|emb|CAB79350.1| serine/threonine kinase-like protein [Arabidopsis thaliana] gi|24030396|gb|AAN41358.1| putative serine/threonine kinase [Arabidopsis thaliana] gi|332659499|gb|AEE84899.1| CBL-interacting serine/threonine-protein kinase 8 [Arabidopsis thaliana]	454	445	0	98.0	93.0	95.8	CBL-interacting serine/threonine-protein kinase 8	gbpln	Arabidopsis thaliana	AT4G24400.1 | Symbols: CIPK8, SnRK3.13, PKS11, ATCIPK8 | CBL-interacting protein kinase 8 | chr4:12617379-12620481 FORWARD LENGTH=445	454	445	0	98.0	93.0	95.8

Rsa1.0_00001.1.g31.t1	ref|NP_567703.4| Rhamnogalacturonate lyase family protein [Arabidopsis thaliana] gi|5051785|emb|CAB45078.1| LG27/30-like gene [Arabidopsis thaliana] gi|7269293|emb|CAB79353.1| LG27/30-like gene [Arabidopsis thaliana] gi|332659503|gb|AEE84903.1| Rhamnogalacturonate lyase family protein [Arabidopsis thaliana]	645	646	0	100.2	88.2	94.0	Rhamnogalacturonate lyase family protein	gbpln	Arabidopsis thaliana	AT4G24430.1 | Symbols: | Rhamnogalacturonate lyase family protein | chr4:12630018-12632975 FORWARD LENGTH=646	645	646	0	100.2	88.2	94.0
Rsa1.0_00001.1.g32.t1	ref|NP_194175.1| transcription initiation factor IIA subunit 2 [Arabidopsis thaliana] gi|30686529|ref|NP_849434.1| transcription initiation factor IIA subunit 2 [Arabidopsis thaliana] gi|297803670|ref|XP_002869719.1| transcription initiation factor IIA gamma chain [Arabidopsis lyrata subsp. lyrata] gi|20141796|sp|Q39236.2|T2AG_ARATH RecName: Full=Transcription initiation factor IIA subunit 2; AltName: Full=General transcription factor IIA subunit 2; AltName: Full=Transcription initiation factor IIA gamma chain; Short=TFIIA-gamma gi|2826882|emb|CAA11524.1| transcription factor IIA small subunit [Arabidopsis thaliana] gi|5051786|emb|CAB45079.1| transcription factor IIA small subunit [Arabidopsis thaliana] gi|7269294|emb|CAB79354.1| transcription factor IIA small subunit [Arabidopsis thaliana] gi|18176271|gb|AAL60014.1| putative transcription factor IIA small subunit [Arabidopsis thaliana] gi|20465319|gb|AAM20063.1| putative transcription factor IIA small subunit [Arabidopsis thaliana] gi|21553865|gb|AAM62958.1| transcription factor IIA small subunit [Arabidopsis thaliana] gi|29545876|gb|AAP28001.1| TFIIA-S [illegible]	106	106	2.00E-55	100.0	99.1	100.0	transcription initiation factor IIA subunit 2	gbpln	Arabidopsis lyrata	AT4G24440.2 | Symbols: | transcription initiation factor IIA gamma chain / TFIIA-gamma (TFIIA-S) | chr4:12633460-12634553 FORWARD LENGTH=106	106	106	3.00E-58	100.0	99.1	100.0
Rsa1.0_00001.1.g33.t6	ref|NP_194177.2| CRT (chloroquine-resistance transporter)-like transporter 2 [Arabidopsis thaliana] gi|334186873|ref|NP_001190820.1| CRT (chloroquine-resistance transporter)-like transporter 2 [Arabidopsis thaliana] gi|119935896|gb|ABM06027.1| At4g24460 [Arabidopsis thaliana] gi|332659507|gb|AEE84907.1| CRT (chloroquine-resistance transporter)-like transporter 2 [Arabidopsis thaliana] gi|332659508|gb|AEE84908.1| CRT (chloroquine-resistance transporter)-like transporter 2 [Arabidopsis thaliana]	432	431	0	99.8	82.2	89.4	CRT (chloroquine-resistance transporter)-like transporter 2	gbpln	Arabidopsis thaliana	AT4G24460.2 | Symbols: CLT2 | CRT (chloroquine-resistance transporter)-like transporter 2 | chr4:12642888-12645310 FORWARD LENGTH=431	432	431	0	99.8	82.2	89.4
Rsa1.0_00001.1.g34.t1	gb|EOA17945.1| hypothetical protein CARUB_v10006355mg [Capsella rubella]	307	312	1.00E-137	101.6	82.4	87.3	hypothetical protein CARUB_v10006355mg	gbpln	Capsella rubella	AT4G24470.2 | Symbols: ZIM, GATA25 | GATA-type zinc finger protein with TIFY domain | chr4:12645785-12647734 FORWARD LENGTH=309	307	309	1.00E-138	100.7	81.4	88.3
Rsa1.0_00001.1.g35.t1	ref|NP_194179.2| protein kinase family protein [Arabidopsis thaliana] gi|332659512|gb|AEE84912.1| protein kinase family protein [Arabidopsis thaliana]	958	956	0	99.8	81.8	88.1	protein kinase family protein	gbpln	Arabidopsis thaliana	AT4G24480.1 | Symbols: | Protein kinase superfamily protein | chr4:12650410-12654755 FORWARD LENGTH=956	958	956	0	99.8	81.8	88.1
Rsa1.0_00001.1.g36.t1	ref|XP_002867663.1| protein binding protein [Arabidopsis lyrata subsp. lyrata] gi|297313499|gb|EFH43922.1| protein binding protein [Arabidopsis lyrata subsp. lyrata]	671	677	0	100.9	82.0	90.2	protein binding protein	gbpln	Arabidopsis lyrata	AT4G24490.2 | Symbols: | RAB geranylgeranyl transferase alpha subunit 1 | chr4:12655330-12658103 REVERSE LENGTH=678	671	678	0	101.0	82.0	90.3

Rsa1.0_00001.1.g37.t1	ref|NP_567704.1| hydroxyproline-rich glycoprotein family protein [Arabidopsis thaliana] gi|334186879|ref|NP_001190822.1| hydroxyproline-rich glycoprotein family protein [Arabidopsis thaliana] gi|4220540|emb|CAA23013.1| hypothetical protein [Arabidopsis thaliana] gi|7269300|emb|CAB79360.1| hypothetical protein [Arabidopsis thaliana] gi|23296316|gb|AAN13039.1| unknown protein [Arabidopsis thaliana] gi|332659515|gb|AEE84915.1| hydroxyproline-rich glycoprotein family protein [Arabidopsis thaliana] gi|332659517|gb|AEE84917.1| hydroxyproline-rich glycoprotein family protein [Arabidopsis thaliana]	329	319	1.00E-114	97.0	72.3	80.2	hydroxyproline-rich glycoprotein family protein	gbpln	Arabidopsis thaliana	AT4G24500.3	Symbols:	hydroxyproline-rich glycoprotein family protein	chr4:12658732-12660211 FORWARD LENGTH=319	329	319	1.00E-117	97.0	72.3	80.2
Rsa1.0_00001.1.g38.t1	ref|XP_002869713.1| hypothetical protein ARALYDRAFT_492392 [Arabidopsis lyrata subsp. lyrata] gi|297315549|gb|EFH45972.1| hypothetical protein ARALYDRAFT_492392 [Arabidopsis lyrata subsp. lyrata]	419	415	1.00E-180	99.0	74.9	85.2	hypothetical protein ARALYDRAFT_492392	gbpln	Arabidopsis lyrata	AT4G24510.1	Symbols: CER2, VC2, VC-2	HXXXD-type acyl-transferase family protein	chr4:12660929-12662537 FORWARD LENGTH=421	419	421	0	100.5	74.0	85.0
Rsa1.0_00001.1.g39.t1	ref|XP_002867662.1| hypothetical protein ARALYDRAFT_492391 [Arabidopsis lyrata subsp. lyrata] gi|297313498|gb|EFH43921.1| hypothetical protein ARALYDRAFT_492391 [Arabidopsis lyrata subsp. lyrata]	707	692	0	97.9	84.7	89.7	hypothetical protein ARALYDRAFT_492391	gbpln	Arabidopsis lyrata	AT4G24520.1	Symbols: ATR1, AR1	P450 reductase 1	chr4:12663065-12667066 REVERSE LENGTH=692	707	692	0	97.9	83.9	89.1
Rsa1.0_00001.1.g40.t1	ref|XP_002867662.1| hypothetical protein ARALYDRAFT_492391 [Arabidopsis lyrata subsp. lyrata] gi|297313498|gb|EFH43921.1| hypothetical protein ARALYDRAFT_492391 [Arabidopsis lyrata subsp. lyrata]	694	692	0	99.7	90.3	95.0	hypothetical protein ARALYDRAFT_492391	gbpln	Arabidopsis lyrata	AT4G24520.1	Symbols: ATR1, AR1	P450 reductase 1	chr4:12663065-12667066 REVERSE LENGTH=692	694	692	0	99.7	89.5	94.7
Rsa1.0_00001.1.g41.t1	gb|EOA18108.1| hypothetical protein CARUB_v10006569mg [Capsella rubella]	518	521	0	100.6	90.3	94.6	hypothetical protein CARUB_v10006569mg	gbpln	Capsella rubella	AT4G24530.1	Symbols:	O-fucosyltransferase family protein	chr4:12667424-12669713 REVERSE LENGTH=519	518	519	0	100.2	88.8	94.0
Rsa1.0_00001.1.g42.t1	gb|AFM77900.1| MADS-box protein AGL24 [Brassica napus]	221	221	1.00E-122	100.0	98.2	99.1	MADS-box protein AGL24	gbpln	Brassica napus	AT4G24540.1	Symbols: AGL24	AGAMOUS-like 24	chr4:12671160-12673645 REVERSE LENGTH=220	221	220	1.00E-106	99.5	86.9	92.8
Rsa1.0_00001.1.g43.t1	ref|XP_002869712.1| clathrin adaptor complexes medium subunit family protein [Arabidopsis lyrata subsp. lyrata] gi|297315548|gb|EFH45971.1| clathrin adaptor complexes medium subunit family protein [Arabidopsis lyrata subsp. lyrata]	453	451	0	99.6	96.2	97.8	clathrin adaptor complexes medium subunit family protein	gbpln	Arabidopsis lyrata	AT4G24550.2	Symbols:	Clathrin adaptor complexes medium subunit family protein	chr4:12675873-12678903 FORWARD LENGTH=451	453	451	0	99.6	95.8	98.0
Rsa1.0_00001.1.g44.t1	ref|XP_002867659.1| ubiquitin-specific protease 16 [Arabidopsis lyrata subsp. lyrata] gi|297313495|gb|EFH43918.1| ubiquitin-specific protease 16 [Arabidopsis lyrata subsp. lyrata]	1024	1007	0	98.3	76.2	84.1	ubiquitin-specific protease 16	gbpln	Arabidopsis lyrata	AT4G24560.1	Symbols: UBP16	ubiquitin-specific protease 16	chr4:12679493-12684528 REVERSE LENGTH=1008	1024	1008	0	98.4	75.6	83.1
Rsa1.0_00001.1.g45.t1	#	#	#	#	#	#	#	-	----	----	#	#	#	#	#	#	#
Rsa1.0_00001.1.g46.t1	ref|XP_002869711.1| hypothetical protein ARALYDRAFT_914130 [Arabidopsis lyrata subsp. lyrata] gi|297315547|gb|EFH45970.1| hypothetical protein ARALYDRAFT_914130 [Arabidopsis lyrata subsp. lyrata]	317	314	1.00E-153	99.1	85.8	90.9	hypothetical protein ARALYDRAFT_914130	gbpln	Arabidopsis lyrata	AT4G24570.1	Symbols: DIC2	dicarboxylate carrier 2	chr4:12686546-12687487 FORWARD LENGTH=313	317	313	1.00E-155	98.7	86.8	92.4
Rsa1.0_00001.1.g47.t2	ref|XP_002867658.1| predicted protein [Arabidopsis lyrata subsp. lyrata] gi|297313494|gb|EFH43917.1| predicted protein [Arabidopsis lyrata subsp. lyrata]	943	925	0	98.1	79.6	86.0	predicted protein	gbpln	Arabidopsis lyrata	AT4G24580.1	Symbols: REN1	Rho GTPase activation protein (RhoGAP) with PH domain	chr4:12687879-12694248 REVERSE LENGTH=933	943	933	0	98.9	78.0	85.0
Rsa1.0_00001.1.g48.t1	#	#	#	#	#	#	#	-	----	----	#	#	#	#	#	#	#

Rsa1.0_00001.1.g49.t1	gb|EOA17092.1| hypothetical protein CARUB_v10005345mg, partial [Capsella rubella]	236	303	2.00E-96	128.4	79.7	88.6	hypothetical protein CARUB_v10005345mg, partial	gbpln	Capsella rubella	AT4G24590.1 | Symbols: | unknown protein; BEST Arabidopsis thaliana protein match is: unknown protein (TAIR:AT5G49710.3); Has 105 Blast hits to 105 proteins in 26 species: Archae - 0; Bacteria - 0; Metazoa - 8; Fungi - 3; Plants - 85; Viruses - 0; Other Eukaryotes - 9 (source: NCBI BLink). | chr4:12696651-12698036 FORWARD LENGTH=241	236	241	4.00E-96	102.1	80.1	84.7
Rsa1.0_00001.1.g50.t1	gb|EOA15925.1| hypothetical protein CARUB_v10004019mg [Capsella rubella]	1147	1152	0	100.4	88.8	93.1	hypothetical protein CARUB_v10004019mg	gbpln	Capsella rubella	AT4G24610.1 | Symbols: | unknown protein; FUNCTIONS IN: molecular_function unknown; INVOLVED IN: biological_process unknown; LOCATED IN: plasma membrane; EXPRESSED IN: 20 plant structures; EXPRESSED DURING: 12 growth stages; BEST Arabidopsis thaliana protein match is: unknown protein (TAIR:AT5G65440.1); Has 820 Blast hits to 264 proteins in 74 species: Archae - 0; Bacteria - 15; Metazoa - 77; Fungi - 83; Plants - 96; Viruses - 0; Other Eukaryotes - 549 (source: NCBI BLink). | chr4:12700837-12707899 REVERSE LENGTH=1150	1147	1150	0	100.3	88.2	92.5
Rsa1.0_00001.1.g51.t1	ref|XP_002869709.1| zinc ion binding protein [Arabidopsis lyrata subsp. lyrata] gi|297315545|gb|EFH45968.1| zinc ion binding protein [Arabidopsis lyrata subsp. lyrata]	420	408	0	97.1	81.0	89.8	zinc ion binding protein	gbpln	Arabidopsis lyrata	AT4G24630.1 | Symbols: | DHHC-type zinc finger family protein | chr4:12714919-12717111 FORWARD LENGTH=407	420	407	0	96.9	80.5	90.0
Rsa1.0_00001.1.g52.t1	gb|AAB48480.1| pollen-specific protein Bnm1 [Brassica napus]	187	182	5.00E-78	97.3	89.3	90.4	pollen-specific protein Bnm1	gbpln	Brassica napus	AT4G24640.1 | Symbols: APPB1 | Plant invertase/pectin methylesterase inhibitor superfamily protein | chr4:12717851-12718804 FORWARD LENGTH=187	187	187	7.00E-72	100.0	79.1	86.6
Rsa1.0_00001.1.g53.t1	dbj|BAJ34347.1| unnamed protein product [Thellungiella halophila]	225	227	2.00E-99	100.9	89.8	93.3	unnamed protein product	----	----	AT4G24660.1 | Symbols: ATHB22, MEE68, HB22, ZHD2 | homeobox protein 22 | chr4:12724851-12725513 REVERSE LENGTH=220	225	220	3.00E-95	97.8	85.8	89.3
Rsa1.0_00001.1.g54.t1	gb|EOA15502.1| hypothetical protein CARUB_v10004822mg [Capsella rubella] gi|482551310|gb|EOA15503.1| hypothetical protein CARUB_v10004822mg [Capsella rubella]	445	441	0	99.1	79.1	86.3	hypothetical protein CARUB_v10004822mg	gbpln	Capsella rubella	AT4G24670.2 | Symbols: TAR2 | tryptophan aminotransferase related 2 | chr4:12727940-12730694 REVERSE LENGTH=440	445	440	0	98.9	78.7	86.1
Rsa1.0_00001.1.g55.t1	#	#	#	#	#	#	#	-	----	----	#	#	#	#	#	#	#
Rsa1.0_00001.1.g56.t1	ref|NP_194199.4| modifier of snc1 protein [Arabidopsis thaliana] gi|332659542|gb|AEE84942.1| modifier of snc1 protein [Arabidopsis thaliana]	1381	1427	0	103.3	74.7	82.2	modifier of snc1 protein	gbpln	Arabidopsis thaliana	AT4G24680.1 | Symbols: MOS1 | modifier of snc1 | chr4:12733425-12739737 FORWARD LENGTH=1427	1381	1427	0	103.3	74.7	82.2
Rsa1.0_00001.1.g57.t1	ref|XP_002869705.1| ubiquitin-associated /TS-N domain-containing protein [Arabidopsis lyrata subsp. lyrata] gi|297315541|gb|EFH45964.1| ubiquitin-associated /TS-N domain-containing protein [Arabidopsis lyrata subsp. lyrata]	700	709	0	101.3	80.7	88.0	ubiquitin-associated /TS-N domain-containing protein	gbpln	Arabidopsis lyrata	AT4G24690.1 | Symbols: | ubiquitin-associated (UBA)/TS-N domain-containing protein / octicosapeptide/Phox/Bemp1 (PB1) domain-containing protein | chr4:12741191-12744202 FORWARD LENGTH=704	700	704	0	100.6	80.9	88.1
Rsa1.0_00001.1.g58.t1	ref|XP_002867652.1| hypothetical protein ARALYDRAFT_492372 [Arabidopsis lyrata subsp. lyrata] gi|297313488|gb|EFH43911.1| hypothetical protein ARALYDRAFT_492372 [Arabidopsis lyrata subsp. lyrata]	137	139	6.00E-43	101.5	77.4	84.7	hypothetical protein ARALYDRAFT_492372	gbpln	Arabidopsis lyrata	AT4G24700.1 | Symbols: | unknown protein; Has 20 Blast hits to 20 proteins in 7 species: Archae - 0; Bacteria - 0; Metazoa - 0; Fungi - 0; Plants - 20; Viruses - 0; Other Eukaryotes - 0 (source: NCBI BLink). | chr4:12744807-12745238 REVERSE LENGTH=143	137	143	1.00E-42	104.4	70.1	73.7
Rsa1.0_00001.1.g59.t1	ref|XP_002867651.1| hypothetical protein ARALYDRAFT_492371 [Arabidopsis lyrata subsp. lyrata] gi|297313487|gb|EFH43910.1| hypothetical protein ARALYDRAFT_492371 [Arabidopsis lyrata subsp. lyrata]	486	477	0	98.1	88.5	92.4	hypothetical protein ARALYDRAFT_492371	gbpln	Arabidopsis lyrata	AT4G24710.1 | Symbols: | P-loop containing nucleoside triphosphate hydrolases superfamily protein | chr4:12745752-12748995 REVERSE LENGTH=475	486	475	0	97.7	87.9	92.2
Rsa1.0_00001.1.g60.t1	gb|EOA17431.1| hypothetical protein CARUB_v10005735mg [Capsella rubella]	208	208	1.00E-82	100.0	71.6	83.2	hypothetical protein CARUB_v10005735mg	gbpln	Capsella rubella	AT4G24730.4 | Symbols: | Calcineurin-like metallo-phosphoesterase superfamily protein | chr4:12749879-12750872 REVERSE LENGTH=231	208	231	2.00E-79	111.1	69.2	79.3
Rsa1.0_00001.1.g61.t21	gb|EOA15516.1| hypothetical protein CARUB_v10004866mg [Capsella rubella]	742	428	0	57.7	53.1	54.9	hypothetical protein CARUB_v10004866mg	gbpln	Capsella rubella	AT4G24740.1 | Symbols: AFC2, AME1, FC2 | FUS3-complementing gene 2 | chr4:12754729-12757653 REVERSE LENGTH=427	742	427	0	57.5	53.0	54.7

Rsa1.0_00001.1.g62.t2	ref|XP_002867646.1| hypothetical protein ARALYDRAFT_492366 [Arabidopsis lyrata subsp. lyrata] gi|297313482|gb|EFH43905.1| hypothetical protein ARALYDRAFT_492366 [Arabidopsis lyrata subsp. lyrata]	366	365	0	99.7	93.7	97.3	hypothetical protein ARALYDRAFT_492366	gbpln	Arabidopsis lyrata	AT4G24760.1 | Symbols: | alpha/beta-Hydrolases superfamily protein | chr4:12761482-12763564 REVERSE LENGTH=365	366	365	0	99.7	93.2	96.7
Rsa1.0_00001.1.g63.t1	ref|XP_002867645.1| RNA-binding protein cp31 [Arabidopsis lyrata subsp. lyrata] gi|297313481|gb|EFH43904.1| RNA-binding protein cp31 [Arabidopsis lyrata subsp. lyrata]	303	305	1.00E-133	100.7	83.5	87.5	RNA-binding protein cp31	gbpln	Arabidopsis lyrata	AT4G24770.1 | Symbols: RBP31, ATRBP31, CP31, ATRBP33 | 31-kDa RNA binding protein | chr4:12766223-12767952 REVERSE LENGTH=329	303	329	1.00E-126	108.6	80.5	87.8
Rsa1.0_00001.1.g64.t1	ref|NP_567707.1| putative pectate lyase 18 [Arabidopsis thaliana] gi|334186896|ref|NP_001190827.1| putative pectate lyase 18 [Arabidopsis thaliana] gi|32129908|sp|Q9C5M8.2|PEL18_ARATH RecName: Full=Probable pectate lyase 18; AltName: Full=Pectate lyase A10; Flags: Precursor gi|57222166|gb|AAW38990.1| At4g24780 [Arabidopsis thaliana] gi|332659555|gb|AEE84955.1| putative pectate lyase 18 [Arabidopsis thaliana] gi|332659556|gb|AEE84956.1| putative pectate lyase 18 [Arabidopsis thaliana]	406	408	0	100.5	93.6	97.8	putative pectate lyase 18	gbpln	Arabidopsis thaliana	AT4G24780.2 | Symbols: | Pectin lyase-like superfamily protein | chr4:12770631-12772227 REVERSE LENGTH=408	406	408	0	100.5	93.6	97.8
Rsa1.0_00001.1.g65.t1	ref|XP_002869702.1| hypothetical protein ARALYDRAFT_329184 [Arabidopsis lyrata subsp. lyrata] gi|297315538|gb|EFH45961.1| hypothetical protein ARALYDRAFT_329184 [Arabidopsis lyrata subsp. lyrata]	706	942	0	133.4	85.7	90.5	hypothetical protein ARALYDRAFT_329184	gbpln	Arabidopsis lyrata	AT4G24800.3 | Symbols: | MA3 domain-containing protein | chr4:12782463-12784902 FORWARD LENGTH=702	706	702	0	99.4	86.4	92.1
Rsa1.0_00001.1.g66.t1	ref|XP_002869702.1| hypothetical protein ARALYDRAFT_329184 [Arabidopsis lyrata subsp. lyrata] gi|297315538|gb|EFH45961.1| hypothetical protein ARALYDRAFT_329184 [Arabidopsis lyrata subsp. lyrata]	243	942	1.00E-113	387.7	88.1	94.2	hypothetical protein ARALYDRAFT_329184	gbpln	Arabidopsis lyrata	AT4G24805.1 | Symbols: | S-adenosyl-L-methionine-dependent methyltransferases superfamily protein | chr4:12785315-12786058 FORWARD LENGTH=247	243	247	1.00E-111	101.6	88.1	94.7
Rsa1.0_00001.1.g67.t1	ref|XP_002867642.1| hypothetical protein ARALYDRAFT_492356 [Arabidopsis lyrata subsp. lyrata] gi|297313478|gb|EFH43901.1| hypothetical protein ARALYDRAFT_492356 [Arabidopsis lyrata subsp. lyrata]	366	387	0	105.7	95.4	97.5	hypothetical protein ARALYDRAFT_492356	gbpln	Arabidopsis lyrata	AT4G24820.2 | Symbols: | 26S proteasome, regulatory subunit Rpn7;Proteasome component (PCI) domain | chr4:12790471-12792599 REVERSE LENGTH=387	366	387	0	105.7	93.7	96.7
Rsa1.0_00001.1.g68.t1	gb|EOA16502.1| hypothetical protein CARUB_v10004661mg [Capsella rubella]	516	494	0	95.7	86.2	88.6	hypothetical protein CARUB_v10004661mg	gbpln	Capsella rubella	AT4G24830.1 | Symbols: | arginosuccinate synthase family | chr4:12793085-12795857 REVERSE LENGTH=494	516	494	0	95.7	88.6	92.8
Rsa1.0_00001.1.g69.t1	gb|EOA16079.1| hypothetical protein CARUB_v10004211mg [Capsella rubella]	758	754	0	99.5	85.4	91.6	hypothetical protein CARUB_v10004211mg	gbpln	Capsella rubella	AT4G24840.1 | Symbols: | FUNCTIONS IN: molecular_function unknown; INVOLVED IN: protein transport, Golgi organization; LOCATED IN: vacuole; EXPRESSED IN: 23 plant structures; EXPRESSED DURING: 13 growth stages; CONTAINS InterPro DOMAIN/s: COG complex component, COG2 (InterPro:IPR009316); Has 30201 Blast hits to 17322 proteins in 780 species: Archae - 12; Bacteria - 1396; Metazoa - 17338; Fungi - 3422; Plants - 5037; Viruses - 0; Other Eukaryotes - 2996 (source: NCBI BLink). | chr4:12796525-12800926 REVERSE LENGTH=756	758	756	0	99.7	85.2	91.4
Rsa1.0_00001.1.g70.t1	gb|EOA16242.1| hypothetical protein CARUB_v10004389mg [Capsella rubella]	616	617	0	100.2	85.7	93.0	hypothetical protein CARUB_v10004389mg	gbpln	Capsella rubella	AT4G24890.1 | Symbols: ATPAP24, PAP24 | purple acid phosphatase 24 | chr4:12811510-12814440 REVERSE LENGTH=615	616	615	0	99.8	84.3	91.2
Rsa1.0_00001.1.g71.t1	ref|NP_194220.2| uncharacterized protein [Arabidopsis thaliana] gi|332659574|gb|AEE84974.1| uncharacterized protein AT4G24900 [Arabidopsis thaliana]	386	421	1.00E-139	109.1	69.9	79.8	uncharacterized protein	gbpln	Arabidopsis thaliana	AT4G24900.1 | Symbols: | unknown protein; Has 119 Blast hits to 96 proteins in 40 species: Archae - 0; Bacteria - 0; Metazoa - 81; Fungi - 0; Plants - 34; Viruses - 0; Other Eukaryotes - 4 (source: NCBI BLink). | chr4:12814867-12817388 FORWARD LENGTH=421	386	421	1.00E-142	109.1	69.9	79.8
Rsa1.0_00001.1.g72.t1	gb|EOA15789.1| hypothetical protein CARUB_v10007135mg [Capsella rubella]	309	317	1.00E-127	102.6	79.9	87.4	hypothetical protein CARUB_v10007135mg	gbpln	Capsella rubella	AT4G24910.1 | Symbols: | Protein of unknown function (DUF579) | chr4:12817954-12818901 REVERSE LENGTH=315	309	315	1.00E-128	101.9	78.3	86.4

Rsa1.0_00001.1.g73.t1	ref|NP_194222.1| protein transport protein sec61 subunit gamma-1 [Arabidopsis thaliana] gi|18423153|ref|NP_568728.1| protein transport protein sec61 subunit gamma-1 [Arabidopsis thaliana] gi|297795847|ref|XP_002865808.1| hypothetical protein ARALYDRAFT_495113 [Arabidopsis lyrata subsp. lyrata] gi|297799504|ref|XP_002867636.1| hypothetical protein ARALYDRAFT_492346 [Arabidopsis lyrata subsp. lyrata] gi|408407792|sp|P0DI74.1|S61G1_ARATH RecName: Full=Protein transport protein Sec61 subunit gamma-1 gi|408407793|sp|P0DI75.1|S61G2_ARATH RecName: Full=Protein transport protein Sec61 subunit gamma-2 gi|13877813|gb|AAK43984.1|AF370169_1 putative protein translocation complex Sec61 gamma chain [Arabidopsis thaliana] gi|4455235|emb|CAB36734.1| PROTEIN TRANSPORT PROTEIN SEC61 GAMMA SUBUNIT-like [Arabidopsis thaliana] gi|7269342|emb|CAB79401.1| PROTEIN TRANSPORT PROTEIN SEC61 GAMMA SUBUNIT-like [Arabidopsis thaliana] gi|9758755|dbj|BAB09131.1| protein translocation complex Sec61 gamma chain [Arabidopsis thaliana] gi|16323486|gb|AAL15237.1| putative	69	69	1.00E-30	100.0	100.0	100.0	protein transport protein sec61 subunit gamma-1	gbpln	Arabidopsis lyrata	AT5G50460.1 | Symbols: | secE/sec61-gamma protein transport protein | chr5:20552168-20552509 REVERSE LENGTH=69	69	69	2.00E-33	100.0	100.0	100.0
Rsa1.0_00001.1.g74.t1	ref|NP_194223.1| thylakoid lumenal protein [Arabidopsis thaliana] gi|17369630|sp|Q9SW33.1|TL1Y_ARATH RecName: Full=Thylakoid lumenal 17.9 kDa protein, chloroplastic; Flags: Precursor gi|4455236|emb|CAB36735.1| hypothetical protein [Arabidopsis thaliana] gi|7269343|emb|CAB79402.1| hypothetical protein [Arabidopsis thaliana] gi|20260598|gb|AAM13197.1| unknown protein [Arabidopsis thaliana] gi|30023698|gb|AAP13382.1| At4g24930 [Arabidopsis thaliana] gi|332659577|gb|AEE84977.1| thylakoid lumenal protein 17.9 [Arabidopsis thaliana]	243	225	5.00E-98	92.6	77.4	84.4	thylakoid lumenal protein	gbpln	Arabidopsis thaliana	AT4G24930.1 | Symbols: | thylakoid lumenal 17.9 kDa protein, chloroplast | chr4:12821496-12822389 REVERSE LENGTH=225	243	225	1.00E-100	92.6	77.4	84.4
Rsa1.0_00001.1.g75.t4	gb|EOA17016.1| hypothetical protein CARUB_v10005251mg [Capsella rubella]	738	322	1.00E-160	43.6	37.9	40.0	hypothetical protein CARUB_v10005251mg	gbpln	Capsella rubella	AT4G24940.1 | Symbols: SAE1A, AT-SAE1-1, ATSAE1A | SUMO-activating enzyme 1A | chr4:12823651-12825971 FORWARD LENGTH=322	738	322	1.00E-161	43.6	37.9	39.3
Rsa1.0_00001.1.g76.t1	dbj|BAJ34072.1| unnamed protein product [Thellungiella halophila]	121	181	1.00E-31	149.6	54.5	56.2	unnamed protein product	----	----	AT4G24960.1 | Symbols: ATHVA22D, HVA22D | HVA22 homologue D | chr4:12828060-12828982 FORWARD LENGTH=135	121	135	5.00E-33	111.6	52.1	56.2
Rsa1.0_00001.1.g77.t1	ref|XP_002869696.1| hypothetical protein ARALYDRAFT_492340 [Arabidopsis lyrata subsp. lyrata] gi|297315532|gb|EFH45955.1| hypothetical protein ARALYDRAFT_492340 [Arabidopsis lyrata subsp. lyrata]	697	709	0	101.7	81.2	89.1	hypothetical protein ARALYDRAFT_492340	gbpln	Arabidopsis lyrata	AT4G24970.1 | Symbols: | Histidine kinase-, DNA gyrase B-, and HSP90-like ATPase family protein | chr4:12831125-12835449 FORWARD LENGTH=707	697	707	0	101.4	79.1	86.5
Rsa1.0_00001.1.g78.t1	ref|NP_974612.1| tapetum determinant 1 [Arabidopsis thaliana] gi|38607340|gb|AAR25553.1| TPD1 [Arabidopsis thaliana] gi|332659584|gb|AEE84984.1| tapetum determinant 1 [Arabidopsis thaliana]	172	176	2.00E-75	102.3	80.8	90.1	tapetum determinant 1	gbpln	Arabidopsis thaliana	AT4G24972.1 | Symbols: TPD1 | tapetum determinant 1 | chr4:12838220-12839619 FORWARD LENGTH=176	172	176	7.00E-78	102.3	80.8	90.1
Rsa1.0_00001.1.g79.t1	ref|XP_002867632.1| hypothetical protein ARALYDRAFT_492339 [Arabidopsis lyrata subsp. lyrata] gi|297313468|gb|EFH43891.1| hypothetical protein ARALYDRAFT_492339 [Arabidopsis lyrata subsp. lyrata] gi|482553486|gb|EOA17679.1| hypothetical protein CARUB_v10006048mg [Capsella rubella]	119	118	7.00E-58	99.2	95.8	96.6	hypothetical protein ARALYDRAFT_492339	gbpln	Arabidopsis lyrata	AT4G24990.1 | Symbols: ATGP4 | Ubiquitin family protein | chr4:12849973-12851249 REVERSE LENGTH=118	119	118	8.00E-60	99.2	94.1	96.6

Rsa1.0_00001.1.g80.t2	gb|AAC33226.1| putative non-LTR retroelement reverse transcriptase [Arabidopsis thaliana]	1760	1529	0	86.9	40.4	53.2	putative non-LTR retroelement reverse transcriptase	gbpln	Arabidopsis thaliana	AT3G24255.1 | Symbols: | RNA-directed DNA polymerase (reverse transcriptase)-related family protein | chr3:8789471-8793208 FORWARD LENGTH=746	1760	746	3.00E-75	42.4	8.2	11.4
Rsa1.0_00001.1.g81.t1	gb|EMJ11389.1| hypothetical protein PRUPE_ppa017790mg [Prunus persica]	1180	1485	0	125.8	49.2	65.7	hypothetical protein PRUPE_ppa017790mg	gbpln	Prunus persica	ATMG00860.1 | Symbols: ORF158 | DNA/RNA polymerases superfamily protein | chrM:235916-236392 FORWARD LENGTH=158	1180	158	5.00E-27	13.4	4.7	7.1
Rsa1.0_00001.1.g82.t1	ref|XP_002867630.1| nodulin MtN3 family protein [Arabidopsis lyrata subsp. lyrata] gi|297313466|gb|EFH43889.1| nodulin MtN3 family protein [Arabidopsis lyrata subsp. lyrata]	268	280	1.00E-127	104.5	86.2	92.9	nodulin MtN3 family protein	gbpln	Arabidopsis lyrata	AT4G25010.1 | Symbols: SWEET14, AtSWEET14 | Nodulin MtN3 family protein | chr4:12854630-12856351 REVERSE LENGTH=281	268	281	1.00E-128	104.9	85.1	91.8
Rsa1.0_00001.1.g83.t1	gb|EOA14264.1| hypothetical protein CARUB_v10027424mg, partial [Capsella rubella]	79	103	3.00E-26	130.4	67.1	82.3	hypothetical protein CARUB_v10027424mg, partial	gbpln	Capsella rubella	AT5G50810.1 | Symbols: TIM8 | translocase inner membrane subunit 8 | chr5:20675875-20676505 REVERSE LENGTH=77	79	77	3.00E-24	97.5	67.1	82.3
Rsa1.0_00001.1.g84.t2	ref|NP_194233.1| uncharacterized protein [Arabidopsis thaliana] gi|42573033|ref|NP_974613.1| uncharacterized protein [Arabidopsis thaliana] gi|4455246|emb|CAB36745.1| putative protein [Arabidopsis thaliana] gi|7269353|emb|CAB79412.1| putative protein [Arabidopsis thaliana] gi|21553767|gb|AAM62860.1| unknown [Arabidopsis thaliana] gi|89000969|gb|ABD59074.1| At4g25030 [Arabidopsis thaliana] gi|110736875|dbj|BAF00395.1| hypothetical protein [Arabidopsis thaliana] gi|222424032|dbj|BAH19977.1| AT4G25030 [Arabidopsis thaliana] gi|332659593|gb|AEE84993.1| uncharacterized protein AT4G25030 [Arabidopsis thaliana] gi|332659594|gb|AEE84994.1| uncharacterized protein AT4G25030 [Arabidopsis thaliana]	361	344	3.00E-59	95.3	32.1	33.5	uncharacterized protein	gbpln	Arabidopsis thaliana	AT4G25030.2 | Symbols: | unknown protein; BEST Arabidopsis thaliana protein match is: unknown protein (TAIR:AT5G45410.3); Has 35333 Blast hits to 34131 proteins in 2444 species: Archae - 798; Bacteria - 22429; Metazoa - 974; Fungi - 991; Plants - 531; Viruses - 0; Other Eukaryotes - 9610 (source: NCBI BLink). | chr4:12865336-12866638 FORWARD LENGTH=344	361	344	7.00E-62	95.3	32.1	33.5
Rsa1.0_00002.1.g85.t1	ref|NP_195981.1| GDSL esterase/lipase [Arabidopsis thaliana] gi|75181338|sp|Q9LZS7.1|GDL71_ARATH RecName: Full=GDSL esterase/lipase At5g03610; AltName: Full=Extracellular lipase At5g03610; Flags: Precursor gi|13430834|gb|AAK26039.1|AF360329_1 unknown protein [Arabidopsis thaliana] gi|7340646|emb|CAB82926.1| putative protein [Arabidopsis thaliana] gi|21537326|gb|AAM61667.1| putative lipase/acylhydrolase [Arabidopsis thaliana] gi|332003250|gb|AED90633.1| GDSL esterase/lipase [Arabidopsis thaliana]	341	359	2.00E-94	105.3	55.1	70.1	GDSL esterase/lipase	gbpln	Arabidopsis thaliana	AT5G03610.1 | Symbols: | GDSL-like Lipase/Acylhydrolase superfamily protein | chr5:915650-918326 FORWARD LENGTH=359	341	359	6.00E-97	105.3	55.1	70.1
Rsa1.0_00002.1.g86.t1	gb|EOA34211.1| hypothetical protein CARUB_v10021720mg [Capsella rubella]	351	352	1.00E-96	100.3	56.7	70.9	hypothetical protein CARUB_v10021720mg	gbpln	Capsella rubella	AT5G03610.1 | Symbols: | GDSL-like Lipase/Acylhydrolase superfamily protein | chr5:915650-918326 FORWARD LENGTH=359	351	359	5.00E-98	102.3	53.3	68.9
Rsa1.0_00002.1.g87.t1	ref|XP_003578788.1| PREDICTED: AP-2 complex subunit sigma-like [Brachypodium distachyon] gi|326523623|dbj|BAJ92982.1| predicted protein [Hordeum vulgare subsp. vulgare] gi|326532016|dbj|BAK01384.1| predicted protein [Hordeum vulgare subsp. vulgare]	84	142	1.00E-27	169.0	95.2	97.6	PREDICTED: AP-2 complex subunit sigma-like	gbpln	Brachypodium distachyon	AT1G47830.1 | Symbols: | SNARE-like superfamily protein | chr1:17613346-17614784 REVERSE LENGTH=142	84	142	3.00E-30	169.0	95.2	96.4
Rsa1.0_00002.1.g88.t1	#	#	#	#	#	#	#	-	----	----	#	#	#	#	#	#	#
Rsa1.0_00002.1.g89.t1	gb|EOA34211.1| hypothetical protein CARUB_v10021720mg [Capsella rubella]	346	352	5.00E-92	101.7	56.4	69.9	hypothetical protein CARUB_v10021720mg	gbpln	Capsella rubella	AT5G03610.1 | Symbols: | GDSL-like Lipase/Acylhydrolase superfamily protein | chr5:915650-918326 FORWARD LENGTH=359	346	359	3.00E-90	103.8	51.4	67.9
Rsa1.0_00002.1.g90.t1	gb|EOA13504.1| hypothetical protein CARUB_v10026565mg [Capsella rubella]	391	391	0	100.0	95.7	98.0	hypothetical protein CARUB_v10026565mg	gbpln	Capsella rubella	AT1G43170.8 | Symbols: RP1 | ribosomal protein 1 | chr1:16266992-16268631 FORWARD LENGTH=389	391	389	0	99.5	92.1	96.2
Rsa1.0_00002.1.g91.t1	gb|EOA12392.1| hypothetical protein CARUB_v10026693mg [Capsella rubella]	352	350	0	99.4	93.5	97.2	hypothetical protein CARUB_v10026693mg	gbpln	Capsella rubella	AT5G42420.1 | Symbols: | Nucleotide-sugar transporter family protein | chr5:16968819-16970225 FORWARD LENGTH=350	352	350	0	99.4	92.3	95.2
Rsa1.0_00002.1.g92.t1	dbj|BAJ34553.1| unnamed protein product [Thellungiella halophila]	391	389	0	99.5	95.9	98.0	unnamed protein product	----	----	AT1G43170.8 | Symbols: RP1 | ribosomal protein 1 | chr1:16266992-16268631 FORWARD LENGTH=389	391	389	0	99.5	92.1	95.9

Rsa1.0_00002.1.g93.t1	ref|NP_199056.1| SAUR-like auxin-responsive protein [Arabidopsis thaliana] gi|9759477|dbj|BAB10482.1| unnamed protein product [Arabidopsis thaliana] gi|332007424|gb|AED94807.1| SAUR-like auxin-responsive protein [Arabidopsis thaliana]	123	111	2.00E-42	90.2	68.3	74.8	SAUR-like auxin-responsive protein	gbpln	Arabidopsis thaliana	AT5G42410.1 | Symbols: | SAUR-like auxin-responsive protein family | chr5:16964764-16965099 FORWARD LENGTH=111	123	111	4.00E-45	90.2	68.3	74.8
Rsa1.0_00002.1.g94.t8	gb|ABV68922.1| SDG25 [Arabidopsis thaliana]	1274	1388	0	108.9	70.1	79.6	SDG25	gbpln	Arabidopsis thaliana	AT5G42400.1 | Symbols: ATXR7, SDG25 | SET domain protein 25 | chr5:16954469-16960671 REVERSE LENGTH=1423	1274	1423	0	111.7	52.8	60.9
Rsa1.0_00002.1.g95.t4	ref|XP_002865532.1| metalloendopeptidase [Arabidopsis lyrata subsp. lyrata] gi|297311367|gb|EFH41791.1| metalloendopeptidase [Arabidopsis lyrata subsp. lyrata]	1279	1275	0	99.7	90.2	94.2	metalloendopeptidase	gbpln	Arabidopsis lyrata	AT5G42390.1 | Symbols: | Insulinase (Peptidase family M16) family protein | chr5:16945308-16952647 FORWARD LENGTH=1265	1279	1265	0	98.9	89.9	94.1
Rsa1.0_00002.1.g96.t1	ref|XP_002863793.1| hypothetical protein ARALYDRAFT_917538 [Arabidopsis lyrata subsp. lyrata] gi|297309628|gb|EFH40052.1| hypothetical protein ARALYDRAFT_917538 [Arabidopsis lyrata subsp. lyrata]	165	185	2.00E-66	112.1	81.8	88.5	hypothetical protein ARALYDRAFT_917538	gbpln	Arabidopsis lyrata	AT5G42380.1 | Symbols: CML39, CML37 | calmodulin like 37 | chr5:16942758-16943315 REVERSE LENGTH=185	165	185	2.00E-68	112.1	81.2	87.9
Rsa1.0_00002.1.g97.t1	ref|XP_002863794.1| predicted protein [Arabidopsis lyrata subsp. lyrata] gi|297309629|gb|EFH40053.1| predicted protein [Arabidopsis lyrata subsp. lyrata]	455	460	0	101.1	83.7	90.3	predicted protein	gbpln	Arabidopsis lyrata	AT5G42370.1 | Symbols: | Calcineurin-like metallo-phosphoesterase superfamily protein | chr5:16939329-16941463 REVERSE LENGTH=447	455	447	0	98.2	82.0	89.5
Rsa1.0_00002.1.g98.t1	ref|XP_002870812.1| predicted protein [Arabidopsis lyrata subsp. lyrata] gi|297316648|gb|EFH47071.1| predicted protein [Arabidopsis lyrata subsp. lyrata]	222	223	6.00E-99	100.5	80.6	89.2	predicted protein	gbpln	Arabidopsis lyrata	AT4G14630.1 | Symbols: GLP9 | germin-like protein 9 | chr4:8392920-8393680 FORWARD LENGTH=222	222	222	4.00E-94	100.0	75.7	84.2
Rsa1.0_00002.1.g99.t2	ref|XP_002865539.1| kelch repeat-containing F-box family protein [Arabidopsis lyrata subsp. lyrata] gi|297311374|gb|EFH41798.1| kelch repeat-containing F-box family protein [Arabidopsis lyrata subsp. lyrata]	556	561	0	100.9	80.6	88.3	kelch repeat-containing F-box family protein	gbpln	Arabidopsis lyrata	AT5G42350.1 | Symbols: | Galactose oxidase/kelch repeat superfamily protein | chr5:16932106-16933797 FORWARD LENGTH=563	556	563	0	101.3	78.2	87.6
Rsa1.0_00002.1.g100.t1	ref|XP_002863798.1| binding protein [Arabidopsis lyrata subsp. lyrata] gi|297309633|gb|EFH40057.1| binding protein [Arabidopsis lyrata subsp. lyrata]	642	660	0	102.8	87.1	93.9	binding protein	gbpln	Arabidopsis lyrata	AT5G42340.1 | Symbols: PUB15 | Plant U-Box 15 | chr5:16928086-16930367 REVERSE LENGTH=660	642	660	0	102.8	86.3	92.8
Rsa1.0_00002.1.g101.t1	gb|EOA13429.1| hypothetical protein CARUB_v10026470mg [Capsella rubella]	433	423	0	97.7	87.3	91.0	hypothetical protein CARUB_v10026470mg	gbpln	Capsella rubella	AT5G42320.1 | Symbols: | Zn-dependent exopeptidases superfamily protein | chr5:16918651-16920845 REVERSE LENGTH=379	433	379	0	87.5	79.0	82.9
Rsa1.0_00002.1.g102.t1	#	#	#	#	#	#	#	–	----	----	#	#	#	#	#	#	#
Rsa1.0_00002.1.g103.t1	ref|NP_198387.2| S-locus lectin protein kinase-like protein [Arabidopsis thaliana] gi|313471768|sp|O65238.2|Y5537_ARATH RecName: Full=G-type lectin S-receptor-like serine/threonine-protein kinase At5g35370; Flags: Precursor gi|332006576|gb|AED93959.1| G-type lectin S-receptor-like serine/threonine-protein kinase [Arabidopsis thaliana]	878	872	0	99.3	83.0	89.3	S-locus lectin protein kinase-like protein	gbpln	Arabidopsis thaliana	AT5G35370.1 | Symbols: | S-locus lectin protein kinase family protein | chr5:13588564-13591182 REVERSE LENGTH=872	878	872	0	99.3	83.0	89.3
Rsa1.0_00002.1.g104.t1	ref|NP_199037.2| ubiquitin family protein [Arabidopsis thaliana] gi|22654985|gb|AAM98085.1| AT5g42220/K5J14_2 [Arabidopsis thaliana] gi|30102508|gb|AAP21172.1| AT5g42220/K5J14_2 [Arabidopsis thaliana] gi|332007398|gb|AED94781.1| ubiquitin family protein [Arabidopsis thaliana]	811	879	0	108.4	75.8	83.4	ubiquitin family protein	gbpln	Arabidopsis thaliana	AT5G42220.1 | Symbols: | Ubiquitin-like superfamily protein | chr5:16872962-16877455 FORWARD LENGTH=879	811	879	0	108.4	75.8	83.4
Rsa1.0_00002.1.g105.t1	ref|XP_002894570.1| HNH endonuclease domain-containing protein [Arabidopsis lyrata subsp. lyrata] gi|297340412|gb|EFH70829.1| HNH endonuclease domain-containing protein [Arabidopsis lyrata subsp. lyrata]	288	283	1.00E-124	98.3	79.9	84.4	HNH endonuclease domain-containing protein	gbpln	Arabidopsis lyrata	AT2G23840.1 | Symbols: | HNH endonuclease | chr2:10153982-10155600 REVERSE LENGTH=284	288	284	1.00E-126	98.6	80.2	85.4

Rsa1.0_00002.1.g106.t1	ref|NP_175851.1| 5'-3' exoribonuclease 4 [Arabidopsis thaliana] gi|75262833|sp|Q9FQ04.1|XRN4_ARATH RecName: Full=5'-3' exoribonuclease 4; AltName: Full=Protein ACC INSENSITIVE 1; AltName: Full=Protein ETHYLENE INSENSITIVE 5; AltName: Full=Protein EXORIBONUCLEASE 4 gi|11875626|gb|AAG40731.1|AF286718_1 XRN4 [Arabidopsis thaliana] gi|17381112|gb|AAL36368.1| putative exonuclease [Arabidopsis thaliana] gi|20259665|gb|AAM14350.1| putative exonuclease [Arabidopsis thaliana] gi|109627646|gb|ABG34298.1| At1g54490 [Arabidopsis thaliana] gi|332194988|gb|AEE33109.1| 5'-3' exoribonuclease 4 [Arabidopsis thaliana]	951	947	0	99.6	84.4	89.1	5'-3' exoribonuclease 4	gbpln	Arabidopsis thaliana	AT1G54490.1 | Symbols: AIN1, EIN5, XRN4, ATXRN4 | exoribonuclease 4 | chr1:20350300-20356650 FORWARD LENGTH=947	951	947	0	99.6	84.4	89.1
Rsa1.0_00002.1.g107.t1	gb|EOA34142.1| hypothetical protein CARUB_v10021644mg [Capsella rubella]	700	649	2.00E-68	92.7	26.9	36.3	hypothetical protein CARUB_v10021644mg	gbpln	Capsella rubella	AT5G22355.1 | Symbols: | Cysteine/Histidine-rich C1 domain family protein | chr5:7401762-7403756 FORWARD LENGTH=664	700	664	5.00E-64	94.9	19.3	23.9
Rsa1.0_00002.1.g108.t1	ref|XP_002894589.1| hypothetical protein ARALYDRAFT_892695 [Arabidopsis lyrata subsp. lyrata] gi|297340431|gb|EFH70848.1| hypothetical protein ARALYDRAFT_892695 [Arabidopsis lyrata subsp. lyrata]	257	239	4.00E-99	93.0	74.3	83.3	hypothetical protein ARALYDRAFT_892695	gbpln	Arabidopsis lyrata	AT1G54540.1 | Symbols: | Late embryogenesis abundant (LEA) hydroxyproline-rich glycoprotein family | chr1:20367702-20368421 REVERSE LENGTH=239	257	239	2.00E-92	93.0	73.9	84.4
Rsa1.0_00002.1.g109.t1	ref|NP_175858.1| Myosin family protein with Dil domain [Arabidopsis thaliana] gi|332194997|gb|AEE33118.1| Myosin family protein with Dil domain [Arabidopsis thaliana]	1517	1529	0	100.8	92.9	97.2	Myosin family protein with Dil domain	gbpln	Arabidopsis thaliana	AT1G54560.1 | Symbols: XIE, ATXIE | Myosin family protein with Dil domain | chr1:20371649-20379745 REVERSE LENGTH=1529	1517	1529	0	100.8	92.9	97.2
Rsa1.0_00002.1.g110.t1	ref|XP_002894592.1| esterase/lipase/thioesterase family protein [Arabidopsis lyrata subsp. lyrata] gi|297340434|gb|EFH70851.1| esterase/lipase/thioesterase family protein [Arabidopsis lyrata subsp. lyrata]	699	703	0	100.6	83.8	91.4	esterase/lipase/thioesterase family protein	gbpln	Arabidopsis lyrata	AT1G54570.1 | Symbols: | Esterase/lipase/thioesterase family protein | chr1:20380649-20384953 REVERSE LENGTH=704	699	704	0	100.7	84.0	90.7
Rsa1.0_00002.1.g111.t1	ref|NP_175862.1| putative serine/threonine-protein kinase [Arabidopsis thaliana] gi|186490957|ref|NP_001117490.1| putative serine/threonine-protein kinase [Arabidopsis thaliana] gi|75339093|sp|Q9ZVM9.1|Y1461_ARATH RecName: Full=Probable serine/threonine-protein kinase At1g54610 gi|13877619|gb|AAK43887.1|AF370510_1 Unknown protein [Arabidopsis thaliana] gi|3776559|gb|AAC64876.1| Strong similarity to gene F14J9.26 gi|3482933 cdc2 protein kinase homolog from A. thaliana BAC gb|AC003970. ESTs gb|Z35332 and gb|F19907 come from this gene [Arabidopsis thaliana] gi|22136480|gb|AAM91318.1| unknown protein [Arabidopsis thaliana] gi|332195002|gb|AEE33123.1| putative serine/threonine-protein kinase [Arabidopsis thaliana] gi|332195004|gb|AEE33125.1| putative serine/threonine-protein kinase [Arabidopsis thaliana]	560	572	0	102.1	89.5	93.9	putative serine/threonine-protein kinase	gbpln	Arabidopsis thaliana	AT1G54610.3 | Symbols: | Protein kinase superfamily protein | chr1:20393962-20396902 REVERSE LENGTH=572	560	572	0	102.1	89.5	93.9

Rsa1.0_00002.1.g112.t1	ref|NP_564663.1| acyl carrier protein 3 [Arabidopsis thaliana] gi|21542384|sp|P25702.2|ACP3_ARATH RecName: Full=Acyl carrier protein 3, chloroplastic; Short=ACP-3; Flags: Precursor gi|3776561|gb|AAC64878.1| Identical to DNA for acyl carrier protein (ACP) gene A2 gb|X57699 from A. thaliana. ESTs gb|W43252, gb|T42821, gb|N65229, gb|N97267, gb|F15491 and gb|AA040955 come from this gene [Arabidopsis thaliana] gi|15451048|gb|AAK96795.1| acyl carrier protein (ACP) gene [Arabidopsis thaliana] gi|18377552|gb|AAL66942.1| acyl carrier protein (ACP) A2 [Arabidopsis thaliana] gi|332195006|gb|AEE33127.1| acyl carrier protein 3 [Arabidopsis thaliana]	131	136	7.00E-51	103.8	81.7	90.1	acyl carrier protein 3	gbpln	Arabidopsis thaliana	AT1G54630.1 | Symbols: ACP3 | acyl carrier protein 3 | chr1:20401642-20402919 REVERSE LENGTH=136	131	136	2.00E-53	103.8	81.7	90.1
Rsa1.0_00002.1.g113.t1	ref|NP_180850.2| DCD (Development and Cell Death) domain protein [Arabidopsis thaliana] gi|330253667|gb|AEC08761.1| DCD (Development and Cell Death) domain protein [Arabidopsis thaliana]	680	691	1.00E-152	101.6	56.3	69.4	DCD (Development and Cell Death) domain protein	gbpln	Arabidopsis thaliana	AT2G32910.1 | Symbols: | DCD (Development and Cell Death) domain protein | chr2:13959685-13961989 FORWARD LENGTH=691	680	691	1.00E-154	101.6	56.3	69.4
Rsa1.0_00002.1.g114.t1	ref|XP_002892399.1| hypothetical protein ARALYDRAFT_470766 [Arabidopsis lyrata subsp. lyrata] gi|297338241|gb|EFH68658.1| hypothetical protein ARALYDRAFT_470766 [Arabidopsis lyrata subsp. lyrata]	265	266	1.00E-125	100.4	81.5	89.1	hypothetical protein ARALYDRAFT_470766	gbpln	Arabidopsis lyrata	AT1G07440.1 | Symbols: | NAD(P)-binding Rossmann-fold superfamily protein | chr1:2286436-2287665 REVERSE LENGTH=266	265	266	1.00E-126	100.4	80.8	88.3
Rsa1.0_00002.1.g115.t1	ref|XP_002894600.1| hypothetical protein ARALYDRAFT_474746 [Arabidopsis lyrata subsp. lyrata] gi|297340442|gb|EFH70859.1| hypothetical protein ARALYDRAFT_474746 [Arabidopsis lyrata subsp. lyrata]	852	931	0	109.3	83.0	89.2	hypothetical protein ARALYDRAFT_474746	gbpln	Arabidopsis lyrata	AT1G54710.1 | Symbols: ATATG18H, ATG18H | homolog of yeast autophagy 18 (ATG18) H | chr1:20417019-20420733 REVERSE LENGTH=927	852	927	0	108.8	81.5	87.6
Rsa1.0_00002.1.g116.t1	ref|NP_563830.1| sugar transporter ERD6 [Arabidopsis thaliana] gi|79317421|ref|NP_001031006.1| sugar transporter ERD6 [Arabidopsis thaliana] gi|117940144|sp|O04036.3|ERD6_ARATH RecName: Full=Sugar transporter ERD6; AltName: Full=Early-responsive to dehydration protein 6; AltName: Full=Sugar transporter-like protein 1 gi|3123712|dbj|BAA25989.1| ERD6 protein [Arabidopsis thaliana] gi|6686825|emb|CAB64732.1| putative sugar transporter [Arabidopsis thaliana] gi|30794056|gb|AAP40473.1| putative zinc finger protein ATZF1 [Arabidopsis thaliana] gi|332190249|gb|AEE28370.1| sugar transporter ERD6 [Arabidopsis thaliana] gi|332190250|gb|AEE28371.1| sugar transporter ERD6 [Arabidopsis thaliana]	343	496	1.00E-149	144.6	74.3	85.7	sugar transporter ERD6	gbpln	Arabidopsis thaliana	AT1G08930.2 | Symbols: ERD6 | Major facilitator superfamily protein | chr1:2873604-2876979 FORWARD LENGTH=496	343	496	1.00E-151	144.6	74.3	85.7
Rsa1.0_00002.1.g117.t1	ref|XP_002894606.1| hypothetical protein ARALYDRAFT_892725 [Arabidopsis lyrata subsp. lyrata] gi|297340448|gb|EFH70865.1| hypothetical protein ARALYDRAFT_892725 [Arabidopsis lyrata subsp. lyrata]	302	320	1.00E-119	106.0	73.5	85.4	hypothetical protein ARALYDRAFT_892725	gbpln	Arabidopsis lyrata	AT1G72210.1 | Symbols: | basic helix-loop-helix (bHLH) DNA-binding superfamily protein | chr1:27180066-27182268 FORWARD LENGTH=320	302	320	6.00E-53	106.0	35.4	47.0
Rsa1.0_00002.1.g118.t1	ref|NP_175886.1| Late embryogenesis abundant-related protein [Arabidopsis thaliana] gi|9857516|gb|AAG00871.1|AC064840_2 Unknown protein [Arabidopsis thaliana] gi|12322166|gb|AAG51122.1|AC069144_19 late embryogenesis protein, putative [Arabidopsis thaliana] gi|332195039|gb|AEE33160.1| Late embryogenesis abundant-related protein [Arabidopsis thaliana]	349	347	1.00E-179	99.4	85.1	92.8	Late embryogenesis abundant-related protein	gbpln	Arabidopsis thaliana	AT1G54890.1 | Symbols: | Late embryogenesis abundant (LEA) protein-related | chr1:20463107-20464407 FORWARD LENGTH=347	349	347	0	99.4	85.1	92.8

Rsa1.0_00002.1.g119.t1	ref|XP_002891917.1| hypothetical protein ARALYDRAFT_474768 [Arabidopsis lyrata subsp. lyrata] gi|297337759|gb|EFH68176.1| hypothetical protein ARALYDRAFT_474768 [Arabidopsis lyrata subsp. lyrata]		865	894	0	103.4	67.4	75.3	hypothetical protein ARALYDRAFT_474768	gbpln	Arabidopsis lyrata	AT1G54920.3 | Symbols: | unknown protein; FUNCTIONS IN: molecular_function unknown; INVOLVED IN: biological_process unknown; EXPRESSED IN: 23 plant structures; EXPRESSED DURING: 15 growth stages. | chr1:20470916-20474898 FORWARD LENGTH=901		865	901	0	104.2	66.7	74.7
Rsa1.0_00002.1.g120.t1	ref|NP_175890.1| GRF zinc finger / Zinc knuckle protein [Arabidopsis thaliana] gi|9857519|gb|AAG00874.1|AC064840_5 Hypothetical protein [Arabidopsis thaliana] gi|12322160|gb|AAG51116.1|AC069144_13 hypothetical protein [Arabidopsis thaliana] gi|332195043|gb|AEE33164.1| GRF zinc finger / Zinc knuckle protein [Arabidopsis thaliana]		368	353	6.00E-57	95.9	43.5	56.3	GRF zinc finger / Zinc knuckle protein	gbpln	Arabidopsis thaliana	AT1G54930.1 | Symbols: | GRF zinc finger / Zinc knuckle protein | chr1:20478267-20479671 REVERSE LENGTH=353		368	353	1.00E-59	95.9	43.5	56.3
Rsa1.0_00002.1.g121.t1	#	#	#	#	#	#	#		-	----	----	#	#	#	#	#	#	#	
Rsa1.0_00002.1.g122.t1	ref|NP_001058585.1| Os06g0715500 [Oryza sativa Japonica Group] gi|53791783|dbj|BAD53577.1| putative SSR alpha subunit [Oryza sativa Japonica Group] gi|113596625|dbj|BAF20499.1| Os06g0715500 [Oryza sativa Japonica Group] gi|125598503|gb|EAZ38283.1| hypothetical protein OsJ_22661 [Oryza sativa Japonica Group] gi|215704734|dbj|BAG94762.1| unnamed protein product [Oryza sativa Japonica Group] gi|215765401|dbj|BAG87098.1| unnamed protein product [Oryza sativa Japonica Group] gi|215765644|dbj|BAG87341.1| unnamed protein product [Oryza sativa Japonica Group]		168	257	3.00E-23	153.0	31.5	39.3	Os06g0715500	gbpln	Oryza sativa	AT2G21160.1 | Symbols: | Translocon-associated protein (TRAP), alpha subunit | chr2:9068428-9070207 FORWARD LENGTH=258		168	258	6.00E-19	153.6	39.9	43.5
Rsa1.0_00002.1.g123.t1	#	#	#	#	#	#	#		-	----	----	#	#	#	#	#	#	#	
Rsa1.0_00002.1.g124.t1	gb|EOA34295.1| hypothetical protein CARUB_v10021810mg [Capsella rubella]		542	559	0	103.1	80.4	89.5	hypothetical protein CARUB_v10021810mg	gbpln	Capsella rubella	AT1G54940.1 | Symbols: PGSIP4 | plant glycogenin-like starch initiation protein 4 | chr1:20481690-20484541 FORWARD LENGTH=557		542	557	0	102.8	78.4	89.3
Rsa1.0_00002.1.g125.t1	#	#	#	#	#	#	#		-	----	----	#	#	#	#	#	#	#	
Rsa1.0_00002.1.g126.t1	ref|XP_002891939.1| hypothetical protein ARALYDRAFT_474779 [Arabidopsis lyrata subsp. lyrata] gi|297337781|gb|EFH68198.1| hypothetical protein ARALYDRAFT_474779 [Arabidopsis lyrata subsp. lyrata]		873	845	0	96.8	75.8	82.0	hypothetical protein ARALYDRAFT_474779	gbpln	Arabidopsis lyrata	AT1G55040.1 | Symbols: | zinc finger (Ran-binding) family protein | chr1:20534895-20538901 FORWARD LENGTH=849		873	849	0	97.3	77.0	82.7
Rsa1.0_00002.1.g127.t2	gb|EOA34587.1| hypothetical protein CARUB_v10022144mg [Capsella rubella]		329	349	1.00E-169	106.1	84.2	94.5	hypothetical protein CARUB_v10022144mg	gbpln	Capsella rubella	AT1G58725.1 | Symbols: | GDSL-like Lipase/Acylhydrolase superfamily protein | chr1:21771935-21773365 REVERSE LENGTH=349		329	349	1.00E-172	106.1	85.1	94.2
Rsa1.0_00002.1.g128.t1	ref|XP_002891941.1| hypothetical protein ARALYDRAFT_474781 [Arabidopsis lyrata subsp. lyrata] gi|297337783|gb|EFH68200.1| hypothetical protein ARALYDRAFT_474781 [Arabidopsis lyrata subsp. lyrata]		801	892	0	111.4	65.0	76.8	hypothetical protein ARALYDRAFT_474781	gbpln	Arabidopsis lyrata	AT1G55050.2 | Symbols: | unknown protein; FUNCTIONS IN: molecular_function unknown; INVOLVED IN: biological_process unknown; LOCATED IN: cellular_component unknown; EXPRESSED IN: cultured cell; BEST Arabidopsis thaliana protein match is: unknown protein (TAIR:AT1G09040.1); Has 30201 Blast hits to 17322 proteins in 780 species: Archae - 12; Bacteria - 1396; Metazoa - 17338; Fungi - 3422; Plants - 5037; Viruses - 0; Other Eukaryotes - 2996 (source: NCBI BLink). | chr1:20542779-20545612 FORWARD LENGTH=915		801	915	0	114.2	61.9	73.8
Rsa1.0_00002.1.g129.t1	ref|XP_002891941.1| hypothetical protein ARALYDRAFT_474781 [Arabidopsis lyrata subsp. lyrata] gi|297337783|gb|EFH68200.1| hypothetical protein ARALYDRAFT_474781 [Arabidopsis lyrata subsp. lyrata]		802	892	0	111.2	66.3	77.8	hypothetical protein ARALYDRAFT_474781	gbpln	Arabidopsis lyrata	AT1G55050.2 | Symbols: | unknown protein; FUNCTIONS IN: molecular_function unknown; INVOLVED IN: biological_process unknown; LOCATED IN: cellular_component unknown; EXPRESSED IN: cultured cell; BEST Arabidopsis thaliana protein match is: unknown protein (TAIR:AT1G09040.1); Has 30201 Blast hits to 17322 proteins in 780 species: Archae - 12; Bacteria - 1396; Metazoa - 17338; Fungi - 3422; Plants - 5037; Viruses - 0; Other Eukaryotes - 2996 (source: NCBI BLink). | chr1:20542779-20545612 FORWARD LENGTH=915		802	915	0	114.1	62.2	74.3

Rsa1.0_00002.1.g130.t1	ref|XP_002888210.1| hypothetical protein ARALYDRAFT_475383 [Arabidopsis lyrata subsp. lyrata] gi|297334051|gb|EFH64469.1| hypothetical protein ARALYDRAFT_475383 [Arabidopsis lyrata subsp. lyrata]	309	350	1.00E-150	113.3	84.5	92.9	hypothetical protein ARALYDRAFT_475383	gbpln	Arabidopsis lyrata	AT1G58725.1 | Symbols: | GDSL-like Lipase/Acylhydrolase superfamily protein | chr1:21771935-21773365 REVERSE LENGTH=349	309	349	1.00E-151	112.9	84.1	92.2
Rsa1.0_00002.1.g131.t1	ref|XP_002894623.1| hypothetical protein ARALYDRAFT_474782 [Arabidopsis lyrata subsp. lyrata] gi|297340465|gb|EFH70882.1| hypothetical protein ARALYDRAFT_474782 [Arabidopsis lyrata subsp. lyrata]	355	355	7.00E-94	100.0	67.9	77.7	hypothetical protein ARALYDRAFT_474782	gbpln	Arabidopsis lyrata	AT1G56210.1 | Symbols: | Heavy metal transport/detoxification superfamily protein | chr1:21035715-21037019 FORWARD LENGTH=364	355	364	2.00E-92	102.5	66.8	77.7
Rsa1.0_00002.1.g132.t1	ref|XP_002891949.1| hypothetical protein ARALYDRAFT_892793 [Arabidopsis lyrata subsp. lyrata] gi|297337791|gb|EFH68208.1| hypothetical protein ARALYDRAFT_892793 [Arabidopsis lyrata subsp. lyrata]	669	691	0	103.3	84.8	91.3	hypothetical protein ARALYDRAFT_892793	gbpln	Arabidopsis lyrata	AT1G56290.1 | Symbols: | CwfJ-like family protein | chr1:21075939-21078608 FORWARD LENGTH=692	669	692	0	103.4	84.6	91.6
Rsa1.0_00002.1.g133.t1	gb|EOA33719.1| hypothetical protein CARUB_v10019911mg [Capsella rubella]	581	668	0	115.0	79.2	86.4	hypothetical protein CARUB_v10019911mg	gbpln	Capsella rubella	AT1G56310.1 | Symbols: | Polynucleotidyl transferase, ribonuclease H-like superfamily protein | chr1:21082863-21085564 FORWARD LENGTH=589	581	589	0	101.4	78.1	87.6
Rsa1.0_00002.1.g134.t1	dbj|BAA78331.1| serine decarboxylase [Brassica napus]	479	490	0	102.3	93.9	96.2	serine decarboxylase	gbpln	Brassica napus	AT1G43710.1 | Symbols: emb1075 | Pyridoxal phosphate (PLP)-dependent transferases superfamily protein | chr1:16486534-16488298 REVERSE LENGTH=482	479	482	0	100.6	89.1	92.7
Rsa1.0_00002.1.g135.t1	sp|O04267.1|SAR1B_BRACM RecName: Full=GTP-binding protein SAR1B gi|2108347|gb|AAC49717.1| small GTP-binding protein Bsar1b [Brassica rapa subsp. oleifera]	195	195	1.00E-109	100.0	99.0	99.5	RecName: Full=GTP-binding protein SAR1B gi|2108347|gb|AAC49717.1| small GTP-binding protein Bsar1b	gbpln	Brassica rapa	AT1G56330.1 | Symbols: SAR1, ATSAR1, ATSARA1B, ATSAR1B, SAR1B | secretion-associated RAS 1B | chr1:21086845-21088478 REVERSE LENGTH=193	195	193	1.00E-108	99.0	97.4	98.5
Rsa1.0_00002.1.g136.t1	ref|XP_002894642.1| pseudouridine synthase family protein [Arabidopsis lyrata subsp. lyrata] gi|297340484|gb|EFH70901.1| pseudouridine synthase family protein [Arabidopsis lyrata subsp. lyrata]	296	321	1.00E-125	108.4	80.1	87.2	pseudouridine synthase family protein	gbpln	Arabidopsis lyrata	AT1G56345.1 | Symbols: | Pseudouridine synthase family protein | chr1:21093409-21094454 REVERSE LENGTH=322	296	322	1.00E-120	108.8	78.4	87.2
Rsa1.0_00002.1.g137.t2	ref|XP_002891952.1| predicted protein [Arabidopsis lyrata subsp. lyrata] gi|297337794|gb|EFH68211.1| predicted protein [Arabidopsis lyrata subsp. lyrata]	434	418	0	96.3	78.6	84.3	predicted protein	gbpln	Arabidopsis lyrata	AT1G56340.1 | Symbols: CRT1, CRT1a, AtCRT1a | calreticulin 1a | chr1:21090059-21092630 REVERSE LENGTH=425	434	425	1.00E-177	97.9	76.7	78.6
Rsa1.0_00002.1.g138.t1	ref|NP_001117508.1| uncharacterized protein [Arabidopsis thaliana] gi|332195272|gb|AEE33393.1| uncharacterized protein AT1G56423 [Arabidopsis thaliana]	270	270	1.00E-126	100.0	82.6	91.9	uncharacterized protein	gbpln	Arabidopsis thaliana	AT1G56423.1 | Symbols: | unknown protein; FUNCTIONS IN: molecular_function unknown; INVOLVED IN: biological_process unknown; LOCATED IN: endomembrane system; Has 30201 Blast hits to 17322 proteins in 780 species: Archae - 12; Bacteria - 1396; Metazoa - 17338; Fungi - 3422; Plants - 5037; Viruses - 0; Other Eukaryotes - 2996 (source: NCBI BLink). | chr1:21129075-21130558 FORWARD LENGTH=270	270	270	1.00E-128	100.0	82.6	91.9
Rsa1.0_00002.1.g139.t1	gb|EOA35645.1| hypothetical protein CARUB_v10020862mg [Capsella rubella]	305	245	1.00E-134	80.3	75.1	78.4	hypothetical protein CARUB_v10020862mg	gbpln	Capsella rubella	AT1G56450.1 | Symbols: PBG1 | 20S proteasome beta subunit G1 | chr1:21141970-21144186 FORWARD LENGTH=246	305	246	1.00E-135	80.7	73.8	77.4
Rsa1.0_00002.1.g140.t1	gb|AAZ67609.1| 80A08_24 [Brassica rapa subsp. pekinensis]	330	475	3.00E-11	143.9	13.0	14.8	80A08_24	gbpln	Brassica rapa	#	#	#	#	#	#	#
Rsa1.0_00002.1.g141.t1	emb|CAB41158.1| putative protein [Arabidopsis thaliana]	645	669	1.00E-177	103.7	54.9	66.4	putative protein	gbpln	Arabidopsis thaliana	AT5G22355.1 | Symbols: | Cysteine/Histidine-rich C1 domain family protein | chr5:7401762-7403756 FORWARD LENGTH=664	645	664	1.00E-172	102.9	52.2	63.7
Rsa1.0_00002.1.g142.t1	ref|XP_002891968.1| hypothetical protein ARALYDRAFT_892829 [Arabidopsis lyrata subsp. lyrata] gi|297337810|gb|EFH68227.1| hypothetical protein ARALYDRAFT_892829 [Arabidopsis lyrata subsp. lyrata]	1062	1058	0	99.6	89.3	93.4	hypothetical protein ARALYDRAFT_892829	gbpln	Arabidopsis lyrata	AT1G56500.1 | Symbols: | haloacid dehalogenase-like hydrolase family protein | chr1:21159775-21167092 FORWARD LENGTH=1055	1062	1055	0	99.3	88.6	92.7

Rsa1.0_00002.1.g143.t1	ref|NP_176050.1| pentatricopeptide repeat-containing protein [Arabidopsis thaliana] gi|75173051|sp|Q9FXA9.1|PPR83_ARATH RecName: Full=Putative pentatricopeptide repeat-containing protein At1g56570 gi|9954755|gb|AAG09106.1|AC009323_17 Hypothetical protein [Arabidopsis thaliana] gi|332195289|gb|AEE33410.1| pentatricopeptide repeat-containing protein [Arabidopsis thaliana]	609	611	0	100.3	88.0	94.1	pentatricopeptide repeat-containing protein	gbpln	Arabidopsis thaliana	AT1G56570.1 | Symbols: | Tetratricopeptide repeat (TPR)-like superfamily protein | chr1:21195804-21197721 FORWARD LENGTH=611	609	611	0	100.3	88.0	94.1
Rsa1.0_00002.1.g144.t1	ref|NP_564720.1| uncharacterized protein [Arabidopsis thaliana] gi|9954754|gb|AAG09105.1|AC009323_16 Unknown protein [Arabidopsis thaliana] gi|16648669|gb|AAL25527.1| At1g56580/F25P12_18 [Arabidopsis thaliana] gi|20856239|gb|AAM26655.1| At1g56580/F25P12_18 [Arabidopsis thaliana] gi|332195290|gb|AEE33411.1| uncharacterized protein AT1G56580 [Arabidopsis thaliana]	155	166	4.00E-75	107.1	90.3	92.3	uncharacterized protein	gbpln	Arabidopsis thaliana	AT1G56580.1 | Symbols: SVB | Protein of unknown function, DUF538 | chr1:21198402-21198902 REVERSE LENGTH=166	155	166	1.00E-77	107.1	90.3	92.3
Rsa1.0_00002.1.g145.t1	ref|NP_176052.3| clathrin adaptor complexes medium subunit-like protein [Arabidopsis thaliana] gi|332195291|gb|AEE33412.1| clathrin adaptor complexes medium subunit-like protein [Arabidopsis thaliana]	435	415	0	95.4	87.4	91.5	clathrin adaptor complexes medium subunit-like protein	gbpln	Arabidopsis thaliana	AT1G56590.1 | Symbols: ZIP4 | Clathrin adaptor complexes medium subunit family protein | chr1:21202250-21204697 REVERSE LENGTH=415	435	415	0	95.4	87.4	91.5
Rsa1.0_00002.1.g146.t1	gb|AAM19710.1|AF499723_1 galactinol synthase-like protein [Eutrema halophilum]	331	337	1.00E-169	101.8	87.9	93.4	galactinol synthase-like protein	gbpln	Eutrema halophilum	AT1G56600.1 | Symbols: AtGolS2, GolS2 | galactinol synthase 2 | chr1:21207620-21209291 FORWARD LENGTH=335	331	335	1.00E-165	101.2	84.9	92.1
Rsa1.0_00002.1.g147.t1	ref|XP_002872685.1| hypothetical protein ARALYDRAFT_490081 [Arabidopsis lyrata subsp. lyrata] gi|297318522|gb|EFH48944.1| hypothetical protein ARALYDRAFT_490081 [Arabidopsis lyrata subsp. lyrata]	449	812	1.00E-109	180.8	53.9	63.7	hypothetical protein ARALYDRAFT_490081	gbpln	Arabidopsis lyrata	AT4G04220.1 | Symbols: AtRLP46, RLP46 | receptor like protein 46 | chr4:2033427-2035946 FORWARD LENGTH=811	449	811	1.00E-103	180.6	53.9	64.6
Rsa1.0_00002.1.g148.t1	#	#	#	#	#	#	#	-	----	----	AT1G56660.1 | Symbols: | unknown protein; Has 665200 Blast hits to 205811 proteins in 4684 species: Archae - 3320; Bacteria - 107592; Metazoa - 249086; Fungi - 76753; Plants - 38542; Viruses - 3008; Other Eukaryotes - 186899 (source: NCBI BLink). | chr1:21238809-21240377 FORWARD LENGTH=522	490	522	2.00E-11	106.5	11.2	14.3
Rsa1.0_00002.1.g149.t1	ref|NP_176059.1| GDSL esterase/lipase LIP-4 [Arabidopsis thaliana] gi|75173058|sp|Q9FXB6.1|LIP4_ARATH RecName: Full=GDSL esterase/lipase LIP-4; AltName: Full=Extracellular lipase LIP-4; Flags: Precursor gi|9954747|gb|AAG09098.1|AC009323_9 Similar to nodulins [Arabidopsis thaliana] gi|332195300|gb|AEE33421.1| GDSL esterase/lipase LIP-4 [Arabidopsis thaliana]	92	373	1.00E-34	405.4	82.6	89.1	GDSL esterase/lipase LIP-4	gbpln	Arabidopsis thaliana	AT1G56670.1 | Symbols: | GDSL-like Lipase/Acylhydrolase superfamily protein | chr1:21241688-21243965 FORWARD LENGTH=373	92	373	2.00E-37	405.4	82.6	89.1
Rsa1.0_00002.1.g150.t1	gb|AAM82604.1|AF525305_2 putative AP endonuclease/reverse transcriptase [Brassica napus]	1260	1214	0	96.3	34.5	52.2	putative AP endonuclease/reverse transcriptase	gbpln	Brassica napus	AT1G43760.1 | Symbols: | DNAse I-like superfamily protein | chr1:16528880-16531065 REVERSE LENGTH=626	1260	626	1.00E-76	49.7	12.5	19.9
Rsa1.0_00002.1.g151.t1	ref|XP_002894685.1| predicted protein [Arabidopsis lyrata subsp. lyrata] gi|297340527|gb|EFH70944.1| predicted protein [Arabidopsis lyrata subsp. lyrata]	62	62	3.00E-21	100.0	75.8	91.9	predicted protein	gbpln	Arabidopsis lyrata	#	#	#	#	#	#	#
Rsa1.0_00002.1.g152.t1	gb|EOA33210.1| hypothetical protein CARUB_v10019892mg [Capsella rubella]	328	692	1.00E-109	211.0	71.6	75.6	hypothetical protein CARUB_v10019892mg	gbpln	Capsella rubella	AT1G57700.1 | Symbols: | Protein kinase superfamily protein | chr1:21371051-21373860 FORWARD LENGTH=692	328	692	1.00E-111	211.0	71.0	75.9

Rsa1.0_00002.1.g153.t1	ref|NP_176081.1| uncharacterized protein [Arabidopsis thaliana] gi|42571907|ref|NP_974044.1| uncharacterized protein [Arabidopsis thaliana] gi|12321352|gb|AAG50748.1|AC079733_16 hypothetical protein [Arabidopsis thaliana] gi|18176030|gb|AAL59971.1| unknown protein [Arabidopsis thaliana] gi|22136846|gb|AAM91767.1| unknown protein [Arabidopsis thaliana] gi|332195330|gb|AEE33451.1| uncharacterized protein AT1G57680 [Arabidopsis thaliana] gi|332195331|gb|AEE33452.1| uncharacterized protein AT1G57680 [Arabidopsis thaliana]	361	362	1.00E-158	100.3	84.5	90.6	uncharacterized protein	gbpln	Arabidopsis thaliana	AT1G57680.2 | Symbols: | FUNCTIONS IN: molecular_function unknown; INVOLVED IN: biological_process unknown; LOCATED IN: endomembrane system; EXPRESSED IN: 24 plant structures; EXPRESSED DURING: 15 growth stages; CONTAINS InterPro DOMAIN/s: Uncharacterised conserved protein UCP031277 (InterPro:IPR016971); Has 35333 Blast hits to 34131 proteins in 2444 species: Archae - 798; Bacteria - 22429; Metazoa - 974; Fungi - 991; Plants - 531; Viruses - 0; Other Eukaryotes - 9610 (source: NCBI BLink). | chr1:21362749-21363837 REVERSE LENGTH=362	361	362	1.00E-160	100.3	84.5	90.6
Rsa1.0_00002.1.g154.t1	ref|XP_002894649.1| hypothetical protein ARALYDRAFT_892826 [Arabidopsis lyrata subsp. lyrata] gi|297340491|gb|EFH70908.1| hypothetical protein ARALYDRAFT_892826 [Arabidopsis lyrata subsp. lyrata]	345	352	1.00E-130	102.0	67.2	78.6	hypothetical protein ARALYDRAFT_892826	gbpln	Arabidopsis lyrata	AT2G19150.1 | Symbols: | Pectin lyase-like superfamily protein | chr2:8305100-8307444 FORWARD LENGTH=339	345	339	2.00E-66	98.3	39.7	51.0
Rsa1.0_00002.1.g155.t1	emb|CAB39638.1| RNA-directed DNA polymerase-like protein [Arabidopsis thaliana] gi|7267666|emb|CAB78094.1| RNA-directed DNA polymerase-like protein [Arabidopsis thaliana]	1969	1274	0	64.7	30.5	41.5	RNA-directed DNA polymerase-like protein	gbpln	Arabidopsis thaliana	AT4G29090.1 | Symbols: | Ribonuclease H-like superfamily protein | chr4:14333528-14335255 FORWARD LENGTH=575	1969	575	1.00E-59	29.2	8.3	13.2
Rsa1.0_00002.1.g156.t1	#	#	#	#	#	#	#	-	----	----	#	#	#	#	#	#	#
Rsa1.0_00002.1.g157.t1	ref|XP_002888226.1| helicase domain-containing protein [Arabidopsis lyrata subsp. lyrata] gi|297334067|gb|EFH64485.1| helicase domain-containing protein [Arabidopsis lyrata subsp. lyrata]	1425	1458	0	102.3	87.4	93.3	helicase domain-containing protein	gbpln	Arabidopsis lyrata	AT1G58060.1 | Symbols: | RNA helicase family protein | chr1:21489480-21501775 REVERSE LENGTH=1459	1425	1459	0	102.4	87.4	93.1
Rsa1.0_00002.1.g158.t1	gb|ACP30602.1| disease resistance protein [Brassica rapa subsp. pekinensis]	291	938	4.00E-27	322.3	24.1	27.5	disease resistance protein	gbpln	Brassica rapa	AT5G18350.1 | Symbols: | Disease resistance protein (TIR-NBS-LRR class) family | chr5:6074069-6078569 REVERSE LENGTH=1245	291	1245	2.00E-13	427.8	17.5	21.0
Rsa1.0_00002.1.g159.t1	gb|EOA34251.1| hypothetical protein CARUB_v10021763mg [Capsella rubella]	236	268	6.00E-84	113.6	78.4	85.6	hypothetical protein CARUB_v10021763mg	gbpln	Capsella rubella	AT1G58070.1 | Symbols: | unknown protein; Has 286 Blast hits to 266 proteins in 81 species: Archae - 2; Bacteria - 25; Metazoa - 90; Fungi - 19; Plants - 78; Viruses - 4; Other Eukaryotes - 68 (source: NCBI BLink). | chr1:21502968-21503822 FORWARD LENGTH=284	236	284	2.00E-83	120.3	78.8	86.4
Rsa1.0_00002.1.g160.t1	gb|AAT74597.1| chloroplast ATP phosphoribosyl transferase [Alyssum montanum]	409	405	0	99.0	88.3	93.2	chloroplast ATP phosphoribosyl transferase	gbpln	Alyssum montanum	AT1G58080.1 | Symbols: ATATP-PRT1, HISN1A, ATP-PRT1 | ATP phosphoribosyl transferase 1 | chr1:21504562-21507429 REVERSE LENGTH=411	409	411	0	100.5	86.6	91.0
Rsa1.0_00002.1.g161.t1	ref|XP_002888231.1| hypothetical protein ARALYDRAFT_475419 [Arabidopsis lyrata subsp. lyrata] gi|297334072|gb|EFH64490.1| hypothetical protein ARALYDRAFT_475419 [Arabidopsis lyrata subsp. lyrata]	394	390	1.00E-126	99.0	74.1	78.9	hypothetical protein ARALYDRAFT_475419	gbpln	Arabidopsis lyrata	AT1G58100.1 | Symbols: | TCP family transcription factor | chr1:21512680-21513885 REVERSE LENGTH=401	394	401	1.00E-128	101.8	75.1	81.0
Rsa1.0_00002.1.g162.t1	dbj|BAJ34015.1| unnamed protein product [Thellungiella halophila]	369	372	1.00E-170	100.8	85.4	91.1	unnamed protein product	----	----	AT1G58110.2 | Symbols: | Basic-leucine zipper (bZIP) transcription factor family protein | chr1:21515919-21517374 REVERSE LENGTH=374	369	374	1.00E-151	101.4	78.3	87.3
Rsa1.0_00002.1.g163.t1	gb|AEM45875.1| hypothetical protein [Brassica rapa subsp. oleifera]	423	419	0	99.1	87.9	92.9	hypothetical protein	gbpln	Brassica rapa	AT1G58120.1 | Symbols: | BEST Arabidopsis thaliana protein match is: methyltransferases (TAIR:AT5G01710.1); Has 93 Blast hits to 93 proteins in 15 species: Archae - 0; Bacteria - 0; Metazoa - 1; Fungi - 0; Plants - 92; Viruses - 0; Other Eukaryotes - 0 (source: NCBI BLink). | chr1:21520962-21522224 FORWARD LENGTH=420	423	420	0	99.3	80.1	87.9
Rsa1.0_00002.1.g164.t1	gb|AAG50957.1|AC073943_7 hypothetical protein [Arabidopsis thaliana]	910	1195	0	131.3	85.2	90.7	hypothetical protein	gbpln	Arabidopsis thaliana	AT1G58210.1 | Symbols: EMB1674 | kinase interacting family protein | chr1:21553621-21558056 FORWARD LENGTH=1246	910	1246	0	136.9	84.7	90.3

Rsa1.0_00002.1.g165.t1	ref|NP_176118.2| myb family transcription factor [Arabidopsis thaliana] gi|26449358|dbj|BAC41806.1| putative MYB-family transcription factor [Arabidopsis thaliana] gi|29029054|gb|AAO64906.1| At1g58220 [Arabidopsis thaliana] gi|41619072|gb|AAS10017.1| MYB transcription factor [Arabidopsis thaliana] gi|332195394|gb|AEE33515.1| myb family transcription factor [Arabidopsis thaliana]	716	834	0	116.5	71.6	81.1	myb family transcription factor	gbpln	Arabidopsis thaliana	AT1G58220.1 | Symbols: | Homeodomain-like superfamily protein | chr1:21558388-21562669 REVERSE LENGTH=834	716	834	0	116.5	71.6	81.1
Rsa1.0_00002.1.g166.t2	ref|XP_002888245.1| hypothetical protein ARALYDRAFT_475439 [Arabidopsis lyrata subsp. lyrata] gi|297334086|gb|EFH64504.1| hypothetical protein ARALYDRAFT_475439 [Arabidopsis lyrata subsp. lyrata]	2608	2607	0	100.0	86.9	92.5	hypothetical protein ARALYDRAFT_475439	gbpln	Arabidopsis lyrata	AT1G58250.1 | Symbols: SAB | Golgi-body localisation protein domain ;RNA pol II promoter Fmp27 protein domain | chr1:21587317-21601373 REVERSE LENGTH=2607	2608	2607	0	100.0	87.0	92.4
Rsa1.0_00002.1.g167.t1	gb|ACR10268.1| sulfotransferase 5b [Brassica rapa subsp. pekinensis]	345	339	1.00E-177	98.3	90.7	94.2	sulfotransferase 5b	gbpln	Brassica rapa	AT1G74090.1 | Symbols: SOT18, ATSOT18, ATST5B | desulfo-glucosinolate sulfotransferase 18 | chr1:27863003-27864055 FORWARD LENGTH=350	345	350	1.00E-158	101.4	77.4	88.1
Rsa1.0_00002.1.g168.t1	gb|ACR10269.1| sulfotransferase 5b [Brassica rapa subsp. pekinensis]	339	342	1.00E-169	100.9	84.4	92.3	sulfotransferase 5b	gbpln	Brassica rapa	AT1G74090.1 | Symbols: SOT18, ATSOT18, ATST5B | desulfo-glucosinolate sulfotransferase 18 | chr1:27863003-27864055 FORWARD LENGTH=350	339	350	1.00E-143	103.2	71.7	84.7
Rsa1.0_00002.1.g169.t3	ref|NP_176126.1| heme oxygenase 4 [Arabidopsis thaliana] gi|75180109|sp|Q9LQC0.1|HMOX4_ARATH RecName: Full=Heme oxygenase 4, chloroplastic; Flags: Precursor gi|8979943|gb|AAF82257.1|AC008051_8 Contains similarity to heme oxygenase 1 (HO1) from Arabidopsis thaliana gb|AF132475 [Arabidopsis thaliana] gi|91805985|gb|ABE65721.1| heme oxygenase [Arabidopsis thaliana] gi|332195412|gb|AEE33533.1| heme oxygenase 4 [Arabidopsis thaliana]	339	283	1.00E-100	83.5	57.5	67.6	heme oxygenase 4	gbpln	Arabidopsis thaliana	AT1G58300.1 | Symbols: HO4 | heme oxygenase 4 | chr1:21628015-21629996 REVERSE LENGTH=283	339	283	1.00E-102	83.5	57.5	67.6
Rsa1.0_00002.1.g170.t1	dbj|BAJ33912.1| unnamed protein product [Thellungiella halophila]	532	539	0	101.3	94.2	96.8	unnamed protein product	----	----	AT1G58290.1 | Symbols: HEMA1 | Glutamyl-tRNA reductase family protein | chr1:21624028-21626051 REVERSE LENGTH=543	532	543	0	102.1	90.6	95.1
Rsa1.0_00002.1.g171.t4	ref|XP_002880114.1| glycosyl hydrolase family 1 protein [Arabidopsis lyrata subsp. lyrata] gi|297325953|gb|EFH56373.1| glycosyl hydrolase family 1 protein [Arabidopsis lyrata subsp. lyrata]	506	507	0	100.2	90.1	94.1	glycosyl hydrolase family 1 protein	gbpln	Arabidopsis lyrata	AT2G44450.1 | Symbols: BGLU15 | beta glucosidase 15 | chr2:18340966-18343744 FORWARD LENGTH=506	506	506	0	100.0	92.1	97.0
Rsa1.0_00002.1.g172.t1	#	#	#	#	#	#	#	-	----	----	#	#	#	#	#	#	#
Rsa1.0_00002.1.g173.t1	#	#	#	#	#	#	#	-	----	----	#	#	#	#	#	#	#
Rsa1.0_00002.1.g174.t1	ref|XP_002888219.1| hypothetical protein ARALYDRAFT_893660 [Arabidopsis lyrata subsp. lyrata] gi|297334060|gb|EFH64478.1| hypothetical protein ARALYDRAFT_893660 [Arabidopsis lyrata subsp. lyrata]	531	532	0	100.2	91.1	95.5	hypothetical protein ARALYDRAFT_893660	gbpln	Arabidopsis lyrata	AT1G58340.1 | Symbols: ZF14 | MATE efflux family protein | chr1:21653162-21655117 FORWARD LENGTH=532	531	532	0	100.2	91.1	95.3
Rsa1.0_00002.1.g175.t1	ref|NP_564732.1| Putative serine esterase family protein [Arabidopsis thaliana] gi|186491769|ref|NP_001117514.1| Putative serine esterase family protein [Arabidopsis thaliana] gi|8979939|gb|AAF82253.1|AC008051_4 Identical to gene ZW18 from Arabidopsis thaliana gb|AB028199 [Arabidopsis thaliana] gi|6520167|dbj|BAA87940.1| ZW18 [Arabidopsis thaliana] gi|110741889|dbj|BAE98886.1| hypothetical protein [Arabidopsis thaliana] gi|332195417|gb|AEE33538.1| Putative serine esterase family protein [Arabidopsis thaliana] gi|332195418|gb|AEE33539.1| Putative serine esterase family protein [Arabidopsis thaliana]	786	794	0	101.0	87.5	93.8	Putative serine esterase family protein	gbpln	Arabidopsis thaliana	AT1G58350.2 | Symbols: ZW18 | Putative serine esterase family protein | chr1:21663131-21666827 REVERSE LENGTH=794	786	794	0	101.0	87.5	93.8
Rsa1.0_00002.1.g176.t1	#	#	#	#	#	#	#	-	----	----	#	#	#	#	#	#	#

Rsa1.0_00002.1.g177.t1	emb|CAD92449.1| amino acid permease 1 [Brassica napus]	509	485	0	95.3	94.1	94.9	amino acid permease 1	gbpln	Brassica napus	AT1G58360.1 | Symbols: AAP1, NAT2 | amino acid permease 1 | chr1:21676623-21680313 FORWARD LENGTH=485	509	485	0	95.3	87.2	92.3
Rsa1.0_00002.1.g178.t1	gb|EOA33499.1| hypothetical protein CARUB_v10019737mg [Capsella rubella]	919	941	0	102.4	88.7	93.5	hypothetical protein CARUB_v10019737mg	gbpln	Capsella rubella	AT1G58370.1 | Symbols: RXF12, ATXYN1 | glycosyl hydrolase family 10 protein / carbohydrate-binding domain-containing protein | chr1:21684751-21688209 FORWARD LENGTH=917	919	917	0	99.8	89.4	93.5
Rsa1.0_00002.1.g179.t1	gb|ACP30587.1| disease resistance protein [Brassica rapa subsp. pekinensis]	913	929	0	101.8	72.5	82.6	disease resistance protein	gbpln	Brassica rapa	AT1G58390.1 | Symbols: | Disease resistance protein (CC-NBS-LRR class) family | chr1:21690962-21693891 REVERSE LENGTH=907	913	907	0	99.3	55.8	68.6
Rsa1.0_00002.1.g180.t1	#	#	#	#	#	#	#	-	----	----	#	#	#	#	#	#	#
Rsa1.0_00002.1.g181.t1	gb|ABD65062.1| hypothetical protein 27.t00126 [Brassica oleracea]	442	578	7.00E-97	130.8	48.9	65.4	hypothetical protein 27.t00126	gbpln	Brassica oleracea	AT4G32200.1 | Symbols: ASY2 | DNA-binding HORMA family protein | chr4:15548840-15554962 FORWARD LENGTH=1399	442	1399	2.00E-20	316.5	10.9	15.4
Rsa1.0_00002.1.g182.t1	#	#	#	#	#	#	#	-	----	----	#	#	#	#	#	#	#
Rsa1.0_00002.1.g183.t1	ref|NP_564733.1| uncharacterized protein [Arabidopsis thaliana] gi|12321051|gb|AAG50647.1|AC082643_11 hypothetical protein [Arabidopsis thaliana] gi|89111882|gb|ABD60713.1| At1g58420 [Arabidopsis thaliana] gi|332195426|gb|AEE33547.1| uncharacterized protein AT1G58420 [Arabidopsis thaliana] gi|343455557|gb|AEM36343.1| At1g58420 [Arabidopsis thaliana]	172	179	7.00E-60	104.1	83.7	90.7	uncharacterized protein	gbpln	Arabidopsis thaliana	AT1G58420.1 | Symbols: | Uncharacterised conserved protein UCP031279 | chr1:21707407-21707946 FORWARD LENGTH=179	172	179	3.00E-62	104.1	83.7	90.7
Rsa1.0_00002.1.g184.t2	ref|XP_002886690.1| hypothetical protein ARALYDRAFT_475389 [Arabidopsis lyrata subsp. lyrata] gi|297332531|gb|EFH62949.1| hypothetical protein ARALYDRAFT_475389 [Arabidopsis lyrata subsp. lyrata]	566	530	0	93.6	85.7	89.6	hypothetical protein ARALYDRAFT_475389	gbpln	Arabidopsis lyrata	AT1G58440.1 | Symbols: XF1, SQE1 | FAD/NAD(P)-binding oxidoreductase family protein | chr1:21714094-21717246 REVERSE LENGTH=531	566	531	0	93.8	84.6	88.5
Rsa1.0_00002.1.g185.t1	ref|XP_002888212.1| hypothetical protein ARALYDRAFT_315379 [Arabidopsis lyrata subsp. lyrata] gi|297334053|gb|EFH64471.1| hypothetical protein ARALYDRAFT_315379 [Arabidopsis lyrata subsp. lyrata]	152	150	4.00E-50	98.7	74.3	78.9	hypothetical protein ARALYDRAFT_315379	gbpln	Arabidopsis lyrata	AT1G58460.1 | Symbols: | unknown protein; Has 35 Blast hits to 35 proteins in 8 species: Archae - 0; Bacteria - 0; Metazoa - 0; Fungi - 0; Plants - 35; Viruses - 0; Other Eukaryotes - 0 (source: NCBI BLink). | chr1:21721404-21722429 FORWARD LENGTH=177	152	177	3.00E-46	116.4	73.0	77.0
Rsa1.0_00002.1.g186.t1	#	#	#	#	#	#	#	-	----	----	#	#	#	#	#	#	#
Rsa1.0_00002.1.g187.t1	gb|EOA35303.1| hypothetical protein CARUB_v10020477mg, partial [Capsella rubella]	362	377	1.00E-129	104.1	68.0	79.3	hypothetical protein CARUB_v10020477mg, partial	gbpln	Capsella rubella	AT1G58470.1 | Symbols: ATRBP1, RBP1 | RNA-binding protein 1 | chr1:21727374-21728539 FORWARD LENGTH=360	362	360	1.00E-131	99.4	68.5	79.3
Rsa1.0_00002.1.g188.t1	ref|NP_176403.1| Mitochondrial transcription termination factor family protein [Arabidopsis thaliana] gi|50253494|gb|AAT71949.1| At1g62120 [Arabidopsis thaliana] gi|53850523|gb|AAU95438.1| At1g62120 [Arabidopsis thaliana] gi|332195804|gb|AEE33925.1| Mitochondrial transcription termination factor family protein [Arabidopsis thaliana]	430	437	1.00E-150	101.6	64.9	79.1	Mitochondrial transcription termination factor family protein	gbpln	Arabidopsis thaliana	AT1G62120.1 | Symbols: | Mitochondrial transcription termination factor family protein | chr1:22960326-22961639 REVERSE LENGTH=437	430	437	1.00E-153	101.6	64.9	79.1
Rsa1.0_00002.1.g189.t1	gb|AEM36358.1| At1g59510 [Arabidopsis thaliana]	447	382	1.00E-151	85.5	62.6	70.9	At1g59510	gbpln	Arabidopsis thaliana	AT1G59510.1 | Symbols: CF9 | Carbohydrate-binding protein | chr1:21859768-21860913 FORWARD LENGTH=381	447	381	1.00E-151	85.2	61.3	70.2
Rsa1.0_00002.1.g190.t1	ref|NP_176162.1| basic leucine-zipper 4 [Arabidopsis thaliana] gi|8778737|gb|AAF79745.1|AC009317_4 T30E16.6 [Arabidopsis thaliana] gi|14475943|gb|AAK62790.1|AC027036_11 G-box binding factor, putative [Arabidopsis thaliana] gi|15278035|gb|AAK94023.1|AF400619_1 transcription factor-like protein bZIP4 [Arabidopsis thaliana] gi|91805987|gb|ABE65722.1| bZIP transcription factor family protein [Arabidopsis thaliana] gi|225898030|dbj|BAH30347.1| hypothetical protein [Arabidopsis thaliana] gi|332195463|gb|AEE33584.1| basic leucine-zipper 4 [Arabidopsis thaliana]	365	148	2.00E-51	40.5	30.1	35.3	basic leucine-zipper 4	gbpln	Arabidopsis thaliana	AT1G59530.1 | Symbols: ATBZIP4, bZIP4 | basic leucine-zipper 4 | chr1:21868262-21868708 FORWARD LENGTH=148	365	148	5.00E-54	40.5	30.1	35.3

Rsa1.0_00002.1.g191.t1	ref|XP_002888202.1| ZCF125 [Arabidopsis lyrata subsp. lyrata] gi|297334043|gb|EFH64461.1| ZCF125 [Arabidopsis lyrata subsp. lyrata]	827	827	0	100.0	87.3	92.1	ZCF125	gbpln	Arabidopsis lyrata	AT1G59540.1 | Symbols: ZCF125 | P-loop containing nucleoside triphosphate hydrolases superfamily protein | chr1:21874083-21879382 FORWARD LENGTH=823	827	823	0	99.5	86.2	92.0
Rsa1.0_00002.1.g192.t1	ref|XP_002864007.1| predicted protein [Arabidopsis lyrata subsp. lyrata] gi|297309842|gb|EFH40266.1| predicted protein [Arabidopsis lyrata subsp. lyrata]	1017	3616	0	355.6	89.9	95.5	predicted protein	gbpln	Arabidopsis lyrata	AT1G55860.2 | Symbols: UPL1 | ubiquitin-protein ligase 1 | chr1:20879900-20892910 REVERSE LENGTH=3681	1017	3681	1.00E-156	361.9	35.6	51.4
Rsa1.0_00003.1.g193.t1	ref|XP_002870026.1| hypothetical protein ARALYDRAFT_914806 [Arabidopsis lyrata subsp. lyrata] gi|297315862|gb|EFH46285.1| hypothetical protein ARALYDRAFT_914806 [Arabidopsis lyrata subsp. lyrata]	375	380	0	101.3	98.9	99.5	hypothetical protein ARALYDRAFT_914806	gbpln	Arabidopsis lyrata	AT4G18710.1 | Symbols: BIN2, DWF12, UCU1, ATSK21, SK21 | Protein kinase superfamily protein | chr4:10296474-10298913 FORWARD LENGTH=380	375	380	0	101.3	98.7	99.5
Rsa1.0_00003.1.g194.t1	ref|NP_191429.1| 60S ribosomal protein L11-2 [Arabidopsis thaliana] gi|18415161|ref|NP_567563.1| 60S ribosomal protein L11-2 [Arabidopsis thaliana] gi|30694822|ref|NP_568649.2| 60S ribosomal protein L11-2 [Arabidopsis thaliana] gi|27735235|sp|P42794.2|RL112_ARATH RecName: Full=60S ribosomal protein L11-2; AltName: Full=L16 gi|11908058|gb|AAG41458.1|AF326876_1 putative ribosomal protein L11 [Arabidopsis thaliana] gi|12642874|gb|AAK00379.1|AF339697_1 putative ribosomal protein L11 [Arabidopsis thaliana] gi|14326537|gb|AAK60313.1|AF385722_1 AT4g18730/F28A21_140 [Arabidopsis thaliana] gi|7630065|emb|CAB88287.1| ribosomal protein L11-like [Arabidopsis thaliana] gi|9758681|dbj|BAB09220.1| ribosomal protein L11-like [Arabidopsis thaliana] gi|14517470|gb|AAK62625.1| AT4g18730/F28A21_140 [Arabidopsis thaliana] gi|18700224|gb|AAL77722.1| AT4g18730/F28A21_140 [Arabidopsis thaliana] gi|21553372|gb|AAM62465.1| ribosomal protein L11, cytosolic [Arabidopsis thaliana] gi|21592421|gb|AAM64372.1| ribosomal protein L11, cytosolic [Arabidopsis thaliana] gi|22136574|gb|AAM91073.1| AT4g18730/F28A21_140 [Arabidopsis thaliana] gi|332007914|gb|AED95297.1|	182	182	1.00E-102	100.0	99.5	100.0	60S ribosomal protein L11-2	gbpln	Arabidopsis thaliana	AT5G45775.2 | Symbols: | Ribosomal L5P family protein | chr5:18565281-18566496 REVERSE LENGTH=182	182	182	1.00E-104	100.0	99.5	100.0
Rsa1.0_00003.1.g195.t1	gb|EOA18242.1| hypothetical protein CARUB_v10006734mg [Capsella rubella]	385	432	1.00E-159	112.2	80.0	87.3	hypothetical protein CARUB_v10006734mg	gbpln	Capsella rubella	AT4G18770.1 | Symbols: MYB98, AtMYB98 | myb domain protein 98 | chr4:10311068-10312557 FORWARD LENGTH=427	385	427	1.00E-157	110.9	77.7	85.5
Rsa1.0_00003.1.g196.t1	gb|ACS68199.1| cellulose synthase 8.2 catalytic subunit [Brassica napus]	985	984	0	99.9	98.4	98.6	cellulose synthase 8.2 catalytic subunit	gbpln	Brassica napus	AT4G18780.1 | Symbols: CESA8, IRX1, ATCESA8, LEW2 | cellulose synthase family protein | chr4:10312846-10316719 REVERSE LENGTH=985	985	985	0	100.0	94.6	96.8
Rsa1.0_00003.1.g197.t2	ref|XP_002867976.1| natural resistance-associated macrophage protein 5 [Arabidopsis lyrata subsp. lyrata] gi|297313812|gb|EFH44235.1| natural resistance-associated macrophage protein 5 [Arabidopsis lyrata subsp. lyrata]	549	529	0	96.4	87.6	90.3	natural resistance-associated macrophage protein 5	gbpln	Arabidopsis lyrata	AT4G18790.1 | Symbols: NRAMP5, ATNRAMP5 | NRAMP metal ion transporter family protein | chr4:10317785-10319660 REVERSE LENGTH=530	549	530	0	96.5	87.4	90.3
Rsa1.0_00003.1.g198.t1	gb|EOA17416.1| hypothetical protein CARUB_v10005719mg [Capsella rubella]	215	214	1.00E-111	99.5	92.1	96.3	hypothetical protein CARUB_v10005719mg	gbpln	Capsella rubella	AT4G18800.1 | Symbols: ATHSGBP, ATRAB11B, ATRABA1D, RABA1d | RAB GTPase homolog A1D | chr4:10320156-10321339 REVERSE LENGTH=214	215	214	1.00E-113	99.5	92.6	96.7
Rsa1.0_00003.1.g199.t1	ref|XP_002887691.1| nucleotide-binding family protein [Arabidopsis lyrata subsp. lyrata] gi|297333532|gb|EFH63950.1| nucleotide-binding family protein [Arabidopsis lyrata subsp. lyrata]	686	355	1.00E-160	51.7	39.5	45.5	nucleotide-binding family protein	gbpln	Arabidopsis lyrata	AT5G50960.1 | Symbols: NBP35, ATNBP35 | nucleotide binding protein 35 | chr5:20734267-20735824 FORWARD LENGTH=350	686	350	1.00E-158	51.0	38.9	45.0
Rsa1.0_00003.1.g200.t1	ref|NP_191844.1| zinc finger protein-like protein [Arabidopsis thaliana] gi|7362765|emb|CAB83135.1| putative protein [Arabidopsis thaliana] gi|332646881|gb|AEE80402.1| zinc finger protein-like protein [Arabidopsis thaliana]	302	472	1.00E-55	156.3	38.4	46.7	zinc finger protein-like protein	gbpln	Arabidopsis thaliana	AT3G62850.1 | Symbols: | zinc finger protein-related | chr3:23237487-23239155 REVERSE LENGTH=472	302	472	3.00E-58	156.3	38.4	46.7

Rsa1.0_00003.1.g201.t1	ref|XP_002883636.1| predicted protein [Arabidopsis lyrata subsp. lyrata] gi|297329476|gb|EFH59895.1| predicted protein [Arabidopsis lyrata subsp. lyrata]	132	720	1.00E-26	545.5	47.0	54.5	predicted protein	gbpln	Arabidopsis lyrata	AT2G04350.2 | Symbols: LACS8 | AMP-dependent synthetase and ligase family protein | chr2:1516086-1519178 FORWARD LENGTH=720	132	720	5.00E-28	545.5	46.2	53.0
Rsa1.0_00003.1.g202.t1	dbj|BAB02990.1| retroelement pol polyprotein-like [Arabidopsis thaliana]	124	1250	3.00E-25	1008.1	58.1	66.1	retroelement pol polyprotein-like	gbpln	Arabidopsis thaliana	#	#	#	#	#	#	#
Rsa1.0_00003.1.g203.t2	ref|XP_002863624.1| pfkB-type carbohydrate kinase family protein [Arabidopsis lyrata subsp. lyrata] gi|297309459|gb|EFH39883.1| pfkB-type carbohydrate kinase family protein [Arabidopsis lyrata subsp. lyrata]	352	365	1.00E-171	103.7	85.5	91.2	pfkB-type carbohydrate kinase family protein	gbpln	Arabidopsis lyrata	AT5G43910.2 | Symbols: | pfkB-like carbohydrate kinase family protein | chr5:17667537-17670085 REVERSE LENGTH=365	352	365	1.00E-166	103.7	84.4	90.6
Rsa1.0_00003.1.g204.t1	gb|EOA12791.1| hypothetical protein CARUB_v10025740mg [Capsella rubella]	1507	1506	0	99.9	94.8	97.3	hypothetical protein CARUB_v10025740mg	gbpln	Capsella rubella	AT5G43900.1 | Symbols: MYA2, ATMYA2, XI-2, XI-6 | myosin 2 | chr5:17657241-17666653 REVERSE LENGTH=1505	1507	1505	0	99.9	94.2	96.9
Rsa1.0_00003.1.g205.t3	gb|AAG50806.1|AC079281_8 unknown protein [Arabidopsis thaliana]	1849	1213	0	65.6	23.7	34.5	unknown protein	gbpln	Arabidopsis thaliana	AT3G24255.1 | Symbols: | RNA-directed DNA polymerase (reverse transcriptase)-related family protein | chr3:8789471-8793208 FORWARD LENGTH=746	1849	746	2.00E-58	40.3	7.0	11.2
Rsa1.0_00003.1.g206.t2	gb|EOA18770.1| hypothetical protein CARUB_v10007367mg [Capsella rubella]	365	335	1.00E-35	91.8	30.1	42.5	hypothetical protein CARUB_v10007367mg	gbpln	Capsella rubella	AT4G19360.1 | Symbols: | SCD6 protein-related | chr4:10564159-10565406 FORWARD LENGTH=268	365	268	4.00E-34	73.4	30.4	41.6
Rsa1.0_00003.1.g207.t1	ref|XP_002867943.1| hypothetical protein ARALYDRAFT_914728 [Arabidopsis lyrata subsp. lyrata] gi|297313779|gb|EFH44202.1| hypothetical protein ARALYDRAFT_914728 [Arabidopsis lyrata subsp. lyrata]	757	725	0	95.8	82.2	88.2	hypothetical protein ARALYDRAFT_914728	gbpln	Arabidopsis lyrata	AT4G19380.1 | Symbols: | Long-chain fatty alcohol dehydrogenase family protein | chr4:10568427-10572288 REVERSE LENGTH=726	757	726	0	95.9	80.8	86.4
Rsa1.0_00003.1.g208.t1	emb|CAB10225.1| retrovirus-related like polyprotein [Arabidopsis thaliana] gi|7268152|emb|CAB78488.1| retrovirus-related like polyprotein [Arabidopsis thaliana]	1428	1489	0	104.3	55.9	70.5	retrovirus-related like polyprotein	gbpln	Arabidopsis thaliana	AT4G23160.1 | Symbols: CRK8 | cysteine-rich RLK (RECEPTOR-like protein kinase) 8 | chr4:12129485-12134086 FORWARD LENGTH=1262	1428	1262	1.00E-163	88.4	19.1	25.9
Rsa1.0_00003.1.g209.t1	dbj|BAB08714.1| non-LTR retroelement reverse transcriptase-like protein [Arabidopsis thaliana]	599	1197	6.00E-83	199.8	30.4	42.6	non-LTR retroelement reverse transcriptase-like protein	gbpln	Arabidopsis thaliana	AT4G29090.1 | Symbols: | Ribonuclease H-like superfamily protein | chr4:14333528-14335255 FORWARD LENGTH=575	599	575	1.00E-38	96.0	21.5	33.4
Rsa1.0_00003.1.g210.t1	gb|AAM62450.1| unknown [Arabidopsis thaliana]	169	160	8.00E-60	94.7	69.8	78.1	unknown	gbpln	Arabidopsis thaliana	AT4G19430.1 | Symbols: | unknown protein; FUNCTIONS IN: molecular_function unknown; INVOLVED IN: biological_process unknown; LOCATED IN: chloroplast; EXPRESSED IN: 17 plant structures; EXPRESSED DURING: 13 growth stages; Has 30201 Blast hits to 17322 proteins in 780 species: Archae - 12; Bacteria - 1396; Metazoa - 17338; Fungi - 3422; Plants - 5037; Viruses - 0; Other Eukaryotes - 2996 (source: NCBI BLink). | chr4:10598606-10599088 REVERSE LENGTH=160	169	160	1.00E-60	94.7	68.6	76.9
Rsa1.0_00003.1.g211.t1	#	#	#	#	#	#	#	-	----	----	AT4G19440.2 | Symbols: | Tetratricopeptide repeat (TPR)-like superfamily protein | chr4:10602006-10604483 REVERSE LENGTH=825	53	825	1.00E-11	1556.6	60.4	67.9
Rsa1.0_00003.1.g212.t1	gb|AAC02664.1| polyprotein [Arabidopsis thaliana]	1410	1451	0	102.9	60.3	74.5	polyprotein	gbpln	Arabidopsis thaliana	AT4G23160.1 | Symbols: CRK8 | cysteine-rich RLK (RECEPTOR-like protein kinase) 8 | chr4:12129485-12134086 FORWARD LENGTH=1262	1410	1262	1.00E-113	89.5	14.2	21.6
Rsa1.0_00003.1.g213.t1	#	#	#	#	#	#	#	-	----	----	#	#	#	#	#	#	#
Rsa1.0_00003.1.g214.t1	gb|EOA16195.1| hypothetical protein CARUB_v10004335mg [Capsella rubella]	657	649	0	98.8	67.1	79.1	hypothetical protein CARUB_v10004335mg	gbpln	Capsella rubella	AT4G23180.1 | Symbols: CRK10, RLK4 | cysteine-rich RLK (RECEPTOR-like protein kinase) 10 | chr4:12138171-12140780 FORWARD LENGTH=669	657	669	0	101.8	62.7	74.9
Rsa1.0_00003.1.g215.t1	gb|EOA16319.1| hypothetical protein CARUB_v10004470mg [Capsella rubella]	572	571	0	99.8	91.4	95.3	hypothetical protein CARUB_v10004470mg	gbpln	Capsella rubella	AT4G19450.1 | Symbols: | Major facilitator superfamily protein | chr4:10606538-10609218 FORWARD LENGTH=572	572	572	0	100.0	93.2	96.5
Rsa1.0_00003.1.g216.t1	gb|EOA16458.1| hypothetical protein CARUB_v10004612mg, partial [Capsella rubella]	492	510	0	103.7	86.6	92.9	hypothetical protein CARUB_v10004612mg, partial	gbpln	Capsella rubella	AT4G19460.1 | Symbols: | UDP-Glycosyltransferase superfamily protein | chr4:10610422-10611972 REVERSE LENGTH=516	492	516	0	104.9	85.4	92.7
Rsa1.0_00003.1.g217.t1	gb|ACP30627.1| disease resistance protein [Brassica rapa subsp. pekinensis]	1225	1226	0	100.1	94.4	97.1	disease resistance protein	gbpln	Brassica rapa	AT4G12010.1 | Symbols: | Disease resistance protein (TIR-NBS-LRR class) family | chr4:7197325-7201393 REVERSE LENGTH=1219	1225	1219	0	99.5	51.6	69.9
Rsa1.0_00003.1.g218.t1	gb|ACP30626.1| disease resistance protein [Brassica rapa subsp. pekinensis]	1366	1276	0	93.4	75.9	82.9	disease resistance protein	gbpln	Brassica rapa	AT4G19500.2 | Symbols: | nucleoside-triphosphatases;transmembrane receptors;nucleotide binding;ATP binding | chr4:10627364-10631532 FORWARD LENGTH=834	1366	834	0	61.1	30.7	38.3

Rsa1.0_00003.1.g219.t1	gb|ACP30626.1| disease resistance protein [Brassica rapa subsp. pekinensis]	130	1276	1.00E-37	981.5	60.8	63.8	disease resistance protein	gbpln	Brassica rapa	AT4G19500.2 | Symbols: | nucleoside-triphosphatases;transmembrane receptors;nucleotide binding;ATP binding | chr4:10627364-10631532 FORWARD LENGTH=834	130	834	8.00E-21	641.5	36.2	50.8
Rsa1.0_00003.1.g220.t1	gb|ACI14393.1| WRKY28-1 transcription factor [Brassica napus]	215	312	5.00E-91	145.1	79.1	87.0	WRKY28-1 transcription factor	gbpln	Brassica napus	AT4G18170.1 | Symbols: WRKY28, ATWRKY28 | WRKY DNA-binding protein 28 | chr4:10061508-10062691 FORWARD LENGTH=318	215	318	2.00E-77	147.9	71.6	81.4
Rsa1.0_00003.1.g221.t1	ref|XP_002869986.1| protein binding protein [Arabidopsis lyrata subsp. lyrata] gi|297315822|gb|EFH46245.1| protein binding protein [Arabidopsis lyrata subsp. lyrata]	1182	1044	0	88.3	41.2	44.8	protein binding protein	gbpln	Arabidopsis lyrata	AT4G19490.2 | Symbols: ATVPS54, VPS54 | VPS54 | chr4:10616017-10622934 FORWARD LENGTH=1034	1182	1034	0	87.5	41.5	44.9
Rsa1.0_00003.1.g222.t1	emb|CAB39638.1| RNA-directed DNA polymerase-like protein [Arabidopsis thaliana] gi|7267666|emb|CAB78094.1| RNA-directed DNA polymerase-like protein [Arabidopsis thaliana]	1911	1274	0	66.7	34.3	45.7	RNA-directed DNA polymerase-like protein	gbpln	Arabidopsis thaliana	AT4G29090.1 | Symbols: | Ribonuclease H-like superfamily protein | chr4:14333528-14335255 FORWARD LENGTH=575	1911	575	3.00E-76	30.1	9.8	14.4
Rsa1.0_00003.1.g223.t1	ref|XP_002867933.1| hypothetical protein ARALYDRAFT_914714 [Arabidopsis lyrata subsp. lyrata] gi|297313769|gb|EFH44192.1| hypothetical protein ARALYDRAFT_914714 [Arabidopsis lyrata subsp. lyrata]	70	71	3.00E-25	101.4	81.4	87.1	hypothetical protein ARALYDRAFT_914714	gbpln	Arabidopsis lyrata	AT4G19520.1 | Symbols: | disease resistance protein (TIR-NBS-LRR class) family | chr4:10639488-10647070 REVERSE LENGTH=1744	70	1744	2.00E-21	2491.4	55.7	57.1
Rsa1.0_00003.1.g224.t1	ref|NP_193687.3| TIR-NBS-LRR class disease resistance protein [Arabidopsis thaliana] gi|332658794|gb|AEE84194.1| TIR-NBS-LRR class disease resistance protein [Arabidopsis thaliana]	1497	1744	0	116.5	62.0	73.3	TIR-NBS-LRR class disease resistance protein	gbpln	Arabidopsis thaliana	AT4G19520.1 | Symbols: | disease resistance protein (TIR-NBS-LRR class) family | chr4:10639488-10647070 REVERSE LENGTH=1744	1497	1744	0	116.5	62.0	73.3
Rsa1.0_00003.1.g225.t1	#	#	#	#	#	#	#	-	----	----	#	#	#	#	#	#	#
Rsa1.0_00003.1.g226.t1	ref|NP_193688.1| TIR-NBS-LRR class disease resistance protein [Arabidopsis thaliana] gi|2853080|emb|CAA16930.1| TMV resistance protein N-like [Arabidopsis thaliana] gi|7268749|emb|CAB78955.1| TMV resistance protein N-like [Arabidopsis thaliana] gi|332658795|gb|AEE84195.1| TIR-NBS-LRR class disease resistance protein [Arabidopsis thaliana]	1202	1167	0	97.1	64.8	73.3	TIR-NBS-LRR class disease resistance protein	gbpln	Arabidopsis thaliana	AT4G19530.1 | Symbols: | disease resistance protein (TIR-NBS-LRR class) family | chr4:10651962-10657090 FORWARD LENGTH=1167	1202	1167	0	97.1	64.8	73.3
Rsa1.0_00003.1.g227.t1	ref|XP_002869958.1| hypothetical protein ARALYDRAFT_914664 [Arabidopsis lyrata subsp. lyrata] gi|297315794|gb|EFH46217.1| hypothetical protein ARALYDRAFT_914664 [Arabidopsis lyrata subsp. lyrata]	422	428	1.00E-121	101.4	57.3	69.9	hypothetical protein ARALYDRAFT_914664	gbpln	Arabidopsis lyrata	AT4G19930.1 | Symbols: | F-box and associated interaction domains-containing protein | chr4:10803558-10804853 FORWARD LENGTH=431	422	431	1.00E-117	102.1	55.7	68.7
Rsa1.0_00003.1.g228.t1	gb|AAF26782.1|AC016829_6 putative trypsin inhibitor [Arabidopsis thaliana]	210	244	5.00E-14	116.2	34.8	42.9	putative trypsin inhibitor	gbpln	Arabidopsis thaliana	AT3G04320.1 | Symbols: | Kunitz family trypsin and protease inhibitor protein | chr3:1143815-1144426 FORWARD LENGTH=203	210	203	2.00E-16	96.7	34.8	42.9
Rsa1.0_00003.1.g229.t3	emb|CAB39638.1| RNA-directed DNA polymerase-like protein [Arabidopsis thaliana] gi|7267666|emb|CAB78094.1| RNA-directed DNA polymerase-like protein [Arabidopsis thaliana]	943	1274	0	135.1	48.0	63.8	RNA-directed DNA polymerase-like protein	gbpln	Arabidopsis thaliana	AT4G29090.1 | Symbols: | Ribonuclease H-like superfamily protein | chr4:14333528-14335255 FORWARD LENGTH=575	943	575	7.00E-76	61.0	17.8	28.8
Rsa1.0_00003.1.g230.t1	ref|XP_002869970.1| iron-responsive transporter 2 [Arabidopsis lyrata subsp. lyrata] gi|297315806|gb|EFH46229.1| iron-responsive transporter 2 [Arabidopsis lyrata subsp. lyrata]	357	349	0	97.8	88.2	93.0	iron-responsive transporter 2	gbpln	Arabidopsis lyrata	AT4G19680.2 | Symbols: IRT2 | iron regulated transporter 2 | chr4:10703385-10704621 FORWARD LENGTH=350	357	350	0	98.0	87.4	93.6
Rsa1.0_00003.1.g231.t1	gb|AAD17352.1| contains similarity to retrovirus-related polyproteins [Arabidopsis thaliana] gi|7267379|emb|CAB77940.1| putative polyprotein [Arabidopsis thaliana]	1443	1366	0	94.7	39.5	54.9	contains similarity to retrovirus-related polyproteins	gbpln	Arabidopsis thaliana	AT4G23160.1 | Symbols: CRK8 | cysteine-rich RLK (RECEPTOR-like protein kinase) 8 | chr4:12129485-12134086 FORWARD LENGTH=1262	1443	1262	1.00E-170	87.5	20.4	27.9
Rsa1.0_00003.1.g232.t1	gb|AFJ05595.1| iron-regulated transporter 1 [Raphanus sativus]	345	345	0	100.0	98.8	99.1	iron-regulated transporter 1	gbpln	Raphanus sativus	AT4G19690.2 | Symbols: IRT1 | iron-regulated transporter 1 | chr4:10707487-10708723 FORWARD LENGTH=347	345	347	1.00E-172	100.6	88.1	93.9
Rsa1.0_00003.1.g233.t1	#	#	#	#	#	#	#	-	----	----	#	#	#	#	#	#	#
Rsa1.0_00003.1.g234.t1	gb|EOA18607.1| hypothetical protein CARUB_v10007180mg [Capsella rubella]	345	345	0	100.0	89.6	96.5	hypothetical protein CARUB_v10007180mg	gbpln	Capsella rubella	AT4G19690.2 | Symbols: IRT1 | iron-regulated transporter 1 | chr4:10707487-10708723 FORWARD LENGTH=347	345	347	1.00E-175	100.6	89.0	95.9
Rsa1.0_00003.1.g235.t1	gb|AAL32681.1| putative protein [Arabidopsis thaliana] gi|21387161|gb|AAM47984.1| putative protein [Arabidopsis thaliana]	271	303	1.00E-103	111.8	74.9	82.7	putative protein	gbpln	Arabidopsis thaliana	AT4G19700.1 | Symbols: RING | SBP (S-ribonuclease binding protein) family protein | chr4:10713633-10714635 REVERSE LENGTH=304	271	304	1.00E-106	112.2	74.9	82.7

Rsa1.0_00003.1.g236.t1	#	#	#	#	#	#	#	-	----	----	#	#	#	#	#	#	#
Rsa1.0_00003.1.g237.t1	ref|NP_193723.3| Intracellular chloride channel-like protein [Arabidopsis thaliana] gi|24030218|gb|AAN41287.1| unknown protein [Arabidopsis thaliana] gi|332658839|gb|AEE84239.1| Intracellular chloride channel-like protein [Arabidopsis thaliana]	361	356	0	98.6	91.4	95.6	Intracellular chloride channel-like protein	gbpln	Arabidopsis thaliana	AT4G19880.1 | Symbols: | Glutathione S-transferase family protein | chr4:10784691-10786376 REVERSE LENGTH=356	361	356	0	98.6	91.4	95.6
Rsa1.0_00003.1.g238.t5	#	#	#	#	#	#	#	-	----	----	#	#	#	#	#	#	#
Rsa1.0_00003.1.g239.t1	ref|XP_002869958.1| hypothetical protein ARALYDRAFT_914664 [Arabidopsis lyrata subsp. lyrata] gi|297315794|gb|EFH46217.1| hypothetical protein ARALYDRAFT_914664 [Arabidopsis lyrata subsp. lyrata]	161	428	9.00E-30	265.8	45.3	64.6	hypothetical protein ARALYDRAFT_914664	gbpln	Arabidopsis lyrata	AT4G19930.1 | Symbols: | F-box and associated interaction domains-containing protein | chr4:10803558-10804853 FORWARD LENGTH=431	161	431	1.00E-25	267.7	44.1	59.0
Rsa1.0_00003.1.g240.t1	ref|NP_001190775.1| K+ uptake permease 9 [Arabidopsis thaliana] gi|38502848|sp|O49423.2|POT9_ARATH RecName: Full=Potassium transporter 9; Short=AtPOT9 gi|332658852|gb|AEE84252.1| K+ uptake permease 9 [Arabidopsis thaliana]	807	807	0	100.0	88.4	93.3	K+ uptake permease 9	gbpln	Arabidopsis thaliana	AT4G19960.2 | Symbols: KUP9 | K+ uptake permease 9 | chr4:10813807-10816997 FORWARD LENGTH=807	807	807	0	100.0	88.4	93.3
Rsa1.0_00003.1.g241.t1	ref|NP_001190775.1| K+ uptake permease 9 [Arabidopsis thaliana] gi|38502848|sp|O49423.2|POT9_ARATH RecName: Full=Potassium transporter 9; Short=AtPOT9 gi|332658852|gb|AEE84252.1| K+ uptake permease 9 [Arabidopsis thaliana]	801	807	0	100.7	85.4	92.0	K+ uptake permease 9	gbpln	Arabidopsis thaliana	AT4G19960.2 | Symbols: KUP9 | K+ uptake permease 9 | chr4:10813807-10816997 FORWARD LENGTH=807	801	807	0	100.7	85.4	92.0
Rsa1.0_00003.1.g242.t1	ref|NP_001190775.1| K+ uptake permease 9 [Arabidopsis thaliana] gi|38502848|sp|O49423.2|POT9_ARATH RecName: Full=Potassium transporter 9; Short=AtPOT9 gi|332658852|gb|AEE84252.1| K+ uptake permease 9 [Arabidopsis thaliana]	730	807	0	110.5	78.8	86.2	K+ uptake permease 9	gbpln	Arabidopsis thaliana	AT4G19960.2 | Symbols: KUP9 | K+ uptake permease 9 | chr4:10813807-10816997 FORWARD LENGTH=807	730	807	0	110.5	78.8	86.2
Rsa1.0_00003.1.g243.t1	#	#	#	#	#	#	#	-	----	----	#	#	#	#	#	#	#
Rsa1.0_00003.1.g244.t1	gb|EOA16117.1| hypothetical protein CARUB_v10004249mg [Capsella rubella]	346	715	1.00E-173	206.6	84.4	88.7	hypothetical protein CARUB_v10004249mg	gbpln	Capsella rubella	AT4G19970.1 | Symbols: | CONTAINS InterPro DOMAIN/s: Nucleotide-diphospho-sugar transferase, predicted (InterPro:IPR005069); BEST Arabidopsis thaliana protein match is: Nucleotide-diphospho-sugar transferase family protein (TAIR:AT5G44820.1); Has 801 Blast hits to 466 proteins in 35 species: Archae - 0; Bacteria - 0; Metazoa - 2; Fungi - 0; Plants - 750; Viruses - 0; Other Eukaryotes - 49 (source: NCBI BLink). | chr4:10818242-10825343 FORWARD LENGTH=715	346	715	1.00E-175	206.6	84.7	89.3
Rsa1.0_00003.1.g245.t1	ref|XP_002869958.1| hypothetical protein ARALYDRAFT_914664 [Arabidopsis lyrata subsp. lyrata] gi|297315794|gb|EFH46217.1| hypothetical protein ARALYDRAFT_914664 [Arabidopsis lyrata subsp. lyrata]	427	428	1.00E-126	100.2	61.1	73.5	hypothetical protein ARALYDRAFT_914664	gbpln	Arabidopsis lyrata	AT4G19930.1 | Symbols: | F-box and associated interaction domains-containing protein | chr4:10803558-10804853 FORWARD LENGTH=431	427	431	1.00E-121	100.9	58.8	70.5
Rsa1.0_00003.1.g246.t1	gb|EOA15545.1| hypothetical protein CARUB_v10004988mg, partial [Capsella rubella]	375	394	1.00E-157	105.1	77.9	84.0	hypothetical protein CARUB_v10004988mg, partial	gbpln	Capsella rubella	AT4G20010.1 | Symbols: PTAC9, OSB2 | plastid transcriptionally active 9 | chr4:10842128-10843989 FORWARD LENGTH=371	375	371	1.00E-157	98.9	76.8	83.5
Rsa1.0_00003.1.g247.t1	ref|NP_567595.1| Pectin lyase-like protein [Arabidopsis thaliana] gi|16226216|gb|AAL16105.1|AF428273_1 AT4g20040/F18F4_140 [Arabidopsis thaliana] gi|22137222|gb|AAM91456.1| AT4g20040/F18F4_140 [Arabidopsis thaliana] gi|332658865|gb|AEE84265.1| Pectin lyase-like protein [Arabidopsis thaliana]	813	483	0	59.4	47.2	51.9	Pectin lyase-like protein	gbpln	Arabidopsis thaliana	AT4G20040.1 | Symbols: | Pectin lyase-like superfamily protein | chr4:10847348-10848963 REVERSE LENGTH=483	813	483	0	59.4	47.2	51.9
Rsa1.0_00004.1.g248.t1	gb|AAG51783.1|AC079679_3 reverse transcriptase, putative; 16838-20266 [Arabidopsis thaliana]	206	1142	3.00E-25	554.4	25.2	33.0	reverse transcriptase, putative; 16838-20266	gbpln	Arabidopsis thaliana	AT4G29090.1 | Symbols: | Ribonuclease H-like superfamily protein | chr4:14333528-14335255 FORWARD LENGTH=575	206	575	2.00E-17	279.1	23.3	37.4
Rsa1.0_00004.1.g249.t1	gb|EOA34452.1| hypothetical protein CARUB_v10021988mg [Capsella rubella]	310	332	1.00E-65	107.1	55.8	63.2	hypothetical protein CARUB_v10021988mg	gbpln	Capsella rubella	AT1G68550.2 | Symbols: | Integrase-type DNA-binding superfamily protein | chr1:25725810-25726784 REVERSE LENGTH=324	310	324	1.00E-67	104.5	53.9	66.1
Rsa1.0_00004.1.g250.t1	#	#	#	#	#	#	#	-	----	----	#	#	#	#	#	#	#

Rsa1.0_00004.1.g251.t1	gb|AAC33226.1| putative non-LTR retroelement reverse transcriptase [Arabidopsis thaliana]	758	1529	0	201.7	46.2	62.4	putative non-LTR retroelement reverse transcriptase	gbpln	Arabidopsis thaliana	AT3G24255.1 | Symbols: | RNA-directed DNA polymerase (reverse transcriptase)-related family protein | chr3:8789471-8793208 FORWARD LENGTH=746	758	746	4.00E-74	98.4	21.0	29.9
Rsa1.0_00004.1.g252.t1	#	#	#	#	#	#	#	-	----	----	#	#	#	#	#	#	#
Rsa1.0_00004.1.g253.t1	ref|NP_177020.1| 3-ketoacyl-CoA synthase 6 [Arabidopsis thaliana] gi|75315046|sp|Q9XF43.1|KCS6_ARATH RecName: Full=3-ketoacyl-CoA synthase 6; Short=KCS-6; AltName: Full=Cuticular protein 1; AltName: Full=Very long-chain fatty acid condensing enzyme 6; Short=VLCFA condensing enzyme 6 gi|5001734|gb|AAD37122.1|AF129511_1 very-long-chain fatty acid condensing enzyme CUT1 [Arabidopsis thaliana] gi|12324880|gb|AAG52390.1|AC011915_4 very-long-chain fatty acid condensing enzyme (CUT1); 56079-54227 [Arabidopsis thaliana] gi|332196683|gb|AEE34804.1| 3-ketoacyl-CoA synthase 6 [Arabidopsis thaliana]	497	497	0	100.0	93.4	96.8	3-ketoacyl-CoA synthase 6	gbpln	Arabidopsis thaliana	AT1G68530.1 | Symbols: CUT1, POP1, CER6, G2, KCS6 | 3-ketoacyl-CoA synthase 6 | chr1:25712881-25714733 REVERSE LENGTH=497	497	497	0	100.0	93.4	96.8
Rsa1.0_00004.1.g254.t1	gb|EOA35226.1| hypothetical protein CARUB_v10020384mg [Capsella rubella]	402	410	1.00E-170	102.0	78.9	88.3	hypothetical protein CARUB_v10020384mg	gbpln	Capsella rubella	AT1G68520.1 | Symbols: | B-box type zinc finger protein with CCT domain | chr1:25709331-25710749 REVERSE LENGTH=406	402	406	1.00E-170	101.0	76.6	86.8
Rsa1.0_00004.1.g255.t1	#	#	#	#	#	#	#	-	----	----	#	#	#	#	#	#	#
Rsa1.0_00004.1.g256.t1	#	#	#	#	#	#	#	-	----	----	#	#	#	#	#	#	#
Rsa1.0_00004.1.g257.t1	#	#	#	#	#	#	#	-	----	----	#	#	#	#	#	#	#
Rsa1.0_00004.1.g258.t1	gb|EOA35949.1| hypothetical protein CARUB_v10021210mg [Capsella rubella]	87	93	7.00E-20	106.9	69.0	81.6	hypothetical protein CARUB_v10021210mg	gbpln	Capsella rubella	AT1G68500.1 | Symbols: | unknown protein; BEST Arabidopsis thaliana protein match is: unknown protein (TAIR:AT1G25422.1); Has 16 Blast hits to 16 proteins in 6 species: Archae - 0; Bacteria - 0; Metazoa - 0; Fungi - 0; Plants - 16; Viruses - 0; Other Eukaryotes - 0 (source: NCBI BLink). | chr1:25702041-25702322 FORWARD LENGTH=93	87	93	3.00E-22	106.9	72.4	82.8
Rsa1.0_00004.1.g259.t1	ref|NP_564931.1| uncharacterized protein [Arabidopsis thaliana] gi|12324888|gb|AAG52398.1|AC011915_12 unknown protein; 35272-36292 [Arabidopsis thaliana] gi|21593205|gb|AAM65154.1| unknown [Arabidopsis thaliana] gi|29028824|gb|AAO64791.1| At1g68490 [Arabidopsis thaliana] gi|110736380|dbj|BAF00159.1| hypothetical protein [Arabidopsis thaliana] gi|332196678|gb|AEE34799.1| uncharacterized protein AT1G68490 [Arabidopsis thaliana]	172	183	8.00E-59	106.4	77.3	83.7	uncharacterized protein	gbpln	Arabidopsis thaliana	AT1G68490.1 | Symbols: | unknown protein; FUNCTIONS IN: molecular_function unknown; INVOLVED IN: biological_process unknown; LOCATED IN: chloroplast; EXPRESSED IN: 22 plant structures; EXPRESSED DURING: 13 growth stages; BEST Arabidopsis thaliana protein match is: unknown protein (TAIR:AT1G13390.2); Has 125 Blast hits to 125 proteins in 18 species: Archae - 0; Bacteria - 0; Metazoa - 0; Fungi - 0; Plants - 125; Viruses - 0; Other Eukaryotes - 0 (source: NCBI BLink). | chr1:25693926-25694946 FORWARD LENGTH=183	172	183	3.00E-61	106.4	77.3	83.7
Rsa1.0_00004.1.g260.t1	ref|XP_002878905.1| predicted protein [Arabidopsis lyrata subsp. lyrata] gi|297324744|gb|EFH55164.1| predicted protein [Arabidopsis lyrata subsp. lyrata]	120	480	8.00E-17	400.0	35.8	52.5	predicted protein	gbpln	Arabidopsis lyrata	#	#	#	#	#	#	#
Rsa1.0_00004.1.g261.t2	gb|AAD32759.1| putative retroelement pol polyprotein [Arabidopsis thaliana]	490	1356	1.00E-133	276.7	50.6	59.6	putative retroelement pol polyprotein	gbpln	Arabidopsis thaliana	AT4G23160.1 | Symbols: CRK8 | cysteine-rich RLK (RECEPTOR-like protein kinase) 8 | chr4:12129485-12134086 FORWARD LENGTH=1262	490	1262	7.00E-38	257.6	22.9	34.7
Rsa1.0_00004.1.g262.t2	ref|XP_002888667.1| hypothetical protein ARALYDRAFT_475967 [Arabidopsis lyrata subsp. lyrata] gi|297334508|gb|EFH64926.1| hypothetical protein ARALYDRAFT_475967 [Arabidopsis lyrata subsp. lyrata]	266	253	1.00E-105	95.1	78.2	82.7	hypothetical protein ARALYDRAFT_475967	gbpln	Arabidopsis lyrata	AT1G68480.1 | Symbols: JAG | C2H2 and C2HC zinc fingers superfamily protein | chr1:25684543-25685932 REVERSE LENGTH=253	266	253	1.00E-105	95.1	76.3	81.6
Rsa1.0_00004.1.g263.t1	#	#	#	#	#	#	#	-	----	----	#	#	#	#	#	#	#
Rsa1.0_00004.1.g264.t1	ref|XP_002882231.1| S-adenosylmethionine decarboxylase [Arabidopsis lyrata subsp. lyrata] gi|297328071|gb|EFH58490.1| S-adenosylmethionine decarboxylase [Arabidopsis lyrata subsp. lyrata]	181	366	1.00E-18	202.2	32.0	39.8	S-adenosylmethionine decarboxylase	gbpln	Arabidopsis lyrata	AT3G02470.4 | Symbols: | S-adenosylmethionine decarboxylase | chr3:510223-511323 FORWARD LENGTH=366	181	366	6.00E-20	202.2	30.4	39.2
Rsa1.0_00004.1.g265.t1	dbj|BAE43826.1| isopentenyltransferase [Brassica rapa subsp. pekinensis]	297	348	1.00E-153	117.2	92.9	95.3	isopentenyltransferase	gbpln	Brassica rapa	AT1G68460.1 | Symbols: ATIPT1, IPT1 | isopentenyltransferase 1 | chr1:25668976-25670049 REVERSE LENGTH=357	297	357	1.00E-130	120.2	78.8	87.5

Rsa1.0_00004.1.g266.t1	gb|EOA34020.1| hypothetical protein CARUB_v10021516mg [Capsella rubella]	147	148	4.00E-49	100.7	70.1	79.6	hypothetical protein CARUB_v10021516mg	gbpln	Capsella rubella	AT1G68450.1 | Symbols: | VQ motif-containing protein | chr1:25661721-25662179 REVERSE LENGTH=152	147	152	3.00E-45	103.4	66.0	78.9
Rsa1.0_00004.1.g267.t1	ref|XP_002868681.1| hypothetical protein ARALYDRAFT_916271 [Arabidopsis lyrata subsp. lyrata] gi|297314517|gb|EFH44940.1| hypothetical protein ARALYDRAFT_916271 [Arabidopsis lyrata subsp. lyrata]	169	1168	4.00E-55	691.1	77.5	81.7	hypothetical protein ARALYDRAFT_916271	gbpln	Arabidopsis lyrata	AT4G10320.1 | Symbols: | tRNA synthetase class I (I, L, M and V) family protein | chr4:6397526-6404509 REVERSE LENGTH=1190	169	1190	3.00E-54	704.1	73.4	81.7
Rsa1.0_00004.1.g268.t1	#	#	#	#	#	#	#	-	----	----	#	#	#	#	#	#	#
Rsa1.0_00004.1.g269.t1	#	#	#	#	#	#	#	-	----	----	#	#	#	#	#	#	#
Rsa1.0_00004.1.g270.t1	ref|NP_564930.1| uncharacterized protein [Arabidopsis thaliana] gi|6714347|gb|AAF26038.1|AC015986_1 unknown protein; 9003-8083 [Arabidopsis thaliana] gi|16648773|gb|AAL25577.1| At1g68440/T2E12_1 [Arabidopsis thaliana] gi|21592921|gb|AAM64871.1| unknown [Arabidopsis thaliana] gi|21700823|gb|AAM70535.1| At1g68440/T2E12_1 [Arabidopsis thaliana] gi|332196673|gb|AEE34794.1| uncharacterized protein AT1G68440 [Arabidopsis thaliana]	312	306	1.00E-103	98.1	73.4	84.3	uncharacterized protein	gbpln	Arabidopsis thaliana	AT1G68440.1 | Symbols: | unknown protein; BEST Arabidopsis thaliana protein match is: unknown protein (TAIR:AT1G25400.2); Has 86 Blast hits to 86 proteins in 29 species: Archae - 0; Bacteria - 6; Metazoa - 27; Fungi - 11; Plants - 24; Viruses - 0; Other Eukaryotes - 18 (source: NCBI BLink). | chr1:25658115-25659035 FORWARD LENGTH=306	312	306	1.00E-105	98.1	73.4	84.3
Rsa1.0_00004.1.g271.t1	ref|XP_002888663.1| hypothetical protein ARALYDRAFT_894609 [Arabidopsis lyrata subsp. lyrata] gi|297334504|gb|EFH64922.1| hypothetical protein ARALYDRAFT_894609 [Arabidopsis lyrata subsp. lyrata]	147	145	3.00E-45	98.6	78.2	83.7	hypothetical protein ARALYDRAFT_894609	gbpln	Arabidopsis lyrata	AT1G68430.1 | Symbols: | unknown protein; FUNCTIONS IN: molecular_function unknown; INVOLVED IN: biological_process unknown; LOCATED IN: chloroplast; EXPRESSED IN: 21 plant structures; EXPRESSED DURING: 13 growth stages; BEST Arabidopsis thaliana protein match is: unknown protein (TAIR:AT1G66890.1); Has 35 Blast hits to 35 proteins in 6 species: Archae - 0; Bacteria - 0; Metazoa - 0; Fungi - 0; Plants - 35; Viruses - 0; Other Eukaryotes - 0 (source: NCBI BLink). | chr1:25654989-25655429 REVERSE LENGTH=146	147	146	2.00E-43	99.3	78.2	84.4
Rsa1.0_00004.1.g272.t2	gb|AAL06846.1| AT3g02470/F16B3_10 [Arabidopsis thaliana]	171	366	5.00E-11	214.0	21.1	23.4	AT3g02470/F16B3_10	gbpln	Arabidopsis thaliana	AT3G02470.4 | Symbols: | S-adenosylmethionine decarboxylase | chr3:510223-511323 FORWARD LENGTH=366	171	366	1.00E-12	214.0	20.5	22.8
Rsa1.0_00004.1.g273.t1	gb|EOA35168.1| hypothetical protein CARUB_v10020313mg [Capsella rubella]	434	437	0	100.7	91.9	95.4	hypothetical protein CARUB_v10020313mg	gbpln	Capsella rubella	AT1G68410.2 | Symbols: | Protein phosphatase 2C family protein | chr1:25650262-25652255 REVERSE LENGTH=436	434	436	0	100.5	91.9	95.2
Rsa1.0_00004.1.g274.t1	ref|XP_002892766.1| predicted protein [Arabidopsis lyrata subsp. lyrata] gi|297338608|gb|EFH69025.1| predicted protein [Arabidopsis lyrata subsp. lyrata]	179	212	4.00E-22	118.4	37.4	52.5	predicted protein	gbpln	Arabidopsis lyrata	AT2G34280.1 | Symbols: | F-box and associated interaction domains-containing protein | chr2:14470335-14471510 REVERSE LENGTH=391	179	391	1.00E-20	218.4	34.6	48.0
Rsa1.0_00004.1.g275.t19	ref|XP_002865187.1| predicted protein [Arabidopsis lyrata subsp. lyrata] gi|297311022|gb|EFH41446.1| predicted protein [Arabidopsis lyrata subsp. lyrata]	95	1162	1.00E-27	1223.2	66.3	78.9	predicted protein	gbpln	Arabidopsis lyrata	AT5G22690.1 | Symbols: | Disease resistance protein (TIR-NBS-LRR class) family | chr5:7541369-7544888 FORWARD LENGTH=1008	95	1008	6.00E-29	1061.1	61.1	80.0
Rsa1.0_00004.1.g276.t1	#	#	#	#	#	#	#	-	----	----	#	#	#	#	#	#	#
Rsa1.0_00004.1.g277.t1	ref|XP_002888661.1| At1g68400/T2E12_5 [Arabidopsis lyrata subsp. lyrata] gi|297334502|gb|EFH64920.1| At1g68400/T2E12_5 [Arabidopsis lyrata subsp. lyrata]	668	669	0	100.1	80.7	85.8	At1g68400/T2E12_5	gbpln	Arabidopsis lyrata	AT1G68400.1 | Symbols: | leucine-rich repeat transmembrane protein kinase family protein | chr1:25646401-25648916 REVERSE LENGTH=670	668	670	0	100.3	81.0	87.0
Rsa1.0_00004.1.g278.t1	ref|XP_002887179.1| hypothetical protein ARALYDRAFT_475958 [Arabidopsis lyrata subsp. lyrata] gi|297333020|gb|EFH63438.1| hypothetical protein ARALYDRAFT_475958 [Arabidopsis lyrata subsp. lyrata]	418	405	1.00E-180	96.9	78.5	84.2	hypothetical protein ARALYDRAFT_475958	gbpln	Arabidopsis lyrata	AT1G68390.1 | Symbols: | Core-2/I-branching beta-1,6-N-acetylglucosaminyltransferase family protein | chr1:25642838-25645484 FORWARD LENGTH=408	418	408	1.00E-170	97.6	78.2	84.0
Rsa1.0_00004.1.g279.t1	gb|AAD32866.1|AC005489_4 F14N23.4 [Arabidopsis thaliana]	186	1161	3.00E-33	624.2	46.2	60.8	F14N23.4	gbpln	Arabidopsis thaliana	AT4G04650.1 | Symbols: | RNA-directed DNA polymerase (reverse transcriptase)-related family protein | chr4:2353989-2355213 REVERSE LENGTH=332	186	332	2.00E-31	178.5	40.9	51.6

Rsa1.0_00004.1.g280.t1	ref|XP_002888659.1| hypothetical protein ARALYDRAFT_894602 [Arabidopsis lyrata subsp. lyrata] gi|297334500|gb|EFH64918.1| hypothetical protein ARALYDRAFT_894602 [Arabidopsis lyrata subsp. lyrata]	302	410	2.00E-94	135.8	57.6	59.9	hypothetical protein ARALYDRAFT_894602	gbpln	Arabidopsis lyrata	AT1G68370.1 | Symbols: ARG1 | Chaperone DnaJ-domain superfamily protein | chr1:25632046-25634527 REVERSE LENGTH=410	302	410	1.00E-95	135.8	56.6	59.9
Rsa1.0_00004.1.g281.t1	gb|AAG51783.1|AC079679_3 reverse transcriptase, putative; 16838-20266 [Arabidopsis thaliana]	227	1142	1.00E-46	503.1	46.3	59.5	reverse transcriptase, putative; 16838-20266	gbpln	Arabidopsis thaliana	AT2G34320.1 | Symbols: | Polynucleotidyl transferase, ribonuclease H-like superfamily protein | chr2:14484959-14485837 FORWARD LENGTH=292	227	292	1.00E-16	128.6	23.8	42.3
Rsa1.0_00004.1.g282.t1	#	#	#	#	#	#	#	-	----	----	#	#	#	#	#	#	#
Rsa1.0_00005.1.g283.t1	emb|CAN79339.1| hypothetical protein VITISV_044312 [Vitis vinifera]	129	354	9.00E-19	274.4	40.3	58.1	hypothetical protein VITISV_044312	gbpln	Vitis vinifera	#	#	#	#	#	#	#
Rsa1.0_00005.1.g284.t1	gb|EOA32296.1| hypothetical protein CARUB_v10015558mg [Capsella rubella]	564	570	0	101.1	95.7	97.5	hypothetical protein CARUB_v10015558mg	gbpln	Capsella rubella	AT3G20840.1 | Symbols: PLT1 | Integrase-type DNA-binding superfamily protein | chr3:7300764-7303370 FORWARD LENGTH=574	564	574	0	101.8	94.3	95.9
Rsa1.0_00005.1.g285.t1	emb|CAB39638.1| RNA-directed DNA polymerase-like protein [Arabidopsis thaliana] gi|7267666|emb|CAB78094.1| RNA-directed DNA polymerase-like protein [Arabidopsis thaliana]	914	1274	0	139.4	45.0	63.9	RNA-directed DNA polymerase-like protein	gbpln	Arabidopsis thaliana	AT4G29090.1 | Symbols: | Ribonuclease H-like superfamily protein | chr4:14333528-14335255 FORWARD LENGTH=575	914	575	3.00E-55	62.9	15.4	27.4
Rsa1.0_00005.1.g286.t1	#	#	#	#	#	#	#	-	----	----	#	#	#	#	#	#	#
Rsa1.0_00005.1.g287.t1	ref|XP_002885407.1| hypothetical protein ARALYDRAFT_898522 [Arabidopsis lyrata subsp. lyrata] gi|297331247|gb|EFH61666.1| hypothetical protein ARALYDRAFT_898522 [Arabidopsis lyrata subsp. lyrata]	64	64	9.00E-14	100.0	64.1	71.9	hypothetical protein ARALYDRAFT_898522	gbpln	Arabidopsis lyrata	AT3G20865.1 | Symbols: AGP40 | arabinogalactan protein 40 | chr3:7308699-7308887 REVERSE LENGTH=62	64	62	2.00E-13	96.9	65.6	70.3
Rsa1.0_00005.1.g288.t1	ref|NP_188724.2| WIP domain protein 4 [Arabidopsis thaliana] gi|18376500|emb|CAC86168.1| WIP4 protein [Arabidopsis thaliana] gi|332642914|gb|AEE76435.1| WIP domain protein 4 [Arabidopsis thaliana]	360	412	1.00E-156	114.4	79.4	84.2	WIP domain protein 4	gbpln	Arabidopsis thaliana	AT3G20880.1 | Symbols: WIP4 | WIP domain protein 4 | chr3:7313759-7315792 REVERSE LENGTH=412	360	412	1.00E-159	114.4	79.4	84.2
Rsa1.0_00005.1.g289.t1	dbj|BAB01899.1| unnamed protein product [Arabidopsis thaliana]	136	135	3.00E-28	99.3	60.3	69.9	unnamed protein product	gbpln	Arabidopsis thaliana	AT3G20898.1 | Symbols: | unknown protein; FUNCTIONS IN: molecular_function unknown; INVOLVED IN: biological_process unknown; LOCATED IN: cellular_component unknown; BEST Arabidopsis thaliana protein match is: unknown protein (TAIR:AT1G51355.1); Has 66 Blast hits to 66 proteins in 10 species: Archae - 0; Bacteria - 0; Metazoa - 0; Fungi - 0; Plants - 66; Viruses - 0; Other Eukaryotes - 0 (source: NCBI BLink). | chr3:7323796-7324203 FORWARD LENGTH=108	136	108	8.00E-31	79.4	56.6	63.2
Rsa1.0_00005.1.g290.t1	#	#	#	#	#	#	#	-	----	----	AT3G20910.1 | Symbols: NF-YA9 | nuclear factor Y, subunit A9 | chr3:7326495-7328369 FORWARD LENGTH=303	110	303	4.00E-12	275.5	50.9	59.1
Rsa1.0_00005.1.g291.t1	gb|EOA30911.1| hypothetical protein CARUB_v10014058mg [Capsella rubella]	337	356	1.00E-175	105.6	90.8	95.5	hypothetical protein CARUB_v10014058mg	gbpln	Capsella rubella	AT3G20920.1 | Symbols: | translocation protein-related | chr3:7328763-7330698 REVERSE LENGTH=365	337	365	1.00E-176	108.3	91.1	95.8
Rsa1.0_00005.1.g292.t1	emb|CAB10225.1| retrovirus-related like polyprotein [Arabidopsis thaliana] gi|7268152|emb|CAB78488.1| retrovirus-related like polyprotein [Arabidopsis thaliana]	366	1489	5.00E-92	406.8	49.5	64.2	retrovirus-related like polyprotein	gbpln	Arabidopsis thaliana	AT1G21280.1 | Symbols: | CONTAINS InterPro DOMAIN/s: Retrotransposon gag protein (InterPro:IPR005162); Has 707 Blast hits to 705 proteins in 25 species: Archae - 0; Bacteria - 0; Metazoa - 4; Fungi - 0; Plants - 703; Viruses - 0; Other Eukaryotes - 0 (source: NCBI BLink). | chr1:7447690-7448403 REVERSE LENGTH=237	366	237	5.00E-23	64.8	15.8	24.6
Rsa1.0_00005.1.g293.t1	ref|XP_002883469.1| hypothetical protein ARALYDRAFT_898931 [Arabidopsis lyrata subsp. lyrata] gi|297329309|gb|EFH59728.1| hypothetical protein ARALYDRAFT_898931 [Arabidopsis lyrata subsp. lyrata]	266	242	5.00E-67	91.0	54.1	66.2	hypothetical protein ARALYDRAFT_898931	gbpln	Arabidopsis lyrata	AT2G16600.1 | Symbols: ROC3 | rotamase CYP 3 | chr2:7200862-7201383 FORWARD LENGTH=173	266	173	2.00E-57	65.0	42.5	49.2
Rsa1.0_00005.1.g294.t1	dbj|BAB01909.1| unnamed protein product [Arabidopsis thaliana]	253	294	2.00E-51	116.2	45.1	62.8	unnamed protein product	gbpln	Arabidopsis thaliana	AT3G20980.1 | Symbols: | Gag-Pol-related retrotransposon family protein | chr3:7355963-7357966 FORWARD LENGTH=405	253	405	1.00E-41	160.1	37.9	57.3
Rsa1.0_00005.1.g295.t1	dbj|BAF01392.1| hypothetical protein [Arabidopsis thaliana]	406	438	3.00E-78	107.9	44.8	64.5	hypothetical protein	gbpln	Arabidopsis thaliana	AT3G21000.1 | Symbols: | Gag-Pol-related retrotransposon family protein | chr3:7363921-7365138 FORWARD LENGTH=405	406	405	1.00E-72	99.8	42.6	60.1

Rsa1.0_00005.1.g296.t1	dbj|BAF01392.1| hypothetical protein [Arabidopsis thaliana]	367	438	1.00E-101	119.3	58.9	73.0	hypothetical protein	gbpln	Arabidopsis thaliana	AT3G21000.1 | Symbols: | Gag-Pol-related retrotransposon family protein | chr3:7363921-7365138 FORWARD LENGTH=405	367	405	3.00E-78	110.4	46.6	66.2
Rsa1.0_00005.1.g297.t1	dbj|BAB01447.1| gag-pol polyprotein-like [Arabidopsis thaliana]	418	420	3.00E-91	100.5	46.2	55.7	gag-pol polyprotein-like	gbpln	Arabidopsis thaliana	AT3G21000.1 | Symbols: | Gag-Pol-related retrotransposon family protein | chr3:7363921-7365138 FORWARD LENGTH=405	418	405	3.00E-50	96.9	31.1	43.1
Rsa1.0_00005.1.g298.t1	ref|XP_002883282.1| hypothetical protein ARALYDRAFT_318845 [Arabidopsis lyrata subsp. lyrata] gi|297329122|gb|EFH59541.1| hypothetical protein ARALYDRAFT_318845 [Arabidopsis lyrata subsp. lyrata]	549	560	0	102.0	89.4	92.9	hypothetical protein ARALYDRAFT_318845	gbpln	Arabidopsis lyrata	AT3G21060.1 | Symbols: | Transducin/WD40 repeat-like superfamily protein | chr3:7377822-7379942 FORWARD LENGTH=547	549	547	0	99.6	86.0	90.3
Rsa1.0_00005.1.g299.t1	ref|NP_974347.1| NAD(H) kinase 1 [Arabidopsis thaliana] gi|94717660|sp|Q56YN3.2|NADK1_ARATH RecName: Full=NAD(H) kinase 1; Short=AtNADK-1 gi|39843359|gb|AAR32133.1| NADK1 [Arabidopsis thaliana] gi|51970406|dbj|BAD43895.1| unnamed protein product [Arabidopsis thaliana] gi|332642935|gb|AEE76456.1| NAD(H) kinase 1 [Arabidopsis thaliana]	580	524	0	90.3	66.6	76.6	NAD(H) kinase 1	gbpln	Arabidopsis thaliana	AT3G21070.2 | Symbols: NADK1 | NAD kinase 1 | chr3:7380434-7383874 REVERSE LENGTH=524	580	524	0	90.3	66.6	76.6
Rsa1.0_00005.1.g300.t1	#	#	#	#	#	#	#	-	----	----	#	#	#	#	#	#	#
Rsa1.0_00005.1.g301.t1	ref|XP_002885419.1| abc transporter family protein [Arabidopsis lyrata subsp. lyrata] gi|297331259|gb|EFH61678.1| abc transporter family protein [Arabidopsis lyrata subsp. lyrata]	83	691	1.00E-14	832.5	60.2	62.7	abc transporter family protein	gbpln	Arabidopsis lyrata	AT3G21090.1 | Symbols: | ABC-2 type transporter family protein | chr3:7391497-7394933 REVERSE LENGTH=691	83	691	2.00E-17	832.5	60.2	62.7
Rsa1.0_00005.1.g302.t1	#	#	#	#	#	#	#	-	----	----	#	#	#	#	#	#	#
Rsa1.0_00005.1.g303.t1	ref|XP_002883283.1| RNA recognition motif-containing protein [Arabidopsis lyrata subsp. lyrata] gi|297329123|gb|EFH59542.1| RNA recognition motif-containing protein [Arabidopsis lyrata subsp. lyrata]	545	607	0	111.4	80.0	87.5	RNA recognition motif-containing protein	gbpln	Arabidopsis lyrata	AT3G21100.2 | Symbols: | RNA-binding (RRM/RBD/RNP motifs) family protein | chr3:7399162-7401870 FORWARD LENGTH=602	545	602	0	110.5	79.6	87.0
Rsa1.0_00005.1.g304.t1	ref|NP_188748.1| phosphoribosylaminoimidazole-succinocarboxamide synthase [Arabidopsis thaliana] gi|79313319|ref|NP_001030739.1| phosphoribosylaminoimidazole-succinocarboxamide synthase [Arabidopsis thaliana] gi|26454658|sp|P38025.2|PUR7_ARATH RecName: Full=Phosphoribosylaminoimidazole-succinocarboxamide synthase, chloroplastic; AltName: Full=SAICAR synthetase; Flags: Precursor gi|11994271|dbj|BAB01454.1| 5'-phosphoribosyl-4-(N-succinocarboxamide)-5- ami noimidazole synthetase [Arabidopsis thaliana] gi|27754219|gb|AAO22563.1| putative phosphoribosyamidoimidazole-succinocarboxamide synthase [Arabidopsis thaliana] gi|332642941|gb|AEE76462.1| phosphoribosylaminoimidazole-succinocarboxamide synthase [Arabidopsis thaliana] gi|332642942|gb|AEE76463.1| phosphoribosylaminoimidazole-succinocarboxamide synthase [Arabidopsis thaliana]	408	411	0	100.7	93.6	95.8	phosphoribosylaminoimidazole-succinocarboxamide synthase	gbpln	Arabidopsis thaliana	AT3G21110.2 | Symbols: PUR7, PURC | purin 7 | chr3:7402696-7405273 REVERSE LENGTH=411	408	411	0	100.7	93.6	95.8
Rsa1.0_00005.1.g305.t1	gb|EOA30727.1| hypothetical protein CARUB_v10013865mg [Capsella rubella]	399	402	0	100.8	90.7	94.0	hypothetical protein CARUB_v10013865mg	gbpln	Capsella rubella	AT1G51540.1 | Symbols: | Galactose oxidase/kelch repeat superfamily protein | chr1:19115702-19117381 FORWARD LENGTH=415	399	415	0	104.0	88.7	94.0
Rsa1.0_00005.1.g306.t1	ref|XP_002885421.1| FMN binding protein [Arabidopsis lyrata subsp. lyrata] gi|297331261|gb|EFH61680.1| FMN binding protein [Arabidopsis lyrata subsp. lyrata]	370	387	1.00E-170	104.6	84.3	87.6	FMN binding protein	gbpln	Arabidopsis lyrata	AT3G21140.1 | Symbols: | Pyridoxamine 5'-phosphate oxidase family protein | chr3:7409696-7412086 REVERSE LENGTH=387	370	387	1.00E-170	104.6	82.7	86.5

Rsa1.0_00005.1.g307.t23	ref|NP_850618.1| GATA transcription factor 24 [Arabidopsis thaliana] gi|14596059|gb|AAK68757.1| Unknown protein [Arabidopsis thaliana] gi|17978695|gb|AAL47341.1| unknown protein [Arabidopsis thaliana] gi|332642950|gb|AEE76471.1| GATA transcription factor 24 [Arabidopsis thaliana]	289	295	1.00E-126	102.1	81.0	87.5	GATA transcription factor 24	gbpln	Arabidopsis thaliana	AT3G21175.2 | Symbols: ZML1, GATA24 | ZIM-like 1 | chr3:7422832-7424598 FORWARD LENGTH=295	289	295	1.00E-129	102.1	81.0	87.5
Rsa1.0_00005.1.g308.t1	ref|XP_002885425.1| hypothetical protein ARALYDRAFT_479639 [Arabidopsis lyrata subsp. lyrata] gi|297331265|gb|EFH61684.1| hypothetical protein ARALYDRAFT_479639 [Arabidopsis lyrata subsp. lyrata]	422	422	0	100.0	91.5	96.4	hypothetical protein ARALYDRAFT_479639	gbpln	Arabidopsis lyrata	AT3G21190.1 | Symbols: | O-fucosyltransferase family protein | chr3:7432579-7434543 REVERSE LENGTH=422	422	422	0	100.0	91.0	96.2
Rsa1.0_00005.1.g309.t1	ref|NP_175576.1| ATP synthase subunit epsilon [Arabidopsis thaliana] gi|2493052|sp|Q96253.3|ATP5E_ARATH RecName: Full=ATP synthase subunit epsilon, mitochondrial; Short=ATPase subunit epsilon gi|12321688|gb|AAG50890.1|AC025294_28 epsilon subunit of mitochondrial F1-ATPase [Arabidopsis thaliana] gi|1655486|dbj|BAA13602.1| epsilon subunit of mitochondrial F1-ATPase [Arabidopsis thaliana] gi|18252167|gb|AAL61916.1| epsilon subunit of mitochondrial F1-ATPase [Arabidopsis thaliana] gi|21386911|gb|AAM47859.1| epsilon subunit of mitochondrial F1-ATPase [Arabidopsis thaliana] gi|332194574|gb|AEE32695.1| ATP synthase subunit epsilon [Arabidopsis thaliana]	70	70	3.00E-30	100.0	88.6	94.3	ATP synthase subunit epsilon	gbpln	Arabidopsis thaliana	AT1G51650.1 | Symbols: | ATP synthase epsilon chain, mitochondrial | chr1:19152680-19153641 FORWARD LENGTH=70	70	70	6.00E-33	100.0	88.6	94.3
Rsa1.0_00005.1.g310.t1	ref|NP_683582.2| RNA recognition motif-containing protein [Arabidopsis thaliana] gi|13605613|gb|AAK32800.1|AF361632_1 At3g21211 [Arabidopsis thaliana] gi|23505943|gb|AAN28831.1| At3g21211/At3g21211 [Arabidopsis thaliana] gi|26451397|dbj|BAC42798.1| unknown protein [Arabidopsis thaliana] gi|110740646|dbj|BAE98426.1| hypothetical protein [Arabidopsis thaliana] gi|222423570|dbj|BAH19754.1| AT3G21215 [Arabidopsis thaliana] gi|332642956|gb|AEE76477.1| RNA recognition motif-containing protein [Arabidopsis thaliana]	349	339	1.00E-142	97.1	88.8	92.0	RNA recognition motif-containing protein	gbpln	Arabidopsis thaliana	AT3G21215.1 | Symbols: | RNA-binding (RRM/RBD/RNP motifs) family protein | chr3:7442873-7445231 REVERSE LENGTH=339	349	339	1.00E-145	97.1	88.8	92.0
Rsa1.0_00005.1.g311.t1	gb|ABF55665.1| double MYC-tagged mitogen activated protein kinase kinase 5 [synthetic construct]	332	380	1.00E-156	114.5	87.0	93.4	double MYC-tagged mitogen activated protein kinase kinase 5	gbenv	synthetic construct	AT3G21220.1 | Symbols: ATMKK5, ATMAP2K_ALPHA, MAP2K_A, MKK5, ATMEK5, MEK5 | MAP kinase kinase 5 | chr3:7445917-7446963 FORWARD LENGTH=348	332	348	1.00E-158	104.8	87.0	93.4
Rsa1.0_00005.1.g312.t1	gb|EOA29726.1| hypothetical protein CARUB_v10012814mg [Capsella rubella] gi|482565538|gb|EOA29727.1| hypothetical protein CARUB_v10012814mg [Capsella rubella]	1494	1464	0	98.0	82.5	89.6	hypothetical protein CARUB_v10012814mg	gbpln	Capsella rubella	AT3G21250.2 | Symbols: MRP6, ABCC8 | multidrug resistance-associated protein 6 | chr3:7457668-7463261 REVERSE LENGTH=1464	1494	1464	0	98.0	81.9	89.0
Rsa1.0_00005.1.g313.t1	ref|XP_002885432.1| hypothetical protein ARALYDRAFT_342275 [Arabidopsis lyrata subsp. lyrata] gi|297331272|gb|EFH61691.1| hypothetical protein ARALYDRAFT_342275 [Arabidopsis lyrata subsp. lyrata]	234	233	1.00E-110	99.6	88.9	94.4	hypothetical protein ARALYDRAFT_342275	gbpln	Arabidopsis lyrata	AT3G21260.3 | Symbols: | Glycolipid transfer protein (GLTP) family protein | chr3:7464132-7465785 REVERSE LENGTH=233	234	233	1.00E-112	99.6	88.5	93.6
Rsa1.0_00005.1.g314.t1	ref|XP_002883294.1| 55 kDa B regulatory subunit of phosphatase 2A [Arabidopsis lyrata subsp. lyrata] gi|297329134|gb|EFH59553.1| 55 kDa B regulatory subunit of phosphatase 2A [Arabidopsis lyrata subsp. lyrata]	511	514	0	100.6	88.3	91.6	55 kDa B regulatory subunit of phosphatase 2A	gbpln	Arabidopsis lyrata	AT1G51690.1 | Symbols: ATB ALPHA, B ALPHA | protein phosphatase 2A 55 kDa regulatory subunit B alpha isoform | chr1:19166218-19169974 FORWARD LENGTH=513	511	513	0	100.4	82.2	89.8

Rsa1.0_00005.1.g315.t1	ref|XP_002883295.1| hypothetical protein ARALYDRAFT_898570 [Arabidopsis lyrata subsp. lyrata] gi|297329135|gb|EFH59554.1| hypothetical protein ARALYDRAFT_898570 [Arabidopsis lyrata subsp. lyrata]	207	199	3.00E-73	96.1	78.7	83.1	hypothetical protein ARALYDRAFT_898570	gbpln	Arabidopsis lyrata	AT3G21270.1 | Symbols: ADOF2, DOF2 | DOF zinc finger protein 2 | chr3:7474934-7475548 FORWARD LENGTH=204	207	204	1.00E-68	98.6	78.7	84.5
Rsa1.0_00005.1.g316.t1	ref|XP_002883297.1| hypothetical protein ARALYDRAFT_479651 [Arabidopsis lyrata subsp. lyrata] gi|297329137|gb|EFH59556.1| hypothetical protein ARALYDRAFT_479651 [Arabidopsis lyrata subsp. lyrata]	99	383	5.00E-32	386.9	72.7	79.8	hypothetical protein ARALYDRAFT_479651	gbpln	Arabidopsis lyrata	AT3G21310.1 | Symbols: | Core-2/I-branching beta-1,6-N-acetylglucosaminyltransferase family protein | chr3:7497774-7499011 FORWARD LENGTH=383	99	383	1.00E-33	386.9	70.7	77.8
Rsa1.0_00005.1.g317.t1	gb|EOA30281.1| hypothetical protein CARUB_v10013409mg [Capsella rubella]	537	538	0	100.2	75.6	84.0	hypothetical protein CARUB_v10013409mg	gbpln	Capsella rubella	AT3G21320.1 | Symbols: | BEST Arabidopsis thaliana protein match is: hydroxyproline-rich glycoprotein family protein (TAIR:AT2G25930.1); Has 63 Blast hits to 58 proteins in 21 species: Archae - 0; Bacteria - 0; Metazoa - 3; Fungi - 0; Plants - 58; Viruses - 0; Other Eukaryotes - 2 (source: NCBI BLink). | chr3:7499408-7501312 REVERSE LENGTH=540	537	540	0	100.6	72.3	81.8
Rsa1.0_00005.1.g318.t1	emb|CAA57913.1| beta-glucosidase [Brassica napus]	338	514	1.00E-172	152.1	88.8	90.5	beta-glucosidase	gbpln	Brassica napus	AT3G21370.1 | Symbols: BGLU19 | beta glucosidase 19 | chr3:7524286-7527579 REVERSE LENGTH=527	338	527	1.00E-170	155.9	86.1	92.6
Rsa1.0_00005.1.g319.t1	dbj|BAB10503.1| retroelement pol polyprotein-like [Arabidopsis thaliana]	1267	1475	0	116.4	37.1	49.5	retroelement pol polyprotein-like	gbpln	Arabidopsis thaliana	AT4G23160.1 | Symbols: CRK8 | cysteine-rich RLK (RECEPTOR-like protein kinase) 8 | chr4:12129485-12134086 FORWARD LENGTH=1262	1267	1262	1.00E-78	99.6	12.3	15.7
Rsa1.0_00005.1.g320.t1	ref|XP_002883303.1| hypothetical protein ARALYDRAFT_479659 [Arabidopsis lyrata subsp. lyrata] gi|297329143|gb|EFH59562.1| hypothetical protein ARALYDRAFT_479659 [Arabidopsis lyrata subsp. lyrata]	407	461	1.00E-177	113.3	81.8	88.9	hypothetical protein ARALYDRAFT_479659	gbpln	Arabidopsis lyrata	AT3G21380.1 | Symbols: | Mannose-binding lectin superfamily protein | chr3:7528478-7530457 FORWARD LENGTH=460	407	460	1.00E-175	113.0	82.3	89.4
Rsa1.0_00005.1.g321.t1	gb|EOA30874.1| hypothetical protein CARUB_v10014020mg [Capsella rubella]	428	364	0	85.0	73.6	76.2	hypothetical protein CARUB_v10014020mg	gbpln	Capsella rubella	AT3G21420.1 | Symbols: | 2-oxoglutarate (2OG) and Fe(II)-dependent oxygenase superfamily protein | chr3:7541579-7543221 FORWARD LENGTH=364	428	364	1.00E-178	85.0	73.1	76.6
Rsa1.0_00005.1.g322.t2	gb|ABD65050.1| hypothetical protein 27.t00005 [Brassica oleracea]	129	236	7.00E-26	182.9	52.7	56.6	hypothetical protein 27.t00005	gbpln	Brassica oleracea	#	#	#	#	#	#	#
Rsa1.0_00005.1.g323.t1	#	#	#	#	#	#	#	-	----	----	#	#	#	#	#	#	#
Rsa1.0_00005.1.g324.t1	ref|XP_002885441.1| electron carrier/ protein disulfide oxidoreductase [Arabidopsis lyrata subsp. lyrata] gi|297331281|gb|EFH61700.1| electron carrier/ protein disulfide oxidoreductase [Arabidopsis lyrata subsp. lyrata]	102	102	4.00E-49	100.0	92.2	97.1	electron carrier/ protein disulfide oxidoreductase	gbpln	Arabidopsis lyrata	AT3G21460.1 | Symbols: | Glutaredoxin family protein | chr3:7557567-7557875 REVERSE LENGTH=102	102	102	1.00E-51	100.0	92.2	97.1
Rsa1.0_00005.1.g325.t1	#	#	#	#	#	#	#	-	----	----	#	#	#	#	#	#	#
Rsa1.0_00005.1.g326.t1	ref|XP_002880880.1| hypothetical protein ARALYDRAFT_481599 [Arabidopsis lyrata subsp. lyrata] gi|297326719|gb|EFH57139.1| hypothetical protein ARALYDRAFT_481599 [Arabidopsis lyrata subsp. lyrata]	201	720	2.00E-19	358.2	40.8	46.3	hypothetical protein ARALYDRAFT_481599	gbpln	Arabidopsis lyrata	AT2G27100.1 | Symbols: SE | C2H2 zinc-finger protein SERRATE (SE) | chr2:11572587-11576357 FORWARD LENGTH=720	201	720	3.00E-20	358.2	40.8	45.8
Rsa1.0_00005.1.g327.t10	ref|XP_002515199.1| hypothetical protein RCOM_1343910 [Ricinus communis] gi|223545679|gb|EEF47183.1| hypothetical protein RCOM_1343910 [Ricinus communis]	224	627	2.00E-23	279.9	30.8	43.3	hypothetical protein RCOM_1343910	gbpln	Ricinus communis	AT3G62200.1 | Symbols: | Putative endonuclease or glycosyl hydrolase | chr3:23023437-23025671 REVERSE LENGTH=673	224	673	1.00E-23	300.4	29.5	44.6
Rsa1.0_00005.1.g328.t1	#	#	#	#	#	#	#	-	----	----	AT3G62210.1 | Symbols: EDA32 | Putative endonuclease or glycosyl hydrolase | chr3:23026910-23028113 REVERSE LENGTH=279	151	279	1.00E-11	184.8	32.5	45.0
Rsa1.0_00005.1.g329.t1	ref|XP_002883306.1| predicted protein [Arabidopsis lyrata subsp. lyrata] gi|297329146|gb|EFH59565.1| predicted protein [Arabidopsis lyrata subsp. lyrata]	137	387	5.00E-11	282.5	29.9	35.0	predicted protein	gbpln	Arabidopsis lyrata	AT3G21465.1 | Symbols: | unknown protein; BEST Arabidopsis thaliana protein match is: unknown protein (TAIR:AT4G15640.1); Has 38 Blast hits to 38 proteins in 14 species: Archae - 0; Bacteria - 0; Metazoa - 0; Fungi - 0; Plants - 38; Viruses - 0; Other Eukaryotes - 0 (source: NCBI BLink). | chr3:7560750-7563202 FORWARD LENGTH=388	137	388	5.00E-12	283.2	28.5	35.0

Rsa1.0_00005.1.g330.t1	gb|EOA29492.1| hypothetical protein CARUB_v10012880mg [Capsella rubella]	999	1044	0	104.5	67.1	77.0	hypothetical protein CARUB_v10012880mg	gbpln	Capsella rubella	AT3G21480.1 | Symbols: | BRCT domain-containing DNA repair protein | chr3:7567730-7571990 FORWARD LENGTH=1041	999	1041	0	104.2	65.4	73.9
Rsa1.0_00005.1.g331.t1	# #	#	#	#	#	#		–	----	----	# #	#	#	#	#	#	
Rsa1.0_00005.1.g332.t1	ref|XP_002885445.1| histidine-containing phosphotransmitter 3 [Arabidopsis lyrata subsp. lyrata] gi|297331285|gb|EFH61704.1| histidine-containing phosphotransmitter 3 [Arabidopsis lyrata subsp. lyrata]	154	154	2.00E-74	100.0	96.1	99.4	histidine-containing phosphotransmitter 3	gbpln	Arabidopsis lyrata	AT3G21510.1 | Symbols: AHP1 | histidine-containing phosphotransmitter 1 | chr3:7578432-7579537 REVERSE LENGTH=154	154	154	4.00E-76	100.0	95.5	98.7
Rsa1.0_00005.1.g333.t1	gb|EOA31454.1| hypothetical protein CARUB_v10014640mg [Capsella rubella]	202	208	5.00E-98	103.0	88.6	93.6	hypothetical protein CARUB_v10014640mg	gbpln	Capsella rubella	AT3G21520.1 | Symbols: DMP1, AtDMP1 | DUF679 domain membrane protein 1 | chr3:7582000-7582623 FORWARD LENGTH=207	202	207	2.00E-99	102.5	90.1	94.6
Rsa1.0_00005.1.g334.t1	ref|XP_002885448.1| hypothetical protein ARALYDRAFT_898603 [Arabidopsis lyrata subsp. lyrata] gi|297331288|gb|EFH61707.1| hypothetical protein ARALYDRAFT_898603 [Arabidopsis lyrata subsp. lyrata]	185	185	8.00E-94	100.0	90.3	96.8	hypothetical protein ARALYDRAFT_898603	gbpln	Arabidopsis lyrata	AT3G21550.1 | Symbols: DMP2, AtDMP2 | DUF679 domain membrane protein 2 | chr3:7591708-7592262 REVERSE LENGTH=184	185	184	9.00E-94	99.5	88.1	95.1
Rsa1.0_00005.1.g335.t1	dbj|BAA77394.1| SAE1-S9-protein [Brassica rapa]	327	255	3.00E-73	78.0	45.9	53.2	SAE1-S9-protein	gbpln	Brassica rapa	AT5G18880.1 | Symbols: | RNA-directed DNA polymerase (reverse transcriptase)-related family protein | chr5:6300535-6301422 REVERSE LENGTH=295	327	295	1.00E-70	90.2	43.4	56.6
Rsa1.0_00005.1.g336.t1	emb|CAS03349.1| sinapate 1-glucosyltransferase [Brassica napus var. napus] gi|226533678|emb|CAS03357.1| sinapate 1-glucosyltransferase [Brassica napus var. napus]	489	497	0	101.6	93.3	96.5	sinapate 1-glucosyltransferase	gbpln	Brassica napus	AT3G21560.1 | Symbols: UGT84A2 | UDP-Glycosyltransferase superfamily protein | chr3:7595884-7597374 FORWARD LENGTH=496	489	496	0	101.4	84.3	92.0
Rsa1.0_00005.1.g337.t1	emb|CAS03358.1| hypothetical protein [Brassica napus var. napus]	139	138	2.00E-57	99.3	90.6	93.5	hypothetical protein	gbpln	Brassica napus	AT3G21570.1 | Symbols: | unknown protein; Has 43 Blast hits to 43 proteins in 13 species: Archae - 0; Bacteria - 0; Metazoa - 0; Fungi - 0; Plants - 43; Viruses - 0; Other Eukaryotes - 0 (source: NCBI BLink). | chr3:7600297-7600707 REVERSE LENGTH=136	139	136	1.00E-55	97.8	77.7	87.8
Rsa1.0_00005.1.g338.t1	ref|NP_001185096.1| phospholipid-translocating ATPase [Arabidopsis thaliana] gi|332192532|gb|AEE30653.1| putative phospholipid-transporting ATPase 12 [Arabidopsis thaliana]	1164	1185	0	101.8	88.0	94.0	phospholipid-translocating ATPase	gbpln	Arabidopsis thaliana	AT1G26130.2 | Symbols: | ATPase E1-E2 type family protein / haloacid dehalogenase-like hydrolase family protein | chr1:9033600-9038246 FORWARD LENGTH=1185	1164	1185	0	101.8	88.0	94.0
Rsa1.0_00005.1.g339.t9	ref|XP_002885459.1| nitrate transporter [Arabidopsis lyrata subsp. lyrata] gi|297331299|gb|EFH61718.1| nitrate transporter [Arabidopsis lyrata subsp. lyrata]	567	590	1.00E-111	104.1	40.4	43.6	nitrate transporter	gbpln	Arabidopsis lyrata	AT3G21670.1 | Symbols: | Major facilitator superfamily protein | chr3:7626942-7628954 REVERSE LENGTH=590	567	590	1.00E-112	104.1	40.2	43.0
Rsa1.0_00005.1.g340.t1	gb|EOA32900.1| hypothetical protein CARUB_v10016225mg [Capsella rubella]	112	125	3.00E-29	111.6	68.8	80.4	hypothetical protein CARUB_v10016225mg	gbpln	Capsella rubella	AT3G21680.1 | Symbols: | unknown protein; FUNCTIONS IN: molecular_function unknown; INVOLVED IN: N-terminal protein myristoylation; LOCATED IN: cellular_component unknown; EXPRESSED IN: root, flower, stamen; EXPRESSED DURING: 4 anthesis, petal differentiation and expansion stage; Has 34 Blast hits to 34 proteins in 7 species: Archae - 0; Bacteria - 0; Metazoa - 0; Fungi - 0; Plants - 34; Viruses - 0; Other Eukaryotes - 0 (source: NCBI BLink). | chr3:7633494-7633853 REVERSE LENGTH=119	112	119	1.00E-28	106.3	75.0	82.1
Rsa1.0_00005.1.g341.t1	dbj|BAJ34540.1| unnamed protein product [Thellungiella halophila]	318	505	1.00E-153	158.8	91.2	95.0	unnamed protein product	----	----	AT3G21690.1 | Symbols: | MATE efflux family protein | chr3:7638750-7641861 FORWARD LENGTH=506	318	506	1.00E-151	159.1	89.6	93.7
Rsa1.0_00005.1.g342.t1	ref|XP_002883313.1| mate efflux family protein [Arabidopsis lyrata subsp. lyrata] gi|297329153|gb|EFH59572.1| mate efflux family protein [Arabidopsis lyrata subsp. lyrata]	156	506	1.00E-68	324.4	87.2	93.6	mate efflux family protein	gbpln	Arabidopsis lyrata	AT3G21690.1 | Symbols: | MATE efflux family protein | chr3:7638750-7641861 FORWARD LENGTH=506	156	506	1.00E-70	324.4	86.5	92.9
Rsa1.0_00005.1.g343.t1	ref|XP_002883313.1| mate efflux family protein [Arabidopsis lyrata subsp. lyrata] gi|297329153|gb|EFH59572.1| mate efflux family protein [Arabidopsis lyrata subsp. lyrata]	548	506	0	92.3	86.1	88.9	mate efflux family protein	gbpln	Arabidopsis lyrata	AT3G21690.1 | Symbols: | MATE efflux family protein | chr3:7638750-7641861 FORWARD LENGTH=506	548	506	0	92.3	85.4	88.3

Rsa1.0_00005.1.g344.t1	ref|NP_850622.1| Ras-related small GTP-binding family protein [Arabidopsis thaliana] gi|11994637|dbj|BAB02832.1| unnamed protein product [Arabidopsis thaliana] gi|22316556|emb|CAD44270.1| monomeric G-protein [Arabidopsis thaliana] gi|332643018|gb|AEE76539.1| Ras-related small GTP-binding family protein [Arabidopsis thaliana]	278	292	1.00E-128	105.0	88.5	93.9	Ras-related small GTP-binding family protein	gbpln	Arabidopsis thaliana	AT3G21700.3 | Symbols: SGP2 | Ras-related small GTP-binding family protein | chr3:7644581-7646190 FORWARD LENGTH=292	278	292	1.00E-131	105.0	88.5	93.9
Rsa1.0_00005.1.g345.t1	gb|EOA31309.1| hypothetical protein CARUB_v10014480mg [Capsella rubella]	333	247	1.00E-52	74.2	37.8	41.7	hypothetical protein CARUB_v10014480mg	gbpln	Capsella rubella	AT3G21710.2 | Symbols: | unknown protein; Has 35333 Blast hits to 34131 proteins in 2444 species: Archae - 798; Bacteria - 22429; Metazoa - 974; Fungi - 991; Plants - 531; Viruses - 0; Other Eukaryotes - 9610 (source: NCBI BLink). | chr3:7648380-7649533 FORWARD LENGTH=211	333	211	2.00E-54	63.4	36.9	40.8
Rsa1.0_00006.1.g346.t1	gb|ABG29728.1| chloroplast chlorophyll a/b binding protein [Brassica napus]	267	267	1.00E-151	100.0	99.3	99.6	chloroplast chlorophyll a/b binding protein	gbpln	Brassica napus	AT1G29930.1 | Symbols: CAB1, AB140, CAB140, LHCB1.3 | chlorophyll A/B binding protein 1 | chr1:10478071-10478874 FORWARD LENGTH=267	267	267	1.00E-150	100.0	97.4	99.3
Rsa1.0_00006.1.g347.t1	ref|XP_002884603.1| hypothetical protein ARALYDRAFT_896807 [Arabidopsis lyrata subsp. lyrata] gi|297330443|gb|EFH60862.1| hypothetical protein ARALYDRAFT_896807 [Arabidopsis lyrata subsp. lyrata]	863	948	0	109.8	64.5	74.7	hypothetical protein ARALYDRAFT_896807	gbpln	Arabidopsis lyrata	AT4G32670.1 | Symbols: | RING/FYVE/PHD zinc finger superfamily protein | chr4:15759527-15762847 REVERSE LENGTH=860	863	860	1.00E-174	99.7	44.6	59.0
Rsa1.0_00006.1.g348.t3	ref|NP_174275.2| Magnesium transporter CorA-like family protein [Arabidopsis thaliana] gi|186479046|ref|NP_001117384.1| Magnesium transporter CorA-like family protein [Arabidopsis thaliana] gi|332193013|gb|AEE31134.1| Magnesium transporter CorA-like family protein [Arabidopsis thaliana] gi|332193014|gb|AEE31135.1| Magnesium transporter CorA-like family protein [Arabidopsis thaliana]	738	540	0	73.2	64.2	68.8	Magnesium transporter CorA-like family protein	gbpln	Arabidopsis thaliana	AT1G29820.2 | Symbols: | Magnesium transporter CorA-like family protein | chr1:10438219-10440625 FORWARD LENGTH=540	738	540	0	73.2	64.2	68.8
Rsa1.0_00006.1.g349.t1	gb|EOA37993.1| hypothetical protein CARUB_v10009462mg [Capsella rubella]	374	376	0	100.5	86.9	94.1	hypothetical protein CARUB_v10009462mg	gbpln	Capsella rubella	AT1G29790.2 | Symbols: | S-adenosyl-L-methionine-dependent methyltransferases superfamily protein | chr1:10430025-10431161 FORWARD LENGTH=378	374	378	0	101.1	87.7	94.1
Rsa1.0_00006.1.g350.t1	gb|EOA36491.1| hypothetical protein CARUB_v10011144mg [Capsella rubella]	106	104	1.00E-29	98.1	65.1	67.9	hypothetical protein CARUB_v10011144mg	gbpln	Capsella rubella	#	#	#	#	#	#	#
Rsa1.0_00006.1.g351.t1	ref|XP_002890830.1| NLI interacting factor family protein [Arabidopsis lyrata subsp. lyrata] gi|297336672|gb|EFH67089.1| NLI interacting factor family protein [Arabidopsis lyrata subsp. lyrata]	230	221	3.00E-98	96.1	79.1	86.5	NLI interacting factor family protein	gbpln	Arabidopsis lyrata	AT1G29780.1 | Symbols: | Haloacid dehalogenase-like hydrolase (HAD) superfamily protein | chr1:10426950-10427615 FORWARD LENGTH=221	230	221	9.00E-97	96.1	76.1	84.3
Rsa1.0_00006.1.g352.t1	ref|XP_002890829.1| NLI interacting factor family protein [Arabidopsis lyrata subsp. lyrata] gi|297336671|gb|EFH67088.1| NLI interacting factor family protein [Arabidopsis lyrata subsp. lyrata]	276	277	1.00E-120	100.4	80.4	87.7	NLI interacting factor family protein	gbpln	Arabidopsis lyrata	AT1G29770.1 | Symbols: | Haloacid dehalogenase-like hydrolase (HAD) superfamily protein | chr1:10424806-10425642 FORWARD LENGTH=278	276	278	1.00E-120	100.7	78.6	86.6
Rsa1.0_00006.1.g353.t1	ref|XP_002890828.1| hypothetical protein ARALYDRAFT_890503 [Arabidopsis lyrata subsp. lyrata] gi|297336670|gb|EFH67087.1| hypothetical protein ARALYDRAFT_890503 [Arabidopsis lyrata subsp. lyrata]	560	527	1.00E-156	94.1	56.3	65.4	hypothetical protein ARALYDRAFT_890503	gbpln	Arabidopsis lyrata	AT1G29760.1 | Symbols: | Putative adipose-regulatory protein (Seipin) | chr1:10422381-10424116 FORWARD LENGTH=526	560	526	1.00E-148	93.9	47.1	54.8
Rsa1.0_00006.1.g354.t1	ref|XP_002893580.1| hypothetical protein ARALYDRAFT_473178 [Arabidopsis lyrata subsp. lyrata] gi|297339422|gb|EFH69839.1| hypothetical protein ARALYDRAFT_473178 [Arabidopsis lyrata subsp. lyrata]	1003	1023	0	102.0	79.9	88.5	hypothetical protein ARALYDRAFT_473178	gbpln	Arabidopsis lyrata	AT1G29750.2 | Symbols: RKF1 | receptor-like kinase in flowers 1 | chr1:10414071-10420469 REVERSE LENGTH=1021	1003	1021	0	101.8	80.4	88.6
Rsa1.0_00006.1.g355.t2	gb|EOA38810.1| hypothetical protein CARUB_v10011128mg [Capsella rubella]	109	1008	2.00E-22	924.8	55.0	69.7	hypothetical protein CARUB_v10011128mg	gbpln	Capsella rubella	AT1G29720.1 | Symbols: | Leucine-rich repeat transmembrane protein kinase | chr1:10393894-10399771 REVERSE LENGTH=1019	109	1019	1.00E-23	934.9	56.0	70.6

Rsa1.0_00006.1.g356.t2	ref|NP_564335.3| Leucine-rich repeat transmembrane protein kinase [Arabidopsis thaliana] gi|325511374|sp|Q9ASQ6.3|Y1972_ARATH RecName: Full=Probable LRR receptor-like serine/threonine-protein kinase At1g29720; Flags: Precursor gi|332193000|gb|AEE31121.1| Leucine-rich repeat transmembrane protein kinase [Arabidopsis thaliana]	973	1019	0	104.7	66.6	74.2	Leucine-rich repeat transmembrane protein kinase	gbpln	Arabidopsis thaliana	AT1G29720.1 | Symbols: | Leucine-rich repeat transmembrane protein kinase | chr1:10393894-10399771 REVERSE LENGTH=1019	973	1019	0	104.7	66.6	74.2
Rsa1.0_00006.1.g357.t12	gb|EOA39568.1| hypothetical protein CARUB_v10008187mg [Capsella rubella]	935	1015	0	108.6	70.9	80.0	hypothetical protein CARUB_v10008187mg	gbpln	Capsella rubella	AT1G29720.1 | Symbols: | Leucine-rich repeat transmembrane protein kinase | chr1:10393894-10399771 REVERSE LENGTH=1019	935	1019	0	109.0	71.2	80.4
Rsa1.0_00006.1.g358.t1	gb|EOA39560.1| hypothetical protein CARUB_v10008178mg [Capsella rubella]	1082	1022	0	94.5	65.3	74.3	hypothetical protein CARUB_v10008178mg	gbpln	Capsella rubella	AT1G29720.1 | Symbols: | Leucine-rich repeat transmembrane protein kinase | chr1:10393894-10399771 REVERSE LENGTH=1019	1082	1019	0	94.2	64.5	72.9
Rsa1.0_00006.1.g359.t5	gb|EOA39560.1| hypothetical protein CARUB_v10008178mg [Capsella rubella]	1945	1022	0	52.5	36.5	41.5	hypothetical protein CARUB_v10008178mg	gbpln	Capsella rubella	AT1G29720.1 | Symbols: | Leucine-rich repeat transmembrane protein kinase | chr1:10393894-10399771 REVERSE LENGTH=1019	1945	1019	0	52.4	35.9	41.2
Rsa1.0_00006.1.g360.t1	ref|XP_002893575.1| hypothetical protein ARALYDRAFT_473173 [Arabidopsis lyrata subsp. lyrata] gi|297339417|gb|EFH69834.1| hypothetical protein ARALYDRAFT_473173 [Arabidopsis lyrata subsp. lyrata]	351	350	1.00E-173	99.7	87.7	93.2	hypothetical protein ARALYDRAFT_473173	gbpln	Arabidopsis lyrata	AT1G29700.1 | Symbols: | Metallo-hydrolase/oxidoreductase superfamily protein | chr1:10385196-10386906 REVERSE LENGTH=350	351	350	1.00E-174	99.7	86.3	92.9
Rsa1.0_00006.1.g361.t1	gb|EOA40030.1| hypothetical protein CARUB_v10008721mg [Capsella rubella]	566	565	0	99.8	92.6	95.9	hypothetical protein CARUB_v10008721mg	gbpln	Capsella rubella	AT1G29690.1 | Symbols: CAD1 | MAC/Perforin domain-containing protein | chr1:10379310-10381861 REVERSE LENGTH=561	566	561	0	99.1	91.5	94.7
Rsa1.0_00006.1.g362.t1	gb|AGJ83937.1| GLIP [Brassica napus]	360	361	0	100.3	95.6	97.5	GLIP	gbpln	Brassica napus	AT1G29670.1 | Symbols: | GDSL-like Lipase/Acylhydrolase superfamily protein | chr1:10375843-10377717 FORWARD LENGTH=363	360	363	0	100.8	86.4	91.7
Rsa1.0_00006.1.g363.t1	ref|NP_174259.1| GDSL esterase/lipase [Arabidopsis thaliana] gi|75169353|sp|Q9C7N5.1|GDL14_ARATH RecName: Full=GDSL esterase/lipase At1g29660; AltName: Full=Extracellular lipase At1g29660; Flags: Precursor gi|12323544|gb|AAG51756.1|AC068667_35 lipase/hydrolase, putative; 114382-116051 [Arabidopsis thaliana] gi|15215768|gb|AAK91429.1| At1g29660/F15D2_21 [Arabidopsis thaliana] gi|22137090|gb|AAM91390.1| At1g29660/F15D2_21 [Arabidopsis thaliana] gi|332192994|gb|AEE31115.1| GDSL esterase/lipase [Arabidopsis thaliana]	386	364	0	94.3	80.3	86.5	GDSL esterase/lipase	gbpln	Arabidopsis thaliana	AT1G29660.1 | Symbols: | GDSL-like Lipase/Acylhydrolase superfamily protein | chr1:10371955-10373624 FORWARD LENGTH=364	386	364	0	94.3	80.3	86.5
Rsa1.0_00006.1.g364.t1	#	#	#	#	#	#	#	–	----	----	#	#	#	#	#	#	#
Rsa1.0_00006.1.g365.t1	ref|NP_174257.1| uncharacterized protein [Arabidopsis thaliana] gi|12323541|gb|AAG51753.1|AC068667_32 hypothetical protein; 98808-98386 [Arabidopsis thaliana] gi|28466825|gb|AAO44021.1| At1g29640 [Arabidopsis thaliana] gi|110743019|dbj|BAE99402.1| hypothetical protein [Arabidopsis thaliana] gi|332192993|gb|AEE31114.1| uncharacterized protein AT1G29640 [Arabidopsis thaliana]	139	140	5.00E-55	100.7	79.9	89.2	uncharacterized protein	gbpln	Arabidopsis thaliana	AT1G29640.1 | Symbols: | Protein of unknown function, DUF584 | chr1:10355959-10356381 REVERSE LENGTH=140	139	140	1.00E-57	100.7	79.9	89.2
Rsa1.0_00006.1.g366.t1	gb|EOA38491.1| hypothetical protein CARUB_v10010257mg [Capsella rubella]	219	219	1.00E-106	100.0	85.4	91.8	hypothetical protein CARUB_v10010257mg	gbpln	Capsella rubella	AT1G17180.1 | Symbols: ATGSTU25, GSTU25 | glutathione S-transferase TAU 25 | chr1:5872208-5872958 FORWARD LENGTH=221	219	221	1.00E-108	100.9	84.0	91.3
Rsa1.0_00006.1.g367.t1	gb|AAG50806.1|AC079281_8 unknown protein [Arabidopsis thaliana]	1600	1213	0	75.8	25.6	38.2	unknown protein	gbpln	Arabidopsis thaliana	AT1G43760.1 | Symbols: | DNAse I-like superfamily protein | chr1:16528880-16531065 REVERSE LENGTH=626	1600	626	1.00E-69	39.1	9.2	13.7
Rsa1.0_00006.1.g368.t1	#	#	#	#	#	#	#	–	----	----	#	#	#	#	#	#	#

Rsa1.0_00006.1.g369.t1	ref|XP_002881330.1| hypothetical protein ARALYDRAFT_482373 [Arabidopsis lyrata subsp. lyrata] gi|297327169|gb|EFH57589.1| hypothetical protein ARALYDRAFT_482373 [Arabidopsis lyrata subsp. lyrata]	235	241	9.00E-17	102.6	42.1	52.8	hypothetical protein ARALYDRAFT_482373	gbpln	Arabidopsis lyrata	AT1G29530.1 | Symbols: | unknown protein; BEST Arabidopsis thaliana protein match is: unknown protein (TAIR:AT2G34310.3); Has 84 Blast hits to 78 proteins in 24 species: Archae - 2; Bacteria - 4; Metazoa - 9; Fungi - 8; Plants - 55; Viruses - 0; Other Eukaryotes - 6 (source: NCBI BLink). | chr1:10325553-10326651 REVERSE LENGTH=236	235	236	3.00E-18	100.4	40.0	47.7
Rsa1.0_00006.1.g370.t1	gb|EOA37440.1| hypothetical protein CARUB_v10011510mg [Capsella rubella]	159	158	2.00E-71	99.4	92.5	95.6	hypothetical protein CARUB_v10011510mg	gbpln	Capsella rubella	AT1G29520.1 | Symbols: | AWPM-19-like family protein | chr1:10323735-10324525 FORWARD LENGTH=158	159	158	1.00E-73	99.4	91.8	96.2
Rsa1.0_00006.1.g371.t1	#	#	#	#	#	#	#	-	----	----	#	#	#	#	#	#	#
Rsa1.0_00006.1.g372.t1	gb|ABV89641.1| auxin-induced protein [Brassica rapa]	89	137	1.00E-17	153.9	49.4	53.9	auxin-induced protein	gbpln	Brassica rapa	AT1G29500.1 | Symbols: | SAUR-like auxin-responsive protein family | chr1:10321290-10321697 FORWARD LENGTH=135	89	135	3.00E-18	151.7	44.9	52.8
Rsa1.0_00006.1.g373.t1	gb|ABV89665.1| auxin-responsive protein [Brassica rapa]	138	139	4.00E-65	100.7	93.5	96.4	auxin-responsive protein	gbpln	Brassica rapa	AT1G29450.1 | Symbols: | SAUR-like auxin-responsive protein family | chr1:10305981-10306406 REVERSE LENGTH=141	138	141	2.00E-56	102.2	77.5	86.2
Rsa1.0_00006.1.g374.t1	ref|XP_002868389.1| hypothetical protein ARALYDRAFT_493566 [Arabidopsis lyrata subsp. lyrata] gi|297314225|gb|EFH44648.1| hypothetical protein ARALYDRAFT_493566 [Arabidopsis lyrata subsp. lyrata]	271	264	1.00E-106	97.4	81.9	86.7	hypothetical protein ARALYDRAFT_493566	gbpln	Arabidopsis lyrata	AT5G32440.1 | Symbols: | Ubiquitin system component Cue protein | chr5:12077014-12078396 FORWARD LENGTH=264	271	264	2.00E-99	97.4	80.8	85.6
Rsa1.0_00006.1.g375.t4	#	#	#	#	#	#	#	-	----	----	#	#	#	#	#	#	#
Rsa1.0_00006.1.g376.t1	gb|EOA36053.1| hypothetical protein CARUB_v10008372mg [Capsella rubella] gi|482571867|gb|EOA36054.1| hypothetical protein CARUB_v10008372mg [Capsella rubella]	704	767	0	108.9	90.6	95.3	hypothetical protein CARUB_v10008372mg	gbpln	Capsella rubella	AT1G29470.2 | Symbols: | S-adenosyl-L-methionine-dependent methyltransferases superfamily protein | chr1:10310424-10313369 REVERSE LENGTH=770	704	770	0	109.4	90.2	95.2
Rsa1.0_00006.1.g377.t1	ref|NP_174231.2| carbohydrate-binding X8 domain-containing protein [Arabidopsis thaliana] gi|49823474|gb|AAT68720.1| hypothetical protein At1g29380 [Arabidopsis thaliana] gi|55740509|gb|AAV63847.1| hypothetical protein At1g29380 [Arabidopsis thaliana] gi|62320282|dbj|BAD94579.1| beta-1,3 glucanase [Arabidopsis thaliana] gi|332192957|gb|AEE31078.1| carbohydrate-binding X8 domain-containing protein [Arabidopsis thaliana]	325	315	2.00E-83	96.9	75.4	79.4	carbohydrate-binding X8 domain-containing protein	gbpln	Arabidopsis thaliana	AT1G29380.1 | Symbols: | Carbohydrate-binding X8 domain superfamily protein | chr1:10284209-10285671 FORWARD LENGTH=315	325	315	6.00E-86	96.9	75.4	79.4
Rsa1.0_00006.1.g378.t1	ref|NP_174229.2| uncharacterized protein [Arabidopsis thaliana] gi|332192954|gb|AEE31075.1| uncharacterized protein AT1G29350 [Arabidopsis thaliana]	865	831	0	96.1	73.2	80.3	uncharacterized protein	gbpln	Arabidopsis thaliana	AT1G29350.1 | Symbols: | Kinase-related protein of unknown function (DUF1296) | chr1:10268761-10273613 REVERSE LENGTH=831	865	831	0	96.1	73.2	80.3
Rsa1.0_00006.1.g379.t1	dbj|BAJ34586.1| unnamed protein product [Thellungiella halophila]	678	724	0	106.8	85.7	92.9	unnamed protein product	----	----	AT1G29340.1 | Symbols: PUB17, ATPUB17 | plant U-box 17 | chr1:10264412-10266601 FORWARD LENGTH=729	678	729	0	107.5	84.7	92.2
Rsa1.0_00006.1.g380.t1	ref|XP_002893557.1| hypothetical protein ARALYDRAFT_473143 [Arabidopsis lyrata subsp. lyrata] gi|297339399|gb|EFH69816.1| hypothetical protein ARALYDRAFT_473143 [Arabidopsis lyrata subsp. lyrata]	215	215	1.00E-118	100.0	95.8	99.1	hypothetical protein ARALYDRAFT_473143	gbpln	Arabidopsis lyrata	AT1G29330.1 | Symbols: ATERD2, AERD2, ERD2 | ER lumen protein retaining receptor family protein | chr1:10258580-10260906 REVERSE LENGTH=215	215	215	1.00E-119	100.0	94.4	98.1
Rsa1.0_00006.1.g381.t1	ref|NP_174226.1| WD40 domain-containing protein [Arabidopsis thaliana] gi|111074196|gb|ABH04471.1| At1g29320 [Arabidopsis thaliana] gi|332192951|gb|AEE31072.1| WD40 domain-containing protein [Arabidopsis thaliana]	444	468	0	105.4	84.2	90.5	WD40 domain-containing protein	gbpln	Arabidopsis thaliana	AT1G29320.1 | Symbols: | Transducin/WD40 repeat-like superfamily protein | chr1:10255355-10258238 FORWARD LENGTH=468	444	468	0	105.4	84.2	90.5
Rsa1.0_00006.1.g382.t1	ref|XP_002893556.1| hypothetical protein ARALYDRAFT_473140 [Arabidopsis lyrata subsp. lyrata] gi|297339398|gb|EFH69815.1| hypothetical protein ARALYDRAFT_473140 [Arabidopsis lyrata subsp. lyrata]	470	459	0	97.7	81.7	89.1	hypothetical protein ARALYDRAFT_473140	gbpln	Arabidopsis lyrata	AT1G29300.1 | Symbols: UNE1 | Plant protein of unknown function (DUF641) | chr1:10248120-10249499 REVERSE LENGTH=459	470	459	0	97.7	81.3	88.1

Rsa1.0_00006.1.g383.t1	ref|XP_002890803.1| hypothetical protein ARALYDRAFT_473139 [Arabidopsis lyrata subsp. lyrata] gi|297336645|gb|EFH67062.1| hypothetical protein ARALYDRAFT_473139 [Arabidopsis lyrata subsp. lyrata]	106	107	1.00E-40	100.9	81.1	90.6	hypothetical protein ARALYDRAFT_473139	gbpln	Arabidopsis lyrata	AT1G29290.1 | Symbols: | unknown protein; FUNCTIONS IN: molecular_function unknown; INVOLVED IN: biological_process unknown; LOCATED IN: endomembrane system; Has 18 Blast hits to 18 proteins in 5 species: Archae - 0; Bacteria - 0; Metazoa - 0; Fungi - 0; Plants - 18; Viruses - 0; Other Eukaryotes - 0 (source: NCBI BLink). | chr1:10245055-10245378 FORWARD LENGTH=107	106	107	2.00E-43	100.9	82.1	90.6
Rsa1.0_00006.1.g384.t1	gb|ACI14404.1| WRKY65-1 transcription factor [Brassica napus]	259	261	1.00E-116	100.8	85.3	88.8	WRKY65-1 transcription factor	gbpln	Brassica napus	AT1G29280.1 | Symbols: WRKY65, ATWRKY65 | WRKY DNA-binding protein 65 | chr1:10236589-10237467 FORWARD LENGTH=259	259	259	1.00E-112	100.0	84.6	89.2
Rsa1.0_00006.1.g385.t1	#	#	#	#	#	#	#	-	----	----	#	#	#	#	#	#	#
Rsa1.0_00006.1.g386.t1	ref|NP_174217.1| CBL-interacting serine/threonine-protein kinase 18 [Arabidopsis thaliana] gi|75335276|sp|Q9LP51.1|CIPKI_ARATH RecName: Full=CBL-interacting serine/threonine-protein kinase 18; AltName: Full=SNF1-related kinase 3.20; AltName: Full=SOS2-like protein kinase PKS22 gi|9502417|gb|AAF88116.1|AC021043_9 Putative protein kinase [Arabidopsis thaliana] gi|14334388|gb|AAK59695.1| CBL-interacting protein kinase 18 [Arabidopsis thaliana] gi|332192942|gb|AEE31063.1| CBL-interacting serine/threonine-protein kinase 18 [Arabidopsis thaliana]	523	520	0	99.4	88.7	93.7	CBL-interacting serine/threonine-protein kinase 18	gbpln	Arabidopsis thaliana	AT1G29230.1 | Symbols: CIPK18, SnRK3.20, ATWL1, WL1, ATCIPK18 | CBL-interacting protein kinase 18 | chr1:10214860-10216422 FORWARD LENGTH=520	523	520	0	99.4	88.7	93.7
Rsa1.0_00006.1.g387.t1	ref|XP_002890798.1| hypothetical protein ARALYDRAFT_890443 [Arabidopsis lyrata subsp. lyrata] gi|297336640|gb|EFH67057.1| hypothetical protein ARALYDRAFT_890443 [Arabidopsis lyrata subsp. lyrata]	500	574	1.00E-168	114.8	69.8	82.0	hypothetical protein ARALYDRAFT_890443	gbpln	Arabidopsis lyrata	AT1G29240.1 | Symbols: | Protein of unknown function (DUF688) | chr1:10217023-10218924 REVERSE LENGTH=577	500	577	1.00E-168	115.4	70.8	81.4
Rsa1.0_00006.1.g388.t1	ref|NP_565781.1| Alba DNA/RNA-binding protein [Arabidopsis thaliana] gi|73921087|sp|O22969.1|Y2416_ARATH RecName: Full=Uncharacterized protein At2g34160 gi|2342735|gb|AAB67633.1| expressed protein [Arabidopsis thaliana] gi|21536653|gb|AAM60985.1| unknown [Arabidopsis thaliana] gi|26450089|dbj|BAC42164.1| unknown protein [Arabidopsis thaliana] gi|111074476|gb|ABH04611.1| At2g34160 [Arabidopsis thaliana] gi|330253832|gb|AEC08926.1| Alba DNA/RNA-binding protein [Arabidopsis thaliana]	130	130	1.00E-59	100.0	89.2	93.1	Alba DNA/RNA-binding protein	gbpln	Arabidopsis thaliana	AT2G34160.1 | Symbols: | Alba DNA/RNA-binding protein | chr2:14426283-14427220 FORWARD LENGTH=130	130	130	2.00E-62	100.0	89.2	93.1
Rsa1.0_00006.1.g389.t1	gb|ABB92566.1| peroxisomal import receptor PTS2 [Brassica napus]	317	317	1.00E-178	100.0	93.7	97.8	peroxisomal import receptor PTS2	gbpln	Brassica napus	AT1G29260.1 | Symbols: PEX7, ATPEX7 | peroxin 7 | chr1:10224923-10225876 FORWARD LENGTH=317	317	317	1.00E-180	100.0	93.1	97.8
Rsa1.0_00006.1.g390.t1	gb|EOA37296.1| hypothetical protein CARUB_v10010917mg [Capsella rubella]	172	184	4.00E-68	107.0	82.0	86.0	hypothetical protein CARUB_v10010917mg	gbpln	Capsella rubella	AT1G29195.1 | Symbols: | unknown protein; FUNCTIONS IN: molecular_function unknown; INVOLVED IN: biological_process unknown; LOCATED IN: chloroplast; EXPRESSED IN: 13 plant structures; EXPRESSED DURING: 4 anthesis, C globular stage, 4 leaf senescence stage, petal differentiation and expansion stage; BEST Arabidopsis thaliana protein match is: unknown protein (TAIR:AT2G30230.1); Has 180 Blast hits to 180 proteins in 14 species: Archae - 0; Bacteria - 0; Metazoa - 0; Fungi - 0; Plants - 180; Viruses - 0; Other Eukaryotes - 0 (source: NCBI BLink). | chr1:10202673-10203254 REVERSE LENGTH=193	172	193	2.00E-69	112.2	82.6	89.0
Rsa1.0_00006.1.g391.t1	ref|NP_001184912.1| uncharacterized protein [Arabidopsis thaliana] gi|332189732|gb|AEE27853.1| uncharacterized protein AT1G05550 [Arabidopsis thaliana]	372	385	7.00E-88	103.5	45.7	58.9	uncharacterized protein	gbpln	Arabidopsis thaliana	AT1G05550.3 | Symbols: | Protein of unknown function (DUF295) | chr1:1641229-1643748 REVERSE LENGTH=385	372	385	2.00E-90	103.5	45.7	58.9

Rsa1.0_00006.1.g392.t1	ref|NP_174212.2| protein SCAR3 [Arabidopsis thaliana] gi|75175509|sp|Q9LP46.1|SCAR3_ARATH RecName: Full=Protein SCAR3; Short=AtSCAR3; AltName: Full=Protein WAVE2 gi|9502422|gb|AAF88121.1|AC021043_14 Hypothetical protein [Arabidopsis thaliana] gi|53801280|gb|AAU93850.1| SCAR3 [Arabidopsis thaliana] gi|332192931|gb|AEE31052.1| protein SCAR3 [Arabidopsis thaliana]	940	1020	0	108.5	72.4	80.7	protein SCAR3	gbpln	Arabidopsis thaliana	AT1G29170.1 | Symbols: WAVE2, ATSCAR3, SCAR3 | SCAR family protein | chr1:10190352-10194900 REVERSE LENGTH=1020	940	1020	0	108.5	72.4	80.7
Rsa1.0_00006.1.g393.t1	gb|EOA37128.1| hypothetical protein CARUB_v10010402mg [Capsella rubella]	179	182	3.00E-79	101.7	86.6	89.9	hypothetical protein CARUB_v10010402mg	gbpln	Capsella rubella	AT1G29160.1 | Symbols: | Dof-type zinc finger DNA-binding family protein | chr1:10183797-10184324 REVERSE LENGTH=175	179	175	4.00E-79	97.8	87.2	90.5
Rsa1.0_00006.1.g394.t1	ref|XP_002890794.1| hypothetical protein ARALYDRAFT_336019 [Arabidopsis lyrata subsp. lyrata] gi|297336636|gb|EFH67053.1| hypothetical protein ARALYDRAFT_336019 [Arabidopsis lyrata subsp. lyrata]	419	621	0	148.2	96.2	96.9	hypothetical protein ARALYDRAFT_336019	gbpln	Arabidopsis lyrata	AT1G29150.1 | Symbols: ATS9, RPN6 | non-ATPase subunit 9 | chr1:10181240-10182499 FORWARD LENGTH=419	419	419	0	100.0	97.6	98.6
Rsa1.0_00006.1.g395.t1	gb|EOA37644.1| hypothetical protein CARUB_v10012122mg [Capsella rubella]	171	171	6.00E-85	100.0	88.9	92.4	hypothetical protein CARUB_v10012122mg	gbpln	Capsella rubella	AT1G29140.1 | Symbols: | Pollen Ole e 1 allergen and extensin family protein | chr1:10179029-10179811 FORWARD LENGTH=171	171	171	2.00E-85	100.0	86.5	91.8
Rsa1.0_00006.1.g396.t1	gb|EOA40380.1| hypothetical protein CARUB_v10009109mg [Capsella rubella]	451	452	0	100.2	89.4	92.7	hypothetical protein CARUB_v10009109mg	gbpln	Capsella rubella	AT1G29120.2 | Symbols: | Hydrolase-like protein family | chr1:10174481-10178143 FORWARD LENGTH=455	451	455	0	100.9	86.9	91.8
Rsa1.0_00006.1.g397.t1	ref|XP_002890790.1| ribosomal protein L34 family protein [Arabidopsis lyrata subsp. lyrata] gi|297336632|gb|EFH67049.1| ribosomal protein L34 family protein [Arabidopsis lyrata subsp. lyrata]	162	159	2.00E-51	98.1	77.8	84.6	ribosomal protein L34 family protein	gbpln	Arabidopsis lyrata	AT1G29070.1 | Symbols: | Ribosomal protein L34 | chr1:10149884-10151155 FORWARD LENGTH=157	162	157	2.00E-53	96.9	80.2	87.0
Rsa1.0_00006.1.g398.t1	ref|NP_567434.1| protein transport protein SFT1 [Arabidopsis thaliana] gi|75248462|sp|Q8VXX9.1|BETL1_ARATH RecName: Full=Bet1-like protein At4g14600 gi|18389246|gb|AAL67066.1| unknown protein [Arabidopsis thaliana] gi|20259643|gb|AAM14339.1| unknown protein [Arabidopsis thaliana] gi|21554084|gb|AAM63165.1| unknown [Arabidopsis thaliana] gi|26452326|dbj|BAC43249.1| unknown protein [Arabidopsis thaliana] gi|332658064|gb|AEE83464.1| Bet1-like protein [Arabidopsis thaliana]	137	137	2.00E-70	100.0	92.7	97.1	protein transport protein SFT1	gbpln	Arabidopsis thaliana	AT4G14600.1 | Symbols: | Target SNARE coiled-coil domain protein | chr4:8376562-8378078 FORWARD LENGTH=137	137	137	6.00E-73	100.0	92.7	97.1
Rsa1.0_00006.1.g399.t1	ref|XP_002893542.1| F1K23.5 [Arabidopsis lyrata subsp. lyrata] gi|297339384|gb|EFH69801.1| F1K23.5 [Arabidopsis lyrata subsp. lyrata]	210	269	2.00E-79	128.1	79.5	85.7	F1K23.5	gbpln	Arabidopsis lyrata	AT1G28960.5 | Symbols: ATNUDT15, ATNUDX15, NUDX15 | nudix hydrolase homolog 15 | chr1:10110135-10111607 REVERSE LENGTH=293	210	293	2.00E-78	139.5	63.8	64.8
Rsa1.0_00006.1.g400.t1	ref|XP_002890784.1| predicted protein [Arabidopsis lyrata subsp. lyrata] gi|297336626|gb|EFH67043.1| predicted protein [Arabidopsis lyrata subsp. lyrata]	119	117	6.00E-42	98.3	71.4	82.4	predicted protein	gbpln	Arabidopsis lyrata	AT1G28815.1 | Symbols: | unknown protein; Has 5 Blast hits to 5 proteins in 2 species: Archae - 0; Bacteria - 0; Metazoa - 0; Fungi - 0; Plants - 5; Viruses - 0; Other Eukaryotes - 0 (source: NCBI BLink). | chr1:10095812-10096244 FORWARD LENGTH=115	119	115	8.00E-40	96.6	63.9	79.0
Rsa1.0_00006.1.g401.t1	ref|NP_174190.2| pentatricopeptide repeat-containing protein [Arabidopsis thaliana] gi|193806502|sp|Q1PFQ9.2|PPR62_ARATH RecName: Full=Pentatricopeptide repeat-containing protein At1g28690, mitochondrial; Flags: Precursor gi|332192893|gb|AEE31014.1| pentatricopeptide repeat-containing protein [Arabidopsis thaliana]	504	520	0	103.2	81.3	90.5	pentatricopeptide repeat-containing protein	gbpln	Arabidopsis thaliana	AT1G28690.1 | Symbols: | Tetratricopeptide repeat (TPR)-like superfamily protein | chr1:10080042-10081604 REVERSE LENGTH=520	504	520	0	103.2	81.3	90.5
Rsa1.0_00006.1.g402.t4	gb|AAX62802.1| lipase 2 [Brassica napus]	241	389	5.00E-98	161.4	72.2	80.9	lipase 2	gbpln	Brassica napus	AT1G28670.1 | Symbols: ARAB-1 | GDSL-like Lipase/Acylhydrolase superfamily protein | chr1:10074669-10076250 REVERSE LENGTH=384	241	384	6.00E-94	159.3	68.5	78.0
Rsa1.0_00006.1.g403.t1	gb|AAC34342.1| Hypothetical protein [Arabidopsis thaliana]	355	374	7.00E-36	105.4	26.2	38.0	Hypothetical protein	gbpln	Arabidopsis thaliana	AT1G10000.1 | Symbols: | Ribonuclease H-like superfamily protein | chr1:3263877-3264788 REVERSE LENGTH=303	355	303	2.00E-38	85.4	26.2	38.0
Rsa1.0_00006.1.g404.t1	#	#	#	#	#	#	#	-	----	----	#	#	#	#	#	#	#

Rsa1.0_00006.1.g405.t1	gb|ABD64987.1| hypothetical protein 26.t00003 [Brassica oleracea]	589	330	6.00E-15	56.0	9.8	11.4	hypothetical protein 26.t00003	gbpln	Brassica oleracea	#	#	#	#	#	#	#
Rsa1.0_00006.1.g406.t2	gb|AAF16748.1|AC010155_1 F3M18.1 [Arabidopsis thaliana] gi|6691212|gb|AAF24550.1|AC007508_13 F1K23.20 [Arabidopsis thaliana]	690	482	1.00E-167	69.9	44.1	51.9	F3M18.1	gbpln	Arabidopsis thaliana	AT1G28560.1 | Symbols: SRD2 | snRNA activating complex family protein | chr1:10038197-10040382 REVERSE LENGTH=375	690	375	1.00E-140	54.3	36.5	42.9
Rsa1.0_00006.1.g407.t1	ref|XP_002890782.1| hypothetical protein ARALYDRAFT_473075 [Arabidopsis lyrata subsp. lyrata] gi|297336624|gb|EFH67041.1| hypothetical protein ARALYDRAFT_473075 [Arabidopsis lyrata subsp. lyrata]	115	104	1.00E-38	90.4	74.8	81.7	hypothetical protein ARALYDRAFT_473075	gbpln	Arabidopsis lyrata	AT1G28540.1 | Symbols: | unknown protein; Has 25 Blast hits to 25 proteins in 10 species: Archae - 0; Bacteria - 0; Metazoa - 0; Fungi - 0; Plants - 25; Viruses - 0; Other Eukaryotes - 0 (source: NCBI BLink). | chr1:10035696-10036007 FORWARD LENGTH=103	115	103	1.00E-40	89.6	74.8	83.5
Rsa1.0_00006.1.g408.t1	gb|EOA37571.1| hypothetical protein CARUB_v10011901mg [Capsella rubella]	609	609	0	100.0	85.1	92.8	hypothetical protein CARUB_v10011901mg	gbpln	Capsella rubella	AT1G28530.2 | Symbols: | unknown protein; INVOLVED IN: biological_process unknown; LOCATED IN: chloroplast; EXPRESSED IN: 19 plant structures; EXPRESSED DURING: 10 growth stages; Has 20 Blast hits to 20 proteins in 6 species: Archae - 0; Bacteria - 0; Metazoa - 0; Fungi - 0; Plants - 20; Viruses - 0; Other Eukaryotes - 0 (source: NCBI BLink). | chr1:10032127-10035496 REVERSE LENGTH=613	609	613	0	100.7	84.1	91.6
Rsa1.0_00006.1.g409.t2	gb|EOA36887.1| hypothetical protein CARUB_v10008982mg [Capsella rubella]	476	486	0	102.1	85.1	88.2	hypothetical protein CARUB_v10008982mg	gbpln	Capsella rubella	AT1G28520.2 | Symbols: VOZ1 | vascular plant one zinc finger protein | chr1:10029713-10031479 FORWARD LENGTH=486	476	486	0	102.1	84.2	87.8
Rsa1.0_00006.1.g410.t6	ref|NP_564311.1| Optic atrophy 3 protein (OPA3) [Arabidopsis thaliana] gi|6560770|gb|AAF16770.1|AC010155_23 F3M18.5 [Arabidopsis thaliana] gi|15724222|gb|AAL06504.1|AF412051_1 At1g28510/F3M18_5 [Arabidopsis thaliana] gi|18252247|gb|AAL62004.1| At1g28510/F3M18_5 [Arabidopsis thaliana] gi|21553821|gb|AAM62914.1| unknown [Arabidopsis thaliana] gi|332192864|gb|AEE30985.1| Optic atrophy 3 protein (OPA3) [Arabidopsis thaliana]	183	171	6.00E-77	93.4	78.7	88.0	Optic atrophy 3 protein (OPA3)	gbpln	Arabidopsis thaliana	AT1G28510.1 | Symbols: | Optic atrophy 3 protein (OPA3) | chr1:10024036-10025939 FORWARD LENGTH=171	183	171	2.00E-79	93.4	78.7	88.0
Rsa1.0_00006.1.g411.t1	ref|XP_002890776.1| hypothetical protein ARALYDRAFT_473063 [Arabidopsis lyrata subsp. lyrata] gi|297336618|gb|EFH67035.1| hypothetical protein ARALYDRAFT_473063 [Arabidopsis lyrata subsp. lyrata]	994	996	0	100.2	83.6	91.3	hypothetical protein ARALYDRAFT_473063	gbpln	Arabidopsis lyrata	AT1G28440.1 | Symbols: HSL1 | HAESA-like 1 | chr1:9996914-10000171 FORWARD LENGTH=996	994	996	0	100.2	83.0	91.6
Rsa1.0_00006.1.g412.t1	ref|XP_002890775.1| HB-1 [Arabidopsis lyrata subsp. lyrata] gi|297336617|gb|EFH67034.1| HB-1 [Arabidopsis lyrata subsp. lyrata]	1572	1705	0	108.5	81.7	88.5	HB-1	gbpln	Arabidopsis lyrata	AT1G28420.1 | Symbols: HB-1 | homeobox-1 | chr1:9979936-9987460 FORWARD LENGTH=1705	1572	1705	0	108.5	80.7	87.3
Rsa1.0_00006.1.g413.t1	ref|XP_002893510.1| hypothetical protein ARALYDRAFT_890356 [Arabidopsis lyrata subsp. lyrata] gi|297339352|gb|EFH69769.1| hypothetical protein ARALYDRAFT_890356 [Arabidopsis lyrata subsp. lyrata]	130	260	3.00E-37	200.0	65.4	69.2	hypothetical protein ARALYDRAFT_890356	gbpln	Arabidopsis lyrata	AT1G28200.1 | Symbols: FIP1 | FH interacting protein 1 | chr1:9850395-9852300 REVERSE LENGTH=259	130	259	6.00E-37	199.2	62.3	66.9

Rsa1.0_00007.1.g414.t1	sp|Q43133.1|GGPPS_SINAL RecName: Full=Geranylgeranyl pyrophosphate synthase, chloroplastic/chromoplastic; Short=GGPP synthase; Short=GGPS; AltName: Full=(2E,6E)-farnesyl diphosphate synthase; AltName: Full=Dimethylallyltranstransferase; AltName: Full=Farnesyl diphosphate synthase; AltName: Full=Farnesyltranstransferase; AltName: Full=Geranyltranstransferase; Flags: Precursor gi|1419758|emb|CAA67330.1| geranylgeranyl pyrophosphate synthase [Sinapis alba]	361	366	1.00E-156	101.4	80.1	89.2	RecName: Full=Geranylgeranyl pyrophosphate synthase, chloroplastic/chromoplastic; Short=GGPP synthase; Short=GGPS; AltName: Full=(2E,6E)-farnesyl diphosphate synthase; AltName: Full=Dimethylallyltransferase; AltName: Full=Farnesyl diphosphate synthase; AltName: Full=Farnesyltranstransferase; AltName: Full=Geranyltranstransferase; Flags: Precursor gi|1419758|emb|CAA67330.1| geranylgeranyl pyrophosphate synthase	gbpln	Sinapis alba	AT4G36810.1 | Symbols: GGPS1 | geranylgeranyl pyrophosphate synthase 1 | chr4:17343513-17344628 FORWARD LENGTH=371	361	371	1.00E-156	102.8	79.2	86.7
Rsa1.0_00007.1.g415.t1	gb|ACI16426.1| RUB1-conjugation enzyme [Brassica juncea]	185	185	1.00E-104	100.0	97.3	98.9	RUB1-conjugation enzyme	gbpln	Brassica juncea	AT4G36800.2 | Symbols: RCE1 | RUB1 conjugating enzyme 1 | chr4:17341237-17342148 REVERSE LENGTH=184	185	184	1.00E-105	99.5	97.3	98.9
Rsa1.0_00007.1.g416.t1	dbj|BAJ34484.1| unnamed protein product [Thellungiella halophila]	640	645	0	100.8	94.5	97.5	unnamed protein product	----	----	AT4G36760.1 | Symbols: ATAPP1, APP1 | aminopeptidase P1 | chr4:17326688-17329979 FORWARD LENGTH=645	640	645	0	100.8	92.2	96.3
Rsa1.0_00007.1.g417.t1	ref|XP_002866994.1| homeobox-leucine zipper protein ATHB-40 [Arabidopsis lyrata subsp. lyrata] gi|297312830|gb|EFH43253.1| homeobox-leucine zipper protein ATHB-40 [Arabidopsis lyrata subsp. lyrata]	221	217	1.00E-91	98.2	78.7	87.8	homeobox-leucine zipper protein ATHB-40	gbpln	Arabidopsis lyrata	AT4G36740.1 | Symbols: HB-5, ATHB40, HB40 | homeobox protein 40 | chr4:17314649-17316314 REVERSE LENGTH=216	221	216	5.00E-94	97.7	77.8	86.9
Rsa1.0_00007.1.g418.t1	#	#	#	#	#	#	#	-	----	----	#	#	#	#	#	#	#
Rsa1.0_00007.1.g419.t4	emb|CAA58774.1| G-box binding factor 1A [Brassica napus]	319	313	1.00E-144	98.1	84.6	87.1	G-box binding factor 1A	gbpln	Brassica napus	AT4G36730.1 | Symbols: GBF1 | G-box binding factor 1 | chr4:17309850-17311752 REVERSE LENGTH=315	319	315	1.00E-141	98.7	83.1	88.1
Rsa1.0_00007.1.g420.t1	ref|XP_002869026.1| hypothetical protein ARALYDRAFT_912692 [Arabidopsis lyrata subsp. lyrata] gi|297314862|gb|EFH45285.1| hypothetical protein ARALYDRAFT_912692 [Arabidopsis lyrata subsp. lyrata]	207	205	7.00E-89	99.0	76.8	88.9	hypothetical protein ARALYDRAFT_912692	gbpln	Arabidopsis lyrata	AT4G36720.1 | Symbols: HVA22K | HVA22-like protein K | chr4:17307769-17309668 FORWARD LENGTH=200	207	200	1.00E-85	96.6	72.9	84.5
Rsa1.0_00007.1.g421.t1	ref|NP_195388.2| cupin family protein [Arabidopsis thaliana] gi|332661291|gb|AEE86691.1| cupin family protein [Arabidopsis thaliana]	579	522	0	90.2	60.4	68.0	cupin family protein	gbpln	Arabidopsis thaliana	AT4G36700.1 | Symbols: | RmlC-like cupins superfamily protein | chr4:17298443-17300337 REVERSE LENGTH=522	579	522	0	90.2	60.4	68.0
Rsa1.0_00007.1.g422.t4	ref|XP_002866997.1| hypothetical protein ARALYDRAFT_490965 [Arabidopsis lyrata subsp. lyrata] gi|297312833|gb|EFH43256.1| hypothetical protein ARALYDRAFT_490965 [Arabidopsis lyrata subsp. lyrata]	560	567	0	101.3	79.5	86.1	hypothetical protein ARALYDRAFT_490965	gbpln	Arabidopsis lyrata	AT4G36690.4 | Symbols: ATU2AF65A | U2 snRNP auxilliary factor, large subunit, splicing factor | chr4:17294139-17297609 REVERSE LENGTH=551	560	551	0	98.4	79.5	85.5
Rsa1.0_00007.1.g423.t1	ref|XP_002866998.1| pentatricopeptide repeat-containing protein [Arabidopsis lyrata subsp. lyrata] gi|297312834|gb|EFH43257.1| pentatricopeptide repeat-containing protein [Arabidopsis lyrata subsp. lyrata]	407	411	0	101.0	76.9	89.4	pentatricopeptide repeat-containing protein	gbpln	Arabidopsis lyrata	AT4G36680.1 | Symbols: | Tetratricopeptide repeat (TPR)-like superfamily protein | chr4:17292479-17293717 REVERSE LENGTH=412	407	412	0	101.2	75.7	88.9
Rsa1.0_00007.1.g424.t1	gb|EOA16170.1| hypothetical protein CARUB_v10004306mg [Capsella rubella]	665	668	0	100.5	82.4	90.5	hypothetical protein CARUB_v10004306mg	gbpln	Capsella rubella	AT4G38030.1 | Symbols: | Rhamnogalacturonate lyase family protein | chr4:17863206-17866730 REVERSE LENGTH=667	665	667	0	100.3	81.7	89.5
Rsa1.0_00007.1.g425.t1	gb|EOA18172.1| hypothetical protein CARUB_v10006648mg [Capsella rubella]	508	493	0	97.0	87.8	93.1	hypothetical protein CARUB_v10006648mg	gbpln	Capsella rubella	AT4G36670.1 | Symbols: | Major facilitator superfamily protein | chr4:17287680-17289483 REVERSE LENGTH=493	508	493	0	97.0	86.6	92.5
Rsa1.0_00007.1.g426.t1	gb|EOA18681.1| hypothetical protein CARUB_v10007257mg [Capsella rubella]	293	293	1.00E-159	100.0	90.4	94.9	hypothetical protein CARUB_v10007257mg	gbpln	Capsella rubella	AT4G36640.1 | Symbols: | Sec14p-like phosphatidylinositol transfer family protein | chr4:17277187-17278447 REVERSE LENGTH=294	293	294	1.00E-153	100.3	85.3	93.2

Rsa1.0_00007.1.g427.t2	ref|NP_195381.6| Vacuolar sorting protein 39 [Arabidopsis thaliana] gi|20466826|gb|AAM20730.1| unknown protein [Arabidopsis thaliana] gi|332661279|gb|AEE86679.1| Vacuolar sorting protein 39 [Arabidopsis thaliana]	565	1000	0	177.0	71.9	81.2	Vacuolar sorting protein 39	gbpln	Arabidopsis thaliana	AT4G36630.1 | Symbols: EMB2754 | Vacuolar sorting protein 39 | chr4:17272088-17276524 REVERSE LENGTH=1000	565	1000	0	177.0	71.9	81.2
Rsa1.0_00007.1.g428.t1	gb|ADP95696.1| class III heme peroxidase [Brassica rapa]	323	296	1.00E-119	91.6	65.0	74.0	class III heme peroxidase	gbpln	Brassica rapa	AT4G36430.1 | Symbols: | Peroxidase superfamily protein | chr4:17204648-17205917 REVERSE LENGTH=331	323	331	1.00E-116	102.5	63.2	74.3
Rsa1.0_00007.1.g429.t1	ref|XP_002886383.1| hypothetical protein ARALYDRAFT_474974 [Arabidopsis lyrata subsp. lyrata] gi|297332224|gb|EFH62642.1| hypothetical protein ARALYDRAFT_474974 [Arabidopsis lyrata subsp. lyrata]	1034	1049	0	101.5	87.3	92.6	hypothetical protein ARALYDRAFT_474974	gbpln	Arabidopsis lyrata	AT1G63810.1 | Symbols: | CONTAINS InterPro DOMAIN/s: Nrap protein (InterPro:IPR005554); Has 396 Blast hits to 382 proteins in 182 species: Archae - 3; Bacteria - 2; Metazoa - 142; Fungi - 146; Plants - 43; Viruses - 0; Other Eukaryotes - 60 (source: NCBI BLink). | chr1:23669690-23675542 REVERSE LENGTH=1053	1034	1053	0	101.8	86.2	92.2
Rsa1.0_00007.1.g430.t1	gb|EOA18974.1| hypothetical protein CARUB_v10007612mg [Capsella rubella]	216	212	4.00E-86	98.1	80.6	87.0	hypothetical protein CARUB_v10007612mg	gbpln	Capsella rubella	AT4G36620.1 | Symbols: GATA19 | GATA transcription factor 19 | chr4:17268906-17269662 REVERSE LENGTH=211	216	211	1.00E-81	97.7	82.9	88.4
Rsa1.0_00007.1.g431.t1	gb|EOA32387.1| hypothetical protein CARUB_v10015657mg [Capsella rubella]	294	313	8.00E-99	106.5	59.5	79.9	hypothetical protein CARUB_v10015657mg	gbpln	Capsella rubella	AT4G36610.1 | Symbols: | alpha/beta-Hydrolases superfamily protein | chr4:17265545-17267274 REVERSE LENGTH=317	294	317	1.00E-100	107.8	60.2	79.3
Rsa1.0_00007.1.g432.t1	gb|EOA18452.1| hypothetical protein CARUB_v10006996mg [Capsella rubella]	456	465	1.00E-128	102.0	65.1	75.0	hypothetical protein CARUB_v10006996mg	gbpln	Capsella rubella	AT4G36600.1 | Symbols: | Late embryogenesis abundant (LEA) protein | chr4:17263666-17264968 FORWARD LENGTH=353	456	353	2.00E-94	77.4	52.2	59.9
Rsa1.0_00007.1.g433.t1	gb|EOA15655.1| hypothetical protein CARUB_v10006084mg, partial [Capsella rubella]	79	105	3.00E-37	132.9	94.9	96.2	hypothetical protein CARUB_v10006084mg, partial	gbpln	Capsella rubella	AT1G75250.1 | Symbols: ATRL6, RSM3, RL6 | RAD-like 6 | chr1:28245073-28245453 REVERSE LENGTH=126	79	126	2.00E-27	159.5	62.0	82.3
Rsa1.0_00007.1.g434.t1	#	#	#	#	#	#	#	-	----	----	#	#	#	#	#	#	#
Rsa1.0_00007.1.g435.t1	ref|XP_002867008.1| U-box domain-containing protein 5 [Arabidopsis lyrata subsp. lyrata] gi|297312844|gb|EFH43267.1| U-box domain-containing protein 5 [Arabidopsis lyrata subsp. lyrata]	748	751	0	100.4	69.7	80.2	U-box domain-containing protein 5	gbpln	Arabidopsis lyrata	AT4G36550.1 | Symbols: | ARM repeat superfamily protein | chr4:17245400-17247926 REVERSE LENGTH=718	748	718	0	96.0	69.1	79.0
Rsa1.0_00007.1.g436.t4	gb|EOA17086.1| hypothetical protein CARUB_v10005334mg [Capsella rubella]	299	304	1.00E-138	101.7	82.3	89.6	hypothetical protein CARUB_v10005334mg	gbpln	Capsella rubella	AT4G36540.1 | Symbols: BEE2 | BR enhanced expression 2 | chr4:17243699-17244965 FORWARD LENGTH=304	299	304	1.00E-139	101.7	81.6	87.6
Rsa1.0_00007.1.g437.t1	#	#	#	#	#	#	#	-	----	----	#	#	#	#	#	#	#
Rsa1.0_00007.1.g438.t1	ref|NP_195368.1| uncharacterized protein [Arabidopsis thaliana] gi|2464932|emb|CAB16811.1| putative protein [Arabidopsis thaliana] gi|7270598|emb|CAB80316.1| putative protein [Arabidopsis thaliana] gi|28393196|gb|AAO42028.1| unknown protein [Arabidopsis thaliana] gi|28827518|gb|AAO50603.1| unknown protein [Arabidopsis thaliana] gi|332661263|gb|AEE86663.1| uncharacterized protein AT4G36500 [Arabidopsis thaliana]	428	122	7.00E-56	28.5	27.3	27.6	uncharacterized protein	gbpln	Arabidopsis thaliana	AT4G36500.1 | Symbols: | unknown protein; FUNCTIONS IN: molecular_function unknown; INVOLVED IN: biological_process unknown; LOCATED IN: mitochondrion; EXPRESSED IN: 22 plant structures; EXPRESSED DURING: 13 growth stages; BEST Arabidopsis thaliana protein match is: unknown protein (TAIR:AT2G18210.1); Has 50 Blast hits to 50 proteins in 7 species: Archae - 0; Bacteria - 0; Metazoa - 0; Fungi - 0; Plants - 50; Viruses - 0; Other Eukaryotes - 0 (source: NCBI BLink). | chr4:17226139-17226507 REVERSE LENGTH=122	428	122	2.00E-58	28.5	27.3	27.6
Rsa1.0_00007.1.g439.t1	ref|NP_195365.1| S-adenosyl-L-methionine-dependent methyltransferase-like protein [Arabidopsis thaliana] gi|4006915|emb|CAB16845.1| hypothetical protein [Arabidopsis thaliana] gi|7270595|emb|CAB80313.1| hypothetical protein [Arabidopsis thaliana] gi|48427662|gb|AAT42380.1| At4g36470 [Arabidopsis thaliana] gi|110741629|dbj|BAE98762.1| hypothetical protein [Arabidopsis thaliana] gi|332661259|gb|AEE86659.1| S-adenosyl-L-methionine-dependent methyltransferase-like protein [Arabidopsis thaliana]	400	371	1.00E-167	92.8	72.0	79.8	S-adenosyl-L-methionine-dependent methyltransferase-like protein	gbpln	Arabidopsis thaliana	AT4G36470.1 | Symbols: | S-adenosyl-L-methionine-dependent methyltransferases superfamily protein | chr4:17215128-17216475 REVERSE LENGTH=371	400	371	1.00E-170	92.8	72.0	79.8

Rsa1.0_00007.1.g440.t1	ref|NP_176033.1| purple acid phosphatase 6 [Arabidopsis thaliana] gi|75268138|sp|Q9C510.1|PPA6_ARATH RecName: Full=Purple acid phosphatase 6; Flags: Precursor gi|12321764|gb|AAG50924.1|AC069159_25 purple acid phosphatase, putative [Arabidopsis thaliana] gi|12323034|gb|AAG51511.1|AC058785_14 purple acid phosphatase, putative [Arabidopsis thaliana] gi|91805975|gb|ABE65716.1| calcineurin-like phosphoesterase family protein [Arabidopsis thaliana] gi|111074186|gb|ABH04466.1| At1g56360 [Arabidopsis thaliana] gi|332195262|gb|AEE33383.1| purple acid phosphatase 6 [Arabidopsis thaliana]	463	466	0	100.6	82.9	90.3	purple acid phosphatase 6	gbpln	Arabidopsis thaliana	AT1G56360.1 | Symbols: PAP6, ATPAP6 | purple acid phosphatase 6 | chr1:21098603-21100842 REVERSE LENGTH=466	463	466	0	100.6	82.9	90.3
Rsa1.0_00007.1.g441.t1	ref|XP_002869039.1| ALDH3F1 [Arabidopsis lyrata subsp. lyrata] gi|297314875|gb|EFH45298.1| ALDH3F1 [Arabidopsis lyrata subsp. lyrata]	488	484	0	99.2	86.5	94.7	ALDH3F1	gbpln	Arabidopsis lyrata	AT4G36250.1 | Symbols: ALDH3F1 | aldehyde dehydrogenase 3F1 | chr4:17151029-17153381 FORWARD LENGTH=484	488	484	0	99.2	85.9	94.3
Rsa1.0_00007.1.g442.t1	gb|ACP30631.1| disease resistance protein [Brassica rapa subsp. pekinensis]	2009	1241	0	61.8	38.6	45.6	disease resistance protein	gbpln	Brassica rapa	AT4G36150.1 | Symbols: | Disease resistance protein (TIR-NBS-LRR class) family | chr4:17104776-17108711 FORWARD LENGTH=1179	2009	1179	0	58.7	38.9	45.2
Rsa1.0_00007.1.g443.t1	gb|ABD65084.1| hypothetical protein 27.t00096 [Brassica oleracea]	158	645	5.00E-28	408.2	38.6	45.6	hypothetical protein 27.t00096	gbpln	Brassica oleracea	#	#	#	#	#	#	#
Rsa1.0_00007.1.g444.t2	gb|EOA23804.1| hypothetical protein CARUB_v10017017mg, partial [Capsella rubella]	1053	521	3.00E-49	49.5	14.4	22.7	hypothetical protein CARUB_v10017017mg, partial	gbpln	Capsella rubella	AT3G31430.1 | Symbols: | unknown protein; FUNCTIONS IN: molecular_function unknown; INVOLVED IN: biological_process unknown; LOCATED IN: endomembrane system; BEST Arabidopsis thaliana protein match is: unknown protein (TAIR:AT5G18636.1); Has 295 Blast hits to 291 proteins in 12 species: Archae - 0; Bacteria - 0; Metazoa - 0; Fungi - 0; Plants - 295; Viruses - 0; Other Eukaryotes - 0 (source: NCBI BLink). | chr3:12795861-12796871 REVERSE LENGTH=336	1053	336	1.00E-14	31.9	5.0	7.8
Rsa1.0_00007.1.g445.t1	#	#	#	#	#	#	#	–	----	----	#	#	#	#	#	#	#
Rsa1.0_00007.1.g446.t1	gb|EOA15885.1| hypothetical protein CARUB_v10003971mg [Capsella rubella]	1622	1947	0	120.0	53.1	62.0	hypothetical protein CARUB_v10003971mg	gbpln	Capsella rubella	AT4G36140.1 | Symbols: | disease resistance protein (TIR-NBS-LRR class), putative | chr4:17098956-17104479 REVERSE LENGTH=1607	1622	1607	0	99.1	47.4	54.8
Rsa1.0_00007.1.g447.t1	gb|ACP30632.1| disease resistance protein [Brassica rapa subsp. pekinensis]	1027	1238	0	120.5	58.9	65.5	disease resistance protein	gbpln	Brassica rapa	AT4G36140.1 | Symbols: | disease resistance protein (TIR-NBS-LRR class), putative | chr4:17098956-17104479 REVERSE LENGTH=1607	1027	1607	0	156.5	54.6	63.1
Rsa1.0_00007.1.g448.t1	gb|ACP30631.1| disease resistance protein [Brassica rapa subsp. pekinensis]	1268	1241	0	97.9	64.9	74.1	disease resistance protein	gbpln	Brassica rapa	AT4G36150.1 | Symbols: | Disease resistance protein (TIR-NBS-LRR class) family | chr4:17104776-17108711 FORWARD LENGTH=1179	1268	1179	0	93.0	61.0	71.4
Rsa1.0_00007.1.g449.t1	gb|EOA15885.1| hypothetical protein CARUB_v10003971mg [Capsella rubella]	598	1947	1.00E-148	325.6	51.8	65.9	hypothetical protein CARUB_v10003971mg	gbpln	Capsella rubella	AT3G44670.2 | Symbols: | Disease resistance protein (TIR-NBS-LRR class) family | chr3:16217242-16221425 FORWARD LENGTH=1219	598	1219	1.00E-127	203.8	39.5	51.3
Rsa1.0_00007.1.g450.t1	gb|EOA15884.1| hypothetical protein CARUB_v10003971mg [Capsella rubella]	1377	1946	0	141.3	33.0	39.0	hypothetical protein CARUB_v10003971mg	gbpln	Capsella rubella	AT4G36140.1 | Symbols: | disease resistance protein (TIR-NBS-LRR class), putative | chr4:17098956-17104479 REVERSE LENGTH=1607	1377	1607	0	116.7	33.0	39.1
Rsa1.0_00007.1.g451.t1	ref|XP_002869043.1| 60S ribosomal protein L8 [Arabidopsis lyrata subsp. lyrata] gi|297314879|gb|EFH45302.1| 60S ribosomal protein L8 [Arabidopsis lyrata subsp. lyrata]	258	258	1.00E-145	100.0	98.1	99.6	60S ribosomal protein L8	gbpln	Arabidopsis lyrata	AT4G36130.1 | Symbols: | Ribosomal protein L2 family | chr4:17097613-17098656 FORWARD LENGTH=258	258	258	1.00E-147	100.0	97.3	99.6
Rsa1.0_00007.1.g452.t1	gb|AAM65223.1| unknown [Arabidopsis thaliana]	349	342	1.00E-133	98.0	80.2	85.1	unknown	gbpln	Arabidopsis thaliana	AT4G24060.1 | Symbols: | Dof-type zinc finger DNA-binding family protein | chr4:12503970-12505417 FORWARD LENGTH=342	349	342	1.00E-131	98.0	80.2	85.7
Rsa1.0_00007.1.g453.t2	ref|XP_002869754.1| UDP-D-glucose/UDP-D-galactose 4-epimerase 2 [Arabidopsis lyrata subsp. lyrata] gi|297315590|gb|EFH46013.1| UDP-D-glucose/UDP-D-galactose 4-epimerase 2 [Arabidopsis lyrata subsp. lyrata]	230	350	7.00E-57	152.2	56.1	63.5	UDP-D-glucose/UDP-D-galactose 4-epimerase 2	gbpln	Arabidopsis lyrata	AT4G23920.1 | Symbols: UGE2, ATUGE2 | UDP-D-glucose/UDP-D-galactose 4-epimerase 2 | chr4:12431416-12433666 FORWARD LENGTH=350	230	350	2.00E-59	152.2	55.7	63.9

Rsa1.0_00007.1.g454.t1	ref|XP_002867686.1| hypothetical protein ARALYDRAFT_492479 [Arabidopsis lyrata subsp. lyrata] gi|297313522|gb|EFH43945.1| hypothetical protein ARALYDRAFT_492479 [Arabidopsis lyrata subsp. lyrata]		194	196	6.00E-72	101.0	75.8	83.0	hypothetical protein ARALYDRAFT_492479	gbpln	Arabidopsis lyrata	AT4G23880.1 | Symbols: | unknown protein; Has 73 Blast hits to 69 proteins in 22 species: Archae - 0; Bacteria - 4; Metazoa - 9; Fungi - 2; Plants - 18; Viruses - 0; Other Eukaryotes - 40 (source: NCBI BLink). | chr4:12415789-12416388 REVERSE LENGTH=199		194	199	2.00E-70	102.6	75.3	83.0
Rsa1.0_00007.1.g455.t1	#	#	#	#	#	#	#		-	----	----	AT4G23770.1 | Symbols: | unknown protein; BEST Arabidopsis thaliana protein match is: unknown protein (TAIR:AT4G23780.1); Has 17 Blast hits to 17 proteins in 6 species: Archae - 0; Bacteria - 2; Metazoa - 0; Fungi - 0; Plants - 13; Viruses - 0; Other Eukaryotes - 2 (source: NCBI BLink). | chr4:12383514-12384050 REVERSE LENGTH=138		132	138	2.00E-12	104.5	37.1	53.0
Rsa1.0_00007.1.g456.t1	#	#	#	#	#	#	#		-	----	----	#	#	#	#	#	#	#	
Rsa1.0_00007.1.g457.t1	#	#	#	#	#	#	#		-	----	----	#	#	#	#	#	#	#	
Rsa1.0_00008.1.g458.t1	gb|EOA30677.1| hypothetical protein CARUB_v10013815mg [Capsella rubella]		203	415	7.00E-78	204.4	73.9	83.7	hypothetical protein CARUB_v10013815mg	gbpln	Capsella rubella	AT3G02340.1 | Symbols: | RING/U-box superfamily protein | chr3:477032-478261 FORWARD LENGTH=409		203	409	1.00E-67	201.5	67.5	78.8
Rsa1.0_00008.1.g459.t1	gb|EOA30007.1| hypothetical protein CARUB_v10013111mg [Capsella rubella]		226	690	2.00E-16	305.3	21.7	23.5	hypothetical protein CARUB_v10013111mg	gbpln	Capsella rubella	AT3G18480.1 | Symbols: AtCASP, CASP | CCAAT-displacement protein alternatively spliced product | chr3:6336924-6341596 FORWARD LENGTH=689		226	689	1.00E-18	304.9	21.7	23.5
Rsa1.0_00008.1.g460.t1	ref|XP_002882224.1| hypothetical protein ARALYDRAFT_477468 [Arabidopsis lyrata subsp. lyrata] gi|297328064|gb|EFH58483.1| hypothetical protein ARALYDRAFT_477468 [Arabidopsis lyrata subsp. lyrata]		544	561	0	103.1	91.4	94.7	hypothetical protein ARALYDRAFT_477468	gbpln	Arabidopsis lyrata	AT3G02350.1 | Symbols: GAUT9 | galacturonosyltransferase 9 | chr3:479248-481178 FORWARD LENGTH=561		544	561	0	103.1	90.8	94.5
Rsa1.0_00008.1.g461.t1	ref|NP_187562.1| RNase H domain-containing protein [Arabidopsis thaliana] gi|6682231|gb|AAF23283.1|AC016661_8 putative non-LTR reverse transcriptase [Arabidopsis thaliana] gi|332641254|gb|AEE74775.1| RNase H domain-containing protein [Arabidopsis thaliana]		343	484	4.00E-62	141.1	35.6	50.1	RNase H domain-containing protein	gbpln	Arabidopsis thaliana	AT3G09510.1 | Symbols: | Ribonuclease H-like superfamily protein | chr3:2921804-2923258 FORWARD LENGTH=484		343	484	1.00E-64	141.1	35.6	50.1
Rsa1.0_00008.1.g462.t1	ref|XP_002863143.1| hypothetical protein ARALYDRAFT_332990 [Arabidopsis lyrata subsp. lyrata] gi|297308977|gb|EFH39402.1| hypothetical protein ARALYDRAFT_332990 [Arabidopsis lyrata subsp. lyrata]		416	428	0	102.9	78.8	89.4	hypothetical protein ARALYDRAFT_332990	gbpln	Arabidopsis lyrata	AT4G13330.1 | Symbols: | S-adenosyl-L-methionine-dependent methyltransferases superfamily protein | chr4:7754248-7755837 REVERSE LENGTH=428		416	428	0	102.9	77.9	89.4
Rsa1.0_00008.1.g463.t1	gb|EOA32415.1| hypothetical protein CARUB_v10015687mg [Capsella rubella]		437	644	1.00E-100	147.4	53.8	67.0	hypothetical protein CARUB_v10015687mg	gbpln	Capsella rubella	AT4G14490.1 | Symbols: | SMAD/FHA domain-containing protein | chr4:8332414-8333574 REVERSE LENGTH=386		437	386	5.00E-67	88.3	41.4	56.3
Rsa1.0_00008.1.g464.t3	ref|XP_002884318.1| hypothetical protein ARALYDRAFT_896208 [Arabidopsis lyrata subsp. lyrata] gi|297330158|gb|EFH60577.1| hypothetical protein ARALYDRAFT_896208 [Arabidopsis lyrata subsp. lyrata]		496	344	1.00E-141	69.4	52.4	55.8	hypothetical protein ARALYDRAFT_896208	gbpln	Arabidopsis lyrata	AT3G02420.1 | Symbols: | unknown protein; FUNCTIONS IN: molecular_function unknown; INVOLVED IN: biological_process unknown; LOCATED IN: membrane; EXPRESSED IN: 25 plant structures; EXPRESSED DURING: 15 growth stages; CONTAINS InterPro DOMAIN/s: Uncharacterised protein family UPF0121 (InterPro:IPR005344); Has 72 Blast hits to 71 proteins in 25 species: Archae - 0; Bacteria - 0; Metazoa - 2; Fungi - 2; Plants - 60; Viruses - 0; Other Eukaryotes - 8 (source: NCBI BLink). | chr3:496179-498772 REVERSE LENGTH=348		496	348	1.00E-141	70.2	52.6	56.0
Rsa1.0_00008.1.g465.t1	ref|NP_186892.1| uncharacterized protein [Arabidopsis thaliana] gi|6957706|gb|AAF32450.1| hypothetical protein [Arabidopsis thaliana] gi|49660133|gb|AAT68357.1| hypothetical protein At3g02430 [Arabidopsis thaliana] gi|50058917|gb|AAT69203.1| hypothetical protein At3g02430 [Arabidopsis thaliana] gi|332640286|gb|AEE73807.1| uncharacterized protein AT3G02430 [Arabidopsis thaliana]		194	219	3.00E-92	112.9	85.6	91.2	uncharacterized protein	gbpln	Arabidopsis thaliana	AT3G02430.1 | Symbols: | Protein of unknown function (DUF679) | chr3:499360-500019 FORWARD LENGTH=219		194	219	1.00E-94	112.9	85.6	91.2

Rsa1.0_00008.1.g466.t1	gb|EOA33068.1| hypothetical protein CARUB_v10016401mg [Capsella rubella]	612	452	0	73.9	58.2	64.1	hypothetical protein CARUB_v10016401mg	gbpln	Capsella rubella	AT3G02440.1 | Symbols: TBL20 | TRICHOME BIREFRINGENCE-LIKE 20 | chr3:500804-502229 REVERSE LENGTH=373	612	373	1.00E-150	60.9	46.1	51.5
Rsa1.0_00008.1.g467.t1	gb|AAS45435.1| S-adenosylmethionine decarboxylase [Brassica juncea]	365	366	0	100.3	97.0	98.1	S-adenosylmethionine decarboxylase	gbpln	Brassica juncea	AT3G02470.4 | Symbols: | S-adenosylmethionine decarboxylase | chr3:510223-511323 FORWARD LENGTH=366	365	366	0	100.3	94.8	97.5
Rsa1.0_00008.1.g468.t1	gb|ABD96872.1| hypothetical protein [Cleome spinosa]	68	69	1.00E-18	101.5	72.1	83.8	hypothetical protein	gbpln	Cleome spinosa	AT3G02480.1 | Symbols: | Late embryogenesis abundant protein (LEA) family protein | chr3:512384-512857 FORWARD LENGTH=68	68	68	7.00E-19	100.0	80.9	91.2
Rsa1.0_00008.1.g469.t1	#	#	#	#	#	#	#	-	----	----	#	#	#	#	#	#	#
Rsa1.0_00008.1.g470.t1	gb|ABD96872.1| hypothetical protein [Cleome spinosa]	68	69	2.00E-18	101.5	70.6	85.3	hypothetical protein	gbpln	Cleome spinosa	AT3G02480.1 | Symbols: | Late embryogenesis abundant protein (LEA) family protein | chr3:512384-512857 FORWARD LENGTH=68	68	68	8.00E-20	100.0	83.8	92.6
Rsa1.0_00008.1.g471.t1	ref|NP_186900.3| regulator of chromosome condensation domain-containing protein [Arabidopsis thaliana] gi|110735845|dbj|BAE99899.1| hypothetical protein [Arabidopsis thaliana] gi|332640299|gb|AEE73820.1| regulator of chromosome condensation domain-containing protein [Arabidopsis thaliana]	392	393	0	100.3	93.6	96.7	regulator of chromosome condensation domain-containing protein	gbpln	Arabidopsis thaliana	AT3G02510.1 | Symbols: | Regulator of chromosome condensation (RCC1) family protein | chr3:522086-524536 REVERSE LENGTH=393	392	393	0	100.3	93.6	96.7
Rsa1.0_00008.1.g472.t2	gb|AAP37970.1| seed specific protein Bn15D17A [Brassica napus]	343	264	1.00E-119	77.0	69.1	70.3	seed specific protein Bn15D17A	gbpln	Brassica napus	AT3G02550.1 | Symbols: LBD41 | LOB domain-containing protein 41 | chr3:536747-537650 REVERSE LENGTH=263	343	263	1.00E-117	76.7	62.1	66.5
Rsa1.0_00008.1.g473.t1	gb|EOA31548.1| hypothetical protein CARUB_v10014739mg [Capsella rubella]	172	185	1.00E-52	107.6	69.8	77.9	hypothetical protein CARUB_v10014739mg	gbpln	Capsella rubella	AT3G02555.1 | Symbols: | unknown protein; BEST Arabidopsis thaliana protein match is: unknown protein (TAIR:AT5G16110.1); Has 130 Blast hits to 130 proteins in 18 species: Archae - 0; Bacteria - 0; Metazoa - 0; Fungi - 0; Plants - 130; Viruses - 0; Other Eukaryotes - 0 (source: NCBI BLink). | chr3:540024-540955 REVERSE LENGTH=162	172	162	6.00E-47	94.2	66.9	74.4
Rsa1.0_00008.1.g474.t1	sp|Q9XH45.1|RS7_BRAOL RecName: Full=40S ribosomal protein S7 gi|5532505|gb|AAD44761.1|AF144752_1 40S ribosomal protein S7 homolog [Brassica oleracea]	191	191	1.00E-104	100.0	97.9	98.4	RecName: Full=40S ribosomal protein S7 gi|5532505|gb|AAD44761.1|AF144752_1 40S ribosomal protein S7 homolog	gbpln	Brassica oleracea	AT3G02560.2 | Symbols: | Ribosomal protein S7e family protein | chr3:542341-543168 FORWARD LENGTH=191	191	191	2.00E-98	100.0	90.1	94.8
Rsa1.0_00008.1.g475.t1	gb|EOA29607.1| hypothetical protein CARUB_v10013753mg [Capsella rubella]	430	432	0	100.5	90.7	95.6	hypothetical protein CARUB_v10013753mg	gbpln	Capsella rubella	AT3G02570.1 | Symbols: MEE31, PMI1 | Mannose-6-phosphate isomerase, type I | chr3:543463-545478 REVERSE LENGTH=432	430	432	0	100.5	89.1	95.1
Rsa1.0_00008.1.g476.t1	gb|AAF32472.1| hypothetical protein [Arabidopsis thaliana]	530	1077	0	203.2	77.5	84.5	hypothetical protein	gbpln	Arabidopsis thaliana	AT3G02645.1 | Symbols: | Plant protein of unknown function (DUF247) | chr3:566271-567860 FORWARD LENGTH=529	530	529	0	99.8	81.3	88.7
Rsa1.0_00008.1.g477.t1	gb|EOA31892.1| hypothetical protein CARUB_v10015120mg, partial [Capsella rubella]	569	582	0	102.3	78.4	86.5	hypothetical protein CARUB_v10015120mg, partial	gbpln	Capsella rubella	AT3G02650.1 | Symbols: | Tetratricopeptide repeat (TPR)-like superfamily protein | chr3:568135-569865 FORWARD LENGTH=576	569	576	0	101.2	78.2	86.8
Rsa1.0_00008.1.g478.t1	ref|XP_002884331.1| tRNA synthetase class I family protein [Arabidopsis lyrata subsp. lyrata] gi|297330171|gb|EFH60590.1| tRNA synthetase class I family protein [Arabidopsis lyrata subsp. lyrata]	509	511	0	100.4	89.8	95.3	tRNA synthetase class I family protein	gbpln	Arabidopsis lyrata	AT3G02660.1 | Symbols: emb2768 | Tyrosyl-tRNA synthetase, class Ib, bacterial/mitochondrial | chr3:570221-571756 REVERSE LENGTH=511	509	511	0	100.4	88.2	94.1
Rsa1.0_00008.1.g479.t1	ref|XP_002882247.1| integral membrane family protein [Arabidopsis lyrata subsp. lyrata] gi|297328087|gb|EFH58506.1| integral membrane family protein [Arabidopsis lyrata subsp. lyrata]	419	424	0	101.2	86.4	92.8	integral membrane family protein	gbpln	Arabidopsis lyrata	AT3G02690.1 | Symbols: | nodulin MtN21 /EamA-like transporter family protein | chr3:579627-581448 FORWARD LENGTH=417	419	417	0	99.5	86.6	93.1
Rsa1.0_00008.1.g480.t1	gb|EOA31304.1| hypothetical protein CARUB_v10014477mg [Capsella rubella]	252	249	1.00E-125	98.8	89.3	94.8	hypothetical protein CARUB_v10014477mg	gbpln	Capsella rubella	AT3G02700.1 | Symbols: | NC domain-containing protein-related | chr3:581727-582813 REVERSE LENGTH=252	252	252	1.00E-127	100.0	87.7	94.8
Rsa1.0_00008.1.g481.t1	gb|EOA30303.1| hypothetical protein CARUB_v10013428mg [Capsella rubella]	504	534	0	106.0	91.5	96.8	hypothetical protein CARUB_v10013428mg	gbpln	Capsella rubella	AT3G02710.1 | Symbols: | ARM repeat superfamily protein | chr3:583184-585780 FORWARD LENGTH=529	504	529	0	105.0	91.9	96.2
Rsa1.0_00008.1.g482.t1	sp|O48897.1|TRXF_BRANA RecName: Full=Thioredoxin F-type, chloroplastic; Short=Trx-F; Flags: Precursor gi|2921094|gb|AAC04671.1| thioredoxin-f [Brassica napus]	182	182	2.00E-93	100.0	95.1	96.7	RecName: Full=Thioredoxin F-type, chloroplastic; Short=Trx-F; Flags: Precursor gi|2921094|gb|AAC04671.1| thioredoxin-f	gbpln	Brassica napus	AT3G02730.1 | Symbols: TRXF1, ATF1 | thioredoxin F-type 1 | chr3:588570-589591 REVERSE LENGTH=178	182	178	2.00E-91	97.8	89.0	94.5

Rsa1.0_00008.1.g483.t1	ref|NP_186923.1| aspartyl protease-like protein [Arabidopsis thaliana] gi|6728988|gb|AAF26986.1|AC018363_31 putative aspartyl protease [Arabidopsis thaliana] gi|21593593|gb|AAM65560.1| putative aspartyl protease [Arabidopsis thaliana] gi|332640332|gb|AEE73853.1| aspartyl protease-like protein [Arabidopsis thaliana]	377	488	1.00E-130	129.4	73.5	82.0	aspartyl protease-like protein	gbpln	Arabidopsis thaliana	AT3G02740.1 | Symbols: | Eukaryotic aspartyl protease family protein | chr3:590561-593089 FORWARD LENGTH=488	377	488	1.00E-132	129.4	73.5	82.0
Rsa1.0_00008.1.g484.t1	ref|XP_002884336.1| phosphatase 2C family protein [Arabidopsis lyrata subsp. lyrata] gi|297330176|gb|EFH60595.1| phosphatase 2C family protein [Arabidopsis lyrata subsp. lyrata]	476	490	0	102.9	87.4	94.5	phosphatase 2C family protein	gbpln	Arabidopsis lyrata	AT3G02750.2 | Symbols: | Protein phosphatase 2C family protein | chr3:593601-595457 REVERSE LENGTH=492	476	492	0	103.4	85.7	93.7
Rsa1.0_00008.1.g485.t1	gb|EOA29853.1| hypothetical protein CARUB_v10012946mg [Capsella rubella]	859	886	0	103.1	77.9	86.7	hypothetical protein CARUB_v10012946mg	gbpln	Capsella rubella	AT3G02760.1 | Symbols: | Class II aaRS and biotin synthetases superfamily protein | chr3:597588-600650 REVERSE LENGTH=883	859	883	0	102.8	77.3	86.3
Rsa1.0_00008.1.g486.t1	ref|XP_002884338.1| dimethylmenaquinone methyltransferase family protein [Arabidopsis lyrata subsp. lyrata] gi|297330178|gb|EFH60597.1| dimethylmenaquinone methyltransferase family protein [Arabidopsis lyrata subsp. lyrata]	166	166	7.00E-88	100.0	97.0	98.8	dimethylmenaquinone methyltransferase family protein	gbpln	Arabidopsis lyrata	AT3G02770.1 | Symbols: | Ribonuclease E inhibitor RraA/Dimethylmenaquinone methyltransferase | chr3:601017-601884 REVERSE LENGTH=166	166	166	2.00E-89	100.0	95.8	98.2
Rsa1.0_00008.1.g487.t1	gb|AAF36996.1|AF236092_1 isopentenyl pyrophosphate:dimethylallyl pyrophosphate isomerase [Brassica oleracea var. botrytis]	261	281	1.00E-133	107.7	90.8	95.8	isopentenyl pyrophosphate:dimethylallyl pyrophosphate isomerase	gbpln	Brassica oleracea	AT3G02780.1 | Symbols: IPP2, IPIAT1, IDI2 | isopentenyl pyrophosphate:dimethylallyl pyrophosphate isomerase 2 | chr3:602578-604648 REVERSE LENGTH=284	261	284	1.00E-128	108.8	87.0	92.3
Rsa1.0_00008.1.g488.t1	gb|AAF26980.1|AC018363_25 unknown protein [Arabidopsis thaliana] gi|34365627|gb|AAQ65125.1| At3g02800 [Arabidopsis thaliana]	204	199	2.00E-99	97.5	83.8	88.2	unknown protein	gbpln	Arabidopsis thaliana	AT3G02800.1 | Symbols: | Tyrosine phosphatase family protein | chr3:606638-607704 REVERSE LENGTH=203	204	203	1.00E-102	99.5	83.8	88.2
Rsa1.0_00008.1.g489.t1	ref|NP_180565.1| mitogen-activated protein kinase kinase kinase 14 [Arabidopsis thaliana] gi|3420047|gb|AAC31848.1| putative protein kinase [Arabidopsis thaliana] gi|24030256|gb|AAN41303.1| putative protein kinase [Arabidopsis thaliana] gi|330253244|gb|AEC08338.1| mitogen-activated protein kinase kinase kinase 14 [Arabidopsis thaliana]	422	463	1.00E-155	109.7	70.6	79.4	mitogen-activated protein kinase kinase kinase 14	gbpln	Arabidopsis thaliana	AT2G30040.1 | Symbols: MAPKKK14 | mitogen-activated protein kinase kinase kinase 14 | chr2:12821747-12823138 FORWARD LENGTH=463	422	463	1.00E-157	109.7	70.6	79.4
Rsa1.0_00008.1.g490.t1	dbj|BAJ34022.1| unnamed protein product [Thellungiella halophila]	370	395	1.00E-164	106.8	84.1	89.5	unnamed protein product	----	----	AT2G30020.1 | Symbols: | Protein phosphatase 2C family protein | chr2:12814437-12815904 FORWARD LENGTH=396	370	396	1.00E-162	107.0	81.4	87.6
Rsa1.0_00008.1.g491.t1	gb|EMT31378.1| Zinc finger CCCH domain-containing protein 30 [Aegilops tauschii]	110	671	2.00E-59	610.0	91.8	93.6	Zinc finger CCCH domain-containing protein 30	gbpln	Aegilops tauschii	AT2G30000.1 | Symbols: | PHF5-like protein | chr2:12804042-12804374 REVERSE LENGTH=110	110	110	6.00E-59	100.0	98.2	99.1
Rsa1.0_00008.1.g492.t1	ref|NP_180560.1| NADH dehydrogenase [Arabidopsis thaliana] gi|75318710|sp|O80874.1|NDA2_ARATH RecName: Full=Alternative NAD(P)H dehydrogenase 2, mitochondrial; Flags: Precursor gi|3420052|gb|AAC31853.1| putative NADH dehydrogenase (ubiquinone oxidoreductase) [Arabidopsis thaliana] gi|330253238|gb|AEC08332.1| alternative NAD(P)H dehydrogenase 2 [Arabidopsis thaliana]	504	508	0	100.8	91.1	94.2	NADH dehydrogenase	gbpln	Arabidopsis thaliana	AT2G29990.1 | Symbols: NDA2 | alternative NAD(P)H dehydrogenase 2 | chr2:12793562-12795913 REVERSE LENGTH=508	504	508	0	100.8	91.1	94.2
Rsa1.0_00008.1.g493.t1	#	#	#	#	#	#	#	-	----	----	#	#	#	#	#	#	#
Rsa1.0_00008.1.g494.t1	#	#	#	#	#	#	#	-	----	----	#	#	#	#	#	#	#
Rsa1.0_00008.1.g495.t1	ref|NP_001190256.1| syntaxin-132 [Arabidopsis thaliana] gi|332003861|gb|AED91244.1| syntaxin-132 [Arabidopsis thaliana]	270	315	5.00E-35	116.7	35.9	38.9	syntaxin-132	gbpln	Arabidopsis thaliana	AT5G08080.3 | Symbols: SYP132 | syntaxin of plants 132 | chr5:2588532-2591106 FORWARD LENGTH=315	270	315	1.00E-37	116.7	35.9	38.9
Rsa1.0_00008.1.g496.t2	emb|CAB39638.1| RNA-directed DNA polymerase-like protein [Arabidopsis thaliana] gi|7267666|emb|CAB78094.1| RNA-directed DNA polymerase-like protein [Arabidopsis thaliana]	2561	1274	0	49.7	24.2	32.7	RNA-directed DNA polymerase-like protein	gbpln	Arabidopsis thaliana	AT3G02990.1 | Symbols: ATHSFA1E, HSFA1E | heat shock transcription factor A1E | chr3:673614-675988 FORWARD LENGTH=468	2561	468	0	18.3	14.7	15.9
Rsa1.0_00008.1.g497.t1	ref|XP_002874698.1| predicted protein [Arabidopsis lyrata subsp. lyrata] gi|297320535|gb|EFH50957.1| predicted protein [Arabidopsis lyrata subsp. lyrata]	295	372	7.00E-49	126.1	42.0	58.0	predicted protein	gbpln	Arabidopsis lyrata	AT4G11580.1 | Symbols: | RNI-like superfamily protein | chr4:7006648-7007738 REVERSE LENGTH=333	295	333	2.00E-45	112.9	40.0	55.3

Rsa1.0_00008.1.g498.t1	gb|EOA31596.1| hypothetical protein CARUB_v10014793mg [Capsella rubella]	170	165	5.00E-78	97.1	88.8	94.7	hypothetical protein CARUB_v10014793mg	gbpln	Capsella rubella	AT3G03000.1 | Symbols: | EF hand calcium-binding protein family | chr3:677388-677885 FORWARD LENGTH=165	170	165	3.00E-79	97.1	88.2	93.5
Rsa1.0_00008.1.g499.t1	gb|EOA32660.1| hypothetical protein CARUB_v10015958mg [Capsella rubella]	175	180	2.00E-74	102.9	85.7	90.9	hypothetical protein CARUB_v10015958mg	gbpln	Capsella rubella	AT3G03010.2 | Symbols: | Peptidyl-tRNA hydrolase II (PTH2) family protein | chr3:678982-680137 REVERSE LENGTH=179	175	179	5.00E-75	102.3	82.3	86.3
Rsa1.0_00008.1.g500.t1	ref|NP_186953.1| F-box/LRR-repeat protein [Arabidopsis thaliana] gi|75264861|sp|Q9M8U4.1|FBL41_ARATH RecName: Full=F-box/LRR-repeat protein At3g03030 gi|6728959|gb|AAF26957.1|AC018363_2 unknown protein [Arabidopsis thaliana] gi|30102686|gb|AAP21261.1| At3g03030 [Arabidopsis thaliana] gi|110735742|dbj|BAE99850.1| hypothetical protein [Arabidopsis thaliana] gi|332640373|gb|AEE73894.1| F-box/LRR-repeat protein [Arabidopsis thaliana]	457	474	1.00E-160	103.7	63.9	77.0	F-box/LRR-repeat protein	gbpln	Arabidopsis thaliana	AT3G03030.1 | Symbols: | F-box/RNI-like superfamily protein | chr3:682252-683850 FORWARD LENGTH=474	457	474	1.00E-163	103.7	63.9	77.0
Rsa1.0_00008.1.g501.t1	gb|EOA30484.1| hypothetical protein CARUB_v10013608mg [Capsella rubella]	413	474	1.00E-130	114.8	60.5	75.5	hypothetical protein CARUB_v10013608mg	gbpln	Capsella rubella	AT3G03030.1 | Symbols: | F-box/RNI-like superfamily protein | chr3:682252-683850 FORWARD LENGTH=474	413	474	1.00E-131	114.8	60.0	75.1
Rsa1.0_00008.1.g502.t1	gb|EOA29758.1| hypothetical protein CARUB_v10012847mg [Capsella rubella]	1160	1147	0	98.9	95.0	97.0	hypothetical protein CARUB_v10012847mg	gbpln	Capsella rubella	AT3G03050.1 | Symbols: CSLD3, KJK, ATCSLD3 | cellulose synthase-like D3 | chr3:687873-691629 FORWARD LENGTH=1145	1160	1145	0	98.7	94.1	96.5
Rsa1.0_00008.1.g503.t1	ref|XP_002882270.1| hypothetical protein ARALYDRAFT_340454 [Arabidopsis lyrata subsp. lyrata] gi|297328110|gb|EFH58529.1| hypothetical protein ARALYDRAFT_340454 [Arabidopsis lyrata subsp. lyrata]	639	642	0	100.5	90.5	93.9	hypothetical protein ARALYDRAFT_340454	gbpln	Arabidopsis lyrata	AT3G03060.1 | Symbols: | P-loop containing nucleoside triphosphate hydrolases superfamily protein | chr3:692188-695424 FORWARD LENGTH=628	639	628	0	98.3	89.2	92.6
Rsa1.0_00008.1.g504.t1	ref|XP_002884353.1| hypothetical protein ARALYDRAFT_896292 [Arabidopsis lyrata subsp. lyrata] gi|297330193|gb|EFH60612.1| hypothetical protein ARALYDRAFT_896292 [Arabidopsis lyrata subsp. lyrata]	111	110	1.00E-54	99.1	91.9	97.3	hypothetical protein ARALYDRAFT_896292	gbpln	Arabidopsis lyrata	AT3G03070.1 | Symbols: | NADH-ubiquinone oxidoreductase-related | chr3:696593-698069 REVERSE LENGTH=110	111	110	2.00E-56	99.1	90.1	96.4
Rsa1.0_00008.1.g505.t17	ref|NP_566192.1| putative NADH dehydrogenase [ubiquinone] 1 alpha subcomplex subunit 12 [Arabidopsis thaliana] gi|12644540|sp|Q9M9M9.1|NDUAC_ARATH RecName: Full=Probable NADH dehydrogenase [ubiquinone] 1 alpha subcomplex subunit 12 gi|6714426|gb|AAF26114.1|AC012328_17 unknown protein [Arabidopsis thaliana] gi|13878173|gb|AAK44164.1|AF370349_1 unknown protein [Arabidopsis thaliana] gi|17104529|gb|AAL34153.1| unknown protein [Arabidopsis thaliana] gi|21593289|gb|AAM65238.1| probable NADH-ubiquinone oxidoreductase subunit B17.2 (Complex I-B17.2) (CI-B17.2) [Arabidopsis thaliana] gi|332640380|gb|AEE73901.1| putative NADH dehydrogenase [ubiquinone] 1 alpha subcomplex subunit 12 [Arabidopsis thaliana]	159	159	8.00E-87	100.0	95.6	98.1	putative NADH dehydrogenase	gbpln	Arabidopsis thaliana	AT3G03100.1 | Symbols: | NADH:ubiquinone oxidoreductase, 17.2kDa subunit | chr3:705564-707578 REVERSE LENGTH=159	159	159	3.00E-89	100.0	95.6	98.1
Rsa1.0_00008.1.g506.t1	ref|XP_002882274.1| hypothetical protein ARALYDRAFT_896304 [Arabidopsis lyrata subsp. lyrata] gi|297328114|gb|EFH58533.1| hypothetical protein ARALYDRAFT_896304 [Arabidopsis lyrata subsp. lyrata]	1099	1076	0	97.9	85.5	91.3	hypothetical protein ARALYDRAFT_896304	gbpln	Arabidopsis lyrata	AT3G03110.1 | Symbols: XPO1B, CRM1B | exportin 1B | chr3:708966-716879 FORWARD LENGTH=1076	1099	1076	0	97.9	85.4	91.2
Rsa1.0_00008.1.g507.t1	dbj|BAA97156.1| unnamed protein product [Arabidopsis thaliana]	174	329	3.00E-24	189.1	38.5	56.3	unnamed protein product	gbpln	Arabidopsis thaliana	AT5G42965.1 | Symbols: | Polynucleotidyl transferase, ribonuclease H-like superfamily protein | chr5:17236736-17237125 FORWARD LENGTH=129	174	129	5.00E-11	74.1	18.4	29.3

Rsa1.0_00008.1.g508.t1	ref|XP_002884360.1| hypothetical protein ARALYDRAFT_477561 [Arabidopsis lyrata subsp. lyrata] gi|297330200|gb|EFH60619.1| hypothetical protein ARALYDRAFT_477561 [Arabidopsis lyrata subsp. lyrata]	517	521	1.00E-126	100.8	63.8	76.2	hypothetical protein ARALYDRAFT_477561	gbpln	Arabidopsis lyrata	AT3G03130.1 | Symbols: | unknown protein; BEST Arabidopsis thaliana protein match is: unknown protein (TAIR:AT5G17160.1); Has 7130 Blast hits to 3690 proteins in 504 species: Archae - 22; Bacteria - 2666; Metazoa - 1654; Fungi - 721; Plants - 246; Viruses - 56; Other Eukaryotes - 1765 (source: NCBI BLink). | chr3:721439-723564 REVERSE LENGTH=520	517	520	1.00E-127	100.6	63.1	74.9
Rsa1.0_00008.1.g509.t1	gb|EOA32118.1| hypothetical protein CARUB_v10015369mg [Capsella rubella]	684	761	0	111.3	78.8	86.4	hypothetical protein CARUB_v10015369mg	gbpln	Capsella rubella	AT3G03140.1 | Symbols: | Tudor/PWWP/MBT superfamily protein | chr3:724348-727093 FORWARD LENGTH=769	684	769	0	112.4	77.5	86.3
Rsa1.0_00008.1.g510.t1	ref|NP_566196.1| uncharacterized protein [Arabidopsis thaliana] gi|89001025|gb|ABD59102.1| At3g03170 [Arabidopsis thaliana] gi|332640388|gb|AEE73909.1| uncharacterized protein AT3G03170 [Arabidopsis thaliana]	154	156	2.00E-65	101.3	87.0	90.9	uncharacterized protein	gbpln	Arabidopsis thaliana	AT3G03170.1 | Symbols: | unknown protein; BEST Arabidopsis thaliana protein match is: unknown protein (TAIR:AT5G24890.1); Has 184 Blast hits to 184 proteins in 18 species: Archae - 0; Bacteria - 0; Metazoa - 0; Fungi - 0; Plants - 184; Viruses - 0; Other Eukaryotes - 0 (source: NCBI BLink). | chr3:732066-732729 FORWARD LENGTH=156	154	156	7.00E-68	101.3	87.0	90.9
Rsa1.0_00008.1.g511.t1	ref|NP_186968.2| Got1/Sft2-like vescicle transport protein [Arabidopsis thaliana] gi|38603916|gb|AAR24703.1| At3g03180 [Arabidopsis thaliana] gi|44681404|gb|AAS47642.1| At3g03180 [Arabidopsis thaliana] gi|332640389|gb|AEE73910.1| Got1/Sft2-like vescicle transport protein [Arabidopsis thaliana]	140	140	5.00E-68	100.0	96.4	97.9	Got1/Sft2-like vescicle transport protein	gbpln	Arabidopsis thaliana	AT3G03180.1 | Symbols: | Got1/Sft2-like vescicle transport protein family | chr3:733531-734421 FORWARD LENGTH=140	140	140	1.00E-70	100.0	96.4	97.9
Rsa1.0_00008.1.g512.t1	ref|XP_002884362.1| predicted protein [Arabidopsis lyrata subsp. lyrata] gi|297330202|gb|EFH60621.1| predicted protein [Arabidopsis lyrata subsp. lyrata]	478	481	0	100.6	87.9	93.7	predicted protein	gbpln	Arabidopsis lyrata	AT3G03200.1 | Symbols: anac045, NAC045 | NAC domain containing protein 45 | chr3:736141-738527 REVERSE LENGTH=479	478	479	0	100.2	88.1	93.1
Rsa1.0_00008.1.g513.t1	#	#	#	#	#	#	#	–	----	----	AT3G03210.1 | Symbols: | unknown protein; FUNCTIONS IN: molecular_function unknown; INVOLVED IN: biological_process unknown; LOCATED IN: endomembrane system; EXPRESSED IN: 24 plant structures; EXPRESSED DURING: 15 growth stages; Has 59 Blast hits to 59 proteins in 17 species: Archae - 0; Bacteria - 0; Metazoa - 0; Fungi - 0; Plants - 56; Viruses - 0; Other Eukaryotes - 3 (source: NCBI BLink). | chr3:740987-742093 FORWARD LENGTH=368	77	368	2.00E-12	477.9	67.5	76.6
Rsa1.0_00008.1.g514.t1	ref|NP_566197.1| expansin A13 [Arabidopsis thaliana] gi|20138030|sp|Q9M9P0.2|EXP13_ARATH RecName: Full=Expansin-A13; Short=AtEXPA13; AltName: Full=Alpha-expansin-13; Short=At-EXP13; Short=AtEx13; AltName: Full=Ath-ExpAlpha-1.22; Flags: Precursor gi|16604575|gb|AAL24089.1| putative expansin precursor protein [Arabidopsis thaliana] gi|21281243|gb|AAM45038.1| putative expansin precursor protein [Arabidopsis thaliana] gi|332640393|gb|AEE73914.1| expansin A13 [Arabidopsis thaliana]	264	266	1.00E-131	100.8	89.0	93.6	expansin A13	gbpln	Arabidopsis thaliana	AT3G03220.1 | Symbols: ATEXPA13, EXP13, ATEXP13, ATHEXP ALPHA 1.22, EXPA13 | expansin A13 | chr3:742655-743975 REVERSE LENGTH=266	264	266	1.00E-134	100.8	89.0	93.6
Rsa1.0_00008.1.g515.t1	ref|XP_002884364.1| esterase/lipase/thioesterase family protein [Arabidopsis lyrata subsp. lyrata] gi|297330204|gb|EFH60623.1| esterase/lipase/thioesterase family protein [Arabidopsis lyrata subsp. lyrata]	583	332	1.00E-158	56.9	46.7	53.3	esterase/lipase/thioesterase family protein	gbpln	Arabidopsis lyrata	AT3G03230.1 | Symbols: | alpha/beta-Hydrolases superfamily protein | chr3:744478-746009 REVERSE LENGTH=333	583	333	1.00E-161	57.1	46.3	53.5
Rsa1.0_00008.1.g516.t1	gb|EOA30501.1| hypothetical protein CARUB_v10013624mg [Capsella rubella]	485	469	0	96.7	91.3	93.2	hypothetical protein CARUB_v10013624mg	gbpln	Capsella rubella	AT3G03250.1 | Symbols: UGP, UGP1, AtUGP1 | UDP-GLUCOSE PYROPHOSPHORYLASE 1 | chr3:749761-754014 REVERSE LENGTH=469	485	469	0	96.7	90.7	92.6
Rsa1.0_00008.1.g517.t1	ref|NP_565646.2| RNA recognition motif-containing protein [Arabidopsis thaliana] gi|106879155|gb|ABF82607.1| At2g27330 [Arabidopsis thaliana] gi|330252880|gb|AEC07974.1| RNA recognition motif-containing protein [Arabidopsis thaliana]	181	116	1.00E-11	64.1	20.4	21.5	RNA recognition motif-containing protein	gbpln	Arabidopsis thaliana	AT2G27330.1 | Symbols: | RNA-binding (RRM/RBD/RNP motifs) family protein | chr2:11695350-11696563 REVERSE LENGTH=116	181	116	4.00E-14	64.1	20.4	21.5

Rsa1.0_00008.1.g518.t1	ref|NP_186976.2| homeobox-leucine zipper protein HDG8 [Arabidopsis thaliana] gi|187471152|sp|Q9M9P4.2|HDG8_ARATH RecName: Full=Homeobox-leucine zipper protein HDG8; AltName: Full=HD-ZIP protein HDG8; AltName: Full=Homeodomain GLABRA 2-like protein 8; AltName: Full=Homeodomain transcription factor HDG8; AltName: Full=Protein HOMEODOMAIN GLABROUS 8 gi|332640397|gb|AEE73918.1| homeobox-leucine zipper protein HDG8 [Arabidopsis thaliana]	657	699	0	106.4	64.2	76.0	homeobox-leucine zipper protein HDG8	gbpln	Arabidopsis thaliana	AT3G03260.1 | Symbols: HDG8 | homeodomain GLABROUS 8 | chr3:755356-759234 REVERSE LENGTH=699	657	699	0	106.4	64.2	76.0
Rsa1.0_00008.1.g519.t1	#	#	#	#	#	#	#	-	----	----	#	#	#	#	#	#	#
Rsa1.0_00008.1.g520.t1	ref|NP_001118559.1| uncharacterized protein [Arabidopsis thaliana] gi|297832980|ref|XP_002884372.1| hypothetical protein ARALYDRAFT_477584 [Arabidopsis lyrata subsp. lyrata] gi|98962219|gb|ABF59439.1| unknown protein [Arabidopsis thaliana] gi|297330212|gb|EFH60631.1| hypothetical protein ARALYDRAFT_477584 [Arabidopsis lyrata subsp. lyrata] gi|332640411|gb|AEE73932.1| uncharacterized protein AT3G03341 [Arabidopsis thaliana] gi|482567721|gb|EOA31910.1| hypothetical protein CARUB_v10015139mg [Capsella rubella]	72	72	5.00E-33	100.0	98.6	100.0	uncharacterized protein	gbpln	Arabidopsis lyrata	AT3G03341.1 | Symbols: | unknown protein; Has 30201 Blast hits to 17322 proteins in 780 species: Archae - 12; Bacteria - 1396; Metazoa - 17338; Fungi - 3422; Plants - 5037; Viruses - 0; Other Eukaryotes - 2996 (source: NCBI BLink). | chr3:790338-790556 REVERSE LENGTH=72	72	72	8.00E-36	100.0	98.6	100.0
Rsa1.0_00008.1.g521.t38	gb|EOA29773.1| hypothetical protein CARUB_v10012864mg [Capsella rubella]	1202	1097	0	91.3	83.0	86.1	hypothetical protein CARUB_v10012864mg	gbpln	Capsella rubella	AT3G03380.1 | Symbols: DegP7 | DegP protease 7 | chr3:799720-808319 FORWARD LENGTH=1097	1202	1097	0	91.3	82.7	85.8
Rsa1.0_00008.1.g522.t1	ref|XP_002884376.1| hypothetical protein ARALYDRAFT_896341 [Arabidopsis lyrata subsp. lyrata] gi|297330216|gb|EFH60635.1| hypothetical protein ARALYDRAFT_896341 [Arabidopsis lyrata subsp. lyrata]	131	148	8.00E-51	113.0	80.2	86.3	hypothetical protein ARALYDRAFT_896341	gbpln	Arabidopsis lyrata	AT3G03400.1 | Symbols: | EF hand calcium-binding protein family | chr3:808745-809158 REVERSE LENGTH=137	131	137	2.00E-46	104.6	69.5	79.4
Rsa1.0_00008.1.g523.t1	gb|EOA32409.1| hypothetical protein CARUB_v10015681mg [Capsella rubella]	131	131	4.00E-44	100.0	67.9	80.2	hypothetical protein CARUB_v10015681mg	gbpln	Capsella rubella	AT3G03410.1 | Symbols: | EF hand calcium-binding protein family | chr3:811324-811719 REVERSE LENGTH=131	131	131	7.00E-44	100.0	64.9	78.6
Rsa1.0_00008.1.g524.t1	gb|EOA31513.1| hypothetical protein CARUB_v10014702mg [Capsella rubella]	195	194	1.00E-107	99.5	94.9	97.4	hypothetical protein CARUB_v10014702mg	gbpln	Capsella rubella	AT3G03420.1 | Symbols: | Ku70-binding family protein | chr3:812527-813644 FORWARD LENGTH=194	195	194	1.00E-109	99.5	94.4	97.4
Rsa1.0_00008.1.g525.t1	pir||S65145 pollen allergen group II (clone 44) - rape	83	83	8.00E-39	100.0	97.6	98.8	pollen allergen group II (clone 44) - rape	----	----	AT3G03430.1 | Symbols: | Calcium-binding EF-hand family protein | chr3:814481-814732 FORWARD LENGTH=83	83	83	1.00E-38	100.0	90.4	95.2
Rsa1.0_00008.1.g526.t1	ref|NP_186994.2| armadillo/beta-catenin-like repeat-containing protein [Arabidopsis thaliana] gi|332640423|gb|AEE73944.1| armadillo/beta-catenin-like repeat-containing protein [Arabidopsis thaliana]	404	408	1.00E-179	101.0	90.1	94.6	armadillo/beta-catenin-like repeat-containing protein	gbpln	Arabidopsis thaliana	AT3G03440.1 | Symbols: | ARM repeat superfamily protein | chr3:815709-818568 FORWARD LENGTH=408	404	408	0	101.0	90.1	94.6
Rsa1.0_00008.1.g527.t1	gb|EOA20745.1| hypothetical protein CARUB_v10001071mg, partial [Capsella rubella]	340	414	2.00E-82	121.8	56.5	65.0	hypothetical protein CARUB_v10001071mg, partial	gbpln	Capsella rubella	AT3G03460.1 | Symbols: | unknown protein; BEST Arabidopsis thaliana protein match is: unknown protein (TAIR:AT5G17510.1); Has 19732 Blast hits to 8747 proteins in 456 species: Archae - 0; Bacteria - 449; Metazoa - 7438; Fungi - 2099; Plants - 1550; Viruses - 53; Other Eukaryotes - 8143 (source: NCBI BLink). | chr3:823069-824485 REVERSE LENGTH=354	340	354	2.00E-83	104.1	56.2	65.9
Rsa1.0_00008.1.g528.t1	ref|NP_187000.1| TatD related DNase [Arabidopsis thaliana] gi|6017102|gb|AAF01585.1|AC009895_6 unknown protein [Arabidopsis thaliana] gi|332640430|gb|AEE73951.1| TatD related DNase [Arabidopsis thaliana]	257	272	1.00E-127	105.8	87.9	92.6	TatD related DNase	gbpln	Arabidopsis thaliana	AT3G03500.1 | Symbols: | TatD related DNase | chr3:832929-834350 FORWARD LENGTH=272	257	272	1.00E-130	105.8	87.9	92.6

Rsa1.0_00008.1.g529.t1	ref|XP_002884391.1| hypothetical protein ARALYDRAFT_317234 [Arabidopsis lyrata subsp. lyrata] gi|297330231|gb|EFH60650.1| hypothetical protein ARALYDRAFT_317234 [Arabidopsis lyrata subsp. lyrata]	539	538	0	99.8	88.5	94.8	hypothetical protein ARALYDRAFT_317234	gbpln	Arabidopsis lyrata	AT3G03530.1 | Symbols: NPC4 | non-specific phospholipase C4 | chr3:842686-844970 REVERSE LENGTH=538	539	538	0	99.8	87.8	93.9
Rsa1.0_00008.1.g530.t1	ref|XP_002881179.1| hypothetical protein ARALYDRAFT_902186 [Arabidopsis lyrata subsp. lyrata] gi|297327018|gb|EFH57438.1| hypothetical protein ARALYDRAFT_902186 [Arabidopsis lyrata subsp. lyrata]	167	561	9.00E-54	335.9	67.7	68.3	hypothetical protein ARALYDRAFT_902186	gbpln	Arabidopsis lyrata	AT2G31510.1 | Symbols: ARI7, ATARI7 | IBR domain-containing protein | chr2:13416991-13421170 REVERSE LENGTH=562	167	562	2.00E-55	336.5	67.1	67.7
Rsa1.0_00008.1.g531.t1	gb|EMJ11865.1| hypothetical protein PRUPE_ppa022462mg [Prunus persica]	206	606	3.00E-39	294.2	40.8	56.8	hypothetical protein PRUPE_ppa022462mg	gbpln	Prunus persica	#	#	#	#	#	#	#
Rsa1.0_00008.1.g532.t1	#	#	#	#	#	#	#	–	----	----	#	#	#	#	#	#	#
Rsa1.0_00008.1.g533.t1	ref|XP_002884394.1| zinc finger family protein [Arabidopsis lyrata subsp. lyrata] gi|297330234|gb|EFH60653.1| zinc finger family protein [Arabidopsis lyrata subsp. lyrata]	350	355	1.00E-119	101.4	73.4	81.4	zinc finger family protein	gbpln	Arabidopsis lyrata	AT3G03550.1 | Symbols: | RING/U-box superfamily protein | chr3:850391-851461 REVERSE LENGTH=356	350	356	1.00E-110	101.7	69.7	77.7
Rsa1.0_00008.1.g534.t1	ref|XP_002882299.1| hypothetical protein ARALYDRAFT_477606 [Arabidopsis lyrata subsp. lyrata] gi|297328139|gb|EFH58558.1| hypothetical protein ARALYDRAFT_477606 [Arabidopsis lyrata subsp. lyrata]	322	322	1.00E-169	100.0	91.6	96.0	hypothetical protein ARALYDRAFT_477606	gbpln	Arabidopsis lyrata	AT3G03610.1 | Symbols: | ELMO/CED-12 family protein | chr3:869479-871687 FORWARD LENGTH=323	322	323	1.00E-171	100.3	91.3	95.7
Rsa1.0_00008.1.g535.t1	gb|EOA30404.1| hypothetical protein CARUB_v10013529mg [Capsella rubella]	529	496	0	93.8	77.7	85.1	hypothetical protein CARUB_v10013529mg	gbpln	Capsella rubella	AT3G03620.1 | Symbols: | MATE efflux family protein | chr3:873904-876252 REVERSE LENGTH=500	529	500	0	94.5	77.3	85.1
Rsa1.0_00008.1.g536.t1	#	#	#	#	#	#	#	–	----	----	#	#	#	#	#	#	#
Rsa1.0_00008.1.g537.t1	ref|XP_002884400.1| hypothetical protein ARALYDRAFT_896375 [Arabidopsis lyrata subsp. lyrata] gi|297330240|gb|EFH60659.1| hypothetical protein ARALYDRAFT_896375 [Arabidopsis lyrata subsp. lyrata]	394	405	1.00E-175	102.8	87.1	91.4	hypothetical protein ARALYDRAFT_896375	gbpln	Arabidopsis lyrata	AT3G03630.1 | Symbols: CS26 | cysteine synthase 26 | chr3:878388-880400 REVERSE LENGTH=404	394	404	1.00E-168	102.5	86.0	90.4
Rsa1.0_00008.1.g538.t1	dbj|BAJ33819.1| unnamed protein product [Thellungiella halophila]	667	528	0	79.2	69.1	73.3	unnamed protein product	----	----	AT3G03640.1 | Symbols: GLUC, BGLU25 | beta glucosidase 25 | chr3:881028-884028 FORWARD LENGTH=531	667	531	0	79.6	67.8	71.4
Rsa1.0_00008.1.g539.t1	ref|NP_001118563.1| WUSCHEL related homeobox 11 [Arabidopsis thaliana] gi|332640448|gb|AEE73969.1| WUSCHEL related homeobox 11 [Arabidopsis thaliana]	301	297	1.00E-121	98.7	84.1	90.7	WUSCHEL related homeobox 11	gbpln	Arabidopsis thaliana	AT3G03660.2 | Symbols: WOX11 | WUSCHEL related homeobox 11 | chr3:889515-892162 REVERSE LENGTH=297	301	297	1.00E-124	98.7	84.1	90.7
Rsa1.0_00008.1.g540.t1	gb|AAG50806.1|AC079281_8 unknown protein [Arabidopsis thaliana]	1810	1213	0	67.0	35.9	46.7	unknown protein	gbpln	Arabidopsis thaliana	AT1G43760.1 | Symbols: | DNAse I-like superfamily protein | chr1:16528880-16531065 REVERSE LENGTH=626	1810	626	5.00E-78	34.6	8.7	14.0
Rsa1.0_00009.1.g541.t1	#	#	#	#	#	#	#	–	----	----	#	#	#	#	#	#	#
Rsa1.0_00009.1.g542.t1	ref|NP_172114.2| uncharacterized protein [Arabidopsis thaliana] gi|24030288|gb|AAN41316.1| unknown protein [Arabidopsis thaliana] gi|332189846|gb|AEE27967.1| uncharacterized protein AT1G06240 [Arabidopsis thaliana]	388	383	0	98.7	84.0	90.2	uncharacterized protein	gbpln	Arabidopsis thaliana	AT1G06240.1 | Symbols: | Protein of unknown function DUF455 | chr1:1911166-1912875 FORWARD LENGTH=383	388	383	0	98.7	84.0	90.2
Rsa1.0_00009.1.g543.t1	gb|EOA39214.1| hypothetical protein CARUB_v10012192mg [Capsella rubella]	448	423	0	94.4	73.2	79.5	hypothetical protein CARUB_v10012192mg	gbpln	Capsella rubella	AT1G06250.1 | Symbols: | alpha/beta-Hydrolases superfamily protein | chr1:1913339-1914850 REVERSE LENGTH=423	448	423	0	94.4	72.1	79.7
Rsa1.0_00009.1.g544.t1	ref|NP_563764.1| cysteine proteinase-like protein [Arabidopsis thaliana] gi|8844131|gb|AAF80223.1|AC025290_12 Contains similarity to a cysteine endopeptidase 1 from Phaseolus vulgaris gb|U52970 and is a member of the papain cysteine protease family PF|00112 [Arabidopsis thaliana] gi|332189848|gb|AEE27969.1| cysteine proteinase-like protein [Arabidopsis thaliana]	343	343	1.00E-171	100.0	82.5	89.8	cysteine proteinase-like protein	gbpln	Arabidopsis thaliana	AT1G06260.1 | Symbols: | Cysteine proteinases superfamily protein | chr1:1916449-1917585 FORWARD LENGTH=343	343	343	1.00E-174	100.0	82.5	89.8

Rsa1.0_00009.1.g545.t1	ref|XP_002892332.1| hypothetical protein ARALYDRAFT_887830 [Arabidopsis lyrata subsp. lyrata] gi|297338174|gb|EFH68591.1| hypothetical protein ARALYDRAFT_887830 [Arabidopsis lyrata subsp. lyrata]	199	205	3.00E-92	103.0	82.4	87.9	hypothetical protein ARALYDRAFT_887830	gbpln	Arabidopsis lyrata	AT1G06280.1 | Symbols: LBD2 | LOB domain-containing protein 2 | chr1:1920327-1920947 REVERSE LENGTH=206	199	206	1.00E-90	103.5	82.9	87.9
Rsa1.0_00009.1.g546.t1	ref|NP_172121.2| uncharacterized protein [Arabidopsis thaliana] gi|91805751|gb|ABE65604.1| hypothetical protein At1g06320 [Arabidopsis thaliana] gi|332189853|gb|AEE27974.1| uncharacterized protein AT1G06320 [Arabidopsis thaliana]	198	195	4.00E-12	98.5	18.2	23.7	uncharacterized protein	gbpln	Arabidopsis thaliana	AT1G06320.1 | Symbols: | unknown protein; Has 24 Blast hits to 24 proteins in 10 species: Archae - 0; Bacteria - 2; Metazoa - 0; Fungi - 0; Plants - 20; Viruses - 0; Other Eukaryotes - 2 (source: NCBI BLink). | chr1:1930602-1931449 FORWARD LENGTH=195	198	195	1.00E-14	98.5	18.2	23.7
Rsa1.0_00009.1.g547.t1	ref|XP_002892336.1| fatty acid desaturase family protein [Arabidopsis lyrata subsp. lyrata] gi|297338178|gb|EFH68595.1| fatty acid desaturase family protein [Arabidopsis lyrata subsp. lyrata]	301	299	1.00E-143	99.3	81.4	88.4	fatty acid desaturase family protein	gbpln	Arabidopsis lyrata	AT1G06350.1 | Symbols: | Fatty acid desaturase family protein | chr1:1935510-1936789 REVERSE LENGTH=300	301	300	1.00E-142	99.7	79.7	88.4
Rsa1.0_00009.1.g548.t1	ref|XP_002892335.1| agenet domain-containing protein [Arabidopsis lyrata subsp. lyrata] gi|297338177|gb|EFH68594.1| agenet domain-containing protein [Arabidopsis lyrata subsp. lyrata]	143	139	6.00E-36	97.2	50.3	67.1	agenet domain-containing protein	gbpln	Arabidopsis lyrata	AT1G06340.1 | Symbols: | Plant Tudor-like protein | chr1:1933583-1933987 REVERSE LENGTH=134	143	134	2.00E-33	93.7	48.3	64.3
Rsa1.0_00009.1.g549.t1	ref|XP_002892337.1| fatty acid desaturase family protein [Arabidopsis lyrata subsp. lyrata] gi|297338179|gb|EFH68596.1| fatty acid desaturase family protein [Arabidopsis lyrata subsp. lyrata]	299	299	1.00E-143	100.0	84.9	91.0	fatty acid desaturase family protein	gbpln	Arabidopsis lyrata	AT1G06360.1 | Symbols: | Fatty acid desaturase family protein | chr1:1939173-1940417 REVERSE LENGTH=299	299	299	1.00E-144	100.0	84.6	89.6
Rsa1.0_00009.1.g550.t1	ref|XP_002889602.1| hypothetical protein ARALYDRAFT_470660 [Arabidopsis lyrata subsp. lyrata] gi|297335444|gb|EFH65861.1| hypothetical protein ARALYDRAFT_470660 [Arabidopsis lyrata subsp. lyrata]	852	851	0	99.9	94.5	97.9	hypothetical protein ARALYDRAFT_470660	gbpln	Arabidopsis lyrata	AT1G06410.1 | Symbols: ATTPS7, TPS7, ATTPSA | trehalose-phosphatase/synthase 7 | chr1:1955413-1958153 FORWARD LENGTH=851	852	851	0	99.9	94.4	97.3
Rsa1.0_00009.1.g551.t3	dbj|BAJ33891.1| unnamed protein product [Thellungiella halophila]	695	693	0	99.7	92.8	95.0	unnamed protein product	----	----	AT1G06430.1 | Symbols: FTSH8 | FTSH protease 8 | chr1:1960214-1962525 REVERSE LENGTH=685	695	685	0	98.6	89.8	92.7
Rsa1.0_00009.1.g552.t1	ref|XP_002889605.1| hypothetical protein ARALYDRAFT_887850 [Arabidopsis lyrata subsp. lyrata] gi|297335447|gb|EFH65864.1| hypothetical protein ARALYDRAFT_887850 [Arabidopsis lyrata subsp. lyrata]	425	414	0	97.4	88.2	91.3	hypothetical protein ARALYDRAFT_887850	gbpln	Arabidopsis lyrata	AT1G06470.2 | Symbols: | Nucleotide/sugar transporter family protein | chr1:1970726-1973540 FORWARD LENGTH=414	425	414	0	97.4	87.3	90.8
Rsa1.0_00009.1.g553.t1	gb|EOA37916.1| hypothetical protein CARUB_v10009384mg [Capsella rubella]	399	392	0	98.2	82.7	89.2	hypothetical protein CARUB_v10009384mg	gbpln	Capsella rubella	AT1G06550.1 | Symbols: | ATP-dependent caseinolytic (Clp) protease/crotonase family protein | chr1:2003834-2006564 REVERSE LENGTH=387	399	387	0	97.0	83.0	88.7
Rsa1.0_00009.1.g554.t12	ref|XP_002889609.1| predicted protein [Arabidopsis lyrata subsp. lyrata] gi|297335451|gb|EFH65868.1| predicted protein [Arabidopsis lyrata subsp. lyrata]	678	599	0	88.3	77.0	81.0	predicted protein	gbpln	Arabidopsis lyrata	AT1G06560.1 | Symbols: | NOL1/NOP2/sun family protein | chr1:2007660-2011824 FORWARD LENGTH=599	678	599	0	88.3	76.4	81.0
Rsa1.0_00009.1.g555.t1	ref|XP_002892352.1| hypothetical protein ARALYDRAFT_887868 [Arabidopsis lyrata subsp. lyrata] gi|297338194|gb|EFH68611.1| hypothetical protein ARALYDRAFT_887868 [Arabidopsis lyrata subsp. lyrata]	487	486	0	99.8	80.5	87.1	hypothetical protein ARALYDRAFT_887868	gbpln	Arabidopsis lyrata	AT1G06660.1 | Symbols: | unknown protein; BEST Arabidopsis thaliana protein match is: unknown protein (TAIR:AT2G30820.2); Has 166 Blast hits to 144 proteins in 35 species: Archae - 0; Bacteria - 17; Metazoa - 13; Fungi - 20; Plants - 104; Viruses - 0; Other Eukaryotes - 12 (source: NCBI BLink). | chr1:2037461-2040148 REVERSE LENGTH=481	487	481	0	98.8	78.2	85.0

Rsa1.0_00009.1.g556.t1	sp|P11594.2|PSBP_SINAL RecName: Full=Oxygen-evolving enhancer protein 2, chloroplastic; Short=OEE2; AltName: Full=23 kDa subunit of oxygen evolving system of photosystem II; AltName: Full=23 kDa thylakoid membrane protein; AltName: Full=OEC 23 kDa subunit; Flags: Precursor gi|21133|emb|CAA35081.1| oxygen-evolving complex of photosystem II [Sinapis alba]	262	260	1.00E-138	99.2	96.6	97.3	RecName: Full=Oxygen-evolving enhancer protein 2, chloroplastic; Short=OEE2; AltName: Full=23 kDa subunit of oxygen evolving system of photosystem II; AltName: Full=23 kDa thylakoid membrane protein; AltName: Full=OEC 23 kDa subunit; Flags: Precursor gi|21133|emb|CAA35081.1| oxygen-evolving complex of photosystem II	gbpln	Sinapis alba	AT1G06680.1 | Symbols: PSBP-1, OEE2, PSII-P, OE23 | photosystem II subunit P-1 | chr1:2047940-2049186 FORWARD LENGTH=263	262	263	1.00E-129	100.4	91.6	96.6
Rsa1.0_00009.1.g557.t1	ref|NP_563770.1| NAD(P)-linked oxidoreductase-like protein [Arabidopsis thaliana] gi|75249772|sp|Q94A68.1|Y1669_ARATH RecName: Full=Uncharacterized oxidoreductase At1g06690, chloroplastic; Flags: Precursor gi|15215594|gb|AAK91342.1| At1g06690/F4H5_17 [Arabidopsis thaliana] gi|24111265|gb|AAN46756.1| At1g06690/F4H5_17 [Arabidopsis thaliana] gi|332189903|gb|AEE28024.1| NAD(P)-linked oxidoreductase-like protein [Arabidopsis thaliana]	358	377	0	105.3	94.1	96.9	NAD(P)-linked oxidoreductase-like protein	gbpln	Arabidopsis thaliana	AT1G06690.1 | Symbols: | NAD(P)-linked oxidoreductase superfamily protein | chr1:2049742-2052039 REVERSE LENGTH=377	358	377	0	105.3	94.1	96.9
Rsa1.0_00009.1.g558.t1	ref|XP_002889618.1| predicted protein [Arabidopsis lyrata subsp. lyrata] gi|297335460|gb|EFH65877.1| predicted protein [Arabidopsis lyrata subsp. lyrata]	203	204	3.00E-84	100.5	76.4	87.7	predicted protein	gbpln	Arabidopsis lyrata	AT1G06790.1 | Symbols: | RNA polymerase Rpb7 N-terminal domain-containing protein | chr1:2087645-2089287 FORWARD LENGTH=204	203	204	1.00E-85	100.5	74.9	86.2
Rsa1.0_00009.1.g559.t1	gb|EOA38195.1| hypothetical protein CARUB_v10009673mg [Capsella rubella] gi|482574009|gb|EOA38196.1| hypothetical protein CARUB_v10009673mg [Capsella rubella]	320	336	1.00E-116	105.0	83.1	85.9	hypothetical protein CARUB_v10009673mg	gbpln	Capsella rubella	AT1G06850.1 | Symbols: AtbZIP52, bZIP52 | basic leucine-zipper 52 | chr1:2105233-2106518 FORWARD LENGTH=337	320	337	1.00E-110	105.3	78.4	83.1
Rsa1.0_00009.1.g560.t3	gb|EOA38106.1| hypothetical protein CARUB_v10009574mg [Capsella rubella]	325	355	1.00E-170	109.2	97.2	99.4	hypothetical protein CARUB_v10009574mg	gbpln	Capsella rubella	AT1G06890.2 | Symbols: | nodulin MtN21 /EamA-like transporter family protein | chr1:2111728-2114038 REVERSE LENGTH=357	325	357	1.00E-171	109.8	97.2	99.1
Rsa1.0_00009.1.g561.t2	ref|NP_172173.2| putative N-arginine dibasic convertase [Arabidopsis thaliana] gi|332189930|gb|AEE28051.1| putative N-arginine dibasic convertase [Arabidopsis thaliana]	997	1024	0	102.7	89.8	94.4	putative N-arginine dibasic convertase	gbpln	Arabidopsis thaliana	AT1G06900.1 | Symbols: | Insulinase (Peptidase family M16) family protein | chr1:2115155-2120635 REVERSE LENGTH=1024	997	1024	0	102.7	89.8	94.4
Rsa1.0_00009.1.g562.t3	ref|NP_172176.1| translocon at the inner envelope membrane of chloroplasts 110 [Arabidopsis thaliana] gi|75157412|sp|Q8LPR9.1|TI110_ARATH RecName: Full=Protein TIC110, chloroplastic; AltName: Full=Translocon at the inner envelope membrane of chloroplasts 110; Short=AtTIC110; Flags: Precursor gi|20453114|gb|AAM19799.1| At1g06940/F4H5_1 [Arabidopsis thaliana] gi|332189936|gb|AEE28057.1| translocon at the inner envelope membrane of chloroplasts 110 [Arabidopsis thaliana]	1029	1016	0	98.7	90.2	94.0	translocon at the inner envelope membrane of chloroplasts 110	gbpln	Arabidopsis thaliana	AT1G06950.1 | Symbols: ATTIC110, TIC110 | translocon at the inner envelope membrane of chloroplasts 110 | chr1:2130303-2135563 REVERSE LENGTH=1016	1029	1016	0	98.7	90.2	94.0
Rsa1.0_00009.1.g563.t1	gb|EOA38940.1| hypothetical protein CARUB_v10011344mg [Capsella rubella]	831	829	0	99.8	81.3	92.1	hypothetical protein CARUB_v10011344mg	gbpln	Capsella rubella	AT1G06970.1 | Symbols: CHX14, ATCHX14 | cation/hydrogen exchanger 14 | chr1:2138109-2140818 FORWARD LENGTH=829	831	829	0	99.8	82.4	91.6

Rsa1.0_00009.1.g564.t1	ref|XP_002892370.1| hypothetical protein ARALYDRAFT_470720 [Arabidopsis lyrata subsp. lyrata] gi|297338212|gb|EFH68629.1| hypothetical protein ARALYDRAFT_470720 [Arabidopsis lyrata subsp. lyrata]	166	168	1.00E-67	101.2	88.0	91.6	hypothetical protein ARALYDRAFT_470720	gbpln	Arabidopsis lyrata	AT1G06980.1 | Symbols: | unknown protein; FUNCTIONS IN: molecular_function unknown; INVOLVED IN: biological_process unknown; LOCATED IN: endomembrane system; EXPRESSED IN: sepal, male gametophyte, carpel; EXPRESSED DURING: 4 anthesis; BEST Arabidopsis thaliana protein match is: unknown protein (TAIR:AT2G30230.1); Has 159 Blast hits to 159 proteins in 13 species: Archae - 0; Bacteria - 0; Metazoa - 0; Fungi - 0; Plants - 159; Viruses - 0; Other Eukaryotes - 0 (source: NCBI BLink). | chr1:2143248-2143757 REVERSE LENGTH=169	166	169	4.00E-68	101.8	86.1	91.0
Rsa1.0_00009.1.g565.t1	gb|AAC19278.1| T14P8.10 [Arabidopsis thaliana] gi|7269009|emb|CAB80742.1| AT4g02490 [Arabidopsis thaliana]	569	657	1.00E-152	115.5	45.5	58.7	T14P8.10	gbpln	Arabidopsis thaliana	AT4G04650.1 | Symbols: | RNA-directed DNA polymerase (reverse transcriptase)-related family protein | chr4:2353989-2355213 REVERSE LENGTH=332	569	332	4.00E-85	58.3	24.6	35.1
Rsa1.0_00009.1.g566.t1	gb|AAF82217.1|AC067971_25 Strong similarity to a mitochondrial carrier protein from Ribes nigrum gb|AJ007580. It contains a mitochondrial carrier protein domain PF|00153. ESTs gb|T46775, gb|R90539, gb|AW029646 and gb|AA605443 come from this gene [Arabidopsis thaliana]	162	781	2.00E-52	482.1	70.4	80.2	Strong similarity to a mitochondrial carrier protein from Ribes nigrum gb|AJ007580. It contains a mitochondrial carrier protein domain PF|00153. ESTs gb|T46775, gb|R90539, gb|AW029646 and gb|AA605443 come from this gene	gbpln	Arabidopsis thaliana	AT1G07020.1 | Symbols: | unknown protein; Has 39 Blast hits to 39 proteins in 17 species: Archae - 0; Bacteria - 0; Metazoa - 6; Fungi - 3; Plants - 28; Viruses - 0; Other Eukaryotes - 2 (source: NCBI BLink). | chr1:2155319-2155884 REVERSE LENGTH=147	162	147	1.00E-53	90.7	70.4	80.2
Rsa1.0_00009.1.g567.t1	ref|XP_002892377.1| predicted protein [Arabidopsis lyrata subsp. lyrata] gi|297338219|gb|EFH68636.1| predicted protein [Arabidopsis lyrata subsp. lyrata]	197	194	9.00E-66	98.5	67.0	78.2	predicted protein	gbpln	Arabidopsis lyrata	AT1G07050.1 | Symbols: | CCT motif family protein | chr1:2164327-2165133 REVERSE LENGTH=195	197	195	1.00E-64	99.0	66.5	78.2
Rsa1.0_00009.1.g568.t1	ref|NP_563779.1| GILT domain-containing protein [Arabidopsis thaliana] gi|15146334|gb|AAK83650.1| At1g07080/F10K1_15 [Arabidopsis thaliana] gi|15809756|gb|AAL06806.1| At1g07080/F10K1_15 [Arabidopsis thaliana] gi|332189954|gb|AEE28075.1| GILT domain-containing protein [Arabidopsis thaliana]	275	265	1.00E-118	96.4	77.1	84.7	GILT domain-containing protein	gbpln	Arabidopsis thaliana	AT1G07080.1 | Symbols: | Thioredoxin superfamily protein | chr1:2170069-2171861 FORWARD LENGTH=265	275	265	1.00E-120	96.4	77.1	84.7
Rsa1.0_00009.1.g569.t4	ref|NP_172191.1| fructose-2,6-bisphosphatase [Arabidopsis thaliana] gi|75192772|sp|Q9MB58.1|F26_ARATH RecName: Full=6-phosphofructo-2-kinase/fructose-2, 6-bisphosphatase; Short=6PF-2-K/Fru-2,6-P2ase; Short=AtF2KP; Short=PFK/FBPase; Includes: RecName: Full=6-phosphofructo-2-kinase; Includes: RecName: Full=Fructose-2,6-bisphosphatase gi|8117172|dbj|BAA96353.1| fructose-6-phosphate,2-kinase/fructose-2, 6-bisphosphatase [Arabidopsis thaliana] gi|332189956|gb|AEE28077.1| fructose-2,6-bisphosphatase [Arabidopsis thaliana]	1150	744	0	64.7	58.5	61.4	fructose-2,6-bisphosphatase	gbpln	Arabidopsis thaliana	AT1G07110.1 | Symbols: F2KP, ATF2KP, FKFBP | fructose-2,6-bisphosphatase | chr1:2178363-2183980 REVERSE LENGTH=744	1150	744	0	64.7	58.5	61.4
Rsa1.0_00009.1.g570.t1	gb|EOA36273.1| hypothetical protein CARUB_v10010524mg [Capsella rubella]	146	158	8.00E-58	108.2	75.3	84.2	hypothetical protein CARUB_v10010524mg	gbpln	Capsella rubella	AT1G07130.1 | Symbols: STN1, ATSTN1 | Nucleic acid-binding, OB-fold-like protein | chr1:2187928-2188410 FORWARD LENGTH=160	146	160	4.00E-56	109.6	69.2	82.2
Rsa1.0_00009.1.g571.t1	ref|XP_002892382.1| hypothetical protein ARALYDRAFT_470734 [Arabidopsis lyrata subsp. lyrata] gi|297338224|gb|EFH68641.1| hypothetical protein ARALYDRAFT_470734 [Arabidopsis lyrata subsp. lyrata]	278	145	8.00E-14	52.2	23.7	26.6	hypothetical protein ARALYDRAFT_470734	gbpln	Arabidopsis lyrata	AT1G07135.1 | Symbols: | glycine-rich protein | chr1:2190157-2190624 REVERSE LENGTH=155	278	155	5.00E-15	55.8	16.5	17.6
Rsa1.0_00009.1.g572.t1	gb|ABB97039.1| unknown [Brassica rapa]	225	225	1.00E-106	100.0	91.1	93.8	unknown	gbpln	Brassica rapa	AT1G07140.1 | Symbols: SIRANBP | Pleckstrin homology (PH) domain superfamily protein | chr1:2192360-2193688 REVERSE LENGTH=228	225	228	2.00E-95	101.3	87.6	91.6

Rsa1.0_00009.1.g573.t1	ref|XP_002889635.1| hypothetical protein ARALYDRAFT_887930 [Arabidopsis lyrata subsp. lyrata] gi|297335477|gb|EFH65894.1| hypothetical protein ARALYDRAFT_887930 [Arabidopsis lyrata subsp. lyrata]	513	509	0	99.2	90.8	94.7	hypothetical protein ARALYDRAFT_887930	gbpln	Arabidopsis lyrata	AT1G07180.1 | Symbols: ATNDI1, NDA1 | alternative NAD(P)H dehydrogenase 1 | chr1:2204414-2206773 FORWARD LENGTH=510	513	510	0	99.4	90.1	94.3
Rsa1.0_00009.1.g574.t1	gb|AAF79564.1|AC022464_22 F22G5.33 [Arabidopsis thaliana]	785	753	0	95.9	60.9	71.1	F22G5.33	gbpln	Arabidopsis thaliana	AT1G07330.1 | Symbols: | unknown protein; BEST Arabidopsis thaliana protein match is: unknown protein (TAIR:AT2G29620.1); Has 597 Blast hits to 536 proteins in 121 species: Archae - 2; Bacteria - 47; Metazoa - 170; Fungi - 43; Plants - 98; Viruses - 0; Other Eukaryotes - 237 (source: NCBI BLink). | chr1:2251131-2253585 FORWARD LENGTH=685	785	685	0	87.3	53.6	62.8
Rsa1.0_00009.1.g575.t1	ref|XP_002889642.1| sugar transport protein 2 [Arabidopsis lyrata subsp. lyrata] gi|297335484|gb|EFH65901.1| sugar transport protein 2 [Arabidopsis lyrata subsp. lyrata]	498	498	0	100.0	84.3	91.0	sugar transport protein 2	gbpln	Arabidopsis lyrata	AT1G07340.1 | Symbols: ATSTP2, STP2 | sugar transporter 2 | chr1:2254873-2256712 FORWARD LENGTH=498	498	498	0	100.0	83.5	90.8
Rsa1.0_00009.1.g576.t1	ref|XP_002892397.1| ceramidase family protein [Arabidopsis lyrata subsp. lyrata] gi|297338239|gb|EFH68656.1| ceramidase family protein [Arabidopsis lyrata subsp. lyrata]	1224	776	0	63.4	52.7	56.0	ceramidase family protein	gbpln	Arabidopsis lyrata	AT1G07380.1 | Symbols: | Neutral/alkaline non-lysosomal ceramidase | chr1:2264829-2268306 REVERSE LENGTH=779	1224	779	0	63.6	52.7	56.0
Rsa1.0_00009.1.g577.t1	gb|EOA37095.1| hypothetical protein CARUB_v10010276mg [Capsella rubella]	216	216	1.00E-119	100.0	96.8	98.6	hypothetical protein CARUB_v10010276mg	gbpln	Capsella rubella	AT1G07410.1 | Symbols: ATRABA2B, RAB-A2B, ATRAB-A2B, RABA2b | RAB GTPase homolog A2B | chr1:2276270-2277154 FORWARD LENGTH=214	216	214	1.00E-119	99.1	96.8	97.7
Rsa1.0_00009.1.g578.t1	ref|XP_002889647.1| sterol 4-alpha-methyl-oxidase 2 [Arabidopsis lyrata subsp. lyrata] gi|297335489|gb|EFH65906.1| sterol 4-alpha-methyl-oxidase 2 [Arabidopsis lyrata subsp. lyrata]	277	266	1.00E-145	96.0	89.2	91.7	sterol 4-alpha-methyl-oxidase 2	gbpln	Arabidopsis lyrata	AT1G07420.1 | Symbols: ATSMO2, SMO2-1 | sterol 4-alpha-methyl-oxidase 2-1 | chr1:2277910-2280033 FORWARD LENGTH=266	277	266	2.33E-156	96.0	88.8	91.3
Rsa1.0_00009.1.g579.t1	gb|AAF79572.1|AC022464_30 F22G5.19 [Arabidopsis thaliana]	321	322	1.00E-122	100.3	77.3	86.3	F22G5.19	gbpln	Arabidopsis thaliana	AT2G29220.1 | Symbols: | Concanavalin A-like lectin protein kinase family protein | chr2:12562781-12564664 REVERSE LENGTH=627	321	627	1.00E-103	195.3	56.7	69.8
Rsa1.0_00009.1.g580.t1	ref|NP_172225.1| tropine dehydrogenase [Arabidopsis thaliana] gi|20258796|gb|AAM13920.1| putative tropinone reductase-I [Arabidopsis thaliana] gi|332190006|gb|AEE28127.1| tropinone reductase-like protein [Arabidopsis thaliana]	253	260	1.00E-109	102.8	75.9	87.4	tropine dehydrogenase	gbpln	Arabidopsis thaliana	AT1G07450.1 | Symbols: | NAD(P)-binding Rossmann-fold superfamily protein | chr1:2288038-2289256 REVERSE LENGTH=260	253	260	1.00E-112	102.8	75.9	87.4
Rsa1.0_00009.1.g581.t1	ref|NP_172225.1| tropine dehydrogenase [Arabidopsis thaliana] gi|20258796|gb|AAM13920.1| putative tropinone reductase-I [Arabidopsis thaliana] gi|332190006|gb|AEE28127.1| tropinone reductase-like protein [Arabidopsis thaliana]	238	260	4.00E-99	109.2	78.6	87.8	tropine dehydrogenase	gbpln	Arabidopsis thaliana	AT1G07450.1 | Symbols: | NAD(P)-binding Rossmann-fold superfamily protein | chr1:2288038-2289256 REVERSE LENGTH=260	238	260	1.00E-101	109.2	78.6	87.8
Rsa1.0_00009.1.g582.t1	dbj|BAJ33917.1| unnamed protein product [Thellungiella halophila]	464	375	1.00E-169	80.8	70.7	74.8	unnamed protein product	----	----	AT1G07480.2 | Symbols: | Transcription factor IIA, alpha/beta subunit | chr1:2296706-2299215 REVERSE LENGTH=375	464	375	1.00E-160	80.8	69.4	73.5
Rsa1.0_00009.1.g583.t1	ref|NP_200759.2| protein rotundifolia like 5 [Arabidopsis thaliana] gi|21553697|gb|AAM62790.1| unknown [Arabidopsis thaliana] gi|42822069|tpg|DAA02289.1| TPA_exp: DVL18 [Arabidopsis thaliana] gi|88900382|gb|ABD57503.1| At5g59510 [Arabidopsis thaliana] gi|332009816|gb|AED97199.1| protein rotundifolia like 5 [Arabidopsis thaliana]	111	144	3.00E-16	129.7	46.8	58.6	protein rotundifolia like 5	gbpln	Arabidopsis thaliana	AT5G59510.1 | Symbols: RTFL5, DVL18 | ROTUNDIFOLIA like 5 | chr5:23990089-23990523 FORWARD LENGTH=144	111	144	4.00E-19	129.7	46.8	58.6
Rsa1.0_00009.1.g584.t1	gb|EOA39018.1| hypothetical protein CARUB_v10011564mg [Capsella rubella]	98	78	5.00E-18	79.6	54.1	64.3	hypothetical protein CARUB_v10011564mg	gbpln	Capsella rubella	#	#	#	#	#	#	#
Rsa1.0_00009.1.g585.t1	dbj|BAJ34044.1| unnamed protein product [Thellungiella halophila]	764	772	0	101.0	83.4	88.4	unnamed protein product	----	----	AT1G07530.1 | Symbols: SCL14, ATGRAS2, GRAS2 | SCARECROW-like 14 | chr1:2313828-2316137 REVERSE LENGTH=769	764	769	0	100.7	80.4	87.4

Rsa1.0_00009.1.g586.t1	ref|NP_172234.2| telomere repeat-binding protein 5 [Arabidopsis thaliana] gi|296439813|sp|Q6R0E3.2|TRP5_ARATH RecName: Full=Telomere repeat-binding protein 5; AltName: Full=Protein TRF-LIKE 2 gi|332190019|gb|AEE28140.1| telomere repeat-binding protein 5 [Arabidopsis thaliana]	603	630	0	104.5	80.3	87.9	telomere repeat-binding protein 5	gbpln	Arabidopsis thaliana	AT1G07540.1 | Symbols: TRFL2 | TRF-like 2 | chr1:2318433-2321048 REVERSE LENGTH=630	603	630	0	104.5	80.3	87.9
Rsa1.0_00009.1.g587.t1	gb|EOA21150.1| hypothetical protein CARUB_v10001497mg [Capsella rubella]	282	315	9.00E-77	111.7	56.4	68.1	hypothetical protein CARUB_v10001497mg	gbpln	Capsella rubella	AT4G05460.1 | Symbols: | RNI-like superfamily protein | chr4:2761106-2762400 REVERSE LENGTH=302	282	302	2.00E-76	107.1	56.4	68.1
Rsa1.0_00009.1.g588.t1	#	#	#	#	#	#	#	–	----	----	#	#	#	#	#	#	#
Rsa1.0_00009.1.g589.t1	gb|EOA24245.1| hypothetical protein CARUB_v10017480mg [Capsella rubella]	336	366	3.00E-81	108.9	47.0	62.8	hypothetical protein CARUB_v10017480mg	gbpln	Capsella rubella	AT5G52930.1 | Symbols: | Protein of unknown function (DUF295) | chr5:21470370-21471449 FORWARD LENGTH=359	336	359	5.00E-80	106.8	43.5	56.8
Rsa1.0_00009.1.g590.t1	gb|EOA24245.1| hypothetical protein CARUB_v10017480mg [Capsella rubella]	420	366	4.00E-88	87.1	44.0	55.5	hypothetical protein CARUB_v10017480mg	gbpln	Capsella rubella	AT5G52930.1 | Symbols: | Protein of unknown function (DUF295) | chr5:21470370-21471449 FORWARD LENGTH=359	420	359	1.00E-80	85.5	39.0	52.1
Rsa1.0_00009.1.g591.t1	gb|EOA38953.1| hypothetical protein CARUB_v10011370mg [Capsella rubella]	871	822	0	94.4	60.7	69.3	hypothetical protein CARUB_v10011370mg	gbpln	Capsella rubella	AT1G51890.2 | Symbols: | Leucine-rich repeat protein kinase family protein | chr1:19274802-19278528 REVERSE LENGTH=828	871	828	0	95.1	44.2	58.9
Rsa1.0_00009.1.g592.t1	#	#	#	#	#	#	#	–	----	----	#	#	#	#	#	#	#
Rsa1.0_00009.1.g593.t1	gb|AAF75096.1|AC007583_32 It is a member of GTP1/OBG family PF|01018 [Arabidopsis thaliana]	489	1029	0	210.4	80.4	89.4	It is a member of GTP1/OBG family PF|01018	gbpln	Arabidopsis thaliana	AT1G07615.1 | Symbols: | GTP-binding protein Obg/CgtA | chr1:2342277-2344200 REVERSE LENGTH=493	489	493	0	100.8	80.2	89.2
Rsa1.0_00009.1.g594.t2	ref|NP_172241.2| GTP-binding protein Obg/CgtA [Arabidopsis thaliana] gi|332190029|gb|AEE28150.1| GTP-binding protein Obg/CgtA [Arabidopsis thaliana]	558	552	0	98.9	71.9	78.3	GTP-binding protein Obg/CgtA	gbpln	Arabidopsis thaliana	AT1G07620.1 | Symbols: ATOBGM | GTP-binding protein Obg/CgtA | chr1:2344556-2346374 REVERSE LENGTH=552	558	552	0	98.9	71.9	78.3
Rsa1.0_00009.1.g595.t1	gb|EOA39848.1| hypothetical protein CARUB_v10008517mg [Capsella rubella]	460	666	0	144.8	84.1	90.9	hypothetical protein CARUB_v10008517mg	gbpln	Capsella rubella	AT1G07630.1 | Symbols: PLL5 | pol-like 5 | chr1:2349189-2351437 FORWARD LENGTH=662	460	662	0	143.9	83.0	90.7
Rsa1.0_00009.1.g596.t1	gb|EOA38961.1| hypothetical protein CARUB_v10011378mg [Capsella rubella]	334	337	1.00E-132	100.9	79.3	85.0	hypothetical protein CARUB_v10011378mg	gbpln	Capsella rubella	AT1G07640.3 | Symbols: OBP2 | Dof-type zinc finger DNA-binding family protein | chr1:2354707-2356012 REVERSE LENGTH=339	334	339	1.00E-128	101.5	76.9	85.0
Rsa1.0_00009.1.g597.t1	ref|NP_172246.3| Ca2+-transporting ATPase [Arabidopsis thaliana] gi|12643934|sp|Q9XES1.2|ECA4_ARATH RecName: Full=Calcium-transporting ATPase 4, endoplasmic reticulum-type gi|8439902|gb|AAF75088.1|AC007583_24 Strong similarity to ER-type calcium pump protein from Arabidopsis thaliana gb|U93845. It is a member of Na+/K+ ATPase C-terminus PF|00690 and a member of E1-E2 ATPase PF|00122 [Arabidopsis thaliana] gi|332190039|gb|AEE28160.1| calcium-transporting ATPase 4 [Arabidopsis thaliana]	1059	1061	0	100.2	94.7	96.6	Ca2+-transporting ATPase	gbpln	Arabidopsis thaliana	AT1G07670.1 | Symbols: ATECA4, ECA4 | endomembrane-type CA-ATPase 4 | chr1:2370305-2374196 REVERSE LENGTH=1061	1059	1061	0	100.2	94.7	96.6
Rsa1.0_00009.1.g598.t1	sp|Q00332.3|RS15A_BRANA RecName: Full=40S ribosomal protein S15a; AltName: Full=PPCB8 gi|17863|emb|CAA42599.1| r-protein BnS15a [Brassica napus] gi|119720818|gb|ABL97979.1| 40S ribosomal protein S15a [Brassica rapa]	130	130	2.00E-69	100.0	100.0	100.0	RecName: Full=40S ribosomal protein S15a; AltName: Full=PPCB8 gi|17863|emb|CAA42599.1| r-protein BnS15a	gbpln	Brassica napus	AT5G59850.1 | Symbols: | Ribosomal protein S8 family protein | chr5:24112499-24113084 REVERSE LENGTH=130	130	130	7.00E-72	100.0	99.2	100.0
Rsa1.0_00009.1.g599.t1	gb|EOA38095.1| hypothetical protein CARUB_v10009562mg [Capsella rubella]	79	356	5.00E-29	450.6	73.4	81.0	hypothetical protein CARUB_v10009562mg	gbpln	Capsella rubella	AT1G07750.1 | Symbols: | RmlC-like cupins superfamily protein | chr1:2404300-2405863 REVERSE LENGTH=356	79	356	3.00E-31	450.6	74.7	79.7
Rsa1.0_00009.1.g600.t1	gb|AAD32898.1| putative retroelement pol polyprotein [Arabidopsis thaliana]	1361	1307	0	96.0	58.6	73.5	putative retroelement pol polyprotein	gbpln	Arabidopsis thaliana	AT4G23160.1 | Symbols: CRK8 | cysteine-rich RLK (RECEPTOR-like protein kinase) 8 | chr4:12129485-12134086 FORWARD LENGTH=1262	1361	1262	1.00E-100	92.7	13.8	22.1
Rsa1.0_00009.1.g601.t1	gb|EOA38095.1| hypothetical protein CARUB_v10009562mg [Capsella rubella]	257	356	1.00E-132	138.5	90.7	93.8	hypothetical protein CARUB_v10009562mg	gbpln	Capsella rubella	AT1G07750.1 | Symbols: | RmlC-like cupins superfamily protein | chr1:2404300-2405863 REVERSE LENGTH=356	257	356	1.00E-132	138.5	88.3	92.6

Rsa1.0_00009.1.g602.t1	ref|NP_172254.2| DNA repair protein RAD51-like 4 [Arabidopsis thaliana] gi|83305360|sp|Q9LQQ2.2|RA51D_ARATH RecName: Full=DNA repair protein RAD51 homolog 4; Short=AtRAD51D gi|57283103|emb|CAD70703.1| putative DNA recombination protein [Arabidopsis thaliana] gi|58430742|dbj|BAD89165.1| AtRAD51D [Arabidopsis thaliana] gi|90969171|gb|ABE02570.1| RAD51D splice variant a [Arabidopsis thaliana] gi|90969179|gb|ABE02658.1| RAD51D splice variant a [Arabidopsis thaliana] gi|332190052|gb|AEE28173.1| DNA repair protein RAD51-like 4 [Arabidopsis thaliana]	67	322	1.00E-14	480.6	56.7	67.2	DNA repair protein RAD51-like 4	gbpln	Arabidopsis thaliana	AT1G07745.1 | Symbols: ATRAD51D, RAD51D, SSN1 | homolog of RAD51 D | chr1:2400797-2402842 REVERSE LENGTH=322	67	322	2.00E-17	480.6	56.7	67.2
Rsa1.0_00009.1.g603.t1	gb|ABW81051.1| tn7 reverse transcriptase [Arabidopsis lyrata subsp. lyrata]	442	441	1.00E-117	99.8	47.3	66.5	tn7 reverse transcriptase	gbpln	Arabidopsis lyrata	AT3G24255.1 | Symbols: | RNA-directed DNA polymerase (reverse transcriptase)-related family protein | chr3:8789471-8793208 FORWARD LENGTH=746	442	746	5.00E-92	168.8	38.2	52.7
Rsa1.0_00009.1.g604.t2	gb|EOA38341.1| hypothetical protein CARUB_v10009852mg, partial [Capsella rubella]	265	304	1.00E-119	114.7	82.3	90.2	hypothetical protein CARUB_v10009852mg, partial	gbpln	Capsella rubella	AT1G07745.2 | Symbols: ATRAD51D, RAD51D, SSN1 | homolog of RAD51 D | chr1:2400797-2402492 REVERSE LENGTH=304	265	304	1.00E-115	114.7	78.9	88.3
Rsa1.0_00010.1.g605.t1	#	#	#	#	#	#	#	-	----	----	#	#	#	#	#	#	#
Rsa1.0_00010.1.g606.t1	#	#	#	#	#	#	#	-	----	----	#	#	#	#	#	#	#
Rsa1.0_00010.1.g607.t1	ref|XP_004245047.1| PREDICTED: uncharacterized protein LOC101267914 [Solanum lycopersicum]	210	175	6.00E-22	83.3	26.7	35.2	PREDICTED: uncharacterized protein LOC101267914	gbpln	Solanum lycopersicum	AT5G21910.1 | Symbols: | unknown protein; Has 30201 Blast hits to 17322 proteins in 780 species: Archae - 12; Bacteria - 1396; Metazoa - 17338; Fungi - 3422; Plants - 5037; Viruses - 0; Other Eukaryotes - 2996 (source: NCBI BLink). | chr5:7240789-7241127 FORWARD LENGTH=112	210	112	3.00E-17	53.3	41.0	42.4
Rsa1.0_00010.1.g608.t1	ref|NP_001142613.1| uncharacterized protein LOC100274882 [Zea mays] gi|195607320|gb|ACG25490.1| hypothetical protein [Zea mays]	253	248	1.00E-126	98.0	90.5	93.7	uncharacterized protein LOC100274882	gbenv/gbpln	Zea mays	AT5G21920.1 | Symbols: YLMG2, ATYLMG2 | YGGT family protein | chr5:7241350-7242765 REVERSE LENGTH=251	253	251	1.00E-115	99.2	81.8	89.7
Rsa1.0_00010.1.g609.t1	ref|XP_002871979.1| hypothetical protein ARALYDRAFT_910174 [Arabidopsis lyrata subsp. lyrata] gi|297317816|gb|EFH48238.1| hypothetical protein ARALYDRAFT_910174 [Arabidopsis lyrata subsp. lyrata]	887	887	0	100.0	91.2	94.9	hypothetical protein ARALYDRAFT_910174	gbpln	Arabidopsis lyrata	AT5G21930.2 | Symbols: PAA2, HMA8 | P-type ATPase of Arabidopsis 2 | chr5:7243129-7248721 FORWARD LENGTH=883	887	883	0	99.5	91.0	95.2
Rsa1.0_00010.1.g610.t1	#	#	#	#	#	#	#	-	----	----	#	#	#	#	#	#	#
Rsa1.0_00010.1.g611.t1	ref|XP_002874038.1| hypothetical protein ARALYDRAFT_910177 [Arabidopsis lyrata subsp. lyrata] gi|297319875|gb|EFH50297.1| hypothetical protein ARALYDRAFT_910177 [Arabidopsis lyrata subsp. lyrata]	229	213	1.00E-80	93.0	72.9	78.2	hypothetical protein ARALYDRAFT_910177	gbpln	Arabidopsis lyrata	AT5G21960.1 | Symbols: | Integrase-type DNA-binding superfamily protein | chr5:7258560-7259210 REVERSE LENGTH=216	229	216	4.00E-82	94.3	72.1	78.2
Rsa1.0_00010.1.g612.t1	ref|NP_680185.1| ubiquitin carboxyl-terminal hydrolase-like protein [Arabidopsis thaliana] gi|13374856|emb|CAC34490.1| putative protein [Arabidopsis thaliana] gi|22531217|gb|AAM97112.1| unknown protein [Arabidopsis thaliana] gi|29294056|gb|AAO73893.1| expressed protein [Arabidopsis thaliana] gi|110742391|dbj|BAE99118.1| hypothetical protein [Arabidopsis thaliana] gi|332005579|gb|AED92962.1| ubiquitin carboxyl-terminal hydrolase-like protein [Arabidopsis thaliana]	446	449	0	100.7	81.4	87.9	ubiquitin carboxyl-terminal hydrolase-like protein	gbpln	Arabidopsis thaliana	AT5G21970.1 | Symbols: | Ubiquitin carboxyl-terminal hydrolase family protein | chr5:7262071-7263420 FORWARD LENGTH=449	446	449	0	100.7	81.4	87.9
Rsa1.0_00010.1.g613.t1	ref|NP_680186.1| hydroxyproline-rich glycoprotein family protein [Arabidopsis thaliana] gi|13374857|emb|CAC34491.1| putative protein [Arabidopsis thaliana] gi|29294058|gb|AAO73895.1| proline-rich protein family [Arabidopsis thaliana] gi|38454174|gb|AAR20781.1| At5g21280 [Arabidopsis thaliana] gi|38604048|gb|AAR24767.1| At5g21280 [Arabidopsis thaliana] gi|332005580|gb|AED92963.1| hydroxyproline-rich glycoprotein family protein [Arabidopsis thaliana]	263	302	1.00E-83	114.8	77.2	83.7	hydroxyproline-rich glycoprotein family protein	gbpln	Arabidopsis thaliana	AT5G21280.1 | Symbols: | hydroxyproline-rich glycoprotein family protein | chr5:7264094-7265514 REVERSE LENGTH=302	263	302	4.00E-86	114.8	77.2	83.7

Rsa1.0_00010.1.g614.t1	ref|NP_680187.2| tetratricopeptide repeat domain-containing protein [Arabidopsis thaliana] gi|357580467|sp|B7ZWR6.1|OEP61_ARATH RecName: Full=Outer envelope protein 61, chloroplastic; AltName: Full=Tetratricopeptide repeat domain-containing protein 7 gi|219291100|gb|ACL13985.1| At5g21990 [Arabidopsis thaliana] gi|332005581|gb|AED92964.1| tetratricopeptide repeat domain-containing protein OEP61 [Arabidopsis thaliana]	613	554	0	90.4	77.0	82.2	tetratricopeptide repeat domain-containing protein	gbpln	Arabidopsis thaliana	AT5G21990.1 | Symbols: TPR7 | Tetratricopeptide repeat (TPR)-like superfamily protein | chr5:7273395-7276318 FORWARD LENGTH=554	613	554	0	90.4	77.0	82.2
Rsa1.0_00010.1.g615.t1	ref|NP_568410.1| E3 ubiquitin-protein ligase RHF2A [Arabidopsis thaliana] gi|30688246|ref|NP_851050.1| E3 ubiquitin-protein ligase RHF2A [Arabidopsis thaliana] gi|75315838|sp|Q9ZT42.1|RHF2A_ARATH RecName: Full=E3 ubiquitin-protein ligase RHF2A; AltName: Full=RING-H2 zinc finger protein RHF2a gi|11762136|gb|AAG40346.1|AF324994_1 CIC7E11 [Arabidopsis thaliana] gi|3790587|gb|AAC69856.1| RING-H2 finger protein RHF2a [Arabidopsis thaliana] gi|13374859|emb|CAC34493.1| RING-H2 finger protein RHF2a [Arabidopsis thaliana] gi|26983856|gb|AAN86180.1| unknown protein [Arabidopsis thaliana] gi|332005583|gb|AED92966.1| E3 ubiquitin-protein ligase RHF2A [Arabidopsis thaliana] gi|332005584|gb|AED92967.1| E3 ubiquitin-protein ligase RHF2A [Arabidopsis thaliana]	387	375	1.00E-167	96.9	83.2	86.8	E3 ubiquitin-protein ligase RHF2A	gbpln	Arabidopsis thaliana	AT5G22000.3 | Symbols: RHF2A | RING-H2 group F2A | chr5:7277436-7279553 FORWARD LENGTH=375	387	375	1.00E-170	96.9	83.2	86.8
Rsa1.0_00010.1.g616.t1	gb|EOA19946.1| hypothetical protein CARUB_v10000196mg [Capsella rubella]	1307	868	0	66.4	59.2	62.4	hypothetical protein CARUB_v10000196mg	gbpln	Capsella rubella	AT5G22030.1 | Symbols: UBP8 | ubiquitin-specific protease 8 | chr5:7290155-7296344 REVERSE LENGTH=913	1307	913	0	69.9	59.4	62.4
Rsa1.0_00010.1.g617.t1	ref|NP_568412.1| chaperone protein dnaJ 2 [Arabidopsis thaliana] gi|21431768|sp|P42825.2|DNAJ2_ARATH RecName: Full=Chaperone protein dnaJ 2; Short=AtDjA2; Flags: Precursor gi|13374865|emb|CAC34499.1| DNAJ PROTEIN HOMOLOG ATJ [Arabidopsis thaliana] gi|26451807|dbj|BAC42997.1| putative DnaJ protein homolog ATJ [Arabidopsis thaliana] gi|111074426|gb|ABH04586.1| At5g22060 [Arabidopsis thaliana] gi|332005594|gb|AED92977.1| chaperone protein dnaJ 2 [Arabidopsis thaliana]	421	419	0	99.5	91.9	96.2	chaperone protein dnaJ 2	gbpln	Arabidopsis thaliana	AT5G22060.1 | Symbols: ATJ2, J2 | DNAJ homologue 2 | chr5:7303798-7305668 REVERSE LENGTH=419	421	419	0	99.5	91.9	96.2
Rsa1.0_00010.1.g618.t1	gb|EOA21400.1| hypothetical protein CARUB_v10001767mg [Capsella rubella]	246	246	1.00E-132	100.0	95.1	97.6	hypothetical protein CARUB_v10001767mg	gbpln	Capsella rubella	AT5G22080.1 | Symbols: | Chaperone DnaJ-domain superfamily protein | chr5:7310600-7313540 REVERSE LENGTH=246	246	246	1.00E-123	100.0	96.3	97.2
Rsa1.0_00010.1.g619.t1	gb|AAM08751.1|AC025098_18 Putative copia-type polyprotein [Oryza sativa Japonica Group]	1367	1803	0	131.9	45.1	61.8	Putative copia-type polyprotein	gbpln	Oryza sativa	AT4G23160.1 | Symbols: CRK8 | cysteine-rich RLK (RECEPTOR-like protein kinase) 8 | chr4:12129485-12134086 FORWARD LENGTH=1262	1367	1262	1.00E-112	92.3	14.6	22.5
Rsa1.0_00010.1.g620.t1	ref|NP_680195.2| uncharacterized protein [Arabidopsis thaliana] gi|334187828|ref|NP_001190360.1| uncharacterized protein [Arabidopsis thaliana] gi|122175037|sp|Q0V865.1|FAFL_ARATH RecName: Full=Protein FAF-like, chloroplastic; Flags: Precursor gi|111074178|gb|ABH04462.1| At5g22090 [Arabidopsis thaliana] gi|332005598|gb|AED92981.1| uncharacterized protein AT5G22090 [Arabidopsis thaliana] gi|332005599|gb|AED92982.1| uncharacterized protein AT5G22090 [Arabidopsis thaliana]	457	463	1.00E-156	101.3	76.6	82.7	uncharacterized protein	gbpln	Arabidopsis thaliana	AT5G22090.2 | Symbols: | Protein of unknown function (DUF3049) | chr5:7315003-7316394 REVERSE LENGTH=463	457	463	1.00E-158	101.3	76.6	82.7
Rsa1.0_00010.1.g621.t1	#	#	#	#	#	#	#	-	----	----	#	#	#	#	#	#	#
Rsa1.0_00010.1.g622.t1	#	#	#	#	#	#	#	-	----	----	#	#	#	#	#	#	#

Rsa1.0_00010.1.g623.t1	ref|XP_002874051.1| pyridine nucleotide-disulfide oxidoreductase family protein [Arabidopsis lyrata subsp. lyrata] gi|297319888|gb|EFH50310.1| pyridine nucleotide-disulfide oxidoreductase family protein [Arabidopsis lyrata subsp. lyrata]	370	365	0	98.6	86.8	92.4	pyridine nucleotide-disulfide oxidoreductase family protein	gbpln	Arabidopsis lyrata	AT5G22140.1 | Symbols: | FAD/NAD(P)-binding oxidoreductase family protein | chr5:7340284-7341657 REVERSE LENGTH=365	370	365	0	98.6	86.2	92.4
Rsa1.0_00010.1.g624.t1	ref|NP_680202.1| uncharacterized protein [Arabidopsis thaliana] gi|13374875|emb|CAC34509.1| putative protein [Arabidopsis thaliana] gi|332005608|gb|AED92991.1| uncharacterized protein AT5G22160 [Arabidopsis thaliana]	178	353	9.00E-39	198.3	50.0	61.8	uncharacterized protein	gbpln	Arabidopsis thaliana	AT5G22160.1 | Symbols: | unknown protein; BEST Arabidopsis thaliana protein match is: unknown protein (TAIR:AT5G22150.1); Has 30201 Blast hits to 17322 proteins in 780 species: Archae - 12; Bacteria - 1396; Metazoa - 17338; Fungi - 3422; Plants - 5037; Viruses - 0; Other Eukaryotes - 2996 (source: NCBI BLink). | chr5:7346944-7349255 FORWARD LENGTH=353	178	353	4.00E-41	198.3	50.0	61.8
Rsa1.0_00010.1.g625.t1	ref|XP_002871991.1| hypothetical protein ARALYDRAFT_910206 [Arabidopsis lyrata subsp. lyrata] gi|297317828|gb|EFH48250.1| hypothetical protein ARALYDRAFT_910206 [Arabidopsis lyrata subsp. lyrata]	225	238	1.00E-38	105.8	44.0	60.9	hypothetical protein ARALYDRAFT_910206	gbpln	Arabidopsis lyrata	AT5G22150.1 | Symbols: | unknown protein; BEST Arabidopsis thaliana protein match is: unknown protein (TAIR:AT5G22160.1); Has 30201 Blast hits to 17322 proteins in 780 species: Archae - 12; Bacteria - 1396; Metazoa - 17338; Fungi - 3422; Plants - 5037; Viruses - 0; Other Eukaryotes - 2996 (source: NCBI BLink). | chr5:7342567-7345956 FORWARD LENGTH=330	225	330	3.00E-36	146.7	36.9	48.9
Rsa1.0_00010.1.g626.t1	gb|EOA22413.1| hypothetical protein CARUB_v10003052mg [Capsella rubella]	208	363	5.00E-23	174.5	34.1	45.7	hypothetical protein CARUB_v10003052mg	gbpln	Capsella rubella	AT5G22160.1 | Symbols: | unknown protein; BEST Arabidopsis thaliana protein match is: unknown protein (TAIR:AT5G22150.1); Has 30201 Blast hits to 17322 proteins in 780 species: Archae - 12; Bacteria - 1396; Metazoa - 17338; Fungi - 3422; Plants - 5037; Viruses - 0; Other Eukaryotes - 2996 (source: NCBI BLink). | chr5:7346944-7349255 FORWARD LENGTH=353	208	353	3.00E-23	169.7	37.0	48.6
Rsa1.0_00010.1.g627.t1	ref|XP_002871991.1| hypothetical protein ARALYDRAFT_910206 [Arabidopsis lyrata subsp. lyrata] gi|297317828|gb|EFH48250.1| hypothetical protein ARALYDRAFT_910206 [Arabidopsis lyrata subsp. lyrata]	234	238	2.00E-65	101.7	57.3	72.2	hypothetical protein ARALYDRAFT_910206	gbpln	Arabidopsis lyrata	AT5G22190.1 | Symbols: | unknown protein; BEST Arabidopsis thaliana protein match is: unknown protein (TAIR:AT5G22160.1); Has 30201 Blast hits to 17322 proteins in 780 species: Archae - 12; Bacteria - 1396; Metazoa - 17338; Fungi - 3422; Plants - 5037; Viruses - 0; Other Eukaryotes - 2996 (source: NCBI BLink). | chr5:7354164-7354688 FORWARD LENGTH=117	234	117	3.00E-22	50.0	24.8	28.2
Rsa1.0_00010.1.g628.t1	gb|EOA21564.1| hypothetical protein CARUB_v10001972mg [Capsella rubella]	207	207	8.00E-84	100.0	73.9	84.5	hypothetical protein CARUB_v10001972mg	gbpln	Capsella rubella	AT3G44220.1 | Symbols: | Late embryogenesis abundant (LEA) hydroxyproline-rich glycoprotein family | chr3:15928216-15929645 FORWARD LENGTH=206	207	206	4.00E-85	99.5	70.0	81.6
Rsa1.0_00010.1.g629.t1	emb|CBI28268.3| unnamed protein product [Vitis vinifera]	80	181	1.00E-26	226.3	72.5	83.8	unnamed protein product	gbpln	Vitis vinifera	AT5G22210.2 | Symbols: | unknown protein; Has 35333 Blast hits to 34131 proteins in 2444 species: Archae - 798; Bacteria - 22429; Metazoa - 974; Fungi - 991; Plants - 531; Viruses - 0; Other Eukaryotes - 9610 (source: NCBI BLink). | chr5:7359549-7359791 FORWARD LENGTH=80	80	80	2.00E-27	100.0	87.5	92.5
Rsa1.0_00010.1.g630.t1	ref|NP_001031921.3| E2F transcription factor 1 [Arabidopsis thaliana] gi|332005616|gb|AED92999.1| E2F transcription factor 1 [Arabidopsis thaliana]	410	466	0	113.7	82.7	88.3	E2F transcription factor 1	gbpln	Arabidopsis thaliana	AT5G22220.3 | Symbols: E2F1, E2FB, ATE2FB | E2F transcription factor 1 | chr5:7360749-7364120 FORWARD LENGTH=466	410	466	0	113.7	82.7	88.3
Rsa1.0_00010.1.g631.t1	ref|XP_002867007.1| hypothetical protein ARALYDRAFT_328110 [Arabidopsis lyrata subsp. lyrata] gi|297312843|gb|EFH43266.1| hypothetical protein ARALYDRAFT_328110 [Arabidopsis lyrata subsp. lyrata]	130	223	5.00E-14	171.5	35.4	52.3	hypothetical protein ARALYDRAFT_328110	gbpln	Arabidopsis lyrata	AT4G36590.1 | Symbols: | MADS-box transcription factor family protein | chr4:17261146-17262189 REVERSE LENGTH=248	130	248	2.00E-15	190.8	35.4	52.3
Rsa1.0_00010.1.g632.t1	gb|EOA21692.1| hypothetical protein CARUB_v10002117mg [Capsella rubella]	149	169	4.00E-35	113.4	49.7	57.0	hypothetical protein CARUB_v10002117mg	gbpln	Capsella rubella	AT5G22240.1 | Symbols: ATOFP10, OFP10 | Ovate family protein | chr5:7364689-7365279 FORWARD LENGTH=196	149	196	1.00E-35	131.5	46.3	52.3
Rsa1.0_00010.1.g633.t1	ref|XP_002874054.1| hypothetical protein ARALYDRAFT_489075 [Arabidopsis lyrata subsp. lyrata] gi|297319891|gb|EFH50313.1| hypothetical protein ARALYDRAFT_489075 [Arabidopsis lyrata subsp. lyrata]	286	274	1.00E-127	95.8	81.5	86.4	hypothetical protein ARALYDRAFT_489075	gbpln	Arabidopsis lyrata	AT5G22250.1 | Symbols: | Polynucleotidyl transferase, ribonuclease H-like superfamily protein | chr5:7365605-7366441 REVERSE LENGTH=278	286	278	1.00E-127	97.2	79.7	86.7

Rsa1.0_00010.1.g634.t1	ref|NP_197618.1| RING/FYVE/PHD zinc finger-containing protein [Arabidopsis thaliana] gi|75262726|sp|Q9FMS5.1|MS1_ARATH RecName: Full=PHD finger protein MALE STERILITY 1 gi|9757806|dbj|BAB08324.1| unnamed protein product [Arabidopsis thaliana] gi|332005619|gb|AED93002.1| PHD finger protein MALE STERILITY 1 [Arabidopsis thaliana]	667	672	0	100.7	88.8	94.5	RING/FYVE/PHD zinc finger-containing protein	gbpln	Arabidopsis thaliana	AT5G22260.1 | Symbols: MS1 | RING/FYVE/PHD zinc finger superfamily protein | chr5:7367707-7370192 REVERSE LENGTH=672	667	672	0	100.7	88.8	94.5
Rsa1.0_00010.1.g635.t1	ref|NP_568414.1| NAC domain containing protein 89 [Arabidopsis thaliana] gi|75165521|sp|Q94F58.1|NAC89_ARATH RecName: Full=NAC domain-containing protein 89; Short=ANAC089; AltName: Full=Protein FRUCTOSE-SENSING QUANTITATIVE TRAIT LOCUS 6 gi|14326465|gb|AAK60278.1|AF385685_1 AT5g22290/MWD9_7 [Arabidopsis thaliana] gi|18700194|gb|AAL77707.1| AT5g22290/MWD9_7 [Arabidopsis thaliana] gi|332005624|gb|AED93007.1| NAC domain containing protein 89 [Arabidopsis thaliana]	446	340	1.00E-147	76.2	56.5	65.5	NAC domain containing protein 89	gbpln	Arabidopsis thaliana	AT5G22290.1 | Symbols: anac089, NAC089 | NAC domain containing protein 89 | chr5:7376044-7377498 REVERSE LENGTH=340	446	340	1.00E-150	76.2	56.5	65.5
Rsa1.0_00010.1.g636.t1	ref|XP_002874061.1| hypothetical protein ARALYDRAFT_489082 [Arabidopsis lyrata subsp. lyrata] gi|297319898|gb|EFH50320.1| hypothetical protein ARALYDRAFT_489082 [Arabidopsis lyrata subsp. lyrata]	490	492	0	100.4	76.1	84.1	hypothetical protein ARALYDRAFT_489082	gbpln	Arabidopsis lyrata	AT5G22310.1 | Symbols: | unknown protein; BEST Arabidopsis thaliana protein match is: unknown protein (TAIR:AT3G11590.1); Has 30201 Blast hits to 17322 proteins in 780 species: Archae - 12; Bacteria - 1396; Metazoa - 17338; Fungi - 3422; Plants - 5037; Viruses - 0; Other Eukaryotes - 2996 (source: NCBI BLink). | chr5:7383742-7385345 REVERSE LENGTH=481	490	481	1.00E-180	98.2	75.7	83.3
Rsa1.0_00010.1.g637.t1	ref|NP_568416.1| leucine-rich repeat-containing protein [Arabidopsis thaliana] gi|9757812|dbj|BAB08330.1| unnamed protein product [Arabidopsis thaliana] gi|15912299|gb|AAL08283.1| AT5g22320/MWD9_11 [Arabidopsis thaliana] gi|19699236|gb|AAL90984.1| AT5g22320/MWD9_11 [Arabidopsis thaliana] gi|332005627|gb|AED93010.1| leucine-rich repeat-containing protein [Arabidopsis thaliana]	458	452	1.00E-178	98.7	81.2	90.6	leucine-rich repeat-containing protein	gbpln	Arabidopsis thaliana	AT5G22320.1 | Symbols: | Leucine-rich repeat (LRR) family protein | chr5:7388175-7390426 REVERSE LENGTH=452	458	452	0	98.7	81.2	90.6
Rsa1.0_00010.1.g638.t1	ref|XP_002874064.1| ATTIP49A/RIN1 [Arabidopsis lyrata subsp. lyrata] gi|297319901|gb|EFH50323.1| ATTIP49A/RIN1 [Arabidopsis lyrata subsp. lyrata]	458	458	0	100.0	98.7	99.6	ATTIP49A/RIN1	gbpln	Arabidopsis lyrata	AT5G22330.1 | Symbols: ATTIP49A, RIN1 | P-loop containing nucleoside triphosphate hydrolases superfamily protein | chr5:7391026-7394071 REVERSE LENGTH=458	458	458	0	100.0	98.3	99.6
Rsa1.0_00010.1.g639.t1	ref|XP_002874065.1| hypothetical protein ARALYDRAFT_910225 [Arabidopsis lyrata subsp. lyrata] gi|297319902|gb|EFH50324.1| hypothetical protein ARALYDRAFT_910225 [Arabidopsis lyrata subsp. lyrata]	421	427	0	101.4	92.4	97.1	hypothetical protein ARALYDRAFT_910225	gbpln	Arabidopsis lyrata	AT5G22350.1 | Symbols: ELM1 | Protein of unknown function (DUF1022) | chr5:7397762-7400746 REVERSE LENGTH=427	421	427	0	101.4	91.4	96.4
Rsa1.0_00010.1.g640.t1	ref|XP_002874066.1| vesicle-associated membrane protein 714 [Arabidopsis lyrata subsp. lyrata] gi|297319903|gb|EFH50325.1| vesicle-associated membrane protein 714 [Arabidopsis lyrata subsp. lyrata]	222	221	1.00E-120	99.5	95.0	97.7	vesicle-associated membrane protein 714	gbpln	Arabidopsis lyrata	AT5G22360.1 | Symbols: ATVAMP714, VAMP714 | vesicle-associated membrane protein 714 | chr5:7404379-7405654 REVERSE LENGTH=221	222	221	1.00E-121	99.5	92.8	97.7
Rsa1.0_00010.1.g641.t1	ref|XP_002874067.1| ANAC090 [Arabidopsis lyrata subsp. lyrata] gi|297319904|gb|EFH50326.1| ANAC090 [Arabidopsis lyrata subsp. lyrata]	235	235	1.00E-115	100.0	85.1	91.1	ANAC090	gbpln	Arabidopsis lyrata	AT5G22380.1 | Symbols: anac090, NAC090 | NAC domain containing protein 90 | chr5:7408924-7410038 REVERSE LENGTH=235	235	235	1.00E-115	100.0	83.0	89.4
Rsa1.0_00010.1.g642.t1	ref|XP_002874068.1| hypothetical protein ARALYDRAFT_489095 [Arabidopsis lyrata subsp. lyrata] gi|297319905|gb|EFH50327.1| hypothetical protein ARALYDRAFT_489095 [Arabidopsis lyrata subsp. lyrata]	186	194	4.00E-65	104.3	74.2	83.3	hypothetical protein ARALYDRAFT_489095	gbpln	Arabidopsis lyrata	AT5G22390.1 | Symbols: | Protein of unknown function (DUF3049) | chr5:7418383-7418991 REVERSE LENGTH=202	186	202	9.00E-64	108.6	68.8	80.1
Rsa1.0_00010.1.g643.t1	gb|EOA12263.1| hypothetical protein CARUB_v10007993mg [Capsella rubella]	173	493	6.00E-63	285.0	66.5	79.2	hypothetical protein CARUB_v10007993mg	gbpln	Capsella rubella	AT3G44550.1 | Symbols: FAR5 | fatty acid reductase 5 | chr3:16138060-16141409 FORWARD LENGTH=496	173	496	3.00E-62	286.7	66.5	78.6

Rsa1.0_00010.1.g644.t3	ref|NP_197634.1| putative fatty acyl-CoA reductase 7 [Arabidopsis thaliana] gi|75171715|sp|Q9FMQ9.1|FACR7_ARATH RecName: Full=Putative fatty acyl-CoA reductase 7 gi|9757823|dbj|BAB08341.1| acyl CoA reductase-like protein [Arabidopsis thaliana] gi|332005641|gb|AED93024.1| putative fatty acyl-CoA reductase 7 [Arabidopsis thaliana]	266	409	1.00E-103	153.8	67.3	81.6	putative fatty acyl-CoA reductase 7	gbpln	Arabidopsis thaliana	AT5G22420.1 | Symbols: FAR7 | fatty acid reductase 7 | chr5:7429903-7432586 FORWARD LENGTH=409	266	409	1.00E-106	153.8	67.3	81.6
Rsa1.0_00010.1.g645.t1	gb|EOA21532.1| hypothetical protein CARUB_v10001937mg [Capsella rubella]	212	217	1.00E-103	102.4	97.2	99.1	hypothetical protein CARUB_v10001937mg	gbpln	Capsella rubella	AT5G22440.2 | Symbols: | Ribosomal protein L1p/L10e family | chr5:7435328-7436486 REVERSE LENGTH=217	212	217	1.00E-106	102.4	97.6	98.1
Rsa1.0_00010.1.g646.t1	dbj|BAB08344.1| unnamed protein product [Arabidopsis thaliana]	1207	1180	0	97.8	82.5	87.9	unnamed protein product	gbpln	Arabidopsis thaliana	AT5G22450.1 | Symbols: | unknown protein; LOCATED IN: chloroplast; EXPRESSED IN: 22 plant structures; EXPRESSED DURING: 13 growth stages; BEST Arabidopsis thaliana protein match is: unknown protein (TAIR:AT2G19390.1); Has 30201 Blast hits to 17322 proteins in 780 species: Archae - 12; Bacteria - 1396; Metazoa - 17338; Fungi - 3422; Plants - 5037; Viruses - 0; Other Eukaryotes - 2996 (source: NCBI BLink). | chr5:7437145-7442856 REVERSE LENGTH=1154	1207	1154	0	95.6	81.9	87.3
Rsa1.0_00010.1.g647.t1	ref|XP_002874071.1| esterase/lipase/thioesterase family protein [Arabidopsis lyrata subsp. lyrata] gi|297319908|gb|EFH50330.1| esterase/lipase/thioesterase family protein [Arabidopsis lyrata subsp. lyrata]	341	340	1.00E-167	99.7	81.5	90.6	esterase/lipase/thioesterase family protein	gbpln	Arabidopsis lyrata	AT5G22460.2 | Symbols: | alpha/beta-Hydrolases superfamily protein | chr5:7443659-7445269 REVERSE LENGTH=340	341	340	1.00E-167	99.7	79.8	90.0
Rsa1.0_00010.1.g648.t1	dbj|BAB09119.1| seed maturation protein PM38 protein [Arabidopsis thaliana]	811	815	0	100.5	92.8	96.8	seed maturation protein PM38 protein	gbpln	Arabidopsis thaliana	AT5G22470.1 | Symbols: | NAD+ ADP-ribosyltransferases;NAD+ ADP-ribosyltransferases | chr5:7447045-7450743 FORWARD LENGTH=814	811	814	0	100.4	92.7	96.7
Rsa1.0_00010.1.g649.t1	ref|XP_002884569.1| hypothetical protein ARALYDRAFT_896743 [Arabidopsis lyrata subsp. lyrata] gi|297330409|gb|EFH60828.1| hypothetical protein ARALYDRAFT_896743 [Arabidopsis lyrata subsp. lyrata]	236	280	1.00E-27	118.6	29.7	48.7	hypothetical protein ARALYDRAFT_896743	gbpln	Arabidopsis lyrata	AT5G17080.1 | Symbols: | Cysteine proteinases superfamily protein | chr5:5619594-5620949 FORWARD LENGTH=298	236	298	2.00E-21	126.3	25.8	40.3
Rsa1.0_00010.1.g650.t1	ref|XP_002889471.1| hypothetical protein ARALYDRAFT_470343 [Arabidopsis lyrata subsp. lyrata] gi|297335313|gb|EFH65730.1| hypothetical protein ARALYDRAFT_470343 [Arabidopsis lyrata subsp. lyrata]	229	219	1.00E-25	95.6	38.4	52.0	hypothetical protein ARALYDRAFT_470343	gbpln	Arabidopsis lyrata	AT1G03710.2 | Symbols: | Cystatin/monellin superfamily protein | chr1:923492-924287 FORWARD LENGTH=202	229	202	2.00E-23	88.2	37.6	50.7
Rsa1.0_00010.1.g651.t3	ref|XP_002874072.1| zinc finger (ZPR1-type) family protein [Arabidopsis lyrata subsp. lyrata] gi|297319909|gb|EFH50331.1| zinc finger (ZPR1-type) family protein [Arabidopsis lyrata subsp. lyrata]	487	493	0	101.2	86.2	89.7	zinc finger (ZPR1-type) family protein	gbpln	Arabidopsis lyrata	AT5G37340.3 | Symbols: | ZPR1 zinc-finger domain protein | chr5:14787488-14792373 REVERSE LENGTH=493	487	493	0	101.2	85.2	88.7
Rsa1.0_00010.1.g652.t1	ref|XP_002872004.1| hypothetical protein ARALYDRAFT_910242 [Arabidopsis lyrata subsp. lyrata] gi|297317841|gb|EFH48263.1| hypothetical protein ARALYDRAFT_910242 [Arabidopsis lyrata subsp. lyrata]	493	491	0	99.6	86.6	94.1	hypothetical protein ARALYDRAFT_910242	gbpln	Arabidopsis lyrata	AT5G22500.1 | Symbols: FAR1 | fatty acid reductase 1 | chr5:7470541-7473916 FORWARD LENGTH=491	493	491	0	99.6	86.0	94.5
Rsa1.0_00011.1.g653.t1	#	#	#	#	#	#	#	-	----	----	#	#	#	#	#	#	#
Rsa1.0_00011.1.g654.t1	gb|EOA17296.1| hypothetical protein CARUB_v10005568mg [Capsella rubella]	263	253	1.00E-116	96.2	79.8	86.7	hypothetical protein CARUB_v10005568mg	gbpln	Capsella rubella	AT4G29260.1 | Symbols: | HAD superfamily, subfamily IIIB acid phosphatase | chr4:14422310-14423409 REVERSE LENGTH=255	263	255	1.00E-117	97.0	78.3	85.2
Rsa1.0_00011.1.g655.t1	gb|EOA16729.1| hypothetical protein CARUB_v10004932mg [Capsella rubella]	406	413	1.00E-175	101.7	84.0	90.1	hypothetical protein CARUB_v10004932mg	gbpln	Capsella rubella	AT4G29240.1 | Symbols: | Leucine-rich repeat (LRR) family protein | chr4:14418826-14420073 FORWARD LENGTH=415	406	415	1.00E-173	102.2	72.2	78.1
Rsa1.0_00011.1.g656.t1	gb|AAG50806.1|AC079281_8 unknown protein [Arabidopsis thaliana]	1166	1213	0	104.0	37.3	55.4	unknown protein	gbpln	Arabidopsis thaliana	AT1G43760.1 | Symbols: | DNAse I-like superfamily protein | chr1:16528880-16531065 REVERSE LENGTH=626	1166	626	2.00E-66	53.7	13.0	21.5

Rsa1.0_00011.1.g657.t1	ref|NP_194652.1| NAC domain containing protein 75 [Arabidopsis thaliana] gi|7269821|emb|CAB79681.1| putative protein [Arabidopsis thaliana] gi|332660205|gb|AEE85605.1| NAC domain containing protein 75 [Arabidopsis thaliana]	523	498	0	95.2	86.4	89.1	NAC domain containing protein 75	gbpln	Arabidopsis thaliana	AT4G29230.1 | Symbols: anac075, NAC075 | NAC domain containing protein 75 | chr4:14410338-14414956 FORWARD LENGTH=498	523	498	0	95.2	86.4	89.1
Rsa1.0_00011.1.g658.t4	gb|AAM91591.1| pyrophosphate-dependent phosphofructo-1-kinase-like protein [Arabidopsis thaliana]	88	473	2.00E-13	537.5	44.3	48.9	pyrophosphate-dependent phosphofructo-1-kinase-like protein	gbpln	Arabidopsis thaliana	AT4G29220.1 | Symbols: PFK1 | phosphofructokinase 1 | chr4:14403621-14406071 REVERSE LENGTH=473	88	473	4.00E-16	537.5	44.3	48.9
Rsa1.0_00011.1.g659.t1	dbj|BAD22536.1| gamma-glutamyl transferase [Raphanus sativus]	639	635	0	99.4	79.3	88.4	gamma-glutamyl transferase	gbpln	Raphanus sativus	AT4G29210.1 | Symbols: GGT3, GGT4 | gamma-glutamyl transpeptidase 4 | chr4:14400990-14403319 FORWARD LENGTH=637	639	637	0	99.7	78.7	87.5
Rsa1.0_00011.1.g660.t1	ref|XP_002330525.1| predicted protein [Populus trichocarpa] gi|222872459|gb|EEF09590.1| predicted protein [Populus trichocarpa]	640	605	1.00E-168	94.5	48.3	63.4	predicted protein	gbpln	Populus trichocarpa	AT4G29210.1 | Symbols: GGT3, GGT4 | gamma-glutamyl transpeptidase 4 | chr4:14400990-14403319 FORWARD LENGTH=637	640	637	1.00E-164	99.5	47.7	61.1
Rsa1.0_00011.1.g661.t1	dbj|BAJ33902.1| unnamed protein product [Thellungiella halophila]	344	365	1.00E-168	106.1	86.6	92.2	unnamed protein product	----	----	AT4G29190.1 | Symbols: | Zinc finger C-x8-C-x5-C-x3-H type family protein | chr4:14392233-14393303 REVERSE LENGTH=356	344	356	1.00E-160	103.5	83.4	88.4
Rsa1.0_00011.1.g662.t1	ref|NP_001190865.1| protein root hair specific 16 [Arabidopsis thaliana] gi|264664523|sp|C0LGR6.2|Y4291_ARATH RecName: Full=Probable LRR receptor-like serine/threonine-protein kinase At4g29180; Flags: Precursor gi|332660199|gb|AEE85599.1| probable LRR receptor-like serine/threonine-protein kinase [Arabidopsis thaliana]	908	913	0	100.6	85.9	92.4	protein root hair specific 16	gbpln	Arabidopsis thaliana	AT4G29180.2 | Symbols: RHS16 | root hair specific 16 | chr4:14385631-14389524 FORWARD LENGTH=913	908	913	0	100.6	85.9	92.4
Rsa1.0_00011.1.g663.t1	ref|NP_194646.2| meiotic nuclear division protein 1-like protein [Arabidopsis thaliana] gi|75151407|sp|Q8GYD2.1|MND1_ARATH RecName: Full=Meiotic nuclear division protein 1 homolog; Short=AtMND1; AltName: Full=Meiotic nuclear division 1-like protein gi|26450501|dbj|BAC42364.1| unknown protein [Arabidopsis thaliana] gi|28973061|gb|AAO63855.1| unknown protein [Arabidopsis thaliana] gi|82408770|gb|ABB73190.1| meiotic nuclear division 1-like protein [Arabidopsis thaliana] gi|82775232|emb|CAJ44238.1| AtMnd1 protein [Arabidopsis thaliana] gi|332660196|gb|AEE85596.1| meiotic nuclear division protein 1-like protein [Arabidopsis thaliana]	256	230	1.00E-100	89.8	75.0	78.5	meiotic nuclear division protein 1-like protein	gbpln	Arabidopsis thaliana	AT4G29170.1 | Symbols: ATMND1 | Mnd1 family protein | chr4:14382985-14385128 FORWARD LENGTH=230	256	230	1.00E-102	89.8	75.0	78.5
Rsa1.0_00011.1.g664.t1	gb|EOA17400.1| hypothetical protein CARUB_v10005703mg [Capsella rubella] gi|482553208|gb|EOA17401.1| hypothetical protein CARUB_v10005703mg [Capsella rubella]	215	218	6.00E-96	101.4	96.3	98.1	hypothetical protein CARUB_v10005703mg	gbpln	Capsella rubella	AT4G29160.3 | Symbols: SNF7.1 | SNF7 family protein | chr4:14381019-14382342 FORWARD LENGTH=219	215	219	2.00E-92	101.9	92.6	94.4
Rsa1.0_00011.1.g665.t1	ref|XP_002869449.1| hypothetical protein ARALYDRAFT_491843 [Arabidopsis lyrata subsp. lyrata] gi|297315285|gb|EFH45708.1| hypothetical protein ARALYDRAFT_491843 [Arabidopsis lyrata subsp. lyrata]	388	385	1.00E-176	99.2	85.8	91.0	hypothetical protein ARALYDRAFT_491843	gbpln	Arabidopsis lyrata	AT4G29150.1 | Symbols: IQD25 | IQ-domain 25 | chr4:14378770-14380340 FORWARD LENGTH=399	388	399	1.00E-174	102.8	85.1	90.2
Rsa1.0_00011.1.g666.t1	#	#	#	#	#	#	#	-	----	----	#	#	#	#	#	#	#
Rsa1.0_00011.1.g667.t1	dbj|BAB08714.1| non-LTR retroelement reverse transcriptase-like protein [Arabidopsis thaliana]	1032	1197	1.00E-110	116.0	18.9	25.3	non-LTR retroelement reverse transcriptase-like protein	gbpln	Arabidopsis thaliana	AT5G36228.1 | Symbols: | nucleic acid binding;zinc ion binding | chr5:14271691-14272776 FORWARD LENGTH=361	1032	361	8.00E-19	35.0	5.4	7.4
Rsa1.0_00011.1.g668.t1	emb|CAB39638.1| RNA-directed DNA polymerase-like protein [Arabidopsis thaliana] gi|7267666|emb|CAB78094.1| RNA-directed DNA polymerase-like protein [Arabidopsis thaliana]	779	1274	0	163.5	49.4	65.2	RNA-directed DNA polymerase-like protein	gbpln	Arabidopsis thaliana	AT4G29090.1 | Symbols: | Ribonuclease H-like superfamily protein | chr4:14333528-14335255 FORWARD LENGTH=575	779	575	1.00E-74	73.8	23.4	35.7
Rsa1.0_00011.1.g669.t1	ref|XP_002869450.1| hypothetical protein ARALYDRAFT_913597 [Arabidopsis lyrata subsp. lyrata] gi|297315286|gb|EFH45709.1| hypothetical protein ARALYDRAFT_913597 [Arabidopsis lyrata subsp. lyrata]	538	533	0	99.1	88.1	93.5	hypothetical protein ARALYDRAFT_913597	gbpln	Arabidopsis lyrata	AT4G29140.1 | Symbols: | MATE efflux family protein | chr4:14369148-14370746 FORWARD LENGTH=532	538	532	0	98.9	85.7	91.6

Rsa1.0_00011.1.g670.t1	#	#	#	#	#	#	#	-	----	----	#	#	#	#	#	#	#
Rsa1.0_00011.1.g671.t1	ref|NP_194640.1| uncharacterized protein [Arabidopsis thaliana] gi|4972057|emb|CAB43925.1| putative protein [Arabidopsis thaliana] gi|7269809|emb|CAB79669.1| putative protein [Arabidopsis thaliana] gi|60547867|gb|AAX23897.1| hypothetical protein At4g29110 [Arabidopsis thaliana] gi|71905525|gb|AAZ52740.1| hypothetical protein At4g29110 [Arabidopsis thaliana] gi|149944337|gb|ABR46211.1| At4g29110 [Arabidopsis thaliana] gi|332660188|gb|AEE85588.1| uncharacterized protein AT4G29110 [Arabidopsis thaliana]	193	200	3.00E-82	103.6	81.9	87.6	uncharacterized protein	gbpln	Arabidopsis thaliana	AT4G29110.1 | Symbols: | unknown protein; FUNCTIONS IN: molecular_function unknown; INVOLVED IN: response to chitin; LOCATED IN: vacuole; BEST Arabidopsis thaliana protein match is: unknown protein (TAIR:AT1G52140.1); Has 109 Blast hits to 109 proteins in 16 species: Archae - 0; Bacteria - 0; Metazoa - 0; Fungi - 0; Plants - 109; Viruses - 0; Other Eukaryotes - 0 (source: NCBI BLink). | chr4:14348866-14349468 FORWARD LENGTH=200	193	200	9.00E-85	103.6	81.9	87.6
Rsa1.0_00011.1.g672.t1	emb|CAC14433.1| putative protein [Brassica napus]	385	389	0	101.0	93.0	95.8	putative protein	gbpln	Brassica napus	AT4G29100.1 | Symbols: | basic helix-loop-helix (bHLH) DNA-binding superfamily protein | chr4:14341140-14344575 FORWARD LENGTH=407	385	407	1.00E-171	105.7	88.1	92.5
Rsa1.0_00011.1.g673.t1	ref|NP_850981.1| aspartyl protease-like protein [Arabidopsis thaliana] gi|17065172|gb|AAL32740.1| nucellin-like protein [Arabidopsis thaliana] gi|24899795|gb|AAN65112.1| nucellin-like protein [Arabidopsis thaliana] gi|332197863|gb|AEE35984.1| aspartyl protease-like protein [Arabidopsis thaliana]	114	466	2.00E-37	408.8	72.8	78.9	aspartyl protease-like protein	gbpln	Arabidopsis thaliana	AT1G77480.1 | Symbols: | Eukaryotic aspartyl protease family protein | chr1:29114705-29117150 REVERSE LENGTH=466	114	466	2.00E-40	408.8	72.8	78.9
Rsa1.0_00011.1.g674.t1	ref|XP_002892897.1| hypothetical protein ARALYDRAFT_471818 [Arabidopsis lyrata subsp. lyrata] gi|297338739|gb|EFH69156.1| hypothetical protein ARALYDRAFT_471818 [Arabidopsis lyrata subsp. lyrata]	70	233	8.00E-26	332.9	77.1	85.7	hypothetical protein ARALYDRAFT_471818	gbpln	Arabidopsis lyrata	AT1G16210.1 | Symbols: | unknown protein; CONTAINS InterPro DOMAIN/s: Protein of unknown function DUF1014 (InterPro:IPR010422); Has 16107 Blast hits to 8386 proteins in 1107 species: Archae - 26; Bacteria - 3370; Metazoa - 4013; Fungi - 1516; Plants - 526; Viruses - 120; Other Eukaryotes - 6536 (source: NCBI BLink). | chr1:5546352-5547447 REVERSE LENGTH=234	70	234	2.00E-27	334.3	74.3	82.9
Rsa1.0_00011.1.g675.t3	emb|CAC14431.1| hypothetical protein [Brassica napus]	203	151	2.00E-50	74.4	51.7	52.7	hypothetical protein	gbpln	Brassica napus	AT4G16295.1 | Symbols: SPH1 | S-protein homologue 1 | chr4:9215680-9216135 REVERSE LENGTH=151	203	151	2.00E-36	74.4	40.4	42.9
Rsa1.0_00011.1.g676.t1	dbj|BAC42653.1| putative glycine-rich protein [Arabidopsis thaliana] gi|28416867|gb|AAO42964.1| At4g29030 [Arabidopsis thaliana]	112	115	6.00E-13	102.7	35.7	39.3	putative glycine-rich protein	gbpln	Arabidopsis thaliana	#	#	#	#	#	#	#
Rsa1.0_00011.1.g677.t1	#	#	#	#	#	#	#	-	----	----	AT4G29020.2 | Symbols: | glycine-rich protein | chr4:14305032-14305508 FORWARD LENGTH=113	152	113	5.00E-13	74.3	22.4	23.0
Rsa1.0_00011.1.g678.t1	ref|NP_194630.1| enoyl-CoA hydratase/3-hydroxyacyl-CoA dehydrogenase [Arabidopsis thaliana] gi|75315624|sp|Q9ZPI6.1|AIM1_ARATH RecName: Full=Peroxisomal fatty acid beta-oxidation multifunctional protein AIM1; AltName: Full=Protein ABNORMAL INFLORESCENCE MERISTEM 1; Short=AtAIM1; Includes: RecName: Full=Enoyl-CoA hydratase/3-2-trans-enoyl-CoA isomerase/3-hydroxybutyryl-CoA epimerase; Includes: RecName: Full=3-hydroxyacyl-CoA dehydrogenase gi|4337025|gb|AAD18041.1| AIM1 protein [Arabidopsis thaliana] gi|4972047|emb|CAB43915.1| AIM1 protein [Arabidopsis thaliana] gi|7269799|emb|CAB79659.1| AIM1 protein [Arabidopsis thaliana] gi|16648891|gb|AAL24297.1| AIM1 protein [Arabidopsis thaliana] gi|18175606|gb|AAL59895.1| putative AIM1 protein [Arabidopsis thaliana] gi|20465649|gb|AAM20293.1| putative AIM1 protein [Arabidopsis thaliana] gi|332660172|gb|AEE85572.1| fatty acid beta-oxidation multifunctional protein AIM1 [Arabidopsis thaliana]	581	721	1.00E-167	124.1	52.2	56.8	enoyl-CoA hydratase/3-hydroxyacyl-CoA dehydrogenase	gbpln	Arabidopsis thaliana	AT4G29010.1 | Symbols: AIM1 | Enoyl-CoA hydratase/isomerase family | chr4:14297312-14302016 REVERSE LENGTH=721	581	721	1.00E-170	124.1	52.2	56.8

Rsa1.0_00011.1.g679.t1	ref|XP_002869459.1| hypothetical protein ARALYDRAFT_491858 [Arabidopsis lyrata subsp. lyrata] gi|297315295|gb|EFH45718.1| hypothetical protein ARALYDRAFT_491858 [Arabidopsis lyrata subsp. lyrata]	525	601	0	114.5	77.5	84.6	hypothetical protein ARALYDRAFT_491858	gbpln	Arabidopsis lyrata	AT4G29000.1 | Symbols: | Tesmin/TSO1-like CXC domain-containing protein | chr4:14293957-14296602 FORWARD LENGTH=603	525	603	0	114.9	75.6	82.7
Rsa1.0_00011.1.g680.t1	ref|NP_194628.1| RNA-binding-related protein [Arabidopsis thaliana] gi|4972045|emb|CAB43913.1| putative protein [Arabidopsis thaliana] gi|7269797|emb|CAB79657.1| putative protein [Arabidopsis thaliana] gi|115646852|gb|ABJ17140.1| At4g28990 [Arabidopsis thaliana] gi|332660169|gb|AEE85569.1| RNA-binding-related protein [Arabidopsis thaliana]	337	347	1.00E-80	103.0	63.5	70.9	RNA-binding-related protein	gbpln	Arabidopsis thaliana	AT4G28990.1 | Symbols: | RNA-binding protein-related | chr4:14291205-14293018 FORWARD LENGTH=347	337	347	3.00E-83	103.0	63.5	70.9
Rsa1.0_00011.1.g681.t1	ref|XP_002517802.1| DNA topoisomerase II, putative [Ricinus communis] gi|223543074|gb|EEF44609.1| DNA topoisomerase II, putative [Ricinus communis]	170	1212	2.00E-16	712.9	35.9	46.5	DNA topoisomerase II, putative	gbpln	Ricinus communis	AT3G23890.2 | Symbols: TOPII, ATTOPII | topoisomerase II | chr3:8624931-8631106 FORWARD LENGTH=1438	170	1438	2.00E-12	845.9	24.7	28.8
Rsa1.0_00011.1.g682.t1	ref|XP_002869463.1| hypothetical protein ARALYDRAFT_491863 [Arabidopsis lyrata subsp. lyrata] gi|297315299|gb|EFH45722.1| hypothetical protein ARALYDRAFT_491863 [Arabidopsis lyrata subsp. lyrata]	350	348	1.00E-167	99.4	82.9	91.1	hypothetical protein ARALYDRAFT_491863	gbpln	Arabidopsis lyrata	AT4G28940.1 | Symbols: | Phosphorylase superfamily protein | chr4:14274414-14276920 FORWARD LENGTH=348	350	348	1.00E-169	99.4	82.6	90.6
Rsa1.0_00011.1.g683.t1	ref|XP_002867431.1| predicted protein [Arabidopsis lyrata subsp. lyrata] gi|297313267|gb|EFH43690.1| predicted protein [Arabidopsis lyrata subsp. lyrata]	330	329	1.00E-121	99.7	67.6	80.3	predicted protein	gbpln	Arabidopsis lyrata	AT4G28920.1 | Symbols: | Protein of unknown function (DUF626) | chr4:14268415-14269885 REVERSE LENGTH=292	330	292	2.00E-65	88.5	44.2	56.1
Rsa1.0_00011.1.g684.t1	ref|XP_002867431.1| predicted protein [Arabidopsis lyrata subsp. lyrata] gi|297313267|gb|EFH43690.1| predicted protein [Arabidopsis lyrata subsp. lyrata]	336	329	1.00E-123	97.9	67.6	81.5	predicted protein	gbpln	Arabidopsis lyrata	AT4G28920.1 | Symbols: | Protein of unknown function (DUF626) | chr4:14268415-14269885 REVERSE LENGTH=292	336	292	6.00E-62	86.9	43.2	53.9
Rsa1.0_00011.1.g685.t1	ref|XP_002869465.1| predicted protein [Arabidopsis lyrata subsp. lyrata] gi|297315301|gb|EFH45724.1| predicted protein [Arabidopsis lyrata subsp. lyrata]	301	280	2.00E-84	93.0	55.8	64.8	predicted protein	gbpln	Arabidopsis lyrata	AT3G19515.2 | Symbols: | CONTAINS InterPro DOMAIN/s: Apoptosis inhibitory 5 (InterPro:IPR008383); BEST Arabidopsis thaliana protein match is: unknown protein (TAIR:AT1G23935.1); Has 546 Blast hits to 394 proteins in 84 species: Archae - 0; Bacteria - 3; Metazoa - 210; Fungi - 15; Plants - 143; Viruses - 0; Other Eukaryotes - 175 (source: NCBI BLink). | chr3:6766872-6771078 REVERSE LENGTH=805	301	805	2.00E-28	267.4	23.9	39.5
Rsa1.0_00011.1.g686.t1	gb|EOA16694.1| hypothetical protein CARUB_v10004886mg [Capsella rubella]	403	424	1.00E-157	105.2	78.4	84.9	hypothetical protein CARUB_v10004886mg	gbpln	Capsella rubella	AT4G28910.3 | Symbols: NINJA | novel interactor of JAZ | chr4:14264330-14265680 REVERSE LENGTH=425	403	425	1.00E-155	105.5	75.4	82.6
Rsa1.0_00011.1.g687.t1	ref|XP_002867435.1| ring-H2 finger protein ATL4J precursor [Arabidopsis lyrata subsp. lyrata] gi|297313271|gb|EFH43694.1| ring-H2 finger protein ATL4J precursor [Arabidopsis lyrata subsp. lyrata]	596	435	1.00E-162	73.0	52.0	56.9	ring-H2 finger protein ATL4J precursor	gbpln	Arabidopsis lyrata	AT4G28890.1 | Symbols: | RING/U-box superfamily protein | chr4:14256437-14257735 REVERSE LENGTH=432	596	432	1.00E-163	72.5	52.3	56.5
Rsa1.0_00011.1.g688.t1	gb|EOA16720.1| hypothetical protein CARUB_v10004924mg [Capsella rubella]	421	415	0	98.6	91.4	94.8	hypothetical protein CARUB_v10004924mg	gbpln	Capsella rubella	AT4G28880.1 | Symbols: ckl3 | casein kinase I-like 3 | chr4:14251351-14254048 FORWARD LENGTH=415	421	415	0	98.6	91.7	94.8
Rsa1.0_00011.1.g689.t1	gb|EOA18729.1| hypothetical protein CARUB_v10007311mg [Capsella rubella]	150	198	2.00E-55	132.0	73.3	79.3	hypothetical protein CARUB_v10007311mg	gbpln	Capsella rubella	AT4G28840.1 | Symbols: | unknown protein; FUNCTIONS IN: molecular_function unknown; INVOLVED IN: biological_process unknown; LOCATED IN: chloroplast; BEST Arabidopsis thaliana protein match is: unknown protein (TAIR:AT2G20080.1); Has 71 Blast hits to 69 proteins in 15 species: Archae - 0; Bacteria - 0; Metazoa - 2; Fungi - 0; Plants - 67; Viruses - 0; Other Eukaryotes - 2 (source: NCBI BLink). | chr4:14240867-14242513 FORWARD LENGTH=193	150	193	7.00E-54	128.7	70.7	76.0
Rsa1.0_00011.1.g690.t1	gb|EOA17434.1| hypothetical protein CARUB_v10005737mg [Capsella rubella]	208	208	1.00E-107	100.0	94.7	98.6	hypothetical protein CARUB_v10005737mg	gbpln	Capsella rubella	AT4G28830.1 | Symbols: | S-adenosyl-L-methionine-dependent methyltransferases superfamily protein | chr4:14232490-14233917 FORWARD LENGTH=208	208	208	1.00E-109	100.0	94.7	98.1

Rsa1.0_00011.1.g691.t1	ref|NP_194611.2| HIT-type zinc finger protein [Arabidopsis thaliana] gi|92856590|gb|ABE77404.1| At4g28820 [Arabidopsis thaliana] gi|332660149|gb|AEE85549.1| HIT-type zinc finger protein [Arabidopsis thaliana]	198	173	9.00E-30	87.4	43.4	50.0	HIT-type zinc finger protein	gbpln	Arabidopsis thaliana	AT4G28820.1 | Symbols: | HIT-type Zinc finger family protein | chr4:14230815-14232287 REVERSE LENGTH=173	198	173	3.00E-32	87.4	43.4	50.0
Rsa1.0_00011.1.g692.t1	ref|XP_002869474.1| expressed protein [Arabidopsis lyrata subsp. lyrata] gi|297315310|gb|EFH45733.1| expressed protein [Arabidopsis lyrata subsp. lyrata]	119	139	1.00E-35	116.8	61.3	78.2	expressed protein	gbpln	Arabidopsis lyrata	AT4G28775.1 | Symbols: | unknown protein; FUNCTIONS IN: molecular_function unknown; INVOLVED IN: biological_process unknown; LOCATED IN: cellular_component unknown; Has 30201 Blast hits to 17322 proteins in 780 species: Archae - 12; Bacteria - 1396; Metazoa - 17338; Fungi - 3422; Plants - 5037; Viruses - 0; Other Eukaryotes - 2996 (source: NCBI BLink). | chr4:14214817-14215233 FORWARD LENGTH=138	119	138	3.00E-37	116.0	61.3	76.5
Rsa1.0_00011.1.g693.t1	gb|EOA30311.1| hypothetical protein CARUB_v10013437mg [Capsella rubella]	544	532	0	97.8	80.9	86.2	hypothetical protein CARUB_v10013437mg	gbpln	Capsella rubella	AT3G24430.1 | Symbols: HCF101 | ATP binding | chr3:8868731-8872154 REVERSE LENGTH=532	544	532	0	97.8	78.9	82.0
Rsa1.0_00011.1.g694.t1	ref|XP_002869474.1| expressed protein [Arabidopsis lyrata subsp. lyrata] gi|297315310|gb|EFH45733.1| expressed protein [Arabidopsis lyrata subsp. lyrata]	138	139	8.00E-46	100.7	68.1	82.6	expressed protein	gbpln	Arabidopsis lyrata	AT4G28775.1 | Symbols: | unknown protein; FUNCTIONS IN: molecular_function unknown; INVOLVED IN: biological_process unknown; LOCATED IN: cellular_component unknown; Has 30201 Blast hits to 17322 proteins in 780 species: Archae - 12; Bacteria - 1396; Metazoa - 17338; Fungi - 3422; Plants - 5037; Viruses - 0; Other Eukaryotes - 2996 (source: NCBI BLink). | chr4:14214817-14215233 FORWARD LENGTH=138	138	138	8.00E-45	100.0	60.9	72.5
Rsa1.0_00011.1.g695.t1	ref|XP_002869476.1| hypothetical protein ARALYDRAFT_913640 [Arabidopsis lyrata subsp. lyrata] gi|297315312|gb|EFH45735.1| hypothetical protein ARALYDRAFT_913640 [Arabidopsis lyrata subsp. lyrata]	338	328	1.00E-164	97.0	86.1	89.6	hypothetical protein ARALYDRAFT_913640	gbpln	Arabidopsis lyrata	AT4G28740.1 | Symbols: | FUNCTIONS IN: molecular_function unknown; INVOLVED IN: biological_process unknown; LOCATED IN: chloroplast; EXPRESSED IN: 21 plant structures; EXPRESSED DURING: 13 growth stages; CONTAINS InterPro DOMAIN/s: Protein of unknown function DUF3493 (InterPro:IPR021883); BEST Arabidopsis thaliana protein match is: tetratricopeptide repeat (TPR)-containing protein (TAIR:AT1G02910.1); Has 30201 Blast hits to 17322 proteins in 780 species: Archae - 12; Bacteria - 1396; Metazoa - 17338; Fungi - 3422; Plants - 5037; Viruses - 0; Other Eukaryotes - 2996 (source: NCBI BLink). | chr4:14201051-14202542 FORWARD LENGTH=347	338	347	1.00E-166	102.7	84.3	89.3
Rsa1.0_00011.1.g696.t1	ref|NP_194602.2| glutaredoxin-C5 [Arabidopsis thaliana] gi|75151040|sp|Q8GWS0.1|GRXC5_ARATH RecName: Full=Glutaredoxin-C5, chloroplastic; Short=AtGrxC5; Flags: Precursor gi|26452363|dbj|BAC43267.1| unknown protein [Arabidopsis thaliana] gi|28372898|gb|AAO39931.1| At4g28730 [Arabidopsis thaliana] gi|332660135|gb|AEE85535.1| glutaredoxin-C5 [Arabidopsis thaliana]	184	174	7.00E-67	94.6	79.3	84.8	glutaredoxin-C5	gbpln	Arabidopsis thaliana	AT4G28730.1 | Symbols: | Glutaredoxin family protein | chr4:14199174-14200712 FORWARD LENGTH=174	184	174	2.00E-69	94.6	79.3	84.8
Rsa1.0_00011.1.g697.t1	ref|XP_002869437.1| hypothetical protein ARALYDRAFT_913571 [Arabidopsis lyrata subsp. lyrata] gi|297315273|gb|EFH45696.1| hypothetical protein ARALYDRAFT_913571 [Arabidopsis lyrata subsp. lyrata]	402	394	5.00E-94	98.0	48.3	61.9	hypothetical protein ARALYDRAFT_913571	gbpln	Arabidopsis lyrata	AT5G39560.1 | Symbols: | Galactose oxidase/kelch repeat superfamily protein | chr5:15841885-15843096 REVERSE LENGTH=403	402	403	1.00E-70	100.2	47.5	61.4
Rsa1.0_00011.1.g698.t1	gb|EOA18879.1| hypothetical protein CARUB_v10007502mg [Capsella rubella]	511	506	0	99.0	89.0	95.3	hypothetical protein CARUB_v10007502mg	gbpln	Capsella rubella	AT4G28700.1 | Symbols: AMT1;4 | ammonium transporter 1;4 | chr4:14161681-14163195 FORWARD LENGTH=504	511	504	0	98.6	87.9	95.3

Rsa1.0_00011.1.g699.t1	ref|NP_567814.1| photosystem II reaction center PSB28 protein [Arabidopsis thaliana] gi|75161816|sp|Q8W0Y8.1|PSB28_ARATH RecName: Full=Photosystem II reaction center PSB28 protein, chloroplastic; AltName: Full=Photosystem II protein W-like; Flags: Precursor gi|18087645|gb|AAL58951.1|AF462867_1 AT4g28660/T5F17_110 [Arabidopsis thaliana] gi|21360421|gb|AAM47326.1| AT4g28660/T5F17_110 [Arabidopsis thaliana] gi|21592461|gb|AAM64412.1| photosystem II protein W-like [Arabidopsis thaliana] gi|332660119|gb|AEE85519.1| photosystem II reaction center PSB28 protein [Arabidopsis thaliana]	152	183	7.00E-72	120.4	88.8	92.1	photosystem II reaction center PSB28 protein	gbpln	Arabidopsis thaliana	AT4G28660.1 | Symbols: PSB28 | photosystem II reaction center PSB28 protein | chr4:14150008-14150933 FORWARD LENGTH=183	152	183	2.00E-74	120.4	88.8	92.1
Rsa1.0_00011.1.g700.t2	dbj|BAJ34152.1| unnamed protein product [Thellungiella halophila]	184	246	3.00E-35	133.7	52.2	56.0	unnamed protein product	----	----	AT4G28640.2 | Symbols: IAA11 | indole-3-acetic acid inducible 11 | chr4:14142288-14143928 FORWARD LENGTH=302	184	302	3.00E-33	164.1	48.9	51.1
Rsa1.0_00011.1.g701.t1	#	#	#	#	#	#	#	-	----	----	#	#	#	#	#	#	#
Rsa1.0_00011.1.g702.t1	ref|XP_002869492.1| hypothetical protein ARALYDRAFT_491906 [Arabidopsis lyrata subsp. lyrata] gi|297315328|gb|EFH45751.1| hypothetical protein ARALYDRAFT_491906 [Arabidopsis lyrata subsp. lyrata]	748	748	0	100.0	82.4	89.8	hypothetical protein ARALYDRAFT_491906	gbpln	Arabidopsis lyrata	AT4G28570.1 | Symbols: | Long-chain fatty alcohol dehydrogenase family protein | chr4:14119548-14121923 FORWARD LENGTH=748	748	748	0	100.0	82.1	88.9
Rsa1.0_00012.1.g703.t1	gb|AAP74222.1| RST1 [Arabidopsis thaliana]	291	1841	4.00E-44	632.6	34.4	38.1	RST1	gbpln	Arabidopsis thaliana	AT3G27670.1 | Symbols: RST1 | ARM repeat superfamily protein | chr3:10245338-10253158 FORWARD LENGTH=1841	291	1841	1.00E-46	632.6	34.4	38.1
Rsa1.0_00012.1.g704.t1	gb|EOA35360.1| hypothetical protein CARUB_v10020554mg [Capsella rubella]	304	353	1.00E-118	116.1	73.0	83.6	hypothetical protein CARUB_v10020554mg	gbpln	Capsella rubella	AT1G69523.1 | Symbols: | S-adenosyl-L-methionine-dependent methyltransferases superfamily protein | chr1:26129827-26131157 FORWARD LENGTH=300	304	300	1.00E-109	98.7	65.5	79.6
Rsa1.0_00012.1.g705.t1	gb|EOA35360.1| hypothetical protein CARUB_v10020554mg [Capsella rubella]	289	353	1.00E-91	122.1	67.8	80.3	hypothetical protein CARUB_v10020554mg	gbpln	Capsella rubella	AT1G69523.1 | Symbols: | S-adenosyl-L-methionine-dependent methyltransferases superfamily protein | chr1:26129827-26131157 FORWARD LENGTH=300	289	300	4.00E-85	103.8	59.2	74.0
Rsa1.0_00012.1.g706.t9	ref|XP_002871663.1| predicted protein [Arabidopsis lyrata subsp. lyrata] gi|297317500|gb|EFH47922.1| predicted protein [Arabidopsis lyrata subsp. lyrata]	568	593	0	104.4	73.1	83.3	predicted protein	gbpln	Arabidopsis lyrata	AT5G15380.1 | Symbols: DRM1 | domains rearranged methylase 1 | chr5:4991347-4994826 FORWARD LENGTH=624	568	624	0	109.9	71.3	82.2
Rsa1.0_00012.1.g707.t1	gb|AFI56994.1| NAC2, partial [Brassica napus]	269	244	1.00E-134	90.7	86.2	87.4	NAC2, partial	gbpln	Brassica napus	AT1G69490.1 | Symbols: NAP, ANAC029, ATNAP | NAC-like, activated by AP3/PI | chr1:26122233-26123222 FORWARD LENGTH=268	269	268	1.00E-131	99.6	84.0	89.6
Rsa1.0_00012.1.g708.t1	ref|XP_002887236.1| predicted protein [Arabidopsis lyrata subsp. lyrata] gi|297333077|gb|EFH63495.1| predicted protein [Arabidopsis lyrata subsp. lyrata]	342	348	1.00E-156	101.8	81.3	86.5	predicted protein	gbpln	Arabidopsis lyrata	AT1G69430.1 | Symbols: | unknown protein; BEST Arabidopsis thaliana protein match is: unknown protein (TAIR:AT1G26650.1); Has 216 Blast hits to 215 proteins in 17 species: Archae - 0; Bacteria - 0; Metazoa - 0; Fungi - 0; Plants - 216; Viruses - 0; Other Eukaryotes - 0 (source: NCBI BLink). | chr1:26098025-26099077 FORWARD LENGTH=350	342	350	1.00E-152	102.3	81.6	86.3
Rsa1.0_00012.1.g709.t1	ref|NP_177107.2| phosphate transporter PHO1-10 [Arabidopsis thaliana] gi|75127834|sp|Q6R8G0.1|PHO1A_ARATH RecName: Full=Phosphate transporter PHO1 homolog 10; AltName: Full=Protein PHO1 homolog 10; Short=AtPHO1;H10 gi|41079301|gb|AAR99492.1| PHO1-like protein [Arabidopsis thaliana] gi|332196809|gb|AEE34930.1| phosphate transporter PHO1-10 [Arabidopsis thaliana]	756	777	0	102.8	85.4	91.3	phosphate transporter PHO1-10	gbpln	Arabidopsis thaliana	AT1G69480.1 | Symbols: | EXS (ERD1/XPR1/SYG1) family protein | chr1:26114187-26117479 REVERSE LENGTH=777	756	777	0	102.8	85.4	91.3

Rsa1.0_00012.1.g710.t1	ref|NP_177105.2| emp24/gp25L/p24 family/GOLD domain-containing protein [Arabidopsis thaliana] gi|75304433|sp|Q8VY92.1|P24DA_ARATH RecName: Full=Transmembrane emp24 domain-containing protein p24delta10; AltName: Full=p24 family protein delta10; Short=p24delta10; AltName: Full=p24 family protein delta2d; Short=p24delta2d; Flags: Precursor gi|18252235|gb|AAL61950.1| unknown protein [Arabidopsis thaliana] gi|21387027|gb|AAM47917.1| unknown protein [Arabidopsis thaliana] gi|332196807|gb|AEE34928.1| emp24/gp25L/p24 family/GOLD domain-containing protein [Arabidopsis thaliana]	216	214	1.00E-105	99.1	83.3	94.0	emp24/gp25L/p24 family/GOLD domain-containing protein	gbpln	Arabidopsis thaliana	AT1G69460.1 | Symbols: | emp24/gp25L/p24 family/GOLD family protein | chr1:26112054-26113160 REVERSE LENGTH=214	216	214	1.00E-108	99.1	83.3	94.0
Rsa1.0_00012.1.g711.t1	gb|EOA33694.1| hypothetical protein CARUB_v10019875mg [Capsella rubella]	720	709	0	98.5	87.4	92.8	hypothetical protein CARUB_v10019875mg	gbpln	Capsella rubella	AT1G69450.2 | Symbols: | Early-responsive to dehydration stress protein (ERD4) | chr1:26107120-26110006 REVERSE LENGTH=711	720	711	0	98.8	86.3	91.9
Rsa1.0_00012.1.g712.t1	ref|NP_177101.2| putative S-acyltransferase [Arabidopsis thaliana] gi|42572047|ref|NP_974114.1| putative S-acyltransferase [Arabidopsis thaliana] gi|223635846|sp|Q9C533.2|ZDHC1_ARATH RecName: Full=Probable S-acyltransferase At1g69420; AltName: Full=Probable palmitoyltransferase At1g69420; AltName: Full=Zinc finger DHHC domain-containing protein At1g69420 gi|332196801|gb|AEE34922.1| putative S-acyltransferase [Arabidopsis thaliana] gi|332196802|gb|AEE34923.1| putative S-acyltransferase [Arabidopsis thaliana]	597	596	0	99.8	89.6	94.5	putative S-acyltransferase	gbpln	Arabidopsis thaliana	AT1G69420.1 | Symbols: | DHHC-type zinc finger family protein | chr1:26093690-26096736 FORWARD LENGTH=596	597	596	0	99.8	89.6	94.5
Rsa1.0_00012.1.g713.t3	ref|NP_177100.1| translation initiation factor eIF-5A [Arabidopsis thaliana] gi|20138712|sp|Q9C505.1|IF5A3_ARATH RecName: Full=Eukaryotic translation initiation factor 5A-3; Short=eIF-5A-3 gi|12325087|gb|AAG52496.1|AC018364_14 putative eukaryotic initiation factor 5A (eIF-5A); 7607-6714 [Arabidopsis thaliana] gi|12597798|gb|AAG60110.1|AC073178_21 Eukaryotic initiation factor 5A , putative [Arabidopsis thaliana] gi|21592652|gb|AAM64601.1| initiation factor 5A-3 (eIF-5A 3) [Arabidopsis thaliana] gi|332196800|gb|AEE34921.1| translation initiation factor eIF-5A [Arabidopsis thaliana]	161	158	2.00E-83	98.1	95.0	96.3	translation initiation factor eIF-5A	gbpln	Arabidopsis thaliana	AT1G69410.1 | Symbols: ATELF5A-3, ELF5A-3 | eukaryotic elongation factor 5A-3 | chr1:26089301-26090194 FORWARD LENGTH=158	161	158	8.00E-86	98.1	95.0	96.3
Rsa1.0_00012.1.g714.t1	gb|AAZ23773.1| plastid division regulator MinE [Brassica napus]	210	224	4.00E-93	106.7	88.1	91.9	plastid division regulator MinE	gbpln	Brassica napus	AT1G69390.1 | Symbols: ATMINE1, MINE1, ARC12 | homologue of bacterial MinE 1 | chr1:26084721-26086284 FORWARD LENGTH=229	210	229	2.00E-86	109.0	81.9	89.5
Rsa1.0_00012.1.g715.t1	ref|XP_002888717.1| hypothetical protein ARALYDRAFT_476065 [Arabidopsis lyrata subsp. lyrata] gi|297334558|gb|EFH64976.1| hypothetical protein ARALYDRAFT_476065 [Arabidopsis lyrata subsp. lyrata]	368	373	1.00E-176	101.4	84.5	90.8	hypothetical protein ARALYDRAFT_476065	gbpln	Arabidopsis lyrata	AT1G69380.1 | Symbols: | Protein of unknown function (DUF155) | chr1:26081729-26083431 REVERSE LENGTH=373	368	373	1.00E-177	101.4	83.2	90.8
Rsa1.0_00012.1.g716.t1	gb|EOA35466.1| hypothetical protein CARUB_v10020675mg [Capsella rubella]	311	317	1.00E-155	101.9	88.4	92.6	hypothetical protein CARUB_v10020675mg	gbpln	Capsella rubella	AT1G69370.1 | Symbols: CM3, cm-3 | chorismate mutase 3 | chr1:26080098-26081559 FORWARD LENGTH=316	311	316	1.00E-152	101.6	86.5	91.0
Rsa1.0_00012.1.g717.t1	ref|XP_002888716.1| predicted protein [Arabidopsis lyrata subsp. lyrata] gi|297334557|gb|EFH64975.1| predicted protein [Arabidopsis lyrata subsp. lyrata]	851	905	0	106.3	70.0	79.6	predicted protein	gbpln	Arabidopsis lyrata	AT1G69360.1 | Symbols: | Plant protein of unknown function (DUF863) | chr1:26072520-26075420 REVERSE LENGTH=896	851	896	0	105.3	69.2	78.3
Rsa1.0_00012.1.g718.t1	ref|XP_002887230.1| hypothetical protein ARALYDRAFT_476061 [Arabidopsis lyrata subsp. lyrata] gi|297333071|gb|EFH63489.1| hypothetical protein ARALYDRAFT_476061 [Arabidopsis lyrata subsp. lyrata]	788	1347	0	170.9	81.6	90.4	hypothetical protein ARALYDRAFT_476061	gbpln	Arabidopsis lyrata	AT1G69350.1 | Symbols: | Tetratricopeptide repeat (TPR)-like superfamily protein | chr1:26069882-26072245 FORWARD LENGTH=787	788	787	0	99.9	80.7	89.5

Rsa1.0_00012.1.g719.t1	ref|XP_002887229.1| appr-1-p processing enzyme family protein [Arabidopsis lyrata subsp. lyrata] gi|297333070|gb|EFH63488.1| appr-1-p processing enzyme family protein [Arabidopsis lyrata subsp. lyrata]	570	562	0	98.6	89.1	91.9	appr-1-p processing enzyme family protein	gbpln	Arabidopsis lyrata	AT1G69340.1 | Symbols: | appr-1-p processing enzyme family protein | chr1:26066298-26069644 FORWARD LENGTH=562	570	562	0	98.6	88.6	91.6
Rsa1.0_00012.1.g720.t1	ref|XP_002888715.1| hypothetical protein ARALYDRAFT_476060 [Arabidopsis lyrata subsp. lyrata] gi|297334556|gb|EFH64974.1| hypothetical protein ARALYDRAFT_476060 [Arabidopsis lyrata subsp. lyrata]	266	264	1.00E-91	99.2	68.8	76.7	hypothetical protein ARALYDRAFT_476060	gbpln	Arabidopsis lyrata	AT1G69330.1 | Symbols: | RING/U-box superfamily protein | chr1:26064138-26064950 REVERSE LENGTH=270	266	270	1.00E-91	101.5	67.7	76.7
Rsa1.0_00012.1.g721.t1	gb|EOA35903.1| hypothetical protein CARUB_v10021160mg, partial [Capsella rubella]	143	121	3.00E-34	84.6	54.5	65.7	hypothetical protein CARUB_v10021160mg, partial	gbpln	Capsella rubella	AT1G69325.1 | Symbols: | Remorin family protein | chr1:26063205-26063736 FORWARD LENGTH=120	143	120	2.00E-30	83.9	51.0	59.4
Rsa1.0_00012.1.g722.t1	ref|NP_564958.2| protein CLAVATA3/ESR-related 10 [Arabidopsis thaliana] gi|75339321|sp|Q4PSX1.1|CLE10_ARATH RecName: Full=CLAVATA3/ESR (CLE)-related protein 10; Contains: RecName: Full=CLE10p; Flags: Precursor gi|67633494|gb|AAY78671.1| putative CLE10 [Arabidopsis thaliana] gi|332196789|gb|AEE34910.1| protein CLAVATA3/ESR-related 10 [Arabidopsis thaliana]	107	107	9.00E-37	100.0	76.6	86.9	protein CLAVATA3/ESR-related 10	gbpln	Arabidopsis thaliana	AT1G69320.1 | Symbols: CLE10 | CLAVATA3/ESR-RELATED 10 | chr1:26061925-26062248 FORWARD LENGTH=107	107	107	1.00E-39	100.0	76.6	86.9
Rsa1.0_00012.1.g723.t1	ref|XP_002888714.1| hypothetical protein ARALYDRAFT_476056 [Arabidopsis lyrata subsp. lyrata] gi|297334555|gb|EFH64973.1| hypothetical protein ARALYDRAFT_476056 [Arabidopsis lyrata subsp. lyrata]	296	285	1.00E-105	96.3	75.0	83.1	hypothetical protein ARALYDRAFT_476056	gbpln	Arabidopsis lyrata	AT1G69310.2 | Symbols: WRKY57, ATWRKY57 | WRKY DNA-binding protein 57 | chr1:26056118-26057909 REVERSE LENGTH=287	296	287	1.00E-105	97.0	76.7	83.1
Rsa1.0_00012.1.g724.t1	gb|EOA33837.1| hypothetical protein CARUB_v10021318mg, partial [Capsella rubella]	280	290	3.00E-32	103.6	25.7	38.6	hypothetical protein CARUB_v10021318mg, partial	gbpln	Capsella rubella	AT4G04650.1 | Symbols: | RNA-directed DNA polymerase (reverse transcriptase)-related family protein | chr4:2353989-2355213 REVERSE LENGTH=332	280	332	2.00E-28	118.6	21.4	32.9
Rsa1.0_00012.1.g725.t1	gb|EOA35673.1| hypothetical protein CARUB_v10020897mg [Capsella rubella]	281	228	1.00E-62	81.1	62.6	70.8	hypothetical protein CARUB_v10020897mg	gbpln	Capsella rubella	AT1G69295.1 | Symbols: PDCB4 | plasmodesmata callose-binding protein 4 | chr1:26050492-26051843 REVERSE LENGTH=222	281	222	2.00E-47	79.0	30.6	33.8
Rsa1.0_00012.1.g726.t3	ref|XP_002888712.1| hypothetical protein ARALYDRAFT_339164 [Arabidopsis lyrata subsp. lyrata] gi|297334553|gb|EFH64971.1| hypothetical protein ARALYDRAFT_339164 [Arabidopsis lyrata subsp. lyrata]	490	1042	4.00E-75	212.7	35.7	40.8	hypothetical protein ARALYDRAFT_339164	gbpln	Arabidopsis lyrata	AT1G69280.1 | Symbols: | unknown protein; BEST Arabidopsis thaliana protein match is: unknown protein (TAIR:AT2G03630.1); Has 885 Blast hits to 474 proteins in 40 species: Archae - 0; Bacteria - 4; Metazoa - 731; Fungi - 9; Plants - 68; Viruses - 0; Other Eukaryotes - 73 (source: NCBI BLink). | chr1:26045290-26046670 REVERSE LENGTH=400	490	400	2.00E-75	81.6	35.1	40.6
Rsa1.0_00012.1.g727.t1	ref|NP_564956.1| Ninja-family protein AFP1 [Arabidopsis thaliana] gi|75180099|sp|Q9LQ98.1|AFP1_ARATH RecName: Full=Ninja-family protein AFP1; AltName: Full=ABI five-binding protein 1; Short=ABI5-binding protein 1 gi|6730641|gb|AAF27062.1|AC008262_11 F4N2.22 [Arabidopsis thaliana] gi|22531038|gb|AAM97023.1| expressed protein [Arabidopsis thaliana] gi|23197982|gb|AAN15518.1| expressed protein [Arabidopsis thaliana] gi|332196782|gb|AEE34903.1| Ninja-family protein AFP1 [Arabidopsis thaliana]	336	345	1.00E-108	102.7	70.5	78.0	Ninja-family protein AFP1	gbpln	Arabidopsis thaliana	AT1G69260.1 | Symbols: AFP1 | ABI five binding protein | chr1:26039314-26040570 FORWARD LENGTH=345	336	345	1.00E-110	102.7	70.5	78.0
Rsa1.0_00012.1.g728.t1	ref|XP_002887226.1| hypothetical protein ARALYDRAFT_894705 [Arabidopsis lyrata subsp. lyrata] gi|297333067|gb|EFH63485.1| hypothetical protein ARALYDRAFT_894705 [Arabidopsis lyrata subsp. lyrata]	442	446	1.00E-104	100.9	52.5	67.4	hypothetical protein ARALYDRAFT_894705	gbpln	Arabidopsis lyrata	AT1G69250.1 | Symbols: | Nuclear transport factor 2 (NTF2) family protein with RNA binding (RRM-RBD-RNP motifs) domain | chr1:26033163-26035301 FORWARD LENGTH=427	442	427	8.00E-99	96.6	48.0	63.8
Rsa1.0_00012.1.g729.t1	#	#	#	#	#	#	#	-	----	----	#	#	#	#	#	#	#
Rsa1.0_00012.1.g730.t1	gb|EOA18595.1| hypothetical protein CARUB_v10007167mg [Capsella rubella]	62	763	1.00E-13	1230.6	58.1	62.9	hypothetical protein CARUB_v10007167mg	gbpln	Capsella rubella	AT4G15430.2 | Symbols: | ERD (early-responsive to dehydration stress) family protein | chr4:8828214-8831354 FORWARD LENGTH=760	62	760	2.00E-16	1225.8	58.1	62.9

Rsa1.0_00012.1.g731.t1	ref|XP_002511619.1| conserved hypothetical protein [Ricinus communis] gi|223548799|gb|EEF50288.1| conserved hypothetical protein [Ricinus communis]	127	276	2.00E-15	217.3	38.6	50.4	conserved hypothetical protein	gbpln	Ricinus communis	#	#	#	#	#	#	#
Rsa1.0_00012.1.g732.t2	ref|XP_002887225.1| pfkB-type carbohydrate kinase family protein [Arabidopsis lyrata subsp. lyrata] gi|297333066|gb|EFH63484.1| pfkB-type carbohydrate kinase family protein [Arabidopsis lyrata subsp. lyrata]	604	612	0	101.3	82.8	88.1	pfkB-type carbohydrate kinase family protein	gbpln	Arabidopsis lyrata	AT1G69200.1 | Symbols: FLN2 | fructokinase-like 2 | chr1:26016018-26018365 FORWARD LENGTH=616	604	616	0	102.0	81.6	88.4
Rsa1.0_00012.1.g733.t3	gb|AAZ23116.1| transcription factor CRC [Brassica juncea] gi|482569951|gb|EOA34139.1| hypothetical protein CARUB_v10021641mg [Capsella rubella]	171	179	2.00E-87	104.7	95.9	97.7	transcription factor CRC	gbpln	Brassica juncea	AT1G69180.1 | Symbols: CRC | Plant-specific transcription factor YABBY family protein | chr1:26007734-26008940 REVERSE LENGTH=181	171	181	4.00E-82	105.8	95.9	97.7
Rsa1.0_00012.1.g734.t1	gb|ACG60678.1| unknown protein [Brassica oleracea var. alboglabra]	325	327	1.00E-101	100.6	70.8	77.5	unknown protein	gbpln	Brassica oleracea	AT1G69170.1 | Symbols: | Squamosa promoter-binding protein-like (SBP domain) transcription factor family protein | chr1:26005626-26007041 FORWARD LENGTH=405	325	405	1.00E-102	124.6	70.8	81.5
Rsa1.0_00012.1.g735.t1	gb|ACG60677.1| hypothetical protein BoB028L01.120 [Brassica oleracea var. alboglabra]	324	322	1.00E-152	99.4	93.8	96.3	hypothetical protein BoB028L01.120	gbpln	Brassica oleracea	AT1G69160.1 | Symbols: | unknown protein; BEST Arabidopsis thaliana protein match is: unknown protein (TAIR:AT3G13980.1); Has 173 Blast hits to 172 proteins in 54 species: Archae - 0; Bacteria - 0; Metazoa - 25; Fungi - 33; Plants - 84; Viruses - 2; Other Eukaryotes - 29 (source: NCBI BLink). | chr1:26000315-26001280 FORWARD LENGTH=321	324	321	1.00E-111	99.1	77.2	83.6
Rsa1.0_00012.1.g736.t1	#	#	#	#	#	#	#	-	----	----	#	#	#	#	#	#	#
Rsa1.0_00012.1.g737.t1	sp|P0DI14.1|AP1_BRARP RecName: Full=Floral homeotic protein APETALA 1; Short=BcpAP1; AltName: Full=Agamous-like MADS-box protein AP1	255	256	1.00E-139	100.4	97.3	99.6	RecName: Full=Floral homeotic protein APETALA 1; Short=BcpAP1; AltName: Full=Agamous-like MADS-box protein AP1	----	----	AT1G69120.1 | Symbols: AP1, AGL7 | K-box region and MADS-box transcription factor family protein | chr1:25982576-25986102 REVERSE LENGTH=256	255	256	1.00E-126	100.4	93.3	98.0
Rsa1.0_00013.1.g738.t1	ref|XP_002879065.1| aldehyde oxidase [Arabidopsis lyrata subsp. lyrata] gi|297324904|gb|EFH55324.1| aldehyde oxidase [Arabidopsis lyrata subsp. lyrata]	1328	1320	0	99.4	81.9	88.9	aldehyde oxidase	gbpln	Arabidopsis lyrata	AT2G27150.2 | Symbols: AAO3, At-AO3, AOdelta, AtAAO3 | abscisic aldehyde oxidase 3 | chr2:11601952-11607014 FORWARD LENGTH=1332	1328	1332	0	100.3	82.0	89.1
Rsa1.0_00013.1.g739.t1	ref|NP_190471.1| FBD-associated F-box protein [Arabidopsis thaliana] gi|75337428|sp|Q9SMT9.1|FBD9_ARATH RecName: Full=FBD-associated F-box protein At3g49020 gi|6522564|emb|CAB62008.1| putative protein [Arabidopsis thaliana] gi|67633680|gb|AAY78764.1| F-box family protein [Arabidopsis thaliana] gi|111074508|gb|ABH04627.1| At3g49020 [Arabidopsis thaliana] gi|332644966|gb|AEE78487.1| FBD-associated F-box protein [Arabidopsis thaliana]	386	447	1.00E-118	115.8	63.5	74.4	FBD-associated F-box protein	gbpln	Arabidopsis thaliana	AT3G49020.1 | Symbols: | FBD, F-box and Leucine Rich Repeat domains containing protein | chr3:18169079-18170660 FORWARD LENGTH=447	386	447	1.00E-120	115.8	63.5	74.4
Rsa1.0_00013.1.g740.t1	ref|XP_002875956.1| F-box family protein [Arabidopsis lyrata subsp. lyrata] gi|297321794|gb|EFH52215.1| F-box family protein [Arabidopsis lyrata subsp. lyrata]	611	448	1.00E-137	73.3	45.2	52.0	F-box family protein	gbpln	Arabidopsis lyrata	AT3G49020.1 | Symbols: | FBD, F-box and Leucine Rich Repeat domains containing protein | chr3:18169079-18170660 FORWARD LENGTH=447	611	447	1.00E-135	73.2	44.5	53.4
Rsa1.0_00013.1.g741.t1	gb|EOA27572.1| hypothetical protein CARUB_v10023709mg, partial [Capsella rubella]	249	304	1.00E-142	122.1	98.8	99.6	hypothetical protein CARUB_v10023709mg, partial	gbpln	Capsella rubella	AT2G27020.1 | Symbols: PAG1 | 20S proteasome alpha subunit G1 | chr2:11528515-11530858 REVERSE LENGTH=249	249	249	1.00E-145	100.0	99.2	99.6
Rsa1.0_00013.1.g742.t1	ref|NP_850095.2| CBL-interacting serine/threonine-protein kinase 3 [Arabidopsis thaliana] gi|330252823|gb|AEC07917.1| CBL-interacting serine/threonine-protein kinase 3 [Arabidopsis thaliana]	440	451	0	102.5	95.9	97.7	CBL-interacting serine/threonine-protein kinase 3	gbpln	Arabidopsis thaliana	AT2G26980.4 | Symbols: CIPK3 | CBL-interacting protein kinase 3 | chr2:11515234-11518426 REVERSE LENGTH=451	440	451	0	102.5	95.9	97.7

Rsa1.0_00013.1.g743.t1	ref|NP_180259.1| ABC transporter G family member 32 [Arabidopsis thaliana] gi|75318727|sp|O81016.1|AB32G_ARATH RecName: Full=ABC transporter G family member 32; Short=ABC transporter ABCG.32; Short=AtABCG32; AltName: Full=Probable pleiotropic drug resistance protein 4 gi|3426037|gb|AAC32236.1| putative ABC transporter [Arabidopsis thaliana] gi|28144347|tpg|DAA00872.1| TPA_exp: PDR4 ABC transporter [Arabidopsis thaliana] gi|330252812|gb|AEC07906.1| ABC transporter G family member 32 [Arabidopsis thaliana]	1420	1420	0	100.0	93.0	97.3	ABC transporter G family member 32	gbpln	Arabidopsis thaliana	AT2G26910.1 | Symbols: PDR4, ATPDR4 | pleiotropic drug resistance 4 | chr2:11481623-11487874 FORWARD LENGTH=1420	1420	1420	0	100.0	93.0	97.3
Rsa1.0_00013.1.g744.t1	ref|XP_002880842.1| phosphoesterase family protein [Arabidopsis lyrata subsp. lyrata] gi|297326681|gb|EFH57101.1| phosphoesterase family protein [Arabidopsis lyrata subsp. lyrata]	519	514	0	99.0	87.5	92.9	phosphoesterase family protein	gbpln	Arabidopsis lyrata	AT2G26870.1 | Symbols: NPC2 | non-specific phospholipase C2 | chr2:11457117-11459355 REVERSE LENGTH=514	519	514	0	99.0	87.1	92.9
Rsa1.0_00013.1.g745.t1	ref|NP_180238.2| protein pinoid2 [Arabidopsis thaliana] gi|75253187|sp|Q64FQ2.1|PID2_ARATH RecName: Full=Protein kinase PINOID 2; AltName: Full=Protein kinase AGC1-10 gi|51944867|gb|AAU14162.1| AGC1-10 [Arabidopsis thaliana] gi|51944869|gb|AAU14163.1| AGC1-10 [Arabidopsis thaliana] gi|330252783|gb|AEC07877.1| protein kinase PINOID 2 [Arabidopsis thaliana]	525	525	0	100.0	89.5	94.1	protein pinoid2	gbpln	Arabidopsis thaliana	AT2G26700.1 | Symbols: PID2 | AGC (cAMP-dependent, cGMP-dependent and protein kinase C) kinase family protein | chr2:11368613-11370951 FORWARD LENGTH=525	525	525	0	100.0	89.5	94.1
Rsa1.0_00013.1.g746.t1	ref|XP_002892763.1| predicted protein [Arabidopsis lyrata subsp. lyrata] gi|297338605|gb|EFH69022.1| predicted protein [Arabidopsis lyrata subsp. lyrata]	150	804	3.00E-21	536.0	46.0	52.0	predicted protein	gbpln	Arabidopsis lyrata	AT1G13630.2 | Symbols: | Tetratricopeptide repeat (TPR)-like superfamily protein | chr1:4670178-4672826 REVERSE LENGTH=798	150	798	3.00E-20	532.0	29.3	34.0
Rsa1.0_00013.1.g747.t1	ref|NP_180241.1| putative inactive receptor kinase [Arabidopsis thaliana] gi|75219509|sp|O48788.1|Y2267_ARATH RecName: Full=Probable inactive receptor kinase At2g26730; Flags: Precursor gi|2760839|gb|AAB95307.1| putative receptor-like protein kinase [Arabidopsis thaliana] gi|60543329|gb|AAX22262.1| At2g26730 [Arabidopsis thaliana] gi|224589527|gb|ACN59297.1| leucine-rich repeat receptor-like protein kinase [Arabidopsis thaliana] gi|330252786|gb|AEC07880.1| putative inactive receptor kinase [Arabidopsis thaliana]	818	658	0	80.4	69.8	73.5	putative inactive receptor kinase	gbpln	Arabidopsis thaliana	AT2G26730.1 | Symbols: | Leucine-rich repeat protein kinase family protein | chr2:11388621-11391286 FORWARD LENGTH=658	818	658	0	80.4	69.8	73.5
Rsa1.0_00013.1.g748.t1	ref|NP_180217.1| WD40 domain-containing protein [Arabidopsis thaliana] gi|2739374|gb|AAC14498.1| En/Spm-like transposon protein [Arabidopsis thaliana] gi|50253512|gb|AAT71958.1| At2g26490 [Arabidopsis thaliana] gi|53850519|gb|AAU95436.1| At2g26490 [Arabidopsis thaliana] gi|330252750|gb|AEC07844.1| WD40 domain-containing protein [Arabidopsis thaliana]	453	465	0	102.6	90.3	94.7	WD40 domain-containing protein	gbpln	Arabidopsis thaliana	AT2G26490.1 | Symbols: | Transducin/WD40 repeat-like superfamily protein | chr2:11268035-11269432 FORWARD LENGTH=465	453	465	0	102.6	90.3	94.7
Rsa1.0_00013.1.g749.t1	emb|CAD30841.1| soluble epoxide hydrolase [Brassica napus]	318	318	1.00E-176	100.0	92.8	97.2	soluble epoxide hydrolase	gbpln	Brassica napus	AT2G26740.1 | Symbols: ATSEH, SEH | soluble epoxide hydrolase | chr2:11393148-11394257 REVERSE LENGTH=321	318	321	1.00E-154	100.9	83.6	94.0
Rsa1.0_00013.1.g750.t1	ref|XP_002878977.1| hypothetical protein ARALYDRAFT_481517 [Arabidopsis lyrata subsp. lyrata] gi|297324816|gb|EFH55236.1| hypothetical protein ARALYDRAFT_481517 [Arabidopsis lyrata subsp. lyrata]	695	794	0	114.2	75.5	81.3	hypothetical protein ARALYDRAFT_481517	gbpln	Arabidopsis lyrata	AT2G26570.1 | Symbols: WEB1 | Plant protein of unknown function (DUF827) | chr2:11299565-11302076 FORWARD LENGTH=807	695	807	0	116.1	75.1	82.4
Rsa1.0_00013.1.g751.t1	gb|ABV89757.1| purple acid phosphatase 12 protein family isoform 7 [Brassica napus] gi|157849936|gb|ABV89759.1| purple acid phosphatase 12 protein family isoform 7 [Brassica napus]	471	469	0	99.6	93.0	97.0	purple acid phosphatase 12 protein family isoform 7	gbpln	Brassica napus	AT2G27190.1 | Symbols: PAP1, ATPAP1, PAP12, ATPAP12 | purple acid phosphatase 12 | chr2:11621400-11623438 REVERSE LENGTH=469	471	469	0	99.6	87.3	91.3

Rsa1.0_00013.1.g752.t1	gb|EOA27281.1| hypothetical protein CARUB_v10023399mg [Capsella rubella]	382	388	1.00E-162	101.6	78.5	85.3	hypothetical protein CARUB_v10023399mg	gbpln	Capsella rubella	AT2G26360.1 | Symbols: | Mitochondrial substrate carrier family protein | chr2:11221603-11223160 REVERSE LENGTH=387	382	387	1.00E-157	101.3	75.9	83.0
Rsa1.0_00013.1.g753.t1	ref|NP_180199.3| chalcone-flavanone isomerase-like protein [Arabidopsis thaliana] gi|487524115|sp|Q84RK2.2|FAP2_ARATH RecName: Full=Fatty-acid-binding protein 2; Short=AtFAP2; AltName: Full=Chalcone-flavanone isomerase family protein 2 gi|61742610|gb|AAX55126.1| hypothetical protein At2g26310 [Arabidopsis thaliana] gi|330252727|gb|AEC07821.1| chalcone-flavanone isomerase-like protein [Arabidopsis thaliana]	381	398	1.00E-154	104.5	74.8	83.5	chalcone-flavanone isomerase-like protein	gbpln	Arabidopsis thaliana	AT2G26310.1 | Symbols: | Chalcone-flavanone isomerase family protein | chr2:11201770-11203919 FORWARD LENGTH=398	381	398	1.00E-156	104.5	74.8	83.5
Rsa1.0_00013.1.g754.t1	ref|NP_180197.1| root-specific kinase 1 [Arabidopsis thaliana] gi|3075390|gb|AAC14522.1| putative protein kinase [Arabidopsis thaliana] gi|330252725|gb|AEC07819.1| root-specific kinase 1 [Arabidopsis thaliana]	424	424	0	100.0	87.5	94.3	root-specific kinase 1	gbpln	Arabidopsis thaliana	AT2G26290.1 | Symbols: ARSK1 | root-specific kinase 1 | chr2:11192237-11194259 REVERSE LENGTH=424	424	424	0	100.0	87.5	94.3
Rsa1.0_00013.1.g755.t1	ref|NP_180705.1| F-box protein DOR [Arabidopsis thaliana] gi|229807544|sp|Q5BPS3.2|DOR_ARATH RecName: Full=F-box protein DOR; AltName: Full=Protein DROUGHT TOLERANCE REPRESSOR gi|4589954|gb|AAD26472.1| hypothetical protein [Arabidopsis thaliana] gi|330253458|gb|AEC08552.1| F-box protein DOR [Arabidopsis thaliana]	406	387	3.00E-90	95.3	47.8	61.1	F-box protein DOR	gbpln	Arabidopsis thaliana	AT2G31470.1 | Symbols: DOR | F-box and associated interaction domains-containing protein | chr2:13407492-13408655 REVERSE LENGTH=387	406	387	8.00E-93	95.3	47.8	61.1
Rsa1.0_00013.1.g756.t1	gb|EOA26805.1| hypothetical protein CARUB_v10022901mg [Capsella rubella] gi|482562617|gb|EOA26807.1| hypothetical protein CARUB_v10022901mg [Capsella rubella] gi|482562618|gb|EOA26808.1| hypothetical protein CARUB_v10022901mg [Capsella rubella]	447	574	1.00E-167	128.4	78.1	85.5	hypothetical protein CARUB_v10022901mg	gbpln	Capsella rubella	AT2G26280.1 | Symbols: CID7 | CTC-interacting domain 7 | chr2:11188143-11190579 REVERSE LENGTH=567	447	567	1.00E-149	126.8	63.1	69.6
Rsa1.0_00013.1.g757.t1	gb|EOA26905.1| hypothetical protein CARUB_v10022994mg [Capsella rubella]	519	525	0	101.2	82.1	87.5	hypothetical protein CARUB_v10022994mg	gbpln	Capsella rubella	AT2G26270.1 | Symbols: | FUNCTIONS IN: molecular_function unknown; INVOLVED IN: biological_process unknown; LOCATED IN: endomembrane system; BEST Arabidopsis thaliana protein match is: BRCT domain-containing DNA repair protein (TAIR:AT3G43930.1); Has 29 Blast hits to 29 proteins in 9 species: Archae - 0; Bacteria - 0; Metazoa - 0; Fungi - 0; Plants - 29; Viruses - 0; Other Eukaryotes - 0 (source: NCBI BLink). | chr2:11184739-11187659 FORWARD LENGTH=470	519	470	0	90.6	68.4	74.4

Rsa1.0_00013.1.g758.t1	ref|NP_180193.1| 3-ketoacyl-CoA synthase 10 [Arabidopsis thaliana] gi|114149941|sp|Q570B4.2|KCS10_ARATH RecName: Full=3-ketoacyl-CoA synthase 10; Short=KCS-10; AltName: Full=Protein FIDDLEHEAD; AltName: Full=Very long-chain fatty acid condensing enzyme 10; Short=VLCFA condensing enzyme 10 gi|8177699|gb|AAF73973.1|AF214490_1 fiddlehead protein [Arabidopsis thaliana] gi|12597467|gb|AAG60062.1|AF337910_1 putative beta-ketoacyl-CoA synthase FIDDLEHEAD [Arabidopsis thaliana] gi|3075394|gb|AAC14526.1| beta-ketoacyl-CoA synthase (FIDDLEHEAD) [Arabidopsis thaliana] gi|3559809|emb|CAA09311.1| fiddlehead protein [Arabidopsis thaliana] gi|14517456|gb|AAK62618.1| At2g26250/T1D16.11 [Arabidopsis thaliana] gi|23506207|gb|AAN31115.1| At2g26250/T1D16.11 [Arabidopsis thaliana] gi|26983882|gb|AAN86193.1| putative beta-ketoacyl-CoA synthase FIDDLEHEAD [Arabidopsis thaliana] gi|330252719|gb|AEC07813.1| 3-ketoacyl-CoA synthase 10 [Arabidopsis thaliana]	550	550	0	100.0	95.3	97.3	3-ketoacyl-CoA synthase 10	gbpln	Arabidopsis thaliana	AT2G26250.1 | Symbols: FDH, KCS10 | 3-ketoacyl-CoA synthase 10 | chr2:11170799-11173059 REVERSE LENGTH=550	550	550	0	100.0	95.3	97.3
Rsa1.0_00013.1.g759.t1	ref|NP_850076.1| Ankyrin repeat family protein [Arabidopsis thaliana] gi|17644149|gb|AAL38772.1| unknown protein [Arabidopsis thaliana] gi|20465459|gb|AAM20189.1| unknown protein [Arabidopsis thaliana] gi|330252714|gb|AEC07808.1| Ankyrin repeat family protein [Arabidopsis thaliana]	161	190	3.00E-65	118.0	79.5	88.8	Ankyrin repeat family protein	gbpln	Arabidopsis thaliana	AT2G26210.1 | Symbols: | Ankyrin repeat family protein | chr2:11157654-11159016 FORWARD LENGTH=190	161	190	1.00E-67	118.0	79.5	88.8
Rsa1.0_00013.1.g760.t1	ref|NP_850075.2| S-adenosyl-L-methionine-dependent methyltransferase domain-containing protein [Arabidopsis thaliana] gi|20197178|gb|AAC14529.2| hypothetical protein [Arabidopsis thaliana] gi|330252713|gb|AEC07807.1| S-adenosyl-L-methionine-dependent methyltransferase domain-containing protein [Arabidopsis thaliana]	532	565	0	106.2	87.2	91.9	S-adenosyl-L-methionine-dependent methyltransferase domain-containing protein	gbpln	Arabidopsis thaliana	AT2G26200.1 | Symbols: | S-adenosyl-L-methionine-dependent methyltransferases superfamily protein | chr2:11152875-11156330 FORWARD LENGTH=565	532	565	0	106.2	87.2	91.9
Rsa1.0_00013.1.g761.t1	ref|NP_565618.1| calmodulin-binding-like protein [Arabidopsis thaliana] gi|16930469|gb|AAL31920.1|AF419588_1 At2g26190/T1D16.17 [Arabidopsis thaliana] gi|3075398|gb|AAC14530.1| expressed protein [Arabidopsis thaliana] gi|20453237|gb|AAM19857.1| At2g26190/T1D16.17 [Arabidopsis thaliana] gi|22135809|gb|AAM91091.1| At2g26190/T1D16.17 [Arabidopsis thaliana] gi|330252712|gb|AEC07806.1| calmodulin-binding-like protein [Arabidopsis thaliana]	531	532	0	100.2	83.6	89.6	calmodulin-binding-like protein	gbpln	Arabidopsis thaliana	AT2G26190.1 | Symbols: | calmodulin-binding family protein | chr2:11147901-11150082 REVERSE LENGTH=532	531	532	0	100.2	83.6	89.6
Rsa1.0_00013.1.g762.t1	ref|NP_001078039.1| uncharacterized protein [Arabidopsis thaliana] gi|330254959|gb|AEC10053.1| uncharacterized protein AT2G41945 [Arabidopsis thaliana]	123	166	2.00E-23	135.0	61.8	69.1	uncharacterized protein	gbpln	Arabidopsis thaliana	AT2G41945.2 | Symbols: | unknown protein; FUNCTIONS IN: molecular_function unknown; INVOLVED IN: biological_process unknown; LOCATED IN: membrane; BEST Arabidopsis thaliana protein match is: unknown protein (TAIR:AT3G04040.1); Has 57 Blast hits to 57 proteins in 12 species: Archae - 0; Bacteria - 0; Metazoa - 0; Fungi - 0; Plants - 57; Viruses - 0; Other Eukaryotes - 0 (source: NCBI BLink). | chr2:17509933-17512898 REVERSE LENGTH=166	123	166	3.00E-26	135.0	61.8	69.1
Rsa1.0_00013.1.g763.t1	ref|XP_002880762.1| IQ-domain 6 [Arabidopsis lyrata subsp. lyrata] gi|297326601|gb|EFH57021.1| IQ-domain 6 [Arabidopsis lyrata subsp. lyrata]	396	417	1.00E-158	105.3	85.4	90.9	IQ-domain 6	gbpln	Arabidopsis lyrata	AT2G26180.1 | Symbols: IQD6 | IQ-domain 6 | chr2:11143433-11144982 REVERSE LENGTH=416	396	416	1.00E-157	105.1	83.8	88.6
Rsa1.0_00013.1.g764.t1	dbj|BAJ33733.1| unnamed protein product [Thellungiella halophila]	720	717	0	99.6	93.1	96.3	unnamed protein product	----	----	AT2G26140.1 | Symbols: ftsh4 | FTSH protease 4 | chr2:11131939-11135126 REVERSE LENGTH=717	720	717	0	99.6	88.9	93.5

Rsa1.0_00013.1.g765.t2	gb|AAD19784.1| putative retroelement pol polyprotein [Arabidopsis thaliana]	1511	1501	0	99.3	32.0	40.2	putative retroelement pol polyprotein	gbpln	Arabidopsis thaliana	AT4G23160.1 | Symbols: CRK8 | cysteine-rich RLK (RECEPTOR-like protein kinase) 8 | chr4:12129485-12134086 FORWARD LENGTH=1262	1511	1262	1.00E-142	83.5	16.5	23.8
Rsa1.0_00013.1.g766.t1	ref|XP_002324769.1| predicted protein [Populus trichocarpa] gi|118482835|gb|ABK93333.1| unknown [Populus trichocarpa] gi|118486784|gb|ABK95227.1| unknown [Populus trichocarpa] gi|222866203|gb|EEF03334.1| predicted protein [Populus trichocarpa]	289	276	3.00E-60	95.5	48.4	65.7	predicted protein	gbpln	Populus trichocarpa	AT5G11890.1 | Symbols: | FUNCTIONS IN: molecular_function unknown; LOCATED IN: plasma membrane; EXPRESSED IN: 12 plant structures; EXPRESSED DURING: 6 growth stages; BEST Arabidopsis thaliana protein match is: Late embryogenesis abundant (LEA) hydroxyproline-rich glycoprotein family (TAIR:AT1G17620.1); Has 1807 Blast hits to 1807 proteins in 277 species: Archae - 0; Bacteria - 0; Metazoa - 736; Fungi - 347; Plants - 385; Viruses - 0; Other Eukaryotes - 339 (source: NCBI BLink). | chr5:3831770-3832633 FORWARD LENGTH=287	289	287	3.00E-50	99.3	43.9	58.5
Rsa1.0_00013.1.g767.t1	gb|AAF79479.1|AC022492_23 F1L3.27 [Arabidopsis thaliana]	300	201	5.00E-71	67.0	49.3	55.0	F1L3.27	gbpln	Arabidopsis thaliana	AT1G17560.1 | Symbols: HLL | Ribosomal protein L14p/L23e family protein | chr1:6037635-6038574 REVERSE LENGTH=196	300	196	6.00E-73	65.3	49.0	54.7
Rsa1.0_00013.1.g768.t1	ref|XP_002880742.1| galactosyltransferase family protein [Arabidopsis lyrata subsp. lyrata] gi|297326581|gb|EFH57001.1| galactosyltransferase family protein [Arabidopsis lyrata subsp. lyrata]	366	367	1.00E-175	100.3	87.7	94.0	galactosyltransferase family protein	gbpln	Arabidopsis lyrata	AT2G26100.1 | Symbols: | Galactosyltransferase family protein | chr2:11116212-11118129 REVERSE LENGTH=371	366	371	1.00E-172	101.4	86.3	93.2
Rsa1.0_00013.1.g769.t1	#	#	#	#	#	#	#	–	----	----	#	#	#	#	#	#	#
Rsa1.0_00013.1.g770.t1	ref|XP_002880729.1| hypothetical protein ARALYDRAFT_481452 [Arabidopsis lyrata subsp. lyrata] gi|297326568|gb|EFH56988.1| hypothetical protein ARALYDRAFT_481452 [Arabidopsis lyrata subsp. lyrata]	711	634	1.00E-126	89.2	35.7	37.4	hypothetical protein ARALYDRAFT_481452	gbpln	Arabidopsis lyrata	AT2G25970.1 | Symbols: | KH domain-containing protein | chr2:11071844-11075604 REVERSE LENGTH=632	711	632	1.00E-125	88.9	35.7	37.7
Rsa1.0_00013.1.g771.t1	ref|NP_565614.1| uncharacterized protein [Arabidopsis thaliana] gi|20197269|gb|AAC31240.2| expressed protein [Arabidopsis thaliana] gi|20197367|gb|AAM15044.1| expressed protein [Arabidopsis thaliana] gi|21593506|gb|AAM65473.1| unknown [Arabidopsis thaliana] gi|29028814|gb|AAO64786.1| At2g25950 [Arabidopsis thaliana] gi|110742984|dbj|BAE99386.1| hypothetical protein [Arabidopsis thaliana] gi|330252682|gb|AEC07776.1| uncharacterized protein AT2G25950 [Arabidopsis thaliana]	224	204	1.00E-99	91.1	82.1	84.8	uncharacterized protein	gbpln	Arabidopsis thaliana	AT2G25950.1 | Symbols: | Protein of unknown function (DUF1000) | chr2:11068735-11070524 FORWARD LENGTH=204	224	204	1.00E-102	91.1	82.1	84.8
Rsa1.0_00013.1.g772.t1	gb|AAG51783.1|AC079679_3 reverse transcriptase, putative; 16838-20266 [Arabidopsis thaliana]	1956	1142	0	58.4	28.3	37.1	reverse transcriptase, putative; 16838-20266	gbpln	Arabidopsis thaliana	AT4G29090.1 | Symbols: | Ribonuclease H-like superfamily protein | chr4:14333528-14335255 FORWARD LENGTH=575	1956	575	1.00E-91	29.4	9.7	15.3
Rsa1.0_00013.1.g773.t1	gb|EOA34405.1| hypothetical protein CARUB_v10021933mg [Capsella rubella]	657	566	0	86.1	51.8	60.3	hypothetical protein CARUB_v10021933mg	gbpln	Capsella rubella	AT1G65630.1 | Symbols: DegP3 | DegP protease 3 | chr1:24406542-24409068 REVERSE LENGTH=559	657	559	0	85.1	51.0	60.9
Rsa1.0_00013.1.g774.t1	ref|XP_002878908.1| hypothetical protein ARALYDRAFT_481449 [Arabidopsis lyrata subsp. lyrata] gi|297324747|gb|EFH55167.1| hypothetical protein ARALYDRAFT_481449 [Arabidopsis lyrata subsp. lyrata]	605	686	1.00E-160	113.4	65.5	74.7	hypothetical protein ARALYDRAFT_481449	gbpln	Arabidopsis lyrata	AT2G25930.1 | Symbols: ELF3, PYK20 | hydroxyproline-rich glycoprotein family protein | chr2:11059459-11063178 FORWARD LENGTH=695	605	695	1.00E-154	114.9	64.8	74.4
Rsa1.0_00013.1.g775.t1	ref|NP_127504.1| ORF I polyprotein [Petunia vein clearing virus] gi|82061579|sp|Q91DM0.1|POLG_PVCV1 RecName: Full=Genome polyprotein; Contains: RecName: Full=Movement protein; Short=MP; Contains: RecName: Full=Capsid protein; Short=CP; Contains: RecName: Full=Aspartic protease; Short=PR; Contains: RecName: Full=Reverse transcriptase; Short=RT gi|14574598|gb|AAK68664.1| ORF I polyprotein [petunia vein clearing virus]	1445	2179	1.00E-137	150.8	16.4	21.9	ORF I polyprotein	gbvrl	Petunia vein	#	#	#	#	#	#	#

Rsa1.0_00013.1.g776.t1	ref|XP_002880724.1| hypothetical protein ARALYDRAFT_481448 [Arabidopsis lyrata subsp. lyrata] gi|297326563|gb|EFH56983.1| hypothetical protein ARALYDRAFT_481448 [Arabidopsis lyrata subsp. lyrata]	333	342	1.00E-178	102.7	91.6	94.9	hypothetical protein ARALYDRAFT_481448	gbpln	Arabidopsis lyrata	AT2G25910.1 | Symbols: | 3'-5' exonuclease domain-containing protein / K homology domain-containing protein / KH domain-containing protein | chr2:11049379-11051997 REVERSE LENGTH=341	333	341	1.00E-180	102.4	90.1	94.3
Rsa1.0_00013.1.g777.t1	ref|XP_002886495.1| hypothetical protein ARALYDRAFT_315184 [Arabidopsis lyrata subsp. lyrata] gi|297332336|gb|EFH62754.1| hypothetical protein ARALYDRAFT_315184 [Arabidopsis lyrata subsp. lyrata]	644	648	0	100.6	67.7	80.0	hypothetical protein ARALYDRAFT_315184	gbpln	Arabidopsis lyrata	AT2G46450.1 | Symbols: ATCNGC12, CNGC12 | cyclic nucleotide-gated channel 12 | chr2:19065845-19068364 FORWARD LENGTH=649	644	649	0	100.8	67.5	79.2
Rsa1.0_00013.1.g778.t1	ref|XP_002878899.1| hypothetical protein ARALYDRAFT_901265 [Arabidopsis lyrata subsp. lyrata] gi|297324738|gb|EFH55158.1| hypothetical protein ARALYDRAFT_901265 [Arabidopsis lyrata subsp. lyrata]	145	143	6.00E-50	98.6	78.6	85.5	hypothetical protein ARALYDRAFT_901265	gbpln	Arabidopsis lyrata	AT2G25890.1 | Symbols: | Oleosin family protein | chr2:11037435-11037884 FORWARD LENGTH=149	145	149	9.00E-49	102.8	73.8	80.7
Rsa1.0_00013.1.g779.t1	ref|XP_002878897.1| hypothetical protein ARALYDRAFT_901259 [Arabidopsis lyrata subsp. lyrata] gi|297324736|gb|EFH55156.1| hypothetical protein ARALYDRAFT_901259 [Arabidopsis lyrata subsp. lyrata]	608	584	0	96.1	85.7	90.1	hypothetical protein ARALYDRAFT_901259	gbpln	Arabidopsis lyrata	AT2G25870.1 | Symbols: | haloacid dehalogenase-like hydrolase family protein | chr2:11031159-11034302 FORWARD LENGTH=584	608	584	0	96.1	84.2	89.8
Rsa1.0_00013.1.g780.t1	ref|NP_187216.1| receptor like protein 32 [Arabidopsis thaliana] gi|6714445|gb|AAF26132.1|AC011620_8 putative disease resistance protein [Arabidopsis thaliana] gi|332640751|gb|AEE74272.1| receptor like protein 32 [Arabidopsis thaliana]	883	868	0	98.3	72.5	80.3	receptor like protein 32	gbpln	Arabidopsis thaliana	AT3G05650.1 | Symbols: AtRLP32, RLP32 | receptor like protein 32 | chr3:1645884-1648490 REVERSE LENGTH=868	883	868	0	98.3	72.5	80.3
Rsa1.0_00013.1.g781.t1	ref|XP_002878896.1| predicted protein [Arabidopsis lyrata subsp. lyrata] gi|297324735|gb|EFH55155.1| predicted protein [Arabidopsis lyrata subsp. lyrata]	180	411	1.00E-50	228.3	51.1	56.7	predicted protein	gbpln	Arabidopsis lyrata	AT2G25840.1 | Symbols: OVA4 | Nucleotidylyl transferase superfamily protein | chr2:11021924-11025158 FORWARD LENGTH=408	180	408	1.00E-50	226.7	48.3	55.6
Rsa1.0_00013.1.g782.t1	ref|NP_175150.1| F-box protein [Arabidopsis thaliana] gi|75268184|sp|Q9C627.1|FB36_ARATH RecName: Full=Putative F-box protein At1g46984 gi|12321006|gb|AAG50624.1|AC083835_9 hypothetical protein [Arabidopsis thaliana] gi|332194012|gb|AEE32133.1| F-box protein [Arabidopsis thaliana]	378	370	5.00E-84	97.9	45.2	61.9	F-box protein	gbpln	Arabidopsis thaliana	AT1G46984.1 | Symbols: | F-box family protein | chr1:17274722-17275834 FORWARD LENGTH=370	378	370	1.00E-86	97.9	45.2	61.9
Rsa1.0_00013.1.g783.t1	ref|XP_002883380.1| hypothetical protein ARALYDRAFT_479795 [Arabidopsis lyrata subsp. lyrata] gi|297329220|gb|EFH59639.1| hypothetical protein ARALYDRAFT_479795 [Arabidopsis lyrata subsp. lyrata]	74	214	2.00E-23	289.2	73.0	79.7	hypothetical protein ARALYDRAFT_479795	gbpln	Arabidopsis lyrata	AT3G22845.1 | Symbols: | emp24/gp25L/p24 family/GOLD family protein | chr3:8087373-8088550 FORWARD LENGTH=214	74	214	4.00E-26	289.2	73.0	79.7
Rsa1.0_00013.1.g784.t4	ref|NP_565611.1| poly(A) polymerase 2 [Arabidopsis thaliana] gi|31747891|gb|AAF66438.2|AF255297_1 nuclear poly(A) polymerase [Arabidopsis thaliana] gi|14532700|gb|AAK64151.1| putative poly(A) polymerase [Arabidopsis thaliana] gi|18491197|gb|AAL69501.1| putative poly(A) polymerase [Arabidopsis thaliana] gi|20197361|gb|AAC42245.2| putative poly(A) polymerase [Arabidopsis thaliana] gi|330252667|gb|AEC07761.1| poly(A) polymerase 2 [Arabidopsis thaliana]	801	800	0	99.9	77.4	84.3	poly(A) polymerase 2	gbpln	Arabidopsis thaliana	AT2G25850.2 | Symbols: PAPS2 | poly(A) polymerase 2 | chr2:11026123-11030440 REVERSE LENGTH=800	801	800	0	99.9	77.4	84.3
Rsa1.0_00013.1.g785.t1	ref|NP_565610.1| YebC-related protein [Arabidopsis thaliana] gi|22096384|sp|O82314.2|U082_ARATH RecName: Full=Probable transcriptional regulatory protein At2g25830 gi|20197365|gb|AAC42247.2| expressed protein [Arabidopsis thaliana] gi|330252663|gb|AEC07757.1| probable transcriptional regulatory protein [Arabidopsis thaliana]	330	331	1.00E-147	100.3	86.4	92.7	YebC-related protein	gbpln	Arabidopsis thaliana	AT2G25830.1 | Symbols: | YebC-related | chr2:11019091-11021665 REVERSE LENGTH=331	330	331	1.00E-150	100.3	86.4	92.7

Rsa1.0_00013.1.g786.t1	#	#	#	#	#	#	#	-	----	----	#	#	#	#	#	#	#
Rsa1.0_00013.1.g787.t1	#	#	#	#	#	#	#	-	----	----	#	#	#	#	#	#	#
Rsa1.0_00014.1.g788.t1	gb|EOA35551.1| hypothetical protein CARUB_v10020757mg [Capsella rubella]	271	280	2.00E-54	103.3	52.8	62.7	hypothetical protein CARUB_v10020757mg	gbpln	Capsella rubella	AT1G75360.1 | Symbols: | unknown protein; Has 74 Blast hits to 71 proteins in 15 species: Archae - 0; Bacteria - 4; Metazoa - 4; Fungi - 0; Plants - 54; Viruses - 0; Other Eukaryotes - 12 (source: NCBI BLink). | chr1:28275084-28275956 FORWARD LENGTH=290	271	290	3.00E-45	107.0	49.1	58.7
Rsa1.0_00014.1.g789.t1	ref|XP_002887591.1| bZIP transcription factor family protein [Arabidopsis lyrata subsp. lyrata] gi|297333432|gb|EFH63850.1| bZIP transcription factor family protein [Arabidopsis lyrata subsp. lyrata]	416	173	4.00E-27	41.6	18.3	21.6	bZIP transcription factor family protein	gbpln	Arabidopsis lyrata	AT1G75390.1 | Symbols: AtbZIP44, bZIP44 | basic leucine-zipper 44 | chr1:28292224-28292745 FORWARD LENGTH=173	416	173	3.00E-29	41.6	17.8	21.2
Rsa1.0_00014.1.g790.t1	gb|EOA33370.1| hypothetical protein CARUB_v10020274mg [Capsella rubella] gi|482569183|gb|EOA33371.1| hypothetical protein CARUB_v10020274mg [Capsella rubella]	479	456	1.00E-180	95.2	77.9	82.7	hypothetical protein CARUB_v10020274mg	gbpln	Capsella rubella	AT1G75400.1 | Symbols: | RING/U-box superfamily protein | chr1:28297609-28299464 FORWARD LENGTH=455	479	455	0	95.0	78.7	82.7
Rsa1.0_00014.1.g791.t1	ref|NP_973745.1| beta glucosidase 11 [Arabidopsis thaliana] gi|332189360|gb|AEE27481.1| beta glucosidase 11 [Arabidopsis thaliana]	149	473	2.00E-25	317.4	41.6	49.0	beta glucosidase 11	gbpln	Arabidopsis thaliana	AT1G02850.3 | Symbols: BGLU11 | beta glucosidase 11 | chr1:630569-633085 FORWARD LENGTH=473	149	473	5.00E-28	317.4	41.6	49.0
Rsa1.0_00014.1.g792.t1	ref|NP_173217.1| PEP1 receptor 2 [Arabidopsis thaliana] gi|75334548|sp|Q9FZ59.1|PEPR2_ARATH RecName: Full=Leucine-rich repeat receptor-like protein kinase PEPR2; AltName: Full=Elicitor peptide 1 receptor 2; Short=PEP1 receptor 2; Flags: Precursor gi|9802748|gb|AAF99817.1|AC034257_9 Unknown protein [Arabidopsis thaliana] gi|224589394|gb|ACN59231.1| leucine-rich repeat receptor-like protein kinase [Arabidopsis thaliana] gi|332191511|gb|AEE29632.1| PEP1 receptor 2 [Arabidopsis thaliana]	92	1088	2.00E-24	1182.6	67.4	78.3	PEP1 receptor 2	gbpln	Arabidopsis thaliana	AT1G17750.1 | Symbols: PEPR2, AtPEPR2 | PEP1 receptor 2 | chr1:6106656-6110008 FORWARD LENGTH=1088	92	1088	3.00E-27	1182.6	67.4	78.3
Rsa1.0_00014.1.g793.t1	ref|NP_177674.1| BEL1-like homeodomain 3 [Arabidopsis thaliana] gi|145327251|ref|NP_001077827.1| BEL1-like homeodomain 3 [Arabidopsis thaliana] gi|75263117|sp|Q9FWS9.1|BLH3_ARATH RecName: Full=BEL1-like homeodomain protein 3; Short=BEL1-like protein 3 gi|10120440|gb|AAG13065.1|AC023754_3 Similar to homeodomain proteins [Arabidopsis thaliana] gi|13877515|gb|AAK43835.1|AF353093_1 BEL1-like homeodomain 3 [Arabidopsis thaliana] gi|21553417|gb|AAM62510.1| homeodomain protein BELL1, putative [Arabidopsis thaliana] gi|47059794|gb|AAT09418.1| BEL1-like homeodomain 3 protein [Arabidopsis thaliana] gi|332197595|gb|AEE35716.1| BEL1-like homeodomain 3 [Arabidopsis thaliana] gi|332197596|gb|AEE35717.1| BEL1-like homeodomain 3 [Arabidopsis thaliana]	505	524	0	103.8	80.8	86.9	BEL1-like homeodomain 3	gbpln	Arabidopsis thaliana	AT1G75410.2 | Symbols: BLH3 | BEL1-like homeodomain 3 | chr1:28300095-28301890 REVERSE LENGTH=524	505	524	0	103.8	80.8	86.9
Rsa1.0_00014.1.g794.t1	gb|AAD22286.1| putative non-LTR retroelement reverse transcriptase [Arabidopsis thaliana]	101	1311	9.00E-13	1298.0	41.6	53.5	putative non-LTR retroelement reverse transcriptase	gbpln	Arabidopsis thaliana	AT4G09490.1 | Symbols: | Polynucleotidyl transferase, ribonuclease H-like superfamily protein | chr4:6015583-6016575 REVERSE LENGTH=170	101	170	6.00E-12	168.3	35.6	49.5
Rsa1.0_00014.1.g795.t1	gb|ADA60976.1| short internode related sequence 5 [Brassica rapa subsp. pekinensis]	336	345	1.00E-159	102.7	93.2	96.1	short internode related sequence 5	gbpln	Brassica rapa	AT1G75520.1 | Symbols: SRS5 | SHI-related sequence 5 | chr1:28351779-28353179 REVERSE LENGTH=346	336	346	1.00E-156	103.0	86.0	89.9
Rsa1.0_00014.1.g796.t1	emb|CAB39638.1| RNA-directed DNA polymerase-like protein [Arabidopsis thaliana] gi|7267666|emb|CAB78094.1| RNA-directed DNA polymerase-like protein [Arabidopsis thaliana]	927	1274	0	137.4	49.3	65.8	RNA-directed DNA polymerase-like protein	gbpln	Arabidopsis thaliana	AT4G29090.1 | Symbols: | Ribonuclease H-like superfamily protein | chr4:14333528-14335255 FORWARD LENGTH=575	927	575	7.00E-59	62.0	17.8	27.3

Rsa1.0_00014.1.g797.t1	ref|NP_177685.1| Forkhead-associated (FHA) domain-containing protein [Arabidopsis thaliana] gi|332197609|gb|AEE35730.1| Forkhead-associated (FHA) domain-containing protein [Arabidopsis thaliana]	548	555	2.33E-156	101.3	58.0	69.7	Forkhead-associated (FHA) domain-containing protein	gbpln	Arabidopsis thaliana	AT1G75530.1 | Symbols: | Forkhead-associated (FHA) domain-containing protein | chr1:28359309-28361815 FORWARD LENGTH=555	548	555	1.00E-149	101.3	58.0	69.7
Rsa1.0_00014.1.g798.t1	pir||S65812 RNA-directed DNA polymerase (EC 2.7.7.49) (clone DW15) - Arabidopsis thaliana retrotransposon Ta11-1 gi|976278|gb|AAA75254.1| reverse transcriptase [Arabidopsis thaliana]	1748	1333	0	76.3	35.4	46.2	RNA-directed DNA polymerase (EC 2.7.7.49) (clone DW15) - Arabidopsis thaliana retrotransposon Ta11-1 gi|976278|gb|AAA75254.1| reverse transcriptase	gbpln	Arabidopsis thaliana	AT4G29090.1 | Symbols: | Ribonuclease H-like superfamily protein | chr4:14333528-14335255 FORWARD LENGTH=575	1748	575	3.00E-97	32.9	11.0	17.0
Rsa1.0_00014.1.g799.t1	ref|NP_177686.1| putative salt tolerance-like protein [Arabidopsis thaliana] gi|17433066|sp|Q9LQZ7.1|STHX_ARATH RecName: Full=Probable salt tolerance-like protein At1g75540 gi|9369377|gb|AAF87126.1|AC006434_22 F10A5.24 [Arabidopsis thaliana] gi|225898084|dbj|BAH30374.1| hypothetical protein [Arabidopsis thaliana] gi|332197610|gb|AEE35731.1| putative salt tolerance-like protein [Arabidopsis thaliana]	319	331	1.00E-134	103.8	82.8	89.3	putative salt tolerance-like protein	gbpln	Arabidopsis thaliana	AT1G75540.1 | Symbols: STH2 | salt tolerance homolog2 | chr1:28366059-28367398 FORWARD LENGTH=331	319	331	1.00E-137	103.8	82.8	89.3
Rsa1.0_00014.1.g800.t1	ref|NP_177687.1| glycine-rich protein [Arabidopsis thaliana] gi|9369369|gb|AAF87118.1|AC006434_14 F10A5.23 [Arabidopsis thaliana] gi|332197611|gb|AEE35732.1| glycine-rich protein [Arabidopsis thaliana]	165	167	4.00E-44	101.2	85.5	90.9	glycine-rich protein	gbpln	Arabidopsis thaliana	AT1G75550.1 | Symbols: | glycine-rich protein | chr1:28369437-28369940 REVERSE LENGTH=167	165	167	1.00E-46	101.2	85.5	90.9
Rsa1.0_00014.1.g801.t1	gb|EOA35562.1| hypothetical protein CARUB_v10020768mg, partial [Capsella rubella]	271	278	1.00E-108	102.6	77.5	80.4	hypothetical protein CARUB_v10020768mg, partial	gbpln	Capsella rubella	AT1G75560.2 | Symbols: | zinc knuckle (CCHC-type) family protein | chr1:28371420-28372717 REVERSE LENGTH=257	271	257	1.00E-103	94.8	76.0	78.6
Rsa1.0_00014.1.g802.t1	ref|XP_002887604.1| hypothetical protein ARALYDRAFT_476705 [Arabidopsis lyrata subsp. lyrata] gi|297333445|gb|EFH63863.1| hypothetical protein ARALYDRAFT_476705 [Arabidopsis lyrata subsp. lyrata]	108	108	2.00E-54	100.0	95.4	96.3	hypothetical protein ARALYDRAFT_476705	gbpln	Arabidopsis lyrata	AT1G75580.1 | Symbols: | SAUR-like auxin-responsive protein family | chr1:28377530-28377856 FORWARD LENGTH=108	108	108	2.00E-56	100.0	94.4	95.4
Rsa1.0_00014.1.g803.t1	#	#	#	#	#	#	#	-	----	----	#	#	#	#	#	#	#
Rsa1.0_00014.1.g804.t1	dbj|BAJ34271.1| unnamed protein product [Thellungiella halophila]	147	152	3.00E-61	103.4	86.4	91.2	unnamed protein product	----	----	AT1G75590.1 | Symbols: | SAUR-like auxin-responsive protein family | chr1:28383250-28383714 REVERSE LENGTH=154	147	154	5.00E-62	104.8	86.4	92.5
Rsa1.0_00014.1.g805.t1	gb|EOA33194.1| hypothetical protein CARUB_v10021300mg [Capsella rubella]	552	547	0	99.1	78.3	86.8	hypothetical protein CARUB_v10021300mg	gbpln	Capsella rubella	AT1G75620.1 | Symbols: | glyoxal oxidase-related protein | chr1:28394951-28396594 REVERSE LENGTH=547	552	547	0	99.1	77.9	87.1
Rsa1.0_00014.1.g806.t1	ref|XP_002889035.1| leucine-rich repeat family protein [Arabidopsis lyrata subsp. lyrata] gi|297334876|gb|EFH65294.1| leucine-rich repeat family protein [Arabidopsis lyrata subsp. lyrata]	1144	1141	0	99.7	86.1	92.4	leucine-rich repeat family protein	gbpln	Arabidopsis lyrata	AT1G75640.1 | Symbols: | Leucine-rich receptor-like protein kinase family protein | chr1:28403600-28407022 REVERSE LENGTH=1140	1144	1140	0	99.7	85.2	91.6
Rsa1.0_00014.1.g807.t2	gb|EOA33199.1| hypothetical protein CARUB_v10019710mg [Capsella rubella]	996	1024	0	102.8	83.5	88.5	hypothetical protein CARUB_v10019710mg	gbpln	Capsella rubella	AT1G75660.1 | Symbols: XRN3, AtXRN3 | 5'-3' exoribonuclease 3 | chr1:28408289-28414825 FORWARD LENGTH=1020	996	1020	0	102.4	84.8	89.8
Rsa1.0_00014.1.g808.t1	gb|AAM63477.1| endo-beta-1,4-glucanase, putative [Arabidopsis thaliana]	527	525	0	99.6	84.6	91.1	endo-beta-1,4-glucanase, putative	gbpln	Arabidopsis thaliana	AT1G75680.1 | Symbols: AtGH9B7, GH9B7 | glycosyl hydrolase 9B7 | chr1:28417215-28419231 REVERSE LENGTH=525	527	525	0	99.6	84.6	91.3
Rsa1.0_00014.1.g809.t1	gb|EOA35847.1| hypothetical protein CARUB_v10021088mg [Capsella rubella]	159	155	5.00E-75	97.5	88.1	93.7	hypothetical protein CARUB_v10021088mg	gbpln	Capsella rubella	AT1G75690.1 | Symbols: | DnaJ/Hsp40 cysteine-rich domain superfamily protein | chr1:28422273-28423170 REVERSE LENGTH=154	159	154	2.00E-76	96.9	89.9	93.1
Rsa1.0_00014.1.g810.t1	ref|NP_177699.4| HVA22-like protein G [Arabidopsis thaliana] gi|334302815|sp|Q9LR09.2|HA22G_ARATH RecName: Full=Putative HVA22-like protein g; Short=AtHVA22g gi|332197626|gb|AEE35747.1| HVA22-like protein G [Arabidopsis thaliana]	187	177	1.00E-81	94.7	75.9	84.5	HVA22-like protein G	gbpln	Arabidopsis thaliana	AT1G75700.1 | Symbols: HVA22G | HVA22-like protein G | chr1:28423956-28424931 FORWARD LENGTH=177	187	177	4.00E-84	94.7	75.9	84.5
Rsa1.0_00014.1.g811.t1	ref|XP_002887608.1| zinc finger family protein [Arabidopsis lyrata subsp. lyrata] gi|297333449|gb|EFH63867.1| zinc finger family protein [Arabidopsis lyrata subsp. lyrata]	456	461	0	101.1	87.3	90.4	zinc finger family protein	gbpln	Arabidopsis lyrata	AT1G75710.1 | Symbols: | C2H2-like zinc finger protein | chr1:28428806-28431128 FORWARD LENGTH=462	456	462	0	101.3	85.3	88.4

Rsa1.0_00014.1.g812.t1	gb|ABK28083.1| unknown [Arabidopsis thaliana]	89	95	3.00E-21	106.7	80.9	88.8	unknown	gbpln	Arabidopsis thaliana	AT1G75717.1 | Symbols: | unknown protein; FUNCTIONS IN: molecular_function unknown; INVOLVED IN: biological_process unknown; LOCATED IN: cellular_component unknown; Has 30201 Blast hits to 17322 proteins in 780 species: Archae - 12; Bacteria - 1396; Metazoa - 17338; Fungi - 3422; Plants - 5037; Viruses - 0; Other Eukaryotes - 2996 (source: NCBI BLink). | chr1:28433977-28434261 FORWARD LENGTH=94	89	94	6.00E-24	105.6	80.9	88.8
Rsa1.0_00014.1.g813.t1	ref|XP_002889039.1| hypothetical protein ARALYDRAFT_316499 [Arabidopsis lyrata subsp. lyrata] gi|297334880|gb|EFH65298.1| hypothetical protein ARALYDRAFT_316499 [Arabidopsis lyrata subsp. lyrata]	227	202	6.00E-65	89.0	63.9	70.5	hypothetical protein ARALYDRAFT_316499	gbpln	Arabidopsis lyrata	AT1G75720.2 | Symbols: | Plant protein of unknown function (DUF827) | chr1:28434648-28435321 REVERSE LENGTH=193	227	193	5.00E-66	85.0	65.6	71.4
Rsa1.0_00014.1.g814.t1	dbj|BAD42907.1| hypothetical protein [Arabidopsis thaliana]	507	589	2.33E-156	116.2	69.0	78.3	hypothetical protein	gbpln	Arabidopsis thaliana	AT1G75730.2 | Symbols: | unknown protein; FUNCTIONS IN: molecular_function unknown; INVOLVED IN: biological_process unknown; EXPRESSED IN: 22 plant structures; EXPRESSED DURING: 12 growth stages. | chr1:28435991-28438983 REVERSE LENGTH=589	507	589	1.00E-148	116.2	69.0	78.3
Rsa1.0_00014.1.g815.t1	ref|NP_565116.1| gibberellin-regulated protein 1 [Arabidopsis thaliana] gi|21431770|sp|P46689.2|GASA1_ARATH RecName: Full=Gibberellin-regulated protein 1; AltName: Full=GAST1 protein homolog 1; Flags: Precursor gi|18087524|gb|AAL58896.1|AF462801_1 At1g75750/F10A5_16 [Arabidopsis thaliana] gi|332197633|gb|AEE35754.1| gibberellin-regulated protein 1 [Arabidopsis thaliana]	98	98	6.00E-34	100.0	88.8	91.8	gibberellin-regulated protein 1	gbpln	Arabidopsis thaliana	AT1G75750.1 | Symbols: GASA1 | GAST1 protein homolog 1 | chr1:28441813-28442284 REVERSE LENGTH=98	98	98	1.00E-36	100.0	88.8	91.8
Rsa1.0_00014.1.g816.t1	gb|ACP30598.1| disease resistance protein [Brassica rapa subsp. pekinensis]	1494	2301	0	154.0	67.9	80.6	disease resistance protein	gbpln	Brassica rapa	AT4G23160.1 | Symbols: CRK8 | cysteine-rich RLK (RECEPTOR-like protein kinase) 8 | chr4:12129485-12134086 FORWARD LENGTH=1262	1494	1262	1.00E-108	84.5	13.1	19.9
Rsa1.0_00014.1.g817.t1	gb|EOA35576.1| hypothetical protein CARUB_v10020786mg [Capsella rubella]	272	272	2.33E-156	100.0	91.5	96.7	hypothetical protein CARUB_v10020786mg	gbpln	Capsella rubella	AT1G75760.1 | Symbols: | ER lumen protein retaining receptor family protein | chr1:28446940-28448421 REVERSE LENGTH=272	272	272	2.33E-156	100.0	89.7	96.0
Rsa1.0_00014.1.g818.t1	ref|XP_002873500.1| predicted protein [Arabidopsis lyrata subsp. lyrata] gi|297319337|gb|EFH49759.1| predicted protein [Arabidopsis lyrata subsp. lyrata]	887	959	0	108.1	69.9	77.5	predicted protein	gbpln	Arabidopsis lyrata	AT5G11210.1 | Symbols: ATGLR2.5, GLR2.5 | glutamate receptor 2.5 | chr5:3571214-3574537 REVERSE LENGTH=829	887	829	0	93.5	67.0	75.6
Rsa1.0_00014.1.g819.t1	#	#	#	#	#	#	#	-	----	----	#	#	#	#	#	#	#
Rsa1.0_00014.1.g820.t1	ref|XP_002887610.1| predicted protein [Arabidopsis lyrata subsp. lyrata] gi|297333451|gb|EFH63869.1| predicted protein [Arabidopsis lyrata subsp. lyrata]	239	236	2.00E-55	98.7	58.2	69.0	predicted protein	gbpln	Arabidopsis lyrata	AT1G75770.1 | Symbols: | unknown protein; Has 6 Blast hits to 5 proteins in 2 species: Archae - 0; Bacteria - 0; Metazoa - 0; Fungi - 0; Plants - 6; Viruses - 0; Other Eukaryotes - 0 (source: NCBI BLink). | chr1:28450022-28450971 FORWARD LENGTH=230	239	230	5.00E-54	96.2	56.9	67.8
Rsa1.0_00014.1.g821.t1	dbj|BAJ33829.1| unnamed protein product [Thellungiella halophila]	447	447	0	100.0	99.6	99.8	unnamed protein product	----	----	AT1G75780.1 | Symbols: TUB1 | tubulin beta-1 chain | chr1:28451378-28453602 REVERSE LENGTH=447	447	447	0	100.0	98.4	99.6
Rsa1.0_00014.1.g822.t1	gb|ABV89616.1| pathogenesis-related thaumatin family protein [Brassica rapa] gi|157849670|gb|ABV89618.1| pathogenesis-related thaumatin family protein [Brassica rapa]	325	321	1.00E-155	98.8	84.3	87.4	pathogenesis-related thaumatin family protein	gbpln	Brassica rapa	AT1G75800.1 | Symbols: | Pathogenesis-related thaumatin superfamily protein | chr1:28458889-28460447 FORWARD LENGTH=330	325	330	1.00E-153	101.5	86.2	89.8
Rsa1.0_00014.1.g823.t1	gb|ADD64789.1| CLAVATA1 [Brassica napus]	980	987	0	100.7	94.9	97.2	CLAVATA1	gbpln	Brassica napus	AT1G75820.1 | Symbols: CLV1, FAS3, FLO5, ATCLV1 | Leucine-rich receptor-like protein kinase family protein | chr1:28463631-28466652 REVERSE LENGTH=980	980	980	0	100.0	87.2	93.1
Rsa1.0_00014.1.g824.t1	sp|O24331.1|DEF4_RAPSA RecName: Full=Defensin-like protein 4; AltName: Full=Cysteine-rich antifungal protein 4; Short=AFP4; Flags: Precursor gi|1655683|emb|CAA65983.1| antifungal protein 4 [Raphanus sativus]	80	80	7.00E-40	100.0	100.0	100.0	RecName: Full=Defensin-like protein 4; AltName: Full=Cysteine-rich antifungal protein 4; Short=AFP4; Flags: Precursor gi|1655683|emb|CAA65983.1| antifungal protein 4	gbpln	Raphanus sativus	AT5G44420.1 | Symbols: PDF1.2, PDF1.2A, LCR77 | plant defensin 1.2 | chr5:17907216-17907550 REVERSE LENGTH=80	80	80	7.00E-37	100.0	83.8	92.5

Rsa1.0_00014.1.g825.t1	emb|CAN68319.1| hypothetical protein VITISV_032191 [Vitis vinifera]	178	312	4.00E-13	175.3	19.1	21.3	hypothetical protein VITISV_032191	gbpln	Vitis vinifera	AT2G18510.1 | Symbols: emb2444 | RNA-binding (RRM/RBD/RNP motifs) family protein | chr2:8031554-8033517 REVERSE LENGTH=363	178	363	2.00E-14	203.9	18.5	20.2
Rsa1.0_00014.1.g826.t1	#	#	#	#	#	#	#	–	----	----	#	#	#	#	#	#	#
Rsa1.0_00014.1.g827.t1	sp|O24332.1|DEF3_RAPSA RecName: Full=Defensin-like protein 3; AltName: Full=Cysteine-rich antifungal protein 3; Short=AFP3; Flags: Precursor gi|1655685|emb|CAA65984.1| antifungal protein 3 [Raphanus sativus]	79	79	2.00E-38	100.0	100.0	100.0	RecName: Full=Defensin-like protein 3; AltName: Full=Cysteine-rich antifungal protein 3; Short=AFP3; Flags: Precursor gi|1655685|emb|CAA65984.1| antifungal protein 3	gbpln	Raphanus sativus	AT5G44420.1 | Symbols: PDF1.2, PDF1.2A, LCR77 | plant defensin 1.2 | chr5:17907216-17907550 REVERSE LENGTH=80	79	80	5.00E-34	101.3	83.5	93.7
Rsa1.0_00014.1.g828.t1	sp|P69241.1|DEF1_RAPSA RecName: Full=Defensin-like protein 1; AltName: Full=Cysteine-rich antifungal protein 1; Short=AFP1; Flags: Precursor gi|609322|gb|AAA69541.1| antifungal protein 1 preprotein [Raphanus sativus] gi|115529964|gb|ABJ09663.1| antifungal rafp1 [Raphanus sativus]	80	80	7.00E-38	100.0	95.0	97.5	RecName: Full=Defensin-like protein 1; AltName: Full=Cysteine-rich antifungal protein 1; Short=AFP1; Flags: Precursor gi|609322|gb|AAA69541.1| antifungal protein 1 preprotein	gbpln	Raphanus sativus	AT5G44420.1 | Symbols: PDF1.2, PDF1.2A, LCR77 | plant defensin 1.2 | chr5:17907216-17907550 REVERSE LENGTH=80	80	80	5.00E-38	100.0	87.5	96.3
Rsa1.0_00014.1.g829.t1	sp|P69241.1|DEF1_RAPSA RecName: Full=Defensin-like protein 1; AltName: Full=Cysteine-rich antifungal protein 1; Short=AFP1; Flags: Precursor gi|609322|gb|AAA69541.1| antifungal protein 1 preprotein [Raphanus sativus] gi|115529964|gb|ABJ09663.1| antifungal rafp1 [Raphanus sativus]	83	80	5.00E-28	96.4	75.9	81.9	RecName: Full=Defensin-like protein 1; AltName: Full=Cysteine-rich antifungal protein 1; Short=AFP1; Flags: Precursor gi|609322|gb|AAA69541.1| antifungal protein 1 preprotein	gbpln	Raphanus sativus	AT5G44420.1 | Symbols: PDF1.2, PDF1.2A, LCR77 | plant defensin 1.2 | chr5:17907216-17907550 REVERSE LENGTH=80	83	80	4.00E-27	96.4	66.3	77.1
Rsa1.0_00014.1.g830.t1	pdb|2NTY|C Chain C, Rop4-Gdp-Prone8 gi|126031217|pdb|2NTY|D Chain D, Rop4-Gdp-Prone8	196	180	1.00E-101	91.8	90.3	91.8	Chain C, Rop4-Gdp-Prone8 gi|126031217|pdb|2NTY|D Chain D, Rop4-Gdp-Prone8	----	----	AT1G75840.1 | Symbols: ARAC5, ATGP3, ROP4, ATROP4 | RAC-like GTP binding protein 5 | chr1:28475964-28477377 FORWARD LENGTH=196	196	196	1.00E-102	100.0	95.9	98.0
Rsa1.0_00014.1.g831.t1	ref|XP_002889047.1| hypothetical protein ARALYDRAFT_895460 [Arabidopsis lyrata subsp. lyrata] gi|297334888|gb|EFH65306.1| hypothetical protein ARALYDRAFT_895460 [Arabidopsis lyrata subsp. lyrata]	1106	791	0	71.5	68.1	69.9	hypothetical protein ARALYDRAFT_895460	gbpln	Arabidopsis lyrata	AT1G75850.1 | Symbols: VPS35B | VPS35 homolog B | chr1:28478053-28483874 REVERSE LENGTH=790	1106	790	0	71.4	68.0	69.8
Rsa1.0_00014.1.g832.t1	#	#	#	#	#	#	#	–	----	----	#	#	#	#	#	#	#
Rsa1.0_00014.1.g833.t1	ref|NP_974149.1| GDSL esterase/lipase EXL1 [Arabidopsis thaliana] gi|28393626|gb|AAO42232.1| putative family II extracellular lipase 1 (EXL1) [Arabidopsis thaliana] gi|28827430|gb|AAO50559.1| putative family II extracellular lipase 1 (EXL1) [Arabidopsis thaliana] gi|332197648|gb|AEE35769.1| GDSL esterase/lipase EXL1 [Arabidopsis thaliana]	340	374	1.00E-173	110.0	84.7	93.5	GDSL esterase/lipase EXL1	gbpln	Arabidopsis thaliana	AT1G75880.2 | Symbols: | SGNH hydrolase-type esterase superfamily protein | chr1:28490564-28492298 FORWARD LENGTH=374	340	374	1.00E-175	110.0	84.7	93.5
Rsa1.0_00014.1.g834.t1	ref|XP_002887618.1| T4O12.12 [Arabidopsis lyrata subsp. lyrata] gi|297333459|gb|EFH63877.1| T4O12.12 [Arabidopsis lyrata subsp. lyrata]	346	673	1.00E-173	194.5	84.4	89.9	T4O12.12	gbpln	Arabidopsis lyrata	AT1G75890.2 | Symbols: | GDSL-like Lipase/Acylhydrolase superfamily protein | chr1:28493051-28495047 FORWARD LENGTH=366	346	366	1.00E-174	105.8	82.9	91.0
Rsa1.0_00015.1.g835.t1	gb|ABV89658.1| UVB-resistance protein UVR8 [Brassica rapa]	151	537	5.00E-31	355.6	71.5	76.8	UVB-resistance protein UVR8	gbpln	Brassica rapa	AT1G19880.1 | Symbols: | Regulator of chromosome condensation (RCC1) family protein | chr1:6900648-6903818 REVERSE LENGTH=538	151	538	2.00E-29	356.3	62.9	72.8
Rsa1.0_00015.1.g836.t1	ref|XP_002893079.1| hypothetical protein ARALYDRAFT_472215 [Arabidopsis lyrata subsp. lyrata] gi|297338921|gb|EFH69338.1| hypothetical protein ARALYDRAFT_472215 [Arabidopsis lyrata subsp. lyrata]	548	549	0	100.2	83.2	90.7	hypothetical protein ARALYDRAFT_472215	gbpln	Arabidopsis lyrata	AT1G19900.1 | Symbols: | glyoxal oxidase-related protein | chr1:6907038-6908684 REVERSE LENGTH=548	548	548	0	100.0	81.0	89.8
Rsa1.0_00015.1.g837.t1	gb|ABV89658.1| UVB-resistance protein UVR8 [Brassica rapa]	535	537	0	100.4	87.9	93.1	UVB-resistance protein UVR8	gbpln	Brassica rapa	AT1G19880.1 | Symbols: | Regulator of chromosome condensation (RCC1) family protein | chr1:6900648-6903818 REVERSE LENGTH=538	535	538	0	100.6	84.7	91.6

Rsa1.0_00015.1.g838.t1	ref|XP_002893077.1| protein IQ-DOMAIN 32 [Arabidopsis lyrata subsp. lyrata] gi|297338919|gb|EFH69336.1| protein IQ-DOMAIN 32 [Arabidopsis lyrata subsp. lyrata]	766	792	0	103.4	73.8	81.9	protein IQ-DOMAIN 32	gbpln	Arabidopsis lyrata	AT1G19870.1 | Symbols: iqd32 | IQ-domain 32 | chr1:6895400-6898539 REVERSE LENGTH=794	766	794	0	103.7	72.8	81.2
Rsa1.0_00015.1.g839.t2	ref|XP_002893076.1| zinc finger (CCCH-type) family protein [Arabidopsis lyrata subsp. lyrata] gi|297338918|gb|EFH69335.1| zinc finger (CCCH-type) family protein [Arabidopsis lyrata subsp. lyrata]	448	411	1.00E-164	91.7	71.7	79.5	zinc finger (CCCH-type) family protein	gbpln	Arabidopsis lyrata	AT1G19860.1 | Symbols: | Zinc finger C-x8-C-x5-C-x3-H type family protein | chr1:6891654-6894400 REVERSE LENGTH=413	448	413	1.00E-164	92.2	71.0	79.9
Rsa1.0_00015.1.g840.t1	dbj|BAJ34343.1| unnamed protein product [Thellungiella halophila]	819	901	0	110.0	89.3	93.8	unnamed protein product	----	----	AT1G19850.1 | Symbols: MP, ARF5, IAA24 | Transcriptional factor B3 family protein / auxin-responsive factor AUX/IAA-related | chr1:6887353-6891182 FORWARD LENGTH=902	819	902	0	110.1	87.3	92.8
Rsa1.0_00015.1.g841.t1	gb|EOA18064.1| hypothetical protein CARUB_v10006510mg, partial [Capsella rubella]	92	555	5.00E-16	603.3	48.9	69.6	hypothetical protein CARUB_v10006510mg, partial	gbpln	Capsella rubella	AT1G52950.1 | Symbols: | Nucleic acid-binding, OB-fold-like protein | chr1:19725483-19728007 FORWARD LENGTH=566	92	566	1.00E-11	615.2	34.8	63.0
Rsa1.0_00015.1.g842.t1	dbj|BAD95408.1| hypothetical protein [Arabidopsis thaliana]	217	478	1.00E-47	220.3	44.7	65.4	hypothetical protein	gbpln	Arabidopsis thaliana	AT3G24255.1 | Symbols: | RNA-directed DNA polymerase (reverse transcriptase)-related family protein | chr3:8789471-8793208 FORWARD LENGTH=746	217	746	3.00E-29	343.8	33.2	47.5
Rsa1.0_00015.1.g843.t2	emb|CAB39638.1| RNA-directed DNA polymerase-like protein [Arabidopsis thaliana] gi|7267666|emb|CAB78094.1| RNA-directed DNA polymerase-like protein [Arabidopsis thaliana]	997	1274	0	127.8	56.0	71.7	RNA-directed DNA polymerase-like protein	gbpln	Arabidopsis thaliana	AT4G29090.1 | Symbols: | Ribonuclease H-like superfamily protein | chr4:14333528-14335255 FORWARD LENGTH=575	997	575	3.00E-61	57.7	15.4	25.8
Rsa1.0_00015.1.g844.t1	sp|Q9SBK6.1|JMT_BRARP RecName: Full=Jasmonate O-methyltransferase; AltName: Full=Floral nectary-specific protein 1; AltName: Full=S-adenosyl-L-methionine:jasmonic acid carboxyl methyltransferase gi|6651395|gb|AAF22289.1|AF179222_1 floral nectary-specific protein [Brassica rapa subsp. pekinensis]	230	392	1.00E-113	170.4	85.7	91.7	RecName: Full=Jasmonate O-methyltransferase; AltName: Full=Floral nectary-specific protein 1; AltName: Full=S-adenosyl-L-methionine:jasmonic acid carboxyl methyltransferase gi|6651395|gb|AAF22289.1|AF179222_1 floral nectary-specific protein	gbpln	Brassica rapa	AT1G19640.1 | Symbols: JMT | jasmonic acid carboxyl methyltransferase | chr1:6789166-6791708 REVERSE LENGTH=389	230	389	1.00E-105	169.1	80.0	88.3
Rsa1.0_00015.1.g845.t1	gb|EOA38300.1| hypothetical protein CARUB_v10009793mg [Capsella rubella]	312	315	1.00E-40	101.0	42.6	57.1	hypothetical protein CARUB_v10009793mg	gbpln	Capsella rubella	AT1G19620.1 | Symbols: | unknown protein; Has 44 Blast hits to 24 proteins in 12 species: Archae - 0; Bacteria - 8; Metazoa - 22; Fungi - 0; Plants - 6; Viruses - 0; Other Eukaryotes - 8 (source: NCBI BLink). | chr1:6784020-6785075 FORWARD LENGTH=317	312	317	7.00E-28	101.6	34.9	44.9
Rsa1.0_00015.1.g846.t1	gb|AAF18538.1|AC006551_24 Very similar to retrotransposon reverse transcriptase [Arabidopsis thaliana]	768	1231	0	160.3	46.9	64.2	Very similar to retrotransposon reverse transcriptase	gbpln	Arabidopsis thaliana	AT4G29090.1 | Symbols: | Ribonuclease H-like superfamily protein | chr4:14333528-14335255 FORWARD LENGTH=575	768	575	5.00E-98	74.9	24.9	38.7
Rsa1.0_00015.1.g847.t1	dbj|BAJ33664.1| unnamed protein product [Thellungiella halophila]	358	355	0	99.2	89.9	94.4	unnamed protein product	----	----	AT1G19600.1 | Symbols: | pfkB-like carbohydrate kinase family protein | chr1:6779085-6780898 FORWARD LENGTH=355	358	355	0	99.2	89.4	93.3
Rsa1.0_00015.1.g848.t1	ref|XP_002890347.1| F18O14.34 [Arabidopsis lyrata subsp. lyrata] gi|297336189|gb|EFH66606.1| F18O14.34 [Arabidopsis lyrata subsp. lyrata]	138	299	9.00E-24	216.7	46.4	55.8	F18O14.34	gbpln	Arabidopsis lyrata	AT1G19580.1 | Symbols: GAMMA CA1 | gamma carbonic anhydrase 1 | chr1:6774937-6777092 FORWARD LENGTH=275	138	275	1.00E-24	199.3	44.9	54.3
Rsa1.0_00015.1.g849.t1	#	#	#	#	#	#	#	-	----	----	#	#	#	#	#	#	#
Rsa1.0_00015.1.g850.t1	ref|XP_002893054.1| hypothetical protein ARALYDRAFT_472181 [Arabidopsis lyrata subsp. lyrata] gi|297338896|gb|EFH69313.1| hypothetical protein ARALYDRAFT_472181 [Arabidopsis lyrata subsp. lyrata]	213	213	1.00E-99	100.0	85.0	90.6	hypothetical protein ARALYDRAFT_472181	gbpln	Arabidopsis lyrata	AT1G19570.1 | Symbols: DHAR1, ATDHAR1, DHAR5 | dehydroascorbate reductase | chr1:6773462-6774413 REVERSE LENGTH=213	213	213	1.00E-101	100.0	84.5	90.1
Rsa1.0_00015.1.g851.t7	ref|XP_002890346.1| hypothetical protein ARALYDRAFT_472180 [Arabidopsis lyrata subsp. lyrata] gi|297336188|gb|EFH66605.1| hypothetical protein ARALYDRAFT_472180 [Arabidopsis lyrata subsp. lyrata]	597	308	1.00E-142	51.6	41.5	46.4	hypothetical protein ARALYDRAFT_472180	gbpln	Arabidopsis lyrata	AT1G19540.1 | Symbols: | NmrA-like negative transcriptional regulator family protein | chr1:6765713-6767247 FORWARD LENGTH=310	597	310	1.00E-142	51.9	41.0	46.6

Rsa1.0_00015.1.g852.t1	gb|EOA38689.1| hypothetical protein CARUB_v10010689mg [Capsella rubella]	110	116	4.00E-49	105.5	88.2	92.7	hypothetical protein CARUB_v10010689mg	gbpln	Capsella rubella	AT1G19530.1 | Symbols: | unknown protein; FUNCTIONS IN: molecular_function unknown; INVOLVED IN: N-terminal protein myristoylation, anaerobic respiration; LOCATED IN: cellular_component unknown; EXPRESSED IN: leaf apex, inflorescence meristem, hypocotyl, root, flower; EXPRESSED DURING: petal differentiation and expansion stage; Has 47 Blast hits to 47 proteins in 13 species: Archae - 0; Bacteria - 0; Metazoa - 0; Fungi - 0; Plants - 47; Viruses - 0; Other Eukaryotes - 0 (source: NCBI BLink). | chr1:6763976-6764686 FORWARD LENGTH=117	110	117	6.00E-42	106.4	80.9	89.1
Rsa1.0_00015.1.g853.t1	ref|NP_564088.2| pentatricopeptide repeat-containing protein [Arabidopsis thaliana] gi|8778424|gb|AAF79432.1|AC025808_14 F18O14.27 [Arabidopsis thaliana] gi|332191742|gb|AEE29863.1| pentatricopeptide repeat-containing protein [Arabidopsis thaliana]	717	725	0	101.1	78.7	87.6	pentatricopeptide repeat-containing protein	gbpln	Arabidopsis thaliana	AT1G19520.1 | Symbols: NFD5 | pentatricopeptide (PPR) repeat-containing protein | chr1:6760032-6762581 FORWARD LENGTH=725	717	725	0	101.1	78.7	87.6
Rsa1.0_00015.1.g854.t1	ref|NP_564087.2| RAD-like 5 protein [Arabidopsis thaliana] gi|75328898|sp|Q8GW75.1|RADL5_ARATH RecName: Full=Protein RADIALIS-like 5; Short=AtRL5; Short=Protein RAD-like 5; AltName: Full=Protein RADIALIS-LIKE SANT/MYB 4; Short=Protein RSM4 gi|26453068|dbj|BAC43610.1| putative myb-related protein [Arabidopsis thaliana] gi|28973505|gb|AAO64077.1| putative myb family transcription factor [Arabidopsis thaliana] gi|41618974|gb|AAS09994.1| MYB transcription factor [Arabidopsis thaliana] gi|87133605|gb|ABD24443.1| RAD-like protein 5 [Arabidopsis thaliana] gi|332191741|gb|AEE29862.1| protein RADIALIS-like 5 [Arabidopsis thaliana]	90	100	2.00E-36	111.1	81.1	85.6	RAD-like 5 protein	gbpln	Arabidopsis thaliana	AT1G19510.1 | Symbols: ATRL5, RSM4, RL5 | RAD-like 5 | chr1:6756483-6757290 REVERSE LENGTH=100	90	100	4.00E-39	111.1	81.1	85.6
Rsa1.0_00015.1.g855.t1	gb|EOA36128.1| hypothetical protein CARUB_v10012246mg, partial [Capsella rubella]	90	88	1.00E-23	97.8	64.4	74.4	hypothetical protein CARUB_v10012246mg, partial	gbpln	Capsella rubella	AT1G19500.1 | Symbols: | unknown protein; FUNCTIONS IN: molecular_function unknown; INVOLVED IN: biological_process unknown; LOCATED IN: endomembrane system; EXPRESSED IN: 9 plant structures; EXPRESSED DURING: L mature pollen stage, M germinated pollen stage, 4 anthesis, C globular stage, petal differentiation and expansion stage; BEST Arabidopsis thaliana protein match is: unknown protein (TAIR:AT2G35765.1); Has 9 Blast hits to 9 proteins in 2 species: Archae - 0; Bacteria - 0; Metazoa - 0; Fungi - 0; Plants - 9; Viruses - 0; Other Eukaryotes - 0 (source: NCBI BLink). | chr1:6754952-6755536 REVERSE LENGTH=80	90	80	1.00E-24	88.9	63.3	72.2
Rsa1.0_00015.1.g856.t1	ref|XP_002893053.1| hypothetical protein ARALYDRAFT_312884 [Arabidopsis lyrata subsp. lyrata] gi|297338895|gb|EFH69312.1| hypothetical protein ARALYDRAFT_312884 [Arabidopsis lyrata subsp. lyrata]	466	647	1.00E-169	138.8	78.3	84.8	hypothetical protein ARALYDRAFT_312884	gbpln	Arabidopsis lyrata	AT1G19490.1 | Symbols: | Basic-leucine zipper (bZIP) transcription factor family protein | chr1:6751953-6753959 REVERSE LENGTH=471	466	471	1.00E-171	101.1	79.4	85.8
Rsa1.0_00015.1.g857.t1	ref|XP_002890343.1| predicted protein [Arabidopsis lyrata subsp. lyrata] gi|297336185|gb|EFH66602.1| predicted protein [Arabidopsis lyrata subsp. lyrata]	811	1262	0	155.6	78.3	86.4	predicted protein	gbpln	Arabidopsis lyrata	AT1G19485.2 | Symbols: | Transducin/WD40 repeat-like superfamily protein | chr1:6747167-6751443 FORWARD LENGTH=815	811	815	0	100.5	76.9	85.2
Rsa1.0_00015.1.g858.t1	gb|AAF79431.1|AC025808_13 F18O14.25 [Arabidopsis thaliana]	362	1314	1.00E-150	363.0	78.7	87.0	F18O14.25	gbpln	Arabidopsis thaliana	AT1G19480.2 | Symbols: | DNA glycosylase superfamily protein | chr1:6744520-6745653 FORWARD LENGTH=377	362	377	1.00E-152	104.1	78.7	87.0

Rsa1.0_00015.1.g859.t1	ref|NP_173377.1| sugar transporter ERD6-like 4 [Arabidopsis thaliana] gi|75331761|sp|Q93YP9.1|ERDL4_ARATH RecName: Full=Sugar transporter ERD6-like 4 gi|16648957|gb|AAL24330.1| similar to integral membrane protein [Arabidopsis thaliana] gi|20259852|gb|AAM13273.1| similar to integral membrane protein [Arabidopsis thaliana] gi|332191732|gb|AEE29853.1| sugar transporter ERD6-like 4 [Arabidopsis thaliana]	487	488	0	100.2	95.7	98.4	sugar transporter ERD6-like 4	gbpln	Arabidopsis thaliana	AT1G19450.1 | Symbols: | Major facilitator superfamily protein | chr1:6731671-6734633 REVERSE LENGTH=488	487	488	0	100.2	95.7	98.4
Rsa1.0_00015.1.g860.t1	gb|EOA37579.1| hypothetical protein CARUB_v10011929mg [Capsella rubella]	515	512	0	99.4	91.8	96.5	hypothetical protein CARUB_v10011929mg	gbpln	Capsella rubella	AT1G19440.1 | Symbols: KCS4 | 3-ketoacyl-CoA synthase 4 | chr1:6729119-6730669 FORWARD LENGTH=516	515	516	0	100.2	91.5	95.7
Rsa1.0_00015.1.g861.t1	ref|NP_564084.1| putative methyltransferase PMT28 [Arabidopsis thaliana] gi|75174900|sp|Q9LN50.1|PMTS_ARATH RecName: Full=Probable methyltransferase PMT28 gi|8778438|gb|AAF79446.1|AC025808_28 F18O14.20 [Arabidopsis thaliana] gi|15810125|gb|AAL07206.1| unknown protein [Arabidopsis thaliana] gi|25054951|gb|AAN71952.1| unknown protein [Arabidopsis thaliana] gi|332191730|gb|AEE29851.1| putative methyltransferase PMT28 [Arabidopsis thaliana]	694	724	0	104.3	90.5	95.0	putative methyltransferase PMT28	gbpln	Arabidopsis thaliana	AT1G19430.1 | Symbols: | S-adenosyl-L-methionine-dependent methyltransferases superfamily protein | chr1:6724669-6727533 REVERSE LENGTH=724	694	724	0	104.3	90.5	95.0
Rsa1.0_00015.1.g862.t1	ref|NP_173373.2| Erythronate-4-phosphate dehydrogenase family protein [Arabidopsis thaliana] gi|30686353|ref|NP_850946.1| Erythronate-4-phosphate dehydrogenase family protein [Arabidopsis thaliana] gi|18176223|gb|AAL60006.1| unknown protein [Arabidopsis thaliana] gi|20465771|gb|AAM20374.1| unknown protein [Arabidopsis thaliana] gi|110740824|dbj|BAE98509.1| hypothetical protein [Arabidopsis thaliana] gi|332191727|gb|AEE29848.1| Erythronate-4-phosphate dehydrogenase family protein [Arabidopsis thaliana] gi|332191728|gb|AEE29849.1| Erythronate-4-phosphate dehydrogenase family protein [Arabidopsis thaliana]	299	303	1.00E-147	101.3	87.6	91.3	Erythronate-4-phosphate dehydrogenase family protein	gbpln	Arabidopsis thaliana	AT1G19400.2 | Symbols: | Erythronate-4-phosphate dehydrogenase family protein | chr1:6712222-6713676 REVERSE LENGTH=303	299	303	1.00E-150	101.3	87.6	91.3
Rsa1.0_00015.1.g863.t1	ref|XP_002893045.1| hypothetical protein ARALYDRAFT_889371 [Arabidopsis lyrata subsp. lyrata] gi|297338887|gb|EFH69304.1| hypothetical protein ARALYDRAFT_889371 [Arabidopsis lyrata subsp. lyrata]	789	791	0	100.3	75.3	86.2	hypothetical protein ARALYDRAFT_889371	gbpln	Arabidopsis lyrata	AT1G19390.1 | Symbols: | Wall-associated kinase family protein | chr1:6700772-6703368 REVERSE LENGTH=788	789	788	0	99.9	74.5	84.5
Rsa1.0_00015.1.g864.t1	ref|XP_002890050.1| hypothetical protein ARALYDRAFT_312432 [Arabidopsis lyrata subsp. lyrata] gi|297335892|gb|EFH66309.1| hypothetical protein ARALYDRAFT_312432 [Arabidopsis lyrata subsp. lyrata]	96	431	1.00E-24	449.0	56.3	60.4	hypothetical protein ARALYDRAFT_312432	gbpln	Arabidopsis lyrata	AT1G14340.1 | Symbols: | RNA-binding (RRM/RBD/RNP motifs) family protein | chr1:4897709-4898776 FORWARD LENGTH=244	96	244	7.00E-27	254.2	59.4	63.5
Rsa1.0_00015.1.g865.t2	ref|XP_002893045.1| hypothetical protein ARALYDRAFT_889371 [Arabidopsis lyrata subsp. lyrata] gi|297338887|gb|EFH69304.1| hypothetical protein ARALYDRAFT_889371 [Arabidopsis lyrata subsp. lyrata]	719	791	0	110.0	75.5	84.6	hypothetical protein ARALYDRAFT_889371	gbpln	Arabidopsis lyrata	AT1G19390.1 | Symbols: | Wall-associated kinase family protein | chr1:6700772-6703368 REVERSE LENGTH=788	719	788	0	109.6	75.1	85.0

Rsa1.0_00015.1.g866.t1	gb|EOA39384.1| hypothetical protein CARUB_v10012465mg [Capsella rubella]	599	609	0	101.7	84.8	92.0	hypothetical protein CARUB_v10012465mg	gbpln	Capsella rubella	AT1G19370.1 | Symbols: | unknown protein; LOCATED IN: endoplasmic reticulum; EXPRESSED IN: 22 plant structures; EXPRESSED DURING: 13 growth stages; BEST Arabidopsis thaliana protein match is: unknown protein (TAIR:AT1G75140.1); Has 45 Blast hits to 43 proteins in 15 species: Archae - 0; Bacteria - 0; Metazoa - 0; Fungi - 0; Plants - 44; Viruses - 0; Other Eukaryotes - 1 (source: NCBI BLink). | chr1:6692904-6694721 REVERSE LENGTH=605	599	605	0	101.0	85.1	91.8
Rsa1.0_00015.1.g867.t1	ref|XP_002893043.1| hypothetical protein ARALYDRAFT_472163 [Arabidopsis lyrata subsp. lyrata] gi|297338885|gb|EFH69302.1| hypothetical protein ARALYDRAFT_472163 [Arabidopsis lyrata subsp. lyrata]	423	428	0	101.2	91.3	95.5	hypothetical protein ARALYDRAFT_472163	gbpln	Arabidopsis lyrata	AT1G19360.1 | Symbols: | Nucleotide-diphospho-sugar transferase family protein | chr1:6690672-6692211 REVERSE LENGTH=428	423	428	0	101.2	91.0	95.0
Rsa1.0_00015.1.g868.t1	dbj|BAJ33837.1| unnamed protein product [Thellungiella halophila]	336	329	1.00E-145	97.9	89.9	92.0	unnamed protein product	----	----	AT1G19350.3 | Symbols: BES1, BZR2 | Brassinosteroid signalling positive regulator (BZR1) family protein | chr1:6688680-6690165 FORWARD LENGTH=357	336	357	1.00E-140	106.3	87.2	91.4
Rsa1.0_00015.1.g869.t1	ref|XP_002890329.1| hypothetical protein ARALYDRAFT_472157 [Arabidopsis lyrata subsp. lyrata] gi|297336171|gb|EFH66588.1| hypothetical protein ARALYDRAFT_472157 [Arabidopsis lyrata subsp. lyrata]	416	410	0	98.6	80.5	87.5	hypothetical protein ARALYDRAFT_472157	gbpln	Arabidopsis lyrata	AT1G19340.1 | Symbols: | Methyltransferase MT-A70 family protein | chr1:6684798-6686798 FORWARD LENGTH=414	416	414	0	99.5	78.8	86.3
Rsa1.0_00015.1.g870.t1	ref|XP_002893042.1| hypothetical protein ARALYDRAFT_472155 [Arabidopsis lyrata subsp. lyrata] gi|297338884|gb|EFH69301.1| hypothetical protein ARALYDRAFT_472155 [Arabidopsis lyrata subsp. lyrata]	244	248	1.00E-117	101.6	88.9	93.0	hypothetical protein ARALYDRAFT_472155	gbpln	Arabidopsis lyrata	AT1G19330.2 | Symbols: | unknown protein; FUNCTIONS IN: molecular_function unknown; INVOLVED IN: biological_process unknown; LOCATED IN: cellular_component unknown; EXPRESSED IN: 22 plant structures; EXPRESSED DURING: 13 growth stages; BEST Arabidopsis thaliana protein match is: unknown protein (TAIR:AT1G75060.1); Has 145 Blast hits to 145 proteins in 43 species: Archae - 0; Bacteria - 0; Metazoa - 40; Fungi - 0; Plants - 104; Viruses - 0; Other Eukaryotes - 1 (source: NCBI BLink). | chr1:6680824-6683290 REVERSE LENGTH=242	244	242	1.00E-117	99.2	89.8	94.3
Rsa1.0_00015.1.g871.t2	ref|XP_002878802.1| predicted protein [Arabidopsis lyrata subsp. lyrata] gi|297324641|gb|EFH55061.1| predicted protein [Arabidopsis lyrata subsp. lyrata]	117	263	2.00E-37	224.8	67.5	76.9	predicted protein	gbpln	Arabidopsis lyrata	AT1G19320.1 | Symbols: | Pathogenesis-related thaumatin superfamily protein | chr1:6679327-6680178 FORWARD LENGTH=247	117	247	2.00E-37	211.1	67.5	73.5
Rsa1.0_00015.1.g872.t2	gb|EOA36204.1| hypothetical protein CARUB_v10010116mg [Capsella rubella]	66	246	1.00E-25	372.7	89.4	93.9	hypothetical protein CARUB_v10010116mg	gbpln	Capsella rubella	AT1G19330.2 | Symbols: | unknown protein; FUNCTIONS IN: molecular_function unknown; INVOLVED IN: biological_process unknown; LOCATED IN: cellular_component unknown; EXPRESSED IN: 22 plant structures; EXPRESSED DURING: 13 growth stages; BEST Arabidopsis thaliana protein match is: unknown protein (TAIR:AT1G75060.1); Has 145 Blast hits to 145 proteins in 43 species: Archae - 0; Bacteria - 0; Metazoa - 40; Fungi - 0; Plants - 104; Viruses - 0; Other Eukaryotes - 1 (source: NCBI BLink). | chr1:6680824-6683290 REVERSE LENGTH=242	66	242	3.00E-27	366.7	84.8	92.4
Rsa1.0_00015.1.g873.t1	ref|XP_002890328.1| hypothetical protein ARALYDRAFT_472154 [Arabidopsis lyrata subsp. lyrata] gi|297336170|gb|EFH66587.1| hypothetical protein ARALYDRAFT_472154 [Arabidopsis lyrata subsp. lyrata]	246	244	6.00E-94	99.2	69.9	78.0	hypothetical protein ARALYDRAFT_472154	gbpln	Arabidopsis lyrata	AT1G19320.1 | Symbols: | Pathogenesis-related thaumatin superfamily protein | chr1:6679327-6680178 FORWARD LENGTH=247	246	247	4.00E-96	100.4	69.9	78.0
Rsa1.0_00015.1.g874.t1	gb|AAD21778.1| putative non-LTR retroelement reverse transcriptase [Arabidopsis thaliana]	1399	1715	0	122.6	42.5	60.3	putative non-LTR retroelement reverse transcriptase	gbpln	Arabidopsis thaliana	AT3G25270.1 | Symbols: | Ribonuclease H-like superfamily protein | chr3:9203934-9204965 REVERSE LENGTH=343	1399	343	1.00E-76	24.5	10.0	15.0
Rsa1.0_00015.1.g875.t1	ref|XP_002893041.1| zinc finger family protein [Arabidopsis lyrata subsp. lyrata] gi|297338883|gb|EFH69300.1| zinc finger family protein [Arabidopsis lyrata subsp. lyrata]	229	226	2.00E-96	98.7	82.5	86.5	zinc finger family protein	gbpln	Arabidopsis lyrata	AT1G19310.1 | Symbols: | RING/U-box superfamily protein | chr1:6676424-6677104 REVERSE LENGTH=226	229	226	5.00E-93	98.7	81.7	86.9

Rsa1.0_00015.1.g876.t1	ref|XP_002893040.1| hypothetical protein ARALYDRAFT_472152 [Arabidopsis lyrata subsp. lyrata] gi|297338882|gb|EFH69299.1| hypothetical protein ARALYDRAFT_472152 [Arabidopsis lyrata subsp. lyrata]	349	350	1.00E-167	100.3	91.4	95.1	hypothetical protein ARALYDRAFT_472152	gbpln	Arabidopsis lyrata	AT1G19300.1 | Symbols: GATL1, PARVUS, GLZ1, ATGATL1 | Nucleotide-diphospho-sugar transferases superfamily protein | chr1:6671451-6672506 REVERSE LENGTH=351	349	351	1.00E-166	100.6	87.7	91.1
Rsa1.0_00015.1.g877.t1	ref|NP_173361.1| protein DA1 [Arabidopsis thaliana] gi|193806611|sp|P0C7Q8.1|DA1_ARATH RecName: Full=Protein DA1; AltName: Full=Protein SUPPRESSOR OF LARGE SEED AND ORGAN PHENOTYPES OF DA1-1 1 gi|332191704|gb|AEE29825.1| protein DA1 [Arabidopsis thaliana]	545	532	0	97.6	86.8	91.6	protein DA1	gbpln	Arabidopsis thaliana	AT1G19270.1 | Symbols: DA1 | DA1 | chr1:6663327-6665845 FORWARD LENGTH=532	545	532	0	97.6	86.8	91.6
Rsa1.0_00015.1.g878.t1	gb|AAF91318.1|AF242848_1 beta-ketoacyl-acyl carrier protein synthase [Brassica napus]	514	543	0	105.6	94.4	96.1	beta-ketoacyl-acyl carrier protein synthase	gbpln	Brassica napus	AT1G74960.3 | Symbols: FAB1 | fatty acid biosynthesis 1 | chr1:28152564-28155948 REVERSE LENGTH=541	514	541	0	105.3	81.5	88.1
Rsa1.0_00015.1.g879.t1	gb|EOA40127.1| hypothetical protein CARUB_v10008834mg [Capsella rubella]	525	528	0	100.6	91.0	95.6	hypothetical protein CARUB_v10008834mg	gbpln	Capsella rubella	AT1G19250.1 | Symbols: FMO1 | flavin-dependent monooxygenase 1 | chr1:6650656-6653053 REVERSE LENGTH=530	525	530	0	101.0	89.5	95.0
Rsa1.0_00015.1.g880.t1	gb|AAM61590.1| unknown [Arabidopsis thaliana]	103	104	3.00E-36	101.0	82.5	93.2	unknown	gbpln	Arabidopsis thaliana	AT1G19240.1 | Symbols: | unknown protein; Has 24 Blast hits to 24 proteins in 11 species: Archae - 0; Bacteria - 0; Metazoa - 0; Fungi - 0; Plants - 24; Viruses - 0; Other Eukaryotes - 0 (source: NCBI BLink). | chr1:6649562-6650384 REVERSE LENGTH=104	103	104	2.00E-38	101.0	81.6	92.2
Rsa1.0_00015.1.g881.t1	ref|XP_002890325.1| hypothetical protein ARALYDRAFT_312861 [Arabidopsis lyrata subsp. lyrata] gi|297336167|gb|EFH66584.1| hypothetical protein ARALYDRAFT_312861 [Arabidopsis lyrata subsp. lyrata]	1009	946	0	93.8	81.9	86.1	hypothetical protein ARALYDRAFT_312861	gbpln	Arabidopsis lyrata	AT1G19230.2 | Symbols: | Riboflavin synthase-like superfamily protein | chr1:6644189-6649149 FORWARD LENGTH=934	1009	934	0	92.6	78.7	83.2
Rsa1.0_00015.1.g882.t1	gb|AFD01318.1| auxin response factor 19-2 [Brassica rapa subsp. pekinensis]	1074	1049	0	97.7	91.9	93.0	auxin response factor 19-2	gbpln	Brassica rapa	AT1G19220.1 | Symbols: ARF19, IAA22, ARF11 | auxin response factor 19 | chr1:6628395-6632779 REVERSE LENGTH=1086	1074	1086	0	101.1	87.9	90.4
Rsa1.0_00015.1.g883.t1	ref|XP_002893033.1| hypothetical protein ARALYDRAFT_472144 [Arabidopsis lyrata subsp. lyrata] gi|297338875|gb|EFH69292.1| hypothetical protein ARALYDRAFT_472144 [Arabidopsis lyrata subsp. lyrata]	161	186	4.00E-53	115.5	70.8	83.9	hypothetical protein ARALYDRAFT_472144	gbpln	Arabidopsis lyrata	AT1G19210.1 | Symbols: | Integrase-type DNA-binding superfamily protein | chr1:6626973-6627530 REVERSE LENGTH=185	161	185	5.00E-55	114.9	71.4	82.0
Rsa1.0_00015.1.g884.t1	ref|NP_567546.1| AT hook motif DNA-binding family protein [Arabidopsis thaliana] gi|15451060|gb|AAK96801.1| putative protein [Arabidopsis thaliana] gi|20148333|gb|AAM10057.1| putative protein [Arabidopsis thaliana] gi|119657370|tpd|FAA00284.1| TPA: AT-hook motif nuclear localized protein 13 [Arabidopsis thaliana] gi|332658571|gb|AEE83971.1| AT hook motif DNA-binding family protein [Arabidopsis thaliana]	294	439	6.00E-41	149.3	42.2	53.7	AT hook motif DNA-binding family protein	gbpln	Arabidopsis thaliana	AT4G17950.1 | Symbols: | AT hook motif DNA-binding family protein | chr4:9967295-9969007 REVERSE LENGTH=439	294	439	2.00E-43	149.3	42.2	53.7
Rsa1.0_00015.1.g885.t1	ref|NP_567546.1| AT hook motif DNA-binding family protein [Arabidopsis thaliana] gi|15451060|gb|AAK96801.1| putative protein [Arabidopsis thaliana] gi|20148333|gb|AAM10057.1| putative protein [Arabidopsis thaliana] gi|119657370|tpd|FAA00284.1| TPA: AT-hook motif nuclear localized protein 13 [Arabidopsis thaliana] gi|332658571|gb|AEE83971.1| AT hook motif DNA-binding family protein [Arabidopsis thaliana]	306	439	3.00E-44	143.5	53.3	66.7	AT hook motif DNA-binding family protein	gbpln	Arabidopsis thaliana	AT4G17950.1 | Symbols: | AT hook motif DNA-binding family protein | chr4:9967295-9969007 REVERSE LENGTH=439	306	439	7.00E-47	143.5	53.3	66.7
Rsa1.0_00015.1.g886.t1	gb|AAF82231.1|AC069143_7 Contains similarity to a hypothetical protein F9E10.21 gi|6646770 from Arabidopsis thaliana BAC F9E10 gb|AC013258 [Arabidopsis thaliana]	225	222	1.00E-54	98.7	56.0	71.1	Contains similarity to a hypothetical protein F9E10.21 gi|6646770 from Arabidopsis thaliana BAC F9E10 gb|AC013258	gbpln	Arabidopsis thaliana	AT1G19200.1 | Symbols: | Protein of unknown function (DUF581) | chr1:6625104-6625856 REVERSE LENGTH=215	225	215	5.00E-57	95.6	56.0	71.1

Rsa1.0_00015.1.g887.t1	ref|NP_173353.1| alpha/beta-hydrolase-like protein [Arabidopsis thaliana] gi|75335190|sp|Q9LMA7.1|CXE1_ARATH RecName: Full=Probable carboxylesterase 1; AltName: Full=AtCXE1 gi|8954057|gb|AAF82230.1|AC069143_6 Contains similarity to a PrMC3 from Pinus radiata gb|AF110333 [Arabidopsis thaliana] gi|119360077|gb|ABL66767.1| At1g19190 [Arabidopsis thaliana] gi|332191695|gb|AEE29816.1| probable carboxylesterase 1 [Arabidopsis thaliana]	320	318	1.00E-125	99.4	69.1	83.1	alpha/beta-hydrolase-like protein	gbpln	Arabidopsis thaliana	AT1G19190.1 | Symbols: | alpha/beta-Hydrolases superfamily protein | chr1:6623876-6624832 FORWARD LENGTH=318	320	318	1.00E-127	99.4	69.1	83.1
Rsa1.0_00015.1.g888.t1	gb|EOA38428.1| hypothetical protein CARUB_v10010018mg [Capsella rubella]	254	268	6.00E-93	105.5	74.8	84.3	hypothetical protein CARUB_v10010018mg	gbpln	Capsella rubella	AT1G19180.1 | Symbols: JAZ1, TIFY10A | jasmonate-zim-domain protein 1 | chr1:6622312-6623271 FORWARD LENGTH=253	254	253	1.00E-93	99.6	76.0	81.5
Rsa1.0_00015.1.g889.t1	ref|NP_564074.1| uncharacterized protein [Arabidopsis thaliana] gi|8954052|gb|AAF82225.1|AC069143_1 Contains similarity to a HSPC326 mRNA from Homo sapiens gb|AF161444. EST gb|AI997162 comes from this gene [Arabidopsis thaliana] gi|332191689|gb|AEE29810.1| uncharacterized protein AT1G19140 [Arabidopsis thaliana]	312	311	1.00E-151	99.7	86.2	91.7	uncharacterized protein	gbpln	Arabidopsis thaliana	AT1G19140.1 | Symbols: | FUNCTIONS IN: molecular_function unknown; INVOLVED IN: ubiquinone biosynthetic process; LOCATED IN: mitochondrion; EXPRESSED IN: 24 plant structures; EXPRESSED DURING: 15 growth stages; CONTAINS InterPro DOMAIN/s: COQ9 (InterPro:IPR013718), Ubiquinone biosynthesis protein COQ9 (InterPro:IPR012762); Has 748 Blast hits to 748 proteins in 260 species: Archae - 0; Bacteria - 218; Metazoa - 126; Fungi - 101; Plants - 39; Viruses - 0; Other Eukaryotes - 264 (source: NCBI BLink). | chr1:6611026-6612414 REVERSE LENGTH=311	312	311	1.00E-154	99.7	86.2	91.7
Rsa1.0_00015.1.g890.t1	ref|NP_173345.2| inter-alpha-trypsin inhibitor heavy chain-like protein [Arabidopsis thaliana] gi|22531102|gb|AAM97055.1| unknown protein [Arabidopsis thaliana] gi|23197960|gb|AAN15507.1| unknown protein [Arabidopsis thaliana] gi|332191682|gb|AEE29803.1| inter-alpha-trypsin inhibitor heavy chain-like protein [Arabidopsis thaliana]	756	754	0	99.7	88.2	94.2	inter-alpha-trypsin inhibitor heavy chain-like protein	gbpln	Arabidopsis thaliana	AT1G19110.1 | Symbols: | inter-alpha-trypsin inhibitor heavy chain-related | chr1:6602270-6605766 FORWARD LENGTH=754	756	754	0	99.7	88.2	94.2
Rsa1.0_00015.1.g891.t1	ref|NP_173344.2| Histidine kinase-, DNA gyrase B-, and HSP90-like ATPase family protein [Arabidopsis thaliana] gi|62320246|dbj|BAD94510.1| hypothetical protein [Arabidopsis thaliana] gi|332191681|gb|AEE29802.1| Histidine kinase-, DNA gyrase B-, and HSP90-like ATPase family protein [Arabidopsis thaliana]	696	663	0	95.3	79.3	84.5	Histidine kinase-, DNA gyrase B-, and HSP90-like ATPase family protein	gbpln	Arabidopsis thaliana	AT1G19100.1 | Symbols: | Histidine kinase-, DNA gyrase B-, and HSP90-like ATPase family protein | chr1:6595560-6601159 FORWARD LENGTH=663	696	663	0	95.3	79.3	84.5
Rsa1.0_00015.1.g892.t1	gb|EOA39914.1| hypothetical protein CARUB_v10008603mg [Capsella rubella]	617	616	0	99.8	80.6	88.2	hypothetical protein CARUB_v10008603mg	gbpln	Capsella rubella	AT1G19090.1 | Symbols: RKF2, CRK1 | receptor-like serine/threonine kinase 2 | chr1:6590350-6592615 FORWARD LENGTH=600	617	600	0	97.2	76.3	84.4
Rsa1.0_00015.1.g893.t1	ref|XP_002893028.1| hypothetical protein ARALYDRAFT_889335 [Arabidopsis lyrata subsp. lyrata] gi|297338870|gb|EFH69287.1| hypothetical protein ARALYDRAFT_889335 [Arabidopsis lyrata subsp. lyrata]	215	209	1.00E-89	97.2	84.7	91.2	hypothetical protein ARALYDRAFT_889335	gbpln	Arabidopsis lyrata	AT1G19050.1 | Symbols: ARR7 | response regulator 7 | chr1:6577919-6579078 REVERSE LENGTH=206	215	206	1.00E-87	95.8	78.6	87.0
Rsa1.0_00015.1.g894.t1	gb|EOA36368.1| hypothetical protein CARUB_v10010768mg [Capsella rubella]	86	87	3.00E-30	101.2	82.6	87.2	hypothetical protein CARUB_v10010768mg	gbpln	Capsella rubella	AT1G19020.1 | Symbols: | unknown protein; BEST Arabidopsis thaliana protein match is: unknown protein (TAIR:AT3G48180.1); Has 88 Blast hits to 88 proteins in 15 species: Archae - 0; Bacteria - 0; Metazoa - 0; Fungi - 0; Plants - 88; Viruses - 0; Other Eukaryotes - 0 (source: NCBI BLink). | chr1:6568142-6568402 FORWARD LENGTH=86	86	86	3.00E-31	100.0	83.7	88.4
Rsa1.0_00015.1.g895.t1	ref|NP_173333.1| uncharacterized protein [Arabidopsis thaliana] gi|8778293|gb|AAF79302.1|AC068602_25 F14D16.14 [Arabidopsis thaliana] gi|27754600|gb|AAO22746.1| unknown protein [Arabidopsis thaliana] gi|332191666|gb|AEE29787.1| uncharacterized protein AT1G18990 [Arabidopsis thaliana]	510	524	0	102.7	84.9	92.4	uncharacterized protein	gbpln	Arabidopsis thaliana	AT1G18990.1 | Symbols: | Protein of unknown function, DUF593 | chr1:6558778-6560432 REVERSE LENGTH=524	510	524	0	102.7	84.9	92.4

Rsa1.0_00015.1.g896.t1	ref|NP_173332.1| germin-like protein subfamily T member 2 [Arabidopsis thaliana] gi|18203242|sp|Q9LMC9.1|GLT2_ARATH RecName: Full=Germin-like protein subfamily T member 2; Flags: Precursor gi|8778294|gb|AAF79303.1|AC068602_26 F14D16.13 [Arabidopsis thaliana] gi|21537092|gb|AAM61433.1| germin, putative [Arabidopsis thaliana] gi|89111904|gb|ABD60724.1| At1g18980 [Arabidopsis thaliana] gi|332191665|gb|AEE29786.1| germin-like protein subfamily T member 2 [Arabidopsis thaliana]	214	220	1.00E-103	102.8	89.3	92.5	germin-like protein subfamily T member 2	gbpln	Arabidopsis thaliana	AT1G18980.1 | Symbols: | RmlC-like cupins superfamily protein | chr1:6557364-6558026 REVERSE LENGTH=220	214	220	1.00E-106	102.8	89.3	92.5
Rsa1.0_00015.1.g897.t1	gb|AAF97281.1|AC010164_3 Hypothetical protein [Arabidopsis thaliana] gi|12324507|gb|AAG52212.1|AC022288_11 putative gag-pol polyprotein; 76173-77576 [Arabidopsis thaliana]	221	467	1.00E-73	211.3	62.4	79.2	Hypothetical protein	gbpln	Arabidopsis thaliana	#	#	#	#	#	#	#
Rsa1.0_00015.1.g898.t3	ref|NP_173329.2| DDT domain-containing protein [Arabidopsis thaliana] gi|332191662|gb|AEE29783.1| DDT domain-containing protein [Arabidopsis thaliana]	815	750	0	92.0	75.6	81.0	DDT domain-containing protein	gbpln	Arabidopsis thaliana	AT1G18950.1 | Symbols: | DDT domain superfamily | chr1:6546539-6551549 FORWARD LENGTH=750	815	750	0	92.0	75.6	81.0
Rsa1.0_00015.1.g899.t1	gb|EOA40010.1| hypothetical protein CARUB_v10008700mg [Capsella rubella]	526	575	0	109.3	86.5	92.8	hypothetical protein CARUB_v10008700mg	gbpln	Capsella rubella	AT1G18940.1 | Symbols: | Nodulin-like / Major Facilitator Superfamily protein | chr1:6543852-6545592 FORWARD LENGTH=526	526	526	0	100.0	86.3	94.1
Rsa1.0_00015.1.g900.t1	ref|XP_002893023.1| F14D16.3 [Arabidopsis lyrata subsp. lyrata] gi|297338865|gb|EFH69282.1| F14D16.3 [Arabidopsis lyrata subsp. lyrata]	1189	1260	0	106.0	81.6	87.7	F14D16.3	gbpln	Arabidopsis lyrata	AT1G18910.1 | Symbols: | zinc ion binding;zinc ion binding | chr1:6532706-6537993 REVERSE LENGTH=1254	1189	1254	0	105.5	80.7	87.6
Rsa1.0_00015.1.g901.t1	ref|NP_173324.1| pentatricopeptide repeat-containing protein [Arabidopsis thaliana] gi|42571539|ref|NP_973860.1| pentatricopeptide repeat-containing protein [Arabidopsis thaliana] gi|75151479|sp|Q8GYP6.1|PPR49_ARATH RecName: Full=Pentatricopeptide repeat-containing protein At1g18900 gi|26450017|dbj|BAC42129.1| unknown protein [Arabidopsis thaliana] gi|28827402|gb|AAO50545.1| unknown protein [Arabidopsis thaliana] gi|332191657|gb|AEE29778.1| pentatricopeptide repeat-containing protein [Arabidopsis thaliana] gi|332191658|gb|AEE29779.1| pentatricopeptide repeat-containing protein [Arabidopsis thaliana]	857	860	0	100.4	83.1	89.0	pentatricopeptide repeat-containing protein	gbpln	Arabidopsis thaliana	AT1G18900.2 | Symbols: | Pentatricopeptide repeat (PPR) superfamily protein | chr1:6529778-6532360 FORWARD LENGTH=860	857	860	0	100.4	83.1	89.0
Rsa1.0_00015.1.g902.t1	dbj|BAJ34189.1| unnamed protein product [Thellungiella halophila]	548	545	0	99.5	90.3	94.3	unnamed protein product	----	----	AT1G18890.1 | Symbols: ATCDPK1, CPK10, CDPK1, AtCPK10 | calcium-dependent protein kinase 1 | chr1:6523468-6525736 REVERSE LENGTH=545	548	545	0	99.5	88.9	94.0
Rsa1.0_00015.1.g903.t1	ref|XP_002890304.1| proton-dependent oligopeptide transport family protein [Arabidopsis lyrata subsp. lyrata] gi|297336146|gb|EFH66563.1| proton-dependent oligopeptide transport family protein [Arabidopsis lyrata subsp. lyrata]	613	587	0	95.8	84.2	87.8	proton-dependent oligopeptide transport family protein	gbpln	Arabidopsis lyrata	AT1G18880.1 | Symbols: | Major facilitator superfamily protein | chr1:6520800-6523241 FORWARD LENGTH=587	613	587	0	95.8	83.2	86.9
Rsa1.0_00015.1.g904.t1	gb|EOA40359.1| hypothetical protein CARUB_v10009088mg, partial [Capsella rubella]	417	457	1.00E-179	109.6	75.5	85.9	hypothetical protein CARUB_v10009088mg, partial	gbpln	Capsella rubella	AT1G18850.1 | Symbols: | unknown protein; FUNCTIONS IN: molecular_function unknown; INVOLVED IN: biological_process unknown; LOCATED IN: nucleolus; EXPRESSED IN: 22 plant structures; EXPRESSED DURING: 13 growth stages; Has 40 Blast hits to 40 proteins in 13 species: Archae - 0; Bacteria - 0; Metazoa - 0; Fungi - 0; Plants - 40; Viruses - 0; Other Eukaryotes - 0 (source: NCBI BLink). | chr1:6504928-6506127 REVERSE LENGTH=399	417	399	1.00E-173	95.7	73.6	83.0
Rsa1.0_00015.1.g905.t2	dbj|BAJ33816.1| unnamed protein product [Thellungiella halophila]	635	571	0	89.9	73.4	79.8	unnamed protein product	----	----	AT1G18840.2 | Symbols: IQD30 | IQ-domain 30 | chr1:6501068-6503435 REVERSE LENGTH=572	635	572	0	90.1	69.8	77.3

Rsa1.0_00015.1.g906.t1	ref|XP_002893019.1| predicted protein [Arabidopsis lyrata subsp. lyrata] gi|297338861|gb|EFH69278.1| predicted protein [Arabidopsis lyrata subsp. lyrata]	94	104	7.00E-27	110.6	80.9	86.2	predicted protein	gbpln	Arabidopsis lyrata	#	#	#	#	#	#	#
Rsa1.0_00015.1.g907.t1	ref|XP_002893018.1| hypothetical protein ARALYDRAFT_472107 [Arabidopsis lyrata subsp. lyrata] gi|297338860|gb|EFH69277.1| hypothetical protein ARALYDRAFT_472107 [Arabidopsis lyrata subsp. lyrata]	88	88	9.00E-39	100.0	89.8	92.0	hypothetical protein ARALYDRAFT_472107	gbpln	Arabidopsis lyrata	AT1G18835.1 | Symbols: MIF3 | mini zinc finger | chr1:6496106-6496372 REVERSE LENGTH=88	88	88	6.00E-37	100.0	90.9	93.2
Rsa1.0_00015.1.g908.t1	dbj|BAC42657.1| unknown protein [Arabidopsis thaliana] gi|28950777|gb|AAO63312.1| At1g18800 [Arabidopsis thaliana]	258	228	1.00E-112	88.4	79.1	83.3	unknown protein	gbpln	Arabidopsis thaliana	AT1G18800.1 | Symbols: NRP2 | NAP1-related protein 2 | chr1:6481466-6483463 REVERSE LENGTH=256	258	256	1.00E-112	99.2	77.5	81.0
Rsa1.0_00015.1.g909.t1	#	#	#	#	#	#	#	-	----	----	#	#	#	#	#	#	#
Rsa1.0_00015.1.g910.t1	gb|EOA36433.1| hypothetical protein CARUB_v10010970mg [Capsella rubella]	166	210	3.00E-23	126.5	42.8	57.8	hypothetical protein CARUB_v10010970mg	gbpln	Capsella rubella	AT1G18760.1 | Symbols: | Zinc finger, C3HC4 type (RING finger) family protein | chr1:6471150-6471824 REVERSE LENGTH=224	166	224	2.00E-17	134.9	39.2	58.4
Rsa1.0_00015.1.g911.t1	gb|ACP30591.1| disease resistance protein [Brassica rapa subsp. pekinensis]	237	1555	7.00E-36	656.1	38.4	48.1	disease resistance protein	gbpln	Brassica rapa	AT4G09490.1 | Symbols: | Polynucleotidyl transferase, ribonuclease H-like superfamily protein | chr4:6015583-6016575 REVERSE LENGTH=170	237	170	2.00E-18	71.7	21.1	32.5
Rsa1.0_00015.1.g912.t1	#	#	#	#	#	#	#	-	----	----	#	#	#	#	#	#	#
Rsa1.0_00015.1.g913.t1	ref|XP_002882812.1| hypothetical protein ARALYDRAFT_341444 [Arabidopsis lyrata subsp. lyrata] gi|297328652|gb|EFH59071.1| hypothetical protein ARALYDRAFT_341444 [Arabidopsis lyrata subsp. lyrata]	147	315	1.00E-19	214.3	43.5	56.5	hypothetical protein ARALYDRAFT_341444	gbpln	Arabidopsis lyrata	AT3G13228.1 | Symbols: | RING/U-box superfamily protein | chr3:4267075-4268052 FORWARD LENGTH=325	147	325	2.00E-15	221.1	40.1	55.8
Rsa1.0_00015.1.g914.t1	ref|XP_002882812.1| hypothetical protein ARALYDRAFT_341444 [Arabidopsis lyrata subsp. lyrata] gi|297328652|gb|EFH59071.1| hypothetical protein ARALYDRAFT_341444 [Arabidopsis lyrata subsp. lyrata]	221	315	1.00E-50	142.5	51.6	65.2	hypothetical protein ARALYDRAFT_341444	gbpln	Arabidopsis lyrata	AT3G13228.1 | Symbols: | RING/U-box superfamily protein | chr3:4267075-4268052 FORWARD LENGTH=325	221	325	2.00E-45	147.1	47.1	61.5
Rsa1.0_00015.1.g915.t1	ref|XP_002890300.1| AGL102 [Arabidopsis lyrata subsp. lyrata] gi|297336142|gb|EFH66559.1| AGL102 [Arabidopsis lyrata subsp. lyrata]	153	383	3.00E-37	250.3	52.9	57.5	AGL102	gbpln	Arabidopsis lyrata	AT1G18750.1 | Symbols: AGL65 | AGAMOUS-like 65 | chr1:6467266-6469640 FORWARD LENGTH=389	153	389	2.00E-39	254.2	52.9	57.5
Rsa1.0_00015.1.g916.t1	ref|XP_002890299.1| hypothetical protein ARALYDRAFT_472100 [Arabidopsis lyrata subsp. lyrata] gi|297336141|gb|EFH66558.1| hypothetical protein ARALYDRAFT_472100 [Arabidopsis lyrata subsp. lyrata]	363	382	1.00E-170	105.2	85.1	91.2	hypothetical protein ARALYDRAFT_472100	gbpln	Arabidopsis lyrata	AT1G18740.1 | Symbols: | Protein of unknown function (DUF793) | chr1:6464125-6465273 FORWARD LENGTH=382	363	382	1.00E-170	105.2	84.8	91.2
Rsa1.0_00015.1.g917.t1	ref|NP_173308.2| NDH dependent flow 6 protein [Arabidopsis thaliana] gi|19310578|gb|AAL85020.1| unknown protein [Arabidopsis thaliana] gi|21436079|gb|AAM51240.1| unknown protein [Arabidopsis thaliana] gi|110741088|dbj|BAE98638.1| hypothetical protein [Arabidopsis thaliana] gi|332191632|gb|AEE29753.1| NDH dependent flow 6 protein [Arabidopsis thaliana]	176	175	2.00E-92	99.4	93.2	95.5	NDH dependent flow 6 protein	gbpln	Arabidopsis thaliana	AT1G18730.1 | Symbols: NDF6 | NDH dependent flow 6 | chr1:6460625-6462107 FORWARD LENGTH=175	176	175	9.00E-95	99.4	93.2	95.5
Rsa1.0_00015.1.g918.t1	dbj|BAD95408.1| hypothetical protein [Arabidopsis thaliana]	447	478	2.00E-83	106.9	33.1	45.9	hypothetical protein	gbpln	Arabidopsis thaliana	AT3G24255.1 | Symbols: | RNA-directed DNA polymerase (reverse transcriptase)-related family protein | chr3:8789471-8793208 FORWARD LENGTH=746	447	746	5.00E-70	166.9	27.7	38.7
Rsa1.0_00015.1.g919.t1	ref|XP_002890296.1| hypothetical protein ARALYDRAFT_335127 [Arabidopsis lyrata subsp. lyrata] gi|297336138|gb|EFH66555.1| hypothetical protein ARALYDRAFT_335127 [Arabidopsis lyrata subsp. lyrata]	266	258	1.00E-97	97.0	69.5	79.3	hypothetical protein ARALYDRAFT_335127	gbpln	Arabidopsis lyrata	AT1G18710.1 | Symbols: AtMYB47, MYB47 | myb domain protein 47 | chr1:6450781-6452986 FORWARD LENGTH=267	266	267	2.00E-70	100.4	54.1	68.8
Rsa1.0_00015.1.g920.t1	#	#	#	#	#	#	#	-	----	----	#	#	#	#	#	#	#
Rsa1.0_00015.1.g921.t1	#	#	#	#	#	#	#	-	----	----	#	#	#	#	#	#	#
Rsa1.0_00015.1.g922.t1	gb|AAC02664.1| polyprotein [Arabidopsis thaliana]	1370	1451	0	105.9	66.6	78.4	polyprotein	gbpln	Arabidopsis thaliana	AT4G23160.1 | Symbols: CRK8 | cysteine-rich RLK (RECEPTOR-like protein kinase) 8 | chr4:12129485-12134086 FORWARD LENGTH=1262	1370	1262	1.00E-110	92.1	14.5	20.8

Rsa1.0_00015.1.g923.t1	ref|NP_173304.2| putative glycosyltransferase 4 [Arabidopsis thaliana] gi|46576343|sp|Q9M9U0.1|GT4_ARATH RecName: Full=Putative glycosyltransferase 4; Short=AtGT4 gi|6730715|gb|AAF27110.1|AC011809_19 Similar to galactosyltransferase [Arabidopsis thaliana] gi|332191625|gb|AEE29746.1| putative glycosyltransferase 4 [Arabidopsis thaliana]	508	513	0	101.0	78.5	83.9	putative glycosyltransferase 4	gbpln	Arabidopsis thaliana	AT1G18690.1 | Symbols: | Galactosyl transferase GMA12/MNN10 family protein | chr1:6435153-6436694 FORWARD LENGTH=513	508	513	0	101.0	78.5	83.9
Rsa1.0_00015.1.g924.t4	ref|XP_002890292.1| zinc finger family protein [Arabidopsis lyrata subsp. lyrata] gi|297336134|gb|EFH66551.1| zinc finger family protein [Arabidopsis lyrata subsp. lyrata]	555	476	0	85.8	78.9	81.3	zinc finger family protein	gbpln	Arabidopsis lyrata	AT1G18660.1 | Symbols: | zinc finger (C3HC4-type RING finger) family protein | chr1:6421433-6425565 FORWARD LENGTH=486	555	486	0	87.6	78.2	80.9
Rsa1.0_00015.1.g925.t17	ref|NP_973858.1| phosphoserine phosphatase [Arabidopsis thaliana] gi|62900892|sp|O82796.2|SERB_ARATH RecName: Full=Phosphoserine phosphatase, chloroplastic; Short=PSP; Short=PSPase; AltName: Full=O-phosphoserine phosphohydrolase; Flags: Precursor gi|9795592|gb|AAF98410.1|AC026238_2 3-phosphoserine phosphatase [Arabidopsis thaliana] gi|17529132|gb|AAL38792.1| putative 3-phosphoserine phosphatase [Arabidopsis thaliana] gi|20465705|gb|AAM20321.1| putative 3-phosphoserine phosphatase [Arabidopsis thaliana] gi|21592986|gb|AAM64935.1| 3-phosphoserine phosphatase [Arabidopsis thaliana] gi|332191617|gb|AEE29738.1| phosphoserine phosphatase [Arabidopsis thaliana]	547	295	1.00E-149	53.9	49.7	51.4	phosphoserine phosphatase	gbpln	Arabidopsis thaliana	AT1G18640.2 | Symbols: PSP | 3-phosphoserine phosphatase | chr1:6416524-6418245 REVERSE LENGTH=295	547	295	1.00E-151	53.9	49.7	51.4
Rsa1.0_00015.1.g926.t1	ref|NP_173298.1| glycine-rich RNA-binding protein 6 [Arabidopsis thaliana] gi|75334552|sp|Q9FZ84.1|RBG6_ARATH RecName: Full=Glycine-rich RNA-binding protein 6, mitochondrial; Short=AtGR-RBP6; AltName: Full=AtRBG6; Flags: Precursor gi|9795594|gb|AAF98412.1|AC026238_4 Similar to glycine-rich RNA-binding proteins [Arabidopsis thaliana] gi|29029110|gb|AAO64934.1| At1g18630 [Arabidopsis thaliana] gi|110743184|dbj|BAE99483.1| hypothetical protein [Arabidopsis thaliana] gi|332191616|gb|AEE29737.1| glycine-rich RNA-binding protein 6 [Arabidopsis thaliana]	149	155	2.00E-47	104.0	61.1	71.1	glycine-rich RNA-binding protein 6	gbpln	Arabidopsis thaliana	AT1G18630.1 | Symbols: GR-RBP6 | glycine-rich RNA-binding protein 6 | chr1:6415226-6416283 FORWARD LENGTH=155	149	155	5.00E-50	104.0	61.1	71.1
Rsa1.0_00015.1.g927.t3	ref|XP_002888967.1| leucine-rich repeat family protein [Arabidopsis lyrata subsp. lyrata] gi|297334808|gb|EFH65226.1| leucine-rich repeat family protein [Arabidopsis lyrata subsp. lyrata]	945	966	0	102.2	63.9	78.1	leucine-rich repeat family protein	gbpln	Arabidopsis lyrata	AT1G74190.1 | Symbols: AtRLP15, RLP15 | receptor like protein 15 | chr1:27902590-27906158 REVERSE LENGTH=965	945	965	0	102.1	62.4	77.7
Rsa1.0_00015.1.g928.t2	ref|XP_002888967.1| leucine-rich repeat family protein [Arabidopsis lyrata subsp. lyrata] gi|297334808|gb|EFH65226.1| leucine-rich repeat family protein [Arabidopsis lyrata subsp. lyrata]	932	966	0	103.6	63.1	76.5	leucine-rich repeat family protein	gbpln	Arabidopsis lyrata	AT1G74190.1 | Symbols: AtRLP15, RLP15 | receptor like protein 15 | chr1:27902590-27906158 REVERSE LENGTH=965	932	965	0	103.5	61.6	76.6
Rsa1.0_00015.1.g929.t1	ref|NP_173296.3| galactose oxidase/kelch repeat domain-containing protein [Arabidopsis thaliana] gi|9795595|gb|AAF98413.1|AC026238_5 Hypothetical protein [Arabidopsis thaliana] gi|332191613|gb|AEE29734.1| galactose oxidase/kelch repeat domain-containing protein [Arabidopsis thaliana]	509	556	0	109.2	84.7	91.6	galactose oxidase/kelch repeat domain-containing protein	gbpln	Arabidopsis thaliana	AT1G18610.1 | Symbols: | Galactose oxidase/kelch repeat superfamily protein | chr1:6405779-6408831 FORWARD LENGTH=556	509	556	0	109.2	84.7	91.6

Rsa1.0_00015.1.g930.t1	ref|NP_564058.1| RHOMBOID-like protein 12 [Arabidopsis thaliana] gi|9795596|gb|AAF98414.1|AC026238_6 Hypothetical protein [Arabidopsis thaliana] gi|13877607|gb|AAK43881.1|AF370504_1 Unknown protein [Arabidopsis thaliana] gi|20148713|gb|AAM10247.1| unknown protein [Arabidopsis thaliana] gi|21592397|gb|AAM64348.1| unknown [Arabidopsis thaliana] gi|332191612|gb|AEE29733.1| RHOMBOID-like protein 12 [Arabidopsis thaliana]	347	336	1.00E-144	96.8	82.1	88.5	RHOMBOID-like protein 12	gbpln	Arabidopsis thaliana	AT1G18600.1 | Symbols: ATRBL12, RBL12 | RHOMBOID-like protein 12 | chr1:6400647-6402587 FORWARD LENGTH=336	347	336	1.00E-147	96.8	82.1	88.5
Rsa1.0_00015.1.g931.t1	#	#	#	#	#	#	#	-	----	----	#	#	#	#	#	#	#
Rsa1.0_00015.1.g932.t1	ref|XP_002862798.1| hypothetical protein ARALYDRAFT_497292 [Arabidopsis lyrata subsp. lyrata] gi|297308526|gb|EFH39056.1| hypothetical protein ARALYDRAFT_497292 [Arabidopsis lyrata subsp. lyrata]	723	842	1.00E-179	116.5	55.5	67.6	hypothetical protein ARALYDRAFT_497292	gbpln	Arabidopsis lyrata	AT1G71400.1 | Symbols: AtRLP12, RLP12 | receptor like protein 12 | chr1:26909905-26912448 FORWARD LENGTH=847	723	847	1.00E-180	117.2	54.8	67.5
Rsa1.0_00015.1.g933.t1	ref|NP_188953.1| receptor like protein 38 [Arabidopsis thaliana] gi|9294202|dbj|BAB02104.1| disease resistance protein [Arabidopsis thaliana] gi|332643199|gb|AEE76720.1| receptor like protein 38 [Arabidopsis thaliana]	744	784	0	105.4	56.0	69.5	receptor like protein 38	gbpln	Arabidopsis thaliana	AT3G23120.1 | Symbols: AtRLP38, RLP38 | receptor like protein 38 | chr3:8227222-8229576 REVERSE LENGTH=784	744	784	0	105.4	56.0	69.5
Rsa1.0_00016.1.g934.t1	ref|NP_196791.1| transcription factor MYB46 [Arabidopsis thaliana] gi|75335627|sp|Q9LXV2.1|MYB46_ARATH RecName: Full=Transcription factor MYB46; AltName: Full=Myb-related protein 46; Short=AtMYB46 gi|7630043|emb|CAB88251.1| putative transcription factor (MYB46) [Arabidopsis thaliana] gi|23306410|gb|AAN17432.1| putative transcription factor (MYB46) [Arabidopsis thaliana] gi|27311887|gb|AAO00909.1| putative transcription factor (MYB46) [Arabidopsis thaliana] gi|41619388|gb|AAS10091.1| MYB transcription factor [Arabidopsis thaliana] gi|332004441|gb|AED91824.1| transcription factor MYB46 [Arabidopsis thaliana]	279	280	1.00E-126	100.4	84.9	90.7	transcription factor MYB46	gbpln	Arabidopsis thaliana	AT5G12870.1 | Symbols: ATMYB46, MYB46 | myb domain protein 46 | chr5:4062939-4064939 REVERSE LENGTH=280	279	280	1.00E-128	100.4	84.9	90.7
Rsa1.0_00016.1.g935.t1	ref|NP_568283.2| dicarboxylate transporter 1 [Arabidopsis thaliana] gi|75180949|sp|Q9LXV3.1|DIT1_ARATH RecName: Full=Dicarboxylate transporter 1, chloroplastic; AltName: Full=2-oxoglutarate/malate translocator 1; Short=AtpOMT1; Flags: Precursor gi|7630042|emb|CAB88250.1| 2-oxoglutarate/malate translocator precursor-like protein [Arabidopsis thaliana] gi|332004439|gb|AED91822.1| dicarboxylate transporter 1 [Arabidopsis thaliana]	596	557	0	93.5	86.9	89.1	dicarboxylate transporter 1	gbpln	Arabidopsis thaliana	AT5G12860.1 | Symbols: DiT1 | dicarboxylate transporter 1 | chr5:4059927-4061919 REVERSE LENGTH=557	596	557	0	93.5	86.9	89.1

Rsa1.0_00016.1.g936.t1	ref|NP_568282.1| nuclear transcription factor Y subunit A-1 [Arabidopsis thaliana] gi|42573353|ref|NP_974773.1| nuclear transcription factor Y subunit A-1 [Arabidopsis thaliana] gi|75180950|sp|Q9LXV5.1|NFYA1_ARATH RecName: Full=Nuclear transcription factor Y subunit A-1; Short=AtNF-YA-1; AltName: Full=Protein EMBRYO DEFECTIVE 2220; AltName: Full=Transcriptional activator HAP2A gi|7630040|emb|CAB88248.1| CCAAT box binding factor/ transcription factor Hap2a [Arabidopsis thaliana] gi|107738403|gb|ABF83691.1| At5g12840 [Arabidopsis thaliana] gi|332004434|gb|AED91817.1| nuclear transcription factor Y subunit A-1 [Arabidopsis thaliana] gi|332004437|gb|AED91820.1| nuclear transcription factor Y subunit A-1 [Arabidopsis thaliana]	290	272	1.00E-116	93.8	76.6	82.1	nuclear transcription factor Y subunit A-1	gbpln	Arabidopsis thaliana	AT5G12840.3 | Symbols: HAP2A, ATHAP2A, NF-YA1 | nuclear factor Y, subunit A1 | chr5:4051147-4052961 REVERSE LENGTH=272	290	272	1.00E-118	93.8	76.6	82.1
Rsa1.0_00016.1.g937.t1	ref|XP_002873580.1| calmodulin-domain protein kinase 7 [Arabidopsis lyrata subsp. lyrata] gi|297319417|gb|EFH49839.1| calmodulin-domain protein kinase 7 [Arabidopsis lyrata subsp. lyrata]	531	535	0	100.8	94.4	97.0	calmodulin-domain protein kinase 7	gbpln	Arabidopsis lyrata	AT5G12480.1 | Symbols: CPK7 | calmodulin-domain protein kinase 7 | chr5:4047817-4050035 REVERSE LENGTH=535	531	535	0	100.8	94.0	96.8
Rsa1.0_00016.1.g938.t1	ref|XP_002873579.1| hypothetical protein ARALYDRAFT_909228 [Arabidopsis lyrata subsp. lyrata] gi|297319416|gb|EFH49838.1| hypothetical protein ARALYDRAFT_909228 [Arabidopsis lyrata subsp. lyrata]	380	385	1.00E-175	101.3	86.1	92.1	hypothetical protein ARALYDRAFT_909228	gbpln	Arabidopsis lyrata	AT5G12470.1 | Symbols: | Protein of unknown function (DUF3411) | chr5:4044950-4047290 REVERSE LENGTH=386	380	386	1.00E-175	101.6	84.2	91.6
Rsa1.0_00016.1.g939.t1	gb|EOA19606.1| hypothetical protein CARUB_v10002857mg [Capsella rubella]	868	442	0	50.9	39.9	43.1	hypothetical protein CARUB_v10002857mg	gbpln	Capsella rubella	AT5G12460.1 | Symbols: | Protein of unknown function (DUF604) | chr5:4043057-4044583 FORWARD LENGTH=441	868	441	0	50.8	38.4	41.9
Rsa1.0_00016.1.g940.t5	ref|XP_002314081.1| predicted protein [Populus trichocarpa] gi|222850489|gb|EEE88036.1| predicted protein [Populus trichocarpa]	926	418	1.00E-134	45.1	24.2	32.0	predicted protein	gbpln	Populus trichocarpa	AT5G12460.1 | Symbols: | Protein of unknown function (DUF604) | chr5:4043057-4044583 FORWARD LENGTH=441	926	441	1.00E-116	47.6	21.9	30.3
Rsa1.0_00016.1.g941.t1	ref|XP_002314081.1| predicted protein [Populus trichocarpa] gi|222850489|gb|EEE88036.1| predicted protein [Populus trichocarpa]	467	418	1.00E-138	89.5	49.5	65.3	predicted protein	gbpln	Populus trichocarpa	AT5G12460.1 | Symbols: | Protein of unknown function (DUF604) | chr5:4043057-4044583 FORWARD LENGTH=441	467	441	1.00E-119	94.4	43.3	60.8
Rsa1.0_00016.1.g942.t1	gb|EOA21902.1| hypothetical protein CARUB_v10002383mg [Capsella rubella]	74	74	1.00E-21	100.0	81.1	89.2	hypothetical protein CARUB_v10002383mg	gbpln	Capsella rubella	#	#	#	#	#	#	#
Rsa1.0_00016.1.g943.t1	gb|EOA20141.1| hypothetical protein CARUB_v10000430mg [Capsella rubella]	639	645	0	100.9	72.0	80.3	hypothetical protein CARUB_v10000430mg	gbpln	Capsella rubella	AT5G12440.3 | Symbols: | CCCH-type zinc fingerfamily protein with RNA-binding domain | chr5:4035891-4038604 REVERSE LENGTH=650	639	650	0	101.7	70.0	78.7
Rsa1.0_00016.1.g944.t1	ref|XP_002873572.1| DNAJ heat shock N-terminal domain-containing protein [Arabidopsis lyrata subsp. lyrata] gi|297319409|gb|EFH49831.1| DNAJ heat shock N-terminal domain-containing protein [Arabidopsis lyrata subsp. lyrata]	1157	1163	0	100.5	76.0	84.4	DNAJ heat shock N-terminal domain-containing protein	gbpln	Arabidopsis lyrata	AT5G12430.1 | Symbols: TPR16 | Heat shock protein DnaJ with tetratricopeptide repeat | chr5:4028475-4034086 REVERSE LENGTH=1165	1157	1165	0	100.7	75.0	83.7
Rsa1.0_00016.1.g945.t1	gb|EOA19414.1| hypothetical protein CARUB_v10001152mg, partial [Capsella rubella]	363	387	1.00E-163	106.6	84.6	90.4	hypothetical protein CARUB_v10001152mg, partial	gbpln	Capsella rubella	AT5G12410.1 | Symbols: | THUMP domain-containing protein | chr5:4021974-4023950 REVERSE LENGTH=376	363	376	1.00E-160	103.6	82.9	90.9
Rsa1.0_00016.1.g946.t12	ref|NP_568273.2| PHD-finger and DNA binding domain-containing protein [Arabidopsis thaliana] gi|332004422|gb|AED91805.1| PHD-finger and DNA binding domain-containing protein [Arabidopsis thaliana]	1191	1602	0	134.5	65.8	76.7	PHD-finger and DNA binding domain-containing protein	gbpln	Arabidopsis thaliana	AT5G12400.1 | Symbols: | DNA binding;zinc ion binding;DNA binding | chr5:4013813-4021018 FORWARD LENGTH=1602	1191	1602	0	134.5	65.8	76.7
Rsa1.0_00016.1.g947.t1	gb|AFO66516.1| putative 17.9 kDa class II heat shock protein [Brassica napus]	154	154	2.00E-71	100.0	94.2	98.7	putative 17.9 kDa class II heat shock protein	gbpln	Brassica napus	AT5G12020.1 | Symbols: HSP17.6II | 17.6 kDa class II heat shock protein | chr5:3882409-3882876 REVERSE LENGTH=155	154	155	3.00E-69	100.6	90.3	95.5

Rsa1.0_00016.1.g948.t1	gb|AFO66517.1| putative kinase [Brassica napus]	503	1266	0	251.7	89.5	92.4	putative kinase	gbpln	Brassica napus	AT5G12010.1 | Symbols: | unknown protein; INVOLVED IN: response to salt stress; LOCATED IN: chloroplast, plasma membrane, membrane; EXPRESSED IN: 23 plant structures; EXPRESSED DURING: 13 growth stages; BEST Arabidopsis thaliana protein match is: unknown protein (TAIR:AT4G29780.1); Has 1807 Blast hits to 1807 proteins in 277 species: Archae - 0; Bacteria - 0; Metazoa - 736; Fungi - 347; Plants - 385; Viruses - 0; Other Eukaryotes - 339 (source: NCBI BLink). | chr5:3877975-3879483 REVERSE LENGTH=502	503	502	0	99.8	83.7	89.5
Rsa1.0_00016.1.g949.t1	gb|AFO66517.1| putative kinase [Brassica napus]	698	1266	0	181.4	93.6	96.3	putative kinase	gbpln	Brassica napus	AT5G12000.1 | Symbols: | Protein kinase protein with adenine nucleotide alpha hydrolases-like domain | chr5:3874151-3876780 REVERSE LENGTH=701	698	701	0	100.4	83.8	90.4
Rsa1.0_00016.1.g950.t1	ref|NP_196760.1| proline-rich family protein [Arabidopsis thaliana] gi|7573366|emb|CAB87672.1| putative protein [Arabidopsis thaliana] gi|332004365|gb|AED91748.1| proline-rich family protein [Arabidopsis thaliana]	249	181	2.00E-26	72.7	52.2	57.4	proline-rich family protein	gbpln	Arabidopsis thaliana	AT5G11990.1 | Symbols: | proline-rich family protein | chr5:3872651-3873196 REVERSE LENGTH=181	249	181	5.00E-29	72.7	52.2	57.4
Rsa1.0_00016.1.g951.t1	gb|AFO66519.1| putative ABC superfamily ATP binding cassette transporter [Brassica napus]	103	356	8.00E-41	345.6	83.5	88.3	putative ABC superfamily ATP binding cassette transporter	gbpln	Brassica napus	AT5G11970.1 | Symbols: | Protein of unknown function (DUF3511) | chr5:3863289-3863606 REVERSE LENGTH=105	103	105	1.00E-41	101.9	86.4	90.3
Rsa1.0_00016.1.g952.t1	gb|EOA20897.1| hypothetical protein CARUB_v10001232mg, partial [Capsella rubella]	384	364	1.00E-141	94.8	67.4	68.2	hypothetical protein CARUB_v10001232mg, partial	gbpln	Capsella rubella	AT5G11960.1 | Symbols: | Protein of unknown function (DUF803) | chr5:3858852-3861462 REVERSE LENGTH=344	384	344	1.00E-141	89.6	66.1	67.2
Rsa1.0_00016.1.g953.t1	gb|AFO66505.1| putative homodimerization protein [Brassica napus]	218	216	1.00E-119	99.1	95.4	97.2	putative homodimerization protein	gbpln	Brassica napus	AT5G11950.2 | Symbols: | Putative lysine decarboxylase family protein | chr5:3855072-3856815 FORWARD LENGTH=216	218	216	1.00E-117	99.1	92.2	96.3
Rsa1.0_00016.1.g954.t1	#	#	#	#	#	#	#	–	----	----	#	#	#	#	#	#	#
Rsa1.0_00016.1.g955.t1	gb|EOA22071.1| hypothetical protein CARUB_v10002611mg [Capsella rubella]	137	147	2.00E-46	107.3	82.5	89.8	hypothetical protein CARUB_v10002611mg	gbpln	Capsella rubella	AT5G11930.1 | Symbols: | Thioredoxin superfamily protein | chr5:3845165-3845611 REVERSE LENGTH=148	137	148	3.00E-48	108.0	81.0	87.6
Rsa1.0_00016.1.g956.t1	gb|AFO66503.1| putative fructan 1-exohydrolase [Brassica napus]	515	524	0	101.7	93.2	95.9	putative fructan 1-exohydrolase	gbpln	Brassica napus	AT5G11920.1 | Symbols: AtcwINV6, cwINV6 | 6-&1-fructan exohydrolase | chr5:3839490-3842206 FORWARD LENGTH=550	515	550	0	106.8	77.5	88.2
Rsa1.0_00016.1.g957.t1	ref|XP_002873537.1| esterase/lipase/thioesterase family protein [Arabidopsis lyrata subsp. lyrata] gi|297319374|gb|EFH49796.1| esterase/lipase/thioesterase family protein [Arabidopsis lyrata subsp. lyrata]	297	297	1.00E-150	100.0	87.9	92.9	esterase/lipase/thioesterase family protein	gbpln	Arabidopsis lyrata	AT5G11910.1 | Symbols: | alpha/beta-Hydrolases superfamily protein | chr5:3836560-3838182 REVERSE LENGTH=297	297	297	2.33E-156	100.0	84.2	91.2
Rsa1.0_00016.1.g958.t1	gb|AFO66502.1| putative eukaryotic translation initiation factor SUI1 family protein [Brassica napus]	200	198	1.00E-105	99.0	97.0	98.0	putative eukaryotic translation initiation factor SUI1 family protein	gbpln	Brassica napus	AT5G11900.1 | Symbols: | Translation initiation factor SUI1 family protein | chr5:3834154-3836023 FORWARD LENGTH=198	200	198	1.00E-106	99.0	95.0	97.5
Rsa1.0_00016.1.g959.t1	gb|AFO66501.1| putative harpin-induced 1 [Brassica napus]	290	284	1.00E-121	97.9	89.0	92.4	putative harpin-induced 1	gbpln	Brassica napus	AT5G11890.1 | Symbols: | FUNCTIONS IN: molecular_function unknown; LOCATED IN: plasma membrane; EXPRESSED IN: 12 plant structures; EXPRESSED DURING: 6 growth stages; BEST Arabidopsis thaliana protein match is: Late embryogenesis abundant (LEA) hydroxyproline-rich glycoprotein family (TAIR:AT1G17620.1); Has 1807 Blast hits to 1807 proteins in 277 species: Archae - 0; Bacteria - 0; Metazoa - 736; Fungi - 347; Plants - 385; Viruses - 0; Other Eukaryotes - 339 (source: NCBI BLink). | chr5:3831770-3832633 FORWARD LENGTH=287	290	287	1.00E-111	99.0	79.7	87.6
Rsa1.0_00016.1.g960.t2	gb|AFO66500.1| putative cf-9 protein precursor [Brassica napus]	1114	919	0	82.5	46.7	49.4	putative cf-9 protein precursor	gbpln	Brassica napus	AT3G05650.1 | Symbols: AtRLP32, RLP32 | receptor like protein 32 | chr3:1645884-1648490 REVERSE LENGTH=868	1114	868	0	77.9	38.1	44.0
Rsa1.0_00016.1.g961.t1	gb|AFO66534.1| putative diaminopimelate decarboxylase [Brassica napus]	485	485	0	100.0	96.3	98.1	putative diaminopimelate decarboxylase	gbpln	Brassica napus	AT5G11880.1 | Symbols: | Pyridoxal-dependent decarboxylase family protein | chr5:3827806-3829942 REVERSE LENGTH=489	485	489	0	100.8	92.2	95.9
Rsa1.0_00016.1.g962.t2	gb|AFO66499.1| putative membrane protein [Brassica napus]	192	202	6.00E-88	105.2	85.4	89.1	putative membrane protein	gbpln	Brassica napus	AT5G11870.1 | Symbols: | Alkaline phytoceramidase (aPHC) | chr5:3825720-3827241 FORWARD LENGTH=262	192	262	6.00E-83	136.5	78.1	85.4

Rsa1.0_00016.1.g963.t2	gb|AFO66533.1| putative NLI interacting factor family protein [Brassica napus]	326	477	1.00E-144	146.3	85.0	89.3	putative NLI interacting factor family protein	gbpln	Brassica napus	AT5G11860.4 | Symbols: | SCP1-like small phosphatase 5 | chr5:3822024-3823395 REVERSE LENGTH=305	326	305	1.00E-144	93.6	83.4	88.3
Rsa1.0_00016.1.g964.t1	gb|AFO66525.1| putative Myo28B1 [Brassica napus]	305	303	1.00E-156	99.3	89.8	95.7	putative Myo28B1	gbpln	Brassica napus	AT5G11810.1 | Symbols: | unknown protein; Has 1807 Blast hits to 1807 proteins in 277 species: Archae - 0; Bacteria - 0; Metazoa - 736; Fungi - 347; Plants - 385; Viruses - 0; Other Eukaryotes - 339 (source: NCBI BLink). | chr5:3808739-3810440 FORWARD LENGTH=306	305	306	1.00E-144	100.3	82.6	91.5
Rsa1.0_00016.1.g965.t1	gb|AFO66532.1| putative potassium ion transmembrane transporter [Brassica napus]	840	580	0	69.0	66.1	66.3	putative potassium ion transmembrane transporter	gbpln	Brassica napus	AT5G11800.1 | Symbols: KEA6, ATKEA6 | K+ efflux antiporter 6 | chr5:3803635-3808069 REVERSE LENGTH=597	840	597	0	71.1	62.4	64.9
Rsa1.0_00016.1.g966.t1	gb|AFO66497.1| putative SNF2 domain-containing protein [Brassica napus]	523	490	0	93.7	79.5	83.6	putative SNF2 domain-containing protein	gbpln	Brassica napus	AT5G11780.1 | Symbols: | unknown protein; Has 37 Blast hits to 37 proteins in 12 species: Archae - 0; Bacteria - 0; Metazoa - 0; Fungi - 3; Plants - 34; Viruses - 0; Other Eukaryotes - 0 (source: NCBI BLink). | chr5:3793928-3795983 FORWARD LENGTH=504	523	504	0	96.4	67.9	77.4
Rsa1.0_00016.1.g967.t1	sp|P42027.1|NDUS7_BRAOL RecName: Full=NADH dehydrogenase [ubiquinone] iron-sulfur protein 7, mitochondrial; Flags: Precursor gi|562282|emb|CAA57725.1| PSST subunit of NADH: ubiquinone oxidoreductase [Brassica oleracea]	217	215	1.00E-121	99.1	97.7	98.6	RecName: Full=NADH dehydrogenase	gbpln	Brassica oleracea	AT5G11770.1 | Symbols: | NADH-ubiquinone oxidoreductase 20 kDa subunit, mitochondrial | chr5:3791148-3792929 REVERSE LENGTH=218	217	218	1.00E-112	100.5	94.9	96.8
Rsa1.0_00016.1.g968.t1	gb|AFO66495.1| hypothetical protein [Brassica napus]	64	59	2.00E-19	92.2	92.2	92.2	hypothetical protein	gbpln	Brassica napus	AT5G11740.1 | Symbols: AGP15, ATAGP15 | arabinogalactan protein 15 | chr5:3784318-3784503 FORWARD LENGTH=61	64	61	1.00E-10	95.3	79.7	85.9
Rsa1.0_00016.1.g969.t1	gb|AFO66494.1| hypothetical protein [Brassica napus]	451	386	0	85.6	80.7	84.0	hypothetical protein	gbpln	Brassica napus	AT5G11730.1 | Symbols: | Core-2/I-branching beta-1,6-N-acetylglucosaminyltransferase family protein | chr5:3780963-3782473 FORWARD LENGTH=386	451	386	0	85.6	76.1	81.8
Rsa1.0_00016.1.g970.t1	ref|NP_001154709.2| uncharacterized protein [Arabidopsis thaliana] gi|332004329|gb|AED91712.1| uncharacterized protein AT5G11700 [Arabidopsis thaliana]	1429	1476	0	103.3	93.0	95.9	uncharacterized protein	gbpln	Arabidopsis thaliana	AT5G11700.2 | Symbols: | BEST Arabidopsis thaliana protein match is: glycine-rich protein (TAIR:AT4G32920.3); Has 8203 Blast hits to 3102 proteins in 389 species: Archae - 3; Bacteria - 5624; Metazoa - 852; Fungi - 139; Plants - 704; Viruses - 77; Other Eukaryotes - 804 (source: NCBI BLink). | chr5:3762961-3771123 REVERSE LENGTH=1476	1429	1476	0	103.3	93.0	95.9
Rsa1.0_00016.1.g971.t1	gb|EOA19413.1| hypothetical protein CARUB_v10001131mg [Capsella rubella]	394	396	0	100.5	90.4	93.1	hypothetical protein CARUB_v10001131mg	gbpln	Capsella rubella	AT5G11650.1 | Symbols: | alpha/beta-Hydrolases superfamily protein | chr5:3745069-3746816 FORWARD LENGTH=390	394	390	0	99.0	88.6	92.1
Rsa1.0_00016.1.g972.t1	ref|NP_196724.3| uncharacterized protein [Arabidopsis thaliana] gi|7573377|emb|CAB87681.1| putative protein [Arabidopsis thaliana] gi|332004320|gb|AED91703.1| uncharacterized protein AT5G11630 [Arabidopsis thaliana]	93	93	7.00E-39	100.0	89.2	95.7	uncharacterized protein	gbpln	Arabidopsis thaliana	AT5G11630.1 | Symbols: | unknown protein; FUNCTIONS IN: molecular_function unknown; INVOLVED IN: biological_process unknown; LOCATED IN: chloroplast; EXPRESSED IN: 22 plant structures; EXPRESSED DURING: 13 growth stages; BEST Arabidopsis thaliana protein match is: unknown protein (TAIR:AT4G17310.1); Has 90 Blast hits to 90 proteins in 10 species: Archae - 0; Bacteria - 0; Metazoa - 0; Fungi - 0; Plants - 90; Viruses - 0; Other Eukaryotes - 0 (source: NCBI BLink). | chr5:3740545-3740909 FORWARD LENGTH=93	93	93	1.00E-41	100.0	89.2	95.7
Rsa1.0_00016.1.g973.t1	ref|XP_002873525.1| hypothetical protein ARALYDRAFT_350357 [Arabidopsis lyrata subsp. lyrata] gi|297319362|gb|EFH49784.1| hypothetical protein ARALYDRAFT_350357 [Arabidopsis lyrata subsp. lyrata]	277	272	1.00E-112	98.2	76.2	84.5	hypothetical protein ARALYDRAFT_350357	gbpln	Arabidopsis lyrata	AT5G11620.1 | Symbols: | SWIM zinc finger family protein / mitogen-activated protein kinase kinase kinase (MAPKKK)-related | chr5:3739076-3740127 REVERSE LENGTH=273	277	273	1.00E-112	98.6	75.5	84.1
Rsa1.0_00016.1.g974.t1	ref|XP_002873524.1| exostosin family protein [Arabidopsis lyrata subsp. lyrata] gi|297319361|gb|EFH49783.1| exostosin family protein [Arabidopsis lyrata subsp. lyrata]	523	546	0	104.4	82.0	90.8	exostosin family protein	gbpln	Arabidopsis lyrata	AT5G11610.1 | Symbols: | Exostosin family protein | chr5:3735569-3737952 REVERSE LENGTH=546	523	546	0	104.4	80.5	88.9
Rsa1.0_00016.1.g975.t1	#	#	#	#	#	#	#	-	----	----	#	#	#	#	#	#	#

Rsa1.0_00016.1.g976.t1	ref|XP_002873522.1| hypothetical protein ARALYDRAFT_487996 [Arabidopsis lyrata subsp. lyrata] gi|297319359|gb|EFH49781.1| hypothetical protein ARALYDRAFT_487996 [Arabidopsis lyrata subsp. lyrata]	234	236	5.00E-96	100.9	79.9	88.5	hypothetical protein ARALYDRAFT_487996	gbpln	Arabidopsis lyrata	AT5G11590.1 | Symbols: TINY2 | Integrase-type DNA-binding superfamily protein | chr5:3727789-3728499 REVERSE LENGTH=236	234	236	1.00E-94	100.9	79.5	88.5
Rsa1.0_00016.1.g977.t1	ref|NP_196717.3| PQQ_DH domain-containing protein [Arabidopsis thaliana] gi|332004312|gb|AED91695.1| PQQ_DH domain-containing protein [Arabidopsis thaliana]	1549	982	0	63.4	57.4	60.4	PQQ_DH domain-containing protein	gbpln	Arabidopsis thaliana	AT5G11560.1 | Symbols: | catalytics | chr5:3709734-3713994 REVERSE LENGTH=982	1549	982	0	63.4	57.4	60.4
Rsa1.0_00016.1.g978.t1	ref|XP_002873518.1| FAD-binding domain-containing protein [Arabidopsis lyrata subsp. lyrata] gi|297319355|gb|EFH49777.1| FAD-binding domain-containing protein [Arabidopsis lyrata subsp. lyrata]	541	584	0	107.9	84.7	91.9	FAD-binding domain-containing protein	gbpln	Arabidopsis lyrata	AT5G11540.1 | Symbols: | D-arabinono-1,4-lactone oxidase family protein | chr5:3703002-3704981 REVERSE LENGTH=585	541	585	0	108.1	83.5	91.9
Rsa1.0_00016.1.g979.t1	gb|EOA19844.1| hypothetical protein CARUB_v10000092mg [Capsella rubella]	1052	1094	2.00E-34	104.0	12.5	16.1	hypothetical protein CARUB_v10000092mg	gbpln	Capsella rubella	AT5G11530.1 | Symbols: EMF1 | embryonic flower 1 (EMF1) | chr5:3697140-3700930 FORWARD LENGTH=1096	1052	1096	3.00E-33	104.2	12.5	16.7
Rsa1.0_00016.1.g980.t1	ref|XP_002871478.1| hypothetical protein ARALYDRAFT_487987 [Arabidopsis lyrata subsp. lyrata] gi|297317315|gb|EFH47737.1| hypothetical protein ARALYDRAFT_487987 [Arabidopsis lyrata subsp. lyrata]	1127	960	0	85.2	63.9	71.1	hypothetical protein ARALYDRAFT_487987	gbpln	Arabidopsis lyrata	AT5G11510.1 | Symbols: MYB3R-4, AtMYB3R4 | myb domain protein 3r-4 | chr5:3680452-3684684 FORWARD LENGTH=961	1127	961	0	85.3	63.0	70.0
Rsa1.0_00016.1.g981.t1	gb|EOA21135.1| hypothetical protein CARUB_v10001479mg [Capsella rubella]	310	318	1.00E-148	102.6	90.3	94.5	hypothetical protein CARUB_v10001479mg	gbpln	Capsella rubella	AT5G11480.1 | Symbols: | P-loop containing nucleoside triphosphate hydrolases superfamily protein | chr5:3669350-3671471 REVERSE LENGTH=318	310	318	1.00E-144	102.6	87.1	92.3
Rsa1.0_00016.1.g982.t1	ref|XP_002873515.1| hypothetical protein ARALYDRAFT_487983 [Arabidopsis lyrata subsp. lyrata] gi|297319352|gb|EFH49774.1| hypothetical protein ARALYDRAFT_487983 [Arabidopsis lyrata subsp. lyrata]	655	695	0	106.1	63.2	75.0	hypothetical protein ARALYDRAFT_487983	gbpln	Arabidopsis lyrata	AT5G11470.1 | Symbols: | bromo-adjacent homology (BAH) domain-containing protein | chr5:3662757-3667041 REVERSE LENGTH=757	655	757	0	115.6	64.4	77.1
Rsa1.0_00016.1.g983.t1	#	#	#	#	#	#	#	-	----	----	#	#	#	#	#	#	#
Rsa1.0_00016.1.g984.t1	#	#	#	#	#	#	#	-	----	----	#	#	#	#	#	#	#
Rsa1.0_00016.1.g985.t1	gb|EOA21007.1| hypothetical protein CARUB_v10001342mg [Capsella rubella]	275	343	2.00E-82	124.7	65.1	77.8	hypothetical protein CARUB_v10001342mg	gbpln	Capsella rubella	AT5G11460.1 | Symbols: | Protein of unknown function (DUF581) | chr5:3657064-3658388 REVERSE LENGTH=344	275	344	2.00E-70	125.1	65.8	76.4
Rsa1.0_00016.1.g986.t2	ref|XP_002871475.1| hypothetical protein ARALYDRAFT_487981 [Arabidopsis lyrata subsp. lyrata] gi|297317312|gb|EFH47734.1| hypothetical protein ARALYDRAFT_487981 [Arabidopsis lyrata subsp. lyrata]	306	297	1.00E-122	97.1	78.8	84.3	hypothetical protein ARALYDRAFT_487981	gbpln	Arabidopsis lyrata	AT5G11450.1 | Symbols: | Mog1/PsbP/DUF1795-like photosystem II reaction center PsbP family protein | chr5:3654475-3656357 FORWARD LENGTH=297	306	297	1.00E-124	97.1	78.8	84.0
Rsa1.0_00016.1.g987.t1	ref|XP_002873513.1| CID5/IPD1 [Arabidopsis lyrata subsp. lyrata] gi|297319350|gb|EFH49772.1| CID5/IPD1 [Arabidopsis lyrata subsp. lyrata]	164	165	2.00E-53	100.6	73.2	82.3	CID5/IPD1	gbpln	Arabidopsis lyrata	AT5G11440.1 | Symbols: CID5, IPD1 | CTC-interacting domain 5 | chr5:3653060-3653607 REVERSE LENGTH=155	164	155	1.00E-54	94.5	72.6	81.7
Rsa1.0_00016.1.g988.t1	gb|EOA19945.1| hypothetical protein CARUB_v10000195mg [Capsella rubella]	901	869	0	96.4	74.6	82.6	hypothetical protein CARUB_v10000195mg	gbpln	Capsella rubella	AT5G11430.1 | Symbols: | SPOC domain / Transcription elongation factor S-II protein | chr5:3648469-3652256 FORWARD LENGTH=873	901	873	0	96.9	73.6	83.1
Rsa1.0_00016.1.g989.t1	ref|XP_002871473.1| hypothetical protein ARALYDRAFT_487977 [Arabidopsis lyrata subsp. lyrata] gi|297317310|gb|EFH47732.1| hypothetical protein ARALYDRAFT_487977 [Arabidopsis lyrata subsp. lyrata]	340	366	0	107.6	92.1	98.2	hypothetical protein ARALYDRAFT_487977	gbpln	Arabidopsis lyrata	AT5G11420.1 | Symbols: | Protein of unknown function, DUF642 | chr5:3644655-3646991 FORWARD LENGTH=366	340	366	0	107.6	91.5	97.9
Rsa1.0_00016.1.g990.t1	ref|NP_196702.1| protein kinase family protein [Arabidopsis thaliana] gi|8953403|emb|CAB96676.1| putative protein [Arabidopsis thaliana] gi|332004291|gb|AED91674.1| protein kinase family protein [Arabidopsis thaliana]	609	336	2.00E-81	55.2	28.4	35.5	protein kinase family protein	gbpln	Arabidopsis thaliana	AT5G11410.1 | Symbols: | Protein kinase superfamily protein | chr5:3638431-3639883 REVERSE LENGTH=336	609	336	6.00E-84	55.2	28.4	35.5
Rsa1.0_00016.1.g991.t1	gb|EOA20067.1| hypothetical protein CARUB_v10000340mg [Capsella rubella]	694	709	0	102.2	80.3	89.0	hypothetical protein CARUB_v10000340mg	gbpln	Capsella rubella	AT5G11390.1 | Symbols: WIT1 | WPP domain-interacting protein 1 | chr5:3633971-3636418 FORWARD LENGTH=703	694	703	0	101.3	80.0	88.6

Rsa1.0_00016.1.g992.t2	ref|NP_196699.1| 1-deoxy-D-xylulose 5-phosphate synthase 3 [Arabidopsis thaliana] gi|8953400|emb|CAB96673.1| 1-D-deoxyxylulose 5-phosphate synthase-like protein [Arabidopsis thaliana] gi|332004286|gb|AED91669.1| 1-deoxy-D-xylulose 5-phosphate synthase 3 [Arabidopsis thaliana]	1465	700	0	47.8	42.4	44.6	1-deoxy-D-xylulose 5-phosphate synthase 3	gbpln	Arabidopsis thaliana	AT5G11380.1 | Symbols: DXPS3 | 1-deoxy-D-xylulose 5-phosphate synthase 3 | chr5:3630172-3633250 FORWARD LENGTH=700	1465	700	0	47.8	42.4	44.6
Rsa1.0_00016.1.g993.t1	ref|XP_002871470.1| hypothetical protein ARALYDRAFT_909096 [Arabidopsis lyrata subsp. lyrata] gi|297317307|gb|EFH47729.1| hypothetical protein ARALYDRAFT_909096 [Arabidopsis lyrata subsp. lyrata]	164	164	6.00E-89	100.0	95.7	99.4	hypothetical protein ARALYDRAFT_909096	gbpln	Arabidopsis lyrata	AT5G11340.1 | Symbols: | Acyl-CoA N-acyltransferases (NAT) superfamily protein | chr5:3619226-3621068 FORWARD LENGTH=164	164	164	4.00E-88	100.0	92.1	97.6
Rsa1.0_00016.1.g994.t1	gb|EOA20763.1| hypothetical protein CARUB_v10001092mg [Capsella rubella]	406	408	0	100.5	84.7	91.4	hypothetical protein CARUB_v10001092mg	gbpln	Capsella rubella	AT5G11330.1 | Symbols: | FAD/NAD(P)-binding oxidoreductase family protein | chr5:3617342-3618861 REVERSE LENGTH=408	406	408	0	100.5	85.0	91.1
Rsa1.0_00016.1.g995.t1	gb|EOA19559.1| hypothetical protein CARUB_v10002580mg [Capsella rubella]	412	412	0	100.0	84.5	88.8	hypothetical protein CARUB_v10002580mg	gbpln	Capsella rubella	AT5G11320.1 | Symbols: YUC4 | Flavin-binding monooxygenase family protein | chr5:3611429-3613361 REVERSE LENGTH=411	412	411	0	99.8	83.3	88.6
Rsa1.0_00016.1.g996.t1	gb|EOA19373.1| hypothetical protein CARUB_v10000508mg [Capsella rubella]	593	605	0	102.0	80.9	88.7	hypothetical protein CARUB_v10000508mg	gbpln	Capsella rubella	AT5G11310.1 | Symbols: | Pentatricopeptide repeat (PPR) superfamily protein | chr5:3606490-3608409 FORWARD LENGTH=602	593	602	0	101.5	79.3	86.3
Rsa1.0_00016.1.g997.t1	ref|XP_002873505.1| hypothetical protein ARALYDRAFT_350326 [Arabidopsis lyrata subsp. lyrata] gi|297319342|gb|EFH49764.1| hypothetical protein ARALYDRAFT_350326 [Arabidopsis lyrata subsp. lyrata]	435	436	0	100.2	84.1	91.5	hypothetical protein ARALYDRAFT_350326	gbpln	Arabidopsis lyrata	AT5G11300.1 | Symbols: CYC3B, CYC2BAT, CYCA2;2 | mitotic-like cyclin 3B from Arabidopsis | chr5:3601811-3604466 REVERSE LENGTH=436	435	436	0	100.2	83.7	90.8
Rsa1.0_00016.1.g998.t1	gb|EOA21558.1| hypothetical protein CARUB_v10001967mg [Capsella rubella]	210	209	1.00E-102	99.5	87.6	95.2	hypothetical protein CARUB_v10001967mg	gbpln	Capsella rubella	AT5G11280.1 | Symbols: | unknown protein; BEST Arabidopsis thaliana protein match is: unknown protein (TAIR:AT1G80200.1); Has 30201 Blast hits to 17322 proteins in 780 species: Archae - 12; Bacteria - 1396; Metazoa - 17338; Fungi - 3422; Plants - 5037; Viruses - 0; Other Eukaryotes - 2996 (source: NCBI BLink). | chr5:3597321-3598347 FORWARD LENGTH=209	210	209	1.00E-103	99.5	86.7	94.3
Rsa1.0_00016.1.g999.t1	ref|XP_002873503.1| hypothetical protein ARALYDRAFT_487959 [Arabidopsis lyrata subsp. lyrata] gi|297319340|gb|EFH49762.1| hypothetical protein ARALYDRAFT_487959 [Arabidopsis lyrata subsp. lyrata]	349	352	1.00E-130	100.9	82.2	88.8	hypothetical protein ARALYDRAFT_487959	gbpln	Arabidopsis lyrata	AT5G11270.1 | Symbols: OCP3 | overexpressor of cationic peroxidase 3 | chr5:3595557-3597076 REVERSE LENGTH=354	349	354	1.00E-116	101.4	75.1	82.8
Rsa1.0_00016.1.g1000.t1	ref|XP_002873502.1| hypothetical protein ARALYDRAFT_909087 [Arabidopsis lyrata subsp. lyrata] gi|297319339|gb|EFH49761.1| hypothetical protein ARALYDRAFT_909087 [Arabidopsis lyrata subsp. lyrata]	169	167	1.00E-66	98.8	88.8	91.7	hypothetical protein ARALYDRAFT_909087	gbpln	Arabidopsis lyrata	AT5G11260.1 | Symbols: HY5, TED 5 | Basic-leucine zipper (bZIP) transcription factor family protein | chr5:3593583-3594800 REVERSE LENGTH=168	169	168	8.00E-66	99.4	89.9	92.3
Rsa1.0_00016.1.g1001.t2	gb|ACP30577.1| disease resistance protein [Brassica rapa subsp. pekinensis]	485	1235	8.00E-91	254.6	48.9	63.3	disease resistance protein	gbpln	Brassica rapa	AT5G11250.1 | Symbols: | Disease resistance protein (TIR-NBS-LRR class) | chr5:3587978-3591960 REVERSE LENGTH=1189	485	1189	5.00E-88	245.2	37.3	43.9
Rsa1.0_00016.1.g1002.t1	gb|EOA20202.1| hypothetical protein CARUB_v10000497mg [Capsella rubella]	606	612	0	101.0	86.3	92.4	hypothetical protein CARUB_v10000497mg	gbpln	Capsella rubella	AT5G11240.1 | Symbols: | transducin family protein / WD-40 repeat family protein | chr5:3582949-3586782 FORWARD LENGTH=615	606	615	0	101.5	86.0	91.4
Rsa1.0_00016.1.g1003.t1	#	#	#	#	#	#	#	-	----	----	#	#	#	#	#	#	#
Rsa1.0_00016.1.g1004.t1	gb|EOA22882.1| hypothetical protein CARUB_v10003614mg [Capsella rubella]	353	349	0	98.9	92.9	96.0	hypothetical protein CARUB_v10003614mg	gbpln	Capsella rubella	AT5G25400.1 | Symbols: | Nucleotide-sugar transporter family protein | chr5:8823283-8824332 FORWARD LENGTH=349	353	349	0	98.9	89.5	94.1
Rsa1.0_00016.1.g1005.t1	ref|XP_002886774.1| hypothetical protein ARALYDRAFT_893811 [Arabidopsis lyrata subsp. lyrata] gi|297332615|gb|EFH63033.1| hypothetical protein ARALYDRAFT_893811 [Arabidopsis lyrata subsp. lyrata]	310	303	1.00E-102	97.7	60.6	73.2	hypothetical protein ARALYDRAFT_893811	gbpln	Arabidopsis lyrata	AT1G64870.1 | Symbols: | unknown protein; BEST Arabidopsis thaliana protein match is: unknown protein (TAIR:AT3G45200.1); Has 99 Blast hits to 91 proteins in 10 species: Archae - 0; Bacteria - 0; Metazoa - 0; Fungi - 0; Plants - 99; Viruses - 0; Other Eukaryotes - 0 (source: NCBI BLink). | chr1:24102917-24103831 FORWARD LENGTH=304	310	304	1.00E-102	98.1	60.6	71.6

Rsa1.0_00016.1.g1006.t1	ref|NP_196670.1| serine-rich protein-like protein [Arabidopsis thaliana] gi|9795161|emb|CAC03457.1| putative protein [Arabidopsis thaliana] gi|21592828|gb|AAM64778.1| serine-rich protein [Arabidopsis thaliana] gi|28466931|gb|AAO44074.1| At5g11090 [Arabidopsis thaliana] gi|110743766|dbj|BAE99719.1| hypothetical protein [Arabidopsis thaliana] gi|332004251|gb|AED91634.1| serine-rich protein-like protein [Arabidopsis thaliana]	271	217	5.00E-76	80.1	62.0	66.4	serine-rich protein-like protein	gbpln	Arabidopsis thaliana	AT5G11090.1 | Symbols: | serine-rich protein-related | chr5:3524796-3525449 FORWARD LENGTH=217	271	217	1.00E-78	80.1	62.0	66.4
Rsa1.0_00016.1.g1007.t1	ref|NP_175758.2| peptidase-S24/S26 domain-containing protein [Arabidopsis thaliana] gi|44681348|gb|AAS47614.1| At1g53530 [Arabidopsis thaliana] gi|45773848|gb|AAS76728.1| At1g53530 [Arabidopsis thaliana] gi|110738199|dbj|BAF01030.1| hypothetical protein [Arabidopsis thaliana] gi|332194831|gb|AEE32952.1| peptidase-S24/S26 domain-containing protein [Arabidopsis thaliana]	182	168	6.00E-79	92.3	81.3	86.3	peptidase-S24/S26 domain-containing protein	gbpln	Arabidopsis thaliana	AT1G53530.1 | Symbols: | Peptidase S24/S26A/S26B/S26C family protein | chr1:19978249-19979778 FORWARD LENGTH=168	182	168	2.00E-81	92.3	81.3	86.3
Rsa1.0_00016.1.g1008.t1	ref|NP_196668.1| uncharacterized protein [Arabidopsis thaliana] gi|9795159|emb|CAC03455.1| putative protein [Arabidopsis thaliana] gi|21593765|gb|AAM65732.1| unknown [Arabidopsis thaliana] gi|110740693|dbj|BAE98448.1| hypothetical protein [Arabidopsis thaliana] gi|332004248|gb|AED91631.1| uncharacterized protein AT5G11070 [Arabidopsis thaliana]	132	152	8.00E-53	115.2	79.5	87.1	uncharacterized protein	gbpln	Arabidopsis thaliana	AT5G11070.1 | Symbols: | unknown protein; Has 1807 Blast hits to 1807 proteins in 277 species: Archae - 0; Bacteria - 0; Metazoa - 736; Fungi - 347; Plants - 385; Viruses - 0; Other Eukaryotes - 339 (source: NCBI BLink). | chr5:3516440-3516898 REVERSE LENGTH=152	132	152	2.00E-55	115.2	79.5	87.1
Rsa1.0_00016.1.g1009.t1	ref|XP_002871450.1| hypothetical protein ARALYDRAFT_487936 [Arabidopsis lyrata subsp. lyrata] gi|297317287|gb|EFH47709.1| hypothetical protein ARALYDRAFT_487936 [Arabidopsis lyrata subsp. lyrata]	1183	1186	0	100.3	95.7	97.4	hypothetical protein ARALYDRAFT_487936	gbpln	Arabidopsis lyrata	AT5G11040.1 | Symbols: TRS120, AtTRS120 | TRS120 | chr5:3495332-3500610 FORWARD LENGTH=1186	1183	1186	0	100.3	95.3	97.0
Rsa1.0_00016.1.g1010.t1	ref|XP_002873494.1| predicted protein [Arabidopsis lyrata subsp. lyrata] gi|297319331|gb|EFH49753.1| predicted protein [Arabidopsis lyrata subsp. lyrata]	588	592	0	100.7	69.0	78.6	predicted protein	gbpln	Arabidopsis lyrata	AT5G11030.2 | Symbols: ALF4 | aberrant lateral root formation 4 | chr5:3490993-3495078 REVERSE LENGTH=602	588	602	0	102.4	66.0	76.2
Rsa1.0_00016.1.g1011.t1	gb|EOA20670.1| hypothetical protein CARUB_v10000982mg [Capsella rubella]	438	438	0	100.0	83.3	89.0	hypothetical protein CARUB_v10000982mg	gbpln	Capsella rubella	AT5G11020.1 | Symbols: | Protein kinase superfamily protein | chr5:3486439-3488983 REVERSE LENGTH=433	438	433	0	98.9	81.1	88.6
Rsa1.0_00016.1.g1012.t1	ref|NP_850805.1| Pre-mRNA cleavage complex II protein family [Arabidopsis thaliana] gi|42573337|ref|NP_974765.1| Pre-mRNA cleavage complex II protein family [Arabidopsis thaliana] gi|75161516|sp|Q8VYP6.1|NOL9_ARATH RecName: Full=Polynucleotide 5'-hydroxyl-kinase NOL9; AltName: Full=Nucleolar protein 9 homolog gi|17979299|gb|AAL49875.1| unknown protein [Arabidopsis thaliana] gi|20465981|gb|AAM20212.1| unknown protein [Arabidopsis thaliana] gi|332004238|gb|AED91621.1| Pre-mRNA cleavage complex II protein family [Arabidopsis thaliana] gi|332004239|gb|AED91622.1| Pre-mRNA cleavage complex II protein family [Arabidopsis thaliana]	370	368	1.00E-170	99.5	80.5	87.8	Pre-mRNA cleavage complex II protein family	gbpln	Arabidopsis thaliana	AT5G11010.3 | Symbols: | Pre-mRNA cleavage complex II protein family | chr5:3484176-3486067 FORWARD LENGTH=368	370	368	1.00E-172	99.5	80.5	87.8
Rsa1.0_00016.1.g1013.t1	gb|EOA21927.1| hypothetical protein CARUB_v10002414mg [Capsella rubella]	222	390	1.00E-52	175.7	62.6	67.6	hypothetical protein CARUB_v10002414mg	gbpln	Capsella rubella	AT5G11000.1 | Symbols: | Plant protein of unknown function (DUF868) | chr5:3479166-3480335 REVERSE LENGTH=389	222	389	1.00E-46	175.2	63.1	69.4
Rsa1.0_00016.1.g1014.t1	gb|EOA19498.1| hypothetical protein CARUB_v10002201mg [Capsella rubella]	148	149	8.00E-71	100.7	89.2	92.6	hypothetical protein CARUB_v10002201mg	gbpln	Capsella rubella	AT5G10990.1 | Symbols: | SAUR-like auxin-responsive protein family | chr5:3476884-3477330 FORWARD LENGTH=148	148	148	2.00E-72	100.0	87.2	90.5
Rsa1.0_00016.1.g1015.t1	gb|EOA22648.1| hypothetical protein CARUB_v10003340mg [Capsella rubella]	251	272	1.00E-101	108.4	76.9	84.5	hypothetical protein CARUB_v10003340mg	gbpln	Capsella rubella	AT5G10970.1 | Symbols: | C2H2 and C2HC zinc fingers superfamily protein | chr5:3469268-3470086 FORWARD LENGTH=272	251	272	1.00E-100	108.4	72.9	82.9

Rsa1.0_00016.1.g1016.t1	ref|XP_002871445.1| hypothetical protein ARALYDRAFT_487926 [Arabidopsis lyrata subsp. lyrata] gi|297317282|gb|EFH47704.1| hypothetical protein ARALYDRAFT_487926 [Arabidopsis lyrata subsp. lyrata]	275	276	1.00E-142	100.4	88.4	96.0	hypothetical protein ARALYDRAFT_487926	gbpln	Arabidopsis lyrata	AT5G10960.1 | Symbols: | Polynucleotidyl transferase, ribonuclease H-like superfamily protein | chr5:3464581-3465414 FORWARD LENGTH=277	275	277	1.00E-143	100.7	88.0	95.3
Rsa1.0_00016.1.g1017.t1	emb|CAB39638.1| RNA-directed DNA polymerase-like protein [Arabidopsis thaliana] gi|7267666|emb|CAB78094.1| RNA-directed DNA polymerase-like protein [Arabidopsis thaliana]	1987	1274	0	64.1	30.5	41.9	RNA-directed DNA polymerase-like protein	gbpln	Arabidopsis thaliana	AT4G29090.1 | Symbols: | Ribonuclease H-like superfamily protein | chr4:14333528-14335255 FORWARD LENGTH=575	1987	575	1.00E-76	28.9	8.9	13.9
Rsa1.0_00016.1.g1018.t1	emb|CAN83015.1| hypothetical protein VITISV_041694 [Vitis vinifera]	243	1099	2.00E-88	452.3	63.4	76.1	hypothetical protein VITISV_041694	gbpln	Vitis vinifera	AT4G23160.1 | Symbols: CRK8 | cysteine-rich RLK (RECEPTOR-like protein kinase) 8 | chr4:12129485-12134086 FORWARD LENGTH=1262	243	1262	5.00E-43	519.3	33.3	52.3
Rsa1.0_00016.1.g1019.t1	ref|XP_002873486.1| hypothetical protein ARALYDRAFT_487925 [Arabidopsis lyrata subsp. lyrata] gi|297319323|gb|EFH49745.1| hypothetical protein ARALYDRAFT_487925 [Arabidopsis lyrata subsp. lyrata]	754	755	0	100.1	89.0	94.3	hypothetical protein ARALYDRAFT_487925	gbpln	Arabidopsis lyrata	AT5G10940.2 | Symbols: | transducin family protein / WD-40 repeat family protein | chr5:3448890-3454127 REVERSE LENGTH=754	754	754	0	100.0	88.9	94.0
Rsa1.0_00016.1.g1020.t1	ref|NP_568241.2| CBL-interacting serine/threonine-protein kinase 5 [Arabidopsis thaliana] gi|75334907|sp|Q9LEU7.1|CIPK5_ARATH RecName: Full=CBL-interacting serine/threonine-protein kinase 5; AltName: Full=SNF1-related kinase 3.24; AltName: Full=SOS2-like protein kinase PKS19 gi|19424694|gb|AAF86504.2|AF285105_1 CBL-interacting protein kinase 5 [Arabidopsis thaliana] gi|8979727|emb|CAB96848.1| serine/threonine protein kinase-like protein [Arabidopsis thaliana] gi|17065378|gb|AAL32843.1| serine/threonine protein kinase-like protein [Arabidopsis thaliana] gi|332004227|gb|AED91610.1| CBL-interacting serine/threonine-protein kinase 5 [Arabidopsis thaliana]	440	445	0	101.1	85.0	90.9	CBL-interacting serine/threonine-protein kinase 5	gbpln	Arabidopsis thaliana	AT5G10930.1 | Symbols: CIPK5, SnRK3.24 | CBL-interacting protein kinase 5 | chr5:3445569-3446906 REVERSE LENGTH=445	440	445	0	101.1	85.0	90.9
Rsa1.0_00016.1.g1021.t3	gb|EOA20415.1| hypothetical protein CARUB_v10000724mg [Capsella rubella]	519	518	0	99.8	91.9	95.2	hypothetical protein CARUB_v10000724mg	gbpln	Capsella rubella	AT5G10920.1 | Symbols: | L-Aspartase-like family protein | chr5:3441805-3443892 FORWARD LENGTH=517	519	517	0	99.6	92.5	96.0
Rsa1.0_00016.1.g1022.t1	ref|XP_002873483.1| predicted protein [Arabidopsis lyrata subsp. lyrata] gi|297319320|gb|EFH49742.1| predicted protein [Arabidopsis lyrata subsp. lyrata]	317	303	6.00E-89	95.6	60.6	73.8	predicted protein	gbpln	Arabidopsis lyrata	AT5G10890.1 | Symbols: | myosin heavy chain-related | chr5:3434030-3435067 REVERSE LENGTH=295	317	295	4.00E-88	93.1	58.4	71.6
Rsa1.0_00016.1.g1023.t1	gb|EOA21983.1| hypothetical protein CARUB_v10002490mg [Capsella rubella]	207	206	1.00E-108	99.5	93.7	96.1	hypothetical protein CARUB_v10002490mg	gbpln	Capsella rubella	AT5G10860.1 | Symbols: | Cystathionine beta-synthase (CBS) family protein | chr5:3429173-3430142 REVERSE LENGTH=206	207	206	1.00E-109	99.5	92.8	95.2
Rsa1.0_00016.1.g1024.t1	#	#	#	#	#	#	#	-	----	----	AT5G10790.1 | Symbols: UBP22 | ubiquitin-specific protease 22 | chr5:3410638-3412559 FORWARD LENGTH=557	134	557	3.00E-12	415.7	22.4	23.9
Rsa1.0_00016.1.g1025.t1	gb|AAF79677.1|AC022314_18 F9C16.26 [Arabidopsis thaliana]	566	1902	3.00E-47	336.0	17.8	25.1	F9C16.26	gbpln	Arabidopsis thaliana	AT5G32613.1 | Symbols: | Zinc knuckle (CCHC-type) family protein | chr5:12263255-12265041 FORWARD LENGTH=457	566	457	1.00E-15	80.7	8.0	11.8
Rsa1.0_00016.1.g1026.t1	gb|EOA20535.1| hypothetical protein CARUB_v10000848mg [Capsella rubella]	140	481	4.00E-17	343.6	42.1	53.6	hypothetical protein CARUB_v10000848mg	gbpln	Capsella rubella	AT5G10760.1 | Symbols: | Eukaryotic aspartyl protease family protein | chr5:3400671-3402165 REVERSE LENGTH=464	140	464	7.00E-18	331.4	44.3	62.9
Rsa1.0_00016.1.g1027.t1	ref|XP_002873476.1| hypothetical protein ARALYDRAFT_325615 [Arabidopsis lyrata subsp. lyrata] gi|297319313|gb|EFH49735.1| hypothetical protein ARALYDRAFT_325615 [Arabidopsis lyrata subsp. lyrata]	479	475	1.00E-178	99.2	70.1	81.6	hypothetical protein ARALYDRAFT_325615	gbpln	Arabidopsis lyrata	AT5G10770.1 | Symbols: | Eukaryotic aspartyl protease family protein | chr5:3403331-3405331 REVERSE LENGTH=474	479	474	1.00E-172	99.0	68.3	77.9
Rsa1.0_00016.1.g1028.t1	ref|XP_002873473.1| hypothetical protein ARALYDRAFT_487908 [Arabidopsis lyrata subsp. lyrata] gi|297319310|gb|EFH49732.1| hypothetical protein ARALYDRAFT_487908 [Arabidopsis lyrata subsp. lyrata]	328	373	1.00E-157	113.7	86.0	92.1	hypothetical protein ARALYDRAFT_487908	gbpln	Arabidopsis lyrata	AT5G10740.1 | Symbols: | Protein phosphatase 2C family protein | chr5:3393797-3395848 REVERSE LENGTH=354	328	354	1.00E-158	107.9	86.6	92.4

Rsa1.0_00016.1.g1029.t1	gb|EOA25676.1| hypothetical protein CARUB_v10019028mg [Capsella rubella]	479	476	0	99.4	76.2	87.5	hypothetical protein CARUB_v10019028mg	gbpln	Capsella rubella	AT3G28600.1 | Symbols: | P-loop containing nucleoside triphosphate hydrolases superfamily protein | chr3:10722437-10723870 FORWARD LENGTH=477	479	477	0	99.6	76.0	86.2
Rsa1.0_00016.1.g1030.t1	ref|XP_002873472.1| catalytic/ coenzyme binding protein [Arabidopsis lyrata subsp. lyrata] gi|297319309|gb|EFH49731.1| catalytic/ coenzyme binding protein [Arabidopsis lyrata subsp. lyrata]	289	287	1.00E-151	99.3	95.2	97.6	catalytic/ coenzyme binding protein	gbpln	Arabidopsis lyrata	AT5G10730.1 | Symbols: | NAD(P)-binding Rossmann-fold superfamily protein | chr5:3390822-3392947 REVERSE LENGTH=287	289	287	1.00E-150	99.3	95.2	97.6
Rsa1.0_00016.1.g1031.t1	ref|XP_002871436.1| hypothetical protein ARALYDRAFT_909030 [Arabidopsis lyrata subsp. lyrata] gi|297317273|gb|EFH47695.1| hypothetical protein ARALYDRAFT_909030 [Arabidopsis lyrata subsp. lyrata]	911	923	0	101.3	87.9	93.3	hypothetical protein ARALYDRAFT_909030	gbpln	Arabidopsis lyrata	AT5G10720.1 | Symbols: AHK5, CKI2, HK5 | histidine kinase 5 | chr5:3386835-3390541 FORWARD LENGTH=922	911	922	0	101.2	87.7	92.9
Rsa1.0_00016.1.g1032.t1	ref|NP_568234.1| uncharacterized protein [Arabidopsis thaliana] gi|19347741|gb|AAL86296.1| unknown protein [Arabidopsis thaliana] gi|24030282|gb|AAN41313.1| unknown protein [Arabidopsis thaliana] gi|332004199|gb|AED91582.1| uncharacterized protein AT5G10695 [Arabidopsis thaliana]	67	67	4.00E-27	100.0	86.6	89.6	uncharacterized protein	gbpln	Arabidopsis thaliana	AT5G10695.1 | Symbols: | unknown protein; BEST Arabidopsis thaliana protein match is: unknown protein (TAIR:AT5G57123.1); Has 30201 Blast hits to 17322 proteins in 780 species: Archae - 12; Bacteria - 1396; Metazoa - 17338; Fungi - 3422; Plants - 5037; Viruses - 0; Other Eukaryotes - 2996 (source: NCBI BLink). | chr5:3377817-3378020 FORWARD LENGTH=67	67	67	7.00E-30	100.0	86.6	89.6
Rsa1.0_00016.1.g1033.t1	ref|NP_196627.1| calmodulin-binding protein-like protein [Arabidopsis thaliana] gi|10129644|emb|CAC08240.1| vacuolar calcium binding protein-like [Arabidopsis thaliana] gi|53828561|gb|AAU94390.1| At5g10660 [Arabidopsis thaliana] gi|55733763|gb|AAV59278.1| At5g10660 [Arabidopsis thaliana] gi|332004195|gb|AED91578.1| calmodulin-binding protein-like protein [Arabidopsis thaliana]	355	407	3.00E-94	114.6	69.0	79.4	calmodulin-binding protein-like protein	gbpln	Arabidopsis thaliana	AT5G10660.1 | Symbols: | calmodulin-binding protein-related | chr5:3370553-3371776 FORWARD LENGTH=407	355	407	7.00E-97	114.6	69.0	79.4
Rsa1.0_00016.1.g1034.t1	ref|NP_196626.2| RING/U-box domain-containing protein [Arabidopsis thaliana] gi|79327651|ref|NP_001031869.1| RING/U-box domain-containing protein [Arabidopsis thaliana] gi|53850555|gb|AAU95454.1| At5g10650 [Arabidopsis thaliana] gi|58652060|gb|AAW80855.1| At5g10650 [Arabidopsis thaliana] gi|332004193|gb|AED91576.1| RING/U-box domain-containing protein [Arabidopsis thaliana] gi|332004194|gb|AED91577.1| RING/U-box domain-containing protein [Arabidopsis thaliana]	521	525	0	100.8	74.7	83.7	RING/U-box domain-containing protein	gbpln	Arabidopsis thaliana	AT5G10650.2 | Symbols: | RING/U-box superfamily protein | chr5:3365237-3367263 REVERSE LENGTH=525	521	525	0	100.8	74.7	83.7
Rsa1.0_00016.1.g1035.t1	#	#	#	#	#	#	#	–	----	----	AT5G10630.2 | Symbols: | Translation elongation factor EF1A/initiation factor IF2gamma family protein | chr5:3360561-3364414 FORWARD LENGTH=668	97	668	2.00E-11	688.7	32.0	35.1
Rsa1.0_00016.1.g1036.t1	#	#	#	#	#	#	#	–	----	----	#	#	#	#	#	#	#
Rsa1.0_00016.1.g1037.t1	emb|CAB89379.1| putative protein [Arabidopsis thaliana]	109	804	5.00E-52	737.6	88.1	91.7	putative protein	gbpln	Arabidopsis thaliana	AT5G10625.1 | Symbols: | BEST Arabidopsis thaliana protein match is: flowering promoting factor 1 (TAIR:AT5G24860.1); Has 35333 Blast hits to 34131 proteins in 2444 species: Archae - 798; Bacteria - 22429; Metazoa - 974; Fungi - 991; Plants - 531; Viruses - 0; Other Eukaryotes - 9610 (source: NCBI BLink). | chr5:3358934-3359272 FORWARD LENGTH=112	109	112	2.00E-53	102.8	91.7	96.3
Rsa1.0_00016.1.g1038.t1	ref|NP_196624.1| putative RNA methyltransferase [Arabidopsis thaliana] gi|32171918|sp|Q9LXB4.1|Y5620_ARATH RecName: Full=Putative RNA methyltransferase At5g10620 gi|7671441|emb|CAB89381.1| putative protein [Arabidopsis thaliana] gi|332004189|gb|AED91572.1| putative RNA methyltransferase [Arabidopsis thaliana]	195	193	6.00E-99	99.0	87.7	91.3	putative RNA methyltransferase	gbpln	Arabidopsis thaliana	AT5G10620.1 | Symbols: | methyltransferases | chr5:3355466-3356615 REVERSE LENGTH=193	195	193	1.00E-101	99.0	87.7	91.3

Rsa1.0_00016.1.g1039.t1	ref|XP_002871428.1| CYP81K1 [Arabidopsis lyrata subsp. lyrata] gi|297317265|gb|EFH47687.1| CYP81K1 [Arabidopsis lyrata subsp. lyrata]	498	500	0	100.4	86.1	92.4	CYP81K1	gbpln	Arabidopsis lyrata	AT5G10610.1 | Symbols: CYP81K1 | cytochrome P450, family 81, subfamily K, polypeptide 1 | chr5:3353518-3355020 FORWARD LENGTH=500	498	500	0	100.4	85.5	92.0
Rsa1.0_00016.1.g1040.t1	ref|XP_002871428.1| CYP81K1 [Arabidopsis lyrata subsp. lyrata] gi|297317265|gb|EFH47687.1| CYP81K1 [Arabidopsis lyrata subsp. lyrata]	499	500	0	100.2	80.6	89.2	CYP81K1	gbpln	Arabidopsis lyrata	AT5G10610.1 | Symbols: CYP81K1 | cytochrome P450, family 81, subfamily K, polypeptide 1 | chr5:3353518-3355020 FORWARD LENGTH=500	499	500	0	100.2	80.0	88.2
Rsa1.0_00016.1.g1041.t1	gb|EOA21978.1| hypothetical protein CARUB_v10002483mg [Capsella rubella]	169	247	3.00E-79	146.2	86.4	93.5	hypothetical protein CARUB_v10002483mg	gbpln	Capsella rubella	AT5G10580.1 | Symbols: | Protein of unknown function, DUF599 | chr5:3347511-3348657 FORWARD LENGTH=246	169	246	2.00E-81	145.6	86.4	92.9
Rsa1.0_00016.1.g1042.t1	gb|EOA21164.1| hypothetical protein CARUB_v10001512mg [Capsella rubella]	302	313	1.00E-110	103.6	68.5	77.2	hypothetical protein CARUB_v10001512mg	gbpln	Capsella rubella	AT5G10440.1 | Symbols: CYCD4;2 | cyclin d4;2 | chr5:3280611-3282342 REVERSE LENGTH=298	302	298	1.00E-109	98.7	69.5	77.2
Rsa1.0_00016.1.g1043.t1	#	#	#	#	#	#	#	-	----	----	#	#	#	#	#	#	#
Rsa1.0_00016.1.g1044.t1	gb|AAZ66942.1| 117M18_23 [Brassica rapa]	483	518	0	107.2	78.3	89.0	117M18_23	gbpln	Brassica rapa	AT5G44050.1 | Symbols: | MATE efflux family protein | chr5:17722484-17726209 FORWARD LENGTH=491	483	491	0	101.7	76.6	86.3
Rsa1.0_00016.1.g1045.t3	gb|AAZ66942.1| 117M18_23 [Brassica rapa]	469	518	0	110.4	86.4	94.2	117M18_23	gbpln	Brassica rapa	AT5G44050.1 | Symbols: | MATE efflux family protein | chr5:17722484-17726209 FORWARD LENGTH=491	469	491	0	104.7	80.4	91.0
Rsa1.0_00016.1.g1046.t1	ref|NP_199218.1| mate efflux domain-containing protein [Arabidopsis thaliana] gi|9758564|dbj|BAB09065.1| unnamed protein product [Arabidopsis thaliana] gi|332007669|gb|AED95052.1| mate efflux domain-containing protein [Arabidopsis thaliana]	453	491	0	108.4	76.4	86.8	mate efflux domain-containing protein	gbpln	Arabidopsis thaliana	AT5G44050.1 | Symbols: | MATE efflux family protein | chr5:17722484-17726209 FORWARD LENGTH=491	453	491	0	108.4	76.4	86.8
Rsa1.0_00016.1.g1047.t1	#	#	#	#	#	#	#	-	----	----	#	#	#	#	#	#	#
Rsa1.0_00016.1.g1048.t1	ref|NP_199218.1| mate efflux domain-containing protein [Arabidopsis thaliana] gi|9758564|dbj|BAB09065.1| unnamed protein product [Arabidopsis thaliana] gi|332007669|gb|AED95052.1| mate efflux domain-containing protein [Arabidopsis thaliana]	506	491	0	97.0	80.0	87.5	mate efflux domain-containing protein	gbpln	Arabidopsis thaliana	AT5G44050.1 | Symbols: | MATE efflux family protein | chr5:17722484-17726209 FORWARD LENGTH=491	506	491	0	97.0	80.0	87.5
Rsa1.0_00016.1.g1049.t1	gb|EOA20878.1| hypothetical protein CARUB_v10001215mg [Capsella rubella]	352	369	1.00E-143	104.8	74.1	84.7	hypothetical protein CARUB_v10001215mg	gbpln	Capsella rubella	AT5G10410.1 | Symbols: | ENTH/ANTH/VHS superfamily protein | chr5:3271952-3273082 FORWARD LENGTH=338	352	338	1.00E-139	96.0	69.0	79.8
Rsa1.0_00016.1.g1050.t1	tpg|DAA55311.1| TPA: histone H3.2 [Zea mays]	136	245	1.00E-72	180.1	100.0	100.0	TPA: histone H3.2	gbenv/gbpln	Zea mays	AT5G65360.1 | Symbols: | Histone superfamily protein | chr5:26120099-26120509 REVERSE LENGTH=136	136	136	5.00E-74	100.0	100.0	100.0
Rsa1.0_00016.1.g1051.t1	gb|AAG51247.1|AC055769_6 copia-type polyprotein, putative; 28768-32772 [Arabidopsis thaliana]	1341	1334	0	99.5	67.3	80.2	copia-type polyprotein, putative; 28768-32772	gbpln	Arabidopsis thaliana	AT4G23160.1 | Symbols: CRK8 | cysteine-rich RLK (RECEPTOR-like protein kinase) 8 | chr4:12129485-12134086 FORWARD LENGTH=1262	1341	1262	4.00E-97	94.1	13.3	21.4
Rsa1.0_00016.1.g1052.t1	ref|XP_002871419.1| hypothetical protein ARALYDRAFT_350245 [Arabidopsis lyrata subsp. lyrata] gi|297317256|gb|EFH47678.1| hypothetical protein ARALYDRAFT_350245 [Arabidopsis lyrata subsp. lyrata]	386	306	3.00E-67	79.3	46.4	54.9	hypothetical protein ARALYDRAFT_350245	gbpln	Arabidopsis lyrata	AT5G10380.1 | Symbols: RING1, ATRING1 | RING/U-box superfamily protein | chr5:3267819-3268724 FORWARD LENGTH=301	386	301	7.00E-65	78.0	45.6	55.2
Rsa1.0_00016.1.g1053.t1	gb|AAZ66938.1| 117M18_19 [Brassica rapa]	1775	1755	0	98.9	82.4	89.7	117M18_19	gbpln	Brassica rapa	AT5G10370.1 | Symbols: | helicase domain-containing protein / IBR domain-containing protein / zinc finger protein-related | chr5:3261245-3267188 FORWARD LENGTH=1775	1775	1775	0	100.0	77.4	87.2
Rsa1.0_00016.1.g1054.t2	gb|AAZ67614.1| 80A08_29 [Brassica rapa subsp. pekinensis]	191	402	3.00E-32	210.5	44.5	51.3	80A08_29	gbpln	Brassica rapa	AT5G10320.1 | Symbols: | unknown protein; Has 54 Blast hits to 53 proteins in 19 species: Archae - 0; Bacteria - 4; Metazoa - 0; Fungi - 0; Plants - 50; Viruses - 0; Other Eukaryotes - 0 (source: NCBI BLink). | chr5:3244180-3246603 REVERSE LENGTH=403	191	403	2.00E-25	211.0	37.2	47.6
Rsa1.0_00016.1.g1055.t1	gb|AAZ66937.1| 117M18_18 [Brassica rapa]	249	249	1.00E-138	100.0	98.4	99.6	117M18_18	gbpln	Brassica rapa	AT5G10360.1 | Symbols: EMB3010, RPS6B | Ribosomal protein S6e | chr5:3258734-3260142 REVERSE LENGTH=249	249	249	1.00E-136	100.0	95.6	97.6
Rsa1.0_00016.1.g1056.t1	gb|AAZ67615.1| 80A08_30 [Brassica rapa subsp. pekinensis]	225	223	1.00E-123	99.1	97.3	97.8	80A08_30	gbpln	Brassica rapa	AT5G65270.1 | Symbols: AtRABA4a, RABA4a | RAB GTPase homolog A4A | chr5:26083437-26084550 FORWARD LENGTH=226	225	226	1.00E-114	100.4	86.7	91.1

Rsa1.0_00016.1.g1057.t1	gb|AAZ67614.1| 80A08_29 [Brassica rapa subsp. pekinensis]	397	402	0	101.3	90.9	94.2	80A08_29	gbpln	Brassica rapa	AT5G10320.1 | Symbols: | unknown protein; Has 54 Blast hits to 53 proteins in 19 species: Archae - 0; Bacteria - 4; Metazoa - 0; Fungi - 0; Plants - 50; Viruses - 0; Other Eukaryotes - 0 (source: NCBI BLink). | chr5:3244180-3246603 REVERSE LENGTH=403	397	403	1.00E-180	101.5	80.4	90.2
Rsa1.0_00016.1.g1058.t1	gb|AAZ67613.1| 80A08_28 [Brassica rapa subsp. pekinensis]	121	120	9.00E-58	99.2	92.6	95.0	80A08_28	gbpln	Brassica rapa	AT5G10310.1 | Symbols: | unknown protein; FUNCTIONS IN: molecular_function unknown; INVOLVED IN: biological_process unknown; LOCATED IN: endomembrane system; EXPRESSED IN: 17 plant structures; EXPRESSED DURING: 10 growth stages; BEST Arabidopsis thaliana protein match is: unknown protein (TAIR:AT3G13898.1); Has 1807 Blast hits to 1807 proteins in 277 species: Archae - 0; Bacteria - 0; Metazoa - 736; Fungi - 347; Plants - 385; Viruses - 0; Other Eukaryotes - 339 (source: NCBI BLink). | chr5:3241666-3242127 REVERSE LENGTH=122	121	122	2.00E-50	100.8	74.4	78.5
Rsa1.0_00016.1.g1059.t1	ref|NP_196592.1| methyl esterase 5 [Arabidopsis thaliana] gi|75334959|sp|Q9LFT6.1|HNL_ARATH RecName: Full=Alpha-hydroxynitrile lyase; Short=AtHNL; AltName: Full=(R)-hydroxynitrile lyase; AltName: Full=(R)-oxynitrilase; AltName: Full=Methylesterase 5; Short=AtMES5 gi|254220946|pdb|3DQZ|A Chain A, Structure Of The Hydroxynitrile Lyase From Arabidopsis Thaliana gi|254220947|pdb|3DQZ|B Chain B, Structure Of The Hydroxynitrile Lyase From Arabidopsis Thaliana gi|254220948|pdb|3DQZ|C Chain C, Structure Of The Hydroxynitrile Lyase From Arabidopsis Thaliana gi|254220949|pdb|3DQZ|D Chain D, Structure Of The Hydroxynitrile Lyase From Arabidopsis Thaliana gi|8953411|emb|CAB96686.1| alpha-hydroxynitrile lyase-like protein [Arabidopsis thaliana] gi|20147249|gb|AAM10338.1| AT5g10300/F18D22_70 [Arabidopsis thaliana] gi|23296322|gb|AAN13041.1| putative alpha-hydroxynitrile lyase [Arabidopsis thaliana] gi|110740625|dbj|BAE98416.1| alpha-hydroxynitrile lyase-like protein [Arabidopsis thaliana] gi|332004135|gb|AED91518.1| methyl esterase 5 [Arabidopsis thaliana]	258	258	1.00E-133	100.0	87.2	93.0	methyl esterase 5	gbpln	Arabidopsis thaliana	AT5G10300.1 | Symbols: ATMES5, MES5, HNL | methyl esterase 5 | chr5:3239684-3240724 FORWARD LENGTH=258	258	258	1.00E-135	100.0	87.2	93.0
Rsa1.0_00016.1.g1060.t1	ref|NP_196590.1| myb domain protein 92 [Arabidopsis thaliana] gi|3941524|gb|AAC83638.1| putative transcription factor [Arabidopsis thaliana] gi|8953409|emb|CAB96684.1| putative transcription factor MYB92 [Arabidopsis thaliana] gi|27754493|gb|AAO22694.1| putative myb family transcription factor [Arabidopsis thaliana] gi|28393977|gb|AAO42396.1| putative myb family transcription factor [Arabidopsis thaliana] gi|41619378|gb|AAS10089.1| MYB transcription factor [Arabidopsis thaliana] gi|332004133|gb|AED91516.1| myb domain protein 92 [Arabidopsis thaliana]	337	334	1.00E-151	99.1	80.4	87.8	myb domain protein 92	gbpln	Arabidopsis thaliana	AT5G10280.1 | Symbols: MYB92, ATMYB92, ATMYB64 | myb domain protein 92 | chr5:3232776-3233956 FORWARD LENGTH=334	337	334	1.00E-154	99.1	80.4	87.8
Rsa1.0_00016.1.g1061.t1	gb|AAZ67608.1| 80A08_23 [Brassica rapa subsp. pekinensis]	510	543	0	106.5	95.5	96.7	80A08_23	gbpln	Brassica rapa	AT5G10270.1 | Symbols: CDKC;1 | cyclin-dependent kinase C;1 | chr5:3221715-3224674 REVERSE LENGTH=505	510	505	0	99.0	86.9	91.6
Rsa1.0_00016.1.g1062.t1	gb|AAZ67607.1| 80A08_22 [Brassica rapa subsp. pekinensis]	205	206	1.00E-110	100.5	97.1	97.6	80A08_22	gbpln	Brassica rapa	AT5G10260.1 | Symbols: AtRABH1e, RABH1e | RAB GTPase homolog H1E | chr5:3219991-3221301 FORWARD LENGTH=207	205	207	1.00E-107	101.0	91.7	97.6
Rsa1.0_00016.1.g1063.t1	gb|AAZ67606.1| 80A08_21 [Brassica rapa subsp. pekinensis]	656	630	0	96.0	88.1	91.9	80A08_21	gbpln	Brassica rapa	AT5G10250.1 | Symbols: DOT3 | Phototropic-responsive NPH3 family protein | chr5:3217028-3219368 REVERSE LENGTH=607	656	607	0	92.5	73.6	81.7

Rsa1.0_00016.1.g1064.t1	gb|AAZ67605.1| 80A08_20 [Brassica rapa subsp. pekinensis]	316	316	0	100.0	97.5	98.7	80A08_20	gbpln	Brassica rapa	AT5G10230.1 | Symbols: ANNAT7, ANN7 | annexin 7 | chr5:3209738-3211396 REVERSE LENGTH=316	316	316	1.00E-170	100.0	89.2	94.6
Rsa1.0_00016.1.g1065.t1	emb|CAB39638.1| RNA-directed DNA polymerase-like protein [Arabidopsis thaliana] gi|7267666|emb|CAB78094.1| RNA-directed DNA polymerase-like protein [Arabidopsis thaliana]	2157	1274	0	59.1	28.2	38.2	RNA-directed DNA polymerase-like protein	gbpln	Arabidopsis thaliana	AT5G10220.1 | Symbols: ANN6, ANNAT6 | annexin 6 | chr5:3206980-3208784 REVERSE LENGTH=318	2157	318	1.00E-151	14.7	11.9	12.9
Rsa1.0_00016.1.g1066.t1	ref|NP_196583.1| uncharacterized protein [Arabidopsis thaliana] gi|7960740|emb|CAB92062.1| putative protein [Arabidopsis thaliana] gi|38564316|gb|AAR23737.1| At5g10210 [Arabidopsis thaliana] gi|38604056|gb|AAR24771.1| At5g10210 [Arabidopsis thaliana] gi|332004125|gb|AED91508.1| uncharacterized protein AT5G10210 [Arabidopsis thaliana]	190	197	8.00E-82	103.7	80.5	91.1	uncharacterized protein	gbpln	Arabidopsis thaliana	AT5G10210.1 | Symbols: | CONTAINS InterPro DOMAIN/s: C2 calcium-dependent membrane targeting (InterPro:IPR000008); BEST Arabidopsis thaliana protein match is: unknown protein (TAIR:AT5G65030.1); Has 1807 Blast hits to 1807 proteins in 277 species: Archae - 0; Bacteria - 0; Metazoa - 736; Fungi - 347; Plants - 385; Viruses - 0; Other Eukaryotes - 339 (source: NCBI BLink). | chr5:3206236-3206829 FORWARD LENGTH=197	190	197	3.00E-84	103.7	80.5	91.1
Rsa1.0_00016.1.g1067.t1	gb|AAZ67602.1| 80A08_17 [Brassica rapa subsp. pekinensis]	633	631	0	99.7	95.6	98.1	80A08_17	gbpln	Brassica rapa	AT5G10200.1 | Symbols: | ARM-repeat/Tetratricopeptide repeat (TPR)-like protein | chr5:3202378-3204367 FORWARD LENGTH=631	633	631	0	99.7	94.6	97.9
Rsa1.0_00017.1.g1068.t1	#	#	#	#	#	#	#	-	----	----	#	#	#	#	#	#	#
Rsa1.0_00017.1.g1069.t1	ref|XP_002869338.1| hypothetical protein ARALYDRAFT_491614 [Arabidopsis lyrata subsp. lyrata] gi|297315174|gb|EFH45597.1| hypothetical protein ARALYDRAFT_491614 [Arabidopsis lyrata subsp. lyrata]	261	227	1.00E-122	87.0	82.8	85.1	hypothetical protein ARALYDRAFT_491614	gbpln	Arabidopsis lyrata	AT4G31290.1 | Symbols: | ChaC-like family protein | chr4:15186851-15188278 FORWARD LENGTH=227	261	227	1.00E-124	87.0	82.8	84.7
Rsa1.0_00017.1.g1070.t1	ref|XP_002869337.1| hypothetical protein ARALYDRAFT_328593 [Arabidopsis lyrata subsp. lyrata] gi|297315173|gb|EFH45596.1| hypothetical protein ARALYDRAFT_328593 [Arabidopsis lyrata subsp. lyrata]	247	234	1.00E-131	94.7	93.9	94.3	hypothetical protein ARALYDRAFT_328593	gbpln	Arabidopsis lyrata	AT4G31300.2 | Symbols: PBA1 | N-terminal nucleophile aminohydrolases (Ntn hydrolases) superfamily protein | chr4:15188927-15190935 FORWARD LENGTH=234	247	234	1.00E-132	94.7	93.1	93.9
Rsa1.0_00017.1.g1071.t1	gb|EOA18510.1| hypothetical protein CARUB_v10007062mg [Capsella rubella]	188	191	7.00E-84	101.6	81.9	87.2	hypothetical protein CARUB_v10007062mg	gbpln	Capsella rubella	AT4G31320.1 | Symbols: | SAUR-like auxin-responsive protein family | chr4:15193993-15194562 REVERSE LENGTH=189	188	189	2.00E-83	100.5	79.8	86.2
Rsa1.0_00017.1.g1072.t2	ref|NP_567872.1| uncharacterized protein [Arabidopsis thaliana] gi|13507569|gb|AAK28647.1|AF360350_1 unknown protein [Arabidopsis thaliana] gi|15293233|gb|AAK93727.1| unknown protein [Arabidopsis thaliana] gi|332660492|gb|AEE85892.1| uncharacterized protein AT4G31330 [Arabidopsis thaliana]	382	239	1.00E-125	62.6	56.5	61.0	uncharacterized protein	gbpln	Arabidopsis thaliana	AT4G31330.1 | Symbols: | Protein of unknown function, DUF599 | chr4:15202354-15203151 FORWARD LENGTH=239	382	239	1.00E-127	62.6	56.5	61.0
Rsa1.0_00017.1.g1073.t1	#	#	#	#	#	#	#	-	----	----	#	#	#	#	#	#	#
Rsa1.0_00017.1.g1074.t2	ref|NP_567873.1| myosin heavy chain-related protein [Arabidopsis thaliana] gi|26983878|gb|AAN86191.1| unknown protein [Arabidopsis thaliana] gi|222423518|dbj|BAH19729.1| AT4G31340 [Arabidopsis thaliana] gi|332660493|gb|AEE85893.1| myosin heavy chain-related protein [Arabidopsis thaliana]	412	437	0	106.1	80.6	88.1	myosin heavy chain-related protein	gbpln	Arabidopsis thaliana	AT4G31340.1 | Symbols: | myosin heavy chain-related | chr4:15205662-15208950 FORWARD LENGTH=437	412	437	0	106.1	80.6	88.1
Rsa1.0_00017.1.g1075.t1	emb|CAA16538.1| predicted protein [Arabidopsis thaliana] gi|7270037|emb|CAB79853.1| predicted protein [Arabidopsis thaliana]	344	711	2.00E-20	206.7	20.3	29.1	predicted protein	gbpln	Arabidopsis thaliana	#	#	#	#	#	#	#
Rsa1.0_00017.1.g1076.t1	#	#	#	#	#	#	#	-	----	----	#	#	#	#	#	#	#

Rsa1.0_00017.1.g1077.t1	ref|NP_001154280.1| Core-2/I-branching beta-1,6-N-acetylglucosaminyltransferase family protein [Arabidopsis thaliana] gi|240256111|ref|NP_194863.4| Core-2/I-branching beta-1,6-N-acetylglucosaminyltransferase family protein [Arabidopsis thaliana] gi|26452247|dbj|BAC43211.1| unknown protein [Arabidopsis thaliana] gi|27311797|gb|AAO00864.1| Unknown protein [Arabidopsis thaliana] gi|30725618|gb|AAP37831.1| At4g31350 [Arabidopsis thaliana] gi|332660495|gb|AEE85895.1| Core-2/I-branching beta-1,6-N-acetylglucosaminyltransferase family protein [Arabidopsis thaliana] gi|332660496|gb|AEE85896.1| Core-2/I-branching beta-1,6-N-acetylglucosaminyltransferase family protein [Arabidopsis thaliana]	376	376	0	100.0	93.1	97.3	Core-2/I-branching beta-1,6-N-acetylglucosaminyltransferase family protein	gbpln	Arabidopsis thaliana	AT4G31350.2 | Symbols: | Core-2/I-branching beta-1,6-N-acetylglucosaminyltransferase family protein | chr4:15213133-15215651 FORWARD LENGTH=376	376	376	0	100.0	93.1	97.3
Rsa1.0_00017.1.g1078.t1	ref|XP_002869331.1| hypothetical protein ARALYDRAFT_913334 [Arabidopsis lyrata subsp. lyrata] gi|297315167|gb|EFH45590.1| hypothetical protein ARALYDRAFT_913334 [Arabidopsis lyrata subsp. lyrata]	187	179	2.00E-58	95.7	73.8	77.5	hypothetical protein ARALYDRAFT_913334	gbpln	Arabidopsis lyrata	AT4G31360.1 | Symbols: | selenium binding | chr4:15221945-15223310 FORWARD LENGTH=186	187	186	1.00E-60	99.5	73.3	77.5
Rsa1.0_00017.1.g1079.t1	ref|NP_194866.2| protein kinase family protein [Arabidopsis thaliana] gi|55978825|gb|AAV68874.1| hypothetical protein AT4G31380 [Arabidopsis thaliana] gi|60547875|gb|AAX23901.1| hypothetical protein At4g31380 [Arabidopsis thaliana] gi|332660502|gb|AEE85902.1| protein kinase family protein [Arabidopsis thaliana]	124	181	3.00E-55	146.0	94.4	97.6	protein kinase family protein	gbpln	Arabidopsis thaliana	AT4G31380.1 | Symbols: FLP1 | FPF1-like protein 1 | chr4:15229837-15230382 FORWARD LENGTH=181	124	181	5.00E-58	146.0	94.4	97.6
Rsa1.0_00017.1.g1080.t1	ref|XP_002869329.1| ABC1 family protein [Arabidopsis lyrata subsp. lyrata] gi|297315165|gb|EFH45588.1| ABC1 family protein [Arabidopsis lyrata subsp. lyrata]	674	682	0	101.2	92.1	95.3	ABC1 family protein	gbpln	Arabidopsis lyrata	AT4G31390.1 | Symbols: | Protein kinase superfamily protein | chr4:15233126-15236764 FORWARD LENGTH=682	674	682	0	101.2	92.1	95.0
Rsa1.0_00017.1.g1081.t1	gb|EOA17077.1| hypothetical protein CARUB_v10005326mg [Capsella rubella]	326	306	1.00E-120	93.9	74.5	79.4	hypothetical protein CARUB_v10005326mg	gbpln	Capsella rubella	AT4G31410.2 | Symbols: | Protein of unknown function (DUF1644) | chr4:15244259-15245474 FORWARD LENGTH=308	326	308	1.00E-122	94.5	74.2	79.8
Rsa1.0_00017.1.g1082.t1	dbj|BAJ34316.1| unnamed protein product [Thellungiella halophila]	396	402	0	101.5	87.4	93.7	unnamed protein product	----	----	AT4G31420.1 | Symbols: | Zinc finger protein 622 | chr4:15245987-15247763 REVERSE LENGTH=404	396	404	0	102.0	83.1	91.9
Rsa1.0_00017.1.g1083.t1	ref|XP_002869326.1| predicted protein [Arabidopsis lyrata subsp. lyrata] gi|297315162|gb|EFH45585.1| predicted protein [Arabidopsis lyrata subsp. lyrata]	529	571	1.00E-151	107.9	70.7	77.5	predicted protein	gbpln	Arabidopsis lyrata	AT4G31430.2 | Symbols: | unknown protein; LOCATED IN: plasma membrane; EXPRESSED IN: 25 plant structures; EXPRESSED DURING: 15 growth stages; Has 35333 Blast hits to 34131 proteins in 2444 species: Archae - 798; Bacteria - 22429; Metazoa - 974; Fungi - 991; Plants - 531; Viruses - 0; Other Eukaryotes - 9610 (source: NCBI BLink). | chr4:15248510-15252204 FORWARD LENGTH=574	529	574	1.00E-145	108.5	66.5	75.8
Rsa1.0_00017.1.g1084.t1	ref|XP_002869325.1| hypothetical protein ARALYDRAFT_491593 [Arabidopsis lyrata subsp. lyrata] gi|297315161|gb|EFH45584.1| hypothetical protein ARALYDRAFT_491593 [Arabidopsis lyrata subsp. lyrata]	382	394	1.00E-148	103.1	74.6	82.5	hypothetical protein ARALYDRAFT_491593	gbpln	Arabidopsis lyrata	AT4G31440.1 | Symbols: | unknown protein; BEST Arabidopsis thaliana protein match is: unknown protein (TAIR:AT2G24530.1); Has 210 Blast hits to 209 proteins in 55 species: Archae - 0; Bacteria - 72; Metazoa - 2; Fungi - 6; Plants - 128; Viruses - 0; Other Eukaryotes - 2 (source: NCBI BLink). | chr4:15253731-15254870 FORWARD LENGTH=379	382	379	1.00E-138	99.2	70.2	77.5
Rsa1.0_00017.1.g1085.t1	#	#	#	#	#	#	#	-	----	----	#	#	#	#	#	#	#
Rsa1.0_00017.1.g1086.t1	gb|EOA22078.1| hypothetical protein CARUB_v10002618mg [Capsella rubella]	164	892	3.00E-17	543.9	31.1	43.9	hypothetical protein CARUB_v10002618mg	gbpln	Capsella rubella	AT4G32670.1 | Symbols: | RING/FYVE/PHD zinc finger superfamily protein | chr4:15759527-15762847 REVERSE LENGTH=860	164	860	1.00E-19	524.4	31.7	44.5

Rsa1.0_00017.1.g1087.t2	ref|XP_002867301.1| ribosomal protein L28 family protein [Arabidopsis lyrata subsp. lyrata] gi|297313137|gb|EFH43560.1| ribosomal protein L28 family protein [Arabidopsis lyrata subsp. lyrata]	217	212	2.00E-92	97.7	81.1	85.7	ribosomal protein L28 family protein	gbpln	Arabidopsis lyrata	AT4G31460.1 | Symbols: | Ribosomal L28 family | chr4:15259773-15260847 REVERSE LENGTH=212	217	212	9.00E-95	97.7	81.1	86.6
Rsa1.0_00017.1.g1088.t1	ref|NP_194876.2| coatomer subunit beta-1 [Arabidopsis thaliana] gi|334187063|ref|NP_001190880.1| coatomer subunit beta-1 [Arabidopsis thaliana] gi|146286090|sp|Q9SV21.2|COPB1_ARATH RecName: Full=Coatomer subunit beta-1; AltName: Full=Beta-coat protein 1; Short=Beta-COP 1 gi|332660518|gb|AEE85918.1| coatomer subunit beta-1 [Arabidopsis thaliana] gi|332660519|gb|AEE85919.1| coatomer subunit beta-1 [Arabidopsis thaliana]	948	948	0	100.0	97.2	98.5	coatomer subunit beta-1	gbpln	Arabidopsis thaliana	AT4G31480.2 | Symbols: | Coatomer, beta subunit | chr4:15264145-15267384 FORWARD LENGTH=948	948	948	0	100.0	97.2	98.5
Rsa1.0_00017.1.g1089.t1	ref|XP_002889985.1| CYP71B2 [Arabidopsis lyrata subsp. lyrata] gi|297335827|gb|EFH66244.1| CYP71B2 [Arabidopsis lyrata subsp. lyrata]	513	502	0	97.9	72.7	83.6	CYP71B2	gbpln	Arabidopsis lyrata	AT1G13080.1 | Symbols: CYP71B2 | cytochrome P450, family 71, subfamily B, polypeptide 2 | chr1:4459212-4460807 FORWARD LENGTH=502	513	502	0	97.9	72.1	82.5
Rsa1.0_00017.1.g1090.t1	ref|XP_002889985.1| CYP71B2 [Arabidopsis lyrata subsp. lyrata] gi|297335827|gb|EFH66244.1| CYP71B2 [Arabidopsis lyrata subsp. lyrata]	510	502	0	98.4	73.7	85.5	CYP71B2	gbpln	Arabidopsis lyrata	AT1G13080.1 | Symbols: CYP71B2 | cytochrome P450, family 71, subfamily B, polypeptide 2 | chr1:4459212-4460807 FORWARD LENGTH=502	510	502	0	98.4	72.4	82.9
Rsa1.0_00017.1.g1091.t1	ref|NP_194882.2| exocyst complex component 7 [Arabidopsis thaliana] gi|32347268|gb|AAP31853.1| EXO70-G1 protein [Arabidopsis thaliana] gi|332660526|gb|AEE85926.1| exocyst subunit exo70 family protein G1 [Arabidopsis thaliana]	673	687	0	102.1	90.6	95.2	exocyst complex component 7	gbpln	Arabidopsis thaliana	AT4G31540.1 | Symbols: ATEXO70G1, EXO70G1 | exocyst subunit exo70 family protein G1 | chr4:15284739-15286802 REVERSE LENGTH=687	673	687	0	102.1	90.6	95.2
Rsa1.0_00017.1.g1092.t1	gb|ACQ76798.1| WRKY transcription factor 11 [Brassica napus]	325	327	1.00E-164	100.6	89.8	94.5	WRKY transcription factor 11	gbpln	Brassica napus	AT4G31550.2 | Symbols: WRKY11, ATWRKY11 | WRKY DNA-binding protein 11 | chr4:15290065-15291458 REVERSE LENGTH=324	325	324	1.00E-149	99.7	79.7	88.0
Rsa1.0_00017.1.g1093.t1	gb|ABD65000.1| hypothetical protein 26.t00020 [Brassica oleracea]	447	302	2.00E-81	67.6	34.9	42.5	hypothetical protein 26.t00020	gbpln	Brassica oleracea	AT3G47680.1 | Symbols: | DNA binding | chr3:17577483-17578391 REVERSE LENGTH=302	447	302	6.00E-58	67.6	27.5	38.0
Rsa1.0_00017.1.g1094.t1	gb|ABD64939.1| hypothetical protein 24.t00017 [Brassica oleracea]	439	442	0	100.7	76.5	86.1	hypothetical protein 24.t00017	gbpln	Brassica oleracea	AT2G13770.1 | Symbols: | CONTAINS InterPro DOMAIN/s: Putative harbinger transposase-derived nuclease (InterPro:IPR006912); Has 451 Blast hits to 352 proteins in 33 species: Archae - 0; Bacteria - 0; Metazoa - 28; Fungi - 8; Plants - 388; Viruses - 0; Other Eukaryotes - 27 (source: NCBI BLink). | chr2:5736603-5737847 FORWARD LENGTH=343	439	343	4.00E-33	78.1	13.7	17.8
Rsa1.0_00017.1.g1095.t1	ref|XP_002862560.1| hypothetical protein ARALYDRAFT_497397 [Arabidopsis lyrata subsp. lyrata] gi|297308158|gb|EFH38818.1| hypothetical protein ARALYDRAFT_497397 [Arabidopsis lyrata subsp. lyrata]	134	137	1.00E-53	102.2	80.6	85.8	hypothetical protein ARALYDRAFT_497397	gbpln	Arabidopsis lyrata	AT4G31560.1 | Symbols: HCF153 | high chlorophyll fluorescence 153 | chr4:15295219-15296028 FORWARD LENGTH=137	134	137	6.00E-56	102.2	79.9	85.8
Rsa1.0_00017.1.g1096.t2	ref|NP_194885.2| uncharacterized protein [Arabidopsis thaliana] gi|332660530|gb|AEE85930.1| uncharacterized protein AT4G31570 [Arabidopsis thaliana]	2708	2730	0	100.8	78.6	86.9	uncharacterized protein	gbpln	Arabidopsis thaliana	AT4G31570.1 | Symbols: | CONTAINS InterPro DOMAIN/s: Prefoldin (InterPro:IPR009053); BEST Arabidopsis thaliana protein match is: unknown protein (TAIR:AT1G24460.1); Has 194354 Blast hits to 66887 proteins in 3244 species: Archae - 3688; Bacteria - 38556; Metazoa - 84828; Fungi - 17265; Plants - 10589; Viruses - 805; Other Eukaryotes - 38623 (source: NCBI BLink). | chr4:15297061-15306036 FORWARD LENGTH=2730	2708	2730	0	100.8	78.6	86.9

Rsa1.0_00017.1.g1097.t1	ref|NP_194887.1| putative xyloglucan glycosyltransferase 5 [Arabidopsis thaliana] gi|75201904|sp|Q9SB75.1|CSLC5_ARATH RecName: Full=Probable xyloglucan glycosyltransferase 5; AltName: Full=Cellulose synthase-like protein C5; Short=AtCslC5 gi|3281868|emb|CAA19764.1| putative protein [Arabidopsis thaliana] gi|7270062|emb|CAB79877.1| putative protein [Arabidopsis thaliana] gi|28058784|gb|AAO29953.1| putative protein [Arabidopsis thaliana] gi|30725520|gb|AAP37782.1| At4g31590 [Arabidopsis thaliana] gi|332660533|gb|AEE85933.1| putative xyloglucan glycosyltransferase 5 [Arabidopsis thaliana]	690	692	0	100.3	95.8	97.8	putative xyloglucan glycosyltransferase 5	gbpln	Arabidopsis thaliana	AT4G31590.1 | Symbols: ATCSLC05, CSLC05, ATCSLC5, CSLC5 | Cellulose-synthase-like C5 | chr4:15309889-15312336 REVERSE LENGTH=692	690	692	0	100.3	95.8	97.8
Rsa1.0_00017.1.g1098.t1	sp|O65098.1|REM1_BRAOB RecName: Full=B3 domain-containing protein REM1; AltName: Full=Protein REPRODUCTIVE MERISTEM 1; Short=BoREM1 gi|3170424|gb|AAC18082.1| reproductive meristem gene 1 [Brassica oleracea var. botrytis]	492	497	1.00E-176	101.0	72.0	80.5	RecName: Full=B3 domain-containing protein REM1; AltName: Full=Protein REPRODUCTIVE MERISTEM 1; Short=BoREM1 gi|3170424|gb|AAC18082.1| reproductive meristem gene 1	gbpln	Brassica oleracea	AT4G31610.1 | Symbols: REM1, ATREM1 | Transcriptional factor B3 family protein | chr4:15317700-15319776 FORWARD LENGTH=517	492	517	1.00E-122	105.1	54.1	68.1
Rsa1.0_00017.1.g1099.t1	sp|O65098.1|REM1_BRAOB RecName: Full=B3 domain-containing protein REM1; AltName: Full=Protein REPRODUCTIVE MERISTEM 1; Short=BoREM1 gi|3170424|gb|AAC18082.1| reproductive meristem gene 1 [Brassica oleracea var. botrytis]	488	497	0	101.8	72.3	81.1	RecName: Full=B3 domain-containing protein REM1; AltName: Full=Protein REPRODUCTIVE MERISTEM 1; Short=BoREM1 gi|3170424|gb|AAC18082.1| reproductive meristem gene 1	gbpln	Brassica oleracea	AT4G31610.1 | Symbols: REM1, ATREM1 | Transcriptional factor B3 family protein | chr4:15317700-15319776 FORWARD LENGTH=517	488	517	1.00E-133	105.9	58.8	72.1
Rsa1.0_00017.1.g1100.t1	gb|EOA16495.1| hypothetical protein CARUB_v10004653mg [Capsella rubella]	530	496	1.00E-165	93.6	59.1	71.3	hypothetical protein CARUB_v10004653mg	gbpln	Capsella rubella	AT4G31630.1 | Symbols: | Transcriptional factor B3 family protein | chr4:15325343-15327220 REVERSE LENGTH=512	530	512	1.00E-157	96.6	57.7	71.1
Rsa1.0_00017.1.g1101.t1	ref|XP_002867286.1| hypothetical protein ARALYDRAFT_913300 [Arabidopsis lyrata subsp. lyrata] gi|297313122|gb|EFH43545.1| hypothetical protein ARALYDRAFT_913300 [Arabidopsis lyrata subsp. lyrata]	507	632	1.00E-115	124.7	44.2	50.9	hypothetical protein ARALYDRAFT_913300	gbpln	Arabidopsis lyrata	AT4G31680.1 | Symbols: | Transcriptional factor B3 family protein | chr4:15340289-15342080 REVERSE LENGTH=462	507	462	1.00E-115	91.1	40.8	50.3
Rsa1.0_00017.1.g1102.t1	dbj|BAD44466.1| unknown protein [Arabidopsis thaliana]	615	631	0	102.6	80.5	87.0	unknown protein	gbpln	Arabidopsis thaliana	AT4G31670.1 | Symbols: UBP18 | ubiquitin-specific protease 18 | chr4:15336007-15339506 REVERSE LENGTH=631	615	631	0	102.6	80.7	87.2
Rsa1.0_00017.1.g1103.t1	ref|XP_002867286.1| hypothetical protein ARALYDRAFT_913300 [Arabidopsis lyrata subsp. lyrata] gi|297313122|gb|EFH43545.1| hypothetical protein ARALYDRAFT_913300 [Arabidopsis lyrata subsp. lyrata]	473	632	1.00E-152	133.6	61.5	73.6	hypothetical protein ARALYDRAFT_913300	gbpln	Arabidopsis lyrata	AT4G31680.1 | Symbols: | Transcriptional factor B3 family protein | chr4:15340289-15342080 REVERSE LENGTH=462	473	462	1.00E-140	97.7	58.4	70.6
Rsa1.0_00017.1.g1104.t1	gb|AAZ66937.1| 117M18_18 [Brassica rapa]	250	249	1.00E-137	99.6	97.2	98.4	117M18_18	gbpln	Brassica rapa	AT5G10360.1 | Symbols: EMB3010, RPS6B | Ribosomal protein S6e | chr5:3258734-3260142 REVERSE LENGTH=249	250	249	1.00E-134	99.6	94.4	96.4

Rsa1.0_00017.1.g1105.t1	ref|NP_194900.1| TBP-associated factor II 15 [Arabidopsis thaliana] gi|30689137|ref|NP_849481.1| TBP-associated factor II 15 [Arabidopsis thaliana] gi|13492648|gb|AAK28290.1|AF344879_1 putative TBP-associated 15 kDa subunit protein [Arabidopsis thaliana] gi|13549156|gb|AAK29671.1|AF352813_1 putative TBP associated factor 15kDa subunit [Arabidopsis thaliana] gi|2072626|emb|CAA73389.1| hypothetical protein [Arabidopsis thaliana] gi|3281856|emb|CAA19751.1| Transcription factor II homolog [Arabidopsis thaliana] gi|7270075|emb|CAB79890.1| Transcription factor II homolog [Arabidopsis thaliana] gi|21554584|gb|AAM63623.1| Transcription factor II homolog [Arabidopsis thaliana] gi|39545934|gb|AAR28030.1| TAF10 [Arabidopsis thaliana] gi|222424014|dbj|BAH19968.1| AT4G31720 [Arabidopsis thaliana] gi|332660549|gb|AEE85949.1| TBP-associated factor II 15 [Arabidopsis thaliana] gi|332660550|gb|AEE85950.1| TBP-associated factor II 15 [Arabidopsis thaliana]	134	134	3.00E-72	100.0	99.3	99.3	TBP-associated factor II 15	gbpln	Arabidopsis thaliana	AT4G31720.1 | Symbols: TAFII15, TAF10, STG1 | TBP-associated factor II 15 | chr4:15354223-15355704 REVERSE LENGTH=134	134	134	7.00E-75	100.0	99.3	99.3
Rsa1.0_00017.1.g1106.t1	gb|EOA15643.1| hypothetical protein CARUB_v10005898mg [Capsella rubella]	166	168	6.00E-60	101.2	69.9	79.5	hypothetical protein CARUB_v10005898mg	gbpln	Capsella rubella	AT4G31730.1 | Symbols: GDU1 | glutamine dumper 1 | chr4:15361207-15361683 FORWARD LENGTH=158	166	158	7.00E-58	95.2	68.7	77.7
Rsa1.0_00017.1.g1107.t1	ref|XP_002869315.1| ADP-ribosylation factor 3 [Arabidopsis lyrata subsp. lyrata] gi|297315151|gb|EFH45574.1| ADP-ribosylation factor 3 [Arabidopsis lyrata subsp. lyrata]	204	182	1.00E-102	89.2	88.7	89.2	ADP-ribosylation factor 3	gbpln	Arabidopsis lyrata	AT2G24765.1 | Symbols: ARF3, ARL1, ATARL1 | ADP-ribosylation factor 3 | chr2:10562822-10564961 FORWARD LENGTH=182	204	182	1.00E-103	89.2	86.3	89.2
Rsa1.0_00017.1.g1108.t1	ref|XP_002867281.1| hypothetical protein ARALYDRAFT_491566 [Arabidopsis lyrata subsp. lyrata] gi|297313117|gb|EFH43540.1| hypothetical protein ARALYDRAFT_491566 [Arabidopsis lyrata subsp. lyrata]	307	311	1.00E-167	101.3	96.1	97.7	hypothetical protein ARALYDRAFT_491566	gbpln	Arabidopsis lyrata	AT4G31750.1 | Symbols: WIN2 | HOPW1-1-interacting 2 | chr4:15364657-15367207 REVERSE LENGTH=311	307	311	1.00E-169	101.3	95.4	97.7
Rsa1.0_00017.1.g1109.t1	ref|NP_194906.1| Monogalactosyldiacylglycerol synthase 1 [Arabidopsis thaliana] gi|75100403|sp|O81770.1|MGDG1_ARATH RecName: Full=Monogalactosyldiacylglycerol synthase 1, chloroplastic; Short=AtMGD1; AltName: Full=MGDG synthase type A; Flags: Precursor gi|3281850|emb|CAA19745.1| monogalactosyldiacylglycerol synthase - like protein [Arabidopsis thaliana] gi|7270081|emb|CAB79896.1| monogalactosyldiacylglycerol synthase-like protein [Arabidopsis thaliana] gi|7621497|gb|AAF65066.1| monogalactosyldiacylglycerol synthase [Arabidopsis thaliana] gi|9927297|dbj|BAB12042.1| MGDG synthase type A [Arabidopsis thaliana] gi|20260132|gb|AAM12964.1| strong similarity to monogalactosyldiacylglycerol synthase, Cucumis sativus, PID:g1805254 [Arabidopsis thaliana] gi|31711946|gb|AAP68329.1| At4g31780 [Arabidopsis thaliana] gi|332660556|gb|AEE85956.1| Monogalactosyldiacylglycerol synthase 1 [Arabidopsis thaliana]	526	533	0	101.3	88.4	93.7	Monogalactosyldiacylglycerol synthase 1	gbpln	Arabidopsis thaliana	AT4G31780.2 | Symbols: MGD1, MGDA | monogalactosyl diacylglycerol synthase 1 | chr4:15374222-15376961 FORWARD LENGTH=533	526	533	0	101.3	88.4	93.7
Rsa1.0_00017.1.g1110.t1	gb|EOA17067.1| hypothetical protein CARUB_v10005307mg, partial [Capsella rubella]	277	310	1.00E-152	111.9	93.5	96.8	hypothetical protein CARUB_v10005307mg, partial	gbpln	Capsella rubella	AT4G31790.2 | Symbols: | Tetrapyrrole (Corrin/Porphyrin) Methylases | chr4:15377479-15378530 FORWARD LENGTH=277	277	277	1.00E-152	100.0	92.1	97.1

Rsa1.0_00017.1.g1111.t1	gb|AAS13378.1| WRKY1 [Brassica rapa subsp. chinensis]	313	308	1.00E-134	98.4	78.3	84.7	WRKY1	gbpln	Brassica rapa	AT4G31800.1 | Symbols: WRKY18, ATWRKY18 | WRKY DNA-binding protein 18 | chr4:15383296-15384812 FORWARD LENGTH=310	313	310	1.00E-132	99.0	78.0	85.3
Rsa1.0_00017.1.g1112.t1	#	#	#	#	#	#	#	-	----	----	#	#	#	#	#	#	#
Rsa1.0_00017.1.g1113.t1	gb|EOA16845.1| hypothetical protein CARUB_v10005068mg [Capsella rubella]	349	372	7.00E-25	106.6	29.8	47.6	hypothetical protein CARUB_v10005068mg	gbpln	Capsella rubella	AT4G14905.2 | Symbols: | Galactose oxidase/kelch repeat superfamily protein | chr4:8526879-8527997 REVERSE LENGTH=372	349	372	1.00E-23	106.6	26.9	47.0
Rsa1.0_00017.1.g1114.t1	ref|NP_189957.1| putative F-box/kelch-repeat protein [Arabidopsis thaliana] gi|75182779|sp|Q9M2B5.1|FBK72_ARATH RecName: Full=Putative F-box/kelch-repeat protein At3g43710 gi|7362796|emb|CAB83072.1| putative protein [Arabidopsis thaliana] gi|332644299|gb|AEE77820.1| putative F-box/kelch-repeat protein [Arabidopsis thaliana]	370	378	1.00E-115	102.2	58.4	70.5	putative F-box/kelch-repeat protein	gbpln	Arabidopsis thaliana	AT3G43710.1 | Symbols: | Galactose oxidase/kelch repeat superfamily protein | chr3:15605608-15606744 FORWARD LENGTH=378	370	378	1.00E-118	102.2	58.4	70.5
Rsa1.0_00017.1.g1115.t1	#	#	#	#	#	#	#	-	----	----	#	#	#	#	#	#	#
Rsa1.0_00017.1.g1116.t1	dbj|BAJ34065.1| unnamed protein product [Thellungiella halophila]	573	579	0	101.0	85.7	89.0	unnamed protein product	----	----	AT4G31820.1 | Symbols: ENP, NPY1, MAB4 | Phototropic-responsive NPH3 family protein | chr4:15390788-15393627 REVERSE LENGTH=571	573	571	0	99.7	83.9	88.3
Rsa1.0_00017.1.g1117.t1	ref|XP_002867277.1| hypothetical protein ARALYDRAFT_913282 [Arabidopsis lyrata subsp. lyrata] gi|297313113|gb|EFH43536.1| hypothetical protein ARALYDRAFT_913282 [Arabidopsis lyrata subsp. lyrata]	100	100	1.00E-48	100.0	96.0	99.0	hypothetical protein ARALYDRAFT_913282	gbpln	Arabidopsis lyrata	AT4G31830.1 | Symbols: | unknown protein; Has 42 Blast hits to 42 proteins in 10 species: Archae - 0; Bacteria - 0; Metazoa - 0; Fungi - 0; Plants - 42; Viruses - 0; Other Eukaryotes - 0 (source: NCBI BLink). | chr4:15400803-15401105 REVERSE LENGTH=100	100	100	3.00E-51	100.0	96.0	98.0
Rsa1.0_00017.1.g1118.t1	ref|XP_002869310.1| hypothetical protein ARALYDRAFT_491553 [Arabidopsis lyrata subsp. lyrata] gi|297315146|gb|EFH45569.1| hypothetical protein ARALYDRAFT_491553 [Arabidopsis lyrata subsp. lyrata]	174	177	8.00E-83	101.7	90.2	97.1	hypothetical protein ARALYDRAFT_491553	gbpln	Arabidopsis lyrata	AT4G31840.1 | Symbols: ENODL15, AtENODL15 | early nodulin-like protein 15 | chr4:15401798-15402426 FORWARD LENGTH=177	174	177	6.00E-83	101.7	88.5	94.8
Rsa1.0_00017.1.g1119.t1	ref|NP_194913.1| proton gradient regulation 3 [Arabidopsis thaliana] gi|75213543|sp|Q9SZ52.1|PP344_ARATH RecName: Full=Pentatricopeptide repeat-containing protein At4g31850, chloroplastic; AltName: Full=Protein PROTON GRADIENT REGULATION 3; Flags: Precursor gi|4584524|emb|CAB40755.1| putative protein [Arabidopsis thaliana] gi|7270088|emb|CAB79903.1| putative protein [Arabidopsis thaliana] gi|332660567|gb|AEE85967.1| proton gradient regulation 3 [Arabidopsis thaliana]	1122	1112	0	99.1	83.7	91.3	proton gradient regulation 3	gbpln	Arabidopsis thaliana	AT4G31850.1 | Symbols: PGR3 | proton gradient regulation 3 | chr4:15403020-15406358 FORWARD LENGTH=1112	1122	1112	0	99.1	83.7	91.3
Rsa1.0_00017.1.g1120.t1	#	#	#	#	#	#	#	-	----	----	#	#	#	#	#	#	#
Rsa1.0_00017.1.g1121.t2	ref|NP_194916.2| uncharacterized protein [Arabidopsis thaliana] gi|26983792|gb|AAN86148.1| unknown protein [Arabidopsis thaliana] gi|332660572|gb|AEE85972.1| uncharacterized protein AT4G31880 [Arabidopsis thaliana]	869	873	0	100.5	63.4	71.3	uncharacterized protein	gbpln	Arabidopsis thaliana	AT4G31880.1 | Symbols: | LOCATED IN: cytosol, chloroplast; EXPRESSED IN: 24 plant structures; EXPRESSED DURING: 14 growth stages; BEST Arabidopsis thaliana protein match is: Tudor/PWWP/MBT superfamily protein (TAIR:AT1G15940.1); Has 137162 Blast hits to 70781 proteins in 2973 species: Archae - 289; Bacteria - 24182; Metazoa - 56725; Fungi - 20130; Plants - 6559; Viruses - 758; Other Eukaryotes - 28519 (source: NCBI BLink). | chr4:15419435-15423939 REVERSE LENGTH=873	869	873	0	100.5	63.4	71.3

Rsa1.0_00017.1.g1122.t1	ref|NP_194917.1| armadillo/beta-catenin-like repeat-containing protein [Arabidopsis thaliana] gi|334187073|ref|NP_001190883.1| armadillo/beta-catenin-like repeat-containing protein [Arabidopsis thaliana] gi|4584528|emb|CAB40759.1| putative protein [Arabidopsis thaliana] gi|7270092|emb|CAB79907.1| putative protein [Arabidopsis thaliana] gi|110736926|dbj|BAF00420.1| hypothetical protein [Arabidopsis thaliana] gi|190341119|gb|ACE74718.1| At4g31890 [Arabidopsis thaliana] gi|332660574|gb|AEE85974.1| armadillo/beta-catenin-like repeat-containing protein [Arabidopsis thaliana] gi|332660575|gb|AEE85975.1| armadillo/beta-catenin-like repeat-containing protein [Arabidopsis thaliana]	509	518	0	101.8	89.0	93.1	armadillo/beta-catenin-like repeat-containing protein	gbpln	Arabidopsis thaliana	AT4G31890.2 | Symbols: | ARM repeat superfamily protein | chr4:15427290-15429049 REVERSE LENGTH=518	509	518	0	101.8	89.0	93.1
Rsa1.0_00017.1.g1123.t1	emb|CAB39638.1| RNA-directed DNA polymerase-like protein [Arabidopsis thaliana] gi|7267666|emb|CAB78094.1| RNA-directed DNA polymerase-like protein [Arabidopsis thaliana]	1913	1274	0	66.6	32.3	44.5	RNA-directed DNA polymerase-like protein	gbpln	Arabidopsis thaliana	AT4G29090.1 | Symbols: | Ribonuclease H-like superfamily protein | chr4:14333528-14335255 FORWARD LENGTH=575	1913	575	1.00E-67	30.1	9.2	14.3
Rsa1.0_00017.1.g1124.t1	ref|XP_002879859.1| hypothetical protein ARALYDRAFT_903307 [Arabidopsis lyrata subsp. lyrata] gi|297325698|gb|EFH56118.1| hypothetical protein ARALYDRAFT_903307 [Arabidopsis lyrata subsp. lyrata]	316	304	1.00E-78	96.2	52.5	64.6	hypothetical protein ARALYDRAFT_903307	gbpln	Arabidopsis lyrata	AT2G20620.1 | Symbols: | Protein of unknown function (DUF626) | chr2:8891929-8893244 REVERSE LENGTH=286	316	286	1.00E-52	90.5	32.9	47.5
Rsa1.0_00017.1.g1125.t4	emb|CAB39638.1| RNA-directed DNA polymerase-like protein [Arabidopsis thaliana] gi|7267666|emb|CAB78094.1| RNA-directed DNA polymerase-like protein [Arabidopsis thaliana]	346	1274	3.00E-77	368.2	46.0	62.1	RNA-directed DNA polymerase-like protein	gbpln	Arabidopsis thaliana	AT1G10000.1 | Symbols: | Ribonuclease H-like superfamily protein | chr1:3263877-3264788 REVERSE LENGTH=303	346	303	7.00E-55	87.6	35.5	49.4
Rsa1.0_00017.1.g1126.t1	dbj|BAJ34619.1| unnamed protein product [Thellungiella halophila]	92	539	2.00E-19	585.9	57.6	58.7	unnamed protein product	----	----	AT4G31920.1 | Symbols: ARR10, RR10 | response regulator 10 | chr4:15444290-15446766 REVERSE LENGTH=552	92	552	3.00E-15	600.0	43.5	52.2
Rsa1.0_00017.1.g1127.t1	gb|EOA18383.1| hypothetical protein CARUB_v10006919mg [Capsella rubella]	232	660	4.00E-91	284.5	75.0	83.6	hypothetical protein CARUB_v10006919mg	gbpln	Capsella rubella	AT4G31980.1 | Symbols: | unknown protein; CONTAINS InterPro DOMAIN/s: Protein of unknown function DUF247, plant (InterPro:IPR004158), Protein of unknown function DUF862, eukaryotic (InterPro:IPR008580); BEST Arabidopsis thaliana protein match is: Plant protein of unknown function (DUF247) (TAIR:AT5G11290.1); Has 1967 Blast hits to 1844 proteins in 183 species: Archae - 0; Bacteria - 6; Metazoa - 223; Fungi - 83; Plants - 1477; Viruses - 0; Other Eukaryotes - 178 (source: NCBI BLink). | chr4:15464905-15469204 FORWARD LENGTH=680	232	680	2.00E-92	293.1	75.0	84.5
Rsa1.0_00017.1.g1128.t1	#	#	#	#	#	#	#	-	----	----	#	#	#	#	#	#	#
Rsa1.0_00017.1.g1129.t3	gb|EOA16711.1| hypothetical protein CARUB_v10004912mg [Capsella rubella]	410	418	0	102.0	87.3	92.9	hypothetical protein CARUB_v10004912mg	gbpln	Capsella rubella	AT4G32000.1 | Symbols: | Protein kinase superfamily protein | chr4:15474083-15476655 REVERSE LENGTH=418	410	418	0	102.0	85.1	90.0
Rsa1.0_00017.1.g1130.t1	gb|EOA16061.1| hypothetical protein CARUB_v10004194mg [Capsella rubella]	131	775	3.00E-51	591.6	74.8	83.2	hypothetical protein CARUB_v10004194mg	gbpln	Capsella rubella	AT4G32010.1 | Symbols: HSL1, HSI2-L1, VAL2 | HSI2-like 1 | chr4:15481231-15484897 FORWARD LENGTH=780	131	780	4.00E-51	595.4	76.3	83.2
Rsa1.0_00017.1.g1131.t1	gb|EOA15616.1| hypothetical protein CARUB_v10005750mg [Capsella rubella]	190	205	2.00E-60	107.9	77.9	85.8	hypothetical protein CARUB_v10005750mg	gbpln	Capsella rubella	AT4G32020.1 | Symbols: | unknown protein; FUNCTIONS IN: molecular_function unknown; INVOLVED IN: biological_process unknown; LOCATED IN: chloroplast; EXPRESSED IN: 24 plant structures; EXPRESSED DURING: 15 growth stages; BEST Arabidopsis thaliana protein match is: unknown protein (TAIR:AT2G25250.1); Has 65 Blast hits to 65 proteins in 19 species: Archae - 0; Bacteria - 0; Metazoa - 3; Fungi - 8; Plants - 54; Viruses - 0; Other Eukaryotes - 0 (source: NCBI BLink). | chr4:15488076-15488621 FORWARD LENGTH=181	190	181	6.00E-49	95.3	64.7	70.5

Rsa1.0_00017.1.g1132.t1	ref|XP_002867262.1| predicted protein [Arabidopsis lyrata subsp. lyrata] gi|297313098|gb|EFH43521.1| predicted protein [Arabidopsis lyrata subsp. lyrata]	82	107	3.00E-16	130.5	54.9	64.6	predicted protein	gbpln	Arabidopsis lyrata	AT4G32100.1 | Symbols: | Beta-1,3-N-Acetylglucosaminyltransferase family protein | chr4:15511757-15512218 REVERSE LENGTH=122	82	122	2.00E-17	148.8	51.2	63.4
Rsa1.0_00017.1.g1133.t1	ref|XP_002867258.1| galactosyltransferase family protein [Arabidopsis lyrata subsp. lyrata] gi|297313094|gb|EFH43517.1| galactosyltransferase family protein [Arabidopsis lyrata subsp. lyrata]	385	345	0	89.6	81.8	85.7	galactosyltransferase family protein	gbpln	Arabidopsis lyrata	AT4G32120.1 | Symbols: | Galactosyltransferase family protein | chr4:15517230-15519687 REVERSE LENGTH=345	385	345	0	89.6	81.0	84.4
Rsa1.0_00017.1.g1134.t5	ref|XP_002867255.1| vesicle-associated membrane protein 7C [Arabidopsis lyrata subsp. lyrata] gi|297313091|gb|EFH43514.1| vesicle-associated membrane protein 7C [Arabidopsis lyrata subsp. lyrata]	540	219	1.00E-103	40.6	33.9	34.6	vesicle-associated membrane protein 7C	gbpln	Arabidopsis lyrata	AT4G32150.1 | Symbols: VAMP711, ATVAMP711 | vesicle-associated membrane protein 711 | chr4:15526407-15527651 REVERSE LENGTH=219	540	219	1.00E-105	40.6	33.5	34.4
Rsa1.0_00017.1.g1135.t6	ref|NP_567888.2| Phox (PX) domain-containing protein [Arabidopsis thaliana] gi|332660612|gb|AEE86012.1| Phox (PX) domain-containing protein [Arabidopsis thaliana]	764	723	0	94.6	82.5	88.7	Phox (PX) domain-containing protein	gbpln	Arabidopsis thaliana	AT4G32160.1 | Symbols: | Phox (PX) domain-containing protein | chr4:15529056-15532892 FORWARD LENGTH=723	764	723	0	94.6	82.5	88.7
Rsa1.0_00017.1.g1136.t1	ref|XP_002867254.1| ATPANK2 [Arabidopsis lyrata subsp. lyrata] gi|297313090|gb|EFH43513.1| ATPANK2 [Arabidopsis lyrata subsp. lyrata]	915	902	0	98.6	94.9	96.7	ATPANK2	gbpln	Arabidopsis lyrata	AT4G32180.1 | Symbols: ATPANK2, PANK2 | pantothenate kinase 2 | chr4:15537724-15543715 REVERSE LENGTH=901	915	901	0	98.5	94.2	96.6
Rsa1.0_00017.1.g1137.t1	gb|EOA16055.1| hypothetical protein CARUB_v10004187mg [Capsella rubella]	758	780	0	102.9	77.4	89.1	hypothetical protein CARUB_v10004187mg	gbpln	Capsella rubella	AT4G32190.1 | Symbols: | Myosin heavy chain-related protein | chr4:15545052-15547689 FORWARD LENGTH=783	758	783	0	103.3	78.4	88.5
Rsa1.0_00017.1.g1138.t1	ref|NP_194953.1| F-type H+-transporting ATPase subunit b [Arabidopsis thaliana] gi|2864617|emb|CAA16964.1| H+-transporting ATP synthase chain9 - like protein [Arabidopsis thaliana] gi|5730141|emb|CAB52473.1| ATP synthase beta chain precursor (subunit II) [Arabidopsis thaliana] gi|7270130|emb|CAB79944.1| H+-transporting ATP synthase chain 9-like protein [Arabidopsis thaliana] gi|16604406|gb|AAL24209.1| AT4g32260/F10M6_100 [Arabidopsis thaliana] gi|21554175|gb|AAM63254.1| H+-transporting ATP synthase chain 9-like protein [Arabidopsis thaliana] gi|332660627|gb|AEE86027.1| F-type H+-transporting ATPase subunit b [Arabidopsis thaliana]	287	219	2.00E-85	76.3	64.1	68.3	F-type H+-transporting ATPase subunit b	gbpln	Arabidopsis thaliana	AT4G32260.1 | Symbols: | ATPase, F0 complex, subunit B/B', bacterial/chloroplast | chr4:15573859-15574586 REVERSE LENGTH=219	287	219	4.00E-88	76.3	64.1	68.3
Rsa1.0_00017.1.g1139.t1	gb|EOA16255.1| hypothetical protein CARUB_v10004401mg [Capsella rubella] gi|482552063|gb|EOA16256.1| hypothetical protein CARUB_v10004401mg [Capsella rubella]	613	611	0	99.7	89.7	94.3	hypothetical protein CARUB_v10004401mg	gbpln	Capsella rubella	AT4G32250.3 | Symbols: | Protein kinase superfamily protein | chr4:15570285-15572528 REVERSE LENGTH=611	613	611	0	99.7	89.2	94.1
Rsa1.0_00017.1.g1140.t2	ref|XP_002867250.1| hypothetical protein ARALYDRAFT_491506 [Arabidopsis lyrata subsp. lyrata] gi|297313086|gb|EFH43509.1| hypothetical protein ARALYDRAFT_491506 [Arabidopsis lyrata subsp. lyrata]	248	237	1.00E-106	95.6	77.8	84.7	hypothetical protein ARALYDRAFT_491506	gbpln	Arabidopsis lyrata	AT4G32270.1 | Symbols: | Ubiquitin-like superfamily protein | chr4:15575015-15576360 REVERSE LENGTH=239	248	239	1.00E-103	96.4	75.4	82.7
Rsa1.0_00017.1.g1141.t1	gb|EOA19749.1| hypothetical protein CARUB_v10003892mg [Capsella rubella]	450	457	6.00E-82	101.6	46.4	61.6	hypothetical protein CARUB_v10003892mg	gbpln	Capsella rubella	AT5G22730.1 | Symbols: | F-box/RNI-like/FBD-like domains-containing protein | chr5:7551632-7553219 REVERSE LENGTH=466	450	466	4.00E-76	103.6	45.3	57.8
Rsa1.0_00017.1.g1142.t1	ref|XP_002869286.1| hypothetical protein ARALYDRAFT_328503 [Arabidopsis lyrata subsp. lyrata] gi|297315122|gb|EFH45545.1| hypothetical protein ARALYDRAFT_328503 [Arabidopsis lyrata subsp. lyrata]	171	845	7.00E-56	494.2	73.1	83.6	hypothetical protein ARALYDRAFT_328503	gbpln	Arabidopsis lyrata	AT4G32280.1 | Symbols: IAA29 | indole-3-acetic acid inducible 29 | chr4:15583479-15584628 FORWARD LENGTH=251	171	251	8.00E-52	146.8	70.8	80.7
Rsa1.0_00017.1.g1143.t1	gb|EOA16210.1| hypothetical protein CARUB_v10004350mg [Capsella rubella]	633	637	0	100.6	90.7	94.2	hypothetical protein CARUB_v10004350mg	gbpln	Capsella rubella	AT4G32285.2 | Symbols: | ENTH/ANTH/VHS superfamily protein | chr4:15586003-15587910 FORWARD LENGTH=635	633	635	0	100.3	89.6	93.7

Rsa1.0_00017.1.g1144.t1	ref|XP_002867248.1| hypothetical protein ARALYDRAFT_491501 [Arabidopsis lyrata subsp. lyrata] gi|297313084|gb|EFH43507.1| hypothetical protein ARALYDRAFT_491501 [Arabidopsis lyrata subsp. lyrata]	384	385	1.00E-177	100.3	81.3	88.0	hypothetical protein ARALYDRAFT_491501	gbpln	Arabidopsis lyrata	AT4G32290.1 | Symbols: | Core-2/I-branching beta-1,6-N-acetylglucosaminyltransferase family protein | chr4:15589839-15590993 REVERSE LENGTH=384	384	384	1.00E-171	100.0	79.2	88.0
Rsa1.0_00017.1.g1145.t1	ref|NP_680756.1| uncharacterized protein [Arabidopsis thaliana] gi|28392894|gb|AAO41883.1| unknown protein [Arabidopsis thaliana] gi|28827750|gb|AAO50719.1| unknown protein [Arabidopsis thaliana] gi|332660636|gb|AEE86036.1| uncharacterized protein AT4G32295 [Arabidopsis thaliana]	265	238	1.00E-106	89.8	79.6	82.6	uncharacterized protein	gbpln	Arabidopsis thaliana	AT4G32295.1 | Symbols: | unknown protein; BEST Arabidopsis thaliana protein match is: unknown protein (TAIR:AT3G24150.1); Has 39 Blast hits to 39 proteins in 10 species: Archae - 0; Bacteria - 0; Metazoa - 0; Fungi - 0; Plants - 39; Viruses - 0; Other Eukaryotes - 0 (source: NCBI BLink). | chr4:15592960-15594080 FORWARD LENGTH=238	265	238	1.00E-109	89.8	79.6	82.6
Rsa1.0_00017.1.g1146.t1	ref|XP_002869284.1| lectin protein kinase family protein [Arabidopsis lyrata subsp. lyrata] gi|297315120|gb|EFH45543.1| lectin protein kinase family protein [Arabidopsis lyrata subsp. lyrata]	828	825	0	99.6	84.5	91.1	lectin protein kinase family protein	gbpln	Arabidopsis lyrata	AT4G32300.1 | Symbols: SD2-5 | S-domain-2 5 | chr4:15599970-15602435 FORWARD LENGTH=821	828	821	0	99.2	84.4	91.1
Rsa1.0_00017.1.g1147.t1	ref|XP_002869284.1| lectin protein kinase family protein [Arabidopsis lyrata subsp. lyrata] gi|297315120|gb|EFH45543.1| lectin protein kinase family protein [Arabidopsis lyrata subsp. lyrata]	247	825	1.00E-60	334.0	55.1	64.4	lectin protein kinase family protein	gbpln	Arabidopsis lyrata	AT4G32300.1 | Symbols: SD2-5 | S-domain-2 5 | chr4:15599970-15602435 FORWARD LENGTH=821	247	821	2.00E-61	332.4	54.3	63.6
Rsa1.0_00017.1.g1148.t1	ref|NP_567893.1| TPX2 (targeting protein for Xklp2) protein family [Arabidopsis thaliana] gi|186515493|ref|NP_001119094.1| TPX2 (targeting protein for Xklp2) protein family [Arabidopsis thaliana] gi|14334556|gb|AAK59686.1| unknown protein [Arabidopsis thaliana] gi|21280971|gb|AAM44942.1| unknown protein [Arabidopsis thaliana] gi|332660640|gb|AEE86040.1| TPX2 (targeting protein for Xklp2) protein family [Arabidopsis thaliana] gi|332660642|gb|AEE86042.1| TPX2 (targeting protein for Xklp2) protein family [Arabidopsis thaliana]	432	437	1.00E-168	101.2	76.4	85.0	TPX2 (targeting protein for Xklp2) protein family	gbpln	Arabidopsis thaliana	AT4G32330.3 | Symbols: | TPX2 (targeting protein for Xklp2) protein family | chr4:15609801-15611867 FORWARD LENGTH=437	432	437	1.00E-170	101.2	76.4	85.0
Rsa1.0_00017.1.g1149.t1	ref|NP_194960.1| tetratricopeptide repeat domain-containing protein-like protein [Arabidopsis thaliana] gi|2864610|emb|CAA16957.1| putative protein [Arabidopsis thaliana] gi|4049336|emb|CAA22561.1| putative protein [Arabidopsis thaliana] gi|7270138|emb|CAB79951.1| putative protein [Arabidopsis thaliana] gi|21592985|gb|AAM64934.1| unknown [Arabidopsis thaliana] gi|23297278|gb|AAN12931.1| unknown protein [Arabidopsis thaliana] gi|332660643|gb|AEE86043.1| tetratricopeptide repeat domain-containing protein-like protein [Arabidopsis thaliana]	244	238	7.00E-77	97.5	72.1	79.9	tetratricopeptide repeat domain-containing protein-like protein	gbpln	Arabidopsis thaliana	AT4G32340.1 | Symbols: | Tetratricopeptide repeat (TPR)-like superfamily protein | chr4:15612741-15614219 REVERSE LENGTH=238	244	238	2.00E-79	97.5	72.1	79.9
Rsa1.0_00017.1.g1150.t1	gb|EOA16104.1| hypothetical protein CARUB_v10004237mg [Capsella rubella]	643	724	0	112.6	73.6	83.0	hypothetical protein CARUB_v10004237mg	gbpln	Capsella rubella	AT4G32350.1 | Symbols: | Regulator of Vps4 activity in the MVB pathway protein | chr4:15617634-15620642 FORWARD LENGTH=732	643	732	0	113.8	72.0	81.6
Rsa1.0_00017.1.g1151.t1	ref|NP_194965.1| Nucleotide-sugar transporter family protein [Arabidopsis thaliana] gi|75209423|sp|Q9SUV2.1|PT432_ARATH RecName: Full=Probable sugar phosphate/phosphate translocator At4g32390 gi|4049341|emb|CAA22566.1| putative protein [Arabidopsis thaliana] gi|7270143|emb|CAB79956.1| putative protein [Arabidopsis thaliana] gi|332660651|gb|AEE86051.1| Nucleotide-sugar transporter family protein [Arabidopsis thaliana]	350	350	0	100.0	95.4	98.0	Nucleotide-sugar transporter family protein	gbpln	Arabidopsis thaliana	AT4G32390.1 | Symbols: | Nucleotide-sugar transporter family protein | chr4:15636550-15637602 FORWARD LENGTH=350	350	350	0	100.0	95.4	98.0

Rsa1.0_00017.1.g1152.t1	ref|XP_002869275.1| mitochondrial substrate carrier family protein [Arabidopsis lyrata subsp. lyrata] gi|297315111|gb|EFH45534.1| mitochondrial substrate carrier family protein [Arabidopsis lyrata subsp. lyrata]	396	391	1.00E−180	98.7	85.6	89.9	mitochondrial substrate carrier family protein	gbpln	Arabidopsis lyrata	AT4G32400.1 | Symbols: EMB104, SHS1, EMB42, ATBT1 | Mitochondrial substrate carrier family protein | chr4:15638686−15640238 FORWARD LENGTH=392	396	392	0	99.0	84.3	88.4
Rsa1.0_00017.1.g1153.t1	gb|ACS68190.1| cellulose synthase 1.2 catalytic subunit [Brassica napus]	1079	1083	0	100.4	97.3	99.1	cellulose synthase 1.2 catalytic subunit	gbpln	Brassica napus	AT4G32410.1 | Symbols: CESA1, RSW1, AtCESA1 | cellulose synthase 1 | chr4:15641009−15646388 REVERSE LENGTH=1081	1079	1081	0	100.2	94.5	97.6
Rsa1.0_00017.1.g1154.t10	ref|XP_002867244.1| hypothetical protein ARALYDRAFT_491484 [Arabidopsis lyrata subsp. lyrata] gi|297313080|gb|EFH43503.1| hypothetical protein ARALYDRAFT_491484 [Arabidopsis lyrata subsp. lyrata]	860	845	1.00E−160	98.3	55.8	62.2	hypothetical protein ARALYDRAFT_491484	gbpln	Arabidopsis lyrata	AT4G32420.3 | Symbols: | Cyclophilin−like peptidyl−prolyl cis−trans isomerase family protein | chr4:15647550−15652103 REVERSE LENGTH=837	860	837	1.00E−128	97.3	38.1	43.7
Rsa1.0_00017.1.g1155.t1	ref|NP_194969.2| pentatricopeptide repeat−containing protein [Arabidopsis thaliana] gi|75147159|sp|Q84MA3.1|PP345_ARATH RecName: Full=Pentatricopeptide repeat−containing protein At4g32430, mitochondrial; Flags: Precursor gi|30102674|gb|AAP21255.1| At4g32430 [Arabidopsis thaliana] gi|110742925|dbj|BAE99358.1| hypothetical protein [Arabidopsis thaliana] gi|332660658|gb|AEE86058.1| pentatricopeptide repeat−containing protein [Arabidopsis thaliana]	769	763	0	99.2	79.7	88.6	pentatricopeptide repeat−containing protein	gbpln	Arabidopsis thaliana	AT4G32430.1 | Symbols: | Pentatricopeptide repeat (PPR) superfamily protein | chr4:15652982−15655273 FORWARD LENGTH=763	769	763	0	99.2	79.7	88.6
Rsa1.0_00017.1.g1156.t1	#	#	#	#	#	#	#	−	−−−−	−−−−	#	#	#	#	#	#	#
Rsa1.0_00017.1.g1157.t1	gb|EOA17836.1| hypothetical protein CARUB_v10006237mg [Capsella rubella]	430	390	1.00E−151	90.7	65.1	68.6	hypothetical protein CARUB_v10006237mg	gbpln	Capsella rubella	AT4G32440.2 | Symbols: | Plant Tudor−like RNA−binding protein | chr4:15657295−15659354 FORWARD LENGTH=393	430	393	1.00E−152	91.4	66.5	72.3
Rsa1.0_00017.1.g1158.t1	dbj|BAD43544.1| putative protein [Arabidopsis thaliana]	557	537	0	96.4	68.9	79.5	putative protein	gbpln	Arabidopsis thaliana	AT4G32450.1 | Symbols: | Pentatricopeptide repeat (PPR) superfamily protein | chr4:15661092−15662705 FORWARD LENGTH=537	557	537	0	96.4	68.9	79.5
Rsa1.0_00017.1.g1159.t1	ref|NP_567894.1| uncharacterized protein [Arabidopsis thaliana] gi|42573129|ref|NP_974661.1| uncharacterized protein [Arabidopsis thaliana] gi|21593257|gb|AAM65206.1| unknown [Arabidopsis thaliana] gi|94442427|gb|ABF19001.1| At4g32460 [Arabidopsis thaliana] gi|332660663|gb|AEE86063.1| uncharacterized protein AT4G32460 [Arabidopsis thaliana] gi|332660664|gb|AEE86064.1| uncharacterized protein AT4G32460 [Arabidopsis thaliana]	365	365	0	100.0	92.6	96.7	uncharacterized protein	gbpln	Arabidopsis thaliana	AT4G32460.2 | Symbols: | Protein of unknown function, DUF642 | chr4:15663036−15664859 REVERSE LENGTH=365	365	365	0	100.0	92.6	96.7
Rsa1.0_00017.1.g1160.t1	gb|EOA17665.1| hypothetical protein CARUB_v10006035mg [Capsella rubella]	122	122	1.00E−57	100.0	89.3	95.9	hypothetical protein CARUB_v10006035mg	gbpln	Capsella rubella	AT4G32470.1 | Symbols: | Cytochrome bd ubiquinol oxidase, 14kDa subunit | chr4:15669641−15671095 REVERSE LENGTH=122	122	122	2.00E−59	100.0	87.7	95.9
Rsa1.0_00017.1.g1161.t1	gb|EOA18796.1| hypothetical protein CARUB_v10007410mg [Capsella rubella]	872	880	0	100.9	88.4	93.1	hypothetical protein CARUB_v10007410mg	gbpln	Capsella rubella	AT4G32500.1 | Symbols: AKT5, KT5 | K+ transporter 5 | chr4:15681122−15685214 FORWARD LENGTH=880	872	880	0	100.9	88.5	92.9
Rsa1.0_00017.1.g1162.t1	gb|EOA16174.1| hypothetical protein CARUB_v10004311mg, partial [Capsella rubella]	674	666	0	98.8	90.2	93.3	hypothetical protein CARUB_v10004311mg, partial	gbpln	Capsella rubella	AT4G32510.1 | Symbols: | HCO3− transporter family | chr4:15685903−15688811 REVERSE LENGTH=673	674	673	0	99.9	90.2	93.9
Rsa1.0_00017.1.g1163.t1	gb|EOA16417.1| hypothetical protein CARUB_v10004571mg [Capsella rubella]	525	529	0	100.8	90.1	94.9	hypothetical protein CARUB_v10004571mg	gbpln	Capsella rubella	AT4G32520.2 | Symbols: SHM3 | serine hydroxymethyltransferase 3 | chr4:15689642−15692334 REVERSE LENGTH=529	525	529	0	100.8	88.6	93.3
Rsa1.0_00017.1.g1164.t1	ref|XP_002880688.1| H+−transporting two−sector ATPase [Arabidopsis lyrata subsp. lyrata] gi|297326527|gb|EFH56947.1| H+−transporting two−sector ATPase [Arabidopsis lyrata subsp. lyrata]	177	179	8.00E−89	101.1	98.9	99.4	H+−transporting two−sector ATPase	gbpln	Arabidopsis lyrata	AT2G25610.1 | Symbols: | ATPase, F0/V0 complex, subunit C protein | chr2:10901585−10902494 REVERSE LENGTH=178	177	178	3.00E−91	100.6	98.3	99.4
Rsa1.0_00017.1.g1165.t1	ref|NP_567826.1| HXXXD−type acyl−transferase−like protein [Arabidopsis thaliana] gi|332660207|gb|AEE85607.1| HXXXD−type acyl−transferase−like protein [Arabidopsis thaliana]	458	460	0	100.4	82.3	88.6	HXXXD−type acyl−transferase−like protein	gbpln	Arabidopsis thaliana	AT4G29250.1 | Symbols: | HXXXD−type acyl−transferase family protein | chr4:14420713−14422177 FORWARD LENGTH=460	458	460	0	100.4	82.3	88.6

Rsa1.0_00017.1.g1166.t1	ref|NP_189279.1| transducin/WD40 domain-containing protein [Arabidopsis thaliana] gi|9294307|dbj|BAB02209.1| unnamed protein product [Arabidopsis thaliana] gi|332643644|gb|AEE77165.1| transducin/WD40 domain-containing protein [Arabidopsis thaliana]	760	764	0	100.5	70.7	81.3	transducin/WD40 domain-containing protein	gbpln	Arabidopsis thaliana	AT3G26480.1 | Symbols: | Transducin family protein / WD-40 repeat family protein | chr3:9687273-9690827 REVERSE LENGTH=764	760	764	0	100.5	70.7	81.3
Rsa1.0_00017.1.g1167.t1	gb|AAC28221.1| similar to reverse transcriptases (PFam: rvt.hmm, score: 60.13) [Arabidopsis thaliana]	221	1164	4.00E-43	526.7	42.1	57.9	similar to reverse transcriptases (PFam: rvt.hmm, score: 60.13)	gbpln	Arabidopsis thaliana	AT3G25720.1 | Symbols: | RNA-directed DNA polymerase (reverse transcriptase)-related family protein | chr3:9380234-9381405 FORWARD LENGTH=282	221	282	4.00E-31	127.6	32.1	38.0
Rsa1.0_00017.1.g1168.t1	ref|XP_002869265.1| hypothetical protein ARALYDRAFT_353573 [Arabidopsis lyrata subsp. lyrata] gi|297315101|gb|EFH45524.1| hypothetical protein ARALYDRAFT_353573 [Arabidopsis lyrata subsp. lyrata]	424	414	0	97.6	84.9	89.9	hypothetical protein ARALYDRAFT_353573	gbpln	Arabidopsis lyrata	AT4G32540.1 | Symbols: YUC, YUC1 | Flavin-binding monooxygenase family protein | chr4:15700904-15702870 FORWARD LENGTH=414	424	414	0	97.6	83.5	89.4
Rsa1.0_00017.1.g1169.t1	gb|AAG50806.1|AC079281_8 unknown protein [Arabidopsis thaliana]	1855	1213	0	65.4	23.9	34.4	unknown protein	gbpln	Arabidopsis thaliana	AT1G43760.1 | Symbols: | DNAse I-like superfamily protein | chr1:16528880-16531065 REVERSE LENGTH=626	1855	626	5.00E-70	33.7	7.8	12.0
Rsa1.0_00017.1.g1170.t1	ref|XP_002869264.1| hypothetical protein ARALYDRAFT_491464 [Arabidopsis lyrata subsp. lyrata] gi|297315100|gb|EFH45523.1| hypothetical protein ARALYDRAFT_491464 [Arabidopsis lyrata subsp. lyrata]	941	920	0	97.8	69.5	73.5	hypothetical protein ARALYDRAFT_491464	gbpln	Arabidopsis lyrata	AT4G32551.1 | Symbols: LUG, RON2 | LisH dimerisation motif;WD40/YVTN repeat-like-containing domain | chr4:15707863-15713359 FORWARD LENGTH=931	941	931	0	98.9	69.1	73.5
Rsa1.0_00017.1.g1171.t20	ref|NP_001031771.1| paramyosin-related protein [Arabidopsis thaliana] gi|227202856|dbj|BAH56901.1| AT4G32560 [Arabidopsis thaliana] gi|332660680|gb|AEE86080.1| paramyosin-related protein [Arabidopsis thaliana]	539	304	1.00E-103	56.4	40.8	43.4	paramyosin-related protein	gbpln	Arabidopsis thaliana	AT4G32560.3 | Symbols: | paramyosin-related | chr4:15713866-15716035 FORWARD LENGTH=304	539	304	1.00E-106	56.4	40.8	43.4
Rsa1.0_00017.1.g1172.t1	ref|NP_567898.1| protein TIFY 8 [Arabidopsis thaliana] gi|75147164|sp|Q84MB2.1|TIF8_ARATH RecName: Full=Protein TIFY 8 gi|30102642|gb|AAP21239.1| At4g32570 [Arabidopsis thaliana] gi|332660681|gb|AEE86081.1| protein TIFY 8 [Arabidopsis thaliana]	360	361	1.00E-129	100.3	75.3	81.7	protein TIFY 8	gbpln	Arabidopsis thaliana	AT4G32570.1 | Symbols: TIFY8 | TIFY domain protein 8 | chr4:15716433-15718702 REVERSE LENGTH=361	360	361	1.00E-132	100.3	75.3	81.7
Rsa1.0_00017.1.g1173.t1	gb|AAG50751.1|AC079733_19 polyprotein, putative [Arabidopsis thaliana]	1447	1468	0	101.5	50.9	67.6	polyprotein, putative	gbpln	Arabidopsis thaliana	AT4G23160.1 | Symbols: CRK8 | cysteine-rich RLK (RECEPTOR-like protein kinase) 8 | chr4:12129485-12134086 FORWARD LENGTH=1262	1447	1262	1.00E-139	87.2	17.6	25.2
Rsa1.0_00017.1.g1174.t1	ref|XP_002869262.1| hypothetical protein ARALYDRAFT_913185 [Arabidopsis lyrata subsp. lyrata] gi|297315098|gb|EFH45521.1| hypothetical protein ARALYDRAFT_913185 [Arabidopsis lyrata subsp. lyrata]	155	175	3.00E-59	112.9	84.5	91.0	hypothetical protein ARALYDRAFT_913185	gbpln	Arabidopsis lyrata	AT4G32590.1 | Symbols: | 2Fe-2S ferredoxin-like superfamily protein | chr4:15721417-15722776 FORWARD LENGTH=173	155	173	4.00E-60	111.6	82.6	89.7
Rsa1.0_00017.1.g1175.t2	ref|XP_002869261.1| hypothetical protein ARALYDRAFT_328473 [Arabidopsis lyrata subsp. lyrata] gi|297315097|gb|EFH45520.1| hypothetical protein ARALYDRAFT_328473 [Arabidopsis lyrata subsp. lyrata]	464	489	0	105.4	82.8	88.6	hypothetical protein ARALYDRAFT_328473	gbpln	Arabidopsis lyrata	AT4G32600.1 | Symbols: | RING/U-box superfamily protein | chr4:15724010-15725737 FORWARD LENGTH=453	464	453	0	97.6	81.5	86.9
Rsa1.0_00017.1.g1176.t1	emb|CAA18600.1| putative protein [Arabidopsis thaliana] gi|7270165|emb|CAB79978.1| putative protein [Arabidopsis thaliana]	167	557	2.00E-71	333.5	88.0	94.0	putative protein	gbpln	Arabidopsis thaliana	AT4G32605.1 | Symbols: | Mitochondrial glycoprotein family protein | chr4:15726429-15727864 REVERSE LENGTH=227	167	227	3.00E-73	135.9	88.0	94.0
Rsa1.0_00017.1.g1177.t1	dbj|BAA97156.1| unnamed protein product [Arabidopsis thaliana]	156	329	1.00E-17	210.9	37.2	52.6	unnamed protein product	gbpln	Arabidopsis thaliana	#	#	#	#	#	#	#
Rsa1.0_00017.1.g1178.t1	ref|NP_194987.2| copper ion binding protein [Arabidopsis thaliana] gi|110743171|dbj|BAE99477.1| hypothetical protein [Arabidopsis thaliana] gi|332660689|gb|AEE86089.1| copper ion binding protein [Arabidopsis thaliana]	318	315	1.00E-65	99.1	74.5	82.4	copper ion binding protein	gbpln	Arabidopsis thaliana	AT4G32610.1 | Symbols: | copper ion binding | chr4:15728376-15729897 REVERSE LENGTH=315	318	315	3.00E-68	99.1	74.5	82.4
Rsa1.0_00017.1.g1179.t1	ref|XP_002869260.1| nucleic acid binding protein [Arabidopsis lyrata subsp. lyrata] gi|297315096|gb|EFH45519.1| nucleic acid binding protein [Arabidopsis lyrata subsp. lyrata]	1409	1550	0	110.0	42.2	46.4	nucleic acid binding protein	gbpln	Arabidopsis lyrata	AT4G32620.2 | Symbols: | Enhancer of polycomb-like transcription factor protein | chr4:15731968-15737222 FORWARD LENGTH=1540	1409	1540	0	109.3	41.4	45.7

Rsa1.0_00017.1.g1180.t1	ref|XP_002869259.1| hypothetical protein ARALYDRAFT_491451 [Arabidopsis lyrata subsp. lyrata] gi|297315095|gb|EFH45518.1| hypothetical protein ARALYDRAFT_491451 [Arabidopsis lyrata subsp. lyrata]	630	631	0	100.2	70.0	78.3	hypothetical protein ARALYDRAFT_491451	gbpln	Arabidopsis lyrata	AT4G32630.2 | Symbols: | ArfGap/RecO-like zinc finger domain-containing protein | chr4:15738315-15741412 FORWARD LENGTH=628	630	628	0	99.7	67.8	77.3
Rsa1.0_00017.1.g1181.t1	dbj|BAM76809.1| Sec24-like transport protein [Arabidopsis thaliana]	1083	1092	0	100.8	89.2	93.3	Sec24-like transport protein	gbpln	Arabidopsis thaliana	AT4G32640.2 | Symbols: | Sec23/Sec24 protein transport family protein | chr4:15742661-15750424 FORWARD LENGTH=1080	1083	1080	0	99.7	88.1	92.1
Rsa1.0_00017.1.g1182.t1	ref|NP_194991.1| Potassium channel KAT3 [Arabidopsis thaliana] gi|44888078|sp|P92960.1|KAT3_ARATH RecName: Full=Potassium channel KAT3; AltName: Full=AKT4; AltName: Full=AtKC1; AltName: Full=Potassium channel TKC gi|1708777|emb|CAB05669.1| potassium channel [Arabidopsis thaliana] gi|3063705|emb|CAA18596.1| potassium channel protein [Arabidopsis thaliana] gi|4090537|gb|AAC98810.1| K+ inward rectifying channel protein [Arabidopsis thaliana] gi|4098949|gb|AAD00503.1| K+ inward rectifying channel [Arabidopsis thaliana] gi|7270169|emb|CAB79982.1| potassium channel protein AtKC [Arabidopsis thaliana] gi|15529218|gb|AAK97703.1| AT4g32650/F4D11_150 [Arabidopsis thaliana] gi|27777708|gb|AAO23890.1| At4g32650/F4D11_150 [Arabidopsis thaliana] gi|332660697|gb|AEE86097.1| Potassium channel KAT3 [Arabidopsis thaliana]	723	662	0	91.6	74.7	82.2	Potassium channel KAT3	gbpln	Arabidopsis thaliana	AT4G32650.1 | Symbols: ATKC1, KAT3, KC1, AtLKT1 | potassium channel in Arabidopsis thaliana 3 | chr4:15751482-15754797 REVERSE LENGTH=662	723	662	0	91.6	74.7	82.2
Rsa1.0_00017.1.g1183.t1	ref|NP_194992.1| serine/threonine-protein kinase AFC3 [Arabidopsis thaliana] gi|5915680|sp|P51568.2|AFC3_ARATH RecName: Full=Serine/threonine-protein kinase AFC3 gi|642134|dbj|BAA08216.1| protein kinase [Arabidopsis thaliana] gi|3063704|emb|CAA18595.1| protein kinase AME3 [Arabidopsis thaliana] gi|7270170|emb|CAB79983.1| protein kinase AME3 [Arabidopsis thaliana] gi|119935963|gb|ABM06046.1| At4g32660 [Arabidopsis thaliana] gi|332660700|gb|AEE86100.1| serine/threonine-protein kinase AFC3 [Arabidopsis thaliana]	398	400	0	100.5	91.5	96.0	serine/threonine-protein kinase AFC3	gbpln	Arabidopsis thaliana	AT4G32660.1 | Symbols: AME3 | Protein kinase superfamily protein | chr4:15756396-15759107 FORWARD LENGTH=400	398	400	0	100.5	91.5	96.0
Rsa1.0_00017.1.g1184.t1	ref|XP_002867231.1| hypothetical protein ARALYDRAFT_491441 [Arabidopsis lyrata subsp. lyrata] gi|297313067|gb|EFH43490.1| hypothetical protein ARALYDRAFT_491441 [Arabidopsis lyrata subsp. lyrata]	741	861	1.00E-152	116.2	36.8	44.5	hypothetical protein ARALYDRAFT_491441	gbpln	Arabidopsis lyrata	AT4G32670.1 | Symbols: | RING/FYVE/PHD zinc finger superfamily protein | chr4:15759527-15762847 REVERSE LENGTH=860	741	860	1.00E-152	116.1	36.6	44.5
Rsa1.0_00017.1.g1185.t1	ref|NP_567900.1| uncharacterized protein [Arabidopsis thaliana] gi|332660703|gb|AEE86103.1| uncharacterized protein AT4G32680 [Arabidopsis thaliana]	280	282	1.00E-103	100.7	75.4	83.9	uncharacterized protein	gbpln	Arabidopsis thaliana	AT4G32680.1 | Symbols: | unknown protein; BEST Arabidopsis thaliana protein match is: unknown protein (TAIR:AT1G52343.1); Has 30201 Blast hits to 17322 proteins in 780 species: Archae - 12; Bacteria - 1396; Metazoa - 17338; Fungi - 3422; Plants - 5037; Viruses - 0; Other Eukaryotes - 2996 (source: NCBI BLink). | chr4:15763462-15765039 REVERSE LENGTH=282	280	282	1.00E-105	100.7	75.4	83.9
Rsa1.0_00017.1.g1186.t1	ref|XP_002869254.1| predicted protein [Arabidopsis lyrata subsp. lyrata] gi|297315090|gb|EFH45513.1| predicted protein [Arabidopsis lyrata subsp. lyrata]	2151	2147	0	99.8	86.5	90.9	predicted protein	gbpln	Arabidopsis lyrata	AT4G32700.2 | Symbols: | helicases;ATP-dependent helicases;nucleic acid binding;ATP binding;DNA-directed DNA polymerases;DNA binding | chr4:15767440-15779185 FORWARD LENGTH=2154	2151	2154	0	100.1	85.7	90.6

Rsa1.0_00017.1.g1187.t1	sp|O65530.1|PEK14_ARATH RecName: Full=Proline-rich receptor-like protein kinase PERK14; AltName: Full=Proline-rich extensin-like receptor kinase 14; Short=AtPERK14 gi|3063699|emb|CAA18590.1| putative protein [Arabidopsis thaliana] gi|7270175|emb|CAB79988.1| putative protein kinase [Arabidopsis thaliana]	705	731	0	103.7	72.5	77.7	RecName: Full=Proline-rich receptor-like protein kinase PERK14; AltName: Full=Proline-rich extensin-like receptor kinase 14; Short=AtPERK14 gi|3063699|emb|CAA18590.1| putative protein	gbpln	Arabidopsis thaliana	AT4G32710.1 | Symbols: | Protein kinase superfamily protein | chr4:15781362-15783242 FORWARD LENGTH=388	705	388	0	55.0	45.7	49.1
Rsa1.0_00017.1.g1188.t1	#	#	#	#	#	#	#	–	----	----	#	#	#	#	#	#	#
Rsa1.0_00017.1.g1189.t1	ref|XP_002869250.1| RNA recognition motif-containing protein [Arabidopsis lyrata subsp. lyrata] gi|297315086|gb|EFH45509.1| RNA recognition motif-containing protein [Arabidopsis lyrata subsp. lyrata]	435	433	0	99.5	87.1	92.4	RNA recognition motif-containing protein	gbpln	Arabidopsis lyrata	AT4G32720.1 | Symbols: AtLa1, La1 | La protein 1 | chr4:15787313-15789683 FORWARD LENGTH=433	435	433	0	99.5	86.7	91.7
Rsa1.0_00017.1.g1190.t1	ref|NP_974718.1| myb-related protein 3R-1 [Arabidopsis thaliana] gi|332660712|gb|AEE86112.1| myb-related protein 3R-1 [Arabidopsis thaliana]	874	995	0	113.8	79.5	85.7	myb-related protein 3R-1	gbpln	Arabidopsis thaliana	AT4G32730.2 | Symbols: PC-MYB1, MYB3R-1, ATMYB3R-1, ATMYB3R1 | Homeodomain-like protein | chr4:15791039-15795643 FORWARD LENGTH=995	874	995	0	113.8	79.5	85.7
Rsa1.0_00017.1.g1191.t1	gb|EOA17128.1| hypothetical protein CARUB_v10005389mg [Capsella rubella]	284	292	1.00E-132	102.8	83.5	90.1	hypothetical protein CARUB_v10005389mg	gbpln	Capsella rubella	AT4G32750.1 | Symbols: | unknown protein; Has 30201 Blast hits to 17322 proteins in 780 species: Archae - 12; Bacteria - 1396; Metazoa - 17338; Fungi - 3422; Plants - 5037; Viruses - 0; Other Eukaryotes - 2996 (source: NCBI BLink). | chr4:15796415-15798091 REVERSE LENGTH=293	284	293	1.00E-133	103.2	83.5	91.2
Rsa1.0_00017.1.g1192.t1	ref|NP_001190892.1| ENTH/VHS/GAT family protein [Arabidopsis thaliana] gi|332660715|gb|AEE86115.1| ENTH/VHS/GAT family protein [Arabidopsis thaliana]	659	676	0	102.6	86.0	90.1	ENTH/VHS/GAT family protein	gbpln	Arabidopsis thaliana	AT4G32760.2 | Symbols: | ENTH/VHS/GAT family protein | chr4:15799376-15803832 FORWARD LENGTH=676	659	676	0	102.6	86.0	90.1
Rsa1.0_00017.1.g1193.t1	gb|EOA16698.1| hypothetical protein CARUB_v10004890mg [Capsella rubella]	550	424	0	77.1	66.2	70.7	hypothetical protein CARUB_v10004890mg	gbpln	Capsella rubella	AT4G32780.1 | Symbols: | phosphoinositide binding | chr4:15808926-15810907 FORWARD LENGTH=270	550	270	1.00E-101	49.1	39.8	42.5
Rsa1.0_00017.1.g1194.t1	ref|NP_195006.1| ethylene-responsive transcription factor ERF043 [Arabidopsis thaliana] gi|75264505|sp|Q9M080.1|ERF43_ARATH RecName: Full=Ethylene-responsive transcription factor ERF043 gi|7270227|emb|CAB79997.1| transcription factor TINY homolog [Arabidopsis thaliana] gi|21618174|gb|AAM67224.1| transcription factor TINY homolog [Arabidopsis thaliana] gi|27754586|gb|AAO22740.1| putative transcription factor TINY [Arabidopsis thaliana] gi|28393859|gb|AAO42337.1| putative transcription factor TINY [Arabidopsis thaliana] gi|332660720|gb|AEE86120.1| ethylene-responsive transcription factor ERF043 [Arabidopsis thaliana]	231	221	9.00E-73	95.7	70.6	79.7	ethylene-responsive transcription factor ERF043	gbpln	Arabidopsis thaliana	AT4G32800.1 | Symbols: | Integrase-type DNA-binding superfamily protein | chr4:15819812-15820477 FORWARD LENGTH=221	231	221	3.00E-75	95.7	70.6	79.7
Rsa1.0_00017.1.g1195.t1	ref|NP_195032.2| peptidyl-prolyl cis-trans isomerase SDCCAG10 [Arabidopsis thaliana] gi|45680878|gb|AAS75308.1| multidomain cyclophilin type peptidyl-prolyl cis-trans isomerase [Arabidopsis thaliana] gi|95147300|gb|ABF57285.1| At4g33060 [Arabidopsis thaliana] gi|332660768|gb|AEE86168.1| peptidyl-prolyl cis-trans isomerase CYP57 [Arabidopsis thaliana]	124	504	7.00E-31	406.5	75.8	83.9	peptidyl-prolyl cis-trans isomerase SDCCAG10	gbpln	Arabidopsis thaliana	AT4G33060.1 | Symbols: | Cyclophilin-like peptidyl-prolyl cis-trans isomerase family protein | chr4:15948535-15951954 FORWARD LENGTH=504	124	504	1.00E-33	406.5	75.8	83.9
Rsa1.0_00017.1.g1196.t1	ref|XP_002869241.1| hypothetical protein ARALYDRAFT_353547 [Arabidopsis lyrata subsp. lyrata] gi|297315077|gb|EFH45500.1| hypothetical protein ARALYDRAFT_353547 [Arabidopsis lyrata subsp. lyrata]	557	570	0	102.3	89.8	94.3	hypothetical protein ARALYDRAFT_353547	gbpln	Arabidopsis lyrata	AT4G32810.1 | Symbols: CCD8, MAX4, ATCCD8 | carotenoid cleavage dioxygenase 8 | chr4:15828348-15831299 FORWARD LENGTH=570	557	570	0	102.3	88.5	93.9

Rsa1.0_00017.1.g1197.t1	ref|NP_195008.6| tetratricopeptide repeat domain-containing protein [Arabidopsis thaliana] gi|332660722|gb|AEE86122.1| tetratricopeptide repeat domain-containing protein [Arabidopsis thaliana]	1868	1863	0	99.7	86.3	91.5	tetratricopeptide repeat domain-containing protein	gbpln	Arabidopsis thaliana	AT4G32820.1 | Symbols: | Tetratricopeptide repeat (TPR)-like superfamily protein | chr4:15831679-15841454 REVERSE LENGTH=1863	1868	1863	0	99.7	86.3	91.5
Rsa1.0_00017.1.g1198.t1	ref|XP_002869240.1| hypothetical protein ARALYDRAFT_913154 [Arabidopsis lyrata subsp. lyrata] gi|297315076|gb|EFH45499.1| hypothetical protein ARALYDRAFT_913154 [Arabidopsis lyrata subsp. lyrata]	295	290	1.00E-161	98.3	94.6	96.3	hypothetical protein ARALYDRAFT_913154	gbpln	Arabidopsis lyrata	AT4G32830.1 | Symbols: AtAUR1, AUR1 | ataurora1 | chr4:15842557-15844354 FORWARD LENGTH=294	295	294	1.00E-161	99.7	93.6	96.9
Rsa1.0_00017.1.g1199.t1	ref|NP_195010.1| 6-phosphofructokinase 6 [Arabidopsis thaliana] gi|75181422|sp|Q9M076.1|K6PF6_ARATH RecName: Full=6-phosphofructokinase 6; AltName: Full=Phosphofructokinase 6; AltName: Full=Phosphohexokinase 6 gi|7270231|emb|CAB80001.1| putative pyrophosphate--fructose-6-phosphate 1-phosphotransferase [Arabidopsis thaliana] gi|29029114|gb|AAO64936.1| At4g32840 [Arabidopsis thaliana] gi|110743163|dbj|BAE99473.1| putative pyrophosphate--fructose-6-phosphate 1-phosphotransferase [Arabidopsis thaliana] gi|332660725|gb|AEE86125.1| 6-phosphofructokinase 6 [Arabidopsis thaliana]	464	462	0	99.6	92.9	96.1	6-phosphofructokinase 6	gbpln	Arabidopsis thaliana	AT4G32840.1 | Symbols: PFK6 | phosphofructokinase 6 | chr4:15845010-15848305 REVERSE LENGTH=462	464	462	0	99.6	92.9	96.1
Rsa1.0_00017.1.g1200.t1	gb|EOA16057.1| hypothetical protein CARUB_v10004188mg [Capsella rubella]	779	780	0	100.1	85.1	89.3	hypothetical protein CARUB_v10004188mg	gbpln	Capsella rubella	AT4G32850.8 | Symbols: nPAP | nuclear poly(a) polymerase | chr4:15850134-15854443 FORWARD LENGTH=765	779	765	0	98.2	84.6	88.6
Rsa1.0_00017.1.g1201.t1	emb|CAB80003.1| putative protein [Arabidopsis thaliana]	188	193	1.00E-84	102.7	84.0	90.4	putative protein	gbpln	Arabidopsis thaliana	AT4G32860.1 | Symbols: | unknown protein; Has 46 Blast hits to 46 proteins in 10 species: Archae - 0; Bacteria - 0; Metazoa - 0; Fungi - 0; Plants - 46; Viruses - 0; Other Eukaryotes - 0 (source: NCBI BLink). | chr4:15856710-15857309 REVERSE LENGTH=199	188	199	6.00E-87	105.9	84.0	90.4
Rsa1.0_00017.1.g1202.t1	ref|XP_002869238.1| hypothetical protein ARALYDRAFT_491409 [Arabidopsis lyrata subsp. lyrata] gi|297315074|gb|EFH45497.1| hypothetical protein ARALYDRAFT_491409 [Arabidopsis lyrata subsp. lyrata]	164	157	3.00E-67	95.7	75.6	86.0	hypothetical protein ARALYDRAFT_491409	gbpln	Arabidopsis lyrata	AT4G32870.1 | Symbols: | Polyketide cyclase/dehydrase and lipid transport superfamily protein | chr4:15862168-15862641 FORWARD LENGTH=157	164	157	6.00E-67	95.7	72.6	84.8
Rsa1.0_00017.1.g1203.t1	ref|NP_195014.1| homeobox-leucine zipper protein ATHB-8 [Arabidopsis thaliana] gi|75220729|sp|Q39123.1|ATHB8_ARATH RecName: Full=Homeobox-leucine zipper protein ATHB-8; AltName: Full=HD-ZIP protein ATHB-8; AltName: Full=Homeodomain transcription factor ATHB-8 gi|1149569|emb|CAA90703.1| HD-zip [Arabidopsis thaliana] gi|7270235|emb|CAB80005.1| HD-zip transcription factor (athb-8) [Arabidopsis thaliana] gi|20152536|emb|CAD29660.1| homeodomain-leucine zipper protein 8 [Arabidopsis thaliana] gi|20466330|gb|AAM20482.1| HD-zip transcription factor (athb-8) [Arabidopsis thaliana] gi|31711762|gb|AAP68237.1| At4g32880 [Arabidopsis thaliana] gi|332660738|gb|AEE86138.1| homeobox-leucine zipper protein ATHB-8 [Arabidopsis thaliana]	833	833	0	100.0	97.0	99.2	homeobox-leucine zipper protein ATHB-8	gbpln	Arabidopsis thaliana	AT4G32880.1 | Symbols: ATHB-8, ATHB8, HB-8 | homeobox gene 8 | chr4:15863587-15867822 REVERSE LENGTH=833	833	833	0	100.0	97.0	99.2

Rsa1.0_00017.1.g1204.t1	ref|NP_195015.1| GATA transcription factor 9 [Arabidopsis thaliana] gi|71159362|sp|O82632.1|GATA9_ARATH RecName: Full=GATA transcription factor 9 gi|3688170|emb|CAA21198.1| putative protein [Arabidopsis thaliana] gi|7270236|emb|CAB80006.1| putative protein [Arabidopsis thaliana] gi|26449440|dbj|BAC41847.1| unknown protein [Arabidopsis thaliana] gi|30725358|gb|AAP37701.1| At4g32890 [Arabidopsis thaliana] gi|332660739|gb|AEE86139.1| GATA transcription factor 9 [Arabidopsis thaliana]	320	308	1.00E-128	96.3	75.0	84.7	GATA transcription factor 9	gbpln	Arabidopsis thaliana	AT4G32890.1 | Symbols: GATA9 | GATA transcription factor 9 | chr4:15875598-15876615 FORWARD LENGTH=308	320	308	1.00E-131	96.3	75.0	84.7
Rsa1.0_00017.1.g1205.t1	gb|EOA17472.1| hypothetical protein CARUB_v10005799mg [Capsella rubella]	193	193	1.00E-91	100.0	85.0	92.7	hypothetical protein CARUB_v10005799mg	gbpln	Capsella rubella	AT4G32900.2 | Symbols: | Peptidyl-tRNA hydrolase II (PTH2) family protein | chr4:15878944-15880979 REVERSE LENGTH=193	193	193	3.00E-92	100.0	84.5	91.2
Rsa1.0_00017.1.g1206.t1	ref|NP_567908.1| uncharacterized protein [Arabidopsis thaliana] gi|75158853|sp|Q8RXH2.1|NUP85_ARATH RecName: Full=Nuclear pore complex protein Nup85; AltName: Full=Nucleoporin Nup85 gi|19698819|gb|AAL91145.1| putative protein [Arabidopsis thaliana] gi|332660742|gb|AEE86142.1| uncharacterized protein AT4G32910 [Arabidopsis thaliana]	814	716	0	88.0	79.1	83.8	uncharacterized protein	gbpln	Arabidopsis thaliana	AT4G32910.1 | Symbols: | CONTAINS InterPro DOMAIN/s: Nuclear pore complex protein, Nucleoporin Nup85-like (InterPro:IPR011502); Has 30201 Blast hits to 17322 proteins in 780 species: Archae - 12; Bacteria - 1396; Metazoa - 17338; Fungi - 3422; Plants - 5037; Viruses - 0; Other Eukaryotes - 2996 (source: NCBI BLink). | chr4:15881364-15885210 FORWARD LENGTH=716	814	716	0	88.0	79.1	83.8
Rsa1.0_00017.1.g1207.t1	ref|XP_002867220.1| glycine-rich protein [Arabidopsis lyrata subsp. lyrata] gi|297313056|gb|EFH43479.1| glycine-rich protein [Arabidopsis lyrata subsp. lyrata]	1435	1424	0	99.2	86.8	92.9	glycine-rich protein	gbpln	Arabidopsis lyrata	AT4G32920.3 | Symbols: | glycine-rich protein | chr4:15888153-15896006 REVERSE LENGTH=1432	1435	1432	0	99.8	86.8	92.8
Rsa1.0_00017.1.g1208.t2	emb|CAA21202.1| putative protein [Arabidopsis thaliana] gi|7270240|emb|CAB80010.1| putative protein [Arabidopsis thaliana]	195	181	8.00E-87	92.8	82.6	86.2	putative protein	gbpln	Arabidopsis thaliana	AT4G32930.1 | Symbols: | unknown protein; CONTAINS InterPro DOMAIN/s: Protein of unknown function DUF866, eukaryotic (InterPro:IPR008584); Has 30201 Blast hits to 17322 proteins in 780 species: Archae - 12; Bacteria - 1396; Metazoa - 17338; Fungi - 3422; Plants - 5037; Viruses - 0; Other Eukaryotes - 2996 (source: NCBI BLink). | chr4:15898878-15900185 FORWARD LENGTH=167	195	167	2.00E-87	85.6	82.1	84.1
Rsa1.0_00017.1.g1209.t1	dbj|BAJ33795.1| unnamed protein product [Thellungiella halophila]	489	478	0	97.8	86.1	91.2	unnamed protein product	----	----	AT4G32940.1 | Symbols: GAMMA-VPE, GAMMAVPE | gamma vacuolar processing enzyme | chr4:15900557-15903161 REVERSE LENGTH=494	489	494	0	101.0	85.5	89.0
Rsa1.0_00017.1.g1210.t1	#	#	#	#	#	#	#	-	----	----	#	#	#	#	#	#	#
Rsa1.0_00017.1.g1211.t1	#	#	#	#	#	#	#	-	----	----	#	#	#	#	#	#	#
Rsa1.0_00017.1.g1212.t1	#	#	#	#	#	#	#	-	----	----	#	#	#	#	#	#	#
Rsa1.0_00017.1.g1213.t1	ref|XP_002867215.1| hypothetical protein ARALYDRAFT_491396 [Arabidopsis lyrata subsp. lyrata] gi|297313051|gb|EFH43474.1| hypothetical protein ARALYDRAFT_491396 [Arabidopsis lyrata subsp. lyrata]	455	472	0	103.7	80.0	86.2	hypothetical protein ARALYDRAFT_491396	gbpln	Arabidopsis lyrata	AT4G32980.1 | Symbols: ATH1 | homeobox gene 1 | chr4:15914865-15916873 REVERSE LENGTH=473	455	473	0	104.0	78.2	85.7
Rsa1.0_00017.1.g1214.t1	gb|EOA17824.1| hypothetical protein CARUB_v10006225mg, partial [Capsella rubella]	195	241	2.00E-95	123.6	88.2	91.8	hypothetical protein CARUB_v10006225mg, partial	gbpln	Capsella rubella	AT4G33000.2 | Symbols: CBL10, SCABP8, ATCBL10 | calcineurin B-like protein 10 | chr4:15924821-15926398 FORWARD LENGTH=246	195	246	7.00E-95	126.2	84.6	90.8
Rsa1.0_00017.1.g1215.t1	ref|XP_002867212.1| hypothetical protein ARALYDRAFT_491390 [Arabidopsis lyrata subsp. lyrata] gi|297313048|gb|EFH43471.1| hypothetical protein ARALYDRAFT_491390 [Arabidopsis lyrata subsp. lyrata]	1043	1037	0	99.4	90.4	94.9	hypothetical protein ARALYDRAFT_491390	gbpln	Arabidopsis lyrata	AT4G33010.1 | Symbols: AtGLDP1, GLDP1 | glycine decarboxylase P-protein 1 | chr4:15926852-15931150 REVERSE LENGTH=1037	1043	1037	0	99.4	90.3	94.5
Rsa1.0_00017.1.g1216.t1	gb|EOA16962.1| hypothetical protein CARUB_v10005191mg [Capsella rubella]	390	340	1.00E-163	87.2	78.7	81.5	hypothetical protein CARUB_v10005191mg	gbpln	Capsella rubella	AT4G33020.1 | Symbols: ZIP9, ATZIP9 | ZIP metal ion transporter family | chr4:15932603-15934267 REVERSE LENGTH=344	390	344	1.00E-163	88.2	77.4	81.3

Rsa1.0_00017.1.g1217.t1	ref|XP_002869229.1| hypothetical protein ARALYDRAFT_491388 [Arabidopsis lyrata subsp. lyrata] gi|297315065|gb|EFH45488.1| hypothetical protein ARALYDRAFT_491388 [Arabidopsis lyrata subsp. lyrata]	482	475	0	98.5	92.9	95.0	hypothetical protein ARALYDRAFT_491388	gbpln	Arabidopsis lyrata	AT4G33030.1 | Symbols: SQD1 | sulfoquinovosyldiacylglycerol 1 | chr4:15936051-15937566 FORWARD LENGTH=477	482	477	0	99.0	91.9	94.8
Rsa1.0_00017.1.g1218.t1	ref|NP_195030.1| glutaredoxin-C6 [Arabidopsis thaliana] gi|119370639|sp|Q8L9S3.2|GRXC6_ARATH RecName: Full=Glutaredoxin-C6; Short=AtGrxC6; AltName: Full=Protein ROXY 21 gi|3688185|emb|CAA21213.1| putative protein [Arabidopsis thaliana] gi|7270251|emb|CAB80021.1| putative protein [Arabidopsis thaliana] gi|18377674|gb|AAL66987.1| unknown protein [Arabidopsis thaliana] gi|20258937|gb|AAM14184.1| unknown protein [Arabidopsis thaliana] gi|226348218|gb|ACO50425.1| glutaredoxin [Arabidopsis thaliana] gi|332660763|gb|AEE86163.1| glutaredoxin-C6 [Arabidopsis thaliana]	144	144	2.00E-67	100.0	91.0	94.4	glutaredoxin-C6	gbpln	Arabidopsis thaliana	AT4G33040.1 | Symbols: | Thioredoxin superfamily protein | chr4:15940779-15941213 REVERSE LENGTH=144	144	144	5.00E-70	100.0	91.0	94.4
Rsa1.0_00017.1.g1219.t1	gb|EOA16526.1| hypothetical protein CARUB_v10004685mg [Capsella rubella]	465	487	0	104.7	90.5	94.2	hypothetical protein CARUB_v10004685mg	gbpln	Capsella rubella	AT4G33050.3 | Symbols: EDA39 | calmodulin-binding family protein | chr4:15944604-15946736 REVERSE LENGTH=488	465	488	0	104.9	90.8	94.8
Rsa1.0_00017.1.g1220.t1	ref|NP_195032.2| peptidyl-prolyl cis-trans isomerase SDCCAG10 [Arabidopsis thaliana] gi|45680878|gb|AAS75308.1| multidomain cyclophilin type peptidyl-prolyl cis-trans isomerase [Arabidopsis thaliana] gi|95147300|gb|ABF57285.1| At4g33060 [Arabidopsis thaliana] gi|332660768|gb|AEE86168.1| peptidyl-prolyl cis-trans isomerase CYP57 [Arabidopsis thaliana]	492	504	0	102.4	87.2	93.5	peptidyl-prolyl cis-trans isomerase SDCCAG10	gbpln	Arabidopsis thaliana	AT4G33060.1 | Symbols: | Cyclophilin-like peptidyl-prolyl cis-trans isomerase family protein | chr4:15948535-15951954 FORWARD LENGTH=504	492	504	0	102.4	87.2	93.5
Rsa1.0_00017.1.g1221.t1	dbj|BAC76896.1| protein kinase [Raphanus sativus]	518	518	0	100.0	99.6	99.6	protein kinase	gbpln	Raphanus sativus	AT4G33080.1 | Symbols: | AGC (cAMP-dependent, cGMP-dependent and protein kinase C) kinase family protein | chr4:15960146-15964296 FORWARD LENGTH=519	518	519	0	100.2	95.4	96.9
Rsa1.0_00017.1.g1222.t1	ref|NP_195035.2| aminopeptidase M1 [Arabidopsis thaliana] gi|17473511|gb|AAL38379.1| AT4g33090/F4I10_20 [Arabidopsis thaliana] gi|24209879|gb|AAN41401.1| aminopeptidase M [Arabidopsis thaliana] gi|29028734|gb|AAO64746.1| At4g33090/F4I10_20 [Arabidopsis thaliana] gi|110742477|dbj|BAE99157.1| aminopeptidase like protein [Arabidopsis thaliana] gi|332660772|gb|AEE86172.1| aminopeptidase M1 [Arabidopsis thaliana]	871	879	0	100.9	93.2	96.7	aminopeptidase M1	gbpln	Arabidopsis thaliana	AT4G33090.1 | Symbols: APM1, ATAPM1 | aminopeptidase M1 | chr4:15965915-15970418 REVERSE LENGTH=879	871	879	0	100.9	93.2	96.7
Rsa1.0_00017.1.g1223.t1	ref|NP_195036.1| uncharacterized protein [Arabidopsis thaliana] gi|9910632|sp|Q9SMZ9.1|Y4331_ARATH RecName: Full=Uncharacterized protein At4g33100 gi|4455324|emb|CAB36784.1| putative protein [Arabidopsis thaliana] gi|7270257|emb|CAB80027.1| putative protein [Arabidopsis thaliana] gi|37202052|gb|AAQ89641.1| At4g33100 [Arabidopsis thaliana] gi|51968658|dbj|BAD43021.1| putative protein [Arabidopsis thaliana] gi|51969898|dbj|BAD43641.1| putative protein [Arabidopsis thaliana] gi|332660773|gb|AEE86173.1| uncharacterized protein AT4G33100 [Arabidopsis thaliana]	84	92	2.00E-35	109.5	86.9	90.5	uncharacterized protein	gbpln	Arabidopsis thaliana	AT4G33100.1 | Symbols: | CONTAINS InterPro DOMAIN/s: Mitochondrial distribution/morphology family 35/apoptosis (InterPro:IPR007918); Has 214 Blast hits to 214 proteins in 102 species: Archae - 0; Bacteria - 0; Metazoa - 110; Fungi - 69; Plants - 29; Viruses - 0; Other Eukaryotes - 6 (source: NCBI BLink). | chr4:15971701-15972342 FORWARD LENGTH=92	84	92	4.00E-38	109.5	86.9	90.5
Rsa1.0_00017.1.g1224.t1	ref|XP_002867201.1| coclaurine N-methyltransferase [Arabidopsis lyrata subsp. lyrata] gi|297313037|gb|EFH43460.1| coclaurine N-methyltransferase [Arabidopsis lyrata subsp. lyrata]	322	355	1.00E-166	110.2	89.1	95.3	coclaurine N-methyltransferase	gbpln	Arabidopsis lyrata	AT4G33110.2 | Symbols: | S-adenosyl-L-methionine-dependent methyltransferases superfamily protein | chr4:15972497-15974531 REVERSE LENGTH=355	322	355	1.00E-165	110.2	86.6	94.4

Rsa1.0_00017.1.g1225.t1	gb|AAC02672.1| polyprotein [Arabidopsis arenosa]	168	1390	1.00E-48	827.4	57.1	70.2	polyprotein	gbpln	Arabidopsis arenosa	#	#	#	#	#	#	#
Rsa1.0_00017.1.g1226.t1	dbj|BAB10876.1| polyprotein [Arabidopsis thaliana]	1546	1429	0	92.4	44.6	54.5	polyprotein	gbpln	Arabidopsis thaliana	AT4G23160.1 | Symbols: CRK8 | cysteine-rich RLK (RECEPTOR-like protein kinase) 8 | chr4:12129485-12134086 FORWARD LENGTH=1262	1546	1262	1.00E-115	81.6	13.3	19.7
Rsa1.0_00017.1.g1227.t1	ref|NP_200901.2| kinesin family member 4/7/21/27 [Arabidopsis thaliana] gi|332010015|gb|AED97398.1| kinesin family member 4/7/21/27 [Arabidopsis thaliana]	719	1294	1.00E-144	180.0	45.3	57.2	kinesin family member 4/7/21/27	gbpln	Arabidopsis thaliana	AT5G60930.1 | Symbols: | P-loop containing nucleoside triphosphate hydrolases superfamily protein | chr5:24515398-24522511 REVERSE LENGTH=1294	719	1294	1.00E-147	180.0	45.3	57.2
Rsa1.0_00017.1.g1228.t4	ref|NP_195039.6| uncharacterized protein [Arabidopsis thaliana] gi|332660777|gb|AEE86177.1| uncharacterized protein AT4G33130 [Arabidopsis thaliana]	225	222	1.00E-101	98.7	82.7	93.8	uncharacterized protein	gbpln	Arabidopsis thaliana	AT4G33130.1 | Symbols: | unknown protein; FUNCTIONS IN: molecular_function unknown; INVOLVED IN: biological_process unknown; LOCATED IN: chloroplast; EXPRESSED IN: 9 plant structures; EXPRESSED DURING: 6 growth stages; BEST Arabidopsis thaliana protein match is: Protein of unknown function, DUF547 (TAIR:AT1G16750.1); Has 192 Blast hits to 192 proteins in 18 species: Archae - 0; Bacteria - 0; Metazoa - 1; Fungi - 0; Plants - 189; Viruses - 0; Other Eukaryotes - 2 (source: NCBI BLink). | chr4:15979437-15980655 REVERSE LENGTH=222	225	222	1.00E-104	98.7	82.7	93.8
Rsa1.0_00017.1.g1229.t1	gb|EOA16925.1| hypothetical protein CARUB_v10005149mg [Capsella rubella]	343	351	1.00E-143	102.3	80.5	86.0	hypothetical protein CARUB_v10005149mg	gbpln	Capsella rubella	AT4G33140.1 | Symbols: | Haloacid dehalogenase-like hydrolase (HAD) superfamily protein | chr4:15981648-15984135 FORWARD LENGTH=353	343	353	1.00E-140	102.9	77.8	82.8
Rsa1.0_00017.1.g1230.t1	gb|AAB96826.1| lysine-ketoglutarate reductase/saccharopine dehydrogenase bifunctional enzyme [Arabidopsis thaliana]	1072	1064	0	99.3	90.9	95.4	lysine-ketoglutarate reductase/saccharopin e dehydrogenase bifunctional enzyme	gbpln	Arabidopsis thaliana	AT4G33150.2 | Symbols: | lysine-ketoglutarate reductase/saccharopine dehydrogenase bifunctional enzyme | chr4:15985479-15991069 REVERSE LENGTH=1064	1072	1064	0	99.3	90.8	95.3
Rsa1.0_00018.1.g1231.t1	gb|EOA38120.1| hypothetical protein CARUB_v10009590mg [Capsella rubella]	287	351	2.00E-75	122.3	55.7	69.3	hypothetical protein CARUB_v10009590mg	gbpln	Capsella rubella	AT2G03810.4 | Symbols: | 18S pre-ribosomal assembly protein gar2-related | chr2:1162703-1164286 FORWARD LENGTH=439	287	439	9.00E-42	153.0	48.8	63.8
Rsa1.0_00018.1.g1232.t1	ref|NP_563930.1| phosphatidylinositol 3- and 4-kinase-like protein [Arabidopsis thaliana] gi|16974596|gb|AAL31202.1| At1g13640/F21F23_7 [Arabidopsis thaliana] gi|23506173|gb|AAN31098.1| At1g13640/F21F23_7 [Arabidopsis thaliana] gi|332190932|gb|AEE29053.1| phosphatidylinositol 3- and 4-kinase-like protein [Arabidopsis thaliana]	617	622	0	100.8	85.7	92.4	phosphatidylinositol 3- and 4-kinase-like protein	gbpln	Arabidopsis thaliana	AT1G13640.1 | Symbols: | Phosphatidylinositol 3- and 4-kinase family protein | chr1:4677276-4679144 REVERSE LENGTH=622	617	622	0	100.8	85.7	92.4
Rsa1.0_00018.1.g1233.t1	gb|EOA36932.1| hypothetical protein CARUB_v10009802mg [Capsella rubella]	302	314	1.00E-148	104.0	91.7	95.0	hypothetical protein CARUB_v10009802mg	gbpln	Capsella rubella	AT1G13635.2 | Symbols: | DNA glycosylase superfamily protein | chr1:4674248-4675784 FORWARD LENGTH=311	302	311	1.00E-144	103.0	89.4	93.0
Rsa1.0_00018.1.g1234.t2	gb|EOA39669.1| hypothetical protein CARUB_v10008310mg [Capsella rubella] gi|482575483|gb|EOA39670.1| hypothetical protein CARUB_v10008310mg [Capsella rubella]	873	835	0	95.6	71.6	81.3	hypothetical protein CARUB_v10008310mg	gbpln	Capsella rubella	AT1G13630.1 | Symbols: | Tetratricopeptide repeat (TPR)-like superfamily protein | chr1:4669784-4672826 REVERSE LENGTH=806	873	806	0	92.3	64.7	73.7
Rsa1.0_00018.1.g1235.t1	gb|EOA36406.1| hypothetical protein CARUB_v10010856mg [Capsella rubella]	104	110	7.00E-26	105.8	60.6	69.2	hypothetical protein CARUB_v10010856mg	gbpln	Capsella rubella	AT1G13620.1 | Symbols: RGF2 | Encodes a root meristem growth factor (RGF). Belongs to a family of functionally redundant homologous peptides that are secreted, tyrosine-sulfated, and expressed mainly in the stem cell area and the innermost layer of central columella cells. RGFs are required for maintenance of the root stem cell niche and transit amplifying cell proliferation. Members of this family include: At5g60810 (RGF1), At1g13620 (RGF2), At2g04025 (RGF3), At3g30350 (RGF4), At5g51451 (RGF5), At4g16515 (RGF6), At3g02240 (RGF7), At2g03830 (RGF8) and At5g64770 (RGF9). | chr1:4668494-4669225 FORWARD LENGTH=109	104	109	9.00E-22	104.8	63.5	70.2
Rsa1.0_00018.1.g1236.t1	ref|XP_002892762.1| hypothetical protein ARALYDRAFT_471522 [Arabidopsis lyrata subsp. lyrata] gi|297338604|gb|EFH69021.1| hypothetical protein ARALYDRAFT_471522 [Arabidopsis lyrata subsp. lyrata]	538	360	1.00E-165	66.9	52.6	57.6	hypothetical protein ARALYDRAFT_471522	gbpln	Arabidopsis lyrata	AT1G13610.1 | Symbols: | alpha/beta-Hydrolases superfamily protein | chr1:4664008-4665975 REVERSE LENGTH=358	538	358	1.00E-161	66.5	51.3	56.3

Rsa1.0_00018.1.g1237.t1	gb|EOA29050.1| hypothetical protein CARUB_v10025304mg [Capsella rubella]	83	105	9.00E-14	126.5	51.8	63.9	hypothetical protein CARUB_v10025304mg	gbpln	Capsella rubella	AT2G25185.1 | Symbols: | Defensin-like (DEFL) family protein | chr2:10731622-10732024 FORWARD LENGTH=102	83	102	2.00E-15	122.9	48.2	60.2
Rsa1.0_00018.1.g1238.t1	gb|EOA29050.1| hypothetical protein CARUB_v10025304mg [Capsella rubella]	83	105	7.00E-13	126.5	49.4	63.9	hypothetical protein CARUB_v10025304mg	gbpln	Capsella rubella	AT2G25185.1 | Symbols: | Defensin-like (DEFL) family protein | chr2:10731622-10732024 FORWARD LENGTH=102	83	102	3.00E-15	122.9	48.2	60.2
Rsa1.0_00018.1.g1239.t1	gb|EOA14018.1| hypothetical protein CARUB_v10027151mg [Capsella rubella]	134	204	9.00E-28	152.2	44.8	47.8	hypothetical protein CARUB_v10027151mg	gbpln	Capsella rubella	AT5G54500.2 | Symbols: FQR1 | flavodoxin-like quinone reductase 1 | chr5:22124674-22126435 FORWARD LENGTH=244	134	244	5.00E-29	182.1	41.8	44.8
Rsa1.0_00018.1.g1240.t1	ref|NP_172817.2| basic leucine-zipper 58 [Arabidopsis thaliana] gi|8920564|gb|AAF81286.1|AC027656_3 Contains similarity to bZIP DNA-binding protein HBF-1 - soybean from Glycine max gb|Y10685. It contains a bZIP transcription factor PF|00170. EST gb|N37717 comes from this gene [Arabidopsis thaliana] gi|9802757|gb|AAF99826.1|AC027134_8 Hypothetical protein [Arabidopsis thaliana] gi|12083268|gb|AAG48793.1|AF332430_1 putative bZIP transcription factor [Arabidopsis thaliana] gi|225897922|dbj|BAH30293.1| hypothetical protein [Arabidopsis thaliana] gi|332190919|gb|AEE29040.1| basic leucine-zipper 58 [Arabidopsis thaliana]	196	196	4.00E-84	100.0	84.2	93.4	basic leucine-zipper 58	gbpln	Arabidopsis thaliana	AT1G13600.1 | Symbols: AtbZIP58, bZIP58 | basic leucine-zipper 58 | chr1:4650787-4651377 REVERSE LENGTH=196	196	196	1.00E-86	100.0	84.2	93.4
Rsa1.0_00018.1.g1241.t1	ref|NP_172816.2| phytosulfokine-beta [Arabidopsis thaliana] gi|41019236|sp|Q9LMY9.3|PSK1_ARATH RecName: Full=Phytosulfokines 1; Short=AtPSK1; Contains: RecName: Full=Phytosulfokine-alpha; Short=PSK-alpha; Short=Phytosulfokine-a; Contains: RecName: Full=Phytosulfokine-beta; Short=PSK-beta; Short=Phytosulfokine-b; Flags: Precursor gi|23466369|tpg|DAA00275.1| TPA_exp: putative phytosulfokine peptide precursor [Arabidopsis thaliana] gi|27529964|dbj|BAB72176.2| phytosulfokine precursor 1 [Arabidopsis thaliana] gi|51970324|dbj|BAD43854.1| hypothetical protein [Arabidopsis thaliana] gi|332190918|gb|AEE29039.1| phytosulfokine-beta [Arabidopsis thaliana]	91	87	6.00E-26	95.6	70.3	80.2	phytosulfokine-beta	gbpln	Arabidopsis thaliana	AT1G13590.1 | Symbols: ATPSK1, PSK1 | phytosulfokine 1 precursor | chr1:4647979-4648612 FORWARD LENGTH=87	91	87	9.00E-29	95.6	70.3	80.2
Rsa1.0_00018.1.g1242.t1	ref|XP_002892759.1| F-box family protein [Arabidopsis lyrata subsp. lyrata] gi|297338601|gb|EFH69018.1| F-box family protein [Arabidopsis lyrata subsp. lyrata]	416	416	0	100.0	92.1	96.9	F-box family protein	gbpln	Arabidopsis lyrata	AT1G13570.1 | Symbols: | F-box/RNI-like superfamily protein | chr1:4642528-4643930 REVERSE LENGTH=416	416	416	0	100.0	90.9	95.7
Rsa1.0_00018.1.g1243.t1	ref|XP_002892758.1| hypothetical protein ARALYDRAFT_471515 [Arabidopsis lyrata subsp. lyrata] gi|297338600|gb|EFH69017.1| hypothetical protein ARALYDRAFT_471515 [Arabidopsis lyrata subsp. lyrata]	389	389	0	100.0	98.5	99.2	hypothetical protein ARALYDRAFT_471515	gbpln	Arabidopsis lyrata	AT1G13560.1 | Symbols: AAPT1, ATAAPT1 | aminoalcoholphosphotransferase 1 | chr1:4638834-4641691 REVERSE LENGTH=389	389	389	0	100.0	97.9	99.2
Rsa1.0_00018.1.g1244.t1	gb|EOA36528.1| hypothetical protein CARUB_v10011549mg [Capsella rubella]	387	385	0	99.5	85.8	90.4	hypothetical protein CARUB_v10011549mg	gbpln	Capsella rubella	AT1G13530.1 | Symbols: | Protein of unknown function (DUF1262) | chr1:4633740-4635129 REVERSE LENGTH=385	387	385	0	99.5	82.4	89.9
Rsa1.0_00018.1.g1245.t1	gb|EOA40223.1| hypothetical protein CARUB_v10008945mg [Capsella rubella]	497	495	0	99.6	83.7	89.7	hypothetical protein CARUB_v10008945mg	gbpln	Capsella rubella	AT1G13460.2 | Symbols: | Protein phosphatase 2A regulatory B subunit family protein | chr1:4616504-4618180 FORWARD LENGTH=492	497	492	0	99.0	84.9	90.7
Rsa1.0_00018.1.g1246.t1	gb|ABV89646.1| DNA binding protein GT-1 [Brassica rapa]	395	399	0	101.0	92.9	94.7	DNA binding protein GT-1	gbpln	Brassica rapa	AT1G13450.1 | Symbols: GT-1 | Homeodomain-like superfamily protein | chr1:4612999-4615115 REVERSE LENGTH=406	395	406	0	102.8	84.3	89.9
Rsa1.0_00018.1.g1247.t3	sp|P04796.2|G3PC_SINAL RecName: Full=Glyceraldehyde-3-phosphate dehydrogenase, cytosolic gi|21143|emb|CAA27844.1| unnamed protein product [Sinapis alba]	579	338	0	58.4	54.9	55.1	RecName: Full=Glyceraldehyde-3-phosphate dehydrogenase, cytosolic gi|21143|emb|CAA27844.1| unnamed protein product	gbpln	Sinapis alba	AT3G04120.1 | Symbols: GAPC, GAPC-1, GAPC1 | glyceraldehyde-3-phosphate dehydrogenase C subunit 1 | chr3:1081077-1083131 FORWARD LENGTH=338	579	338	0	58.4	53.5	54.6

Rsa1.0_00018.1.g1248.t1	ref|XP_002892660.1| pentatricopeptide repeat-containing protein [Arabidopsis lyrata subsp. lyrata] gi|297338502|gb|EFH68919.1| pentatricopeptide repeat-containing protein [Arabidopsis lyrata subsp. lyrata]	348	367	1.00E-141	105.5	74.7	84.2	pentatricopeptide repeat-containing protein	gbpln	Arabidopsis lyrata	AT1G11900.1 | Symbols: | Tetratricopeptide repeat (TPR)-like superfamily protein | chr1:4013166-4014630 REVERSE LENGTH=367	348	367	1.00E-143	105.5	73.9	83.3
Rsa1.0_00018.1.g1249.t1	ref|XP_002869525.1| pentatricopeptide repeat-containing protein [Arabidopsis lyrata subsp. lyrata] gi|297315361|gb|EFH45784.1| pentatricopeptide repeat-containing protein [Arabidopsis lyrata subsp. lyrata]	686	707	0	103.1	75.2	86.6	pentatricopeptide repeat-containing protein	gbpln	Arabidopsis lyrata	AT4G28010.1 | Symbols: | Tetratricopeptide repeat (TPR)-like superfamily protein | chr4:13930379-13932493 FORWARD LENGTH=704	686	704	0	102.6	75.1	86.9
Rsa1.0_00018.1.g1250.t1	ref|XP_002880840.1| F-box family protein [Arabidopsis lyrata subsp. lyrata] gi|297326679|gb|EFH57099.1| F-box family protein [Arabidopsis lyrata subsp. lyrata]	371	371	1.00E-177	100.0	80.3	89.2	F-box family protein	gbpln	Arabidopsis lyrata	AT2G32560.1 | Symbols: | F-box family protein | chr2:13824820-13826761 FORWARD LENGTH=371	371	371	1.00E-177	100.0	81.9	89.8
Rsa1.0_00018.1.g1251.t1	ref|NP_564794.1| octicosapeptide/Phox/Be.1 domain-containing protein [Arabidopsis thaliana] gi|7940289|gb|AAF70848.1|AC003113_15 F2401.12 [Arabidopsis thaliana] gi|19423968|gb|AAL87265.1| unknown protein [Arabidopsis thaliana] gi|21281131|gb|AAM45044.1| unknown protein [Arabidopsis thaliana] gi|332195840|gb|AEE33961.1| octicosapeptide/Phox/Be.1 domain-containing protein [Arabidopsis thaliana]	740	751	0	101.5	75.9	84.2	octicosapeptide/Phox/Be.1 domain-containing protein	gbpln	Arabidopsis thaliana	AT1G62390.1 | Symbols: Phox2 | Octicosapeptide/Phox/Bem1p (PB1) domain-containing protein / tetratricopeptide repeat (TPR)-containing protein | chr1:23084632-23086887 REVERSE LENGTH=751	740	751	0	101.5	75.9	84.2
Rsa1.0_00018.1.g1252.t1	#	#	#	#	#	#	#	-	----	----	#	#	#	#	#	#	#
Rsa1.0_00018.1.g1253.t1	ref|XP_002889903.1| hypothetical protein ARALYDRAFT_471341 [Arabidopsis lyrata subsp. lyrata] gi|297335745|gb|EFH66162.1| hypothetical protein ARALYDRAFT_471341 [Arabidopsis lyrata subsp. lyrata]	234	329	1.00E-112	140.6	91.5	96.2	hypothetical protein ARALYDRAFT_471341	gbpln	Arabidopsis lyrata	AT1G11915.1 | Symbols: | unknown protein; FUNCTIONS IN: molecular_function unknown; INVOLVED IN: biological_process unknown; LOCATED IN: endomembrane system; EXPRESSED IN: root; BEST Arabidopsis thaliana protein match is: unknown protein (TAIR:AT3G17350.1); Has 261 Blast hits to 261 proteins in 13 species: Archae - 0; Bacteria - 0; Metazoa - 0; Fungi - 0; Plants - 261; Viruses - 0; Other Eukaryotes - 0 (source: NCBI BLink). | chr1:4021830-4023084 FORWARD LENGTH=329	234	329	1.00E-113	140.6	89.7	95.7
Rsa1.0_00018.1.g1254.t1	gb|EOA36500.1| hypothetical protein CARUB_v10011187mg [Capsella rubella]	372	385	1.00E-168	103.5	78.2	85.2	hypothetical protein CARUB_v10011187mg	gbpln	Capsella rubella	AT1G11920.1 | Symbols: | Pectin lyase-like superfamily protein | chr1:4023667-4025097 REVERSE LENGTH=384	372	384	1.00E-170	103.2	78.2	85.5
Rsa1.0_00018.1.g1255.t1	gb|EOA36500.1| hypothetical protein CARUB_v10011187mg [Capsella rubella]	384	385	0	100.3	91.9	95.6	hypothetical protein CARUB_v10011187mg	gbpln	Capsella rubella	AT1G11920.1 | Symbols: | Pectin lyase-like superfamily protein | chr1:4023667-4025097 REVERSE LENGTH=384	384	384	0	100.0	91.7	96.1
Rsa1.0_00018.1.g1256.t1	gb|EOA38666.1| hypothetical protein CARUB_v10010604mg [Capsella rubella]	140	140	1.00E-51	100.0	72.1	83.6	hypothetical protein CARUB_v10010604mg	gbpln	Capsella rubella	AT1G11925.1 | Symbols: | Stigma-specific Stig1 family protein | chr1:4026195-4026617 REVERSE LENGTH=140	140	140	4.00E-52	100.0	70.7	82.1
Rsa1.0_00018.1.g1257.t1	ref|NP_172660.5| putative ERD4 protein [Arabidopsis thaliana] gi|204324149|gb|ACI01072.1| putative membrane protein [Arabidopsis thaliana] gi|332190700|gb|AEE28821.1| putative ERD4 protein [Arabidopsis thaliana]	768	771	0	100.4	90.2	96.0	putative ERD4 protein	gbpln	Arabidopsis thaliana	AT1G11960.1 | Symbols: | ERD (early-responsive to dehydration stress) family protein | chr1:4039871-4043143 REVERSE LENGTH=771	768	771	0	100.4	90.2	96.0
Rsa1.0_00018.1.g1258.t1	ref|NP_172662.1| NEDD8-like protein RUB3 [Arabidopsis thaliana] gi|38258406|sp|O65381.1|RUB3_ARATH RecName: Full=NEDD8-like protein RUB3; AltName: Full=Ubiquitin-related protein 3; Short=AtRUB3; Flags: Precursor gi|3157940|gb|AAC17623.1| Contains similarity to Ubiquitin-like protein NEDD8 gb|D10918 from Mus musculus [Arabidopsis thaliana] gi|332190702|gb|AEE28823.1| NEDD8-like protein RUB3 [Arabidopsis thaliana]	90	78	2.00E-26	86.7	67.8	74.4	NEDD8-like protein RUB3	gbpln	Arabidopsis thaliana	AT1G11980.1 | Symbols: RUB3 | ubiquitin-related protein 3 | chr1:4045619-4045855 FORWARD LENGTH=78	90	78	4.00E-29	86.7	67.8	74.4
Rsa1.0_00018.1.g1259.t1	gb|AAC17628.1| Contains similarity to axi 1 gene gb|X80301 from Nicotiana tabacum [Arabidopsis thaliana]	622	627	0	100.8	78.3	86.3	Contains similarity to axi 1 gene gb|X80301 from Nicotiana tabacum	gbpln	Arabidopsis thaliana	AT1G11990.1 | Symbols: | O-fucosyltransferase family protein | chr1:4046246-4049060 REVERSE LENGTH=590	622	590	0	94.9	74.3	81.2

Rsa1.0_00018.1.g1260.t1	gb|EOA40028.1| hypothetical protein CARUB_v10008719mg [Capsella rubella]	567	567	0	100.0	95.6	97.9	hypothetical protein CARUB_v10008719mg	gbpln	Capsella rubella	AT1G12000.1 | Symbols: | Phosphofructokinase family protein | chr1:4050159-4053727 REVERSE LENGTH=566	567	566	0	99.8	95.6	97.5
Rsa1.0_00018.1.g1261.t7	dbj|BAJ33770.1| unnamed protein product [Thellungiella halophila]	278	416	1.00E-53	149.6	43.2	46.0	unnamed protein product	----	----	AT5G57630.1 | Symbols: CIPK21, SnRK3.4 | CBL-interacting protein kinase 21 | chr5:23341092-23343143 REVERSE LENGTH=416	278	416	2.00E-53	149.6	41.0	46.4
Rsa1.0_00018.1.g1262.t1	emb|CAA57285.1| ACC oxidase [Brassica oleracea]	320	321	1.00E-179	100.3	95.0	97.5	ACC oxidase	gbpln	Brassica oleracea	AT1G12010.1 | Symbols: | 2-oxoglutarate (2OG) and Fe(II)-dependent oxygenase superfamily protein | chr1:4056274-4057670 FORWARD LENGTH=320	320	320	1.00E-175	100.0	90.9	94.7
Rsa1.0_00018.1.g1263.t1	gb|EOA37066.1| hypothetical protein CARUB_v10010193mg [Capsella rubella]	231	231	5.00E-80	100.0	73.6	82.3	hypothetical protein CARUB_v10010193mg	gbpln	Capsella rubella	AT1G12020.1 | Symbols: | unknown protein; BEST Arabidopsis thaliana protein match is: unknown protein (TAIR:AT1G62422.1); Has 89 Blast hits to 88 proteins in 16 species: Archae - 0; Bacteria - 0; Metazoa - 0; Fungi - 0; Plants - 87; Viruses - 0; Other Eukaryotes - 2 (source: NCBI BLink). | chr1:4062625-4063305 REVERSE LENGTH=226	231	226	1.00E-78	97.8	71.4	78.8
Rsa1.0_00018.1.g1264.t1	gb|EOA38371.1| hypothetical protein CARUB_v10009904mg [Capsella rubella]	292	291	1.00E-116	99.7	78.1	83.9	hypothetical protein CARUB_v10009904mg	gbpln	Capsella rubella	AT1G12030.1 | Symbols: | Protein of unknown function (DUF506) | chr1:4064751-4066319 REVERSE LENGTH=295	292	295	1.00E-110	101.0	75.0	83.9
Rsa1.0_00018.1.g1265.t2	ref|XP_002889911.1| hypothetical protein ARALYDRAFT_471353 [Arabidopsis lyrata subsp. lyrata] gi|297335753|gb|EFH66170.1| hypothetical protein ARALYDRAFT_471353 [Arabidopsis lyrata subsp. lyrata]	450	421	0	93.6	84.0	86.2	hypothetical protein ARALYDRAFT_471353	gbpln	Arabidopsis lyrata	AT1G12050.1 | Symbols: | fumarylacetoacetase, putative | chr1:4072904-4075856 FORWARD LENGTH=421	450	421	0	93.6	82.2	86.0
Rsa1.0_00018.1.g1266.t1	gb|EOA37704.1| hypothetical protein CARUB_v10012423mg [Capsella rubella]	311	223	1.00E-106	71.7	64.3	67.8	hypothetical protein CARUB_v10012423mg	gbpln	Capsella rubella	AT1G12070.1 | Symbols: | Immunoglobulin E-set superfamily protein | chr1:4078913-4080106 REVERSE LENGTH=223	311	223	1.00E-107	71.7	63.7	67.8
Rsa1.0_00018.1.g1267.t1	#	#	#	#	#	#	#	-	----	----	#	#	#	#	#	#	#
Rsa1.0_00018.1.g1268.t1	#	#	#	#	#	#	#	-	----	----	#	#	#	#	#	#	#
Rsa1.0_00018.1.g1269.t1	gb|AAK30571.1|AF346659_1 extensin-like protein [Brassica napus]	137	137	3.00E-46	100.0	94.2	97.1	extensin-like protein	gbpln	Brassica napus	AT1G12090.1 | Symbols: ELP | extensin-like protein | chr1:4090176-4090589 REVERSE LENGTH=137	137	137	2.00E-45	100.0	95.6	97.8
Rsa1.0_00018.1.g1270.t1	#	#	#	#	#	#	#	-	----	----	#	#	#	#	#	#	#
Rsa1.0_00018.1.g1271.t1	gb|AEA35415.1| nitrate transporter [Brassica rapa subsp. chinensis]	590	589	0	99.8	93.4	97.3	nitrate transporter	gbpln	Brassica rapa	AT1G12110.1 | Symbols: NRT1.1, CHL1-1, NRT1, B-1, ATNRT1, CHL1 | nitrate transporter 1.1 | chr1:4105341-4109290 FORWARD LENGTH=590	590	590	0	100.0	92.2	95.9
Rsa1.0_00018.1.g1272.t1	gb|EOA40154.1| hypothetical protein CARUB_v10008871mg, partial [Capsella rubella]	410	516	1.00E-123	125.9	69.3	77.6	hypothetical protein CARUB_v10008871mg, partial	gbpln	Capsella rubella	AT1G12120.1 | Symbols: | Plant protein of unknown function (DUF863) | chr1:4114933-4116469 REVERSE LENGTH=483	410	483	1.00E-119	117.8	68.3	75.1
Rsa1.0_00018.1.g1273.t1	ref|NP_172679.1| uncharacterized protein [Arabidopsis thaliana] gi|75172923|sp|Q9FWW5.1|Y1215_ARATH RecName: Full=WEB family protein At1g12150 gi|10086518|gb|AAG12578.1|AC022522_11 Hypothetical protein [Arabidopsis thaliana] gi|332190722|gb|AEE28843.1| uncharacterized protein AT1G12150 [Arabidopsis thaliana]	63	548	2.00E-12	869.8	58.7	61.9	uncharacterized protein	gbpln	Arabidopsis thaliana	AT1G12150.1 | Symbols: | Plant protein of unknown function (DUF827) | chr1:4123530-4125328 REVERSE LENGTH=548	63	548	3.00E-15	869.8	58.7	61.9
Rsa1.0_00018.1.g1274.t1	ref|NP_172684.1| dimethylaniline monooxygenase (N-oxide forming) [Arabidopsis thaliana] gi|75172924|sp|Q9FWW9.1|GSXL2_ARATH RecName: Full=Flavin-containing monooxygenase FMO GS-OX-like 2; AltName: Full=Flavin-monooxygenase glucosinolate S-oxygenase-like 2 gi|10086514|gb|AAG12574.1|AC022522_7 Unknown protein [Arabidopsis thaliana] gi|20258836|gb|AAM13900.1| unknown protein [Arabidopsis thaliana] gi|22136992|gb|AAM91725.1| unknown protein [Arabidopsis thaliana] gi|332190727|gb|AEE28848.1| flavin-containing monooxygenase FMO GS-OX-like 2 [Arabidopsis thaliana]	463	465	0	100.4	90.9	95.2	dimethylaniline monooxygenase (N-oxide forming)	gbpln	Arabidopsis thaliana	AT1G12200.1 | Symbols: | Flavin-binding monooxygenase family protein | chr1:4137627-4139835 FORWARD LENGTH=465	463	465	0	100.4	90.9	95.2

Rsa1.0_00018.1.g1275.t1	ref|NP_563900.1| transaldolase [Arabidopsis thaliana] gi|10086511|gb|AAG12571.1|AC022522_4 Similar to transaldolase [Arabidopsis thaliana] gi|25083140|gb|AAN72047.1| expressed protein [Arabidopsis thaliana] gi|30725648|gb|AAP37846.1| At1g12230 [Arabidopsis thaliana] gi|332190732|gb|AEE28853.1| transaldolase [Arabidopsis thaliana]	409	405	0	99.0	91.0	96.6	transaldolase	gbpln	Arabidopsis thaliana	AT1G12230.1 | Symbols: | Aldolase superfamily protein | chr1:4148050-4150708 FORWARD LENGTH=405	409	405	0	99.0	91.0	96.6
Rsa1.0_00018.1.g1276.t2	gb|AAG36943.1|AF274299_1 acid invertase [Brassica oleracea]	665	662	0	99.5	95.6	97.0	acid invertase	gbpln	Brassica oleracea	AT1G12240.1 | Symbols: ATBETAFRUCT4, VAC-INV | Glycosyl hydrolases family 32 protein | chr1:4153699-4157457 FORWARD LENGTH=664	665	664	0	99.8	88.6	93.7
Rsa1.0_00018.1.g1277.t1	ref|XP_002892680.1| hypothetical protein ARALYDRAFT_471376 [Arabidopsis lyrata subsp. lyrata] gi|297338522|gb|EFH68939.1| hypothetical protein ARALYDRAFT_471376 [Arabidopsis lyrata subsp. lyrata]	396	395	0	99.7	88.1	91.2	hypothetical protein ARALYDRAFT_471376	gbpln	Arabidopsis lyrata	AT1G12260.1 | Symbols: VND4, EMB2749, ANAC007, NAC007 | NAC 007 | chr1:4163058-4164486 REVERSE LENGTH=395	396	395	0	99.7	88.1	91.2
Rsa1.0_00018.1.g1278.t1	#	#	#	#	#	#	#	-	----	----	#	#	#	#	#	#	#
Rsa1.0_00018.1.g1279.t1	#	#	#	#	#	#	#	-	----	----	#	#	#	#	#	#	#
Rsa1.0_00018.1.g1280.t1	ref|NP_172686.1| disease resistance protein RPS5 [Arabidopsis thaliana] gi|334182494|ref|NP_001184970.1| disease resistance protein RPS5 [Arabidopsis thaliana] gi|46396675|sp|O64973.2|RPS5_ARATH RecName: Full=Disease resistance protein RPS5; AltName: Full=Resistance to Pseudomonas syringae protein 5; AltName: Full=pNd3/pNd10 gi|10086512|gb|AAG12572.1|AC022522_5 resistance to Pseudomonas syringae protein 5 [Arabidopsis thaliana] gi|3309620|gb|AAC26126.1| resistance to Pseudomonas syringae protein 5 [Arabidopsis thaliana] gi|34849895|gb|AAQ82844.1| At1g12220 [Arabidopsis thaliana] gi|62319935|dbj|BAD94018.1| NBS/LRR disease resistance protein [Arabidopsis thaliana] gi|77632414|gb|ABB00204.1| disease resistance protein [Arabidopsis thaliana] gi|332190730|gb|AEE28851.1| disease resistance protein RPS5 [Arabidopsis thaliana] gi|332190731|gb|AEE28852.1| disease resistance protein RPS5 [Arabidopsis thaliana]	327	889	3.00E-67	271.9	41.6	52.6	disease resistance protein RPS5	gbpln	Arabidopsis thaliana	AT1G12220.2 | Symbols: RPS5 | Disease resistance protein (CC-NBS-LRR class) family | chr1:4145011-4147680 FORWARD LENGTH=889	327	889	9.00E-70	271.9	41.6	52.6
Rsa1.0_00018.1.g1281.t1	ref|XP_002879162.1| hypothetical protein ARALYDRAFT_320641 [Arabidopsis lyrata subsp. lyrata] gi|297325001|gb|EFH55421.1| hypothetical protein ARALYDRAFT_320641 [Arabidopsis lyrata subsp. lyrata]	279	709	3.00E-47	254.1	35.8	52.7	hypothetical protein ARALYDRAFT_320641	gbpln	Arabidopsis lyrata	AT1G12220.2 | Symbols: RPS5 | Disease resistance protein (CC-NBS-LRR class) family | chr1:4145011-4147680 FORWARD LENGTH=889	279	889	1.00E-47	318.6	39.1	50.5
Rsa1.0_00018.1.g1282.t1	gb|ACP30557.1| disease resistance protein [Brassica rapa subsp. pekinensis]	885	886	0	100.1	67.3	78.8	disease resistance protein	gbpln	Brassica rapa	AT1G12210.1 | Symbols: RFL1 | RPS5-like 1 | chr1:4140948-4143605 FORWARD LENGTH=885	885	885	0	100.0	66.1	78.0
Rsa1.0_00018.1.g1283.t1	gb|AAF79658.1|AC025416_32 F5O11.4 [Arabidopsis thaliana]	148	975	1.00E-78	658.8	96.6	98.6	F5O11.4	gbpln	Arabidopsis thaliana	AT1G12310.1 | Symbols: | Calcium-binding EF-hand family protein | chr1:4187500-4187946 REVERSE LENGTH=148	148	148	2.00E-80	100.0	97.3	99.3
Rsa1.0_00018.1.g1284.t1	ref|XP_002889928.1| hypothetical protein ARALYDRAFT_471382 [Arabidopsis lyrata subsp. lyrata] gi|297335770|gb|EFH66187.1| hypothetical protein ARALYDRAFT_471382 [Arabidopsis lyrata subsp. lyrata]	506	504	0	99.6	86.6	91.1	hypothetical protein ARALYDRAFT_471382	gbpln	Arabidopsis lyrata	AT1G12330.1 | Symbols: | unknown protein; INVOLVED IN: biological_process unknown; LOCATED IN: chloroplast; EXPRESSED IN: 21 plant structures; EXPRESSED DURING: 12 growth stages; BEST Arabidopsis thaliana protein match is: unknown protein (TAIR:AT5G12900.1); Has 249 Blast hits to 249 proteins in 27 species: Archae - 0; Bacteria - 0; Metazoa - 7; Fungi - 14; Plants - 217; Viruses - 0; Other Eukaryotes - 11 (source: NCBI BLink). | chr1:4194673-4196627 FORWARD LENGTH=505	506	505	0	99.8	86.6	90.7
Rsa1.0_00018.1.g1285.t6	gb|EOA38925.1| hypothetical protein CARUB_v10011323mg [Capsella rubella]	699	666	0	95.3	87.3	90.4	hypothetical protein CARUB_v10011323mg	gbpln	Capsella rubella	AT1G12360.1 | Symbols: KEU | Sec1/munc18-like (SM) proteins superfamily | chr1:4201172-4206144 FORWARD LENGTH=666	699	666	0	95.3	86.4	90.0

Rsa1.0_00018.1.g1286.t1	#	#	#	#	#	#	#	-	----	----	#	#	#	#	#	#	#
Rsa1.0_00018.1.g1287.t1	ref|XP_002892685.1| hypothetical protein ARALYDRAFT_888561 [Arabidopsis lyrata subsp. lyrata] gi|297338527|gb|EFH68944.1| hypothetical protein ARALYDRAFT_888561 [Arabidopsis lyrata subsp. lyrata]	769	792	0	103.0	86.0	90.8	hypothetical protein ARALYDRAFT_888561	gbpln	Arabidopsis lyrata	AT1G12380.1 | Symbols: | unknown protein; BEST Arabidopsis thaliana protein match is: unknown protein (TAIR:AT1G62870.1); Has 173 Blast hits to 170 proteins in 34 species: Archae - 0; Bacteria - 4; Metazoa - 25; Fungi - 8; Plants - 123; Viruses - 7; Other Eukaryotes - 6 (source: NCBI BLink). | chr1:4214499-4216880 REVERSE LENGTH=793	769	793	0	103.1	86.0	91.5
Rsa1.0_00018.1.g1288.t2	ref|XP_002889932.1| cornichon family protein [Arabidopsis lyrata subsp. lyrata] gi|297335774|gb|EFH66191.1| cornichon family protein [Arabidopsis lyrata subsp. lyrata]	137	137	9.00E-69	100.0	93.4	96.4	cornichon family protein	gbpln	Arabidopsis lyrata	AT1G12390.1 | Symbols: | Cornichon family protein | chr1:4220347-4221481 FORWARD LENGTH=137	137	137	5.00E-71	100.0	92.7	95.6
Rsa1.0_00018.1.g1289.t1	gb|EOA38397.1| hypothetical protein CARUB_v10009963mg [Capsella rubella]	279	279	1.00E-156	100.0	95.0	97.8	hypothetical protein CARUB_v10009963mg	gbpln	Capsella rubella	AT1G12410.1 | Symbols: CLPR2, NCLPP2, CLP2 | CLP protease proteolytic subunit 2 | chr1:4223099-4224954 FORWARD LENGTH=279	279	279	1.00E-158	100.0	94.6	97.8
Rsa1.0_00018.1.g1290.t1	gb|EOA40435.1| hypothetical protein CARUB_v10009162mg [Capsella rubella]	441	441	0	100.0	93.7	95.9	hypothetical protein CARUB_v10009162mg	gbpln	Capsella rubella	AT1G12420.1 | Symbols: ACR8 | ACT domain repeat 8 | chr1:4226673-4228917 REVERSE LENGTH=441	441	441	0	100.0	93.7	95.9
Rsa1.0_00018.1.g1291.t1	ref|XP_002887985.1| hypothetical protein ARALYDRAFT_893174 [Arabidopsis lyrata subsp. lyrata] gi|297333826|gb|EFH64244.1| hypothetical protein ARALYDRAFT_893174 [Arabidopsis lyrata subsp. lyrata]	267	300	1.00E-128	112.4	85.0	92.9	hypothetical protein ARALYDRAFT_893174	gbpln	Arabidopsis lyrata	AT1G63000.1 | Symbols: NRS/ER, UER1 | nucleotide-rhamnose synthase/epimerase-reductase | chr1:23342510-23343859 FORWARD LENGTH=301	267	301	1.00E-127	112.7	85.0	92.9
Rsa1.0_00018.1.g1292.t1	gb|AAF88098.1|AC025417_26 T12C24.11 [Arabidopsis thaliana]	535	549	0	102.6	92.7	96.6	T12C24.11	gbpln	Arabidopsis thaliana	AT1G12570.1 | Symbols: | Glucose-methanol-choline (GMC) oxidoreductase family protein | chr1:4278192-4280753 REVERSE LENGTH=572	535	572	0	106.9	92.7	96.6
Rsa1.0_00018.1.g1293.t1	ref|NP_172721.1| dehydration-responsive element-binding protein 1F [Arabidopsis thaliana] gi|47605747|sp|Q9LN86.1|DRE1F_ARATH RecName: Full=Dehydration-responsive element-binding protein 1F; Short=Protein DREB1F gi|9502389|gb|AAF88096.1|AC025417_24 T12C24.14 [Arabidopsis thaliana] gi|48479384|gb|AAT44959.1| putative AP2/EREBP transcription factor [Arabidopsis thaliana] gi|110736358|dbj|BAF00148.1| hypothetical protein [Arabidopsis thaliana] gi|332190780|gb|AEE28901.1| dehydration-responsive element-binding protein 1F [Arabidopsis thaliana]	231	209	4.00E-83	90.5	73.6	77.9	dehydration-responsive element-binding protein 1F	gbpln	Arabidopsis thaliana	AT1G12610.1 | Symbols: DDF1 | Integrase-type DNA-binding superfamily protein | chr1:4290205-4290834 REVERSE LENGTH=209	231	209	1.00E-85	90.5	73.6	77.9
Rsa1.0_00018.1.g1294.t1	ref|NP_172724.2| MBOAT (membrane bound O-acyl transferase)-like protein [Arabidopsis thaliana] gi|332190783|gb|AEE28904.1| MBOAT (membrane bound O-acyl transferase)-like protein [Arabidopsis thaliana]	465	462	0	99.4	92.3	95.9	MBOAT (membrane bound O-acyl transferase)-like protein	gbpln	Arabidopsis thaliana	AT1G12640.1 | Symbols: | MBOAT (membrane bound O-acyl transferase) family protein | chr1:4303586-4305666 REVERSE LENGTH=462	465	462	0	99.4	92.3	95.9
Rsa1.0_00018.1.g1295.t1	gb|EOA37663.1| hypothetical protein CARUB_v10012242mg, partial [Capsella rubella]	133	137	6.00E-18	103.0	33.8	46.6	hypothetical protein CARUB_v10012242mg, partial	gbpln	Capsella rubella	AT1G12660.1 | Symbols: | Predicted to encode a PR (pathogenesis-related) protein. Belongs to the plant thionin (PR-13) family with the following members: At1g66100, At5g36910, At1g72260, At2g15010, At1g12663, At1g12660. | chr1:4308943-4309583 FORWARD LENGTH=137	133	137	2.00E-12	103.0	28.6	38.3
Rsa1.0_00018.1.g1296.t1	gb|EOA40316.1| hypothetical protein CARUB_v10009041mg [Capsella rubella]	451	470	0	104.2	86.9	91.6	hypothetical protein CARUB_v10009041mg	gbpln	Capsella rubella	AT1G12680.1 | Symbols: PEPKR2 | phosphoenolpyruvate carboxylase-related kinase 2 | chr1:4320123-4322269 REVERSE LENGTH=470	451	470	0	104.2	85.8	90.5
Rsa1.0_00018.1.g1297.t1	gb|EOA36152.1| hypothetical protein CARUB_v10009895mg [Capsella rubella]	284	294	1.00E-135	103.5	83.5	88.7	hypothetical protein CARUB_v10009895mg	gbpln	Capsella rubella	AT1G12710.1 | Symbols: AtPP2-A12, PP2-A12 | phloem protein 2-A12 | chr1:4326980-4328324 REVERSE LENGTH=291	284	291	1.00E-136	102.5	83.8	88.4
Rsa1.0_00018.1.g1298.t1	ref|XP_002889964.1| T12C24.27 [Arabidopsis lyrata subsp. lyrata] gi|297335806|gb|EFH66223.1| T12C24.27 [Arabidopsis lyrata subsp. lyrata]	478	478	0	100.0	92.3	95.2	T12C24.27	gbpln	Arabidopsis lyrata	AT1G12740.1 | Symbols: CYP87A2 | cytochrome P450, family 87, subfamily A, polypeptide 2 | chr1:4342462-4344569 FORWARD LENGTH=472	478	472	0	98.7	91.6	94.6
Rsa1.0_00018.1.g1299.t1	gb|EOA37577.1| hypothetical protein CARUB_v10011917mg [Capsella rubella]	304	304	1.00E-138	100.0	78.3	89.1	hypothetical protein CARUB_v10011917mg	gbpln	Capsella rubella	AT1G12750.3 | Symbols: RBL6 | RHOMBOID-like protein 6 | chr1:4345100-4346688 REVERSE LENGTH=307	304	307	1.00E-133	101.0	73.0	86.2

Rsa1.0_00018.1.g1300.t1	gb|EOA37885.1| hypothetical protein CARUB_v10009353mg [Capsella rubella]	407	397	1.00E-163	97.5	80.1	84.8	hypothetical protein CARUB_v10009353mg	gbpln	Capsella rubella	AT1G12760.1 | Symbols: | Zinc finger, C3HC4 type (RING finger) family protein | chr1:4348728-4350512 FORWARD LENGTH=408	407	408	1.00E-161	100.2	77.4	81.1
Rsa1.0_00018.1.g1301.t1	gb|EOA38122.1| hypothetical protein CARUB_v10009592mg [Capsella rubella]	351	351	0	100.0	96.0	98.3	hypothetical protein CARUB_v10009592mg	gbpln	Capsella rubella	AT1G12780.1 | Symbols: UGE1, ATUGE1 | UDP-D-glucose/UDP-D-galactose 4-epimerase 1 | chr1:4356124-4358120 REVERSE LENGTH=351	351	351	0	100.0	95.4	97.4
Rsa1.0_00018.1.g1302.t1	gb|AAM65961.1| DNA ligase-like protein [Arabidopsis thaliana]	219	250	3.00E-97	114.2	84.9	92.2	DNA ligase-like protein	gbpln	Arabidopsis thaliana	AT1G12790.1 | Symbols: | CONTAINS InterPro DOMAIN/s: RuvA domain 2-like (InterPro:IPR010994); Has 29 Blast hits to 29 proteins in 9 species: Archae - 0; Bacteria - 2; Metazoa - 0; Fungi - 0; Plants - 27; Viruses - 0; Other Eukaryotes - 0 (source: NCBI BLink). | chr1:4359597-4361222 REVERSE LENGTH=250	219	250	2.00E-98	114.2	84.5	91.3
Rsa1.0_00019.1.g1303.t2	ref|NP_195532.1| cellulose synthase-like protein D4 [Arabidopsis thaliana] gi|75213628|sp|Q9SZL9.1|CSLD4_ARATH RecName: Full=Cellulose synthase-like protein D4; Short=AtCslD4 gi|4467125|emb|CAB37559.1| putative protein [Arabidopsis thaliana] gi|7270803|emb|CAB80484.1| putative protein [Arabidopsis thaliana] gi|332661491|gb|AEE86891.1| cellulose synthase-like protein D4 [Arabidopsis thaliana]	1232	1111	0	90.2	87.7	88.7	cellulose synthase-like protein D4	gbpln	Arabidopsis thaliana	AT4G38190.1 | Symbols: ATCSLD4, CSLD4 | cellulose synthase like D4 | chr4:17910096-17913641 REVERSE LENGTH=1111	1232	1111	0	90.2	87.7	88.7
Rsa1.0_00019.1.g1304.t1	gb|EOA15893.1| hypothetical protein CARUB_v10003980mg [Capsella rubella]	1749	1688	0	96.5	86.8	91.7	hypothetical protein CARUB_v10003980mg	gbpln	Capsella rubella	AT4G38200.1 | Symbols: | SEC7-like guanine nucleotide exchange family protein | chr4:17915293-17922502 FORWARD LENGTH=1687	1749	1687	0	96.5	86.8	91.1
Rsa1.0_00019.1.g1305.t1	ref|XP_002868837.1| ATEXPA20 [Arabidopsis lyrata subsp. lyrata] gi|297314673|gb|EFH45096.1| ATEXPA20 [Arabidopsis lyrata subsp. lyrata]	256	256	1.00E-135	100.0	93.0	95.7	ATEXPA20	gbpln	Arabidopsis lyrata	AT4G38210.1 | Symbols: ATEXPA20, EXP20, ATEXP20, ATHEXP ALPHA 1.23, EXPA20 | expansin A20 | chr4:17922975-17923821 REVERSE LENGTH=256	256	256	1.00E-137	100.0	92.6	95.3
Rsa1.0_00019.1.g1306.t2	ref|NP_568036.2| Peptidase M20/M25/M40 family protein [Arabidopsis thaliana] gi|18650600|gb|AAL75900.1| AT4g38220/F20D10_340 [Arabidopsis thaliana] gi|332661494|gb|AEE86894.1| Peptidase M20/M25/M40 family protein [Arabidopsis thaliana]	559	430	0	76.9	63.1	66.7	Peptidase M20/M25/M40 family protein	gbpln	Arabidopsis thaliana	AT4G38220.1 | Symbols: | Peptidase M20/M25/M40 family protein | chr4:17925251-17926919 FORWARD LENGTH=430	559	430	0	76.9	63.1	66.7
Rsa1.0_00019.1.g1307.t1	gb|EOA18945.1| hypothetical protein CARUB_v10007579mg [Capsella rubella]	467	483	0	103.4	87.8	91.9	hypothetical protein CARUB_v10007579mg	gbpln	Capsella rubella	AT4G38360.2 | Symbols: LAZ1 | Protein of unknown function (DUF300) | chr4:17967389-17969798 FORWARD LENGTH=485	467	485	0	103.9	87.8	91.2
Rsa1.0_00019.1.g1308.t1	gb|EOA17245.1| hypothetical protein CARUB_v10005519mg [Capsella rubella]	269	263	1.00E-123	97.8	80.7	88.5	hypothetical protein CARUB_v10005519mg	gbpln	Capsella rubella	AT3G45970.1 | Symbols: ATEXLA1, EXPL1, ATEXPL1, ATHEXP BETA 2.1, EXLA1 | expansin-like A1 | chr3:16896238-16897189 FORWARD LENGTH=265	269	265	1.00E-121	98.5	82.9	89.2

Rsa1.0_00019.1.g1309.t1	ref|NP_195558.1| geranylgeranyl diphosphate synthase, type II [Arabidopsis thaliana] gi|85680919|sp|Q39108.2|GGR_ARATH RecName: Full=Heterodimeric geranylgeranyl pyrophosphate synthase small subunit, chloroplastic; Flags: Precursor gi|11908118|gb|AAG41488.1|AF326906_1 putative geranylgeranyl pyrophosphate synthase-related protein [Arabidopsis thaliana] gi|12642930|gb|AAK00407.1|AF339725_1 putative geranylgeranyl pyrophosphate synthase-related protein [Arabidopsis thaliana] gi|13926334|gb|AAK49631.1|AF372915_1 AT4g38460/F20M13_20 [Arabidopsis thaliana] gi|4467133|emb|CAB37502.1| geranylgeranyl pyrophosphate synthase-related protein [Arabidopsis thaliana] gi|7270829|emb|CAB80510.1| geranylgeranyl pyrophosphate synthase-related protein [Arabidopsis thaliana] gi|16323260|gb|AAL15364.1| AT4g38460/F20M13_20 [Arabidopsis thaliana] gi|21555524|gb|AAM63877.1| geranylgeranyl pyrophosphate synthase-related protein [Arabidopsis thaliana] gi|332661530|gb|AEE86930.1| geranylgeranyl reductase [Arabidopsis thaliana]	329	326	1.00E-126	99.1	76.3	84.5	geranylgeranyl diphosphate synthase, type II	gbpln	Arabidopsis thaliana	AT4G38460.1 | Symbols: GGR | geranylgeranyl reductase | chr4:17994849-17995974 FORWARD LENGTH=326	329	326	1.00E-129	99.1	76.3	84.5
Rsa1.0_00019.1.g1310.t1	ref|NP_568041.1| ACT-like protein tyrosine kinase family protein [Arabidopsis thaliana] gi|332661531|gb|AEE86931.1| ACT-like protein tyrosine kinase family protein [Arabidopsis thaliana]	564	575	0	102.0	85.5	91.1	ACT-like protein tyrosine kinase family protein	gbpln	Arabidopsis thaliana	AT4G38470.1 | Symbols: | ACT-like protein tyrosine kinase family protein | chr4:17999432-18003551 FORWARD LENGTH=575	564	575	0	102.0	85.5	91.1
Rsa1.0_00019.1.g1311.t1	gb|EOA18127.1| hypothetical protein CARUB_v10006588mg [Capsella rubella]	117	123	9.00E-47	105.1	79.5	87.2	hypothetical protein CARUB_v10006588mg	gbpln	Capsella rubella	AT4G38495.1 | Symbols: | CONTAINS InterPro DOMAIN/s: YL1 nuclear, C-terminal (InterPro:IPR013272); Has 279 Blast hits to 279 proteins in 147 species: Archae - 0; Bacteria - 0; Metazoa - 94; Fungi - 133; Plants - 35; Viruses - 0; Other Eukaryotes - 17 (source: NCBI BLink). | chr4:18007466-18008025 REVERSE LENGTH=124	117	124	1.00E-47	106.0	76.9	86.3
Rsa1.0_00019.1.g1312.t2	gb|EOA17176.1| hypothetical protein CARUB_v10005442mg [Capsella rubella]	273	281	1.00E-139	102.9	90.8	93.4	hypothetical protein CARUB_v10005442mg	gbpln	Capsella rubella	AT4G38670.3 | Symbols: | Pathogenesis-related thaumatin superfamily protein | chr4:18069856-18071227 REVERSE LENGTH=281	273	281	1.00E-140	102.9	89.4	92.7
Rsa1.0_00019.1.g1313.t1	gb|EOA18006.1| hypothetical protein CARUB_v10006441mg [Capsella rubella]	98	98	3.00E-40	100.0	88.8	94.9	hypothetical protein CARUB_v10006441mg	gbpln	Capsella rubella	AT4G38840.1 | Symbols: | SAUR-like auxin-responsive protein family | chr4:18125174-18125473 REVERSE LENGTH=99	98	99	7.00E-39	101.0	83.7	93.9
Rsa1.0_00019.1.g1314.t1	gb|EOA15659.1| hypothetical protein CARUB_v10006088mg [Capsella rubella]	105	105	4.00E-53	100.0	96.2	100.0	hypothetical protein CARUB_v10006088mg	gbpln	Capsella rubella	AT4G38860.1 | Symbols: | SAUR-like auxin-responsive protein family | chr4:18130357-18130674 FORWARD LENGTH=105	105	105	4.00E-55	100.0	95.2	99.0
Rsa1.0_00019.1.g1315.t1	ref|NP_195599.1| amidophosphoribosyltransferase [Arabidopsis thaliana] gi|75213783|sp|Q9T0J5.1|ASE3_ARATH RecName: Full=Amidophosphoribosyltransferase 3, chloroplastic; Short=AtATase3; Short=PRPP3; AltName: Full=Glutamine phosphoribosylpyrophosphate amidotransferase 3; Short=AtGPRAT3; Flags: Precursor gi|4490340|emb|CAB38622.1| amidophosphoribosyltransferase-like protein [Arabidopsis thaliana] gi|7270871|emb|CAB80551.1| amidophosphoribosyltransferase-like protein [Arabidopsis thaliana] gi|332661586|gb|AEE86986.1| amidophosphoribosyltransferase 3 [Arabidopsis thaliana]	531	532	0	100.2	84.9	91.1	amidophosphoribosyltransferase	gbpln	Arabidopsis thaliana	AT4G38880.1 | Symbols: ATASE3, ASE3 | GLN phosphoribosyl pyrophosphate amidotransferase 3 | chr4:18134116-18135714 FORWARD LENGTH=532	531	532	0	100.2	84.9	91.1
Rsa1.0_00019.1.g1316.t1	ref|XP_002868869.1| dihydrouridine synthase family protein [Arabidopsis lyrata subsp. lyrata] gi|297314705|gb|EFH45128.1| dihydrouridine synthase family protein [Arabidopsis lyrata subsp. lyrata]	692	691	0	99.9	83.2	88.2	dihydrouridine synthase family protein	gbpln	Arabidopsis lyrata	AT4G38890.1 | Symbols: | FMN-linked oxidoreductases superfamily protein | chr4:18135909-18139100 REVERSE LENGTH=691	692	691	0	99.9	82.9	88.2

Rsa1.0_00019.1.g1317.t1	gb|ABU63288.1| GAGA-motif binding transcriptional activator, partial [Cardamine pratensis]	265	275	1.00E-123	103.8	80.8	90.9	GAGA-motif binding transcriptional activator, partial	gbpln	Cardamine pratensis	AT4G38910.2 | Symbols: BPC5 | basic pentacysteine 5 | chr4:18145478-18146429 REVERSE LENGTH=283	265	283	1.00E-116	106.8	76.6	87.5
Rsa1.0_00019.1.g1318.t1	ref|NP_195606.2| ATP binding microtubule motor family protein [Arabidopsis thaliana] gi|186517568|ref|NP_001119143.1| ATP binding microtubule motor family protein [Arabidopsis thaliana] gi|332661597|gb|AEE86997.1| ATP binding microtubule motor family protein [Arabidopsis thaliana] gi|332661598|gb|AEE86998.1| ATP binding microtubule motor family protein [Arabidopsis thaliana]	800	836	0	104.5	85.8	91.3	ATP binding microtubule motor family protein	gbpln	Arabidopsis thaliana	AT4G38950.2 | Symbols: | ATP binding microtubule motor family protein | chr4:18154606-18158461 REVERSE LENGTH=836	800	836	0	104.5	85.8	91.3
Rsa1.0_00019.1.g1319.t1	ref|XP_002868874.1| hypothetical protein ARALYDRAFT_490667 [Arabidopsis lyrata subsp. lyrata] gi|297314710|gb|EFH45133.1| hypothetical protein ARALYDRAFT_490667 [Arabidopsis lyrata subsp. lyrata]	237	393	5.00E-72	165.8	54.4	56.1	hypothetical protein ARALYDRAFT_490667	gbpln	Arabidopsis lyrata	AT4G38970.1 | Symbols: FBA2 | fructose-bisphosphate aldolase 2 | chr4:18163714-18165659 REVERSE LENGTH=398	237	398	1.00E-73	167.9	55.3	56.5
Rsa1.0_00019.1.g1320.t1	gb|EOA18196.1| hypothetical protein CARUB_v10006679mg [Capsella rubella]	443	493	0	111.3	81.0	87.1	hypothetical protein CARUB_v10006679mg	gbpln	Capsella rubella	AT4G39000.1 | Symbols: AtGH9B17, GH9B17 | glycosyl hydrolase 9B17 | chr4:18171722-18173797 REVERSE LENGTH=493	443	493	0	111.3	76.5	86.7
Rsa1.0_00019.1.g1321.t1	gb|EOA16494.1| hypothetical protein CARUB_v10004652mg [Capsella rubella]	472	496	0	105.1	92.2	95.8	hypothetical protein CARUB_v10004652mg	gbpln	Capsella rubella	AT4G39010.1 | Symbols: AtGH9B18, GH9B18 | glycosyl hydrolase 9B18 | chr4:18176162-18179102 REVERSE LENGTH=497	472	497	0	105.3	92.6	95.3
Rsa1.0_00019.1.g1322.t1	ref|XP_002866828.1| hypothetical protein ARALYDRAFT_490671 [Arabidopsis lyrata subsp. lyrata] gi|297312664|gb|EFH43087.1| hypothetical protein ARALYDRAFT_490671 [Arabidopsis lyrata subsp. lyrata]	514	541	0	105.3	86.4	92.6	hypothetical protein ARALYDRAFT_490671	gbpln	Arabidopsis lyrata	AT4G39030.1 | Symbols: EDS5, SID1 | MATE efflux family protein | chr4:18185740-18188898 FORWARD LENGTH=543	514	543	0	105.6	86.4	91.4
Rsa1.0_00019.1.g1323.t1	ref|XP_002868882.1| zinc finger (B-box type) family protein [Arabidopsis lyrata subsp. lyrata] gi|297314718|gb|EFH45141.1| zinc finger (B-box type) family protein [Arabidopsis lyrata subsp. lyrata]	234	238	2.00E-97	101.7	83.3	88.0	zinc finger (B-box type) family protein	gbpln	Arabidopsis lyrata	AT4G39070.1 | Symbols: | B-box zinc finger family protein | chr4:18205061-18206421 REVERSE LENGTH=242	234	242	1.00E-100	103.4	82.9	88.5
Rsa1.0_00019.1.g1324.t1	gb|EOA19105.1| hypothetical protein CARUB_v10007773mg [Capsella rubella]	794	819	0	103.1	92.7	96.9	hypothetical protein CARUB_v10007773mg	gbpln	Capsella rubella	AT4G39080.1 | Symbols: VHA-A3 | vacuolar proton ATPase A3 | chr4:18209513-18214752 FORWARD LENGTH=821	794	821	0	103.4	90.7	95.0
Rsa1.0_00019.1.g1325.t1	ref|XP_002868883.1| hypothetical protein ARALYDRAFT_490677 [Arabidopsis lyrata subsp. lyrata] gi|297314719|gb|EFH45142.1| hypothetical protein ARALYDRAFT_490677 [Arabidopsis lyrata subsp. lyrata]	371	368	0	99.2	87.9	92.2	hypothetical protein ARALYDRAFT_490677	gbpln	Arabidopsis lyrata	AT4G39090.1 | Symbols: RD19, RD19A | Papain family cysteine protease | chr4:18215826-18217326 REVERSE LENGTH=368	371	368	0	99.2	85.4	90.8
Rsa1.0_00019.1.g1326.t1	gb|EOA18823.1| hypothetical protein CARUB_v10007437mg [Capsella rubella]	110	110	2.00E-38	100.0	83.6	90.9	hypothetical protein CARUB_v10007437mg	gbpln	Capsella rubella	#	#	#	#	#	#	#
Rsa1.0_00019.1.g1327.t1	gb|EOA18763.1| hypothetical protein CARUB_v10007359mg [Capsella rubella]	877	882	0	100.6	92.4	95.9	hypothetical protein CARUB_v10007359mg	gbpln	Capsella rubella	AT4G39110.1 | Symbols: | Malectin/receptor-like protein kinase family protein | chr4:18222483-18225119 REVERSE LENGTH=878	877	878	0	100.1	91.6	95.3
Rsa1.0_00019.1.g1328.t1	ref|NP_195624.1| Dehydrin family protein [Arabidopsis thaliana] gi|4914425|emb|CAB43628.1| putative protein [Arabidopsis thaliana] gi|7270896|emb|CAB80576.1| putative protein [Arabidopsis thaliana] gi|21592637|gb|AAM64586.1| unknown [Arabidopsis thaliana] gi|332661621|gb|AEE87021.1| Dehydrin family protein [Arabidopsis thaliana]	277	151	2.00E-44	54.5	34.3	40.8	Dehydrin family protein	gbpln	Arabidopsis thaliana	AT4G39130.1 | Symbols: | Dehydrin family protein | chr4:18228183-18228844 REVERSE LENGTH=151	277	151	7.00E-47	54.5	34.3	40.8
Rsa1.0_00019.1.g1329.t1	gb|EOA15554.1| hypothetical protein CARUB_v10005169mg [Capsella rubella] gi|482551362|gb|EOA15555.1| hypothetical protein CARUB_v10005169mg [Capsella rubella]	344	344	0	100.0	88.4	95.6	hypothetical protein CARUB_v10005169mg	gbpln	Capsella rubella	AT4G39150.2 | Symbols: | DNAJ heat shock N-terminal domain-containing protein | chr4:18233651-18235740 REVERSE LENGTH=345	344	345	1.00E-180	100.3	87.5	95.3

Rsa1.0_00019.1.g1330.t1	ref|NP_195631.1| 40S ribosomal protein S25-4 [Arabidopsis thaliana] gi|30580493|sp|Q9T029.1|RS254_ARATH RecName: Full=40S ribosomal protein S25-4 gi|4914432|emb|CAB43635.1| ribosomal protein S25 [Arabidopsis thaliana] gi|7270903|emb|CAB80583.1| ribosomal protein S25 [Arabidopsis thaliana] gi|14335026|gb|AAK59777.1| AT4g39200/T22F8_100 [Arabidopsis thaliana] gi|16323232|gb|AAL15350.1| AT4g39200/T22F8_100 [Arabidopsis thaliana] gi|21553704|gb|AAM62797.1| ribosomal protein S25 [Arabidopsis thaliana] gi|332661636|gb|AEE87036.1| 40S ribosomal protein S25-4 [Arabidopsis thaliana] gi|482553510|gb|EOA17703.1| hypothetical protein CARUB_v10006075mg [Capsella rubella]	108	108	3.00E-55	100.0	99.1	99.1	40S ribosomal protein S25-4	gbpln	Arabidopsis thaliana	AT4G39200.1 | Symbols: | Ribosomal protein S25 family protein | chr4:18257464-18258464 FORWARD LENGTH=108	108	108	5.00E-58	100.0	99.1	99.1
Rsa1.0_00019.1.g1331.t1	gb|EOA17728.1| hypothetical protein CARUB_v10006110mg [Capsella rubella]	82	100	2.00E-30	122.0	89.0	95.1	hypothetical protein CARUB_v10006110mg	gbpln	Capsella rubella	AT4G39250.1 | Symbols: ATRL1, RSM2, RL1 | RAD-like 1 | chr4:18271457-18271857 REVERSE LENGTH=100	82	100	1.00E-32	122.0	86.6	91.5
Rsa1.0_00019.1.g1332.t1	sp|Q05966.1|GRP10_BRANA RecName: Full=Glycine-rich RNA-binding protein 10 gi|17819|emb|CAA78513.1| glycine-rich RNA binding protein [Brassica napus]	163	169	5.00E-40	103.7	48.5	50.3	RecName: Full=Glycine-rich RNA-binding protein 10 gi|17819|emb|CAA78513.1| glycine-rich RNA binding protein	gbpln	Brassica napus	AT4G39260.3 | Symbols: CCR1, ATGRP8, GR-RBP8, GRP8 | cold, circadian rhythm, and RNA binding 1 | chr4:18274166-18274958 REVERSE LENGTH=92	163	92	2.00E-43	56.4	49.7	54.0
Rsa1.0_00019.1.g1333.t1	ref|XP_002866840.1| hypothetical protein ARALYDRAFT_912391 [Arabidopsis lyrata subsp. lyrata] gi|297312676|gb|EFH43099.1| hypothetical protein ARALYDRAFT_912391 [Arabidopsis lyrata subsp. lyrata]	152	173	1.00E-39	113.8	65.1	78.3	hypothetical protein ARALYDRAFT_912391	gbpln	Arabidopsis lyrata	AT4G39320.1 | Symbols: | microtubule-associated protein-related | chr4:18289102-18289702 FORWARD LENGTH=166	152	166	4.00E-36	109.2	59.9	73.7
Rsa1.0_00019.1.g1334.t1	ref|NP_195644.1| uncharacterized protein [Arabidopsis thaliana] gi|75213705|sp|Q9T039.1|EC14_ARATH RecName: Full=Egg cell-secreted protein 1.4; Flags: Precursor gi|4914446|emb|CAB43649.1| hypothetical protein [Arabidopsis thaliana] gi|7270918|emb|CAB80597.1| hypothetical protein [Arabidopsis thaliana] gi|91805627|gb|ABE65542.1| hypothetical protein At4g39340 [Arabidopsis thaliana] gi|332661658|gb|AEE87058.1| uncharacterized protein AT4G39340 [Arabidopsis thaliana]	129	127	6.00E-53	98.4	79.8	86.8	uncharacterized protein	gbpln	Arabidopsis thaliana	AT4G39340.1 | Symbols: | Protein of unknown function (DUF1278) | chr4:18293129-18293512 REVERSE LENGTH=127	129	127	1.00E-55	98.4	79.8	86.8
Rsa1.0_00019.1.g1335.t1	gb|EOA18118.1| hypothetical protein CARUB_v10006579mg [Capsella rubella]	115	136	4.00E-29	118.3	62.6	75.7	hypothetical protein CARUB_v10006579mg	gbpln	Capsella rubella	AT4G39360.1 | Symbols: | unknown protein; BEST Arabidopsis thaliana protein match is: unknown protein (TAIR:AT2G21780.1); Has 13 Blast hits to 13 proteins in 2 species: Archae - 0; Bacteria - 0; Metazoa - 0; Fungi - 0; Plants - 13; Viruses - 0; Other Eukaryotes - 0 (source: NCBI BLink). | chr4:18303031-18303435 FORWARD LENGTH=134	115	134	1.00E-26	116.5	62.6	73.9
Rsa1.0_00019.1.g1336.t1	ref|NP_568059.1| nucleotide sugar transporter-KT 1 [Arabidopsis thaliana] gi|30692341|ref|NP_849527.1| nucleotide sugar transporter-KT 1 [Arabidopsis thaliana] gi|20259516|gb|AAM13878.1| unknown protein [Arabidopsis thaliana] gi|23296523|gb|AAN13117.1| unknown protein [Arabidopsis thaliana] gi|332661666|gb|AEE87066.1| nucleotide sugar transporter-KT 1 [Arabidopsis thaliana] gi|332661667|gb|AEE87067.1| nucleotide sugar transporter-KT 1 [Arabidopsis thaliana]	339	337	1.00E-171	99.4	92.3	95.0	nucleotide sugar transporter-KT 1	gbpln	Arabidopsis thaliana	AT4G39390.2 | Symbols: ATNST-KT1, NST-K1 | nucleotide sugar transporter-KT 1 | chr4:18316278-18317854 FORWARD LENGTH=337	339	337	1.00E-174	99.4	92.3	95.0
Rsa1.0_00019.1.g1337.t1	ref|XP_002866847.1| hypothetical protein ARALYDRAFT_912402 [Arabidopsis lyrata subsp. lyrata] gi|297312683|gb|EFH43106.1| hypothetical protein ARALYDRAFT_912402 [Arabidopsis lyrata subsp. lyrata]	1193	1195	0	100.2	89.3	93.6	hypothetical protein ARALYDRAFT_912402	gbpln	Arabidopsis lyrata	AT4G39400.1 | Symbols: BRI1, CBB2, DWF2, BIN1, ATBRI1 | Leucine-rich receptor-like protein kinase family protein | chr4:18324826-18328416 FORWARD LENGTH=1196	1193	1196	0	100.3	88.5	93.1

Rsa1.0_00019.1.g1338.t1	ref|NP_195651.1| putative WRKY transcription factor 13 [Arabidopsis thaliana] gi|29839686|sp|Q9SVB7.1|WRK13_ARATH RecName: Full=Probable WRKY transcription factor 13; AltName: Full=WRKY DNA-binding protein 13 gi|15991730|gb|AAL13042.1|AF421153_1 WRKY transcription factor 13 [Arabidopsis thaliana] gi|5042157|emb|CAB44676.1| putative WRKY DNA-binding protein [Arabidopsis thaliana] gi|7270925|emb|CAB80604.1| putative WRKY DNA-binding protein [Arabidopsis thaliana] gi|225898873|dbj|BAH30567.1| hypothetical protein [Arabidopsis thaliana] gi|332661671|gb|AEE87071.1| putative WRKY transcription factor 13 [Arabidopsis thaliana]	295	304	1.00E-102	103.1	73.9	82.7	putative WRKY transcription factor 13	gbpln	Arabidopsis thaliana	AT4G39410.1 | Symbols: WRKY13, ATWRKY13 | WRKY DNA-binding protein 13 | chr4:18332937-18334789 REVERSE LENGTH=304	295	304	1.00E-105	103.1	73.9	82.7
Rsa1.0_00019.1.g1339.t8	ref|NP_001154293.2| uncharacterized protein [Arabidopsis thaliana] gi|332661673|gb|AEE87073.1| uncharacterized protein AT4G39420 [Arabidopsis thaliana]	3143	3184	0	101.3	87.0	92.2	uncharacterized protein	gbpln	Arabidopsis thaliana	AT4G39420.2 | Symbols: | unknown protein; INVOLVED IN: biological_process unknown; LOCATED IN: cellular_component unknown; EXPRESSED IN: leaf; EXPRESSED DURING: LP.04 four leaves visible, LP.02 two leaves visible; Has 20 Blast hits to 19 proteins in 8 species: Archae - 0; Bacteria - 0; Metazoa - 0; Fungi - 0; Plants - 20; Viruses - 0; Other Eukaryotes - 0 (source: NCBI BLink). | chr4:18339731-18354701 FORWARD LENGTH=3184	3143	3184	0	101.3	87.0	92.2
Rsa1.0_00019.1.g1340.t1	gb|EOA12720.1| hypothetical protein CARUB_v10028124mg [Capsella rubella]	507	514	0	101.4	69.6	84.6	hypothetical protein CARUB_v10028124mg	gbpln	Capsella rubella	AT4G32170.1 | Symbols: CYP96A2 | cytochrome P450, family 96, subfamily A, polypeptide 2 | chr4:15533772-15535292 FORWARD LENGTH=506	507	506	0	99.8	65.9	79.7
Rsa1.0_00019.1.g1341.t1	ref|NP_195661.1| cytochrome P450, family 96, subfamily A, polypeptide 12 [Arabidopsis thaliana] gi|5042167|emb|CAB44686.1| cytochrome P450-like protein [Arabidopsis thaliana] gi|7270935|emb|CAB80614.1| cytochrome P450-like protein [Arabidopsis thaliana] gi|16604438|gb|AAL24225.1| AT4g39510/F23K16_140 [Arabidopsis thaliana] gi|27764938|gb|AAO23590.1| At4g39510/F23K16_140 [Arabidopsis thaliana] gi|332661680|gb|AEE87080.1| cytochrome P450, family 96, subfamily A, polypeptide 12 [Arabidopsis thaliana]	500	508	0	101.6	76.8	88.2	cytochrome P450, family 96, subfamily A, polypeptide 12	gbpln	Arabidopsis thaliana	AT4G39510.1 | Symbols: CYP96A12 | cytochrome P450, family 96, subfamily A, polypeptide 12 | chr4:18368945-18370471 REVERSE LENGTH=508	500	508	0	101.6	76.8	88.2
Rsa1.0_00019.1.g1342.t1	ref|NP_195662.1| GTP-binding protein-relatedlike protein [Arabidopsis thaliana] gi|5042168|emb|CAB44687.1| GTP-binding-like protein [Arabidopsis thaliana] gi|7270936|emb|CAB80615.1| GTP-binding-like protein [Arabidopsis thaliana] gi|14334722|gb|AAK59539.1| putative GTP-binding protein [Arabidopsis thaliana] gi|332661681|gb|AEE87081.1| GTP-binding protein-relatedlike protein [Arabidopsis thaliana]	369	369	0	100.0	97.0	98.4	GTP-binding protein-relatedlike protein	gbpln	Arabidopsis thaliana	AT4G39520.1 | Symbols: | GTP-binding protein-related | chr4:18371329-18374000 REVERSE LENGTH=369	369	369	0	100.0	97.0	98.4
Rsa1.0_00019.1.g1343.t1	ref|NP_195664.2| shikimate kinase 2 [Arabidopsis thaliana] gi|42573237|ref|NP_974715.1| shikimate kinase 2 [Arabidopsis thaliana] gi|75151384|sp|Q8GY88.1|SK2_ARATH RecName: Full=Shikimate kinase 2, chloroplastic; Short=AtSK2; Flags: Precursor gi|26450651|dbj|BAC42436.1| putative shikimate kinase [Arabidopsis thaliana] gi|28950863|gb|AAO63355.1| At4g39540 [Arabidopsis thaliana] gi|332661683|gb|AEE87083.1| shikimate kinase 2 [Arabidopsis thaliana] gi|332661684|gb|AEE87084.1| shikimate kinase 2 [Arabidopsis thaliana]	256	300	1.00E-111	117.2	84.4	89.8	shikimate kinase 2	gbpln	Arabidopsis thaliana	AT4G39540.2 | Symbols: SK2, ATSK2 | shikimate kinase 2 | chr4:18378561-18380253 FORWARD LENGTH=300	256	300	1.00E-113	117.2	84.4	89.8

Rsa1.0_00019.1.g1344.t1	ref|XP_002868913.1| kelch repeat-containing F-box family protein [Arabidopsis lyrata subsp. lyrata] gi|297314749|gb|EFH45172.1| kelch repeat-containing F-box family protein [Arabidopsis lyrata subsp. lyrata]	400	389	1.00E-119	97.3	56.8	69.5	kelch repeat-containing F-box family protein	gbpln	Arabidopsis lyrata	AT4G39550.1 | Symbols: | Galactose oxidase/kelch repeat superfamily protein | chr4:18380681-18381859 REVERSE LENGTH=392	400	392	1.00E-121	98.0	60.5	72.8
Rsa1.0_00019.1.g1345.t1	ref|XP_002868918.1| hypothetical protein ARALYDRAFT_490739 [Arabidopsis lyrata subsp. lyrata] gi|297314754|gb|EFH45177.1| hypothetical protein ARALYDRAFT_490739 [Arabidopsis lyrata subsp. lyrata]	391	400	7.00E-93	102.3	53.2	66.0	hypothetical protein ARALYDRAFT_490739	gbpln	Arabidopsis lyrata	AT4G39590.1 | Symbols: | Galactose oxidase/kelch repeat superfamily protein | chr4:18387515-18388723 REVERSE LENGTH=402	391	402	4.00E-90	102.8	52.2	63.4
Rsa1.0_00019.1.g1346.t1	#	#	#	#	#	#	#	-	----	----	#	#	#	#	#	#	#
Rsa1.0_00019.1.g1347.t1	gb|EOA39864.1| hypothetical protein CARUB_v10008540mg [Capsella rubella] gi|482575678|gb|EOA39865.1| hypothetical protein CARUB_v10008540mg [Capsella rubella] gi|482575679|gb|EOA39866.1| hypothetical protein CARUB_v10008540mg [Capsella rubella]	111	654	6.00E-11	589.2	39.6	46.8	hypothetical protein CARUB_v10008540mg	gbpln	Capsella rubella	AT1G14380.3 | Symbols: IQD28 | IQ-domain 28 | chr1:4918279-4920761 REVERSE LENGTH=664	111	664	3.00E-13	598.2	39.6	45.9
Rsa1.0_00019.1.g1348.t1	gb|EOA34391.1| hypothetical protein CARUB_v10021918mg [Capsella rubella]	407	403	7.00E-96	99.0	52.8	65.6	hypothetical protein CARUB_v10021918mg	gbpln	Capsella rubella	AT1G61540.1 | Symbols: | Galactose oxidase/kelch repeat superfamily protein | chr1:22702498-22703706 FORWARD LENGTH=402	407	402	2.00E-96	98.8	53.1	63.9
Rsa1.0_00019.1.g1349.t1	ref|NP_195665.1| F-box/kelch-repeat protein [Arabidopsis thaliana] gi|75210850|sp|Q9SVA3.1|FBK98_ARATH RecName: Full=F-box/kelch-repeat protein At4g39550 gi|5042171|emb|CAB44690.1| putative protein [Arabidopsis thaliana] gi|7270939|emb|CAB80618.1| putative protein [Arabidopsis thaliana] gi|27808596|gb|AAO24578.1| At4g39550 [Arabidopsis thaliana] gi|110736223|dbj|BAF00082.1| hypothetical protein [Arabidopsis thaliana] gi|332661686|gb|AEE87086.1| F-box/kelch-repeat protein [Arabidopsis thaliana]	392	392	1.00E-138	100.0	69.4	80.1	F-box/kelch-repeat protein	gbpln	Arabidopsis thaliana	AT4G39550.1 | Symbols: | Galactose oxidase/kelch repeat superfamily protein | chr4:18380681-18381859 REVERSE LENGTH=392	392	392	1.00E-140	100.0	69.4	80.1
Rsa1.0_00019.1.g1350.t1	ref|XP_002868918.1| hypothetical protein ARALYDRAFT_490739 [Arabidopsis lyrata subsp. lyrata] gi|297314754|gb|EFH45177.1| hypothetical protein ARALYDRAFT_490739 [Arabidopsis lyrata subsp. lyrata]	385	400	1.00E-93	103.9	49.4	65.5	hypothetical protein ARALYDRAFT_490739	gbpln	Arabidopsis lyrata	AT4G39590.1 | Symbols: | Galactose oxidase/kelch repeat superfamily protein | chr4:18387515-18388723 REVERSE LENGTH=402	385	402	3.00E-94	104.4	51.4	66.5
Rsa1.0_00019.1.g1351.t1	gb|EOA34391.1| hypothetical protein CARUB_v10021918mg [Capsella rubella]	412	403	5.00E-93	97.8	50.2	63.8	hypothetical protein CARUB_v10021918mg	gbpln	Capsella rubella	AT1G61540.1 | Symbols: | Galactose oxidase/kelch repeat superfamily protein | chr1:22702498-22703706 FORWARD LENGTH=402	412	402	2.00E-93	97.6	50.0	64.3
Rsa1.0_00019.1.g1352.t1	#	#	#	#	#	#	#	-	----	----	#	#	#	#	#	#	#
Rsa1.0_00019.1.g1353.t1	ref|NP_179734.1| coatomer subunit alpha-2 [Arabidopsis thaliana] gi|75337326|sp|Q9SJT9.1|COPA2_ARATH RecName: Full=Coatomer subunit alpha-2; AltName: Full=Alpha-coat protein 2; Short=Alpha-COP 2 gi|4567286|gb|AAD23699.1| coatomer alpha subunit [Arabidopsis thaliana] gi|110737300|dbj|BAF00596.1| coatomer alpha subunit [Arabidopsis thaliana] gi|330252079|gb|AEC07173.1| coatomer subunit alpha-2 [Arabidopsis thaliana]	1136	1218	0	107.2	79.2	86.4	coatomer subunit alpha-2	gbpln	Arabidopsis thaliana	AT2G21390.1 | Symbols: | Coatomer, alpha subunit | chr2:9152428-9156577 FORWARD LENGTH=1218	1136	1218	0	107.2	79.2	86.4
Rsa1.0_00019.1.g1354.t1	gb|EOA16490.1| hypothetical protein CARUB_v10004648mg [Capsella rubella]	500	497	0	99.4	85.6	90.4	hypothetical protein CARUB_v10004648mg	gbpln	Capsella rubella	AT4G39620.1 | Symbols: EMB2453, ATPPR5 | Tetratricopeptide repeat (TPR)-like superfamily protein | chr4:18395294-18397578 FORWARD LENGTH=563	500	563	0	112.6	83.8	88.8
Rsa1.0_00019.1.g1355.t1	gb|ACJ65004.1| myo-inositol 1-phosphate synthase type 2 [Brassica napus]	510	510	0	100.0	98.8	99.6	myo-inositol 1-phosphate synthase type 2	gbpln	Brassica napus	AT4G39800.1 | Symbols: MI-1-P SYNTHASE, MIPS1, ATMIPS1, ATIPS1 | myo-inositol-1-phosphate synthase 1 | chr4:18469659-18471893 REVERSE LENGTH=511	510	511	0	100.2	95.1	98.2
Rsa1.0_00019.1.g1356.t1	#	#	#	#	#	#	#	-	----	----	#	#	#	#	#	#	#

Rsa1.0_00019.1.g1357.t1	ref|XP_002866887.1| hypothetical protein ARALYDRAFT_490769 [Arabidopsis lyrata subsp. lyrata] gi|297312723|gb|EFH43146.1| hypothetical protein ARALYDRAFT_490769 [Arabidopsis lyrata subsp. lyrata]	446	449	1.00E-166	100.7	83.2	88.3	hypothetical protein ARALYDRAFT_490769	gbpln	Arabidopsis lyrata	AT4G39840.1 | Symbols: | unknown protein; FUNCTIONS IN: molecular_function unknown; INVOLVED IN: biological_process unknown; LOCATED IN: endomembrane system; EXPRESSED IN: 23 plant structures; EXPRESSED DURING: 13 growth stages; Has 20719 Blast hits to 6096 proteins in 607 species: Archae - 22; Bacteria - 3243; Metazoa - 4364; Fungi - 2270; Plants - 237; Viruses - 128; Other Eukaryotes - 10455 (source: NCBI BLink). | chr4:18485268-18486623 FORWARD LENGTH=451	446	451	1.00E-156	101.1	83.4	89.2
Rsa1.0_00019.1.g1358.t1	ref|XP_002866889.1| hypothetical protein ARALYDRAFT_912474 [Arabidopsis lyrata subsp. lyrata] gi|297312725|gb|EFH43148.1| hypothetical protein ARALYDRAFT_912474 [Arabidopsis lyrata subsp. lyrata]	621	297	1.00E-152	47.8	43.6	45.4	hypothetical protein ARALYDRAFT_912474	gbpln	Arabidopsis lyrata	AT4G39860.1 | Symbols: | unknown protein; BEST Arabidopsis thaliana protein match is: unknown protein (TAIR:AT2G22270.1); Has 152 Blast hits to 146 proteins in 19 species: Archae - 0; Bacteria - 0; Metazoa - 0; Fungi - 2; Plants - 146; Viruses - 0; Other Eukaryotes - 4 (source: NCBI BLink). | chr4:18499909-18501472 FORWARD LENGTH=299	621	299	1.00E-150	48.1	43.5	45.4
Rsa1.0_00019.1.g1359.t1	gb|AAM63197.1| unknown [Arabidopsis thaliana]	180	178	2.00E-81	98.9	87.2	92.8	unknown	gbpln	Arabidopsis thaliana	AT4G39880.1 | Symbols: | Ribosomal protein L23/L15e family protein | chr4:18504601-18505137 FORWARD LENGTH=178	180	178	2.00E-76	98.9	85.0	90.6
Rsa1.0_00019.1.g1360.t1	ref|XP_002868935.1| hypothetical protein ARALYDRAFT_912479 [Arabidopsis lyrata subsp. lyrata] gi|297314771|gb|EFH45194.1| hypothetical protein ARALYDRAFT_912479 [Arabidopsis lyrata subsp. lyrata]	231	244	2.00E-90	105.6	78.4	87.4	hypothetical protein ARALYDRAFT_912479	gbpln	Arabidopsis lyrata	AT4G39900.1 | Symbols: | unknown protein; Has 30201 Blast hits to 17322 proteins in 780 species: Archae - 12; Bacteria - 1396; Metazoa - 17338; Fungi - 3422; Plants - 5037; Viruses - 0; Other Eukaryotes - 2996 (source: NCBI BLink). | chr4:18508569-18510289 REVERSE LENGTH=245	231	245	9.00E-92	106.1	77.1	84.4
Rsa1.0_00019.1.g1361.t1	ref|NP_568074.1| ubiquitin carboxyl-terminal hydrolase 3 [Arabidopsis thaliana] gi|62901126|sp|O24454.1|UBP3_ARATH RecName: Full=Ubiquitin carboxyl-terminal hydrolase 3; AltName: Full=Deubiquitinating enzyme 3; Short=AtUBP3; AltName: Full=Ubiquitin thioesterase 3; AltName: Full=Ubiquitin-specific-processing protease 3 gi|2347098|gb|AAB67966.1| ubiquitin-specific protease [Arabidopsis thaliana] gi|4490742|emb|CAB38904.1| ubiquitin-specific protease (AtUBP3) [Arabidopsis thaliana] gi|7271046|emb|CAB80654.1| ubiquitin-specific protease (AtUBP3) [Arabidopsis thaliana] gi|16604539|gb|AAL24275.1| AT4g39910/T5J17_80 [Arabidopsis thaliana] gi|18958042|gb|AAL79594.1| AT4g39910/T5J17_80 [Arabidopsis thaliana] gi|332661736|gb|AEE87136.1| ubiquitin carboxyl-terminal hydrolase 3 [Arabidopsis thaliana]	370	371	0	100.3	95.7	97.8	ubiquitin carboxyl-terminal hydrolase 3	gbpln	Arabidopsis thaliana	AT4G39910.1 | Symbols: ATUBP3, UBP3 | ubiquitin-specific protease 3 | chr4:18511858-18514139 REVERSE LENGTH=371	370	371	0	100.3	95.7	97.8
Rsa1.0_00019.1.g1362.t1	ref|XP_002866895.1| aps-kinase 2 [Arabidopsis lyrata subsp. lyrata] gi|297312731|gb|EFH43154.1| aps-kinase 2 [Arabidopsis lyrata subsp. lyrata]	293	292	1.00E-147	99.7	87.0	92.2	aps-kinase 2	gbpln	Arabidopsis lyrata	AT4G39940.1 | Symbols: AKN2, APK2 | APS-kinase 2 | chr4:18519787-18521276 FORWARD LENGTH=293	293	293	1.00E-148	100.0	87.7	92.8
Rsa1.0_00019.1.g1363.t1	gb|AAN76810.1|AF453287_1 cytochrome P450 [Brassica napus]	540	540	0	100.0	98.0	98.7	cytochrome P450	gbpln	Brassica napus	AT4G39950.1 | Symbols: CYP79B2 | cytochrome P450, family 79, subfamily B, polypeptide 2 | chr4:18525311-18527284 FORWARD LENGTH=541	540	541	0	100.2	93.5	97.4
Rsa1.0_00019.1.g1364.t1	ref|NP_680777.2| pentatricopeptide repeat-containing protein [Arabidopsis thaliana] gi|223635624|sp|Q3E9N1.2|PP359_ARATH RecName: Full=Pentatricopeptide repeat-containing protein At4g39952, mitochondrial; Flags: Precursor gi|332661744|gb|AEE87144.1| pentatricopeptide repeat-containing protein [Arabidopsis thaliana]	781	775	0	99.2	82.1	89.4	pentatricopeptide repeat-containing protein	gbpln	Arabidopsis thaliana	AT4G39952.1 | Symbols: | Pentatricopeptide repeat (PPR) superfamily protein | chr4:18527680-18530007 FORWARD LENGTH=775	781	775	0	99.2	82.1	89.4
Rsa1.0_00019.1.g1365.t1	gb|ACP30598.1| disease resistance protein [Brassica rapa subsp. pekinensis]	1459	2301	0	157.7	82.5	89.1	disease resistance protein	gbpln	Brassica rapa	AT4G23160.1 | Symbols: CRK8 | cysteine-rich RLK (RECEPTOR-like protein kinase) 8 | chr4:12129485-12134086 FORWARD LENGTH=1262	1459	1262	1.00E-108	86.5	13.9	21.2

Rsa1.0_00019.1.g1366.t1	ref|XP_002866900.1| DNAJ heat shock family protein [Arabidopsis lyrata subsp. lyrata] gi|297312736|gb|EFH43159.1| DNAJ heat shock family protein [Arabidopsis lyrata subsp. lyrata]	450	447	0	99.3	93.1	95.3	DNAJ heat shock family protein	gbpln	Arabidopsis lyrata	AT4G39960.1 | Symbols: | Molecular chaperone Hsp40/DnaJ family protein | chr4:18534194-18536320 FORWARD LENGTH=447	450	447	0	99.3	92.9	95.3
Rsa1.0_00019.1.g1367.t1	ref|XP_002866901.1| 3-deoxy-D-arabino-heptulosonate 7-phosphate synthase 1 [Arabidopsis lyrata subsp. lyrata] gi|297312737|gb|EFH43160.1| 3-deoxy-D-arabino-heptulosonate 7-phosphate synthase 1 [Arabidopsis lyrata subsp. lyrata]	528	528	0	100.0	93.9	95.8	3-deoxy-D-arabino-heptulosonate 7-phosphate synthase 1	gbpln	Arabidopsis lyrata	AT4G39980.1 | Symbols: DHS1 | 3-deoxy-D-arabino-heptulosonate 7-phosphate synthase 1 | chr4:18539654-18541832 FORWARD LENGTH=525	528	525	0	99.4	93.6	95.3
Rsa1.0_00019.1.g1368.t1	gb|EOA17314.1| hypothetical protein CARUB_v10005586mg, partial [Capsella rubella]	224	250	1.00E-114	111.6	88.4	93.8	hypothetical protein CARUB_v10005586mg, partial	gbpln	Capsella rubella	AT4G39990.1 | Symbols: ATRABA4B, ATRAB11G, ATGB3, RABA4B | RAB GTPase homolog A4B | chr4:18542722-18543779 FORWARD LENGTH=224	224	224	1.00E-116	100.0	86.6	93.3
Rsa1.0_00019.1.g1369.t1	ref|XP_002865966.1| hypothetical protein ARALYDRAFT_357572 [Arabidopsis lyrata subsp. lyrata] gi|297311801|gb|EFH42225.1| hypothetical protein ARALYDRAFT_357572 [Arabidopsis lyrata subsp. lyrata]	429	455	1.00E-74	106.1	44.1	55.0	hypothetical protein ARALYDRAFT_357572	gbpln	Arabidopsis lyrata	AT1G30790.1 | Symbols: | F-box and associated interaction domains-containing protein | chr1:10932713-10933912 FORWARD LENGTH=399	429	399	6.00E-62	93.0	34.5	45.0
Rsa1.0_00019.1.g1370.t1	ref|XP_002866878.1| hypothetical protein ARALYDRAFT_490751 [Arabidopsis lyrata subsp. lyrata] gi|297312714|gb|EFH43137.1| hypothetical protein ARALYDRAFT_490751 [Arabidopsis lyrata subsp. lyrata]	133	657	1.00E-12	494.0	45.9	64.7	hypothetical protein ARALYDRAFT_490751	gbpln	Arabidopsis lyrata	AT4G39690.1 | Symbols: | FUNCTIONS IN: molecular_function unknown; INVOLVED IN: biological_process unknown; LOCATED IN: mitochondrion; EXPRESSED IN: 26 plant structures; EXPRESSED DURING: 14 growth stages; CONTAINS InterPro DOMAIN/s: Mitochondrial inner membrane protein Mitofilin (InterPro:IPR019133); Has 30201 Blast hits to 17322 proteins in 780 species: Archae - 12; Bacteria - 1396; Metazoa - 17338; Fungi - 3422; Plants - 5037; Viruses - 0; Other Eukaryotes - 2996 (source: NCBI BLink). | chr4:18417755-18421633 FORWARD LENGTH=650	133	650	3.00E-14	488.7	45.9	63.2
Rsa1.0_00019.1.g1371.t1	gb|EOA37553.1| hypothetical protein CARUB_v10011820mg, partial [Capsella rubella]	380	374	1.00E-70	98.4	40.8	51.8	hypothetical protein CARUB_v10011820mg, partial	gbpln	Capsella rubella	AT1G30790.1 | Symbols: | F-box and associated interaction domains-containing protein | chr1:10932713-10933912 FORWARD LENGTH=399	380	399	2.00E-64	105.0	39.2	51.6
Rsa1.0_00019.1.g1372.t1	ref|NP_195710.3| S-adenosyl-L-methionine-dependent methyltransferase-like protein [Arabidopsis thaliana] gi|332661751|gb|AEE87151.1| S-adenosyl-L-methionine-dependent methyltransferase-like protein [Arabidopsis thaliana]	747	783	0	104.8	81.4	88.9	S-adenosyl-L-methionine-dependent methyltransferase-like protein	gbpln	Arabidopsis thaliana	AT4G40000.1 | Symbols: | S-adenosyl-L-methionine-dependent methyltransferases superfamily protein | chr4:18543989-18547443 REVERSE LENGTH=783	747	783	0	104.8	81.4	88.9
Rsa1.0_00019.1.g1373.t1	ref|XP_002868939.1| hypothetical protein ARALYDRAFT_353011 [Arabidopsis lyrata subsp. lyrata] gi|297314775|gb|EFH45198.1| hypothetical protein ARALYDRAFT_353011 [Arabidopsis lyrata subsp. lyrata]	350	350	1.00E-168	100.0	87.1	92.0	hypothetical protein ARALYDRAFT_353011	gbpln	Arabidopsis lyrata	AT4G40010.1 | Symbols: SNRK2-7, SNRK2.7, SRK2F | SNF1-related protein kinase 2.7 | chr4:18548704-18551056 REVERSE LENGTH=350	350	350	1.00E-165	100.0	88.0	91.4
Rsa1.0_00019.1.g1374.t1	ref|XP_002868940.1| hypothetical protein ARALYDRAFT_912494 [Arabidopsis lyrata subsp. lyrata] gi|297314776|gb|EFH45199.1| hypothetical protein ARALYDRAFT_912494 [Arabidopsis lyrata subsp. lyrata]	591	618	0	104.6	73.3	85.8	hypothetical protein ARALYDRAFT_912494	gbpln	Arabidopsis lyrata	AT4G40020.1 | Symbols: | Myosin heavy chain-related protein | chr4:18553453-18555300 REVERSE LENGTH=615	591	615	0	104.1	71.9	84.9
Rsa1.0_00019.1.g1375.t1	gb|AFW86017.1| histone H3 [Zea mays]	149	227	7.00E-73	152.3	91.3	91.9	histone H3	gbenv/gbpln	Zea mays	AT4G40030.2 | Symbols: | Histone superfamily protein | chr4:18555840-18556827 REVERSE LENGTH=164	149	164	5.00E-75	110.1	91.3	92.6
Rsa1.0_00019.1.g1376.t1	gb|EOA17741.1| hypothetical protein CARUB_v10006125mg [Capsella rubella]	91	92	6.00E-37	101.1	81.3	85.7	hypothetical protein CARUB_v10006125mg	gbpln	Capsella rubella	AT4G40042.1 | Symbols: | Microsomal signal peptidase 12 kDa subunit (SPC12) | chr4:18559289-18559570 FORWARD LENGTH=93	91	93	3.00E-37	102.2	76.9	82.4

Rsa1.0_00019.1.g1377.t1	ref|NP_568078.1| uncharacterized protein [Arabidopsis thaliana] gi|15450922|gb|AAK96732.1| Unknown protein [Arabidopsis thaliana] gi|17978747|gb|AAL47367.1| unknown protein [Arabidopsis thaliana] gi|21592335|gb|AAM64286.1| unknown [Arabidopsis thaliana] gi|110742774|dbj|BAE99292.1| hypothetical protein [Arabidopsis thaliana] gi|332661761|gb|AEE87161.1| uncharacterized protein AT4G40045 [Arabidopsis thaliana]	141	140	2.00E-52	99.3	80.1	87.2	uncharacterized protein	gbpln	Arabidopsis thaliana	AT4G40045.1 | Symbols: | unknown protein; Has 30201 Blast hits to 17322 proteins in 780 species: Archae - 12; Bacteria - 1396; Metazoa - 17338; Fungi - 3422; Plants - 5037; Viruses - 0; Other Eukaryotes - 2996 (source: NCBI BLink). | chr4:18559926-18560348 REVERSE LENGTH=140	141	140	4.00E-55	99.3	80.1	87.2
Rsa1.0_00019.1.g1378.t1	ref|XP_002866906.1| hypothetical protein ARALYDRAFT_490794 [Arabidopsis lyrata subsp. lyrata] gi|297312742|gb|EFH43165.1| hypothetical protein ARALYDRAFT_490794 [Arabidopsis lyrata subsp. lyrata]	595	603	0	101.3	90.4	95.0	hypothetical protein ARALYDRAFT_490794	gbpln	Arabidopsis lyrata	AT4G40050.1 | Symbols: | Protein of unknown function (DUF3550/UPF0682) | chr4:18561206-18563727 REVERSE LENGTH=599	595	599	0	100.7	88.4	94.3
Rsa1.0_00020.1.g1379.t1	#	#	#	#	#	#	#	-	----	----	#	#	#	#	#	#	#
Rsa1.0_00020.1.g1380.t1	#	#	#	#	#	#	#	-	----	----	#	#	#	#	#	#	#
Rsa1.0_00020.1.g1381.t1	ref|NP_172777.1| actin-related protein 3 [Arabidopsis thaliana] gi|75313139|sp|Q9SAF1.1|ARP3_ARATH RecName: Full=Actin-related protein 3; AltName: Full=Protein DISTORTED TRICHOMES 1 gi|4850401|gb|AAD31071.1|AC007357_20 Strong similarity to gb|U29610 Actin-like protein (Arp3) from Acanthamoeba castellanii and is a member of the PF|00022 Actin family [Arabidopsis thaliana] gi|21427461|gb|AAM53243.1|AF507911_1 actin-related protein 3 [Arabidopsis thaliana] gi|20260500|gb|AAM13148.1| similar to actin-like protein [Arabidopsis thaliana] gi|21489929|tpg|DAA00033.1| TPA_exp: actin-related protein 3; AtARP3 [Arabidopsis thaliana] gi|30387525|gb|AAP31928.1| At1g13180 [Arabidopsis thaliana] gi|332190859|gb|AEE28980.1| actin-related protein 3 [Arabidopsis thaliana]	427	427	0	100.0	95.1	97.2	actin-related protein 3	gbpln	Arabidopsis thaliana	AT1G13180.1 | Symbols: DIS1, ARP3, ATARP3 | Actin-like ATPase superfamily protein | chr1:4495394-4498204 FORWARD LENGTH=427	427	427	0	100.0	95.1	97.2
Rsa1.0_00020.1.g1382.t1	gb|AAK31592.1| cytochrome P450 [Brassica rapa subsp. pekinensis]	526	525	0	99.8	91.1	95.1	cytochrome P450	gbpln	Brassica rapa	AT1G13150.1 | Symbols: CYP86C4 | cytochrome P450, family 86, subfamily C, polypeptide 4 | chr1:4481995-4483584 REVERSE LENGTH=529	526	529	0	100.6	86.1	92.8
Rsa1.0_00020.1.g1383.t1	ref|NP_172767.1| cytochrome P450 71B2 [Arabidopsis thaliana] gi|38503391|sp|O65788.2|C71B2_ARATH RecName: Full=Cytochrome P450 71B2 gi|4850391|gb|AAD31061.1|AC007357_10 Identical to gb|D78605 cytochrome P450 monooxygenase from Arabidopsis thaliana and is a member of the PF|00067 Cytochrome P450 family. ESTs gb|Z18072, gb|Z35218 and gb|T43466 come from this gene [Arabidopsis thaliana] gi|28392854|gb|AAO41864.1| putative cytochrome P450 monooxygenase [Arabidopsis thaliana] gi|332190846|gb|AEE28967.1| cytochrome P450 71B2 [Arabidopsis thaliana]	502	502	0	100.0	81.7	92.0	cytochrome P450 71B2	gbpln	Arabidopsis thaliana	AT1G13080.1 | Symbols: CYP71B2 | cytochrome P450, family 71, subfamily B, polypeptide 2 | chr1:4459212-4460807 FORWARD LENGTH=502	502	502	0	100.0	81.7	92.0
Rsa1.0_00020.1.g1384.t1	gb|EOA40203.1| hypothetical protein CARUB_v10008923mg [Capsella rubella]	462	502	0	108.7	76.2	86.4	hypothetical protein CARUB_v10008923mg	gbpln	Capsella rubella	AT1G13080.1 | Symbols: CYP71B2 | cytochrome P450, family 71, subfamily B, polypeptide 2 | chr1:4459212-4460807 FORWARD LENGTH=502	462	502	0	108.7	75.5	86.1

Rsa1.0_00020.1.g1385.t1	ref|NP_172767.1| cytochrome P450 71B2 [Arabidopsis thaliana] gi|38503391|sp|O65788.2|C71B2_ARATH RecName: Full=Cytochrome P450 71B2 gi|4850391|gb|AAD31061.1|AC007357_10 Identical to gb|D78605 cytochrome P450 monooxygenase from Arabidopsis thaliana and is a member of the PF|00067 Cytochrome P450 family. ESTs gb|Z18072, gb|Z35218 and gb|T43466 come from this gene [Arabidopsis thaliana] gi|28392854|gb|AAO41864.1| putative cytochrome P450 monooxygenase [Arabidopsis thaliana] gi|332190846|gb|AEE28967.1| cytochrome P450 71B2 [Arabidopsis thaliana]	476	502	0	105.5	77.5	88.7	cytochrome P450 71B2	gbpln	Arabidopsis thaliana	AT1G13080.1 | Symbols: CYP71B2 | cytochrome P450, family 71, subfamily B, polypeptide 2 | chr1:4459212-4460807 FORWARD LENGTH=502	476	502	0	105.5	77.5	88.7
Rsa1.0_00020.1.g1386.t1	ref|NP_189265.1| proteasome subunit beta type-5-B [Arabidopsis thaliana] gi|75273433|sp|Q9LIP2.1|PSB5B_ARATH RecName: Full=Proteasome subunit beta type-5-B; AltName: Full=20S proteasome beta subunit E-2; AltName: Full=Proteasome epsilon-2 chain; Flags: Precursor gi|16612308|gb|AAL27514.1|AF439846_1 AT3g26340/F20C19_6 [Arabidopsis thaliana] gi|9294292|dbj|BAB02194.1| proteasome epsilon chain precursor [Arabidopsis thaliana] gi|21553804|gb|AAM62897.1| 26S proteasome beta subunit, putative [Arabidopsis thaliana] gi|21928101|gb|AAM78079.1| AT3g26340/F20C19_6 [Arabidopsis thaliana] gi|332643626|gb|AEE77147.1| proteasome subunit beta type-5-B [Arabidopsis thaliana]	274	273	1.00E-149	99.6	93.8	96.0	proteasome subunit beta type-5-B	gbpln	Arabidopsis thaliana	AT3G26340.1 | Symbols: | N-terminal nucleophile aminohydrolases (Ntn hydrolases) superfamily protein | chr3:9650600-9652572 REVERSE LENGTH=273	274	273	1.00E-152	99.6	93.8	96.0
Rsa1.0_00020.1.g1387.t1	gb|EOA36472.1| hypothetical protein CARUB_v10011087mg [Capsella rubella]	320	317	1.00E-137	99.1	81.3	87.5	hypothetical protein CARUB_v10011087mg	gbpln	Capsella rubella	AT1G13050.1 | Symbols: | unknown protein; BEST Arabidopsis thaliana protein match is: unknown protein (TAIR:AT3G26350.1); Has 538 Blast hits to 510 proteins in 88 species: Archae - 0; Bacteria - 23; Metazoa - 81; Fungi - 36; Plants - 361; Viruses - 8; Other Eukaryotes - 29 (source: NCBI BLink). | chr1:4450568-4451521 FORWARD LENGTH=317	320	317	1.00E-134	99.1	82.2	88.1
Rsa1.0_00020.1.g1388.t1	ref|XP_002889980.1| pentatricopeptide repeat-containing protein [Arabidopsis lyrata subsp. lyrata] gi|297335822|gb|EFH66239.1| pentatricopeptide repeat-containing protein [Arabidopsis lyrata subsp. lyrata]	520	517	0	99.4	88.3	94.4	pentatricopeptide repeat-containing protein	gbpln	Arabidopsis lyrata	AT1G13040.1 | Symbols: | Pentatricopeptide repeat (PPR-like) superfamily protein | chr1:4447647-4449200 FORWARD LENGTH=517	520	517	0	99.4	87.7	94.2
Rsa1.0_00020.1.g1389.t1	gb|EOA39000.1| hypothetical protein CARUB_v10011529mg [Capsella rubella]	519	519	0	100.0	91.1	96.0	hypothetical protein CARUB_v10011529mg	gbpln	Capsella rubella	AT1G12950.1 | Symbols: RSH2 | root hair specific 2 | chr1:4419849-4422462 FORWARD LENGTH=522	519	522	0	100.6	88.8	94.6
Rsa1.0_00020.1.g1390.t1	#	#	#	#	#	#	#	-	----	----	#	#	#	#	#	#	#
Rsa1.0_00020.1.g1391.t1	gb|EOA40211.1| hypothetical protein CARUB_v10008932mg [Capsella rubella]	503	500	0	99.4	92.2	96.2	hypothetical protein CARUB_v10008932mg	gbpln	Capsella rubella	AT1G12940.1 | Symbols: ATNRT2.5, NRT2.5 | nitrate transporter2.5 | chr1:4416405-4418337 FORWARD LENGTH=502	503	502	0	99.8	90.5	95.8
Rsa1.0_00020.1.g1392.t1	gb|ACZ71034.1| eukaryotic release factor 1-2 [Brassica oleracea var. botrytis]	435	435	0	100.0	99.8	100.0	eukaryotic release factor 1-2	gbpln	Brassica oleracea	AT1G12920.1 | Symbols: ERF1-2 | eukaryotic release factor 1-2 | chr1:4396555-4397859 REVERSE LENGTH=434	435	434	0	99.8	97.0	98.6
Rsa1.0_00020.1.g1393.t1	gb|EOA40353.1| hypothetical protein CARUB_v10009081mg [Capsella rubella]	446	461	1.00E-175	103.4	78.9	85.0	hypothetical protein CARUB_v10009081mg	gbpln	Capsella rubella	AT1G12860.1 | Symbols: SCRM2, ICE2 | basic helix-loop-helix (bHLH) DNA-binding superfamily protein | chr1:4384758-4386392 FORWARD LENGTH=450	446	450	1.00E-168	100.9	78.3	84.5
Rsa1.0_00020.1.g1394.t1	ref|NP_563918.1| phosphoglycerate mutase-like protein [Arabidopsis thaliana] gi|14517376|gb|AAK62579.1| At1g12850/F13K23_8 [Arabidopsis thaliana] gi|21360535|gb|AAM47383.1| At1g12850/F13K23_8 [Arabidopsis thaliana] gi|332190817|gb|AEE28938.1| phosphoglycerate mutase-like protein [Arabidopsis thaliana]	403	405	1.00E-173	100.5	85.6	89.3	phosphoglycerate mutase-like protein	gbpln	Arabidopsis thaliana	AT1G12850.1 | Symbols: | Phosphoglycerate mutase family protein | chr1:4380022-4381577 REVERSE LENGTH=405	403	405	1.00E-175	100.5	85.6	89.3

Rsa1.0_00020.1.g1395.t1	ref|XP_002892717.1| hypothetical protein ARALYDRAFT_334590 [Arabidopsis lyrata subsp. lyrata] gi|297338559|gb|EFH68976.1| hypothetical protein ARALYDRAFT_334590 [Arabidopsis lyrata subsp. lyrata]	108	563	4.00E-35	521.3	74.1	79.6	hypothetical protein ARALYDRAFT_334590	gbpln	Arabidopsis lyrata	AT1G12845.1 | Symbols: | unknown protein; FUNCTIONS IN: molecular_function unknown; INVOLVED IN: biological_process unknown; LOCATED IN: endomembrane system; EXPRESSED IN: 22 plant structures; EXPRESSED DURING: 13 growth stages; Has 8 Blast hits to 8 proteins in 4 species: Archae - 0; Bacteria - 0; Metazoa - 0; Fungi - 0; Plants - 8; Viruses - 0; Other Eukaryotes - 0 (source: NCBI BLink). | chr1:4379023-4379382 REVERSE LENGTH=119	108	119	1.00E-35	110.2	72.2	77.8
Rsa1.0_00020.1.g1396.t1	ref|NP_563916.1| V-type proton ATPase subunit C [Arabidopsis thaliana] gi|12585488|sp|Q9SDS7.1|VATC_ARATH RecName: Full=V-type proton ATPase subunit C; Short=V-ATPase subunit C; AltName: Full=Vacuolar H(+)-ATPase subunit C; AltName: Full=Vacuolar proton pump subunit C gi|6636332|gb|AAF20146.1|AF208261_1 vacuolar ATP synthase subunit C [Arabidopsis thaliana] gi|8698731|gb|AAF78489.1|AC012187_9 Identical to vacuolar ATP sythase subunit C (DET3) from Arabidopsis thaliana gb|AF208261. ESTs gb|AA067533, gb|Z37481, gb|AA721838, gb|Z37180, gb|T21206 come from this gene [Arabidopsis thaliana] gi|12248023|gb|AAG50103.1|AF334725_1 putative vacuolar ATP synthase subunit C [Arabidopsis thaliana] gi|16649005|gb|AAL24354.1| Identical to vacuolar ATP sythase subunit C (DET3) [Arabidopsis thaliana] gi|20259972|gb|AAM13333.1| vacuolar ATP sythase subunit C [Arabidopsis thaliana] gi|225897918|dbj|BAH30291.1| hypothetical protein [Arabidopsis thaliana] gi|332190815|gb|AEE28936.1| V-type proton ATPase subunit C [Arabidopsis thaliana]	376	375	0	99.7	94.9	97.6	V-type proton ATPase subunit C	gbpln	Arabidopsis thaliana	AT1G12840.1 | Symbols: DET3, ATVHA-C | vacuolar ATP synthase subunit C (VATC) / V-ATPase C subunit / vacuolar proton pump C subunit (DET3) | chr1:4375584-4378220 FORWARD LENGTH=375	376	375	0	99.7	94.9	97.6
Rsa1.0_00020.1.g1397.t1	ref|XP_002892716.1| hypothetical protein ARALYDRAFT_471437 [Arabidopsis lyrata subsp. lyrata] gi|297338558|gb|EFH68975.1| hypothetical protein ARALYDRAFT_471437 [Arabidopsis lyrata subsp. lyrata]	192	212	2.00E-38	110.4	64.1	77.6	hypothetical protein ARALYDRAFT_471437	gbpln	Arabidopsis lyrata	AT1G12830.1 | Symbols: | unknown protein; FUNCTIONS IN: molecular_function unknown; INVOLVED IN: biological_process unknown; LOCATED IN: cellular_component unknown; EXPRESSED IN: 23 plant structures; EXPRESSED DURING: 13 growth stages; Has 39778 Blast hits to 22088 proteins in 1060 species: Archae - 152; Bacteria - 6161; Metazoa - 14109; Fungi - 6144; Plants - 2156; Viruses - 601; Other Eukaryotes - 10455 (source: NCBI BLink). | chr1:4374412-4375053 REVERSE LENGTH=213	192	213	5.00E-39	110.9	64.6	73.4
Rsa1.0_00020.1.g1398.t1	dbj|BAB09379.1| non-LTR retroelement reverse transcriptase-like protein [Arabidopsis thaliana]	1594	1223	0	76.7	34.3	47.0	non-LTR retroelement reverse transcriptase-like protein	gbpln	Arabidopsis thaliana	AT3G24255.1 | Symbols: | RNA-directed DNA polymerase (reverse transcriptase)-related family protein | chr3:8789471-8793208 FORWARD LENGTH=746	1594	746	1.00E-84	46.8	10.6	14.8
Rsa1.0_00020.1.g1399.t1	gb|ABQ50554.1| hypothetical protein [Brassica rapa]	595	590	0	99.2	93.4	95.5	hypothetical protein	gbpln	Brassica rapa	AT1G12820.1 | Symbols: AFB3 | auxin signaling F-box 3 | chr1:4368879-4370780 REVERSE LENGTH=577	595	577	0	97.0	86.1	91.9
Rsa1.0_00020.1.g1400.t1	#	#	#	#	#	#	#	-	----	----	#	#	#	#	#	#	#
Rsa1.0_00020.1.g1401.t1	#	#	#	#	#	#	#	-	----	----	#	#	#	#	#	#	#
Rsa1.0_00020.1.g1402.t1	dbj|BAH20409.1| AT1G12800 [Arabidopsis thaliana]	80	525	3.00E-25	656.3	76.3	80.0	AT1G12800	gbpln	Arabidopsis thaliana	AT1G12800.1 | Symbols: | Nucleic acid-binding, OB-fold-like protein | chr1:4361778-4365189 REVERSE LENGTH=767	80	767	6.00E-28	958.8	76.3	80.0
Rsa1.0_00020.1.g1403.t1	#	#	#	#	#	#	#	-	----	----	#	#	#	#	#	#	#
Rsa1.0_00020.1.g1404.t1	gb|ABQ50560.1| hypothetical protein [Brassica rapa]	73	74	5.00E-30	101.4	86.3	94.5	hypothetical protein	gbpln	Brassica rapa	AT1G12805.1 | Symbols: | nucleotide binding | chr1:4366728-4366955 FORWARD LENGTH=75	73	75	1.00E-23	102.7	68.5	83.6
Rsa1.0_00020.1.g1405.t1	ref|XP_002892712.1| UDP-D-glucose/UDP-D-galactose 4-epimerase 1 [Arabidopsis lyrata subsp. lyrata] gi|297338554|gb|EFH68971.1| UDP-D-glucose/UDP-D-galactose 4-epimerase 1 [Arabidopsis lyrata subsp. lyrata]	351	351	0	100.0	94.9	96.9	UDP-D-glucose/UDP-D-galactose 4-epimerase 1	gbpln	Arabidopsis lyrata	AT1G12780.1 | Symbols: UGE1, ATUGE1 | UDP-D-glucose/UDP-D-galactose 4-epimerase 1 | chr1:4356124-4358120 REVERSE LENGTH=351	351	351	0	100.0	94.3	96.3

Rsa1.0_00020.1.g1406.t1	gb|ABQ50546.1| hypothetical protein [Brassica rapa]	1163	650	0	55.9	37.2	45.8	hypothetical protein	gbpln	Brassica rapa	AT1G12770.1 | Symbols: ISE1, EMB1586 | P-loop containing nucleoside triphosphate hydrolases superfamily protein | chr1:4351888-4353543 FORWARD LENGTH=551	1163	551	0	47.4	39.1	40.8
Rsa1.0_00020.1.g1407.t1	gb|EOA36065.1| hypothetical protein CARUB_v10008555mg [Capsella rubella]	634	641	0	101.1	71.6	83.6	hypothetical protein CARUB_v10008555mg	gbpln	Capsella rubella	AT1G12300.1 | Symbols: | Tetratricopeptide repeat (TPR)-like superfamily protein | chr1:4184163-4186076 REVERSE LENGTH=637	634	637	0	100.5	69.6	82.0
Rsa1.0_00020.1.g1408.t1	ref|NP_172694.1| pentatricopeptide repeat-containing protein [Arabidopsis thaliana] gi|122242333|sp|Q0WKV3.1|PPR36_ARATH RecName: Full=Pentatricopeptide repeat-containing protein At1g12300, mitochondrial; Flags: Precursor gi|110741411|dbj|BAF02254.1| hypothetical protein [Arabidopsis thaliana] gi|332190743|gb|AEE28864.1| pentatricopeptide repeat-containing protein [Arabidopsis thaliana]	566	637	0	112.5	67.3	82.5	pentatricopeptide repeat-containing protein	gbpln	Arabidopsis thaliana	AT1G12300.1 | Symbols: | Tetratricopeptide repeat (TPR)-like superfamily protein | chr1:4184163-4186076 REVERSE LENGTH=637	566	637	0	112.5	67.3	82.5
Rsa1.0_00020.1.g1409.t1	gb|EOA37885.1| hypothetical protein CARUB_v10009353mg [Capsella rubella]	402	397	1.00E-162	98.8	83.3	87.1	hypothetical protein CARUB_v10009353mg	gbpln	Capsella rubella	AT1G12760.1 | Symbols: | Zinc finger, C3HC4 type (RING finger) family protein | chr1:4348728-4350512 FORWARD LENGTH=408	402	408	1.00E-160	101.5	77.9	81.3
Rsa1.0_00020.1.g1410.t1	gb|EOA40316.1| hypothetical protein CARUB_v10009041mg [Capsella rubella]	467	470	0	100.6	89.9	94.6	hypothetical protein CARUB_v10009041mg	gbpln	Capsella rubella	AT1G12680.1 | Symbols: PEPKR2 | phosphoenolpyruvate carboxylase-related kinase 2 | chr1:4320123-4322269 REVERSE LENGTH=470	467	470	0	100.6	87.8	92.9
Rsa1.0_00020.1.g1411.t1	ref|XP_002889943.1| DNA binding protein [Arabidopsis lyrata subsp. lyrata] gi|297335785|gb|EFH66202.1| DNA binding protein [Arabidopsis lyrata subsp. lyrata]	191	192	2.00E-67	100.5	77.5	86.4	DNA binding protein	gbpln	Arabidopsis lyrata	AT1G12630.1 | Symbols: | Integrase-type DNA-binding superfamily protein | chr1:4298897-4299475 FORWARD LENGTH=192	191	192	8.00E-62	100.5	74.9	83.8
Rsa1.0_00020.1.g1412.t1	ref|XP_002885504.1| pentatricopeptide repeat-containing protein [Arabidopsis lyrata subsp. lyrata] gi|297331344|gb|EFH61763.1| pentatricopeptide repeat-containing protein [Arabidopsis lyrata subsp. lyrata]	603	618	0	102.5	67.7	82.6	pentatricopeptide repeat-containing protein	gbpln	Arabidopsis lyrata	AT1G12300.1 | Symbols: | Tetratricopeptide repeat (TPR)-like superfamily protein | chr1:4184163-4186076 REVERSE LENGTH=637	603	637	0	105.6	68.7	83.7
Rsa1.0_00020.1.g1413.t1	ref|NP_563911.1| pentatricopeptide repeat-containing protein [Arabidopsis thaliana] gi|75167758|sp|Q9ASZ8.1|PPR37_ARATH RecName: Full=Pentatricopeptide repeat-containing protein At1g12620 gi|13605505|gb|AAK32746.1|AF361578_1 At1g12620/T12C24_25 [Arabidopsis thaliana] gi|24111307|gb|AAN46777.1| At1g12620/T12C24_25 [Arabidopsis thaliana] gi|332190781|gb|AEE28902.1| pentatricopeptide repeat-containing protein [Arabidopsis thaliana]	580	621	0	107.1	67.9	82.6	pentatricopeptide repeat-containing protein	gbpln	Arabidopsis thaliana	AT1G12620.1 | Symbols: | Pentatricopeptide repeat (PPR) superfamily protein | chr1:4294883-4296748 REVERSE LENGTH=621	580	621	0	107.1	67.9	82.6
Rsa1.0_00020.1.g1414.t1	ref|NP_172721.1| dehydration-responsive element-binding protein 1F [Arabidopsis thaliana] gi|47605747|sp|Q9LN86.1|DRE1F_ARATH RecName: Full=Dehydration-responsive element-binding protein 1F; Short=Protein DREB1F gi|9502389|gb|AAF88096.1|AC025417_24 T12C24.14 [Arabidopsis thaliana] gi|48479384|gb|AAT44959.1| putative AP2/EREBP transcription factor [Arabidopsis thaliana] gi|110736358|dbj|BAF00148.1| hypothetical protein [Arabidopsis thaliana] gi|332190780|gb|AEE28901.1| dehydration-responsive element-binding protein 1F [Arabidopsis thaliana]	202	209	4.00E-84	103.5	82.2	87.6	dehydration-responsive element-binding protein 1F	gbpln	Arabidopsis thaliana	AT1G12610.1 | Symbols: DDF1 | Integrase-type DNA-binding superfamily protein | chr1:4290205-4290834 REVERSE LENGTH=209	202	209	1.00E-86	103.5	82.2	87.6

Rsa1.0_00020.1.g1415.t3	ref|NP_172720.1| UDP-N-acetylglucosamine transporter-like protein [Arabidopsis thaliana] gi|75173863|sp|Q9LDX3.1|UTR4_ARATH RecName: Full=UDP-galactose/UDP-glucose transporter 4; Short=AtUTr4 gi|8778639|gb|AAF79647.1|AC025416_21 F5O11.33 [Arabidopsis thaliana] gi|9502390|gb|AAF88097.1|AC025417_25 T12C24.13 [Arabidopsis thaliana] gi|55978703|gb|AAV68813.1| hypothetical protein AT1G12600 [Arabidopsis thaliana] gi|60547555|gb|AAX23741.1| hypothetical protein At1g12600 [Arabidopsis thaliana] gi|332190779|gb|AEE28900.1| UDP-galactose/UDP-glucose transporter 4 [Arabidopsis thaliana]	536	349	1.00E-177	65.1	59.5	61.0	UDP-N-acetylglucosamine transporter-like protein	gbpln	Arabidopsis thaliana	AT1G12600.1 | Symbols: | UDP-N-acetylglucosamine (UAA) transporter family | chr1:4286414-4288859 REVERSE LENGTH=349	536	349	1.00E-179	65.1	59.5	61.0
Rsa1.0_00020.1.g1416.t1	ref|XP_002889942.1| hypothetical protein ARALYDRAFT_471407 [Arabidopsis lyrata subsp. lyrata] gi|297335784|gb|EFH66201.1| hypothetical protein ARALYDRAFT_471407 [Arabidopsis lyrata subsp. lyrata]	533	522	0	97.9	86.7	91.6	hypothetical protein ARALYDRAFT_471407	gbpln	Arabidopsis lyrata	AT1G12580.1 | Symbols: PEPKR1 | phosphoenolpyruvate carboxylase-related kinase 1 | chr1:4283635-4285675 FORWARD LENGTH=522	533	522	0	97.9	86.1	91.7
Rsa1.0_00020.1.g1417.t1	gb|AAF79648.1|AC025416_22 F5O11.31 [Arabidopsis thaliana]	537	539	0	100.4	93.1	96.6	F5O11.31	gbpln	Arabidopsis thaliana	AT1G12570.1 | Symbols: | Glucose-methanol-choline (GMC) oxidoreductase family protein | chr1:4278192-4280753 REVERSE LENGTH=572	537	572	0	106.5	93.1	96.6
Rsa1.0_00020.1.g1418.t1	gb|EOA36118.1| hypothetical protein CARUB_v10011828mg [Capsella rubella]	263	263	1.00E-144	100.0	92.8	97.7	hypothetical protein CARUB_v10011828mg	gbpln	Capsella rubella	AT1G12560.1 | Symbols: ATEXPA7, EXP7, ATEXP7, ATHEXP ALPHA 1.26, EXPA7 | expansin A7 | chr1:4276557-4277693 FORWARD LENGTH=262	263	262	1.00E-140	99.6	90.5	94.7
Rsa1.0_00020.1.g1419.t1	ref|XP_002892693.1| Cu/Zn-superoxide dismutase copper chaperone precursor [Arabidopsis lyrata subsp. lyrata] gi|297338535|gb|EFH68952.1| Cu/Zn-superoxide dismutase copper chaperone precursor [Arabidopsis lyrata subsp. lyrata]	316	318	1.00E-148	100.6	80.7	84.5	Cu/Zn-superoxide dismutase copper chaperone precursor	gbpln	Arabidopsis lyrata	AT1G12520.1 | Symbols: ATCCS, CCS | copper chaperone for SOD1 | chr1:4267277-4268900 REVERSE LENGTH=320	316	320	1.00E-145	101.3	87.7	91.8
Rsa1.0_00020.1.g1420.t1	ref|NP_172712.1| Nucleotide-sugar transporter family protein [Arabidopsis thaliana] gi|75173828|sp|Q9LDH3.1|PT112_ARATH RecName: Full=Probable sugar phosphate/phosphate translocator At1g12500 gi|8778643|gb|AAF79651.1|AC025416_25 F5O11.25 [Arabidopsis thaliana] gi|9502394|gb|AAF88101.1|AC025417_29 T12C24.5 [Arabidopsis thaliana] gi|26449593|dbj|BAC41922.1| unknown protein [Arabidopsis thaliana] gi|332190769|gb|AEE28890.1| Nucleotide-sugar transporter family protein [Arabidopsis thaliana]	361	361	0	100.0	96.7	98.9	Nucleotide-sugar transporter family protein	gbpln	Arabidopsis thaliana	AT1G12500.1 | Symbols: | Nucleotide-sugar transporter family protein | chr1:4263542-4264957 REVERSE LENGTH=361	361	361	0	100.0	96.7	98.9
Rsa1.0_00020.1.g1421.t1	gb|EOA39582.1| hypothetical protein CARUB_v10008200mg [Capsella rubella]	1148	988	0	86.1	80.1	83.2	hypothetical protein CARUB_v10008200mg	gbpln	Capsella rubella	AT1G12470.1 | Symbols: | zinc ion binding | chr1:4251359-4257201 FORWARD LENGTH=988	1148	988	0	86.1	79.2	82.8
Rsa1.0_00020.1.g1422.t1	ref|NP_172707.1| SNARE associated Golgi protein family [Arabidopsis thaliana] gi|34222066|gb|AAQ62869.1| At1g12450 [Arabidopsis thaliana] gi|62320872|dbj|BAD93843.1| hypothetical protein [Arabidopsis thaliana] gi|332190763|gb|AEE28884.1| SNARE associated Golgi protein family [Arabidopsis thaliana]	290	303	2.33E-156	104.5	90.0	95.5	SNARE associated Golgi protein family	gbpln	Arabidopsis thaliana	AT1G12450.1 | Symbols: | SNARE associated Golgi protein family | chr1:4244496-4245808 FORWARD LENGTH=303	290	303	1.00E-148	104.5	90.0	95.5
Rsa1.0_00020.1.g1423.t1	ref|NP_172704.1| ACT domain repeat 8 protein [Arabidopsis thaliana] gi|8778647|gb|AAF79655.1|AC025416_29 F5O11.14 [Arabidopsis thaliana] gi|22138106|gb|AAM93433.1| ACR8 [Arabidopsis thaliana] gi|110737291|dbj|BAF00592.1| hypothetical protein [Arabidopsis thaliana] gi|111074504|gb|ABH04625.1| At1g12420 [Arabidopsis thaliana] gi|332190757|gb|AEE28878.1| ACT domain repeat 8 protein [Arabidopsis thaliana]	441	441	0	100.0	89.8	95.2	ACT domain repeat 8 protein	gbpln	Arabidopsis thaliana	AT1G12420.1 | Symbols: ACR8 | ACT domain repeat 8 | chr1:4226673-4228917 REVERSE LENGTH=441	441	441	0	100.0	89.8	95.2

Rsa1.0_00020.1.g1424.t1	ref|NP_172701.2| protein cornichon-4 [Arabidopsis thaliana] gi|75148636|sp|Q84W04.1|CNIH4_ARATH RecName: Full=Protein cornichon homolog 4 gi|28416577|gb|AAO42819.1| At1g12390 [Arabidopsis thaliana] gi|110743211|dbj|BAE99496.1| hypothetical protein [Arabidopsis thaliana] gi|332190753|gb|AEE28874.1| protein cornichon-4 [Arabidopsis thaliana]	219	137	5.00E-63	62.6	55.3	58.0	protein cornichon-4	gbpln	Arabidopsis thaliana	AT1G12390.1 | Symbols: | Cornichon family protein | chr1:4220347-4221481 FORWARD LENGTH=137	219	137	2.00E-65	62.6	55.3	58.0
Rsa1.0_00020.1.g1425.t1	ref|XP_002892685.1| hypothetical protein ARALYDRAFT_888561 [Arabidopsis lyrata subsp. lyrata] gi|297338527|gb|EFH68944.1| hypothetical protein ARALYDRAFT_888561 [Arabidopsis lyrata subsp. lyrata]	765	792	0	103.5	86.1	90.3	hypothetical protein ARALYDRAFT_888561	gbpln	Arabidopsis lyrata	AT1G12380.1 | Symbols: | unknown protein; BEST Arabidopsis thaliana protein match is: unknown protein (TAIR:AT1G62870.1); Has 173 Blast hits to 170 proteins in 34 species: Archae - 0; Bacteria - 4; Metazoa - 25; Fungi - 8; Plants - 123; Viruses - 7; Other Eukaryotes - 6 (source: NCBI BLink). | chr1:4214499-4216880 REVERSE LENGTH=793	765	793	0	103.7	87.3	92.2
Rsa1.0_00020.1.g1426.t2	ref|XP_002892684.1| hypothetical protein ARALYDRAFT_471385 [Arabidopsis lyrata subsp. lyrata] gi|297338526|gb|EFH68943.1| hypothetical protein ARALYDRAFT_471385 [Arabidopsis lyrata subsp. lyrata]	493	496	0	100.6	89.5	93.9	hypothetical protein ARALYDRAFT_471385	gbpln	Arabidopsis lyrata	AT1G12370.2 | Symbols: PHR1, UVR2 | photolyase 1 | chr1:4206500-4208842 REVERSE LENGTH=496	493	496	0	100.6	88.4	93.5
Rsa1.0_00020.1.g1427.t1	ref|XP_002876578.1| hypothetical protein ARALYDRAFT_486544 [Arabidopsis lyrata subsp. lyrata] gi|297322416|gb|EFH52837.1| hypothetical protein ARALYDRAFT_486544 [Arabidopsis lyrata subsp. lyrata]	252	287	1.00E-112	113.9	80.6	82.5	hypothetical protein ARALYDRAFT_486544	gbpln	Arabidopsis lyrata	AT3G60720.1 | Symbols: PDLP8 | plasmodesmata-located protein 8 | chr3:22442035-22443608 FORWARD LENGTH=279	252	279	1.00E-113	110.7	81.0	82.9
Rsa1.0_00020.1.g1428.t1	ref|XP_002889928.1| hypothetical protein ARALYDRAFT_471382 [Arabidopsis lyrata subsp. lyrata] gi|297335770|gb|EFH66187.1| hypothetical protein ARALYDRAFT_471382 [Arabidopsis lyrata subsp. lyrata]	514	504	0	98.1	79.4	86.6	hypothetical protein ARALYDRAFT_471382	gbpln	Arabidopsis lyrata	AT1G12330.1 | Symbols: | unknown protein; INVOLVED IN: biological_process unknown; LOCATED IN: chloroplast; EXPRESSED IN: 21 plant structures; EXPRESSED DURING: 12 growth stages; BEST Arabidopsis thaliana protein match is: unknown protein (TAIR:AT5G12900.1); Has 249 Blast hits to 249 proteins in 27 species: Archae - 0; Bacteria - 0; Metazoa - 7; Fungi - 14; Plants - 217; Viruses - 0; Other Eukaryotes - 11 (source: NCBI BLink). | chr1:4194673-4196627 FORWARD LENGTH=505	514	505	0	98.2	79.4	86.2
Rsa1.0_00020.1.g1429.t1	ref|XP_002889927.1| hypothetical protein ARALYDRAFT_471381 [Arabidopsis lyrata subsp. lyrata] gi|297335769|gb|EFH66186.1| hypothetical protein ARALYDRAFT_471381 [Arabidopsis lyrata subsp. lyrata]	218	213	4.00E-95	97.7	84.4	90.4	hypothetical protein ARALYDRAFT_471381	gbpln	Arabidopsis lyrata	AT1G12320.1 | Symbols: | Protein of unknown function (DUF1442) | chr1:4189424-4190209 FORWARD LENGTH=212	218	212	6.00E-93	97.2	83.0	89.4
Rsa1.0_00020.1.g1430.t1	gb|AAF79658.1|AC025416_32 F5O11.4 [Arabidopsis thaliana]	148	975	5.00E-78	658.8	95.9	98.6	F5O11.4	gbpln	Arabidopsis thaliana	AT1G12310.1 | Symbols: | Calcium-binding EF-hand family protein | chr1:4187500-4187946 REVERSE LENGTH=148	148	148	1.00E-79	100.0	96.6	99.3
Rsa1.0_00020.1.g1431.t1	ref|NP_172690.1| NAC domain-containing protein 7 [Arabidopsis thaliana] gi|238065075|sp|Q9FWX2.2|NAC7_ARATH RecName: Full=NAC domain-containing protein 7; Short=ANAC007; AltName: Full=Protein EMBRYO DEFECTIVE 2749; AltName: Full=Protein VASCULAR RELATED NAC-DOMAIN 4 gi|119935909|gb|ABM06031.1| At1g12260 [Arabidopsis thaliana] gi|332190738|gb|AEE28859.1| NAC domain-containing protein 7 [Arabidopsis thaliana]	391	395	0	101.0	89.3	92.6	NAC domain-containing protein 7	gbpln	Arabidopsis thaliana	AT1G12260.1 | Symbols: VND4, EMB2749, ANAC007, NAC007 | NAC 007 | chr1:4163058-4164486 REVERSE LENGTH=395	391	395	0	101.0	89.3	92.6
Rsa1.0_00020.1.g1432.t1	#	#	#	#	#	#	#	-	----	----	#	#	#	#	#	#	#
Rsa1.0_00020.1.g1433.t1	gb|AAK30571.1|AF346659_1 extensin-like protein [Brassica napus]	137	137	7.00E-47	100.0	96.4	98.5	extensin-like protein	gbpln	Brassica napus	AT1G12090.1 | Symbols: ELP | extensin-like protein | chr1:4090176-4090589 REVERSE LENGTH=137	137	137	4.00E-45	100.0	95.6	98.5

Rsa1.0_00021.1.g1434.t1	ref|NP_564293.1| transcription factor bHLH54 [Arabidopsis thaliana] gi|75301354|sp|Q8LEG1.1|BH054_ARATH RecName: Full=Transcription factor bHLH54; AltName: Full=Basic helix-loop-helix protein 54; Short=AtbHLH54; Short=bHLH 54; AltName: Full=Transcription factor EN 114; AltName: Full=bHLH transcription factor bHLH054 gi|21553570|gb|AAM62663.1| unknown [Arabidopsis thaliana] gi|225897974|dbj|BAH30319.1| hypothetical protein [Arabidopsis thaliana] gi|332192750|gb|AEE30871.1| transcription factor bHLH54 [Arabidopsis thaliana]	255	258	1.00E-120	101.2	88.6	92.9	transcription factor bHLH54	gbpln	Arabidopsis thaliana	AT1G27740.1 | Symbols: RSL4 | root hair defective 6-like 4 | chr1:9654753-9655806 FORWARD LENGTH=258	255	258	1.00E-122	101.2	88.6	92.9
Rsa1.0_00021.1.g1435.t2	gb|AAF24949.1|AC012375_12 T22C5.20 [Arabidopsis thaliana]	1115	1840	0	165.0	73.1	78.5	T22C5.20	gbpln	Arabidopsis thaliana	AT1G27750.1 | Symbols: | nucleic acid binding | chr1:9657425-9661750 FORWARD LENGTH=1075	1115	1075	0	96.4	65.5	69.5
Rsa1.0_00021.1.g1436.t1	ref|XP_002890749.1| hypothetical protein ARALYDRAFT_472983 [Arabidopsis lyrata subsp. lyrata] gi|297336591|gb|EFH67008.1| hypothetical protein ARALYDRAFT_472983 [Arabidopsis lyrata subsp. lyrata]	443	442	0	99.8	87.6	93.0	hypothetical protein ARALYDRAFT_472983	gbpln	Arabidopsis lyrata	AT1G27760.3 | Symbols: SAT32, ATSAT32 | interferon-related developmental regulator family protein / IFRD protein family | chr1:9668895-9671322 FORWARD LENGTH=441	443	441	0	99.5	87.1	92.3
Rsa1.0_00021.1.g1437.t1	ref|XP_002893491.1| ATCSA-1 [Arabidopsis lyrata subsp. lyrata] gi|297339333|gb|EFH69750.1| ATCSA-1 [Arabidopsis lyrata subsp. lyrata]	460	450	0	97.8	85.7	91.7	ATCSA-1	gbpln	Arabidopsis lyrata	AT1G27840.1 | Symbols: ATCSA-1 | Transducin/WD40 repeat-like superfamily protein | chr1:9693332-9696257 REVERSE LENGTH=450	460	450	0	97.8	84.6	90.9
Rsa1.0_00021.1.g1438.t1	gb|EOA36789.1| hypothetical protein CARUB_v10008135mg [Capsella rubella]	1038	1149	0	110.7	50.8	53.9	hypothetical protein CARUB_v10008135mg	gbpln	Capsella rubella	AT1G27850.1 | Symbols: | unknown protein; FUNCTIONS IN: molecular_function unknown; INVOLVED IN: biological_process unknown; LOCATED IN: plasma membrane; EXPRESSED IN: 22 plant structures; EXPRESSED DURING: 13 growth stages; BEST Arabidopsis thaliana protein match is: unknown protein (TAIR:AT2G40070.1); Has 9215 Blast hits to 5316 proteins in 473 species: Archae - 6; Bacteria - 773; Metazoa - 3392; Fungi - 1710; Plants - 539; Viruses - 143; Other Eukaryotes - 2652 (source: NCBI BLink). | chr1:9699265-9703701 FORWARD LENGTH=1148	1038	1148	0	110.6	50.6	53.3
Rsa1.0_00021.1.g1439.t1	gb|EOA39726.1| hypothetical protein CARUB_v10008370mg [Capsella rubella]	770	768	0	99.7	90.5	94.7	hypothetical protein CARUB_v10008370mg	gbpln	Capsella rubella	AT1G27910.1 | Symbols: PUB45, ATPUB45 | plant U-box 45 | chr1:9720962-9723975 REVERSE LENGTH=768	770	768	0	99.7	89.9	94.9
Rsa1.0_00021.1.g1440.t2	gb|AAG51477.1|AC069471_8 hypothetical protein [Arabidopsis thaliana]	584	592	0	101.4	81.2	86.5	hypothetical protein	gbpln	Arabidopsis thaliana	AT1G27920.1 | Symbols: MAP65-8 | microtubule-associated protein 65-8 | chr1:9727106-9729848 FORWARD LENGTH=562	584	562	0	96.2	79.1	84.1
Rsa1.0_00021.1.g1441.t1	gb|AAM61740.1| unknown [Arabidopsis thaliana]	289	289	1.00E-150	100.0	91.0	95.5	unknown	gbpln	Arabidopsis thaliana	AT1G27930.1 | Symbols: | Protein of unknown function (DUF579) | chr1:9731510-9732379 REVERSE LENGTH=289	289	289	1.00E-153	100.0	91.0	95.2
Rsa1.0_00021.1.g1442.t1	ref|XP_002893495.1| P-glycoprotein 13 [Arabidopsis lyrata subsp. lyrata] gi|297339337|gb|EFH69754.1| P-glycoprotein 13 [Arabidopsis lyrata subsp. lyrata]	1167	1246	0	106.8	89.4	94.0	P-glycoprotein 13	gbpln	Arabidopsis lyrata	AT1G27940.1 | Symbols: PGP13 | P-glycoprotein 13 | chr1:9733597-9738129 REVERSE LENGTH=1245	1167	1245	0	106.7	88.9	93.6
Rsa1.0_00021.1.g1443.t1	ref|XP_002890752.1| hypothetical protein ARALYDRAFT_890328 [Arabidopsis lyrata subsp. lyrata] gi|297336594|gb|EFH67011.1| hypothetical protein ARALYDRAFT_890328 [Arabidopsis lyrata subsp. lyrata]	194	197	5.00E-78	101.5	82.0	89.2	hypothetical protein ARALYDRAFT_890328	gbpln	Arabidopsis lyrata	AT1G27950.1 | Symbols: LTPG1 | glycosylphosphatidylinositol-anchored lipid protein transfer 1 | chr1:9740740-9741991 FORWARD LENGTH=193	194	193	6.00E-76	99.5	77.3	86.6
Rsa1.0_00021.1.g1444.t1	#	#	#	#	#	#	#	-	----	----	#	#	#	#	#	#	#
Rsa1.0_00021.1.g1445.t1	ref|NP_174118.1| nuclear transport factor 2B [Arabidopsis thaliana] gi|15214148|sp|Q9C7F5.1|NTF2_ARATH RecName: Full=Nuclear transport factor 2; Short=NTF-2 gi|12323001|gb|AAG51491.1|AC069471_22 nuclear transport factor 2, putative [Arabidopsis thaliana] gi|98961065|gb|ABF59016.1| At1g27970 [Arabidopsis thaliana] gi|332192775|gb|AEE30896.1| nuclear transport factor 2B [Arabidopsis thaliana]	128	126	8.00E-66	98.4	93.8	96.1	nuclear transport factor 2B	gbpln	Arabidopsis thaliana	AT1G27970.1 | Symbols: NTF2B | nuclear transport factor 2B | chr1:9746921-9747787 FORWARD LENGTH=126	128	126	2.00E-68	98.4	93.8	96.1

Rsa1.0_00021.1.g1446.t1	gb|EOA37762.1| hypothetical protein CARUB_v10012601mg [Capsella rubella]	187	544	4.00E-34	290.9	55.1	62.0	hypothetical protein CARUB_v10012601mg	gbpln	Capsella rubella	AT1G27980.1 | Symbols: DPL1, ATDPL1 | dihydrosphingosine phosphate lyase | chr1:9748812-9752618 FORWARD LENGTH=544	187	544	6.00E-36	290.9	54.5	61.5
Rsa1.0_00021.1.g1447.t1	gb|EOA37464.1| hypothetical protein CARUB_v10011574mg [Capsella rubella]	271	270	1.00E-134	99.6	89.7	94.5	hypothetical protein CARUB_v10011574mg	gbpln	Capsella rubella	AT1G27990.1 | Symbols: | unknown protein; BEST Arabidopsis thaliana protein match is: unknown protein (TAIR:AT5G52420.1); Has 86 Blast hits to 86 proteins in 15 species: Archae - 0; Bacteria - 0; Metazoa - 0; Fungi - 0; Plants - 84; Viruses - 0; Other Eukaryotes - 2 (source: NCBI BLink). | chr1:9752799-9753919 REVERSE LENGTH=271	271	271	1.00E-132	100.0	89.3	94.8
Rsa1.0_00021.1.g1448.t1	gb|EOA17180.1| hypothetical protein CARUB_v10005451mg [Capsella rubella]	289	278	9.00E-76	96.2	51.6	67.5	hypothetical protein CARUB_v10005451mg	gbpln	Capsella rubella	AT4G17700.1 | Symbols: | unknown protein; BEST Arabidopsis thaliana protein match is: unknown protein (TAIR:AT4G17990.1); Has 30201 Blast hits to 17322 proteins in 780 species: Archae - 12; Bacteria - 1396; Metazoa - 17338; Fungi - 3422; Plants - 5037; Viruses - 0; Other Eukaryotes - 2996 (source: NCBI BLink). | chr4:9854322-9855227 FORWARD LENGTH=164	289	164	7.00E-43	56.7	28.7	38.4
Rsa1.0_00021.1.g1449.t1	sp|Q9C7F1.2|PPR61_ARATH RecName: Full=Putative pentatricopeptide repeat-containing protein At1g28020	564	566	1.00E-169	100.4	57.8	69.5	RecName: Full=Putative pentatricopeptide repeat-containing protein At1g28020	----	----	AT1G28020.1 | Symbols: | Tetratricopeptide repeat (TPR)-like superfamily protein | chr1:9768395-9771157 REVERSE LENGTH=612	564	612	1.00E-163	108.5	55.1	66.7
Rsa1.0_00021.1.g1450.t1	gb|AAG51783.1|AC079679_3 reverse transcriptase, putative; 16838-20266 [Arabidopsis thaliana]	141	1142	1.00E-25	809.9	44.0	49.6	reverse transcriptase, putative; 16838-20266	gbpln	Arabidopsis thaliana	#	#	#	#	#	#	#
Rsa1.0_00021.1.g1451.t1	gb|EOA39175.1| hypothetical protein CARUB_v10012135mg [Capsella rubella]	423	410	1.00E-120	96.9	56.0	67.1	hypothetical protein CARUB_v10012135mg	gbpln	Capsella rubella	AT1G28000.1 | Symbols: | Pentatricopeptide repeat (PPR) superfamily protein | chr1:9756674-9757947 REVERSE LENGTH=388	423	388	1.00E-118	91.7	51.3	61.9
Rsa1.0_00021.1.g1452.t1	gb|EOA39175.1| hypothetical protein CARUB_v10012135mg [Capsella rubella]	304	410	1.00E-84	134.9	53.0	62.8	hypothetical protein CARUB_v10012135mg	gbpln	Capsella rubella	AT1G28000.1 | Symbols: | Pentatricopeptide repeat (PPR) superfamily protein | chr1:9756674-9757947 REVERSE LENGTH=388	304	388	7.00E-84	127.6	53.9	62.8
Rsa1.0_00021.1.g1453.t1	gb|EOA36857.1| hypothetical protein CARUB_v10008803mg [Capsella rubella]	515	536	9.00E-68	104.1	23.9	31.3	hypothetical protein CARUB_v10008803mg	gbpln	Capsella rubella	AT5G28823.1 | Symbols: | FUNCTIONS IN: molecular_function unknown; INVOLVED IN: biological_process unknown; LOCATED IN: cellular_component unknown; EXPRESSED IN: cultured cell; BEST Arabidopsis thaliana protein match is: Zinc knuckle (CCHC-type) family protein (TAIR:AT2G07760.1); Has 30201 Blast hits to 17322 proteins in 780 species: Archae - 12; Bacteria - 1396; Metazoa - 17338; Fungi - 3422; Plants - 5037; Viruses - 0; Other Eukaryotes - 2996 (source: NCBI BLink). | chr5:10837849-10839826 REVERSE LENGTH=568	515	568	8.00E-47	110.3	26.8	38.1
Rsa1.0_00021.1.g1454.t1	#	#	#	#	#	#	#	-	----	----	#	#	#	#	#	#	#
Rsa1.0_00021.1.g1455.t1	ref|XP_002890755.1| P-glycoprotein 14 [Arabidopsis lyrata subsp. lyrata] gi|297336597|gb|EFH67014.1| P-glycoprotein 14 [Arabidopsis lyrata subsp. lyrata]	1243	1248	0	100.4	88.2	94.0	P-glycoprotein 14	gbpln	Arabidopsis lyrata	AT1G28010.1 | Symbols: PGP14, ATABCB14, ABCB14 | P-glycoprotein 14 | chr1:9763436-9767917 FORWARD LENGTH=1247	1243	1247	0	100.3	87.4	93.7
Rsa1.0_00021.1.g1456.t1	ref|XP_002868101.1| hypothetical protein ARALYDRAFT_915040 [Arabidopsis lyrata subsp. lyrata] gi|297313937|gb|EFH44360.1| hypothetical protein ARALYDRAFT_915040 [Arabidopsis lyrata subsp. lyrata]	277	495	4.00E-70	178.7	52.7	69.7	hypothetical protein ARALYDRAFT_915040	gbpln	Arabidopsis lyrata	AT1G28020.1 | Symbols: | Tetratricopeptide repeat (TPR)-like superfamily protein | chr1:9768395-9771157 REVERSE LENGTH=612	277	612	4.00E-49	220.9	39.0	47.7
Rsa1.0_00021.1.g1457.t1	gb|ABD64987.1| hypothetical protein 26.t00003 [Brassica oleracea]	468	330	2.00E-15	70.5	11.8	13.0	hypothetical protein 26.t00003	gbpln	Brassica oleracea	#	#	#	#	#	#	#
Rsa1.0_00021.1.g1458.t1	ref|XP_002893501.1| predicted protein [Arabidopsis lyrata subsp. lyrata] gi|297339343|gb|EFH69760.1| predicted protein [Arabidopsis lyrata subsp. lyrata]	395	667	1.00E-112	168.9	61.0	72.7	predicted protein	gbpln	Arabidopsis lyrata	AT1G28020.1 | Symbols: | Tetratricopeptide repeat (TPR)-like superfamily protein | chr1:9768395-9771157 REVERSE LENGTH=612	395	612	1.00E-110	154.9	58.7	69.4
Rsa1.0_00021.1.g1459.t1	gb|EOA39175.1| hypothetical protein CARUB_v10012135mg [Capsella rubella]	375	410	1.00E-108	109.3	56.8	68.8	hypothetical protein CARUB_v10012135mg	gbpln	Capsella rubella	AT1G28000.1 | Symbols: | Pentatricopeptide repeat (PPR) superfamily protein | chr1:9756674-9757947 REVERSE LENGTH=388	375	388	1.00E-105	103.5	50.1	60.5
Rsa1.0_00021.1.g1460.t1	gb|AAG50751.1|AC079733_19 polyprotein, putative [Arabidopsis thaliana]	1407	1468	0	104.3	55.4	70.5	polyprotein, putative	gbpln	Arabidopsis thaliana	AT4G23160.1 | Symbols: CRK8 | cysteine-rich RLK (RECEPTOR-like protein kinase) 8 | chr4:12129485-12134086 FORWARD LENGTH=1262	1407	1262	1.00E-140	89.7	17.2	25.3

Rsa1.0_00021.1.g1461.t1	gb|EOA39702.1| hypothetical protein CARUB_v10008347mg [Capsella rubella]	773	787	0	101.8	74.9	82.7	hypothetical protein CARUB_v10008347mg	gbpln	Capsella rubella	AT1G28060.1 | Symbols: | Pre-mRNA-splicing factor 3 | chr1:9779167-9782486 FORWARD LENGTH=786	773	786	0	101.7	74.3	82.3
Rsa1.0_00021.1.g1462.t1	ref|NP_174128.1| uncharacterized protein [Arabidopsis thaliana] gi|12323005|gb|AAG51495.1|AC069471_26 hypothetical protein [Arabidopsis thaliana] gi|44681470|gb|AAS47675.1| At1g28070 [Arabidopsis thaliana] gi|62318580|dbj|BAD94977.1| hypothetical protein [Arabidopsis thaliana] gi|332192787|gb|AEE30908.1| uncharacterized protein AT1G28070 [Arabidopsis thaliana]	149	176	4.00E-38	118.1	63.1	77.2	uncharacterized protein	gbpln	Arabidopsis thaliana	AT1G28070.1 | Symbols: | unknown protein; BEST Arabidopsis thaliana protein match is: unknown protein (TAIR:AT2G33510.1); Has 85 Blast hits to 77 proteins in 15 species: Archae - 0; Bacteria - 0; Metazoa - 0; Fungi - 0; Plants - 85; Viruses - 0; Other Eukaryotes - 0 (source: NCBI BLink). | chr1:9783082-9783864 FORWARD LENGTH=176	149	176	1.00E-40	118.1	63.1	77.2
Rsa1.0_00021.1.g1463.t1	emb|CAN60947.1| hypothetical protein VITISV_015758 [Vitis vinifera]	336	1306	1.00E-102	388.7	61.9	69.3	hypothetical protein VITISV_015758	gbpln	Vitis vinifera	#	#	#	#	#	#	#
Rsa1.0_00021.1.g1464.t1	ref|XP_002890758.1| hypothetical protein ARALYDRAFT_313512 [Arabidopsis lyrata subsp. lyrata] gi|297336600|gb|EFH67017.1| hypothetical protein ARALYDRAFT_313512 [Arabidopsis lyrata subsp. lyrata]	258	296	3.00E-91	114.7	71.7	81.4	hypothetical protein ARALYDRAFT_313512	gbpln	Arabidopsis lyrata	AT1G28080.1 | Symbols: | RING finger protein | chr1:9789826-9790918 FORWARD LENGTH=291	258	291	7.00E-86	112.8	65.9	76.0
Rsa1.0_00021.1.g1465.t1	gb|EOA40088.1| hypothetical protein CARUB_v10008782mg [Capsella rubella]	540	543	0	100.6	86.3	94.1	hypothetical protein CARUB_v10008782mg	gbpln	Capsella rubella	AT1G28090.1 | Symbols: | Polynucleotide adenylyltransferase family protein | chr1:9795826-9798891 FORWARD LENGTH=541	540	541	0	100.2	85.0	93.7
Rsa1.0_00021.1.g1466.t1	gb|EOA36155.1| hypothetical protein CARUB_v10009948mg [Capsella rubella]	337	282	1.00E-119	83.7	66.2	71.5	hypothetical protein CARUB_v10009948mg	gbpln	Capsella rubella	AT1G28100.2 | Symbols: | unknown protein; Has 64 Blast hits to 64 proteins in 27 species: Archae - 0; Bacteria - 14; Metazoa - 0; Fungi - 6; Plants - 42; Viruses - 0; Other Eukaryotes - 2 (source: NCBI BLink). | chr1:9802120-9803981 FORWARD LENGTH=282	337	282	1.00E-121	83.7	66.2	71.8
Rsa1.0_00021.1.g1467.t1	gb|EOA37308.1| hypothetical protein CARUB_v10010972mg [Capsella rubella]	428	459	0	107.2	94.6	97.0	hypothetical protein CARUB_v10010972mg	gbpln	Capsella rubella	AT1G28110.2 | Symbols: SCPL45 | serine carboxypeptidase-like 45 | chr1:9804153-9806832 REVERSE LENGTH=461	428	461	0	107.7	93.7	96.5
Rsa1.0_00021.1.g1468.t1	#	#	#	#	#	#	#	-	----	----	AT1G30890.2 | Symbols: | Integral membrane HRF1 family protein | chr1:10994890-10995999 FORWARD LENGTH=269	231	269	1.00E-12	116.5	13.4	13.9
Rsa1.0_00021.1.g1469.t1	#	#	#	#	#	#	#	-	----	----	#	#	#	#	#	#	#
Rsa1.0_00021.1.g1470.t1	gb|EOA36105.1| hypothetical protein CARUB_v10011293mg [Capsella rubella]	595	608	0	102.2	94.3	98.2	hypothetical protein CARUB_v10011293mg	gbpln	Capsella rubella	AT1G28130.1 | Symbols: GH3.17 | Auxin-responsive GH3 family protein | chr1:9825431-9827883 FORWARD LENGTH=609	595	609	0	102.4	94.1	98.7
Rsa1.0_00021.1.g1471.t3	gb|AAG51484.1|AC069471_15 unknown protein [Arabidopsis thaliana]	292	295	1.00E-122	101.0	80.1	87.0	unknown protein	gbpln	Arabidopsis thaliana	AT1G28140.1 | Symbols: | unknown protein; FUNCTIONS IN: molecular_function unknown; INVOLVED IN: biological_process unknown; LOCATED IN: chloroplast; EXPRESSED IN: 22 plant structures; EXPRESSED DURING: 13 growth stages; CONTAINS InterPro DOMAIN/s: Protein of unknown function DUF2301, transmembrane (InterPro:IPR019275); Has 140 Blast hits to 140 proteins in 72 species: Archae - 0; Bacteria - 86; Metazoa - 10; Fungi - 0; Plants - 41; Viruses - 0; Other Eukaryotes - 3 (source: NCBI BLink). | chr1:9833029-9834390 REVERSE LENGTH=280	292	280	1.00E-124	95.9	80.1	87.0
Rsa1.0_00021.1.g1472.t1	ref|NP_174138.1| ethylene-responsive transcription factor ERF087 [Arabidopsis thaliana] gi|75263257|sp|Q9FZ90.1|ERF87_ARATH RecName: Full=Ethylene-responsive transcription factor ERF087 gi|9795622|gb|AAF98440.1|AC021044_19 Hypothetical protein [Arabidopsis thaliana] gi|12323000|gb|AAG51490.1|AC069471_21 AP2 domain transcription factor, putative [Arabidopsis thaliana] gi|91805861|gb|ABE65659.1| ethylene-responsive element-binding family protein [Arabidopsis thaliana] gi|332192805|gb|AEE30926.1| ethylene-responsive transcription factor ERF087 [Arabidopsis thaliana]	242	245	1.00E-97	101.2	82.2	88.4	ethylene-responsive transcription factor ERF087	gbpln	Arabidopsis thaliana	AT1G28160.1 | Symbols: | Integrase-type DNA-binding superfamily protein | chr1:9839387-9840124 FORWARD LENGTH=245	242	245	1.00E-100	101.2	82.2	88.4

Rsa1.0_00021.1.g1473.t1	ref|XP_002893509.1| hypothetical protein ARALYDRAFT_890355 [Arabidopsis lyrata subsp. lyrata] gi|297339351|gb|EFH69768.1| hypothetical protein ARALYDRAFT_890355 [Arabidopsis lyrata subsp. lyrata]	315	263	1.00E-88	83.5	59.7	66.3	hypothetical protein ARALYDRAFT_890355	gbpln	Arabidopsis lyrata	AT1G28190.1 | Symbols: | unknown protein; BEST Arabidopsis thaliana protein match is: unknown protein (TAIR:AT5G12340.1); Has 166 Blast hits to 162 proteins in 36 species: Archae - 0; Bacteria - 2; Metazoa - 15; Fungi - 5; Plants - 124; Viruses - 0; Other Eukaryotes - 20 (source: NCBI BLink). | chr1:9847015-9847815 REVERSE LENGTH=266	315	266	8.00E-89	84.4	54.6	60.3
Rsa1.0_00021.1.g1474.t1	ref|XP_002873866.1| hypothetical protein ARALYDRAFT_909803 [Arabidopsis lyrata subsp. lyrata] gi|297319703|gb|EFH50125.1| hypothetical protein ARALYDRAFT_909803 [Arabidopsis lyrata subsp. lyrata]	369	384	1.00E-79	104.1	51.2	64.8	hypothetical protein ARALYDRAFT_909803	gbpln	Arabidopsis lyrata	AT5G18160.1 | Symbols: | F-box and associated interaction domains-containing protein | chr5:6002770-6003909 FORWARD LENGTH=379	369	379	4.00E-56	102.7	38.2	54.2
Rsa1.0_00021.1.g1475.t1	ref|XP_002873866.1| hypothetical protein ARALYDRAFT_909803 [Arabidopsis lyrata subsp. lyrata] gi|297319703|gb|EFH50125.1| hypothetical protein ARALYDRAFT_909803 [Arabidopsis lyrata subsp. lyrata]	376	384	6.00E-53	102.1	39.4	52.7	hypothetical protein ARALYDRAFT_909803	gbpln	Arabidopsis lyrata	AT1G50870.1 | Symbols: | F-box and associated interaction domains-containing protein | chr1:18855147-18856337 FORWARD LENGTH=396	376	396	3.00E-37	105.3	31.1	44.4
Rsa1.0_00022.1.g1476.t1	dbj|BAJ33863.1| unnamed protein product [Thellungiella halophila]	101	196	9.00E-32	194.1	69.3	77.2	unnamed protein product	----	----	AT5G65060.1 | Symbols: MAF3, FCL3, AGL70 | K-box region and MADS-box transcription factor family protein | chr5:25987527-25991065 FORWARD LENGTH=196	101	196	1.00E-30	194.1	64.4	73.3
Rsa1.0_00022.1.g1477.t1	dbj|BAJ33863.1| unnamed protein product [Thellungiella halophila]	62	196	8.00E-24	316.1	88.7	95.2	unnamed protein product	----	----	AT5G65050.2 | Symbols: AGL31, MAF2 | AGAMOUS-like 31 | chr5:25982415-25985743 FORWARD LENGTH=178	62	178	7.00E-25	287.1	85.5	91.9
Rsa1.0_00022.1.g1478.t1	gb|EOA14245.1| hypothetical protein CARUB_v10027399mg [Capsella rubella]	115	114	3.00E-44	99.1	76.5	85.2	hypothetical protein CARUB_v10027399mg	gbpln	Capsella rubella	AT5G65040.1 | Symbols: | Protein of unknown function (DUF581) | chr5:25977864-25978350 REVERSE LENGTH=113	115	113	2.00E-45	98.3	75.7	84.3
Rsa1.0_00022.1.g1479.t1	#	#	#	#	#	#	#	-	----	----	AT5G19270.1 | Symbols: | unknown protein; BEST Arabidopsis thaliana protein match is: unknown protein (TAIR:AT4G03566.1); Has 30201 Blast hits to 17322 proteins in 780 species: Archae - 12; Bacteria - 1396; Metazoa - 17338; Fungi - 3422; Plants - 5037; Viruses - 0; Other Eukaryotes - 2996 (source: NCBI BLink). | chr5:6485617-6487009 REVERSE LENGTH=365	154	365	7.00E-12	237.0	28.6	39.0
Rsa1.0_00022.1.g1480.t1	gb|ABD47519.1| annexin 2 [Brassica juncea] gi|124001973|gb|ABM87935.1| annexin 2 [Brassica juncea]	316	316	1.00E-173	100.0	93.4	97.2	annexin 2	gbpln	Brassica juncea	AT5G65020.1 | Symbols: ANNAT2 | annexin 2 | chr5:25973915-25975554 FORWARD LENGTH=317	316	317	1.00E-170	100.3	89.9	96.2
Rsa1.0_00022.1.g1481.t1	gb|EOA15086.1| hypothetical protein CARUB_v10028454mg [Capsella rubella]	414	427	0	103.1	84.5	91.5	hypothetical protein CARUB_v10028454mg	gbpln	Capsella rubella	AT5G64970.1 | Symbols: | Mitochondrial substrate carrier family protein | chr5:25958806-25960443 REVERSE LENGTH=428	414	428	0	103.4	82.4	89.4
Rsa1.0_00022.1.g1482.t1	ref|NP_201301.1| cyclin-dependent kinase C-2 [Arabidopsis thaliana] gi|152013386|sp|Q8W4P1.2|CDKC2_ARATH RecName: Full=Cyclin-dependent kinase C-2; Short=CDKC;2 gi|13430444|gb|AAK25844.1|AF360134_1 putative cdc2 protein kinase [Arabidopsis thaliana] gi|8843760|dbj|BAA97308.1| cdc2-like protein kinase [Arabidopsis thaliana] gi|14532732|gb|AAK64067.1| putative cdc2 protein kinase [Arabidopsis thaliana] gi|332010592|gb|AED97975.1| cyclin-dependent kinase C-2 [Arabidopsis thaliana]	574	513	0	89.4	82.8	84.5	cyclin-dependent kinase C-2	gbpln	Arabidopsis thaliana	AT5G64960.1 | Symbols: CDKC2, CDKC;2 | cyclin dependent kinase group C2 | chr5:25955497-25958427 FORWARD LENGTH=513	574	513	0	89.4	82.8	84.5
Rsa1.0_00022.1.g1483.t1	ref|NP_569001.1| elicitor peptide 1 [Arabidopsis thaliana] gi|75180610|sp|Q9LV87.1|PEP1_ARATH RecName: Full=Elicitor peptide 1; Flags: Precursor gi|8843754|dbj|BAA97302.1| unnamed protein product [Arabidopsis thaliana] gi|17065508|gb|AAL32908.1| Unknown protein [Arabidopsis thaliana] gi|20148667|gb|AAM10224.1| unknown protein [Arabidopsis thaliana] gi|332010583|gb|AED97966.1| elicitor peptide 1 [Arabidopsis thaliana]	109	92	8.00E-20	84.4	53.2	67.9	elicitor peptide 1	gbpln	Arabidopsis thaliana	AT5G64900.1 | Symbols: PROPEP1, ATPEP1, PEP1 | precursor of peptide 1 | chr5:25937253-25937939 FORWARD LENGTH=92	109	92	1.00E-22	84.4	53.2	67.9

Rsa1.0_00022.1.g1484.t1	ref|XP_002866640.1| hypothetical protein ARALYDRAFT_496707 [Arabidopsis lyrata subsp. lyrata] gi|297312475|gb|EFH42899.1| hypothetical protein ARALYDRAFT_496707 [Arabidopsis lyrata subsp. lyrata]	471	479	0	101.7	89.6	95.5	hypothetical protein ARALYDRAFT_496707	gbpln	Arabidopsis lyrata	AT5G64870.1 | Symbols: | SPFH/Band 7/PHB domain-containing membrane-associated protein family | chr5:25929776-25931308 REVERSE LENGTH=479	471	479	0	101.7	91.3	96.6
Rsa1.0_00022.1.g1485.t1	ref|NP_201291.1| 4-alpha-glucanotransferase-like protein [Arabidopsis thaliana] gi|75180614|sp|Q9LV91.1|DPE1_ARATH RecName: Full=4-alpha-glucanotransferase DPE1, chloroplastic/amyloplastic; AltName: Full=Amylomaltase; AltName: Full=Disproportionating enzyme; Short=D-enzyme; AltName: Full=Protein DISPROPORTIONATING ENZYME 1; Flags: Precursor gi|8843750|dbj|BAA97298.1| 4-alpha-glucanotransferase [Arabidopsis thaliana] gi|14335104|gb|AAK59831.1| AT5g64860/MXK3_9 [Arabidopsis thaliana] gi|19548067|gb|AAL87397.1| AT5g64860/MXK3_9 [Arabidopsis thaliana] gi|332010579|gb|AED97962.1| 4-alpha-glucanotransferase DPE1 [Arabidopsis thaliana]	94	576	2.00E-27	612.8	64.9	69.1	4-alpha-glucanotransferase-like protein	gbpln	Arabidopsis thaliana	AT5G64860.1 | Symbols: DPE1 | disproportionating enzyme | chr5:25925373-25928788 REVERSE LENGTH=576	94	576	4.00E-30	612.8	64.9	69.1
Rsa1.0_00022.1.g1486.t1	ref|XP_002864926.1| hypothetical protein ARALYDRAFT_496705 [Arabidopsis lyrata subsp. lyrata] gi|297310761|gb|EFH41185.1| hypothetical protein ARALYDRAFT_496705 [Arabidopsis lyrata subsp. lyrata]	119	114	4.00E-31	95.8	63.9	70.6	hypothetical protein ARALYDRAFT_496705	gbpln	Arabidopsis lyrata	AT5G64850.1 | Symbols: | FUNCTIONS IN: molecular_function unknown; INVOLVED IN: biological_process unknown; LOCATED IN: plasma membrane; EXPRESSED IN: 22 plant structures; EXPRESSED DURING: 13 growth stages; CONTAINS InterPro DOMAIN/s: RPM1-interacting protein 4, defence response (InterPro:IPR008700); BEST Arabidopsis thaliana protein match is: unknown protein (TAIR:AT5G09960.1); Has 30201 Blast hits to 17322 proteins in 780 species: Archae - 12; Bacteria - 1396; Metazoa - 17338; Fungi - 3422; Plants - 5037; Viruses - 0; Other Eukaryotes - 2996 (source: NCBI BLink). | chr5:25921585-25923038 FORWARD LENGTH=114	119	114	6.00E-33	95.8	58.0	63.9
Rsa1.0_00022.1.g1487.t1	ref|XP_002866638.1| ATGCN5 [Arabidopsis lyrata subsp. lyrata] gi|297312473|gb|EFH42897.1| ATGCN5 [Arabidopsis lyrata subsp. lyrata]	690	694	0	100.6	93.2	97.4	ATGCN5	gbpln	Arabidopsis lyrata	AT5G64840.1 | Symbols: GCN5, ATGCN5 | general control non-repressible 5 | chr5:25916956-25919693 REVERSE LENGTH=692	690	692	0	100.3	92.2	97.0
Rsa1.0_00022.1.g1488.t1	ref|XP_002866636.1| hypothetical protein ARALYDRAFT_332703 [Arabidopsis lyrata subsp. lyrata] gi|297312471|gb|EFH42895.1| hypothetical protein ARALYDRAFT_332703 [Arabidopsis lyrata subsp. lyrata]	143	149	7.00E-61	104.2	86.7	90.9	hypothetical protein ARALYDRAFT_332703	gbpln	Arabidopsis lyrata	AT5G64820.1 | Symbols: | unknown protein; FUNCTIONS IN: molecular_function unknown; INVOLVED IN: biological_process unknown; LOCATED IN: endomembrane system; BEST Arabidopsis thaliana protein match is: unknown protein (TAIR:AT1G16850.1); Has 1807 Blast hits to 1807 proteins in 277 species: Archae - 0; Bacteria - 0; Metazoa - 736; Fungi - 347; Plants - 385; Viruses - 0; Other Eukaryotes - 339 (source: NCBI BLink). | chr5:25913999-25914523 REVERSE LENGTH=145	143	145	1.00E-59	101.4	82.5	89.5
Rsa1.0_00022.1.g1489.t1	ref|XP_002864921.1| glycosyl hydrolase family 17 protein [Arabidopsis lyrata subsp. lyrata] gi|297310756|gb|EFH41180.1| glycosyl hydrolase family 17 protein [Arabidopsis lyrata subsp. lyrata]	671	485	0	72.3	60.8	65.9	glycosyl hydrolase family 17 protein	gbpln	Arabidopsis lyrata	AT5G64790.1 | Symbols: | O-Glycosyl hydrolases family 17 protein | chr5:25902713-25904559 FORWARD LENGTH=485	671	485	0	72.3	60.8	65.9
Rsa1.0_00022.1.g1490.t1	ref|NP_201282.1| uncharacterized protein [Arabidopsis thaliana] gi|75170417|sp|Q9FGF6.1|RGF9_ARATH RecName: Full=Root meristem growth factor 9; Short=AtRGF9; Flags: Precursor gi|10177208|dbj|BAB10310.1| unnamed protein product [Arabidopsis thaliana] gi|27311821|gb|AAO00876.1| unknown protein [Arabidopsis thaliana] gi|30102780|gb|AAP21308.1| At5g64770 [Arabidopsis thaliana] gi|332010566|gb|AED97949.1| uncharacterized protein AT5G64770 [Arabidopsis thaliana]	79	79	5.00E-25	100.0	72.2	83.5	uncharacterized protein	gbpln	Arabidopsis thaliana	AT5G64770.1 | Symbols: RGF9 | Encodes a root meristem growth factor (RGF). Belongs to a family of functionally redundant homologous peptides that are secreted, tyrosine-sulfated, and expressed mainly in the stem cell area and the innermost layer of central columella cells. RGFs are required for maintenance of the root stem cell niche and transit amplifying cell proliferation. Members of this family include: At5g60810 (RGF1), At1g13620 (RGF2), At2g04025 (RGF3), At3g30350 (RGF4), At5g51451 (RGF5), At4g16515 (RGF6), At3g02240 (RGF7), At2g03830 (RGF8) and At5g64770 (RGF9). | chr5:25897681-25898085 REVERSE LENGTH=79	79	79	8.00E-28	100.0	72.2	83.5

Rsa1.0_00022.1.g1491.t1	ref|XP_002864920.1| hypothetical protein ARALYDRAFT_496690 [Arabidopsis lyrata subsp. lyrata] gi|297310755|gb|EFH41179.1| hypothetical protein ARALYDRAFT_496690 [Arabidopsis lyrata subsp. lyrata]	380	392	1.00E-122	103.2	78.4	83.4	hypothetical protein ARALYDRAFT_496690	gbpln	Arabidopsis lyrata	AT5G64750.1 | Symbols: ABR1 | Integrase-type DNA-binding superfamily protein | chr5:25891679-25893656 FORWARD LENGTH=391	380	391	1.00E-118	102.9	77.1	81.6
Rsa1.0_00022.1.g1492.t1	ref|NP_201279.1| cellulose synthase A catalytic subunit 6 [UDP-forming] [Arabidopsis thaliana] gi|73917714|sp|Q94JQ6.2|CESA6_ARATH RecName: Full=Cellulose synthase A catalytic subunit 6 [UDP-forming]; Short=AtCesA6; AltName: Full=AraxCelA; AltName: Full=Isoxaben-resistant protein 2; AltName: Full=Protein PROCUSTE 1; AltName: Full=Protein QUILL gi|10177205|dbj|BAB10307.1| cellulose synthase catalytic subunit [Arabidopsis thaliana] gi|332010562|gb|AED97945.1| cellulose synthase A catalytic subunit 6 [UDP-forming] [Arabidopsis thaliana]	1079	1084	0	100.5	96.4	98.6	cellulose synthase A catalytic subunit 6	gbpln	Arabidopsis thaliana	AT5G64740.1 | Symbols: CESA6, IXR2, E112, PRC1 | cellulose synthase 6 | chr5:25881555-25886333 FORWARD LENGTH=1084	1079	1084	0	100.5	96.4	98.6
Rsa1.0_00022.1.g1493.t1	ref|XP_002864917.1| hypothetical protein ARALYDRAFT_919795 [Arabidopsis lyrata subsp. lyrata] gi|297310752|gb|EFH41176.1| hypothetical protein ARALYDRAFT_919795 [Arabidopsis lyrata subsp. lyrata]	721	860	0	119.3	64.9	80.0	hypothetical protein ARALYDRAFT_919795	gbpln	Arabidopsis lyrata	AT5G64710.1 | Symbols: | Putative endonuclease or glycosyl hydrolase | chr5:25869368-25872092 FORWARD LENGTH=841	721	841	0	116.6	64.4	77.8
Rsa1.0_00022.1.g1494.t1	#	#	#	#	#	#	#	-	----	----	#	#	#	#	#	#	#
Rsa1.0_00022.1.g1495.t1	gb|EOA12539.1| hypothetical protein CARUB_v10026529mg [Capsella rubella]	440	400	4.00E-82	90.9	54.8	64.5	hypothetical protein CARUB_v10026529mg	gbpln	Capsella rubella	AT5G64690.1 | Symbols: | neurofilament triplet H protein-related | chr5:25862969-25864521 FORWARD LENGTH=344	440	344	3.00E-64	78.2	40.5	49.1
Rsa1.0_00022.1.g1496.t1	ref|NP_201273.1| uncharacterized protein [Arabidopsis thaliana] gi|30698059|ref|NP_851270.1| uncharacterized protein [Arabidopsis thaliana] gi|10178071|dbj|BAB11435.1| unnamed protein product [Arabidopsis thaliana] gi|26452974|dbj|BAC43563.1| unknown protein [Arabidopsis thaliana] gi|28973531|gb|AAO64090.1| unknown protein [Arabidopsis thaliana] gi|332010553|gb|AED97936.1| uncharacterized protein AT5G64680 [Arabidopsis thaliana] gi|332010554|gb|AED97937.1| uncharacterized protein AT5G64680 [Arabidopsis thaliana]	200	203	2.00E-43	101.5	50.0	63.5	uncharacterized protein	gbpln	Arabidopsis thaliana	AT5G64680.2 | Symbols: | unknown protein; FUNCTIONS IN: molecular_function unknown; INVOLVED IN: biological_process unknown; LOCATED IN: nucleolus; EXPRESSED IN: 23 plant structures; EXPRESSED DURING: 13 growth stages; Has 1807 Blast hits to 1807 proteins in 277 species: Archae - 0; Bacteria - 0; Metazoa - 736; Fungi - 347; Plants - 385; Viruses - 0; Other Eukaryotes - 339 (source: NCBI BLink). | chr5:25854433-25855331 FORWARD LENGTH=203	200	203	9.00E-46	101.5	50.0	63.5
Rsa1.0_00022.1.g1497.t1	dbj|BAE44362.1| alpha-L-arabinofuranosidase [Raphanus sativus]	778	780	0	100.3	91.0	94.5	alpha-L-arabinofuranosidase	gbpln	Raphanus sativus	AT5G64570.1 | Symbols: XYL4, ATBXL4 | beta-D-xylosidase 4 | chr5:25810227-25813309 REVERSE LENGTH=784	778	784	0	100.8	89.5	94.2
Rsa1.0_00022.1.g1498.t2	ref|XP_002866621.1| hypothetical protein ARALYDRAFT_496668 [Arabidopsis lyrata subsp. lyrata] gi|297312456|gb|EFH42880.1| hypothetical protein ARALYDRAFT_496668 [Arabidopsis lyrata subsp. lyrata]	660	395	1.00E-155	59.8	45.0	49.4	hypothetical protein ARALYDRAFT_496668	gbpln	Arabidopsis lyrata	AT5G64560.1 | Symbols: MGT9, MRS2-2, ATMGT9 | magnesium transporter 9 | chr5:25807025-25809395 REVERSE LENGTH=394	660	394	1.00E-152	59.7	44.8	49.2
Rsa1.0_00022.1.g1499.t1	ref|XP_002866619.1| hypothetical protein ARALYDRAFT_358659 [Arabidopsis lyrata subsp. lyrata] gi|297312454|gb|EFH42878.1| hypothetical protein ARALYDRAFT_358659 [Arabidopsis lyrata subsp. lyrata]	574	1082	0	188.5	89.9	93.4	hypothetical protein ARALYDRAFT_358659	gbpln	Arabidopsis lyrata	AT5G64550.1 | Symbols: | loricrin-related | chr5:25801794-25803698 REVERSE LENGTH=634	574	634	0	110.5	89.2	93.7
Rsa1.0_00022.1.g1500.t1	ref|NP_201258.2| xylem NAC domain 1 [Arabidopsis thaliana] gi|26452583|dbj|BAC43376.1| unknown protein [Arabidopsis thaliana] gi|107738104|gb|ABF83636.1| At5g64530 [Arabidopsis thaliana] gi|332010530|gb|AED97913.1| xylem NAC domain 1 [Arabidopsis thaliana]	185	187	1.00E-88	101.1	89.7	94.6	xylem NAC domain 1	gbpln	Arabidopsis thaliana	AT5G64530.1 | Symbols: ANAC104, XND1 | xylem NAC domain 1 | chr5:25795360-25796699 FORWARD LENGTH=187	185	187	5.00E-91	101.1	89.7	94.6

Rsa1.0_00022.1.g1501.t1	ref|XP_002864749.1| hypothetical protein ARALYDRAFT_496339 [Arabidopsis lyrata subsp. lyrata] gi|297310584|gb|EFH41008.1| hypothetical protein ARALYDRAFT_496339 [Arabidopsis lyrata subsp. lyrata]	390	390	6.00E-90	100.0	48.7	62.6	hypothetical protein ARALYDRAFT_496339	gbpln	Arabidopsis lyrata	AT5G61720.1 | Symbols: | Protein of unknown function (DUF1216) | chr5:24801621-24803006 FORWARD LENGTH=390	390	390	5.00E-91	100.0	47.7	63.1
Rsa1.0_00022.1.g1502.t2	ref|XP_002864902.1| N-acylethanolamine amidohydrolase [Arabidopsis lyrata subsp. lyrata] gi|297310737|gb|EFH41161.1| N-acylethanolamine amidohydrolase [Arabidopsis lyrata subsp. lyrata]	632	607	0	96.0	81.2	88.9	N-acylethanolamine amidohydrolase	gbpln	Arabidopsis lyrata	AT5G64440.1 | Symbols: AtFAAH, FAAH | fatty acid amide hydrolase | chr5:25766229-25770260 FORWARD LENGTH=607	632	607	0	96.0	80.9	88.0
Rsa1.0_00022.1.g1503.t1	gb|EOA12745.1| hypothetical protein CARUB_v10028367mg [Capsella rubella]	486	503	0	103.5	77.2	84.0	hypothetical protein CARUB_v10028367mg	gbpln	Capsella rubella	AT5G64430.1 | Symbols: | Octicosapeptide/Phox/Bem1p family protein | chr5:25762540-25764081 REVERSE LENGTH=513	486	513	1.00E-174	105.6	79.2	85.6
Rsa1.0_00022.1.g1504.t1	gb|EOA12796.1| hypothetical protein CARUB_v10025747mg [Capsella rubella]	178	1306	2.00E-37	733.7	41.6	47.8	hypothetical protein CARUB_v10025747mg	gbpln	Capsella rubella	AT5G64420.1 | Symbols: | DNA polymerase V family | chr5:25756416-25761122 FORWARD LENGTH=1306	178	1306	1.00E-39	733.7	40.4	47.2
Rsa1.0_00022.1.g1505.t1	gb|AAM64614.1| Isp4-like protein [Arabidopsis thaliana]	729	729	0	100.0	94.1	97.4	Isp4-like protein	gbpln	Arabidopsis thaliana	AT5G64410.1 | Symbols: ATOPT4, OPT4 | oligopeptide transporter 4 | chr5:25750921-25754974 REVERSE LENGTH=729	729	729	0	100.0	94.1	97.1
Rsa1.0_00022.1.g1506.t1	gb|EOA12386.1| hypothetical protein CARUB_v10026348mg [Capsella rubella] gi|482548193|gb|EOA12387.1| hypothetical protein CARUB_v10026348mg [Capsella rubella]	381	469	1.00E-138	123.1	74.3	82.7	hypothetical protein CARUB_v10026348mg	gbpln	Capsella rubella	AT5G64360.4 | Symbols: | Chaperone DnaJ-domain superfamily protein | chr5:25737208-25738602 REVERSE LENGTH=464	381	464	1.00E-132	121.8	73.2	80.8
Rsa1.0_00022.1.g1507.t1	dbj|BAB11643.1| unnamed protein product [Arabidopsis thaliana]	675	711	0	105.3	54.5	67.9	unnamed protein product	gbpln	Arabidopsis thaliana	AT5G37210.1 | Symbols: | Cysteine/Histidine-rich C1 domain family protein | chr5:14729225-14731508 REVERSE LENGTH=677	675	677	0	100.3	54.5	66.8
Rsa1.0_00022.1.g1508.t1	#	#	#	#	#	#	#	-	----	----	#	#	#	#	#	#	#
Rsa1.0_00022.1.g1509.t1	ref|XP_002866611.1| FK506-binding protein 12 kD [Arabidopsis lyrata subsp. lyrata] gi|297312446|gb|EFH42870.1| FK506-binding protein 12 kD [Arabidopsis lyrata subsp. lyrata]	126	112	3.00E-42	88.9	65.9	68.3	FK506-binding protein 12 kD	gbpln	Arabidopsis lyrata	AT5G64350.1 | Symbols: FKBP12, ATFKBP12 | FK506-binding protein 12 | chr5:25734810-25735990 REVERSE LENGTH=112	126	112	4.00E-43	88.9	64.3	65.9
Rsa1.0_00022.1.g1510.t1	dbj|BAJ34099.1| unnamed protein product [Thellungiella halophila]	270	329	6.00E-88	121.9	73.0	84.8	unnamed protein product	----	----	AT5G64340.1 | Symbols: SAC51 | sequence-specific DNA binding transcription factors;transcription regulators | chr5:25730890-25731936 REVERSE LENGTH=348	270	348	1.00E-87	128.9	74.8	85.6
Rsa1.0_00022.1.g1511.t1	gb|EOA12443.1| hypothetical protein CARUB_v10028516mg [Capsella rubella]	818	728	0	89.0	69.8	77.5	hypothetical protein CARUB_v10028516mg	gbpln	Capsella rubella	AT5G64320.1 | Symbols: | Pentatricopeptide repeat (PPR) superfamily protein | chr5:25723247-25725439 REVERSE LENGTH=730	818	730	0	89.2	69.3	76.8
Rsa1.0_00022.1.g1512.t1	ref|NP_201233.1| dicarboxylate transporter 2.2 [Arabidopsis thaliana] gi|75171657|sp|Q9FMF8.1|DIT22_ARATH RecName: Full=Dicarboxylate transporter 2.2, chloroplastic; AltName: Full=AtpDCT2; Flags: Precursor gi|9759404|dbj|BAB09859.1| 2-oxoglutarate/malate translocator [Arabidopsis thaliana] gi|15028073|gb|AAK76567.1| putative 2-oxoglutarate/malate translocator protein [Arabidopsis thaliana] gi|22136830|gb|AAM91759.1| putative 2-oxoglutarate/malate translocator protein [Arabidopsis thaliana] gi|332010482|gb|AED97865.1| dicarboxylate transporter 2.2 [Arabidopsis thaliana]	558	549	0	98.4	85.8	91.9	dicarboxylate transporter 2.2	gbpln	Arabidopsis thaliana	AT5G64280.1 | Symbols: DiT2.2 | dicarboxylate transporter 2.2 | chr5:25711330-25713411 REVERSE LENGTH=549	558	549	0	98.4	85.8	91.9
Rsa1.0_00022.1.g1513.t1	gb|ABD65112.1| hypothetical protein 31.t00014 [Brassica oleracea]	102	122	8.00E-18	119.6	49.0	66.7	hypothetical protein 31.t00014	gbpln	Brassica oleracea	#	#	#	#	#	#	#
Rsa1.0_00022.1.g1514.t1	dbj|BAJ33879.1| unnamed protein product [Thellungiella halophila]	301	304	2.33E-156	101.0	86.0	91.0	unnamed protein product	----	----	AT5G64260.1 | Symbols: EXL2 | EXORDIUM like 2 | chr5:25703980-25704897 FORWARD LENGTH=305	301	305	2.33E-156	101.3	84.1	90.0
Rsa1.0_00022.1.g1515.t1	gb|ABD64939.1| hypothetical protein 24.t00017 [Brassica oleracea]	348	442	1.00E-136	127.0	69.0	77.9	hypothetical protein 24.t00017	gbpln	Brassica oleracea	AT2G13770.1 | Symbols: | CONTAINS InterPro DOMAIN/s: Putative harbinger transposase-derived nuclease (InterPro:IPR006912); Has 451 Blast hits to 352 proteins in 33 species: Archae - 0; Bacteria - 0; Metazoa - 28; Fungi - 8; Plants - 388; Viruses - 0; Other Eukaryotes - 27 (source: NCBI BLink). | chr2:5736603-5737847 FORWARD LENGTH=343	348	343	1.00E-32	98.6	17.5	22.7

Rsa1.0_00022.1.g1516.t1	ref|NP_201229.1| metacaspase 3 [Arabidopsis thaliana] gi|75262715|sp|Q9FMG1.1|MCA3_ARATH RecName: Full=Metacaspase-3; Short=AtMC3; AltName: Full=Metacaspase 1a; Short=AtMCP1a gi|9759400|dbj|BAB09855.1| unnamed protein product [Arabidopsis thaliana] gi|21592565|gb|AAM64514.1| latex-abundant protein, putative [Arabidopsis thaliana] gi|27311579|gb|AAO00755.1| putative protein [Arabidopsis thaliana] gi|32482816|gb|AAP84708.1| metacaspase 3 [Arabidopsis thaliana] gi|34098865|gb|AAQ56815.1| At5g64240 [Arabidopsis thaliana] gi|332010477|gb|AED97860.1| metacaspase 3 [Arabidopsis thaliana]	386	362	1.00E-158	93.8	72.3	79.0	metacaspase 3	gbpln	Arabidopsis thaliana	AT5G64240.2 | Symbols: AtMC3, MC3 | metacaspase 3 | chr5:25695836-25697249 FORWARD LENGTH=362	386	362	1.00E-160	93.8	72.3	79.0
Rsa1.0_00022.1.g1517.t1	ref|XP_002870617.1| hypothetical protein ARALYDRAFT_916024 [Arabidopsis lyrata subsp. lyrata] gi|297316453|gb|EFH46876.1| hypothetical protein ARALYDRAFT_916024 [Arabidopsis lyrata subsp. lyrata]	679	704	0	103.7	76.4	86.7	hypothetical protein ARALYDRAFT_916024	gbpln	Arabidopsis lyrata	AT5G41770.1 | Symbols: | crooked neck protein, putative / cell cycle protein, putative | chr5:16718021-16720936 FORWARD LENGTH=705	679	705	0	103.8	76.7	86.9
Rsa1.0_00022.1.g1518.t1	ref|NP_568987.2| uncharacterized protein [Arabidopsis thaliana] gi|332010475|gb|AED97858.1| uncharacterized protein AT5G64230 [Arabidopsis thaliana]	371	379	1.00E-150	102.2	77.6	86.0	uncharacterized protein	gbpln	Arabidopsis thaliana	AT5G64230.1 | Symbols: | unknown protein; FUNCTIONS IN: molecular_function unknown; INVOLVED IN: biological_process unknown; LOCATED IN: endomembrane system; EXPRESSED IN: 9 plant structures; EXPRESSED DURING: 4 anthesis, petal differentiation and expansion stage; BEST Arabidopsis thaliana protein match is: unknown protein (TAIR:AT3G19920.1); Has 35333 Blast hits to 34131 proteins in 2444 species: Archae - 798; Bacteria - 22429; Metazoa - 974; Fungi - 991; Plants - 531; Viruses - 0; Other Eukaryotes - 9610 (source: NCBI BLink). | chr5:25692572-25693909 FORWARD LENGTH=379	371	379	1.00E-153	102.2	77.6	86.0
Rsa1.0_00022.1.g1519.t1	ref|XP_002866597.1| hypothetical protein ARALYDRAFT_496610 [Arabidopsis lyrata subsp. lyrata] gi|297312432|gb|EFH42856.1| hypothetical protein ARALYDRAFT_496610 [Arabidopsis lyrata subsp. lyrata]	113	118	2.00E-47	104.4	88.5	91.2	hypothetical protein ARALYDRAFT_496610	gbpln	Arabidopsis lyrata	AT5G64130.1 | Symbols: | cAMP-regulated phosphoprotein 19-related protein | chr5:25664547-25665339 REVERSE LENGTH=115	113	115	5.00E-50	101.8	87.6	90.3
Rsa1.0_00022.1.g1520.t2	#	#	#	#	#	#	#	-	----	----	#	#	#	#	#	#	#
Rsa1.0_00022.1.g1521.t1	gb|EOA13703.1| hypothetical protein CARUB_v10026774mg [Capsella rubella]	328	328	1.00E-163	100.0	86.3	91.8	hypothetical protein CARUB_v10026774mg	gbpln	Capsella rubella	AT5G64120.1 | Symbols: | Peroxidase superfamily protein | chr5:25659551-25660946 REVERSE LENGTH=328	328	328	1.00E-163	100.0	84.8	91.5
Rsa1.0_00023.1.g1522.t2	ref|NP_564728.3| WD40 and Beach domain-containing protein [Arabidopsis thaliana] gi|332195397|gb|AEE33518.1| WD40 and Beach domain-containing protein [Arabidopsis thaliana]	2615	2604	0	99.6	90.6	94.7	WD40 and Beach domain-containing protein	gbpln	Arabidopsis thaliana	AT1G58230.1 | Symbols: | binding | chr1:21566331-21578865 FORWARD LENGTH=2604	2615	2604	0	99.6	90.6	94.7
Rsa1.0_00023.1.g1523.t1	#	#	#	#	#	#	#	-	----	----	#	#	#	#	#	#	#
Rsa1.0_00023.1.g1524.t1	#	#	#	#	#	#	#	-	----	----	#	#	#	#	#	#	#
Rsa1.0_00023.1.g1525.t1	gb|ACG60682.1| transposon-like ORF [Brassica oleracea var. alboglabra]	553	704	1.00E-104	127.3	32.7	34.4	transposon-like ORF	gbpln	Brassica oleracea	#	#	#	#	#	#	#
Rsa1.0_00023.1.g1526.t1	gb|ABD65073.1| hypothetical protein 27.t00059 [Brassica oleracea]	334	341	7.00E-21	102.1	14.4	17.4	hypothetical protein 27.t00059	gbpln	Brassica oleracea	#	#	#	#	#	#	#
Rsa1.0_00023.1.g1527.t1	emb|CAB39638.1| RNA-directed DNA polymerase-like protein [Arabidopsis thaliana] gi|7267666|emb|CAB78094.1| RNA-directed DNA polymerase-like protein [Arabidopsis thaliana]	508	1274	1.00E-131	250.8	51.0	63.4	RNA-directed DNA polymerase-like protein	gbpln	Arabidopsis thaliana	AT4G29090.1 | Symbols: | Ribonuclease H-like superfamily protein | chr4:14333528-14335255 FORWARD LENGTH=575	508	575	1.00E-61	113.2	32.1	49.0
Rsa1.0_00023.1.g1528.t1	gb|AAG50957.1|AC073943_7 hypothetical protein [Arabidopsis thaliana]	940	1195	0	127.1	80.4	87.1	hypothetical protein	gbpln	Arabidopsis thaliana	AT1G58210.1 | Symbols: EMB1674 | kinase interacting family protein | chr1:21553621-21558056 FORWARD LENGTH=1246	940	1246	0	132.6	79.9	86.7
Rsa1.0_00023.1.g1529.t1	ref|XP_002886709.1| EMB1674 [Arabidopsis lyrata subsp. lyrata] gi|297332550|gb|EFH62968.1| EMB1674 [Arabidopsis lyrata subsp. lyrata]	271	1287	2.00E-63	474.9	52.8	64.2	EMB1674	gbpln	Arabidopsis lyrata	AT1G58210.1 | Symbols: EMB1674 | kinase interacting family protein | chr1:21553621-21558056 FORWARD LENGTH=1246	271	1246	2.00E-62	459.8	53.9	67.5

Rsa1.0_00023.1.g1530.t2	ref|XP_002888236.1| hypothetical protein ARALYDRAFT_475431 [Arabidopsis lyrata subsp. lyrata] gi|297334077|gb|EFH64495.1| hypothetical protein ARALYDRAFT_475431 [Arabidopsis lyrata subsp. lyrata]	618	671	0	108.6	84.1	89.8	hypothetical protein ARALYDRAFT_475431	gbpln	Arabidopsis lyrata	AT1G58200.2 | Symbols: MSL3 | MSCS-like 3 | chr1:21548370-21552488 REVERSE LENGTH=678	618	678	0	109.7	82.4	89.0
Rsa1.0_00023.1.g1531.t1	gb|AAG50756.1|AC079131_1 hypothetical protein [Arabidopsis thaliana]	831	1784	0	214.7	73.3	82.4	hypothetical protein	gbpln	Arabidopsis thaliana	AT1G58190.1 | Symbols: AtRLP9, RLP9 | receptor like protein 9 | chr1:21540720-21544330 FORWARD LENGTH=932	831	932	0	112.2	73.3	82.4
Rsa1.0_00023.1.g1532.t1	gb|AAD15321.1| putative reverse transcriptase [Arabidopsis thaliana] gi|7270688|emb|CAB77850.1| putative reverse transcriptase [Arabidopsis thaliana]	358	839	2.00E-27	234.4	19.8	30.4	putative reverse transcriptase	gbpln	Arabidopsis thaliana	#	#	#	#	#	#	#
Rsa1.0_00023.1.g1533.t1	#	#	#	#	#	#	#	-	----	----	#	#	#	#	#	#	#
Rsa1.0_00023.1.g1534.t1	gb|EOA35530.1| hypothetical protein CARUB_v10020737mg [Capsella rubella]	290	290	1.00E-151	100.0	87.6	94.5	hypothetical protein CARUB_v10020737mg	gbpln	Capsella rubella	AT1G58180.4 | Symbols: BCA6 | beta carbonic anhydrase 6 | chr1:21538010-21539711 REVERSE LENGTH=290	290	290	1.00E-151	100.0	86.6	92.4
Rsa1.0_00023.1.g1535.t1	gb|EOA34327.1| hypothetical protein CARUB_v10021847mg [Capsella rubella]	420	422	0	100.5	86.4	91.4	hypothetical protein CARUB_v10021847mg	gbpln	Capsella rubella	AT1G58120.1 | Symbols: | BEST Arabidopsis thaliana protein match is: methyltransferases (TAIR:AT5G01710.1); Has 93 Blast hits to 93 proteins in 15 species: Archae - 0; Bacteria - 0; Metazoa - 1; Fungi - 0; Plants - 92; Viruses - 0; Other Eukaryotes - 0 (source: NCBI BLink). | chr1:21520962-21522224 FORWARD LENGTH=420	420	420	0	100.0	86.9	92.4
Rsa1.0_00023.1.g1536.t2	ref|XP_002888232.1| predicted protein [Arabidopsis lyrata subsp. lyrata] gi|297334073|gb|EFH64491.1| predicted protein [Arabidopsis lyrata subsp. lyrata]	359	372	1.00E-131	103.6	75.8	82.7	predicted protein	gbpln	Arabidopsis lyrata	AT1G58110.2 | Symbols: | Basic-leucine zipper (bZIP) transcription factor family protein | chr1:21515919-21517374 REVERSE LENGTH=374	359	374	1.00E-133	104.2	76.0	82.7
Rsa1.0_00023.1.g1537.t2	ref|NP_191684.1| myb domain protein 17 [Arabidopsis thaliana] gi|6850892|emb|CAB71055.1| putative transcription factor (MYB17) [Arabidopsis thaliana] gi|41619302|gb|AAS10071.1| MYB transcription factor [Arabidopsis thaliana] gi|193885153|gb|ACF28390.1| At3g61250 [Arabidopsis thaliana] gi|332646658|gb|AEE80179.1| myb domain protein 17 [Arabidopsis thaliana]	436	299	3.00E-65	68.6	35.3	43.1	myb domain protein 17	gbpln	Arabidopsis thaliana	AT3G61250.1 | Symbols: AtMYB17, MYB17 | myb domain protein 17 | chr3:22671306-22672551 FORWARD LENGTH=299	436	299	8.00E-68	68.6	35.3	43.1
Rsa1.0_00023.1.g1538.t1	gb|EOA34940.1| hypothetical protein CARUB_v10020024mg [Capsella rubella]	576	592	0	102.8	68.8	77.3	hypothetical protein CARUB_v10020024mg	gbpln	Capsella rubella	AT1G58025.1 | Symbols: | DNA-binding bromodomain-containing protein | chr1:21458219-21461757 REVERSE LENGTH=572	576	572	0	99.3	67.7	76.2
Rsa1.0_00023.1.g1539.t1	ref|XP_002879419.1| hypothetical protein ARALYDRAFT_902347 [Arabidopsis lyrata subsp. lyrata] gi|297325258|gb|EFH55678.1| hypothetical protein ARALYDRAFT_902347 [Arabidopsis lyrata subsp. lyrata]	173	336	2.00E-33	194.2	56.1	65.9	hypothetical protein ARALYDRAFT_902347	gbpln	Arabidopsis lyrata	AT2G32820.1 | Symbols: | Transcription elongation factor (TFIIS) family protein | chr2:13926037-13926786 FORWARD LENGTH=249	173	249	3.00E-35	143.9	56.1	71.7
Rsa1.0_00023.1.g1540.t1	#	#	#	#	#	#	#	-	----	----	#	#	#	#	#	#	#
Rsa1.0_00023.1.g1541.t1	ref|NP_176081.1| uncharacterized protein [Arabidopsis thaliana] gi|42571907|ref|NP_974044.1| uncharacterized protein [Arabidopsis thaliana] gi|12321352|gb|AAG50748.1|AC079733_16 hypothetical protein [Arabidopsis thaliana] gi|18176030|gb|AAL59971.1| unknown protein [Arabidopsis thaliana] gi|22136846|gb|AAM91767.1| unknown protein [Arabidopsis thaliana] gi|332195330|gb|AEE33451.1| uncharacterized protein AT1G57680 [Arabidopsis thaliana] gi|332195331|gb|AEE33452.1| uncharacterized protein AT1G57680 [Arabidopsis thaliana]	354	362	1.00E-159	102.3	87.6	93.8	uncharacterized protein	gbpln	Arabidopsis thaliana	AT1G57680.2 | Symbols: | FUNCTIONS IN: molecular_function unknown; INVOLVED IN: biological_process unknown; LOCATED IN: endomembrane system; EXPRESSED IN: 24 plant structures; EXPRESSED DURING: 15 growth stages; CONTAINS InterPro DOMAIN/s: Uncharacterised conserved protein UCP031277 (InterPro:IPR016971); Has 35333 Blast hits to 34131 proteins in 2444 species: Archae - 798; Bacteria - 22429; Metazoa - 974; Fungi - 991; Plants - 531; Viruses - 0; Other Eukaryotes - 9610 (source: NCBI BLink). | chr1:21362749-21363837 REVERSE LENGTH=362	354	362	1.00E-161	102.3	87.6	93.8
Rsa1.0_00023.1.g1542.t1	ref|NP_176083.2| protein kinase-like protein [Arabidopsis thaliana] gi|332195333|gb|AEE33454.1| protein kinase-like protein [Arabidopsis thaliana]	683	692	0	101.3	84.6	91.1	protein kinase-like protein	gbpln	Arabidopsis thaliana	AT1G57700.1 | Symbols: | Protein kinase superfamily protein | chr1:21371051-21373860 FORWARD LENGTH=692	683	692	0	101.3	84.6	91.1

Rsa1.0_00023.1.g1543.t1	ref|NP_176084.1| elongation factor EF-1 gamma subunit [Arabidopsis thaliana] gi|79320145|ref|NP_001031202.1| elongation factor EF-1 gamma subunit [Arabidopsis thaliana] gi|13626393|sp|Q9FVT2.1|EF1G2_ARATH RecName: Full=Probable elongation factor 1-gamma 2; Short=EF-1-gamma 2; AltName: Full=eEF-1B gamma 2 gi|12321359|gb|AAG50755.1|AC079733_23 elongation factor 1B gamma, putative [Arabidopsis thaliana] gi|13877603|gb|AAK43879.1|AF370502_1 Unknown protein [Arabidopsis thaliana] gi|15983511|gb|AAL11623.1|AF424630_1 At1g57720/T8L23_18 [Arabidopsis thaliana] gi|16226841|gb|AAL16277.1|AF428347_1 At1g57720/T8L23_18 [Arabidopsis thaliana] gi|17978699|gb|AAL47343.1| unknown protein [Arabidopsis thaliana] gi|21360471|gb|AAM47351.1| At1g57720/T8L23_18 [Arabidopsis thaliana] gi|21553395|gb|AAM62488.1| elongation factor 1B gamma, putative [Arabidopsis thaliana] gi|24030437|gb|AAN41373.1| putative elongation factor 1B gamma [Arabidopsis thaliana] gi|332195334|gb|AEE33455.1| elongation factor EF-1 gamma subunit [Arabidopsis thaliana] gi|332195335|gb|AEE33456.1| elongation factor EF-1 gamma subunit	413	413	0	100.0	93.0	96.6	elongation factor EF-1 gamma subunit	gbpln	Arabidopsis thaliana	AT1G57720.2 | Symbols: | Translation elongation factor EF1B, gamma chain | chr1:21377873-21380114 FORWARD LENGTH=413	413	413	0	100.0	93.0	96.6
Rsa1.0_00023.1.g1544.t1	gb|AAC97237.1| putative TNP2-like transposon protein [Arabidopsis thaliana]	107	889	3.00E-30	830.8	55.1	71.0	putative TNP2-like transposon protein	gbpln	Arabidopsis thaliana	#	#	#	#	#	#	#
Rsa1.0_00023.1.g1545.t1	ref|NP_187098.2| endoribonuclease [Arabidopsis thaliana] gi|332640566|gb|AEE74087.1| endoribonuclease [Arabidopsis thaliana]	379	718	1.00E-101	189.4	49.3	51.5	endoribonuclease	gbpln	Arabidopsis thaliana	AT3G04480.1 | Symbols: | endoribonucleases | chr3:1193988-1197320 REVERSE LENGTH=718	379	718	1.00E-104	189.4	49.3	51.5
Rsa1.0_00023.1.g1546.t1	ref|XP_002884447.1| hypothetical protein ARALYDRAFT_896485 [Arabidopsis lyrata subsp. lyrata] gi|297330287|gb|EFH60706.1| hypothetical protein ARALYDRAFT_896485 [Arabidopsis lyrata subsp. lyrata]	227	371	6.00E-28	163.4	31.7	39.2	hypothetical protein ARALYDRAFT_896485	gbpln	Arabidopsis lyrata	AT5G19170.1 | Symbols: | Protein of Unknown Function (DUF239) | chr5:6445245-6447305 FORWARD LENGTH=391	227	391	1.00E-24	172.2	25.6	35.2
Rsa1.0_00023.1.g1547.t1	ref|NP_564725.1| F-box protein [Arabidopsis thaliana] gi|75172668|sp|Q9FVS1.1|FBK23_ARATH RecName: Full=F-box/kelch-repeat protein At1g57790 gi|11079521|gb|AAG29231.1|AC079732_2 hypothetical protein [Arabidopsis thaliana] gi|20466201|gb|AAM20418.1| unknown protein [Arabidopsis thaliana] gi|30984578|gb|AAP42752.1| At1g57790 [Arabidopsis thaliana] gi|332195346|gb|AEE33467.1| F-box protein [Arabidopsis thaliana]	331	352	1.00E-134	106.3	68.6	82.5	F-box protein	gbpln	Arabidopsis thaliana	AT1G57790.1 | Symbols: | F-box family protein | chr1:21404578-21405636 REVERSE LENGTH=352	331	352	1.00E-136	106.3	68.6	82.5
Rsa1.0_00023.1.g1548.t1	gb|EOA25688.1| hypothetical protein CARUB_v10019041mg [Capsella rubella]	236	203	8.00E-67	86.0	52.5	59.3	hypothetical protein CARUB_v10019041mg	gbpln	Capsella rubella	AT2G03300.1 | Symbols: | Toll-Interleukin-Resistance (TIR) domain family protein | chr2:1003468-1004222 REVERSE LENGTH=203	236	203	8.00E-69	86.0	55.5	61.9
Rsa1.0_00023.1.g1549.t3	gb|AAF67380.1| Hypothetical protein T15F17.l [Arabidopsis thaliana]	998	1141	1.00E-158	114.3	27.4	32.3	Hypothetical protein T15F17.l	gbpln	Arabidopsis thaliana	AT4G23160.1 | Symbols: CRK8 | cysteine-rich RLK (RECEPTOR-like protein kinase) 8 | chr4:12129485-12134086 FORWARD LENGTH=1262	998	1262	1.00E-14	126.5	5.8	9.9
Rsa1.0_00023.1.g1550.t1	#	#	#	#	#	#	#	-	----	----	#	#	#	#	#	#	#
Rsa1.0_00023.1.g1551.t1	gb|EOA35713.1| hypothetical protein CARUB_v10020940mg [Capsella rubella]	215	215	1.00E-101	100.0	80.9	87.9	hypothetical protein CARUB_v10020940mg	gbpln	Capsella rubella	AT1G57620.1 | Symbols: | emp24/gp25L/p24 family/GOLD family protein | chr1:21342863-21344581 FORWARD LENGTH=212	215	212	1.00E-102	98.6	82.3	90.2

Rsa1.0_00023.1.g1552.t1	ref|NP_176074.2| uncharacterized protein [Arabidopsis thaliana] gi|42571905|ref|NP_974043.1| uncharacterized protein [Arabidopsis thaliana] gi|17979163|gb|AAL49777.1| unknown protein [Arabidopsis thaliana] gi|332195320|gb|AEE33441.1| uncharacterized protein AT1G57610 [Arabidopsis thaliana] gi|332195321|gb|AEE33442.1| uncharacterized protein AT1G57610 [Arabidopsis thaliana]	292	293	1.00E-125	100.3	79.8	87.3	uncharacterized protein	gbpln	Arabidopsis thaliana	AT1G57610.2 | Symbols: | Protein of unknown function (DUF607) | chr1:21337449-21338412 REVERSE LENGTH=293	292	293	1.00E-128	100.3	79.8	87.3
Rsa1.0_00023.1.g1553.t1	gb|EOA35193.1| hypothetical protein CARUB_v10020343mg [Capsella rubella]	391	425	1.00E-167	108.7	73.1	80.8	hypothetical protein CARUB_v10020343mg	gbpln	Capsella rubella	AT1G57590.1 | Symbols: | Pectinacetylesterase family protein | chr1:21327458-21329707 REVERSE LENGTH=444	391	444	1.00E-164	113.6	71.1	79.8
Rsa1.0_00024.1.g1554.t1	gb|EOA13735.1| hypothetical protein CARUB_v10026813mg [Capsella rubella]	312	312	1.00E-162	100.0	89.4	94.2	hypothetical protein CARUB_v10026813mg	gbpln	Capsella rubella	AT5G57330.1 | Symbols: | Galactose mutarotase-like superfamily protein | chr5:23218392-23220664 FORWARD LENGTH=312	312	312	1.00E-163	100.0	88.1	93.6
Rsa1.0_00024.1.g1555.t1	gb|EOA15125.1| hypothetical protein CARUB_v10028500mg [Capsella rubella]	965	982	0	101.8	86.8	93.0	hypothetical protein CARUB_v10028500mg	gbpln	Capsella rubella	AT5G57320.1 | Symbols: VLN5 | villin, putative | chr5:23212690-23217549 FORWARD LENGTH=962	965	962	0	99.7	86.4	92.2
Rsa1.0_00024.1.g1556.t1	ref|XP_002866199.1| hypothetical protein ARALYDRAFT_918901 [Arabidopsis lyrata subsp. lyrata] gi|297312034|gb|EFH42458.1| hypothetical protein ARALYDRAFT_918901 [Arabidopsis lyrata subsp. lyrata]	120	120	2.00E-30	100.0	94.2	95.8	hypothetical protein ARALYDRAFT_918901	gbpln	Arabidopsis lyrata	AT5G57290.1 | Symbols: | 60S acidic ribosomal protein family | chr5:23207049-23207835 REVERSE LENGTH=120	120	120	2.00E-31	100.0	93.3	95.0
Rsa1.0_00024.1.g1557.t1	gb|EOA13062.1| hypothetical protein CARUB_v10026062mg [Capsella rubella]	269	646	8.00E-59	240.1	40.5	54.3	hypothetical protein CARUB_v10026062mg	gbpln	Capsella rubella	AT5G60250.1 | Symbols: | zinc finger (C3HC4-type RING finger) family protein | chr5:24252226-24254710 FORWARD LENGTH=655	269	655	2.00E-60	243.5	40.9	53.2
Rsa1.0_00024.1.g1558.t1	gb|EOA22836.1| hypothetical protein CARUB_v10003560mg [Capsella rubella]	162	406	2.00E-26	250.6	50.6	64.8	hypothetical protein CARUB_v10003560mg	gbpln	Capsella rubella	AT4G11000.1 | Symbols: | Ankyrin repeat family protein | chr4:6731020-6732464 FORWARD LENGTH=406	162	406	3.00E-28	250.6	48.8	64.2
Rsa1.0_00024.1.g1559.t1	emb|CAR63886.1| cytochrome P450 monooxygenase [Arabidopsis thaliana]	493	491	0	99.6	87.0	93.3	cytochrome P450 monooxygenase	gbpln	Arabidopsis thaliana	AT5G57220.1 | Symbols: CYP81F2 | cytochrome P450, family 81, subfamily F, polypeptide 2 | chr5:23187911-23189681 FORWARD LENGTH=491	493	491	0	99.6	86.6	93.1
Rsa1.0_00024.1.g1560.t1	gb|AAC33958.1| contains similarity to Zea mays embryogenesis transmembrane protein (GB:X97570) [Arabidopsis thaliana]	475	417	1.00E-142	87.8	56.6	68.0	contains similarity to Zea mays embryogenesis transmembrane protein (GB:X97570)	gbpln	Arabidopsis thaliana	AT4G11000.1 | Symbols: | Ankyrin repeat family protein | chr4:6731020-6732464 FORWARD LENGTH=406	475	406	1.00E-106	85.5	46.3	58.5
Rsa1.0_00024.1.g1561.t4	gb|EOA14132.1| hypothetical protein CARUB_v10027277mg [Capsella rubella]	259	160	6.00E-33	61.8	26.3	28.2	hypothetical protein CARUB_v10027277mg	gbpln	Capsella rubella	AT5G57230.1 | Symbols: | Thioredoxin superfamily protein | chr5:23190572-23191485 FORWARD LENGTH=160	259	160	2.00E-35	61.8	26.3	27.8
Rsa1.0_00024.1.g1562.t1	ref|XP_002864506.1| CYP81F2 [Arabidopsis lyrata subsp. lyrata] gi|297310341|gb|EFH40765.1| CYP81F2 [Arabidopsis lyrata subsp. lyrata]	493	497	0	100.8	89.9	94.5	CYP81F2	gbpln	Arabidopsis lyrata	AT5G57220.1 | Symbols: CYP81F2 | cytochrome P450, family 81, subfamily F, polypeptide 2 | chr5:23187911-23189681 FORWARD LENGTH=491	493	491	0	99.6	88.2	93.7
Rsa1.0_00024.1.g1563.t1	ref|XP_002864505.1| predicted protein [Arabidopsis lyrata subsp. lyrata] gi|297310340|gb|EFH40764.1| predicted protein [Arabidopsis lyrata subsp. lyrata]	594	590	0	99.3	91.2	95.3	predicted protein	gbpln	Arabidopsis lyrata	AT5G57200.1 | Symbols: | ENTH/ANTH/VHS superfamily protein | chr5:23177696-23180601 FORWARD LENGTH=591	594	591	0	99.5	89.9	93.8
Rsa1.0_00024.1.g1564.t1	#	#	#	#	#	#	#	-	----	----	#	#	#	#	#	#	#
Rsa1.0_00024.1.g1565.t1	ref|XP_002864503.1| hypothetical protein ARALYDRAFT_495815 [Arabidopsis lyrata subsp. lyrata] gi|297310338|gb|EFH40762.1| hypothetical protein ARALYDRAFT_495815 [Arabidopsis lyrata subsp. lyrata]	373	426	7.00E-94	114.2	63.5	77.2	hypothetical protein ARALYDRAFT_495815	gbpln	Arabidopsis lyrata	AT5G57180.2 | Symbols: CIA2 | chloroplast import apparatus 2 | chr5:23168393-23170763 FORWARD LENGTH=435	373	435	2.00E-95	116.6	63.3	77.5
Rsa1.0_00024.1.g1566.t3	#	#	#	#	#	#	#	-	----	----	#	#	#	#	#	#	#
Rsa1.0_00024.1.g1567.t1	ref|XP_002864502.1| basic helix-loop-helix family protein [Arabidopsis lyrata subsp. lyrata] gi|297310337|gb|EFH40761.1| basic helix-loop-helix family protein [Arabidopsis lyrata subsp. lyrata]	244	247	1.00E-125	101.2	92.6	96.7	basic helix-loop-helix family protein	gbpln	Arabidopsis lyrata	AT5G57150.1 | Symbols: | basic helix-loop-helix (bHLH) DNA-binding superfamily protein | chr5:23152361-23154872 FORWARD LENGTH=247	244	247	1.00E-127	101.2	92.6	96.7
Rsa1.0_00024.1.g1568.t1	ref|XP_002864501.1| ATPAP28/PAP28 [Arabidopsis lyrata subsp. lyrata] gi|297310336|gb|EFH40760.1| ATPAP28/PAP28 [Arabidopsis lyrata subsp. lyrata]	426	400	1.00E-172	93.9	69.2	76.5	ATPAP28/PAP28	gbpln	Arabidopsis lyrata	AT5G57140.1 | Symbols: ATPAP28, PAP28 | purple acid phosphatase 28 | chr5:23149926-23151370 FORWARD LENGTH=397	426	397	1.00E-171	93.2	68.5	75.6

Rsa1.0_00024.1.g1569.t1	gb|EOA13758.1| hypothetical protein CARUB_v10026851mg [Capsella rubella]	364	304	3.00E-79	83.5	54.9	65.7	hypothetical protein CARUB_v10026851mg	gbpln	Capsella rubella	AT5G57120.1 | Symbols: | FUNCTIONS IN: molecular_function unknown; INVOLVED IN: biological_process unknown; LOCATED IN: nucleolus; EXPRESSED IN: 21 plant structures; EXPRESSED DURING: 13 growth stages; CONTAINS InterPro DOMAIN/s: LisH dimerisation motif (InterPro:IPR006594), SRP40, C-terminal (InterPro:IPR007718); Has 101969 Blast hits to 55488 proteins in 2506 species: Archae - 424; Bacteria - 13843; Metazoa - 37674; Fungi - 9726; Plants - 4941; Viruses - 569; Other Eukaryotes - 34792 (source: NCBI BLink). | chr5:23122767-23124400 REVERSE LENGTH=330	364	330	4.00E-73	90.7	59.6	71.7
Rsa1.0_00024.1.g1570.t1	#	#	#	#	#	#	#	-	----	----	#	#	#	#	#	#	#
Rsa1.0_00024.1.g1571.t4	dbj|BAJ34212.1| unnamed protein product [Thellungiella halophila]	1131	1073	0	94.9	89.4	92.0	unnamed protein product	----	----	AT5G57110.2 | Symbols: ACA8, AT-ACA8 | autoinhibited Ca2+ -ATPase, isoform 8 | chr5:23109729-23116857 REVERSE LENGTH=1074	1131	1074	0	95.0	87.9	91.1
Rsa1.0_00024.1.g1572.t1	gb|EOA13053.1| hypothetical protein CARUB_v10026053mg [Capsella rubella]	636	655	0	103.0	95.0	97.5	hypothetical protein CARUB_v10026053mg	gbpln	Capsella rubella	AT5G57090.1 | Symbols: EIR1, WAV6, ATPIN2, PIN2, AGR, AGR1 | Auxin efflux carrier family protein | chr5:23100765-23104456 FORWARD LENGTH=647	636	647	0	101.7	91.5	95.1
Rsa1.0_00024.1.g1573.t1	#	#	#	#	#	#	#	-	----	----	#	#	#	#	#	#	#
Rsa1.0_00024.1.g1574.t1	ref|NP_200517.2| hydroxyproline-rich glycoprotein family protein [Arabidopsis thaliana] gi|332009460|gb|AED96843.1| hydroxyproline-rich glycoprotein family protein [Arabidopsis thaliana]	83	575	2.00E-27	692.8	72.3	80.7	hydroxyproline-rich glycoprotein family protein	gbpln	Arabidopsis thaliana	AT5G57070.1 | Symbols: | hydroxyproline-rich glycoprotein family protein | chr5:23096221-23097948 FORWARD LENGTH=575	83	575	3.00E-30	692.8	72.3	80.7
Rsa1.0_00024.1.g1575.t1	ref|NP_180929.1| DEAD-box ATP-dependent RNA helicase 21 [Arabidopsis thaliana] gi|75319077|sp|P93008.1|RH21_ARATH RecName: Full=DEAD-box ATP-dependent RNA helicase 21 gi|1707017|gb|AAC69128.1| putative U5 small nuclear ribonucleoprotein, an RNA helicase [Arabidopsis thaliana] gi|17473908|gb|AAL38370.1| putative U5 small nuclear ribonucleoprotein, an RNA helicase [Arabidopsis thaliana] gi|20259792|gb|AAM13243.1| putative U5 small nuclear ribonucleoprotein, an RNA helicase [Arabidopsis thaliana] gi|330253781|gb|AEC08875.1| DEAD-box ATP-dependent RNA helicase 21 [Arabidopsis thaliana]	1187	733	7.00E-69	61.8	12.0	13.6	DEAD-box ATP-dependent RNA helicase 21	gbpln	Arabidopsis thaliana	AT2G33730.1 | Symbols: | P-loop containing nucleoside triphosphate hydrolases superfamily protein | chr2:14265679-14267880 REVERSE LENGTH=733	1187	733	2.00E-71	61.8	12.0	13.6
Rsa1.0_00024.1.g1576.t1	dbj|BAA97357.1| unnamed protein product [Arabidopsis thaliana]	372	607	1.00E-101	163.2	72.6	81.2	unnamed protein product	gbpln	Arabidopsis thaliana	AT5G57070.1 | Symbols: | hydroxyproline-rich glycoprotein family protein | chr5:23096221-23097948 FORWARD LENGTH=575	372	575	1.00E-103	154.6	72.6	81.2
Rsa1.0_00024.1.g1577.t1	#	#	#	#	#	#	#	-	----	----	#	#	#	#	#	#	#
Rsa1.0_00024.1.g1578.t1	gb|EOA15144.1| hypothetical protein CARUB_v10028520mg [Capsella rubella]	420	435	0	103.6	91.0	94.8	hypothetical protein CARUB_v10028520mg	gbpln	Capsella rubella	AT5G57015.1 | Symbols: ckl12 | casein kinase I-like 12 | chr5:23071508-23074577 FORWARD LENGTH=435	420	435	0	103.6	90.5	94.5
Rsa1.0_00024.1.g1579.t1	gb|EOA13247.1| hypothetical protein CARUB_v10026276mg [Capsella rubella]	511	499	0	97.7	83.6	90.2	hypothetical protein CARUB_v10026276mg	gbpln	Capsella rubella	AT5G57010.1 | Symbols: | calmodulin-binding family protein | chr5:23068207-23070203 FORWARD LENGTH=495	511	495	0	96.9	83.8	89.6
Rsa1.0_00024.1.g1580.t1	gb|EOA14040.1| hypothetical protein CARUB_v10027175mg [Capsella rubella]	192	195	8.00E-64	101.6	72.9	81.8	hypothetical protein CARUB_v10027175mg	gbpln	Capsella rubella	AT5G57000.1 | Symbols: | unknown protein; FUNCTIONS IN: molecular_function unknown; INVOLVED IN: biological_process unknown; LOCATED IN: cellular_component unknown; EXPRESSED IN: 24 plant structures; EXPRESSED DURING: 15 growth stages; BEST Arabidopsis thaliana protein match is: unknown protein (TAIR:AT1G72690.1); Has 153 Blast hits to 116 proteins in 29 species: Archae - 0; Bacteria - 6; Metazoa - 33; Fungi - 7; Plants - 82; Viruses - 0; Other Eukaryotes - 25 (source: NCBI BLink). | chr5:23063870-23065203 REVERSE LENGTH=187	192	187	2.00E-56	97.4	68.8	77.6

Rsa1.0_00024.1.g1581.t1	ref|XP_002866176.1| AT5g56980/MHM17_10 [Arabidopsis lyrata subsp. lyrata] gi|297312011|gb|EFH42435.1| AT5g56980/MHM17_10 [Arabidopsis lyrata subsp. lyrata]	371	375	1.00E-150	101.1	82.5	88.9	AT5g56980/MHM17_10	gbpln	Arabidopsis lyrata	AT5G56980.1 | Symbols: | unknown protein; FUNCTIONS IN: molecular_function unknown; INVOLVED IN: biological_process unknown; LOCATED IN: endomembrane system; EXPRESSED IN: 18 plant structures; EXPRESSED DURING: 12 growth stages; BEST Arabidopsis thaliana protein match is: unknown protein (TAIR:AT4G26130.1); Has 30201 Blast hits to 17322 proteins in 780 species: Archae - 12; Bacteria - 1396; Metazoa - 17338; Fungi - 3422; Plants - 5037; Viruses - 0; Other Eukaryotes - 2996 (source: NCBI BLink). | chr5:23056574-23057713 REVERSE LENGTH=379	371	379	1.00E-149	102.2	83.6	88.7
Rsa1.0_00024.1.g1582.t1	gb|AAG09097.1|AC009323_8 Putative retroelement polyprotein [Arabidopsis thaliana]	362	1486	3.00E-60	410.5	35.9	51.7	Putative retroelement polyprotein	gbpln	Arabidopsis thaliana	AT1G21280.1 | Symbols: | CONTAINS InterPro DOMAIN/s: Retrotransposon gag protein (InterPro:IPR005162); Has 707 Blast hits to 705 proteins in 25 species: Archae - 0; Bacteria - 0; Metazoa - 4; Fungi - 0; Plants - 703; Viruses - 0; Other Eukaryotes - 0 (source: NCBI BLink). | chr1:7447690-7448403 REVERSE LENGTH=237	362	237	3.00E-22	65.5	14.6	25.1
Rsa1.0_00025.1.g1583.t1	ref|NP_565516.1| uncharacterized protein [Arabidopsis thaliana] gi|20198097|gb|AAM15398.1| Expressed protein [Arabidopsis thaliana] gi|88900416|gb|ABD57520.1| At2g21655 [Arabidopsis thaliana] gi|91806234|gb|ABE65845.1| unknown [Arabidopsis thaliana] gi|330252114|gb|AEC07208.1| uncharacterized protein AT2G21655 [Arabidopsis thaliana]	161	156	2.00E-28	96.9	37.3	58.4	uncharacterized protein	gbpln	Arabidopsis thaliana	AT2G21655.1 | Symbols: | Protein of unknown function (DUF784) | chr2:9263584-9264054 FORWARD LENGTH=156	161	156	8.00E-31	96.9	37.3	58.4
Rsa1.0_00025.1.g1584.t25	gb|AAG51069.1|AC069472_9 unknown protein; 47773-46410 [Arabidopsis thaliana] gi|15795151|dbj|BAB03139.1| unnamed protein product [Arabidopsis thaliana]	154	238	6.00E-50	154.5	66.9	70.8	unknown protein; 47773-46410	gbpln	Arabidopsis thaliana	AT3G12300.1 | Symbols: | unknown protein; FUNCTIONS IN: molecular_function unknown; INVOLVED IN: biological_process unknown; LOCATED IN: endomembrane system; EXPRESSED IN: 23 plant structures; EXPRESSED DURING: 15 growth stages; CONTAINS InterPro DOMAIN/s: Protein of unknown function DUF667 (InterPro:IPR007714); Has 373 Blast hits to 371 proteins in 116 species: Archae - 0; Bacteria - 0; Metazoa - 213; Fungi - 4; Plants - 71; Viruses - 0; Other Eukaryotes - 85 (source: NCBI BLink). | chr3:3921787-3923092 REVERSE LENGTH=190	154	190	9.00E-52	123.4	66.2	70.1
Rsa1.0_00025.1.g1585.t1	ref|XP_002868305.1| binding protein [Arabidopsis lyrata subsp. lyrata] gi|297314141|gb|EFH44564.1| binding protein [Arabidopsis lyrata subsp. lyrata]	494	502	0	101.6	71.1	81.6	binding protein	gbpln	Arabidopsis lyrata	AT4G14190.1 | Symbols: | Pentatricopeptide repeat (PPR) superfamily protein | chr4:8183594-8185180 REVERSE LENGTH=501	494	501	0	101.4	72.5	81.6
Rsa1.0_00025.1.g1586.t1	ref|XP_002884895.1| complex 1 family protein [Arabidopsis lyrata subsp. lyrata] gi|297330735|gb|EFH61154.1| complex 1 family protein [Arabidopsis lyrata subsp. lyrata]	133	133	2.00E-71	100.0	95.5	100.0	complex 1 family protein	gbpln	Arabidopsis lyrata	AT3G12260.1 | Symbols: | LYR family of Fe/S cluster biogenesis protein | chr3:3909252-3910337 REVERSE LENGTH=133	133	133	2.00E-73	100.0	94.7	99.2
Rsa1.0_00025.1.g1587.t2	ref|NP_566415.3| transcription factor TGA6 [Arabidopsis thaliana] gi|42572393|ref|NP_974292.1| transcription factor TGA6 [Arabidopsis thaliana] gi|44888355|sp|Q39140.2|TGA6_ARATH RecName: Full=Transcription factor TGA6; AltName: Full=bZIP transcription factor 45; Short=AtbZIP45 gi|12322056|gb|AAG51079.1|AC069472_19 transcription factor HBP-1B-like; 31032-33264 [Arabidopsis thaliana] gi|14571607|emb|CAC42807.1| transcription factor TGA6 [Arabidopsis thaliana] gi|119360085|gb|ABL66771.1| At3g12250 [Arabidopsis thaliana] gi|225898635|dbj|BAH30448.1| hypothetical protein [Arabidopsis thaliana] gi|332641651|gb|AEE75172.1| transcription factor TGA6 [Arabidopsis thaliana] gi|332641652|gb|AEE75173.1| transcription factor TGA6 [Arabidopsis thaliana]	321	330	1.00E-165	102.8	95.6	96.9	transcription factor TGA6	gbpln	Arabidopsis thaliana	AT3G12250.2 | Symbols: TGA6, BZIP45 | TGACG motif-binding factor 6 | chr3:3906351-3908583 FORWARD LENGTH=330	321	330	1.00E-167	102.8	95.6	96.9

Rsa1.0_00025.1.g1588.t1	ref|XP_002884894.1| hypothetical protein ARALYDRAFT_897434 [Arabidopsis lyrata subsp. lyrata] gi|297330734|gb|EFH61153.1| hypothetical protein ARALYDRAFT_897434 [Arabidopsis lyrata subsp. lyrata]	711	435	1.00E-132	61.2	34.9	40.2	hypothetical protein ARALYDRAFT_897434	gbpln	Arabidopsis lyrata	AT3G12240.1 | Symbols: SCPL15 | serine carboxypeptidase-like 15 | chr3:3902436-3904918 REVERSE LENGTH=436	711	436	1.00E-134	61.3	35.0	39.2
Rsa1.0_00025.1.g1589.t1	#	#	#	#	#	#	#	–	----	----	#	#	#	#	#	#	#
Rsa1.0_00025.1.g1590.t6	ref|XP_002884894.1| hypothetical protein ARALYDRAFT_897434 [Arabidopsis lyrata subsp. lyrata] gi|297330734|gb|EFH61153.1| hypothetical protein ARALYDRAFT_897434 [Arabidopsis lyrata subsp. lyrata]	983	435	1.00E-177	44.3	31.4	36.1	hypothetical protein ARALYDRAFT_897434	gbpln	Arabidopsis lyrata	AT3G12240.1 | Symbols: SCPL15 | serine carboxypeptidase-like 15 | chr3:3902436-3904918 REVERSE LENGTH=436	983	436	1.00E-177	44.4	31.0	36.1
Rsa1.0_00025.1.g1591.t2	ref|XP_002884892.1| kinase family protein [Arabidopsis lyrata subsp. lyrata] gi|297330732|gb|EFH61151.1| kinase family protein [Arabidopsis lyrata subsp. lyrata]	530	570	0	107.5	81.1	87.4	kinase family protein	gbpln	Arabidopsis lyrata	AT3G12200.1 | Symbols: AtNek7, Nek7 | NIMA-related kinase 7 | chr3:3887173-3890550 REVERSE LENGTH=571	530	571	0	107.7	81.3	87.4
Rsa1.0_00025.1.g1592.t1	ref|XP_002882768.1| DNAJ heat shock N-terminal domain-containing protein [Arabidopsis lyrata subsp. lyrata] gi|297328608|gb|EFH59027.1| DNAJ heat shock N-terminal domain-containing protein [Arabidopsis lyrata subsp. lyrata]	396	262	1.00E-123	66.2	56.1	60.4	DNAJ heat shock N-terminal domain-containing protein	gbpln	Arabidopsis lyrata	AT3G12170.1 | Symbols: | Chaperone DnaJ-domain superfamily protein | chr3:3881021-3882655 FORWARD LENGTH=262	396	262	1.00E-125	66.2	55.6	60.4
Rsa1.0_00025.1.g1593.t1	ref|NP_187823.1| RAB GTPase-like protein A4D [Arabidopsis thaliana] gi|297834018|ref|XP_002884891.1| predicted protein [Arabidopsis lyrata subsp. lyrata] gi|75334990|sp|Q9LH50.1|RAA4D_ARATH RecName: Full=Ras-related protein RABA4d; Short=AtRABA4d gi|12322042|gb|AAG51065.1|AC069472_5 ras-related GTP-binding protein; 5118-4176 [Arabidopsis thaliana] gi|9294115|dbj|BAB01966.1| GTP-binding protein-like [Arabidopsis thaliana] gi|67633628|gb|AAY78738.1| Ras-related GTP-binding family protein [Arabidopsis thaliana] gi|297330731|gb|EFH61150.1| predicted protein [Arabidopsis lyrata subsp. lyrata] gi|332641639|gb|AEE75160.1| RAB GTPase-like protein A4D [Arabidopsis thaliana] gi|482567210|gb|EOA31399.1| hypothetical protein CARUB_v10014578mg [Capsella rubella]	222	222	1.00E-125	100.0	97.7	99.5	RAB GTPase-like protein A4D	gbpln	Arabidopsis lyrata	AT3G12160.1 | Symbols: ATRABA4D, RABA4D | RAB GTPase homolog A4D | chr3:3879495-3880437 REVERSE LENGTH=222	222	222	1.00E-128	100.0	97.7	99.5
Rsa1.0_00025.1.g1594.t1	gb|ACX42230.1| polygalacturonase inhibitory protein [Brassica rapa subsp. oleifera]	325	325	1.00E-169	100.0	93.2	97.5	polygalacturonase inhibitory protein	gbpln	Brassica rapa	AT3G12145.1 | Symbols: FLR1, FLOR1 | Leucine-rich repeat (LRR) family protein | chr3:3874764-3876075 REVERSE LENGTH=325	325	325	1.00E-152	100.0	80.3	89.2
Rsa1.0_00025.1.g1595.t1	gb|AAF63175.1|AC010657_11 T5E21.11 [Arabidopsis thaliana]	141	1115	2.00E-36	790.8	58.2	66.7	T5E21.11	gbpln	Arabidopsis thaliana	AT1G14610.1 | Symbols: TWN2, VALRS | valyl-tRNA synthetase / valine--tRNA ligase (VALRS) | chr1:5008502-5014486 REVERSE LENGTH=1108	141	1108	2.00E-38	785.8	56.7	65.2
Rsa1.0_00025.1.g1596.t1	gb|EOA31025.1| hypothetical protein CARUB_v10014171mg [Capsella rubella]	328	327	1.00E-149	99.7	86.3	91.2	hypothetical protein CARUB_v10014171mg	gbpln	Capsella rubella	AT3G12140.2 | Symbols: | Emsy N Terminus (ENT)/ plant Tudor-like domains-containing protein | chr3:3868947-3870964 REVERSE LENGTH=327	328	327	1.00E-151	99.7	86.0	90.9
Rsa1.0_00025.1.g1597.t1	dbj|BAJ34617.1| unnamed protein product [Thellungiella halophila]	258	244	1.00E-115	94.6	89.9	92.2	unnamed protein product	----	----	AT3G12130.1 | Symbols: | KH domain-containing protein / zinc finger (CCCH type) family protein | chr3:3864486-3866406 REVERSE LENGTH=248	258	248	1.00E-112	96.1	85.7	91.9
Rsa1.0_00025.1.g1598.t1	gb|AFC41106.1| FAD2-1 [Brassica oleracea]	386	384	0	99.5	90.2	95.9	FAD2-1	gbpln	Brassica oleracea	AT3G12120.2 | Symbols: FAD2 | fatty acid desaturase 2 | chr3:3860592-3861743 REVERSE LENGTH=383	386	383	0	99.2	85.0	92.5
Rsa1.0_00025.1.g1599.t1	gb|AAC19278.1| T14P8.10 [Arabidopsis thaliana] gi|7269009|emb|CAB80742.1| AT4g02490 [Arabidopsis thaliana]	841	657	0	78.1	43.0	53.3	T14P8.10	gbpln	Arabidopsis thaliana	AT1G43760.1 | Symbols: | DNAse I-like superfamily protein | chr1:16528880-16531065 REVERSE LENGTH=626	841	626	4.00E-83	74.4	17.8	25.9
Rsa1.0_00025.1.g1600.t1	dbj|BAB01957.1| senescence-associated protein-like [Arabidopsis thaliana]	282	270	1.00E-141	95.7	88.7	93.6	senescence-associated protein-like	gbpln	Arabidopsis thaliana	AT3G12090.1 | Symbols: TET6 | tetraspanin6 | chr3:3852326-3853714 REVERSE LENGTH=282	282	282	1.00E-143	100.0	92.9	97.9

Rsa1.0_00025.1.g1601.t1	ref|XP_002882764.1| predicted protein [Arabidopsis lyrata subsp. lyrata] gi|297328604|gb|EFH59023.1| predicted protein [Arabidopsis lyrata subsp. lyrata]	524	559	0	106.7	75.8	85.5	predicted protein	gbpln	Arabidopsis lyrata	AT3G12060.1 | Symbols: TBL1 | Plant protein of unknown function (DUF828) | chr3:3843142-3845150 FORWARD LENGTH=556	524	556	0	106.1	74.8	84.9
Rsa1.0_00025.1.g1602.t1	ref|XP_002871226.1| hypothetical protein ARALYDRAFT_908596 [Arabidopsis lyrata subsp. lyrata] gi|297317063|gb|EFH47485.1| hypothetical protein ARALYDRAFT_908596 [Arabidopsis lyrata subsp. lyrata]	196	196	8.00E-96	100.0	93.9	98.0	hypothetical protein ARALYDRAFT_908596	gbpln	Arabidopsis lyrata	AT5G06660.1 | Symbols: | Protein of unknown function DUF106, transmembrane | chr5:2046863-2047453 FORWARD LENGTH=196	196	196	7.00E-98	100.0	93.9	98.0
Rsa1.0_00025.1.g1603.t1	gb|ABO14927.1| male sterility 2 [Brassica rapa subsp. chinensis]	610	616	0	101.0	95.9	97.7	male sterility 2	gbpln	Brassica rapa	AT3G11980.1 | Symbols: MS2, FAR2 | Jojoba acyl CoA reductase-related male sterility protein | chr3:3814484-3816927 FORWARD LENGTH=616	610	616	0	101.0	90.5	95.4
Rsa1.0_00025.1.g1604.t1	ref|NP_187803.4| RNA binding protein [Arabidopsis thaliana] gi|332641610|gb|AEE75131.1| RNA binding protein [Arabidopsis thaliana]	1913	1896	0	99.1	84.9	91.2	RNA binding protein	gbpln	Arabidopsis thaliana	AT3G11964.1 | Symbols: | RNA binding;RNA binding | chr3:3793957-3806626 REVERSE LENGTH=1896	1913	1896	0	99.1	84.9	91.2
Rsa1.0_00025.1.g1605.t1	gb|EOA31455.1| hypothetical protein CARUB_v10014641mg [Capsella rubella]	207	207	1.00E-114	100.0	97.1	98.6	hypothetical protein CARUB_v10014641mg	gbpln	Capsella rubella	AT2G37270.2 | Symbols: ATRPS5B, RPS5B | ribosomal protein 5B | chr2:15647883-15649042 REVERSE LENGTH=207	207	207	1.00E-116	100.0	96.1	99.0
Rsa1.0_00025.1.g1606.t1	ref|NP_001030675.1| adenine nucleotide alpha hydrolases-like protein [Arabidopsis thaliana] gi|332641601|gb|AEE75122.1| adenine nucleotide alpha hydrolases-like protein [Arabidopsis thaliana]	200	201	5.00E-96	100.5	85.5	94.5	adenine nucleotide alpha hydrolases-like protein	gbpln	Arabidopsis thaliana	AT3G11930.4 | Symbols: | Adenine nucleotide alpha hydrolases-like superfamily protein | chr3:3776371-3777393 FORWARD LENGTH=201	200	201	2.00E-98	100.5	85.5	94.5
Rsa1.0_00025.1.g1607.t1	#	#	#	#	#	#	#	-	----	----	#	#	#	#	#	#	#
Rsa1.0_00025.1.g1608.t1	#	#	#	#	#	#	#	-	----	----	#	#	#	#	#	#	#
Rsa1.0_00025.1.g1609.t2	ref|XP_002882755.1| predicted protein [Arabidopsis lyrata subsp. lyrata] gi|297328595|gb|EFH59014.1| predicted protein [Arabidopsis lyrata subsp. lyrata]	612	629	0	102.8	73.0	80.7	predicted protein	gbpln	Arabidopsis lyrata	AT3G11920.1 | Symbols: | glutaredoxin-related | chr3:3772311-3774887 FORWARD LENGTH=630	612	630	0	102.9	72.5	79.7
Rsa1.0_00025.1.g1610.t1	ref|NP_187791.1| uncharacterized protein [Arabidopsis thaliana] gi|42572387|ref|NP_974289.1| uncharacterized protein [Arabidopsis thaliana] gi|6671941|gb|AAF23201.1|AC016795_14 hypothetical protein [Arabidopsis thaliana] gi|20466598|gb|AAM20616.1| unknown protein [Arabidopsis thaliana] gi|22136430|gb|AAM91293.1| unknown protein [Arabidopsis thaliana] gi|332641587|gb|AEE75108.1| uncharacterized protein AT3G11850 [Arabidopsis thaliana] gi|332641588|gb|AEE75109.1| uncharacterized protein AT3G11850 [Arabidopsis thaliana]	410	504	1.00E-106	122.9	58.3	71.7	uncharacterized protein	gbpln	Arabidopsis thaliana	AT3G11850.2 | Symbols: | Protein of unknown function, DUF593 | chr3:3739222-3741101 REVERSE LENGTH=504	410	504	1.00E-109	122.9	58.3	71.7
Rsa1.0_00025.1.g1611.t1	ref|NP_566402.1| plant U-box 24 protein [Arabidopsis thaliana] gi|332641586|gb|AEE75107.1| E3 ubiquitin-protein ligase PUB24 [Arabidopsis thaliana]	447	470	0	105.1	76.5	88.6	plant U-box 24 protein	gbpln	Arabidopsis thaliana	AT3G11840.1 | Symbols: PUB24 | plant U-box 24 | chr3:3736578-3738250 REVERSE LENGTH=470	447	470	0	105.1	76.5	88.6
Rsa1.0_00025.1.g1612.t1	ref|NP_187789.1| TCP-1/cpn60 chaperonin family protein [Arabidopsis thaliana] gi|6671939|gb|AAF23199.1|AC016795_12 putative T-complex protein 1, ETA subunit [Arabidopsis thaliana] gi|17979243|gb|AAL49938.1| AT3g11830/F26K24_12 [Arabidopsis thaliana] gi|20857172|gb|AAM26704.1| AT3g11830/F26K24_12 [Arabidopsis thaliana] gi|332641584|gb|AEE75105.1| TCP-1/cpn60 chaperonin family protein [Arabidopsis thaliana]	548	557	0	101.6	94.7	95.4	TCP-1/cpn60 chaperonin family protein	gbpln	Arabidopsis thaliana	AT3G11830.1 | Symbols: | TCP-1/cpn60 chaperonin family protein | chr3:3732734-3736156 FORWARD LENGTH=557	548	557	0	101.6	94.7	95.4
Rsa1.0_00025.1.g1613.t1	ref|XP_002884864.1| hypothetical protein ARALYDRAFT_897381 [Arabidopsis lyrata subsp. lyrata] gi|297330704|gb|EFH61123.1| hypothetical protein ARALYDRAFT_897381 [Arabidopsis lyrata subsp. lyrata]	235	200	7.00E-97	85.1	74.0	78.3	hypothetical protein ARALYDRAFT_897381	gbpln	Arabidopsis lyrata	AT3G11770.1 | Symbols: | Polynucleotidyl transferase, ribonuclease H-like superfamily protein | chr3:3721490-3722092 REVERSE LENGTH=200	235	200	3.00E-98	85.1	72.8	77.4

Rsa1.0_00025.1.g1614.t1	gb|EOA32949.1| hypothetical protein CARUB_v10016277mg [Capsella rubella]	706	704	0	99.7	82.7	87.7	hypothetical protein CARUB_v10016277mg	gbpln	Capsella rubella	AT3G11760.1 | Symbols: | unknown protein; FUNCTIONS IN: molecular_function unknown; INVOLVED IN: biological_process unknown; LOCATED IN: cellular_component unknown; EXPRESSED IN: 23 plant structures; EXPRESSED DURING: 14 growth stages; BEST Arabidopsis thaliana protein match is: unknown protein (TAIR:AT5G04860.1); Has 84 Blast hits to 73 proteins in 13 species: Archae - 0; Bacteria - 0; Metazoa - 0; Fungi - 0; Plants - 84; Viruses - 0; Other Eukaryotes - 0 (source: NCBI BLink). | chr3:3718529-3721123 FORWARD LENGTH=702	706	702	0	99.4	81.7	87.1
Rsa1.0_00025.1.g1615.t1	ref|XP_002884863.1| hypothetical protein ARALYDRAFT_478521 [Arabidopsis lyrata subsp. lyrata] gi|297330703|gb|EFH61122.1| hypothetical protein ARALYDRAFT_478521 [Arabidopsis lyrata subsp. lyrata]	153	146	2.00E-60	95.4	77.8	86.3	hypothetical protein ARALYDRAFT_478521	gbpln	Arabidopsis lyrata	AT3G11750.1 | Symbols: FOLB1 | Dihydroneopterin aldolase | chr3:3715071-3715904 REVERSE LENGTH=146	153	146	1.00E-61	95.4	76.5	86.3
Rsa1.0_00025.1.g1616.t1	ref|XP_002882745.1| hypothetical protein ARALYDRAFT_478520 [Arabidopsis lyrata subsp. lyrata] gi|297328585|gb|EFH59004.1| hypothetical protein ARALYDRAFT_478520 [Arabidopsis lyrata subsp. lyrata]	194	193	5.00E-99	99.5	88.7	95.4	hypothetical protein ARALYDRAFT_478520	gbpln	Arabidopsis lyrata	AT3G11740.1 | Symbols: | Protein of unknown function (DUF567) | chr3:3712427-3713389 FORWARD LENGTH=194	194	194	1.00E-100	100.0	87.6	95.4
Rsa1.0_00025.1.g1617.t1	ref|NP_187779.1| Ras-related protein RABD1 [Arabidopsis thaliana] gi|75338904|sp|Q9ZRE2.1|RABD1_ARATH RecName: Full=Ras-related protein RABD1; Short=AtRABD1; AltName: Full=Ras-related protein ATFP8 gi|6671929|gb|AAF23189.1|AC016795_2 putative GTP-binding protein (ATFP8) [Arabidopsis thaliana] gi|4097557|gb|AAD00111.1| ATFP8 [Arabidopsis thaliana] gi|26451349|dbj|BAC42775.1| putative GTP-binding protein ATFP8 [Arabidopsis thaliana] gi|28973343|gb|AAO63996.1| putative GTP-binding protein (ATFP8) [Arabidopsis thaliana] gi|332641569|gb|AEE75090.1| Ras-related protein RABD1 [Arabidopsis thaliana]	206	205	1.00E-109	99.5	93.2	95.6	Ras-related protein RABD1	gbpln	Arabidopsis thaliana	AT3G11730.1 | Symbols: ATFP8, ATRABD1, RABD1 | Ras-related small GTP-binding family protein | chr3:3709490-3711397 REVERSE LENGTH=205	206	205	1.00E-112	99.5	93.2	95.6
Rsa1.0_00025.1.g1618.t1	ref|XP_002884859.1| hypothetical protein ARALYDRAFT_478516 [Arabidopsis lyrata subsp. lyrata] gi|297330699|gb|EFH61118.1| hypothetical protein ARALYDRAFT_478516 [Arabidopsis lyrata subsp. lyrata]	605	625	0	103.3	90.9	95.0	hypothetical protein ARALYDRAFT_478516	gbpln	Arabidopsis lyrata	AT3G11710.1 | Symbols: ATKRS-1 | lysyl-tRNA synthetase 1 | chr3:3702359-3705613 REVERSE LENGTH=626	605	626	0	103.5	87.9	93.9
Rsa1.0_00025.1.g1619.t1	gb|EOA30537.1| hypothetical protein CARUB_v10013670mg [Capsella rubella]	443	457	0	103.2	91.6	94.4	hypothetical protein CARUB_v10013670mg	gbpln	Capsella rubella	AT3G11700.1 | Symbols: FLA18 | FASCICLIN-like arabinogalactan protein 18 precursor | chr3:3698992-3700971 FORWARD LENGTH=462	443	462	0	104.3	92.3	94.1
Rsa1.0_00025.1.g1620.t1	ref|XP_002882743.1| hypothetical protein ARALYDRAFT_478514 [Arabidopsis lyrata subsp. lyrata] gi|297328583|gb|EFH59002.1| hypothetical protein ARALYDRAFT_478514 [Arabidopsis lyrata subsp. lyrata]	259	251	3.00E-80	96.9	74.9	83.4	hypothetical protein ARALYDRAFT_478514	gbpln	Arabidopsis lyrata	AT3G11690.1 | Symbols: | unknown protein; FUNCTIONS IN: molecular_function unknown; INVOLVED IN: biological_process unknown; LOCATED IN: chloroplast; EXPRESSED IN: 24 plant structures; EXPRESSED DURING: 15 growth stages; BEST Arabidopsis thaliana protein match is: unknown protein (TAIR:AT5G06380.1); Has 84 Blast hits to 84 proteins in 12 species: Archae - 0; Bacteria - 0; Metazoa - 0; Fungi - 0; Plants - 84; Viruses - 0; Other Eukaryotes - 0 (source: NCBI BLink). | chr3:3695484-3696242 FORWARD LENGTH=252	259	252	2.00E-77	97.3	69.5	76.4
Rsa1.0_00025.1.g1621.t1	gb|EOA32513.1| hypothetical protein CARUB_v10015793mg [Capsella rubella]	500	487	0	97.4	78.8	85.8	hypothetical protein CARUB_v10015793mg	gbpln	Capsella rubella	AT3G11680.1 | Symbols: | Aluminium activated malate transporter family protein | chr3:3686995-3689329 REVERSE LENGTH=488	500	488	0	97.6	77.6	85.6
Rsa1.0_00025.1.g1622.t1	gb|EOA33837.1| hypothetical protein CARUB_v10021318mg, partial [Capsella rubella]	261	290	2.00E-49	111.1	42.5	60.9	hypothetical protein CARUB_v10021318mg, partial	gbpln	Capsella rubella	AT4G04650.1 | Symbols: | RNA-directed DNA polymerase (reverse transcriptase)-related family protein | chr4:2353989-2355213 REVERSE LENGTH=332	261	332	9.00E-49	127.2	35.6	54.8

Rsa1.0_00025.1.g1623.t1	ref|NP_566396.1| NDR1/HIN1-Like protein 1 [Arabidopsis thaliana] gi|6041819|gb|AAF02134.1|AC009918_6 unknown protein [Arabidopsis thaliana] gi|9502172|gb|AAF88021.1|AF264697_1 NDR1/HIN1-Like protein 1 [Arabidopsis thaliana] gi|21595601|gb|AAM66116.1| harpin-induced protein-like [Arabidopsis thaliana] gi|56381911|gb|AAV85674.1| At3g11660 [Arabidopsis thaliana] gi|58331799|gb|AAW70397.1| At3g11660 [Arabidopsis thaliana] gi|332641559|gb|AEE75080.1| NDR1/HIN1-Like protein 1 [Arabidopsis thaliana]	211	209	1.00E-102	99.1	85.3	91.0	NDR1/HIN1-Like protein 1	gbpln	Arabidopsis thaliana	AT3G11660.1 | Symbols: NHL1 | NDR1/HIN1-like 1 | chr3:3679031-3679660 REVERSE LENGTH=209	211	209	1.00E-104	99.1	85.3	91.0
Rsa1.0_00025.1.g1624.t1	gb|EOA32680.1| hypothetical protein CARUB_v10015978mg [Capsella rubella]	490	442	0	90.2	74.9	80.4	hypothetical protein CARUB_v10015978mg	gbpln	Capsella rubella	AT3G11490.1 | Symbols: | rac GTPase activating protein | chr3:3617523-3619567 REVERSE LENGTH=435	490	435	0	88.8	75.9	80.4
Rsa1.0_00025.1.g1625.t1	ref|XP_002882732.1| hypothetical protein ARALYDRAFT_478481 [Arabidopsis lyrata subsp. lyrata] gi|297328572|gb|EFH58991.1| hypothetical protein ARALYDRAFT_478481 [Arabidopsis lyrata subsp. lyrata]	500	551	0	110.2	74.8	80.6	hypothetical protein ARALYDRAFT_478481	gbpln	Arabidopsis lyrata	AT3G11440.1 | Symbols: ATMYB65, MYB65 | myb domain protein 65 | chr3:3603056-3604929 FORWARD LENGTH=553	500	553	0	110.6	73.0	80.0
Rsa1.0_00025.1.g1626.t1	ref|XP_002882731.1| glycerol-3-phosphate acyltransferase 5 [Arabidopsis lyrata subsp. lyrata] gi|297328571|gb|EFH58990.1| glycerol-3-phosphate acyltransferase 5 [Arabidopsis lyrata subsp. lyrata]	500	502	0	100.4	87.8	94.4	glycerol-3-phosphate acyltransferase 5	gbpln	Arabidopsis lyrata	AT3G11430.1 | Symbols: ATGPAT5, GPAT5 | glycerol-3-phosphate acyltransferase 5 | chr3:3595911-3597678 FORWARD LENGTH=502	500	502	0	100.4	87.2	93.8
Rsa1.0_00025.1.g1627.t1	emb|CAB39638.1| RNA-directed DNA polymerase-like protein [Arabidopsis thaliana] gi|7267666|emb|CAB78094.1| RNA-directed DNA polymerase-like protein [Arabidopsis thaliana]	1306	1274	0	97.5	43.3	58.3	RNA-directed DNA polymerase-like protein	gbpln	Arabidopsis thaliana	AT4G29090.1 | Symbols: | Ribonuclease H-like superfamily protein | chr4:14333528-14335255 FORWARD LENGTH=575	1306	575	7.00E-74	44.0	13.7	21.8
Rsa1.0_00025.1.g1628.t1	ref|XP_002882730.1| hypothetical protein ARALYDRAFT_478479 [Arabidopsis lyrata subsp. lyrata] gi|297328570|gb|EFH58989.1| hypothetical protein ARALYDRAFT_478479 [Arabidopsis lyrata subsp. lyrata]	508	505	0	99.4	84.4	90.7	hypothetical protein ARALYDRAFT_478479	gbpln	Arabidopsis lyrata	AT3G11420.1 | Symbols: | Protein of unknown function (DUF604) | chr3:3591834-3594323 FORWARD LENGTH=505	508	505	0	99.4	83.7	91.1
Rsa1.0_00025.1.g1629.t1	#	#	#	#	#	#	#	–	----	----	#	#	#	#	#	#	#
Rsa1.0_00025.1.g1630.t1	ref|XP_002884844.1| AHG3/ATPP2CA [Arabidopsis lyrata subsp. lyrata] gi|297330684|gb|EFH61103.1| AHG3/ATPP2CA [Arabidopsis lyrata subsp. lyrata]	388	396	1.00E-160	102.1	80.7	88.9	AHG3/ATPP2CA	gbpln	Arabidopsis lyrata	AT3G11410.1 | Symbols: ATPP2CA, AHG3, PP2CA | protein phosphatase 2CA | chr3:3584181-3585649 REVERSE LENGTH=399	388	399	1.00E-160	102.8	80.2	88.1
Rsa1.0_00025.1.g1631.t1	ref|NP_187747.1| translation initiation factor eIF-3 subunit 4 [Arabidopsis thaliana] gi|12322907|gb|AAG51445.1|AC008153_18 putative eukaryotic translation initiation factor 3 subunit; 21071-22901 [Arabidopsis thaliana] gi|16226341|gb|AAL16140.1|AF428308_1 AT3g11400/F24K9_7 [Arabidopsis thaliana] gi|9755847|emb|CAC01929.1| translation initiation factor 3, subunit g (eIF3g) [Arabidopsis thaliana] gi|18377870|gb|AAL67121.1| AT3g11400/F24K9_7 [Arabidopsis thaliana] gi|22137220|gb|AAM91455.1| AT3g11400/F24K9_7 [Arabidopsis thaliana] gi|332641520|gb|AEE75041.1| translation initiation factor eIF-3 subunit 4 [Arabidopsis thaliana]	296	294	1.00E-157	99.3	91.2	94.9	translation initiation factor eIF-3 subunit 4	gbpln	Arabidopsis thaliana	AT3G11400.1 | Symbols: EIF3G1, ATEIF3G1 | eukaryotic translation initiation factor 3G1 | chr3:3578536-3580366 FORWARD LENGTH=294	296	294	1.00E-159	99.3	91.2	94.9
Rsa1.0_00025.1.g1632.t2	gb|EOA32620.1| hypothetical protein CARUB_v10015914mg [Capsella rubella]	385	293	3.00E-59	76.1	29.9	33.0	hypothetical protein CARUB_v10015914mg	gbpln	Capsella rubella	AT2G15710.1 | Symbols: | TRAF-like family protein | chr2:6842648-6845103 FORWARD LENGTH=365	385	365	2.00E-50	94.8	26.2	31.2
Rsa1.0_00025.1.g1633.t1	ref|NP_566390.1| uncharacterized protein [Arabidopsis thaliana] gi|110736642|dbj|BAF00285.1| hypothetical protein [Arabidopsis thaliana] gi|332641505|gb|AEE75026.1| uncharacterized protein AT3G11290 [Arabidopsis thaliana]	499	460	1.00E-176	92.2	64.1	76.8	uncharacterized protein	gbpln	Arabidopsis thaliana	AT3G11290.1 | Symbols: | unknown protein; BEST Arabidopsis thaliana protein match is: unknown protein (TAIR:AT3G11310.1); Has 720 Blast hits to 435 proteins in 28 species: Archae - 0; Bacteria - 2; Metazoa - 0; Fungi - 32; Plants - 682; Viruses - 0; Other Eukaryotes - 4 (source: NCBI BLink). | chr3:3535766-3537295 REVERSE LENGTH=460	499	460	1.00E-179	92.2	64.1	76.8

Rsa1.0_00025.1.g1634.t1	ref|NP_187737.1| duplicated SANT DNA-binding domain-containing protein [Arabidopsis thaliana] gi|30681667|ref|NP_850558.1| duplicated SANT DNA-binding domain-containing protein [Arabidopsis thaliana] gi|12321867|gb|AAG50963.1|AC073395_5 MYB-family transcription factor, putative; alternative splicing isoform 1 of 2;71559-70643 [Arabidopsis thaliana] gi|12321868|gb|AAG50964.1|AC073395_6 MYB-family transcription factor, putative; alternative splicing isoform 2 of 2;71559-70643 [Arabidopsis thaliana] gi|15810283|gb|AAL07029.1| putative MYB-family transcription factor [Arabidopsis thaliana] gi|20258977|gb|AAM14204.1| putative MYB-family transcription factor [Arabidopsis thaliana] gi|45357120|gb|AAS58519.1| MYB transcription factor [Arabidopsis thaliana] gi|332641503|gb|AEE75024.1| duplicated SANT DNA-binding domain-containing protein [Arabidopsis thaliana] gi|332641504|gb|AEE75025.1| duplicated SANT DNA-binding domain-containing protein [Arabidopsis thaliana]	259	263	1.00E-120	101.5	81.9	87.3	duplicated SANT DNA-binding domain-containing protein	gbpln	Arabidopsis thaliana	AT3G11280.2 | Symbols: | Duplicated homeodomain-like superfamily protein | chr3:3533477-3534393 REVERSE LENGTH=263	259	263	1.00E-123	101.5	81.9	87.3
Rsa1.0_00025.1.g1635.t1	ref|NP_567015.1| splicing factor 3B subunit 3 [Arabidopsis thaliana] gi|18410226|ref|NP_567016.1| putative splicing factor [Arabidopsis thaliana] gi|7019653|emb|CAB75754.1| spliceosomal-like protein [Arabidopsis thaliana] gi|7019655|emb|CAB75756.1| spliceosomal-like protein [Arabidopsis thaliana] gi|332645831|gb|AEE79352.1| spliceosomal associated protein 130A [Arabidopsis thaliana] gi|332645833|gb|AEE79354.1| spliceosome-associated protein 130B [Arabidopsis thaliana]	1214	1214	0	100.0	95.4	98.4	splicing factor 3B subunit 3	gbpln	Arabidopsis thaliana	AT3G55200.1 | Symbols: | Cleavage and polyadenylation specificity factor (CPSF) A subunit protein | chr3:20460533-20464361 FORWARD LENGTH=1214	1214	1214	0	100.0	95.4	98.4
Rsa1.0_00025.1.g1636.t1	ref|NP_001078135.1| yippee-like protein [Arabidopsis thaliana] gi|332641497|gb|AEE75018.1| yippee-like protein [Arabidopsis thaliana]	129	162	4.00E-61	125.6	87.6	93.8	yippee-like protein	gbpln	Arabidopsis thaliana	AT3G11230.2 | Symbols: | Yippee family putative zinc-binding protein | chr3:3516683-3518193 FORWARD LENGTH=162	129	162	9.00E-64	125.6	87.6	93.8
Rsa1.0_00026.1.g1637.t1	dbj|BAJ33984.1| unnamed protein product [Thellungiella halophila]	696	699	0	100.4	96.7	99.1	unnamed protein product	----	----	AT5G56000.1 | Symbols: Hsp81.4, AtHsp90.4 | HEAT SHOCK PROTEIN 81.4 | chr5:22677602-22680067 REVERSE LENGTH=699	696	699	0	100.4	94.5	98.9
Rsa1.0_00026.1.g1638.t1	gb|EOA12588.1| hypothetical protein CARUB_v10027067mg [Capsella rubella]	226	226	1.00E-124	100.0	96.5	98.7	hypothetical protein CARUB_v10027067mg	gbpln	Capsella rubella	AT5G55990.1 | Symbols: CBL2, ATCBL2 | calcineurin B-like protein 2 | chr5:22672189-22673579 FORWARD LENGTH=226	226	226	1.00E-126	100.0	96.0	98.2
Rsa1.0_00026.1.g1639.t1	gb|EOA12638.1| hypothetical protein CARUB_v10027572mg [Capsella rubella]	753	758	0	100.7	92.8	97.5	hypothetical protein CARUB_v10027572mg	gbpln	Capsella rubella	AT5G55930.1 | Symbols: ATOPT1, OPT1 | oligopeptide transporter 1 | chr5:22652988-22655827 FORWARD LENGTH=755	753	755	0	100.3	92.4	96.7
Rsa1.0_00026.1.g1640.t1	ref|NP_850927.1| S-adenosyl-L-methionine-dependent methyltransferase-like protein [Arabidopsis thaliana] gi|9758212|dbj|BAB08657.1| nucleolar protein-like [Arabidopsis thaliana] gi|332009316|gb|AED96699.1| S-adenosyl-L-methionine-dependent methyltransferase-like protein [Arabidopsis thaliana]	663	682	0	102.9	75.9	87.0	S-adenosyl-L-methionine-dependent methyltransferase-like protein	gbpln	Arabidopsis thaliana	AT5G55920.1 | Symbols: OLI2 | S-adenosyl-L-methionine-dependent methyltransferases superfamily protein | chr5:22645742-22649383 REVERSE LENGTH=682	663	682	0	102.9	75.9	87.0
Rsa1.0_00026.1.g1641.t1	dbj|BAJ33984.1| unnamed protein product [Thellungiella halophila]	696	699	0	100.4	96.4	99.0	unnamed protein product	----	----	AT5G56000.1 | Symbols: Hsp81.4, AtHsp90.4 | HEAT SHOCK PROTEIN 81.4 | chr5:22677602-22680067 REVERSE LENGTH=699	696	699	0	100.4	94.4	98.9
Rsa1.0_00026.1.g1642.t1	ref|XP_002864413.1| hypothetical protein ARALYDRAFT_495666 [Arabidopsis lyrata subsp. lyrata] gi|297310248|gb|EFH40672.1| hypothetical protein ARALYDRAFT_495666 [Arabidopsis lyrata subsp. lyrata]	71	102	3.00E-28	143.7	87.3	91.5	hypothetical protein ARALYDRAFT_495666	gbpln	Arabidopsis lyrata	AT5G55850.1 | Symbols: NOI | RPM1-interacting protein 4 (RIN4) family protein | chr5:22603617-22604951 FORWARD LENGTH=114	71	114	8.00E-31	160.6	85.9	90.1
Rsa1.0_00026.1.g1643.t1	#	#	#	#	#	#	#	-	----	----	#	#	#	#	#	#	#

Rsa1.0_00026.1.g1644.t2	gb|EOA13370.1| hypothetical protein CARUB_v10026407mg, partial [Capsella rubella]	407	443	1.00E-167	108.8	81.6	90.7	hypothetical protein CARUB_v10026407mg, partial	gbpln	Capsella rubella	AT5G55730.2 | Symbols: FLA1 | FASCICLIN-like arabinogalactan 1 | chr5:22558375-22560392 REVERSE LENGTH=424	407	424	1.00E-163	104.2	79.4	86.0
Rsa1.0_00026.1.g1645.t1	ref|XP_002864408.1| pectate lyase family protein [Arabidopsis lyrata subsp. lyrata] gi|297310243|gb|EFH40667.1| pectate lyase family protein [Arabidopsis lyrata subsp. lyrata]	385	391	0	101.6	87.8	93.8	pectate lyase family protein	gbpln	Arabidopsis lyrata	AT5G55720.1 | Symbols: | Pectin lyase-like superfamily protein | chr5:22556047-22557496 FORWARD LENGTH=392	385	392	0	101.8	85.5	92.7
Rsa1.0_00026.1.g1646.t1	gb|EOA14333.1| hypothetical protein CARUB_v10027511mg [Capsella rubella]	211	209	4.00E-97	99.1	88.2	91.5	hypothetical protein CARUB_v10027511mg	gbpln	Capsella rubella	AT5G55710.1 | Symbols: | FUNCTIONS IN: molecular_function unknown; INVOLVED IN: biological_process unknown; LOCATED IN: chloroplast thylakoid membrane, chloroplast; EXPRESSED IN: 23 plant structures; EXPRESSED DURING: 14 growth stages; BEST Arabidopsis thaliana protein match is: Uncharacterised conserved protein ycf60 (TAIR:AT2G47840.1); Has 1807 Blast hits to 1807 proteins in 277 species: Archae - 0; Bacteria - 0; Metazoa - 736; Fungi - 347; Plants - 385; Viruses - 0; Other Eukaryotes - 339 (source: NCBI BLink). | chr5:22554995-22555624 REVERSE LENGTH=209	211	209	2.00E-97	99.1	87.2	90.5
Rsa1.0_00026.1.g1647.t1	ref|NP_568829.2| inactive beta-amylase 4 [Arabidopsis thaliana] gi|75334054|sp|Q9FM68.1|BAM4_ARATH RecName: Full=Inactive beta-amylase 4, chloroplastic; AltName: Full=Inactive beta-amylase 6; Flags: Precursor gi|9758604|dbj|BAB09237.1| beta-amylase [Arabidopsis thaliana] gi|332009286|gb|AED96669.1| inactive beta-amylase 4 [Arabidopsis thaliana]	534	531	0	99.4	90.1	94.2	inactive beta-amylase 4	gbpln	Arabidopsis thaliana	AT5G55700.1 | Symbols: BMY6, BAM4 | beta-amylase 4 | chr5:22551873-22554702 FORWARD LENGTH=531	534	531	0	99.4	90.1	94.2
Rsa1.0_00026.1.g1648.t5	gb|EOA12471.1| hypothetical protein CARUB_v10025902mg [Capsella rubella]	1233	804	0	65.2	40.2	46.9	hypothetical protein CARUB_v10025902mg	gbpln	Capsella rubella	AT5G55660.1 | Symbols: | DEK domain-containing chromatin associated protein | chr5:22539375-22543142 FORWARD LENGTH=778	1233	778	1.00E-174	63.1	38.9	44.5
Rsa1.0_00026.1.g1649.t1	ref|NP_200370.1| pectinesterase QRT1 [Arabidopsis thaliana] gi|75171621|sp|Q9FM79.1|PME62_ARATH RecName: Full=Pectinesterase QRT1; Short=AtQRT1; Short=PE QRT1; AltName: Full=Pectin methylesterase 62; Short=AtPME62; AltName: Full=Pectin methylesterase QRT1; AltName: Full=Protein QUARTET 1; Flags: Precursor gi|9758593|dbj|BAB09226.1| unnamed protein product [Arabidopsis thaliana] gi|115490609|gb|ABI97858.1| quartet1 [Arabidopsis thaliana] gi|332009270|gb|AED96653.1| pectinesterase QRT1 [Arabidopsis thaliana]	392	380	1.00E-137	96.9	64.5	77.6	pectinesterase QRT1	gbpln	Arabidopsis thaliana	AT5G55590.1 | Symbols: QRT1 | Pectin lyase-like superfamily protein | chr5:22519911-22521699 FORWARD LENGTH=380	392	380	1.00E-140	96.9	64.5	77.6
Rsa1.0_00026.1.g1650.t1	gb|AAC33963.1| contains similarity to reverse transcriptases (Pfam; rvt.hmm, score: 11.19) [Arabidopsis thaliana]	1916	1633	0	85.2	51.0	59.0	contains similarity to reverse transcriptases (Pfam; rvt.hmm, score: 11.19)	gbpln	Arabidopsis thaliana	AT4G23160.1 | Symbols: CRK8 | cysteine-rich RLK (RECEPTOR-like protein kinase) 8 | chr4:12129485-12134086 FORWARD LENGTH=1262	1916	1262	1.00E-179	65.9	15.3	20.0
Rsa1.0_00026.1.g1651.t1	ref|NP_201434.1| leucine-rich repeat-containing protein [Arabidopsis thaliana] gi|10177427|dbj|BAB10712.1| unnamed protein product [Arabidopsis thaliana] gi|17529198|gb|AAL38825.1| unknown protein [Arabidopsis thaliana] gi|21436283|gb|AAM51280.1| unknown protein [Arabidopsis thaliana] gi|332010817|gb|AED98200.1| leucine-rich repeat-containing protein [Arabidopsis thaliana]	360	418	1.00E-134	116.1	69.7	76.9	leucine-rich repeat-containing protein	gbpln	Arabidopsis thaliana	AT5G66330.1 | Symbols: | Leucine-rich repeat (LRR) family protein | chr5:26500531-26501787 REVERSE LENGTH=418	360	418	1.00E-137	116.1	69.7	76.9
Rsa1.0_00026.1.g1652.t1	#	#	#	#	#	#	#	-	----	----	#	#	#	#	#	#	#
Rsa1.0_00026.1.g1653.t1	gb|EOA12552.1| hypothetical protein CARUB_v10026802mg [Capsella rubella]	308	316	1.00E-149	102.6	83.4	88.3	hypothetical protein CARUB_v10026802mg	gbpln	Capsella rubella	AT5G55560.1 | Symbols: | Protein kinase superfamily protein | chr5:22506477-22507757 REVERSE LENGTH=314	308	314	1.00E-149	101.9	82.1	87.3

Rsa1.0_00026.1.g1654.t1	ref|XP_002866100.1| predicted protein [Arabidopsis lyrata subsp. lyrata] gi|297311935|gb|EFH42359.1| predicted protein [Arabidopsis lyrata subsp. lyrata]	788	790	0	100.3	77.8	84.8	predicted protein	gbpln	Arabidopsis lyrata	AT5G55520.2 | Symbols: | CONTAINS InterPro DOMAIN/s: Kinesin-related protein (InterPro:IPR010544); BEST Arabidopsis thaliana protein match is: unknown protein (TAIR:AT4G26660.1); Has 31032 Blast hits to 19733 proteins in 1535 species: Archae - 330; Bacteria - 3150; Metazoa - 16413; Fungi - 2511; Plants - 1475; Viruses - 48; Other Eukaryotes - 7105 (source: NCBI BLink). | chr5:22488205-22491187 REVERSE LENGTH=801	788	801	0	101.6	77.8	85.3
Rsa1.0_00026.1.g1655.t1	ref|NP_174635.1| terpene cyclase, C1 domain-containing protein [Arabidopsis thaliana] gi|317412196|sp|Q9LQ27.2|TPS22_ARATH RecName: Full=Terpenoid synthase 22; Short=AtTPS22 gi|332193499|gb|AEE31620.1| terpenoid synthase 22 [Arabidopsis thaliana]	655	603	0	92.1	67.0	77.4	terpene cyclase, C1 domain-containing protein	gbpln	Arabidopsis thaliana	AT1G33750.1 | Symbols: | Terpenoid cyclases/Protein prenyltransferases superfamily protein | chr1:12233769-12236348 FORWARD LENGTH=603	655	603	0	92.1	67.0	77.4
Rsa1.0_00026.1.g1656.t1	gb|AAZ66923.1| 117M18_4 [Brassica rapa]	380	424	1.00E-129	111.6	65.5	78.4	117M18_4	gbpln	Brassica rapa	AT5G10010.1 | Symbols: | unknown protein; FUNCTIONS IN: molecular_function unknown; INVOLVED IN: biological_process unknown; LOCATED IN: nucleolus; EXPRESSED IN: 25 plant structures; EXPRESSED DURING: 15 growth stages; BEST Arabidopsis thaliana protein match is: unknown protein (TAIR:AT5G64910.1); Has 33260 Blast hits to 16857 proteins in 1270 species: Archae - 88; Bacteria - 3040; Metazoa - 11915; Fungi - 3137; Plants - 1371; Viruses - 424; Other Eukaryotes - 13285 (source: NCBI BLink). | chr5:3128098-3131452 FORWARD LENGTH=434	380	434	1.00E-121	114.2	57.1	67.9
Rsa1.0_00026.1.g1657.t1	gb|EOA14260.1| hypothetical protein CARUB_v10027418mg [Capsella rubella]	118	106	6.00E-25	89.8	50.0	68.6	hypothetical protein CARUB_v10027418mg	gbpln	Capsella rubella	AT5G55450.1 | Symbols: | Bifunctional inhibitor/lipid-transfer protein/seed storage 2S albumin superfamily protein | chr5:22467560-22467874 FORWARD LENGTH=104	118	104	2.00E-25	88.1	54.2	67.8
Rsa1.0_00026.1.g1658.t1	ref|XP_002864390.1| protease inhibitor/seed storage/lipid transfer protein family protein [Arabidopsis lyrata subsp. lyrata] gi|297310225|gb|EFH40649.1| protease inhibitor/seed storage/lipid transfer protein family protein [Arabidopsis lyrata subsp. lyrata]	107	107	3.00E-40	100.0	75.7	83.2	protease inhibitor/seed storage/lipid transfer protein family protein	gbpln	Arabidopsis lyrata	AT5G55410.1 | Symbols: | Bifunctional inhibitor/lipid-transfer protein/seed storage 2S albumin superfamily protein | chr5:22460677-22461081 FORWARD LENGTH=107	107	107	7.00E-42	100.0	72.9	81.3
Rsa1.0_00026.1.g1659.t1	gb|EOA12364.1| hypothetical protein CARUB_v10025745mg, partial [Capsella rubella]	1325	1349	0	101.8	74.7	84.6	hypothetical protein CARUB_v10025745mg, partial	gbpln	Capsella rubella	AT5G55390.2 | Symbols: EDM2 | ENHANCED DOWNY MILDEW 2 | chr5:22448152-22454414 REVERSE LENGTH=1297	1325	1297	0	97.9	74.5	83.6
Rsa1.0_00026.1.g1660.t1	ref|XP_002866093.1| hypothetical protein ARALYDRAFT_495621 [Arabidopsis lyrata subsp. lyrata] gi|297311928|gb|EFH42352.1| hypothetical protein ARALYDRAFT_495621 [Arabidopsis lyrata subsp. lyrata]	347	346	1.00E-152	99.7	76.7	84.1	hypothetical protein ARALYDRAFT_495621	gbpln	Arabidopsis lyrata	AT5G55380.1 | Symbols: | MBOAT (membrane bound O-acyl transferase) family protein | chr5:22446846-22447871 REVERSE LENGTH=341	347	341	1.00E-148	98.3	75.5	83.3
Rsa1.0_00026.1.g1661.t1	ref|NP_200348.1| putative long-chain-alcohol O-fatty-acyltransferase 2 [Arabidopsis thaliana] gi|75170973|sp|Q9FJ73.1|WAXS2_ARATH RecName: Full=Probable long-chain-alcohol O-fatty-acyltransferase 2; AltName: Full=Wax synthase 2 gi|9758169|dbj|BAB08554.1| wax synthase-like protein [Arabidopsis thaliana] gi|332009238|gb|AED96621.1| putative long-chain-alcohol O-fatty-acyltransferase 2 [Arabidopsis thaliana]	489	343	1.00E-138	70.1	50.1	57.7	putative long-chain-alcohol O-fatty-acyltransferase 2	gbpln	Arabidopsis thaliana	AT5G55370.1 | Symbols: | MBOAT (membrane bound O-acyl transferase) family protein | chr5:22445085-22446116 REVERSE LENGTH=343	489	343	1.00E-140	70.1	50.1	57.7
Rsa1.0_00026.1.g1662.t1	#	#	#	#	#	#	#	-	----	----	#	#	#	#	#	#	#
Rsa1.0_00026.1.g1663.t1	gb|EOA25182.1| hypothetical protein CARUB_v10018494mg [Capsella rubella]	181	282	7.00E-25	155.8	41.4	53.0	hypothetical protein CARUB_v10018494mg	gbpln	Capsella rubella	AT4G09490.1 | Symbols: | Polynucleotidyl transferase, ribonuclease H-like superfamily protein | chr4:6015583-6016575 REVERSE LENGTH=170	181	170	1.00E-19	93.9	29.3	40.9
Rsa1.0_00026.1.g1664.t1	ref|XP_002882965.1| hypothetical protein ARALYDRAFT_897881 [Arabidopsis lyrata subsp. lyrata] gi|297328805|gb|EFH59224.1| hypothetical protein ARALYDRAFT_897881 [Arabidopsis lyrata subsp. lyrata]	235	301	4.00E-95	128.1	71.5	83.4	hypothetical protein ARALYDRAFT_897881	gbpln	Arabidopsis lyrata	AT3G15820.1 | Symbols: ROD1 | phosphatidic acid phosphatase-related / PAP2-related | chr3:5351217-5353573 FORWARD LENGTH=301	235	301	2.00E-96	128.1	70.6	82.1

Rsa1.0_00026.1.g1665.t1	emb|CAB39638.1| RNA-directed DNA polymerase-like protein [Arabidopsis thaliana] gi|7267666|emb|CAB78094.1| RNA-directed DNA polymerase-like protein [Arabidopsis thaliana]	812	1274	4.00E-90	156.9	25.4	36.2	RNA-directed DNA polymerase-like protein	gbpln	Arabidopsis thaliana	AT5G55340.1 | Symbols: | MBOAT (membrane bound O-acyl transferase) family protein | chr5:22439985-22440986 REVERSE LENGTH=333	812	333	4.00E-57	41.0	13.7	17.9
Rsa1.0_00026.1.g1666.t1	ref|XP_002866092.1| hypothetical protein ARALYDRAFT_495620 [Arabidopsis lyrata subsp. lyrata] gi|297311927|gb|EFH42351.1| hypothetical protein ARALYDRAFT_495620 [Arabidopsis lyrata subsp. lyrata]	339	345	1.00E-121	101.8	63.7	75.8	hypothetical protein ARALYDRAFT_495620	gbpln	Arabidopsis lyrata	AT5G55350.1 | Symbols: | MBOAT (membrane bound O-acyl transferase) family protein | chr5:22442356-22443393 REVERSE LENGTH=345	339	345	1.00E-122	101.8	62.8	76.1
Rsa1.0_00026.1.g1667.t6	gb|EOA12470.1| hypothetical protein CARUB_v10025842mg [Capsella rubella]	1720	923	0	53.7	39.5	44.6	hypothetical protein CARUB_v10025842mg	gbpln	Capsella rubella	AT5G55300.2 | Symbols: TOP1ALPHA | DNA topoisomerase I alpha | chr5:22424701-22429045 REVERSE LENGTH=933	1720	933	0	54.2	39.5	44.5
Rsa1.0_00026.1.g1668.t1	gb|ACG70179.1| chloroplast FtsZ1-1 [Brassica oleracea var. botrytis]	425	425	0	100.0	92.5	94.8	chloroplast FtsZ1-1	gbpln	Brassica oleracea	AT5G55280.1 | Symbols: FTSZ1-1, ATFTSZ1-1, CPFTSZ | homolog of bacterial cytokinesis Z-ring protein FTSZ 1-1 | chr5:22420740-22422527 REVERSE LENGTH=433	425	433	0	101.9	90.1	94.4
Rsa1.0_00026.1.g1669.t1	ref|XP_002864385.1| atmap65-1 [Arabidopsis lyrata subsp. lyrata] gi|297310220|gb|EFH40644.1| atmap65-1 [Arabidopsis lyrata subsp. lyrata]	584	587	0	100.5	91.6	94.2	atmap65-1	gbpln	Arabidopsis lyrata	AT5G55230.1 | Symbols: ATMAP65-1, MAP65-1 | microtubule-associated proteins 65-1 | chr5:22402716-22405182 FORWARD LENGTH=587	584	587	0	100.5	90.8	94.0
Rsa1.0_00026.1.g1670.t1	ref|XP_002864384.1| trigger factor type chaperone family protein [Arabidopsis lyrata subsp. lyrata] gi|297310219|gb|EFH40643.1| trigger factor type chaperone family protein [Arabidopsis lyrata subsp. lyrata]	548	551	0	100.5	85.6	92.0	trigger factor type chaperone family protein	gbpln	Arabidopsis lyrata	AT5G55220.1 | Symbols: | trigger factor type chaperone family protein | chr5:22397677-22400678 FORWARD LENGTH=547	548	547	0	99.8	85.6	92.2
Rsa1.0_00026.1.g1671.t1	#	#	#	#	#	#	#	-	----	----	#	#	#	#	#	#	#
Rsa1.0_00026.1.g1672.t1	gb|EMJ23759.1| hypothetical protein PRUPE_ppa008371mg [Prunus persica]	221	335	1.00E-130	151.6	99.1	99.5	hypothetical protein PRUPE_ppa008371mg	gbpln	Prunus persica	AT5G55190.1 | Symbols: RAN3, ATRAN3 | RAN GTPase 3 | chr5:22392285-22393957 FORWARD LENGTH=221	221	221	1.00E-130	100.0	99.1	99.5
Rsa1.0_00026.1.g1673.t1	ref|XP_002862436.1| predicted protein [Arabidopsis lyrata subsp. lyrata] gi|297307949|gb|EFH38694.1| predicted protein [Arabidopsis lyrata subsp. lyrata]	544	530	1.00E-40	97.4	18.9	25.7	predicted protein	gbpln	Arabidopsis lyrata	AT2G07760.1 | Symbols: | Zinc knuckle (CCHC-type) family protein | chr2:3584420-3586278 REVERSE LENGTH=530	544	530	7.00E-17	97.4	7.7	13.2
Rsa1.0_00026.1.g1674.t1	gb|AAG50806.1|AC079281_8 unknown protein [Arabidopsis thaliana]	1177	1213	0	103.1	38.2	56.2	unknown protein	gbpln	Arabidopsis thaliana	AT1G43760.1 | Symbols: | DNAse I-like superfamily protein | chr1:16528880-16531065 REVERSE LENGTH=626	1177	626	4.00E-69	53.2	13.3	20.2
Rsa1.0_00026.1.g1675.t1	ref|XP_002864380.1| glycosyl hydrolase family 17 protein [Arabidopsis lyrata subsp. lyrata] gi|297310215|gb|EFH40639.1| glycosyl hydrolase family 17 protein [Arabidopsis lyrata subsp. lyrata]	475	461	0	97.1	81.7	87.4	glycosyl hydrolase family 17 protein	gbpln	Arabidopsis lyrata	AT5G55180.1 | Symbols: | O-Glycosyl hydrolases family 17 protein | chr5:22388834-22390550 FORWARD LENGTH=460	475	460	0	96.8	83.8	90.5
Rsa1.0_00026.1.g1676.t3	ref|XP_002866078.1| F-box family protein [Arabidopsis lyrata subsp. lyrata] gi|297311913|gb|EFH42337.1| F-box family protein [Arabidopsis lyrata subsp. lyrata]	381	375	1.00E-140	98.4	68.8	78.0	F-box family protein	gbpln	Arabidopsis lyrata	AT5G55150.1 | Symbols: | Protein of unknown function (DUF295) | chr5:22382275-22383392 REVERSE LENGTH=360	381	360	1.00E-132	94.5	65.4	74.8
Rsa1.0_00026.1.g1677.t2	ref|NP_680438.1| uncharacterized protein [Arabidopsis thaliana] gi|91807044|gb|ABE66249.1| hypothetical protein At5g55135 [Arabidopsis thaliana] gi|332009207|gb|AED96590.1| uncharacterized protein AT5G55135 [Arabidopsis thaliana]	112	112	2.00E-33	100.0	68.8	81.3	uncharacterized protein	gbpln	Arabidopsis thaliana	AT5G55135.1 | Symbols: | unknown protein; Has 30201 Blast hits to 17322 proteins in 780 species: Archae - 12; Bacteria - 1396; Metazoa - 17338; Fungi - 3422; Plants - 5037; Viruses - 0; Other Eukaryotes - 2996 (source: NCBI BLink). | chr5:22379959-22380386 FORWARD LENGTH=112	112	112	3.00E-36	100.0	68.8	81.3
Rsa1.0_00026.1.g1678.t1	#	#	#	#	#	#	#	-	----	----	#	#	#	#	#	#	#
Rsa1.0_00026.1.g1679.t1	gb|EOA14782.1| hypothetical protein CARUB_v10028082mg [Capsella rubella]	57	76	1.00E-13	133.3	64.9	66.7	hypothetical protein CARUB_v10028082mg	gbpln	Capsella rubella	AT5G55125.2 | Symbols: | Ribosomal protein L31 | chr5:22372737-22372967 FORWARD LENGTH=76	57	76	2.00E-15	133.3	63.2	64.9
Rsa1.0_00026.1.g1680.t1	emb|CAN62821.1| hypothetical protein VITISV_013041 [Vitis vinifera]	1091	1357	1.00E-145	124.4	35.2	54.6	hypothetical protein VITISV_013041	gbpln	Vitis vinifera	ATMG00860.1 | Symbols: ORF158 | DNA/RNA polymerases superfamily protein | chrM:235916-236392 FORWARD LENGTH=158	1091	158	2.00E-28	14.5	5.6	7.0
Rsa1.0_00026.1.g1681.t1	ref|NP_192687.1| RNase H domain-containing protein [Arabidopsis thaliana] gi|7267644|emb|CAB78072.1| putative proteins [Arabidopsis thaliana] gi|332657356|gb|AEE82756.1| RNase H domain-containing protein [Arabidopsis thaliana]	90	170	8.00E-12	188.9	45.6	56.7	RNase H domain-containing protein	gbpln	Arabidopsis thaliana	AT4G09490.1 | Symbols: | Polynucleotidyl transferase, ribonuclease H-like superfamily protein | chr4:6015583-6016575 REVERSE LENGTH=170	90	170	1.00E-14	188.9	45.6	56.7
Rsa1.0_00026.1.g1682.t1	gb|EOA14764.1| hypothetical protein CARUB_v10028061mg [Capsella rubella]	345	164	3.00E-59	47.5	34.8	36.5	hypothetical protein CARUB_v10028061mg	gbpln	Capsella rubella	AT5G55110.1 | Symbols: | Stigma-specific Stig1 family protein | chr5:22367870-22368361 FORWARD LENGTH=163	345	163	6.00E-59	47.2	32.5	34.5

Rsa1.0_00026.1.g1683.t1	ref|XP_002866076.1| swap (Suppressor-of-White-APricot)/surp domain-containing protein [Arabidopsis lyrata subsp. lyrata] gi|297311911|gb|EFH42335.1| swap (Suppressor-of-White-APricot)/surp domain-containing protein [Arabidopsis lyrata subsp. lyrata]	868	852	0	98.2	76.3	83.6	swap (Suppressor-of-White-APricot)/surp domain-containing protein	gbpln	Arabidopsis lyrata	AT5G55100.2 | Symbols: | SWAP (Suppressor-of-White-APricot)/surp domain-containing protein | chr5:22361401-22364664 REVERSE LENGTH=844	868	844	0	97.2	74.8	82.7
Rsa1.0_00026.1.g1684.t1	#	#	#	#	#	#	#	-	----	----	#	#	#	#	#	#	#
Rsa1.0_00026.1.g1685.t1	ref|NP_200320.1| mitogen-activated protein kinase kinase kinase 15 [Arabidopsis thaliana] gi|9758106|dbj|BAB08578.1| unnamed protein product [Arabidopsis thaliana] gi|332009196|gb|AED96579.1| mitogen-activated protein kinase kinase kinase 15 [Arabidopsis thaliana]	448	448	1.00E-176	100.0	73.2	81.3	mitogen-activated protein kinase kinase kinase 15	gbpln	Arabidopsis thaliana	AT5G55090.1 | Symbols: MAPKKK15 | mitogen-activated protein kinase kinase kinase 15 | chr5:22356852-22358198 REVERSE LENGTH=448	448	448	1.00E-179	100.0	73.2	81.3
Rsa1.0_00026.1.g1686.t3	gb|EOA13330.1| hypothetical protein CARUB_v10026364mg [Capsella rubella]	531	462	0	87.0	79.3	82.1	hypothetical protein CARUB_v10026364mg	gbpln	Capsella rubella	AT5G55070.1 | Symbols: | Dihydrolipoamide succinyltransferase | chr5:22347637-22350409 FORWARD LENGTH=464	531	464	0	87.4	76.8	79.5
Rsa1.0_00026.1.g1687.t1	#	#	#	#	#	#	#	-	----	----	#	#	#	#	#	#	#
Rsa1.0_00026.1.g1688.t1	ref|XP_002864371.1| GDSL-motif lipase/hydrolase family protein [Arabidopsis lyrata subsp. lyrata] gi|297310206|gb|EFH40630.1| GDSL-motif lipase/hydrolase family protein [Arabidopsis lyrata subsp. lyrata]	136	376	3.00E-50	276.5	67.6	77.9	GDSL-motif lipase/hydrolase family protein	gbpln	Arabidopsis lyrata	AT5G55050.1 | Symbols: | GDSL-like Lipase/Acylhydrolase superfamily protein | chr5:22337745-22339741 FORWARD LENGTH=376	136	376	2.00E-50	276.5	64.0	77.2
Rsa1.0_00026.1.g1689.t1	gb|EOA13535.1| hypothetical protein CARUB_v10026598mg [Capsella rubella]	377	378	1.00E-171	100.3	79.0	89.7	hypothetical protein CARUB_v10026598mg	gbpln	Capsella rubella	AT5G55050.1 | Symbols: | GDSL-like Lipase/Acylhydrolase superfamily protein | chr5:22337745-22339741 FORWARD LENGTH=376	377	376	1.00E-168	99.7	78.8	90.7
Rsa1.0_00026.1.g1690.t1	ref|NP_200310.1| RING/U-box domain-containing protein [Arabidopsis thaliana] gi|9758279|dbj|BAB08778.1| unnamed protein product [Arabidopsis thaliana] gi|332009183|gb|AED96566.1| RING/U-box domain-containing protein [Arabidopsis thaliana]	282	226	1.00E-47	80.1	43.6	52.5	RING/U-box domain-containing protein	gbpln	Arabidopsis thaliana	AT5G54990.1 | Symbols: | RING/U-box superfamily protein | chr5:22317505-22318185 FORWARD LENGTH=226	282	226	4.00E-50	80.1	43.6	52.5
Rsa1.0_00026.1.g1691.t1	ref|NP_200310.1| RING/U-box domain-containing protein [Arabidopsis thaliana] gi|9758279|dbj|BAB08778.1| unnamed protein product [Arabidopsis thaliana] gi|332009183|gb|AED96566.1| RING/U-box domain-containing protein [Arabidopsis thaliana]	249	226	7.00E-24	90.8	34.9	47.0	RING/U-box domain-containing protein	gbpln	Arabidopsis thaliana	AT5G54990.1 | Symbols: | RING/U-box superfamily protein | chr5:22317505-22318185 FORWARD LENGTH=226	249	226	2.00E-26	90.8	34.9	47.0
Rsa1.0_00026.1.g1692.t1	dbj|BAJ33777.1| unnamed protein product [Thellungiella halophila]	569	552	0	97.0	85.4	88.9	unnamed protein product	----	----	AT3G02020.1 | Symbols: AK3 | aspartate kinase 3 | chr3:340739-343410 REVERSE LENGTH=559	569	559	0	98.2	82.8	88.9
Rsa1.0_00026.1.g1693.t2	ref|NP_568814.2| DTW domain-containing protein [Arabidopsis thaliana] gi|9758268|dbj|BAB08767.1| unnamed protein product [Arabidopsis thaliana] gi|15450589|gb|AAK96566.1| AT5g54880/MBG8_15 [Arabidopsis thaliana] gi|24797048|gb|AAN64536.1| At5g54880/MBG8_15 [Arabidopsis thaliana] gi|332009169|gb|AED96552.1| DTW domain-containing protein [Arabidopsis thaliana]	313	394	1.00E-124	125.9	77.6	86.9	DTW domain-containing protein	gbpln	Arabidopsis thaliana	AT5G54880.1 | Symbols: | DTW domain-containing protein | chr5:22291920-22293104 FORWARD LENGTH=394	313	394	1.00E-126	125.9	77.6	86.9
Rsa1.0_00026.1.g1694.t1	gb|EOA13149.1| hypothetical protein CARUB_v10026167mg, partial [Capsella rubella]	530	563	0	106.2	89.4	93.2	hypothetical protein CARUB_v10026167mg, partial	gbpln	Capsella rubella	AT5G54870.1 | Symbols: | unknown protein; FUNCTIONS IN: molecular_function unknown; INVOLVED IN: biological_process unknown; LOCATED IN: vacuole; EXPRESSED IN: 24 plant structures; EXPRESSED DURING: 15 growth stages; BEST Arabidopsis thaliana protein match is: unknown protein (TAIR:AT4G27020.1); Has 1807 Blast hits to 1807 proteins in 277 species: Archae - 0; Bacteria - 0; Metazoa - 736; Fungi - 347; Plants - 385; Viruses - 0; Other Eukaryotes - 339 (source: NCBI BLink). | chr5:22289149-22291604 FORWARD LENGTH=531	530	531	0	100.2	87.4	91.1
Rsa1.0_00026.1.g1695.t1	gb|AAC63844.1| putative non-LTR retroelement reverse transcriptase [Arabidopsis thaliana]	1769	1231	0	69.6	36.8	45.8	putative non-LTR retroelement reverse transcriptase	gbpln	Arabidopsis thaliana	AT2G01050.1 | Symbols: | zinc ion binding;nucleic acid binding | chr2:68337-69884 REVERSE LENGTH=515	1769	515	1.00E-124	29.1	13.6	18.1
Rsa1.0_00026.1.g1696.t1	gb|AAD21699.1| Contains reverse transcriptase domain (rvt) PF|00078 [Arabidopsis thaliana]	226	1253	3.00E-28	554.4	24.3	35.4	Contains reverse transcriptase domain (rvt) PF|00078	gbpln	Arabidopsis thaliana	AT1G60720.1 | Symbols: | RNA-directed DNA polymerase (reverse transcriptase)-related family protein | chr1:22357072-22357941 FORWARD LENGTH=289	226	289	2.00E-27	127.9	24.3	33.6

Rsa1.0_00026.1.g1697.t2	emb|CAB81573.1| putative protein [Arabidopsis thaliana]	321	510	7.00E-22	158.9	17.4	25.2	putative protein	gbpln	Arabidopsis thaliana	AT2G07760.1 | Symbols: | Zinc knuckle (CCHC-type) family protein | chr2:3584420-3586278 REVERSE LENGTH=530	321	530	1.00E-12	165.1	13.1	21.5
Rsa1.0_00026.1.g1698.t2	gb|AAG51873.1|AC079678_3 disease resistance protein, putative; 11609-15699 [Arabidopsis thaliana]	1057	1068	0	101.0	63.6	73.4	disease resistance protein, putative; 11609-15699	gbpln	Arabidopsis thaliana	AT1G74170.1 | Symbols: AtRLP13, RLP13 | receptor like protein 13 | chr1:27891555-27895441 REVERSE LENGTH=1000	1057	1000	0	94.6	59.6	68.6
Rsa1.0_00027.1.g1699.t1	gb|EOA36095.1| hypothetical protein CARUB_v10010848mg, partial [Capsella rubella]	134	134	9.00E-14	100.0	32.1	50.0	hypothetical protein CARUB_v10010848mg, partial	gbpln	Capsella rubella	AT4G09490.1 | Symbols: | Polynucleotidyl transferase, ribonuclease H-like superfamily protein | chr4:6015583-6016575 REVERSE LENGTH=170	134	170	1.00E-11	126.9	25.4	40.3
Rsa1.0_00027.1.g1700.t1	gb|EOA18568.1| hypothetical protein CARUB_v10007127mg [Capsella rubella]	973	969	0	99.6	93.7	96.9	hypothetical protein CARUB_v10007127mg	gbpln	Capsella rubella	AT4G30160.1 | Symbols: VLN4, ATVLN4 | villin 4 | chr4:14754528-14759511 FORWARD LENGTH=974	973	974	0	100.1	92.3	96.6
Rsa1.0_00027.1.g1701.t1	gb|EOA17560.1| hypothetical protein CARUB_v10005921mg [Capsella rubella]	159	159	2.00E-43	100.0	69.2	78.0	hypothetical protein CARUB_v10005921mg	gbpln	Capsella rubella	AT2G18969.1 | Symbols: | BEST Arabidopsis thaliana protein match is: sequence-specific DNA binding transcription factors;transcription regulators (TAIR:AT4G30180.1); Has 30201 Blast hits to 17322 proteins in 780 species: Archae - 12; Bacteria - 1396; Metazoa - 17338; Fungi - 3422; Plants - 5037; Viruses - 0; Other Eukaryotes - 2996 (source: NCBI BLink). | chr2:8229047-8229574 REVERSE LENGTH=175	159	175	3.00E-42	110.1	60.4	73.6
Rsa1.0_00027.1.g1702.t1	ref|NP_194748.1| H(+)-ATPase 2 [Arabidopsis thaliana] gi|114335|sp|P19456.2|PMA2_ARATH RecName: Full=ATPase 2, plasma membrane-type; AltName: Full=Proton pump 2 gi|166629|gb|AAA32751.1| H+-ATPase [Arabidopsis thaliana] gi|5730129|emb|CAB52463.1| H+-transporting ATPase type 2, plasma membrane [Arabidopsis thaliana] gi|7269919|emb|CAB81012.1| H+-transporting ATPase type 2, plasma membrane [Arabidopsis thaliana] gi|14334804|gb|AAK59580.1| putative H+-transporting ATPase [Arabidopsis thaliana] gi|23397277|gb|AAN31920.1| putative H+-transporting ATPase type 2 [Arabidopsis thaliana] gi|25055009|gb|AAN71968.1| putative H+-transporting ATPase [Arabidopsis thaliana] gi|332660331|gb|AEE85731.1| H(+)-ATPase 2 [Arabidopsis thaliana]	947	948	0	100.1	97.5	99.0	H(+)-ATPase 2	gbpln	Arabidopsis thaliana	AT4G30190.1 | Symbols: AHA2, PMA2, HA2 | H(+)-ATPase 2 | chr4:14770820-14775920 REVERSE LENGTH=948	947	948	0	100.1	97.5	99.0
Rsa1.0_00027.1.g1703.t1	ref|NP_194749.1| vernalization5/VIN3-like protein [Arabidopsis thaliana] gi|5730130|emb|CAB52464.1| putative protein [Arabidopsis thaliana] gi|7269920|emb|CAB81013.1| putative protein [Arabidopsis thaliana] gi|11177137|dbj|BAB17836.1| nuclear coiled-coil protein AT4g30200 [Arabidopsis thaliana] gi|118566356|gb|ABL01539.1| VIN3-like 2 protein [Arabidopsis thaliana] gi|332660335|gb|AEE85735.1| vernalization5/VIN3-like protein [Arabidopsis thaliana]	670	714	0	106.6	80.3	88.5	vernalization5/VIN3-like protein	gbpln	Arabidopsis thaliana	AT4G30200.2 | Symbols: VEL1, VIL2 | vernalization5/VIN3-like | chr4:14786852-14790070 REVERSE LENGTH=714	670	714	0	106.6	80.3	88.5
Rsa1.0_00027.1.g1704.t1	gb|AAB88009.1| heat shock cognate protein HSC70 [Brassica napus]	647	645	0	99.7	96.3	98.6	heat shock cognate protein HSC70	gbpln	Brassica napus	AT5G02500.1 | Symbols: HSC70-1, HSP70-1, AT-HSC70-1, HSC70 | heat shock cognate protein 70-1 | chr5:554055-556334 REVERSE LENGTH=651	647	651	0	100.6	96.1	98.6
Rsa1.0_00027.1.g1705.t2	dbj|BAJ33878.1| unnamed protein product [Thellungiella halophila]	244	711	2.00E-73	291.4	65.2	73.4	unnamed protein product	----	----	AT4G30210.2 | Symbols: ATR2, AR2 | P450 reductase 2 | chr4:14796900-14800578 FORWARD LENGTH=711	244	711	3.00E-71	291.4	59.0	69.3

Rsa1.0_00027.1.g1706.t1	ref|NP_194751.1| small nuclear ribonucleoprotein F [Arabidopsis thaliana] gi|297798954|ref|XP_002867361.1| hypothetical protein ARALYDRAFT_913456 [Arabidopsis lyrata subsp. lyrata] gi|297836060|ref|XP_002885912.1| hypothetical protein ARALYDRAFT_899645 [Arabidopsis lyrata subsp. lyrata] gi|10720264|sp|Q9SUM2.1|RUXF_ARATH RecName: Full=Probable small nuclear ribonucleoprotein F; Short=snRNP-F; AltName: Full=Sm protein F; Short=Sm-F; Short=SmF gi|5730132|emb|CAB52466.1| snRNP Sm protein F-like [Arabidopsis thaliana] gi|7269922|emb|CAB81015.1| snRNP Sm protein F-like [Arabidopsis thaliana] gi|21593208|gb|AAM65157.1| snRNP Sm protein F-like [Arabidopsis thaliana] gi|30102544|gb|AAP21190.1| At4g30220 [Arabidopsis thaliana] gi|110743810|dbj|BAE99740.1| snRNP Sm protein F - like [Arabidopsis thaliana] gi|297313197|gb|EFH43620.1| hypothetical protein ARALYDRAFT_913456 [Arabidopsis lyrata subsp. lyrata] gi|297331752|gb|EFH62171.1| hypothetical protein ARALYDRAFT_899645 [Arabidopsis lyrata subsp. lyrata]	88	88	8.00E-43	100.0	97.7	98.9	small nuclear ribonucleoprotein F	gbpln	Arabidopsis lyrata	AT4G30220.1 | Symbols: RUXF | small nuclear ribonucleoprotein F | chr4:14803100-14804259 REVERSE LENGTH=88	88	88	1.00E-45	100.0	97.7	98.9
Rsa1.0_00027.1.g1707.t1	ref|NP_567842.1| Syntaxin/t-SNARE family protein [Arabidopsis thaliana] gi|16226657|gb|AAL16225.1|AF428456_1 AT4g30240/F9N11_90 [Arabidopsis thaliana] gi|22137126|gb|AAM91408.1| At4g30240/F9N11_90 [Arabidopsis thaliana] gi|332660342|gb|AEE85742.1| Syntaxin/t-SNARE family protein [Arabidopsis thaliana]	278	300	1.00E-108	107.9	73.7	83.8	Syntaxin/t-SNARE family protein	gbpln	Arabidopsis thaliana	AT4G30240.1 | Symbols: | Syntaxin/t-SNARE family protein | chr4:14808359-14809745 REVERSE LENGTH=300	278	300	1.00E-110	107.9	73.7	83.8
Rsa1.0_00027.1.g1708.t1	gb|EOA15588.1| hypothetical protein CARUB_v10005446mg [Capsella rubella]	284	280	1.00E-117	98.6	91.2	94.0	hypothetical protein CARUB_v10005446mg	gbpln	Capsella rubella	AT2G18840.1 | Symbols: | Integral membrane Yip1 family protein | chr2:8158288-8159848 FORWARD LENGTH=281	284	281	1.00E-115	98.9	86.3	90.1
Rsa1.0_00027.1.g1709.t1	gb|AEX07595.1| xyloglucosyl transferase 2, partial [Brassica juncea]	282	265	1.00E-144	94.0	85.8	89.4	xyloglucosyl transferase 2, partial	gbpln	Brassica juncea	AT4G30270.1 | Symbols: MERI5B, MERI-5, XTH24, SEN4 | xyloglucan endotransglucosylase/hydrolase 24 | chr4:14819445-14820448 REVERSE LENGTH=269	282	269	1.00E-142	95.4	84.8	89.4
Rsa1.0_00027.1.g1710.t1	emb|CAB39638.1| RNA-directed DNA polymerase-like protein [Arabidopsis thaliana] gi|7267666|emb|CAB78094.1| RNA-directed DNA polymerase-like protein [Arabidopsis thaliana]	345	1274	2.00E-51	369.3	35.1	51.0	RNA-directed DNA polymerase-like protein	gbpln	Arabidopsis thaliana	AT4G29090.1 | Symbols: | Ribonuclease H-like superfamily protein | chr4:14333528-14335255 FORWARD LENGTH=575	345	575	9.00E-30	166.7	26.4	47.2
Rsa1.0_00027.1.g1711.t1	ref|NP_176710.1| xyloglucan:xyloglucosyl transferase [Arabidopsis thaliana] gi|38605119|sp|O80803.1|XTH17_ARATH RecName: Full=Probable xyloglucan endotransglucosylase/hydrolase protein 17; Short=At-XTH17; Short=XTH-17; Flags: Precursor gi|13877725|gb|AAK43940.1|AF370621_1 xylglucan endo-transglycolsylase-like protein [Arabidopsis thaliana] gi|3335340|gb|AAC27142.1| Strong similarity to xylglucan endo-transglycolsylase (TCH4) gene gb|U27609, first exon contains strong similarity to meri 5 gene gb|Z17989 from A. thaliana. EST gb|N37583 comes from this gene [Arabidopsis thaliana] gi|124300988|gb|ABN04746.1| At1g65310 [Arabidopsis thaliana] gi|332196236|gb|AEE34357.1| probable xyloglucan endotransglucosylase/hydrolase protein 17 [Arabidopsis thaliana]	97	282	2.00E-44	290.7	86.6	91.8	xyloglucan:xyloglucosyl transferase	gbpln	Arabidopsis thaliana	AT1G65310.1 | Symbols: ATXTH17, XTH17 | xyloglucan endotransglucosylase/hydrolase 17 | chr1:24257262-24258319 FORWARD LENGTH=282	97	282	3.00E-47	290.7	86.6	91.8

Rsa1.0_00027.1.g1712.t1	ref|NP_199618.1| xyloglucan endotransglucosylase/hydrolase protein 20 [Arabidopsis thaliana] gi|38605469|sp|Q9FI31.1|XTH20_ARATH RecName: Full=Xyloglucan endotransglucosylase/hydrolase protein 20; Short=At-XTH20; Short=XTH-20; Flags: Precursor gi|10177758|dbj|BAB11071.1| xyloglucan endo-1,4-beta-D-glucanase [Arabidopsis thaliana] gi|45825157|gb|AAS77486.1| At5g48070 [Arabidopsis thaliana] gi|62320256|dbj|BAD94531.1| xyloglucan endo-1,4-beta-D-glucanase [Arabidopsis thaliana] gi|332008233|gb|AED95616.1| xyloglucan endotransglucosylase/hydrolase protein 20 [Arabidopsis thaliana]	280	282	1.00E-133	100.7	81.8	87.5	xyloglucan endotransglucosylase/ hydrolase protein 20	gbpln	Arabidopsis thaliana	AT5G48070.1 | Symbols: ATXTH20, XTH20 | xyloglucan endotransglucosylase/hydrolase 20 | chr5:19482158-19483231 FORWARD LENGTH=282	280	282	1.00E-136	100.7	81.8	87.5
Rsa1.0_00027.1.g1713.t1	ref|XP_002867352.1| zinc finger family protein [Arabidopsis lyrata subsp. lyrata] gi|297313188|gb|EFH43611.1| zinc finger family protein [Arabidopsis lyrata subsp. lyrata]	153	169	2.00E-37	110.5	64.7	74.5	zinc finger family protein	gbpln	Arabidopsis lyrata	AT4G30370.1 | Symbols: | RING/U-box superfamily protein | chr4:14858743-14859273 REVERSE LENGTH=176	153	176	3.00E-39	115.0	64.1	75.8
Rsa1.0_00027.1.g1714.t3	#	#	#	#	#	#	#	-	----	----	#	#	#	#	#	#	#
Rsa1.0_00027.1.g1715.t1	gb|EOA17519.1| hypothetical protein CARUB_v10005852mg [Capsella rubella]	180	180	1.00E-81	100.0	87.2	95.0	hypothetical protein CARUB_v10005852mg	gbpln	Capsella rubella	AT4G30410.1 | Symbols: | sequence-specific DNA binding transcription factors | chr4:14871309-14871851 REVERSE LENGTH=180	180	180	2.00E-81	100.0	88.9	94.4
Rsa1.0_00027.1.g1716.t1	#	#	#	#	#	#	#	-	----	----	#	#	#	#	#	#	#
Rsa1.0_00027.1.g1717.t3	ref|NP_194771.1| nodulin MtN21 /EamA-like transporter family protein [Arabidopsis thaliana] gi|75181441|sp|Q9M0B8.1|WTR37_ARATH RecName: Full=WAT1-related protein At4g30420 gi|7269943|emb|CAB79760.1| nodulin-like protein [Arabidopsis thaliana] gi|332660363|gb|AEE85763.1| nodulin MtN21 /EamA-like transporter family protein [Arabidopsis thaliana]	390	373	1.00E-179	95.6	80.5	85.9	nodulin MtN21 /EamA-like transporter family protein	gbpln	Arabidopsis thaliana	AT4G30420.1 | Symbols: | nodulin MtN21 /EamA-like transporter family protein | chr4:14877069-14878914 FORWARD LENGTH=373	390	373	0	95.6	80.5	85.9
Rsa1.0_00027.1.g1718.t1	gb|AAD22330.1| putative non-LTR retroelement reverse transcriptase [Arabidopsis thaliana]	213	631	1.00E-30	296.2	34.7	53.1	putative non-LTR retroelement reverse transcriptase	gbpln	Arabidopsis thaliana	AT3G24255.1 | Symbols: | RNA-directed DNA polymerase (reverse transcriptase)-related family protein | chr3:8789471-8793208 FORWARD LENGTH=746	213	746	9.00E-29	350.2	32.9	43.7
Rsa1.0_00027.1.g1719.t1	ref|NP_194782.1| class I glutamine amidotransferase domain-containing protein [Arabidopsis thaliana] gi|15294152|gb|AAK95253.1|AF410267_1 AT4g30530/F17I23_130 [Arabidopsis thaliana] gi|7269954|emb|CAB79771.1| putative protein [Arabidopsis thaliana] gi|20147269|gb|AAM10348.1| AT4g30530/F17I23_130 [Arabidopsis thaliana] gi|332660377|gb|AEE85777.1| class I glutamine amidotransferase domain-containing protein [Arabidopsis thaliana]	250	250	1.00E-129	100.0	88.8	93.6	class I glutamine amidotransferase domain-containing protein	gbpln	Arabidopsis thaliana	AT4G30530.1 | Symbols: | Class I glutamine amidotransferase-like superfamily protein | chr4:14920605-14922286 FORWARD LENGTH=250	250	250	1.00E-132	100.0	88.8	93.6
Rsa1.0_00027.1.g1720.t1	gb|EOA17311.1| hypothetical protein CARUB_v10005590mg [Capsella rubella]	253	249	1.00E-124	98.4	83.4	91.3	hypothetical protein CARUB_v10005590mg	gbpln	Capsella rubella	AT4G30550.1 | Symbols: | Class I glutamine amidotransferase-like superfamily protein | chr4:14925618-14926713 FORWARD LENGTH=249	253	249	1.00E-125	98.4	85.4	91.7
Rsa1.0_00027.1.g1721.t1	gb|EOA18329.1| hypothetical protein CARUB_v10006848mg [Capsella rubella]	191	187	6.00E-68	97.9	71.7	78.5	hypothetical protein CARUB_v10006848mg	gbpln	Capsella rubella	AT4G30590.1 | Symbols: ENODL12, AtENODL12 | early nodulin-like protein 12 | chr4:14935760-14936469 REVERSE LENGTH=190	191	190	1.00E-64	99.5	73.3	80.6
Rsa1.0_00027.1.g1722.t1	ref|XP_002867336.1| hypothetical protein ARALYDRAFT_913413 [Arabidopsis lyrata subsp. lyrata] gi|297313172|gb|EFH43595.1| hypothetical protein ARALYDRAFT_913413 [Arabidopsis lyrata subsp. lyrata]	182	181	2.00E-85	99.5	91.8	94.5	hypothetical protein ARALYDRAFT_913413	gbpln	Arabidopsis lyrata	AT4G30620.1 | Symbols: | Uncharacterised BCR, YbaB family COG0718 | chr4:14948724-14950035 REVERSE LENGTH=180	182	180	5.00E-87	98.9	90.1	92.9
Rsa1.0_00027.1.g1723.t1	gb|EOA18905.1| hypothetical protein CARUB_v10007535mg [Capsella rubella]	301	301	1.00E-133	100.0	74.8	84.7	hypothetical protein CARUB_v10007535mg	gbpln	Capsella rubella	AT4G30640.1 | Symbols: | RNI-like superfamily protein | chr4:14952670-14953682 FORWARD LENGTH=301	301	301	1.00E-127	100.0	70.8	83.1
Rsa1.0_00027.1.g1724.t1	#	#	#	#	#	#	#	-	----	----	#	#	#	#	#	#	#

Rsa1.0_00027.1.g1725.t1	ref|XP_002869411.1| predicted protein [Arabidopsis lyrata subsp. lyrata] gi|297315247|gb|EFH45670.1| predicted protein [Arabidopsis lyrata subsp. lyrata]	272	280	2.00E-72	102.9	51.8	69.5	predicted protein	gbpln	Arabidopsis lyrata	AT4G29760.1 | Symbols: | unknown protein; BEST Arabidopsis thaliana protein match is: unknown protein (TAIR:AT4G29770.2); Has 63 Blast hits to 63 proteins in 2 species: Archae - 0; Bacteria - 0; Metazoa - 0; Fungi - 0; Plants - 63; Viruses - 0; Other Eukaryotes - 0 (source: NCBI BLink). | chr4:14574114-14575148 FORWARD LENGTH=280	272	280	1.00E-71	102.9	50.7	68.0
Rsa1.0_00027.1.g1726.t1	ref|XP_002869411.1| predicted protein [Arabidopsis lyrata subsp. lyrata] gi|297315247|gb|EFH45670.1| predicted protein [Arabidopsis lyrata subsp. lyrata]	270	280	2.00E-81	103.7	57.8	72.2	predicted protein	gbpln	Arabidopsis lyrata	AT4G29760.1 | Symbols: | unknown protein; BEST Arabidopsis thaliana protein match is: unknown protein (TAIR:AT4G29770.2); Has 63 Blast hits to 63 proteins in 2 species: Archae - 0; Bacteria - 0; Metazoa - 0; Fungi - 0; Plants - 63; Viruses - 0; Other Eukaryotes - 0 (source: NCBI BLink). | chr4:14574114-14575148 FORWARD LENGTH=280	270	280	1.00E-79	103.7	56.7	71.5
Rsa1.0_00027.1.g1727.t6	ref|XP_002867329.1| hypothetical protein ARALYDRAFT_913400 [Arabidopsis lyrata subsp. lyrata] gi|297313165|gb|EFH43588.1| hypothetical protein ARALYDRAFT_913400 [Arabidopsis lyrata subsp. lyrata]	149	157	9.00E-67	105.4	87.9	91.9	hypothetical protein ARALYDRAFT_913400	gbpln	Arabidopsis lyrata	AT4G30770.1 | Symbols: | Putative membrane lipoprotein | chr4:14986140-14986613 REVERSE LENGTH=157	149	157	2.00E-61	105.4	86.6	90.6
Rsa1.0_00027.1.g1728.t1	gb|EOA16289.1| hypothetical protein CARUB_v10004435mg [Capsella rubella]	543	586	0	107.9	82.3	89.7	hypothetical protein CARUB_v10004435mg	gbpln	Capsella rubella	AT4G30780.1 | Symbols: | unknown protein; BEST Arabidopsis thaliana protein match is: unknown protein (TAIR:AT2G24100.1); Has 109 Blast hits to 109 proteins in 18 species: Archae - 0; Bacteria - 0; Metazoa - 1; Fungi - 0; Plants - 95; Viruses - 0; Other Eukaryotes - 13 (source: NCBI BLink). | chr4:14990523-14992855 FORWARD LENGTH=589	543	589	0	108.5	81.6	89.9
Rsa1.0_00027.1.g1729.t1	#	#	#	#	#	#	#	-	----	----	#	#	#	#	#	#	#
Rsa1.0_00027.1.g1730.t2	gb|EOA15927.1| hypothetical protein CARUB_v10004021mg [Capsella rubella]	267	1147	9.00E-34	429.6	31.8	41.6	hypothetical protein CARUB_v10004021mg	gbpln	Capsella rubella	AT4G30790.1 | Symbols: | INVOLVED IN: autophagy; EXPRESSED IN: 24 plant structures; EXPRESSED DURING: 15 growth stages; CONTAINS InterPro DOMAIN/s: Autophagy-related protein 17 (InterPro:IPR007240), Autophagy-related protein 11 (InterPro:IPR019460); Has 8793 Blast hits to 6268 proteins in 570 species: Archae - 89; Bacteria - 657; Metazoa - 4844; Fungi - 808; Plants - 441; Viruses - 15; Other Eukaryotes - 1939 (source: NCBI BLink). | chr4:14993383-14997785 REVERSE LENGTH=1148	267	1148	2.00E-31	430.0	33.0	41.9
Rsa1.0_00027.1.g1731.t1	ref|NP_567854.1| carboxypeptidase D [Arabidopsis thaliana] gi|75332010|sp|Q949Q7.1|SCP29_ARATH RecName: Full=Serine carboxypeptidase-like 29; Flags: Precursor gi|15293049|gb|AAK93635.1| putative serine carboxypeptidase II [Arabidopsis thaliana] gi|22136958|gb|AAM91708.1| putative serine carboxypeptidase II [Arabidopsis thaliana] gi|332660411|gb|AEE85811.1| serine carboxypeptidase-like 29 [Arabidopsis thaliana]	472	479	0	101.5	85.6	92.2	carboxypeptidase D	gbpln	Arabidopsis thaliana	AT4G30810.1 | Symbols: scpl29 | serine carboxypeptidase-like 29 | chr4:15003474-15006017 FORWARD LENGTH=479	472	479	0	101.5	85.6	92.2
Rsa1.0_00027.1.g1732.t1	gb|EOA23552.1| hypothetical protein CARUB_v10016746mg [Capsella rubella]	502	722	2.00E-98	143.8	49.0	61.2	hypothetical protein CARUB_v10016746mg	gbpln	Capsella rubella	AT5G11250.1 | Symbols: | Disease resistance protein (TIR-NBS-LRR class) | chr5:3587978-3591960 REVERSE LENGTH=1189	502	1189	1.00E-97	236.9	46.2	60.6
Rsa1.0_00027.1.g1733.t1	gb|AAD32898.1| putative retroelement pol polyprotein [Arabidopsis thaliana]	1382	1307	0	94.6	58.1	72.8	putative retroelement pol polyprotein	gbpln	Arabidopsis thaliana	AT4G23160.1 | Symbols: CRK8 | cysteine-rich RLK (RECEPTOR-like protein kinase) 8 | chr4:12129485-12134086 FORWARD LENGTH=1262	1382	1262	1.00E-103	91.3	14.5	22.5
Rsa1.0_00027.1.g1734.t1	ref|XP_002870849.1| predicted protein [Arabidopsis lyrata subsp. lyrata] gi|297316685|gb|EFH47108.1| predicted protein [Arabidopsis lyrata subsp. lyrata]	394	1225	1.00E-143	310.9	61.9	74.4	predicted protein	gbpln	Arabidopsis lyrata	AT5G11250.1 | Symbols: | Disease resistance protein (TIR-NBS-LRR class) | chr5:3587978-3591960 REVERSE LENGTH=1189	394	1189	1.00E-140	301.8	57.9	72.1
Rsa1.0_00027.1.g1735.t1	#	#	#	#	#	#	#	-	----	----	#	#	#	#	#	#	#

Rsa1.0_00027.1.g1736.t1	ref|XP_002869360.1| hypothetical protein ARALYDRAFT_491672 [Arabidopsis lyrata subsp. lyrata] gi|297315196|gb|EFH45619.1| hypothetical protein ARALYDRAFT_491672 [Arabidopsis lyrata subsp. lyrata]	183	182	3.00E-73	99.5	74.9	80.9	hypothetical protein ARALYDRAFT_491672	gbpln	Arabidopsis lyrata	AT4G30820.3 | Symbols: | cyclin-dependent kinase-activating kinase assembly factor-related / CDK-activating kinase assembly factor-related | chr4:15007144-15008378 FORWARD LENGTH=178	183	178	6.00E-67	97.3	71.6	79.2
Rsa1.0_00027.1.g1737.t1	ref|XP_002869358.1| SET domain-containing protein [Arabidopsis lyrata subsp. lyrata] gi|297315194|gb|EFH45617.1| SET domain-containing protein [Arabidopsis lyrata subsp. lyrata]	503	497	0	98.8	84.5	89.7	SET domain-containing protein	gbpln	Arabidopsis lyrata	AT4G30860.1 | Symbols: ASHR3, SDG4 | SET domain group 4 | chr4:15024546-15027427 FORWARD LENGTH=497	503	497	0	98.8	82.9	88.7
Rsa1.0_00027.1.g1738.t1	gb|EOA17813.1| hypothetical protein CARUB_v10006211mg [Capsella rubella]	110	109	8.00E-46	99.1	82.7	90.9	hypothetical protein CARUB_v10006211mg	gbpln	Capsella rubella	AT4G30880.1 | Symbols: | Bifunctional inhibitor/lipid-transfer protein/seed storage 2S albumin superfamily protein | chr4:15035229-15035750 FORWARD LENGTH=109	110	109	3.00E-47	99.1	82.7	87.3
Rsa1.0_00027.1.g1739.t3	gb|EOA16186.1| hypothetical protein CARUB_v10004326mg [Capsella rubella]	569	551	0	96.8	79.1	84.5	hypothetical protein CARUB_v10004326mg	gbpln	Capsella rubella	AT4G30890.3 | Symbols: UBP24 | ubiquitin-specific protease 24 | chr4:15036383-15038825 REVERSE LENGTH=551	569	551	0	96.8	76.4	82.2
Rsa1.0_00027.1.g1740.t1	ref|XP_002867320.1| hypothetical protein ARALYDRAFT_328625 [Arabidopsis lyrata subsp. lyrata] gi|297313156|gb|EFH43579.1| hypothetical protein ARALYDRAFT_328625 [Arabidopsis lyrata subsp. lyrata]	732	770	0	105.2	70.4	76.6	hypothetical protein ARALYDRAFT_328625	gbpln	Arabidopsis lyrata	AT4G30935.1 | Symbols: WRKY32, ATWRKY32 | WRKY DNA-binding protein 32 | chr4:15051916-15053908 REVERSE LENGTH=466	732	466	1.00E-159	63.7	42.8	46.9
Rsa1.0_00027.1.g1741.t1	ref|NP_178356.1| RNA-directed DNA polymerase (reverse transcriptase)-related family protein [Arabidopsis thaliana] gi|3184287|gb|AAC18934.1| putative non-LTR retroelement reverse transcriptase [Arabidopsis thaliana] gi|330250497|gb|AEC05591.1| RNA-directed DNA polymerase (reverse transcriptase)-related family protein [Arabidopsis thaliana]	195	211	1.00E-41	108.2	40.0	57.9	RNA-directed DNA polymerase (reverse transcriptase)-related family protein	gbpln	Arabidopsis thaliana	AT2G02520.1 | Symbols: | RNA-directed DNA polymerase (reverse transcriptase)-related family protein | chr2:676771-678145 REVERSE LENGTH=211	195	211	4.00E-44	108.2	40.0	57.9
Rsa1.0_00027.1.g1742.t1	sp|P48627.1|FAD6C_BRANA RecName: Full=Omega-6 fatty acid desaturase, chloroplastic; Flags: Precursor gi|457631|gb|AAA50157.1| omega-6 desaturase [Brassica napus]	446	443	0	99.3	95.7	97.8	RecName: Full=Omega-6 fatty acid desaturase, chloroplastic; Flags: Precursor gi|457631|gb|AAA50157.1| omega-6 desaturase	gbpln	Brassica napus	AT4G30950.1 | Symbols: FAD6, FADC, SFD4 | fatty acid desaturase 6 | chr4:15057278-15059673 REVERSE LENGTH=448	446	448	0	100.4	91.7	96.4
Rsa1.0_00027.1.g1743.t1	ref|XP_002864803.1| predicted protein [Arabidopsis lyrata subsp. lyrata] gi|297310638|gb|EFH41062.1| predicted protein [Arabidopsis lyrata subsp. lyrata]	158	607	2.00E-12	384.2	31.6	35.4	predicted protein	gbpln	Arabidopsis lyrata	AT5G62580.1 | Symbols: | ARM repeat superfamily protein | chr5:25120010-25122259 FORWARD LENGTH=615	158	615	7.00E-15	389.2	29.7	32.3
Rsa1.0_00027.1.g1744.t1	#	#	#	#	#	#	#	-	----	----	#	#	#	#	#	#	#
Rsa1.0_00027.1.g1745.t1	#	#	#	#	#	#	#	-	----	----	#	#	#	#	#	#	#
Rsa1.0_00027.1.g1746.t1	gb|AAG52094.1|AC012680_5 putative Mutator-like transposase; 12516-14947 [Arabidopsis thaliana]	771	761	0	98.7	49.8	63.6	putative Mutator-like transposase; 12516-14947	gbpln	Arabidopsis thaliana	AT3G30370.1 | Symbols: | CONTAINS InterPro DOMAIN/s: Transposase, MuDR, plant (InterPro:IPR004332); BEST Arabidopsis thaliana protein match is: SWAP (Suppressor-of-White-APricot)/surp RNA-binding domain-containing protein (TAIR:AT1G18050.1); Has 35 Blast hits to 35 proteins in 4 species: Archae - 0; Bacteria - 0; Metazoa - 0; Fungi - 0; Plants - 35; Viruses - 0; Other Eukaryotes - 0 (source: NCBI BLink). | chr3:11965180-11965719 REVERSE LENGTH=179	771	179	5.00E-20	23.2	7.1	10.0
Rsa1.0_00027.1.g1747.t1	gb|EOA18341.1| hypothetical protein CARUB_v10006862mg [Capsella rubella]	424	448	0	105.7	92.0	96.2	hypothetical protein CARUB_v10006862mg	gbpln	Capsella rubella	AT4G30960.1 | Symbols: CIPK6, SIP3, SNRK3.14, ATCIPK6 | SOS3-interacting protein 3 | chr4:15067400-15068725 FORWARD LENGTH=441	424	441	0	104.0	92.0	96.2
Rsa1.0_00027.1.g1748.t2	ref|XP_002884524.1| protein binding protein [Arabidopsis lyrata subsp. lyrata] gi|297330364|gb|EFH60783.1| protein binding protein [Arabidopsis lyrata subsp. lyrata]	573	442	1.00E-170	77.1	56.4	60.4	protein binding protein	gbpln	Arabidopsis lyrata	AT3G05675.1 | Symbols: | BTB/POZ domain-containing protein | chr3:1658425-1659859 REVERSE LENGTH=441	573	441	1.00E-169	77.0	54.3	59.3
Rsa1.0_00027.1.g1749.t2	gb|ACP30603.1| disease resistance protein [Brassica rapa subsp. pekinensis]	1111	1056	0	95.0	68.0	75.4	disease resistance protein	gbpln	Brassica rapa	AT5G18370.1 | Symbols: | Disease resistance protein (TIR-NBS-LRR class) family | chr5:6085036-6088926 REVERSE LENGTH=1210	1111	1210	0	108.9	48.7	61.7
Rsa1.0_00027.1.g1750.t1	#	#	#	#	#	#	#	-	----	----	#	#	#	#	#	#	#

Rsa1.0_00027.1.g1751.t3	gb|ACP30603.1| disease resistance protein [Brassica rapa subsp. pekinensis]	938	1056	0	112.6	66.2	74.3	disease resistance protein	gbpln	Brassica rapa	AT5G44510.1 | Symbols: TAO1 | target of AVRB operation1 | chr5:17929673-17934188 REVERSE LENGTH=1187	938	1187	1.00E-132	126.5	36.6	49.5
Rsa1.0_00027.1.g1752.t1	ref|XP_002882430.1| hypothetical protein ARALYDRAFT_477858 [Arabidopsis lyrata subsp. lyrata] gi|297328270|gb|EFH58689.1| hypothetical protein ARALYDRAFT_477858 [Arabidopsis lyrata subsp. lyrata]	860	869	0	101.0	77.4	84.2	hypothetical protein ARALYDRAFT_477858	gbpln	Arabidopsis lyrata	AT3G05670.1 | Symbols: | RING/U-box protein | chr3:1653888-1657022 FORWARD LENGTH=883	860	883	0	102.7	77.8	85.2
Rsa1.0_00027.1.g1753.t1	ref|NP_683531.2| tetratricopeptide repeat-containing protein [Arabidopsis thaliana] gi|27311659|gb|AAO00795.1| Unknown protein [Arabidopsis thaliana] gi|30023732|gb|AAP13399.1| At3g05625 [Arabidopsis thaliana] gi|332640747|gb|AEE74268.1| tetratricopeptide repeat-containing protein [Arabidopsis thaliana]	259	257	1.00E-135	99.2	88.4	94.6	tetratricopeptide repeat-containing protein	gbpln	Arabidopsis thaliana	AT3G05625.1 | Symbols: | Tetratricopeptide repeat (TPR)-like superfamily protein | chr3:1632408-1633912 FORWARD LENGTH=257	259	257	1.00E-137	99.2	88.4	94.6
Rsa1.0_00027.1.g1754.t1	gb|EOA32651.1| hypothetical protein CARUB_v10015948mg [Capsella rubella]	772	702	0	90.9	71.0	78.0	hypothetical protein CARUB_v10015948mg	gbpln	Capsella rubella	AT3G05610.1 | Symbols: | Plant invertase/pectin methylesterase inhibitor superfamily | chr3:1625876-1627976 REVERSE LENGTH=669	772	669	0	86.7	68.8	76.2
Rsa1.0_00027.1.g1755.t1	ref|XP_002882427.1| hypothetical protein ARALYDRAFT_477850 [Arabidopsis lyrata subsp. lyrata] gi|297328267|gb|EFH58686.1| hypothetical protein ARALYDRAFT_477850 [Arabidopsis lyrata subsp. lyrata]	187	187	1.00E-100	100.0	97.3	98.9	hypothetical protein ARALYDRAFT_477850	gbpln	Arabidopsis lyrata	AT3G05590.1 | Symbols: RPL18 | ribosomal protein L18 | chr3:1621511-1622775 FORWARD LENGTH=187	187	187	1.00E-102	100.0	97.3	98.4
Rsa1.0_00027.1.g1756.t1	gb|EOA31060.1| hypothetical protein CARUB_v10014212mg [Capsella rubella]	203	318	1.00E-109	156.7	94.1	98.5	hypothetical protein CARUB_v10014212mg	gbpln	Capsella rubella	AT3G05580.1 | Symbols: | Calcineurin-like metallo-phosphoesterase superfamily protein | chr3:1618216-1619850 REVERSE LENGTH=318	203	318	1.00E-112	156.7	93.6	98.5
Rsa1.0_00028.1.g1757.t1	gb|EOA12688.1| hypothetical protein CARUB_v10027918mg [Capsella rubella]	267	515	2.00E-97	192.9	71.9	79.0	hypothetical protein CARUB_v10027918mg	gbpln	Capsella rubella	AT5G48830.1 | Symbols: | unknown protein; FUNCTIONS IN: molecular_function unknown; INVOLVED IN: biological_process unknown; LOCATED IN: chloroplast; EXPRESSED IN: 22 plant structures; EXPRESSED DURING: 13 growth stages; Has 30201 Blast hits to 17322 proteins in 780 species: Archae - 12; Bacteria - 1396; Metazoa - 17338; Fungi - 3422; Plants - 5037; Viruses - 0; Other Eukaryotes - 2996 (source: NCBI BLink). | chr5:19800269-19802625 REVERSE LENGTH=511	267	511	4.00E-78	191.4	66.3	76.4
Rsa1.0_00028.1.g1758.t1	gb|EOA12563.1| hypothetical protein CARUB_v10026830mg [Capsella rubella]	117	308	1.00E-24	263.2	47.0	52.1	hypothetical protein CARUB_v10026830mg	gbpln	Capsella rubella	AT5G48850.1 | Symbols: ATSDI1 | Tetratricopeptide repeat (TPR)-like superfamily protein | chr5:19805576-19807699 REVERSE LENGTH=306	117	306	3.00E-21	261.5	37.6	41.9
Rsa1.0_00028.1.g1759.t1	gb|EOA12563.1| hypothetical protein CARUB_v10026830mg [Capsella rubella]	382	308	1.00E-145	80.6	64.1	68.1	hypothetical protein CARUB_v10026830mg	gbpln	Capsella rubella	AT5G48850.1 | Symbols: ATSDI1 | Tetratricopeptide repeat (TPR)-like superfamily protein | chr5:19805576-19807699 REVERSE LENGTH=306	382	306	2.33E-156	80.1	66.8	69.6
Rsa1.0_00028.1.g1760.t1	#	#	#	#	#	#	#	-	----	----	#	#	#	#	#	#	#
Rsa1.0_00028.1.g1761.t1	ref|NP_199698.1| U6 snRNA-associated Sm-like protein LSm5 [Arabidopsis thaliana] gi|9758886|dbj|BAB09440.1| U6 snRNA-associated Sm-like protein-like [Arabidopsis thaliana] gi|16554971|gb|AAK61592.1| Sm-like protein [Arabidopsis thaliana] gi|106879147|gb|ABF82603.1| At5g48870 [Arabidopsis thaliana] gi|332008352|gb|AED95735.1| U6 snRNA-associated Sm-like protein LSm5 [Arabidopsis thaliana] gi|386305005|gb|AFJ05005.1| hypothetical protein [Arabidopsis thaliana]	141	88	1.00E-31	62.4	56.7	58.9	U6 snRNA-associated Sm-like protein LSm5	gbpln	Arabidopsis thaliana	AT5G48870.1 | Symbols: SAD1 | Small nuclear ribonucleoprotein family protein | chr5:19813407-19814362 FORWARD LENGTH=88	141	88	4.00E-34	62.4	56.7	58.9
Rsa1.0_00028.1.g1762.t1	ref|XP_002865685.1| KAT5/PKT1/PKT2 peroxisomal 3-keto-acyl-CoA thiolase [Arabidopsis lyrata subsp. lyrata] gi|297311520|gb|EFH41944.1| KAT5/PKT1/PKT2 peroxisomal 3-keto-acyl-CoA thiolase [Arabidopsis lyrata subsp. lyrata]	461	457	0	99.1	89.2	94.1	KAT5/PKT1/PKT2 peroxisomal 3-keto-acyl-CoA thiolase	gbpln	Arabidopsis lyrata	AT5G48880.3 | Symbols: | peroxisomal 3-keto-acyl-CoA thiolase 2 | chr5:19814576-19817021 REVERSE LENGTH=457	461	457	0	99.1	89.2	93.9

Rsa1.0_00028.1.g1763.t1	ref|NP_199700.1| C2H2-like zinc finger protein [Arabidopsis thaliana] gi|9758888|dbj|BAB09442.1| unnamed protein product [Arabidopsis thaliana] gi|332008356|gb|AED95739.1| C2H2-like zinc finger protein [Arabidopsis thaliana]	167	173	1.00E-54	103.6	72.5	79.6	C2H2-like zinc finger protein	gbpln	Arabidopsis thaliana	AT5G48890.1 | Symbols: | C2H2-like zinc finger protein | chr5:19820353-19820874 FORWARD LENGTH=173	167	173	6.00E-57	103.6	72.5	79.6
Rsa1.0_00028.1.g1764.t1	gb|EOA13438.1| hypothetical protein CARUB_v10026482mg [Capsella rubella]	417	417	0	100.0	93.5	95.4	hypothetical protein CARUB_v10026482mg	gbpln	Capsella rubella	AT5G48900.1 | Symbols: | Pectin lyase-like superfamily protein | chr5:19825240-19828909 FORWARD LENGTH=417	417	417	0	100.0	94.7	97.4
Rsa1.0_00028.1.g1765.t1	ref|XP_002876919.1| hypothetical protein ARALYDRAFT_904719 [Arabidopsis lyrata subsp. lyrata] gi|297322757|gb|EFH53178.1| hypothetical protein ARALYDRAFT_904719 [Arabidopsis lyrata subsp. lyrata]	328	330	2.00E-63	100.6	44.2	55.5	hypothetical protein ARALYDRAFT_904719	gbpln	Arabidopsis lyrata	AT3G19520.2 | Symbols: | Protein of unknown function (DUF626) | chr3:6772773-6774209 FORWARD LENGTH=312	328	312	3.00E-50	95.1	35.7	51.8
Rsa1.0_00028.1.g1766.t1	#	#	#	#	#	#	#	-	----	----	#	#	#	#	#	#	#
Rsa1.0_00028.1.g1767.t3	emb|CAA54075.1| phytochrome E [Arabidopsis thaliana] gi|5816999|emb|CAB53654.1| phytochrome E [Arabidopsis thaliana] gi|7268606|emb|CAB78815.1| phytochrome E [Arabidopsis thaliana]	262	1112	6.00E-33	424.4	32.4	34.4	phytochrome E	gbpln	Arabidopsis thaliana	AT4G18130.1 | Symbols: PHYE | phytochrome E | chr4:10042312-10045948 REVERSE LENGTH=1112	262	1112	2.00E-35	424.4	32.4	34.4
Rsa1.0_00028.1.g1768.t1	gb|AAL32602.1| Unknown protein [Arabidopsis thaliana]	607	642	0	105.8	94.2	96.5	Unknown protein	gbpln	Arabidopsis thaliana	AT5G48960.1 | Symbols: | HAD-superfamily hydrolase, subfamily IG, 5'-nucleotidase | chr5:19849645-19853382 FORWARD LENGTH=642	607	642	0	105.8	94.2	96.5
Rsa1.0_00028.1.g1769.t1	ref|XP_002865696.1| hypothetical protein ARALYDRAFT_917838 [Arabidopsis lyrata subsp. lyrata] gi|297311531|gb|EFH41955.1| hypothetical protein ARALYDRAFT_917838 [Arabidopsis lyrata subsp. lyrata]	107	107	9.00E-50	100.0	86.0	95.3	hypothetical protein ARALYDRAFT_917838	gbpln	Arabidopsis lyrata	AT5G24860.1 | Symbols: FPF1, ATFPF1 | flowering promoting factor 1 | chr5:8541822-8542154 FORWARD LENGTH=110	107	110	1.00E-31	102.8	58.9	77.6
Rsa1.0_00028.1.g1770.t1	ref|NP_199713.2| histone-arginine methyltransferase CARM1 [Arabidopsis thaliana] gi|152013379|sp|A3KPF2.1|ANM14_ARATH RecName: Full=Probable histone-arginine methyltransferase 1.4; Short=AtPRMT14; AltName: Full=Coactivator-associated methyltransferase 1B; AltName: Full=Protein arginine N-methyltransferase 4A; Short=AtPRMT4A gi|126352276|gb|ABO09883.1| At5g49020 [Arabidopsis thaliana] gi|126352282|gb|ABO09886.1| At5g49020 [Arabidopsis thaliana] gi|332008378|gb|AED95761.1| protein arginine methyltransferase 4A [Arabidopsis thaliana]	526	528	0	100.4	89.0	92.0	histone-arginine methyltransferase CARM1	gbpln	Arabidopsis thaliana	AT5G49020.1 | Symbols: ATPRMT4A, PRMT4A | protein arginine methyltransferase 4A | chr5:19871341-19874683 FORWARD LENGTH=528	526	528	0	100.4	89.0	92.0
Rsa1.0_00028.1.g1771.t1	ref|NP_001190497.1| isoleucyl-tRNA synthetase [Arabidopsis thaliana] gi|332008381|gb|AED95764.1| isoleucyl-tRNA synthetase [Arabidopsis thaliana]	1048	1279	0	122.0	93.4	97.2	isoleucyl-tRNA synthetase	gbpln	Arabidopsis thaliana	AT5G49030.3 | Symbols: OVA2 | tRNA synthetase class I (I, L, M and V) family protein | chr5:19875091-19883251 REVERSE LENGTH=1279	1048	1279	0	122.0	93.4	97.2
Rsa1.0_00028.1.g1772.t1	ref|XP_002863949.1| hypothetical protein ARALYDRAFT_917855 [Arabidopsis lyrata subsp. lyrata] gi|297309784|gb|EFH40208.1| hypothetical protein ARALYDRAFT_917855 [Arabidopsis lyrata subsp. lyrata]	167	167	3.00E-79	100.0	85.6	92.8	hypothetical protein ARALYDRAFT_917855	gbpln	Arabidopsis lyrata	AT5G49050.1 | Symbols: | FUNCTIONS IN: molecular_function unknown; INVOLVED IN: response to stress; LOCATED IN: cellular_component unknown; CONTAINS InterPro DOMAIN/s: UspA (InterPro:IPR006016), Rossmann-like alpha/beta/alpha sandwich fold (InterPro:IPR014729); BEST Arabidopsis thaliana protein match is: Adenine nucleotide alpha hydrolases-like superfamily protein (TAIR:AT2G47710.1); Has 1807 Blast hits to 1807 proteins in 277 species: Archae - 0; Bacteria - 0; Metazoa - 736; Fungi - 347; Plants - 385; Viruses - 0; Other Eukaryotes - 339 (source: NCBI BLink). | chr5:19883929-19884381 FORWARD LENGTH=150	167	150	1.00E-54	89.8	58.7	65.9
Rsa1.0_00028.1.g1773.t1	gb|EOA14459.1| hypothetical protein CARUB_v10027668mg [Capsella rubella]	404	465	0	115.1	84.9	93.3	hypothetical protein CARUB_v10027668mg	gbpln	Capsella rubella	AT5G49070.1 | Symbols: KCS21 | 3-ketoacyl-CoA synthase 21 | chr5:19888751-19890145 REVERSE LENGTH=464	404	464	0	114.9	83.9	93.3

Rsa1.0_00028.1.g1774.t1	ref|XP_002865709.1| hypothetical protein ARALYDRAFT_917876 [Arabidopsis lyrata subsp. lyrata] gi|297311544|gb|EFH41968.1| hypothetical protein ARALYDRAFT_917876 [Arabidopsis lyrata subsp. lyrata]	372	384	1.00E-109	103.2	71.0	78.2	hypothetical protein ARALYDRAFT_917876	gbpln	Arabidopsis lyrata	AT5G49100.1 | Symbols: | unknown protein; BEST Arabidopsis thaliana protein match is: unknown protein (TAIR:AT3G06868.1); Has 30201 Blast hits to 17322 proteins in 780 species: Archae - 12; Bacteria - 1396; Metazoa - 17338; Fungi - 3422; Plants - 5037; Viruses - 0; Other Eukaryotes - 2996 (source: NCBI BLink). | chr5:19897700-19898890 REVERSE LENGTH=396	372	396	1.00E-107	106.5	72.6	81.2
Rsa1.0_00028.1.g1775.t2	ref|XP_002863960.1| hypothetical protein ARALYDRAFT_917877 [Arabidopsis lyrata subsp. lyrata] gi|297309795|gb|EFH40219.1| hypothetical protein ARALYDRAFT_917877 [Arabidopsis lyrata subsp. lyrata]	1444	1450	0	100.4	83.2	90.1	hypothetical protein ARALYDRAFT_917877	gbpln	Arabidopsis lyrata	AT5G49110.1 | Symbols: | unknown protein; INVOLVED IN: biological_process unknown; LOCATED IN: cellular_component unknown; EXPRESSED IN: cultured cell; Has 30201 Blast hits to 17322 proteins in 780 species: Archae - 12; Bacteria - 1396; Metazoa - 17338; Fungi - 3422; Plants - 5037; Viruses - 0; Other Eukaryotes - 2996 (source: NCBI BLink). | chr5:19900203-19905925 FORWARD LENGTH=1469	1444	1469	0	101.7	82.7	90.1
Rsa1.0_00028.1.g1776.t1	ref|XP_002863963.1| hypothetical protein ARALYDRAFT_917882 [Arabidopsis lyrata subsp. lyrata] gi|297309798|gb|EFH40222.1| hypothetical protein ARALYDRAFT_917882 [Arabidopsis lyrata subsp. lyrata]	1086	921	0	84.8	70.2	76.2	hypothetical protein ARALYDRAFT_917882	gbpln	Arabidopsis lyrata	AT5G49150.1 | Symbols: ATGEX2, GEX2 | gamete expressed 2 | chr5:19924760-19928527 FORWARD LENGTH=914	1086	914	0	84.2	69.0	75.2
Rsa1.0_00028.1.g1777.t1	ref|NP_199728.1| uncharacterized protein [Arabidopsis thaliana] gi|110737324|dbj|BAF00608.1| hypothetical protein [Arabidopsis thaliana] gi|332008395|gb|AED95778.1| uncharacterized protein AT5G49170 [Arabidopsis thaliana]	175	185	1.00E-53	105.7	69.1	75.4	uncharacterized protein	gbpln	Arabidopsis thaliana	AT5G49170.1 | Symbols: | unknown protein; BEST Arabidopsis thaliana protein match is: unknown protein (TAIR:AT3G06840.1); Has 1807 Blast hits to 1807 proteins in 277 species: Archae - 0; Bacteria - 0; Metazoa - 736; Fungi - 347; Plants - 385; Viruses - 0; Other Eukaryotes - 339 (source: NCBI BLink). | chr5:19938839-19939482 REVERSE LENGTH=185	175	185	6.00E-56	105.7	69.1	75.4
Rsa1.0_00028.1.g1778.t1	gb|ABB97028.1| unknown [Brassica rapa]	198	193	5.00E-89	97.5	82.8	91.4	unknown	gbpln	Brassica rapa	AT5G49210.2 | Symbols: | unknown protein; Has 30201 Blast hits to 17322 proteins in 780 species: Archae - 12; Bacteria - 1396; Metazoa - 17338; Fungi - 3422; Plants - 5037; Viruses - 0; Other Eukaryotes - 2996 (source: NCBI BLink). | chr5:19950093-19951464 FORWARD LENGTH=195	198	195	1.00E-76	98.5	72.2	85.9
Rsa1.0_00028.1.g1779.t1	ref|NP_680409.1| glycoside hydrolase family 28 protein / polygalacturonase (pectinase) family protein [Arabidopsis thaliana] gi|20258821|gb|AAM14020.1| unknown protein [Arabidopsis thaliana] gi|22136814|gb|AAM91751.1| unknown protein [Arabidopsis thaliana] gi|332008401|gb|AED95784.1| glycoside hydrolase family 28 protein / polygalacturonase (pectinase) family protein [Arabidopsis thaliana]	444	449	0	101.1	83.1	89.2	glycoside hydrolase family 28 protein / polygalacturonase (pectinase) family protein	gbpln	Arabidopsis thaliana	AT5G49215.1 | Symbols: | Pectin lyase-like superfamily protein | chr5:19953670-19955874 FORWARD LENGTH=449	444	449	0	101.1	83.1	89.2
Rsa1.0_00028.1.g1780.t1	ref|XP_002863975.1| hypothetical protein ARALYDRAFT_494985 [Arabidopsis lyrata subsp. lyrata] gi|297309810|gb|EFH40234.1| hypothetical protein ARALYDRAFT_494985 [Arabidopsis lyrata subsp. lyrata]	409	418	2.33E-156	102.2	75.1	81.4	hypothetical protein ARALYDRAFT_494985	gbpln	Arabidopsis lyrata	AT5G49220.1 | Symbols: | Protein of unknown function (DUF789) | chr5:19956627-19958453 FORWARD LENGTH=409	409	409	1.00E-144	100.0	72.1	79.0
Rsa1.0_00028.1.g1781.t1	ref|XP_002863976.1| hypothetical protein ARALYDRAFT_357174 [Arabidopsis lyrata subsp. lyrata] gi|297309811|gb|EFH40235.1| hypothetical protein ARALYDRAFT_357174 [Arabidopsis lyrata subsp. lyrata]	496	280	4.00E-78	56.5	31.9	40.1	hypothetical protein ARALYDRAFT_357174	gbpln	Arabidopsis lyrata	AT5G49240.1 | Symbols: APRR4, PRR4 | pseudo-response regulator 4 | chr5:19962934-19964351 FORWARD LENGTH=292	496	292	2.00E-77	58.9	30.0	38.7
Rsa1.0_00028.1.g1782.t1	ref|XP_002865724.1| hypothetical protein ARALYDRAFT_494989 [Arabidopsis lyrata subsp. lyrata] gi|297311559|gb|EFH41983.1| hypothetical protein ARALYDRAFT_494989 [Arabidopsis lyrata subsp. lyrata]	167	161	2.00E-24	96.4	72.5	76.0	hypothetical protein ARALYDRAFT_494989	gbpln	Arabidopsis lyrata	AT5G49280.1 | Symbols: | hydroxyproline-rich glycoprotein family protein | chr5:19976842-19977330 REVERSE LENGTH=162	167	162	1.00E-12	97.0	18.6	19.8
Rsa1.0_00028.1.g1783.t1	#	#	#	#	#	#	#	-	----	----	#	#	#	#	#	#	#

Rsa1.0_00028.1.g1784.t1	ref|NP_199741.1| GATA transcription factor 16 [Arabidopsis thaliana] gi|71660826|sp|Q9FJ10.1|GAT16_ARATH RecName: Full=GATA transcription factor 16 gi|10177159|dbj|BAB10348.1| unnamed protein product [Arabidopsis thaliana] gi|117168139|gb|ABK32152.1| At5g49300 [Arabidopsis thaliana] gi|225879100|dbj|BAH30620.1| hypothetical protein [Arabidopsis thaliana] gi|332008411|gb|AED95794.1| GATA transcription factor 16 [Arabidopsis thaliana]	136	139	2.00E-46	102.2	77.9	83.8	GATA transcription factor 16	gbpln	Arabidopsis thaliana	AT5G49300.1 | Symbols: GATA16 | GATA transcription factor 16 | chr5:19984849-19985475 REVERSE LENGTH=139	136	139	4.00E-49	102.2	77.9	83.8
Rsa1.0_00028.1.g1785.t1	ref|XP_002882488.1| AT-IMP [Arabidopsis lyrata subsp. lyrata] gi|297328328|gb|EFH58747.1| AT-IMP [Arabidopsis lyrata subsp. lyrata]	533	532	0	99.8	70.0	80.9	AT-IMP	gbpln	Arabidopsis lyrata	AT3G06720.2 | Symbols: AT-IMP, ATKAP ALPHA, AIMP ALPHA | importin alpha isoform 1 | chr3:2120559-2123555 FORWARD LENGTH=532	533	532	0	99.8	69.4	80.9
Rsa1.0_00028.1.g1786.t1	ref|XP_002865728.1| hypothetical protein ARALYDRAFT_917912 [Arabidopsis lyrata subsp. lyrata] gi|297311563|gb|EFH41987.1| hypothetical protein ARALYDRAFT_917912 [Arabidopsis lyrata subsp. lyrata]	257	257	1.00E-136	100.0	93.0	96.1	hypothetical protein ARALYDRAFT_917912	gbpln	Arabidopsis lyrata	AT5G49320.1 | Symbols: | Protein of unknown function (DUF1218) | chr5:19995083-19996214 REVERSE LENGTH=257	257	257	1.00E-137	100.0	92.6	95.3
Rsa1.0_00028.1.g1787.t1	ref|XP_002865730.1| hypothetical protein ARALYDRAFT_331346 [Arabidopsis lyrata subsp. lyrata] gi|297311565|gb|EFH41989.1| hypothetical protein ARALYDRAFT_331346 [Arabidopsis lyrata subsp. lyrata]	444	459	0	103.4	85.8	91.0	hypothetical protein ARALYDRAFT_331346	gbpln	Arabidopsis lyrata	AT5G49340.1 | Symbols: TBL4 | TRICHOME BIREFRINGENCE-LIKE 4 | chr5:20007348-20009038 REVERSE LENGTH=457	444	457	0	102.9	84.5	91.7
Rsa1.0_00028.1.g1788.t1	#	#	#	#	#	#	#	-	----	----	#	#	#	#	#	#	#
Rsa1.0_00028.1.g1789.t1	ref|NP_199747.1| beta-xylosidase 1 [Arabidopsis thaliana] gi|75262458|sp|Q9FGY1.1|BXL1_ARATH RecName: Full=Beta-D-xylosidase 1; Short=AtBXL1; AltName: Full=Alpha-L-arabinofuranosidase; Flags: Precursor gi|9759419|dbj|BAB09906.1| xylosidase [Arabidopsis thaliana] gi|21539545|gb|AAM53325.1| xylosidase [Arabidopsis thaliana] gi|332008419|gb|AED95802.1| beta-xylosidase 1 [Arabidopsis thaliana]	772	774	0	100.3	92.7	96.9	beta-xylosidase 1	gbpln	Arabidopsis thaliana	AT5G49360.1 | Symbols: BXL1, ATBXL1 | beta-xylosidase 1 | chr5:20012179-20016659 REVERSE LENGTH=774	772	774	0	100.3	92.7	96.9
Rsa1.0_00028.1.g1790.t1	ref|XP_002884605.1| binding protein [Arabidopsis lyrata subsp. lyrata] gi|297330445|gb|EFH60864.1| binding protein [Arabidopsis lyrata subsp. lyrata]	750	864	0	115.2	67.6	79.7	binding protein	gbpln	Arabidopsis lyrata	AT3G06670.1 | Symbols: | binding | chr3:2105908-2113136 REVERSE LENGTH=865	750	865	0	115.3	68.1	79.9
Rsa1.0_00028.1.g1791.t1	ref|XP_002314930.1| predicted protein [Populus trichocarpa] gi|222863970|gb|EEF01101.1| predicted protein [Populus trichocarpa]	277	258	9.00E-60	93.1	49.8	56.0	predicted protein	gbpln	Populus trichocarpa	AT5G49400.1 | Symbols: | zinc knuckle (CCHC-type) family protein | chr5:20031154-20031981 FORWARD LENGTH=275	277	275	1.00E-54	99.3	42.6	45.8
Rsa1.0_00028.1.g1792.t1	ref|XP_002863981.1| hypothetical protein ARALYDRAFT_357185 [Arabidopsis lyrata subsp. lyrata] gi|297309816|gb|EFH40240.1| hypothetical protein ARALYDRAFT_357185 [Arabidopsis lyrata subsp. lyrata]	142	143	2.00E-65	100.7	88.0	91.5	hypothetical protein ARALYDRAFT_357185	gbpln	Arabidopsis lyrata	AT5G49410.2 | Symbols: | unknown protein; BEST Arabidopsis thaliana protein match is: unknown protein (TAIR:AT1G73940.1); Has 58 Blast hits to 58 proteins in 12 species: Archae - 0; Bacteria - 0; Metazoa - 0; Fungi - 0; Plants - 58; Viruses - 0; Other Eukaryotes - 0 (source: NCBI BLink). | chr5:20032901-20033751 FORWARD LENGTH=145	142	145	2.00E-65	102.1	88.0	92.3
Rsa1.0_00028.1.g1793.t1	gb|EOA39471.1| hypothetical protein CARUB_v10008066mg [Capsella rubella]	81	2170	1.00E-31	2679.0	85.2	88.9	hypothetical protein CARUB_v10008066mg	gbpln	Capsella rubella	AT1G20960.2 | Symbols: emb1507 | U5 small nuclear ribonucleoprotein helicase, putative | chr1:7302591-7309914 REVERSE LENGTH=2171	81	2171	3.00E-33	2680.2	82.7	86.4
Rsa1.0_00028.1.g1794.t1	ref|XP_002865733.1| transducin family protein [Arabidopsis lyrata subsp. lyrata] gi|297311568|gb|EFH41992.1| transducin family protein [Arabidopsis lyrata subsp. lyrata]	460	1662	1.00E-45	361.3	24.8	27.4	transducin family protein	gbpln	Arabidopsis lyrata	AT5G49430.1 | Symbols: | WD40/YVTN repeat-like-containing domain;Bromodomain | chr5:20037338-20045454 REVERSE LENGTH=1677	460	1677	8.00E-48	364.6	25.2	27.4
Rsa1.0_00028.1.g1795.t1	gb|EOA12770.1| hypothetical protein CARUB_v10028626mg [Capsella rubella]	504	608	0	120.6	86.5	90.1	hypothetical protein CARUB_v10028626mg	gbpln	Capsella rubella	AT5G49460.1 | Symbols: ACLB-2 | ATP citrate lyase subunit B 2 | chr5:20055048-20058195 FORWARD LENGTH=608	504	608	0	120.6	86.7	89.9
Rsa1.0_00028.1.g1796.t1	#	#	#	#	#	#	#	-	----	----	AT1G06900.1 | Symbols: | Insulinase (Peptidase family M16) family protein | chr1:2115155-2120635 REVERSE LENGTH=1024	236	1024	3.00E-12	433.9	27.1	45.8
Rsa1.0_00028.1.g1797.t2	#	#	#	#	#	#	#	-	----	----	#	#	#	#	#	#	#

Rsa1.0_00028.1.g1798.t1	#	#	#	#	#	#	#	–	----	----	AT1G10430.1 | Symbols: PP2A-2 | protein phosphatase 2A-2 | chr1:3428705-3430437 REVERSE LENGTH=306	141	306	8.00E-11	217.0	35.5	44.0
Rsa1.0_00028.1.g1799.t3	#	#	#	#	#	#	#	–	----	----	#	#	#	#	#	#	#
Rsa1.0_00028.1.g1800.t1	ref|NP_199763.1| putative WRKY transcription factor 48 [Arabidopsis thaliana] gi|20978785|sp|Q9FGZ4.1|WRK48_ARATH RecName: Full=Probable WRKY transcription factor 48; AltName: Full=WRKY DNA-binding protein 48 gi|17064166|gb|AAL35290.1|AF442397_1 WRKY transcription factor 48 [Arabidopsis thaliana] gi|10177618|dbj|BAB10765.1| unnamed protein product [Arabidopsis thaliana] gi|17380792|gb|AAL36226.1| unknown protein [Arabidopsis thaliana] gi|20259613|gb|AAM14163.1| unknown protein [Arabidopsis thaliana] gi|332008441|gb|AED95824.1| putative WRKY transcription factor 48 [Arabidopsis thaliana]	390	399	1.00E-125	102.3	77.4	85.9	putative WRKY transcription factor 48	gbpln	Arabidopsis thaliana	AT5G49520.1 | Symbols: WRKY48, ATWRKY48 | WRKY DNA-binding protein 48 | chr5:20090890-20092867 FORWARD LENGTH=399	390	399	1.00E-128	102.3	77.4	85.9
Rsa1.0_00028.1.g1801.t2	gb|EOA12473.1| hypothetical protein CARUB_v10025904mg [Capsella rubella]	613	800	5.00E-92	130.5	32.0	37.7	hypothetical protein CARUB_v10025904mg	gbpln	Capsella rubella	AT5G59560.2 | Symbols: SRR1 | sensitivity to red light reduced protein (SRR1) | chr5:24000747-24001574 REVERSE LENGTH=275	613	275	6.00E-94	44.9	28.1	33.3
Rsa1.0_00028.1.g1802.t1	ref|XP_002864001.1| hypothetical protein ARALYDRAFT_917948 [Arabidopsis lyrata subsp. lyrata] gi|297309836|gb|EFH40260.1| hypothetical protein ARALYDRAFT_917948 [Arabidopsis lyrata subsp. lyrata]	172	172	1.00E-72	100.0	82.0	89.5	hypothetical protein ARALYDRAFT_917948	gbpln	Arabidopsis lyrata	AT5G49600.1 | Symbols: | Protein of unknown function, DUF538 | chr5:20130780-20131295 FORWARD LENGTH=171	172	171	4.00E-72	99.4	76.7	81.4
Rsa1.0_00028.1.g1803.t1	gb|AAM60856.1| unknown [Arabidopsis thaliana]	359	359	1.00E-172	100.0	83.3	89.1	unknown	gbpln	Arabidopsis thaliana	AT5G49610.1 | Symbols: | F-box family protein | chr5:20131448-20132527 REVERSE LENGTH=359	359	359	1.00E-174	100.0	83.3	89.1
Rsa1.0_00028.1.g1804.t1	#	#	#	#	#	#	#	–	----	----	#	#	#	#	#	#	#
Rsa1.0_00028.1.g1805.t1	ref|XP_002865745.1| AtMYB78 [Arabidopsis lyrata subsp. lyrata] gi|297311580|gb|EFH42004.1| AtMYB78 [Arabidopsis lyrata subsp. lyrata]	318	306	1.00E-119	96.2	74.2	84.9	AtMYB78	gbpln	Arabidopsis lyrata	AT5G49620.1 | Symbols: AtMYB78, MYB78 | myb domain protein 78 | chr5:20137491-20139384 REVERSE LENGTH=307	318	307	1.00E-118	96.5	71.1	83.0
Rsa1.0_00028.1.g1806.t1	#	#	#	#	#	#	#	–	----	----	#	#	#	#	#	#	#
Rsa1.0_00028.1.g1807.t1	ref|XP_002872333.1| predicted protein [Arabidopsis lyrata subsp. lyrata] gi|297318170|gb|EFH48592.1| predicted protein [Arabidopsis lyrata subsp. lyrata]	238	238	1.00E-129	100.0	91.2	96.6	predicted protein	gbpln	Arabidopsis lyrata	AT5G29560.1 | Symbols: | caleosin-related family protein | chr5:11190543-11191963 REVERSE LENGTH=220	238	220	1.00E-111	92.4	80.3	87.8
Rsa1.0_00028.1.g1808.t1	ref|NP_568512.3| myb family transcription factor [Arabidopsis thaliana] gi|75328846|sp|Q8GUN5.1|PHL1_ARATH RecName: Full=Protein PHR1-LIKE 1; AltName: Full=Myb-like transcription factor 1 gi|26983850|gb|AAN86177.1| unknown protein [Arabidopsis thaliana] gi|332006474|gb|AED93857.1| myb family transcription factor [Arabidopsis thaliana]	410	413	1.00E-159	100.7	77.1	84.1	myb family transcription factor	gbpln	Arabidopsis thaliana	AT5G29000.2 | Symbols: PHL1 | Homeodomain-like superfamily protein | chr5:11022336-11024229 REVERSE LENGTH=413	410	413	1.00E-161	100.7	77.1	84.1
Rsa1.0_00028.1.g1809.t1	ref|NP_568509.1| protein phosphatase 2 (formerly 2A), regulatory subunit B'' [Arabidopsis thaliana] gi|42740922|gb|AAS44557.1| protein phosphatase 2A beta [Arabidopsis thaliana] gi|332006463|gb|AED93846.1| protein phosphatase 2A B' beta [Arabidopsis thaliana]	458	536	0	117.0	77.5	86.5	protein phosphatase 2 (formerly 2A), regulatory subunit B''	gbpln	Arabidopsis thaliana	AT5G28850.2 | Symbols: | Calcium-binding EF-hand family protein | chr5:10877360-10881278 REVERSE LENGTH=536	458	536	0	117.0	77.5	86.5
Rsa1.0_00028.1.g1810.t7	ref|XP_002872330.1| calcium-binding EF hand family protein [Arabidopsis lyrata subsp. lyrata] gi|297318167|gb|EFH48589.1| calcium-binding EF hand family protein [Arabidopsis lyrata subsp. lyrata]	625	536	0	85.8	70.9	75.2	calcium-binding EF hand family protein	gbpln	Arabidopsis lyrata	AT5G28900.1 | Symbols: | Calcium-binding EF-hand family protein | chr5:10925852-10929892 FORWARD LENGTH=536	625	536	0	85.8	70.6	75.0
Rsa1.0_00028.1.g1811.t1	gb|AET14213.1| GDP-D-mannose-3,5-epimerase [Brassica rapa subsp. chinensis]	379	379	0	100.0	96.6	98.4	GDP-D-mannose-3,5-epimerase	gbpln	Brassica rapa	AT5G28840.2 | Symbols: GME | GDP-D-mannose 3',5'-epimerase | chr5:10862472-10864024 REVERSE LENGTH=377	379	377	0	99.5	96.3	97.6
Rsa1.0_00028.1.g1812.t1	dbj|BAJ34351.1| unnamed protein product [Thellungiella halophila]	354	319	1.00E-136	90.1	75.1	79.1	unnamed protein product	----	----	AT5G28770.2 | Symbols: BZO2H3 | bZIP transcription factor family protein | chr5:10796648-10798147 REVERSE LENGTH=314	354	314	1.00E-125	88.7	71.5	74.0

Rsa1.0_00028.1.g1813.t1	gb|EOA19859.1| hypothetical protein CARUB_v10000110mg, partial [Capsella rubella]	844	1050	0	124.4	71.4	74.4	hypothetical protein CARUB_v10000110mg, partial	gbpln	Capsella rubella	AT5G28740.1 | Symbols: | Tetratricopeptide repeat (TPR)-like superfamily protein | chr5:10780774-10783772 FORWARD LENGTH=917	844	917	0	108.6	71.4	74.6
Rsa1.0_00028.1.g1814.t1	gb|EOA22561.1| hypothetical protein CARUB_v10003221mg [Capsella rubella]	854	859	0	100.6	88.5	94.6	hypothetical protein CARUB_v10003221mg	gbpln	Capsella rubella	AT5G28680.1 | Symbols: ANX2 | Malectin/receptor-like protein kinase family protein | chr5:10719437-10722013 REVERSE LENGTH=858	854	858	0	100.5	87.2	94.1
Rsa1.0_00029.1.g1815.t2	ref|XP_002881778.1| predicted protein [Arabidopsis lyrata subsp. lyrata] gi|297327617|gb|EFH58037.1| predicted protein [Arabidopsis lyrata subsp. lyrata]	428	326	1.00E-166	76.2	65.4	69.6	predicted protein	gbpln	Arabidopsis lyrata	AT2G41480.1 | Symbols: | Peroxidase superfamily protein | chr2:17296986-17298729 REVERSE LENGTH=341	428	341	1.00E-167	79.7	65.4	69.4
Rsa1.0_00029.1.g1816.t1	ref|XP_002879932.1| apurinic endonuclease-redox protein [Arabidopsis lyrata subsp. lyrata] gi|297325771|gb|EFH56191.1| apurinic endonuclease-redox protein [Arabidopsis lyrata subsp. lyrata]	522	550	0	105.4	81.8	89.3	apurinic endonuclease-redox protein	gbpln	Arabidopsis lyrata	AT2G41460.1 | Symbols: ARP | apurinic endonuclease-redox protein | chr2:17285731-17288769 FORWARD LENGTH=536	522	536	0	102.7	80.1	87.9
Rsa1.0_00029.1.g1817.t1	gb|ADP37978.1| ERD15 protein [Brassica napus]	143	157	2.00E-61	109.8	87.4	94.4	ERD15 protein	gbpln	Brassica napus	AT2G41430.5 | Symbols: ERD15 | dehydration-induced protein (ERD15) | chr2:17269736-17270314 FORWARD LENGTH=163	143	163	1.00E-52	114.0	83.2	88.8
Rsa1.0_00029.1.g1818.t1	#	#	#	#	#	#	#	-	----	----	#	#	#	#	#	#	#
Rsa1.0_00029.1.g1819.t1	ref|XP_002492787.1| Protein involved in positive regulation of both 1,3-beta-glucan synthesis and the Pkc1p-MAPK pathway [Komagataella pastoris GS115] gi|238032585|emb|CAY70608.1| Protein involved in positive regulation of both 1,3-beta-glucan synthesis and the Pkc1p-MAPK pathway [Komagataella pastoris GS115]	77	1338	5.00E-11	1737.7	48.1	48.1	Protein involved in positive regulation of both 1,3-beta-glucan synthesis and the Pkc1p-MAPK pathway	gbpln	Komagataella pastoris	#	#	#	#	#	#	#
Rsa1.0_00029.1.g1820.t1	#	#	#	#	#	#	#	-	----	----	#	#	#	#	#	#	#
Rsa1.0_00029.1.g1821.t1	gb|EOA29383.1| hypothetical protein CARUB_v10025670mg [Capsella rubella]	295	298	1.00E-155	101.0	91.5	96.6	hypothetical protein CARUB_v10025670mg	gbpln	Capsella rubella	AT2G41350.1 | Symbols: | unknown protein; BEST Arabidopsis thaliana protein match is: unknown protein (TAIR:AT2G21980.1); Has 188 Blast hits to 188 proteins in 66 species: Archae - 0; Bacteria - 12; Metazoa - 90; Fungi - 15; Plants - 54; Viruses - 0; Other Eukaryotes - 17 (source: NCBI BLink). | chr2:17231565-17233458 FORWARD LENGTH=298	295	298	1.00E-156	101.0	90.8	96.3
Rsa1.0_00029.1.g1822.t1	gb|ABK28027.1| unknown [Arabidopsis thaliana]	82	83	5.00E-26	101.2	76.8	81.7	unknown	gbpln	Arabidopsis thaliana	AT2G41342.1 | Symbols: | unknown protein; FUNCTIONS IN: molecular_function unknown; INVOLVED IN: biological_process unknown; LOCATED IN: cellular_component unknown; Has 30201 Blast hits to 17322 proteins in 780 species: Archae - 12; Bacteria - 1396; Metazoa - 17338; Fungi - 3422; Plants - 5037; Viruses - 0; Other Eukaryotes - 2996 (source: NCBI BLink). | chr2:17230650-17230898 REVERSE LENGTH=82	82	82	4.00E-28	100.0	75.6	80.5
Rsa1.0_00029.1.g1823.t1	ref|XP_002879920.1| hypothetical protein ARALYDRAFT_903442 [Arabidopsis lyrata subsp. lyrata] gi|297325759|gb|EFH56179.1| hypothetical protein ARALYDRAFT_903442 [Arabidopsis lyrata subsp. lyrata]	49	67	4.00E-13	136.7	75.5	77.6	hypothetical protein ARALYDRAFT_903442	gbpln	Arabidopsis lyrata	AT3G57062.2 | Symbols: | unknown protein; FUNCTIONS IN: molecular_function unknown; INVOLVED IN: biological_process unknown; LOCATED IN: endomembrane system. | chr3:21121443-21121589 FORWARD LENGTH=48	49	48	1.00E-12	98.0	61.2	73.5
Rsa1.0_00029.1.g1824.t1	ref|XP_002881767.1| hypothetical protein ARALYDRAFT_321816 [Arabidopsis lyrata subsp. lyrata] gi|297327606|gb|EFH58026.1| hypothetical protein ARALYDRAFT_321816 [Arabidopsis lyrata subsp. lyrata]	369	370	0	100.3	88.3	94.9	hypothetical protein ARALYDRAFT_321816	gbpln	Arabidopsis lyrata	AT2G41300.1 | Symbols: SSL1 | strictosidine synthase-like 1 | chr2:17214312-17216292 REVERSE LENGTH=394	369	394	1.00E-171	106.8	77.0	81.8
Rsa1.0_00029.1.g1825.t1	ref|XP_002881766.1| hypothetical protein ARALYDRAFT_483200 [Arabidopsis lyrata subsp. lyrata] gi|297327605|gb|EFH58025.1| hypothetical protein ARALYDRAFT_483200 [Arabidopsis lyrata subsp. lyrata]	375	376	0	100.3	84.0	92.0	hypothetical protein ARALYDRAFT_483200	gbpln	Arabidopsis lyrata	AT2G41290.1 | Symbols: SSL2 | strictosidine synthase-like 2 | chr2:17210599-17212904 REVERSE LENGTH=376	375	376	0	100.3	83.2	90.1

Rsa1.0_00029.1.g1826.t1	ref|XP_002879918.1| hypothetical protein ARALYDRAFT_345920 [Arabidopsis lyrata subsp. lyrata] gi|297325757|gb|EFH56177.1| hypothetical protein ARALYDRAFT_345920 [Arabidopsis lyrata subsp. lyrata]	207	156	5.00E-18	75.4	37.7	43.5	hypothetical protein ARALYDRAFT_345920	gbpln	Arabidopsis lyrata	AT2G41280.1 | Symbols: M10, ATM10 | late embryogenesis abundant protein (M10) / LEA protein M10 | chr2:17209763-17210211 FORWARD LENGTH=107	207	107	1.00E-10	51.7	31.4	36.2
Rsa1.0_00029.1.g1827.t1	#	#	#	#	#	#	#	-	----	----	#	#	#	#	#	#	#
Rsa1.0_00029.1.g1828.t1	gb|AAF97969.1|AC000103_19 F21J9.30 [Arabidopsis thaliana]	1632	1270	0	77.8	34.5	47.1	F21J9.30	gbpln	Arabidopsis thaliana	AT4G29090.1 | Symbols: | Ribonuclease H-like superfamily protein | chr4:14333528-14335255 FORWARD LENGTH=575	1632	575	6.00E-81	35.2	10.5	16.8
Rsa1.0_00029.1.g1829.t1	#	#	#	#	#	#	#	-	----	----	#	#	#	#	#	#	#
Rsa1.0_00029.1.g1830.t1	#	#	#	#	#	#	#	-	----	----	#	#	#	#	#	#	#
Rsa1.0_00029.1.g1831.t1	gb|EOA30627.1| hypothetical protein CARUB_v10013762mg [Capsella rubella]	284	428	5.00E-42	150.7	31.7	34.9	hypothetical protein CARUB_v10013762mg	gbpln	Capsella rubella	AT3G03950.3 | Symbols: ECT1 | evolutionarily conserved C-terminal region 1 | chr3:1021502-1023767 FORWARD LENGTH=428	284	428	4.00E-44	150.7	31.7	33.8
Rsa1.0_00029.1.g1832.t1	ref|XP_002879915.1| phosphatidylinositol-4-phosphate 5-kinase family protein [Arabidopsis lyrata subsp. lyrata] gi|297325754|gb|EFH56174.1| phosphatidylinositol-4-phosphate 5-kinase family protein [Arabidopsis lyrata subsp. lyrata]	773	772	0	99.9	93.0	97.4	phosphatidylinositol-4-phosphate 5-kinase family protein	gbpln	Arabidopsis lyrata	AT2G41210.1 | Symbols: PIP5K5 | phosphatidylinositol- 4-phosphate 5-kinase 5 | chr2:17173627-17176575 FORWARD LENGTH=772	773	772	0	99.9	93.3	97.7
Rsa1.0_00029.1.g1833.t1	ref|XP_002879914.1| hypothetical protein ARALYDRAFT_483189 [Arabidopsis lyrata subsp. lyrata] gi|297325753|gb|EFH56173.1| hypothetical protein ARALYDRAFT_483189 [Arabidopsis lyrata subsp. lyrata]	140	161	3.00E-68	115.0	89.3	95.7	hypothetical protein ARALYDRAFT_483189	gbpln	Arabidopsis lyrata	AT2G41200.1 | Symbols: | unknown protein; Has 26 Blast hits to 26 proteins in 11 species: Archae - 0; Bacteria - 0; Metazoa - 0; Fungi - 0; Plants - 26; Viruses - 0; Other Eukaryotes - 0 (source: NCBI BLink). | chr2:17171657-17172929 FORWARD LENGTH=161	140	161	2.00E-63	115.0	88.6	95.7
Rsa1.0_00029.1.g1834.t1	#	#	#	#	#	#	#	-	----	----	AT3G18000.1 | Symbols: NMT1, XPL1, PEAMT | S-adenosyl-L-methionine-dependent methyltransferases superfamily protein | chr3:6154578-6157331 FORWARD LENGTH=491	136	491	2.00E-11	361.0	23.5	27.9
Rsa1.0_00029.1.g1835.t1	ref|NP_030664.1| transmembrane amino acid transporter-like protein [Arabidopsis thaliana] gi|14532708|gb|AAK64155.1| unknown protein [Arabidopsis thaliana] gi|19310729|gb|AAL85095.1| unknown protein [Arabidopsis thaliana] gi|20197120|gb|AAD11993.2| expressed protein [Arabidopsis thaliana] gi|330254848|gb|AEC09942.1| transmembrane amino acid transporter-like protein [Arabidopsis thaliana]	544	536	0	98.5	78.5	83.1	transmembrane amino acid transporter-like protein	gbpln	Arabidopsis thaliana	AT2G41190.1 | Symbols: | Transmembrane amino acid transporter family protein | chr2:17167561-17170145 REVERSE LENGTH=536	544	536	0	98.5	78.5	83.1
Rsa1.0_00029.1.g1836.t1	ref|XP_002879913.1| hypothetical protein ARALYDRAFT_483187 [Arabidopsis lyrata subsp. lyrata] gi|297325752|gb|EFH56172.1| hypothetical protein ARALYDRAFT_483187 [Arabidopsis lyrata subsp. lyrata]	138	140	3.00E-52	101.4	80.4	87.0	hypothetical protein ARALYDRAFT_483187	gbpln	Arabidopsis lyrata	AT2G41180.1 | Symbols: | VQ motif-containing protein | chr2:17165242-17165667 FORWARD LENGTH=141	138	141	7.00E-52	102.2	77.5	84.1
Rsa1.0_00029.1.g1837.t2	gb|ABO36622.1| copia LTR rider [Solanum lycopersicum] gi|133711819|gb|ABO36636.1| copia LTR rider [Solanum lycopersicum]	957	1307	0	136.6	59.6	69.4	copia LTR rider	gbpln	Solanum lycopersicum	AT4G23160.1 | Symbols: CRK8 | cysteine-rich RLK (RECEPTOR-like protein kinase) 8 | chr4:12129485-12134086 FORWARD LENGTH=1262	957	1262	2.00E-52	131.9	12.4	18.8
Rsa1.0_00029.1.g1838.t1	ref|NP_181647.1| CDPK-related kinase 1 [Arabidopsis thaliana] gi|75318680|sp|O80673.1|CAMK1_ARATH RecName: Full=CDPK-related kinase 1; Short=AtCRK1; AltName: Full=Calcium/calmodulin-dependent protein kinase 3; AltName: Full=Calmodulin-binding protein kinase 3; Short=AtCBK3; Short=CaM-binding protein kinase 3 gi|5020366|gb|AAD38058.1|AF153351_1 CDPK-related kinase 1 [Arabidopsis thaliana] gi|16904218|gb|AAL30816.1|AF435448_1 calcium/calmodulin-dependent protein kinase CaMK3 [Arabidopsis thaliana] gi|3402722|gb|AAD12016.1| CPDK-related protein kinase [Arabidopsis thaliana] gi|330254842|gb|AEC09936.1| CDPK-related kinase 1 [Arabidopsis thaliana]	520	576	0	110.8	94.8	96.7	CDPK-related kinase 1	gbpln	Arabidopsis thaliana	AT2G41140.1 | Symbols: CRK1, ATCRK1, ATCBK3 | CDPK-related kinase 1 | chr2:17150492-17153378 FORWARD LENGTH=576	520	576	0	110.8	94.8	96.7

Rsa1.0_00029.1.g1839.t1	dbj|BAB09923.1| copia-like retrotransposable element [Arabidopsis thaliana]	1534	1342	0	87.5	43.5	60.6	copia-like retrotransposable element	gbpln	Arabidopsis thaliana	AT4G23160.1 | Symbols: CRK8 | cysteine-rich RLK (RECEPTOR-like protein kinase) 8 | chr4:12129485-12134086 FORWARD LENGTH=1262	1534	1262	2.00E-87	82.3	12.3	17.4
Rsa1.0_00029.1.g1840.t1	ref|XP_002879910.1| basic helix-loop-helix family protein [Arabidopsis lyrata subsp. lyrata] gi|297325749|gb|EFH56169.1| basic helix-loop-helix family protein [Arabidopsis lyrata subsp. lyrata]	263	253	1.00E-110	96.2	85.2	90.9	basic helix-loop-helix family protein	gbpln	Arabidopsis lyrata	AT2G41130.1 | Symbols: | basic helix-loop-helix (bHLH) DNA-binding superfamily protein | chr2:17143360-17144686 FORWARD LENGTH=253	263	253	1.00E-111	96.2	84.0	90.9
Rsa1.0_00029.1.g1841.t1	gb|EOA26366.1| hypothetical protein CARUB_v10023283mg [Capsella rubella]	383	422	1.00E-144	110.2	67.9	79.4	hypothetical protein CARUB_v10023283mg	gbpln	Capsella rubella	AT2G40910.1 | Symbols: | F-box and associated interaction domains-containing protein | chr2:17069938-17071554 REVERSE LENGTH=449	383	449	1.00E-138	117.2	62.9	78.9
Rsa1.0_00029.1.g1842.t1	#	#	#	#	#	#	#	-	----	----	#	#	#	#	#	#	#
Rsa1.0_00029.1.g1843.t2	gb|EOA26366.1| hypothetical protein CARUB_v10023283mg [Capsella rubella]	179	422	4.00E-28	235.8	41.3	53.6	hypothetical protein CARUB_v10023283mg	gbpln	Capsella rubella	AT2G40910.1 | Symbols: | F-box and associated interaction domains-containing protein | chr2:17069938-17071554 REVERSE LENGTH=449	179	449	1.00E-29	250.8	35.8	45.3
Rsa1.0_00029.1.g1844.t1	#	#	#	#	#	#	#	-	----	----	#	#	#	#	#	#	#
Rsa1.0_00029.1.g1845.t1	ref|XP_002881746.1| hypothetical protein ARALYDRAFT_321778 [Arabidopsis lyrata subsp. lyrata] gi|297327585|gb|EFH58005.1| hypothetical protein ARALYDRAFT_321778 [Arabidopsis lyrata subsp. lyrata]	385	449	1.00E-134	116.6	62.6	77.4	hypothetical protein ARALYDRAFT_321778	gbpln	Arabidopsis lyrata	AT2G40910.1 | Symbols: | F-box and associated interaction domains-containing protein | chr2:17069938-17071554 REVERSE LENGTH=449	385	449	1.00E-132	116.6	61.0	77.7
Rsa1.0_00029.1.g1846.t1	ref|NP_181623.2| F-box protein [Arabidopsis thaliana] gi|122215015|sp|Q3EBI7.1|FB130_ARATH RecName: Full=F-box protein At2g40910 gi|330254802|gb|AEC09896.1| F-box protein [Arabidopsis thaliana]	391	449	1.00E-82	114.8	43.0	54.5	F-box protein	gbpln	Arabidopsis thaliana	AT2G40910.1 | Symbols: | F-box and associated interaction domains-containing protein | chr2:17069938-17071554 REVERSE LENGTH=449	391	449	3.00E-85	114.8	43.0	54.5
Rsa1.0_00029.1.g1847.t1	dbj|BAA97099.1| retroelement pol polyprotein-like [Arabidopsis thaliana]	361	1098	2.00E-58	304.2	30.5	35.5	retroelement pol polyprotein-like	gbpln	Arabidopsis thaliana	AT1G21280.1 | Symbols: | CONTAINS InterPro DOMAIN/s: Retrotransposon gag protein (InterPro:IPR005162); Has 707 Blast hits to 705 proteins in 25 species: Archae - 0; Bacteria - 0; Metazoa - 4; Fungi - 0; Plants - 703; Viruses - 0; Other Eukaryotes - 0 (source: NCBI BLink). | chr1:7447690-7448403 REVERSE LENGTH=237	361	237	8.00E-23	65.7	16.3	24.7
Rsa1.0_00029.1.g1848.t1	gb|EOA26681.1| hypothetical protein CARUB_v10022762mg [Capsella rubella]	780	696	0	89.2	76.9	81.8	hypothetical protein CARUB_v10022762mg	gbpln	Capsella rubella	AT2G40980.1 | Symbols: | Protein kinase superfamily protein | chr2:17102090-17104443 FORWARD LENGTH=617	780	617	0	79.1	57.1	60.0
Rsa1.0_00029.1.g1849.t1	ref|NP_850337.1| cytochrome P450 98A3 [Arabidopsis thaliana] gi|5915859|sp|O22203.1|C98A3_ARATH RecName: Full=Cytochrome P450 98A3; AltName: Full=Protein REDUCED EPIDERMAL FLUORESCENCE 8; AltName: Full=p-coumaroylshikimate/quinate 3'-hydrolxylase; Short=C3'H gi|330254799|gb|AEC09893.1| cytochrome P450 98A3 [Arabidopsis thaliana]	508	508	0	100.0	95.1	97.2	cytochrome P450 98A3	gbpln	Arabidopsis thaliana	AT2G40890.1 | Symbols: CYP98A3 | cytochrome P450, family 98, subfamily A, polypeptide 3 | chr2:17058291-17060532 REVERSE LENGTH=508	508	508	0	100.0	95.1	97.2
Rsa1.0_00029.1.g1850.t1	gb|AAL86314.1| putative cysteine proteinase inhibitor cystatin B [Arabidopsis thaliana]	116	116	5.00E-19	100.0	42.2	44.0	putative cysteine proteinase inhibitor cystatin B	gbpln	Arabidopsis thaliana	AT2G40880.1 | Symbols: FL3-27, ATCYSA, CYSA | cystatin A | chr2:17057463-17057930 FORWARD LENGTH=125	116	125	1.00E-21	107.8	42.2	44.0
Rsa1.0_00029.1.g1851.t2	dbj|BAJ33673.1| unnamed protein product [Thellungiella halophila]	684	654	0	95.6	84.8	89.2	unnamed protein product	----	----	AT2G40860.1 | Symbols: | protein kinase family protein / protein phosphatase 2C (PP2C) family protein | chr2:17053747-17057108 REVERSE LENGTH=658	684	658	0	96.2	83.6	89.5
Rsa1.0_00029.1.g1852.t1	ref|NP_181617.1| phosphoinositide 4-kinase gamma 1 [Arabidopsis thaliana] gi|2623299|gb|AAB86445.1| hypothetical protein [Arabidopsis thaliana] gi|330254796|gb|AEC09890.1| phosphoinositide 4-kinase gamma 1 [Arabidopsis thaliana]	541	561	0	103.7	86.5	91.9	phosphoinositide 4-kinase gamma 1	gbpln	Arabidopsis thaliana	AT2G40850.1 | Symbols: ATPI4K GAMMA 1, PI4K GAMMA 1 | phosphoinositide 4-kinase gamma 1 | chr2:17051575-17053260 FORWARD LENGTH=561	541	561	0	103.7	86.5	91.9
Rsa1.0_00029.1.g1853.t1	ref|XP_002879893.1| ring-H2 finger C1A [Arabidopsis lyrata subsp. lyrata] gi|297325732|gb|EFH56152.1| ring-H2 finger C1A [Arabidopsis lyrata subsp. lyrata]	310	329	1.00E-132	106.1	85.2	90.3	ring-H2 finger C1A	gbpln	Arabidopsis lyrata	AT2G40830.3 | Symbols: RHC1A | RING-H2 finger C1A | chr2:17043642-17044628 FORWARD LENGTH=328	310	328	1.00E-133	105.8	82.9	88.7

Rsa1.0_00029.1.g1854.t1	gb|AAB86442.1| hypothetical protein [Arabidopsis thaliana]	392	945	2.00E-97	241.1	63.0	71.9	hypothetical protein	gbpln	Arabidopsis thaliana	AT2G40815.1 | Symbols: | Calcium-dependent lipid-binding (CaLB domain) family protein | chr2:17034736-17036110 REVERSE LENGTH=367	392	367	3.00E-68	93.6	30.9	34.2
Rsa1.0_00029.1.g1855.t1	#	#	#	#	#	#	#	-	----	----	#	#	#	#	#	#	#
Rsa1.0_00029.1.g1856.t1	gb|EOA28930.1| hypothetical protein CARUB_v10025177mg [Capsella rubella]	331	322	2.00E-70	97.3	41.4	48.0	hypothetical protein CARUB_v10025177mg	gbpln	Capsella rubella	AT2G40815.1 | Symbols: | Calcium-dependent lipid-binding (CaLB domain) family protein | chr2:17034736-17036110 REVERSE LENGTH=367	331	367	3.00E-68	110.9	44.4	51.1
Rsa1.0_00029.1.g1857.t2	ref|XP_002879696.1| predicted protein [Arabidopsis lyrata subsp. lyrata] gi|297325535|gb|EFH55955.1| predicted protein [Arabidopsis lyrata subsp. lyrata]	194	389	8.00E-12	200.5	21.1	22.7	predicted protein	gbpln	Arabidopsis lyrata	AT3G56440.1 | Symbols: ATATG18D, ATG18D | homolog of yeast autophagy 18 (ATG18) D | chr3:20926874-20928797 FORWARD LENGTH=391	194	391	2.00E-12	201.5	19.6	21.1
Rsa1.0_00030.1.g1858.t1	ref|XP_002882450.1| basic helix-loop-helix family protein [Arabidopsis lyrata subsp. lyrata] gi|297328290|gb|EFH58709.1| basic helix-loop-helix family protein [Arabidopsis lyrata subsp. lyrata]	206	202	1.00E-100	98.1	88.8	93.7	basic helix-loop-helix family protein	gbpln	Arabidopsis lyrata	AT3G06120.1 | Symbols: MUTE | basic helix-loop-helix (bHLH) DNA-binding superfamily protein | chr3:1846531-1848016 FORWARD LENGTH=202	206	202	1.00E-101	98.1	89.3	94.2
Rsa1.0_00030.1.g1859.t1	gb|EOA30708.1| hypothetical protein CARUB_v10013845mg [Capsella rubella]	394	407	0	103.3	89.1	95.2	hypothetical protein CARUB_v10013845mg	gbpln	Capsella rubella	AT3G06190.1 | Symbols: ATBPM2, BPM2 | BTB-POZ and MATH domain 2 | chr3:1874577-1876575 REVERSE LENGTH=406	394	406	0	103.0	88.6	94.4
Rsa1.0_00030.1.g1860.t1	ref|NP_187269.1| ribosomal protein L34e-like protein [Arabidopsis thaliana] gi|6862922|gb|AAF30311.1|AC018907_11 unknown protein [Arabidopsis thaliana] gi|62867643|gb|AAY17425.1| At3g06180 [Arabidopsis thaliana] gi|90962960|gb|ABE02404.1| At3g06180 [Arabidopsis thaliana] gi|332640836|gb|AEE74357.1| ribosomal protein L34e-like protein [Arabidopsis thaliana]	203	241	4.00E-73	118.7	87.2	93.1	ribosomal protein L34e-like protein	gbpln	Arabidopsis thaliana	AT3G06180.1 | Symbols: | Ribosomal protein L34e superfamily protein | chr3:1871705-1873554 FORWARD LENGTH=241	203	241	1.00E-75	118.7	87.2	93.1
Rsa1.0_00030.1.g1861.t1	gb|EOA29649.1| hypothetical protein CARUB_v10015151mg [Capsella rubella]	351	351	0	100.0	92.9	96.3	hypothetical protein CARUB_v10015151mg	gbpln	Capsella rubella	AT3G06260.1 | Symbols: GATL4 | galacturonosyltransferase-like 4 | chr3:1893804-1894859 REVERSE LENGTH=351	351	351	0	100.0	93.2	96.3
Rsa1.0_00030.1.g1862.t1	ref|XP_002882459.1| hypothetical protein ARALYDRAFT_477927 [Arabidopsis lyrata subsp. lyrata] gi|297328299|gb|EFH58718.1| hypothetical protein ARALYDRAFT_477927 [Arabidopsis lyrata subsp. lyrata]	351	348	1.00E-180	99.1	89.5	92.6	hypothetical protein ARALYDRAFT_477927	gbpln	Arabidopsis lyrata	AT3G06270.1 | Symbols: | Protein phosphatase 2C family protein | chr3:1896763-1897887 FORWARD LENGTH=348	351	348	0	99.1	88.6	92.0
Rsa1.0_00030.1.g1863.t1	ref|XP_002882462.1| hypothetical protein ARALYDRAFT_896744 [Arabidopsis lyrata subsp. lyrata] gi|297328302|gb|EFH58721.1| hypothetical protein ARALYDRAFT_896744 [Arabidopsis lyrata subsp. lyrata]	410	425	1.00E-132	103.7	61.5	76.3	hypothetical protein ARALYDRAFT_896744	gbpln	Arabidopsis lyrata	AT1G03710.2 | Symbols: | Cystatin/monellin superfamily protein | chr1:923492-924287 FORWARD LENGTH=202	410	202	3.00E-28	49.3	17.6	25.6
Rsa1.0_00030.1.g1864.t1	gb|EOA30590.1| hypothetical protein CARUB_v10013722mg [Capsella rubella]	436	440	1.00E-169	100.9	77.8	83.3	hypothetical protein CARUB_v10013722mg	gbpln	Capsella rubella	AT3G06330.1 | Symbols: | RING/U-box superfamily protein | chr3:1917334-1919709 FORWARD LENGTH=426	436	426	1.00E-169	97.7	75.5	82.1
Rsa1.0_00030.1.g1865.t1	ref|XP_002884573.1| EMB3004/MEE32 [Arabidopsis lyrata subsp. lyrata] gi|297330413|gb|EFH60832.1| EMB3004/MEE32 [Arabidopsis lyrata subsp. lyrata]	604	603	0	99.8	85.6	91.2	EMB3004/MEE32	gbpln	Arabidopsis lyrata	AT3G06350.1 | Symbols: EMB3004, MEE32 | dehydroquinate dehydratase, putative / shikimate dehydrogenase, putative | chr3:1924536-1927701 REVERSE LENGTH=603	604	603	0	99.8	86.3	90.6
Rsa1.0_00030.1.g1866.t1	gb|EOA31716.1| hypothetical protein CARUB_v10014924mg [Capsella rubella]	126	126	4.00E-26	100.0	60.3	65.9	hypothetical protein CARUB_v10014924mg	gbpln	Capsella rubella	AT3G06360.1 | Symbols: AGP27, ATAGP27 | arabinogalactan protein 27 | chr3:1928976-1929353 FORWARD LENGTH=125	126	125	8.00E-28	99.2	73.8	83.3
Rsa1.0_00030.1.g1867.t1	dbj|BAF01676.1| hypothetical protein [Arabidopsis thaliana]	223	450	3.00E-46	201.8	47.1	65.0	hypothetical protein	gbpln	Arabidopsis thaliana	AT1G26540.1 | Symbols: | Agenet domain-containing protein | chr1:9167783-9170795 REVERSE LENGTH=695	223	695	1.00E-48	311.7	47.1	65.0
Rsa1.0_00030.1.g1868.t1	ref|NP_182245.1| uncharacterized protein [Arabidopsis thaliana] gi|2275201|gb|AAB63823.1| unknown protein [Arabidopsis thaliana] gi|330255722|gb|AEC10816.1| uncharacterized protein AT2G47230 [Arabidopsis thaliana]	417	701	3.00E-91	168.1	50.8	61.4	uncharacterized protein	gbpln	Arabidopsis thaliana	AT2G47230.1 | Symbols: DUF6, ATDUF6 | DOMAIN OF UNKNOWN FUNCTION 724 6 | chr2:19387126-19390011 FORWARD LENGTH=701	417	701	8.00E-94	168.1	50.8	61.4
Rsa1.0_00030.1.g1869.t5	gb|EOA32811.1| hypothetical protein CARUB_v10016122mg [Capsella rubella]	616	1058	0	171.8	83.4	85.9	hypothetical protein CARUB_v10016122mg	gbpln	Capsella rubella	AT3G06400.1 | Symbols: CHR11 | chromatin-remodeling protein 11 | chr3:1941066-1946700 FORWARD LENGTH=1055	616	1055	0	171.3	83.9	86.2

Rsa1.0_00030.1.g1870.t1	gb|ADK63414.1| CCCH type zinc finger protein [Brassica rapa]	467	455	0	97.4	80.9	87.6	CCCH type zinc finger protein	gbpln	Brassica rapa	AT3G06410.1 | Symbols: | Zinc finger C-x8-C-x5-C-x3-H type family protein | chr3:1947471-1949528 REVERSE LENGTH=462	467	462	1.00E-175	98.9	81.8	87.6
Rsa1.0_00030.1.g1871.t1	emb|CAB39638.1| RNA-directed DNA polymerase-like protein [Arabidopsis thaliana] gi|7267666|emb|CAB78094.1| RNA-directed DNA polymerase-like protein [Arabidopsis thaliana]	511	1274	2.00E-92	249.3	38.2	55.6	RNA-directed DNA polymerase-like protein	gbpln	Arabidopsis thaliana	AT4G29090.1 | Symbols: | Ribonuclease H-like superfamily protein | chr4:14333528-14335255 FORWARD LENGTH=575	511	575	8.00E-62	112.5	30.5	47.7
Rsa1.0_00030.1.g1872.t1	ref|XP_002882368.1| hypothetical protein ARALYDRAFT_896506 [Arabidopsis lyrata subsp. lyrata] gi|297328208|gb|EFH58627.1| hypothetical protein ARALYDRAFT_896506 [Arabidopsis lyrata subsp. lyrata]	181	392	2.00E-32	216.6	45.9	58.6	hypothetical protein ARALYDRAFT_896506	gbpln	Arabidopsis lyrata	AT4G29970.1 | Symbols: | F-box and associated interaction domains-containing protein | chr4:14662765-14663917 REVERSE LENGTH=283	181	283	1.00E-24	156.4	34.8	48.6
Rsa1.0_00030.1.g1873.t1	gb|EOA31508.1| hypothetical protein CARUB_v10014696mg [Capsella rubella]	183	196	7.00E-35	107.1	45.4	60.1	hypothetical protein CARUB_v10014696mg	gbpln	Capsella rubella	AT3G06435.1 | Symbols: | Expressed protein | chr3:1970102-1970704 REVERSE LENGTH=200	183	200	3.00E-37	109.3	45.9	59.6
Rsa1.0_00030.1.g1874.t14	ref|NP_566284.1| putative beta-1,3-galactosyltransferase 16 [Arabidopsis thaliana] gi|75167739|sp|Q9ASW1.1|B3GTG_ARATH RecName: Full=Probable beta-1,3-galactosyltransferase 16 gi|13605629|gb|AAK32808.1|AF361640_1 AT3g06440/F24P17_7 [Arabidopsis thaliana] gi|25090104|gb|AAN72229.1| At3g06440/F24P17_7 [Arabidopsis thaliana] gi|332640872|gb|AEE74393.1| putative beta-1,3-galactosyltransferase 16 [Arabidopsis thaliana]	959	619	0	64.5	47.5	51.9	putative beta-1,3-galactosyltransferase 16	gbpln	Arabidopsis thaliana	AT3G06440.1 | Symbols: | Galactosyltransferase family protein | chr3:1972913-1975272 REVERSE LENGTH=619	959	619	0	64.5	47.5	51.9
Rsa1.0_00030.1.g1875.t1	ref|NP_187298.1| GNS1/SUR4 membrane-like protein [Arabidopsis thaliana] gi|6437542|gb|AAF08569.1|AC011623_2 unknown protein [Arabidopsis thaliana] gi|332640878|gb|AEE74399.1| GNS1/SUR4 membrane-like protein [Arabidopsis thaliana]	273	278	1.00E-130	101.8	88.6	93.0	GNS1/SUR4 membrane-like protein	gbpln	Arabidopsis thaliana	AT3G06470.1 | Symbols: | GNS1/SUR4 membrane protein family | chr3:1984206-1985042 FORWARD LENGTH=278	273	278	1.00E-132	101.8	88.6	93.0
Rsa1.0_00030.1.g1876.t1	ref|XP_002882473.1| GNS1/SUR4 membrane family protein [Arabidopsis lyrata subsp. lyrata] gi|297328313|gb|EFH58732.1| GNS1/SUR4 membrane family protein [Arabidopsis lyrata subsp. lyrata]	294	278	1.00E-115	94.6	72.1	79.3	GNS1/SUR4 membrane family protein	gbpln	Arabidopsis lyrata	AT3G06470.1 | Symbols: | GNS1/SUR4 membrane protein family | chr3:1984206-1985042 FORWARD LENGTH=278	294	278	1.00E-116	94.6	72.1	79.6
Rsa1.0_00030.1.g1877.t1	ref|NP_187298.1| GNS1/SUR4 membrane-like protein [Arabidopsis thaliana] gi|6437542|gb|AAF08569.1|AC011623_2 unknown protein [Arabidopsis thaliana] gi|332640878|gb|AEE74399.1| GNS1/SUR4 membrane-like protein [Arabidopsis thaliana]	236	278	8.00E-91	117.8	76.3	86.0	GNS1/SUR4 membrane-like protein	gbpln	Arabidopsis thaliana	AT3G06470.1 | Symbols: | GNS1/SUR4 membrane protein family | chr3:1984206-1985042 FORWARD LENGTH=278	236	278	2.00E-93	117.8	76.3	86.0
Rsa1.0_00030.1.g1878.t1	#	#	#	#	#	#	#	-	----	----	#	#	#	#	#	#	#
Rsa1.0_00030.1.g1879.t1	ref|XP_002882474.1| botrytis-susceptible1, myb domain protein 108 [Arabidopsis lyrata subsp. lyrata] gi|297328314|gb|EFH58733.1| botrytis-susceptible1, myb domain protein 108 [Arabidopsis lyrata subsp. lyrata]	311	322	1.00E-150	103.5	89.4	94.5	botrytis-susceptible1, myb domain protein 108	gbpln	Arabidopsis lyrata	AT3G06490.1 | Symbols: MYB108, AtMYB108, BOS1 | myb domain protein 108 | chr3:2004298-2006358 FORWARD LENGTH=323	311	323	1.00E-144	103.9	88.7	94.2
Rsa1.0_00030.1.g1880.t1	ref|NP_566285.1| beta-glucosidase-like SFR2 [Arabidopsis thaliana] gi|75305863|sp|Q93Y07.1|SFR2_ARATH RecName: Full=Beta-glucosidase-like SFR2, chloroplastic; AltName: Full=Protein SENSITIVE TO FREEZING 2; Short=AtSFR2 gi|16649039|gb|AAL24371.1| beta-glucosidase, putative [Arabidopsis thaliana] gi|22077112|emb|CAD36512.1| putative beta-glycosidase [Arabidopsis thaliana] gi|25083735|gb|AAN72111.1| beta-glucosidase, putative [Arabidopsis thaliana] gi|332640883|gb|AEE74404.1| beta-glucosidase-like SFR2 [Arabidopsis thaliana]	570	622	0	109.1	84.4	91.6	beta-glucosidase-like SFR2	gbpln	Arabidopsis thaliana	AT3G06510.1 | Symbols: SFR2, ATSFR2 | Glycosyl hydrolase superfamily protein | chr3:2016450-2019533 FORWARD LENGTH=622	570	622	0	109.1	84.4	91.6

Rsa1.0_00030.1.g1881.t1	ref|XP_002884591.1| hypothetical protein ARALYDRAFT_477961 [Arabidopsis lyrata subsp. lyrata] gi|297330431|gb|EFH60850.1| hypothetical protein ARALYDRAFT_477961 [Arabidopsis lyrata subsp. lyrata]	559	545	0	97.5	89.3	92.5	hypothetical protein ARALYDRAFT_477961	gbpln	Arabidopsis lyrata	AT3G06550.2 | Symbols: | O-acetyltransferase family protein | chr3:2039408-2042664 REVERSE LENGTH=568	559	568	0	101.6	88.7	92.7
Rsa1.0_00030.1.g1882.t1	ref|XP_002884593.1| hypothetical protein ARALYDRAFT_896791 [Arabidopsis lyrata subsp. lyrata] gi|297330433|gb|EFH60852.1| hypothetical protein ARALYDRAFT_896791 [Arabidopsis lyrata subsp. lyrata]	496	496	0	100.0	91.5	95.6	hypothetical protein ARALYDRAFT_896791	gbpln	Arabidopsis lyrata	AT3G06580.1 | Symbols: GAL1, GALK | Mevalonate/galactokinase family protein | chr3:2049141-2051867 REVERSE LENGTH=496	496	496	0	100.0	91.1	95.2
Rsa1.0_00030.1.g1883.t1	ref|XP_002884594.1| hypothetical protein ARALYDRAFT_477966 [Arabidopsis lyrata subsp. lyrata] gi|297330434|gb|EFH60853.1| hypothetical protein ARALYDRAFT_477966 [Arabidopsis lyrata subsp. lyrata]	229	217	8.00E-68	94.8	69.4	80.3	hypothetical protein ARALYDRAFT_477966	gbpln	Arabidopsis lyrata	AT3G06590.2 | Symbols: | basic helix-loop-helix (bHLH) DNA-binding superfamily protein | chr3:2054647-2055312 REVERSE LENGTH=221	229	221	2.00E-64	96.5	69.4	79.9
Rsa1.0_00030.1.g1884.t1	ref|NP_187312.1| uncharacterized protein [Arabidopsis thaliana] gi|186509849|ref|NP_001118593.1| uncharacterized protein [Arabidopsis thaliana] gi|12322684|gb|AAG51336.1|AC020580_16 hypothetical protein; 25909-26809 [Arabidopsis thaliana] gi|49660141|gb|AAT68361.1| hypothetical protein At3g06600 [Arabidopsis thaliana] gi|50058941|gb|AAT69215.1| hypothetical protein At3g06600 [Arabidopsis thaliana] gi|332640899|gb|AEE74420.1| uncharacterized protein AT3G06600 [Arabidopsis thaliana] gi|332640900|gb|AEE74421.1| uncharacterized protein AT3G06600 [Arabidopsis thaliana]	193	193	4.00E-64	100.0	73.1	82.9	uncharacterized protein	gbpln	Arabidopsis thaliana	AT3G06600.2 | Symbols: | unknown protein; FUNCTIONS IN: molecular_function unknown; INVOLVED IN: biological_process unknown; LOCATED IN: cellular_component unknown; EXPRESSED IN: 15 plant structures; EXPRESSED DURING: 7 growth stages; Has 30201 Blast hits to 17322 proteins in 780 species: Archae - 12; Bacteria - 1396; Metazoa - 17338; Fungi - 3422; Plants - 5037; Viruses - 0; Other Eukaryotes - 2996 (source: NCBI BLink). | chr3:2059558-2060458 REVERSE LENGTH=193	193	193	1.00E-66	100.0	73.1	82.9
Rsa1.0_00030.1.g1885.t1	gb|EOA31742.1| hypothetical protein CARUB_v10014957mg [Capsella rubella]	115	115	2.00E-52	100.0	93.0	95.7	hypothetical protein CARUB_v10014957mg	gbpln	Capsella rubella	AT3G06610.1 | Symbols: | DNA-binding enhancer protein-related | chr3:2060837-2061845 FORWARD LENGTH=115	115	115	6.00E-55	100.0	91.3	93.9
Rsa1.0_00030.1.g1886.t2	ref|NP_187314.1| PAS domain-containing tyrosine kinase-like protein [Arabidopsis thaliana] gi|12322680|gb|AAG51332.1|AC020580_12 protein kinase, putative; 19229-23534 [Arabidopsis thaliana] gi|20258844|gb|AAM13904.1| putative protein kinase [Arabidopsis thaliana] gi|21689823|gb|AAM67555.1| putative protein kinase [Arabidopsis thaliana] gi|110741529|dbj|BAE98714.1| putative protein kinase [Arabidopsis thaliana] gi|332640902|gb|AEE74423.1| PAS domain-containing tyrosine kinase-like protein [Arabidopsis thaliana]	766	773	0	100.9	87.3	93.5	PAS domain-containing tyrosine kinase-like protein	gbpln	Arabidopsis thaliana	AT3G06620.1 | Symbols: | PAS domain-containing protein tyrosine kinase family protein | chr3:2062833-2067138 REVERSE LENGTH=773	766	773	0	100.9	87.3	93.5
Rsa1.0_00030.1.g1887.t2	ref|NP_187314.1| PAS domain-containing tyrosine kinase-like protein [Arabidopsis thaliana] gi|12322680|gb|AAG51332.1|AC020580_12 protein kinase, putative; 19229-23534 [Arabidopsis thaliana] gi|20258844|gb|AAM13904.1| putative protein kinase [Arabidopsis thaliana] gi|21689823|gb|AAM67555.1| putative protein kinase [Arabidopsis thaliana] gi|110741529|dbj|BAE98714.1| putative protein kinase [Arabidopsis thaliana] gi|332640902|gb|AEE74423.1| PAS domain-containing tyrosine kinase-like protein [Arabidopsis thaliana]	170	773	5.00E-53	454.7	61.8	70.0	PAS domain-containing tyrosine kinase-like protein	gbpln	Arabidopsis thaliana	AT3G06620.1 | Symbols: | PAS domain-containing protein tyrosine kinase family protein | chr3:2062833-2067138 REVERSE LENGTH=773	170	773	2.00E-55	454.7	61.8	70.0
Rsa1.0_00030.1.g1888.t1	ref|NP_683536.1| plant invertase/pectin methylesterase inhibitor domain-containing protein [Arabidopsis thaliana] gi|332640914|gb|AEE74435.1| plant invertase/pectin methylesterase inhibitor domain-containing protein [Arabidopsis thaliana]	329	264	4.00E-69	80.2	53.5	59.9	plant invertase/pectin methylesterase inhibitor domain-containing protein	gbpln	Arabidopsis thaliana	AT3G36659.1 | Symbols: | Plant invertase/pectin methylesterase inhibitor superfamily protein | chr3:2100189-2100983 FORWARD LENGTH=264	329	264	1.00E-71	80.2	53.5	59.9

Rsa1.0_00030.1.g1889.t1	ref|NP_566289.1| PAPA-1-like and zinc finger-HIT domain-containing protein [Arabidopsis thaliana] gi|14334462|gb|AAK59429.1| unknown protein [Arabidopsis thaliana] gi|21436325|gb|AAM51332.1| unknown protein [Arabidopsis thaliana] gi|332640915|gb|AEE74436.1| PAPA-1-like and zinc finger-HIT domain-containing protein [Arabidopsis thaliana]	391	427	1.00E-125	109.2	72.1	79.5	PAPA-1-like and zinc finger-HIT domain-containing protein	gbpln	Arabidopsis thaliana	AT3G06660.1 | Symbols: | PAPA-1-like family protein / zinc finger (HIT type) family protein | chr3:2103060-2104776 FORWARD LENGTH=427	391	427	1.00E-127	109.2	72.1	79.5
Rsa1.0_00030.1.g1890.t1	ref|XP_002884605.1| binding protein [Arabidopsis lyrata subsp. lyrata] gi|297330445|gb|EFH60864.1| binding protein [Arabidopsis lyrata subsp. lyrata]	868	864	0	99.5	91.5	95.6	binding protein	gbpln	Arabidopsis lyrata	AT3G06670.1 | Symbols: | binding | chr3:2105908-2113136 REVERSE LENGTH=865	868	865	0	99.7	91.2	95.5
Rsa1.0_00030.1.g1891.t1	ref|XP_002880967.1| 60S ribosomal protein L29 [Arabidopsis lyrata subsp. lyrata] gi|297326806|gb|EFH57226.1| 60S ribosomal protein L29 [Arabidopsis lyrata subsp. lyrata]	61	61	1.00E-25	100.0	95.1	100.0	60S ribosomal protein L29	gbpln	Arabidopsis lyrata	AT3G06700.3 | Symbols: | Ribosomal L29e protein family | chr3:2117519-2117800 REVERSE LENGTH=61	61	61	4.00E-28	100.0	95.1	98.4
Rsa1.0_00030.1.g1892.t1	ref|XP_002882489.1| hypothetical protein ARALYDRAFT_477988 [Arabidopsis lyrata subsp. lyrata] gi|297328329|gb|EFH58748.1| hypothetical protein ARALYDRAFT_477988 [Arabidopsis lyrata subsp. lyrata]	186	183	4.00E-84	98.4	84.4	90.9	hypothetical protein ARALYDRAFT_477988	gbpln	Arabidopsis lyrata	AT3G06730.1 | Symbols: TRX P, TRX z | Thioredoxin z | chr3:2124276-2125845 FORWARD LENGTH=183	186	183	1.00E-85	98.4	82.8	90.3
Rsa1.0_00030.1.g1893.t1	ref|NP_566290.1| GATA transcription factor 15 [Arabidopsis thaliana] gi|71660789|sp|Q8LG10.2|GAT15_ARATH RecName: Full=GATA transcription factor 15 gi|17380940|gb|AAL36282.1| unknown protein [Arabidopsis thaliana] gi|20258947|gb|AAM14189.1| unknown protein [Arabidopsis thaliana] gi|332640929|gb|AEE74450.1| GATA transcription factor 15 [Arabidopsis thaliana]	142	149	8.00E-50	104.9	78.2	85.9	GATA transcription factor 15	gbpln	Arabidopsis thaliana	AT3G06740.1 | Symbols: GATA15 | GATA transcription factor 15 | chr3:2126658-2127265 FORWARD LENGTH=149	142	149	2.00E-52	104.9	78.2	85.9
Rsa1.0_00030.1.g1894.t1	gb|EOA31388.1| hypothetical protein CARUB_v10014564mg [Capsella rubella]	187	226	1.00E-57	120.9	77.0	83.4	hypothetical protein CARUB_v10014564mg	gbpln	Capsella rubella	AT3G06760.1 | Symbols: | Drought-responsive family protein | chr3:2132971-2134355 FORWARD LENGTH=224	187	224	2.00E-56	119.8	74.3	81.3
Rsa1.0_00030.1.g1895.t1	ref|XP_002884609.1| heat shock protein binding protein [Arabidopsis lyrata subsp. lyrata] gi|297330449|gb|EFH60868.1| heat shock protein binding protein [Arabidopsis lyrata subsp. lyrata]	226	228	1.00E-106	100.9	83.6	90.3	heat shock protein binding protein	gbpln	Arabidopsis lyrata	AT3G06778.1 | Symbols: | Chaperone DnaJ-domain superfamily protein | chr3:2140249-2141437 REVERSE LENGTH=229	226	229	1.00E-107	101.3	82.7	88.9
Rsa1.0_00030.1.g1896.t1	ref|XP_002882368.1| hypothetical protein ARALYDRAFT_896506 [Arabidopsis lyrata subsp. lyrata] gi|297328208|gb|EFH58627.1| hypothetical protein ARALYDRAFT_896506 [Arabidopsis lyrata subsp. lyrata]	371	392	1.00E-114	105.7	61.5	74.4	hypothetical protein ARALYDRAFT_896506	gbpln	Arabidopsis lyrata	AT3G04660.1 | Symbols: | F-box and associated interaction domains-containing protein | chr3:1264794-1265966 FORWARD LENGTH=390	371	390	2.00E-78	105.1	47.7	62.0
Rsa1.0_00030.1.g1897.t1	gb|EOA31285.1| hypothetical protein CARUB_v10014458mg, partial [Capsella rubella]	216	253	9.00E-76	117.1	69.4	73.1	hypothetical protein CARUB_v10014458mg, partial	gbpln	Capsella rubella	AT3G06790.2 | Symbols: | plastid developmental protein DAG, putative | chr3:2144564-2145743 REVERSE LENGTH=244	216	244	1.00E-73	113.0	65.7	69.4
Rsa1.0_00030.1.g1898.t1	ref|XP_002882496.1| hypothetical protein ARALYDRAFT_478003 [Arabidopsis lyrata subsp. lyrata] gi|297328336|gb|EFH58755.1| hypothetical protein ARALYDRAFT_478003 [Arabidopsis lyrata subsp. lyrata]	187	187	2.00E-70	100.0	78.6	88.2	hypothetical protein ARALYDRAFT_478003	gbpln	Arabidopsis lyrata	AT3G06840.1 | Symbols: | unknown protein; BEST Arabidopsis thaliana protein match is: unknown protein (TAIR:AT5G49170.1); Has 33 Blast hits to 33 proteins in 9 species: Archae - 0; Bacteria - 0; Metazoa - 0; Fungi - 0; Plants - 33; Viruses - 0; Other Eukaryotes - 0 (source: NCBI BLink). | chr3:2156930-2157624 FORWARD LENGTH=187	187	187	3.00E-72	100.0	80.2	88.8
Rsa1.0_00030.1.g1899.t3	ref|XP_002884612.1| DIN3/LTA1 [Arabidopsis lyrata subsp. lyrata] gi|297330452|gb|EFH60871.1| DIN3/LTA1 [Arabidopsis lyrata subsp. lyrata]	229	484	1.00E-65	211.4	68.1	73.4	DIN3/LTA1	gbpln	Arabidopsis lyrata	AT3G06850.2 | Symbols: BCE2, LTA1, DIN3 | 2-oxoacid dehydrogenases acyltransferase family protein | chr3:2158212-2160465 REVERSE LENGTH=483	229	483	2.00E-64	210.9	65.5	72.1

Rsa1.0_00030.1.g1900.t1	sp|O49809.2|MFPA_BRANA RecName: Full=Glyoxysomal fatty acid beta-oxidation multifunctional protein MFP-a; Includes: RecName: Full=Enoyl-CoA hydratase/3-2-trans-enoyl-CoA isomerase/3-hydroxybutyryl-CoA epimerase; Includes: RecName: Full=3-hydroxyacyl-CoA dehydrogenase gi|2832898|emb|CAA04386.1| Tetrafunctional protein of glyoxysomal fatty acid beta-oxidation [Brassica napus]	726	725	0	99.9	90.9	95.9	RecName: Full=Glyoxysomal fatty acid beta-oxidation multifunctional protein MFP-a; Includes: RecName: Full=Enoyl-CoA hydratase/3-2-trans-enoyl-CoA isomerase/3-hydroxybutyryl-CoA epimerase; Includes: RecName: Full=3-hydroxyacyl-CoA dehydrogenase gi|2832898|emb|CAA04386.1| Tetrafunctional protein of glyoxysomal fatty acid beta-oxidation	gbpln	Brassica napus	AT3G06860.1 | Symbols: MFP2, ATMFP2 | multifunctional protein 2 | chr3:2161926-2166009 FORWARD LENGTH=725	726	725	0	99.9	89.0	93.9
Rsa1.0_00030.1.g1901.t1	ref|XP_002882498.1| hypothetical protein ARALYDRAFT_478007 [Arabidopsis lyrata subsp. lyrata] gi|297328338|gb|EFH58757.1| hypothetical protein ARALYDRAFT_478007 [Arabidopsis lyrata subsp. lyrata]	352	372	1.00E-136	105.7	82.7	88.6	hypothetical protein ARALYDRAFT_478007	gbpln	Arabidopsis lyrata	AT3G06868.1 | Symbols: | unknown protein; FUNCTIONS IN: molecular_function unknown; INVOLVED IN: biological_process unknown; LOCATED IN: cellular_component unknown; BEST Arabidopsis thaliana protein match is: unknown protein (TAIR:AT5G49100.1); Has 30201 Blast hits to 17322 proteins in 780 species: Archae - 12; Bacteria - 1396; Metazoa - 17338; Fungi - 3422; Plants - 5037; Viruses - 0; Other Eukaryotes - 2996 (source: NCBI BLink). | chr3:2167872-2168981 FORWARD LENGTH=369	352	369	1.00E-137	104.8	84.7	91.2
Rsa1.0_00030.1.g1902.t1	gb|EOA30495.1| hypothetical protein CARUB_v10013619mg [Capsella rubella]	439	471	1.00E-154	107.3	67.7	78.1	hypothetical protein CARUB_v10013619mg	gbpln	Capsella rubella	AT3G06910.1 | Symbols: ULP1A, ELS1, AtULP1a | UB-like protease 1A | chr3:2178905-2181188 REVERSE LENGTH=502	439	502	1.00E-153	114.4	66.7	77.7
Rsa1.0_00030.1.g1903.t1	ref|NP_566296.1| protein pigment defective 320 [Arabidopsis thaliana] gi|6729003|gb|AAF27000.1|AC016827_11 hypothetical protein [Arabidopsis thaliana] gi|14194139|gb|AAK56264.1|AF367275_1 AT3g06960/F17A9_11 [Arabidopsis thaliana] gi|16323342|gb|AAL15384.1| AT3g06960/F17A9_11 [Arabidopsis thaliana] gi|332640959|gb|AEE74480.1| protein pigment defective 320 [Arabidopsis thaliana]	464	479	0	103.2	84.5	90.3	protein pigment defective 320	gbpln	Arabidopsis thaliana	AT3G06960.1 | Symbols: PDE320, TGD4 | pigment defective 320 | chr3:2194379-2196504 REVERSE LENGTH=479	464	479	0	103.2	84.5	90.3
Rsa1.0_00030.1.g1904.t1	ref|XP_002882502.1| predicted protein [Arabidopsis lyrata subsp. lyrata] gi|98961639|gb|ABF59149.1| unknown protein [Arabidopsis thaliana] gi|297328342|gb|EFH58761.1| predicted protein [Arabidopsis lyrata subsp. lyrata]	69	69	4.00E-23	100.0	87.0	91.3	predicted protein	gbpln	Arabidopsis lyrata	#	#	#	#	#	#	#
Rsa1.0_00030.1.g1905.t2	gb|EOA30082.1| hypothetical protein CARUB_v10013187mg [Capsella rubella]	631	642	0	101.7	86.4	92.6	hypothetical protein CARUB_v10013187mg	gbpln	Capsella rubella	AT3G07020.2 | Symbols: | UDP-Glycosyltransferase superfamily protein | chr3:2218120-2221590 REVERSE LENGTH=637	631	637	0	101.0	85.6	90.0
Rsa1.0_00030.1.g1906.t1	gb|AAC99466.1| disease resistance gene homolog 9N [Brassica napus]	903	926	0	102.5	89.5	94.7	disease resistance gene homolog 9N	gbpln	Brassica napus	AT3G07040.1 | Symbols: RPM1, RPS3 | NB-ARC domain-containing disease resistance protein | chr3:2226244-2229024 REVERSE LENGTH=926	903	926	0	102.5	82.5	89.8
Rsa1.0_00030.1.g1907.t1	ref|XP_002884623.1| GTP-binding family protein [Arabidopsis lyrata subsp. lyrata] gi|297330463|gb|EFH60882.1| GTP-binding family protein [Arabidopsis lyrata subsp. lyrata]	588	582	0	99.0	79.1	84.2	GTP-binding family protein	gbpln	Arabidopsis lyrata	AT3G07050.1 | Symbols: | GTP-binding family protein | chr3:2229602-2232279 REVERSE LENGTH=582	588	582	0	99.0	81.1	85.5
Rsa1.0_00030.1.g1908.t1	ref|NP_566298.1| protein kinase domain-containing protein [Arabidopsis thaliana] gi|75337179|sp|Q9SFT7.1|Y3707_ARATH RecName: Full=Serine/threonine-protein kinase At3g07070 gi|6642658|gb|AAF20239.1|AC012395_26 putative protein kinase [Arabidopsis thaliana] gi|332640974|gb|AEE74495.1| serine/threonine-protein kinase [Arabidopsis thaliana]	443	414	0	93.5	83.7	88.7	protein kinase domain-containing protein	gbpln	Arabidopsis thaliana	AT3G07070.1 | Symbols: | Protein kinase superfamily protein | chr3:2238455-2240074 FORWARD LENGTH=414	443	414	0	93.5	83.7	88.7

Rsa1.0_00030.1.g1909.t1	ref|XP_002884625.1| predicted protein [Arabidopsis lyrata subsp. lyrata] gi|297330465|gb|EFH60884.1| predicted protein [Arabidopsis lyrata subsp. lyrata]	253	265	1.00E-116	104.7	81.8	88.9	predicted protein	gbpln	Arabidopsis lyrata	AT3G07090.1 | Symbols: | PPPDE putative thiol peptidase family protein | chr3:2243153-2244476 REVERSE LENGTH=265	253	265	1.00E-118	104.7	81.4	90.5
Rsa1.0_00030.1.g1910.t1	ref|NP_187366.2| sec24-like transport protein [Arabidopsis thaliana] gi|78099801|sp|Q9SFU0.2|SC24A_ARATH RecName: Full=Protein transport protein Sec24-like At3g07100 gi|22531076|gb|AAM97042.1| putative Sec24-like COPII protein [Arabidopsis thaliana] gi|23197930|gb|AAN15492.1| putative Sec24-like COPII protein [Arabidopsis thaliana] gi|332640977|gb|AEE74498.1| sec24-like transport protein [Arabidopsis thaliana]	1002	1038	0	103.6	87.1	91.7	sec24-like transport protein	gbpln	Arabidopsis thaliana	AT3G07100.1 | Symbols: ERMO2, SEC24A | Sec23/Sec24 protein transport family protein | chr3:2245689-2250077 REVERSE LENGTH=1038	1002	1038	0	103.6	87.1	91.7
Rsa1.0_00030.1.g1911.t1	ref|NP_187368.1| RING/U-box domain-containing protein [Arabidopsis thaliana] gi|6642653|gb|AAF20234.1|AC012395_21 putative RING zinc finger protein [Arabidopsis thaliana] gi|56381891|gb|AAV85664.1| At3g07120 [Arabidopsis thaliana] gi|58531344|gb|AAW78594.1| At3g07120 [Arabidopsis thaliana] gi|332640980|gb|AEE74501.1| RING/U-box domain-containing protein [Arabidopsis thaliana]	348	360	1.00E-107	103.4	72.7	82.5	RING/U-box domain-containing protein	gbpln	Arabidopsis thaliana	AT3G07120.1 | Symbols: | RING/U-box superfamily protein | chr3:2254560-2255642 FORWARD LENGTH=360	348	360	1.00E-109	103.4	72.7	82.5
Rsa1.0_00030.1.g1912.t1	gb|EOA30308.1| hypothetical protein CARUB_v10013434mg [Capsella rubella]	541	532	0	98.3	76.9	86.0	hypothetical protein CARUB_v10013434mg	gbpln	Capsella rubella	AT3G07130.1 | Symbols: ATPAP15, PAP15 | purple acid phosphatase 15 | chr3:2255763-2257981 REVERSE LENGTH=532	541	532	0	98.3	76.9	86.0
Rsa1.0_00030.1.g1913.t1	ref|NP_187372.5| callose synthase [Arabidopsis thaliana] gi|378405154|sp|Q9SFU6.2|CALS9_ARATH RecName: Full=Callose synthase 9; AltName: Full=1,3-beta-glucan synthase; AltName: Full=Protein GLUCAN SYNTHASE-LIKE 10 gi|332640985|gb|AEE74506.1| callose synthase 10 [Arabidopsis thaliana]	1864	1890	0	101.4	80.5	88.1	callose synthase	gbpln	Arabidopsis thaliana	AT3G07160.1 | Symbols: ATGSL10, gsl10, CALS9 | glucan synthase-like 10 | chr3:2265142-2279383 REVERSE LENGTH=1890	1864	1890	0	101.4	80.5	88.1
Rsa1.0_00030.1.g1914.t1	gb|EOA31542.1| hypothetical protein CARUB_v10014744mg [Capsella rubella]	154	183	8.00E-38	118.8	54.5	62.3	hypothetical protein CARUB_v10014744mg	gbpln	Capsella rubella	AT3G07200.2 | Symbols: | RING/U-box superfamily protein | chr3:2291343-2292223 FORWARD LENGTH=182	154	182	2.00E-38	118.2	53.2	62.3
Rsa1.0_00030.1.g1915.t1	ref|XP_002884637.1| hypothetical protein ARALYDRAFT_340917 [Arabidopsis lyrata subsp. lyrata] gi|297330477|gb|EFH60896.1| hypothetical protein ARALYDRAFT_340917 [Arabidopsis lyrata subsp. lyrata]	421	286	3.00E-77	67.9	41.3	47.7	hypothetical protein ARALYDRAFT_340917	gbpln	Arabidopsis lyrata	AT3G07250.1 | Symbols: | nuclear transport factor 2 (NTF2) family protein / RNA recognition motif (RRM)-containing protein | chr3:2300579-2308305 REVERSE LENGTH=1294	421	1294	2.00E-71	307.4	38.5	45.6
Rsa1.0_00031.1.g1916.t1	#	#	#	#	#	#	#	–	----	----	#	#	#	#	#	#	#
Rsa1.0_00031.1.g1917.t1	ref|XP_002878467.1| hypothetical protein ARALYDRAFT_907836 [Arabidopsis lyrata subsp. lyrata] gi|297324305|gb|EFH54726.1| hypothetical protein ARALYDRAFT_907836 [Arabidopsis lyrata subsp. lyrata]	285	380	3.00E-91	133.3	76.5	84.2	hypothetical protein ARALYDRAFT_907836	gbpln	Arabidopsis lyrata	AT3G62630.1 | Symbols: | Protein of unknown function (DUF1645) | chr3:23163937-23165079 REVERSE LENGTH=380	285	380	3.00E-92	133.3	75.4	82.8
Rsa1.0_00031.1.g1918.t1	gb|AAD32898.1| putative retroelement pol polyprotein [Arabidopsis thaliana]	1366	1307	0	95.7	59.7	73.9	putative retroelement pol polyprotein	gbpln	Arabidopsis thaliana	AT4G23160.1 | Symbols: CRK8 | cysteine-rich RLK (RECEPTOR-like protein kinase) 8 | chr4:12129485-12134086 FORWARD LENGTH=1262	1366	1262	1.00E-100	92.4	14.2	22.5
Rsa1.0_00031.1.g1919.t1	ref|NP_191824.1| uncharacterized protein [Arabidopsis thaliana] gi|42572765|ref|NP_974478.1| uncharacterized protein [Arabidopsis thaliana] gi|7362745|emb|CAB83115.1| putative protein [Arabidopsis thaliana] gi|15450964|gb|AAK96753.1| putative protein [Arabidopsis thaliana] gi|17978723|gb|AAL47355.1| putative protein [Arabidopsis thaliana] gi|332646854|gb|AEE80375.1| uncharacterized protein AT3G62650 [Arabidopsis thaliana] gi|332646855|gb|AEE80376.1| uncharacterized protein AT3G62650 [Arabidopsis thaliana]	147	152	5.00E-48	103.4	75.5	83.7	uncharacterized protein	gbpln	Arabidopsis thaliana	AT3G62650.1 | Symbols: | unknown protein; BEST Arabidopsis thaliana protein match is: unknown protein (TAIR:AT2G47485.1); Has 57 Blast hits to 57 proteins in 13 species: Archae - 0; Bacteria - 0; Metazoa - 0; Fungi - 0; Plants - 57; Viruses - 0; Other Eukaryotes - 0 (source: NCBI BLink). | chr3:23168531-23168989 FORWARD LENGTH=152	147	152	1.00E-50	103.4	75.5	83.7

Rsa1.0_00031.1.g1920.t1	#	#	#	#	#	#	#	-	----	----	#	#	#	#	#	#	#
Rsa1.0_00031.1.g1921.t1	ref|XP_002891930.1| hypothetical protein ARALYDRAFT_474772 [Arabidopsis lyrata subsp. lyrata] gi|297337772|gb|EFH68189.1| hypothetical protein ARALYDRAFT_474772 [Arabidopsis lyrata subsp. lyrata]	300	334	1.00E-88	111.3	76.7	81.3	hypothetical protein ARALYDRAFT_474772	gbpln	Arabidopsis lyrata	AT1G54970.1 | Symbols: ATPRP1, RHS7, PRP1 | proline-rich protein 1 | chr1:20505000-20506238 FORWARD LENGTH=335	300	335	2.00E-90	111.7	74.3	80.7
Rsa1.0_00031.1.g1922.t1	gb|AAF64548.1|AF110985_1 proline-rich protein 1 [Arabidopsis thaliana]	322	335	2.00E-25	104.0	39.8	43.2	proline-rich protein 1	gbpln	Arabidopsis thaliana	AT1G54970.1 | Symbols: ATPRP1, RHS7, PRP1 | proline-rich protein 1 | chr1:20505000-20506238 FORWARD LENGTH=335	322	335	3.00E-27	104.0	37.3	41.0
Rsa1.0_00031.1.g1923.t1	#	#	#	#	#	#	#	-	----	----	#	#	#	#	#	#	#
Rsa1.0_00031.1.g1924.t1	ref|NP_191830.1| glycosyl hydrolase-like protein [Arabidopsis thaliana] gi|7362751|emb|CAB83121.1| beta-D-glucan exohydrolase-like protein [Arabidopsis thaliana] gi|17065160|gb|AAL32734.1| beta-D-glucan exohydrolase-like protein [Arabidopsis thaliana] gi|20259922|gb|AAM13308.1| beta-D-glucan exohydrolase-like protein [Arabidopsis thaliana] gi|332646861|gb|AEE80382.1| glycosyl hydrolase-like protein [Arabidopsis thaliana]	642	650	0	101.2	82.1	89.1	glycosyl hydrolase-like protein	gbpln	Arabidopsis thaliana	AT3G62710.1 | Symbols: | Glycosyl hydrolase family protein | chr3:23197739-23200559 REVERSE LENGTH=650	642	650	0	101.2	82.1	89.1
Rsa1.0_00031.1.g1925.t1	ref|NP_191831.1| xyloglucan 6-xylosyltransferase [Arabidopsis thaliana] gi|79316129|ref|NP_001030917.1| xyloglucan 6-xylosyltransferase [Arabidopsis thaliana] gi|46577293|sp|Q9LZJ3.1|XT1_ARATH RecName: Full=Xyloglucan 6-xylosyltransferase; Short=AtXT1 gi|15983426|gb|AAL11581.1|AF424587_1 AT3g62720/F26K9_150 [Arabidopsis thaliana] gi|7362752|emb|CAB83122.1| alpha galactosyltransferase-like protein [Arabidopsis thaliana] gi|25141201|gb|AAN73295.1| At3g62720/F26K9_150 [Arabidopsis thaliana] gi|222424476|dbj|BAH20193.1| AT3G62720 [Arabidopsis thaliana] gi|332646862|gb|AEE80383.1| xyloglucan 6-xylosyltransferase [Arabidopsis thaliana] gi|332646863|gb|AEE80384.1| xyloglucan 6-xylosyltransferase [Arabidopsis thaliana]	463	460	0	99.4	89.4	93.1	xyloglucan 6-xylosyltransferase	gbpln	Arabidopsis thaliana	AT3G62720.2 | Symbols: ATXT1, XT1, XXT1 | xylosyltransferase 1 | chr3:23201326-23202708 FORWARD LENGTH=460	463	460	0	99.4	89.4	93.1
Rsa1.0_00031.1.g1926.t1	dbj|BAJ34035.1| unnamed protein product [Thellungiella halophila]	498	506	0	101.6	77.9	86.7	unnamed protein product	----	----	AT4G27830.1 | Symbols: BGLU10 | beta glucosidase 10 | chr4:13861794-13864489 REVERSE LENGTH=508	498	508	0	102.0	76.7	88.2
Rsa1.0_00031.1.g1927.t1	dbj|BAJ34035.1| unnamed protein product [Thellungiella halophila]	501	506	0	101.0	76.0	84.0	unnamed protein product	----	----	AT4G27830.1 | Symbols: BGLU10 | beta glucosidase 10 | chr4:13861794-13864489 REVERSE LENGTH=508	501	508	0	101.4	73.1	84.8
Rsa1.0_00031.1.g1928.t1	ref|NP_191835.1| Glutathione S-transferase-like protein [Arabidopsis thaliana] gi|75335711|sp|Q9LZI9.1|GSTFD_ARATH RecName: Full=Glutathione S-transferase F13; Short=AtGSTF13; AltName: Full=GST class-phi member 13 gi|7362756|emb|CAB83126.1| Glutathione transferase III-like protein [Arabidopsis thaliana] gi|332646867|gb|AEE80388.1| glutathione S-transferase F13 [Arabidopsis thaliana]	219	219	1.00E-115	100.0	90.0	95.9	Glutathione S-transferase-like protein	gbpln	Arabidopsis thaliana	AT3G62760.1 | Symbols: ATGSTF13 | Glutathione S-transferase family protein | chr3:23217425-23218246 REVERSE LENGTH=219	219	219	1.00E-118	100.0	90.0	95.9
Rsa1.0_00031.1.g1929.t1	gb|EOA24065.1| hypothetical protein CARUB_v10017285mg [Capsella rubella]	421	435	0	103.3	88.8	93.6	hypothetical protein CARUB_v10017285mg	gbpln	Capsella rubella	AT3G62770.1 | Symbols: AtATG18a | Transducin/WD40 repeat-like superfamily protein | chr3:23218858-23221110 REVERSE LENGTH=425	421	425	0	101.0	85.7	90.7

Rsa1.0_00031.1.g1930.t1	ref|NP_191837.1| calcium-dependent lipid-binding domain-containing protein [Arabidopsis thaliana] gi|7362758|emb|CAB83128.1| shock protein SRC2-like [Arabidopsis thaliana] gi|42600565|gb|AAS21129.1| At3g62780 [Arabidopsis thaliana] gi|45773884|gb|AAS76746.1| At3g62780 [Arabidopsis thaliana] gi|332646870|gb|AEE80391.1| calcium-dependent lipid-binding domain-containing protein [Arabidopsis thaliana]	273	298	3.00E-99	109.2	75.8	80.2	calcium-dependent lipid-binding domain-containing protein	gbpln	Arabidopsis thaliana	AT3G62780.1 | Symbols: | Calcium-dependent lipid-binding (CaLB domain) family protein | chr3:23222029-23222925 REVERSE LENGTH=298	273	298	1.00E-102	109.2	75.8	80.2
Rsa1.0_00031.1.g1931.t1	gb|EOA24988.1| hypothetical protein CARUB_v10018285mg [Capsella rubella] gi|482560798|gb|EOA24989.1| hypothetical protein CARUB_v10018285mg [Capsella rubella]	106	106	2.00E-48	100.0	91.5	96.2	hypothetical protein CARUB_v10018285mg	gbpln	Capsella rubella	AT3G62810.1 | Symbols: | complex 1 family protein / LVR family protein | chr3:23227763-23228180 FORWARD LENGTH=106	106	106	4.00E-50	100.0	90.6	96.2
Rsa1.0_00031.1.g1932.t1	ref|NP_191842.1| UDP-glucuronic acid decarboxylase [Arabidopsis thaliana] gi|186511349|ref|NP_001118893.1| UDP-glucuronic acid decarboxylase [Arabidopsis thaliana] gi|75335709|sp|Q9LZI2.1|UXS2_ARATH RecName: Full=UDP-glucuronic acid decarboxylase 2; AltName: Full=UDP-XYL synthase 2; AltName: Full=UDP-glucuronate decarboxylase 2; Short=UGD; Short=UXS-2; AltName: Full=dTDP-glucose 4-6-dehydratase homolog D18 gi|7362763|emb|CAB83133.1| dTDP-glucose 4-6-dehydratase homolog D18 [Arabidopsis thaliana] gi|23505953|gb|AAN28836.1| At3g62830/F26K9_260 [Arabidopsis thaliana] gi|332646877|gb|AEE80398.1| UDP-glucuronic acid decarboxylase [Arabidopsis thaliana] gi|332646878|gb|AEE80399.1| UDP-glucuronic acid decarboxylase [Arabidopsis thaliana]	423	445	0	105.2	89.6	94.1	UDP-glucuronic acid decarboxylase	gbpln	Arabidopsis thaliana	AT3G62830.2 | Symbols: UXS2, ATUXS2 | NAD(P)-binding Rossmann-fold superfamily protein | chr3:23232539-23235353 FORWARD LENGTH=445	423	445	0	105.2	89.6	94.1
Rsa1.0_00031.1.g1933.t4	ref|NP_850477.1| small nuclear ribonucleoprotein D2 [Arabidopsis thaliana] gi|42571273|ref|NP_973710.1| small nuclear ribonucleoprotein D2 [Arabidopsis thaliana] gi|145332931|ref|NP_001078331.1| small nuclear ribonucleoprotein D2 [Arabidopsis thaliana] gi|7362764|emb|CAB83134.1| small nuclear ribonucleoprotein-like protein [Arabidopsis thaliana] gi|28466881|gb|AAO44049.1| At2g47640 [Arabidopsis thaliana] gi|110743879|dbj|BAE99774.1| putative small nuclear ribonucleoprotein D2 [Arabidopsis thaliana] gi|330255774|gb|AEC10868.1| small nuclear ribonucleoprotein D2 [Arabidopsis thaliana] gi|330255775|gb|AEC10869.1| small nuclear ribonucleoprotein D2 [Arabidopsis thaliana] gi|332646880|gb|AEE80401.1| small nuclear ribonucleoprotein D2 [Arabidopsis thaliana] gi|482563987|gb|EOA28177.1| hypothetical protein CARUB_v10024367mg [Capsella rubella]	104	108	3.00E-53	103.8	100.0	100.0	small nuclear ribonucleoprotein D2	gbpln	Arabidopsis thaliana	AT3G62840.2 | Symbols: | Small nuclear ribonucleoprotein family protein | chr3:23235727-23236615 REVERSE LENGTH=108	104	108	5.00E-56	103.8	100.0	100.0
Rsa1.0_00031.1.g1934.t1	gb|EOA27436.1| hypothetical protein CARUB_v10023573mg [Capsella rubella]	256	338	1.00E-141	132.0	96.9	97.7	hypothetical protein CARUB_v10023573mg	gbpln	Capsella rubella	AT3G62870.1 | Symbols: | Ribosomal protein L7Ae/L30e/S12e/Gadd45 family protein | chr3:23242862-23244273 REVERSE LENGTH=256	256	256	1.00E-140	100.0	94.1	97.3
Rsa1.0_00031.1.g1935.t1	gb|EOA26198.1| hypothetical protein CARUB_v10019636mg [Capsella rubella]	142	134	5.00E-50	94.4	78.2	85.9	hypothetical protein CARUB_v10019636mg	gbpln	Capsella rubella	AT3G62880.2 | Symbols: ATOEP16-4 | Mitochondrial import inner membrane translocase subunit Tim17/Tim22/Tim23 family protein | chr3:23245103-23246002 REVERSE LENGTH=136	142	136	2.00E-49	95.8	74.6	82.4

Rsa1.0_00031.1.g1936.t1	gb|EOA24179.1| hypothetical protein CARUB_v10017414mg [Capsella rubella]	392	391	1.00E-172	99.7	78.8	86.0	hypothetical protein CARUB_v10017414mg	gbpln	Capsella rubella	AT1G21150.1 | Symbols: | Mitochondrial transcription termination factor family protein | chr1:7406406-7407578 REVERSE LENGTH=390	392	390	1.00E-124	99.5	60.2	74.7
Rsa1.0_00031.1.g1937.t1	gb|EOA24095.1| hypothetical protein CARUB_v10017325mg [Capsella rubella]	411	422	0	102.7	92.0	95.9	hypothetical protein CARUB_v10017325mg	gbpln	Capsella rubella	AT3G62910.1 | Symbols: APG3 | Peptide chain release factor 1 | chr3:23257661-23260386 REVERSE LENGTH=422	411	422	0	102.7	91.5	95.9
Rsa1.0_00031.1.g1938.t1	gb|EOA23343.1| hypothetical protein CARUB_v10019414mg [Capsella rubella]	102	102	2.00E-49	100.0	93.1	98.0	hypothetical protein CARUB_v10019414mg	gbpln	Capsella rubella	AT3G62930.1 | Symbols: | Thioredoxin superfamily protein | chr3:23261538-23261846 REVERSE LENGTH=102	102	102	1.00E-50	100.0	89.2	95.1
Rsa1.0_00031.1.g1939.t1	ref|XP_002878488.1| hypothetical protein ARALYDRAFT_486799 [Arabidopsis lyrata subsp. lyrata] gi|297324326|gb|EFH54747.1| hypothetical protein ARALYDRAFT_486799 [Arabidopsis lyrata subsp. lyrata]	314	326	1.00E-138	103.8	84.1	91.1	hypothetical protein ARALYDRAFT_486799	gbpln	Arabidopsis lyrata	AT3G62940.3 | Symbols: | Cysteine proteinases superfamily protein | chr3:23263106-23264245 REVERSE LENGTH=332	314	332	1.00E-138	105.7	86.9	93.0
Rsa1.0_00031.1.g1940.t1	ref|NP_191854.1| glutaredoxin-C11 [Arabidopsis thaliana] gi|297817626|ref|XP_002876696.1| glutaredoxin family protein [Arabidopsis lyrata subsp. lyrata] gi|75264377|sp|Q9LYC6.1|GRC11_ARATH RecName: Full=Glutaredoxin-C11; Short=AtGrxC11; AltName: Full=Protein ROXY 4 gi|7573424|emb|CAB87740.1| glutaredoxin-like protein [Arabidopsis thaliana] gi|50253460|gb|AAT71932.1| At3g62950 [Arabidopsis thaliana] gi|51972084|gb|AAU15146.1| At3g62950 [Arabidopsis thaliana] gi|226348186|gb|ACO50409.1| glutaredoxin [Arabidopsis thaliana] gi|297322534|gb|EFH52955.1| glutaredoxin family protein [Arabidopsis lyrata subsp. lyrata] gi|332646895|gb|AEE80416.1| glutaredoxin-C11 [Arabidopsis thaliana]	103	103	4.00E-53	100.0	99.0	100.0	glutaredoxin-C11	gbpln	Arabidopsis lyrata	AT3G62950.1 | Symbols: | Thioredoxin superfamily protein | chr3:23266303-23266614 FORWARD LENGTH=103	103	103	6.00E-56	100.0	99.0	100.0
Rsa1.0_00031.1.g1941.t1	ref|NP_191855.1| glutaredoxin-C14 [Arabidopsis thaliana] gi|75181054|sp|Q9LYC5.1|GRC14_ARATH RecName: Full=Glutaredoxin-C14; Short=AtGrxC14; AltName: Full=Protein ROXY 8 gi|7573425|emb|CAB87741.1| glutaredoxin-like protein [Arabidopsis thaliana] gi|88900350|gb|ABD57487.1| At3g62960 [Arabidopsis thaliana] gi|226348194|gb|ACO50413.1| glutaredoxin [Arabidopsis thaliana] gi|332646896|gb|AEE80417.1| glutaredoxin-C14 [Arabidopsis thaliana] gi|482560809|gb|EOA25000.1| hypothetical protein CARUB_v10018297mg [Capsella rubella]	102	102	1.00E-51	100.0	97.1	98.0	glutaredoxin-C14	gbpln	Arabidopsis thaliana	AT3G62960.1 | Symbols: | Thioredoxin superfamily protein | chr3:23268780-23269088 FORWARD LENGTH=102	102	102	2.00E-54	100.0	97.1	98.0
Rsa1.0_00031.1.g1942.t1	ref|NP_001190481.1| phosphatidylinositol glycan, class X [Arabidopsis thaliana] gi|332008052|gb|AED95435.1| uncharacterized protein AT5G46850 [Arabidopsis thaliana]	132	325	5.00E-28	246.2	53.0	65.9	phosphatidylinositol glycan, class X	gbpln	Arabidopsis thaliana	AT5G46850.2 | Symbols: | FUNCTIONS IN: molecular_function unknown; INVOLVED IN: GPI anchor biosynthetic process; LOCATED IN: endoplasmic reticulum membrane; EXPRESSED IN: 22 plant structures; EXPRESSED DURING: 13 growth stages; CONTAINS InterPro DOMAIN/s: PIG-X/PBN1 (InterPro:IPR013233). | chr5:19010714-19012010 FORWARD LENGTH=325	132	325	1.00E-30	246.2	53.0	65.9
Rsa1.0_00031.1.g1943.t1	gb|EOA23690.1| hypothetical protein CARUB_v10016894mg [Capsella rubella]	597	594	0	99.5	93.6	96.5	hypothetical protein CARUB_v10016894mg	gbpln	Capsella rubella	AT3G62980.1 | Symbols: TIR1 | F-box/RNI-like superfamily protein | chr3:23273479-23276181 REVERSE LENGTH=594	597	594	0	99.5	92.6	96.5
Rsa1.0_00031.1.g1944.t1	#	#	#	#	#	#	#	–	----	----	#	#	#	#	#	#	#
Rsa1.0_00031.1.g1945.t1	gb|EOA24114.1| hypothetical protein CARUB_v10017347mg [Capsella rubella]	414	413	0	99.8	90.1	96.9	hypothetical protein CARUB_v10017347mg	gbpln	Capsella rubella	AT3G63000.1 | Symbols: NPL41 | NPL4-like protein 1 | chr3:23283836-23285357 FORWARD LENGTH=413	414	413	0	99.8	90.3	96.9
Rsa1.0_00031.1.g1946.t1	gb|ADT78692.1| gibberellin receptor 1B [Brassica napus]	364	358	0	98.4	91.8	95.6	gibberellin receptor 1B	gbpln	Brassica napus	AT3G63010.1 | Symbols: ATGID1B, GID1B | alpha/beta-Hydrolases superfamily protein | chr3:23289717-23290998 FORWARD LENGTH=358	364	358	0	98.4	87.6	93.1

Rsa1.0_00031.1.g1947.t1	ref|XP_002876703.1| methyl-CpG-binding domain 4 [Arabidopsis lyrata subsp. lyrata] gi|297322541|gb|EFH52962.1| methyl-CpG-binding domain 4 [Arabidopsis lyrata subsp. lyrata]	327	182	7.00E-71	55.7	37.9	42.8	methyl-CpG-binding domain 4	gbpln	Arabidopsis lyrata	AT3G63030.1 | Symbols: MBD4 | methyl-CPG-binding domain 4 | chr3:23295394-23296425 FORWARD LENGTH=186	327	186	1.00E-71	56.9	36.4	41.6
Rsa1.0_00031.1.g1948.t1	gb|EOA25199.1| hypothetical protein CARUB_v10018512mg [Capsella rubella]	117	179	1.00E-12	153.0	41.0	52.1	hypothetical protein CARUB_v10018512mg	gbpln	Capsella rubella	AT3G63040.1 | Symbols: | unknown protein; FUNCTIONS IN: molecular_function unknown; INVOLVED IN: biological_process unknown; LOCATED IN: endomembrane system; Has 5 Blast hits to 5 proteins in 2 species: Archae - 0; Bacteria - 0; Metazoa - 0; Fungi - 0; Plants - 5; Viruses - 0; Other Eukaryotes - 0 (source: NCBI BLink). | chr3:23296646-23297209 REVERSE LENGTH=187	117	187	4.00E-14	159.8	40.2	49.6
Rsa1.0_00031.1.g1949.t1	ref|XP_002878491.1| hypothetical protein ARALYDRAFT_486808 [Arabidopsis lyrata subsp. lyrata] gi|297324329|gb|EFH54750.1| hypothetical protein ARALYDRAFT_486808 [Arabidopsis lyrata subsp. lyrata]	131	135	2.00E-23	103.1	55.7	70.2	hypothetical protein ARALYDRAFT_486808	gbpln	Arabidopsis lyrata	AT3G63050.1 | Symbols: | unknown protein; BEST Arabidopsis thaliana protein match is: unknown protein (TAIR:AT2G48075.1); Has 30201 Blast hits to 17322 proteins in 780 species: Archae - 12; Bacteria - 1396; Metazoa - 17338; Fungi - 3422; Plants - 5037; Viruses - 0; Other Eukaryotes - 2996 (source: NCBI BLink). | chr3:23297950-23298372 REVERSE LENGTH=140	131	140	9.00E-26	106.9	56.5	70.2
Rsa1.0_00031.1.g1950.t1	gb|AAF79749.1|AC009317_8 T30E16.12 [Arabidopsis thaliana]	351	341	1.00E-100	97.2	54.7	68.4	T30E16.12	gbpln	Arabidopsis thaliana	AT1G59560.1 | Symbols: ZCF61, DAL2 | E3 Ubiquitin ligase family protein | chr1:21881741-21883632 FORWARD LENGTH=338	351	338	1.00E-101	96.3	54.7	68.4
Rsa1.0_00031.1.g1951.t1	gb|EOA25934.1| hypothetical protein CARUB_v10019315mg [Capsella rubella]	1583	1353	0	85.5	60.7	68.3	hypothetical protein CARUB_v10019315mg	gbpln	Capsella rubella	AT2G48160.1 | Symbols: | Tudor/PWWP/MBT domain-containing protein | chr2:19689784-19696584 REVERSE LENGTH=1366	1583	1366	0	86.3	51.0	60.5
Rsa1.0_00031.1.g1952.t1	ref|NP_001078333.1| tetratricopeptide repeat-containing protein-like protein [Arabidopsis thaliana] gi|332646914|gb|AEE80435.1| tetratricopeptide repeat-containing protein-like protein [Arabidopsis thaliana]	258	250	6.00E-16	96.9	15.9	17.8	tetratricopeptide repeat-containing protein-like protein	gbpln	Arabidopsis thaliana	AT3G63095.1 | Symbols: | Tetratricopeptide repeat (TPR)-like superfamily protein | chr3:23315506-23316258 FORWARD LENGTH=250	258	250	2.00E-18	96.9	15.9	17.8
Rsa1.0_00031.1.g1953.t1	ref|XP_002878496.1| ATIPT3 [Arabidopsis lyrata subsp. lyrata] gi|297324334|gb|EFH54755.1| ATIPT3 [Arabidopsis lyrata subsp. lyrata]	334	336	1.00E-167	100.6	86.8	92.8	ATIPT3	gbpln	Arabidopsis lyrata	AT3G63110.1 | Symbols: ATIPT3, IPT3 | isopentenyltransferase 3 | chr3:23318437-23319447 REVERSE LENGTH=336	334	336	1.00E-168	100.6	85.0	93.1
Rsa1.0_00031.1.g1954.t1	gb|EOA25999.1| hypothetical protein CARUB_v10019395mg [Capsella rubella]	528	535	0	101.3	80.3	89.0	hypothetical protein CARUB_v10019395mg	gbpln	Capsella rubella	AT3G63130.2 | Symbols: RANGAP1 | RAN GTPase activating protein 1 | chr3:23325108-23326715 FORWARD LENGTH=535	528	535	0	101.3	79.5	88.1
Rsa1.0_00031.1.g1955.t1	gb|EOA24136.1| hypothetical protein CARUB_v10017370mg [Capsella rubella]	449	404	0	90.0	78.4	81.3	hypothetical protein CARUB_v10017370mg	gbpln	Capsella rubella	AT3G63140.1 | Symbols: CSP41A | chloroplast stem-loop binding protein of 41 kDa | chr3:23327006-23328620 REVERSE LENGTH=406	449	406	0	90.4	78.8	82.0
Rsa1.0_00031.1.g1956.t1	ref|NP_567139.1| MIRO-related GTP-ase 2 [Arabidopsis thaliana] gi|332646920|gb|AEE80441.1| MIRO-related GTP-ase 2 [Arabidopsis thaliana]	646	643	0	99.5	90.4	94.9	MIRO-related GTP-ase 2	gbpln	Arabidopsis thaliana	AT3G63150.1 | Symbols: MIRO2, ATCBG | MIRO-related GTP-ase 2 | chr3:23329200-23332692 REVERSE LENGTH=643	646	643	0	99.5	90.4	94.9
Rsa1.0_00031.1.g1957.t1	ref|XP_002876711.1| hypothetical protein ARALYDRAFT_486819 [Arabidopsis lyrata subsp. lyrata] gi|297322549|gb|EFH52970.1| hypothetical protein ARALYDRAFT_486819 [Arabidopsis lyrata subsp. lyrata]	283	279	1.00E-132	98.6	83.0	90.5	hypothetical protein ARALYDRAFT_486819	gbpln	Arabidopsis lyrata	AT3G63170.1 | Symbols: | Chalcone-flavanone isomerase family protein | chr3:23334675-23335993 FORWARD LENGTH=279	283	279	1.00E-128	98.6	83.7	90.8
Rsa1.0_00032.1.g1958.t2	#	#	#	#	#	#	#	-	----	----	#	#	#	#	#	#	#
Rsa1.0_00032.1.g1959.t2	ref|XP_002882851.1| hypothetical protein ARALYDRAFT_478784 [Arabidopsis lyrata subsp. lyrata] gi|297328691|gb|EFH59110.1| hypothetical protein ARALYDRAFT_478784 [Arabidopsis lyrata subsp. lyrata]	498	507	0	101.8	81.7	89.2	hypothetical protein ARALYDRAFT_478784	gbpln	Arabidopsis lyrata	AT3G13810.1 | Symbols: AtIDD11, IDD11 | indeterminate(ID)-domain 11 | chr3:4544941-4547300 FORWARD LENGTH=513	498	513	0	103.0	80.7	87.3
Rsa1.0_00032.1.g1960.t1	gb|EOA31706.1| hypothetical protein CARUB_v10014912mg [Capsella rubella]	70	130	2.00E-13	185.7	88.6	90.0	hypothetical protein CARUB_v10014912mg	gbpln	Capsella rubella	#	#	#	#	#	#	#

Rsa1.0_00032.1.g1961.t1	ref|NP_188001.1| LOB domain-containing protein 22 [Arabidopsis thaliana] gi|29428016|sp|Q9LRW1.1|LBD22_ARATH RecName: Full=LOB domain-containing protein 22; AltName: Full=ASYMMETRIC LEAVES 2-like protein 30; Short=AS2-like protein 30 gi|9294609|dbj|BAB02910.1| unnamed protein product [Arabidopsis thaliana] gi|219807136|dbj|BAH10574.1| ASYMMETRIC LEAVES2-like 30 protein [Arabidopsis thaliana] gi|332641904|gb|AEE75425.1| LOB domain-containing protein 22 [Arabidopsis thaliana]	259	268	1.00E-112	103.5	80.3	90.3	LOB domain-containing protein 22	gbpln	Arabidopsis thaliana	AT3G13850.1 | Symbols: LBD22 | LOB domain-containing protein 22 | chr3:4559910-4560716 FORWARD LENGTH=268	259	268	1.00E-115	103.5	80.3	90.3
Rsa1.0_00032.1.g1962.t2	ref|NP_188003.1| Root hair defective 3 GTP-binding protein RHD3 [Arabidopsis thaliana] gi|75279781|sp|P93042.1|RHD3_ARATH RecName: Full=Protein ROOT HAIR DEFECTIVE 3; AltName: Full=Protein FRAGILE FIBER 4; AltName: Full=Protein SEY1 homolog 1 gi|1839188|gb|AAB58375.1| root hair defective 3 [Arabidopsis thaliana] gi|20260178|gb|AAM12987.1| unknown protein [Arabidopsis thaliana] gi|22136246|gb|AAM91201.1| unknown protein [Arabidopsis thaliana] gi|332641908|gb|AEE75429.1| Root hair defective 3 GTP-binding protein RHD3 [Arabidopsis thaliana]	851	802	0	94.2	86.0	90.4	Root hair defective 3 GTP-binding protein RHD3	gbpln	Arabidopsis thaliana	AT3G13870.1 | Symbols: RHD3 | Root hair defective 3 GTP-binding protein (RHD3) | chr3:4565762-4571109 REVERSE LENGTH=802	851	802	0	94.2	86.0	90.4
Rsa1.0_00032.1.g1963.t1	ref|XP_002882857.1| structural constituent of ribosome [Arabidopsis lyrata subsp. lyrata] gi|297328697|gb|EFH59116.1| structural constituent of ribosome [Arabidopsis lyrata subsp. lyrata]	147	147	8.00E-65	100.0	89.1	92.5	structural constituent of ribosome	gbpln	Arabidopsis lyrata	AT3G13882.1 | Symbols: | Ribosomal protein L34 | chr3:4574600-4575852 FORWARD LENGTH=147	147	147	4.00E-66	100.0	87.1	91.8
Rsa1.0_00032.1.g1964.t2	gb|EOA32487.1| hypothetical protein CARUB_v10015767mg [Capsella rubella]	346	357	1.00E-128	103.2	77.7	84.4	hypothetical protein CARUB_v10015767mg	gbpln	Capsella rubella	AT3G13890.2 | Symbols: MYB26 | myb domain protein 26 | chr3:4576744-4578027 REVERSE LENGTH=358	346	358	1.00E-128	103.5	75.4	82.1
Rsa1.0_00032.1.g1965.t7	gb|AAC95175.1| putative non-LTR retroelement reverse transcriptase [Arabidopsis thaliana]	1663	1352	1.00E-180	81.3	23.6	34.2	putative non-LTR retroelement reverse transcriptase	gbpln	Arabidopsis thaliana	AT3G24255.1 | Symbols: | RNA-directed DNA polymerase (reverse transcriptase)-related family protein | chr3:8789471-8793208 FORWARD LENGTH=746	1663	746	5.00E-50	44.9	7.6	10.6
Rsa1.0_00032.1.g1966.t1	#	#	#	#	#	#	#	-	----	----	#	#	#	#	#	#	#
Rsa1.0_00032.1.g1967.t1	ref|XP_002893400.1| predicted protein [Arabidopsis lyrata subsp. lyrata] gi|297339242|gb|EFH69659.1| predicted protein [Arabidopsis lyrata subsp. lyrata]	222	196	1.00E-52	88.3	48.2	52.3	predicted protein	gbpln	Arabidopsis lyrata	AT2G46230.1 | Symbols: | PIN domain-like family protein | chr2:18984459-18985850 REVERSE LENGTH=196	222	196	2.00E-52	88.3	45.9	50.9
Rsa1.0_00032.1.g1968.t4	ref|XP_002882859.1| hypothetical protein ARALYDRAFT_341522 [Arabidopsis lyrata subsp. lyrata] gi|297328699|gb|EFH59118.1| hypothetical protein ARALYDRAFT_341522 [Arabidopsis lyrata subsp. lyrata]	1253	1243	0	99.2	92.6	95.7	hypothetical protein ARALYDRAFT_341522	gbpln	Arabidopsis lyrata	AT3G13900.1 | Symbols: | ATPase E1-E2 type family protein / haloacid dehalogenase-like hydrolase family protein | chr3:4586151-4590681 FORWARD LENGTH=1243	1253	1243	0	99.2	92.2	95.7
Rsa1.0_00032.1.g1969.t1	gb|EOA31757.1| hypothetical protein CARUB_v10014974mg [Capsella rubella]	92	106	1.00E-33	115.2	84.8	91.3	hypothetical protein CARUB_v10014974mg	gbpln	Capsella rubella	AT3G13910.1 | Symbols: | Protein of unknown function (DUF3511) | chr3:4591124-4591432 REVERSE LENGTH=102	92	102	5.00E-32	110.9	69.6	72.8
Rsa1.0_00032.1.g1970.t1	gb|EOA30691.1| hypothetical protein CARUB_v10013828mg [Capsella rubella]	422	412	0	97.6	96.0	97.2	hypothetical protein CARUB_v10013828mg	gbpln	Capsella rubella	AT3G13920.1 | Symbols: EIF4A1, RH4, TIF4A1 | eukaryotic translation initiation factor 4A1 | chr3:4592635-4594128 REVERSE LENGTH=412	422	412	0	97.6	95.0	97.2

Rsa1.0_00032.1.g1971.t15	ref|NP_566470.1| dihydrolipoyllysine-residue acetyltransferase component 2 of pyruvate dehydrogenase complex [Arabidopsis thaliana] gi|118573090|sp|Q8RWN9.2|OPD22_ARATH RecName: Full=Dihydrolipoyllysine-residue acetyltransferase component 2 of pyruvate dehydrogenase complex, mitochondrial; AltName: Full=Dihydrolipoamide S-acetyltransferase component 2 of pyruvate dehydrogenase complex; AltName: Full=Pyruvate dehydrogenase complex component E2 2; Short=PDC-E2 2; Short=PDCE2 2; Flags: Precursor gi|13605807|gb|AAK32889.1|AF367302_1 AT3g13930/MDC16_5 [Arabidopsis thaliana] gi|20147147|gb|AAM10290.1| AT3g13930/MDC16_5 [Arabidopsis thaliana] gi|23306388|gb|AAN17421.1| putative acetyltransferase [Arabidopsis thaliana] gi|23397124|gb|AAN31846.1| putative acetyltransferase [Arabidopsis thaliana] gi|24899791|gb|AAN65110.1| putative acetyltransferase [Arabidopsis thaliana] gi|332641921|gb|AEE75442.1| dihydrolipoyllysine-residue acetyltransferase component 2 of pyruvate dehydrogenase complex [Arabidopsis thaliana]	868	539	0	62.1	49.1	52.4	dihydrolipoyllysine-residue acetyltransferase component 2 of pyruvate dehydrogenase complex	gbpln	Arabidopsis thaliana	AT3G13930.1 | Symbols: | Dihydrolipoamide acetyltransferase, long form protein | chr3:4596240-4600143 FORWARD LENGTH=539	868	539	0	62.1	49.1	52.4
Rsa1.0_00032.1.g1972.t1	#	#	#	#	#	#	#	-	----	----	#	#	#	#	#	#	#
Rsa1.0_00032.1.g1973.t1	ref|XP_002862273.1| predicted protein [Arabidopsis lyrata subsp. lyrata] gi|297307605|gb|EFH38531.1| predicted protein [Arabidopsis lyrata subsp. lyrata]	203	219	3.00E-68	107.9	64.0	68.5	predicted protein	gbpln	Arabidopsis lyrata	AT3G13950.1 | Symbols: | unknown protein; BEST Arabidopsis thaliana protein match is: unknown protein (TAIR:AT4G13266.1); Has 339 Blast hits to 265 proteins in 12 species: Archae - 0; Bacteria - 0; Metazoa - 0; Fungi - 0; Plants - 339; Viruses - 0; Other Eukaryotes - 0 (source: NCBI BLink). | chr3:4604149-4605195 FORWARD LENGTH=207	203	207	4.00E-69	102.0	62.6	69.0
Rsa1.0_00032.1.g1974.t1	gb|EOA30723.1| hypothetical protein CARUB_v10013860mg [Capsella rubella]	405	403	1.00E-175	99.5	81.5	88.9	hypothetical protein CARUB_v10013860mg	gbpln	Capsella rubella	AT3G13960.1 | Symbols: AtGRF5, GRF5 | growth-regulating factor 5 | chr3:4608526-4610160 FORWARD LENGTH=397	405	397	1.00E-154	98.0	80.2	88.1
Rsa1.0_00032.1.g1975.t1	ref|XP_002884999.1| hypothetical protein ARALYDRAFT_318183 [Arabidopsis lyrata subsp. lyrata] gi|297330839|gb|EFH61258.1| hypothetical protein ARALYDRAFT_318183 [Arabidopsis lyrata subsp. lyrata]	177	180	7.00E-22	101.7	58.2	63.3	hypothetical protein ARALYDRAFT_318183	gbpln	Arabidopsis lyrata	#	#	#	#	#	#	#
Rsa1.0_00032.1.g1976.t1	gb|EOA37234.1| hypothetical protein CARUB_v10010750mg [Capsella rubella]	94	94	8.00E-45	100.0	93.6	95.7	hypothetical protein CARUB_v10010750mg	gbpln	Capsella rubella	AT3G13970.1 | Symbols: APG12, APG12B | Ubiquitin-like superfamily protein | chr3:4613696-4614794 REVERSE LENGTH=94	94	94	3.00E-46	100.0	90.4	95.7
Rsa1.0_00032.1.g1977.t1	#	#	#	#	#	#	#	-	----	----	#	#	#	#	#	#	#
Rsa1.0_00032.1.g1978.t1	ref|NP_188014.1| uncharacterized protein [Arabidopsis thaliana] gi|11994369|dbj|BAB02328.1| unnamed protein product [Arabidopsis thaliana] gi|332641926|gb|AEE75447.1| uncharacterized protein AT3G13980 [Arabidopsis thaliana]	371	357	1.00E-108	96.2	73.9	81.4	uncharacterized protein	gbpln	Arabidopsis thaliana	AT3G13980.1 | Symbols: | unknown protein; BEST Arabidopsis thaliana protein match is: unknown protein (TAIR:AT1G54200.1); Has 1485 Blast hits to 418 proteins in 98 species: Archae - 0; Bacteria - 6; Metazoa - 246; Fungi - 61; Plants - 107; Viruses - 6; Other Eukaryotes - 1059 (source: NCBI BLink). | chr3:4619189-4620262 REVERSE LENGTH=357	371	357	1.00E-110	96.2	73.9	81.4
Rsa1.0_00032.1.g1979.t17	ref|NP_001078149.1| uncharacterized protein [Arabidopsis thaliana] gi|110742569|dbj|BAE99198.1| hypothetical protein [Arabidopsis thaliana] gi|332641928|gb|AEE75449.1| uncharacterized protein AT3G13990 [Arabidopsis thaliana]	934	847	0	90.7	70.6	77.3	uncharacterized protein	gbpln	Arabidopsis thaliana	AT3G13990.2 | Symbols: | Kinase-related protein of unknown function (DUF1296) | chr3:4625708-4630383 REVERSE LENGTH=847	934	847	0	90.7	70.6	77.3

Rsa1.0_00032.1.g1980.t1	ref|NP_188016.2| protein Brevis radix-like 2 [Arabidopsis thaliana] gi|42572427|ref|NP_974309.1| protein Brevis radix-like 2 [Arabidopsis thaliana] gi|75151461|sp|Q8GYL9.1|BRXL2_ARATH RecName: Full=Protein Brevis radix-like 2; Short=AtBRXL2 gi|26450131|dbj|BAC42185.1| unknown protein [Arabidopsis thaliana] gi|115311461|gb|ABI93911.1| At3g14000 [Arabidopsis thaliana] gi|332641929|gb|AEE75450.1| protein Brevis radix-like 2 [Arabidopsis thaliana] gi|332641930|gb|AEE75451.1| protein Brevis radix-like 2 [Arabidopsis thaliana]	377	374	1.00E-160	99.2	81.2	89.1	protein Brevis radix-like 2	gbpln	Arabidopsis thaliana	AT3G14000.2 | Symbols: ATBRXL2, BRX-LIKE2 | DZC (Disease resistance/zinc finger/chromosome condensation-like region) domain containing protein | chr3:4631170-4633353 REVERSE LENGTH=374	377	374	1.00E-162	99.2	81.2	89.1
Rsa1.0_00032.1.g1981.t19	dbj|BAB02332.1| unnamed protein product [Arabidopsis thaliana]	1337	596	1.00E-114	44.6	16.8	19.7	unnamed protein product	gbpln	Arabidopsis thaliana	AT3G14010.3 | Symbols: CID4 | CTC-interacting domain 4 | chr3:4637164-4640691 FORWARD LENGTH=595	1337	595	1.00E-114	44.5	16.8	19.7
Rsa1.0_00032.1.g1982.t1	gb|AFP97582.1| nodulation signaling pathway 1-like protein [Brassica ruvo]	513	509	0	99.2	92.4	95.9	nodulation signaling pathway 1-like protein	gbpln	Brassica ruvo	AT3G13840.1 | Symbols: | GRAS family transcription factor | chr3:4555305-4556837 REVERSE LENGTH=510	513	510	0	99.4	77.0	86.0
Rsa1.0_00032.1.g1983.t1	ref|XP_002885006.1| hypothetical protein ARALYDRAFT_897663 [Arabidopsis lyrata subsp. lyrata] gi|297330846|gb|EFH61265.1| hypothetical protein ARALYDRAFT_897663 [Arabidopsis lyrata subsp. lyrata]	678	714	0	105.3	88.2	92.9	hypothetical protein ARALYDRAFT_897663	gbpln	Arabidopsis lyrata	AT3G14050.1 | Symbols: RSH2, AT-RSH2, ATRSH2 | RELA/SPOT homolog 2 | chr3:4650902-4653514 REVERSE LENGTH=709	678	709	0	104.6	87.5	91.6
Rsa1.0_00032.1.g1984.t1	ref|XP_002885009.1| subtilase family protein [Arabidopsis lyrata subsp. lyrata] gi|297330849|gb|EFH61268.1| subtilase family protein [Arabidopsis lyrata subsp. lyrata]	778	777	0	99.9	92.5	95.2	subtilase family protein	gbpln	Arabidopsis lyrata	AT3G14067.1 | Symbols: | Subtilase family protein | chr3:4658421-4660754 REVERSE LENGTH=777	778	777	0	99.9	92.2	95.2
Rsa1.0_00032.1.g1985.t1	gb|EOA32738.1| hypothetical protein CARUB_v10016043mg [Capsella rubella]	638	643	0	100.8	85.6	93.7	hypothetical protein CARUB_v10016043mg	gbpln	Capsella rubella	AT3G14070.1 | Symbols: CAX9, CCX3, ATCCX3 | cation exchanger 9 | chr3:4661143-4663074 REVERSE LENGTH=643	638	643	0	100.8	84.5	93.3
Rsa1.0_00032.1.g1986.t1	ref|XP_002882867.1| hypothetical protein ARALYDRAFT_478821 [Arabidopsis lyrata subsp. lyrata] gi|297328707|gb|EFH59126.1| hypothetical protein ARALYDRAFT_478821 [Arabidopsis lyrata subsp. lyrata] gi|482567523|gb|EOA31712.1| hypothetical protein CARUB_v10014920mg [Capsella rubella]	129	128	1.00E-65	99.2	96.1	97.7	hypothetical protein ARALYDRAFT_478821	gbpln	Arabidopsis lyrata	AT3G14080.2 | Symbols: | Small nuclear ribonucleoprotein family protein | chr3:4667717-4668989 FORWARD LENGTH=128	129	128	2.00E-67	99.2	94.6	97.7
Rsa1.0_00032.1.g1987.t1	ref|NP_001147016.1| protein binding protein [Zea mays] gi|195606494|gb|ACG25077.1| protein binding protein [Zea mays]	617	618	0	100.2	87.5	91.2	protein binding protein	gbenv/gbpln	Zea mays	AT3G14090.1 | Symbols: ATEXO70D3, EXO70D3 | exocyst subunit exo70 family protein D3 | chr3:4669508-4671379 REVERSE LENGTH=623	617	623	0	101.0	81.7	88.0
Rsa1.0_00032.1.g1988.t1	ref|NP_566478.1| flourescent in blue light protein [Arabidopsis thaliana] gi|75331838|sp|Q940U6.1|FLU_ARATH RecName: Full=Protein FLUORESCENT IN BLUE LIGHT, chloroplastic; Flags: Precursor gi|17386154|gb|AAL38623.1|AF446890_1 AT3g14110/MAG2_6 [Arabidopsis thaliana] gi|15450619|gb|AAK96581.1| AT3g14110/MAG2_6 [Arabidopsis thaliana] gi|21593122|gb|AAM65071.1| unknown [Arabidopsis thaliana] gi|332641949|gb|AEE75470.1| flourescent in blue light protein [Arabidopsis thaliana]	312	316	1.00E-127	101.3	76.3	86.5	flourescent in blue light protein	gbpln	Arabidopsis thaliana	AT3G14110.1 | Symbols: FLU | Tetratricopeptide repeat (TPR)-like superfamily protein | chr3:4676222-4677602 REVERSE LENGTH=316	312	316	1.00E-129	101.3	76.3	86.5
Rsa1.0_00032.1.g1989.t1	gb|EOA32951.1| hypothetical protein CARUB_v10016280mg [Capsella rubella]	1072	1077	0	100.5	90.8	95.0	hypothetical protein CARUB_v10016280mg	gbpln	Capsella rubella	AT3G14120.1 | Symbols: | FUNCTIONS IN: molecular_function unknown; INVOLVED IN: transport; LOCATED IN: nuclear pore; EXPRESSED IN: 23 plant structures; EXPRESSED DURING: 13 growth stages; CONTAINS InterPro DOMAIN/s: Nuclear pore protein 84/107 (InterPro:IPR007252); Has 5399 Blast hits to 5001 proteins in 612 species: Archae - 19; Bacteria - 730; Metazoa - 2186; Fungi - 823; Plants - 382; Viruses - 37; Other Eukaryotes - 1222 (source: NCBI BLink). | chr3:4677993-4685455 FORWARD LENGTH=1101	1072	1101	0	102.7	90.4	94.8

<table>
<tr><th></th><th></th><th></th><th></th><th></th><th></th><th></th><th></th><th></th><th></th><th></th><th></th><th></th><th></th><th></th><th></th><th></th><th></th></tr>
<tr><td>Rsa1.0_00032.1.g1990.t1</td><td>ref|NP_566479.5| 2-oxoglutarate-dependent dioxygenase family protein [Arabidopsis thaliana] gi|9294641|dbj|BAB02980.1| unnamed protein product [Arabidopsis thaliana] gi|332641958|gb|AEE75479.1| 2-oxoglutarate-dependent dioxygenase family protein [Arabidopsis thaliana]</td><td>457</td><td>455</td><td>1.00E-148</td><td>99.6</td><td>65.0</td><td>74.6</td><td>2-oxoglutarate-dependent dioxygenase family protein</td><td>gbpln</td><td>Arabidopsis thaliana</td><td>AT3G14160.1 | Symbols: | 2-oxoglutarate-dependent dioxygenase family protein | chr3:4693495-4695198 FORWARD LENGTH=455</td><td>457</td><td>455</td><td>1.00E-151</td><td>99.6</td><td>65.0</td><td>74.6</td></tr>
<tr><td>Rsa1.0_00032.1.g1991.t1</td><td>ref|XP_002885015.1| hypothetical protein ARALYDRAFT_478830 [Arabidopsis lyrata subsp. lyrata] gi|297330855|gb|EFH61274.1| hypothetical protein ARALYDRAFT_478830 [Arabidopsis lyrata subsp. lyrata]</td><td>543</td><td>506</td><td>0</td><td>93.2</td><td>74.4</td><td>82.3</td><td>hypothetical protein ARALYDRAFT_478830</td><td>gbpln</td><td>Arabidopsis lyrata</td><td>AT3G14170.1 | Symbols: | Plant protein of unknown function (DUF936) | chr3:4696115-4697989 REVERSE LENGTH=505</td><td>543</td><td>505</td><td>0</td><td>93.0</td><td>72.6</td><td>81.2</td></tr>
<tr><td>Rsa1.0_00032.1.g1992.t1</td><td>ref|XP_002885016.1| hypothetical protein ARALYDRAFT_478831 [Arabidopsis lyrata subsp. lyrata] gi|297330856|gb|EFH61275.1| hypothetical protein ARALYDRAFT_478831 [Arabidopsis lyrata subsp. lyrata]</td><td>1161</td><td>1256</td><td>0</td><td>108.2</td><td>79.3</td><td>86.3</td><td>hypothetical protein ARALYDRAFT_478831</td><td>gbpln</td><td>Arabidopsis lyrata</td><td>AT3G14172.1 | Symbols: | FUNCTIONS IN: molecular_function unknown; INVOLVED IN: biological_process unknown; LOCATED IN: cellular_component unknown; BEST Arabidopsis thaliana protein match is: COP1-interacting protein-related (TAIR:AT1G72410.1); Has 30201 Blast hits to 17322 proteins in 780 species: Archae - 12; Bacteria - 1396; Metazoa - 17338; Fungi - 3422; Plants - 5037; Viruses - 0; Other Eukaryotes - 2996 (source: NCBI BLink). | chr3:4698860-4704773 REVERSE LENGTH=1270</td><td>1161</td><td>1270</td><td>0</td><td>109.4</td><td>78.5</td><td>85.3</td></tr>
<tr><td>Rsa1.0_00033.1.g1993.t1</td><td>gb|EOA17573.1| hypothetical protein CARUB_v10005934mg, partial [Capsella rubella]</td><td>138</td><td>154</td><td>7.00E-44</td><td>111.6</td><td>67.4</td><td>77.5</td><td>hypothetical protein CARUB_v10005934mg, partial</td><td>gbpln</td><td>Capsella rubella</td><td>AT4G15990.1 | Symbols: | unknown protein; BEST Arabidopsis thaliana protein match is: unknown protein (TAIR:AT4G16024.1); Has 14 Blast hits to 14 proteins in 2 species: Archae - 0; Bacteria - 0; Metazoa - 0; Fungi - 0; Plants - 14; Viruses - 0; Other Eukaryotes - 0 (source: NCBI BLink). | chr4:9061269-9061676 REVERSE LENGTH=135</td><td>138</td><td>135</td><td>3.00E-46</td><td>97.8</td><td>65.9</td><td>78.3</td></tr>
<tr><td>Rsa1.0_00033.1.g1994.t1</td><td>gb|EOA18428.1| hypothetical protein CARUB_v10006972mg [Capsella rubella]</td><td>522</td><td>641</td><td>0</td><td>122.8</td><td>63.8</td><td>76.8</td><td>hypothetical protein CARUB_v10006972mg</td><td>gbpln</td><td>Capsella rubella</td><td>AT4G13992.1 | Symbols: | Cysteine/Histidine-rich C1 domain family protein | chr4:8087340-8089268 FORWARD LENGTH=642</td><td>522</td><td>642</td><td>0</td><td>123.0</td><td>62.5</td><td>76.1</td></tr>
<tr><td>Rsa1.0_00033.1.g1995.t1</td><td>ref|NP_193008.1| nudix hydrolase 7 [Arabidopsis thaliana] gi|30682217|ref|NP_849367.1| nudix hydrolase 7 [Arabidopsis thaliana] gi|30682219|ref|NP_849368.1| nudix hydrolase 7 [Arabidopsis thaliana] gi|68565949|sp|Q9SU14.1|NUDT7_ARATH RecName: Full=Nudix hydrolase 7; Short=AtNUDT7; AltName: Full=ADP-ribose pyrophosphatase; AltName: Full=NADH pyrophosphatase; AltName: Full=Protein GROWTH FACTOR GENE 1 gi|13272467|gb|AAK17172.1|AF325104_1 growth factor like protein [Arabidopsis thaliana] gi|13877893|gb|AAK44024.1|AF370209_1 putative growth factor [Arabidopsis thaliana] gi|4586248|emb|CAB40989.1| growth factor like protein [Arabidopsis thaliana] gi|7267973|emb|CAB78314.1| growth factor like protein [Arabidopsis thaliana] gi|15810611|gb|AAL07193.1| putative growth factor [Arabidopsis thaliana] gi|332657767|gb|AEE83167.1| nudix hydrolase 7 [Arabidopsis thaliana] gi|332657768|gb|AEE83168.1| nudix hydrolase 7 [Arabidopsis thaliana] gi|332657769|gb|AEE83169.1| nudix hydrolase 7 [Arabidopsis thaliana]</td><td>278</td><td>282</td><td>1.00E-128</td><td>101.4</td><td>75.2</td><td>87.8</td><td>nudix hydrolase 7</td><td>gbpln</td><td>Arabidopsis thaliana</td><td>AT4G12720.3 | Symbols: AtNUDT7, GFG1, ATNUDX7 | MutT/nudix family protein | chr4:7487716-7489557 FORWARD LENGTH=282</td><td>278</td><td>282</td><td>1.00E-131</td><td>101.4</td><td>75.2</td><td>87.8</td></tr>
<tr><td>Rsa1.0_00033.1.g1996.t1</td><td>dbj|BAJ34567.1| unnamed protein product [Thellungiella halophila]</td><td>99</td><td>97</td><td>2.00E-39</td><td>98.0</td><td>82.8</td><td>87.9</td><td>unnamed protein product</td><td>----</td><td>----</td><td>AT4G15910.1 | Symbols: ATDI21, DI21 | drought-induced 21 | chr4:9028657-9029269 FORWARD LENGTH=104</td><td>99</td><td>104</td><td>7.00E-40</td><td>105.1</td><td>83.8</td><td>85.9</td></tr>
<tr><td>Rsa1.0_00033.1.g1997.t1</td><td>gb|EOA32197.1| hypothetical protein CARUB_v10015453mg [Capsella rubella]</td><td>102</td><td>552</td><td>9.00E-20</td><td>541.2</td><td>42.2</td><td>66.7</td><td>hypothetical protein CARUB_v10015453mg</td><td>gbpln</td><td>Capsella rubella</td><td>AT1G52950.1 | Symbols: | Nucleic acid-binding, OB-fold-like protein | chr1:19725483-19728007 FORWARD LENGTH=566</td><td>102</td><td>566</td><td>5.00E-14</td><td>554.9</td><td>33.3</td><td>52.0</td></tr>
</table>

Rsa1.0_00033.1.g1998.t2	ref|XP_002870205.1| hypothetical protein ARALYDRAFT_493299 [Arabidopsis lyrata subsp. lyrata] gi|297316041|gb|EFH46464.1| hypothetical protein ARALYDRAFT_493299 [Arabidopsis lyrata subsp. lyrata]	519	522	0	100.6	79.4	87.5	hypothetical protein ARALYDRAFT_493299	gbpln	Arabidopsis lyrata	AT4G15850.1 | Symbols: ATRH1, RH1 | RNA helicase 1 | chr4:9001426-9004534 FORWARD LENGTH=522	519	522	0	100.6	79.0	87.3
Rsa1.0_00033.1.g1999.t1	ref|NP_568712.1| FAD/NAD(P)-binding oxidoreductase family protein [Arabidopsis thaliana] gi|332008446|gb|AED95829.1| FAD/NAD(P)-binding oxidoreductase family protein [Arabidopsis thaliana]	566	556	0	98.2	89.6	93.5	FAD/NAD(P)-binding oxidoreductase family protein	gbpln	Arabidopsis thaliana	AT5G49555.1 | Symbols: | FAD/NAD(P)-binding oxidoreductase family protein | chr5:20107411-20110602 REVERSE LENGTH=556	566	556	0	98.2	89.6	93.5
Rsa1.0_00033.1.g2000.t1	ref|NP_199767.1| Putative methyltransferase family protein [Arabidopsis thaliana] gi|10177622|dbj|BAB10769.1| unnamed protein product [Arabidopsis thaliana] gi|332008447|gb|AED95830.1| Putative methyltransferase family protein [Arabidopsis thaliana]	284	274	1.00E-114	96.5	75.0	83.8	Putative methyltransferase family protein	gbpln	Arabidopsis thaliana	AT5G49560.1 | Symbols: | Putative methyltransferase family protein | chr5:20110961-20111785 REVERSE LENGTH=274	284	274	1.00E-117	96.5	75.0	83.8
Rsa1.0_00033.1.g2001.t3	ref|XP_002868178.1| hypothetical protein ARALYDRAFT_355185 [Arabidopsis lyrata subsp. lyrata] gi|297314014|gb|EFH44437.1| hypothetical protein ARALYDRAFT_355185 [Arabidopsis lyrata subsp. lyrata]	1197	1153	0	96.3	57.0	68.3	hypothetical protein ARALYDRAFT_355185	gbpln	Arabidopsis lyrata	AT4G15810.1 | Symbols: | P-loop containing nucleoside triphosphate hydrolases superfamily protein | chr4:8989162-8992591 REVERSE LENGTH=918	1197	918	1.00E-164	76.7	32.3	40.2
Rsa1.0_00033.1.g2002.t1	ref|NP_567475.5| uncharacterized protein [Arabidopsis thaliana] gi|18175846|gb|AAL59938.1| unknown protein [Arabidopsis thaliana] gi|21689873|gb|AAM67497.1| unknown protein [Arabidopsis thaliana] gi|332658252|gb|AEE83652.1| uncharacterized protein AT4G15790 [Arabidopsis thaliana]	146	147	7.00E-56	100.7	82.2	89.7	uncharacterized protein	gbpln	Arabidopsis thaliana	AT4G15790.1 | Symbols: | unknown protein; INVOLVED IN: biological_process unknown; LOCATED IN: chloroplast; EXPRESSED IN: 21 plant structures; EXPRESSED DURING: 13 growth stages; Has 411 Blast hits to 380 proteins in 121 species: Archae - 4; Bacteria - 107; Metazoa - 85; Fungi - 29; Plants - 59; Viruses - 0; Other Eukaryotes - 127 (source: NCBI BLink). | chr4:8982390-8984069 FORWARD LENGTH=147	146	147	2.00E-58	100.7	82.2	89.7
Rsa1.0_00033.1.g2003.t1	ref|NP_197566.1| vacuolar protein / peptidase dimerization domain-containing protein [Arabidopsis thaliana] gi|110741500|dbj|BAE98701.1| 24 kDa vacuolar protein - like [Arabidopsis thaliana] gi|332005489|gb|AED92872.1| vacuolar protein / peptidase dimerization domain-containing protein [Arabidopsis thaliana]	911	910	0	99.9	82.7	92.3	vacuolar protein / peptidase dimerization domain-containing protein	gbpln	Arabidopsis thaliana	AT5G20660.1 | Symbols: | Zn-dependent exopeptidases superfamily protein | chr5:6986402-6990947 FORWARD LENGTH=910	911	910	0	99.9	82.7	92.3
Rsa1.0_00033.1.g2004.t1	ref|XP_002868180.1| hypothetical protein ARALYDRAFT_915207 [Arabidopsis lyrata subsp. lyrata] gi|297314016|gb|EFH44439.1| hypothetical protein ARALYDRAFT_915207 [Arabidopsis lyrata subsp. lyrata]	222	222	1.00E-124	100.0	96.4	98.2	hypothetical protein ARALYDRAFT_915207	gbpln	Arabidopsis lyrata	AT4G15780.1 | Symbols: ATVAMP724, VAMP724 | vesicle-associated membrane protein 724 | chr4:8980081-8981697 REVERSE LENGTH=222	222	222	1.00E-118	100.0	96.8	98.6
Rsa1.0_00033.1.g2005.t1	gb|EOA15832.1| hypothetical protein CARUB_v10007453mg [Capsella rubella]	187	187	1.00E-99	100.0	93.6	96.8	hypothetical protein CARUB_v10007453mg	gbpln	Capsella rubella	AT4G15770.1 | Symbols: | RNA binding | chr4:8978060-8978623 FORWARD LENGTH=187	187	187	1.00E-100	100.0	92.0	95.7
Rsa1.0_00033.1.g2006.t1	gb|EOA19124.1| hypothetical protein CARUB_v10007793mg [Capsella rubella]	344	511	1.00E-151	148.5	75.0	86.0	hypothetical protein CARUB_v10007793mg	gbpln	Capsella rubella	AT5G24950.1 | Symbols: CYP71A15 | cytochrome P450, family 71, subfamily A, polypeptide 15 | chr5:8595209-8597761 REVERSE LENGTH=496	344	496	1.00E-119	144.2	62.5	75.6
Rsa1.0_00033.1.g2007.t1	gb|EOA19124.1| hypothetical protein CARUB_v10007793mg [Capsella rubella]	145	511	5.00E-43	352.4	57.2	62.1	hypothetical protein CARUB_v10007793mg	gbpln	Capsella rubella	AT4G13310.2 | Symbols: CYP71A20 | cytochrome P450, family 71, subfamily A, polypeptide 20 | chr4:7750453-7751856 FORWARD LENGTH=390	145	390	5.00E-45	269.0	58.6	62.8
Rsa1.0_00033.1.g2008.t1	gb|EOA19124.1| hypothetical protein CARUB_v10007793mg [Capsella rubella]	489	511	0	104.5	75.3	84.0	hypothetical protein CARUB_v10007793mg	gbpln	Capsella rubella	AT5G24950.1 | Symbols: CYP71A15 | cytochrome P450, family 71, subfamily A, polypeptide 15 | chr5:8595209-8597761 REVERSE LENGTH=496	489	496	0	101.4	63.4	76.3
Rsa1.0_00033.1.g2009.t1	gb|EOA18110.1| hypothetical protein CARUB_v10006571mg [Capsella rubella]	440	457	1.00E-163	103.9	68.4	78.9	hypothetical protein CARUB_v10006571mg	gbpln	Capsella rubella	AT4G15740.1 | Symbols: | Calcium-dependent lipid-binding (CaLB domain) family protein | chr4:8964912-8966318 REVERSE LENGTH=468	440	468	1.00E-163	106.4	66.1	76.1

Rsa1.0_00033.1.g2010.t1	ref|XP_002870231.1| hypothetical protein ARALYDRAFT_915246 [Arabidopsis lyrata subsp. lyrata] gi|297316067|gb|EFH46490.1| hypothetical protein ARALYDRAFT_915246 [Arabidopsis lyrata subsp. lyrata]	334	335	1.00E-163	100.3	85.0	91.9	hypothetical protein ARALYDRAFT_915246	gbpln	Arabidopsis lyrata	AT4G15545.1 | Symbols: | unknown protein; BEST Arabidopsis thaliana protein match is: unknown protein (TAIR:AT1G16520.1); Has 30201 Blast hits to 17322 proteins in 780 species: Archae - 12; Bacteria - 1396; Metazoa - 17338; Fungi - 3422; Plants - 5037; Viruses - 0; Other Eukaryotes - 2996 (source: NCBI BLink). | chr4:8875932-8877567 FORWARD LENGTH=337	334	337	1.00E-164	100.9	85.0	91.6
Rsa1.0_00033.1.g2011.t1	dbj|BAJ34577.1| unnamed protein product [Thellungiella halophila]	325	325	1.00E-169	100.0	88.0	95.7	unnamed protein product	----	----	AT3G59380.1 | Symbols: FTA, PLP, ATFTA, PFT/PGGT-IALPHA | farnesyltransferase A | chr3:21944209-21945781 FORWARD LENGTH=326	325	326	1.00E-165	100.3	87.7	94.5
Rsa1.0_00033.1.g2012.t1	ref|NP_849391.2| pyruvate, phosphate dikinase 1 [Arabidopsis thaliana] gi|145333150|ref|NP_001078395.1| pyruvate, phosphate dikinase 1 [Arabidopsis thaliana] gi|222423813|dbj|BAH19872.1| AT4G15530 [Arabidopsis thaliana] gi|332658217|gb|AEE83617.1| pyruvate, phosphate dikinase 1 [Arabidopsis thaliana] gi|332658219|gb|AEE83619.1| pyruvate, phosphate dikinase 1 [Arabidopsis thaliana]	92	956	8.00E-26	1039.1	62.0	73.9	pyruvate, phosphate dikinase 1	gbpln	Arabidopsis thaliana	AT4G15530.4 | Symbols: PPDK | pyruvate orthophosphate dikinase | chr4:8864828-8870727 REVERSE LENGTH=956	92	956	1.00E-28	1039.1	62.0	73.9
Rsa1.0_00033.1.g2013.t1	ref|NP_193288.2| pyruvate, phosphate dikinase 1 [Arabidopsis thaliana] gi|222424574|dbj|BAH20242.1| AT4G15530 [Arabidopsis thaliana] gi|332658216|gb|AEE83616.1| pyruvate, phosphate dikinase 1 [Arabidopsis thaliana]	875	875	0	100.0	94.9	98.5	pyruvate, phosphate dikinase 1	gbpln	Arabidopsis thaliana	AT4G15530.2 | Symbols: PPDK | pyruvate orthophosphate dikinase | chr4:8864828-8869183 REVERSE LENGTH=875	875	875	0	100.0	94.9	98.5
Rsa1.0_00033.1.g2014.t2	ref|NP_567468.1| PsbP domain-containing protein 1 [Arabidopsis thaliana] gi|13959580|sp|O23403.1|PPD1_ARATH RecName: Full=PsbP domain-containing protein 1, chloroplastic; AltName: Full=OEC23-like protein 3; AltName: Full=PsbP-related thylakoid lumenal protein 1; Flags: Precursor gi|2244908|emb|CAB10329.1| hypothetical protein [Arabidopsis thaliana] gi|7268298|emb|CAB78593.1| hypothetical protein [Arabidopsis thaliana] gi|332658213|gb|AEE83613.1| PsbP domain-containing protein 1 [Arabidopsis thaliana]	270	287	1.00E-115	106.3	80.7	85.9	PsbP domain-containing protein 1	gbpln	Arabidopsis thaliana	AT4G15510.1 | Symbols: | Photosystem II reaction center PsbP family protein | chr4:8860701-8862529 FORWARD LENGTH=287	270	287	1.00E-118	106.3	80.7	85.9
Rsa1.0_00033.1.g2015.t1	ref|XP_002885447.1| transducin family protein [Arabidopsis lyrata subsp. lyrata] gi|297331287|gb|EFH61706.1| transducin family protein [Arabidopsis lyrata subsp. lyrata]	955	954	0	99.9	83.1	91.5	transducin family protein	gbpln	Arabidopsis lyrata	AT3G21540.1 | Symbols: | transducin family protein / WD-40 repeat family protein | chr3:7586100-7590856 REVERSE LENGTH=955	955	955	0	100.0	82.3	91.1
Rsa1.0_00033.1.g2016.t1	ref|XP_002870258.1| hypothetical protein ARALYDRAFT_493374 [Arabidopsis lyrata subsp. lyrata] gi|297316094|gb|EFH46517.1| hypothetical protein ARALYDRAFT_493374 [Arabidopsis lyrata subsp. lyrata]	330	451	2.00E-43	136.7	26.1	26.7	hypothetical protein ARALYDRAFT_493374	gbpln	Arabidopsis lyrata	AT4G15160.2 | Symbols: | Bifunctional inhibitor/lipid-transfer protein/seed storage 2S albumin superfamily protein | chr4:8646192-8647019 FORWARD LENGTH=193	330	193	2.00E-42	58.5	25.8	26.7
Rsa1.0_00033.1.g2017.t1	ref|NP_567450.1| SNARE associated Golgi protein [Arabidopsis thaliana] gi|53749170|gb|AAU90070.1| At4g14950 [Arabidopsis thaliana] gi|332658128|gb|AEE83528.1| SNARE associated Golgi protein [Arabidopsis thaliana]	441	416	0	94.3	82.8	85.9	SNARE associated Golgi protein	gbpln	Arabidopsis thaliana	AT4G14950.1 | Symbols: | SNARE associated Golgi protein family | chr4:8545517-8547722 FORWARD LENGTH=416	441	416	0	94.3	82.8	85.9
Rsa1.0_00033.1.g2018.t1	gb|AAL47166.1|AF449459_1 diamine oxidase [Brassica juncea]	649	649	0	100.0	96.6	98.6	diamine oxidase	gbpln	Brassica juncea	AT4G14940.1 | Symbols: ATAO1, AO1 | amine oxidase 1 | chr4:8541879-8544304 FORWARD LENGTH=650	649	650	0	100.2	90.1	95.4
Rsa1.0_00033.1.g2019.t1	ref|NP_567449.1| 5'-nucleotidase [Arabidopsis thaliana] gi|21593317|gb|AAM65266.1| unknown [Arabidopsis thaliana] gi|27311791|gb|AAO00861.1| expressed protein [Arabidopsis thaliana] gi|30984528|gb|AAP42727.1| At4g14930 [Arabidopsis thaliana] gi|332658124|gb|AEE83524.1| survival protein SurE-like phosphatase/nucleotidase [Arabidopsis thaliana]	296	315	1.00E-138	106.4	81.1	89.9	5'-nucleotidase	gbpln	Arabidopsis thaliana	AT4G14930.1 | Symbols: | Survival protein SurE-like phosphatase/nucleotidase | chr4:8538831-8541205 FORWARD LENGTH=315	296	315	1.00E-140	106.4	81.1	89.9

Rsa1.0_00033.1.g2020.t1	ref|NP_194638.1| Ribonuclease H-like protein [Arabidopsis thaliana] gi|4972055|emb|CAB43923.1| putative protein [Arabidopsis thaliana] gi|7269807|emb|CAB79667.1| putative protein [Arabidopsis thaliana] gi|67633766|gb|AAY78807.1| putative reverse transcriptase/RNA-dependent DNA polymerase [Arabidopsis thaliana] gi|332660185|gb|AEE85585.1| Ribonuclease H-like protein [Arabidopsis thaliana]	108	575	4.00E-16	532.4	35.2	43.5	Ribonuclease H-like protein	gbpln	Arabidopsis thaliana	AT4G29090.1 | Symbols: | Ribonuclease H-like superfamily protein | chr4:14333528-14335255 FORWARD LENGTH=575	108	575	6.00E-19	532.4	35.2	43.5
Rsa1.0_00033.1.g2021.t1	ref|XP_002876895.1| hypothetical protein ARALYDRAFT_484284 [Arabidopsis lyrata subsp. lyrata] gi|297322733|gb|EFH53154.1| hypothetical protein ARALYDRAFT_484284 [Arabidopsis lyrata subsp. lyrata]	335	333	1.00E-166	99.4	84.5	91.3	hypothetical protein ARALYDRAFT_484284	gbpln	Arabidopsis lyrata	AT2G03750.1 | Symbols: | P-loop containing nucleoside triphosphate hydrolases superfamily protein | chr2:1147968-1149023 REVERSE LENGTH=351	335	351	1.00E-161	104.8	82.1	90.1
Rsa1.0_00033.1.g2022.t1	emb|CAB10271.1| hypothetical protein [Arabidopsis thaliana] gi|7268238|emb|CAB78534.1| hypothetical protein [Arabidopsis thaliana]	420	1040	1.00E-106	247.6	64.0	75.0	hypothetical protein	gbpln	Arabidopsis thaliana	AT4G14920.1 | Symbols: | Acyl-CoA N-acyltransferase with RING/FYVE/PHD-type zinc finger protein | chr4:8531157-8535842 REVERSE LENGTH=1138	420	1138	1.00E-108	271.0	64.0	75.0
Rsa1.0_00033.1.g2023.t1	ref|NP_001147025.1| ferredoxin [Zea mays] gi|195606586|gb|ACG25123.1| ferredoxin [Zea mays]	151	151	1.00E-75	100.0	92.7	96.0	ferredoxin	gbenv/gbpln	Zea mays	AT4G14890.1 | Symbols: FdC2 | 2Fe-2S ferredoxin-like superfamily protein | chr4:8520887-8521351 FORWARD LENGTH=154	151	154	3.00E-72	102.0	88.7	94.0
Rsa1.0_00033.1.g2024.t1	gb|ACX70136.1| O-acetylserine(thiol)lyase isoform A6 [Brassica rapa subsp. chinensis]	322	322	1.00E-180	100.0	98.1	98.8	O-acetylserine(thiol)lyase isoform A6	gbpln	Brassica rapa	AT4G14880.4 | Symbols: OASA1 | O-acetylserine (thiol) lyase (OAS-TL) isoform A1 | chr4:8518209-8520050 REVERSE LENGTH=322	322	322	1.00E-167	100.0	96.3	97.8
Rsa1.0_00033.1.g2025.t1	gb|EOA18180.1| hypothetical protein CARUB_v10006658mg [Capsella rubella]	371	177	3.00E-70	47.7	38.5	41.2	hypothetical protein CARUB_v10006658mg	gbpln	Capsella rubella	AT4G14870.1 | Symbols: SECE1 | secE/sec61-gamma protein transport protein | chr4:8517248-8517781 FORWARD LENGTH=177	371	177	1.00E-69	47.7	37.7	39.4
Rsa1.0_00033.1.g2026.t1	ref|NP_193218.1| pentatricopeptide repeat-containing protein [Arabidopsis thaliana] gi|75274931|sp|O23337.1|PP311_ARATH RecName: Full=Pentatricopeptide repeat-containing protein At4g14820 gi|2244839|emb|CAB10261.1| hypothetical protein [Arabidopsis thaliana] gi|7268228|emb|CAB78524.1| hypothetical protein [Arabidopsis thaliana] gi|332658106|gb|AEE83506.1| pentatricopeptide repeat-containing protein [Arabidopsis thaliana]	628	722	0	115.0	78.5	88.5	pentatricopeptide repeat-containing protein	gbpln	Arabidopsis thaliana	AT4G14820.1 | Symbols: | Pentatricopeptide repeat (PPR) superfamily protein | chr4:8507794-8510038 REVERSE LENGTH=722	628	722	0	115.0	78.5	88.5
Rsa1.0_00033.1.g2027.t1	gb|EOA18115.1| hypothetical protein CARUB_v10006576mg [Capsella rubella]	106	106	2.00E-45	100.0	84.9	87.7	hypothetical protein CARUB_v10006576mg	gbpln	Capsella rubella	AT4G14819.1 | Symbols: | Protein of unknown function (DUF1677) | chr4:8507365-8507679 FORWARD LENGTH=104	106	104	1.00E-46	98.1	83.0	87.7
Rsa1.0_00033.1.g2028.t1	ref|XP_002870280.1| predicted protein [Arabidopsis lyrata subsp. lyrata] gi|297316116|gb|EFH46539.1| predicted protein [Arabidopsis lyrata subsp. lyrata]	165	155	1.00E-53	93.9	67.3	75.2	predicted protein	gbpln	Arabidopsis lyrata	AT4G14815.1 | Symbols: | Bifunctional inhibitor/lipid-transfer protein/seed storage 2S albumin superfamily protein | chr4:8505122-8505760 FORWARD LENGTH=156	165	156	1.00E-52	94.5	64.2	73.9
Rsa1.0_00033.1.g2029.t1	ref|XP_002885514.1| hypothetical protein ARALYDRAFT_898737 [Arabidopsis lyrata subsp. lyrata] gi|297331354|gb|EFH61773.1| hypothetical protein ARALYDRAFT_898737 [Arabidopsis lyrata subsp. lyrata]	204	204	1.00E-111	100.0	94.1	99.5	hypothetical protein ARALYDRAFT_898737	gbpln	Arabidopsis lyrata	AT3G22630.1 | Symbols: PBD1, PRCGB | 20S proteasome beta subunit D1 | chr3:8009709-8010774 REVERSE LENGTH=204	204	204	1.00E-113	100.0	93.6	99.0
Rsa1.0_00033.1.g2030.t1	ref|NP_193215.2| ATP-dependent RNA helicase, mitochondrial (SUV3) [Arabidopsis thaliana] gi|5823579|emb|CAB53782.1| mitochondrial RNA helicase [Arabidopsis thaliana] gi|22655093|gb|AAM98137.1| RNA helicase like protein [Arabidopsis thaliana] gi|31711986|gb|AAP68349.1| At4g14790 [Arabidopsis thaliana] gi|332658099|gb|AEE83499.1| ATP-dependent RNA helicase, mitochondrial (SUV3) [Arabidopsis thaliana]	599	571	0	95.3	80.6	86.5	ATP-dependent RNA helicase, mitochondrial (SUV3)	gbpln	Arabidopsis thaliana	AT4G14790.1 | Symbols: ATSUV3, EDA15 | ATP-dependent RNA helicase, mitochondrial (SUV3) | chr4:8496351-8499829 REVERSE LENGTH=571	599	571	0	95.3	80.6	86.5

Rsa1.0_00033.1.g2031.t1	ref|NP_201386.1| F-box protein [Arabidopsis thaliana] gi|75170652|sp|Q9FHP3.1|FB300_ARATH RecName: Full=F-box protein At5g65850 gi|9759569|dbj|BAB11132.1| unnamed protein product [Arabidopsis thaliana] gi|50253490|gb|AAT71947.1| At5g65850 [Arabidopsis thaliana] gi|51972136|gb|AAU15172.1| At5g65850 [Arabidopsis thaliana] gi|332010731|gb|AED98114.1| F-box protein [Arabidopsis thaliana]	388	392	1.00E-81	101.0	46.6	63.4	F-box protein	gbpln	Arabidopsis thaliana	AT5G65850.1 | Symbols: | F-box and associated interaction domains-containing protein | chr5:26346317-26347495 FORWARD LENGTH=392	388	392	2.00E-84	101.0	46.6	63.4
Rsa1.0_00033.1.g2032.t1	ref|NP_180705.1| F-box protein DOR [Arabidopsis thaliana] gi|229807544|sp|Q5BPS3.2|DOR_ARATH RecName: Full=F-box protein DOR; AltName: Full=Protein DROUGHT TOLERANCE REPRESSOR gi|4589954|gb|AAD26472.1| hypothetical protein [Arabidopsis thaliana] gi|330253458|gb|AEC08552.1| F-box protein DOR [Arabidopsis thaliana]	427	387	8.00E-77	90.6	43.1	56.7	F-box protein DOR	gbpln	Arabidopsis thaliana	AT2G31470.1 | Symbols: DOR | F-box and associated interaction domains-containing protein | chr2:13407492-13408655 REVERSE LENGTH=387	427	387	2.00E-79	90.6	43.1	56.7
Rsa1.0_00033.1.g2033.t3	gb|AAC63844.1| putative non-LTR retroelement reverse transcriptase [Arabidopsis thaliana]	1332	1231	0	92.4	58.0	70.9	putative non-LTR retroelement reverse transcriptase	gbpln	Arabidopsis thaliana	AT5G42905.1 | Symbols: | Polynucleotidyl transferase, ribonuclease H-like superfamily protein | chr5:17201414-17202323 REVERSE LENGTH=258	1332	258	6.00E-81	19.4	10.8	13.2
Rsa1.0_00033.1.g2034.t1	gb|EOA15412.1| hypothetical protein CARUB_v10007448mg [Capsella rubella]	647	675	0	104.3	75.0	83.3	hypothetical protein CARUB_v10007448mg	gbpln	Capsella rubella	AT4G14770.1 | Symbols: TCX2, ATTCX2 | TESMIN/TSO1-like CXC 2 | chr4:8481522-8484825 REVERSE LENGTH=674	647	674	0	104.2	75.4	83.6
Rsa1.0_00033.1.g2035.t1	gb|EOA15328.1| hypothetical protein CARUB_v10003978mg [Capsella rubella]	1606	1717	0	106.9	77.1	87.9	hypothetical protein CARUB_v10003978mg	gbpln	Capsella rubella	AT4G14760.1 | Symbols: | kinase interacting (KIP1-like) family protein | chr4:8475718-8481094 FORWARD LENGTH=1710	1606	1710	0	106.5	77.0	87.4
Rsa1.0_00033.1.g2036.t1	gb|AAL86322.1| unknown protein [Arabidopsis thaliana]	373	409	1.00E-150	109.7	78.8	86.3	unknown protein	gbpln	Arabidopsis thaliana	AT4G14750.1 | Symbols: IQD19 | IQ-domain 19 | chr4:8470449-8471903 FORWARD LENGTH=387	373	387	1.00E-152	103.8	78.8	86.3
Rsa1.0_00033.1.g2037.t1	ref|XP_002868255.1| hypothetical protein ARALYDRAFT_493423 [Arabidopsis lyrata subsp. lyrata] gi|297314091|gb|EFH44514.1| hypothetical protein ARALYDRAFT_493423 [Arabidopsis lyrata subsp. lyrata]	201	214	1.00E-77	106.5	73.1	83.6	hypothetical protein ARALYDRAFT_493423	gbpln	Arabidopsis lyrata	AT4G14746.1 | Symbols: | CONTAINS InterPro DOMAIN/s: EGF-like (InterPro:IPR006210); Has 259 Blast hits to 234 proteins in 55 species: Archae - 0; Bacteria - 0; Metazoa - 184; Fungi - 0; Plants - 69; Viruses - 0; Other Eukaryotes - 6 (source: NCBI BLink). | chr4:8462720-8464326 REVERSE LENGTH=212	201	212	2.00E-64	105.5	71.1	82.6
Rsa1.0_00033.1.g2038.t1	ref|NP_193210.2| uncharacterized protein [Arabidopsis thaliana] gi|42572905|ref|NP_974549.1| uncharacterized protein [Arabidopsis thaliana] gi|18377642|gb|AAL66971.1| unknown protein [Arabidopsis thaliana] gi|23297568|gb|AAN12896.1| unknown protein [Arabidopsis thaliana] gi|332658090|gb|AEE83490.1| uncharacterized protein AT4G14740 [Arabidopsis thaliana] gi|332658091|gb|AEE83491.1| uncharacterized protein AT4G14740 [Arabidopsis thaliana]	472	475	0	100.6	91.7	94.9	uncharacterized protein	gbpln	Arabidopsis thaliana	AT4G14740.2 | Symbols: | Plant protein of unknown function (DUF828) with plant pleckstrin homology-like region | chr4:8450567-8453946 REVERSE LENGTH=475	472	475	0	100.6	91.7	94.9
Rsa1.0_00033.1.g2039.t1	gb|ACJ68113.1| hypothetical protein [Brassica napus]	239	239	1.00E-104	100.0	88.7	95.4	hypothetical protein	gbpln	Brassica napus	AT4G14730.1 | Symbols: | Bax inhibitor-1 family protein | chr4:8448607-8450001 FORWARD LENGTH=235	239	235	4.00E-89	98.3	71.1	84.1
Rsa1.0_00033.1.g2040.t1	ref|NP_001031641.1| epidermal patterning factor-like protein 4 [Arabidopsis thaliana] gi|122209356|sp|Q2V3I3.1|EPFL4_ARATH RecName: Full=EPIDERMAL PATTERNING FACTOR-like protein 4; Short=EPF-like protein 4; Flags: Precursor gi|98961833|gb|ABF59246.1| unknown protein [Arabidopsis thaliana] gi|332658088|gb|AEE83488.1| epidermal patterning factor-like protein 4 [Arabidopsis thaliana]	117	109	1.00E-32	93.2	62.4	67.5	epidermal patterning factor-like protein 4	gbpln	Arabidopsis thaliana	AT4G14723.1 | Symbols: | BEST Arabidopsis thaliana protein match is: allergen-related (TAIR:AT3G22820.1); Has 35333 Blast hits to 34131 proteins in 2444 species: Archae - 798; Bacteria - 22429; Metazoa - 974; Fungi - 991; Plants - 531; Viruses - 0; Other Eukaryotes - 9610 (source: NCBI BLink). | chr4:8440167-8440693 FORWARD LENGTH=109	117	109	2.00E-35	93.2	62.4	67.5
Rsa1.0_00033.1.g2041.t1	gb|AAR03591.1| ARD-like protein [Brassica juncea]	204	195	1.00E-102	95.6	87.7	89.7	ARD-like protein	gbpln	Brassica juncea	AT4G14710.2 | Symbols: ATARD2 | RmlC-like cupins superfamily protein | chr4:8424897-8426360 REVERSE LENGTH=199	204	199	1.00E-99	97.5	83.3	86.8

Rsa1.0_00033.1.g2042.t1	ref|NP_567438.1| early light-inducible protein 2 [Arabidopsis thaliana] gi|13878007|gb|AAK44081.1|AF370266_1 unknown protein [Arabidopsis thaliana] gi|17104737|gb|AAL34257.1| unknown protein [Arabidopsis thaliana] gi|332658075|gb|AEE83475.1| early light-inducible protein 2 [Arabidopsis thaliana]	127	193	6.00E-55	152.0	84.3	88.2	early light-inducible protein 2	gbpln	Arabidopsis thaliana	AT4G14690.1 | Symbols: ELIP2 | Chlorophyll A-B binding family protein | chr4:8418373-8419129 FORWARD LENGTH=193	127	193	1.00E-57	152.0	84.3	88.2
Rsa1.0_00033.1.g2043.t1	gb|AAR03591.1| ARD-like protein [Brassica juncea]	195	195	1.00E-110	100.0	97.9	99.0	ARD-like protein	gbpln	Brassica juncea	AT4G14710.2 | Symbols: ATARD2 | RmlC-like cupins superfamily protein | chr4:8424897-8426360 REVERSE LENGTH=199	195	199	1.00E-106	102.1	93.3	96.9
Rsa1.0_00033.1.g2044.t1	gb|EOA18123.1| hypothetical protein CARUB_v10006584mg [Capsella rubella]	191	191	2.00E-80	100.0	85.3	90.1	hypothetical protein CARUB_v10006584mg	gbpln	Capsella rubella	AT4G14690.1 | Symbols: ELIP2 | Chlorophyll A-B binding family protein | chr4:8418373-8419129 FORWARD LENGTH=193	191	193	2.00E-79	101.0	85.3	90.6
Rsa1.0_00033.1.g2045.t1	gb|ACV04807.1| ATP sulfurylase 3 [Brassica napus]	862	465	0	53.9	50.5	51.5	ATP sulfurylase 3	gbpln	Brassica napus	AT4G14680.1 | Symbols: APS3 | Pseudouridine synthase/archaeosine transglycosylase-like family protein | chr4:8413443-8415311 REVERSE LENGTH=465	862	465	0	53.9	47.8	50.7
Rsa1.0_00033.1.g2046.t1	ref|NP_193202.2| DNA-directed RNA polymerase II subunit G [Arabidopsis thaliana] gi|149944323|gb|ABR46204.1| At4g14660 [Arabidopsis thaliana] gi|332658072|gb|AEE83472.1| DNA-directed RNA polymerase NRPE7 [Arabidopsis thaliana]	178	178	1.00E-78	100.0	77.0	91.0	DNA-directed RNA polymerase II subunit G	gbpln	Arabidopsis thaliana	AT4G14660.1 | Symbols: NRPE7 | RNA polymerase Rpb7-like, N-terminal domain | chr4:8406500-8407036 REVERSE LENGTH=178	178	178	4.00E-81	100.0	77.0	91.0
Rsa1.0_00033.1.g2047.t1	ref|XP_002868271.1| hypothetical protein ARALYDRAFT_493441 [Arabidopsis lyrata subsp. lyrata] gi|297314107|gb|EFH44530.1| hypothetical protein ARALYDRAFT_493441 [Arabidopsis lyrata subsp. lyrata]	299	604	5.00E-26	202.0	28.1	33.8	hypothetical protein ARALYDRAFT_493441	gbpln	Arabidopsis lyrata	AT4G14650.1 | Symbols: | unknown protein; FUNCTIONS IN: molecular_function unknown; INVOLVED IN: N-terminal protein myristoylation; EXPRESSED IN: 6 plant structures; EXPRESSED DURING: petal differentiation and expansion stage; Has 2407 Blast hits to 1833 proteins in 310 species: Archae - 19; Bacteria - 223; Metazoa - 708; Fungi - 285; Plants - 140; Viruses - 42; Other Eukaryotes - 990 (source: NCBI BLink). | chr4:8400760-8402817 REVERSE LENGTH=632	299	632	1.00E-25	211.4	26.1	33.1
Rsa1.0_00033.1.g2048.t1	ref|NP_188935.1| ADP-ribosylation factor C1 [Arabidopsis thaliana] gi|334185556|ref|NP_001189951.1| ADP-ribosylation factor C1 [Arabidopsis thaliana] gi|297788976|ref|XP_002862508.1| ADP-ribosylation factor C1 [Arabidopsis lyrata subsp. lyrata] gi|11994726|dbj|BAB03042.1| unnamed protein product [Arabidopsis thaliana] gi|17381004|gb|AAL36314.1| putative ADP-ribosylation factor [Arabidopsis thaliana] gi|20465873|gb|AAM20041.1| putative ADP-ribosylation factor [Arabidopsis thaliana] gi|21592454|gb|AAM64405.1| ADP-ribosylation factor, putative [Arabidopsis thaliana] gi|297308070|gb|EFH38766.1| ADP-ribosylation factor C1 [Arabidopsis lyrata subsp. lyrata] gi|332643174|gb|AEE76695.1| ADP-ribosylation factor C1 [Arabidopsis thaliana] gi|332643175|gb|AEE76696.1| ADP-ribosylation factor C1 [Arabidopsis thaliana]	183	183	1.00E-98	100.0	95.1	97.3	ADP-ribosylation factor C1	gbpln	Arabidopsis lyrata	AT3G22950.2 | Symbols: ARFC1 | ADP-ribosylation factor C1 | chr3:8136364-8137513 REVERSE LENGTH=183	183	183	1.00E-101	100.0	95.1	97.3
Rsa1.0_00033.1.g2049.t1	ref|NP_193198.2| uncharacterized protein [Arabidopsis thaliana] gi|22136618|gb|AAM91628.1| unknown protein [Arabidopsis thaliana] gi|332658067|gb|AEE83467.1| uncharacterized protein AT4G14620 [Arabidopsis thaliana]	362	341	1.00E-125	94.2	68.2	77.9	uncharacterized protein	gbpln	Arabidopsis thaliana	AT4G14620.1 | Symbols: | Protein of unknown function (DUF506) | chr4:8389246-8390448 REVERSE LENGTH=341	362	341	1.00E-127	94.2	68.2	77.9
Rsa1.0_00033.1.g2050.t1	gb|EOA18498.1| hypothetical protein CARUB_v10007049mg [Capsella rubella]	489	488	0	99.8	85.1	90.4	hypothetical protein CARUB_v10007049mg	gbpln	Capsella rubella	AT4G14605.1 | Symbols: | Mitochondrial transcription termination factor family protein | chr4:8378815-8380564 FORWARD LENGTH=493	489	493	0	100.8	84.9	90.4
Rsa1.0_00033.1.g2051.t2	emb|CAB10171.1| disease resistance Cf-2 like protein [Arabidopsis thaliana] gi|7268096|emb|CAB78434.1| disease resistance Cf-2 like protein [Arabidopsis thaliana]	243	869	1.00E-45	357.6	44.9	52.7	disease resistance Cf-2 like protein	gbpln	Arabidopsis thaliana	AT4G13920.1 | Symbols: AtRLP50, RLP50 | receptor like protein 50 | chr4:8043861-8046536 FORWARD LENGTH=891	243	891	4.00E-48	366.7	44.9	52.7

Rsa1.0_00033.1.g2052.t1	gb|EOA15994.1| hypothetical protein CARUB_v10004113mg [Capsella rubella]	844	866	0	102.6	63.6	77.4	hypothetical protein CARUB_v10004113mg	gbpln	Capsella rubella	AT4G13810.1 | Symbols: AtRLP47, RLP47 | receptor like protein 47 | chr4:8005062-8007287 REVERSE LENGTH=741	844	741	0	87.8	55.8	67.8
Rsa1.0_00034.1.g2053.t1	gb|EOA38545.1| hypothetical protein CARUB_v10010350mg [Capsella rubella]	133	196	6.00E-72	147.4	99.2	100.0	hypothetical protein CARUB_v10010350mg	gbpln	Capsella rubella	AT1G20090.1 | Symbols: ARAC4, ROP2, ATROP2, ATRAC4 | RHO-related protein from plants 2 | chr1:6967223-6968603 FORWARD LENGTH=195	133	195	2.00E-74	146.6	99.2	100.0
Rsa1.0_00034.1.g2054.t1	ref|XP_002893089.1| hypothetical protein ARALYDRAFT_472236 [Arabidopsis lyrata subsp. lyrata] gi|297338931|gb|EFH69348.1| hypothetical protein ARALYDRAFT_472236 [Arabidopsis lyrata subsp. lyrata]	269	285	2.00E-69	105.9	67.3	77.7	hypothetical protein ARALYDRAFT_472236	gbpln	Arabidopsis lyrata	AT1G20100.1 | Symbols: | unknown protein; BEST Arabidopsis thaliana protein match is: unknown protein (TAIR:AT1G75860.1); Has 471 Blast hits to 438 proteins in 92 species: Archae - 0; Bacteria - 14; Metazoa - 217; Fungi - 43; Plants - 91; Viruses - 1; Other Eukaryotes - 105 (source: NCBI BLink). | chr1:6969314-6970699 REVERSE LENGTH=285	269	285	2.00E-62	105.9	64.3	73.2
Rsa1.0_00034.1.g2055.t1	ref|NP_564103.1| RING/FYVE/PHD zinc finger-containing protein [Arabidopsis thaliana] gi|13605833|gb|AAK32902.1|AF367315_1 At1g20110/T20H2_10 [Arabidopsis thaliana] gi|16226228|gb|AAL16109.1|AF428277_1 At1g20110/T20H2_10 [Arabidopsis thaliana] gi|23506043|gb|AAN28881.1| At1g20110/T20H2_10 [Arabidopsis thaliana] gi|332191816|gb|AEE29937.1| RING/FYVE/PHD zinc finger-containing protein [Arabidopsis thaliana]	584	601	0	102.9	77.9	82.4	RING/FYVE/PHD zinc finger-containing protein	gbpln	Arabidopsis thaliana	AT1G20110.1 | Symbols: | RING/FYVE/PHD zinc finger superfamily protein | chr1:6971554-6974578 FORWARD LENGTH=601	584	601	0	102.9	77.9	82.4
Rsa1.0_00034.1.g2056.t1	gb|EOA37331.1| hypothetical protein CARUB_v10011033mg [Capsella rubella]	321	389	1.00E-101	121.2	53.6	66.4	hypothetical protein CARUB_v10011033mg	gbpln	Capsella rubella	AT1G20132.1 | Symbols: | GDSL-like Lipase/Acylhydrolase superfamily protein | chr1:6981358-6983495 FORWARD LENGTH=383	321	383	1.00E-101	119.3	52.6	64.8
Rsa1.0_00034.1.g2057.t1	ref|NP_173445.2| uncharacterized protein [Arabidopsis thaliana] gi|158706505|sp|Q6DYE5.2|U496K_ARATH RecName: Full=UPF0496 protein At1g20180 gi|8778981|gb|AAF79896.1|AC022472_5 Contains similarity to At14a protein from Arabidopsis thaliana gi|4589123 [Arabidopsis thaliana] gi|91805813|gb|ABE65635.1| hypothetical protein At1g20180 [Arabidopsis thaliana] gi|332191826|gb|AEE29947.1| uncharacterized protein AT1G20180 [Arabidopsis thaliana]	390	390	1.00E-150	100.0	77.7	85.4	uncharacterized protein	gbpln	Arabidopsis thaliana	AT1G20180.1 | Symbols: | Protein of unknown function (DUF677) | chr1:6996354-6997779 FORWARD LENGTH=390	390	390	1.00E-153	100.0	77.7	85.4
Rsa1.0_00034.1.g2058.t1	ref|XP_002893092.1| Alpha-expansin 11 precursor [Arabidopsis lyrata subsp. lyrata] gi|297338934|gb|EFH69351.1| Alpha-expansin 11 precursor [Arabidopsis lyrata subsp. lyrata]	213	252	1.00E-101	118.3	90.6	95.3	Alpha-expansin 11 precursor	gbpln	Arabidopsis lyrata	AT1G20190.1 | Symbols: ATEXPA11, EXP11, ATEXP11, ATHEXP ALPHA 1.14, EXPA11 | expansin 11 | chr1:6998762-6999710 REVERSE LENGTH=252	213	252	7.00E-99	118.3	91.1	95.3
Rsa1.0_00034.1.g2059.t1	ref|NP_173447.1| 26S proteasome regulatory subunit N3 [Arabidopsis thaliana] gi|30316389|sp|Q9LNU4.3|PSD31_ARATH RecName: Full=Probable 26S proteasome non-ATPase regulatory subunit 3a; Short=26S proteasome subunit S3-a; AltName: Full=26S proteasome regulatory subunit RPN3a; AltName: Full=Protein EMBRYO DEFECTIVE 2719 gi|32700016|gb|AAP86658.1| 26S proteasome subunit RPN3a [Arabidopsis thaliana] gi|332191829|gb|AEE29950.1| probable 26S proteasome non-ATPase regulatory subunit 3a [Arabidopsis thaliana]	463	488	0	105.4	95.0	97.6	26S proteasome regulatory subunit N3	gbpln	Arabidopsis thaliana	AT1G20200.1 | Symbols: EMB2719, HAP15 | PAM domain (PCI/PINT associated module) protein | chr1:7001409-7004154 REVERSE LENGTH=488	463	488	0	105.4	95.0	97.6
Rsa1.0_00034.1.g2060.t1	ref|XP_002893094.1| hypothetical protein ARALYDRAFT_312946 [Arabidopsis lyrata subsp. lyrata] gi|297338936|gb|EFH69353.1| hypothetical protein ARALYDRAFT_312946 [Arabidopsis lyrata subsp. lyrata]	369	529	1.00E-72	143.4	52.8	57.7	hypothetical protein ARALYDRAFT_312946	gbpln	Arabidopsis lyrata	AT1G76010.1 | Symbols: | Alba DNA/RNA-binding protein | chr1:28528505-28530488 REVERSE LENGTH=350	369	350	5.00E-73	94.9	53.4	60.2

Rsa1.0_00034.1.g2061.t2	ref|XP_002890376.1| hypothetical protein ARALYDRAFT_472256 [Arabidopsis lyrata subsp. lyrata] gi|297336218|gb|EFH66635.1| hypothetical protein ARALYDRAFT_472256 [Arabidopsis lyrata subsp. lyrata]	486	487	0	100.2	98.1	98.8	hypothetical protein ARALYDRAFT_472256	gbpln	Arabidopsis lyrata	AT1G20260.1 | Symbols: | ATPase, V1 complex, subunit B protein | chr1:7016971-7020290 FORWARD LENGTH=487	486	487	0	100.2	97.9	98.6
Rsa1.0_00034.1.g2062.t1	gb|EOA38379.1| hypothetical protein CARUB_v10009921mg [Capsella rubella]	289	287	1.00E-155	99.3	91.3	96.5	hypothetical protein CARUB_v10009921mg	gbpln	Capsella rubella	AT1G20270.1 | Symbols: | 2-oxoglutarate (2OG) and Fe(II)-dependent oxygenase superfamily protein | chr1:7021383-7022923 REVERSE LENGTH=287	289	287	1.00E-157	99.3	91.0	96.2
Rsa1.0_00034.1.g2063.t1	dbj|BAJ33832.1| unnamed protein product [Thellungiella halophila]	165	167	5.00E-75	101.2	92.1	96.4	unnamed protein product	----	----	AT1G20340.1 | Symbols: DRT112, PETE2 | Cupredoxin superfamily protein | chr1:7042770-7043273 REVERSE LENGTH=167	165	167	6.00E-71	101.2	85.5	92.7
Rsa1.0_00034.1.g2064.t1	gb|EOA37077.1| hypothetical protein CARUB_v10010213mg, partial [Capsella rubella]	219	229	1.00E-74	104.6	68.9	78.1	hypothetical protein CARUB_v10010213mg, partial	gbpln	Capsella rubella	AT1G20350.1 | Symbols: ATTIM17-1, TIM17-1 | translocase inner membrane subunit 17-1 | chr1:7043737-7044393 FORWARD LENGTH=218	219	218	2.00E-69	99.5	63.5	76.7
Rsa1.0_00034.1.g2065.t1	gb|EOA37077.1| hypothetical protein CARUB_v10010213mg, partial [Capsella rubella]	220	229	2.00E-60	104.1	58.6	70.9	hypothetical protein CARUB_v10010213mg, partial	gbpln	Capsella rubella	AT1G20350.1 | Symbols: ATTIM17-1, TIM17-1 | translocase inner membrane subunit 17-1 | chr1:7043737-7044393 FORWARD LENGTH=218	220	218	4.00E-54	99.1	53.6	66.8
Rsa1.0_00034.1.g2066.t1	ref|NP_173472.1| 4-coumarate--CoA ligase-like 2 [Arabidopsis thaliana] gi|158564049|sp|Q84P25.2|4CLL2_ARATH RecName: Full=4-coumarate--CoA ligase-like 2 gi|332191856|gb|AEE29977.1| 4-coumarate--CoA ligase-like 2 [Arabidopsis thaliana]	556	565	0	101.6	80.6	89.0	4-coumarate--CoA ligase-like 2	gbpln	Arabidopsis thaliana	AT1G20480.1 | Symbols: | AMP-dependent synthetase and ligase family protein | chr1:7094978-7097073 REVERSE LENGTH=565	556	565	0	101.6	80.6	89.0
Rsa1.0_00034.1.g2067.t1	gb|AAP03015.1| 4-coumarate-CoA ligase-like protein [Arabidopsis thaliana]	534	550	0	103.0	76.2	86.9	4-coumarate-CoA ligase-like protein	gbpln	Arabidopsis thaliana	AT5G38120.1 | Symbols: 4CL8 | AMP-dependent synthetase and ligase family protein | chr5:15213773-15216137 FORWARD LENGTH=550	534	550	0	103.0	76.0	86.7
Rsa1.0_00034.1.g2068.t1	ref|XP_002893114.1| opc-8:0 CoA ligase1 [Arabidopsis lyrata subsp. lyrata] gi|297338956|gb|EFH69373.1| opc-8:0 CoA ligase1 [Arabidopsis lyrata subsp. lyrata]	548	546	0	99.6	92.2	96.4	opc-8:0 CoA ligase1	gbpln	Arabidopsis lyrata	AT1G20510.1 | Symbols: OPCL1 | OPC-8:0 CoA ligase1 | chr1:7103645-7105856 REVERSE LENGTH=546	548	546	0	99.6	91.2	95.8
Rsa1.0_00034.1.g2069.t1	gb|EOA36143.1| hypothetical protein CARUB_v10012650mg [Capsella rubella]	230	233	1.00E-100	101.3	80.0	91.3	hypothetical protein CARUB_v10012650mg	gbpln	Capsella rubella	AT1G20520.1 | Symbols: | Arabidopsis protein of unknown function (DUF241) | chr1:7106922-7107617 REVERSE LENGTH=231	230	231	1.00E-102	100.4	82.6	91.7
Rsa1.0_00034.1.g2070.t1	ref|XP_004291296.1| PREDICTED: small nuclear ribonucleoprotein Sm D3-like [Fragaria vesca subsp. vesca]	130	131	1.00E-61	100.8	90.8	94.6	PREDICTED: small nuclear ribonucleoprotein Sm D3-like	gbpln	Fragaria vesca	AT1G20580.1 | Symbols: | Small nuclear ribonucleoprotein family protein | chr1:7128979-7130371 FORWARD LENGTH=131	130	131	7.00E-60	100.8	95.4	96.2
Rsa1.0_00034.1.g2071.t1	ref|NP_173485.1| cyclin-B2-3 [Arabidopsis thaliana] gi|147743080|sp|Q9LDM4.2|CCB23_ARATH RecName: Full=Cyclin-B2-3; AltName: Full=G2/mitotic-specific cyclin-B2-3; Short=CycB2;3 gi|119935845|gb|ABM06010.1| At1g20610 [Arabidopsis thaliana] gi|332191872|gb|AEE29993.1| cyclin-B2-3 [Arabidopsis thaliana]	421	429	0	101.9	83.4	91.2	cyclin-B2-3	gbpln	Arabidopsis thaliana	AT1G20610.1 | Symbols: CYCB2;3 | Cyclin B2;3 | chr1:7135073-7137273 REVERSE LENGTH=429	421	429	0	101.9	83.4	91.2
Rsa1.0_00034.1.g2072.t1	gb|AAD30291.2|AF139538_1 catalase 2 [Raphanus sativus]	492	492	0	100.0	99.8	99.8	catalase 2	gbpln	Raphanus sativus	AT1G20620.1 | Symbols: CAT3, SEN2, ATCAT3 | catalase 3 | chr1:7143142-7146193 FORWARD LENGTH=492	492	492	0	100.0	98.6	99.4
Rsa1.0_00034.1.g2073.t1	#	#	#	#	#	#	#	-	----	----	#	#	#	#	#	#	#
Rsa1.0_00034.1.g2074.t1	gb|EOA39657.1| hypothetical protein CARUB_v10008300mg [Capsella rubella]	780	844	0	108.2	85.8	91.3	hypothetical protein CARUB_v10008300mg	gbpln	Capsella rubella	AT1G20640.2 | Symbols: | Plant regulator RWP-RK family protein | chr1:7155201-7157986 FORWARD LENGTH=844	780	844	0	108.2	86.0	91.0
Rsa1.0_00034.1.g2075.t1	ref|NP_173489.2| protein kinase-like protein [Arabidopsis thaliana] gi|8778594|gb|AAF79602.1|AC027665_3 F5M15.3 [Arabidopsis thaliana] gi|8886951|gb|AAF80637.1|AC069251_30 F2D10.13 [Arabidopsis thaliana] gi|51971194|dbj|BAD44289.1| unknown protein [Arabidopsis thaliana] gi|332191880|gb|AEE30001.1| protein kinase-like protein [Arabidopsis thaliana]	377	381	0	101.1	92.6	95.0	protein kinase-like protein	gbpln	Arabidopsis thaliana	AT1G20650.1 | Symbols: | Protein kinase superfamily protein | chr1:7158422-7160022 REVERSE LENGTH=381	377	381	0	101.1	92.6	95.0

Rsa1.0_00034.1.g2076.t2	gb|EOA39820.1| hypothetical protein CARUB_v10008487mg, partial [Capsella rubella]	667	685	0	102.7	80.5	85.9	hypothetical protein CARUB_v10008487mg, partial	gbpln	Capsella rubella	AT1G20670.1 | Symbols: | DNA-binding bromodomain-containing protein | chr1:7164537-7167933 REVERSE LENGTH=652	667	652	0	97.8	77.4	83.1
Rsa1.0_00034.1.g2077.t2	ref|NP_173499.1| calcium-binding EF hand-containing protein [Arabidopsis thaliana] gi|8886934|gb|AAF80620.1|AC069251_13 F2D10.25 [Arabidopsis thaliana] gi|110742187|dbj|BAE99021.1| hypothetical protein [Arabidopsis thaliana] gi|332191898|gb|AEE30019.1| calcium-binding EF hand-containing protein [Arabidopsis thaliana]	1035	1019	0	98.5	84.5	88.2	calcium-binding EF hand-containing protein	gbpln	Arabidopsis thaliana	AT1G20760.1 | Symbols: | Calcium-binding EF hand family protein | chr1:7209515-7214773 FORWARD LENGTH=1019	1035	1019	0	98.5	84.5	88.2
Rsa1.0_00034.1.g2078.t1	ref|NP_173500.1| uncharacterized protein [Arabidopsis thaliana] gi|8886946|gb|AAF80632.1|AC069251_25 F2D10.26 [Arabidopsis thaliana] gi|38454150|gb|AAR20769.1| At1g20770 [Arabidopsis thaliana] gi|41349930|gb|AAS00350.1| At1g20770 [Arabidopsis thaliana] gi|332191899|gb|AEE30020.1| uncharacterized protein AT1G20770 [Arabidopsis thaliana]	223	215	9.00E-88	96.4	78.9	83.9	uncharacterized protein	gbpln	Arabidopsis thaliana	AT1G20770.1 | Symbols: | unknown protein; Has 59 Blast hits to 59 proteins in 24 species: Archae - 0; Bacteria - 0; Metazoa - 26; Fungi - 4; Plants - 29; Viruses - 0; Other Eukaryotes - 0 (source: NCBI BLink). | chr1:7215345-7216648 REVERSE LENGTH=215	223	215	3.00E-90	96.4	78.9	83.9
Rsa1.0_00034.1.g2079.t1	gb|EOA39693.1| hypothetical protein CARUB_v10008332mg [Capsella rubella]	870	801	0	92.1	76.2	84.4	hypothetical protein CARUB_v10008332mg	gbpln	Capsella rubella	AT1G20780.1 | Symbols: PUB44, ATPUB44, SAUL1 | senescence-associated E3 ubiquitin ligase 1 | chr1:7217812-7220609 FORWARD LENGTH=801	870	801	0	92.1	76.7	83.6
Rsa1.0_00034.1.g2080.t1	ref|NP_173502.2| F-box protein [Arabidopsis thaliana] gi|75264017|sp|Q9LM75.1|FBK6_ARATH RecName: Full=Putative F-box/kelch-repeat protein At1g20790 gi|8886945|gb|AAF80631.1|AC069251_24 F2D10.28 [Arabidopsis thaliana] gi|332191901|gb|AEE30022.1| putative F-box/kelch-repeat protein [Arabidopsis thaliana]	274	435	5.00E-64	158.8	50.4	64.2	F-box protein	gbpln	Arabidopsis thaliana	AT1G20790.1 | Symbols: | F-box family protein | chr1:7220961-7222268 REVERSE LENGTH=435	274	435	1.00E-66	158.8	50.4	64.2
Rsa1.0_00034.1.g2081.t1	ref|NP_173503.1| F-box family protein [Arabidopsis thaliana] gi|332191903|gb|AEE30024.1| F-box family protein [Arabidopsis thaliana]	150	492	4.00E-22	328.0	51.3	64.0	F-box family protein	gbpln	Arabidopsis thaliana	AT1G20800.1 | Symbols: | F-box family protein | chr1:7228718-7231600 REVERSE LENGTH=492	150	492	1.00E-24	328.0	51.3	64.0
Rsa1.0_00034.1.g2082.t1	gb|EOA36226.1| hypothetical protein CARUB_v10010177mg [Capsella rubella]	194	234	1.00E-81	120.6	85.1	92.3	hypothetical protein CARUB_v10010177mg	gbpln	Capsella rubella	AT1G20823.1 | Symbols: | RING/U-box superfamily protein | chr1:7238880-7239473 FORWARD LENGTH=197	194	197	4.00E-82	101.5	87.1	91.8
Rsa1.0_00034.1.g2083.t1	ref|NP_173507.1| multiple chloroplast division site 1 [Arabidopsis thaliana] gi|26452945|dbj|BAC43549.1| unknown protein [Arabidopsis thaliana] gi|29824121|gb|AAP04021.1| unknown protein [Arabidopsis thaliana] gi|332191908|gb|AEE30029.1| multiple chloroplast division site 1 [Arabidopsis thaliana]	347	349	1.00E-163	100.6	82.1	88.8	multiple chloroplast division site 1	gbpln	Arabidopsis thaliana	AT1G20830.1 | Symbols: MCD1 | multiple chloroplast division site 1 | chr1:7242933-7244736 FORWARD LENGTH=349	347	349	1.00E-166	100.6	82.1	88.8
Rsa1.0_00034.1.g2084.t1	dbj|BAJ33724.1| unnamed protein product [Thellungiella halophila]	730	733	0	100.4	92.7	95.8	unnamed protein product	----	----	AT1G20840.1 | Symbols: TMT1 | tonoplast monosaccharide transporter1 | chr1:7245107-7247674 REVERSE LENGTH=734	730	734	0	100.5	91.5	95.6
Rsa1.0_00034.1.g2085.t1	ref|XP_002890413.1| hypothetical protein ARALYDRAFT_472321 [Arabidopsis lyrata subsp. lyrata] gi|297336255|gb|EFH66672.1| hypothetical protein ARALYDRAFT_472321 [Arabidopsis lyrata subsp. lyrata]	356	357	0	100.3	92.4	94.7	hypothetical protein ARALYDRAFT_472321	gbpln	Arabidopsis lyrata	AT1G20850.1 | Symbols: XCP2 | xylem cysteine peptidase 2 | chr1:7252208-7253537 FORWARD LENGTH=356	356	356	0	100.0	92.4	95.5
Rsa1.0_00034.1.g2086.t1	ref|XP_002893134.1| phosphate transporter family protein [Arabidopsis lyrata subsp. lyrata] gi|297338976|gb|EFH69393.1| phosphate transporter family protein [Arabidopsis lyrata subsp. lyrata]	540	536	0	99.3	88.7	93.3	phosphate transporter family protein	gbpln	Arabidopsis lyrata	AT1G20860.1 | Symbols: PHT1;8 | phosphate transporter 1;8 | chr1:7254007-7258677 REVERSE LENGTH=534	540	534	0	98.9	86.7	90.7
Rsa1.0_00034.1.g2087.t1	gb|EOA35544.1| hypothetical protein CARUB_v10020749mg [Capsella rubella]	262	285	1.00E-113	108.8	79.0	88.9	hypothetical protein CARUB_v10020749mg	gbpln	Capsella rubella	AT1G76460.1 | Symbols: | RNA-binding (RRM/RBD/RNP motifs) family protein | chr1:28686932-28688506 REVERSE LENGTH=285	262	285	1.00E-113	108.8	77.9	87.4
Rsa1.0_00034.1.g2088.t1	gb|AAB68964.1| trypsin inhibitor propeptide [Brassica oleracea]	173	214	6.00E-29	123.7	48.0	61.3	trypsin inhibitor propeptide	gbpln	Brassica oleracea	AT1G73260.1 | Symbols: ATKTI1, KTI1 | kunitz trypsin inhibitor 1 | chr1:27547410-27548057 REVERSE LENGTH=215	173	215	5.00E-19	124.3	38.7	56.1
Rsa1.0_00034.1.g2089.t2	#	#	#	#	#	#	#	-	----	----	#	#	#	#	#	#	#

Rsa1.0_00034.1.g2090.t1	ref|NP_173514.1| putative DNA-binding protein ESCAROLA [Arabidopsis thaliana] gi|20532086|sp|Q9S7C9.1|ESCA_ARATH RecName: Full=Putative DNA-binding protein ESCAROLA gi|4836899|gb|AAD30602.1|AC007369_12 Unknown protein [Arabidopsis thaliana] gi|6319180|gb|AAF07197.1|AF194974_1 ESCAROLA [Arabidopsis thaliana] gi|30102700|gb|AAP21268.1| At1g20900 [Arabidopsis thaliana] gi|110736548|dbj|BAF00240.1| putative DNA-binding protein [Arabidopsis thaliana] gi|119657398|tpd|FAA00298.1| TPA: AT-hook motif nuclear localized protein 27 [Arabidopsis thaliana] gi|225897950|dbj|BAH30307.1| hypothetical protein [Arabidopsis thaliana] gi|332191917|gb|AEE30038.1| putative DNA-binding protein ESCAROLA [Arabidopsis thaliana]	314	311	1.00E-120	99.0	94.3	94.9	putative DNA-binding protein ESCAROLA	gbpln	Arabidopsis thaliana	AT1G20900.1 | Symbols: ESC, ORE7, AHL27 | Predicted AT-hook DNA-binding family protein | chr1:7273024-7273959 FORWARD LENGTH=311	314	311	1.00E-123	99.0	94.3	94.9
Rsa1.0_00034.1.g2091.t1	gb|EOA40571.1| hypothetical protein CARUB_v10009301mg [Capsella rubella]	374	410	1.00E-142	109.6	73.0	80.7	hypothetical protein CARUB_v10009301mg	gbpln	Capsella rubella	AT1G20910.1 | Symbols: | ARID/BRIGHT DNA-binding domain-containing protein | chr1:7277209-7280635 REVERSE LENGTH=398	374	398	1.00E-140	106.4	71.7	79.9
Rsa1.0_00034.1.g2092.t1	gb|EOA39882.1| hypothetical protein CARUB_v10008559mg, partial [Capsella rubella]	619	639	0	103.2	94.0	97.1	hypothetical protein CARUB_v10008559mg, partial	gbpln	Capsella rubella	AT1G20950.1 | Symbols: | Phosphofructokinase family protein | chr1:7297467-7301336 REVERSE LENGTH=614	619	614	0	99.2	94.0	97.1
Rsa1.0_00034.1.g2093.t1	ref|XP_002890421.1| zinc-binding family protein [Arabidopsis lyrata subsp. lyrata] gi|297336263|gb|EFH66680.1| zinc-binding family protein [Arabidopsis lyrata subsp. lyrata]	243	246	1.00E-132	101.2	91.4	97.1	zinc-binding family protein	gbpln	Arabidopsis lyrata	AT1G21000.2 | Symbols: | PLATZ transcription factor family protein | chr1:7338013-7339088 FORWARD LENGTH=243	243	243	1.00E-132	100.0	90.1	95.9
Rsa1.0_00034.1.g2094.t1	gb|EOA36953.1| hypothetical protein CARUB_v10009886mg [Capsella rubella]	209	296	4.00E-81	141.6	79.9	89.0	hypothetical protein CARUB_v10009886mg	gbpln	Capsella rubella	AT1G21010.1 | Symbols: | unknown protein; FUNCTIONS IN: molecular_function unknown; INVOLVED IN: N-terminal protein myristoylation; LOCATED IN: cellular_component unknown; EXPRESSED IN: 24 plant structures; EXPRESSED DURING: 14 growth stages; BEST Arabidopsis thaliana protein match is: unknown protein (TAIR:AT1G76600.1); Has 206 Blast hits to 206 proteins in 13 species: Archae - 0; Bacteria - 0; Metazoa - 0; Fungi - 0; Plants - 206; Viruses - 0; Other Eukaryotes - 0 (source: NCBI BLink). | chr1:7346239-7346871 FORWARD LENGTH=210	209	210	3.00E-80	100.5	78.0	87.1
Rsa1.0_00034.1.g2095.t1	dbj|BAJ33623.1| unnamed protein product [Thellungiella halophila]	471	471	0	100.0	89.2	94.3	unnamed protein product	----	----	AT1G21400.1 | Symbols: | Thiamin diphosphate-binding fold (THDP-binding) superfamily protein | chr1:7493492-7496240 FORWARD LENGTH=472	471	472	0	100.2	88.1	93.8
Rsa1.0_00034.1.g2096.t1	ref|NP_564139.1| F-box protein SKP2A [Arabidopsis thaliana] gi|75177240|sp|Q9LPL4.1|SKP2A_ARATH RecName: Full=F-box protein SKP2A; AltName: Full=FBL5-like protein; Short=AtFBL5; AltName: Full=SKP2-like protein 1; Short=AtSKP2;1 gi|9454572|gb|AAF87895.1|AC015447_5 Unknown protein [Arabidopsis thaliana] gi|16604366|gb|AAL24189.1| At1g21410/F24J8_17 [Arabidopsis thaliana] gi|19699206|gb|AAL90969.1| At1g21410/F24J8_17 [Arabidopsis thaliana] gi|332191979|gb|AEE30100.1| F-box protein SKP2A [Arabidopsis thaliana]	284	360	1.00E-145	126.8	91.2	93.3	F-box protein SKP2A	gbpln	Arabidopsis thaliana	AT1G21410.1 | Symbols: SKP2A | F-box/RNI-like superfamily protein | chr1:7497479-7499386 FORWARD LENGTH=360	284	360	1.00E-148	126.8	91.2	93.3

Rsa1.0_00034.1.g2097.t1	ref|NP_564140.1| Nodulin MtN3-like protein [Arabidopsis thaliana] gi|75154590|sp|Q8L9J7.1|SWET1_ARATH RecName: Full=Bidirectional sugar transporter SWEET1; Short=AtSWEET1 gi|21594011|gb|AAM65929.1| unknown [Arabidopsis thaliana] gi|28393568|gb|AAO42204.1| unknown protein [Arabidopsis thaliana] gi|28973143|gb|AAO63896.1| unknown protein [Arabidopsis thaliana] gi|332191983|gb|AEE30104.1| bidirectional sugar transporter SWEET1 [Arabidopsis thaliana]	250	247	1.00E-128	98.8	92.0	95.6	Nodulin MtN3-like protein	gbpln	Arabidopsis thaliana	AT1G21460.1 | Symbols: SWEET1, AtSWEET1 | Nodulin MtN3 family protein | chr1:7512030-7513281 REVERSE LENGTH=247	250	247	1.00E-130	98.8	92.0	95.6
Rsa1.0_00034.1.g2098.t1	#	#	#	#	#	#	#	–	----	----	#	#	#	#	#	#	#
Rsa1.0_00034.1.g2099.t1	ref|NP_564141.1| exostosin-like protein [Arabidopsis thaliana] gi|332191985|gb|AEE30106.1| exostosin-like protein [Arabidopsis thaliana]	466	462	0	99.1	92.7	95.5	exostosin-like protein	gbpln	Arabidopsis thaliana	AT1G21480.1 | Symbols: | Exostosin family protein | chr1:7519116-7521426 REVERSE LENGTH=462	466	462	0	99.1	92.7	95.5
Rsa1.0_00034.1.g2100.t1	gb|EMJ00939.1| hypothetical protein PRUPE_ppa002697mg [Prunus persica]	642	644	0	100.3	73.7	85.4	hypothetical protein PRUPE_ppa002697mg	gbpln	Prunus persica	AT1G77130.1 | Symbols: PGSIP2, GUX3 | plant glycogenin-like starch initiation protein 2 | chr1:28979066-28981228 REVERSE LENGTH=618	642	618	0	96.3	73.4	82.7
Rsa1.0_00034.1.g2101.t1	#	#	#	#	#	#	#	–	----	----	#	#	#	#	#	#	#
Rsa1.0_00034.1.g2102.t1	ref|XP_002893172.1| hypothetical protein ARALYDRAFT_472389 [Arabidopsis lyrata subsp. lyrata] gi|297339014|gb|EFH69431.1| hypothetical protein ARALYDRAFT_472389 [Arabidopsis lyrata subsp. lyrata]	73	71	2.00E-13	97.3	60.3	72.6	hypothetical protein ARALYDRAFT_472389	gbpln	Arabidopsis lyrata	#	#	#	#	#	#	#
Rsa1.0_00035.1.g2103.t2	ref|NP_176277.1| AP-1 complex subunit mu [Arabidopsis thaliana] gi|2462748|gb|AAB71967.1| putative Clathrin Coat Assembly protein [Arabidopsis thaliana] gi|20466372|gb|AAM20503.1| clathrin adaptor medium chain protein MU1B, putative [Arabidopsis thaliana] gi|25084014|gb|AAN72155.1| clathrin adaptor medium chain protein MU1B, putative [Arabidopsis thaliana] gi|332195610|gb|AEE33731.1| protein HAPLESS 13 [Arabidopsis thaliana]	888	428	0	48.2	45.9	46.7	AP-1 complex subunit mu	gbpln	Arabidopsis thaliana	AT1G60780.1 | Symbols: HAP13 | Clathrin adaptor complexes medium subunit family protein | chr1:22369289-22371885 REVERSE LENGTH=428	888	428	0	48.2	45.9	46.7
Rsa1.0_00035.1.g2104.t2	#	#	#	#	#	#	#	–	----	----	#	#	#	#	#	#	#
Rsa1.0_00035.1.g2105.t1	gb|AFO67891.1| leucine-rich repeat receptor-like kinase (mitochondrion) [Brassica rapa subsp. oleifera]	635	635	0	100.0	96.4	98.6	leucine-rich repeat receptor-like kinase (mitochondrion)	gbpln	Brassica rapa	AT1G60800.1 | Symbols: NIK3 | NSP-interacting kinase 3 | chr1:22383601-22386931 REVERSE LENGTH=632	635	632	0	99.5	91.8	96.2
Rsa1.0_00035.1.g2106.t1	gb|AAG51783.1|AC079679_3 reverse transcriptase, putative; 16838-20266 [Arabidopsis thaliana]	260	1142	5.00E-55	439.2	40.0	55.0	reverse transcriptase, putative; 16838-20266	gbpln	Arabidopsis thaliana	AT4G29090.1 | Symbols: | Ribonuclease H-like superfamily protein | chr4:14333528-14335255 FORWARD LENGTH=575	260	575	3.00E-25	221.2	29.2	42.7
Rsa1.0_00035.1.g2107.t1	ref|XP_002884699.1| D-alanine--D-alanine ligase family [Arabidopsis lyrata subsp. lyrata] gi|297330539|gb|EFH60958.1| D-alanine--D-alanine ligase family [Arabidopsis lyrata subsp. lyrata]	950	943	0	99.3	79.9	87.8	D-alanine--D-alanine ligase family	gbpln	Arabidopsis lyrata	AT3G08840.2 | Symbols: | D-alanine--D-alanine ligase family | chr3:2679648-2686157 REVERSE LENGTH=937	950	937	0	98.6	79.7	88.3
Rsa1.0_00035.1.g2108.t1	gb|EOA34924.1| hypothetical protein CARUB_v10020009mg [Capsella rubella]	612	603	0	98.5	60.8	63.1	hypothetical protein CARUB_v10020009mg	gbpln	Capsella rubella	AT1G60900.1 | Symbols: | U2 snRNP auxilliary factor, large subunit, splicing factor | chr1:22424008-22427806 FORWARD LENGTH=589	612	589	0	96.2	60.0	62.6
Rsa1.0_00035.1.g2109.t1	gb|AAM67112.1| putative serine/threonine-protein kinase [Arabidopsis thaliana]	352	361	1.00E-176	102.6	89.8	93.8	putative serine/threonine-protein kinase	gbpln	Arabidopsis thaliana	AT1G60940.2 | Symbols: SNRK2-10, SNRK2.10, SRK2B | SNF1-related protein kinase 2.10 | chr1:22439398-22441896 REVERSE LENGTH=361	352	361	1.00E-178	102.6	89.5	93.5
Rsa1.0_00035.1.g2110.t1	gb|EOA37821.1| hypothetical protein CARUB_v10011995mg [Capsella rubella]	598	589	0	98.5	88.1	91.5	hypothetical protein CARUB_v10011995mg	gbpln	Capsella rubella	AT1G10950.1 | Symbols: TMN1, AtTMN1 | transmembrane nine 1 | chr1:3659322-3663622 FORWARD LENGTH=589	598	589	0	98.5	87.3	91.3
Rsa1.0_00035.1.g2111.t1	dbj|BAJ33728.1| unnamed protein product [Thellungiella halophila]	148	148	3.00E-75	100.0	92.6	97.3	unnamed protein product	----	----	AT1G60950.1 | Symbols: FED A, ATFD2 | 2Fe-2S ferredoxin-like superfamily protein | chr1:22444565-22445011 FORWARD LENGTH=148	148	148	7.00E-77	100.0	92.6	96.6
Rsa1.0_00035.1.g2112.t1	gb|AAK69430.1|AF275752_1 ZIP-like zinc transporter [Noccaea caerulescens]	416	422	0	101.4	86.5	90.4	ZIP-like zinc transporter	gbpln	Noccaea caerulescens	AT1G60960.1 | Symbols: IRT3, ATIRT3 | iron regulated transporter 3 | chr1:22445410-22447060 REVERSE LENGTH=425	416	425	0	102.2	85.3	90.6

Rsa1.0_00035.1.g2113.t1	ref|NP_568221.1| Protein kinase family protein [Arabidopsis thaliana] gi|14334482|gb|AAK59439.1| putative protein kinase [Arabidopsis thaliana] gi|15147869|dbj|BAB62845.1| Ndr kinase [Arabidopsis thaliana] gi|16323450|gb|AAL15219.1| putative protein kinase [Arabidopsis thaliana] gi|332004078|gb|AED91461.1| Protein kinase family protein [Arabidopsis thaliana]	525	515	0	98.1	90.1	93.5	Protein kinase family protein	gbpln	Arabidopsis thaliana	AT5G09890.1 | Symbols: | Protein kinase family protein | chr5:3085810-3088842 REVERSE LENGTH=515	525	515	0	98.1	90.1	93.5
Rsa1.0_00035.1.g2114.t1	ref|NP_176294.1| gibberellin 20-oxidase 4 [Arabidopsis thaliana] gi|12323345|gb|AAG51653.1|AC018908_19 putative gibberellin 20-oxidase; 47658-49225 [Arabidopsis thaliana] gi|67633472|gb|AAY78660.1| putative gibberellin 20-oxidase [Arabidopsis thaliana] gi|332195634|gb|AEE33755.1| gibberellin 20-oxidase 4 [Arabidopsis thaliana]	369	376	1.00E-126	101.9	61.5	69.1	gibberellin 20-oxidase 4	gbpln	Arabidopsis thaliana	AT1G60980.1 | Symbols: ATGA20OX4, GA20OX4 | gibberellin 20-oxidase 4 | chr1:22452573-22454140 FORWARD LENGTH=376	369	376	1.00E-129	101.9	61.5	69.1
Rsa1.0_00035.1.g2115.t2	gb|ACY30421.1| C.BANa [Brassica napus]	135	338	2.00E-29	250.4	60.7	69.6	C.BANa	gbpln	Brassica napus	AT1G61720.1 | Symbols: BAN | NAD(P)-binding Rossmann-fold superfamily protein | chr1:22791326-22792757 REVERSE LENGTH=340	135	340	2.00E-29	251.9	57.0	67.4
Rsa1.0_00035.1.g2116.t1	gb|EOA34094.1| hypothetical protein CARUB_v10021594mg [Capsella rubella]	439	441	1.00E-175	100.5	76.3	83.8	hypothetical protein CARUB_v10021594mg	gbpln	Capsella rubella	AT1G61050.1 | Symbols: | alpha 1,4-glycosyltransferase family protein | chr1:22486736-22488043 FORWARD LENGTH=435	439	435	1.00E-171	99.1	76.8	84.1
Rsa1.0_00035.1.g2117.t1	ref|XP_002888111.1| At1g61040/T7P1_17 [Arabidopsis lyrata subsp. lyrata] gi|297333952|gb|EFH64370.1| At1g61040/T7P1_17 [Arabidopsis lyrata subsp. lyrata]	643	643	0	100.0	86.0	89.6	At1g61040/T7P1_17	gbpln	Arabidopsis lyrata	AT1G61040.1 | Symbols: VIP5 | plus-3 domain-containing protein | chr1:22483817-22485748 FORWARD LENGTH=643	643	643	0	100.0	85.7	89.9
Rsa1.0_00035.1.g2118.t1	gb|EOA33209.1| hypothetical protein CARUB_v10019868mg [Capsella rubella]	693	725	0	104.6	94.5	97.1	hypothetical protein CARUB_v10019868mg	gbpln	Capsella rubella	AT1G61010.3 | Symbols: CPSF73-I | cleavage and polyadenylation specificity factor 73-I | chr1:22474954-22477660 REVERSE LENGTH=693	693	693	0	100.0	93.7	96.5
Rsa1.0_00035.1.g2119.t1	ref|XP_002886570.1| hypothetical protein ARALYDRAFT_475227 [Arabidopsis lyrata subsp. lyrata] gi|297332411|gb|EFH62829.1| hypothetical protein ARALYDRAFT_475227 [Arabidopsis lyrata subsp. lyrata]	618	623	0	100.8	88.0	91.9	hypothetical protein ARALYDRAFT_475227	gbpln	Arabidopsis lyrata	AT1G60995.1 | Symbols: | FUNCTIONS IN: molecular_function unknown; INVOLVED IN: biological_process unknown; LOCATED IN: cellular_component unknown; EXPRESSED IN: 20 plant structures; EXPRESSED DURING: 10 growth stages; CONTAINS InterPro DOMAIN/s: Membralin (InterPro:IPR019144); Has 172 Blast hits to 170 proteins in 70 species: Archae - 0; Bacteria - 0; Metazoa - 110; Fungi - 0; Plants - 38; Viruses - 0; Other Eukaryotes - 24 (source: NCBI BLink). | chr1:22465768-22470575 REVERSE LENGTH=623	618	623	0	100.8	87.9	91.6
Rsa1.0_00035.1.g2120.t3	ref|NP_176295.3| aminomethyltransferase [Arabidopsis thaliana] gi|145326078|ref|NP_001077748.1| aminomethyltransferase [Arabidopsis thaliana] gi|186492130|ref|NP_001117522.1| aminomethyltransferase [Arabidopsis thaliana] gi|51969110|dbj|BAD43247.1| unnamed protein product [Arabidopsis thaliana] gi|332195639|gb|AEE33760.1| aminomethyltransferase [Arabidopsis thaliana] gi|332195640|gb|AEE33761.1| aminomethyltransferase [Arabidopsis thaliana] gi|332195641|gb|AEE33762.1| aminomethyltransferase [Arabidopsis thaliana]	418	432	0	103.3	87.3	91.1	aminomethyltransferase	gbpln	Arabidopsis thaliana	AT1G60990.3 | Symbols: | Glycine cleavage T-protein family | chr1:22462771-22465416 REVERSE LENGTH=432	418	432	0	103.3	87.3	91.1
Rsa1.0_00035.1.g2121.t1	gb|EOA39455.1| hypothetical protein CARUB_v10012652mg [Capsella rubella]	340	320	1.00E-140	94.1	72.1	82.4	hypothetical protein CARUB_v10012652mg	gbpln	Capsella rubella	AT1G05250.1 | Symbols: | Peroxidase superfamily protein | chr1:1525924-1527169 REVERSE LENGTH=325	340	325	1.00E-132	95.6	65.0	77.4

Rsa1.0_00035.1.g2122.t1	ref|NP_001031213.1| defensin-like protein 245 [Arabidopsis thaliana] gi|55584174|sp|P82623.2|DF245_ARATH RecName: Full=Defensin-like protein 245; AltName: Full=S locus cysteine-rich-like protein 4; Short=Protein SCRL4; Short=SCR-like protein 4; Flags: Precursor gi|113204420|gb|ABI34004.1| unknown [Arabidopsis thaliana] gi|332195637|gb|AEE33758.1| defensin-like protein 245 [Arabidopsis thaliana]	96	97	9.00E-30	101.0	68.8	79.2	defensin-like protein 245	gbpln	Arabidopsis thaliana	AT1G60986.1 | Symbols: SCRL4 | SCR-like 4 | chr1:22459072-22459468 FORWARD LENGTH=97	96	97	2.00E-32	101.0	68.8	79.2
Rsa1.0_00035.1.g2123.t1	ref|XP_002886566.1| protease inhibitor II [Arabidopsis lyrata subsp. lyrata] gi|297332407|gb|EFH62825.1| protease inhibitor II [Arabidopsis lyrata subsp. lyrata]	77	76	2.00E-25	98.7	74.0	87.0	protease inhibitor II	gbpln	Arabidopsis lyrata	AT2G02130.1 | Symbols: PDF2.3, LCR68 | low-molecular-weight cysteine-rich 68 | chr2:540071-540407 FORWARD LENGTH=77	77	77	1.00E-25	100.0	64.9	74.0
Rsa1.0_00035.1.g2124.t1	ref|XP_002886565.1| hypothetical protein ARALYDRAFT_475218 [Arabidopsis lyrata subsp. lyrata] gi|297332406|gb|EFH62824.1| hypothetical protein ARALYDRAFT_475218 [Arabidopsis lyrata subsp. lyrata]	858	879	1.00E-127	102.4	33.2	34.6	hypothetical protein ARALYDRAFT_475218	gbpln	Arabidopsis lyrata	AT1G61080.1 | Symbols: | Hydroxyproline-rich glycoprotein family protein | chr1:22493194-22497019 REVERSE LENGTH=907	858	907	1.00E-129	105.7	32.3	33.8
Rsa1.0_00035.1.g2125.t1	dbj|BAJ34413.1| unnamed protein product [Thellungiella halophila]	770	809	0	105.1	80.1	87.0	unnamed protein product	----	----	AT1G61100.2 | Symbols: | disease resistance protein (TIR class), putative | chr1:22508831-22512122 REVERSE LENGTH=807	770	807	0	104.8	77.8	85.6
Rsa1.0_00035.1.g2126.t1	ref|NP_974060.1| Toll-Interleukin-Resistance (TIR) domain-containing protein [Arabidopsis thaliana] gi|332195661|gb|AEE33782.1| Toll-Interleukin-Resistance (TIR) domain-containing protein [Arabidopsis thaliana]	216	188	4.00E-81	87.0	67.1	74.5	Toll-Interleukin-Resistance (TIR) domain-containing protein	gbpln	Arabidopsis thaliana	AT1G61105.1 | Symbols: | Toll-Interleukin-Resistance (TIR) domain family protein | chr1:22513616-22514492 REVERSE LENGTH=188	216	188	1.00E-83	87.0	67.1	74.5
Rsa1.0_00035.1.g2127.t2	gb|EOA34398.1| hypothetical protein CARUB_v10021926mg [Capsella rubella]	448	462	0	103.1	80.6	87.9	hypothetical protein CARUB_v10021926mg	gbpln	Capsella rubella	AT1G61130.1 | Symbols: SCPL32 | serine carboxypeptidase-like 32 | chr1:22529024-22531784 REVERSE LENGTH=463	448	463	0	103.3	73.7	83.5
Rsa1.0_00035.1.g2128.t1	gb|EOA11936.1| hypothetical protein CARUB_v10016547mg, partial [Capsella rubella]	116	149	4.00E-24	128.4	42.2	63.8	hypothetical protein CARUB_v10016547mg, partial	gbpln	Capsella rubella	AT2G34320.1 | Symbols: | Polynucleotidyl transferase, ribonuclease H-like superfamily protein | chr2:14484959-14485837 FORWARD LENGTH=292	116	292	3.00E-12	251.7	30.2	51.7
Rsa1.0_00035.1.g2129.t1	ref|NP_176309.2| SNF2, helicase and zinc finger domain-containing protein [Arabidopsis thaliana] gi|332195665|gb|AEE33786.1| SNF2, helicase and zinc finger domain-containing protein [Arabidopsis thaliana]	1173	1280	0	109.1	74.9	83.8	SNF2, helicase and zinc finger domain-containing protein	gbpln	Arabidopsis thaliana	AT1G61140.1 | Symbols: EDA16 | SNF2 domain-containing protein / helicase domain-containing protein / zinc finger protein-related | chr1:22535038-22540610 REVERSE LENGTH=1280	1173	1280	0	109.1	74.9	83.8
Rsa1.0_00035.1.g2130.t1	gb|EOA33443.1| hypothetical protein CARUB_v10020867mg [Capsella rubella]	226	226	1.00E-124	100.0	96.9	100.0	hypothetical protein CARUB_v10020867mg	gbpln	Capsella rubella	AT1G61150.6 | Symbols: | LisH and RanBPM domains containing protein | chr1:22542979-22544355 FORWARD LENGTH=226	226	226	1.00E-125	100.0	95.1	99.1
Rsa1.0_00035.1.g2131.t2	ref|NP_001154324.1| uncharacterized protein [Arabidopsis thaliana] gi|332190567|gb|AEE28688.1| uncharacterized protein AT1G11120 [Arabidopsis thaliana]	109	161	7.00E-26	147.7	63.3	78.0	uncharacterized protein	gbpln	Arabidopsis thaliana	AT1G11120.2 | Symbols: | unknown protein; FUNCTIONS IN: molecular_function unknown; INVOLVED IN: biological_process unknown; LOCATED IN: cellular_component unknown; EXPRESSED IN: 10 plant structures; EXPRESSED DURING: 4 anthesis, F mature embryo stage, petal differentiation and expansion stage, E expanded cotyledon stage, D bilateral stage; BEST Arabidopsis thaliana protein match is: unknown protein (TAIR:AT4G28170.1); Has 94 Blast hits to 94 proteins in 13 species: Archae - 0; Bacteria - 0; Metazoa - 0; Fungi - 0; Plants - 94; Viruses - 0; Other Eukaryotes - 0 (source: NCBI BLink). | chr1:3715229-3717320 FORWARD LENGTH=161	109	161	1.00E-28	147.7	63.3	78.0
Rsa1.0_00035.1.g2132.t1	#	#	#	#	#	#	#	-	----	----	#	#	#	#	#	#	#

Rsa1.0_00035.1.g2133.t1	ref|NP_564773.1| uncharacterized protein [Arabidopsis thaliana] gi|2443885|gb|AAB71478.1| Unknown protein [Arabidopsis thaliana] gi|30017227|gb|AAP12847.1| At1g61170 [Arabidopsis thaliana] gi|110735914|dbj|BAE99932.1| hypothetical protein [Arabidopsis thaliana] gi|332195677|gb|AEE33798.1| uncharacterized protein AT1G61170 [Arabidopsis thaliana]	245	251	2.00E-82	102.4	78.4	84.1	uncharacterized protein	gbpln	Arabidopsis thaliana	AT1G61170.1 | Symbols: | unknown protein; FUNCTIONS IN: molecular_function unknown; INVOLVED IN: N-terminal protein myristoylation; LOCATED IN: cellular_component unknown; EXPRESSED IN: 19 plant structures; EXPRESSED DURING: 12 growth stages; BEST Arabidopsis thaliana protein match is: unknown protein (TAIR:AT1G11125.1); Has 54 Blast hits to 54 proteins in 15 species: Archae - 0; Bacteria - 0; Metazoa - 17; Fungi - 0; Plants - 34; Viruses - 1; Other Eukaryotes - 2 (source: NCBI BLink). | chr1:22549718-22550473 FORWARD LENGTH=251	245	251	6.00E-85	102.4	78.4	84.1
Rsa1.0_00035.1.g2134.t1	#	#	#	#	#	#	#	-	----	----	#	#	#	#	#	#	#
Rsa1.0_00036.1.g2135.t1	ref|XP_002887662.1| F-box family protein [Arabidopsis lyrata subsp. lyrata] gi|297333503|gb|EFH63921.1| F-box family protein [Arabidopsis lyrata subsp. lyrata]	366	374	0	102.2	90.4	94.0	F-box family protein	gbpln	Arabidopsis lyrata	AT1G76920.1 | Symbols: | F-box family protein | chr1:28892295-28893419 FORWARD LENGTH=374	366	374	0	102.2	89.6	94.0
Rsa1.0_00036.1.g2136.t1	gb|EOA36857.1| hypothetical protein CARUB_v10008803mg [Capsella rubella]	600	536	1.00E-86	89.3	32.0	41.0	hypothetical protein CARUB_v10008803mg	gbpln	Capsella rubella	AT2G07760.1 | Symbols: | Zinc knuckle (CCHC-type) family protein | chr2:3584420-3586278 REVERSE LENGTH=530	600	530	9.00E-50	88.3	16.5	20.3
Rsa1.0_00036.1.g2137.t1	ref|NP_177835.2| peroxidase 13 [Arabidopsis thaliana] gi|2829914|gb|AAC00622.1| putative peroxidase [Arabidopsis thaliana] gi|332197814|gb|AEE35935.1| peroxidase 13 [Arabidopsis thaliana]	319	336	1.00E-152	105.3	81.8	89.0	peroxidase 13	gbpln	Arabidopsis thaliana	AT1G77100.1 | Symbols: | Peroxidase superfamily protein | chr1:28965772-28967066 REVERSE LENGTH=336	319	336	1.00E-154	105.3	81.8	89.0
Rsa1.0_00036.1.g2138.t1	emb|CAB39638.1| RNA-directed DNA polymerase-like protein [Arabidopsis thaliana] gi|7267666|emb|CAB78094.1| RNA-directed DNA polymerase-like protein [Arabidopsis thaliana]	242	1274	1.00E-29	526.4	40.1	53.3	RNA-directed DNA polymerase-like protein	gbpln	Arabidopsis thaliana	AT1G10000.1 | Symbols: | Ribonuclease H-like superfamily protein | chr1:3263877-3264788 REVERSE LENGTH=303	242	303	2.00E-18	125.2	31.0	48.3
Rsa1.0_00036.1.g2139.t1	#	#	#	#	#	#	#	-	----	----	#	#	#	#	#	#	#
Rsa1.0_00036.1.g2140.t1	ref|XP_002887671.1| hypothetical protein ARALYDRAFT_476877 [Arabidopsis lyrata subsp. lyrata] gi|297333512|gb|EFH63930.1| hypothetical protein ARALYDRAFT_476877 [Arabidopsis lyrata subsp. lyrata]	569	569	0	100.0	96.7	98.1	hypothetical protein ARALYDRAFT_476877	gbpln	Arabidopsis lyrata	AT1G77140.1 | Symbols: VPS45, ATVPS45 | vacuolar protein sorting 45 | chr1:28984163-28987681 FORWARD LENGTH=569	569	569	0	100.0	95.8	97.9
Rsa1.0_00036.1.g2141.t1	ref|XP_002889128.1| hypothetical protein ARALYDRAFT_476882 [Arabidopsis lyrata subsp. lyrata] gi|297334969|gb|EFH65387.1| hypothetical protein ARALYDRAFT_476882 [Arabidopsis lyrata subsp. lyrata]	496	610	0	123.0	78.0	86.3	hypothetical protein ARALYDRAFT_476882	gbpln	Arabidopsis lyrata	AT1G77180.2 | Symbols: SKIP | chromatin protein family | chr1:28999791-29001632 REVERSE LENGTH=613	496	613	0	123.6	74.8	84.9
Rsa1.0_00036.1.g2142.t1	ref|XP_002889129.1| hypothetical protein ARALYDRAFT_476884 [Arabidopsis lyrata subsp. lyrata] gi|297334970|gb|EFH65388.1| hypothetical protein ARALYDRAFT_476884 [Arabidopsis lyrata subsp. lyrata]	224	247	6.00E-82	110.3	83.5	86.6	hypothetical protein ARALYDRAFT_476884	gbpln	Arabidopsis lyrata	AT1G77200.1 | Symbols: | Integrase-type DNA-binding superfamily protein | chr1:29004357-29005091 REVERSE LENGTH=244	224	244	9.00E-81	108.9	79.5	83.0
Rsa1.0_00036.1.g2143.t1	ref|XP_002890504.1| hypothetical protein ARALYDRAFT_889727 [Arabidopsis lyrata subsp. lyrata] gi|297336346|gb|EFH66763.1| hypothetical protein ARALYDRAFT_889727 [Arabidopsis lyrata subsp. lyrata]	419	426	1.00E-100	101.7	53.5	69.7	hypothetical protein ARALYDRAFT_889727	gbpln	Arabidopsis lyrata	AT1G25150.1 | Symbols: | F-box family protein | chr1:8821027-8822328 FORWARD LENGTH=433	419	433	1.00E-102	103.3	53.2	68.0
Rsa1.0_00036.1.g2144.t1	ref|XP_002893432.1| predicted protein [Arabidopsis lyrata subsp. lyrata] gi|297339274|gb|EFH69691.1| predicted protein [Arabidopsis lyrata subsp. lyrata]	412	414	5.00E-88	100.5	50.0	64.6	predicted protein	gbpln	Arabidopsis lyrata	AT1G25150.1 | Symbols: | F-box family protein | chr1:8821027-8822328 FORWARD LENGTH=433	412	433	1.00E-89	105.1	50.0	63.6

Rsa1.0_00036.1.g2145.t1	ref|NP_001117347.1| F-box protein [Arabidopsis thaliana] gi|186478848|ref|NP_001117348.1| F-box protein [Arabidopsis thaliana] gi|186478854|ref|NP_001117350.1| F-box protein [Arabidopsis thaliana] gi|186478863|ref|NP_001117352.1| F-box protein [Arabidopsis thaliana] gi|334182847|ref|NP_001185091.1| F-box family protein [Arabidopsis thaliana] gi|380876887|sp|P0DI02.1|FBK10_ARATH RecName: Full=F-box/kelch-repeat protein At1g24800 gi|380876888|sp|P0DI03.1|FBK11_ARATH RecName: Full=F-box/kelch-repeat protein At1g24881 gi|380876889|sp|P0DI04.1|FBK12_ARATH RecName: Full=F-box/kelch-repeat protein At1g25055 gi|380876890|sp|P0DI05.1|FBK13_ARATH RecName: Full=F-box/kelch-repeat protein At1g25150 gi|380876891|sp|P0DI06.1|FBK14_ARATH RecName: Full=F-box/kelch-repeat protein At1g25211 gi|23297235|gb|AAN12922.1| unknown protein [Arabidopsis thaliana] gi|26452089|dbj|BAC43134.1| unknown protein [Arabidopsis thaliana] gi|33942045|gb|AAQ55275.1| At1g24880 [Arabidopsis thaliana] gi|62320789|dbj|BAD93716.1| hypothetical protein [Arabidopsis thaliana] gi|110735803|dbj|BAE99878.1|	422	433	1.00E-105	102.6	55.7	70.4	F-box protein	gbpln	Arabidopsis thaliana	AT1G25150.1 | Symbols: | F-box family protein | chr1:8821027-8822328 FORWARD LENGTH=433	422	433	1.00E-107	102.6	55.7	70.4
Rsa1.0_00036.1.g2146.t1	gb|AAG50806.1|AC079281_8 unknown protein [Arabidopsis thaliana]	317	1213	9.00E-35	382.6	27.8	42.3	unknown protein	gbpln	Arabidopsis thaliana	AT1G43760.1 | Symbols: | DNAse I-like superfamily protein | chr1:16528880-16531065 REVERSE LENGTH=626	317	626	1.00E-29	197.5	21.8	38.2
Rsa1.0_00036.1.g2147.t1	ref|XP_002863741.1| hypothetical protein ARALYDRAFT_917455 [Arabidopsis lyrata subsp. lyrata] gi|297309576|gb|EFH40000.1| hypothetical protein ARALYDRAFT_917455 [Arabidopsis lyrata subsp. lyrata]	186	390	8.00E-31	209.7	38.2	54.8	hypothetical protein ARALYDRAFT_917455	gbpln	Arabidopsis lyrata	AT4G04650.1 | Symbols: | RNA-directed DNA polymerase (reverse transcriptase)-related family protein | chr4:2353989-2355213 REVERSE LENGTH=332	186	332	1.00E-26	178.5	32.3	52.7
Rsa1.0_00036.1.g2148.t1	ref|XP_002887672.1| hypothetical protein ARALYDRAFT_895607 [Arabidopsis lyrata subsp. lyrata] gi|297333513|gb|EFH63931.1| hypothetical protein ARALYDRAFT_895607 [Arabidopsis lyrata subsp. lyrata]	487	484	0	99.4	89.1	94.7	hypothetical protein ARALYDRAFT_895607	gbpln	Arabidopsis lyrata	AT1G77220.1 | Symbols: | Protein of unknown function (DUF300) | chr1:29013232-29015530 FORWARD LENGTH=484	487	484	0	99.4	88.5	94.3
Rsa1.0_00036.1.g2149.t1	emb|CAA64328.1| amp-binding protein [Brassica napus]	553	552	0	99.8	94.9	96.7	amp-binding protein	gbpln	Brassica napus	AT1G77240.1 | Symbols: | AMP-dependent synthetase and ligase family protein | chr1:29017958-29019595 REVERSE LENGTH=545	553	545	0	98.6	85.2	91.3
Rsa1.0_00036.1.g2150.t1	#	#	#	#	#	#	#	-	----	----	#	#	#	#	#	#	#
Rsa1.0_00036.1.g2151.t1	gb|EOA33584.1| hypothetical protein CARUB_v10019729mg [Capsella rubella]	547	969	1.00E-90	177.1	44.4	61.2	hypothetical protein CARUB_v10019729mg	gbpln	Capsella rubella	AT1G77270.1 | Symbols: | unknown protein; BEST Arabidopsis thaliana protein match is: unknown protein (TAIR:AT3G07730.1); Has 428 Blast hits to 388 proteins in 92 species: Archae - 0; Bacteria - 31; Metazoa - 189; Fungi - 10; Plants - 97; Viruses - 0; Other Eukaryotes - 101 (source: NCBI BLink). | chr1:29028342-29031068 FORWARD LENGTH=682	547	682	5.00E-86	124.7	42.2	58.0
Rsa1.0_00036.1.g2152.t1	ref|NP_177852.2| adenine nucleotide alpha hydrolases-domain containing protein kinase [Arabidopsis thaliana] gi|332197836|gb|AEE35957.1| adenine nucleotide alpha hydrolases-domain containing protein kinase [Arabidopsis thaliana]	723	794	0	109.8	80.5	88.1	adenine nucleotide alpha hydrolases-domain containing protein kinase	gbpln	Arabidopsis thaliana	AT1G77280.1 | Symbols: | Protein kinase protein with adenine nucleotide alpha hydrolases-like domain | chr1:29031468-29035882 REVERSE LENGTH=794	723	794	0	109.8	80.5	88.1
Rsa1.0_00036.1.g2153.t1	#	#	#	#	#	#	#	-	----	----	#	#	#	#	#	#	#
Rsa1.0_00036.1.g2154.t1	gb|EOA33259.1| hypothetical protein CARUB_v10022136mg [Capsella rubella]	136	266	5.00E-25	195.6	56.6	64.0	hypothetical protein CARUB_v10022136mg	gbpln	Capsella rubella	AT1G77290.2 | Symbols: | Glutathione S-transferase family protein | chr1:29038917-29039815 FORWARD LENGTH=266	136	266	6.00E-27	195.6	56.6	64.0

Rsa1.0_00036.1.g2155.t1	ref|NP_177854.6| histone-lysine N-methyltransferase SETD2 [Arabidopsis thaliana] gi|157734196|gb|ABV68921.1| SDG8 [Arabidopsis thaliana] gi|332197839|gb|AEE35960.1| histone-lysine N-methyltransferase SETD2 [Arabidopsis thaliana]	1527	1805	0	118.2	72.1	79.2	histone-lysine N-methyltransferase SETD2	gbpln	Arabidopsis thaliana	AT1G77300.1 | Symbols: EFS, SDG8, CCR1, ASHH2, LAZ2 | histone methyltransferases(H3-K4 specific);histone methyltransferases(H3-K36 specific) | chr1:29040160-29048810 REVERSE LENGTH=1805	1527	1805	0	118.2	72.1	79.2
Rsa1.0_00036.1.g2156.t1	gb|EOA15566.1| hypothetical protein CARUB_v10005224mg [Capsella rubella]	187	329	1.00E-41	175.9	62.0	72.2	hypothetical protein CARUB_v10005224mg	gbpln	Capsella rubella	AT4G28430.1 | Symbols: | Reticulon family protein | chr4:14057846-14059772 FORWARD LENGTH=457	187	457	9.00E-39	244.4	56.7	66.3
Rsa1.0_00036.1.g2157.t1	gb|EJP69747.1| hypothetical protein BBA_01712 [Beauveria bassiana ARSEF 2860]	251	318	2.00E-67	126.7	48.6	68.9	hypothetical protein BBA_01712	gbenv/gbpln	Beauveria bassiana	#	#	#	#	#	#	#
Rsa1.0_00036.1.g2158.t2	gb|AAC34358.1| Hypothetical protein [Arabidopsis thaliana]	98	1767	3.00E-19	1803.1	53.1	55.1	Hypothetical protein	gbpln	Arabidopsis thaliana	AT1G77300.1 | Symbols: EFS, SDG8, CCR1, ASHH2, LAZ2 | histone methyltransferases(H3-K4 specific);histone methyltransferases(H3-K36 specific) | chr1:29040160-29048810 REVERSE LENGTH=1805	98	1805	4.00E-22	1841.8	53.1	55.1
Rsa1.0_00036.1.g2159.t2	ref|NP_177855.4| uncharacterized protein [Arabidopsis thaliana] gi|332197841|gb|AEE35962.1| uncharacterized protein AT1G77310 [Arabidopsis thaliana]	670	717	0	107.0	65.8	76.0	uncharacterized protein	gbpln	Arabidopsis thaliana	AT1G77310.1 | Symbols: | BEST Arabidopsis thaliana protein match is: wound-responsive family protein (TAIR:AT1G21610.1); Has 493 Blast hits to 482 proteins in 163 species: Archae - 0; Bacteria - 100; Metazoa - 172; Fungi - 66; Plants - 65; Viruses - 7; Other Eukaryotes - 83 (source: NCBI BLink). | chr1:29051671-29056179 FORWARD LENGTH=717	670	717	0	107.0	65.8	76.0
Rsa1.0_00036.1.g2160.t1	ref|NP_565154.1| aminocyclopropanecarboxylate oxidase [Arabidopsis thaliana] gi|122246564|sp|Q0WPW4.1|ACCO5_ARATH RecName: Full=1-aminocyclopropane-1-carboxylate oxidase 5; Short=ACC oxidase 5; Short=AtACO5 gi|110737793|dbj|BAF00835.1| hypothetical protein [Arabidopsis thaliana] gi|111074356|gb|ABH04551.1| At1g77330 [Arabidopsis thaliana] gi|332197844|gb|AEE35965.1| 1-aminocyclopropane-1-carboxylate oxidase 5 [Arabidopsis thaliana]	314	307	1.00E-170	97.8	93.3	96.2	aminocyclopropanecarboxylate oxidase	gbpln	Arabidopsis thaliana	AT1G77330.1 | Symbols: | 2-oxoglutarate (2OG) and Fe(II)-dependent oxygenase superfamily protein | chr1:29063215-29064447 REVERSE LENGTH=307	314	307	1.00E-173	97.8	93.3	96.2
Rsa1.0_00036.1.g2161.t1	#	#	#	#	#	#	#	-	----	----	#	#	#	#	#	#	#
Rsa1.0_00036.1.g2162.t1	gb|EJP69747.1| hypothetical protein BBA_01712 [Beauveria bassiana ARSEF 2860]	248	318	5.00E-66	128.2	48.4	67.7	hypothetical protein BBA_01712	gbenv/gbpln	Beauveria bassiana	#	#	#	#	#	#	#
Rsa1.0_00036.1.g2163.t1	ref|NP_194638.1| Ribonuclease H-like protein [Arabidopsis thaliana] gi|4972055|emb|CAB43923.1| putative protein [Arabidopsis thaliana] gi|7269807|emb|CAB79667.1| putative protein [Arabidopsis thaliana] gi|67633766|gb|AAY78807.1| putative reverse transcriptase/RNA-dependent DNA polymerase [Arabidopsis thaliana] gi|332660185|gb|AEE85585.1| Ribonuclease H-like protein [Arabidopsis thaliana]	300	575	5.00E-30	191.7	33.0	51.0	Ribonuclease H-like protein	gbpln	Arabidopsis thaliana	AT4G29090.1 | Symbols: | Ribonuclease H-like superfamily protein | chr4:14333528-14335255 FORWARD LENGTH=575	300	575	1.00E-32	191.7	33.0	51.0
Rsa1.0_00036.1.g2164.t1	gb|AAG29201.1|AC078898_11 hypothetical protein [Arabidopsis thaliana]	476	481	0	101.1	86.3	93.5	hypothetical protein	gbpln	Arabidopsis thaliana	AT1G77360.1 | Symbols: | Tetratricopeptide repeat (TPR)-like superfamily protein | chr1:29071983-29073536 REVERSE LENGTH=517	476	517	0	108.6	86.3	93.5
Rsa1.0_00036.1.g2165.t1	ref|NP_177864.1| uncharacterized protein [Arabidopsis thaliana] gi|11079486|gb|AAG29198.1|AC078898_8 hypothetical protein [Arabidopsis thaliana] gi|28466875|gb|AAO44046.1| At1g77400 [Arabidopsis thaliana] gi|51971947|dbj|BAD44638.1| At1g77400 [Arabidopsis thaliana] gi|110736099|dbj|BAF00022.1| hypothetical protein [Arabidopsis thaliana] gi|332197852|gb|AEE35973.1| uncharacterized protein AT1G77400 [Arabidopsis thaliana]	190	232	6.00E-50	122.1	83.2	85.3	uncharacterized protein	gbpln	Arabidopsis thaliana	AT1G77400.1 | Symbols: | CONTAINS InterPro DOMAIN/s: Protein of unknown function DUF688 (InterPro:IPR007789); BEST Arabidopsis thaliana protein match is: hydroxyproline-rich glycoprotein family protein (TAIR:AT1G21695.1); Has 328 Blast hits to 314 proteins in 61 species: Archae - 0; Bacteria - 12; Metazoa - 130; Fungi - 28; Plants - 92; Viruses - 10; Other Eukaryotes - 56 (source: NCBI BLink). | chr1:29085881-29086579 FORWARD LENGTH=232	190	232	2.00E-52	122.1	83.2	85.3
Rsa1.0_00036.1.g2166.t1	ref|XP_002887681.1| binding protein [Arabidopsis lyrata subsp. lyrata] gi|297333522|gb|EFH63940.1| binding protein [Arabidopsis lyrata subsp. lyrata]	447	459	0	102.7	86.6	91.5	binding protein	gbpln	Arabidopsis lyrata	AT1G77405.1 | Symbols: | Pentatricopeptide repeat (PPR) superfamily protein | chr1:29087145-29088521 FORWARD LENGTH=458	447	458	0	102.5	85.2	90.2

Rsa1.0_00036.1.g2167.t1	ref|XP_002889142.1| hypothetical protein ARALYDRAFT_476906 [Arabidopsis lyrata subsp. lyrata] gi|297334983|gb|EFH65401.1| hypothetical protein ARALYDRAFT_476906 [Arabidopsis lyrata subsp. lyrata]	818	818	0	100.0	84.5	91.1	hypothetical protein ARALYDRAFT_476906	gbpln	Arabidopsis lyrata	AT1G77410.1 | Symbols: BGAL16 | beta-galactosidase 16 | chr1:29088771-29093148 REVERSE LENGTH=815	818	815	0	99.6	83.6	90.6
Rsa1.0_00036.1.g2168.t1	gb|EOA35294.1| hypothetical protein CARUB_v10020464mg [Capsella rubella]	394	382	0	97.0	80.2	87.6	hypothetical protein CARUB_v10020464mg	gbpln	Capsella rubella	AT1G77420.1 | Symbols: | alpha/beta-Hydrolases superfamily protein | chr1:29093641-29095442 FORWARD LENGTH=382	394	382	0	97.0	79.9	86.8
Rsa1.0_00036.1.g2169.t1	gb|AAP35055.1| NAC-domain protein 14 [Brassica napus]	261	260	1.00E-142	99.6	93.9	96.6	NAC-domain protein 14	gbpln	Brassica napus	AT1G77450.1 | Symbols: anac032, NAC032 | NAC domain containing protein 32 | chr1:29100029-29100981 FORWARD LENGTH=253	261	253	1.00E-119	96.9	83.9	87.0
Rsa1.0_00036.1.g2170.t1	gb|EOA30101.1| hypothetical protein CARUB_v10013211mg, partial [Capsella rubella]	92	628	5.00E-33	682.6	75.0	87.0	hypothetical protein CARUB_v10013211mg, partial	gbpln	Capsella rubella	AT3G10050.1 | Symbols: OMR1 | L-O-methylthreonine resistant 1 | chr3:3099164-3101741 REVERSE LENGTH=592	92	592	9.00E-36	643.5	73.9	87.0
Rsa1.0_00036.1.g2171.t1	ref|XP_002889143.1| hypothetical protein ARALYDRAFT_476911 [Arabidopsis lyrata subsp. lyrata] gi|297334984|gb|EFH65402.1| hypothetical protein ARALYDRAFT_476911 [Arabidopsis lyrata subsp. lyrata]	368	369	0	100.3	91.8	94.3	hypothetical protein ARALYDRAFT_476911	gbpln	Arabidopsis lyrata	AT1G77470.1 | Symbols: RFC3, RFC5 | replication factor C subunit 3 | chr1:29112194-29114323 REVERSE LENGTH=369	368	369	0	100.3	89.1	93.5
Rsa1.0_00036.1.g2172.t1	gb|EJP69747.1| hypothetical protein BBA_01712 [Beauveria bassiana ARSEF 2860]	251	318	2.00E-62	126.7	48.2	64.9	hypothetical protein BBA_01712	gbenv/gbpln	Beauveria bassiana	#	#	#	#	#	#	#
Rsa1.0_00036.1.g2173.t1	gb|ABB17025.1| protein disulfide isomerase [Brassica carinata]	511	509	0	99.6	84.9	91.6	protein disulfide isomerase	gbpln	Brassica carinata	AT1G77510.1 | Symbols: ATPDIL1-2, PDI6, ATPDI6, PDIL1-2 | PDI-like 1-2 | chr1:29126742-29129433 FORWARD LENGTH=508	511	508	0	99.4	82.0	89.2
Rsa1.0_00036.1.g2174.t1	ref|NP_177876.1| O-methyltransferase family protein [Arabidopsis thaliana] gi|12323395|gb|AAG51676.1|AC010704_20 putative caffeic acid 3-O-methyltransferase; 41078-42528 [Arabidopsis thaliana] gi|332197867|gb|AEE35988.1| O-methyltransferase family protein [Arabidopsis thaliana]	339	381	1.00E-152	112.4	80.2	87.0	O-methyltransferase family protein	gbpln	Arabidopsis thaliana	AT1G77520.1 | Symbols: | O-methyltransferase family protein | chr1:29130557-29132007 FORWARD LENGTH=381	339	381	1.00E-155	112.4	80.2	87.0
Rsa1.0_00037.1.g2175.t1	ref|XP_002867032.1| hypothetical protein ARALYDRAFT_491017 [Arabidopsis lyrata subsp. lyrata] gi|297312868|gb|EFH43291.1| hypothetical protein ARALYDRAFT_491017 [Arabidopsis lyrata subsp. lyrata]	377	376	0	99.7	85.9	92.3	hypothetical protein ARALYDRAFT_491017	gbpln	Arabidopsis lyrata	AT4G36160.1 | Symbols: ANAC076, VND2, NAC076 | NAC domain containing protein 76 | chr4:17110877-17112891 REVERSE LENGTH=377	377	377	0	100.0	85.9	91.0
Rsa1.0_00037.1.g2176.t2	#	#	#	#	#	#	#	-	----	----	#	#	#	#	#	#	#
Rsa1.0_00037.1.g2177.t1	ref|XP_002867031.1| hypothetical protein ARALYDRAFT_491015 [Arabidopsis lyrata subsp. lyrata] gi|297312867|gb|EFH43290.1| hypothetical protein ARALYDRAFT_491015 [Arabidopsis lyrata subsp. lyrata]	1120	1132	0	101.1	88.5	93.8	hypothetical protein ARALYDRAFT_491015	gbpln	Arabidopsis lyrata	AT4G36180.1 | Symbols: | Leucine-rich receptor-like protein kinase family protein | chr4:17120209-17123698 REVERSE LENGTH=1136	1120	1136	0	101.4	87.5	93.8
Rsa1.0_00037.1.g2178.t1	gb|EOA16518.1| hypothetical protein CARUB_v10004677mg [Capsella rubella]	473	489	0	103.4	88.2	93.0	hypothetical protein CARUB_v10004677mg	gbpln	Capsella rubella	AT4G36195.3 | Symbols: | Serine carboxypeptidase S28 family protein | chr4:17127202-17129787 FORWARD LENGTH=488	473	488	0	103.2	87.3	94.7
Rsa1.0_00037.1.g2179.t1	ref|NP_001078500.2| uncharacterized protein [Arabidopsis thaliana] gi|332661235|gb|AEE86635.1| uncharacterized protein AT4G36210 [Arabidopsis thaliana]	676	672	0	99.4	90.1	95.4	uncharacterized protein	gbpln	Arabidopsis thaliana	AT4G36210.3 | Symbols: | Protein of unknown function (DUF726) | chr4:17130354-17134302 FORWARD LENGTH=672	676	672	0	99.4	90.1	95.4
Rsa1.0_00037.1.g2180.t1	gb|ACP30591.1| disease resistance protein [Brassica rapa subsp. pekinensis]	106	1555	5.00E-17	1467.0	49.1	61.3	disease resistance protein	gbpln	Brassica rapa	#	#	#	#	#	#	#
Rsa1.0_00037.1.g2181.t1	gb|AAG14961.1|AF214007_1 cytochrome p450-dependent monooxygenase [Brassica napus]	520	520	0	100.0	97.3	98.7	cytochrome p450-dependent monooxygenase	gbpln	Brassica napus	AT4G36220.1 | Symbols: FAH1, CYP84A1 | ferulic acid 5-hydroxylase 1 | chr4:17137584-17139619 REVERSE LENGTH=520	520	520	0	100.0	92.9	96.7

Rsa1.0_00037.1.g2182.t1	ref|NP_195346.1| uncharacterized protein [Arabidopsis thaliana] gi|2961382|emb|CAA18129.1| putative glycine-rich cell wall protein [Arabidopsis thaliana] gi|7270576|emb|CAB80294.1| putative glycine-rich cell wall protein [Arabidopsis thaliana] gi|332661237|gb|AEE86637.1| uncharacterized protein AT4G36230 [Arabidopsis thaliana]	220	221	3.00E-11	100.5	20.0	25.0	uncharacterized protein	gbpln	Arabidopsis thaliana	AT4G36230.1 | Symbols: | unknown protein; FUNCTIONS IN: molecular_function unknown; INVOLVED IN: biological_process unknown; LOCATED IN: endomembrane system; EXPRESSED IN: leaf apex, hypocotyl, flower; EXPRESSED DURING: petal differentiation and expansion stage; Has 250444 Blast hits to 52918 proteins in 2611 species: Archae - 614; Bacteria - 68464; Metazoa - 90603; Fungi - 16312; Plants - 24752; Viruses - 3701; Other Eukaryotes - 45998 (source: NCBI BLink). | chr4:17145586-17146251 FORWARD LENGTH=221	220	221	9.00E-14	100.5	20.0	25.0
Rsa1.0_00037.1.g2183.t1	ref|NP_195347.1| GATA transcription factor 7 [Arabidopsis thaliana] gi|71660767|sp|O65515.1|GATA7_ARATH RecName: Full=GATA transcription factor 7 gi|2961383|emb|CAA18130.1| putative protein [Arabidopsis thaliana] gi|7270577|emb|CAB80295.1| putative protein [Arabidopsis thaliana] gi|18252999|gb|AAL62426.1| putative protein [Arabidopsis thaliana] gi|21389681|gb|AAM48039.1| putative protein [Arabidopsis thaliana] gi|332661238|gb|AEE86638.1| GATA transcription factor 7 [Arabidopsis thaliana]	236	238	2.00E-91	100.8	74.2	80.1	GATA transcription factor 7	gbpln	Arabidopsis thaliana	AT4G36240.1 | Symbols: GATA7 | GATA transcription factor 7 | chr4:17147376-17148211 REVERSE LENGTH=238	236	238	7.00E-94	100.8	74.2	80.1
Rsa1.0_00037.1.g2184.t1	gb|EOA16534.1| hypothetical protein CARUB_v10004694mg [Capsella rubella]	481	485	0	100.8	87.1	95.8	hypothetical protein CARUB_v10004694mg	gbpln	Capsella rubella	AT4G36250.1 | Symbols: ALDH3F1 | aldehyde dehydrogenase 3F1 | chr4:17151029-17153381 FORWARD LENGTH=484	481	484	0	100.6	85.7	94.6
Rsa1.0_00037.1.g2185.t1	gb|ADA60974.1| stylish 2b [Brassica rapa subsp. pekinensis]	312	321	1.00E-125	102.9	88.5	92.6	stylish 2b	gbpln	Brassica rapa	AT4G36260.1 | Symbols: STY2, SRS2 | Lateral root primordium (LRP) protein-related | chr4:17155705-17157006 REVERSE LENGTH=322	312	322	1.00E-119	103.2	86.9	91.0
Rsa1.0_00037.1.g2186.t2	ref|XP_002867022.1| predicted protein [Arabidopsis lyrata subsp. lyrata] gi|297312858|gb|EFH43281.1| predicted protein [Arabidopsis lyrata subsp. lyrata]	1325	631	0	47.6	38.4	42.4	predicted protein	gbpln	Arabidopsis lyrata	AT4G36290.1 | Symbols: CRT1 | compromised recognition of TCV 1 | chr4:17169669-17173187 REVERSE LENGTH=635	1325	635	0	47.9	37.1	41.5
Rsa1.0_00037.1.g2187.t1	dbj|BAJ34544.1| unnamed protein product [Thellungiella halophila]	846	856	0	101.2	92.6	96.0	unnamed protein product	----	----	AT4G36360.1 | Symbols: BGAL3 | beta-galactosidase 3 | chr4:17176840-17181143 REVERSE LENGTH=856	846	856	0	101.2	91.6	95.6
Rsa1.0_00037.1.g2188.t1	#	#	#	#	#	#	#	-	----	----	#	#	#	#	#	#	#
Rsa1.0_00037.1.g2189.t1	#	#	#	#	#	#	#	-	----	----	#	#	#	#	#	#	#
Rsa1.0_00037.1.g2190.t1	ref|XP_002867019.1| cytochrome P450 [Arabidopsis lyrata subsp. lyrata] gi|297312855|gb|EFH43278.1| cytochrome P450 [Arabidopsis lyrata subsp. lyrata]	527	525	0	99.6	86.3	92.4	cytochrome P450	gbpln	Arabidopsis lyrata	AT4G36380.1 | Symbols: ROT3 | Cytochrome P450 superfamily protein | chr4:17187973-17192202 REVERSE LENGTH=524	527	524	0	99.4	85.6	91.3
Rsa1.0_00037.1.g2191.t1	#	#	#	#	#	#	#	-	----	----	#	#	#	#	#	#	#
Rsa1.0_00037.1.g2192.t1	emb|CAA18234.1| putative protein [Arabidopsis thaliana] gi|7269488|emb|CAB79491.1| putative protein [Arabidopsis thaliana]	368	1141	1.00E-58	310.1	34.5	52.7	putative protein	gbpln	Arabidopsis thaliana	AT1G43760.1 | Symbols: | DNAse I-like superfamily protein | chr1:16528880-16531065 REVERSE LENGTH=626	368	626	1.00E-37	170.1	25.8	38.9
Rsa1.0_00037.1.g2193.t1	ref|NP_001190937.1| Splicing factor U2af large subunit A [Arabidopsis thaliana] gi|332661290|gb|AEE86690.1| Splicing factor U2af large subunit A [Arabidopsis thaliana]	525	551	0	105.0	83.4	88.8	Splicing factor U2af large subunit A	gbpln	Arabidopsis thaliana	AT4G36690.4 | Symbols: ATU2AF65A | U2 snRNP auxilliary factor, large subunit, splicing factor | chr4:17294139-17297609 REVERSE LENGTH=551	525	551	0	105.0	83.4	88.8
Rsa1.0_00037.1.g2194.t4	gb|EOA16541.1| hypothetical protein CARUB_v10004700mg [Capsella rubella]	685	483	0	70.5	63.4	66.3	hypothetical protein CARUB_v10004700mg	gbpln	Capsella rubella	AT4G36710.1 | Symbols: | GRAS family transcription factor | chr4:17306060-17307520 FORWARD LENGTH=486	685	486	0	70.9	62.5	65.7
Rsa1.0_00037.1.g2195.t1	emb|CAA58774.1| G-box binding factor 1A [Brassica napus]	311	313	1.00E-155	100.6	92.6	95.2	G-box binding factor 1A	gbpln	Brassica napus	AT4G36730.1 | Symbols: GBF1 | G-box binding factor 1 | chr4:17309850-17311752 REVERSE LENGTH=315	311	315	1.00E-147	101.3	86.8	92.6
Rsa1.0_00037.1.g2196.t1	ref|XP_002866994.1| homeobox-leucine zipper protein ATHB-40 [Arabidopsis lyrata subsp. lyrata] gi|297312830|gb|EFH43253.1| homeobox-leucine zipper protein ATHB-40 [Arabidopsis lyrata subsp. lyrata]	217	217	1.00E-106	100.0	90.8	95.4	homeobox-leucine zipper protein ATHB-40	gbpln	Arabidopsis lyrata	AT4G36740.1 | Symbols: HB-5, ATHB40, HB40 | homeobox protein 40 | chr4:17314649-17316314 REVERSE LENGTH=216	217	216	1.00E-103	99.5	88.0	94.5
Rsa1.0_00037.1.g2197.t1	gb|EOA33837.1| hypothetical protein CARUB_v10021318mg, partial [Capsella rubella]	194	290	1.00E-48	149.5	52.1	67.0	hypothetical protein CARUB_v10021318mg, partial	gbpln	Capsella rubella	AT4G04650.1 | Symbols: | RNA-directed DNA polymerase (reverse transcriptase)-related family protein | chr4:2353989-2355213 REVERSE LENGTH=332	194	332	3.00E-41	171.1	41.8	53.1

Rsa1.0_00037.1.g2198.t1	gb|EOA18652.1| hypothetical protein CARUB_v10007228mg [Capsella rubella]	257	273	1.00E-123	106.2	88.7	92.2	hypothetical protein CARUB_v10007228mg	gbpln	Capsella rubella	AT4G36750.1 | Symbols: | Quinone reductase family protein | chr4:17324642-17326215 FORWARD LENGTH=273	257	273	1.00E-119	106.2	85.6	89.1
Rsa1.0_00037.1.g2199.t1	dbj|BAJ34484.1| unnamed protein product [Thellungiella halophila]	583	645	0	110.6	89.2	93.7	unnamed protein product	----	----	AT4G36760.1 | Symbols: ATAPP1, APP1 | aminopeptidase P1 | chr4:17326688-17329979 FORWARD LENGTH=645	583	645	0	110.6	87.5	92.5
Rsa1.0_00037.1.g2200.t1	ref|XP_002866993.1| UDP-glucoronosyl/UDP-glucosyl transferase family protein [Arabidopsis lyrata subsp. lyrata] gi|297312829|gb|EFH43252.1| UDP-glucoronosyl/UDP-glucosyl transferase family protein [Arabidopsis lyrata subsp. lyrata]	462	457	0	98.9	83.8	91.1	UDP-glucoronosyl/UDP-glucosyl transferase family protein	gbpln	Arabidopsis lyrata	AT4G36770.1 | Symbols: | UDP-Glycosyltransferase superfamily protein | chr4:17330217-17331590 REVERSE LENGTH=457	462	457	0	98.9	83.5	90.0
Rsa1.0_00037.1.g2201.t1	#	#	#	#	#	#	#	-	----	----	#	#	#	#	#	#	#
Rsa1.0_00037.1.g2202.t1	gb|EOA17023.1| hypothetical protein CARUB_v10005257mg [Capsella rubella]	312	322	1.00E-135	103.2	89.4	92.3	hypothetical protein CARUB_v10005257mg	gbpln	Capsella rubella	AT4G36780.1 | Symbols: BEH2 | BES1/BZR1 homolog 2 | chr4:17332989-17334212 REVERSE LENGTH=265	312	265	5.00E-78	84.9	59.6	60.9
Rsa1.0_00037.1.g2203.t1	ref|XP_002869023.1| predicted protein [Arabidopsis lyrata subsp. lyrata] gi|297314859|gb|EFH45282.1| predicted protein [Arabidopsis lyrata subsp. lyrata]	433	489	0	112.9	93.3	96.5	predicted protein	gbpln	Arabidopsis lyrata	AT4G36790.1 | Symbols: | Major facilitator superfamily protein | chr4:17336360-17338304 FORWARD LENGTH=489	433	489	0	112.9	92.6	95.8
Rsa1.0_00037.1.g2204.t1	gb|ACI16426.1| RUB1-conjugation enzyme [Brassica juncea]	185	185	1.00E-103	100.0	96.8	98.9	RUB1-conjugation enzyme	gbpln	Brassica juncea	AT4G36800.2 | Symbols: RCE1 | RUB1 conjugating enzyme 1 | chr4:17341237-17342148 REVERSE LENGTH=184	185	184	1.00E-105	99.5	97.8	98.9
Rsa1.0_00037.1.g2205.t1	sp|Q43133.1|GGPPS_SINAL RecName: Full=Geranylgeranyl pyrophosphate synthase, chloroplastic/chromoplastic; Short=GGPP synthase; Short=GGPS; AltName: Full=(2E,6E)-farnesyl diphosphate synthase; AltName: Full=Dimethylallyltranstransferase; AltName: Full=Farnesyl diphosphate synthase; AltName: Full=Farnesyltranstransferase; AltName: Full=Geranyltranstransferase; Flags: Precursor gi|1419758|emb|CAA67330.1| geranylgeranyl pyrophosphate synthase [Sinapis alba]	368	366	0	99.5	92.7	97.3	RecName: Full=Geranylgeranyl pyrophosphate synthase, chloroplastic/chromoplastic; Short=GGPP synthase; Short=GGPS; AltName: Full=(2E,6E)-farnesyl diphosphate synthase; AltName: Full=Dimethylallyltransferase; AltName: Full=Farnesyl diphosphate synthase; AltName: Full=Farnesyltranstransferase; AltName: Full=Geranyltranstransferase; Flags: Precursor gi|1419758|emb|CAA67330.1| geranylgeranyl pyrophosphate synthase	gbpln	Sinapis alba	AT4G36810.1 | Symbols: GGPS1 | geranylgeranyl pyrophosphate synthase 1 | chr4:17343513-17344628 FORWARD LENGTH=371	368	371	1.00E-173	100.8	86.1	91.6
Rsa1.0_00037.1.g2206.t1	#	#	#	#	#	#	#	-	----	----	#	#	#	#	#	#	#
Rsa1.0_00037.1.g2207.t2	gb|EOA16793.1| hypothetical protein CARUB_v10005013mg, partial [Capsella rubella]	313	388	1.00E-139	124.0	79.6	87.5	hypothetical protein CARUB_v10005013mg, partial	gbpln	Capsella rubella	AT4G36820.1 | Symbols: | Protein of unknown function (DUF607) | chr4:17346889-17347994 FORWARD LENGTH=338	313	338	1.00E-131	108.0	74.1	85.3
Rsa1.0_00037.1.g2208.t1	ref|NP_568009.5| PQ-loop repeat family protein / transmembrane family protein [Arabidopsis thaliana] gi|15028159|gb|AAK76703.1| unknown protein [Arabidopsis thaliana] gi|332661309|gb|AEE86709.1| PQ-loop repeat family protein / transmembrane family protein [Arabidopsis thaliana]	384	392	0	102.1	88.3	92.2	PQ-loop repeat family protein / transmembrane family protein	gbpln	Arabidopsis thaliana	AT4G36850.1 | Symbols: | PQ-loop repeat family protein / transmembrane family protein | chr4:17353415-17355822 REVERSE LENGTH=392	384	392	0	102.1	88.3	92.2
Rsa1.0_00037.1.g2209.t3	ref|XP_002866988.1| hypothetical protein ARALYDRAFT_328082 [Arabidopsis lyrata subsp. lyrata] gi|297312824|gb|EFH43247.1| hypothetical protein ARALYDRAFT_328082 [Arabidopsis lyrata subsp. lyrata]	584	558	0	95.5	87.8	92.5	hypothetical protein ARALYDRAFT_328082	gbpln	Arabidopsis lyrata	AT4G36860.1 | Symbols: | LIM domain-containing protein | chr4:17358580-17361189 REVERSE LENGTH=553	584	553	0	94.7	86.5	91.1
Rsa1.0_00037.1.g2210.t2	gb|AAD51349.1| bel1-like homeodomain 2 [Arabidopsis thaliana] gi|15215857|gb|AAK91472.1| AT4g36870/C7A10_490 [Arabidopsis thaliana]	636	739	0	116.2	84.0	87.6	bel1-like homeodomain 2	gbpln	Arabidopsis thaliana	AT4G36870.2 | Symbols: BLH2, SAW1 | BEL1-like homeodomain 2 | chr4:17369423-17373723 FORWARD LENGTH=739	636	739	0	116.2	83.8	87.4

Rsa1.0_00037.1.g2211.t1	ref|XP_002867047.1| hypothetical protein ARALYDRAFT_328161 [Arabidopsis lyrata subsp. lyrata] gi|297312883|gb|EFH43306.1| hypothetical protein ARALYDRAFT_328161 [Arabidopsis lyrata subsp. lyrata]	457	459	0	100.4	85.3	91.9	hypothetical protein ARALYDRAFT_328161	gbpln	Arabidopsis lyrata	AT4G35910.1 | Symbols: | Adenine nucleotide alpha hydrolases-like superfamily protein | chr4:17008817-17011014 REVERSE LENGTH=458	457	458	0	100.2	84.0	91.2
Rsa1.0_00037.1.g2212.t1	#	#	#	#	#	#	#	–	----	----	#	#	#	#	#	#	#
Rsa1.0_00037.1.g2213.t1	gb|EOA15637.1| hypothetical protein CARUB_v10005881mg [Capsella rubella]	171	173	2.00E-67	101.2	82.5	87.7	hypothetical protein CARUB_v10005881mg	gbpln	Capsella rubella	AT4G37240.1 | Symbols: | unknown protein; FUNCTIONS IN: molecular_function unknown; INVOLVED IN: N-terminal protein myristoylation; LOCATED IN: cellular_component unknown; EXPRESSED IN: 22 plant structures; EXPRESSED DURING: 13 growth stages; BEST Arabidopsis thaliana protein match is: unknown protein (TAIR:AT2G23690.1); Has 30201 Blast hits to 17322 proteins in 780 species: Archae - 12; Bacteria - 1396; Metazoa - 17338; Fungi - 3422; Plants - 5037; Viruses - 0; Other Eukaryotes - 2996 (source: NCBI BLink). | chr4:17524546-17525052 FORWARD LENGTH=168	171	168	9.00E-63	98.2	79.5	87.1
Rsa1.0_00037.1.g2214.t1	gb|AAM08751.1|AC025098_18 Putative copia-type polyprotein [Oryza sativa Japonica Group]	1074	1803	0	167.9	45.7	60.2	Putative copia-type polyprotein	gbpln	Oryza sativa	AT4G23160.1 | Symbols: CRK8 | cysteine-rich RLK (RECEPTOR-like protein kinase) 8 | chr4:12129485-12134086 FORWARD LENGTH=1262	1074	1262	2.00E-85	117.5	15.7	23.1
Rsa1.0_00037.1.g2215.t1	ref|XP_002866981.1| tetratricopeptide repeat-containing protein [Arabidopsis lyrata subsp. lyrata] gi|297312817|gb|EFH43240.1| tetratricopeptide repeat-containing protein [Arabidopsis lyrata subsp. lyrata]	450	498	1.00E-161	110.7	76.4	85.3	tetratricopeptide repeat-containing protein	gbpln	Arabidopsis lyrata	AT4G37210.1 | Symbols: | Tetratricopeptide repeat (TPR)-like superfamily protein | chr4:17512376-17514421 FORWARD LENGTH=492	450	492	1.00E-153	109.3	73.8	82.2
Rsa1.0_00037.1.g2216.t3	gb|EOA17278.1| hypothetical protein CARUB_v10005573mg [Capsella rubella]	294	252	1.00E-107	85.7	68.7	73.5	hypothetical protein CARUB_v10005573mg	gbpln	Capsella rubella	AT4G37200.1 | Symbols: HCF164 | Thioredoxin superfamily protein | chr4:17509836-17511230 REVERSE LENGTH=261	294	261	1.00E-106	88.8	68.0	73.1
Rsa1.0_00037.1.g2217.t1	ref|NP_568018.1| myb family transcription factor [Arabidopsis thaliana] gi|115311427|gb|ABI93894.1| At4g37180 [Arabidopsis thaliana] gi|332661363|gb|AEE86763.1| myb family transcription factor [Arabidopsis thaliana]	342	356	1.00E-127	104.1	76.9	86.0	myb family transcription factor	gbpln	Arabidopsis thaliana	AT4G37180.1 | Symbols: | Homeodomain-like superfamily protein | chr4:17504648-17506107 FORWARD LENGTH=356	342	356	1.00E-130	104.1	76.9	86.0
Rsa1.0_00037.1.g2218.t1	ref|XP_002869013.1| pentatricopeptide repeat-containing protein [Arabidopsis lyrata subsp. lyrata] gi|297314849|gb|EFH45272.1| pentatricopeptide repeat-containing protein [Arabidopsis lyrata subsp. lyrata]	1202	693	0	57.7	48.0	52.5	pentatricopeptide repeat-containing protein	gbpln	Arabidopsis lyrata	AT4G37170.1 | Symbols: | Pentatricopeptide repeat (PPR) superfamily protein | chr4:17498580-17500655 REVERSE LENGTH=691	1202	691	0	57.5	47.8	52.0
Rsa1.0_00037.1.g2219.t1	ref|XP_002869010.1| hypothetical protein ARALYDRAFT_912657 [Arabidopsis lyrata subsp. lyrata] gi|297314846|gb|EFH45269.1| hypothetical protein ARALYDRAFT_912657 [Arabidopsis lyrata subsp. lyrata]	257	261	1.00E-124	101.6	82.1	90.3	hypothetical protein ARALYDRAFT_912657	gbpln	Arabidopsis lyrata	AT4G37150.1 | Symbols: ATMES9, MES9 | methyl esterase 9 | chr4:17492985-17494057 REVERSE LENGTH=256	257	256	1.00E-116	99.6	75.5	85.2
Rsa1.0_00037.1.g2220.t1	ref|NP_195430.2| hydroxyproline-rich glycoprotein family protein [Arabidopsis thaliana] gi|20260346|gb|AAM13071.1| unknown protein [Arabidopsis thaliana] gi|31711924|gb|AAP68318.1| At4g37130 [Arabidopsis thaliana] gi|332661357|gb|AEE86757.1| hydroxyproline-rich glycoprotein family protein [Arabidopsis thaliana]	510	513	0	100.6	75.9	79.8	hydroxyproline-rich glycoprotein family protein	gbpln	Arabidopsis thaliana	AT4G37130.1 | Symbols: | hydroxyproline-rich glycoprotein family protein | chr4:17489567-17491773 FORWARD LENGTH=513	510	513	0	100.6	75.9	79.8
Rsa1.0_00037.1.g2221.t1	gb|EOA16987.1| hypothetical protein CARUB_v10005219mg [Capsella rubella]	323	332	1.00E-140	102.8	75.9	87.0	hypothetical protein CARUB_v10005219mg	gbpln	Capsella rubella	AT4G25900.1 | Symbols: | Galactose mutarotase-like superfamily protein | chr4:13161487-13163397 FORWARD LENGTH=318	323	318	1.00E-135	98.5	73.4	83.3
Rsa1.0_00037.1.g2222.t1	ref|XP_002869009.1| hypothetical protein ARALYDRAFT_490927 [Arabidopsis lyrata subsp. lyrata] gi|297314845|gb|EFH45268.1| hypothetical protein ARALYDRAFT_490927 [Arabidopsis lyrata subsp. lyrata]	410	430	1.00E-150	104.9	72.4	82.4	hypothetical protein ARALYDRAFT_490927	gbpln	Arabidopsis lyrata	AT4G37110.1 | Symbols: | Zinc-finger domain of monoamine-oxidase A repressor R1 | chr4:17484343-17486197 REVERSE LENGTH=417	410	417	1.00E-138	101.7	67.3	79.0

Rsa1.0_00037.1.g2223.t1	ref|NP_195427.1| catalytic/ pyridoxal phosphate binding protein [Arabidopsis thaliana] gi|4006867|emb|CAB16785.1| putative protein [Arabidopsis thaliana] gi|7270659|emb|CAB80376.1| putative protein [Arabidopsis thaliana] gi|332661354|gb|AEE86754.1| catalytic/ pyridoxal phosphate binding protein [Arabidopsis thaliana]	882	896	0	101.6	84.8	90.8	catalytic/ pyridoxal phosphate binding protein	gbpln	Arabidopsis thaliana	AT4G37100.1 | Symbols: | Pyridoxal phosphate (PLP)-dependent transferases superfamily protein | chr4:17479173-17481863 REVERSE LENGTH=896	882	896	0	101.6	84.8	90.8
Rsa1.0_00037.1.g2224.t2	gb|EOA16265.1| hypothetical protein CARUB_v10004409mg [Capsella rubella]	590	605	0	102.5	87.8	91.9	hypothetical protein CARUB_v10004409mg	gbpln	Capsella rubella	AT4G37080.3 | Symbols: | Protein of unknown function, DUF547 | chr4:17473719-17476716 FORWARD LENGTH=610	590	610	0	103.4	88.1	91.5
Rsa1.0_00037.1.g2225.t1	gb|EOA18418.1| hypothetical protein CARUB_v10006962mg [Capsella rubella]	408	414	0	101.5	83.6	93.9	hypothetical protein CARUB_v10006962mg	gbpln	Capsella rubella	AT4G37070.2 | Symbols: PLP1, PLA IVA, AtPLAIVA | Acyl transferase/acyl hydrolase/lysophospholipase superfamily protein | chr4:17464916-17467058 REVERSE LENGTH=414	408	414	0	101.5	82.6	93.1
Rsa1.0_00037.1.g2226.t1	gb|EOA18418.1| hypothetical protein CARUB_v10006962mg [Capsella rubella]	336	414	1.00E-105	123.2	56.0	64.3	hypothetical protein CARUB_v10006962mg	gbpln	Capsella rubella	AT4G37070.2 | Symbols: PLP1, PLA IVA, AtPLAIVA | Acyl transferase/acyl hydrolase/lysophospholipase superfamily protein | chr4:17464916-17467058 REVERSE LENGTH=414	336	414	1.00E-106	123.2	55.4	65.2
Rsa1.0_00037.1.g2227.t1	ref|XP_002869006.1| hypothetical protein ARALYDRAFT_490916 [Arabidopsis lyrata subsp. lyrata] gi|297314842|gb|EFH45265.1| hypothetical protein ARALYDRAFT_490916 [Arabidopsis lyrata subsp. lyrata]	412	412	0	100.0	77.4	87.9	hypothetical protein ARALYDRAFT_490916	gbpln	Arabidopsis lyrata	AT4G37050.1 | Symbols: PLP4, PLA V, AtPLAIVC | PATATIN-like protein 4 | chr4:17457261-17459642 REVERSE LENGTH=428	412	428	0	103.9	76.7	86.7
Rsa1.0_00037.1.g2228.t1	ref|XP_002866973.1| hypothetical protein ARALYDRAFT_912645 [Arabidopsis lyrata subsp. lyrata] gi|297312809|gb|EFH43232.1| hypothetical protein ARALYDRAFT_912645 [Arabidopsis lyrata subsp. lyrata]	351	350	0	99.7	91.2	95.4	hypothetical protein ARALYDRAFT_912645	gbpln	Arabidopsis lyrata	AT4G37040.1 | Symbols: MAP1D | methionine aminopeptidase 1D | chr4:17455175-17457085 FORWARD LENGTH=350	351	350	0	99.7	90.3	94.9
Rsa1.0_00037.1.g2229.t1	gb|ABV89640.1| heat shock factor 4 [Brassica rapa]	277	285	1.00E-138	102.9	93.1	94.9	heat shock factor 4	gbpln	Brassica rapa	AT4G36990.1 | Symbols: HSF4, HSFB1, AT-HSFB1, ATHSF4 | heat shock factor 4 | chr4:17440660-17441706 FORWARD LENGTH=284	277	284	1.00E-117	102.5	80.9	88.1
Rsa1.0_00037.1.g2230.t3	ref|XP_002869003.1| hypothetical protein ARALYDRAFT_490906 [Arabidopsis lyrata subsp. lyrata] gi|297314839|gb|EFH45262.1| hypothetical protein ARALYDRAFT_490906 [Arabidopsis lyrata subsp. lyrata]	547	505	0	92.3	75.5	80.6	hypothetical protein ARALYDRAFT_490906	gbpln	Arabidopsis lyrata	AT4G36980.4 | Symbols: | FUNCTIONS IN: molecular_function unknown; INVOLVED IN: biological_process unknown; EXPRESSED IN: 22 plant structures; EXPRESSED DURING: 13 growth stages; CONTAINS InterPro DOMAIN/s: Splicing factor, suppressor of white apricot (InterPro:IPR019147). | chr4:17433861-17437012 REVERSE LENGTH=556	547	556	0	101.6	75.1	80.6
Rsa1.0_00038.1.g2231.t1	ref|XP_002891171.1| predicted protein [Arabidopsis lyrata subsp. lyrata] gi|297337013|gb|EFH67430.1| predicted protein [Arabidopsis lyrata subsp. lyrata]	133	151	2.00E-45	113.5	67.7	75.2	predicted protein	gbpln	Arabidopsis lyrata	AT1G35260.1 | Symbols: MLP165 | MLP-like protein 165 | chr1:12937059-12937689 REVERSE LENGTH=152	133	152	2.00E-45	114.3	65.4	77.4
Rsa1.0_00038.1.g2232.t1	ref|XP_002875142.1| pectate lyase family protein [Arabidopsis lyrata subsp. lyrata] gi|297320980|gb|EFH51401.1| pectate lyase family protein [Arabidopsis lyrata subsp. lyrata]	459	455	0	99.1	81.0	88.9	pectate lyase family protein	gbpln	Arabidopsis lyrata	AT2G02720.1 | Symbols: | Pectate lyase family protein | chr2:763173-764834 FORWARD LENGTH=455	459	455	0	99.1	81.3	88.5
Rsa1.0_00038.1.g2233.t14	ref|NP_178378.1| pentatricopeptide repeat-containing protein [Arabidopsis thaliana] gi|218546778|sp|Q1PFA6.2|PP144_ARATH RecName: Full=Pentatricopeptide repeat-containing protein At2g02750 gi|2947066|gb|AAC05347.1| hypothetical protein [Arabidopsis thaliana] gi|330250526|gb|AEC05620.1| pentatricopeptide repeat-containing protein [Arabidopsis thaliana]	638	613	0	96.1	76.3	84.2	pentatricopeptide repeat-containing protein	gbpln	Arabidopsis thaliana	AT2G02750.1 | Symbols: | Pentatricopeptide repeat (PPR) superfamily protein | chr2:771641-773482 REVERSE LENGTH=613	638	613	0	96.1	76.3	84.2

Rsa1.0_00038.1.g2234.t1	ref|NP_565289.1| ubiquitin-conjugating enzyme E2 A [Arabidopsis thaliana] gi|297814522|ref|XP_002875144.1| ubiquiting-conjugating enzyme 2 [Arabidopsis lyrata subsp. lyrata] gi|1174844|sp|P42745.1|UBC2_ARATH RecName: Full=Ubiquitin-conjugating enzyme E2 2; AltName: Full=Ubiquitin carrier protein 2; AltName: Full=Ubiquitin-conjugating enzyme E2-17 kDa 2; AltName: Full=Ubiquitin-protein ligase 2 gi|13899127|gb|AAK48985.1|AF370558_1 putative ubiquitin-conjugating enzyme E2 [Arabidopsis thaliana] gi|431264|gb|AAA32899.1| ubiquitin conjugating enzyme [Arabidopsis thaliana] gi|2689243|emb|CAA73476.1| ubiquitin conjugating enzyme [Arabidopsis thaliana] gi|2947065|gb|AAC05346.1| E2, ubiquitin-conjugating enzyme 2 (UBC2) [Arabidopsis thaliana] gi|18377456|gb|AAL66894.1| putative ubiquitin-conjugating enzyme E2 [Arabidopsis thaliana] gi|66354400|gb|AAY44843.1| ubiquitinating enzyme [Arabidopsis thaliana] gi|118197458|gb|ABK78692.1| ubiquitin conjugating enzyme [Brassica rapa] gi|297320982|gb|EFH51403.1| ubiquiting-conjugating enzyme 2 [Arabidopsis lyrata subsp. lyrata] gi|312281529|dbj|BAJ33630.1| unnamed	126	152	9.00E-64	120.6	100.0	100.0	ubiquitin-conjugating enzyme E2 A	gbpln	Arabidopsis lyrata	AT2G02760.1 | Symbols: ATUBC2, UBC2 | ubiquiting-conjugating enzyme 2 | chr2:774271-775149 FORWARD LENGTH=152	126	152	2.00E-66	120.6	100.0	100.0
Rsa1.0_00038.1.g2235.t1	ref|NP_178382.2| protein IQ-domain 29 [Arabidopsis thaliana] gi|330250530|gb|AEC05624.1| protein IQ-domain 29 [Arabidopsis thaliana]	595	636	0	106.9	71.6	81.8	protein IQ-domain 29	gbpln	Arabidopsis thaliana	AT2G02790.1 | Symbols: IQD29 | IQ-domain 29 | chr2:788708-790946 FORWARD LENGTH=636	595	636	0	106.9	71.6	81.8
Rsa1.0_00038.1.g2236.t1	gb|EOA23949.1| hypothetical protein CARUB_v10017166mg [Capsella rubella]	418	472	0	112.9	87.8	93.1	hypothetical protein CARUB_v10017166mg	gbpln	Capsella rubella	AT2G02870.3 | Symbols: | Galactose oxidase/kelch repeat superfamily protein | chr2:838378-839781 FORWARD LENGTH=467	418	467	0	111.7	87.6	93.5
Rsa1.0_00038.1.g2237.t1	gb|AAD38033.1|AF149053_1 phytochrome kinase substrate 1 [Arabidopsis thaliana]	401	439	1.00E-138	109.5	73.1	81.5	phytochrome kinase substrate 1	gbpln	Arabidopsis thaliana	AT2G02950.1 | Symbols: PKS1 | phytochrome kinase substrate 1 | chr2:855149-856468 REVERSE LENGTH=439	401	439	1.00E-140	109.5	72.8	81.5
Rsa1.0_00038.1.g2238.t1	ref|XP_002875185.1| predicted protein [Arabidopsis lyrata subsp. lyrata] gi|297321023|gb|EFH51444.1| predicted protein [Arabidopsis lyrata subsp. lyrata]	416	376	1.00E-159	90.4	71.6	76.7	predicted protein	gbpln	Arabidopsis lyrata	AT2G03060.2 | Symbols: AGL30 | AGAMOUS-like 30 | chr2:901614-903569 FORWARD LENGTH=386	416	386	1.00E-154	92.8	71.4	76.9
Rsa1.0_00038.1.g2239.t1	gb|EOA24327.1| hypothetical protein CARUB_v10017566mg [Capsella rubella]	339	344	1.00E-171	101.5	91.4	95.0	hypothetical protein CARUB_v10017566mg	gbpln	Capsella rubella	AT2G03120.1 | Symbols: ATSPP, SPP | signal peptide peptidase | chr2:937554-940083 FORWARD LENGTH=344	339	344	1.00E-173	101.5	91.2	94.1
Rsa1.0_00038.1.g2240.t1	ref|XP_002875191.1| EMB1579 [Arabidopsis lyrata subsp. lyrata] gi|297321029|gb|EFH51450.1| EMB1579 [Arabidopsis lyrata subsp. lyrata]	1285	1333	0	103.7	53.3	58.3	EMB1579	gbpln	Arabidopsis lyrata	AT2G03150.1 | Symbols: emb1579 | ATP/GTP-binding protein family | chr2:952313-959004 FORWARD LENGTH=1340	1285	1340	0	104.3	53.0	58.8
Rsa1.0_00038.1.g2241.t1	gb|AAF82795.1|AF274864_1 SKP1gamma1 protein [Brassica napus]	156	159	3.00E-59	101.9	73.1	82.7	SKP1gamma1 protein	gbpln	Brassica napus	AT2G03170.1 | Symbols: ASK14, SK14 | SKP1-like 14 | chr2:961322-961771 FORWARD LENGTH=149	156	149	4.00E-45	95.5	59.0	73.7
Rsa1.0_00038.1.g2242.t2	ref|XP_002875234.1| hypothetical protein ARALYDRAFT_484298 [Arabidopsis lyrata subsp. lyrata] gi|297321072|gb|EFH51493.1| hypothetical protein ARALYDRAFT_484298 [Arabidopsis lyrata subsp. lyrata]	181	99	3.00E-43	54.7	49.7	50.3	hypothetical protein ARALYDRAFT_484298	gbpln	Arabidopsis lyrata	AT2G03870.1 | Symbols: | Small nuclear ribonucleoprotein family protein | chr2:1180306-1181256 FORWARD LENGTH=99	181	99	3.00E-45	54.7	49.2	49.2
Rsa1.0_00038.1.g2243.t1	ref|XP_002875236.1| phosphatidylinositol 3-and 4-kinase family protein [Arabidopsis lyrata subsp. lyrata] gi|297321074|gb|EFH51495.1| phosphatidylinositol 3-and 4-kinase family protein [Arabidopsis lyrata subsp. lyrata]	627	650	0	103.7	86.6	92.3	phosphatidylinositol 3-and 4-kinase family protein	gbpln	Arabidopsis lyrata	AT2G03890.1 | Symbols: ATPI4K GAMMA 7, UBDK GAMMA 7, PI4K GAMMA 7 | phosphoinositide 4-kinase gamma 7 | chr2:1186199-1188151 FORWARD LENGTH=650	627	650	0	103.7	86.3	92.3
Rsa1.0_00038.1.g2244.t1	#	#	#	#	#	#	#	-	----	----	#	#	#	#	#	#	#

Rsa1.0_00038.1.g2245.t1	ref|XP_002875239.1| GDSL-motif lipase/hydrolase family protein [Arabidopsis lyrata subsp. lyrata] gi|297321077|gb|EFH51498.1| GDSL-motif lipase/hydrolase family protein [Arabidopsis lyrata subsp. lyrata]	365	366	1.00E-159	100.3	74.5	81.6	GDSL-motif lipase/hydrolase family protein	gbpln	Arabidopsis lyrata	AT2G03980.1 | Symbols: | GDSL-like Lipase/Acylhydrolase superfamily protein | chr2:1260907-1262408 FORWARD LENGTH=367	365	367	1.00E-157	100.5	70.7	81.9
Rsa1.0_00038.1.g2246.t1	ref|XP_002875239.1| GDSL-motif lipase/hydrolase family protein [Arabidopsis lyrata subsp. lyrata] gi|297321077|gb|EFH51498.1| GDSL-motif lipase/hydrolase family protein [Arabidopsis lyrata subsp. lyrata]	365	366	1.00E-159	100.3	74.0	82.7	GDSL-motif lipase/hydrolase family protein	gbpln	Arabidopsis lyrata	AT2G03980.1 | Symbols: | GDSL-like Lipase/Acylhydrolase superfamily protein | chr2:1260907-1262408 FORWARD LENGTH=367	365	367	1.00E-155	100.5	69.9	81.4
Rsa1.0_00038.1.g2247.t1	gb|EOA25349.1| hypothetical protein CARUB_v10018672mg [Capsella rubella]	779	781	0	100.3	92.8	95.3	hypothetical protein CARUB_v10018672mg	gbpln	Capsella rubella	AT2G04030.1 | Symbols: CR88, EMB1956, HSP90.5, Hsp88.1, AtHsp90.5 | Chaperone protein htpG family protein | chr2:1281983-1285909 FORWARD LENGTH=780	779	780	0	100.1	91.8	94.4
Rsa1.0_00038.1.g2248.t1	#	#	#	#	#	#	#	-	----	----	#	#	#	#	#	#	#
Rsa1.0_00038.1.g2249.t1	ref|NP_178489.1| basic leucine-zipper 48 [Arabidopsis thaliana] gi|20198170|gb|AAM15441.1| bZIP protein (AtbZIP48) [Arabidopsis thaliana] gi|225898102|dbj|BAH30383.1| hypothetical protein [Arabidopsis thaliana] gi|330250693|gb|AEC05787.1| basic leucine-zipper 48 [Arabidopsis thaliana]	185	166	1.00E-54	89.7	77.8	82.7	basic leucine-zipper 48	gbpln	Arabidopsis thaliana	AT2G04038.1 | Symbols: AtbZIP48, bZIP48 | basic leucine-zipper 48 | chr2:1331919-1332419 FORWARD LENGTH=166	185	166	4.00E-57	89.7	77.8	82.7
Rsa1.0_00038.1.g2250.t1	gb|EOA24583.1| hypothetical protein CARUB_v10017846mg [Capsella rubella]	174	265	6.00E-47	152.3	60.9	70.1	hypothetical protein CARUB_v10017846mg	gbpln	Capsella rubella	AT3G25545.1 | Symbols: | unknown protein; FUNCTIONS IN: molecular_function unknown; INVOLVED IN: biological_process unknown; LOCATED IN: cellular_component unknown; EXPRESSED IN: 23 plant structures; EXPRESSED DURING: 13 growth stages; Has 38 Blast hits to 38 proteins in 14 species: Archae - 0; Bacteria - 0; Metazoa - 0; Fungi - 0; Plants - 38; Viruses - 0; Other Eukaryotes - 0 (source: NCBI BLink). | chr3:9277637-9278440 FORWARD LENGTH=267	174	267	1.00E-48	153.4	63.2	68.4
Rsa1.0_00038.1.g2251.t1	gb|ADK13078.1| phytocystatin 5-3 [Brassica rapa subsp. pekinensis]	120	120	9.00E-52	100.0	80.8	93.3	phytocystatin 5-3	gbpln	Brassica rapa	AT5G47550.1 | Symbols: | Cystatin/monellin superfamily protein | chr5:19286596-19286964 REVERSE LENGTH=122	120	122	4.00E-31	101.7	50.8	67.5
Rsa1.0_00038.1.g2252.t1	ref|XP_002875263.1| hypothetical protein ARALYDRAFT_904723 [Arabidopsis lyrata subsp. lyrata] gi|297321101|gb|EFH51522.1| hypothetical protein ARALYDRAFT_904723 [Arabidopsis lyrata subsp. lyrata]	330	423	1.00E-117	128.2	78.8	85.2	hypothetical protein ARALYDRAFT_904723	gbpln	Arabidopsis lyrata	AT3G25590.1 | Symbols: | unknown protein; Has 149 Blast hits to 140 proteins in 44 species: Archae - 0; Bacteria - 6; Metazoa - 40; Fungi - 6; Plants - 39; Viruses - 0; Other Eukaryotes - 58 (source: NCBI BLink). | chr3:9302271-9303542 FORWARD LENGTH=423	330	423	1.00E-115	128.2	76.1	82.1
Rsa1.0_00038.1.g2253.t1	ref|NP_189188.4| putative calcium-binding protein CML16 [Arabidopsis thaliana] gi|332278174|sp|Q9LI84.2|CML16_ARATH RecName: Full=Probable calcium-binding protein CML16; AltName: Full=Calmodulin-like protein 16 gi|28393082|gb|AAO41975.1| putative calmodulin [Arabidopsis thaliana] gi|48310609|gb|AAT41852.1| At3g25600 [Arabidopsis thaliana] gi|332643520|gb|AEE77041.1| putative calcium-binding protein CML16 [Arabidopsis thaliana]	161	161	7.00E-83	100.0	93.8	96.9	putative calcium-binding protein CML16	gbpln	Arabidopsis thaliana	AT3G25600.1 | Symbols: | Calcium-binding EF-hand family protein | chr3:9307367-9307852 FORWARD LENGTH=161	161	161	3.00E-85	100.0	93.8	96.9
Rsa1.0_00038.1.g2254.t1	ref|NP_189194.1| Amidase family protein [Arabidopsis thaliana] gi|75273321|sp|Q9LI77.1|GATA_ARATH RecName: Full=Glutamyl-tRNA(Gln) amidotransferase subunit A, chloroplastic/mitochondrial; Short=Glu-AdT subunit A; Flags: Precursor gi|11994757|dbj|BAB03086.1| glutamyl tRNA amidotransferase, subunit A [Arabidopsis thaliana] gi|15010726|gb|AAK74022.1| AT3g25660/T5M7_8 [Arabidopsis thaliana] gi|23308481|gb|AAN18210.1| At3g25660/T5M7_8 [Arabidopsis thaliana] gi|110741094|dbj|BAE98641.1| putative glutamyl-tRNA amidotransferase subunit A [Arabidopsis thaliana] gi|332643527|gb|AEE77048.1| Amidase family protein [Arabidopsis thaliana]	542	537	0	99.1	91.0	95.8	Amidase family protein	gbpln	Arabidopsis thaliana	AT3G25660.1 | Symbols: | Amidase family protein | chr3:9339640-9342044 REVERSE LENGTH=537	542	537	0	99.1	91.0	95.8

Rsa1.0_00038.1.g2255.t1	ref|XP_002875269.1| hypothetical protein ARALYDRAFT_904734 [Arabidopsis lyrata subsp. lyrata] gi|297321107|gb|EFH51528.1| hypothetical protein ARALYDRAFT_904734 [Arabidopsis lyrata subsp. lyrata]	552	558	0	101.1	78.1	88.4	hypothetical protein ARALYDRAFT_904734	gbpln	Arabidopsis lyrata	AT3G25680.1 | Symbols: | FUNCTIONS IN: molecular_function unknown; INVOLVED IN: biological_process unknown; LOCATED IN: chloroplast; EXPRESSED IN: 21 plant structures; EXPRESSED DURING: 13 growth stages; CONTAINS InterPro DOMAIN/s: S-layer homology domain (InterPro:IPR001119); BEST Arabidopsis thaliana protein match is: unknown protein (TAIR:AT5G23890.1); Has 2454 Blast hits to 2065 proteins in 355 species: Archae - 39; Bacteria - 284; Metazoa - 1081; Fungi - 166; Plants - 264; Viruses - 45; Other Eukaryotes - 575 (source: NCBI BLink). | chr3:9349693-9352258 FORWARD LENGTH=558	552	558	0	101.1	76.4	87.0
Rsa1.0_00038.1.g2256.t1	ref|NP_850631.2| protein rotundifolia like 16 [Arabidopsis thaliana] gi|42822045|tpg|DAA02277.1| TPA_exp: DVL6 [Arabidopsis thaliana] gi|332643537|gb|AEE77058.1| protein rotundifolia like 16 [Arabidopsis thaliana]	40	40	4.00E-13	100.0	97.5	100.0	protein rotundifolia like 16	gbpln	Arabidopsis thaliana	AT3G25717.1 | Symbols: RTFL16, DVL6 | ROTUNDIFOLIA like 16 | chr3:9375472-9375594 REVERSE LENGTH=40	40	40	7.00E-16	100.0	97.5	100.0
Rsa1.0_00038.1.g2257.t1	gb|EOA24619.1| hypothetical protein CARUB_v10017888mg [Capsella rubella]	253	254	1.00E-121	100.4	86.2	92.9	hypothetical protein CARUB_v10017888mg	gbpln	Capsella rubella	AT3G25770.1 | Symbols: AOC2 | allene oxide cyclase 2 | chr3:9406975-9407839 FORWARD LENGTH=253	253	253	1.00E-121	100.0	85.0	91.7
Rsa1.0_00038.1.g2258.t1	gb|ACY74379.1| allene oxidase cyclase [Brassica napus]	253	253	1.00E-139	100.0	95.7	97.2	allene oxidase cyclase	gbpln	Brassica napus	AT3G25780.1 | Symbols: AOC3 | allene oxide cyclase 3 | chr3:9409362-9410409 FORWARD LENGTH=258	253	258	1.00E-121	102.0	89.3	93.3
Rsa1.0_00038.1.g2259.t1	ref|XP_002875280.1| myb family transcription factor [Arabidopsis lyrata subsp. lyrata] gi|297321118|gb|EFH51539.1| myb family transcription factor [Arabidopsis lyrata subsp. lyrata]	366	354	1.00E-132	96.7	68.9	80.1	myb family transcription factor	gbpln	Arabidopsis lyrata	AT3G25790.1 | Symbols: | myb-like transcription factor family protein | chr3:9413196-9414951 FORWARD LENGTH=357	366	357	1.00E-130	97.5	68.3	79.0
Rsa1.0_00038.1.g2260.t3	gb|EMJ15110.1| hypothetical protein PRUPE_ppa005492mg [Prunus persica]	268	458	3.00E-26	170.9	20.9	21.6	hypothetical protein PRUPE_ppa005492mg	gbpln	Prunus persica	AT1G30270.1 | Symbols: CIPK23, SnRK3.23, ATCIPK23, LKS1 | CBL-interacting protein kinase 23 | chr1:10655270-10658524 FORWARD LENGTH=482	268	482	9.00E-29	179.9	20.9	21.3
Rsa1.0_00038.1.g2261.t1	ref|XP_002876929.1| hypothetical protein ARALYDRAFT_904748 [Arabidopsis lyrata subsp. lyrata] gi|297322767|gb|EFH53188.1| hypothetical protein ARALYDRAFT_904748 [Arabidopsis lyrata subsp. lyrata]	586	587	0	100.2	96.1	99.0	hypothetical protein ARALYDRAFT_904748	gbpln	Arabidopsis lyrata	AT3G25800.1 | Symbols: PDF1, PR 65, PP2AA2 | protein phosphatase 2A subunit A2 | chr3:9422822-9425783 REVERSE LENGTH=587	586	587	0	100.2	95.6	98.8
Rsa1.0_00038.1.g2262.t1	ref|NP_566779.1| uncharacterized protein [Arabidopsis thaliana] gi|13877955|gb|AAK44055.1|AF370240_1 unknown protein [Arabidopsis thaliana] gi|17065628|gb|AAL33808.1| unknown protein [Arabidopsis thaliana] gi|332643550|gb|AEE77071.1| uncharacterized protein AT3G25805 [Arabidopsis thaliana]	338	335	1.00E-156	99.1	80.5	87.6	uncharacterized protein	gbpln	Arabidopsis thaliana	AT3G25805.1 | Symbols: | unknown protein; Has 98 Blast hits to 98 proteins in 45 species: Archae - 0; Bacteria - 51; Metazoa - 0; Fungi - 0; Plants - 43; Viruses - 0; Other Eukaryotes - 4 (source: NCBI BLink). | chr3:9426354-9428169 REVERSE LENGTH=335	338	335	1.00E-158	99.1	80.5	87.6
Rsa1.0_00038.1.g2263.t2	gb|EOA23468.1| hypothetical protein CARUB_v10016659mg [Capsella rubella]	929	916	0	98.6	74.8	81.5	hypothetical protein CARUB_v10016659mg	gbpln	Capsella rubella	AT3G25840.1 | Symbols: | Protein kinase superfamily protein | chr3:9452993-9457446 REVERSE LENGTH=935	929	935	0	100.6	75.1	81.9
Rsa1.0_00038.1.g2264.t1	ref|NP_189215.1| 2-oxoacid dehydrogenases acyltransferase family protein [Arabidopsis thaliana] gi|5881963|gb|AAD55139.1|AF066079_1 dihydrolipoamide S-acetyltransferase [Arabidopsis thaliana] gi|14335166|gb|AAK59863.1| AT3g25860/MPE11_1 [Arabidopsis thaliana] gi|26983848|gb|AAN86176.1| putative dihydrolipoamide S-acetyltransferase [Arabidopsis thaliana] gi|332643559|gb|AEE77080.1| pyruvate decarboxylase E2 subunit [Arabidopsis thaliana]	479	480	0	100.2	85.2	89.4	2-oxoacid dehydrogenases acyltransferase family protein	gbpln	Arabidopsis thaliana	AT3G25860.1 | Symbols: LTA2, PLE2 | 2-oxoacid dehydrogenases acyltransferase family protein | chr3:9460632-9462585 FORWARD LENGTH=480	479	480	0	100.2	85.2	89.4
Rsa1.0_00038.1.g2265.t1	ref|XP_002893783.1| predicted protein [Arabidopsis lyrata subsp. lyrata] gi|297339625|gb|EFH70042.1| predicted protein [Arabidopsis lyrata subsp. lyrata]	296	276	2.00E-31	93.2	30.1	38.2	predicted protein	gbpln	Arabidopsis lyrata	AT3G25882.1 | Symbols: NIMIN-2 | NIM1-interacting 2 | chr3:9470521-9470889 REVERSE LENGTH=122	296	122	1.00E-28	41.2	24.3	28.4
Rsa1.0_00038.1.g2266.t1	gb|EOA24563.1| hypothetical protein CARUB_v10017825mg [Capsella rubella]	272	273	1.00E-125	100.4	86.4	91.5	hypothetical protein CARUB_v10017825mg	gbpln	Capsella rubella	AT3G25920.1 | Symbols: RPL15 | ribosomal protein L15 | chr3:9491268-9492558 REVERSE LENGTH=277	272	277	1.00E-121	101.8	81.3	87.5

Rsa1.0_00038.1.g2267.t1	ref|NP_189227.1| mitotic spindle checkpoint protein MAD2 [Arabidopsis thaliana] gi|12230292|sp|Q9LU93.1|MAD2_ARATH RecName: Full=Mitotic spindle checkpoint protein MAD2 gi|9279603|dbj|BAB01061.1| cell cycle checkpoint protein MAD2-like [Arabidopsis thaliana] gi|37202030|gb|AAQ89630.1| At3g25980 [Arabidopsis thaliana] gi|51968734|dbj|BAD43059.1| putative mitotic checkpoint protein [Arabidopsis thaliana] gi|51970868|dbj|BAD44126.1| putative mitotic checkpoint protein [Arabidopsis thaliana] gi|332643576|gb|AEE77097.1| mitotic spindle checkpoint protein MAD2 [Arabidopsis thaliana]	209	209	1.00E-115	100.0	95.7	99.0	mitotic spindle checkpoint protein MAD2	gbpln	Arabidopsis thaliana	AT3G25980.1 | Symbols: MAD2 | DNA-binding HORMA family protein | chr3:9503232-9504402 FORWARD LENGTH=209	209	209	1.00E-118	100.0	95.7	99.0
Rsa1.0_00038.1.g2268.t1	gb|EOA24007.1| hypothetical protein CARUB_v10017220mg [Capsella rubella] gi|482559817|gb|EOA24008.1| hypothetical protein CARUB_v10017220mg [Capsella rubella]	418	455	1.00E-167	108.9	78.2	85.9	hypothetical protein CARUB_v10017220mg	gbpln	Capsella rubella	AT3G26000.1 | Symbols: | Ribonuclease inhibitor | chr3:9507042-9508542 REVERSE LENGTH=453	418	453	1.00E-155	108.4	72.0	81.8
Rsa1.0_00038.1.g2269.t1	dbj|BAB01067.1| acetyltranferase-like protein [Arabidopsis thaliana]	458	455	0	99.3	82.3	90.6	acetyltranferase-like protein	gbpln	Arabidopsis thaliana	AT3G26040.1 | Symbols: | HXXXD-type acyl-transferase family protein | chr3:9519741-9521069 FORWARD LENGTH=442	458	442	0	96.5	79.9	88.0
Rsa1.0_00038.1.g2270.t1	ref|NP_189238.2| regulator of G-protein signaling 1 [Arabidopsis thaliana] gi|75299756|sp|Q8H1F2.1|RGS1_ARATH RecName: Full=Regulator of G-protein signaling 1; Short=AtRGS1; AltName: Full=Putative uncharacterized protein At3g26090 gi|23297446|gb|AAN12971.1| unknown protein [Arabidopsis thaliana] gi|332643595|gb|AEE77116.1| regulator of G-protein signaling 1 [Arabidopsis thaliana]	470	459	0	97.7	79.8	86.4	regulator of G-protein signaling 1	gbpln	Arabidopsis thaliana	AT3G26090.1 | Symbols: RGS1, ATRGS1 | G-protein coupled receptors;GTPase activators | chr3:9532883-9535410 FORWARD LENGTH=459	470	459	0	97.7	79.8	86.4
Rsa1.0_00038.1.g2271.t1	ref|XP_002875307.1| regulator of chromosome condensation family protein [Arabidopsis lyrata subsp. lyrata] gi|297321145|gb|EFH51566.1| regulator of chromosome condensation family protein [Arabidopsis lyrata subsp. lyrata]	541	533	0	98.5	88.0	93.7	regulator of chromosome condensation family protein	gbpln	Arabidopsis lyrata	AT3G26100.2 | Symbols: | Regulator of chromosome condensation (RCC1) family protein | chr3:9538063-9540149 FORWARD LENGTH=532	541	532	0	98.3	86.7	93.0
Rsa1.0_00038.1.g2272.t1	ref|XP_002875308.1| hypothetical protein ARALYDRAFT_484375 [Arabidopsis lyrata subsp. lyrata] gi|297321146|gb|EFH51567.1| hypothetical protein ARALYDRAFT_484375 [Arabidopsis lyrata subsp. lyrata]	121	128	2.00E-17	105.8	60.3	66.9	hypothetical protein ARALYDRAFT_484375	gbpln	Arabidopsis lyrata	AT3G26110.1 | Symbols: | Anther-specific protein agp1-like | chr3:9541573-9541959 FORWARD LENGTH=128	121	128	6.00E-17	105.8	61.2	67.8
Rsa1.0_00038.1.g2273.t1	gb|ABD64939.1| hypothetical protein 24.t00017 [Brassica oleracea]	139	442	3.00E-35	318.0	56.8	71.9	hypothetical protein 24.t00017	gbpln	Brassica oleracea	#	#	#	#	#	#	#
Rsa1.0_00038.1.g2274.t3	gb|ABD65099.1| hypothetical protein 31.t00074 [Brassica oleracea]	365	258	4.00E-46	70.7	27.9	34.2	hypothetical protein 31.t00074	gbpln	Brassica oleracea	AT5G41220.1 | Symbols: ATGSTT3, GST10C, GSTT3 | glutathione S-transferase THETA 3 | chr5:16494560-16496969 REVERSE LENGTH=590	365	590	2.00E-41	161.6	24.7	29.6
Rsa1.0_00038.1.g2275.t1	ref|NP_189243.1| cytochrome P450, family 86, subfamily C, polypeptide 2 [Arabidopsis thaliana] gi|11994429|dbj|BAB02431.1| cytochrome P-450-like protein [Arabidopsis thaliana] gi|332643602|gb|AEE77123.1| cytochrome P450, family 86, subfamily C, polypeptide 2 [Arabidopsis thaliana]	533	541	0	101.5	89.1	94.4	cytochrome P450, family 86, subfamily C, polypeptide 2	gbpln	Arabidopsis thaliana	AT3G26125.1 | Symbols: CYP86C2 | cytochrome P450, family 86, subfamily C, polypeptide 2 | chr3:9551812-9553437 FORWARD LENGTH=541	533	541	0	101.5	89.1	94.4
Rsa1.0_00038.1.g2276.t1	ref|XP_002876956.1| CYP71B4 [Arabidopsis lyrata subsp. lyrata] gi|297322794|gb|EFH53215.1| CYP71B4 [Arabidopsis lyrata subsp. lyrata]	499	502	0	100.6	69.5	81.2	CYP71B4	gbpln	Arabidopsis lyrata	AT3G26280.1 | Symbols: CYP71B4 | cytochrome P450, family 71, subfamily B, polypeptide 4 | chr3:9630358-9631970 REVERSE LENGTH=504	499	504	0	101.0	69.9	82.2
Rsa1.0_00038.1.g2277.t1	ref|XP_002876956.1| CYP71B4 [Arabidopsis lyrata subsp. lyrata] gi|297322794|gb|EFH53215.1| CYP71B4 [Arabidopsis lyrata subsp. lyrata]	499	502	0	100.6	71.3	82.8	CYP71B4	gbpln	Arabidopsis lyrata	AT3G26280.1 | Symbols: CYP71B4 | cytochrome P450, family 71, subfamily B, polypeptide 4 | chr3:9630358-9631970 REVERSE LENGTH=504	499	504	0	101.0	70.1	83.0
Rsa1.0_00038.1.g2278.t1	gb|EOA26964.1| hypothetical protein CARUB_v10023060mg [Capsella rubella]	247	502	1.00E-71	203.2	55.9	66.8	hypothetical protein CARUB_v10023060mg	gbpln	Capsella rubella	AT2G38110.1 | Symbols: ATGPAT6, GPAT6 | glycerol-3-phosphate acyltransferase 6 | chr2:15952816-15955364 REVERSE LENGTH=501	247	501	5.00E-73	202.8	57.1	68.8

Rsa1.0_00038.1.g2279.t1	ref|NP_189260.1| cytochrome P450 71B26 [Arabidopsis thaliana] gi|13878380|sp|Q9LTL0.1|C71BQ_ARATH RecName: Full=Cytochrome P450 71B26 gi|11994450|dbj|BAB02452.1| cytochrome P450 [Arabidopsis thaliana] gi|22136028|gb|AAM91596.1| cytochrome P450, putative [Arabidopsis thaliana] gi|31711908|gb|AAP68310.1| At3g26290 [Arabidopsis thaliana] gi|332643621|gb|AEE77142.1| cytochrome P450 71B26 [Arabidopsis thaliana]	500	500	0	100.0	78.6	86.6	cytochrome P450 71B26	gbpln	Arabidopsis thaliana	AT3G26290.1 | Symbols: CYP71B26 | cytochrome P450, family 71, subfamily B, polypeptide 26 | chr3:9632770-9634439 REVERSE LENGTH=500	500	500	0	100.0	78.6	86.6
Rsa1.0_00038.1.g2280.t1	gb|ABD64987.1| hypothetical protein 26.t00003 [Brassica oleracea]	234	330	1.00E-20	141.0	23.1	25.2	hypothetical protein 26.t00003	gbpln	Brassica oleracea	AT1G10000.1 | Symbols: | Ribonuclease H-like superfamily protein | chr1:3263877-3264788 REVERSE LENGTH=303	234	303	7.00E-17	129.5	22.6	31.2
Rsa1.0_00038.1.g2281.t1	gb|EOA25173.1| hypothetical protein CARUB_v10018485mg [Capsella rubella]	235	499	5.00E-71	212.3	57.0	61.7	hypothetical protein CARUB_v10018485mg	gbpln	Capsella rubella	AT3G26330.1 | Symbols: CYP71B37 | cytochrome P450, family 71, subfamily B, polypeptide 37 | chr3:9646873-9648536 REVERSE LENGTH=500	235	500	8.00E-68	212.8	53.2	58.7
Rsa1.0_00038.1.g2282.t4	ref|NP_177511.1| mate efflux-like protein [Arabidopsis thaliana] gi|12324218|gb|AAG52084.1|AC012679_22 putative integral membrane protein; 47574-45498 [Arabidopsis thaliana] gi|332197378|gb|AEE35499.1| mate efflux-like protein [Arabidopsis thaliana]	462	476	1.00E-128	103.0	55.2	57.4	mate efflux-like protein	gbpln	Arabidopsis thaliana	AT1G73700.1 | Symbols: | MATE efflux family protein | chr1:27717554-27719630 REVERSE LENGTH=476	462	476	1.00E-131	103.0	55.2	57.4
Rsa1.0_00039.1.g2283.t1	gb|EOA14133.1| hypothetical protein CARUB_v10027278mg [Capsella rubella]	161	160	3.00E-78	99.4	87.6	93.2	hypothetical protein CARUB_v10027278mg	gbpln	Capsella rubella	AT5G63070.1 | Symbols: | Ribosomal protein S19 family protein | chr5:25299178-25299660 REVERSE LENGTH=160	161	160	5.00E-78	99.4	85.7	91.9
Rsa1.0_00039.1.g2284.t1	#	#	#	#	#	#	#	-	----	----	#	#	#	#	#	#	#
Rsa1.0_00039.1.g2285.t1	ref|XP_002864835.1| predicted protein [Arabidopsis lyrata subsp. lyrata] gi|297310670|gb|EFH41094.1| predicted protein [Arabidopsis lyrata subsp. lyrata]	80	82	2.00E-18	102.5	68.8	80.0	predicted protein	gbpln	Arabidopsis lyrata	AT5G63085.1 | Symbols: | Plant thionin family protein | chr5:25302862-25303230 FORWARD LENGTH=81	80	81	3.00E-14	101.3	61.3	70.0
Rsa1.0_00039.1.g2286.t1	gb|EOA13869.1| hypothetical protein CARUB_v10026971mg [Capsella rubella]	247	263	1.00E-125	106.5	90.7	96.0	hypothetical protein CARUB_v10026971mg	gbpln	Capsella rubella	AT5G63060.1 | Symbols: | Sec14p-like phosphatidylinositol transfer family protein | chr5:25295082-25296714 REVERSE LENGTH=263	247	263	1.00E-125	106.5	89.1	96.4
Rsa1.0_00039.1.g2287.t1	gb|EOA13639.1| hypothetical protein CARUB_v10026710mg [Capsella rubella]	346	345	1.00E-173	99.7	81.5	91.3	hypothetical protein CARUB_v10026710mg	gbpln	Capsella rubella	AT5G63050.1 | Symbols: EMB2759 | embryo defective 2759 | chr5:25293109-25294799 FORWARD LENGTH=345	346	345	1.00E-170	99.7	79.8	89.6
Rsa1.0_00039.1.g2288.t1	ref|XP_002884098.1| hypothetical protein ARALYDRAFT_343424 [Arabidopsis lyrata subsp. lyrata] gi|297329938|gb|EFH60357.1| hypothetical protein ARALYDRAFT_343424 [Arabidopsis lyrata subsp. lyrata]	355	407	1.00E-144	114.6	75.8	81.1	hypothetical protein ARALYDRAFT_343424	gbpln	Arabidopsis lyrata	AT5G63040.1 | Symbols: | unknown protein; FUNCTIONS IN: molecular_function unknown; INVOLVED IN: biological_process unknown; LOCATED IN: chloroplast; EXPRESSED IN: 25 plant structures; EXPRESSED DURING: 15 growth stages; BEST Arabidopsis thaliana protein match is: unknown protein (TAIR:AT1G48460.1); Has 30201 Blast hits to 17322 proteins in 780 species: Archae - 12; Bacteria - 1396; Metazoa - 17338; Fungi - 3422; Plants - 5037; Viruses - 0; Other Eukaryotes - 2996 (source: NCBI BLink). | chr5:25288504-25290326 FORWARD LENGTH=366	355	366	1.00E-143	103.1	76.9	82.3
Rsa1.0_00039.1.g2289.t1	ref|XP_002866529.1| hypothetical protein ARALYDRAFT_919585 [Arabidopsis lyrata subsp. lyrata] gi|297312364|gb|EFH42788.1| hypothetical protein ARALYDRAFT_919585 [Arabidopsis lyrata subsp. lyrata]	125	125	2.00E-65	100.0	96.8	98.4	hypothetical protein ARALYDRAFT_919585	gbpln	Arabidopsis lyrata	AT5G63030.1 | Symbols: | Thioredoxin superfamily protein | chr5:25286352-25287517 FORWARD LENGTH=125	125	125	6.00E-66	100.0	92.8	98.4
Rsa1.0_00039.1.g2290.t1	ref|XP_002864830.1| EMB1692 [Arabidopsis lyrata subsp. lyrata] gi|297310665|gb|EFH41089.1| EMB1692 [Arabidopsis lyrata subsp. lyrata]	488	492	0	100.8	82.8	90.4	EMB1692	gbpln	Arabidopsis lyrata	AT5G62990.1 | Symbols: emb1692 | Ubiquitin carboxyl-terminal hydrolase family protein | chr5:25277828-25279312 FORWARD LENGTH=494	488	494	0	101.2	78.5	86.3
Rsa1.0_00039.1.g2291.t1	#	#	#	#	#	#	#	-	----	----	#	#	#	#	#	#	#
Rsa1.0_00039.1.g2292.t1	ref|XP_002866526.1| predicted protein [Arabidopsis lyrata subsp. lyrata] gi|297312361|gb|EFH42785.1| predicted protein [Arabidopsis lyrata subsp. lyrata]	100	177	9.00E-23	177.0	57.0	66.0	predicted protein	gbpln	Arabidopsis lyrata	#	#	#	#	#	#	#

Rsa1.0_00039.1.g2293.t1	dbj|BAB08839.1| unnamed protein product [Arabidopsis thaliana]	334	317	1.00E-133	94.9	77.2	80.8	unnamed protein product	gbpln	Arabidopsis thaliana	AT5G62960.1 | Symbols: | unknown protein; BEST Arabidopsis thaliana protein match is: unknown protein (TAIR:AT1G10660.1); Has 35333 Blast hits to 34131 proteins in 2444 species: Archae - 798; Bacteria - 22429; Metazoa - 974; Fungi - 991; Plants - 531; Viruses - 0; Other Eukaryotes - 9610 (source: NCBI BLink). | chr5:25269444-25271533 FORWARD LENGTH=347	334	347	1.00E-127	103.9	66.2	69.2
Rsa1.0_00039.1.g2294.t1	ref|NP_201100.1| RNA polymerase II, Rpb4, core protein [Arabidopsis thaliana] gi|334188590|ref|NP_001190602.1| RNA polymerase II, Rpb4, core protein [Arabidopsis thaliana] gi|32815911|gb|AAP88340.1| At5g62950 [Arabidopsis thaliana] gi|110743157|dbj|BAE99470.1| hypothetical protein [Arabidopsis thaliana] gi|332010294|gb|AED97677.1| RNA polymerase II, Rpb4, core protein [Arabidopsis thaliana] gi|332010296|gb|AED97679.1| RNA polymerase II, Rpb4, core protein [Arabidopsis thaliana]	160	139	2.00E-32	86.9	53.1	61.3	RNA polymerase II, Rpb4, core protein	gbpln	Arabidopsis thaliana	AT5G62950.3 | Symbols: | RNA polymerase II, Rpb4, core protein | chr5:25262355-25263033 REVERSE LENGTH=139	160	139	9.00E-35	86.9	53.1	61.3
Rsa1.0_00039.1.g2295.t1	ref|XP_002866523.1| hypothetical protein ARALYDRAFT_919576 [Arabidopsis lyrata subsp. lyrata] gi|297312358|gb|EFH42782.1| hypothetical protein ARALYDRAFT_919576 [Arabidopsis lyrata subsp. lyrata]	394	375	1.00E-120	95.2	62.7	68.5	hypothetical protein ARALYDRAFT_919576	gbpln	Arabidopsis lyrata	AT5G62940.1 | Symbols: HCA2, DOF5.6 | Dof-type zinc finger DNA-binding family protein | chr5:25257183-25258504 REVERSE LENGTH=372	394	372	1.00E-117	94.4	63.2	68.5
Rsa1.0_00039.1.g2296.t3	ref|NP_201094.1| nucleobase-ascorbate transporter 6 [Arabidopsis thaliana] gi|79600443|ref|NP_851251.2| nucleobase-ascorbate transporter 6 [Arabidopsis thaliana] gi|145323680|ref|NP_001032127.2| nucleobase-ascorbate transporter 6 [Arabidopsis thaliana] gi|122064607|sp|Q27GI3.2|NAT6_ARATH RecName: Full=Nucleobase-ascorbate transporter 6; Short=AtNAT6 gi|21326025|gb|AAM47573.1|AF466198_1 putative permease 1 [Arabidopsis thaliana] gi|10177467|dbj|BAB10858.1| permease 1 [Arabidopsis thaliana] gi|332010285|gb|AED97668.1| nucleobase-ascorbate transporter 6 [Arabidopsis thaliana] gi|332010286|gb|AED97669.1| nucleobase-ascorbate transporter 6 [Arabidopsis thaliana] gi|332010287|gb|AED97670.1| nucleobase-ascorbate transporter 6 [Arabidopsis thaliana]	563	532	0	94.5	89.3	90.6	nucleobase-ascorbate transporter 6	gbpln	Arabidopsis thaliana	AT5G62890.3 | Symbols: | Xanthine/uracil permease family protein | chr5:25243723-25247075 FORWARD LENGTH=532	563	532	0	94.5	89.3	90.6
Rsa1.0_00039.1.g2297.t1	ref|XP_002866520.1| hypothetical protein ARALYDRAFT_919569 [Arabidopsis lyrata subsp. lyrata] gi|297312355|gb|EFH42779.1| hypothetical protein ARALYDRAFT_919569 [Arabidopsis lyrata subsp. lyrata]	1500	1597	0	106.5	78.7	86.0	hypothetical protein ARALYDRAFT_919569	gbpln	Arabidopsis lyrata	AT3G48050.2 | Symbols: | BAH domain ;TFIIS helical bundle-like domain | chr3:17734270-17739412 REVERSE LENGTH=1613	1500	1613	0	107.5	26.9	32.7
Rsa1.0_00039.1.g2298.t1	ref|XP_002866520.1| hypothetical protein ARALYDRAFT_919569 [Arabidopsis lyrata subsp. lyrata] gi|297312355|gb|EFH42779.1| hypothetical protein ARALYDRAFT_919569 [Arabidopsis lyrata subsp. lyrata]	1398	1597	0	114.2	47.7	52.8	hypothetical protein ARALYDRAFT_919569	gbpln	Arabidopsis lyrata	AT3G48050.2 | Symbols: | BAH domain ;TFIIS helical bundle-like domain | chr3:17734270-17739412 REVERSE LENGTH=1613	1398	1613	0	115.4	26.0	28.5

Rsa1.0_00039.1.g2299.t1	ref|NP_201093.1| Rac-like GTP-binding protein ARAC10 [Arabidopsis thaliana] gi|297793877|ref|XP_002864823.1| hypothetical protein ARALYDRAFT_496470 [Arabidopsis lyrata subsp. lyrata] gi|51701730|sp|O82481.1|RAC10_ARATH RecName: Full=Rac-like GTP-binding protein ARAC10; AltName: Full=GTPase protein ROP11 gi|7211193|gb|AAF40238.1|AF115467_1 Arac10 [Arabidopsis thaliana] gi|3702964|gb|AAC63014.1| rac GTP binding protein Arac10 [Arabidopsis thaliana] gi|10177466|dbj|BAB10857.1| rac GTP binding protein Arac10 [Arabidopsis thaliana] gi|27754724|gb|AAO22805.1| putative GTP binding protein Arac10 [Arabidopsis thaliana] gi|28394091|gb|AAO42453.1| putative GTP binding protein Arac10 [Arabidopsis thaliana] gi|51971983|dbj|BAD44656.1| Arac10 [Arabidopsis thaliana] gi|297310658|gb|EFH41082.1| hypothetical protein ARALYDRAFT_496470 [Arabidopsis lyrata subsp. lyrata] gi|332010284|gb|AED97667.1| Rac-like GTP-binding protein ARAC10 [Arabidopsis thaliana]	216	215	1.00E-113	99.5	97.7	99.5	Rac-like GTP-binding protein ARAC10	gbpln	Arabidopsis lyrata	AT5G62880.1 | Symbols: ARAC10, ATRAC10, ATROP11, RAC10 | RAC-like 10 | chr5:25237236-25238939 FORWARD LENGTH=215	216	215	1.00E-116	99.5	97.7	99.5
Rsa1.0_00039.1.g2300.t1	ref|NP_201091.2| nodulin MtN3-like protein [Arabidopsis thaliana] gi|322967651|sp|Q9FM10.2|SWET5_ARATH RecName: Full=Bidirectional sugar transporter SWEET5; Short=AtSWEET5; AltName: Full=Protein VEGETATIVE CELL EXPRESSED 1; Short=AtVEX1 gi|332010281|gb|AED97664.1| bidirectional sugar transporter SWEET5 [Arabidopsis thaliana]	240	240	1.00E-123	100.0	87.9	95.8	nodulin MtN3-like protein	gbpln	Arabidopsis thaliana	AT5G62850.1 | Symbols: AtVEX1, SWEET5, AtSWEET5 | Nodulin MtN3 family protein | chr5:25230204-25231527 REVERSE LENGTH=240	240	240	1.00E-126	100.0	87.9	95.8
Rsa1.0_00039.1.g2301.t1	ref|XP_002864820.1| hypothetical protein ARALYDRAFT_496464 [Arabidopsis lyrata subsp. lyrata] gi|297310655|gb|EFH41079.1| hypothetical protein ARALYDRAFT_496464 [Arabidopsis lyrata subsp. lyrata]	493	515	0	104.5	79.5	86.8	hypothetical protein ARALYDRAFT_496464	gbpln	Arabidopsis lyrata	AT5G62810.1 | Symbols: PEX14, ATPEX14, PED2 | peroxin 14 | chr5:25220323-25223571 FORWARD LENGTH=507	493	507	0	102.8	79.5	85.8
Rsa1.0_00039.1.g2302.t2	ref|XP_002866510.1| 1-deoxy-D-xylulose-5-phosphate reductoisomerase [Arabidopsis lyrata subsp. lyrata] gi|297312345|gb|EFH42769.1| 1-deoxy-D-xylulose-5-phosphate reductoisomerase [Arabidopsis lyrata subsp. lyrata]	472	476	0	100.8	96.0	98.1	1-deoxy-D-xylulose-5-phosphate reductoisomerase	gbpln	Arabidopsis lyrata	AT5G62790.1 | Symbols: DXR, PDE129 | 1-deoxy-D-xylulose 5-phosphate reductoisomerase | chr5:25214358-25217292 REVERSE LENGTH=477	472	477	0	101.1	96.4	98.3
Rsa1.0_00039.1.g2303.t1	dbj|BAE99884.1| hypothetical protein [Arabidopsis thaliana]	223	268	4.00E-71	120.2	72.6	80.7	hypothetical protein	gbpln	Arabidopsis thaliana	AT5G62770.1 | Symbols: | Protein of unknown function (DUF1645) | chr5:25210564-25211370 FORWARD LENGTH=268	223	268	5.00E-65	120.2	72.6	81.2
Rsa1.0_00039.1.g2304.t1	gb|EOA13897.1| hypothetical protein CARUB_v10027013mg [Capsella rubella]	255	243	8.00E-98	95.3	77.6	84.7	hypothetical protein CARUB_v10027013mg	gbpln	Capsella rubella	AT5G62720.1 | Symbols: | Integral membrane HPP family protein | chr5:25191969-25194845 FORWARD LENGTH=243	255	243	1.00E-99	95.3	76.1	83.9
Rsa1.0_00039.1.g2305.t2	ref|NP_201077.2| leucine-rich repeat protein kinase-like protein [Arabidopsis thaliana] gi|26451750|dbj|BAC42970.1| putative receptor like protein kinase [Arabidopsis thaliana] gi|224589741|gb|ACN59402.1| leucine-rich repeat receptor-like protein kinase [Arabidopsis thaliana] gi|332010262|gb|AED97645.1| leucine-rich repeat protein kinase-like protein [Arabidopsis thaliana]	193	604	5.00E-32	313.0	37.3	38.9	leucine-rich repeat protein kinase-like protein	gbpln	Arabidopsis thaliana	AT5G62710.1 | Symbols: | Leucine-rich repeat protein kinase family protein | chr5:25187438-25190325 FORWARD LENGTH=604	193	604	2.00E-34	313.0	37.3	38.9

Rsa1.0_00039.1.g2306.t1	ref|NP_201074.1| putative peptide/nitrate transporter [Arabidopsis thaliana] gi|75180586|sp|Q9LV10.1|PTR53_ARATH RecName: Full=Probable peptide/nitrate transporter At5g62680 gi|8809664|dbj|BAA97215.1| peptide transporter [Arabidopsis thaliana] gi|222423295|dbj|BAH19623.1| AT5G62680 [Arabidopsis thaliana] gi|332010259|gb|AED97642.1| probable peptide/nitrate transporter [Arabidopsis thaliana]	600	616	0	102.7	86.8	93.2	putative peptide/nitrate transporter	gbpln	Arabidopsis thaliana	AT5G62680.1 | Symbols: | Major facilitator superfamily protein | chr5:25165430-25167822 REVERSE LENGTH=616	600	616	0	102.7	86.8	93.2
Rsa1.0_00039.1.g2307.t1	#	#	#	#	#	#	#	-	----	----	#	#	#	#	#	#	#
Rsa1.0_00039.1.g2308.t1	gb|EOA13406.1| hypothetical protein CARUB_v10026444mg, partial [Capsella rubella]	416	430	1.00E-114	103.4	55.8	68.0	hypothetical protein CARUB_v10026444mg, partial	gbpln	Capsella rubella	AT5G62660.1 | Symbols: | F-box and associated interaction domains-containing protein | chr5:25156326-25157465 REVERSE LENGTH=379	416	379	1.00E-112	91.1	50.7	62.7
Rsa1.0_00039.1.g2309.t1	ref|XP_002866500.1| basic helix-loop-helix family protein [Arabidopsis lyrata subsp. lyrata] gi|297312335|gb|EFH42759.1| basic helix-loop-helix family protein [Arabidopsis lyrata subsp. lyrata]	118	282	3.00E-28	239.0	54.2	59.3	basic helix-loop-helix family protein	gbpln	Arabidopsis lyrata	AT5G62610.1 | Symbols: | basic helix-loop-helix (bHLH) DNA-binding superfamily protein | chr5:25133117-25134600 REVERSE LENGTH=281	118	281	4.00E-30	238.1	53.4	58.5
Rsa1.0_00039.1.g2310.t1	ref|XP_002886534.1| hypothetical protein ARALYDRAFT_315221 [Arabidopsis lyrata subsp. lyrata] gi|297332375|gb|EFH62793.1| hypothetical protein ARALYDRAFT_315221 [Arabidopsis lyrata subsp. lyrata]	586	586	0	100.0	84.8	91.3	hypothetical protein ARALYDRAFT_315221	gbpln	Arabidopsis lyrata	AT1G61560.1 | Symbols: MLO6, ATMLO6 | Seven transmembrane MLO family protein | chr1:22708875-22712032 REVERSE LENGTH=583	586	583	0	99.5	84.6	91.3
Rsa1.0_00039.1.g2311.t1	#	#	#	#	#	#	#	-	----	----	#	#	#	#	#	#	#
Rsa1.0_00039.1.g2312.t1	ref|XP_002886531.1| hypothetical protein ARALYDRAFT_893358 [Arabidopsis lyrata subsp. lyrata] gi|297332372|gb|EFH62790.1| hypothetical protein ARALYDRAFT_893358 [Arabidopsis lyrata subsp. lyrata]	75	80	3.00E-18	106.7	62.7	73.3	hypothetical protein ARALYDRAFT_893358	gbpln	Arabidopsis lyrata	AT1G61566.1 | Symbols: RALFL9 | ralf-like 9 | chr1:22717265-22717492 REVERSE LENGTH=75	75	75	1.00E-20	100.0	64.0	70.7
Rsa1.0_00039.1.g2313.t1	gb|EOA35572.1| hypothetical protein CARUB_v10020781mg [Capsella rubella]	271	273	1.00E-140	100.7	95.9	98.2	hypothetical protein CARUB_v10020781mg	gbpln	Capsella rubella	AT1G61520.3 | Symbols: LHCA3 | photosystem I light harvesting complex gene 3 | chr1:22700152-22701149 FORWARD LENGTH=273	271	273	1.00E-141	100.7	94.5	98.2
Rsa1.0_00039.1.g2314.t1	ref|NP_176343.2| S-locus lectin protein kinase-like protein [Arabidopsis thaliana] gi|313471784|sp|O64771.2|Y1148_ARATH RecName: Full=G-type lectin S-receptor-like serine/threonine-protein kinase At1g61480; Flags: Precursor gi|332195722|gb|AEE33843.1| G-type lectin S-receptor-like serine/threonine-protein kinase [Arabidopsis thaliana]	806	809	0	100.4	77.9	86.6	S-locus lectin protein kinase-like protein	gbpln	Arabidopsis thaliana	AT1G61480.1 | Symbols: | S-locus lectin protein kinase family protein | chr1:22681420-22684404 REVERSE LENGTH=809	806	809	0	100.4	77.9	86.6
Rsa1.0_00039.1.g2315.t1	ref|XP_004295385.1| PREDICTED: uncharacterized protein LOC101312912 [Fragaria vesca subsp. vesca]	1628	1583	0	97.2	47.5	62.0	PREDICTED: uncharacterized protein LOC101312912	gbpln	Fragaria vesca	AT1G61490.1 | Symbols: | S-locus lectin protein kinase family protein | chr1:22685154-22688267 REVERSE LENGTH=804	1628	804	0	49.4	37.6	41.6
Rsa1.0_00039.1.g2316.t1	ref|NP_176334.1| putative S-locus protein kinase [Arabidopsis thaliana] gi|75099193|sp|O64781.1|Y1639_ARATH RecName: Full=G-type lectin S-receptor-like serine/threonine-protein kinase At1g61390; Flags: Precursor gi|3056591|gb|AAC13902.1|AAC13902 T1F9.12 [Arabidopsis thaliana] gi|332195709|gb|AEE33830.1| putative S-locus protein kinase [Arabidopsis thaliana]	783	831	0	106.1	83.1	90.5	putative S-locus protein kinase	gbpln	Arabidopsis thaliana	AT1G61390.1 | Symbols: | S-locus lectin protein kinase family protein | chr1:22650338-22653639 REVERSE LENGTH=831	783	831	0	106.1	83.1	90.5

Rsa1.0_00039.1.g2317.t1	ref|NP_564775.1| protein S-domain-1 29 [Arabidopsis thaliana] gi|75099194|sp|O64782.1|SD129_ARATH RecName: Full=G-type lectin S-receptor-like serine/threonine-protein kinase SD1-29; AltName: Full=S-domain-1 (SD1) receptor kinase 29; Short=SD1-29; Flags: Precursor gi|3056592|gb|AAC13903.1|AAC13903 T1F9.13 [Arabidopsis thaliana] gi|19699075|gb|AAL90905.1| At1g61380/T1F9_13 [Arabidopsis thaliana] gi|23308469|gb|AAN18204.1| At1g61380/T1F9_13 [Arabidopsis thaliana] gi|332195708|gb|AEE33829.1| G-type lectin S-receptor-like serine/threonine-protein kinase SD1-29 [Arabidopsis thaliana]	813	805	0	99.0	78.8	86.3	protein S-domain-1 29	gbpln	Arabidopsis thaliana	AT1G61380.1 | Symbols: SD1-29 | S-domain-1 29 | chr1:22646277-22649401 REVERSE LENGTH=805	813	805	0	99.0	78.8	86.3
Rsa1.0_00039.1.g2318.t1	ref|XP_002888089.1| armadillo/beta-catenin repeat family protein [Arabidopsis lyrata subsp. lyrata] gi|297333930|gb|EFH64348.1| armadillo/beta-catenin repeat family protein [Arabidopsis lyrata subsp. lyrata]	650	572	0	88.0	73.5	81.1	armadillo/beta-catenin repeat family protein	gbpln	Arabidopsis lyrata	AT1G61350.1 | Symbols: | ARM repeat superfamily protein | chr1:22634099-22635820 FORWARD LENGTH=573	650	573	0	88.2	73.4	80.5
Rsa1.0_00039.1.g2319.t1	ref|XP_002886553.1| hypothetical protein ARALYDRAFT_893390 [Arabidopsis lyrata subsp. lyrata] gi|297332394|gb|EFH62812.1| hypothetical protein ARALYDRAFT_893390 [Arabidopsis lyrata subsp. lyrata]	127	352	4.00E-52	277.2	81.9	86.6	hypothetical protein ARALYDRAFT_893390	gbpln	Arabidopsis lyrata	AT1G61260.1 | Symbols: | Protein of unknown function (DUF761) | chr1:22593756-22594986 REVERSE LENGTH=344	127	344	1.00E-54	270.9	81.9	87.4
Rsa1.0_00039.1.g2320.t1	gb|EOA35839.1| hypothetical protein CARUB_v10021081mg [Capsella rubella]	113	158	3.00E-22	139.8	76.1	80.5	hypothetical protein CARUB_v10021081mg	gbpln	Capsella rubella	AT1G61255.1 | Symbols: | BEST Arabidopsis thaliana protein match is: glycine-rich protein (TAIR:AT4G21620.2); Has 58 Blast hits to 58 proteins in 12 species: Archae - 0; Bacteria - 2; Metazoa - 0; Fungi - 0; Plants - 56; Viruses - 0; Other Eukaryotes - 0 (source: NCBI BLink). | chr1:22589260-22589691 REVERSE LENGTH=143	113	143	3.00E-19	126.5	56.6	61.1
Rsa1.0_00039.1.g2321.t1	ref|NP_176320.1| secretory carrier-associated membrane protein 3 [Arabidopsis thaliana] gi|75185088|sp|Q9M5P2.1|SCAM3_ARATH RecName: Full=Secretory carrier-associated membrane protein 3; Short=AtSC3; Short=Secretory carrier membrane protein 3 gi|7109228|gb|AAF36686.1|AF225920_1 secretory carrier membrane protein [Arabidopsis thaliana] gi|15027863|gb|AAK76462.1| putative secretory carrier membrane protein [Arabidopsis thaliana] gi|19310673|gb|AAL85067.1| putative secretory carrier membrane protein [Arabidopsis thaliana] gi|21555163|gb|AAM63793.1| secretory carrier membrane protein [Arabidopsis thaliana] gi|332195690|gb|AEE33811.1| secretory carrier-associated membrane protein 3 [Arabidopsis thaliana]	292	289	1.00E-154	99.0	91.1	95.2	secretory carrier-associated membrane protein 3	gbpln	Arabidopsis thaliana	AT1G61250.1 | Symbols: SC3 | secretory carrier 3 | chr1:22586035-22588664 FORWARD LENGTH=289	292	289	1.00E-156	99.0	91.1	95.2
Rsa1.0_00039.1.g2322.t1	ref|XP_002888099.1| predicted protein [Arabidopsis lyrata subsp. lyrata] gi|297333940|gb|EFH64358.1| predicted protein [Arabidopsis lyrata subsp. lyrata]	365	588	1.00E-160	161.1	75.3	85.8	predicted protein	gbpln	Arabidopsis lyrata	AT1G57570.1 | Symbols: | Mannose-binding lectin superfamily protein | chr1:21321717-21324284 FORWARD LENGTH=614	365	614	1.00E-148	168.2	69.6	82.7
Rsa1.0_00039.1.g2323.t1	gb|EOA33627.1| hypothetical protein CARUB_v10019789mg [Capsella rubella]	861	846	0	98.3	61.8	73.4	hypothetical protein CARUB_v10019789mg	gbpln	Capsella rubella	AT1G61190.1 | Symbols: | LRR and NB-ARC domains-containing disease resistance protein | chr1:22557602-22560687 FORWARD LENGTH=967	861	967	0	112.3	63.4	74.2
Rsa1.0_00039.1.g2324.t1	#	#	#	#	#	#	#	-	----	----	#	#	#	#	#	#	#
Rsa1.0_00039.1.g2325.t3	gb|AAD24652.1| putative non-LTR retroelement reverse transcriptase [Arabidopsis thaliana]	994	977	4.00E-75	98.3	20.4	29.9	putative non-LTR retroelement reverse transcriptase	gbpln	Arabidopsis thaliana	AT5G41550.1 | Symbols: | Disease resistance protein (TIR-NBS-LRR class) family | chr5:16617232-16620785 REVERSE LENGTH=1085	994	1085	2.00E-64	109.2	11.6	14.1
Rsa1.0_00039.1.g2326.t16	#	#	#	#	#	#	#	-	----	----	#	#	#	#	#	#	#
Rsa1.0_00039.1.g2327.t1	gb|AAG50806.1|AC079281_8 unknown protein [Arabidopsis thaliana]	104	1213	7.00E-15	1166.3	45.2	68.3	unknown protein	gbpln	Arabidopsis thaliana	AT1G60720.1 | Symbols: | RNA-directed DNA polymerase (reverse transcriptase)-related family protein | chr1:22357072-22357941 FORWARD LENGTH=289	104	289	6.00E-16	277.9	40.4	64.4

Rsa1.0_00039.1.g2328.t1	ref|XP_002888109.1| ANAC025 [Arabidopsis lyrata subsp. lyrata] gi|297333950|gb|EFH64368.1| ANAC025 [Arabidopsis lyrata subsp. lyrata]	319	326	1.00E-161	102.2	93.7	97.2	ANAC025	gbpln	Arabidopsis lyrata	AT1G61110.1 | Symbols: anac025, NAC025 | NAC domain containing protein 25 | chr1:22516730-22518055 FORWARD LENGTH=323	319	323	1.00E-163	101.3	94.7	97.8
Rsa1.0_00039.1.g2329.t1	#	#	#	#	#	#	#	-	----	----	AT3G61860.1 | Symbols: ATRSP31, RSP31, At-RS31, RS31 | RNA-binding (RRM/RBD/RNP motifs) family protein | chr3:22900311-22902159 REVERSE LENGTH=264	191	264	4.00E-11	138.2	16.8	19.4
Rsa1.0_00039.1.g2330.t1	ref|NP_974060.1| Toll-Interleukin-Resistance (TIR) domain-containing protein [Arabidopsis thaliana] gi|332195661|gb|AEE33782.1| Toll-Interleukin-Resistance (TIR) domain-containing protein [Arabidopsis thaliana]	198	188	3.00E-83	94.9	76.3	83.3	Toll-Interleukin-Resistance (TIR) domain-containing protein	gbpln	Arabidopsis thaliana	AT1G61105.1 | Symbols: | Toll-Interleukin-Resistance (TIR) domain family protein | chr1:22513616-22514492 REVERSE LENGTH=188	198	188	9.00E-86	94.9	76.3	83.3
Rsa1.0_00039.1.g2331.t1	#	#	#	#	#	#	#	-	----	----	#	#	#	#	#	#	#
Rsa1.0_00039.1.g2332.t1	ref|XP_002317226.1| predicted protein [Populus trichocarpa] gi|222860291|gb|EEE97838.1| predicted protein [Populus trichocarpa]	911	792	1.00E-170	86.9	45.0	58.2	predicted protein	gbpln	Populus trichocarpa	AT1G61080.1 | Symbols: | Hydroxyproline-rich glycoprotein family protein | chr1:22493194-22497019 REVERSE LENGTH=907	911	907	1.00E-142	99.6	33.2	34.5
Rsa1.0_00039.1.g2333.t1	gb|EOA34094.1| hypothetical protein CARUB_v10021594mg [Capsella rubella]	440	441	1.00E-178	100.2	78.6	86.6	hypothetical protein CARUB_v10021594mg	gbpln	Capsella rubella	AT1G61050.1 | Symbols: | alpha 1,4-glycosyltransferase family protein | chr1:22486736-22488043 FORWARD LENGTH=435	440	435	1.00E-176	98.9	78.4	86.6
Rsa1.0_00039.1.g2334.t1	ref|NP_564767.1| coatomer subunit zeta-1 [Arabidopsis thaliana] gi|75163941|sp|Q940S5.1|COPZ1_ARATH RecName: Full=Coatomer subunit zeta-1; AltName: Full=Zeta-1-coat protein; AltName: Full=Zeta-COP 1 gi|15450727|gb|AAK96635.1| At1g60970/T7P1_11 [Arabidopsis thaliana] gi|22137000|gb|AAM91345.1| At1g60970/T7P1_11 [Arabidopsis thaliana] gi|332195633|gb|AEE33754.1| coatomer subunit zeta-1 [Arabidopsis thaliana]	177	177	8.00E-89	100.0	90.4	95.5	coatomer subunit zeta-1	gbpln	Arabidopsis thaliana	AT1G60970.1 | Symbols: | SNARE-like superfamily protein | chr1:22448008-22449387 REVERSE LENGTH=177	177	177	3.00E-91	100.0	90.4	95.5
Rsa1.0_00039.1.g2335.t1	ref|XP_002886573.1| hypothetical protein ARALYDRAFT_315258 [Arabidopsis lyrata subsp. lyrata] gi|297332414|gb|EFH62832.1| hypothetical protein ARALYDRAFT_315258 [Arabidopsis lyrata subsp. lyrata]	372	429	1.00E-177	115.3	90.6	95.7	hypothetical protein ARALYDRAFT_315258	gbpln	Arabidopsis lyrata	AT1G60960.1 | Symbols: IRT3, ATIRT3 | iron regulated transporter 3 | chr1:22445410-22447060 REVERSE LENGTH=425	372	425	1.00E-176	114.2	88.7	93.8
Rsa1.0_00039.1.g2336.t1	#	#	#	#	#	#	#	-	----	----	#	#	#	#	#	#	#
Rsa1.0_00039.1.g2337.t1	dbj|BAJ33728.1| unnamed protein product [Thellungiella halophila]	148	148	2.00E-74	100.0	91.9	96.6	unnamed protein product	----	----	AT1G60950.1 | Symbols: FED A, ATFD2 | 2Fe-2S ferredoxin-like superfamily protein | chr1:22444565-22445011 FORWARD LENGTH=148	148	148	4.00E-76	100.0	91.9	95.9
Rsa1.0_00039.1.g2338.t1	gb|EOA37821.1| hypothetical protein CARUB_v10011995mg [Capsella rubella]	668	589	0	88.2	78.3	81.7	hypothetical protein CARUB_v10011995mg	gbpln	Capsella rubella	AT1G10950.1 | Symbols: TMN1, AtTMN1 | transmembrane nine 1 | chr1:3659322-3663622 FORWARD LENGTH=589	668	589	0	88.2	77.5	81.6
Rsa1.0_00040.1.g2339.t1	ref|XP_002873716.1| predicted protein [Arabidopsis lyrata subsp. lyrata] gi|297319553|gb|EFH49975.1| predicted protein [Arabidopsis lyrata subsp. lyrata]	219	685	7.00E-76	312.8	68.9	77.2	predicted protein	gbpln	Arabidopsis lyrata	AT5G15250.1 | Symbols: FTSH6, ATFTSH6 | FTSH protease 6 | chr5:4950411-4952777 REVERSE LENGTH=688	219	688	2.00E-77	314.2	67.6	76.7
Rsa1.0_00040.1.g2340.t1	ref|XP_002873717.1| structural constituent of ribosome [Arabidopsis lyrata subsp. lyrata] gi|297319554|gb|EFH49976.1| structural constituent of ribosome [Arabidopsis lyrata subsp. lyrata]	215	235	1.00E-86	109.3	79.1	86.5	structural constituent of ribosome	gbpln	Arabidopsis lyrata	AT5G15260.1 | Symbols: | Ribosomal protein L34e superfamily protein | chr5:4953646-4954350 REVERSE LENGTH=234	215	234	2.00E-87	108.8	78.1	87.0
Rsa1.0_00040.1.g2341.t4	ref|NP_001078588.1| uncharacterized protein [Arabidopsis thaliana] gi|62319943|dbj|BAD94032.1| hypothetical protein [Arabidopsis thaliana] gi|332004757|gb|AED92140.1| uncharacterized protein AT5G15265 [Arabidopsis thaliana]	59	83	4.00E-16	140.7	67.8	81.4	uncharacterized protein	gbpln	Arabidopsis thaliana	AT5G15265.1 | Symbols: | unknown protein; FUNCTIONS IN: molecular_function unknown; INVOLVED IN: biological_process unknown; LOCATED IN: endomembrane system; Has 5 Blast hits to 5 proteins in 3 species: Archae - 0; Bacteria - 0; Metazoa - 0; Fungi - 0; Plants - 5; Viruses - 0; Other Eukaryotes - 0 (source: NCBI BLink). | chr5:4956505-4957351 FORWARD LENGTH=83	59	83	7.00E-19	140.7	67.8	81.4

Rsa1.0_00040.1.g2342.t1	ref|XP_002873718.1| pentatricopeptide repeat-containing protein [Arabidopsis lyrata subsp. lyrata] gi|297319555|gb|EFH49977.1| pentatricopeptide repeat-containing protein [Arabidopsis lyrata subsp. lyrata]	537	548	0	102.0	85.8	93.3	pentatricopeptide repeat-containing protein	gbpln	Arabidopsis lyrata	AT5G15300.1 | Symbols: | Pentatricopeptide repeat (PPR) superfamily protein | chr5:4968384-4970030 REVERSE LENGTH=548	537	548	0	102.0	85.8	93.3
Rsa1.0_00040.1.g2343.t1	dbj|BAJ34432.1| unnamed protein product [Thellungiella halophila]	314	326	1.00E-147	103.8	87.3	91.7	unnamed protein product	----	----	AT5G15310.1 | Symbols: ATMYB16, ATMIXTA, MYB16 | myb domain protein 16 | chr5:4974894-4976106 FORWARD LENGTH=326	314	326	1.00E-142	103.8	84.4	90.1
Rsa1.0_00040.1.g2344.t1	ref|NP_197036.2| uncharacterized protein [Arabidopsis thaliana] gi|26451598|dbj|BAC42896.1| GPI-anchored protein [Arabidopsis thaliana] gi|88010805|gb|ABD38855.1| At5g15320 [Arabidopsis thaliana] gi|332004765|gb|AED92148.1| uncharacterized protein AT5G15320 [Arabidopsis thaliana]	51	53	6.00E-12	103.9	78.4	86.3	uncharacterized protein	gbpln	Arabidopsis thaliana	AT5G15320.1 | Symbols: | unknown protein; FUNCTIONS IN: molecular_function unknown; INVOLVED IN: biological_process unknown; LOCATED IN: endomembrane system; EXPRESSED IN: 23 plant structures; EXPRESSED DURING: 15 growth stages; BEST Arabidopsis thaliana protein match is: unknown protein (TAIR:AT3G01130.1); Has 64 Blast hits to 64 proteins in 11 species: Archae - 0; Bacteria - 0; Metazoa - 0; Fungi - 0; Plants - 64; Viruses - 0; Other Eukaryotes - 0 (source: NCBI BLink). | chr5:4977647-4978658 FORWARD LENGTH=53	51	53	1.00E-14	103.9	78.4	86.3
Rsa1.0_00040.1.g2345.t1	ref|XP_002882199.1| hypothetical protein ARALYDRAFT_477417 [Arabidopsis lyrata subsp. lyrata] gi|297328039|gb|EFH58458.1| hypothetical protein ARALYDRAFT_477417 [Arabidopsis lyrata subsp. lyrata]	417	564	1.00E-176	135.3	72.2	81.8	hypothetical protein ARALYDRAFT_477417	gbpln	Arabidopsis lyrata	AT3G01120.1 | Symbols: MTO1, CGS, ATCYS1, CGS1 | Pyridoxal phosphate (PLP)-dependent transferases superfamily protein | chr3:39234-41865 REVERSE LENGTH=563	417	563	1.00E-179	135.0	71.9	82.0
Rsa1.0_00040.1.g2346.t1	gb|EOA21138.1| hypothetical protein CARUB_v10001482mg [Capsella rubella]	320	318	1.00E-154	99.4	87.5	92.2	hypothetical protein CARUB_v10001482mg	gbpln	Capsella rubella	AT5G15330.1 | Symbols: ATSPX4, SPX4 | SPX domain gene 4 | chr5:4980595-4982043 FORWARD LENGTH=318	320	318	1.00E-153	99.4	87.2	92.5
Rsa1.0_00040.1.g2347.t1	ref|NP_197038.1| pentatricopeptide repeat-containing protein [Arabidopsis thaliana] gi|75180838|sp|Q9LXE8.1|PP386_ARATH RecName: Full=Pentatricopeptide repeat-containing protein At5g15340, mitochondrial; Flags: Precursor gi|7671503|emb|CAB89344.1| putative protein [Arabidopsis thaliana] gi|332004768|gb|AED92151.1| pentatricopeptide repeat-containing protein [Arabidopsis thaliana]	774	623	0	80.5	68.0	72.9	pentatricopeptide repeat-containing protein	gbpln	Arabidopsis thaliana	AT5G15340.1 | Symbols: | Pentatricopeptide repeat (PPR) superfamily protein | chr5:4982273-4984144 REVERSE LENGTH=623	774	623	0	80.5	68.0	72.9
Rsa1.0_00040.1.g2348.t1	ref|NP_197043.1| tRNA/rRNA methyltransferase (SpoU) family protein [Arabidopsis thaliana] gi|7671508|emb|CAB89349.1| rRNA methylase-like protein [Arabidopsis thaliana] gi|110737570|dbj|BAF00727.1| rRNA methylase - like protein [Arabidopsis thaliana] gi|332004772|gb|AED92155.1| tRNA/rRNA methyltransferase (SpoU) family protein [Arabidopsis thaliana]	364	350	1.00E-145	96.2	76.1	81.0	tRNA/rRNA methyltransferase (SpoU) family protein	gbpln	Arabidopsis thaliana	AT5G15390.1 | Symbols: | tRNA/rRNA methyltransferase (SpoU) family protein | chr5:4995333-4997255 FORWARD LENGTH=350	364	350	1.00E-147	96.2	76.1	81.0
Rsa1.0_00040.1.g2349.t1	gb|ABG89128.1| UFD2 [synthetic construct]	1077	1037	0	96.3	87.3	91.6	UFD2	gbenv	synthetic construct	AT5G15400.1 | Symbols: | U-box domain-containing protein | chr5:4997764-5002907 REVERSE LENGTH=1038	1077	1038	0	96.4	87.3	91.6
Rsa1.0_00040.1.g2350.t1	dbj|BAJ33868.1| unnamed protein product [Thellungiella halophila]	733	722	0	98.5	92.1	94.8	unnamed protein product	----	----	AT5G15410.1 | Symbols: DND1, ATCNGC2, CNGC2 | Cyclic nucleotide-regulated ion channel family protein | chr5:5003460-5006763 REVERSE LENGTH=726	733	726	0	99.0	91.5	95.1
Rsa1.0_00040.1.g2351.t1	ref|NP_197051.2| alpha-1,4-galacturonosyltransferase [Arabidopsis thaliana] gi|75151049|sp|Q8GWT1.1|GAUTE_ARATH RecName: Full=Probable galacturonosyltransferase 14 gi|26452322|dbj|BAC43247.1| unknown protein [Arabidopsis thaliana] gi|29028910|gb|AAO64834.1| At5g15470 [Arabidopsis thaliana] gi|332004782|gb|AED92165.1| probable galacturonosyltransferase 14 [Arabidopsis thaliana]	562	532	0	94.7	85.1	89.3	alpha-1,4-galacturonosyltransferase	gbpln	Arabidopsis thaliana	AT5G15470.1 | Symbols: GAUT14 | galacturonosyltransferase 14 | chr5:5021433-5024168 REVERSE LENGTH=532	562	532	0	94.7	85.1	89.3

Rsa1.0_00040.1.g2352.t1	gb|EOA20540.1| hypothetical protein CARUB_v10000855mg [Capsella rubella] gi|482556349|gb|EOA20541.1| hypothetical protein CARUB_v10000855mg [Capsella rubella] gi|482556350|gb|EOA20542.1| hypothetical protein CARUB_v10000855mg [Capsella rubella]	480	480	0	100.0	98.5	99.4	hypothetical protein CARUB_v10000855mg	gbpln	Capsella rubella	AT5G15490.1 | Symbols: | UDP-glucose 6-dehydrogenase family protein | chr5:5027872-5029314 REVERSE LENGTH=480	480	480	0	100.0	97.3	99.2
Rsa1.0_00040.1.g2353.t1	gb|AEI16468.1| acetyl-CoA carboxylase biotin-containing subunit [Brassica rapa]	248	257	1.00E-117	103.6	90.7	94.8	acetyl-CoA carboxylase biotin-containing subunit	gbpln	Brassica rapa	AT5G15530.1 | Symbols: BCCP2, CAC1-B | biotin carboxyl carrier protein 2 | chr5:5038955-5040437 FORWARD LENGTH=255	248	255	3.00E-94	102.8	78.2	84.7
Rsa1.0_00040.1.g2354.t1	ref|XP_002873731.1| sister chromatid cohesion 2 [Arabidopsis lyrata subsp. lyrata] gi|297319568|gb|EFH49990.1| sister chromatid cohesion 2 [Arabidopsis lyrata subsp. lyrata]	1842	1847	0	100.3	86.5	91.5	sister chromatid cohesion 2	gbpln	Arabidopsis lyrata	AT5G15540.1 | Symbols: EMB2773, ATSCC2, SCC2 | PHD finger family protein | chr5:5047887-5057411 REVERSE LENGTH=1846	1842	1846	0	100.2	85.5	90.8
Rsa1.0_00040.1.g2355.t1	#	#	#	#	#	#	#	-	----	----	#	#	#	#	#	#	#
Rsa1.0_00040.1.g2356.t1	gb|EMJ16100.1| hypothetical protein PRUPE_ppa000160mg [Prunus persica]	846	1582	4.00E-56	187.0	20.9	27.8	hypothetical protein PRUPE_ppa000160mg	gbpln	Prunus persica	AT5G07980.1 | Symbols: | dentin sialophosphoprotein-related | chr5:2549432-2554669 REVERSE LENGTH=1501	846	1501	9.00E-44	177.4	12.5	18.4
Rsa1.0_00040.1.g2357.t1	ref|NP_197059.1| transducin/WD40 domain-containing protein [Arabidopsis thaliana] gi|9755810|emb|CAC01754.1| putative protein [Arabidopsis thaliana] gi|17381110|gb|AAL36367.1| unknown protein [Arabidopsis thaliana] gi|20258961|gb|AAM14196.1| unknown protein [Arabidopsis thaliana] gi|332004793|gb|AED92176.1| transducin/WD40 domain-containing protein [Arabidopsis thaliana]	414	433	0	104.6	82.9	90.8	transducin/WD40 domain-containing protein	gbpln	Arabidopsis thaliana	AT5G15550.1 | Symbols: | Transducin/WD40 repeat-like superfamily protein | chr5:5059051-5062003 REVERSE LENGTH=433	414	433	0	104.6	82.9	90.8
Rsa1.0_00040.1.g2358.t1	ref|XP_002871671.1| hypothetical protein ARALYDRAFT_909531 [Arabidopsis lyrata subsp. lyrata] gi|297317508|gb|EFH47930.1| hypothetical protein ARALYDRAFT_909531 [Arabidopsis lyrata subsp. lyrata]	362	380	1.00E-147	105.0	75.4	83.4	hypothetical protein ARALYDRAFT_909531	gbpln	Arabidopsis lyrata	AT5G15570.1 | Symbols: | Bromodomain transcription factor | chr5:5066851-5067996 FORWARD LENGTH=381	362	381	1.00E-148	105.2	74.9	83.1
Rsa1.0_00040.1.g2359.t1	gb|EOA19910.1| hypothetical protein CARUB_v10000160mg [Capsella rubella]	913	931	0	102.0	83.7	90.5	hypothetical protein CARUB_v10000160mg	gbpln	Capsella rubella	AT5G15580.1 | Symbols: LNG1 | longifolia1 | chr5:5068495-5071930 REVERSE LENGTH=927	913	927	0	101.5	81.9	89.3
Rsa1.0_00040.1.g2360.t1	ref|NP_198644.1| PR5-like receptor kinase [Arabidopsis thaliana] gi|10177803|dbj|BAB11294.1| receptor serine/threonine kinase [Arabidopsis thaliana] gi|332006906|gb|AED94289.1| PR5-like receptor kinase [Arabidopsis thaliana]	948	665	0	70.1	37.3	44.7	PR5-like receptor kinase	gbpln	Arabidopsis thaliana	AT5G38280.1 | Symbols: PR5K | PR5-like receptor kinase | chr5:15293325-15295838 REVERSE LENGTH=665	948	665	0	70.1	37.3	44.7
Rsa1.0_00040.1.g2361.t1	gb|EOA21793.1| hypothetical protein CARUB_v10002258mg [Capsella rubella] gi|482557602|gb|EOA21794.1| hypothetical protein CARUB_v10002258mg [Capsella rubella]	130	127	3.00E-40	97.7	78.5	86.9	hypothetical protein CARUB_v10002258mg	gbpln	Capsella rubella	AT5G15600.1 | Symbols: SP1L4 | SPIRAL1-like4 | chr5:5078405-5078863 FORWARD LENGTH=127	130	127	1.00E-42	97.7	80.8	86.2
Rsa1.0_00040.1.g2362.t1	ref|NP_197069.1| reversibly glycosylated polypeptide 2 [Arabidopsis thaliana] gi|75174193|sp|Q9LFW1.1|RGP2_ARATH RecName: Full=UDP-arabinopyranose mutase 2; AltName: Full=Reversibly glycosylated polypeptide 2; Short=AtRGP2; AltName: Full=UDP-L-arabinose mutase 2 gi|9755610|emb|CAC01764.1| reversibly glycosylated polypeptide-2 (AtRGB) [Arabidopsis thaliana] gi|14532444|gb|AAK63950.1| AT5g15650/F14F8_30 [Arabidopsis thaliana] gi|21464559|gb|AAM52234.1| AT5g15650/F14F8_30 [Arabidopsis thaliana] gi|332004805|gb|AED92188.1| reversibly glycosylated polypeptide 2 [Arabidopsis thaliana]	357	360	0	100.8	96.6	98.6	reversibly glycosylated polypeptide 2	gbpln	Arabidopsis thaliana	AT5G15650.1 | Symbols: RGP2, ATRGP2 | reversibly glycosylated polypeptide 2 | chr5:5092203-5094093 FORWARD LENGTH=360	357	360	0	100.8	96.6	98.6
Rsa1.0_00040.1.g2363.t1	ref|XP_002890654.1| F-box family protein [Arabidopsis lyrata subsp. lyrata] gi|297336496|gb|EFH66913.1| F-box family protein [Arabidopsis lyrata subsp. lyrata]	398	665	2.00E-80	167.1	44.0	58.8	F-box family protein	gbpln	Arabidopsis lyrata	AT5G15660.1 | Symbols: | F-box and associated interaction domains-containing protein | chr5:5095652-5097041 FORWARD LENGTH=438	398	438	9.00E-82	110.1	46.5	61.6

Rsa1.0_00040.1.g2364.t1	ref|NP_197070.1| putative F-box protein [Arabidopsis thaliana] gi|75174192|sp|Q9LFW0.1|FB257_ARATH RecName: Full=Putative F-box protein At5g15660 gi|9755611|emb|CAC01765.1| putative protein [Arabidopsis thaliana] gi|332004806|gb|AED92189.1| putative F-box protein [Arabidopsis thaliana]	386	438	1.00E-90	113.5	49.7	66.6	putative F-box protein	gbpln	Arabidopsis thaliana	AT5G15660.1 | Symbols: | F-box and associated interaction domains-containing protein | chr5:5095652-5097041 FORWARD LENGTH=438	386	438	2.00E-93	113.5	49.7	66.6
Rsa1.0_00040.1.g2365.t1	ref|XP_002871678.1| F-box family protein [Arabidopsis lyrata subsp. lyrata] gi|297317515|gb|EFH47937.1| F-box family protein [Arabidopsis lyrata subsp. lyrata]	440	430	8.00E-77	97.7	39.8	52.3	F-box family protein	gbpln	Arabidopsis lyrata	AT5G15660.1 | Symbols: | F-box and associated interaction domains-containing protein | chr5:5095652-5097041 FORWARD LENGTH=438	440	438	3.00E-79	99.5	39.8	54.5
Rsa1.0_00040.1.g2366.t1	ref|XP_002877486.1| predicted protein [Arabidopsis lyrata subsp. lyrata] gi|297323324|gb|EFH53745.1| predicted protein [Arabidopsis lyrata subsp. lyrata]	461	362	1.00E-108	78.5	45.8	53.1	predicted protein	gbpln	Arabidopsis lyrata	AT1G09650.1 | Symbols: | F-box and associated interaction domains-containing protein | chr1:3125978-3127126 FORWARD LENGTH=382	461	382	1.00E-102	82.9	48.4	58.1
Rsa1.0_00040.1.g2367.t1	gb|EOA20674.1| hypothetical protein CARUB_v10000986mg [Capsella rubella]	452	437	0	96.7	87.4	92.3	hypothetical protein CARUB_v10000986mg	gbpln	Capsella rubella	AT5G15730.2 | Symbols: CRLK2, AtCRLK2 | Protein kinase superfamily protein | chr5:5131284-5133046 FORWARD LENGTH=436	452	436	0	96.5	86.5	89.4
Rsa1.0_00040.1.g2368.t1	ref|NP_197078.2| O-fucosyltransferase family protein [Arabidopsis thaliana] gi|66792694|gb|AAY56449.1| At5g15740 [Arabidopsis thaliana] gi|110743719|dbj|BAE99696.1| hypothetical protein [Arabidopsis thaliana] gi|332004817|gb|AED92200.1| O-fucosyltransferase family protein [Arabidopsis thaliana]	509	508	0	99.8	91.4	96.3	O-fucosyltransferase family protein	gbpln	Arabidopsis thaliana	AT5G15740.1 | Symbols: | O-fucosyltransferase family protein | chr5:5134788-5136956 REVERSE LENGTH=508	509	508	0	99.8	91.4	96.3
Rsa1.0_00040.1.g2369.t1	ref|XP_002871683.1| hypothetical protein ARALYDRAFT_488420 [Arabidopsis lyrata subsp. lyrata] gi|297317520|gb|EFH47942.1| hypothetical protein ARALYDRAFT_488420 [Arabidopsis lyrata subsp. lyrata]	174	183	3.00E-67	105.2	81.6	87.9	hypothetical protein ARALYDRAFT_488420	gbpln	Arabidopsis lyrata	AT5G15760.1 | Symbols: | Ribosomal protein PSRP-3/Ycf65 | chr5:5143100-5143905 FORWARD LENGTH=183	174	183	4.00E-68	105.2	79.3	86.8
Rsa1.0_00040.1.g2370.t1	ref|XP_002873740.1| hypothetical protein ARALYDRAFT_909552 [Arabidopsis lyrata subsp. lyrata] gi|297319577|gb|EFH49999.1| hypothetical protein ARALYDRAFT_909552 [Arabidopsis lyrata subsp. lyrata]	159	149	6.00E-66	93.7	76.1	84.9	hypothetical protein ARALYDRAFT_909552	gbpln	Arabidopsis lyrata	AT5G15770.1 | Symbols: AtGNA1, GNA1 | glucose-6-phosphate acetyltransferase 1 | chr5:5144093-5144542 REVERSE LENGTH=149	159	149	8.00E-67	93.7	74.8	83.6
Rsa1.0_00040.1.g2371.t1	ref|XP_002873740.1| hypothetical protein ARALYDRAFT_909552 [Arabidopsis lyrata subsp. lyrata] gi|297319577|gb|EFH49999.1| hypothetical protein ARALYDRAFT_909552 [Arabidopsis lyrata subsp. lyrata]	160	149	1.00E-65	93.1	74.4	84.4	hypothetical protein ARALYDRAFT_909552	gbpln	Arabidopsis lyrata	AT5G15770.1 | Symbols: AtGNA1, GNA1 | glucose-6-phosphate acetyltransferase 1 | chr5:5144093-5144542 REVERSE LENGTH=149	160	149	4.00E-67	93.1	73.8	83.8
Rsa1.0_00040.1.g2372.t1	ref|XP_002873741.1| hypothetical protein ARALYDRAFT_488422 [Arabidopsis lyrata subsp. lyrata] gi|297319578|gb|EFH50000.1| hypothetical protein ARALYDRAFT_488422 [Arabidopsis lyrata subsp. lyrata]	361	363	2.00E-92	100.6	55.1	61.5	hypothetical protein ARALYDRAFT_488422	gbpln	Arabidopsis lyrata	AT5G15780.1 | Symbols: | Pollen Ole e 1 allergen and extensin family protein | chr5:5144898-5146297 REVERSE LENGTH=401	361	401	2.00E-87	111.1	44.0	47.9
Rsa1.0_00040.1.g2373.t1	ref|NP_197083.1| RING/U-box domain-containing protein [Arabidopsis thaliana] gi|42573377|ref|NP_974785.1| RING/U-box domain-containing protein [Arabidopsis thaliana] gi|9755624|emb|CAC01778.1| putative protein [Arabidopsis thaliana] gi|22655214|gb|AAM98197.1| unknown protein [Arabidopsis thaliana] gi|30023690|gb|AAP13378.1| At5g15790 [Arabidopsis thaliana] gi|110738178|dbj|BAF01020.1| hypothetical protein [Arabidopsis thaliana] gi|332004822|gb|AED92205.1| RING/U-box domain-containing protein [Arabidopsis thaliana] gi|332004823|gb|AED92206.1| RING/U-box domain-containing protein [Arabidopsis thaliana]	231	232	4.00E-98	100.4	79.7	90.9	RING/U-box domain-containing protein	gbpln	Arabidopsis thaliana	AT5G15790.2 | Symbols: | RING/U-box superfamily protein | chr5:5149800-5150964 FORWARD LENGTH=232	231	232	1.00E-100	100.4	79.7	90.9

Rsa1.0_00040.1.g2374.t1	gb|AAA32732.1| transcription factor [Arabidopsis thaliana] gi|52547968|gb|AAU81987.1| SEPALLATA1 [Arabidopsis thaliana] gi|52547972|gb|AAU81989.1| SEPALLATA1 [Arabidopsis thaliana] gi|52547974|gb|AAU81990.1| SEPALLATA1 [Arabidopsis thaliana] gi|52547976|gb|AAU81991.1| SEPALLATA1 [Arabidopsis thaliana] gi|52547978|gb|AAU81992.1| SEPALLATA1 [Arabidopsis thaliana] gi|52547980|gb|AAU81993.1| SEPALLATA1 [Arabidopsis thaliana] gi|52547982|gb|AAU81994.1| SEPALLATA1 [Arabidopsis thaliana] gi|52547984|gb|AAU81995.1| SEPALLATA1 [Arabidopsis thaliana] gi|52547986|gb|AAU81996.1| SEPALLATA1 [Arabidopsis thaliana] gi|52547988|gb|AAU81997.1| SEPALLATA1 [Arabidopsis thaliana] gi|52547990|gb|AAU81998.1| SEPALLATA1 [Arabidopsis thaliana] gi|52547992|gb|AAU81999.1| SEPALLATA1 [Arabidopsis thaliana] gi|52547994|gb|AAU82000.1| SEPALLATA1 [Arabidopsis thaliana] gi|52547996|gb|AAU82001.1| SEPALLATA1 [Arabidopsis thaliana] gi|52547998|gb|AAU82002.1| SEPALLATA1 [Arabidopsis thaliana] gi|52548002|gb|AAU82004.1| [illegible]	105	248	3.00E-49	236.2	90.5	94.3	transcription factor	gbpln	Arabidopsis thaliana	AT5G15800.1 | Symbols: SEP1, AGL2 | K-box region and MADS-box transcription factor family protein | chr5:5151594-5153767 REVERSE LENGTH=251	105	251	4.00E-42	239.0	92.4	96.2
Rsa1.0_00040.1.g2375.t1	gb|ADR83588.1| Sepallata 1-like protein [Platanus x acerifolia]	62	244	1.00E-27	393.5	98.4	98.4	Sepallata 1-like protein	gbpln	Platanus x	AT2G03710.3 | Symbols: SEP4, AGL3 | K-box region and MADS-box transcription factor family protein | chr2:1129622-1131242 FORWARD LENGTH=187	62	187	5.00E-29	301.6	91.9	96.8
Rsa1.0_00040.1.g2376.t1	ref|NP_850827.1| uncharacterized protein [Arabidopsis thaliana] gi|297811719|ref|XP_002873743.1| hypothetical protein ARALYDRAFT_326030 [Arabidopsis lyrata subsp. lyrata] gi|26450077|dbj|BAC42158.1| unknown protein [Arabidopsis thaliana] gi|28827744|gb|AAO50716.1| unknown protein [Arabidopsis thaliana] gi|297319580|gb|EFH50002.1| hypothetical protein ARALYDRAFT_326030 [Arabidopsis lyrata subsp. lyrata] gi|332004826|gb|AED92209.1| uncharacterized protein AT5G15802 [Arabidopsis thaliana]	101	99	1.00E-41	98.0	86.1	90.1	uncharacterized protein	gbpln	Arabidopsis lyrata	AT5G15802.1 | Symbols: | unknown protein; FUNCTIONS IN: molecular_function unknown; INVOLVED IN: biological_process unknown; LOCATED IN: endomembrane system; Has 35 Blast hits to 35 proteins in 15 species: Archae - 0; Bacteria - 4; Metazoa - 0; Fungi - 0; Plants - 31; Viruses - 0; Other Eukaryotes - 0 (source: NCBI BLink). | chr5:5156578-5157520 REVERSE LENGTH=99	101	99	2.00E-44	98.0	86.1	90.1
Rsa1.0_00040.1.g2377.t1	gb|EOA20072.1| hypothetical protein CARUB_v10000341mg [Capsella rubella]	589	709	0	120.4	89.8	93.7	hypothetical protein CARUB_v10000341mg	gbpln	Capsella rubella	AT5G15810.1 | Symbols: | N2,N2-dimethylguanosine tRNA methyltransferase | chr5:5157798-5161121 FORWARD LENGTH=691	589	691	0	117.3	90.0	94.4
Rsa1.0_00040.1.g2378.t1	gb|EOA21044.1| hypothetical protein CARUB_v10001383mg [Capsella rubella]	334	336	2.00E-44	100.6	43.7	57.8	hypothetical protein CARUB_v10001383mg	gbpln	Capsella rubella	AT5G15820.1 | Symbols: | RING/U-box superfamily protein | chr5:5161787-5162833 FORWARD LENGTH=348	334	348	5.00E-36	104.2	39.5	52.7
Rsa1.0_00040.1.g2379.t1	dbj|BAJ33807.1| unnamed protein product [Thellungiella halophila]	189	190	7.00E-77	100.5	81.0	90.5	unnamed protein product	----	----	AT5G15830.1 | Symbols: AtbZIP3, bZIP3 | basic leucine-zipper 3 | chr5:5168591-5169151 FORWARD LENGTH=186	189	186	4.00E-69	98.4	78.8	88.9
Rsa1.0_00040.1.g2380.t1	#	#	#	#	#	#	#	-	----	----	#	#	#	#	#	#	#
Rsa1.0_00040.1.g2381.t1	gb|AAG27546.1| constans-like protein [Brassica nigra]	375	380	0	101.3	92.5	96.3	constans-like protein	gbpln	Brassica nigra	AT5G15840.1 | Symbols: CO, FG | B-box type zinc finger protein with CCT domain | chr5:5171343-5172697 REVERSE LENGTH=373	375	373	1.00E-151	99.5	75.2	84.0
Rsa1.0_00040.1.g2382.t1	gb|AAN09808.1| COL1 protein [Brassica nigra] gi|22854912|gb|AAN09811.1| COL1 protein [Brassica nigra] gi|22854914|gb|AAN09812.1| COL1 protein [Brassica nigra] gi|22854918|gb|AAN09814.1| COL1 protein [Brassica nigra]	354	348	1.00E-175	98.3	89.5	93.2	COL1 protein	gbpln	Brassica nigra	AT5G15850.1 | Symbols: COL1, ATCOL1 | CONSTANS-like 1 | chr5:5176297-5177473 REVERSE LENGTH=355	354	355	1.00E-131	100.3	74.3	81.9
Rsa1.0_00040.1.g2383.t1	gb|AAP42646.1| putative beta-glucan elicitor receptor [Brassica napus]	759	752	0	99.1	93.1	95.7	putative beta-glucan elicitor receptor	gbpln	Brassica napus	AT5G15870.1 | Symbols: | glycosyl hydrolase family 81 protein | chr5:5182641-5184878 REVERSE LENGTH=745	759	745	0	98.2	83.1	89.5

Rsa1.0_00040.1.g2384.t1	ref|NP_197092.1| uncharacterized protein [Arabidopsis thaliana] gi|9755634|emb|CAC01787.1| putative protein [Arabidopsis thaliana] gi|20466278|gb|AAM20456.1| putative protein [Arabidopsis thaliana] gi|31711832|gb|AAP68272.1| At5g15880 [Arabidopsis thaliana] gi|332004837|gb|AED92220.1| uncharacterized protein AT5G15880 [Arabidopsis thaliana]	216	348	5.00E-48	161.1	61.6	67.1	uncharacterized protein	gbpln	Arabidopsis thaliana	AT5G15880.1 | Symbols: | unknown protein; Has 1807 Blast hits to 1807 proteins in 277 species: Archae - 0; Bacteria - 0; Metazoa - 736; Fungi - 347; Plants - 385; Viruses - 0; Other Eukaryotes - 339 (source: NCBI BLink). | chr5:5185788-5187461 FORWARD LENGTH=348	216	348	2.00E-50	161.1	61.6	67.1
Rsa1.0_00040.1.g2385.t1	gb|EOA22444.1| hypothetical protein CARUB_v10003090mg [Capsella rubella]	460	517	0	112.4	79.8	88.7	hypothetical protein CARUB_v10003090mg	gbpln	Capsella rubella	AT5G15890.1 | Symbols: TBL21 | TRICHOME BIREFRINGENCE-LIKE 21 | chr5:5187687-5189348 REVERSE LENGTH=526	460	526	0	114.3	78.3	88.0
Rsa1.0_00040.1.g2386.t1	ref|XP_002873749.1| hypothetical protein ARALYDRAFT_488438 [Arabidopsis lyrata subsp. lyrata] gi|297319586|gb|EFH50008.1| hypothetical protein ARALYDRAFT_488438 [Arabidopsis lyrata subsp. lyrata]	425	426	0	100.2	83.1	92.0	hypothetical protein ARALYDRAFT_488438	gbpln	Arabidopsis lyrata	AT5G15900.1 | Symbols: TBL19 | TRICHOME BIREFRINGENCE-LIKE 19 | chr5:5189524-5192070 REVERSE LENGTH=426	425	426	0	100.2	81.2	90.4
Rsa1.0_00040.1.g2387.t2	gb|AAR15894.1| S-adenosyl-L-methionine decarboxylase [Brassica juncea]	617	369	0	59.8	54.1	56.2	S-adenosyl-L-methionine decarboxylase	gbpln	Brassica juncea	AT5G15950.2 | Symbols: | Adenosylmethionine decarboxylase family protein | chr5:5206706-5207794 FORWARD LENGTH=362	617	362	0	58.7	52.4	55.4
Rsa1.0_00040.1.g2388.t1	gb|AAG13408.1|AF297472_1 BN28b [Brassica napus]	65	65	4.00E-25	100.0	92.3	98.5	BN28b	gbpln	Brassica napus	#	#	#	#	#	#	#
Rsa1.0_00040.1.g2389.t1	gb|EOA20115.1| hypothetical protein CARUB_v10000394mg [Capsella rubella]	662	670	0	101.2	73.0	84.0	hypothetical protein CARUB_v10000394mg	gbpln	Capsella rubella	AT5G15980.1 | Symbols: | Pentatricopeptide repeat (PPR) superfamily protein | chr5:5213290-5215296 FORWARD LENGTH=668	662	668	0	100.9	71.8	82.6
Rsa1.0_00040.1.g2390.t1	ref|XP_002871697.1| nsp-interacting kinase 1 [Arabidopsis lyrata subsp. lyrata] gi|297317534|gb|EFH47956.1| nsp-interacting kinase 1 [Arabidopsis lyrata subsp. lyrata]	645	638	0	98.9	91.3	94.3	nsp-interacting kinase 1	gbpln	Arabidopsis lyrata	AT5G16000.1 | Symbols: NIK1 | NSP-interacting kinase 1 | chr5:5224264-5227003 FORWARD LENGTH=638	645	638	0	98.9	91.3	93.6
Rsa1.0_00040.1.g2391.t1	gb|EOA34948.1| hypothetical protein CARUB_v10020033mg [Capsella rubella]	604	591	0	97.8	81.1	89.4	hypothetical protein CARUB_v10020033mg	gbpln	Capsella rubella	AT1G72550.1 | Symbols: | tRNA synthetase beta subunit family protein | chr1:27319947-27323908 REVERSE LENGTH=598	604	598	0	99.0	78.8	88.9
Rsa1.0_00040.1.g2392.t1	ref|XP_002871699.1| hypothetical protein ARALYDRAFT_488440 [Arabidopsis lyrata subsp. lyrata] gi|297317536|gb|EFH47958.1| hypothetical protein ARALYDRAFT_488440 [Arabidopsis lyrata subsp. lyrata]	938	640	0	68.2	56.6	60.7	hypothetical protein ARALYDRAFT_488440	gbpln	Arabidopsis lyrata	AT5G16020.1 | Symbols: GEX3 | gamete-expressed 3 | chr5:5229692-5232241 FORWARD LENGTH=641	938	641	0	68.3	54.7	59.1
Rsa1.0_00040.1.g2393.t1	ref|XP_002871700.1| predicted protein [Arabidopsis lyrata subsp. lyrata] gi|297317537|gb|EFH47959.1| predicted protein [Arabidopsis lyrata subsp. lyrata]	350	338	1.00E-135	96.6	74.9	86.0	predicted protein	gbpln	Arabidopsis lyrata	AT5G16030.1 | Symbols: | unknown protein; BEST Arabidopsis thaliana protein match is: unknown protein (TAIR:AT3G02500.1); Has 30201 Blast hits to 17322 proteins in 780 species: Archae - 12; Bacteria - 1396; Metazoa - 17338; Fungi - 3422; Plants - 5037; Viruses - 0; Other Eukaryotes - 2996 (source: NCBI BLink). | chr5:5238242-5240578 FORWARD LENGTH=339	350	339	1.00E-136	96.9	76.0	86.0
Rsa1.0_00040.1.g2394.t1	gb|EOA19527.1| hypothetical protein CARUB_v10002448mg [Capsella rubella]	413	396	0	95.9	89.6	92.0	hypothetical protein CARUB_v10002448mg	gbpln	Capsella rubella	AT5G16040.1 | Symbols: | Regulator of chromosome condensation (RCC1) family protein | chr5:5240860-5243308 REVERSE LENGTH=396	413	396	0	95.9	89.6	91.8
Rsa1.0_00040.1.g2395.t1	ref|XP_002871701.1| hypothetical protein ARALYDRAFT_488456 [Arabidopsis lyrata subsp. lyrata] gi|297317538|gb|EFH47960.1| hypothetical protein ARALYDRAFT_488456 [Arabidopsis lyrata subsp. lyrata]	96	95	3.00E-44	99.0	90.6	93.8	hypothetical protein ARALYDRAFT_488456	gbpln	Arabidopsis lyrata	AT5G16060.1 | Symbols: | Cytochrome c oxidase biogenesis protein Cmc1-like | chr5:5246161-5247209 FORWARD LENGTH=95	96	95	2.00E-46	99.0	89.6	92.7
Rsa1.0_00040.1.g2396.t1	ref|XP_002884325.1| hypothetical protein ARALYDRAFT_477487 [Arabidopsis lyrata subsp. lyrata] gi|297330165|gb|EFH60584.1| hypothetical protein ARALYDRAFT_477487 [Arabidopsis lyrata subsp. lyrata]	536	535	0	99.8	97.2	99.1	hypothetical protein ARALYDRAFT_477487	gbpln	Arabidopsis lyrata	AT5G16070.1 | Symbols: | TCP-1/cpn60 chaperonin family protein | chr5:5247549-5251050 REVERSE LENGTH=535	536	535	0	99.8	97.9	98.7

Rsa1.0_00040.1.g2397.t1	ref|XP_002873755.1| hypothetical protein ARALYDRAFT_488458 [Arabidopsis lyrata subsp. lyrata] gi|297319592|gb|EFH50014.1| hypothetical protein ARALYDRAFT_488458 [Arabidopsis lyrata subsp. lyrata]	338	343	1.00E-170	101.5	87.6	93.2	hypothetical protein ARALYDRAFT_488458	gbpln	Arabidopsis lyrata	AT5G16080.1 | Symbols: AtCXE17, CXE17 | carboxyesterase 17 | chr5:5252533-5253567 REVERSE LENGTH=344	338	344	1.00E-170	101.8	85.8	92.6
Rsa1.0_00040.1.g2398.t1	gb|ABY66298.1| RAD23-like protein [Brassica napus]	70	357	3.00E-15	510.0	52.9	57.1	RAD23-like protein	gbpln	Brassica napus	#	#	#	#	#	#	#
Rsa1.0_00040.1.g2399.t1	gb|ABY66298.1| RAD23-like protein [Brassica napus]	310	357	9.00E-98	115.2	66.1	76.5	RAD23-like protein	gbpln	Brassica napus	AT5G38470.2 | Symbols: RAD23D | Rad23 UV excision repair protein family | chr5:15404720-15407251 FORWARD LENGTH=332	310	332	1.00E-38	107.1	36.1	51.0
Rsa1.0_00040.1.g2400.t3	gb|ABY66298.1| RAD23-like protein [Brassica napus]	420	357	1.00E-114	85.0	58.8	69.8	RAD23-like protein	gbpln	Brassica napus	AT5G38470.1 | Symbols: RAD23D | Rad23 UV excision repair protein family | chr5:15404720-15407500 FORWARD LENGTH=378	420	378	6.00E-37	90.0	32.9	46.9
Rsa1.0_00040.1.g2401.t5	gb|ABY66299.1| DNA repair protein RAD23 [Brassica napus]	236	327	3.00E-53	138.6	64.4	75.0	DNA repair protein RAD23	gbpln	Brassica napus	AT5G38470.2 | Symbols: RAD23D | Rad23 UV excision repair protein family | chr5:15404720-15407251 FORWARD LENGTH=332	236	332	8.00E-20	140.7	23.7	29.2
Rsa1.0_00040.1.g2402.t1	gb|EOA12107.1| hypothetical protein CARUB_v10000012mg, partial [Capsella rubella]	189	238	3.00E-66	125.9	82.0	86.2	hypothetical protein CARUB_v10000012mg, partial	gbpln	Capsella rubella	AT5G16110.1 | Symbols: | unknown protein; FUNCTIONS IN: molecular_function unknown; INVOLVED IN: biological_process unknown; LOCATED IN: chloroplast; EXPRESSED IN: 24 plant structures; EXPRESSED DURING: 15 growth stages; BEST Arabidopsis thaliana protein match is: unknown protein (TAIR:AT3G02555.1); Has 133 Blast hits to 133 proteins in 18 species: Archae - 0; Bacteria - 0; Metazoa - 0; Fungi - 0; Plants - 133; Viruses - 0; Other Eukaryotes - 0 (source: NCBI BLink). | chr5:5261171-5262668 REVERSE LENGTH=244	189	244	9.00E-68	129.1	81.0	86.2
Rsa1.0_00040.1.g2403.t3	ref|NP_197117.1| 40S ribosomal protein S7-3 [Arabidopsis thaliana] gi|115311860|sp|Q8LD03.2|RS73_ARATH RecName: Full=40S ribosomal protein S7-3 gi|9755823|emb|CAC01854.1| 40S ribosomal protein S7-like [Arabidopsis thaliana] gi|90568000|gb|ABD94070.1| At5g16130 [Arabidopsis thaliana] gi|110742740|dbj|BAE99279.1| 40S ribosomal protein S7-like [Arabidopsis thaliana] gi|332004867|gb|AED92250.1| 40S ribosomal protein S7-3 [Arabidopsis thaliana]	190	190	1.00E-100	100.0	93.7	96.8	40S ribosomal protein S7-3	gbpln	Arabidopsis thaliana	AT5G16130.1 | Symbols: | Ribosomal protein S7e family protein | chr5:5268984-5269912 FORWARD LENGTH=190	190	190	1.00E-103	100.0	93.7	96.8
Rsa1.0_00040.1.g2404.t1	gb|EOA21457.1| hypothetical protein CARUB_v10001847mg [Capsella rubella]	241	239	1.00E-125	99.2	90.0	95.0	hypothetical protein CARUB_v10001847mg	gbpln	Capsella rubella	AT5G16140.2 | Symbols: | Peptidyl-tRNA hydrolase family protein | chr5:5270308-5271517 REVERSE LENGTH=240	241	240	1.00E-123	99.6	88.8	92.9
Rsa1.0_00040.1.g2405.t1	ref|XP_002871705.1| GLT1 [Arabidopsis lyrata subsp. lyrata] gi|297317542|gb|EFH47964.1| GLT1 [Arabidopsis lyrata subsp. lyrata]	530	545	0	102.8	93.0	95.3	GLT1	gbpln	Arabidopsis lyrata	AT5G16150.3 | Symbols: GLT1, PGLCT | plastidic GLC translocator | chr5:5272904-5275678 FORWARD LENGTH=546	530	546	0	103.0	92.8	95.7
Rsa1.0_00040.1.g2406.t1	gb|EOA20743.1| hypothetical protein CARUB_v10001069mg [Capsella rubella]	312	414	1.00E-155	132.7	86.5	92.9	hypothetical protein CARUB_v10001069mg	gbpln	Capsella rubella	AT5G16170.1 | Symbols: | Core-2/I-branching beta-1,6-N-acetylglucosaminyltransferase family protein | chr5:5277926-5279751 FORWARD LENGTH=411	312	411	1.00E-154	131.7	83.7	91.3
Rsa1.0_00040.1.g2407.t4	gb|EOA20451.1| hypothetical protein CARUB_v10000762mg [Capsella rubella]	505	507	0	100.4	88.1	94.5	hypothetical protein CARUB_v10000762mg	gbpln	Capsella rubella	AT4G13410.1 | Symbols: ATCSLA15, CSLA15 | Nucleotide-diphospho-sugar transferases superfamily protein | chr4:7792219-7795824 REVERSE LENGTH=537	505	537	0	106.3	82.6	90.5
Rsa1.0_00041.1.g2408.t1	gb|EOA31480.1| hypothetical protein CARUB_v10014673mg [Capsella rubella]	203	200	4.00E-78	98.5	79.8	84.2	hypothetical protein CARUB_v10014673mg	gbpln	Capsella rubella	AT3G04510.1 | Symbols: LSH2 | Protein of unknown function (DUF640) | chr3:1215812-1216417 REVERSE LENGTH=201	203	201	9.00E-76	99.0	79.3	83.7
Rsa1.0_00041.1.g2409.t1	gb|EOA31608.1| hypothetical protein CARUB_v10014805mg, partial [Capsella rubella]	103	161	1.00E-33	156.3	74.8	77.7	hypothetical protein CARUB_v10014805mg, partial	gbpln	Capsella rubella	AT3G04400.1 | Symbols: emb2171 | Ribosomal protein L14p/L23e family protein | chr3:1167339-1168308 FORWARD LENGTH=140	103	140	4.00E-36	135.9	74.8	77.7
Rsa1.0_00041.1.g2410.t1	gb|EOA31608.1| hypothetical protein CARUB_v10014805mg, partial [Capsella rubella]	140	161	1.00E-74	115.0	99.3	100.0	hypothetical protein CARUB_v10014805mg, partial	gbpln	Capsella rubella	AT3G04400.1 | Symbols: emb2171 | Ribosomal protein L14p/L23e family protein | chr3:1167339-1168308 FORWARD LENGTH=140	140	140	1.00E-76	100.0	99.3	100.0
Rsa1.0_00041.1.g2411.t1	gb|EOA31980.1| hypothetical protein CARUB_v10015223mg [Capsella rubella]	564	567	0	100.5	84.8	91.8	hypothetical protein CARUB_v10015223mg	gbpln	Capsella rubella	AT3G04350.1 | Symbols: | Plant protein of unknown function (DUF946) | chr3:1153972-1156469 REVERSE LENGTH=567	564	567	0	100.5	86.5	93.8

Rsa1.0_00041.1.g2412.t1	gb|EOA30270.1| hypothetical protein CARUB_v10013391mg [Capsella rubella]	552	548	0	99.3	88.9	94.0	hypothetical protein CARUB_v10013391mg	gbpln	Capsella rubella	AT5G18480.1 | Symbols: PGSIP6 | plant glycogenin-like starch initiation protein 6 | chr5:6131307-6133787 REVERSE LENGTH=537	552	537	0	97.3	80.6	88.2
Rsa1.0_00041.1.g2413.t1	ref|NP_187076.2| plastid transcriptionally active 3 [Arabidopsis thaliana] gi|332640537|gb|AEE74058.1| plastid transcriptionally active 3 [Arabidopsis thaliana]	908	910	0	100.2	91.3	95.0	plastid transcriptionally active 3	gbpln	Arabidopsis thaliana	AT3G04260.1 | Symbols: PTAC3 | plastid transcriptionally active 3 | chr3:1123231-1127515 REVERSE LENGTH=910	908	910	0	100.2	91.3	95.0
Rsa1.0_00041.1.g2414.t1	gb|AAN08437.1| hypothetical protein [Arabidopsis thaliana]	620	538	1.00E-133	86.8	43.2	53.4	hypothetical protein	gbpln	Arabidopsis thaliana	AT2G43220.1 | Symbols: | Cysteine/Histidine-rich C1 domain family protein | chr2:17964340-17965956 FORWARD LENGTH=538	620	538	1.00E-135	86.8	42.6	52.6
Rsa1.0_00041.1.g2415.t1	ref|NP_187074.1| putative UDP-N-acetylglucosamine--peptide N-acetylglucosaminyltransferase SEC [Arabidopsis thaliana] gi|75336082|sp|Q9M8Y0.1|SEC_ARATH RecName: Full=Probable UDP-N-acetylglucosamine--peptide N-acetylglucosaminyltransferase SEC; AltName: Full=Protein SECRET AGENT gi|6721161|gb|AAF26789.1|AC016829_13 putative O-linked GlcNAc transferase [Arabidopsis thaliana] gi|18139887|gb|AAL60196.1|AF441079_1 O-linked N-acetyl glucosamine transferase [Arabidopsis thaliana] gi|20259324|gb|AAM13988.1| putative O-linked GlcNAc transferase [Arabidopsis thaliana] gi|21436429|gb|AAM51415.1| putative O-linked GlcNAc transferase [Arabidopsis thaliana] gi|110742062|dbj|BAE98963.1| O-linked GlcNAc transferase like protein [Arabidopsis thaliana] gi|332640535|gb|AEE74056.1| putative UDP-N-acetylglucosamine--peptide N-acetylglucosaminyltransferase SEC [Arabidopsis thaliana]	980	977	0	99.7	93.5	96.0	putative UDP-N-acetylglucosamine--peptide N-acetylglucosaminyltransferase SEC	gbpln	Arabidopsis thaliana	AT3G04240.1 | Symbols: SEC | Tetratricopeptide repeat (TPR)-like superfamily protein | chr3:1114187-1120722 REVERSE LENGTH=977	980	977	0	99.7	93.5	96.0
Rsa1.0_00041.1.g2416.t1	gb|EOA17608.1| hypothetical protein CARUB_v10005968mg [Capsella rubella] gi|482557559|gb|EOA21751.1| hypothetical protein CARUB_v10002207mg [Capsella rubella]	146	146	5.00E-74	100.0	93.2	95.2	hypothetical protein CARUB_v10005968mg	gbpln	Capsella rubella	AT5G18380.1 | Symbols: | Ribosomal protein S5 domain 2-like superfamily protein | chr5:6090253-6090693 REVERSE LENGTH=146	146	146	6.00E-76	100.0	92.5	95.2
Rsa1.0_00041.1.g2417.t1	#	#	#	#	#	#	#	-	----	----	#	#	#	#	#	#	#
Rsa1.0_00041.1.g2418.t1	dbj|BAJ33926.1| unnamed protein product [Thellungiella halophila]	338	338	0	100.0	96.7	98.5	unnamed protein product	----	----	AT3G04120.1 | Symbols: GAPC, GAPC-1, GAPC1 | glyceraldehyde-3-phosphate dehydrogenase C subunit 1 | chr3:1081077-1083131 FORWARD LENGTH=338	338	338	0	100.0	95.6	98.2
Rsa1.0_00041.1.g2419.t2	ref|NP_187061.1| glutamate receptor 1.1 [Arabidopsis thaliana] gi|41017234|sp|Q9M8W7.1|GLR11_ARATH RecName: Full=Glutamate receptor 1.1; Short=AtGLR1; AltName: Full=Ligand-gated ion channel 1.1; Flags: Precursor gi|6721174|gb|AAF26802.1|AC016829_26 putative glutamate receptor (GLR1) [Arabidopsis thaliana] gi|26450250|dbj|BAC42242.1| putative glutamate receptor GLR1 [Arabidopsis thaliana] gi|332640517|gb|AEE74038.1| glutamate receptor 1.1 [Arabidopsis thaliana]	796	808	0	101.5	70.1	80.9	glutamate receptor 1.1	gbpln	Arabidopsis thaliana	AT3G04110.1 | Symbols: GLR1, ATGLR1.1, GLR1.1 | glutamate receptor 1.1 | chr3:1077361-1080236 FORWARD LENGTH=808	796	808	0	101.5	70.1	80.9
Rsa1.0_00041.1.g2420.t1	ref|XP_002882329.1| MADS-box family protein [Arabidopsis lyrata subsp. lyrata] gi|297328169|gb|EFH58588.1| MADS-box family protein [Arabidopsis lyrata subsp. lyrata]	187	205	2.00E-45	109.6	56.7	70.6	MADS-box family protein	gbpln	Arabidopsis lyrata	AT3G04100.1 | Symbols: AGL57 | AGAMOUS-like 57 | chr3:1075299-1075922 FORWARD LENGTH=207	187	207	1.00E-43	110.7	51.9	67.9
Rsa1.0_00041.1.g2421.t1	ref|NP_197329.4| nucleoside-triphosphatase [Arabidopsis thaliana] gi|332005149|gb|AED92532.1| nucleoside-triphosphatase [Arabidopsis thaliana]	749	578	0	77.2	56.2	61.8	nucleoside-triphosphatase	gbpln	Arabidopsis thaliana	AT5G18280.1 | Symbols: ATAPY2, APY2 | apyrase 2 | chr5:6050799-6054875 REVERSE LENGTH=578	749	578	0	77.2	56.2	61.8

Rsa1.0_00041.1.g2422.t1	ref|NP_187057.2| NAC domain containing protein 47 [Arabidopsis thaliana] gi|29029082|gb|AAO64920.1| At3g04070 [Arabidopsis thaliana] gi|110743057|dbj|BAE99421.1| NAM-like protein [Arabidopsis thaliana] gi|332640512|gb|AEE74033.1| NAC domain containing protein 47 [Arabidopsis thaliana]	356	359	1.00E-166	100.8	82.6	89.0	NAC domain containing protein 47	gbpln	Arabidopsis thaliana	AT3G04070.1 | Symbols: anac047, NAC047 | NAC domain containing protein 47 | chr3:1061573-1062976 REVERSE LENGTH=359	356	359	1.00E-168	100.8	82.6	89.0
Rsa1.0_00041.1.g2423.t1	ref|XP_002884425.1| ANAC046 [Arabidopsis lyrata subsp. lyrata] gi|297330265|gb|EFH60684.1| ANAC046 [Arabidopsis lyrata subsp. lyrata]	342	342	1.00E-164	100.0	82.5	88.6	ANAC046	gbpln	Arabidopsis lyrata	AT3G04060.1 | Symbols: anac046, NAC046 | NAC domain containing protein 46 | chr3:1053625-1054952 REVERSE LENGTH=338	342	338	1.00E-164	98.8	82.5	87.1
Rsa1.0_00041.1.g2424.t1	#	#	#	#	#	#	#	–	----	----	#	#	#	#	#	#	#
Rsa1.0_00041.1.g2425.t1	ref|XP_002882328.1| hypothetical protein ARALYDRAFT_340564 [Arabidopsis lyrata subsp. lyrata] gi|297328168|gb|EFH58587.1| hypothetical protein ARALYDRAFT_340564 [Arabidopsis lyrata subsp. lyrata]	487	510	0	104.7	80.3	88.7	hypothetical protein ARALYDRAFT_340564	gbpln	Arabidopsis lyrata	AT3G04050.1 | Symbols: | Pyruvate kinase family protein | chr3:1049795-1051522 FORWARD LENGTH=510	487	510	0	104.7	79.9	88.5
Rsa1.0_00041.1.g2426.t7	ref|XP_002863119.1| predicted protein [Arabidopsis lyrata subsp. lyrata] gi|297308943|gb|EFH39378.1| predicted protein [Arabidopsis lyrata subsp. lyrata]	1394	1121	0	80.4	43.7	51.9	predicted protein	gbpln	Arabidopsis lyrata	AT5G11250.1 | Symbols: | Disease resistance protein (TIR-NBS-LRR class) | chr5:3587978-3591960 REVERSE LENGTH=1189	1394	1189	0	85.3	42.0	51.6
Rsa1.0_00041.1.g2427.t1	ref|XP_002863119.1| predicted protein [Arabidopsis lyrata subsp. lyrata] gi|297308943|gb|EFH39378.1| predicted protein [Arabidopsis lyrata subsp. lyrata]	1100	1121	0	101.9	56.9	70.1	predicted protein	gbpln	Arabidopsis lyrata	AT5G11250.1 | Symbols: | Disease resistance protein (TIR-NBS-LRR class) | chr5:3587978-3591960 REVERSE LENGTH=1189	1100	1189	0	108.1	56.4	69.7
Rsa1.0_00041.1.g2428.t1	ref|NP_187048.1| 3-oxoacyl-[acyl-carrier protein] reductase [Arabidopsis thaliana] gi|6223643|gb|AAF05857.1|AC011698_8 putative short-chain type dehydrogenase/reductase [Arabidopsis thaliana] gi|332640501|gb|AEE74022.1| NAD(P)-binding Rossmann-fold domain-containing protein [Arabidopsis thaliana]	263	270	1.00E-126	102.7	86.3	93.5	3-oxoacyl-	gbpln	Arabidopsis thaliana	AT3G03980.1 | Symbols: | NAD(P)-binding Rossmann-fold superfamily protein | chr3:1031786-1033081 FORWARD LENGTH=270	263	270	1.00E-129	102.7	86.3	93.5
Rsa1.0_00041.1.g2429.t1	gb|ACP30572.1| disease resistance protein [Brassica rapa subsp. pekinensis]	1266	1184	0	93.5	44.6	56.8	disease resistance protein	gbpln	Brassica rapa	AT5G11250.1 | Symbols: | Disease resistance protein (TIR-NBS-LRR class) | chr5:3587978-3591960 REVERSE LENGTH=1189	1266	1189	0	93.9	46.1	57.7
Rsa1.0_00041.1.g2430.t1	ref|NP_196686.1| TIR-NBS-LRR class disease resistance protein [Arabidopsis thaliana] gi|8953387|emb|CAB96660.1| RPP1 disease resistance protein-like [Arabidopsis thaliana] gi|332004269|gb|AED91652.1| TIR-NBS-LRR class disease resistance protein [Arabidopsis thaliana]	1644	1189	0	72.3	41.0	50.5	TIR-NBS-LRR class disease resistance protein	gbpln	Arabidopsis thaliana	AT5G11250.1 | Symbols: | Disease resistance protein (TIR-NBS-LRR class) | chr5:3587978-3591960 REVERSE LENGTH=1189	1644	1189	0	72.3	41.0	50.5
Rsa1.0_00041.1.g2431.t1	gb|EOA32860.1| hypothetical protein CARUB_v10016174mg [Capsella rubella]	567	549	0	96.8	93.3	94.9	hypothetical protein CARUB_v10016174mg	gbpln	Capsella rubella	AT3G03960.1 | Symbols: | TCP-1/cpn60 chaperonin family protein | chr3:1024432-1027604 FORWARD LENGTH=549	567	549	0	96.8	92.9	94.9
Rsa1.0_00041.1.g2432.t1	ref|XP_002882321.1| hypothetical protein ARALYDRAFT_477648 [Arabidopsis lyrata subsp. lyrata] gi|297328161|gb|EFH58580.1| hypothetical protein ARALYDRAFT_477648 [Arabidopsis lyrata subsp. lyrata]	415	428	0	103.1	84.8	90.8	hypothetical protein ARALYDRAFT_477648	gbpln	Arabidopsis lyrata	AT3G03950.3 | Symbols: ECT1 | evolutionarily conserved C-terminal region 1 | chr3:1021502-1023767 FORWARD LENGTH=428	415	428	0	103.1	83.1	89.4
Rsa1.0_00041.1.g2433.t1	ref|NP_196686.1| TIR-NBS-LRR class disease resistance protein [Arabidopsis thaliana] gi|8953387|emb|CAB96660.1| RPP1 disease resistance protein-like [Arabidopsis thaliana] gi|332004269|gb|AED91652.1| TIR-NBS-LRR class disease resistance protein [Arabidopsis thaliana]	1078	1189	0	110.3	57.4	71.5	TIR-NBS-LRR class disease resistance protein	gbpln	Arabidopsis thaliana	AT5G11250.1 | Symbols: | Disease resistance protein (TIR-NBS-LRR class) | chr5:3587978-3591960 REVERSE LENGTH=1189	1078	1189	0	110.3	57.4	71.5
Rsa1.0_00041.1.g2434.t1	ref|NP_187044.1| protein kinase-like protein [Arabidopsis thaliana] gi|6223639|gb|AAF05853.1|AC011698_4 putative casein kinase [Arabidopsis thaliana] gi|332640493|gb|AEE74014.1| protein kinase-like protein [Arabidopsis thaliana]	704	701	0	99.6	92.6	96.3	protein kinase-like protein	gbpln	Arabidopsis thaliana	AT3G03940.1 | Symbols: | Protein kinase family protein | chr3:1014412-1018244 REVERSE LENGTH=701	704	701	0	99.6	92.6	96.3

Rsa1.0_00041.1.g2435.t1	ref|NP_566217.1| H/ACA ribonucleoprotein complex subunit 1 [Arabidopsis thaliana] gi|68565918|sp|Q8VZT0.1|NLAL1_ARATH RecName: Full=Putative H/ACA ribonucleoprotein complex subunit 1-like protein 1 gi|17380836|gb|AAL36230.1| putative GAR1 protein [Arabidopsis thaliana] gi|20259633|gb|AAM14173.1| putative GAR1 protein [Arabidopsis thaliana] gi|332640491|gb|AEE74012.1| H/ACA ribonucleoprotein complex, subunit Gar1/Naf1 protein [Arabidopsis thaliana]	208	202	4.00E-51	97.1	58.2	59.6	H/ACA ribonucleoprotein complex subunit 1	gbpln	Arabidopsis thaliana	AT3G03920.1 | Symbols: | H/ACA ribonucleoprotein complex, subunit Gar1/Naf1 protein | chr3:1009123-1010379 REVERSE LENGTH=202	208	202	1.00E-53	97.1	58.2	59.6
Rsa1.0_00041.1.g2436.t1	#	#	#	#	#	#	#	-	----	----	AT3G03910.1 | Symbols: GDH3 | glutamate dehydrogenase 3 | chr3:1006913-1008846 FORWARD LENGTH=411	37	411	5.00E-13	1110.8	81.1	83.8
Rsa1.0_00041.1.g2437.t1	gb|EOA32962.1| hypothetical protein CARUB_v10016292mg [Capsella rubella]	411	411	0	100.0	94.6	98.3	hypothetical protein CARUB_v10016292mg	gbpln	Capsella rubella	AT3G03910.1 | Symbols: GDH3 | glutamate dehydrogenase 3 | chr3:1006913-1008846 FORWARD LENGTH=411	411	411	0	100.0	93.9	98.1
Rsa1.0_00041.1.g2438.t1	#	#	#	#	#	#	#	-	----	----	#	#	#	#	#	#	#
Rsa1.0_00041.1.g2439.t1	gb|EOA31260.1| hypothetical protein CARUB_v10014432mg [Capsella rubella]	286	260	5.00E-92	90.9	64.3	72.7	hypothetical protein CARUB_v10014432mg	gbpln	Capsella rubella	AT3G03870.2 | Symbols: | unknown protein; BEST Arabidopsis thaliana protein match is: unknown protein (TAIR:AT5G18130.1); Has 47 Blast hits to 47 proteins in 13 species: Archae - 0; Bacteria - 0; Metazoa - 2; Fungi - 0; Plants - 45; Viruses - 0; Other Eukaryotes - 0 (source: NCBI BLink). | chr3:995520-997180 FORWARD LENGTH=266	286	266	2.00E-91	93.0	65.7	72.7
Rsa1.0_00041.1.g2440.t1	ref|XP_002882315.1| hypothetical protein ARALYDRAFT_477638 [Arabidopsis lyrata subsp. lyrata] gi|297328155|gb|EFH58574.1| hypothetical protein ARALYDRAFT_477638 [Arabidopsis lyrata subsp. lyrata]	302	300	1.00E-137	99.3	80.5	89.4	hypothetical protein ARALYDRAFT_477638	gbpln	Arabidopsis lyrata	AT3G03860.1 | Symbols: ATAPRL5, APRL5 | APR-like 5 | chr3:992465-994315 FORWARD LENGTH=300	302	300	1.00E-140	99.3	80.5	89.4
Rsa1.0_00041.1.g2441.t1	ref|XP_002884412.1| hypothetical protein ARALYDRAFT_477634 [Arabidopsis lyrata subsp. lyrata] gi|297330252|gb|EFH60671.1| hypothetical protein ARALYDRAFT_477634 [Arabidopsis lyrata subsp. lyrata]	656	656	0	100.0	91.8	95.9	hypothetical protein ARALYDRAFT_477634	gbpln	Arabidopsis lyrata	AT3G03810.1 | Symbols: EDA30 | O-fucosyltransferase family protein | chr3:972190-975901 REVERSE LENGTH=656	656	656	0	100.0	91.2	95.4
Rsa1.0_00041.1.g2442.t1	ref|NP_187030.1| syntaxin 1B/2/3 [Arabidopsis thaliana] gi|28380139|sp|Q9SRV7.1|SY131_ARATH RecName: Full=Putative syntaxin-131; Short=AtSYP131 gi|6006872|gb|AAF00648.1|AC009540_25 s-syntaxin-like protein [Arabidopsis thaliana] gi|332640474|gb|AEE73995.1| putative syntaxin-131 [Arabidopsis thaliana]	278	306	1.00E-135	110.1	91.7	95.7	syntaxin 1B/2/3	gbpln	Arabidopsis thaliana	AT3G03800.1 | Symbols: SYP131, ATSYP131 | syntaxin of plants 131 | chr3:969314-971460 FORWARD LENGTH=306	278	306	1.00E-137	110.1	91.7	95.7
Rsa1.0_00041.1.g2443.t1	gb|ACP30600.1| disease resistance protein [Brassica rapa subsp. pekinensis]	645	909	1.00E-167	140.9	49.9	66.7	disease resistance protein	gbpln	Brassica rapa	AT5G18360.1 | Symbols: | Disease resistance protein (TIR-NBS-LRR class) family | chr5:6080049-6083027 REVERSE LENGTH=900	645	900	1.00E-169	139.5	50.1	66.8
Rsa1.0_00041.1.g2444.t1	ref|NP_683525.2| HSP20-like chaperon-like protein [Arabidopsis thaliana] gi|73921112|sp|Q6ID70.1|Y3377_ARATH RecName: Full=Uncharacterized protein At3g03773 gi|48310264|gb|AAT41786.1| At3g03773 [Arabidopsis thaliana] gi|49823520|gb|AAT68743.1| hypothetical protein At3g03773 [Arabidopsis thaliana] gi|50198936|gb|AAT70471.1| At3g03773 [Arabidopsis thaliana] gi|61742697|gb|AAX55169.1| hypothetical protein At3g03773 [Arabidopsis thaliana] gi|332640465|gb|AEE73986.1| HSP20-like chaperon-like protein [Arabidopsis thaliana]	150	150	8.00E-75	100.0	90.7	96.0	HSP20-like chaperon-like protein	gbpln	Arabidopsis thaliana	AT3G03773.1 | Symbols: | HSP20-like chaperones superfamily protein | chr3:951885-953490 FORWARD LENGTH=150	150	150	3.00E-77	100.0	90.7	96.0
Rsa1.0_00041.1.g2445.t1	ref|XP_002877469.1| predicted protein [Arabidopsis lyrata subsp. lyrata] gi|297323307|gb|EFH53728.1| predicted protein [Arabidopsis lyrata subsp. lyrata]	336	328	1.00E-43	97.6	27.1	37.2	predicted protein	gbpln	Arabidopsis lyrata	AT1G60720.1 | Symbols: | RNA-directed DNA polymerase (reverse transcriptase)-related family protein | chr1:22357072-22357941 FORWARD LENGTH=289	336	289	1.00E-31	86.0	22.3	28.6

Rsa1.0_00041.1.g2446.t1	ref|XP_002882305.1| LOB domain protein 20 [Arabidopsis lyrata subsp. lyrata] gi|297328145|gb|EFH58564.1| LOB domain protein 20 [Arabidopsis lyrata subsp. lyrata]	273	281	1.00E-117	102.9	83.9	87.5	LOB domain protein 20	gbpln	Arabidopsis lyrata	AT3G03760.1 | Symbols: LBD20 | LOB domain-containing protein 20 | chr3:943632-944673 FORWARD LENGTH=273	273	273	1.00E-116	100.0	86.4	89.4
Rsa1.0_00042.1.g2447.t1	gb|AAD10829.1| putative inositol polyphosphate 5-phosphatase At5P2 [Arabidopsis thaliana]	646	646	0	100.0	86.8	90.7	putative inositol polyphosphate 5-phosphatase At5P2	gbpln	Arabidopsis thaliana	AT4G18010.1 | Symbols: IP5PII, AT5PTASE2, 5PTASE2 | myo-inositol polyphosphate 5-phosphatase 2 | chr4:9991194-9994099 REVERSE LENGTH=646	646	646	0	100.0	86.8	90.7
Rsa1.0_00042.1.g2448.t1	gb|EMJ12169.1| hypothetical protein PRUPE_ppa022548mg [Prunus persica]	792	495	1.00E-167	62.5	38.4	45.1	hypothetical protein PRUPE_ppa022548mg	gbpln	Prunus persica	AT5G46570.1 | Symbols: BSK2 | BR-signaling kinase 2 | chr5:18894687-18897198 FORWARD LENGTH=489	792	489	1.00E-169	61.7	38.6	46.1
Rsa1.0_00042.1.g2449.t1	ref|XP_002868027.1| predicted protein [Arabidopsis lyrata subsp. lyrata] gi|297313863|gb|EFH44286.1| predicted protein [Arabidopsis lyrata subsp. lyrata]	286	261	1.00E-126	91.3	78.0	81.1	predicted protein	gbpln	Arabidopsis lyrata	AT4G17980.1 | Symbols: anac071, NAC071 | NAC domain containing protein 71 | chr4:9978850-9980038 REVERSE LENGTH=262	286	262	1.00E-120	91.6	75.2	79.0
Rsa1.0_00042.1.g2450.t1	#	#	#	#	#	#	#	–	----	----	#	#	#	#	#	#	#
Rsa1.0_00042.1.g2451.t1	ref|NP_193531.1| aluminum-activated, malate transporter 12 [Arabidopsis thaliana] gi|75219677|sp|O49696.1|ALMTC_ARATH RecName: Full=Aluminum-activated malate transporter 12; Short=AtALMT12; AltName: Full=Quick anion channel 1 gi|2894606|emb|CAA17140.1| putative protein [Arabidopsis thaliana] gi|7268549|emb|CAB78799.1| putative protein [Arabidopsis thaliana] gi|332658573|gb|AEE83973.1| aluminum-activated, malate transporter 12 [Arabidopsis thaliana]	536	560	0	104.5	90.5	94.2	aluminum-activated, malate transporter 12	gbpln	Arabidopsis thaliana	AT4G17970.1 | Symbols: ALMT12, ATALMT12 | aluminum-activated, malate transporter 12 | chr4:9975482-9977722 FORWARD LENGTH=560	536	560	0	104.5	90.5	94.2
Rsa1.0_00042.1.g2452.t1	ref|NP_567546.1| AT hook motif DNA-binding family protein [Arabidopsis thaliana] gi|15451060|gb|AAK96801.1| putative protein [Arabidopsis thaliana] gi|20148333|gb|AAM10057.1| putative protein [Arabidopsis thaliana] gi|119657370|tpd|FAA00284.1| TPA: AT-hook motif nuclear localized protein 13 [Arabidopsis thaliana] gi|332658571|gb|AEE83971.1| AT hook motif DNA-binding family protein [Arabidopsis thaliana]	346	439	5.00E-57	126.9	48.0	58.7	AT hook motif DNA-binding family protein	gbpln	Arabidopsis thaliana	AT4G17950.1 | Symbols: | AT hook motif DNA-binding family protein | chr4:9967295-9969007 REVERSE LENGTH=439	346	439	1.00E-59	126.9	48.0	58.7
Rsa1.0_00042.1.g2453.t1	ref|XP_002885669.1| hypothetical protein ARALYDRAFT_319176 [Arabidopsis lyrata subsp. lyrata] gi|297331509|gb|EFH61928.1| hypothetical protein ARALYDRAFT_319176 [Arabidopsis lyrata subsp. lyrata]	485	467	1.00E-132	96.3	52.2	63.5	hypothetical protein ARALYDRAFT_319176	gbpln	Arabidopsis lyrata	AT3G62850.1 | Symbols: | zinc finger protein-related | chr3:23237487-23239155 REVERSE LENGTH=472	485	472	1.00E-132	97.3	49.7	61.6
Rsa1.0_00042.1.g2454.t1	ref|NP_567546.1| AT hook motif DNA-binding family protein [Arabidopsis thaliana] gi|15451060|gb|AAK96801.1| putative protein [Arabidopsis thaliana] gi|20148333|gb|AAM10057.1| putative protein [Arabidopsis thaliana] gi|119657370|tpd|FAA00284.1| TPA: AT-hook motif nuclear localized protein 13 [Arabidopsis thaliana] gi|332658571|gb|AEE83971.1| AT hook motif DNA-binding family protein [Arabidopsis thaliana]	306	439	4.00E-57	143.5	54.2	67.6	AT hook motif DNA-binding family protein	gbpln	Arabidopsis thaliana	AT4G17950.1 | Symbols: | AT hook motif DNA-binding family protein | chr4:9967295-9969007 REVERSE LENGTH=439	306	439	1.00E-59	143.5	54.2	67.6

Rsa1.0_00042.1.g2455.t2	ref|NP_195449.1| cytochrome P450, family 81, subfamily D, polypeptide 5 [Arabidopsis thaliana] gi|4376088|emb|CAB16770.1| cytochrome P450-like protein [Arabidopsis thaliana] gi|7270715|emb|CAB80398.1| cytochrome P450-like protein [Arabidopsis thaliana] gi|27754487|gb|AAO22691.1| putative cytochrome p450 family protein [Arabidopsis thaliana] gi|28394071|gb|AAO42443.1| putative cytochrome p450 family protein [Arabidopsis thaliana] gi|332661381|gb|AEE86781.1| cytochrome P450, family 81, subfamily D, polypeptide 5 [Arabidopsis thaliana]	474	495	0	104.4	79.3	87.6	cytochrome P450, family 81, subfamily D, polypeptide 5	gbpln	Arabidopsis thaliana	AT4G37320.1 | Symbols: CYP81D5 | cytochrome P450, family 81, subfamily D, polypeptide 5 | chr4:17559742-17561690 REVERSE LENGTH=495	474	495	0	104.4	79.3	87.6
Rsa1.0_00042.1.g2456.t1	ref|XP_002868029.1| zinc finger family protein [Arabidopsis lyrata subsp. lyrata] gi|297313865|gb|EFH44288.1| zinc finger family protein [Arabidopsis lyrata subsp. lyrata]	298	294	1.00E-118	98.7	73.5	78.9	zinc finger family protein	gbpln	Arabidopsis lyrata	AT4G17920.1 | Symbols: | RING/U-box superfamily protein | chr4:9963221-9964090 REVERSE LENGTH=289	298	289	1.00E-116	97.0	74.8	81.2
Rsa1.0_00042.1.g2457.t1	sp|P0C043.1|PP318_ARATH RecName: Full=Putative pentatricopeptide repeat-containing protein At4g17915	464	463	0	99.8	88.8	95.3	RecName: Full=Putative pentatricopeptide repeat-containing protein At4g17915	----	----	AT5G46680.1 | Symbols: | Pentatricopeptide repeat (PPR-like) superfamily protein | chr5:18941118-18942524 FORWARD LENGTH=468	464	468	0	100.9	68.5	84.1
Rsa1.0_00042.1.g2458.t1	gb|EOA17250.1| hypothetical protein CARUB_v10005524mg, partial [Capsella rubella]	350	262	1.00E-118	74.9	58.3	60.0	hypothetical protein CARUB_v10005524mg, partial	gbpln	Capsella rubella	AT4G17900.1 | Symbols: | PLATZ transcription factor family protein | chr4:9946046-9947697 FORWARD LENGTH=227	350	227	1.00E-120	64.9	58.3	59.4
Rsa1.0_00042.1.g2459.t1	#	#	#	#	#	#	#	-	----	----	#	#	#	#	#	#	#
Rsa1.0_00042.1.g2460.t1	ref|XP_002870079.1| hypothetical protein ARALYDRAFT_329738 [Arabidopsis lyrata subsp. lyrata] gi|297315915|gb|EFH46338.1| hypothetical protein ARALYDRAFT_329738 [Arabidopsis lyrata subsp. lyrata]	685	1082	0	158.0	58.7	68.2	hypothetical protein ARALYDRAFT_329738	gbpln	Arabidopsis lyrata	AT4G17895.1 | Symbols: UBP20 | ubiquitin-specific protease 20 | chr4:9939914-9942698 FORWARD LENGTH=695	685	695	1.00E-175	101.5	57.5	68.3
Rsa1.0_00042.1.g2461.t1	ref|NP_567543.1| putative ADP-ribosylation factor GTPase-activating protein AGD8 [Arabidopsis thaliana] gi|75244593|sp|Q8H100.1|AGD8_ARATH RecName: Full=Probable ADP-ribosylation factor GTPase-activating protein AGD8; Short=ARF GAP AGD8; AltName: Full=Protein ARF-GAP DOMAIN 8; Short=AtAGD8 gi|24030421|gb|AAN41368.1| unknown protein [Arabidopsis thaliana] gi|51970716|dbj|BAD44050.1| unknown protein [Arabidopsis thaliana] gi|51971433|dbj|BAD44381.1| unknown protein [Arabidopsis thaliana] gi|62319827|dbj|BAD93852.1| hypothetical protein [Arabidopsis thaliana] gi|62320091|dbj|BAD94263.1| hypothetical protein [Arabidopsis thaliana] gi|110739292|dbj|BAF01559.1| hypothetical protein [Arabidopsis thaliana] gi|332658561|gb|AEE83961.1| putative ADP-ribosylation factor GTPase-activating protein AGD8 [Arabidopsis thaliana]	409	413	0	101.0	86.6	92.2	putative ADP-ribosylation factor GTPase-activating protein AGD8	gbpln	Arabidopsis thaliana	AT4G17890.1 | Symbols: AGD8 | ARF-GAP domain 8 | chr4:9937121-9939146 FORWARD LENGTH=413	409	413	0	101.0	86.6	92.2
Rsa1.0_00042.1.g2462.t2	#	#	#	#	#	#	#	-	----	----	#	#	#	#	#	#	#
Rsa1.0_00042.1.g2463.t1	#	#	#	#	#	#	#	-	----	----	#	#	#	#	#	#	#

Rsa1.0_00042.1.g2464.t1	gb|ABD65174.1| hypothetical protein 40.t00061 [Brassica oleracea]	404	407	0	100.7	90.6	92.8	hypothetical protein 40.t00061	gbpln	Brassica oleracea	AT4G17840.1 | Symbols: | FUNCTIONS IN: molecular_function unknown; INVOLVED IN: biological_process unknown; LOCATED IN: chloroplast, membrane; EXPRESSED IN: 23 plant structures; EXPRESSED DURING: 13 growth stages; CONTAINS InterPro DOMAIN/s: Abortive infection protein (InterPro:IPR003675); BEST Arabidopsis thaliana protein match is: unknown protein (TAIR:AT2G35260.1); Has 30201 Blast hits to 17322 proteins in 780 species: Archae - 12; Bacteria - 1396; Metazoa - 17338; Fungi - 3422; Plants - 5037; Viruses - 0; Other Eukaryotes - 2996 (source: NCBI BLink). | chr4:9918222-9920190 REVERSE LENGTH=422	404	422	0	104.5	87.9	91.6
Rsa1.0_00042.1.g2465.t1	gb|ABD65175.1| Streptomyces cyclase/dehydrase family protein [Brassica oleracea]	191	191	1.00E-105	100.0	96.3	98.4	Streptomyces cyclase/dehydrase family protein	gbpln	Brassica oleracea	AT4G17870.1 | Symbols: PYR1, RCAR11 | Polyketide cyclase/dehydrase and lipid transport superfamily protein | chr4:9928792-9929367 FORWARD LENGTH=191	191	191	1.00E-101	100.0	90.1	94.8
Rsa1.0_00042.1.g2466.t1	gb|ABD65173.1| acetylornithine deacetylase, putative [Brassica oleracea]	438	437	0	99.8	98.6	99.1	acetylornithine deacetylase, putative	gbpln	Brassica oleracea	AT4G17830.1 | Symbols: | Peptidase M20/M25/M40 family protein | chr4:9915916-9918049 FORWARD LENGTH=440	438	440	0	100.5	90.4	95.7
Rsa1.0_00042.1.g2467.t1	gb|EOA38195.1| hypothetical protein CARUB_v10009673mg [Capsella rubella] gi|482574009|gb|EOA38196.1| hypothetical protein CARUB_v10009673mg [Capsella rubella]	87	336	2.00E-15	386.2	66.7	71.3	hypothetical protein CARUB_v10009673mg	gbpln	Capsella rubella	AT1G06850.1 | Symbols: AtbZIP52, bZIP52 | basic leucine-zipper 52 | chr1:2105233-2106518 FORWARD LENGTH=337	87	337	4.00E-16	387.4	62.1	67.8
Rsa1.0_00042.1.g2468.t1	gb|ABD65171.1| zinc finger (C2H2 type) containing protein [Brassica oleracea]	206	200	1.00E-107	97.1	90.3	92.7	zinc finger (C2H2 type) containing protein	gbpln	Brassica oleracea	AT4G17810.1 | Symbols: | C2H2 and C2HC zinc fingers superfamily protein | chr4:9906918-9907532 FORWARD LENGTH=204	206	204	1.00E-93	99.0	81.6	87.4
Rsa1.0_00042.1.g2469.t1	#	#	#	#	#	#	#	-	----	----	#	#	#	#	#	#	#
Rsa1.0_00042.1.g2470.t1	#	#	#	#	#	#	#	-	----	----	#	#	#	#	#	#	#
Rsa1.0_00042.1.g2471.t1	ref|XP_002863741.1| hypothetical protein ARALYDRAFT_917455 [Arabidopsis lyrata subsp. lyrata] gi|297309576|gb|EFH40000.1| hypothetical protein ARALYDRAFT_917455 [Arabidopsis lyrata subsp. lyrata]	469	390	2.00E-80	83.2	37.1	49.0	hypothetical protein ARALYDRAFT_917455	gbpln	Arabidopsis lyrata	AT3G24255.1 | Symbols: | RNA-directed DNA polymerase (reverse transcriptase)-related family protein | chr3:8789471-8793208 FORWARD LENGTH=746	469	746	4.00E-63	159.1	28.4	37.5
Rsa1.0_00042.1.g2472.t1	gb|ABD65169.1| hypothetical protein 40.t00056 [Brassica oleracea]	270	293	1.00E-139	108.5	95.2	95.6	hypothetical protein 40.t00056	gbpln	Brassica oleracea	AT4G17800.1 | Symbols: | Predicted AT-hook DNA-binding family protein | chr4:9895549-9896427 REVERSE LENGTH=292	270	292	1.00E-101	108.1	90.0	91.5
Rsa1.0_00042.1.g2473.t1	gb|ABD65614.1| hypothetical protein 23.t00026 [Brassica oleracea]	264	257	1.00E-125	97.3	88.3	91.3	hypothetical protein 23.t00026	gbpln	Brassica oleracea	AT4G17790.1 | Symbols: | SNARE associated Golgi protein family | chr4:9891425-9892675 FORWARD LENGTH=264	264	264	1.00E-125	100.0	85.2	89.0
Rsa1.0_00042.1.g2474.t1	gb|ABD65167.1| myb family transcription factor [Brassica oleracea]	341	337	1.00E-168	98.8	85.0	90.0	myb family transcription factor	gbpln	Brassica oleracea	AT4G17785.1 | Symbols: MYB39 | myb domain protein 39 | chr4:9881867-9883368 REVERSE LENGTH=360	341	360	1.00E-150	105.6	78.6	85.9
Rsa1.0_00042.1.g2475.t1	gb|ABD65165.1| trehalose-6-phosphate synthase, putative [Brassica oleracea]	861	857	0	99.5	95.0	96.9	trehalose-6-phosphate synthase, putative	gbpln	Brassica oleracea	AT4G17770.1 | Symbols: ATTPS5, TPS5 | trehalose phosphatase/synthase 5 | chr4:9877055-9880084 FORWARD LENGTH=862	861	862	0	100.1	92.5	96.1
Rsa1.0_00042.1.g2476.t1	gb|ABD65164.1| hypothetical protein 40.t00049 [Brassica oleracea]	297	297	1.00E-169	100.0	97.0	97.6	hypothetical protein 40.t00049	gbpln	Brassica oleracea	AT4G17760.1 | Symbols: | damaged DNA binding;exodeoxyribonuclease IIIs | chr4:9871820-9873257 FORWARD LENGTH=300	297	300	1.00E-167	101.0	93.9	95.3
Rsa1.0_00042.1.g2477.t1	ref|XP_002868041.1| hypothetical protein ARALYDRAFT_329753 [Arabidopsis lyrata subsp. lyrata] gi|297313877|gb|EFH44300.1| hypothetical protein ARALYDRAFT_329753 [Arabidopsis lyrata subsp. lyrata]	510	515	0	101.0	91.0	95.1	hypothetical protein ARALYDRAFT_329753	gbpln	Arabidopsis lyrata	AT4G17740.1 | Symbols: | Peptidase S41 family protein | chr4:9867088-9869719 REVERSE LENGTH=515	510	515	0	101.0	90.6	94.3
Rsa1.0_00042.1.g2478.t1	gb|ABD65159.1| syntaxin, putative [Brassica oleracea]	281	303	1.00E-130	107.8	90.0	93.6	syntaxin, putative	gbpln	Brassica oleracea	AT4G17730.2 | Symbols: SYP23, ATSYP23 | syntaxin of plants 23 | chr4:9865351-9866717 FORWARD LENGTH=262	281	262	1.00E-114	93.2	78.3	85.1
Rsa1.0_00042.1.g2479.t2	gb|ABD65158.1| RNA recognition motif (RRM)-containing protein [Brassica oleracea]	312	300	1.00E-151	96.2	87.8	88.5	RNA recognition motif (RRM)-containing protein	gbpln	Brassica oleracea	AT4G17720.1 | Symbols: | RNA-binding (RRM/RBD/RNP motifs) family protein | chr4:9862660-9864498 REVERSE LENGTH=313	312	313	1.00E-152	100.3	87.5	90.4

Rsa1.0_00042.1.g2480.t1	gb|ABD65155.1| hypothetical protein 40.t00037 [Brassica oleracea] gi|89257700|gb|ABD65187.1| hypothetical protein 40.t00083 [Brassica oleracea]	99	101	2.00E-28	102.0	67.7	73.7	hypothetical protein 40.t00037	gbpln	Brassica oleracea	AT4G17718.1 | Symbols: | Defensin-like (DEFL) family protein | chr4:9861596-9861995 REVERSE LENGTH=101	99	101	1.00E-28	102.0	59.6	73.7
Rsa1.0_00042.1.g2481.t1	gb|ABD65154.1| hypothetical protein 40.t00036 [Brassica oleracea] gi|89257699|gb|ABD65186.1| hypothetical protein 40.t00081 [Brassica oleracea]	95	161	1.00E-30	169.5	71.6	75.8	hypothetical protein 40.t00036	gbpln	Brassica oleracea	AT4G17718.1 | Symbols: | Defensin-like (DEFL) family protein | chr4:9861596-9861995 REVERSE LENGTH=101	95	101	9.00E-29	106.3	66.3	74.7
Rsa1.0_00042.1.g2482.t1	gb|ABD65151.1| peroxidase, putative [Brassica oleracea]	329	329	0	100.0	96.0	98.5	peroxidase, putative	gbpln	Brassica oleracea	AT4G17690.1 | Symbols: | Peroxidase superfamily protein | chr4:9846127-9847107 FORWARD LENGTH=326	329	326	1.00E-160	99.1	82.4	92.1
Rsa1.0_00043.1.g2483.t1	gb|ABE65512.1| hypothetical protein At4g04650 [Arabidopsis thaliana]	225	296	2.00E-52	131.6	47.6	68.0	hypothetical protein At4g04650	gbpln	Arabidopsis thaliana	AT1G43730.1 | Symbols: | RNA-directed DNA polymerase (reverse transcriptase)-related family protein | chr1:16508723-16509784 REVERSE LENGTH=320	225	320	2.00E-54	142.2	46.2	65.3
Rsa1.0_00043.1.g2484.t1	gb|EOA38594.1| hypothetical protein CARUB_v10010427mg [Capsella rubella]	176	178	1.00E-68	101.1	75.6	83.5	hypothetical protein CARUB_v10010427mg	gbpln	Capsella rubella	AT1G32583.1 | Symbols: | FUNCTIONS IN: molecular_function unknown; INVOLVED IN: biological_process unknown; LOCATED IN: endomembrane system; BEST Arabidopsis thaliana protein match is: tapetum determinant 1 (TAIR:AT4G24972.1); Has 35333 Blast hits to 34131 proteins in 2444 species: Archae - 798; Bacteria - 22429; Metazoa - 974; Fungi - 991; Plants - 531; Viruses - 0; Other Eukaryotes - 9610 (source: NCBI BLink). | chr1:11787351-11788103 FORWARD LENGTH=179	176	179	3.00E-65	101.7	71.0	81.8
Rsa1.0_00043.1.g2485.t1	ref|XP_004288454.1| PREDICTED: MATH domain-containing protein At5g43560-like [Fragaria vesca subsp. vesca]	410	1138	1.00E-121	277.6	54.4	66.8	PREDICTED: MATH domain-containing protein At5g43560-like	gbpln	Fragaria vesca	AT1G04300.1 | Symbols: | TRAF-like superfamily protein | chr1:1148818-1153895 REVERSE LENGTH=1074	410	1074	1.00E-118	262.0	55.4	64.9
Rsa1.0_00043.1.g2486.t1	ref|NP_174539.1| hydroxyproline-rich glycoprotein-like protein [Arabidopsis thaliana] gi|79319084|ref|NP_001031129.1| hydroxyproline-rich glycoprotein-like protein [Arabidopsis thaliana] gi|6714285|gb|AAF25981.1|AC017118_18 F6N18.2 [Arabidopsis thaliana] gi|34146860|gb|AAQ62438.1| At1g32610 [Arabidopsis thaliana] gi|51969172|dbj|BAD43278.1| unknown protein [Arabidopsis thaliana] gi|51971371|dbj|BAD44350.1| unknown protein [Arabidopsis thaliana] gi|332193389|gb|AEE31510.1| hydroxyproline-rich glycoprotein-like protein [Arabidopsis thaliana] gi|332193390|gb|AEE31511.1| hydroxyproline-rich glycoprotein-like protein [Arabidopsis thaliana]	199	291	5.00E-15	146.2	49.2	57.3	hydroxyproline-rich glycoprotein-like protein	gbpln	Arabidopsis thaliana	AT1G32610.2 | Symbols: | hydroxyproline-rich glycoprotein family protein | chr1:11795892-11796767 REVERSE LENGTH=291	199	291	2.00E-17	146.2	49.2	57.3
Rsa1.0_00043.1.g2487.t1	gb|EOA37193.1| hypothetical protein CARUB_v10010595mg [Capsella rubella]	155	141	1.00E-27	91.0	63.2	73.5	hypothetical protein CARUB_v10010595mg	gbpln	Capsella rubella	AT1G32630.1 | Symbols: | unknown protein; Has 18 Blast hits to 18 proteins in 10 species: Archae - 0; Bacteria - 0; Metazoa - 2; Fungi - 2; Plants - 12; Viruses - 0; Other Eukaryotes - 2 (source: NCBI BLink). | chr1:11797942-11798343 FORWARD LENGTH=133	155	133	7.00E-26	85.8	56.1	66.5
Rsa1.0_00043.1.g2488.t1	#	#	#	#	#	#	#	-	----	----	#	#	#	#	#	#	#
Rsa1.0_00043.1.g2489.t1	gb|ABS11038.1| MYC [Brassica oleracea var. gemmifera]	614	610	0	99.3	93.5	95.8	MYC	gbpln	Brassica oleracea	AT1G32640.1 | Symbols: ATMYC2, RD22BP1, JAI1, JIN1, MYC2, ZBF1 | Basic helix-loop-helix (bHLH) DNA-binding family protein | chr1:11799042-11800913 REVERSE LENGTH=623	614	623	0	101.5	88.1	93.3
Rsa1.0_00043.1.g2490.t1	#	#	#	#	#	#	#	-	----	----	#	#	#	#	#	#	#
Rsa1.0_00043.1.g2491.t1	emb|CAB39638.1| RNA-directed DNA polymerase-like protein [Arabidopsis thaliana] gi|7267666|emb|CAB78094.1| RNA-directed DNA polymerase-like protein [Arabidopsis thaliana]	1930	1274	0	66.0	30.8	43.5	RNA-directed DNA polymerase-like protein	gbpln	Arabidopsis thaliana	AT4G29090.1 | Symbols: | Ribonuclease H-like superfamily protein | chr4:14333528-14335255 FORWARD LENGTH=575	1930	575	1.00E-69	29.8	9.3	14.5
Rsa1.0_00043.1.g2492.t1	#	#	#	#	#	#	#	-	----	----	#	#	#	#	#	#	#
Rsa1.0_00043.1.g2493.t1	#	#	#	#	#	#	#	-	----	----	#	#	#	#	#	#	#

Rsa1.0_00043.1.g2494.t1	gb|AAF82236.1|AC069143_12 Contains similarity to a transposable element Tip100 protein for transposase from Ipomoea purpurea gb|4063769 and is a member of the transmembrane 4 family PF|00335 [Arabidopsis thaliana]	621	811	0	130.6	69.2	82.8	Contains similarity to a transposable element Tip100 protein for transposase from Ipomoea purpurea gb|4063769 and is a member of the transmembrane 4 family PF|00335	gbpln	Arabidopsis thaliana	AT1G19260.1 | Symbols: | TTF-type zinc finger protein with HAT dimerisation domain | chr1:6657260-6659569 REVERSE LENGTH=769	621	769	0	123.8	69.2	82.8
Rsa1.0_00043.1.g2495.t1	#	#	#	#	#	#	#	-	----	----	#	#	#	#	#	#	#
Rsa1.0_00043.1.g2496.t1	ref|NP_174546.1| uncharacterized protein [Arabidopsis thaliana] gi|38566628|gb|AAR24204.1| At1g32690 [Arabidopsis thaliana] gi|40824115|gb|AAR92349.1| At1g32690 [Arabidopsis thaliana] gi|332193396|gb|AEE31517.1| uncharacterized protein AT1G32690 [Arabidopsis thaliana]	197	200	3.00E-69	101.5	80.2	87.8	uncharacterized protein	gbpln	Arabidopsis thaliana	AT1G32690.1 | Symbols: | unknown protein; FUNCTIONS IN: molecular_function unknown; INVOLVED IN: biological_process unknown; LOCATED IN: plasma membrane; EXPRESSED IN: 21 plant structures; EXPRESSED DURING: 11 growth stages; BEST Arabidopsis thaliana protein match is: unknown protein (TAIR:AT2G35200.1); Has 45 Blast hits to 45 proteins in 8 species: Archae - 0; Bacteria - 0; Metazoa - 0; Fungi - 0; Plants - 45; Viruses - 0; Other Eukaryotes - 0 (source: NCBI BLink). | chr1:11821079-11821681 REVERSE LENGTH=200	197	200	1.00E-71	101.5	80.2	87.8
Rsa1.0_00043.1.g2497.t1	ref|NP_199370.1| Ulp1 protease family protein [Arabidopsis thaliana] gi|10177930|dbj|BAB11195.1| unnamed protein product [Arabidopsis thaliana] gi|332007886|gb|AED95269.1| Ulp1 protease family protein [Arabidopsis thaliana]	126	921	6.00E-17	731.0	27.8	46.8	Ulp1 protease family protein	gbpln	Arabidopsis thaliana	AT5G45570.1 | Symbols: | Ulp1 protease family protein | chr5:18472301-18475574 FORWARD LENGTH=921	126	921	1.00E-19	731.0	27.8	46.8
Rsa1.0_00043.1.g2498.t1	ref|NP_564407.1| uncharacterized protein [Arabidopsis thaliana] gi|6714274|gb|AAF25970.1|AC017118_7 F6N18.11 [Arabidopsis thaliana] gi|21536980|gb|AAM61321.1| unknown [Arabidopsis thaliana] gi|225897996|dbj|BAH30330.1| hypothetical protein [Arabidopsis thaliana] gi|332193401|gb|AEE31522.1| uncharacterized protein AT1G32730 [Arabidopsis thaliana]	260	327	2.00E-93	125.8	72.7	83.8	uncharacterized protein	gbpln	Arabidopsis thaliana	AT1G32730.1 | Symbols: | unknown protein; LOCATED IN: chloroplast; EXPRESSED IN: 12 plant structures; EXPRESSED DURING: 6 growth stages; CONTAINS InterPro DOMAIN/s: Protein of unknown function DUF702 (InterPro:IPR007818); Has 120 Blast hits to 118 proteins in 39 species: Archae - 0; Bacteria - 8; Metazoa - 63; Fungi - 4; Plants - 33; Viruses - 0; Other Eukaryotes - 12 (source: NCBI BLink). | chr1:11841077-11842956 FORWARD LENGTH=327	260	327	6.00E-96	125.8	72.7	83.8
Rsa1.0_00043.1.g2499.t1	#	#	#	#	#	#	#	-	----	----	#	#	#	#	#	#	#
Rsa1.0_00043.1.g2500.t1	gb|EOA37570.1| hypothetical protein CARUB_v10011900mg [Capsella rubella]	360	368	1.00E-174	102.2	87.5	91.9	hypothetical protein CARUB_v10011900mg	gbpln	Capsella rubella	AT1G32770.1 | Symbols: ANAC012, SND1, NST3, NAC012 | NAC domain containing protein 12 | chr1:11865343-11866950 REVERSE LENGTH=358	360	358	1.00E-173	99.4	85.3	89.2
Rsa1.0_00043.1.g2501.t1	gb|EOA37905.1| hypothetical protein CARUB_v10009372mg [Capsella rubella]	394	394	0	100.0	90.9	95.7	hypothetical protein CARUB_v10009372mg	gbpln	Capsella rubella	AT1G32780.1 | Symbols: | GroES-like zinc-binding dehydrogenase family protein | chr1:11869977-11872595 REVERSE LENGTH=394	394	394	0	100.0	90.9	94.4
Rsa1.0_00043.1.g2502.t1	#	#	#	#	#	#	#	-	----	----	#	#	#	#	#	#	#
Rsa1.0_00043.1.g2503.t2	ref|XP_002891012.1| ubiquitin carboxyl-terminal hydrolase family protein [Arabidopsis lyrata subsp. lyrata] gi|297336854|gb|EFH67271.1| ubiquitin carboxyl-terminal hydrolase family protein [Arabidopsis lyrata subsp. lyrata]	833	894	0	107.3	72.3	82.5	ubiquitin carboxyl-terminal hydrolase family protein	gbpln	Arabidopsis lyrata	AT1G32850.1 | Symbols: UBP11 | ubiquitin-specific protease 11 | chr1:11902629-11906900 FORWARD LENGTH=892	833	892	0	107.1	69.7	81.0
Rsa1.0_00043.1.g2504.t1	ref|NP_174566.1| granule-bound starch synthase [Arabidopsis thaliana] gi|29337142|sp|Q9MAQ0.1|SSG1_ARATH RecName: Full=Probable granule-bound starch synthase 1, chloroplastic/amyloplastic; AltName: Full=Granule-bound starch synthase I; Short=GBSS-I; Flags: Precursor gi|6910568|gb|AAF31273.1|AC006424_2 granule-bound starch synthase [Arabidopsis thaliana] gi|20453077|gb|AAM19783.1| At1g32900/F9L11_8 [Arabidopsis thaliana] gi|21595154|gb|AAM66076.1| starch synthase, putative [Arabidopsis thaliana] gi|21703108|gb|AAM74496.1| At1g32900/F9L11_8 [Arabidopsis thaliana] gi|23506181|gb|AAN31102.1| At1g32900/F9L11_8 [Arabidopsis thaliana] gi|332193416|gb|AEE31537.1| granule-bound starch synthase [Arabidopsis thaliana]	608	610	0	100.3	90.0	93.9	granule-bound starch synthase	gbpln	Arabidopsis thaliana	AT1G32900.1 | Symbols: | UDP-Glycosyltransferase superfamily protein | chr1:11920582-11923506 REVERSE LENGTH=610	608	610	0	100.3	90.0	93.9

Rsa1.0_00043.1.g2505.t1	ref|NP_199780.1| UDP-glycosyltransferase-like protein [Arabidopsis thaliana] gi|75264223|sp|Q9LTA3.1|U91C1_ARATH RecName: Full=UDP-glycosyltransferase 91C1 gi|8978266|dbj|BAA98157.1| anthocyanidin-3-glucoside rhamnosyltransferase-like [Arabidopsis thaliana] gi|26449402|dbj|BAC41828.1| putative anthocyanidin-3-glucoside rhamnosyltransferase [Arabidopsis thaliana] gi|28951061|gb|AAO63454.1| At5g49690 [Arabidopsis thaliana] gi|332008462|gb|AED95845.1| UDP-glycosyltransferase 91C1 [Arabidopsis thaliana]	453	460	0	101.5	75.1	83.7	UDP-glycosyltransferase-like protein	gbpln	Arabidopsis thaliana	AT5G49690.1 | Symbols: | UDP-Glycosyltransferase superfamily protein | chr5:20189968-20191350 REVERSE LENGTH=460	453	460	0	101.5	75.1	83.7
Rsa1.0_00043.1.g2506.t1	gb|EMJ11389.1| hypothetical protein PRUPE_ppa017790mg [Prunus persica]	1466	1485	0	101.3	41.3	54.4	hypothetical protein PRUPE_ppa017790mg	gbpln	Prunus persica	ATMG00860.1 | Symbols: ORF158 | DNA/RNA polymerases superfamily protein | chrM:235916-236392 FORWARD LENGTH=158	1466	158	7.00E-28	10.8	3.8	5.5
Rsa1.0_00043.1.g2507.t1	ref|XP_002893492.1| hypothetical protein ARALYDRAFT_890322 [Arabidopsis lyrata subsp. lyrata] gi|297339334|gb|EFH69751.1| hypothetical protein ARALYDRAFT_890322 [Arabidopsis lyrata subsp. lyrata]	334	322	1.00E-81	96.4	49.1	68.3	hypothetical protein ARALYDRAFT_890322	gbpln	Arabidopsis lyrata	AT1G61470.1 | Symbols: | Polynucleotidyl transferase, ribonuclease H-like superfamily protein | chr1:22678185-22679021 FORWARD LENGTH=278	334	278	4.00E-78	83.2	41.6	55.7
Rsa1.0_00043.1.g2508.t1	ref|XP_002891017.1| hypothetical protein ARALYDRAFT_473474 [Arabidopsis lyrata subsp. lyrata] gi|297336859|gb|EFH67276.1| hypothetical protein ARALYDRAFT_473474 [Arabidopsis lyrata subsp. lyrata]	78	82	7.00E-20	105.1	73.1	80.8	hypothetical protein ARALYDRAFT_473474	gbpln	Arabidopsis lyrata	AT1G32928.1 | Symbols: | unknown protein; BEST Arabidopsis thaliana protein match is: unknown protein (TAIR:AT1G32920.1); Has 35333 Blast hits to 34131 proteins in 2444 species: Archae - 798; Bacteria - 22429; Metazoa - 974; Fungi - 991; Plants - 531; Viruses - 0; Other Eukaryotes - 9610 (source: NCBI BLink). | chr1:11931332-11931586 FORWARD LENGTH=84	78	84	2.00E-22	107.7	75.6	82.1
Rsa1.0_00043.1.g2509.t1	#	#	#	#	#	#	#	-	----	----	#	#	#	#	#	#	#
Rsa1.0_00043.1.g2510.t1	gb|EOA37890.1| hypothetical protein CARUB_v10009358mg [Capsella rubella]	79	397	5.00E-18	502.5	63.3	74.7	hypothetical protein CARUB_v10009358mg	gbpln	Capsella rubella	AT1G32930.1 | Symbols: | Galactosyltransferase family protein | chr1:11931980-11934399 REVERSE LENGTH=399	79	399	6.00E-20	505.1	63.3	73.4
Rsa1.0_00043.1.g2511.t1	ref|XP_002872503.1| predicted protein [Arabidopsis lyrata subsp. lyrata] gi|297318340|gb|EFH48762.1| predicted protein [Arabidopsis lyrata subsp. lyrata]	86	89	1.00E-30	103.5	76.7	86.0	predicted protein	gbpln	Arabidopsis lyrata	AT4G10270.1 | Symbols: | Wound-responsive family protein | chr4:6374805-6375077 FORWARD LENGTH=90	86	90	1.00E-29	104.7	74.4	82.6
Rsa1.0_00043.1.g2512.t1	#	#	#	#	#	#	#	-	----	----	#	#	#	#	#	#	#
Rsa1.0_00043.1.g2513.t1	gb|EOA35150.1| hypothetical protein CARUB_v10020289mg, partial [Capsella rubella]	556	449	1.00E-139	80.8	45.5	52.0	hypothetical protein CARUB_v10020289mg, partial	gbpln	Capsella rubella	AT1G73810.1 | Symbols: | Core-2/I-branching beta-1,6-N-acetylglucosaminyltransferase family protein | chr1:27752506-27755208 REVERSE LENGTH=418	556	418	1.00E-138	75.2	45.3	50.5
Rsa1.0_00043.1.g2514.t1	ref|NP_174606.2| tetratricopeptide repeat-containing protein [Arabidopsis thaliana] gi|332193468|gb|AEE31589.1| tetratricopeptide repeat-containing protein [Arabidopsis thaliana]	832	798	0	95.9	78.4	85.9	tetratricopeptide repeat-containing protein	gbpln	Arabidopsis thaliana	AT1G33400.1 | Symbols: TPR9 | Tetratricopeptide repeat (TPR)-like superfamily protein | chr1:12104891-12109488 REVERSE LENGTH=798	832	798	0	95.9	78.4	85.9
Rsa1.0_00043.1.g2515.t1	ref|NP_001185129.1| suppressor of auxin resistance1 protein [Arabidopsis thaliana] gi|332193470|gb|AEE31591.1| suppressor of auxin resistance1 protein [Arabidopsis thaliana]	1468	1504	0	102.5	82.2	89.1	suppressor of auxin resistance1 protein	gbpln	Arabidopsis thaliana	AT1G33410.2 | Symbols: SAR1 | SUPPRESSOR OF AUXIN RESISTANCE1 | chr1:12109769-12118828 REVERSE LENGTH=1504	1468	1504	0	102.5	82.2	89.1
Rsa1.0_00043.1.g2516.t1	#	#	#	#	#	#	#	-	----	----	#	#	#	#	#	#	#
Rsa1.0_00043.1.g2517.t1	gb|EOA37671.1| hypothetical protein CARUB_v10012280mg [Capsella rubella]	720	704	0	97.8	76.8	84.4	hypothetical protein CARUB_v10012280mg	gbpln	Capsella rubella	AT1G33420.1 | Symbols: | RING/FYVE/PHD zinc finger superfamily protein | chr1:12121063-12123346 REVERSE LENGTH=697	720	697	0	96.8	77.1	83.5
Rsa1.0_00043.1.g2518.t1	ref|XP_002885337.1| hypothetical protein ARALYDRAFT_898378 [Arabidopsis lyrata subsp. lyrata] gi|297331177|gb|EFH61596.1| hypothetical protein ARALYDRAFT_898378 [Arabidopsis lyrata subsp. lyrata]	374	392	1.00E-104	104.8	55.6	73.0	hypothetical protein ARALYDRAFT_898378	gbpln	Arabidopsis lyrata	AT3G20030.1 | Symbols: | F-box and associated interaction domains-containing protein | chr3:6990254-6991462 FORWARD LENGTH=402	374	402	3.00E-97	107.5	55.6	70.3
Rsa1.0_00043.1.g2519.t1	#	#	#	#	#	#	#	-	----	----	#	#	#	#	#	#	#

Rsa1.0_00043.1.g2520.t1	gb|EOA37666.1| hypothetical protein CARUB_v10012254mg [Capsella rubella]	389	401	0	103.1	94.9	97.2	hypothetical protein CARUB_v10012254mg	gbpln	Capsella rubella	AT1G33430.1 | Symbols: | Galactosyltransferase family protein | chr1:12124438-12126052 REVERSE LENGTH=395	389	395	0	101.5	93.8	96.9
Rsa1.0_00043.1.g2521.t1	dbj|BAE79546.1| carotenoid isomerase [Chrysanthemum x morifolium]	66	641	2.00E-30	971.2	92.4	98.5	carotenoid isomerase	gbpln	Chrysanthemum x	AT1G06820.1 | Symbols: CRTISO, CCR2 | carotenoid isomerase | chr1:2093145-2096220 REVERSE LENGTH=595	66	595	3.00E-25	901.5	72.7	75.8
Rsa1.0_00044.1.g2522.t1	gb|EOA20241.1| hypothetical protein CARUB_v10000542mg [Capsella rubella]	584	588	0	100.7	94.5	97.3	hypothetical protein CARUB_v10000542mg	gbpln	Capsella rubella	AT5G25880.1 | Symbols: ATNADP-ME3, NADP-ME3 | NADP-malic enzyme 3 | chr5:9024549-9028260 FORWARD LENGTH=588	584	588	0	100.7	93.5	96.6
Rsa1.0_00044.1.g2523.t1	#	#	#	#	#	#	#	-	----	----	#	#	#	#	#	#	#
Rsa1.0_00044.1.g2524.t1	ref|NP_568478.1| auxin-responsive protein IAA28 [Arabidopsis thaliana] gi|11131406|sp|Q9XFM0.1|IAA28_ARATH RecName: Full=Auxin-responsive protein IAA28; AltName: Full=Indoleacetic acid-induced protein 28 gi|4929493|gb|AAD34019.1|AF149816_1 IAA28 [Arabidopsis thaliana] gi|23306354|gb|AAN17404.1| putative protein [Arabidopsis thaliana] gi|25084149|gb|AAN72186.1| putative protein [Arabidopsis thaliana] gi|49616373|gb|AAT67083.1| IAA28 [Arabidopsis thaliana] gi|110736579|dbj|BAF00255.1| IAA28 [Arabidopsis thaliana] gi|284794587|gb|ADB93666.1| indole-3-acetic acid inducible 28 [Arabidopsis thaliana] gi|284794589|gb|ADB93667.1| indole-3-acetic acid inducible 28 [Arabidopsis thaliana] gi|284794591|gb|ADB93668.1| indole-3-acetic acid inducible 28 [Arabidopsis thaliana] gi|304322548|gb|ADL70761.1| indole-3-acetic acid inducible 28 [Arabidopsis thaliana] gi|304322560|gb|ADL70767.1| indole-3-acetic acid inducible 28 [Arabidopsis thaliana] gi|304322566|gb|ADL70770.1| indole-3-acetic acid inducible 28 [Arabidopsis thaliana] gi|304322570|gb|ADL70772.1| indole-3-acetic acid inducible 28 [Arabidopsis	174	175	2.00E-77	100.6	89.7	93.1	auxin-responsive protein IAA28	gbpln	Arabidopsis thaliana	AT5G25890.1 | Symbols: IAA28, IAR2 | indole-3-acetic acid inducible 28 | chr5:9033480-9034554 FORWARD LENGTH=175	174	175	6.00E-80	100.6	89.7	93.1
Rsa1.0_00044.1.g2525.t1	dbj|BAJ33740.1| unnamed protein product [Thellungiella halophila]	506	506	0	100.0	91.3	95.3	unnamed protein product	----	----	AT5G25900.1 | Symbols: GA3, CYP701A3, ATKO1 | GA requiring 3 | chr5:9036073-9038278 FORWARD LENGTH=509	506	509	0	100.6	89.7	95.3
Rsa1.0_00044.1.g2526.t1	ref|XP_002872190.1| leucine-rich repeat family protein [Arabidopsis lyrata subsp. lyrata] gi|297318027|gb|EFH48449.1| leucine-rich repeat family protein [Arabidopsis lyrata subsp. lyrata]	1005	1005	0	100.0	81.7	87.7	leucine-rich repeat family protein	gbpln	Arabidopsis lyrata	AT5G25930.1 | Symbols: | Protein kinase family protein with leucine-rich repeat domain | chr5:9050880-9053978 FORWARD LENGTH=1005	1005	1005	0	100.0	81.1	87.3
Rsa1.0_00044.1.g2527.t1	ref|NP_197965.1| Protein kinase family protein with leucine-rich repeat domain [Arabidopsis thaliana] gi|5107831|gb|AAD40144.1|AF149413_25 contains similarity to protein kinase domains (Pfam F00069, Score=162.6, E=6.8e-45, N=1) and leucien rich repeats (Pfam PF00560, Score=210.7, E=2.2e-59, N=10) [Arabidopsis thaliana] gi|28393326|gb|AAO42089.1| putative receptor protein kinase [Arabidopsis thaliana] gi|224589685|gb|ACN59374.1| leucine-rich repeat receptor-like protein kinase [Arabidopsis thaliana] gi|332006119|gb|AED93502.1| Protein kinase family protein with leucine-rich repeat domain [Arabidopsis thaliana]	371	1005	2.00E-56	270.9	29.4	35.3	Protein kinase family protein with leucine-rich repeat domain	gbpln	Arabidopsis thaliana	AT5G25930.1 | Symbols: | Protein kinase family protein with leucine-rich repeat domain | chr5:9050880-9053978 FORWARD LENGTH=1005	371	1005	5.00E-59	270.9	29.4	35.3
Rsa1.0_00044.1.g2528.t1	ref|XP_002874288.1| hypothetical protein ARALYDRAFT_351609 [Arabidopsis lyrata subsp. lyrata] gi|297320125|gb|EFH50547.1| hypothetical protein ARALYDRAFT_351609 [Arabidopsis lyrata subsp. lyrata]	412	413	0	100.2	79.4	87.6	hypothetical protein ARALYDRAFT_351609	gbpln	Arabidopsis lyrata	AT5G25950.1 | Symbols: | Protein of Unknown Function (DUF239) | chr5:9057929-9059860 REVERSE LENGTH=432	412	432	0	104.9	75.5	86.4
Rsa1.0_00044.1.g2529.t1	gb|EOA19883.1| hypothetical protein CARUB_v10000132mg [Capsella rubella]	160	1006	1.00E-55	628.8	66.3	78.1	hypothetical protein CARUB_v10000132mg	gbpln	Capsella rubella	AT5G25930.1 | Symbols: | Protein kinase family protein with leucine-rich repeat domain | chr5:9050880-9053978 FORWARD LENGTH=1005	160	1005	1.00E-57	628.1	65.0	78.1

Rsa1.0_00044.1.g2530.t1	ref|XP_002874288.1| hypothetical protein ARALYDRAFT_351609 [Arabidopsis lyrata subsp. lyrata] gi|297320125|gb|EFH50547.1| hypothetical protein ARALYDRAFT_351609 [Arabidopsis lyrata subsp. lyrata]	412	413	0	100.2	79.6	89.3	hypothetical protein ARALYDRAFT_351609	gbpln	Arabidopsis lyrata	AT5G25950.1 | Symbols: | Protein of Unknown Function (DUF239) | chr5:9057929-9059860 REVERSE LENGTH=432	412	432	0	104.9	74.0	86.2
Rsa1.0_00044.1.g2531.t1	ref|XP_002872192.1| hypothetical protein ARALYDRAFT_910666 [Arabidopsis lyrata subsp. lyrata] gi|297318029|gb|EFH48451.1| hypothetical protein ARALYDRAFT_910666 [Arabidopsis lyrata subsp. lyrata]	448	386	0	86.2	73.7	79.0	hypothetical protein ARALYDRAFT_910666	gbpln	Arabidopsis lyrata	AT5G25970.1 | Symbols: | Core-2/I-branching beta-1,6-N-acetylglucosaminyltransferase family protein | chr5:9066697-9067974 FORWARD LENGTH=387	448	387	0	86.4	72.5	78.1
Rsa1.0_00044.1.g2532.t1	gb|EOA23016.1| hypothetical protein CARUB_v10003772mg [Capsella rubella]	338	331	1.00E-157	97.9	82.0	89.1	hypothetical protein CARUB_v10003772mg	gbpln	Capsella rubella	AT5G26010.1 | Symbols: | Protein phosphatase 2C family protein | chr5:9085512-9087372 REVERSE LENGTH=331	338	331	1.00E-158	97.9	83.7	89.6
Rsa1.0_00044.1.g2533.t1	ref|XP_002872194.1| ferrochelatase I [Arabidopsis lyrata subsp. lyrata] gi|297318031|gb|EFH48453.1| ferrochelatase I [Arabidopsis lyrata subsp. lyrata]	466	466	0	100.0	86.9	91.4	ferrochelatase I	gbpln	Arabidopsis lyrata	AT5G26030.2 | Symbols: FC1, FC-I, ATFC-I | ferrochelatase 1 | chr5:9096675-9098752 FORWARD LENGTH=466	466	466	0	100.0	85.4	91.2
Rsa1.0_00044.1.g2534.t1	ref|NP_568480.2| histone deacetylase 2 [Arabidopsis thaliana] gi|145558941|sp|Q944K3.2|HDA2_ARATH RecName: Full=Histone deacetylase 2 gi|332006134|gb|AED93517.1| histone deacetylase 2 [Arabidopsis thaliana]	349	387	0	110.9	88.8	93.4	histone deacetylase 2	gbpln	Arabidopsis thaliana	AT5G26040.2 | Symbols: HDA2 | histone deacetylase 2 | chr5:9099321-9101598 REVERSE LENGTH=387	349	387	0	110.9	88.8	93.4
Rsa1.0_00044.1.g2535.t1	gb|AAD40130.1|AF149413_11 T1N24.10 gene product [Arabidopsis thaliana]	157	375	5.00E-69	238.9	80.3	90.4	T1N24.10 gene product	gbpln	Arabidopsis thaliana	AT5G26050.1 | Symbols: | Plant self-incompatibility protein S1 family | chr5:9103395-9103877 REVERSE LENGTH=160	157	160	3.00E-70	101.9	80.3	90.4
Rsa1.0_00044.1.g2536.t1	ref|NP_568481.1| self-incompatibility S1 family protein [Arabidopsis thaliana] gi|51969422|dbj|BAD43403.1| unknown protein [Arabidopsis thaliana] gi|51969814|dbj|BAD43599.1| unknown protein [Arabidopsis thaliana] gi|91806910|gb|ABE66182.1| S1 self-incompatibility protein-like protein [Arabidopsis thaliana] gi|332006136|gb|AED93519.1| self-incompatibility S1 family protein [Arabidopsis thaliana]	155	154	2.00E-70	99.4	79.4	92.3	self-incompatibility S1 family protein	gbpln	Arabidopsis thaliana	AT5G26060.1 | Symbols: | Plant self-incompatibility protein S1 family | chr5:9105013-9105477 REVERSE LENGTH=154	155	154	5.00E-73	99.4	79.4	92.3
Rsa1.0_00044.1.g2537.t1	ref|NP_197979.1| hydroxyproline-rich glycoprotein family protein [Arabidopsis thaliana] gi|67633826|gb|AAY78837.1| hydroxyproline-rich glycoprotein family protein [Arabidopsis thaliana] gi|332006137|gb|AED93520.1| hydroxyproline-rich glycoprotein family protein [Arabidopsis thaliana]	166	102	6.00E-13	61.4	47.0	49.4	hydroxyproline-rich glycoprotein family protein	gbpln	Arabidopsis thaliana	AT5G26070.1 | Symbols: | hydroxyproline-rich glycoprotein family protein | chr5:9106341-9106649 FORWARD LENGTH=102	166	102	2.00E-15	61.4	47.0	49.4
Rsa1.0_00044.1.g2538.t1	gb|EOA19542.1| hypothetical protein CARUB_v10002514mg [Capsella rubella]	194	398	1.00E-68	205.2	64.4	71.6	hypothetical protein CARUB_v10002514mg	gbpln	Capsella rubella	AT5G26090.1 | Symbols: | Plant self-incompatibility protein S1 family | chr5:9109026-9113235 REVERSE LENGTH=401	194	401	3.00E-68	206.7	59.8	68.6
Rsa1.0_00044.1.g2539.t1	ref|XP_002886410.1| ATP binding protein [Arabidopsis lyrata subsp. lyrata] gi|297332251|gb|EFH62669.1| ATP binding protein [Arabidopsis lyrata subsp. lyrata]	386	226	1.00E-110	58.5	51.0	53.4	ATP binding protein	gbpln	Arabidopsis lyrata	AT5G26110.1 | Symbols: | Protein kinase superfamily protein | chr5:9118239-9119352 REVERSE LENGTH=226	386	226	1.00E-112	58.5	51.0	52.8
Rsa1.0_00044.1.g2540.t1	emb|CAB39638.1| RNA-directed DNA polymerase-like protein [Arabidopsis thaliana] gi|7267666|emb|CAB78094.1| RNA-directed DNA polymerase-like protein [Arabidopsis thaliana]	111	1274	3.00E-24	1147.7	49.5	63.1	RNA-directed DNA polymerase-like protein	gbpln	Arabidopsis thaliana	#	#	#	#	#	#	#
Rsa1.0_00044.1.g2541.t1	ref|NP_197988.3| uncharacterized protein [Arabidopsis thaliana] gi|30793967|gb|AAP40435.1| unknown protein [Arabidopsis thaliana] gi|110736920|dbj|BAF00417.1| hypothetical protein [Arabidopsis thaliana] gi|332006148|gb|AED93531.1| uncharacterized protein AT5G26160 [Arabidopsis thaliana]	926	976	0	105.4	74.4	83.2	uncharacterized protein	gbpln	Arabidopsis thaliana	AT5G26160.1 | Symbols: | unknown protein; BEST Arabidopsis thaliana protein match is: unknown protein (TAIR:AT5G20610.1); Has 197 Blast hits to 158 proteins in 44 species: Archae - 0; Bacteria - 14; Metazoa - 28; Fungi - 15; Plants - 117; Viruses - 2; Other Eukaryotes - 21 (source: NCBI BLink). | chr5:9143269-9146312 FORWARD LENGTH=976	926	976	0	105.4	74.4	83.2

Rsa1.0_00044.1.g2542.t1	ref|NP_197989.2| putative WRKY transcription factor 50 [Arabidopsis thaliana] gi|29839580|sp|Q8VWQ5.1|WRK50_ARATH RecName: Full=Probable WRKY transcription factor 50; AltName: Full=WRKY DNA-binding protein 50 gi|18252117|gb|AAL61857.1| WRKY transcription factor 50 [Arabidopsis thaliana] gi|225898933|dbj|BAH30597.1| hypothetical protein [Arabidopsis thaliana] gi|332006149|gb|AED93532.1| putative WRKY transcription factor 50 [Arabidopsis thaliana]	177	173	3.00E-67	97.7	78.0	85.3	putative WRKY transcription factor 50	gbpln	Arabidopsis thaliana	AT5G26170.1 | Symbols: WRKY50, ATWRKY50 | WRKY DNA-binding protein 50 | chr5:9147176-9148128 REVERSE LENGTH=173	177	173	1.00E-69	97.7	78.0	85.3
Rsa1.0_00044.1.g2543.t1	#	#	#	#	#	#	#	-	----	----	#	#	#	#	#	#	#
Rsa1.0_00044.1.g2544.t1	gb|EOA22853.1| hypothetical protein CARUB_v10003583mg [Capsella rubella]	340	344	1.00E-174	101.2	87.9	94.7	hypothetical protein CARUB_v10003583mg	gbpln	Capsella rubella	AT5G26200.1 | Symbols: | Mitochondrial substrate carrier family protein | chr5:9157268-9158296 FORWARD LENGTH=342	340	342	1.00E-174	100.6	86.5	94.7
Rsa1.0_00044.1.g2545.t1	ref|XP_002516111.1| DNA binding protein, putative [Ricinus communis] gi|223544597|gb|EEF46113.1| DNA binding protein, putative [Ricinus communis]	260	251	1.00E-107	96.5	79.6	88.5	DNA binding protein, putative	gbpln	Ricinus communis	AT5G26210.1 | Symbols: AL4 | alfin-like 4 | chr5:9158566-9160221 REVERSE LENGTH=255	260	255	5.00E-97	98.1	88.8	93.5
Rsa1.0_00044.1.g2546.t1	ref|NP_198719.1| putative receptor-like protein kinase [Arabidopsis thaliana] gi|75333904|sp|Q9FID5.1|Y5393_ARATH RecName: Full=Probable receptor-like protein kinase At5g39030; Flags: Precursor gi|10177548|dbj|BAB10827.1| receptor protein kinase-like protein [Arabidopsis thaliana] gi|332007006|gb|AED94389.1| putative receptor-like protein kinase [Arabidopsis thaliana]	780	806	0	103.3	64.5	76.4	putative receptor-like protein kinase	gbpln	Arabidopsis thaliana	AT5G39030.1 | Symbols: | Protein kinase superfamily protein | chr5:15620066-15622486 FORWARD LENGTH=806	780	806	0	103.3	64.5	76.4
Rsa1.0_00044.1.g2547.t1	#	#	#	#	#	#	#	-	----	----	#	#	#	#	#	#	#
Rsa1.0_00044.1.g2548.t1	ref|NP_197995.1| uncharacterized protein [Arabidopsis thaliana] gi|122214260|sp|Q3E936.1|MAKR1_ARATH RecName: Full=Probable membrane-associated kinase regulator 1 gi|332006156|gb|AED93539.1| uncharacterized protein AT5G26230 [Arabidopsis thaliana]	331	341	1.00E-105	103.0	78.5	85.8	uncharacterized protein	gbpln	Arabidopsis thaliana	AT5G26230.1 | Symbols: | unknown protein; FUNCTIONS IN: molecular_function unknown; INVOLVED IN: biological_process unknown; LOCATED IN: chloroplast; EXPRESSED IN: 22 plant structures; EXPRESSED DURING: 13 growth stages; Has 1807 Blast hits to 1807 proteins in 277 species: Archae - 0; Bacteria - 0; Metazoa - 736; Fungi - 347; Plants - 385; Viruses - 0; Other Eukaryotes - 339 (source: NCBI BLink). | chr5:9173517-9174542 REVERSE LENGTH=341	331	341	1.00E-108	103.0	78.5	85.8
Rsa1.0_00044.1.g2549.t1	#	#	#	#	#	#	#	-	----	----	#	#	#	#	#	#	#
Rsa1.0_00044.1.g2550.t1	ref|NP_197996.1| chloride channel protein CLC-d [Arabidopsis thaliana] gi|41688458|sp|P92943.2|CLCD_ARATH RecName: Full=Chloride channel protein CLC-d; Short=AtCLC-d; AltName: Full=CBS domain-containing protein CBSCLC2 gi|332006157|gb|AED93540.1| chloride channel protein CLC-d [Arabidopsis thaliana]	793	792	0	99.9	96.3	97.9	chloride channel protein CLC-d	gbpln	Arabidopsis thaliana	AT5G26240.1 | Symbols: CLC-D, ATCLC-D | chloride channel D | chr5:9189622-9194347 FORWARD LENGTH=792	793	792	0	99.9	96.3	97.9
Rsa1.0_00044.1.g2551.t1	gb|EOA22969.1| hypothetical protein CARUB_v10003709mg [Capsella rubella]	499	507	0	101.6	83.6	92.0	hypothetical protein CARUB_v10003709mg	gbpln	Capsella rubella	AT5G26250.1 | Symbols: | Major facilitator superfamily protein | chr5:9196758-9198681 FORWARD LENGTH=507	499	507	0	101.6	83.6	92.0
Rsa1.0_00044.1.g2552.t1	ref|XP_002872211.1| hypothetical protein ARALYDRAFT_489472 [Arabidopsis lyrata subsp. lyrata] gi|297318048|gb|EFH48470.1| hypothetical protein ARALYDRAFT_489472 [Arabidopsis lyrata subsp. lyrata]	350	350	1.00E-174	100.0	86.0	89.7	hypothetical protein ARALYDRAFT_489472	gbpln	Arabidopsis lyrata	AT5G26280.1 | Symbols: | TRAF-like family protein | chr5:9208724-9210403 FORWARD LENGTH=350	350	350	1.00E-176	100.0	85.4	90.0
Rsa1.0_00044.1.g2553.t3	ref|XP_002872211.1| hypothetical protein ARALYDRAFT_489472 [Arabidopsis lyrata subsp. lyrata] gi|297318048|gb|EFH48470.1| hypothetical protein ARALYDRAFT_489472 [Arabidopsis lyrata subsp. lyrata]	357	350	1.00E-158	98.0	79.0	85.2	hypothetical protein ARALYDRAFT_489472	gbpln	Arabidopsis lyrata	AT5G26280.1 | Symbols: | TRAF-like family protein | chr5:9208724-9210403 FORWARD LENGTH=350	357	350	1.00E-157	98.0	76.8	84.6

Rsa1.0_00044.1.g2554.t1	ref|NP_198006.1| sugar transport protein 13 [Arabidopsis thaliana] gi|85701281|sp|Q94AZ2.2|STP13_ARATH RecName: Full=Sugar transport protein 13; AltName: Full=Hexose transporter 13; AltName: Full=Multicopy suppressor of snf4 deficiency protein 1 gi|9965739|gb|AAG10146.1|AF250340_1 putative hexose transporter MSS1 [Arabidopsis thaliana] gi|3319354|gb|AAC26243.1| contains similarity to sugar transporters (Pfam: sugar_tr.hmm, score: 395.39) [Arabidopsis thaliana] gi|15450649|gb|AAK96596.1| AT5g26340/F9D12_17 [Arabidopsis thaliana] gi|15487258|emb|CAC69074.1| STP13 protein [Arabidopsis thaliana] gi|332006170|gb|AED93553.1| sugar transport protein 13 [Arabidopsis thaliana]	349	526	1.00E-153	150.7	76.5	81.4	sugar transport protein 13	gbpln	Arabidopsis thaliana	AT5G26340.1 | Symbols: MSS1, STP13, ATSTP13 | Major facilitator superfamily protein | chr5:9243851-9246994 REVERSE LENGTH=526	349	526	1.00E-155	150.7	76.5	81.4
Rsa1.0_00044.1.g2555.t1	gb|EOA20395.1| hypothetical protein CARUB_v10000707mg [Capsella rubella]	196	525	6.00E-92	267.9	88.8	92.9	hypothetical protein CARUB_v10000707mg	gbpln	Capsella rubella	AT5G26340.1 | Symbols: MSS1, STP13, ATSTP13 | Major facilitator superfamily protein | chr5:9243851-9246994 REVERSE LENGTH=526	196	526	3.00E-92	268.4	85.7	90.3
Rsa1.0_00044.1.g2556.t8	gb|AAD21778.1| putative non-LTR retroelement reverse transcriptase [Arabidopsis thaliana]	1831	1715	0	93.7	32.4	46.5	putative non-LTR retroelement reverse transcriptase	gbpln	Arabidopsis thaliana	AT5G26667.3 | Symbols: PYR6 | P-loop containing nucleoside triphosphate hydrolases superfamily protein | chr5:9276659-9278091 FORWARD LENGTH=202	1831	202	1.00E-105	11.0	10.3	10.6
Rsa1.0_00044.1.g2557.t1	emb|CAA09196.1| RNA helicase [Arabidopsis thaliana]	813	748	0	92.0	83.8	87.2	RNA helicase	gbpln	Arabidopsis thaliana	AT5G26742.2 | Symbols: emb1138 | DEAD box RNA helicase (RH3) | chr5:9285540-9288871 REVERSE LENGTH=748	813	748	0	92.0	83.8	87.2
Rsa1.0_00044.1.g2558.t1	gb|EOA20719.1| hypothetical protein CARUB_v10001039mg [Capsella rubella]	420	422	0	100.5	92.6	94.5	hypothetical protein CARUB_v10001039mg	gbpln	Capsella rubella	AT5G26740.3 | Symbols: | Protein of unknown function (DUF300) | chr5:9292436-9294407 FORWARD LENGTH=422	420	422	0	100.5	91.7	94.0
Rsa1.0_00044.1.g2559.t1	gb|AAM64547.1| unknown [Arabidopsis thaliana]	101	99	6.00E-22	98.0	60.4	64.4	unknown	gbpln	Arabidopsis thaliana	AT5G26731.1 | Symbols: | unknown protein; Has 30201 Blast hits to 17322 proteins in 780 species: Archae - 12; Bacteria - 1396; Metazoa - 17338; Fungi - 3422; Plants - 5037; Viruses - 0; Other Eukaryotes - 2996 (source: NCBI BLink). | chr5:9295676-9295975 FORWARD LENGTH=99	101	99	1.00E-24	98.0	60.4	64.4
Rsa1.0_00044.1.g2560.t1	gb|EOA19490.1| hypothetical protein CARUB_v10002163mg, partial [Capsella rubella]	100	158	3.00E-37	158.0	80.0	93.0	hypothetical protein CARUB_v10002163mg, partial	gbpln	Capsella rubella	AT3G05936.1 | Symbols: | unknown protein; FUNCTIONS IN: molecular_function unknown; INVOLVED IN: biological_process unknown; LOCATED IN: cellular_component unknown; Has 30201 Blast hits to 17322 proteins in 780 species: Archae - 12; Bacteria - 1396; Metazoa - 17338; Fungi - 3422; Plants - 5037; Viruses - 0; Other Eukaryotes - 2996 (source: NCBI BLink). | chr3:1774062-1774403 REVERSE LENGTH=113	100	113	1.00E-28	113.0	61.0	75.0
Rsa1.0_00044.1.g2561.t1	ref|NP_850872.1| Fasciclin-like arabinogalactan family protein [Arabidopsis thaliana] gi|332006188|gb|AED93571.1| Fasciclin-like arabinogalactan family protein [Arabidopsis thaliana]	105	268	3.00E-40	255.2	89.5	95.2	Fasciclin-like arabinogalactan family protein	gbpln	Arabidopsis thaliana	AT5G26730.1 | Symbols: | Fasciclin-like arabinogalactan family protein | chr5:9300422-9301990 FORWARD LENGTH=268	105	268	4.00E-43	255.2	89.5	95.2
Rsa1.0_00044.1.g2562.t1	ref|XP_002872222.1| hypothetical protein ARALYDRAFT_351658 [Arabidopsis lyrata subsp. lyrata] gi|297318059|gb|EFH48481.1| hypothetical protein ARALYDRAFT_351658 [Arabidopsis lyrata subsp. lyrata]	147	285	1.00E-52	193.9	66.7	85.0	hypothetical protein ARALYDRAFT_351658	gbpln	Arabidopsis lyrata	AT5G26730.1 | Symbols: | Fasciclin-like arabinogalactan family protein | chr5:9300422-9301990 FORWARD LENGTH=268	147	268	7.00E-55	182.3	66.7	85.0
Rsa1.0_00044.1.g2563.t2	ref|NP_850873.1| uncharacterized protein [Arabidopsis thaliana] gi|26451568|dbj|BAC42881.1| unknown protein [Arabidopsis thaliana] gi|28827232|gb|AAO50460.1| unknown protein [Arabidopsis thaliana] gi|332006189|gb|AED93572.1| uncharacterized protein AT5G26720 [Arabidopsis thaliana]	376	134	3.00E-25	35.6	23.4	28.5	uncharacterized protein	gbpln	Arabidopsis thaliana	AT5G26720.1 | Symbols: | unknown protein; Has 15 Blast hits to 15 proteins in 5 species: Archae - 0; Bacteria - 0; Metazoa - 0; Fungi - 0; Plants - 15; Viruses - 0; Other Eukaryotes - 0 (source: NCBI BLink). | chr5:9304105-9304509 FORWARD LENGTH=134	376	134	7.00E-28	35.6	23.4	28.5
Rsa1.0_00044.1.g2564.t1	gb|AAM61454.1| unknown [Arabidopsis thaliana]	126	126	1.00E-28	100.0	56.3	73.0	unknown	gbpln	Arabidopsis thaliana	AT3G05920.1 | Symbols: | Heavy metal transport/detoxification superfamily protein | chr3:1768991-1769522 REVERSE LENGTH=126	126	126	3.00E-31	100.0	56.3	73.0

Rsa1.0_00044.1.g2565.t1	ref|XP_002874315.1| hypothetical protein ARALYDRAFT_489493 [Arabidopsis lyrata subsp. lyrata] gi|297320152|gb|EFH50574.1| hypothetical protein ARALYDRAFT_489493 [Arabidopsis lyrata subsp. lyrata]	417	448	0	107.4	90.9	95.2	hypothetical protein ARALYDRAFT_489493	gbpln	Arabidopsis lyrata	AT5G26680.2 | Symbols: | 5'-3' exonuclease family protein | chr5:9311882-9315458 REVERSE LENGTH=383	417	383	0	91.8	82.5	85.4
Rsa1.0_00044.1.g2566.t1	ref|NP_850878.2| Pectinacetylesterase family protein [Arabidopsis thaliana] gi|51536518|gb|AAU05497.1| At5g26670 [Arabidopsis thaliana] gi|332006195|gb|AED93578.1| Pectinacetylesterase family protein [Arabidopsis thaliana]	416	416	0	100.0	92.8	96.6	Pectinacetylesterase family protein	gbpln	Arabidopsis thaliana	AT5G26670.1 | Symbols: | Pectinacetylesterase family protein | chr5:9318456-9320816 FORWARD LENGTH=416	416	416	0	100.0	92.8	96.6
Rsa1.0_00044.1.g2567.t1	gb|AAG50806.1|AC079281_8 unknown protein [Arabidopsis thaliana]	1755	1213	0	69.1	25.2	37.9	unknown protein	gbpln	Arabidopsis thaliana	AT1G43760.1 | Symbols: | DNAse I-like superfamily protein | chr1:16528880-16531065 REVERSE LENGTH=626	1755	626	2.00E-62	35.7	9.2	15.2
Rsa1.0_00044.1.g2568.t1	ref|NP_850879.1| transcription factor MYB86 [Arabidopsis thaliana] gi|56749347|sp|Q8LPH6.1|MYB86_ARATH RecName: Full=Transcription factor MYB86; AltName: Full=Myb homolog 4; Short=AtMyb4; AltName: Full=Myb-related protein 86; Short=AtMYB86 gi|20466622|gb|AAM20628.1| transcription factor ATMYB4 [Arabidopsis thaliana] gi|30984580|gb|AAP42753.1| At5g26655 [Arabidopsis thaliana] gi|41619424|gb|AAS10099.1| MYB transcription factor [Arabidopsis thaliana] gi|332006197|gb|AED93580.1| transcription factor MYB86 [Arabidopsis thaliana]	355	352	1.00E-159	99.2	85.9	90.4	transcription factor MYB86	gbpln	Arabidopsis thaliana	AT5G26660.1 | Symbols: ATMYB86, MYB86 | myb domain protein 86 | chr5:9331775-9333044 REVERSE LENGTH=352	355	352	1.00E-162	99.2	85.9	90.4
Rsa1.0_00044.1.g2569.t2	gb|AAD21687.1| Strong similarity to gi|3600044 T12H20.12 protease homolog from Arabidopsis thaliana BAC gb|AF080119 and is a member of the reverse transcriptase family PF|00078 [Arabidopsis thaliana]	1188	1415	0	119.1	37.4	44.9	Strong similarity to gi|3600044 T12H20.12 protease homolog from Arabidopsis thaliana BAC gb|AF080119 and is a member of the reverse transcriptase family PF|00078	gbpln	Arabidopsis thaliana	AT4G23160.1 | Symbols: CRK8 | cysteine-rich RLK (RECEPTOR-like protein kinase) 8 | chr4:12129485-12134086 FORWARD LENGTH=1262	1188	1262	9.00E-86	106.2	14.3	21.9
Rsa1.0_00044.1.g2570.t1	ref|NP_174444.1| agamous-like MADS-box protein AGL86 [Arabidopsis thaliana] gi|75333444|sp|Q9C6V3.1|AGL86_ARATH RecName: Full=Agamous-like MADS-box protein AGL86 gi|12597831|gb|AAG60141.1|AC074360_6 MADS-box protein, putative [Arabidopsis thaliana] gi|67633410|gb|AAY78630.1| MADS-box family protein [Arabidopsis thaliana] gi|332193257|gb|AEE31378.1| agamous-like MADS-box protein AGL86 [Arabidopsis thaliana]	364	339	7.00E-45	93.1	35.7	49.2	agamous-like MADS-box protein AGL86	gbpln	Arabidopsis thaliana	AT1G31630.1 | Symbols: AGL86 | AGAMOUS-like 86 | chr1:11318528-11319547 REVERSE LENGTH=339	364	339	2.00E-47	93.1	35.7	49.2
Rsa1.0_00044.1.g2571.t1	gb|EAY96485.1| hypothetical protein OsI_18385 [Oryza sativa Indica Group] gi|222630128|gb|EEE62260.1| hypothetical protein OsJ_17047 [Oryza sativa Japonica Group]	62	92	3.00E-19	148.4	77.4	83.9	hypothetical protein OsI_18385	gbpln	Oryza sativa	AT3G05880.1 | Symbols: RCI2A | Low temperature and salt responsive protein family | chr3:1756195-1756494 REVERSE LENGTH=54	62	54	2.00E-18	87.1	67.7	75.8
Rsa1.0_00044.1.g2572.t1	ref|XP_004287934.1| PREDICTED: anaphase-promoting complex subunit 11-like isoform 2 [Fragaria vesca subsp. vesca]	95	92	9.00E-42	96.8	87.4	92.6	PREDICTED: anaphase-promoting complex subunit 11-like isoform 2	gbpln	Fragaria vesca	AT3G05870.2 | Symbols: APC11 | anaphase-promoting complex/cyclosome 11 | chr3:1753961-1754802 FORWARD LENGTH=87	95	87	7.00E-44	91.6	88.4	90.5
Rsa1.0_00044.1.g2573.t1	gb|EOA19465.1| hypothetical protein CARUB_v10001920mg [Capsella rubella]	218	220	1.00E-48	100.9	51.8	68.3	hypothetical protein CARUB_v10001920mg	gbpln	Capsella rubella	AT5G26630.1 | Symbols: | MADS-box transcription factor family protein | chr5:9350815-9351471 FORWARD LENGTH=218	218	218	2.00E-47	100.0	48.6	66.5
Rsa1.0_00044.1.g2574.t1	ref|XP_002883989.1| hypothetical protein ARALYDRAFT_899939 [Arabidopsis lyrata subsp. lyrata] gi|297329829|gb|EFH60248.1| hypothetical protein ARALYDRAFT_899939 [Arabidopsis lyrata subsp. lyrata]	240	352	3.00E-33	146.7	32.5	44.6	hypothetical protein ARALYDRAFT_899939	gbpln	Arabidopsis lyrata	AT3G19515.2 | Symbols: | CONTAINS InterPro DOMAIN/s: Apoptosis inhibitory 5 (InterPro:IPR008383); BEST Arabidopsis thaliana protein match is: unknown protein (TAIR:AT1G23935.1); Has 546 Blast hits to 394 proteins in 84 species: Archae - 0; Bacteria - 3; Metazoa - 210; Fungi - 15; Plants - 143; Viruses - 0; Other Eukaryotes - 175 (source: NCBI BLink). | chr3:6766872-6771078 REVERSE LENGTH=805	240	805	2.00E-11	335.4	22.9	38.3
Rsa1.0_00044.1.g2575.t1	#	#	#	#	#	#	#	-	----	----	#	#	#	#	#	#	#

Rsa1.0_00044.1.g2576.t1	gb|ABE65560.1| hypothetical protein At5g26620 [Arabidopsis thaliana]	86	88	2.00E-35	102.3	90.7	94.2	hypothetical protein At5g26620	gbpln	Arabidopsis thaliana	AT3G05858.1 | Symbols: | unknown protein; FUNCTIONS IN: molecular_function unknown; INVOLVED IN: biological_process unknown; LOCATED IN: cellular_component unknown; BEST Arabidopsis thaliana protein match is: unknown protein (TAIR:AT5G26620.1); Has 30201 Blast hits to 17322 proteins in 780 species: Archae - 12; Bacteria - 1396; Metazoa - 17338; Fungi - 3422; Plants - 5037; Viruses - 0; Other Eukaryotes - 2996 (source: NCBI BLink). | chr3:1749464-1749736 FORWARD LENGTH=90	86	90	6.00E-30	104.7	80.2	86.0
Rsa1.0_00044.1.g2577.t1	ref|XP_002886053.1| hypothetical protein ARALYDRAFT_899963 [Arabidopsis lyrata subsp. lyrata] gi|297331893|gb|EFH62312.1| hypothetical protein ARALYDRAFT_899963 [Arabidopsis lyrata subsp. lyrata]	320	288	9.00E-49	90.0	36.9	50.0	hypothetical protein ARALYDRAFT_899963	gbpln	Arabidopsis lyrata	AT1G22090.1 | Symbols: emb2204 | Protein of unknown function (DUF626) | chr1:7795721-7797252 FORWARD LENGTH=329	320	329	3.00E-32	102.8	29.7	43.4
Rsa1.0_00044.1.g2578.t1	gb|EOA20562.1| hypothetical protein CARUB_v10000875mg [Capsella rubella]	475	473	0	99.6	83.8	90.5	hypothetical protein CARUB_v10000875mg	gbpln	Capsella rubella	AT5G26600.1 | Symbols: | Pyridoxal phosphate (PLP)-dependent transferases superfamily protein | chr5:9377455-9378882 FORWARD LENGTH=475	475	475	0	100.0	82.7	90.3
Rsa1.0_00044.1.g2579.t1	gb|EOA22823.1| hypothetical protein CARUB_v10003541mg [Capsella rubella]	406	409	0	100.7	96.6	97.3	hypothetical protein CARUB_v10003541mg	gbpln	Capsella rubella	AT5G26751.1 | Symbols: ATSK11, SK 11 | shaggy-related kinase 11 | chr5:9399582-9401839 REVERSE LENGTH=405	406	405	0	99.8	96.3	97.3
Rsa1.0_00044.1.g2580.t1	ref|NP_974839.1| uncharacterized protein [Arabidopsis thaliana] gi|380877125|sp|F4K1B1.1|RPAP2_ARATH RecName: Full=Putative RNA polymerase II subunit B1 CTD phosphatase RPAP2 homolog; AltName: Full=RNA polymerase II-associated protein 2 homolog gi|332006215|gb|AED93598.1| uncharacterized protein AT5G26760 [Arabidopsis thaliana]	82	735	1.00E-22	896.3	63.4	75.6	uncharacterized protein	gbpln	Arabidopsis thaliana	AT5G26760.2 | Symbols: | unknown protein; CONTAINS InterPro DOMAIN/s: Protein of unknown function DUF408 (InterPro:IPR007308); Has 35333 Blast hits to 34131 proteins in 2444 species: Archae - 798; Bacteria - 22429; Metazoa - 974; Fungi - 991; Plants - 531; Viruses - 0; Other Eukaryotes - 9610 (source: NCBI BLink). | chr5:9403869-9407170 REVERSE LENGTH=735	82	735	2.00E-25	896.3	63.4	75.6
Rsa1.0_00044.1.g2581.t1	ref|NP_568487.1| uncharacterized protein [Arabidopsis thaliana] gi|42573487|ref|NP_974840.1| uncharacterized protein [Arabidopsis thaliana] gi|332006216|gb|AED93599.1| uncharacterized protein AT5G26770 [Arabidopsis thaliana] gi|332006217|gb|AED93600.1| uncharacterized protein AT5G26770 [Arabidopsis thaliana]	331	335	1.00E-158	101.2	84.9	92.7	uncharacterized protein	gbpln	Arabidopsis thaliana	AT5G26770.1 | Symbols: | unknown protein; BEST Arabidopsis thaliana protein match is: unknown protein (TAIR:AT3G05830.1); Has 30201 Blast hits to 17322 proteins in 780 species: Archae - 12; Bacteria - 1396; Metazoa - 17338; Fungi - 3422; Plants - 5037; Viruses - 0; Other Eukaryotes - 2996 (source: NCBI BLink). | chr5:9407981-9409735 REVERSE LENGTH=335	331	335	1.00E-160	101.2	84.9	92.7
Rsa1.0_00045.1.g2582.t1	ref|XP_002874373.1| zinc finger family protein [Arabidopsis lyrata subsp. lyrata] gi|297320210|gb|EFH50632.1| zinc finger family protein [Arabidopsis lyrata subsp. lyrata]	358	370	1.00E-141	103.4	72.9	79.9	zinc finger family protein	gbpln	Arabidopsis lyrata	AT5G27420.1 | Symbols: CNI1, ATL31 | carbon/nitrogen insensitive 1 | chr5:9684119-9685225 FORWARD LENGTH=368	358	368	1.00E-138	102.8	70.7	77.7
Rsa1.0_00045.1.g2583.t1	ref|NP_001190410.1| branched-chain-amino-acid aminotransferase-like protein 2 [Arabidopsis thaliana] gi|332006301|gb|AED93684.1| branched-chain-amino-acid aminotransferase-like protein 2 [Arabidopsis thaliana]	528	936	0	177.3	83.3	90.9	branched-chain-amino-acid aminotransferase-like protein 2	gbpln	Arabidopsis thaliana	AT5G27410.2 | Symbols: | D-aminoacid aminotransferase-like PLP-dependent enzymes superfamily protein | chr5:9676362-9682468 FORWARD LENGTH=936	528	936	0	177.3	83.3	90.9
Rsa1.0_00045.1.g2584.t1	tpg|DAA63922.1| TPA: hypothetical protein ZEAMMB73_644873 [Zea mays]	92	194	1.00E-11	210.9	41.3	57.6	TPA: hypothetical protein ZEAMMB73_644873	gbenv/gbpln	Zea mays	AT5G27395.2 | Symbols: | Mitochondrial inner membrane translocase complex, subunit Tim44-related protein | chr5:9671845-9674085 REVERSE LENGTH=296	92	296	6.00E-14	321.7	40.2	41.3
Rsa1.0_00045.1.g2585.t1	sp|O23732.1|GSHB_BRAJU RecName: Full=Glutathione synthetase, chloroplastic; Short=GSH synthetase; Short=GSH-S; Short=Glutathione synthase; Flags: Precursor gi|2243118|emb|CAA71878.1| glutathione synthetase [Brassica juncea]	530	530	0	100.0	94.0	95.8	RecName: Full=Glutathione synthetase, chloroplastic; Short=GSH synthetase; Short=GSH-S; Short=Glutathione synthase; Flags: Precursor gi|2243118|emb|CAA71878.1| glutathione synthetase	gbpln	Brassica juncea	AT5G27380.1 | Symbols: GSH2, GSHB | glutathione synthetase 2 | chr5:9668211-9670912 REVERSE LENGTH=539	530	539	0	101.7	77.2	84.9

Rsa1.0_00045.1.g2586.t3	ref|NP_187148.2| DHHC-type zinc finger-containing protein [Arabidopsis thaliana] gi|122214962|sp|Q3EBC2.1|ZDHC5_ARATH RecName: Full=Probable S-acyltransferase At3g04970; AltName: Full=Probable palmitoyltransferase At3g04970; AltName: Full=Zinc finger DHHC domain-containing protein At3g04970 gi|332640648|gb|AEE74169.1| DHHC-type zinc finger-containing protein [Arabidopsis thaliana]	381	397	1.00E-167	104.2	77.2	86.1	DHHC-type zinc finger-containing protein	gbpln	Arabidopsis thaliana	AT3G04970.1 | Symbols: | DHHC-type zinc finger family protein | chr3:1376240-1378339 FORWARD LENGTH=397	381	397	1.00E-169	104.2	77.2	86.1
Rsa1.0_00045.1.g2587.t1	ref|NP_198076.1| DNAJ heat shock N-terminal domain-containing protein [Arabidopsis thaliana] gi|332006279|gb|AED93662.1| DNAJ heat shock N-terminal domain-containing protein [Arabidopsis thaliana]	1063	1104	0	103.9	63.1	75.2	DNAJ heat shock N-terminal domain-containing protein	gbpln	Arabidopsis thaliana	AT5G27240.1 | Symbols: | DNAJ heat shock N-terminal domain-containing protein | chr5:9597552-9600866 FORWARD LENGTH=1104	1063	1104	0	103.9	63.1	75.2
Rsa1.0_00045.1.g2588.t1	gb|EOA19886.1| hypothetical protein CARUB_v10000134mg [Capsella rubella]	866	1000	0	115.5	63.0	77.4	hypothetical protein CARUB_v10000134mg	gbpln	Capsella rubella	AT5G27230.1 | Symbols: | Frigida-like protein | chr5:9584255-9587838 FORWARD LENGTH=948	866	948	0	109.5	62.4	77.4
Rsa1.0_00045.1.g2589.t1	ref|XP_002874392.1| hypothetical protein ARALYDRAFT_910868 [Arabidopsis lyrata subsp. lyrata] gi|297320229|gb|EFH50651.1| hypothetical protein ARALYDRAFT_910868 [Arabidopsis lyrata subsp. lyrata]	855	994	0	116.3	65.7	79.3	hypothetical protein ARALYDRAFT_910868	gbpln	Arabidopsis lyrata	AT5G27230.1 | Symbols: | Frigida-like protein | chr5:9584255-9587838 FORWARD LENGTH=948	855	948	0	110.9	65.3	78.0
Rsa1.0_00045.1.g2590.t1	ref|XP_002874393.1| predicted protein [Arabidopsis lyrata subsp. lyrata] gi|297320230|gb|EFH50652.1| predicted protein [Arabidopsis lyrata subsp. lyrata]	1192	1191	0	99.9	56.9	72.0	predicted protein	gbpln	Arabidopsis lyrata	AT5G27220.1 | Symbols: | Frigida-like protein | chr5:9578757-9582752 FORWARD LENGTH=1181	1192	1181	0	99.1	54.6	69.6
Rsa1.0_00045.1.g2591.t2	gb|AAC33961.1| contains similarity to reverse trancriptase (Pfam: rvt.hmm, score: 42.57) [Arabidopsis thaliana]	1154	1662	0	144.0	37.2	55.4	contains similarity to reverse trancriptase (Pfam: rvt.hmm, score: 42.57)	gbpln	Arabidopsis thaliana	AT4G29090.1 | Symbols: | Ribonuclease H-like superfamily protein | chr4:14333528-14335255 FORWARD LENGTH=575	1154	575	3.00E-37	49.8	6.4	10.7
Rsa1.0_00045.1.g2592.t1	#	#	#	#	#	#	#	–	––––	––––	#	#	#	#	#	#	#
Rsa1.0_00045.1.g2593.t2	ref|XP_002874393.1| predicted protein [Arabidopsis lyrata subsp. lyrata] gi|297320230|gb|EFH50652.1| predicted protein [Arabidopsis lyrata subsp. lyrata]	561	1191	8.00E-40	212.3	19.1	22.8	predicted protein	gbpln	Arabidopsis lyrata	AT5G27220.1 | Symbols: | Frigida-like protein | chr5:9578757-9582752 FORWARD LENGTH=1181	561	1181	3.00E-42	210.5	18.7	23.0
Rsa1.0_00045.1.g2594.t1	ref|XP_002866462.1| hypothetical protein ARALYDRAFT_496364 [Arabidopsis lyrata subsp. lyrata] gi|297312297|gb|EFH42721.1| hypothetical protein ARALYDRAFT_496364 [Arabidopsis lyrata subsp. lyrata]	146	400	1.00E-25	274.0	37.7	43.8	hypothetical protein ARALYDRAFT_496364	gbpln	Arabidopsis lyrata	AT5G61930.2 | Symbols: APO3 | Arabidopsis thaliana protein of unknown function (DUF794) | chr5:24866230-24867665 REVERSE LENGTH=402	146	402	1.00E-27	275.3	37.0	43.2
Rsa1.0_00045.1.g2595.t6	gb|EOA13406.1| hypothetical protein CARUB_v10026444mg, partial [Capsella rubella]	929	430	1.00E-107	46.3	24.4	29.9	hypothetical protein CARUB_v10026444mg, partial	gbpln	Capsella rubella	AT5G62660.1 | Symbols: | F-box and associated interaction domains-containing protein | chr5:25156326-25157465 REVERSE LENGTH=379	929	379	1.00E-101	40.8	21.6	26.6
Rsa1.0_00045.1.g2596.t25	ref|XP_002874217.1| hypothetical protein ARALYDRAFT_489332 [Arabidopsis lyrata subsp. lyrata] gi|297320054|gb|EFH50476.1| hypothetical protein ARALYDRAFT_489332 [Arabidopsis lyrata subsp. lyrata]	1281	1376	0	107.4	84.9	89.0	hypothetical protein ARALYDRAFT_489332	gbpln	Arabidopsis lyrata	AT5G24710.1 | Symbols: | Transducin/WD40 repeat-like superfamily protein | chr5:8459148-8467920 REVERSE LENGTH=1377	1281	1377	0	107.5	84.9	89.1
Rsa1.0_00045.1.g2597.t1	#	#	#	#	#	#	#	–	––––	––––	#	#	#	#	#	#	#
Rsa1.0_00045.1.g2598.t1	ref|NP_198074.1| Frigida-like protein [Arabidopsis thaliana] gi|2191186|gb|AAB61071.1| contains similarity to tropomyosin and kinesin [Arabidopsis thaliana] gi|332006276|gb|AED93659.1| Frigida-like protein [Arabidopsis thaliana]	1421	1181	1.00E-177	83.1	35.4	48.9	Frigida-like protein	gbpln	Arabidopsis thaliana	AT5G27220.1 | Symbols: | Frigida-like protein | chr5:9578757-9582752 FORWARD LENGTH=1181	1421	1181	1.00E-180	83.1	35.4	48.9
Rsa1.0_00045.1.g2599.t1	ref|XP_002874393.1| predicted protein [Arabidopsis lyrata subsp. lyrata] gi|297320230|gb|EFH50652.1| predicted protein [Arabidopsis lyrata subsp. lyrata]	869	1191	0	137.1	51.9	69.6	predicted protein	gbpln	Arabidopsis lyrata	AT5G27220.1 | Symbols: | Frigida-like protein | chr5:9578757-9582752 FORWARD LENGTH=1181	869	1181	0	135.9	50.7	67.9

Rsa1.0_00045.1.g2600.t1	ref|NP_198073.1| uncharacterized protein [Arabidopsis thaliana] gi|332006275|gb|AED93658.1| uncharacterized protein AT5G27210 [Arabidopsis thaliana]	331	297	1.00E-140	89.7	77.9	82.5	uncharacterized protein	gbpln	Arabidopsis thaliana	AT5G27210.1 | Symbols: | Protein of unknown function, transmembrane-40 | chr5:9572333-9574075 REVERSE LENGTH=297	331	297	1.00E-143	89.7	77.9	82.5
Rsa1.0_00045.1.g2601.t1	gb|ACZ92142.1| Na+/H+ antiporter [Brassica napus]	546	542	0	99.3	96.3	97.8	Na+/H+ antiporter	gbpln	Brassica napus	AT5G27150.1 | Symbols: NHX1, ATNHX, AT-NHX1, ATNHX1 | Na+/H+ exchanger 1 | chr5:9554251-9557266 FORWARD LENGTH=538	546	538	0	98.5	90.7	95.1
Rsa1.0_00045.1.g2602.t1	ref|NP_198064.1| putative nucleolar protein 5-1 [Arabidopsis thaliana] gi|147744575|sp|O04658.2|NOP5A_ARATH RecName: Full=Probable nucleolar protein 5-1; AltName: Full=MAR-binding NOP56/58 homolog 1; AltName: Full=NOP58-like protein F108; AltName: Full=Nucleolar protein 58-1 gi|11878185|gb|AAG40836.1|AF302490_1 NOP58-like protein F108 [Arabidopsis thaliana] gi|18377656|gb|AAL66978.1| putative SAR DNA-binding protein [Arabidopsis thaliana] gi|20465699|gb|AAM20318.1| putative SAR DNA-binding protein [Arabidopsis thaliana] gi|332006269|gb|AED93652.1| putative nucleolar protein 5-1 [Arabidopsis thaliana]	532	533	0	100.2	75.6	79.9	putative nucleolar protein 5-1	gbpln	Arabidopsis thaliana	AT5G27120.1 | Symbols: | NOP56-like pre RNA processing ribonucleoprotein | chr5:9541287-9543684 FORWARD LENGTH=533	532	533	0	100.2	75.6	79.9
Rsa1.0_00045.1.g2603.t1	ref|XP_002874409.1| seed maturation family protein [Arabidopsis lyrata subsp. lyrata] gi|297320246|gb|EFH50668.1| seed maturation family protein [Arabidopsis lyrata subsp. lyrata]	185	192	1.00E-73	103.8	87.0	90.8	seed maturation family protein	gbpln	Arabidopsis lyrata	AT5G27980.1 | Symbols: | Seed maturation protein | chr5:10015883-10016676 REVERSE LENGTH=192	185	192	9.00E-76	103.8	87.0	90.8
Rsa1.0_00045.1.g2604.t1	gb|EOA21519.1| hypothetical protein CARUB_v10001923mg, partial [Capsella rubella]	194	220	8.00E-58	113.4	63.4	76.3	hypothetical protein CARUB_v10001923mg, partial	gbpln	Capsella rubella	AT5G27990.1 | Symbols: | Pre-rRNA-processing protein TSR2, conserved region | chr5:10017358-10018265 FORWARD LENGTH=184	194	184	2.00E-56	94.8	59.8	70.6
Rsa1.0_00045.1.g2605.t1	emb|CAA71799.1| O-acetylserine(thiol) lyase [Brassica juncea]	323	345	1.00E-163	106.8	94.4	97.8	O-acetylserine(thiol) lyase	gbpln	Brassica juncea	AT5G28020.6 | Symbols: CYSD2, ATCYSD2 | cysteine synthase D2 | chr5:10026395-10028166 REVERSE LENGTH=323	323	323	1.00E-156	100.0	88.2	94.7
Rsa1.0_00045.1.g2606.t1	dbj|BAJ34422.1| unnamed protein product [Thellungiella halophila]	323	323	1.00E-161	100.0	93.5	98.5	unnamed protein product	----	----	AT5G28030.1 | Symbols: DES1 | L-cysteine desulfhydrase 1 | chr5:10030493-10032285 REVERSE LENGTH=323	323	323	1.00E-154	100.0	89.5	95.4
Rsa1.0_00045.1.g2607.t1	dbj|BAC42632.1| unknown protein [Arabidopsis thaliana] gi|28950993|gb|AAO63420.1| At5g28040 [Arabidopsis thaliana]	387	427	1.00E-132	110.3	72.9	81.7	unknown protein	gbpln	Arabidopsis thaliana	AT5G28040.1 | Symbols: | DNA-binding storekeeper protein-related transcriptional regulator | chr5:10037654-10038937 REVERSE LENGTH=427	387	427	1.00E-135	110.3	72.9	82.2
Rsa1.0_00045.1.g2608.t1	ref|XP_002882383.1| 40S ribosomal protein S24 [Arabidopsis lyrata subsp. lyrata] gi|297328223|gb|EFH58642.1| 40S ribosomal protein S24 [Arabidopsis lyrata subsp. lyrata]	133	133	8.00E-69	100.0	99.2	99.2	40S ribosomal protein S24	gbpln	Arabidopsis lyrata	AT3G04920.1 | Symbols: | Ribosomal protein S24e family protein | chr3:1360989-1362065 FORWARD LENGTH=133	133	133	1.00E-70	100.0	98.5	98.5
Rsa1.0_00045.1.g2609.t1	gb|EOA20484.1| hypothetical protein CARUB_v10000796mg [Capsella rubella]	446	498	1.00E-150	111.7	67.7	78.9	hypothetical protein CARUB_v10000796mg	gbpln	Capsella rubella	AT3G04910.1 | Symbols: WNK1, ZIK4, ATWNK1 | with no lysine (K) kinase 1 | chr3:1355084-1358057 FORWARD LENGTH=700	446	700	1.00E-121	157.0	45.3	50.9
Rsa1.0_00045.1.g2610.t1	gb|EOA12246.1| hypothetical protein CARUB_v10012686mg [Capsella rubella]	417	417	0	100.0	95.7	97.4	hypothetical protein CARUB_v10012686mg	gbpln	Capsella rubella	AT5G30510.1 | Symbols: RPS1, ARRPS1 | ribosomal protein S1 | chr5:11619262-11621223 REVERSE LENGTH=416	417	416	0	99.8	96.2	98.6
Rsa1.0_00045.1.g2611.t1	gb|EOA12248.1| hypothetical protein CARUB_v10012688mg [Capsella rubella]	241	237	3.00E-71	98.3	78.0	83.0	hypothetical protein CARUB_v10012688mg	gbpln	Capsella rubella	AT5G30490.2 | Symbols: | FUNCTIONS IN: molecular_function unknown; INVOLVED IN: biological_process unknown; LOCATED IN: cellular_component unknown; EXPRESSED IN: 22 plant structures; EXPRESSED DURING: 13 growth stages; CONTAINS InterPro DOMAIN/s: Craniofacial development protein 1/Bucentaur (InterPro:IPR011421); Has 333 Blast hits to 324 proteins in 149 species: Archae - 0; Bacteria - 18; Metazoa - 117; Fungi - 96; Plants - 49; Viruses - 0; Other Eukaryotes - 53 (source: NCBI BLink). | chr5:11612199-11614271 FORWARD LENGTH=236	241	236	1.00E-73	97.9	77.2	82.6
Rsa1.0_00045.1.g2612.t1	ref|XP_002872334.1| hypothetical protein ARALYDRAFT_489677 [Arabidopsis lyrata subsp. lyrata] gi|297318171|gb|EFH48593.1| hypothetical protein ARALYDRAFT_489677 [Arabidopsis lyrata subsp. lyrata]	198	196	4.00E-84	99.0	77.3	87.9	hypothetical protein ARALYDRAFT_489677	gbpln	Arabidopsis lyrata	AT5G30495.1 | Symbols: | Fcf2 pre-rRNA processing protein | chr5:11380190-11381391 FORWARD LENGTH=196	198	196	3.00E-85	99.0	75.8	87.4

Rsa1.0_00045.1.g2613.t1	ref|NP_568512.3| myb family transcription factor [Arabidopsis thaliana] gi|75328846|sp|Q8GUN5.1|PHL1_ARATH RecName: Full=Protein PHR1-LIKE 1; AltName: Full=Myb-like transcription factor 1 gi|26983850|gb|AAN86177.1| unknown protein [Arabidopsis thaliana] gi|332006474|gb|AED93857.1| myb family transcription factor [Arabidopsis thaliana]	429	413	1.00E-174	96.3	75.1	81.4	myb family transcription factor	gbpln	Arabidopsis thaliana	AT5G29000.2 | Symbols: PHL1 | Homeodomain-like superfamily protein | chr5:11022336-11024229 REVERSE LENGTH=413	429	413	1.00E-177	96.3	75.1	81.4
Rsa1.0_00045.1.g2614.t1	ref|XP_002874460.1| hypothetical protein ARALYDRAFT_489671 [Arabidopsis lyrata subsp. lyrata] gi|297320297|gb|EFH50719.1| hypothetical protein ARALYDRAFT_489671 [Arabidopsis lyrata subsp. lyrata]	531	533	0	100.4	83.8	91.7	hypothetical protein ARALYDRAFT_489671	gbpln	Arabidopsis lyrata	AT5G28910.2 | Symbols: | unknown protein; FUNCTIONS IN: molecular_function unknown; INVOLVED IN: biological_process unknown; BEST Arabidopsis thaliana protein match is: unknown protein (TAIR:AT5G28960.1); Has 82 Blast hits to 80 proteins in 14 species: Archae - 0; Bacteria - 0; Metazoa - 1; Fungi - 0; Plants - 78; Viruses - 0; Other Eukaryotes - 3 (source: NCBI BLink). | chr5:10930633-10932575 REVERSE LENGTH=535	531	535	0	100.8	83.6	90.8
Rsa1.0_00045.1.g2615.t1	ref|XP_002884444.1| hypothetical protein ARALYDRAFT_896477 [Arabidopsis lyrata subsp. lyrata] gi|297330284|gb|EFH60703.1| hypothetical protein ARALYDRAFT_896477 [Arabidopsis lyrata subsp. lyrata]	634	640	0	100.9	78.5	86.3	hypothetical protein ARALYDRAFT_896477	gbpln	Arabidopsis lyrata	AT3G04470.1 | Symbols: | Ankyrin repeat family protein | chr3:1189841-1191853 REVERSE LENGTH=640	634	640	0	100.9	75.7	82.6
Rsa1.0_00045.1.g2616.t1	ref|XP_002872330.1| calcium-binding EF hand family protein [Arabidopsis lyrata subsp. lyrata] gi|297318167|gb|EFH48589.1| calcium-binding EF hand family protein [Arabidopsis lyrata subsp. lyrata]	509	536	0	105.3	88.6	95.3	calcium-binding EF hand family protein	gbpln	Arabidopsis lyrata	AT5G28900.1 | Symbols: | Calcium-binding EF-hand family protein | chr5:10925852-10929892 FORWARD LENGTH=536	509	536	0	105.3	87.4	94.9
Rsa1.0_00045.1.g2617.t1	gb|EOA21064.1| hypothetical protein CARUB_v10001403mg [Capsella rubella]	330	331	1.00E-141	100.3	76.4	87.6	hypothetical protein CARUB_v10001403mg	gbpln	Capsella rubella	AT5G28830.1 | Symbols: | calcium-binding EF hand family protein | chr5:10856801-10858394 FORWARD LENGTH=324	330	324	1.00E-140	98.2	77.0	87.3
Rsa1.0_00045.1.g2618.t1	gb|EOA21752.1| hypothetical protein CARUB_v10002208mg [Capsella rubella]	142	146	8.00E-50	102.8	90.1	91.5	hypothetical protein CARUB_v10002208mg	gbpln	Capsella rubella	AT5G28750.1 | Symbols: | Bacterial sec-independent translocation protein mttA/Hcf106 | chr5:10784142-10785677 REVERSE LENGTH=147	142	147	1.00E-45	103.5	71.1	73.9
Rsa1.0_00045.1.g2619.t1	ref|NP_187113.1| Alba DNA/RNA-binding protein [Arabidopsis thaliana] gi|6175166|gb|AAF04892.1|AC011437_7 unknown protein [Arabidopsis thaliana] gi|38454036|gb|AAR20712.1| At3g04620 [Arabidopsis thaliana] gi|38604012|gb|AAR24749.1| At3g04620 [Arabidopsis thaliana] gi|332640586|gb|AEE74107.1| Alba DNA/RNA-binding protein [Arabidopsis thaliana]	159	164	6.00E-45	103.1	61.6	67.9	Alba DNA/RNA-binding protein	gbpln	Arabidopsis thaliana	AT3G04620.1 | Symbols: | Alba DNA/RNA-binding protein | chr3:1255660-1256739 REVERSE LENGTH=164	159	164	2.00E-47	103.1	61.6	67.9
Rsa1.0_00045.1.g2620.t1	gb|AAF88010.1| Hypothetical protein T10I18.2 [Arabidopsis thaliana]	143	131	3.00E-25	91.6	67.8	78.3	Hypothetical protein T10I18.2	gbpln	Arabidopsis thaliana	AT5G28610.1 | Symbols: | BEST Arabidopsis thaliana protein match is: glycine-rich protein (TAIR:AT5G28630.1); Has 1536 Blast hits to 1202 proteins in 136 species: Archae - 0; Bacteria - 8; Metazoa - 888; Fungi - 120; Plants - 71; Viruses - 39; Other Eukaryotes - 410 (source: NCBI BLink). | chr5:10610976-10611347 FORWARD LENGTH=123	143	123	6.00E-24	86.0	63.6	73.4
Rsa1.0_00045.1.g2621.t1	ref|XP_002874429.1| hypothetical protein ARALYDRAFT_489653 [Arabidopsis lyrata subsp. lyrata] gi|297320266|gb|EFH50688.1| hypothetical protein ARALYDRAFT_489653 [Arabidopsis lyrata subsp. lyrata]	159	217	3.00E-25	136.5	41.5	46.5	hypothetical protein ARALYDRAFT_489653	gbpln	Arabidopsis lyrata	AT5G28590.1 | Symbols: | DNA-binding family protein | chr5:10579446-10581559 REVERSE LENGTH=216	159	216	2.00E-25	135.8	41.5	45.3
Rsa1.0_00045.1.g2622.t1	#	#	#	#	#	#	#	-	----	----	#	#	#	#	#	#	#
Rsa1.0_00045.1.g2623.t2	gb|AAG51783.1|AC079679_3 reverse transcriptase, putative; 16838-20266 [Arabidopsis thaliana]	473	1142	1.00E-38	241.4	20.3	29.6	reverse transcriptase, putative; 16838-20266	gbpln	Arabidopsis thaliana	AT2G22440.1 | Symbols: | BEST Arabidopsis thaliana protein match is: Ribonuclease H-like superfamily protein (TAIR:AT4G29090.1); Has 208 Blast hits to 191 proteins in 10 species: Archae - 0; Bacteria - 0; Metazoa - 0; Fungi - 0; Plants - 208; Viruses - 0; Other Eukaryotes - 0 (source: NCBI BLink). | chr2:9528910-9529917 FORWARD LENGTH=256	473	256	2.00E-17	54.1	11.4	15.6
Rsa1.0_00045.1.g2624.t1	#	#	#	#	#	#	#	-	----	----	#	#	#	#	#	#	#

Rsa1.0_00045.1.g2625.t2	gb|AAF18538.1|AC006551_24 Very similar to retrotransposon reverse transcriptase [Arabidopsis thaliana]	358	1231	7.00E-72	343.9	44.7	62.8	Very similar to retrotransposon reverse transcriptase	gbpln	Arabidopsis thaliana	AT4G29090.1 | Symbols: | Ribonuclease H-like superfamily protein | chr4:14333528-14335255 FORWARD LENGTH=575	358	575	3.00E-42	160.6	27.1	45.5
Rsa1.0_00045.1.g2626.t1	ref|XP_002874426.1| light-dependent short hypocotyls 1 [Arabidopsis lyrata subsp. lyrata] gi|297320263|gb|EFH50685.1| light-dependent short hypocotyls 1 [Arabidopsis lyrata subsp. lyrata]	192	192	6.00E-81	100.0	88.5	92.7	light-dependent short hypocotyls 1	gbpln	Arabidopsis lyrata	AT5G28490.1 | Symbols: LSH1 | Protein of unknown function (DUF640) | chr5:10454541-10455113 REVERSE LENGTH=190	192	190	3.00E-82	99.0	89.6	93.8
Rsa1.0_00045.1.g2627.t1	emb|CAB75932.1| putative protein [Arabidopsis thaliana]	1175	1339	0	114.0	72.3	79.2	putative protein	gbpln	Arabidopsis thaliana	AT4G23160.1 | Symbols: CRK8 | cysteine-rich RLK (RECEPTOR-like protein kinase) 8 | chr4:12129485-12134086 FORWARD LENGTH=1262	1175	1262	7.00E-58	107.4	10.2	15.7
Rsa1.0_00045.1.g2628.t1	ref|NP_568711.1| uncharacterized protein [Arabidopsis thaliana] gi|363579829|sp|F4K657.1|BL1S2_ARATH RecName: Full=Biogenesis of lysosome-related organelles complex 1 subunit 2; Short=BLOC-1 subunit 2 gi|332008445|gb|AED95828.1| uncharacterized protein AT5G49550 [Arabidopsis thaliana]	126	127	3.00E-55	100.8	88.9	93.7	uncharacterized protein	gbpln	Arabidopsis thaliana	AT5G49550.1 | Symbols: BLOS2 | Putative homolog of mammalian BLOC-1 Subunit 2. Protein - protein interaction with BLOS1. | chr5:20106702-20107156 REVERSE LENGTH=127	126	127	6.00E-58	100.8	88.9	93.7
Rsa1.0_00045.1.g2629.t1	gb|EOA31472.1| hypothetical protein CARUB_v10014658mg [Capsella rubella]	194	204	4.00E-60	105.2	62.4	75.8	hypothetical protein CARUB_v10014658mg	gbpln	Capsella rubella	AT3G04320.1 | Symbols: | Kunitz family trypsin and protease inhibitor protein | chr3:1143815-1144426 FORWARD LENGTH=203	194	203	5.00E-58	104.6	57.2	72.7
Rsa1.0_00045.1.g2630.t2	ref|NP_187083.1| Kunitz family trypsin and protease inhibitor protein [Arabidopsis thaliana] gi|6721153|gb|AAF26781.1|AC016829_5 putative trypsin inhibitor [Arabidopsis thaliana] gi|332640546|gb|AEE74067.1| Kunitz family trypsin and protease inhibitor protein [Arabidopsis thaliana]	295	202	3.00E-43	68.5	33.9	42.0	Kunitz family trypsin and protease inhibitor protein	gbpln	Arabidopsis thaliana	AT3G04330.1 | Symbols: | Kunitz family trypsin and protease inhibitor protein | chr3:1145757-1146365 FORWARD LENGTH=202	295	202	7.00E-46	68.5	33.9	42.0
Rsa1.0_00045.1.g2631.t1	gb|EOA14246.1| hypothetical protein CARUB_v10027400mg [Capsella rubella]	124	114	2.00E-51	91.9	83.1	85.5	hypothetical protein CARUB_v10027400mg	gbpln	Capsella rubella	AT5G49540.1 | Symbols: | Rab5-interacting family protein | chr5:20104804-20105609 REVERSE LENGTH=114	124	114	7.00E-53	91.9	80.6	83.9
Rsa1.0_00045.1.g2632.t1	ref|XP_002863991.1| hypothetical protein ARALYDRAFT_917932 [Arabidopsis lyrata subsp. lyrata] gi|297309826|gb|EFH40250.1| hypothetical protein ARALYDRAFT_917932 [Arabidopsis lyrata subsp. lyrata]	382	396	1.00E-118	103.7	78.5	85.9	hypothetical protein ARALYDRAFT_917932	gbpln	Arabidopsis lyrata	AT5G49520.1 | Symbols: WRKY48, ATWRKY48 | WRKY DNA-binding protein 48 | chr5:20090890-20092867 FORWARD LENGTH=399	382	399	1.00E-118	104.5	73.3	79.1
Rsa1.0_00045.1.g2633.t3	#	#	#	#	#	#	#	-	----	----	#	#	#	#	#	#	#
Rsa1.0_00045.1.g2634.t1	ref|XP_002863987.1| hypothetical protein ARALYDRAFT_495005 [Arabidopsis lyrata subsp. lyrata] gi|297309822|gb|EFH40246.1| hypothetical protein ARALYDRAFT_495005 [Arabidopsis lyrata subsp. lyrata]	161	161	8.00E-61	100.0	82.0	85.1	hypothetical protein ARALYDRAFT_495005	gbpln	Arabidopsis lyrata	AT5G49480.1 | Symbols: ATCP1, CP1 | Ca2+-binding protein 1 | chr5:20070812-20071294 FORWARD LENGTH=160	161	160	5.00E-63	99.4	80.7	83.9
Rsa1.0_00045.1.g2635.t1	#	#	#	#	#	#	#	-	----	----	#	#	#	#	#	#	#
Rsa1.0_00045.1.g2636.t1	ref|NP_001190501.1| PAS domain-containing protein tyrosine kinase [Arabidopsis thaliana] gi|332008435|gb|AED95818.1| PAS domain-containing protein tyrosine kinase [Arabidopsis thaliana]	624	770	0	123.4	89.1	95.0	PAS domain-containing protein tyrosine kinase	gbpln	Arabidopsis thaliana	AT5G49470.3 | Symbols: | PAS domain-containing protein tyrosine kinase family protein | chr5:20063616-20068311 FORWARD LENGTH=770	624	770	0	123.4	89.1	95.0
Rsa1.0_00045.1.g2637.t1	ref|XP_002863985.1| kinase family protein [Arabidopsis lyrata subsp. lyrata] gi|297309820|gb|EFH40244.1| kinase family protein [Arabidopsis lyrata subsp. lyrata]	130	856	3.00E-41	658.5	76.9	81.5	kinase family protein	gbpln	Arabidopsis lyrata	AT5G49470.2 | Symbols: | PAS domain-containing protein tyrosine kinase family protein | chr5:20063616-20068311 FORWARD LENGTH=831	130	831	6.00E-44	639.2	75.4	80.8
Rsa1.0_00045.1.g2638.t1	ref|XP_002863984.1| ATP-citrate lyase B-2 [Arabidopsis lyrata subsp. lyrata] gi|297309819|gb|EFH40243.1| ATP-citrate lyase B-2 [Arabidopsis lyrata subsp. lyrata]	608	608	0	100.0	99.2	99.8	ATP-citrate lyase B-2	gbpln	Arabidopsis lyrata	AT5G49460.1 | Symbols: ACLB-2 | ATP citrate lyase subunit B 2 | chr5:20055048-20058195 FORWARD LENGTH=608	608	608	0	100.0	98.5	99.5
Rsa1.0_00045.1.g2639.t1	#	#	#	#	#	#	#	-	----	----	#	#	#	#	#	#	#
Rsa1.0_00045.1.g2640.t1	dbj|BAJ33845.1| unnamed protein product [Thellungiella halophila]	140	141	4.00E-59	100.7	85.7	92.9	unnamed protein product	----	----	AT5G49450.1 | Symbols: AtbZIP1, bZIP1 | basic leucine-zipper 1 | chr5:20052145-20052582 FORWARD LENGTH=145	140	145	1.00E-58	103.6	83.6	90.7
Rsa1.0_00045.1.g2641.t1	#	#	#	#	#	#	#	-	----	----	#	#	#	#	#	#	#

Rsa1.0_00045.1.g2642.t1	ref|NP_199747.1| beta-xylosidase 1 [Arabidopsis thaliana] gi|75262458|sp|Q9FGY1.1|BXL1_ARATH RecName: Full=Beta-D-xylosidase 1; Short=AtBXL1; AltName: Full=Alpha-L-arabinofuranosidase; Flags: Precursor gi|9759419|dbj|BAB09906.1| xylosidase [Arabidopsis thaliana] gi|21539545|gb|AAM53325.1| xylosidase [Arabidopsis thaliana] gi|332008419|gb|AED95802.1| beta-xylosidase 1 [Arabidopsis thaliana]	562	774	0	137.7	90.9	94.5	beta-xylosidase 1	gbpln	Arabidopsis thaliana	AT5G49360.1 | Symbols: BXL1, ATBXL1 | beta-xylosidase 1 | chr5:20012179-20016659 REVERSE LENGTH=774	562	774	0	137.7	90.9	94.5
Rsa1.0_00045.1.g2643.t1	gb|ABD65099.1| hypothetical protein 31.t00074 [Brassica oleracea]	261	258	5.00E-57	98.9	51.7	67.8	hypothetical protein 31.t00074	gbpln	Brassica oleracea	AT3G47680.1 | Symbols: | DNA binding | chr3:17577483-17578391 REVERSE LENGTH=302	261	302	2.00E-47	115.7	43.7	64.0
Rsa1.0_00045.1.g2644.t1	gb|ABD64939.1| hypothetical protein 24.t00017 [Brassica oleracea]	611	442	1.00E-157	72.3	43.5	51.2	hypothetical protein 24.t00017	gbpln	Brassica oleracea	AT5G49360.1 | Symbols: BXL1, ATBXL1 | beta-xylosidase 1 | chr5:20012179-20016659 REVERSE LENGTH=774	611	774	1.00E-111	126.7	31.8	34.0
Rsa1.0_00045.1.g2645.t1	ref|XP_002865730.1| hypothetical protein ARALYDRAFT_331346 [Arabidopsis lyrata subsp. lyrata] gi|297311565|gb|EFH41989.1| hypothetical protein ARALYDRAFT_331346 [Arabidopsis lyrata subsp. lyrata]	279	459	4.00E-63	164.5	44.8	50.5	hypothetical protein ARALYDRAFT_331346	gbpln	Arabidopsis lyrata	AT5G49340.1 | Symbols: TBL4 | TRICHOME BIREFRINGENCE-LIKE 4 | chr5:20007348-20009038 REVERSE LENGTH=457	279	457	8.00E-64	163.8	43.0	50.5
Rsa1.0_00045.1.g2646.t1	gb|ADZ98865.1| MYB domain protein 111 [Brassica rapa subsp. rapa]	341	343	1.00E-149	100.6	79.2	86.2	MYB domain protein 111	gbpln	Brassica rapa	AT5G49330.1 | Symbols: MYB111, ATMYB111, PFG3 | myb domain protein 111 | chr5:19999147-20001293 REVERSE LENGTH=342	341	342	1.00E-143	100.3	77.1	83.9
Rsa1.0_00045.1.g2647.t1	gb|EOA14118.1| hypothetical protein CARUB_v10027260mg [Capsella rubella]	164	166	2.00E-25	101.2	69.5	76.8	hypothetical protein CARUB_v10027260mg	gbpln	Capsella rubella	AT5G49280.1 | Symbols: | hydroxyproline-rich glycoprotein family protein | chr5:19976842-19977330 REVERSE LENGTH=162	164	162	1.00E-12	98.8	18.3	20.7
Rsa1.0_00045.1.g2648.t1	ref|XP_002865723.1| hypothetical protein ARALYDRAFT_494988 [Arabidopsis lyrata subsp. lyrata] gi|297311558|gb|EFH41982.1| hypothetical protein ARALYDRAFT_494988 [Arabidopsis lyrata subsp. lyrata]	663	661	0	99.7	85.4	91.6	hypothetical protein ARALYDRAFT_494988	gbpln	Arabidopsis lyrata	AT5G49270.1 | Symbols: SHV2, DER9, MRH4, COBL9 | COBRA-like extracellular glycosyl-phosphatidyl inositol-anchored protein family | chr5:19972021-19974398 REVERSE LENGTH=663	663	663	0	100.0	85.8	92.2
Rsa1.0_00045.1.g2649.t1	ref|NP_196810.5| ARMADILLO BTB protein 1 [Arabidopsis thaliana] gi|325529879|sp|B7U179.1|ABAP1_ARATH RecName: Full=ARMADILLO BTB ARABIDOPSIS PROTEIN 1; Short=ABAP1 gi|213391123|gb|ACJ46331.1| ABAP1 [Arabidopsis thaliana] gi|332004463|gb|AED91846.1| ARMADILLO BTB protein 1 [Arabidopsis thaliana]	717	737	0	102.8	84.4	90.9	ARMADILLO BTB protein 1	gbpln	Arabidopsis thaliana	AT5G13060.1 | Symbols: ABAP1 | ARMADILLO BTB protein 1 | chr5:4142958-4146952 FORWARD LENGTH=737	717	737	0	102.8	84.4	90.9
Rsa1.0_00045.1.g2650.t1	gb|AEK67480.1| ZZ [Arabidopsis thaliana]	154	206	2.00E-46	133.8	73.4	82.5	ZZ	gbpln	Arabidopsis thaliana	AT5G49230.1 | Symbols: HRB1 | Drought-responsive family protein | chr5:19959048-19960422 REVERSE LENGTH=211	154	211	2.00E-48	137.0	73.4	82.5
Rsa1.0_00045.1.g2651.t1	#	#	#	#	#	#	#	-	----	----	#	#	#	#	#	#	#
Rsa1.0_00045.1.g2652.t1	ref|XP_002863962.1| predicted protein [Arabidopsis lyrata subsp. lyrata] gi|297309797|gb|EFH40221.1| predicted protein [Arabidopsis lyrata subsp. lyrata]	414	501	1.00E-139	121.0	60.9	63.3	predicted protein	gbpln	Arabidopsis lyrata	AT5G49130.1 | Symbols: | MATE efflux family protein | chr5:19915904-19917525 FORWARD LENGTH=502	414	502	1.00E-138	121.3	59.2	62.6
Rsa1.0_00045.1.g2653.t1	gb|AAG50806.1|AC079281_8 unknown protein [Arabidopsis thaliana]	386	1213	3.00E-92	314.2	48.7	65.3	unknown protein	gbpln	Arabidopsis thaliana	AT1G60720.1 | Symbols: | RNA-directed DNA polymerase (reverse transcriptase)-related family protein | chr1:22357072-22357941 FORWARD LENGTH=289	386	289	1.00E-51	74.9	28.5	41.5
Rsa1.0_00046.1.g2654.t1	gb|AAB68964.1| trypsin inhibitor propeptide [Brassica oleracea]	209	214	8.00E-62	102.4	64.1	73.2	trypsin inhibitor propeptide	gbpln	Brassica oleracea	AT1G73260.1 | Symbols: ATKTI1, KTI1 | kunitz trypsin inhibitor 1 | chr1:27547410-27548057 REVERSE LENGTH=215	209	215	3.00E-54	102.9	58.4	67.9
Rsa1.0_00046.1.g2655.t1	gb|AAB68964.1| trypsin inhibitor propeptide [Brassica oleracea]	211	214	3.00E-66	101.4	66.4	73.9	trypsin inhibitor propeptide	gbpln	Brassica oleracea	AT1G73260.1 | Symbols: ATKTI1, KTI1 | kunitz trypsin inhibitor 1 | chr1:27547410-27548057 REVERSE LENGTH=215	211	215	9.00E-56	101.9	55.5	70.1
Rsa1.0_00046.1.g2656.t1	ref|NP_565061.1| kunitz trypsin inhibitor 1 [Arabidopsis thaliana] gi|19698953|gb|AAL91212.1| putative trypsin inhibitor [Arabidopsis thaliana] gi|27311885|gb|AAO00908.1| putative trypsin inhibitor [Arabidopsis thaliana] gi|110740417|dbj|BAF02103.1| putative trypsin inhibitor [Arabidopsis thaliana] gi|332197314|gb|AEE35435.1| kunitz trypsin inhibitor 1 [Arabidopsis thaliana]	202	215	2.00E-89	106.4	78.7	88.6	kunitz trypsin inhibitor 1	gbpln	Arabidopsis thaliana	AT1G73260.1 | Symbols: ATKTI1, KTI1 | kunitz trypsin inhibitor 1 | chr1:27547410-27548057 REVERSE LENGTH=215	202	215	7.00E-92	106.4	78.7	88.6

Rsa1.0_00046.1.g2657.t1	ref|XP_002888917.1| GDP-4-keto-6-deoxymannose-3,5-epimerase-4-reductase 1 [Arabidopsis lyrata subsp. lyrata] gi|297334758|gb|EFH65176.1| GDP-4-keto-6-deoxymannose-3,5-epimerase-4-reductase 1 [Arabidopsis lyrata subsp. lyrata]	313	323	1.00E-171	103.2	91.4	95.8	GDP-4-keto-6-deoxymannose-3,5-epimerase-4-reductase 1	gbpln	Arabidopsis lyrata	AT1G73250.1 | Symbols: ATFX, GER1 | GDP-4-keto-6-deoxymannose-3,5-epimerase-4-reductase 1 | chr1:27545213-27546360 REVERSE LENGTH=323	313	323	1.00E-174	103.2	91.4	95.8
Rsa1.0_00046.1.g2658.t1	ref|XP_002888916.1| hypothetical protein ARALYDRAFT_476456 [Arabidopsis lyrata subsp. lyrata] gi|297334757|gb|EFH65175.1| hypothetical protein ARALYDRAFT_476456 [Arabidopsis lyrata subsp. lyrata]	507	519	0	102.4	86.4	93.5	hypothetical protein ARALYDRAFT_476456	gbpln	Arabidopsis lyrata	AT1G73240.1 | Symbols: | CONTAINS InterPro DOMAIN/s: Nucleoporin protein Ndc1-Nup (InterPro:IPR019049); Has 36 Blast hits to 36 proteins in 17 species: Archae - 0; Bacteria - 0; Metazoa - 1; Fungi - 0; Plants - 35; Viruses - 0; Other Eukaryotes - 0 (source: NCBI BLink). | chr1:27542397-27544668 REVERSE LENGTH=519	507	519	0	102.4	86.2	93.1
Rsa1.0_00046.1.g2659.t1	ref|NP_177466.1| nascent polypeptide-associated complex subunit beta [Arabidopsis thaliana] gi|297842067|ref|XP_002888915.1| nascent polypeptide-associated complex domain-containing protein [Arabidopsis lyrata subsp. lyrata] gi|12324314|gb|AAG52123.1|AC010556_5 putative transcription factor BTF3 (RNA polymerase B transcription factor 3); 26343-27201 [Arabidopsis thaliana] gi|13877981|gb|AAK44068.1|AF370253_1 putative RNA polymerase B transcription factor BTF3 [Arabidopsis thaliana] gi|17104709|gb|AAL34243.1| putative RNA polymerase B transcription factor 3 [Arabidopsis thaliana] gi|297334756|gb|EFH65174.1| nascent polypeptide-associated complex domain-containing protein [Arabidopsis lyrata subsp. lyrata] gi|332197311|gb|AEE35432.1| nascent polypeptide-associated complex subunit beta [Arabidopsis thaliana]	167	165	1.00E-73	98.8	91.6	94.0	nascent polypeptide-associated complex subunit beta	gbpln	Arabidopsis lyrata	AT1G73230.1 | Symbols: | Nascent polypeptide-associated complex NAC | chr1:27540506-27541364 REVERSE LENGTH=165	167	165	5.00E-76	98.8	91.6	94.0
Rsa1.0_00046.1.g2660.t1	ref|NP_565059.2| organic cation/carnitine transporter1 [Arabidopsis thaliana] gi|75262291|sp|Q9CAT6.1|OCT1_ARATH RecName: Full=Organic cation/carnitine transporter 1; Short=AtOCT1 gi|12324316|gb|AAG52125.1|AC010556_7 putative transporter; 29320-27598 [Arabidopsis thaliana] gi|27311699|gb|AAO00815.1| putative transporter [Arabidopsis thaliana] gi|31711954|gb|AAP68333.1| At1g73220 [Arabidopsis thaliana] gi|332197310|gb|AEE35431.1| organic cation/carnitine transporter1 [Arabidopsis thaliana]	556	539	0	96.9	83.6	89.6	organic cation/carnitine transporter1	gbpln	Arabidopsis thaliana	AT1G73220.1 | Symbols: AtOCT1, 1-Oct | organic cation/carnitine transporter1 | chr1:27538387-27540109 FORWARD LENGTH=539	556	539	0	96.9	83.6	89.6
Rsa1.0_00046.1.g2661.t1	ref|NP_177464.1| uncharacterized protein [Arabidopsis thaliana] gi|12324319|gb|AAG52128.1|AC010556_10 hypothetical protein; 37254-39279 [Arabidopsis thaliana] gi|53828629|gb|AAU94424.1| At1g73210 [Arabidopsis thaliana] gi|332197308|gb|AEE35429.1| uncharacterized protein AT1G73210 [Arabidopsis thaliana]	312	314	1.00E-157	100.6	86.9	92.9	uncharacterized protein	gbpln	Arabidopsis thaliana	AT1G73210.1 | Symbols: | Protein of unknown function (DUF789) | chr1:27528428-27530453 REVERSE LENGTH=314	312	314	1.00E-159	100.6	86.9	92.9
Rsa1.0_00046.1.g2662.t1	ref|XP_002888912.1| hypothetical protein ARALYDRAFT_895179 [Arabidopsis lyrata subsp. lyrata] gi|297334753|gb|EFH65171.1| hypothetical protein ARALYDRAFT_895179 [Arabidopsis lyrata subsp. lyrata]	763	779	0	102.1	79.4	87.8	hypothetical protein ARALYDRAFT_895179	gbpln	Arabidopsis lyrata	AT1G73200.1 | Symbols: | Putative integral membrane protein conserved region (DUF2404) | chr1:27523454-27526573 REVERSE LENGTH=779	763	779	0	102.1	78.5	87.3
Rsa1.0_00046.1.g2663.t1	gb|EOA34775.1| hypothetical protein CARUB_v10022350mg [Capsella rubella]	267	265	1.00E-130	99.3	93.3	97.0	hypothetical protein CARUB_v10022350mg	gbpln	Capsella rubella	AT1G73190.1 | Symbols: ALPHA-TIP, TIP3;1 | Aquaporin-like superfamily protein | chr1:27522155-27523171 FORWARD LENGTH=268	267	268	1.00E-129	100.4	91.4	95.9

Rsa1.0_00046.1.g2664.t1	ref|NP_177455.1| uncharacterized protein [Arabidopsis thaliana] gi|12324324|gb|AAG52133.1|AC010556_15 hypothetical protein; 69822-70342 [Arabidopsis thaliana] gi|26449662|dbj|BAC41955.1| unknown protein [Arabidopsis thaliana] gi|28416835|gb|AAO42948.1| At1g73120 [Arabidopsis thaliana] gi|332197295|gb|AEE35416.1| uncharacterized protein AT1G73120 [Arabidopsis thaliana]	110	109	7.00E-46	99.1	81.8	90.0	uncharacterized protein	gbpln	Arabidopsis thaliana	AT1G73120.1 | Symbols: | unknown protein; FUNCTIONS IN: molecular_function unknown; INVOLVED IN: response to oxidative stress; EXPRESSED IN: root, cultured cell; Has 36 Blast hits to 36 proteins in 8 species: Archae - 0; Bacteria - 0; Metazoa - 0; Fungi - 0; Plants - 36; Viruses - 0; Other Eukaryotes - 0 (source: NCBI BLink). | chr1:27497365-27497885 REVERSE LENGTH=109	110	109	1.00E-48	99.1	81.8	90.0
Rsa1.0_00046.1.g2665.t2	ref|NP_177454.1| putative Rubisco activase 2 [Arabidopsis thaliana] gi|13605706|gb|AAK32846.1|AF361834_1 At1g73110/F3N23_39 [Arabidopsis thaliana] gi|18700270|gb|AAL77745.1| At1g73110/F3N23_39 [Arabidopsis thaliana] gi|26452888|dbj|BAC43522.1| unknown protein [Arabidopsis thaliana] gi|332197294|gb|AEE35415.1| putative Rubisco activase 2 [Arabidopsis thaliana]	435	432	0	99.3	90.6	94.0	putative Rubisco activase 2	gbpln	Arabidopsis thaliana	AT1G73110.1 | Symbols: | P-loop containing nucleoside triphosphate hydrolases superfamily protein | chr1:27494344-27496844 REVERSE LENGTH=432	435	432	0	99.3	90.6	94.0
Rsa1.0_00046.1.g2666.t1	ref|XP_002887472.1| hypothetical protein ARALYDRAFT_895166 [Arabidopsis lyrata subsp. lyrata] gi|297333313|gb|EFH63731.1| hypothetical protein ARALYDRAFT_895166 [Arabidopsis lyrata subsp. lyrata]	672	669	0	99.6	81.7	88.1	hypothetical protein ARALYDRAFT_895166	gbpln	Arabidopsis lyrata	AT1G73100.1 | Symbols: SUVH3, SDG19 | SU(VAR)3-9 homolog 3 | chr1:27491970-27493979 FORWARD LENGTH=669	672	669	0	99.6	79.9	86.9
Rsa1.0_00046.1.g2667.t1	ref|NP_001117591.1| Leucine-rich repeat family protein [Arabidopsis thaliana] gi|5903096|gb|AAD55654.1|AC008017_27 Highly similar to receptor-like protein kinase [Arabidopsis thaliana] gi|26449861|dbj|BAC42053.1| unknown protein [Arabidopsis thaliana] gi|332197289|gb|AEE35410.1| Leucine-rich repeat family protein [Arabidopsis thaliana]	951	598	0	62.9	49.8	54.4	Leucine-rich repeat family protein	gbpln	Arabidopsis thaliana	AT1G73066.1 | Symbols: | Leucine-rich repeat family protein | chr1:27481785-27483581 FORWARD LENGTH=598	951	598	0	62.9	49.8	54.4
Rsa1.0_00046.1.g2668.t1	gb|EOA33724.1| hypothetical protein CARUB_v10019915mg [Capsella rubella]	660	664	0	100.6	91.5	95.9	hypothetical protein CARUB_v10019915mg	gbpln	Capsella rubella	AT1G73020.2 | Symbols: | unknown protein; FUNCTIONS IN: molecular_function unknown; INVOLVED IN: biological_process unknown; LOCATED IN: cellular_component unknown; EXPRESSED IN: 22 plant structures; EXPRESSED DURING: 13 growth stages; CONTAINS InterPro DOMAIN/s: Protein of unknown function DUF590 (InterPro:IPR007632). | chr1:27469714-27473181 REVERSE LENGTH=665	660	665	0	100.8	90.5	95.2
Rsa1.0_00046.1.g2669.t1	ref|XP_002888902.1| hypothetical protein ARALYDRAFT_476431 [Arabidopsis lyrata subsp. lyrata] gi|297334743|gb|EFH65161.1| hypothetical protein ARALYDRAFT_476431 [Arabidopsis lyrata subsp. lyrata]	292	294	1.00E-139	100.7	86.0	91.8	hypothetical protein ARALYDRAFT_476431	gbpln	Arabidopsis lyrata	AT1G73010.1 | Symbols: ATPS2, PS2 | phosphate starvation-induced gene 2 | chr1:27464780-27466180 REVERSE LENGTH=295	292	295	1.00E-139	101.0	84.2	90.8
Rsa1.0_00046.1.g2670.t1	gb|EOA20703.1| hypothetical protein CARUB_v10001020mg [Capsella rubella]	145	427	7.00E-32	294.5	52.4	55.2	hypothetical protein CARUB_v10001020mg	gbpln	Capsella rubella	AT5G25220.1 | Symbols: KNAT3 | KNOTTED1-like homeobox gene 3 | chr5:8736208-8738115 FORWARD LENGTH=431	145	431	2.00E-34	297.2	52.4	55.2
Rsa1.0_00046.1.g2671.t1	gb|EOA33560.1| hypothetical protein CARUB_v10019685mg [Capsella rubella]	1159	1170	0	100.9	84.8	92.1	hypothetical protein CARUB_v10019685mg	gbpln	Capsella rubella	AT1G72960.1 | Symbols: | Root hair defective 3 GTP-binding protein (RHD3) | chr1:27446263-27450862 FORWARD LENGTH=795	1159	795	0	68.6	61.8	65.3
Rsa1.0_00046.1.g2672.t5	gb|EOA31387.1| hypothetical protein CARUB_v10014563mg [Capsella rubella]	129	226	2.00E-16	175.2	51.9	61.2	hypothetical protein CARUB_v10014563mg	gbpln	Capsella rubella	AT3G16770.1 | Symbols: RAP2.3, ATEBP, ERF72, EBP | ethylene-responsive element binding protein | chr3:5705784-5706768 FORWARD LENGTH=248	129	248	7.00E-13	192.2	26.4	27.9
Rsa1.0_00046.1.g2673.t1	gb|ACP30576.1| disease resistance protein [Brassica rapa subsp. pekinensis]	437	426	0	97.5	81.7	87.6	disease resistance protein	gbpln	Brassica rapa	AT1G72890.1 | Symbols: | Disease resistance protein (TIR-NBS class) | chr1:27429947-27431717 FORWARD LENGTH=438	437	438	1.00E-173	100.2	71.2	83.1
Rsa1.0_00046.1.g2674.t2	#	#	#	#	#	#	#	-	----	----	#	#	#	#	#	#	#
Rsa1.0_00046.1.g2675.t1	gb|EOA35268.1| hypothetical protein CARUB_v10020451mg [Capsella rubella]	381	385	0	101.0	86.4	92.9	hypothetical protein CARUB_v10020451mg	gbpln	Capsella rubella	AT1G72880.1 | Symbols: | Survival protein SurE-like phosphatase/nucleotidase | chr1:27423678-27425928 REVERSE LENGTH=385	381	385	0	101.0	86.1	91.6

Rsa1.0_00046.1.g2676.t2	ref|XP_002887456.1| hypothetical protein ARALYDRAFT_895140 [Arabidopsis lyrata subsp. lyrata] gi|297333297|gb|EFH63715.1| hypothetical protein ARALYDRAFT_895140 [Arabidopsis lyrata subsp. lyrata]	676	409	1.00E-68	60.5	22.2	28.6	hypothetical protein ARALYDRAFT_895140	gbpln	Arabidopsis lyrata	AT1G72870.1 | Symbols: | Disease resistance protein (TIR-NBS class) | chr1:27421086-27422999 FORWARD LENGTH=512	676	512	2.00E-54	75.7	24.4	37.4
Rsa1.0_00046.1.g2677.t1	ref|XP_002888896.1| predicted protein [Arabidopsis lyrata subsp. lyrata] gi|297334737|gb|EFH65155.1| predicted protein [Arabidopsis lyrata subsp. lyrata]	154	1169	1.00E-66	759.1	81.8	89.6	predicted protein	gbpln	Arabidopsis lyrata	AT1G72860.1 | Symbols: | Disease resistance protein (TIR-NBS-LRR class) family | chr1:27417096-27420778 REVERSE LENGTH=1163	154	1163	1.00E-48	755.2	55.8	71.4
Rsa1.0_00046.1.g2678.t1	ref|XP_002888896.1| predicted protein [Arabidopsis lyrata subsp. lyrata] gi|297334737|gb|EFH65155.1| predicted protein [Arabidopsis lyrata subsp. lyrata]	922	1169	0	126.8	74.0	83.9	predicted protein	gbpln	Arabidopsis lyrata	AT4G09360.1 | Symbols: | NB-ARC domain-containing disease resistance protein | chr4:5940186-5943280 FORWARD LENGTH=853	922	853	0	92.5	56.6	66.6
Rsa1.0_00046.1.g2679.t1	ref|NP_192680.1| TIR-NBS class of disease resistance protein [Arabidopsis thaliana] gi|7267584|emb|CAB78065.1| putative protein [Arabidopsis thaliana] gi|21689643|gb|AAM67443.1| unknown protein [Arabidopsis thaliana] gi|332657346|gb|AEE82746.1| TIR-NBS class of disease resistance protein [Arabidopsis thaliana]	450	457	1.00E-126	101.6	57.6	73.1	TIR-NBS class of disease resistance protein	gbpln	Arabidopsis thaliana	AT4G09420.1 | Symbols: | Disease resistance protein (TIR-NBS class) | chr4:5962319-5963776 REVERSE LENGTH=457	450	457	1.00E-129	101.6	57.6	73.1
Rsa1.0_00046.1.g2680.t1	ref|NP_177427.2| TIR-NBS-LRR class disease resistance protein [Arabidopsis thaliana] gi|110737528|dbj|BAF00706.1| hypothetical protein [Arabidopsis thaliana] gi|332197259|gb|AEE35380.1| TIR-NBS-LRR class disease resistance protein [Arabidopsis thaliana]	1017	1042	0	102.5	53.2	65.0	TIR-NBS-LRR class disease resistance protein	gbpln	Arabidopsis thaliana	AT1G72840.1 | Symbols: | Disease resistance protein (TIR-NBS-LRR class) | chr1:27410020-27413485 REVERSE LENGTH=1042	1017	1042	0	102.5	53.2	65.0
Rsa1.0_00046.1.g2681.t1	ref|NP_565049.1| nuclear transcription factor Y subunit A-3 [Arabidopsis thaliana] gi|81174954|sp|Q93ZH2.2|NFYA3_ARATH RecName: Full=Nuclear transcription factor Y subunit A-3; Short=AtNF-YA-3; AltName: Full=Transcriptional activator HAP2C gi|5903072|gb|AAD55630.1|AC008017_3 Transcription Factor [Arabidopsis thaliana] gi|22655158|gb|AAM98169.1| CCAAT-binding factor B subunit-like protein, putative [Arabidopsis thaliana] gi|31711816|gb|AAP68264.1| At1g72830 [Arabidopsis thaliana] gi|332197257|gb|AEE35378.1| nuclear transcription factor Y subunit A-3 [Arabidopsis thaliana]	302	340	1.00E-137	112.6	84.8	91.1	nuclear transcription factor Y subunit A-3	gbpln	Arabidopsis thaliana	AT1G72830.1 | Symbols: HAP2C, ATHAP2C, NF-YA3 | nuclear factor Y, subunit A3 | chr1:27405699-27407088 REVERSE LENGTH=340	302	340	1.00E-140	112.6	84.8	91.1
Rsa1.0_00046.1.g2682.t1	ref|NP_565049.1| nuclear transcription factor Y subunit A-3 [Arabidopsis thaliana] gi|81174954|sp|Q93ZH2.2|NFYA3_ARATH RecName: Full=Nuclear transcription factor Y subunit A-3; Short=AtNF-YA-3; AltName: Full=Transcriptional activator HAP2C gi|5903072|gb|AAD55630.1|AC008017_3 Transcription Factor [Arabidopsis thaliana] gi|22655158|gb|AAM98169.1| CCAAT-binding factor B subunit-like protein, putative [Arabidopsis thaliana] gi|31711816|gb|AAP68264.1| At1g72830 [Arabidopsis thaliana] gi|332197257|gb|AEE35378.1| nuclear transcription factor Y subunit A-3 [Arabidopsis thaliana]	116	340	4.00E-37	293.1	75.9	83.6	nuclear transcription factor Y subunit A-3	gbpln	Arabidopsis thaliana	AT1G72830.1 | Symbols: HAP2C, ATHAP2C, NF-YA3 | nuclear factor Y, subunit A3 | chr1:27405699-27407088 REVERSE LENGTH=340	116	340	6.00E-40	293.1	75.9	83.6
Rsa1.0_00046.1.g2683.t1	gb|EOA35384.1| hypothetical protein CARUB_v10020581mg [Capsella rubella] gi|482571197|gb|EOA35385.1| hypothetical protein CARUB_v10020581mg [Capsella rubella]	350	346	0	98.9	91.1	94.3	hypothetical protein CARUB_v10020581mg	gbpln	Capsella rubella	AT1G72820.1 | Symbols: | Mitochondrial substrate carrier family protein | chr1:27403457-27404506 FORWARD LENGTH=349	350	349	1.00E-179	99.7	91.1	94.9
Rsa1.0_00046.1.g2684.t1	gb|EOA35815.1| hypothetical protein CARUB_v10021053mg [Capsella rubella] gi|482571628|gb|EOA35816.1| hypothetical protein CARUB_v10021053mg [Capsella rubella]	156	166	2.00E-68	106.4	84.6	89.7	hypothetical protein CARUB_v10021053mg	gbpln	Capsella rubella	AT1G72510.2 | Symbols: | Protein of unknown function (DUF1677) | chr1:27303906-27304403 FORWARD LENGTH=165	156	165	1.00E-70	105.8	84.0	91.0

Rsa1.0_00047.1.g2685.t1	ref|NP_568344.2| myb family transcription factor [Arabidopsis thaliana] gi|332005027|gb|AED92410.1| myb family transcription factor [Arabidopsis thaliana]	372	387	1.00E-132	104.0	75.5	82.3	myb family transcription factor	gbpln	Arabidopsis thaliana	AT5G17300.1 | Symbols: RVE1 | Homeodomain-like superfamily protein | chr5:5690435-5692435 REVERSE LENGTH=387	372	387	1.00E-135	104.0	75.5	82.3
Rsa1.0_00047.1.g2686.t1	ref|NP_197231.1| autophagy protein 5 [Arabidopsis thaliana] gi|75170235|sp|Q9FFI2.1|ATG5_ARATH RecName: Full=Autophagy protein 5; AltName: Full=Protein autophagy 5; Short=AtAPG5 gi|10177074|dbj|BAB10516.1| APG5 (autophagy 5)-like protein [Arabidopsis thaliana] gi|26450228|dbj|BAC42232.1| APG5 (autophagy 5) like protein [Arabidopsis thaliana] gi|28827578|gb|AAO50633.1| putative APG5 (autophagy 5) protein [Arabidopsis thaliana] gi|332005026|gb|AED92409.1| autophagy protein 5 [Arabidopsis thaliana]	334	337	1.00E-175	100.9	89.2	95.2	autophagy protein 5	gbpln	Arabidopsis thaliana	AT5G17290.1 | Symbols: ATG5, APG5, ATATG5 | autophagy protein Apg5 family | chr5:5687160-5689821 REVERSE LENGTH=337	334	337	1.00E-177	100.9	89.2	95.2
Rsa1.0_00047.1.g2687.t5	gb|EOA22499.1| hypothetical protein CARUB_v10003154mg [Capsella rubella]	134	143	2.00E-25	106.7	63.4	68.7	hypothetical protein CARUB_v10003154mg	gbpln	Capsella rubella	AT5G17280.1 | Symbols: | CONTAINS InterPro DOMAIN/s: Oxidoreductase-like, N-terminal (InterPro:IPR019180); Has 1807 Blast hits to 1807 proteins in 277 species: Archae - 0; Bacteria - 0; Metazoa - 736; Fungi - 347; Plants - 385; Viruses - 0; Other Eukaryotes - 339 (source: NCBI BLink). | chr5:5686355-5686762 FORWARD LENGTH=135	134	135	2.00E-26	100.7	58.2	65.7
Rsa1.0_00047.1.g2688.t1	gb|EOA23087.1| hypothetical protein CARUB_v10003869mg [Capsella rubella]	498	489	0	98.2	78.9	85.1	hypothetical protein CARUB_v10003869mg	gbpln	Capsella rubella	AT5G17260.1 | Symbols: anac086, NAC086 | NAC domain containing protein 86 | chr5:5675323-5677912 REVERSE LENGTH=476	498	476	0	95.6	76.7	83.5
Rsa1.0_00047.1.g2689.t1	ref|NP_197227.4| alkaline-phosphatase-like protein [Arabidopsis thaliana] gi|332005021|gb|AED92404.1| alkaline-phosphatase-like protein [Arabidopsis thaliana]	1088	925	0	85.0	74.2	78.4	alkaline-phosphatase-like protein	gbpln	Arabidopsis thaliana	AT5G17250.1 | Symbols: | Alkaline-phosphatase-like family protein | chr5:5668993-5673516 REVERSE LENGTH=925	1088	925	0	85.0	74.2	78.4
Rsa1.0_00047.1.g2690.t1	gb|AEX31286.1| phytoene synthase [Brassica oleracea] gi|371486488|gb|AEX31289.1| phytoene synthase [Brassica napus]	423	424	0	100.2	95.3	97.6	phytoene synthase	gbpln	Brassica napus	AT5G17230.2 | Symbols: PSY | PHYTOENE SYNTHASE | chr5:5659839-5662087 REVERSE LENGTH=422	423	422	0	99.8	89.4	92.2
Rsa1.0_00047.1.g2691.t1	dbj|BAJ34302.1| unnamed protein product [Thellungiella halophila]	213	214	1.00E-108	100.5	87.8	93.9	unnamed protein product	----	----	AT5G17220.1 | Symbols: ATGSTF12, GST26, TT19, GSTF12 | glutathione S-transferase phi 12 | chr5:5658528-5659322 FORWARD LENGTH=214	213	214	1.00E-104	100.5	83.1	92.0
Rsa1.0_00047.1.g2692.t1	ref|XP_002871754.1| hypothetical protein ARALYDRAFT_488583 [Arabidopsis lyrata subsp. lyrata] gi|297317591|gb|EFH48013.1| hypothetical protein ARALYDRAFT_488583 [Arabidopsis lyrata subsp. lyrata]	215	212	4.00E-91	98.6	74.9	87.4	hypothetical protein ARALYDRAFT_488583	gbpln	Arabidopsis lyrata	AT5G17210.1 | Symbols: | Protein of unknown function (DUF1218) | chr5:5656519-5657970 FORWARD LENGTH=209	215	209	8.00E-92	97.2	73.0	85.1
Rsa1.0_00047.1.g2693.t1	ref|NP_197222.1| polygalacturonase [Arabidopsis thaliana] gi|10177065|dbj|BAB10507.1| polygalacturonase-like protein [Arabidopsis thaliana] gi|332005012|gb|AED92395.1| pectin lyase-like superfamily protein [Arabidopsis thaliana]	428	421	1.00E-161	98.4	68.0	78.7	polygalacturonase	gbpln	Arabidopsis thaliana	AT5G17200.1 | Symbols: | Pectin lyase-like superfamily protein | chr5:5653524-5655336 REVERSE LENGTH=421	428	421	1.00E-164	98.4	68.0	78.7
Rsa1.0_00047.1.g2694.t1	ref|NP_197220.1| uncharacterized protein [Arabidopsis thaliana] gi|10177063|dbj|BAB10505.1| unnamed protein product [Arabidopsis thaliana] gi|117168077|gb|ABK32121.1| At5g17180 [Arabidopsis thaliana] gi|332005010|gb|AED92393.1| uncharacterized protein AT5G17180 [Arabidopsis thaliana]	124	123	8.00E-14	99.2	37.9	48.4	uncharacterized protein	gbpln	Arabidopsis thaliana	AT5G17180.1 | Symbols: | unknown protein; Has 1807 Blast hits to 1807 proteins in 277 species: Archae - 0; Bacteria - 0; Metazoa - 736; Fungi - 347; Plants - 385; Viruses - 0; Other Eukaryotes - 339 (source: NCBI BLink). | chr5:5651436-5651807 FORWARD LENGTH=123	124	123	2.00E-16	99.2	37.9	48.4

Rsa1.0_00047.1_g2695.t1	ref|XP_002873815.1| hypothetical protein ARALYDRAFT_909708 [Arabidopsis lyrata subsp. lyrata] gi|297319652|gb|EFH50074.1| hypothetical protein ARALYDRAFT_909708 [Arabidopsis lyrata subsp. lyrata]	123	120	1.00E-46	97.6	78.0	85.4	hypothetical protein ARALYDRAFT_909708	gbpln	Arabidopsis lyrata	AT5G17165.1 | Symbols: | unknown protein; FUNCTIONS IN: molecular_function unknown; INVOLVED IN: biological_process unknown; LOCATED IN: cellular_component unknown; BEST Arabidopsis thaliana protein match is: unknown protein (TAIR:AT3G03150.1); Has 39 Blast hits to 39 proteins in 11 species: Archae - 0; Bacteria - 0; Metazoa - 0; Fungi - 0; Plants - 39; Viruses - 0; Other Eukaryotes - 0 (source: NCBI BLink). | chr5:5643143-5647702 REVERSE LENGTH=116	123	116	5.00E-45	94.3	73.2	83.7
Rsa1.0_00047.1_g2696.t1	ref|XP_002873814.1| hypothetical protein ARALYDRAFT_488576 [Arabidopsis lyrata subsp. lyrata] gi|297319651|gb|EFH50073.1| hypothetical protein ARALYDRAFT_488576 [Arabidopsis lyrata subsp. lyrata]	767	568	1.00E-111	74.1	34.2	40.3	hypothetical protein ARALYDRAFT_488576	gbpln	Arabidopsis lyrata	AT5G17160.1 | Symbols: | unknown protein; BEST Arabidopsis thaliana protein match is: unknown protein (TAIR:AT3G03130.1); Has 14330 Blast hits to 10381 proteins in 896 species: Archae - 94; Bacteria - 2881; Metazoa - 4019; Fungi - 1576; Plants - 515; Viruses - 110; Other Eukaryotes - 5135 (source: NCBI BLink). | chr5:5639843-5642427 REVERSE LENGTH=569	767	569	1.00E-113	74.2	33.4	40.4
Rsa1.0_00047.1_g2697.t1	gb|AAM19705.1|AF499718_1 protein phosphatase 2c-like protein [Eutrema halophilum]	233	378	8.00E-16	162.2	22.3	26.6	protein phosphatase 2c-like protein	gbpln	Eutrema halophilum	AT3G51370.2 | Symbols: | Protein phosphatase 2C family protein | chr3:19070387-19071975 FORWARD LENGTH=294	233	294	1.00E-15	126.2	20.6	25.3
Rsa1.0_00047.1_g2698.t3	ref|XP_002871747.1| hypothetical protein ARALYDRAFT_488569 [Arabidopsis lyrata subsp. lyrata] gi|297317584|gb|EFH48006.1| hypothetical protein ARALYDRAFT_488569 [Arabidopsis lyrata subsp. lyrata] gi|482557425|gb|EOA21617.1| hypothetical protein CARUB_v10002030mg [Capsella rubella]	211	192	1.00E-105	91.0	90.0	91.0	hypothetical protein ARALYDRAFT_488569	gbpln	Arabidopsis lyrata	AT5G17060.1 | Symbols: ATARFB1B, ARFB1B | ADP-ribosylation factor B1B | chr5:5611056-5612639 FORWARD LENGTH=192	211	192	1.00E-106	91.0	89.6	90.5
Rsa1.0_00047.1_g2699.t6	ref|XP_002871746.1| hypothetical protein ARALYDRAFT_909689 [Arabidopsis lyrata subsp. lyrata] gi|297317583|gb|EFH48005.1| hypothetical protein ARALYDRAFT_909689 [Arabidopsis lyrata subsp. lyrata]	1072	1076	0	100.4	94.5	96.5	hypothetical protein ARALYDRAFT_909689	gbpln	Arabidopsis lyrata	AT5G17020.1 | Symbols: XPO1A, ATCRM1, ATXPO1, XPO1, HIT2 | exportin 1A | chr5:5594904-5602467 FORWARD LENGTH=1075	1072	1075	0	100.3	94.2	96.4
Rsa1.0_00047.1_g2700.t1	dbj|BAJ33830.1| unnamed protein product [Thellungiella halophila]	352	351	0	99.7	90.3	96.0	unnamed protein product	----	----	AT5G16970.1 | Symbols: AT-AER, AER | alkenal reductase | chr5:5576291-5578001 REVERSE LENGTH=345	352	345	1.00E-178	98.0	84.4	90.9
Rsa1.0_00047.1_g2701.t1	ref|NP_197195.2| AAA-type ATPase family protein [Arabidopsis thaliana] gi|19424059|gb|AAL87351.1| unknown protein [Arabidopsis thaliana] gi|22136966|gb|AAM91712.1| unknown protein [Arabidopsis thaliana] gi|332004976|gb|AED92359.1| AAA-type ATPase family protein [Arabidopsis thaliana]	663	644	0	97.1	87.6	92.0	AAA-type ATPase family protein	gbpln	Arabidopsis thaliana	AT5G16930.1 | Symbols: | AAA-type ATPase family protein | chr5:5568578-5571565 FORWARD LENGTH=644	663	644	0	97.1	87.6	92.0
Rsa1.0_00047.1_g2702.t1	ref|XP_002871742.1| hypothetical protein ARALYDRAFT_488554 [Arabidopsis lyrata subsp. lyrata] gi|297317579|gb|EFH48001.1| hypothetical protein ARALYDRAFT_488554 [Arabidopsis lyrata subsp. lyrata]	1135	1143	0	100.7	89.1	93.5	hypothetical protein ARALYDRAFT_488554	gbpln	Arabidopsis lyrata	AT5G16910.1 | Symbols: ATCSLD2, CSLD2 | cellulose-synthase like D2 | chr5:5561679-5565290 FORWARD LENGTH=1145	1135	1145	0	100.9	87.8	92.4
Rsa1.0_00047.1_g2703.t1	gb|EOA22278.1| hypothetical protein CARUB_v10002876mg [Capsella rubella]	505	513	0	101.6	86.7	91.7	hypothetical protein CARUB_v10002876mg	gbpln	Capsella rubella	AT5G16890.1 | Symbols: | Exostosin family protein | chr5:5551664-5554741 FORWARD LENGTH=511	505	511	0	101.2	84.8	89.3
Rsa1.0_00047.1_g2704.t1	ref|XP_002870649.1| hypothetical protein ARALYDRAFT_355852 [Arabidopsis lyrata subsp. lyrata] gi|297316485|gb|EFH46908.1| hypothetical protein ARALYDRAFT_355852 [Arabidopsis lyrata subsp. lyrata]	756	634	1.00E-121	83.9	37.0	45.4	hypothetical protein ARALYDRAFT_355852	gbpln	Arabidopsis lyrata	AT5G41690.1 | Symbols: | RNA-binding (RRM/RBD/RNP motifs) family protein | chr5:16670126-16674189 REVERSE LENGTH=567	756	567	1.00E-106	75.0	32.8	41.4

Rsa1.0_00047.1.g2705.t1	ref|NP_197190.1| Target of Myb protein 1 [Arabidopsis thaliana] gi|30686076|ref|NP_850833.1| Target of Myb protein 1 [Arabidopsis thaliana] gi|9755689|emb|CAC01701.1| TOM (target of myb1)-like protein [Arabidopsis thaliana] gi|15983761|gb|AAL10477.1| AT5g16880/F2K13_30 [Arabidopsis thaliana] gi|17065128|gb|AAL32718.1| TOM (target of myb1)-like protein [Arabidopsis thaliana] gi|21537352|gb|AAM61693.1| TOM (target of myb1)-like protein [Arabidopsis thaliana] gi|27311895|gb|AAO00913.1| TOM (target of myb1)-like protein [Arabidopsis thaliana] gi|332004970|gb|AED92353.1| Target of Myb protein 1 [Arabidopsis thaliana] gi|332004971|gb|AED92354.1| Target of Myb protein 1 [Arabidopsis thaliana]	408	407	0	99.8	89.7	93.9	Target of Myb protein 1	gbpln	Arabidopsis thaliana	AT5G16880.2 | Symbols: | Target of Myb protein 1 | chr5:5549658-5551274 FORWARD LENGTH=407	408	407	0	99.8	89.7	93.9
Rsa1.0_00047.1.g2706.t2	ref|NP_568340.1| Peptidyl-tRNA hydrolase II (PTH2) family protein [Arabidopsis thaliana] gi|98960911|gb|ABF58939.1| At5g16870 [Arabidopsis thaliana] gi|332004968|gb|AED92351.1| Peptidyl-tRNA hydrolase II (PTH2) family protein [Arabidopsis thaliana]	175	169	7.00E-79	96.6	89.7	90.9	Peptidyl-tRNA hydrolase II (PTH2) family protein	gbpln	Arabidopsis thaliana	AT5G16870.1 | Symbols: | Peptidyl-tRNA hydrolase II (PTH2) family protein | chr5:5546975-5548542 REVERSE LENGTH=169	175	169	3.00E-81	96.6	89.7	90.9
Rsa1.0_00047.1.g2707.t1	ref|XP_002871739.1| pentatricopeptide repeat-containing protein [Arabidopsis lyrata subsp. lyrata] gi|297317576|gb|EFH47998.1| pentatricopeptide repeat-containing protein [Arabidopsis lyrata subsp. lyrata]	837	850	0	101.6	85.7	92.5	pentatricopeptide repeat-containing protein	gbpln	Arabidopsis lyrata	AT5G16860.1 | Symbols: | Tetratricopeptide repeat (TPR)-like superfamily protein | chr5:5543834-5546386 FORWARD LENGTH=850	837	850	0	101.6	84.9	91.8
Rsa1.0_00047.1.g2708.t2	gb|AAD54276.1|AF172097_1 telomerase reverse transcriptase catalytic subunit [Arabidopsis thaliana]	1066	1123	0	105.3	74.2	81.9	telomerase reverse transcriptase catalytic subunit	gbpln	Arabidopsis thaliana	AT5G16850.1 | Symbols: ATTERT, TERT | telomerase reverse transcriptase | chr5:5538323-5543444 REVERSE LENGTH=1123	1066	1123	0	105.3	74.2	81.9
Rsa1.0_00047.1.g2709.t1	gb|EOA19437.1| hypothetical protein CARUB_v10001749mg [Capsella rubella]	262	260	1.00E-118	99.2	84.7	90.5	hypothetical protein CARUB_v10001749mg	gbpln	Capsella rubella	AT5G16840.1 | Symbols: BPA1 | binding partner of acd11 1 | chr5:5536042-5538026 FORWARD LENGTH=259	262	259	1.00E-119	98.9	85.9	92.4
Rsa1.0_00047.1.g2710.t1	ref|XP_002887653.1| 12-oxophytodienoate reductase 1 [Arabidopsis lyrata subsp. lyrata] gi|297333494|gb|EFH63912.1| 12-oxophytodienoate reductase 1 [Arabidopsis lyrata subsp. lyrata]	369	372	0	100.8	89.7	93.8	12-oxophytodienoate reductase 1	gbpln	Arabidopsis lyrata	AT1G76680.1 | Symbols: OPR1, ATOPR1 | 12-oxophytodienoate reductase 1 | chr1:28776982-28778271 FORWARD LENGTH=372	369	372	0	100.8	88.6	93.0
Rsa1.0_00047.1.g2711.t1	gb|ABB83612.1| syntaxin-like protein [Brassica oleracea]	281	276	1.00E-141	98.2	89.3	92.9	syntaxin-like protein	gbpln	Brassica oleracea	AT5G16830.1 | Symbols: SYP21, ATSYP21, PEP12, ATPEP12, PEP12P | syntaxin of plants 21 | chr5:5533076-5535152 REVERSE LENGTH=279	281	279	1.00E-130	99.3	81.1	89.0
Rsa1.0_00047.1.g2712.t1	gb|EOA22596.1| hypothetical protein CARUB_v10003259mg [Capsella rubella]	468	478	0	102.1	73.5	82.5	hypothetical protein CARUB_v10003259mg	gbpln	Capsella rubella	AT5G16820.2 | Symbols: HSF3, HSFA1B, ATHSFA1B | heat shock factor 3 | chr5:5530446-5532497 FORWARD LENGTH=481	468	481	1.00E-179	102.8	72.0	80.1
Rsa1.0_00047.1.g2713.t2	ref|NP_974793.1| GCN5-related N-acetyltransferase (GNAT) family protein [Arabidopsis thaliana] gi|44681356|gb|AAS47618.1| At5g16800 [Arabidopsis thaliana] gi|45773876|gb|AAS76742.1| At5g16800 [Arabidopsis thaliana] gi|332004956|gb|AED92339.1| GCN5-related N-acetyltransferase (GNAT) family protein [Arabidopsis thaliana]	257	270	1.00E-132	105.1	88.3	93.8	GCN5-related N-acetyltransferase (GNAT) family protein	gbpln	Arabidopsis thaliana	AT5G16800.2 | Symbols: | Acyl-CoA N-acyltransferases (NAT) superfamily protein | chr5:5524509-5526295 REVERSE LENGTH=270	257	270	1.00E-134	105.1	88.3	93.8
Rsa1.0_00047.1.g2714.t1	ref|XP_002873791.1| hypothetical protein ARALYDRAFT_488536 [Arabidopsis lyrata subsp. lyrata] gi|297319628|gb|EFH50050.1| hypothetical protein ARALYDRAFT_488536 [Arabidopsis lyrata subsp. lyrata]	812	812	0	100.0	82.8	89.5	hypothetical protein ARALYDRAFT_488536	gbpln	Arabidopsis lyrata	AT5G16780.1 | Symbols: DOT2, MDF | SART-1 family | chr5:5517784-5521566 REVERSE LENGTH=820	812	820	0	101.0	82.4	89.2
Rsa1.0_00047.1.g2715.t1	emb|CAC01841.1| putative transcription factor (MYB9) [Arabidopsis thaliana]	328	325	1.00E-156	99.1	83.8	89.6	putative transcription factor (MYB9)	gbpln	Arabidopsis thaliana	AT5G16770.2 | Symbols: AtMYB9, MYB9 | myb domain protein 9 | chr5:5515209-5516734 FORWARD LENGTH=336	328	336	1.00E-158	102.4	83.8	89.6

Rsa1.0_00047.1.g2716.t1	ref|NP_197178.1| inositol-tetrakisphosphate 1-kinase 1 [Arabidopsis thaliana] gi|75202063|sp|Q9SBA5.1|ITPK1_ARATH RecName: Full=Inositol-tetrakisphosphate 1-kinase 1; AltName: Full=Inositol 1,3,4-trisphosphate 5/6-kinase 1; Short=AtItpk-1; Short=Inositol-triphosphate 5/6-kinase 1; Short=Ins(1,3,4)P(3) 5/6-kinase 1 gi|3660465|emb|CAA04976.1| Inositol 1,3,4-Trisphosphate 5/6 kinase [Arabidopsis thaliana] gi|9755728|emb|CAC01840.1| Inositol 1, 3, 4-Trisphosphate 5/6 kinase [Arabidopsis thaliana] gi|18176069|gb|AAL59978.1| putative Inositol 1,3,4-Trisphosphate 5/6 kinase [Arabidopsis thaliana] gi|20465297|gb|AAM20052.1| putative inositol 1,3,4-trisphosphate 5/6 kinase [Arabidopsis thaliana] gi|21592636|gb|AAM64585.1| inositol 1,3,4-Trisphosphate 5/6 kinase [Arabidopsis thaliana] gi|332004951|gb|AED92334.1| inositol-tetrakisphosphate 1-kinase 1 [Arabidopsis thaliana	326	319	1.00E-158	97.9	86.5	92.3	inositol-tetrakisphosphate 1-kinase 1	gbpln	Arabidopsis thaliana	AT5G16760.1 | Symbols: | Inositol 1,3,4-trisphosphate 5/6-kinase family protein | chr5:5509890-5510849 FORWARD LENGTH=319	326	319	1.00E-161	97.9	86.5	92.3
Rsa1.0_00047.1.g2717.t1	gb|EOA19941.1| hypothetical protein CARUB_v10000193mg [Capsella rubella]	872	876	0	100.5	92.3	96.3	hypothetical protein CARUB_v10000193mg	gbpln	Capsella rubella	AT5G16750.1 | Symbols: TOZ | Transducin family protein / WD-40 repeat family protein | chr5:5504541-5509266 REVERSE LENGTH=876	872	876	0	100.5	91.2	95.6
Rsa1.0_00047.1.g2718.t1	ref|NP_178413.2| alpha/beta-hydrolases-like protein [Arabidopsis thaliana] gi|330250573|gb|AEC05667.1| alpha/beta-hydrolases-like protein [Arabidopsis thaliana]	1754	1797	0	102.5	73.2	83.1	alpha/beta-hydrolases-like protein	gbpln	Arabidopsis thaliana	AT2G03140.1 | Symbols: | alpha/beta-Hydrolases superfamily protein | chr2:942000-949337 FORWARD LENGTH=1797	1754	1797	0	102.5	73.2	83.1
Rsa1.0_00047.1.g2719.t1	gb|EOA19951.1| hypothetical protein CARUB_v10000202mg [Capsella rubella]	816	857	0	105.0	78.2	89.7	hypothetical protein CARUB_v10000202mg	gbpln	Capsella rubella	AT5G16730.1 | Symbols: | Plant protein of unknown function (DUF827) | chr5:5497890-5500775 FORWARD LENGTH=853	816	853	0	104.5	76.7	87.5
Rsa1.0_00047.1.g2720.t1	ref|XP_002871733.1| hypothetical protein ARALYDRAFT_909657 [Arabidopsis lyrata subsp. lyrata] gi|297317570|gb|EFH47992.1| hypothetical protein ARALYDRAFT_909657 [Arabidopsis lyrata subsp. lyrata]	684	688	0	100.6	74.9	83.5	hypothetical protein ARALYDRAFT_909657	gbpln	Arabidopsis lyrata	AT5G16720.1 | Symbols: | Protein of unknown function, DUF593 | chr5:5494309-5496562 FORWARD LENGTH=675	684	675	0	98.7	74.4	83.6
Rsa1.0_00047.1.g2721.t1	gb|EOA19901.1| hypothetical protein CARUB_v10000149mg [Capsella rubella]	1284	975	0	75.9	71.7	73.5	hypothetical protein CARUB_v10000149mg	gbpln	Capsella rubella	AT5G16715.1 | Symbols: EMB2247 | ATP binding;valine-tRNA ligases;aminoacyl-tRNA ligases;nucleotide binding;ATP binding;aminoacyl-tRNA ligases | chr5:5485353-5493229 FORWARD LENGTH=974	1284	974	0	75.9	71.4	73.4
Rsa1.0_00047.1.g2722.t1	ref|NP_197172.1| glycosyl hydrolase family 5 protein [Arabidopsis thaliana] gi|9755722|emb|CAC01834.1| putative protein [Arabidopsis thaliana] gi|332004944|gb|AED92327.1| glycosyl hydrolase family 5 protein [Arabidopsis thaliana]	291	488	1.00E-113	167.7	72.2	78.7	glycosyl hydrolase family 5 protein	gbpln	Arabidopsis thaliana	AT5G16700.1 | Symbols: | Glycosyl hydrolase superfamily protein | chr5:5480763-5483045 FORWARD LENGTH=488	291	488	1.00E-116	167.7	72.2	78.7
Rsa1.0_00048.1.g2723.t1	ref|XP_002869161.1| hypothetical protein ARALYDRAFT_912968 [Arabidopsis lyrata subsp. lyrata] gi|297314997|gb|EFH45420.1| hypothetical protein ARALYDRAFT_912968 [Arabidopsis lyrata subsp. lyrata]	723	844	0	116.7	89.6	95.6	hypothetical protein ARALYDRAFT_912968	gbpln	Arabidopsis lyrata	AT4G34260.1 | Symbols: FUC95A | 1,2-alpha-L-fucosidases | chr4:16398130-16401591 FORWARD LENGTH=843	723	843	0	116.6	90.0	95.4
Rsa1.0_00048.1.g2724.t1	ref|XP_002869160.1| hypothetical protein ARALYDRAFT_491242 [Arabidopsis lyrata subsp. lyrata] gi|297314996|gb|EFH45419.1| hypothetical protein ARALYDRAFT_491242 [Arabidopsis lyrata subsp. lyrata]	101	94	8.00E-34	93.1	77.2	82.2	hypothetical protein ARALYDRAFT_491242	gbpln	Arabidopsis lyrata	AT2G15000.5 | Symbols: | unknown protein; FUNCTIONS IN: molecular_function unknown; INVOLVED IN: biological_process unknown; LOCATED IN: chloroplast; EXPRESSED IN: 22 plant structures; EXPRESSED DURING: 13 growth stages; BEST Arabidopsis thaliana protein match is: unknown protein (TAIR:AT4G34265.2); Has 80 Blast hits to 80 proteins in 11 species: Archae - 0; Bacteria - 0; Metazoa - 0; Fungi - 0; Plants - 80; Viruses - 0; Other Eukaryotes - 0 (source: NCBI BLink). | chr2:6481839-6482316 REVERSE LENGTH=101	101	101	1.00E-35	100.0	75.2	87.1

Rsa1.0_00048.1.g2725.t1	ref|XP_002867124.1| hypothetical protein ARALYDRAFT_491240 [Arabidopsis lyrata subsp. lyrata] gi|297312960|gb|EFH43383.1| hypothetical protein ARALYDRAFT_491240 [Arabidopsis lyrata subsp. lyrata]	290	290	1.00E-150	100.0	89.3	94.5	hypothetical protein ARALYDRAFT_491240	gbpln	Arabidopsis lyrata	AT4G34270.1 | Symbols: | TIP41-like family protein | chr4:16404138-16406153 REVERSE LENGTH=290	290	290	1.00E-150	100.0	87.6	94.1
Rsa1.0_00048.1.g2726.t1	gb|EOA24746.1| hypothetical protein CARUB_v10018023mg [Capsella rubella]	210	210	1.00E-109	100.0	91.0	96.2	hypothetical protein CARUB_v10018023mg	gbpln	Capsella rubella	AT3G25580.1 | Symbols: | Thioredoxin superfamily protein | chr3:9292557-9294148 FORWARD LENGTH=210	210	210	1.00E-108	100.0	89.0	95.2
Rsa1.0_00048.1.g2727.t1	ref|XP_002869159.1| transducin family protein [Arabidopsis lyrata subsp. lyrata] gi|297314995|gb|EFH45418.1| transducin family protein [Arabidopsis lyrata subsp. lyrata]	739	785	0	106.2	79.8	88.4	transducin family protein	gbpln	Arabidopsis lyrata	AT4G34280.1 | Symbols: | transducin family protein / WD-40 repeat family protein | chr4:16407084-16410644 FORWARD LENGTH=783	739	783	0	106.0	78.9	87.4
Rsa1.0_00048.1.g2728.t1	gb|EOA19159.1| hypothetical protein CARUB_v10007836mg [Capsella rubella]	139	150	4.00E-56	107.9	84.2	91.4	hypothetical protein CARUB_v10007836mg	gbpln	Capsella rubella	AT4G34290.1 | Symbols: | SWIB/MDM2 domain superfamily protein | chr4:16410883-16412122 FORWARD LENGTH=144	139	144	8.00E-56	103.6	81.3	87.8
Rsa1.0_00048.1.g2729.t1	ref|XP_002869156.1| binding protein [Arabidopsis lyrata subsp. lyrata] gi|297314992|gb|EFH45415.1| binding protein [Arabidopsis lyrata subsp. lyrata]	1221	1228	0	100.6	91.0	94.1	binding protein	gbpln	Arabidopsis lyrata	AT4G34310.1 | Symbols: | alpha/beta-Hydrolases superfamily protein | chr4:16414113-16419542 FORWARD LENGTH=1228	1221	1228	0	100.6	90.2	93.9
Rsa1.0_00048.1.g2730.t1	gb|AAB82639.1| putative non-LTR retroelement reverse transcriptase [Arabidopsis thaliana]	1327	1374	0	103.5	54.6	72.6	putative non-LTR retroelement reverse transcriptase	gbpln	Arabidopsis thaliana	AT4G29090.1 | Symbols: | Ribonuclease H-like superfamily protein | chr4:14333528-14335255 FORWARD LENGTH=575	1327	575	1.00E-180	43.3	21.9	31.3
Rsa1.0_00048.1.g2731.t1	ref|XP_002867123.1| hypothetical protein ARALYDRAFT_491232 [Arabidopsis lyrata subsp. lyrata] gi|297312959|gb|EFH43382.1| hypothetical protein ARALYDRAFT_491232 [Arabidopsis lyrata subsp. lyrata]	355	355	1.00E-125	100.0	73.5	83.1	hypothetical protein ARALYDRAFT_491232	gbpln	Arabidopsis lyrata	AT4G34340.1 | Symbols: TAF8 | TBP-associated factor 8 | chr4:16426771-16427832 REVERSE LENGTH=353	355	353	1.00E-126	99.4	72.4	82.8
Rsa1.0_00048.1.g2732.t1	ref|XP_002885880.1| hypothetical protein ARALYDRAFT_899583 [Arabidopsis lyrata subsp. lyrata] gi|297331720|gb|EFH62139.1| hypothetical protein ARALYDRAFT_899583 [Arabidopsis lyrata subsp. lyrata]	166	183	1.00E-11	110.2	30.1	41.0	hypothetical protein ARALYDRAFT_899583	gbpln	Arabidopsis lyrata	AT5G33330.1 | Symbols: | Polynucleotidyl transferase, ribonuclease H-like superfamily protein | chr5:12591754-12592896 REVERSE LENGTH=183	166	183	2.00E-11	110.2	21.1	31.3
Rsa1.0_00048.1.g2733.t1	gb|EOA16580.1| hypothetical protein CARUB_v10004745mg [Capsella rubella]	466	467	0	100.2	93.6	97.4	hypothetical protein CARUB_v10004745mg	gbpln	Capsella rubella	AT4G34350.1 | Symbols: CLB6, ISPH, HDR | 4-hydroxy-3-methylbut-2-enyl diphosphate reductase | chr4:16428681-16431038 REVERSE LENGTH=466	466	466	0	100.0	92.5	96.4
Rsa1.0_00048.1.g2734.t1	gb|EOA17276.1| hypothetical protein CARUB_v10005548mg [Capsella rubella]	257	256	1.00E-129	99.6	87.9	93.4	hypothetical protein CARUB_v10005548mg	gbpln	Capsella rubella	AT4G34360.1 | Symbols: | S-adenosyl-L-methionine-dependent methyltransferases superfamily protein | chr4:16432011-16433752 FORWARD LENGTH=248	257	248	1.00E-127	96.5	85.6	89.5
Rsa1.0_00048.1.g2735.t1	ref|XP_002869152.1| IBR domain-containing protein [Arabidopsis lyrata subsp. lyrata] gi|297314988|gb|EFH45411.1| IBR domain-containing protein [Arabidopsis lyrata subsp. lyrata]	592	597	0	100.8	83.6	91.7	IBR domain-containing protein	gbpln	Arabidopsis lyrata	AT4G34370.1 | Symbols: ATARI1, ARI1 | RING/U-box superfamily protein | chr4:16434547-16437037 FORWARD LENGTH=597	592	597	0	100.8	82.4	91.7
Rsa1.0_00048.1.g2736.t1	gb|EOA18995.1| hypothetical protein CARUB_v10007640mg [Capsella rubella]	527	492	0	93.4	75.9	83.5	hypothetical protein CARUB_v10007640mg	gbpln	Capsella rubella	AT4G34380.1 | Symbols: | Transducin/WD40 repeat-like superfamily protein | chr4:16438835-16440322 FORWARD LENGTH=495	527	495	0	93.9	74.6	82.5
Rsa1.0_00048.1.g2737.t1	ref|XP_002869151.1| extra-large GTP-binding protein 2 [Arabidopsis lyrata subsp. lyrata] gi|297314987|gb|EFH45410.1| extra-large GTP-binding protein 2 [Arabidopsis lyrata subsp. lyrata]	842	859	0	102.0	76.7	85.6	extra-large GTP-binding protein 2	gbpln	Arabidopsis lyrata	AT4G34390.1 | Symbols: XLG2 | extra-large GTP-binding protein 2 | chr4:16441579-16444840 FORWARD LENGTH=861	842	861	0	102.3	77.3	85.3
Rsa1.0_00048.1.g2738.t1	gb|EOA15869.1| hypothetical protein CARUB_v10007825mg [Capsella rubella]	276	272	1.00E-100	98.6	80.4	87.7	hypothetical protein CARUB_v10007825mg	gbpln	Capsella rubella	AT4G34410.1 | Symbols: RRTF1 | redox responsive transcription factor 1 | chr4:16451992-16452798 FORWARD LENGTH=268	276	268	1.00E-92	97.1	79.0	85.5
Rsa1.0_00048.1.g2739.t1	gb|EOA17538.1| hypothetical protein CARUB_v10005889mg [Capsella rubella]	98	172	3.00E-38	175.5	78.6	89.8	hypothetical protein CARUB_v10005889mg	gbpln	Capsella rubella	AT4G34412.1 | Symbols: | CONTAINS InterPro DOMAIN/s: Kinase binding protein CGI-121 (InterPro:IPR013926); Has 275 Blast hits to 275 proteins in 139 species: Archae - 0; Bacteria - 5; Metazoa - 98; Fungi - 109; Plants - 42; Viruses - 0; Other Eukaryotes - 21 (source: NCBI BLink). | chr4:16453099-16454515 REVERSE LENGTH=172	98	172	5.00E-40	175.5	78.6	88.8

Rsa1.0_00048.1.g2740.t1	ref|NP_001190912.1| uncharacterized protein [Arabidopsis thaliana] gi|51970964|dbj|BAD44174.1| hypothetical protein [Arabidopsis thaliana] gi|332660971|gb|AEE86371.1| uncharacterized protein AT4G34419 [Arabidopsis thaliana]	129	129	5.00E-61	100.0	87.6	93.0	uncharacterized protein	gbpln	Arabidopsis thaliana	AT4G34419.1 | Symbols: | unknown protein; BEST Arabidopsis thaliana protein match is: unknown protein (TAIR:AT3G19200.1). | chr4:16456597-16456986 FORWARD LENGTH=129	129	129	1.00E-63	100.0	87.6	93.0
Rsa1.0_00048.1.g2741.t1	gb|EOA15557.1| hypothetical protein CARUB_v10005172mg [Capsella rubella]	343	344	0	100.3	88.6	94.2	hypothetical protein CARUB_v10005172mg	gbpln	Capsella rubella	AT4G34420.1 | Symbols: | P-loop containing nucleoside triphosphate hydrolases superfamily protein | chr4:16458430-16460526 FORWARD LENGTH=342	343	342	0	99.7	87.8	93.6
Rsa1.0_00048.1.g2742.t1	ref|XP_002869145.1| predicted protein [Arabidopsis lyrata subsp. lyrata] gi|297314981|gb|EFH45404.1| predicted protein [Arabidopsis lyrata subsp. lyrata]	677	669	0	98.8	83.0	89.1	predicted protein	gbpln	Arabidopsis lyrata	AT4G34440.1 | Symbols: | Protein kinase superfamily protein | chr4:16466008-16468748 FORWARD LENGTH=670	677	670	0	99.0	80.9	87.7
Rsa1.0_00048.1.g2743.t1	ref|NP_195171.2| coatomer subunit gamma [Arabidopsis thaliana] gi|146286094|sp|Q0WW26.2|COPG_ARATH RecName: Full=Coatomer subunit gamma; AltName: Full=Gamma-coat protein; Short=Gamma-COP gi|222424705|dbj|BAH20306.1| AT4G34450 [Arabidopsis thaliana] gi|332660978|gb|AEE86378.1| coatomer subunit gamma [Arabidopsis thaliana]	886	886	0	100.0	96.5	98.8	coatomer subunit gamma	gbpln	Arabidopsis thaliana	AT4G34450.1 | Symbols: | coatomer gamma-2 subunit, putative / gamma-2 coat protein, putative / gamma-2 COP, putative | chr4:16471956-16476795 FORWARD LENGTH=886	886	886	0	100.0	96.5	98.8
Rsa1.0_00048.1.g2744.t1	dbj|BAJ34135.1| unnamed protein product [Thellungiella halophila]	411	377	0	91.7	88.3	90.3	unnamed protein product	----	----	AT4G34460.1 | Symbols: AGB1, ELK4, ATAGB1 | GTP binding protein beta 1 | chr4:16477393-16479266 REVERSE LENGTH=377	411	377	0	91.7	86.1	89.1
Rsa1.0_00048.1.g2745.t1	ref|NP_195174.6| glucan endo-1,3-beta-glucosidase 7 [Arabidopsis thaliana] gi|259016223|sp|Q9M069.2|E137_ARATH RecName: Full=Glucan endo-1,3-beta-glucosidase 7; AltName: Full=(1-3)-beta-glucan endohydrolase 7; Short=(1-3)-beta-glucanase 7; AltName: Full=Beta-1,3-endoglucanase 7; Short=Beta-1,3-glucanase 7; Flags: Precursor gi|332660984|gb|AEE86384.1| glucan endo-1,3-beta-glucosidase 7 [Arabidopsis thaliana]	509	504	0	99.0	81.7	87.6	glucan endo-1,3-beta-glucosidase 7	gbpln	Arabidopsis thaliana	AT4G34480.1 | Symbols: | O-Glycosyl hydrolases family 17 protein | chr4:16481147-16483988 REVERSE LENGTH=504	509	504	0	99.0	81.7	87.6
Rsa1.0_00048.1.g2746.t1	ref|NP_195175.1| cyclase associated protein 1 [Arabidopsis thaliana] gi|3096918|emb|CAA18828.1| putative cyclase associated protein CAP [Arabidopsis thaliana] gi|3169136|dbj|BAA28621.1| Atcap1 [Arabidopsis thaliana] gi|7270399|emb|CAB80166.1| putative cyclase associated protein CAP [Arabidopsis thaliana] gi|115646727|gb|ABJ17096.1| At4g34490 [Arabidopsis thaliana] gi|332660985|gb|AEE86385.1| cyclase associated protein 1 [Arabidopsis thaliana]	479	476	0	99.4	93.3	97.3	cyclase associated protein 1	gbpln	Arabidopsis thaliana	AT4G34490.1 | Symbols: ATCAP1, CAP 1, CAP1 | cyclase associated protein 1 | chr4:16484896-16487355 REVERSE LENGTH=476	479	476	0	99.4	93.3	97.3
Rsa1.0_00048.1.g2747.t1	gb|EOA16482.1| hypothetical protein CARUB_v10004641mg, partial [Capsella rubella]	442	499	0	112.9	85.7	90.3	hypothetical protein CARUB_v10004641mg, partial	gbpln	Capsella rubella	AT4G34500.1 | Symbols: | Protein kinase superfamily protein | chr4:16488005-16490792 REVERSE LENGTH=437	442	437	0	98.9	87.3	91.6
Rsa1.0_00048.1.g2748.t1	gb|AAG51228.1|AC035249_3 Tam3-like transposon protein; 93317-95488 [Arabidopsis thaliana] gi|12323055|gb|AAG51515.1|AC068324_3 hAT-element transposase, putative [Arabidopsis thaliana]	691	723	0	104.6	57.0	73.8	Tam3-like transposon protein; 93317-95488	gbpln	Arabidopsis thaliana	AT3G42170.1 | Symbols: | BED zinc finger ;hAT family dimerisation domain | chr3:14321838-14323928 FORWARD LENGTH=696	691	696	7.00E-45	100.7	19.7	37.6
Rsa1.0_00048.1.g2749.t1	ref|NP_195180.1| NmrA-like negative transcriptional regulator family protein [Arabidopsis thaliana] gi|3641839|emb|CAA18833.1| isoflavone reductase-like protein [Arabidopsis thaliana] gi|7270404|emb|CAB80171.1| isoflavone reductase-like protein [Arabidopsis thaliana] gi|332660990|gb|AEE86390.1| NmrA-like negative transcriptional regulator family protein [Arabidopsis thaliana]	672	306	2.33E-156	45.5	38.1	41.5	NmrA-like negative transcriptional regulator family protein	gbpln	Arabidopsis thaliana	AT4G34540.1 | Symbols: | NmrA-like negative transcriptional regulator family protein | chr4:16500506-16501932 FORWARD LENGTH=306	672	306	1.00E-149	45.5	38.1	41.5

Rsa1.0_00048.1.g2750.t1	gb|EOA17396.1| hypothetical protein CARUB_v10005690mg [Capsella rubella]	216	221	3.00E-75	102.3	75.5	85.2	hypothetical protein CARUB_v10005690mg	gbpln	Capsella rubella	AT4G34560.1 | Symbols: | unknown protein; BEST Arabidopsis thaliana protein match is: unknown protein (TAIR:AT5G66440.1); Has 67 Blast hits to 66 proteins in 11 species: Archae - 0; Bacteria - 0; Metazoa - 0; Fungi - 0; Plants - 67; Viruses - 0; Other Eukaryotes - 0 (source: NCBI BLink). | chr4:16507923-16508588 FORWARD LENGTH=221	216	221	2.00E-69	102.3	73.1	81.9
Rsa1.0_00048.1.g2751.t1	ref|NP_195183.2| thymidylate synthase [Arabidopsis thaliana] gi|150421542|sp|Q05763.2|DRTS2_ARATH RecName: Full=Bifunctional dihydrofolate reductase-thymidylate synthase 2; Short=DHFR-TS 2; Includes: RecName: Full=Dihydrofolate reductase; Includes: RecName: Full=Thymidylate synthase gi|110743754|dbj|BAE99713.1| dihydrofolate reductase-thymidylate synthase [Arabidopsis thaliana] gi|332660994|gb|AEE86394.1| thymidylate synthase [Arabidopsis thaliana]	559	565	0	101.1	87.8	93.0	thymidylate synthase	gbpln	Arabidopsis thaliana	AT4G34570.1 | Symbols: THY-2 | thymidylate synthase 2 | chr4:16511129-16514110 REVERSE LENGTH=565	559	565	0	101.1	87.8	93.0
Rsa1.0_00048.1.g2752.t1	#	#	#	#	#	#	#	-	----	----	#	#	#	#	#	#	#
Rsa1.0_00048.1.g2753.t1	ref|XP_002883443.1| hypothetical protein ARALYDRAFT_342502 [Arabidopsis lyrata subsp. lyrata] gi|297329283|gb|EFH59702.1| hypothetical protein ARALYDRAFT_342502 [Arabidopsis lyrata subsp. lyrata]	326	454	2.00E-19	139.3	21.8	35.0	hypothetical protein ARALYDRAFT_342502	gbpln	Arabidopsis lyrata	AT1G01355.1 | Symbols: | Putative endonuclease or glycosyl hydrolase | chr1:138513-139568 FORWARD LENGTH=228	326	228	4.00E-12	69.9	19.0	27.6
Rsa1.0_00048.1.g2754.t2	ref|NP_195184.2| Sec14p-like phosphatidylinositol transfer family protein [Arabidopsis thaliana] gi|332660995|gb|AEE86395.1| Sec14p-like phosphatidylinositol transfer family protein [Arabidopsis thaliana]	551	554	0	100.5	91.1	95.3	Sec14p-like phosphatidylinositol transfer family protein	gbpln	Arabidopsis thaliana	AT4G34580.1 | Symbols: COW1, SRH1 | Sec14p-like phosphatidylinositol transfer family protein | chr4:16515422-16518527 FORWARD LENGTH=554	551	554	0	100.5	91.1	95.3
Rsa1.0_00048.1.g2755.t1	gb|EOA17614.1| hypothetical protein CARUB_v10005978mg [Capsella rubella]	154	143	1.00E-59	92.9	77.3	81.2	hypothetical protein CARUB_v10005978mg	gbpln	Capsella rubella	AT4G34590.1 | Symbols: ATB2, GBF6, AtbZIP11, BZIP11 | G-box binding factor 6 | chr4:16522449-16522928 FORWARD LENGTH=159	154	159	1.00E-59	103.2	74.7	81.2
Rsa1.0_00048.1.g2756.t2	#	#	#	#	#	#	#	-	----	----	#	#	#	#	#	#	#
Rsa1.0_00048.1.g2757.t1	ref|NP_195186.1| uncharacterized protein [Arabidopsis thaliana] gi|3096929|emb|CAA18839.1| putative protein [Arabidopsis thaliana] gi|7270410|emb|CAB80177.1| putative protein [Arabidopsis thaliana] gi|38566572|gb|AAR24176.1| At4g34600 [Arabidopsis thaliana] gi|47550671|gb|AAT35234.1| At4g34600 [Arabidopsis thaliana] gi|332660998|gb|AEE86398.1| uncharacterized protein AT4G34600 [Arabidopsis thaliana]	80	83	2.00E-28	103.8	83.8	92.5	uncharacterized protein	gbpln	Arabidopsis thaliana	AT4G34600.1 | Symbols: | unknown protein; FUNCTIONS IN: molecular_function unknown; INVOLVED IN: pollen tube growth; LOCATED IN: endomembrane system; EXPRESSED IN: 11 plant structures; EXPRESSED DURING: M germinated pollen stage, LP.04 four leaves visible, 4 anthesis, petal differentiation and expansion stage; BEST Arabidopsis thaliana protein match is: unknown protein (TAIR:AT2G16385.1); Has 17 Blast hits to 17 proteins in 4 species: Archae - 0; Bacteria - 0; Metazoa - 0; Fungi - 0; Plants - 17; Viruses - 0; Other Eukaryotes - 0 (source: NCBI BLink). | chr4:16529280-16529958 FORWARD LENGTH=83	80	83	3.00E-31	103.8	83.8	92.5
Rsa1.0_00048.1.g2758.t1	gb|EOA39417.1| hypothetical protein CARUB_v10012524mg [Capsella rubella]	169	535	7.00E-25	316.6	33.7	40.8	hypothetical protein CARUB_v10012524mg	gbpln	Capsella rubella	AT1G48920.1 | Symbols: ATNUC-L1, PARL1, NUC-L1 | nucleolin like 1 | chr1:18098186-18101422 FORWARD LENGTH=557	169	557	2.00E-26	329.6	34.3	41.4
Rsa1.0_00048.1.g2759.t1	#	#	#	#	#	#	#	-	----	----	#	#	#	#	#	#	#
Rsa1.0_00048.1.g2760.t1	ref|XP_002867110.1| hypothetical protein ARALYDRAFT_912923 [Arabidopsis lyrata subsp. lyrata] gi|297312946|gb|EFH43369.1| hypothetical protein ARALYDRAFT_912923 [Arabidopsis lyrata subsp. lyrata]	113	113	7.00E-58	100.0	94.7	99.1	hypothetical protein ARALYDRAFT_912923	gbpln	Arabidopsis lyrata	AT4G34620.1 | Symbols: SSR16 | small subunit ribosomal protein 16 | chr4:16535084-16536092 REVERSE LENGTH=113	113	113	2.00E-57	100.0	89.4	96.5
Rsa1.0_00048.1.g2761.t1	ref|XP_002867109.1| hypothetical protein ARALYDRAFT_491192 [Arabidopsis lyrata subsp. lyrata] gi|297312945|gb|EFH43368.1| hypothetical protein ARALYDRAFT_491192 [Arabidopsis lyrata subsp. lyrata]	221	210	4.00E-67	95.0	67.9	76.0	hypothetical protein ARALYDRAFT_491192	gbpln	Arabidopsis lyrata	AT4G34630.1 | Symbols: | unknown protein; Has 30 Blast hits to 30 proteins in 10 species: Archae - 0; Bacteria - 2; Metazoa - 0; Fungi - 0; Plants - 24; Viruses - 0; Other Eukaryotes - 4 (source: NCBI BLink). | chr4:16537093-16537692 REVERSE LENGTH=199	221	199	2.00E-56	90.0	61.5	70.6

Rsa1.0_00048.1.g2762.t1	ref|XP_002869133.1| hypothetical protein ARALYDRAFT_912921 [Arabidopsis lyrata subsp. lyrata] gi|297314969|gb|EFH45392.1| hypothetical protein ARALYDRAFT_912921 [Arabidopsis lyrata subsp. lyrata]	396	412	0	104.0	81.3	84.8	hypothetical protein ARALYDRAFT_912921	gbpln	Arabidopsis lyrata	AT4G34640.1 | Symbols: SQS1, ERG9 | squalene synthase 1 | chr4:16538489-16541655 FORWARD LENGTH=410	396	410	0	103.5	79.8	84.1
Rsa1.0_00048.1.g2763.t1	ref|NP_195191.2| squalene synthase 2 [Arabidopsis thaliana] gi|5678626|emb|CAA18844.2| putative squalene synthase [Arabidopsis thaliana] gi|7270415|emb|CAB80182.1| putative squalene synthase [Arabidopsis thaliana] gi|332661004|gb|AEE86404.1| squalene synthase 2 [Arabidopsis thaliana]	416	413	0	99.3	84.1	91.3	squalene synthase 2	gbpln	Arabidopsis thaliana	AT4G34650.1 | Symbols: SQS2 | squalene synthase 2 | chr4:16542377-16544915 FORWARD LENGTH=413	416	413	0	99.3	84.1	91.3
Rsa1.0_00048.1.g2764.t1	gb|EOA16836.1| hypothetical protein CARUB_v10005057mg [Capsella rubella]	375	377	0	100.5	94.4	98.1	hypothetical protein CARUB_v10005057mg	gbpln	Capsella rubella	AT4G34660.1 | Symbols: | SH3 domain-containing protein | chr4:16545595-16548294 REVERSE LENGTH=368	375	368	0	98.1	92.3	96.0
Rsa1.0_00048.1.g2765.t1	gb|EOA17249.1| hypothetical protein CARUB_v10005523mg [Capsella rubella]	262	262	1.00E-138	100.0	93.9	98.5	hypothetical protein CARUB_v10005523mg	gbpln	Capsella rubella	AT4G34670.1 | Symbols: | Ribosomal protein S3Ae | chr4:16548724-16550222 FORWARD LENGTH=262	262	262	1.00E-139	100.0	93.9	96.9
Rsa1.0_00048.1.g2766.t2	gb|EOA17609.1| hypothetical protein CARUB_v10005980mg, partial [Capsella rubella]	143	140	4.00E-56	97.9	75.5	77.6	hypothetical protein CARUB_v10005980mg, partial	gbpln	Capsella rubella	AT4G34700.1 | Symbols: CIB22, AtCIB22 | LYR family of Fe/S cluster biogenesis protein | chr4:16556874-16558362 FORWARD LENGTH=117	143	117	4.00E-58	81.8	73.4	74.1
Rsa1.0_00048.1.g2767.t1	gb|AAR08423.1| arginine decarboxylase 2 [Pringlea antiscorbutica]	275	711	1.00E-120	258.5	81.5	88.7	arginine decarboxylase 2	gbpln	Pringlea antiscorbutica	AT4G34710.2 | Symbols: ADC2, SPE2, ATADC2 | arginine decarboxylase 2 | chr4:16560315-16562450 REVERSE LENGTH=711	275	711	1.00E-114	258.5	78.9	86.5
Rsa1.0_00049.1.g2768.t1	dbj|BAJ33888.1| unnamed protein product [Thellungiella halophila]	243	245	1.00E-81	100.8	90.1	93.0	unnamed protein product	----	----	AT4G20260.3 | Symbols: ATPCAP1, PCAP1 | plasma-membrane associated cation-binding protein 1 | chr4:10941593-10943227 FORWARD LENGTH=225	243	225	1.00E-76	92.6	79.0	83.1
Rsa1.0_00049.1.g2769.t1	gb|EOA19016.1| hypothetical protein CARUB_v10007667mg [Capsella rubella]	595	557	0	93.6	66.6	73.6	hypothetical protein CARUB_v10007667mg	gbpln	Capsella rubella	AT4G20200.1 | Symbols: | Terpenoid cyclases/Protein prenyltransferases superfamily protein | chr4:10908672-10911151 REVERSE LENGTH=604	595	604	0	101.5	50.6	64.5
Rsa1.0_00049.1.g2770.t2	ref|XP_002867888.1| hypothetical protein ARALYDRAFT_492849 [Arabidopsis lyrata subsp. lyrata] gi|297313724|gb|EFH44147.1| hypothetical protein ARALYDRAFT_492849 [Arabidopsis lyrata subsp. lyrata]	393	394	1.00E-174	100.3	84.5	91.1	hypothetical protein ARALYDRAFT_492849	gbpln	Arabidopsis lyrata	AT4G20190.1 | Symbols: | unknown protein; BEST Arabidopsis thaliana protein match is: unknown protein (TAIR:AT5G44660.1); Has 271 Blast hits to 209 proteins in 52 species: Archae - 0; Bacteria - 15; Metazoa - 63; Fungi - 14; Plants - 48; Viruses - 3; Other Eukaryotes - 128 (source: NCBI BLink). | chr4:10906508-10907677 REVERSE LENGTH=389	393	389	1.00E-174	99.0	83.2	89.3
Rsa1.0_00049.1.g2771.t1	ref|NP_193750.1| uncharacterized protein [Arabidopsis thaliana] gi|2982434|emb|CAA18242.1| putative protein [Arabidopsis thaliana] gi|7268812|emb|CAB79017.1| putative protein [Arabidopsis thaliana] gi|332658882|gb|AEE84282.1| uncharacterized protein AT4G20170 [Arabidopsis thaliana]	504	504	0	100.0	84.9	90.9	uncharacterized protein	gbpln	Arabidopsis thaliana	AT4G20170.1 | Symbols: | Domain of unknown function (DUF23) | chr4:10896810-10898794 FORWARD LENGTH=504	504	504	0	100.0	84.9	90.9
Rsa1.0_00049.1.g2772.t1	ref|NP_193749.1| uncharacterized protein [Arabidopsis thaliana] gi|2982433|emb|CAA18241.1| Glu-rich protein [Arabidopsis thaliana] gi|7268811|emb|CAB79016.1| Glu-rich protein [Arabidopsis thaliana] gi|332658881|gb|AEE84281.1| uncharacterized protein AT4G20160 [Arabidopsis thaliana]	1223	1188	0	97.1	57.4	67.9	uncharacterized protein	gbpln	Arabidopsis thaliana	AT4G20160.1 | Symbols: | LOCATED IN: chloroplast; EXPRESSED IN: 6 plant structures; EXPRESSED DURING: L mature pollen stage, M germinated pollen stage, 4 anthesis, petal differentiation and expansion stage; BEST Arabidopsis thaliana protein match is: RING/U-box superfamily protein (TAIR:AT1G30860.1); Has 64917 Blast hits to 39974 proteins in 1949 species: Archae - 279; Bacteria - 7425; Metazoa - 26604; Fungi - 5788; Plants - 3193; Viruses - 275; Other Eukaryotes - 21353 (source: NCBI BLink). | chr4:10890983-10894971 FORWARD LENGTH=1188	1223	1188	0	97.1	57.4	67.9
Rsa1.0_00049.1.g2773.t2	ref|XP_002869946.1| hypothetical protein ARALYDRAFT_354732 [Arabidopsis lyrata subsp. lyrata] gi|297315782|gb|EFH46205.1| hypothetical protein ARALYDRAFT_354732 [Arabidopsis lyrata subsp. lyrata]	1144	1252	0	109.4	41.6	44.0	hypothetical protein ARALYDRAFT_354732	gbpln	Arabidopsis lyrata	AT4G20140.1 | Symbols: GSO1 | Leucine-rich repeat transmembrane protein kinase | chr4:10884220-10888045 FORWARD LENGTH=1249	1144	1249	0	109.2	40.9	43.9

Rsa1.0_00049.1.g2774.t1	ref|XP_002869946.1| hypothetical protein ARALYDRAFT_354732 [Arabidopsis lyrata subsp. lyrata] gi|297315782|gb|EFH46205.1| hypothetical protein ARALYDRAFT_354732 [Arabidopsis lyrata subsp. lyrata]	1255	1252	0	99.8	87.6	92.6	hypothetical protein ARALYDRAFT_354732	gbpln	Arabidopsis lyrata	AT4G20140.1 | Symbols: GSO1 | Leucine-rich repeat transmembrane protein kinase | chr4:10884220-10888045 FORWARD LENGTH=1249	1255	1249	0	99.5	85.9	91.4
Rsa1.0_00049.1.g2775.t1	ref|XP_002869948.1| hypothetical protein ARALYDRAFT_492857 [Arabidopsis lyrata subsp. lyrata] gi|297315784|gb|EFH46207.1| hypothetical protein ARALYDRAFT_492857 [Arabidopsis lyrata subsp. lyrata]	631	625	0	99.0	89.2	95.4	hypothetical protein ARALYDRAFT_492857	gbpln	Arabidopsis lyrata	AT4G20110.1 | Symbols: VSR7, VSR3;1, BP80-3;1 | VACUOLAR SORTING RECEPTOR 7 | chr4:10875567-10878545 FORWARD LENGTH=625	631	625	0	99.0	88.7	95.1
Rsa1.0_00049.1.g2776.t1	ref|XP_002867892.1| EMB1025 [Arabidopsis lyrata subsp. lyrata] gi|297313728|gb|EFH44151.1| EMB1025 [Arabidopsis lyrata subsp. lyrata]	612	658	0	107.5	81.5	89.4	EMB1025	gbpln	Arabidopsis lyrata	AT4G20090.1 | Symbols: EMB1025 | Pentatricopeptide repeat (PPR) superfamily protein | chr4:10868400-10870382 REVERSE LENGTH=660	612	660	0	107.8	80.6	88.9
Rsa1.0_00049.1.g2777.t1	ref|XP_002867893.1| EMB1895 [Arabidopsis lyrata subsp. lyrata] gi|297313729|gb|EFH44152.1| EMB1895 [Arabidopsis lyrata subsp. lyrata]	1131	1134	0	100.3	79.0	87.5	EMB1895	gbpln	Arabidopsis lyrata	AT4G20060.1 | Symbols: EMB1895 | ARM repeat superfamily protein | chr4:10854790-10859330 REVERSE LENGTH=1134	1131	1134	0	100.3	78.7	87.5
Rsa1.0_00049.1.g2778.t1	gb|AFL69960.1| polygalacturonase [Brassica napus]	475	475	0	100.0	93.5	95.6	polygalacturonase	gbpln	Brassica napus	AT4G20050.2 | Symbols: QRT3 | Pectin lyase-like superfamily protein | chr4:10849911-10852090 REVERSE LENGTH=481	475	481	0	101.3	87.4	94.9
Rsa1.0_00049.1.g2779.t2	gb|EOA18460.1| hypothetical protein CARUB_v10007004mg [Capsella rubella]	466	485	0	104.1	81.8	90.1	hypothetical protein CARUB_v10007004mg	gbpln	Capsella rubella	AT4G20040.1 | Symbols: | Pectin lyase-like superfamily protein | chr4:10847348-10848963 REVERSE LENGTH=483	466	483	0	103.6	79.8	87.6
Rsa1.0_00049.1.g2780.t1	gb|EOA17585.1| hypothetical protein CARUB_v10005947mg [Capsella rubella]	154	152	6.00E-68	98.7	83.8	90.9	hypothetical protein CARUB_v10005947mg	gbpln	Capsella rubella	AT4G20030.1 | Symbols: | RNA-binding (RRM/RBD/RNP motifs) family protein | chr4:10846362-10847246 FORWARD LENGTH=152	154	152	3.00E-69	98.7	82.5	89.6
Rsa1.0_00049.1.g2781.t1	ref|NP_193735.1| uncharacterized protein [Arabidopsis thaliana] gi|2827656|emb|CAA16610.1| DAG-like protein [Arabidopsis thaliana] gi|7268797|emb|CAB79002.1| DAG-like protein [Arabidopsis thaliana] gi|27754272|gb|AAO22589.1| putative DAG protein [Arabidopsis thaliana] gi|332658862|gb|AEE84262.1| uncharacterized protein AT4G20020 [Arabidopsis thaliana]	381	419	1.00E-154	110.0	78.5	85.3	uncharacterized protein	gbpln	Arabidopsis thaliana	AT4G20020.1 | Symbols: | unknown protein; BEST Arabidopsis thaliana protein match is: unknown protein (TAIR:AT5G44780.1); Has 28928 Blast hits to 16023 proteins in 1033 species: Archae - 4; Bacteria - 4155; Metazoa - 15463; Fungi - 2938; Plants - 3091; Viruses - 205; Other Eukaryotes - 3072 (source: NCBI BLink). | chr4:10844433-10846085 REVERSE LENGTH=419	381	419	1.00E-157	110.0	78.5	85.3
Rsa1.0_00049.1.g2782.t1	ref|XP_002867898.1| VQ motif-containing protein [Arabidopsis lyrata subsp. lyrata] gi|297313734|gb|EFH44157.1| VQ motif-containing protein [Arabidopsis lyrata subsp. lyrata]	211	209	9.00E-73	99.1	72.5	82.0	VQ motif-containing protein	gbpln	Arabidopsis lyrata	AT4G20000.1 | Symbols: | VQ motif-containing protein | chr4:10838367-10838993 FORWARD LENGTH=208	211	208	4.00E-67	98.6	67.8	78.2
Rsa1.0_00049.1.g2783.t1	dbj|BAJ33925.1| unnamed protein product [Thellungiella halophila]	858	752	0	87.6	69.2	74.9	unnamed protein product	----	----	AT4G19990.2 | Symbols: FRS1 | FAR1-related sequence 1 | chr4:10832848-10835559 FORWARD LENGTH=774	858	774	0	90.2	66.0	71.7
Rsa1.0_00049.1.g2784.t1	ref|XP_002867900.1| hypothetical protein ARALYDRAFT_492876 [Arabidopsis lyrata subsp. lyrata] gi|297313736|gb|EFH44159.1| hypothetical protein ARALYDRAFT_492876 [Arabidopsis lyrata subsp. lyrata]	240	236	9.00E-99	98.3	79.2	82.5	hypothetical protein ARALYDRAFT_492876	gbpln	Arabidopsis lyrata	AT4G19985.1 | Symbols: | Acyl-CoA N-acyltransferases (NAT) superfamily protein | chr4:10830542-10831711 REVERSE LENGTH=237	240	237	4.00E-95	98.8	75.8	80.0
Rsa1.0_00049.1.g2785.t1	gb|EOA17660.1| hypothetical protein CARUB_v10006027mg [Capsella rubella]	127	125	3.00E-46	98.4	79.5	81.1	hypothetical protein CARUB_v10006027mg	gbpln	Capsella rubella	AT4G19980.1 | Symbols: | unknown protein; FUNCTIONS IN: molecular_function unknown; INVOLVED IN: biological_process unknown; LOCATED IN: chloroplast; EXPRESSED IN: root, pedicel; EXPRESSED DURING: 4 anthesis; Has 30201 Blast hits to 17322 proteins in 780 species: Archae - 12; Bacteria - 1396; Metazoa - 17338; Fungi - 3422; Plants - 5037; Viruses - 0; Other Eukaryotes - 2996 (source: NCBI BLink). | chr4:10828153-10828536 REVERSE LENGTH=127	127	127	2.00E-43	100.0	74.0	81.1

Rsa1.0_00049.1.g2786.t1	dbj|BAJ34335.1| unnamed protein product [Thellungiella halophila]	374	374	0	100.0	89.8	94.7	unnamed protein product	----	----	AT4G19970.1 | Symbols: | CONTAINS InterPro DOMAIN/s: Nucleotide-diphospho-sugar transferase, predicted (InterPro:IPR005069); BEST Arabidopsis thaliana protein match is: Nucleotide-diphospho-sugar transferase family protein (TAIR:AT5G44820.1); Has 801 Blast hits to 466 proteins in 35 species: Archae - 0; Bacteria - 0; Metazoa - 2; Fungi - 0; Plants - 750; Viruses - 0; Other Eukaryotes - 49 (source: NCBI BLink). | chr4:10818242-10825343 FORWARD LENGTH=715	374	715	0	191.2	86.1	90.9
Rsa1.0_00049.1.g2787.t2	gb|EOA16028.1| hypothetical protein CARUB_v10004157mg [Capsella rubella]	812	812	0	100.0	87.9	92.7	hypothetical protein CARUB_v10004157mg	gbpln	Capsella rubella	AT4G19960.2 | Symbols: KUP9 | K+ uptake permease 9 | chr4:10813807-10816997 FORWARD LENGTH=807	812	807	0	99.4	87.9	92.1
Rsa1.0_00049.1.g2788.t1	ref|NP_193728.2| uncharacterized protein [Arabidopsis thaliana] gi|50253430|gb|AAT71917.1| At4g19950 [Arabidopsis thaliana] gi|51972070|gb|AAU15139.1| At4g19950 [Arabidopsis thaliana] gi|332658850|gb|AEE84250.1| uncharacterized protein AT4G19950 [Arabidopsis thaliana]	318	321	1.00E-147	100.9	85.5	93.1	uncharacterized protein	gbpln	Arabidopsis thaliana	AT4G19950.1 | Symbols: | unknown protein; BEST Arabidopsis thaliana protein match is: unknown protein (TAIR:AT5G44860.1); Has 338 Blast hits to 330 proteins in 72 species: Archae - 2; Bacteria - 94; Metazoa - 7; Fungi - 0; Plants - 232; Viruses - 0; Other Eukaryotes - 3 (source: NCBI BLink). | chr4:10809977-10810942 FORWARD LENGTH=321	318	321	1.00E-150	100.9	85.5	93.1
Rsa1.0_00049.1.g2789.t1	ref|NP_001190774.1| Pentatricopeptide repeat domain-containing protein [Arabidopsis thaliana] gi|223635614|sp|P0C8Q3.1|PP326_ARATH RecName: Full=Pentatricopeptide repeat-containing protein At4g19890 gi|332658842|gb|AEE84242.1| Pentatricopeptide repeat domain-containing protein [Arabidopsis thaliana]	700	701	0	100.1	89.4	94.1	Pentatricopeptide repeat domain-containing protein	gbpln	Arabidopsis thaliana	AT4G19890.1 | Symbols: | Pentatricopeptide repeat (PPR-like) superfamily protein | chr4:10786948-10789053 REVERSE LENGTH=701	700	701	0	100.1	89.4	94.1
Rsa1.0_00049.1.g2790.t2	gb|EOA33274.1| hypothetical protein CARUB_v10019647mg [Capsella rubella]	2355	2361	0	100.3	97.9	98.9	hypothetical protein CARUB_v10019647mg	gbpln	Capsella rubella	AT1G80070.1 | Symbols: SUS2, EMB33, EMB177, EMB14 | Pre-mRNA-processing-splicing factor | chr1:30118052-30127574 FORWARD LENGTH=2359	2355	2359	0	100.2	97.6	98.6
Rsa1.0_00049.1.g2791.t1	ref|XP_002867917.1| lecithin:cholesterol acyltransferase family protein [Arabidopsis lyrata subsp. lyrata] gi|297313753|gb|EFH44176.1| lecithin:cholesterol acyltransferase family protein [Arabidopsis lyrata subsp. lyrata]	533	535	0	100.4	90.6	95.1	lecithin:cholesterol acyltransferase family protein	gbpln	Arabidopsis lyrata	AT4G19860.1 | Symbols: | alpha/beta-Hydrolases superfamily protein | chr4:10777655-10780260 REVERSE LENGTH=535	533	535	0	100.4	90.2	95.1
Rsa1.0_00049.1.g2792.t1	ref|NP_001154254.2| protein PHLOEM protein 2-LIKE A2 [Arabidopsis thaliana] gi|332658834|gb|AEE84234.1| protein PHLOEM protein 2-LIKE A2 [Arabidopsis thaliana]	221	220	3.00E-66	99.5	62.0	68.3	protein PHLOEM protein 2-LIKE A2	gbpln	Arabidopsis thaliana	AT4G19850.2 | Symbols: | lectin-related | chr4:10776657-10777588 FORWARD LENGTH=220	221	220	1.00E-68	99.5	62.0	68.3
Rsa1.0_00049.1.g2793.t1	ref|NP_193719.1| protein PHLOEM protein 2-LIKE A1 [Arabidopsis thaliana] gi|75100453|sp|O81865.1|P2A01_ARATH RecName: Full=Protein PHLOEM PROTEIN 2-LIKE A1; Short=AtPP2-A1 gi|3250693|emb|CAA19701.1| lectin like protein [Arabidopsis thaliana] gi|7268780|emb|CAB78986.1| lectin like protein [Arabidopsis thaliana] gi|19699298|gb|AAL91260.1| AT4g19840/T16H5_200 [Arabidopsis thaliana] gi|21689635|gb|AAM67439.1| AT4g19840/T16H5_200 [Arabidopsis thaliana] gi|332658832|gb|AEE84232.1| protein PHLOEM protein 2-LIKE A1 [Arabidopsis thaliana]	217	246	2.00E-73	113.4	71.4	80.6	protein PHLOEM protein 2-LIKE A1	gbpln	Arabidopsis thaliana	AT4G19840.1 | Symbols: ATPP2-A1, ATPP2A-1, PP2-A1 | phloem protein 2-A1 | chr4:10774336-10775701 FORWARD LENGTH=246	217	246	7.00E-76	113.4	71.4	80.6
Rsa1.0_00049.1.g2794.t1	gb|EOA16822.1| hypothetical protein CARUB_v10005044mg [Capsella rubella]	380	380	0	100.0	90.0	94.5	hypothetical protein CARUB_v10005044mg	gbpln	Capsella rubella	AT4G19810.1 | Symbols: | Glycosyl hydrolase family protein with chitinase insertion domain | chr4:10764151-10765753 REVERSE LENGTH=379	380	379	0	99.7	89.5	93.7
Rsa1.0_00049.1.g2795.t1	gb|EOA15450.1| hypothetical protein CARUB_v10004092mg [Capsella rubella]	923	920	0	99.7	94.1	96.5	hypothetical protein CARUB_v10004092mg	gbpln	Capsella rubella	AT4G19710.2 | Symbols: AK-HSDH II, AK-HSDH | aspartate kinase-homoserine dehydrogenase ii | chr4:10725229-10729536 FORWARD LENGTH=916	923	916	0	99.2	93.4	96.3
Rsa1.0_00049.1.g2796.t1	#	#	#	#	#	#		-	----	----	#	#	#	#	#	#	#
Rsa1.0_00050.1.g2797.t1	ref|XP_002893450.1| protein binding protein [Arabidopsis lyrata subsp. lyrata] gi|297339292|gb|EFH69709.1| protein binding protein [Arabidopsis lyrata subsp. lyrata]	253	251	1.00E-133	99.2	91.3	95.3	protein binding protein	gbpln	Arabidopsis lyrata	AT1G24440.1 | Symbols: | RING/U-box superfamily protein | chr1:8662340-8663777 FORWARD LENGTH=251	253	251	1.00E-135	99.2	90.9	94.9

Rsa1.0_00050.1.g2798.t1	ref|XP_002893449.1| hypothetical protein ARALYDRAFT_472903 [Arabidopsis lyrata subsp. lyrata] gi|297339291|gb|EFH69708.1| hypothetical protein ARALYDRAFT_472903 [Arabidopsis lyrata subsp. lyrata]	190	190	3.00E-73	100.0	74.7	85.3	hypothetical protein ARALYDRAFT_472903	gbpln	Arabidopsis lyrata	AT1G24450.1 | Symbols: NFD2 | Ribonuclease III family protein | chr1:8664201-8665077 FORWARD LENGTH=191	190	191	2.00E-74	100.5	74.7	85.3
Rsa1.0_00050.1.g2799.t2	gb|EOA37586.1| hypothetical protein CARUB_v10011938mg [Capsella rubella]	1792	1772	0	98.9	76.0	85.8	hypothetical protein CARUB_v10011938mg	gbpln	Capsella rubella	AT1G24460.1 | Symbols: | unknown protein; FUNCTIONS IN: molecular_function unknown; INVOLVED IN: biological_process unknown; LOCATED IN: cellular_component unknown; EXPRESSED IN: 23 plant structures; EXPRESSED DURING: 14 growth stages; BEST Arabidopsis thaliana protein match is: unknown protein (TAIR:AT4G31570.1); Has 181008 Blast hits to 85359 proteins in 3551 species: Archae - 3290; Bacteria - 48304; Metazoa - 70793; Fungi - 13943; Plants - 10118; Viruses - 785; Other Eukaryotes - 33775 (source: NCBI BLink). | chr1:8666072-8672338 FORWARD LENGTH=1807	1792	1807	0	100.8	75.3	84.8
Rsa1.0_00050.1.g2800.t1	gb|EOA38309.1| hypothetical protein CARUB_v10009809mg [Capsella rubella]	318	313	1.00E-151	98.4	84.3	88.1	hypothetical protein CARUB_v10009809mg	gbpln	Capsella rubella	AT1G24470.1 | Symbols: KCR2, ATKCR2 | beta-ketoacyl reductase 2 | chr1:8674056-8676277 FORWARD LENGTH=312	318	312	1.00E-152	98.1	84.3	88.1
Rsa1.0_00050.1.g2801.t1	#	#	#	#	#	#	#	–	----	----	#	#	#	#	#	#	#
Rsa1.0_00050.1.g2802.t1	gb|AAD19784.1| putative retroelement pol polyprotein [Arabidopsis thaliana]	1389	1501	0	108.1	57.0	71.6	putative retroelement pol polyprotein	gbpln	Arabidopsis thaliana	AT4G23160.1 | Symbols: CRK8 | cysteine-rich RLK (RECEPTOR-like protein kinase) 8 | chr4:12129485-12134086 FORWARD LENGTH=1262	1389	1262	1.00E-130	90.9	16.4	24.7
Rsa1.0_00050.1.g2803.t1	ref|XP_002880790.1| ania-6a type cyclin [Arabidopsis lyrata subsp. lyrata] gi|297326629|gb|EFH57049.1| ania-6a type cyclin [Arabidopsis lyrata subsp. lyrata]	413	415	0	100.5	86.9	91.8	ania-6a type cyclin	gbpln	Arabidopsis lyrata	AT2G26430.1 | Symbols: RCY1, ATRCY1 | arginine-rich cyclin 1 | chr2:11243433-11245504 REVERSE LENGTH=416	413	416	0	100.7	82.3	89.1
Rsa1.0_00050.1.g2804.t1	ref|NP_173858.5| ALBINO3-like protein 1 [Arabidopsis thaliana] gi|223590242|sp|Q9FYL3.3|ALB31_ARATH RecName: Full=ALBINO3-like protein 1, chloroplastic; AltName: Full=Arabidopsis thaliana envelope membrane integrase; AltName: Full=Protein ALBINA 4; Short=Ath4; AltName: Full=Protein ARTEMIS; Flags: Precursor gi|91680591|emb|CAJ45566.1| Alb protein [Arabidopsis thaliana] gi|332192418|gb|AEE30539.1| ALBINO3-like protein 1 [Arabidopsis thaliana]	485	499	0	102.9	86.0	92.2	ALBINO3-like protein 1	gbpln	Arabidopsis thaliana	AT1G24490.1 | Symbols: ALB4, ARTEMIS | OxaA/YidC-like membrane insertion protein | chr1:8682364-8684966 FORWARD LENGTH=499	485	499	0	102.9	86.0	92.2
Rsa1.0_00050.1.g2805.t1	ref|NP_173859.1| T-complex protein 1 subunit epsilon [Arabidopsis thaliana] gi|3024697|sp|O04450.1|TCPE_ARATH RecName: Full=T-complex protein 1 subunit epsilon; Short=TCP-1-epsilon; AltName: Full=CCT-epsilon gi|9743353|gb|AAF97977.1|AC000103_27 F21J9.17 [Arabidopsis thaliana] gi|19715605|gb|AAL91625.1| At1g24510/F21J9_150 [Arabidopsis thaliana] gi|23463047|gb|AAN33193.1| At1g24510/F21J9_150 [Arabidopsis thaliana] gi|332192420|gb|AEE30541.1| T-complex protein 1 subunit epsilon [Arabidopsis thaliana]	535	535	0	100.0	97.6	99.4	T-complex protein 1 subunit epsilon	gbpln	Arabidopsis thaliana	AT1G24510.1 | Symbols: | TCP-1/cpn60 chaperonin family protein | chr1:8685504-8688101 REVERSE LENGTH=535	535	535	0	100.0	97.6	99.4
Rsa1.0_00050.1.g2806.t1	emb|CAA48292.1| bgp1 [Brassica rapa] gi|448272|prf||1916399A Bgp1 gene	108	119	3.00E-12	110.2	62.0	70.4	bgp1	gbpln	Brassica rapa	AT1G24520.1 | Symbols: BCP1 | homolog of Brassica campestris pollen protein 1 | chr1:8688699-8689058 FORWARD LENGTH=119	108	119	7.00E-13	110.2	51.9	62.0
Rsa1.0_00050.1.g2807.t1	ref|XP_002893441.1| hypothetical protein ARALYDRAFT_472892 [Arabidopsis lyrata subsp. lyrata] gi|297339283|gb|EFH69700.1| hypothetical protein ARALYDRAFT_472892 [Arabidopsis lyrata subsp. lyrata]	700	679	0	97.0	82.0	88.9	hypothetical protein ARALYDRAFT_472892	gbpln	Arabidopsis lyrata	AT1G24560.1 | Symbols: | unknown protein; BEST Arabidopsis thaliana protein match is: unknown protein (TAIR:AT3G49055.1); Has 71940 Blast hits to 38124 proteins in 2481 species: Archae - 1358; Bacteria - 12126; Metazoa - 32861; Fungi - 6786; Plants - 4024; Viruses - 195; Other Eukaryotes - 14590 (source: NCBI BLink). | chr1:8702644-8705640 FORWARD LENGTH=678	700	678	0	96.9	82.0	88.3

Rsa1.0_00050.1.g2808.t5	ref|XP_002893440.1| hypothetical protein ARALYDRAFT_313424 [Arabidopsis lyrata subsp. lyrata] gi|297339282|gb|EFH69699.1| hypothetical protein ARALYDRAFT_313424 [Arabidopsis lyrata subsp. lyrata]	396	363	0	91.7	77.8	84.1	hypothetical protein ARALYDRAFT_313424	gbpln	Arabidopsis lyrata	AT1G24570.1 | Symbols: | Protein of unknown function (DUF707) | chr1:8707197-8709491 FORWARD LENGTH=381	396	381	0	96.2	79.3	86.6
Rsa1.0_00050.1.g2809.t1	ref|NP_564220.1| uncharacterized protein [Arabidopsis thaliana] gi|21593326|gb|AAM65275.1| unknown [Arabidopsis thaliana] gi|56236092|gb|AAV84502.1| At1g24575 [Arabidopsis thaliana] gi|57222124|gb|AAW38969.1| At1g24575 [Arabidopsis thaliana] gi|88900286|gb|ABD57455.1| At1g24575 [Arabidopsis thaliana] gi|332192428|gb|AEE30549.1| uncharacterized protein AT1G24575 [Arabidopsis thaliana]	78	88	1.00E-11	112.8	48.7	62.8	uncharacterized protein	gbpln	Arabidopsis thaliana	AT1G24575.1 | Symbols: | unknown protein; Has 7 Blast hits to 7 proteins in 2 species: Archae - 0; Bacteria - 0; Metazoa - 0; Fungi - 0; Plants - 7; Viruses - 0; Other Eukaryotes - 0 (source: NCBI BLink). | chr1:8711211-8711477 REVERSE LENGTH=88	78	88	2.00E-14	112.8	48.7	62.8
Rsa1.0_00050.1.g2810.t1	ref|XP_002893438.1| hypothetical protein ARALYDRAFT_890209 [Arabidopsis lyrata subsp. lyrata] gi|297339280|gb|EFH69697.1| hypothetical protein ARALYDRAFT_890209 [Arabidopsis lyrata subsp. lyrata]	70	72	3.00E-14	102.9	65.7	77.1	hypothetical protein ARALYDRAFT_890209	gbpln	Arabidopsis lyrata	AT1G24600.1 | Symbols: | unknown protein; BEST Arabidopsis thaliana protein match is: unknown protein (TAIR:AT1G67920.1); Has 18 Blast hits to 18 proteins in 5 species: Archae - 0; Bacteria - 0; Metazoa - 0; Fungi - 0; Plants - 18; Viruses - 0; Other Eukaryotes - 0 (source: NCBI BLink). | chr1:8720375-8720593 FORWARD LENGTH=72	70	72	9.00E-15	102.9	61.4	74.3
Rsa1.0_00050.1.g2811.t1	gb|EOA40117.1| hypothetical protein CARUB_v10008819mg, partial [Capsella rubella] gi|482575931|gb|EOA40118.1| hypothetical protein CARUB_v10008819mg, partial [Capsella rubella]	475	532	0	112.0	86.1	93.3	hypothetical protein CARUB_v10008819mg, partial	gbpln	Capsella rubella	AT1G24610.1 | Symbols: | Rubisco methyltransferase family protein | chr1:8720906-8722711 REVERSE LENGTH=476	475	476	0	100.2	87.6	94.3
Rsa1.0_00050.1.g2812.t1	ref|NP_173866.1| putative calcium-binding protein CML25 [Arabidopsis thaliana] gi|75334522|sp|Q9FYK2.1|CML25_ARATH RecName: Full=Probable calcium-binding protein CML25; AltName: Full=Calmodulin-like protein 25 gi|9743349|gb|AAF97973.1|AC000103_23 F21J9.28 [Arabidopsis thaliana] gi|91805849|gb|ABE65653.1| polcalcin [Arabidopsis thaliana] gi|332192433|gb|AEE30554.1| putative calcium-binding protein CML25 [Arabidopsis thaliana]	187	186	6.00E-87	99.5	89.3	90.9	putative calcium-binding protein CML25	gbpln	Arabidopsis thaliana	AT1G24620.1 | Symbols: | EF hand calcium-binding protein family | chr1:8723893-8724453 REVERSE LENGTH=186	187	186	2.00E-89	99.5	89.3	90.9
Rsa1.0_00050.1.g2813.t1	#	#	#	#	#	#	#	–	----	----	#	#	#	#	#	#	#
Rsa1.0_00050.1.g2814.t1	ref|XP_002890697.1| At1g68060/T23K23_9 [Arabidopsis lyrata subsp. lyrata] gi|297336539|gb|EFH66956.1| At1g68060/T23K23_9 [Arabidopsis lyrata subsp. lyrata]	632	634	0	100.3	91.3	94.9	At1g68060/T23K23_9	gbpln	Arabidopsis lyrata	AT1G24764.1 | Symbols: ATMAP70-2, MAP70-2 | microtubule-associated proteins 70-2 | chr1:8760001-8763256 REVERSE LENGTH=634	632	634	0	100.3	90.3	94.6
Rsa1.0_00050.1.g2815.t1	ref|NP_173893.1| anthranilate synthase beta subunit 1 [Arabidopsis thaliana] gi|11067285|gb|AAG28813.1|AC079374_16 anthranilate synthase beta subunit [Arabidopsis thaliana] gi|403434|gb|AAA32742.1| anthranilate synthase beta subunit [Arabidopsis thaliana] gi|20466736|gb|AAM20685.1| anthranilate synthase beta subunit [Arabidopsis thaliana] gi|30023756|gb|AAP13411.1| At1g25220 [Arabidopsis thaliana] gi|110741096|dbj|BAE98642.1| hypothetical protein [Arabidopsis thaliana] gi|332192468|gb|AEE30589.1| anthranilate synthase beta subunit 1 [Arabidopsis thaliana]	262	276	1.00E-122	105.3	87.0	92.0	anthranilate synthase beta subunit 1	gbpln	Arabidopsis thaliana	AT1G25220.1 | Symbols: ASB1, TRP4, WEI7 | anthranilate synthase beta subunit 1 | chr1:8837430-8839478 REVERSE LENGTH=276	262	276	1.00E-125	105.3	87.0	92.0
Rsa1.0_00050.1.g2816.t1	ref|XP_002893431.1| hypothetical protein ARALYDRAFT_890187 [Arabidopsis lyrata subsp. lyrata] gi|297339273|gb|EFH69690.1| hypothetical protein ARALYDRAFT_890187 [Arabidopsis lyrata subsp. lyrata]	475	376	0	79.2	68.6	73.9	hypothetical protein ARALYDRAFT_890187	gbpln	Arabidopsis lyrata	AT1G25240.1 | Symbols: | ENTH/VHS/GAT family protein | chr1:8845231-8846361 FORWARD LENGTH=376	475	376	0	79.2	67.8	73.7

Rsa1.0_00050.1.g2817.t1	ref|XP_002893430.1| predicted protein [Arabidopsis lyrata subsp. lyrata] gi|297339272|gb|EFH69689.1| predicted protein [Arabidopsis lyrata subsp. lyrata]	382	385	1.00E-170	100.8	81.4	88.0	predicted protein	gbpln	Arabidopsis lyrata	AT1G25250.1 | Symbols: AtIDD16, IDD16 | indeterminate(ID)-domain 16 | chr1:8849549-8851520 FORWARD LENGTH=362	382	362	1.00E-156	94.8	73.6	79.8
Rsa1.0_00050.1.g2818.t8	gb|AAD21687.1| Strong similarity to gi|3600044 T12H20.12 protease homolog from Arabidopsis thaliana BAC gb|AF080119 and is a member of the reverse transcriptase family PF|00078 [Arabidopsis thaliana]	241	1415	4.00E-65	587.1	53.5	68.0	Strong similarity to gi|3600044 T12H20.12 protease homolog from Arabidopsis thaliana BAC gb|AF080119 and is a member of the reverse transcriptase family PF|00078	gbpln	Arabidopsis thaliana	#	#	#	#	#	#	#
Rsa1.0_00050.1.g2819.t1	ref|NP_564226.1| Ribosomal protein L10 family protein [Arabidopsis thaliana] gi|15028333|gb|AAK76643.1| unknown protein [Arabidopsis thaliana] gi|19310645|gb|AAL85053.1| unknown protein [Arabidopsis thaliana] gi|21593993|gb|AAM65913.1| unknown [Arabidopsis thaliana] gi|332192473|gb|AEE30594.1| Ribosomal protein L10 family protein [Arabidopsis thaliana]	287	235	1.00E-106	81.9	67.2	71.4	Ribosomal protein L10 family protein	gbpln	Arabidopsis thaliana	AT1G25260.1 | Symbols: | Ribosomal protein L10 family protein | chr1:8854163-8855766 REVERSE LENGTH=235	287	235	1.00E-109	81.9	67.2	71.4
Rsa1.0_00050.1.g2820.t1	ref|NP_173898.2| putative MtN21 nodulin protein [Arabidopsis thaliana] gi|75339341|sp|Q4PT23.1|WTR6_ARATH RecName: Full=WAT1-related protein At1g25270 gi|67633390|gb|AAY78620.1| nodulin MtN21 family protein [Arabidopsis thaliana] gi|332192474|gb|AEE30595.1| WAT1-related protein [Arabidopsis thaliana]	364	355	1.00E-160	97.5	76.1	87.9	putative MtN21 nodulin protein	gbpln	Arabidopsis thaliana	AT1G25270.1 | Symbols: | nodulin MtN21 /EamA-like transporter family protein | chr1:8857726-8859909 FORWARD LENGTH=355	364	355	1.00E-163	97.5	76.1	87.9
Rsa1.0_00050.1.g2821.t1	gb|EOA40428.1| hypothetical protein CARUB_v10009152mg [Capsella rubella]	442	442	0	100.0	88.7	92.8	hypothetical protein CARUB_v10009152mg	gbpln	Capsella rubella	AT1G25280.1 | Symbols: AtTLP10, TLP10 | tubby like protein 10 | chr1:8864961-8866608 FORWARD LENGTH=445	442	445	0	100.7	87.3	92.3
Rsa1.0_00050.1.g2822.t1	gb|EOA40428.1| hypothetical protein CARUB_v10009152mg [Capsella rubella]	209	442	1.00E-24	211.5	28.2	28.7	hypothetical protein CARUB_v10009152mg	gbpln	Capsella rubella	AT1G25280.3 | Symbols: AtTLP10, TLP10 | tubby like protein 10 | chr1:8865676-8866608 FORWARD LENGTH=267	209	267	2.00E-23	127.8	27.3	27.8
Rsa1.0_00050.1.g2823.t1	gb|AAG50806.1|AC079281_8 unknown protein [Arabidopsis thaliana]	1167	1213	0	103.9	37.0	56.7	unknown protein	gbpln	Arabidopsis thaliana	AT1G43760.1 | Symbols: | DNAse I-like superfamily protein | chr1:16528880-16531065 REVERSE LENGTH=626	1167	626	1.00E-60	53.6	11.5	20.4
Rsa1.0_00050.1.g2824.t1	ref|XP_002862436.1| predicted protein [Arabidopsis lyrata subsp. lyrata] gi|297307949|gb|EFH38694.1| predicted protein [Arabidopsis lyrata subsp. lyrata]	647	530	3.00E-39	81.9	12.2	17.5	predicted protein	gbpln	Arabidopsis lyrata	AT2G07760.1 | Symbols: | Zinc knuckle (CCHC-type) family protein | chr2:3584420-3586278 REVERSE LENGTH=530	647	530	2.00E-12	81.9	5.7	11.3
Rsa1.0_00050.1.g2825.t1	gb|EOA39802.1| hypothetical protein CARUB_v10008465mg [Capsella rubella]	700	700	0	100.0	84.7	92.4	hypothetical protein CARUB_v10008465mg	gbpln	Capsella rubella	AT1G25320.1 | Symbols: | Leucine-rich repeat protein kinase family protein | chr1:8877988-8880180 FORWARD LENGTH=702	700	702	0	100.3	85.7	92.3
Rsa1.0_00050.1.g2826.t1	ref|NP_564230.1| myb domain protein 116 [Arabidopsis thaliana] gi|15375295|gb|AAK25748.2|AF334815_1 putative transcription factor MYB116 [Arabidopsis thaliana] gi|41619120|gb|AAS10028.1| MYB transcription factor [Arabidopsis thaliana] gi|91805853|gb|ABE65655.1| myb family transcription factor [Arabidopsis thaliana] gi|332192487|gb|AEE30608.1| myb domain protein 116 [Arabidopsis thaliana]	287	283	1.00E-120	98.6	75.3	84.0	myb domain protein 116	gbpln	Arabidopsis thaliana	AT1G25340.1 | Symbols: MYB116, AtMYB116 | myb domain protein 116 | chr1:8885210-8886271 FORWARD LENGTH=283	287	283	1.00E-122	98.6	75.3	84.0
Rsa1.0_00050.1.g2827.t1	ref|NP_173906.2| putative tRNA-glutamine synthetase [Arabidopsis thaliana] gi|17065036|gb|AAL32672.1| Unknown protein [Arabidopsis thaliana] gi|21387137|gb|AAM47972.1| unknown protein [Arabidopsis thaliana] gi|332192489|gb|AEE30610.1| putative tRNA-glutamine synthetase [Arabidopsis thaliana]	818	795	0	97.2	86.6	91.2	putative tRNA-glutamine synthetase	gbpln	Arabidopsis thaliana	AT1G25350.1 | Symbols: OVA9 | glutamine-tRNA ligase, putative / glutaminyl-tRNA synthetase, putative / GlnRS, putative | chr1:8889280-8894205 REVERSE LENGTH=795	818	795	0	97.2	86.6	91.2
Rsa1.0_00050.1.g2828.t1	pir||F86383 hypothetical protein F4F7.22 - Arabidopsis thaliana	277	767	8.00E-62	276.9	57.8	65.3	hypothetical protein F4F7.22 - Arabidopsis thaliana	----	----	AT1G25370.1 | Symbols: | Protein of unknown function (DUF1639) | chr1:8898046-8898934 REVERSE LENGTH=263	277	263	1.00E-63	94.9	57.8	65.3

Rsa1.0_00050.1.g2829.t1	ref|NP_564232.1| Metallo-beta-lactamase domain-containing protein [Arabidopsis thaliana] gi|51968784|dbj|BAD43084.1| unknown protein [Arabidopsis thaliana] gi|332192493|gb|AEE30614.1| Metallo-beta-lactamase domain-containing protein [Arabidopsis thaliana]	589	524	0	89.0	70.5	76.7	Metallo-beta-lactamase domain-containing protein	gbpln	Arabidopsis thaliana	AT1G25375.1 | Symbols: | Metallo-hydrolase/oxidoreductase superfamily protein | chr1:8900279-8903220 REVERSE LENGTH=524	589	524	0	89.0	70.5	76.7
Rsa1.0_00050.1.g2830.t1	ref|XP_002893428.1| mitochondrial substrate carrier family protein [Arabidopsis lyrata subsp. lyrata] gi|297339270|gb|EFH69687.1| mitochondrial substrate carrier family protein [Arabidopsis lyrata subsp. lyrata]	360	364	1.00E-171	101.1	87.5	95.3	mitochondrial substrate carrier family protein	gbpln	Arabidopsis lyrata	AT1G25380.1 | Symbols: ATNDT2, NDT2 | NAD+ transporter 2 | chr1:8903726-8905818 FORWARD LENGTH=363	360	363	1.00E-171	100.8	86.1	94.4
Rsa1.0_00050.1.g2831.t1	ref|NP_173910.1| protein kinase-like protein [Arabidopsis thaliana] gi|12321510|gb|AAG50813.1|AC079281_15 wall-associated kinase, putative [Arabidopsis thaliana] gi|332192495|gb|AEE30616.1| protein kinase-like protein [Arabidopsis thaliana]	609	629	0	103.3	77.8	85.4	protein kinase-like protein	gbpln	Arabidopsis thaliana	AT1G25390.1 | Symbols: | Protein kinase superfamily protein | chr1:8906640-8908800 REVERSE LENGTH=629	609	629	0	103.3	77.8	85.4
Rsa1.0_00050.1.g2832.t1	ref|XP_002887289.1| hypothetical protein ARALYDRAFT_476148 [Arabidopsis lyrata subsp. lyrata] gi|297333130|gb|EFH63548.1| hypothetical protein ARALYDRAFT_476148 [Arabidopsis lyrata subsp. lyrata]	687	801	1.00E-120	116.6	33.2	38.0	hypothetical protein ARALYDRAFT_476148	gbpln	Arabidopsis lyrata	AT1G25390.1 | Symbols: | Protein kinase superfamily protein | chr1:8906640-8908800 REVERSE LENGTH=629	687	629	1.00E-118	91.6	35.7	47.6
Rsa1.0_00050.1.g2833.t1	gb|ABK28015.1| unknown [Arabidopsis thaliana]	84	108	3.00E-21	128.6	60.7	67.9	unknown	gbpln	Arabidopsis thaliana	AT1G25422.1 | Symbols: | unknown protein; FUNCTIONS IN: molecular_function unknown; INVOLVED IN: biological_process unknown; LOCATED IN: cellular_component unknown; BEST Arabidopsis thaliana protein match is: unknown protein (TAIR:AT1G68500.1); Has 16 Blast hits to 16 proteins in 6 species: Archae - 0; Bacteria - 0; Metazoa - 0; Fungi - 0; Plants - 16; Viruses - 0; Other Eukaryotes - 0 (source: NCBI BLink). | chr1:8919087-8919410 FORWARD LENGTH=107	84	107	5.00E-24	127.4	60.7	67.9
Rsa1.0_00050.1.g2834.t1	gb|ABK28016.1| unknown [Arabidopsis thaliana]	96	97	3.00E-31	101.0	77.1	88.5	unknown	gbpln	Arabidopsis thaliana	AT1G25425.1 | Symbols: CLE43 | CLAVATA3/ESR-RELATED 43 | chr1:8922832-8923122 FORWARD LENGTH=96	96	96	6.00E-34	100.0	77.1	88.5
Rsa1.0_00050.1.g2835.t1	ref|NP_173915.2| zinc finger protein CONSTANS-LIKE 16 [Arabidopsis thaliana] gi|52782786|sp|Q8RWD0.2|COL16_ARATH RecName: Full=Zinc finger protein CONSTANS-LIKE 16 gi|53749166|gb|AAU90068.1| At1g25440 [Arabidopsis thaliana] gi|110740969|dbj|BAE98579.1| hypothetical protein [Arabidopsis thaliana] gi|332192504|gb|AEE30625.1| zinc finger protein CONSTANS-LIKE 16 [Arabidopsis thaliana]	414	417	1.00E-179	100.7	82.4	89.1	zinc finger protein CONSTANS-LIKE 16	gbpln	Arabidopsis thaliana	AT1G25440.1 | Symbols: | B-box type zinc finger protein with CCT domain | chr1:8933939-8935284 REVERSE LENGTH=417	414	417	0	100.7	82.4	89.1
Rsa1.0_00051.1.g2836.t1	ref|NP_197839.1| large subunit ribosomal protein LP1 [Arabidopsis thaliana] gi|10177851|dbj|BAB11203.1| 60s acidic ribosomal protein P1 [Arabidopsis thaliana] gi|67633816|gb|AAY78832.1| putative 60S acidic ribosomal protein P1 [Arabidopsis thaliana] gi|332005935|gb|AED93318.1| 60S acidic ribosomal protein family [Arabidopsis thaliana]	111	111	5.00E-38	100.0	72.1	82.0	large subunit ribosomal protein LP1	gbpln	Arabidopsis thaliana	AT5G24510.1 | Symbols: | 60S acidic ribosomal protein family | chr5:8369295-8369866 REVERSE LENGTH=111	111	111	8.00E-41	100.0	72.1	82.0
Rsa1.0_00051.1.g2837.t1	ref|NP_001154738.1| DNA-binding protein BIN4 [Arabidopsis thaliana] gi|332005958|gb|AED93341.1| DNA-binding protein BIN4 [Arabidopsis thaliana]	419	446	1.00E-107	106.4	62.3	71.8	DNA-binding protein BIN4	gbpln	Arabidopsis thaliana	AT5G24630.5 | Symbols: BIN4 | double-stranded DNA binding | chr5:8432523-8435682 REVERSE LENGTH=446	419	446	1.00E-109	106.4	62.3	71.8

Rsa1.0_00051.1.g2838.t1	ref|NP_193753.1| terpene cyclase, C1 domain-containing protein [Arabidopsis thaliana] gi|317412200|sp|O65434.2|TPS07_ARATH RecName: Full=Putative terpenoid synthase 7; Short=AtTPS07 gi|332658884|gb|AEE84284.1| putative terpenoid synthase 7 [Arabidopsis thaliana]	559	604	1.00E-179	108.1	57.2	72.3	terpene cyclase, C1 domain-containing protein	gbpln	Arabidopsis thaliana	AT4G20200.1 | Symbols: | Terpenoid cyclases/Protein prenyltransferases superfamily protein | chr4:10908672-10911151 REVERSE LENGTH=604	559	604	0	108.1	57.2	72.3
Rsa1.0_00051.1.g2839.t1	ref|XP_002874216.1| hypothetical protein ARALYDRAFT_910514 [Arabidopsis lyrata subsp. lyrata] gi|297320053|gb|EFH50475.1| hypothetical protein ARALYDRAFT_910514 [Arabidopsis lyrata subsp. lyrata]	535	520	0	97.2	83.0	89.2	hypothetical protein ARALYDRAFT_910514	gbpln	Arabidopsis lyrata	AT5G24690.1 | Symbols: | Protein of unknown function (DUF3411) | chr5:8455783-8458513 REVERSE LENGTH=521	535	521	0	97.4	82.8	88.8
Rsa1.0_00051.1.g2840.t3	ref|XP_002874217.1| hypothetical protein ARALYDRAFT_489332 [Arabidopsis lyrata subsp. lyrata] gi|297320054|gb|EFH50476.1| hypothetical protein ARALYDRAFT_489332 [Arabidopsis lyrata subsp. lyrata]	1419	1376	0	97.0	84.9	88.9	hypothetical protein ARALYDRAFT_489332	gbpln	Arabidopsis lyrata	AT5G24710.1 | Symbols: | Transducin/WD40 repeat-like superfamily protein | chr5:8459148-8467920 REVERSE LENGTH=1377	1419	1377	0	97.0	84.6	88.7
Rsa1.0_00051.1.g2841.t2	sp|Q84KJ5.2|CRYD_ARATH RecName: Full=Cryptochrome DASH, chloroplastic/mitochondrial; AltName: Full=Cryptochrome-3; Flags: Precursor	128	569	2.00E-47	444.5	75.0	78.9	RecName: Full=Cryptochrome DASH, chloroplastic/mitochondrial; AltName: Full=Cryptochrome-3; Flags: Precursor	----	----	AT5G24850.1 | Symbols: CRY3 | cryptochrome 3 | chr5:8535399-8538016 REVERSE LENGTH=526	128	526	4.00E-50	410.9	75.0	78.9
Rsa1.0_00051.1.g2842.t1	gb|EOA23031.1| hypothetical protein CARUB_v10003797mg [Capsella rubella]	108	108	7.00E-51	100.0	90.7	95.4	hypothetical protein CARUB_v10003797mg	gbpln	Capsella rubella	AT5G24860.1 | Symbols: FPF1, ATFPF1 | flowering promoting factor 1 | chr5:8541822-8542154 FORWARD LENGTH=110	108	110	1.00E-51	101.9	89.8	94.4
Rsa1.0_00051.1.g2843.t1	gb|EOA21407.1| hypothetical protein CARUB_v10001780mg [Capsella rubella]	206	255	2.00E-65	123.8	77.7	85.4	hypothetical protein CARUB_v10001780mg	gbpln	Capsella rubella	AT5G24890.1 | Symbols: | unknown protein; FUNCTIONS IN: molecular_function unknown; INVOLVED IN: biological_process unknown; LOCATED IN: cellular_component unknown; EXPRESSED IN: 24 plant structures; EXPRESSED DURING: 13 growth stages; BEST Arabidopsis thaliana protein match is: unknown protein (TAIR:AT2G24550.1); Has 1807 Blast hits to 1807 proteins in 277 species: Archae - 0; Bacteria - 0; Metazoa - 736; Fungi - 347; Plants - 385; Viruses - 0; Other Eukaryotes - 339 (source: NCBI BLink). | chr5:8558357-8559172 REVERSE LENGTH=240	206	240	7.00E-67	116.5	76.2	84.0
Rsa1.0_00051.1.g2844.t1	ref|XP_002872134.1| hypothetical protein ARALYDRAFT_489353 [Arabidopsis lyrata subsp. lyrata] gi|297317971|gb|EFH48393.1| hypothetical protein ARALYDRAFT_489353 [Arabidopsis lyrata subsp. lyrata]	348	360	1.00E-165	103.4	89.7	94.3	hypothetical protein ARALYDRAFT_489353	gbpln	Arabidopsis lyrata	AT5G24930.1 | Symbols: ATCOL4, COL4 | CONSTANS-like 4 | chr5:8589325-8590949 FORWARD LENGTH=406	348	406	1.00E-165	116.7	89.7	94.5
Rsa1.0_00051.1.g2845.t1	ref|XP_002874237.1| RNA recognition motif-containing protein [Arabidopsis lyrata subsp. lyrata] gi|297320074|gb|EFH50496.1| RNA recognition motif-containing protein [Arabidopsis lyrata subsp. lyrata]	947	948	0	100.1	87.8	93.9	RNA recognition motif-containing protein	gbpln	Arabidopsis lyrata	AT5G25060.1 | Symbols: | RNA recognition motif (RRM)-containing protein | chr5:8634219-8639984 REVERSE LENGTH=946	947	946	0	99.9	87.9	93.8
Rsa1.0_00051.1.g2846.t1	ref|NP_197890.1| Sas10/Utp3/C1D family protein [Arabidopsis thaliana] gi|14190385|gb|AAK55673.1|AF378870_1 AT5g25080/T11H3_90 [Arabidopsis thaliana] gi|15215877|gb|AAK91482.1| AT5g25080/T11H3_90 [Arabidopsis thaliana] gi|21593008|gb|AAM64957.1| unknown [Arabidopsis thaliana] gi|332006014|gb|AED93397.1| Sas10/Utp3/C1D family protein [Arabidopsis thaliana]	181	217	5.00E-72	119.9	71.8	83.4	Sas10/Utp3/C1D family protein	gbpln	Arabidopsis thaliana	AT5G25080.1 | Symbols: | Sas10/Utp3/C1D family | chr5:8643782-8646306 FORWARD LENGTH=217	181	217	2.00E-74	119.9	71.8	83.4
Rsa1.0_00051.1.g2847.t1	gb|EOA21643.1| hypothetical protein CARUB_v10002061mg [Capsella rubella]	187	184	3.00E-68	98.4	76.5	81.8	hypothetical protein CARUB_v10002061mg	gbpln	Capsella rubella	AT5G25090.1 | Symbols: ENODL13, AtENODL13 | early nodulin-like protein 13 | chr5:8647117-8647755 REVERSE LENGTH=186	187	186	1.00E-62	99.5	74.3	82.9
Rsa1.0_00051.1.g2848.t1	#	#	#	#	#	#	#	-	----	----	#	#	#	#	#	#	#
Rsa1.0_00051.1.g2849.t1	ref|XP_002872140.1| CYP71B11 [Arabidopsis lyrata subsp. lyrata] gi|297317977|gb|EFH48399.1| CYP71B11 [Arabidopsis lyrata subsp. lyrata]	468	496	0	106.0	77.4	87.8	CYP71B11	gbpln	Arabidopsis lyrata	AT5G25140.1 | Symbols: CYP71B13 | cytochrome P450, family 71, subfamily B, polypeptide 13 | chr5:8672989-8674557 FORWARD LENGTH=496	468	496	0	106.0	77.8	87.6

Rsa1.0_00051.1.g2850.t1	gb|EOA19571.1| hypothetical protein CARUB_v10002639mg [Capsella rubella]	674	668	0	99.1	92.0	95.3	hypothetical protein CARUB_v10002639mg	gbpln	Capsella rubella	AT5G25150.1 | Symbols: TAF5 | TBP-associated factor 5 | chr5:8677117-8682058 FORWARD LENGTH=669	674	669	0	99.3	91.4	95.0
Rsa1.0_00051.1.g2851.t1	ref|XP_002872142.1| hypothetical protein ARALYDRAFT_910560 [Arabidopsis lyrata subsp. lyrata] gi|297317979|gb|EFH48401.1| hypothetical protein ARALYDRAFT_910560 [Arabidopsis lyrata subsp. lyrata]	247	232	6.00E-91	93.9	69.6	78.9	hypothetical protein ARALYDRAFT_910560	gbpln	Arabidopsis lyrata	AT5G25160.1 | Symbols: ZFP3 | zinc finger protein 3 | chr5:8687524-8688231 FORWARD LENGTH=235	247	235	7.00E-88	95.1	69.2	78.9
Rsa1.0_00051.1.g2852.t1	ref|XP_002874244.1| hypothetical protein ARALYDRAFT_489371 [Arabidopsis lyrata subsp. lyrata] gi|297320081|gb|EFH50503.1| hypothetical protein ARALYDRAFT_489371 [Arabidopsis lyrata subsp. lyrata]	180	183	2.00E-70	101.7	81.7	87.2	hypothetical protein ARALYDRAFT_489371	gbpln	Arabidopsis lyrata	AT5G25190.1 | Symbols: | Integrase-type DNA-binding superfamily protein | chr5:8707007-8707655 REVERSE LENGTH=181	180	181	2.00E-71	100.6	77.2	82.8
Rsa1.0_00051.1.g2853.t1	ref|NP_197906.1| uncharacterized protein [Arabidopsis thaliana] gi|26450052|dbj|BAC42146.1| unknown protein [Arabidopsis thaliana] gi|28827680|gb|AAO50684.1| unknown protein [Arabidopsis thaliana] gi|332006033|gb|AED93416.1| uncharacterized protein AT5G25240 [Arabidopsis thaliana]	124	131	3.00E-44	105.6	77.4	87.1	uncharacterized protein	gbpln	Arabidopsis thaliana	AT5G25240.1 | Symbols: | unknown protein; FUNCTIONS IN: molecular_function unknown; INVOLVED IN: biological_process unknown; LOCATED IN: cellular_component unknown; EXPRESSED IN: 23 plant structures; EXPRESSED DURING: 13 growth stages; Has 1807 Blast hits to 1807 proteins in 277 species: Archae - 0; Bacteria - 0; Metazoa - 736; Fungi - 347; Plants - 385; Viruses - 0; Other Eukaryotes - 339 (source: NCBI BLink). | chr5:8746779-8747174 REVERSE LENGTH=131	124	131	5.00E-47	105.6	77.4	87.1
Rsa1.0_00051.1.g2854.t1	#	#	#	#	#	#	#	–	----	----	#	#	#	#	#	#	#
Rsa1.0_00051.1.g2855.t1	ref|XP_002874247.1| hypothetical protein ARALYDRAFT_910571 [Arabidopsis lyrata subsp. lyrata] gi|297320084|gb|EFH50506.1| hypothetical protein ARALYDRAFT_910571 [Arabidopsis lyrata subsp. lyrata]	357	367	0	102.8	89.4	95.0	hypothetical protein ARALYDRAFT_910571	gbpln	Arabidopsis lyrata	AT5G25265.1 | Symbols: | unknown protein; FUNCTIONS IN: molecular_function unknown; INVOLVED IN: biological_process unknown; LOCATED IN: plasma membrane, membrane; EXPRESSED IN: cultured cell, leaf; BEST Arabidopsis thaliana protein match is: unknown protein (TAIR:AT2G25260.1); Has 30201 Blast hits to 17322 proteins in 780 species: Archae - 12; Bacteria - 1396; Metazoa - 17338; Fungi - 3422; Plants - 5037; Viruses - 0; Other Eukaryotes - 2996 (source: NCBI BLink). | chr5:8754794-8756855 REVERSE LENGTH=366	357	366	0	102.5	89.4	94.4
Rsa1.0_00051.1.g2856.t1	ref|NP_566112.1| uncharacterized protein [Arabidopsis thaliana] gi|75100577|sp|O82251.1|TI202_ARATH RecName: Full=Protein TIC 20-II, chloroplastic; AltName: Full=Translocon at the inner envelope membrane of chloroplasts 20-II; Short=AtTIC20-II; Flags: Precursor gi|3738296|gb|AAC63638.1| expressed protein [Arabidopsis thaliana] gi|330255801|gb|AEC10895.1| protein TIC 20-II [Arabidopsis thaliana]	206	208	3.00E-96	101.0	89.3	93.2	uncharacterized protein	gbpln	Arabidopsis thaliana	AT2G47840.1 | Symbols: | Uncharacterised conserved protein ycf60 | chr2:19594331-19594957 REVERSE LENGTH=208	206	208	1.00E-98	101.0	89.3	93.2
Rsa1.0_00051.1.g2857.t1	#	#	#	#	#	#	#	–	----	----	#	#	#	#	#	#	#
Rsa1.0_00051.1.g2858.t1	gb|ABL97972.1| serine-rich protein [Brassica rapa]	200	193	1.00E-78	96.5	85.0	88.5	serine-rich protein	gbpln	Brassica rapa	AT5G25280.2 | Symbols: | serine-rich protein-related | chr5:8773882-8774544 FORWARD LENGTH=220	200	220	1.00E-69	110.0	66.0	70.5
Rsa1.0_00051.1.g2859.t2	ref|NP_197917.1| EIN3-binding F-box protein 2 [Arabidopsis thaliana] gi|75325708|sp|Q708Y0.1|EBF2_ARATH RecName: Full=EIN3-binding F-box protein 2 gi|38705081|gb|AAR27072.1| EIN3-binding F-box protein 2 [Arabidopsis thaliana] gi|40641627|emb|CAE75865.1| F-box protein [Arabidopsis thaliana] gi|110735710|dbj|BAE99835.1| leucine-rich repeats containing protein [Arabidopsis thaliana] gi|332006046|gb|AED93429.1| EIN3-binding F-box protein 2 [Arabidopsis thaliana]	600	623	0	103.8	86.8	93.5	EIN3-binding F-box protein 2	gbpln	Arabidopsis thaliana	AT5G25350.1 | Symbols: EBF2 | EIN3-binding F box protein 2 | chr5:8794842-8796882 REVERSE LENGTH=623	600	623	0	103.8	86.8	93.5

Rsa1.0_00051.1.g2860.t1	ref|XP_002874256.1| hypothetical protein ARALYDRAFT_326806 [Arabidopsis lyrata subsp. lyrata] gi|297320093|gb|EFH50515.1| hypothetical protein ARALYDRAFT_326806 [Arabidopsis lyrata subsp. lyrata]	185	189	1.00E-82	102.2	83.8	88.6	hypothetical protein ARALYDRAFT_326806	gbpln	Arabidopsis lyrata	AT5G25390.2 | Symbols: SHN2 | Integrase-type DNA-binding superfamily protein | chr5:8820637-8821741 FORWARD LENGTH=189	185	189	1.00E-84	102.2	82.7	88.6
Rsa1.0_00051.1.g2861.t1	ref|XP_002869301.1| predicted protein [Arabidopsis lyrata subsp. lyrata] gi|297315137|gb|EFH45560.1| predicted protein [Arabidopsis lyrata subsp. lyrata]	418	677	1.00E-154	162.0	67.9	79.2	predicted protein	gbpln	Arabidopsis lyrata	AT4G31980.1 | Symbols: | unknown protein; CONTAINS InterPro DOMAIN/s: Protein of unknown function DUF247, plant (InterPro:IPR004158), Protein of unknown function DUF862, eukaryotic (InterPro:IPR008580); BEST Arabidopsis thaliana protein match is: Plant protein of unknown function (DUF247) (TAIR:AT5G11290.1); Has 1967 Blast hits to 1844 proteins in 183 species: Archae - 0; Bacteria - 6; Metazoa - 223; Fungi - 83; Plants - 1477; Viruses - 0; Other Eukaryotes - 178 (source: NCBI BLink). | chr4:15464905-15469204 FORWARD LENGTH=680	418	680	1.00E-153	162.7	67.5	78.0
Rsa1.0_00051.1.g2862.t1	ref|XP_002869301.1| predicted protein [Arabidopsis lyrata subsp. lyrata] gi|297315137|gb|EFH45560.1| predicted protein [Arabidopsis lyrata subsp. lyrata]	421	677	1.00E-161	160.8	67.9	80.8	predicted protein	gbpln	Arabidopsis lyrata	AT4G31980.1 | Symbols: | unknown protein; CONTAINS InterPro DOMAIN/s: Protein of unknown function DUF247, plant (InterPro:IPR004158), Protein of unknown function DUF862, eukaryotic (InterPro:IPR008580); BEST Arabidopsis thaliana protein match is: Plant protein of unknown function (DUF247) (TAIR:AT5G11290.1); Has 1967 Blast hits to 1844 proteins in 183 species: Archae - 0; Bacteria - 6; Metazoa - 223; Fungi - 83; Plants - 1477; Viruses - 0; Other Eukaryotes - 178 (source: NCBI BLink). | chr4:15464905-15469204 FORWARD LENGTH=680	421	680	1.00E-157	161.5	66.3	79.6
Rsa1.0_00051.1.g2863.t1	ref|XP_002874258.1| CYCA2_1 [Arabidopsis lyrata subsp. lyrata] gi|297320095|gb|EFH50517.1| CYCA2_1 [Arabidopsis lyrata subsp. lyrata]	104	433	4.00E-16	416.3	55.8	65.4	CYCA2_1	gbpln	Arabidopsis lyrata	AT5G25380.1 | Symbols: CYCA2;1 | cyclin a2;1 | chr5:8815230-8817566 FORWARD LENGTH=437	104	437	8.00E-18	420.2	53.8	64.4
Rsa1.0_00051.1.g2864.t1	gb|EOA18383.1| hypothetical protein CARUB_v10006919mg [Capsella rubella]	437	660	1.00E-141	151.0	57.7	67.0	hypothetical protein CARUB_v10006919mg	gbpln	Capsella rubella	AT4G31980.1 | Symbols: | unknown protein; CONTAINS InterPro DOMAIN/s: Protein of unknown function DUF247, plant (InterPro:IPR004158), Protein of unknown function DUF862, eukaryotic (InterPro:IPR008580); BEST Arabidopsis thaliana protein match is: Plant protein of unknown function (DUF247) (TAIR:AT5G11290.1); Has 1967 Blast hits to 1844 proteins in 183 species: Archae - 0; Bacteria - 6; Metazoa - 223; Fungi - 83; Plants - 1477; Viruses - 0; Other Eukaryotes - 178 (source: NCBI BLink). | chr4:15464905-15469204 FORWARD LENGTH=680	437	680	1.00E-138	155.6	56.1	65.4
Rsa1.0_00051.1.g2865.t10	ref|XP_002874259.1| predicted protein [Arabidopsis lyrata subsp. lyrata] gi|297320096|gb|EFH50518.1| predicted protein [Arabidopsis lyrata subsp. lyrata]	1459	547	0	37.5	32.1	34.0	predicted protein	gbpln	Arabidopsis lyrata	AT1G65550.1 | Symbols: | Xanthine/uracil permease family protein | chr1:24367694-24370800 REVERSE LENGTH=541	1459	541	0	37.1	31.3	33.2
Rsa1.0_00051.1.g2866.t1	gb|EOA13406.1| hypothetical protein CARUB_v10026444mg, partial [Capsella rubella]	411	430	1.00E-108	104.6	58.2	68.6	hypothetical protein CARUB_v10026444mg, partial	gbpln	Capsella rubella	AT5G62510.1 | Symbols: | F-box family protein | chr5:25095373-25096635 REVERSE LENGTH=420	411	420	1.00E-100	102.2	53.8	64.5
Rsa1.0_00051.1.g2867.t1	ref|XP_002874263.1| predicted protein [Arabidopsis lyrata subsp. lyrata] gi|297320100|gb|EFH50522.1| predicted protein [Arabidopsis lyrata subsp. lyrata]	258	280	9.00E-70	108.5	59.3	71.7	predicted protein	gbpln	Arabidopsis lyrata	AT5G25475.4 | Symbols: | AP2/B3-like transcriptional factor family protein | chr5:8867920-8869492 REVERSE LENGTH=324	258	324	6.00E-71	125.6	58.1	74.0
Rsa1.0_00051.1.g2868.t1	ref|XP_002874266.1| hypothetical protein ARALYDRAFT_489409 [Arabidopsis lyrata subsp. lyrata] gi|297320103|gb|EFH50525.1| hypothetical protein ARALYDRAFT_489409 [Arabidopsis lyrata subsp. lyrata]	492	499	0	101.4	85.4	92.7	hypothetical protein ARALYDRAFT_489409	gbpln	Arabidopsis lyrata	AT5G25510.1 | Symbols: | Protein phosphatase 2A regulatory B subunit family protein | chr5:8882728-8884325 REVERSE LENGTH=500	492	500	0	101.6	86.6	93.9
Rsa1.0_00051.1.g2869.t1	gb|EOA20666.1| hypothetical protein CARUB_v10000978mg, partial [Capsella rubella]	439	439	0	100.0	83.6	88.4	hypothetical protein CARUB_v10000978mg, partial	gbpln	Capsella rubella	AT5G25620.2 | Symbols: YUC6 | Flavin-binding monooxygenase family protein | chr5:8935312-8938200 REVERSE LENGTH=426	439	426	0	97.0	83.4	86.6

Rsa1.0_00051.1.g2870.t1	gb|EOA19383.1| hypothetical protein CARUB_v10000581mg [Capsella rubella]	571	571	0	100.0	87.2	92.5	hypothetical protein CARUB_v10000581mg	gbpln	Capsella rubella	AT5G25630.1 | Symbols: | Tetratricopeptide repeat (TPR)-like superfamily protein | chr5:8947426-8949424 FORWARD LENGTH=574	571	574	0	100.5	85.8	92.1
Rsa1.0_00051.1.g2871.t1	ref|XP_002874282.1| predicted protein [Arabidopsis lyrata subsp. lyrata] gi|297320119|gb|EFH50541.1| predicted protein [Arabidopsis lyrata subsp. lyrata]	925	402	0	43.5	33.3	36.2	predicted protein	gbpln	Arabidopsis lyrata	AT5G25770.3 | Symbols: | alpha/beta-Hydrolases superfamily protein | chr5:8969308-8971806 REVERSE LENGTH=421	925	421	0	45.5	32.5	36.3
Rsa1.0_00052.1.g2872.t13	gb|EOA30455.1| hypothetical protein CARUB_v10013580mg [Capsella rubella]	770	481	0	62.5	50.8	53.8	hypothetical protein CARUB_v10013580mg	gbpln	Capsella rubella	AT3G22810.1 | Symbols: | Plant protein of unknown function (DUF828) with plant pleckstrin homology-like region | chr3:8068595-8071559 FORWARD LENGTH=472	770	472	0	61.3	49.1	52.5
Rsa1.0_00052.1.g2873.t1	#	#	#	#	#	#	#	-	----	----	#	#	#	#	#	#	#
Rsa1.0_00052.1.g2874.t1	ref|XP_002885525.1| hypothetical protein ARALYDRAFT_479793 [Arabidopsis lyrata subsp. lyrata] gi|297331365|gb|EFH61784.1| hypothetical protein ARALYDRAFT_479793 [Arabidopsis lyrata subsp. lyrata]	117	108	2.00E-32	92.3	60.7	70.1	hypothetical protein ARALYDRAFT_479793	gbpln	Arabidopsis lyrata	AT3G22820.1 | Symbols: | allergen-related | chr3:8073451-8074061 REVERSE LENGTH=107	117	107	3.00E-32	91.5	63.2	71.8
Rsa1.0_00052.1.g2875.t1	gb|AAF97279.1|AC010164_1 Hypothetical protein [Arabidopsis thaliana]	117	352	7.00E-13	300.9	34.2	52.1	Hypothetical protein	gbpln	Arabidopsis thaliana	#	#	#	#	#	#	#
Rsa1.0_00052.1.g2876.t4	gb|AAF06087.1|AC007918_11 Similar to gi|4325351 T25H8.2 TNP2 protein homolog from Arabidopsis thaliana BAC gb|AF128394 [Arabidopsis thaliana]	307	1121	3.00E-45	365.1	42.0	58.0	Similar to gi|4325351 T25H8.2 TNP2 protein homolog from Arabidopsis thaliana BAC gb|AF128394	gbpln	Arabidopsis thaliana	#	#	#	#	#	#	#
Rsa1.0_00052.1.g2877.t1	gb|ABD65636.1| hypothetical protein 23.t00055 [Brassica oleracea]	412	414	1.00E-104	100.5	58.0	64.3	hypothetical protein 23.t00055	gbpln	Brassica oleracea	#	#	#	#	#	#	#
Rsa1.0_00052.1.g2878.t1	gb|EOA30671.1| hypothetical protein CARUB_v10013808mg [Capsella rubella]	398	417	1.00E-163	104.8	76.6	86.7	hypothetical protein CARUB_v10013808mg	gbpln	Capsella rubella	AT3G22830.1 | Symbols: AT-HSFA6B, HSFA6B | heat shock transcription factor A6B | chr3:8078981-8080895 FORWARD LENGTH=406	398	406	1.00E-164	102.0	78.1	86.4
Rsa1.0_00052.1.g2879.t1	#	#	#	#	#	#	#	-	----	----	#	#	#	#	#	#	#
Rsa1.0_00052.1.g2880.t1	gb|AAR11456.1| ELIP [Brassica rapa subsp. pekinensis]	196	194	3.00E-94	99.0	96.4	96.9	ELIP	gbpln	Brassica rapa	AT3G22840.1 | Symbols: ELIP1, ELIP | Chlorophyll A-B binding family protein | chr3:8084628-8085445 REVERSE LENGTH=195	196	195	5.00E-84	99.5	88.3	90.8
Rsa1.0_00052.1.g2881.t1	gb|EOA26720.1| hypothetical protein CARUB_v10022808mg [Capsella rubella]	71	645	9.00E-15	908.5	64.8	73.2	hypothetical protein CARUB_v10022808mg	gbpln	Capsella rubella	AT2G35610.1 | Symbols: XEG113 | xyloglucanase 113 | chr2:14947617-14951106 REVERSE LENGTH=644	71	644	4.00E-16	907.0	66.2	73.2
Rsa1.0_00052.1.g2882.t1	ref|NP_188924.3| emp24/gp25L/p24 family/GOLD domain-containing protein [Arabidopsis thaliana] gi|75273406|sp|Q9LIL4.1|P24B3_ARATH RecName: Full=Transmembrane emp24 domain-containing protein p24beta3; AltName: Full=p24 family protein beta2; Short=p24beta2; AltName: Full=p24 family protein beta3; Short=p24beta3; Flags: Precursor gi|11994713|dbj|BAB03029.1| coated vesicle membrane protein-like [Arabidopsis thaliana] gi|17979492|gb|AAL50082.1| AT3g22845/MWI23_22 [Arabidopsis thaliana] gi|20147305|gb|AAM10366.1| AT3g22845/MWI23_22 [Arabidopsis thaliana] gi|332643162|gb|AEE76683.1| emp24/gp25L/p24 family/GOLD domain-containing protein [Arabidopsis thaliana]	218	214	1.00E-114	98.2	91.3	95.9	emp24/gp25L/p24 family/GOLD domain-containing protein	gbpln	Arabidopsis thaliana	AT3G22845.1 | Symbols: | emp24/gp25L/p24 family/GOLD family protein | chr3:8087373-8088550 FORWARD LENGTH=214	218	214	1.00E-116	98.2	91.3	95.9
Rsa1.0_00052.1.g2883.t1	#	#	#	#	#	#	#	-	----	----	#	#	#	#	#	#	#
Rsa1.0_00052.1.g2884.t1	gb|EOA32024.1| hypothetical protein CARUB_v10015267mg [Capsella rubella]	402	729	6.00E-62	181.3	41.3	55.0	hypothetical protein CARUB_v10015267mg	gbpln	Capsella rubella	AT3G14950.1 | Symbols: TTL2 | tetratricopetide-repeat thioredoxin-like 2 | chr3:5030216-5032892 REVERSE LENGTH=721	402	721	1.00E-63	179.4	39.8	53.0
Rsa1.0_00052.1.g2885.t1	ref|XP_002883384.1| RNA polymerase Rpb7 N-terminal domain-containing protein [Arabidopsis lyrata subsp. lyrata] gi|297329224|gb|EFH59643.1| RNA polymerase Rpb7 N-terminal domain-containing protein [Arabidopsis lyrata subsp. lyrata]	175	174	8.00E-65	99.4	68.6	82.9	RNA polymerase Rpb7 N-terminal domain-containing protein	gbpln	Arabidopsis lyrata	AT3G22900.1 | Symbols: NRPD7 | RNA polymerase Rpb7-like, N-terminal domain | chr3:8115645-8116169 FORWARD LENGTH=174	175	174	9.00E-67	99.4	68.6	82.9

Rsa1.0_00052.1.g2886.t1	ref|XP_002885529.1| hypothetical protein ARALYDRAFT_898774 [Arabidopsis lyrata subsp. lyrata] gi|297331369|gb|EFH61788.1| hypothetical protein ARALYDRAFT_898774 [Arabidopsis lyrata subsp. lyrata]	1021	1022	0	100.1	91.7	95.6	hypothetical protein ARALYDRAFT_898774	gbpln	Arabidopsis lyrata	AT3G22910.1 | Symbols: | ATPase E1-E2 type family protein / haloacid dehalogenase-like hydrolase family protein | chr3:8116335-8119388 REVERSE LENGTH=1017	1021	1017	0	99.6	90.7	94.5
Rsa1.0_00052.1.g2887.t1	ref|NP_188933.1| calmodulin-like protein 11 [Arabidopsis thaliana] gi|75335042|sp|Q9LIK5.1|CML11_ARATH RecName: Full=Calmodulin-like protein 11 gi|16226344|gb|AAL16141.1|AF428309_1 AT3g22930/F5N5_10 [Arabidopsis thaliana] gi|11994722|dbj|BAB03038.1| unnamed protein product [Arabidopsis thaliana] gi|21436047|gb|AAM51601.1| AT3g22930/F5N5_10 [Arabidopsis thaliana] gi|332643171|gb|AEE76692.1| calmodulin-like protein 11 [Arabidopsis thaliana]	205	173	4.00E-75	84.4	68.3	70.2	calmodulin-like protein 11	gbpln	Arabidopsis thaliana	AT3G22930.1 | Symbols: CML11 | calmodulin-like 11 | chr3:8124286-8125835 REVERSE LENGTH=173	205	173	1.00E-77	84.4	68.3	70.2
Rsa1.0_00052.1.g2888.t1	ref|NP_191135.1| uncharacterized protein [Arabidopsis thaliana] gi|30694316|ref|NP_850708.1| uncharacterized protein [Arabidopsis thaliana] gi|334186001|ref|NP_001190098.1| uncharacterized protein [Arabidopsis thaliana] gi|58652076|gb|AAW80863.1| At3g55760 [Arabidopsis thaliana] gi|332645910|gb|AEE79431.1| uncharacterized protein AT3G55760 [Arabidopsis thaliana] gi|332645911|gb|AEE79432.1| uncharacterized protein AT3G55760 [Arabidopsis thaliana] gi|332645912|gb|AEE79433.1| uncharacterized protein AT3G55760 [Arabidopsis thaliana]	566	578	0	102.1	82.9	89.6	uncharacterized protein	gbpln	Arabidopsis thaliana	AT3G55760.3 | Symbols: | unknown protein; EXPRESSED IN: 16 plant structures; EXPRESSED DURING: 10 growth stages; BEST Arabidopsis thaliana protein match is: unknown protein (TAIR:AT1G42430.2). | chr3:20700648-20702886 FORWARD LENGTH=578	566	578	0	102.1	82.9	89.6
Rsa1.0_00052.1.g2889.t1	ref|NP_850746.1| G-protein gamma subunit 2 [Arabidopsis thaliana] gi|75163105|sp|Q93V47.1|GG2_ARATH RecName: Full=Guanine nucleotide-binding protein subunit gamma 2; AltName: Full=Ggamma-subunit 2; AltName: Full=Heterotrimeric G protein gamma-subunit 2; Short=AtAGG2; Flags: Precursor gi|14625852|gb|AAK71536.1|AF347077_1 heterotrimeric G-protein gamma subunit 2 [Arabidopsis thaliana] gi|14625854|gb|AAK71537.1|AF347078_1 heterotrimeric G-protein gamma subunit 2 [Arabidopsis thaliana] gi|88900340|gb|ABD57482.1| At3g22942 [Arabidopsis thaliana] gi|110736567|dbj|BAF00249.1| hypothetical protein [Arabidopsis thaliana] gi|332643173|gb|AEE76694.1| G-protein gamma subunit 2 [Arabidopsis thaliana]	143	100	6.00E-35	69.9	59.4	61.5	G-protein gamma subunit 2	gbpln	Arabidopsis thaliana	AT3G22942.1 | Symbols: AGG2 | G-protein gamma subunit 2 | chr3:8134475-8135667 FORWARD LENGTH=100	143	100	2.00E-37	69.9	59.4	61.5
Rsa1.0_00052.1.g2890.t1	ref|NP_566720.1| Pyruvate kinase family protein [Arabidopsis thaliana] gi|75311203|sp|Q9LIK0.1|PKP1_ARATH RecName: Full=Plastidial pyruvate kinase 1, chloroplastic; Short=PK1; Short=PKp1; AltName: Full=Pyruvate kinase II; AltName: Full=Pyruvate kinase isozyme A; Short=PKP-ALPHA; Flags: Precursor gi|11994727|dbj|BAB03043.1| pyruvate kinase [Arabidopsis thaliana] gi|15983775|gb|AAL10484.1| AT3g22960/F5N5_15 [Arabidopsis thaliana] gi|16604372|gb|AAL24192.1| AT3g22960/F5N5_15 [Arabidopsis thaliana] gi|26983820|gb|AAN86162.1| putative pyruvate kinase [Arabidopsis thaliana] gi|332643176|gb|AEE76697.1| Pyruvate kinase family protein [Arabidopsis thaliana]	606	596	0	98.3	85.1	89.3	Pyruvate kinase family protein	gbpln	Arabidopsis thaliana	AT3G22960.1 | Symbols: PKP1, PKP-ALPHA | Pyruvate kinase family protein | chr3:8139369-8141771 FORWARD LENGTH=596	606	596	0	98.3	85.1	89.3

Rsa1.0_00052.1.g2891.t1	gb|EOA20393.1| hypothetical protein CARUB_v10000704mg [Capsella rubella]	517	525	0	101.5	74.3	84.7	hypothetical protein CARUB_v10000704mg	gbpln	Capsella rubella	AT5G22110.1 | Symbols: ATDPB2, CYL2, DPB2 | DNA polymerase epsilon subunit B2 | chr5:7331071-7334178 REVERSE LENGTH=526	517	526	0	101.7	73.3	83.2
Rsa1.0_00052.1.g2892.t1	ref|NP_001118679.1| uncharacterized protein [Arabidopsis thaliana] gi|11994728|dbj|BAB03044.1| unnamed protein product [Arabidopsis thaliana] gi|332643177|gb|AEE76698.1| uncharacterized protein AT3G22961 [Arabidopsis thaliana]	285	223	8.00E-28	78.2	31.9	44.9	uncharacterized protein	gbpln	Arabidopsis thaliana	AT3G22961.1 | Symbols: | Paired amphipathic helix (PAH2) superfamily protein | chr3:8142138-8142809 FORWARD LENGTH=223	285	223	2.00E-30	78.2	31.9	44.9
Rsa1.0_00052.1.g2893.t1	#	#	#	#	#	#	#	–	----	----	#	#	#	#	#	#	#
Rsa1.0_00052.1.g2894.t1	ref|NP_188940.1| CBL-interacting serine/threonine-protein kinase 7 [Arabidopsis thaliana] gi|75338665|sp|Q9XIW0.1|CIPK7_ARATH RecName: Full=CBL-interacting serine/threonine-protein kinase 7; AltName: Full=SNF1-related kinase 3.10; AltName: Full=SOS2-like protein kinase PKS7; AltName: Full=Serine/threonine-protein kinase SR2; Short=AtSR2; Short=AtSRPK1 gi|13249113|gb|AAK16682.1|AF290192_1 CBL-interacting protein kinase 7 [Arabidopsis thaliana] gi|13448039|gb|AAK26846.1|AF339148_1 SOS2-like protein kinase PKS7 [Arabidopsis thaliana] gi|5478791|dbj|BAA77716.2| SNF1 related protein kinase [Arabidopsis thaliana] gi|9294189|dbj|BAB02091.1| SNF1 related protein kinase [Arabidopsis thaliana] gi|57222172|gb|AAW38993.1| At3g23000 [Arabidopsis thaliana] gi|114213519|gb|ABI54342.1| At3g23000 [Arabidopsis thaliana] gi|332643183|gb|AEE76704.1| CBL-interacting serine/threonine-protein kinase 7 [Arabidopsis thaliana]	413	429	0	103.9	82.6	90.3	CBL-interacting serine/threonine-protein kinase 7	gbpln	Arabidopsis thaliana	AT3G23000.1 | Symbols: CIPK7, SnRK3.10, PKS7, ATSRPK1, ATSR2 | CBL-interacting protein kinase 7 | chr3:8172654-8173943 FORWARD LENGTH=429	413	429	0	103.9	82.6	90.3
Rsa1.0_00052.1.g2895.t1	emb|CCD74510.1| unknown, partial [Arabidopsis halleri subsp. halleri]	172	300	3.00E-27	174.4	35.5	52.9	unknown, partial	gbpln	Arabidopsis halleri	AT4G29090.1 | Symbols: | Ribonuclease H-like superfamily protein | chr4:14333528-14335255 FORWARD LENGTH=575	172	575	6.00E-12	334.3	26.2	45.9
Rsa1.0_00052.1.g2896.t1	ref|XP_002885542.1| indoleacetic acid-induced protein 2 [Arabidopsis lyrata subsp. lyrata] gi|297331382|gb|EFH61801.1| indoleacetic acid-induced protein 2 [Arabidopsis lyrata subsp. lyrata]	172	174	2.00E-79	101.2	89.0	91.9	indoleacetic acid-induced protein 2	gbpln	Arabidopsis lyrata	AT3G23030.1 | Symbols: IAA2 | indole-3-acetic acid inducible 2 | chr3:8181069-8181685 REVERSE LENGTH=174	172	174	2.00E-81	101.2	87.2	91.9
Rsa1.0_00053.1.g2897.t1	ref|XP_002886390.1| hypothetical protein ARALYDRAFT_893070 [Arabidopsis lyrata subsp. lyrata] gi|297332231|gb|EFH62649.1| hypothetical protein ARALYDRAFT_893070 [Arabidopsis lyrata subsp. lyrata]	262	298	6.00E-90	113.7	61.8	79.0	hypothetical protein ARALYDRAFT_893070	gbpln	Arabidopsis lyrata	AT2G42470.1 | Symbols: | TRAF-like family protein | chr2:17679887-17685187 REVERSE LENGTH=898	262	898	2.00E-73	342.7	53.1	73.3
Rsa1.0_00053.1.g2898.t1	ref|NP_198882.1| ATP synthase mitochondrial F1 complex assembly factor 2 [Arabidopsis thaliana] gi|14517426|gb|AAK62603.1| AT5g40660/MNF13_180 [Arabidopsis thaliana] gi|22655442|gb|AAM98313.1| At5g40660/MNF13_180 [Arabidopsis thaliana] gi|332007196|gb|AED94579.1| ATP12 protein-related [Arabidopsis thaliana]	114	325	1.00E-35	285.1	71.1	75.4	ATP synthase mitochondrial F1 complex assembly factor 2	gbpln	Arabidopsis thaliana	AT5G40660.1 | Symbols: | ATP12 protein-related | chr5:16283650-16285161 REVERSE LENGTH=325	114	325	2.00E-38	285.1	71.1	75.4
Rsa1.0_00053.1.g2899.t1	gb|EOA35049.1| hypothetical protein CARUB_v10020154mg [Capsella rubella]	399	514	2.00E-38	128.8	20.1	24.8	hypothetical protein CARUB_v10020154mg	gbpln	Capsella rubella	AT1G80710.1 | Symbols: DRS1 | DROUGHT SENSITIVE 1 | chr1:30333499-30335796 REVERSE LENGTH=516	399	516	7.00E-41	129.3	20.3	24.6
Rsa1.0_00053.1.g2900.t1	ref|NP_564758.2| Pectin lyase-like protein [Arabidopsis thaliana] gi|2462753|gb|AAB71972.1| putative polygalacturonase [Arabidopsis thaliana] gi|332195579|gb|AEE33700.1| Pectin lyase-like protein [Arabidopsis thaliana]	538	540	0	100.4	83.6	88.8	Pectin lyase-like protein	gbpln	Arabidopsis thaliana	AT1G60590.1 | Symbols: | Pectin lyase-like superfamily protein | chr1:22314426-22316867 REVERSE LENGTH=540	538	540	0	100.4	83.6	88.8

Rsa1.0_00053.1.g2901.t1	ref|XP_002886390.1| hypothetical protein ARALYDRAFT_893070 [Arabidopsis lyrata subsp. lyrata] gi|297332231|gb|EFH62649.1| hypothetical protein ARALYDRAFT_893070 [Arabidopsis lyrata subsp. lyrata]	318	298	3.00E-97	93.7	55.7	73.9	hypothetical protein ARALYDRAFT_893070	gbpln	Arabidopsis lyrata	AT2G42470.1 | Symbols: | TRAF-like family protein | chr2:17679887-17685187 REVERSE LENGTH=898	318	898	2.00E-80	282.4	49.7	67.6
Rsa1.0_00053.1.g2902.t1	ref|NP_176255.2| naphthoate synthase [Arabidopsis thaliana] gi|327488386|sp|Q8GYN9.2|MENB_ARATH RecName: Full=1,4-Dihydroxy-2-naphthoyl-CoA synthase, peroxisomal; Short=DHNS; AltName: Full=Enoyl-CoA hydratase/isomerase D; Short=ECHID; AltName: Full=Naphthoate synthase gi|192571726|gb|ACF04808.1| At1g60550 [Arabidopsis thaliana] gi|332195575|gb|AEE33696.1| 1,4-Dihydroxy-2-naphthoyl-CoA synthase [Arabidopsis thaliana]	338	337	1.00E-174	99.7	87.3	93.5	naphthoate synthase	gbpln	Arabidopsis thaliana	AT1G60550.1 | Symbols: ECHID, DHNS | enoyl-CoA hydratase/isomerase D | chr1:22305988-22308092 REVERSE LENGTH=337	338	337	1.00E-177	99.7	87.3	93.5
Rsa1.0_00053.1.g2903.t1	ref|XP_002888139.1| dynamin family protein [Arabidopsis lyrata subsp. lyrata] gi|297333980|gb|EFH64398.1| dynamin family protein [Arabidopsis lyrata subsp. lyrata]	659	659	0	100.0	86.0	91.0	dynamin family protein	gbpln	Arabidopsis lyrata	AT1G60500.1 | Symbols: DRP4C | Dynamin related protein 4C | chr1:22291582-22293822 FORWARD LENGTH=669	659	669	0	101.5	82.5	90.0
Rsa1.0_00053.1.g2904.t1	ref|XP_002888139.1| dynamin family protein [Arabidopsis lyrata subsp. lyrata] gi|297333980|gb|EFH64398.1| dynamin family protein [Arabidopsis lyrata subsp. lyrata]	677	659	0	97.3	85.5	90.3	dynamin family protein	gbpln	Arabidopsis lyrata	AT1G60500.1 | Symbols: DRP4C | Dynamin related protein 4C | chr1:22291582-22293822 FORWARD LENGTH=669	677	669	0	98.8	81.2	89.1
Rsa1.0_00053.1.g2905.t10	emb|CBI39638.3| unnamed protein product [Vitis vinifera]	181	591	1.00E-102	326.5	98.9	99.4	unnamed protein product	gbpln	Vitis vinifera	AT1G10630.1 | Symbols: ATARFA1F, ARFA1F | ADP-ribosylation factor A1F | chr1:3513189-3514230 REVERSE LENGTH=181	181	181	1.00E-104	100.0	98.9	100.0
Rsa1.0_00053.1.g2906.t1	gb|EOA35424.1| hypothetical protein CARUB_v10020626mg [Capsella rubella]	332	334	1.00E-177	100.6	90.4	95.2	hypothetical protein CARUB_v10020626mg	gbpln	Capsella rubella	AT1G60470.1 | Symbols: AtGolS4, GolS4 | galactinol synthase 4 | chr1:22279181-22280454 REVERSE LENGTH=334	332	334	1.00E-179	100.6	91.0	95.5
Rsa1.0_00053.1.g2907.t1	gb|AAC24075.1| Strong similarity to water stress-induced protein, WSI76 isolog T08I13.2 gb|2275196 from A. thaliana BAC gb|AC002337 [Arabidopsis thaliana]	49	345	6.00E-16	704.1	73.5	85.7	Strong similarity to water stress-induced protein, WSI76 isolog T08I13.2 gb|2275196 from A. thaliana BAC gb|AC002337	gbpln	Arabidopsis thaliana	AT1G60450.1 | Symbols: AtGolS7, GolS7 | galactinol synthase 7 | chr1:22271226-22273214 REVERSE LENGTH=332	49	332	1.00E-18	677.6	73.5	85.7
Rsa1.0_00053.1.g2908.t11	ref|XP_002886619.1| ATCOAA/ATPANK1 [Arabidopsis lyrata subsp. lyrata] gi|297332460|gb|EFH62878.1| ATCOAA/ATPANK1 [Arabidopsis lyrata subsp. lyrata]	607	384	0	63.3	55.4	57.7	ATCOAA/ATPANK1	gbpln	Arabidopsis lyrata	AT1G60440.1 | Symbols: ATCOAA, ATPANK1, PANK1 | pantothenate kinase 1 | chr1:22266653-22269070 REVERSE LENGTH=383	607	383	0	63.1	56.0	58.0
Rsa1.0_00053.1.g2909.t1	#	#	#	#	#	#	#	-	----	----	#	#	#	#	#	#	#
Rsa1.0_00053.1.g2910.t1	#	#	#	#	#	#	#	-	----	----	#	#	#	#	#	#	#
Rsa1.0_00053.1.g2911.t1	ref|XP_002889769.1| MYB transcription factor [Arabidopsis lyrata subsp. lyrata] gi|297335611|gb|EFH66028.1| MYB transcription factor [Arabidopsis lyrata subsp. lyrata]	230	844	1.00E-102	367.0	84.3	90.4	MYB transcription factor	gbpln	Arabidopsis lyrata	AT1G09770.1 | Symbols: ATCDC5, CDC5, ATMYBCDC5 | cell division cycle 5 | chr1:3162002-3165122 FORWARD LENGTH=844	230	844	1.00E-104	367.0	84.3	90.4
Rsa1.0_00053.1.g2912.t1	emb|CCD74525.1| polygalacturonase 1 [Arabidopsis halleri subsp. halleri]	625	626	0	100.2	81.9	89.9	polygalacturonase 1	gbpln	Arabidopsis halleri	AT1G60390.1 | Symbols: PG1 | polygalacturonase 1 | chr1:22247611-22249556 REVERSE LENGTH=624	625	624	0	99.8	81.0	89.0
Rsa1.0_00053.1.g2913.t1	ref|XP_002886632.1| radical SAM domain-containing protein [Arabidopsis lyrata subsp. lyrata] gi|297332473|gb|EFH62891.1| radical SAM domain-containing protein [Arabidopsis lyrata subsp. lyrata]	447	458	0	102.5	90.4	95.1	radical SAM domain-containing protein	gbpln	Arabidopsis lyrata	AT1G60230.1 | Symbols: | Radical SAM superfamily protein | chr1:22212223-22214346 REVERSE LENGTH=458	447	458	0	102.5	89.5	94.2
Rsa1.0_00053.1.g2914.t2	gb|EOA34334.1| hypothetical protein CARUB_v10021854mg [Capsella rubella]	593	593	0	100.0	64.8	77.1	hypothetical protein CARUB_v10021854mg	gbpln	Capsella rubella	AT1G60220.1 | Symbols: OTS1, ULP1D | UB-like protease 1D | chr1:22208332-22211910 FORWARD LENGTH=584	593	584	0	98.5	65.6	75.9
Rsa1.0_00053.1.g2915.t1	#	#	#	#	#	#	#	-	----	----	#	#	#	#	#	#	#
Rsa1.0_00053.1.g2916.t1	ref|XP_002886636.1| predicted protein [Arabidopsis lyrata subsp. lyrata] gi|297332477|gb|EFH62895.1| predicted protein [Arabidopsis lyrata subsp. lyrata]	301	302	1.00E-153	100.3	89.4	93.7	predicted protein	gbpln	Arabidopsis lyrata	AT1G60080.1 | Symbols: | 3'-5'-exoribonuclease family protein | chr1:22152605-22154339 REVERSE LENGTH=302	301	302	1.00E-145	100.3	87.7	93.4
Rsa1.0_00053.1.g2917.t1	gb|AAC63844.1| putative non-LTR retroelement reverse transcriptase [Arabidopsis thaliana]	1769	1231	0	69.6	40.4	50.2	putative non-LTR retroelement reverse transcriptase	gbpln	Arabidopsis thaliana	AT2G01050.1 | Symbols: | zinc ion binding;nucleic acid binding | chr2:68337-69884 REVERSE LENGTH=515	1769	515	1.00E-117	29.1	13.7	18.4

Rsa1.0_00053.1.g2918.t1	ref|NP_564753.1| serine/threonine-protein kinase WNK (With No lysine)-related protein [Arabidopsis thaliana] gi|4249385|gb|AAD14482.1|AAD14482 T2K10.11 [Arabidopsis thaliana] gi|21537011|gb|AAM61352.1| unknown [Arabidopsis thaliana] gi|225898034|dbj|BAH30349.1| hypothetical protein [Arabidopsis thaliana] gi|332195533|gb|AEE33654.1| serine/threonine-protein kinase WNK (With No lysine)-related protein [Arabidopsis thaliana]	364	386	1.00E-159	106.0	79.7	82.4	serine/threonine-protein kinase WNK (With No lysine)-related protein	gbpln	Arabidopsis thaliana	AT1G60060.1 | Symbols: | Serine/threonine-protein kinase WNK (With No Lysine)-related | chr1:22139282-22141585 FORWARD LENGTH=386	364	386	1.00E-161	106.0	79.7	82.4
Rsa1.0_00053.1.g2919.t1	#	#	#	#	#	#	#	-	----	----	#	#	#	#	#	#	#
Rsa1.0_00053.1.g2920.t1	ref|XP_002886638.1| hypothetical protein ARALYDRAFT_315326 [Arabidopsis lyrata subsp. lyrata] gi|297332479|gb|EFH62897.1| hypothetical protein ARALYDRAFT_315326 [Arabidopsis lyrata subsp. lyrata]	373	373	0	100.0	89.3	93.0	hypothetical protein ARALYDRAFT_315326	gbpln	Arabidopsis lyrata	AT1G60050.1 | Symbols: | Nodulin MtN21 /EamA-like transporter family protein | chr1:22121550-22123702 REVERSE LENGTH=374	373	374	0	100.3	90.6	94.1
Rsa1.0_00053.1.g2921.t1	ref|NP_564752.1| uncharacterized protein [Arabidopsis thaliana] gi|4249380|gb|AAD14477.1| ESTs gb|Z37637, gb|AA042498 and gb|AA042269 come from this gene [Arabidopsis thaliana] gi|26450481|dbj|BAC42354.1| unknown protein [Arabidopsis thaliana] gi|28827334|gb|AAO50511.1| unknown protein [Arabidopsis thaliana] gi|332195529|gb|AEE33650.1| uncharacterized protein AT1G60010 [Arabidopsis thaliana]	169	173	3.00E-74	102.4	82.2	92.9	uncharacterized protein	gbpln	Arabidopsis thaliana	AT1G60010.1 | Symbols: | unknown protein; FUNCTIONS IN: molecular_function unknown; INVOLVED IN: N-terminal protein myristoylation; LOCATED IN: cellular_component unknown; EXPRESSED IN: 22 plant structures; EXPRESSED DURING: 13 growth stages; BEST Arabidopsis thaliana protein match is: unknown protein (TAIR:AT1G10530.1); Has 185 Blast hits to 185 proteins in 18 species: Archae - 0; Bacteria - 0; Metazoa - 0; Fungi - 3; Plants - 180; Viruses - 0; Other Eukaryotes - 2 (source: NCBI BLink). | chr1:22095660-22096434 REVERSE LENGTH=173	169	173	1.00E-76	102.4	82.2	92.9
Rsa1.0_00053.1.g2922.t1	gb|EOA35601.1| hypothetical protein CARUB_v10020814mg [Capsella rubella]	255	262	1.00E-116	102.7	84.3	90.2	hypothetical protein CARUB_v10020814mg	gbpln	Capsella rubella	AT1G60000.1 | Symbols: | RNA-binding (RRM/RBD/RNP motifs) family protein | chr1:22093678-22094540 REVERSE LENGTH=258	255	258	1.00E-118	101.2	82.7	89.0
Rsa1.0_00053.1.g2923.t1	#	#	#	#	#	#	#	-	----	----	#	#	#	#	#	#	#
Rsa1.0_00053.1.g2924.t1	ref|XP_002888160.1| hypothetical protein ARALYDRAFT_475312 [Arabidopsis lyrata subsp. lyrata] gi|297334001|gb|EFH64419.1| hypothetical protein ARALYDRAFT_475312 [Arabidopsis lyrata subsp. lyrata]	597	418	1.00E-177	70.0	53.8	56.1	hypothetical protein ARALYDRAFT_475312	gbpln	Arabidopsis lyrata	AT1G59980.1 | Symbols: ATDJC39, ARL2 | ARG1-like 2 | chr1:22081069-22083491 FORWARD LENGTH=414	597	414	1.00E-175	69.3	52.8	55.4
Rsa1.0_00053.1.g2925.t1	ref|XP_002891550.1| hypothetical protein ARALYDRAFT_474131 [Arabidopsis lyrata subsp. lyrata] gi|297337392|gb|EFH67809.1| hypothetical protein ARALYDRAFT_474131 [Arabidopsis lyrata subsp. lyrata]	429	422	2.00E-96	98.4	49.7	65.7	hypothetical protein ARALYDRAFT_474131	gbpln	Arabidopsis lyrata	AT1G49990.1 | Symbols: | F-box family protein | chr1:18513696-18514988 FORWARD LENGTH=430	429	430	2.00E-97	100.2	49.2	66.2
Rsa1.0_00053.1.g2926.t1	ref|XP_002888162.1| predicted protein [Arabidopsis lyrata subsp. lyrata] gi|297334003|gb|EFH64421.1| predicted protein [Arabidopsis lyrata subsp. lyrata]	359	351	1.00E-152	97.8	74.9	83.8	predicted protein	gbpln	Arabidopsis lyrata	AT1G59970.1 | Symbols: | Matrixin family protein | chr1:22073601-22074683 FORWARD LENGTH=360	359	360	1.00E-154	100.3	73.8	84.1
Rsa1.0_00053.1.g2927.t1	ref|NP_176204.1| putative Aldo/keto reductase [Arabidopsis thaliana] gi|5080826|gb|AAD39335.1|AC007258_24 Putative Aldo/keto reductase [Arabidopsis thaliana] gi|15451098|gb|AAK96820.1| Putative Aldo/keto reductase [Arabidopsis thaliana] gi|18377508|gb|AAL66920.1| putative aldo/keto reductase [Arabidopsis thaliana] gi|332195523|gb|AEE33644.1| putative Aldo/keto reductase [Arabidopsis thaliana]	323	326	1.00E-162	100.9	85.8	92.9	putative Aldo/keto reductase	gbpln	Arabidopsis thaliana	AT1G59960.1 | Symbols: | NAD(P)-linked oxidoreductase superfamily protein | chr1:22071410-22073067 REVERSE LENGTH=326	323	326	1.00E-164	100.9	85.8	92.9

Rsa1.0_00053.1_g2928.t2	ref|NP_683446.1| putative disease resistance protein RDL5/RF45 [Arabidopsis thaliana] gi|387942479|sp|F4IBE4.1|DRL10_ARATH RecName: Full=Probable disease resistance protein RF45 gi|387942481|sp|P0DI16.1|DRL44_ARATH RecName: Full=Probable disease resistance protein RDL5 gi|332195441|gb|AEE33562.1| putative disease resistance protein RDL5/RF45 [Arabidopsis thaliana]	1129	1017	0	90.1	50.5	64.0	putative disease resistance protein RDL5/RF45	gbpln	Arabidopsis thaliana	AT1G58807.1 | Symbols: | Disease resistance protein (CC-NBS-LRR class) family | chr1:21780574-21783793 FORWARD LENGTH=1017	1129	1017	0	90.1	50.5	64.0
Rsa1.0_00053.1_g2929.t1	ref|XP_002886657.1| predicted protein [Arabidopsis lyrata subsp. lyrata] gi|297332498|gb|EFH62916.1| predicted protein [Arabidopsis lyrata subsp. lyrata]	334	608	2.00E-72	182.0	49.4	60.8	predicted protein	gbpln	Arabidopsis lyrata	AT5G35450.1 | Symbols: | Disease resistance protein (CC-NBS-LRR class) family | chr5:13667809-13670685 FORWARD LENGTH=901	334	901	5.00E-75	269.8	46.7	60.2
Rsa1.0_00053.1_g2930.t1	gb|EOA33570.1| hypothetical protein CARUB_v10019704mg [Capsella rubella]	379	1046	0	276.0	92.9	95.5	hypothetical protein CARUB_v10019704mg	gbpln	Capsella rubella	AT1G59760.1 | Symbols: | RNA helicase, ATP-dependent, SK12/DOB1 protein | chr1:21984571-21990110 REVERSE LENGTH=988	379	988	0	260.7	91.8	95.8
Rsa1.0_00053.1_g2931.t1	gb|EOA33570.1| hypothetical protein CARUB_v10019704mg [Capsella rubella]	564	1046	0	185.5	92.6	96.3	hypothetical protein CARUB_v10019704mg	gbpln	Capsella rubella	AT1G59760.1 | Symbols: | RNA helicase, ATP-dependent, SK12/DOB1 protein | chr1:21984571-21990110 REVERSE LENGTH=988	564	988	0	175.2	93.4	97.2
Rsa1.0_00053.1_g2932.t1	ref|XP_002888185.1| proton-dependent oligopeptide transport family protein [Arabidopsis lyrata subsp. lyrata] gi|297334026|gb|EFH64444.1| proton-dependent oligopeptide transport family protein [Arabidopsis lyrata subsp. lyrata]	739	592	0	80.1	71.6	75.0	proton-dependent oligopeptide transport family protein	gbpln	Arabidopsis lyrata	AT1G59740.1 | Symbols: | Major facilitator superfamily protein | chr1:21968227-21972312 FORWARD LENGTH=591	739	591	0	80.0	71.3	74.8
Rsa1.0_00053.1_g2933.t1	ref|XP_002886664.1| thioredoxin H-type 7 [Arabidopsis lyrata subsp. lyrata] gi|297332505|gb|EFH62923.1| thioredoxin H-type 7 [Arabidopsis lyrata subsp. lyrata]	128	128	3.00E-61	100.0	89.1	93.8	thioredoxin H-type 7	gbpln	Arabidopsis lyrata	AT1G59730.1 | Symbols: ATH7, TH7 | thioredoxin H-type 7 | chr1:21952759-21953392 REVERSE LENGTH=129	128	129	5.00E-61	100.8	87.5	91.4
Rsa1.0_00053.1_g2934.t1	ref|NP_172506.1| putative DNAJ heat-shock protein [Arabidopsis thaliana] gi|4914337|gb|AAD32885.1|AC005489_23 F14N23.23 [Arabidopsis thaliana] gi|13430680|gb|AAK25962.1|AF360252_1 putative heat-shock protein [Arabidopsis thaliana] gi|14532888|gb|AAK64126.1| putative heat-shock protein [Arabidopsis thaliana] gi|332190448|gb|AEE28569.1| putative DNAJ heat-shock protein [Arabidopsis thaliana]	424	349	1.00E-121	82.3	57.8	66.7	putative DNAJ heat-shock protein	gbpln	Arabidopsis thaliana	AT1G10350.1 | Symbols: | DNAJ heat shock family protein | chr1:3393595-3394860 REVERSE LENGTH=349	424	349	1.00E-123	82.3	57.8	66.7
Rsa1.0_00053.1_g2935.t1	gb|EOA35526.1| hypothetical protein CARUB_v10020734mg [Capsella rubella]	291	293	1.00E-154	100.7	91.1	95.5	hypothetical protein CARUB_v10020734mg	gbpln	Capsella rubella	AT1G59710.1 | Symbols: | Protein of unknown function (DUF569) | chr1:21938623-21939673 FORWARD LENGTH=300	291	300	1.00E-143	103.1	89.3	95.2
Rsa1.0_00053.1_g2936.t1	ref|NP_176178.1| glutathione S-transferase TAU 16 [Arabidopsis thaliana] gi|75338642|sp|Q9XIF8.1|GSTUG_ARATH RecName: Full=Glutathione S-transferase U16; Short=AtGSTU16; AltName: Full=GST class-tau member 16 gi|5080803|gb|AAD39312.1|AC007258_1 Similar to glutathione transferase [Arabidopsis thaliana] gi|8778752|gb|AAF79760.1|AC009317_19 T30E16.30 [Arabidopsis thaliana] gi|48310666|gb|AAT41863.1| At1g59700 [Arabidopsis thaliana] gi|332195484|gb|AEE33605.1| glutathione S-transferase TAU 16 [Arabidopsis thaliana]	267	234	1.00E-107	87.6	74.2	81.3	glutathione S-transferase TAU 16	gbpln	Arabidopsis thaliana	AT1G59700.1 | Symbols: ATGSTU16, GSTU16 | glutathione S-transferase TAU 16 | chr1:21936459-21937763 FORWARD LENGTH=234	267	234	1.00E-110	87.6	74.2	81.3
Rsa1.0_00053.1_g2937.t1	gb|EOA13425.1| hypothetical protein CARUB_v10026465mg [Capsella rubella] gi|482549232|gb|EOA13426.1| hypothetical protein CARUB_v10026465mg [Capsella rubella]	112	423	2.00E-12	377.7	33.0	48.2	hypothetical protein CARUB_v10026465mg	gbpln	Capsella rubella	AT5G56420.2 | Symbols: | F-box/RNI-like/FBD-like domains-containing protein | chr5:22850863-22852334 REVERSE LENGTH=422	112	422	1.00E-13	376.8	32.1	47.3

Rsa1.0_00053.1.g2938.t1	ref|NP_189971.1| uncharacterized protein [Arabidopsis thaliana] gi|7362785|emb|CAB83157.1| putative protein [Arabidopsis thaliana] gi|332644314|gb|AEE77835.1| uncharacterized protein AT3G43850 [Arabidopsis thaliana]	134	270	4.00E-33	201.5	53.0	60.4	uncharacterized protein	gbpln	Arabidopsis thaliana	AT3G43850.1 | Symbols: | unknown protein; FUNCTIONS IN: molecular_function unknown; INVOLVED IN: biological_process unknown; LOCATED IN: vacuole; BEST Arabidopsis thaliana protein match is: unknown protein (TAIR:AT5G21940.1); Has 215 Blast hits to 215 proteins in 19 species: Archae - 0; Bacteria - 0; Metazoa - 0; Fungi - 0; Plants - 213; Viruses - 0; Other Eukaryotes - 2 (source: NCBI BLink). | chr3:15702085-15704152 REVERSE LENGTH=270	134	270	1.00E-35	201.5	53.0	60.4
Rsa1.0_00053.1.g2939.t1	dbj|BAJ34445.1| unnamed protein product [Thellungiella halophila]	231	229	1.00E-101	99.1	77.5	87.9	unnamed protein product	----	----	AT1G59700.1 | Symbols: ATGSTU16, GSTU16 | glutathione S-transferase TAU 16 | chr1:21936459-21937763 FORWARD LENGTH=234	231	234	1.00E-102	101.3	75.3	84.8
Rsa1.0_00053.1.g2940.t1	gb|EOA34239.1| hypothetical protein CARUB_v10021751mg, partial [Capsella rubella]	970	978	0	100.8	61.9	70.7	hypothetical protein CARUB_v10021751mg, partial	gbpln	Capsella rubella	AT1G59660.1 | Symbols: | Nucleoporin autopeptidase | chr1:21924693-21929233 FORWARD LENGTH=997	970	997	0	102.8	60.5	68.9
Rsa1.0_00053.1.g2941.t1	gb|EOA35051.1| hypothetical protein CARUB_v10020162mg [Capsella rubella]	511	511	0	100.0	89.0	92.6	hypothetical protein CARUB_v10020162mg	gbpln	Capsella rubella	AT1G59650.1 | Symbols: CW14 | Protein of unknown function (DUF1336) | chr1:21920099-21922670 FORWARD LENGTH=492	511	492	0	96.3	86.5	89.8
Rsa1.0_00053.1.g2942.t6	gb|AEM36350.1| At1g58602 [Arabidopsis thaliana]	1190	1133	0	95.2	40.2	51.8	At1g58602	gbpln	Arabidopsis thaliana	AT1G58602.2 | Symbols: | LRR and NB-ARC domains-containing disease resistance protein | chr1:21760167-21763765 FORWARD LENGTH=1138	1190	1138	0	95.6	40.1	51.7
Rsa1.0_00054.1.g2943.t1	#	#	#	#	#	#	#	-	----	----	#	#	#	#	#	#	#
Rsa1.0_00054.1.g2944.t1	gb|EOA29338.1| hypothetical protein CARUB_v10025620mg [Capsella rubella]	208	198	3.00E-32	95.2	40.9	57.7	hypothetical protein CARUB_v10025620mg	gbpln	Capsella rubella	AT3G28620.1 | Symbols: | Zinc finger, C3HC4 type (RING finger) family protein | chr3:10727826-10728461 FORWARD LENGTH=211	208	211	2.00E-30	101.4	39.9	55.3
Rsa1.0_00054.1.g2945.t1	ref|XP_002880588.1| ATRPA2/ROR1/RPA2 [Arabidopsis lyrata subsp. lyrata] gi|297326427|gb|EFH56847.1| ATRPA2/ROR1/RPA2 [Arabidopsis lyrata subsp. lyrata]	273	279	1.00E-132	102.2	85.0	92.7	ATRPA2/ROR1/RPA2	gbpln	Arabidopsis lyrata	AT2G24490.2 | Symbols: RPA2, ATRPA2, ROR1, ATRPA32A, RPA32A | replicon protein A2 | chr2:10398651-10400416 REVERSE LENGTH=279	273	279	1.00E-132	102.2	83.5	91.6
Rsa1.0_00054.1.g2946.t2	gb|ACP30611.1| disease resistance protein [Brassica rapa subsp. pekinensis]	1069	1050	0	98.2	38.4	49.8	disease resistance protein	gbpln	Brassica rapa	AT5G22690.1 | Symbols: | Disease resistance protein (TIR-NBS-LRR class) family | chr5:7541369-7544888 FORWARD LENGTH=1008	1069	1008	0	94.3	37.3	48.1
Rsa1.0_00054.1.g2947.t1	dbj|BAJ34463.1| unnamed protein product [Thellungiella halophila]	382	403	1.00E-172	105.5	85.3	90.6	unnamed protein product	----	----	AT2G24500.1 | Symbols: FZF | Zinc finger protein 622 | chr2:10401259-10402805 REVERSE LENGTH=395	382	395	1.00E-167	103.4	82.2	87.2
Rsa1.0_00054.1.g2948.t1	gb|ACG60669.1| copia-type polyprotein-like protein [Brassica oleracea var. alboglabra]	112	196	2.00E-40	175.0	71.4	83.0	copia-type polyprotein-like protein	gbpln	Brassica oleracea	#	#	#	#	#	#	#
Rsa1.0_00054.1.g2949.t1	ref|XP_002878766.1| hypothetical protein ARALYDRAFT_900997 [Arabidopsis lyrata subsp. lyrata] gi|297324605|gb|EFH55025.1| hypothetical protein ARALYDRAFT_900997 [Arabidopsis lyrata subsp. lyrata]	328	409	1.00E-108	124.7	72.0	81.7	hypothetical protein ARALYDRAFT_900997	gbpln	Arabidopsis lyrata	AT2G24530.1 | Symbols: | unknown protein; BEST Arabidopsis thaliana protein match is: unknown protein (TAIR:AT4G31440.1); Has 30201 Blast hits to 17322 proteins in 780 species: Archae - 12; Bacteria - 1396; Metazoa - 17338; Fungi - 3422; Plants - 5037; Viruses - 0; Other Eukaryotes - 2996 (source: NCBI BLink). | chr2:10422597-10423820 FORWARD LENGTH=407	328	407	1.00E-110	124.1	70.4	81.4
Rsa1.0_00054.1.g2950.t1	gb|EOA29338.1| hypothetical protein CARUB_v10025620mg [Capsella rubella]	210	198	4.00E-34	94.3	41.0	57.6	hypothetical protein CARUB_v10025620mg	gbpln	Capsella rubella	AT2G24480.1 | Symbols: | Zinc finger, C3HC4 type (RING finger) family protein | chr2:10397588-10398184 FORWARD LENGTH=198	210	198	4.00E-34	94.3	39.5	58.6
Rsa1.0_00054.1.g2951.t1	ref|XP_002878766.1| hypothetical protein ARALYDRAFT_900997 [Arabidopsis lyrata subsp. lyrata] gi|297324605|gb|EFH55025.1| hypothetical protein ARALYDRAFT_900997 [Arabidopsis lyrata subsp. lyrata]	264	409	9.00E-54	154.9	46.6	53.0	hypothetical protein ARALYDRAFT_900997	gbpln	Arabidopsis lyrata	AT2G24530.1 | Symbols: | unknown protein; BEST Arabidopsis thaliana protein match is: unknown protein (TAIR:AT4G31440.1); Has 30201 Blast hits to 17322 proteins in 780 species: Archae - 12; Bacteria - 1396; Metazoa - 17338; Fungi - 3422; Plants - 5037; Viruses - 0; Other Eukaryotes - 2996 (source: NCBI BLink). | chr2:10422597-10423820 FORWARD LENGTH=407	264	407	2.00E-55	154.2	45.8	52.7
Rsa1.0_00054.1.g2952.t1	gb|EOA29338.1| hypothetical protein CARUB_v10025620mg [Capsella rubella]	210	198	4.00E-34	94.3	41.4	58.1	hypothetical protein CARUB_v10025620mg	gbpln	Capsella rubella	AT2G24480.1 | Symbols: | Zinc finger, C3HC4 type (RING finger) family protein | chr2:10397588-10398184 FORWARD LENGTH=198	210	198	6.00E-32	94.3	38.6	56.7

Rsa1.0_00054.1.g2953.t1	ref|XP_002880584.1| hypothetical protein ARALYDRAFT_481301 [Arabidopsis lyrata subsp. lyrata] gi|297326423|gb|EFH56843.1| hypothetical protein ARALYDRAFT_481301 [Arabidopsis lyrata subsp. lyrata]	288	279	1.00E-118	96.9	81.6	89.9	hypothetical protein ARALYDRAFT_481301	gbpln	Arabidopsis lyrata	AT2G24450.1 | Symbols: FLA3 | FASCICLIN-like arabinogalactan protein 3 precursor | chr2:10393019-10393861 REVERSE LENGTH=280	288	280	1.00E-114	97.2	80.6	88.5
Rsa1.0_00054.1.g2954.t1	ref|NP_565570.1| selenium binding protein [Arabidopsis thaliana] gi|4337201|gb|AAD18115.1| expressed protein [Arabidopsis thaliana] gi|21553632|gb|AAM62725.1| unknown [Arabidopsis thaliana] gi|21805670|gb|AAM76749.1| hypothetical protein [Arabidopsis thaliana] gi|50058859|gb|AAT69174.1| hypothetical protein At2g24440 [Arabidopsis thaliana] gi|330252484|gb|AEC07578.1| selenium binding protein [Arabidopsis thaliana]	191	183	8.00E-32	95.8	37.2	44.0	selenium binding protein	gbpln	Arabidopsis thaliana	AT2G24440.1 | Symbols: | selenium binding | chr2:10391124-10392492 FORWARD LENGTH=183	191	183	3.00E-34	95.8	37.2	44.0
Rsa1.0_00054.1.g2955.t1	gb|EOA12673.1| hypothetical protein CARUB_v10027827mg, partial [Capsella rubella]	497	438	1.00E-137	88.1	52.9	66.0	hypothetical protein CARUB_v10027827mg, partial	gbpln	Capsella rubella	AT3G49020.1 | Symbols: | FBD, F-box and Leucine Rich Repeat domains containing protein | chr3:18169079-18170660 FORWARD LENGTH=447	497	447	1.00E-136	89.9	53.9	65.6
Rsa1.0_00054.1.g2956.t1	gb|EOA29236.1| hypothetical protein CARUB_v10025508mg [Capsella rubella]	317	318	1.00E-172	100.3	94.3	97.2	hypothetical protein CARUB_v10025508mg	gbpln	Capsella rubella	AT2G24430.2 | Symbols: ANAC039 | NAC domain containing protein 38 | chr2:10383517-10386386 REVERSE LENGTH=316	317	316	1.00E-172	99.7	94.3	96.5
Rsa1.0_00054.1.g2957.t1	ref|NP_200800.1| cysteine/histidine-rich C1 domain-containing protein [Arabidopsis thaliana] gi|17225050|gb|AAL37222.1|AF321919_1 ULI3 [Arabidopsis thaliana] gi|332009868|gb|AED97251.1| cysteine/histidine-rich C1 domain-containing protein [Arabidopsis thaliana]	716	710	0	99.2	50.8	62.2	cysteine/histidine-rich C1 domain-containing protein	gbpln	Arabidopsis thaliana	AT5G59920.1 | Symbols: ULI3 | Cysteine/Histidine-rich C1 domain family protein | chr5:24128756-24130888 FORWARD LENGTH=710	716	710	0	99.2	50.8	62.2
Rsa1.0_00054.1.g2958.t1	#	#	#	#	#	#	#	-	----	----	AT4G23630.1 | Symbols: BTI1, RTNLB1 | VIRB2-interacting protein 1 | chr4:12318070-12319574 FORWARD LENGTH=275	88	275	7.00E-12	312.5	35.2	43.2
Rsa1.0_00054.1.g2959.t1	dbj|BAB08360.1| Ta11-like non-LTR retroelement protein-like [Arabidopsis thaliana]	648	655	0	101.1	57.3	71.3	Ta11-like non-LTR retroelement protein-like	gbpln	Arabidopsis thaliana	AT5G59920.1 | Symbols: ULI3 | Cysteine/Histidine-rich C1 domain family protein | chr5:24128756-24130888 FORWARD LENGTH=710	648	710	0	109.6	55.7	69.6
Rsa1.0_00054.1.g2960.t1	#	#	#	#	#	#	#	-	----	----	#	#	#	#	#	#	#
Rsa1.0_00054.1.g2961.t1	ref|NP_180016.1| SAUR-like auxin-responsive protein [Arabidopsis thaliana] gi|4337198|gb|AAD18112.1| putative auxin-regulated protein [Arabidopsis thaliana] gi|38566584|gb|AAR24182.1| At2g24400 [Arabidopsis thaliana] gi|40824022|gb|AAR92327.1| At2g24400 [Arabidopsis thaliana] gi|330252478|gb|AEC07572.1| SAUR-like auxin-responsive protein [Arabidopsis thaliana]	178	178	7.00E-80	100.0	86.0	91.6	SAUR-like auxin-responsive protein	gbpln	Arabidopsis thaliana	AT2G24400.1 | Symbols: | SAUR-like auxin-responsive protein family | chr2:10377993-10378529 REVERSE LENGTH=178	178	178	3.00E-82	100.0	86.0	91.6
Rsa1.0_00054.1.g2962.t1	ref|NP_850052.1| chaperone protein dnaJ-related protein [Arabidopsis thaliana] gi|44917527|gb|AAS49088.1| At2g24395 [Arabidopsis thaliana] gi|62321035|dbj|BAD94100.1| hypothetical protein [Arabidopsis thaliana] gi|330252477|gb|AEC07571.1| chaperone protein dnaJ-related protein [Arabidopsis thaliana]	132	132	5.00E-44	100.0	70.5	81.8	chaperone protein dnaJ-related protein	gbpln	Arabidopsis thaliana	AT2G24395.1 | Symbols: | chaperone protein dnaJ-related | chr2:10375497-10376081 FORWARD LENGTH=132	132	132	1.00E-46	100.0	70.5	81.8
Rsa1.0_00054.1.g2963.t1	gb|EOA27992.1| hypothetical protein CARUB_v10024168mg [Capsella rubella]	153	175	2.00E-71	114.4	82.4	90.2	hypothetical protein CARUB_v10024168mg	gbpln	Capsella rubella	AT2G24390.1 | Symbols: | AIG2-like (avirulence induced gene) family protein | chr2:10373796-10374654 FORWARD LENGTH=152	153	152	1.00E-70	99.3	79.1	89.5
Rsa1.0_00054.1.g2964.t1	gb|EOA29306.1| hypothetical protein CARUB_v10025585mg [Capsella rubella]	763	806	0	105.6	90.0	96.6	hypothetical protein CARUB_v10025585mg	gbpln	Capsella rubella	AT2G24370.1 | Symbols: | Protein kinase protein with adenine nucleotide alpha hydrolases-like domain | chr2:10369979-10373063 REVERSE LENGTH=788	763	788	0	103.3	88.2	94.6

Rsa1.0_00054.1.g2965.t1	sp|Q9ZSR8.1|RSSA_BRANA RecName: Full=40S ribosomal protein SA; AltName: Full=Laminin receptor-like protein; AltName: Full=p40 gi|3769681|gb|AAC97937.1| laminin receptor-like protein [Brassica napus]	292	292	2.33E-156	100.0	87.3	91.4	RecName: Full=40S ribosomal protein SA; AltName: Full=Laminin receptor-like protein; AltName: Full=p40 gi|3769681|gb|AAC97937.1| laminin receptor-like protein	gbpln	Brassica napus	AT1G72370.2 | Symbols: P40, AP40, RP40, RPSAA | 40s ribosomal protein SA | chr1:27243148-27244842 REVERSE LENGTH=294	292	294	1.00E-134	100.7	77.4	81.5
Rsa1.0_00054.1.g2966.t1	#	#	#	#	#	#	#	-	----	----	#	#	#	#	#	#	#
Rsa1.0_00054.1.g2967.t1	ref|XP_002893652.1| protein binding protein [Arabidopsis lyrata subsp. lyrata] gi|297339494|gb|EFH69911.1| protein binding protein [Arabidopsis lyrata subsp. lyrata]	208	725	3.00E-62	348.6	53.8	57.2	protein binding protein	gbpln	Arabidopsis lyrata	AT1G30860.1 | Symbols: | RING/U-box superfamily protein | chr1:10986696-10989246 REVERSE LENGTH=730	208	730	3.00E-61	351.0	51.0	55.3
Rsa1.0_00054.1.g2968.t1	ref|XP_002866147.1| F-box family protein [Arabidopsis lyrata subsp. lyrata] gi|297311982|gb|EFH42406.1| F-box family protein [Arabidopsis lyrata subsp. lyrata]	462	423	4.00E-64	91.6	35.3	51.1	F-box family protein	gbpln	Arabidopsis lyrata	AT5G56420.2 | Symbols: | F-box/RNI-like/FBD-like domains-containing protein | chr5:22850863-22852334 REVERSE LENGTH=422	462	422	4.00E-65	91.3	35.3	51.3
Rsa1.0_00054.1.g2969.t1	gb|EOA40378.1| hypothetical protein CARUB_v10009105mg [Capsella rubella]	455	453	0	99.6	76.9	84.6	hypothetical protein CARUB_v10009105mg	gbpln	Capsella rubella	AT1G01430.1 | Symbols: TBL25 | TRICHOME BIREFRINGENCE-LIKE 25 | chr1:156953-158536 REVERSE LENGTH=456	455	456	0	100.2	76.0	84.6
Rsa1.0_00054.1.g2970.t1	ref|XP_002864982.1| F-box family protein [Arabidopsis lyrata subsp. lyrata] gi|297310817|gb|EFH41241.1| F-box family protein [Arabidopsis lyrata subsp. lyrata]	242	392	2.00E-37	162.0	43.4	59.1	F-box family protein	gbpln	Arabidopsis lyrata	AT5G65850.1 | Symbols: | F-box and associated interaction domains-containing protein | chr5:26346317-26347495 FORWARD LENGTH=392	242	392	5.00E-39	162.0	43.8	59.5
Rsa1.0_00054.1.g2971.t1	gb|EOA27207.1| hypothetical protein CARUB_v10023317mg [Capsella rubella]	411	412	0	100.2	89.1	94.2	hypothetical protein CARUB_v10023317mg	gbpln	Capsella rubella	AT4G31170.3 | Symbols: | Protein kinase superfamily protein | chr4:15153499-15154846 REVERSE LENGTH=412	411	412	0	100.2	83.5	90.3
Rsa1.0_00054.1.g2972.t1	ref|XP_002878748.1| RNA recognition motif-containing protein [Arabidopsis lyrata subsp. lyrata] gi|297324587|gb|EFH55007.1| RNA recognition motif-containing protein [Arabidopsis lyrata subsp. lyrata]	580	534	1.00E-171	92.1	59.0	71.0	RNA recognition motif-containing protein	gbpln	Arabidopsis lyrata	AT2G24350.1 | Symbols: | RNA binding (RRM/RBD/RNP motifs) family protein | chr2:10361239-10364223 FORWARD LENGTH=537	580	537	1.00E-168	92.6	57.4	69.8
Rsa1.0_00054.1.g2973.t1	ref|XP_002879925.1| F-box family protein [Arabidopsis lyrata subsp. lyrata] gi|297325764|gb|EFH56184.1| F-box family protein [Arabidopsis lyrata subsp. lyrata]	379	371	3.00E-52	97.9	42.5	54.4	F-box family protein	gbpln	Arabidopsis lyrata	AT2G41360.1 | Symbols: | Galactose oxidase/kelch repeat superfamily protein | chr2:17234374-17235956 FORWARD LENGTH=387	379	387	1.00E-45	102.1	36.7	46.4
Rsa1.0_00054.1.g2974.t1	ref|XP_002880573.1| hypothetical protein ARALYDRAFT_900957 [Arabidopsis lyrata subsp. lyrata] gi|297326412|gb|EFH56832.1| hypothetical protein ARALYDRAFT_900957 [Arabidopsis lyrata subsp. lyrata]	406	411	1.00E-178	101.2	76.8	85.5	hypothetical protein ARALYDRAFT_900957	gbpln	Arabidopsis lyrata	AT2G24330.1 | Symbols: | Protein of unknown function (DUF2296) | chr2:10347340-10349566 REVERSE LENGTH=408	406	408	1.00E-174	100.5	75.4	83.7
Rsa1.0_00054.1.g2975.t1	ref|XP_002880572.1| hypothetical protein ARALYDRAFT_320242 [Arabidopsis lyrata subsp. lyrata] gi|297326411|gb|EFH56831.1| hypothetical protein ARALYDRAFT_320242 [Arabidopsis lyrata subsp. lyrata]	295	301	1.00E-147	102.0	82.7	94.2	hypothetical protein ARALYDRAFT_320242	gbpln	Arabidopsis lyrata	AT2G24320.1 | Symbols: | alpha/beta-Hydrolases superfamily protein | chr2:10345659-10346829 REVERSE LENGTH=286	295	286	1.00E-147	96.9	80.7	91.9
Rsa1.0_00054.1.g2976.t1	ref|XP_002878745.1| hypothetical protein ARALYDRAFT_481288 [Arabidopsis lyrata subsp. lyrata] gi|297324584|gb|EFH55004.1| hypothetical protein ARALYDRAFT_481288 [Arabidopsis lyrata subsp. lyrata]	287	345	2.00E-43	120.2	57.8	67.9	hypothetical protein ARALYDRAFT_481288	gbpln	Arabidopsis lyrata	AT2G24310.1 | Symbols: | unknown protein; BEST Arabidopsis thaliana protein match is: unknown protein (TAIR:AT5G47170.1); Has 9168 Blast hits to 2316 proteins in 376 species: Archae - 2; Bacteria - 1905; Metazoa - 1700; Fungi - 1409; Plants - 76; Viruses - 48; Other Eukaryotes - 4028 (source: NCBI BLink). | chr2:10344361-10345329 FORWARD LENGTH=322	287	322	1.00E-38	112.2	51.9	64.5
Rsa1.0_00054.1.g2977.t1	ref|XP_002878744.1| calmodulin-binding protein [Arabidopsis lyrata subsp. lyrata] gi|297324583|gb|EFH55003.1| calmodulin-binding protein [Arabidopsis lyrata subsp. lyrata]	601	599	0	99.7	89.5	94.3	calmodulin-binding protein	gbpln	Arabidopsis lyrata	AT2G24300.2 | Symbols: | Calmodulin-binding protein | chr2:10340913-10343457 FORWARD LENGTH=599	601	599	0	99.7	90.0	94.2
Rsa1.0_00054.1.g2978.t1	#	#	#	#	#	#	#	-	----	----	#	#	#	#	#	#	#
Rsa1.0_00054.1.g2979.t1	dbj|BAJ33970.1| unnamed protein product [Thellungiella halophila]	494	494	0	100.0	94.1	97.4	unnamed protein product	----	----	AT2G24280.1 | Symbols: | alpha/beta-Hydrolases superfamily protein | chr2:10334622-10336927 FORWARD LENGTH=494	494	494	0	100.0	91.1	95.1
Rsa1.0_00054.1.g2980.t1	gb|ABD64940.1| hypothetical protein 24.t00018 [Brassica oleracea]	134	380	2.00E-27	283.6	50.0	61.2	hypothetical protein 24.t00018	gbpln	Brassica oleracea	AT3G47680.1 | Symbols: | DNA binding | chr3:17577483-17578391 REVERSE LENGTH=302	134	302	2.00E-19	225.4	38.8	55.2
Rsa1.0_00054.1.g2981.t1	#	#	#	#	#	#	#	-	----	----	#	#	#	#	#	#	#

Rsa1.0_00055.1.g2982.t1	gb|EOA31608.1| hypothetical protein CARUB_v10014805mg, partial [Capsella rubella]	135	161	6.00E-72	119.3	100.0	100.0	hypothetical protein CARUB_v10014805mg, partial	gbpln	Capsella rubella	AT3G04400.1 | Symbols: emb2171 | Ribosomal protein L14p/L23e family protein | chr3:1167339-1168308 FORWARD LENGTH=140	135	140	4.00E-74	103.7	100.0	100.0
Rsa1.0_00055.1.g2983.t1	gb|EOA30756.1| hypothetical protein CARUB_v10013898mg [Capsella rubella]	393	393	0	100.0	93.6	97.5	hypothetical protein CARUB_v10013898mg	gbpln	Capsella rubella	AT3G04460.1 | Symbols: PEX12, ATPEX12, APM4 | peroxin-12 | chr3:1186641-1189094 FORWARD LENGTH=393	393	393	0	100.0	92.9	96.7
Rsa1.0_00055.1.g2984.t1	gb|EOA29968.1| hypothetical protein CARUB_v10013071mg [Capsella rubella]	711	721	0	101.4	85.8	92.8	hypothetical protein CARUB_v10013071mg	gbpln	Capsella rubella	AT3G04480.1 | Symbols: | endoribonucleases | chr3:1193988-1197320 REVERSE LENGTH=718	711	718	0	101.0	85.7	92.1
Rsa1.0_00055.1.g2985.t1	ref|XP_002878332.1| hypothetical protein ARALYDRAFT_907560 [Arabidopsis lyrata subsp. lyrata] gi|297324170|gb|EFH54591.1| hypothetical protein ARALYDRAFT_907560 [Arabidopsis lyrata subsp. lyrata]	388	741	1.00E-57	191.0	35.1	38.4	hypothetical protein ARALYDRAFT_907560	gbpln	Arabidopsis lyrata	AT3G60380.1 | Symbols: | FUNCTIONS IN: molecular_function unknown; INVOLVED IN: biological_process unknown; LOCATED IN: cellular_component unknown; EXPRESSED IN: 24 plant structures; EXPRESSED DURING: 13 growth stages; BEST Arabidopsis thaliana protein match is: hydroxyproline-rich glycoprotein family protein (TAIR:AT4G16790.1); Has 6102 Blast hits to 3981 proteins in 424 species: Archae - 6; Bacteria - 372; Metazoa - 2603; Fungi - 655; Plants - 291; Viruses - 28; Other Eukaryotes - 2147 (source: NCBI BLink). | chr3:22316913-22319144 REVERSE LENGTH=743	388	743	2.00E-46	191.5	34.0	37.4
Rsa1.0_00055.1.g2986.t1	gb|EOA31480.1| hypothetical protein CARUB_v10014673mg [Capsella rubella]	191	200	2.00E-78	104.7	86.9	90.6	hypothetical protein CARUB_v10014673mg	gbpln	Capsella rubella	AT3G04510.1 | Symbols: LSH2 | Protein of unknown function (DUF640) | chr3:1215812-1216417 REVERSE LENGTH=201	191	201	1.00E-77	105.2	84.3	89.0
Rsa1.0_00055.1.g2987.t1	gb|AAG51783.1|AC079679_3 reverse transcriptase, putative; 16838-20266 [Arabidopsis thaliana]	818	1142	0	139.6	47.6	61.7	reverse transcriptase, putative; 16838-20266	gbpln	Arabidopsis thaliana	AT2G34320.1 | Symbols: | Polynucleotidyl transferase, ribonuclease H-like superfamily protein | chr2:14484959-14485837 FORWARD LENGTH=292	818	292	8.00E-32	35.7	9.8	16.3
Rsa1.0_00055.1.g2988.t1	ref|XP_002882364.1| tRNA synthetase class I family protein [Arabidopsis lyrata subsp. lyrata] gi|297328204|gb|EFH58623.1| tRNA synthetase class I family protein [Arabidopsis lyrata subsp. lyrata]	155	402	5.00E-80	259.4	94.8	98.1	tRNA synthetase class I family protein	gbpln	Arabidopsis lyrata	AT3G04600.3 | Symbols: | Nucleotidylyl transferase superfamily protein | chr3:1243152-1245958 FORWARD LENGTH=402	155	402	2.00E-81	259.4	93.5	98.1
Rsa1.0_00055.1.g2989.t1	dbj|BAB08360.1| Ta11-like non-LTR retroelement protein-like [Arabidopsis thaliana]	643	655	0	101.9	59.3	70.8	Ta11-like non-LTR retroelement protein-like	gbpln	Arabidopsis thaliana	AT5G59920.1 | Symbols: ULI3 | Cysteine/Histidine-rich C1 domain family protein | chr5:24128756-24130888 FORWARD LENGTH=710	643	710	0	110.4	57.2	68.4
Rsa1.0_00055.1.g2990.t1	gb|EOA14327.1| hypothetical protein CARUB_v10027503mg [Capsella rubella]	542	655	1.00E-123	120.8	47.2	56.1	hypothetical protein CARUB_v10027503mg	gbpln	Capsella rubella	AT5G59920.1 | Symbols: ULI3 | Cysteine/Histidine-rich C1 domain family protein | chr5:24128756-24130888 FORWARD LENGTH=710	542	710	1.00E-125	131.0	46.5	55.4
Rsa1.0_00055.1.g2991.t1	gb|EOA29585.1| hypothetical protein CARUB_v10013041mg [Capsella rubella]	746	750	0	100.5	91.4	95.7	hypothetical protein CARUB_v10013041mg	gbpln	Capsella rubella	AT1G64260.1 | Symbols: | MuDR family transposase | chr1:23847756-23849915 FORWARD LENGTH=719	746	719	9.00E-42	96.4	17.2	29.9
Rsa1.0_00055.1.g2992.t1	gb|EOA30196.1| hypothetical protein CARUB_v10013313mg [Capsella rubella]	577	582	0	100.9	74.2	83.7	hypothetical protein CARUB_v10013313mg	gbpln	Capsella rubella	AT1G03670.1 | Symbols: | ankyrin repeat family protein | chr1:914222-916222 REVERSE LENGTH=616	577	616	3.00E-48	106.8	25.8	44.0
Rsa1.0_00055.1.g2993.t1	gb|EOA30206.1| hypothetical protein CARUB_v10013322mg [Capsella rubella]	637	577	0	90.6	72.7	78.3	hypothetical protein CARUB_v10013322mg	gbpln	Capsella rubella	AT3G04610.1 | Symbols: FLK | RNA-binding KH domain-containing protein | chr3:1250762-1254272 REVERSE LENGTH=577	637	577	0	90.6	64.7	73.3
Rsa1.0_00055.1.g2994.t1	ref|XP_002884455.1| nucleic acid binding protein [Arabidopsis lyrata subsp. lyrata] gi|297330295|gb|EFH60714.1| nucleic acid binding protein [Arabidopsis lyrata subsp. lyrata]	203	164	4.00E-53	80.8	66.5	71.9	nucleic acid binding protein	gbpln	Arabidopsis lyrata	AT3G04620.1 | Symbols: | Alba DNA/RNA-binding protein | chr3:1255660-1256739 REVERSE LENGTH=164	203	164	5.00E-55	80.8	66.0	71.9
Rsa1.0_00055.1.g2995.t1	dbj|BAH19881.1| AT3G04630 [Arabidopsis thaliana]	279	286	1.00E-109	102.5	79.6	87.5	AT3G04630	gbpln	Arabidopsis thaliana	AT3G04630.3 | Symbols: WDL1 | WVD2-like 1 | chr3:1259231-1260652 FORWARD LENGTH=286	279	286	1.00E-111	102.5	79.2	87.1
Rsa1.0_00055.1.g2996.t1	gb|EOA30431.1| hypothetical protein CARUB_v10013555mg [Capsella rubella]	491	487	0	99.2	90.6	94.7	hypothetical protein CARUB_v10013555mg	gbpln	Capsella rubella	AT3G04650.1 | Symbols: | FAD/NAD(P)-binding oxidoreductase family protein | chr3:1262017-1264343 FORWARD LENGTH=486	491	486	0	99.0	89.0	92.5
Rsa1.0_00055.1.g2997.t1	dbj|BAJ34400.1| unnamed protein product [Thellungiella halophila]	320	330	1.00E-150	103.1	85.6	90.9	unnamed protein product	----	----	AT3G04670.1 | Symbols: WRKY39, ATWRKY39 | WRKY DNA-binding protein 39 | chr3:1266530-1267691 REVERSE LENGTH=330	320	330	1.00E-151	103.1	85.0	91.3
Rsa1.0_00055.1.g2998.t1	gb|EOA33128.1| hypothetical protein CARUB_v10016466mg [Capsella rubella]	459	445	0	96.9	85.4	91.9	hypothetical protein CARUB_v10016466mg	gbpln	Capsella rubella	AT3G04680.2 | Symbols: CLPS3 | CLP-similar protein 3 | chr3:1270053-1272415 REVERSE LENGTH=444	459	444	0	96.7	85.6	91.5

Rsa1.0_00055.1.g2999.t1	ref|XP_002882370.1| hypothetical protein ARALYDRAFT_477736 [Arabidopsis lyrata subsp. lyrata] gi|297328210|gb|EFH58629.1| hypothetical protein ARALYDRAFT_477736 [Arabidopsis lyrata subsp. lyrata]	192	191	1.00E-77	99.5	81.8	88.5	hypothetical protein ARALYDRAFT_477736	gbpln	Arabidopsis lyrata	AT3G04700.1	Symbols:	Protein of unknown function (DUF1685)	chr3:1276948-1277607 FORWARD LENGTH=191	192	191	5.00E-79	99.5	80.2	87.5
Rsa1.0_00055.1.g3000.t1	gb|EOA30549.1| hypothetical protein CARUB_v10013671mg [Capsella rubella]	251	456	1.00E-118	181.7	84.5	90.8	hypothetical protein CARUB_v10013671mg	gbpln	Capsella rubella	AT3G04710.3	Symbols: TPR10	ankyrin repeat family protein	chr3:1276948-1280942 FORWARD LENGTH=680	251	680	1.00E-119	270.9	83.3	90.0
Rsa1.0_00055.1.g3001.t1	ref|NP_001189808.1| ankyrin repeat family protein [Arabidopsis thaliana] gi|332640603|gb|AEE74124.1| ankyrin repeat family protein [Arabidopsis thaliana]	249	680	2.00E-93	273.1	72.7	77.5	ankyrin repeat family protein	gbpln	Arabidopsis thaliana	AT3G04710.3	Symbols: TPR10	ankyrin repeat family protein	chr3:1276948-1280942 FORWARD LENGTH=680	249	680	7.00E-96	273.1	72.7	77.5
Rsa1.0_00055.1.g3002.t1	gb|EOA31668.1| hypothetical protein CARUB_v10014871mg [Capsella rubella]	141	141	1.00E-71	100.0	90.1	96.5	hypothetical protein CARUB_v10014871mg	gbpln	Capsella rubella	AT3G04720.1	Symbols: PR4, HEL, PR-4	pathogenesis-related 4	chr3:1285691-1286531 REVERSE LENGTH=212	141	212	5.00E-47	150.4	58.9	68.8
Rsa1.0_00055.1.g3003.t1	gb|AAN23106.2| PR4-type protein [Brassica rapa subsp. pekinensis] gi|195536976|dbj|BAG68208.1| PR4 protein [Brassica rapa subsp. chinensis] gi|244539681|dbj|BAH82748.1| pathogenesis related protein 4 [Brassica rapa subsp. chinensis]	141	140	3.00E-72	99.3	95.0	96.5	PR4-type protein	gbpln	Brassica rapa	AT3G04720.1	Symbols: PR4, HEL, PR-4	pathogenesis-related 4	chr3:1285691-1286531 REVERSE LENGTH=212	141	212	5.00E-51	150.4	63.8	72.3
Rsa1.0_00055.1.g3004.t1	dbj|BAJ33708.1| unnamed protein product [Thellungiella halophila]	213	214	1.00E-104	100.5	85.9	90.6	unnamed protein product	----	----	AT3G04720.1	Symbols: PR4, HEL, PR-4	pathogenesis-related 4	chr3:1285691-1286531 REVERSE LENGTH=212	213	212	1.00E-100	99.5	87.8	92.0
Rsa1.0_00055.1.g3005.t1	ref|XP_002882372.1| hypothetical protein ARALYDRAFT_477743 [Arabidopsis lyrata subsp. lyrata] gi|297328212|gb|EFH58631.1| hypothetical protein ARALYDRAFT_477743 [Arabidopsis lyrata subsp. lyrata]	283	1702	1.00E-61	601.4	51.6	54.1	hypothetical protein ARALYDRAFT_477743	gbpln	Arabidopsis lyrata	AT3G04740.1	Symbols: SWP, MED14, ATMED14	RNA polymerase II transcription mediators	chr3:1294037-1300555 FORWARD LENGTH=1703	283	1703	2.00E-60	601.8	50.2	53.0
Rsa1.0_00055.1.g3006.t1	gb|EOA39027.1| hypothetical protein CARUB_v10011597mg [Capsella rubella]	173	186	2.00E-11	107.5	27.2	48.0	hypothetical protein CARUB_v10011597mg	gbpln	Capsella rubella	#	#	#	#	#	#	#
Rsa1.0_00055.1.g3007.t1	gb|ABD65090.1| hypothetical protein 27.t00116 [Brassica oleracea]	250	484	1.00E-82	193.6	65.2	78.4	hypothetical protein 27.t00116	gbpln	Brassica oleracea	AT1G14800.1	Symbols:	Nucleic acid-binding, OB-fold-like protein	chr1:5099572-5102558 FORWARD LENGTH=384	250	384	4.00E-11	153.6	13.6	26.4
Rsa1.0_00055.1.g3008.t1	ref|XP_002882372.1| hypothetical protein ARALYDRAFT_477743 [Arabidopsis lyrata subsp. lyrata] gi|297328212|gb|EFH58631.1| hypothetical protein ARALYDRAFT_477743 [Arabidopsis lyrata subsp. lyrata]	119	1702	1.00E-11	1430.3	34.5	39.5	hypothetical protein ARALYDRAFT_477743	gbpln	Arabidopsis lyrata	AT3G04740.1	Symbols: SWP, MED14, ATMED14	RNA polymerase II transcription mediators	chr3:1294037-1300555 FORWARD LENGTH=1703	119	1703	2.00E-14	1431.1	34.5	39.5
Rsa1.0_00055.1.g3009.t1	ref|NP_566238.1| uncharacterized protein [Arabidopsis thaliana] gi|85701293|sp|Q9SQZ9.2|PITH1_ARATH RecName: Full=PITH domain-containing protein At3g04780 gi|16648681|gb|AAL25533.1| AT3g04780/F7O18_27 [Arabidopsis thaliana] gi|20147247|gb|AAM10337.1| AT3g04780/F7O18_27 [Arabidopsis thaliana] gi|21593799|gb|AAM65766.1| unknown [Arabidopsis thaliana] gi|332640614|gb|AEE74135.1| uncharacterized protein AT3G04780 [Arabidopsis thaliana]	64	176	9.00E-18	275.0	75.0	76.6	uncharacterized protein	gbpln	Arabidopsis thaliana	AT3G04780.1	Symbols:	Protein of unknown function (DUF1000)	chr3:1311447-1313013 REVERSE LENGTH=176	64	176	1.00E-20	275.0	75.0	76.6

Rsa1.0_00055.1.g3010.t1	ref|NP_187132.1| serine/threonine-protein kinase Nek2 [Arabidopsis thaliana] gi|79607883|ref|NP_974221.2| serine/threonine-protein kinase Nek2 [Arabidopsis thaliana] gi|75333670|sp|Q9CAU7.1|NEK2_ARATH RecName: Full=Serine/threonine-protein kinase Nek2; AltName: Full=NimA-related protein kinase 2; Short=AtNek2 gi|12322861|gb|AAG51423.1|AC009465_23 putative kinase; 86849-83844 [Arabidopsis thaliana] gi|24030188|gb|AAN41275.1| putative kinase [Arabidopsis thaliana] gi|332640617|gb|AEE74138.1| serine/threonine-protein kinase Nek2 [Arabidopsis thaliana] gi|332640618|gb|AEE74139.1| serine/threonine-protein kinase Nek2 [Arabidopsis thaliana]	572	606	0	105.9	92.0	94.8	serine/threonine-protein kinase Nek2	gbpln	Arabidopsis thaliana	AT3G04810.2 | Symbols: ATNEK2, NEK2 | NIMA-related kinase 2 | chr3:1318096-1321101 FORWARD LENGTH=606	572	606	0	105.9	92.0	94.8
Rsa1.0_00055.1.g3011.t1	ref|NP_187133.3| pseudouridine synthase [Arabidopsis thaliana] gi|52354299|gb|AAU44470.1| hypothetical protein AT3G04820 [Arabidopsis thaliana] gi|61742699|gb|AAX55170.1| hypothetical protein At3g04820 [Arabidopsis thaliana] gi|332640619|gb|AEE74140.1| pseudouridine synthase [Arabidopsis thaliana]	663	715	0	107.8	88.7	94.0	pseudouridine synthase	gbpln	Arabidopsis thaliana	AT3G04820.1 | Symbols: | Pseudouridine synthase family protein | chr3:1321453-1325949 REVERSE LENGTH=715	663	715	0	107.8	88.7	94.0
Rsa1.0_00055.1.g3012.t1	gb|EOA31103.1| hypothetical protein CARUB_v10014256mg [Capsella rubella]	302	304	1.00E-166	100.7	94.7	98.3	hypothetical protein CARUB_v10014256mg	gbpln	Capsella rubella	AT3G04830.1 | Symbols: | Protein prenylyltransferase superfamily protein | chr3:1326289-1329132 FORWARD LENGTH=303	302	303	1.00E-166	100.3	92.7	97.7
Rsa1.0_00055.1.g3013.t3	ref|XP_002882386.1| hypothetical protein ARALYDRAFT_477772 [Arabidopsis lyrata subsp. lyrata] gi|297328226|gb|EFH58645.1| hypothetical protein ARALYDRAFT_477772 [Arabidopsis lyrata subsp. lyrata]	250	230	1.00E-101	92.0	75.6	80.8	hypothetical protein ARALYDRAFT_477772	gbpln	Arabidopsis lyrata	AT3G04950.1 | Symbols: | CONTAINS InterPro DOMAIN/s: SEC-C motif (InterPro:IPR004027); Has 583 Blast hits to 583 proteins in 248 species: Archae - 0; Bacteria - 488; Metazoa - 0; Fungi - 0; Plants - 34; Viruses - 0; Other Eukaryotes - 61 (source: NCBI BLink). | chr3:1371786-1373300 FORWARD LENGTH=231	250	231	1.00E-104	92.4	76.8	81.2
Rsa1.0_00055.1.g3014.t1	emb|CAA31519.1| ACP preprotein [Brassica napus]	133	134	1.00E-55	100.8	85.7	91.7	ACP preprotein	gbpln	Brassica napus	AT3G05020.1 | Symbols: ACP1, ACP | acyl carrier protein 1 | chr3:1391863-1392878 REVERSE LENGTH=137	133	137	2.00E-52	103.0	82.0	90.2
Rsa1.0_00055.1.g3015.t1	ref|XP_002882392.1| hypothetical protein ARALYDRAFT_477790 [Arabidopsis lyrata subsp. lyrata] gi|297328232|gb|EFH58651.1| hypothetical protein ARALYDRAFT_477790 [Arabidopsis lyrata subsp. lyrata]	140	144	5.00E-56	102.9	83.6	91.4	hypothetical protein ARALYDRAFT_477790	gbpln	Arabidopsis lyrata	AT3G05070.1 | Symbols: | CONTAINS InterPro DOMAIN/s: mRNA splicing factor, Cwf18 (InterPro:IPR013169); Has 292 Blast hits to 292 proteins in 153 species: Archae - 0; Bacteria - 0; Metazoa - 118; Fungi - 83; Plants - 38; Viruses - 11; Other Eukaryotes - 42 (source: NCBI BLink). | chr3:1416480-1417533 FORWARD LENGTH=144	140	144	3.00E-57	102.9	82.1	91.4
Rsa1.0_00055.1.g3016.t2	gb|ACY38730.1| salt-induced WD protein 1 [Brassica napus]	757	754	0	99.6	94.6	97.5	salt-induced WD protein 1	gbpln	Brassica napus	AT3G05090.2 | Symbols: LRS1 | Transducin/WD40 repeat-like superfamily protein | chr3:1418573-1422723 REVERSE LENGTH=753	757	753	0	99.5	91.3	95.5
Rsa1.0_00055.1.g3017.t1	ref|XP_002882393.1| hypothetical protein ARALYDRAFT_317378 [Arabidopsis lyrata subsp. lyrata] gi|297328233|gb|EFH58652.1| hypothetical protein ARALYDRAFT_317378 [Arabidopsis lyrata subsp. lyrata]	369	341	1.00E-159	92.4	77.2	82.4	hypothetical protein ARALYDRAFT_317378	gbpln	Arabidopsis lyrata	AT3G05100.1 | Symbols: | S-adenosyl-L-methionine-dependent methyltransferases superfamily protein | chr3:1424155-1425667 FORWARD LENGTH=336	369	336	1.00E-156	91.1	76.4	81.6
Rsa1.0_00055.1.g3018.t1	dbj|BAJ33907.1| unnamed protein product [Thellungiella halophila]	552	595	0	107.8	91.5	93.1	unnamed protein product	----	----	AT5G60790.1 | Symbols: ATGCN1, GCN1 | ABC transporter family protein | chr5:24453760-24455767 REVERSE LENGTH=595	552	595	0	107.8	89.9	92.2
Rsa1.0_00055.1.g3019.t9	ref|XP_002866393.1| hypothetical protein ARALYDRAFT_332324 [Arabidopsis lyrata subsp. lyrata] gi|297312228|gb|EFH42652.1| hypothetical protein ARALYDRAFT_332324 [Arabidopsis lyrata subsp. lyrata]	394	1109	1.00E-172	281.5	80.2	84.8	hypothetical protein ARALYDRAFT_332324	gbpln	Arabidopsis lyrata	AT5G60740.1 | Symbols: | ABC transporter family protein | chr5:24425824-24430269 REVERSE LENGTH=1109	394	1109	1.00E-174	281.5	79.9	84.5
Rsa1.0_00055.1.g3020.t1	gb|EOA20112.1| hypothetical protein CARUB_v10000391mg [Capsella rubella]	416	672	1.00E-139	161.5	64.4	75.5	hypothetical protein CARUB_v10000391mg	gbpln	Capsella rubella	AT3G62310.1 | Symbols: | RNA helicase family protein | chr3:23057516-23060561 REVERSE LENGTH=726	416	726	1.00E-137	174.5	63.2	76.9

Rsa1.0_00055.1.g3021.t1	gb|EOA32865.1| hypothetical protein CARUB_v10016180mg [Capsella rubella]	71	922	1.00E-13	1298.6	62.0	74.6	hypothetical protein CARUB_v10016180mg	gbpln	Capsella rubella	AT3G03710.1 | Symbols: RIF10, PNP | polyribonucleotide nucleotidyltransferase, putative | chr3:919542-924906 FORWARD LENGTH=922	71	922	1.00E-13	1298.6	63.4	70.4
Rsa1.0_00055.1.g3022.t1	#	#	#	#	#	#	#	–	----	----	#	#	#	#	#	#	#
Rsa1.0_00055.1.g3023.t1	ref|XP_002884492.1| kinase family protein [Arabidopsis lyrata subsp. lyrata] gi|297330332|gb|EFH60751.1| kinase family protein [Arabidopsis lyrata subsp. lyrata]	501	469	0	93.6	79.6	86.0	kinase family protein	gbpln	Arabidopsis lyrata	AT3G05140.1 | Symbols: RBK2 | ROP binding protein kinases 2 | chr3:1435817-1437800 REVERSE LENGTH=460	501	460	0	91.8	77.8	84.6
Rsa1.0_00055.1.g3024.t1	ref|NP_566249.1| E3 ubiquitin-protein ligase ATL6 [Arabidopsis thaliana] gi|68565231|sp|Q8RXX9.2|ATL6_ARATH RecName: Full=E3 ubiquitin-protein ligase ATL6; AltName: Full=RING-H2 finger protein ATL6; Flags: Precursor gi|70905101|gb|AAZ14076.1| At3g05200 [Arabidopsis thaliana] gi|332640683|gb|AEE74204.1| E3 ubiquitin-protein ligase ATL6 [Arabidopsis thaliana]	384	398	0	103.6	87.2	91.7	E3 ubiquitin-protein ligase ATL6	gbpln	Arabidopsis thaliana	AT3G05200.1 | Symbols: ATL6 | RING/U-box superfamily protein | chr3:1477377-1478573 FORWARD LENGTH=398	384	398	0	103.6	87.2	91.7
Rsa1.0_00055.1.g3025.t1	gb|EOA30233.1| hypothetical protein CARUB_v10013356mg [Capsella rubella]	501	565	1.00E-55	112.8	63.1	68.7	hypothetical protein CARUB_v10013356mg	gbpln	Capsella rubella	AT3G05220.1 | Symbols: | Heavy metal transport/detoxification superfamily protein | chr3:1488808-1491171 FORWARD LENGTH=577	501	577	8.00E-43	115.2	23.8	25.7
Rsa1.0_00055.1.g3026.t1	ref|XP_002882405.1| signal peptidase subunit family protein [Arabidopsis lyrata subsp. lyrata] gi|297328245|gb|EFH58664.1| signal peptidase subunit family protein [Arabidopsis lyrata subsp. lyrata]	167	167	8.00E-89	100.0	92.2	97.0	signal peptidase subunit family protein	gbpln	Arabidopsis lyrata	AT3G05230.1 | Symbols: | Signal peptidase subunit | chr3:1492304-1493452 FORWARD LENGTH=167	167	167	3.00E-90	100.0	91.0	97.0
Rsa1.0_00055.1.g3027.t1	ref|NP_566558.1| F-box/kelch-repeat protein [Arabidopsis thaliana] gi|75274278|sp|Q9LUQ9.1|FBK56_ARATH RecName: Full=F-box/kelch-repeat protein At3g16740 gi|11994629|dbj|BAB02766.1| unnamed protein product [Arabidopsis thaliana] gi|28466967|gb|AAO44092.1| At3g16740 [Arabidopsis thaliana] gi|110743773|dbj|BAE99722.1| hypothetical protein [Arabidopsis thaliana] gi|332642338|gb|AEE75859.1| F-box/kelch-repeat protein [Arabidopsis thaliana]	396	391	1.00E-122	98.7	59.1	74.0	F-box/kelch-repeat protein	gbpln	Arabidopsis thaliana	AT3G16740.1 | Symbols: | F-box and associated interaction domains-containing protein | chr3:5699476-5700651 FORWARD LENGTH=391	396	391	1.00E-124	98.7	59.1	74.0
Rsa1.0_00055.1.g3028.t1	ref|NP_187179.2| Integral membrane Yip1-like protein [Arabidopsis thaliana] gi|26452921|dbj|BAC43538.1| unknown protein [Arabidopsis thaliana] gi|28973139|gb|AAO63894.1| unknown protein [Arabidopsis thaliana] gi|332640694|gb|AEE74215.1| Integral membrane Yip1-like protein [Arabidopsis thaliana]	445	281	1.00E-149	63.1	60.2	62.2	Integral membrane Yip1-like protein	gbpln	Arabidopsis thaliana	AT3G05280.1 | Symbols: | Integral membrane Yip1 family protein | chr3:1503992-1505554 REVERSE LENGTH=281	445	281	1.00E-152	63.1	60.2	62.2
Rsa1.0_00055.1.g3029.t1	ref|XP_002884499.1| mitochondrial substrate carrier family protein [Arabidopsis lyrata subsp. lyrata] gi|297330339|gb|EFH60758.1| mitochondrial substrate carrier family protein [Arabidopsis lyrata subsp. lyrata]	321	322	1.00E-156	100.3	87.9	93.1	mitochondrial substrate carrier family protein	gbpln	Arabidopsis lyrata	AT3G05290.1 | Symbols: PNC1 | peroxisomal adenine nucleotide carrier 1 | chr3:1506129-1507614 REVERSE LENGTH=322	321	322	1.00E-158	100.3	87.2	93.1
Rsa1.0_00055.1.g3030.t3	ref|XP_002882407.1| hypothetical protein ARALYDRAFT_477819 [Arabidopsis lyrata subsp. lyrata] gi|297328247|gb|EFH58666.1| hypothetical protein ARALYDRAFT_477819 [Arabidopsis lyrata subsp. lyrata]	641	647	0	100.9	62.1	78.0	hypothetical protein ARALYDRAFT_477819	gbpln	Arabidopsis lyrata	AT3G05310.1 | Symbols: MIRO3 | MIRO-related GTP-ase 3 | chr3:1510160-1513301 FORWARD LENGTH=648	641	648	0	101.1	61.0	77.7
Rsa1.0_00055.1.g3031.t1	ref|XP_002882412.1| acyl-CoA binding protein 4, acyl-CoA-binding domain 4 [Arabidopsis lyrata subsp. lyrata] gi|297328252|gb|EFH58671.1| acyl-CoA binding protein 4, acyl-CoA-binding domain 4 [Arabidopsis lyrata subsp. lyrata]	267	669	1.00E-127	250.6	80.9	87.6	acyl-CoA binding protein 4, acyl-CoA-binding domain 4	gbpln	Arabidopsis lyrata	AT3G05420.1 | Symbols: ACBP4 | acyl-CoA binding protein 4 | chr3:1561880-1567047 FORWARD LENGTH=668	267	668	1.00E-128	250.2	80.1	88.0
Rsa1.0_00055.1.g3032.t3	gb|ACP30603.1| disease resistance protein [Brassica rapa subsp. pekinensis]	2468	1056	0	42.8	31.5	34.8	disease resistance protein	gbpln	Brassica rapa	AT5G18370.1 | Symbols: | Disease resistance protein (TIR-NBS-LRR class) family | chr5:6085036-6088926 REVERSE LENGTH=1210	2468	1210	0	49.0	22.6	29.1

Rsa1.0_00055.1.g3033.t22	ref|NP_187219.5| embryo defective 2016 protein [Arabidopsis thaliana] gi|332640756|gb|AEE74277.1| embryo defective 2016 protein [Arabidopsis thaliana]	2601	2138	0	82.2	71.4	75.3	embryo defective 2016 protein	gbpln	Arabidopsis thaliana	AT3G05680.1 | Symbols: EMB2016 | embryo defective 2016 | chr3:1660802-1672015 REVERSE LENGTH=2138	2601	2138	0	82.2	71.4	75.3
Rsa1.0_00055.1.g3034.t1	emb|CAA74049.1| Transcription factor [Arabidopsis thaliana]	286	295	1.00E-128	103.1	80.1	86.4	Transcription factor	gbpln	Arabidopsis thaliana	AT3G05690.1 | Symbols: UNE8, ATHAP2B, HAP2B, NF-YA2 | nuclear factor Y, subunit A2 | chr3:1676922-1678324 REVERSE LENGTH=295	286	295	1.00E-130	103.1	80.1	86.4
Rsa1.0_00055.1.g3035.t1	gb|EOA32343.1| hypothetical protein CARUB_v10015609mg [Capsella rubella]	454	444	3.00E-56	97.8	33.5	43.6	hypothetical protein CARUB_v10015609mg	gbpln	Capsella rubella	AT3G05770.1 | Symbols: | unknown protein; BEST Arabidopsis thaliana protein match is: unknown protein (TAIR:AT1G54300.1); Has 105 Blast hits to 105 proteins in 16 species: Archae - 0; Bacteria - 0; Metazoa - 0; Fungi - 0; Plants - 99; Viruses - 0; Other Eukaryotes - 6 (source: NCBI BLink). | chr3:1710328-1712165 REVERSE LENGTH=410	454	410	3.00E-54	90.3	30.6	35.5
Rsa1.0_00055.1.g3036.t1	ref|NP_566260.1| transcription factor bHLH150 [Arabidopsis thaliana] gi|75191236|sp|Q9M9L6.1|BH150_ARATH RecName: Full=Transcription factor bHLH150; AltName: Full=ATBS1 interacting factor 1; AltName: Full=Basic helix-loop-helix protein 150; Short=AtbHLH150; Short=bHLH 150; AltName: Full=Transcription factor EN 145; AltName: Full=bHLH transcription factor bHLH150 gi|6714393|gb|AAF26082.1|AC012393_8 hypothetical protein [Arabidopsis thaliana] gi|21554259|gb|AAM63334.1| unknown [Arabidopsis thaliana] gi|51971903|dbj|BAD44616.1| unknown protein [Arabidopsis thaliana] gi|94442469|gb|ABF19022.1| At3g05800 [Arabidopsis thaliana] gi|225898621|dbj|BAH30441.1| hypothetical protein [Arabidopsis thaliana] gi|332640777|gb|AEE74298.1| transcription factor bHLH150 [Arabidopsis thaliana]	182	211	2.00E-61	115.9	78.0	84.1	transcription factor bHLH150	gbpln	Arabidopsis thaliana	AT3G05800.1 | Symbols: AIF1 | AtBS1(activation-tagged BRI1 suppressor 1)-interacting factor 1 | chr3:1727476-1728111 FORWARD LENGTH=211	182	211	8.00E-64	115.9	78.0	84.1
Rsa1.0_00055.1.g3037.t1	#	#	#	#	#	#	#	-	----	----	#	#	#	#	#	#	#
Rsa1.0_00055.1.g3038.t1	ref|XP_002882438.1| hypothetical protein ARALYDRAFT_477878 [Arabidopsis lyrata subsp. lyrata] gi|297328278|gb|EFH58697.1| hypothetical protein ARALYDRAFT_477878 [Arabidopsis lyrata subsp. lyrata]	476	409	0	85.9	80.3	82.6	hypothetical protein ARALYDRAFT_477878	gbpln	Arabidopsis lyrata	AT3G05840.1 | Symbols: ATSK12 | Protein kinase superfamily protein | chr3:1740793-1742927 FORWARD LENGTH=409	476	409	0	85.9	79.8	82.4
Rsa1.0_00055.1.g3039.t1	ref|XP_002882439.1| hypothetical protein ARALYDRAFT_896682 [Arabidopsis lyrata subsp. lyrata] gi|297328279|gb|EFH58698.1| hypothetical protein ARALYDRAFT_896682 [Arabidopsis lyrata subsp. lyrata]	90	90	1.00E-39	100.0	93.3	95.6	hypothetical protein ARALYDRAFT_896682	gbpln	Arabidopsis lyrata	AT3G05858.1 | Symbols: | unknown protein; FUNCTIONS IN: molecular_function unknown; INVOLVED IN: biological_process unknown; LOCATED IN: cellular_component unknown; BEST Arabidopsis thaliana protein match is: unknown protein (TAIR:AT5G26620.1); Has 30201 Blast hits to 17322 proteins in 780 species: Archae - 12; Bacteria - 1396; Metazoa - 17338; Fungi - 3422; Plants - 5037; Viruses - 0; Other Eukaryotes - 2996 (source: NCBI BLink). | chr3:1749464-1749736 FORWARD LENGTH=90	90	90	1.00E-41	100.0	92.2	93.3
Rsa1.0_00055.1.g3040.t1	#	#	#	#	#	#	#	-	----	----	#	#	#	#	#	#	#
Rsa1.0_00055.1.g3041.t1	gb|AFC41201.1| PM-YC3.6-Lti6b [Binary expression vector PM-YC3.6-LTI6b]	83	726	5.00E-15	874.7	49.4	62.7	PM-YC3.6-Lti6b	----	----	AT3G05890.1 | Symbols: RCI2B | Low temperature and salt responsive protein family | chr3:1758179-1758452 REVERSE LENGTH=54	83	54	2.00E-14	65.1	43.4	50.6
Rsa1.0_00055.1.g3042.t1	gb|AFC41201.1| PM-YC3.6-Lti6b [Binary expression vector PM-YC3.6-LTI6b]	55	726	6.00E-22	1320.0	85.5	92.7	PM-YC3.6-Lti6b	----	----	AT3G05890.1 | Symbols: RCI2B | Low temperature and salt responsive protein family | chr3:1758179-1758452 REVERSE LENGTH=54	55	54	7.00E-21	98.2	85.5	92.7

Rsa1.0_00055.1.g3043.t1	ref|NP_566263.1| Pectin lyase-like protein [Arabidopsis thaliana] gi|6671966|gb|AAF23225.1|AC013454_12 putative pectinacetylesterase [Arabidopsis thaliana] gi|16226325|gb|AAL16135.1|AF428303_1 AT3g05910/F2O10_3 [Arabidopsis thaliana] gi|15292827|gb|AAK92782.1| putative pectinacetylesterase [Arabidopsis thaliana] gi|20465793|gb|AAM20385.1| putative pectinacetylesterase [Arabidopsis thaliana] gi|332640793|gb|AEE74314.1| Pectin lyase-like protein [Arabidopsis thaliana]	416	415	0	99.8	93.0	96.6	Pectin lyase-like protein	gbpln	Arabidopsis thaliana	AT3G05910.1 | Symbols: | Pectinacetylesterase family protein | chr3:1764509-1767240 REVERSE LENGTH=415	416	415	0	99.8	93.0	96.6
Rsa1.0_00055.1.g3044.t1	ref|XP_002882441.1| hypothetical protein ARALYDRAFT_896692 [Arabidopsis lyrata subsp. lyrata] gi|297328281|gb|EFH58700.1| hypothetical protein ARALYDRAFT_896692 [Arabidopsis lyrata subsp. lyrata]	218	219	9.00E-97	100.5	80.7	88.5	hypothetical protein ARALYDRAFT_896692	gbpln	Arabidopsis lyrata	AT3G05930.1 | Symbols: GLP8 | germin-like protein 8 | chr3:1770377-1771183 FORWARD LENGTH=219	218	219	2.00E-98	100.5	80.3	88.5
Rsa1.0_00055.1.g3045.t1	ref|XP_002884544.1| hypothetical protein ARALYDRAFT_896695 [Arabidopsis lyrata subsp. lyrata] gi|297330384|gb|EFH60803.1| hypothetical protein ARALYDRAFT_896695 [Arabidopsis lyrata subsp. lyrata]	103	101	2.00E-21	98.1	80.6	82.5	hypothetical protein ARALYDRAFT_896695	gbpln	Arabidopsis lyrata	AT5G26731.1 | Symbols: | unknown protein; Has 30201 Blast hits to 17322 proteins in 780 species: Archae - 12; Bacteria - 1396; Metazoa - 17338; Fungi - 3422; Plants - 5037; Viruses - 0; Other Eukaryotes - 2996 (source: NCBI BLink). | chr5:9295676-9295975 FORWARD LENGTH=99	103	99	4.00E-17	96.1	44.7	53.4
Rsa1.0_00055.1.g3046.t1	ref|NP_198008.1| TCP-1/cpn60 chaperonin family protein [Arabidopsis thaliana] gi|27754225|gb|AAO22566.1| putative chaperonin gamma chain [Arabidopsis thaliana] gi|332006171|gb|AED93554.1| TCP-1/cpn60 chaperonin family protein [Arabidopsis thaliana]	559	555	0	99.3	94.1	97.5	TCP-1/cpn60 chaperonin family protein	gbpln	Arabidopsis thaliana	AT5G26360.1 | Symbols: | TCP-1/cpn60 chaperonin family protein | chr5:9255561-9258891 REVERSE LENGTH=555	559	555	0	99.3	94.1	97.5
Rsa1.0_00055.1.g3047.t1	ref|NP_187247.1| sugar transport protein 6 [Arabidopsis thaliana] gi|75337175|sp|Q9SFG0.1|STP6_ARATH RecName: Full=Sugar transport protein 6; AltName: Full=Hexose transporter 6 gi|6671961|gb|AAF23220.1|AC013454_7 putative hexose transporter [Arabidopsis thaliana] gi|15487256|emb|CAC69073.1| STP6 protein [Arabidopsis thaliana] gi|332640802|gb|AEE74323.1| sugar transport protein 6 [Arabidopsis thaliana]	508	507	0	99.8	89.0	94.7	sugar transport protein 6	gbpln	Arabidopsis thaliana	AT3G05960.1 | Symbols: ATSTP6, STP6 | sugar transporter 6 | chr3:1783587-1785334 REVERSE LENGTH=507	508	507	0	99.8	89.0	94.7
Rsa1.0_00055.1.g3048.t1	emb|CAA96522.1| AMP-binding protein [Brassica napus]	686	677	0	98.7	95.0	96.8	AMP-binding protein	gbpln	Brassica napus	AT3G05970.1 | Symbols: LACS6, ATLACS6 | long-chain acyl-CoA synthetase 6 | chr3:1786510-1791746 REVERSE LENGTH=701	686	701	0	102.2	92.9	95.5
Rsa1.0_00055.1.g3049.t1	ref|XP_002882446.1| hypothetical protein ARALYDRAFT_896708 [Arabidopsis lyrata subsp. lyrata] gi|297328286|gb|EFH58705.1| hypothetical protein ARALYDRAFT_896708 [Arabidopsis lyrata subsp. lyrata]	246	299	5.00E-63	121.5	69.5	78.5	hypothetical protein ARALYDRAFT_896708	gbpln	Arabidopsis lyrata	AT3G06020.1 | Symbols: | Protein of unknown function (DUF3049) | chr3:1813267-1814169 FORWARD LENGTH=300	246	300	3.00E-64	122.0	72.0	81.7
Rsa1.0_00055.1.g3050.t1	ref|NP_566267.1| glycoprotein membrane precursor GPI-anchored protein [Arabidopsis thaliana] gi|34395875|sp|Q84MC0.1|UGPI4_ARATH RecName: Full=Uncharacterized GPI-anchored protein At3g06035; Flags: Precursor gi|30102608|gb|AAP21222.1| At3g06035 [Arabidopsis thaliana] gi|110735967|dbj|BAE99958.1| hypothetical protein [Arabidopsis thaliana] gi|332640812|gb|AEE74333.1| glycoprotein membrane precursor GPI-anchored protein [Arabidopsis thaliana]	342	200	2.00E-70	58.5	38.3	44.2	glycoprotein membrane precursor GPI-anchored protein	gbpln	Arabidopsis thaliana	AT3G06035.1 | Symbols: | Glycoprotein membrane precursor GPI-anchored | chr3:1823172-1824110 REVERSE LENGTH=200	342	200	6.00E-73	58.5	38.3	44.2

Rsa1.0_00055.1.g3051.t1	ref|XP_002884557.1| predicted protein [Arabidopsis lyrata subsp. lyrata] gi|297330397|gb|EFH60816.1| predicted protein [Arabidopsis lyrata subsp. lyrata]	148	152	9.00E-52	102.7	75.0	87.2	predicted protein	gbpln	Arabidopsis lyrata	AT3G06070.1 | Symbols: | unknown protein; BEST Arabidopsis thaliana protein match is: unknown protein (TAIR:AT5G19190.1); Has 33 Blast hits to 33 proteins in 12 species: Archae - 0; Bacteria - 0; Metazoa - 1; Fungi - 0; Plants - 32; Viruses - 0; Other Eukaryotes - 0 (source: NCBI BLink). | chr3:1831305-1831846 REVERSE LENGTH=151	148	151	4.00E-52	102.0	74.3	85.8
Rsa1.0_00055.1.g3052.t1	gb|EOA30487.1| hypothetical protein CARUB_v10013611mg [Capsella rubella]	458	473	0	103.3	84.9	91.5	hypothetical protein CARUB_v10013611mg	gbpln	Capsella rubella	AT3G06080.2 | Symbols: | Plant protein of unknown function (DUF828) | chr3:1834959-1837524 REVERSE LENGTH=469	458	469	0	102.4	83.8	90.4
Rsa1.0_00055.1.g3053.t1	ref|NP_187263.1| transcription factor MUTE [Arabidopsis thaliana] gi|75312289|sp|Q9M8K6.1|MUTE_ARATH RecName: Full=Transcription factor MUTE; AltName: Full=Basic helix-loop-helix protein 45; Short=AtbHLH45; Short=bHLH 45; AltName: Full=Transcription factor EN 20; AltName: Full=bHLH transcription factor bHLH045 gi|6862916|gb|AAF30305.1|AC018907_5 putative helix-loop-helix DNA-binding protein [Arabidopsis thaliana] gi|91806385|gb|ABE65920.1| basic helix-loop-helix family protein [Arabidopsis thaliana] gi|113206517|gb|ABI34465.1| basic helix-loop-helix protein [Arabidopsis thaliana] gi|114446460|gb|ABI74926.1| helix-loop-helix protein [Arabidopsis thaliana] gi|332640826|gb|AEE74347.1| transcription factor MUTE [Arabidopsis thaliana]	205	202	1.00E-102	98.5	91.2	95.1	transcription factor MUTE	gbpln	Arabidopsis thaliana	AT3G06120.1 | Symbols: MUTE | basic helix-loop-helix (bHLH) DNA-binding superfamily protein | chr3:1846531-1848016 FORWARD LENGTH=202	205	202	1.00E-104	98.5	91.2	95.1
Rsa1.0_00055.1.g3054.t1	ref|XP_002884560.1| hypothetical protein ARALYDRAFT_477915 [Arabidopsis lyrata subsp. lyrata] gi|297330400|gb|EFH60819.1| hypothetical protein ARALYDRAFT_477915 [Arabidopsis lyrata subsp. lyrata]	469	445	1.00E-68	94.9	38.0	41.6	hypothetical protein ARALYDRAFT_477915	gbpln	Arabidopsis lyrata	AT3G06130.2 | Symbols: | Heavy metal transport/detoxification superfamily protein | chr3:1853193-1854673 REVERSE LENGTH=349	469	349	5.00E-66	74.4	36.7	40.5
Rsa1.0_00055.1.g3055.t1	ref|NP_187266.1| uncharacterized protein [Arabidopsis thaliana] gi|6862919|gb|AAF30308.1|AC018907_8 unknown protein [Arabidopsis thaliana] gi|332640832|gb|AEE74353.1| uncharacterized protein AT3G06150 [Arabidopsis thaliana]	586	594	0	101.4	80.5	89.8	uncharacterized protein	gbpln	Arabidopsis thaliana	AT3G06150.1 | Symbols: | unknown protein; BEST Arabidopsis thaliana protein match is: unknown protein (TAIR:AT5G19060.1); Has 61 Blast hits to 59 proteins in 10 species: Archae - 0; Bacteria - 0; Metazoa - 3; Fungi - 0; Plants - 58; Viruses - 0; Other Eukaryotes - 0 (source: NCBI BLink). | chr3:1861971-1863755 REVERSE LENGTH=594	586	594	0	101.4	80.5	89.8
Rsa1.0_00055.1.g3056.t1	gb|EOA30708.1| hypothetical protein CARUB_v10013845mg [Capsella rubella]	400	407	0	101.8	93.0	96.5	hypothetical protein CARUB_v10013845mg	gbpln	Capsella rubella	AT3G06190.1 | Symbols: ATBPM2, BPM2 | BTB-POZ and MATH domain 2 | chr3:1874577-1876575 REVERSE LENGTH=406	400	406	0	101.5	91.5	95.5
Rsa1.0_00055.1.g3057.t1	ref|NP_566277.2| F-box/kelch-repeat protein [Arabidopsis thaliana] gi|75151183|sp|Q8GXC7.1|FBK50_ARATH RecName: Full=F-box/kelch-repeat protein At3g06240 gi|26451648|dbj|BAC42921.1| unknown protein [Arabidopsis thaliana] gi|29824145|gb|AAP04033.1| putative F-box protein family [Arabidopsis thaliana] gi|332640843|gb|AEE74364.1| F-box/kelch-repeat protein [Arabidopsis thaliana]	405	427	1.00E-127	105.4	61.7	74.6	F-box/kelch-repeat protein	gbpln	Arabidopsis thaliana	AT3G06240.1 | Symbols: | F-box family protein | chr3:1887336-1888619 FORWARD LENGTH=427	405	427	1.00E-129	105.4	61.7	74.6
Rsa1.0_00055.1.g3058.t1	ref|NP_566277.2| F-box/kelch-repeat protein [Arabidopsis thaliana] gi|75151183|sp|Q8GXC7.1|FBK50_ARATH RecName: Full=F-box/kelch-repeat protein At3g06240 gi|26451648|dbj|BAC42921.1| unknown protein [Arabidopsis thaliana] gi|29824145|gb|AAP04033.1| putative F-box protein family [Arabidopsis thaliana] gi|332640843|gb|AEE74364.1| F-box/kelch-repeat protein [Arabidopsis thaliana]	411	427	1.00E-144	103.9	67.9	78.8	F-box/kelch-repeat protein	gbpln	Arabidopsis thaliana	AT3G06240.1 | Symbols: | F-box family protein | chr3:1887336-1888619 FORWARD LENGTH=427	411	427	2.33E-156	103.9	67.9	78.8

Rsa1.0_00055.1.g3059.t1	ref|XP_002869401.1| aspartyl protease family [Arabidopsis lyrata subsp. lyrata] gi|297315237|gb|EFH45660.1| aspartyl protease family [Arabidopsis lyrata subsp. lyrata]	399	438	1.00E-35	109.8	28.3	42.9	aspartyl protease family	gbpln	Arabidopsis lyrata	AT4G30030.1 | Symbols: | Eukaryotic aspartyl protease family protein | chr4:14682210-14683484 REVERSE LENGTH=424	399	424	6.00E-31	106.3	25.6	41.9
Rsa1.0_00055.1.g3060.t1	dbj|BAB08714.1| non-LTR retroelement reverse transcriptase-like protein [Arabidopsis thaliana]	137	1197	2.00E-13	873.7	27.7	46.7	non-LTR retroelement reverse transcriptase-like protein	gbpln	Arabidopsis thaliana	#	#	#	#	#	#	#
Rsa1.0_00055.1.g3061.t1	gb|AAD22286.1| putative non-LTR retroelement reverse transcriptase [Arabidopsis thaliana]	153	1311	1.00E-14	856.9	30.1	43.8	putative non-LTR retroelement reverse transcriptase	gbpln	Arabidopsis thaliana	AT1G10000.1 | Symbols: | Ribonuclease H-like superfamily protein | chr1:3263877-3264788 REVERSE LENGTH=303	153	303	2.00E-11	198.0	27.5	44.4
Rsa1.0_00055.1.g3062.t1	ref|XP_002882653.1| predicted protein [Arabidopsis lyrata subsp. lyrata] gi|297328493|gb|EFH58912.1| predicted protein [Arabidopsis lyrata subsp. lyrata]	260	173	4.00E-20	66.5	26.9	36.2	predicted protein	gbpln	Arabidopsis lyrata	#	#	#	#	#	#	#
Rsa1.0_00055.1.g3063.t1	ref|NP_187318.2| fip1 motif-containing protein [Arabidopsis thaliana] gi|334185148|ref|NP_001189830.1| fip1 motif-containing protein [Arabidopsis thaliana] gi|332640906|gb|AEE74427.1| fip1 motif-containing protein [Arabidopsis thaliana] gi|332640907|gb|AEE74428.1| fip1 motif-containing protein [Arabidopsis thaliana]	991	997	0	100.6	58.1	68.5	fip1 motif-containing protein	gbpln	Arabidopsis thaliana	AT3G66652.2 | Symbols: | fip1 motif-containing protein | chr3:2084030-2087556 FORWARD LENGTH=997	991	997	0	100.6	58.1	68.5
Rsa1.0_00056.1.g3064.t1	dbj|BAH56865.1| AT5G61660 [Arabidopsis thaliana]	141	113	1.00E-25	80.1	62.4	64.5	AT5G61660	gbpln	Arabidopsis thaliana	AT5G61660.1 | Symbols: | glycine-rich protein | chr5:24779458-24779862 REVERSE LENGTH=134	141	134	1.00E-27	95.0	42.6	44.7
Rsa1.0_00056.1.g3065.t1	gb|ADF57182.1| AP2/ERF transcription factor [Brassica napus]	204	200	2.00E-87	98.0	83.8	89.2	AP2/ERF transcription factor	gbpln	Brassica napus	AT5G61590.1 | Symbols: | Integrase-type DNA-binding superfamily protein | chr5:24764653-24765258 REVERSE LENGTH=201	204	201	1.00E-84	98.5	83.3	87.7
Rsa1.0_00056.1.g3066.t1	gb|EOA14619.1| hypothetical protein CARUB_v10027877mg [Capsella rubella]	264	849	1.00E-142	321.6	92.0	95.1	hypothetical protein CARUB_v10027877mg	gbpln	Capsella rubella	AT5G61550.1 | Symbols: | U-box domain-containing protein kinase family protein | chr5:24748325-24751558 FORWARD LENGTH=845	264	845	1.00E-139	320.1	88.6	93.6
Rsa1.0_00056.1.g3067.t7	gb|EOA14619.1| hypothetical protein CARUB_v10027877mg [Capsella rubella]	794	849	0	106.9	81.4	87.8	hypothetical protein CARUB_v10027877mg	gbpln	Capsella rubella	AT5G61550.2 | Symbols: | U-box domain-containing protein kinase family protein | chr5:24748325-24751805 FORWARD LENGTH=860	794	860	0	108.3	78.2	86.9
Rsa1.0_00056.1.g3068.t1	ref|XP_002864736.1| small G protein family protein [Arabidopsis lyrata subsp. lyrata] gi|297310571|gb|EFH40995.1| small G protein family protein [Arabidopsis lyrata subsp. lyrata]	376	376	0	100.0	93.4	96.8	small G protein family protein	gbpln	Arabidopsis lyrata	AT5G61530.1 | Symbols: | small G protein family protein / RhoGAP family protein | chr5:24742630-24744586 FORWARD LENGTH=376	376	376	0	100.0	91.5	96.0
Rsa1.0_00056.1.g3069.t2	gb|EOA15037.1| hypothetical protein CARUB_v10028392mg [Capsella rubella]	411	400	0	97.3	85.2	90.5	hypothetical protein CARUB_v10028392mg	gbpln	Capsella rubella	AT5G61510.1 | Symbols: | GroES-like zinc-binding alcohol dehydrogenase family protein | chr5:24737084-24738975 REVERSE LENGTH=406	411	406	0	98.8	86.6	90.8
Rsa1.0_00056.1.g3070.t2	gb|AAO85536.1| terpene synthase [Arabidopsis thaliana]	546	603	0	110.4	60.4	79.7	terpene synthase	gbpln	Arabidopsis thaliana	AT3G29410.1 | Symbols: | Terpenoid cyclases/Protein prenyltransferases superfamily protein | chr3:11302574-11305060 REVERSE LENGTH=603	546	603	0	110.4	61.0	78.8
Rsa1.0_00056.1.g3071.t1	gb|ACR48179.1| MYB domain protein 28-2 [Brassica rapa subsp. pekinensis]	361	357	0	98.9	90.6	93.1	MYB domain protein 28-2	gbpln	Brassica rapa	AT5G61420.2 | Symbols: MYB28, HAG1 | myb domain protein 28 | chr5:24689475-24690795 REVERSE LENGTH=366	361	366	1.00E-166	101.4	80.9	88.6
Rsa1.0_00056.1.g3072.t1	#	#	#	#	#	#	#	-	----	----	AT5G61412.1 | Symbols: | unknown protein; FUNCTIONS IN: molecular_function unknown; INVOLVED IN: biological_process unknown; LOCATED IN: cellular_component unknown; BEST Arabidopsis thaliana protein match is: unknown protein (TAIR:AT4G33145.1); Has 30201 Blast hits to 17322 proteins in 780 species: Archae - 12; Bacteria - 1396; Metazoa - 17338; Fungi - 3422; Plants - 5037; Viruses - 0; Other Eukaryotes - 2996 (source: NCBI BLink). | chr5:24686761-24686994 REVERSE LENGTH=77	76	77	3.00E-13	101.3	43.4	46.1
Rsa1.0_00056.1.g3073.t1	ref|XP_002864731.1| pentatricopeptide repeat-containing protein [Arabidopsis lyrata subsp. lyrata] gi|297310566|gb|EFH40990.1| pentatricopeptide repeat-containing protein [Arabidopsis lyrata subsp. lyrata]	645	732	0	113.5	76.4	86.7	pentatricopeptide repeat-containing protein	gbpln	Arabidopsis lyrata	AT5G61400.1 | Symbols: | Pentatricopeptide repeat (PPR) superfamily protein | chr5:24681550-24683514 FORWARD LENGTH=654	645	654	0	101.4	76.0	85.4
Rsa1.0_00056.1.g3074.t1	#	#	#	#	#	#	#	-	----	----	#	#	#	#	#	#	#

Rsa1.0_00056.1.g3075.t1	ref|NP_200947.3| exonuclease-like protein [Arabidopsis thaliana] gi|111074224|gb|ABH04485.1| At5g61390 [Arabidopsis thaliana] gi|332010078|gb|AED97461.1| exonuclease-like protein [Arabidopsis thaliana]	335	487	1.00E-145	145.4	77.3	84.8	exonuclease-like protein	gbpln	Arabidopsis thaliana	AT5G61390.1 | Symbols: | Polynucleotidyl transferase, ribonuclease H-like superfamily protein | chr5:24678802-24680857 REVERSE LENGTH=487	335	487	1.00E-148	145.4	77.3	84.8
Rsa1.0_00056.1.g3076.t1	gb|ADA58344.1| pseudo-response regulator 1b [Brassica rapa]	549	509	0	92.7	83.6	86.2	pseudo-response regulator 1b	gbpln	Brassica rapa	AT5G61380.1 | Symbols: TOC1, APRR1, PRR1, AtTOC1 | CCT motif -containing response regulator protein | chr5:24675540-24678176 FORWARD LENGTH=618	549	618	0	112.6	84.3	89.8
Rsa1.0_00056.1.g3077.t1	gb|EOA14352.1| hypothetical protein CARUB_v10027533mg [Capsella rubella]	836	842	0	100.7	88.2	93.9	hypothetical protein CARUB_v10027533mg	gbpln	Capsella rubella	AT5G61350.1 | Symbols: | Protein kinase superfamily protein | chr5:24667973-24670501 FORWARD LENGTH=842	836	842	0	100.7	87.6	92.9
Rsa1.0_00056.1.g3078.t1	gb|EOA13720.1| hypothetical protein CARUB_v10026789mg [Capsella rubella]	319	322	1.00E-131	100.9	80.3	89.7	hypothetical protein CARUB_v10026789mg	gbpln	Capsella rubella	AT5G61340.1 | Symbols: | unknown protein; BEST Arabidopsis thaliana protein match is: unknown protein (TAIR:AT1G26650.1); Has 30201 Blast hits to 17322 proteins in 780 species: Archae - 12; Bacteria - 1396; Metazoa - 17338; Fungi - 3422; Plants - 5037; Viruses - 0; Other Eukaryotes - 2996 (source: NCBI BLink). | chr5:24662064-24663044 REVERSE LENGTH=326	319	326	1.00E-132	102.2	82.4	89.7
Rsa1.0_00056.1.g3079.t1	gb|EOA15044.1| hypothetical protein CARUB_v10028402mg [Capsella rubella]	190	447	2.00E-19	235.3	23.7	24.7	hypothetical protein CARUB_v10028402mg	gbpln	Capsella rubella	AT5G61330.1 | Symbols: | rRNA processing protein-related | chr5:24657287-24660103 REVERSE LENGTH=436	190	436	3.00E-21	229.5	23.2	23.7
Rsa1.0_00056.1.g3080.t1	gb|EOA13427.1| hypothetical protein CARUB_v10026466mg [Capsella rubella]	390	423	1.00E-82	108.5	51.5	65.4	hypothetical protein CARUB_v10026466mg	gbpln	Capsella rubella	AT5G62510.1 | Symbols: | F-box family protein | chr5:25095373-25096635 REVERSE LENGTH=420	390	420	3.00E-80	107.7	49.2	62.6
Rsa1.0_00056.1.g3081.t1	#	#	#	#	#	#	#	-	----	----	#	#	#	#	#	#	#
Rsa1.0_00056.1.g3082.t1	gb|AGJ70091.1| LRR-like disease resistance protein [Brassica oleracea var. italica]	327	326	1.00E-178	99.7	93.3	95.4	LRR-like disease resistance protein	gbpln	Brassica oleracea	AT5G61240.1 | Symbols: | Leucine-rich repeat (LRR) family protein | chr5:24629485-24631958 FORWARD LENGTH=326	327	326	1.00E-179	99.7	92.0	95.1
Rsa1.0_00056.1.g3083.t1	gb|EOA14101.1| hypothetical protein CARUB_v10027241mg [Capsella rubella]	241	173	4.00E-81	71.8	60.2	63.1	hypothetical protein CARUB_v10027241mg	gbpln	Capsella rubella	AT5G61230.1 | Symbols: | Ankyrin repeat family protein | chr5:24628254-24628778 FORWARD LENGTH=174	241	174	5.00E-81	72.2	58.1	61.4
Rsa1.0_00056.1.g3084.t2	ref|XP_002864722.1| hypothetical protein ARALYDRAFT_496271 [Arabidopsis lyrata subsp. lyrata] gi|297310557|gb|EFH40981.1| hypothetical protein ARALYDRAFT_496271 [Arabidopsis lyrata subsp. lyrata]	707	955	1.00E-165	135.1	59.1	68.7	hypothetical protein ARALYDRAFT_496271	gbpln	Arabidopsis lyrata	AT5G61190.1 | Symbols: | putative endonuclease or glycosyl hydrolase with C2H2-type zinc finger domain | chr5:24615480-24619886 FORWARD LENGTH=995	707	995	1.00E-143	140.7	50.4	59.7
Rsa1.0_00056.1.g3085.t1	gb|EOA14082.1| hypothetical protein CARUB_v10027219mg, partial [Capsella rubella]	143	180	1.00E-75	125.9	95.8	96.5	hypothetical protein CARUB_v10027219mg, partial	gbpln	Capsella rubella	AT5G61170.1 | Symbols: | Ribosomal protein S19e family protein | chr5:24611158-24612202 FORWARD LENGTH=143	143	143	9.00E-78	100.0	96.5	96.5
Rsa1.0_00056.1.g3086.t2	gb|EOA13076.1| hypothetical protein CARUB_v10026081mg [Capsella rubella]	661	626	0	94.7	76.7	83.1	hypothetical protein CARUB_v10026081mg	gbpln	Capsella rubella	AT5G61150.2 | Symbols: VIP4 | leo1-like family protein | chr5:24603846-24607528 REVERSE LENGTH=623	661	623	0	94.3	75.6	82.6
Rsa1.0_00056.1.g3087.t1	gb|EOA14034.1| hypothetical protein CARUB_v10027167mg [Capsella rubella]	199	198	3.00E-78	99.5	76.4	84.4	hypothetical protein CARUB_v10027167mg	gbpln	Capsella rubella	AT5G61130.1 | Symbols: PDCB1 | plasmodesmata callose-binding protein 1 | chr5:24587438-24589369 REVERSE LENGTH=201	199	201	1.00E-67	101.0	74.4	80.4
Rsa1.0_00056.1.g3088.t1	ref|XP_002866412.1| hypothetical protein ARALYDRAFT_919345 [Arabidopsis lyrata subsp. lyrata] gi|297312247|gb|EFH42671.1| hypothetical protein ARALYDRAFT_919345 [Arabidopsis lyrata subsp. lyrata]	681	660	0	96.9	79.0	85.5	hypothetical protein ARALYDRAFT_919345	gbpln	Arabidopsis lyrata	AT5G61060.1 | Symbols: HDA05, HDA5, ATHDA5 | histone deacetylase 5 | chr5:24567137-24570917 REVERSE LENGTH=660	681	660	0	96.9	78.1	85.3
Rsa1.0_00056.1.g3089.t1	ref|XP_002866412.1| hypothetical protein ARALYDRAFT_919345 [Arabidopsis lyrata subsp. lyrata] gi|297312247|gb|EFH42671.1| hypothetical protein ARALYDRAFT_919345 [Arabidopsis lyrata subsp. lyrata]	223	660	8.00E-96	296.0	73.1	84.3	hypothetical protein ARALYDRAFT_919345	gbpln	Arabidopsis lyrata	AT5G61060.2 | Symbols: HDA05 | histone deacetylase 5 | chr5:24567137-24570917 REVERSE LENGTH=664	223	664	3.00E-98	297.8	74.0	84.3
Rsa1.0_00056.1.g3090.t1	gb|EOA13791.1| hypothetical protein CARUB_v10026886mg [Capsella rubella]	254	295	5.00E-55	116.1	41.3	44.9	hypothetical protein CARUB_v10026886mg	gbpln	Capsella rubella	AT5G61030.1 | Symbols: GR-RBP3 | glycine-rich RNA-binding protein 3 | chr5:24560870-24562152 FORWARD LENGTH=309	254	309	2.00E-55	121.7	41.3	45.7

Rsa1.0_00056.1.g3091.t1	ref|NP_200908.1| Replication factor-A protein 1-like protein [Arabidopsis thaliana] gi|10177323|dbj|BAB10649.1| replication protein A1 [Arabidopsis thaliana] gi|28393124|gb|AAO41995.1| putative RNA helicase [Arabidopsis thaliana] gi|332010026|gb|AED97409.1| Replication factor-A protein 1-like protein [Arabidopsis thaliana]	626	629	0	100.5	90.3	95.7	Replication factor-A protein 1-like protein	gbpln	Arabidopsis thaliana	AT5G61000.1 | Symbols: RPA70D, ATRPA70D | Replication factor-A protein 1-related | chr5:24549682-24552641 REVERSE LENGTH=629	626	629	0	100.5	90.3	95.7
Rsa1.0_00056.1.g3092.t1	ref|NP_200906.2| nuclear transport factor 2 and RNA recognition motif domain-containing protein [Arabidopsis thaliana] gi|10177321|dbj|BAB10647.1| unnamed protein product [Arabidopsis thaliana] gi|17063173|gb|AAL32982.1| AT5g60980/MSL3_100 [Arabidopsis thaliana] gi|27764908|gb|AAO23575.1| At5g60980/MSL3_100 [Arabidopsis thaliana] gi|332010024|gb|AED97407.1| nuclear transport factor 2 and RNA recognition motif domain-containing protein [Arabidopsis thaliana]	460	460	1.00E-171	100.0	77.8	84.3	nuclear transport factor 2 and RNA recognition motif domain-containing protein	gbpln	Arabidopsis thaliana	AT5G60980.2 | Symbols: | Nuclear transport factor 2 (NTF2) family protein with RNA binding (RRM-RBD-RNP motifs) domain | chr5:24543988-24546027 FORWARD LENGTH=460	460	460	1.00E-173	100.0	77.8	84.3
Rsa1.0_00056.1.g3093.t1	gb|EOA13591.1| hypothetical protein CARUB_v10026656mg [Capsella rubella]	341	359	1.00E-145	105.3	83.0	88.3	hypothetical protein CARUB_v10026656mg	gbpln	Capsella rubella	AT5G60970.1 | Symbols: TCP5 | TEOSINTE BRANCHED 1, cycloidea and PCF transcription factor 5 | chr5:24535570-24536652 REVERSE LENGTH=360	341	360	1.00E-138	105.6	80.6	86.8
Rsa1.0_00056.1.g3094.t1	gb|EOA14836.1| hypothetical protein CARUB_v10028146mg [Capsella rubella]	206	204	3.00E-89	99.0	75.7	82.5	hypothetical protein CARUB_v10028146mg	gbpln	Capsella rubella	AT5G60950.1 | Symbols: COBL5 | COBRA-like protein 5 precursor | chr5:24527157-24528005 REVERSE LENGTH=204	206	204	3.00E-89	99.0	74.8	84.0
Rsa1.0_00056.1.g3095.t1	gb|EOA13179.1| hypothetical protein CARUB_v10026206mg, partial [Capsella rubella]	456	537	0	117.8	92.8	96.3	hypothetical protein CARUB_v10026206mg, partial	gbpln	Capsella rubella	AT5G60920.1 | Symbols: COB | COBRA-like extracellular glycosyl-phosphatidyl inositol-anchored protein family | chr5:24511466-24513932 REVERSE LENGTH=456	456	456	0	100.0	90.8	94.1
Rsa1.0_00056.1.g3096.t3	ref|XP_002889590.1| hypothetical protein ARALYDRAFT_887817 [Arabidopsis lyrata subsp. lyrata] gi|297335432|gb|EFH65849.1| hypothetical protein ARALYDRAFT_887817 [Arabidopsis lyrata subsp. lyrata]	431	341	2.00E-53	79.1	32.5	37.6	hypothetical protein ARALYDRAFT_887817	gbpln	Arabidopsis lyrata	#	#	#	#	#	#	#
Rsa1.0_00056.1.g3097.t1	gb|AAC28221.1| similar to reverse transcriptases (PFam: rvt.hmm, score: 60.13) [Arabidopsis thaliana]	522	1164	1.00E-105	223.0	37.4	54.6	similar to reverse transcriptases (PFam: rvt.hmm, score: 60.13)	gbpln	Arabidopsis thaliana	AT3G24255.1 | Symbols: | RNA-directed DNA polymerase (reverse transcriptase)-related family protein | chr3:8789471-8793208 FORWARD LENGTH=746	522	746	2.00E-51	142.9	23.9	36.4
Rsa1.0_00056.1.g3098.t1	emb|CAD47852.1| MADS-box protein FUL-d [Brassica oleracea var. botrytis] gi|89279392|gb|ABD67165.1| Ful-like protein [Brassica napus]	162	242	4.00E-85	149.4	98.1	98.1	MADS-box protein FUL-d	gbpln	Brassica napus	AT5G60910.1 | Symbols: AGL8, FUL | AGAMOUS-like 8 | chr5:24502736-24506013 REVERSE LENGTH=242	162	242	2.00E-86	149.4	96.3	97.5
Rsa1.0_00056.1.g3099.t1	sp|Q39202.2|RLK1_ARATH RecName: Full=G-type lectin S-receptor-like serine/threonine-protein kinase RLK1; AltName: Full=Receptor-like protein kinase 1; Flags: Precursor	821	832	0	101.3	68.5	79.4	RecName: Full=G-type lectin S-receptor-like serine/threonine-protein kinase RLK1; AltName: Full=Receptor-like protein kinase 1; Flags: Precursor	----	----	AT5G60900.1 | Symbols: RLK1 | receptor-like protein kinase 1 | chr5:24498467-24501494 REVERSE LENGTH=748	821	748	0	91.1	64.3	74.1
Rsa1.0_00056.1.g3100.t1	ref|XP_002866401.1| hypothetical protein ARALYDRAFT_496241 [Arabidopsis lyrata subsp. lyrata] gi|297312236|gb|EFH42660.1| hypothetical protein ARALYDRAFT_496241 [Arabidopsis lyrata subsp. lyrata]	467	758	1.00E-162	162.3	64.5	73.4	hypothetical protein ARALYDRAFT_496241	gbpln	Arabidopsis lyrata	AT5G60900.1 | Symbols: RLK1 | receptor-like protein kinase 1 | chr5:24498467-24501494 REVERSE LENGTH=748	467	748	1.00E-152	160.2	60.2	70.4
Rsa1.0_00056.1.g3101.t1	#	#	#	#	#	#	#	-	----	----	#	#	#	#	#	#	#
Rsa1.0_00056.1.g3102.t1	gb|ACR48184.1| MYB domain protein 34-1 [Brassica rapa subsp. pekinensis]	299	302	1.00E-143	101.0	87.0	91.6	MYB domain protein 34-1	gbpln	Brassica rapa	AT5G60890.1 | Symbols: ATMYB34, ATR1, MYB34 | myb domain protein 34 | chr5:24495029-24496220 FORWARD LENGTH=295	299	295	1.00E-117	98.7	75.3	82.6
Rsa1.0_00056.1.g3103.t1	ref|XP_002866400.1| hypothetical protein ARALYDRAFT_919327 [Arabidopsis lyrata subsp. lyrata] gi|297312235|gb|EFH42659.1| hypothetical protein ARALYDRAFT_919327 [Arabidopsis lyrata subsp. lyrata]	255	264	7.00E-91	103.5	80.0	85.9	hypothetical protein ARALYDRAFT_919327	gbpln	Arabidopsis lyrata	AT5G60880.1 | Symbols: BASL | breaking of asymmetry in the stomatal lineage | chr5:24488553-24489703 REVERSE LENGTH=262	255	262	4.00E-92	102.7	76.9	83.9

Rsa1.0_00056.1.g3104.t1	ref|NP_200893.1| Dof zinc finger protein DOF5.4 [Arabidopsis thaliana] gi|55584037|sp|Q8LDR0.2|DOF54_ARATH RecName: Full=Dof zinc finger protein DOF5.4; Short=AtDOF5.4; AltName: Full=OBF-binding protein 4 gi|10176912|dbj|BAB10105.1| zinc finger protein [Arabidopsis thaliana] gi|15912271|gb|AAL08269.1| AT5g60850/mae1_100 [Arabidopsis thaliana] gi|19547997|gb|AAL87362.1| AT5g60850/mae1_100 [Arabidopsis thaliana] gi|332010003|gb|AED97386.1| Dof zinc finger protein DOF5.4 [Arabidopsis thaliana]	252	307	2.00E-77	121.8	74.6	81.0	Dof zinc finger protein DOF5.4	gbpln	Arabidopsis thaliana	AT5G60850.1 | Symbols: OBP4 | OBF binding protein 4 | chr5:24480578-24481501 FORWARD LENGTH=307	252	307	5.00E-80	121.8	74.6	81.0
Rsa1.0_00056.1.g3105.t1	gb|EOA14747.1| hypothetical protein CARUB_v10028041mg [Capsella rubella]	183	204	1.00E-64	111.5	72.1	82.5	hypothetical protein CARUB_v10028041mg	gbpln	Capsella rubella	AT5G60830.1 | Symbols: AtbZIP70, bZIP70 | basic leucine-zipper 70 | chr5:24472639-24473319 FORWARD LENGTH=206	183	206	2.00E-65	112.6	72.1	82.0
Rsa1.0_00056.1.g3106.t1	ref|XP_002872033.1| hypothetical protein ARALYDRAFT_910314 [Arabidopsis lyrata subsp. lyrata] gi|297317870|gb|EFH48292.1| hypothetical protein ARALYDRAFT_910314 [Arabidopsis lyrata subsp. lyrata]	251	281	1.00E-101	112.0	74.5	83.3	hypothetical protein ARALYDRAFT_910314	gbpln	Arabidopsis lyrata	AT5G23070.1 | Symbols: | Thymidine kinase | chr5:7741979-7742827 FORWARD LENGTH=282	251	282	1.00E-101	112.4	75.3	83.7
Rsa1.0_00056.1.g3107.t1	ref|XP_002874111.1| hypothetical protein ARALYDRAFT_489162 [Arabidopsis lyrata subsp. lyrata] gi|297319948|gb|EFH50370.1| hypothetical protein ARALYDRAFT_489162 [Arabidopsis lyrata subsp. lyrata]	942	944	0	100.2	78.3	83.9	hypothetical protein ARALYDRAFT_489162	gbpln	Arabidopsis lyrata	AT5G23080.1 | Symbols: TGH | SWAP (Suppressor-of-White-APricot)/surp domain-containing protein | chr5:7743226-7748889 REVERSE LENGTH=930	942	930	0	98.7	80.4	86.7
Rsa1.0_00056.1.g3108.t1	ref|NP_001031927.1| protein Dr1-like protein [Arabidopsis thaliana] gi|332005738|gb|AED93121.1| protein Dr1-like protein [Arabidopsis thaliana]	163	158	9.00E-81	96.9	92.0	93.9	protein Dr1-like protein	gbpln	Arabidopsis thaliana	AT5G23090.4 | Symbols: NF-YB13 | nuclear factor Y, subunit B13 | chr5:7749391-7750203 FORWARD LENGTH=158	163	158	3.00E-83	96.9	92.0	93.9
Rsa1.0_00056.1.g3109.t1	ref|XP_002872035.1| hypothetical protein ARALYDRAFT_910318 [Arabidopsis lyrata subsp. lyrata] gi|297317872|gb|EFH48294.1| hypothetical protein ARALYDRAFT_910318 [Arabidopsis lyrata subsp. lyrata]	253	278	1.00E-113	109.9	87.4	92.9	hypothetical protein ARALYDRAFT_910318	gbpln	Arabidopsis lyrata	AT5G23100.1 | Symbols: | Protein of unknown function, DUF617 | chr5:7753557-7754390 FORWARD LENGTH=277	253	277	1.00E-111	109.5	86.6	90.1
Rsa1.0_00056.1.g3110.t1	#	#	#	#	#	#	#	-	----	----	#	#	#	#	#	#	#
Rsa1.0_00056.1.g3111.t1	#	#	#	#	#	#	#	-	----	----	#	#	#	#	#	#	#
Rsa1.0_00056.1.g3112.t1	gb|ABD64939.1| hypothetical protein 24.t00017 [Brassica oleracea]	664	442	1.00E-153	66.6	39.8	47.4	hypothetical protein 24.t00017	gbpln	Brassica oleracea	AT2G13770.1 | Symbols: | CONTAINS InterPro DOMAIN/s: Putative harbinger transposase-derived nuclease (InterPro:IPR006912); Has 451 Blast hits to 352 proteins in 33 species: Archae - 0; Bacteria - 0; Metazoa - 28; Fungi - 8; Plants - 388; Viruses - 0; Other Eukaryotes - 27 (source: NCBI BLink). | chr2:5736603-5737847 FORWARD LENGTH=343	664	343	2.00E-32	51.7	9.2	11.6
Rsa1.0_00056.1.g3113.t1	#	#	#	#	#	#	#	-	----	----	#	#	#	#	#	#	#
Rsa1.0_00056.1.g3114.t1	gb|AEF32147.1| MADS-box DNA-binding domain transcription factor [Brassica napus]	231	244	1.00E-105	105.6	86.6	91.8	MADS-box DNA-binding domain transcription factor	gbpln	Brassica napus	AT5G23260.3 | Symbols: TT16, ABS, AGL32 | K-box region and MADS-box transcription factor family protein | chr5:7836442-7838340 FORWARD LENGTH=238	231	238	1.00E-99	103.0	77.1	90.0
Rsa1.0_00056.1.g3115.t1	ref|NP_197719.1| transcription factor TCP7 [Arabidopsis thaliana] gi|75171749|sp|Q9FMX2.1|TCP7_ARATH RecName: Full=Transcription factor TCP7 gi|10177817|dbj|BAB11183.1| unnamed protein product [Arabidopsis thaliana] gi|332005764|gb|AED93147.1| transcription factor TCP7 [Arabidopsis thaliana]	209	250	9.00E-81	119.6	82.3	86.6	transcription factor TCP7	gbpln	Arabidopsis thaliana	AT5G23280.1 | Symbols: | TCP family transcription factor | chr5:7843017-7843769 FORWARD LENGTH=250	209	250	3.00E-83	119.6	82.3	86.6
Rsa1.0_00056.1.g3116.t1	gb|EOA21748.1| hypothetical protein CARUB_v10002195mg [Capsella rubella]	153	151	6.00E-72	98.7	89.5	94.8	hypothetical protein CARUB_v10002195mg	gbpln	Capsella rubella	AT5G23290.1 | Symbols: PFD5 | prefoldin 5 | chr5:7846144-7847428 FORWARD LENGTH=151	153	151	2.00E-74	98.7	89.5	94.8
Rsa1.0_00056.1.g3117.t1	gb|EOA20609.1| hypothetical protein CARUB_v10000920mg [Capsella rubella]	459	460	0	100.2	94.1	96.1	hypothetical protein CARUB_v10000920mg	gbpln	Capsella rubella	AT5G23300.1 | Symbols: PYRD | pyrimidine d | chr5:7847792-7850243 REVERSE LENGTH=460	459	460	0	100.2	93.2	96.1

Rsa1.0_00056.1.g3118.t1	ref|XP_002872050.1| hypothetical protein ARALYDRAFT_489192 [Arabidopsis lyrata subsp. lyrata] gi|297317887|gb|EFH48309.1| hypothetical protein ARALYDRAFT_489192 [Arabidopsis lyrata subsp. lyrata]	488	263	4.00E-33	53.9	21.1	23.6	hypothetical protein ARALYDRAFT_489192	gbpln	Arabidopsis lyrata	AT5G23310.1 | Symbols: FSD3 | Fe superoxide dismutase 3 | chr5:7850624-7852241 FORWARD LENGTH=263	488	263	2.00E-34	53.9	14.1	14.8
Rsa1.0_00056.1.g3119.t1	ref|XP_002874125.1| hypothetical protein ARALYDRAFT_910349 [Arabidopsis lyrata subsp. lyrata] gi|297319962|gb|EFH50384.1| hypothetical protein ARALYDRAFT_910349 [Arabidopsis lyrata subsp. lyrata]	213	219	1.00E-104	102.8	87.8	94.4	hypothetical protein ARALYDRAFT_910349	gbpln	Arabidopsis lyrata	AT5G23370.1 | Symbols: | GRAM domain-containing protein / ABA-responsive protein-related | chr5:7863542-7864201 REVERSE LENGTH=219	213	219	1.00E-103	102.8	85.0	92.0
Rsa1.0_00056.1.g3120.t1	ref|NP_197729.1| uncharacterized protein [Arabidopsis thaliana] gi|9758537|dbj|BAB08944.1| unnamed protein product [Arabidopsis thaliana] gi|332005774|gb|AED93157.1| uncharacterized protein AT5G23380 [Arabidopsis thaliana]	283	301	2.00E-76	106.4	56.2	73.1	uncharacterized protein	gbpln	Arabidopsis thaliana	AT5G23380.1 | Symbols: | Protein of unknown function (DUF789) | chr5:7866742-7870098 FORWARD LENGTH=301	283	301	6.00E-79	106.4	56.2	73.1
Rsa1.0_00056.1.g3121.t1	#	#	#	#	#	#	#	–	----	----	#	#	#	#	#	#	#
Rsa1.0_00056.1.g3122.t1	dbj|BAJ34388.1| unnamed protein product [Thellungiella halophila]	583	587	0	100.7	83.4	90.9	unnamed protein product	----	----	AT5G23400.1 | Symbols: | Leucine-rich repeat (LRR) family protein | chr5:7880603-7882372 FORWARD LENGTH=589	583	589	0	101.0	80.6	89.7
Rsa1.0_00056.1.g3123.t1	gb|EOA13406.1| hypothetical protein CARUB_v10026444mg, partial [Capsella rubella]	402	430	1.00E-105	107.0	56.7	69.2	hypothetical protein CARUB_v10026444mg, partial	gbpln	Capsella rubella	AT5G62660.1 | Symbols: | F-box and associated interaction domains-containing protein | chr5:25156326-25157465 REVERSE LENGTH=379	402	379	1.00E-101	94.3	51.5	62.2
Rsa1.0_00056.1.g3124.t1	gb|EOA21466.1| hypothetical protein CARUB_v10001858mg [Capsella rubella]	350	236	1.00E-78	67.4	52.9	57.4	hypothetical protein CARUB_v10001858mg	gbpln	Capsella rubella	AT5G23420.1 | Symbols: HMGB6 | high-mobility group box 6 | chr5:7888712-7890111 REVERSE LENGTH=241	350	241	2.00E-65	68.9	46.9	51.4
Rsa1.0_00056.1.g3125.t1	ref|NP_200428.1| LORELEI-LIKE-GPI-ANCHORED PROTEIN 1 [Arabidopsis thaliana] gi|9758637|dbj|BAB09299.1| unnamed protein product [Arabidopsis thaliana] gi|23306374|gb|AAN17414.1| putative protein [Arabidopsis thaliana] gi|24899659|gb|AAN65044.1| putative protein [Arabidopsis thaliana] gi|332009346|gb|AED96729.1| LORELEI-LIKE-GPI-ANCHORED PROTEIN 1 [Arabidopsis thaliana]	127	168	1.00E-28	132.3	48.8	60.6	LORELEI-LIKE-GPI-ANCHORED PROTEIN 1	gbpln	Arabidopsis thaliana	AT5G56170.1 | Symbols: LLG1 | LORELEI-LIKE-GPI-ANCHORED PROTEIN 1 | chr5:22736072-22737108 FORWARD LENGTH=168	127	168	2.00E-31	132.3	48.8	60.6
Rsa1.0_00056.1.g3126.t1	ref|NP_197740.2| uncharacterized protein [Arabidopsis thaliana] gi|332005792|gb|AED93175.1| uncharacterized protein AT5G23490 [Arabidopsis thaliana]	741	729	0	98.4	75.4	83.8	uncharacterized protein	gbpln	Arabidopsis thaliana	AT5G23490.1 | Symbols: | unknown protein; BEST Arabidopsis thaliana protein match is: unknown protein (TAIR:AT5G08440.1); Has 202 Blast hits to 197 proteins in 48 species: Archae - 0; Bacteria - 13; Metazoa - 25; Fungi - 9; Plants - 109; Viruses - 0; Other Eukaryotes - 46 (source: NCBI BLink). | chr5:7919831-7926499 FORWARD LENGTH=729	741	729	0	98.4	75.4	83.8
Rsa1.0_00056.1.g3127.t1	ref|NP_197744.1| carboxyesterase 18 [Arabidopsis thaliana] gi|75335430|sp|Q9LT10.1|CXE18_ARATH RecName: Full=Probable carboxylesterase 18; AltName: Full=AtCXE18 gi|8809707|dbj|BAA97248.1| unnamed protein product [Arabidopsis thaliana] gi|110742010|dbj|BAE98944.1| hypothetical protein [Arabidopsis thaliana] gi|114050665|gb|ABI49482.1| At5g23530 [Arabidopsis thaliana] gi|332005796|gb|AED93179.1| carboxyesterase 18 [Arabidopsis thaliana]	332	335	1.00E-164	100.9	83.7	92.8	carboxyesterase 18	gbpln	Arabidopsis thaliana	AT5G23530.1 | Symbols: AtCXE18, CXE18 | carboxyesterase 18 | chr5:7933366-7934373 REVERSE LENGTH=335	332	335	1.00E-167	100.9	83.7	92.8

Rsa1.0_00056.1.g3128.t1	ref|NP_680212.1| 50S ribosomal protein L24-like protein [Arabidopsis thaliana] gi|297808355|ref|XP_002872061.1| KOW domain-containing protein [Arabidopsis lyrata subsp. lyrata] gi|8809706|dbj|BAA97247.1| 50S ribosomal protein L24 [Arabidopsis thaliana] gi|44681334|gb|AAS47607.1| At5g23535 [Arabidopsis thaliana] gi|45773844|gb|AAS76726.1| At5g23535 [Arabidopsis thaliana] gi|110735795|dbj|BAE99874.1| 50S ribosomal protein L24 [Arabidopsis thaliana] gi|297317898|gb|EFH48320.1| KOW domain-containing protein [Arabidopsis lyrata subsp. lyrata] gi|332005797|gb|AED93180.1| 50S ribosomal protein L24-like protein [Arabidopsis thaliana]	201	159	6.00E-86	79.1	78.6	78.6	50S ribosomal protein L24-like protein	gbpln	Arabidopsis lyrata	AT5G23535.1 | Symbols: | KOW domain-containing protein | chr5:7935871-7937127 FORWARD LENGTH=159	201	159	2.00E-88	79.1	78.6	78.6
Rsa1.0_00056.1.g3129.t1	ref|NP_197745.1| 26S proteasome non-ATPase regulatory subunit 14 [Arabidopsis thaliana] gi|51701846|sp|Q9LT08.1|PSDE_ARATH RecName: Full=26S proteasome non-ATPase regulatory subunit 14; AltName: Full=26S proteasome regulatory subunit rpn11 gi|8809705|dbj|BAA97246.1| 26S proteasome, non-ATPase regulatory subunit [Arabidopsis thaliana] gi|17979145|gb|AAL49768.1| putative 26S proteasome, non-ATPase regulatory subunit [Arabidopsis thaliana] gi|20259105|gb|AAM14268.1| putative 26S proteasome, non-ATPase regulatory subunit [Arabidopsis thaliana] gi|32700040|gb|AAP86670.1| 26S proteasome subunit RPN11A [Arabidopsis thaliana] gi|32700042|gb|AAP86671.1| 26S proteasome subunit RPN11a [Arabidopsis thaliana] gi|32700044|gb|AAP86672.1| 26S proteasome subunit RPN11 [Arabidopsis thaliana] gi|110737189|dbj|BAF00543.1| 26S proteasome, non-ATPase regulatory subunit [Arabidopsis thaliana] gi|332005798|gb|AED93181.1| 26S proteasome non-ATPase regulatory subunit 14 [Arabidopsis thaliana]	308	308	0	100.0	100.0	100.0	26S proteasome non-ATPase regulatory subunit 14	gbpln	Arabidopsis thaliana	AT5G23540.1 | Symbols: | Mov34/MPN/PAD-1 family protein | chr5:7937772-7939339 FORWARD LENGTH=308	308	308	0	100.0	100.0	100.0
Rsa1.0_00056.1.g3130.t4	gb|EOA19564.1| hypothetical protein CARUB_v10002585mg [Capsella rubella]	210	175	1.00E-75	83.3	73.3	77.6	hypothetical protein CARUB_v10002585mg	gbpln	Capsella rubella	AT5G23550.1 | Symbols: | Got1/Sft2-like vescicle transport protein family | chr5:7939914-7941479 REVERSE LENGTH=175	210	175	7.00E-76	83.3	71.4	77.1
Rsa1.0_00056.1.g3131.t1	gb|EOA20190.1| hypothetical protein CARUB_v10000486mg [Capsella rubella]	331	616	1.00E-33	186.1	34.4	47.7	hypothetical protein CARUB_v10000486mg	gbpln	Capsella rubella	AT5G23570.1 | Symbols: SGS3, ATSGS3 | XS domain-containing protein / XS zinc finger domain-containing protein-related | chr5:7943621-7945874 FORWARD LENGTH=625	331	625	1.00E-28	188.8	19.9	24.8
Rsa1.0_00056.1.g3132.t3	ref|NP_680213.1| transmembrane CLPTM1 family protein [Arabidopsis thaliana] gi|17381232|gb|AAL36035.1| AT5g08500/MAH20_6 [Arabidopsis thaliana] gi|23506007|gb|AAN28863.1| At5g08500/MAH20_6 [Arabidopsis thaliana] gi|332005802|gb|AED93185.1| transmembrane CLPTM1 family protein [Arabidopsis thaliana]	519	593	1.00E-179	114.3	70.5	78.4	transmembrane CLPTM1 family protein	gbpln	Arabidopsis thaliana	AT5G23575.1 | Symbols: | Transmembrane CLPTM1 family protein | chr5:7946563-7950041 FORWARD LENGTH=593	519	593	0	114.3	70.5	78.4
Rsa1.0_00056.1.g3133.t1	ref|XP_002874139.1| calcium-dependent protein kinase 9 [Arabidopsis lyrata subsp. lyrata] gi|297319976|gb|EFH50398.1| calcium-dependent protein kinase 9 [Arabidopsis lyrata subsp. lyrata]	96	489	1.00E-39	509.4	84.4	95.8	calcium-dependent protein kinase 9	gbpln	Arabidopsis lyrata	AT5G23580.1 | Symbols: CDPK9, ATCDPK9, CPK12, ATCPK12 | calmodulin-like domain protein kinase 9 | chr5:7950388-7952433 REVERSE LENGTH=490	96	490	2.00E-42	510.4	84.4	95.8
Rsa1.0_00056.1.g3134.t1	gb|AAD15471.1| putative non-LTR retroelement reverse transcriptase [Arabidopsis thaliana]	230	1277	1.00E-24	555.2	24.3	26.5	putative non-LTR retroelement reverse transcriptase	gbpln	Arabidopsis thaliana	AT3G24255.1 | Symbols: | RNA-directed DNA polymerase (reverse transcriptase)-related family protein | chr3:8789471-8793208 FORWARD LENGTH=746	230	746	4.00E-26	324.3	22.2	26.5
Rsa1.0_00057.1.g3135.t1	#	#	#	#	#	#	#	-	----	----	#	#	#	#	#	#	#

Rsa1.0_00057.1.g3136.t1	gb|EOA14510.1| hypothetical protein CARUB_v10027730mg [Capsella rubella]	417	237	7.00E-75	56.8	36.0	45.1	hypothetical protein CARUB_v10027730mg	gbpln	Capsella rubella	AT5G43650.1 | Symbols: BHLH92 | basic helix-loop-helix (bHLH) DNA-binding superfamily protein | chr5:17533310-17534758 REVERSE LENGTH=247	417	247	3.00E-77	59.2	37.9	45.8
Rsa1.0_00057.1.g3137.t1	#	#	#	#	#	#	#	–	----	----	#	#	#	#	#	#	#
Rsa1.0_00057.1.g3138.t1	#	#	#	#	#	#	#	–	----	----	#	#	#	#	#	#	#
Rsa1.0_00057.1.g3139.t1	gb|EOA12931.1| hypothetical protein CARUB_v10025910mg [Capsella rubella] gi|482548738|gb|EOA12932.1| hypothetical protein CARUB_v10025910mg [Capsella rubella]	754	791	0	104.9	88.1	93.8	hypothetical protein CARUB_v10025910mg	gbpln	Capsella rubella	AT5G43670.1 | Symbols: | Sec23/Sec24 protein transport family protein | chr5:17539025-17541733 REVERSE LENGTH=794	754	794	0	105.3	88.3	93.8
Rsa1.0_00057.1.g3140.t1	#	#	#	#	#	#	#	–	----	----	#	#	#	#	#	#	#
Rsa1.0_00057.1.g3141.t1	#	#	#	#	#	#	#	–	----	----	#	#	#	#	#	#	#
Rsa1.0_00057.1.g3142.t1	emb|CAN60947.1| hypothetical protein VITISV_015758 [Vitis vinifera]	275	1306	5.00E-61	474.9	48.0	64.7	hypothetical protein VITISV_015758	gbpln	Vitis vinifera	#	#	#	#	#	#	#
Rsa1.0_00057.1.g3143.t1	gb|AAG10810.1|AC018460_4 Hypothetical protein [Arabidopsis thaliana] gi|12320956|gb|AAG50601.1|AC079605_6 hypothetical protein [Arabidopsis thaliana]	156	484	1.00E-55	310.3	63.5	71.8	Hypothetical protein	gbpln	Arabidopsis thaliana	#	#	#	#	#	#	#
Rsa1.0_00057.1.g3144.t5	emb|CAB39638.1| RNA-directed DNA polymerase-like protein [Arabidopsis thaliana] gi|7267666|emb|CAB78094.1| RNA-directed DNA polymerase-like protein [Arabidopsis thaliana]	1785	1274	0	71.4	30.5	42.4	RNA-directed DNA polymerase-like protein	gbpln	Arabidopsis thaliana	AT5G43680.2 | Symbols: | unknown protein; EXPRESSED IN: 21 plant structures; EXPRESSED DURING: 13 growth stages. | chr5:17544074-17545354 FORWARD LENGTH=232	1785	232	2.00E-79	13.0	8.7	9.9
Rsa1.0_00057.1.g3145.t1	ref|XP_002865424.1| hypothetical protein ARALYDRAFT_494656 [Arabidopsis lyrata subsp. lyrata] gi|297311259|gb|EFH41683.1| hypothetical protein ARALYDRAFT_494656 [Arabidopsis lyrata subsp. lyrata]	174	379	4.00E-35	217.8	54.6	66.7	hypothetical protein ARALYDRAFT_494656	gbpln	Arabidopsis lyrata	AT5G43420.1 | Symbols: | RING/U-box superfamily protein | chr5:17451790-17452917 FORWARD LENGTH=375	174	375	2.00E-35	215.5	51.1	64.4
Rsa1.0_00057.1.g3146.t4	gb|AAF99785.1|AC012463_2 T2E6.4 [Arabidopsis thaliana]	569	740	7.00E-46	130.1	22.3	29.5	T2E6.4	gbpln	Arabidopsis thaliana	#	#	#	#	#	#	#
Rsa1.0_00057.1.g3147.t1	gb|EOA13697.1| hypothetical protein CARUB_v10026768mg, partial [Capsella rubella]	346	330	1.00E-130	95.4	73.7	78.9	hypothetical protein CARUB_v10026768mg, partial	gbpln	Capsella rubella	AT5G43720.1 | Symbols: | Protein of unknown function (DUF2361) | chr5:17557431-17559238 REVERSE LENGTH=329	346	329	1.00E-127	95.1	73.1	76.0
Rsa1.0_00057.1.g3148.t1	ref|XP_002882671.1| hypothetical protein ARALYDRAFT_897225 [Arabidopsis lyrata subsp. lyrata] gi|297328511|gb|EFH58930.1| hypothetical protein ARALYDRAFT_897225 [Arabidopsis lyrata subsp. lyrata]	262	136	4.00E-25	51.9	21.8	28.6	hypothetical protein ARALYDRAFT_897225	gbpln	Arabidopsis lyrata	AT1G09245.1 | Symbols: | Plant self-incompatibility protein S1 family | chr1:2986948-2987367 REVERSE LENGTH=139	262	139	2.00E-15	53.1	15.3	22.1
Rsa1.0_00057.1.g3149.t1	dbj|BAB09269.1| unnamed protein product [Arabidopsis thaliana]	554	422	1.00E-115	76.2	36.8	50.2	unnamed protein product	gbpln	Arabidopsis thaliana	AT3G24255.1 | Symbols: | RNA-directed DNA polymerase (reverse transcriptase)-related family protein | chr3:8789471-8793208 FORWARD LENGTH=746	554	746	3.00E-27	134.7	13.4	24.4
Rsa1.0_00057.1.g3150.t1	gb|AAF79618.1|AC027665_19 F5M15.26 [Arabidopsis thaliana]	258	1838	2.00E-59	712.4	43.0	50.4	F5M15.26	gbpln	Arabidopsis thaliana	#	#	#	#	#	#	#
Rsa1.0_00057.1.g3151.t1	ref|NP_199189.1| 3-ketoacyl-CoA synthase 19 [Arabidopsis thaliana] gi|75262422|sp|Q9FG87.1|KCS19_ARATH RecName: Full=3-ketoacyl-CoA synthase 19; Short=KCS-19; AltName: Full=Very long-chain fatty acid condensing enzyme 19; Short=VLCFA condensing enzyme 19 gi|15983491|gb|AAL11613.1|AF424620_1 AT5g43760/MQD19_11 [Arabidopsis thaliana] gi|10177945|dbj|BAB11304.1| beta-ketoacyl-CoA synthase [Arabidopsis thaliana] gi|23297625|gb|AAN12994.1| beta-ketoacyl-CoA synthase [Arabidopsis thaliana] gi|332007624|gb|AED95007.1| 3-ketoacyl-CoA synthase 19 [Arabidopsis thaliana]	704	529	0	75.1	69.9	71.9	3-ketoacyl-CoA synthase 19	gbpln	Arabidopsis thaliana	AT5G43760.1 | Symbols: KCS20 | 3-ketoacyl-CoA synthase 20 | chr5:17585903-17588486 FORWARD LENGTH=529	704	529	0	75.1	69.9	71.9
Rsa1.0_00057.1.g3152.t1	emb|CAA11416.1| ATP sulfurylase [Brassica juncea]	469	470	0	100.2	95.9	97.7	ATP sulfurylase	gbpln	Brassica juncea	AT5G43780.1 | Symbols: APS4 | Pseudouridine synthase/archaeosine transglycosylase-like family protein | chr5:17589631-17591480 REVERSE LENGTH=469	469	469	0	100.0	88.7	93.4

Rsa1.0_00057.1.g3153.t1	dbj|BAB08714.1| non-LTR retroelement reverse transcriptase-like protein [Arabidopsis thaliana]	377	1197	3.00E-37	317.5	27.6	39.5	non-LTR retroelement reverse transcriptase-like protein	gbpln	Arabidopsis thaliana	AT2G06845.1 | Symbols: | Beta-galactosidase related protein | chr2:2754666-2756008 FORWARD LENGTH=315	377	315	3.00E-29	83.6	21.0	26.5
Rsa1.0_00057.1.g3154.t1	#	#	#	#	#	#	#	–	----	----	#	#	#	#	#	#	#
Rsa1.0_00057.1.g3155.t1	gb|AAF18641.1|AC006228_12 F5J5.16 [Arabidopsis thaliana]	124	1024	6.00E-15	825.8	45.2	57.3	F5J5.16	gbpln	Arabidopsis thaliana	#	#	#	#	#	#	#
Rsa1.0_00057.1.g3156.t1	#	#	#	#	#	#	#	–	----	----	#	#	#	#	#	#	#
Rsa1.0_00057.1.g3157.t2	gb|AAF69169.1|AC007915_21 F27F5.21 [Arabidopsis thaliana]	536	1023	4.00E-65	190.9	26.5	35.6	F27F5.21	gbpln	Arabidopsis thaliana	AT4G29090.1 | Symbols: | Ribonuclease H-like superfamily protein | chr4:14333528-14335255 FORWARD LENGTH=575	536	575	4.00E-25	107.3	13.4	18.7
Rsa1.0_00057.1.g3158.t1	ref|XP_002865401.1| hypothetical protein ARALYDRAFT_917259 [Arabidopsis lyrata subsp. lyrata] gi|297311236|gb|EFH41660.1| hypothetical protein ARALYDRAFT_917259 [Arabidopsis lyrata subsp. lyrata]	984	983	0	99.9	94.1	96.3	hypothetical protein ARALYDRAFT_917259	gbpln	Arabidopsis lyrata	AT5G43810.2 | Symbols: ZLL | Stabilizer of iron transporter SufD / Polynucleotidyl transferase | chr5:17611939-17616562 FORWARD LENGTH=988	984	988	0	100.4	93.8	96.3
Rsa1.0_00057.1.g3159.t1	dbj|BAB11311.1| unnamed protein product [Arabidopsis thaliana]	731	680	0	93.0	73.5	82.1	unnamed protein product	gbpln	Arabidopsis thaliana	AT5G43820.1 | Symbols: | Pentatricopeptide repeat (PPR) superfamily protein | chr5:17618948-17620588 FORWARD LENGTH=546	731	546	0	74.7	58.5	65.0
Rsa1.0_00058.1.g3160.t1	ref|XP_002862428.1| predicted protein [Arabidopsis lyrata subsp. lyrata] gi|297307936|gb|EFH38686.1| predicted protein [Arabidopsis lyrata subsp. lyrata]	209	218	2.00E-76	104.3	77.0	83.3	predicted protein	gbpln	Arabidopsis lyrata	AT5G08480.2 | Symbols: | VQ motif-containing protein | chr5:2744486-2745007 FORWARD LENGTH=173	209	173	4.00E-70	82.8	71.3	75.6
Rsa1.0_00058.1.g3161.t1	gb|AAG44817.1| peroxisome biogenesis protein PEX1 [Arabidopsis thaliana]	1125	1119	0	99.5	88.0	93.2	peroxisome biogenesis protein PEX1	gbpln	Arabidopsis thaliana	AT5G08470.1 | Symbols: PEX1 | peroxisome 1 | chr5:2735925-2742731 FORWARD LENGTH=1130	1125	1130	0	100.4	88.0	93.2
Rsa1.0_00058.1.g3162.t1	ref|XP_002871339.1| predicted protein [Arabidopsis lyrata subsp. lyrata] gi|297317176|gb|EFH47598.1| predicted protein [Arabidopsis lyrata subsp. lyrata]	373	383	0	102.7	86.1	91.2	predicted protein	gbpln	Arabidopsis lyrata	AT5G08460.1 | Symbols: | GDSL-like Lipase/Acylhydrolase superfamily protein | chr5:2733220-2735351 FORWARD LENGTH=385	373	385	0	103.2	85.3	91.4
Rsa1.0_00058.1.g3163.t1	ref|NP_196462.2| uncharacterized protein [Arabidopsis thaliana] gi|30682392|ref|NP_851033.1| uncharacterized protein [Arabidopsis thaliana] gi|79327334|ref|NP_001031857.1| uncharacterized protein [Arabidopsis thaliana] gi|20259547|gb|AAM13893.1| unknown protein [Arabidopsis thaliana] gi|21689693|gb|AAM67468.1| unknown protein [Arabidopsis thaliana] gi|332003920|gb|AED91303.1| uncharacterized protein AT5G08450 [Arabidopsis thaliana] gi|332003921|gb|AED91304.1| uncharacterized protein AT5G08450 [Arabidopsis thaliana] gi|332003922|gb|AED91305.1| uncharacterized protein AT5G08450 [Arabidopsis thaliana]	474	918	1.00E-180	193.7	66.7	69.8	uncharacterized protein	gbpln	Arabidopsis thaliana	AT5G08450.3 | Symbols: | FUNCTIONS IN: molecular_function unknown; INVOLVED IN: biological_process unknown; LOCATED IN: cellular_component unknown; EXPRESSED IN: 25 plant structures; EXPRESSED DURING: 15 growth stages; CONTAINS InterPro DOMAIN/s: Histone deacetylation protein Rxt3 (InterPro:IPR013951); Has 34444 Blast hits to 20801 proteins in 1175 species: Archae - 64; Bacteria - 2390; Metazoa - 15568; Fungi - 3729; Plants - 1886; Viruses - 208; Other Eukaryotes - 10599 (source: NCBI BLink). | chr5:2727970-2732572 REVERSE LENGTH=918	474	918	0	193.7	66.7	69.8
Rsa1.0_00058.1.g3164.t1	ref|XP_003534058.1| PREDICTED: uncharacterized protein LOC100811756 [Glycine max]	453	481	1.00E-133	106.2	57.0	73.3	PREDICTED: uncharacterized protein LOC100811756	gbenv/gbpln	Glycine max	AT5G41980.1 | Symbols: | CONTAINS InterPro DOMAIN/s: Putative harbinger transposase-derived nuclease (InterPro:IPR006912); BEST Arabidopsis thaliana protein match is: unknown protein (TAIR:AT1G43722.1); Has 1807 Blast hits to 1807 proteins in 277 species: Archae - 0; Bacteria - 0; Metazoa - 736; Fungi - 347; Plants - 385; Viruses - 0; Other Eukaryotes - 339 (source: NCBI BLink). | chr5:16793765-16794889 FORWARD LENGTH=374	453	374	1.00E-58	82.6	31.6	42.8
Rsa1.0_00058.1.g3165.t1	ref|XP_002871327.1| hypothetical protein ARALYDRAFT_325441 [Arabidopsis lyrata subsp. lyrata] gi|297317164|gb|EFH47586.1| hypothetical protein ARALYDRAFT_325441 [Arabidopsis lyrata subsp. lyrata]	720	724	0	100.6	83.6	89.2	hypothetical protein ARALYDRAFT_325441	gbpln	Arabidopsis lyrata	AT5G08440.1 | Symbols: | unknown protein; FUNCTIONS IN: molecular_function unknown; INVOLVED IN: biological_process unknown; LOCATED IN: plasma membrane; EXPRESSED IN: 21 plant structures; EXPRESSED DURING: 13 growth stages; BEST Arabidopsis thaliana protein match is: unknown protein (TAIR:AT5G23490.1); Has 141 Blast hits to 139 proteins in 35 species: Archae - 0; Bacteria - 9; Metazoa - 21; Fungi - 6; Plants - 94; Viruses - 0; Other Eukaryotes - 11 (source: NCBI BLink). | chr5:2721037-2726970 FORWARD LENGTH=726	720	726	0	100.8	84.0	90.0

Rsa1.0_00058.1.g3166.t1	ref|XP_002871325.1| hypothetical protein ARALYDRAFT_487679 [Arabidopsis lyrata subsp. lyrata] gi|297317162|gb|EFH47584.1| hypothetical protein ARALYDRAFT_487679 [Arabidopsis lyrata subsp. lyrata]	554	391	1.00E-153	70.6	53.6	60.8	hypothetical protein ARALYDRAFT_487679	gbpln	Arabidopsis lyrata	AT5G08420.1 | Symbols: | RNA-binding KH domain-containing protein | chr5:2713555-2716064 FORWARD LENGTH=391	554	391	1.00E-152	70.6	55.1	61.4
Rsa1.0_00058.1.g3167.t1	gb|EOA21459.1| hypothetical protein CARUB_v10001850mg [Capsella rubella]	230	238	8.00E-91	103.5	83.5	88.3	hypothetical protein CARUB_v10001850mg	gbpln	Capsella rubella	AT5G08330.1 | Symbols: | TCP family transcription factor | chr5:2680828-2681547 FORWARD LENGTH=239	230	239	1.00E-88	103.9	80.9	85.2
Rsa1.0_00058.1.g3168.t1	gb|AAG51783.1|AC079679_3 reverse transcriptase, putative; 16838-20266 [Arabidopsis thaliana]	145	1142	6.00E-26	787.6	49.7	62.1	reverse transcriptase, putative; 16838-20266	gbpln	Arabidopsis thaliana	#	#	#	#	#	#	#
Rsa1.0_00058.1.g3169.t1	gb|EOA15137.1| hypothetical protein CARUB_v10028512mg [Capsella rubella]	80	81	3.00E-27	101.3	73.8	85.0	hypothetical protein CARUB_v10028512mg	gbpln	Capsella rubella	AT5G08315.1 | Symbols: | Defensin-like (DEFL) family protein | chr5:2676177-2676580 REVERSE LENGTH=82	80	82	5.00E-28	102.5	72.5	82.5
Rsa1.0_00058.1.g3170.t1	gb|EOA19347.1| hypothetical protein CARUB_v10000227mg [Capsella rubella]	892	830	0	93.0	74.0	82.4	hypothetical protein CARUB_v10000227mg	gbpln	Capsella rubella	AT5G08310.1 | Symbols: | Tetratricopeptide repeat (TPR)-like superfamily protein | chr5:2672756-2675254 REVERSE LENGTH=832	892	832	0	93.3	74.7	82.3
Rsa1.0_00058.1.g3171.t1	ref|XP_002871316.1| hypothetical protein ARALYDRAFT_487662 [Arabidopsis lyrata subsp. lyrata] gi|297317153|gb|EFH47575.1| hypothetical protein ARALYDRAFT_487662 [Arabidopsis lyrata subsp. lyrata]	345	347	0	100.6	95.9	98.3	hypothetical protein ARALYDRAFT_487662	gbpln	Arabidopsis lyrata	AT5G08300.1 | Symbols: | Succinyl-CoA ligase, alpha subunit | chr5:2667579-2669672 FORWARD LENGTH=347	345	347	0	100.6	95.7	98.3
Rsa1.0_00058.1.g3172.t1	gb|EEC67208.1| hypothetical protein OsI_34094 [Oryza sativa Indica Group]	142	274	9.00E-79	193.0	96.5	100.0	hypothetical protein OsI_34094	gbpln	Oryza sativa	AT5G08290.1 | Symbols: YLS8 | mRNA splicing factor, thioredoxin-like U5 snRNP | chr5:2666043-2666936 FORWARD LENGTH=142	142	142	7.00E-81	100.0	97.9	100.0
Rsa1.0_00058.1.g3173.t2	ref|XP_002873350.1| hypothetical protein ARALYDRAFT_908776 [Arabidopsis lyrata subsp. lyrata] gi|297319187|gb|EFH49609.1| hypothetical protein ARALYDRAFT_908776 [Arabidopsis lyrata subsp. lyrata]	379	382	0	100.8	92.6	95.5	hypothetical protein ARALYDRAFT_908776	gbpln	Arabidopsis lyrata	AT5G08280.1 | Symbols: HEMC | hydroxymethylbilane synthase | chr5:2663763-2665596 REVERSE LENGTH=382	379	382	0	100.8	91.3	95.0
Rsa1.0_00058.1.g3174.t1	ref|XP_002871313.1| hypothetical protein ARALYDRAFT_908774 [Arabidopsis lyrata subsp. lyrata] gi|297317150|gb|EFH47572.1| hypothetical protein ARALYDRAFT_908774 [Arabidopsis lyrata subsp. lyrata]	825	479	0	58.1	46.5	50.4	hypothetical protein ARALYDRAFT_908774	gbpln	Arabidopsis lyrata	AT5G08260.1 | Symbols: scpl35 | serine carboxypeptidase-like 35 | chr5:2657236-2661272 FORWARD LENGTH=480	825	480	0	58.2	45.8	50.1
Rsa1.0_00058.1.g3175.t2	gb|EOA20528.1| hypothetical protein CARUB_v10000841mg [Capsella rubella]	560	483	1.00E-125	86.3	40.4	45.7	hypothetical protein CARUB_v10000841mg	gbpln	Capsella rubella	AT5G08260.1 | Symbols: scpl35 | serine carboxypeptidase-like 35 | chr5:2657236-2661272 FORWARD LENGTH=480	560	480	1.00E-126	85.7	39.8	45.2
Rsa1.0_00058.1.g3176.t1	ref|XP_002873348.1| predicted protein [Arabidopsis lyrata subsp. lyrata] gi|297319185|gb|EFH49607.1| predicted protein [Arabidopsis lyrata subsp. lyrata]	263	252	4.00E-96	95.8	70.7	81.0	predicted protein	gbpln	Arabidopsis lyrata	AT5G08240.1 | Symbols: | unknown protein; BEST Arabidopsis thaliana protein match is: unknown protein (TAIR:AT5G23160.1); Has 69 Blast hits to 69 proteins in 10 species: Archae - 0; Bacteria - 1; Metazoa - 0; Fungi - 0; Plants - 68; Viruses - 0; Other Eukaryotes - 0 (source: NCBI BLink). | chr5:2651382-2652336 REVERSE LENGTH=258	263	258	2.00E-98	98.1	72.2	83.3
Rsa1.0_00058.1.g3177.t1	gb|EOA19452.1| hypothetical protein CARUB_v10001842mg [Capsella rubella]	238	240	1.00E-124	100.8	90.3	95.4	hypothetical protein CARUB_v10001842mg	gbpln	Capsella rubella	AT5G23140.1 | Symbols: CLPP2, NCLPP7 | nuclear-encoded CLP protease P7 | chr5:7783811-7784826 FORWARD LENGTH=241	238	241	1.00E-126	101.3	89.5	94.1
Rsa1.0_00058.1.g3178.t1	gb|EOA19796.1| hypothetical protein CARUB_v10000043mg [Capsella rubella] gi|482555605|gb|EOA19797.1| hypothetical protein CARUB_v10000043mg [Capsella rubella]	1399	1392	0	99.5	57.8	67.3	hypothetical protein CARUB_v10000043mg	gbpln	Capsella rubella	AT5G08230.1 | Symbols: | Tudor/PWWP/MBT domain-containing protein | chr5:2643846-2649788 REVERSE LENGTH=1445	1399	1445	0	103.3	49.0	55.2
Rsa1.0_00058.1.g3179.t1	ref|XP_002871312.1| hypothetical protein ARALYDRAFT_487656 [Arabidopsis lyrata subsp. lyrata] gi|297317149|gb|EFH47571.1| hypothetical protein ARALYDRAFT_487656 [Arabidopsis lyrata subsp. lyrata]	419	407	0	97.1	85.2	90.7	hypothetical protein ARALYDRAFT_487656	gbpln	Arabidopsis lyrata	AT5G08200.1 | Symbols: | peptidoglycan-binding LysM domain-containing protein | chr5:2638385-2640508 FORWARD LENGTH=409	419	409	0	97.6	84.7	90.0
Rsa1.0_00058.1.g3180.t1	gb|EOA21718.1| hypothetical protein CARUB_v10002155mg [Capsella rubella]	164	160	2.00E-79	97.6	86.0	92.1	hypothetical protein CARUB_v10002155mg	gbpln	Capsella rubella	AT5G23090.1 | Symbols: NF-YB13 | nuclear factor Y, subunit B13 | chr5:7749391-7750203 FORWARD LENGTH=159	164	159	1.00E-80	97.0	86.0	91.5

Rsa1.0_00058.1.g3181.t1	ref|XP_002873346.1| ribosomal protein L7Ae/L30e/S12e/Gadd45 family protein [Arabidopsis lyrata subsp. lyrata] gi|297319183|gb|EFH49605.1| ribosomal protein L7Ae/L30e/S12e/Gadd45 family protein [Arabidopsis lyrata subsp. lyrata]	156	156	3.00E-77	100.0	92.3	97.4	ribosomal protein L7Ae/L30e/S12e/Gadd45 family protein	gbpln	Arabidopsis lyrata	AT5G08180.2 | Symbols: | Ribosomal protein L7Ae/L30e/S12e/Gadd45 family protein | chr5:2631843-2633374 REVERSE LENGTH=156	156	156	3.00E-79	100.0	91.7	96.8
Rsa1.0_00058.1.g3182.t1	ref|NP_196433.1| serine/threonine protein kinase 3 [Arabidopsis thaliana] gi|11762274|gb|AAG40409.1|AF325057_1 AT5g08160 [Arabidopsis thaliana] gi|2109293|gb|AAB69123.1| serine/threonine protein kinase [Arabidopsis thaliana] gi|8346553|emb|CAB93717.1| serine/threonine protein kinase [Arabidopsis thaliana] gi|24417498|gb|AAN60359.1| unknown [Arabidopsis thaliana] gi|94442405|gb|ABF18990.1| At5g08160 [Arabidopsis thaliana] gi|332003876|gb|AED91259.1| serine/threonine protein kinase 3 [Arabidopsis thaliana]	340	347	0	102.1	93.2	95.9	serine/threonine protein kinase 3	gbpln	Arabidopsis thaliana	AT5G08160.1 | Symbols: ATPK3, PK3 | serine/threonine protein kinase 3 | chr5:2625903-2627942 REVERSE LENGTH=347	340	347	0	102.1	93.2	95.9
Rsa1.0_00058.1.g3183.t1	ref|XP_002873343.1| hypothetical protein ARALYDRAFT_908765 [Arabidopsis lyrata subsp. lyrata] gi|297319180|gb|EFH49602.1| hypothetical protein ARALYDRAFT_908765 [Arabidopsis lyrata subsp. lyrata]	134	140	4.00E-31	104.5	75.4	83.6	hypothetical protein ARALYDRAFT_908765	gbpln	Arabidopsis lyrata	AT5G08150.1 | Symbols: SOB5 | suppressor of phytochrome b 5 | chr5:2622164-2622598 REVERSE LENGTH=144	134	144	8.00E-26	107.5	71.6	80.6
Rsa1.0_00058.1.g3184.t1	emb|CAB93715.1| putative protein [Arabidopsis thaliana]	379	530	5.00E-93	139.8	59.9	71.8	putative protein	gbpln	Arabidopsis thaliana	AT5G08139.1 | Symbols: | RING/U-box superfamily protein | chr5:2616487-2617617 FORWARD LENGTH=376	379	376	2.00E-94	99.2	60.2	72.0
Rsa1.0_00058.1.g3185.t1	ref|XP_002873342.1| hypothetical protein ARALYDRAFT_908762 [Arabidopsis lyrata subsp. lyrata] gi|297319179|gb|EFH49601.1| hypothetical protein ARALYDRAFT_908762 [Arabidopsis lyrata subsp. lyrata]	551	540	0	98.0	82.4	86.8	hypothetical protein ARALYDRAFT_908762	gbpln	Arabidopsis lyrata	AT5G08130.4 | Symbols: BIM1 | basic helix-loop-helix (bHLH) DNA-binding superfamily protein | chr5:2606655-2609571 REVERSE LENGTH=529	551	529	0	96.0	79.7	84.6
Rsa1.0_00058.1.g3186.t1	ref|NP_196429.1| movement protein binding protein 2C [Arabidopsis thaliana] gi|13877923|gb|AAK44039.1|AF370224_1 putative myosin heavy chain [Arabidopsis thaliana] gi|8346549|emb|CAB93713.1| myosin heavy chain-like protein [Arabidopsis thaliana] gi|21436331|gb|AAM51335.1| putative myosin heavy chain [Arabidopsis thaliana] gi|332003866|gb|AED91249.1| movement protein binding protein 2C [Arabidopsis thaliana]	329	326	1.00E-157	99.1	83.3	92.7	movement protein binding protein 2C	gbpln	Arabidopsis thaliana	AT5G08120.1 | Symbols: MPB2C | movement protein binding protein 2C | chr5:2600743-2602678 REVERSE LENGTH=326	329	326	1.00E-160	99.1	83.3	92.7
Rsa1.0_00058.1.g3187.t1	gb|EOA22920.1| hypothetical protein CARUB_v10003654mg [Capsella rubella]	1498	1153	0	77.0	56.9	64.3	hypothetical protein CARUB_v10003654mg	gbpln	Capsella rubella	AT5G08110.1 | Symbols: | nucleic acid binding;ATP-dependent helicases;ATP binding;helicases;ATP-dependent helicases | chr5:2594854-2600114 REVERSE LENGTH=1141	1498	1141	0	76.2	54.9	62.2
Rsa1.0_00058.1.g3188.t1	gb|EOA21213.1| hypothetical protein CARUB_v10001560mg [Capsella rubella]	465	304	1.00E-101	65.4	46.7	49.2	hypothetical protein CARUB_v10001560mg	gbpln	Capsella rubella	AT5G08080.1 | Symbols: SYP132, ATSYP132 | syntaxin of plants 132 | chr5:2588532-2591106 FORWARD LENGTH=304	465	304	1.00E-102	65.4	46.0	49.0
Rsa1.0_00058.1.g3189.t1	gb|AAM64394.1| unknown [Arabidopsis thaliana]	130	131	2.00E-60	100.8	83.1	93.1	unknown	gbpln	Arabidopsis thaliana	AT5G08060.1 | Symbols: | unknown protein; FUNCTIONS IN: molecular_function unknown; INVOLVED IN: biological_process unknown; LOCATED IN: mitochondrion; EXPRESSED IN: 24 plant structures; EXPRESSED DURING: 16 growth stages; Has 42 Blast hits to 42 proteins in 17 species: Archae - 0; Bacteria - 0; Metazoa - 0; Fungi - 0; Plants - 41; Viruses - 0; Other Eukaryotes - 1 (source: NCBI BLink). | chr5:2580588-2580983 FORWARD LENGTH=131	130	131	6.00E-63	100.8	83.1	93.1
Rsa1.0_00058.1.g3190.t1	dbj|BAF81519.1| glycerophosphodiester phosphodiesterase [Brassica rapa]	379	372	0	98.2	94.7	95.8	glycerophosphodiester phosphodiesterase	gbpln	Brassica rapa	AT5G08030.1 | Symbols: | PLC-like phosphodiesterases superfamily protein | chr5:2575152-2576770 REVERSE LENGTH=372	379	372	0	98.2	84.4	89.7

Rsa1.0_00058.1.g3191.t1	gb|EOA20180.1| hypothetical protein CARUB_v10000503mg [Capsella rubella]	423	607	1.00E-180	143.5	73.3	78.5	hypothetical protein CARUB_v10000503mg	gbpln	Capsella rubella	AT5G08020.1 | Symbols: ATRPA70B, RPA70B | RPA70-kDa subunit B | chr5:2572107-2574879 FORWARD LENGTH=604	423	604	0	142.8	73.3	78.7
Rsa1.0_00058.1.g3192.t1	ref|XP_002871301.1| hypothetical protein ARALYDRAFT_487634 [Arabidopsis lyrata subsp. lyrata] gi|297317138|gb|EFH47560.1| hypothetical protein ARALYDRAFT_487634 [Arabidopsis lyrata subsp. lyrata]	573	571	1.00E-175	99.7	70.0	78.5	hypothetical protein ARALYDRAFT_487634	gbpln	Arabidopsis lyrata	AT5G08010.1 | Symbols: | unknown protein; BEST Arabidopsis thaliana protein match is: unknown protein (TAIR:AT5G61040.1); Has 5732 Blast hits to 4319 proteins in 440 species: Archae - 66; Bacteria - 397; Metazoa - 2437; Fungi - 292; Plants - 238; Viruses - 35; Other Eukaryotes - 2267 (source: NCBI BLink). | chr5:2569606-2571613 FORWARD LENGTH=566	573	566	1.00E-169	98.8	68.8	76.6
Rsa1.0_00058.1.g3193.t2	ref|XP_002871299.1| hypothetical protein ARALYDRAFT_350051 [Arabidopsis lyrata subsp. lyrata] gi|297317136|gb|EFH47558.1| hypothetical protein ARALYDRAFT_350051 [Arabidopsis lyrata subsp. lyrata]	198	194	6.00E-75	98.0	81.8	87.9	hypothetical protein ARALYDRAFT_350051	gbpln	Arabidopsis lyrata	AT5G08000.1 | Symbols: E13L3, PDCB2 | glucan endo-1,3-beta-glucosidase-like protein 3 | chr5:2563684-2565282 FORWARD LENGTH=194	198	194	4.00E-73	98.0	76.3	82.8
Rsa1.0_00058.1.g3194.t1	gb|ABC58722.1| flavonoid 3'-hydroxylase [Brassica napus] gi|84380743|gb|ABC58723.1| flavonoid 3'-hydroxylase [Brassica napus]	511	511	0	100.0	97.7	98.8	flavonoid 3'-hydroxylase	gbpln	Brassica napus	AT5G07990.1 | Symbols: TT7, CYP75B1, D501 | Cytochrome P450 superfamily protein | chr5:2560437-2562859 FORWARD LENGTH=513	511	513	0	100.4	89.0	94.3
Rsa1.0_00059.1.g3195.t1	ref|NP_177241.3| MLP-like protein 31 [Arabidopsis thaliana] gi|148872485|sp|Q941R6.2|MLP31_ARATH RecName: Full=MLP-like protein 31 gi|19424013|gb|AAL87294.1| unknown protein [Arabidopsis thaliana] gi|21689799|gb|AAM67543.1| unknown protein [Arabidopsis thaliana] gi|110740998|dbj|BAE98593.1| hypothetical protein [Arabidopsis thaliana] gi|332197005|gb|AEE35126.1| MLP-like protein 31 [Arabidopsis thaliana]	182	171	2.00E-60	94.0	61.5	75.3	MLP-like protein 31	gbpln	Arabidopsis thaliana	AT1G70840.1 | Symbols: MLP31 | MLP-like protein 31 | chr1:26713170-26714014 REVERSE LENGTH=171	182	171	9.00E-63	94.0	61.5	75.3
Rsa1.0_00059.1.g3196.t1	#	#	#	#	#	#	#	-	----	----	#	#	#	#	#	#	#
Rsa1.0_00059.1.g3197.t1	gb|EOA39173.1| hypothetical protein CARUB_v10012133mg [Capsella rubella]	632	631	0	99.8	94.5	98.1	hypothetical protein CARUB_v10012133mg	gbpln	Capsella rubella	AT1G23090.1 | Symbols: AST91, SULTR3;3 | sulfate transporter 91 | chr1:8185238-8188954 REVERSE LENGTH=631	632	631	0	99.8	93.4	97.5
Rsa1.0_00059.1.g3198.t1	ref|XP_002890553.1| hypothetical protein ARALYDRAFT_472555 [Arabidopsis lyrata subsp. lyrata] gi|297336395|gb|EFH66812.1| hypothetical protein ARALYDRAFT_472555 [Arabidopsis lyrata subsp. lyrata]	136	158	7.00E-35	116.2	77.2	83.8	hypothetical protein ARALYDRAFT_472555	gbpln	Arabidopsis lyrata	AT1G23050.1 | Symbols: | hydroxyproline-rich glycoprotein family protein | chr1:8168631-8169116 FORWARD LENGTH=161	136	161	1.00E-32	118.4	73.5	81.6
Rsa1.0_00059.1.g3199.t1	ref|NP_564187.1| hydroxyproline-rich glycoprotein family protein [Arabidopsis thaliana] gi|2462823|gb|AAB72158.1| unknown [Arabidopsis thaliana] gi|2829907|gb|AAC00615.1| Unknown protein [Arabidopsis thaliana] gi|98960877|gb|ABF58922.1| At1g23040 [Arabidopsis thaliana] gi|110738822|dbj|BAF01334.1| predicted GPI-anchored protein [Arabidopsis thaliana] gi|332192205|gb|AEE30326.1| hydroxyproline-rich glycoprotein family protein [Arabidopsis thaliana]	144	144	4.00E-41	100.0	80.6	86.8	hydroxyproline-rich glycoprotein family protein	gbpln	Arabidopsis thaliana	AT1G23040.1 | Symbols: | hydroxyproline-rich glycoprotein family protein | chr1:8165025-8165459 FORWARD LENGTH=144	144	144	1.00E-43	100.0	80.6	86.8
Rsa1.0_00059.1.g3200.t1	ref|XP_002890550.1| armadillo/beta-catenin repeat family protein [Arabidopsis lyrata subsp. lyrata] gi|297336392|gb|EFH66809.1| armadillo/beta-catenin repeat family protein [Arabidopsis lyrata subsp. lyrata]	613	612	0	99.8	89.7	94.1	armadillo/beta-catenin repeat family protein	gbpln	Arabidopsis lyrata	AT1G23030.1 | Symbols: | ARM repeat superfamily protein | chr1:8156745-8158842 FORWARD LENGTH=612	613	612	0	99.8	88.9	93.5
Rsa1.0_00059.1.g3201.t1	gb|ABG81320.1| low-phosphate root 1 [Arabidopsis thaliana]	566	581	0	102.7	88.7	93.1	low-phosphate root 1	gbpln	Arabidopsis thaliana	AT1G23010.1 | Symbols: LPR1 | Cupredoxin superfamily protein | chr1:8147353-8149580 FORWARD LENGTH=581	566	581	0	102.7	88.7	93.1
Rsa1.0_00059.1.g3202.t1	ref|NP_173713.1| heavy-metal-associated domain-containing protein [Arabidopsis thaliana] gi|124301010|gb|ABN04757.1| At1g23000 [Arabidopsis thaliana] gi|332192199|gb|AEE30320.1| heavy-metal-associated domain-containing protein [Arabidopsis thaliana]	356	358	1.00E-103	100.6	72.8	81.5	heavy-metal-associated domain-containing protein	gbpln	Arabidopsis thaliana	AT1G23000.1 | Symbols: | Heavy metal transport/detoxification superfamily protein | chr1:8143614-8144865 FORWARD LENGTH=358	356	358	1.00E-106	100.6	72.8	81.5

Rsa1.0_00059.1.g3203.t1	gb|AAN23108.2| putative farnesylated protein [Brassica rapa subsp. pekinensis]	152	152	1.00E-82	100.0	97.4	98.7	putative farnesylated protein	gbpln	Brassica rapa	AT1G22990.1 | Symbols: HIPP22 | Heavy metal transport/detoxification superfamily protein | chr1:8139221-8140045 FORWARD LENGTH=152	152	152	7.00E-83	100.0	93.4	98.0
Rsa1.0_00059.1.g3204.t1	ref|XP_002893254.1| hypothetical protein ARALYDRAFT_472548 [Arabidopsis lyrata subsp. lyrata] gi|297339096|gb|EFH69513.1| hypothetical protein ARALYDRAFT_472548 [Arabidopsis lyrata subsp. lyrata]	356	355	1.00E-161	99.7	83.4	90.7	hypothetical protein ARALYDRAFT_472548	gbpln	Arabidopsis lyrata	AT1G22970.1 | Symbols: | unknown protein; BEST Arabidopsis thaliana protein match is: unknown protein (TAIR:AT1G71150.1); Has 134 Blast hits to 132 proteins in 48 species: Archae - 0; Bacteria - 0; Metazoa - 66; Fungi - 10; Plants - 48; Viruses - 0; Other Eukaryotes - 10 (source: NCBI BLink). | chr1:8130840-8132199 REVERSE LENGTH=357	356	357	1.00E-159	100.3	82.9	90.7
Rsa1.0_00059.1.g3205.t1	gb|EOA36666.1| hypothetical protein CARUB_v10011979mg [Capsella rubella]	748	720	0	96.3	67.1	77.8	hypothetical protein CARUB_v10011979mg	gbpln	Capsella rubella	AT1G22960.1 | Symbols: | Pentatricopeptide repeat (PPR) superfamily protein | chr1:8128086-8130242 REVERSE LENGTH=718	748	718	0	96.0	68.2	78.6
Rsa1.0_00059.1.g3206.t2	ref|NP_173708.3| uncharacterized PKHD-type hydroxylase [Arabidopsis thaliana] gi|193806639|sp|Q3ED68.2|Y1295_ARATH RecName: Full=Uncharacterized PKHD-type hydroxylase At1g22950 gi|332192193|gb|AEE30314.1| uncharacterized PKHD-type hydroxylase [Arabidopsis thaliana]	389	397	1.00E-176	102.1	77.9	87.7	uncharacterized PKHD-type hydroxylase	gbpln	Arabidopsis thaliana	AT1G22950.1 | Symbols: | 2-oxoglutarate (2OG) and Fe(II)-dependent oxygenase superfamily protein | chr1:8125291-8127168 REVERSE LENGTH=397	389	397	1.00E-179	102.1	77.9	87.7
Rsa1.0_00059.1.g3207.t1	sp|O48881.1|TPS1_BRANA RecName: Full=Thiamine biosynthetic bifunctional enzyme BTH1, chloroplastic; Includes: RecName: Full=Thiamine-phosphate synthase; Short=TP synthase; Short=TPS; AltName: Full=Thiamine-phosphate pyrophosphorylase; Short=TMP pyrophosphorylase; Short=TMP-PPase; Includes: RecName: Full=Hydroxymethylpyrimidine kinase; Short=HMP kinase; Flags: Precursor gi|2746079|gb|AAC31298.1| BTH1 [Brassica napus]	526	523	0	99.4	94.1	97.0	RecName: Full=Thiamine biosynthetic bifunctional enzyme BTH1, chloroplastic; Includes: RecName: Full=Thiamine-phosphate synthase; Short=TP synthase; Short=TPS; AltName: Full=Thiamine-phosphate pyrophosphorylase; Short=TMP pyrophosphorylase; Short=TMP-PPase; Includes: RecName: Full=Hydroxymethylpyrimidine kinase; Short=HMP kinase; Flags: Precursor gi|2746079|gb|AAC31298.1| BTH1	gbpln	Brassica napus	AT1G22940.1 | Symbols: TH1, TH-1, THIE | thiamin biosynthesis protein, putative | chr1:8122384-8124908 FORWARD LENGTH=522	526	522	0	99.2	90.5	94.9
Rsa1.0_00059.1.g3208.t1	ref|XP_002890962.1| hypothetical protein ARALYDRAFT_890756 [Arabidopsis lyrata subsp. lyrata] gi|297336804|gb|EFH67221.1| hypothetical protein ARALYDRAFT_890756 [Arabidopsis lyrata subsp. lyrata]	160	159	2.00E-53	99.4	67.5	76.9	hypothetical protein ARALYDRAFT_890756	gbpln	Arabidopsis lyrata	AT1G56270.1 | Symbols: | unknown protein; Has 38 Blast hits to 38 proteins in 3 species: Archae - 0; Bacteria - 0; Metazoa - 0; Fungi - 0; Plants - 38; Viruses - 0; Other Eukaryotes - 0 (source: NCBI BLink). | chr1:21071148-21071636 REVERSE LENGTH=162	160	162	1.00E-22	101.3	40.0	57.5
Rsa1.0_00059.1.g3209.t1	ref|XP_002890545.1| T-complex protein 11 [Arabidopsis lyrata subsp. lyrata] gi|297336387|gb|EFH66804.1| T-complex protein 11 [Arabidopsis lyrata subsp. lyrata]	1203	1130	0	93.9	78.3	84.3	T-complex protein 11	gbpln	Arabidopsis lyrata	AT1G22930.1 | Symbols: | T-complex protein 11 | chr1:8117521-8121854 FORWARD LENGTH=1131	1203	1131	0	94.0	77.4	83.2
Rsa1.0_00059.1.g3210.t2	ref|XP_002893252.1| hypothetical protein ARALYDRAFT_472541 [Arabidopsis lyrata subsp. lyrata] gi|297339094|gb|EFH69511.1| hypothetical protein ARALYDRAFT_472541 [Arabidopsis lyrata subsp. lyrata]	384	357	0	93.0	85.4	88.5	hypothetical protein ARALYDRAFT_472541	gbpln	Arabidopsis lyrata	AT1G22920.1 | Symbols: CSN5A, JAB1, AJH1 | COP9 signalosome 5A | chr1:8109882-8111895 REVERSE LENGTH=357	384	357	1.00E-180	93.0	84.1	87.8
Rsa1.0_00059.1.g3211.t1	#	#	#	#	#	#	#	-	----	----	#	#	#	#	#	#	#
Rsa1.0_00059.1.g3212.t1	ref|XP_002890544.1| hypothetical protein ARALYDRAFT_889804 [Arabidopsis lyrata subsp. lyrata] gi|297336386|gb|EFH66803.1| hypothetical protein ARALYDRAFT_889804 [Arabidopsis lyrata subsp. lyrata]	244	252	1.00E-109	103.3	88.1	91.4	hypothetical protein ARALYDRAFT_889804	gbpln	Arabidopsis lyrata	AT1G22910.1 | Symbols: | RNA-binding (RRM/RBD/RNP motifs) family protein | chr1:8105808-8107659 FORWARD LENGTH=249	244	249	1.00E-111	102.0	88.5	91.0
Rsa1.0_00059.1.g3213.t1	#	#	#	#	#	#	#	-	----	----	#	#	#	#	#	#	#

Rsa1.0_00059.1.g3214.t1	ref|XP_002863772.1| disease resistance-responsive family protein [Arabidopsis lyrata subsp. lyrata] gi|297791781|ref|XP_002863775.1| disease resistance-responsive family protein [Arabidopsis lyrata subsp. lyrata] gi|297309607|gb|EFH40031.1| disease resistance-responsive family protein [Arabidopsis lyrata subsp. lyrata] gi|297309610|gb|EFH40034.1| disease resistance-responsive family protein [Arabidopsis lyrata subsp. lyrata]	185	185	8.00E-82	100.0	80.5	88.1	disease resistance-responsive family protein	gbpln	Arabidopsis lyrata	AT5G42500.1 | Symbols: | Disease resistance-responsive (dirigent-like protein) family protein | chr5:16994359-16994916 REVERSE LENGTH=185	185	185	1.00E-79	100.0	80.5	89.2
Rsa1.0_00059.1.g3215.t1	ref|XP_002863772.1| disease resistance-responsive family protein [Arabidopsis lyrata subsp. lyrata] gi|297791781|ref|XP_002863775.1| disease resistance-responsive family protein [Arabidopsis lyrata subsp. lyrata] gi|297309607|gb|EFH40031.1| disease resistance-responsive family protein [Arabidopsis lyrata subsp. lyrata] gi|297309610|gb|EFH40034.1| disease resistance-responsive family protein [Arabidopsis lyrata subsp. lyrata]	185	185	1.00E-74	100.0	76.8	83.8	disease resistance-responsive family protein	gbpln	Arabidopsis lyrata	AT5G42500.1 | Symbols: | Disease resistance-responsive (dirigent-like protein) family protein | chr5:16994359-16994916 REVERSE LENGTH=185	185	185	1.00E-69	100.0	75.1	83.2
Rsa1.0_00059.1.g3216.t1	gb|EOA39826.1| hypothetical protein CARUB_v10008493mg [Capsella rubella]	680	681	0	100.1	74.1	81.5	hypothetical protein CARUB_v10008493mg	gbpln	Capsella rubella	AT1G22882.1 | Symbols: | Galactose-binding protein | chr1:8099150-8101220 FORWARD LENGTH=660	680	660	0	97.1	71.6	79.3
Rsa1.0_00059.1.g3217.t8	gb|EOA36793.1| hypothetical protein CARUB_v10008204mg [Capsella rubella]	1812	983	0	54.2	48.0	50.8	hypothetical protein CARUB_v10008204mg	gbpln	Capsella rubella	AT1G22860.1 | Symbols: | Vacuolar sorting protein 39 | chr1:8083400-8088867 FORWARD LENGTH=961	1812	961	0	53.0	46.1	49.1
Rsa1.0_00059.1.g3218.t1	gb|EOA38177.1| hypothetical protein CARUB_v10009653mg [Capsella rubella]	326	340	1.00E-124	104.3	81.0	85.0	hypothetical protein CARUB_v10009653mg	gbpln	Capsella rubella	AT1G22850.1 | Symbols: | SNARE associated Golgi protein family | chr1:8080671-8082816 REVERSE LENGTH=344	326	344	1.00E-126	105.5	82.2	86.5
Rsa1.0_00059.1.g3219.t1	gb|EOA38639.1| hypothetical protein CARUB_v10010544mg, partial [Capsella rubella]	112	154	7.00E-59	137.5	97.3	99.1	hypothetical protein CARUB_v10010544mg, partial	gbpln	Capsella rubella	AT1G22840.1 | Symbols: CYTC-1, ATCYTC-A | CYTOCHROME C-1 | chr1:8079384-8080286 FORWARD LENGTH=114	112	114	8.00E-60	101.8	94.6	98.2
Rsa1.0_00059.1.g3220.t1	gb|EOA37595.1| hypothetical protein CARUB_v10011955mg [Capsella rubella]	130	147	4.00E-58	113.1	88.5	96.2	hypothetical protein CARUB_v10011955mg	gbpln	Capsella rubella	AT1G22810.1 | Symbols: | Integrase-type DNA-binding superfamily protein | chr1:8074535-8074969 REVERSE LENGTH=144	130	144	2.00E-57	110.8	86.2	93.1
Rsa1.0_00059.1.g3221.t2	ref|XP_002890535.1| hypothetical protein ARALYDRAFT_889792 [Arabidopsis lyrata subsp. lyrata] gi|297336377|gb|EFH66794.1| hypothetical protein ARALYDRAFT_889792 [Arabidopsis lyrata subsp. lyrata]	357	355	1.00E-177	99.4	86.6	92.4	hypothetical protein ARALYDRAFT_889792	gbpln	Arabidopsis lyrata	AT1G22800.1 | Symbols: | S-adenosyl-L-methionine-dependent methyltransferases superfamily protein | chr1:8072020-8074039 FORWARD LENGTH=355	357	355	1.00E-179	99.4	87.1	91.9
Rsa1.0_00059.1.g3222.t1	ref|XP_002890534.1| hypothetical protein ARALYDRAFT_889791 [Arabidopsis lyrata subsp. lyrata] gi|297336376|gb|EFH66793.1| hypothetical protein ARALYDRAFT_889791 [Arabidopsis lyrata subsp. lyrata]	214	214	5.00E-55	100.0	65.4	72.0	hypothetical protein ARALYDRAFT_889791	gbpln	Arabidopsis lyrata	AT1G22790.2 | Symbols: | unknown protein; FUNCTIONS IN: molecular_function unknown; INVOLVED IN: biological_process unknown; LOCATED IN: plasma membrane; EXPRESSED IN: 22 plant structures; EXPRESSED DURING: 13 growth stages; BEST Arabidopsis thaliana protein match is: unknown protein (TAIR:AT1G34010.1); Has 35333 Blast hits to 34131 proteins in 2444 species: Archae - 798; Bacteria - 22429; Metazoa - 974; Fungi - 991; Plants - 531; Viruses - 0; Other Eukaryotes - 9610 (source: NCBI BLink). | chr1:8070271-8071292 FORWARD LENGTH=216	214	216	1.00E-53	100.9	64.0	70.1
Rsa1.0_00059.1.g3223.t1	gb|AEB33730.1| GIGANTEA [Brassica rapa]	1177	1183	0	100.5	95.7	97.0	GIGANTEA	gbpln	Brassica rapa	AT1G22770.1 | Symbols: GI, FB | gigantea protein (GI) | chr1:8062398-8067447 FORWARD LENGTH=1173	1177	1173	0	99.7	91.8	94.2
Rsa1.0_00059.1.g3224.t2	ref|XP_002890530.1| hypothetical protein ARALYDRAFT_472519 [Arabidopsis lyrata subsp. lyrata] gi|297336372|gb|EFH66789.1| hypothetical protein ARALYDRAFT_472519 [Arabidopsis lyrata subsp. lyrata]	451	256	1.00E-113	56.8	45.7	48.8	hypothetical protein ARALYDRAFT_472519	gbpln	Arabidopsis lyrata	AT1G22750.1 | Symbols: | unknown protein; FUNCTIONS IN: molecular_function unknown; INVOLVED IN: biological_process unknown; LOCATED IN: vacuole; EXPRESSED IN: 23 plant structures; EXPRESSED DURING: 15 growth stages; CONTAINS InterPro DOMAIN/s: Protein of unknown function DUF1475 (InterPro:IPR009943); Has 185 Blast hits to 155 proteins in 21 species: Archae - 0; Bacteria - 8; Metazoa - 3; Fungi - 0; Plants - 64; Viruses - 0; Other Eukaryotes - 110 (source: NCBI BLink). | chr1:8050911-8052618 FORWARD LENGTH=244	451	244	1.00E-114	54.1	45.7	48.3

Rsa1.0_00059.1.g3225.t1	ref|NP_173687.1| MA3 domain-containing protein [Arabidopsis thaliana] gi|3287683|gb|AAC25511.1| Similar to apoptosis protein MA-3 gb|D50465 from Mus musculus [Arabidopsis thaliana] gi|14334598|gb|AAK59477.1| putative topoisomerase [Arabidopsis thaliana] gi|22136820|gb|AAM91754.1| putative topoisomerase [Arabidopsis thaliana] gi|332192157|gb|AEE30278.1| MA3 domain-containing protein [Arabidopsis thaliana]	687	693	0	100.9	82.5	88.9	MA3 domain-containing protein	gbpln	Arabidopsis thaliana	AT1G22730.1 | Symbols: | MA3 domain-containing protein | chr1:8046511-8048769 FORWARD LENGTH=693	687	693	0	100.9	82.5	88.9
Rsa1.0_00059.1.g3226.t1	ref|XP_002893745.1| hypothetical protein ARALYDRAFT_336372 [Arabidopsis lyrata subsp. lyrata] gi|297339587|gb|EFH70004.1| hypothetical protein ARALYDRAFT_336372 [Arabidopsis lyrata subsp. lyrata]	176	180	4.00E-75	102.3	82.4	90.9	hypothetical protein ARALYDRAFT_336372	gbpln	Arabidopsis lyrata	AT3G03773.1 | Symbols: | HSP20-like chaperones superfamily protein | chr3:951885-953490 FORWARD LENGTH=150	176	150	3.00E-29	85.2	33.5	46.0
Rsa1.0_00060.1.g3227.t1	gb|EOA23115.1| hypothetical protein CARUB_v10003908mg [Capsella rubella]	531	1282	5.00E-73	241.4	31.6	52.0	hypothetical protein CARUB_v10003908mg	gbpln	Capsella rubella	AT4G09420.1 | Symbols: | Disease resistance protein (TIR-NBS class) | chr4:5962319-5963776 REVERSE LENGTH=457	531	457	1.00E-72	86.1	35.0	50.5
Rsa1.0_00060.1.g3228.t1	#	#	#	#	#	#	#	-	----	----	#	#	#	#	#	#	#
Rsa1.0_00060.1.g3229.t1	gb|AAD49099.1|AF177535_3 contains similarity to maize transposon MuDR (GB:M76978) [Arabidopsis thaliana]	619	664	5.00E-60	107.3	26.8	41.7	contains similarity to maize transposon MuDR (GB:M76978)	gbpln	Arabidopsis thaliana	AT1G49920.1 | Symbols: | MuDR family transposase | chr1:18481798-18484233 REVERSE LENGTH=785	619	785	7.00E-19	126.8	11.1	19.2
Rsa1.0_00060.1.g3230.t1	gb|AAG51783.1|AC079679_3 reverse transcriptase, putative; 16838-20266 [Arabidopsis thaliana]	350	1142	3.00E-88	326.3	48.3	63.7	reverse transcriptase, putative; 16838-20266	gbpln	Arabidopsis thaliana	AT4G29090.1 | Symbols: | Ribonuclease H-like superfamily protein | chr4:14333528-14335255 FORWARD LENGTH=575	350	575	3.00E-34	164.3	29.1	48.6
Rsa1.0_00060.1.g3231.t1	ref|XP_002888896.1| predicted protein [Arabidopsis lyrata subsp. lyrata] gi|297334737|gb|EFH65155.1| predicted protein [Arabidopsis lyrata subsp. lyrata]	1140	1169	0	102.5	50.2	64.7	predicted protein	gbpln	Arabidopsis lyrata	AT1G72840.2 | Symbols: | Disease resistance protein (TIR-NBS-LRR class) | chr1:27409504-27413485 REVERSE LENGTH=1183	1140	1183	0	103.8	48.1	62.8
Rsa1.0_00060.1.g3232.t1	ref|XP_002887455.1| predicted protein [Arabidopsis lyrata subsp. lyrata] gi|297333296|gb|EFH63714.1| predicted protein [Arabidopsis lyrata subsp. lyrata]	419	412	1.00E-145	98.3	65.9	77.1	predicted protein	gbpln	Arabidopsis lyrata	AT1G72850.1 | Symbols: | Disease resistance protein (TIR-NBS class) | chr1:27413971-27415335 FORWARD LENGTH=422	419	422	1.00E-130	100.7	59.9	73.7
Rsa1.0_00060.1.g3233.t1	ref|NP_001185386.1| TIR-NBS-LRR class disease resistance protein [Arabidopsis thaliana] gi|332197260|gb|AEE35381.1| TIR-NBS-LRR class disease resistance protein [Arabidopsis thaliana]	1087	1183	0	108.8	52.3	66.1	TIR-NBS-LRR class disease resistance protein	gbpln	Arabidopsis thaliana	AT1G72840.2 | Symbols: | Disease resistance protein (TIR-NBS-LRR class) | chr1:27409504-27413485 REVERSE LENGTH=1183	1087	1183	0	108.8	52.3	66.1
Rsa1.0_00060.1.g3234.t1	emb|CAA74050.1| Transcription factor [Arabidopsis thaliana]	336	340	1.00E-134	101.2	76.5	83.0	Transcription factor	gbpln	Arabidopsis thaliana	AT1G72830.1 | Symbols: HAP2C, ATHAP2C, NF-YA3 | nuclear factor Y, subunit A3 | chr1:27405699-27407088 REVERSE LENGTH=340	336	340	1.00E-137	101.2	76.8	83.6
Rsa1.0_00060.1.g3235.t1	gb|EOA35384.1| hypothetical protein CARUB_v10020581mg [Capsella rubella] gi|482571197|gb|EOA35385.1| hypothetical protein CARUB_v10020581mg [Capsella rubella]	342	346	0	101.2	91.8	95.0	hypothetical protein CARUB_v10020581mg	gbpln	Capsella rubella	AT1G72820.1 | Symbols: | Mitochondrial substrate carrier family protein | chr1:27403457-27404506 FORWARD LENGTH=349	342	349	0	102.0	93.6	96.5
Rsa1.0_00060.1.g3236.t1	gb|EOA34998.1| hypothetical protein CARUB_v10020090mg [Capsella rubella]	532	550	1.00E-166	103.4	74.6	82.3	hypothetical protein CARUB_v10020090mg	gbpln	Capsella rubella	AT1G72790.1 | Symbols: | hydroxyproline-rich glycoprotein family protein | chr1:27394848-27396533 FORWARD LENGTH=561	532	561	1.00E-166	105.5	73.9	81.4

Rsa1.0_00060.1.g3237.t1	ref|NP_177417.1| translation initiation factor 4A-3 [Arabidopsis thaliana] gi|75333652|sp|Q9CAI7.1|IF4A3_ARATH RecName: Full=Eukaryotic initiation factor 4A-3; Short=eIF-4A-3; AltName: Full=ATP-dependent RNA helicase eIF4A-3; AltName: Full=DEAD-box ATP-dependent RNA helicase 23 gi|12323781|gb|AAG51861.1|AC010926_24 putative Eukaryotic initiation factor 4A; 30924-32477 [Arabidopsis thaliana] gi|16974629|gb|AAL31217.1| At1g72730/F28P22_8 [Arabidopsis thaliana] gi|21593752|gb|AAM65719.1| putative Eukaryotic initiation factor 4A [Arabidopsis thaliana] gi|22655476|gb|AAM98330.1| At1g72730/F28P22_8 [Arabidopsis thaliana] gi|332197244|gb|AEE35365.1| translation initiation factor 4A-3 [Arabidopsis thaliana]	108	414	5.00E-17	383.3	35.2	40.7	translation initiation factor 4A-3	gbpln	Arabidopsis thaliana	AT1G72730.1 | Symbols: | DEA(D/H)-box RNA helicase family protein | chr1:27378040-27379593 REVERSE LENGTH=414	108	414	8.00E-20	383.3	35.2	40.7
Rsa1.0_00060.1.g3238.t1	ref|NP_177411.1| IQ-domain 8 protein [Arabidopsis thaliana] gi|12323773|gb|AAG51853.1|AC010926_16 hypothetical protein; 51860-53619 [Arabidopsis thaliana] gi|38016019|gb|AAR07516.1| At1g72670 [Arabidopsis thaliana] gi|51969960|dbj|BAD43672.1| unknown protein [Arabidopsis thaliana] gi|332197238|gb|AEE35359.1| IQ-domain 8 protein [Arabidopsis thaliana]	165	414	2.00E-28	250.9	58.8	68.5	IQ-domain 8 protein	gbpln	Arabidopsis thaliana	AT1G72670.1 | Symbols: iqd8 | IQ-domain 8 | chr1:27356898-27358657 REVERSE LENGTH=414	165	414	6.00E-31	250.9	58.8	68.5
Rsa1.0_00060.1.g3239.t1	ref|XP_002887444.1| hypothetical protein ARALYDRAFT_476400 [Arabidopsis lyrata subsp. lyrata] gi|297333285|gb|EFH63703.1| hypothetical protein ARALYDRAFT_476400 [Arabidopsis lyrata subsp. lyrata]	576	627	0	108.9	70.7	78.5	hypothetical protein ARALYDRAFT_476400	gbpln	Arabidopsis lyrata	AT1G72650.2 | Symbols: TRFL6 | TRF-like 6 | chr1:27350253-27353483 FORWARD LENGTH=630	576	630	0	109.4	69.1	78.3
Rsa1.0_00060.1.g3240.t1	gb|EOA33816.1| hypothetical protein CARUB_v10021290mg [Capsella rubella]	337	338	1.00E-159	100.3	81.0	92.3	hypothetical protein CARUB_v10021290mg	gbpln	Capsella rubella	AT1G72620.1 | Symbols: | alpha/beta-Hydrolases superfamily protein | chr1:27341079-27342401 FORWARD LENGTH=335	337	335	1.00E-159	99.4	80.7	91.7
Rsa1.0_00060.1.g3241.t1	ref|NP_177405.1| germin-like protein subfamily 3 member 1 [Arabidopsis thaliana] gi|18202460|sp|P94040.1|GL31_ARATH RecName: Full=Germin-like protein subfamily 3 member 1; Short=At-GERM1; Short=AtGER1; Short=AtGLP1; Flags: Precursor gi|12323768|gb|AAG51848.1|AC010926_11 germin-like protein; 70589-71215 [Arabidopsis thaliana] gi|1755156|gb|AAB51567.1| germin-like protein [Arabidopsis thaliana] gi|1755158|gb|AAB51568.1| germin-like protein [Arabidopsis thaliana] gi|1755170|gb|AAB51574.1| germin-like protein [Arabidopsis thaliana] gi|1755172|gb|AAB51575.1| germin-like protein [Arabidopsis thaliana] gi|1755180|gb|AAB51579.1| germin-like protein [Arabidopsis thaliana] gi|1755190|gb|AAB51584.1| germin-like protein [Arabidopsis thaliana] gi|1934728|gb|AAB51751.1| germin-like protein [Arabidopsis thaliana] gi|4154285|gb|AAD05223.1| germin-like protein 1 [Arabidopsis thaliana] gi|4666248|dbj|BAA77207.1| germin-like protein precursor [Arabidopsis thaliana] gi|21554080|gb|AAM63161.1| germin-like protein [Arabidopsis thaliana] gi|24417314|gb|AAN60267.1| unknown [Arabidopsis thaliana] gi|332197227|gb|AEE35248.1| germin-	208	208	5.00E-98	100.0	91.8	94.7	germin-like protein subfamily 3 member 1	gbpln	Arabidopsis thaliana	AT1G72610.1 | Symbols: GLP1, ATGER1, GER1 | germin-like protein 1 | chr1:27339302-27339928 REVERSE LENGTH=208	208	208	1.00E-100	100.0	91.8	94.7
Rsa1.0_00060.1.g3242.t1	gb|EOA34366.1| hypothetical protein CARUB_v10021890mg [Capsella rubella]	392	448	0	114.3	86.2	94.1	hypothetical protein CARUB_v10021890mg	gbpln	Capsella rubella	AT1G72540.1 | Symbols: | Protein kinase superfamily protein | chr1:27314932-27316669 REVERSE LENGTH=450	392	450	0	114.8	87.2	94.9

Rsa1.0_00060.1.g3243.t1	gb|EOA35762.1| hypothetical protein CARUB_v10020995mg [Capsella rubella]	295	188	7.00E-54	63.7	34.6	37.6	hypothetical protein CARUB_v10020995mg	gbpln	Capsella rubella	AT1G72530.1 | Symbols: | plastid developmental protein DAG, putative | chr1:27312999-27313937 FORWARD LENGTH=188	295	188	2.00E-55	63.7	33.2	38.0
Rsa1.0_00060.1.g3244.t1	gb|AAD32898.1| putative retroelement pol polyprotein [Arabidopsis thaliana]	1362	1307	0	96.0	60.4	74.0	putative retroelement pol polyprotein	gbpln	Arabidopsis thaliana	AT4G23160.1 | Symbols: CRK8 | cysteine-rich RLK (RECEPTOR-like protein kinase) 8 | chr4:12129485-12134086 FORWARD LENGTH=1262	1362	1262	1.00E-102	92.7	14.3	22.5
Rsa1.0_00060.1.g3245.t1	gb|EOA35532.1| hypothetical protein CARUB_v10020740mg [Capsella rubella]	291	288	1.00E-135	99.0	85.6	90.7	hypothetical protein CARUB_v10020740mg	gbpln	Capsella rubella	AT1G72490.1 | Symbols: | unknown protein; BEST Arabidopsis thaliana protein match is: unknown protein (TAIR:AT1G17400.1); Has 75 Blast hits to 75 proteins in 21 species: Archae - 0; Bacteria - 0; Metazoa - 3; Fungi - 4; Plants - 58; Viruses - 0; Other Eukaryotes - 10 (source: NCBI BLink). | chr1:27289435-27290732 REVERSE LENGTH=287	291	287	1.00E-134	98.6	83.5	89.7
Rsa1.0_00060.1.g3246.t1	ref|NP_177391.1| exocyst subunit exo70-D1 [Arabidopsis thaliana] gi|12325284|gb|AAG52591.1|AC016529_22 unknown protein; 29470-27569 [Arabidopsis thaliana] gi|332197207|gb|AEE35328.1| exocyst subunit exo70-D1 [Arabidopsis thaliana]	629	633	0	100.6	89.7	96.0	exocyst subunit exo70-D1	gbpln	Arabidopsis thaliana	AT1G72470.1 | Symbols: ATEXO70D1, EXO70D1 | exocyst subunit exo70 family protein D1 | chr1:27283759-27285660 FORWARD LENGTH=633	629	633	0	100.6	89.7	96.0
Rsa1.0_00060.1.g3247.t1	gb|EOA34527.1| hypothetical protein CARUB_v10022071mg [Capsella rubella]	649	644	0	99.2	75.7	86.1	hypothetical protein CARUB_v10022071mg	gbpln	Capsella rubella	AT1G72460.1 | Symbols: | Leucine-rich repeat protein kinase family protein | chr1:27279510-27281533 FORWARD LENGTH=644	649	644	0	99.2	74.7	85.7
Rsa1.0_00060.1.g3248.t1	gb|AFC38438.1| small auxin up RNA protein [Sinapis alba]	118	118	5.00E-62	100.0	98.3	99.2	small auxin up RNA protein	gbpln	Sinapis alba	AT1G72430.1 | Symbols: | SAUR-like auxin-responsive protein family | chr1:27265150-27265509 REVERSE LENGTH=119	118	119	3.00E-61	100.8	94.9	97.5
Rsa1.0_00060.1.g3249.t1	sp|Q9ZSR8.1|RSSA_BRANA RecName: Full=40S ribosomal protein SA; AltName: Full=Laminin receptor-like protein; AltName: Full=p40 gi|3769681|gb|AAC97937.1| laminin receptor-like protein [Brassica napus]	350	292	1.00E-160	83.4	78.9	79.7	RecName: Full=40S ribosomal protein SA; AltName: Full=Laminin receptor-like protein; AltName: Full=p40 gi|3769681|gb|AAC97937.1| laminin receptor-like protein	gbpln	Brassica napus	AT1G72370.1 | Symbols: P40, AP40, RP40, RPSAA | 40s ribosomal protein SA | chr1:27243148-27244842 REVERSE LENGTH=298	350	298	1.00E-137	85.1	67.4	68.9
Rsa1.0_00060.1.g3250.t1	ref|NP_001117587.1| ethylene-responsive transcription factor ERF073 [Arabidopsis thaliana] gi|332197191|gb|AEE35312.1| ethylene-responsive transcription factor ERF073 [Arabidopsis thaliana]	262	260	4.00E-61	99.2	55.7	69.1	ethylene-responsive transcription factor ERF073	gbpln	Arabidopsis thaliana	AT1G72360.3 | Symbols: HRE1 | Integrase-type DNA-binding superfamily protein | chr1:27241904-27242777 FORWARD LENGTH=260	262	260	1.00E-63	99.2	55.7	69.1
Rsa1.0_00060.1.g3251.t1	gb|EOA34308.1| hypothetical protein CARUB_v10021825mg [Capsella rubella]	213	222	1.00E-71	104.2	65.3	75.6	hypothetical protein CARUB_v10021825mg	gbpln	Capsella rubella	AT1G72350.1 | Symbols: | MADS-box transcription factor family protein | chr1:27239273-27239947 REVERSE LENGTH=224	213	224	1.00E-70	105.2	67.1	77.0
Rsa1.0_00060.1.g3252.t1	gb|EOA33336.1| hypothetical protein CARUB_v10020102mg [Capsella rubella]	181	543	7.00E-22	300.0	31.5	33.7	hypothetical protein CARUB_v10020102mg	gbpln	Capsella rubella	AT1G72330.3 | Symbols: ALAAT2 | alanine aminotransferase 2 | chr1:27233637-27236571 FORWARD LENGTH=553	181	553	3.00E-24	305.5	31.5	33.1
Rsa1.0_00060.1.g3253.t1	ref|XP_002887428.1| hypothetical protein ARALYDRAFT_476359 [Arabidopsis lyrata subsp. lyrata] gi|297333269|gb|EFH63687.1| hypothetical protein ARALYDRAFT_476359 [Arabidopsis lyrata subsp. lyrata]	163	324	1.00E-26	198.8	46.0	54.0	hypothetical protein ARALYDRAFT_476359	gbpln	Arabidopsis lyrata	AT1G72310.1 | Symbols: ATL3 | RING/U-box superfamily protein | chr1:27226405-27227379 FORWARD LENGTH=324	163	324	7.00E-25	198.8	40.5	47.2
Rsa1.0_00060.1.g3254.t1	emb|CAB39638.1| RNA-directed DNA polymerase-like protein [Arabidopsis thaliana] gi|7267666|emb|CAB78094.1| RNA-directed DNA polymerase-like protein [Arabidopsis thaliana]	1357	1274	0	93.9	45.3	61.2	RNA-directed DNA polymerase-like protein	gbpln	Arabidopsis thaliana	AT4G29090.1 | Symbols: | Ribonuclease H-like superfamily protein | chr4:14333528-14335255 FORWARD LENGTH=575	1357	575	2.00E-64	42.4	12.3	19.5

Rsa1.0_00060.1.g3255.t1	ref|NP_177366.1| transcription factor bHLH96 [Arabidopsis thaliana] gi|75308860|sp|Q9C7T4.1|BH096_ARATH RecName: Full=Transcription factor bHLH96; AltName: Full=Basic helix-loop-helix protein 96; Short=AtbHLH96; Short=bHLH 96; AltName: Full=Transcription factor EN 15; AltName: Full=bHLH transcription factor bHLH096 gi|12323671|gb|AAG51804.1|AC067754_20 unknown protein; 44011-46213 [Arabidopsis thaliana] gi|20520637|emb|CAD30833.1| basic-helix-loop-helix transcription factor [Arabidopsis thaliana] gi|28392970|gb|AAO41920.1| putative bHLH protein [Arabidopsis thaliana] gi|29824221|gb|AAP04071.1| putative bHLH protein [Arabidopsis thaliana] gi|332197168|gb|AEE35289.1| transcription factor bHLH96 [Arabidopsis thaliana]	323	320	1.00E-142	99.1	81.4	89.2	transcription factor bHLH96	gbpln	Arabidopsis thaliana	AT1G72210.1 | Symbols: | basic helix-loop-helix (bHLH) DNA-binding superfamily protein | chr1:27180066-27182268 FORWARD LENGTH=320	323	320	1.00E-144	99.1	81.4	89.2
Rsa1.0_00060.1.g3256.t1	gb|EOA34977.1| hypothetical protein CARUB_v10020065mg [Capsella rubella]	525	567	0	108.0	81.1	86.5	hypothetical protein CARUB_v10020065mg	gbpln	Capsella rubella	AT1G72150.1 | Symbols: PATL1 | PATELLIN 1 | chr1:27148558-27150652 FORWARD LENGTH=573	525	573	0	109.1	77.0	83.6
Rsa1.0_00060.1.g3257.t1	gb|EOA34991.1| hypothetical protein CARUB_v10020083mg [Capsella rubella]	552	556	0	100.7	81.2	90.9	hypothetical protein CARUB_v10020083mg	gbpln	Capsella rubella	AT1G72140.1 | Symbols: | Major facilitator superfamily protein | chr1:27141877-27144346 FORWARD LENGTH=555	552	555	0	100.5	81.7	90.6
Rsa1.0_00060.1.g3258.t1	ref|XP_002887414.1| hypothetical protein ARALYDRAFT_316182 [Arabidopsis lyrata subsp. lyrata] gi|297333255|gb|EFH63673.1| hypothetical protein ARALYDRAFT_316182 [Arabidopsis lyrata subsp. lyrata]	527	535	0	101.5	76.7	87.5	hypothetical protein ARALYDRAFT_316182	gbpln	Arabidopsis lyrata	AT1G72130.1 | Symbols: | Major facilitator superfamily protein | chr1:27137201-27139223 FORWARD LENGTH=538	527	538	0	102.1	74.8	87.3
Rsa1.0_00060.1.g3259.t1	ref|XP_002887414.1| hypothetical protein ARALYDRAFT_316182 [Arabidopsis lyrata subsp. lyrata] gi|297333255|gb|EFH63673.1| hypothetical protein ARALYDRAFT_316182 [Arabidopsis lyrata subsp. lyrata]	529	535	0	101.1	73.5	84.5	hypothetical protein ARALYDRAFT_316182	gbpln	Arabidopsis lyrata	AT1G72130.1 | Symbols: | Major facilitator superfamily protein | chr1:27137201-27139223 FORWARD LENGTH=538	529	538	0	101.7	73.0	84.7
Rsa1.0_00060.1.g3260.t1	ref|XP_002887414.1| hypothetical protein ARALYDRAFT_316182 [Arabidopsis lyrata subsp. lyrata] gi|297333255|gb|EFH63673.1| hypothetical protein ARALYDRAFT_316182 [Arabidopsis lyrata subsp. lyrata]	547	535	0	97.8	75.7	82.6	hypothetical protein ARALYDRAFT_316182	gbpln	Arabidopsis lyrata	AT1G72130.1 | Symbols: | Major facilitator superfamily protein | chr1:27137201-27139223 FORWARD LENGTH=538	547	538	0	98.4	72.6	81.7
Rsa1.0_00060.1.g3261.t1	ref|NP_001117585.1| putative peptide/nitrate transporter [Arabidopsis thaliana] gi|310947338|sp|Q0WSZ6.2|PTR23_ARATH RecName: Full=Probable peptide/nitrate transporter At1g72125 gi|332197156|gb|AEE35277.1| probable peptide/nitrate transporter [Arabidopsis thaliana]	557	561	0	100.7	81.3	89.2	putative peptide/nitrate transporter	gbpln	Arabidopsis thaliana	AT1G72125.1 | Symbols: | Major facilitator superfamily protein | chr1:27134168-27136257 FORWARD LENGTH=561	557	561	0	100.7	81.3	89.2
Rsa1.0_00060.1.g3262.t1	dbj|BAJ33896.1| unnamed protein product [Thellungiella halophila]	479	467	0	97.5	81.0	88.7	unnamed protein product	----	----	AT1G72110.1 | Symbols: | O-acyltransferase (WSD1-like) family protein | chr1:27129253-27131354 REVERSE LENGTH=479	479	479	0	100.0	78.9	89.4
Rsa1.0_00060.1.g3263.t2	gb|EOA34693.1| hypothetical protein CARUB_v10022259mg [Capsella rubella]	402	401	1.00E-176	99.8	78.4	85.1	hypothetical protein CARUB_v10022259mg	gbpln	Capsella rubella	AT1G72050.2 | Symbols: TFIIIA | transcription factor IIIA | chr1:27115082-27117228 FORWARD LENGTH=412	402	412	1.00E-178	102.5	77.6	84.1
Rsa1.0_00060.1.g3264.t1	ref|XP_002868530.1| predicted protein [Arabidopsis lyrata subsp. lyrata] gi|297314366|gb|EFH44789.1| predicted protein [Arabidopsis lyrata subsp. lyrata]	731	851	0	116.4	70.6	81.4	predicted protein	gbpln	Arabidopsis lyrata	AT1G15890.1 | Symbols: | Disease resistance protein (CC-NBS-LRR class) family | chr1:5461406-5463961 FORWARD LENGTH=851	731	851	0	116.4	66.3	76.2
Rsa1.0_00060.1.g3265.t1	gb|AET08927.1| sucrose transporter [Brassica napus]	447	514	0	115.0	92.8	94.9	sucrose transporter	gbpln	Brassica napus	AT1G71880.1 | Symbols: SUC1, ATSUC1 | sucrose-proton symporter 1 | chr1:27054334-27056100 FORWARD LENGTH=513	447	513	0	114.8	84.3	90.6
Rsa1.0_00060.1.g3266.t1	emb|CAB39638.1| RNA-directed DNA polymerase-like protein [Arabidopsis thaliana] gi|7267666|emb|CAB78094.1| RNA-directed DNA polymerase-like protein [Arabidopsis thaliana]	261	1274	5.00E-21	488.1	31.0	42.5	RNA-directed DNA polymerase-like protein	gbpln	Arabidopsis thaliana	#	#	#	#	#	#	#

Rsa1.0_00060.1.g3267.t1	ref|NP_173802.1| AP-1 complex subunit gamma-1 [Arabidopsis thaliana] gi|30688616|ref|NP_849701.1| AP-1 complex subunit gamma-1 [Arabidopsis thaliana] gi|75146766|sp|Q84K16.1|AP1G1_ARATH RecName: Full=AP-1 complex subunit gamma-1; AltName: Full=Adapter-related protein complex 1 subunit gamma-1; AltName: Full=Adaptor protein complex AP-1 large subunit gamma-1; AltName: Full=Clathrin assembly protein complex 1 gamma-1 large chain; Short=At-g-Ad; Short=At-gamma-Ad; AltName: Full=Gamma-adaptin 1 gi|28393791|gb|AAO42305.1| putative gamma-adaptin [Arabidopsis thaliana] gi|28973305|gb|AAO63977.1| putative gamma-adaptin [Arabidopsis thaliana] gi|332192327|gb|AEE30448.1| AP-1 complex subunit gamma-1 [Arabidopsis thaliana] gi|332192328|gb|AEE30449.1| AP-1 complex subunit gamma-1 [Arabidopsis thaliana]	160	876	4.00E-28	547.5	40.6	42.5	AP-1 complex subunit gamma-1	gbpln	Arabidopsis thaliana	AT1G23900.2 | Symbols: GAMMA-ADAPTIN 1 | gamma-adaptin 1 | chr1:8441379-8447152 FORWARD LENGTH=876	160	876	2.00E-30	547.5	40.6	42.5
Rsa1.0_00060.1.g3268.t1	ref|NP_849877.1| uncharacterized protein [Arabidopsis thaliana] gi|26453012|dbj|BAC43582.1| unknown protein [Arabidopsis thaliana] gi|28973253|gb|AAO63951.1| unknown protein [Arabidopsis thaliana] gi|332197123|gb|AEE35244.1| uncharacterized protein AT1G71865 [Arabidopsis thaliana]	101	99	2.00E-32	98.0	88.1	89.1	uncharacterized protein	gbpln	Arabidopsis thaliana	AT1G71865.1 | Symbols: | unknown protein; FUNCTIONS IN: molecular_function unknown; INVOLVED IN: biological_process unknown; LOCATED IN: chloroplast; Has 33 Blast hits to 33 proteins in 11 species: Archae - 0; Bacteria - 0; Metazoa - 0; Fungi - 0; Plants - 33; Viruses - 0; Other Eukaryotes - 0 (source: NCBI BLink). | chr1:27030117-27030416 FORWARD LENGTH=99	101	99	3.00E-35	98.0	88.1	89.1
Rsa1.0_00060.1.g3269.t1	gb|AAF43237.1|AC012654_21 Contains similarity to the vegetative incompatibility protein HET-E-1 from Podospora ansering gi|3023956; It contains 5 WD40 domains PF|00400 [Arabidopsis thaliana]	119	370	3.00E-32	310.9	58.0	61.3	Contains similarity to the vegetative incompatibility protein HET-E-1 from Podospora ansering gi|3023956; It contains 5 WD40 domains PF|00400	gbpln	Arabidopsis thaliana	AT1G71840.1 | Symbols: | transducin family protein / WD-40 repeat family protein | chr1:27022424-27024380 FORWARD LENGTH=407	119	407	6.00E-35	342.0	58.0	61.3
Rsa1.0_00060.1.g3270.t1	ref|NP_850465.1| cytochrome P450, family 709, subfamily B, polypeptide 1 [Arabidopsis thaliana] gi|330255683|gb|AEC10777.1| cytochrome P450, family 709, subfamily B, polypeptide 1 [Arabidopsis thaliana]	200	403	5.00E-89	201.5	75.5	87.5	cytochrome P450, family 709, subfamily B, polypeptide 1	gbpln	Arabidopsis thaliana	AT2G46960.1 | Symbols: CYP709B1 | cytochrome P450, family 709, subfamily B, polypeptide 1 | chr2:19292295-19293765 REVERSE LENGTH=403	200	403	2.00E-91	201.5	75.5	87.5
Rsa1.0_00060.1.g3271.t3	gb|AAF43237.1|AC012654_21 Contains similarity to the vegetative incompatibility protein HET-E-1 from Podospora ansering gi|3023956; It contains 5 WD40 domains PF|00400 [Arabidopsis thaliana]	379	370	0	97.6	86.0	92.9	Contains similarity to the vegetative incompatibility protein HET-E-1 from Podospora ansering gi|3023956; It contains 5 WD40 domains PF|00400	gbpln	Arabidopsis thaliana	AT1G71840.1 | Symbols: | transducin family protein / WD-40 repeat family protein | chr1:27022424-27024380 FORWARD LENGTH=407	379	407	0	107.4	85.2	91.8
Rsa1.0_00060.1.g3272.t1	ref|XP_002887391.1| hypothetical protein ARALYDRAFT_476305 [Arabidopsis lyrata subsp. lyrata] gi|297333232|gb|EFH63650.1| hypothetical protein ARALYDRAFT_476305 [Arabidopsis lyrata subsp. lyrata]	851	785	0	92.2	85.1	87.7	hypothetical protein ARALYDRAFT_476305	gbpln	Arabidopsis lyrata	AT1G71820.1 | Symbols: SEC6 | SEC6 | chr1:27010022-27016745 FORWARD LENGTH=752	851	752	0	88.4	84.3	86.1
Rsa1.0_00060.1.g3273.t1	gb|EOA34351.1| hypothetical protein CARUB_v10021875mg [Capsella rubella]	453	458	0	101.1	85.9	91.2	hypothetical protein CARUB_v10021875mg	gbpln	Capsella rubella	AT1G71800.1 | Symbols: CSTF64 | cleavage stimulating factor 64 | chr1:26999606-27001850 FORWARD LENGTH=461	453	461	0	101.8	82.6	89.2
Rsa1.0_00060.1.g3274.t1	gb|EOA35753.1| hypothetical protein CARUB_v10020985mg [Capsella rubella]	233	194	1.00E-46	83.3	40.8	45.1	hypothetical protein CARUB_v10020985mg	gbpln	Capsella rubella	AT1G71780.1 | Symbols: | unknown protein; FUNCTIONS IN: molecular_function unknown; INVOLVED IN: biological_process unknown; LOCATED IN: endoplasmic reticulum; EXPRESSED IN: 25 plant structures; EXPRESSED DURING: 15 growth stages; Has 34 Blast hits to 34 proteins in 11 species: Archae - 0; Bacteria - 0; Metazoa - 0; Fungi - 0; Plants - 34; Viruses - 0; Other Eukaryotes - 0 (source: NCBI BLink). | chr1:26995406-26996638 REVERSE LENGTH=197	233	197	9.00E-47	84.5	39.9	45.1

Rsa1.0_00060.1.g3275.t1	ref|XP_002888846.1| hypothetical protein ARALYDRAFT_339396 [Arabidopsis lyrata subsp. lyrata] gi|297334687|gb|EFH65105.1| hypothetical protein ARALYDRAFT_339396 [Arabidopsis lyrata subsp. lyrata]	662	685	0	103.5	83.1	89.6	hypothetical protein ARALYDRAFT_339396	gbpln	Arabidopsis lyrata	AT1G71770.2 | Symbols: PAB5 | poly(A)-binding protein 5 | chr1:26990777-26993489 REVERSE LENGTH=682	662	682	0	103.0	79.6	87.5
Rsa1.0_00060.1.g3276.t1	ref|NP_177295.1| receptor like protein 11 [Arabidopsis thaliana] gi|12323717|gb|AAG51813.1|AC016163_2 putative disease resistance protein; 69620-67266 [Arabidopsis thaliana] gi|332197075|gb|AEE35196.1| receptor like protein 11 [Arabidopsis thaliana]	740	784	0	105.9	68.9	79.5	receptor like protein 11	gbpln	Arabidopsis thaliana	AT1G71390.1 | Symbols: AtRLP11, RLP11 | receptor like protein 11 | chr1:26906453-26908807 FORWARD LENGTH=784	740	784	0	105.9	68.9	79.5
Rsa1.0_00060.1.g3277.t1	gb|EOA15757.1| hypothetical protein CARUB_v10006835mg [Capsella rubella]	441	438	0	99.3	93.0	96.1	hypothetical protein CARUB_v10006835mg	gbpln	Capsella rubella	AT5G42090.1 | Symbols: | Lung seven transmembrane receptor family protein | chr5:16826830-16828149 FORWARD LENGTH=439	441	439	0	99.5	91.4	95.5
Rsa1.0_00060.1.g3278.t1	ref|XP_002887372.1| hypothetical protein ARALYDRAFT_476271 [Arabidopsis lyrata subsp. lyrata] gi|297333213|gb|EFH63631.1| hypothetical protein ARALYDRAFT_476271 [Arabidopsis lyrata subsp. lyrata]	206	832	3.00E-76	403.9	71.8	81.1	hypothetical protein ARALYDRAFT_476271	gbpln	Arabidopsis lyrata	AT1G71390.1 | Symbols: AtRLP11, RLP11 | receptor like protein 11 | chr1:26906453-26908807 FORWARD LENGTH=784	206	784	4.00E-73	380.6	68.0	80.1
Rsa1.0_00060.1.g3279.t1	gb|EOA34941.1| hypothetical protein CARUB_v10020025mg [Capsella rubella]	569	592	0	104.0	75.4	83.1	hypothetical protein CARUB_v10020025mg	gbpln	Capsella rubella	AT1G71360.1 | Symbols: | Galactose-binding protein | chr1:26892214-26894166 REVERSE LENGTH=596	569	596	0	104.7	72.9	82.6
Rsa1.0_00060.1.g3280.t1	ref|NP_851080.1| serine hydroxymethyltransferase 2 [Arabidopsis thaliana] gi|227202628|dbj|BAH56787.1| AT5G26780 [Arabidopsis thaliana] gi|332006219|gb|AED93602.1| serine hydroxymethyltransferase 2 [Arabidopsis thaliana]	539	517	0	95.9	88.3	91.3	serine hydroxymethyltransferase 2	gbpln	Arabidopsis thaliana	AT5G26780.1 | Symbols: SHM2 | serine hydroxymethyltransferase 2 | chr5:9418299-9421725 FORWARD LENGTH=517	539	517	0	95.9	88.3	91.3
Rsa1.0_00060.1.g3281.t1	ref|XP_002887361.1| hydrolase/ zinc ion binding protein [Arabidopsis lyrata subsp. lyrata] gi|297333202|gb|EFH63620.1| hydrolase/ zinc ion binding protein [Arabidopsis lyrata subsp. lyrata]	420	400	0	95.2	77.6	82.4	hydrolase/ zinc ion binding protein	gbpln	Arabidopsis lyrata	AT5G24670.1 | Symbols: | Cytidine/deoxycytidylate deaminase family protein | chr5:8448880-8451046 FORWARD LENGTH=400	420	400	0	95.2	75.2	81.2
Rsa1.0_00060.1.g3282.t1	ref|NP_177287.1| cobalt ion binding protein [Arabidopsis thaliana] gi|30698791|ref|NP_849876.1| cobalt ion binding protein [Arabidopsis thaliana] gi|12323832|gb|AAG51886.1|AC016162_7 hypothetical protein; 63020-64147 [Arabidopsis thaliana] gi|22135986|gb|AAM91575.1| unknown protein [Arabidopsis thaliana] gi|23198344|gb|AAN15699.1| unknown protein [Arabidopsis thaliana] gi|332197065|gb|AEE35186.1| cobalt ion binding protein [Arabidopsis thaliana] gi|332197066|gb|AEE35187.1| cobalt ion binding protein [Arabidopsis thaliana]	179	176	3.00E-79	98.3	81.0	87.7	cobalt ion binding protein	gbpln	Arabidopsis thaliana	AT1G71310.1 | Symbols: | cobalt ion binding | chr1:26878717-26879844 REVERSE LENGTH=176	179	176	1.00E-81	98.3	81.0	87.7
Rsa1.0_00060.1.g3283.t1	ref|XP_002887358.1| predicted protein [Arabidopsis lyrata subsp. lyrata] gi|297333199|gb|EFH63617.1| predicted protein [Arabidopsis lyrata subsp. lyrata]	347	374	1.00E-163	107.8	83.3	91.4	predicted protein	gbpln	Arabidopsis lyrata	AT1G71250.1 | Symbols: | GDSL-like Lipase/Acylhydrolase superfamily protein | chr1:26860125-26861582 FORWARD LENGTH=374	347	374	1.00E-165	107.8	81.8	91.1

Rsa1.0_00061.1.g3284.t1	ref|NP_192117.1| GTP-binding protein SAR1A [Arabidopsis thaliana] gi|3334323|sp|O04834.1|SAR1A_ARATH RecName: Full=GTP-binding protein SAR1A gi|1314860|gb|AAA99827.1| Sar1 homolog [Arabidopsis thaliana] gi|2104532|gb|AAC78700.1| SAR1/GTP-binding secretory factor [Arabidopsis thaliana] gi|2104550|gb|AAB57799.1| AGAA.4 [Arabidopsis thaliana] gi|7268592|emb|CAB80701.1| SAR1/GTP-binding secretory factor [Arabidopsis thaliana] gi|17529144|gb|AAL38798.1| putative SAR1/GTP-binding secretory factor [Arabidopsis thaliana] gi|20465729|gb|AAM20333.1| putative SAR1/GTP-binding secretory factor [Arabidopsis thaliana] gi|21618030|gb|AAM67080.1| SAR1/GTP-binding secretory factor [Arabidopsis thaliana] gi|332656722|gb|AEE82122.1| GTP-binding protein SAR1A [Arabidopsis thaliana]	458	193	1.00E-101	42.1	41.3	41.7	GTP-binding protein SAR1A	gbpln	Arabidopsis thaliana	AT4G02080.1 | Symbols: ASAR1, ATSARA1C, ATSAR2, SAR2 | secretion-associated RAS super family 2 | chr4:921554-922547 FORWARD LENGTH=193	458	193	1.00E-104	42.1	41.3	41.7
Rsa1.0_00061.1.g3285.t1	sp|P34794.1|RUB2_BRANA RecName: Full=RuBisCO large subunit-binding protein subunit alpha, chloroplastic; AltName: Full=60 kDa chaperonin subunit alpha; AltName: Full=CPN-60 alpha; Flags: Precursor gi|415925|emb|CAA81736.1| chaperonin-60 alpha subunit [Brassica napus]	183	583	5.00E-60	318.6	63.9	66.7	RecName: Full=RuBisCO large subunit-binding protein subunit alpha, chloroplastic; AltName: Full=60 kDa chaperonin subunit alpha; AltName: Full=CPN-60 alpha; Flags: Precursor gi|415925|emb|CAA81736.1| chaperonin-60 alpha subunit	gbpln	Brassica napus	AT2G28000.1 | Symbols: CPN60A, CH-CPN60A, SLP | chaperonin-60alpha | chr2:11926603-11929184 FORWARD LENGTH=586	183	586	4.00E-55	320.2	57.9	60.7
Rsa1.0_00061.1.g3286.t1	#	#	#	#	#	#	#	-	----	----	#	#	#	#	#	#	#
Rsa1.0_00061.1.g3287.t1	#	#	#	#	#	#	#	-	----	----	#	#	#	#	#	#	#
Rsa1.0_00061.1.g3288.t1	gb|EOA21485.1| hypothetical protein CARUB_v10001882mg [Capsella rubella]	214	230	1.00E-71	107.5	76.6	86.4	hypothetical protein CARUB_v10001882mg	gbpln	Capsella rubella	AT4G02075.1 | Symbols: PIT1 | RING/FYVE/PHD zinc finger superfamily protein | chr4:913555-916414 REVERSE LENGTH=218	214	218	4.00E-74	101.9	77.6	86.9
Rsa1.0_00061.1.g3289.t1	dbj|BAJ33893.1| unnamed protein product [Thellungiella halophila]	718	716	0	99.7	96.2	97.9	unnamed protein product	----	----	AT4G02060.2 | Symbols: PRL | Minichromosome maintenance (MCM2/3/5) family protein | chr4:901484-905297 FORWARD LENGTH=716	718	716	0	99.7	94.7	97.4
Rsa1.0_00061.1.g3290.t1	ref|XP_002872859.1| hypothetical protein ARALYDRAFT_327595 [Arabidopsis lyrata subsp. lyrata] gi|297318696|gb|EFH49118.1| hypothetical protein ARALYDRAFT_327595 [Arabidopsis lyrata subsp. lyrata]	511	515	0	100.8	92.4	96.5	hypothetical protein ARALYDRAFT_327595	gbpln	Arabidopsis lyrata	AT4G02050.1 | Symbols: STP7 | sugar transporter protein 7 | chr4:898387-900095 REVERSE LENGTH=513	511	513	0	100.4	92.0	95.9
Rsa1.0_00061.1.g3291.t1	ref|XP_002874944.1| hypothetical protein ARALYDRAFT_490376 [Arabidopsis lyrata subsp. lyrata] gi|297320781|gb|EFH51203.1| hypothetical protein ARALYDRAFT_490376 [Arabidopsis lyrata subsp. lyrata]	152	154	6.00E-35	101.3	59.9	71.7	hypothetical protein ARALYDRAFT_490376	gbpln	Arabidopsis lyrata	AT4G02040.1 | Symbols: | unknown protein; FUNCTIONS IN: molecular_function unknown; INVOLVED IN: biological_process unknown; LOCATED IN: chloroplast; Has 30 Blast hits to 30 proteins in 6 species: Archae - 0; Bacteria - 0; Metazoa - 0; Fungi - 0; Plants - 30; Viruses - 0; Other Eukaryotes - 0 (source: NCBI BLink). | chr4:897563-898021 FORWARD LENGTH=152	152	152	3.00E-34	100.0	57.2	70.4
Rsa1.0_00061.1.g3292.t1	gb|EOA19997.1| hypothetical protein CARUB_v10000264mg [Capsella rubella]	759	786	0	103.6	91.0	94.5	hypothetical protein CARUB_v10000264mg	gbpln	Capsella rubella	AT4G02030.1 | Symbols: | Vps51/Vps67 family (components of vesicular transport) protein | chr4:892262-897175 FORWARD LENGTH=780	759	780	0	102.8	90.8	94.3
Rsa1.0_00061.1.g3293.t6	ref|XP_002874946.1| swinger [Arabidopsis lyrata subsp. lyrata] gi|297320783|gb|EFH51205.1| swinger [Arabidopsis lyrata subsp. lyrata]	908	846	0	93.2	69.6	75.3	swinger	gbpln	Arabidopsis lyrata	AT4G02020.1 | Symbols: EZA1, SWN, SDG10 | SET domain-containing protein | chr4:886693-891743 FORWARD LENGTH=856	908	856	0	94.3	69.2	76.1
Rsa1.0_00061.1.g3294.t1	gb|ADQ20113.1| stachyose synthase [Brassica napus]	874	873	0	99.9	92.6	97.0	stachyose synthase	gbpln	Brassica napus	AT4G01970.1 | Symbols: AtSTS, STS | stachyose synthase | chr4:854073-856953 REVERSE LENGTH=876	874	876	0	100.2	84.8	93.1

Rsa1.0_00061.1.g3295.t1	ref|NP_192105.1| uncharacterized protein [Arabidopsis thaliana] gi|4558565|gb|AAD22658.1|AC007138_22 predicted protein of unknown function [Arabidopsis thaliana] gi|7268580|emb|CAB80689.1| predicted protein of unknown function [Arabidopsis thaliana] gi|21537343|gb|AAM61684.1| unknown [Arabidopsis thaliana] gi|88196763|gb|ABD43024.1| At4g01960 [Arabidopsis thaliana] gi|110738203|dbj|BAF01032.1| hypothetical protein [Arabidopsis thaliana] gi|332656705|gb|AEE82105.1| uncharacterized protein AT4G01960 [Arabidopsis thaliana]	229	236	2.00E-81	103.1	80.8	89.1	uncharacterized protein	gbpln	Arabidopsis thaliana	AT4G01960.1	Symbols:	unknown protein; BEST Arabidopsis thaliana protein match is: unknown protein (TAIR:AT1G02380.1); Has 67 Blast hits to 67 proteins in 11 species: Archae - 0; Bacteria - 0; Metazoa - 0; Fungi - 0; Plants - 67; Viruses - 0; Other Eukaryotes - 0 (source: NCBI BLink).	chr4:851387-852441 REVERSE LENGTH=236	229	236	7.00E-84	103.1	80.8	89.1
Rsa1.0_00061.1.g3296.t1	gb|AAG51783.1|AC079679_3 reverse transcriptase, putative; 16838-20266 [Arabidopsis thaliana]	1454	1142	0	78.5	22.8	32.1	reverse transcriptase, putative; 16838-20266	gbpln	Arabidopsis thaliana	AT4G29090.1	Symbols:	Ribonuclease H-like superfamily protein	chr4:14333528-14335255 FORWARD LENGTH=575	1454	575	1.00E-40	39.5	5.8	9.1
Rsa1.0_00061.1.g3297.t1	dbj|BAJ34109.1| unnamed protein product [Thellungiella halophila]	521	521	0	100.0	88.1	93.7	unnamed protein product	----	----	AT4G01950.1	Symbols: ATGPAT3, GPAT3	glycerol-3-phosphate acyltransferase 3	chr4:844597-846710 REVERSE LENGTH=520	521	520	0	99.8	87.1	93.5
Rsa1.0_00061.1.g3298.t1	gb|EOA22935.1| hypothetical protein CARUB_v10003671mg, partial [Capsella rubella]	517	145	5.00E-17	28.0	9.9	13.3	hypothetical protein CARUB_v10003671mg, partial	gbpln	Capsella rubella	#	#	#	#	#	#	#
Rsa1.0_00061.1.g3299.t1	gb|EOA30146.1| hypothetical protein CARUB_v10013253mg [Capsella rubella]	104	609	4.00E-44	585.6	83.7	87.5	hypothetical protein CARUB_v10013253mg	gbpln	Capsella rubella	AT3G18060.1	Symbols:	transducin family protein / WD-40 repeat family protein	chr3:6183880-6186788 FORWARD LENGTH=609	104	609	7.00E-47	585.6	83.7	86.5
Rsa1.0_00061.1.g3300.t1	gb|AAF98181.1|AC000107_4 F17F8.5 [Arabidopsis thaliana]	301	872	7.00E-70	289.7	49.2	67.4	F17F8.5	gbpln	Arabidopsis thaliana	AT3G24255.1	Symbols:	RNA-directed DNA polymerase (reverse transcriptase)-related family protein	chr3:8789471-8793208 FORWARD LENGTH=746	301	746	4.00E-46	247.8	38.5	54.2
Rsa1.0_00061.1.g3301.t1	ref|XP_002872902.1| hypothetical protein ARALYDRAFT_912111 [Arabidopsis lyrata subsp. lyrata] gi|297318739|gb|EFH49161.1| hypothetical protein ARALYDRAFT_912111 [Arabidopsis lyrata subsp. lyrata]	348	356	1.00E-144	102.3	74.7	81.9	hypothetical protein ARALYDRAFT_912111	gbpln	Arabidopsis lyrata	AT4G01220.1	Symbols:	Nucleotide-diphospho-sugar transferase family protein	chr4:513431-515648 REVERSE LENGTH=360	348	360	1.00E-144	103.4	70.4	77.3
Rsa1.0_00061.1.g3302.t1	#	#	#	#	#	#	#	-	----	----	#	#	#	#	#	#	#
Rsa1.0_00061.1.g3303.t1	ref|XP_002872902.1| hypothetical protein ARALYDRAFT_912111 [Arabidopsis lyrata subsp. lyrata] gi|297318739|gb|EFH49161.1| hypothetical protein ARALYDRAFT_912111 [Arabidopsis lyrata subsp. lyrata]	342	356	1.00E-145	104.1	72.8	78.9	hypothetical protein ARALYDRAFT_912111	gbpln	Arabidopsis lyrata	AT4G01220.1	Symbols:	Nucleotide-diphospho-sugar transferase family protein	chr4:513431-515648 REVERSE LENGTH=360	342	360	1.00E-145	105.3	71.9	78.7
Rsa1.0_00061.1.g3304.t2	ref|XP_002874954.1| hypothetical protein ARALYDRAFT_327611 [Arabidopsis lyrata subsp. lyrata] gi|297320791|gb|EFH51213.1| hypothetical protein ARALYDRAFT_327611 [Arabidopsis lyrata subsp. lyrata]	386	226	3.00E-93	58.5	47.4	49.5	hypothetical protein ARALYDRAFT_327611	gbpln	Arabidopsis lyrata	AT4G01883.1	Symbols:	Polyketide cyclase / dehydrase and lipid transport protein	chr4:813162-814800 FORWARD LENGTH=224	386	224	1.00E-90	58.0	45.9	49.2
Rsa1.0_00061.1.g3305.t1	ref|NP_192095.2| transducin/WD40 domain-containing protein [Arabidopsis thaliana] gi|30678983|ref|NP_849536.1| transducin/WD40 domain-containing protein [Arabidopsis thaliana] gi|19699003|gb|AAL91237.1| unknown protein [Arabidopsis thaliana] gi|32306503|gb|AAP78935.1| At4g01860 [Arabidopsis thaliana] gi|332656686|gb|AEE82086.1| transducin/WD40 domain-containing protein [Arabidopsis thaliana] gi|332656687|gb|AEE82087.1| transducin/WD40 domain-containing protein [Arabidopsis thaliana]	1615	1308	0	81.0	67.2	72.3	transducin/WD40 domain-containing protein	gbpln	Arabidopsis thaliana	AT4G01860.2	Symbols:	Transducin family protein / WD-40 repeat family protein	chr4:801713-808018 REVERSE LENGTH=1308	1615	1308	0	81.0	67.2	72.3

Rsa1.0_00061.1.g3306.t1	sp|Q5DNB1.1|METK_BRARP RecName: Full=S-adenosylmethionine synthase; Short=AdoMet synthase; AltName: Full=Methionine adenosyltransferase; Short=MAT gi|75306462|sp|Q94FA4.1|METK5_BRAJU RecName: Full=S-adenosylmethionine synthase 5; Short=AdoMet synthase 5; AltName: Full=Methionine adenosyltransferase 5; Short=MAT 5 gi|14600072|gb|AAK71235.1| S-adenosylmethionine synthetase [Brassica juncea] gi|56130947|gb|AAV80205.1| S-adenosyl-L-methionine synthetase [Brassica rapa subsp. pekinensis]	393	393	0	100.0	99.7	100.0	RecName: Full=S-adenosylmethionine synthase; Short=AdoMet synthase; AltName: Full=Methionine adenosyltransferase; Short=MAT gi|75306462|sp|Q94FA4.1|METK5_BRAJU RecName: Full=S-adenosylmethionine synthase 5; Short=AdoMet synthase 5; AltName: Full=Methionine adenosyltransferase 5; Short=MAT 5 gi|14600072|gb|AAK71235.1| S-adenosylmethionine synthetase	gbpln	Brassica juncea	AT4G01850.2 | Symbols: SAM-2, MAT2, SAM2, AtSAM2 | S-adenosylmethionine synthetase 2 | chr4:796298-797479 REVERSE LENGTH=393	393	393	0	100.0	99.2	99.7
Rsa1.0_00061.1.g3307.t1	gb|EOA20795.1| hypothetical protein CARUB_v10001130mg [Capsella rubella]	401	396	0	98.8	84.0	89.0	hypothetical protein CARUB_v10001130mg	gbpln	Capsella rubella	AT4G01840.1 | Symbols: KCO5, ATTPK5, ATKCO5, TPK5 | Ca2+ activated outward rectifying K+ channel 5 | chr4:793403-794933 FORWARD LENGTH=408	401	408	1.00E-178	101.7	81.5	87.5
Rsa1.0_00061.1.g3308.t1	ref|XP_002872875.1| hypothetical protein ARALYDRAFT_490398 [Arabidopsis lyrata subsp. lyrata] gi|297318712|gb|EFH49134.1| hypothetical protein ARALYDRAFT_490398 [Arabidopsis lyrata subsp. lyrata]	651	882	0	135.5	67.7	69.3	hypothetical protein ARALYDRAFT_490398	gbpln	Arabidopsis lyrata	AT4G01810.1 | Symbols: | Sec23/Sec24 protein transport family protein | chr4:776734-779802 REVERSE LENGTH=880	651	880	0	135.2	67.6	69.3
Rsa1.0_00061.1.g3309.t1	gb|EOA22935.1| hypothetical protein CARUB_v10003671mg, partial [Capsella rubella]	600	145	7.00E-19	24.2	9.2	14.3	hypothetical protein CARUB_v10003671mg, partial	gbpln	Capsella rubella	#	#	#	#	#	#	#
Rsa1.0_00061.1.g3310.t1	ref|NP_192081.1| putative WRKY transcription factor 47 [Arabidopsis thaliana] gi|20978798|sp|Q9ZSI7.2|WRK47_ARATH RecName: Full=Probable WRKY transcription factor 47; AltName: Full=WRKY DNA-binding protein 47 gi|19172392|gb|AAL85881.1|AF480165_1 WRKY transcription factor 47 [Arabidopsis thaliana] gi|7268215|emb|CAB77742.1| putative DNA-binding protein [Arabidopsis thaliana] gi|30794136|gb|AAP40510.1| putative WRKY family transcription factor [Arabidopsis thaliana] gi|110738911|dbj|BAF01377.1| putative DNA-binding protein [Arabidopsis thaliana] gi|115311505|gb|ABI93933.1| At4g01720 [Arabidopsis thaliana] gi|332656669|gb|AEE82069.1| putative WRKY transcription factor 47 [Arabidopsis thaliana]	488	489	0	100.2	79.9	87.5	putative WRKY transcription factor 47	gbpln	Arabidopsis thaliana	AT4G01720.1 | Symbols: WRKY47, AtWRKY47 | WRKY family transcription factor | chr4:744949-748219 FORWARD LENGTH=489	488	489	0	100.2	79.9	87.5
Rsa1.0_00062.1.g3311.t1	#	#	#	#	#	#	#	-	----	----	#	#	#	#	#	#	#
Rsa1.0_00062.1.g3312.t1	gb|AAF24531.1|AC007534_12 F7F22.17 [Arabidopsis thaliana]	629	1799	7.00E-92	286.0	29.1	39.7	F7F22.17	gbpln	Arabidopsis thaliana	#	#	#	#	#	#	#
Rsa1.0_00062.1.g3313.t1	#	#	#	#	#	#	#	-	----	----	#	#	#	#	#	#	#
Rsa1.0_00062.1.g3314.t1	gb|EOA28006.1| hypothetical protein CARUB_v10024183mg [Capsella rubella]	69	170	2.00E-19	246.4	69.6	72.5	hypothetical protein CARUB_v10024183mg	gbpln	Capsella rubella	AT5G37070.1 | Symbols: | Protein of unknown function, DUF538 | chr5:14651091-14652147 FORWARD LENGTH=170	69	170	8.00E-22	246.4	66.7	75.4
Rsa1.0_00062.1.g3315.t1	dbj|BAJ33618.1| unnamed protein product [Thellungiella halophila]	508	510	0	100.4	90.4	93.3	unnamed protein product	----	----	AT3G50660.1 | Symbols: DWF4, CYP90B1, CLM, SNP2, SAV1, PSC1 | Cytochrome P450 superfamily protein | chr3:18814262-18817168 REVERSE LENGTH=513	508	513	0	101.0	87.6	90.9
Rsa1.0_00062.1.g3316.t1	gb|AAG50806.1|AC079281_8 unknown protein [Arabidopsis thaliana]	1878	1213	0	64.6	31.1	40.7	unknown protein	gbpln	Arabidopsis thaliana	AT1G43760.1 | Symbols: | DNAse I-like superfamily protein | chr1:16528880-16531065 REVERSE LENGTH=626	1878	626	5.00E-74	33.3	7.9	12.1
Rsa1.0_00062.1.g3317.t1	#	#	#	#	#	#	#	-	----	----	#	#	#	#	#	#	#
Rsa1.0_00062.1.g3318.t1	dbj|BAB01972.1| copia-like retrotransposable element [Arabidopsis thaliana]	169	1499	4.00E-25	887.0	35.5	50.3	copia-like retrotransposable element	gbpln	Arabidopsis thaliana	#	#	#	#	#	#	#

Rsa1.0_00062.1.g3319.t1	ref|XP_002877765.1| UDP-glucosyl transferase 72E1 [Arabidopsis lyrata subsp. lyrata] gi|297323603|gb|EFH54024.1| UDP-glucosyl transferase 72E1 [Arabidopsis lyrata subsp. lyrata]	488	487	0	99.8	76.6	86.9	UDP-glucosyl transferase 72E1	gbpln	Arabidopsis lyrata	AT3G50740.1 | Symbols: UGT72E1 | UDP-glucosyl transferase 72E1 | chr3:18855348-18856811 REVERSE LENGTH=487	488	487	0	99.8	75.4	86.9
Rsa1.0_00062.1.g3320.t1	#	#	#	#	#	#	#	-	----	----	#	#	#	#	#	#	#
Rsa1.0_00062.1.g3321.t1	ref|NP_566938.1| coniferyl-alcohol glucosyltransferase [Arabidopsis thaliana] gi|75306318|sp|Q94A84.1|U72E1_ARATH RecName: Full=UDP-glycosyltransferase 72E1 gi|15146272|gb|AAK83619.1| AT3g50740/T3A5_120 [Arabidopsis thaliana] gi|53749150|gb|AAU90060.1| At3g50740 [Arabidopsis thaliana] gi|332645182|gb|AEE78703.1| UDP-glucosyl transferase 72E1 [Arabidopsis thaliana]	482	487	0	101.0	80.1	90.0	coniferyl-alcohol glucosyltransferase	gbpln	Arabidopsis thaliana	AT3G50740.1 | Symbols: UGT72E1 | UDP-glucosyl transferase 72E1 | chr3:18855348-18856811 REVERSE LENGTH=487	482	487	0	101.0	80.1	90.0
Rsa1.0_00062.1.g3322.t1	#	#	#	#	#	#	#	-	----	----	AT2G23450.1 | Symbols: | Protein kinase superfamily protein | chr2:9988926-9991244 REVERSE LENGTH=708	62	708	2.00E-11	1141.9	54.8	67.7
Rsa1.0_00062.1.g3323.t1	#	#	#	#	#	#	#	-	----	----	#	#	#	#	#	#	#
Rsa1.0_00062.1.g3324.t1	ref|NP_001154670.1| PLATZ transcription factor family protein [Arabidopsis thaliana] gi|254801606|sp|P0CB19.1|Y3081_ARATH RecName: Full=Uncharacterized protein At3g50808 gi|332645191|gb|AEE78712.1| PLATZ transcription factor family protein [Arabidopsis thaliana]	226	110	5.00E-27	48.7	25.2	36.3	PLATZ transcription factor family protein	gbpln	Arabidopsis thaliana	AT3G50808.1 | Symbols: | PLATZ transcription factor family protein | chr3:18887614-18888047 REVERSE LENGTH=110	226	110	2.00E-29	48.7	25.2	36.3
Rsa1.0_00062.1.g3325.t1	#	#	#	#	#	#	#	-	----	----	#	#	#	#	#	#	#
Rsa1.0_00062.1.g3326.t1	ref|XP_002877774.1| photosystem II subunit O-2 [Arabidopsis lyrata subsp. lyrata] gi|297323612|gb|EFH54033.1| photosystem II subunit O-2 [Arabidopsis lyrata subsp. lyrata]	333	331	0	99.4	96.4	97.6	photosystem II subunit O-2	gbpln	Arabidopsis lyrata	AT3G50820.1 | Symbols: PSBO2, PSBO-2, OEC33 | photosystem II subunit O-2 | chr3:18891008-18892311 REVERSE LENGTH=331	333	331	0	99.4	95.5	97.0
Rsa1.0_00062.1.g3327.t1	gb|AAG51228.1|AC035249_3 Tam3-like transposon protein; 93317-95488 [Arabidopsis thaliana] gi|12323055|gb|AAG51515.1|AC068324_3 hAT-element transposase, putative [Arabidopsis thaliana]	689	723	0	104.9	56.6	74.0	Tam3-like transposon protein; 93317-95488	gbpln	Arabidopsis thaliana	AT3G42170.1 | Symbols: | BED zinc finger ;hAT family dimerisation domain | chr3:14321838-14323928 FORWARD LENGTH=696	689	696	5.00E-46	101.0	21.2	37.7
Rsa1.0_00062.1.g3328.t1	#	#	#	#	#	#	#	-	----	----	#	#	#	#	#	#	#
Rsa1.0_00062.1.g3329.t1	#	#	#	#	#	#	#	-	----	----	#	#	#	#	#	#	#
Rsa1.0_00062.1.g3330.t1	ref|XP_002876050.1| predicted protein [Arabidopsis lyrata subsp. lyrata] gi|297321888|gb|EFH52309.1| predicted protein [Arabidopsis lyrata subsp. lyrata]	283	294	1.00E-113	103.9	80.9	87.3	predicted protein	gbpln	Arabidopsis lyrata	AT3G50870.1 | Symbols: MNP, HAN, GATA18 | GATA type zinc finger transcription factor family protein | chr3:18911112-18912369 FORWARD LENGTH=295	283	295	1.00E-115	104.2	79.5	86.6
Rsa1.0_00062.1.g3331.t1	ref|XP_002876053.1| hypothetical protein ARALYDRAFT_906429 [Arabidopsis lyrata subsp. lyrata] gi|297321891|gb|EFH52312.1| hypothetical protein ARALYDRAFT_906429 [Arabidopsis lyrata subsp. lyrata]	108	114	7.00E-40	105.6	77.8	85.2	hypothetical protein ARALYDRAFT_906429	gbpln	Arabidopsis lyrata	AT3G50900.1 | Symbols: | unknown protein; BEST Arabidopsis thaliana protein match is: unknown protein (TAIR:AT5G66490.1); Has 45 Blast hits to 45 proteins in 7 species: Archae - 0; Bacteria - 0; Metazoa - 0; Fungi - 0; Plants - 45; Viruses - 0; Other Eukaryotes - 0 (source: NCBI BLink). | chr3:18918122-18918466 FORWARD LENGTH=114	108	114	2.00E-42	105.6	75.9	84.3
Rsa1.0_00062.1.g3332.t1	gb|EOA23929.1| hypothetical protein CARUB_v10017143mg [Capsella rubella]	199	444	6.00E-33	223.1	37.7	43.2	hypothetical protein CARUB_v10017143mg	gbpln	Capsella rubella	AT3G50910.1 | Symbols: | unknown protein; BEST Arabidopsis thaliana protein match is: unknown protein (TAIR:AT5G66480.1); Has 76 Blast hits to 75 proteins in 28 species: Archae - 0; Bacteria - 10; Metazoa - 7; Fungi - 2; Plants - 49; Viruses - 0; Other Eukaryotes - 8 (source: NCBI BLink). | chr3:18920189-18921999 FORWARD LENGTH=447	199	447	1.00E-32	224.6	36.2	41.7
Rsa1.0_00062.1.g3333.t1	#	#	#	#	#	#	#	-	----	----	#	#	#	#	#	#	#

Rsa1.0_00062.1.g3334.t1	ref|XP_002876055.1| hypothetical protein ARALYDRAFT_906432 [Arabidopsis lyrata subsp. lyrata] gi|297321893|gb|EFH52314.1| hypothetical protein ARALYDRAFT_906432 [Arabidopsis lyrata subsp. lyrata]	222	268	2.00E-60	120.7	66.7	75.2	hypothetical protein ARALYDRAFT_906432	gbpln	Arabidopsis lyrata	AT5G66440.1 | Symbols: | unknown protein; BEST Arabidopsis thaliana protein match is: unknown protein (TAIR:AT4G34560.1); Has 1807 Blast hits to 1807 proteins in 277 species: Archae - 0; Bacteria - 0; Metazoa - 736; Fungi - 347; Plants - 385; Viruses - 0; Other Eukaryotes - 339 (source: NCBI BLink). | chr5:26530274-26531032 REVERSE LENGTH=252	222	252	2.00E-51	113.5	54.1	68.0
Rsa1.0_00062.1.g3335.t1	ref|NP_568344.2| myb family transcription factor [Arabidopsis thaliana] gi|332005027|gb|AED92410.1| myb family transcription factor [Arabidopsis thaliana]	378	387	1.00E-147	102.4	78.8	85.2	myb family transcription factor	gbpln	Arabidopsis thaliana	AT5G17300.1 | Symbols: RVE1 | Homeodomain-like superfamily protein | chr5:5690435-5692435 REVERSE LENGTH=387	378	387	1.00E-149	102.4	78.8	85.2
Rsa1.0_00062.1.g3336.t1	#	#	#	#	#	#	#	-	----	----	#	#	#	#	#	#	#
Rsa1.0_00062.1.g3337.t1	gb|EOA19553.1| hypothetical protein CARUB_v10002574mg [Capsella rubella]	1061	724	0	68.2	41.0	49.3	hypothetical protein CARUB_v10002574mg	gbpln	Capsella rubella	AT5G17320.1 | Symbols: HDG9 | homeodomain GLABROUS 9 | chr5:5703380-5707637 REVERSE LENGTH=718	1061	718	0	67.7	40.1	48.6
Rsa1.0_00062.1.g3338.t3	gb|ACB59199.1| copia-like protein [Brassica oleracea]	829	975	1.00E-121	117.6	31.7	40.4	copia-like protein	gbpln	Brassica oleracea	AT4G23160.1 | Symbols: CRK8 | cysteine-rich RLK (RECEPTOR-like protein kinase) 8 | chr4:12129485-12134086 FORWARD LENGTH=1262	829	1262	5.00E-37	152.2	11.8	18.3
Rsa1.0_00062.1.g3339.t1	gb|EOA20468.1| hypothetical protein CARUB_v10000781mg [Capsella rubella]	195	502	4.00E-58	257.4	64.1	70.3	hypothetical protein CARUB_v10000781mg	gbpln	Capsella rubella	AT5G17330.1 | Symbols: GAD, GAD1 | glutamate decarboxylase | chr5:5711141-5714839 FORWARD LENGTH=502	195	502	9.00E-60	257.4	62.6	70.8
Rsa1.0_00062.1.g3340.t2	gb|ABA70759.1| baby boom interacting protein 1B [Brassica napus]	116	711	6.00E-20	612.9	39.7	44.8	baby boom interacting protein 1B	gbpln	Brassica napus	AT1G73360.1 | Symbols: HDG11, EDT1, ATHDG11 | homeodomain GLABROUS 11 | chr1:27578893-27581820 REVERSE LENGTH=722	116	722	1.00E-21	622.4	37.9	44.8
Rsa1.0_00062.1.g3341.t1	ref|NP_197236.1| Putative membrane lipoprotein [Arabidopsis thaliana] gi|10177079|dbj|BAB10521.1| unnamed protein product [Arabidopsis thaliana] gi|62867603|gb|AAY17405.1| At5g17340 [Arabidopsis thaliana] gi|66841346|gb|AAY57310.1| At5g17340 [Arabidopsis thaliana] gi|332005032|gb|AED92415.1| Putative membrane lipoprotein [Arabidopsis thaliana]	164	160	6.00E-52	97.6	70.7	82.9	Putative membrane lipoprotein	gbpln	Arabidopsis thaliana	AT5G17340.1 | Symbols: | Putative membrane lipoprotein | chr5:5715736-5716218 REVERSE LENGTH=160	164	160	2.00E-54	97.6	70.7	82.9
Rsa1.0_00062.1.g3342.t1	#	#	#	#	#	#	#	-	----	----	#	#	#	#	#	#	#
Rsa1.0_00062.1.g3343.t1	ref|NP_197237.1| uncharacterized protein [Arabidopsis thaliana] gi|9755758|emb|CAC01730.1| putative protein [Arabidopsis thaliana] gi|21537084|gb|AAM61425.1| unknown [Arabidopsis thaliana] gi|110737524|dbj|BAF00704.1| hypothetical protein [Arabidopsis thaliana] gi|332005033|gb|AED92416.1| uncharacterized protein AT5G17350 [Arabidopsis thaliana]	186	183	5.00E-69	98.4	78.0	84.4	uncharacterized protein	gbpln	Arabidopsis thaliana	AT5G17350.1 | Symbols: | unknown protein; FUNCTIONS IN: molecular_function unknown; INVOLVED IN: N-terminal protein myristoylation; LOCATED IN: cellular_component unknown; EXPRESSED IN: 15 plant structures; EXPRESSED DURING: 8 growth stages; BEST Arabidopsis thaliana protein match is: unknown protein (TAIR:AT3G03280.1); Has 1807 Blast hits to 1807 proteins in 277 species: Archae - 0; Bacteria - 0; Metazoa - 736; Fungi - 347; Plants - 385; Viruses - 0; Other Eukaryotes - 339 (source: NCBI BLink). | chr5:5718853-5719404 FORWARD LENGTH=183	186	183	2.00E-71	98.4	78.0	84.4
Rsa1.0_00062.1.g3344.t1	gb|EOA20498.1| hypothetical protein CARUB_v10000811mg [Capsella rubella]	536	492	0	91.8	69.8	78.7	hypothetical protein CARUB_v10000811mg	gbpln	Capsella rubella	AT5G17370.1 | Symbols: | Transducin/WD40 repeat-like superfamily protein | chr5:5721801-5724719 REVERSE LENGTH=529	536	529	0	98.7	69.6	77.8
Rsa1.0_00062.1.g3345.t5	gb|ACS68197.1| cellulose synthase 7.1 catalytic subunit [Brassica napus]	1016	1031	0	101.5	98.2	99.0	cellulose synthase 7.1 catalytic subunit	gbpln	Brassica napus	AT5G17420.1 | Symbols: IRX3, CESA7, ATCESA7, MUR10 | Cellulose synthase family protein | chr5:5736859-5741407 REVERSE LENGTH=1026	1016	1026	0	101.0	95.2	97.2
Rsa1.0_00062.1.g3346.t1	ref|XP_002873828.1| predicted protein [Arabidopsis lyrata subsp. lyrata] gi|297319665|gb|EFH50087.1| predicted protein [Arabidopsis lyrata subsp. lyrata]	579	587	0	101.4	80.1	86.2	predicted protein	gbpln	Arabidopsis lyrata	AT5G17430.1 | Symbols: BBM | Integrase-type DNA-binding superfamily protein | chr5:5742542-5745568 REVERSE LENGTH=584	579	584	0	100.9	77.4	83.4
Rsa1.0_00062.1.g3347.t1	ref|NP_568347.1| LUC7 related protein [Arabidopsis thaliana] gi|15450599|gb|AAK96571.1| AT5g17440/K3M16_10 [Arabidopsis thaliana] gi|17380624|gb|AAL36075.1| AT5g17440/K3M16_10 [Arabidopsis thaliana] gi|332005043|gb|AED92426.1| LUC7 related protein [Arabidopsis thaliana]	406	404	1.00E-174	99.5	73.4	76.4	LUC7 related protein	gbpln	Arabidopsis thaliana	AT5G17440.1 | Symbols: | LUC7 related protein | chr5:5749849-5753415 FORWARD LENGTH=404	406	404	1.00E-177	99.5	73.4	76.4

Rsa1.0_00062.1.g3348.t2	gb|EOA37750.1| hypothetical protein CARUB_v10012565mg [Capsella rubella]	549	679	1.00E-114	123.7	45.9	66.1	hypothetical protein CARUB_v10012565mg	gbpln	Capsella rubella	AT3G42170.1 | Symbols: | BED zinc finger ;hAT family dimerisation domain | chr3:14321838-14323928 FORWARD LENGTH=696	549	696	3.00E-21	126.8	14.4	23.7
Rsa1.0_00062.1.g3349.t2	ref|NP_197247.1| heavy metal transport/detoxification domain-containing protein [Arabidopsis thaliana] gi|297807767|ref|XP_002871767.1| hypothetical protein ARALYDRAFT_488611 [Arabidopsis lyrata subsp. lyrata] gi|9755769|emb|CAC01889.1| farnesylated protein ATFP6-like protein [Arabidopsis thaliana] gi|117168109|gb|ABK32137.1| At5g17450 [Arabidopsis thaliana] gi|297317604|gb|EFH48026.1| hypothetical protein ARALYDRAFT_488611 [Arabidopsis lyrata subsp. lyrata] gi|332005044|gb|AED92427.1| heavy metal transport/detoxification domain-containing protein [Arabidopsis thaliana] gi|482555303|gb|EOA19495.1| hypothetical protein CARUB_v10002198mg [Capsella rubella]	149	149	4.00E-73	100.0	94.6	96.0	heavy metal transport/detoxification domain-containing protein	gbpln	Arabidopsis lyrata	AT5G17450.1 | Symbols: HIPP21 | Heavy metal transport/detoxification superfamily protein | chr5:5755386-5756743 FORWARD LENGTH=149	149	149	1.00E-75	100.0	94.6	96.0
Rsa1.0_00062.1.g3350.t1	ref|XP_002873829.1| hypothetical protein ARALYDRAFT_909741 [Arabidopsis lyrata subsp. lyrata] gi|297319666|gb|EFH50088.1| hypothetical protein ARALYDRAFT_909741 [Arabidopsis lyrata subsp. lyrata]	305	309	1.00E-135	101.3	87.5	90.8	hypothetical protein ARALYDRAFT_909741	gbpln	Arabidopsis lyrata	AT5G17460.1 | Symbols: | unknown protein; FUNCTIONS IN: molecular_function unknown; INVOLVED IN: response to salt stress; LOCATED IN: mitochondrion; Has 30201 Blast hits to 17322 proteins in 780 species: Archae - 12; Bacteria - 1396; Metazoa - 17338; Fungi - 3422; Plants - 5037; Viruses - 0; Other Eukaryotes - 2996 (source: NCBI BLink). | chr5:5757582-5758867 REVERSE LENGTH=309	305	309	1.00E-137	101.3	86.9	90.8
Rsa1.0_00062.1.g3351.t1	sp|P69196.1|POLC1_BRANA RecName: Full=Polcalcin Bra n 1; AltName: Full=Calcium-binding pollen allergen Bra n 1; AltName: Allergen=Bra n 1 gi|59800144|sp|P69197.1|POLC1_BRARA RecName: Full=Polcalcin Bra r 1; AltName: Full=Calcium-binding pollen allergen Bra r 1; AltName: Allergen=Bra r 1 gi|1255536|dbj|BAA09632.1| calcium-binding protein [Brassica napus] gi|1255540|dbj|BAA09634.1| calcium-binding protein [Brassica rapa] gi|7415719|dbj|BAA93509.1| pollen calcium-binding protein [Brassica rapa]	80	79	9.00E-34	98.8	91.3	93.8	RecName: Full=Polcalcin Bra n 1; AltName: Full=Calcium-binding pollen allergen Bra n 1; AltName: Allergen=Bra n 1 gi|59800144|sp|P69197.1|POLC1_BRARA RecName: Full=Polcalcin Bra r 1; AltName: Full=Calcium-binding pollen allergen Bra r 1; AltName: Allergen=Bra r 1 gi|1255536|dbj|BAA09632.1| calcium-binding protein	gbpln	Brassica napus	AT5G17480.1 | Symbols: APC1, PC1 | pollen calcium-binding protein 1 | chr5:5762689-5762940 FORWARD LENGTH=83	80	83	4.00E-33	103.8	83.8	90.0
Rsa1.0_00063.1.g3352.t1	ref|NP_172188.1| 60S ribosomal protein L35a-1 [Arabidopsis thaliana] gi|297843500|ref|XP_002889631.1| 60S ribosomal protein L35a [Arabidopsis lyrata subsp. lyrata] gi|75174783|sp|Q9LMK0.1|R35A1_ARATH RecName: Full=60S ribosomal protein L35a-1 gi|8954039|gb|AAF82213.1|AC067971_21 Strong similarity to a ribosomal protein from Arabidopsis thaliana gb|AL161667. It contains a ribosomal protein L35Ae domain PF|01247 [Arabidopsis thaliana] gi|21555376|gb|AAM63844.1| ribosomal protein, putative [Arabidopsis thaliana] gi|26453120|dbj|BAC43636.1| unknown protein [Arabidopsis thaliana] gi|28416859|gb|AAO42960.1| At1g07070 [Arabidopsis thaliana] gi|297335473|gb|EFH65890.1| 60S ribosomal protein L35a [Arabidopsis lyrata subsp. lyrata] gi|332189953|gb|AEE28074.1| 60S ribosomal protein L35a-1 [Arabidopsis thaliana]	242	112	4.00E-57	46.3	43.4	44.6	60S ribosomal protein L35a-1	gbpln	Arabidopsis lyrata	AT1G07070.1 | Symbols: | Ribosomal protein L35Ae family protein | chr1:2168652-2169703 FORWARD LENGTH=112	242	112	1.00E-59	46.3	43.4	44.6

Rsa1.0_00063.1.g3353.t1	gb|EOA33304.1| hypothetical protein CARUB_v10019953mg [Capsella rubella]	619	634	0	102.4	78.8	87.4	hypothetical protein CARUB_v10019953mg	gbpln	Capsella rubella	AT1G74250.1 | Symbols: | DNAJ heat shock N-terminal domain-containing protein | chr1:27920328-27922414 FORWARD LENGTH=630	619	630	0	101.8	76.7	88.0
Rsa1.0_00063.1.g3354.t1	ref|XP_002887533.1| hypothetical protein ARALYDRAFT_339621 [Arabidopsis lyrata subsp. lyrata] gi|297333374|gb|EFH63792.1| hypothetical protein ARALYDRAFT_339621 [Arabidopsis lyrata subsp. lyrata]	333	364	1.00E-131	109.3	73.3	80.2	hypothetical protein ARALYDRAFT_339621	gbpln	Arabidopsis lyrata	AT1G74240.1 | Symbols: | Mitochondrial substrate carrier family protein | chr1:27917437-27919987 FORWARD LENGTH=364	333	364	1.00E-126	109.3	66.7	71.2
Rsa1.0_00063.1.g3355.t1	gb|ABB97027.1| unknown [Brassica rapa]	261	208	2.00E-56	79.7	41.0	41.8	unknown	gbpln	Brassica rapa	AT1G74230.1 | Symbols: GR-RBP5 | glycine-rich RNA-binding protein 5 | chr1:27915346-27916857 FORWARD LENGTH=289	261	289	1.00E-55	110.7	40.2	41.8
Rsa1.0_00063.1.g3356.t3	ref|XP_002888967.1| leucine-rich repeat family protein [Arabidopsis lyrata subsp. lyrata] gi|297334808|gb|EFH65226.1| leucine-rich repeat family protein [Arabidopsis lyrata subsp. lyrata]	689	966	1.00E-134	140.2	46.3	56.9	leucine-rich repeat family protein	gbpln	Arabidopsis lyrata	AT1G74190.1 | Symbols: AtRLP15, RLP15 | receptor like protein 15 | chr1:27902590-27906158 REVERSE LENGTH=965	689	965	1.00E-132	140.1	45.7	56.2
Rsa1.0_00063.1.g3357.t1	ref|NP_565084.1| putative LRR receptor-like serine/threonine-protein kinase [Arabidopsis thaliana] gi|264664459|sp|C0LGJ1.1|Y1743_ARATH RecName: Full=Probable LRR receptor-like serine/threonine-protein kinase At1g74360; Flags: Precursor gi|224589485|gb|ACN59276.1| leucine-rich repeat receptor-like protein kinase [Arabidopsis thaliana] gi|332197461|gb|AEE35582.1| putative LRR receptor-like serine/threonine-protein kinase [Arabidopsis thaliana]	771	1106	0	143.5	70.7	78.1	putative LRR receptor-like serine/threonine-protein kinase	gbpln	Arabidopsis thaliana	AT1G74360.1 | Symbols: | Leucine-rich repeat protein kinase family protein | chr1:27954299-27957911 FORWARD LENGTH=1106	771	1106	0	143.5	70.7	78.1
Rsa1.0_00063.1.g3358.t2	ref|XP_002888967.1| leucine-rich repeat family protein [Arabidopsis lyrata subsp. lyrata] gi|297334808|gb|EFH65226.1| leucine-rich repeat family protein [Arabidopsis lyrata subsp. lyrata]	932	966	0	103.6	77.3	87.4	leucine-rich repeat family protein	gbpln	Arabidopsis lyrata	AT1G74190.1 | Symbols: AtRLP15, RLP15 | receptor like protein 15 | chr1:27902590-27906158 REVERSE LENGTH=965	932	965	0	103.5	76.1	85.7
Rsa1.0_00063.1.g3359.t1	ref|XP_002888967.1| leucine-rich repeat family protein [Arabidopsis lyrata subsp. lyrata] gi|297334808|gb|EFH65226.1| leucine-rich repeat family protein [Arabidopsis lyrata subsp. lyrata]	136	966	2.00E-39	710.3	69.9	80.9	leucine-rich repeat family protein	gbpln	Arabidopsis lyrata	AT1G54470.1 | Symbols: RPP27 | RNI-like superfamily protein | chr1:20344738-20347339 FORWARD LENGTH=457	136	457	7.00E-38	336.0	58.1	71.3
Rsa1.0_00063.1.g3360.t1	ref|NP_177559.1| receptor like protein 15 [Arabidopsis thaliana] gi|12323812|gb|AAG51871.1|AC079678_1 disease resistance protein, putative; 1096-4664 [Arabidopsis thaliana] gi|332197443|gb|AEE35564.1| receptor like protein 15 [Arabidopsis thaliana]	594	965	1.00E-150	162.5	52.2	64.6	receptor like protein 15	gbpln	Arabidopsis thaliana	AT1G74190.1 | Symbols: AtRLP15, RLP15 | receptor like protein 15 | chr1:27902590-27906158 REVERSE LENGTH=965	594	965	1.00E-153	162.5	52.2	64.6
Rsa1.0_00063.1.g3361.t1	#	#	#	#	#	#	#	-	----	----	#	#	#	#	#	#	#
Rsa1.0_00063.1.g3362.t1	#	#	#	#	#	#	#	-	----	----	#	#	#	#	#	#	#
Rsa1.0_00063.1.g3363.t1	gb|EOA33578.1| hypothetical protein CARUB_v10019719mg, partial [Capsella rubella]	212	984	9.00E-64	464.2	63.2	72.6	hypothetical protein CARUB_v10019719mg, partial	gbpln	Capsella rubella	AT2G25470.1 | Symbols: AtRLP21, RLP21 | receptor like protein 21 | chr2:10838420-10841881 FORWARD LENGTH=935	212	935	2.00E-54	441.0	52.8	65.1
Rsa1.0_00063.1.g3364.t1	ref|XP_002888967.1| leucine-rich repeat family protein [Arabidopsis lyrata subsp. lyrata] gi|297334808|gb|EFH65226.1| leucine-rich repeat family protein [Arabidopsis lyrata subsp. lyrata]	788	966	0	122.6	56.6	67.1	leucine-rich repeat family protein	gbpln	Arabidopsis lyrata	AT1G74190.1 | Symbols: AtRLP15, RLP15 | receptor like protein 15 | chr1:27902590-27906158 REVERSE LENGTH=965	788	965	0	122.5	56.7	66.1
Rsa1.0_00063.1.g3365.t1	ref|XP_002887529.1| hypothetical protein ARALYDRAFT_476557 [Arabidopsis lyrata subsp. lyrata] gi|297333370|gb|EFH63788.1| hypothetical protein ARALYDRAFT_476557 [Arabidopsis lyrata subsp. lyrata]	1007	1029	0	102.2	81.9	87.4	hypothetical protein ARALYDRAFT_476557	gbpln	Arabidopsis lyrata	AT1G74160.1 | Symbols: | unknown protein; BEST Arabidopsis thaliana protein match is: unknown protein (TAIR:AT1G18620.1); Has 3407 Blast hits to 2217 proteins in 314 species: Archae - 0; Bacteria - 264; Metazoa - 1296; Fungi - 318; Plants - 346; Viruses - 34; Other Eukaryotes - 1149 (source: NCBI BLink). | chr1:27887164-27891151 FORWARD LENGTH=1025	1007	1025	0	101.8	81.5	86.8
Rsa1.0_00063.1.g3366.t1	ref|XP_002887528.1| hypothetical protein ARALYDRAFT_316360 [Arabidopsis lyrata subsp. lyrata] gi|297333369|gb|EFH63787.1| hypothetical protein ARALYDRAFT_316360 [Arabidopsis lyrata subsp. lyrata]	486	567	0	116.7	70.4	77.8	hypothetical protein ARALYDRAFT_316360	gbpln	Arabidopsis lyrata	AT1G74150.1 | Symbols: | Galactose oxidase/kelch repeat superfamily protein | chr1:27880528-27883626 FORWARD LENGTH=569	486	569	0	117.1	70.2	77.0
Rsa1.0_00063.1.g3367.t2	gb|EOA34985.1| hypothetical protein CARUB_v10020076mg [Capsella rubella]	429	561	1.00E-141	130.8	56.9	63.9	hypothetical protein CARUB_v10020076mg	gbpln	Capsella rubella	AT1G74150.1 | Symbols: | Galactose oxidase/kelch repeat superfamily protein | chr1:27880528-27883626 FORWARD LENGTH=569	429	569	1.00E-143	132.6	57.3	64.1

Rsa1.0_00063.1.g3368.t1	gb|ADW54463.1| sulfotransferase [Brassica oleracea var. botrytis]	337	337	0	100.0	95.3	97.6	sulfotransferase	gbpln	Brassica oleracea	AT1G74100.1 | Symbols: SOT16, ATSOT16, CORI-7, ATST5A | sulfotransferase 16 | chr1:27864489-27865505 REVERSE LENGTH=338	337	338	0	100.3	94.1	96.1
Rsa1.0_00063.1.g3369.t1	gb|ADW54463.1| sulfotransferase [Brassica oleracea var. botrytis]	339	337	0	99.4	90.9	95.6	sulfotransferase	gbpln	Brassica oleracea	AT1G74100.1 | Symbols: SOT16, ATSOT16, CORI-7, ATST5A | sulfotransferase 16 | chr1:27864489-27865505 REVERSE LENGTH=338	339	338	0	99.7	89.7	95.0
Rsa1.0_00063.1.g3370.t1	#	#	#	#	#	#	#	-	----	----	#	#	#	#	#	#	#
Rsa1.0_00063.1.g3371.t1	ref|NP_177548.1| myb domain protein 122 [Arabidopsis thaliana] gi|12325142|gb|AAG52518.1|AC016662_12 putative transcription factor; 17206-15746 [Arabidopsis thaliana] gi|14161427|gb|AAK54746.1|AF371983_1 putative transcription factor MYB122 [Arabidopsis thaliana] gi|114050657|gb|ABI49478.1| At1g74080 [Arabidopsis thaliana] gi|225898078|dbj|BAH30371.1| hypothetical protein [Arabidopsis thaliana] gi|332197425|gb|AEE35546.1| myb domain protein 122 [Arabidopsis thaliana]	106	333	3.00E-25	314.2	56.6	64.2	myb domain protein 122	gbpln	Arabidopsis thaliana	AT1G74080.1 | Symbols: ATMYB122, MYB122 | myb domain protein 122 | chr1:27855972-27857432 FORWARD LENGTH=333	106	333	4.00E-28	314.2	56.6	64.2
Rsa1.0_00063.1.g3372.t2	gb|AAD15534.1| putative retroelement pol polyprotein [Arabidopsis thaliana]	759	1664	1.00E-180	219.2	45.7	58.4	putative retroelement pol polyprotein	gbpln	Arabidopsis thaliana	AT4G23160.1 | Symbols: CRK8 | cysteine-rich RLK (RECEPTOR-like protein kinase) 8 | chr4:12129485-12134086 FORWARD LENGTH=1262	759	1262	4.00E-24	166.3	7.4	10.0
Rsa1.0_00063.1.g3373.t1	#	#	#	#	#	#	#	-	----	----	#	#	#	#	#	#	#
Rsa1.0_00063.1.g3374.t1	gb|AAD32898.1| putative retroelement pol polyprotein [Arabidopsis thaliana]	921	1307	1.00E-166	141.9	33.3	42.2	putative retroelement pol polyprotein	gbpln	Arabidopsis thaliana	AT4G23160.1 | Symbols: CRK8 | cysteine-rich RLK (RECEPTOR-like protein kinase) 8 | chr4:12129485-12134086 FORWARD LENGTH=1262	921	1262	8.00E-71	137.0	17.5	28.2
Rsa1.0_00063.1.g3375.t1	gb|AAC28221.1| similar to reverse transcriptases (PFam: rvt.hmm, score: 60.13) [Arabidopsis thaliana]	1272	1164	1.00E-180	91.5	28.4	39.4	similar to reverse transcriptases (PFam: rvt.hmm, score: 60.13)	gbpln	Arabidopsis thaliana	AT1G43760.1 | Symbols: | DNAse I-like superfamily protein | chr1:16528880-16531065 REVERSE LENGTH=626	1272	626	5.00E-51	49.2	8.4	13.8
Rsa1.0_00063.1.g3376.t1	ref|NP_177548.1| myb domain protein 122 [Arabidopsis thaliana] gi|12325142|gb|AAG52518.1|AC016662_12 putative transcription factor; 17206-15746 [Arabidopsis thaliana] gi|14161427|gb|AAK54746.1|AF371983_1 putative transcription factor MYB122 [Arabidopsis thaliana] gi|114050657|gb|ABI49478.1| At1g74080 [Arabidopsis thaliana] gi|225898078|dbj|BAH30371.1| hypothetical protein [Arabidopsis thaliana] gi|332197425|gb|AEE35546.1| myb domain protein 122 [Arabidopsis thaliana]	334	333	1.00E-130	99.7	76.3	84.1	myb domain protein 122	gbpln	Arabidopsis thaliana	AT1G74080.1 | Symbols: ATMYB122, MYB122 | myb domain protein 122 | chr1:27855972-27857432 FORWARD LENGTH=333	334	333	1.00E-132	99.7	76.3	84.1
Rsa1.0_00063.1.g3377.t1	gb|EOA35264.1| hypothetical protein CARUB_v10020433mg [Capsella rubella]	322	362	1.00E-138	112.4	80.1	87.6	hypothetical protein CARUB_v10020433mg	gbpln	Capsella rubella	AT1G74070.1 | Symbols: | Cyclophilin-like peptidyl-prolyl cis-trans isomerase family protein | chr1:27851749-27852861 REVERSE LENGTH=317	322	317	1.00E-139	98.4	82.0	89.1
Rsa1.0_00063.1.g3378.t1	#	#	#	#	#	#	#	-	----	----	#	#	#	#	#	#	#
Rsa1.0_00063.1.g3379.t1	gb|EOA33232.1| hypothetical protein CARUB_v10021263mg [Capsella rubella]	321	233	1.00E-109	72.6	66.0	69.2	hypothetical protein CARUB_v10021263mg	gbpln	Capsella rubella	AT1G74050.1 | Symbols: | Ribosomal protein L6 family protein | chr1:27847256-27848680 REVERSE LENGTH=233	321	233	1.00E-109	72.6	66.4	68.5
Rsa1.0_00063.1.g3380.t1	dbj|BAJ34366.1| unnamed protein product [Thellungiella halophila]	328	328	1.00E-169	100.0	93.6	97.3	unnamed protein product	----	----	AT1G74020.1 | Symbols: SS2 | strictosidine synthase 2 | chr1:27835289-27837277 REVERSE LENGTH=335	328	335	1.00E-169	102.1	88.7	91.5
Rsa1.0_00063.1.g3381.t1	ref|XP_002888957.1| strictosidine synthase family protein [Arabidopsis lyrata subsp. lyrata] gi|297334798|gb|EFH65216.1| strictosidine synthase family protein [Arabidopsis lyrata subsp. lyrata]	327	326	1.00E-154	99.7	86.2	91.7	strictosidine synthase family protein	gbpln	Arabidopsis lyrata	AT1G74010.1 | Symbols: | Calcium-dependent phosphotriesterase superfamily protein | chr1:27832432-27834017 REVERSE LENGTH=325	327	325	1.00E-153	99.4	85.3	90.2
Rsa1.0_00063.1.g3382.t1	ref|XP_002888956.1| hypothetical protein ARALYDRAFT_476540 [Arabidopsis lyrata subsp. lyrata] gi|297334797|gb|EFH65215.1| hypothetical protein ARALYDRAFT_476540 [Arabidopsis lyrata subsp. lyrata]	325	327	1.00E-145	100.6	79.1	85.8	hypothetical protein ARALYDRAFT_476540	gbpln	Arabidopsis lyrata	AT1G74000.1 | Symbols: SS3 | strictosidine synthase 3 | chr1:27829266-27831401 REVERSE LENGTH=329	325	329	1.00E-144	101.2	76.9	85.2
Rsa1.0_00063.1.g3383.t1	gb|EOA23321.1| hypothetical protein CARUB_v10019219mg [Capsella rubella]	431	444	1.00E-112	103.0	50.1	63.8	hypothetical protein CARUB_v10019219mg	gbpln	Capsella rubella	AT2G40920.1 | Symbols: | F-box and associated interaction domains-containing protein | chr2:17072586-17074012 REVERSE LENGTH=436	431	436	3.00E-72	101.2	37.4	53.4

Rsa1.0_00064.1.g3384.t1	ref|XP_002880263.1| hypothetical protein ARALYDRAFT_346489 [Arabidopsis lyrata subsp. lyrata] gi|297326102|gb|EFH56522.1| hypothetical protein ARALYDRAFT_346489 [Arabidopsis lyrata subsp. lyrata]	328	334	1.00E-112	101.8	76.2	80.8	hypothetical protein ARALYDRAFT_346489	gbpln	Arabidopsis lyrata	AT2G46780.1 | Symbols: | RNA-binding (RRM/RBD/RNP motifs) family protein | chr2:19229516-19231043 FORWARD LENGTH=336	328	336	1.00E-115	102.4	83.8	87.2
Rsa1.0_00064.1.g3385.t1	dbj|BAJ34373.1| unnamed protein product [Thellungiella halophila]	431	486	0	112.8	81.2	87.7	unnamed protein product	----	----	AT2G46790.1 | Symbols: APRR9, PRR9, TL1 | pseudo-response regulator 9 | chr2:19232874-19234901 FORWARD LENGTH=468	431	468	1.00E-173	108.6	75.9	85.4
Rsa1.0_00064.1.g3386.t1	gb|AAO83658.1| cation-efflux transporter [Brassica juncea]	389	387	1.00E-180	99.5	88.4	90.7	cation-efflux transporter	gbpln	Brassica juncea	AT2G46800.2 | Symbols: ZAT, ATMTP1, MTP1, ZAT1, ATCDF1 | zinc transporter of Arabidopsis thaliana | chr2:19238128-19239324 FORWARD LENGTH=398	389	398	1.00E-172	102.3	84.3	87.9
Rsa1.0_00064.1.g3387.t1	#	#	#	#	#	#	#	–	----	----	AT2G47000.1 | Symbols: MDR4, PGP4, ABCB4, ATPGP4 | ATP binding cassette subfamily B4 | chr2:19310008-19314750 REVERSE LENGTH=1286	100	1286	9.00E-12	1286.0	34.0	41.0
Rsa1.0_00064.1.g3388.t3	ref|XP_002864330.1| hypothetical protein ARALYDRAFT_495521 [Arabidopsis lyrata subsp. lyrata] gi|297310165|gb|EFH40589.1| hypothetical protein ARALYDRAFT_495521 [Arabidopsis lyrata subsp. lyrata]	559	356	1.00E-115	63.7	38.5	46.3	hypothetical protein ARALYDRAFT_495521	gbpln	Arabidopsis lyrata	AT5G54330.1 | Symbols: | Protein of unknown function (DUF295) | chr5:22064993-22066063 FORWARD LENGTH=356	559	356	1.00E-118	63.7	38.5	46.3
Rsa1.0_00064.1.g3389.t1	ref|NP_001189765.1| uncharacterized protein [Arabidopsis thaliana] gi|330255688|gb|AEC10782.1| uncharacterized protein AT2G46980 [Arabidopsis thaliana]	119	790	4.00E-23	663.9	52.1	54.6	uncharacterized protein	gbpln	Arabidopsis thaliana	AT2G46980.3 | Symbols: | unknown protein; FUNCTIONS IN: molecular_function unknown; INVOLVED IN: biological_process unknown; LOCATED IN: cellular_component unknown. | chr2:19299868-19303429 REVERSE LENGTH=790	119	790	7.00E-26	663.9	52.1	54.6
Rsa1.0_00064.1.g3390.t1	ref|NP_001189765.1| uncharacterized protein [Arabidopsis thaliana] gi|330255688|gb|AEC10782.1| uncharacterized protein AT2G46980 [Arabidopsis thaliana]	401	790	1.00E-45	197.0	34.4	39.4	uncharacterized protein	gbpln	Arabidopsis thaliana	AT2G46980.3 | Symbols: | unknown protein; FUNCTIONS IN: molecular_function unknown; INVOLVED IN: biological_process unknown; LOCATED IN: cellular_component unknown. | chr2:19299868-19303429 REVERSE LENGTH=790	401	790	3.00E-48	197.0	34.4	39.4
Rsa1.0_00064.1.g3391.t1	ref|NP_001031550.1| homeobox-leucine zipper protein ATHB-7 [Arabidopsis thaliana] gi|330255646|gb|AEC10740.1| homeobox-leucine zipper protein ATHB-7 [Arabidopsis thaliana]	71	256	1.00E-27	360.6	85.9	91.5	homeobox-leucine zipper protein ATHB-7	gbpln	Arabidopsis thaliana	AT2G46680.2 | Symbols: ATHB-7, ATHB7, HB-7 | homeobox 7 | chr2:19165777-19166773 REVERSE LENGTH=256	71	256	2.00E-30	360.6	85.9	91.5
Rsa1.0_00064.1.g3392.t1	ref|NP_200244.1| uncharacterized protein [Arabidopsis thaliana] gi|9759506|dbj|BAB10756.1| unnamed protein product [Arabidopsis thaliana] gi|93007389|gb|ABE97197.1| hypothetical protein At5g54330 [Arabidopsis thaliana] gi|332009102|gb|AED96485.1| uncharacterized protein AT5G54330 [Arabidopsis thaliana]	380	356	1.00E-113	93.7	56.8	68.7	uncharacterized protein	gbpln	Arabidopsis thaliana	AT5G54330.1 | Symbols: | Protein of unknown function (DUF295) | chr5:22064993-22066063 FORWARD LENGTH=356	380	356	1.00E-115	93.7	56.8	68.7
Rsa1.0_00064.1.g3393.t1	dbj|BAM14091.1| basic helix-loop-helix DNA-binding superfamily protein [Arabidopsis thaliana]	325	359	1.00E-110	110.5	75.1	86.2	basic helix-loop-helix DNA-binding superfamily protein	gbpln	Arabidopsis thaliana	AT2G46810.1 | Symbols: | basic helix-loop-helix (bHLH) DNA-binding superfamily protein | chr2:19239694-19242373 FORWARD LENGTH=371	325	371	1.00E-109	114.2	72.3	85.2
Rsa1.0_00064.1.g3394.t1	gb|EOA27989.1| hypothetical protein CARUB_v10024165mg [Capsella rubella]	181	175	4.00E-67	96.7	76.8	87.3	hypothetical protein CARUB_v10024165mg	gbpln	Capsella rubella	AT2G46820.2 | Symbols: PTAC8, TMP14, PSAP, PSI-P | photosystem I P subunit | chr2:19243729-19244870 FORWARD LENGTH=174	181	174	1.00E-54	96.1	71.8	83.4
Rsa1.0_00064.1.g3395.t2	ref|XP_002880270.1| hypothetical protein ARALYDRAFT_483853 [Arabidopsis lyrata subsp. lyrata] gi|297326109|gb|EFH56529.1| hypothetical protein ARALYDRAFT_483853 [Arabidopsis lyrata subsp. lyrata]	220	216	1.00E-118	98.2	93.6	95.9	hypothetical protein ARALYDRAFT_483853	gbpln	Arabidopsis lyrata	AT2G46860.1 | Symbols: AtPPa3, PPa3 | pyrophosphorylase 3 | chr2:19253843-19255060 FORWARD LENGTH=216	220	216	1.00E-119	98.2	92.3	95.0

Rsa1.0_00064.1.g3396.t1	ref|NP_566089.1| B3 domain-containing transcription factor NGA1 [Arabidopsis thaliana] gi|75100798|sp|O82799.1|NGA1_ARATH RecName: Full=B3 domain-containing transcription factor NGA1; AltName: Full=Protein NGATHA 1 gi|3522951|gb|AAC34233.1| putative RAV-like B3 domain DNA binding protein [Arabidopsis thaliana] gi|20197317|gb|AAM15018.1| putative RAV-like B3 domain DNA binding protein [Arabidopsis thaliana] gi|26451145|dbj|BAC42676.1| putative RAV-like B3 domain DNA binding protein [Arabidopsis thaliana] gi|30793817|gb|AAP40361.1| putative RAV B3 domain DNA binding protein [Arabidopsis thaliana] gi|330255671|gb|AEC10765.1| B3 domain-containing transcription factor NGA1 [Arabidopsis thaliana]	298	310	1.00E-118	104.0	78.9	85.2	B3 domain-containing transcription factor NGA1	gbpln	Arabidopsis thaliana	AT2G46870.1 | Symbols: NGA1 | AP2/B3-like transcriptional factor family protein | chr2:19261313-19262245 FORWARD LENGTH=310	298	310	1.00E-120	104.0	78.9	85.2
Rsa1.0_00064.1.g3397.t1	ref|NP_182211.2| purple acid phosphatase 14 [Arabidopsis thaliana] gi|75272080|sp|Q84LR6.1|PPA14_ARATH RecName: Full=Probable inactive purple acid phosphatase 14; Flags: Precursor gi|30267807|gb|AAP21684.1| hypothetical protein [Arabidopsis thaliana] gi|50058953|gb|AAT69221.1| hypothetical protein At2g46880 [Arabidopsis thaliana] gi|330255673|gb|AEC10767.1| purple acid phosphatase 14 [Arabidopsis thaliana]	394	401	1.00E-179	101.8	79.4	87.3	purple acid phosphatase 14	gbpln	Arabidopsis thaliana	AT2G46880.1 | Symbols: ATPAP14, PAP14 | purple acid phosphatase 14 | chr2:19264910-19266412 REVERSE LENGTH=401	394	401	0	101.8	79.4	87.3
Rsa1.0_00064.1.g3398.t1	ref|XP_002882091.1| hypothetical protein ARALYDRAFT_904159 [Arabidopsis lyrata subsp. lyrata] gi|297327930|gb|EFH58350.1| hypothetical protein ARALYDRAFT_904159 [Arabidopsis lyrata subsp. lyrata]	325	322	1.00E-165	99.1	90.2	95.1	hypothetical protein ARALYDRAFT_904159	gbpln	Arabidopsis lyrata	AT2G46890.1 | Symbols: | Protein of unknown function (DUF1295) | chr2:19266879-19268134 REVERSE LENGTH=322	325	322	1.00E-160	99.1	88.3	93.5
Rsa1.0_00064.1.g3399.t1	ref|NP_566090.1| uncharacterized protein [Arabidopsis thaliana] gi|3522948|gb|AAC34230.1| expressed protein [Arabidopsis thaliana] gi|14532608|gb|AAK64032.1| unknown protein [Arabidopsis thaliana] gi|23296678|gb|AAN13144.1| unknown protein [Arabidopsis thaliana] gi|330255675|gb|AEC10769.1| uncharacterized protein AT2G46900 [Arabidopsis thaliana]	108	627	2.00E-28	580.6	59.3	65.7	uncharacterized protein	gbpln	Arabidopsis thaliana	AT2G46900.1 | Symbols: | CONTAINS InterPro DOMAIN/s: Basic helix-loop-helix, Nulp1-type (InterPro:IPR006994); Has 2929 Blast hits to 2464 proteins in 333 species: Archae - 2; Bacteria - 151; Metazoa - 913; Fungi - 372; Plants - 141; Viruses - 47; Other Eukaryotes - 1303 (source: NCBI BLink). | chr2:19269678-19271987 FORWARD LENGTH=627	108	627	3.00E-31	580.6	59.3	65.7
Rsa1.0_00064.1.g3400.t1	ref|XP_002885669.1| hypothetical protein ARALYDRAFT_319176 [Arabidopsis lyrata subsp. lyrata] gi|297331509|gb|EFH61928.1| hypothetical protein ARALYDRAFT_319176 [Arabidopsis lyrata subsp. lyrata]	448	467	1.00E-113	104.2	51.3	62.5	hypothetical protein ARALYDRAFT_319176	gbpln	Arabidopsis lyrata	AT3G62850.1 | Symbols: | zinc finger protein-related | chr3:23237487-23239155 REVERSE LENGTH=472	448	472	1.00E-112	105.4	49.1	61.2
Rsa1.0_00064.1.g3401.t1	ref|XP_002880272.1| hypothetical protein ARALYDRAFT_483858 [Arabidopsis lyrata subsp. lyrata] gi|297326111|gb|EFH56531.1| hypothetical protein ARALYDRAFT_483858 [Arabidopsis lyrata subsp. lyrata]	577	634	0	109.9	74.4	84.6	hypothetical protein ARALYDRAFT_483858	gbpln	Arabidopsis lyrata	AT2G46900.1 | Symbols: | CONTAINS InterPro DOMAIN/s: Basic helix-loop-helix, Nulp1-type (InterPro:IPR006994); Has 2929 Blast hits to 2464 proteins in 333 species: Archae - 2; Bacteria - 151; Metazoa - 913; Fungi - 372; Plants - 141; Viruses - 47; Other Eukaryotes - 1303 (source: NCBI BLink). | chr2:19269678-19271987 FORWARD LENGTH=627	577	627	0	108.7	72.1	82.8
Rsa1.0_00064.1.g3402.t1	ref|XP_002880273.1| plastid-lipid associated protein pap [Arabidopsis lyrata subsp. lyrata] gi|297326112|gb|EFH56532.1| plastid-lipid associated protein pap [Arabidopsis lyrata subsp. lyrata]	281	283	1.00E-143	100.7	88.6	94.3	plastid-lipid associated protein pap	gbpln	Arabidopsis lyrata	AT2G46910.1 | Symbols: | Plastid-lipid associated protein PAP / fibrillin family protein | chr2:19272427-19273856 FORWARD LENGTH=284	281	284	1.00E-141	101.1	87.2	93.6
Rsa1.0_00064.1.g3403.t2	gb|EOA26563.1| hypothetical protein CARUB_v10022621mg [Capsella rubella]	854	858	0	100.5	91.1	95.1	hypothetical protein CARUB_v10022621mg	gbpln	Capsella rubella	AT2G46920.2 | Symbols: POL | Protein phosphatase 2C family protein | chr2:19278106-19280921 REVERSE LENGTH=856	854	856	0	100.2	89.8	94.8

Rsa1.0_00064.1.g3404.t1	ref|XP_002880274.1| hypothetical protein ARALYDRAFT_483863 [Arabidopsis lyrata subsp. lyrata] gi|297326113|gb|EFH56533.1| hypothetical protein ARALYDRAFT_483863 [Arabidopsis lyrata subsp. lyrata]	419	417	0	99.5	85.4	90.5	hypothetical protein ARALYDRAFT_483863	gbpln	Arabidopsis lyrata	AT2G46930.1 | Symbols: | Pectinacetylesterase family protein | chr2:19283625-19286090 FORWARD LENGTH=416	419	416	0	99.3	84.0	89.7
Rsa1.0_00064.1.g3405.t1	ref|NP_182217.4| uncharacterized protein [Arabidopsis thaliana] gi|330255681|gb|AEC10775.1| uncharacterized protein AT2G46940 [Arabidopsis thaliana]	249	252	4.00E-81	101.2	74.7	80.7	uncharacterized protein	gbpln	Arabidopsis thaliana	AT2G46940.1 | Symbols: | unknown protein; BEST Arabidopsis thaliana protein match is: unknown protein (TAIR:AT3G62070.1); Has 143 Blast hits to 141 proteins in 14 species: Archae - 0; Bacteria - 0; Metazoa - 4; Fungi - 0; Plants - 139; Viruses - 0; Other Eukaryotes - 0 (source: NCBI BLink). | chr2:19286658-19287505 REVERSE LENGTH=252	249	252	1.00E-83	101.2	74.7	80.7
Rsa1.0_00064.1.g3406.t1	ref|XP_002882095.1| predicted protein [Arabidopsis lyrata subsp. lyrata] gi|297327934|gb|EFH58354.1| predicted protein [Arabidopsis lyrata subsp. lyrata]	520	517	0	99.4	81.5	91.0	predicted protein	gbpln	Arabidopsis lyrata	AT2G46950.1 | Symbols: CYP709B2 | cytochrome P450, family 709, subfamily B, polypeptide 2 | chr2:19289466-19291541 REVERSE LENGTH=572	520	572	0	110.0	79.8	89.8
Rsa1.0_00064.1.g3407.t2	ref|XP_002882098.1| hypothetical protein ARALYDRAFT_904169 [Arabidopsis lyrata subsp. lyrata] gi|297327937|gb|EFH58357.1| hypothetical protein ARALYDRAFT_904169 [Arabidopsis lyrata subsp. lyrata]	384	413	1.00E-153	107.6	74.5	84.6	hypothetical protein ARALYDRAFT_904169	gbpln	Arabidopsis lyrata	AT2G46970.1 | Symbols: PIL1 | phytochrome interacting factor 3-like 1 | chr2:19295617-19297678 REVERSE LENGTH=416	384	416	1.00E-154	108.3	71.9	81.0
Rsa1.0_00064.1.g3408.t1	ref|XP_002889561.1| hypothetical protein ARALYDRAFT_470580 [Arabidopsis lyrata subsp. lyrata] gi|297335403|gb|EFH65820.1| hypothetical protein ARALYDRAFT_470580 [Arabidopsis lyrata subsp. lyrata]	132	149	3.00E-30	112.9	48.5	51.5	hypothetical protein ARALYDRAFT_470580	gbpln	Arabidopsis lyrata	AT1G05730.1 | Symbols: | Eukaryotic protein of unknown function (DUF842) | chr1:1719107-1720159 FORWARD LENGTH=149	132	149	8.00E-33	112.9	48.5	51.5
Rsa1.0_00064.1.g3409.t1	gb|EOA33837.1| hypothetical protein CARUB_v10021318mg, partial [Capsella rubella]	197	290	1.00E-48	147.2	48.2	62.9	hypothetical protein CARUB_v10021318mg, partial	gbpln	Capsella rubella	AT4G04650.1 | Symbols: | RNA-directed DNA polymerase (reverse transcriptase)-related family protein | chr4:2353989-2355213 REVERSE LENGTH=332	197	332	5.00E-39	168.5	37.1	50.3
Rsa1.0_00064.1.g3410.t1	gb|EOA28335.1| hypothetical protein CARUB_v10024536mg [Capsella rubella]	437	441	2.00E-74	100.9	43.7	58.8	hypothetical protein CARUB_v10024536mg	gbpln	Capsella rubella	AT3G60960.1 | Symbols: | Tetratricopeptide repeat (TPR)-like superfamily protein | chr3:22535729-22536940 FORWARD LENGTH=403	437	403	9.00E-71	92.2	39.6	55.8
Rsa1.0_00064.1.g3411.t1	ref|XP_002882048.1| hypothetical protein ARALYDRAFT_904059 [Arabidopsis lyrata subsp. lyrata] gi|297327887|gb|EFH58307.1| hypothetical protein ARALYDRAFT_904059 [Arabidopsis lyrata subsp. lyrata]	436	448	9.00E-70	102.8	33.7	40.1	hypothetical protein ARALYDRAFT_904059	gbpln	Arabidopsis lyrata	AT1G10270.1 | Symbols: GRP23 | glutamine-rich protein 23 | chr1:3363535-3366276 FORWARD LENGTH=913	436	913	2.00E-21	209.4	15.1	22.0
Rsa1.0_00064.1.g3412.t7	ref|NP_850466.2| uncharacterized protein [Arabidopsis thaliana] gi|110736851|dbj|BAF00383.1| hypothetical protein [Arabidopsis thaliana] gi|330255687|gb|AEC10781.1| uncharacterized protein AT2G46980 [Arabidopsis thaliana]	872	793	0	90.9	67.9	75.0	uncharacterized protein	gbpln	Arabidopsis thaliana	AT2G46980.2 | Symbols: | unknown protein; Has 3995 Blast hits to 3325 proteins in 374 species: Archae - 19; Bacteria - 205; Metazoa - 2201; Fungi - 364; Plants - 149; Viruses - 22; Other Eukaryotes - 1035 (source: NCBI BLink). | chr2:19299868-19303429 REVERSE LENGTH=793	872	793	0	90.9	67.9	75.0
Rsa1.0_00064.1.g3413.t1	ref|XP_002882100.1| P-glycoprotein 4, P-glycoprotein4 [Arabidopsis lyrata subsp. lyrata] gi|297327939|gb|EFH58359.1| P-glycoprotein 4, P-glycoprotein4 [Arabidopsis lyrata subsp. lyrata]	1262	1286	0	101.9	93.1	97.4	P-glycoprotein 4, P-glycoprotein4	gbpln	Arabidopsis lyrata	AT2G47000.1 | Symbols: MDR4, PGP4, ABCB4, ATPGP4 | ATP binding cassette subfamily B4 | chr2:19310008-19314750 REVERSE LENGTH=1286	1262	1286	0	101.9	93.6	97.3
Rsa1.0_00064.1.g3414.t1	ref|XP_002880277.1| At2g47010/F14M4.16 [Arabidopsis lyrata subsp. lyrata] gi|297326116|gb|EFH56536.1| At2g47010/F14M4.16 [Arabidopsis lyrata subsp. lyrata]	478	493	0	103.1	88.3	93.3	At2g47010/F14M4.16	gbpln	Arabidopsis lyrata	AT2G47010.2 | Symbols: | unknown protein; FUNCTIONS IN: molecular_function unknown; INVOLVED IN: biological_process unknown; LOCATED IN: endomembrane system; EXPRESSED IN: 17 plant structures; EXPRESSED DURING: 10 growth stages; BEST Arabidopsis thaliana protein match is: unknown protein (TAIR:AT1G17030.1); Has 72 Blast hits to 72 proteins in 13 species: Archae - 0; Bacteria - 0; Metazoa - 0; Fungi - 0; Plants - 71; Viruses - 0; Other Eukaryotes - 1 (source: NCBI BLink). | chr2:19317505-19319252 FORWARD LENGTH=493	478	493	0	103.1	86.8	92.9
Rsa1.0_00064.1.g3415.t1	gb|EOA27206.1| hypothetical protein CARUB_v10023316mg [Capsella rubella]	418	412	0	98.6	84.9	90.2	hypothetical protein CARUB_v10023316mg	gbpln	Capsella rubella	AT2G47020.1 | Symbols: | Peptide chain release factor 1 | chr2:19320090-19322279 REVERSE LENGTH=413	418	413	0	98.8	84.9	89.7

Rsa1.0_00064.1.g3416.t1	gb|EOA28312.1| hypothetical protein CARUB_v10024512mg [Capsella rubella]	595	595	0	100.0	86.1	93.9	hypothetical protein CARUB_v10024512mg	gbpln	Capsella rubella	AT2G47040.1 | Symbols: VGD1 | Plant invertase/pectin methylesterase inhibitor superfamily | chr2:19328186-19330060 REVERSE LENGTH=595	595	595	0	100.0	86.1	93.6
Rsa1.0_00064.1.g3417.t1	gb|EOA28856.1| hypothetical protein CARUB_v10025101mg [Capsella rubella]	216	216	1.00E-106	100.0	84.3	92.6	hypothetical protein CARUB_v10025101mg	gbpln	Capsella rubella	AT2G47050.1 | Symbols: | Plant invertase/pectin methylesterase inhibitor superfamily protein | chr2:19331927-19332577 REVERSE LENGTH=216	216	216	1.00E-108	100.0	84.7	92.1
Rsa1.0_00064.1.g3418.t1	gb|EOA27227.1| hypothetical protein CARUB_v10023336mg, partial [Capsella rubella]	362	407	0	112.4	95.6	97.0	hypothetical protein CARUB_v10023336mg, partial	gbpln	Capsella rubella	AT2G47060.4 | Symbols: | Protein kinase superfamily protein | chr2:19333116-19334759 REVERSE LENGTH=397	362	397	0	109.7	93.1	95.6
Rsa1.0_00064.1.g3419.t1	ref|XP_002880278.1| hypothetical protein ARALYDRAFT_483883 [Arabidopsis lyrata subsp. lyrata] gi|297326117|gb|EFH56537.1| hypothetical protein ARALYDRAFT_483883 [Arabidopsis lyrata subsp. lyrata]	925	899	0	97.2	82.5	89.0	hypothetical protein ARALYDRAFT_483883	gbpln	Arabidopsis lyrata	AT2G47070.1 | Symbols: SPL1 | squamosa promoter binding protein-like 1 | chr2:19337144-19340552 FORWARD LENGTH=881	925	881	0	95.2	81.1	87.1
Rsa1.0_00064.1.g3420.t1	gb|EOA27267.1| hypothetical protein CARUB_v10023387mg [Capsella rubella]	391	391	0	100.0	98.7	99.5	hypothetical protein CARUB_v10023387mg	gbpln	Capsella rubella	AT1G02500.2 | Symbols: SAM1, SAM-1, MAT1, AtSAM1 | S-adenosylmethionine synthetase 1 | chr1:519037-520218 FORWARD LENGTH=393	391	393	0	100.5	93.1	96.4
Rsa1.0_00064.1.g3421.t1	ref|XP_002882106.1| nucleic acid binding protein [Arabidopsis lyrata subsp. lyrata] gi|297327945|gb|EFH58365.1| nucleic acid binding protein [Arabidopsis lyrata subsp. lyrata]	681	735	0	107.9	72.4	80.8	nucleic acid binding protein	gbpln	Arabidopsis lyrata	AT2G47090.1 | Symbols: | zinc ion binding;nucleic acid binding | chr2:19341444-19344297 REVERSE LENGTH=766	681	766	0	112.5	73.3	82.1
Rsa1.0_00064.1.g3422.t1	ref|XP_002882109.1| predicted protein [Arabidopsis lyrata subsp. lyrata] gi|297327948|gb|EFH58368.1| predicted protein [Arabidopsis lyrata subsp. lyrata]	262	257	1.00E-130	98.1	87.8	93.9	predicted protein	gbpln	Arabidopsis lyrata	AT2G47140.1 | Symbols: | NAD(P)-binding Rossmann-fold superfamily protein | chr2:19350970-19352059 REVERSE LENGTH=257	262	257	1.00E-131	98.1	86.6	92.7
Rsa1.0_00064.1.g3423.t1	ref|NP_850737.1| putative protein phosphatase 2C 49 [Arabidopsis thaliana] gi|122214683|sp|Q3EAF9.1|P2C49_ARATH RecName: Full=Probable protein phosphatase 2C 49; Short=AtPP2C49 gi|332646807|gb|AEE80328.1| putative protein phosphatase 2C 49 [Arabidopsis thaliana]	387	384	1.00E-143	99.2	67.7	80.4	putative protein phosphatase 2C 49	gbpln	Arabidopsis thaliana	AT3G62260.2 | Symbols: | Protein phosphatase 2C family protein | chr3:23038516-23040391 REVERSE LENGTH=384	387	384	1.00E-145	99.2	67.7	80.4
Rsa1.0_00064.1.g3424.t1	#	#	#	#	#	#	#	-	----	----	#	#	#	#	#	#	#
Rsa1.0_00064.1.g3425.t1	ref|NP_182241.1| myb domain protein 2 [Arabidopsis thaliana] gi|506189|dbj|BAA03534.1| ATMYB2 [Arabidopsis thaliana] gi|2275197|gb|AAB63819.1| MYB transcription factor (Atmyb2) [Arabidopsis thaliana] gi|14970986|dbj|BAB62128.1| MYB transcription factor Atmyb2 [Arabidopsis thaliana] gi|38603964|gb|AAR24727.1| At2g47190 [Arabidopsis thaliana] gi|41619210|gb|AAS10049.1| MYB transcription factor [Arabidopsis thaliana] gi|44681444|gb|AAS47662.1| At2g47190 [Arabidopsis thaliana] gi|110738166|dbj|BAF01014.1| MYB transcription factor [Arabidopsis thaliana] gi|330255718|gb|AEC10812.1| myb domain protein 2 [Arabidopsis thaliana]	275	273	1.00E-135	99.3	86.5	93.5	myb domain protein 2	gbpln	Arabidopsis thaliana	AT2G47190.1 | Symbols: ATMYB2, MYB2 | myb domain protein 2 | chr2:19376284-19377297 FORWARD LENGTH=273	275	273	1.00E-138	99.3	86.5	93.5
Rsa1.0_00064.1.g3426.t1	ref|XP_002882113.1| hypothetical protein ARALYDRAFT_322367 [Arabidopsis lyrata subsp. lyrata] gi|297327952|gb|EFH58372.1| hypothetical protein ARALYDRAFT_322367 [Arabidopsis lyrata subsp. lyrata]	86	89	2.00E-27	103.5	74.4	82.6	hypothetical protein ARALYDRAFT_322367	gbpln	Arabidopsis lyrata	AT2G47200.1 | Symbols: | unknown protein; Has 3 Blast hits to 3 proteins in 1 species: Archae - 0; Bacteria - 0; Metazoa - 0; Fungi - 0; Plants - 3; Viruses - 0; Other Eukaryotes - 0 (source: NCBI BLink). | chr2:19377884-19378150 REVERSE LENGTH=88	86	88	1.00E-27	102.3	68.6	79.1
Rsa1.0_00064.1.g3427.t1	dbj|BAJ34105.1| unnamed protein product [Thellungiella halophila]	433	439	0	101.4	93.5	96.5	unnamed protein product	----	----	AT2G47210.1 | Symbols: | myb-like transcription factor family protein | chr2:19379039-19382204 FORWARD LENGTH=441	433	441	0	101.8	91.5	95.2
Rsa1.0_00064.1.g3428.t1	gb|EOA40254.1| hypothetical protein CARUB_v10008975mg [Capsella rubella]	482	487	1.00E-140	101.0	62.4	72.4	hypothetical protein CARUB_v10008975mg	gbpln	Capsella rubella	AT1G48400.1 | Symbols: | F-box/RNI-like/FBD-like domains-containing protein | chr1:17882136-17883855 REVERSE LENGTH=513	482	513	1.00E-138	106.4	61.4	72.6

Rsa1.0_00064.1.g3429.t1	gb|EOA37307.1| hypothetical protein CARUB_v10010977mg [Capsella rubella]	151	711	1.00E-28	470.9	51.7	72.2	hypothetical protein CARUB_v10010977mg	gbpln	Capsella rubella	AT1G26540.1 | Symbols: | Agenet domain-containing protein | chr1:9167783-9170795 REVERSE LENGTH=695	151	695	1.00E-29	460.3	49.7	70.9
Rsa1.0_00064.1.g3430.t3	gb|EOA36732.1| hypothetical protein CARUB_v10012522mg [Capsella rubella]	416	617	4.00E-48	148.3	21.9	26.2	hypothetical protein CARUB_v10012522mg	gbpln	Capsella rubella	AT1G26540.1 | Symbols: | Agenet domain-containing protein | chr1:9167783-9170795 REVERSE LENGTH=695	416	695	2.00E-50	167.1	21.2	26.2
Rsa1.0_00064.1.g3431.t2	ref|XP_002880290.1| long-chain-fatty-acid--CoA ligase family protein [Arabidopsis lyrata subsp. lyrata] gi|297326129|gb|EFH56549.1| long-chain-fatty-acid--CoA ligase family protein [Arabidopsis lyrata subsp. lyrata]	636	660	0	103.8	82.1	88.4	long-chain-fatty-acid--CoA ligase family protein	gbpln	Arabidopsis lyrata	AT2G47240.2 | Symbols: LACS1 | AMP-dependent synthetase and ligase family protein | chr2:19393835-19397616 FORWARD LENGTH=660	636	660	0	103.8	79.7	86.5
Rsa1.0_00064.1.g3432.t1	ref|XP_002882114.1| predicted protein [Arabidopsis lyrata subsp. lyrata] gi|297327953|gb|EFH58373.1| predicted protein [Arabidopsis lyrata subsp. lyrata]	317	337	1.00E-121	106.3	81.1	87.7	predicted protein	gbpln	Arabidopsis lyrata	AT2G47260.1 | Symbols: WRKY23, ATWRKY23 | WRKY DNA-binding protein 23 | chr2:19405045-19406446 REVERSE LENGTH=337	317	337	1.00E-114	106.3	80.4	86.8
Rsa1.0_00064.1.g3433.t1	ref|NP_566098.1| transcription factor bHLH151 [Arabidopsis thaliana] gi|75097628|sp|O22901.1|BH151_ARATH RecName: Full=Transcription factor UPBEAT1; AltName: Full=Basic helix-loop-helix protein 151; Short=AtbHLH151; Short=bHLH 151; AltName: Full=Transcription factor EN 146; AltName: Full=Transcription factor bHLH151; AltName: Full=bHLH transcription factor bHLH151 gi|2275205|gb|AAB63827.1| expressed protein [Arabidopsis thaliana] gi|89111836|gb|ABD60690.1| At2g47270 [Arabidopsis thaliana] gi|225898607|dbj|BAH30434.1| hypothetical protein [Arabidopsis thaliana] gi|330255728|gb|AEC10822.1| transcription factor bHLH151 [Arabidopsis thaliana]	102	102	5.00E-47	100.0	90.2	98.0	transcription factor bHLH151	gbpln	Arabidopsis thaliana	AT2G47270.1 | Symbols: UPB1 | sequence-specific DNA binding transcription factors;transcription regulators | chr2:19411741-19412049 REVERSE LENGTH=102	102	102	9.00E-50	100.0	90.2	98.0
Rsa1.0_00064.1.g3434.t1	ref|NP_566099.1| DEAD-box ATP-dependent RNA helicase 24 [Arabidopsis thaliana] gi|75318047|sp|O22907.2|RH24_ARATH RecName: Full=DEAD-box ATP-dependent RNA helicase 24 gi|16323192|gb|AAL15330.1| At2g47330/T8I13.17 [Arabidopsis thaliana] gi|20196880|gb|AAB63833.2| putative ATP-dependent RNA helicase [Arabidopsis thaliana] gi|21700913|gb|AAM70580.1| At2g47330/T8I13.17 [Arabidopsis thaliana] gi|330255734|gb|AEC10828.1| DEAD-box ATP-dependent RNA helicase 24 [Arabidopsis thaliana]	727	760	0	104.5	88.3	92.8	DEAD-box ATP-dependent RNA helicase 24	gbpln	Arabidopsis thaliana	AT2G47330.1 | Symbols: | P-loop containing nucleoside triphosphate hydrolases superfamily protein | chr2:19429083-19431617 REVERSE LENGTH=760	727	760	0	104.5	88.3	92.8
Rsa1.0_00064.1.g3435.t1	ref|XP_002880296.1| hypothetical protein ARALYDRAFT_904210 [Arabidopsis lyrata subsp. lyrata] gi|297326135|gb|EFH56555.1| hypothetical protein ARALYDRAFT_904210 [Arabidopsis lyrata subsp. lyrata]	472	487	0	103.2	77.8	86.2	hypothetical protein ARALYDRAFT_904210	gbpln	Arabidopsis lyrata	AT2G47350.1 | Symbols: | HIT zinc finger ;PAPA-1-like conserved region | chr2:19434588-19437045 FORWARD LENGTH=486	472	486	0	103.0	76.9	84.7
Rsa1.0_00064.1.g3436.t1	ref|XP_002880297.1| hypothetical protein ARALYDRAFT_904211 [Arabidopsis lyrata subsp. lyrata] gi|297326136|gb|EFH56556.1| hypothetical protein ARALYDRAFT_904211 [Arabidopsis lyrata subsp. lyrata]	308	305	1.00E-124	99.0	83.4	89.9	hypothetical protein ARALYDRAFT_904211	gbpln	Arabidopsis lyrata	AT2G47360.1 | Symbols: | unknown protein; BEST Arabidopsis thaliana protein match is: unknown protein (TAIR:AT1G02570.1); Has 58 Blast hits to 55 proteins in 6 species: Archae - 0; Bacteria - 0; Metazoa - 0; Fungi - 0; Plants - 58; Viruses - 0; Other Eukaryotes - 0 (source: NCBI BLink). | chr2:19437820-19438731 FORWARD LENGTH=303	308	303	1.00E-125	98.4	83.4	89.9

Rsa1.0_00064.1.g3437.t1	ref|NP_565392.1| calcium-dependent phosphotriesterase-like protein [Arabidopsis thaliana] gi|4581119|gb|AAD24609.1| expressed protein [Arabidopsis thaliana] gi|21537036|gb|AAM61377.1| unknown [Arabidopsis thaliana] gi|26451541|dbj|BAC42868.1| unknown protein [Arabidopsis thaliana] gi|28973387|gb|AAO64018.1| unknown protein [Arabidopsis thaliana] gi|330251441|gb|AEC06535.1| calcium-dependent phosphotriesterase-like protein [Arabidopsis thaliana]	334	327	1.00E-161	97.9	85.9	92.2	calcium-dependent phosphotriesterase-like protein	gbpln	Arabidopsis thaliana	AT2G16760.1 | Symbols: | Calcium-dependent phosphotriesterase superfamily protein | chr2:7275739-7277122 FORWARD LENGTH=327	334	327	1.00E-163	97.9	85.9	92.2
Rsa1.0_00064.1.g3438.t2	gb|EOA34962.1| hypothetical protein CARUB_v10020048mg [Capsella rubella] gi|482570775|gb|EOA34963.1| hypothetical protein CARUB_v10020048mg [Capsella rubella]	716	575	0	80.3	52.5	59.8	hypothetical protein CARUB_v10020048mg	gbpln	Capsella rubella	AT3G09360.1 | Symbols: | Cyclin/Brf1-like TBP-binding protein | chr3:2873796-2878432 FORWARD LENGTH=604	716	604	1.00E-164	84.4	43.4	53.2
Rsa1.0_00064.1.g3439.t1	ref|NP_182264.1| dimethyladenosine transferase [Arabidopsis thaliana] gi|2529685|gb|AAC62868.1| putative dimethyladenosine transferase [Arabidopsis thaliana] gi|14532650|gb|AAK64053.1| putative dimethyladenosine transferase [Arabidopsis thaliana] gi|21280901|gb|AAM44912.1| putative dimethyladenosine transferase [Arabidopsis thaliana] gi|330255745|gb|AEC10839.1| dimethyladenosine transferase [Arabidopsis thaliana]	347	353	1.00E-166	101.7	91.1	94.5	dimethyladenosine transferase	gbpln	Arabidopsis thaliana	AT2G47420.1 | Symbols: | Ribosomal RNA adenine dimethylase family protein | chr2:19457574-19458777 FORWARD LENGTH=353	347	353	1.00E-169	101.7	91.1	94.5
Rsa1.0_00064.1.g3440.t1	ref|NP_182265.1| histidine kinase CKI1 [Arabidopsis thaliana] gi|75097398|sp|O22267.1|CKI1_ARATH RecName: Full=Histidine kinase CKI1; AltName: Full=Protein CYTOKININ-INDEPENDENT 1 gi|1679803|dbj|BAA13416.1| histidine kinase homolog [Arabidopsis thaliana] gi|2529684|gb|AAC62867.1| putative histidine kinase [Arabidopsis thaliana] gi|330255746|gb|AEC10840.1| histidine kinase CKI1 [Arabidopsis thaliana]	1093	1122	0	102.7	72.0	80.6	histidine kinase CKI1	gbpln	Arabidopsis thaliana	AT2G47430.1 | Symbols: CKI1 | Signal transduction histidine kinase | chr2:19459167-19463122 REVERSE LENGTH=1122	1093	1122	0	102.7	72.0	80.6
Rsa1.0_00065.1.g3441.t1	ref|XP_002892917.1| hypothetical protein ARALYDRAFT_471862 [Arabidopsis lyrata subsp. lyrata] gi|297338759|gb|EFH69176.1| hypothetical protein ARALYDRAFT_471862 [Arabidopsis lyrata subsp. lyrata]	830	823	0	99.2	62.3	73.1	hypothetical protein ARALYDRAFT_471862	gbpln	Arabidopsis lyrata	AT1G16630.1 | Symbols: | unknown protein; LOCATED IN: chloroplast; BEST Arabidopsis thaliana protein match is: unknown protein (TAIR:AT2G16270.1); Has 10587 Blast hits to 5736 proteins in 617 species: Archae - 88; Bacteria - 963; Metazoa - 3686; Fungi - 820; Plants - 541; Viruses - 438; Other Eukaryotes - 4051 (source: NCBI BLink). | chr1:5682212-5684749 REVERSE LENGTH=845	830	845	0	101.8	62.5	72.5
Rsa1.0_00065.1.g3442.t1	ref|XP_002890176.1| hypothetical protein ARALYDRAFT_889054 [Arabidopsis lyrata subsp. lyrata] gi|297336018|gb|EFH66435.1| hypothetical protein ARALYDRAFT_889054 [Arabidopsis lyrata subsp. lyrata]	813	770	0	94.7	77.4	82.5	hypothetical protein ARALYDRAFT_889054	gbpln	Arabidopsis lyrata	AT1G79090.2 | Symbols: | FUNCTIONS IN: molecular_function unknown; INVOLVED IN: biological_process unknown; LOCATED IN: cellular_component unknown; EXPRESSED IN: 25 plant structures; EXPRESSED DURING: 15 growth stages; CONTAINS InterPro DOMAIN/s: Topoisomerase II-associated protein PAT1 (InterPro:IPR019167); BEST Arabidopsis thaliana protein match is: Topoisomerase II-associated protein PAT1 (TAIR:AT3G22270.1); Has 1260 Blast hits to 1163 proteins in 186 species: Archae - 0; Bacteria - 32; Metazoa - 596; Fungi - 277; Plants - 212; Viruses - 0; Other Eukaryotes - 143 (source: NCBI BLink). | chr1:29749551-29752945 REVERSE LENGTH=793	813	793	0	97.5	65.6	75.5
Rsa1.0_00065.1.g3443.t2	gb|EOA40545.1| hypothetical protein CARUB_v10009275mg [Capsella rubella] gi|482576359|gb|EOA40546.1| hypothetical protein CARUB_v10009275mg [Capsella rubella]	459	416	2.00E-82	90.6	43.8	46.0	hypothetical protein CARUB_v10009275mg	gbpln	Capsella rubella	AT1G16610.3 | Symbols: SR45 | arginine/serine-rich 45 | chr1:5675925-5678686 REVERSE LENGTH=425	459	425	2.00E-72	92.6	37.3	39.4
Rsa1.0_00065.1.g3444.t1	gb|EOA39738.1| hypothetical protein CARUB_v10008384mg [Capsella rubella]	728	761	0	104.5	80.6	89.3	hypothetical protein CARUB_v10008384mg	gbpln	Capsella rubella	AT1G78960.1 | Symbols: ATLUP2, LUP2 | lupeol synthase 2 | chr1:29696722-29701024 FORWARD LENGTH=763	728	763	0	104.8	74.2	85.3

Rsa1.0_00065.1.g3445.t1	ref|XP_002892915.1| predicted protein [Arabidopsis lyrata subsp. lyrata] gi|297338757|gb|EFH69174.1| predicted protein [Arabidopsis lyrata subsp. lyrata]	457	465	0	101.8	93.7	96.7	predicted protein	gbpln	Arabidopsis lyrata	AT1G16570.1 | Symbols: | UDP-Glycosyltransferase superfamily protein | chr1:5670763-5673423 REVERSE LENGTH=465	457	465	0	101.8	92.8	95.6
Rsa1.0_00065.1.g3446.t2	gb|EOA38074.1| hypothetical protein CARUB_v10009543mg, partial [Capsella rubella]	344	358	1.00E-179	104.1	88.4	93.3	hypothetical protein CARUB_v10009543mg, partial	gbpln	Capsella rubella	AT1G16560.3 | Symbols: | Per1-like family protein | chr1:5668945-5670343 FORWARD LENGTH=342	344	342	1.00E-173	99.4	84.6	90.7
Rsa1.0_00065.1.g3447.t1	ref|NP_564001.1| Molybdenum cofactor sulfurase [Arabidopsis thaliana] gi|75169006|sp|Q9C5X8.1|MOCOS_ARATH RecName: Full=Molybdenum cofactor sulfurase; Short=MOS; Short=MoCo sulfurase; AltName: Full=Abscisic acid protein 3; AltName: Full=Low expression of osmotically expressive genes protein 5; AltName: Full=Molybdenum cofactor sulfurtransferase gi|13123673|gb|AAK12939.1|AF325457_1 molybdenum cofactor sulfurase [Arabidopsis thaliana] gi|15407262|gb|AAK58888.1| molybdenum cofactor sulfurase [Arabidopsis thaliana] gi|332191346|gb|AEE29467.1| Molybdenum cofactor sulfurase [Arabidopsis thaliana]	821	819	0	99.8	86.4	92.2	Molybdenum cofactor sulfurase	gbpln	Arabidopsis thaliana	AT1G16540.1 | Symbols: SIR3, LOS5, ABA3, ATABA3, ACI2 | molybdenum cofactor sulfurase (LOS5) (ABA3) | chr1:5659465-5665201 FORWARD LENGTH=819	821	819	0	99.8	86.4	92.2
Rsa1.0_00065.1.g3448.t1	ref|XP_002892914.1| predicted protein [Arabidopsis lyrata subsp. lyrata] gi|297338756|gb|EFH69173.1| predicted protein [Arabidopsis lyrata subsp. lyrata]	166	164	5.00E-77	98.8	83.1	89.8	predicted protein	gbpln	Arabidopsis lyrata	AT1G16530.1 | Symbols: LBD3, ASL9 | ASYMMETRIC LEAVES 2-like 9 | chr1:5651470-5652839 REVERSE LENGTH=165	166	165	1.00E-74	99.4	78.9	87.3
Rsa1.0_00065.1.g3449.t1	ref|XP_002890170.1| hypothetical protein ARALYDRAFT_471850 [Arabidopsis lyrata subsp. lyrata] gi|297336012|gb|EFH66429.1| hypothetical protein ARALYDRAFT_471850 [Arabidopsis lyrata subsp. lyrata]	319	325	1.00E-152	101.9	86.8	90.9	hypothetical protein ARALYDRAFT_471850	gbpln	Arabidopsis lyrata	AT1G16520.1 | Symbols: | unknown protein; FUNCTIONS IN: molecular_function unknown; INVOLVED IN: biological_process unknown; LOCATED IN: plasma membrane; EXPRESSED IN: 22 plant structures; EXPRESSED DURING: 13 growth stages; BEST Arabidopsis thaliana protein match is: unknown protein (TAIR:AT1G56080.1); Has 243 Blast hits to 234 proteins in 69 species: Archae - 2; Bacteria - 2; Metazoa - 61; Fungi - 9; Plants - 125; Viruses - 0; Other Eukaryotes - 44 (source: NCBI BLink). | chr1:5648904-5650998 FORWARD LENGTH=325	319	325	1.00E-152	101.9	88.4	92.5
Rsa1.0_00065.1.g3450.t1	#	#	#	#	#	#	#	-	----	----	#	#	#	#	#	#	#
Rsa1.0_00065.1.g3451.t1	gb|ADP37974.1| auxin-responsive family protein [Brassica napus]	145	145	6.00E-76	100.0	97.9	98.6	auxin-responsive family protein	gbpln	Brassica napus	AT1G16510.1 | Symbols: | SAUR-like auxin-responsive protein family | chr1:5644784-5645227 REVERSE LENGTH=147	145	147	2.00E-67	101.4	87.6	93.1
Rsa1.0_00065.1.g3452.t1	ref|XP_002863230.1| RNA recognition motif-containing protein [Arabidopsis lyrata subsp. lyrata] gi|297309064|gb|EFH39489.1| RNA recognition motif-containing protein [Arabidopsis lyrata subsp. lyrata]	802	817	0	101.9	76.6	83.9	RNA recognition motif-containing protein	gbpln	Arabidopsis lyrata	AT4G12640.1 | Symbols: | RNA recognition motif (RRM)-containing protein | chr4:7462697-7467630 FORWARD LENGTH=823	802	823	0	102.6	74.8	83.2
Rsa1.0_00065.1.g3453.t1	ref|XP_002889235.1| hypothetical protein ARALYDRAFT_316812 [Arabidopsis lyrata subsp. lyrata] gi|297335076|gb|EFH65494.1| hypothetical protein ARALYDRAFT_316812 [Arabidopsis lyrata subsp. lyrata]	249	238	7.00E-71	95.6	63.5	70.7	hypothetical protein ARALYDRAFT_316812	gbpln	Arabidopsis lyrata	AT1G16500.1 | Symbols: | unknown protein; BEST Arabidopsis thaliana protein match is: unknown protein (TAIR:AT1G79160.1); Has 136 Blast hits to 134 proteins in 17 species: Archae - 0; Bacteria - 0; Metazoa - 0; Fungi - 4; Plants - 131; Viruses - 1; Other Eukaryotes - 0 (source: NCBI BLink). | chr1:5639079-5639995 FORWARD LENGTH=259	249	259	2.00E-70	104.0	66.7	77.5
Rsa1.0_00065.1.g3454.t1	gb|AAM62687.1| putative myb-related transcription factor [Arabidopsis thaliana]	326	272	5.00E-98	83.4	53.4	59.2	putative myb-related transcription factor	gbpln	Arabidopsis thaliana	AT1G16490.1 | Symbols: ATMYB58, MYB58 | myb domain protein 58 | chr1:5629859-5630844 REVERSE LENGTH=274	326	274	1.00E-100	84.0	53.4	59.2
Rsa1.0_00065.1.g3455.t1	gb|ACB59214.1| cytoplasmic thiosulfate:cyanide sulfur transferase [Brassica oleracea]	378	379	0	100.3	93.9	98.4	cytoplasmic thiosulfate:cyanide sulfur transferase	gbpln	Brassica oleracea	AT1G79230.1 | Symbols: ST1, ATMST1, MST1, ATRDH1, STR1 | mercaptopyruvate sulfurtransferase 1 | chr1:29800824-29803679 FORWARD LENGTH=379	378	379	1.00E-178	100.3	78.6	87.8
Rsa1.0_00065.1.g3456.t1	ref|NP_173094.4| protein root hair specific 3 [Arabidopsis thaliana] gi|332191330|gb|AEE29451.1| protein root hair specific 3 [Arabidopsis thaliana]	646	499	0	77.2	62.5	67.3	protein root hair specific 3	gbpln	Arabidopsis thaliana	AT1G16440.1 | Symbols: RSH3 | root hair specific 3 | chr1:5615841-5617632 FORWARD LENGTH=499	646	499	0	77.2	62.5	67.3

Rsa1.0_00065.1.g3457.t1	gb|ACR10252.1| cytochrome P450 79f1 [Brassica rapa subsp. pekinensis]	540	537	0	99.4	95.9	98.0	cytochrome P450 79f1	gbpln	Brassica rapa	AT1G16410.1 | Symbols: CYP79F1, BUS1, SPS1 | cytochrome p450 79f1 | chr1:5608862-5611118 FORWARD LENGTH=538	540	538	0	99.6	83.1	90.2
Rsa1.0_00065.1.g3458.t1	gb|ACB59211.1| antiporter family protein [Brassica oleracea]	790	756	0	95.7	90.9	93.0	antiporter family protein	gbpln	Brassica oleracea	AT1G16380.1 | Symbols: ATCHX1, CHX1 | Cation/hydrogen exchanger family protein | chr1:5598453-5601367 REVERSE LENGTH=785	790	785	0	99.4	85.6	91.6
Rsa1.0_00065.1.g3459.t1	ref|NP_173087.1| organic cation/carnitine transporter 6 [Arabidopsis thaliana] gi|75265503|sp|Q9SA36.1|OCT6_ARATH RecName: Full=Organic cation/carnitine transporter 6; Short=AtOCT6 gi|4966358|gb|AAD34689.1|AC006341_17 Is a member of the PF|00083 sugar transporter family [Arabidopsis thaliana] gi|110738583|dbj|BAF01217.1| putative transport protein [Arabidopsis thaliana] gi|332191322|gb|AEE29443.1| organic cation/carnitine transporter 6 [Arabidopsis thaliana]	521	521	0	100.0	85.4	91.9	organic cation/carnitine transporter 6	gbpln	Arabidopsis thaliana	AT1G16370.1 | Symbols: ATOCT6, OCT6 | organic cation/carnitine transporter 6 | chr1:5596762-5598327 FORWARD LENGTH=521	521	521	0	100.0	85.4	91.9
Rsa1.0_00065.1.g3460.t1	dbj|BAJ33639.1| unnamed protein product [Thellungiella halophila]	119	176	2.00E-11	147.9	31.1	37.8	unnamed protein product	----	----	#	#	#	#	#	#	#
Rsa1.0_00065.1.g3461.t1	ref|NP_173087.1| organic cation/carnitine transporter 6 [Arabidopsis thaliana] gi|75265503|sp|Q9SA36.1|OCT6_ARATH RecName: Full=Organic cation/carnitine transporter 6; Short=AtOCT6 gi|4966358|gb|AAD34689.1|AC006341_17 Is a member of the PF|00083 sugar transporter family [Arabidopsis thaliana] gi|110738583|dbj|BAF01217.1| putative transport protein [Arabidopsis thaliana] gi|332191322|gb|AEE29443.1| organic cation/carnitine transporter 6 [Arabidopsis thaliana]	524	521	0	99.4	80.5	90.3	organic cation/carnitine transporter 6	gbpln	Arabidopsis thaliana	AT1G16370.1 | Symbols: ATOCT6, OCT6 | organic cation/carnitine transporter 6 | chr1:5596762-5598327 FORWARD LENGTH=521	524	521	0	99.4	80.5	90.3
Rsa1.0_00065.1.g3462.t1	ref|NP_173085.1| IMP dehydrogenase [Arabidopsis thaliana] gi|14194878|sp|Q9SA34.1|IMDH2_ARATH RecName: Full=Inosine-5'-monophosphate dehydrogenase 2; Short=IMP dehydrogenase 2; Short=IMPD 2; Short=IMPDH 2 gi|4966356|gb|AAD34687.1|AC006341_15 Strong similarity to gb|L34684 inosine monophosphate dehydrogenase (IMPDH) from Arabidopsis thaliana and is a member of the PF|00478 IMP dehydrogenase family [Arabidopsis thaliana] gi|332191320|gb|AEE29441.1| IMP dehydrogenase [Arabidopsis thaliana]	521	502	0	96.4	90.6	93.9	IMP dehydrogenase	gbpln	Arabidopsis thaliana	AT1G16350.1 | Symbols: | Aldolase-type TIM barrel family protein | chr1:5590951-5592872 FORWARD LENGTH=502	521	502	0	96.4	90.6	93.9
Rsa1.0_00065.1.g3463.t1	gb|EOA37727.1| hypothetical protein CARUB_v10012501mg [Capsella rubella]	608	646	0	106.3	76.8	84.7	hypothetical protein CARUB_v10012501mg	gbpln	Capsella rubella	AT1G16330.1 | Symbols: CYCB3;1 | cyclin b3;1 | chr1:5582387-5587391 REVERSE LENGTH=648	608	648	0	106.6	75.3	84.0
Rsa1.0_00065.1.g3464.t1	gb|EOA38416.1| hypothetical protein CARUB_v10009990mg [Capsella rubella]	275	274	1.00E-120	99.6	81.1	90.5	hypothetical protein CARUB_v10009990mg	gbpln	Capsella rubella	AT1G16320.1 | Symbols: | Uncharacterized conserved protein (DUF2358) | chr1:5580951-5581932 FORWARD LENGTH=273	275	273	1.00E-122	99.3	81.8	90.5
Rsa1.0_00065.1.g3465.t1	gb|AAD34683.1|AC006341_11 F3O9.11 [Arabidopsis thaliana] gi|17529248|gb|AAL38851.1| unknown protein [Arabidopsis thaliana]	398	398	0	100.0	95.0	97.5	F3O9.11	gbpln	Arabidopsis thaliana	AT1G16310.1 | Symbols: | Cation efflux family protein | chr1:5578435-5580585 FORWARD LENGTH=428	398	428	0	107.5	95.0	97.5
Rsa1.0_00065.1.g3466.t1	ref|XP_002890157.1| GAPCP-2 [Arabidopsis lyrata subsp. lyrata] gi|297335999|gb|EFH66416.1| GAPCP-2 [Arabidopsis lyrata subsp. lyrata]	420	420	0	100.0	93.6	96.7	GAPCP-2	gbpln	Arabidopsis lyrata	AT1G16300.1 | Symbols: GAPCP-2 | glyceraldehyde-3-phosphate dehydrogenase of plastid 2 | chr1:5574433-5577406 FORWARD LENGTH=420	420	420	0	100.0	94.0	96.9

Rsa1.0_00065.1.g3467.t1	ref|XP_002892901.1| hypothetical protein ARALYDRAFT_312606 [Arabidopsis lyrata subsp. lyrata] gi|297338743|gb|EFH69160.1| hypothetical protein ARALYDRAFT_312606 [Arabidopsis lyrata subsp. lyrata]	433	458	0	105.8	88.0	92.6	hypothetical protein ARALYDRAFT_312606	gbpln	Arabidopsis lyrata	AT1G16290.1 | Symbols: | FUNCTIONS IN: molecular_function unknown; INVOLVED IN: biological_process unknown; LOCATED IN: chloroplast, vacuole; EXPRESSED IN: 19 plant structures; EXPRESSED DURING: 11 growth stages; CONTAINS InterPro DOMAIN/s: Lytic transglycosylase-like, catalytic (InterPro:IPR008258); Has 171 Blast hits to 155 proteins in 40 species: Archae - 0; Bacteria - 54; Metazoa - 0; Fungi - 0; Plants - 55; Viruses - 0; Other Eukaryotes - 62 (source: NCBI BLink). | chr1:5570938-5573538 REVERSE LENGTH=419	433	419	0	96.8	84.1	90.3
Rsa1.0_00065.1.g3468.t1	gb|EOA16203.1| hypothetical protein CARUB_v10004342mg [Capsella rubella]	294	637	8.00E-29	216.7	25.5	31.0	hypothetical protein CARUB_v10004342mg	gbpln	Capsella rubella	AT4G12670.1 | Symbols: | Homeodomain-like superfamily protein | chr4:7472530-7474519 FORWARD LENGTH=499	294	499	9.00E-29	169.7	22.1	25.5
Rsa1.0_00065.1.g3469.t1	gb|EOA36788.1| hypothetical protein CARUB_v10008134mg [Capsella rubella]	1130	1156	0	102.3	80.3	85.9	hypothetical protein CARUB_v10008134mg	gbpln	Capsella rubella	AT1G16270.2 | Symbols: | Protein kinase superfamily protein with octicosapeptide/Phox/Bem1p domain | chr1:5563890-5568145 FORWARD LENGTH=1147	1130	1147	0	101.5	80.0	85.2
Rsa1.0_00065.1.g3470.t1	ref|NP_173075.3| F-box/kelch-repeat protein [Arabidopsis thaliana] gi|122242689|sp|Q0WW40.1|FBK5_ARATH RecName: Full=F-box/kelch-repeat protein At1g16250 gi|110741130|dbj|BAE98658.1| hypothetical protein [Arabidopsis thaliana] gi|119360151|gb|ABL66804.1| At1g16250 [Arabidopsis thaliana] gi|332191305|gb|AEE29426.1| F-box/kelch-repeat protein [Arabidopsis thaliana]	435	383	1.00E-156	88.0	66.2	73.3	F-box/kelch-repeat protein	gbpln	Arabidopsis thaliana	AT1G16250.1 | Symbols: | Galactose oxidase/kelch repeat superfamily protein | chr1:5557214-5558697 FORWARD LENGTH=383	435	383	1.00E-159	88.0	66.2	73.3
Rsa1.0_00065.1.g3471.t1	ref|NP_563994.1| syntaxin-51 [Arabidopsis thaliana] gi|79318036|ref|NP_001031054.1| syntaxin-51 [Arabidopsis thaliana] gi|28380164|sp|Q9SA23.1|SYP51_ARATH RecName: Full=Syntaxin-51; Short=AtSYP51 gi|4966344|gb|AAD34675.1|AC006341_3 ESTs gb|F15498, gb|H37515, gb|T41906, gb|T22448, gb|W43356 and gb|T20739 come from this gene [Arabidopsis thaliana] gi|13811644|gb|AAK40223.1|AF355755_1 syntaxin of plants 51 [Arabidopsis thaliana] gi|18650630|gb|AAL75885.1| At1g16240/F3O9_4 [Arabidopsis thaliana] gi|20453319|gb|AAM19898.1| At1g16240/F3O9_4 [Arabidopsis thaliana] gi|21553458|gb|AAM62551.1| unknown [Arabidopsis thaliana] gi|222424478|dbj|BAH20194.1| AT1G16240 [Arabidopsis thaliana] gi|332191302|gb|AEE29423.1| syntaxin-51 [Arabidopsis thaliana] gi|332191303|gb|AEE29424.1| syntaxin-51 [Arabidopsis thaliana]	233	232	1.00E-116	99.6	88.0	95.7	syntaxin-51	gbpln	Arabidopsis thaliana	AT1G16240.2 | Symbols: SYP51, ATSYP51 | syntaxin of plants 51 | chr1:5555183-5556340 REVERSE LENGTH=232	233	232	1.00E-118	99.6	88.0	95.7
Rsa1.0_00065.1.g3472.t1	ref|NP_173072.1| putative protein phosphatase 2C 6 [Arabidopsis thaliana] gi|75200039|sp|Q9SA22.1|P2C06_ARATH RecName: Full=Probable protein phosphatase 2C 6; Short=AtPP2C06 gi|4966343|gb|AAD34674.1|AC006341_2 Is a member of PF|00481 Protein phosphatase 2C family [Arabidopsis thaliana] gi|332191299|gb|AEE29420.1| putative protein phosphatase 2C 6 [Arabidopsis thaliana]	492	491	0	99.8	84.3	90.0	putative protein phosphatase 2C 6	gbpln	Arabidopsis thaliana	AT1G16220.1 | Symbols: | Protein phosphatase 2C family protein | chr1:5548653-5550553 FORWARD LENGTH=491	492	491	0	99.8	84.3	90.0
Rsa1.0_00065.1.g3473.t1	gb|EOA39107.1| hypothetical protein CARUB_v10011879mg [Capsella rubella]	366	368	1.00E-174	100.5	85.5	91.5	hypothetical protein CARUB_v10011879mg	gbpln	Capsella rubella	AT1G16190.1 | Symbols: RAD23A | Rad23 UV excision repair protein family | chr1:5543267-5545892 FORWARD LENGTH=368	366	368	1.00E-174	100.5	85.5	91.5
Rsa1.0_00065.1.g3474.t1	gb|EOA14759.1| hypothetical protein CARUB_v10028056mg [Capsella rubella]	258	270	9.00E-49	104.7	40.3	47.7	hypothetical protein CARUB_v10028056mg	gbpln	Capsella rubella	AT5G45220.1 | Symbols: | Disease resistance protein (TIR-NBS-LRR class) family | chr5:18298926-18301069 REVERSE LENGTH=546	258	546	1.00E-35	211.6	30.2	41.1

Rsa1.0_00065.1.g3475.t2	gb|EOA40567.1| hypothetical protein CARUB_v10009295mg [Capsella rubella]	185	412	7.00E-50	222.7	69.2	73.0	hypothetical protein CARUB_v10009295mg	gbpln	Capsella rubella	AT1G16180.2 | Symbols: | Serinc-domain containing serine and sphingolipid biosynthesis protein | chr1:5540905-5542670 FORWARD LENGTH=412	185	412	1.00E-50	222.7	69.2	73.5
Rsa1.0_00065.1.g3476.t1	gb|EOA33804.1| hypothetical protein CARUB_v10021275mg [Capsella rubella]	91	93	1.00E-30	102.2	76.9	90.1	hypothetical protein CARUB_v10021275mg	gbpln	Capsella rubella	AT1G79660.1 | Symbols: | unknown protein; BEST Arabidopsis thaliana protein match is: unknown protein (TAIR:AT1G16170.1); Has 30201 Blast hits to 17322 proteins in 780 species: Archae - 12; Bacteria - 1396; Metazoa - 17338; Fungi - 3422; Plants - 5037; Viruses - 0; Other Eukaryotes - 2996 (source: NCBI BLink). | chr1:29976294-29976575 FORWARD LENGTH=93	91	93	3.00E-33	102.2	76.9	87.9
Rsa1.0_00065.1.g3477.t1	gb|EOA33654.1| hypothetical protein CARUB_v10019823mg [Capsella rubella]	716	794	0	110.9	59.9	71.8	hypothetical protein CARUB_v10019823mg	gbpln	Capsella rubella	AT1G79670.1 | Symbols: RFO1, WAKL22 | Wall-associated kinase family protein | chr1:29976887-29979337 REVERSE LENGTH=751	716	751	0	104.9	57.5	71.8
Rsa1.0_00065.1.g3478.t1	gb|EOA38313.1| hypothetical protein CARUB_v10009813mg [Capsella rubella]	310	312	1.00E-155	100.6	88.4	94.5	hypothetical protein CARUB_v10009813mg	gbpln	Capsella rubella	AT1G16080.1 | Symbols: | unknown protein; LOCATED IN: apoplast, chloroplast stroma, chloroplast, chloroplast envelope; EXPRESSED IN: 23 plant structures; EXPRESSED DURING: 14 growth stages; Has 81 Blast hits to 81 proteins in 28 species: Archae - 0; Bacteria - 2; Metazoa - 0; Fungi - 0; Plants - 62; Viruses - 0; Other Eukaryotes - 17 (source: NCBI BLink). | chr1:5514394-5515761 FORWARD LENGTH=313	310	313	1.00E-151	101.0	87.1	92.6
Rsa1.0_00065.1.g3479.t1	ref|XP_002892894.1| hypothetical protein ARALYDRAFT_471808 [Arabidopsis lyrata subsp. lyrata] gi|297338736|gb|EFH69153.1| hypothetical protein ARALYDRAFT_471808 [Arabidopsis lyrata subsp. lyrata]	398	397	0	99.7	89.4	95.0	hypothetical protein ARALYDRAFT_471808	gbpln	Arabidopsis lyrata	AT1G16070.1 | Symbols: AtTLP8, TLP8 | tubby like protein 8 | chr1:5511899-5513779 REVERSE LENGTH=397	398	397	0	99.7	88.2	94.0
Rsa1.0_00065.1.g3480.t1	ref|XP_002890145.1| hypothetical protein ARALYDRAFT_471806 [Arabidopsis lyrata subsp. lyrata] gi|297335987|gb|EFH66404.1| hypothetical protein ARALYDRAFT_471806 [Arabidopsis lyrata subsp. lyrata]	338	332	2.33E-156	98.2	84.0	90.8	hypothetical protein ARALYDRAFT_471806	gbpln	Arabidopsis lyrata	AT1G16060.1 | Symbols: ADAP | ARIA-interacting double AP2 domain protein | chr1:5508563-5511609 FORWARD LENGTH=345	338	345	1.00E-149	102.1	84.3	92.0
Rsa1.0_00065.1.g3481.t1	ref|XP_002890143.1| predicted protein [Arabidopsis lyrata subsp. lyrata] gi|297335985|gb|EFH66402.1| predicted protein [Arabidopsis lyrata subsp. lyrata]	231	228	4.00E-93	98.7	74.9	84.0	predicted protein	gbpln	Arabidopsis lyrata	AT1G16040.1 | Symbols: | FUNCTIONS IN: molecular_function unknown; INVOLVED IN: GPI anchor biosynthetic process; LOCATED IN: integral to membrane, endoplasmic reticulum membrane; EXPRESSED IN: 24 plant structures; EXPRESSED DURING: 13 growth stages; CONTAINS InterPro DOMAIN/s: GPI biosynthesis protein Pig-F (InterPro:IPR009580); Has 280 Blast hits to 280 proteins in 133 species: Archae - 0; Bacteria - 0; Metazoa - 113; Fungi - 111; Plants - 44; Viruses - 0; Other Eukaryotes - 12 (source: NCBI BLink). | chr1:5504747-5506456 FORWARD LENGTH=226	231	226	3.00E-81	97.8	68.8	81.8
Rsa1.0_00065.1.g3482.t1	dbj|BAJ34597.1| unnamed protein product [Thellungiella halophila]	649	647	0	99.7	94.9	97.7	unnamed protein product	----	----	AT1G16030.1 | Symbols: Hsp70b | heat shock protein 70B | chr1:5502386-5504326 REVERSE LENGTH=646	649	646	0	99.5	93.1	97.4
Rsa1.0_00065.1.g3483.t1	ref|NP_173051.1| cyclic nucleotide gated channel 7 [Arabidopsis thaliana] gi|332191272|gb|AEE29393.1| cyclic nucleotide gated channel 7 [Arabidopsis thaliana]	713	709	0	99.4	94.2	97.2	cyclic nucleotide gated channel 7	gbpln	Arabidopsis thaliana	AT1G15990.1 | Symbols: ATCNGC7, CNGC7 | cyclic nucleotide gated channel 7 | chr1:5491304-5493772 REVERSE LENGTH=709	713	709	0	99.4	94.2	97.2
Rsa1.0_00065.1.g3484.t1	ref|NP_563986.1| NDH-dependent cyclic electron flow 1 [Arabidopsis thaliana] gi|6587804|gb|AAF18495.1|AC010924_8 ESTs gb|T43280, gb|AA395195 come from this gene [Arabidopsis thaliana] gi|16323198|gb|AAL15333.1| At1g15980/T24D18_8 [Arabidopsis thaliana] gi|21537186|gb|AAM61527.1| unknown [Arabidopsis thaliana] gi|21700921|gb|AAM70584.1| At1g15980/T24D18_8 [Arabidopsis thaliana] gi|332191271|gb|AEE29392.1| NDH-dependent cyclic electron flow 1 [Arabidopsis thaliana]	461	461	0	100.0	89.4	94.1	NDH-dependent cyclic electron flow 1	gbpln	Arabidopsis thaliana	AT1G15980.1 | Symbols: NDF1, NDH48 | NDH-dependent cyclic electron flow 1 | chr1:5489314-5491199 FORWARD LENGTH=461	461	461	0	100.0	89.4	94.1
Rsa1.0_00065.1.g3485.t1	# #	#	#	#	#	#		-	----	----	# #	#	#	#	#	#	
Rsa1.0_00065.1.g3486.t1	# #	#	#	#	#	#		-	----	----	# #	#	#	#	#	#	

Rsa1.0_00065.1.g3487.t5	ref|NP_173048.3| metal transporter Nramp6 [Arabidopsis thaliana] gi|30173130|sp|Q9S9N8.2|NRAM6_ARATH RecName: Full=Metal transporter Nramp6; Short=AtNramp6 gi|12666983|emb|CAC28123.1| putative metal transporter [Arabidopsis thaliana] gi|332191269|gb|AEE29390.1| metal transporter Nramp6 [Arabidopsis thaliana]	475	527	0	110.9	92.2	94.9	metal transporter Nramp6	gbpln	Arabidopsis thaliana	AT1G15960.1 | Symbols: NRAMP6, ATNRAMP6 | NRAMP metal ion transporter 6 | chr1:5482202-5485066 REVERSE LENGTH=527	475	527	0	110.9	92.2	94.9
Rsa1.0_00065.1.g3488.t1	gb|ADC40029.1| cinnamoyl-CoA reductase [Isatis tinctoria]	344	341	0	99.1	94.2	96.5	cinnamoyl-CoA reductase	gbpln	Isatis tinctoria	AT1G15950.1 | Symbols: CCR1, IRX4, ATCCR1 | cinnamoyl coa reductase 1 | chr1:5478855-5481915 FORWARD LENGTH=344	344	344	0	100.0	95.3	96.8
Rsa1.0_00065.1.g3489.t1	ref|NP_173046.2| aspartyl beta-hydroxylase N-terminal region domain-containing protein [Arabidopsis thaliana] gi|6587800|gb|AAF18491.1|AC010924_4 T24D18.4 [Arabidopsis thaliana] gi|332191266|gb|AEE29387.1| aspartyl beta-hydroxylase N-terminal region domain-containing protein [Arabidopsis thaliana]	836	990	0	118.4	50.5	62.4	aspartyl beta-hydroxylase N-terminal region domain-containing protein	gbpln	Arabidopsis thaliana	AT1G15940.1 | Symbols: | Tudor/PWWP/MBT superfamily protein | chr1:5473672-5478050 FORWARD LENGTH=990	836	990	0	118.4	50.5	62.4
Rsa1.0_00065.1.g3490.t1	gb|EOA36301.1| hypothetical protein CARUB_v10010589mg [Capsella rubella]	144	142	5.00E-71	98.6	95.8	97.9	hypothetical protein CARUB_v10010589mg	gbpln	Capsella rubella	AT2G32060.2 | Symbols: | Ribosomal protein L7Ae/L30e/S12e/Gadd45 family protein | chr2:13639228-13640104 REVERSE LENGTH=144	144	144	6.00E-63	100.0	84.0	91.7
Rsa1.0_00065.1.g3491.t5	ref|XP_002890135.1| hypothetical protein ARALYDRAFT_312580 [Arabidopsis lyrata subsp. lyrata] gi|297335977|gb|EFH66394.1| hypothetical protein ARALYDRAFT_312580 [Arabidopsis lyrata subsp. lyrata]	119	113	4.00E-41	95.0	82.4	89.1	hypothetical protein ARALYDRAFT_312580	gbpln	Arabidopsis lyrata	AT1G15900.1 | Symbols: | unknown protein; Has 19 Blast hits to 19 proteins in 10 species: Archae - 0; Bacteria - 2; Metazoa - 0; Fungi - 0; Plants - 15; Viruses - 0; Other Eukaryotes - 2 (source: NCBI BLink). | chr1:5464356-5464697 FORWARD LENGTH=113	119	113	1.00E-42	95.0	81.5	86.6
Rsa1.0_00065.1.g3492.t1	ref|NP_173041.1| CC-NBS-LRR class disease resistance protein [Arabidopsis thaliana] gi|374095383|sp|Q9LMP6.2|DRL3_ARATH RecName: Full=Probable disease resistance protein At1g15890 gi|332191259|gb|AEE29380.1| CC-NBS-LRR class disease resistance protein [Arabidopsis thaliana]	867	851	0	98.2	67.7	78.9	CC-NBS-LRR class disease resistance protein	gbpln	Arabidopsis thaliana	AT1G15890.1 | Symbols: | Disease resistance protein (CC-NBS-LRR class) family | chr1:5461406-5463961 FORWARD LENGTH=851	867	851	0	98.2	67.7	78.9
Rsa1.0_00065.1.g3493.t1	gb|EOA36249.1| hypothetical protein CARUB_v10010239mg [Capsella rubella]	263	223	2.00E-95	84.8	67.3	69.2	hypothetical protein CARUB_v10010239mg	gbpln	Capsella rubella	AT1G15880.1 | Symbols: GOS11, atgos11 | golgi snare 11 | chr1:5458718-5460089 REVERSE LENGTH=223	263	223	1.00E-97	84.8	66.9	69.6
Rsa1.0_00065.1.g3494.t1	ref|NP_001154344.1| uncharacterized protein [Arabidopsis thaliana] gi|240254082|ref|NP_563983.4| uncharacterized protein [Arabidopsis thaliana] gi|32815875|gb|AAP88334.1| At1g15860 [Arabidopsis thaliana] gi|332191253|gb|AEE29374.1| uncharacterized protein AT1G15860 [Arabidopsis thaliana] gi|332191254|gb|AEE29375.1| uncharacterized protein AT1G15860 [Arabidopsis thaliana]	229	227	1.00E-110	99.1	82.5	87.8	uncharacterized protein	gbpln	Arabidopsis thaliana	AT1G15860.2 | Symbols: | Domain of unknown function (DUF298) | chr1:5455055-5456741 FORWARD LENGTH=227	229	227	1.00E-112	99.1	82.5	87.8
Rsa1.0_00065.1.g3495.t1	gb|EOA38586.1| hypothetical protein CARUB_v10010411mg [Capsella rubella]	197	181	6.00E-39	91.9	51.8	70.6	hypothetical protein CARUB_v10010411mg	gbpln	Capsella rubella	AT4G00420.2 | Symbols: | Double-stranded RNA-binding domain (DsRBD)-containing protein | chr4:181518-182629 REVERSE LENGTH=190	197	190	1.00E-39	96.4	49.2	62.4
Rsa1.0_00065.1.g3496.t1	gb|AAF82153.1|AC034256_17 Contains similarity to extensin (atExt1) from Arabidopsis thaliana gb|U43627 and is rich in proline and glycine. EST gb|Z37262 comes from this gene [Arabidopsis thaliana]	711	1006	4.00E-72	141.5	34.7	39.7	Contains similarity to extensin (atExt1) from Arabidopsis thaliana gb|U43627 and is rich in proline and glycine. EST gb|Z37262 comes from this gene	gbpln	Arabidopsis thaliana	AT1G15825.1 | Symbols: | hydroxyproline-rich glycoprotein family protein | chr1:5448149-5448529 REVERSE LENGTH=126	711	126	2.00E-37	17.7	13.1	14.8
Rsa1.0_00065.1.g3497.t1	ref|XP_002892885.1| Lhcb6 protein [Arabidopsis lyrata subsp. lyrata] gi|297338727|gb|EFH69144.1| Lhcb6 protein [Arabidopsis lyrata subsp. lyrata]	256	256	1.00E-134	100.0	94.1	96.5	Lhcb6 protein	gbpln	Arabidopsis lyrata	AT1G15820.1 | Symbols: LHCB6, CP24 | light harvesting complex photosystem II subunit 6 | chr1:5446685-5447676 REVERSE LENGTH=258	256	258	1.00E-134	100.8	93.0	95.7

Rsa1.0_00065.1.g3498.t1	ref|NP_563982.1| S15/NS1, RNA-binding protein [Arabidopsis thaliana] gi|8927660|gb|AAF82151.1|AC034256_15 Contains similarity to an unknown protein T21F11.5 gi|6730725 from Arabidopsis thaliana BAC T21F11 gb|AC018849 and contains a Ribosomal Protein S15 PF|00312 domain [Arabidopsis thaliana] gi|13605577|gb|AAK32782.1|AF361614_1 At1g15810/F7H2_23 [Arabidopsis thaliana] gi|22137170|gb|AAM91430.1| At1g15810/F7H2_23 [Arabidopsis thaliana] gi|332191247|gb|AEE29368.1| S15/NS1, RNA-binding protein [Arabidopsis thaliana]	418	419	1.00E-124	100.2	64.6	73.9	S15/NS1, RNA-binding protein	gbpln	Arabidopsis thaliana	AT1G15810.1 | Symbols: | S15/NS1, RNA-binding protein | chr1:5444496-5446271 FORWARD LENGTH=419	418	419	1.00E-127	100.2	64.6	73.9
Rsa1.0_00065.1.g3499.t1	ref|XP_002892884.1| hypothetical protein ARALYDRAFT_312568 [Arabidopsis lyrata subsp. lyrata] gi|297338726|gb|EFH69143.1| hypothetical protein ARALYDRAFT_312568 [Arabidopsis lyrata subsp. lyrata]	143	308	6.00E-35	215.4	62.9	67.8	hypothetical protein ARALYDRAFT_312568	gbpln	Arabidopsis lyrata	AT1G15800.1 | Symbols: | unknown protein; BEST Arabidopsis thaliana protein match is: unknown protein (TAIR:AT1G80610.1); Has 56 Blast hits to 52 proteins in 9 species: Archae - 0; Bacteria - 0; Metazoa - 0; Fungi - 0; Plants - 56; Viruses - 0; Other Eukaryotes - 0 (source: NCBI BLink). | chr1:5441425-5443584 REVERSE LENGTH=207	143	207	4.00E-33	144.8	59.4	62.9
Rsa1.0_00065.1.g3500.t1	dbj|BAM73280.1| gibberellin 3 oxidase 1 [Raphanus sativus]	359	359	0	100.0	99.2	99.4	gibberellin 3 oxidase 1	gbpln	Raphanus sativus	AT1G15550.1 | Symbols: GA4, ATGA3OX1, GA3OX1 | gibberellin 3-oxidase 1 | chr1:5344569-5346078 REVERSE LENGTH=358	359	358	0	99.7	88.0	92.8
Rsa1.0_00065.1.g3501.t1	ref|XP_002890109.1| predicted protein [Arabidopsis lyrata subsp. lyrata] gi|297335951|gb|EFH66368.1| predicted protein [Arabidopsis lyrata subsp. lyrata]	322	322	1.00E-166	100.0	84.2	92.9	predicted protein	gbpln	Arabidopsis lyrata	AT1G15540.1 | Symbols: | 2-oxoglutarate (2OG) and Fe(II)-dependent oxygenase superfamily protein | chr1:5342587-5343705 FORWARD LENGTH=320	322	320	1.00E-160	99.4	85.7	92.2
Rsa1.0_00065.1.g3502.t1	ref|NP_173005.1| ABC transporter G family member 40 [Arabidopsis thaliana] gi|75336094|sp|Q9M9E1.1|AB40G_ARATH RecName: Full=ABC transporter G family member 40; Short=ABC transporter ABCG.40; Short=AtABCG40; AltName: Full=Pleiotropic drug resistance protein 12 gi|8072390|gb|AAF71978.1|AC013453_3 Putative ABC transporter [Arabidopsis thaliana] gi|28144321|tpg|DAA00880.1| TPA_exp: PDR12 ABC transporter [Arabidopsis thaliana] gi|332191211|gb|AEE29332.1| ABC transporter G family member 40 [Arabidopsis thaliana]	1414	1423	0	100.6	94.2	97.8	ABC transporter G family member 40	gbpln	Arabidopsis thaliana	AT1G15520.1 | Symbols: PDR12, ATPDR12, ABCG40, ATABCG40 | pleiotropic drug resistance 12 | chr1:5331993-5338175 REVERSE LENGTH=1423	1414	1423	0	100.6	94.2	97.8
Rsa1.0_00065.1.g3503.t1	ref|NP_173004.1| pentatricopeptide repeat-containing protein [Arabidopsis thaliana] gi|75191104|sp|Q9M9E2.1|PPR45_ARATH RecName: Full=Pentatricopeptide repeat-containing protein At1g15510, chloroplastic; Flags: Precursor gi|8072389|gb|AAF71977.1|AC013453_2 Hypothetical protein [Arabidopsis thaliana] gi|300825685|gb|ADK35876.1| chloroplast vanilla cream 1 [Arabidopsis thaliana] gi|332191210|gb|AEE29331.1| pentatricopeptide repeat-containing protein [Arabidopsis thaliana]	865	866	0	100.1	89.2	95.1	pentatricopeptide repeat-containing protein	gbpln	Arabidopsis thaliana	AT1G15510.1 | Symbols: ATECB2, ECB2, VAC1 | Tetratricopeptide repeat (TPR)-like superfamily protein | chr1:5329111-5331711 FORWARD LENGTH=866	865	866	0	100.1	89.2	95.1
Rsa1.0_00065.1.g3504.t1	gb|EOA39910.1| hypothetical protein CARUB_v10008598mg [Capsella rubella]	608	618	0	101.6	92.4	95.7	hypothetical protein CARUB_v10008598mg	gbpln	Capsella rubella	AT1G15500.1 | Symbols: ATNTT2 | TLC ATP/ADP transporter | chr1:5326426-5328688 FORWARD LENGTH=618	608	618	0	101.6	92.8	95.2
Rsa1.0_00065.1.g3505.t1	ref|NP_173002.1| alpha/beta-hydrolase domain-containing protein [Arabidopsis thaliana] gi|5103847|gb|AAD39677.1|AC007591_42 Contains PF|00561 alpha/beta hydrolase fold [Arabidopsis thaliana] gi|17529298|gb|AAL38876.1| unknown protein [Arabidopsis thaliana] gi|26983890|gb|AAN86197.1| unknown protein [Arabidopsis thaliana] gi|332191208|gb|AEE29329.1| alpha/beta-hydrolase domain-containing protein [Arabidopsis thaliana]	636	648	0	101.9	85.4	89.0	alpha/beta-hydrolase domain-containing protein	gbpln	Arabidopsis thaliana	AT1G15490.1 | Symbols: | alpha/beta-Hydrolases superfamily protein | chr1:5320859-5323422 REVERSE LENGTH=648	636	648	0	101.9	85.4	89.0

Rsa1.0_00065.1.g3506.t1	ref|NP_563978.1| serine-threonine kinase receptor-associated protein [Arabidopsis thaliana] gi|5103845|gb|AAD39675.1|AC007591_40 Strong similarity to gb|AF096285 serine-threonine kinase receptor-associated protein from Mus musculus and contains 5 PF|00400 WD40, G-beta repeat domains. EST gb|F14050 comes from this gene [Arabidopsis thaliana] gi|21536836|gb|AAM61168.1| Similar to unr-interacting protein and contains WD40 domains [Arabidopsis thaliana] gi|24030339|gb|AAN41335.1| unknown protein [Arabidopsis thaliana] gi|332191206|gb|AEE29327.1| transducin/WD40 repeat-like superfamily protein [Arabidopsis thaliana]	335	333	0	99.4	94.0	96.7	serine-threonine kinase receptor-associated protein	gbpln	Arabidopsis thaliana	AT1G15470.1 | Symbols: | Transducin/WD40 repeat-like superfamily protein | chr1:5315838-5317696 FORWARD LENGTH=333	335	333	0	99.4	94.0	96.7
Rsa1.0_00065.1.g3507.t1	ref|NP_172999.1| boron transporter 4 [Arabidopsis thaliana] gi|75215622|sp|Q9XI23.1|BOR4_ARATH RecName: Full=Boron transporter 4 gi|5103843|gb|AAD39673.1|AC007591_38 Is a member of the PF|00955 Anion exchanger family [Arabidopsis thaliana] gi|17978949|gb|AAL47440.1| At1g15460/T16N11_24 [Arabidopsis thaliana] gi|332191205|gb|AEE29326.1| boron transporter 4 [Arabidopsis thaliana]	678	683	0	100.7	91.4	95.4	boron transporter 4	gbpln	Arabidopsis thaliana	AT1G15460.1 | Symbols: ATBOR4, BOR4 | HCO3- transporter family | chr1:5310196-5313376 REVERSE LENGTH=683	678	683	0	100.7	91.4	95.4
Rsa1.0_00065.1.g3508.t1	gb|EOA39629.1| hypothetical protein CARUB_v10008260mg [Capsella rubella]	888	900	0	101.4	89.6	95.0	hypothetical protein CARUB_v10008260mg	gbpln	Capsella rubella	AT1G15440.1 | Symbols: PWP2, ATPWP2 | periodic tryptophan protein 2 | chr1:5306159-5309460 REVERSE LENGTH=900	888	900	0	101.4	88.3	95.3
Rsa1.0_00065.1.g3509.t1	gb|EOA36181.1| hypothetical protein CARUB_v10010051mg [Capsella rubella]	268	260	1.00E-100	97.0	71.3	81.3	hypothetical protein CARUB_v10010051mg	gbpln	Capsella rubella	AT1G15430.2 | Symbols: | Protein of unknown function (DUF1644) | chr1:5305011-5305790 FORWARD LENGTH=259	268	259	1.00E-98	96.6	68.3	79.9
Rsa1.0_00065.1.g3510.t1	ref|NP_172994.1| uncharacterized protein [Arabidopsis thaliana] gi|5103841|gb|AAD39671.1|AC007591_36 F9L1.37 [Arabidopsis thaliana] gi|332191199|gb|AEE29320.1| uncharacterized protein AT1G15415 [Arabidopsis thaliana]	101	96	6.00E-36	95.0	78.2	86.1	uncharacterized protein	gbpln	Arabidopsis thaliana	AT1G15415.1 | Symbols: | The protein encoded by this gene was identified as a part of pollen proteome by mass spec analysis. It has weak homology to LEA (late embryo abundant) proteins. Encodes protein phosphatase 2A (PP2A) B'gamma subunit. Targeted to nucleus and cytosol. | chr1:5300931-5301412 REVERSE LENGTH=96	101	96	1.00E-38	95.0	78.2	86.1
Rsa1.0_00065.1.g3511.t1	ref|NP_172993.1| aspartate-glutamate racemase-like protein [Arabidopsis thaliana] gi|5103839|gb|AAD39669.1|AC007591_34 Is a member of the PF|01177 Aspartate-glutamate racemase family. EST gb|T43554 comes from this gene [Arabidopsis thaliana] gi|26983862|gb|AAN86183.1| unknown protein [Arabidopsis thaliana] gi|332191198|gb|AEE29319.1| aspartate-glutamate racemase-like protein [Arabidopsis thaliana]	330	330	1.00E-158	100.0	83.9	89.7	aspartate-glutamate racemase-like protein	gbpln	Arabidopsis thaliana	AT1G15410.1 | Symbols: | aspartate-glutamate racemase family | chr1:5299577-5300681 FORWARD LENGTH=330	330	330	1.00E-160	100.0	83.9	89.7
Rsa1.0_00065.1.g3512.t1	#	#	#	#	#	#	#	-	----	----	#	#	#	#	#	#	#
Rsa1.0_00065.1.g3513.t1	gb|EOA37299.1| hypothetical protein CARUB_v10010935mg [Capsella rubella]	145	148	4.00E-50	102.1	74.5	85.5	hypothetical protein CARUB_v10010935mg	gbpln	Capsella rubella	AT1G15400.3 | Symbols: | unknown protein; FUNCTIONS IN: molecular_function unknown; INVOLVED IN: biological_process unknown; LOCATED IN: plasma membrane; EXPRESSED IN: 23 plant structures; EXPRESSED DURING: 13 growth stages; BEST Arabidopsis thaliana protein match is: unknown protein (TAIR:AT1G80180.1); Has 84 Blast hits to 84 proteins in 17 species: Archae - 0; Bacteria - 6; Metazoa - 0; Fungi - 0; Plants - 75; Viruses - 0; Other Eukaryotes - 3 (source: NCBI BLink). | chr1:5296019-5296824 REVERSE LENGTH=148	145	148	2.00E-46	102.1	66.9	77.2

Rsa1.0_00065.1.g3514.t1	ref|XP_002890102.1| hypothetical protein ARALYDRAFT_334826 [Arabidopsis lyrata subsp. lyrata] gi|297335944|gb|EFH66361.1| hypothetical protein ARALYDRAFT_334826 [Arabidopsis lyrata subsp. lyrata]	265	270	1.00E-121	101.9	84.5	90.6	hypothetical protein ARALYDRAFT_334826	gbpln	Arabidopsis lyrata	AT1G15390.1 | Symbols: PDF1A, ATDEF1 | peptide deformylase 1A | chr1:5294653-5295625 FORWARD LENGTH=269	265	269	1.00E-123	101.5	83.8	89.8
Rsa1.0_00065.1.g3515.t1	gb|ABD65019.1| Sulfite oxidase, putative [Brassica oleracea]	389	404	0	103.9	95.9	99.2	Sulfite oxidase, putative	gbpln	Brassica oleracea	AT3G01910.1 | Symbols: SOX, AT-SO, AtSO | sulfite oxidase | chr3:314919-317274 REVERSE LENGTH=393	389	393	0	101.0	91.8	97.2
Rsa1.0_00065.1.g3516.t1	ref|XP_002892864.1| hypothetical protein ARALYDRAFT_888930 [Arabidopsis lyrata subsp. lyrata] gi|297338706|gb|EFH69123.1| hypothetical protein ARALYDRAFT_888930 [Arabidopsis lyrata subsp. lyrata]	74	76	9.00E-22	102.7	73.0	74.3	hypothetical protein ARALYDRAFT_888930	gbpln	Arabidopsis lyrata	AT1G15385.1 | Symbols: | unknown protein; CONTAINS InterPro DOMAIN/s: Protein of unknown function DUF761, plant (InterPro:IPR008480); Has 6 Blast hits to 6 proteins in 3 species: Archae - 0; Bacteria - 0; Metazoa - 0; Fungi - 0; Plants - 6; Viruses - 0; Other Eukaryotes - 0 (source: NCBI BLink). | chr1:5292976-5293197 REVERSE LENGTH=73	74	73	4.00E-22	98.6	67.6	70.3
Rsa1.0_00065.1.g3517.t1	gb|EOA38607.1| hypothetical protein CARUB_v10010450mg [Capsella rubella]	174	174	1.00E-87	100.0	88.5	94.3	hypothetical protein CARUB_v10010450mg	gbpln	Capsella rubella	AT1G15380.2 | Symbols: | Lactoylglutathione lyase / glyoxalase I family protein | chr1:5290955-5292287 FORWARD LENGTH=174	174	174	8.00E-87	100.0	86.2	93.1
Rsa1.0_00066.1.g3518.t1	ref|XP_002876205.1| hypothetical protein ARALYDRAFT_485718 [Arabidopsis lyrata subsp. lyrata] gi|297322043|gb|EFH52464.1| hypothetical protein ARALYDRAFT_485718 [Arabidopsis lyrata subsp. lyrata]	504	515	1.00E-133	102.2	61.5	71.2	hypothetical protein ARALYDRAFT_485718	gbpln	Arabidopsis lyrata	AT3G53440.2 | Symbols: | Homeodomain-like superfamily protein | chr3:19810936-19812474 FORWARD LENGTH=512	504	512	1.00E-126	101.6	59.3	69.8
Rsa1.0_00066.1.g3519.t1	gb|EOA25801.1| hypothetical protein CARUB_v10019168mg [Capsella rubella]	216	215	1.00E-114	99.5	93.1	96.8	hypothetical protein CARUB_v10019168mg	gbpln	Capsella rubella	AT3G53450.1 | Symbols: | Putative lysine decarboxylase family protein | chr3:19812977-19815430 REVERSE LENGTH=215	216	215	1.00E-117	99.5	93.5	96.3
Rsa1.0_00066.1.g3520.t1	dbj|BAA06519.1| RNA-binding protein cp29 [Arabidopsis thaliana]	330	326	1.00E-110	98.8	78.2	83.3	RNA-binding protein cp29	gbpln	Arabidopsis thaliana	AT3G53460.3 | Symbols: CP29 | chloroplast RNA-binding protein 29 | chr3:19819738-19821423 REVERSE LENGTH=342	330	342	1.00E-110	103.6	75.8	80.6
Rsa1.0_00066.1.g3521.t1	ref|NP_190915.1| uncharacterized protein [Arabidopsis thaliana] gi|6729498|emb|CAB67654.1| putative protein [Arabidopsis thaliana] gi|26450564|dbj|BAC42394.1| unknown protein [Arabidopsis thaliana] gi|28416887|gb|AAO42974.1| At3g53470 [Arabidopsis thaliana] gi|332645573|gb|AEE79094.1| uncharacterized protein AT3G53470 [Arabidopsis thaliana]	134	135	6.00E-44	100.7	77.6	84.3	uncharacterized protein	gbpln	Arabidopsis thaliana	AT3G53470.1 | Symbols: | unknown protein; FUNCTIONS IN: molecular_function unknown; INVOLVED IN: biological_process unknown; LOCATED IN: chloroplast thylakoid membrane, chloroplast; EXPRESSED IN: 22 plant structures; EXPRESSED DURING: 13 growth stages; Has 35 Blast hits to 35 proteins in 13 species: Archae - 0; Bacteria - 0; Metazoa - 0; Fungi - 0; Plants - 35; Viruses - 0; Other Eukaryotes - 0 (source: NCBI BLink). | chr3:19822813-19823298 FORWARD LENGTH=135	134	135	1.00E-46	100.7	77.6	84.3
Rsa1.0_00066.1.g3522.t1	ref|NP_190918.3| RNA recognition motif and CCHC-type zinc finger domain-containing protein [Arabidopsis thaliana] gi|75334513|sp|Q9FYB7.1|RSZ32_ARATH RecName: Full=Serine/arginine-rich splicing factor RS2Z32; AltName: Full=RS-containing zinc finger protein 32; Short=At-RS2Z32; Short=At-RSZ32 gi|9795142|emb|CAB67657.2| splicing factor-like protein [Arabidopsis thaliana] gi|222422875|dbj|BAH19424.1| AT3G53500 [Arabidopsis thaliana] gi|332645577|gb|AEE79098.1| serine/arginine-rich splicing factor RS2Z32 [Arabidopsis thaliana]	316	284	1.00E-114	89.9	81.6	85.1	RNA recognition motif and CCHC-type zinc finger domain-containing protein	gbpln	Arabidopsis thaliana	AT3G53500.2 | Symbols: RSZ32, RS2Z32, At-RS2Z | RNA-binding (RRM/RBD/RNP motifs) family protein with retrovirus zinc finger-like domain | chr3:19834557-19836507 REVERSE LENGTH=284	316	284	1.00E-116	89.9	81.6	85.1
Rsa1.0_00066.1.g3523.t1	gb|EOA26043.1| hypothetical protein CARUB_v10019457mg, partial [Capsella rubella]	419	489	0	116.7	95.9	97.6	hypothetical protein CARUB_v10019457mg, partial	gbpln	Capsella rubella	AT3G53520.1 | Symbols: UXS1, ATUXS1 | UDP-glucuronic acid decarboxylase 1 | chr3:19841635-19844057 FORWARD LENGTH=435	419	435	0	103.8	95.0	97.1
Rsa1.0_00066.1.g3524.t1	emb|CAB67660.1| putative protein [Arabidopsis thaliana]	256	250	1.00E-103	97.7	76.6	83.6	putative protein	gbpln	Arabidopsis thaliana	AT3G53530.1 | Symbols: NAKR3 | Chloroplast-targeted copper chaperone protein | chr3:19845199-19846583 FORWARD LENGTH=247	256	247	1.00E-103	96.5	75.4	82.4

Rsa1.0_00066.1.g3525.t1	ref|NP_190922.3| uncharacterized protein [Arabidopsis thaliana] gi|332645585|gb|AEE79106.1| uncharacterized protein AT3G53540 [Arabidopsis thaliana]	897	924	0	103.0	78.6	86.6	uncharacterized protein	gbpln	Arabidopsis thaliana	AT3G53540.1 | Symbols: | unknown protein; LOCATED IN: plasma membrane; EXPRESSED IN: 24 plant structures; EXPRESSED DURING: 14 growth stages; CONTAINS InterPro DOMAIN/s: Protein of unknown function DUF3741 (InterPro:IPR022212); BEST Arabidopsis thaliana protein match is: Protein of unknown function (DUF3741) (TAIR:AT4G28760.2); Has 1710 Blast hits to 868 proteins in 206 species: Archae - 2; Bacteria - 409; Metazoa - 304; Fungi - 204; Plants - 304; Viruses - 2; Other Eukaryotes - 485 (source: NCBI BLink). | chr3:19846805-19850670 REVERSE LENGTH=924	897	924	0	103.0	78.6	86.6
Rsa1.0_00066.1.g3526.t1	gb|EOA22416.1| hypothetical protein CARUB_v10003055mg, partial [Capsella rubella]	512	466	1.00E-110	91.0	47.1	58.2	hypothetical protein CARUB_v10003055mg, partial	gbpln	Capsella rubella	AT1G22000.1 | Symbols: | FBD, F-box and Leucine Rich Repeat domains containing protein | chr1:7744189-7747123 FORWARD LENGTH=717	512	717	1.00E-98	140.0	43.4	56.1
Rsa1.0_00066.1.g3527.t1	emb|CAB67663.1| putative protein [Arabidopsis thaliana]	324	388	4.00E-94	119.8	62.7	78.7	putative protein	gbpln	Arabidopsis thaliana	AT3G53560.1 | Symbols: | Tetratricopeptide repeat (TPR)-like superfamily protein | chr3:19859954-19860976 REVERSE LENGTH=340	324	340	1.00E-96	104.9	62.7	78.7
Rsa1.0_00066.1.g3528.t15	ref|XP_002877927.1| fus3-complementing gene 1 [Arabidopsis lyrata subsp. lyrata] gi|297323765|gb|EFH54186.1| fus3-complementing gene 1 [Arabidopsis lyrata subsp. lyrata]	496	469	0	94.6	88.7	90.9	fus3-complementing gene 1	gbpln	Arabidopsis lyrata	AT3G53570.4 | Symbols: AFC1, AME2, FC1 | FUS3-complementing gene 1 | chr3:19861449-19864125 REVERSE LENGTH=467	496	467	0	94.2	89.3	91.1
Rsa1.0_00066.1.g3529.t1	ref|NP_190926.1| diaminopimelate epimerase [Arabidopsis thaliana] gi|75263858|sp|Q9LFG2.1|DAPF_ARATH RecName: Full=Diaminopimelate epimerase, chloroplastic; Short=DAP epimerase; Flags: Precursor gi|6729509|emb|CAB67665.1| diaminopimelate epimerase-like protein [Arabidopsis thaliana] gi|22022530|gb|AAM83223.1| AT3g53580/F4P12_280 [Arabidopsis thaliana] gi|23505901|gb|AAN28810.1| At3g53580/F4P12_280 [Arabidopsis thaliana] gi|332645592|gb|AEE79113.1| diaminopimelate epimerase [Arabidopsis thaliana]	366	362	0	98.9	87.7	92.3	diaminopimelate epimerase	gbpln	Arabidopsis thaliana	AT3G53580.1 | Symbols: | diaminopimelate epimerase family protein | chr3:19864784-19866907 FORWARD LENGTH=362	366	362	0	98.9	87.7	92.3
Rsa1.0_00066.1.g3530.t1	gb|EOA16369.1| hypothetical protein CARUB_v10004519mg [Capsella rubella]	541	550	0	101.7	81.3	89.8	hypothetical protein CARUB_v10004519mg	gbpln	Capsella rubella	AT4G31180.2 | Symbols: | Class II aminoacyl-tRNA and biotin synthetases superfamily protein | chr4:15156696-15159362 FORWARD LENGTH=558	541	558	0	103.1	79.5	88.4
Rsa1.0_00066.1.g3531.t6	emb|CAA90080.1| small GTP-binding protein [Pisum sativum]	216	216	1.00E-116	100.0	92.1	98.1	small GTP-binding protein	gbpln	Pisum sativum	AT3G53610.3 | Symbols: ATRAB8, RAB8 | RAB GTPase homolog 8 | chr3:19876531-19878264 REVERSE LENGTH=216	216	216	1.00E-115	100.0	91.7	94.0
Rsa1.0_00066.1.g3532.t1	ref|NP_190930.1| pyrophosphorylase 4 [Arabidopsis thaliana] gi|6729513|emb|CAB67669.1| inorganic pyrophosphatase-like protein [Arabidopsis thaliana] gi|110741493|dbj|BAE98700.1| inorganic pyrophosphatase -like protein [Arabidopsis thaliana] gi|332645599|gb|AEE79120.1| pyrophosphorylase 4 [Arabidopsis thaliana]	217	216	1.00E-116	99.5	93.5	96.8	pyrophosphorylase 4	gbpln	Arabidopsis thaliana	AT3G53620.1 | Symbols: AtPPa4, PPa4 | pyrophosphorylase 4 | chr3:19881491-19883308 FORWARD LENGTH=216	217	216	1.00E-119	99.5	93.5	96.8
Rsa1.0_00066.1.g3533.t1	ref|XP_002876217.1| hypothetical protein ARALYDRAFT_485749 [Arabidopsis lyrata subsp. lyrata] gi|297322055|gb|EFH52476.1| hypothetical protein ARALYDRAFT_485749 [Arabidopsis lyrata subsp. lyrata]	187	192	1.00E-66	102.7	81.3	87.2	hypothetical protein ARALYDRAFT_485749	gbpln	Arabidopsis lyrata	AT3G53630.1 | Symbols: | unknown protein; BEST Arabidopsis thaliana protein match is: unknown protein (TAIR:AT4G18692.1); Has 42 Blast hits to 42 proteins in 10 species: Archae - 0; Bacteria - 0; Metazoa - 0; Fungi - 0; Plants - 42; Viruses - 0; Other Eukaryotes - 0 (source: NCBI BLink). | chr3:19883986-19885568 FORWARD LENGTH=194	187	194	3.00E-68	103.7	80.7	86.6
Rsa1.0_00066.1.g3534.t1	ref|XP_002532621.1| histone h2b, putative [Ricinus communis] gi|223527641|gb|EEF29752.1| histone h2b, putative [Ricinus communis]	138	141	3.00E-62	102.2	93.5	98.6	histone h2b, putative	gbpln	Ricinus communis	AT2G37470.1 | Symbols: | Histone superfamily protein | chr2:15736832-15737248 FORWARD LENGTH=138	138	138	5.00E-64	100.0	95.7	97.8

Rsa1.0_00066.1.g3535.t1	ref|NP_190935.1| uncharacterized protein [Arabidopsis thaliana] gi|334185954|ref|NP_001190081.1| uncharacterized protein [Arabidopsis thaliana] gi|14423470|gb|AAK62417.1|AF386972_1 putative protein [Arabidopsis thaliana] gi|6729518|emb|CAB67674.1| putative protein [Arabidopsis thaliana] gi|18377574|gb|AAL66953.1| putative protein [Arabidopsis thaliana] gi|332645603|gb|AEE79124.1| uncharacterized protein AT3G53670 [Arabidopsis thaliana] gi|332645604|gb|AEE79125.1| uncharacterized protein AT3G53670 [Arabidopsis thaliana]	209	203	1.00E-56	97.1	67.0	76.1	uncharacterized protein	gbpln	Arabidopsis thaliana	AT3G53670.2 | Symbols: | unknown protein; FUNCTIONS IN: molecular_function unknown; INVOLVED IN: biological_process unknown; LOCATED IN: cellular_component unknown; EXPRESSED IN: sperm cell, male gametophyte, pollen tube; EXPRESSED DURING: L mature pollen stage, M germinated pollen stage; BEST Arabidopsis thaliana protein match is: unknown protein (TAIR:AT2G37480.1). | chr3:19891104-19892214 FORWARD LENGTH=203	209	203	3.00E-59	97.1	67.0	76.1
Rsa1.0_00066.1.g3536.t1	ref|NP_190936.2| Acyl-CoA N-acyltransferase with RING/FYVE/PHD-type zinc finger domain-containing protein [Arabidopsis thaliana] gi|225898713|dbj|BAH30487.1| hypothetical protein [Arabidopsis thaliana] gi|332645606|gb|AEE79127.1| Acyl-CoA N-acyltransferase with RING/FYVE/PHD-type zinc finger domain-containing protein [Arabidopsis thaliana]	858	841	0	98.0	68.2	74.6	Acyl-CoA N-acyltransferase with RING/FYVE/PHD-type zinc finger domain-containing protein	gbpln	Arabidopsis thaliana	AT3G53680.1 | Symbols: | Acyl-CoA N-acyltransferase with RING/FYVE/PHD-type zinc finger domain | chr3:19892863-19897412 REVERSE LENGTH=841	858	841	0	98.0	68.2	74.6
Rsa1.0_00066.1.g3537.t1	ref|NP_190940.1| cation/H(+) antiporter 20 [Arabidopsis thaliana] gi|75311818|sp|Q9M353.1|CHX20_ARATH RecName: Full=Cation/H(+) antiporter 20; AltName: Full=Protein CATION/H+ EXCHANGER 20; Short=AtCHX20 gi|7629992|emb|CAB88334.1| putative protein [Arabidopsis thaliana] gi|27311847|gb|AAO00889.1| putative protein [Arabidopsis thaliana] gi|38603806|gb|AAR24648.1| At3g53720 [Arabidopsis thaliana] gi|110742710|dbj|BAE99266.1| hypothetical protein [Arabidopsis thaliana] gi|332645611|gb|AEE79132.1| cation/H(+) antiporter 20 [Arabidopsis thaliana]	834	842	0	101.0	90.0	94.6	cation/H(+) antiporter 20	gbpln	Arabidopsis thaliana	AT3G53720.1 | Symbols: ATCHX20, CHX20 | cation/H+ exchanger 20 | chr3:19905826-19910027 REVERSE LENGTH=842	834	842	0	101.0	90.0	94.6
Rsa1.0_00066.1.g3538.t1	#	#	#	#	#	#	#	-	----	----	#	#	#	#	#	#	#
Rsa1.0_00066.1.g3539.t1	gb|AAC28221.1| similar to reverse transcriptases (PFam: rvt.hmm, score: 60.13) [Arabidopsis thaliana]	583	1164	2.00E-84	199.7	26.9	40.8	similar to reverse transcriptases (PFam: rvt.hmm, score: 60.13)	gbpln	Arabidopsis thaliana	AT3G24255.1 | Symbols: | RNA-directed DNA polymerase (reverse transcriptase)-related family protein | chr3:8789471-8793208 FORWARD LENGTH=746	583	746	3.00E-64	128.0	26.4	38.9
Rsa1.0_00066.1.g3540.t1	tpg|DAA40122.1| TPA: histone H4.3 [Zea mays]	103	248	3.00E-52	240.8	100.0	100.0	TPA: histone H4.3	gbenv/gbpln	Zea mays	AT5G59970.1 | Symbols: | Histone superfamily protein | chr5:24146352-24146663 REVERSE LENGTH=103	103	103	2.00E-52	100.0	100.0	100.0
Rsa1.0_00066.1.g3541.t1	ref|NP_850697.1| 60S ribosomal protein L36-2 [Arabidopsis thaliana] gi|79314916|ref|NP_001030855.1| 60S ribosomal protein L36-2 [Arabidopsis thaliana] gi|145332827|ref|NP_001078279.1| 60S ribosomal protein L36-2 [Arabidopsis thaliana] gi|17865567|sp|Q9M352.1|RL362_ARATH RecName: Full=60S ribosomal protein L36-2 gi|7629994|emb|CAB88336.1| 60S RIBOSOMAL PROTEIN L36 homolog [Arabidopsis thaliana] gi|17065430|gb|AAL32869.1| 60S RIBOSOMAL PROTEIN L36 homolog [Arabidopsis thaliana] gi|20148501|gb|AAM10141.1| 60S ribosomal protein L36-like protein [Arabidopsis thaliana] gi|332645614|gb|AEE79135.1| 60S ribosomal protein L36-2 [Arabidopsis thaliana] gi|332645615|gb|AEE79136.1| 60S ribosomal protein L36-2 [Arabidopsis thaliana] gi|332645616|gb|AEE79137.1| 60S ribosomal protein L36-2 [Arabidopsis thaliana]	112	112	7.00E-52	100.0	94.6	99.1	60S ribosomal protein L36-2	gbpln	Arabidopsis thaliana	AT3G53740.4 | Symbols: | Ribosomal protein L36e family protein | chr3:19913921-19914813 REVERSE LENGTH=112	112	112	1.00E-54	100.0	94.6	99.1

Rsa1.0_00066.1.g3542.t1	ref|NP_566988.1| actin 3 [Arabidopsis thaliana] gi|30687201|ref|NP_850284.1| actin 1 [Arabidopsis thaliana] gi|79324605|ref|NP_001031504.1| actin 1 [Arabidopsis thaliana] gi|460415910|ref|XP_004253296.1| PREDICTED: actin-1-like [Solanum lycopersicum] gi|317374840|sp|P0CJ46.1|ACT1_ARATH RecName: Full=Actin-1 gi|317374841|sp|P0CJ47.1|ACT3_ARATH RecName: Full=Actin-3 gi|1145695|gb|AAA98562.1| actin 3 [Arabidopsis thaliana] gi|15146304|gb|AAK83635.1| AT3g53750/F5K20_50 [Arabidopsis thaliana] gi|18650648|gb|AAL75893.1| At2g37620/F13M22.12 [Arabidopsis thaliana] gi|20147381|gb|AAM10400.1| At2g37620/F13M22.12 [Arabidopsis thaliana] gi|21554576|gb|AAM63620.1| actin (ACT3) [Arabidopsis thaliana] gi|25090278|gb|AAN72268.1| At3g53750/F5K20_50 [Arabidopsis thaliana] gi|330254332|gb|AEC09426.1| actin 1 [Arabidopsis thaliana] gi|330254333|gb|AEC09427.1| actin 1 [Arabidopsis thaliana] gi|332645617|gb|AEE79138.1| actin 3 [Arabidopsis thaliana]	377	377	0	100.0	99.5	99.7	actin 3	gbpln	Arabidopsis thaliana	AT3G53750.1 | Symbols: ACT3 | actin 3 | chr3:19915924-19917371 FORWARD LENGTH=377	377	377	0	100.0	99.5	99.7
Rsa1.0_00066.1.g3543.t1	gb|EOA23531.1| hypothetical protein CARUB_v10016729mg [Capsella rubella]	748	745	0	99.6	92.1	94.9	hypothetical protein CARUB_v10016729mg	gbpln	Capsella rubella	AT3G53760.1 | Symbols: GCP4, ATGCP4 | GAMMA-TUBULIN COMPLEX PROTEIN 4 | chr3:19918182-19922264 REVERSE LENGTH=745	748	745	0	99.6	91.6	94.8
Rsa1.0_00066.1.g3544.t1	gb|EOA24166.1| hypothetical protein CARUB_v10017398mg [Capsella rubella]	392	396	0	101.0	90.6	95.4	hypothetical protein CARUB_v10017398mg	gbpln	Capsella rubella	AT3G53780.2 | Symbols: ATRBL4, RBL4 | RHOMBOID-like protein 4 | chr3:19924729-19926648 REVERSE LENGTH=394	392	394	0	100.5	88.5	94.6
Rsa1.0_00066.1.g3545.t1	ref|NP_190948.1| hsp70-interacting protein [Arabidopsis thaliana] gi|7630000|emb|CAB88342.1| putative protein [Arabidopsis thaliana] gi|21593297|gb|AAM65246.1| unknown [Arabidopsis thaliana] gi|332645624|gb|AEE79145.1| hsp70-interacting protein FES1B-like protein [Arabidopsis thaliana]	363	363	0	100.0	88.7	93.7	hsp70-interacting protein	gbpln	Arabidopsis thaliana	AT3G53800.1 | Symbols: Fes1B | Fes1B | chr3:19930942-19932719 FORWARD LENGTH=363	363	363	0	100.0	88.7	93.7
Rsa1.0_00066.1.g3546.t1	gb|EOA25977.1| hypothetical protein CARUB_v10019366mg [Capsella rubella]	677	723	0	106.8	85.2	92.0	hypothetical protein CARUB_v10019366mg	gbpln	Capsella rubella	AT3G53810.1 | Symbols: | Concanavalin A-like lectin protein kinase family protein | chr3:19933153-19935186 REVERSE LENGTH=677	677	677	0	100.0	84.6	91.6
Rsa1.0_00066.1.g3547.t1	gb|AAC33226.1| putative non-LTR retroelement reverse transcriptase [Arabidopsis thaliana]	553	1529	4.00E-99	276.5	38.5	55.0	putative non-LTR retroelement reverse transcriptase	gbpln	Arabidopsis thaliana	AT3G24255.1 | Symbols: | RNA-directed DNA polymerase (reverse transcriptase)-related family protein | chr3:8789471-8793208 FORWARD LENGTH=746	553	746	3.00E-63	134.9	27.1	36.5
Rsa1.0_00066.1.g3548.t1	ref|XP_002876227.1| zinc finger family protein [Arabidopsis lyrata subsp. lyrata] gi|297322065|gb|EFH52486.1| zinc finger family protein [Arabidopsis lyrata subsp. lyrata]	180	142	5.00E-53	78.9	57.8	67.8	zinc finger family protein	gbpln	Arabidopsis lyrata	AT3G53820.1 | Symbols: | C2H2 and C2HC zinc fingers superfamily protein | chr3:19938706-19939134 FORWARD LENGTH=142	180	142	2.00E-54	78.9	57.8	66.7
Rsa1.0_00066.1.g3549.t1	gb|EOA23633.1| hypothetical protein CARUB_v10016831mg [Capsella rubella]	644	638	0	99.1	82.9	89.1	hypothetical protein CARUB_v10016831mg	gbpln	Capsella rubella	AT3G53840.1 | Symbols: | Protein kinase superfamily protein | chr3:19945571-19947719 FORWARD LENGTH=639	644	639	0	99.2	81.5	87.7
Rsa1.0_00066.1.g3550.t1	ref|NP_566990.1| uncharacterized protein [Arabidopsis thaliana] gi|75164334|sp|Q945M8.1|CSPLI_ARATH RecName: Full=CASP-like protein At3g53850 gi|15724236|gb|AAL06511.1|AF412058_1 AT4g31460/F3L17_30 [Arabidopsis thaliana] gi|21464571|gb|AAM52240.1| AT4g31460/F3L17_30 [Arabidopsis thaliana] gi|332645629|gb|AEE79150.1| uncharacterized protein AT3G53850 [Arabidopsis thaliana]	167	154	5.00E-42	92.2	55.7	64.7	uncharacterized protein	gbpln	Arabidopsis thaliana	AT3G53850.1 | Symbols: | Uncharacterised protein family (UPF0497) | chr3:19948262-19948994 REVERSE LENGTH=154	167	154	2.00E-44	92.2	55.7	64.7
Rsa1.0_00066.1.g3551.t1	gb|EOA24417.1| hypothetical protein CARUB_v10017668mg [Capsella rubella]	289	315	1.00E-150	109.0	93.4	96.2	hypothetical protein CARUB_v10017668mg	gbpln	Capsella rubella	AT3G53880.1 | Symbols: | NAD(P)-linked oxidoreductase superfamily protein | chr3:19953238-19955171 FORWARD LENGTH=315	289	315	1.00E-151	109.0	91.3	95.8
Rsa1.0_00066.1.g3552.t1	gb|EOA25028.1| hypothetical protein CARUB_v10018334mg [Capsella rubella]	82	82	1.00E-39	100.0	95.1	97.6	hypothetical protein CARUB_v10018334mg	gbpln	Capsella rubella	AT5G27700.1 | Symbols: | Ribosomal protein S21e | chr5:9807541-9808048 REVERSE LENGTH=82	82	82	1.00E-41	100.0	93.9	96.3

Rsa1.0_00066.1.g3553.t1	ref|XP_002877946.1| hypothetical protein ARALYDRAFT_323928 [Arabidopsis lyrata subsp. lyrata] gi|297323784|gb|EFH54205.1| hypothetical protein ARALYDRAFT_323928 [Arabidopsis lyrata subsp. lyrata]	328	295	1.00E-131	89.9	75.3	79.9	hypothetical protein ARALYDRAFT_323928	gbpln	Arabidopsis lyrata	AT3G53900.2 | Symbols: UPP, PYRR | uracil phosphoribosyltransferase | chr3:19956914-19959006 REVERSE LENGTH=296	328	296	1.00E-133	90.2	75.0	80.2
Rsa1.0_00066.1.g3554.t1	gb|EOA23672.1| hypothetical protein CARUB_v10016879mg [Capsella rubella]	593	602	0	101.5	83.3	92.4	hypothetical protein CARUB_v10016879mg	gbpln	Capsella rubella	AT3G53960.1 | Symbols: | Major facilitator superfamily protein | chr3:19978306-19980886 REVERSE LENGTH=602	593	602	0	101.5	82.3	91.7
Rsa1.0_00067.1.g3555.t1	ref|NP_173609.1| ethylene-responsive transcription factor ERF012 [Arabidopsis thaliana] gi|75265793|sp|Q9SFE4.1|ERF12_ARATH RecName: Full=Ethylene-responsive transcription factor ERF012 gi|6552733|gb|AAF16532.1|AC013482_6 T26F17.14 [Arabidopsis thaliana] gi|51971555|dbj|BAD44442.1| TINY like protein [Arabidopsis thaliana] gi|88196743|gb|ABD43014.1| At1g21910 [Arabidopsis thaliana] gi|332192050|gb|AEE30171.1| ethylene-responsive transcription factor ERF012 [Arabidopsis thaliana]	227	230	2.00E-70	101.3	74.4	84.6	ethylene-responsive transcription factor ERF012	gbpln	Arabidopsis thaliana	AT1G21910.1 | Symbols: DREB26 | Integrase-type DNA-binding superfamily protein | chr1:7696655-7697347 FORWARD LENGTH=230	227	230	5.00E-73	101.3	74.4	84.6
Rsa1.0_00067.1.g3556.t1	gb|AAC28221.1| similar to reverse transcriptases (PFam: rvt.hmm, score: 60.13) [Arabidopsis thaliana]	142	1164	6.00E-11	819.7	26.1	39.4	similar to reverse transcriptases (PFam: rvt.hmm, score: 60.13)	gbpln	Arabidopsis thaliana	#	#	#	#	#	#	#
Rsa1.0_00067.1.g3557.t1	ref|XP_002893188.1| emp24/gp25L/p24 family protein [Arabidopsis lyrata subsp. lyrata] gi|297339030|gb|EFH69447.1| emp24/gp25L/p24 family protein [Arabidopsis lyrata subsp. lyrata]	224	216	1.00E-103	96.4	80.8	87.1	emp24/gp25L/p24 family protein	gbpln	Arabidopsis lyrata	AT1G21900.1 | Symbols: | emp24/gp25L/p24 family/GOLD family protein | chr1:7691165-7692327 REVERSE LENGTH=216	224	216	1.00E-103	96.4	79.5	84.8
Rsa1.0_00067.1.g3558.t1	gb|EOA40579.1| hypothetical protein CARUB_v10009309mg, partial [Capsella rubella]	387	409	0	105.7	90.7	95.1	hypothetical protein CARUB_v10009309mg, partial	gbpln	Capsella rubella	AT1G21890.1 | Symbols: | nodulin MtN21 /EamA-like transporter family protein | chr1:7682808-7685581 REVERSE LENGTH=389	387	389	0	100.5	89.4	93.3
Rsa1.0_00067.1.g3559.t1	ref|NP_564153.1| LysM domain-containing GPI-anchored protein 1 [Arabidopsis thaliana] gi|38258218|sp|Q93ZH0.1|LYM1_ARATH RecName: Full=LysM domain-containing GPI-anchored protein 1; Flags: Precursor gi|15982870|gb|AAL09782.1| At1g21880/T26F17_5 [Arabidopsis thaliana] gi|53749174|gb|AAU90072.1| At1g21880 [Arabidopsis thaliana] gi|332192047|gb|AEE30168.1| LysM domain-containing GPI-anchored protein 1 [Arabidopsis thaliana]	447	416	0	93.1	81.4	87.9	LysM domain-containing GPI-anchored protein 1	gbpln	Arabidopsis thaliana	AT1G21880.2 | Symbols: LYM1 | lysm domain GPI-anchored protein 1 precursor | chr1:7680689-7682526 FORWARD LENGTH=416	447	416	0	93.1	81.4	87.9
Rsa1.0_00067.1.g3560.t1	ref|XP_002893185.1| hypothetical protein ARALYDRAFT_313074 [Arabidopsis lyrata subsp. lyrata] gi|297339027|gb|EFH69444.1| hypothetical protein ARALYDRAFT_313074 [Arabidopsis lyrata subsp. lyrata]	273	205	9.00E-73	75.1	60.4	62.6	hypothetical protein ARALYDRAFT_313074	gbpln	Arabidopsis lyrata	AT1G21830.1 | Symbols: | unknown protein; CONTAINS InterPro DOMAIN/s: Protein of unknown function DUF740 (InterPro:IPR008004); BEST Arabidopsis thaliana protein match is: unknown protein (TAIR:AT1G44608.1); Has 49 Blast hits to 49 proteins in 12 species: Archae - 0; Bacteria - 0; Metazoa - 0; Fungi - 0; Plants - 49; Viruses - 0; Other Eukaryotes - 0 (source: NCBI BLink). | chr1:7661429-7662521 REVERSE LENGTH=206	273	206	6.00E-73	75.5	59.7	62.6
Rsa1.0_00067.1.g3561.t1	ref|XP_002890465.1| hypothetical protein ARALYDRAFT_472418 [Arabidopsis lyrata subsp. lyrata] gi|297336307|gb|EFH66724.1| hypothetical protein ARALYDRAFT_472418 [Arabidopsis lyrata subsp. lyrata]	280	288	1.00E-144	102.9	90.7	93.9	hypothetical protein ARALYDRAFT_472418	gbpln	Arabidopsis lyrata	AT1G21790.1 | Symbols: | TRAM, LAG1 and CLN8 (TLC) lipid-sensing domain containing protein | chr1:7654357-7655691 FORWARD LENGTH=288	280	288	1.00E-144	102.9	90.0	93.6
Rsa1.0_00067.1.g3562.t1	ref|XP_002890464.1| BTB/POZ domain-containing protein [Arabidopsis lyrata subsp. lyrata] gi|297336306|gb|EFH66723.1| BTB/POZ domain-containing protein [Arabidopsis lyrata subsp. lyrata]	384	326	0	84.9	80.2	82.8	BTB/POZ domain-containing protein	gbpln	Arabidopsis lyrata	AT1G21780.2 | Symbols: | BTB/POZ domain-containing protein | chr1:7652476-7653866 FORWARD LENGTH=326	384	326	0	84.9	79.9	82.6
Rsa1.0_00067.1.g3563.t1	gb|EOA38692.1| hypothetical protein CARUB_v10010702mg [Capsella rubella]	104	110	7.00E-42	105.8	82.7	91.3	hypothetical protein CARUB_v10010702mg	gbpln	Capsella rubella	AT1G21770.1 | Symbols: | Acyl-CoA N-acyltransferases (NAT) superfamily protein | chr1:7651843-7652249 REVERSE LENGTH=111	104	111	2.00E-43	106.7	81.7	88.5

Rsa1.0_00067.1.g3564.t1	ref|XP_002890463.1| F-box family protein [Arabidopsis lyrata subsp. lyrata] gi|297336305|gb|EFH66722.1| F-box family protein [Arabidopsis lyrata subsp. lyrata]	323	328	1.00E-179	101.5	94.7	96.9	F-box family protein	gbpln	Arabidopsis lyrata	AT1G21760.1 | Symbols: ATFBP7, FBP7 | F-box protein 7 | chr1:7649324-7651613 FORWARD LENGTH=328	323	328	1.00E-179	101.5	92.6	96.9
Rsa1.0_00067.1.g3565.t1	tpg|DAA63284.1| TPA: hypothetical protein ZEAMMB73_434507 [Zea mays]	292	704	3.00E-25	241.1	30.1	54.8	TPA: hypothetical protein ZEAMMB73_434507	gbenv/gbpln	Zea mays	AT5G19820.1 | Symbols: emb2734 | ARM repeat superfamily protein | chr5:6695731-6701247 REVERSE LENGTH=1116	292	1116	2.00E-26	382.2	30.1	55.1
Rsa1.0_00067.1.g3566.t1	ref|XP_002890462.1| hypothetical protein ARALYDRAFT_889647 [Arabidopsis lyrata subsp. lyrata] gi|297336304|gb|EFH66721.1| hypothetical protein ARALYDRAFT_889647 [Arabidopsis lyrata subsp. lyrata]	495	501	0	101.2	80.6	88.3	hypothetical protein ARALYDRAFT_889647	gbpln	Arabidopsis lyrata	AT1G21750.1 | Symbols: ATPDIL1-1, ATPDI5, PDI5, PDIL1-1 | PDI-like 1-1 | chr1:7645767-7648514 FORWARD LENGTH=501	495	501	0	101.2	79.6	87.5
Rsa1.0_00067.1.g3567.t1	ref|XP_002890461.1| hypothetical protein ARALYDRAFT_472413 [Arabidopsis lyrata subsp. lyrata] gi|297336303|gb|EFH66720.1| hypothetical protein ARALYDRAFT_472413 [Arabidopsis lyrata subsp. lyrata]	934	942	0	100.9	81.9	88.0	hypothetical protein ARALYDRAFT_472413	gbpln	Arabidopsis lyrata	AT1G21740.1 | Symbols: | Protein of unknown function (DUF630 and DUF632) | chr1:7641580-7645078 FORWARD LENGTH=953	934	953	0	102.0	82.0	88.3
Rsa1.0_00067.1.g3568.t1	ref|NP_173592.3| P-loop containing nucleoside triphosphate hydrolase-like protein [Arabidopsis thaliana] gi|16902292|dbj|BAB71851.1| kinesin-related protein [Arabidopsis thaliana] gi|332192027|gb|AEE30148.1| P-loop containing nucleoside triphosphate hydrolase-like protein [Arabidopsis thaliana]	925	890	0	96.2	83.4	87.8	P-loop containing nucleoside triphosphate hydrolase-like protein	gbpln	Arabidopsis thaliana	AT1G21730.1 | Symbols: | P-loop containing nucleoside triphosphate hydrolases superfamily protein | chr1:7630365-7636247 FORWARD LENGTH=890	925	890	0	96.2	83.4	87.8
Rsa1.0_00067.1.g3569.t1	gb|EOA36141.1| hypothetical protein CARUB_v10012612mg [Capsella rubella]	224	229	8.00E-86	102.2	77.7	87.5	hypothetical protein CARUB_v10012612mg	gbpln	Capsella rubella	AT1G21722.1 | Symbols: | unknown protein; FUNCTIONS IN: molecular_function unknown; INVOLVED IN: biological_process unknown; LOCATED IN: cellular_component unknown; BEST Arabidopsis thaliana protein match is: unknown protein (TAIR:AT1G78922.1); Has 47 Blast hits to 47 proteins in 13 species: Archae - 0; Bacteria - 0; Metazoa - 1; Fungi - 1; Plants - 42; Viruses - 0; Other Eukaryotes - 3 (source: NCBI BLink). | chr1:7628456-7629252 FORWARD LENGTH=233	224	233	1.00E-86	104.0	77.7	86.6
Rsa1.0_00067.1.g3570.t1	ref|NP_564149.1| proteasome subunit beta type-3-A [Arabidopsis thaliana] gi|288558823|sp|Q9XI05.2|PSB3A_ARATH RecName: Full=Proteasome subunit beta type-3-A; AltName: Full=20S proteasome beta subunit C-1; AltName: Full=Proteasome component T gi|17473525|gb|AAL38246.1| proteasome subunit [Arabidopsis thaliana] gi|21387087|gb|AAM47947.1| proteasome subunit [Arabidopsis thaliana] gi|110740894|dbj|BAE98543.1| 20S proteasome beta subunit PBC2 like protein [Arabidopsis thaliana] gi|332192025|gb|AEE30146.1| proteasome subunit beta type-3-A [Arabidopsis thaliana]	204	204	1.00E-116	100.0	99.0	100.0	proteasome subunit beta type-3-A	gbpln	Arabidopsis thaliana	AT1G21720.1 | Symbols: PBC1 | proteasome beta subunit C1 | chr1:7626394-7628070 FORWARD LENGTH=204	204	204	1.00E-118	100.0	99.0	100.0
Rsa1.0_00067.1.g3571.t1	gb|EOA36733.1| hypothetical protein CARUB_v10012527mg, partial [Capsella rubella]	778	833	0	107.1	82.3	89.2	hypothetical protein CARUB_v10012527mg, partial	gbpln	Capsella rubella	AT1G21700.1 | Symbols: ATSWI3C, CHB4, SWI3C | SWITCH/sucrose nonfermenting 3C | chr1:7620156-7623978 REVERSE LENGTH=807	778	807	0	103.7	82.6	88.6
Rsa1.0_00067.1.g3572.t1	ref|XP_002890454.1| hypothetical protein ARALYDRAFT_472409 [Arabidopsis lyrata subsp. lyrata] gi|297336296|gb|EFH66713.1| hypothetical protein ARALYDRAFT_472409 [Arabidopsis lyrata subsp. lyrata]	211	205	7.00E-36	97.2	55.0	64.0	hypothetical protein ARALYDRAFT_472409	gbpln	Arabidopsis lyrata	AT1G21695.1 | Symbols: | hydroxyproline-rich glycoprotein family protein | chr1:7619132-7619785 FORWARD LENGTH=217	211	217	1.00E-29	102.8	53.6	63.0
Rsa1.0_00067.1.g3573.t1	ref|XP_002890453.1| EMB1968 [Arabidopsis lyrata subsp. lyrata] gi|297336295|gb|EFH66712.1| EMB1968 [Arabidopsis lyrata subsp. lyrata]	339	339	0	100.0	93.2	96.8	EMB1968	gbpln	Arabidopsis lyrata	AT1G21690.1 | Symbols: EMB1968, RFC4 | ATPase family associated with various cellular activities (AAA) | chr1:7615675-7618362 FORWARD LENGTH=339	339	339	0	100.0	92.9	96.5

Rsa1.0_00067.1.g3574.t1	ref|XP_002890452.1| hypothetical protein ARALYDRAFT_472406 [Arabidopsis lyrata subsp. lyrata] gi|297336294|gb|EFH66711.1| hypothetical protein ARALYDRAFT_472406 [Arabidopsis lyrata subsp. lyrata]	665	706	0	106.2	86.5	92.8	hypothetical protein ARALYDRAFT_472406	gbpln	Arabidopsis lyrata	AT1G21680.1 | Symbols: | DPP6 N-terminal domain-like protein | chr1:7613028-7615148 FORWARD LENGTH=706	665	706	0	106.2	85.6	91.3
Rsa1.0_00067.1.g3575.t1	dbj|BAF01687.1| hypothetical protein [Arabidopsis thaliana]	657	1072	1.00E-134	163.2	39.9	57.2	hypothetical protein	gbpln	Arabidopsis thaliana	AT3G24255.1 | Symbols: | RNA-directed DNA polymerase (reverse transcriptase)-related family protein | chr3:8789471-8793208 FORWARD LENGTH=746	657	746	2.00E-60	113.5	21.3	31.5
Rsa1.0_00067.1.g3576.t1	#	#	#	#	#	#	#	-	----	----	#	#	#	#	#	#	#
Rsa1.0_00067.1.g3577.t2	gb|AAG51081.1|AC027032_1 hypothetical protein [Arabidopsis thaliana]	1327	1678	0	126.5	38.4	49.9	hypothetical protein	gbpln	Arabidopsis thaliana	AT3G51690.1 | Symbols: | PIF1 helicase | chr3:19176731-19178107 REVERSE LENGTH=331	1327	331	5.00E-57	24.9	8.4	11.5
Rsa1.0_00067.1.g3578.t1	ref|XP_002890451.1| hypothetical protein ARALYDRAFT_472405 [Arabidopsis lyrata subsp. lyrata] gi|297336293|gb|EFH66710.1| hypothetical protein ARALYDRAFT_472405 [Arabidopsis lyrata subsp. lyrata]	705	703	0	99.7	78.9	87.1	hypothetical protein ARALYDRAFT_472405	gbpln	Arabidopsis lyrata	AT1G21670.1 | Symbols: | LOCATED IN: cell wall, plant-type cell wall; EXPRESSED IN: 23 plant structures; EXPRESSED DURING: 14 growth stages; CONTAINS InterPro DOMAIN/s: WD40-like Beta Propeller (InterPro:IPR011659), Six-bladed beta-propeller, TolB-like (InterPro:IPR011042); BEST Arabidopsis thaliana protein match is: DPP6 N-terminal domain-like protein (TAIR:AT1G21680.1); Has 8461 Blast hits to 5060 proteins in 1257 species: Archae - 79; Bacteria - 5567; Metazoa - 37; Fungi - 70; Plants - 117; Viruses - 0; Other Eukaryotes - 2591 (source: NCBI BLink). | chr1:7610409-7612520 FORWARD LENGTH=703	705	703	0	99.7	76.6	85.1
Rsa1.0_00067.1.g3579.t9	gb|EOA40087.1| hypothetical protein CARUB_v10008781mg [Capsella rubella]	539	544	1.00E-176	100.9	67.7	76.3	hypothetical protein CARUB_v10008781mg	gbpln	Capsella rubella	AT1G21660.1 | Symbols: | Chaperone DnaJ-domain superfamily protein | chr1:7605924-7608835 FORWARD LENGTH=523	539	523	1.00E-170	97.0	65.9	74.0
Rsa1.0_00067.1.g3580.t1	gb|EOA35901.1| hypothetical protein CARUB_v10021156mg [Capsella rubella]	147	121	2.00E-49	82.3	71.4	78.9	hypothetical protein CARUB_v10021156mg	gbpln	Capsella rubella	AT1G77350.2 | Symbols: | unknown protein; FUNCTIONS IN: molecular_function unknown; INVOLVED IN: biological_process unknown; LOCATED IN: endomembrane system; EXPRESSED IN: 23 plant structures; EXPRESSED DURING: 13 growth stages; CONTAINS InterPro DOMAIN/s: Protein of unknown function KRTCAP2 (InterPro:IPR018614); Has 35333 Blast hits to 34131 proteins in 2444 species: Archae - 798; Bacteria - 22429; Metazoa - 974; Fungi - 991; Plants - 531; Viruses - 0; Other Eukaryotes - 9610 (source: NCBI BLink). | chr1:29070497-29071545 FORWARD LENGTH=122	147	122	2.00E-51	83.0	72.8	80.3
Rsa1.0_00067.1.g3581.t1	gb|EOA39091.1| hypothetical protein CARUB_v10011823mg [Capsella rubella]	806	1799	0	223.2	73.6	83.9	hypothetical protein CARUB_v10011823mg	gbpln	Capsella rubella	AT1G21650.3 | Symbols: SECA2 | Preprotein translocase SecA family protein | chr1:7592891-7604152 REVERSE LENGTH=1805	806	1805	0	223.9	73.4	84.9
Rsa1.0_00067.1.g3582.t1	ref|XP_002890449.1| hypothetical protein ARALYDRAFT_889629 [Arabidopsis lyrata subsp. lyrata] gi|297336291|gb|EFH66708.1| hypothetical protein ARALYDRAFT_889629 [Arabidopsis lyrata subsp. lyrata]	1009	983	0	97.4	85.5	90.4	hypothetical protein ARALYDRAFT_889629	gbpln	Arabidopsis lyrata	AT1G21640.1 | Symbols: NADK2, ATNADK2 | NAD kinase 2 | chr1:7588726-7592669 FORWARD LENGTH=985	1009	985	0	97.6	84.9	89.6
Rsa1.0_00067.1.g3583.t1	ref|NP_173582.2| calcium-binding EF-hand-containing protein [Arabidopsis thaliana] gi|332192007|gb|AEE30128.1| calcium-binding EF-hand-containing protein [Arabidopsis thaliana]	1228	1218	0	99.2	83.0	87.5	calcium-binding EF-hand-containing protein	gbpln	Arabidopsis thaliana	AT1G21630.1 | Symbols: | Calcium-binding EF hand family protein | chr1:7581457-7587796 FORWARD LENGTH=1218	1228	1218	0	99.2	83.0	87.5
Rsa1.0_00067.1.g3584.t2	ref|XP_002893178.1| hypothetical protein ARALYDRAFT_472396 [Arabidopsis lyrata subsp. lyrata] gi|297339020|gb|EFH69437.1| hypothetical protein ARALYDRAFT_472396 [Arabidopsis lyrata subsp. lyrata]	334	328	1.00E-162	98.2	90.1	92.2	hypothetical protein ARALYDRAFT_472396	gbpln	Arabidopsis lyrata	AT1G21600.2 | Symbols: PTAC6 | plastid transcriptionally active 6 | chr1:7571514-7573782 REVERSE LENGTH=328	334	328	1.00E-150	98.2	85.0	87.7
Rsa1.0_00067.1.g3585.t3	ref|NP_173577.2| zinc finger CCCH domain-containing protein [Arabidopsis thaliana] gi|332191999|gb|AEE30120.1| zinc finger CCCH domain-containing protein [Arabidopsis thaliana]	2016	2166	0	107.4	64.7	74.6	zinc finger CCCH domain-containing protein	gbpln	Arabidopsis thaliana	AT1G21580.1 | Symbols: | Zinc finger C-x8-C-x5-C-x3-H type family protein | chr1:7557822-7565655 REVERSE LENGTH=2166	2016	2166	0	107.4	64.7	74.6

Rsa1.0_00067.1.g3586.t1	ref|XP_002890445.1| hypothetical protein ARALYDRAFT_889619 [Arabidopsis lyrata subsp. lyrata] gi|297336287|gb|EFH66704.1| hypothetical protein ARALYDRAFT_889619 [Arabidopsis lyrata subsp. lyrata]	379	394	7.00E-86	104.0	53.3	64.4	hypothetical protein ARALYDRAFT_889619	gbpln	Arabidopsis lyrata	AT1G21560.3 | Symbols: | unknown protein; FUNCTIONS IN: molecular_function unknown; INVOLVED IN: biological_process unknown; LOCATED IN: cellular_component unknown; EXPRESSED IN: 22 plant structures; EXPRESSED DURING: 13 growth stages; BEST Arabidopsis thaliana protein match is: unknown protein (TAIR:AT4G01170.1). | chr1:7555208-7556789 FORWARD LENGTH=399	379	399	5.00E-83	105.3	51.7	64.1
Rsa1.0_00067.1.g3587.t1	gb|EOA39012.1| hypothetical protein CARUB_v10011559mg [Capsella rubella]	142	159	1.00E-56	112.0	78.2	85.9	hypothetical protein CARUB_v10011559mg	gbpln	Capsella rubella	AT1G21550.1 | Symbols: | Calcium-binding EF-hand family protein | chr1:7553317-7553784 REVERSE LENGTH=155	142	155	2.00E-58	109.2	76.1	87.3
Rsa1.0_00067.1.g3588.t1	ref|XP_002893174.1| hypothetical protein ARALYDRAFT_889616 [Arabidopsis lyrata subsp. lyrata] gi|297339016|gb|EFH69433.1| hypothetical protein ARALYDRAFT_889616 [Arabidopsis lyrata subsp. lyrata]	550	550	0	100.0	94.4	97.6	hypothetical protein ARALYDRAFT_889616	gbpln	Arabidopsis lyrata	AT1G21540.1 | Symbols: | AMP-dependent synthetase and ligase family protein | chr1:7548758-7550521 REVERSE LENGTH=550	550	550	0	100.0	92.7	97.3
Rsa1.0_00067.1.g3589.t1	gb|EOA36652.1| hypothetical protein CARUB_v10011926mg [Capsella rubella]	69	72	1.00E-14	104.3	71.0	78.3	hypothetical protein CARUB_v10011926mg	gbpln	Capsella rubella	AT1G21528.1 | Symbols: | unknown protein; Has 2 Blast hits to 2 proteins in 1 species: Archae - 0; Bacteria - 0; Metazoa - 0; Fungi - 0; Plants - 2; Viruses - 0; Other Eukaryotes - 0 (source: NCBI BLink). | chr1:7542830-7543039 REVERSE LENGTH=69	69	69	7.00E-16	100.0	65.2	75.4
Rsa1.0_00067.1.g3590.t1	ref|XP_002893172.1| hypothetical protein ARALYDRAFT_472389 [Arabidopsis lyrata subsp. lyrata] gi|297339014|gb|EFH69431.1| hypothetical protein ARALYDRAFT_472389 [Arabidopsis lyrata subsp. lyrata]	70	71	3.00E-16	101.4	68.6	81.4	hypothetical protein ARALYDRAFT_472389	gbpln	Arabidopsis lyrata	#	#	#	#	#	#	#
Rsa1.0_00067.1.g3591.t1	ref|XP_002893172.1| hypothetical protein ARALYDRAFT_472389 [Arabidopsis lyrata subsp. lyrata] gi|297339014|gb|EFH69431.1| hypothetical protein ARALYDRAFT_472389 [Arabidopsis lyrata subsp. lyrata]	73	71	2.00E-19	97.3	72.6	79.5	hypothetical protein ARALYDRAFT_472389	gbpln	Arabidopsis lyrata	#	#	#	#	#	#	#
Rsa1.0_00067.1.g3592.t1	ref|XP_002893171.1| hypothetical protein ARALYDRAFT_889613 [Arabidopsis lyrata subsp. lyrata] gi|297339013|gb|EFH69430.1| hypothetical protein ARALYDRAFT_889613 [Arabidopsis lyrata subsp. lyrata]	59	66	5.00E-12	111.9	72.9	79.7	hypothetical protein ARALYDRAFT_889613	gbpln	Arabidopsis lyrata	AT1G21520.1 | Symbols: | unknown protein; Has 9 Blast hits to 9 proteins in 2 species: Archae - 0; Bacteria - 0; Metazoa - 0; Fungi - 0; Plants - 9; Viruses - 0; Other Eukaryotes - 0 (source: NCBI BLink). | chr1:7534533-7534733 REVERSE LENGTH=66	59	66	2.00E-13	111.9	71.2	76.3
Rsa1.0_00067.1.g3593.t1	ref|XP_002893170.1| hypothetical protein ARALYDRAFT_472387 [Arabidopsis lyrata subsp. lyrata] gi|297339012|gb|EFH69429.1| hypothetical protein ARALYDRAFT_472387 [Arabidopsis lyrata subsp. lyrata]	325	326	1.00E-117	100.3	75.7	84.0	hypothetical protein ARALYDRAFT_472387	gbpln	Arabidopsis lyrata	AT1G21510.1 | Symbols: | unknown protein; FUNCTIONS IN: molecular_function unknown; INVOLVED IN: N-terminal protein myristoylation; LOCATED IN: cellular_component unknown; EXPRESSED IN: cotyledon; Has 380 Blast hits to 286 proteins in 63 species: Archae - 0; Bacteria - 6; Metazoa - 254; Fungi - 44; Plants - 13; Viruses - 5; Other Eukaryotes - 58 (source: NCBI BLink). | chr1:7531534-7532505 REVERSE LENGTH=323	325	323	1.00E-110	99.4	71.7	82.5
Rsa1.0_00067.1.g3594.t9	sp|Q9LNF6.3|FB48_ARATH RecName: Full=F-box protein At1g48060	256	365	2.00E-30	142.6	39.5	59.8	RecName: Full=F-box protein At1g48060	----	----	AT1G48060.1 | Symbols: | F-box and associated interaction domains-containing protein | chr1:17728295-17729448 FORWARD LENGTH=353	256	353	7.00E-33	137.9	39.5	59.8
Rsa1.0_00067.1.g3595.t1	gb|EOA39248.1| hypothetical protein CARUB_v10012244mg [Capsella rubella]	114	253	4.00E-12	221.9	43.0	51.8	hypothetical protein CARUB_v10012244mg	gbpln	Capsella rubella	#	#	#	#	#	#	#
Rsa1.0_00067.1.g3596.t1	ref|NP_564141.1| exostosin-like protein [Arabidopsis thaliana] gi|332191985|gb|AEE30106.1| exostosin-like protein [Arabidopsis thaliana]	445	462	0	103.8	92.6	96.2	exostosin-like protein	gbpln	Arabidopsis thaliana	AT1G21480.1 | Symbols: | Exostosin family protein | chr1:7519116-7521426 REVERSE LENGTH=462	445	462	0	103.8	92.6	96.2
Rsa1.0_00067.1.g3597.t1	ref|XP_002893163.1| nodulin MtN3 family protein [Arabidopsis lyrata subsp. lyrata] gi|297339005|gb|EFH69422.1| nodulin MtN3 family protein [Arabidopsis lyrata subsp. lyrata]	247	247	1.00E-126	100.0	91.1	95.1	nodulin MtN3 family protein	gbpln	Arabidopsis lyrata	AT1G21460.1 | Symbols: SWEET1, AtSWEET1 | Nodulin MtN3 family protein | chr1:7512030-7513281 REVERSE LENGTH=247	247	247	1.00E-126	100.0	89.5	94.3
Rsa1.0_00067.1.g3598.t1	dbj|BAJ33657.1| unnamed protein product [Thellungiella halophila]	571	595	0	104.2	87.4	93.3	unnamed protein product	----	----	AT1G21450.1 | Symbols: SCL1 | SCARECROW-like 1 | chr1:7509721-7511502 FORWARD LENGTH=593	571	593	0	103.9	85.1	91.6

Rsa1.0_00067.1.g3599.t1	ref|NP_191713.1| peptidase family protein [Arabidopsis thaliana] gi|6850845|emb|CAB71084.1| prolyl aminopeptidase-like protein [Arabidopsis thaliana] gi|15450667|gb|AAK96605.1| AT3g61540/F2A19_140 [Arabidopsis thaliana] gi|17380608|gb|AAL36067.1| AT3g61540/F2A19_140 [Arabidopsis thaliana] gi|332646699|gb|AEE80220.1| peptidase family protein [Arabidopsis thaliana]	93	515	9.00E-36	553.8	78.5	82.8	peptidase family protein	gbpln	Arabidopsis thaliana	AT3G61540.1 | Symbols: | alpha/beta-Hydrolases superfamily protein | chr3:22773399-22775699 FORWARD LENGTH=515	93	515	1.00E-38	553.8	78.5	82.8
Rsa1.0_00067.1.g3600.t1	gb|EOA38182.1| hypothetical protein CARUB_v10009659mg [Capsella rubella]	352	338	1.00E-159	96.0	81.0	84.9	hypothetical protein CARUB_v10009659mg	gbpln	Capsella rubella	AT1G21440.1 | Symbols: | Phosphoenolpyruvate carboxylase family protein | chr1:7502325-7504103 REVERSE LENGTH=336	352	336	1.00E-161	95.5	81.0	84.7
Rsa1.0_00067.1.g3601.t1	gb|EOA37849.1| hypothetical protein CARUB_v10011440mg [Capsella rubella]	382	390	0	102.1	82.2	89.8	hypothetical protein CARUB_v10011440mg	gbpln	Capsella rubella	AT1G21430.1 | Symbols: YUC11 | Flavin-binding monooxygenase family protein | chr1:7500845-7502186 FORWARD LENGTH=391	382	391	0	102.4	79.3	87.4
Rsa1.0_00067.1.g3602.t1	dbj|BAJ33623.1| unnamed protein product [Thellungiella halophila]	472	471	0	99.8	91.5	94.1	unnamed protein product	----	----	AT1G21400.1 | Symbols: | Thiamin diphosphate-binding fold (THDP-binding) superfamily protein | chr1:7493492-7496240 FORWARD LENGTH=472	472	472	0	100.0	91.1	94.7
Rsa1.0_00067.1.g3603.t3	gb|EOA38425.1| hypothetical protein CARUB_v10010005mg, partial [Capsella rubella]	215	271	3.00E-66	126.0	65.1	76.3	hypothetical protein CARUB_v10010005mg, partial	gbpln	Capsella rubella	AT1G21390.1 | Symbols: emb2170 | embryo defective 2170 | chr1:7489385-7490554 REVERSE LENGTH=248	215	248	3.00E-64	115.3	65.6	74.9
Rsa1.0_00067.1.g3604.t1	ref|XP_002890439.1| hypothetical protein ARALYDRAFT_472372 [Arabidopsis lyrata subsp. lyrata] gi|297336281|gb|EFH66698.1| hypothetical protein ARALYDRAFT_472372 [Arabidopsis lyrata subsp. lyrata]	334	341	1.00E-163	102.1	83.8	91.3	hypothetical protein ARALYDRAFT_472372	gbpln	Arabidopsis lyrata	AT1G21370.2 | Symbols: | unknown protein; CONTAINS InterPro DOMAIN/s: Protein of unknown function DUF218 (InterPro:IPR003848); Has 35333 Blast hits to 34131 proteins in 2444 species: Archae - 798; Bacteria - 22429; Metazoa - 974; Fungi - 991; Plants - 531; Viruses - 0; Other Eukaryotes - 9610 (source: NCBI BLink). | chr1:7484250-7485451 FORWARD LENGTH=342	334	342	1.00E-161	102.4	82.3	91.0
Rsa1.0_00067.1.g3605.t1	ref|XP_002890438.1| predicted protein [Arabidopsis lyrata subsp. lyrata] gi|297336280|gb|EFH66697.1| predicted protein [Arabidopsis lyrata subsp. lyrata]	222	222	1.00E-109	100.0	86.5	91.9	predicted protein	gbpln	Arabidopsis lyrata	AT1G21360.1 | Symbols: GLTP2 | glycolipid transfer protein 2 | chr1:7481365-7483237 FORWARD LENGTH=223	222	223	1.00E-109	100.5	84.2	91.0
Rsa1.0_00067.1.g3606.t1	ref|XP_002893159.1| electron carrier/ oxidoreductase [Arabidopsis lyrata subsp. lyrata] gi|297339001|gb|EFH69418.1| electron carrier/ oxidoreductase [Arabidopsis lyrata subsp. lyrata]	254	253	1.00E-121	99.6	84.3	89.4	electron carrier/ oxidoreductase	gbpln	Arabidopsis lyrata	AT1G21350.3 | Symbols: | Thioredoxin superfamily protein | chr1:7477376-7479012 REVERSE LENGTH=252	254	252	1.00E-119	99.2	81.5	89.0
Rsa1.0_00067.1.g3607.t1	gb|EOA38986.1| hypothetical protein CARUB_v10011507mg [Capsella rubella]	256	260	1.00E-116	101.6	84.0	90.6	hypothetical protein CARUB_v10011507mg	gbpln	Capsella rubella	AT1G21340.1 | Symbols: | Dof-type zinc finger DNA-binding family protein | chr1:7476087-7476869 FORWARD LENGTH=260	256	260	1.00E-118	101.6	84.4	89.5
Rsa1.0_00067.1.g3608.t1	gb|EOA36400.1| hypothetical protein CARUB_v10010842mg [Capsella rubella]	204	237	1.00E-43	116.2	64.2	75.5	hypothetical protein CARUB_v10010842mg	gbpln	Capsella rubella	AT1G21326.1 | Symbols: | VQ motif-containing protein | chr1:7469002-7469721 REVERSE LENGTH=239	204	239	8.00E-37	117.2	57.8	70.1
Rsa1.0_00067.1.g3609.t1	ref|NP_001154354.1| nucleic acid/nucleotide binding protein [Arabidopsis thaliana] gi|332191964|gb|AEE30085.1| nucleic acid/nucleotide binding protein [Arabidopsis thaliana]	230	253	4.00E-80	110.0	73.5	82.2	nucleic acid/nucleotide binding protein	gbpln	Arabidopsis thaliana	AT1G21320.2 | Symbols: | nucleotide binding;nucleic acid binding | chr1:7462834-7466164 REVERSE LENGTH=253	230	253	1.00E-82	110.0	73.5	82.2
Rsa1.0_00067.1.g3610.t1	ref|NP_173553.1| extensin 3 [Arabidopsis thaliana] gi|334302912|sp|Q9FS16.3|EXTN3_ARATH RecName: Full=Extensin-3; Short=AtExt3; Short=AtExt5; Flags: Precursor gi|8920638|gb|AAF81360.1|AC036104_9 Contains similarity to Extensin (atExt1) from Arabidopsis thaliana gb|U43627 and contains 12 concatamers of 28 amino acids rich in proline. ESTs gb|AA597816, gb|AA712635, gb|N65860, gb|AA598180, gb|H77085, gb|AA394416, gb|AA394413, gb|AA650774, gb|AA650748, gb|Z25975, gb|AA597958, gb|AA597955 come from this gene [Arabidopsis thaliana] gi|332191962|gb|AEE30083.1| extensin 3 [Arabidopsis thaliana]	449	431	1.00E-98	96.0	94.4	94.9	extensin 3	gbpln	Arabidopsis thaliana	AT1G21310.1 | Symbols: ATEXT3, RSH, EXT3 | extensin 3 | chr1:7453693-7454988 REVERSE LENGTH=431	449	431	1.00E-101	96.0	94.4	94.9

Rsa1.0_00067.1.g3611.t1	ref|NP_173550.1| uncharacterized protein [Arabidopsis thaliana] gi|28393025|gb|AAO41947.1| unknown protein [Arabidopsis thaliana] gi|28827240|gb|AAO50464.1| unknown protein [Arabidopsis thaliana] gi|55740501|gb|AAV63843.1| hypothetical protein At1g21280 [Arabidopsis thaliana] gi|332191960|gb|AEE30081.1| uncharacterized protein AT1G21280 [Arabidopsis thaliana]	170	237	4.00E-32	139.4	50.6	64.7	uncharacterized protein	gbpln	Arabidopsis thaliana	AT1G21280.1 | Symbols: | CONTAINS InterPro DOMAIN/s: Retrotransposon gag protein (InterPro:IPR005162); Has 707 Blast hits to 705 proteins in 25 species: Archae - 0; Bacteria - 0; Metazoa - 4; Fungi - 0; Plants - 703; Viruses - 0; Other Eukaryotes - 0 (source: NCBI BLink). | chr1:7447690-7448403 REVERSE LENGTH=237	170	237	2.00E-34	139.4	50.6	64.7
Rsa1.0_00067.1.g3612.t1	ref|NP_173546.1| wall-associated receptor kinase 5 [Arabidopsis thaliana] gi|75174802|sp|Q9LMN7.1|WAK5_ARATH RecName: Full=Wall-associated receptor kinase 5; Flags: Precursor gi|8920637|gb|AAF81359.1|AC036104_8 Strong similarity to wall-associated kinase 1 from Arabidopsis thaliana gb|AJ009696 and contains Eukaryotic protein kinase PF|00069 and EGF-like PF|00008 domains [Arabidopsis thaliana] gi|332191955|gb|AEE30076.1| wall-associated receptor kinase 5 [Arabidopsis thaliana]	737	733	0	99.5	70.7	81.3	wall-associated receptor kinase 5	gbpln	Arabidopsis thaliana	AT1G21230.1 | Symbols: WAK5 | wall associated kinase 5 | chr1:7429980-7432346 FORWARD LENGTH=733	737	733	0	99.5	70.7	81.3
Rsa1.0_00067.1.g3613.t1	gb|EOA39743.1| hypothetical protein CARUB_v10008400mg [Capsella rubella]	738	740	0	100.3	72.1	82.4	hypothetical protein CARUB_v10008400mg	gbpln	Capsella rubella	AT1G21270.1 | Symbols: WAK2 | wall-associated kinase 2 | chr1:7444997-7447345 FORWARD LENGTH=732	738	732	0	99.2	69.5	80.4
Rsa1.0_00067.1.g3614.t1	gb|EOA39743.1| hypothetical protein CARUB_v10008400mg [Capsella rubella]	690	740	0	107.2	73.6	83.3	hypothetical protein CARUB_v10008400mg	gbpln	Capsella rubella	AT1G21230.1 | Symbols: WAK5 | wall associated kinase 5 | chr1:7429980-7432346 FORWARD LENGTH=733	690	733	0	106.2	70.1	81.6
Rsa1.0_00067.1.g3615.t1	gb|EOA39743.1| hypothetical protein CARUB_v10008400mg [Capsella rubella]	737	740	0	100.4	72.5	82.8	hypothetical protein CARUB_v10008400mg	gbpln	Capsella rubella	AT1G21270.1 | Symbols: WAK2 | wall-associated kinase 2 | chr1:7444997-7447345 FORWARD LENGTH=732	737	732	0	99.3	70.1	80.7
Rsa1.0_00067.1.g3616.t2	gb|EOA39743.1| hypothetical protein CARUB_v10008400mg [Capsella rubella]	717	740	0	103.2	80.6	90.2	hypothetical protein CARUB_v10008400mg	gbpln	Capsella rubella	AT1G21270.1 | Symbols: WAK2 | wall-associated kinase 2 | chr1:7444997-7447345 FORWARD LENGTH=732	717	732	0	102.1	72.2	82.4
Rsa1.0_00067.1.g3617.t1	gb|EOA40426.1| hypothetical protein CARUB_v10009151mg [Capsella rubella] gi|482576240|gb|EOA40427.1| hypothetical protein CARUB_v10009151mg [Capsella rubella]	416	442	0	106.3	84.6	91.3	hypothetical protein CARUB_v10009151mg	gbpln	Capsella rubella	AT1G21200.1 | Symbols: | sequence-specific DNA binding transcription factors | chr1:7421483-7422814 FORWARD LENGTH=443	416	443	1.00E-180	106.5	82.9	90.4
Rsa1.0_00067.1.g3618.t1	ref|XP_002893156.1| hypothetical protein ARALYDRAFT_472357 [Arabidopsis lyrata subsp. lyrata] gi|297338998|gb|EFH69415.1| hypothetical protein ARALYDRAFT_472357 [Arabidopsis lyrata subsp. lyrata]	292	98	8.00E-32	33.6	24.3	24.3	hypothetical protein ARALYDRAFT_472357	gbpln	Arabidopsis lyrata	AT1G21190.1 | Symbols: | Small nuclear ribonucleoprotein family protein | chr1:7419989-7420856 REVERSE LENGTH=97	292	97	3.00E-34	33.2	24.0	24.3
Rsa1.0_00067.1.g3619.t1	ref|NP_189952.1| vacuolar iron transporter-like protein [Arabidopsis thaliana] gi|75182792|sp|Q9M2C0.1|VITH4_ARATH RecName: Full=Vacuolar iron transporter homolog 4; AltName: Full=Protein NODULIN-LIKE 4 gi|7362791|emb|CAB83067.1| nodulin-like protein [Arabidopsis thaliana] gi|34365643|gb|AAQ65133.1| At3g43660 [Arabidopsis thaliana] gi|51970300|dbj|BAD43842.1| nodulin -like protein [Arabidopsis thaliana] gi|332644295|gb|AEE77816.1| vacuolar iron transporter homolog 4 [Arabidopsis thaliana]	201	198	3.00E-92	98.5	83.1	91.0	vacuolar iron transporter-like protein	gbpln	Arabidopsis thaliana	AT3G43660.1 | Symbols: | Vacuolar iron transporter (VIT) family protein | chr3:15565332-15565928 FORWARD LENGTH=198	201	198	1.00E-94	98.5	83.1	91.0
Rsa1.0_00067.1.g3620.t1	gb|AAM67269.1| O-methyltransferase, putative [Arabidopsis thaliana]	345	373	1.00E-173	108.1	91.6	95.7	O-methyltransferase, putative	gbpln	Arabidopsis thaliana	AT1G21130.1 | Symbols: | O-methyltransferase family protein | chr1:7399170-7400470 REVERSE LENGTH=373	345	373	1.00E-175	108.1	91.3	95.1

Rsa1.0_00067.1.g3621.t1	ref|NP_173537.1| O-methyltransferase-like protein [Arabidopsis thaliana] gi|8886988|gb|AAF80648.1|AC012190_4 Contains similarity to caffeic acid 3-O-Methyltransferase from Saccharum officinarum gb|AJ231133. It is a member of O-methyltransferase family. ESTs gb|AI994592 and gb|T20793 come from this gene [Arabidopsis thaliana] gi|332191946|gb|AEE30067.1| O-methyltransferase-like protein [Arabidopsis thaliana]	373	373	0	100.0	93.6	96.5	O-methyltransferase-like protein	gbpln	Arabidopsis thaliana	AT1G21130.1 | Symbols: | O-methyltransferase family protein | chr1:7399170-7400470 REVERSE LENGTH=373	373	373	0	100.0	93.6	96.5
Rsa1.0_00067.1.g3622.t1	#	#	#	#	#	#	#	-	----	----	#	#	#	#	#	#	#
Rsa1.0_00067.1.g3623.t1	ref|NP_173537.1| O-methyltransferase-like protein [Arabidopsis thaliana] gi|8886988|gb|AAF80648.1|AC012190_4 Contains similarity to caffeic acid 3-O-Methyltransferase from Saccharum officinarum gb|AJ231133. It is a member of O-methyltransferase family. ESTs gb|AI994592 and gb|T20793 come from this gene [Arabidopsis thaliana] gi|332191946|gb|AEE30067.1| O-methyltransferase-like protein [Arabidopsis thaliana]	369	373	0	101.1	91.9	95.9	O-methyltransferase-like protein	gbpln	Arabidopsis thaliana	AT1G21130.1 | Symbols: | O-methyltransferase family protein | chr1:7399170-7400470 REVERSE LENGTH=373	369	373	0	101.1	91.9	95.9
Rsa1.0_00067.1.g3624.t1	ref|NP_173537.1| O-methyltransferase-like protein [Arabidopsis thaliana] gi|8886988|gb|AAF80648.1|AC012190_4 Contains similarity to caffeic acid 3-O-Methyltransferase from Saccharum officinarum gb|AJ231133. It is a member of O-methyltransferase family. ESTs gb|AI994592 and gb|T20793 come from this gene [Arabidopsis thaliana] gi|332191946|gb|AEE30067.1| O-methyltransferase-like protein [Arabidopsis thaliana]	372	373	0	100.3	91.9	94.6	O-methyltransferase-like protein	gbpln	Arabidopsis thaliana	AT1G21130.1 | Symbols: | O-methyltransferase family protein | chr1:7399170-7400470 REVERSE LENGTH=373	372	373	0	100.3	91.9	94.6
Rsa1.0_00067.1.g3625.t1	ref|NP_564135.1| cupredoxin-like protein [Arabidopsis thaliana] gi|111074298|gb|ABH04522.1| At1g21090 [Arabidopsis thaliana] gi|332191942|gb|AEE30063.1| cupredoxin-like protein [Arabidopsis thaliana]	243	242	1.00E-109	99.6	85.2	88.9	cupredoxin-like protein	gbpln	Arabidopsis thaliana	AT1G21090.1 | Symbols: | Cupredoxin superfamily protein | chr1:7384854-7386199 FORWARD LENGTH=242	243	242	1.00E-111	99.6	85.2	88.9
Rsa1.0_00067.1.g3626.t1	gb|AAW50121.1| DnaJ-like protein [Brassica juncea]	368	390	1.00E-178	106.0	86.4	90.2	DnaJ-like protein	gbpln	Brassica juncea	AT1G21080.3 | Symbols: | DNAJ heat shock N-terminal domain-containing protein | chr1:7378822-7382275 REVERSE LENGTH=400	368	400	1.00E-177	108.7	83.7	87.0
Rsa1.0_00067.1.g3627.t1	ref|XP_002890424.1| hypothetical protein ARALYDRAFT_472343 [Arabidopsis lyrata subsp. lyrata] gi|297336266|gb|EFH66683.1| hypothetical protein ARALYDRAFT_472343 [Arabidopsis lyrata subsp. lyrata]	726	505	0	69.6	51.8	57.3	hypothetical protein ARALYDRAFT_472343	gbpln	Arabidopsis lyrata	AT1G21060.1 | Symbols: | Protein of unknown function, DUF547 | chr1:7371799-7374085 FORWARD LENGTH=505	726	505	0	69.6	51.2	57.0
Rsa1.0_00067.1.g3628.t1	ref|XP_002890423.1| hypothetical protein ARALYDRAFT_889559 [Arabidopsis lyrata subsp. lyrata] gi|297336265|gb|EFH66682.1| hypothetical protein ARALYDRAFT_889559 [Arabidopsis lyrata subsp. lyrata]	249	244	1.00E-104	98.0	86.7	89.2	hypothetical protein ARALYDRAFT_889559	gbpln	Arabidopsis lyrata	AT1G21050.1 | Symbols: | Protein of unknown function, DUF617 | chr1:7366859-7367596 FORWARD LENGTH=245	249	245	1.00E-104	98.4	83.5	89.2
Rsa1.0_00067.1.g3629.t1	#	#	#	#	#	#	#	-	----	----	#	#	#	#	#	#	#
Rsa1.0_00068.1.g3630.t3	ref|NP_178398.1| pentatricopeptide repeat-containing protein [Arabidopsis thaliana] gi|218546779|sp|Q8LK93.2|PP145_ARATH RecName: Full=Pentatricopeptide repeat-containing protein At2g02980 gi|3461822|gb|AAC32916.1| hypothetical protein [Arabidopsis thaliana] gi|330250557|gb|AEC05651.1| pentatricopeptide repeat-containing protein [Arabidopsis thaliana]	362	603	1.00E-108	166.6	51.1	55.8	pentatricopeptide repeat-containing protein	gbpln	Arabidopsis thaliana	AT2G02980.1 | Symbols: OTP85 | Pentatricopeptide repeat (PPR) superfamily protein | chr2:868468-870279 FORWARD LENGTH=603	362	603	1.00E-110	166.6	51.1	55.8
Rsa1.0_00068.1.g3631.t1	#	#	#	#	#	#	#	-	----	----	#	#	#	#	#	#	#

Rsa1.0_00068.1.g3632.t1	gb|EOA25480.1| hypothetical protein CARUB_v10018819mg [Capsella rubella]	101	191	2.00E-35	189.1	81.2	91.1	hypothetical protein CARUB_v10018819mg	gbpln	Capsella rubella	AT2G01580.1 | Symbols: | unknown protein; BEST Arabidopsis thaliana protein match is: unknown protein (TAIR:AT3G07510.2); Has 129 Blast hits to 129 proteins in 12 species: Archae - 0; Bacteria - 0; Metazoa - 0; Fungi - 0; Plants - 129; Viruses - 0; Other Eukaryotes - 0 (source: NCBI BLink). | chr2:265181-265756 REVERSE LENGTH=191	101	191	5.00E-34	189.1	72.3	79.2
Rsa1.0_00068.1.g3633.t1	ref|NP_973517.1| serine carboxypeptidase-like 9 [Arabidopsis thaliana] gi|330252302|gb|AEC07396.1| serine carboxypeptidase-like 9 [Arabidopsis thaliana]	163	437	5.00E-67	268.1	75.5	87.1	serine carboxypeptidase-like 9	gbpln	Arabidopsis thaliana	AT2G23010.2 | Symbols: SCPL9 | serine carboxypeptidase-like 9 | chr2:9798843-9802485 FORWARD LENGTH=437	163	437	2.00E-69	268.1	75.5	87.1
Rsa1.0_00068.1.g3634.t5	#	#	#	#	#	#	#	-	----	----	#	#	#	#	#	#	#
Rsa1.0_00068.1.g3635.t1	ref|NP_179884.1| serine carboxypeptidase-like 9 [Arabidopsis thaliana] gi|75099209|sp|O64811.1|SCP9_ARATH RecName: Full=Serine carboxypeptidase-like 9; AltName: Full=Sinapoylglucose--sinapoylglucose O-sinapoyltransferase; Short=SST; AltName: Full=Sinapoylglucose--sinapoylglucose acyltransferase; Flags: Precursor gi|3169175|gb|AAC17818.1| putative serine carboxypeptidase I [Arabidopsis thaliana] gi|330252303|gb|AEC07397.1| serine carboxypeptidase-like 9 [Arabidopsis thaliana]	158	437	5.00E-47	276.6	55.1	59.5	serine carboxypeptidase-like 9	gbpln	Arabidopsis thaliana	AT2G23010.1 | Symbols: SCPL9 | serine carboxypeptidase-like 9 | chr2:9798843-9802485 FORWARD LENGTH=437	158	437	2.00E-49	276.6	55.1	59.5
Rsa1.0_00068.1.g3636.t1	ref|XP_002875182.1| pentatricopeptide repeat-containing protein [Arabidopsis lyrata subsp. lyrata] gi|297321020|gb|EFH51441.1| pentatricopeptide repeat-containing protein [Arabidopsis lyrata subsp. lyrata]	607	605	0	99.7	82.9	89.0	pentatricopeptide repeat-containing protein	gbpln	Arabidopsis lyrata	AT2G02980.1 | Symbols: OTP85 | Pentatricopeptide repeat (PPR) superfamily protein | chr2:868468-870279 FORWARD LENGTH=603	607	603	0	99.3	80.4	89.1
Rsa1.0_00068.1.g3637.t1	gb|AFI98882.1| phytochrome kinase substrate [Brassica juncea var. tumida]	434	376	1.00E-171	86.6	74.2	76.5	phytochrome kinase substrate	gbpln	Brassica juncea	AT2G02950.1 | Symbols: PKS1 | phytochrome kinase substrate 1 | chr2:855149-856468 REVERSE LENGTH=439	434	439	1.00E-150	101.2	70.7	80.6
Rsa1.0_00068.1.g3638.t1	gb|AAD32898.1| putative retroelement pol polyprotein [Arabidopsis thaliana]	1320	1307	0	99.0	60.2	75.0	putative retroelement pol polyprotein	gbpln	Arabidopsis thaliana	AT4G23160.1 | Symbols: CRK8 | cysteine-rich RLK (RECEPTOR-like protein kinase) 8 | chr4:12129485-12134086 FORWARD LENGTH=1262	1320	1262	2.00E-91	95.6	13.0	19.9
Rsa1.0_00068.1.g3639.t1	ref|XP_002876849.1| hypothetical protein ARALYDRAFT_484201 [Arabidopsis lyrata subsp. lyrata] gi|297322687|gb|EFH53108.1| hypothetical protein ARALYDRAFT_484201 [Arabidopsis lyrata subsp. lyrata]	472	468	0	99.2	88.1	94.1	hypothetical protein ARALYDRAFT_484201	gbpln	Arabidopsis lyrata	AT2G02910.1 | Symbols: | Protein of unknown function (DUF616) | chr2:847335-849371 REVERSE LENGTH=460	472	460	0	97.5	85.8	91.3
Rsa1.0_00068.1.g3640.t1	gb|EOA24405.1| hypothetical protein CARUB_v10017655mg, partial [Capsella rubella]	344	321	1.00E-128	93.3	71.8	78.5	hypothetical protein CARUB_v10017655mg, partial	gbpln	Capsella rubella	AT2G02880.1 | Symbols: | mucin-related | chr2:841303-843151 FORWARD LENGTH=314	344	314	1.00E-126	91.3	71.8	77.3

Rsa1.0_00068.1.g3641.t1	ref|NP_178390.1| F-box/kelch-repeat protein SKIP11 [Arabidopsis thaliana] gi|79316620|ref|NP_001030959.1| F-box/kelch-repeat protein SKIP11 [Arabidopsis thaliana] gi|79316634|ref|NP_001030960.1| F-box/kelch-repeat protein SKIP11 [Arabidopsis thaliana] gi|142994697|sp|Q8L736.2|SKI11_ARATH RecName: Full=F-box/kelch-repeat protein SKIP11; AltName: Full=SKP1-interacting partner 11 gi|3461814|gb|AAC32908.1| predicted by genefinder and genscan [Arabidopsis thaliana] gi|16974560|gb|AAL31196.1| At2g02870/T17M13.4 [Arabidopsis thaliana] gi|25090100|gb|AAN72228.1| At2g02870/T17M13.4 [Arabidopsis thaliana] gi|222423827|dbj|BAH19879.1| AT2G02870 [Arabidopsis thaliana] gi|330250542|gb|AEC05636.1| F-box/kelch-repeat protein SKIP11 [Arabidopsis thaliana] gi|330250543|gb|AEC05637.1| F-box/kelch-repeat protein SKIP11 [Arabidopsis thaliana] gi|330250544|gb|AEC05638.1| F-box/kelch-repeat protein SKIP11 [Arabidopsis thaliana]	441	467	0	105.9	85.3	90.0	F-box/kelch-repeat protein SKIP11	gbpln	Arabidopsis thaliana	AT2G02870.3 | Symbols: | Galactose oxidase/kelch repeat superfamily protein | chr2:838378-839781 FORWARD LENGTH=467	441	467	0	105.9	85.3	90.0
Rsa1.0_00068.1.g3642.t2	ref|NP_178389.1| sucrose transport protein SUC3 [Arabidopsis thaliana] gi|75223209|sp|O80605.1|SUC3_ARATH RecName: Full=Sucrose transport protein SUC3; AltName: Full=Sucrose permease 3; AltName: Full=Sucrose transporter 2; AltName: Full=Sucrose-proton symporter 3 gi|3461813|gb|AAC32907.1| putative sucrose/H+ symporter [Arabidopsis thaliana] gi|8052190|emb|CAB92307.1| sucrose transporter [Arabidopsis thaliana] gi|110742223|dbj|BAE99038.1| Sucrose transporter [Arabidopsis thaliana] gi|330250541|gb|AEC05635.1| sucrose transport protein SUC3 [Arabidopsis thaliana]	592	594	0	100.3	87.0	91.6	sucrose transport protein SUC3	gbpln	Arabidopsis thaliana	AT2G02860.1 | Symbols: SUT2, ATSUC3, SUC3, ATSUT2 | sucrose transporter 2 | chr2:828546-832296 REVERSE LENGTH=594	592	594	0	100.3	87.0	91.6
Rsa1.0_00068.1.g3643.t1	ref|XP_002876842.1| predicted protein [Arabidopsis lyrata subsp. lyrata] gi|297322680|gb|EFH53101.1| predicted protein [Arabidopsis lyrata subsp. lyrata]	129	129	1.00E-58	100.0	82.9	91.5	predicted protein	gbpln	Arabidopsis lyrata	AT2G02850.1 | Symbols: ARPN | plantacyanin | chr2:826630-827720 REVERSE LENGTH=129	129	129	5.00E-60	100.0	81.4	88.4
Rsa1.0_00068.1.g3644.t1	#	#	#	#	#	#	#	-	----	----	#	#	#	#	#	#	#
Rsa1.0_00068.1.g3645.t1	gb|AAC19278.1| T14P8.10 [Arabidopsis thaliana] gi|7269009|emb|CAB80742.1| AT4g02490 [Arabidopsis thaliana]	312	657	3.00E-17	210.6	19.6	28.8	T14P8.10	gbpln	Arabidopsis thaliana	AT1G43760.1 | Symbols: | DNAse I-like superfamily protein | chr1:16528880-16531065 REVERSE LENGTH=626	312	626	7.00E-13	200.6	13.1	20.8
Rsa1.0_00068.1.g3646.t1	#	#	#	#	#	#	#	-	----	----	#	#	#	#	#	#	#
Rsa1.0_00068.1.g3647.t1	gb|EOA23904.1| hypothetical protein CARUB_v10017119mg [Capsella rubella]	465	483	0	103.9	77.8	86.2	hypothetical protein CARUB_v10017119mg	gbpln	Capsella rubella	AT2G02820.2 | Symbols: MYB88 | myb domain protein 88 | chr2:804716-807139 REVERSE LENGTH=484	465	484	0	104.1	80.0	88.4
Rsa1.0_00068.1.g3648.t1	gb|EOA24258.1| hypothetical protein CARUB_v10017499mg [Capsella rubella]	332	363	1.00E-180	109.3	95.2	97.9	hypothetical protein CARUB_v10017499mg	gbpln	Capsella rubella	AT2G02810.1 | Symbols: ATUTR1, UTR1 | UDP-galactose transporter 1 | chr2:801643-803289 FORWARD LENGTH=332	332	332	0	100.0	94.3	97.9
Rsa1.0_00068.1.g3649.t1	ref|XP_002876829.1| hypothetical protein ARALYDRAFT_484186 [Arabidopsis lyrata subsp. lyrata] gi|297322667|gb|EFH53088.1| hypothetical protein ARALYDRAFT_484186 [Arabidopsis lyrata subsp. lyrata]	472	426	0	90.3	86.0	88.1	hypothetical protein ARALYDRAFT_484186	gbpln	Arabidopsis lyrata	AT2G02800.2 | Symbols: APK2B | protein kinase 2B | chr2:796889-799250 REVERSE LENGTH=426	472	426	0	90.3	85.4	88.1
Rsa1.0_00068.1.g3650.t1	gb|EOA23708.1| hypothetical protein CARUB_v10016916mg [Capsella rubella]	565	584	0	103.4	77.2	87.1	hypothetical protein CARUB_v10016916mg	gbpln	Capsella rubella	AT2G02790.1 | Symbols: IQD29 | IQ-domain 29 | chr2:788708-790946 FORWARD LENGTH=636	565	636	0	112.6	76.5	86.0
Rsa1.0_00068.1.g3651.t1	#	#	#	#	#	#	#	-	----	----	#	#	#	#	#	#	#
Rsa1.0_00068.1.g3652.t1	ref|NP_196645.2| endomembrane family protein 70 [Arabidopsis thaliana] gi|332004219|gb|AED91602.1| endomembrane family protein 70 [Arabidopsis thaliana]	606	648	0	106.9	93.4	96.7	endomembrane family protein 70	gbpln	Arabidopsis thaliana	AT5G10840.1 | Symbols: | Endomembrane protein 70 protein family | chr5:3424910-3427797 REVERSE LENGTH=648	606	648	0	106.9	93.4	96.7

Rsa1.0_00068.1.g3653.t1	ref|NP_565289.1| ubiquitin-conjugating enzyme E2 A [Arabidopsis thaliana] gi|297814522|ref|XP_002875144.1| ubiquiting-conjugating enzyme 2 [Arabidopsis lyrata subsp. lyrata] gi|1174844|sp|P42745.1|UBC2_ARATH RecName: Full=Ubiquitin-conjugating enzyme E2 2; AltName: Full=Ubiquitin carrier protein 2; AltName: Full=Ubiquitin-conjugating enzyme E2-17 kDa 2; AltName: Full=Ubiquitin-protein ligase 2 gi|13899127|gb|AAK48985.1|AF370558_1 putative ubiquitin-conjugating enzyme E2 [Arabidopsis thaliana] gi|431264|gb|AAA32899.1| ubiquitin conjugating enzyme [Arabidopsis thaliana] gi|2689243|emb|CAA73476.1| ubiquitin conjugating enzyme [Arabidopsis thaliana] gi|2947065|gb|AAC05346.1| E2, ubiquitin-conjugating enzyme 2 (UBC2) [Arabidopsis thaliana] gi|18377456|gb|AAL66894.1| putative ubiquitin-conjugating enzyme E2 [Arabidopsis thaliana] gi|66354400|gb|AAY44843.1| ubiquitinating enzyme [Arabidopsis thaliana] gi|118197458|gb|ABK78692.1| ubiquitin conjugating enzyme [Brassica rapa] gi|297320982|gb|EFH51403.1| ubiquiting-conjugating enzyme 2 [Arabidopsis lyrata subsp. lyrata] gi|312281529|dbj|BAJ33630.1| unnamed	152	152	3.00E-84	100.0	99.3	100.0	ubiquitin-conjugating enzyme E2 A	gbpln	Arabidopsis lyrata	AT2G02760.1 | Symbols: ATUBC2, UBC2 | ubiquiting-conjugating enzyme 2 | chr2:774271-775149 FORWARD LENGTH=152	152	152	1.00E-86	100.0	99.3	100.0
Rsa1.0_00068.1.g3654.t1	#	#	#	#	#	#	#	-	----	----	#	#	#	#	#	#	#
Rsa1.0_00068.1.g3655.t1	ref|NP_178375.1| putative pectate lyase 6 [Arabidopsis thaliana] gi|32129832|sp|O64510.1|PEL6_ARATH RecName: Full=Probable pectate lyase 6; Flags: Precursor gi|2947069|gb|AAC05350.1| putative pectate lyase [Arabidopsis thaliana] gi|28973690|gb|AAO64162.1| putative pectate lyase [Arabidopsis thaliana] gi|54606856|gb|AAV34776.1| At2g02720 [Arabidopsis thaliana] gi|330250521|gb|AEC05615.1| putative pectate lyase 6 [Arabidopsis thaliana]	457	455	0	99.6	83.8	90.6	putative pectate lyase 6	gbpln	Arabidopsis thaliana	AT2G02720.1 | Symbols: | Pectate lyase family protein | chr2:763173-764834 FORWARD LENGTH=455	457	455	0	99.6	83.8	90.6
Rsa1.0_00068.1.g3656.t1	ref|NP_564190.1| polyketide cyclase/dehydrase and lipid transport-like protein [Arabidopsis thaliana] gi|2829899|gb|AAC00607.1| similar to ripening-induced protein, gp|AJ001449|2465015 and major#latex protein, gp|X91961|1107495 [Arabidopsis thaliana] gi|14517378|gb|AAK62580.1| At1g23130/T26J12_10 [Arabidopsis thaliana] gi|15450533|gb|AAK96444.1| At1g23130/T26J12_10 [Arabidopsis thaliana] gi|332192222|gb|AEE30343.1| polyketide cyclase/dehydrase and lipid transport-like protein [Arabidopsis thaliana]	161	160	4.00E-58	99.4	67.1	83.2	polyketide cyclase/dehydrase and lipid transport-like protein	gbpln	Arabidopsis thaliana	AT1G23130.1 | Symbols: | Polyketide cyclase/dehydrase and lipid transport superfamily protein | chr1:8200434-8200997 FORWARD LENGTH=160	161	160	1.00E-60	99.4	67.1	83.2
Rsa1.0_00069.1.g3657.t3	ref|NP_563620.1| L-fucokinase/GDP-L-fucose pyrophosphorylase [Arabidopsis thaliana] gi|332313388|sp|Q9LNJ9.2|FKGP_ARATH RecName: Full=Bifunctional fucokinase/fucose pyrophosphorylase; Short=AtFKGP; Includes: RecName: Full=L-fucokinase; Includes: RecName: Full=Fucose-1-phosphate guanylyltransferase; AltName: Full=GDP-fucose pyrophosphorylase gi|332189134|gb|AEE27255.1| L-fucokinase/GDP-L-fucose pyrophosphorylase [Arabidopsis thaliana]	1369	1055	0	77.1	70.5	73.3	L-fucokinase/GDP-L-fucose pyrophosphorylase	gbpln	Arabidopsis thaliana	AT1G01220.1 | Symbols: FKGP, AtFKGP | L-fucokinase/GDP-L-fucose pyrophosphorylase | chr1:91750-95552 FORWARD LENGTH=1055	1369	1055	0	77.1	70.5	73.3
Rsa1.0_00069.1.g3658.t1	ref|XP_002892079.1| ORMDL family protein [Arabidopsis lyrata subsp. lyrata] gi|297337921|gb|EFH68338.1| ORMDL family protein [Arabidopsis lyrata subsp. lyrata]	157	157	7.00E-80	100.0	88.5	94.9	ORMDL family protein	gbpln	Arabidopsis lyrata	AT1G01230.1 | Symbols: | ORMDL family protein | chr1:97620-99046 FORWARD LENGTH=157	157	157	5.00E-82	100.0	87.9	94.9

Rsa1.0_00069.1_g3659.t1	ref|XP_002892078.1| hypothetical protein ARALYDRAFT_470152 [Arabidopsis lyrata subsp. lyrata] gi|297337920|gb|EFH68337.1| hypothetical protein ARALYDRAFT_470152 [Arabidopsis lyrata subsp. lyrata]	345	334	1.00E-127	96.8	76.8	85.8	hypothetical protein ARALYDRAFT_470152	gbpln	Arabidopsis lyrata	AT1G01240.3 | Symbols: | unknown protein; BEST Arabidopsis thaliana protein match is: unknown protein (TAIR:AT2G46550.1); Has 35333 Blast hits to 34131 proteins in 2444 species: Archae - 798; Bacteria - 22429; Metazoa - 974; Fungi - 991; Plants - 531; Viruses - 0; Other Eukaryotes - 9610 (source: NCBI BLink). | chr1:100683-101678 FORWARD LENGTH=331	345	331	1.00E-129	95.9	77.7	85.8
Rsa1.0_00069.1_g3660.t1	#	#	#	#	#	#	#	-	----	----	#	#	#	#	#	#	#
Rsa1.0_00069.1_g3661.t1	ref|NP_188299.1| ethylene-responsive transcription factor RAP2-3 [Arabidopsis thaliana] gi|27735162|sp|P42736.2|RAP23_ARATH RecName: Full=Ethylene-responsive transcription factor RAP2-3; AltName: Full=Cadmium-induced protein AS30; AltName: Full=Ethylene response factor 72; Short=ERF72; AltName: Full=Ethylene-responsive element binding protein; Short=AtEBP; AltName: Full=Protein RELATED TO APETALA2 3; Short=Related to AP2 3 gi|2281631|gb|AAC49769.1| AP2 domain containing protein RAP2.3 [Arabidopsis thaliana] gi|11994632|dbj|BAB02769.1| AP2 domain transcription factor RAP2.3 [Arabidopsis thaliana] gi|14334854|gb|AAK59605.1| putative AP2 domain containing protein RAP2.3 [Arabidopsis thaliana] gi|16649095|gb|AAL24399.1| AP2 domain transcription factor RAP2.3 [Arabidopsis thaliana] gi|21593082|gb|AAM65031.1| AP2 domain containing protein RAP2.3 [Arabidopsis thaliana] gi|23296611|gb|AAN13131.1| putative AP2 domain containing protein RAP2.3 [Arabidopsis thaliana] gi|332642342|gb|AEE75863.1| ethylene-responsive transcription factor RAP2-3 [Arabidopsis thaliana]	155	248	7.00E-28	160.0	49.7	51.6	ethylene-responsive transcription factor RAP2-3	gbpln	Arabidopsis thaliana	AT3G16770.1 | Symbols: RAP2.3, ATEBP, ERF72, EBP | ethylene-responsive element binding protein | chr3:5705784-5706768 FORWARD LENGTH=248	155	248	2.00E-30	160.0	49.7	51.6
Rsa1.0_00069.1_g3662.t1	dbj|BAJ34192.1| unnamed protein product [Thellungiella halophila]	184	197	6.00E-81	107.1	89.1	94.6	unnamed protein product	----	----	AT1G01250.1 | Symbols: | Integrase-type DNA-binding superfamily protein | chr1:104731-105309 REVERSE LENGTH=192	184	192	6.00E-80	104.3	86.4	90.8
Rsa1.0_00069.1_g3663.t1	gb|EOA38419.1| hypothetical protein CARUB_v10009994mg, partial [Capsella rubella] gi|482574233|gb|EOA38420.1| hypothetical protein CARUB_v10009994mg, partial [Capsella rubella] gi|482574234|gb|EOA38421.1| hypothetical protein CARUB_v10009994mg, partial [Capsella rubella]	238	273	1.00E-109	114.7	86.1	93.7	hypothetical protein CARUB_v10009994mg, partial	gbpln	Capsella rubella	AT1G01290.2 | Symbols: CNX3 | cofactor of nitrate reductase and xanthine dehydrogenase 3 | chr1:114299-115296 FORWARD LENGTH=270	238	270	1.00E-108	113.4	84.5	90.8
Rsa1.0_00069.1_g3664.t1	ref|XP_002892072.1| hypothetical protein ARALYDRAFT_470143 [Arabidopsis lyrata subsp. lyrata] gi|297337914|gb|EFH68331.1| hypothetical protein ARALYDRAFT_470143 [Arabidopsis lyrata subsp. lyrata]	240	241	3.00E-93	100.4	74.2	80.8	hypothetical protein ARALYDRAFT_470143	gbpln	Arabidopsis lyrata	AT1G01310.1 | Symbols: | CAP (Cysteine-rich secretory proteins, Antigen 5, and Pathogenesis-related 1 protein) superfamily protein | chr1:120221-120946 FORWARD LENGTH=241	240	241	8.00E-91	100.4	72.9	79.6
Rsa1.0_00069.1_g3665.t1	gb|EOA38162.1| hypothetical protein CARUB_v10009639mg [Capsella rubella]	327	342	1.00E-166	104.6	89.3	94.8	hypothetical protein CARUB_v10009639mg	gbpln	Capsella rubella	AT1G01350.1 | Symbols: | Zinc finger (CCCH-type/C3HC4-type RING finger) family protein | chr1:136732-137970 FORWARD LENGTH=343	327	343	1.00E-159	104.9	88.1	93.3
Rsa1.0_00069.1_g3666.t1	dbj|BAF49733.1| centromeric histone H3-like protein_1 [Raphanus sativus]	178	178	7.00E-98	100.0	100.0	100.0	centromeric histone H3-like protein_1	gbpln	Raphanus sativus	AT1G01370.2 | Symbols: HTR12, CENH3 | Histone superfamily protein | chr1:143773-145400 FORWARD LENGTH=178	178	178	2.00E-65	100.0	70.2	83.7
Rsa1.0_00069.1_g3667.t1	gb|EOA38963.1| hypothetical protein CARUB_v10011382mg [Capsella rubella]	79	85	6.00E-30	107.6	79.7	86.1	hypothetical protein CARUB_v10011382mg	gbpln	Capsella rubella	AT4G01060.1 | Symbols: ETC3, CPL3 | CAPRICE-like MYB3 | chr4:460534-460967 FORWARD LENGTH=77	79	77	2.00E-26	97.5	72.2	84.8
Rsa1.0_00069.1_g3668.t1	ref|XP_002889366.1| predicted protein [Arabidopsis lyrata subsp. lyrata] gi|297335208|gb|EFH65625.1| predicted protein [Arabidopsis lyrata subsp. lyrata]	689	970	0	140.8	83.3	91.1	predicted protein	gbpln	Arabidopsis lyrata	AT1G01450.1 | Symbols: | Protein kinase superfamily protein | chr1:164105-165517 REVERSE LENGTH=470	689	470	0	68.2	52.2	57.2

Rsa1.0_00069.1_g3669.t1	gb|AAF81309.1|AC061957_5 Contains a weak similarity to a farnesylated protein GMFP5 mRNA from Glycine max gb|U64916. ESTs gb|AI993148, gb|T44360 come from this gene [Arabidopsis thaliana]	162	203	2.00E-32	125.3	83.3	85.2	Contains a weak similarity to a farnesylated protein GMFP5 mRNA from Glycine max gb|U64916. ESTs gb|AI993148, gb|T44360 come from this gene	gbpln	Arabidopsis thaliana	AT1G01490.2 | Symbols: | Heavy metal transport/detoxification superfamily protein | chr1:180401-182066 REVERSE LENGTH=177	162	177	3.00E-34	109.3	83.3	85.2
Rsa1.0_00069.1_g3670.t1	#	#	#	#	#	#	#	-	----	----	#	#	#	#	#	#	#
Rsa1.0_00069.1_g3671.t1	ref|XP_002892061.1| hypothetical protein ARALYDRAFT_311286 [Arabidopsis lyrata subsp. lyrata] gi|297337903|gb|EFH68320.1| hypothetical protein ARALYDRAFT_311286 [Arabidopsis lyrata subsp. lyrata]	315	1248	1.00E-136	396.2	79.4	83.8	hypothetical protein ARALYDRAFT_311286	gbpln	Arabidopsis lyrata	AT1G01500.1 | Symbols: | Erythronate-4-phosphate dehydrogenase family protein | chr1:185260-186573 FORWARD LENGTH=327	315	327	1.00E-134	103.8	78.1	83.2
Rsa1.0_00069.1_g3672.t1	ref|NP_563629.1| Rossmann-fold NAD(P)-binding domain-containing protein [Arabidopsis thaliana] gi|75274951|sp|O23702.1|CTBP_ARATH RecName: Full=C-terminal binding protein AN; Short=CtBP; AltName: Full=Protein ANGUSTIFOLIA gi|2505877|emb|CAA73306.1| dehydrogenase [Arabidopsis thaliana] gi|332189177|gb|AEE27298.1| C-terminal binding protein AN [Arabidopsis thaliana]	628	636	0	101.3	90.6	94.1	Rossmann-fold NAD(P)-binding domain-containing protein	gbpln	Arabidopsis thaliana	AT1G01510.1 | Symbols: AN | NAD(P)-binding Rossmann-fold superfamily protein | chr1:187235-189836 FORWARD LENGTH=636	628	636	0	101.3	90.6	94.1
Rsa1.0_00069.1_g3673.t1	ref|XP_002892058.1| kinase family protein [Arabidopsis lyrata subsp. lyrata] gi|297337900|gb|EFH68317.1| kinase family protein [Arabidopsis lyrata subsp. lyrata]	397	472	0	118.9	93.7	95.7	kinase family protein	gbpln	Arabidopsis lyrata	AT1G01540.2 | Symbols: | Protein kinase superfamily protein | chr1:195980-198383 FORWARD LENGTH=472	397	472	0	118.9	93.5	95.5
Rsa1.0_00069.1_g3674.t1	gb|EOA38922.1| hypothetical protein CARUB_v10011320mg [Capsella rubella]	111	568	4.00E-37	511.7	73.9	79.3	hypothetical protein CARUB_v10011320mg	gbpln	Capsella rubella	AT1G04890.1 | Symbols: | Protein of unknown function, DUF593 | chr1:1381116-1382546 REVERSE LENGTH=411	111	411	2.00E-36	370.3	75.7	82.0
Rsa1.0_00069.1_g3675.t1	gb|AAC19278.1| T14P8.10 [Arabidopsis thaliana] gi|7269009|emb|CAB80742.1| AT4g02490 [Arabidopsis thaliana]	682	657	0	96.3	49.9	64.1	T14P8.10	gbpln	Arabidopsis thaliana	AT4G04650.1 | Symbols: | RNA-directed DNA polymerase (reverse transcriptase)-related family protein | chr4:2353989-2355213 REVERSE LENGTH=332	682	332	1.00E-56	48.7	15.5	22.3
Rsa1.0_00069.1_g3676.t1	gb|AAD32866.1|AC005489_4 F14N23.4 [Arabidopsis thaliana]	813	1161	3.00E-99	142.8	22.1	30.9	F14N23.4	gbpln	Arabidopsis thaliana	AT1G43760.1 | Symbols: | DNAse I-like superfamily protein | chr1:16528880-16531065 REVERSE LENGTH=626	813	626	1.00E-74	77.0	19.2	28.3
Rsa1.0_00069.1_g3677.t1	ref|XP_002892053.1| hypothetical protein ARALYDRAFT_470119 [Arabidopsis lyrata subsp. lyrata] gi|297337895|gb|EFH68312.1| hypothetical protein ARALYDRAFT_470119 [Arabidopsis lyrata subsp. lyrata]	707	725	0	102.5	85.4	92.1	hypothetical protein ARALYDRAFT_470119	gbpln	Arabidopsis lyrata	AT1G01580.1 | Symbols: FRO2, FRD1, ATFRO2 | ferric reduction oxidase 2 | chr1:209395-212810 FORWARD LENGTH=725	707	725	0	102.5	84.3	91.1
Rsa1.0_00069.1_g3678.t2	gb|AFH02721.1| sn-glycerol-3-phosphate acyltransferase 4 isoform C1, partial [Brassica napus] gi|383289233|gb|AFH02724.1| sn-glycerol-3-phosphate acyltransferase 4 isoform C1, partial [Brassica napus]	298	505	1.00E-158	169.5	96.3	97.0	sn-glycerol-3-phosphate acyltransferase 4 isoform C1, partial	gbpln	Brassica napus	AT4G00400.1 | Symbols: GPAT8, AtGPAT8 | glycerol-3-phosphate acyltransferase 8 | chr4:174312-176734 REVERSE LENGTH=500	298	500	1.00E-157	167.8	89.6	92.3
Rsa1.0_00069.1_g3679.t1	dbj|BAJ34288.1| unnamed protein product [Thellungiella halophila]	286	286	1.00E-160	100.0	96.5	99.7	unnamed protein product	----	----	AT4G00430.1 | Symbols: TMP-C, PIP1;4, PIP1E | plasma membrane intrinsic protein 1;4 | chr4:186143-187531 REVERSE LENGTH=287	286	287	1.00E-160	100.3	96.2	99.3
Rsa1.0_00069.1_g3680.t1	ref|XP_002892050.1| hypothetical protein ARALYDRAFT_470113 [Arabidopsis lyrata subsp. lyrata] gi|297337892|gb|EFH68309.1| hypothetical protein ARALYDRAFT_470113 [Arabidopsis lyrata subsp. lyrata]	268	256	1.00E-123	95.5	76.5	84.7	hypothetical protein ARALYDRAFT_470113	gbpln	Arabidopsis lyrata	AT1G01630.1 | Symbols: | Sec14p-like phosphatidylinositol transfer family protein | chr1:229206-230675 FORWARD LENGTH=255	268	255	1.00E-125	95.1	76.1	84.3
Rsa1.0_00069.1_g3681.t1	ref|XP_002889358.1| peptidase [Arabidopsis lyrata subsp. lyrata] gi|297335200|gb|EFH65617.1| peptidase [Arabidopsis lyrata subsp. lyrata]	516	540	0	104.7	92.4	95.3	peptidase	gbpln	Arabidopsis lyrata	AT1G01650.1 | Symbols: SPPL4, ATSPPL4 | SIGNAL PEPTIDE PEPTIDASE-LIKE 4 | chr1:233188-237647 REVERSE LENGTH=540	516	540	0	104.7	91.9	94.8
Rsa1.0_00069.1_g3682.t1	ref|XP_002453623.1| hypothetical protein SORBIDRAFT_04g009135 [Sorghum bicolor] gi|241933454|gb|EES06599.1| hypothetical protein SORBIDRAFT_04g009135 [Sorghum bicolor]	147	801	3.00E-21	544.9	39.5	49.7	hypothetical protein SORBIDRAFT_04g009135	gbpln	Sorghum bicolor	ATMG00810.1 | Symbols: ORF240B | DNA/RNA polymerases superfamily protein | chrM:227709-228431 REVERSE LENGTH=240	147	240	1.00E-13	163.3	23.8	33.3

Rsa1.0_00069.1.g3683.t1	ref|NP_001117211.1| Phosphatidylinositol N-acetyglucosaminlytransferase subunit P-like protein [Arabidopsis thaliana] gi|332189202|gb|AEE27323.1| Phosphatidylinositol N-acetyglucosaminlytransferase subunit P-like protein [Arabidopsis thaliana]	437	433	1.00E-90	99.1	50.8	64.8	Phosphatidylinositol N-acetyglucosaminlytransferase subunit P-like protein	gbpln	Arabidopsis thaliana	AT1G01695.1 | Symbols: | Phosphatidylinositol N-acetyglucosaminlytransferase subunit P-related | chr1:252947-254495 FORWARD LENGTH=433	437	433	3.00E-93	99.1	50.8	64.8
Rsa1.0_00069.1.g3684.t1	gb|EOA37536.1| hypothetical protein CARUB_v10011760mg [Capsella rubella]	368	140	3.00E-73	38.0	35.9	37.0	hypothetical protein CARUB_v10011760mg	gbpln	Capsella rubella	AT1G01750.1 | Symbols: ADF11 | actin depolymerizing factor 11 | chr1:275528-276126 FORWARD LENGTH=140	368	140	6.00E-75	38.0	35.3	37.0
Rsa1.0_00069.1.g3685.t1	#	#	#	#	#	#	#	-	----	----	#	#	#	#	#	#	#
Rsa1.0_00069.1.g3686.t1	ref|XP_002892044.1| hypothetical protein ARALYDRAFT_470098 [Arabidopsis lyrata subsp. lyrata] gi|297337886|gb|EFH68303.1| hypothetical protein ARALYDRAFT_470098 [Arabidopsis lyrata subsp. lyrata]	686	634	0	92.4	80.5	85.4	hypothetical protein ARALYDRAFT_470098	gbpln	Arabidopsis lyrata	AT1G01770.1 | Symbols: | unknown protein; CONTAINS InterPro DOMAIN/s: Protein of unknown function DUF1446 (InterPro:IPR010839); Has 1597 Blast hits to 1509 proteins in 306 species: Archae - 4; Bacteria - 843; Metazoa - 22; Fungi - 131; Plants - 31; Viruses - 0; Other Eukaryotes - 566 (source: NCBI BLink). | chr1:278759-282490 FORWARD LENGTH=632	686	632	0	92.1	80.0	84.8
Rsa1.0_00069.1.g3687.t1	gb|EOA37281.1| hypothetical protein CARUB_v10010886mg [Capsella rubella]	205	205	1.00E-111	100.0	93.7	96.6	hypothetical protein CARUB_v10010886mg	gbpln	Capsella rubella	AT1G01780.1 | Symbols: | GATA type zinc finger transcription factor family protein | chr1:282919-284082 FORWARD LENGTH=205	205	205	1.00E-112	100.0	92.7	96.1
Rsa1.0_00069.1.g3688.t1	#	#	#	#	#	#	#	-	----	----	#	#	#	#	#	#	#
Rsa1.0_00069.1.g3689.t1	ref|XP_002892042.1| hypothetical protein ARALYDRAFT_311263 [Arabidopsis lyrata subsp. lyrata] gi|297337884|gb|EFH68301.1| hypothetical protein ARALYDRAFT_311263 [Arabidopsis lyrata subsp. lyrata]	1167	1171	0	100.3	83.6	89.4	hypothetical protein ARALYDRAFT_311263	gbpln	Arabidopsis lyrata	AT1G01790.1 | Symbols: KEA1, ATKEA1 | K+ efflux antiporter 1 | chr1:284781-290869 FORWARD LENGTH=1193	1167	1193	0	102.2	83.3	89.3
Rsa1.0_00069.1.g3690.t1	ref|XP_002889352.1| peroxisomal biogenesis factor 11 family protein [Arabidopsis lyrata subsp. lyrata] gi|297335194|gb|EFH65611.1| peroxisomal biogenesis factor 11 family protein [Arabidopsis lyrata subsp. lyrata]	237	235	1.00E-128	99.2	94.1	97.0	peroxisomal biogenesis factor 11 family protein	gbpln	Arabidopsis lyrata	AT1G01820.1 | Symbols: PEX11C | peroxin 11c | chr1:296213-297723 REVERSE LENGTH=235	237	235	1.00E-129	99.2	92.8	96.6
Rsa1.0_00069.1.g3691.t1	ref|NP_171690.1| Ribosomal RNA adenine dimethylase family protein [Arabidopsis thaliana] gi|3005590|gb|AAC09322.1| dimethyladenosine transferase [Arabidopsis thaliana] gi|26449914|dbj|BAC42078.1| putative dimethyladenosine transferase [Arabidopsis thaliana] gi|28827572|gb|AAO50630.1| putative dimethyladenosine transferase [Arabidopsis thaliana] gi|332189223|gb|AEE27344.1| Ribosomal RNA adenine dimethylase family protein [Arabidopsis thaliana]	374	343	1.00E-149	91.7	72.7	76.7	Ribosomal RNA adenine dimethylase family protein	gbpln	Arabidopsis thaliana	AT1G01860.1 | Symbols: PFC1 | Ribosomal RNA adenine dimethylase family protein | chr1:304439-306275 REVERSE LENGTH=343	374	343	1.00E-151	91.7	72.7	76.7
Rsa1.0_00069.1.g3692.t1	gb|EOA36590.1| hypothetical protein CARUB_v10011785mg [Capsella rubella]	599	597	0	99.7	77.8	86.6	hypothetical protein CARUB_v10011785mg	gbpln	Capsella rubella	AT1G01880.1 | Symbols: | 5'-3' exonuclease family protein | chr1:306558-308991 REVERSE LENGTH=599	599	599	0	100.0	77.6	85.5
Rsa1.0_00069.1.g3693.t1	gb|EOA38113.1| hypothetical protein CARUB_v10009581mg [Capsella rubella]	358	353	1.00E-175	98.6	89.4	92.2	hypothetical protein CARUB_v10009581mg	gbpln	Capsella rubella	AT1G01910.4 | Symbols: | P-loop containing nucleoside triphosphate hydrolases superfamily protein | chr1:313595-315831 REVERSE LENGTH=353	358	353	1.00E-177	98.6	88.5	92.2
Rsa1.0_00069.1.g3694.t1	ref|NP_171694.3| SET domain-containing protein [Arabidopsis thaliana] gi|332189232|gb|AEE27353.1| SET domain-containing protein [Arabidopsis thaliana]	547	572	0	104.6	80.8	88.5	SET domain-containing protein	gbpln	Arabidopsis thaliana	AT1G01920.1 | Symbols: | SET domain-containing protein | chr1:316204-319507 FORWARD LENGTH=572	547	572	0	104.6	80.8	88.5
Rsa1.0_00069.1.g3695.t1	ref|XP_002889345.1| hypothetical protein ARALYDRAFT_470081 [Arabidopsis lyrata subsp. lyrata] gi|297335187|gb|EFH65604.1| hypothetical protein ARALYDRAFT_470081 [Arabidopsis lyrata subsp. lyrata]	582	579	0	99.5	80.2	86.9	hypothetical protein ARALYDRAFT_470081	gbpln	Arabidopsis lyrata	AT1G01930.1 | Symbols: | zinc finger protein-related | chr1:320041-322809 REVERSE LENGTH=580	582	580	0	99.7	78.7	84.7

Rsa1.0_00069.1.g3696.t1	ref|XP_002892036.1| peptidyl-prolyl cis-trans isomerase cyclophilin-type family protein [Arabidopsis lyrata subsp. lyrata] gi|297337878|gb|EFH68295.1| peptidyl-prolyl cis-trans isomerase cyclophilin-type family protein [Arabidopsis lyrata subsp. lyrata]	160	160	5.00E-88	100.0	96.9	98.8	peptidyl-prolyl cis-trans isomerase cyclophilin-type family protein	gbpln	Arabidopsis lyrata	AT1G01940.1 | Symbols: | Cyclophilin-like peptidyl-prolyl cis-trans isomerase family protein | chr1:323082-324719 FORWARD LENGTH=160	160	160	3.00E-90	100.0	96.9	98.1
Rsa1.0_00069.1.g3697.t1	ref|NP_171697.3| armadillo repeat-containing kinesin-like protein 2 [Arabidopsis thaliana] gi|193806751|sp|Q9LPC6.2|ARK2_ARATH RecName: Full=Armadillo repeat-containing kinesin-like protein 2 gi|162958325|dbj|BAF95586.1| armadillo repeat kinesin2 [Arabidopsis thaliana] gi|332189236|gb|AEE27357.1| armadillo repeat-containing kinesin-like protein 2 [Arabidopsis thaliana]	898	894	0	99.6	94.9	96.9	armadillo repeat-containing kinesin-like protein 2	gbpln	Arabidopsis thaliana	AT1G01950.1 | Symbols: ARK2 | armadillo repeat kinesin 2 | chr1:325473-330403 FORWARD LENGTH=894	898	894	0	99.6	94.9	96.9
Rsa1.0_00069.1.g3698.t1	gb|EOA36031.1| hypothetical protein CARUB_v10008079mg [Capsella rubella]	1754	1780	0	101.5	94.0	97.0	hypothetical protein CARUB_v10008079mg	gbpln	Capsella rubella	AT1G01960.1 | Symbols: EDA10 | SEC7-like guanine nucleotide exchange family protein | chr1:330830-337582 REVERSE LENGTH=1750	1754	1750	0	99.8	93.4	96.3
Rsa1.0_00069.1.g3699.t1	dbj|BAB09923.1| copia-like retrotransposable element [Arabidopsis thaliana]	1186	1342	0	113.2	49.6	67.1	copia-like retrotransposable element	gbpln	Arabidopsis thaliana	AT4G23160.1 | Symbols: CRK8 | cysteine-rich RLK (RECEPTOR-like protein kinase) 8 | chr4:12129485-12134086 FORWARD LENGTH=1262	1186	1262	1.00E-57	106.4	11.4	16.9
Rsa1.0_00069.1.g3700.t2	dbj|BAJ34466.1| unnamed protein product [Thellungiella halophila]	689	675	0	98.0	90.9	93.8	unnamed protein product	----	----	AT1G02010.1 | Symbols: SEC1A | secretory 1A | chr1:348046-352197 FORWARD LENGTH=673	689	673	0	97.7	90.9	93.8
Rsa1.0_00069.1.g3701.t1	ref|XP_002889340.1| hypothetical protein ARALYDRAFT_333453 [Arabidopsis lyrata subsp. lyrata] gi|297335182|gb|EFH65599.1| hypothetical protein ARALYDRAFT_333453 [Arabidopsis lyrata subsp. lyrata]	258	264	6.00E-97	102.3	80.2	89.5	hypothetical protein ARALYDRAFT_333453	gbpln	Arabidopsis lyrata	AT1G02040.1 | Symbols: | C2H2-type zinc finger family protein | chr1:358104-359078 REVERSE LENGTH=324	258	324	2.00E-99	125.6	81.8	88.4
Rsa1.0_00069.1.g3702.t1	ref|XP_002892031.1| hypothetical protein ARALYDRAFT_470069 [Arabidopsis lyrata subsp. lyrata] gi|297337873|gb|EFH68290.1| hypothetical protein ARALYDRAFT_470069 [Arabidopsis lyrata subsp. lyrata]	337	323	1.00E-100	95.8	64.1	70.3	hypothetical protein ARALYDRAFT_470069	gbpln	Arabidopsis lyrata	AT1G02065.1 | Symbols: SPL8 | squamosa promoter binding protein-like 8 | chr1:365625-367149 FORWARD LENGTH=333	337	333	7.00E-95	98.8	62.3	70.9
Rsa1.0_00069.1.g3703.t3	#	#	#	#	#	#	#	-	----	----	#	#	#	#	#	#	#
Rsa1.0_00069.1.g3704.t1	gb|ABD65090.1| hypothetical protein 27.t00116 [Brassica oleracea]	584	484	3.00E-91	82.9	28.3	34.8	hypothetical protein 27.t00116	gbpln	Brassica oleracea	AT1G52950.1 | Symbols: | Nucleic acid-binding, OB-fold-like protein | chr1:19725483-19728007 FORWARD LENGTH=566	584	566	2.00E-12	96.9	15.6	29.5
Rsa1.0_00069.1.g3705.t1	gb|EOA39027.1| hypothetical protein CARUB_v10011597mg [Capsella rubella]	186	186	2.00E-15	100.0	26.9	45.2	hypothetical protein CARUB_v10011597mg	gbpln	Capsella rubella	AT5G53910.1 | Symbols: | RING/U-box superfamily protein | chr5:21890643-21891335 FORWARD LENGTH=230	186	230	6.00E-11	123.7	14.5	18.8
Rsa1.0_00069.1.g3706.t1	ref|XP_003609124.1| ATP-dependent DNA helicase PIF1 [Medicago truncatula] gi|355510179|gb|AES91321.1| ATP-dependent DNA helicase PIF1 [Medicago truncatula]	1509	1558	0	103.2	44.1	60.8	ATP-dependent DNA helicase PIF1	gbpln	Medicago truncatula	AT5G28780.1 | Symbols: | PIF1 helicase | chr5:10812907-10814173 REVERSE LENGTH=337	1509	337	5.00E-70	22.3	8.7	12.2
Rsa1.0_00069.1.g3707.t2	#	#	#	#	#	#	#	-	----	----	#	#	#	#	#	#	#
Rsa1.0_00069.1.g3708.t1	gb|AAG10809.1|AC018460_3 Similar to mutator transposase [Arabidopsis thaliana]	436	884	7.00E-17	202.8	19.0	30.5	Similar to mutator transposase	gbpln	Arabidopsis thaliana	#	#	#	#	#	#	#
Rsa1.0_00069.1.g3709.t1	#	#	#	#	#	#	#	-	----	----	#	#	#	#	#	#	#
Rsa1.0_00069.1.g3710.t1	gb|ADK13090.1| annexin 1 [Brassica napus]	317	317	0	100.0	98.1	99.4	annexin 1	gbpln	Brassica napus	AT1G35720.1 | Symbols: ANNAT1, OXY5, ATOXY5 | annexin 1 | chr1:13225304-13226939 FORWARD LENGTH=317	317	317	1.00E-176	100.0	93.4	97.8
Rsa1.0_00069.1.g3711.t1	gb|EOA39555.1| hypothetical protein CARUB_v10008173mg [Capsella rubella]	1037	1027	0	99.0	74.4	85.3	hypothetical protein CARUB_v10008173mg	gbpln	Capsella rubella	AT4G08850.1 | Symbols: | Leucine-rich repeat receptor-like protein kinase family protein | chr4:5636693-5640496 REVERSE LENGTH=1045	1037	1045	0	100.8	70.6	83.7
Rsa1.0_00069.1.g3712.t4	#	#	#	#	#	#	#	-	----	----	#	#	#	#	#	#	#

Rsa1.0_00069.1.g3713.t1	ref|NP_174807.1| calcium-dependent protein kinase 2 [Arabidopsis thaliana] gi|332278169|sp|Q39016.2|CDPKB_ARATH RecName: Full=Calcium-dependent protein kinase 11; AltName: Full=Calcium-dependent protein kinase isoform CDPK2; Short=AtCDPK2 gi|15293095|gb|AAK93658.1| putative calcium-dependent protein kinase [Arabidopsis thaliana] gi|21281141|gb|AAM45034.1| putative calcium-dependent protein kinase [Arabidopsis thaliana] gi|332193701|gb|AEE31822.1| calcium-dependent protein kinase 2 [Arabidopsis thaliana]	497	495	0	99.6	97.4	98.2	calcium-dependent protein kinase 2	gbpln	Arabidopsis thaliana	AT1G35670.1 | Symbols: ATCDPK2, CPK11, ATCPK11, CDPK2 | calcium-dependent protein kinase 2 | chr1:13205456-13208058 FORWARD LENGTH=495	497	495	0	99.6	97.4	98.2
Rsa1.0_00069.1.g3714.t1	gb|EOA40451.1| hypothetical protein CARUB_v10009176mg [Capsella rubella]	468	438	0	93.6	78.6	84.6	hypothetical protein CARUB_v10009176mg	gbpln	Capsella rubella	AT1G35620.1 | Symbols: ATPDIL5-2, ATPDI8, PDI8, PDIL5-2 | PDI-like 5-2 | chr1:13156504-13158280 FORWARD LENGTH=440	468	440	0	94.0	78.0	84.0
Rsa1.0_00070.1.g3715.t1	#	#	#	#	#	#	#	–	----	----	AT4G24590.1 | Symbols: | unknown protein; BEST Arabidopsis thaliana protein match is: unknown protein (TAIR:AT5G49710.3); Has 105 Blast hits to 105 proteins in 26 species: Archae - 0; Bacteria - 0; Metazoa - 8; Fungi - 3; Plants - 85; Viruses - 0; Other Eukaryotes - 9 (source: NCBI BLink). | chr4:12696651-12698036 FORWARD LENGTH=241	71	241	2.00E-11	339.4	47.9	52.1
Rsa1.0_00070.1.g3716.t1	gb|EOA15984.1| hypothetical protein CARUB_v10004098mg [Capsella rubella]	897	907	0	101.1	77.5	83.5	hypothetical protein CARUB_v10004098mg	gbpln	Capsella rubella	AT4G24580.1 | Symbols: REN1 | Rho GTPase activation protein (RhoGAP) with PH domain | chr4:12687879-12694248 REVERSE LENGTH=933	897	933	0	104.0	79.3	86.1
Rsa1.0_00070.1.g3717.t1	#	#	#	#	#	#	#	–	----	----	#	#	#	#	#	#	#
Rsa1.0_00070.1.g3718.t1	ref|XP_002867659.1| ubiquitin-specific protease 16 [Arabidopsis lyrata subsp. lyrata] gi|297313495|gb|EFH43918.1| ubiquitin-specific protease 16 [Arabidopsis lyrata subsp. lyrata]	1009	1007	0	99.8	77.6	85.7	ubiquitin-specific protease 16	gbpln	Arabidopsis lyrata	AT4G24560.1 | Symbols: UBP16 | ubiquitin-specific protease 16 | chr4:12679493-12684528 REVERSE LENGTH=1008	1009	1008	0	99.9	78.0	85.5
Rsa1.0_00070.1.g3719.t1	gb|AFM77897.1| MADS-box protein AGL24 [Brassica napus]	233	221	1.00E-119	94.8	91.4	94.0	MADS-box protein AGL24	gbpln	Brassica napus	AT4G24540.1 | Symbols: AGL24 | AGAMOUS-like 24 | chr4:12671160-12673645 REVERSE LENGTH=220	233	220	1.00E-102	94.4	80.3	88.0
Rsa1.0_00070.1.g3720.t1	#	#	#	#	#	#	#	–	----	----	#	#	#	#	#	#	#
Rsa1.0_00070.1.g3721.t1	gb|EOA23805.1| hypothetical protein CARUB_v10017019mg [Capsella rubella]	160	520	5.00E-13	325.0	25.6	29.4	hypothetical protein CARUB_v10017019mg	gbpln	Capsella rubella	AT4G24530.1 | Symbols: | O-fucosyltransferase family protein | chr4:12667424-12669713 REVERSE LENGTH=519	160	519	2.00E-14	324.4	21.3	21.3
Rsa1.0_00070.1.g3722.t1	ref|XP_002867662.1| hypothetical protein ARALYDRAFT_492391 [Arabidopsis lyrata subsp. lyrata] gi|297313498|gb|EFH43921.1| hypothetical protein ARALYDRAFT_492391 [Arabidopsis lyrata subsp. lyrata]	695	692	0	99.6	91.2	94.8	hypothetical protein ARALYDRAFT_492391	gbpln	Arabidopsis lyrata	AT4G24520.1 | Symbols: ATR1, AR1 | P450 reductase 1 | chr4:12663065-12667066 REVERSE LENGTH=692	695	692	0	99.6	89.8	94.2
Rsa1.0_00070.1.g3723.t1	#	#	#	#	#	#	#	–	----	----	#	#	#	#	#	#	#
Rsa1.0_00070.1.g3724.t6	gb|EOA37111.1| hypothetical protein CARUB_v10010324mg [Capsella rubella] gi|482572925|gb|EOA37112.1| hypothetical protein CARUB_v10010324mg [Capsella rubella]	211	203	4.00E-75	96.2	69.7	77.3	hypothetical protein CARUB_v10010324mg	gbpln	Capsella rubella	AT1G07700.1 | Symbols: | Thioredoxin superfamily protein | chr1:2380214-2381127 FORWARD LENGTH=204	211	204	3.00E-73	96.7	65.4	73.9
Rsa1.0_00070.1.g3725.t1	gb|EOA17945.1| hypothetical protein CARUB_v10006355mg [Capsella rubella]	131	312	1.00E-53	238.2	76.3	85.5	hypothetical protein CARUB_v10006355mg	gbpln	Capsella rubella	AT4G24470.2 | Symbols: ZIM, GATA25 | GATA-type zinc finger protein with TIFY domain | chr4:12645785-12647734 FORWARD LENGTH=309	131	309	2.00E-55	235.9	76.3	86.3
Rsa1.0_00070.1.g3726.t2	ref|XP_002869716.1| zinc-finger protein expressed in inflorescence meristem [Arabidopsis lyrata subsp. lyrata] gi|297315552|gb|EFH45975.1| zinc-finger protein expressed in inflorescence meristem [Arabidopsis lyrata subsp. lyrata]	183	311	5.00E-54	169.9	67.2	72.1	zinc-finger protein expressed in inflorescence meristem	gbpln	Arabidopsis lyrata	AT4G24470.2 | Symbols: ZIM, GATA25 | GATA-type zinc finger protein with TIFY domain | chr4:12645785-12647734 FORWARD LENGTH=309	183	309	9.00E-54	168.9	67.2	72.1
Rsa1.0_00070.1.g3727.t1	gb|EOA17710.1| hypothetical protein CARUB_v10006082mg [Capsella rubella]	106	106	6.00E-55	100.0	98.1	100.0	hypothetical protein CARUB_v10006082mg	gbpln	Capsella rubella	AT4G24440.2 | Symbols: | transcription initiation factor IIA gamma chain / TFIIA-gamma (TFIIA-S) | chr4:12633460-12634553 FORWARD LENGTH=106	106	106	3.00E-57	100.0	97.2	100.0
Rsa1.0_00070.1.g3728.t1	gb|EOA29907.1| hypothetical protein CARUB_v10013001mg [Capsella rubella]	1093	803	3.00E-75	73.5	18.4	23.6	hypothetical protein CARUB_v10013001mg	gbpln	Capsella rubella	AT3G02930.2 | Symbols: | Plant protein of unknown function (DUF827) | chr3:655158-658319 FORWARD LENGTH=804	1093	804	1.00E-70	73.6	17.6	22.6

Rsa1.0_00070.1.g3729.t3	ref|NP_194164.1| uncharacterized protein [Arabidopsis thaliana] gi|5051775|emb|CAB45068.1| putative protein [Arabidopsis thaliana] gi|7269283|emb|CAB79343.1| putative protein [Arabidopsis thaliana] gi|58652054|gb|AAW80852.1| At4g24330 [Arabidopsis thaliana] gi|332659490|gb|AEE84890.1| uncharacterized protein AT4G24330 [Arabidopsis thaliana]	477	478	0	100.2	81.1	87.4	uncharacterized protein	gbpln	Arabidopsis thaliana	AT4G24330.1 | Symbols: | Protein of unknown function (DUF1682) | chr4:12603848-12606229 REVERSE LENGTH=478	477	478	0	100.2	81.1	87.4
Rsa1.0_00070.1.g3730.t1	ref|XP_002869727.1| cphsc70-1 [Arabidopsis lyrata subsp. lyrata] gi|297315563|gb|EFH45986.1| cphsc70-1 [Arabidopsis lyrata subsp. lyrata]	713	718	0	100.7	93.8	96.4	cphsc70-1	gbpln	Arabidopsis lyrata	AT4G24280.1 | Symbols: cpHsc70-1 | chloroplast heat shock protein 70-1 | chr4:12590094-12593437 FORWARD LENGTH=718	713	718	0	100.7	88.9	91.4
Rsa1.0_00070.1.g3731.t1	gb|EOA15374.1| hypothetical protein CARUB_v10006044mg [Capsella rubella]	125	121	1.00E-27	96.8	65.6	75.2	hypothetical protein CARUB_v10006044mg	gbpln	Capsella rubella	AT4G24275.1 | Symbols: | Identified as a screen for stress-responsive genes. | chr4:12588579-12588959 FORWARD LENGTH=126	125	126	1.00E-26	100.8	71.2	80.8
Rsa1.0_00070.1.g3732.t1	gb|EOA15955.1| hypothetical protein CARUB_v10004057mg [Capsella rubella]	843	1002	0	118.9	64.1	70.9	hypothetical protein CARUB_v10004057mg	gbpln	Capsella rubella	AT4G24200.1 | Symbols: | Transcription elongation factor (TFIIS) family protein | chr4:12556927-12559929 REVERSE LENGTH=1000	843	1000	0	118.6	63.9	70.6
Rsa1.0_00070.1.g3733.t1	#	#	#	#	#	#	#	-	----	----	#	#	#	#	#	#	#
Rsa1.0_00070.1.g3734.t1	ref|NP_194157.1| endoglucanase 21 [Arabidopsis thaliana] gi|75266338|sp|Q9STW8.1|GUN21_ARATH RecName: Full=Endoglucanase 21; AltName: Full=Endo-1,4-beta glucanase 21 gi|5051768|emb|CAB45061.1| endo-1, 4-beta-glucanase like protein [Arabidopsis thaliana] gi|7269276|emb|CAB79336.1| endo-1, 4-beta-glucanase like protein [Arabidopsis thaliana] gi|332659479|gb|AEE84879.1| endoglucanase 21 [Arabidopsis thaliana]	643	620	0	96.4	73.7	82.7	endoglucanase 21	gbpln	Arabidopsis thaliana	AT4G24260.1 | Symbols: ATGH9A3, KOR3, GH9A3 | glycosyl hydrolase 9A3 | chr4:12577871-12580133 REVERSE LENGTH=620	643	620	0	96.4	73.7	82.7
Rsa1.0_00070.1.g3735.t1	ref|XP_002867673.1| hypothetical protein ARALYDRAFT_914165 [Arabidopsis lyrata subsp. lyrata] gi|297313509|gb|EFH43932.1| hypothetical protein ARALYDRAFT_914165 [Arabidopsis lyrata subsp. lyrata]	449	482	0	107.3	87.1	92.0	hypothetical protein ARALYDRAFT_914165	gbpln	Arabidopsis lyrata	AT4G24250.1 | Symbols: MLO13, ATMLO13 | Seven transmembrane MLO family protein | chr4:12575008-12577517 REVERSE LENGTH=478	449	478	0	106.5	86.2	91.8
Rsa1.0_00070.1.g3736.t1	gb|ACQ76809.1| WRKY transcription factor 7 [Brassica napus]	352	348	1.00E-170	98.9	90.1	92.6	WRKY transcription factor 7	gbpln	Brassica napus	AT4G24240.1 | Symbols: WRKY7, ATWRKY7 | WRKY DNA-binding protein 7 | chr4:12571930-12573446 FORWARD LENGTH=353	352	353	1.00E-139	100.3	76.4	83.5
Rsa1.0_00070.1.g3737.t1	ref|NP_194154.1| acyl-CoA-binding domain 3 [Arabidopsis thaliana] gi|145333919|ref|NP_001078439.1| acyl-CoA-binding domain 3 [Arabidopsis thaliana] gi|145333930|ref|NP_001078440.1| acyl-CoA-binding domain 3 [Arabidopsis thaliana] gi|145333941|ref|NP_001078441.1| acyl-CoA-binding domain 3 [Arabidopsis thaliana] gi|75207746|sp|Q9STX1.1|ACBP3_ARATH RecName: Full=Acyl-CoA-binding domain-containing protein 3; Short=Acyl-CoA binding protein 3; Flags: Precursor gi|5051765|emb|CAB45058.1| putative protein [Arabidopsis thaliana] gi|7269273|emb|CAB79333.1| putative protein [Arabidopsis thaliana] gi|110742400|dbj|BAE99122.1| hypothetical protein [Arabidopsis thaliana] gi|332659471|gb|AEE84871.1| acyl-CoA-binding domain 3 [Arabidopsis thaliana] gi|332659472|gb|AEE84872.1| acyl-CoA-binding domain 3 [Arabidopsis thaliana] gi|332659473|gb|AEE84873.1| acyl-CoA-binding domain 3 [Arabidopsis thaliana] gi|332659474|gb|AEE84874.1| acyl-CoA-binding domain 3 [Arabidopsis thaliana]	368	362	2.00E-88	98.4	61.4	72.0	acyl-CoA-binding domain 3	gbpln	Arabidopsis thaliana	AT4G24230.5 | Symbols: ACBP3 | acyl-CoA-binding domain 3 | chr4:12567240-12568754 REVERSE LENGTH=362	368	362	5.00E-91	98.4	61.4	72.0

Rsa1.0_00070.1.g3738.t1	gb|AFD96485.1| putative enone 5-beta-reductase [Brassica oleracea var. gemmifera]	380	382	0	100.5	91.1	96.6	putative enone 5-beta-reductase	gbpln	Brassica oleracea	AT4G24220.1 | Symbols: VEP1, AWI31 | NAD(P)-binding Rossmann-fold superfamily protein | chr4:12565219-12566474 FORWARD LENGTH=388	380	388	1.00E-178	102.1	78.7	90.3
Rsa1.0_00070.1.g3739.t1	gb|AAD09508.1| ATFP4, partial [Arabidopsis thaliana]	159	179	6.00E-33	112.6	65.4	72.3	ATFP4, partial	gbpln	Arabidopsis thaliana	AT3G07600.1 | Symbols: | Heavy metal transport/detoxification superfamily protein | chr3:2424300-2424954 REVERSE LENGTH=157	159	157	4.00E-35	98.7	65.4	72.3
Rsa1.0_00070.1.g3740.t2	ref|XP_002891519.1| hypothetical protein ARALYDRAFT_337099 [Arabidopsis lyrata subsp. lyrata] gi|297337361|gb|EFH67778.1| hypothetical protein ARALYDRAFT_337099 [Arabidopsis lyrata subsp. lyrata]	643	1163	4.00E-40	180.9	23.5	36.5	hypothetical protein ARALYDRAFT_337099	gbpln	Arabidopsis lyrata	AT1G67370.1 | Symbols: ASY1, ATASY1 | DNA-binding HORMA family protein | chr1:25239347-25243713 REVERSE LENGTH=596	643	596	4.00E-33	92.7	12.8	14.5
Rsa1.0_00070.1.g3741.t1	ref|XP_002862875.1| hypothetical protein ARALYDRAFT_920182 [Arabidopsis lyrata subsp. lyrata] gi|297308637|gb|EFH39134.1| hypothetical protein ARALYDRAFT_920182 [Arabidopsis lyrata subsp. lyrata]	310	315	3.00E-31	101.6	32.3	46.5	hypothetical protein ARALYDRAFT_920182	gbpln	Arabidopsis lyrata	AT1G02770.1 | Symbols: | Protein of unknown function (DUF626) | chr1:605071-606624 REVERSE LENGTH=316	310	316	7.00E-34	101.9	31.9	49.4
Rsa1.0_00070.1.g3742.t1	ref|XP_002867675.1| hypothetical protein ARALYDRAFT_329238 [Arabidopsis lyrata subsp. lyrata] gi|297313511|gb|EFH43934.1| hypothetical protein ARALYDRAFT_329238 [Arabidopsis lyrata subsp. lyrata]	121	157	3.00E-40	129.8	68.6	81.8	hypothetical protein ARALYDRAFT_329238	gbpln	Arabidopsis lyrata	AT4G24204.3 | Symbols: | RING/U-box superfamily protein | chr4:12562069-12562815 REVERSE LENGTH=178	121	178	1.00E-42	147.1	70.2	81.0
Rsa1.0_00070.1.g3743.t1	ref|XP_002867676.1| hypothetical protein ARALYDRAFT_492440 [Arabidopsis lyrata subsp. lyrata] gi|297313512|gb|EFH43935.1| hypothetical protein ARALYDRAFT_492440 [Arabidopsis lyrata subsp. lyrata]	986	1002	0	101.6	79.5	87.3	hypothetical protein ARALYDRAFT_492440	gbpln	Arabidopsis lyrata	AT4G24200.1 | Symbols: | Transcription elongation factor (TFIIS) family protein | chr4:12556927-12559929 REVERSE LENGTH=1000	986	1000	0	101.4	77.9	86.0
Rsa1.0_00070.1.g3744.t1	ref|XP_002867677.1| hypothetical protein ARALYDRAFT_492441 [Arabidopsis lyrata subsp. lyrata] gi|297313513|gb|EFH43936.1| hypothetical protein ARALYDRAFT_492441 [Arabidopsis lyrata subsp. lyrata]	804	823	0	102.4	93.5	96.5	hypothetical protein ARALYDRAFT_492441	gbpln	Arabidopsis lyrata	AT4G24190.1 | Symbols: SHD, HSP90.7, AtHsp90.7, AtHsp90-7 | Chaperone protein htpG family protein | chr4:12551902-12555851 REVERSE LENGTH=823	804	823	0	102.4	92.2	95.9
Rsa1.0_00070.1.g3745.t1	emb|CAB45053.1| thaumatin-like protein [Arabidopsis thaliana] gi|7269268|emb|CAB79328.1| thaumatin-like protein [Arabidopsis thaliana]	256	255	1.00E-130	99.6	89.1	94.1	thaumatin-like protein	gbpln	Arabidopsis thaliana	AT4G24180.1 | Symbols: TLP1, ATTLP1 | THAUMATIN-LIKE PROTEIN 1 | chr4:12550356-12551221 REVERSE LENGTH=260	256	260	1.00E-131	101.6	89.1	94.1
Rsa1.0_00070.1.g3746.t1	ref|XP_002869734.1| hydrolase, alpha/beta fold family protein [Arabidopsis lyrata subsp. lyrata] gi|297315570|gb|EFH45993.1| hydrolase, alpha/beta fold family protein [Arabidopsis lyrata subsp. lyrata]	407	417	0	102.5	88.2	94.1	hydrolase, alpha/beta fold family protein	gbpln	Arabidopsis lyrata	AT4G24160.1 | Symbols: | alpha/beta-Hydrolases superfamily protein | chr4:12539871-12542210 FORWARD LENGTH=418	407	418	0	102.7	88.7	94.1
Rsa1.0_00070.1.g3747.t1	ref|XP_002869735.1| hypothetical protein ARALYDRAFT_354358 [Arabidopsis lyrata subsp. lyrata] gi|297315571|gb|EFH45994.1| hypothetical protein ARALYDRAFT_354358 [Arabidopsis lyrata subsp. lyrata]	435	431	1.00E-139	99.1	68.0	76.3	hypothetical protein ARALYDRAFT_354358	gbpln	Arabidopsis lyrata	AT4G24150.1 | Symbols: AtGRF8, GRF8 | growth-regulating factor 8 | chr4:12535972-12539387 FORWARD LENGTH=493	435	493	1.00E-134	113.3	62.8	71.5
Rsa1.0_00070.1.g3748.t1	gb|EOA16234.1| hypothetical protein CARUB_v10004382mg [Capsella rubella]	591	622	0	105.2	74.6	82.7	hypothetical protein CARUB_v10004382mg	gbpln	Capsella rubella	AT4G24260.1 | Symbols: ATGH9A3, KOR3, GH9A3 | glycosyl hydrolase 9A3 | chr4:12577871-12580133 REVERSE LENGTH=620	591	620	0	104.9	75.0	83.9
Rsa1.0_00070.1.g3749.t1	#	#	#	#	#	#	#	-	----	----	#	#	#	#	#	#	#
Rsa1.0_00070.1.g3750.t1	gb|EOA17566.1| hypothetical protein CARUB_v10005927mg [Capsella rubella]	157	157	7.00E-73	100.0	83.4	91.7	hypothetical protein CARUB_v10005927mg	gbpln	Capsella rubella	AT4G24130.1 | Symbols: | Protein of unknown function, DUF538 | chr4:12527861-12528423 FORWARD LENGTH=157	157	157	1.00E-74	100.0	83.4	91.7
Rsa1.0_00070.1.g3751.t1	gb|EOA16163.1| hypothetical protein CARUB_v10004301mg [Capsella rubella]	403	672	0	166.7	91.6	96.8	hypothetical protein CARUB_v10004301mg	gbpln	Capsella rubella	AT4G24120.1 | Symbols: YSL1, ATYSL1 | YELLOW STRIPE like 1 | chr4:12524581-12527023 FORWARD LENGTH=673	403	673	0	167.0	90.6	96.0
Rsa1.0_00071.1.g3752.t1	gb|EOA14930.1| hypothetical protein CARUB_v10028273mg [Capsella rubella]	1265	1269	0	100.3	92.0	95.6	hypothetical protein CARUB_v10028273mg	gbpln	Capsella rubella	AT5G64270.1 | Symbols: | splicing factor, putative | chr5:25706909-25710718 FORWARD LENGTH=1269	1265	1269	0	100.3	90.8	94.9

Rsa1.0_00071.1.g3753.t1	ref|NP_201234.1| dicarboxylate transport 2.1 [Arabidopsis thaliana] gi|75171656|sp|Q9FMF7.1|DIT21_ARATH RecName: Full=Dicarboxylate transporter 2.1, chloroplastic; AltName: Full=AtpDCT1; AltName: Full=Glutamate/malate translocator; Flags: Precursor gi|9759405|dbj|BAB09860.1| 2-oxoglutarate/malate translocator [Arabidopsis thaliana] gi|15810581|gb|AAL07178.1| putative 2-oxoglutarate/malate translocator protein [Arabidopsis thaliana] gi|23397031|gb|AAN31801.1| putative 2-oxoglutarate/malate translocator [Arabidopsis thaliana] gi|53850569|gb|AAU95461.1| At5g64290 [Arabidopsis thaliana] gi|332010483|gb|AED97866.1| dicarboxylate transport 2.1 [Arabidopsis thaliana]	560	563	0	100.5	93.4	96.4	dicarboxylate transport 2.1	gbpln	Arabidopsis thaliana	AT5G64290.1 | Symbols: DCT, DIT2.1 | dicarboxylate transport 2.1 | chr5:25714495-25716642 REVERSE LENGTH=563	560	563	0	100.5	93.4	96.4
Rsa1.0_00071.1.g3754.t1	gb|EOA12507.1| hypothetical protein CARUB_v10026209mg [Capsella rubella]	520	534	0	102.7	86.5	90.2	hypothetical protein CARUB_v10026209mg	gbpln	Capsella rubella	AT5G64300.1 | Symbols: ATGCH, GCH, ATRIBA1, RFD1 | GTP cyclohydrolase II | chr5:25718459-25720790 FORWARD LENGTH=543	520	543	0	104.4	86.0	90.4
Rsa1.0_00071.1.g3755.t4	dbj|BAB09864.1| non-phototropic hypocotyl 3 [Arabidopsis thaliana]	1146	745	0	65.0	54.5	56.7	non-phototropic hypocotyl 3	gbpln	Arabidopsis thaliana	AT5G64330.1 | Symbols: NPH3, RPT3, JK218 | Phototropic-responsive NPH3 family protein | chr5:25727568-25730225 FORWARD LENGTH=746	1146	746	0	65.1	54.5	56.6
Rsa1.0_00071.1.g3756.t1	ref|XP_002269289.1| PREDICTED: uncharacterized protein C6C3.02c [Vitis vinifera] gi|296088792|emb|CBI38242.3| unnamed protein product [Vitis vinifera]	162	145	5.00E-38	89.5	59.9	69.8	PREDICTED: uncharacterized protein C6C3.02c	gbpln	Vitis vinifera	AT5G09570.1 | Symbols: | Cox19-like CHCH family protein | chr5:2970733-2971970 FORWARD LENGTH=139	162	139	3.00E-40	85.8	52.5	63.0
Rsa1.0_00071.1.g3757.t1	gb|EOA13529.1| hypothetical protein CARUB_v10026593mg [Capsella rubella] gi|482549336|gb|EOA13530.1| hypothetical protein CARUB_v10026593mg [Capsella rubella]	360	379	1.00E-157	105.3	76.7	83.6	hypothetical protein CARUB_v10026593mg	gbpln	Capsella rubella	AT5G64520.1 | Symbols: XRCC2, ATXRCC2 | homolog of X-ray repair cross complementing 2 (XRCC2) | chr5:25788069-25790204 FORWARD LENGTH=372	360	372	1.00E-152	103.3	76.7	84.7
Rsa1.0_00071.1.g3758.t1	ref|XP_002864908.1| ANAC104/XND1 [Arabidopsis lyrata subsp. lyrata] gi|297310743|gb|EFH41167.1| ANAC104/XND1 [Arabidopsis lyrata subsp. lyrata]	188	188	1.00E-89	100.0	92.6	96.8	ANAC104/XND1	gbpln	Arabidopsis lyrata	AT5G64530.1 | Symbols: ANAC104, XND1 | xylem NAC domain 1 | chr5:25795360-25796699 FORWARD LENGTH=187	188	187	7.00E-91	99.5	89.4	94.1
Rsa1.0_00071.1.g3759.t1	ref|XP_002866619.1| hypothetical protein ARALYDRAFT_358659 [Arabidopsis lyrata subsp. lyrata] gi|297312454|gb|EFH42878.1| hypothetical protein ARALYDRAFT_358659 [Arabidopsis lyrata subsp. lyrata]	513	1082	0	210.9	86.4	90.3	hypothetical protein ARALYDRAFT_358659	gbpln	Arabidopsis lyrata	AT5G64550.1 | Symbols: | loricrin-related | chr5:25801794-25803698 REVERSE LENGTH=634	513	634	0	123.6	86.0	89.7
Rsa1.0_00071.1.g3760.t1	gb|AAD12028.1| putative non-LTR retroelement reverse transcriptase [Arabidopsis thaliana]	331	1447	5.00E-37	437.2	30.5	50.5	putative non-LTR retroelement reverse transcriptase	gbpln	Arabidopsis thaliana	AT5G18880.1 | Symbols: | RNA-directed DNA polymerase (reverse transcriptase)-related family protein | chr5:6300535-6301422 REVERSE LENGTH=295	331	295	1.00E-32	89.1	26.9	45.0
Rsa1.0_00071.1.g3761.t1	gb|EOA12936.1| hypothetical protein CARUB_v10025915mg [Capsella rubella]	778	784	0	100.8	89.2	94.0	hypothetical protein CARUB_v10025915mg	gbpln	Capsella rubella	AT5G64570.1 | Symbols: XYL4, ATBXL4 | beta-D-xylosidase 4 | chr5:25810227-25813309 REVERSE LENGTH=784	778	784	0	100.8	87.4	93.2
Rsa1.0_00071.1.g3762.t1	ref|XP_002864909.1| hypothetical protein ARALYDRAFT_496672 [Arabidopsis lyrata subsp. lyrata] gi|297310744|gb|EFH41168.1| hypothetical protein ARALYDRAFT_496672 [Arabidopsis lyrata subsp. lyrata]	639	521	0	81.5	66.7	70.6	hypothetical protein ARALYDRAFT_496672	gbpln	Arabidopsis lyrata	AT5G64600.1 | Symbols: | O-fucosyltransferase family protein | chr5:25825178-25827931 FORWARD LENGTH=522	639	522	0	81.7	66.0	69.8
Rsa1.0_00071.1.g3763.t1	gb|EOA13365.1| hypothetical protein CARUB_v10026402mg [Capsella rubella]	438	445	0	101.6	92.7	95.4	hypothetical protein CARUB_v10026402mg	gbpln	Capsella rubella	AT5G64610.1 | Symbols: HAM1 | histone acetyltransferase of the MYST family 1 | chr5:25828333-25830503 REVERSE LENGTH=445	438	445	0	101.6	92.2	94.5
Rsa1.0_00071.1.g3764.t2	gb|AAC28221.1| similar to reverse transcriptases (PFam: rvt.hmm, score: 60.13) [Arabidopsis thaliana]	728	1164	1.00E-110	159.9	26.6	40.5	similar to reverse transcriptases (PFam: rvt.hmm, score: 60.13)	gbpln	Arabidopsis thaliana	AT5G64620.1 | Symbols: C/VIF2, ATC/VIF2 | cell wall / vacuolar inhibitor of fructosidase 2 | chr5:25831875-25832417 FORWARD LENGTH=180	728	180	7.00E-61	24.7	15.9	17.2
Rsa1.0_00071.1.g3765.t1	ref|XP_002864912.1| pectinesterase family protein [Arabidopsis lyrata subsp. lyrata] gi|297310747|gb|EFH41171.1| pectinesterase family protein [Arabidopsis lyrata subsp. lyrata]	570	602	0	105.6	85.3	91.6	pectinesterase family protein	gbpln	Arabidopsis lyrata	AT5G64640.1 | Symbols: | Plant invertase/pectin methylesterase inhibitor superfamily | chr5:25836820-25839053 FORWARD LENGTH=602	570	602	0	105.6	84.2	91.1

Rsa1.0_00071.1.g3766.t1	gb|EOA21717.1| hypothetical protein CARUB_v10002154mg [Capsella rubella]	161	160	9.00E-81	99.4	91.3	93.8	hypothetical protein CARUB_v10002154mg	gbpln	Capsella rubella	AT5G64650.1 | Symbols: | Ribosomal protein L17 family protein | chr5:25839542-25840775 REVERSE LENGTH=160	161	160	7.00E-82	99.4	90.1	93.2
Rsa1.0_00071.1.g3767.t1	ref|XP_002866628.1| ribosomal protein L15 family protein [Arabidopsis lyrata subsp. lyrata] gi|297312463|gb|EFH42887.1| ribosomal protein L15 family protein [Arabidopsis lyrata subsp. lyrata]	286	281	1.00E-145	98.3	91.3	93.7	ribosomal protein L15 family protein	gbpln	Arabidopsis lyrata	AT5G64670.1 | Symbols: | Ribosomal protein L18e/L15 superfamily protein | chr5:25852535-25853880 REVERSE LENGTH=281	286	281	2.33E-156	98.3	90.6	93.4
Rsa1.0_00071.1.g3768.t1	ref|NP_201273.1| uncharacterized protein [Arabidopsis thaliana] gi|30698059|ref|NP_851270.1| uncharacterized protein [Arabidopsis thaliana] gi|10178071|dbj|BAB11435.1| unnamed protein product [Arabidopsis thaliana] gi|26452974|dbj|BAC43563.1| unknown protein [Arabidopsis thaliana] gi|28973531|gb|AAO64090.1| unknown protein [Arabidopsis thaliana] gi|332010553|gb|AED97936.1| uncharacterized protein AT5G64680 [Arabidopsis thaliana] gi|332010554|gb|AED97937.1| uncharacterized protein AT5G64680 [Arabidopsis thaliana]	145	203	1.00E-21	140.0	49.0	62.1	uncharacterized protein	gbpln	Arabidopsis thaliana	AT5G64680.2 | Symbols: | unknown protein; FUNCTIONS IN: molecular_function unknown; INVOLVED IN: biological_process unknown; LOCATED IN: nucleolus; EXPRESSED IN: 23 plant structures; EXPRESSED DURING: 13 growth stages; Has 1807 Blast hits to 1807 proteins in 277 species: Archae - 0; Bacteria - 0; Metazoa - 736; Fungi - 347; Plants - 385; Viruses - 0; Other Eukaryotes - 339 (source: NCBI BLink). | chr5:25854433-25855331 FORWARD LENGTH=203	145	203	3.00E-24	140.0	49.0	62.1
Rsa1.0_00071.1.g3769.t1	ref|XP_002866629.1| hypothetical protein ARALYDRAFT_332687 [Arabidopsis lyrata subsp. lyrata] gi|297312464|gb|EFH42888.1| hypothetical protein ARALYDRAFT_332687 [Arabidopsis lyrata subsp. lyrata]	109	95	8.00E-33	87.2	72.5	79.8	hypothetical protein ARALYDRAFT_332687	gbpln	Arabidopsis lyrata	AT5G09830.1 | Symbols: | BolA-like family protein | chr5:3057816-3058769 REVERSE LENGTH=93	109	93	3.00E-32	85.3	64.2	72.5
Rsa1.0_00071.1.g3770.t1	gb|EOA12539.1| hypothetical protein CARUB_v10026529mg [Capsella rubella]	399	400	6.00E-94	100.3	63.9	76.2	hypothetical protein CARUB_v10026529mg	gbpln	Capsella rubella	AT5G64690.1 | Symbols: | neurofilament triplet H protein-related | chr5:25862969-25864521 FORWARD LENGTH=344	399	344	3.00E-75	86.2	53.1	66.7
Rsa1.0_00071.1.g3771.t1	ref|NP_201275.1| nodulin MtN21 /EamA-like transporter family protein [Arabidopsis thaliana] gi|75170420|sp|Q9FGG3.1|WTR45_ARATH RecName: Full=WAT1-related protein At5g64700 gi|10177201|dbj|BAB10303.1| nodulin-like protein [Arabidopsis thaliana] gi|332010557|gb|AED97940.1| nodulin MtN21 /EamA-like transporter family protein [Arabidopsis thaliana]	354	359	1.00E-163	101.4	87.9	92.4	nodulin MtN21 /EamA-like transporter family protein	gbpln	Arabidopsis thaliana	AT5G64700.1 | Symbols: | nodulin MtN21 /EamA-like transporter family protein | chr5:25865190-25866845 REVERSE LENGTH=359	354	359	1.00E-166	101.4	87.9	92.4
Rsa1.0_00071.1.g3772.t1	gb|EOA14833.1| hypothetical protein CARUB_v10028143mg [Capsella rubella]	157	158	2.00E-64	100.6	75.2	86.0	hypothetical protein CARUB_v10028143mg	gbpln	Capsella rubella	AT5G64720.1 | Symbols: | Protein of unknown function (DUF1278) | chr5:25872346-25872813 REVERSE LENGTH=155	157	155	3.00E-66	98.7	73.9	85.4
Rsa1.0_00071.1.g3773.t1	#	#	#	#	#	#	#	-	----	----	#	#	#	#	#	#	#
Rsa1.0_00071.1.g3774.t1	gb|AAG50806.1|AC079281_8 unknown protein [Arabidopsis thaliana]	1770	1213	0	68.5	37.9	48.1	unknown protein	gbpln	Arabidopsis thaliana	AT1G60720.1 | Symbols: | RNA-directed DNA polymerase (reverse transcriptase)-related family protein | chr1:22357072-22357941 FORWARD LENGTH=289	1770	289	2.00E-79	16.3	8.0	10.5
Rsa1.0_00071.1.g3775.t2	gb|ACS68196.1| cellulose synthase 6.1 catalytic subunit [Brassica napus]	1082	1084	0	100.2	99.1	99.4	cellulose synthase 6.1 catalytic subunit	gbpln	Brassica napus	AT5G64740.1 | Symbols: CESA6, IXR2, E112, PRC1 | cellulose synthase 6 | chr5:25881555-25886333 FORWARD LENGTH=1084	1082	1084	0	100.2	95.7	98.0
Rsa1.0_00071.1.g3776.t1	gb|AFI57747.1| ethylene-responsive transcription factor ABR1 [Brassica juncea]	318	381	1.00E-106	119.8	70.4	77.7	ethylene-responsive transcription factor ABR1	gbpln	Brassica juncea	AT5G64750.1 | Symbols: ABR1 | Integrase-type DNA-binding superfamily protein | chr5:25891679-25893656 FORWARD LENGTH=391	318	391	1.00E-104	123.0	78.6	83.0
Rsa1.0_00071.1.g3777.t3	dbj|BAF00918.1| putative reverse transcriptase [Arabidopsis thaliana]	1164	910	0	78.2	28.0	37.5	putative reverse transcriptase	gbpln	Arabidopsis thaliana	AT1G43760.1 | Symbols: | DNAse I-like superfamily protein | chr1:16528880-16531065 REVERSE LENGTH=626	1164	626	2.00E-68	53.8	10.7	15.6
Rsa1.0_00071.1.g3778.t1	ref|XP_002864811.1| hypothetical protein ARALYDRAFT_358470 [Arabidopsis lyrata subsp. lyrata] gi|297310646|gb|EFH41070.1| hypothetical protein ARALYDRAFT_358470 [Arabidopsis lyrata subsp. lyrata]	348	556	2.00E-78	159.8	47.7	56.9	hypothetical protein ARALYDRAFT_358470	gbpln	Arabidopsis lyrata	AT5G62660.1 | Symbols: | F-box and associated interaction domains-containing protein | chr5:25156326-25157465 REVERSE LENGTH=379	348	379	9.00E-74	108.9	45.7	54.6
Rsa1.0_00071.1.g3779.t1	ref|XP_002864921.1| glycosyl hydrolase family 17 protein [Arabidopsis lyrata subsp. lyrata] gi|297310756|gb|EFH41180.1| glycosyl hydrolase family 17 protein [Arabidopsis lyrata subsp. lyrata]	485	485	0	100.0	87.0	92.0	glycosyl hydrolase family 17 protein	gbpln	Arabidopsis lyrata	AT5G64790.1 | Symbols: | O-Glycosyl hydrolases family 17 protein | chr5:25902713-25904559 FORWARD LENGTH=485	485	485	0	100.0	85.8	92.4

Rsa1.0_00071.1.g3780.t1	ref|XP_002864923.1| WRKY DNA-binding protein 51 [Arabidopsis lyrata subsp. lyrata] gi|297310758|gb|EFH41182.1| WRKY DNA-binding protein 51 [Arabidopsis lyrata subsp. lyrata]	166	194	2.00E-68	116.9	81.3	84.9	WRKY DNA-binding protein 51	gbpln	Arabidopsis lyrata	AT5G64810.1 | Symbols: WRKY51, ATWRKY51 | WRKY DNA-binding protein 51 | chr5:25908415-25909687 FORWARD LENGTH=194	166	194	8.00E-70	116.9	79.5	84.9
Rsa1.0_00071.1.g3781.t1	ref|NP_568996.1| Ras-related small GTP-binding family protein [Arabidopsis thaliana] gi|29839609|sp|Q9C5J9.1|Y5483_ARATH RecName: Full=Uncharacterized GTP-binding protein At5g64813 gi|13430582|gb|AAK25913.1|AF360203_1 unknown protein [Arabidopsis thaliana] gi|14532852|gb|AAK64108.1| unknown protein [Arabidopsis thaliana] gi|332010571|gb|AED97954.1| Ras-related small GTP-binding family protein [Arabidopsis thaliana]	323	342	1.00E-165	105.9	92.9	96.3	Ras-related small GTP-binding family protein	gbpln	Arabidopsis thaliana	AT5G64813.1 | Symbols: LIP1 | Ras-related small GTP-binding family protein | chr5:25910836-25912625 FORWARD LENGTH=342	323	342	1.00E-168	105.9	92.9	96.3
Rsa1.0_00071.1.g3782.t1	#	#	#	#	#	#	#	-	----	----	#	#	#	#	#	#	#
Rsa1.0_00071.1.g3783.t1	ref|XP_002864926.1| hypothetical protein ARALYDRAFT_496705 [Arabidopsis lyrata subsp. lyrata] gi|297310761|gb|EFH41185.1| hypothetical protein ARALYDRAFT_496705 [Arabidopsis lyrata subsp. lyrata]	141	114	3.00E-40	80.9	63.1	65.2	hypothetical protein ARALYDRAFT_496705	gbpln	Arabidopsis lyrata	AT5G64850.1 | Symbols: | FUNCTIONS IN: molecular_function unknown; INVOLVED IN: biological_process unknown; LOCATED IN: plasma membrane; EXPRESSED IN: 22 plant structures; EXPRESSED DURING: 13 growth stages; CONTAINS InterPro DOMAIN/s: RPM1-interacting protein 4, defence response (InterPro:IPR008700); BEST Arabidopsis thaliana protein match is: unknown protein (TAIR:AT5G09960.1); Has 30201 Blast hits to 17322 proteins in 780 species: Archae - 12; Bacteria - 1396; Metazoa - 17338; Fungi - 3422; Plants - 5037; Viruses - 0; Other Eukaryotes - 2996 (source: NCBI BLink). | chr5:25921585-25923038 FORWARD LENGTH=114	141	114	1.00E-41	80.9	61.7	65.2
Rsa1.0_00071.1.g3784.t3	ref|NP_201291.1| 4-alpha-glucanotransferase-like protein [Arabidopsis thaliana] gi|75180614|sp|Q9LV91.1|DPE1_ARATH RecName: Full=4-alpha-glucanotransferase DPE1, chloroplastic/amyloplastic; AltName: Full=Amylomaltase; AltName: Full=Disproportionating enzyme; Short=D-enzyme; AltName: Full=Protein DISPROPORTIONATING ENZYME 1; Flags: Precursor gi|8843750|dbj|BAA97298.1| 4-alpha-glucanotransferase [Arabidopsis thaliana] gi|14335104|gb|AAK59831.1| AT5g64860/MXK3_9 [Arabidopsis thaliana] gi|19548067|gb|AAL87397.1| AT5g64860/MXK3_9 [Arabidopsis thaliana] gi|332010579|gb|AED97962.1| 4-alpha-glucanotransferase DPE1 [Arabidopsis thaliana]	579	576	0	99.5	85.3	89.8	4-alpha-glucanotransferase-like protein	gbpln	Arabidopsis thaliana	AT5G64860.1 | Symbols: DPE1 | disproportionating enzyme | chr5:25925373-25928788 REVERSE LENGTH=576	579	576	0	99.5	85.3	89.8
Rsa1.0_00071.1.g3785.t1	gb|EOA14807.1| hypothetical protein CARUB_v10028114mg [Capsella rubella]	297	335	1.00E-102	112.8	73.4	80.5	hypothetical protein CARUB_v10028114mg	gbpln	Capsella rubella	AT5G64920.1 | Symbols: CIP8 | COP1-interacting protein 8 | chr5:25944338-25945342 REVERSE LENGTH=334	297	334	2.00E-98	112.5	76.4	82.2
Rsa1.0_00071.1.g3786.t1	gb|EOA13275.1| hypothetical protein CARUB_v10026304mg [Capsella rubella]	483	486	0	100.6	88.0	94.0	hypothetical protein CARUB_v10026304mg	gbpln	Capsella rubella	AT5G65380.1 | Symbols: | MATE efflux family protein | chr5:26123241-26126352 REVERSE LENGTH=486	483	486	0	100.6	87.2	93.0
Rsa1.0_00071.1.g3787.t1	#	#	#	#	#	#	#	-	----	----	#	#	#	#	#	#	#
Rsa1.0_00071.1.g3788.t1	ref|XP_002866670.1| predicted protein [Arabidopsis lyrata subsp. lyrata] gi|297312505|gb|EFH42929.1| predicted protein [Arabidopsis lyrata subsp. lyrata]	288	305	1.00E-137	105.9	84.7	90.3	predicted protein	gbpln	Arabidopsis lyrata	AT5G65420.1 | Symbols: CYCD4;1 | CYCLIN D4;1 | chr5:26141592-26143750 REVERSE LENGTH=308	288	308	1.00E-134	106.9	84.4	88.9
Rsa1.0_00071.1.g3789.t1	gb|EOA13890.1| hypothetical protein CARUB_v10026999mg [Capsella rubella]	248	248	1.00E-136	100.0	94.4	98.0	hypothetical protein CARUB_v10026999mg	gbpln	Capsella rubella	AT5G65430.1 | Symbols: GRF8, 14-3-3KAPPA, GF14 KAPPA | general regulatory factor 8 | chr5:26148546-26150255 REVERSE LENGTH=248	248	248	1.00E-138	100.0	94.4	98.0

Rsa1.0_00071.1.g3790.t1	gb|EOA12401.1| hypothetical protein CARUB_v10027313mg [Capsella rubella]	165	149	1.00E-30	90.3	53.3	60.0	hypothetical protein CARUB_v10027313mg	gbpln	Capsella rubella	AT5G65480.1 | Symbols: | unknown protein; FUNCTIONS IN: molecular_function unknown; INVOLVED IN: biological_process unknown; LOCATED IN: chloroplast; EXPRESSED IN: 24 plant structures; EXPRESSED DURING: 15 growth stages; BEST Arabidopsis thaliana protein match is: unknown protein (TAIR:AT4G38060.2); Has 1807 Blast hits to 1807 proteins in 277 species: Archae - 0; Bacteria - 0; Metazoa - 736; Fungi - 347; Plants - 385; Viruses - 0; Other Eukaryotes - 339 (source: NCBI BLink). | chr5:26176827-26177434 FORWARD LENGTH=153	165	153	2.00E-31	92.7	49.1	53.3
Rsa1.0_00071.1.g3791.t1	gb|EOA12374.1| hypothetical protein CARUB_v10025893mg, partial [Capsella rubella]	788	825	0	104.7	84.0	91.9	hypothetical protein CARUB_v10025893mg, partial	gbpln	Capsella rubella	AT5G65500.1 | Symbols: | U-box domain-containing protein kinase family protein | chr5:26181093-26183997 REVERSE LENGTH=791	788	791	0	100.4	81.6	90.1
Rsa1.0_00071.1.g3792.t1	ref|NP_201355.1| tetratricopeptide repeat domain-containing protein [Arabidopsis thaliana] gi|8978280|dbj|BAA98171.1| unnamed protein product [Arabidopsis thaliana] gi|26453066|dbj|BAC43609.1| unknown protein [Arabidopsis thaliana] gi|28973455|gb|AAO64052.1| unknown protein [Arabidopsis thaliana] gi|332010683|gb|AED98066.1| tetratricopeptide repeat domain-containing protein [Arabidopsis thaliana]	181	206	9.00E-94	113.8	93.4	97.8	tetratricopeptide repeat domain-containing protein	gbpln	Arabidopsis thaliana	AT5G65520.1 | Symbols: | Tetratricopeptide repeat (TPR)-like superfamily protein | chr5:26189876-26190496 REVERSE LENGTH=206	181	206	4.00E-96	113.8	93.4	97.8
Rsa1.0_00071.1.g3793.t1	dbj|BAJ34054.1| unnamed protein product [Thellungiella halophila]	153	650	1.00E-16	424.8	35.3	39.2	unnamed protein product	----	----	AT5G02500.1 | Symbols: HSC70-1, HSP70-1, AT-HSC70-1, HSC70 | heat shock cognate protein 70-1 | chr5:554055-556334 REVERSE LENGTH=651	153	651	2.00E-18	425.5	33.3	37.3
Rsa1.0_00071.1.g3794.t2	ref|NP_201353.4| U-box domain-containing protein kinase family protein [Arabidopsis thaliana] gi|332010681|gb|AED98064.1| U-box domain-containing protein kinase family protein [Arabidopsis thaliana]	180	791	1.00E-12	439.4	20.0	23.3	U-box domain-containing protein kinase family protein	gbpln	Arabidopsis thaliana	AT5G65500.1 | Symbols: | U-box domain-containing protein kinase family protein | chr5:26181093-26183997 REVERSE LENGTH=791	180	791	4.00E-15	439.4	20.0	23.3
Rsa1.0_00071.1.g3795.t1	#	#	#	#	#	#	#	-	----	----	#	#	#	#	#	#	#
Rsa1.0_00071.1.g3796.t1	#	#	#	#	#	#	#	-	----	----	#	#	#	#	#	#	#
Rsa1.0_00071.1.g3797.t1	ref|XP_002864981.1| hypothetical protein ARALYDRAFT_496816 [Arabidopsis lyrata subsp. lyrata] gi|297310816|gb|EFH41240.1| hypothetical protein ARALYDRAFT_496816 [Arabidopsis lyrata subsp. lyrata]	361	374	1.00E-169	103.6	86.4	89.2	hypothetical protein ARALYDRAFT_496816	gbpln	Arabidopsis lyrata	AT5G65790.1 | Symbols: ATMYB68, MYB68 | myb domain protein 68 | chr5:26323035-26324385 FORWARD LENGTH=374	361	374	1.00E-164	103.6	85.9	88.4
Rsa1.0_00071.1.g3798.t1	ref|XP_002866689.1| hypothetical protein ARALYDRAFT_332796 [Arabidopsis lyrata subsp. lyrata] gi|297312524|gb|EFH42948.1| hypothetical protein ARALYDRAFT_332796 [Arabidopsis lyrata subsp. lyrata]	470	470	0	100.0	96.2	98.3	hypothetical protein ARALYDRAFT_332796	gbpln	Arabidopsis lyrata	AT5G65800.1 | Symbols: ACS5, CIN5, ETO2, ATACS5 | ACC synthase 5 | chr5:26331096-26332698 REVERSE LENGTH=470	470	470	0	100.0	96.2	98.7
Rsa1.0_00071.1.g3799.t1	gb|AAM65141.1| unknown [Arabidopsis thaliana]	261	258	1.00E-135	98.9	88.5	96.2	unknown	gbpln	Arabidopsis thaliana	AT5G65810.1 | Symbols: | unknown protein; BEST Arabidopsis thaliana protein match is: unknown protein (TAIR:AT3G49720.2); Has 30201 Blast hits to 17322 proteins in 780 species: Archae - 12; Bacteria - 1396; Metazoa - 17338; Fungi - 3422; Plants - 5037; Viruses - 0; Other Eukaryotes - 2996 (source: NCBI BLink). | chr5:26337833-26339144 REVERSE LENGTH=258	261	258	1.00E-137	98.9	88.1	95.8
Rsa1.0_00071.1.g3800.t1	gb|EOA24531.1| hypothetical protein CARUB_v10017786mg [Capsella rubella]	281	282	1.00E-107	100.4	73.3	81.1	hypothetical protein CARUB_v10017786mg	gbpln	Capsella rubella	AT5G65830.1 | Symbols: ATRLP57, RLP57 | receptor like protein 57 | chr5:26342396-26343235 REVERSE LENGTH=279	281	279	1.00E-106	99.3	75.1	84.3
Rsa1.0_00071.1.g3801.t1	ref|XP_002866692.1| hypothetical protein ARALYDRAFT_496819 [Arabidopsis lyrata subsp. lyrata] gi|297312527|gb|EFH42951.1| hypothetical protein ARALYDRAFT_496819 [Arabidopsis lyrata subsp. lyrata]	267	265	1.00E-115	99.3	77.2	85.0	hypothetical protein ARALYDRAFT_496819	gbpln	Arabidopsis lyrata	AT5G65840.1 | Symbols: | Thioredoxin superfamily protein | chr5:26344097-26346057 REVERSE LENGTH=275	267	275	1.00E-114	103.0	76.4	84.3

Rsa1.0_00071.1.g3802.t1	ref|NP_201393.1| U-box domain-containing protein 31 [Arabidopsis thaliana] gi|75170648|sp|Q9FHN9.1|PUB31_ARATH RecName: Full=U-box domain-containing protein 31; AltName: Full=Plant U-box protein 31 gi|9759576|dbj|BAB11139.1| unnamed protein product [Arabidopsis thaliana] gi|332010740|gb|AED98123.1| U-box domain-containing protein 31 [Arabidopsis thaliana]	442	444	0	100.5	84.2	90.0	U-box domain-containing protein 31	gbpln	Arabidopsis thaliana	AT5G65920.1 | Symbols: | ARM repeat superfamily protein | chr5:26364323-26365657 REVERSE LENGTH=444	442	444	0	100.5	84.2	90.0
Rsa1.0_00071.1.g3803.t1	ref|NP_201563.1| uncharacterized protein [Arabidopsis thaliana] gi|9757883|dbj|BAB08470.1| unnamed protein product [Arabidopsis thaliana] gi|32815941|gb|AAP88355.1| At5g67620 [Arabidopsis thaliana] gi|110736456|dbj|BAF00196.1| hypothetical protein [Arabidopsis thaliana] gi|332010985|gb|AED98368.1| uncharacterized protein AT5G67620 [Arabidopsis thaliana]	177	182	3.00E-73	102.8	83.6	90.4	uncharacterized protein	gbpln	Arabidopsis thaliana	AT5G67620.1 | Symbols: | unknown protein; FUNCTIONS IN: molecular_function unknown; INVOLVED IN: N-terminal protein myristoylation; LOCATED IN: microtubule; BEST Arabidopsis thaliana protein match is: unknown protein (TAIR:AT5G62900.1); Has 1807 Blast hits to 1807 proteins in 277 species: Archae - 0; Bacteria - 0; Metazoa - 736; Fungi - 347; Plants - 385; Viruses - 0; Other Eukaryotes - 339 (source: NCBI BLink). | chr5:26964891-26965720 REVERSE LENGTH=182	177	182	1.00E-75	102.8	83.6	90.4
Rsa1.0_00071.1.g3804.t1	ref|XP_002866696.1| hypothetical protein ARALYDRAFT_358798 [Arabidopsis lyrata subsp. lyrata] gi|297312531|gb|EFH42955.1| hypothetical protein ARALYDRAFT_358798 [Arabidopsis lyrata subsp. lyrata]	156	154	2.00E-75	98.7	91.0	94.2	hypothetical protein ARALYDRAFT_358798	gbpln	Arabidopsis lyrata	AT5G67590.1 | Symbols: FRO1 | NADH-ubiquinone oxidoreductase-related | chr5:26958073-26959356 FORWARD LENGTH=154	156	154	2.00E-75	98.7	88.5	92.3
Rsa1.0_00071.1.g3805.t1	gb|EOA13780.1| hypothetical protein CARUB_v10026874mg [Capsella rubella]	115	299	1.00E-30	260.0	70.4	75.7	hypothetical protein CARUB_v10026874mg	gbpln	Capsella rubella	AT5G67580.2 | Symbols: TRB2, ATTRB2, TBP3, ATTBP3 | Homeodomain-like/winged-helix DNA-binding family protein | chr5:26955843-26957073 REVERSE LENGTH=299	115	299	4.00E-27	260.0	65.2	70.4
Rsa1.0_00071.1.g3806.t1	ref|NP_201556.2| uncharacterized protein [Arabidopsis thaliana] gi|91807108|gb|ABE66281.1| unknown [Arabidopsis thaliana] gi|332010976|gb|AED98359.1| uncharacterized protein AT5G67550 [Arabidopsis thaliana]	509	509	0	100.0	81.9	89.8	uncharacterized protein	gbpln	Arabidopsis thaliana	AT5G67550.1 | Symbols: | unknown protein; FUNCTIONS IN: molecular_function unknown; INVOLVED IN: biological_process unknown; LOCATED IN: endomembrane system; EXPRESSED IN: flower; EXPRESSED DURING: 4 anthesis; BEST Arabidopsis thaliana protein match is: unknown protein (TAIR:AT1G71110.1); Has 161 Blast hits to 154 proteins in 16 species: Archae - 0; Bacteria - 0; Metazoa - 0; Fungi - 0; Plants - 161; Viruses - 0; Other Eukaryotes - 0 (source: NCBI BLink). | chr5:26946908-26949112 REVERSE LENGTH=509	509	509	0	100.0	81.9	89.8
Rsa1.0_00071.1.g3807.t1	ref|XP_002863741.1| hypothetical protein ARALYDRAFT_917455 [Arabidopsis lyrata subsp. lyrata] gi|297309576|gb|EFH40000.1| hypothetical protein ARALYDRAFT_917455 [Arabidopsis lyrata subsp. lyrata]	136	390	7.00E-27	286.8	44.9	61.8	hypothetical protein ARALYDRAFT_917455	gbpln	Arabidopsis lyrata	AT5G18880.1 | Symbols: | RNA-directed DNA polymerase (reverse transcriptase)-related family protein | chr5:6300535-6301422 REVERSE LENGTH=295	136	295	6.00E-12	216.9	32.4	50.7
Rsa1.0_00071.1.g3808.t1	gb|AAW22622.1| porin-like protein [Brassica napus]	276	276	1.00E-152	100.0	97.5	98.9	porin-like protein	gbpln	Brassica napus	AT5G67500.1 | Symbols: VDAC2, ATVDAC2 | voltage dependent anion channel 2 | chr5:26935223-26937123 FORWARD LENGTH=276	276	276	1.00E-144	100.0	89.9	95.7
Rsa1.0_00071.1.g3809.t1	gb|AAB53260.1| zinc-finger protein-1 [Brassica rapa subsp. oleifera]	236	235	5.00E-86	99.6	79.2	84.3	zinc-finger protein-1	gbpln	Brassica rapa	AT5G67450.1 | Symbols: AZF1, ZF1 | zinc-finger protein 1 | chr5:26919142-26919879 REVERSE LENGTH=245	236	245	1.00E-82	103.8	76.3	79.7

Rsa1.0_00072.1.g3810.t1	ref|NP_564548.1| uncharacterized protein [Arabidopsis thaliana] gi|5430751|gb|AAD43151.1|AC007504_6 Unknown protein [Arabidopsis thaliana] gi|13926179|gb|AAK49571.1|AF370563_1 Unknown protein [Arabidopsis thaliana] gi|17381288|gb|AAL36062.1| At1g49500/F13F21_6 [Arabidopsis thaliana] gi|20453373|gb|AAM19925.1| At1g49500/F13F21_6 [Arabidopsis thaliana] gi|20466376|gb|AAM20505.1| unknown protein [Arabidopsis thaliana] gi|21592836|gb|AAM64786.1| unknown [Arabidopsis thaliana] gi|22136326|gb|AAM91241.1| unknown protein [Arabidopsis thaliana] gi|25083427|gb|AAN72075.1| expressed protein [Arabidopsis thaliana] gi|332194315|gb|AEE32436.1| uncharacterized protein AT1G49500 [Arabidopsis thaliana]	63	63	2.00E-21	100.0	79.4	93.7	uncharacterized protein	gbpln	Arabidopsis thaliana	AT1G49500.1 | Symbols: | unknown protein; FUNCTIONS IN: molecular_function unknown; INVOLVED IN: biological_process unknown; LOCATED IN: endomembrane system; EXPRESSED IN: 19 plant structures; EXPRESSED DURING: 10 growth stages; BEST Arabidopsis thaliana protein match is: unknown protein (TAIR:AT3G19030.1); Has 24 Blast hits to 24 proteins in 2 species: Archae - 0; Bacteria - 0; Metazoa - 0; Fungi - 0; Plants - 24; Viruses - 0; Other Eukaryotes - 0 (source: NCBI BLink). | chr1:18321988-18322179 REVERSE LENGTH=63	63	63	4.00E-24	100.0	79.4	93.7
Rsa1.0_00072.1.g3811.t1	ref|XP_002893547.1| peptidase C1A papain family protein [Arabidopsis lyrata subsp. lyrata] gi|297339389|gb|EFH69806.1| peptidase C1A papain family protein [Arabidopsis lyrata subsp. lyrata]	106	345	1.00E-39	325.5	70.8	81.1	peptidase C1A papain family protein	gbpln	Arabidopsis lyrata	AT1G29080.1 | Symbols: | Papain family cysteine protease | chr1:10157494-10158674 REVERSE LENGTH=346	106	346	1.00E-40	326.4	67.9	80.2
Rsa1.0_00072.1.g3812.t1	#	#	#	#	#	#	#	-	----	----	#	#	#	#	#	#	#
Rsa1.0_00072.1.g3813.t1	#	#	#	#	#	#	#	-	----	----	#	#	#	#	#	#	#
Rsa1.0_00072.1.g3814.t1	#	#	#	#	#	#	#	-	----	----	#	#	#	#	#	#	#
Rsa1.0_00072.1.g3815.t1	#	#	#	#	#	#	#	-	----	----	AT5G08380.1 | Symbols: AtAGAL1, AGAL1 | alpha-galactosidase 1 | chr5:2694851-2697616 REVERSE LENGTH=410	40	410	1.00E-12	1025.0	75.0	82.5
Rsa1.0_00072.1.g3816.t1	#	#	#	#	#	#	#	-	----	----	#	#	#	#	#	#	#
Rsa1.0_00072.1.g3817.t1	gb|EOA32197.1| hypothetical protein CARUB_v10015453mg [Capsella rubella]	208	552	1.00E-24	265.4	25.0	39.9	hypothetical protein CARUB_v10015453mg	gbpln	Capsella rubella	AT1G52950.1 | Symbols: | Nucleic acid-binding, OB-fold-like protein | chr1:19725483-19728007 FORWARD LENGTH=566	208	566	5.00E-18	272.1	22.1	34.6
Rsa1.0_00072.1.g3818.t1	pir||S65812 RNA-directed DNA polymerase (EC 2.7.7.49) (clone DW15) - Arabidopsis thaliana retrotransposon Ta11-1 gi|976278|gb|AAA75254.1| reverse transcriptase [Arabidopsis thaliana]	409	1333	2.00E-77	325.9	42.1	58.4	RNA-directed DNA polymerase (EC 2.7.7.49) (clone DW15) - Arabidopsis thaliana retrotransposon Ta11-1 gi|976278|gb|AAA75254.1| reverse transcriptase	gbpln	Arabidopsis thaliana	AT1G43760.1 | Symbols: | DNAse I-like superfamily protein | chr1:16528880-16531065 REVERSE LENGTH=626	409	626	2.00E-19	153.1	19.6	32.0
Rsa1.0_00072.1.g3819.t1	ref|XP_002893547.1| peptidase C1A papain family protein [Arabidopsis lyrata subsp. lyrata] gi|297339389|gb|EFH69806.1| peptidase C1A papain family protein [Arabidopsis lyrata subsp. lyrata]	300	345	1.00E-107	115.0	63.0	78.0	peptidase C1A papain family protein	gbpln	Arabidopsis lyrata	AT1G29080.1 | Symbols: | Papain family cysteine protease | chr1:10157494-10158674 REVERSE LENGTH=346	300	346	1.00E-108	115.3	62.7	78.7
Rsa1.0_00072.1.g3820.t1	gb|AAZ41812.1| 01P13-2 [Brassica rapa subsp. pekinensis]	340	558	1.00E-75	164.1	48.2	63.5	01P13-2	gbpln	Brassica rapa	#	#	#	#	#	#	#
Rsa1.0_00072.1.g3821.t1	gb|AAG10812.1|AC018460_6 Putative retroelement polyprotein [Arabidopsis thaliana]	664	1404	0	211.4	64.5	77.0	Putative retroelement polyprotein	gbpln	Arabidopsis thaliana	AT4G23160.1 | Symbols: CRK8 | cysteine-rich RLK (RECEPTOR-like protein kinase) 8 | chr4:12129485-12134086 FORWARD LENGTH=1262	664	1262	2.00E-94	190.1	29.1	40.1
Rsa1.0_00072.1.g3822.t1	ref|XP_002894196.1| nucleotide binding protein [Arabidopsis lyrata subsp. lyrata] gi|297340038|gb|EFH70455.1| nucleotide binding protein [Arabidopsis lyrata subsp. lyrata]	838	839	0	100.1	88.8	93.6	nucleotide binding protein	gbpln	Arabidopsis lyrata	AT1G49540.2 | Symbols: ELP2, AtELP2 | elongator protein 2 | chr1:18333767-18337382 REVERSE LENGTH=840	838	840	0	100.2	87.8	92.6
Rsa1.0_00072.1.g3823.t1	#	#	#	#	#	#	#	-	----	----	#	#	#	#	#	#	#
Rsa1.0_00072.1.g3824.t1	ref|XP_002891521.1| predicted protein [Arabidopsis lyrata subsp. lyrata] gi|297337363|gb|EFH67780.1| predicted protein [Arabidopsis lyrata subsp. lyrata]	628	350	1.00E-176	55.7	48.9	51.9	predicted protein	gbpln	Arabidopsis lyrata	AT1G49570.1 | Symbols: | Peroxidase superfamily protein | chr1:18347077-18348712 FORWARD LENGTH=350	628	350	1.00E-178	55.7	48.9	51.8

Rsa1.0_00072.1.g3825.t1	ref|NP_175381.1| putative calcium-dependent protein kinase [Arabidopsis thaliana] gi|75334455|sp|Q9FX86.1|CAMK8_ARATH RecName: Full=CDPK-related kinase 8; Short=AtCRK8; AltName: Full=Calcium/calmodulin-dependent protein kinase CRK8 gi|10120419|gb|AAG13044.1|AC011807_3 Putative CDPK-related protein kinase [Arabidopsis thaliana] gi|37202008|gb|AAQ89619.1| At1g49580 [Arabidopsis thaliana] gi|110738705|dbj|BAF01277.1| putative CDPK-related protein kinase [Arabidopsis thaliana] gi|332194323|gb|AEE32444.1| CDPK-related kinase 8 [Arabidopsis thaliana]	594	606	0	102.0	89.9	93.8	putative calcium-dependent protein kinase	gbpln	Arabidopsis thaliana	AT1G49580.1 | Symbols: | Calcium-dependent protein kinase (CDPK) family protein | chr1:18351611-18354384 FORWARD LENGTH=606	594	606	0	102.0	89.9	93.8
Rsa1.0_00072.1.g3826.t1	dbj|BAB09912.1| MADS-box protein-like [Arabidopsis thaliana]	347	368	6.00E-47	106.1	32.0	43.5	MADS-box protein-like	gbpln	Arabidopsis thaliana	AT5G49420.1 | Symbols: | MADS-box transcription factor family protein | chr5:20035166-20036170 REVERSE LENGTH=334	347	334	2.00E-49	96.3	32.0	43.5
Rsa1.0_00072.1.g3827.t1	gb|AAG50652.1|AC073433_4 transposase, putative [Arabidopsis thaliana]	81	659	2.00E-23	813.6	66.7	82.7	transposase, putative	gbpln	Arabidopsis thaliana	#	#	#	#	#	#	#
Rsa1.0_00072.1.g3828.t1	#	#	#	#	#	#	#	–	----	----	#	#	#	#	#	#	#
Rsa1.0_00072.1.g3829.t1	gb|ABD65636.1| hypothetical protein 23.t00055 [Brassica oleracea]	215	414	1.00E-17	192.6	20.9	23.7	hypothetical protein 23.t00055	gbpln	Brassica oleracea	#	#	#	#	#	#	#
Rsa1.0_00072.1.g3830.t1	gb|EOA14700.1| hypothetical protein CARUB_v10027975mg [Capsella rubella]	396	766	1.00E-124	193.4	59.8	75.5	hypothetical protein CARUB_v10027975mg	gbpln	Capsella rubella	AT1G19260.1 | Symbols: | TTF-type zinc finger protein with HAT dimerisation domain | chr1:6657260-6659569 REVERSE LENGTH=769	396	769	1.00E-126	194.2	60.6	75.0
Rsa1.0_00072.1.g3831.t1	ref|XP_002894219.1| hypothetical protein ARALYDRAFT_474111 [Arabidopsis lyrata subsp. lyrata] gi|297340061|gb|EFH70478.1| hypothetical protein ARALYDRAFT_474111 [Arabidopsis lyrata subsp. lyrata]	112	102	4.00E-32	91.1	67.0	76.8	hypothetical protein ARALYDRAFT_474111	gbpln	Arabidopsis lyrata	AT1G49800.1 | Symbols: | unknown protein; FUNCTIONS IN: molecular_function unknown; INVOLVED IN: biological_process unknown; LOCATED IN: endomembrane system; EXPRESSED IN: sepal, male gametophyte, stamen; EXPRESSED DURING: 4 anthesis; Has 12 Blast hits to 12 proteins in 5 species: Archae - 0; Bacteria - 0; Metazoa - 0; Fungi - 0; Plants - 12; Viruses - 0; Other Eukaryotes - 0 (source: NCBI BLink). | chr1:18438550-18438876 REVERSE LENGTH=108	112	108	2.00E-29	96.4	65.2	80.4
Rsa1.0_00072.1.g3832.t1	gb|AAP53216.2| retrotransposon protein, putative, Ty1-copia subclass [Oryza sativa Japonica Group]	440	1262	1.00E-64	286.8	35.0	43.6	retrotransposon protein, putative, Ty1-copia subclass	gbpln	Oryza sativa	AT4G23160.1 | Symbols: CRK8 | cysteine-rich RLK (RECEPTOR-like protein kinase) 8 | chr4:12129485-12134086 FORWARD LENGTH=1262	440	1262	2.00E-23	286.8	16.4	24.3
Rsa1.0_00072.1.g3833.t1	emb|CAB39638.1| RNA-directed DNA polymerase-like protein [Arabidopsis thaliana] gi|7267666|emb|CAB78094.1| RNA-directed DNA polymerase-like protein [Arabidopsis thaliana]	1667	1274	0	76.4	29.8	41.6	RNA-directed DNA polymerase-like protein	gbpln	Arabidopsis thaliana	AT4G29090.1 | Symbols: | Ribonuclease H-like superfamily protein | chr4:14333528-14335255 FORWARD LENGTH=575	1667	575	1.00E-48	34.5	6.5	10.4
Rsa1.0_00072.1.g3834.t1	ref|XP_002894216.1| U-box domain-containing protein [Arabidopsis lyrata subsp. lyrata] gi|297340058|gb|EFH70475.1| U-box domain-containing protein [Arabidopsis lyrata subsp. lyrata]	424	421	0	99.3	88.9	95.0	U-box domain-containing protein	gbpln	Arabidopsis lyrata	AT1G49780.1 | Symbols: PUB26 | plant U-box 26 | chr1:18429024-18430289 REVERSE LENGTH=421	424	421	0	99.3	87.7	94.6
Rsa1.0_00072.1.g3835.t1	gb|AAG51754.1|AC068667_33 reverse transcriptase, putative; 100033-105622 [Arabidopsis thaliana]	318	1557	2.00E-15	489.6	17.6	30.5	reverse transcriptase, putative; 100033-105622	gbpln	Arabidopsis thaliana	#	#	#	#	#	#	#
Rsa1.0_00072.1.g3836.t1	ref|NP_178819.1| Zinc knuckle (CCHC-type) family protein [Arabidopsis thaliana] gi|3327395|gb|AAC26677.1| putative Ta11-like non-LTR retroelement protein [Arabidopsis thaliana] gi|330251037|gb|AEC06131.1| Zinc knuckle (CCHC-type) family protein [Arabidopsis thaliana]	338	530	4.00E-42	156.8	23.4	30.5	Zinc knuckle (CCHC-type) family protein	gbpln	Arabidopsis thaliana	AT2G07760.1 | Symbols: | Zinc knuckle (CCHC-type) family protein | chr2:3584420-3586278 REVERSE LENGTH=530	338	530	1.00E-44	156.8	23.4	30.5
Rsa1.0_00072.1.g3837.t1	ref|NP_188563.1| leucine-rich repeat-containing protein [Arabidopsis thaliana] gi|11994454|dbj|BAB02456.1| unnamed protein product [Arabidopsis thaliana] gi|332642702|gb|AEE76223.1| leucine-rich repeat-containing protein [Arabidopsis thaliana]	489	493	1.00E-165	100.8	68.7	77.1	leucine-rich repeat-containing protein	gbpln	Arabidopsis thaliana	AT3G19320.1 | Symbols: | Leucine-rich repeat (LRR) family protein | chr3:6696395-6698073 REVERSE LENGTH=493	489	493	1.00E-168	100.8	68.7	77.1

Rsa1.0_00073.1.g3838.t1	gb|AAC35532.1| contains similarity to proteases [Arabidopsis thaliana]	1351	1392	0	103.0	55.5	70.0	contains similarity to proteases	gbpln	Arabidopsis thaliana	AT4G23160.1 | Symbols: CRK8 | cysteine-rich RLK (RECEPTOR-like protein kinase) 8 | chr4:12129485-12134086 FORWARD LENGTH=1262	1351	1262	1.00E-112	93.4	13.8	22.3
Rsa1.0_00073.1.g3839.t1	ref|NP_001190617.1| K-box region and MADS-box transcription factor family protein [Arabidopsis thaliana] gi|332010614|gb|AED97997.1| K-box region and MADS-box transcription factor family protein [Arabidopsis thaliana]	67	232	7.00E-22	346.3	74.6	89.6	K-box region and MADS-box transcription factor family protein	gbpln	Arabidopsis thaliana	AT5G65070.3 | Symbols: MAF4 | K-box region and MADS-box transcription factor family protein | chr5:25992310-25995930 FORWARD LENGTH=232	67	232	1.00E-24	346.3	74.6	89.6
Rsa1.0_00073.1.g3840.t1	gb|EOA35746.1| hypothetical protein CARUB_v10020979mg [Capsella rubella]	173	197	1.00E-30	113.9	48.0	59.5	hypothetical protein CARUB_v10020979mg	gbpln	Capsella rubella	AT5G65070.1 | Symbols: MAF4, FCL4, AGL69 | K-box region and MADS-box transcription factor family protein | chr5:25992310-25995930 FORWARD LENGTH=200	173	200	4.00E-32	115.6	48.6	58.4
Rsa1.0_00073.1.g3841.t1	#	#	#	#	#	#	#	–	----	----	#	#	#	#	#	#	#
Rsa1.0_00073.1.g3842.t5	gb|EOA12728.1| hypothetical protein CARUB_v10028173mg [Capsella rubella]	211	197	5.00E-72	93.4	69.2	80.1	hypothetical protein CARUB_v10028173mg	gbpln	Capsella rubella	AT5G65070.1 | Symbols: MAF4, FCL4, AGL69 | K-box region and MADS-box transcription factor family protein | chr5:25992310-25995930 FORWARD LENGTH=200	211	200	2.00E-73	94.8	68.2	79.1
Rsa1.0_00073.1.g3843.t2	ref|XP_002864939.1| hypothetical protein ARALYDRAFT_332725 [Arabidopsis lyrata subsp. lyrata] gi|297310774|gb|EFH41198.1| hypothetical protein ARALYDRAFT_332725 [Arabidopsis lyrata subsp. lyrata]	554	527	0	95.1	80.3	85.0	hypothetical protein ARALYDRAFT_332725	gbpln	Arabidopsis lyrata	AT5G65090.1 | Symbols: MRH3, BST1, DER4 | DNAse I-like superfamily protein | chr5:26004282-26006656 FORWARD LENGTH=529	554	529	0	95.5	79.2	84.1
Rsa1.0_00073.1.g3844.t1	ref|XP_002864941.1| hypothetical protein ARALYDRAFT_919839 [Arabidopsis lyrata subsp. lyrata] gi|297310776|gb|EFH41200.1| hypothetical protein ARALYDRAFT_919839 [Arabidopsis lyrata subsp. lyrata]	272	286	5.00E-98	105.1	69.1	80.5	hypothetical protein ARALYDRAFT_919839	gbpln	Arabidopsis lyrata	AT5G65130.1 | Symbols: | Integrase-type DNA-binding superfamily protein | chr5:26017403-26018236 FORWARD LENGTH=277	272	277	6.00E-98	101.8	71.3	80.9
Rsa1.0_00073.1.g3845.t1	gb|EOA13558.1| hypothetical protein CARUB_v10026620mg [Capsella rubella]	375	369	0	98.4	84.5	90.4	hypothetical protein CARUB_v10026620mg	gbpln	Capsella rubella	AT5G65140.1 | Symbols: TPPJ | Haloacid dehalogenase-like hydrolase (HAD) superfamily protein | chr5:26019878-26022077 REVERSE LENGTH=370	375	370	0	98.7	85.3	90.1
Rsa1.0_00073.1.g3846.t1	#	#	#	#	#	#	#	–	----	----	#	#	#	#	#	#	#
Rsa1.0_00073.1.g3847.t1	gb|EOA14828.1| hypothetical protein CARUB_v10028138mg [Capsella rubella]	174	175	1.00E-80	100.6	79.9	85.6	hypothetical protein CARUB_v10028138mg	gbpln	Capsella rubella	AT5G65158.1 | Symbols: | Lipase/lipooxygenase, PLAT/LH2 family protein | chr5:26030390-26030704 FORWARD LENGTH=104	174	104	3.00E-56	59.8	56.9	58.6
Rsa1.0_00073.1.g3848.t1	ref|NP_680465.2| succinate dehydrogenase [ubiquinone] iron-sulfur subunit 3 [Arabidopsis thaliana] gi|75262571|sp|Q9FJP9.1|DHSB3_ARATH RecName: Full=Succinate dehydrogenase [ubiquinone] iron-sulfur subunit 3, mitochondrial; AltName: Full=Iron-sulfur subunit of complex II; Short=Ip; Flags: Precursor gi|10178178|dbj|BAB11652.1| succinate dehydrogenase iron-sulfur protein-like [Arabidopsis thaliana] gi|12049602|emb|CAC19857.1| mitochondrial succinate dehydrogenase iron-sulphur subunit [Arabidopsis thaliana] gi|332010628|gb|AED98011.1| succinate dehydrogenase [ubiquinone] iron-sulfur subunit 3 [Arabidopsis thaliana]	306	309	1.00E-163	101.0	89.5	93.8	succinate dehydrogenase	gbpln	Arabidopsis thaliana	AT5G65165.1 | Symbols: SDH2-3 | succinate dehydrogenase 2-3 | chr5:26034515-26035922 REVERSE LENGTH=309	306	309	1.00E-165	101.0	89.5	93.8
Rsa1.0_00073.1.g3849.t1	ref|NP_201321.1| VQ motif-containing protein [Arabidopsis thaliana] gi|10178179|dbj|BAB11653.1| unnamed protein product [Arabidopsis thaliana] gi|44917435|gb|AAS49042.1| At5g65170 [Arabidopsis thaliana] gi|46931270|gb|AAT06439.1| At5g65170 [Arabidopsis thaliana] gi|332010630|gb|AED98013.1| VQ motif-containing protein [Arabidopsis thaliana]	335	362	1.00E-105	108.1	70.4	78.5	VQ motif-containing protein	gbpln	Arabidopsis thaliana	AT5G65170.1 | Symbols: | VQ motif-containing protein | chr5:26041218-26042306 FORWARD LENGTH=362	335	362	1.00E-107	108.1	70.4	78.5

Rsa1.0_00073.1.g3850.t1	ref|XP_002866656.1| armadillo/beta-catenin repeat family protein [Arabidopsis lyrata subsp. lyrata] gi|297312491|gb|EFH42915.1| armadillo/beta-catenin repeat family protein [Arabidopsis lyrata subsp. lyrata]	544	547	0	100.6	83.3	89.7	armadillo/beta-catenin repeat family protein	gbpln	Arabidopsis lyrata	AT5G65200.1 | Symbols: PUB38, ATPUB38 | plant U-box 38 | chr5:26048173-26049843 REVERSE LENGTH=556	544	556	0	102.2	83.5	90.8
Rsa1.0_00073.1.g3851.t1	ref|XP_002866657.1| short-chain dehydrogenase/reductase family protein [Arabidopsis lyrata subsp. lyrata] gi|297312492|gb|EFH42916.1| short-chain dehydrogenase/reductase family protein [Arabidopsis lyrata subsp. lyrata]	285	285	1.00E-145	100.0	87.4	92.6	short-chain dehydrogenase/reductase family protein	gbpln	Arabidopsis lyrata	AT5G65205.1 | Symbols: | NAD(P)-binding Rossmann-fold superfamily protein | chr5:26050926-26052017 REVERSE LENGTH=285	285	285	1.00E-144	100.0	85.6	91.6
Rsa1.0_00073.1.g3852.t1	ref|NP_680466.1| uncharacterized protein [Arabidopsis thaliana] gi|19347918|gb|AAL85980.1| unknown protein [Arabidopsis thaliana] gi|21689793|gb|AAM67540.1| unknown protein [Arabidopsis thaliana] gi|26453218|dbj|BAC43683.1| unknown protein [Arabidopsis thaliana] gi|332010635|gb|AED98018.1| uncharacterized protein AT5G65207 [Arabidopsis thaliana]	72	73	3.00E-21	101.4	72.2	84.7	uncharacterized protein	gbpln	Arabidopsis thaliana	AT5G65207.1 | Symbols: | unknown protein; BEST Arabidopsis thaliana protein match is: unknown protein (TAIR:AT5G10040.1); Has 30201 Blast hits to 17322 proteins in 780 species: Archae - 12; Bacteria - 1396; Metazoa - 17338; Fungi - 3422; Plants - 5037; Viruses - 0; Other Eukaryotes - 2996 (source: NCBI BLink). | chr5:26052364-26052585 REVERSE LENGTH=73	72	73	4.00E-24	101.4	72.2	84.7
Rsa1.0_00073.1.g3853.t1	#	#	#	#	#	#	#	–	----	----	#	#	#	#	#	#	#
Rsa1.0_00073.1.g3854.t1	gb|ACU32463.1| TGA2 protein [Brassica rapa subsp. pekinensis]	370	367	0	99.2	89.7	93.8	TGA2 protein	gbpln	Brassica rapa	AT5G65210.5 | Symbols: TGA1 | bZIP transcription factor family protein | chr5:26059031-26060749 FORWARD LENGTH=368	370	368	0	99.5	87.6	94.3
Rsa1.0_00073.1.g3855.t1	ref|XP_004288191.1| PREDICTED: 50S ribosomal protein L29, chloroplastic-like [Fragaria vesca subsp. vesca]	94	174	2.00E-16	185.1	45.7	59.6	PREDICTED: 50S ribosomal protein L29, chloroplastic-like	gbpln	Fragaria vesca	AT5G65220.1 | Symbols: | Ribosomal L29 family protein | chr5:26061301-26062506 FORWARD LENGTH=173	94	173	2.00E-18	184.0	47.9	57.4
Rsa1.0_00073.1.g3856.t1	ref|XP_002866660.1| hypothetical protein ARALYDRAFT_496756 [Arabidopsis lyrata subsp. lyrata] gi|297312495|gb|EFH42919.1| hypothetical protein ARALYDRAFT_496756 [Arabidopsis lyrata subsp. lyrata]	279	299	1.00E-108	107.2	77.4	84.6	hypothetical protein ARALYDRAFT_496756	gbpln	Arabidopsis lyrata	AT5G65250.1 | Symbols: | unknown protein; FUNCTIONS IN: molecular_function unknown; INVOLVED IN: biological_process unknown; LOCATED IN: chloroplast; EXPRESSED IN: 22 plant structures; EXPRESSED DURING: 13 growth stages; Has 30201 Blast hits to 17322 proteins in 780 species: Archae - 12; Bacteria - 1396; Metazoa - 17338; Fungi - 3422; Plants - 5037; Viruses - 0; Other Eukaryotes - 2996 (source: NCBI BLink). | chr5:26078699-26080051 REVERSE LENGTH=300	279	300	1.00E-110	107.5	77.1	85.3
Rsa1.0_00073.1.g3857.t1	gb|EOA12395.1| hypothetical protein CARUB_v10027046mg, partial [Capsella rubella]	211	233	1.00E-105	110.4	89.6	93.4	hypothetical protein CARUB_v10027046mg, partial	gbpln	Capsella rubella	AT5G65260.1 | Symbols: | RNA-binding (RRM/RBD/RNP motifs) family protein | chr5:26080501-26082984 REVERSE LENGTH=220	211	220	1.00E-105	104.3	91.5	96.2
Rsa1.0_00073.1.g3858.t1	ref|XP_002864951.1| LMBR1 integral membrane family protein [Arabidopsis lyrata subsp. lyrata] gi|297310786|gb|EFH41210.1| LMBR1 integral membrane family protein [Arabidopsis lyrata subsp. lyrata]	733	733	0	100.0	91.8	95.8	LMBR1 integral membrane family protein	gbpln	Arabidopsis lyrata	AT5G65290.1 | Symbols: | LMBR1-like membrane protein | chr5:26089814-26094337 FORWARD LENGTH=733	733	733	0	100.0	91.8	95.5
Rsa1.0_00073.1.g3859.t1	ref|XP_002866663.1| hypothetical protein ARALYDRAFT_496763 [Arabidopsis lyrata subsp. lyrata] gi|297312498|gb|EFH42922.1| hypothetical protein ARALYDRAFT_496763 [Arabidopsis lyrata subsp. lyrata]	149	150	4.00E-37	100.7	65.1	76.5	hypothetical protein ARALYDRAFT_496763	gbpln	Arabidopsis lyrata	AT5G65300.1 | Symbols: | unknown protein; Has 30201 Blast hits to 17322 proteins in 780 species: Archae - 12; Bacteria - 1396; Metazoa - 17338; Fungi - 3422; Plants - 5037; Viruses - 0; Other Eukaryotes - 2996 (source: NCBI BLink). | chr5:26095266-26095718 REVERSE LENGTH=150	149	150	9.00E-30	100.7	61.7	73.8
Rsa1.0_00073.1.g3860.t1	gb|EOA13730.1| hypothetical protein CARUB_v10026808mg [Capsella rubella]	312	315	1.00E-133	101.0	83.3	88.5	hypothetical protein CARUB_v10026808mg	gbpln	Capsella rubella	AT5G65310.1 | Symbols: ATHB5, ATHB-5, HB5 | homeobox protein 5 | chr5:26102457-26104217 REVERSE LENGTH=312	312	312	1.00E-135	100.0	82.7	89.4
Rsa1.0_00073.1.g3861.t1	gb|EOA14656.1| hypothetical protein CARUB_v10027920mg [Capsella rubella]	257	293	3.00E-86	114.0	68.9	82.1	hypothetical protein CARUB_v10027920mg	gbpln	Capsella rubella	AT5G65320.1 | Symbols: | basic helix-loop-helix (bHLH) DNA-binding superfamily protein | chr5:26107368-26108640 REVERSE LENGTH=296	257	296	1.00E-82	115.2	69.3	80.9
Rsa1.0_00073.1.g3862.t1	ref|NP_201336.1| protein agamous-like 78 [Arabidopsis thaliana] gi|9759613|dbj|BAB11555.1| unnamed protein product [Arabidopsis thaliana] gi|32402412|gb|AAN52788.1| MADS-box protein AGL78 [Arabidopsis thaliana] gi|332010657|gb|AED98040.1| protein agamous-like 78 [Arabidopsis thaliana]	339	341	1.00E-71	100.6	50.7	63.4	protein agamous-like 78	gbpln	Arabidopsis thaliana	AT5G65330.1 | Symbols: AGL78 | AGAMOUS-like 78 | chr5:26110326-26111351 FORWARD LENGTH=341	339	341	3.00E-74	100.6	50.7	63.4
Rsa1.0_00073.1.g3863.t1	gb|AAD32950.1| putative non-LTR retroelement reverse transcriptase [Arabidopsis thaliana]	258	773	3.00E-26	299.6	29.1	43.8	putative non-LTR retroelement reverse transcriptase	gbpln	Arabidopsis thaliana	AT4G29090.1 | Symbols: | Ribonuclease H-like superfamily protein | chr4:14333528-14335255 FORWARD LENGTH=575	258	575	2.00E-15	222.9	29.8	45.7

Rsa1.0_00073.1.g3864.t1	ref|NP_201337.1| uncharacterized protein [Arabidopsis thaliana] gi|9759614|dbj|BAB11556.1| unnamed protein product [Arabidopsis thaliana] gi|34146816|gb|AAQ62416.1| At5g65340 [Arabidopsis thaliana] gi|51969418|dbj|BAD43401.1| putative protein [Arabidopsis thaliana] gi|332010658|gb|AED98041.1| uncharacterized protein AT5G65340 [Arabidopsis thaliana]	251	253	1.00E-118	100.8	86.1	94.0	uncharacterized protein	gbpln	Arabidopsis thaliana	AT5G65340.1 | Symbols: | Protein of unknown function, DUF617 | chr5:26113684-26114445 REVERSE LENGTH=253	251	253	1.00E-121	100.8	86.1	94.0
Rsa1.0_00073.1.g3865.t1	tpg|DAA55311.1| TPA: histone H3.2 [Zea mays]	136	245	1.00E-72	180.1	100.0	100.0	TPA: histone H3.2	gbenv/gbpln	Zea mays	AT5G65360.1 | Symbols: | Histone superfamily protein | chr5:26120099-26120509 REVERSE LENGTH=136	136	136	5.00E-74	100.0	100.0	100.0
Rsa1.0_00073.1.g3866.t1	ref|XP_002864953.1| hypothetical protein ARALYDRAFT_358736 [Arabidopsis lyrata subsp. lyrata] gi|297310788|gb|EFH41212.1| hypothetical protein ARALYDRAFT_358736 [Arabidopsis lyrata subsp. lyrata]	293	295	1.00E-122	100.7	73.0	84.0	hypothetical protein ARALYDRAFT_358736	gbpln	Arabidopsis lyrata	AT5G65370.1 | Symbols: | ENTH/ANTH/VHS superfamily protein | chr5:26121708-26122749 FORWARD LENGTH=295	293	295	1.00E-118	100.7	70.6	80.9
Rsa1.0_00073.1.g3867.t1	gb|EOA13275.1| hypothetical protein CARUB_v10026304mg [Capsella rubella]	301	486	1.00E-139	161.5	84.1	92.0	hypothetical protein CARUB_v10026304mg	gbpln	Capsella rubella	AT5G65380.1 | Symbols: | MATE efflux family protein | chr5:26123241-26126352 REVERSE LENGTH=486	301	486	1.00E-141	161.5	84.1	92.0
Rsa1.0_00073.1.g3868.t1	#	#	#	#	#	#	#	-	----	----	#	#	#	#	#	#	#
Rsa1.0_00073.1.g3869.t1	gb|EOA13872.1| hypothetical protein CARUB_v10026973mg [Capsella rubella]	258	261	1.00E-110	101.2	77.9	86.4	hypothetical protein CARUB_v10026973mg	gbpln	Capsella rubella	AT5G65400.1 | Symbols: | alpha/beta-Hydrolases superfamily protein | chr5:26131448-26133062 FORWARD LENGTH=252	258	252	1.00E-106	97.7	77.5	86.4
Rsa1.0_00073.1.g3870.t1	dbj|BAJ34581.1| unnamed protein product [Thellungiella halophila]	262	286	3.00E-98	109.2	82.4	88.2	unnamed protein product	----	----	AT5G65410.1 | Symbols: ATHB25, ZFHD2, HB25, ZHD1 | homeobox protein 25 | chr5:26136179-26137018 FORWARD LENGTH=279	262	279	8.00E-92	106.5	77.5	83.2
Rsa1.0_00073.1.g3871.t3	gb|AAD22286.1| putative non-LTR retroelement reverse transcriptase [Arabidopsis thaliana]	356	1311	1.00E-28	368.3	33.7	43.3	putative non-LTR retroelement reverse transcriptase	gbpln	Arabidopsis thaliana	AT1G10000.1 | Symbols: | Ribonuclease H-like superfamily protein | chr1:3263877-3264788 REVERSE LENGTH=303	356	303	5.00E-26	85.1	21.1	34.3
Rsa1.0_00073.1.g3872.t1	ref|XP_002864958.1| hypothetical protein ARALYDRAFT_496780 [Arabidopsis lyrata subsp. lyrata] gi|297310793|gb|EFH41217.1| hypothetical protein ARALYDRAFT_496780 [Arabidopsis lyrata subsp. lyrata]	506	504	0	99.6	91.5	94.9	hypothetical protein ARALYDRAFT_496780	gbpln	Arabidopsis lyrata	AT5G65470.1 | Symbols: | O-fucosyltransferase family protein | chr5:26172432-26174904 FORWARD LENGTH=504	506	504	0	99.6	90.7	94.9
Rsa1.0_00073.1.g3873.t1	gb|AAL87313.1| unknown protein [Arabidopsis thaliana]	89	645	9.00E-24	724.7	60.7	64.0	unknown protein	gbpln	Arabidopsis thaliana	AT5G65490.1 | Symbols: | FUNCTIONS IN: molecular_function unknown; INVOLVED IN: biological_process unknown; LOCATED IN: chloroplast; EXPRESSED IN: 22 plant structures; EXPRESSED DURING: 13 growth stages; CONTAINS InterPro DOMAIN/s: SGT1 (InterPro:IPR010770); Has 1807 Blast hits to 1807 proteins in 277 species: Archae - 0; Bacteria - 0; Metazoa - 736; Fungi - 347; Plants - 385; Viruses - 0; Other Eukaryotes - 339 (source: NCBI BLink). | chr5:26178003-26180309 REVERSE LENGTH=643	89	643	1.00E-26	722.5	60.7	64.0
Rsa1.0_00073.1.g3874.t1	gb|EOA30867.1| hypothetical protein CARUB_v10014010mg [Capsella rubella]	403	366	1.00E-148	90.8	65.0	73.9	hypothetical protein CARUB_v10014010mg	gbpln	Capsella rubella	AT3G57640.1 | Symbols: | Protein kinase superfamily protein | chr3:21344921-21345991 REVERSE LENGTH=356	403	356	3.00E-86	88.3	45.9	57.6
Rsa1.0_00073.1.g3875.t1	ref|NP_201356.2| protein kinase family protein [Arabidopsis thaliana] gi|332010684|gb|AED98067.1| protein kinase family protein [Arabidopsis thaliana]	470	456	0	97.0	84.9	90.6	protein kinase family protein	gbpln	Arabidopsis thaliana	AT5G65530.1 | Symbols: | Protein kinase superfamily protein | chr5:26190844-26192826 REVERSE LENGTH=456	470	456	0	97.0	84.9	90.6
Rsa1.0_00073.1.g3876.t1	ref|NP_201362.1| Dof zinc finger protein DOF5.7 [Arabidopsis thaliana] gi|55583993|sp|Q9LSL6.1|DOF57_ARATH RecName: Full=Dof zinc finger protein DOF5.7; Short=AtDOF5.7 gi|8978287|dbj|BAA98178.1| DOF zinc finger protein-like [Arabidopsis thaliana] gi|50198797|gb|AAT70432.1| At5g65590 [Arabidopsis thaliana] gi|52421291|gb|AAU45215.1| At4g35930 [Arabidopsis thaliana] gi|225879166|dbj|BAH30653.1| hypothetical protein [Arabidopsis thaliana] gi|332010691|gb|AED98074.1| Dof zinc finger protein DOF5.7 [Arabidopsis thaliana]	288	316	1.00E-119	109.7	80.6	87.2	Dof zinc finger protein DOF5.7	gbpln	Arabidopsis thaliana	AT5G65590.1 | Symbols: | Dof-type zinc finger DNA-binding family protein | chr5:26212084-26213034 REVERSE LENGTH=316	288	316	1.00E-122	109.7	80.6	87.2

Rsa1.0_00073.1_g3877.t1	#	#	#	#	#	#	#	–	----	----	#	#	#	#	#	#	#
Rsa1.0_00074.1_g3878.t1	dbj|BAB11288.1| unnamed protein product [Arabidopsis thaliana]	955	978	0	102.4	66.0	76.3	unnamed protein product	gbpln	Arabidopsis thaliana	AT1G66880.1 | Symbols: | Protein kinase superfamily protein | chr1:24946928-24955438 FORWARD LENGTH=1296	955	1296	0	135.7	53.5	67.1
Rsa1.0_00074.1_g3879.t1	gb|AAC26241.1| F9D12.15 gene product [Arabidopsis thaliana]	789	850	1.00E-111	107.7	34.5	49.3	F9D12.15 gene product	gbpln	Arabidopsis thaliana	#	#	#	#	#	#	#
Rsa1.0_00074.1_g3880.t1	#	#	#	#	#	#	#	–	----	----	#	#	#	#	#	#	#
Rsa1.0_00074.1_g3881.t1	ref|XP_002870862.1| hypothetical protein ARALYDRAFT_494160 [Arabidopsis lyrata subsp. lyrata] gi|297316698|gb|EFH47121.1| hypothetical protein ARALYDRAFT_494160 [Arabidopsis lyrata subsp. lyrata]	427	436	0	102.1	90.4	96.5	hypothetical protein ARALYDRAFT_494160	gbpln	Arabidopsis lyrata	AT5G38200.1 | Symbols: | Class I glutamine amidotransferase-like superfamily protein | chr5:15256383-15258474 FORWARD LENGTH=436	427	436	0	102.1	89.9	96.3
Rsa1.0_00074.1_g3882.t1	ref|NP_198631.2| plastid movement impaired 15 protein [Arabidopsis thaliana] gi|334302885|sp|Q9FF41.3|PMI15_ARATH RecName: Full=Protein PLASTID MOVEMENT IMPAIRED 15 gi|332006890|gb|AED94273.1| plastid movement impaired 15 protein [Arabidopsis thaliana]	573	579	1.00E-157	101.0	60.9	75.9	plastid movement impaired 15 protein	gbpln	Arabidopsis thaliana	AT5G38150.1 | Symbols: PMI15 | Plant protein of unknown function (DUF827) | chr5:15223116-15224950 REVERSE LENGTH=579	573	579	1.00E-160	101.0	60.9	75.9
Rsa1.0_00074.1_g3883.t1	gb|AAC33226.1| putative non-LTR retroelement reverse transcriptase [Arabidopsis thaliana]	1225	1529	0	124.8	34.6	46.7	putative non-LTR retroelement reverse transcriptase	gbpln	Arabidopsis thaliana	AT3G24255.1 | Symbols: | RNA-directed DNA polymerase (reverse transcriptase)-related family protein | chr3:8789471-8793208 FORWARD LENGTH=746	1225	746	1.00E-86	60.9	13.8	19.0
Rsa1.0_00074.1_g3884.t1	ref|NP_198630.2| nuclear transcription factor Y subunit C-10 [Arabidopsis thaliana] gi|75221594|sp|Q58CM8.1|NFYCA_ARATH RecName: Full=Nuclear transcription factor Y subunit C-10; Short=AtNF-YC-10 gi|61656131|gb|AAX49368.1| At5g38140 [Arabidopsis thaliana] gi|107738227|gb|ABF83665.1| At5g38140 [Arabidopsis thaliana] gi|332006889|gb|AED94272.1| nuclear transcription factor Y subunit C-10 [Arabidopsis thaliana]	218	195	3.00E-56	89.4	54.1	64.7	nuclear transcription factor Y subunit C-10	gbpln	Arabidopsis thaliana	AT5G38140.1 | Symbols: NF-YC12 | nuclear factor Y, subunit C12 | chr5:15220377-15222025 REVERSE LENGTH=195	218	195	9.00E-59	89.4	54.1	64.7
Rsa1.0_00074.1_g3885.t1	#	#	#	#	#	#	#	–	----	----	#	#	#	#	#	#	#
Rsa1.0_00074.1_g3886.t1	dbj|BAA85462.1| transposon-like ORF [Brassica rapa]	436	703	8.00E-70	161.2	30.7	36.0	transposon-like ORF	gbpln	Brassica rapa	#	#	#	#	#	#	#
Rsa1.0_00074.1_g3887.t1	gb|EOA26039.1| hypothetical protein CARUB_v10019447mg [Capsella rubella]	437	442	0	101.1	74.8	84.4	hypothetical protein CARUB_v10019447mg	gbpln	Capsella rubella	AT5G38130.1 | Symbols: | HXXXD-type acyl-transferase family protein | chr5:15217956-15219344 REVERSE LENGTH=462	437	462	0	105.7	74.1	85.1
Rsa1.0_00074.1_g3888.t1	#	#	#	#	#	#	#	–	----	----	#	#	#	#	#	#	#
Rsa1.0_00074.1_g3889.t1	gb|ABV21212.1| Ty1_Copia-element protein [Arabidopsis thaliana]	473	438	1.00E-85	92.6	35.7	53.1	Ty1_Copia-element protein	gbpln	Arabidopsis thaliana	AT3G24255.1 | Symbols: | RNA-directed DNA polymerase (reverse transcriptase)-related family protein | chr3:8789471-8793208 FORWARD LENGTH=746	473	746	1.00E-55	157.7	28.1	42.9
Rsa1.0_00074.1_g3890.t1	#	#	#	#	#	#	#	–	----	----	#	#	#	#	#	#	#
Rsa1.0_00074.1_g3891.t1	dbj|BAB02625.1| unnamed protein product [Arabidopsis thaliana]	395	420	2.00E-85	106.3	47.8	61.8	unnamed protein product	gbpln	Arabidopsis thaliana	AT3G28223.1 | Symbols: | F-box family protein | chr3:10527669-10528921 REVERSE LENGTH=391	395	391	2.00E-76	99.0	42.0	58.2
Rsa1.0_00074.1_g3892.t1	dbj|BAC41800.2| putative disease resistance protein [Arabidopsis thaliana]	435	977	9.00E-91	224.6	46.9	62.8	putative disease resistance protein	gbpln	Arabidopsis thaliana	AT5G40910.1 | Symbols: | Disease resistance protein (TIR-NBS-LRR class) family | chr5:16395507-16399129 FORWARD LENGTH=1104	435	1104	2.00E-93	253.8	46.9	62.8
Rsa1.0_00074.1_g3893.t1	ref|XP_002868590.1| predicted protein [Arabidopsis lyrata subsp. lyrata] gi|297314426|gb|EFH44849.1| predicted protein [Arabidopsis lyrata subsp. lyrata]	786	624	0	79.4	57.8	66.4	predicted protein	gbpln	Arabidopsis lyrata	AT5G41550.1 | Symbols: | Disease resistance protein (TIR-NBS-LRR class) family | chr5:16617232-16620785 REVERSE LENGTH=1085	786	1085	0	138.0	56.0	64.5
Rsa1.0_00074.1_g3894.t1	ref|XP_002870580.1| hypothetical protein ARALYDRAFT_915957 [Arabidopsis lyrata subsp. lyrata] gi|297316416|gb|EFH46839.1| hypothetical protein ARALYDRAFT_915957 [Arabidopsis lyrata subsp. lyrata]	329	286	4.00E-71	86.9	40.4	50.2	hypothetical protein ARALYDRAFT_915957	gbpln	Arabidopsis lyrata	AT5G37890.1 | Symbols: | Protein with RING/U-box and TRAF-like domains | chr5:15090512-15091822 REVERSE LENGTH=286	329	286	9.00E-70	86.9	38.6	49.2
Rsa1.0_00074.1_g3895.t1	gb|AAC95173.1| putative retroelement pol polyprotein [Arabidopsis thaliana]	1231	1200	0	97.5	47.8	59.1	putative retroelement pol polyprotein	gbpln	Arabidopsis thaliana	AT4G23160.1 | Symbols: CRK8 | cysteine-rich RLK (RECEPTOR-like protein kinase) 8 | chr4:12129485-12134086 FORWARD LENGTH=1262	1231	1262	9.00E-44	102.5	7.2	11.5

Rsa1.0_00074.1.g3896.t1	sp|O65780.1|GALE1_CYATE RecName: Full=UDP-glucose 4-epimerase GEPI42; AltName: Full=Galactowaldenase; AltName: Full=UDP-galactose 4-epimerase gi|3021355|emb|CAA06338.1| UDP-galactose 4-epimerase [Cyamopsis tetragonoloba]	73	354	2.00E-21	484.9	64.4	69.9	RecName: Full=UDP-glucose 4-epimerase GEPI42; AltName: Full=Galactowaldenase; AltName: Full=UDP-galactose 4-epimerase gi|3021355|emb|CAA06338.1| UDP-galactose 4-epimerase	gbpln	Cyamopsis tetragonoloba	AT1G12780.1 | Symbols: UGE1, ATUGE1 | UDP-D-glucose/UDP-D-galactose 4-epimerase 1 | chr1:4356124-4358120 REVERSE LENGTH=351	73	351	6.00E-23	480.8	63.0	68.5
Rsa1.0_00074.1.g3897.t1	ref|XP_002870573.1| hypothetical protein ARALYDRAFT_355742 [Arabidopsis lyrata subsp. lyrata] gi|297316409|gb|EFH46832.1| hypothetical protein ARALYDRAFT_355742 [Arabidopsis lyrata subsp. lyrata]	242	267	2.00E-91	110.3	78.9	86.8	hypothetical protein ARALYDRAFT_355742	gbpln	Arabidopsis lyrata	AT5G37860.1 | Symbols: | Heavy metal transport/detoxification superfamily protein | chr5:15069578-15070555 REVERSE LENGTH=262	242	262	1.00E-93	108.3	78.1	86.4
Rsa1.0_00074.1.g3898.t1	gb|ABE73472.1| putative pyridoxal kinase splice variant 1 [Brassica napus]	331	334	1.00E-171	100.9	91.2	92.7	putative pyridoxal kinase splice variant 1	gbpln	Brassica napus	AT5G37850.2 | Symbols: SOS4 | pfkB-like carbohydrate kinase family protein | chr5:15065621-15068783 FORWARD LENGTH=309	331	309	1.00E-168	93.4	88.5	91.8
Rsa1.0_00074.1.g3899.t2	dbj|BAB10363.1| unnamed protein product [Arabidopsis thaliana]	213	219	1.00E-65	102.8	64.8	72.3	unnamed protein product	gbpln	Arabidopsis thaliana	AT5G37840.1 | Symbols: | BEST Arabidopsis thaliana protein match is: plastid movement impaired 2 (TAIR:AT1G66480.1); Has 1807 Blast hits to 1807 proteins in 277 species: Archae - 0; Bacteria - 0; Metazoa - 736; Fungi - 347; Plants - 385; Viruses - 0; Other Eukaryotes - 339 (source: NCBI BLink). | chr5:15062192-15063327 REVERSE LENGTH=214	213	214	4.00E-68	100.5	64.8	71.8
Rsa1.0_00075.1.g3900.t1	dbj|BAB59032.1| cytokinin synthase [Arabidopsis thaliana]	334	330	1.00E-172	98.8	87.7	94.9	cytokinin synthase	gbpln	Arabidopsis thaliana	AT5G19040.1 | Symbols: ATIPT5, IPT5 | isopentenyltransferase 5 | chr5:6362389-6363381 REVERSE LENGTH=330	334	330	1.00E-174	98.8	87.4	94.9
Rsa1.0_00075.1.g3901.t1	ref|XP_002880309.1| hypothetical protein ARALYDRAFT_483931 [Arabidopsis lyrata subsp. lyrata] gi|297326148|gb|EFH56568.1| hypothetical protein ARALYDRAFT_483931 [Arabidopsis lyrata subsp. lyrata]	74	492	4.00E-14	664.9	50.0	51.4	hypothetical protein ARALYDRAFT_483931	gbpln	Arabidopsis lyrata	AT2G47510.2 | Symbols: FUM1 | fumarase 1 | chr2:19498614-19502020 FORWARD LENGTH=492	74	492	7.00E-17	664.9	50.0	51.4
Rsa1.0_00075.1.g3902.t1	ref|NP_187269.1| ribosomal protein L34e-like protein [Arabidopsis thaliana] gi|6862922|gb|AAF30311.1|AC018907_11 unknown protein [Arabidopsis thaliana] gi|62867643|gb|AAY17425.1| At3g06180 [Arabidopsis thaliana] gi|90962960|gb|ABE02404.1| At3g06180 [Arabidopsis thaliana] gi|332640836|gb|AEE74357.1| ribosomal protein L34e-like protein [Arabidopsis thaliana]	221	241	1.00E-58	109.0	68.3	74.7	ribosomal protein L34e-like protein	gbpln	Arabidopsis thaliana	AT3G06180.1 | Symbols: | Ribosomal protein L34e superfamily protein | chr3:1871705-1873554 FORWARD LENGTH=241	221	241	3.00E-61	109.0	68.3	74.7
Rsa1.0_00075.1.g3903.t1	ref|NP_197403.2| mitochondrial editing factor 18 [Arabidopsis thaliana] gi|223635651|sp|P0C8Q8.1|PP394_ARATH RecName: Full=Pentatricopeptide repeat-containing protein At5g19020, mitochondrial; Flags: Precursor gi|332005257|gb|AED92640.1| mitochondrial editing factor 18 [Arabidopsis thaliana]	696	685	0	98.4	82.8	90.7	mitochondrial editing factor 18	gbpln	Arabidopsis thaliana	AT5G19020.1 | Symbols: MEF18 | mitochondrial editing factor 18 | chr5:6352771-6354828 REVERSE LENGTH=685	696	685	0	98.4	82.8	90.7
Rsa1.0_00075.1.g3904.t1	ref|NP_197402.1| mitogen-activated protein kinase 16 [Arabidopsis thaliana] gi|114152844|sp|Q8W4J2.2|MPK16_ARATH RecName: Full=Mitogen-activated protein kinase 16; Short=AtMPK16; Short=MAP kinase 16 gi|332005256|gb|AED92639.1| mitogen-activated protein kinase 16 [Arabidopsis thaliana]	600	567	0	94.5	88.5	90.0	mitogen-activated protein kinase 16	gbpln	Arabidopsis thaliana	AT5G19010.1 | Symbols: MPK16 | mitogen-activated protein kinase 16 | chr5:6345096-6347676 REVERSE LENGTH=567	600	567	0	94.5	88.5	90.0

Rsa1.0_00075.1.g3905.t1	ref|NP_197401.2| BTB-POZ and math domain-containing protein [Arabidopsis thaliana] gi|75301130|sp|Q8L765.1|BPM1_ARATH RecName: Full=BTB/POZ and MATH domain-containing protein 1; AltName: Full=Protein BTB-POZ AND MATH DOMAIN 1; Short=AtBPM1 gi|22531225|gb|AAM97116.1| putative protein [Arabidopsis thaliana] gi|27311975|gb|AAO00953.1| putative protein [Arabidopsis thaliana] gi|332005254|gb|AED92637.1| BTB-POZ and math domain-containing protein [Arabidopsis thaliana]	425	407	0	95.8	76.0	81.6	BTB-POZ and math domain-containing protein	gbpln	Arabidopsis thaliana	AT5G19000.1 | Symbols: ATBPM1, BPM1 | BTB-POZ and MATH domain 1 | chr5:6342563-6344641 FORWARD LENGTH=407	425	407	0	95.8	76.0	81.6
Rsa1.0_00075.1.g3906.t1	#	#	#	#	#	#	#	-	----	----	#	#	#	#	#	#	#
Rsa1.0_00075.1.g3907.t1	gb|EOA19340.1| hypothetical protein CARUB_v10000220mg [Capsella rubella]	834	835	0	100.1	88.0	93.2	hypothetical protein CARUB_v10000220mg	gbpln	Capsella rubella	AT5G18980.1 | Symbols: | ARM repeat superfamily protein | chr5:6334944-6337943 REVERSE LENGTH=835	834	835	0	100.1	87.5	92.9
Rsa1.0_00075.1.g3908.t1	gb|EOA21686.1| hypothetical protein CARUB_v10002111mg [Capsella rubella]	170	170	9.00E-86	100.0	92.9	95.9	hypothetical protein CARUB_v10002111mg	gbpln	Capsella rubella	AT5G18970.1 | Symbols: | AWPM-19-like family protein | chr5:6333714-6334539 REVERSE LENGTH=171	170	171	6.00E-86	100.6	91.8	95.3
Rsa1.0_00075.1.g3909.t1	gb|EOA22563.1| hypothetical protein CARUB_v10003224mg [Capsella rubella]	507	486	0	95.9	70.4	81.7	hypothetical protein CARUB_v10003224mg	gbpln	Capsella rubella	AT5G18950.1 | Symbols: | Tetratricopeptide repeat (TPR)-like superfamily protein | chr5:6328519-6329970 REVERSE LENGTH=483	507	483	0	95.3	68.4	80.1
Rsa1.0_00075.1.g3910.t3	#	#	#	#	#	#	#	-	----	----	#	#	#	#	#	#	#
Rsa1.0_00075.1.g3911.t1	#	#	#	#	#	#	#	-	----	----	#	#	#	#	#	#	#
Rsa1.0_00075.1.g3912.t1	gb|EOA22928.1| hypothetical protein CARUB_v10003664mg [Capsella rubella]	350	347	1.00E-170	99.1	86.0	93.1	hypothetical protein CARUB_v10003664mg	gbpln	Capsella rubella	AT5G18930.1 | Symbols: BUD2, SAMDC4 | Adenosylmethionine decarboxylase family protein | chr5:6312172-6313215 REVERSE LENGTH=347	350	347	1.00E-173	99.1	86.0	91.4
Rsa1.0_00075.1.g3913.t1	gb|EOA19930.1| hypothetical protein CARUB_v10000179mg [Capsella rubella]	1011	893	0	88.3	73.8	79.5	hypothetical protein CARUB_v10000179mg	gbpln	Capsella rubella	AT5G18860.1 | Symbols: | inosine-uridine preferring nucleoside hydrolase family protein | chr5:6291023-6295581 FORWARD LENGTH=890	1011	890	0	88.0	72.9	78.5
Rsa1.0_00075.1.g3914.t1	gb|EOA22613.1| hypothetical protein CARUB_v10003281mg [Capsella rubella]	62	96	2.00E-17	154.8	67.7	75.8	hypothetical protein CARUB_v10003281mg	gbpln	Capsella rubella	AT5G18850.1 | Symbols: | unknown protein; FUNCTIONS IN: molecular_function unknown; INVOLVED IN: biological_process unknown; LOCATED IN: endomembrane system; EXPRESSED IN: 23 plant structures; EXPRESSED DURING: 13 growth stages; Has 1807 Blast hits to 1807 proteins in 277 species: Archae - 0; Bacteria - 0; Metazoa - 736; Fungi - 347; Plants - 385; Viruses - 0; Other Eukaryotes - 339 (source: NCBI BLink). | chr5:6286896-6287272 FORWARD LENGTH=99	62	99	1.00E-18	159.7	69.4	74.2
Rsa1.0_00075.1.g3915.t1	ref|NP_568367.1| sugar transporter ERD6-like 16 [Arabidopsis thaliana] gi|118572295|sp|Q8LBI9.2|EDL16_ARATH RecName: Full=Sugar transporter ERD6-like 16 gi|17381265|gb|AAL36051.1| AT5g18840/F17K4_90 [Arabidopsis thaliana] gi|23309019|gb|AAN18269.1| At5g18840/F17K4_90 [Arabidopsis thaliana] gi|332005237|gb|AED92620.1| sugar transporter ERD6-like 16 [Arabidopsis thaliana]	488	482	0	98.8	88.7	92.0	sugar transporter ERD6-like 16	gbpln	Arabidopsis thaliana	AT5G18840.1 | Symbols: | Major facilitator superfamily protein | chr5:6282954-6286399 FORWARD LENGTH=482	488	482	0	98.8	88.7	92.0
Rsa1.0_00075.1.g3916.t1	dbj|BAJ34638.1| unnamed protein product [Thellungiella halophila]	784	803	0	102.4	87.0	93.0	unnamed protein product	----	----	AT5G18830.1 | Symbols: SPL7, ATSPL7 | squamosa promoter binding protein-like 7 | chr5:6276116-6280227 FORWARD LENGTH=801	784	801	0	102.2	83.9	90.4
Rsa1.0_00075.1.g3917.t1	#	#	#	#	#	#	#	-	----	----	AT5G16620.1 | Symbols: PDE120, TIC40, ATTIC40 | hydroxyproline-rich glycoprotein family protein | chr5:5450808-5454256 FORWARD LENGTH=447	53	447	2.00E-12	843.4	64.2	69.8

Rsa1.0_00075.1.g3918.t1	ref|NP_197381.1| NADH dehydrogenase (ubiquinone) 1 alpha subcomplex 8 [Arabidopsis thaliana] gi|30687007|ref|NP_850849.1| NADH dehydrogenase (ubiquinone) 1 alpha subcomplex 8 [Arabidopsis thaliana] gi|297807915|ref|XP_002871841.1| NADH-ubiquinone oxidoreductase 19 kDa subunit family protein [Arabidopsis lyrata subsp. lyrata] gi|75156052|sp|Q8LGE7.1|NDA8B_ARATH RecName: Full=NADH dehydrogenase [ubiquinone] 1 alpha subcomplex subunit 8-B gi|21536569|gb|AAM60901.1| NADH:ubiquinone oxidoreductase-like protein [Arabidopsis thaliana] gi|28466879|gb|AAO44048.1| At5g18800 [Arabidopsis thaliana] gi|110735767|dbj|BAE99861.1| NADH:ubiquinone oxidoreductase like protein [Arabidopsis thaliana] gi|297317678|gb|EFH48100.1| NADH-ubiquinone oxidoreductase 19 kDa subunit family protein [Arabidopsis lyrata subsp. lyrata] gi|332005230|gb|AED92613.1| NADH dehydrogenase (ubiquinone) 1 alpha subcomplex 8 [Arabidopsis thaliana] gi|332005231|gb|AED92614.1| NADH dehydrogenase (ubiquinone) 1 alpha subcomplex 8 [Arabidopsis thaliana] gi|482557653|gb|EOA21845.1| hypothetical protein	106	106	6.00E-56	100.0	99.1	100.0	NADH dehydrogenase (ubiquinone) 1 alpha subcomplex 8	gbpln	Arabidopsis lyrata	AT5G18800.2 | Symbols: | Cox19-like CHCH family protein | chr5:6267304-6268393 FORWARD LENGTH=106	106	106	1.00E-58	100.0	99.1	100.0
Rsa1.0_00075.1.g3919.t1	gb|EOA32347.1| hypothetical protein CARUB_v10015614mg [Capsella rubella]	119	97	3.00E-26	81.5	48.7	51.3	hypothetical protein CARUB_v10015614mg	gbpln	Capsella rubella	AT5G18790.1 | Symbols: | Ribosomal protein L33 family protein | chr5:6266324-6266500 REVERSE LENGTH=58	119	58	4.00E-28	48.7	47.9	48.7
Rsa1.0_00075.1.g3920.t1	gb|EOA19923.1| hypothetical protein CARUB_v10000173mg [Capsella rubella]	858	903	0	105.2	66.4	77.9	hypothetical protein CARUB_v10000173mg	gbpln	Capsella rubella	AT5G18750.1 | Symbols: | DNAJ heat shock N-terminal domain-containing protein | chr5:6255177-6257831 FORWARD LENGTH=884	858	884	0	103.0	66.0	78.4
Rsa1.0_00075.1.g3921.t1	gb|EOA20118.1| hypothetical protein CARUB_v10000401mg [Capsella rubella]	560	666	0	118.9	61.6	72.7	hypothetical protein CARUB_v10000401mg	gbpln	Capsella rubella	AT5G18730.1 | Symbols: | CONTAINS InterPro DOMAIN/s: Molecular chaperone, heat shock protein, Hsp40, DnaJ (InterPro:IPR015609); BEST Arabidopsis thaliana protein match is: Domain of unknown function (DUF3444) (TAIR:AT5G18740.1); Has 30201 Blast hits to 17322 proteins in 780 species: Archae - 12; Bacteria - 1396; Metazoa - 17338; Fungi - 3422; Plants - 5037; Viruses - 0; Other Eukaryotes - 2996 (source: NCBI BLink). | chr5:6247611-6249197 FORWARD LENGTH=528	560	528	0	94.3	62.1	72.0
Rsa1.0_00075.1.g3922.t1	gb|EOA20118.1| hypothetical protein CARUB_v10000401mg [Capsella rubella]	1010	666	1.00E-179	65.9	31.2	37.4	hypothetical protein CARUB_v10000401mg	gbpln	Capsella rubella	AT5G18730.1 | Symbols: | CONTAINS InterPro DOMAIN/s: Molecular chaperone, heat shock protein, Hsp40, DnaJ (InterPro:IPR015609); BEST Arabidopsis thaliana protein match is: Domain of unknown function (DUF3444) (TAIR:AT5G18740.1); Has 30201 Blast hits to 17322 proteins in 780 species: Archae - 12; Bacteria - 1396; Metazoa - 17338; Fungi - 3422; Plants - 5037; Viruses - 0; Other Eukaryotes - 2996 (source: NCBI BLink). | chr5:6247611-6249197 FORWARD LENGTH=528	1010	528	1.00E-173	52.3	30.7	36.0
Rsa1.0_00075.1.g3923.t1	gb|EOA22541.1| hypothetical protein CARUB_v10003196mg [Capsella rubella]	532	554	0	104.1	67.3	78.2	hypothetical protein CARUB_v10003196mg	gbpln	Capsella rubella	AT5G18730.1 | Symbols: | CONTAINS InterPro DOMAIN/s: Molecular chaperone, heat shock protein, Hsp40, DnaJ (InterPro:IPR015609); BEST Arabidopsis thaliana protein match is: Domain of unknown function (DUF3444) (TAIR:AT5G18740.1); Has 30201 Blast hits to 17322 proteins in 780 species: Archae - 12; Bacteria - 1396; Metazoa - 17338; Fungi - 3422; Plants - 5037; Viruses - 0; Other Eukaryotes - 2996 (source: NCBI BLink). | chr5:6247611-6249197 FORWARD LENGTH=528	532	528	1.00E-176	99.2	58.8	70.7

Rsa1.0_00075.1.g3924.t1	ref|XP_002871829.1| hypothetical protein ARALYDRAFT_488759 [Arabidopsis lyrata subsp. lyrata] gi|297317666|gb|EFH48088.1| hypothetical protein ARALYDRAFT_488759 [Arabidopsis lyrata subsp. lyrata]	587	566	0	96.4	65.1	74.1	hypothetical protein ARALYDRAFT_488759	gbpln	Arabidopsis lyrata	AT5G18730.1 | Symbols: | CONTAINS InterPro DOMAIN/s: Molecular chaperone, heat shock protein, Hsp40, DnaJ (InterPro:IPR015609); BEST Arabidopsis thaliana protein match is: Domain of unknown function (DUF3444) (TAIR:AT5G18740.1); Has 30201 Blast hits to 17322 proteins in 780 species: Archae - 12; Bacteria - 1396; Metazoa - 17338; Fungi - 3422; Plants - 5037; Viruses - 0; Other Eukaryotes - 2996 (source: NCBI BLink). | chr5:6247611-6249197 FORWARD LENGTH=528	587	528	1.00E-122	89.9	41.4	55.5
Rsa1.0_00075.1.g3925.t1	ref|XP_002873912.1| EMB3013 [Arabidopsis lyrata subsp. lyrata] gi|297319749|gb|EFH50171.1| EMB3013 [Arabidopsis lyrata subsp. lyrata]	1362	1366	0	100.3	91.6	95.8	EMB3013	gbpln	Arabidopsis lyrata	AT5G18700.1 | Symbols: RUK, EMB3013 | Protein kinase family protein with ARM repeat domain | chr5:6235387-6240733 REVERSE LENGTH=1366	1362	1366	0	100.3	90.3	95.0
Rsa1.0_00075.1.g3926.t1	gb|EOA19299.1| hypothetical protein CARUB_v10002269mg [Capsella rubella]	133	122	2.00E-21	91.7	63.9	70.7	hypothetical protein CARUB_v10002269mg	gbpln	Capsella rubella	#	#	#	#	#	#	#
Rsa1.0_00075.1.g3927.t1	ref|NP_197369.2| Tubby-like F-box protein 11 [Arabidopsis thaliana] gi|75223283|sp|Q6NPQ1.1|TLP11_ARATH RecName: Full=Tubby-like F-box protein 11; Short=AtTLP11 gi|38564318|gb|AAR23738.1| At5g18680 [Arabidopsis thaliana] gi|58652098|gb|AAW80874.1| At5g18680 [Arabidopsis thaliana] gi|225898921|dbj|BAH30591.1| hypothetical protein [Arabidopsis thaliana] gi|332005215|gb|AED92598.1| Tubby-like F-box protein 11 [Arabidopsis thaliana]	393	389	0	99.0	87.5	92.1	Tubby-like F-box protein 11	gbpln	Arabidopsis thaliana	AT5G18680.1 | Symbols: AtTLP11, TLP11 | tubby like protein 11 | chr5:6228358-6230426 REVERSE LENGTH=389	393	389	0	99.0	87.5	92.1
Rsa1.0_00075.1.g3928.t1	ref|NP_197368.1| inactive beta-amylase 9 [Arabidopsis thaliana] gi|75331155|sp|Q8VYW2.1|BAM9_ARATH RecName: Full=Inactive beta-amylase 9; AltName: Full=1,4-alpha-D-glucan maltohydrolase; AltName: Full=Inactive beta-amylase 3 gi|17978935|gb|AAL47434.1| AT5g18670/T1A4_50 [Arabidopsis thaliana] gi|21592648|gb|AAM64597.1| beta-amylase-like proten [Arabidopsis thaliana] gi|22655358|gb|AAM98271.1| At5g18670/T1A4_50 [Arabidopsis thaliana] gi|332005214|gb|AED92597.1| inactive beta-amylase 9 [Arabidopsis thaliana]	536	536	0	100.0	85.4	90.3	inactive beta-amylase 9	gbpln	Arabidopsis thaliana	AT5G18670.1 | Symbols: BMY3, BAM9 | beta-amylase 3 | chr5:6226138-6227999 FORWARD LENGTH=536	536	536	0	100.0	85.4	90.3
Rsa1.0_00075.1.g3929.t5	#	#	#	#	#	#	#	-	----	----	#	#	#	#	#	#	#
Rsa1.0_00075.1.g3930.t1	ref|XP_002873908.1| pale-green and chlorophyll B reduced 2 [Arabidopsis lyrata subsp. lyrata] gi|297319745|gb|EFH50167.1| pale-green and chlorophyll B reduced 2 [Arabidopsis lyrata subsp. lyrata]	419	417	0	99.5	83.5	90.2	pale-green and chlorophyll B reduced 2	gbpln	Arabidopsis lyrata	AT5G18660.1 | Symbols: PCB2 | NAD(P)-binding Rossmann-fold superfamily protein | chr5:6220872-6222125 REVERSE LENGTH=417	419	417	0	99.5	82.6	90.2
Rsa1.0_00075.1.g3931.t2	ref|XP_002871827.1| zinc finger family protein [Arabidopsis lyrata subsp. lyrata] gi|297317664|gb|EFH48086.1| zinc finger family protein [Arabidopsis lyrata subsp. lyrata]	258	267	1.00E-137	103.5	91.1	96.1	zinc finger family protein	gbpln	Arabidopsis lyrata	AT5G18650.1 | Symbols: | CHY-type/CTCHY-type/RING-type Zinc finger protein | chr5:6218260-6220374 FORWARD LENGTH=267	258	267	1.00E-138	103.5	90.3	95.0
Rsa1.0_00076.1.g3932.t1	ref|XP_002880944.1| hypothetical protein ARALYDRAFT_481690 [Arabidopsis lyrata subsp. lyrata] gi|297326783|gb|EFH57203.1| hypothetical protein ARALYDRAFT_481690 [Arabidopsis lyrata subsp. lyrata]	284	276	1.00E-110	97.2	78.9	84.2	hypothetical protein ARALYDRAFT_481690	gbpln	Arabidopsis lyrata	AT2G28200.1 | Symbols: | C2H2-type zinc finger family protein | chr2:12024321-12025181 FORWARD LENGTH=286	284	286	1.00E-111	100.7	79.2	85.2
Rsa1.0_00076.1.g3933.t1	#	#	#	#	#	#	#	-	----	----	#	#	#	#	#	#	#
Rsa1.0_00076.1.g3934.t1	gb|AAC28221.1| similar to reverse transcriptases (PFam: rvt.hmm, score: 60.13) [Arabidopsis thaliana]	703	1164	0	165.6	56.9	71.0	similar to reverse transcriptases (PFam: rvt.hmm, score: 60.13)	gbpln	Arabidopsis thaliana	AT3G24255.1 | Symbols: | RNA-directed DNA polymerase (reverse transcriptase)-related family protein | chr3:8789471-8793208 FORWARD LENGTH=746	703	746	1.00E-63	106.1	19.3	27.3
Rsa1.0_00076.1.g3935.t1	gb|EOA27882.1| hypothetical protein CARUB_v10024052mg [Capsella rubella]	213	213	1.00E-115	100.0	95.3	98.6	hypothetical protein CARUB_v10024052mg	gbpln	Capsella rubella	AT2G28305.1 | Symbols: LOG1, ATLOG1 | Putative lysine decarboxylase family protein | chr2:12081186-12084307 FORWARD LENGTH=213	213	213	1.00E-116	100.0	94.4	98.1

Rsa1.0_00076.1.g3936.t3	ref|XP_002880936.1| hypothetical protein ARALYDRAFT_481676 [Arabidopsis lyrata subsp. lyrata] gi|297326775|gb|EFH57195.1| hypothetical protein ARALYDRAFT_481676 [Arabidopsis lyrata subsp. lyrata]	379	381	0	100.5	91.0	92.1	hypothetical protein ARALYDRAFT_481676	gbpln	Arabidopsis lyrata	AT2G28310.3 | Symbols: | Protein of unknown function (DUF707) | chr2:12085773-12087888 FORWARD LENGTH=374	379	374	0	98.7	90.2	92.3
Rsa1.0_00076.1.g3937.t1	ref|XP_002880935.1| hypothetical protein ARALYDRAFT_320561 [Arabidopsis lyrata subsp. lyrata] gi|297326774|gb|EFH57194.1| hypothetical protein ARALYDRAFT_320561 [Arabidopsis lyrata subsp. lyrata]	341	342	0	100.3	96.8	98.2	hypothetical protein ARALYDRAFT_320561	gbpln	Arabidopsis lyrata	AT2G28315.1 | Symbols: | Nucleotide/sugar transporter family protein | chr2:12088896-12090570 FORWARD LENGTH=342	341	342	0	100.3	96.5	98.2
Rsa1.0_00076.1.g3938.t1	ref|XP_002880933.1| hypothetical protein ARALYDRAFT_901675 [Arabidopsis lyrata subsp. lyrata] gi|297326772|gb|EFH57192.1| hypothetical protein ARALYDRAFT_901675 [Arabidopsis lyrata subsp. lyrata]	290	320	1.00E-77	110.3	57.9	72.1	hypothetical protein ARALYDRAFT_901675	gbpln	Arabidopsis lyrata	AT1G08010.2 | Symbols: GATA11 | GATA transcription factor 11 | chr1:2486202-2487402 REVERSE LENGTH=303	290	303	5.00E-71	104.5	53.8	67.2
Rsa1.0_00076.1.g3939.t2	ref|NP_180403.4| SIT4 phosphatase-associated-like protein [Arabidopsis thaliana] gi|330253018|gb|AEC08112.1| SIT4 phosphatase-associated-like protein [Arabidopsis thaliana]	798	788	0	98.7	82.2	88.0	SIT4 phosphatase-associated-like protein	gbpln	Arabidopsis thaliana	AT2G28360.1 | Symbols: | SIT4 phosphatase-associated family protein | chr2:12124604-12129879 REVERSE LENGTH=788	798	788	0	98.7	82.2	88.0
Rsa1.0_00076.1.g3940.t1	ref|NP_565671.1| uncharacterized protein [Arabidopsis thaliana] gi|75206504|sp|Q9SKN3.1|CSPL6_ARATH RecName: Full=CASP-like protein At2g28370 gi|14030701|gb|AAK53025.1|AF375441_1 At2g28370/T1B3.11 [Arabidopsis thaliana] gi|4432840|gb|AAD20689.1| expressed protein [Arabidopsis thaliana] gi|21553379|gb|AAM62472.1| unknown [Arabidopsis thaliana] gi|23506069|gb|AAN28894.1| At2g28370/T1B3.11 [Arabidopsis thaliana] gi|330253019|gb|AEC08113.1| uncharacterized protein AT2G28370 [Arabidopsis thaliana]	185	179	2.00E-82	96.8	89.7	93.5	uncharacterized protein	gbpln	Arabidopsis thaliana	AT2G28370.1 | Symbols: | Uncharacterised protein family (UPF0497) | chr2:12132092-12133447 FORWARD LENGTH=179	185	179	6.00E-85	96.8	89.7	93.5
Rsa1.0_00076.1.g3941.t1	gb|EOA27124.1| hypothetical protein CARUB_v10023224mg [Capsella rubella]	436	443	0	101.6	78.7	85.1	hypothetical protein CARUB_v10023224mg	gbpln	Capsella rubella	AT2G28380.1 | Symbols: DRB2 | dsRNA-binding protein 2 | chr2:12134098-12135915 REVERSE LENGTH=434	436	434	1.00E-180	99.5	78.2	84.9
Rsa1.0_00076.1.g3942.t1	#	#	#	#	#	#	#	-	----	----	#	#	#	#	#	#	#
Rsa1.0_00076.1.g3943.t1	ref|XP_002879098.1| hypothetical protein ARALYDRAFT_481665 [Arabidopsis lyrata subsp. lyrata] gi|297324937|gb|EFH55357.1| hypothetical protein ARALYDRAFT_481665 [Arabidopsis lyrata subsp. lyrata]	162	164	4.00E-59	101.2	78.4	87.0	hypothetical protein ARALYDRAFT_481665	gbpln	Arabidopsis lyrata	AT2G28400.1 | Symbols: | Protein of unknown function, DUF584 | chr2:12148513-12149001 REVERSE LENGTH=162	162	162	2.00E-59	100.0	77.8	86.4
Rsa1.0_00076.1.g3944.t1	#	#	#	#	#	#	#	-	----	----	#	#	#	#	#	#	#
Rsa1.0_00076.1.g3945.t1	gb|EOA28155.1| hypothetical protein CARUB_v10024345mg [Capsella rubella]	116	118	2.00E-44	101.7	77.6	83.6	hypothetical protein CARUB_v10024345mg	gbpln	Capsella rubella	AT2G28410.1 | Symbols: | unknown protein; Has 18 Blast hits to 18 proteins in 5 species: Archae - 0; Bacteria - 0; Metazoa - 0; Fungi - 0; Plants - 18; Viruses - 0; Other Eukaryotes - 0 (source: NCBI BLink). | chr2:12156202-12156549 FORWARD LENGTH=115	116	115	6.00E-43	99.1	70.7	81.0
Rsa1.0_00076.1.g3946.t2	#	#	#	#	#	#	#	-	----	----	#	#	#	#	#	#	#
Rsa1.0_00076.1.g3947.t1	ref|NP_180411.1| proline-rich protein [Arabidopsis thaliana] gi|4432833|gb|AAD20682.1| En/Spm-like transposon protein [Arabidopsis thaliana] gi|67633550|gb|AAY78699.1| proline-rich family protein [Arabidopsis thaliana] gi|330253029|gb|AEC08123.1| proline-rich protein [Arabidopsis thaliana]	168	268	3.00E-23	159.5	53.6	61.3	proline-rich protein	gbpln	Arabidopsis thaliana	AT2G28440.1 | Symbols: | proline-rich family protein | chr2:12161226-12162032 FORWARD LENGTH=268	168	268	1.00E-25	159.5	53.6	61.3
Rsa1.0_00076.1.g3948.t1	gb|AAC98462.1| putative chloroplast nucleoid DNA binding protein [Arabidopsis thaliana] gi|20197956|gb|AAM15330.1| putative chloroplast nucleoid DNA binding protein [Arabidopsis thaliana]	429	389	1.00E-138	90.7	58.7	70.6	putative chloroplast nucleoid DNA binding protein	gbpln	Arabidopsis thaliana	AT2G28040.1 | Symbols: | Eukaryotic aspartyl protease family protein | chr2:11936203-11937390 REVERSE LENGTH=395	429	395	1.00E-141	92.1	58.7	70.6
Rsa1.0_00076.1.g3949.t1	#	#	#	#	#	#	#	-	----	----	#	#	#	#	#	#	#
Rsa1.0_00076.1.g3950.t1	#	#	#	#	#	#	#	-	----	----	#	#	#	#	#	#	#

Rsa1.0_00076.1.g3951.t1	ref|NP_180417.2| LOB domain-containing protein 11 [Arabidopsis thaliana] gi|334302828|sp|Q9SK08.2|LBD11_ARATH RecName: Full=LOB domain-containing protein 11; AltName: Full=ASYMMETRIC LEAVES 2-like protein 7; Short=AS2-like protein 7 gi|330253037|gb|AEC08131.1| LOB domain-containing protein 11 [Arabidopsis thaliana]	236	232	1.00E-101	98.3	89.8	93.2	LOB domain-containing protein 11	gbpln	Arabidopsis thaliana	AT2G28500.1 | Symbols: LBD11 | LOB domain-containing protein 11 | chr2:12186603-12188110 REVERSE LENGTH=232	236	232	1.00E-103	98.3	89.8	93.2
Rsa1.0_00076.1.g3952.t3	#	#	#	#	#	#	#	-	----	----	#	#	#	#	#	#	#
Rsa1.0_00077.1.g3953.t1	ref|XP_002894568.1| transferase family protein [Arabidopsis lyrata subsp. lyrata] gi|297340410|gb|EFH70827.1| transferase family protein [Arabidopsis lyrata subsp. lyrata]	180	433	5.00E-48	240.6	61.1	67.8	transferase family protein	gbpln	Arabidopsis lyrata	AT5G48930.1 | Symbols: HCT | hydroxycinnamoyl-CoA shikimate/quinate hydroxycinnamoyl transferase | chr5:19836654-19838092 REVERSE LENGTH=433	180	433	3.00E-49	240.6	58.3	65.6
Rsa1.0_00077.1.g3954.t1	ref|XP_002870182.1| hypothetical protein ARALYDRAFT_915160 [Arabidopsis lyrata subsp. lyrata] gi|297316018|gb|EFH46441.1| hypothetical protein ARALYDRAFT_915160 [Arabidopsis lyrata subsp. lyrata]	652	676	0	103.7	80.7	86.7	hypothetical protein ARALYDRAFT_915160	gbpln	Arabidopsis lyrata	AT4G16110.1 | Symbols: ARR2, RR2 | response regulator 2 | chr4:9112979-9115785 FORWARD LENGTH=664	652	664	0	101.8	77.8	84.0
Rsa1.0_00077.1.g3955.t1	ref|XP_002868161.1| ATSEB1 [Arabidopsis lyrata subsp. lyrata] gi|297313997|gb|EFH44420.1| ATSEB1 [Arabidopsis lyrata subsp. lyrata]	660	658	0	99.7	83.6	89.4	ATSEB1	gbpln	Arabidopsis lyrata	AT4G16120.1 | Symbols: ATSEB1, COBL7, SEB1 | COBRA-like protein-7 precursor | chr4:9116591-9119138 REVERSE LENGTH=661	660	661	0	100.2	84.5	91.8
Rsa1.0_00077.1.g3956.t13	ref|XP_002870181.1| hypothetical protein ARALYDRAFT_355153 [Arabidopsis lyrata subsp. lyrata] gi|297316017|gb|EFH46440.1| hypothetical protein ARALYDRAFT_355153 [Arabidopsis lyrata subsp. lyrata]	1027	989	0	96.3	90.2	93.6	hypothetical protein ARALYDRAFT_355153	gbpln	Arabidopsis lyrata	AT4G16130.1 | Symbols: ARA1, ISA1, ATISA1 | arabinose kinase | chr4:9120875-9127656 FORWARD LENGTH=1039	1027	1039	0	101.2	90.4	93.4
Rsa1.0_00077.1.g3957.t1	gb|EOA17545.1| hypothetical protein CARUB_v10005904mg [Capsella rubella]	177	166	3.00E-20	93.8	33.9	36.7	hypothetical protein CARUB_v10005904mg	gbpln	Capsella rubella	#	#	#	#	#	#	#
Rsa1.0_00077.1.g3958.t1	#	#	#	#	#	#	#	-	----	----	#	#	#	#	#	#	#
Rsa1.0_00077.1.g3959.t1	gb|EOA17527.1| hypothetical protein CARUB_v10005860mg [Capsella rubella]	184	178	4.00E-57	96.7	72.8	84.2	hypothetical protein CARUB_v10005860mg	gbpln	Capsella rubella	AT4G16141.1 | Symbols: | GATA type zinc finger transcription factor family protein | chr4:9131872-9132893 REVERSE LENGTH=197	184	197	2.00E-58	107.1	75.5	84.8
Rsa1.0_00077.1.g3960.t1	gb|EOA18223.1| hypothetical protein CARUB_v10006712mg [Capsella rubella]	528	533	0	100.9	92.8	95.5	hypothetical protein CARUB_v10006712mg	gbpln	Capsella rubella	AT4G16143.2 | Symbols: IMPA-2 | importin alpha isoform 2 | chr4:9134450-9137134 REVERSE LENGTH=535	528	535	0	101.3	92.6	95.8
Rsa1.0_00077.1.g3961.t1	gb|AAV85709.1| At4g16144 [Arabidopsis thaliana]	530	507	0	95.7	87.0	90.8	At4g16144	gbpln	Arabidopsis thaliana	AT4G16144.1 | Symbols: AMSH3 | associated molecule with the SH3 domain of STAM 3 | chr4:9138053-9141422 REVERSE LENGTH=507	530	507	0	95.7	87.0	90.6
Rsa1.0_00077.1.g3962.t1	gb|EOA17716.1| hypothetical protein CARUB_v10006097mg [Capsella rubella]	117	103	2.00E-27	88.0	59.0	65.8	hypothetical protein CARUB_v10006097mg	gbpln	Capsella rubella	AT4G16146.1 | Symbols: | cAMP-regulated phosphoprotein 19-related protein | chr4:9142663-9144356 REVERSE LENGTH=102	117	102	9.00E-29	87.2	55.6	63.2
Rsa1.0_00077.1.g3963.t2	ref|XP_002867797.1| hypothetical protein ARALYDRAFT_329412 [Arabidopsis lyrata subsp. lyrata] gi|297313633|gb|EFH44056.1| hypothetical protein ARALYDRAFT_329412 [Arabidopsis lyrata subsp. lyrata]	718	732	0	101.9	87.5	93.9	hypothetical protein ARALYDRAFT_329412	gbpln	Arabidopsis lyrata	AT4G04910.1 | Symbols: NSF | AAA-type ATPase family protein | chr4:2489696-2495666 REVERSE LENGTH=742	718	742	0	103.3	75.6	85.8
Rsa1.0_00077.1.g3964.t1	gb|EOA16331.1| hypothetical protein CARUB_v10004481mg [Capsella rubella]	563	564	0	100.2	94.0	96.6	hypothetical protein CARUB_v10004481mg	gbpln	Capsella rubella	AT4G16155.1 | Symbols: | dihydrolipoyl dehydrogenases | chr4:9153570-9157322 REVERSE LENGTH=630	563	630	0	111.9	94.8	96.4
Rsa1.0_00077.1.g3965.t3	gb|EOA15607.1| hypothetical protein CARUB_v10005679mg [Capsella rubella] gi|482551415|gb|EOA15608.1| hypothetical protein CARUB_v10005679mg [Capsella rubella]	229	224	1.00E-80	97.8	75.1	81.7	hypothetical protein CARUB_v10005679mg	gbpln	Capsella rubella	AT3G17100.2 | Symbols: | sequence-specific DNA binding transcription factors | chr3:5831552-5832244 FORWARD LENGTH=230	229	230	6.00E-58	100.4	62.0	74.7

Rsa1.0_00077.1_g3966.t1	ref|XP_002868153.1| predicted protein [Arabidopsis lyrata subsp. lyrata] gi|297313989|gb|EFH44412.1| predicted protein [Arabidopsis lyrata subsp. lyrata]	820	822	0	100.2	93.5	96.6	predicted protein	gbpln	Arabidopsis lyrata	AT4G16180.2 | Symbols: | unknown protein; FUNCTIONS IN: molecular_function unknown; INVOLVED IN: biological_process unknown; LOCATED IN: plasma membrane; BEST Arabidopsis thaliana protein match is: unknown protein (TAIR:AT3G28720.1); Has 5 Blast hits to 5 proteins in 3 species: Archae - 0; Bacteria - 0; Metazoa - 0; Fungi - 0; Plants - 4; Viruses - 0; Other Eukaryotes - 1 (source: NCBI BLink). | chr4:9165365-9170323 REVERSE LENGTH=820	820	820	0	100.0	91.2	94.3
Rsa1.0_00077.1_g3967.t1	gb|EOA16872.1| hypothetical protein CARUB_v10005098mg [Capsella rubella]	368	365	0	99.2	86.1	91.0	hypothetical protein CARUB_v10005098mg	gbpln	Capsella rubella	AT4G16190.1 | Symbols: | Papain family cysteine protease | chr4:9171512-9172877 FORWARD LENGTH=373	368	373	0	101.4	86.7	91.6
Rsa1.0_00077.1_g3968.t1	#	#	#	#	#	#	#	–	----	----	#	#	#	#	#	#	#
Rsa1.0_00077.1_g3969.t1	gb|EOA17085.1| hypothetical protein CARUB_v10005333mg [Capsella rubella]	307	305	1.00E-147	99.3	83.7	89.3	hypothetical protein CARUB_v10005333mg	gbpln	Capsella rubella	AT3G17000.1 | Symbols: UBC32 | ubiquitin-conjugating enzyme 32 | chr3:5797296-5799467 FORWARD LENGTH=309	307	309	1.00E-119	100.7	70.0	81.4
Rsa1.0_00077.1_g3970.t1	ref|NP_567490.1| RNA polymerases M/15 Kd subunit [Arabidopsis thaliana] gi|297800522|ref|XP_002868145.1| hypothetical protein ARALYDRAFT_493259 [Arabidopsis lyrata subsp. lyrata] gi|21537166|gb|AAM61507.1| RNA Polymerase II subunit 14.5 kD, putative [Arabidopsis thaliana] gi|114050701|gb|ABI49500.1| At4g16265 [Arabidopsis thaliana] gi|297313981|gb|EFH44404.1| hypothetical protein ARALYDRAFT_493259 [Arabidopsis lyrata subsp. lyrata] gi|332658323|gb|AEE83723.1| RNA polymerases M/15 Kd subunit [Arabidopsis thaliana] gi|482553494|gb|EOA17687.1| hypothetical protein CARUB_v10006057mg [Capsella rubella]	114	114	1.00E-61	100.0	99.1	99.1	RNA polymerases M/15 Kd subunit	gbpln	Arabidopsis lyrata	AT4G16265.1 | Symbols: NRPB9B, NRPD9B, NRPE9B | RNA polymerases M/15 Kd subunit | chr4:9202529-9203975 REVERSE LENGTH=114	114	114	2.00E-64	100.0	99.1	99.1
Rsa1.0_00077.1_g3971.t1	ref|XP_002870166.1| peroxidase 40 [Arabidopsis lyrata subsp. lyrata] gi|297316002|gb|EFH46425.1| peroxidase 40 [Arabidopsis lyrata subsp. lyrata]	359	362	1.00E-179	100.8	87.2	90.5	peroxidase 40	gbpln	Arabidopsis lyrata	AT4G16270.1 | Symbols: | Peroxidase superfamily protein | chr4:9205038-9206483 FORWARD LENGTH=362	359	362	1.00E-173	100.8	82.7	86.4
Rsa1.0_00077.1_g3972.t1	gb|AAL61622.1|AF414188_1 FCA gamma [Brassica napus]	572	715	0	125.0	89.0	92.1	FCA gamma	gbpln	Brassica napus	AT4G16280.4 | Symbols: | RNA binding;abscisic acid binding | chr4:9207164-9214187 REVERSE LENGTH=672	572	672	0	117.5	76.7	81.6
Rsa1.0_00077.1_g3973.t1	emb|CAB41488.2| putative FCA orthologue [Brassica napus]	266	384	3.00E-47	144.4	39.5	42.1	putative FCA orthologue	gbpln	Brassica napus	AT4G16280.2 | Symbols: FCA | RNA binding;abscisic acid binding | chr4:9207164-9214412 REVERSE LENGTH=747	266	747	3.00E-26	280.8	28.9	33.8
Rsa1.0_00077.1_g3974.t1	ref|XP_002883592.1| hypothetical protein ARALYDRAFT_342698 [Arabidopsis lyrata subsp. lyrata] gi|297329432|gb|EFH59851.1| hypothetical protein ARALYDRAFT_342698 [Arabidopsis lyrata subsp. lyrata]	288	369	2.00E-85	128.1	61.1	75.3	hypothetical protein ARALYDRAFT_342698	gbpln	Arabidopsis lyrata	AT3G19470.2 | Symbols: | F-box and associated interaction domains-containing protein | chr3:6749766-6750914 FORWARD LENGTH=382	288	382	1.00E-78	132.6	59.4	72.2
Rsa1.0_00077.1_g3975.t1	ref|XP_002868142.1| hypothetical protein ARALYDRAFT_355126 [Arabidopsis lyrata subsp. lyrata] gi|297313978|gb|EFH44401.1| hypothetical protein ARALYDRAFT_355126 [Arabidopsis lyrata subsp. lyrata]	342	338	1.00E-168	98.8	83.9	91.5	hypothetical protein ARALYDRAFT_355126	gbpln	Arabidopsis lyrata	AT4G16330.2 | Symbols: | 2-oxoglutarate (2OG) and Fe(II)-dependent oxygenase superfamily protein | chr4:9226298-9227914 REVERSE LENGTH=364	342	364	1.00E-156	106.4	76.9	82.7
Rsa1.0_00077.1_g3976.t2	ref|XP_002868141.1| hypothetical protein ARALYDRAFT_493252 [Arabidopsis lyrata subsp. lyrata] gi|297313977|gb|EFH44400.1| hypothetical protein ARALYDRAFT_493252 [Arabidopsis lyrata subsp. lyrata]	1826	1830	0	100.2	96.4	98.0	hypothetical protein ARALYDRAFT_493252	gbpln	Arabidopsis lyrata	AT4G16340.1 | Symbols: SPK1 | guanyl-nucleotide exchange factors;GTPase binding;GTP binding | chr4:9228773-9241060 REVERSE LENGTH=1830	1826	1830	0	100.2	95.8	97.8
Rsa1.0_00077.1_g3977.t2	ref|XP_002868140.1| hypothetical protein ARALYDRAFT_493251 [Arabidopsis lyrata subsp. lyrata] gi|297313976|gb|EFH44399.1| hypothetical protein ARALYDRAFT_493251 [Arabidopsis lyrata subsp. lyrata]	263	226	1.00E-90	85.9	61.6	69.2	hypothetical protein ARALYDRAFT_493251	gbpln	Arabidopsis lyrata	AT4G16350.1 | Symbols: CBL6, SCABP2 | calcineurin B-like protein 6 | chr4:9242505-9243669 REVERSE LENGTH=226	263	226	9.00E-92	85.9	60.5	68.1

Rsa1.0_00077.1.g3978.t1	ref|NP_974559.5| metal ion binding protein [Arabidopsis thaliana] gi|332658339|gb|AEE83739.1| metal ion binding protein [Arabidopsis thaliana]	271	254	3.00E-67	93.7	71.6	75.6	metal ion binding protein	gbpln	Arabidopsis thaliana	AT4G16380.1 | Symbols: | Heavy metal transport/detoxification superfamily protein | chr4:9254638-9255955 FORWARD LENGTH=254	271	254	8.00E-70	93.7	71.6	75.6
Rsa1.0_00077.1.g3979.t1	emb|CAC01940.1| 67kD chloroplastic RNA-binding protein, P67.1 [Raphanus sativus]	701	700	0	99.9	98.4	99.1	67kD chloroplastic RNA-binding protein, P67.1	gbpln	Raphanus sativus	AT4G16390.1 | Symbols: SVR7 | pentatricopeptide (PPR) repeat-containing protein | chr4:9257985-9260093 FORWARD LENGTH=702	701	702	0	100.1	86.7	92.7
Rsa1.0_00077.1.g3980.t1	emb|CAB10417.1| hypothetical protein [Arabidopsis thaliana] gi|7268389|emb|CAB78682.1| hypothetical protein [Arabidopsis thaliana]	96	209	6.00E-18	217.7	52.1	66.7	hypothetical protein	gbpln	Arabidopsis thaliana	AT4G16400.1 | Symbols: | unknown protein; BEST Arabidopsis thaliana protein match is: unknown protein (TAIR:AT3G13175.1); Has 30201 Blast hits to 17322 proteins in 780 species: Archae - 12; Bacteria - 1396; Metazoa - 17338; Fungi - 3422; Plants - 5037; Viruses - 0; Other Eukaryotes - 2996 (source: NCBI BLink). | chr4:9260564-9262102 FORWARD LENGTH=218	96	218	1.00E-20	227.1	52.1	66.7
Rsa1.0_00077.1.g3981.t1	ref|XP_002870160.1| hypothetical protein ARALYDRAFT_329873 [Arabidopsis lyrata subsp. lyrata] gi|297315996|gb|EFH46419.1| hypothetical protein ARALYDRAFT_329873 [Arabidopsis lyrata subsp. lyrata]	88	192	2.00E-39	218.2	85.2	98.9	hypothetical protein ARALYDRAFT_329873	gbpln	Arabidopsis lyrata	AT4G16400.1 | Symbols: | unknown protein; BEST Arabidopsis thaliana protein match is: unknown protein (TAIR:AT3G13175.1); Has 30201 Blast hits to 17322 proteins in 780 species: Archae - 12; Bacteria - 1396; Metazoa - 17338; Fungi - 3422; Plants - 5037; Viruses - 0; Other Eukaryotes - 2996 (source: NCBI BLink). | chr4:9260564-9262102 FORWARD LENGTH=218	88	218	3.00E-41	247.7	83.0	96.6
Rsa1.0_00077.1.g3982.t1	ref|NP_193376.1| transcription factor bHLH3 [Arabidopsis thaliana] gi|75318117|sp|O23487.1|BH003_ARATH RecName: Full=Transcription factor bHLH3; AltName: Full=Basic helix-loop-helix protein 3; Short=AtbHLH3; Short=bHLH 3; AltName: Full=Transcription factor EN 34; AltName: Full=bHLH transcription factor bHLH003 gi|16226919|gb|AAL16298.1|AF428368_1 AT4g16430/dl4240w [Arabidopsis thaliana] gi|18026954|gb|AAL55710.1|AF251688_1 putative transcription factor BHLH3 [Arabidopsis thaliana] gi|2244999|emb|CAB10419.1| transcription factor like protein [Arabidopsis thaliana] gi|7268393|emb|CAB78685.1| transcription factor like protein [Arabidopsis thaliana] gi|16323045|gb|AAL15257.1| AT4g16430/dl4240w [Arabidopsis thaliana] gi|25141207|gb|AAN73298.1| At4g16430/dl4240w [Arabidopsis thaliana] gi|332658347|gb|AEE83747.1| transcription factor bHLH3 [Arabidopsis thaliana]	465	467	0	100.4	86.0	91.2	transcription factor bHLH3	gbpln	Arabidopsis thaliana	AT4G16430.1 | Symbols: | basic helix-loop-helix (bHLH) DNA-binding superfamily protein | chr4:9267599-9269002 FORWARD LENGTH=467	465	467	0	100.4	86.0	91.2
Rsa1.0_00077.1.g3983.t1	ref|XP_002868134.1| predicted protein [Arabidopsis lyrata subsp. lyrata] gi|297313970|gb|EFH44393.1| predicted protein [Arabidopsis lyrata subsp. lyrata]	468	474	0	101.3	88.9	96.2	predicted protein	gbpln	Arabidopsis lyrata	AT4G16440.1 | Symbols: | ferredoxin hydrogenases | chr4:9269263-9271510 REVERSE LENGTH=474	468	474	0	101.3	89.1	96.2
Rsa1.0_00077.1.g3984.t1	ref|XP_002868133.1| integral membrane family protein [Arabidopsis lyrata subsp. lyrata] gi|341958538|sp|D7M9V1.1|CSPL7_ARALL RecName: Full=CASP-like protein ARALYDRAFT_915107 gi|297313969|gb|EFH44392.1| integral membrane family protein [Arabidopsis lyrata subsp. lyrata]	206	182	5.00E-72	88.3	63.1	76.7	integral membrane family protein	gbpln	Arabidopsis lyrata	AT4G16442.1 | Symbols: | Uncharacterised protein family (UPF0497) | chr4:9272042-9272970 REVERSE LENGTH=182	206	182	5.00E-74	88.3	62.6	76.2

Rsa1.0_00077.1.g3985.t1	ref|XP_002867712.1| hypothetical protein ARALYDRAFT_492539 [Arabidopsis lyrata subsp. lyrata] gi|297313548|gb|EFH43971.1| hypothetical protein ARALYDRAFT_492539 [Arabidopsis lyrata subsp. lyrata]	365	1003	1.00E-126	274.8	63.8	75.1	hypothetical protein ARALYDRAFT_492539	gbpln	Arabidopsis lyrata	AT4G23370.1 | Symbols: | unknown protein; FUNCTIONS IN: molecular_function unknown; INVOLVED IN: biological_process unknown; LOCATED IN: endomembrane system; CONTAINS InterPro DOMAIN/s: Protein of unknown function DUF239, plant (InterPro:IPR004314); BEST Arabidopsis thaliana protein match is: unknown protein (TAIR:AT4G23360.1); Has 2365 Blast hits to 710 proteins in 27 species: Archae - 0; Bacteria - 35; Metazoa - 0; Fungi - 30; Plants - 2300; Viruses - 0; Other Eukaryotes - 0 (source: NCBI BLink). | chr4:12206362-12213435 REVERSE LENGTH=1038	365	1038	1.00E-120	284.4	58.6	74.5
Rsa1.0_00078.1.g3986.t1	gb|AAM63759.1| unknown [Arabidopsis thaliana]	187	187	2.00E-83	100.0	84.0	90.4	unknown	gbpln	Arabidopsis thaliana	AT3G02910.1 | Symbols: | AIG2-like (avirulence induced gene) family protein | chr3:649986-650549 FORWARD LENGTH=187	187	187	3.00E-85	100.0	83.4	89.3
Rsa1.0_00078.1.g3987.t1	ref|NP_186943.1| uncharacterized protein [Arabidopsis thaliana] gi|75186537|sp|Q9M8T5.1|Y3293_ARATH RecName: Full=WEB family protein At3g02930, chloroplastic; Flags: Precursor gi|6728968|gb|AAF26966.1|AC018363_11 unknown protein [Arabidopsis thaliana] gi|332640360|gb|AEE73881.1| uncharacterized protein AT3G02930 [Arabidopsis thaliana]	740	806	0	108.9	70.0	84.1	uncharacterized protein	gbpln	Arabidopsis thaliana	AT3G02930.1 | Symbols: | Plant protein of unknown function (DUF827) | chr3:655306-658319 FORWARD LENGTH=806	740	806	0	108.9	70.0	84.1
Rsa1.0_00078.1.g3988.t1	ref|XP_002882262.1| MYB107 [Arabidopsis lyrata subsp. lyrata] gi|297328102|gb|EFH58521.1| MYB107 [Arabidopsis lyrata subsp. lyrata]	312	322	1.00E-142	103.2	87.2	94.2	MYB107	gbpln	Arabidopsis lyrata	AT3G02940.1 | Symbols: MYB107, AtMYB107 | myb domain protein 107 | chr3:662141-663830 FORWARD LENGTH=321	312	321	1.00E-142	102.9	86.2	92.9
Rsa1.0_00078.1.g3989.t5	ref|NP_566189.1| Tho complex subunit 7/Mft1p [Arabidopsis thaliana] gi|6728966|gb|AAF26964.1|AC018363_9 unknown protein [Arabidopsis thaliana] gi|15983444|gb|AAL11590.1|AF424596_1 AT3g02950/F13E7_10 [Arabidopsis thaliana] gi|48310661|gb|AAT41862.1| At3g02950 [Arabidopsis thaliana] gi|332640363|gb|AEE73884.1| Tho complex subunit 7/Mft1p [Arabidopsis thaliana]	236	236	1.00E-109	100.0	88.1	94.5	Tho complex subunit 7/Mft1p	gbpln	Arabidopsis thaliana	AT3G02950.1 | Symbols: THO7, AtTHO7 | Tho complex subunit 7/Mft1p | chr3:665631-666439 REVERSE LENGTH=236	236	236	1.00E-111	100.0	88.1	94.5
Rsa1.0_00078.1.g3990.t2	ref|NP_186947.1| protein exordium like 6 [Arabidopsis thaliana] gi|6728964|gb|AAF26962.1|AC018363_7 phi-1-like protein [Arabidopsis thaliana] gi|332640365|gb|AEE73886.1| protein exordium like 6 [Arabidopsis thaliana]	603	332	1.00E-144	55.1	42.0	45.6	protein exordium like 6	gbpln	Arabidopsis thaliana	AT3G02970.1 | Symbols: EXL6 | EXORDIUM like 6 | chr3:669217-670495 REVERSE LENGTH=332	603	332	2.33E-156	55.1	42.0	45.6
Rsa1.0_00078.1.g3991.t1	gb|EOA30505.1| hypothetical protein CARUB_v10013628mg [Capsella rubella]	462	468	0	101.3	80.3	86.8	hypothetical protein CARUB_v10013628mg	gbpln	Capsella rubella	AT3G02990.1 | Symbols: ATHSFA1E, HSFA1E | heat shock transcription factor A1E | chr3:673614-675988 FORWARD LENGTH=468	462	468	0	101.3	81.4	88.3
Rsa1.0_00078.1.g3992.t1	ref|NP_186924.1| putative protein phosphatase 2C 33 [Arabidopsis thaliana] gi|79295498|ref|NP_001030624.1| putative protein phosphatase 2C 33 [Arabidopsis thaliana] gi|75186497|sp|Q9M8R7.1|P2C33_ARATH RecName: Full=Probable protein phosphatase 2C 33; Short=AtPP2C33; AltName: Full=AtPPC6;1 gi|6728987|gb|AAF26985.1|AC018363_30 putative protein phosphatase-2C (PP2C) [Arabidopsis thaliana] gi|58652052|gb|AAW80851.1| At3g02750 [Arabidopsis thaliana] gi|115311509|gb|ABI93935.1| At3g02750 [Arabidopsis thaliana] gi|332640333|gb|AEE73854.1| putative protein phosphatase 2C 33 [Arabidopsis thaliana] gi|332640334|gb|AEE73855.1| putative protein phosphatase 2C 33 [Arabidopsis thaliana]	193	492	5.00E-40	254.9	54.4	58.0	putative protein phosphatase 2C 33	gbpln	Arabidopsis thaliana	AT3G02750.2 | Symbols: | Protein phosphatase 2C family protein | chr3:593601-595457 REVERSE LENGTH=492	193	492	2.00E-42	254.9	54.4	58.0

Rsa1.0_00078.1.g3993.t1	ref|NP_186923.1| aspartyl protease-like protein [Arabidopsis thaliana] gi|6728988|gb|AAF26986.1|AC018363_31 putative aspartyl protease [Arabidopsis thaliana] gi|21593593|gb|AAM65560.1| putative aspartyl protease [Arabidopsis thaliana] gi|332640332|gb|AEE73853.1| aspartyl protease-like protein [Arabidopsis thaliana]	490	488	0	99.6	83.9	90.6	aspartyl protease-like protein	gbpln	Arabidopsis thaliana	AT3G02740.1 | Symbols: | Eukaryotic aspartyl protease family protein | chr3:590561-593089 FORWARD LENGTH=488	490	488	0	99.6	83.9	90.6
Rsa1.0_00078.1.g3994.t1	ref|XP_002884335.1| thioredoxin F-type 1 [Arabidopsis lyrata subsp. lyrata] gi|297330175|gb|EFH60594.1| thioredoxin F-type 1 [Arabidopsis lyrata subsp. lyrata]	182	178	4.00E-82	97.8	84.1	87.9	thioredoxin F-type 1	gbpln	Arabidopsis lyrata	AT3G02730.1 | Symbols: TRXF1, ATF1 | thioredoxin F-type 1 | chr3:588570-589591 REVERSE LENGTH=178	182	178	4.00E-83	97.8	82.4	87.9
Rsa1.0_00078.1.g3995.t2	gb|EOA14628.1| hypothetical protein CARUB_v10027900mg [Capsella rubella]	524	550	0	105.0	64.3	76.0	hypothetical protein CARUB_v10027900mg	gbpln	Capsella rubella	AT5G54050.1 | Symbols: | Cysteine/Histidine-rich C1 domain family protein | chr5:21933663-21935970 REVERSE LENGTH=580	524	580	0	110.7	66.2	80.0
Rsa1.0_00078.1.g3996.t1	ref|XP_002882249.1| hypothetical protein ARALYDRAFT_896249 [Arabidopsis lyrata subsp. lyrata] gi|297328089|gb|EFH58508.1| hypothetical protein ARALYDRAFT_896249 [Arabidopsis lyrata subsp. lyrata]	388	388	0	100.0	92.0	95.4	hypothetical protein ARALYDRAFT_896249	gbpln	Arabidopsis lyrata	AT3G02720.1 | Symbols: | Class I glutamine amidotransferase-like superfamily protein | chr3:586336-588088 FORWARD LENGTH=388	388	388	0	100.0	91.0	94.8
Rsa1.0_00078.1.g3997.t1	ref|NP_566174.1| 14-3-3-like protein GF14 nu [Arabidopsis thaliana] gi|3023217|sp|Q96300.1|14337_ARATH RecName: Full=14-3-3-like protein GF14 nu; AltName: Full=General regulatory factor 7 gi|5802792|gb|AAD51782.1|AF145299_1 14-3-3 protein GF14 nu [Arabidopsis thaliana] gi|1531631|gb|AAB49335.1| GF14 nu [Arabidopsis thaliana] gi|6957715|gb|AAF32459.1| putative 14-3-3 protein [Arabidopsis thaliana] gi|17529080|gb|AAL38750.1| putative 14-3-3 protein GF14nu (grf7) [Arabidopsis thaliana] gi|20465433|gb|AAM20176.1| putative 14-3-3 protein [Arabidopsis thaliana] gi|21593311|gb|AAM65260.1| 14-3-3 protein GF14nu (grf7) [Arabidopsis thaliana] gi|332640301|gb|AEE73822.1| 14-3-3-like protein GF14 nu [Arabidopsis thaliana]	261	265	1.00E-141	101.5	95.0	98.1	14-3-3-like protein GF14 nu	gbpln	Arabidopsis thaliana	AT3G02520.1 | Symbols: GRF7, GF14 NU | general regulatory factor 7 | chr3:526800-527915 REVERSE LENGTH=265	261	265	1.00E-144	101.5	95.0	98.1
Rsa1.0_00078.1.g3998.t1	ref|XP_002884325.1| hypothetical protein ARALYDRAFT_477487 [Arabidopsis lyrata subsp. lyrata] gi|297330165|gb|EFH60584.1| hypothetical protein ARALYDRAFT_477487 [Arabidopsis lyrata subsp. lyrata]	535	535	0	100.0	98.7	99.8	hypothetical protein ARALYDRAFT_477487	gbpln	Arabidopsis lyrata	AT3G02530.1 | Symbols: | TCP-1/cpn60 chaperonin family protein | chr3:528806-532457 REVERSE LENGTH=535	535	535	0	100.0	98.1	99.6
Rsa1.0_00078.1.g3999.t1	sp|Q9XH45.1|RS7_BRAOL RecName: Full=40S ribosomal protein S7 gi|5532505|gb|AAD44761.1|AF144752_1 40S ribosomal protein S7 homolog [Brassica oleracea]	202	191	1.00E-100	94.6	87.6	91.6	RecName: Full=40S ribosomal protein S7 gi|5532505|gb|AAD44761.1|AF144752_1 40S ribosomal protein S7 homolog	gbpln	Brassica oleracea	AT3G02560.2 | Symbols: | Ribosomal protein S7e family protein | chr3:542341-543168 FORWARD LENGTH=191	202	191	1.00E-100	94.6	85.1	91.1
Rsa1.0_00078.1.g4000.t2	ref|NP_186906.1| mannose-6-phosphate isomerase [Arabidopsis thaliana] gi|75336067|sp|Q9M884.1|MPI1_ARATH RecName: Full=Mannose-6-phosphate isomerase 1; AltName: Full=Phosphohexomutase 1; AltName: Full=Phosphomannose isomerase 1; Short=PMI1; AltName: Full=Protein MATERNAL EFFECT EMBRYO ARREST 31 gi|6957720|gb|AAF32464.1| putative mannose-6-phosphate isomerase [Arabidopsis thaliana] gi|17979416|gb|AAL49850.1| putative mannose-6-phosphate isomerase [Arabidopsis thaliana] gi|20465985|gb|AAM20214.1| putative mannose-6-phosphate isomerase [Arabidopsis thaliana] gi|332640310|gb|AEE73831.1| mannose-6-phosphate isomerase [Arabidopsis thaliana]	427	432	0	101.2	92.0	95.6	mannose-6-phosphate isomerase	gbpln	Arabidopsis thaliana	AT3G02570.1 | Symbols: MEE31, PMI1 | Mannose-6-phosphate isomerase, type I | chr3:543463-545478 REVERSE LENGTH=432	427	432	0	101.2	92.0	95.6

Rsa1.0_00078.1.g4001.t1	ref|NP_186907.1| delta(7)-sterol-C5(6)-desaturase 1 [Arabidopsis thaliana] gi|33301592|sp|Q39208.2|SC5D1_ARATH RecName: Full=Delta(7)-sterol-C5(6)-desaturase 1; AltName: Full=Delta(7)-sterol-C5-desaturase 1; AltName: Full=Delta-7-C-5 sterol desaturase 1; AltName: Full=Protein STEROL 1 gi|4234768|gb|AAD12944.1| sterol-C5-desaturase [Arabidopsis thaliana] gi|6957721|gb|AAF32465.1| sterol-C5-desaturase [Arabidopsis thaliana] gi|51971639|dbj|BAD44484.1| sterol-C5-desaturase [Arabidopsis thaliana] gi|105829795|gb|ABF74704.1| At3g02580 [Arabidopsis thaliana] gi|332640311|gb|AEE73832.1| delta(7)-sterol-C5(6)-desaturase 1 [Arabidopsis thaliana]	276	281	1.00E-144	101.8	89.1	94.2	delta(7)-sterol-C5(6)-desaturase 1	gbpln	Arabidopsis thaliana	AT3G02580.1 | Symbols: STE1, DWF7, BUL1 | sterol 1 | chr3:547048-548615 FORWARD LENGTH=281	276	281	1.00E-147	101.8	89.1	94.2
Rsa1.0_00078.1.g4002.t4	gb|EOA30892.1| hypothetical protein CARUB_v10014038mg [Capsella rubella]	374	360	0	96.3	84.0	86.6	hypothetical protein CARUB_v10014038mg	gbpln	Capsella rubella	AT3G02600.1 | Symbols: LPP3, ATLPP3 | lipid phosphate phosphatase 3 | chr3:551534-554411 FORWARD LENGTH=364	374	364	0	97.3	82.4	86.1
Rsa1.0_00078.1.g4003.t1	ref|NP_186910.2| acyl-[acyl-carrier-protein] desaturase [Arabidopsis thaliana] gi|332640318|gb|AEE73839.1| acyl-[acyl-carrier-protein] desaturase [Arabidopsis thaliana]	409	413	0	101.0	90.0	92.9	acyl-	gbpln	Arabidopsis thaliana	AT3G02610.1 | Symbols: | Plant stearoyl-acyl-carrier-protein desaturase family protein | chr3:555665-557458 FORWARD LENGTH=413	409	413	0	101.0	90.0	92.9
Rsa1.0_00078.1.g4004.t1	gb|AAM65632.1| PhD-finger protein, putative [Arabidopsis thaliana]	684	429	1.00E-104	62.7	34.4	42.8	PhD-finger protein, putative	gbpln	Arabidopsis thaliana	AT1G14770.2 | Symbols: | RING/FYVE/PHD zinc finger superfamily protein | chr1:5086867-5088437 REVERSE LENGTH=429	684	429	1.00E-106	62.7	34.4	42.8
Rsa1.0_00078.1.g4005.t1	gb|ADG57793.1| S-adenosyl-l-methionine decarboxylase 3-2 [Brassica napus]	364	368	0	101.1	96.2	98.6	S-adenosyl-l-methionine decarboxylase 3-2	gbpln	Brassica napus	AT3G02470.4 | Symbols: | S-adenosylmethionine decarboxylase | chr3:510223-511323 FORWARD LENGTH=366	364	366	0	100.5	93.4	97.5
Rsa1.0_00078.1.g4006.t2	gb|EOA30699.1| hypothetical protein CARUB_v10013836mg [Capsella rubella]	387	409	0	105.7	86.8	92.8	hypothetical protein CARUB_v10013836mg	gbpln	Capsella rubella	AT3G02410.1 | Symbols: ICME-LIKE2 | alpha/beta-Hydrolases superfamily protein | chr3:492118-494877 REVERSE LENGTH=422	387	422	0	109.0	85.8	92.0
Rsa1.0_00078.1.g4007.t1	gb|EOA30433.1| hypothetical protein CARUB_v10013557mg [Capsella rubella]	488	486	0	99.6	96.3	98.4	hypothetical protein CARUB_v10013557mg	gbpln	Capsella rubella	AT3G02360.1 | Symbols: | 6-phosphogluconate dehydrogenase family protein | chr3:482498-483958 FORWARD LENGTH=486	488	486	0	99.6	95.7	98.4
Rsa1.0_00078.1.g4008.t2	#	#	#	#	#	#	#	–	----	----	#	#	#	#	#	#	#
[truncated: 710,713 more chars]
