# Supplementary material for: Draft Sequences of the Radish (Raphanus sativus L.) Genome
Source: DNA Res. 2014 May 16;21(5):481–90. doi: 10.1093/dnares/dsu014 (PMC4195494; doi:10.1093/dnares/dsu014)
Supplement: Supplementary Data [file supp_dsu014_dsu014supp_tables5.pdf]

Table S5 Gene prediction by Augustus 2.7 with *A. thaliana* training set

Groups	Number of CDSs
Transposable Element	15,545
(partial, psudo, short)	
Intrinsic genes	45,002
Partial	16,570
Psudo and short genes	3,404
Total	80,521

Table S6. Numbers of predicted tRNAs

	R. sativus	*B. rapa*	*A. thaliana*
	RSA_r1.0	BRAD, v1.2	TAIR10
Ala	81	72	33
Arg	89	78	39
Asn	43	31	19
Asp	71	54	28
Cys	33	33	17
Gln	40	35	19
Glu	66	57	27
Gly	109	94	43
His	29	33	12
Ile	65	48	25
Leu	99	85	45
Lys	71	68	33
Met	54	52	31
Phe	42	36	17
Pro	112	101	68
Ser	83	75	72
Thr	48	53	26
Trp	28	30	16
Tyr	51	38	83
Val	66	65	32
Pseudo	52	38	13
SeC	0	1	0
Sup	0	1	0
Undet	3	2	1
Total	1,335	1,180	699

Pseudo; Predicted pseudogenes

SeC; Selenocysteine tRNAs

Sup; Possible suppressor tRNAs

Undet; tRNAs with undetermined/unknown isotypes

Table S7 Blast search of RSA_r1.0 with RadishBase

Query	Subject	Length (query)	Length (subject)	E-value	Identity	Aligned length	Identity%	Strand	Start (query)	End (query)	Start (subject)	End (subject)
UN00001	Rsa1.0_03223.1	853	24512	0	725	767	94	+	90	851	12771	13536
UN00002	#	#	#	#	#	#	#	#	#	#	#	#
UN00003	Rsa1.0_04281.1	640	18107	0	443	467	94	+	175	640	14748	15204
UN00004	Rsa1.0_00773.1	848	64839	0	523	560	93	+	266	813	25939	26496
UN00005	Rsa1.0_00265.1	941	220635	0	421	428	98	−	6	433	162036	162463
UN00006	Rsa1.0_06990.1	988	10060	0	853	916	93	+	88	988	4389	5287
UN00007	Rsa1.0_01338.1	959	155644	1.00E−166	344	359	95	−	509	867	148865	149220
UN00008	Rsa1.0_00652.1	750	70089	0	727	742	97	+	3	744	34069	34809
UN00009	Rsa1.0_01278.1	1423	49370	0	1103	1231	89	+	195	1420	9095	10323
UN00010	Rsa1.0_00008.1	750	358124	5.00E−98	185	186	99	+	3	188	306470	306655
UN00011	Rsa1.0_02570.1	770	30000	0	743	769	96	−	5	770	5790	6551
UN00012	Rsa1.0_01561.1	770	43227	1.00E−166	303	305	99	+	214	518	30907	31211
UN00013	Rsa1.0_02039.1	1016	35699	1.00E−120	343	380	90	+	3	378	899	1259
UN00014	Rsa1.0_03440.1	863	22928	0	843	891	94	−	3	863	8551	9434
UN00015	Rsa1.0_02079.1	1084	35306	0	365	375	97	−	710	1084	3738	4111
UN00016	Rsa1.0_01502.1	947	44519	0	515	532	96	−	3	531	36621	37147
UN00017	Rsa1.0_00200.1	776	162016	0	440	463	95	+	2	464	113402	113852
UN00018	Rsa1.0_00655.1	804	195840	1.00E−67	161	171	94	+	387	557	164863	165033
UN00019	Rsa1.0_07105.1	654	9886	1.00E−167	329	338	97	−	317	654	1509	1845
UN00020	Rsa1.0_00556.1	788	207212	0	780	786	99	−	3	788	135257	136036
UN00021	Rsa1.0_03348.1	906	23559	0	601	637	94	+	285	906	9013	9640
UN00022	Rsa1.0_00254.1	1193	102600	1.00E−139	251	251	100	+	3	253	87897	88147
UN00023	Rsa1.0_04106.1	730	19018	1.00E−178	328	332	98	−	2	333	6894	7225
UN00024	Rsa1.0_01847.1	776	38346	0	392	393	99	+	1	393	985	1377
UN00025	Rsa1.0_39229.1	1069	573	1.00E−179	349	355	98	+	716	1069	57	409
UN00026	Rsa1.0_00651.1	714	247766	1.00E−108	216	219	98	+	460	677	200722	200939
UN00027	Rsa1.0_00108.1	700	478491	0	576	603	95	−	14	616	460317	460913
UN00028	Rsa1.0_01166.1	815	51877	0	423	438	96	+	379	815	26786	27222
UN00029	Rsa1.0_01251.1	911	49873	2.00E−88	256	286	89	+	628	911	33589	33856
UN00030	Rsa1.0_00385.1	476	89160	1.00E−122	238	242	98	+	3	244	30406	30646
UN00031	Rsa1.0_00824.1	1129	188447	0	473	478	98	−	650	1125	144181	144658
UN00032	Rsa1.0_00559.1	927	190486	0	378	389	97	−	392	779	2470	2858
UN00033	Rsa1.0_00676.1	1303	69166	0	489	519	94	−	166	669	54623	55138
UN00034	Rsa1.0_00707.1	1226	190112	0	622	660	94	−	587	1226	139912	140571
UN00035	Rsa1.0_01758.1	1169	39747	0	612	618	99	−	552	1169	34996	35612
UN00036	Rsa1.0_00066.1	871	152418	0	462	489	94	−	392	871	37136	37622
UN00037	Rsa1.0_00248.1	769	163568	0	359	366	98	−	389	754	40157	40522

UN00038	Rsa1.0_03413.1	794	23137	0	684	740	92	+	3	725	15902	16631
UN00039	Rsa1.0_16170.1	924	2258	0	333	335	99	−	591	924	1208	1542
UN00040	Rsa1.0_01896.1	951	37512	0	917	951	96	−	2	951	31690	32640
UN00041	Rsa1.0_00972.1	1053	57937	0	491	510	96	−	549	1053	52689	53193
UN00042	Rsa1.0_00035.1	1355	176449	0	901	952	94	−	4	952	125394	126333
UN00043	Rsa1.0_00418.1	1380	215377	0	1250	1296	96	−	95	1380	24897	26192
UN00044	Rsa1.0_00031.1	1346	179862	0	486	504	96	−	82	585	82448	82951
UN00045	Rsa1.0_00707.1	885	190112	0	874	885	98	−	1	885	8145	9028
UN00046	Rsa1.0_00387.1	572	495407	0	413	431	95	+	3	433	468202	468632
UN00047	Rsa1.0_00716.1	562	67078	0	417	426	97	−	137	562	37489	37913
UN00048	Rsa1.0_01029.1	1045	256254	1.00E−133	308	329	93	+	724	1042	245893	246219
UN00049	Rsa1.0_00104.1	1112	368722	0	1074	1111	96	−	3	1112	71831	72940
UN00050	Rsa1.0_03944.1	661	19926	1.00E−140	331	356	92	+	11	359	9333	9679
UN00051	Rsa1.0_29195.1	621	789	6.00E−57	129	133	96	+	482	613	551	683
UN00052	Rsa1.0_20338.1	1125	1316	1.00E−139	355	387	91	−	722	1106	930	1316
UN00053	Rsa1.0_04786.1	1088	15988	0	656	686	95	+	2	687	11442	12126
UN00054	Rsa1.0_00816.1	988	403350	1.00E−120	283	301	94	+	692	988	370202	370496
UN00055	Rsa1.0_07851.1	681	8423	1.00E−141	270	275	98	+	407	681	4308	4582
UN00056	Rsa1.0_00876.1	1346	61328	1.00E−120	276	295	93	+	73	361	49382	49676
UN00057	Rsa1.0_00390.1	1112	88681	1.00E−151	275	276	99	+	787	1062	35462	35737
UN00058	Rsa1.0_00939.1	1462	185227	1.00E−175	321	324	99	−	733	1056	178977	179300
UN00059	Rsa1.0_01245.1	1403	149868	0	593	635	93	−	5	638	37644	38260
UN00060	Rsa1.0_00145.1	1500	261008	1.00E−124	248	255	97	−	415	669	39995	40249
UN00061	Rsa1.0_02071.1	842	35409	1.00E−113	318	355	89	−	1	355	33313	33667
UN00062	Rsa1.0_00079.1	791	606987	1.00E−102	214	221	96	−	521	740	464325	464545
UN00063	Rsa1.0_35376.1	1129	640	1.00E−105	289	324	89	−	420	722	164	487
UN00064	Rsa1.0_00278.1	1295	231896	1.00E−107	219	226	96	+	808	1033	148696	148921
UN00065	Rsa1.0_10637.1	1028	5774	1.00E−180	374	392	95	+	633	1024	2793	3184
UN00066	Rsa1.0_00090.1	825	273859	1.00E−126	273	287	95	−	1	279	41355	41641
UN00067	Rsa1.0_06390.1	1239	11106	0	609	693	87	−	73	765	6070	6756
UN00068	Rsa1.0_00229.1	798	105471	0	669	692	96	+	114	798	82848	83539
UN00069	Rsa1.0_00203.1	1012	109129	0	632	667	94	+	349	1011	38683	39345
UN00070	Rsa1.0_03295.1	508	23970	1.00E−152	282	285	98	+	224	508	7379	7663
UN00071	Rsa1.0_01958.1	804	261128	1.00E−172	322	327	98	−	57	383	131033	131359
UN00072	Rsa1.0_09116.1	1544	6637	0	1061	1071	99	−	3	1073	182	1252
UN00073	Rsa1.0_02185.1	1181	34150	0	500	523	95	−	199	716	21129	21651
UN00074	Rsa1.0_00466.1	860	82132	0	395	403	98	−	459	860	21576	21978
UN00075	Rsa1.0_02241.1	1265	33520	1.00E−121	224	225	99	+	793	1017	23921	24145
UN00076	Rsa1.0_01382.1	832	46877	0	483	514	93	+	330	832	13890	14398

UN00077	Rsa1.0_01466.1	750	45161	1.00E-111	252	260	96	+	497	749	28487	28746
UN00078	Rsa1.0_00048.1	914	167185	1.00E-92	224	237	94	+	672	906	33623	33858
UN00079	Rsa1.0_00454.1	1316	216970	0	384	395	97	-	615	1009	191029	191423
UN00080	Rsa1.0_03662.1	1284	21492	0	1209	1299	93	+	1	1284	12730	14005
UN00081	Rsa1.0_03740.1	1385	21065	0	413	418	98	-	466	883	9526	9943
UN00082	Rsa1.0_00944.1	539	58733	0	450	469	95	+	76	539	35701	36167
UN00083	Rsa1.0_00476.1	1330	280966	0	553	561	98	-	6	566	212345	212904
UN00084	Rsa1.0_00857.1	885	61904	0	526	555	94	-	331	885	44571	45114
UN00085	Rsa1.0_00115.1	1093	131321	1.00E-153	335	353	94	+	717	1066	48839	49191
UN00086	Rsa1.0_00218.1	694	201480	1.00E-134	246	247	99	+	1	247	52227	52473
UN00087	Rsa1.0_03646.1	1041	21598	1.00E-147	307	321	95	-	477	797	11101	11421
UN00088	Rsa1.0_00008.1	707	358124	0	491	534	91	+	122	644	142943	143470
UN00089	Rsa1.0_07479.1	948	9281	1.00E-154	277	277	100	+	71	347	3570	3846
UN00090	Rsa1.0_00454.1	781	216970	0	586	604	97	+	119	722	155884	156487
UN00091	Rsa1.0_06124.1	1048	11681	0	997	1076	92	+	2	1041	6612	7678
UN00092	Rsa1.0_01002.1	1167	57063	0	505	513	98	+	655	1167	18269	18781
UN00093	Rsa1.0_00166.1	1071	240912	0	513	521	98	-	2	522	221531	222051
UN00094	Rsa1.0_00360.1	718	91190	2.00E-79	166	171	97	+	239	409	46323	46493
UN00095	Rsa1.0_00541.1	869	76863	1.00E-161	315	324	97	+	377	700	34580	34903
UN00096	Rsa1.0_04806.1	575	15898	0	355	356	99	+	220	575	7284	7639
UN00097	Rsa1.0_02173.1	784	34318	1.00E-110	219	223	98	+	1	223	15516	15737
UN00098	Rsa1.0_05367.1	479	14010	0	383	397	96	+	73	467	4072	4467
UN00099	Rsa1.0_00487.1	1298	80674	1.00E-161	343	359	95	-	938	1294	37993	38350
UN00100	Rsa1.0_01603.1	919	42468	0	858	945	90	+	3	917	8931	9856
UN00101	Rsa1.0_01905.1	694	37450	8.00E-63	204	233	87	+	181	413	21327	21543
UN00102	Rsa1.0_00115.1	1171	131321	1.00E-178	371	388	95	-	748	1134	49272	49653
UN00103	Rsa1.0_00009.1	802	232558	1.00E-137	262	267	98	-	145	411	132659	132925
UN00104	Rsa1.0_03404.1	1489	23177	0	939	967	97	+	2	962	10088	11054
UN00105	Rsa1.0_02028.1	780	35857	1.00E-140	317	336	94	-	9	344	5808	6140
UN00106	Rsa1.0_01867.1	698	37960	0	414	415	99	-	282	695	19649	20063
UN00107	Rsa1.0_00295.1	1412	152597	0	431	460	93	-	951	1410	48279	48731
UN00108	Rsa1.0_00881.1	966	61260	1.00E-178	693	810	85	+	160	961	47054	47857
UN00109	Rsa1.0_00245.1	1087	103569	0	433	436	99	+	653	1087	80498	80933
UN00110	Rsa1.0_01978.1	823	36546	0	390	398	97	-	1	398	5240	5637
UN00111	Rsa1.0_00451.1	768	322533	0	360	369	97	-	3	371	309261	309629
UN00112	Rsa1.0_00757.1	422	65559	1.00E-75	183	194	94	-	216	405	12485	12678
UN00113	Rsa1.0_40781.1	1447	550	2.33E-156	267	268	99	+	1180	1447	1	268
UN00114	Rsa1.0_06901.1	1047	10186	0	467	489	95	-	7	495	9698	10186
UN00115	Rsa1.0_01519.1	764	44144	1.00E-125	251	259	96	-	337	595	40675	40933

UN00116	Rsa1.0_01666.1	874	173972	0	340	345	98	+	431	775	135570	135914
UN00117	Rsa1.0_01978.1	1397	36546	0	712	722	98	+	678	1397	28038	28759
UN00118	Rsa1.0_00734.1	825	66508	0	465	493	94	−	3	494	44653	45142
UN00119	Rsa1.0_03054.1	838	25738	0	501	501	100	+	1	501	17279	17779
UN00120	Rsa1.0_01563.1	1372	170823	1.00E−161	289	289	100	+	1084	1372	19906	20194
UN00121	Rsa1.0_00028.1	775	293984	1.00E−132	275	287	95	+	489	775	281443	281729
UN00122	Rsa1.0_00149.1	532	305196	1.00E−141	313	328	95	+	204	528	102071	102395
UN00123	Rsa1.0_01985.1	365	36491	2.00E−52	130	137	94	−	229	365	1883	2018
UN00124	Rsa1.0_12214.1	802	5287	1.00E−165	319	327	97	+	262	588	2639	2965
UN00125	Rsa1.0_00552.1	1160	206757	0	594	623	95	−	534	1149	67991	68611
UN00126	Rsa1.0_05632.1	801	13120	0	474	487	97	+	315	801	9527	10010
UN00127	Rsa1.0_04236.1	1562	18370	0	828	830	99	+	733	1562	5917	6746
UN00128	Rsa1.0_24457.1	1183	987	1.00E−105	264	284	92	−	321	595	647	928
UN00129	Rsa1.0_00856.1	1274	62037	7.00E−80	215	237	90	+	1017	1252	16952	17176
UN00130	Rsa1.0_00673.1	1299	69325	1.00E−150	378	410	92	−	897	1299	53354	53747
UN00131	Rsa1.0_00654.1	1356	70012	0	784	811	96	−	31	838	35270	36076
UN00132	Rsa1.0_00832.1	875	62622	0	363	376	96	+	500	875	33766	34139
UN00133	Rsa1.0_00075.1	253	146680	1.00E−120	242	249	97	−	7	253	69250	69498
UN00134	Rsa1.0_02949.1	918	26506	1.00E−111	212	213	99	+	707	918	4000	4212
UN00135	Rsa1.0_00061.1	975	218685	0	547	570	95	+	129	698	99303	99866
UN00136	Rsa1.0_05963.1	689	12090	1.00E−138	269	274	98	+	73	346	2744	3016
UN00137	Rsa1.0_00230.1	1357	105389	0	1169	1217	96	−	151	1357	82518	83733
UN00138	Rsa1.0_00237.1	809	235974	8.00E−94	175	175	100	−	212	386	19068	19242
UN00139	Rsa1.0_01569.1	1312	43029	1.00E−129	262	271	96	+	426	696	37369	37639
UN00140	Rsa1.0_01393.1	1299	46661	1.00E−163	321	331	96	−	975	1299	41309	41639
UN00141	Rsa1.0_00911.1	1024	59943	0	986	1025	96	−	1	1023	56165	57185
UN00142	Rsa1.0_00111.1	1446	390088	7.00E−68	171	179	95	−	1273	1446	33632	33810
UN00143	Rsa1.0_01092.1	978	54348	1.00E−117	243	252	96	+	3	251	50206	50457
UN00144	Rsa1.0_02381.1	668	31972	1.00E−121	242	249	97	+	282	530	25042	25290
UN00145	Rsa1.0_04333.1	844	17865	4.00E−62	131	134	97	−	68	201	13294	13427
UN00146	Rsa1.0_07542.1	729	9160	5.00E−55	110	110	100	+	511	620	2709	2818
UN00147	Rsa1.0_03747.1	796	21046	1.00E−126	252	260	96	−	1	254	20332	20591
UN00148	Rsa1.0_02068.1	587	35432	1.00E−133	348	383	90	−	188	558	15245	15611
UN00149	Rsa1.0_00469.1	610	82084	0	498	506	98	−	105	610	20497	21002
UN00150	Rsa1.0_01789.1	1325	39250	0	450	462	97	+	859	1319	5483	5943
UN00151	Rsa1.0_00092.1	736	659932	1.00E−86	249	276	90	−	463	736	627152	627409
UN00152	Rsa1.0_00416.1	954	86772	1.00E−110	209	211	99	−	743	953	47497	47707
UN00153	Rsa1.0_02958.1	885	26443	0	859	904	95	+	1	885	8747	9650
UN00154	Rsa1.0_02012.1	853	36084	3.00E−87	302	350	86	+	2	351	15973	16286

UN00155	Rsa1.0_05140.1	756	14710	1.00E-111	227	233	97	-	48	280	12012	12243
UN00156	Rsa1.0_02079.1	747	35306	0	476	500	95	-	248	747	16731	17229
UN00157	Rsa1.0_01221.1	835	50421	9.00E-63	126	127	99	+	471	597	47186	47312
UN00158	Rsa1.0_00127.1	793	127446	3.00E-78	152	153	99	-	512	664	30829	30981
UN00159	Rsa1.0_01491.1	1342	44718	0	454	465	97	-	870	1334	42118	42580
UN00160	Rsa1.0_03228.1	784	24471	0	448	463	96	+	326	784	20972	21434
UN00161	Rsa1.0_00732.1	759	66631	1.00E-104	205	209	98	-	348	556	4101	4309
UN00162	Rsa1.0_00469.1	1126	82084	1.00E-144	272	275	98	+	850	1124	69947	70220
UN00163	Rsa1.0_01451.1	900	45459	0	800	812	98	+	1	811	15133	15944
UN00164	Rsa1.0_00545.1	912	189649	0	588	594	98	-	311	904	25078	25671
UN00165	Rsa1.0_00502.1	1430	204180	0	1182	1194	98	-	243	1430	32125	33318
UN00166	Rsa1.0_03488.1	874	22556	0	321	321	100	+	112	432	13317	13637
UN00167	Rsa1.0_00651.1	706	247766	5.00E-67	293	337	86	-	352	681	90205	90524
UN00168	Rsa1.0_03434.1	587	22960	2.33E-156	310	323	95	-	272	587	12154	12476
UN00169	Rsa1.0_01234.1	744	50183	1.00E-104	226	237	95	-	9	245	11958	12194
UN00170	Rsa1.0_00380.1	1251	89748	0	528	582	90	-	669	1250	74642	75200
UN00171	Rsa1.0_06853.1	1391	10264	0	862	902	95	-	2	899	1	899
UN00172	Rsa1.0_16783.1	899	2037	0	392	393	99	+	507	899	1540	1932
UN00173	Rsa1.0_02688.1	789	28644	2.00E-60	148	157	94	-	3	156	13635	13791
UN00174	Rsa1.0_00133.1	670	123768	0	351	358	98	+	1	358	27299	27654
UN00175	Rsa1.0_00876.1	2855	61328	0	1086	1119	97	+	1574	2691	35058	36167
UN00176	Rsa1.0_39807.1	848	564	1.00E-61	162	172	94	-	675	846	296	463
UN00177	Rsa1.0_01422.1	1061	46172	1.00E-136	341	368	92	-	701	1056	24766	25133
UN00178	Rsa1.0_01674.1	2037	168300	1.00E-165	304	307	99	+	554	860	22938	23244
UN00179	Rsa1.0_21410.1	1030	1207	1.00E-103	376	434	86	+	484	913	340	768
UN00180	Rsa1.0_03573.1	1221	22013	1.00E-163	298	300	99	-	1	300	12429	12728
UN00181	Rsa1.0_00611.1	1055	72410	1.00E-111	286	311	91	+	736	1036	1963	2269
UN00182	Rsa1.0_03346.1	589	23589	0	562	586	95	+	1	586	10511	11095
UN00183	Rsa1.0_00017.1	1505	752458	5.00E-78	155	157	98	+	635	791	28933	29089
UN00184	Rsa1.0_01026.1	1301	171624	0	580	605	95	-	700	1299	30310	30906
UN00185	Rsa1.0_00238.1	608	193193	0	459	475	96	+	138	608	190030	190504
UN00186	Rsa1.0_01168.1	685	153765	1.00E-115	283	304	93	+	382	683	41780	42069
UN00187	#	#	#	#	#	#	#	#	#	#	#	#
UN00188	Rsa1.0_02223.1	717	152421	1.00E-141	311	326	95	+	392	715	129665	129988
UN00189	Rsa1.0_00608.1	980	172672	0	352	358	98	+	623	980	100163	100515
UN00190	Rsa1.0_00121.1	1289	278029	1.00E-87	171	173	98	+	489	661	77428	77600
UN00191	Rsa1.0_03467.1	1017	22687	0	636	668	95	+	352	1017	10718	11378
UN00192	Rsa1.0_00172.1	1357	115476	0	906	910	99	-	5	914	58471	59380
UN00193	Rsa1.0_00388.1	1138	88890	1.00E-96	205	212	96	-	882	1092	63459	63670

UN00194	Rsa1.0_00136.1	753	122464	1.00E-46	108	112	96	+	206	317	28826	28937
UN00195	Rsa1.0_01280.1	1024	49321	2.00E-64	132	134	98	−	550	683	38226	38359
UN00196	Rsa1.0_00203.1	915	109129	0	434	445	97	+	3	447	54534	54975
UN00197	Rsa1.0_00123.1	1110	325650	0	650	689	94	+	418	1105	240510	241183
UN00198	Rsa1.0_00127.1	783	127446	0	520	536	97	−	3	538	64912	65446
UN00199	Rsa1.0_04319.1	1323	17944	1.00E-145	264	265	99	+	66	330	16768	17032
UN00200	Rsa1.0_00954.1	890	189536	0	683	692	98	+	199	890	31151	31842
UN00201	Rsa1.0_03274.1	1106	24159	0	615	632	97	+	144	769	14951	15577
UN00202	Rsa1.0_00529.1	1193	77487	1.00E-115	229	235	97	+	609	843	71526	71760
UN00203	Rsa1.0_00042.1	783	171507	2.00E-73	153	157	97	−	386	542	88765	88921
UN00204	Rsa1.0_11811.1	529	5401	8.00E-84	176	182	96	+	48	229	2094	2275
UN00205	Rsa1.0_10543.1	1201	5808	1.00E-178	404	434	93	+	736	1169	4342	4767
UN00206	Rsa1.0_00018.1	1272	410899	0	360	366	98	+	275	640	67806	68169
UN00207	Rsa1.0_00211.1	1836	228189	0	563	599	93	−	432	1006	8748	9346
UN00208	Rsa1.0_00900.1	898	60367	0	475	479	99	−	420	898	19867	20345
UN00209	Rsa1.0_01322.1	1308	48166	0	568	598	94	−	710	1306	7334	7931
UN00210	Rsa1.0_03471.1	620	22678	1.00E-111	227	234	97	−	168	398	14969	15202
UN00211	Rsa1.0_01245.1	894	149868	0	570	587	97	+	309	894	45960	46539
UN00212	Rsa1.0_00437.1	1237	84879	3.00E-97	193	197	97	−	687	883	13824	14020
UN00213	Rsa1.0_02198.1	664	33989	2.00E-75	188	203	92	−	462	664	14016	14207
UN00214	Rsa1.0_01493.1	978	44670	1.00E-110	245	258	94	+	721	978	7846	8101
UN00215	Rsa1.0_03786.1	1361	20879	0	1305	1364	95	+	2	1361	13533	14896
UN00216	Rsa1.0_01768.1	1252	138463	1.00E-133	248	250	99	+	414	663	109251	109500
UN00217	Rsa1.0_00305.1	1035	97308	0	395	405	97	−	599	1003	63923	64326
UN00218	Rsa1.0_11906.1	784	5374	1.00E-133	241	241	100	−	221	461	2650	2890
UN00219	Rsa1.0_00665.1	816	69539	0	621	627	99	−	135	756	64646	65272
UN00220	Rsa1.0_04200.1	1264	18490	1.00E-135	271	280	96	−	3	282	13777	14056
UN00221	Rsa1.0_00111.1	1784	390088	0	370	375	98	+	62	436	332775	333148
UN00222	Rsa1.0_00202.1	1086	185895	0	1040	1050	99	+	39	1086	84872	85920
UN00223	Rsa1.0_00020.1	1218	283099	0	1184	1221	96	+	3	1218	13362	14582
UN00224	Rsa1.0_00805.1	949	63724	1.00E-138	372	409	90	−	545	945	35477	35874
UN00225	Rsa1.0_04961.1	760	15316	0	737	762	96	+	4	760	1405	2163
UN00226	Rsa1.0_00053.1	1349	263459	1.00E-141	312	329	94	+	962	1288	62436	62762
UN00227	Rsa1.0_00222.1	776	172263	3.00E-84	171	175	97	−	170	344	59424	59598
UN00228	Rsa1.0_01762.1	1325	39682	1.00E-103	218	227	96	−	7	233	10878	11104
UN00229	Rsa1.0_00123.1	802	325650	0	605	624	96	−	2	625	295974	296597
UN00230	Rsa1.0_04553.1	873	16877	0	357	365	97	−	509	873	5964	6328
UN00231	Rsa1.0_01504.1	787	189978	1.00E-114	275	296	92	−	10	297	171988	172278
UN00232	Rsa1.0_00655.1	936	195840	0	387	397	97	+	541	936	174971	175367

UN00233	Rsa1.0_01880.1	851	37745	0	815	852	95	−	4	849	35125	35976
UN00234	Rsa1.0_04307.1	1245	18001	0	563	572	98	+	4	575	8159	8730
UN00235	Rsa1.0_00327.1	719	94892	0	707	720	98	−	1	719	70658	71372
UN00236	Rsa1.0_01641.1	436	188213	0	420	449	93	−	2	436	161037	161478
UN00237	Rsa1.0_01562.1	921	43196	1.00E−77	148	148	100	+	649	796	9608	9755
UN00238	Rsa1.0_00048.1	1011	167185	0	386	388	99	+	218	605	120284	120671
UN00239	Rsa1.0_00051.1	762	258225	0	414	426	97	−	340	762	47637	48062
UN00240	Rsa1.0_00190.1	1186	318240	1.00E−102	296	329	89	−	845	1169	196798	197113
UN00241	Rsa1.0_01559.1	755	43262	1.00E−148	309	322	95	+	3	324	3009	3329
UN00242	Rsa1.0_00306.1	990	146760	1.00E−139	390	438	89	−	166	573	133202	133639
UN00243	Rsa1.0_70420.1	1067	325	1.00E−109	220	224	98	−	845	1067	11	232
UN00244	Rsa1.0_00975.1	711	57865	0	439	456	96	+	123	578	55583	56038
UN00245	Rsa1.0_00198.1	652	222963	1.00E−155	326	340	95	+	296	631	107094	107433
UN00246	Rsa1.0_00017.1	1795	752458	0	600	657	91	+	3	658	158694	159335
UN00247	Rsa1.0_04533.1	1113	16953	0	607	617	98	+	497	1113	14028	14644
UN00248	Rsa1.0_00608.1	1087	172672	0	1019	1034	98	+	54	1087	111863	112896
UN00249	Rsa1.0_00358.1	1220	91293	0	552	554	99	−	223	776	56559	57112
UN00250	Rsa1.0_00254.1	1055	102600	0	389	408	95	−	126	533	12642	13039
UN00251	Rsa1.0_08831.1	680	6887	1.00E−95	226	242	93	−	109	350	5156	5397
UN00252	Rsa1.0_02600.1	767	29657	8.00E−63	156	167	93	−	493	659	1755	1921
UN00253	Rsa1.0_00080.1	876	190437	1.00E−133	265	273	97	−	152	424	34258	34530
UN00254	Rsa1.0_00792.1	591	64163	1.00E−79	226	247	91	−	14	248	7358	7603
UN00255	Rsa1.0_01567.1	321	43090	1.00E−115	228	234	97	+	88	321	26025	26258
UN00256	Rsa1.0_00037.1	1290	307838	0	477	501	95	+	1	499	241158	241654
UN00257	Rsa1.0_00067.1	1026	415055	1.00E−148	353	376	93	+	41	411	138300	138672
UN00258	Rsa1.0_03982.1	788	19723	1.00E−132	285	296	96	−	1	289	1	295
UN00259	Rsa1.0_00466.1	538	82132	0	521	532	97	+	1	532	17636	18163
UN00260	Rsa1.0_04298.1	909	147466	1.00E−125	258	267	96	+	52	318	5562	5827
UN00261	Rsa1.0_00007.1	840	239325	0	423	426	99	+	1	424	119516	119941
UN00262	Rsa1.0_00381.1	788	89718	0	400	420	95	+	367	786	75168	75586
UN00263	Rsa1.0_00087.1	1164	761073	1.00E−154	337	353	95	+	2	352	300618	300969
UN00264	Rsa1.0_00030.1	504	250988	3.00E−86	264	294	89	+	79	356	149353	149644
UN00265	Rsa1.0_02515.1	1364	30616	1.00E−151	322	340	94	+	1023	1362	29932	30258
UN00266	Rsa1.0_02386.1	742	31895	0	720	739	97	+	1	739	10448	11185
UN00267	Rsa1.0_03539.1	915	22199	9.00E−91	179	182	98	−	499	680	6376	6557
UN00268	Rsa1.0_00697.1	776	67913	0	752	775	97	−	3	774	46048	46821
UN00269	Rsa1.0_03413.1	732	23137	0	678	753	90	−	1	732	16105	16841
UN00270	Rsa1.0_00131.1	961	432233	1.00E−89	174	176	98	−	785	960	260552	260727
UN00271	Rsa1.0_02355.1	1099	32260	0	621	637	97	+	1	637	6910	7545

UN00272	Rsa1.0_00960.1	937	58199	0	540	562	96	−	254	812	19414	19969
UN00273	Rsa1.0_00253.1	717	231292	1.00E-140	428	485	88	+	1	476	210298	210760
UN00274	Rsa1.0_00288.1	933	98659	1.00E-132	307	328	93	−	523	845	51618	51944
UN00275	Rsa1.0_00045.1	1237	328694	1.00E-130	259	265	97	+	630	894	140951	141214
UN00276	Rsa1.0_01708.1	498	40622	0	495	497	99	+	2	498	19467	19963
UN00277	Rsa1.0_00420.1	1026	86496	0	807	858	94	+	1	833	39871	40728
UN00278	Rsa1.0_00093.1	1005	214962	1.00E-153	348	368	94	−	2	363	48722	49089
UN00279	Rsa1.0_00079.1	712	606987	0	700	712	98	−	1	712	57261	57972
UN00280	Rsa1.0_00477.1	901	81556	1.00E-160	299	302	99	+	13	314	40152	40452
UN00281	Rsa1.0_02004.1	1736	169138	0	1673	1741	96	+	1	1734	148491	150218
UN00282	Rsa1.0_00060.1	1175	502678	0	454	475	95	+	714	1173	144937	145411
UN00283	Rsa1.0_00195.1	1470	251884	0	666	673	98	−	803	1470	205123	205795
UN00284	Rsa1.0_03401.1	1265	23191	1.00E-152	294	301	97	+	575	875	5986	6286
UN00285	Rsa1.0_00281.1	565	99908	2.33E-156	287	295	97	+	269	563	71665	71959
UN00286	Rsa1.0_00339.1	807	222974	4.00E-80	158	160	98	+	566	725	63385	63544
UN00287	Rsa1.0_02585.1	944	29865	0	463	470	98	+	473	941	25874	26343
UN00288	Rsa1.0_06936.1	822	10143	0	804	826	97	−	1	822	77	901
UN00289	Rsa1.0_01149.1	699	160459	0	409	412	99	+	3	414	14501	14912
UN00290	Rsa1.0_01136.1	920	52724	0	636	665	95	−	1	665	35476	36140
UN00291	#	#	#	#	#	#	#	#	#	#	#	#
UN00292	Rsa1.0_03163.1	1401	24894	0	496	512	96	−	2	511	22793	23303
UN00293	Rsa1.0_01756.1	1015	39779	1.00E-120	255	267	95	+	1	262	8898	9164
UN00294	Rsa1.0_04146.1	900	18833	0	730	768	95	+	1	768	18073	18826
UN00295	Rsa1.0_03040.1	992	25812	7.00E-98	238	257	92	+	249	505	3854	4104
UN00296	Rsa1.0_00209.1	994	108531	0	470	470	100	−	1	470	62516	62985
UN00297	Rsa1.0_00738.1	585	66322	1.00E-70	152	156	97	−	7	162	28985	29139
UN00298	Rsa1.0_00273.1	1060	326186	3.00E-97	202	209	96	+	514	722	132133	132341
UN00299	Rsa1.0_01151.1	572	52268	0	352	357	98	+	22	378	26285	26641
UN00300	Rsa1.0_00037.1	828	307838	1.00E-102	210	214	98	−	1	213	307235	307447
UN00301	Rsa1.0_00277.1	969	100132	0	958	969	98	+	1	967	31286	32254
UN00302	Rsa1.0_00734.1	1002	66508	1.00E-56	116	117	99	−	499	615	15185	15301
UN00303	Rsa1.0_00023.1	700	194764	0	678	698	97	+	5	700	37236	37933
UN00304	Rsa1.0_05266.1	1272	14320	0	333	333	100	−	940	1272	7089	7421
UN00305	Rsa1.0_08430.1	829	7367	0	391	406	96	−	7	412	2169	2566
UN00306	Rsa1.0_00273.1	1326	326186	0	379	391	96	+	1	389	38763	39150
UN00307	Rsa1.0_01730.1	942	40223	0	896	944	94	−	3	941	35591	36517
UN00308	Rsa1.0_01482.1	805	44855	0	411	421	97	+	384	803	12466	12886
UN00309	Rsa1.0_00058.1	1308	159921	1.00E-140	253	253	100	+	738	990	22511	22763
UN00310	Rsa1.0_02185.1	1202	34150	0	508	541	93	+	467	1006	21129	21651

UN00311	Rsa1.0_00409.1	541	146788	0	358	368	97	+	170	537	1659	2026
UN00312	Rsa1.0_02496.1	757	30814	3.00E-78	158	161	98	-	514	674	10513	10673
UN00313	Rsa1.0_00055.1	1349	520193	1.00E-145	264	265	99	+	253	517	99465	99729
UN00314	Rsa1.0_01326.1	1763	48072	0	532	548	97	-	954	1501	38848	39389
UN00315	Rsa1.0_00228.1	736	105511	0	665	696	95	+	1	692	48976	49664
UN00316	Rsa1.0_00697.1	854	67913	1.00E-125	292	311	93	+	1	308	36874	37179
UN00317	Rsa1.0_00031.1	1321	179862	0	395	404	97	+	918	1321	161267	161670
UN00318	Rsa1.0_07695.1	1262	8770	0	422	433	97	-	283	715	2664	3095
UN00319	Rsa1.0_00457.1	1059	337301	1.00E-139	261	263	99	+	1	262	288149	288411
UN00320	Rsa1.0_00067.1	1095	415055	0	438	460	95	-	1	457	301029	301485
UN00321	Rsa1.0_00185.1	1085	248445	0	486	498	97	-	588	1085	215144	215641
UN00322	Rsa1.0_00278.1	1215	231896	0	364	372	97	-	10	379	60144	60512
UN00323	Rsa1.0_00453.1	1061	83031	1.00E-84	166	168	98	+	631	798	70878	71045
UN00324	Rsa1.0_00032.1	1317	306326	4.00E-60	142	149	95	+	25	173	177729	177874
UN00325	Rsa1.0_00104.1	779	368722	0	749	789	94	+	1	778	205882	206658
UN00326	Rsa1.0_00420.1	1540	86496	1.00E-127	294	314	93	+	1237	1538	45120	45433
UN00327	Rsa1.0_00015.1	736	561803	0	391	410	95	-	322	731	375314	375723
UN00328	Rsa1.0_06516.1	728	10866	0	398	409	97	-	6	411	8524	8932
UN00329	Rsa1.0_01026.1	996	171624	0	593	614	96	+	1	610	165278	165890
UN00330	Rsa1.0_07049.1	819	9983	0	749	793	94	-	36	819	308	1099
UN00331	Rsa1.0_00073.1	948	240924	1.00E-156	301	307	98	-	633	939	187854	188159
UN00332	Rsa1.0_04490.1	1224	17144	0	1127	1151	97	-	30	1176	12309	13459
UN00333	Rsa1.0_01087.1	746	54508	1.00E-154	311	321	96	-	5	324	19557	19877
UN00334	Rsa1.0_02954.1	1111	26485	0	1032	1073	96	+	52	1109	16486	17556
UN00335	Rsa1.0_21366.1	704	1211	1.00E-131	248	250	99	-	3	252	1	249
UN00336	Rsa1.0_00326.1	706	171955	0	415	431	96	+	2	431	66166	66589
UN00337	Rsa1.0_00077.1	870	145005	0	826	880	93	-	1	870	33244	34109
UN00338	Rsa1.0_00009.1	864	232558	0	422	430	98	-	162	591	129957	130386
UN00339	Rsa1.0_05815.1	988	12563	1.00E-115	253	267	94	-	409	675	8883	9149
UN00340	Rsa1.0_00546.1	756	76523	0	455	472	96	+	109	580	60591	61062
UN00341	Rsa1.0_00003.1	795	340054	0	427	430	99	-	1	430	87090	87517
UN00342	Rsa1.0_00171.1	903	115622	1.00E-157	282	282	100	-	4	285	36495	36776
UN00343	Rsa1.0_14764.1	795	3084	0	583	602	96	+	3	603	281	882
UN00344	Rsa1.0_00038.1	984	314568	1.00E-132	246	248	99	+	37	284	47930	48177
UN00345	Rsa1.0_00078.1	1331	175053	0	405	428	94	-	444	868	101013	101438
UN00346	Rsa1.0_07456.1	837	9360	0	733	836	87	-	1	823	4378	5213
UN00347	Rsa1.0_01586.1	1053	42804	1.00E-119	245	254	96	-	615	868	28243	28496
UN00348	Rsa1.0_00733.1	804	66583	0	557	575	96	+	174	744	46236	46809
UN00349	Rsa1.0_41608.1	808	540	1.00E-155	296	302	98	-	431	732	1	302

UN00350	Rsa1.0_03650.1	1309	21583	1.00E-159	294	297	98	-	1	297	9550	9846
UN00351	Rsa1.0_00965.1	1206	58139	1.00E-118	257	270	95	+	936	1204	9265	9527
UN00352	Rsa1.0_00129.1	759	126831	5.00E-92	175	176	99	+	3	178	43923	44098
UN00353	Rsa1.0_00067.1	1712	415055	0	418	418	100	-	468	885	391301	391718
UN00354	Rsa1.0_01144.1	1287	52431	1.00E-174	362	376	96	+	8	378	22890	23265
UN00355	Rsa1.0_06373.1	1122	11138	0	960	1010	95	-	125	1120	3623	4618
UN00356	Rsa1.0_00964.1	711	316648	3.00E-93	223	237	94	+	477	711	165354	165589
UN00357	Rsa1.0_02673.1	1286	28877	3.00E-94	223	239	93	-	1046	1284	25637	25864
UN00358	Rsa1.0_04870.1	975	135142	0	386	392	98	-	584	975	164	554
UN00359	Rsa1.0_08339.1	1202	7509	0	960	985	97	-	121	1100	10	991
UN00360	Rsa1.0_00255.1	883	204967	0	699	731	95	-	3	729	178141	178870
UN00361	Rsa1.0_00006.1	759	396176	0	752	759	99	+	3	759	157555	158313
UN00362	Rsa1.0_00596.1	980	73578	0	541	596	90	-	370	944	39243	39833
UN00363	Rsa1.0_00140.1	436	121541	1.00E-155	288	290	99	-	147	436	56161	56449
UN00364	Rsa1.0_02585.1	1079	29865	0	404	411	98	+	3	413	7721	8131
UN00365	Rsa1.0_00542.1	959	76831	0	473	503	94	-	457	959	36174	36661
UN00366	Rsa1.0_01626.1	1193	41989	0	526	527	99	+	667	1193	12907	13433
UN00367	Rsa1.0_02467.1	990	31141	0	375	388	96	-	4	388	7529	7915
UN00368	Rsa1.0_03348.1	921	23559	0	593	630	94	+	292	921	9013	9618
UN00369	Rsa1.0_03318.1	820	23780	0	473	492	96	-	3	493	4296	4786
UN00370	Rsa1.0_03743.1	1285	21062	0	692	709	97	-	411	1119	18007	18714
UN00371	Rsa1.0_03501.1	980	22458	0	531	532	99	+	1	532	18907	19438
UN00372	Rsa1.0_03097.1	939	25434	1.00E-142	312	331	94	+	361	679	19280	19610
UN00373	Rsa1.0_04369.1	1760	17703	0	423	429	98	+	1	428	1707	2134
UN00374	Rsa1.0_03162.1	992	24895	0	398	401	99	+	592	992	19790	20190
UN00375	Rsa1.0_25520.1	935	933	5.00E-71	143	145	98	+	650	794	4	148
UN00376	Rsa1.0_11859.1	808	5389	0	627	629	99	+	4	632	4393	5021
UN00377	Rsa1.0_00389.1	779	88795	1.00E-111	205	205	100	+	3	207	74105	74309
UN00378	Rsa1.0_00087.1	1368	761073	0	1289	1371	94	+	2	1368	94677	96043
UN00379	Rsa1.0_00161.1	1231	116891	0	356	360	98	-	626	985	25631	25990
UN00380	Rsa1.0_00377.1	977	90038	0	732	772	94	-	34	804	80774	81545
UN00381	Rsa1.0_00341.1	784	249309	1.00E-77	166	172	96	-	249	420	103263	103434
UN00382	Rsa1.0_03297.1	566	23969	1.00E-120	309	336	91	-	226	559	14221	14552
UN00383	Rsa1.0_00123.1	799	325650	0	632	649	97	-	2	650	176132	176780
UN00384	Rsa1.0_00301.1	1218	365315	0	495	527	93	-	268	776	194651	195173
UN00385	Rsa1.0_08511.1	922	7252	0	440	473	93	-	457	916	1797	2266
UN00386	Rsa1.0_00141.1	1222	121494	0	1065	1101	96	-	2	1102	60110	61210
UN00387	Rsa1.0_04325.1	1417	17885	1.00E-133	260	266	97	+	460	725	14508	14773
UN00388	Rsa1.0_02281.1	933	292576	1.00E-117	253	263	96	-	510	771	264132	264388

UN00389	Rsa1.0_05389.1	1165	13943	0	455	475	95	+	690	1162	8760	9230
UN00390	Rsa1.0_00336.1	1169	445051	0	364	367	99	+	698	1064	156223	156589
UN00391	Rsa1.0_04342.1	1184	17828	0	499	509	98	−	70	578	355	863
UN00392	Rsa1.0_01403.1	815	46453	0	414	428	96	+	1	426	2564	2987
UN00393	Rsa1.0_00447.1	1071	84051	1.00E−169	332	341	97	+	733	1071	33914	34254
UN00394	Rsa1.0_04065.1	974	19275	1.00E−167	317	323	98	−	6	328	8566	8888
UN00395	Rsa1.0_00002.1	1331	601661	4.00E−29	88	95	92	−	994	1088	140996	141090
UN00396	Rsa1.0_00671.1	1151	397884	0	608	628	96	+	3	624	184881	185506
UN00397	Rsa1.0_03191.1	842	145762	1.00E−150	361	386	93	+	463	842	16325	16698
UN00398	Rsa1.0_03809.1	1092	20731	0	738	787	93	+	312	1092	19080	19863
UN00399	Rsa1.0_01373.1	564	47052	0	391	397	98	+	1	397	31451	31847
UN00400	Rsa1.0_00745.1	961	66067	1.00E−129	251	256	98	−	75	327	23665	23920
UN00401	Rsa1.0_04200.1	761	18490	1.00E−142	268	272	98	−	3	274	10181	10452
UN00402	Rsa1.0_00671.1	1937	397884	0	504	504	100	−	1433	1936	267940	268443
UN00403	Rsa1.0_00322.1	1231	212233	1.00E−123	231	233	99	−	833	1065	80161	80393
UN00404	Rsa1.0_23843.1	1155	1022	0	610	633	96	+	524	1155	93	723
UN00405	Rsa1.0_00083.1	970	442773	1.00E−99	197	201	98	−	770	970	361205	361405
UN00406	Rsa1.0_25269.1	983	944	0	400	411	97	−	450	860	1	408
UN00407	Rsa1.0_00516.1	1458	78417	0	1243	1308	95	+	151	1453	73599	74901
UN00408	Rsa1.0_00040.1	779	324196	0	627	666	94	+	113	777	166240	166904
UN00409	Rsa1.0_00446.1	617	167829	0	608	612	99	+	5	616	153047	153658
UN00410	Rsa1.0_01437.1	1226	45887	0	548	552	99	−	675	1226	31902	32453
UN00411	Rsa1.0_29875.1	1145	769	0	412	417	98	−	727	1143	23	439
UN00412	Rsa1.0_05874.1	693	12351	0	548	562	97	−	3	564	9390	9951
UN00413	Rsa1.0_10218.1	1032	5935	0	637	640	99	+	393	1032	3887	4526
UN00414	Rsa1.0_00134.1	766	390219	1.00E−150	298	306	97	−	1	305	157266	157571
UN00415	Rsa1.0_00828.1	1206	62744	0	389	402	96	−	2	403	60815	61216
UN00416	Rsa1.0_11943.1	709	5365	1.00E−158	348	367	94	+	351	707	570	934
UN00417	Rsa1.0_37225.1	1293	605	0	560	563	99	−	731	1293	28	590
UN00418	Rsa1.0_00694.1	826	68074	0	735	759	96	−	44	794	61705	62461
UN00419	Rsa1.0_00296.1	1094	97854	0	330	330	100	+	765	1094	89589	89918
UN00420	Rsa1.0_05324.1	935	14145	0	397	411	96	+	197	607	11922	12332
UN00421	Rsa1.0_00037.1	1353	307838	0	478	494	96	−	810	1301	268658	269150
UN00422	Rsa1.0_00026.1	943	327835	1.00E−136	261	266	98	−	678	943	177093	177358
UN00423	Rsa1.0_00387.1	819	495407	1.00E−169	366	388	94	+	393	780	410022	410403
UN00424	Rsa1.0_00027.1	835	283362	0	792	839	94	−	2	835	42794	43629
UN00425	Rsa1.0_00032.1	933	306326	0	375	386	97	−	549	933	265504	265888
UN00426	Rsa1.0_37242.1	954	605	1.00E−163	328	339	96	−	589	923	1	337
UN00427	Rsa1.0_02052.1	1007	35555	0	449	469	95	−	546	1007	32962	33429

UN00428	Rsa1.0_00107.1	970	431659	4.00E-96	191	195	97	-	442	636	8283	8477
UN00429	Rsa1.0_16726.1	1174	2053	3.00E-85	329	389	84	+	259	647	306	652
UN00430	Rsa1.0_03547.1	927	22167	0	489	493	99	+	4	495	815	1307
UN00431	Rsa1.0_00679.1	1184	68971	1.00E-149	272	273	99	+	912	1184	34534	34806
UN00432	Rsa1.0_02180.1	1307	34176	1.00E-154	287	289	99	+	3	290	15721	16009
UN00433	Rsa1.0_00375.1	787	90207	1.00E-176	362	377	96	+	411	787	2405	2780
UN00434	Rsa1.0_04243.1	1344	18321	3.00E-82	180	188	95	-	791	978	3909	4096
UN00435	Rsa1.0_03631.1	1038	21647	0	509	540	94	+	433	972	9124	9638
UN00436	Rsa1.0_00243.1	1682	103651	0	1156	1207	95	+	442	1642	85850	87048
UN00437	Rsa1.0_00089.1	523	406142	1.00E-177	357	369	96	-	157	523	210291	210658
UN00438	Rsa1.0_00367.1	872	90750	1.00E-137	262	266	98	+	3	265	25507	25772
UN00439	Rsa1.0_00469.1	1207	82084	0	676	710	95	+	489	1182	40678	41385
UN00440	Rsa1.0_01435.1	763	45942	1.00E-123	267	276	96	+	492	763	40444	40719
UN00441	Rsa1.0_06179.1	1537	11548	0	527	538	97	-	415	952	9266	9803
UN00442	Rsa1.0_07104.1	1146	9889	0	681	693	98	-	227	919	7558	8249
UN00443	Rsa1.0_04213.1	1382	18437	7.00E-77	153	155	98	+	531	685	18130	18284
UN00444	Rsa1.0_00027.1	878	283362	0	330	333	99	+	374	706	101858	102190
UN00445	Rsa1.0_00017.1	1381	752458	4.00E-94	191	196	97	+	391	586	38117	38312
UN00446	Rsa1.0_41660.1	1207	539	0	380	383	99	+	203	585	157	539
UN00447	Rsa1.0_01356.1	1440	47287	0	750	751	99	+	689	1439	8186	8936
UN00448	Rsa1.0_01570.1	1487	43015	0	337	340	99	-	1148	1486	10785	11124
UN00449	Rsa1.0_00113.1	1357	131574	0	770	774	99	-	583	1356	123303	124076
UN00450	Rsa1.0_01613.1	718	182542	1.00E-165	300	302	99	+	250	551	28807	29108
UN00451	Rsa1.0_00211.1	774	228189	1.00E-105	223	232	96	-	465	693	146603	146834
UN00452	Rsa1.0_01803.1	631	173846	1.00E-177	315	315	100	-	317	631	18839	19153
UN00453	Rsa1.0_01282.1	1058	49304	4.00E-87	287	329	87	-	746	1056	28018	28331
UN00454	Rsa1.0_00891.1	1362	60724	1.00E-130	323	346	93	+	1021	1362	22254	22596
UN00455	Rsa1.0_01423.1	779	46170	2.00E-57	142	150	94	-	3	151	4788	4937
UN00456	Rsa1.0_04395.1	486	17570	1.00E-144	271	275	98	+	212	486	9572	9846
UN00457	Rsa1.0_00154.1	1306	189461	1.00E-180	344	352	97	+	396	747	165258	165609
UN00458	Rsa1.0_02810.1	1690	27671	1.00E-100	240	261	91	-	1444	1689	23912	24172
UN00459	Rsa1.0_02126.1	701	34765	0	473	480	98	+	222	701	1828	2307
UN00460	Rsa1.0_00015.1	822	561803	1.00E-157	344	365	94	+	451	806	401951	402314
UN00461	Rsa1.0_00425.1	1000	147080	0	623	669	93	-	347	990	125711	126375
UN00462	Rsa1.0_00483.1	1333	81102	0	467	467	100	+	94	560	75313	75779
UN00463	Rsa1.0_14932.1	543	2956	1.00E-121	224	225	99	+	318	542	620	844
UN00464	Rsa1.0_01634.1	1030	41865	1.00E-172	413	442	93	-	589	1028	1124	1551
UN00465	Rsa1.0_02169.1	1055	34367	0	800	839	95	-	188	1009	21213	22051
UN00466	Rsa1.0_04080.1	1227	19198	0	676	741	91	+	362	1097	9176	9912

UN00467	Rsa1.0_03894.1	830	20207	1.00E-140	259	261	99	-	1	261	19453	19713
UN00468	Rsa1.0_13384.1	867	4873	7.00E-73	140	140	100	+	92	231	573	712
UN00469	Rsa1.0_00294.1	1381	144860	1.00E-139	289	299	96	+	1035	1333	23941	24237
UN00470	Rsa1.0_02518.1	553	30586	1.00E-147	274	277	98	-	277	553	25561	25837
UN00471	Rsa1.0_08511.1	934	7252	0	448	472	94	-	461	921	1798	2266
UN00472	Rsa1.0_05457.1	1219	13687	1.00E-169	367	388	94	+	17	401	2461	2848
UN00473	Rsa1.0_01595.1	981	175383	1.00E-135	262	268	97	-	313	580	2704	2971
UN00474	Rsa1.0_00273.1	699	326186	1.00E-161	347	366	94	-	282	644	113973	114338
UN00475	Rsa1.0_01696.1	928	301850	4.00E-68	141	144	97	+	97	240	291366	291509
UN00476	Rsa1.0_00057.1	855	160473	2.33E-156	266	267	99	+	3	269	69734	70000
UN00477	Rsa1.0_02946.1	1479	26537	0	938	967	97	+	515	1475	857	1821
UN00478	Rsa1.0_00018.1	905	410899	0	481	483	99	-	423	905	57046	57528
UN00479	Rsa1.0_02120.1	1178	34819	0	1117	1159	96	+	2	1159	19889	21046
UN00480	Rsa1.0_00523.1	617	178770	1.00E-161	288	288	100	+	3	290	141283	141570
UN00481	Rsa1.0_02494.1	903	132923	0	576	593	97	-	311	903	6102	6692
UN00482	Rsa1.0_01719.1	902	40388	8.00E-82	206	218	94	-	690	902	27252	27464
UN00483	Rsa1.0_00397.1	1364	88189	0	1007	1061	94	-	1	1035	4783	5838
UN00484	Rsa1.0_00049.1	1161	167018	0	420	425	98	-	688	1112	146693	147117
UN00485	Rsa1.0_03209.1	859	24592	0	386	404	95	+	5	408	16842	17241
UN00486	Rsa1.0_02749.1	1117	28241	0	695	716	97	-	3	713	26791	27506
UN00487	Rsa1.0_00145.1	991	261008	0	827	936	88	+	57	958	75205	76105
UN00488	Rsa1.0_03682.1	1550	21396	0	368	380	96	+	579	958	12324	12703
UN00489	Rsa1.0_00749.1	841	65933	0	824	840	98	-	2	841	27966	28805
UN00490	Rsa1.0_03015.1	1510	26006	0	499	508	98	+	1003	1510	20793	21300
UN00491	Rsa1.0_00262.1	716	101906	1.00E-176	322	325	99	+	391	715	46959	47283
UN00492	Rsa1.0_00366.1	969	90880	0	939	967	97	-	2	966	51559	52525
UN00493	Rsa1.0_07021.1	1067	10016	1.00E-172	327	334	97	+	339	672	7093	7426
UN00494	Rsa1.0_00338.1	612	94109	8.00E-81	261	293	89	-	320	612	34848	35117
UN00495	Rsa1.0_00219.1	993	106983	1.00E-132	267	274	97	-	721	992	29590	29862
UN00496	Rsa1.0_06683.1	1029	10549	1.00E-179	321	322	99	+	1	322	7871	8192
UN00497	Rsa1.0_00039.1	865	296927	0	771	789	97	-	75	863	133718	134505
UN00498	Rsa1.0_03790.1	825	20844	0	638	667	95	-	89	745	8413	9071
UN00499	Rsa1.0_04881.1	1266	15608	0	869	934	93	-	305	1228	8384	9311
UN00500	Rsa1.0_02235.1	1031	33615	0	473	474	99	-	558	1031	17608	18081
UN00501	Rsa1.0_00654.1	983	70012	0	921	944	97	+	5	937	9140	10081
UN00502	Rsa1.0_01071.1	1090	166914	0	1032	1092	94	+	3	1085	12837	13926
UN00503	Rsa1.0_18158.1	1114	1655	0	721	735	98	-	379	1113	921	1655
UN00504	Rsa1.0_05522.1	1130	13441	1.00E-161	445	500	89	+	279	754	12962	13441
UN00505	Rsa1.0_00247.1	850	103408	1.00E-128	298	316	94	-	290	604	8483	8796

UN00506	Rsa1.0_01685.1	1024	175033	0	449	465	96	+	560	1023	15769	16230
UN00507	Rsa1.0_02265.1	1279	33233	0	1106	1116	99	−	163	1277	10854	11969
UN00508	Rsa1.0_01285.1	1341	172709	0	1321	1338	98	+	2	1339	39423	40760
UN00509	Rsa1.0_00398.1	812	88007	1.00E−169	346	360	96	−	2	358	42461	42820
UN00510	Rsa1.0_02518.1	1001	30586	1.00E−118	279	296	94	−	687	981	27798	28091
UN00511	Rsa1.0_02536.1	829	30368	0	793	824	96	+	8	827	28663	29486
UN00512	Rsa1.0_02579.1	830	29923	0	401	403	99	−	377	779	14697	15099
UN00513	Rsa1.0_00251.1	509	303968	2.00E−90	253	276	91	−	242	509	120948	121221
UN00514	Rsa1.0_00819.1	1226	62918	0	359	366	98	−	861	1225	46856	47221
UN00515	Rsa1.0_20310.1	858	1318	0	378	389	97	−	473	857	904	1292
UN00516	Rsa1.0_02266.1	1216	33195	0	1159	1227	94	+	3	1210	28532	29757
UN00517	Rsa1.0_07232.1	1804	9684	1.00E−168	353	365	96	+	1436	1799	1213	1570
UN00518	Rsa1.0_00121.1	937	278029	0	367	369	99	−	569	937	9592	9960
UN00519	Rsa1.0_00017.1	395	752458	1.00E−78	217	238	91	+	156	391	271555	271789
UN00520	Rsa1.0_02286.1	1199	32993	0	868	868	100	−	332	1199	24136	25003
UN00521	Rsa1.0_06814.1	1250	10309	0	660	678	97	−	572	1249	6129	6806
UN00522	Rsa1.0_01718.1	541	40389	1.00E−136	294	309	95	−	236	538	35336	35644
UN00523	Rsa1.0_00119.1	2101	467509	0	863	901	95	−	119	1010	346643	347543
UN00524	Rsa1.0_00818.1	997	62939	1.00E−178	388	410	94	−	95	504	40497	40902
UN00525	Rsa1.0_00036.1	1453	176186	0	783	789	99	+	652	1440	3281	4069
UN00526	Rsa1.0_09945.1	882	6076	0	444	456	97	+	427	882	303	758
UN00527	Rsa1.0_03207.1	969	24604	0	503	510	98	+	454	962	15790	16299
UN00528	Rsa1.0_00037.1	839	307838	0	380	381	99	−	459	839	266929	267309
UN00529	Rsa1.0_01373.1	875	47052	7.00E−76	162	167	97	+	223	389	2046	2209
UN00530	Rsa1.0_02269.1	1068	33168	0	555	566	98	−	109	674	23759	24324
UN00531	Rsa1.0_00065.1	1504	454616	0	439	454	96	−	2	450	213563	214016
UN00532	Rsa1.0_00208.1	983	108565	0	513	524	97	−	3	526	104420	104943
UN00533	Rsa1.0_06900.1	604	131268	1.00E−140	312	324	96	−	280	598	129932	130254
UN00534	Rsa1.0_02622.1	879	142402	1.00E−150	322	338	95	+	3	329	6671	7008
UN00535	Rsa1.0_00784.1	1090	64359	0	582	589	98	+	2	589	52062	52650
UN00536	Rsa1.0_00192.1	1234	278200	0	572	587	97	+	364	950	73828	74414
UN00537	Rsa1.0_27704.1	1449	842	1.00E−179	319	319	100	+	640	958	47	365
UN00538	Rsa1.0_03722.1	1032	21184	0	641	689	93	+	3	679	19983	20668
UN00539	Rsa1.0_00145.1	1267	261008	1.00E−140	253	253	100	−	1015	1267	93718	93970
UN00540	Rsa1.0_05241.1	1057	14405	0	356	364	97	−	687	1046	3058	3421
UN00541	Rsa1.0_02252.1	981	33375	0	376	383	98	−	179	561	31510	31892
UN00542	Rsa1.0_00032.1	866	306326	1.00E−121	251	263	95	+	615	866	279058	279320
UN00543	Rsa1.0_01105.1	886	53958	1.00E−92	185	189	97	+	102	290	10357	10545
UN00544	Rsa1.0_00383.1	1148	398431	0	761	816	93	+	286	1083	147000	147797

UN00545	Rsa1.0_00220.1	842	192936	5.00E-92	231	248	93	-	594	829	143213	143460
UN00546	Rsa1.0_05691.1	1729	12918	0	1716	1728	99	-	2	1729	351	2071
UN00547	Rsa1.0_02310.1	1284	32735	0	379	382	99	+	4	385	27360	27741
UN00548	Rsa1.0_04057.1	849	19333	1.00E-156	291	295	98	+	128	422	1571	1865
UN00549	Rsa1.0_02758.1	1059	28186	0	463	473	97	+	591	1059	13847	14319
UN00550	Rsa1.0_00572.1	925	74969	0	856	899	95	+	12	904	41155	42039
UN00551	Rsa1.0_01285.1	811	172709	3.00E-84	192	202	95	-	256	455	123780	123981
UN00552	Rsa1.0_00105.1	910	134138	0	829	853	97	+	2	852	62588	63440
UN00553	Rsa1.0_00782.1	805	64468	0	579	616	93	+	187	793	18764	19376
UN00554	Rsa1.0_15748.1	987	2448	9.00E-45	96	97	98	-	421	517	1488	1584
UN00555	Rsa1.0_01186.1	799	51418	0	794	806	98	+	1	799	31990	32795
UN00556	Rsa1.0_02725.1	807	28404	0	533	552	96	+	256	807	1939	2490
UN00557	Rsa1.0_00227.1	552	105849	0	514	536	95	+	2	534	35727	36258
UN00558	Rsa1.0_00017.1	816	752458	0	540	579	93	-	3	579	474193	474755
UN00559	Rsa1.0_00971.1	802	57973	9.00E-23	59	60	98	+	18	77	43498	43557
UN00560	Rsa1.0_00293.1	936	339294	1.00E-74	229	254	90	-	430	683	307382	307621
UN00561	Rsa1.0_00273.1	972	326186	1.00E-102	214	221	96	-	646	866	9437	9656
UN00562	Rsa1.0_01998.1	1086	36276	0	804	854	94	+	234	1082	10886	11722
UN00563	Rsa1.0_01141.1	799	52530	0	653	711	91	-	2	711	42680	43390
UN00564	Rsa1.0_00224.1	1015	327530	0	986	1020	96	+	1	1012	184453	185471
UN00565	Rsa1.0_00347.1	946	93048	1.00E-167	411	448	91	+	3	438	90999	91440
UN00566	Rsa1.0_00190.1	1017	318240	0	690	714	96	+	286	994	191430	192143
UN00567	Rsa1.0_21317.1	780	1216	0	602	623	96	-	145	764	183	805
UN00568	Rsa1.0_00277.1	1492	100132	1.00E-114	236	245	96	-	199	443	90130	90374
UN00569	Rsa1.0_04819.1	1785	140505	1.00E-160	320	331	96	-	1332	1662	5636	5966
UN00570	Rsa1.0_00925.1	649	59481	1.00E-142	286	296	96	+	290	585	38881	39176
UN00571	Rsa1.0_08285.1	1449	7606	0	548	563	97	-	382	944	2625	3187
UN00572	Rsa1.0_01435.1	891	45942	1.00E-136	277	285	97	+	3	287	24555	24835
UN00573	Rsa1.0_00087.1	1222	761073	1.00E-133	279	289	96	+	934	1220	223283	223571
UN00574	Rsa1.0_04126.1	1038	18939	0	384	386	99	+	3	388	18318	18703
UN00575	Rsa1.0_02682.1	546	28711	1.00E-128	257	261	98	-	287	546	9616	9874
UN00576	Rsa1.0_00539.1	1119	76926	1.00E-137	287	298	96	+	822	1118	28381	28675
UN00577	Rsa1.0_05848.1	875	12432	0	435	436	99	-	439	874	10367	10802
UN00578	Rsa1.0_02874.1	1542	27070	0	941	948	99	+	65	1009	4410	5357
UN00579	Rsa1.0_00336.1	847	445051	0	825	845	97	+	3	846	146873	147717
UN00580	Rsa1.0_01286.1	839	49209	1.00E-148	278	282	98	-	102	383	41233	41514
UN00581	Rsa1.0_03750.1	1314	21027	0	704	753	93	+	2	747	5247	5988
UN00582	Rsa1.0_00979.1	1416	57733	1.00E-178	371	384	96	-	1027	1405	40233	40616
UN00583	Rsa1.0_07865.1	1118	8400	1.00E-140	253	253	100	-	760	1012	3836	4088

UN00584	Rsa1.0_00017.1	1526	752458	0	409	428	95	+	2	426	412620	413043
UN00585	Rsa1.0_04415.1	1041	147889	1.00E-120	238	244	97	-	254	497	141887	142130
UN00586	Rsa1.0_00577.1	1329	174827	0	438	473	92	+	112	566	149759	150231
UN00587	Rsa1.0_03216.1	828	24540	1.00E-138	276	284	97	-	3	286	13319	13600
UN00588	Rsa1.0_00595.1	1310	341648	0	575	585	98	+	6	590	2389	2973
UN00589	Rsa1.0_00100.1	1225	249232	0	337	338	99	-	3	340	180836	181173
UN00590	Rsa1.0_00014.1	927	348985	0	679	707	96	-	208	909	36913	37612
UN00591	Rsa1.0_00727.1	890	66724	0	390	401	97	+	3	396	4925	5325
UN00592	Rsa1.0_00419.1	973	86562	0	451	454	99	+	380	833	79787	80240
UN00593	Rsa1.0_00224.1	708	327530	0	687	724	94	+	1	707	192374	193096
UN00594	Rsa1.0_00200.1	853	162016	1.00E-68	164	173	94	-	1	173	103491	103657
UN00595	Rsa1.0_01046.1	673	152989	1.00E-163	333	347	95	-	327	673	116451	116797
UN00596	Rsa1.0_01751.1	587	39806	1.00E-116	275	296	92	-	291	586	30350	30634
UN00597	Rsa1.0_00381.1	755	89718	1.00E-175	392	417	94	-	6	418	75167	75583
UN00598	#	#	#	#	#	#	#	#	#	#	#	#
UN00599	Rsa1.0_19257.1	1552	1455	0	433	433	100	-	1117	1549	699	1131
UN00600	Rsa1.0_03583.1	857	21946	0	494	505	97	+	361	855	20939	21443
UN00601	Rsa1.0_02179.1	1358	34188	0	479	482	99	+	877	1358	7052	7533
UN00602	Rsa1.0_00371.1	1126	90463	1.00E-119	239	246	97	-	153	398	13675	13920
UN00603	Rsa1.0_09236.1	899	6540	0	868	874	99	-	26	899	88	961
UN00604	Rsa1.0_13240.1	1547	4960	0	337	337	100	-	1164	1500	1	337
UN00605	Rsa1.0_04005.1	1015	19586	0	551	566	97	+	454	1015	18748	19313
UN00606	Rsa1.0_00046.1	2328	320315	0	668	691	96	+	1637	2327	67411	68101
UN00607	Rsa1.0_00960.1	778	58199	0	491	515	95	+	3	502	20997	21510
UN00608	Rsa1.0_00061.1	800	218685	0	774	809	95	-	1	800	856	1660
UN00609	Rsa1.0_13693.1	769	4469	0	706	782	90	+	2	769	2608	3362
UN00610	Rsa1.0_02723.1	1202	28408	0	627	656	95	-	546	1201	7665	8303
UN00611	Rsa1.0_01509.1	1131	44258	0	836	858	97	-	278	1131	35523	36380
UN00612	Rsa1.0_03746.1	1083	21050	0	490	505	97	+	160	664	19838	20342
UN00613	Rsa1.0_00131.1	598	432233	1.00E-85	176	181	97	-	122	302	274061	274241
UN00614	Rsa1.0_00440.1	1234	84723	1.00E-171	314	317	99	+	761	1077	24477	24793
UN00615	Rsa1.0_00067.1	1257	415055	0	820	820	100	-	438	1257	38266	39085
UN00616	Rsa1.0_01026.1	702	171624	0	606	606	100	-	97	702	14047	14652
UN00617	Rsa1.0_52552.1	855	433	0	431	433	99	-	333	765	1	433
UN00618	Rsa1.0_02658.1	1191	28978	0	355	362	98	-	832	1190	21972	22330
UN00619	Rsa1.0_03524.1	1283	22297	0	760	769	98	-	517	1283	4388	5155
UN00620	Rsa1.0_00146.1	1211	149343	1.00E-172	364	380	95	-	3	378	73067	73445
UN00621	Rsa1.0_01537.1	1554	187285	0	422	434	97	+	883	1316	16266	16699
UN00622	Rsa1.0_00354.1	937	232005	1.00E-115	267	281	95	-	659	937	192326	192602

UN00623	Rsa1.0_00006.1	1147	396176	0	417	423	98	+	725	1147	201166	201588
UN00624	Rsa1.0_00837.1	1411	62568	1.00E-136	247	247	100	-	3	249	53363	53609
UN00625	Rsa1.0_00271.1	920	101125	0	622	657	94	+	268	920	75163	75818
UN00626	Rsa1.0_01121.1	880	53407	0	840	871	96	+	1	865	7235	8105
UN00627	Rsa1.0_00740.1	1092	215972	0	857	865	99	-	228	1092	199540	200404
UN00628	Rsa1.0_00079.1	815	606987	2.33E-156	312	327	95	-	494	815	464220	464545
UN00629	Rsa1.0_06419.1	832	11055	0	670	767	87	-	9	774	1489	2253
UN00630	Rsa1.0_00129.1	748	126831	1.00E-147	402	443	90	+	316	741	113976	114410
UN00631	Rsa1.0_00129.1	760	126831	1.00E-156	301	307	98	-	83	389	45712	46016
UN00632	Rsa1.0_01955.1	1176	36850	1.00E-144	415	463	89	-	474	917	13073	13535
UN00633	Rsa1.0_02616.1	1364	29542	0	613	662	92	+	668	1317	1279	1931
UN00634	Rsa1.0_00046.1	578	320315	3.00E-52	135	144	93	+	435	576	75134	75277
UN00635	Rsa1.0_01373.1	1046	47052	0	396	400	99	-	336	732	21559	21958
UN00636	Rsa1.0_00190.1	1026	318240	1.00E-167	365	383	95	+	43	421	134842	135221
UN00637	Rsa1.0_02322.1	682	32555	1.00E-107	225	233	96	-	1	233	26970	27201
UN00638	Rsa1.0_02634.1	1041	29299	0	439	470	93	-	582	1039	1428	1897
UN00639	Rsa1.0_04169.1	1133	18712	1.00E-139	327	347	94	+	790	1129	2537	2880
UN00640	Rsa1.0_00097.1	1355	138858	0	636	643	98	+	710	1352	34058	34700
UN00641	Rsa1.0_01974.1	1221	36583	0	419	437	95	-	778	1211	11606	12039
UN00642	Rsa1.0_02694.1	1055	28610	0	503	528	95	-	4	528	5002	5524
UN00643	Rsa1.0_00228.1	1396	105511	0	1345	1404	95	+	1	1394	27844	29218
UN00644	Rsa1.0_00714.1	1000	67187	3.00E-91	338	390	86	-	5	384	14248	14623
UN00645	Rsa1.0_00250.1	1176	273771	2.00E-86	199	211	94	+	1	211	60373	60583
UN00646	Rsa1.0_01857.1	1304	38103	0	392	405	96	+	851	1254	22856	23259
UN00647	Rsa1.0_03005.1	1654	26082	1.00E-104	243	260	93	-	1	254	10402	10656
UN00648	Rsa1.0_00294.1	845	144860	0	809	837	96	-	2	838	81600	82431
UN00649	Rsa1.0_00180.1	762	114581	1.00E-111	240	250	96	+	1	245	16501	16750
UN00650	Rsa1.0_03477.1	841	22635	0	813	832	97	+	1	832	6174	7005
UN00651	Rsa1.0_05874.1	990	12351	0	880	906	97	+	1	906	9254	10158
UN00652	Rsa1.0_00151.1	1441	119541	0	545	575	94	+	1	567	55775	56339
UN00653	Rsa1.0_00068.1	857	151813	1.00E-121	280	298	93	+	1	296	112707	113001
UN00654	Rsa1.0_04238.1	717	18352	0	410	417	98	-	1	415	8709	9123
UN00655	Rsa1.0_00548.1	1079	76518	1.00E-113	239	249	95	+	7	252	60513	60761
UN00656	Rsa1.0_00344.1	787	191774	0	367	371	98	-	2	372	182012	182382
UN00657	Rsa1.0_20130.1	386	1337	1.00E-144	361	391	92	-	1	383	217	607
UN00658	Rsa1.0_00752.1	1495	65756	0	452	469	96	+	1032	1494	21393	21855
UN00659	Rsa1.0_00040.1	959	324196	1.00E-133	254	258	98	+	252	509	154828	155085
UN00660	Rsa1.0_00868.1	1386	61630	0	736	736	100	+	1	736	43876	44611
UN00661	Rsa1.0_00151.1	1617	119541	1.00E-141	362	399	90	-	2	383	45328	45719

UN00662	Rsa1.0_00130.1	899	125737	0	716	716	100	−	2	717	8316	9031
UN00663	Rsa1.0_02413.1	2322	31628	0	469	469	100	−	831	1299	16457	16925
UN00664	Rsa1.0_00279.1	864	100039	1.00E−174	325	330	98	−	1	330	25208	25537
UN00665	Rsa1.0_03238.1	708	24432	1.00E−109	254	265	95	−	1	261	18219	18482
UN00666	Rsa1.0_05074.1	972	14925	0	380	383	99	+	2	384	10125	10506
UN00667	Rsa1.0_00140.1	720	121541	1.00E−136	298	314	94	−	405	718	21983	22295
UN00668	Rsa1.0_04126.1	1099	18939	0	488	489	99	−	1	489	17402	17890
UN00669	Rsa1.0_00597.1	499	73445	1.00E−166	347	363	95	−	144	499	10945	11307
UN00670	Rsa1.0_00901.1	1518	60363	0	1509	1516	99	−	1	1516	57966	59481
UN00671	Rsa1.0_00108.1	1007	478491	1.00E−147	292	300	97	−	1	299	277523	277822
UN00672	Rsa1.0_00271.1	1223	101125	1.00E−155	330	341	96	−	16	351	49646	49985
UN00673	Rsa1.0_03847.1	758	20479	0	740	759	97	−	1	758	5002	5760
UN00674	Rsa1.0_06482.1	713	10937	1.00E−132	296	315	93	+	388	702	8153	8456
UN00675	Rsa1.0_00848.1	724	152362	7.00E−94	205	215	95	+	1	215	39215	39429
UN00676	Rsa1.0_04375.1	610	141717	0	606	608	99	−	1	608	8395	9002
UN00677	Rsa1.0_05173.1	796	14606	0	583	606	96	+	4	608	8132	8730
UN00678	Rsa1.0_26431.1	793	892	0	496	506	98	−	2	507	370	872
UN00679	Rsa1.0_02248.1	1060	33414	0	467	470	99	+	356	825	12478	12947
UN00680	Rsa1.0_00176.1	970	240984	1.00E−43	121	131	92	+	812	942	18131	18261
UN00681	Rsa1.0_00039.1	1296	296927	0	685	691	99	+	1	691	254847	255531
UN00682	Rsa1.0_00716.1	801	67078	4.00E−65	291	343	84	+	1	341	51491	51833
UN00683	Rsa1.0_00880.1	616	61271	0	599	616	97	−	1	616	47645	48256
UN00684	Rsa1.0_00112.1	1320	132199	0	719	746	96	+	4	749	83967	84710
UN00685	Rsa1.0_00633.1	809	71250	0	468	471	99	−	1	471	5178	5648
UN00686	Rsa1.0_00782.1	955	64468	0	805	835	96	+	123	954	47530	48364
UN00687	Rsa1.0_00070.1	831	150321	0	509	527	96	+	1	520	59090	59616
UN00688	Rsa1.0_00458.1	911	82717	1.00E−126	248	254	97	+	412	665	68138	68391
UN00689	Rsa1.0_01506.1	471	44370	1.00E−115	274	293	93	−	183	471	17888	18180
UN00690	Rsa1.0_01549.1	754	43543	1.00E−175	338	347	97	−	408	754	696	1042
UN00691	Rsa1.0_02867.1	889	27121	0	887	887	100	+	1	887	705	1591
UN00692	Rsa1.0_00486.1	529	80875	1.00E−85	227	249	91	−	249	497	34925	35163
UN00693	Rsa1.0_00037.1	1426	307838	0	457	461	99	+	1	458	193408	193868
UN00694	Rsa1.0_00464.1	995	82253	1.00E−105	219	227	96	+	135	361	37374	37600
UN00695	Rsa1.0_04819.1	930	140505	0	353	354	99	+	1	354	5613	5966
UN00696	Rsa1.0_00387.1	1269	495407	0	422	441	95	+	1	439	401061	401493
UN00697	Rsa1.0_08190.1	736	7764	1.00E−167	317	322	98	−	1	322	4617	4937
UN00698	Rsa1.0_00205.1	551	193401	1.00E−133	283	293	96	+	1	291	79344	79635
UN00699	Rsa1.0_39485.1	817	569	1.00E−132	239	239	100	−	1	239	1	239
UN00700	Rsa1.0_02239.1	536	33534	1.00E−125	349	387	90	+	1	376	28368	28743

UN00701	Rsa1.0_00157.1	485	245832	2.00E-47	141	154	91	-	15	163	136953	137106
UN00702	Rsa1.0_00032.1	826	306326	0	596	627	95	+	1	625	284776	285401
UN00703	Rsa1.0_21164.1	779	1229	1.00E-178	353	363	97	-	3	365	836	1194
UN00704	Rsa1.0_02650.1	1187	29047	1.00E-169	310	313	99	+	578	890	11720	12032
UN00705	Rsa1.0_00116.1	1180	365886	1.00E-179	324	326	99	+	6	331	36657	36982
UN00706	Rsa1.0_03873.1	157	20360	6.00E-57	121	124	97	+	1	124	9834	9957
UN00707	Rsa1.0_46918.1	658	481	1.00E-165	301	303	99	-	356	658	176	478
UN00708	Rsa1.0_03664.1	539	21487	1.00E-116	219	221	99	-	296	516	7043	7263
UN00709	Rsa1.0_00714.1	159	67187	3.00E-68	150	155	96	-	1	155	3659	3812
UN00710	Rsa1.0_00320.1	831	95784	0	446	477	93	+	361	824	36827	37294
UN00711	Rsa1.0_01825.1	715	38725	0	356	363	98	+	304	666	27981	28343
UN00712	Rsa1.0_00556.1	1317	207212	0	549	597	91	+	4	590	37977	38572
UN00713	Rsa1.0_01683.1	757	41059	0	680	744	91	-	1	732	24130	24844
UN00714	Rsa1.0_00664.1	1010	69573	0	429	451	95	+	1	440	16651	17101
UN00715	Rsa1.0_01118.1	1323	53460	1.00E-135	244	244	100	+	486	729	13629	13872
UN00716	Rsa1.0_17967.1	939	1699	0	930	944	98	-	1	937	517	1460
UN00717	Rsa1.0_00837.1	1267	62568	0	346	349	99	+	1	349	50675	51023
UN00718	Rsa1.0_03606.1	704	21818	1.00E-124	277	291	95	-	5	294	4426	4706
UN00719	Rsa1.0_00018.1	797	410899	0	399	406	98	+	393	797	403692	404095
UN00720	Rsa1.0_01704.1	1340	40711	0	1268	1337	94	-	1	1311	31433	32769
UN00721	Rsa1.0_00928.1	711	59293	1.00E-151	287	291	98	-	419	709	29451	29740
UN00722	Rsa1.0_00113.1	642	131574	0	439	477	92	+	160	630	4781	5256
UN00723	Rsa1.0_19822.1	890	1378	0	790	828	95	-	72	890	336	1163
UN00724	Rsa1.0_03073.1	1222	25574	0	780	816	95	-	120	935	86	897
UN00725	Rsa1.0_10486.1	821	5830	4.00E-74	157	162	96	+	553	714	393	554
UN00726	Rsa1.0_00265.1	855	220635	1.00E-89	174	176	98	-	306	481	172356	172531
UN00727	Rsa1.0_02703.1	1305	28544	1.00E-138	283	294	96	+	620	913	9049	9342
UN00728	Rsa1.0_00233.1	749	104918	1.00E-115	246	255	96	-	497	749	86427	86681
UN00729	Rsa1.0_00027.1	854	283362	1.00E-128	248	253	98	-	132	384	169310	169562
UN00730	Rsa1.0_00253.1	1325	231292	1.00E-119	236	242	97	+	846	1087	123819	124060
UN00731	Rsa1.0_03396.1	821	23242	0	586	624	93	+	175	795	8871	9471
UN00732	Rsa1.0_00424.1	1715	86005	0	681	697	97	+	374	1070	66771	67467
UN00733	Rsa1.0_03393.1	455	23247	1.00E-120	270	287	94	-	179	455	946	1232
UN00734	Rsa1.0_04753.1	1361	16124	0	566	589	96	+	753	1339	15339	15924
UN00735	Rsa1.0_01986.1	611	36489	1.00E-95	206	214	96	-	398	611	27726	27938
UN00736	Rsa1.0_00631.1	838	71277	0	414	439	94	-	2	426	18179	18617
UN00737	Rsa1.0_03001.1	924	26108	1.00E-122	229	231	99	-	272	502	13258	13488
UN00738	Rsa1.0_00087.1	880	761073	1.00E-154	343	359	95	+	526	879	225356	225712
UN00739	Rsa1.0_00049.1	1151	167018	0	594	597	99	-	2	598	49432	50028

UN00740	Rsa1.0_00253.1	715	231292	1.00E-129	428	493	86	-	73	565	196884	197364
UN00741	Rsa1.0_00006.1	1193	396176	0	348	352	98	+	624	975	83435	83786
UN00742	Rsa1.0_08042.1	776	8020	0	743	777	95	+	5	776	1090	1865
UN00743	Rsa1.0_16567.1	850	2109	1.00E-101	196	199	98	+	414	612	1316	1514
UN00744	Rsa1.0_02224.1	937	33758	1.00E-132	243	244	99	-	263	506	30415	30658
UN00745	Rsa1.0_00454.1	629	216970	1.00E-101	326	366	89	+	270	629	142994	143349
UN00746	Rsa1.0_00970.1	1084	57994	0	491	517	94	-	582	1084	30952	31466
UN00747	Rsa1.0_07208.1	2156	9719	0	1314	1374	95	-	788	2155	6630	7993
UN00748	Rsa1.0_02247.1	920	33427	1.00E-118	267	282	94	+	584	864	28646	28924
UN00749	Rsa1.0_02234.1	873	33618	0	482	508	94	-	366	873	4042	4534
UN00750	Rsa1.0_01246.1	726	49968	0	704	722	97	-	3	723	34409	35125
UN00751	Rsa1.0_00713.1	554	158017	1.00E-137	341	368	92	+	152	511	153615	153982
UN00752	Rsa1.0_09117.1	1005	6637	1.00E-169	333	346	96	-	132	465	2458	2803
UN00753	Rsa1.0_02658.1	1841	28978	0	393	413	95	-	1447	1841	5435	5846
UN00754	Rsa1.0_00697.1	1320	67913	0	953	988	96	-	330	1313	37664	38651
UN00755	Rsa1.0_04301.1	831	18022	0	478	520	91	+	3	517	3138	3629
UN00756	Rsa1.0_00100.1	472	249232	0	434	468	92	-	4	458	181624	182082
UN00757	Rsa1.0_16374.1	961	2173	0	734	776	94	-	29	804	976	1735
UN00758	Rsa1.0_00566.1	755	75325	1.00E-145	371	404	91	-	38	427	25336	25729
UN00759	Rsa1.0_00609.1	1158	72785	0	435	449	96	+	568	1015	70202	70650
UN00760	Rsa1.0_01136.1	598	52724	1.00E-127	268	277	96	+	321	596	22713	22985
UN00761	Rsa1.0_00921.1	1057	59599	1.00E-125	240	244	98	+	738	981	2810	3053
UN00762	Rsa1.0_01309.1	1294	48556	0	533	543	98	-	755	1292	16161	16703
UN00763	Rsa1.0_00388.1	654	88890	1.00E-134	243	243	100	+	125	367	54976	55218
UN00764	Rsa1.0_01080.1	747	54752	0	539	544	99	-	203	745	7932	8475
UN00765	Rsa1.0_02269.1	1395	33168	1.00E-177	355	367	96	+	628	993	3346	3712
UN00766	Rsa1.0_00457.1	638	337301	7.00E-66	131	132	99	+	394	525	179228	179359
UN00767	Rsa1.0_00833.1	791	62621	4.00E-68	164	174	94	-	1	171	7472	7645
UN00768	Rsa1.0_00001.1	895	363028	1.00E-106	199	200	99	-	2	201	305974	306173
UN00769	Rsa1.0_01669.1	763	41286	0	737	775	95	+	1	756	38600	39374
UN00770	Rsa1.0_00055.1	1112	520193	0	680	689	98	+	424	1112	98332	99020
UN00771	Rsa1.0_25516.1	1015	933	0	619	627	98	-	391	1013	23	649
UN00772	Rsa1.0_00300.1	1432	97747	1.00E-114	306	338	90	-	1079	1416	43860	44197
UN00773	Rsa1.0_00173.1	779	288743	0	388	401	96	+	380	779	203686	204082
UN00774	Rsa1.0_20572.1	1248	1292	0	1111	1141	97	+	108	1248	1	1139
UN00775	Rsa1.0_01119.1	1240	53459	0	1116	1179	94	+	11	1178	3953	5114
UN00776	Rsa1.0_05583.1	655	13268	1.00E-79	169	175	96	-	124	298	1012	1186
UN00777	Rsa1.0_00069.1	1658	403561	0	1195	1226	97	-	1	1224	208564	209779
UN00778	Rsa1.0_02916.1	838	26809	1.00E-161	406	444	91	+	407	838	5584	6013

UN00779	Rsa1.0_00391.1	1095	183853	0	465	481	96	−	615	1093	45950	46427
UN00780	Rsa1.0_00188.1	1125	112020	0	449	463	96	−	221	683	74179	74638
UN00781	Rsa1.0_02907.1	1191	26861	0	549	593	92	−	610	1191	15213	15794
UN00782	Rsa1.0_03034.1	1359	157864	0	939	942	99	−	158	1099	3881	4822
UN00783	Rsa1.0_00060.1	1338	502678	0	325	325	100	−	1014	1338	252641	252965
UN00784	Rsa1.0_07676.1	1040	8827	0	496	498	99	+	544	1040	763	1260
UN00785	Rsa1.0_00392.1	1128	88523	0	569	621	91	+	493	1088	65484	66095
UN00786	Rsa1.0_02600.1	1109	29657	1.00E−118	341	383	89	−	740	1097	1277	1658
UN00787	Rsa1.0_00421.1	734	86365	1.00E−104	280	310	90	−	437	734	14724	15033
UN00788	Rsa1.0_00127.1	1182	127446	1.00E−130	352	384	91	+	423	800	36967	37345
UN00789	Rsa1.0_00979.1	1043	57733	1.00E−115	261	277	94	−	167	440	41925	42201
UN00790	Rsa1.0_00002.1	945	601661	0	541	554	97	+	278	831	231774	232327
UN00791	Rsa1.0_00731.1	716	66638	0	705	711	99	−	5	715	12893	13603
UN00792	Rsa1.0_04689.1	697	16376	0	450	461	97	+	1	461	715	1175
UN00793	Rsa1.0_05153.1	894	14663	1.00E−115	269	285	94	+	600	878	2743	3023
UN00794	Rsa1.0_04920.1	1237	15434	0	1182	1206	98	−	44	1237	412	1617
UN00795	Rsa1.0_06553.1	772	10795	1.00E−122	229	231	99	−	196	426	5503	5733
UN00796	Rsa1.0_01239.1	713	50107	1.00E−136	270	276	97	+	3	278	17129	17400
UN00797	Rsa1.0_00092.1	1077	659932	1.00E−119	230	234	98	−	253	486	444940	445173
UN00798	Rsa1.0_00965.1	233	58139	6.00E−30	81	85	95	+	89	173	7327	7408
UN00799	Rsa1.0_03395.1	1084	23243	0	428	450	95	+	642	1084	21042	21487
UN00800	Rsa1.0_33048.1	764	687	7.00E−79	294	338	86	−	373	707	239	576
UN00801	Rsa1.0_00085.1	1052	142226	0	371	384	96	−	670	1052	62439	62819
UN00802	Rsa1.0_00102.1	1295	135497	2.00E−46	102	104	98	+	882	985	8020	8123
UN00803	Rsa1.0_21386.1	1044	1209	0	835	876	95	−	172	1044	131	1006
UN00804	Rsa1.0_01245.1	850	149868	0	516	544	94	+	326	850	45960	46503
UN00805	Rsa1.0_00003.1	1665	340054	0	750	766	97	−	898	1663	8201	8966
UN00806	Rsa1.0_00103.1	720	135304	0	697	718	97	−	3	720	22154	22869
UN00807	Rsa1.0_00981.1	803	57700	1.00E−141	360	396	90	−	4	372	50690	51085
UN00808	Rsa1.0_00673.1	736	69325	0	732	736	99	−	3	736	32994	33729
UN00809	Rsa1.0_43878.1	832	512	0	503	511	98	+	1	508	2	511
UN00810	Rsa1.0_00451.1	503	322533	1.00E−119	284	300	94	+	205	500	301659	301956
UN00811	Rsa1.0_01523.1	793	43959	1.00E−159	285	285	100	−	508	792	2751	3035
UN00812	Rsa1.0_00374.1	1043	165738	0	402	418	96	+	630	1043	5585	5999
UN00813	Rsa1.0_00092.1	1192	659932	0	399	416	95	+	784	1191	505878	506292
UN00814	Rsa1.0_00255.1	1400	204967	0	836	844	99	+	557	1399	70989	71832
UN00815	Rsa1.0_02492.1	571	30859	1.00E−120	291	309	94	+	264	571	12178	12480
UN00816	Rsa1.0_00012.1	917	222148	0	586	603	97	+	319	917	130843	131441
UN00817	Rsa1.0_00273.1	765	326186	1.00E−166	419	450	93	−	322	765	26633	27078

UN00818	Rsa1.0_00625.1	739	71672	5.00E-89	188	195	96	-	476	670	31476	31670
UN00819	Rsa1.0_11393.1	1290	5521	0	397	418	94	-	3	419	205	619
UN00820	Rsa1.0_01804.1	797	39038	0	420	430	97	+	370	797	34084	34510
UN00821	Rsa1.0_00467.1	832	156542	0	785	815	96	+	3	810	92931	93745
UN00822	Rsa1.0_00022.1	1166	200461	0	815	856	95	+	18	873	43338	44178
UN00823	Rsa1.0_00082.1	857	237584	1.00E-117	259	274	94	-	48	315	56446	56719
UN00824	Rsa1.0_00778.1	1031	196751	9.00E-82	158	159	99	-	400	558	7426	7584
UN00825	Rsa1.0_02625.1	885	139104	1.00E-160	367	393	93	+	2	388	11405	11796
UN00826	Rsa1.0_00023.1	785	194764	6.00E-67	133	134	99	+	218	351	69517	69650
UN00827	Rsa1.0_26169.1	1063	903	1.00E-150	272	273	99	+	200	472	551	823
UN00828	Rsa1.0_01545.1	908	43599	0	518	533	97	-	3	535	27015	27539
UN00829	Rsa1.0_00544.1	409	76787	0	380	394	96	-	2	392	8466	8859
UN00830	Rsa1.0_00136.1	947	122464	0	393	398	98	-	3	400	68244	68638
UN00831	Rsa1.0_00045.1	1033	328694	0	495	514	96	-	525	1032	184204	184717
UN00832	Rsa1.0_00087.1	1216	761073	0	643	693	92	-	536	1215	733567	734242
UN00833	Rsa1.0_03005.1	1721	26082	1.00E-112	215	218	98	+	17	234	7720	7937
UN00834	Rsa1.0_02362.1	799	32190	1.00E-176	348	356	97	-	434	785	24790	25145
UN00835	Rsa1.0_21931.1	1369	1162	0	665	682	97	-	599	1280	4	685
UN00836	Rsa1.0_02473.1	621	31083	0	600	623	96	-	3	621	9004	9620
UN00837	Rsa1.0_01061.1	1052	55349	1.00E-156	283	284	99	+	769	1052	38814	39097
UN00838	Rsa1.0_01412.1	433	46311	1.00E-94	306	348	87	-	119	433	10270	10614
UN00839	Rsa1.0_41478.1	1734	542	0	398	416	95	+	545	960	35	450
UN00840	Rsa1.0_00277.1	1042	100132	0	478	516	92	+	437	946	2808	3323
UN00841	Rsa1.0_01168.1	1317	153765	0	1117	1181	94	+	159	1317	20893	22072
UN00842	Rsa1.0_01390.1	831	46695	0	434	449	96	+	387	831	24733	25181
UN00843	Rsa1.0_02041.1	867	163634	0	830	867	95	+	1	867	157069	157935
UN00844	Rsa1.0_02358.1	949	32213	1.00E-166	325	335	97	-	427	761	384	712
UN00845	Rsa1.0_00179.1	1918	393324	0	387	391	98	-	715	1105	51783	52173
UN00846	Rsa1.0_00035.1	769	176449	1.00E-128	241	244	98	-	3	246	5459	5702
UN00847	Rsa1.0_04230.1	903	18384	0	461	474	97	+	4	474	10531	11003
UN00848	Rsa1.0_00644.1	750	136881	2.00E-57	123	126	97	+	231	356	30581	30706
UN00849	Rsa1.0_04945.1	972	15370	0	877	891	98	+	81	971	8767	9656
UN00850	Rsa1.0_00051.1	684	258225	0	491	513	95	+	3	515	113688	114193
UN00851	Rsa1.0_00262.1	680	101906	1.00E-112	227	234	97	+	7	240	46434	46663
UN00852	Rsa1.0_00362.1	798	299497	1.00E-154	292	297	98	-	3	299	270726	271022
UN00853	Rsa1.0_03982.1	884	19723	0	682	719	94	-	176	884	11052	11767
UN00854	Rsa1.0_00354.1	1409	232005	0	561	563	99	-	342	904	230428	230990
UN00855	Rsa1.0_00457.1	939	337301	1.00E-155	296	302	98	+	637	938	2588	2889
UN00856	Rsa1.0_00393.1	1032	158674	0	569	588	96	+	1	588	135770	136351

UN00857	Rsa1.0_00740.1	897	215972	0	887	913	97	+	4	897	43427	44336
UN00858	Rsa1.0_01601.1	1230	42519	1.00E-124	236	238	99	−	991	1227	17159	17396
UN00859	Rsa1.0_01989.1	1568	36439	1.00E-173	345	355	97	+	7	357	8731	9085
UN00860	Rsa1.0_03887.1	1064	20247	0	394	396	99	−	3	398	4347	4742
UN00861	Rsa1.0_00273.1	928	326186	5.00E-83	166	169	98	−	6	174	162185	162353
UN00862	Rsa1.0_01000.1	765	57140	1.00E-64	129	130	99	−	69	198	39653	39782
UN00863	Rsa1.0_00308.1	798	96915	0	385	405	95	+	357	760	81326	81724
UN00864	Rsa1.0_04160.1	929	18795	0	454	456	99	+	474	928	9896	10351
UN00865	Rsa1.0_02794.1	772	27820	1.00E-148	267	267	100	−	1	267	2429	2695
UN00866	Rsa1.0_01116.1	1140	142088	1.00E-167	308	310	99	−	831	1140	119147	119455
UN00867	Rsa1.0_20459.1	814	1303	0	455	483	94	−	334	811	349	828
UN00868	Rsa1.0_00895.1	1366	60615	2.33E-156	293	303	96	−	245	547	41770	42072
UN00869	Rsa1.0_00245.1	1209	103569	0	662	687	96	+	528	1209	100778	101459
UN00870	Rsa1.0_00017.1	1126	752458	1.00E-111	238	248	95	+	882	1125	450189	450434
UN00871	Rsa1.0_00790.1	733	148693	1.00E-136	267	274	97	−	10	283	57272	57545
UN00872	Rsa1.0_00139.1	751	357786	1.00E-171	449	492	91	−	241	730	45669	46158
UN00873	Rsa1.0_13182.1	1247	4980	0	499	538	92	−	704	1240	3911	4436
UN00874	Rsa1.0_02573.1	1228	29955	0	1216	1231	98	+	5	1227	10723	11953
UN00875	Rsa1.0_05319.1	957	14158	0	677	685	98	−	6	690	5065	5749
UN00876	Rsa1.0_00878.1	1109	330303	1.00E-154	348	372	93	−	741	1109	9130	9489
UN00877	Rsa1.0_00727.1	856	66724	5.00E-89	167	167	100	+	690	856	33639	33805
UN00878	Rsa1.0_17985.1	1279	1695	1.00E-106	219	225	97	−	4	228	540	763
UN00879	Rsa1.0_00033.1	881	515811	0	421	439	95	−	354	792	485172	485610
UN00880	Rsa1.0_01896.1	914	37512	0	883	934	94	+	1	914	19194	20127
UN00881	Rsa1.0_00074.1	947	147591	0	922	930	99	+	2	930	90669	91598
UN00882	Rsa1.0_01170.1	763	161131	0	452	485	93	−	304	761	117423	117907
UN00883	Rsa1.0_00748.1	824	65983	1.00E-147	277	281	98	+	413	693	58948	59228
UN00884	Rsa1.0_02039.1	1113	35699	1.00E-147	350	377	92	+	2	358	883	1259
UN00885	Rsa1.0_00999.1	658	57162	0	507	533	95	+	1	530	24609	25141
UN00886	Rsa1.0_03305.1	1167	147552	0	467	486	96	+	683	1165	141859	142342
UN00887	Rsa1.0_06160.1	1293	11596	0	473	485	97	+	184	668	10937	11421
UN00888	Rsa1.0_00224.1	630	327530	1.00E-161	287	287	100	+	343	629	195632	195918
UN00889	Rsa1.0_00030.1	1300	250988	0	626	684	91	−	650	1299	124921	125598
UN00890	Rsa1.0_06032.1	906	11886	1.00E-151	284	288	98	−	619	906	9059	9346
UN00891	Rsa1.0_00974.1	927	57893	0	361	361	100	−	225	585	38890	39250
UN00892	Rsa1.0_00240.1	1197	104175	0	383	395	96	+	802	1196	18059	18453
UN00893	Rsa1.0_03640.1	1018	21624	0	487	506	96	−	438	933	15487	15992
UN00894	Rsa1.0_00200.1	1500	162016	0	379	394	96	−	98	488	150563	150956
UN00895	Rsa1.0_01478.1	685	44991	1.00E-73	144	145	99	+	42	186	13009	13153

UN00896	Rsa1.0_10601.1	951	5789	1.00E-143	316	332	95	−		625	951	5284	5615
UN00897	Rsa1.0_01339.1	724	47813	1.00E-123	310	336	92	+		238	573	14806	15133
UN00898	Rsa1.0_00123.1	1180	325650	1.00E-142	256	256	100	+		925	1180	175498	175753
UN00899	Rsa1.0_01454.1	716	45440	1.00E-101	344	400	86	−		20	417	23564	23938
UN00900	Rsa1.0_00491.1	769	80219	1.00E-148	340	364	93	+		408	769	78437	78794
UN00901	Rsa1.0_01042.1	1227	293264	0	615	631	97	+		604	1225	137345	137974
UN00902	Rsa1.0_00447.1	793	84051	1.00E-113	223	228	97	−		293	520	57123	57350
UN00903	Rsa1.0_01233.1	802	50184	1.00E-157	285	286	99	−		3	288	30435	30720
UN00904	Rsa1.0_00437.1	785	84879	0	380	386	98	+		354	739	39662	40047
UN00905	Rsa1.0_01389.1	1094	46716	3.00E-97	190	193	98	+		536	728	26528	26720
UN00906	Rsa1.0_01971.1	1355	36641	1.00E-122	238	243	97	−		1104	1346	25146	25388
UN00907	Rsa1.0_00275.1	568	210104	1.00E-159	301	304	99	−		263	566	154366	154667
UN00908	#	#	#	#	#	#	#	#	#	#	#	#	
UN00909	Rsa1.0_00018.1	713	410899	2.00E-97	184	185	99	+		124	308	258616	258800
UN00910	Rsa1.0_00632.1	684	183777	0	653	693	94	−		2	684	145375	146055
UN00911	Rsa1.0_02212.1	1018	33840	1.00E-83	164	166	98	−		446	611	28024	28189
UN00912	Rsa1.0_09577.1	1325	6275	0	929	975	95	−		303	1265	961	1935
UN00913	Rsa1.0_02550.1	910	291051	0	370	371	99	−		540	910	18189	18559
UN00914	Rsa1.0_02041.1	731	163634	0	430	443	97	−		3	445	14539	14972
UN00915	Rsa1.0_00013.1	1788	292129	0	633	635	99	+		1153	1787	168710	169343
UN00916	Rsa1.0_00411.1	1282	86944	0	497	508	97	+		1	508	3204	3711
UN00917	Rsa1.0_35196.1	1023	643	0	412	417	98	+		151	567	227	643
UN00918	Rsa1.0_01668.1	975	41299	1.00E-113	355	401	88	+		587	975	19391	19783
UN00919	Rsa1.0_05662.1	804	13011	0	367	371	98	−		434	804	5288	5658
UN00920	Rsa1.0_01096.1	814	54266	1.00E-170	309	311	99	−		3	313	10256	10566
UN00921	Rsa1.0_00690.1	739	140527	1.00E-128	256	264	96	+		3	266	57035	57298
UN00922	Rsa1.0_03239.1	403	24430	1.00E-136	249	250	99	+		2	251	12175	12424
UN00923	Rsa1.0_00093.1	921	214962	0	402	409	98	+		518	921	13902	14310
UN00924	Rsa1.0_00893.1	1062	60626	0	388	395	98	−		2	396	4394	4788
UN00925	Rsa1.0_00512.1	1193	200277	0	399	400	99	+		794	1193	30514	30913
UN00926	Rsa1.0_01503.1	624	44467	1.00E-152	293	300	97	+		190	489	43714	44013
UN00927	Rsa1.0_02872.1	831	27080	1.00E-145	302	313	96	−		519	831	9164	9469
UN00928	Rsa1.0_13514.1	1059	4757	0	701	723	96	+		341	1059	836	1552
UN00929	Rsa1.0_05522.1	517	13441	4.00E-76	168	175	96	+		1	175	8265	8436
UN00930	#	#	#	#	#	#	#	#	#	#	#	#	
UN00931	Rsa1.0_04404.1	866	17510	0	614	626	98	+		147	772	9475	10100
UN00932	Rsa1.0_00538.1	827	77149	0	373	381	97	+		163	543	65814	66194
UN00933	Rsa1.0_00714.1	1002	67187	1.00E-129	260	269	96	+		4	272	22282	22544
UN00934	Rsa1.0_00214.1	1030	107508	1.00E-126	350	391	89	−		644	1030	28880	29247

UN00935	Rsa1.0_01504.1		779	189978	1.00E-147	285	291	97	-	8	296	171988	172278
UN00936	Rsa1.0_01561.1		525	43227	0	489	524	93	+	1	524	33388	33880
UN00937	Rsa1.0_00327.1		939	94892	0	662	686	96	-	261	939	70626	71308
UN00938	Rsa1.0_17015.1		1049	1953	1.00E-103	237	251	94	-	2	244	874	1121
UN00939	Rsa1.0_00121.1		998	278029	0	982	999	98	+	2	998	103336	104334
UN00940	Rsa1.0_00924.1		1636	59524	1.00E-137	254	256	99	-	1020	1275	38046	38301
UN00941	Rsa1.0_01946.1		852	36982	1.00E-120	219	219	100	+	246	464	31309	31527
UN00942	Rsa1.0_03034.1		1501	157864	0	496	516	96	+	982	1496	134541	135055
UN00943	Rsa1.0_01416.1		1273	46262	1.00E-131	281	293	95	-	980	1272	28739	29028
UN00944	Rsa1.0_00251.1		760	303968	1.00E-138	316	338	93	+	3	339	130075	130403
UN00945	Rsa1.0_00078.1		1032	175053	1.00E-172	378	401	94	+	608	1008	139900	140298
UN00946	#	#	#	#	#	#	#	#	#	#	#	#	
UN00947	Rsa1.0_00136.1		959	122464	0	347	353	98	-	385	737	112510	112862
UN00948	Rsa1.0_01803.1		801	173846	8.00E-91	182	185	98	+	116	300	136431	136613
UN00949	Rsa1.0_00038.1		775	314568	1.00E-144	259	259	100	+	49	307	166217	166475
UN00950	Rsa1.0_08124.1		1017	7869	0	723	729	99	-	142	870	5	733
UN00951	Rsa1.0_00008.1		874	358124	1.00E-130	239	240	99	-	7	246	200510	200749
UN00952	Rsa1.0_00632.1		603	183777	1.00E-141	285	293	97	-	309	601	149149	149438
UN00953	Rsa1.0_00467.1		1143	156542	0	1086	1135	95	-	3	1137	118192	119311
UN00954	Rsa1.0_00105.1		1189	134138	0	568	624	91	-	279	879	1925	2540
UN00955	Rsa1.0_00119.1		888	467509	1.00E-121	277	293	94	+	592	882	332175	332467
UN00956	Rsa1.0_01696.1		749	301850	1.00E-77	154	156	98	-	359	514	299403	299558
UN00957	Rsa1.0_01329.1		862	47982	0	857	862	99	+	2	862	6363	7222
UN00958	Rsa1.0_25074.1		943	952	1.00E-163	297	299	99	-	645	943	654	952
UN00959	Rsa1.0_00253.1		851	231292	0	668	716	93	-	120	823	79564	80274
UN00960	Rsa1.0_00304.1		948	97429	0	542	565	95	+	398	946	57550	58113
UN00961	Rsa1.0_03323.1		523	23751	2.00E-75	159	164	96	-	230	393	15045	15208
UN00962	Rsa1.0_00117.1		850	213118	0	835	846	98	-	5	850	128766	129611
UN00963	Rsa1.0_01179.1		131	51549	2.00E-16	80	92	86	+	1	92	42489	42580
UN00964	Rsa1.0_06333.1		902	11217	1.00E-139	255	256	99	+	520	775	224	479
UN00965	Rsa1.0_00073.1		1480	240924	0	736	748	98	-	1	743	109830	110575
UN00966	Rsa1.0_00365.1		1222	90902	1.00E-129	247	251	98	-	971	1221	41879	42129
UN00967	Rsa1.0_01862.1		671	38030	1.00E-125	227	227	100	-	1	227	14590	14816
UN00968	Rsa1.0_01060.1		1285	55368	1.00E-162	319	326	97	-	957	1281	20960	21284
UN00969	Rsa1.0_01411.1		964	46326	1.00E-164	326	337	96	+	626	962	7472	7808
UN00970	Rsa1.0_02252.1		921	33375	0	378	383	98	+	368	750	31510	31892
UN00971	Rsa1.0_00089.1		137	406142	4.00E-42	123	135	91	-	3	137	163364	163498
UN00972	Rsa1.0_01997.1		892	158416	5.00E-89	167	167	100	-	1	167	16696	16862
UN00973	Rsa1.0_00744.1		827	66084	1.00E-102	211	218	96	+	601	818	58901	59118

UN00974	Rsa1.0_00129.1	1023	126831	0	416	441	94	+	1	439	113601	114039
UN00975	Rsa1.0_01000.1	880	57140	1.00E-104	268	289	92	+	3	290	16819	17100
UN00976	Rsa1.0_00307.1	1006	199003	0	762	825	92	+	8	832	155929	156753
UN00977	Rsa1.0_41025.1	1303	547	0	402	402	100	-	17	418	146	547
UN00978	Rsa1.0_00350.1	906	92346	6.00E-89	251	276	90	-	5	278	10176	10447
UN00979	Rsa1.0_03354.1	1067	23491	0	852	869	98	-	1	869	17878	18737
UN00980	Rsa1.0_09141.1	1687	6612	0	498	498	100	+	73	570	974	1471
UN00981	Rsa1.0_54193.1	1349	421	0	420	421	99	-	797	1217	1	421
UN00982	Rsa1.0_00131.1	686	432233	1.00E-149	268	268	100	+	419	686	395234	395501
UN00983	Rsa1.0_00900.1	719	60367	1.00E-144	301	312	96	+	7	318	25861	26170
UN00984	Rsa1.0_00327.1	922	94892	0	526	528	99	+	290	817	70549	71076
UN00985	Rsa1.0_03962.1	1152	19860	0	566	598	94	+	1	584	14046	14639
UN00986	Rsa1.0_02496.1	786	30814	0	427	443	96	+	347	786	28985	29425
UN00987	Rsa1.0_07000.1	903	10049	6.00E-92	220	232	94	-	3	232	8619	8844
UN00988	Rsa1.0_00179.1	1197	393324	5.00E-90	214	229	93	-	1	229	218140	218368
UN00989	Rsa1.0_05618.1	461	13165	3.00E-98	254	274	92	+	7	278	6819	7091
UN00990	Rsa1.0_02859.1	1104	162118	0	377	380	99	-	1	380	144252	144631
UN00991	Rsa1.0_00006.1	949	396176	0	474	518	91	+	7	508	368527	369036
UN00992	Rsa1.0_04450.1	824	17297	1.00E-166	348	364	95	-	462	824	12622	12985
UN00993	Rsa1.0_01091.1	865	54383	0	506	516	98	+	286	801	8775	9290
UN00994	Rsa1.0_02990.1	1046	26180	1.00E-141	266	269	98	+	1	269	18344	18610
UN00995	Rsa1.0_01014.1	751	56762	7.00E-82	218	240	90	+	1	238	39994	40217
UN00996	Rsa1.0_02438.1	1187	31392	1.00E-118	222	224	99	+	644	867	5578	5801
UN00997	Rsa1.0_00079.1	1554	606987	0	509	518	98	+	1034	1548	190876	191388
UN00998	Rsa1.0_00322.1	1460	212233	1.00E-122	229	231	99	-	1	231	73895	74125
UN00999	Rsa1.0_00160.1	751	346678	1.00E-117	251	263	95	-	452	714	32107	32369
UN01000	Rsa1.0_13949.1	748	4028	1.00E-169	347	361	96	-	387	746	1108	1468
UN01001	Rsa1.0_00089.1	1147	406142	0	424	434	97	+	1	431	390229	390659
UN01002	Rsa1.0_01779.1	871	39448	0	422	430	98	-	1	430	25472	25897
UN01003	Rsa1.0_01392.1	1569	46665	0	1301	1309	99	-	2	1310	24719	26026
UN01004	Rsa1.0_05359.1	1385	14054	1.00E-176	329	333	98	+	1	333	10246	10577
UN01005	Rsa1.0_04819.1	738	140505	0	703	749	93	+	1	738	10808	11547
UN01006	Rsa1.0_00387.1	1154	495407	1.00E-136	309	327	94	-	827	1152	384412	384737
UN01007	Rsa1.0_02575.1	841	29944	0	499	503	99	-	2	504	3747	4249
UN01008	Rsa1.0_01545.1	432	43599	2.00E-77	187	199	93	+	1	198	25184	25382
UN01009	Rsa1.0_02274.1	845	33109	1.00E-174	338	345	97	-	3	345	22370	22714
UN01010	Rsa1.0_16067.1	1002	2300	0	383	393	97	-	611	998	311	703
UN01011	Rsa1.0_01489.1	1489	44764	0	433	434	99	-	1	433	1590	2023
UN01012	Rsa1.0_00332.1	903	94575	0	492	504	97	-	335	838	54514	55017

UN01013	Rsa1.0_05581.1	935	13272	0	652	659	98	−	1	659	5335	5990
UN01014	Rsa1.0_00375.1	200	90207	8.00E−63	180	198	90	−	3	200	2417	2613
UN01015	Rsa1.0_00092.1	680	659932	0	420	444	94	+	16	457	242851	243294
UN01016	Rsa1.0_09594.1	681	6263	1.00E−115	230	235	97	+	447	681	872	1105
UN01017	Rsa1.0_01190.1	1264	194501	0	733	770	95	+	7	757	44595	45363
UN01018	Rsa1.0_01071.1	984	166914	0	364	367	99	+	617	983	27109	27475
UN01019	Rsa1.0_00925.1	675	59481	0	638	674	94	−	1	669	21081	21749
UN01020	Rsa1.0_01308.1	778	48596	0	385	387	99	−	389	775	21408	21794
UN01021	Rsa1.0_00332.1	1080	94575	0	379	381	99	−	1	381	73577	73957
UN01022	Rsa1.0_00016.1	945	831256	2.00E−67	195	211	92	+	25	234	265984	266181
UN01023	Rsa1.0_05025.1	657	15097	0	651	654	99	+	1	654	13034	13687
UN01024	Rsa1.0_03867.1	461	20369	0	435	457	95	−	1	457	15318	15773
UN01025	Rsa1.0_00047.1	1222	168582	0	460	494	93	−	2	493	67922	68415
UN01026	Rsa1.0_01803.1	834	173846	1.00E−136	270	278	97	−	113	390	34629	34906
UN01027	Rsa1.0_03904.1	898	20162	0	498	503	99	+	396	898	4565	5067
UN01028	Rsa1.0_00202.1	778	185895	0	568	587	96	+	191	775	136773	137356
UN01029	Rsa1.0_00045.1	1331	328694	0	1323	1330	99	+	3	1330	35877	37206
UN01030	Rsa1.0_00338.1	902	94109	0	852	884	96	+	3	885	89856	90736
UN01031	Rsa1.0_00200.1	894	162016	1.00E−132	267	275	97	+	4	278	148988	149260
UN01032	Rsa1.0_01478.1	760	44991	3.00E−65	127	127	100	−	221	347	15709	15835
UN01033	Rsa1.0_00579.1	702	148308	0	410	423	96	+	1	415	63787	64208
UN01034	Rsa1.0_00290.1	788	225096	1.00E−158	301	307	98	−	482	788	3869	4175
UN01035	Rsa1.0_21578.1	1399	1190	1.00E−166	302	304	99	+	1	304	526	829
UN01036	Rsa1.0_05431.1	597	13780	1.00E−147	286	293	97	+	4	296	8196	8488
UN01037	Rsa1.0_02018.1	729	35994	0	711	730	97	−	4	729	30090	30817
UN01038	Rsa1.0_02275.1	1015	33107	0	496	502	98	−	2	500	30103	30604
UN01039	Rsa1.0_02144.1	1246	34630	0	376	383	98	+	862	1244	23103	23484
UN01040	Rsa1.0_01803.1	560	173846	0	354	360	98	−	31	390	37469	37825
UN01041	Rsa1.0_02183.1	773	34170	0	389	392	99	+	381	771	13946	14337
UN01042	Rsa1.0_00263.1	1250	224968	0	490	506	96	+	5	510	186882	187381
UN01043	Rsa1.0_00296.1	753	97854	1.00E−118	237	244	97	−	1	244	53004	53247
UN01044	Rsa1.0_03834.1	1407	20572	0	1385	1409	98	−	1	1407	18529	19937
UN01045	Rsa1.0_02955.1	454	26484	2.00E−77	159	163	97	−	237	399	13393	13555
UN01046	Rsa1.0_01763.1	774	39659	8.00E−57	223	257	86	−	298	548	37646	37901
UN01047	Rsa1.0_02412.1	923	31643	0	741	760	97	−	4	759	6107	6862
UN01048	Rsa1.0_01286.1	584	49209	0	516	534	96	+	1	533	3993	4521
UN01049	Rsa1.0_00602.1	1263	73302	0	630	648	97	−	1	647	29630	30276
UN01050	Rsa1.0_02209.1	1066	33873	1.00E−169	337	347	97	+	1	344	26612	26957
UN01051	Rsa1.0_02803.1	840	27715	1.00E−162	299	302	99	−	180	481	3172	3473

UN01052	Rsa1.0_04762.1	1454	16101	0	934	939	99	+	510	1448	4259	5197
UN01053	Rsa1.0_05160.1	1400	14637	0	594	600	99	+	613	1212	13452	14051
UN01054	Rsa1.0_01536.1	872	43747	0	857	873	98	+	1	870	18854	19724
UN01055	Rsa1.0_17015.1	1148	1953	1.00E-130	237	237	100	+	912	1148	874	1110
UN01056	Rsa1.0_03735.1	681	21087	0	549	566	96	-	1	561	5153	5715
UN01057	Rsa1.0_00035.1	795	176449	0	510	513	99	+	1	513	41112	41624
UN01058	Rsa1.0_00288.1	954	98659	0	384	398	96	+	155	551	82911	83305
UN01059	Rsa1.0_00131.1	1366	432233	1.00E-118	325	351	92	-	20	359	412408	412754
UN01060	Rsa1.0_00167.1	628	116000	1.00E-116	246	257	95	-	1	257	14352	14608
UN01061	Rsa1.0_02928.1	788	26680	0	386	400	96	+	387	783	20709	21108
UN01062	Rsa1.0_02780.1	1140	27965	0	588	589	99	-	552	1140	24650	25238
UN01063	Rsa1.0_02564.1	1378	30047	0	508	513	99	-	538	1048	5427	5939
UN01064	Rsa1.0_00152.1	1369	257730	0	646	663	97	-	4	665	230931	231593
UN01065	Rsa1.0_00312.1	1606	96765	1.00E-131	241	242	99	-	1365	1606	77943	78184
UN01066	Rsa1.0_02496.1	710	30814	1.00E-151	308	318	96	-	392	708	14669	14984
UN01067	Rsa1.0_00248.1	1100	163568	2.00E-89	197	207	95	+	90	290	52500	52706
UN01068	Rsa1.0_02553.1	633	30190	1.00E-118	235	240	97	+	395	633	18667	18906
UN01069	Rsa1.0_04385.1	694	17627	1.00E-135	245	245	100	-	450	694	16884	17128
UN01070	Rsa1.0_04955.1	771	15337	0	614	646	95	+	125	767	8724	9366
UN01071	Rsa1.0_00577.1	1337	174827	1.00E-164	320	330	96	+	1015	1336	118729	119058
UN01072	Rsa1.0_02114.1	909	34873	1.00E-113	211	212	99	+	207	418	664	875
UN01073	Rsa1.0_00548.1	1483	76518	0	809	841	96	-	645	1478	63650	64478
UN01074	Rsa1.0_00402.1	1360	385315	0	628	661	95	+	3	643	119044	119698
UN01075	Rsa1.0_00098.1	1033	349239	0	446	447	99	+	3	449	292716	293162
UN01076	Rsa1.0_01168.1	930	153765	1.00E-168	392	423	92	+	63	479	38497	38919
UN01077	Rsa1.0_00115.1	777	131321	0	592	610	97	+	169	777	34041	34648
UN01078	Rsa1.0_00385.1	912	89160	0	368	372	98	+	541	912	9411	9779
UN01079	Rsa1.0_02811.1	1293	27669	0	478	482	99	-	812	1293	7890	8371
UN01080	Rsa1.0_02976.1	589	26315	1.00E-178	341	349	97	-	240	588	15693	16041
UN01081	Rsa1.0_03385.1	769	23280	1.00E-167	305	306	99	+	465	769	13870	14175
UN01082	Rsa1.0_04250.1	1193	18264	0	827	828	99	+	1	828	13122	13949
UN01083	Rsa1.0_01168.1	1525	153765	0	849	902	94	+	45	934	38497	39389
UN01084	Rsa1.0_02677.1	1470	167801	1.00E-66	159	169	94	-	1302	1470	11977	12139
UN01085	Rsa1.0_12087.1	882	5317	0	858	887	96	-	3	882	4202	5088
UN01086	Rsa1.0_00108.1	1380	478491	1.00E-109	224	230	97	+	1147	1375	420682	420911
UN01087	Rsa1.0_00008.1	812	358124	0	618	639	96	-	177	812	50867	51499
UN01088	Rsa1.0_00272.1	572	447836	1.00E-152	284	288	98	+	285	572	83843	84130
UN01089	Rsa1.0_06073.1	772	11807	0	385	386	99	+	279	664	3893	4278
UN01090	Rsa1.0_01732.1	1346	40194	0	1215	1279	94	-	80	1344	24250	25521

UN01091	Rsa1.0_00048.1	1136	167185	0	1125	1136	99	+	5	1136	125219	126354
UN01092	Rsa1.0_07134.1	1328	9854	7.00E-77	153	155	98	−	673	827	7276	7430
UN01093	Rsa1.0_05467.1	725	13649	3.00E-84	174	179	97	−	544	722	9353	9531
UN01094	Rsa1.0_00223.1	746	106231	1.00E-145	267	269	99	−	303	571	79400	79668
UN01095	Rsa1.0_00420.1	1222	86496	2.33E-156	296	306	96	+	919	1222	11931	12236
UN01096	Rsa1.0_00483.1	1061	81102	0	504	523	96	−	539	1059	27457	27970
UN01097	Rsa1.0_02053.1	507	35554	0	376	382	98	+	127	507	32532	32910
UN01098	Rsa1.0_00307.1	1156	199003	1.00E-124	265	277	95	−	24	300	184304	184577
UN01099	Rsa1.0_00016.1	1257	831256	1.00E-142	284	294	96	−	964	1257	522418	522704
UN01100	Rsa1.0_00191.1	872	316976	1.00E-156	279	279	100	+	333	611	145775	146053
UN01101	Rsa1.0_03257.1	1268	24305	0	388	393	98	−	3	395	15536	15928
UN01102	Rsa1.0_01933.1	817	37172	1.00E-132	274	282	97	+	207	488	11409	11685
UN01103	Rsa1.0_00017.1	863	752458	1.00E-61	220	246	89	+	622	863	462733	462968
UN01104	Rsa1.0_00104.1	1133	368722	5.00E-93	183	186	98	−	3	188	336882	337067
UN01105	Rsa1.0_01960.1	648	36766	1.00E-103	214	222	96	+	128	349	8003	8224
UN01106	Rsa1.0_00697.1	1104	67913	1.00E-135	248	249	99	+	488	736	57657	57905
UN01107	Rsa1.0_00098.1	959	349239	0	365	373	97	−	17	384	269214	269585
UN01108	Rsa1.0_02045.1	964	35650	0	922	961	95	−	2	962	15172	16104
UN01109	Rsa1.0_00944.1	870	58733	0	503	512	98	−	1	512	12555	13066
UN01110	Rsa1.0_02939.1	1114	26595	1.00E-164	372	397	93	−	693	1083	20157	20553
UN01111	Rsa1.0_00614.1	659	72276	1.00E-131	280	293	95	−	3	295	44076	44366
UN01112	Rsa1.0_00323.1	1035	159650	0	1030	1035	99	−	3	1035	53000	54034
UN01113	Rsa1.0_00658.1	881	69740	1.00E-123	224	224	100	−	188	411	18542	18765
UN01114	Rsa1.0_00293.1	1395	339294	0	749	761	98	−	635	1395	318346	319106
UN01115	Rsa1.0_00501.1	804	79545	0	548	602	91	−	219	800	41893	42484
UN01116	Rsa1.0_01537.1	860	187285	0	464	470	98	+	1	469	14844	15313
UN01117	Rsa1.0_00069.1	1219	403561	1.00E-102	219	229	95	+	515	743	224222	224450
UN01118	Rsa1.0_02711.1	768	28475	1.00E-129	290	303	95	+	462	761	24537	24838
UN01119	Rsa1.0_00226.1	609	105884	1.00E-165	310	315	98	−	2	316	22862	23176
UN01120	Rsa1.0_02606.1	1074	29635	1.00E-115	217	219	99	−	142	360	19085	19303
UN01121	Rsa1.0_00185.1	1298	248445	0	724	771	93	−	48	812	241787	242545
UN01122	Rsa1.0_03811.1	1622	20721	1.00E-157	284	285	99	+	1338	1622	19707	19991
UN01123	Rsa1.0_00914.1	1379	59880	1.00E-152	305	313	97	+	1067	1379	41830	42140
UN01124	Rsa1.0_00566.1	772	75325	0	363	365	99	−	409	772	24496	24860
UN01125	Rsa1.0_03900.1	800	133221	0	762	784	97	−	1	777	12231	13006
UN01126	Rsa1.0_04168.1	1746	18712	0	853	857	99	+	890	1746	13950	14806
UN01127	Rsa1.0_00658.1	827	69740	1.00E-165	309	313	98	−	515	827	15719	16029
UN01128	Rsa1.0_04823.1	689	15840	9.00E-75	152	155	98	+	197	351	13954	14108
UN01129	Rsa1.0_00049.1	1395	167018	1.00E-134	322	350	92	+	1	347	131911	132243

UN01130	Rsa1.0_05978.1	1182	12053	1.00E-139	297	309	96	−	872	1175	1955	2262
UN01131	Rsa1.0_00342.1	819	93695	1.00E-130	236	236	100	−	184	419	32256	32491
UN01132	Rsa1.0_00108.1	1183	478491	0	382	400	95	+	582	981	283803	284202
UN01133	Rsa1.0_00092.1	484	659932	1.00E-126	286	304	94	−	163	466	190047	190338
UN01134	Rsa1.0_01654.1	880	41532	0	845	898	94	−	1	880	20338	21226
UN01135	Rsa1.0_10332.1	876	5886	1.00E-43	97	99	97	−	623	721	1004	1102
UN01136	Rsa1.0_01393.1	944	46661	0	561	588	95	−	369	944	25512	26099
UN01137	Rsa1.0_02630.1	605	29349	0	428	448	95	+	155	602	28217	28664
UN01138	Rsa1.0_00571.1	917	329018	0	910	915	99	−	3	917	173859	174771
UN01139	Rsa1.0_61778.1	1083	371	3.00E-88	173	174	99	−	852	1024	130	303
UN01140	Rsa1.0_45370.1	745	497	1.00E-109	234	243	96	−	101	339	251	493
UN01141	Rsa1.0_03450.1	922	22835	0	396	398	99	−	9	406	6175	6572
UN01142	Rsa1.0_12096.1	701	5315	0	371	382	97	+	318	699	456	837
UN01143	Rsa1.0_00705.1	943	67505	1.00E-175	393	415	94	+	530	943	30393	30799
UN01144	Rsa1.0_03728.1	1443	21147	0	438	467	93	−	980	1443	631	1092
UN01145	Rsa1.0_01076.1	841	54835	0	836	839	99	−	3	841	19165	20003
UN01146	Rsa1.0_07330.1	1035	9548	0	653	656	99	−	160	815	1	656
UN01147	Rsa1.0_00471.1	1345	81897	0	713	735	97	−	609	1343	36591	37325
UN01148	Rsa1.0_00055.1	1173	520193	1.00E-174	354	366	96	+	809	1173	106239	106603
UN01149	Rsa1.0_13970.1	772	3973	1.00E-164	393	422	93	+	145	559	3553	3969
UN01150	Rsa1.0_00171.1	603	115622	1.00E-155	287	290	98	+	2	291	58910	59199
UN01151	Rsa1.0_00381.1	473	89718	0	463	474	97	+	1	472	79917	80390
UN01152	Rsa1.0_00241.1	1097	104000	0	588	593	99	+	506	1097	88938	89530
UN01153	Rsa1.0_00134.1	1003	390219	1.00E-177	439	480	91	−	524	1000	45226	45694
UN01154	Rsa1.0_01785.1	2011	39368	0	748	759	98	+	3	758	19322	20080
UN01155	Rsa1.0_00006.1	882	396176	1.00E-145	267	269	99	+	270	538	222130	222398
UN01156	Rsa1.0_00009.1	854	232558	1.00E-71	165	175	94	+	670	844	231969	232135
UN01157	Rsa1.0_03319.1	722	23767	0	514	526	97	−	2	527	1513	2037
UN01158	Rsa1.0_01340.1	1093	47805	0	560	560	100	+	534	1093	32618	33177
UN01159	Rsa1.0_00145.1	1151	261008	0	431	445	96	−	637	1081	27389	27833
UN01160	Rsa1.0_00299.1	938	97770	1.00E-117	259	273	94	+	142	414	26265	26534
UN01161	Rsa1.0_00197.1	1349	109714	0	433	437	99	−	914	1349	99274	99709
UN01162	Rsa1.0_05090.1	1372	14879	0	808	851	94	+	215	1065	2190	3027
UN01163	Rsa1.0_01424.1	472	46139	1.00E-115	271	291	93	+	25	315	42149	42439
UN01164	Rsa1.0_02545.1	1307	30286	0	464	479	96	−	828	1303	13644	14116
UN01165	Rsa1.0_03935.1	1356	19983	1.00E-135	291	303	96	−	1055	1355	7778	8078
UN01166	Rsa1.0_00086.1	897	439854	0	557	562	99	+	178	739	282513	283074
UN01167	Rsa1.0_00473.1	778	212204	1.00E-167	346	361	95	+	7	365	6968	7328
UN01168	Rsa1.0_00092.1	1367	659932	0	751	754	99	+	306	1059	198767	199520

UN01169	Rsa1.0_02872.1	817	27080	1.00E-137	290	300	96	−	520	817	9175	9469
UN01170	Rsa1.0_03100.1	1003	25411	0	423	428	98	+	577	1003	1871	2297
UN01171	Rsa1.0_00409.1	735	146788	0	582	632	92	+	118	735	80588	81199
UN01172	Rsa1.0_26747.1	1287	879	0	447	449	99	−	2	449	1	449
UN01173	Rsa1.0_00991.1	1376	143101	0	682	702	97	+	678	1376	137872	138569
UN01174	Rsa1.0_00001.1	1099	363028	0	1022	1022	100	−	7	1028	77607	78628
UN01175	Rsa1.0_01348.1	1102	47569	0	610	687	88	−	419	1098	45195	45867
UN01176	Rsa1.0_01631.1	575	41882	0	567	570	99	+	6	575	11514	12081
UN01177	Rsa1.0_02688.1	273	28644	7.00E-64	133	136	97	−	3	138	13636	13771
UN01178	Rsa1.0_00964.1	613	316648	0	597	613	97	−	1	613	277546	278158
UN01179	Rsa1.0_01592.1	780	42685	1.00E-165	313	318	98	+	138	453	25582	25899
UN01180	Rsa1.0_01156.1	1422	52147	0	376	376	100	+	1047	1422	32397	32772
UN01181	Rsa1.0_00686.1	615	68511	0	611	614	99	−	4	615	52392	53004
UN01182	Rsa1.0_04065.1	1206	19275	1.00E-137	281	292	96	+	146	437	745	1036
UN01183	Rsa1.0_00017.1	1162	752458	1.00E-123	257	265	96	+	902	1162	450189	450450
UN01184	Rsa1.0_00213.1	1235	108167	0	431	450	95	−	784	1225	36364	36811
UN01185	Rsa1.0_06450.1	930	10998	1.00E-173	314	316	99	−	261	576	5882	6197
UN01186	Rsa1.0_03209.1	1326	24592	0	1182	1226	96	−	118	1326	21123	22348
UN01187	Rsa1.0_00006.1	787	396176	1.00E-140	256	257	99	−	326	582	107662	107918
UN01188	Rsa1.0_00453.1	777	83031	2.00E-94	194	200	97	+	246	445	72257	72456
UN01189	Rsa1.0_04646.1	1337	16541	0	421	423	99	+	506	928	929	1351
UN01190	Rsa1.0_13353.1	720	4894	3.00E-99	208	216	96	+	117	332	561	776
UN01191	Rsa1.0_03544.1	1413	22182	0	592	606	97	−	199	803	3965	4567
UN01192	Rsa1.0_04004.1	1273	19594	0	494	495	99	−	29	523	4915	5409
UN01193	Rsa1.0_00003.1	766	340054	0	399	416	95	+	6	421	163906	164311
UN01194	Rsa1.0_01966.1	1305	36681	1.00E-164	324	333	97	−	979	1305	33698	34029
UN01195	Rsa1.0_02671.1	813	28887	0	424	427	99	+	226	652	7972	8398
UN01196	Rsa1.0_01387.1	864	46745	4.00E-68	144	148	97	−	614	761	31423	31570
UN01197	Rsa1.0_03350.1	1444	23522	0	358	367	97	−	1028	1394	19941	20307
UN01198	Rsa1.0_02053.1	773	35554	0	381	395	96	+	6	396	31827	32221
UN01199	Rsa1.0_01313.1	1005	48387	0	384	386	99	−	621	1005	1418	1803
UN01200	Rsa1.0_14712.1	879	3134	1.00E-138	341	367	92	−	516	877	1514	1869
UN01201	Rsa1.0_00837.1	811	62568	0	402	423	95	−	396	811	57200	57622
UN01202	Rsa1.0_01552.1	476	43396	0	369	371	99	+	106	476	24239	24607
UN01203	Rsa1.0_03390.1	1044	23270	0	433	438	98	−	163	600	17528	17965
UN01204	Rsa1.0_04193.1	667	18571	1.00E-167	342	354	96	−	316	667	9142	9495
UN01205	Rsa1.0_00001.1	1223	363028	0	538	601	89	−	3	596	266567	267140
UN01206	Rsa1.0_01539.1	495	43698	0	488	495	98	−	3	495	29264	29758
UN01207	Rsa1.0_03336.1	1212	23648	0	1134	1165	97	−	48	1212	13919	15064

UN01208	Rsa1.0_08437.1	798	7357	0	749	770	97	−	3	771	2852	3618
UN01209	Rsa1.0_00851.1	805	62181	0	789	812	97	−	2	805	20274	21085
UN01210	Rsa1.0_00127.1	475	127446	1.00E−148	290	297	97	+	179	475	35697	35992
UN01211	Rsa1.0_04402.1	973	17511	1.00E−160	322	333	96	+	1	333	14790	15119
UN01212	Rsa1.0_01443.1	1132	45705	2.00E−30	160	188	85	+	912	1098	34823	35008
UN01213	Rsa1.0_00134.1	1345	390219	0	346	348	99	−	998	1345	230194	230541
UN01214	Rsa1.0_00213.1	1009	108167	1.00E−144	274	279	98	+	244	522	44341	44619
UN01215	Rsa1.0_03156.1	933	24956	0	358	364	98	−	570	932	3524	3887
UN01216	Rsa1.0_00392.1	1244	88523	0	552	564	97	−	678	1239	6450	7011
UN01217	Rsa1.0_00351.1	1012	413096	0	790	814	97	+	158	971	124165	124977
UN01218	Rsa1.0_04118.1	1375	18968	0	348	348	100	−	61	408	4094	4441
UN01219	Rsa1.0_14327.1	626	3518	0	400	415	96	+	212	626	233	641
UN01220	Rsa1.0_05321.1	1958	14154	0	343	349	98	+	1244	1592	6131	6479
UN01221	Rsa1.0_00453.1	588	83031	1.00E−160	299	302	99	+	287	588	1355	1655
UN01222	Rsa1.0_05019.1	1575	15114	1.00E−161	294	296	99	−	3	298	14348	14643
UN01223	Rsa1.0_00485.1	1094	80937	0	845	877	96	−	52	925	71868	72738
UN01224	Rsa1.0_01340.1	1286	47805	0	409	416	98	−	871	1286	24298	24712
UN01225	Rsa1.0_00381.1	741	89718	0	351	351	100	−	3	353	75167	75517
UN01226	Rsa1.0_01848.1	1244	38312	0	600	602	99	+	255	856	25724	26325
UN01227	Rsa1.0_01760.1	857	39730	1.00E−118	370	416	88	+	3	402	11016	11423
UN01228	Rsa1.0_00222.1	1330	172263	3.00E−85	167	169	98	−	608	776	58615	58783
UN01229	Rsa1.0_00075.1	614	146680	1.00E−139	326	346	94	−	268	612	82352	82688
UN01230	Rsa1.0_02841.1	1270	27383	1.00E−119	227	230	98	+	1041	1270	8321	8550
UN01231	Rsa1.0_02377.1	635	31994	0	594	632	93	−	13	633	19526	20157
UN01232	Rsa1.0_08285.1	766	7606	0	726	786	92	−	3	759	2983	3764
UN01233	Rsa1.0_00788.1	858	64237	1.00E−130	248	251	98	+	3	253	32049	32297
UN01234	Rsa1.0_00383.1	1020	398431	0	379	380	99	+	1	380	25098	25477
UN01235	Rsa1.0_01719.1	886	40388	8.00E−85	182	187	97	−	698	883	27280	27464
UN01236	Rsa1.0_00111.1	716	390088	0	472	480	98	−	237	716	200994	201473
UN01237	Rsa1.0_02128.1	1399	34758	1.00E−151	328	345	95	−	6	345	16324	16668
UN01238	Rsa1.0_00067.1	1218	415055	0	695	715	97	−	437	1151	38371	39085
UN01239	Rsa1.0_05162.1	1158	14631	0	474	525	90	−	480	984	5857	6369
UN01240	Rsa1.0_07471.1	1311	9307	0	711	744	95	−	125	867	2	745
UN01241	Rsa1.0_08622.1	863	7122	1.00E−132	239	239	100	+	3	241	3836	4074
UN01242	Rsa1.0_00016.1	1195	831256	0	457	477	95	−	349	822	253037	253513
UN01243	Rsa1.0_03303.1	1102	23890	0	628	628	100	−	475	1102	9623	10250
UN01244	Rsa1.0_00104.1	1278	368722	1.00E−161	322	331	97	+	940	1270	205076	205402
UN01245	Rsa1.0_02191.1	929	34078	0	493	494	99	−	3	496	23661	24154
UN01246	Rsa1.0_00041.1	1243	186197	0	449	452	99	−	793	1243	56655	57106

UN01247	Rsa1.0_06803.1	1169	10330	3.00E-85	170	173	98	-	537	709	7999	8171
UN01248	Rsa1.0_01504.1	1938	189978	0	712	749	95	-	282	1011	25772	26517
UN01249	Rsa1.0_00426.1	1329	85668	0	593	626	94	-	3	615	73386	74001
UN01250	Rsa1.0_01540.1	825	151890	1.00E-176	322	325	99	+	198	522	149398	149722
UN01251	Rsa1.0_09692.1	577	6209	0	430	440	97	+	3	440	689	1128
UN01252	Rsa1.0_16459.1	758	2148	0	460	469	98	+	292	758	1564	2032
UN01253	Rsa1.0_02637.1	761	29244	0	462	477	96	-	1	477	24077	24553
UN01254	Rsa1.0_00144.1	851	120708	0	804	830	96	-	5	831	63828	64653
UN01255	Rsa1.0_00769.1	763	64911	1.00E-141	261	263	99	-	327	589	21293	21555
UN01256	Rsa1.0_13641.1	931	4588	0	878	897	97	-	1	896	2306	3202
UN01257	Rsa1.0_00079.1	1181	606987	1.00E-173	309	309	100	-	871	1179	551378	551686
UN01258	Rsa1.0_02212.1	1088	33840	0	1041	1084	96	-	2	1084	25081	26159
UN01259	Rsa1.0_00251.1	816	303968	1.00E-144	263	264	99	+	1	264	111514	111777
UN01260	Rsa1.0_00353.1	928	92242	0	511	512	99	-	1	512	83624	84135
UN01261	Rsa1.0_02097.1	1078	35036	1.00E-150	277	278	99	+	802	1078	10310	10587
UN01262	Rsa1.0_00045.1	617	328694	1.00E-132	293	311	94	-	172	482	131911	132215
UN01263	Rsa1.0_00004.1	1081	253996	0	656	687	95	-	392	1075	69089	69773
UN01264	Rsa1.0_01104.1	1319	285480	0	511	542	94	+	776	1316	274158	274692
UN01265	Rsa1.0_00026.1	1418	327835	0	821	833	98	-	588	1418	100567	101399
UN01266	Rsa1.0_00761.1	856	65133	1.00E-128	313	335	93	-	1	325	62157	62485
UN01267	Rsa1.0_00571.1	585	329018	1.00E-143	274	277	98	-	114	389	35755	36030
UN01268	Rsa1.0_00621.1	1589	71840	1.00E-148	273	275	99	-	294	568	50769	51043
UN01269	Rsa1.0_00741.1	718	66117	0	698	729	95	-	3	718	33724	34452
UN01270	Rsa1.0_00159.1	909	431686	0	901	902	99	-	3	904	244943	245843
UN01271	Rsa1.0_02939.1	813	26595	0	555	608	91	+	25	623	19909	20492
UN01272	Rsa1.0_00454.1	783	216970	0	729	766	95	+	3	761	187143	187900
UN01273	Rsa1.0_02820.1	603	27629	0	598	602	99	+	2	603	22422	23023
UN01274	Rsa1.0_00977.1	806	198001	0	768	782	98	-	3	776	189713	190494
UN01275	Rsa1.0_00017.1	724	752458	0	455	458	99	+	1	458	409133	409589
UN01276	Rsa1.0_00686.1	404	68511	2.00E-99	187	188	99	+	157	344	61089	61276
UN01277	Rsa1.0_03925.1	728	20025	1.00E-135	324	344	94	-	2	338	3816	4155
UN01278	Rsa1.0_05473.1	942	13628	0	660	704	93	-	9	700	11376	12077
UN01279	Rsa1.0_00913.1	815	59914	1.00E-123	280	300	93	-	1	291	32123	32422
UN01280	Rsa1.0_03253.1	858	24321	0	744	804	92	+	88	858	13179	13977
UN01281	Rsa1.0_00343.1	683	93570	0	449	482	93	-	1	472	2493	2967
UN01282	Rsa1.0_01992.1	783	36424	0	750	781	96	+	4	782	5477	6257
UN01283	Rsa1.0_02780.1	977	27965	0	458	474	96	+	1	469	8341	8809
UN01284	Rsa1.0_03356.1	1839	23487	0	706	742	95	-	434	1166	6339	7080
UN01285	Rsa1.0_01071.1	714	166914	0	433	433	100	-	7	439	24488	24920

UN01286	Rsa1.0_03058.1	833	25715	0	728	746	97	+	88	831	4691	5436
UN01287	Rsa1.0_00711.1	776	67223	0	434	434	100	+	2	435	23860	24293
UN01288	Rsa1.0_01491.1	568	44718	0	563	566	99	−	3	568	33365	33930
UN01289	Rsa1.0_00772.1	767	64856	5.00E−64	163	175	93	−	55	229	7619	7790
UN01290	Rsa1.0_03294.1	757	23971	0	473	497	95	−	148	635	17160	17653
UN01291	Rsa1.0_00269.1	816	341668	0	449	455	98	−	312	764	291208	291662
UN01292	Rsa1.0_01701.1	1079	162121	1.00E−173	312	313	99	+	1	313	20618	20930
UN01293	Rsa1.0_02684.1	740	28676	0	448	453	98	−	1	453	27458	27909
UN01294	Rsa1.0_07290.1	921	9608	0	425	435	97	+	4	438	2139	2570
UN01295	Rsa1.0_07534.1	585	9184	1.00E−177	349	358	97	−	222	578	7359	7714
UN01296	Rsa1.0_04380.1	1025	17644	8.00E−76	226	253	89	+	568	820	9371	9623
UN01297	Rsa1.0_02299.1	611	32857	0	423	430	98	−	183	611	13717	14146
UN01298	Rsa1.0_01519.1	1419	44144	0	815	850	95	+	571	1419	37306	38141
UN01299	Rsa1.0_00193.1	1257	194198	0	493	505	97	+	11	515	2816	3320
UN01300	Rsa1.0_09702.1	1031	6201	0	703	746	94	−	299	1031	830	1573
UN01301	Rsa1.0_02550.1	1527	291051	0	1340	1377	97	−	3	1370	2209	3585
UN01302	Rsa1.0_32375.1	770	703	1.00E−143	264	265	99	−	8	271	7	271
UN01303	Rsa1.0_00686.1	896	68511	0	356	357	99	+	1	356	53125	53481
UN01304	Rsa1.0_00158.1	686	117999	0	567	600	94	+	17	611	101331	101928
UN01305	Rsa1.0_00421.1	621	86365	1.00E−73	165	172	95	+	448	619	34765	34934
UN01306	Rsa1.0_00344.1	364	191774	1.00E−159	342	360	95	−	1	360	155630	155988
UN01307	Rsa1.0_12579.1	997	5185	0	462	467	98	+	125	591	1	467
UN01308	Rsa1.0_00197.1	931	109714	0	355	359	98	+	158	514	99351	99709
UN01309	Rsa1.0_00118.1	809	130637	0	461	468	98	+	287	753	90339	90806
UN01310	Rsa1.0_03209.1	1041	24592	0	478	478	100	+	563	1040	20232	20709
UN01311	Rsa1.0_01016.1	924	181422	9.00E−88	207	221	93	−	276	496	3576	3796
UN01312	Rsa1.0_04646.1	1380	16541	0	411	423	97	−	918	1340	929	1351
UN01313	Rsa1.0_01468.1	914	45146	0	585	591	98	−	1	591	15030	15619
UN01314	Rsa1.0_02481.1	584	31035	0	558	586	95	+	1	584	17753	18335
UN01315	Rsa1.0_01222.1	735	50398	0	555	577	96	+	1	574	2217	2793
UN01316	Rsa1.0_00121.1	1128	278029	0	486	495	98	+	1	495	37383	37875
UN01317	Rsa1.0_00773.1	691	64839	0	680	691	98	−	1	691	20228	20915
UN01318	Rsa1.0_00832.1	830	62622	1.00E−157	331	341	97	−	3	341	41378	41715
UN01319	Rsa1.0_00048.1	718	167185	0	676	678	99	−	1	678	125701	126378
UN01320	Rsa1.0_01503.1	762	44467	0	759	761	99	−	3	761	26720	27480
UN01321	Rsa1.0_01774.1	1852	39528	0	982	1004	97	−	37	1037	7477	8477
UN01322	Rsa1.0_02690.1	910	28629	1.00E−132	249	251	99	−	2	252	17941	18190
UN01323	Rsa1.0_02681.1	1218	28711	0	917	936	97	+	1	935	12438	13370
UN01324	Rsa1.0_07695.1	1244	8770	0	423	435	97	−	311	745	2661	3095

UN01325	Rsa1.0_00005.1	857	353093	0	618	620	99	+	1	620	143656	144275
UN01326	Rsa1.0_00678.1	825	193163	0	360	366	98	-	406	771	165336	165700
UN01327	Rsa1.0_04273.1	522	18144	1.00E-106	238	252	94	-	5	256	12147	12398
UN01328	Rsa1.0_02668.1	1497	28895	1.00E-174	390	414	94	-	1	413	12485	12892
UN01329	Rsa1.0_03638.1	543	21634	0	366	369	99	+	1	367	20034	20402
UN01330	Rsa1.0_01779.1	1254	39448	0	555	557	99	+	2	558	33270	33826
UN01331	Rsa1.0_00079.1	875	606987	0	449	465	96	+	1	457	186517	186977
UN01332	Rsa1.0_01835.1	562	38542	0	369	372	99	+	190	561	20857	21228
UN01333	Rsa1.0_00284.1	1593	323267	1.00E-107	266	289	92	+	3	280	237070	237358
UN01334	Rsa1.0_00260.1	855	102021	1.00E-169	304	305	99	-	454	758	3365	3669
UN01335	#	#	#	#	#	#	#	#	#	#	#	#
UN01336	Rsa1.0_21616.1	1592	1186	0	727	727	100	-	674	1400	265	991
UN01337	Rsa1.0_00060.1	1471	502678	0	608	610	99	-	6	615	41528	42137
UN01338	Rsa1.0_00304.1	1094	97429	0	331	333	99	-	1	333	8237	8569
UN01339	Rsa1.0_04193.1	1113	18571	1.00E-107	220	225	97	+	3	225	6657	6881
UN01340	Rsa1.0_00467.1	840	156542	0	448	475	94	+	2	472	125592	126061
UN01341	Rsa1.0_01569.1	1164	43029	1.00E-105	210	215	97	-	2	216	9452	9666
UN01342	Rsa1.0_00001.1	863	363028	0	410	434	94	+	19	448	314832	315259
UN01343	Rsa1.0_00429.1	717	85559	0	697	717	97	-	2	717	20481	21197
UN01344	Rsa1.0_00111.1	694	390088	0	669	697	95	+	1	694	200286	200979
UN01345	Rsa1.0_00722.1	1443	66840	0	353	356	99	+	1	356	35937	36290
UN01346	Rsa1.0_08230.1	161	7688	3.00E-49	141	155	90	-	3	157	5431	5585
UN01347	Rsa1.0_00275.1	780	210104	0	765	785	97	-	1	779	16930	17714
UN01348	Rsa1.0_00205.1	1436	193401	0	331	331	100	-	10	340	33441	33771
UN01349	Rsa1.0_04275.1	830	18136	1.00E-121	274	289	94	+	57	345	9487	9773
UN01350	Rsa1.0_00134.1	1309	390219	1.00E-176	342	349	97	+	1	348	230194	230541
UN01351	Rsa1.0_00213.1	974	108167	1.00E-164	367	389	94	-	29	414	46415	46798
UN01352	Rsa1.0_00196.1	737	109960	0	576	588	97	+	153	735	28104	28691
UN01353	Rsa1.0_00040.1	472	324196	3.00E-89	232	251	92	-	37	286	17904	18153
UN01354	Rsa1.0_00285.1	1236	149638	1.00E-178	350	361	96	+	1	361	143051	143411
UN01355	Rsa1.0_05618.1	945	13165	1.00E-114	218	221	98	+	471	691	7388	7608
UN01356	Rsa1.0_01848.1	708	38312	0	700	707	99	-	2	708	26830	27536
UN01357	Rsa1.0_01760.1	928	39730	1.00E-114	219	222	98	-	1	222	13353	13574
UN01358	Rsa1.0_00331.1	761	176754	1.00E-177	333	339	98	+	1	339	107341	107679
UN01359	Rsa1.0_10134.1	1029	5984	1.00E-139	252	252	100	-	254	505	3960	4211
UN01360	Rsa1.0_00069.1	717	403561	1.00E-136	267	273	97	+	36	308	185324	185594
UN01361	Rsa1.0_02997.1	1119	26150	0	1101	1116	98	+	5	1119	3288	4403
UN01362	Rsa1.0_00195.1	884	251884	1.00E-89	210	221	95	-	1	215	241757	241977
UN01363	Rsa1.0_06160.1	1371	11596	0	474	485	97	+	245	729	10937	11421

UN01364	Rsa1.0_04029.1	1552	19491	0	582	592	98	−	962	1552	8373	8963
UN01365	Rsa1.0_02222.1	1396	152798	1.00E−166	302	304	99	−	1093	1396	122191	122494
UN01366	Rsa1.0_00090.1	1142	273859	0	1067	1114	95	−	35	1142	87904	89002
UN01367	Rsa1.0_02215.1	1382	33822	0	541	584	92	−	3	586	9505	10064
UN01368	Rsa1.0_01303.1	832	48853	0	405	426	95	+	249	674	15257	15682
UN01369	Rsa1.0_00079.1	1200	606987	1.00E−165	332	343	96	+	859	1200	71467	71809
UN01370	Rsa1.0_03408.1	1081	23154	0	663	679	97	+	405	1081	12765	13442
UN01371	Rsa1.0_00321.1	1037	95671	0	1021	1040	98	−	1	1037	56895	57927
UN01372	Rsa1.0_03878.1	988	20305	0	581	597	97	+	386	980	1652	2244
UN01373	Rsa1.0_00087.1	808	761073	1.00E−166	318	324	98	+	486	808	83067	83390
UN01374	Rsa1.0_00008.1	1265	358124	1.00E−106	222	228	97	+	1037	1261	308529	308756
UN01375	Rsa1.0_05344.1	971	14103	0	727	729	99	+	237	965	4842	5570
UN01376	Rsa1.0_00744.1	941	66084	0	511	517	98	−	426	941	3356	3872
UN01377	Rsa1.0_00847.1	640	62270	1.00E−116	227	232	97	+	141	372	52454	52685
UN01378	Rsa1.0_00060.1	410	502678	1.00E−159	287	288	99	+	123	410	54986	55273
UN01379	Rsa1.0_01510.1	918	44237	1.00E−119	362	402	90	+	525	918	19080	19474
UN01380	Rsa1.0_00033.1	1285	515811	0	533	538	99	+	741	1278	135945	136481
UN01381	Rsa1.0_16075.1	1310	2295	2.00E−58	125	128	97	−	1183	1310	2065	2192
UN01382	Rsa1.0_00798.1	931	63945	1.00E−178	429	463	92	+	473	931	43764	44220
UN01383	Rsa1.0_00118.1	621	130637	0	547	574	95	+	2	575	27468	28041
UN01384	Rsa1.0_00217.1	829	107079	1.00E−176	322	325	99	−	9	333	95136	95460
UN01385	Rsa1.0_00016.1	765	831256	0	372	381	97	−	385	765	570152	570531
UN01386	Rsa1.0_00245.1	962	103569	0	544	557	97	−	407	962	52410	52966
UN01387	Rsa1.0_01209.1	916	50668	1.00E−121	251	260	96	−	23	280	47015	47274
UN01388	Rsa1.0_02872.1	842	27080	1.00E−160	340	358	94	−	18	375	12112	12463
UN01389	Rsa1.0_00142.1	852	121474	0	429	447	95	+	374	815	62984	63430
UN01390	Rsa1.0_47641.1	778	475	1.00E−179	389	410	94	−	376	778	66	475
UN01391	Rsa1.0_00163.1	844	116847	1.00E−135	268	276	97	−	545	820	25937	26212
UN01392	Rsa1.0_04111.1	887	18991	2.33E−156	308	321	95	−	3	323	13843	14159
UN01393	Rsa1.0_00839.1	909	62533	0	552	591	93	−	278	867	46420	47010
UN01394	Rsa1.0_01413.1	891	46275	0	398	398	100	−	494	891	28609	29006
UN01395	Rsa1.0_00207.1	1289	248736	0	335	335	100	+	3	337	216909	217243
UN01396	Rsa1.0_00384.1	1342	89278	0	441	450	98	−	350	799	88013	88461
UN01397	Rsa1.0_00263.1	1158	224968	0	405	413	98	−	747	1157	47421	47832
UN01398	Rsa1.0_01234.1	993	50183	0	941	997	94	+	1	991	15511	16507
UN01399	Rsa1.0_00651.1	993	247766	1.00E−126	281	296	94	−	699	993	90236	90524
UN01400	Rsa1.0_00092.1	950	659932	1.00E−148	287	294	97	+	373	666	358012	358305
UN01401	Rsa1.0_19998.1	757	1354	1.00E−178	319	320	99	+	3	322	141	460
UN01402	Rsa1.0_03418.1	822	147293	1.00E−80	162	165	98	−	3	167	11737	11901

UN01403	Rsa1.0_00065.1	1076	454616	0	355	355	100	−	422	776	417752	418106
UN01404	Rsa1.0_00101.1	978	267193	1.00E−114	280	306	91	+	648	936	208637	208942
UN01405	Rsa1.0_01089.1	751	142191	0	339	339	100	−	306	644	101822	102160
UN01406	Rsa1.0_00065.1	956	454616	0	602	627	96	+	322	948	254496	255122
UN01407	Rsa1.0_00577.1	789	174827	0	746	778	95	+	4	772	494	1269
UN01408	Rsa1.0_00273.1	1180	326186	0	587	620	94	+	579	1180	1356	1975
UN01409	Rsa1.0_00879.1	754	61297	1.00E−114	221	225	98	−	40	264	29418	29642
UN01410	Rsa1.0_00342.1	682	93695	4.00E−49	119	124	95	+	388	511	32256	32378
UN01411	Rsa1.0_01461.1	817	45240	0	422	423	99	+	396	817	7012	7434
UN01412	Rsa1.0_07113.1	1318	9877	1.00E−109	224	232	96	−	27	251	228	459
UN01413	Rsa1.0_01012.1	928	56836	0	486	525	92	−	168	689	30849	31372
UN01414	Rsa1.0_02694.1	1275	28610	0	392	402	97	+	423	821	6542	6943
UN01415	Rsa1.0_03366.1	734	23401	0	415	417	99	−	3	419	12024	12440
UN01416	Rsa1.0_04855.1	1433	15708	0	419	423	99	−	1009	1431	10580	10999
UN01417	Rsa1.0_00341.1	1027	249309	0	807	883	91	+	62	932	179827	180671
UN01418	Rsa1.0_00960.1	1373	58199	0	462	487	94	−	889	1373	14526	15007
UN01419	Rsa1.0_00002.1	981	601661	1.00E−138	304	324	93	+	670	981	270307	270627
UN01420	Rsa1.0_02376.1	912	32011	0	456	494	92	+	222	706	13930	14420
UN01421	Rsa1.0_03475.1	803	22653	9.00E−60	153	161	95	−	3	159	22098	22258
UN01422	Rsa1.0_00243.1	729	103651	1.00E−116	243	250	97	−	479	728	35555	35799
UN01423	Rsa1.0_01146.1	1034	163455	0	592	594	99	+	441	1034	148849	149442
UN01424	Rsa1.0_01026.1	964	171624	0	643	662	97	−	302	962	165230	165890
UN01425	Rsa1.0_02191.1	786	34078	0	425	425	100	+	362	786	17613	18037
UN01426	Rsa1.0_00017.1	627	752458	0	477	499	95	+	132	627	537785	538275
UN01427	Rsa1.0_01188.1	1209	51362	0	504	514	98	+	3	516	17186	17699
UN01428	Rsa1.0_01591.1	887	42718	0	870	883	98	+	2	883	5393	6275
UN01429	Rsa1.0_00127.1	640	127446	1.00E−116	236	244	96	−	154	397	4782	5025
UN01430	Rsa1.0_00966.1	984	58136	1.00E−120	294	312	94	−	19	324	53466	53774
UN01431	Rsa1.0_00077.1	1270	145005	0	535	537	99	−	591	1127	89653	90189
UN01432	Rsa1.0_00437.1	959	84879	0	444	446	99	−	514	959	70164	70609
UN01433	Rsa1.0_00089.1	851	406142	0	575	576	99	+	276	851	275428	276003
UN01434	Rsa1.0_00096.1	719	198773	1.00E−108	231	241	95	+	1	241	171687	171924
UN01435	Rsa1.0_01156.1	1690	52147	1.00E−130	280	292	95	+	1401	1690	38895	39186
UN01436	Rsa1.0_07694.1	941	8776	0	385	399	96	+	543	941	8243	8636
UN01437	Rsa1.0_04744.1	1193	16154	1.00E−145	336	354	94	+	847	1193	7259	7609
UN01438	Rsa1.0_00055.1	736	520193	1.00E−171	316	320	98	−	417	736	211371	211690
UN01439	Rsa1.0_00048.1	711	167185	1.00E−101	222	231	96	+	280	507	148073	148303
UN01440	Rsa1.0_01008.1	1393	56924	0	1374	1391	98	−	3	1391	14315	15703
UN01441	Rsa1.0_02825.1	945	27568	0	879	911	96	−	1	902	17441	18345

UN01442	Rsa1.0_00838.1	884	153820	0	357	359	99	−	527	884	147715	148073
UN01443	Rsa1.0_00131.1	1326	432233	0	1158	1202	96	+	123	1324	44958	46158
UN01444	Rsa1.0_20002.1	1054	1353	0	333	336	99	+	3	338	612	944
UN01445	Rsa1.0_10275.1	722	5907	3.00E−81	169	174	97	+	251	424	887	1060
UN01446	Rsa1.0_03543.1	1291	22185	0	493	509	96	−	130	634	3871	4379
UN01447	Rsa1.0_00199.1	849	249604	0	557	562	99	−	3	563	13361	13921
UN01448	Rsa1.0_00318.1	788	96094	1.00E−133	251	253	99	+	301	553	75462	75713
UN01449	Rsa1.0_03469.1	857	22681	1.00E−148	285	291	97	+	337	627	20324	20614
UN01450	Rsa1.0_01971.1	267	36641	7.00E−95	243	268	90	−	2	254	23472	23736
UN01451	Rsa1.0_01429.1	988	46061	0	967	987	97	−	3	988	17004	17987
UN01452	Rsa1.0_02091.1	1421	35157	0	1292	1360	95	+	58	1414	4073	5413
UN01453	Rsa1.0_04538.1	1293	16940	0	1278	1296	98	+	1	1293	5887	7181
UN01454	Rsa1.0_00213.1	1205	108167	1.00E−161	439	483	90	−	741	1204	36347	36811
UN01455	Rsa1.0_04450.1	1093	17297	0	697	710	98	+	1	710	13778	14487
UN01456	Rsa1.0_00156.1	897	118383	0	361	368	98	−	1	368	13496	13863
UN01457	Rsa1.0_02999.1	913	26135	0	908	915	99	+	3	913	13715	14629
UN01458	Rsa1.0_00642.1	912	201230	0	391	391	100	−	520	910	162508	162898
UN01459	Rsa1.0_00866.1	681	61676	1.00E−64	129	130	99	+	194	323	56046	56175
UN01460	Rsa1.0_10126.1	805	5987	0	760	806	94	+	5	800	3716	4506
UN01461	Rsa1.0_09588.1	772	6267	1.00E−121	227	229	99	+	313	541	5405	5633
UN01462	Rsa1.0_00340.1	644	305782	0	628	642	97	+	3	644	289427	290068
UN01463	Rsa1.0_03281.1	827	143349	0	445	452	98	+	379	827	136448	136899
UN01464	Rsa1.0_00507.1	812	79074	1.00E−131	331	362	91	−	28	385	20321	20676
UN01465	Rsa1.0_01360.1	994	47246	0	582	615	94	−	389	992	23905	24514
UN01466	Rsa1.0_03464.1	947	22715	8.00E−79	150	150	100	+	798	947	12662	12811
UN01467	Rsa1.0_00185.1	1424	248445	0	907	920	98	−	504	1423	233983	234902
UN01468	Rsa1.0_04029.1	894	19491	0	383	396	96	−	470	859	10003	10398
UN01469	Rsa1.0_00111.1	1017	390088	0	466	472	98	−	546	1017	197737	198208
UN01470	Rsa1.0_00001.1	752	363028	3.00E−78	155	157	98	−	248	404	29910	30066
UN01471	Rsa1.0_00021.1	1555	263655	1.00E−136	437	496	88	+	1014	1506	199080	199572
UN01472	Rsa1.0_00744.1	698	66084	8.00E−69	148	153	96	+	345	497	48388	48540
UN01473	Rsa1.0_00946.1	1557	58666	1.00E−133	283	294	96	+	1266	1553	52830	53122
UN01474	Rsa1.0_01728.1	1258	40239	0	921	962	95	+	310	1258	3010	3970
UN01475	Rsa1.0_00108.1	1222	478491	2.33E−156	269	271	99	+	534	804	463378	463648
UN01476	Rsa1.0_06421.1	958	11053	1.00E−177	381	399	95	−	560	958	3836	4231
UN01477	Rsa1.0_54086.1	827	422	2.00E−60	128	131	97	−	362	492	147	277
UN01478	Rsa1.0_00708.1	1301	67366	0	460	492	93	+	343	831	41786	42277
UN01479	Rsa1.0_04098.1	802	19078	1.00E−134	306	326	93	+	488	802	10291	10616
UN01480	Rsa1.0_00123.1	1318	325650	0	794	848	93	+	466	1310	109752	110582

UN01481	Rsa1.0_02029.1	1362	35857	0	713	762	93	+	3	764	25120	25866
UN01482	Rsa1.0_00676.1	707	69166	1.00E-175	312	312	100	+	396	707	19280	19591
UN01483	Rsa1.0_00216.1	1240	197563	0	674	685	98	+	1	685	99017	99700
UN01484	Rsa1.0_02269.1	772	33168	5.00E-55	123	126	97	+	288	412	15955	16080
UN01485	Rsa1.0_00741.1	801	66117	3.00E-75	293	342	85	−	180	521	32766	33104
UN01486	Rsa1.0_00439.1	787	142776	0	412	423	97	+	3	422	57722	58144
UN01487	Rsa1.0_04591.1	1306	16717	0	1217	1223	99	+	85	1306	2892	4113
UN01488	Rsa1.0_30952.1	1394	740	2.00E-80	242	269	89	−	966	1233	383	650
UN01489	Rsa1.0_00512.1	824	200277	1.00E-108	228	236	96	+	4	238	194177	194412
UN01490	Rsa1.0_06369.1	913	11141	0	394	399	98	+	343	741	6231	6629
UN01491	Rsa1.0_07149.1	1107	9824	0	387	402	96	−	716	1107	844	1245
UN01492	Rsa1.0_05947.1	983	12142	0	401	419	95	+	263	678	2699	3111
UN01493	Rsa1.0_00247.1	1173	103408	0	530	531	99	−	1	531	95495	96025
UN01494	Rsa1.0_01835.1	859	38542	1.00E-124	226	226	100	−	632	857	17025	17250
UN01495	Rsa1.0_03141.1	985	25061	0	698	755	92	+	263	985	12076	12827
UN01496	Rsa1.0_00458.1	1253	82717	0	417	436	95	−	818	1253	75544	75964
UN01497	Rsa1.0_05159.1	997	14642	0	559	576	97	−	422	997	7615	8179
UN01498	Rsa1.0_00631.1	466	71277	1.00E-122	251	258	97	−	209	466	59025	59280
UN01499	Rsa1.0_00414.1	1564	202190	0	516	534	96	+	862	1392	172207	172734
UN01500	Rsa1.0_03132.1	1250	25142	0	604	639	94	−	615	1250	7901	8530
UN01501	Rsa1.0_04189.1	1012	18584	1.00E-116	272	290	93	+	730	1010	4224	4512
UN01502	Rsa1.0_04510.1	845	17033	2.00E-94	208	216	96	+	548	762	12403	12617
UN01503	Rsa1.0_01591.1	1536	42718	1.00E-162	406	447	90	−	1099	1517	26831	27277
UN01504	Rsa1.0_00048.1	840	167185	5.00E-80	167	172	97	+	139	310	71654	71825
UN01505	Rsa1.0_01731.1	1425	40208	0	1097	1141	96	−	295	1425	30987	32114
UN01506	Rsa1.0_00495.1	777	79934	3.00E-62	150	158	94	+	151	308	9240	9396
UN01507	Rsa1.0_00518.1	765	78360	5.00E-98	194	198	97	−	395	592	19614	19811
UN01508	Rsa1.0_02036.1	1094	35731	0	1090	1105	98	−	3	1094	2907	4011
UN01509	Rsa1.0_00163.1	1125	116847	1.00E-174	340	350	97	−	308	657	30602	30951
UN01510	Rsa1.0_00092.1	1233	659932	1.00E-62	123	123	100	+	1	123	439120	439242
UN01511	Rsa1.0_01601.1	765	42519	0	415	434	95	−	146	579	12367	12797
UN01512	Rsa1.0_00913.1	744	59914	1.00E-67	140	143	97	−	415	557	20658	20800
UN01513	Rsa1.0_00759.1	1220	65465	0	839	873	96	+	358	1220	55681	56553
UN01514	Rsa1.0_15043.1	1231	2872	1.00E-117	227	230	98	−	3	232	323	550
UN01515	Rsa1.0_00513.1	1455	78579	0	401	402	99	−	1054	1455	46560	46961
UN01516	Rsa1.0_02574.1	1230	29954	0	596	600	99	+	629	1228	7851	8450
UN01517	Rsa1.0_00603.1	1172	73299	2.00E-76	249	285	87	−	852	1136	18990	19254
UN01518	Rsa1.0_03471.1	1147	22678	0	976	991	98	+	121	1105	296	1284
UN01519	Rsa1.0_00336.1	1380	445051	1.00E-162	296	298	99	−	2	299	441311	441608

UN01520	Rsa1.0_11496.1	1355	5485	0	1327	1366	97	+	2	1355	1374	2739
UN01521	Rsa1.0_00047.1	956	168582	1.00E-124	229	230	99	−	726	955	53787	54016
UN01522	Rsa1.0_00112.1	703	132199	0	423	442	95	+	257	693	55783	56220
UN01523	Rsa1.0_02299.1	1220	32857	0	441	441	100	−	1	441	4421	4861
UN01524	Rsa1.0_00139.1	841	357786	2.00E-63	153	164	93	−	1	164	292098	292252
UN01525	Rsa1.0_00006.1	858	396176	0	818	845	96	−	2	842	376370	377214
UN01526	Rsa1.0_00108.1	668	478491	1.00E-176	316	317	99	+	352	668	270627	270943
UN01527	Rsa1.0_06488.1	963	141774	0	381	383	99	+	582	963	2452	2834
UN01528	Rsa1.0_00085.1	674	142226	0	375	379	98	+	297	674	56009	56387
UN01529	Rsa1.0_01220.1	1310	161775	2.00E-92	193	199	96	−	949	1144	122655	122853
UN01530	Rsa1.0_00884.1	794	61150	1.00E-141	261	262	99	+	485	745	19478	19739
UN01531	Rsa1.0_00602.1	1437	73302	0	968	975	99	+	465	1437	33512	34486
UN01532	Rsa1.0_05593.1	777	13235	0	592	616	96	+	148	759	1134	1748
UN01533	Rsa1.0_02181.1	809	34172	1.00E-137	273	279	97	+	192	470	18160	18435
UN01534	Rsa1.0_00439.1	892	142776	0	882	892	98	+	2	892	123194	124083
UN01535	Rsa1.0_00415.1	748	86795	1.00E-134	246	247	99	+	332	578	72219	72465
UN01536	Rsa1.0_08366.1	735	7466	0	383	401	95	+	335	735	5695	6081
UN01537	Rsa1.0_00361.1	836	91181	1.00E-150	273	274	99	−	423	696	12177	12450
UN01538	Rsa1.0_00364.1	846	90951	0	829	856	96	−	1	846	76974	77829
UN01539	Rsa1.0_01043.1	1181	55893	0	523	552	94	−	631	1181	34936	35473
UN01540	Rsa1.0_00145.1	1041	261008	0	653	661	98	+	368	1028	17556	18215
UN01541	Rsa1.0_12370.1	935	5243	0	597	597	100	−	1	597	878	1474
UN01542	Rsa1.0_00301.1	974	365315	0	966	967	99	−	8	974	25981	26947
UN01543	Rsa1.0_00653.1	699	70029	0	384	390	98	+	3	392	33896	34283
UN01544	Rsa1.0_02625.1	799	139104	0	525	525	100	+	20	544	138580	139104
UN01545	Rsa1.0_02454.1	1164	31263	0	893	896	99	+	3	897	6909	7804
UN01546	Rsa1.0_00327.1	1451	94892	0	550	568	96	−	674	1240	62360	62927
UN01547	Rsa1.0_00105.1	653	134138	7.00E-94	190	195	97	+	221	415	91498	91692
UN01548	Rsa1.0_00024.1	1304	188018	1.00E-141	267	271	98	−	844	1114	5016	5286
UN01549	Rsa1.0_01459.1	1233	45277	0	1171	1243	94	−	2	1231	35841	37070
UN01550	Rsa1.0_01411.1	1030	46326	0	350	351	99	+	3	353	22913	23263
UN01551	Rsa1.0_00739.1	1606	66155	1.00E-120	237	243	97	−	757	999	42962	43204
UN01552	Rsa1.0_14912.1	793	2970	6.00E-73	177	189	93	+	567	751	1944	2132
UN01553	Rsa1.0_00360.1	1017	91190	0	522	536	97	+	31	566	75819	76354
UN01554	Rsa1.0_00041.1	835	186197	0	493	513	96	+	3	514	71394	71906
UN01555	Rsa1.0_00520.1	814	78226	0	376	378	99	−	1	378	37747	38124
UN01556	Rsa1.0_14737.1	940	3115	0	321	321	100	+	275	595	2055	2375
UN01557	Rsa1.0_02091.1	647	35157	0	625	646	96	−	4	647	4771	5413
UN01558	Rsa1.0_01175.1	739	51674	1.00E-101	187	187	100	−	1	187	38400	38586

UN01559	Rsa1.0_00949.1	1408	58549	0	437	440	99	+	747	1185	15414	15851
UN01560	Rsa1.0_00893.1	982	60626	2.00E-15	63	70	90	−	913	982	24296	24358
UN01561	Rsa1.0_04415.1	816	147889	0	575	579	99	+	2	580	137359	137935
UN01562	Rsa1.0_00167.1	606	116000	1.00E-122	244	250	97	+	1	249	90120	90369
UN01563	Rsa1.0_01712.1	1206	154210	1.00E-149	272	273	99	+	682	954	2819	3091
UN01564	Rsa1.0_01434.1	1473	45951	0	1144	1182	96	−	3	1174	5302	6480
UN01565	Rsa1.0_13979.1	828	3964	1.00E-145	264	265	99	−	386	650	2589	2853
UN01566	Rsa1.0_00260.1	908	102021	0	415	419	99	+	249	667	72077	72495
UN01567	Rsa1.0_03055.1	261	25730	1.00E-123	247	255	96	−	3	257	2665	2919
UN01568	Rsa1.0_00698.1	1074	67816	1.00E-119	263	278	94	−	464	741	46344	46616
UN01569	Rsa1.0_06603.1	923	10682	1.00E-137	301	320	94	−	1	307	5476	5794
UN01570	Rsa1.0_00245.1	718	103569	0	685	686	99	−	34	718	11734	12419
UN01571	Rsa1.0_02179.1	708	34188	0	687	710	96	−	1	707	20218	20927
UN01572	Rsa1.0_00028.1	1413	293984	1.00E-130	240	241	99	−	1	241	44903	45143
UN01573	Rsa1.0_01377.1	1135	46967	1.00E-117	292	315	92	−	1	309	21582	21895
UN01574	Rsa1.0_13250.1	676	4955	1.00E-129	235	235	100	+	58	292	1	235
UN01575	Rsa1.0_00497.1	813	79830	0	472	472	100	−	1	472	76695	77166
UN01576	Rsa1.0_01530.1	1069	43820	1.00E-132	266	273	97	−	1	272	5090	5359
UN01577	Rsa1.0_02041.1	1060	163634	1.00E-153	318	331	96	+	1	330	18706	19032
UN01578	Rsa1.0_00075.1	995	146680	1.00E-117	220	222	99	+	1	222	66201	66422
UN01579	Rsa1.0_00087.1	708	761073	0	664	708	93	−	2	708	182815	183499
UN01580	Rsa1.0_00254.1	1149	102600	1.00E-148	306	318	96	+	10	326	74046	74363
UN01581	Rsa1.0_00034.1	762	260119	1.00E-122	262	275	95	+	116	390	246416	246690
UN01582	Rsa1.0_00632.1	860	183777	1.00E-136	298	312	95	−	3	310	3462	3772
UN01583	Rsa1.0_17297.1	1704	1867	0	494	524	94	+	1144	1667	1344	1867
UN01584	Rsa1.0_02377.1	622	31994	0	576	625	92	+	8	615	19545	20165
UN01585	Rsa1.0_02654.1	1228	29028	0	1018	1059	96	−	1	1053	21338	22382
UN01586	Rsa1.0_16075.1	426	2295	8.00E-37	97	103	94	+	3	105	2090	2192
UN01587	Rsa1.0_01725.1	1059	40290	0	579	590	98	−	412	1001	1697	2286
UN01588	Rsa1.0_00051.1	750	258225	0	505	507	99	+	3	509	2656	3162
UN01589	Rsa1.0_00044.1	451	271658	1.00E-127	297	319	93	+	1	319	176730	177048
UN01590	Rsa1.0_00646.1	1319	70473	1.00E-129	238	239	99	−	807	1045	8478	8716
UN01591	Rsa1.0_02481.1	810	31035	0	375	384	97	+	198	581	18627	19010
UN01592	Rsa1.0_08805.1	825	6906	0	643	650	98	+	176	822	531	1180
UN01593	Rsa1.0_00649.1	1283	70263	0	1261	1282	98	+	2	1282	4280	5561
UN01594	Rsa1.0_03001.1	883	26108	0	441	442	99	+	7	448	5384	5824
UN01595	Rsa1.0_00269.1	764	341668	0	734	764	96	−	1	764	17129	17884
UN01596	Rsa1.0_02299.1	659	32857	0	434	435	99	+	1	435	1464	1898
UN01597	Rsa1.0_00005.1	1237	353093	1.00E-135	247	248	99	+	3	250	116631	116878

UN01598	Rsa1.0_00006.1	1220	396176	0	740	750	98	+	1	750	375321	376070
UN01599	Rsa1.0_01313.1	951	48387	1.00E-142	299	313	95	−	1	313	16151	16463
UN01600	Rsa1.0_00398.1	1080	88007	0	670	697	96	+	2	698	70672	71367
UN01601	Rsa1.0_00884.1	836	61150	0	694	714	97	−	3	715	20224	20935
UN01602	Rsa1.0_00976.1	866	57809	1.00E-170	303	303	100	−	1	303	49516	49818
UN01603	Rsa1.0_13975.1	936	3968	0	801	807	99	−	1	807	2406	3209
UN01604	Rsa1.0_02433.1	338	158318	1.00E-148	323	338	95	+	1	337	20008	20343
UN01605	Rsa1.0_14458.1	1233	3368	1.00E-160	295	298	98	+	821	1118	1277	1574
UN01606	Rsa1.0_02874.1	802	27070	2.00E-97	195	199	97	−	602	800	1695	1890
UN01607	Rsa1.0_00576.1	771	178949	0	376	380	98	−	2	381	23674	24053
UN01608	Rsa1.0_03469.1	826	22681	1.00E-137	302	319	94	−	9	325	21947	22265
UN01609	Rsa1.0_00285.1	1157	149638	1.00E-157	309	316	97	−	1	315	97144	97457
UN01610	Rsa1.0_03471.1	821	22678	0	803	820	97	−	3	821	915	1731
UN01611	Rsa1.0_00892.1	775	60703	0	448	457	98	−	5	460	21370	21826
UN01612	Rsa1.0_02130.1	826	34755	0	492	515	95	+	318	825	30534	31048
UN01613	Rsa1.0_01572.1	1004	42995	0	812	828	98	−	190	1004	6475	7302
UN01614	Rsa1.0_00234.1	1193	197060	0	401	415	96	+	367	781	144150	144564
UN01615	Rsa1.0_00275.1	838	210104	1.00E-108	233	247	94	+	391	637	119346	119579
UN01616	Rsa1.0_00131.1	1453	432233	0	490	545	89	−	380	915	395234	395750
UN01617	Rsa1.0_00001.1	919	363028	0	462	474	97	+	448	919	209188	209657
UN01618	Rsa1.0_02772.1	1247	28047	0	389	390	99	−	858	1247	17226	17615
UN01619	Rsa1.0_00105.1	922	134138	0	491	492	99	−	429	920	10801	11292
UN01620	Rsa1.0_01387.1	1005	46745	1.00E-154	309	319	96	−	435	753	39359	39675
UN01621	Rsa1.0_40505.1	869	554	1.00E-166	306	309	99	−	392	700	1	309
UN01622	Rsa1.0_00179.1	430	393324	1.00E-156	323	336	96	−	103	430	352408	352740
UN01623	Rsa1.0_00388.1	1184	88890	1.00E-138	261	265	98	−	918	1182	85714	85978
UN01624	Rsa1.0_00005.1	1602	353093	1.00E-151	423	476	88	−	1138	1591	71259	71734
UN01625	Rsa1.0_00010.1	874	294827	1.00E-124	226	226	100	−	67	292	248372	248597
UN01626	Rsa1.0_01569.1	883	43029	0	609	613	99	−	260	872	24201	24811
UN01627	Rsa1.0_01465.1	881	45175	0	605	615	98	+	206	817	28909	29523
UN01628	Rsa1.0_02325.1	1485	160025	0	508	514	98	−	970	1483	140806	141319
UN01629	Rsa1.0_00542.1	915	76831	0	391	400	97	+	118	517	71903	72302
UN01630	Rsa1.0_01134.1	1478	52829	0	1022	1052	97	−	420	1470	25945	26996
UN01631	Rsa1.0_03473.1	816	22665	0	812	814	99	−	3	816	9420	10233
UN01632	Rsa1.0_00251.1	636	303968	0	566	573	98	+	67	636	139709	140281
UN01633	Rsa1.0_00872.1	1169	61495	1.00E-172	390	415	93	+	746	1159	17030	17443
UN01634	Rsa1.0_01717.1	1162	143162	0	380	387	98	−	1	385	6372	6758
UN01635	Rsa1.0_03375.1	1022	23332	2.00E-82	191	199	95	+	3	198	13164	13361
UN01636	Rsa1.0_04065.1	1308	19275	0	659	660	99	+	3	661	15393	16052

UN01637	Rsa1.0_12729.1	1084	5142	1.00E-154	292	296	98	+	2	297	2468	2762
UN01638	Rsa1.0_01357.1	1030	47280	0	460	463	99	-	567	1029	2522	2983
UN01639	Rsa1.0_07003.1	963	10045	1.00E-165	301	303	99	+	661	963	1	303
UN01640	Rsa1.0_00278.1	1089	231896	1.00E-121	225	226	99	+	59	284	148696	148921
UN01641	Rsa1.0_04646.1	780	16541	0	749	779	96	+	1	779	10792	11564
UN01642	Rsa1.0_00773.1	1573	64839	0	622	642	96	-	656	1297	22429	23070
UN01643	Rsa1.0_00001.1	1059	363028	0	909	945	96	-	13	947	173735	174678
UN01644	Rsa1.0_00067.1	737	415055	0	431	440	97	+	2	439	313442	313881
UN01645	Rsa1.0_01565.1	2035	155447	0	1721	1754	98	-	265	2010	147188	148935
UN01646	Rsa1.0_00759.1	664	65465	0	401	403	99	+	1	402	53702	54104
UN01647	Rsa1.0_00525.1	761	78030	0	469	470	99	+	1	470	46658	47127
UN01648	Rsa1.0_02428.1	1400	31542	0	334	334	100	-	37	370	17805	18138
UN01649	Rsa1.0_00012.1	1126	222148	0	974	978	99	+	145	1121	110290	111267
UN01650	Rsa1.0_01595.1	801	175383	0	750	766	97	-	27	791	37756	38520
UN01651	Rsa1.0_01095.1	724	54276	3.00E-84	224	246	91	-	3	240	35225	35462
UN01652	Rsa1.0_00383.1	946	398431	1.00E-142	335	360	93	-	598	943	182048	182404
UN01653	Rsa1.0_00092.1	832	659932	1.00E-177	330	335	98	+	246	580	13028	13362
UN01654	Rsa1.0_00012.1	996	222148	0	601	635	94	+	270	898	110484	111109
UN01655	Rsa1.0_02110.1	998	34919	4.00E-93	222	239	92	-	773	998	23653	23891
UN01656	Rsa1.0_00301.1	1328	365315	0	533	539	98	+	788	1326	275569	276107
UN01657	Rsa1.0_00913.1	1185	59914	0	414	415	99	-	2	416	32123	32537
UN01658	Rsa1.0_04479.1	792	17181	4.00E-31	73	74	98	-	719	792	7907	7980
UN01659	Rsa1.0_00541.1	932	76863	0	428	431	99	-	4	434	74721	75151
UN01660	Rsa1.0_00105.1	894	134138	4.00E-99	196	200	98	+	148	347	67944	68143
UN01661	Rsa1.0_01154.1	901	52249	0	445	446	99	-	456	901	9037	9482
UN01662	Rsa1.0_01530.1	951	43820	0	474	491	96	+	212	702	5965	6455
UN01663	Rsa1.0_01141.1	636	52530	0	615	634	97	+	3	636	38330	38960
UN01664	Rsa1.0_05599.1	1358	143653	0	401	412	97	+	914	1320	4266	4677
UN01665	Rsa1.0_00914.1	1334	59880	0	767	772	99	-	3	774	53485	54256
UN01666	Rsa1.0_00286.1	704	98709	1.00E-102	237	252	94	+	404	655	13001	13250
UN01667	Rsa1.0_02867.1	825	27121	0	471	490	96	+	338	825	12695	13183
UN01668	Rsa1.0_02398.1	1044	31766	0	1038	1046	99	-	1	1043	16724	17769
UN01669	Rsa1.0_00749.1	923	65933	0	609	630	96	-	302	923	44807	45434
UN01670	Rsa1.0_00678.1	808	193163	1.00E-137	310	330	93	-	489	805	129966	130295
UN01671	Rsa1.0_00116.1	941	365886	0	543	544	99	+	398	941	144907	145450
UN01672	Rsa1.0_05897.1	1098	12302	0	864	868	99	-	3	870	4216	5083
UN01673	Rsa1.0_00066.1	1444	152418	0	496	499	99	+	946	1444	83634	84132
UN01674	Rsa1.0_02449.1	1219	31292	0	1219	1219	100	+	1	1219	23811	25029
UN01675	Rsa1.0_01246.1	873	49968	0	495	499	99	+	43	541	26897	27395

UN01676	Rsa1.0_02226.1	768	33726	2.00E-54	115	117	98	-	311	427	11062	11178
UN01677	Rsa1.0_00621.1	1222	71840	0	725	752	96	-	1	743	52315	53061
UN01678	Rsa1.0_00816.1	994	403350	0	974	996	97	+	1	994	394006	394999
UN01679	Rsa1.0_00363.1	1084	90968	0	521	521	100	-	563	1083	9260	9780
UN01680	Rsa1.0_02947.1	574	26531	1.00E-106	280	313	89	+	172	456	22870	23181
UN01681	Rsa1.0_03413.1	1170	23137	0	542	543	99	+	628	1170	8284	8826
UN01682	Rsa1.0_02095.1	897	35071	2.00E-98	538	645	83	+	255	895	6233	6865
UN01683	Rsa1.0_02032.1	1159	35830	0	783	801	97	+	2	802	2941	3741
UN01684	Rsa1.0_00070.1	770	150321	0	731	790	92	+	5	770	53680	54460
UN01685	Rsa1.0_03943.1	1175	19931	0	405	407	99	+	578	984	8436	8842
UN01686	Rsa1.0_00131.1	990	432233	0	697	714	97	+	181	893	205248	205959
UN01687	Rsa1.0_00019.1	1936	301669	0	480	502	95	+	1448	1936	99010	99510
UN01688	Rsa1.0_01105.1	742	53958	1.00E-73	147	149	98	-	378	526	22860	23008
UN01689	Rsa1.0_00193.1	1209	194198	0	1149	1162	98	+	51	1208	10121	11282
UN01690	Rsa1.0_16268.1	613	2219	0	571	610	93	+	1	600	42	636
UN01691	Rsa1.0_05623.1	1248	13141	2.00E-33	220	266	82	-	823	1081	1359	1624
UN01692	Rsa1.0_03529.1	665	22277	0	392	403	97	-	1	403	15606	16008
UN01693	Rsa1.0_00471.1	1029	81897	0	473	478	98	-	550	1027	72216	72693
UN01694	Rsa1.0_01682.1	1385	41090	1.00E-180	412	439	93	+	949	1385	10171	10607
UN01695	Rsa1.0_00123.1	1140	325650	1.00E-140	253	253	100	-	655	907	216452	216704
UN01696	Rsa1.0_05545.1	896	13379	0	866	892	97	-	3	894	11086	11969
UN01697	Rsa1.0_03833.1	829	20575	2.00E-67	140	143	97	+	591	733	9936	10078
UN01698	Rsa1.0_06630.1	825	10633	0	419	438	95	-	5	442	2463	2896
UN01699	Rsa1.0_01976.1	1401	36570	0	653	656	99	-	2	657	9059	9714
UN01700	Rsa1.0_06705.1	1337	10507	1.00E-126	281	301	93	-	1038	1337	5994	6277
UN01701	Rsa1.0_01112.1	807	310601	1.00E-156	305	313	97	+	16	328	174755	175064
UN01702	Rsa1.0_00030.1	1523	250988	0	1498	1527	98	-	3	1523	12137	13662
UN01703	Rsa1.0_00044.1	1430	271658	0	429	438	97	-	993	1430	176611	177048
UN01704	Rsa1.0_04616.1	922	16627	1.00E-163	362	383	94	+	447	827	342	723
UN01705	Rsa1.0_02748.1	700	28242	0	690	701	98	+	3	700	17531	18229
UN01706	Rsa1.0_00094.1	1097	139042	0	513	515	99	-	583	1097	25949	26463
UN01707	Rsa1.0_00140.1	1317	121541	0	496	497	99	-	820	1316	30925	31421
UN01708	Rsa1.0_07243.1	742	9673	1.00E-109	207	209	99	+	234	442	979	1187
UN01709	Rsa1.0_31900.1	1396	715	0	703	716	98	-	317	1032	1	715
UN01710	Rsa1.0_00205.1	809	193401	1.00E-114	238	246	96	+	324	569	98206	98450
UN01711	Rsa1.0_00200.1	1342	162016	0	783	808	96	+	3	810	145180	145987
UN01712	Rsa1.0_03460.1	1319	22747	0	650	682	95	-	640	1316	12216	12897
UN01713	Rsa1.0_00049.1	1489	167018	0	589	614	95	+	574	1187	81876	82477
UN01714	Rsa1.0_06213.1	527	11478	4.00E-79	174	181	96	+	15	195	9833	10006

UN01715	Rsa1.0_00632.1	652	183777	0	340	345	98	−	307	650	149094	149438
UN01716	Rsa1.0_01076.1	759	54835	0	346	346	100	−	87	432	5707	6052
UN01717	Rsa1.0_09709.1	600	6198	0	572	600	95	−	3	597	238	825
UN01718	Rsa1.0_00383.1	1252	398431	0	592	607	97	−	3	607	271063	271668
UN01719	Rsa1.0_01146.1	770	163455	1.00E−122	241	247	97	−	524	770	20552	20793
UN01720	Rsa1.0_00017.1	1403	752458	0	388	397	97	−	4	398	455551	455945
UN01721	Rsa1.0_02003.1	1498	36171	1.00E−117	273	291	93	−	45	335	33665	33949
UN01722	Rsa1.0_14158.1	1216	3726	0	1206	1217	99	+	1	1216	713	1925
UN01723	Rsa1.0_10555.1	609	5802	1.00E−103	389	456	85	+	163	606	3361	3816
UN01724	Rsa1.0_00805.1	1263	63724	1.00E−164	320	325	98	−	1	323	45608	45930
UN01725	Rsa1.0_00049.1	927	167018	1.00E−147	352	377	93	+	3	378	131874	132243
UN01726	Rsa1.0_04580.1	1188	16773	1.00E−175	346	356	97	−	834	1188	7350	7705
UN01727	Rsa1.0_00281.1	1496	99908	0	429	449	95	+	4	449	24092	24539
UN01728	Rsa1.0_06872.1	662	10231	1.00E−112	231	237	97	−	4	240	755	989
UN01729	Rsa1.0_02008.1	917	36109	1.00E−89	240	262	91	−	671	917	16939	17196
UN01730	Rsa1.0_01326.1	758	48072	0	410	415	98	−	345	757	43752	44166
UN01731	Rsa1.0_00008.1	1319	358124	0	770	793	97	+	528	1319	81	864
UN01732	Rsa1.0_01400.1	720	46560	0	350	354	98	+	6	357	11245	11598
UN01733	Rsa1.0_06807.1	1045	10324	1.00E−148	269	270	99	−	32	301	7844	8113
UN01734	Rsa1.0_00100.1	599	249232	5.00E−79	206	222	92	+	380	599	115618	115834
UN01735	Rsa1.0_07373.1	1338	9503	0	731	734	99	−	605	1338	8770	9503
UN01736	Rsa1.0_37707.1	888	597	0	596	597	99	−	201	797	1	597
UN01737	Rsa1.0_00062.1	1069	299291	1.00E−118	216	216	100	+	853	1068	283841	284056
UN01738	Rsa1.0_01456.1	695	45372	0	481	498	96	−	198	695	27565	28060
UN01739	Rsa1.0_02788.1	870	27852	0	852	870	97	−	1	870	1460	2329
UN01740	Rsa1.0_00049.1	1108	167018	1.00E−157	288	289	99	−	821	1108	7082	7370
UN01741	Rsa1.0_00437.1	1260	84879	0	477	483	98	+	3	485	22654	23136
UN01742	Rsa1.0_04518.1	919	17005	1.00E−108	236	247	95	−	609	855	11964	12209
UN01743	Rsa1.0_00476.1	1064	280966	0	748	776	96	−	22	794	221860	222633
UN01744	Rsa1.0_00716.1	1275	67078	1.00E−123	233	236	98	−	1040	1275	27760	27995
UN01745	Rsa1.0_00407.1	1281	191544	0	426	426	100	−	418	843	52956	53381
UN01746	Rsa1.0_01617.1	1213	42127	0	1210	1213	99	−	2	1213	2288	3500
UN01747	Rsa1.0_01985.1	782	36491	5.00E−61	202	228	88	−	291	506	1795	2018
UN01748	Rsa1.0_00778.1	853	196751	0	365	371	98	+	2	372	177849	178219
UN01749	Rsa1.0_00382.1	1051	251946	0	659	665	99	−	388	1051	172909	173573
UN01750	Rsa1.0_01143.1	1305	52456	0	496	503	98	+	86	588	44825	45327
UN01751	Rsa1.0_00168.1	1241	115870	0	1239	1239	100	+	3	1241	16028	17266
UN01752	Rsa1.0_00029.1	1177	272992	0	614	640	95	+	560	1177	96813	97452
UN01753	Rsa1.0_02282.1	1021	163951	0	509	512	99	+	27	538	149562	150072

UN01754	Rsa1.0_12341.1	848	5250	0	488	497	98	−	141	635	2120	2615
UN01755	Rsa1.0_01485.1	721	44797	1.00E−112	221	225	98	−	495	719	1279	1502
UN01756	Rsa1.0_00278.1	1441	231896	1.00E−167	312	314	99	−	562	873	71054	71367
UN01757	Rsa1.0_00101.1	843	267193	0	785	787	99	+	3	787	88976	89762
UN01758	Rsa1.0_01310.1	1069	164719	1.00E−141	270	275	98	+	2	272	11679	11953
UN01759	Rsa1.0_00002.1	1907	601661	0	433	440	98	−	1466	1905	124252	124691
UN01760	Rsa1.0_08220.1	918	7701	0	858	887	96	+	29	914	767	1653
UN01761	Rsa1.0_21221.1	1166	1224	0	572	601	95	−	413	1010	552	1149
UN01762	Rsa1.0_06581.1	774	10722	0	463	474	97	+	306	774	1	474
UN01763	Rsa1.0_01604.1	840	42461	0	457	476	96	−	359	821	40151	40626
UN01764	Rsa1.0_00101.1	1177	267193	0	1123	1132	99	−	2	1133	253253	254383
UN01765	Rsa1.0_01199.1	820	163576	0	439	444	98	−	3	446	154725	155168
UN01766	Rsa1.0_03953.1	1092	19889	1.00E−114	246	260	94	−	824	1083	220	469
UN01767	Rsa1.0_00269.1	1047	341668	0	503	519	96	+	528	1045	18901	19419
UN01768	Rsa1.0_02910.1	835	26843	0	831	835	99	+	1	834	15208	16042
UN01769	Rsa1.0_04059.1	812	19323	1.00E−178	338	345	97	+	2	346	13812	14156
UN01770	Rsa1.0_00053.1	1056	263459	1.00E−126	274	288	95	+	3	283	49103	49390
UN01771	Rsa1.0_02071.1	800	35409	1.00E−154	280	281	99	+	3	283	5478	5758
UN01772	Rsa1.0_00714.1	1082	67187	0	525	536	97	+	352	886	19675	20210
UN01773	Rsa1.0_00296.1	729	97854	1.00E−128	260	269	96	−	131	399	46769	47037
UN01774	Rsa1.0_08595.1	720	7147	0	719	719	100	−	2	720	4709	5427
UN01775	Rsa1.0_01070.1	1033	55099	6.00E−49	100	100	100	−	391	490	33925	34024
UN01776	Rsa1.0_02609.1	745	29600	0	668	728	91	+	20	735	3130	3857
UN01777	Rsa1.0_00128.1	1256	228675	1.00E−169	372	395	94	+	3	397	1241	1626
UN01778	Rsa1.0_00484.1	789	203294	1.00E−156	283	284	99	−	48	331	154484	154767
UN01779	Rsa1.0_02562.1	988	30061	1.00E−160	329	342	96	+	645	986	27953	28293
UN01780	Rsa1.0_11318.1	720	5544	0	714	719	99	+	2	720	661	1379
UN01781	Rsa1.0_03341.1	872	23604	0	487	498	97	−	2	499	2950	3447
UN01782	Rsa1.0_02711.1	1599	28475	0	845	883	95	−	713	1595	8767	9649
UN01783	Rsa1.0_10304.1	919	5896	1.00E−163	321	330	97	−	22	348	4798	5127
UN01784	Rsa1.0_00554.1	786	150953	0	769	781	98	−	3	778	53260	54040
UN01785	Rsa1.0_00341.1	1139	249309	0	546	557	98	+	3	557	21522	22077
UN01786	Rsa1.0_03890.1	1010	20223	0	983	1016	96	+	3	1010	1388	2400
UN01787	Rsa1.0_00701.1	1282	67691	0	632	636	99	−	646	1281	40825	41457
UN01788	Rsa1.0_00859.1	836	61795	0	468	483	96	+	292	774	19663	20145
UN01789	Rsa1.0_07524.1	1319	9199	1.00E−123	275	292	94	+	362	653	7175	7466
UN01790	Rsa1.0_00010.1	1099	294827	0	1094	1100	99	−	2	1099	195490	196589
UN01791	Rsa1.0_32202.1	1090	707	0	436	442	98	−	98	537	266	707
UN01792	Rsa1.0_00896.1	524	60509	1.00E−102	189	189	100	+	336	524	52074	52262

UN01793	Rsa1.0_00740.1	606	215972	1.00E-135	316	337	93	+	1	334	48443	48776
UN01794	Rsa1.0_05762.1	1335	12715	0	1294	1329	97	+	8	1335	8820	10148
UN01795	Rsa1.0_04027.1	1480	19501	0	935	983	95	+	510	1479	14735	15716
UN01796	Rsa1.0_00029.1	1077	272992	0	587	591	99	+	487	1077	3110	3700
UN01797	Rsa1.0_05611.1	711	13193	1.00E-107	217	222	97	−	7	228	8443	8663
UN01798	Rsa1.0_00165.1	665	116466	1.00E-86	196	205	95	−	5	205	27427	27631
UN01799	Rsa1.0_02053.1	1226	35554	0	1225	1226	99	−	1	1226	6025	7250
UN01800	Rsa1.0_01951.1	772	36941	9.00E-69	133	133	100	+	1	133	8473	8605
UN01801	Rsa1.0_00015.1	639	561803	1.00E-155	498	579	86	+	79	635	483083	483641
UN01802	Rsa1.0_00758.1	1280	65477	0	1264	1284	98	−	2	1280	38804	40087
UN01803	Rsa1.0_02054.1	690	35548	0	526	565	93	+	11	563	32519	33062
UN01804	Rsa1.0_00122.1	824	265937	1.00E-165	322	329	97	−	6	331	223063	223390
UN01805	Rsa1.0_00060.1	785	502678	9.00E-63	126	127	99	+	248	374	493391	493517
UN01806	Rsa1.0_00454.1	876	216970	0	676	684	98	+	193	876	155884	156567
UN01807	Rsa1.0_01193.1	951	51147	0	324	324	100	+	628	951	18602	18925
UN01808	Rsa1.0_04934.1	521	143962	0	459	494	92	−	3	494	141943	142435
UN01809	Rsa1.0_01762.1	1039	39682	0	399	412	96	−	519	928	6859	7267
UN01810	Rsa1.0_13863.1	838	4186	0	561	579	96	+	261	837	3143	3719
UN01811	Rsa1.0_05013.1	803	15131	0	390	396	98	−	364	756	6261	6656
UN01812	Rsa1.0_00387.1	817	495407	1.00E-125	233	235	99	−	471	705	340053	340287
UN01813	Rsa1.0_01211.1	1292	50646	0	692	697	99	+	596	1292	41800	42496
UN01814	Rsa1.0_05162.1	798	14631	1.00E-122	254	263	96	+	516	778	4180	4441
UN01815	Rsa1.0_05293.1	764	14237	1.00E-168	321	327	98	−	3	329	8559	8884
UN01816	Rsa1.0_00825.1	730	62855	0	616	624	98	+	110	730	940	1563
UN01817	Rsa1.0_19408.1	676	1436	1.00E-104	241	257	93	+	418	674	391	638
UN01818	Rsa1.0_00747.1	775	66030	1.00E-143	267	270	98	−	206	475	36804	37073
UN01819	Rsa1.0_05428.1	1246	13789	1.00E-135	273	281	97	−	967	1246	7932	8212
UN01820	Rsa1.0_00108.1	890	478491	0	427	441	96	+	34	471	458911	459346
UN01821	Rsa1.0_02802.1	1323	27736	0	665	705	94	+	1	696	9984	10678
UN01822	Rsa1.0_03985.1	904	19704	0	879	909	96	−	1	903	15045	15953
UN01823	Rsa1.0_02939.1	1974	26595	0	559	604	92	+	989	1592	13646	14222
UN01824	Rsa1.0_00128.1	1143	228675	0	467	477	97	+	283	759	69485	69961
UN01825	Rsa1.0_00321.1	770	95671	0	767	769	99	+	2	770	9617	10384
UN01826	Rsa1.0_06164.1	1281	11584	0	440	444	99	−	1	442	3193	3635
UN01827	Rsa1.0_01134.1	800	52829	0	778	799	97	+	2	800	25944	26739
UN01828	Rsa1.0_02320.1	815	32575	9.00E-66	143	148	96	−	83	230	24899	25046
UN01829	Rsa1.0_00286.1	787	98709	1.00E-102	237	252	94	−	50	301	13001	13250
UN01830	Rsa1.0_00025.1	1282	277312	0	495	495	100	+	301	795	97736	98230
UN01831	Rsa1.0_20851.1	1200	1263	6.00E-40	181	212	85	+	809	1011	616	824

UN01832	Rsa1.0_22307.1	802	1132	0	342	344	99	+	459	802	26	369
UN01833	Rsa1.0_01517.1	1201	44170	0	850	866	98	−	1	866	16545	17410
UN01834	Rsa1.0_00157.1	1444	245832	1.00E−167	342	352	97	−	2	351	16951	17298
UN01835	Rsa1.0_06803.1	866	10330	0	412	423	97	+	9	430	9670	10084
UN01836	Rsa1.0_00300.1	1286	97747	0	539	562	95	−	662	1214	81513	82074
UN01837	Rsa1.0_00374.1	848	165738	0	361	369	97	−	1	369	131321	131684
UN01838	Rsa1.0_00007.1	795	239325	1.00E−120	222	223	99	+	530	752	117634	117856
UN01839	Rsa1.0_00011.1	827	225044	0	660	662	99	+	2	661	213213	213874
UN01840	Rsa1.0_00320.1	285	95784	3.00E−85	163	164	99	−	121	284	37735	37898
UN01841	Rsa1.0_00034.1	1469	260119	0	456	459	99	+	1008	1466	28435	28892
UN01842	Rsa1.0_00198.1	1035	222963	0	428	429	99	−	605	1033	139103	139531
UN01843	Rsa1.0_00276.1	1243	100160	0	407	410	99	+	348	757	61194	61603
UN01844	Rsa1.0_01020.1	1147	56619	1.00E−122	248	255	97	+	894	1147	20374	20628
UN01845	Rsa1.0_01515.1	726	170864	0	690	744	92	+	1	725	126735	127470
UN01846	Rsa1.0_00069.1	884	403561	0	879	892	98	−	1	884	48378	49269
UN01847	Rsa1.0_00210.1	1027	108424	0	429	432	99	−	594	1025	50678	51109
UN01848	Rsa1.0_03391.1	828	23251	1.00E−165	300	302	99	+	1	302	7096	7397
UN01849	Rsa1.0_00793.1	985	64103	1.00E−132	242	243	99	−	743	985	13030	13272
UN01850	Rsa1.0_04845.1	1405	15747	1.00E−111	220	225	97	+	171	395	4169	4393
UN01851	Rsa1.0_00185.1	979	248445	3.00E−60	122	123	99	+	701	823	2901	3023
UN01852	Rsa1.0_31152.1	1022	735	0	615	620	99	−	307	925	116	735
UN01853	Rsa1.0_00442.1	1086	84596	0	359	360	99	−	3	362	63588	63947
UN01854	Rsa1.0_00675.1	725	145590	0	445	449	99	+	4	452	45278	45726
UN01855	Rsa1.0_14950.1	843	2940	0	835	851	98	+	1	843	1295	2144
UN01856	Rsa1.0_02474.1	862	31076	1.00E−178	363	374	97	−	1	372	29475	29844
UN01857	Rsa1.0_00190.1	751	318240	0	521	551	94	−	3	549	20540	21082
UN01858	Rsa1.0_02314.1	798	32642	0	781	799	97	+	3	797	18742	19540
UN01859	Rsa1.0_02946.1	672	26537	0	470	472	99	+	185	656	26012	26483
UN01860	Rsa1.0_01136.1	678	52724	1.00E−115	213	214	99	−	249	462	49049	49262
UN01861	Rsa1.0_05122.1	1149	14791	0	379	379	100	−	411	789	7708	8086
UN01862	Rsa1.0_00133.1	785	123768	0	464	468	99	−	3	470	25129	25596
UN01863	Rsa1.0_00361.1	924	91181	1.00E−138	270	277	97	+	161	437	12174	12450
UN01864	Rsa1.0_01134.1	1198	52829	0	402	404	99	+	793	1196	43276	43679
UN01865	Rsa1.0_00363.1	494	90968	1.00E−149	298	308	96	−	10	317	22565	22872
UN01866	Rsa1.0_01319.1	979	48273	1.00E−157	355	382	92	−	596	977	10916	11279
UN01867	Rsa1.0_18598.1	763	1567	0	566	629	89	−	63	666	258	885
UN01868	Rsa1.0_02303.1	1333	32818	4.00E−32	198	240	82	+	111	350	3298	3537
UN01869	Rsa1.0_04247.1	1083	18295	0	898	971	92	+	112	1078	378	1334
UN01870	Rsa1.0_01388.1	1256	46741	0	472	474	99	−	362	835	20000	20473

UN01871	Rsa1.0_03767.1	1410	20969	0	1405	1409	99	−	1	1409	9206	10610
UN01872	Rsa1.0_00029.1	1124	272992	0	719	721	99	−	112	832	248566	249286
UN01873	Rsa1.0_00107.1	1038	431659	1.00E−154	291	295	98	+	742	1036	355792	356084
UN01874	Rsa1.0_05880.1	740	12343	0	434	446	97	−	1	446	5495	5940
UN01875	Rsa1.0_29122.1	1537	792	0	394	397	99	+	988	1384	396	792
UN01876	Rsa1.0_02787.1	922	27872	0	907	931	97	−	2	922	542	1466
UN01877	Rsa1.0_01768.1	774	138463	1.00E−174	313	314	99	−	1	314	119313	119626
UN01878	Rsa1.0_00556.1	1233	207212	1.00E−145	406	454	89	+	3	439	33418	33859
UN01879	Rsa1.0_00875.1	827	61371	0	825	844	97	−	3	827	3319	4162
UN01880	Rsa1.0_03781.1	826	20891	1.00E−119	255	267	95	−	307	570	13363	13629
UN01881	Rsa1.0_00185.1	1067	248445	0	906	970	93	−	1	960	204443	205411
UN01882	Rsa1.0_05611.1	819	13193	0	574	575	99	+	2	576	5087	5661
UN01883	Rsa1.0_01296.1	458	49035	1.00E−51	150	167	89	−	87	253	8439	8595
UN01884	Rsa1.0_00377.1	788	90038	0	423	434	97	−	3	434	12014	12443
UN01885	Rsa1.0_03964.1	1193	19835	0	873	907	96	−	293	1193	7210	8113
UN01886	Rsa1.0_01303.1	899	48853	1.00E−125	333	367	90	+	179	544	16242	16598
UN01887	Rsa1.0_00536.1	827	77199	1.00E−150	330	351	94	+	7	357	42224	42557
UN01888	Rsa1.0_00835.1	891	278058	0	574	574	100	+	3	576	138159	138732
UN01889	Rsa1.0_06253.1	911	11385	0	367	373	98	+	539	911	7588	7960
UN01890	Rsa1.0_00813.1	855	63202	1.00E−129	289	306	94	+	1	300	10858	11162
UN01891	Rsa1.0_00807.1	790	63607	7.00E−85	169	172	98	−	494	665	46630	46801
UN01892	Rsa1.0_03524.1	720	22297	0	351	353	99	+	1	353	18608	18959
UN01893	Rsa1.0_18047.1	1313	1682	0	370	371	99	−	1	370	162	532
UN01894	Rsa1.0_03602.1	777	21847	0	376	385	97	−	42	425	6953	7336
UN01895	Rsa1.0_00527.1	1119	77733	1.00E−134	345	375	92	+	228	598	54819	55189
UN01896	Rsa1.0_00157.1	542	245832	1.00E−126	241	244	98	−	1	244	5489	5730
UN01897	Rsa1.0_00481.1	709	81316	1.00E−112	236	245	96	+	20	260	36809	37051
UN01898	Rsa1.0_03236.1	732	24441	0	518	531	97	+	202	732	5413	5943
UN01899	Rsa1.0_01719.1	860	40388	0	789	810	97	+	2	810	31928	32737
UN01900	Rsa1.0_00066.1	1277	152418	1.00E−133	244	245	99	+	1	245	98253	98497
UN01901	Rsa1.0_00652.1	1111	70089	0	554	582	95	−	1	582	47920	48498
UN01902	Rsa1.0_01937.1	716	37147	1.00E−103	197	199	98	+	1	199	30210	30408
UN01903	Rsa1.0_00431.1	516	85391	0	489	509	96	−	8	516	6275	6783
UN01904	Rsa1.0_03774.1	529	20932	1.00E−123	310	335	92	+	1	323	9822	10154
UN01905	Rsa1.0_05110.1	1119	14814	1.00E−154	303	312	97	+	242	553	5291	5602
UN01906	Rsa1.0_00367.1	1076	90750	0	1068	1075	99	+	1	1075	80559	81633
UN01907	Rsa1.0_00306.1	1108	146760	0	509	519	98	−	1	517	126933	127445
UN01908	Rsa1.0_02398.1	704	31766	0	394	394	100	+	309	702	9658	10051
UN01909	Rsa1.0_01416.1	988	46262	0	442	448	98	+	3	449	21659	22105

UN01910	Rsa1.0_00182.1	1380	142305	0	1045	1061	98	-	317	1377	52241	53301
UN01911	Rsa1.0_01371.1	942	47079	0	917	942	97	+	1	942	19897	20838
UN01912	Rsa1.0_00140.1	765	121541	0	638	640	99	-	8	647	60097	60736
UN01913	Rsa1.0_00007.1	737	239325	1.00E-180	338	344	98	-	360	703	118158	118501
UN01914	Rsa1.0_00045.1	891	328694	1.00E-140	277	285	97	-	434	718	162174	162458
UN01915	Rsa1.0_00016.1	989	831256	1.00E-161	311	318	97	-	100	414	429113	429430
UN01916	Rsa1.0_00673.1	1072	69325	1.00E-16	68	74	91	-	98	171	15476	15548
UN01917	Rsa1.0_02050.1	724	35570	1.00E-141	325	346	93	-	377	721	9337	9675
UN01918	Rsa1.0_01675.1	1576	41155	1.00E-177	327	331	98	+	1242	1572	9381	9711
UN01919	Rsa1.0_06125.1	817	11680	1.00E-27	154	182	84	+	1	179	3542	3722
UN01920	Rsa1.0_00132.1	1185	124299	0	731	755	96	+	434	1185	89996	90747
UN01921	Rsa1.0_00047.1	458	168582	1.00E-91	202	211	95	+	247	456	166425	166635
UN01922	Rsa1.0_05278.1	1409	14282	0	467	475	98	+	3	476	8860	9330
UN01923	Rsa1.0_01184.1	1255	51452	1.00E-167	365	387	94	-	12	391	42359	42732
UN01924	Rsa1.0_00221.1	958	106972	4.00E-90	172	173	99	-	195	367	64560	64732
UN01925	Rsa1.0_00251.1	1004	303968	2.00E-89	177	180	98	-	707	886	126181	126360
UN01926	Rsa1.0_03809.1	1178	20731	0	771	823	93	-	1	816	19080	19901
UN01927	Rsa1.0_00273.1	769	326186	5.00E-98	212	222	95	+	286	507	308381	308602
UN01928	Rsa1.0_00139.1	946	357786	0	325	325	100	+	620	944	275224	275548
UN01929	Rsa1.0_02764.1	920	28142	0	429	439	97	-	3	439	24175	24610
UN01930	Rsa1.0_01293.1	1259	49139	1.00E-136	250	251	99	+	388	638	32799	33049
UN01931	Rsa1.0_00041.1	611	186197	1.00E-147	273	276	98	-	2	277	33784	34059
UN01932	Rsa1.0_00175.1	732	166121	0	451	461	97	-	1	461	67110	67569
UN01933	Rsa1.0_03711.1	1285	21237	1.00E-118	228	232	98	-	667	898	12134	12365
UN01934	Rsa1.0_00377.1	779	90038	0	511	528	96	+	6	530	79430	79953
UN01935	Rsa1.0_00706.1	1097	291570	0	521	528	98	-	1	527	182172	182699
UN01936	Rsa1.0_01080.1	1528	54752	0	1281	1305	98	+	1	1303	33269	34573
UN01937	Rsa1.0_05267.1	842	14319	1.00E-166	315	321	98	+	254	574	1130	1450
UN01938	Rsa1.0_00687.1	808	68494	0	686	696	98	+	3	698	42407	43102
UN01939	Rsa1.0_17040.1	568	1944	0	400	417	95	+	154	566	1207	1623
UN01940	Rsa1.0_03738.1	742	21071	0	710	749	94	-	6	742	19581	20321
UN01941	Rsa1.0_00861.1	571	61759	1.00E-126	251	256	98	+	49	302	1779	2033
UN01942	Rsa1.0_05412.1	573	13836	0	568	573	99	-	1	573	10164	10736
UN01943	Rsa1.0_33576.1	713	675	0	428	434	98	+	196	626	1	434
UN01944	Rsa1.0_00693.1	476	161311	0	460	479	96	+	1	476	47054	47526
UN01945	Rsa1.0_03488.1	1154	22556	1.00E-179	318	318	100	-	681	998	13813	14130
UN01946	Rsa1.0_05700.1	539	12886	0	376	390	96	+	80	464	3841	4229
UN01947	Rsa1.0_01174.1	863	51696	0	511	530	96	-	132	654	12826	13354
UN01948	Rsa1.0_00449.1	1296	83863	0	760	787	96	-	510	1295	40367	41153

UN01949	Rsa1.0_02431.1	689	31504	0	410	415	98	-	7	418	26607	27021
UN01950	Rsa1.0_03565.1	955	22062	1.00E-105	234	246	95	+	709	954	3911	4154
UN01951	Rsa1.0_00518.1	1246	78360	0	635	681	93	-	1	666	76228	76895
UN01952	Rsa1.0_05815.1	295	12563	5.00E-93	219	231	94	-	41	266	9748	9978
UN01953	Rsa1.0_20439.1	1148	1305	0	464	488	95	+	1	488	696	1167
UN01954	Rsa1.0_00083.1	1083	442773	0	836	839	99	-	1	839	319657	320495
UN01955	Rsa1.0_04714.1	867	146738	0	513	528	97	-	46	573	139509	140034
UN01956	Rsa1.0_00796.1	808	63997	1.00E-125	239	242	98	+	145	386	58148	58387
UN01957	Rsa1.0_01595.1	1160	175383	1.00E-161	291	292	99	-	1	292	40568	40859
UN01958	Rsa1.0_00040.1	1034	324196	0	598	640	93	-	1	629	246545	247183
UN01959	Rsa1.0_02345.1	472	32337	2.00E-77	187	200	93	-	8	198	17017	17216
UN01960	Rsa1.0_02803.1	441	27715	2.00E-56	143	152	94	-	197	347	26242	26393
UN01961	Rsa1.0_00106.1	728	133916	0	438	454	96	+	7	460	5590	6037
UN01962	Rsa1.0_00689.1	1570	68305	0	1539	1572	97	+	1	1570	33607	35176
UN01963	Rsa1.0_01046.1	966	152989	1.00E-127	280	296	94	-	71	366	139680	139965
UN01964	Rsa1.0_00039.1	892	296927	0	574	585	98	+	311	886	229887	230470
UN01965	Rsa1.0_01243.1	738	50061	0	366	366	100	-	373	738	49696	50061
UN01966	Rsa1.0_00848.1	607	152362	0	590	601	98	+	6	606	31053	31653
UN01967	Rsa1.0_00175.1	1183	166121	1.00E-158	353	374	94	+	3	373	139880	140253
UN01968	Rsa1.0_02872.1	415	27080	1.00E-133	295	312	94	+	1	312	9164	9469
UN01969	Rsa1.0_01910.1	1062	37405	0	547	555	98	-	2	555	9739	10292
UN01970	Rsa1.0_00322.1	1130	212233	1.00E-132	322	347	92	+	5	349	92240	92586
UN01971	Rsa1.0_37242.1	697	605	1.00E-156	325	337	96	+	83	413	1	337
UN01972	Rsa1.0_02146.1	781	34625	1.00E-129	318	343	92	+	1	342	30083	30420
UN01973	Rsa1.0_06482.1	911	10937	0	580	604	96	+	287	889	8154	8752
UN01974	Rsa1.0_02017.1	1442	35999	0	557	585	95	-	5	565	24178	24762
UN01975	Rsa1.0_00472.1	958	398134	1.00E-148	333	354	94	+	7	356	306510	306856
UN01976	Rsa1.0_13353.1	798	4894	1.00E-125	257	267	96	-	202	468	2419	2685
UN01977	Rsa1.0_01172.1	1197	51734	0	676	747	90	-	469	1191	42903	43637
UN01978	Rsa1.0_00162.1	1627	239935	0	502	514	97	+	822	1332	19100	19613
UN01979	Rsa1.0_03415.1	885	23117	0	844	887	95	+	1	884	20176	21060
UN01980	Rsa1.0_00281.1	587	99908	1.00E-158	323	335	96	+	5	339	68711	69044
UN01981	Rsa1.0_00022.1	884	200461	0	472	497	94	+	1	494	86012	86504
UN01982	Rsa1.0_00265.1	1175	220635	0	419	438	95	+	78	514	126859	127296
UN01983	Rsa1.0_04789.1	987	15981	0	929	969	95	-	1	962	14387	15354
UN01984	Rsa1.0_00010.1	1595	294827	0	749	833	89	+	15	825	210886	211689
UN01985	Rsa1.0_03521.1	1271	396204	1.00E-133	318	336	94	-	4	337	136464	136793
UN01986	Rsa1.0_00451.1	765	322533	1.00E-118	267	286	93	+	6	290	48180	48450
UN01987	Rsa1.0_00019.1	413	301669	1.00E-130	307	328	93	+	89	408	286292	286616

UN01988	Rsa1.0_01377.1	900	46967	0	545	558	97	+	2	557	10095	10647
UN01989	Rsa1.0_00454.1	852	216970	0	842	852	98	−	1	851	10432	11280
UN01990	Rsa1.0_00942.1	894	58873	0	442	474	93	+	1	452	21602	22075
UN01991	Rsa1.0_00017.1	1410	752458	1.00E−152	294	301	97	−	449	749	542268	542568
UN01992	Rsa1.0_03609.1	1047	21798	0	676	696	97	+	360	1046	17383	18078
UN01993	Rsa1.0_01220.1	1079	161775	1.00E−116	312	348	89	−	8	355	7326	7649
UN01994	Rsa1.0_00090.1	631	273859	0	589	641	91	−	6	631	232559	233183
UN01995	Rsa1.0_00895.1	645	60615	1.00E−168	327	335	97	−	7	341	39714	40047
UN01996	Rsa1.0_00018.1	1179	410899	0	544	549	99	−	340	887	83637	84184
UN01997	Rsa1.0_02766.1	673	28128	0	502	528	95	−	146	673	13420	13947
UN01998	Rsa1.0_04399.1	938	17543	0	414	417	99	−	13	429	12162	12578
UN01999	Rsa1.0_00119.1	876	467509	4.00E−68	159	168	94	−	464	631	403899	404066
UN02000	Rsa1.0_00175.1	1525	166121	1.00E−103	251	270	92	−	3	272	28735	28992
UN02001	Rsa1.0_00046.1	1540	320315	1.00E−136	369	406	90	−	1136	1538	124579	124974
UN02002	Rsa1.0_00130.1	1249	125737	0	586	588	99	+	661	1247	88567	89154
UN02003	Rsa1.0_00602.1	1394	73302	0	951	996	95	+	95	1086	30579	31574
UN02004	Rsa1.0_02481.1	651	31035	1.00E−128	301	324	92	+	1	324	17776	18099
UN02005	Rsa1.0_00205.1	1122	193401	1.00E−113	226	231	97	+	8	237	130115	130345
UN02006	Rsa1.0_04319.1	974	17944	9.00E−82	158	159	99	+	814	972	16954	17112
UN02007	Rsa1.0_00144.1	962	120708	0	404	411	98	−	1	407	68637	69047
UN02008	Rsa1.0_02280.1	894	33063	0	389	403	96	−	490	892	106	508
UN02009	Rsa1.0_02911.1	1463	142461	0	618	632	97	−	1	627	23404	24034
UN02010	Rsa1.0_00505.1	1239	79323	1.00E−125	237	240	98	−	839	1078	74685	74924
UN02011	Rsa1.0_03088.1	545	25476	0	400	411	97	−	1	409	507	917
UN02012	Rsa1.0_06720.1	907	10473	0	698	719	97	+	145	860	3962	4677
UN02013	Rsa1.0_03436.1	671	22958	0	500	526	95	+	48	573	3709	4216
UN02014	Rsa1.0_07381.1	913	9488	0	906	912	99	+	1	911	8152	9063
UN02015	Rsa1.0_00923.1	1052	153678	1.00E−159	297	301	98	−	3	303	21396	21696
UN02016	Rsa1.0_01700.1	944	40745	0	920	942	97	−	3	943	18560	19501
UN02017	Rsa1.0_00077.1	1067	145005	1.00E−150	308	321	95	+	2	317	75404	75724
UN02018	Rsa1.0_00773.1	1403	64839	0	1272	1334	95	−	73	1395	8132	9456
UN02019	Rsa1.0_00009.1	2096	232558	0	658	676	97	+	1281	1956	164054	164723
UN02020	Rsa1.0_14271.1	1381	3591	0	1012	1055	95	+	2	1056	2537	3591
UN02021	Rsa1.0_00056.1	866	332604	1.00E−159	395	433	91	+	451	866	206057	206478
UN02022	Rsa1.0_04734.1	349	16184	1.00E−177	342	350	97	−	1	349	4018	4367
UN02023	Rsa1.0_00330.1	1251	94666	0	1202	1263	95	−	1	1247	34830	36092
UN02024	Rsa1.0_05730.1	1127	12798	1.00E−34	153	178	85	−	439	616	12287	12461
UN02025	Rsa1.0_00240.1	2114	104175	0	1039	1045	99	−	1075	2113	72543	73587
UN02026	Rsa1.0_00321.1	917	95671	0	352	354	99	−	1	353	40671	41024

UN02027	Rsa1.0_01140.1	1288	52586	1.00E-172	336	344	97	+	946	1286	33897	34238
UN02028	Rsa1.0_00059.1	1127	159650	1.00E-119	252	260	96	-	869	1127	147770	148025
UN02029	Rsa1.0_00870.1	548	61606	0	451	470	95	+	3	470	26357	26825
UN02030	Rsa1.0_01269.1	720	49622	0	673	693	97	+	28	720	13153	13844
UN02031	Rsa1.0_00071.1	972	345714	1.00E-172	365	382	95	-	591	972	71154	71533
UN02032	Rsa1.0_11907.1	830	5374	0	437	455	96	-	1	455	1101	1551
UN02033	Rsa1.0_02051.1	748	35564	1.00E-129	305	324	94	+	3	319	22594	22913
UN02034	Rsa1.0_00102.1	1121	135497	0	946	975	97	-	154	1120	60039	61013
UN02035	Rsa1.0_00294.1	1119	144860	0	360	367	98	-	2	368	68768	69134
UN02036	Rsa1.0_00123.1	1022	325650	0	633	647	97	+	296	942	272132	272778
UN02037	Rsa1.0_06019.1	1433	11916	0	567	571	99	+	171	740	1918	2488
UN02038	Rsa1.0_01228.1	545	50238	0	349	352	99	-	194	545	1700	2051
UN02039	Rsa1.0_03394.1	731	23247	0	375	391	95	-	54	443	1931	2321
UN02040	Rsa1.0_28959.1	789	798	0	513	530	96	+	3	532	273	798
UN02041	Rsa1.0_00703.1	1158	67597	0	412	421	97	-	738	1158	30616	31035
UN02042	Rsa1.0_00857.1	972	61904	0	607	640	94	+	2	638	44482	45110
UN02043	Rsa1.0_02159.1	634	34471	0	535	540	99	-	1	540	32493	33028
UN02044	Rsa1.0_00121.1	589	278029	6.00E-88	171	173	98	+	240	412	263661	263833
UN02045	Rsa1.0_03297.1	680	23969	0	473	478	98	+	3	476	8203	8680
UN02046	Rsa1.0_02400.1	555	31750	0	541	555	97	+	1	555	12369	12910
UN02047	Rsa1.0_01431.1	1140	167877	2.00E-79	163	167	97	+	445	611	16242	16408
UN02048	Rsa1.0_04139.1	707	18883	1.00E-110	375	429	87	-	287	704	13205	13619
UN02049	Rsa1.0_02347.1	1355	32332	0	355	356	99	-	2	356	25631	25986
UN02050	Rsa1.0_05547.1	1159	13369	0	564	583	96	+	578	1159	919	1489
UN02051	Rsa1.0_06578.1	1545	10726	0	486	486	100	-	1060	1545	1059	1544
UN02052	Rsa1.0_00142.1	867	121474	0	430	450	95	+	368	812	62984	63430
UN02053	Rsa1.0_05737.1	813	12778	1.00E-174	442	474	93	+	349	813	307	776
UN02054	Rsa1.0_00336.1	764	445051	0	392	412	95	-	3	413	161052	161459
UN02055	Rsa1.0_34294.1	1470	660	1.00E-173	318	321	99	-	1075	1395	1	321
UN02056	Rsa1.0_03028.1	1061	25872	1.00E-159	285	285	100	-	704	988	20174	20458
UN02057	Rsa1.0_00694.1	1454	68074	1.00E-125	228	228	100	+	1227	1454	4300	4527
UN02058	Rsa1.0_00377.1	958	90038	0	490	503	97	-	1	502	80774	81276
UN02059	Rsa1.0_00088.1	1076	141341	0	694	716	96	+	1	711	45804	46517
UN02060	Rsa1.0_00039.1	1265	296927	8.00E-86	180	186	96	+	842	1027	113194	113379
UN02061	Rsa1.0_00119.1	1103	467509	0	377	377	100	-	727	1103	107065	107441
UN02062	Rsa1.0_16460.1	739	2148	0	564	582	96	+	151	729	1	582
UN02063	Rsa1.0_03024.1	841	25931	1.00E-119	295	322	91	+	125	437	25281	25593
UN02064	Rsa1.0_03357.1	1598	23478	0	554	560	98	+	1042	1598	19472	20031
UN02065	Rsa1.0_00291.1	774	172129	0	739	773	95	+	1	770	31588	32350

UN02066	Rsa1.0_02012.1	651	36084	0	531	537	98	−	3	539	20591	21127
UN02067	Rsa1.0_14744.1	834	3111	1.00E−151	426	475	89	−	337	809	724	1198
UN02068	Rsa1.0_01431.1	720	167877	1.00E−178	371	386	96	+	340	720	12009	12393
UN02069	Rsa1.0_00100.1	1287	249232	1.00E−173	373	392	95	+	896	1286	179062	179452
UN02070	Rsa1.0_00449.1	701	83863	0	693	700	99	+	2	701	40332	41031
UN02071	Rsa1.0_13356.1	335	4890	2.00E−12	89	106	83	−	65	170	110	215
UN02072	Rsa1.0_01154.1	623	52249	0	348	351	99	−	274	623	3672	4022
UN02073	Rsa1.0_00437.1	553	84879	1.00E−128	253	260	97	+	294	553	54610	54869
UN02074	Rsa1.0_03191.1	1128	145762	0	902	910	99	+	222	1128	137789	138697
UN02075	Rsa1.0_27709.1	1537	842	0	834	841	99	−	630	1468	2	842
UN02076	Rsa1.0_01104.1	1112	285480	1.00E−166	402	439	91	+	3	426	266546	266983
UN02077	Rsa1.0_00998.1	1118	57168	0	368	373	98	+	747	1118	31843	32214
UN02078	Rsa1.0_00492.1	1382	80178	0	400	406	98	+	694	1099	70497	70900
UN02079	Rsa1.0_01081.1	466	499226	0	460	465	98	−	2	466	42806	43270
UN02080	Rsa1.0_08143.1	1233	7836	0	1166	1266	92	−	5	1232	3715	4965
UN02081	Rsa1.0_00094.1	722	139042	0	365	372	98	−	353	722	77038	77409
UN02082	Rsa1.0_00373.1	1218	90296	0	780	802	97	−	415	1216	79717	80516
UN02083	Rsa1.0_01071.1	1113	166914	0	358	366	97	−	748	1113	14042	14407
UN02084	Rsa1.0_03439.1	651	22930	1.00E−152	320	333	96	+	327	651	21322	21650
UN02085	Rsa1.0_00342.1	1571	93695	0	1550	1575	98	+	3	1571	49202	50776
UN02086	Rsa1.0_02675.1	724	28852	2.00E−72	142	143	99	−	334	476	17929	18071
UN02087	Rsa1.0_05815.1	1039	12563	0	458	493	92	+	150	631	8659	9149
UN02088	Rsa1.0_01539.1	1248	43698	0	653	692	94	+	563	1247	4615	5296
UN02089	Rsa1.0_00968.1	1106	58020	0	423	432	97	−	295	726	51850	52281
UN02090	Rsa1.0_00905.1	997	60110	0	931	941	98	−	56	996	7778	8718
UN02091	Rsa1.0_34683.1	956	653	1.00E−108	199	199	100	+	758	956	270	468
UN02092	Rsa1.0_01034.1	1294	56046	0	730	757	96	+	238	994	19416	20172
UN02093	Rsa1.0_09707.1	697	6199	1.00E−156	304	311	97	+	389	697	946	1256
UN02094	Rsa1.0_02803.1	738	27715	1.00E−106	309	346	89	−	181	523	8863	9208
UN02095	Rsa1.0_00273.1	758	326186	0	440	458	96	+	3	449	123679	124133
UN02096	Rsa1.0_01682.1	699	41090	1.00E−123	260	268	97	+	3	270	38026	38290
UN02097	Rsa1.0_02513.1	816	30643	0	418	424	98	+	3	426	9835	10256
UN02098	Rsa1.0_01719.1	576	40388	1.00E−109	209	212	98	+	365	576	30549	30760
UN02099	Rsa1.0_02548.1	1096	30228	0	366	372	98	+	729	1096	10107	10478
UN02100	Rsa1.0_11534.1	1079	5477	0	1006	1047	96	+	2	1039	674	1720
UN02101	Rsa1.0_01592.1	938	42685	1.00E−150	277	278	99	+	661	938	40225	40501
UN02102	Rsa1.0_01263.1	947	49703	1.00E−113	280	301	93	−	628	922	30578	30878
UN02103	Rsa1.0_00055.1	839	520193	1.00E−86	189	197	95	+	2	195	87168	87364
UN02104	Rsa1.0_01339.1	1347	47813	0	375	388	96	+	965	1347	29690	30077

UN02105	Rsa1.0_00420.1	864	86496	1.00E-154	276	276	100	+	1	276	42749	43024
UN02106	Rsa1.0_02345.1	785	32337	1.00E-104	293	324	90	+	465	785	15392	15705
UN02107	Rsa1.0_01792.1	505	39227	1.00E-152	339	359	94	-	150	505	7076	7434
UN02108	Rsa1.0_02851.1	1199	27295	0	354	362	97	-	844	1197	8835	9196
UN02109	Rsa1.0_04784.1	849	15995	0	642	665	96	-	192	848	9057	9721
UN02110	Rsa1.0_00628.1	1618	214360	0	453	459	98	+	263	721	201979	202437
UN02111	Rsa1.0_00097.1	758	138858	0	655	678	96	+	68	742	29953	30621
UN02112	Rsa1.0_02660.1	691	28969	0	682	691	98	-	1	690	17619	18309
UN02113	Rsa1.0_00676.1	1228	69166	0	496	519	95	-	184	699	54623	55138
UN02114	Rsa1.0_00336.1	1016	445051	1.00E-117	318	354	89	-	667	1016	162016	162354
UN02115	Rsa1.0_00949.1	1471	58549	1.00E-173	415	446	93	-	284	720	15414	15851
UN02116	Rsa1.0_03418.1	919	147293	1.00E-143	258	258	100	-	660	917	10080	10337
UN02117	Rsa1.0_01591.1	878	42718	0	452	452	100	-	3	454	24117	24568
UN02118	Rsa1.0_04065.1	1834	19275	0	379	382	99	+	1452	1833	3173	3553
UN02119	Rsa1.0_01081.1	916	499226	1.00E-162	310	316	98	-	604	916	16506	16821
UN02120	Rsa1.0_00512.1	639	200277	1.00E-107	225	234	96	+	4	237	194182	194415
UN02121	Rsa1.0_01282.1	1084	49304	2.00E-49	119	125	95	-	513	637	33427	33551
UN02122	Rsa1.0_07504.1	879	9237	1.00E-173	311	312	99	-	568	879	7983	8294
UN02123	Rsa1.0_14203.1	1181	3670	0	433	441	98	+	336	776	1903	2342
UN02124	Rsa1.0_16020.1	1461	2321	1.00E-173	350	361	96	-	1	359	1410	1770
UN02125	Rsa1.0_03415.1	1169	23117	0	762	798	95	-	385	1169	21162	21959
UN02126	Rsa1.0_00088.1	957	141341	1.00E-136	274	282	97	+	675	956	108001	108281
UN02127	Rsa1.0_02221.1	991	33796	0	526	541	97	-	3	543	18658	19197
UN02128	Rsa1.0_01836.1	722	38503	0	368	377	97	+	3	379	1705	2078
UN02129	Rsa1.0_00836.1	1058	185968	1.00E-132	297	313	94	-	747	1053	17849	18161
UN02130	Rsa1.0_03569.1	614	22041	2.00E-75	212	228	92	+	388	614	10002	10225
UN02131	Rsa1.0_09769.1	1185	6159	0	487	531	91	-	530	1059	3945	4475
UN02132	Rsa1.0_00082.1	1033	237584	1.00E-131	310	330	93	-	705	1032	14748	15066
UN02133	Rsa1.0_00226.1	816	105884	1.00E-175	321	324	99	-	151	474	22862	23185
UN02134	Rsa1.0_00206.1	479	109040	1.00E-125	293	312	93	-	171	479	74215	74522
UN02135	Rsa1.0_01413.1	941	46275	1.00E-139	514	602	85	+	82	677	20235	20836
UN02136	Rsa1.0_04282.1	750	18100	0	437	450	97	+	302	750	17329	17778
UN02137	Rsa1.0_00965.1	552	58139	8.00E-53	112	114	98	+	98	211	9005	9118
UN02138	Rsa1.0_05890.1	625	12317	5.00E-70	215	240	89	-	404	625	739	976
UN02139	Rsa1.0_02471.1	797	31104	1.00E-122	244	250	97	+	200	449	17799	18047
UN02140	Rsa1.0_00706.1	1068	291570	0	432	451	95	+	3	453	244671	245121
UN02141	Rsa1.0_02605.1	634	29637	1.00E-67	223	247	90	+	396	633	13844	14084
UN02142	Rsa1.0_00090.1	612	273859	0	420	444	94	+	169	612	231858	232289
UN02143	Rsa1.0_04203.1	960	18479	0	445	461	96	-	3	463	17886	18342

UN02144	Rsa1.0_00416.1	943	86772	1.00E-166	366	387	94	+	556	941	50850	51234
UN02145	Rsa1.0_10577.1	834	5796	0	486	488	99	+	346	833	535	1022
UN02146	Rsa1.0_05864.1	1150	12387	1.00E-147	354	381	92	−	11	383	11077	11443
UN02147	Rsa1.0_04188.1	1184	18586	0	1183	1185	99	−	1	1184	6452	7636
UN02148	Rsa1.0_54279.1	1189	420	0	420	420	100	+	514	933	1	420
UN02149	Rsa1.0_01895.1	1247	37512	0	1027	1050	97	+	198	1247	23772	24821
UN02150	Rsa1.0_02099.1	652	35030	0	640	643	99	+	4	646	9869	10511
UN02151	Rsa1.0_00067.1	1742	415055	0	421	443	95	−	1313	1742	46411	46853
UN02152	Rsa1.0_00451.1	949	322533	0	925	950	97	−	1	949	153403	154352
UN02153	Rsa1.0_01293.1	1217	49139	0	997	1029	96	+	172	1200	2686	3713
UN02154	Rsa1.0_00296.1	711	97854	0	710	711	99	+	1	711	80056	80766
UN02155	Rsa1.0_00082.1	896	237584	0	485	497	97	+	123	619	227517	228013
UN02156	Rsa1.0_00163.1	1101	116847	1.00E-149	268	268	100	+	693	960	62264	62531
UN02157	Rsa1.0_00530.1	1009	77389	0	516	524	98	−	3	524	15978	16501
UN02158	Rsa1.0_03292.1	600	23977	1.00E-161	319	328	97	+	2	329	7755	8081
UN02159	Rsa1.0_02222.1	682	152798	0	381	386	98	−	3	388	12231	12613
UN02160	Rsa1.0_00810.1	490	63289	1.00E-88	239	268	89	−	222	489	18821	19064
UN02161	Rsa1.0_00600.1	745	73395	0	463	472	98	+	274	745	68561	69025
UN02162	Rsa1.0_02080.1	735	35297	0	598	615	97	−	10	620	13926	14540
UN02163	Rsa1.0_03672.1	856	21452	1.00E-61	148	157	94	+	248	404	6740	6896
UN02164	Rsa1.0_01924.1	927	37226	0	442	446	99	+	482	927	23204	23649
UN02165	Rsa1.0_06720.1	1379	10473	0	837	886	94	−	470	1349	4306	5191
UN02166	Rsa1.0_00911.1	1392	59943	0	635	636	99	+	3	637	14801	15436
UN02167	Rsa1.0_00341.1	1406	249309	0	1007	1054	95	−	363	1405	209413	210459
UN02168	Rsa1.0_00031.1	1214	179862	0	492	492	100	+	723	1214	72788	73279
UN02169	Rsa1.0_00306.1	973	146760	0	428	459	93	−	533	973	5781	6239
UN02170	Rsa1.0_00325.1	1232	95371	1.00E-105	256	276	92	+	288	563	79215	79487
UN02171	Rsa1.0_02446.1	823	31330	0	662	670	98	−	2	671	3867	4536
UN02172	Rsa1.0_02365.1	670	32118	1.00E-83	222	239	92	+	425	660	16962	17196
UN02173	Rsa1.0_00016.1	931	831256	0	447	463	96	+	3	458	264139	264600
UN02174	Rsa1.0_04243.1	991	18321	0	658	679	96	−	314	990	12991	13668
UN02175	Rsa1.0_00016.1	921	831256	1.00E-167	340	353	96	+	1	350	809657	810009
UN02176	Rsa1.0_00171.1	1218	115622	0	941	977	96	+	2	966	78507	79481
UN02177	Rsa1.0_01182.1	1046	167304	0	503	504	99	+	369	872	165655	166158
UN02178	Rsa1.0_00782.1	958	64468	1.00E-113	298	328	90	−	1	328	53873	54200
UN02179	Rsa1.0_13580.1	688	4711	2.00E-81	166	170	97	−	228	397	4004	4173
UN02180	Rsa1.0_00245.1	1250	103569	0	511	527	96	−	722	1248	100095	100620
UN02181	Rsa1.0_00607.1	1500	73146	0	574	574	100	−	564	1137	21023	21596
UN02182	Rsa1.0_00065.1	495	454616	1.00E-165	315	319	98	−	11	329	448185	448501

UN02183	Rsa1.0_00168.1	485	115870	1.00E-167	356	374	95	+	2	374	90845	91213
UN02184	Rsa1.0_00237.1	945	235974	2.00E-88	202	214	94	−	625	838	4758	4971
UN02185	Rsa1.0_02365.1	1134	32118	0	567	607	93	+	2	607	29263	29863
UN02186	Rsa1.0_00867.1	686	61635	1.00E-104	225	233	96	+	39	270	24840	25071
UN02187	Rsa1.0_05127.1	829	147533	0	828	829	99	−	1	829	8292	9119
UN02188	Rsa1.0_00817.1	1163	62961	0	424	431	98	−	1	431	37547	37977
UN02189	Rsa1.0_00838.1	761	153820	0	573	600	95	−	95	691	32152	32748
UN02190	Rsa1.0_04835.1	1049	15766	0	427	438	97	+	1	438	9141	9578
UN02191	Rsa1.0_00878.1	978	330303	1.00E-116	212	212	100	+	765	976	327480	327691
UN02192	Rsa1.0_00131.1	1229	432233	0	488	513	95	−	79	591	289092	289601
UN02193	Rsa1.0_44391.1	839	507	1.00E-137	322	341	94	−	1	329	1	340
UN02194	Rsa1.0_00773.1	997	64839	1.00E-121	300	322	93	−	660	972	10122	10440
UN02195	Rsa1.0_04085.1	824	19168	7.00E-76	195	205	95	+	10	209	1697	1901
UN02196	Rsa1.0_02012.1	1338	36084	1.00E-149	309	325	95	−	1012	1336	15975	16286
UN02197	Rsa1.0_01674.1	867	168300	0	820	864	94	−	1	864	17121	17984
UN02198	Rsa1.0_04110.1	718	18998	1.00E-111	249	264	94	−	453	716	4839	5102
UN02199	Rsa1.0_05766.1	838	12708	1.00E-152	282	285	98	+	3	287	7055	7339
UN02200	Rsa1.0_00006.1	1406	396176	6.00E-28	137	161	85	−	1089	1249	17231	17391
UN02201	Rsa1.0_00211.1	1007	228189	0	990	1012	97	−	1	1006	168784	169795
UN02202	Rsa1.0_01907.1	841	37431	1.00E-126	259	268	96	−	452	719	22948	23212
UN02203	Rsa1.0_05382.1	1001	13963	0	394	411	95	+	447	857	423	833
UN02204	Rsa1.0_00876.1	1249	61328	0	1023	1079	94	+	1	1073	8255	9332
UN02205	Rsa1.0_03366.1	416	23401	2.00E-65	151	158	95	−	142	299	21234	21389
UN02206	Rsa1.0_00527.1	1027	77733	1.00E-126	284	301	94	+	437	734	58084	58381
UN02207	Rsa1.0_01674.1	826	168300	1.00E-142	283	291	97	+	30	320	3324	3611
UN02208	Rsa1.0_00744.1	683	66084	1.00E-157	367	390	94	−	1	374	53238	53625
UN02209	Rsa1.0_00043.1	832	264937	0	788	822	95	+	12	830	88499	89313
UN02210	Rsa1.0_00111.1	1078	390088	1.00E-138	253	254	99	+	723	976	349143	349396
UN02211	Rsa1.0_00018.1	1269	410899	0	898	940	95	+	1	936	79091	80027
UN02212	Rsa1.0_00517.1	826	142790	3.00E-81	208	224	92	−	606	825	113495	113713
UN02213	Rsa1.0_01294.1	1280	49090	1.00E-172	356	371	95	+	10	379	42100	42470
UN02214	Rsa1.0_02969.1	1126	26390	0	498	532	93	−	5	523	6035	6560
UN02215	Rsa1.0_00030.1	974	250988	0	504	549	91	+	234	768	212998	213530
UN02216	Rsa1.0_00706.1	1269	291570	0	1221	1262	96	+	6	1267	60183	61440
UN02217	Rsa1.0_00505.1	1206	79323	1.00E-118	282	298	94	−	2	297	76389	76680
UN02218	Rsa1.0_07450.1	1062	9373	0	497	543	91	−	1	532	2274	2813
UN02219	Rsa1.0_07213.1	869	9711	0	837	864	96	+	12	869	4293	5156
UN02220	Rsa1.0_00257.1	828	102407	1.00E-171	367	388	94	−	5	392	57801	58188
UN02221	Rsa1.0_04323.1	1059	17915	1.00E-99	260	281	92	+	9	287	13905	14184

UN02222	Rsa1.0_01371.1	1241	47079	0	865	960	90	−	28	985	42892	43850
UN02223	Rsa1.0_01563.1	913	170823	1.00E−119	241	249	96	−	376	624	38793	39041
UN02224	Rsa1.0_00253.1	1081	231292	0	404	419	96	+	55	473	204548	204960
UN02225	Rsa1.0_04224.1	906	18402	1.00E−168	334	343	97	−	1	342	704	1045
UN02226	Rsa1.0_06762.1	1173	10401	0	535	546	97	+	451	996	4911	5456
UN02227	Rsa1.0_01341.1	1176	47764	0	383	398	96	+	1	398	226	618
UN02228	Rsa1.0_00017.1	361	752458	1.00E−139	333	359	92	+	3	361	369612	369969
UN02229	Rsa1.0_04161.1	590	18788	1.00E−144	268	271	98	−	183	453	9629	9899
UN02230	Rsa1.0_01375.1	854	47038	2.00E−94	199	204	97	+	113	316	11241	11442
UN02231	Rsa1.0_02586.1	793	29856	0	661	675	97	−	62	736	15528	16201
UN02232	Rsa1.0_06073.1	1337	11807	0	647	664	97	−	12	675	4362	5024
UN02233	Rsa1.0_74475.1	1479	308	1.00E−114	266	285	93	+	5	285	30	308
UN02234	Rsa1.0_00009.1	701	232558	0	684	696	98	+	6	701	130900	131595
UN02235	Rsa1.0_09048.1	746	6683	0	640	670	95	+	76	738	5245	5913
UN02236	Rsa1.0_07909.1	535	8315	1.00E−156	313	324	96	−	209	532	6038	6361
UN02237	Rsa1.0_04411.1	1115	142979	1.00E−145	261	261	100	+	852	1112	137462	137722
UN02238	Rsa1.0_04563.1	590	16837	1.00E−152	327	344	95	+	1	344	1547	1889
UN02239	Rsa1.0_04651.1	963	16516	1.00E−101	206	212	97	+	708	919	14952	15163
UN02240	Rsa1.0_00301.1	642	365315	0	468	490	95	−	123	612	27314	27803
UN02241	Rsa1.0_01236.1	607	50156	1.00E−101	279	309	90	−	7	297	1967	2275
UN02242	Rsa1.0_03290.1	1271	24011	1.00E−176	351	363	96	+	3	362	7193	7555
UN02243	Rsa1.0_00283.1	1076	183797	1.00E−122	334	376	88	−	720	1074	10947	11313
UN02244	Rsa1.0_00079.1	1270	606987	0	770	771	99	+	500	1270	4648	5418
UN02245	Rsa1.0_02871.1	761	27085	3.00E−99	187	188	99	−	227	414	17885	18072
UN02246	Rsa1.0_00469.1	625	82084	0	494	506	97	+	35	539	20501	21002
UN02247	Rsa1.0_04455.1	1214	17266	1.00E−166	392	419	93	+	805	1212	12968	13386
UN02248	Rsa1.0_00187.1	1585	393093	0	448	456	98	−	1134	1585	155736	156191
UN02249	Rsa1.0_00526.1	562	169279	1.00E−149	321	339	94	−	224	562	148192	148530
UN02250	Rsa1.0_05653.1	1062	13043	0	982	1048	93	+	16	1060	11416	12437
UN02251	Rsa1.0_00633.1	948	71250	0	524	524	100	−	1	524	24436	24959
UN02252	Rsa1.0_08154.1	609	7823	1.00E−92	194	201	96	−	255	455	5891	6091
UN02253	Rsa1.0_01265.1	1165	49686	0	979	1124	87	+	5	1120	36157	37250
UN02254	Rsa1.0_00377.1	1034	90038	1.00E−148	332	353	94	+	121	473	46253	46603
UN02255	Rsa1.0_00219.1	721	106983	1.00E−110	243	254	95	+	1	252	29610	29862
UN02256	Rsa1.0_00342.1	1415	93695	0	686	736	93	+	11	732	12438	13164
UN02257	Rsa1.0_00344.1	815	191774	0	746	812	91	+	20	810	128705	129513
UN02258	Rsa1.0_14565.1	674	3261	4.00E−18	164	199	82	−	414	610	1394	1585
UN02259	Rsa1.0_00855.1	454	62047	0	434	453	95	−	1	453	42812	43250
UN02260	Rsa1.0_00376.1	925	90101	0	323	323	100	+	603	925	34364	34686

UN02261	Rsa1.0_00065.1	920	454616	1.00E-130	296	312	94	-	1	309	414187	414497
UN02262	Rsa1.0_03244.1	937	24413	0	412	439	93	-	500	926	10841	11276
UN02263	Rsa1.0_06102.1	1395	11740	1.00E-118	264	280	94	+	452	720	5202	5481
UN02264	Rsa1.0_00111.1	1670	390088	0	921	984	93	-	248	1212	56276	57249
UN02265	Rsa1.0_15326.1	738	2683	0	678	680	99	+	14	693	659	1338
UN02266	Rsa1.0_09314.1	1036	6461	0	603	629	95	-	412	1036	4153	4776
UN02267	Rsa1.0_01143.1	777	52456	0	387	392	98	-	1	392	6081	6472
UN02268	Rsa1.0_00556.1	785	207212	0	352	360	97	-	337	696	52529	52888
UN02269	Rsa1.0_00616.1	933	195713	0	346	351	98	-	448	798	158877	159227
UN02270	Rsa1.0_07208.1	1237	9719	0	389	393	98	-	845	1237	7601	7993
UN02271	Rsa1.0_00387.1	1290	495407	0	688	718	95	+	573	1287	377227	377943
UN02272	Rsa1.0_02660.1	988	28969	0	459	464	98	-	1	464	26558	27021
UN02273	Rsa1.0_00064.1	686	240927	1.00E-124	264	272	97	-	1	266	60195	60466
UN02274	Rsa1.0_76117.1	608	301	1.00E-113	264	279	94	+	1	271	24	301
UN02275	Rsa1.0_00179.1	799	393324	0	481	489	98	+	302	790	360380	360868
UN02276	Rsa1.0_00446.1	921	167829	0	611	630	96	+	4	620	104797	105424
UN02277	Rsa1.0_01300.1	717	48942	1.00E-131	274	285	96	+	13	297	47254	47536
UN02278	Rsa1.0_24087.1	1030	1008	0	502	544	92	+	494	1028	238	781
UN02279	Rsa1.0_02939.1	1115	26595	1.00E-114	219	222	98	-	681	902	3410	3631
UN02280	Rsa1.0_18568.1	1185	1573	8.00E-92	256	285	89	-	817	1084	453	736
UN02281	Rsa1.0_03241.1	1116	24423	0	924	1014	91	+	43	1041	5633	6646
UN02282	Rsa1.0_10032.1	890	6032	0	490	490	100	-	1	490	4495	4984
UN02283	Rsa1.0_01411.1	909	46326	0	383	397	96	+	2	398	6377	6771
UN02284	Rsa1.0_09091.1	796	6656	0	783	797	98	-	1	796	2338	3134
UN02285	Rsa1.0_02210.1	1303	33857	0	460	489	94	-	837	1303	22536	23023
UN02286	Rsa1.0_00446.1	1179	167829	2.00E-61	121	121	100	-	724	844	159190	159310
UN02287	Rsa1.0_00178.1	983	114861	0	936	988	94	+	1	980	82214	83200
UN02288	Rsa1.0_00007.1	971	239325	2.00E-70	160	167	95	-	687	853	12607	12771
UN02289	Rsa1.0_00096.1	1606	198773	0	786	787	99	-	737	1523	117615	118401
UN02290	Rsa1.0_00010.1	1025	294827	1.00E-165	447	491	91	-	537	1024	81154	81627
UN02291	Rsa1.0_00453.1	1355	83031	1.00E-127	274	286	95	-	1069	1353	79184	79466
UN02292	Rsa1.0_00070.1	864	150321	0	443	448	98	-	14	461	55838	56285
UN02293	Rsa1.0_01945.1	1083	36989	1.00E-169	359	373	96	-	1	368	6140	6507
UN02294	Rsa1.0_37056.1	1146	608	0	390	406	96	-	748	1144	34	439
UN02295	Rsa1.0_02937.1	937	26605	1.00E-159	310	317	97	-	61	377	26140	26455
UN02296	Rsa1.0_07693.1	890	8776	0	367	376	97	+	206	581	7062	7437
UN02297	Rsa1.0_02622.1	1162	142402	0	647	669	96	-	3	670	9528	10196
UN02298	Rsa1.0_00476.1	463	280966	1.00E-111	287	311	92	+	5	312	236919	237223
UN02299	Rsa1.0_00470.1	657	81968	1.00E-159	359	381	94	+	1	381	27995	28369

UN02300	Rsa1.0_01987.1	947	36446	0	895	949	94	−	5	945	14414	15341
UN02301	Rsa1.0_00419.1	1085	86562	1.00E−148	321	338	94	−	1	333	53124	53460
UN02302	Rsa1.0_00079.1	536	606987	1.00E−135	341	370	92	+	11	370	83974	84338
UN02303	Rsa1.0_03566.1	1072	22050	0	605	628	96	−	2	621	7757	8382
UN02304	Rsa1.0_13204.1	891	4970	1.00E−161	321	330	97	−	3	331	777	1103
UN02305	Rsa1.0_00571.1	970	329018	0	488	500	97	−	469	968	156814	157313
UN02306	Rsa1.0_03016.1	406	26000	1.00E−177	386	408	94	+	1	404	5053	5460
UN02307	Rsa1.0_01046.1	1018	152989	1.00E−160	311	319	97	+	213	531	127932	128250
UN02308	Rsa1.0_01679.1	1021	146876	4.00E−87	230	249	92	+	80	323	135000	135247
UN02309	Rsa1.0_02456.1	706	31222	9.00E−78	218	237	91	+	21	249	23772	24004
UN02310	Rsa1.0_02400.1	738	31750	1.00E−132	268	276	97	−	253	527	4467	4742
UN02311	Rsa1.0_02922.1	1370	26753	0	1198	1215	98	−	1	1208	1658	2872
UN02312	Rsa1.0_03149.1	448	24983	1.00E−117	256	270	94	+	1	270	15431	15700
UN02313	Rsa1.0_14292.1	1355	3569	0	486	500	97	−	832	1331	1	500
UN02314	Rsa1.0_00227.1	991	105849	1.00E−178	327	329	99	+	661	989	2883	3210
UN02315	Rsa1.0_00341.1	779	249309	1.00E−106	218	224	97	−	541	763	122671	122894
UN02316	Rsa1.0_01357.1	987	47280	1.00E−142	272	277	98	−	706	982	2515	2791
UN02317	Rsa1.0_06701.1	1054	10514	0	508	526	96	−	466	986	707	1232
UN02318	Rsa1.0_01453.1	990	45449	1.00E−110	318	361	88	−	650	981	25082	25442
UN02319	Rsa1.0_01042.1	941	293264	0	532	557	95	+	364	917	141476	142031
UN02320	Rsa1.0_00094.1	1177	139042	1.00E−175	354	364	97	−	1	363	5876	6237
UN02321	Rsa1.0_00642.1	764	201230	1.00E−107	213	218	97	+	48	265	196262	196479
UN02322	Rsa1.0_02444.1	1482	31350	0	1446	1481	97	−	5	1479	21304	22778
UN02323	Rsa1.0_71705.1	1079	320	4.00E−84	226	245	92	+	1	235	80	319
UN02324	Rsa1.0_00197.1	1384	109714	0	860	893	96	−	491	1382	22224	23109
UN02325	Rsa1.0_00016.1	1214	831256	1.00E−139	306	321	95	+	157	475	353893	354210
UN02326	Rsa1.0_06955.1	811	10111	0	381	391	97	+	230	620	7836	8226
UN02327	Rsa1.0_00002.1	1362	601661	0	872	908	96	+	445	1349	145814	146721
UN02328	Rsa1.0_02070.1	1238	158516	0	622	641	97	+	3	636	8444	9081
UN02329	Rsa1.0_00265.1	878	220635	1.00E−105	194	194	100	+	504	697	147088	147281
UN02330	Rsa1.0_13580.1	791	4711	3.00E−84	168	171	98	−	619	789	2294	2464
UN02331	Rsa1.0_02400.1	899	31750	0	675	724	93	−	19	711	4467	5190
UN02332	Rsa1.0_11795.1	926	5406	1.00E−110	235	246	95	−	246	491	5051	5296
UN02333	Rsa1.0_02041.1	548	163634	0	424	434	97	−	115	548	157407	157838
UN02334	Rsa1.0_01123.1	893	53359	0	461	486	94	−	42	526	21946	22422
UN02335	Rsa1.0_00131.1	1174	432233	0	393	396	99	+	360	755	194264	194659
UN02336	Rsa1.0_00971.1	1049	57973	0	676	703	96	+	352	1047	14832	15533
UN02337	Rsa1.0_00007.1	1310	239325	0	554	580	95	+	733	1309	58424	58999
UN02338	Rsa1.0_01819.1	1604	38834	1.00E−134	247	248	99	−	1357	1604	26434	26681

UN02339	Rsa1.0_00657.1	1236	69858	1.00E-157	307	313	98	+	925	1236	9457	9768
UN02340	Rsa1.0_04128.1	820	18927	1.00E-80	197	208	94	+	4	208	10980	11186
UN02341	Rsa1.0_00181.1	1967	114269	0	961	990	97	−	6	994	94058	95036
UN02342	Rsa1.0_00017.1	887	752458	1.00E-173	361	377	95	+	5	380	40792	41168
UN02343	Rsa1.0_01245.1	1363	149868	0	539	578	93	−	3	577	37644	38206
UN02344	Rsa1.0_03880.1	1213	20293	1.00E-136	307	322	95	+	816	1132	19678	19999
UN02345	Rsa1.0_01405.1	811	46445	1.00E-103	205	210	97	−	219	428	39329	39538
UN02346	Rsa1.0_04456.1	709	17260	7.00E-91	213	225	94	+	426	649	7706	7928
UN02347	Rsa1.0_03069.1	1256	25615	0	539	568	94	−	355	921	18568	19131
UN02348	Rsa1.0_03490.1	1177	22545	0	681	706	96	−	149	853	13983	14688
UN02349	Rsa1.0_00108.1	925	478491	1.00E-65	140	144	97	−	607	750	81949	82092
UN02350	Rsa1.0_02246.1	891	33435	0	861	894	96	−	1	891	8821	9705
UN02351	Rsa1.0_01405.1	722	46445	0	699	727	96	−	3	721	14749	15472
UN02352	Rsa1.0_02222.1	795	152798	1.00E-108	260	279	93	−	14	292	124886	125157
UN02353	Rsa1.0_01270.1	1103	49574	0	535	549	97	−	5	553	29988	30536
UN02354	Rsa1.0_01536.1	1071	43747	0	757	802	94	−	289	1071	31883	32683
UN02355	Rsa1.0_01016.1	1051	181422	0	502	508	98	+	358	863	18736	19243
UN02356	Rsa1.0_01843.1	722	265131	1.00E-135	289	304	95	+	100	403	9831	10134
UN02357	Rsa1.0_00394.1	801	148426	1.00E-131	326	353	92	−	3	344	137048	137399
UN02358	Rsa1.0_01104.1	977	285480	0	504	512	98	+	442	952	14355	14866
UN02359	Rsa1.0_00303.1	1454	97468	0	518	558	92	+	911	1454	30230	30776
UN02360	Rsa1.0_01633.1	1220	41868	0	715	763	93	+	11	764	18063	18813
UN02361	Rsa1.0_00758.1	636	65477	0	589	596	98	−	3	598	36765	37359
UN02362	Rsa1.0_01531.1	1421	43778	0	515	531	96	+	883	1413	12110	12639
UN02363	Rsa1.0_00196.1	1123	109960	1.00E-179	373	389	95	+	736	1123	77908	78294
UN02364	Rsa1.0_03813.1	1031	20708	6.00E-83	193	204	94	+	3	206	170	371
UN02365	Rsa1.0_10936.1	712	5671	1.00E-110	211	214	98	+	322	535	5458	5671
UN02366	Rsa1.0_01561.1	832	43227	0	527	546	96	−	4	549	20971	21516
UN02367	Rsa1.0_01675.1	1537	41155	1.00E-161	327	338	96	−	2	336	9381	9717
UN02368	Rsa1.0_00055.1	1173	520193	0	686	720	95	−	459	1173	385953	386668
UN02369	#	#	#	#	#	#	#	#	#	#	#	#
UN02370	Rsa1.0_03408.1	966	23154	0	672	694	96	+	272	965	12750	13442
UN02371	Rsa1.0_00034.1	1433	260119	1.00E-165	332	343	96	+	240	581	233596	233938
UN02372	Rsa1.0_01416.1	1080	46262	0	1046	1084	96	+	3	1080	44990	46072
UN02373	Rsa1.0_04040.1	1145	19439	0	402	415	96	−	2	414	9830	10244
UN02374	Rsa1.0_02079.1	953	35306	1.00E-138	268	274	97	−	611	884	21522	21795
UN02375	Rsa1.0_04886.1	1742	15597	0	1106	1111	99	+	73	1182	6460	7570
UN02376	Rsa1.0_00031.1	1018	179862	0	483	504	95	−	94	597	82448	82951
UN02377	Rsa1.0_01959.1	420	36798	0	417	418	99	−	3	420	31362	31779

UN02378	Rsa1.0_01433.1	1426	45976	0	520	569	91	−	325	893	6422	6990
UN02379	Rsa1.0_01214.1	1272	50599	1.00E−158	389	420	92	+	868	1272	46274	46685
UN02380	Rsa1.0_02831.1	1116	27533	0	906	916	98	+	37	950	13241	14156
UN02381	Rsa1.0_00008.1	628	358124	1.00E−101	259	282	91	+	276	554	29181	29462
UN02382	Rsa1.0_00724.1	806	66789	0	600	625	96	+	2	613	4424	5045
UN02383	Rsa1.0_00583.1	741	74309	1.00E−168	312	316	98	−	281	596	13328	13643
UN02384	Rsa1.0_02722.1	893	28417	4.00E−90	211	225	93	+	669	893	16664	16888
UN02385	Rsa1.0_07440.1	1309	9389	0	1193	1259	94	+	32	1285	4094	5348
UN02386	Rsa1.0_02831.1	978	27533	0	554	574	96	+	405	978	13977	14548
UN02387	Rsa1.0_13713.1	934	4437	1.00E−170	319	324	98	+	611	934	1	319
UN02388	Rsa1.0_00142.1	743	121474	1.00E−140	261	264	98	−	140	403	64267	64530
UN02389	Rsa1.0_00034.1	723	260119	0	632	655	96	−	69	723	32921	33569
UN02390	Rsa1.0_00502.1	779	204180	0	529	550	96	−	231	779	181101	181649
UN02391	Rsa1.0_00179.1	829	393324	6.00E−70	162	171	94	+	305	475	210557	210727
UN02392	Rsa1.0_00044.1	1087	271658	0	479	513	93	−	590	1087	68722	69225
UN02393	Rsa1.0_00332.1	1182	94575	0	546	558	97	+	625	1182	37359	37915
UN02394	Rsa1.0_00123.1	767	325650	1.00E−127	285	298	95	+	470	766	153387	153680
UN02395	Rsa1.0_03672.1	1774	21452	1.00E−115	229	235	97	+	3	237	4200	4434
UN02396	Rsa1.0_28582.1	762	810	2.00E−97	181	181	100	−	49	229	630	810
UN02397	Rsa1.0_00020.1	743	283099	0	715	738	96	+	3	737	197113	197847
UN02398	Rsa1.0_02871.1	479	27085	7.00E−90	180	184	97	+	39	222	17889	18072
UN02399	Rsa1.0_04563.1	1275	16837	0	654	673	97	−	258	930	1988	2660
UN02400	Rsa1.0_00183.1	1354	113277	0	1288	1367	94	−	2	1352	96744	98100
UN02401	Rsa1.0_01991.1	560	36425	2.00E−50	147	163	90	−	76	229	8629	8787
UN02402	Rsa1.0_00979.1	1232	57733	3.00E−97	251	275	91	−	84	346	41925	42199
UN02403	Rsa1.0_09645.1	846	6234	1.00E−108	248	258	96	+	1	255	5803	6056
UN02404	Rsa1.0_00454.1	793	216970	1.00E−132	326	349	93	−	454	792	184249	184595
UN02405	Rsa1.0_02043.1	306	163754	8.00E−61	177	195	90	+	3	196	136195	136374
UN02406	Rsa1.0_09270.1	799	6513	0	748	800	93	−	6	792	5397	6177
UN02407	Rsa1.0_07616.1	573	9016	0	538	572	94	−	1	570	5317	5870
UN02408	Rsa1.0_05168.1	1316	14621	0	653	689	94	−	90	773	8801	9486
UN02409	Rsa1.0_00131.1	798	432233	1.00E−125	282	298	94	+	503	797	409494	409791
UN02410	Rsa1.0_25015.1	1235	956	0	471	485	97	−	409	893	475	956
UN02411	Rsa1.0_05271.1	715	14306	1.00E−108	217	223	97	+	359	581	14071	14293
UN02412	Rsa1.0_01559.1	696	43262	1.00E−139	281	291	96	+	392	682	2268	2558
UN02413	Rsa1.0_02353.1	806	32300	1.00E−159	312	321	97	−	239	559	31549	31869
UN02414	Rsa1.0_10792.1	752	5719	1.00E−107	213	217	98	−	304	520	2743	2958
UN02415	Rsa1.0_01580.1	1256	42852	1.00E−149	305	314	97	+	945	1253	37442	37755
UN02416	Rsa1.0_02946.1	1495	26537	0	918	938	97	−	74	1010	857	1790

UN02417	Rsa1.0_01516.1	710	44196	6.00E-79	156	158	98	-	5	162	26319	26476
UN02418	Rsa1.0_01273.1	955	49464	0	439	439	100	-	465	903	41505	41943
UN02419	Rsa1.0_16547.1	547	2117	0	438	473	92	+	58	529	973	1445
UN02420	Rsa1.0_00059.1	1560	159650	0	1317	1330	99	-	231	1560	38603	39932
UN02421	Rsa1.0_15326.1	871	2683	0	706	727	97	-	145	866	611	1334
UN02422	Rsa1.0_07877.1	1047	8373	3.00E-72	148	151	98	-	474	624	5171	5321
UN02423	Rsa1.0_00790.1	776	148693	1.00E-157	343	362	94	-	1	348	58714	59073
UN02424	Rsa1.0_01967.1	1119	36672	0	555	568	97	-	513	1080	24386	24953
UN02425	Rsa1.0_05900.1	1234	12295	1.00E-163	359	377	95	-	3	370	5008	5381
UN02426	Rsa1.0_08961.1	814	6750	1.00E-108	233	244	95	-	13	256	5289	5532
UN02427	Rsa1.0_00403.1	848	87701	0	432	447	96	-	3	449	44934	45371
UN02428	Rsa1.0_03783.1	1133	20890	1.00E-150	316	326	96	-	804	1128	17825	18147
UN02429	Rsa1.0_01169.1	612	51829	0	514	528	97	+	4	531	23073	23598
UN02430	Rsa1.0_00145.1	812	261008	0	493	508	97	+	304	806	244127	244628
UN02431	Rsa1.0_00571.1	1146	329018	0	1108	1170	94	-	1	1145	173584	174742
UN02432	Rsa1.0_01434.1	1637	45951	0	450	474	94	-	16	483	39461	39934
UN02433	Rsa1.0_02812.1	1109	27660	0	364	374	97	-	3	376	16559	16931
UN02434	Rsa1.0_02846.1	1200	27338	0	822	860	95	-	235	1094	20814	21673
UN02435	Rsa1.0_05352.1	889	14079	1.00E-175	318	320	99	+	63	382	1030	1349
UN02436	Rsa1.0_00451.1	800	322533	1.00E-104	202	205	98	-	216	420	23958	24162
UN02437	Rsa1.0_01203.1	1162	50848	1.00E-110	220	226	97	+	608	833	39552	39777
UN02438	Rsa1.0_00487.1	1175	80674	0	672	673	99	+	503	1175	24259	24930
UN02439	Rsa1.0_01570.1	832	43015	2.00E-57	123	126	97	-	424	549	12707	12832
UN02440	Rsa1.0_00977.1	552	198001	0	406	429	94	+	79	507	50790	51216
UN02441	Rsa1.0_01789.1	937	39250	0	580	581	99	-	357	937	6028	6607
UN02442	Rsa1.0_02045.1	596	35650	6.00E-97	186	188	98	-	208	395	23658	23845
UN02443	Rsa1.0_01015.1	691	56752	0	466	505	92	-	25	518	45893	46396
UN02444	Rsa1.0_00129.1	908	126831	1.00E-144	301	310	97	+	2	306	113574	113883
UN02445	Rsa1.0_01169.1	1126	51829	0	502	510	98	-	618	1126	49042	49551
UN02446	Rsa1.0_07914.1	775	8294	0	717	781	91	+	8	775	472	1250
UN02447	Rsa1.0_16978.1	973	1964	3.00E-81	163	166	98	+	330	495	206	371
UN02448	Rsa1.0_01371.1	775	47079	0	676	681	99	-	97	775	43553	44233
UN02449	Rsa1.0_00301.1	1245	365315	1.00E-154	305	313	97	-	463	774	276755	277067
UN02450	Rsa1.0_01140.1	850	52586	7.00E-76	145	145	100	+	4	148	3354	3498
UN02451	Rsa1.0_00963.1	601	58176	0	584	601	97	-	1	601	776	1367
UN02452	Rsa1.0_01144.1	1342	52431	1.00E-149	322	339	94	+	54	389	22927	23265
UN02453	Rsa1.0_25755.1	665	922	0	554	562	98	+	100	661	1	562
UN02454	Rsa1.0_00190.1	1208	318240	0	381	390	97	+	32	421	116678	117067
UN02455	Rsa1.0_00600.1	919	73395	0	807	887	90	-	43	917	9873	10759

UN02456	Rsa1.0_00144.1	1012	120708	0	617	637	96	−	323	957	47699	48335
UN02457	Rsa1.0_02896.1	721	26970	1.00E−167	487	555	87	+	3	530	11184	11720
UN02458	Rsa1.0_00002.1	951	601661	1.00E−93	184	187	98	+	505	691	255311	255497
UN02459	Rsa1.0_02499.1	940	30774	1.00E−114	235	242	97	−	624	864	1181	1422
UN02460	Rsa1.0_02611.1	1346	29586	1.00E−118	308	338	91	−	1012	1346	19152	19473
UN02461	Rsa1.0_01146.1	664	163455	3.00E−90	220	234	94	−	433	664	41256	41485
UN02462	Rsa1.0_02043.1	1306	163754	1.00E−164	338	353	95	+	1	353	140832	141184
UN02463	Rsa1.0_00050.1	817	237174	1.00E−168	306	308	99	−	510	817	188989	189296
UN02464	Rsa1.0_00003.1	785	340054	1.00E−106	201	203	99	+	219	421	85507	85709
UN02465	Rsa1.0_00955.1	1355	58358	0	505	510	99	−	846	1355	8320	8826
UN02466	Rsa1.0_00324.1	964	218805	1.00E−141	314	331	94	+	633	963	182790	183113
UN02467	Rsa1.0_00007.1	1006	239325	1.00E−159	347	365	95	+	642	1004	13416	13780
UN02468	Rsa1.0_00684.1	1382	141701	0	1002	1043	96	−	2	1044	9100	10142
UN02469	Rsa1.0_05482.1	1022	13595	1.00E−161	328	341	96	−	199	539	12266	12606
UN02470	Rsa1.0_00093.1	1357	214962	0	368	377	97	+	395	771	133459	133835
UN02471	Rsa1.0_00748.1	884	65983	0	497	544	91	−	3	542	63996	64525
UN02472	Rsa1.0_01371.1	733	47079	0	713	737	96	+	3	732	30775	31511
UN02473	Rsa1.0_04118.1	777	18968	0	564	566	99	−	212	777	15468	16033
UN02474	Rsa1.0_03994.1	1448	19659	0	351	357	98	+	1091	1446	1755	2110
UN02475	Rsa1.0_00419.1	1108	86562	1.00E−121	315	350	90	+	766	1108	53124	53454
UN02476	Rsa1.0_03795.1	1035	20818	1.00E−115	263	274	95	+	756	1027	15481	15750
UN02477	Rsa1.0_00280.1	1479	99966	0	468	470	99	−	1010	1479	15032	15501
UN02478	Rsa1.0_00243.1	927	103651	0	768	807	95	−	5	808	79074	79880
UN02479	Rsa1.0_04323.1	1086	17915	1.00E−142	284	293	96	−	794	1086	13892	14184
UN02480	Rsa1.0_00803.1	1140	144228	1.00E−173	341	351	97	+	790	1140	15466	15813
UN02481	Rsa1.0_00653.1	717	70029	1.00E−173	357	372	95	−	309	680	37130	37500
UN02482	Rsa1.0_00281.1	1101	99908	1.00E−151	316	328	96	+	775	1101	6793	7119
UN02483	Rsa1.0_00311.1	1296	96847	0	507	519	97	−	7	523	35682	36200
UN02484	Rsa1.0_00122.1	849	265937	0	473	477	99	−	373	849	49369	49845
UN02485	Rsa1.0_00128.1	817	228675	0	745	764	97	−	45	806	203869	204627
UN02486	Rsa1.0_00623.1	1283	207630	0	850	888	95	−	340	1212	152309	153196
UN02487	Rsa1.0_00697.1	1112	67913	1.00E−159	307	313	98	+	617	928	58544	58856
UN02488	Rsa1.0_09946.1	955	6076	1.00E−178	316	316	100	+	3	318	493	808
UN02489	Rsa1.0_00021.1	855	263655	1.00E−169	326	333	97	+	209	540	179134	179466
UN02490	Rsa1.0_19149.1	791	1472	1.00E−117	281	309	90	−	235	521	257	565
UN02491	Rsa1.0_00642.1	1291	201230	1.00E−173	415	452	91	−	840	1268	62521	62971
UN02492	Rsa1.0_01060.1	850	55368	1.00E−162	315	322	97	−	8	328	50250	50571
UN02493	Rsa1.0_04561.1	677	16849	3.00E−65	136	139	97	+	147	285	9223	9361
UN02494	Rsa1.0_01184.1	1135	51452	1.00E−179	378	400	94	+	741	1135	50577	50965

UN02495	Rsa1.0_00326.1	1054	171955	1.00E-105	238	251	94	-	532	777	62166	62416
UN02496	Rsa1.0_03572.1	574	146346	1.00E-70	207	231	89	-	69	293	137177	137407
UN02497	Rsa1.0_00032.1	931	306326	0	896	956	93	+	1	931	144544	145489
UN02498	Rsa1.0_00699.1	1238	184398	1.00E-163	328	339	96	+	886	1224	11508	11844
UN02499	Rsa1.0_00028.1	1427	293984	1.00E-60	126	128	98	-	907	1034	21632	21759
UN02500	Rsa1.0_00664.1	1006	69573	0	387	398	97	-	608	1004	16704	17101
UN02501	Rsa1.0_00086.1	685	439854	1.00E-148	307	320	95	-	366	685	296403	296719
UN02502	Rsa1.0_04622.1	1281	16618	0	614	638	96	-	644	1281	13865	14502
UN02503	Rsa1.0_01244.1	1262	50061	0	498	499	99	-	759	1257	4518	5016
UN02504	Rsa1.0_03953.1	1025	19889	0	795	831	95	-	209	1023	6197	7021
UN02505	Rsa1.0_00471.1	832	81897	0	384	395	97	-	3	396	4146	4539
UN02506	Rsa1.0_01422.1	1155	46172	1.00E-172	328	334	98	+	814	1147	28595	28926
UN02507	Rsa1.0_20596.1	797	1289	1.00E-175	330	335	98	+	325	659	12	345
UN02508	Rsa1.0_00301.1	1139	365315	0	489	513	95	+	604	1116	304665	305171
UN02509	Rsa1.0_00163.1	1443	116847	0	1172	1218	96	-	226	1443	7919	9130
UN02510	Rsa1.0_37892.1	1578	595	1.00E-116	213	213	100	-	473	685	374	586
UN02511	Rsa1.0_00045.1	997	328694	0	700	705	99	+	293	997	229947	230651
UN02512	Rsa1.0_14656.1	875	3183	1.00E-122	261	274	95	-	592	865	1368	1641
UN02513	Rsa1.0_03765.1	1261	20971	0	757	762	99	+	1	762	14012	14773
UN02514	Rsa1.0_01464.1	1043	45193	3.00E-94	214	226	94	-	623	848	16256	16478
UN02515	Rsa1.0_00162.1	1266	239935	0	707	751	94	+	1	735	38536	39286
UN02516	Rsa1.0_03613.1	801	21773	1.00E-134	281	292	96	+	1	291	16649	16932
UN02517	Rsa1.0_01833.1	992	38555	1.00E-107	211	214	98	-	76	289	16525	16737
UN02518	Rsa1.0_07757.1	1038	8633	0	910	971	93	+	46	1011	4136	5094
UN02519	Rsa1.0_01113.1	1125	53616	1.00E-115	263	278	94	-	849	1123	2899	3171
UN02520	Rsa1.0_00286.1	1232	98709	1.00E-159	425	469	90	-	9	450	26258	26726
UN02521	Rsa1.0_00944.1	791	58733	0	708	766	92	-	1	756	32281	33034
UN02522	Rsa1.0_00020.1	482	283099	0	466	482	96	-	1	482	74904	75385
UN02523	Rsa1.0_00544.1	684	76787	1.00E-135	244	244	100	+	1	244	57829	58072
UN02524	Rsa1.0_02157.1	1432	149652	0	531	533	99	-	901	1432	130569	131101
UN02525	Rsa1.0_00039.1	1240	296927	0	639	650	98	+	1	650	254868	255508
UN02526	Rsa1.0_00017.1	1184	752458	0	1030	1064	96	-	1	1063	317267	318329
UN02527	Rsa1.0_00353.1	842	92242	1.00E-58	119	120	99	+	243	362	56357	56476
UN02528	Rsa1.0_00046.1	770	320315	1.00E-159	369	396	93	-	1	374	29574	29969
UN02529	Rsa1.0_00275.1	650	210104	0	554	602	92	+	53	650	189716	190316
UN02530	Rsa1.0_00318.1	752	96094	0	443	447	99	-	209	655	20836	21282
UN02531	Rsa1.0_00243.1	1328	103651	0	1224	1281	95	-	3	1281	85850	87116
UN02532	Rsa1.0_00306.1	1214	146760	3.00E-82	198	212	93	-	626	837	101654	101865
UN02533	Rsa1.0_00170.1	402	179538	1.00E-118	261	276	94	+	1	276	20767	21038

UN02534	Rsa1.0_03955.1	968	19884	1.00E-164	324	335	96	+	1	327	5792	6126
UN02535	Rsa1.0_02955.1	729	26484	1.00E-120	280	298	93	−	1	290	21585	21882
UN02536	Rsa1.0_16489.1	996	2137	0	915	921	99	+	75	993	7	927
UN02537	Rsa1.0_02648.1	810	29073	1.00E-172	340	350	97	+	74	423	25877	26225
UN02538	Rsa1.0_02157.1	535	149652	1.00E-180	370	387	95	−	65	451	11588	11963
UN02539	Rsa1.0_01281.1	1388	49313	0	1318	1351	97	−	36	1386	4296	5645
UN02540	Rsa1.0_00816.1	1235	403350	0	668	682	97	+	1	682	139120	139801
UN02541	Rsa1.0_01322.1	301	48166	9.00E-76	238	263	90	+	45	301	28203	28460
UN02542	Rsa1.0_26227.1	1143	900	0	531	551	96	−	212	759	351	900
UN02543	Rsa1.0_07011.1	840	10034	0	511	525	97	−	314	838	9512	10034
UN02544	Rsa1.0_00127.1	1359	127446	1.00E-160	368	390	94	+	1	385	110336	110716
UN02545	Rsa1.0_20596.1	1459	1289	1.00E-173	329	336	97	−	802	1137	12	347
UN02546	Rsa1.0_01818.1	1269	38837	1.00E-175	331	337	98	−	2	338	31247	31580
UN02547	Rsa1.0_01405.1	995	46445	0	574	614	93	+	11	611	32628	33235
UN02548	Rsa1.0_06109.1	1337	11728	0	596	623	95	−	1	620	5921	6539
UN02549	Rsa1.0_04836.1	1335	15765	0	550	585	94	−	5	589	13779	14363
UN02550	Rsa1.0_03299.1	1273	23949	0	631	654	96	+	1	654	15163	15816
UN02551	Rsa1.0_00114.1	716	131330	0	480	488	98	−	3	490	62925	63412
UN02552	Rsa1.0_00065.1	1417	454616	1.00E-112	229	235	97	−	3	237	448185	448418
UN02553	Rsa1.0_04379.1	665	17646	0	550	568	96	+	100	665	4214	4780
UN02554	Rsa1.0_00075.1	623	146680	9.00E-81	197	209	94	+	173	379	29535	29743
UN02555	Rsa1.0_01281.1	896	49313	0	502	553	90	−	1	532	30590	31131
UN02556	Rsa1.0_02321.1	1079	32572	0	712	776	91	−	1	770	20709	21466
UN02557	Rsa1.0_02266.1	794	33195	0	351	355	98	+	1	355	16287	16637
UN02558	Rsa1.0_00706.1	524	291570	1.00E-106	277	305	90	+	4	308	229288	229580
UN02559	Rsa1.0_00568.1	877	75219	1.00E-170	312	315	99	+	8	322	16072	16386
UN02560	Rsa1.0_00273.1	916	326186	0	487	504	96	+	3	506	8093	8595
UN02561	Rsa1.0_00272.1	972	447836	1.00E-171	363	377	96	+	1	371	425350	425723
UN02562	Rsa1.0_00985.1	811	161950	0	768	787	97	+	2	783	27018	27802
UN02563	Rsa1.0_00107.1	987	431659	0	414	418	99	−	88	504	369243	369659
UN02564	Rsa1.0_00636.1	621	71113	0	616	621	99	+	1	621	7710	8329
UN02565	Rsa1.0_00002.1	1238	601661	0	1231	1237	99	−	1	1237	490426	491662
UN02566	Rsa1.0_01959.1	844	36798	0	787	839	93	+	5	836	4352	5187
UN02567	Rsa1.0_05748.1	784	12749	0	399	407	98	+	1	400	6545	6951
UN02568	Rsa1.0_00744.1	682	66084	1.00E-167	377	402	93	−	1	392	53238	53638
UN02569	Rsa1.0_00377.1	1071	90038	0	917	936	97	−	1	936	33637	34569
UN02570	Rsa1.0_01882.1	633	37697	9.00E-84	223	242	92	−	67	302	20903	21138
UN02571	Rsa1.0_09069.1	1422	6671	0	469	480	97	+	415	894	2813	3292
UN02572	Rsa1.0_01696.1	992	301850	0	585	615	95	+	1	615	160677	161284

UN02573	Rsa1.0_00019.1	1033	301669	0	1011	1036	97	−	1	1031	83127	84162
UN02574	Rsa1.0_02789.1	1136	27850	1.00E−129	248	251	98	−	1	250	24794	25044
UN02575	Rsa1.0_00108.1	964	478491	1.00E−104	267	286	93	+	125	402	67382	67663
UN02576	Rsa1.0_10698.1	831	5753	1.00E−120	248	257	96	+	156	412	217	470
UN02577	Rsa1.0_05683.1	1084	12944	0	461	474	97	+	610	1083	4930	5394
UN02578	Rsa1.0_07922.1	1045	8254	2.00E−45	118	126	93	+	211	336	4202	4327
UN02579	Rsa1.0_01103.1	1149	53974	0	480	488	98	−	138	618	37812	38299
UN02580	Rsa1.0_00604.1	1132	146282	0	649	656	98	+	263	916	86846	87500
UN02581	Rsa1.0_06861.1	1160	10246	0	727	729	99	+	1	729	4901	5629
UN02582	Rsa1.0_02157.1	1207	149652	6.00E−31	111	122	90	+	144	265	143782	143901
UN02583	Rsa1.0_02078.1	1325	35308	0	492	508	96	+	817	1324	22705	23212
UN02584	Rsa1.0_01724.1	954	40292	0	485	488	99	+	1	486	356	843
UN02585	Rsa1.0_05229.1	812	14437	0	780	818	95	+	2	808	8166	8983
UN02586	Rsa1.0_00154.1	690	189461	0	497	524	94	−	1	511	171027	171548
UN02587	Rsa1.0_06869.1	838	10234	1.00E−135	251	253	99	+	586	838	448	700
UN02588	Rsa1.0_06169.1	1617	11578	0	675	678	99	+	940	1617	7905	8581
UN02589	Rsa1.0_03695.1	1325	21329	6.00E−99	233	246	94	+	89	332	9883	10125
UN02590	Rsa1.0_00494.1	1304	79967	0	711	720	98	−	326	1045	20834	21553
UN02591	Rsa1.0_01516.1	934	44196	1.00E−160	341	358	95	+	492	848	24091	24448
UN02592	Rsa1.0_10606.1	1145	5787	0	1075	1149	93	+	1	1143	838	1969
UN02593	Rsa1.0_01064.1	1286	55295	0	495	518	95	+	619	1133	51704	52221
UN02594	Rsa1.0_01355.1	1362	47303	0	1257	1398	89	−	1	1361	39411	40772
UN02595	Rsa1.0_00331.1	1339	176754	0	836	878	95	−	477	1336	22215	23092
UN02596	Rsa1.0_00123.1	796	325650	0	594	628	94	−	14	641	240510	241126
UN02597	Rsa1.0_01601.1	1154	42519	1.00E−128	236	237	99	+	5	241	17160	17396
UN02598	Rsa1.0_00015.1	1300	561803	0	404	417	96	−	1	417	371807	372219
UN02599	Rsa1.0_00886.1	871	167350	0	841	877	95	+	2	871	41687	42545
UN02600	Rsa1.0_01343.1	554	47735	1.00E−138	284	293	96	−	15	305	30311	30602
UN02601	Rsa1.0_00015.1	1341	561803	0	1031	1038	99	+	306	1340	254336	255373
UN02602	Rsa1.0_00111.1	867	390088	1.00E−123	331	363	91	−	335	685	55562	55911
UN02603	Rsa1.0_04458.1	1215	17255	1.00E−167	314	319	98	+	666	984	7725	8043
UN02604	Rsa1.0_07606.1	695	9035	0	642	653	98	+	1	650	1974	2623
UN02605	Rsa1.0_00122.1	733	265937	0	700	722	96	−	8	728	30875	31593
UN02606	Rsa1.0_01088.1	1205	54416	0	857	881	97	−	329	1204	7393	8259
UN02607	Rsa1.0_02348.1	787	32316	0	513	530	96	+	255	782	5536	6062
UN02608	Rsa1.0_00644.1	1416	136881	1.00E−178	332	337	98	−	1	337	27149	27485
UN02609	Rsa1.0_00327.1	895	94892	0	463	472	98	+	1	472	24984	25448
UN02610	Rsa1.0_01492.1	914	44704	1.00E−105	249	266	93	−	8	261	35636	35901
UN02611	Rsa1.0_00608.1	1075	172672	0	755	807	93	−	2	787	113434	114240

UN02612	Rsa1.0_00027.1	1022	283362	1.00E-175	327	332	98	−	321	652	99118	99449
UN02613	Rsa1.0_02585.1	1090	29865	0	382	397	96	−	685	1078	7735	8131
UN02614	Rsa1.0_00518.1	1284	78360	0	708	731	96	−	1	729	76155	76882
UN02615	Rsa1.0_09359.1	1419	6427	0	751	769	97	−	650	1418	305	1073
UN02616	Rsa1.0_00504.1	1454	79334	0	1453	1454	99	−	1	1454	70259	71712
UN02617	Rsa1.0_00035.1	1223	176449	0	557	562	99	−	1	562	98646	99207
UN02618	Rsa1.0_01298.1	793	48999	1.00E-127	275	294	93	+	3	296	6696	6971
UN02619	Rsa1.0_00457.1	780	337301	1.00E-155	287	290	98	+	161	450	9577	9866
UN02620	Rsa1.0_04958.1	1292	15327	0	407	409	99	+	1	409	12484	12892
UN02621	Rsa1.0_01450.1	1020	144251	0	523	556	94	+	48	603	41518	42073
UN02622	Rsa1.0_21131.1	1339	1232	1.00E-133	251	254	98	−	1084	1337	228	481
UN02623	Rsa1.0_00660.1	875	69688	1.00E-71	141	142	99	+	502	643	53034	53175
UN02624	Rsa1.0_00306.1	1483	146760	0	748	804	93	−	662	1459	88781	89563
UN02625	Rsa1.0_02606.1	1014	29635	1.00E-105	204	207	98	−	637	843	10487	10693
UN02626	Rsa1.0_01897.1	1419	37499	0	824	865	95	−	483	1338	8386	9249
UN02627	Rsa1.0_01803.1	762	173846	1.00E-175	395	416	94	−	1	413	137460	137869
UN02628	Rsa1.0_03694.1	867	21336	0	748	771	97	−	96	866	20089	20858
UN02629	Rsa1.0_00067.1	757	415055	0	534	536	99	−	16	551	220023	220558
UN02630	Rsa1.0_00341.1	679	249309	4.00E-77	153	155	98	−	377	531	122984	123138
UN02631	Rsa1.0_01537.1	636	187285	1.00E-163	357	375	95	−	1	373	839	1207
UN02632	Rsa1.0_00111.1	230	390088	2.00E-85	200	212	94	+	4	215	45742	45952
UN02633	Rsa1.0_00714.1	1448	67187	9.00E-92	242	262	92	−	1122	1376	13175	13433
UN02634	Rsa1.0_00127.1	1163	127446	0	1003	1087	92	+	1	1068	16351	17408
UN02635	#	#	#	#	#	#	#	#	#	#	#	#
UN02636	Rsa1.0_01184.1	953	51452	0	381	383	99	−	345	727	26949	27331
UN02637	Rsa1.0_02257.1	1159	33318	1.00E-115	229	235	97	+	728	962	21475	21709
UN02638	Rsa1.0_02791.1	1277	27832	0	529	540	97	−	2	539	7700	8234
UN02639	Rsa1.0_01517.1	1181	44170	3.00E-94	218	230	94	+	3	223	25298	25527
UN02640	Rsa1.0_04450.1	771	17297	1.00E-143	288	298	96	−	474	771	12688	12985
UN02641	Rsa1.0_03828.1	1226	20588	0	382	399	95	+	1	392	5984	6381
UN02642	Rsa1.0_01039.1	753	55946	1.00E-147	271	273	99	+	481	753	3862	4134
UN02643	Rsa1.0_03001.1	717	26108	0	482	492	97	+	1	492	11731	12222
UN02644	Rsa1.0_02820.1	1128	27629	0	506	527	96	−	590	1104	19486	20011
UN02645	Rsa1.0_09273.1	1036	6511	7.00E-98	226	239	94	+	790	1024	1960	2197
UN02646	Rsa1.0_00583.1	703	74309	1.00E-165	324	333	97	+	250	579	71950	72282
UN02647	Rsa1.0_04428.1	1123	17399	0	1059	1126	94	+	1	1123	8438	9531
UN02648	Rsa1.0_02270.1	505	249821	0	499	505	98	−	1	505	6888	7392
UN02649	Rsa1.0_00056.1	1211	332604	0	1121	1146	97	+	70	1211	306623	307767
UN02650	Rsa1.0_00376.1	1240	90101	1.00E-118	309	338	91	−	870	1203	2720	3057

UN02651	Rsa1.0_02086.1	860	35198	0	841	861	97	+	1	860	27642	28502
UN02652	Rsa1.0_01099.1	689	158481	1.00E-117	289	312	92	-	1	303	132505	132816
UN02653	Rsa1.0_00332.1	1311	94575	6.00E-68	197	220	89	-	1098	1308	40447	40666
UN02654	Rsa1.0_00060.1	912	502678	1.00E-150	295	302	97	+	1	302	252665	252965
UN02655	Rsa1.0_04741.1	667	16164	0	514	542	94	-	1	541	6532	7070
UN02656	Rsa1.0_01139.1	619	52601	0	381	390	97	-	1	389	50365	50754
UN02657	Rsa1.0_01389.1	744	46716	1.00E-106	258	278	92	+	421	695	13119	13396
UN02658	Rsa1.0_36749.1	892	613	1.00E-168	331	340	97	+	174	512	186	525
UN02659	Rsa1.0_00582.1	818	74333	3.00E-81	157	158	99	-	268	425	18123	18280
UN02660	Rsa1.0_00910.1	1092	59975	0	509	534	95	+	1	532	55819	56351
UN02661	Rsa1.0_02064.1	1017	35476	0	473	492	96	+	1	491	16539	17023
UN02662	Rsa1.0_58913.1	784	388	5.00E-95	198	205	96	+	117	321	26	230
UN02663	#	#	#	#	#	#	#	#	#	#	#	#
UN02664	Rsa1.0_00286.1	1238	98709	0	421	439	95	+	800	1237	26258	26691
UN02665	Rsa1.0_04271.1	923	18148	0	911	922	98	+	2	923	14139	15060
UN02666	Rsa1.0_05847.1	1303	12433	0	432	444	97	-	429	872	2039	2478
UN02667	Rsa1.0_01146.1	631	163455	1.00E-85	204	215	94	+	329	538	162766	162980
UN02668	Rsa1.0_02287.1	982	32986	0	961	981	97	+	1	981	23736	24715
UN02669	Rsa1.0_14741.1	807	3112	0	462	470	98	+	336	805	1341	1810
UN02670	Rsa1.0_00011.1	811	225044	1.00E-175	386	412	93	+	412	809	207085	207496
UN02671	Rsa1.0_00296.1	633	97854	1.00E-132	295	309	95	+	1	303	70450	70757
UN02672	Rsa1.0_01081.1	986	499226	0	884	904	97	+	84	986	42018	42920
UN02673	Rsa1.0_04455.1	1210	17266	0	407	423	96	+	780	1198	12968	13390
UN02674	Rsa1.0_07870.1	979	8384	0	365	369	98	+	168	535	5013	5381
UN02675	Rsa1.0_00311.1	733	96847	1.00E-112	235	245	95	+	87	331	33654	33892
UN02676	Rsa1.0_00111.1	688	390088	0	597	626	95	+	7	624	338227	338850
UN02677	Rsa1.0_01434.1	1374	45951	1.00E-176	338	346	97	-	505	850	28843	29188
UN02678	Rsa1.0_00703.1	983	67597	0	952	981	97	+	3	983	40091	41071
UN02679	Rsa1.0_05230.1	728	14437	7.00E-51	109	111	98	+	339	449	14327	14437
UN02680	Rsa1.0_00552.1	1300	206757	8.00E-92	202	212	95	+	859	1070	187055	187266
UN02681	Rsa1.0_00607.1	963	73146	4.00E-93	235	258	91	+	2	241	69522	69779
UN02682	Rsa1.0_01061.1	1442	55349	1.00E-104	217	223	97	+	6	227	40608	40827
UN02683	Rsa1.0_00549.1	1679	76473	0	547	594	92	+	3	593	23871	24461
UN02684	Rsa1.0_02959.1	1244	26441	0	374	374	100	+	35	408	20224	20597
UN02685	Rsa1.0_01247.1	706	49934	0	671	699	95	+	3	701	24345	25034
UN02686	Rsa1.0_00348.1	1385	92818	0	365	377	96	-	12	380	65410	65786
UN02687	Rsa1.0_00066.1	1410	152418	1.00E-137	372	411	90	+	853	1252	66553	66951
UN02688	Rsa1.0_00236.1	1376	104771	0	643	649	99	+	728	1376	58279	58927
UN02689	Rsa1.0_00251.1	897	303968	0	333	337	98	+	561	897	66488	66824

UN02690	Rsa1.0_00128.1	973	228675	3.00E-57	120	122	98	-	522	643	151246	151367
UN02691	Rsa1.0_11463.1	494	5500	0	345	346	99	+	149	494	258	602
UN02692	Rsa1.0_02991.1	933	26178	1.00E-109	287	309	92	-	619	921	6920	7224
UN02693	Rsa1.0_00867.1	673	61635	1.00E-108	297	329	90	-	3	325	27352	27668
UN02694	Rsa1.0_00077.1	1917	145005	1.00E-141	311	326	95	+	1595	1917	31241	31565
UN02695	Rsa1.0_25195.1	1039	947	1.00E-148	286	292	97	+	745	1036	261	548
UN02696	Rsa1.0_01587.1	1364	42789	0	378	384	98	-	731	1114	29407	29790
UN02697	Rsa1.0_01420.1	1412	46210	1.00E-163	295	296	99	+	3	298	6598	6893
UN02698	Rsa1.0_00745.1	1433	66067	0	490	513	95	+	927	1432	52944	53451
UN02699	Rsa1.0_01306.1	1348	48720	0	710	728	97	-	2	729	3824	4551
UN02700	Rsa1.0_01016.1	873	181422	0	469	497	94	+	378	873	177066	177557
UN02701	Rsa1.0_00512.1	842	200277	0	349	350	99	-	491	840	18455	18804
UN02702	Rsa1.0_00092.1	1106	659932	0	1079	1137	94	-	3	1106	175633	176766
UN02703	Rsa1.0_02506.1	1215	30700	1.00E-165	345	361	95	-	416	773	6452	6812
UN02704	Rsa1.0_09897.1	1063	6100	0	981	1048	93	+	49	1059	3765	4797
UN02705	Rsa1.0_01513.1	878	44234	1.00E-122	337	366	92	-	497	858	17141	17498
UN02706	Rsa1.0_00398.1	1280	88007	1.00E-150	594	697	85	-	8	683	76377	77049
UN02707	Rsa1.0_00844.1	1190	62403	0	530	545	97	-	463	1006	20009	20553
UN02708	Rsa1.0_00218.1	1033	201480	0	441	456	96	-	578	1033	101432	101883
UN02709	Rsa1.0_01958.1	829	261128	0	401	416	96	-	51	463	131033	131446
UN02710	Rsa1.0_00371.1	942	90463	0	367	372	98	-	571	942	73399	73770
UN02711	Rsa1.0_07397.1	1276	9464	0	1021	1025	99	+	241	1263	636	1659
UN02712	Rsa1.0_00305.1	787	97308	1.00E-122	275	289	95	-	500	784	39294	39582
UN02713	Rsa1.0_00077.1	1153	145005	0	1127	1156	97	+	2	1153	129159	130314
UN02714	Rsa1.0_00829.1	678	158305	1.00E-101	206	211	97	+	467	676	40842	41052
UN02715	Rsa1.0_00522.1	779	78103	0	421	435	96	-	29	457	38920	39354
UN02716	Rsa1.0_00300.1	1330	97747	0	554	577	96	+	78	654	81510	82074
UN02717	Rsa1.0_00699.1	697	184398	1.00E-76	302	355	85	+	246	592	127771	128125
UN02718	Rsa1.0_01248.1	699	154103	1.00E-125	234	236	99	+	274	509	49463	49698
UN02719	Rsa1.0_04218.1	633	18423	0	450	482	93	-	164	633	15799	16280
UN02720	Rsa1.0_03125.1	1398	147978	0	389	391	99	-	35	424	143840	144230
UN02721	Rsa1.0_00930.1	802	59224	2.00E-85	201	209	96	+	555	759	48406	48614
UN02722	Rsa1.0_00107.1	1515	431659	1.00E-151	280	283	98	-	516	798	330676	330958
UN02723	Rsa1.0_01037.1	1343	55969	0	577	581	99	+	764	1343	36234	36814
UN02724	Rsa1.0_05634.1	1032	13114	0	997	1033	96	+	3	1032	7011	8043
UN02725	Rsa1.0_07056.1	1026	9969	0	715	766	93	+	280	1026	6091	6849
UN02726	Rsa1.0_00130.1	739	125737	1.00E-126	250	254	98	-	487	739	103390	103642
UN02727	Rsa1.0_07657.1	1047	8896	0	358	362	98	+	684	1044	3658	4019
UN02728	Rsa1.0_00595.1	1411	341648	0	1354	1414	95	-	9	1405	25673	27084

UN02729	Rsa1.0_00951.1	714	164588	0	688	724	95	−	3	714	122835	123556
UN02730	Rsa1.0_00702.1	1291	67600	1.00E−105	307	343	89	−	32	373	28141	28483
UN02731	Rsa1.0_00581.1	1038	163575	0	365	365	100	+	247	611	26641	27005
UN02732	Rsa1.0_00092.1	816	659932	0	513	519	98	+	65	583	199002	199520
UN02733	Rsa1.0_01447.1	600	45654	1.00E−171	311	312	99	−	288	598	4527	4838
UN02734	Rsa1.0_00202.1	608	185895	9.00E−87	215	231	93	+	3	227	11683	11913
UN02735	Rsa1.0_00372.1	1016	90418	1.00E−171	351	363	96	+	653	1014	45301	45659
UN02736	Rsa1.0_00019.1	1103	301669	1.00E−119	262	276	94	−	416	691	238714	238986
UN02737	Rsa1.0_02707.1	751	28520	0	330	332	99	+	416	747	11082	11413
UN02738	Rsa1.0_05036.1	1369	15058	0	1339	1394	96	+	3	1368	13114	14503
UN02739	Rsa1.0_06403.1	1667	11078	0	547	589	92	+	16	584	5658	6237
UN02740	Rsa1.0_01440.1	695	45807	0	353	357	98	−	339	695	31768	32121
UN02741	Rsa1.0_02528.1	841	30452	0	839	841	99	−	1	841	22261	23101
UN02742	Rsa1.0_13940.1	1009	4047	0	961	1007	95	+	2	1008	433	1430
UN02743	Rsa1.0_00907.1	1074	60062	0	850	887	95	+	3	884	17643	18520
UN02744	Rsa1.0_00343.1	949	93570	1.00E−31	83	87	95	+	1	87	11039	11125
UN02745	Rsa1.0_03264.1	756	145959	1.00E−180	366	381	96	+	2	382	141562	141939
UN02746	Rsa1.0_00131.1	739	432233	1.00E−149	284	288	98	−	432	719	189370	189656
UN02747	Rsa1.0_02705.1	1234	28535	0	1117	1203	92	+	41	1231	1566	2742
UN02748	Rsa1.0_02710.1	1397	28492	1.00E−150	294	302	97	−	1	302	25405	25706
UN02749	Rsa1.0_00734.1	1207	66508	0	370	370	100	+	837	1206	6510	6879
UN02750	Rsa1.0_00350.1	741	92346	1.00E−103	223	230	96	+	1	227	12235	12464
UN02751	Rsa1.0_01989.1	1012	36439	0	471	500	94	+	3	494	12040	12536
UN02752	Rsa1.0_06728.1	1007	10455	1.00E−138	357	391	91	−	625	1000	9062	9448
UN02753	Rsa1.0_00121.1	942	278029	1.00E−132	277	288	96	+	3	289	5597	5884
UN02754	Rsa1.0_09314.1	996	6461	0	510	529	96	+	9	535	5586	6112
UN02755	Rsa1.0_00005.1	895	353093	1.00E−151	284	288	98	+	509	796	129667	129954
UN02756	Rsa1.0_00057.1	1252	160473	1.00E−122	338	373	90	+	889	1248	143476	143836
UN02757	Rsa1.0_02329.1	1133	32474	0	749	769	97	+	368	1132	10253	11017
UN02758	Rsa1.0_24740.1	783	969	1.00E−137	355	391	90	+	393	783	476	866
UN02759	Rsa1.0_05940.1	934	12169	0	440	440	100	−	1	440	9960	10399
UN02760	Rsa1.0_00010.1	941	294827	0	362	375	96	−	484	858	132283	132657
UN02761	Rsa1.0_00442.1	763	84596	0	647	661	97	+	46	706	9550	10210
UN02762	Rsa1.0_00157.1	1091	245832	1.00E−115	227	231	98	+	663	893	185692	185921
UN02763	Rsa1.0_02314.1	1038	32642	0	516	533	96	−	508	1037	4774	5305
UN02764	Rsa1.0_05204.1	635	14516	0	619	638	97	+	3	635	2020	2657
UN02765	Rsa1.0_00015.1	804	561803	1.00E−112	308	345	89	+	28	348	370530	370874
UN02766	Rsa1.0_00706.1	1019	291570	1.00E−138	294	309	95	−	712	1019	229281	229580
UN02767	Rsa1.0_00437.1	1147	84879	0	505	521	96	+	583	1101	50510	51028

UN02768	Rsa1.0_00092.1	1073	659932	1.00E-120	241	248	97	-	409	656	127591	127838
UN02769	Rsa1.0_01931.1	806	37187	1.00E-114	248	258	96	-	550	806	28491	28747
UN02770	Rsa1.0_00190.1	720	318240	0	371	374	99	-	345	717	91428	91801
UN02771	Rsa1.0_04741.1	957	16164	1.00E-103	200	203	98	+	467	669	5949	6151
UN02772	Rsa1.0_00057.1	1661	160473	0	535	552	96	-	3	548	101205	101756
UN02773	Rsa1.0_01376.1	1345	47004	1.00E-174	385	410	93	+	936	1340	21956	22349
UN02774	Rsa1.0_04450.1	1368	17297	0	1333	1374	97	+	2	1368	11747	13119
UN02775	Rsa1.0_00487.1	1641	80674	0	429	442	97	-	1202	1640	12797	13238
UN02776	Rsa1.0_03252.1	1118	24338	1.00E-151	287	292	98	-	23	314	2166	2457
UN02777	Rsa1.0_36775.1	1002	613	1.00E-116	231	237	97	-	751	987	184	420
UN02778	Rsa1.0_14183.1	901	132309	0	749	773	96	+	2	768	1473	2245
UN02779	Rsa1.0_06603.1	783	10682	1.00E-125	271	285	95	+	3	287	6408	6684
UN02780	Rsa1.0_29978.1	1306	766	1.00E-129	297	315	94	+	993	1306	160	473
UN02781	Rsa1.0_01285.1	900	172709	0	522	525	99	-	376	900	153445	153969
UN02782	Rsa1.0_08192.1	967	7762	1.00E-142	294	304	96	+	630	931	6351	6654
UN02783	Rsa1.0_02490.1	1170	30885	0	376	377	99	-	794	1170	5484	5860
UN02784	Rsa1.0_00152.1	985	257730	0	948	983	96	-	3	985	201916	202890
UN02785	Rsa1.0_01502.1	1180	44519	0	480	493	97	-	4	496	36621	37111
UN02786	Rsa1.0_00218.1	927	201480	0	492	543	90	+	3	540	152508	153020
UN02787	Rsa1.0_01464.1	1006	45193	1.00E-166	360	381	94	+	50	424	25050	25427
UN02788	Rsa1.0_02532.1	1238	30403	0	905	924	97	+	52	973	26787	27710
UN02789	Rsa1.0_00213.1	1190	108167	1.00E-172	423	455	92	-	748	1189	36360	36807
UN02790	Rsa1.0_00648.1	907	70351	3.00E-87	225	244	92	+	622	865	24878	25115
UN02791	Rsa1.0_01523.1	921	43959	6.00E-15	109	131	83	+	330	460	20026	20156
UN02792	Rsa1.0_00074.1	1111	147591	1.00E-137	270	276	97	-	836	1111	135631	135905
UN02793	Rsa1.0_01458.1	603	175719	2.00E-81	177	184	96	-	6	186	415	598
UN02794	Rsa1.0_00045.1	1723	328694	0	621	634	97	+	1000	1633	289123	289756
UN02795	Rsa1.0_02756.1	659	28202	1.00E-70	148	152	97	+	508	659	17942	18093
UN02796	Rsa1.0_03155.1	1727	24958	0	461	472	97	+	1252	1723	508	978
UN02797	Rsa1.0_02188.1	999	34104	0	497	517	96	-	486	999	2422	2933
UN02798	Rsa1.0_30387.1	893	755	4.00E-90	175	177	98	-	261	437	313	489
UN02799	Rsa1.0_01829.1	717	166063	0	457	474	96	+	247	717	154869	155339
UN02800	Rsa1.0_00050.1	1130	237174	0	390	391	99	+	740	1130	19315	19705
UN02801	Rsa1.0_01306.1	1751	48720	0	402	424	94	+	1336	1751	755	1178
UN02802	Rsa1.0_00323.1	939	159650	1.00E-158	410	446	91	+	509	939	43841	44285
UN02803	Rsa1.0_00007.1	1024	239325	8.00E-70	296	344	86	-	653	993	14222	14555
UN02804	Rsa1.0_03132.1	845	25142	0	415	427	97	-	64	490	22052	22478
UN02805	Rsa1.0_01899.1	1007	37474	0	819	837	97	+	172	1007	3081	3916
UN02806	Rsa1.0_00751.1	877	65759	0	385	403	95	+	280	679	41458	41860

UN02807	Rsa1.0_39553.1	729	568	0	423	438	96	+	236	672	1	438
UN02808	Rsa1.0_00071.1	760	345714	1.00E-150	289	293	98	−	470	760	324444	324736
UN02809	Rsa1.0_00618.1	522	72195	0	514	517	99	−	4	520	20170	20686
UN02810	Rsa1.0_00101.1	789	267193	0	616	670	91	+	76	728	89099	89762
UN02811	Rsa1.0_00008.1	1476	358124	0	423	434	97	−	13	446	56542	56975
UN02812	Rsa1.0_00577.1	893	174827	2.00E-94	248	269	92	−	626	893	168313	168571
UN02813	Rsa1.0_02358.1	908	32213	0	453	485	93	−	426	908	247	712
UN02814	Rsa1.0_20008.1	827	1353	0	490	505	97	−	330	827	849	1353
UN02815	Rsa1.0_00429.1	1831	85559	0	816	945	86	−	670	1607	44086	45014
UN02816	Rsa1.0_16459.1	818	2148	1.00E-142	412	464	88	+	22	485	1642	2105
UN02817	Rsa1.0_03264.1	1158	145959	1.00E-139	275	283	97	+	376	658	134142	134424
UN02818	Rsa1.0_13693.1	875	4469	0	594	650	91	+	235	875	2608	3233
UN02819	Rsa1.0_02494.1	1606	132923	0	922	928	99	+	450	1377	4498	5425
UN02820	Rsa1.0_00960.1	1344	58199	0	476	478	99	−	860	1337	14530	15007
UN02821	Rsa1.0_00682.1	766	68680	1.00E-159	291	293	99	+	7	299	14059	14351
UN02822	Rsa1.0_00513.1	879	78579	1.00E-127	246	251	98	+	142	392	57763	58013
UN02823	Rsa1.0_04831.1	1043	15799	1.00E-28	127	146	86	+	392	536	12656	12801
UN02824	Rsa1.0_21334.1	1130	1214	0	688	746	92	+	307	1052	236	981
UN02825	Rsa1.0_00030.1	931	250988	0	547	573	95	+	2	573	157138	157688
UN02826	Rsa1.0_14984.1	1088	2912	1.00E-153	290	294	98	+	205	498	2296	2588
UN02827	Rsa1.0_10606.1	1151	5787	0	1118	1169	95	+	1	1149	838	2005
UN02828	Rsa1.0_00611.1	915	72410	0	544	587	92	+	335	912	7150	7732
UN02829	Rsa1.0_11079.1	1008	5624	0	408	409	99	−	359	767	4076	4484
UN02830	Rsa1.0_00671.1	1051	397884	0	1022	1064	96	+	2	1049	245073	246135
UN02831	Rsa1.0_02023.1	937	35922	0	552	583	94	−	1	581	26292	26874
UN02832	Rsa1.0_08686.1	667	7041	0	650	665	97	−	3	667	4483	5147
UN02833	Rsa1.0_04126.1	834	18939	0	460	480	95	−	1	478	17402	17876
UN02834	Rsa1.0_00436.1	1130	85008	1.00E-154	292	297	98	+	2	298	7435	7731
UN02835	Rsa1.0_01924.1	1071	37226	1.00E-116	324	358	90	−	407	752	1369	1723
UN02836	Rsa1.0_00001.1	1434	363028	0	969	1022	94	−	1	1003	144755	145776
UN02837	Rsa1.0_03785.1	1120	20883	0	379	380	99	−	7	386	15659	16038
UN02838	Rsa1.0_00865.1	1616	61686	0	580	586	98	−	605	1190	45315	45900
UN02839	Rsa1.0_00009.1	657	232558	2.33E-156	271	274	98	+	384	657	207990	208263
UN02840	Rsa1.0_00192.1	877	278200	1.00E-138	261	265	98	−	122	386	231729	231993
UN02841	Rsa1.0_00390.1	632	88681	1.00E-116	296	325	91	+	247	569	41294	41596
UN02842	Rsa1.0_01835.1	1232	38542	0	765	812	94	−	430	1220	15417	16222
UN02843	Rsa1.0_00148.1	1677	120234	0	865	895	96	−	1	878	63550	64444
UN02844	Rsa1.0_04646.1	1111	16541	0	655	665	98	+	1	665	4309	4973
UN02845	Rsa1.0_02006.1	633	36135	8.00E-78	233	260	89	−	76	334	7421	7680

UN02846	Rsa1.0_01220.1	595	161775	1.00E-136	322	351	91	−	3	353	7326	7660
UN02847	Rsa1.0_02254.1	1007	33344	1.00E-165	313	319	98	+	684	1002	12021	12339
UN02848	Rsa1.0_02641.1	1208	29191	0	459	472	97	−	735	1206	21757	22219
UN02849	Rsa1.0_01286.1	856	49209	0	453	484	93	+	377	854	25577	26059
UN02850	Rsa1.0_00789.1	1222	64233	0	526	546	96	−	2	542	34406	34951
UN02851	Rsa1.0_00712.1	1008	67217	0	504	523	96	−	484	1006	46782	47304
UN02852	Rsa1.0_00973.1	1368	57913	1.00E-152	273	273	100	−	933	1205	5428	5700
UN02853	Rsa1.0_00197.1	898	109714	0	701	706	99	+	194	897	84124	84829
UN02854	Rsa1.0_00041.1	762	186197	2.00E-75	175	184	95	+	509	691	175346	175529
UN02855	Rsa1.0_01808.1	891	39007	0	533	547	97	+	347	889	29595	30141
UN02856	Rsa1.0_00157.1	611	245832	0	412	412	100	−	1	412	160498	160909
UN02857	Rsa1.0_09577.1	792	6275	1.00E-131	309	334	92	+	468	792	1281	1614
UN02858	Rsa1.0_00471.1	599	81897	0	591	601	98	−	1	599	23991	24591
UN02859	Rsa1.0_07814.1	1463	8487	1.00E-117	214	214	100	+	1250	1463	1175	1388
UN02860	Rsa1.0_06303.1	1289	11268	0	591	615	96	−	137	751	1748	2362
UN02861	Rsa1.0_00016.1	1202	831256	1.00E-168	336	348	96	−	577	924	428671	429018
UN02862	Rsa1.0_02265.1	1295	33233	0	359	362	99	+	1	361	17366	17727
UN02863	Rsa1.0_26026.1	1127	909	0	779	801	97	−	1	801	1	800
UN02864	Rsa1.0_06092.1	1015	11756	0	412	425	96	−	7	430	2681	3104
UN02865	Rsa1.0_00027.1	1213	283362	1.00E-118	235	241	97	−	564	804	90878	91118
UN02866	Rsa1.0_00713.1	805	158017	0	788	805	97	+	1	805	24033	24837
UN02867	Rsa1.0_00027.1	889	283362	0	599	601	99	+	2	602	56405	57005
UN02868	Rsa1.0_00188.1	167	112020	8.00E-75	160	165	96	−	1	163	23385	23549
UN02869	Rsa1.0_71754.1	809	319	1.00E-162	306	310	98	−	1	310	1	309
UN02870	Rsa1.0_00833.1	1538	62621	0	511	543	94	−	574	1104	28121	28663
UN02871	Rsa1.0_04557.1	1183	145048	1.00E-129	364	397	91	+	2	379	135337	135723
UN02872	Rsa1.0_41904.1	1663	536	0	524	536	97	+	139	663	1	536
UN02873	#	#	#	#	#	#	#	#	#	#	#	#
UN02874	Rsa1.0_00829.1	1782	158305	0	938	954	98	+	829	1782	29034	29986
UN02875	Rsa1.0_00876.1	649	61328	0	375	376	99	−	26	401	28187	28562
UN02876	Rsa1.0_00671.1	934	397884	3.00E-81	248	281	88	+	4	260	192679	192958
UN02877	Rsa1.0_00220.1	710	192936	0	573	594	96	−	1	586	4428	5020
UN02878	Rsa1.0_35031.1	707	646	0	447	449	99	+	65	513	1	449
UN02879	Rsa1.0_00762.1	990	165246	0	357	360	99	+	2	361	28941	29300
UN02880	Rsa1.0_01363.1	1321	47195	1.00E-170	334	344	97	−	976	1319	20177	20520
UN02881	Rsa1.0_05324.1	1138	14145	0	438	456	96	−	1	454	13120	13569
UN02882	Rsa1.0_17832.1	1125	1729	0	329	329	100	−	1	329	123	451
UN02883	Rsa1.0_02988.1	1131	26190	0	366	372	98	+	253	624	17445	17812
UN02884	Rsa1.0_05168.1	596	14621	0	566	607	93	+	2	596	8077	8675

UN02885	Rsa1.0_00387.1	762	495407	0	745	747	99	−	16	762	349647	350393
UN02886	Rsa1.0_01401.1	1189	142441	0	640	667	95	+	368	1034	132155	132821
UN02887	Rsa1.0_07385.1	1027	9485	1.00E-170	321	326	98	+	702	1027	5009	5332
UN02888	Rsa1.0_00409.1	1385	146788	0	1330	1396	95	−	1	1383	123784	125176
UN02889	Rsa1.0_00236.1	839	104771	0	835	839	99	−	1	839	70007	70844
UN02890	Rsa1.0_00408.1	1548	176752	0	420	426	98	+	457	882	133299	133724
UN02891	Rsa1.0_05618.1	1018	13165	1.00E-114	218	221	98	+	471	691	7388	7608
UN02892	Rsa1.0_00660.1	743	69688	0	396	400	99	+	1	398	52547	52946
UN02893	Rsa1.0_00122.1	522	265937	0	438	473	92	−	56	522	224808	225273
UN02894	Rsa1.0_00359.1	497	91256	0	445	470	94	−	27	496	22278	22733
UN02895	Rsa1.0_03137.1	500	25105	1.00E-169	322	327	98	−	1	323	9465	9791
UN02896	Rsa1.0_03237.1	797	24434	0	718	734	97	−	10	743	1749	2480
UN02897	Rsa1.0_00193.1	1181	194198	1.00E-150	297	306	97	+	530	835	103702	104007
UN02898	Rsa1.0_12558.1	1137	5193	0	1008	1058	95	−	1	1050	1	1056
UN02899	Rsa1.0_00039.1	650	296927	0	529	579	91	+	1	575	75433	75994
UN02900	Rsa1.0_00895.1	1079	60615	3.00E-35	108	117	92	+	309	425	19320	19435
UN02901	Rsa1.0_00297.1	1185	97836	1.00E-169	348	361	96	+	113	473	33840	34198
UN02902	Rsa1.0_02294.1	1206	32940	1.00E-164	300	302	99	+	836	1137	15437	15738
UN02903	Rsa1.0_00280.1	1368	99966	0	408	408	100	+	876	1283	1487	1894
UN02904	Rsa1.0_00405.1	434	202864	0	377	385	97	+	2	386	59823	60207
UN02905	Rsa1.0_00068.1	544	151813	0	527	547	96	−	1	544	104387	104931
UN02906	Rsa1.0_27174.1	1130	863	0	483	486	99	−	1	486	132	617
UN02907	Rsa1.0_01943.1	759	37042	1.00E-103	249	262	95	+	1	259	4846	5105
UN02908	Rsa1.0_01296.1	845	49035	1.00E-137	248	248	100	−	1	248	13384	13631
UN02909	Rsa1.0_00595.1	1247	341648	1.00E-123	243	249	97	+	1	249	142025	142273
UN02910	Rsa1.0_00180.1	219	114581	4.00E-80	200	215	93	+	5	219	34742	34950
UN02911	Rsa1.0_00441.1	1105	84607	0	620	627	98	−	1	627	61230	61856
UN02912	Rsa1.0_03347.1	662	23588	4.00E-92	183	186	98	+	2	187	20400	20582
UN02913	Rsa1.0_00175.1	1512	166121	0	686	705	97	+	808	1511	85418	86122
UN02914	Rsa1.0_03246.1	399	24379	1.00E-151	274	275	99	+	1	275	9287	9561
UN02915	Rsa1.0_02406.1	820	145276	1.00E-170	332	339	97	−	1	338	1739	2076
UN02916	Rsa1.0_01775.1	1134	39519	1.00E-166	306	309	99	+	431	739	2567	2875
UN02917	Rsa1.0_01243.1	1243	50061	0	777	790	98	+	279	1064	33078	33867
UN02918	Rsa1.0_01768.1	1169	138463	0	1082	1114	97	+	3	1111	112732	113845
UN02919	Rsa1.0_00025.1	888	277312	0	853	885	96	+	3	886	67909	68787
UN02920	Rsa1.0_01121.1	874	53407	0	750	753	99	+	121	873	19076	19828
UN02921	Rsa1.0_15052.1	954	2866	0	948	952	99	−	1	952	582	1532
UN02922	Rsa1.0_01190.1	571	194501	1.00E-113	207	207	100	+	202	408	5041	5247
UN02923	Rsa1.0_05546.1	859	13379	1.00E-163	301	303	99	+	375	676	7592	7894

UN02924	Rsa1.0_03037.1		956	25825	1.00E-173	343	353	97	+	603	954	5163	5515
UN02925	Rsa1.0_25367.1		1276	939	1.00E-170	310	312	99	+	628	939	457	768
UN02926	Rsa1.0_00021.1		1366	263655	0	733	747	98	-	621	1366	30406	31146
UN02927	#	#	#	#	#	#	#	#	#	#	#	#	
UN02928	Rsa1.0_03341.1		1230	23604	0	793	815	97	+	345	1159	2950	3752
UN02929	Rsa1.0_39619.1		778	567	0	444	459	96	-	310	768	1	459
UN02930	Rsa1.0_01722.1		699	40320	0	651	724	89	+	1	697	36420	37139
UN02931	Rsa1.0_01763.1		857	39659	0	440	444	99	-	416	857	38145	38588
UN02932	Rsa1.0_03364.1		893	23446	0	781	800	97	+	2	800	18681	19479
UN02933	Rsa1.0_01294.1		860	49090	1.00E-114	328	365	89	-	7	352	26968	27329
UN02934	Rsa1.0_02114.1		752	34873	0	499	509	98	-	2	505	16049	16557
UN02935	Rsa1.0_01791.1		1083	160383	0	750	759	98	+	1	758	13646	14404
UN02936	Rsa1.0_00015.1		866	561803	1.00E-145	279	284	98	-	3	283	363481	363764
UN02937	Rsa1.0_00761.1		1407	65133	0	865	870	99	-	327	1196	12489	13358
UN02938	Rsa1.0_18025.1		1368	1687	1.00E-178	482	533	90	-	147	663	1162	1687
UN02939	Rsa1.0_03822.1		1386	20640	0	659	666	98	+	3	668	6396	7060
UN02940	Rsa1.0_00828.1		1218	62744	0	757	785	96	+	434	1218	15688	16470
UN02941	Rsa1.0_00149.1		1369	305196	0	550	562	97	-	208	769	200113	200674
UN02942	Rsa1.0_13838.1		746	4236	0	618	648	95	-	1	644	3281	3920
UN02943	Rsa1.0_00080.1		964	190437	0	642	654	98	-	4	657	112626	113277
UN02944	Rsa1.0_00538.1		1131	77149	7.00E-15	73	83	87	+	501	583	11743	11825
UN02945	Rsa1.0_01989.1		900	36439	0	869	898	96	-	4	899	25043	25939
UN02946	Rsa1.0_02493.1		797	30854	0	581	594	97	+	1	589	26849	27442
UN02947	Rsa1.0_00796.1		830	63997	7.00E-39	95	99	95	+	407	505	2078	2176
UN02948	Rsa1.0_00237.1		681	235974	0	616	621	99	-	61	679	14746	15366
UN02949	Rsa1.0_09517.1		1084	6319	0	688	708	97	-	70	777	2293	3000
UN02950	Rsa1.0_00681.1		591	68779	0	386	401	96	+	1	398	42467	42866
UN02951	Rsa1.0_00034.1		1240	260119	1.00E-118	229	233	98	+	510	742	30072	30304
UN02952	Rsa1.0_00125.1		661	253043	1.00E-168	329	336	97	-	4	339	168487	168820
UN02953	Rsa1.0_00135.1		1396	178077	0	694	757	91	-	296	1029	165548	166298
UN02954	Rsa1.0_00371.1		1139	90463	0	742	774	95	+	365	1137	11545	12312
UN02955	Rsa1.0_06041.1		871	11861	0	741	787	94	+	87	870	9352	10120
UN02956	Rsa1.0_03952.1		1127	19891	0	415	420	98	+	213	632	19139	19558
UN02957	Rsa1.0_00054.1		1049	162053	0	705	716	98	+	209	924	140724	141439
UN02958	Rsa1.0_00290.1		1182	225096	1.00E-127	246	251	98	-	930	1180	188600	188850
UN02959	Rsa1.0_39984.1		1177	562	0	380	397	95	+	141	536	7	402
UN02960	Rsa1.0_00087.1		1048	761073	0	494	505	97	+	1	496	56053	56557
UN02961	Rsa1.0_01352.1		693	47443	0	658	689	95	-	6	692	45244	45924
UN02962	Rsa1.0_01617.1		1224	42127	0	1115	1227	90	+	4	1217	2291	3498

UN02963	Rsa1.0_01480.1	607	44926	0	439	452	97	+	1	451	36814	37264
UN02964	Rsa1.0_00180.1	997	114581	1.00E-139	318	337	94	+	1	334	34738	35066
UN02965	Rsa1.0_00826.1	1007	166736	1.00E-115	292	317	92	−	1	305	156527	156841
UN02966	Rsa1.0_19085.1	1262	1482	0	603	642	93	−	573	1199	843	1482
UN02967	Rsa1.0_06482.1	840	10937	0	768	814	94	+	2	805	7954	8752
UN02968	Rsa1.0_02028.1	792	35857	1.00E-111	223	229	97	−	450	678	34453	34681
UN02969	Rsa1.0_00096.1	1032	198773	0	1005	1030	97	−	1	1030	97540	98569
UN02970	Rsa1.0_03046.1	1048	25784	1.00E-144	291	303	96	+	751	1043	15386	15688
UN02971	Rsa1.0_02865.1	1339	27133	0	467	480	97	+	867	1339	12296	12774
UN02972	Rsa1.0_00462.1	782	82288	1.00E-151	278	280	99	−	318	597	37812	38091
UN02973	Rsa1.0_04882.1	1000	15606	1.00E-175	314	315	99	−	345	659	7553	7867
UN02974	Rsa1.0_00891.1	1092	60724	0	1036	1063	97	−	2	1063	50532	51594
UN02975	Rsa1.0_00002.1	766	601661	1.00E-134	272	282	96	+	325	606	555743	556024
UN02976	Rsa1.0_04881.1	821	15608	0	651	667	97	−	1	663	8460	9126
UN02977	Rsa1.0_00973.1	1369	57913	0	838	842	99	+	524	1365	5808	6648
UN02978	Rsa1.0_00056.1	1142	332604	0	351	353	99	+	790	1142	313940	314292
UN02979	Rsa1.0_03529.1	2000	22277	0	1010	1010	100	−	728	1737	15606	16615
UN02980	Rsa1.0_00960.1	792	58199	1.00E-160	299	303	98	+	490	792	29999	30301
UN02981	Rsa1.0_00821.1	1066	62891	0	615	630	97	+	31	660	54395	55021
UN02982	Rsa1.0_00659.1	657	69733	1.00E-128	296	321	92	+	356	655	20560	20879
UN02983	Rsa1.0_00169.1	1813	274298	0	1773	1812	97	−	17	1810	68797	70605
UN02984	Rsa1.0_03367.1	1574	23387	1.00E-173	308	308	100	−	243	550	147	454
UN02985	Rsa1.0_02245.1	965	33478	0	887	904	98	−	16	919	11294	12191
UN02986	Rsa1.0_03005.1	923	26082	1.00E-160	353	373	94	+	559	923	10402	10774
UN02987	Rsa1.0_01859.1	1360	38073	0	645	682	94	+	685	1350	6242	6911
UN02988	Rsa1.0_00740.1	725	215972	1.00E-164	342	357	95	−	370	725	11871	12222
UN02989	Rsa1.0_00480.1	1077	81325	0	382	383	99	+	3	385	3549	3931
UN02990	Rsa1.0_08192.1	990	7762	1.00E-126	289	309	93	+	664	958	6351	6658
UN02991	Rsa1.0_00416.1	1265	86772	2.00E-77	161	164	98	−	1102	1264	4755	4918
UN02992	Rsa1.0_01124.1	1739	53295	0	1005	1027	97	+	509	1534	33250	34276
UN02993	Rsa1.0_00414.1	1384	202190	0	1064	1084	98	−	1	1081	187714	188797
UN02994	Rsa1.0_00069.1	933	403561	0	891	920	96	−	2	921	231100	232012
UN02995	Rsa1.0_02551.1	1300	30196	1.00E-130	246	249	98	−	348	596	26138	26386
UN02996	Rsa1.0_00336.1	1391	445051	0	843	854	98	−	538	1391	159561	160414
UN02997	Rsa1.0_00360.1	1742	91190	1.00E-122	242	248	97	−	152	399	7457	7704
UN02998	Rsa1.0_18326.1	508	1620	0	413	430	96	+	38	467	1196	1620
UN02999	Rsa1.0_30513.1	1302	752	0	550	565	97	−	692	1256	1	558
UN03000	Rsa1.0_02174.1	1318	34316	1.00E-126	278	294	94	+	1036	1316	13678	13970
UN03001	Rsa1.0_03590.1	1549	21889	0	1020	1088	93	+	3	1063	2552	3634

UN03002	Rsa1.0_01696.1	765	301850	0	516	541	95	−	199	730	159111	159651
UN03003	Rsa1.0_00564.1	1286	75428	0	501	526	95	−	712	1236	46259	46784
UN03004	Rsa1.0_08617.1	688	7124	0	665	701	94	−	1	688	313	1013
UN03005	Rsa1.0_30865.1	810	742	9.00E−97	228	242	94	+	553	792	1	240
UN03006	Rsa1.0_00197.1	638	109714	1.00E−124	247	254	97	+	5	258	78041	78294
UN03007	Rsa1.0_03946.1	988	19912	0	447	463	96	+	530	988	16589	17051
UN03008	Rsa1.0_00549.1	774	76473	1.00E−178	334	339	98	+	1	339	26156	26492
UN03009	Rsa1.0_01696.1	1001	301850	2.33E−156	263	263	100	−	3	265	32637	32899
UN03010	Rsa1.0_08644.1	1595	7095	1.00E−123	240	245	97	−	236	480	150	394
UN03011	Rsa1.0_01724.1	1072	40292	1.00E−105	210	215	97	−	510	724	10412	10626
UN03012	Rsa1.0_06869.1	821	10234	1.00E−103	207	210	98	+	614	821	448	657
UN03013	Rsa1.0_20968.1	927	1249	0	340	346	98	−	159	504	904	1249
UN03014	Rsa1.0_00078.1	1221	175053	0	400	407	98	+	815	1221	93430	93835
UN03015	Rsa1.0_00234.1	1122	197060	0	404	413	97	+	395	807	144150	144562
UN03016	Rsa1.0_00480.1	775	81325	0	768	776	98	+	3	775	3543	4318
UN03017	Rsa1.0_52639.1	795	433	0	432	433	99	−	103	535	1	433
UN03018	Rsa1.0_03606.1	685	21818	1.00E−110	237	247	95	−	58	304	4426	4666
UN03019	Rsa1.0_04209.1	1373	18442	0	443	447	99	+	926	1372	2017	2463
UN03020	Rsa1.0_00019.1	969	301669	1.00E−134	255	259	98	+	480	738	25352	25610
UN03021	Rsa1.0_03603.1	809	21845	0	404	421	95	+	49	463	21425	21845
UN03022	Rsa1.0_00284.1	658	323267	0	596	631	94	−	3	630	232430	233057
UN03023	Rsa1.0_00546.1	1327	76523	0	384	384	100	+	944	1327	51920	52303
UN03024	Rsa1.0_00075.1	581	146680	1.00E−161	287	287	100	−	295	581	82402	82688
UN03025	Rsa1.0_00902.1	909	60241	0	484	489	98	−	421	909	15521	16009
UN03026	Rsa1.0_00586.1	727	163802	0	439	452	97	−	284	727	154651	155101
UN03027	Rsa1.0_02505.1	1129	30703	0	905	923	98	+	205	1117	14331	15252
UN03028	Rsa1.0_15081.1	1398	2848	0	490	516	94	−	801	1315	728	1238
UN03029	Rsa1.0_00674.1	1203	193753	0	571	574	99	+	631	1202	181840	182413
UN03030	Rsa1.0_01121.1	889	53407	0	708	747	94	+	153	881	19076	19822
UN03031	Rsa1.0_13997.1	807	3934	1.00E−126	248	254	97	+	14	267	2399	2652
UN03032	Rsa1.0_04625.1	1189	16613	0	1142	1184	96	+	3	1186	8427	9603
UN03033	Rsa1.0_02859.1	999	162118	1.00E−101	227	242	93	+	2	243	142766	142996
UN03034	Rsa1.0_01385.1	1474	46782	0	795	797	99	−	673	1467	13282	14078
UN03035	Rsa1.0_04022.1	870	19534	0	629	633	99	−	194	825	7539	8171
UN03036	Rsa1.0_03885.1	1496	20256	0	709	724	97	+	772	1494	9632	10353
UN03037	Rsa1.0_00470.1	622	81968	1.00E−119	217	217	100	−	405	621	7043	7259
UN03038	Rsa1.0_03632.1	1140	21645	2.33E−156	303	315	96	−	792	1106	2985	3298
UN03039	Rsa1.0_10922.1	1109	5677	0	392	399	98	+	665	1063	1	399
UN03040	Rsa1.0_03665.1	1142	21487	2.00E−61	192	217	88	−	745	952	12880	13096

UN03041	Rsa1.0_04517.1	1818	17015	1.00E-155	306	314	97	-	1503	1815	16702	17015
UN03042	Rsa1.0_05690.1	990	12921	0	573	601	95	-	306	897	12321	12921
UN03043	Rsa1.0_00088.1	432	141341	0	423	436	97	-	1	432	47375	47810
UN03044	Rsa1.0_07868.1	1573	8387	0	765	767	99	+	805	1571	6215	6981
UN03045	Rsa1.0_06127.1	1324	11675	0	364	370	98	-	3	369	3128	3497
UN03046	Rsa1.0_01158.1	1338	52121	0	575	615	93	+	506	1120	31439	32053
UN03047	Rsa1.0_00504.1	815	79334	0	458	461	99	+	3	461	55826	56286
UN03048	Rsa1.0_00420.1	1488	86496	1.00E-152	277	278	99	+	358	635	10516	10793
UN03049	Rsa1.0_00685.1	651	68515	4.00E-89	209	223	93	+	311	533	8208	8430
UN03050	Rsa1.0_07898.1	1098	8339	0	565	578	97	+	521	1098	6826	7403
UN03051	Rsa1.0_00350.1	903	92346	1.00E-103	197	199	98	-	705	903	79680	79878
UN03052	Rsa1.0_00427.1	1878	189678	0	573	593	96	-	1	590	110678	111270
UN03053	Rsa1.0_01668.1	1748	41299	0	459	465	98	-	1285	1748	33632	34096
UN03054	Rsa1.0_00277.1	568	100132	1.00E-124	320	349	91	+	237	568	10173	10521
UN03055	Rsa1.0_09363.1	516	6425	2.00E-81	154	154	100	+	361	514	3801	3954
UN03056	Rsa1.0_00224.1	834	327530	1.00E-141	270	275	98	+	380	654	208584	208858
UN03057	Rsa1.0_05063.1	1119	14951	1.00E-152	295	302	97	-	2	303	12766	13067
UN03058	Rsa1.0_00180.1	965	114581	1.00E-129	280	294	95	-	674	964	34776	35066
UN03059	Rsa1.0_46705.1	754	483	1.00E-121	231	233	99	+	469	700	235	467
UN03060	Rsa1.0_00655.1	833	195840	2.00E-57	114	114	100	-	5	118	134770	134883
UN03061	Rsa1.0_00097.1	1404	138858	0	352	352	100	+	161	512	50294	50645
UN03062	Rsa1.0_00190.1	1600	318240	0	983	987	99	-	72	1058	126608	127594
UN03063	Rsa1.0_00101.1	1183	267193	0	357	357	100	+	825	1181	235547	235903
UN03064	Rsa1.0_00901.1	1152	60363	0	618	618	100	-	533	1150	15440	16057
UN03065	Rsa1.0_00253.1	1146	231292	1.00E-151	302	311	97	-	837	1146	208323	208633
UN03066	Rsa1.0_01147.1	1194	52391	0	372	379	98	+	269	647	37794	38171
UN03067	Rsa1.0_03638.1	818	21634	0	392	397	98	+	422	817	1353	1748
UN03068	Rsa1.0_00974.1	1335	57893	0	418	427	97	+	907	1333	32774	33200
UN03069	Rsa1.0_08348.1	777	7495	1.00E-107	200	201	99	-	577	777	1921	2121
UN03070	Rsa1.0_00152.1	803	257730	0	341	344	99	+	3	346	244035	244378
UN03071	Rsa1.0_05139.1	1398	14718	1.00E-134	270	279	96	+	596	874	5752	6030
UN03072	Rsa1.0_01061.1	1010	55349	0	499	505	98	-	328	832	29738	30241
UN03073	Rsa1.0_06041.1	1089	11861	0	675	709	95	-	381	1089	9861	10551
UN03074	Rsa1.0_01570.1	1275	43015	2.00E-95	287	323	88	-	972	1275	10807	11124
UN03075	Rsa1.0_00192.1	1374	278200	0	846	892	94	-	490	1374	98204	99091
UN03076	Rsa1.0_01156.1	1590	52147	1.00E-174	326	331	98	+	40	370	18198	18528
UN03077	Rsa1.0_02717.1	843	28458	1.00E-130	245	247	99	-	420	664	13199	13445
UN03078	Rsa1.0_00678.1	643	193163	2.00E-78	163	167	97	+	477	640	24101	24267
UN03079	Rsa1.0_00040.1	1209	324196	1.00E-164	414	451	91	-	1	441	175165	175594

UN03080	Rsa1.0_00336.1	582	445051	0	502	526	95	−	1	522	221235	221748
UN03081	Rsa1.0_00198.1	1069	222963	1.00E−167	328	338	97	−	609	946	139194	139531
UN03082	Rsa1.0_00336.1	813	445051	1.00E−103	213	219	97	−	503	720	164725	164943
UN03083	Rsa1.0_01679.1	882	146876	1.00E−179	372	390	95	+	2	381	132696	133080
UN03084	Rsa1.0_00191.1	955	316976	1.00E−174	322	326	98	+	3	328	140937	141262
UN03085	Rsa1.0_00698.1	1382	67816	0	479	488	98	−	2	489	65744	66230
UN03086	Rsa1.0_02356.1	1199	141500	0	1046	1078	97	−	118	1194	128737	129811
UN03087	Rsa1.0_00420.1	946	86496	1.00E−65	137	140	97	−	499	638	67496	67635
UN03088	Rsa1.0_02157.1	1260	149652	0	447	449	99	+	731	1179	147090	147538
UN03089	Rsa1.0_08562.1	919	7193	0	374	379	98	+	5	380	333	711
UN03090	Rsa1.0_00018.1	1324	410899	0	436	457	95	+	872	1318	68738	69191
UN03091	Rsa1.0_01321.1	1234	48248	0	375	389	96	+	33	419	34818	35202
UN03092	Rsa1.0_02682.1	907	28711	0	861	906	95	+	4	901	9620	10525
UN03093	Rsa1.0_01882.1	1265	37697	0	919	956	96	−	1	949	23907	24859
UN03094	Rsa1.0_07850.1	1634	8423	0	1630	1636	99	−	1	1634	3704	5339
UN03095	Rsa1.0_00534.1	917	77361	0	338	340	99	+	1	340	28353	28692
UN03096	Rsa1.0_00184.1	960	218748	0	461	464	99	−	496	959	51747	52210
UN03097	Rsa1.0_00027.1	813	283362	1.00E−154	295	301	98	−	415	715	115850	116150
UN03098	Rsa1.0_00048.1	937	167185	1.00E−96	234	248	94	−	8	254	33623	33868
UN03099	Rsa1.0_01313.1	1243	48387	1.00E−111	217	221	98	+	930	1150	19753	19973
UN03100	Rsa1.0_03870.1	864	20362	0	815	860	94	−	1	859	266	1097
UN03101	Rsa1.0_10304.1	1227	5896	0	507	510	99	+	2	511	5359	5868
UN03102	Rsa1.0_03873.1	841	20360	1.00E−159	293	296	98	+	546	841	4285	4580
UN03103	Rsa1.0_01580.1	1242	42852	1.00E−175	354	364	97	−	170	531	31149	31511
UN03104	Rsa1.0_01521.1	995	142147	1.00E−145	281	286	98	+	8	292	6842	7127
UN03105	Rsa1.0_00323.1	787	159650	0	411	413	99	−	6	418	148152	148564
UN03106	Rsa1.0_02755.1	1098	28212	0	1006	1109	90	−	1	1089	2152	3258
UN03107	Rsa1.0_00191.1	549	316976	1.00E−162	309	314	98	−	6	318	142530	142843
UN03108	Rsa1.0_02015.1	1218	36008	0	467	482	96	+	499	980	3355	3836
UN03109	Rsa1.0_04425.1	1925	17406	0	859	861	99	−	1	861	1	861
UN03110	Rsa1.0_01309.1	513	48556	3.00E−21	56	57	98	−	26	82	35344	35400
UN03111	Rsa1.0_01016.1	831	181422	0	649	653	99	−	178	829	158103	158755
UN03112	Rsa1.0_04140.1	1034	18879	0	1013	1033	98	−	1	1032	14391	15423
UN03113	Rsa1.0_01579.1	826	42860	0	485	500	97	−	16	514	23243	23733
UN03114	Rsa1.0_08630.1	478	7104	0	453	482	93	−	2	478	5852	6333
UN03115	Rsa1.0_04540.1	1317	16934	0	1290	1322	97	−	1	1317	7370	8690
UN03116	Rsa1.0_01282.1	1263	49304	1.00E−126	291	313	92	−	39	351	8878	9176
UN03117	Rsa1.0_41755.1	1391	538	2.00E−96	200	206	97	−	164	366	333	538
UN03118	Rsa1.0_01448.1	481	45645	0	442	468	94	−	3	460	16268	16734

UN03119	Rsa1.0_01101.1	890	54031	1.00E-132	260	267	97	−	4	270	41580	41846
UN03120	Rsa1.0_02201.1	1208	33981	0	339	342	99	−	1	342	23386	23727
UN03121	Rsa1.0_02836.1	1332	27481	0	519	538	96	+	527	1064	5020	5556
UN03122	Rsa1.0_07823.1	885	8468	1.00E-144	296	308	96	−	225	532	4793	5100
UN03123	Rsa1.0_00595.1	270	341648	7.00E-61	192	218	88	+	62	269	193427	193634
UN03124	Rsa1.0_00125.1	1000	253043	0	778	808	96	+	186	983	203048	203854
UN03125	Rsa1.0_01147.1	784	52391	0	549	565	97	+	1	558	17652	18214
UN03126	Rsa1.0_11641.1	1171	5453	0	482	486	99	−	2	487	230	715
UN03127	Rsa1.0_04181.1	960	18615	0	950	960	98	−	1	959	16411	17370
UN03128	Rsa1.0_00005.1	1395	353093	0	672	713	94	+	336	1046	275726	276436
UN03129	Rsa1.0_00038.1	927	314568	1.00E-144	331	358	92	−	1	340	176457	176813
UN03130	Rsa1.0_00564.1	1303	75428	1.00E-149	298	308	96	+	63	370	46351	46658
UN03131	Rsa1.0_04109.1	615	19000	0	552	571	96	−	1	569	8583	9150
UN03132	Rsa1.0_00020.1	1407	283099	0	1389	1407	98	−	1	1407	176342	177745
UN03133	Rsa1.0_01732.1	1462	40194	0	1276	1277	99	−	186	1462	24245	25520
UN03134	Rsa1.0_02705.1	670	28535	0	643	660	97	−	3	662	2205	2861
UN03135	Rsa1.0_03247.1	1145	24371	0	808	838	96	+	1	836	11707	12544
UN03136	Rsa1.0_00329.1	1249	222544	0	539	569	94	+	22	589	72488	73053
UN03137	Rsa1.0_11201.1	565	5585	1.00E-131	332	361	91	−	19	375	3824	4181
UN03138	Rsa1.0_01269.1	1335	49622	1.00E-132	281	294	95	+	1	294	14013	14304
UN03139	Rsa1.0_00068.1	1043	151813	1.00E-116	248	260	95	−	782	1041	147810	148063
UN03140	Rsa1.0_24850.1	458	964	1.00E-167	343	358	95	−	5	362	261	618
UN03141	Rsa1.0_00012.1	965	222148	0	355	355	100	−	2	356	210339	210693
UN03142	Rsa1.0_02064.1	1058	35476	1.00E-158	320	332	96	+	283	614	5466	5797
UN03143	Rsa1.0_00503.1	779	79468	0	684	687	99	−	3	688	3854	4540
UN03144	Rsa1.0_00013.1	1151	292129	0	799	903	88	−	12	904	22577	23477
UN03145	Rsa1.0_06720.1	1156	10473	0	1122	1156	97	+	1	1155	3867	5009
UN03146	Rsa1.0_00273.1	962	326186	1.00E-154	276	276	100	+	1	276	249003	249278
UN03147	Rsa1.0_05384.1	123	13960	2.00E-49	111	115	96	+	9	123	1088	1202
UN03148	Rsa1.0_00238.1	1007	193193	0	537	555	96	+	2	548	192075	192627
UN03149	Rsa1.0_00152.1	897	257730	0	458	462	99	+	1	462	85532	85993
UN03150	Rsa1.0_01124.1	1298	53295	0	1205	1256	95	+	1	1243	50470	51721
UN03151	Rsa1.0_11740.1	752	5423	0	539	570	94	+	188	751	4551	5119
UN03152	Rsa1.0_02142.1	1114	34633	0	446	464	96	+	652	1112	20960	21423
UN03153	Rsa1.0_01768.1	950	138463	1.00E-163	439	480	91	−	1	465	123667	124145
UN03154	Rsa1.0_01147.1	1001	52391	0	641	651	98	−	1	651	1559	2209
UN03155	Rsa1.0_01316.1	844	48325	0	843	844	99	−	1	843	5709	6552
UN03156	Rsa1.0_02750.1	893	28239	1.00E-145	262	262	100	+	3	264	22863	23124
UN03157	Rsa1.0_00413.1	1566	86921	0	762	791	96	−	758	1548	71434	72224

UN03158	Rsa1.0_00844.1	562	62403	0	364	375	97	−	189	562	19081	19455
UN03159	Rsa1.0_00037.1	1459	307838	0	679	691	98	−	3	685	261462	262152
UN03160	Rsa1.0_01190.1	1014	194501	0	822	876	93	+	1	863	37247	38111
UN03161	Rsa1.0_00207.1	1447	248736	0	849	850	99	−	3	852	194940	195789
UN03162	Rsa1.0_00020.1	1162	283099	0	450	459	98	+	3	461	102867	103322
UN03163	Rsa1.0_00972.1	672	57937	9.00E−75	165	171	96	−	183	352	52543	52713
UN03164	Rsa1.0_00419.1	777	86562	0	753	767	98	−	11	777	56772	57535
UN03165	Rsa1.0_02038.1	1822	35715	0	631	631	100	−	1086	1716	7868	8498
UN03166	Rsa1.0_07875.1	1321	8374	0	562	563	99	+	434	996	2487	3049
UN03167	Rsa1.0_00673.1	750	69325	1.00E−148	272	274	99	+	3	276	48658	48931
UN03168	Rsa1.0_00275.1	1050	210104	0	605	618	97	−	411	1028	154998	155614
UN03169	Rsa1.0_00207.1	1463	248736	0	939	948	99	+	1	948	52530	53477
UN03170	Rsa1.0_01951.1	1331	36941	0	1330	1333	99	−	1	1331	5929	7261
UN03171	Rsa1.0_00139.1	1021	357786	1.00E−114	280	301	93	+	726	1018	275224	275524
UN03172	Rsa1.0_00991.1	420	143101	1.00E−123	224	224	100	−	1	224	138346	138569
UN03173	Rsa1.0_00491.1	1134	80219	0	1023	1093	93	−	1	1076	75459	76542
UN03174	Rsa1.0_00223.1	1157	106231	0	572	578	98	+	440	1017	71134	71711
UN03175	Rsa1.0_06564.1	1393	10765	0	836	870	96	−	523	1387	9799	10659
UN03176	Rsa1.0_03728.1	898	21147	0	492	522	94	+	2	523	3494	4003
UN03177	Rsa1.0_00910.1	885	59975	0	860	893	96	−	2	883	27250	28142
UN03178	Rsa1.0_00197.1	834	109714	0	358	360	99	−	1	360	93155	93514
UN03179	Rsa1.0_01098.1	1035	54215	2.00E−52	142	154	92	−	880	1033	40131	40284
UN03180	Rsa1.0_01696.1	1087	301850	1.00E−129	250	254	98	−	1	253	293333	293586
UN03181	Rsa1.0_03452.1	1008	22817	0	1002	1010	99	−	1	1007	2642	3651
UN03182	Rsa1.0_02239.1	707	33534	0	397	410	96	+	1	407	14846	15252
UN03183	Rsa1.0_00044.1	1064	271658	0	550	571	96	−	1	570	6694	7258
UN03184	Rsa1.0_13906.1	377	4114	1.00E−100	262	284	92	+	2	285	1190	1469
UN03185	Rsa1.0_00631.1	1004	71277	0	539	552	97	−	1	552	48255	48806
UN03186	Rsa1.0_06133.1	1068	11663	0	1013	1026	98	+	43	1067	8486	9501
UN03187	Rsa1.0_20514.1	2228	1297	1.00E−135	246	246	100	+	67	312	50	295
UN03188	Rsa1.0_01739.1	2010	40068	0	1144	1204	95	+	821	2010	8211	9405
UN03189	Rsa1.0_00628.1	1169	214360	0	904	906	99	+	264	1169	8480	9385
UN03190	Rsa1.0_04066.1	812	19262	1.00E−24	68	71	95	−	1	71	8483	8553
UN03191	Rsa1.0_00523.1	735	178770	0	507	524	96	+	2	522	29584	30106
UN03192	Rsa1.0_00222.1	1381	172263	0	606	625	96	−	763	1381	39667	40291
UN03193	Rsa1.0_00225.1	1166	105892	1.00E−173	324	329	98	−	811	1139	53905	54233
UN03194	Rsa1.0_01933.1	1336	37172	1.00E−169	311	314	99	−	3	316	13625	13938
UN03195	Rsa1.0_06932.1	825	10145	1.00E−165	294	294	100	+	532	825	142	435
UN03196	Rsa1.0_00469.1	1036	82084	1.00E−179	328	331	99	−	320	650	66858	67188

UN03197	Rsa1.0_00475.1	722	81678	2.00E-35	148	167	88	+	63	224	674	839
UN03198	Rsa1.0_30366.1	1311	756	0	732	756	96	−	302	1055	1	756
UN03199	Rsa1.0_01527.1	803	43905	1.00E-163	292	292	100	−	511	802	19317	19608
UN03200	Rsa1.0_06309.1	811	11263	1.00E-135	268	275	97	−	3	275	7759	8033
UN03201	Rsa1.0_18610.1	1195	1565	1.00E-103	229	240	95	+	555	791	1220	1457
UN03202	Rsa1.0_00082.1	925	237584	1.00E-175	374	390	95	+	1	376	55282	55670
UN03203	Rsa1.0_00237.1	750	235974	0	387	392	98	+	2	393	214301	214689
UN03204	Rsa1.0_00034.1	1805	260119	0	453	473	95	+	1335	1805	28435	28901
UN03205	Rsa1.0_00033.1	1385	515811	0	763	836	91	+	1	816	36184	36994
UN03206	Rsa1.0_00587.1	1494	73982	0	867	892	97	−	2	886	22418	23307
UN03207	Rsa1.0_00060.1	714	502678	1.00E-109	265	286	92	−	423	705	499397	499682
UN03208	Rsa1.0_02970.1	404	26372	0	398	404	98	−	1	404	8468	8870
UN03209	Rsa1.0_00032.1	1096	306326	0	605	635	95	−	463	1085	284767	285401
UN03210	Rsa1.0_01634.1	1098	41865	0	434	452	96	−	647	1096	1106	1551
UN03211	Rsa1.0_08019.1	998	8075	0	355	355	100	+	304	658	3265	3619
UN03212	Rsa1.0_02955.1	1398	26484	0	618	651	94	−	756	1395	17256	17899
UN03213	Rsa1.0_04744.1	1216	16154	1.00E-142	332	352	94	+	869	1216	7259	7609
UN03214	Rsa1.0_01140.1	1265	52586	0	633	656	96	+	167	822	31183	31838
UN03215	Rsa1.0_00337.1	818	143174	3.00E-90	221	240	92	−	187	426	141486	141710
UN03216	Rsa1.0_00065.1	1605	454616	0	1026	1032	99	−	527	1558	443700	444731
UN03217	Rsa1.0_00241.1	1245	104000	0	1041	1051	99	−	1	1051	89741	90788
UN03218	Rsa1.0_00040.1	1182	324196	0	864	900	96	+	288	1182	230200	231098
UN03219	Rsa1.0_01110.1	706	195852	0	409	424	96	−	1	424	185543	185966
UN03220	Rsa1.0_01936.1	533	37151	2.00E-99	449	532	84	−	22	533	18243	18755
UN03221	Rsa1.0_01462.1	571	45231	1.00E-134	278	292	95	−	280	571	25445	25725
UN03222	Rsa1.0_00507.1	958	79074	0	935	961	97	+	2	958	77176	78135
UN03223	Rsa1.0_00065.1	928	454616	0	424	431	98	−	498	928	444301	444731
UN03224	Rsa1.0_01249.1	191	188432	6.00E-45	147	163	90	+	29	191	150849	151008
UN03225	Rsa1.0_00738.1	687	66322	0	390	393	99	−	1	393	26036	26428
UN03226	Rsa1.0_00104.1	826	368722	1.00E-147	264	264	100	+	1	264	333196	333459
UN03227	Rsa1.0_15193.1	802	2768	0	371	371	100	+	3	373	1298	1668
UN03228	Rsa1.0_02203.1	1038	164532	0	548	588	93	+	349	935	148518	149102
UN03229	Rsa1.0_02523.1	585	30527	6.00E-88	171	173	98	−	3	175	10966	11138
UN03230	Rsa1.0_02074.1	733	35370	0	433	445	97	−	289	733	12083	12527
UN03231	Rsa1.0_00645.1	1431	189716	0	420	421	99	+	2	422	33293	33713
UN03232	Rsa1.0_01245.1	1034	149868	0	475	493	96	−	541	1033	40921	41413
UN03233	Rsa1.0_01791.1	1182	160383	0	455	463	98	−	719	1181	155276	155735
UN03234	Rsa1.0_11079.1	454	5624	0	432	451	95	−	4	454	5077	5525
UN03235	Rsa1.0_03315.1	692	23797	1.00E-107	198	198	100	+	3	200	924	1121

UN03236	Rsa1.0_01039.1	912	55946	1.00E-106	202	204	99	+	3	206	26961	27164
UN03237	Rsa1.0_00094.1	913	139042	0	506	531	95	−	2	532	25774	26304
UN03238	Rsa1.0_02550.1	538	291051	0	531	537	98	−	2	537	282468	283001
UN03239	Rsa1.0_04948.1	1050	15356	0	721	753	95	−	4	756	12946	13698
UN03240	Rsa1.0_00304.1	818	97429	0	393	402	97	−	417	818	31748	32145
UN03241	Rsa1.0_00706.1	796	291570	4.00E-80	174	180	96	+	3	182	160967	161145
UN03242	Rsa1.0_04191.1	1011	18578	0	435	439	99	−	3	441	7426	7864
UN03243	Rsa1.0_01101.1	1366	54031	1.00E-141	264	267	98	−	883	1149	22500	22766
UN03244	Rsa1.0_00497.1	1134	79830	0	904	936	96	−	3	934	6527	7459
UN03245	Rsa1.0_00711.1	984	67223	0	354	354	100	−	1	354	59902	60255
UN03246	Rsa1.0_06274.1	688	11343	1.00E-174	369	385	95	+	172	554	7108	7486
UN03247	Rsa1.0_11186.1	969	5590	0	394	397	99	+	146	542	436	832
UN03248	Rsa1.0_00127.1	941	127446	0	449	480	93	−	462	926	56170	56649
UN03249	Rsa1.0_03074.1	1134	25568	0	426	426	100	−	426	851	16751	17176
UN03250	Rsa1.0_00205.1	606	193401	0	348	353	98	+	254	606	38432	38784
UN03251	Rsa1.0_00115.1	1378	131321	1.00E-169	379	399	94	+	845	1238	68836	69232
UN03252	Rsa1.0_00427.1	839	189678	2.00E-91	177	179	98	−	257	435	183909	184087
UN03253	Rsa1.0_16916.1	718	1990	0	623	639	97	−	64	702	8	641
UN03254	Rsa1.0_03169.1	1000	24826	1.00E-147	302	314	96	−	16	325	18033	18346
UN03255	Rsa1.0_01377.1	1069	46967	4.00E-81	169	174	97	+	776	949	21582	21755
UN03256	Rsa1.0_13611.1	1410	4641	0	621	642	96	−	742	1364	4000	4641
UN03257	Rsa1.0_04203.1	755	18479	0	450	460	97	−	3	462	17886	18342
UN03258	Rsa1.0_01530.1	921	43820	0	710	718	98	−	2	719	18245	18961
UN03259	Rsa1.0_00740.1	780	215972	0	669	702	95	−	4	705	9972	10664
UN03260	Rsa1.0_03569.1	1416	22041	0	352	356	98	−	4	359	14787	15142
UN03261	Rsa1.0_00529.1	438	77487	1.00E-69	186	199	93	−	240	436	25100	25293
UN03262	Rsa1.0_01105.1	682	53958	1.00E-119	244	252	96	−	410	659	7729	7980
UN03263	Rsa1.0_00816.1	711	403350	2.00E-88	169	170	99	+	541	710	273442	273611
UN03264	Rsa1.0_05339.1	1731	14114	0	1670	1746	95	−	1	1731	7135	8879
UN03265	Rsa1.0_00329.1	1092	222544	1.00E-160	302	307	98	−	784	1090	79574	79880
UN03266	Rsa1.0_00271.1	1305	101125	1.00E-127	249	255	97	+	787	1041	25866	26120
UN03267	Rsa1.0_00176.1	981	240984	0	973	981	99	+	1	981	1936	2915
UN03268	Rsa1.0_00268.1	1048	101470	1.00E-101	312	360	86	−	4	344	61574	61913
UN03269	Rsa1.0_00121.1	1606	278029	0	413	421	98	+	284	704	41474	41894
UN03270	Rsa1.0_01879.1	959	37748	0	362	373	97	+	587	959	23051	23422
UN03271	Rsa1.0_02344.1	1916	32369	0	1764	1881	93	+	2	1880	27164	29041
UN03272	Rsa1.0_00094.1	1801	139042	0	553	578	95	+	12	588	90538	91115
UN03273	Rsa1.0_00998.1	1140	57168	1.00E-149	290	297	97	+	422	718	9918	10214
UN03274	Rsa1.0_00048.1	817	167185	0	722	724	99	−	2	725	44608	45331

UN03275	Rsa1.0_00306.1	1073	146760	0	546	552	98	−	1	552	136226	136777
UN03276	Rsa1.0_00009.1	1017	232558	0	449	461	97	+	1	460	121559	122019
UN03277	Rsa1.0_00017.1	781	752458	0	661	672	98	+	110	781	695063	695734
UN03278	Rsa1.0_05618.1	1145	13165	1.00E−116	219	221	99	−	516	736	7388	7608
UN03279	Rsa1.0_02518.1	459	30586	8.00E−59	122	124	98	−	119	242	25939	26062
UN03280	Rsa1.0_01200.1	740	50895	0	390	407	95	−	337	740	30781	31187
UN03281	Rsa1.0_01493.1	881	44670	0	491	502	97	+	3	504	5423	5924
UN03282	Rsa1.0_00067.1	992	415055	0	884	917	96	−	78	992	152049	152965
UN03283	Rsa1.0_00042.1	766	171507	1.00E−149	316	332	95	−	1	332	144822	145153
UN03284	Rsa1.0_06247.1	607	11395	1.00E−73	156	161	96	+	3	163	8715	8875
UN03285	Rsa1.0_01161.1	739	52029	1.00E−150	288	294	97	+	446	739	19876	20169
UN03286	Rsa1.0_00314.1	721	96472	1.00E−123	248	256	96	−	466	721	15658	15913
UN03287	Rsa1.0_00683.1	396	68638	3.00E−79	182	192	94	−	25	213	59008	59199
UN03288	Rsa1.0_00350.1	1339	92346	0	1321	1351	97	+	1	1339	57739	59086
UN03289	Rsa1.0_00821.1	944	62891	0	379	381	99	+	565	944	18256	18636
UN03290	Rsa1.0_05289.1	1357	14259	0	1182	1195	98	−	165	1356	2088	3281
UN03291	Rsa1.0_07208.1	792	9719	0	762	786	96	+	3	785	6506	7291
UN03292	Rsa1.0_00025.1	843	277312	4.00E−99	281	308	91	−	527	820	134694	134996
UN03293	Rsa1.0_00387.1	1236	495407	0	486	490	99	−	747	1236	354494	354981
UN03294	Rsa1.0_00336.1	1020	445051	1.00E−111	242	253	95	−	504	756	164694	164945
UN03295	Rsa1.0_07006.1	1527	10043	2.00E−86	166	167	99	+	1135	1301	9174	9340
UN03296	Rsa1.0_00979.1	1390	57733	0	713	713	100	−	676	1388	1000	1712
UN03297	Rsa1.0_00894.1	664	269700	0	650	664	97	−	2	664	101028	101688
UN03298	Rsa1.0_00855.1	808	62047	0	417	420	99	−	82	501	5071	5490
UN03299	Rsa1.0_00246.1	1129	103541	4.00E−87	170	172	98	+	618	789	75837	76008
UN03300	Rsa1.0_00360.1	765	91190	1.00E−162	302	306	98	−	460	765	49982	50287
UN03301	Rsa1.0_00341.1	1138	249309	1.00E−139	338	364	92	−	750	1110	125794	126149
UN03302	Rsa1.0_04594.1	771	16713	0	526	532	98	−	237	765	4770	5301
UN03303	Rsa1.0_00097.1	844	138858	0	344	347	99	−	493	839	113687	114033
UN03304	Rsa1.0_01224.1	921	50388	9.00E−20	110	127	86	−	26	150	39776	39902
UN03305	Rsa1.0_01980.1	1330	36544	0	645	650	99	+	6	655	15880	16529
UN03306	Rsa1.0_00115.1	680	131321	1.00E−175	343	351	97	−	332	680	97416	97765
UN03307	Rsa1.0_00310.1	796	96852	1.00E−114	212	213	99	−	584	796	39207	39419
UN03308	Rsa1.0_03105.1	1164	25373	0	663	671	98	−	3	670	17351	18021
UN03309	Rsa1.0_00867.1	918	61635	0	449	451	99	−	1	451	33487	33937
UN03310	Rsa1.0_01924.1	965	37226	1.00E−163	344	355	96	+	449	798	1369	1723
UN03311	Rsa1.0_01389.1	1061	46716	0	816	850	96	+	213	1061	45323	46169
UN03312	Rsa1.0_00594.1	1248	73729	0	488	501	97	+	744	1242	36888	37386
UN03313	Rsa1.0_05755.1	789	12730	0	622	623	99	+	83	705	11775	12397

UN03314	Rsa1.0_00159.1	866	431686	1.00E-176	352	364	96	-	15	378	210507	210867
UN03315	Rsa1.0_00748.1	754	65983	2.00E-75	156	160	97	-	332	491	16394	16553
UN03316	Rsa1.0_01844.1	1023	38421	1.00E-168	309	312	99	+	153	464	8557	8868
UN03317	Rsa1.0_00808.1	1132	63351	0	1120	1130	99	-	3	1131	29216	30345
UN03318	Rsa1.0_01767.1	1012	39615	4.00E-87	182	188	96	-	461	648	14604	14791
UN03319	Rsa1.0_02224.1	1060	33758	1.00E-135	245	245	100	-	203	447	23489	23733
UN03320	Rsa1.0_00169.1	1426	274298	0	909	949	95	-	473	1414	62059	63006
UN03321	Rsa1.0_00380.1	845	89748	1.00E-113	237	248	95	-	226	464	78451	78698
UN03322	Rsa1.0_04652.1	960	16515	3.00E-97	196	201	97	+	109	309	2822	3022
UN03323	Rsa1.0_06549.1	896	10800	0	575	591	97	-	289	874	5361	5951
UN03324	Rsa1.0_04080.1	1934	19198	0	752	814	92	+	1124	1933	9679	10485
UN03325	Rsa1.0_02935.1	548	26621	1.00E-112	228	234	97	-	3	235	25753	25981
UN03326	Rsa1.0_00324.1	1182	218805	0	382	382	100	-	444	825	168953	169334
UN03327	Rsa1.0_01236.1	811	50156	1.00E-101	196	199	98	-	611	809	40403	40601
UN03328	Rsa1.0_00714.1	669	67187	0	514	514	100	+	3	516	58147	58660
UN03329	Rsa1.0_01206.1	784	50772	0	681	703	96	-	89	783	27319	28020
UN03330	Rsa1.0_00018.1	761	410899	0	751	765	98	+	1	761	228726	229488
UN03331	Rsa1.0_04134.1	906	149811	0	725	746	97	+	3	747	5762	6507
UN03332	Rsa1.0_00566.1	781	75325	1.00E-139	276	283	97	+	491	772	23290	23572
UN03333	Rsa1.0_02071.1	1216	35409	0	640	659	97	+	561	1205	15282	15940
UN03334	Rsa1.0_06412.1	1509	11060	0	346	347	99	-	767	1113	1969	2315
UN03335	Rsa1.0_04323.1	928	17915	2.00E-94	206	216	95	-	3	218	15646	15861
UN03336	Rsa1.0_03448.1	1744	22844	1.00E-166	312	317	98	-	1148	1464	9284	9600
UN03337	Rsa1.0_00570.1	2162	75002	0	634	637	99	-	1526	2162	14738	15374
UN03338	Rsa1.0_01011.1	802	56883	0	800	802	99	-	3	802	7360	8161
UN03339	Rsa1.0_04254.1	1311	18258	0	1178	1209	97	-	85	1290	15216	16420
UN03340	Rsa1.0_02803.1	750	27715	0	342	342	100	-	199	540	10374	10715
UN03341	Rsa1.0_01249.1	1405	188432	0	399	406	98	-	7	410	174715	175117
UN03342	Rsa1.0_02666.1	1207	28915	0	859	867	99	-	341	1207	5610	6476
UN03343	Rsa1.0_01747.1	797	39881	2.33E-156	294	303	97	-	491	792	23641	23943
UN03344	Rsa1.0_00104.1	1047	368722	0	400	407	98	+	235	641	279741	280147
UN03345	Rsa1.0_03565.1	1168	22062	1.00E-170	355	371	95	-	2	372	3775	4143
UN03346	Rsa1.0_01049.1	380	55655	1.00E-125	248	255	97	+	1	255	39587	39841
UN03347	Rsa1.0_01695.1	742	40848	0	471	478	98	-	267	742	9080	9557
UN03348	Rsa1.0_00086.1	1663	439854	0	401	412	97	+	641	1052	241976	242387
UN03349	Rsa1.0_00302.1	1000	97521	0	425	432	98	+	104	535	86355	86785
UN03350	Rsa1.0_00122.1	1494	265937	0	467	502	93	-	988	1482	247379	247866
UN03351	Rsa1.0_00654.1	902	70012	1.00E-150	329	348	94	+	295	639	41118	41465
UN03352	Rsa1.0_02070.1	885	158516	0	405	420	96	+	7	426	138395	138814

UN03353	Rsa1.0_00259.1	1202	163204	0	1188	1204	98	−	1	1202	154262	155465
UN03354	Rsa1.0_00661.1	619	213041	2.00E−94	265	291	91	−	37	323	195844	196134
UN03355	Rsa1.0_13906.1	786	4114	1.00E−153	408	452	90	−	228	661	1932	2377
UN03356	Rsa1.0_00007.1	1449	239325	0	462	486	95	+	972	1449	29997	30482
UN03357	Rsa1.0_00031.1	592	179862	1.00E−130	265	274	96	−	305	578	73545	73816
UN03358	Rsa1.0_07133.1	766	9856	0	713	728	97	+	1	728	7050	7765
UN03359	Rsa1.0_00258.1	710	102381	1.00E−106	220	227	96	−	482	708	45419	45643
UN03360	Rsa1.0_18647.1	847	1560	0	347	348	99	−	498	845	585	932
UN03361	Rsa1.0_00684.1	1800	141701	0	902	925	97	−	432	1356	9291	10215
UN03362	Rsa1.0_00176.1	1426	240984	1.00E−111	217	221	98	−	713	933	4524	4744
UN03363	Rsa1.0_02462.1	1412	31158	0	839	840	99	+	3	841	5537	6376
UN03364	Rsa1.0_00134.1	922	390219	1.00E−133	300	313	95	−	22	328	154581	154893
UN03365	Rsa1.0_05713.1	1233	12855	1.00E−114	209	209	100	+	1024	1232	1	209
UN03366	Rsa1.0_01585.1	1122	42806	0	473	481	98	+	593	1065	15623	16103
UN03367	Rsa1.0_01154.1	732	52249	0	443	465	95	−	41	504	38105	38568
UN03368	Rsa1.0_02570.1	842	30000	0	822	837	98	+	5	840	5724	6556
UN03369	Rsa1.0_01182.1	758	167304	1.00E−163	317	324	97	−	1	324	14177	14499
UN03370	Rsa1.0_02514.1	1274	30642	0	980	989	99	−	286	1274	8753	9739
UN03371	Rsa1.0_00153.1	1255	192931	0	367	369	99	+	889	1255	170695	171063
UN03372	Rsa1.0_00010.1	1200	294827	0	614	634	96	−	568	1200	116378	116996
UN03373	Rsa1.0_00359.1	1528	91256	0	703	734	95	−	794	1527	76255	76982
UN03374	Rsa1.0_17830.1	1017	1729	0	611	620	98	−	104	720	904	1523
UN03375	#	#	#	#	#	#	#	#	#	#	#	#
UN03376	Rsa1.0_00017.1	1028	752458	0	490	499	98	+	363	861	392621	393119
UN03377	Rsa1.0_00577.1	1244	174827	1.00E−173	309	309	100	+	3	311	136416	136724
UN03378	Rsa1.0_00363.1	1305	90968	0	563	603	93	−	1	598	63275	63876
UN03379	Rsa1.0_02733.1	1068	28354	0	494	507	97	−	18	524	21873	22377
UN03380	Rsa1.0_03446.1	1003	22876	0	372	374	99	+	1	374	18481	18854
UN03381	Rsa1.0_01305.1	1366	48730	1.00E−129	237	238	99	+	352	589	12354	12591
UN03382	Rsa1.0_05900.1	1087	12295	1.00E−104	216	223	96	+	671	891	4712	4934
UN03383	Rsa1.0_00186.1	967	112669	0	497	503	98	+	3	503	58882	59382
UN03384	Rsa1.0_00779.1	1371	64568	0	461	466	98	+	2	467	20592	21057
UN03385	Rsa1.0_00457.1	531	337301	0	422	434	97	−	6	439	197550	197983
UN03386	Rsa1.0_00438.1	714	84861	0	420	420	100	−	10	429	76921	77340
UN03387	Rsa1.0_00568.1	1057	75219	0	719	720	99	+	2	721	26634	27353
UN03388	Rsa1.0_54382.1	1336	419	0	406	424	95	+	307	720	1	419
UN03389	Rsa1.0_00049.1	1257	167018	0	878	915	95	−	347	1257	78769	79681
UN03390	Rsa1.0_02238.1	893	33540	0	887	892	99	+	1	890	17205	18096
UN03391	Rsa1.0_00566.1	151	75325	8.00E−47	119	127	93	−	23	149	61498	61624

UN03392	Rsa1.0_03728.1	1236	21147	0	407	427	95	+	9	435	3578	4003
UN03393	Rsa1.0_05969.1	1290	12072	0	617	656	94	-	243	895	6377	7011
UN03394	Rsa1.0_00755.1	1330	192559	0	589	621	94	-	1	610	138958	139563
UN03395	Rsa1.0_08807.1	746	6906	0	736	746	98	-	1	746	2629	3374
UN03396	Rsa1.0_00864.1	1412	61702	0	459	460	99	-	953	1412	6569	7028
UN03397	Rsa1.0_00571.1	841	329018	3.00E-90	231	245	94	-	374	614	39794	40037
UN03398	Rsa1.0_00001.1	804	363028	0	781	807	96	-	1	804	147907	148708
UN03399	Rsa1.0_00222.1	763	172263	0	427	448	95	+	287	734	85716	86157
UN03400	Rsa1.0_01420.1	1214	46210	0	756	777	97	+	435	1211	10987	11754
UN03401	Rsa1.0_02812.1	917	27660	1.00E-173	349	361	96	+	540	899	16559	16919
UN03402	Rsa1.0_01260.1	762	49750	0	396	414	95	-	1	411	33751	34164
UN03403	Rsa1.0_00284.1	909	323267	1.00E-136	427	492	86	-	3	471	275018	275487
UN03404	Rsa1.0_01115.1	1077	183545	0	1003	1069	93	+	8	1069	168083	169149
UN03405	Rsa1.0_02029.1	487	35857	0	472	518	91	-	1	487	26144	26661
UN03406	Rsa1.0_03639.1	1022	21629	6.00E-80	195	211	92	-	3	203	4990	5200
UN03407	Rsa1.0_02931.1	1212	26654	0	1177	1214	96	-	1	1212	19523	20730
UN03408	Rsa1.0_01027.1	858	56402	0	439	441	99	+	1	440	44990	45430
UN03409	Rsa1.0_00576.1	1146	178949	0	1078	1116	96	+	30	1145	28622	29735
UN03410	Rsa1.0_00097.1	624	138858	6.00E-14	98	117	83	-	185	301	106303	106419
UN03411	Rsa1.0_01054.1	973	55470	0	960	989	97	+	1	971	23570	24556
UN03412	Rsa1.0_00738.1	1030	66322	1.00E-136	294	308	95	-	722	1027	27060	27359
UN03413	Rsa1.0_17682.1	976	1765	0	862	943	91	-	55	973	37	971
UN03414	Rsa1.0_04026.1	3219	19508	0	865	866	99	+	1	866	6275	7140
UN03415	Rsa1.0_00415.1	692	86795	0	600	621	96	+	72	692	8987	9607
UN03416	Rsa1.0_02981.1	672	26285	1.00E-140	274	280	97	+	4	283	5774	6052
UN03417	Rsa1.0_00231.1	943	105381	0	377	378	99	-	1	377	26766	27143
UN03418	Rsa1.0_03427.1	822	22992	0	425	434	97	+	107	540	1	434
UN03419	Rsa1.0_01724.1	535	40292	1.00E-130	301	322	93	+	1	312	10005	10323
UN03420	Rsa1.0_01007.1	534	56933	0	520	524	99	-	10	533	46916	47439
UN03421	Rsa1.0_05572.1	510	13291	1.00E-124	232	234	99	+	250	483	11854	12087
UN03422	Rsa1.0_00190.1	1777	318240	1.00E-179	405	431	93	-	1349	1777	300505	300925
UN03423	Rsa1.0_00173.1	727	288743	0	455	496	91	-	3	495	64738	65232
UN03424	Rsa1.0_00382.1	762	251946	0	541	549	98	-	1	546	155766	156313
UN03425	Rsa1.0_06136.1	1342	11659	0	1335	1346	99	+	1	1342	3199	4544
UN03426	Rsa1.0_01523.1	736	43959	0	589	615	95	+	4	616	26567	27173
UN03427	Rsa1.0_00635.1	648	175826	1.00E-135	329	354	92	+	1	352	123372	123721
UN03428	Rsa1.0_01951.1	690	36941	0	363	367	98	+	3	369	2054	2418
UN03429	Rsa1.0_00064.1	1436	240927	0	1265	1280	98	-	2	1277	7959	9238
UN03430	Rsa1.0_23614.1	838	1037	0	731	808	90	+	1	800	20	821

UN03431	Rsa1.0_00203.1	875	109129	1.00E-125	286	303	94	+	2	302	27426	27728
UN03432	Rsa1.0_16058.1	758	2302	1.00E-152	285	289	98	+	177	465	1139	1427
UN03433	Rsa1.0_00108.1	729	478491	1.00E-109	224	232	96	+	116	347	119259	119490
UN03434	Rsa1.0_03951.1	1035	19894	1.00E-158	291	292	99	+	2	292	2811	3102
UN03435	Rsa1.0_00734.1	829	66508	1.00E-150	321	336	95	+	2	335	50875	51209
UN03436	Rsa1.0_01642.1	789	41772	1.00E-117	228	232	98	+	3	230	20716	20947
UN03437	Rsa1.0_00451.1	1092	322533	1.00E-134	249	251	99	−	1	251	293086	293336
UN03438	Rsa1.0_00453.1	791	83031	1.00E-177	358	371	96	+	6	370	54730	55100
UN03439	Rsa1.0_04355.1	761	146733	0	555	582	95	+	3	579	3253	3831
UN03440	Rsa1.0_01735.1	1100	40128	0	736	765	96	+	336	1099	33599	34351
UN03441	Rsa1.0_08548.1	1379	7212	0	796	810	98	−	573	1379	2923	3732
UN03442	Rsa1.0_03036.1	811	25826	5.00E-95	193	197	97	−	616	811	13406	13602
UN03443	Rsa1.0_01485.1	824	44797	0	382	387	98	−	3	386	11613	11999
UN03444	Rsa1.0_00056.1	914	332604	0	445	470	94	−	4	468	96611	97079
UN03445	Rsa1.0_02353.1	1344	32300	0	603	639	94	+	1	633	30075	30701
UN03446	Rsa1.0_00453.1	835	83031	0	731	777	94	−	8	782	75551	76302
UN03447	Rsa1.0_01806.1	1065	39021	0	1060	1066	99	−	1	1065	34815	35880
UN03448	Rsa1.0_02865.1	861	27133	0	390	408	95	−	2	399	12296	12703
UN03449	Rsa1.0_02080.1	1366	35297	0	1360	1366	99	−	1	1366	13168	14533
UN03450	Rsa1.0_00740.1	1345	215972	0	612	629	97	+	716	1344	198503	199128
UN03451	Rsa1.0_00284.1	956	323267	0	581	612	94	+	1	612	287184	287792
UN03452	Rsa1.0_00471.1	1670	81897	0	329	329	100	−	1	329	10228	10556
UN03453	Rsa1.0_02957.1	897	26479	1.00E-25	178	213	83	+	416	626	14087	14298
UN03454	Rsa1.0_03093.1	892	25445	1.00E-111	234	243	96	−	1	238	3055	3295
UN03455	Rsa1.0_10193.1	1565	5950	0	384	392	97	+	510	901	661	1052
UN03456	Rsa1.0_01232.1	1301	155185	1.00E-112	219	223	98	−	288	510	131817	132039
UN03457	Rsa1.0_00067.1	1193	415055	0	1185	1192	99	−	1	1192	156898	158088
UN03458	Rsa1.0_00345.1	1169	93226	0	557	594	93	−	578	1168	54790	55365
UN03459	Rsa1.0_00380.1	789	89748	0	777	791	98	+	2	789	56563	57353
UN03460	Rsa1.0_00167.1	705	116000	0	541	559	96	+	114	672	33824	34381
UN03461	Rsa1.0_05669.1	705	12993	1.00E-178	347	356	97	−	39	394	6854	7208
UN03462	Rsa1.0_00065.1	926	454616	1.00E-126	291	306	95	−	3	306	173385	173684
UN03463	Rsa1.0_00409.1	1268	146788	0	762	804	94	−	5	808	9529	10325
UN03464	Rsa1.0_07567.1	1665	9117	0	595	604	98	+	3	606	837	1439
UN03465	Rsa1.0_00708.1	905	67366	0	488	492	99	−	1	492	63092	63580
UN03466	Rsa1.0_04126.1	772	18939	0	387	393	98	−	4	396	17496	17888
UN03467	Rsa1.0_00510.1	1366	78718	2.33E-156	347	375	92	−	87	446	28307	28681
UN03468	Rsa1.0_48789.1	1352	464	0	356	357	99	−	426	782	73	429
UN03469	Rsa1.0_00009.1	978	232558	0	460	463	99	+	1	462	29413	29875

UN03470	Rsa1.0_02787.1	786	27872	0	776	785	98	−	1	785	684	1467
UN03471	Rsa1.0_00391.1	819	183853	9.00E−66	140	144	97	+	1	144	30672	30815
UN03472	Rsa1.0_00922.1	1292	59588	5.00E−59	150	161	93	−	824	984	20072	20232
UN03473	Rsa1.0_02805.1	988	27698	1.00E−110	208	210	99	−	2	211	2636	2845
UN03474	Rsa1.0_03636.1	654	21638	1.00E−104	208	213	97	−	1	213	17912	18124
UN03475	Rsa1.0_00190.1	733	318240	0	636	691	92	+	3	677	286420	287104
UN03476	Rsa1.0_06102.1	530	11740	1.00E−173	320	323	99	+	1	321	4421	4743
UN03477	Rsa1.0_00658.1	936	69740	1.00E−162	322	328	98	+	11	336	15706	16029
UN03478	Rsa1.0_00071.1	435	345714	1.00E−125	258	267	96	+	2	267	324470	324736
UN03479	Rsa1.0_00377.1	1388	90038	0	424	428	99	−	8	435	47697	48124
UN03480	Rsa1.0_05701.1	764	12884	0	758	764	99	+	3	764	9899	10662
UN03481	Rsa1.0_00111.1	899	390088	0	419	440	95	−	453	892	4781	5217
UN03482	Rsa1.0_10645.1	1038	5771	1.00E−147	275	277	99	+	1	276	5495	5771
UN03483	Rsa1.0_01024.1	768	56569	3.00E−44	111	116	95	−	651	766	6288	6402
UN03484	Rsa1.0_03225.1	1070	24500	0	475	509	93	−	6	512	14578	15068
UN03485	Rsa1.0_00224.1	969	327530	1.00E−177	324	327	99	+	1	327	228495	228821
UN03486	Rsa1.0_00015.1	1357	561803	0	455	456	99	+	1	456	178658	179113
UN03487	Rsa1.0_11431.1	993	5510	0	392	407	96	+	2	404	839	1245
UN03488	Rsa1.0_02513.1	1031	30643	0	1021	1028	99	−	3	1029	18779	19806
UN03489	Rsa1.0_02411.1	914	31655	0	466	472	98	+	356	827	27638	28109
UN03490	Rsa1.0_02681.1	690	28711	1.00E−158	307	315	97	−	58	372	20439	20753
UN03491	Rsa1.0_02296.1	886	32920	9.00E−57	181	200	90	−	640	839	12164	12359
UN03492	Rsa1.0_11032.1	928	5640	0	569	571	99	+	71	641	5070	5640
UN03493	Rsa1.0_01403.1	743	46453	0	730	743	98	−	1	743	24737	25479
UN03494	Rsa1.0_02091.1	1408	35157	0	1213	1275	95	−	98	1367	4066	5322
UN03495	Rsa1.0_01327.1	975	48033	0	963	968	99	+	1	968	47066	48033
UN03496	Rsa1.0_00644.1	958	136881	0	367	368	99	−	1	368	31373	31740
UN03497	Rsa1.0_05354.1	801	14073	1.00E−148	310	324	95	+	341	661	5027	5350
UN03498	Rsa1.0_02622.1	1153	142402	0	652	666	97	+	488	1151	9528	10193
UN03499	Rsa1.0_05795.1	1070	12609	0	604	605	99	+	2	606	7660	8264
UN03500	Rsa1.0_14886.1	1306	2991	0	1116	1146	97	−	1	1143	1501	2637
UN03501	Rsa1.0_01139.1	1273	52601	0	468	470	99	+	802	1271	18673	19142
UN03502	Rsa1.0_00054.1	1419	162053	0	734	772	95	+	516	1272	15265	16036
UN03503	Rsa1.0_02099.1	1379	35030	0	450	450	100	+	925	1374	2291	2740
UN03504	Rsa1.0_01213.1	973	50616	0	967	971	99	−	1	971	6836	7806
UN03505	Rsa1.0_09522.1	835	6316	0	809	822	98	−	1	822	1	819
UN03506	Rsa1.0_04444.1	2120	17320	0	488	532	91	+	1	518	8362	8888
UN03507	Rsa1.0_00014.1	1213	348985	0	552	618	89	−	596	1213	331473	332065
UN03508	Rsa1.0_01073.1	1235	55030	0	1173	1235	94	−	2	1231	6030	7237

UN03509	Rsa1.0_00098.1	937	349239	2.00E-67	137	139	98	-	276	414	314363	314501
UN03510	Rsa1.0_00548.1	1070	76518	1.00E-120	232	236	98	-	3	238	62227	62462
UN03511	Rsa1.0_00112.1	944	132199	0	944	945	99	+	1	944	98359	99303
UN03512	Rsa1.0_01800.1	1037	39109	0	395	397	99	+	11	407	28395	28791
UN03513	Rsa1.0_27162.1	1448	863	1.00E-117	267	286	93	+	1170	1446	506	791
UN03514	Rsa1.0_07466.1	1149	9321	8.00E-24	100	114	87	+	1009	1122	4059	4172
UN03515	Rsa1.0_13685.1	1238	4481	3.00E-14	77	86	89	-	617	700	3795	3880
UN03516	Rsa1.0_00128.1	960	228675	1.00E-164	315	322	97	+	211	532	116359	116680
UN03517	Rsa1.0_00088.1	645	141341	1.00E-100	195	198	98	+	7	204	56606	56803
UN03518	Rsa1.0_12091.1	1148	5316	0	366	381	96	-	1	381	3852	4232
UN03519	Rsa1.0_00577.1	792	174827	0	426	447	95	+	148	591	149759	150199
UN03520	Rsa1.0_00152.1	1056	257730	0	528	541	97	+	519	1055	240049	240585
UN03521	Rsa1.0_02869.1	1279	27109	0	1275	1279	99	+	1	1279	13911	15189
UN03522	Rsa1.0_02722.1	849	28417	5.00E-92	175	176	99	+	241	416	15840	16015
UN03523	Rsa1.0_00875.1	792	61371	0	350	357	98	-	4	360	60272	60628
UN03524	Rsa1.0_05900.1	1400	12295	0	402	415	96	-	692	1103	3737	4151
UN03525	Rsa1.0_00484.1	1606	203294	0	364	365	99	-	1243	1606	135710	136074
UN03526	Rsa1.0_00048.1	629	167185	5.00E-79	237	267	88	+	2	263	32133	32381
UN03527	Rsa1.0_05413.1	1147	13834	0	750	759	98	-	182	940	4335	5093
UN03528	Rsa1.0_01400.1	696	46560	1.00E-147	311	325	95	+	3	322	11274	11598
UN03529	Rsa1.0_03148.1	1638	24989	1.00E-171	377	400	94	+	1177	1576	21012	21408
UN03530	Rsa1.0_01184.1	1236	51452	0	496	502	98	+	737	1236	50577	51078
UN03531	Rsa1.0_00022.1	1202	200461	0	740	766	96	+	189	951	162335	163100
UN03532	Rsa1.0_01228.1	1181	50238	0	437	443	98	-	2	444	2671	3113
UN03533	Rsa1.0_11552.1	1460	5472	0	942	944	99	-	517	1460	4529	5472
UN03534	Rsa1.0_02969.1	592	26390	1.00E-167	329	338	97	-	255	592	10013	10349
UN03535	Rsa1.0_00312.1	1334	96765	1.00E-124	236	239	98	+	1093	1331	67188	67426
UN03536	Rsa1.0_12829.1	780	5111	1.00E-148	336	358	93	-	3	348	1801	2158
UN03537	Rsa1.0_00833.1	899	62621	1.00E-173	308	308	100	-	592	899	8598	8905
UN03538	Rsa1.0_00222.1	1271	172263	0	1189	1259	94	-	32	1271	22592	23839
UN03539	Rsa1.0_02882.1	1144	27039	1.00E-161	313	320	97	-	824	1143	3418	3736
UN03540	Rsa1.0_02860.1	956	27207	9.00E-54	114	116	98	-	602	717	9938	10053
UN03541	Rsa1.0_02682.1	1218	28711	0	935	971	96	-	129	1096	9789	10759
UN03542	Rsa1.0_03728.1	1468	21147	1.00E-157	360	387	93	-	976	1349	3617	4003
UN03543	Rsa1.0_00487.1	1739	80674	0	1017	1046	97	-	93	1138	4125	5170
UN03544	Rsa1.0_00593.1	964	143463	1.00E-175	312	312	100	-	653	964	57024	57335
UN03545	Rsa1.0_01866.1	620	37961	1.00E-141	283	291	97	-	1	290	1850	2140
UN03546	Rsa1.0_05278.1	778	14282	2.00E-85	167	169	98	-	456	624	9732	9900
UN03547	Rsa1.0_01143.1	899	52456	0	739	755	97	-	137	891	20604	21352

UN03548	Rsa1.0_00042.1	938	171507	0	428	442	96	−	483	923	143977	144412
UN03549	Rsa1.0_02320.1	787	32575	1.00E−164	355	375	94	+	1	375	22073	22444
UN03550	Rsa1.0_00350.1	1052	92346	4.00E−87	164	164	100	−	651	814	79954	80117
UN03551	Rsa1.0_00770.1	612	64900	0	603	609	99	+	4	612	3697	4304
UN03552	Rsa1.0_00363.1	685	90968	0	424	437	97	−	249	685	13482	13913
UN03553	Rsa1.0_02750.1	984	28239	2.00E−82	190	198	95	−	59	252	23986	24182
UN03554	Rsa1.0_06701.1	886	10514	0	436	447	97	+	3	449	5788	6234
UN03555	Rsa1.0_00016.1	861	831256	1.00E−109	233	242	96	−	618	859	204654	204890
UN03556	Rsa1.0_06535.1	1411	10827	0	1340	1406	95	+	6	1410	2660	4045
UN03557	Rsa1.0_03790.1	1652	20844	1.00E−178	336	341	98	−	51	390	8731	9071
UN03558	Rsa1.0_00273.1	897	326186	0	547	547	100	−	350	896	26532	27078
UN03559	Rsa1.0_00183.1	1464	113277	0	749	781	95	+	695	1464	24804	25583
UN03560	Rsa1.0_02754.1	1058	28213	4.00E−87	189	197	95	−	1	193	2089	2285
UN03561	Rsa1.0_00055.1	811	520193	1.00E−165	388	409	94	+	387	785	163773	164181
UN03562	Rsa1.0_01923.1	820	37233	3.00E−84	168	171	98	+	98	268	35356	35526
UN03563	Rsa1.0_05026.1	1474	15095	0	432	436	99	+	1039	1474	9795	10230
UN03564	Rsa1.0_02523.1	1336	30527	0	921	928	99	−	221	1145	8432	9359
UN03565	Rsa1.0_00163.1	884	116847	1.00E−120	251	262	95	−	254	515	11140	11395
UN03566	Rsa1.0_00113.1	1251	131574	0	518	533	97	+	719	1251	130815	131338
UN03567	Rsa1.0_01092.1	910	54348	1.00E−155	281	282	99	+	628	909	51572	51853
UN03568	Rsa1.0_00556.1	1299	207212	0	466	489	95	+	822	1297	47334	47820
UN03569	Rsa1.0_21301.1	932	1217	1.00E−152	273	273	100	+	3	275	105	377
UN03570	Rsa1.0_00100.1	862	249232	1.00E−137	301	319	94	+	1	313	11198	11516
UN03571	Rsa1.0_20105.1	1006	1340	0	388	390	99	+	618	1006	457	846
UN03572	Rsa1.0_03420.1	1365	23090	0	627	632	99	−	734	1365	18006	18637
UN03573	Rsa1.0_02139.1	813	34686	1.00E−114	241	246	97	−	535	776	5667	5912
UN03574	Rsa1.0_19040.1	737	1491	1.00E−147	361	392	92	+	284	674	953	1344
UN03575	Rsa1.0_02686.1	1411	28666	2.00E−28	102	114	89	−	129	242	24224	24337
UN03576	Rsa1.0_01344.1	1075	142535	0	1027	1032	99	+	3	1033	127388	128419
UN03577	Rsa1.0_00107.1	543	431659	1.00E−141	258	259	99	+	79	337	84232	84490
UN03578	Rsa1.0_00033.1	1054	515811	0	564	570	98	+	2	571	144223	144788
UN03579	Rsa1.0_01556.1	1223	163858	0	614	640	95	−	288	927	142924	143557
UN03580	Rsa1.0_13949.1	731	4028	1.00E−144	268	271	98	+	461	731	2439	2709
UN03581	Rsa1.0_16086.1	917	2289	1.00E−124	238	242	98	+	676	917	1078	1319
UN03582	Rsa1.0_20678.1	1212	1280	1.00E−105	198	199	99	+	521	719	372	570
UN03583	Rsa1.0_07475.1	697	9292	0	457	463	98	+	3	462	6531	6993
UN03584	Rsa1.0_03280.1	877	145083	0	446	446	100	−	432	877	1611	2056
UN03585	Rsa1.0_01634.1	1318	41865	0	358	362	98	+	958	1318	34189	34550
UN03586	Rsa1.0_00566.1	820	75325	1.00E−132	276	283	97	+	495	773	23290	23572

UN03587	Rsa1.0_01975.1	1184	36578	1.00E-118	223	225	99	−	2	226	34503	34727
UN03588	Rsa1.0_00049.1	1100	167018	3.00E-17	230	291	79	+	129	419	19574	19864
UN03589	Rsa1.0_00929.1	1322	137796	1.00E-133	248	249	99	−	1	249	99706	99953
UN03590	Rsa1.0_28856.1	635	801	0	504	544	92	+	81	620	255	797
UN03591	Rsa1.0_04557.1	1117	145048	7.00E-58	127	131	96	−	136	266	137585	137715
UN03592	Rsa1.0_07444.1	710	9386	0	704	709	99	+	2	710	8109	8817
UN03593	Rsa1.0_01719.1	914	40388	1.00E-107	198	198	100	−	717	914	27267	27464
UN03594	Rsa1.0_00018.1	1536	410899	2.00E-96	199	204	97	+	57	259	122671	122874
UN03595	Rsa1.0_00066.1	1171	152418	0	496	539	92	−	635	1171	14990	15508
UN03596	Rsa1.0_00111.1	873	390088	1.00E-127	280	291	96	−	577	863	76129	76419
UN03597	Rsa1.0_01506.1	1419	44370	0	351	356	98	+	4	358	28343	28698
UN03598	Rsa1.0_01845.1	1234	38415	1.00E-155	284	286	99	+	949	1234	8035	8320
UN03599	Rsa1.0_00108.1	1168	478491	0	552	576	95	+	583	1151	77201	77775
UN03600	Rsa1.0_00019.1	737	301669	0	717	733	97	−	6	737	192346	193078
UN03601	Rsa1.0_31506.1	1191	725	0	700	745	93	+	382	1117	1	725
UN03602	Rsa1.0_00571.1	1199	329018	0	656	676	97	+	446	1119	204751	205423
UN03603	Rsa1.0_03465.1	1840	22700	0	941	982	95	−	186	1163	16546	17526
UN03604	Rsa1.0_00960.1	738	58199	0	454	454	100	+	3	456	21057	21510
UN03605	Rsa1.0_00713.1	1310	158017	0	473	489	96	−	725	1213	1180	1668
UN03606	Rsa1.0_02165.1	728	34423	1.00E-159	300	305	98	−	239	543	23089	23393
UN03607	Rsa1.0_01232.1	1207	155185	1.00E-112	219	223	98	+	678	900	131817	132039
UN03608	Rsa1.0_00088.1	742	141341	0	452	469	96	+	3	468	10088	10550
UN03609	Rsa1.0_01614.1	804	42238	1.00E-157	305	312	97	−	493	804	18880	19189
UN03610	Rsa1.0_00566.1	989	75325	1.00E-161	303	308	98	+	265	572	66997	67304
UN03611	Rsa1.0_00213.1	538	108167	7.00E-81	261	294	88	+	255	523	39818	40110
UN03612	Rsa1.0_00532.1	880	77384	0	432	433	99	−	1	432	52841	53273
UN03613	Rsa1.0_02631.1	1514	29343	0	416	419	99	+	826	1244	22559	22977
UN03614	Rsa1.0_00110.1	1271	132719	0	691	772	89	−	501	1269	49779	50543
UN03615	Rsa1.0_02826.1	959	27560	1.00E-143	286	294	97	−	666	959	14788	15075
UN03616	Rsa1.0_00678.1	600	193163	4.00E-24	73	78	93	−	160	237	26963	27040
UN03617	Rsa1.0_03761.1	760	20982	0	697	711	98	−	56	760	11391	12101
UN03618	Rsa1.0_00056.1	1319	332604	1.00E-176	479	531	90	−	800	1319	114291	114791
UN03619	Rsa1.0_00567.1	1991	75260	0	412	413	99	−	1	413	60996	61408
UN03620	Rsa1.0_64105.1	720	358	1.00E-104	211	217	97	−	291	507	120	336
UN03621	Rsa1.0_00243.1	1285	103651	0	893	921	96	−	368	1284	20396	21316
UN03622	Rsa1.0_00610.1	1963	72740	0	734	763	96	−	28	790	44345	45104
UN03623	Rsa1.0_00289.1	1249	98620	0	537	543	98	+	707	1249	25075	25617
UN03624	Rsa1.0_11393.1	1317	5521	0	417	420	99	−	4	423	205	624
UN03625	Rsa1.0_00270.1	848	101236	3.00E-75	153	156	98	−	89	244	1852	2007

UN03626	Rsa1.0_02389.1	2050	146428	0	1028	1040	98	+	1008	2047	128372	129411
UN03627	Rsa1.0_00516.1	1357	78417	0	538	555	96	-	24	578	7335	7889
UN03628	Rsa1.0_00337.1	795	143174	1.00E-92	229	247	92	-	179	425	141486	141719
UN03629	Rsa1.0_01156.1	1401	52147	0	540	581	92	-	776	1347	48765	49327
UN03630	Rsa1.0_01539.1	879	43698	6.00E-98	197	202	97	-	524	725	38016	38217
UN03631	Rsa1.0_00338.1	873	94109	0	671	685	97	-	3	687	90088	90771
UN03632	Rsa1.0_01095.1	1302	54276	0	439	451	97	-	2	449	27903	28353
UN03633	Rsa1.0_47443.1	1179	476	1.00E-71	197	222	88	+	1	222	264	464
UN03634	Rsa1.0_01146.1	576	163455	4.00E-70	165	175	94	-	158	332	41964	42138
UN03635	Rsa1.0_04040.1	778	19439	0	437	451	96	-	214	664	9830	10280
UN03636	Rsa1.0_02611.1	1136	29586	0	408	411	99	-	335	745	1337	1747
UN03637	Rsa1.0_00196.1	834	109960	1.00E-176	365	382	95	+	4	385	10148	10529
UN03638	Rsa1.0_12445.1	1651	5224	0	524	527	99	+	1125	1651	3411	3937
UN03639	Rsa1.0_01360.1	1178	47246	1.00E-162	321	330	97	-	848	1177	21073	21401
UN03640	#	#	#	#	#	#	#	#	#	#	#	#
UN03641	Rsa1.0_53010.1	1212	430	4.00E-81	173	178	97	-	915	1091	1	178
UN03642	Rsa1.0_08072.1	857	7955	0	695	730	95	-	131	857	2130	2858
UN03643	Rsa1.0_01640.1	1056	41822	0	504	518	97	+	2	516	36325	36839
UN03644	Rsa1.0_03506.1	1552	22416	0	684	711	96	+	3	710	7178	7878
UN03645	Rsa1.0_02093.1	793	35125	0	395	406	97	+	131	536	20234	20639
UN03646	Rsa1.0_00125.1	1197	253043	0	532	597	89	-	227	823	770	1363
UN03647	Rsa1.0_01363.1	1109	47195	0	764	793	96	-	324	1107	11032	11818
UN03648	Rsa1.0_02707.1	1338	28520	0	777	787	98	-	552	1338	16576	17360
UN03649	Rsa1.0_02411.1	791	31655	0	419	430	97	-	3	432	29042	29469
UN03650	Rsa1.0_00625.1	1294	71672	1.00E-142	308	321	95	-	975	1294	30706	31024
UN03651	Rsa1.0_00116.1	815	365886	0	751	802	93	+	3	796	211736	212531
UN03652	Rsa1.0_01799.1	1456	39127	0	580	612	94	+	1	597	2429	3040
UN03653	Rsa1.0_03028.1	996	25872	0	442	447	98	+	2	448	2851	3296
UN03654	Rsa1.0_04379.1	1338	17646	0	581	590	98	-	663	1249	4214	4803
UN03655	Rsa1.0_00039.1	995	296927	0	369	378	97	+	589	966	290916	291293
UN03656	Rsa1.0_00920.1	1256	601703	0	840	844	99	+	1	844	436590	437433
UN03657	Rsa1.0_00436.1	875	85008	1.00E-102	328	369	88	-	1	358	34234	34599
UN03658	Rsa1.0_00361.1	1318	91181	0	484	484	100	+	835	1318	79919	80402
UN03659	Rsa1.0_01559.1	642	43262	1.00E-120	253	262	96	-	1	260	2270	2530
UN03660	Rsa1.0_00816.1	720	403350	1.00E-134	267	273	97	-	1	273	154309	154578
UN03661	Rsa1.0_00661.1	879	213041	1.00E-144	295	307	96	+	573	879	203672	203978
UN03662	Rsa1.0_01333.1	1009	47929	0	563	563	100	-	3	565	29681	30243
UN03663	Rsa1.0_00412.1	1270	86935	1.00E-126	316	342	92	-	5	343	49294	49631
UN03664	Rsa1.0_00075.1	953	146680	0	916	953	96	-	1	950	28329	29281

UN03665	Rsa1.0_00839.1	939	62533	0	467	469	99	+	3	470	29345	29813
UN03666	Rsa1.0_01580.1	1091	42852	1.00E-166	387	416	93	−	1	405	40018	40433
UN03667	Rsa1.0_02914.1	519	26815	0	352	358	98	−	160	517	26110	26467
UN03668	Rsa1.0_04374.1	191	17670	4.00E-80	178	187	95	−	1	187	8052	8238
UN03669	Rsa1.0_02939.1	829	26595	0	479	510	93	+	1	498	16774	17273
UN03670	Rsa1.0_00006.1	1700	396176	1.00E-155	298	302	98	+	1398	1698	305844	306143
UN03671	Rsa1.0_03007.1	938	26070	3.00E-66	181	195	92	−	16	205	17499	17693
UN03672	Rsa1.0_01230.1	1150	50221	1.00E-121	230	233	98	+	1	233	22666	22898
UN03673	Rsa1.0_00417.1	685	395068	1.00E-152	300	309	97	+	369	677	221978	222286
UN03674	Rsa1.0_04588.1	1089	16739	1.00E-153	274	274	100	−	1	274	15917	16190
UN03675	Rsa1.0_00094.1	947	139042	1.00E-120	310	341	90	−	11	344	41073	41398
UN03676	Rsa1.0_04482.1	1709	17175	0	1313	1356	96	−	3	1353	6014	7358
UN03677	Rsa1.0_01825.1	1426	38725	0	361	362	99	−	1063	1424	21288	21649
UN03678	Rsa1.0_07638.1	1240	8967	1.00E-114	209	209	100	−	1031	1239	4347	4555
UN03679	Rsa1.0_00039.1	1043	296927	0	750	827	90	+	216	1042	219101	219927
UN03680	Rsa1.0_01344.1	946	142535	0	456	461	98	−	3	461	130981	131441
UN03681	Rsa1.0_01221.1	949	50421	0	890	949	93	−	3	948	18208	19152
UN03682	Rsa1.0_00592.1	954	73788	1.00E-118	294	318	92	+	62	370	22125	22442
UN03683	Rsa1.0_01111.1	947	53669	0	360	361	99	+	3	363	31370	31730
UN03684	Rsa1.0_02295.1	1034	149884	2.00E-98	186	187	99	−	1	187	146923	147109
UN03685	Rsa1.0_00092.1	849	659932	0	641	649	98	+	1	647	126051	126699
UN03686	Rsa1.0_02654.1	1208	29028	0	460	469	98	−	741	1207	22466	22934
UN03687	Rsa1.0_02513.1	1035	30643	0	580	602	96	+	439	1031	29577	30178
UN03688	Rsa1.0_02942.1	904	26577	0	595	626	95	−	1	620	5773	6396
UN03689	Rsa1.0_00232.1	1024	104936	1.00E-156	311	322	96	−	2	323	38388	38703
UN03690	Rsa1.0_08740.1	1008	6980	0	779	781	99	+	1	781	6200	6980
UN03691	Rsa1.0_01123.1	1137	53359	0	407	425	95	−	712	1136	29822	30246
UN03692	Rsa1.0_00145.1	813	261008	0	813	813	100	−	1	813	233358	234170
UN03693	Rsa1.0_00410.1	1633	86957	0	475	491	96	+	1150	1632	69069	69559
UN03694	Rsa1.0_02555.1	1180	30146	2.33E-156	341	365	93	−	6	368	2219	2571
UN03695	Rsa1.0_00077.1	1219	145005	0	1184	1212	97	−	10	1217	105855	107065
UN03696	Rsa1.0_16602.1	1123	2094	0	680	683	99	+	442	1123	1	683
UN03697	Rsa1.0_03553.1	1044	22150	1.00E-166	320	327	97	+	1	324	14901	15227
UN03698	Rsa1.0_00329.1	1366	222544	0	1326	1361	97	+	3	1363	60715	62072
UN03699	Rsa1.0_00116.1	868	365886	1.00E-122	238	242	98	+	19	260	239019	239259
UN03700	Rsa1.0_00336.1	1229	445051	0	780	795	98	−	434	1228	224609	225403
UN03701	Rsa1.0_01156.1	879	52147	0	433	436	99	+	4	439	18093	18528
UN03702	Rsa1.0_00476.1	548	280966	0	398	398	100	+	1	398	223006	223403
UN03703	Rsa1.0_00001.1	923	363028	0	533	537	99	−	6	542	333079	333615

UN03704	Rsa1.0_01803.1	841	173846	0	444	451	98	−	1	451	37469	37915
UN03705	Rsa1.0_02442.1	563	31355	0	398	398	100	+	1	398	24462	24859
UN03706	Rsa1.0_00061.1	635	218685	1.00E−158	304	310	98	−	2	309	100416	100725
UN03707	Rsa1.0_00079.1	814	606987	0	694	712	97	+	3	713	53242	53953
UN03708	Rsa1.0_02630.1	1087	29349	0	625	677	92	−	5	676	26496	27172
UN03709	Rsa1.0_00437.1	1349	84879	0	478	501	95	−	1	486	37648	38148
UN03710	Rsa1.0_06225.1	1301	11443	0	363	363	100	+	275	637	8432	8794
UN03711	Rsa1.0_01302.1	1264	48907	1.00E−158	293	296	98	−	5	300	2932	3227
UN03712	Rsa1.0_03345.1	847	23590	0	447	448	99	−	7	454	21924	22371
UN03713	Rsa1.0_02274.1	581	33109	0	356	361	98	−	2	360	22370	22730
UN03714	Rsa1.0_01753.1	1650	39794	0	602	624	96	+	111	734	21547	22170
UN03715	Rsa1.0_00307.1	1351	199003	1.00E−172	310	311	99	+	1040	1350	184644	184954
UN03716	Rsa1.0_00875.1	1327	61371	0	469	471	99	+	1	471	58161	58631
UN03717	Rsa1.0_03686.1	778	21387	1.00E−110	206	207	99	+	229	435	9134	9340
UN03718	Rsa1.0_02265.1	834	33233	0	574	591	97	+	2	585	9638	10228
UN03719	Rsa1.0_00322.1	716	212233	0	359	362	99	+	355	716	208091	208452
UN03720	Rsa1.0_04411.1	1468	142979	0	412	418	98	+	1	418	134958	135375
UN03721	Rsa1.0_02702.1	1175	28572	1.00E−152	353	381	92	−	3	364	6909	7288
UN03722	Rsa1.0_00502.1	1305	204180	0	431	440	97	−	865	1304	72582	73021
UN03723	Rsa1.0_00505.1	877	79323	0	864	875	98	−	2	876	27550	28424
UN03724	Rsa1.0_00005.1	889	353093	0	393	399	98	−	1	398	347680	348076
UN03725	Rsa1.0_00532.1	1197	77384	0	565	566	99	−	1	566	7546	8111
UN03726	Rsa1.0_05117.1	1096	14801	0	411	413	99	+	682	1094	13785	14197
UN03727	Rsa1.0_00069.1	971	403561	1.00E−159	291	293	99	+	593	885	179274	179566
UN03728	Rsa1.0_04411.1	861	142979	1.00E−150	280	282	99	−	1	282	128710	128990
UN03729	Rsa1.0_02045.1	1394	35650	0	334	335	99	−	1	335	22741	23075
UN03730	Rsa1.0_01069.1	1023	55113	0	1016	1020	99	+	3	1021	16393	17410
UN03731	Rsa1.0_00355.1	556	92038	0	493	499	98	+	1	499	77899	78397
UN03732	Rsa1.0_03393.1	801	23247	0	567	577	98	−	224	800	22595	23169
UN03733	Rsa1.0_00050.1	927	237174	0	805	819	98	+	1	819	85006	85823
UN03734	Rsa1.0_00639.1	1726	165873	0	526	542	97	+	842	1383	144584	145125
UN03735	Rsa1.0_13762.1	820	4370	0	533	534	99	+	1	534	3837	4370
UN03736	Rsa1.0_02798.1	886	27792	0	591	612	96	−	3	606	26142	26750
UN03737	Rsa1.0_00121.1	597	278029	1.00E−132	257	263	97	−	273	535	114461	114723
UN03738	Rsa1.0_09471.1	722	6345	1.00E−121	224	225	99	−	498	722	522	746
UN03739	Rsa1.0_00595.1	911	341648	1.00E−148	272	274	99	+	2	275	142000	142273
UN03740	Rsa1.0_08741.1	719	6977	1.00E−103	249	270	92	−	449	718	5630	5884
UN03741	Rsa1.0_00130.1	875	125737	0	530	572	92	−	2	557	28245	28815
UN03742	Rsa1.0_02902.1	1239	26907	1.00E−176	325	328	99	−	1	326	1	328

UN03743	Rsa1.0_00039.1	1389	296927	1.00E-124	257	266	96	+	8	271	210823	211088
UN03744	Rsa1.0_00282.1	1477	177799	0	611	614	99	−	4	617	172464	173077
UN03745	Rsa1.0_00931.1	727	59210	0	451	475	94	+	1	474	26244	26698
UN03746	Rsa1.0_03281.1	1332	143349	0	419	424	98	+	7	430	2518	2941
UN03747	Rsa1.0_00276.1	836	100160	0	819	822	99	+	1	822	13658	14479
UN03748	Rsa1.0_00646.1	944	70473	1.00E-154	284	285	99	+	1	284	5970	6254
UN03749	Rsa1.0_06696.1	567	10526	0	497	502	99	+	1	502	10025	10526
UN03750	Rsa1.0_03256.1	812	24305	0	486	486	100	+	4	489	22632	23117
UN03751	Rsa1.0_00116.1	864	365886	0	588	631	93	−	250	859	323520	324147
UN03752	Rsa1.0_00092.1	1218	659932	0	650	658	98	−	561	1218	510944	511601
UN03753	Rsa1.0_00381.1	572	89718	0	507	507	100	+	1	507	75991	76497
UN03754	Rsa1.0_01553.1	889	43379	0	499	515	96	−	26	538	2477	2991
UN03755	Rsa1.0_00510.1	895	78718	0	787	887	88	+	8	885	37753	38626
UN03756	Rsa1.0_00002.1	954	601661	1.00E-130	237	237	100	−	3	239	255585	255821
UN03757	Rsa1.0_00726.1	699	66734	0	681	701	97	−	1	698	4113	4813
UN03758	Rsa1.0_00305.1	1733	97308	0	1695	1729	98	−	5	1733	15688	17402
UN03759	Rsa1.0_36697.1	942	614	0	406	411	98	−	8	418	2	411
UN03760	Rsa1.0_08593.1	1441	7148	0	998	1006	99	+	2	1005	110	1114
UN03761	Rsa1.0_00377.1	1164	90038	0	701	707	99	−	456	1162	13288	13994
UN03762	Rsa1.0_00974.1	766	57893	1.00E-172	332	340	97	+	186	525	50839	51175
UN03763	Rsa1.0_00037.1	751	307838	0	745	750	99	+	1	750	33032	33781
UN03764	Rsa1.0_00268.1	1013	101470	0	835	840	99	−	1	836	52913	53752
UN03765	Rsa1.0_00913.1	878	59914	0	537	540	99	−	341	878	10711	11250
UN03766	Rsa1.0_00636.1	685	71113	7.00E-94	197	203	97	+	26	228	14419	14620
UN03767	Rsa1.0_64289.1	960	357	0	357	357	100	+	348	704	1	357
UN03768	Rsa1.0_00979.1	1245	57733	0	338	338	100	+	685	1022	31552	31889
UN03769	Rsa1.0_00012.1	1146	222148	0	338	339	99	−	806	1144	206533	206871
UN03770	Rsa1.0_00071.1	748	345714	0	685	689	99	+	1	689	214892	215579
UN03771	Rsa1.0_00026.1	996	327835	3.00E-94	211	219	96	+	2	216	3987	4205
UN03772	Rsa1.0_00190.1	863	318240	6.00E-98	188	190	98	−	372	561	119860	120049
UN03773	Rsa1.0_00589.1	141	209965	1.00E-51	130	139	93	+	1	139	196776	196914
UN03774	Rsa1.0_00059.1	1027	159650	1.00E-123	251	259	96	+	2	260	147993	148249
UN03775	Rsa1.0_01005.1	849	56967	1.00E-142	262	264	99	−	399	662	19291	19554
UN03776	Rsa1.0_00799.1	1214	63918	1.00E-149	268	268	100	−	945	1212	18668	18935
UN03777	Rsa1.0_02335.1	1285	32400	0	677	693	97	+	3	695	19673	20362
UN03778	Rsa1.0_00520.1	779	78226	0	747	774	96	−	4	777	19282	20045
UN03779	Rsa1.0_00157.1	1168	245832	0	786	789	99	+	379	1167	21355	22143
UN03780	Rsa1.0_01114.1	573	53602	0	553	556	99	−	11	564	14620	15175
UN03781	Rsa1.0_00327.1	526	94892	0	357	365	97	+	3	367	42263	42627

UN03782	Rsa1.0_00201.1	755	232851	2.00E-91	171	171	100	+	562	732	71075	71245
UN03783	Rsa1.0_02171.1	837	167557	0	407	421	96	-	416	836	11696	12116
UN03784	Rsa1.0_00136.1	987	122464	0	609	610	99	-	267	876	15000	15609
UN03785	Rsa1.0_14130.1	896	3769	5.00E-80	207	230	90	-	395	624	2861	3071
UN03786	Rsa1.0_00028.1	848	293984	0	813	853	95	-	1	848	71978	72812
UN03787	Rsa1.0_02547.1	916	30255	9.00E-91	193	200	96	-	717	916	24858	25054
UN03788	Rsa1.0_23467.1	1140	1047	0	350	352	99	-	787	1138	696	1047
UN03789	Rsa1.0_01029.1	1052	256254	0	466	484	96	-	1	480	32633	33116
UN03790	Rsa1.0_00240.1	808	104175	0	548	553	99	+	1	553	46919	47471
UN03791	Rsa1.0_00215.1	1328	201775	0	600	603	99	-	722	1324	62205	62807
UN03792	Rsa1.0_07810.1	1259	8503	0	736	744	98	+	1	744	149	892
UN03793	Rsa1.0_07849.1	1013	8425	0	532	552	96	+	461	1011	4228	4776
UN03794	Rsa1.0_01390.1	902	46695	0	426	426	100	+	477	902	16856	17281
UN03795	Rsa1.0_01771.1	1031	172709	1.00E-152	320	335	95	-	3	334	33281	33615
UN03796	Rsa1.0_00303.1	1319	97468	0	574	641	89	+	687	1319	43995	44598
UN03797	Rsa1.0_00017.1	706	752458	0	692	707	97	-	1	706	416279	416985
UN03798	Rsa1.0_00070.1	1403	150321	1.00E-124	226	226	100	+	422	647	30421	30646
UN03799	Rsa1.0_00273.1	878	326186	0	529	550	96	+	14	557	8056	8597
UN03800	Rsa1.0_02201.1	957	33981	1.00E-127	238	240	99	+	272	511	30288	30527
UN03801	Rsa1.0_02234.1	709	33618	0	371	378	98	+	332	709	1295	1672
UN03802	Rsa1.0_01924.1	993	37226	7.00E-95	195	201	97	+	367	567	1523	1723
UN03803	Rsa1.0_46626.1	653	484	2.33E-156	266	267	99	+	348	614	215	481
UN03804	Rsa1.0_00092.1	930	659932	0	537	606	88	+	267	869	502432	503004
UN03805	Rsa1.0_01600.1	853	42537	1.00E-142	260	261	99	+	2	262	24281	24541
UN03806	Rsa1.0_00838.1	1040	153820	2.00E-58	208	235	88	-	181	414	144717	144948
UN03807	Rsa1.0_06482.1	1319	10937	1.00E-157	365	396	92	-	943	1319	4040	4434
UN03808	Rsa1.0_00060.1	767	502678	0	411	417	98	+	1	417	390371	390786
UN03809	Rsa1.0_01012.1	1216	56836	0	487	524	92	-	418	938	30849	31372
UN03810	Rsa1.0_05877.1	768	12345	1.00E-147	274	277	98	+	47	323	10511	10787
UN03811	Rsa1.0_00943.1	642	58862	0	437	449	97	-	194	642	6634	7081
UN03812	Rsa1.0_04937.1	1297	15401	0	575	605	95	+	3	597	10428	11032
UN03813	Rsa1.0_00056.1	1291	332604	0	1084	1086	99	+	206	1291	47781	48865
UN03814	Rsa1.0_00414.1	815	202190	3.00E-78	202	219	92	-	160	366	79780	79997
UN03815	Rsa1.0_01026.1	1381	171624	0	500	500	100	-	3	502	155677	156176
UN03816	Rsa1.0_00482.1	1354	81162	0	402	430	93	-	773	1202	30959	31372
UN03817	Rsa1.0_00806.1	967	63674	1.00E-130	236	236	100	+	254	489	21955	22190
UN03818	Rsa1.0_00192.1	830	278200	0	340	342	99	-	261	602	226553	226894
UN03819	Rsa1.0_00911.1	1363	59943	0	1139	1155	98	-	211	1363	56165	57318
UN03820	Rsa1.0_08182.1	1754	7776	0	390	390	100	-	3	392	5212	5601

UN03821	Rsa1.0_02777.1	948	27986	1.00E-149	268	268	100	-	311	578	6968	7235
UN03822	Rsa1.0_22896.1	1267	1085	0	370	373	99	+	172	544	1	373
UN03823	Rsa1.0_00786.1	1000	64285	7.00E-95	195	201	97	+	366	566	41596	41796
UN03824	Rsa1.0_00403.1	1064	87701	0	488	498	97	+	159	656	26353	26847
UN03825	Rsa1.0_01803.1	811	173846	0	396	396	100	+	3	398	35610	36005
UN03826	Rsa1.0_00382.1	881	251946	0	387	394	98	+	4	397	155225	155618
UN03827	Rsa1.0_03059.1	1111	25711	1.00E-158	312	319	97	-	716	1031	6699	7017
UN03828	Rsa1.0_05081.1	1520	136103	1.00E-169	308	310	99	-	1211	1520	1453	1762
UN03829	Rsa1.0_00272.1	528	447836	1.00E-171	346	358	96	+	172	528	83797	84151
UN03830	Rsa1.0_01786.1	1062	133324	1.00E-99	247	267	92	-	803	1062	106779	107039
UN03831	Rsa1.0_02446.1	844	31330	0	735	818	89	-	27	844	5783	6587
UN03832	Rsa1.0_00116.1	1104	365886	1.00E-127	238	240	99	-	321	560	37389	37628
UN03833	Rsa1.0_01881.1	548	37721	0	536	543	98	-	3	545	8551	9093
UN03834	Rsa1.0_00484.1	1957	203294	1.00E-148	279	283	98	+	604	886	41096	41378
UN03835	Rsa1.0_00001.1	660	363028	0	345	346	99	+	304	649	119840	120185
UN03836	Rsa1.0_14712.1	848	3134	0	361	362	99	-	487	848	1508	1869
UN03837	Rsa1.0_01506.1	1133	44370	1.00E-172	319	323	98	-	810	1132	20975	21297
UN03838	Rsa1.0_04957.1	793	15330	1.00E-153	308	315	97	-	482	793	1601	1915
UN03839	Rsa1.0_04991.1	1181	15215	0	797	797	100	+	385	1181	9687	10483
UN03840	Rsa1.0_26193.1	1260	902	0	394	395	99	+	863	1257	1	395
UN03841	Rsa1.0_00148.1	1688	120234	0	1258	1283	98	-	417	1686	30640	31922
UN03842	Rsa1.0_00953.1	1339	145012	0	993	1159	85	-	188	1338	113868	115012
UN03843	Rsa1.0_00148.1	536	120234	0	526	537	97	+	3	534	30665	31201
UN03844	Rsa1.0_00405.1	1085	202864	0	392	399	98	-	400	794	185736	186134
UN03845	Rsa1.0_00323.1	1245	159650	0	441	441	100	+	804	1244	43841	44281
UN03846	Rsa1.0_00032.1	896	306326	0	894	894	100	-	3	896	267495	268388
UN03847	Rsa1.0_03410.1	792	23147	0	566	573	98	+	218	790	10247	10816
UN03848	Rsa1.0_00581.1	1758	163575	1.00E-112	215	218	98	+	296	513	33184	33401
UN03849	Rsa1.0_00847.1	1543	62270	0	478	481	99	+	1032	1509	50627	51107
UN03850	Rsa1.0_00745.1	799	66067	0	770	805	95	+	3	799	19659	20447
UN03851	Rsa1.0_00423.1	1670	86138	0	672	672	100	-	123	794	39541	40212
UN03852	Rsa1.0_00737.1	1132	66432	1.00E-151	365	396	92	-	745	1130	61662	62048
UN03853	Rsa1.0_00054.1	1265	162053	0	464	479	96	+	789	1265	136337	136815
UN03854	Rsa1.0_01476.1	1121	172928	1.00E-170	309	311	99	+	811	1121	9187	9497
UN03855	Rsa1.0_04474.1	711	17191	2.00E-88	176	178	98	-	535	711	15334	15511
UN03856	Rsa1.0_01419.1	1219	167402	0	1020	1052	96	-	171	1219	159720	160768
UN03857	Rsa1.0_00087.1	1725	761073	0	689	689	100	-	1036	1724	527869	528557
UN03858	Rsa1.0_00342.1	1599	93695	0	764	764	100	-	44	807	14710	15473
UN03859	Rsa1.0_01247.1	1202	49934	5.00E-96	203	211	96	+	992	1202	31079	31289

UN03860	Rsa1.0_00356.1	1101	91521	0	545	547	99	−	1	547	39827	40373
UN03861	Rsa1.0_00848.1	1309	152362	1.00E−113	248	259	95	−	50	306	19591	19848
UN03862	Rsa1.0_02254.1	809	33344	0	417	418	99	+	392	809	4778	5194
UN03863	Rsa1.0_01296.1	805	49035	1.00E−102	201	205	98	−	562	766	9970	10174
UN03864	Rsa1.0_00301.1	715	365315	0	414	430	96	−	18	443	57406	57835
UN03865	Rsa1.0_01130.1	746	52929	1.00E−111	216	220	98	+	281	500	33004	33223
UN03866	Rsa1.0_00421.1	1561	86365	1.00E−145	331	350	94	−	1203	1552	75833	76174
UN03867	Rsa1.0_02978.1	736	26304	0	519	566	91	+	1	564	21308	21873
UN03868	Rsa1.0_00024.1	790	188018	1.00E−165	347	367	94	+	21	375	84731	85097
UN03869	Rsa1.0_00234.1	1061	197060	0	593	611	97	+	2	611	3831	4441
UN03870	Rsa1.0_00010.1	883	294827	8.00E−85	245	272	90	−	606	867	135567	135837
UN03871	Rsa1.0_02914.1	506	26815	0	352	359	98	−	148	504	26109	26467
UN03872	Rsa1.0_03399.1	843	23204	0	840	841	99	−	3	843	11022	11862
UN03873	Rsa1.0_00200.1	1004	162016	1.00E−111	210	212	99	−	97	308	80374	80585
UN03874	Rsa1.0_08791.1	790	6918	1.00E−140	267	271	98	−	512	782	4600	4867
UN03875	Rsa1.0_06032.1	732	11886	2.00E−78	239	269	88	−	16	275	10750	11012
UN03876	Rsa1.0_00345.1	1543	93226	0	415	430	96	−	549	978	25584	26013
UN03877	Rsa1.0_02128.1	1068	34758	0	451	496	90	−	3	469	25609	26104
UN03878	Rsa1.0_13838.1	1807	4236	0	524	528	99	+	3	528	1110	1637
UN03879	Rsa1.0_02528.1	958	30452	1.00E−163	307	311	98	−	650	958	136	446
UN03880	Rsa1.0_00472.1	503	398134	0	493	502	98	+	1	502	283481	283978
UN03881	Rsa1.0_01412.1	743	46311	0	370	373	99	−	363	735	10246	10616
UN03882	Rsa1.0_00101.1	831	267193	1.00E−149	303	312	97	−	480	789	194779	195090
UN03883	Rsa1.0_25317.1	1455	941	0	626	644	97	−	813	1455	296	939
UN03884	Rsa1.0_03100.1	882	25411	0	656	660	99	−	223	882	19524	20183
UN03885	Rsa1.0_03543.1	1222	22185	0	614	625	98	−	469	1093	15844	16464
UN03886	Rsa1.0_00094.1	1335	139042	1.00E−103	218	226	96	+	263	485	134442	134667
UN03887	Rsa1.0_06990.1	878	10060	0	829	874	94	+	7	861	4341	5204
UN03888	Rsa1.0_01204.1	1436	50843	0	1406	1428	98	+	10	1436	7030	8457
UN03889	Rsa1.0_02874.1	786	27070	1.00E−117	221	223	99	−	110	332	770	992
UN03890	Rsa1.0_02276.1	786	33098	0	525	540	97	−	239	773	23905	24441
UN03891	Rsa1.0_00016.1	1012	831256	0	579	586	98	−	21	606	6600	7183
UN03892	Rsa1.0_00419.1	771	86562	0	412	414	99	−	358	771	15419	15832
UN03893	Rsa1.0_02210.1	1339	33857	0	476	490	97	−	854	1339	22539	23023
UN03894	Rsa1.0_02141.1	1238	34677	0	711	714	99	+	527	1238	13105	13818
UN03895	Rsa1.0_00630.1	1049	71281	1.00E−126	262	273	95	−	777	1049	34386	34658
UN03896	Rsa1.0_00079.1	1181	606987	0	645	660	97	−	3	662	4651	5310
UN03897	Rsa1.0_01929.1	1109	37191	0	339	341	99	+	251	591	9108	9448
UN03898	Rsa1.0_00092.1	1231	659932	0	1068	1078	99	−	125	1199	21129	22206

UN03899	Rsa1.0_01768.1	1201	138463	1.00E-120	257	268	95	-	932	1199	120034	120300
UN03900	Rsa1.0_02375.1	832	32026	1.00E-77	211	232	90	+	1	232	29150	29381
UN03901	Rsa1.0_04387.1	1204	17625	1.00E-148	267	267	100	+	495	761	15904	16170
UN03902	Rsa1.0_00777.1	1820	191865	0	402	403	99	+	119	521	180529	180931
UN03903	Rsa1.0_02070.1	856	158516	3.00E-87	176	180	97	-	677	856	140096	140275
UN03904	Rsa1.0_08426.1	717	7373	1.00E-129	235	235	100	+	483	717	6644	6878
UN03905	Rsa1.0_19744.1	1089	1389	1.00E-105	209	211	99	-	22	230	1179	1389
UN03906	Rsa1.0_02478.1	1195	150183	0	918	957	95	-	249	1193	28837	29791
UN03907	Rsa1.0_02232.1	1331	33659	0	674	695	96	+	643	1331	15141	15835
UN03908	Rsa1.0_01882.1	827	37697	0	642	651	98	-	178	825	13442	14092
UN03909	Rsa1.0_00250.1	696	273771	0	415	436	95	-	261	696	231503	231935
UN03910	Rsa1.0_01727.1	640	40243	1.00E-172	313	315	99	+	221	535	23645	23959
UN03911	Rsa1.0_00265.1	853	220635	0	510	510	100	+	337	846	176079	176588
UN03912	Rsa1.0_05384.1	2023	13960	0	841	841	100	-	3	843	2553	3393
UN03913	Rsa1.0_00628.1	775	214360	1.00E-130	239	240	99	-	202	441	66081	66320
UN03914	Rsa1.0_00045.1	1166	328694	1.00E-123	311	342	90	+	3	340	91541	91864
UN03915	Rsa1.0_03109.1	1268	25336	0	678	702	96	+	567	1268	3130	3818
UN03916	Rsa1.0_00178.1	1201	114861	5.00E-93	226	243	93	-	31	273	32804	33037
UN03917	Rsa1.0_00039.1	1292	296927	0	372	372	100	-	321	692	16707	17078
UN03918	Rsa1.0_02314.1	1189	32642	0	651	661	98	-	4	660	5396	6056
UN03919	Rsa1.0_00497.1	816	79830	0	373	373	100	+	3	375	75694	76066
UN03920	Rsa1.0_02765.1	1474	28141	0	408	416	98	-	1065	1474	16290	16705
UN03921	Rsa1.0_00025.1	1264	277312	3.00E-45	106	110	96	-	1058	1167	47859	47968
UN03922	Rsa1.0_00175.1	1530	166121	3.00E-70	142	144	98	+	1029	1172	28296	28439
UN03923	Rsa1.0_06075.1	818	11805	0	440	445	98	-	374	818	3939	4383
UN03924	Rsa1.0_01104.1	1653	285480	0	414	417	99	+	1238	1653	268402	268818
UN03925	Rsa1.0_42244.1	1694	531	1.00E-116	213	213	100	+	12	224	319	531
UN03926	Rsa1.0_00974.1	1151	57893	1.00E-158	327	342	95	-	514	849	6312	6653
UN03927	Rsa1.0_01495.1	1358	44613	0	1270	1320	96	+	3	1314	15583	16900
UN03928	Rsa1.0_00133.1	1024	123768	0	331	331	100	+	205	535	33145	33475
UN03929	Rsa1.0_00727.1	651	66724	1.00E-122	270	281	96	-	367	641	14960	15240
UN03930	Rsa1.0_00789.1	1322	64233	0	519	548	94	+	781	1320	34406	34948
UN03931	Rsa1.0_35196.1	731	643	0	405	427	94	+	148	555	217	643
UN03932	Rsa1.0_00196.1	893	109960	1.00E-107	234	244	95	+	607	849	72827	73068
UN03933	Rsa1.0_00706.1	865	291570	1.00E-160	305	311	98	+	135	445	32346	32656
UN03934	Rsa1.0_00751.1	1184	65759	0	487	493	98	-	222	714	46552	47044
UN03935	Rsa1.0_01586.1	1077	42804	0	407	426	95	+	1	420	30183	30604
UN03936	Rsa1.0_00975.1	890	57865	0	855	894	95	-	1	890	55313	56203
UN03937	Rsa1.0_00964.1	716	316648	0	700	716	97	+	1	716	304594	305309

UN03938	Rsa1.0_00451.1	1266	322533	5.00E-87	211	229	92	+	754	982	284872	285088
UN03939	Rsa1.0_00409.1	860	146788	0	459	461	99	−	398	858	84601	85061
UN03940	Rsa1.0_01707.1	933	40686	0	388	390	99	−	330	719	18514	18903
UN03941	Rsa1.0_00016.1	879	831256	0	409	417	98	+	461	877	795321	795737
UN03942	Rsa1.0_00152.1	1277	257730	0	583	618	94	−	1	618	118021	118629
UN03943	Rsa1.0_00013.1	571	292129	1.00E-150	369	396	93	+	160	548	2789	3180
UN03944	Rsa1.0_00383.1	670	398431	0	663	665	99	−	4	668	186021	186685
UN03945	Rsa1.0_00044.1	1272	271658	0	445	452	98	+	434	885	270014	270465
UN03946	Rsa1.0_00437.1	1024	84879	0	607	614	98	−	14	627	39452	40065
UN03947	Rsa1.0_00354.1	919	232005	1.00E-122	265	278	95	−	643	919	192325	192602
UN03948	Rsa1.0_01434.1	2901	45951	0	1149	1191	96	−	3	1189	5302	6491
UN03949	Rsa1.0_04067.1	715	19256	1.00E-118	222	224	99	+	155	378	17557	17780
UN03950	Rsa1.0_02434.1	806	31464	1.00E-159	312	317	98	−	2	316	6189	6504
UN03951	Rsa1.0_00890.1	830	323870	0	826	832	99	−	1	830	216005	216836
UN03952	Rsa1.0_03633.1	1345	21644	1.00E-162	326	333	97	+	947	1275	4854	5185
UN03953	Rsa1.0_01625.1	724	42019	1.00E-147	274	277	98	−	448	724	20456	20732
UN03954	Rsa1.0_02020.1	1115	173211	0	1071	1111	96	−	5	1113	156792	157898
UN03955	Rsa1.0_02179.1	736	34188	0	690	727	94	+	12	734	15454	16176
UN03956	Rsa1.0_07104.1	876	9889	0	365	366	99	+	1	366	4549	4914
UN03957	Rsa1.0_00994.1	1252	165189	5.00E-96	205	211	97	−	79	287	316	526
UN03958	Rsa1.0_18896.1	838	1515	0	819	841	97	−	1	838	64	896
UN03959	Rsa1.0_00910.1	745	59975	1.00E-159	284	284	100	+	1	284	16201	16484
UN03960	Rsa1.0_01626.1	718	41989	0	692	711	97	+	8	718	15832	16537
UN03961	Rsa1.0_17750.1	1028	1750	0	374	385	97	−	141	525	1366	1750
UN03962	Rsa1.0_01511.1	981	44235	0	680	687	98	+	2	686	29365	30051
UN03963	Rsa1.0_00450.1	629	139397	1.00E-147	288	295	97	−	3	297	22106	22397
UN03964	Rsa1.0_09306.1	1091	6468	0	902	910	99	−	127	1036	436	1345
UN03965	Rsa1.0_00201.1	383	232851	4.00E-94	215	224	95	−	162	383	109854	110072
UN03966	Rsa1.0_00075.1	1334	146680	0	692	728	95	−	1	723	141185	141903
UN03967	Rsa1.0_08421.1	1076	7380	0	350	356	98	−	1	354	4123	4478
UN03968	Rsa1.0_00709.1	1673	67329	0	566	577	98	−	901	1477	9314	9890
UN03969	Rsa1.0_02692.1	831	28626	1.00E-138	330	357	92	+	437	793	21962	22318
UN03970	Rsa1.0_03614.1	665	21773	0	585	589	99	+	1	589	15021	15609
UN03971	Rsa1.0_00199.1	829	249604	1.00E-156	332	346	95	−	1	339	13561	13904
UN03972	Rsa1.0_01239.1	2645	50107	0	1569	1603	97	+	1043	2645	18949	20546
UN03973	Rsa1.0_01026.1	789	171624	0	579	625	92	+	21	624	159949	160566
UN03974	Rsa1.0_02205.1	1107	33923	0	979	1056	92	+	50	1103	26261	27298
UN03975	Rsa1.0_01717.1	1279	143162	0	1270	1282	99	−	1	1278	127661	128942
UN03976	Rsa1.0_00464.1	1370	82253	0	968	978	98	+	396	1370	5075	6051

UN03977	Rsa1.0_01460.1	1456	45255	0	490	507	96	+	2	502	32136	32642
UN03978	Rsa1.0_00293.1	1263	339294	0	1203	1217	98	+	3	1219	170623	171838
UN03979	Rsa1.0_02573.1	1081	29955	0	352	355	99	+	717	1071	19028	19382
UN03980	Rsa1.0_02707.1	563	28520	0	500	531	94	+	32	562	9469	9995
UN03981	Rsa1.0_01380.1	937	155003	0	539	541	99	−	237	777	25543	26083
UN03982	Rsa1.0_14764.1	980	3084	0	412	434	94	−	11	433	1483	1916
UN03983	Rsa1.0_37757.1	1561	597	0	478	478	100	−	303	780	1	478
UN03984	Rsa1.0_00560.1	964	75685	0	359	365	98	−	297	661	58221	58584
UN03985	Rsa1.0_03074.1	600	25568	1.00E-152	383	416	92	−	182	594	16245	16644
UN03986	Rsa1.0_00792.1	669	64163	0	497	529	93	−	147	669	3293	3821
UN03987	Rsa1.0_00343.1	1427	93570	0	391	392	99	−	1034	1425	30764	31155
UN03988	Rsa1.0_00668.1	993	69421	0	587	610	96	−	1	598	23289	23898
UN03989	Rsa1.0_00154.1	1390	189461	1.00E-123	294	314	93	+	2	313	129855	130164
UN03990	Rsa1.0_00537.1	1298	186980	0	835	891	93	−	346	1233	16481	17354
UN03991	Rsa1.0_03267.1	756	24230	2.00E-94	179	180	99	+	10	189	13680	13859
UN03992	Rsa1.0_01573.1	854	42964	1.00E-163	368	390	94	−	7	388	4044	4431
UN03993	Rsa1.0_00741.1	998	66117	1.00E-170	325	331	98	−	12	341	50868	51198
UN03994	Rsa1.0_00169.1	1392	274298	0	1297	1383	93	+	10	1369	270701	272079
UN03995	Rsa1.0_03999.1	1428	19645	0	405	412	98	+	725	1136	8850	9261
UN03996	Rsa1.0_16067.1	929	2300	0	380	382	99	−	292	673	783	1164
UN03997	Rsa1.0_00539.1	1308	76926	0	661	669	98	−	1	669	15980	16647
UN03998	Rsa1.0_00032.1	1860	306326	0	468	482	97	+	438	919	238497	238972
UN03999	Rsa1.0_01182.1	1067	167304	1.00E-103	254	267	95	−	782	1044	145	409
UN04000	Rsa1.0_04563.1	1696	16837	1.00E-128	258	263	98	+	1438	1696	16117	16378
UN04001	Rsa1.0_00058.1	669	159921	0	353	359	98	−	1	359	89801	90155
UN04002	Rsa1.0_10423.1	953	5854	0	733	753	97	−	7	759	1	752
UN04003	Rsa1.0_11593.1	1041	5464	0	1010	1041	97	+	1	1041	1942	2965
UN04004	Rsa1.0_01460.1	1069	45255	0	1048	1066	98	+	3	1068	41716	42776
UN04005	Rsa1.0_02858.1	1367	27246	0	1154	1205	95	−	1	1205	22920	24115
UN04006	Rsa1.0_00211.1	913	228189	1.00E-154	276	276	100	+	636	911	5857	6132
UN04007	Rsa1.0_00260.1	962	102021	0	622	633	98	−	328	960	40005	40637
UN04008	Rsa1.0_03162.1	782	24895	1.00E-100	197	200	98	−	17	214	19790	19989
UN04009	Rsa1.0_01292.1	843	175783	1.00E-133	251	253	99	+	590	841	149086	149338
UN04010	Rsa1.0_00224.1	513	327530	2.00E-84	181	187	96	−	1	186	195632	195818
UN04011	Rsa1.0_00108.1	1367	478491	0	628	631	99	+	494	1124	305436	306066
UN04012	Rsa1.0_00487.1	885	80674	2.33E-156	343	371	92	+	523	883	75851	76212
UN04013	Rsa1.0_00728.1	1140	66704	0	486	515	94	−	502	1016	32892	33389
UN04014	Rsa1.0_00770.1	1060	64900	0	972	1005	96	−	3	1005	53553	54555
UN04015	Rsa1.0_01538.1	837	43733	1.00E-159	297	301	98	−	105	405	40507	40807

UN04016	Rsa1.0_00435.1	576	317579	1.00E-169	352	369	95	-	218	576	70712	71080
UN04017	Rsa1.0_02621.1	1631	156120	0	548	548	100	-	1084	1631	12899	13446
UN04018	Rsa1.0_01396.1	1053	151624	0	428	447	95	+	608	1053	141509	141952
UN04019	Rsa1.0_01001.1	970	57077	1.00E-86	178	183	97	+	422	604	24046	24228
UN04020	Rsa1.0_07129.1	1056	9861	0	828	865	95	-	9	856	5653	6517
UN04021	Rsa1.0_01626.1	1365	41989	1.00E-103	281	303	92	-	1029	1317	8489	8789
UN04022	Rsa1.0_00136.1	1498	122464	0	469	491	95	+	1007	1497	65300	65784
UN04023	Rsa1.0_01326.1	1207	48072	0	784	812	96	+	405	1205	44398	45204
UN04024	Rsa1.0_01309.1	1463	48556	0	566	567	99	+	897	1463	41209	41775
UN04025	Rsa1.0_00198.1	1158	222963	0	450	456	98	+	3	458	128659	129114
UN04026	Rsa1.0_03216.1	944	24540	0	939	945	99	+	1	944	19438	20382
UN04027	Rsa1.0_01724.1	783	40292	1.00E-77	151	152	99	-	1	152	13121	13272
UN04028	Rsa1.0_00303.1	604	97468	3.00E-74	154	158	97	+	220	377	57321	57478
UN04029	Rsa1.0_00134.1	1154	390219	0	561	597	93	+	8	599	45107	45694
UN04030	Rsa1.0_04422.1	598	17423	0	577	597	96	-	2	598	8953	9544
UN04031	Rsa1.0_00392.1	870	88523	7.00E-70	147	151	97	-	364	514	15492	15642
UN04032	Rsa1.0_04297.1	752	18030	1.00E-125	292	310	94	+	359	665	17379	17687
UN04033	Rsa1.0_13850.1	1325	4205	0	559	560	99	+	1	560	2896	3455
UN04034	Rsa1.0_00107.1	1227	431659	0	670	673	99	+	1	672	251010	251682
UN04035	Rsa1.0_00265.1	1051	220635	1.00E-119	217	217	100	+	818	1034	190433	190649
UN04036	Rsa1.0_05168.1	987	14621	0	632	648	97	-	1	648	12471	13117
UN04037	Rsa1.0_01422.1	1197	46172	0	492	511	96	-	688	1197	10154	10663
UN04038	Rsa1.0_00386.1	1177	89134	0	450	458	98	+	437	894	53821	54278
UN04039	Rsa1.0_01674.1	798	168300	0	439	445	98	+	3	444	1848	2289
UN04040	Rsa1.0_02060.1	943	35528	0	535	563	95	+	388	941	17139	17694
UN04041	Rsa1.0_00005.1	1547	353093	0	368	368	100	+	1178	1545	121587	121954
UN04042	Rsa1.0_00579.1	1369	148308	1.00E-172	361	379	95	+	1	379	66641	67019
UN04043	Rsa1.0_33464.1	963	678	1.00E-148	278	282	98	+	437	718	154	435
UN04044	Rsa1.0_04016.1	731	19570	0	492	498	98	-	3	499	18111	18608
UN04045	Rsa1.0_00051.1	364	258225	7.00E-34	204	238	85	+	18	244	119357	119588
UN04046	Rsa1.0_00524.1	1342	169613	0	1138	1142	99	+	201	1341	17154	18295
UN04047	Rsa1.0_00094.1	1442	139042	0	556	557	99	-	884	1440	60519	61075
UN04048	Rsa1.0_01223.1	1312	50392	0	634	665	95	+	1	665	29039	29700
UN04049	#	#	#	#	#	#	#	#	#	#	#	#
UN04050	Rsa1.0_07908.1	753	8319	0	517	517	100	+	1	517	206	722
UN04051	Rsa1.0_00473.1	1217	212204	0	445	465	95	+	2	458	64237	64697
UN04052	Rsa1.0_00879.1	958	61297	0	412	427	96	-	533	958	34194	34620
UN04053	Rsa1.0_00001.1	1436	363028	0	938	964	97	+	476	1436	147746	148704
UN04054	Rsa1.0_00757.1	1677	65559	0	679	724	93	-	957	1677	34361	35065

UN04055	Rsa1.0_01602.1	696	42503	1.00E-130	269	279	96	+	416	694	1873	2144
UN04056	Rsa1.0_02817.1	1679	27644	0	1291	1370	94	-	179	1530	9695	11043
UN04057	Rsa1.0_01171.1	953	51752	0	448	466	96	-	467	932	35729	36194
UN04058	Rsa1.0_01146.1	630	163455	2.00E-78	196	208	94	-	424	630	41281	41485
UN04059	Rsa1.0_04835.1	925	15766	0	584	592	98	+	329	920	2483	3074
UN04060	Rsa1.0_02409.1	885	31663	1.00E-150	374	405	92	-	486	879	8883	9279
UN04061	Rsa1.0_10722.1	1317	5745	0	699	701	99	-	617	1317	4278	4977
UN04062	Rsa1.0_00278.1	1557	231896	0	587	603	97	+	711	1313	205275	205877
UN04063	Rsa1.0_00871.1	1005	61539	1.00E-127	327	354	92	+	3	346	8039	8382
UN04064	Rsa1.0_01981.1	662	36537	0	591	600	98	-	42	637	3891	4487
UN04065	Rsa1.0_00782.1	1067	64468	0	401	420	95	+	560	979	30043	30462
UN04066	Rsa1.0_00561.1	467	75653	1.00E-103	257	279	92	-	190	467	16292	16556
UN04067	Rsa1.0_03119.1	504	25270	0	499	508	98	+	2	504	15752	16257
UN04068	Rsa1.0_02528.1	1166	30452	1.00E-130	305	326	93	+	5	324	18618	18940
UN04069	Rsa1.0_08988.1	1382	6732	0	479	531	90	-	854	1369	727	1243
UN04070	Rsa1.0_00136.1	927	122464	6.00E-89	182	187	97	-	2	188	70440	70626
UN04071	Rsa1.0_02756.1	998	28202	4.00E-62	125	126	99	+	300	425	13570	13695
UN04072	Rsa1.0_01214.1	1257	50599	0	397	414	95	+	855	1257	46274	46685
UN04073	Rsa1.0_03114.1	1049	25318	0	608	624	97	-	387	1008	10053	10676
UN04074	Rsa1.0_02506.1	871	30700	1.00E-105	209	213	98	-	1	211	26272	26484
UN04075	Rsa1.0_00262.1	1196	101906	1.00E-135	245	245	100	+	86	330	48780	49024
UN04076	Rsa1.0_01127.1	1406	53124	0	450	450	100	+	600	1049	51280	51729
UN04077	Rsa1.0_00101.1	823	267193	0	710	763	93	+	64	819	89099	89846
UN04078	Rsa1.0_00027.1	805	283362	1.00E-141	338	368	91	-	450	802	279797	280163
UN04079	Rsa1.0_03544.1	2702	22182	1.00E-160	453	504	89	+	1001	1501	11920	12409
UN04080	Rsa1.0_00045.1	1211	328694	0	355	360	98	+	1	360	160663	161022
UN04081	Rsa1.0_02268.1	577	33172	2.00E-38	98	102	96	-	334	435	32461	32561
UN04082	Rsa1.0_01149.1	1334	160459	1.00E-149	274	276	99	-	1059	1334	149685	149960
UN04083	Rsa1.0_00278.1	1493	231896	0	401	408	98	+	491	898	23233	23640
UN04084	Rsa1.0_00915.1	958	194446	0	535	552	96	-	3	546	181873	182421
UN04085	Rsa1.0_02402.1	1294	31710	0	429	441	97	+	833	1273	30607	31044
UN04086	Rsa1.0_39987.1	764	562	1.00E-136	249	250	99	+	365	614	313	562
UN04087	Rsa1.0_00038.1	1381	314568	0	767	814	94	-	2	781	239043	239856
UN04088	Rsa1.0_00715.1	1046	67116	0	413	422	97	-	127	548	17550	17969
UN04089	Rsa1.0_01283.1	1075	49257	1.00E-172	327	334	97	-	181	514	19047	19380
UN04090	Rsa1.0_00773.1	957	64839	0	672	714	94	+	260	957	50259	50967
UN04091	Rsa1.0_01705.1	1038	40710	1.00E-68	220	247	89	+	746	991	38585	38823
UN04092	Rsa1.0_00305.1	980	97308	1.00E-142	273	277	98	-	704	979	65771	66047
UN04093	Rsa1.0_00599.1	1356	73418	0	351	353	99	+	322	674	46750	47102

UN04094	Rsa1.0_00773.1	1205	64839	0	471	481	97	−	1	480	53064	53544
UN04095	Rsa1.0_01281.1	713	49313	0	678	712	95	+	3	712	26768	27468
UN04096	Rsa1.0_01220.1	1188	161775	0	454	456	99	−	2	457	147689	148144
UN04097	Rsa1.0_00254.1	1169	102600	0	593	618	95	−	543	1159	52242	52845
UN04098	Rsa1.0_00123.1	1044	325650	1.00E−99	223	235	94	+	3	234	173067	173301
UN04099	Rsa1.0_00579.1	799	148308	6.00E−70	141	143	98	−	57	199	68688	68830
UN04100	Rsa1.0_03185.1	944	24739	0	436	448	97	−	49	496	11026	11473
UN04101	Rsa1.0_01115.1	1196	183545	0	703	705	99	−	4	708	168513	169217
UN04102	Rsa1.0_60278.1	1238	380	1.00E−109	202	202	100	−	3	204	85	286
UN04103	Rsa1.0_03833.1	1008	20575	0	893	905	98	−	104	1007	17249	18153
UN04104	Rsa1.0_06443.1	810	11008	1.00E−135	320	343	93	+	3	342	5740	6081
UN04105	Rsa1.0_00149.1	839	305196	2.00E−51	140	152	92	−	511	662	11665	11816
UN04106	Rsa1.0_00370.1	864	90472	0	378	395	95	−	357	751	4902	5296
UN04107	Rsa1.0_00318.1	1138	96094	1.00E−133	277	288	96	+	853	1138	70330	70617
UN04108	Rsa1.0_00041.1	675	186197	1.00E−107	248	264	93	+	415	675	150099	150358
UN04109	Rsa1.0_01172.1	1024	51734	0	371	381	97	−	9	386	6903	7283
UN04110	Rsa1.0_03007.1	507	26070	1.00E−116	227	232	97	+	276	507	22442	22673
UN04111	Rsa1.0_00208.1	1265	108565	0	788	795	99	−	2	796	21769	22563
UN04112	Rsa1.0_00108.1	1025	478491	1.00E−160	295	298	98	+	728	1025	51602	51899
UN04113	Rsa1.0_00275.1	1203	210104	1.00E−109	207	209	99	+	31	239	176533	176741
UN04114	Rsa1.0_00033.1	1167	515811	0	545	555	98	+	613	1167	135945	136498
UN04115	Rsa1.0_03964.1	685	19835	4.00E−55	189	216	87	−	481	685	13128	13337
UN04116	Rsa1.0_02041.1	681	163634	1.00E−126	307	330	93	−	17	346	20074	20401
UN04117	Rsa1.0_00842.1	1300	62493	1.00E−114	215	217	99	+	1084	1300	32618	32834
UN04118	Rsa1.0_00978.1	673	57739	1.00E−175	526	596	88	+	30	611	26146	26730
UN04119	Rsa1.0_00372.1	866	90418	0	452	460	98	−	3	462	25938	26392
UN04120	Rsa1.0_00216.1	1119	197563	0	424	434	97	+	295	728	24584	25017
UN04121	Rsa1.0_06683.1	1577	10549	0	446	451	98	+	253	703	7742	8192
UN04122	Rsa1.0_03310.1	781	23851	0	372	374	99	+	3	376	7955	8328
UN04123	Rsa1.0_02896.1	827	26970	0	350	361	96	+	322	672	16798	17158
UN04124	Rsa1.0_02540.1	745	30344	0	658	678	97	−	2	678	22062	22739
UN04125	Rsa1.0_00030.1	591	250988	1.00E−135	247	248	99	+	343	590	124651	124898
UN04126	Rsa1.0_00963.1	1072	58176	0	998	1054	94	−	6	1051	31115	32148
UN04127	Rsa1.0_10636.1	1021	5774	0	863	868	99	+	154	1021	3793	4660
UN04128	Rsa1.0_00001.1	1127	363028	1.00E−131	259	265	97	−	862	1126	253441	253703
UN04129	Rsa1.0_06323.1	1801	11236	0	623	642	97	−	1160	1800	7497	8138
UN04130	Rsa1.0_00190.1	867	318240	0	813	830	97	−	30	859	179081	179909
UN04131	Rsa1.0_00483.1	1114	81102	0	1087	1113	97	−	3	1114	17104	18206
UN04132	Rsa1.0_11690.1	942	5440	1.00E−116	367	415	88	+	3	417	5021	5421

UN04133	Rsa1.0_00190.1	997	318240	1.00E-167	362	378	95	-	623	997	134846	135221
UN04134	Rsa1.0_01397.1	699	183276	0	520	541	96	-	3	543	11118	11658
UN04135	Rsa1.0_00925.1	773	59481	0	520	522	99	-	3	524	25571	26092
UN04136	Rsa1.0_06062.1	890	11836	1.00E-152	317	329	96	-	556	881	5210	5534
UN04137	Rsa1.0_00744.1	1425	66084	0	338	338	100	-	1	338	50865	51202
UN04138	Rsa1.0_04709.1	1363	16285	0	714	719	99	-	1	718	6049	6764
UN04139	Rsa1.0_00759.1	765	65465	0	371	378	98	+	392	765	54383	54759
UN04140	Rsa1.0_00205.1	868	193401	0	386	401	96	+	467	867	77712	78101
UN04141	Rsa1.0_00396.1	1572	88242	0	380	385	98	-	3	387	32410	32794
UN04142	Rsa1.0_01045.1	656	55853	0	393	404	97	-	253	656	34043	34443
UN04143	Rsa1.0_00679.1	1202	68971	1.00E-118	275	293	93	-	1	287	34537	34829
UN04144	Rsa1.0_00310.1	1352	96852	0	413	419	98	-	2	418	36360	36778
UN04145	Rsa1.0_05547.1	1206	13369	0	501	519	96	-	683	1201	12677	13195
UN04146	Rsa1.0_00994.1	457	165189	0	453	455	99	+	3	457	12459	12913
UN04147	Rsa1.0_00541.1	1191	76863	3.00E-45	278	342	81	+	846	1174	1692	2033
UN04148	Rsa1.0_05679.1	785	12958	1.00E-106	199	200	99	+	253	452	917	1116
UN04149	Rsa1.0_00160.1	1058	346678	0	826	848	97	+	22	863	28090	28936
UN04150	Rsa1.0_04667.1	901	16460	0	341	341	100	-	2	342	15632	15972
UN04151	Rsa1.0_14624.1	956	3217	1.00E-110	289	312	92	+	652	956	1334	1639
UN04152	Rsa1.0_02289.1	926	32981	7.00E-64	148	155	95	-	225	376	21690	21844
UN04153	Rsa1.0_15662.1	1086	2495	2.00E-73	194	211	91	-	730	940	2257	2459
UN04154	Rsa1.0_00012.1	1340	222148	0	1306	1309	99	-	4	1312	194825	196133
UN04155	Rsa1.0_05596.1	853	137912	4.00E-22	64	67	95	-	9	75	6636	6702
UN04156	Rsa1.0_00816.1	1157	403350	0	559	585	95	+	571	1155	133188	133770
UN04157	Rsa1.0_02118.1	842	34839	0	496	503	98	-	18	519	8900	9402
UN04158	Rsa1.0_02411.1	1024	31655	1.00E-135	458	534	85	+	123	656	27947	28456
UN04159	Rsa1.0_01860.1	762	38057	0	467	491	95	-	282	760	567	1057
UN04160	Rsa1.0_00148.1	921	120234	0	516	537	96	-	386	919	36560	37093
UN04161	Rsa1.0_00896.1	1223	60509	6.00E-34	160	187	85	-	534	720	3275	3460
UN04162	Rsa1.0_03860.1	1202	20407	0	420	444	94	+	16	445	15873	16316
UN04163	Rsa1.0_00548.1	1252	76518	0	1129	1208	93	+	1	1200	18922	20114
UN04164	Rsa1.0_02207.1	961	33914	0	510	513	99	+	3	515	18537	19049
UN04165	Rsa1.0_02396.1	926	31783	1.00E-106	231	242	95	-	597	838	18648	18886
UN04166	Rsa1.0_00015.1	791	561803	1.00E-159	321	331	96	-	1	331	304577	304904
UN04167	Rsa1.0_00517.1	874	142790	0	419	431	97	-	2	432	113059	113487
UN04168	Rsa1.0_00228.1	506	105511	0	379	393	96	+	49	439	13156	13545
UN04169	Rsa1.0_01060.1	1236	55368	1.00E-109	222	229	96	-	925	1153	38736	38964
UN04170	Rsa1.0_07823.1	1171	8468	0	1012	1061	95	-	3	1060	4559	5615
UN04171	Rsa1.0_02173.1	1136	34318	0	470	491	95	-	382	872	30684	31174

UN04172	Rsa1.0_00101.1	1384	267193	0	1212	1284	94	−	3	1273	89099	90372
UN04173	Rsa1.0_07877.1	1048	8373	3.00E−72	145	147	98	−	696	842	4515	4661
UN04174	Rsa1.0_02161.1	1078	34459	0	435	467	93	−	589	1052	32620	33077
UN04175	Rsa1.0_00297.1	1151	97836	0	441	477	92	+	7	478	26263	26734
UN04176	Rsa1.0_01102.1	907	53987	0	669	697	95	−	1	695	51418	52114
UN04177	Rsa1.0_01173.1	979	51727	0	397	411	96	−	415	825	29674	30084
UN04178	Rsa1.0_10201.1	1282	5946	0	1170	1210	96	+	86	1278	389	1597
UN04179	Rsa1.0_00377.1	879	90038	1.00E−101	291	315	92	+	31	340	28082	28387
UN04180	Rsa1.0_00100.1	1142	249232	0	743	788	94	−	2	773	126923	127710
UN04181	Rsa1.0_00121.1	715	278029	0	707	711	99	+	5	715	269852	270562
UN04182	Rsa1.0_00675.1	1041	145590	1.00E−163	428	469	91	+	1	463	45279	45726
UN04183	Rsa1.0_00351.1	1314	413096	1.00E−155	281	282	99	+	587	868	133363	133644
UN04184	Rsa1.0_02920.1	1195	26779	1.00E−109	246	257	95	+	1	254	20299	20554
UN04185	Rsa1.0_03981.1	433	19724	1.00E−81	255	285	89	−	39	319	17580	17854
UN04186	Rsa1.0_00427.1	933	189678	0	927	929	99	+	1	929	129289	130217
UN04187	Rsa1.0_01156.1	606	52147	1.00E−118	278	302	92	+	1	284	33024	33324
UN04188	Rsa1.0_03634.1	593	145192	1.00E−135	350	388	90	+	1	374	133529	133901
UN04189	Rsa1.0_01112.1	1144	310601	0	534	569	93	−	2	569	31685	32250
UN04190	Rsa1.0_00078.1	801	175053	0	781	828	94	+	1	801	78730	79556
UN04191	Rsa1.0_00376.1	1053	90101	1.00E−134	252	255	98	−	797	1051	46615	46869
UN04192	Rsa1.0_00800.1	1222	63868	1.00E−107	219	226	96	−	648	873	60809	61034
UN04193	Rsa1.0_06795.1	1134	10346	0	371	383	96	−	502	884	5026	5408
UN04194	Rsa1.0_14298.1	993	3561	2.00E−89	183	188	97	+	641	828	3278	3465
UN04195	Rsa1.0_00299.1	1203	97770	0	468	499	93	−	3	501	58798	59284
UN04196	Rsa1.0_01151.1	1287	52268	1.00E−151	328	346	94	−	207	549	25416	25761
UN04197	Rsa1.0_00630.1	850	71281	0	511	534	95	+	1	528	63615	64143
UN04198	Rsa1.0_00339.1	1059	222974	1.00E−102	202	206	98	−	2	207	18437	18642
UN04199	Rsa1.0_01381.1	909	46908	1.00E−132	252	256	98	−	1	256	36596	36851
UN04200	Rsa1.0_02329.1	703	32474	0	678	709	95	−	1	703	10329	11034
UN04201	Rsa1.0_00589.1	685	209965	1.00E−119	243	250	97	+	240	488	200359	200608
UN04202	Rsa1.0_00451.1	746	322533	0	722	749	96	+	1	746	9191	9929
UN04203	Rsa1.0_18598.1	794	1567	0	653	687	95	−	1	670	258	943
UN04204	Rsa1.0_01790.1	1224	39248	0	429	431	99	+	1	431	17097	17526
UN04205	Rsa1.0_00147.1	1468	249503	0	978	1060	92	−	408	1467	240267	241326
UN04206	Rsa1.0_09223.1	741	6545	0	500	511	97	−	229	739	5163	5669
UN04207	Rsa1.0_08700.1	1160	7028	0	797	807	98	−	1	806	4858	5664
UN04208	Rsa1.0_11740.1	1253	5423	3.00E−88	312	356	87	+	761	1112	4636	4989
UN04209	Rsa1.0_01398.1	1209	149922	1.00E−161	340	354	96	−	9	360	139454	139803
UN04210	Rsa1.0_00048.1	1095	167185	0	1011	1090	92	−	8	1089	97236	98284

UN04211	Rsa1.0_02582.1	754	29889	0	735	754	97	+	1	754	4209	4962
UN04212	Rsa1.0_03916.1	1541	20117	0	574	582	98	+	557	1138	18468	19049
UN04213	Rsa1.0_01745.1	1457	39905	0	531	547	97	+	923	1455	13001	13547
UN04214	Rsa1.0_00058.1	889	159921	0	602	627	96	+	1	627	153366	153990
UN04215	Rsa1.0_10503.1	1069	5824	1.00E-130	262	269	97	+	3	270	1994	2262
UN04216	Rsa1.0_00542.1	1036	76831	0	553	584	94	+	15	597	74742	75324
UN04217	Rsa1.0_00039.1	879	296927	2.00E-61	195	221	88	−	196	414	223486	223694
UN04218	Rsa1.0_01005.1	882	56967	0	715	752	95	+	1	737	4934	5682
UN04219	Rsa1.0_07757.1	1052	8633	0	626	649	96	+	53	700	864	1512
UN04220	Rsa1.0_05305.1	1681	14207	0	570	589	96	+	306	891	2999	3582
UN04221	Rsa1.0_00409.1	858	146788	0	533	545	97	−	312	856	86809	87353
UN04222	Rsa1.0_00420.1	1152	86496	0	1011	1068	94	+	2	1064	39871	40930
UN04223	Rsa1.0_61123.1	669	375	1.00E-158	323	335	96	−	339	667	41	375
UN04224	Rsa1.0_00296.1	1117	97854	0	486	515	94	−	1	514	17737	18250
UN04225	Rsa1.0_03187.1	1412	24726	0	545	593	91	−	829	1398	19186	19770
UN04226	Rsa1.0_01081.1	952	499226	1.00E-99	269	294	91	+	1	291	323186	323476
UN04227	Rsa1.0_00002.1	1055	601661	0	883	930	94	−	127	1054	536726	537655
UN04228	Rsa1.0_00058.1	898	159921	0	522	548	95	−	2	549	71365	71895
UN04229	Rsa1.0_00092.1	1130	659932	1.00E-120	285	308	92	+	821	1128	340621	340915
UN04230	Rsa1.0_08902.1	910	6805	0	519	536	96	+	262	796	4643	5178
UN04231	Rsa1.0_00004.1	1026	253996	0	615	646	95	+	1	636	69131	69773
UN04232	Rsa1.0_27229.1	820	861	0	397	420	94	+	8	414	108	527
UN04233	Rsa1.0_00528.1	862	164755	0	626	646	96	+	1	646	109377	110022
UN04234	Rsa1.0_34634.1	846	654	3.00E-16	81	93	87	−	354	446	167	259
UN04235	Rsa1.0_05360.1	1049	14053	0	588	589	99	−	456	1044	4843	5431
UN04236	Rsa1.0_00035.1	836	176449	1.00E-176	417	457	91	−	378	834	158955	159389
UN04237	Rsa1.0_03981.1	369	19724	5.00E-81	332	390	85	+	2	365	18253	18628
UN04238	Rsa1.0_00713.1	1013	158017	0	466	489	95	+	437	925	1180	1668
UN04239	Rsa1.0_00561.1	1298	75653	0	864	926	93	+	1	921	4139	5053
UN04240	Rsa1.0_02281.1	478	292576	1.00E-112	230	238	96	+	228	465	263640	263877
UN04241	Rsa1.0_00420.1	280	86496	2.00E-55	131	138	94	+	119	256	37486	37623
UN04242	Rsa1.0_00044.1	844	271658	0	545	550	99	−	1	550	22375	22924
UN04243	Rsa1.0_00221.1	842	106972	0	555	563	98	−	72	633	53911	54473
UN04244	Rsa1.0_00569.1	982	75060	0	546	548	99	+	1	548	72168	72715
UN04245	Rsa1.0_03606.1	922	21818	1.00E-113	210	211	99	−	472	682	847	1057
UN04246	Rsa1.0_00082.1	968	237584	1.00E-118	296	321	92	+	663	968	38549	38864
UN04247	Rsa1.0_00227.1	514	105849	0	510	511	99	−	2	512	35727	36237
UN04248	Rsa1.0_00556.1	822	207212	0	516	540	95	+	180	717	51917	52456
UN04249	Rsa1.0_00551.1	1263	76227	0	520	545	95	−	723	1261	54753	55293

UN04250	Rsa1.0_00122.1	691	265937	1.00E-110	258	275	93	-	39	312	223063	223337
UN04251	Rsa1.0_00120.1	863	277288	1.00E-153	296	302	98	+	562	863	216704	217003
UN04252	Rsa1.0_02240.1	681	153768	1.00E-135	257	260	98	-	141	399	145616	145875
UN04253	Rsa1.0_02478.1	1008	150183	0	911	967	94	+	42	994	28834	29791
UN04254	Rsa1.0_03767.1	738	20969	0	659	684	96	+	4	684	9886	10566
UN04255	Rsa1.0_01638.1	1411	41857	0	810	860	94	-	1	848	2718	3576
UN04256	Rsa1.0_00813.1	741	63202	5.00E-98	200	206	97	-	411	616	14295	14500
UN04257	Rsa1.0_00623.1	1280	207630	0	493	502	98	-	648	1149	164086	164587
UN04258	Rsa1.0_05570.1	980	13301	0	448	473	94	+	1	473	10572	11032
UN04259	Rsa1.0_03981.1	1133	19724	1.00E-118	448	512	87	+	4	495	18219	18729
UN04260	Rsa1.0_00899.1	1491	60404	1.00E-145	290	298	97	+	1197	1491	10658	10952
UN04261	Rsa1.0_05267.1	916	14319	1.00E-159	398	430	92	+	11	439	7441	7863
UN04262	Rsa1.0_03257.1	1566	24305	0	1164	1166	99	+	3	1168	17594	18759
UN04263	Rsa1.0_00152.1	1068	257730	0	334	335	99	-	734	1068	200189	200523
UN04264	Rsa1.0_04772.1	968	16040	6.00E-86	194	201	96	-	2	198	13400	13600
UN04265	Rsa1.0_00997.1	1114	57190	0	436	440	99	-	1	439	52204	52640
UN04266	Rsa1.0_14744.1	1327	3111	0	457	475	96	+	853	1327	724	1198
UN04267	Rsa1.0_01012.1	946	56836	0	828	834	99	-	2	835	51667	52500
UN04268	Rsa1.0_00374.1	1501	165738	0	969	1015	95	-	502	1500	100943	101952
UN04269	Rsa1.0_02490.1	619	30885	1.00E-121	249	258	96	-	362	619	13643	13896
UN04270	Rsa1.0_00201.1	1405	232851	0	571	586	97	-	820	1405	81190	81766
UN04271	Rsa1.0_01309.1	483	48556	4.00E-82	215	235	91	-	115	349	2155	2389
UN04272	Rsa1.0_00437.1	1179	84879	1.00E-99	194	197	98	-	654	850	13824	14020
UN04273	Rsa1.0_01035.1	958	56019	0	420	435	96	+	4	433	33719	34153
UN04274	Rsa1.0_04563.1	1190	16837	8.00E-95	180	181	99	+	3	183	12914	13094
UN04275	Rsa1.0_00193.1	966	194198	0	812	853	95	-	125	965	23793	24642
UN04276	Rsa1.0_03319.1	1426	23767	0	481	481	100	-	5	485	18145	18625
UN04277	Rsa1.0_05217.1	607	142938	0	591	615	96	-	3	604	134906	135520
UN04278	Rsa1.0_00990.1	788	57445	1.00E-107	198	198	100	-	211	408	10576	10773
UN04279	Rsa1.0_01691.1	655	40921	0	600	657	91	+	1	655	29173	29823
UN04280	Rsa1.0_00089.1	885	406142	1.00E-180	366	380	96	-	506	885	233428	233806
UN04281	Rsa1.0_01778.1	920	39509	1.00E-101	251	267	94	+	657	920	9400	9660
UN04282	Rsa1.0_31020.1	772	738	1.00E-92	219	231	94	+	1	229	108	335
UN04283	Rsa1.0_06229.1	906	11439	0	467	490	95	+	421	905	8749	9238
UN04284	Rsa1.0_00491.1	1167	80219	0	1032	1141	90	-	58	1165	64152	65277
UN04285	Rsa1.0_00089.1	1054	406142	1.00E-160	330	342	96	+	22	362	161996	162336
UN04286	Rsa1.0_00047.1	1022	168582	1.00E-120	241	248	97	-	6	253	166424	166671
UN04287	Rsa1.0_01078.1	846	316910	0	753	754	99	-	93	846	280152	280905
UN04288	Rsa1.0_00398.1	1096	88007	0	661	709	93	-	403	1096	70663	71367

UN04289	Rsa1.0_08617.1	1339	7124	0	1167	1240	94	−	143	1339	309	1548
UN04290	Rsa1.0_01890.1	1297	37542	0	335	337	99	+	748	1084	12677	13013
UN04291	Rsa1.0_00044.1	556	271658	0	546	555	98	−	3	556	109390	109944
UN04292	Rsa1.0_02377.1	631	31994	0	604	648	93	−	3	631	19525	20171
UN04293	Rsa1.0_00228.1	1210	105511	0	399	400	99	−	537	936	81684	82083
UN04294	Rsa1.0_02966.1	952	135126	0	460	475	96	−	22	487	17972	18446
UN04295	Rsa1.0_02748.1	1013	28242	0	394	406	97	+	606	1011	11199	11604
UN04296	Rsa1.0_01414.1	755	149508	2.00E−85	161	161	100	+	69	229	8113	8273
UN04297	Rsa1.0_00889.1	621	60978	0	616	629	97	−	3	621	55959	56587
UN04298	Rsa1.0_00825.1	663	62855	1.00E−108	211	215	98	+	155	369	53331	53545
UN04299	Rsa1.0_12642.1	1382	5165	0	493	506	97	−	161	664	18	523
UN04300	Rsa1.0_00153.1	1140	192931	1.00E−101	263	289	91	−	3	276	170695	170981
UN04301	Rsa1.0_00467.1	1867	156542	0	580	581	99	+	8	587	142267	142847
UN04302	Rsa1.0_00135.1	1079	178077	1.00E−161	291	292	99	+	380	671	103755	104046
UN04303	Rsa1.0_01273.1	880	49464	1.00E−103	194	195	99	+	74	268	30606	30800
UN04304	Rsa1.0_00354.1	690	232005	1.00E−107	221	229	96	−	184	412	198867	199095
UN04305	Rsa1.0_00655.1	727	195840	1.00E−92	185	189	97	+	155	343	131412	131600
UN04306	Rsa1.0_02748.1	966	28242	1.00E−142	300	314	95	−	480	793	10342	10652
UN04307	Rsa1.0_03443.1	1892	22917	0	965	982	98	−	11	991	5065	6035
UN04308	Rsa1.0_41105.1	1313	546	1.00E−123	287	307	93	+	3	298	164	469
UN04309	Rsa1.0_03252.1	1498	24338	0	437	443	98	−	1056	1498	391	833
UN04310	Rsa1.0_00363.1	674	90968	1.00E−121	260	271	95	−	15	285	31971	32237
UN04311	Rsa1.0_01611.1	907	42269	1.00E−114	339	386	87	−	3	377	17493	17863
UN04312	Rsa1.0_28787.1	931	803	0	365	371	98	−	561	931	269	639
UN04313	Rsa1.0_15993.1	631	2329	1.00E−134	377	421	89	+	217	622	1900	2308
UN04314	Rsa1.0_04155.1	674	134772	0	462	464	99	+	210	673	119046	119509
UN04315	Rsa1.0_00071.1	728	345714	1.00E−178	344	353	97	+	34	386	102837	103189
UN04316	Rsa1.0_02004.1	1117	169138	0	882	911	96	+	2	912	167715	168625
UN04317	Rsa1.0_06892.1	981	10197	1.00E−136	313	335	93	−	645	975	9719	10053
UN04318	Rsa1.0_01435.1	695	45942	0	417	441	94	+	259	695	28028	28467
UN04319	Rsa1.0_00060.1	1273	502678	0	921	965	95	+	154	1110	484225	485189
UN04320	Rsa1.0_00211.1	756	228189	1.00E−113	228	235	97	−	143	377	149262	149496
UN04321	Rsa1.0_01220.1	1514	161775	0	446	470	94	−	1032	1500	122390	122853
UN04322	Rsa1.0_01338.1	1038	155644	0	404	418	96	−	350	767	143018	143435
UN04323	Rsa1.0_00110.1	1150	132719	0	468	494	94	+	660	1150	6856	7340
UN04324	Rsa1.0_00017.1	952	752458	9.00E−85	184	192	95	+	597	788	3792	3983
UN04325	Rsa1.0_02120.1	738	34819	1.00E−125	286	304	94	+	55	358	10757	11055
UN04326	Rsa1.0_00301.1	1318	365315	0	1309	1320	99	−	1	1318	47664	48983
UN04327	Rsa1.0_06303.1	989	11268	1.00E−112	236	246	95	+	544	789	9882	10127

UN04328	Rsa1.0_03085.1	1520	25500	0	693	712	97	+	812	1520	18211	18922
UN04329	Rsa1.0_00056.1	1231	332604	0	998	1071	93	−	167	1229	84538	85600
UN04330	Rsa1.0_01142.1	1756	52464	0	358	361	99	+	1396	1756	50939	51297
UN04331	Rsa1.0_03643.1	1161	21607	1.00E−168	300	300	100	−	3	302	1398	1697
UN04332	Rsa1.0_00347.1	803	93048	1.00E−108	262	281	93	+	427	704	86405	86679
UN04333	Rsa1.0_00699.1	1591	184398	0	637	662	96	−	929	1589	31005	31665
UN04334	Rsa1.0_01110.1	735	195852	0	399	410	97	−	325	733	185048	185457
UN04335	Rsa1.0_14734.1	1376	3116	0	505	505	100	−	5	509	139	643
UN04336	Rsa1.0_03463.1	1319	22724	0	410	413	99	+	1	411	18384	18796
UN04337	Rsa1.0_00127.1	1060	127446	1.00E−137	286	296	96	−	1	293	78175	78466
UN04338	Rsa1.0_00440.1	841	84723	1.00E−140	281	289	97	+	3	291	25111	25398
UN04339	Rsa1.0_16337.1	468	2191	1.00E−137	259	263	98	−	206	468	574	836
UN04340	Rsa1.0_04405.1	699	17508	1.00E−180	405	429	94	−	269	694	14200	14619
UN04341	Rsa1.0_00306.1	862	146760	0	453	479	94	+	24	501	122222	122685
UN04342	Rsa1.0_00062.1	1269	299291	0	986	1020	96	−	204	1220	266762	267781
UN04343	Rsa1.0_00596.1	793	73578	5.00E−95	210	220	95	+	575	793	28854	29067
UN04344	Rsa1.0_00611.1	1358	72410	0	898	899	99	−	1	899	6791	7689
UN04345	Rsa1.0_35196.1	1325	643	0	627	649	96	+	113	761	1	643
UN04346	Rsa1.0_01412.1	794	46311	0	458	482	95	−	1	465	9158	9634
UN04347	Rsa1.0_03166.1	870	24863	1.00E−122	240	246	97	−	467	712	6047	6292
UN04348	Rsa1.0_22732.1	644	1099	6.00E−82	173	179	96	−	152	330	916	1094
UN04349	Rsa1.0_01387.1	844	46745	1.00E−144	271	275	98	−	18	292	46152	46426
UN04350	Rsa1.0_00069.1	1331	403561	1.00E−160	305	310	98	+	1	310	185286	185594
UN04351	Rsa1.0_00002.1	768	601661	1.00E−112	215	218	98	+	303	520	149395	149612
UN04352	Rsa1.0_00951.1	1227	164588	1.00E−93	178	179	99	−	377	555	50502	50680
UN04353	Rsa1.0_05945.1	1146	12149	0	376	376	100	+	3	378	11742	12117
UN04354	Rsa1.0_22443.1	1306	1120	0	330	332	99	+	412	743	750	1081
UN04355	Rsa1.0_04288.1	1411	18077	2.00E−86	163	163	100	−	802	964	9680	9842
UN04356	Rsa1.0_03172.1	1235	24816	0	492	492	100	+	652	1143	13722	14213
UN04357	Rsa1.0_00068.1	1013	151813	0	489	494	98	−	520	1013	97326	97819
UN04358	Rsa1.0_06757.1	921	10411	0	540	555	97	−	3	555	4331	4882
UN04359	Rsa1.0_04282.1	1502	18100	1.00E−138	366	401	91	+	946	1340	8780	9167
UN04360	Rsa1.0_00328.1	940	224661	0	860	880	97	−	6	879	140444	141323
UN04361	Rsa1.0_00341.1	1137	249309	0	414	418	99	−	1	418	227346	227763
UN04362	Rsa1.0_00440.1	777	84723	5.00E−86	203	213	95	−	541	750	25187	25398
UN04363	Rsa1.0_01292.1	781	175783	2.00E−79	193	207	93	+	534	735	2295	2501
UN04364	Rsa1.0_00124.1	586	262503	0	442	451	98	+	5	455	27047	27497
UN04365	Rsa1.0_01585.1	1034	42806	1.00E−157	287	289	99	+	590	878	15623	15911
UN04366	Rsa1.0_00037.1	858	307838	2.00E−70	170	179	94	−	680	856	181437	181614

UN04367	Rsa1.0_20133.1	687	1337	1.00E-98	246	269	91	-	432	687	1072	1337
UN04368	Rsa1.0_00731.1	1560	66638	1.00E-176	323	326	99	-	867	1192	1000	1325
UN04369	Rsa1.0_12457.1	1064	5222	7.00E-98	194	198	97	-	2	199	4765	4962
UN04370	Rsa1.0_00545.1	821	189649	1.00E-124	266	277	96	+	515	791	2122	2390
UN04371	Rsa1.0_03263.1	524	24268	1.00E-154	365	390	93	-	135	524	15033	15411
UN04372	Rsa1.0_00205.1	1066	193401	0	433	462	93	+	543	1000	127625	128084
UN04373	Rsa1.0_00739.1	1362	66155	0	338	341	99	-	3	343	61085	61424
UN04374	Rsa1.0_00509.1	689	78829	0	676	687	98	-	1	687	8715	9400
UN04375	Rsa1.0_02167.1	816	34401	0	561	573	97	+	204	775	26786	27358
UN04376	Rsa1.0_00155.1	770	118465	1.00E-106	217	224	96	+	283	506	104178	104401
UN04377	Rsa1.0_01000.1	1237	57140	0	392	413	94	-	804	1212	2343	2750
UN04378	Rsa1.0_00483.1	959	81102	0	550	555	99	+	340	894	2359	2913
UN04379	Rsa1.0_00275.1	1225	210104	0	550	580	94	+	3	577	206418	206995
UN04380	Rsa1.0_00042.1	816	171507	1.00E-123	252	260	96	-	1	260	157980	158238
UN04381	Rsa1.0_00341.1	829	249309	0	551	557	98	+	2	558	80699	81254
UN04382	Rsa1.0_00007.1	904	239325	1.00E-162	310	317	97	+	583	899	108872	109188
UN04383	Rsa1.0_00066.1	461	152418	0	438	457	95	+	3	458	90303	90755
UN04384	Rsa1.0_39148.1	828	575	0	553	577	95	+	28	603	1	575
UN04385	Rsa1.0_00159.1	887	431686	1.00E-131	249	253	98	+	635	887	427719	427971
UN04386	Rsa1.0_00077.1	897	145005	1.00E-178	323	325	99	+	573	897	31241	31565
UN04387	Rsa1.0_00633.1	846	71250	0	431	436	98	+	306	741	23460	23895
UN04388	Rsa1.0_00021.1	1633	263655	1.00E-154	280	281	99	-	3	283	19978	20258
UN04389	Rsa1.0_39426.1	1036	570	0	389	392	99	-	3	394	57	448
UN04390	Rsa1.0_00028.1	775	293984	0	752	781	96	+	1	775	71401	72175
UN04391	Rsa1.0_02884.1	953	27034	1.00E-169	410	444	92	+	517	953	12730	13172
UN04392	Rsa1.0_00163.1	1491	116847	0	446	476	93	+	1018	1490	83512	83979
UN04393	Rsa1.0_01434.1	766	45951	0	453	476	95	-	13	482	39461	39934
UN04394	Rsa1.0_00522.1	1477	78103	1.00E-152	314	325	96	+	63	384	48418	48742
UN04395	Rsa1.0_09223.1	1125	6545	0	796	823	96	+	280	1094	4476	5298
UN04396	Rsa1.0_02888.1	1220	27020	0	993	1029	96	+	3	1030	5412	6438
UN04397	Rsa1.0_01092.1	1474	54348	0	532	550	96	-	934	1474	22694	23240
UN04398	Rsa1.0_02943.1	1227	153598	0	510	525	97	-	632	1156	128989	129513
UN04399	Rsa1.0_06581.1	834	10722	1.00E-95	199	206	96	-	294	499	8954	9159
UN04400	Rsa1.0_07576.1	1071	9098	0	589	607	97	+	467	1071	2476	3081
UN04401	Rsa1.0_00289.1	1332	98620	1.00E-140	273	277	98	+	1054	1330	97044	97318
UN04402	Rsa1.0_04511.1	998	17032	1.00E-115	329	368	89	+	652	997	4974	5339
UN04403	Rsa1.0_01177.1	753	51572	0	386	392	98	-	162	553	9103	9494
UN04404	Rsa1.0_04646.1	868	16541	0	843	872	96	+	2	866	10692	11563
UN04405	Rsa1.0_01964.1	944	36702	0	560	602	93	-	361	944	35629	36221

UN04406	Rsa1.0_00037.1	1397	307838	0	804	804	100	−	587	1390	296672	297475
UN04407	Rsa1.0_00163.1	847	116847	1.00E−145	292	302	96	+	80	381	66573	66874
UN04408	Rsa1.0_03808.1	837	20732	0	797	818	97	−	2	814	715	1529
UN04409	Rsa1.0_00065.1	1369	454616	1.00E−151	366	394	92	−	984	1369	417285	417667
UN04410	Rsa1.0_02562.1	789	30061	4.00E−83	175	181	96	+	609	789	27886	28066
UN04411	Rsa1.0_00016.1	1385	831256	0	465	468	99	−	1	468	202082	202547
UN04412	Rsa1.0_00028.1	798	293984	0	428	451	94	+	71	521	111859	112305
UN04413	Rsa1.0_03916.1	1044	20117	0	458	464	98	−	583	1043	13461	13924
UN04414	Rsa1.0_00104.1	1065	368722	0	373	377	98	+	3	376	3957	4333
UN04415	Rsa1.0_04387.1	1228	17625	0	524	531	98	+	703	1228	7587	8117
UN04416	Rsa1.0_04142.1	1242	18861	0	499	541	92	+	416	954	1699	2239
UN04417	Rsa1.0_00585.1	964	74164	0	431	447	96	−	3	445	53696	54141
UN04418	Rsa1.0_00744.1	1049	66084	0	368	379	97	−	3	381	58901	59277
UN04419	Rsa1.0_01789.1	899	39250	0	520	536	97	−	365	898	6077	6607
UN04420	Rsa1.0_00006.1	1535	396176	0	1447	1481	97	−	55	1535	298181	299661
UN04421	Rsa1.0_00990.1	935	57445	4.00E−62	125	126	99	−	764	889	27028	27153
UN04422	Rsa1.0_01411.1	948	46326	1.00E−179	334	339	98	−	610	948	6419	6757
UN04423	Rsa1.0_00143.1	858	121313	0	828	857	96	+	1	856	32761	33608
UN04424	Rsa1.0_00037.1	1663	307838	0	387	403	96	+	245	647	218766	219165
UN04425	Rsa1.0_00175.1	929	166121	1.00E−107	237	246	96	+	1	245	56315	56558
UN04426	Rsa1.0_00379.1	1029	89865	1.00E−84	166	168	98	+	239	406	51509	51676
UN04427	Rsa1.0_53918.1	702	423	6.00E−48	98	98	100	+	369	466	1	98
UN04428	Rsa1.0_02056.1	1138	35543	0	577	593	97	+	399	991	34959	35543
UN04429	Rsa1.0_07885.1	1023	8359	0	712	772	92	+	1	765	6554	7323
UN04430	Rsa1.0_00453.1	339	83031	1.00E−148	325	340	95	−	1	336	73800	74138
UN04431	Rsa1.0_00181.1	1177	114269	1.00E−179	325	327	99	−	697	1023	43259	43585
UN04432	Rsa1.0_00042.1	1246	171507	1.00E−166	329	338	97	+	901	1234	159615	159952
UN04433	Rsa1.0_00338.1	1401	94109	1.00E−103	245	260	94	−	3	261	85638	85896
UN04434	Rsa1.0_02675.1	795	28852	1.00E−138	310	326	95	−	471	795	18809	19132
UN04435	Rsa1.0_01814.1	1273	38917	0	416	432	96	+	8	434	5754	6180
UN04436	Rsa1.0_02020.1	710	173211	1.00E−109	209	212	98	−	470	681	168234	168445
UN04437	Rsa1.0_01668.1	838	41299	0	468	473	98	+	365	837	30426	30898
UN04438	Rsa1.0_14737.1	801	3115	1.00E−164	314	321	97	−	352	672	2055	2375
UN04439	Rsa1.0_00374.1	1034	165738	0	422	439	96	+	595	1033	95144	95576
UN04440	Rsa1.0_02181.1	1207	34172	0	467	469	99	+	740	1206	20277	20745
UN04441	Rsa1.0_00100.1	806	249232	1.00E−169	310	313	99	−	2	314	176030	176342
UN04442	Rsa1.0_00096.1	930	198773	0	482	483	99	+	2	484	106464	106946
UN04443	Rsa1.0_03702.1	1082	21292	0	501	503	99	−	1	503	8205	8707
UN04444	Rsa1.0_00223.1	1713	106231	0	441	462	95	−	3	457	6079	6540

UN04445	Rsa1.0_01098.1	1098	54215	0	341	346	98	+	1	344	9648	9990
UN04446	Rsa1.0_00015.1	791	561803	0	520	546	95	−	61	601	13424	13965
UN04447	Rsa1.0_00051.1	927	258225	0	921	927	99	−	1	927	143003	143929
UN04448	Rsa1.0_00290.1	827	225096	1.00E−141	295	306	96	−	5	309	190482	190785
UN04449	Rsa1.0_00166.1	1452	240912	0	726	727	99	+	726	1452	129055	129781
UN04450	Rsa1.0_02139.1	688	34686	5.00E−70	230	256	89	+	1	250	5578	5831
UN04451	Rsa1.0_00197.1	840	109714	0	518	540	95	−	165	704	64829	65368
UN04452	Rsa1.0_01629.1	1364	41917	0	1362	1365	99	−	1	1363	5375	6739
UN04453	Rsa1.0_03380.1	897	23320	1.00E−153	287	291	98	+	2	292	6043	6333
UN04454	Rsa1.0_00325.1	774	95371	0	371	371	100	−	369	739	57594	57964
UN04455	Rsa1.0_01007.1	784	56933	0	765	781	97	+	4	784	6770	7540
UN04456	Rsa1.0_00589.1	1107	209965	0	1077	1107	97	−	1	1105	207003	208109
UN04457	Rsa1.0_03746.1	1092	21050	0	489	505	96	−	428	932	19838	20342
UN04458	Rsa1.0_10313.1	611	5892	0	590	610	96	+	1	609	2653	3261
UN04459	Rsa1.0_00250.1	775	273771	0	497	545	91	+	4	524	4460	5000
UN04460	Rsa1.0_00695.1	1237	68019	0	564	590	95	+	2	589	58750	59336
UN04461	Rsa1.0_00648.1	1110	70351	1.00E−148	345	375	92	+	64	420	12134	12505
UN04462	Rsa1.0_07749.1	452	8648	0	445	452	98	−	1	450	5793	6244
UN04463	Rsa1.0_00089.1	878	406142	1.00E−127	246	251	98	−	161	411	360701	360951
UN04464	Rsa1.0_01277.1	1295	49375	0	375	376	99	+	918	1293	8346	8721
UN04465	#	#	#	#	#	#	#	#	#	#	#	#
UN04466	Rsa1.0_00621.1	724	71840	0	337	340	99	+	1	340	41500	41839
UN04467	Rsa1.0_01588.1	763	42724	6.00E−73	165	173	95	−	17	185	41412	41584
UN04468	Rsa1.0_00030.1	377	250988	7.00E−74	192	204	94	+	31	229	149441	149644
UN04469	Rsa1.0_00258.1	1692	102381	0	1100	1100	100	+	2	1101	93318	94417
UN04470	Rsa1.0_02133.1	1101	34726	1.00E−93	228	245	93	+	181	422	31515	31759
UN04471	Rsa1.0_05162.1	971	14631	1.00E−167	350	366	95	−	611	971	5143	5507
UN04472	Rsa1.0_31253.1	1228	732	0	711	732	97	−	50	781	1	732
UN04473	Rsa1.0_00006.1	1109	396176	0	615	631	97	−	51	681	299033	299663
UN04474	Rsa1.0_00588.1	1259	73939	0	496	537	92	−	54	582	54483	55005
UN04475	Rsa1.0_01172.1	304	51734	3.00E−48	116	122	95	−	182	303	49798	49919
UN04476	Rsa1.0_00975.1	883	57865	0	824	857	96	−	1	854	55325	56177
UN04477	Rsa1.0_00378.1	1252	89892	0	685	721	95	+	8	722	47722	48441
UN04478	Rsa1.0_01727.1	530	40243	0	390	408	95	−	1	406	27900	28306
UN04479	Rsa1.0_00964.1	2062	316648	0	553	584	94	−	1483	2062	261721	262289
UN04480	Rsa1.0_02564.1	810	30047	1.00E−111	242	252	96	−	60	310	6841	7091
UN04481	Rsa1.0_01077.1	678	54772	0	417	417	100	+	3	419	51213	51629
UN04482	Rsa1.0_00120.1	870	277288	1.00E−77	239	266	89	+	605	865	119581	119846
UN04483	Rsa1.0_00273.1	966	326186	1.00E−148	307	319	96	+	22	340	46018	46335

UN04484	Rsa1.0_05505.1	1162	13500	0	969	1014	95	−	157	1162	12418	13420
UN04485	Rsa1.0_03727.1	931	21158	0	598	653	91	−	254	891	2879	3516
UN04486	Rsa1.0_00008.1	1218	358124	0	926	991	93	+	77	1064	28476	29460
UN04487	Rsa1.0_00037.1	1012	307838	1.00E−175	409	446	91	+	587	1012	239665	240101
UN04488	Rsa1.0_10770.1	1214	5723	2.00E−92	234	256	91	−	961	1213	570	813
UN04489	Rsa1.0_00341.1	914	249309	0	436	445	97	+	1	445	118056	118500
UN04490	Rsa1.0_21219.1	901	1224	1.00E−178	362	374	96	−	32	400	138	511
UN04491	Rsa1.0_00111.1	1924	390088	0	378	391	96	−	1218	1608	381110	381500
UN04492	Rsa1.0_00706.1	1127	291570	0	628	657	95	−	474	1125	23308	23961
UN04493	Rsa1.0_00299.1	719	97770	1.00E−150	272	273	99	−	294	566	19658	19930
UN04494	Rsa1.0_00018.1	1449	410899	0	1259	1288	97	+	167	1449	6100	7387
UN04495	Rsa1.0_00008.1	789	358124	1.00E−178	319	320	99	+	468	787	277916	278235
UN04496	Rsa1.0_03618.1	1540	21744	0	681	681	100	−	860	1540	5900	6580
UN04497	Rsa1.0_14912.1	927	2970	1.00E−131	319	342	93	+	594	927	1944	2285
UN04498	Rsa1.0_00016.1	1001	831256	0	451	462	97	−	91	552	569300	569761
UN04499	Rsa1.0_00335.1	1144	94371	1.00E−164	353	373	94	+	29	401	42163	42535
UN04500	Rsa1.0_00037.1	769	307838	2.00E−94	185	188	98	+	505	692	21411	21598
UN04501	Rsa1.0_02402.1	1803	31710	0	727	763	95	−	1045	1803	23950	24706
UN04502	Rsa1.0_00792.1	1073	64163	1.00E−116	251	261	96	−	1	260	7358	7617
UN04503	Rsa1.0_02769.1	888	28107	1.00E−150	301	309	97	−	228	534	20934	21241
UN04504	Rsa1.0_11276.1	1016	5560	1.00E−167	346	362	95	+	92	453	3	364
UN04505	Rsa1.0_00307.1	983	199003	2.00E−89	258	280	92	+	3	278	87390	87662
UN04506	Rsa1.0_01144.1	1028	52431	0	590	638	92	−	87	718	36426	37057
UN04507	Rsa1.0_05124.1	995	14790	0	954	995	95	−	2	993	307	1294
UN04508	Rsa1.0_01192.1	1376	51150	0	829	846	97	+	7	850	5907	6748
UN04509	Rsa1.0_00928.1	639	59293	1.00E−111	225	232	96	+	406	637	30313	30544
UN04510	Rsa1.0_00187.1	1046	393093	0	924	972	95	+	76	1044	212078	213045
UN04511	Rsa1.0_00183.1	1188	113277	0	439	453	96	−	724	1176	62771	63223
UN04512	Rsa1.0_00086.1	1541	439854	1.00E−144	266	268	99	−	1	268	373324	373591
UN04513	Rsa1.0_01487.1	1357	44784	0	372	388	95	−	330	717	27939	28326
UN04514	Rsa1.0_00323.1	967	159650	0	486	494	98	−	474	967	138320	138813
UN04515	Rsa1.0_04610.1	831	16653	1.00E−113	274	294	93	+	513	803	995	1288
UN04516	Rsa1.0_00698.1	283	67816	1.00E−111	243	255	95	−	35	283	12515	12769
UN04517	Rsa1.0_00374.1	877	165738	0	446	465	95	−	413	873	154901	155362
UN04518	Rsa1.0_00301.1	735	365315	1.00E−166	325	332	97	−	3	334	215181	215509
UN04519	Rsa1.0_05442.1	1287	13741	0	399	418	95	−	1	415	1778	2184
UN04520	Rsa1.0_01151.1	1192	52268	1.00E−147	271	273	99	+	600	872	10761	11033
UN04521	Rsa1.0_01244.1	1519	50061	1.00E−177	384	406	94	−	1	392	30687	31088
UN04522	Rsa1.0_04422.1	1373	17423	0	507	520	97	+	5	524	10123	10641

UN04523	Rsa1.0_00476.1	1069	280966	1.00E-179	343	351	97	−	3	353	220252	220602
UN04524	Rsa1.0_00235.1	749	154696	1.00E-150	272	273	99	+	346	618	98741	99013
UN04525	Rsa1.0_01560.1	833	43256	1.00E-118	288	306	94	+	525	824	30828	31132
UN04526	Rsa1.0_02036.1	732	35731	0	528	533	99	−	198	730	9713	10242
UN04527	Rsa1.0_00414.1	1128	202190	1.00E-169	351	365	96	−	423	785	200762	201126
UN04528	Rsa1.0_00160.1	779	346678	8.00E-97	180	180	100	−	398	577	318676	318855
UN04529	Rsa1.0_02569.1	916	30002	0	565	571	98	+	215	785	2463	3032
UN04530	Rsa1.0_01477.1	1212	45008	0	827	844	97	−	369	1212	42698	43540
UN04531	Rsa1.0_01081.1	835	499226	0	786	818	96	+	33	835	315447	316264
UN04532	Rsa1.0_11694.1	1255	131228	0	764	807	94	−	460	1255	127881	128687
UN04533	Rsa1.0_05076.1	1107	14916	0	1092	1108	98	−	1	1107	12234	13341
UN04534	Rsa1.0_02215.1	806	33822	1.00E-153	281	283	99	+	522	804	20760	21042
UN04535	Rsa1.0_02735.1	722	28344	0	405	423	95	+	12	427	11423	11845
UN04536	Rsa1.0_00246.1	800	103541	0	770	811	94	−	2	800	30122	30932
UN04537	Rsa1.0_00230.1	781	105389	1.00E-144	299	312	95	−	1	307	88061	88372
UN04538	Rsa1.0_01077.1	1185	54772	0	722	750	96	+	3	747	37847	38596
UN04539	Rsa1.0_00092.1	814	659932	0	503	538	93	−	291	814	313009	313534
UN04540	Rsa1.0_00067.1	1153	415055	1.00E-150	279	282	98	+	863	1144	244191	244472
UN04541	Rsa1.0_12731.1	1349	5142	1.00E-168	346	361	95	−	1	352	130	490
UN04542	Rsa1.0_00921.1	1246	59599	0	1186	1237	95	−	33	1244	4232	5464
UN04543	Rsa1.0_02128.1	1066	34758	0	434	458	94	−	83	540	23212	23665
UN04544	Rsa1.0_03792.1	778	20835	5.00E-98	251	274	91	−	418	691	10857	11130
UN04545	Rsa1.0_00015.1	1021	561803	1.00E-150	389	430	90	−	53	478	526505	526905
UN04546	Rsa1.0_01755.1	943	155581	0	884	930	95	−	1	929	11036	11959
UN04547	Rsa1.0_05347.1	670	14096	0	498	526	94	−	7	530	9260	9767
UN04548	Rsa1.0_00284.1	1062	323267	1.00E-157	329	340	96	+	4	339	281438	281775
UN04549	Rsa1.0_01606.1	962	42432	0	500	518	96	+	440	957	9028	9545
UN04550	Rsa1.0_00002.1	1603	601661	0	1492	1492	100	−	1	1492	367891	369382
UN04551	Rsa1.0_20494.1	1449	1299	0	524	550	95	+	351	892	356	899
UN04552	Rsa1.0_04762.1	1357	16101	0	400	412	97	+	1	410	9039	9450
UN04553	Rsa1.0_01154.1	1420	52249	1.00E-173	311	312	99	−	1004	1315	997	1308
UN04554	Rsa1.0_26696.1	826	881	0	501	514	97	+	2	514	368	881
UN04555	Rsa1.0_02383.1	792	31931	0	555	601	92	+	3	591	15278	15878
UN04556	Rsa1.0_01387.1	1106	46745	0	510	543	93	−	1	543	41689	42216
UN04557	Rsa1.0_04418.1	1120	17434	0	358	358	100	−	692	1049	11947	12304
UN04558	Rsa1.0_02641.1	1355	29191	0	429	429	100	−	1	429	16435	16863
UN04559	Rsa1.0_00010.1	815	294827	0	459	464	98	−	5	468	65435	65898
UN04560	Rsa1.0_00040.1	641	324196	1.00E-107	291	322	90	−	23	344	261455	261757
UN04561	Rsa1.0_00944.1	1143	58733	1.00E-149	271	272	99	+	283	554	34376	34647

UN04562	Rsa1.0_07695.1	1168	8770	0	458	477	96	+	3	479	1421	1897
UN04563	Rsa1.0_07066.1	707	9954	5.00E-70	144	147	97	−	160	306	8112	8258
UN04564	Rsa1.0_00051.1	1133	258225	0	518	551	94	−	592	1133	47526	48062
UN04565	Rsa1.0_00660.1	1390	69688	1.00E-126	242	245	98	−	1146	1390	61247	61490
UN04566	Rsa1.0_00356.1	1133	91521	0	386	405	95	−	372	776	80712	81097
UN04567	Rsa1.0_02900.1	731	26928	1.00E-104	286	313	91	+	413	723	16858	17165
UN04568	Rsa1.0_01696.1	1074	301850	7.00E-55	116	118	98	+	112	229	291386	291503
UN04569	Rsa1.0_00005.1	943	353093	0	484	505	95	+	415	919	260758	261250
UN04570	Rsa1.0_03296.1	1463	23969	0	705	710	99	−	454	1163	7643	8352
UN04571	Rsa1.0_00189.1	841	111830	0	594	595	99	+	247	841	110540	111134
UN04572	Rsa1.0_00139.1	1281	357786	0	392	410	95	+	1	404	257345	257754
UN04573	Rsa1.0_03122.1	504	25207	0	363	373	97	−	1	373	22678	23050
UN04574	Rsa1.0_00048.1	830	167185	1.00E-133	271	280	96	+	443	720	8087	8366
UN04575	Rsa1.0_00909.1	903	59976	1.00E-151	347	375	92	+	1	374	38203	38554
UN04576	Rsa1.0_03367.1	1657	23387	3.00E-86	196	207	94	+	257	463	1583	1789
UN04577	Rsa1.0_36749.1	919	613	1.00E-180	335	340	98	+	237	576	186	525
UN04578	Rsa1.0_01269.1	1103	49622	0	493	503	98	+	1	502	32580	33079
UN04579	Rsa1.0_20566.1	828	1292	1.00E-107	234	246	95	+	513	758	809	1054
UN04580	Rsa1.0_00054.1	938	162053	0	458	469	97	+	4	472	40324	40792
UN04581	Rsa1.0_01569.1	1092	43029	0	978	1001	97	+	88	1088	23798	24798
UN04582	Rsa1.0_01223.1	956	50392	1.00E-130	270	281	96	−	675	955	32546	32826
UN04583	Rsa1.0_00132.1	768	124299	1.00E-120	243	248	97	+	1	247	108496	108742
UN04584	Rsa1.0_01016.1	635	181422	1.00E-138	288	301	95	+	264	564	44377	44671
UN04585	Rsa1.0_00067.1	991	415055	0	984	984	100	+	6	989	400763	401746
UN04586	Rsa1.0_02429.1	717	31527	0	493	542	90	−	144	679	9754	10295
UN04587	Rsa1.0_01594.1	1282	42612	0	406	415	97	−	3	414	9290	9704
UN04588	Rsa1.0_05769.1	1416	12694	1.00E-168	309	312	99	−	447	758	8306	8617
UN04589	Rsa1.0_00007.1	757	239325	1.00E-100	204	210	97	−	541	750	41360	41569
UN04590	Rsa1.0_14767.1	538	3079	0	387	402	96	+	1	402	2511	2909
UN04591	Rsa1.0_05398.1	1146	13885	0	1110	1161	95	+	2	1144	9272	10420
UN04592	Rsa1.0_00307.1	1399	199003	1.00E-161	306	312	98	−	1	312	50440	50746
UN04593	Rsa1.0_01006.1	930	163670	0	489	503	97	+	3	491	135844	136346
UN04594	Rsa1.0_01442.1	734	45776	0	555	567	97	−	168	734	26177	26743
UN04595	Rsa1.0_02157.1	1026	149652	1.00E-121	289	313	92	+	1	310	143023	143319
UN04596	Rsa1.0_05621.1	654	13145	0	642	652	98	+	3	654	2047	2696
UN04597	Rsa1.0_00073.1	1260	240924	0	1255	1261	99	−	1	1260	79248	80508
UN04598	Rsa1.0_00111.1	1238	390088	0	410	426	96	−	505	930	199287	199712
UN04599	Rsa1.0_00816.1	844	403350	2.00E-91	171	171	100	+	542	712	271244	271414
UN04600	Rsa1.0_11057.1	639	5631	0	588	600	98	−	40	639	4116	4715

UN04601	Rsa1.0_00104.1	980	368722	3.00E-94	239	260	91	+	590	849	201374	201633
UN04602	Rsa1.0_01569.1	661	43029	0	458	462	99	-	200	661	19163	19624
UN04603	Rsa1.0_05701.1	1157	12884	0	604	604	100	-	1	604	1	604
UN04604	Rsa1.0_15024.1	976	2885	1.00E-142	289	298	96	-	679	974	2298	2592
UN04605	Rsa1.0_01806.1	1231	39021	0	516	541	95	-	1	535	24515	25049
UN04606	Rsa1.0_00094.1	903	139042	0	694	734	94	+	5	724	119268	119990
UN04607	Rsa1.0_04910.1	877	15484	1.00E-151	286	291	98	-	581	871	1909	2199
UN04608	Rsa1.0_05704.1	954	12883	0	591	623	94	+	2	624	2517	3136
UN04609	Rsa1.0_02246.1	1726	33435	0	598	598	100	-	810	1407	23284	23881
UN04610	Rsa1.0_05776.1	1269	12664	0	866	889	97	+	304	1192	132	1018
UN04611	Rsa1.0_00274.1	1070	210119	1.00E-140	278	286	97	-	1	286	98748	99029
UN04612	Rsa1.0_01656.1	1538	41494	0	548	569	96	-	769	1337	35614	36181
UN04613	Rsa1.0_00161.1	862	116891	0	773	809	95	+	58	862	54521	55311
UN04614	Rsa1.0_00057.1	1161	160473	0	396	408	97	-	55	462	100728	101135
UN04615	Rsa1.0_00960.1	713	58199	2.00E-94	188	192	97	-	265	456	13844	14035
UN04616	Rsa1.0_00391.1	299	183853	1.00E-139	287	299	95	-	1	299	87034	87332
UN04617	Rsa1.0_01730.1	952	40223	0	916	951	96	-	2	952	35582	36519
UN04618	Rsa1.0_00707.1	987	190112	0	354	364	97	+	621	984	55985	56346
UN04619	Rsa1.0_00293.1	991	339294	0	425	435	97	-	556	990	300404	300837
UN04620	Rsa1.0_01285.1	944	172709	0	915	947	96	-	1	944	150287	151230
UN04621	Rsa1.0_00833.1	980	62621	1.00E-132	249	252	98	-	1	252	7472	7723
UN04622	Rsa1.0_00201.1	1790	232851	1.00E-173	344	352	97	+	3	354	122097	122444
UN04623	Rsa1.0_00212.1	756	108350	1.00E-134	318	338	94	+	1	334	59942	60278
UN04624	Rsa1.0_00628.1	756	214360	1.00E-116	270	289	93	-	467	755	202724	203012
UN04625	Rsa1.0_00119.1	1341	467509	1.00E-153	324	339	95	-	1	339	302973	303310
UN04626	Rsa1.0_00789.1	751	64233	1.00E-70	152	155	98	-	3	157	44999	45150
UN04627	Rsa1.0_00758.1	603	65477	1.00E-135	260	265	98	-	334	598	11258	11522
UN04628	Rsa1.0_00276.1	1144	100160	0	736	749	98	+	393	1141	65711	66459
UN04629	Rsa1.0_02139.1	1204	34686	0	535	545	98	-	661	1204	24870	25414
UN04630	Rsa1.0_04786.1	1336	15988	0	632	663	95	-	73	725	14357	15007
UN04631	Rsa1.0_00247.1	761	103408	0	744	757	98	-	3	758	2156	2910
UN04632	Rsa1.0_21931.1	1702	1162	0	1147	1155	99	-	459	1613	1	1155
UN04633	Rsa1.0_03756.1	1032	143061	0	360	362	99	-	2	362	138726	139087
UN04634	Rsa1.0_08255.1	646	7652	1.00E-171	354	371	95	-	274	638	3887	4257
UN04635	Rsa1.0_03943.1	1164	19931	0	429	455	94	+	3	454	9444	9893
UN04636	Rsa1.0_00059.1	629	159650	1.00E-112	281	301	93	-	331	626	123662	123957
UN04637	Rsa1.0_13662.1	1111	4542	0	914	942	97	+	169	1107	3404	4345
UN04638	Rsa1.0_03244.1	796	24413	1.00E-168	402	439	91	+	12	429	10841	11276
UN04639	Rsa1.0_05241.1	1391	14405	0	528	545	96	+	851	1391	918	1461

UN04640	Rsa1.0_00159.1	1504	431686	0	804	808	99	−	691	1498	232519	233326
UN04641	Rsa1.0_02044.1	690	35651	1.00E−117	273	296	92	−	211	503	10568	10845
UN04642	Rsa1.0_01167.1	791	51859	0	756	787	96	−	3	789	48979	49746
UN04643	Rsa1.0_07017.1	768	10024	1.00E−129	272	282	96	−	3	283	5214	5489
UN04644	Rsa1.0_00955.1	1327	58358	1.00E−117	214	214	100	−	214	427	35701	35914
UN04645	Rsa1.0_22781.1	929	1095	1.00E−132	240	240	100	−	689	928	386	625
UN04646	Rsa1.0_02289.1	1361	32981	0	892	892	100	−	470	1361	11023	11914
UN04647	Rsa1.0_01730.1	1725	40223	0	1400	1436	97	+	295	1725	35176	36595
UN04648	Rsa1.0_07590.1	775	9068	0	756	777	97	+	1	775	2421	3184
UN04649	Rsa1.0_13893.1	661	4139	0	637	658	96	+	4	661	789	1446
UN04650	Rsa1.0_04428.1	748	17399	1.00E−170	326	331	98	−	3	332	11778	12107
UN04651	Rsa1.0_00894.1	605	269700	0	598	604	99	+	2	605	6004	6604
UN04652	Rsa1.0_01539.1	473	43698	0	354	357	99	−	117	473	29321	29677
UN04653	Rsa1.0_04411.1	1247	142979	1.00E−139	267	271	98	−	976	1245	6356	6626
UN04654	Rsa1.0_01343.1	1376	47735	0	356	357	99	−	8	364	9134	9490
UN04655	Rsa1.0_00385.1	2654	89160	9.00E−81	169	174	97	+	250	423	50842	51015
UN04656	Rsa1.0_01701.1	412	162121	1.00E−165	328	339	96	+	74	412	13837	14175
UN04657	Rsa1.0_00059.1	1279	159650	0	782	829	94	+	435	1240	63397	64222
UN04658	Rsa1.0_00468.1	1063	82095	0	1047	1054	99	+	2	1055	16354	17407
UN04659	Rsa1.0_01344.1	626	142535	4.00E−98	191	194	98	−	12	205	112174	112367
UN04660	Rsa1.0_00138.1	1321	247040	0	343	343	100	+	309	651	228322	228664
UN04661	Rsa1.0_00380.1	947	89748	1.00E−106	234	248	94	−	223	461	78451	78698
UN04662	Rsa1.0_03001.1	844	26108	1.00E−161	320	331	96	−	520	844	9377	9707
UN04663	Rsa1.0_00037.1	817	307838	1.00E−160	318	326	97	+	1	324	291540	291864
UN04664	Rsa1.0_00798.1	845	63945	0	425	438	97	−	154	591	45476	45913
UN04665	Rsa1.0_00886.1	1317	167350	1.00E−173	330	337	97	−	567	903	33611	33947
UN04666	Rsa1.0_00899.1	1489	60404	0	367	377	97	+	3	376	49106	49480
UN04667	Rsa1.0_07091.1	1017	9907	1.00E−129	262	271	96	−	60	330	1444	1714
UN04668	Rsa1.0_01115.1	1037	183545	1.00E−154	381	411	92	+	640	1037	6514	6921
UN04669	Rsa1.0_00052.1	1393	162771	0	471	477	98	+	476	952	119971	120447
UN04670	Rsa1.0_01220.1	1160	161775	1.00E−155	307	316	97	−	574	889	151183	151495
UN04671	Rsa1.0_00828.1	1047	62744	0	363	365	99	−	3	367	9576	9940
UN04672	Rsa1.0_00045.1	1154	328694	1.00E−137	266	271	98	+	885	1153	93515	93785
UN04673	Rsa1.0_00019.1	1209	301669	0	508	514	98	−	444	954	55803	56316
UN04674	Rsa1.0_00039.1	807	296927	1.00E−160	315	323	97	−	452	774	12676	12997
UN04675	Rsa1.0_00339.1	1429	222974	0	768	800	96	−	3	779	81609	82408
UN04676	Rsa1.0_00152.1	849	257730	0	742	795	93	−	44	824	41453	42242
UN04677	Rsa1.0_01186.1	1832	51418	0	748	766	97	−	828	1593	10845	11609
UN04678	Rsa1.0_00979.1	1152	57733	1.00E−130	266	275	96	−	180	451	41925	42199

UN04679	Rsa1.0_02950.1	977	26506	1.00E-123	266	280	95	-	462	741	6577	6856
UN04680	Rsa1.0_00253.1	1004	231292	0	642	659	97	+	348	1004	97765	98413
UN04681	Rsa1.0_01575.1	985	42924	0	531	545	97	-	263	807	32336	32880
UN04682	Rsa1.0_03450.1	927	22835	0	385	398	96	-	14	411	6175	6569
UN04683	Rsa1.0_00124.1	1227	262503	0	782	841	92	-	173	1006	194245	195065
UN04684	Rsa1.0_00037.1	818	307838	1.00E-113	225	231	97	-	215	445	290183	290413
UN04685	Rsa1.0_00582.1	714	74333	1.00E-126	257	264	97	+	3	262	61533	61796
UN04686	Rsa1.0_00533.1	770	77375	1.00E-130	271	282	96	-	223	504	66091	66369
UN04687	Rsa1.0_00528.1	1187	164755	0	612	624	98	+	1	621	100715	101332
UN04688	Rsa1.0_04284.1	920	18085	1.00E-138	328	350	93	-	23	366	10452	10800
UN04689	Rsa1.0_03789.1	683	20847	0	659	680	96	-	2	681	15957	16632
UN04690	Rsa1.0_29592.1	847	778	0	764	772	98	+	2	773	7	778
UN04691	Rsa1.0_00253.1	1540	231292	0	469	483	97	+	954	1431	161204	161686
UN04692	Rsa1.0_00009.1	924	232558	0	867	871	99	-	54	924	12145	13013
UN04693	Rsa1.0_00386.1	428	89134	0	420	427	98	+	2	428	62834	63256
UN04694	Rsa1.0_06803.1	1153	10330	2.00E-83	164	166	98	-	257	422	8465	8630
UN04695	Rsa1.0_00006.1	989	396176	0	932	999	93	+	2	983	385740	386718
UN04696	Rsa1.0_04214.1	1153	18437	0	566	568	99	+	3	570	2964	3530
UN04697	Rsa1.0_01606.1	837	42432	1.00E-142	263	265	99	-	1	265	7853	8117
UN04698	Rsa1.0_00131.1	1268	432233	0	427	434	98	-	1	431	11596	12029
UN04699	Rsa1.0_01375.1	792	47038	1.00E-122	293	322	90	+	3	324	11143	11442
UN04700	Rsa1.0_01673.1	1228	41197	0	405	410	98	+	211	620	8507	8916
UN04701	Rsa1.0_14756.1	1316	3096	8.00E-86	184	190	96	+	1	190	1777	1965
UN04702	Rsa1.0_00846.1	1208	62342	0	393	394	99	+	10	402	44296	44689
UN04703	Rsa1.0_00627.1	919	71566	0	535	605	88	+	184	776	25176	25747
UN04704	Rsa1.0_02599.1	1021	29660	0	605	633	95	-	6	631	4092	4721
UN04705	Rsa1.0_00562.1	1314	75565	0	1156	1187	97	+	1	1172	23864	25047
UN04706	Rsa1.0_21131.1	1317	1232	1.00E-115	212	212	100	-	965	1176	482	693
UN04707	Rsa1.0_00051.1	969	258225	0	936	991	94	-	1	967	113691	114675
UN04708	Rsa1.0_04537.1	1027	16946	1.00E-122	226	227	99	-	799	1025	129	355
UN04709	Rsa1.0_00221.1	925	106972	1.00E-174	459	510	90	-	391	876	96286	96790
UN04710	Rsa1.0_00027.1	1285	283362	0	1074	1110	96	+	37	1146	37638	38735
UN04711	Rsa1.0_00745.1	253	66067	1.00E-52	225	260	86	+	1	253	46551	46802
UN04712	Rsa1.0_00019.1	1147	301669	0	463	492	94	+	464	952	283135	283626
UN04713	Rsa1.0_00317.1	1158	218290	0	401	415	96	+	742	1156	93301	93715
UN04714	Rsa1.0_02792.1	1196	27829	6.00E-77	240	269	89	-	481	746	18518	18785
UN04715	Rsa1.0_00640.1	584	70979	0	531	553	96	-	1	553	49402	49953
UN04716	Rsa1.0_02171.1	1047	167557	9.00E-39	241	291	82	-	535	824	11741	12030
UN04717	Rsa1.0_00498.1	1063	79746	0	379	394	96	-	677	1061	65592	65985

UN04718	Rsa1.0_02360.1	776	32203	0	531	562	94	−	6	555	28271	28827
UN04719	Rsa1.0_00051.1	1313	258225	0	656	665	98	+	649	1313	56038	56702
UN04720	Rsa1.0_31253.1	1176	732	0	715	732	97	−	35	766	1	732
UN04721	Rsa1.0_00451.1	1042	322533	0	559	590	94	+	19	607	261509	262097
UN04722	Rsa1.0_02448.1	926	31315	0	592	614	96	−	1	609	24478	25090
UN04723	Rsa1.0_00661.1	1089	213041	0	502	516	97	+	11	525	57222	57737
UN04724	Rsa1.0_04557.1	764	145048	0	397	419	94	+	25	439	135308	135723
UN04725	Rsa1.0_00551.1	1194	76227	1.00E−125	228	228	100	−	593	820	49962	50189
UN04726	Rsa1.0_49294.1	988	460	0	427	428	99	+	560	987	1	428
UN04727	Rsa1.0_00087.1	988	761073	4.00E−96	185	187	98	−	173	359	50644	50830
UN04728	Rsa1.0_00265.1	642	220635	1.00E−172	348	362	96	+	279	640	158595	158956
UN04729	Rsa1.0_00369.1	736	266462	4.00E−83	209	224	93	−	108	331	10326	10541
UN04730	Rsa1.0_18758.1	843	1540	0	617	633	97	−	213	838	902	1532
UN04731	Rsa1.0_00173.1	799	288743	1.00E−108	257	277	92	+	162	424	41089	41362
UN04732	Rsa1.0_01411.1	383	46326	0	378	381	99	−	1	381	29718	30098
UN04733	Rsa1.0_00466.1	1073	82132	0	689	697	98	+	3	698	7931	8624
UN04734	Rsa1.0_02754.1	1494	28213	0	500	500	100	−	1	500	14522	15021
UN04735	Rsa1.0_00140.1	1033	121541	0	536	541	99	−	7	545	60172	60712
UN04736	Rsa1.0_00394.1	1218	148426	0	560	561	99	+	3	563	135130	135689
UN04737	Rsa1.0_09922.1	1338	6089	0	759	765	99	+	1	765	1229	1993
UN04738	Rsa1.0_07613.1	1278	9021	0	724	726	99	−	4	729	2805	3530
UN04739	Rsa1.0_00016.1	1132	831256	0	496	531	93	−	532	1061	356909	357430
UN04740	Rsa1.0_00014.1	961	348985	0	582	590	98	+	1	587	282720	283307
UN04741	Rsa1.0_00237.1	928	235974	1.00E−103	261	281	92	−	650	926	4692	4971
UN04742	Rsa1.0_01397.1	563	183276	2.00E−81	169	174	97	−	325	498	171632	171805
UN04743	Rsa1.0_00104.1	1001	368722	1.00E−59	271	322	84	+	15	336	186977	187298
UN04744	Rsa1.0_00018.1	1070	410899	1.00E−136	276	286	96	−	114	399	68738	69023
UN04745	Rsa1.0_01110.1	1007	195852	0	437	447	97	+	1	447	31836	32280
UN04746	Rsa1.0_00408.1	1216	176752	0	705	732	96	+	1	728	105869	106593
UN04747	Rsa1.0_06607.1	983	10674	0	406	424	95	+	562	983	10219	10636
UN04748	Rsa1.0_03826.1	1320	20614	1.00E−151	287	292	98	−	232	523	14877	15168
UN04749	Rsa1.0_00451.1	850	322533	1.00E−165	335	346	96	−	224	568	156753	157097
UN04750	Rsa1.0_04235.1	839	18373	0	835	842	99	−	2	839	16353	17194
UN04751	Rsa1.0_01377.1	1302	46967	0	803	817	98	−	1	817	9190	10006
UN04752	Rsa1.0_01544.1	1120	185602	1.00E−113	368	420	87	−	12	430	165822	166241
UN04753	Rsa1.0_00191.1	571	316976	2.33E−156	302	310	97	−	2	308	293050	293358
UN04754	Rsa1.0_00092.1	675	659932	1.00E−177	363	374	97	+	77	448	457311	457682
UN04755	Rsa1.0_07292.1	1082	9605	1.00E−131	247	249	99	+	364	610	2153	2401
UN04756	Rsa1.0_00387.1	1243	495407	1.00E−161	325	337	96	−	906	1242	396974	397310

UN04757	Rsa1.0_02696.1	900	28600	1.00E-166	312	317	98	+	1	317	17258	17574
UN04758	Rsa1.0_00039.1	944	296927	9.00E-54	342	420	81	+	439	858	229897	230316
UN04759	Rsa1.0_01293.1	1214	49139	1.00E-158	283	283	100	-	244	526	23051	23333
UN04760	Rsa1.0_00484.1	788	203294	0	680	689	98	+	1	689	156462	157147
UN04761	Rsa1.0_15815.1	1018	2409	0	474	511	92	-	38	526	1687	2196
UN04762	Rsa1.0_04199.1	1016	18501	0	519	530	97	+	1	528	5304	5833
UN04763	Rsa1.0_04250.1	944	18264	1.00E-179	325	327	99	-	1	327	37	363
UN04764	Rsa1.0_00652.1	905	70089	0	431	431	100	-	475	905	46873	47303
UN04765	Rsa1.0_00632.1	653	183777	1.00E-121	309	340	90	-	307	633	149100	149438
UN04766	Rsa1.0_01769.1	756	39568	1.00E-142	352	384	91	+	190	573	30783	31166
UN04767	Rsa1.0_00305.1	778	97308	1.00E-166	296	296	100	+	2	297	94401	94696
UN04768	Rsa1.0_00652.1	1305	70089	0	580	586	98	+	49	632	18064	18649
UN04769	Rsa1.0_18699.1	771	1549	0	444	451	98	+	2	451	778	1228
UN04770	Rsa1.0_00596.1	665	73578	0	661	665	99	+	1	665	70145	70809
UN04771	Rsa1.0_00724.1	949	66789	0	769	782	98	-	13	792	58679	59460
UN04772	Rsa1.0_00519.1	753	197864	0	739	754	98	+	1	752	35029	35782
UN04773	Rsa1.0_00527.1	857	77733	0	408	417	97	+	441	857	54019	54435
UN04774	Rsa1.0_02649.1	833	29072	0	809	846	95	-	2	831	12637	13481
UN04775	Rsa1.0_02753.1	669	28215	0	633	688	92	+	1	667	8048	8728
UN04776	Rsa1.0_03586.1	1069	21913	0	888	923	96	-	149	1069	11244	12161
UN04777	Rsa1.0_01121.1	846	53407	0	662	699	94	-	6	698	19076	19773
UN04778	Rsa1.0_00038.1	827	314568	1.00E-100	266	289	92	-	23	301	16997	17282
UN04779	Rsa1.0_03837.1	591	20559	1.00E-152	434	478	90	-	125	591	14785	15242
UN04780	Rsa1.0_00473.1	1078	212204	0	400	402	99	+	1	402	66299	66700
UN04781	Rsa1.0_01112.1	1012	310601	0	531	552	96	-	20	569	31685	32233
UN04782	Rsa1.0_00038.1	1500	314568	0	359	362	99	-	2	362	45790	46151
UN04783	Rsa1.0_00017.1	1148	752458	4.00E-50	239	282	84	-	122	396	647716	647997
UN04784	Rsa1.0_17407.1	895	1838	1.00E-15	146	175	83	-	369	541	1277	1448
UN04785	Rsa1.0_04039.1	962	19467	0	560	573	97	-	3	568	18509	19081
UN04786	Rsa1.0_01373.1	638	47052	0	637	638	99	-	1	638	31440	32077
UN04787	Rsa1.0_00058.1	867	159921	0	419	445	94	-	428	867	118367	118804
UN04788	Rsa1.0_01679.1	1260	146876	2.00E-80	232	257	90	+	18	272	135000	135247
UN04789	Rsa1.0_02744.1	329	28268	1.00E-160	322	331	97	-	3	329	21574	21903
UN04790	Rsa1.0_02347.1	1332	32332	0	538	544	98	+	1	542	18586	19129
UN04791	Rsa1.0_01719.1	1177	40388	0	529	548	96	+	4	551	31915	32459
UN04792	Rsa1.0_10873.1	1205	5694	1.00E-169	421	457	92	+	1	440	2548	3002
UN04793	Rsa1.0_02474.1	1116	31076	1.00E-144	282	289	97	+	831	1116	21211	21499
UN04794	Rsa1.0_01644.1	1385	41754	2.00E-68	143	145	98	-	1132	1275	32706	32850
UN04795	Rsa1.0_44152.1	1301	509	1.00E-160	317	326	97	+	865	1189	184	509

UN04796	Rsa1.0_01809.1	1282	39004	0	427	438	97	+	1	438	27652	28085
UN04797	Rsa1.0_32801.1	1052	694	0	418	423	98	+	406	828	1	423
UN04798	Rsa1.0_02955.1	1353	26484	1.00E-155	308	315	97	−	1038	1351	1308	1621
UN04799	Rsa1.0_03860.1	1377	20407	1.00E-143	273	278	98	+	653	930	16711	16988
UN04800	Rsa1.0_00002.1	1468	601661	0	322	322	100	+	1	322	398039	398360
UN04801	Rsa1.0_03974.1	802	19770	0	787	803	98	−	1	802	703	1505
UN04802	Rsa1.0_00015.1	1571	561803	0	1121	1191	94	+	2	1163	412690	413872
UN04803	Rsa1.0_02950.1	1207	26506	0	536	538	99	−	247	784	5272	5809
UN04804	Rsa1.0_00109.1	1601	132735	0	612	612	100	+	986	1597	87248	87859
UN04805	Rsa1.0_00180.1	1041	114581	1.00E-113	270	289	93	+	1	283	34782	35066
UN04806	Rsa1.0_02182.1	907	34172	0	501	505	99	+	1	505	21484	21987
UN04807	Rsa1.0_00116.1	1097	365886	0	595	661	90	+	438	1091	28071	28706
UN04808	Rsa1.0_01511.1	1506	44235	0	674	680	99	+	1	678	18470	19149
UN04809	Rsa1.0_04203.1	1295	18479	0	477	501	95	−	1	500	14421	14915
UN04810	Rsa1.0_02223.1	1301	152421	0	387	387	100	−	915	1301	152035	152421
UN04811	Rsa1.0_02085.1	1307	35198	0	1184	1254	94	+	1	1244	18331	19557
UN04812	Rsa1.0_01604.1	1345	42461	0	800	808	99	−	538	1345	38433	39240
UN04813	Rsa1.0_00016.1	1437	831256	0	511	558	91	−	805	1349	17399	17944
UN04814	Rsa1.0_01042.1	817	293264	1.00E-172	306	306	100	−	223	528	152814	153119
UN04815	Rsa1.0_00271.1	907	101125	1.00E-178	455	501	90	+	107	591	26584	27065
UN04816	Rsa1.0_00026.1	810	327835	0	649	655	99	+	155	809	145834	146488
UN04817	Rsa1.0_05586.1	1072	13263	1.00E-153	288	291	98	+	647	936	2007	2297
UN04818	Rsa1.0_00036.1	1106	176186	0	360	369	97	+	308	676	102434	102802
UN04819	Rsa1.0_02178.1	922	156197	0	753	802	93	−	31	821	150274	151074
UN04820	Rsa1.0_00916.1	1162	59789	1.00E-144	363	394	92	+	700	1082	30361	30751
UN04821	Rsa1.0_00473.1	1206	212204	0	607	634	95	−	20	652	81059	81682
UN04822	Rsa1.0_00179.1	825	393324	0	747	752	99	−	1	752	390831	391582
UN04823	Rsa1.0_02215.1	1099	33822	0	587	610	96	−	38	643	9505	10108
UN04824	Rsa1.0_05800.1	734	141392	0	394	400	98	−	100	499	134991	135390
UN04825	Rsa1.0_00585.1	675	74164	0	589	591	99	−	84	674	52712	53302
UN04826	Rsa1.0_59968.1	695	382	1.00E-143	273	278	98	+	358	635	104	381
UN04827	Rsa1.0_00297.1	1147	97836	0	450	470	95	−	678	1147	26265	26734
UN04828	Rsa1.0_00731.1	1263	66638	0	554	577	96	−	680	1256	14123	14699
UN04829	Rsa1.0_01537.1	676	187285	1.00E-162	407	445	91	+	235	676	839	1260
UN04830	Rsa1.0_00683.1	1526	68638	0	1376	1400	98	−	129	1524	31148	32547
UN04831	Rsa1.0_02210.1	1112	33857	1.00E-175	311	311	100	−	802	1112	12095	12405
UN04832	Rsa1.0_19209.1	1231	1464	0	377	391	96	+	834	1223	927	1317
UN04833	Rsa1.0_01133.1	1357	52916	1.00E-164	300	302	99	+	1056	1357	49183	49484
UN04834	Rsa1.0_00339.1	1401	222974	1.00E-177	424	454	93	+	955	1401	66593	67042

UN04835	Rsa1.0_00634.1	947	71222	0	323	323	100	-	3	325	70500	70822
UN04836	Rsa1.0_01077.1	993	54772	1.00E-87	168	169	99	+	563	731	18326	18494
UN04837	Rsa1.0_03091.1	1272	25454	0	426	437	97	-	837	1272	417	853
UN04838	Rsa1.0_02248.1	787	33414	0	753	783	96	-	3	771	32009	32788
UN04839	Rsa1.0_02020.1	700	173211	1.00E-126	248	254	97	-	3	256	21498	21751
UN04840	Rsa1.0_01920.1	1727	158533	0	470	490	95	+	1202	1687	128072	128554
UN04841	Rsa1.0_00398.1	1691	88007	0	535	554	96	-	1138	1691	10261	10807
UN04842	Rsa1.0_06775.1	606	10379	1.00E-121	280	297	94	-	4	300	3644	3938
UN04843	Rsa1.0_02433.1	1236	158318	1.00E-161	294	296	99	-	939	1234	13335	13630
UN04844	Rsa1.0_00187.1	1039	393093	1.00E-141	270	275	98	-	592	866	34497	34771
UN04845	Rsa1.0_05338.1	768	144511	1.00E-116	241	249	96	-	8	256	10748	10995
UN04846	Rsa1.0_00538.1	1023	77149	1.00E-87	165	165	100	+	772	936	69811	69975
UN04847	Rsa1.0_01512.1	1584	44234	0	500	516	96	+	596	1111	17477	17992
UN04848	Rsa1.0_04603.1	1095	16682	0	568	572	99	+	524	1095	7586	8157
UN04849	Rsa1.0_02266.1	1006	33195	1.00E-99	195	197	98	-	811	1006	30769	30965
UN04850	Rsa1.0_04735.1	1109	16180	0	703	704	99	+	407	1109	8132	8835
UN04851	Rsa1.0_00012.1	1103	222148	0	402	423	95	-	685	1103	10177	10599
UN04852	Rsa1.0_01026.1	801	171624	1.00E-123	269	281	95	+	11	289	158746	159026
UN04853	Rsa1.0_00325.1	1406	95371	1.00E-128	263	273	96	+	303	575	79215	79487
UN04854	Rsa1.0_09720.1	1475	6190	0	359	360	99	-	1116	1475	4163	4522
UN04855	Rsa1.0_03027.1	833	25883	8.00E-48	134	146	91	-	609	754	11931	12076
UN04856	Rsa1.0_02128.1	1150	34758	0	344	345	99	+	806	1150	16324	16668
UN04857	Rsa1.0_02224.1	729	33758	0	362	364	99	-	366	729	4588	4951
UN04858	Rsa1.0_02725.1	844	28404	1.00E-125	227	227	100	+	3	229	6530	6756
UN04859	Rsa1.0_01762.1	744	39682	1.00E-180	359	372	96	+	165	536	1493	1864
UN04860	Rsa1.0_08776.1	840	6932	1.00E-142	275	281	97	+	559	839	2706	2986
UN04861	Rsa1.0_02720.1	732	28427	0	456	480	95	+	253	732	11937	12408
UN04862	Rsa1.0_00008.1	1202	358124	0	571	575	99	+	16	589	293999	294573
UN04863	Rsa1.0_00377.1	1417	90038	1.00E-133	348	384	90	-	1047	1417	51756	52136
UN04864	Rsa1.0_01091.1	1006	54383	0	416	441	94	-	514	954	11466	11894
UN04865	Rsa1.0_01951.1	1070	36941	1.00E-149	333	354	94	-	509	862	11135	11485
UN04866	Rsa1.0_01092.1	830	54348	6.00E-67	136	138	98	+	430	567	51202	51339
UN04867	Rsa1.0_00813.1	1114	63202	0	530	550	96	-	520	1063	23119	23668
UN04868	Rsa1.0_04238.1	1164	18352	0	399	413	96	+	751	1160	8709	9119
UN04869	Rsa1.0_00967.1	1149	58049	0	801	850	94	-	99	945	18214	19035
UN04870	Rsa1.0_00360.1	1240	91190	0	1201	1241	96	+	1	1240	60201	61423
UN04871	Rsa1.0_00925.1	1087	59481	0	446	464	96	+	2	464	5621	6084
UN04872	Rsa1.0_00657.1	1227	69858	8.00E-95	192	197	97	-	1029	1225	58014	58210
UN04873	Rsa1.0_09414.1	830	6386	0	692	744	93	-	3	737	607	1349

UN04874	Rsa1.0_00122.1		822	265937	0	807	822	98	−		1	822	258766	259575
UN04875	Rsa1.0_00740.1		1135	215972	0	425	427	99	+		709	1134	212344	212769
UN04876	Rsa1.0_00632.1		790	183777	1.00E−126	230	230	100	+		431	660	156932	157161
UN04877	Rsa1.0_00482.1		1668	81162	0	657	680	96	−		1	672	41277	41955
UN04878	Rsa1.0_00585.1		784	74164	1.00E−177	323	326	99	−		335	660	49228	49553
UN04879	Rsa1.0_00985.1		836	161950	1.00E−163	319	327	97	−		3	328	28899	29225
UN04880	Rsa1.0_00004.1		1076	253996	0	660	683	96	−		399	1076	69092	69773
UN04881	Rsa1.0_01263.1		782	49703	7.00E−48	111	114	97	−		627	739	30765	30878
UN04882	Rsa1.0_15481.1		1444	2582	0	603	626	96	−		835	1443	1066	1691
UN04883	Rsa1.0_04065.1		805	19275	2.33E−156	358	381	93	+		425	801	3173	3543
UN04884	Rsa1.0_08862.1		1435	6847	0	406	408	99	+		691	1098	6440	6847
UN04885	Rsa1.0_06164.1		1347	11584	1.00E−148	322	338	95	+		5	340	1546	1882
UN04886	Rsa1.0_04957.1		812	15330	1.00E−104	204	208	98	−		259	466	563	770
UN04887	Rsa1.0_00260.1		902	102021	1.00E−166	401	436	91	+		282	711	63807	64242
UN04888	Rsa1.0_01273.1		756	49464	1.00E−104	214	221	96	−		186	406	40519	40739
UN04889	Rsa1.0_03166.1		1901	24863	0	1254	1306	96	−		3	1305	2873	4176
UN04890	Rsa1.0_00218.1		1603	201480	0	456	479	95	+		1122	1600	180348	180822
UN04891	Rsa1.0_00646.1		1193	70473	0	430	445	96	+		26	470	22394	22838
UN04892	#	#	#	#	#	#	#		#	#	#	#	#	
UN04893	Rsa1.0_02171.1		805	167557	1.00E−177	325	327	99	−		479	805	12971	13296
UN04894	Rsa1.0_04197.1		888	18515	0	639	642	99	+		247	888	7981	8621
UN04895	Rsa1.0_00190.1		808	318240	1.00E−160	289	290	99	+		2	291	10015	10304
UN04896	Rsa1.0_00506.1		1551	79106	1.00E−105	204	207	98	−		433	639	13459	13665
UN04897	Rsa1.0_01302.1		998	48907	0	590	613	96	+		180	792	1159	1767
UN04898	Rsa1.0_00203.1		1482	109129	0	790	815	96	+		2	816	37274	38088
UN04899	Rsa1.0_00377.1		1220	90038	1.00E−107	224	233	96	−		982	1214	85916	86148
UN04900	Rsa1.0_07815.1		1573	8487	1.00E−100	186	186	100	+		1388	1573	1245	1430
UN04901	Rsa1.0_02575.1		1734	29944	0	1243	1306	95	−		20	1303	19483	20787
UN04902	Rsa1.0_02220.1		1096	176031	0	1090	1096	99	−		1	1096	146	1241
UN04903	Rsa1.0_00744.1		813	66084	1.00E−148	307	319	96	−		316	633	3557	3875
UN04904	Rsa1.0_08511.1		891	7252	0	459	469	97	−		411	878	1798	2266
UN04905	Rsa1.0_08961.1		961	6750	0	398	414	96	−		551	961	4341	4752
UN04906	Rsa1.0_23397.1		983	1052	0	505	521	96	−		108	628	492	1012
UN04907	Rsa1.0_02200.1		697	33981	0	442	449	98	+		203	651	24074	24522
UN04908	Rsa1.0_00178.1		1893	114861	0	1059	1065	99	−		829	1893	93333	94397
UN04909	Rsa1.0_01149.1		726	160459	0	543	554	98	−		3	556	17137	17680
UN04910	Rsa1.0_00092.1		1165	659932	2.33E−156	275	279	98	−		3	281	340621	340899
UN04911	Rsa1.0_13911.1		481	4104	1.00E−123	245	252	97	−		230	481	2642	2893
UN04912	Rsa1.0_00051.1		1112	258225	0	397	398	99	−		715	1112	151335	151732

UN04913	Rsa1.0_01100.1	1570	54113	0	436	436	100	−	1125	1560	171	606
UN04914	Rsa1.0_00189.1	683	111830	0	385	397	96	−	288	683	71029	71425
UN04915	Rsa1.0_01595.1	1501	175383	0	412	423	97	−	1079	1501	17815	18237
UN04916	Rsa1.0_00078.1	817	175053	1.00E−123	225	225	100	+	120	344	54810	55034
UN04917	Rsa1.0_01016.1	883	181422	0	355	362	98	+	396	755	26071	26432
UN04918	Rsa1.0_70187.1	1128	326	1.00E−170	304	304	100	−	742	1045	1	304
UN04919	Rsa1.0_01121.1	854	53407	0	849	852	99	−	1	852	28959	29810
UN04920	Rsa1.0_00768.1	678	64969	1.00E−73	159	165	96	−	289	453	52219	52383
UN04921	Rsa1.0_05482.1	943	13595	0	366	366	100	−	104	469	12266	12631
UN04922	Rsa1.0_00055.1	952	520193	0	426	438	97	−	1	435	58688	59125
UN04923	Rsa1.0_00089.1	554	406142	0	355	363	97	+	3	365	80796	81158
UN04924	Rsa1.0_26100.1	913	906	0	645	647	99	−	240	885	1	647
UN04925	Rsa1.0_22847.1	836	1089	0	378	384	98	−	453	835	706	1089
UN04926	Rsa1.0_00061.1	1372	218685	1.00E−134	320	347	92	−	1026	1372	84200	84527
UN04927	Rsa1.0_33701.1	819	673	0	388	398	97	−	1	398	2	397
UN04928	Rsa1.0_01649.1	766	41624	7.00E−88	174	177	98	+	262	438	36122	36298
UN04929	Rsa1.0_01920.1	787	158533	0	390	394	98	−	10	403	25105	25497
UN04930	Rsa1.0_01606.1	747	42432	1.00E−107	223	230	96	+	4	233	3851	4075
UN04931	Rsa1.0_00330.1	725	94666	1.00E−138	337	364	92	+	3	366	85362	85721
UN04932	Rsa1.0_11786.1	880	5409	0	876	876	100	−	3	878	3020	3895
UN04933	Rsa1.0_00302.1	902	97521	0	892	899	99	−	4	902	91995	92892
UN04934	Rsa1.0_00017.1	975	752458	0	706	759	93	−	216	965	510949	511702
UN04935	Rsa1.0_03487.1	596	22564	0	593	594	99	+	3	596	8625	9218
UN04936	Rsa1.0_00035.1	862	176449	2.00E−76	152	154	98	+	223	376	155795	155948
UN04937	Rsa1.0_04342.1	1487	17828	1.00E−137	269	276	97	−	1207	1482	7828	8103
UN04938	Rsa1.0_00037.1	965	307838	1.00E−142	277	284	97	+	546	829	239665	239948
UN04939	Rsa1.0_02205.1	1174	33923	1.00E−167	307	310	99	−	733	1042	14306	14615
UN04940	Rsa1.0_08097.1	651	7914	1.00E−110	227	234	97	−	2	234	1390	1623
UN04941	Rsa1.0_01663.1	780	168589	0	499	517	96	+	3	516	69	582
UN04942	Rsa1.0_01779.1	1167	39448	0	1010	1062	95	−	3	1063	16345	17394
UN04943	Rsa1.0_00808.1	1185	63351	0	640	673	95	−	534	1185	39465	40137
UN04944	Rsa1.0_00192.1	861	278200	0	380	380	100	+	482	861	223102	223481
UN04945	Rsa1.0_01275.1	512	49405	1.00E−132	266	275	96	−	238	512	8117	8391
UN04946	Rsa1.0_00087.1	950	761073	1.00E−148	377	407	92	−	531	932	615104	615505
UN04947	Rsa1.0_01660.1	864	41400	0	380	394	96	+	440	833	27246	27639
UN04948	Rsa1.0_00074.1	1099	147591	0	1001	1094	91	+	2	1094	13948	15017
UN04949	Rsa1.0_06047.1	994	11854	0	384	400	96	−	593	987	305	704
UN04950	Rsa1.0_02722.1	1167	28417	1.00E−152	301	310	97	+	513	822	959	1268
UN04951	Rsa1.0_02895.1	982	26979	0	959	979	97	+	3	981	6988	7966

UN04952	Rsa1.0_00752.1	870	65756	1.00E-148	324	339	95	+	533	870	14186	14522
UN04953	Rsa1.0_00011.1	914	225044	0	437	449	97	-	3	449	146662	147109
UN04954	Rsa1.0_10147.1	1216	5976	0	440	446	98	+	3	448	658	1103
UN04955	Rsa1.0_01388.1	1130	46741	0	429	442	97	+	283	724	8787	9225
UN04956	Rsa1.0_01400.1	1313	46560	0	403	417	96	+	898	1312	13162	13575
UN04957	Rsa1.0_03330.1	861	23671	1.00E-123	230	232	99	-	3	234	20283	20514
UN04958	Rsa1.0_00416.1	963	86772	0	502	517	97	+	453	963	43593	44109
UN04959	Rsa1.0_11046.1	1311	5635	1.00E-151	324	339	95	+	978	1309	3092	3429
UN04960	Rsa1.0_06039.1	1157	11867	7.00E-86	237	260	91	-	890	1145	11496	11750
UN04961	Rsa1.0_01760.1	1269	39730	0	553	573	96	-	700	1269	5789	6361
UN04962	Rsa1.0_12094.1	1502	5316	1.00E-91	172	172	100	-	1288	1459	1	172
UN04963	Rsa1.0_01216.1	1424	50580	0	1422	1427	99	-	2	1424	3394	4820
UN04964	Rsa1.0_03838.1	1389	20544	0	1384	1391	99	+	1	1389	4478	5868
UN04965	Rsa1.0_02681.1	667	28711	1.00E-157	294	298	98	-	225	522	14013	14310
UN04966	Rsa1.0_04735.1	1017	16180	0	482	513	93	+	495	999	10738	11242
UN04967	Rsa1.0_00015.1	1182	561803	0	595	606	98	-	34	638	478288	478892
UN04968	Rsa1.0_00626.1	1328	71582	0	412	417	98	+	914	1328	32116	32532
UN04969	Rsa1.0_01543.1	670	43645	0	652	670	97	+	3	669	5907	6576
UN04970	Rsa1.0_00745.1	424	66067	2.00E-49	235	272	86	+	97	359	51155	51410
UN04971	Rsa1.0_01811.1	1258	38977	1.00E-145	866	1050	82	-	213	1254	28552	29592
UN04972	Rsa1.0_00045.1	748	328694	0	359	366	98	-	383	748	133097	133462
UN04973	Rsa1.0_03502.1	1088	22446	0	384	391	98	-	696	1086	4184	4574
UN04974	Rsa1.0_00907.1	1008	60062	0	919	953	96	+	55	1007	48727	49679
UN04975	Rsa1.0_02550.1	1116	291051	1.00E-151	281	283	99	+	1	282	19916	20198
UN04976	Rsa1.0_04142.1	1460	18861	0	512	541	94	-	401	941	1699	2239
UN04977	Rsa1.0_03972.1	781	19787	0	770	782	98	-	2	781	33	812
UN04978	Rsa1.0_00058.1	1591	159921	0	454	472	96	+	1121	1591	119077	119545
UN04979	Rsa1.0_01633.1	611	41868	0	395	411	96	-	1	411	3039	3448
UN04980	Rsa1.0_00332.1	671	94575	0	651	671	97	-	3	671	38899	39561
UN04981	Rsa1.0_01913.1	594	37363	0	476	481	98	-	1	480	4208	4688
UN04982	Rsa1.0_00299.1	2246	97770	0	1786	1829	97	+	432	2245	42542	44370
UN04983	Rsa1.0_08114.1	1328	7883	0	476	502	94	+	534	1035	6483	6984
UN04984	Rsa1.0_03846.1	651	20481	0	644	644	100	+	3	646	19667	20310
UN04985	Rsa1.0_00131.1	1161	432233	0	359	361	99	+	4	364	151892	152252
UN04986	Rsa1.0_16514.1	828	2127	0	453	469	96	-	373	828	1245	1713
UN04987	Rsa1.0_47414.1	699	477	0	472	477	98	-	207	683	1	477
UN04988	Rsa1.0_13708.1	808	4447	1.00E-131	271	281	96	-	528	808	3599	3877
UN04989	Rsa1.0_00417.1	656	395068	0	484	496	97	-	4	499	239633	240126
UN04990	Rsa1.0_02207.1	1504	33914	0	952	954	99	-	79	1032	29585	30538

UN04991	Rsa1.0_01543.1	1024	43645	0	984	1025	96	−	2	1022	24614	25625
UN04992	Rsa1.0_00836.1	795	185968	0	707	710	99	+	3	712	7908	8617
UN04993	Rsa1.0_00033.1	805	515811	0	780	813	95	+	2	804	355685	356491
UN04994	Rsa1.0_00079.1	1276	606987	0	805	836	96	+	2	837	450551	451377
UN04995	Rsa1.0_02300.1	775	32847	1.00E−174	315	317	99	−	459	775	15424	15740
UN04996	Rsa1.0_04121.1	738	18951	0	475	499	95	−	5	503	13882	14379
UN04997	Rsa1.0_00314.1	793	96472	0	495	513	96	+	282	793	63022	63534
UN04998	Rsa1.0_00306.1	707	146760	0	399	413	96	+	295	707	87667	88079
UN04999	Rsa1.0_16246.1	489	2228	0	454	481	94	−	8	488	1133	1611
UN05000	Rsa1.0_01593.1	1064	150996	0	509	523	97	+	1	515	126016	126538
UN05001	Rsa1.0_00410.1	815	86957	1.00E−169	357	372	95	−	1	362	21084	21455
UN05002	Rsa1.0_03842.1	865	20503	0	474	495	95	−	1	493	17620	18107
UN05003	Rsa1.0_01116.1	870	142088	0	416	419	99	+	450	868	102212	102630
UN05004	Rsa1.0_06035.1	300	11874	1.00E−104	191	191	100	−	109	299	8517	8707
UN05005	Rsa1.0_02343.1	920	32374	0	903	934	96	+	3	920	24183	25116
UN05006	Rsa1.0_07345.1	791	9526	0	423	425	99	+	1	424	6237	6661
UN05007	Rsa1.0_00210.1	767	108424	0	480	484	99	−	283	766	55835	56318
UN05008	Rsa1.0_00118.1	951	130637	0	901	964	93	+	3	951	39945	40908
UN05009	Rsa1.0_00581.1	927	163575	0	404	404	100	−	192	595	142441	142844
UN05010	Rsa1.0_06546.1	995	10801	1.00E−107	237	250	94	+	498	747	4874	5123
UN05011	Rsa1.0_00069.1	848	403561	0	837	848	98	−	3	847	29695	30541
UN05012	Rsa1.0_00083.1	1107	442773	2.00E−89	177	180	98	−	577	756	138429	138608
UN05013	Rsa1.0_00116.1	775	365886	7.00E−79	181	190	95	+	3	191	314582	314771
UN05014	Rsa1.0_03568.1	912	22048	0	679	700	97	+	214	912	18563	19251
UN05015	Rsa1.0_04153.1	477	18807	1.00E−150	269	269	100	+	209	477	16149	16417
UN05016	Rsa1.0_01834.1	709	38550	0	588	598	98	+	112	709	24064	24654
UN05017	Rsa1.0_03501.1	969	22458	0	841	847	99	+	122	968	21034	21880
UN05018	Rsa1.0_00708.1	864	67366	0	358	358	100	+	1	358	60723	61080
UN05019	Rsa1.0_13204.1	997	4970	0	697	699	99	−	299	997	1453	2151
UN05020	Rsa1.0_00305.1	1204	97308	0	718	745	96	−	465	1204	92432	93174
UN05021	Rsa1.0_01176.1	848	51638	1.00E−111	210	212	99	−	89	300	48438	48649
UN05022	Rsa1.0_00271.1	1357	101125	0	778	795	97	+	2	794	13400	14193
UN05023	Rsa1.0_00909.1	637	59976	0	545	601	90	+	50	637	55626	56224
UN05024	Rsa1.0_02099.1	853	35030	0	366	368	99	−	3	370	18345	18712
UN05025	Rsa1.0_00171.1	607	115622	1.00E−143	301	314	95	+	3	316	50108	50420
UN05026	Rsa1.0_00085.1	745	142226	0	488	515	94	−	11	517	15550	16061
UN05027	Rsa1.0_00013.1	818	292129	0	614	632	97	+	189	818	177634	178263
UN05028	Rsa1.0_02248.1	1131	33414	1.00E−169	301	301	100	−	3	303	9846	10146
UN05029	Rsa1.0_04399.1	1041	17543	1.00E−165	391	418	93	+	623	1036	9928	10344

UN05030	Rsa1.0_00071.1	1149	345714	1.00E-53	238	277	85	−	2	274	137828	138101
UN05031	Rsa1.0_00247.1	1510	103408	0	714	826	86	−	716	1486	19877	20689
UN05032	Rsa1.0_01206.1	2019	50772	0	798	830	96	+	1183	2009	25627	26453
UN05033	Rsa1.0_00131.1	1373	432233	0	521	530	98	+	3	531	70980	71509
UN05034	Rsa1.0_00023.1	1471	194764	0	474	488	97	+	518	996	117583	118070
UN05035	Rsa1.0_00244.1	1629	103590	0	570	573	99	−	1058	1627	83585	84157
UN05036	Rsa1.0_00339.1	1019	222974	0	540	547	98	+	467	1013	66593	67135
UN05037	Rsa1.0_00649.1	1720	70263	0	1692	1720	98	+	1	1719	13699	15416
UN05038	Rsa1.0_07588.1	776	9070	1.00E-111	205	205	100	+	7	211	3496	3700
UN05039	Rsa1.0_01034.1	1420	56046	0	1165	1202	96	−	220	1420	20989	22188
UN05040	Rsa1.0_01075.1	1124	156953	1.00E-143	258	258	100	+	700	957	144150	144407
UN05041	Rsa1.0_18589.1	1036	1570	0	914	1005	90	+	50	1036	1	999
UN05042	Rsa1.0_03132.1	780	25142	0	399	416	95	−	57	472	22052	22467
UN05043	Rsa1.0_00568.1	1124	75219	0	544	547	99	+	564	1110	42305	42851
UN05044	Rsa1.0_23954.1	1342	1015	0	542	548	98	+	129	676	1	548
UN05045	Rsa1.0_32404.1	1495	703	0	596	598	99	−	581	1177	81	677
UN05046	Rsa1.0_00556.1	818	207212	0	508	528	96	+	212	737	51917	52444
UN05047	Rsa1.0_00451.1	1358	322533	1.00E-118	219	220	99	−	269	488	284872	285091
UN05048	Rsa1.0_16475.1	804	2143	1.00E-110	221	226	97	+	519	743	1084	1309
UN05049	Rsa1.0_00448.1	1362	84048	1.00E-165	298	299	99	−	1064	1362	62168	62466
UN05050	Rsa1.0_02192.1	1273	171291	0	916	935	97	−	2	933	152494	153423
UN05051	Rsa1.0_00112.1	602	132199	1.00E-124	254	263	96	+	224	486	71227	71486
UN05052	Rsa1.0_00411.1	857	86944	0	592	610	97	+	1	609	64634	65237
UN05053	Rsa1.0_02283.1	1019	33024	0	399	401	99	−	292	692	30355	30755
UN05054	Rsa1.0_01017.1	1168	56714	0	455	459	99	−	237	692	33346	33804
UN05055	Rsa1.0_00382.1	987	251946	0	950	989	96	−	1	987	108610	109598
UN05056	Rsa1.0_05047.1	1264	15032	0	653	653	100	−	5	657	11264	11916
UN05057	Rsa1.0_00863.1	1046	254535	0	1008	1042	96	+	5	1046	209268	210288
UN05058	Rsa1.0_02881.1	1236	27041	0	861	873	98	+	364	1235	20939	21810
UN05059	Rsa1.0_03671.1	1078	21459	1.00E-117	295	316	93	+	724	1026	9842	10157
UN05060	Rsa1.0_02790.1	872	27844	0	866	872	99	+	1	872	11309	12180
UN05061	Rsa1.0_01452.1	386	45459	0	364	371	98	−	3	373	44828	45198
UN05062	Rsa1.0_05733.1	739	12785	1.00E-166	322	328	98	+	412	738	4105	4430
UN05063	Rsa1.0_00173.1	1018	288743	0	418	419	99	+	3	421	109178	109596
UN05064	Rsa1.0_02602.1	904	29653	0	763	802	95	−	1	801	28254	29055
UN05065	Rsa1.0_00269.1	1108	341668	1.00E-47	101	102	99	−	29	130	42739	42840
UN05066	Rsa1.0_00410.1	1160	86957	1.00E-157	287	289	99	−	872	1160	16810	17098
UN05067	Rsa1.0_02645.1	955	29158	0	882	903	97	−	51	949	17506	18408
UN05068	Rsa1.0_05969.1	791	12072	1.00E-114	218	221	98	+	3	223	4249	4469

UN05069	Rsa1.0_00527.1	814	77733	1.00E-100	271	298	90	-	274	565	58084	58381
UN05070	Rsa1.0_00097.1	704	138858	0	369	380	97	-	325	704	16249	16628
UN05071	Rsa1.0_00706.1	994	291570	0	441	461	95	-	264	721	14249	14709
UN05072	Rsa1.0_00007.1	893	239325	1.00E-113	226	231	97	-	628	857	65927	66157
UN05073	Rsa1.0_01571.1	1091	43000	1.00E-158	284	284	100	+	401	684	12885	13168
UN05074	Rsa1.0_00064.1	1249	240927	1.00E-148	273	275	99	-	598	872	125727	126001
UN05075	Rsa1.0_00139.1	1001	357786	0	353	353	100	-	649	1001	284190	284542
UN05076	Rsa1.0_02550.1	758	291051	1.00E-178	320	321	99	+	100	420	4853	5173
UN05077	Rsa1.0_01930.1	1072	37189	0	346	350	98	+	1	350	18743	19092
UN05078	Rsa1.0_00285.1	1989	149638	0	573	578	99	+	799	1375	141606	142183
UN05079	Rsa1.0_23508.1	1032	1043	0	519	530	97	+	1	530	3	525
UN05080	Rsa1.0_13891.1	423	4144	1.00E-141	384	423	90	-	6	423	765	1175
UN05081	Rsa1.0_00137.1	1081	122457	0	724	754	96	+	162	915	90926	91667
UN05082	Rsa1.0_00707.1	878	190112	1.00E-142	269	273	98	-	172	444	188378	188650
UN05083	Rsa1.0_00092.1	757	659932	0	458	468	97	+	297	757	503777	504244
UN05084	Rsa1.0_00146.1	704	149343	1.00E-131	243	245	99	+	460	704	82075	82319
UN05085	Rsa1.0_14850.1	1348	3014	0	549	555	98	+	511	1065	542	1094
UN05086	Rsa1.0_01785.1	837	39368	0	420	431	97	+	4	432	24586	25016
UN05087	Rsa1.0_00105.1	810	134138	1.00E-167	386	413	93	+	403	810	91785	92184
UN05088	Rsa1.0_16170.1	407	2258	1.00E-102	200	204	98	-	204	407	1771	1974
UN05089	Rsa1.0_01612.1	1124	42255	0	1113	1124	99	-	3	1124	34901	36023
UN05090	Rsa1.0_00441.1	893	84607	9.00E-88	177	181	97	+	65	245	67739	67919
UN05091	Rsa1.0_00802.1	1259	63846	0	858	862	99	-	398	1259	49603	50463
UN05092	Rsa1.0_00732.1	1302	66631	0	396	408	97	+	896	1302	59691	60098
UN05093	Rsa1.0_08902.1	1157	6805	0	1096	1137	96	-	5	1129	4688	5824
UN05094	Rsa1.0_01365.1	1358	47142	0	730	741	98	-	128	865	6938	7678
UN05095	Rsa1.0_06346.1	1209	11203	0	1169	1189	98	-	23	1209	2204	3392
UN05096	#	#	#	#	#	#	#	#	#	#	#	#
UN05097	Rsa1.0_00065.1	729	454616	1.00E-144	290	300	96	-	111	410	231797	232096
UN05098	Rsa1.0_01384.1	1490	46803	0	445	456	97	+	1038	1490	20756	21208
UN05099	Rsa1.0_00381.1	965	89718	1.00E-102	199	202	98	+	363	564	16185	16386
UN05100	Rsa1.0_00107.1	1197	431659	0	658	669	98	-	4	672	257715	258382
UN05101	Rsa1.0_00037.1	1691	307838	0	422	429	98	+	1264	1690	46810	47238
UN05102	Rsa1.0_00502.1	548	204180	0	473	482	98	-	68	548	63892	64372
UN05103	Rsa1.0_00351.1	524	413096	1.00E-153	347	369	94	-	3	363	234781	235143
UN05104	Rsa1.0_03380.1	870	23320	1.00E-142	269	273	98	+	458	730	6687	6959
UN05105	Rsa1.0_08078.1	839	7944	1.00E-125	316	344	91	+	500	839	2693	3025
UN05106	Rsa1.0_01059.1	1165	55410	1.00E-176	348	359	96	-	104	462	14840	15195
UN05107	Rsa1.0_03943.1	1086	19931	0	413	429	96	-	1	425	5337	5765

UN05108	Rsa1.0_00352.1	1158	92266	0	724	749	96	−	24	772	65700	66445
UN05109	Rsa1.0_00059.1	1128	159650	0	1117	1127	99	−	3	1128	1405	2531
UN05110	Rsa1.0_03327.1	647	23707	0	331	331	100	+	3	333	16577	16907
UN05111	Rsa1.0_03985.1	982	19704	0	976	981	99	+	2	982	3902	4880
UN05112	Rsa1.0_01619.1	1123	42117	1.00E−106	225	232	96	+	893	1122	1	232
UN05113	Rsa1.0_00539.1	1406	76926	1.00E−151	275	276	99	−	1131	1406	32802	33077
UN05114	Rsa1.0_01734.1	1592	40183	0	1582	1589	99	+	6	1592	17248	18836
UN05115	Rsa1.0_00683.1	884	68638	0	878	884	99	−	1	884	66525	67408
UN05116	Rsa1.0_00645.1	519	189716	0	483	499	96	+	21	518	31549	32047
UN05117	Rsa1.0_03209.1	925	24592	0	380	395	96	−	534	924	19393	19778
UN05118	Rsa1.0_03674.1	827	21442	0	822	828	99	−	2	826	5200	6026
UN05119	Rsa1.0_00522.1	1166	78103	0	1132	1181	95	+	1	1164	38671	39840
UN05120	Rsa1.0_02678.1	1215	135053	1.00E−161	298	301	99	+	908	1208	112508	112808
UN05121	Rsa1.0_00018.1	1055	410899	1.00E−104	263	285	92	+	778	1055	262610	262890
UN05122	Rsa1.0_03413.1	1005	23137	0	499	544	91	+	497	1005	8284	8826
UN05123	Rsa1.0_00725.1	949	66757	0	944	947	99	+	3	949	45579	46524
UN05124	Rsa1.0_04194.1	1170	132734	2.00E−36	97	103	94	−	644	746	118157	118259
UN05125	Rsa1.0_25669.1	1034	926	0	599	602	99	−	433	1034	76	677
UN05126	Rsa1.0_00030.1	1041	250988	0	984	1039	94	+	3	1041	64475	65512
UN05127	Rsa1.0_16090.1	882	2289	0	708	739	95	−	8	743	941	1678
UN05128	Rsa1.0_00306.1	680	146760	0	580	632	91	+	3	631	75343	75968
UN05129	Rsa1.0_00081.1	816	143261	0	459	463	99	−	1	461	87969	88431
UN05130	Rsa1.0_00081.1	1251	143261	0	1145	1174	97	−	78	1251	77391	78564
UN05131	Rsa1.0_00410.1	889	86957	0	388	402	96	+	3	404	19648	20045
UN05132	Rsa1.0_05774.1	913	12667	0	714	746	95	−	2	747	3452	4190
UN05133	Rsa1.0_00651.1	1039	247766	0	355	355	100	+	1	355	90170	90524
UN05134	Rsa1.0_00745.1	1486	66067	0	492	520	94	+	971	1485	52944	53451
UN05135	Rsa1.0_03623.1	807	21714	0	483	504	95	−	303	805	3310	3803
UN05136	Rsa1.0_00881.1	917	61260	0	631	636	99	+	246	881	18614	19248
UN05137	Rsa1.0_01608.1	978	170540	0	361	366	98	−	347	712	35343	35708
UN05138	Rsa1.0_03792.1	1356	20835	1.00E−126	263	274	95	−	447	720	10857	11130
UN05139	Rsa1.0_00126.1	485	127534	1.00E−118	295	319	92	−	176	485	113999	114314
UN05140	Rsa1.0_02802.1	1463	27736	0	703	714	98	−	337	1050	13800	14513
UN05141	Rsa1.0_00916.1	2213	59789	0	1292	1333	96	+	469	1795	31531	32860
UN05142	Rsa1.0_00173.1	998	288743	1.00E−86	197	207	95	−	301	507	284813	285018
UN05143	Rsa1.0_00016.1	814	831256	0	414	419	98	+	395	813	681896	682314
UN05144	Rsa1.0_13514.1	916	4757	0	563	587	95	+	330	916	836	1419
UN05145	Rsa1.0_04358.1	1444	17759	0	1417	1455	97	−	3	1444	10686	12133
UN05146	Rsa1.0_04273.1	915	18144	1.00E−106	199	200	99	+	399	598	11608	11807

UN05147	Rsa1.0_01102.1	1400	53987	0	1382	1396	98	+	6	1400	10087	11482
UN05148	Rsa1.0_00071.1	865	345714	0	375	382	98	+	143	524	311950	312331
UN05149	Rsa1.0_07588.1	997	9070	0	832	870	95	−	23	880	5044	5913
UN05150	Rsa1.0_00045.1	829	328694	1.00E−112	259	273	94	+	1	272	245137	245406
UN05151	Rsa1.0_09157.1	644	6602	1.00E−121	238	244	97	+	5	248	5288	5531
UN05152	Rsa1.0_01061.1	891	55349	1.00E−167	406	432	93	+	1	423	21819	22249
UN05153	Rsa1.0_02673.1	1358	28877	1.00E−112	254	269	94	+	9	269	25599	25867
UN05154	Rsa1.0_06194.1	739	11516	0	663	688	96	+	67	736	5194	5878
UN05155	Rsa1.0_07636.1	868	8971	0	526	535	98	+	3	537	7136	7668
UN05156	Rsa1.0_00867.1	720	61635	0	362	370	97	−	5	374	31869	32235
UN05157	Rsa1.0_06407.1	1076	11071	0	858	884	97	+	190	1073	9341	10224
UN05158	Rsa1.0_05849.1	1463	12425	0	433	434	99	−	447	880	8382	8815
UN05159	Rsa1.0_01620.1	1121	42091	1.00E−160	295	298	98	−	1	298	22880	23177
UN05160	Rsa1.0_00240.1	931	104175	1.00E−118	265	278	95	+	24	296	53559	53836
UN05161	Rsa1.0_02987.1	736	26196	1.00E−162	330	341	96	−	396	732	25859	26196
UN05162	Rsa1.0_08293.1	749	7593	0	363	364	99	+	386	749	2270	2633
UN05163	Rsa1.0_00017.1	812	752458	0	760	784	96	−	1	778	555179	555948
UN05164	Rsa1.0_08617.1	774	7124	0	756	773	97	+	1	773	291	1063
UN05165	Rsa1.0_08707.1	1319	7018	0	734	749	97	+	501	1249	1	749
UN05166	Rsa1.0_00284.1	797	323267	1.00E−151	287	291	98	+	1	291	134882	135170
UN05167	Rsa1.0_00056.1	723	332604	0	430	439	97	+	2	440	19450	19888
UN05168	Rsa1.0_68740.1	985	334	1.00E−164	311	317	98	+	1	317	18	334
UN05169	Rsa1.0_02855.1	961	27271	0	455	492	92	−	2	480	10770	11254
UN05170	Rsa1.0_04611.1	1139	16647	0	471	482	97	−	1	477	2562	3043
UN05171	Rsa1.0_14400.1	1003	3430	0	594	637	93	−	15	645	1	634
UN05172	Rsa1.0_00810.1	934	63289	0	922	930	99	+	2	931	60504	61430
UN05173	Rsa1.0_00247.1	219	103408	6.00E−33	109	120	90	+	100	219	66830	66948
UN05174	Rsa1.0_00369.1	1380	266462	0	1351	1405	96	−	3	1376	263081	264481
UN05175	Rsa1.0_02700.1	1067	28584	0	383	384	99	−	1	383	15011	15394
UN05176	Rsa1.0_00416.1	1116	86772	0	394	402	98	−	519	916	43719	44120
UN05177	Rsa1.0_00253.1	1045	231292	1.00E−144	325	343	94	+	24	360	126609	126949
UN05178	Rsa1.0_01306.1	1860	48720	0	572	594	96	−	2	590	33628	34220
UN05179	Rsa1.0_61642.1	579	372	1.00E−169	313	317	98	−	137	453	56	372
UN05180	Rsa1.0_00137.1	1213	122457	0	1003	1009	99	−	205	1213	90659	91667
UN05181	Rsa1.0_03284.1	652	24069	0	641	649	98	−	7	652	7230	7878
UN05182	Rsa1.0_00001.1	748	363028	0	730	749	97	−	5	746	33667	34414
UN05183	Rsa1.0_02276.1	745	33098	0	733	752	97	+	3	745	22945	23696
UN05184	Rsa1.0_00012.1	843	222148	0	710	710	100	+	2	711	85333	86042
UN05185	Rsa1.0_00119.1	1209	467509	0	422	426	99	+	1	426	46341	46766

UN05186	Rsa1.0_00344.1	1317	191774	0	758	769	98	+	548	1315	120085	120853
UN05187	Rsa1.0_06042.1	740	11860	0	737	739	99	−	2	740	661	1399
UN05188	Rsa1.0_03133.1	815	25135	8.00E-91	179	182	98	+	357	538	9682	9863
UN05189	Rsa1.0_04028.1	395	19493	7.00E-99	198	203	97	+	1	203	2386	2588
UN05190	Rsa1.0_00070.1	694	150321	0	692	694	99	+	2	694	47124	47817
UN05191	Rsa1.0_05979.1	1499	12051	0	503	503	100	−	1	503	10020	10522
UN05192	Rsa1.0_15269.1	779	2719	1.00E-155	317	330	96	−	6	325	296	625
UN05193	Rsa1.0_00157.1	1405	245832	1.00E-176	320	321	99	−	1086	1405	188327	188647
UN05194	Rsa1.0_00016.1	833	831256	3.00E-87	220	235	93	+	92	317	809775	810009
UN05195	Rsa1.0_00955.1	683	58358	0	484	492	98	−	1	492	42045	42534
UN05196	Rsa1.0_00214.1	889	107508	3.00E-87	342	394	86	+	5	373	28862	29247
UN05197	Rsa1.0_04372.1	1397	17692	0	854	856	99	−	3	858	9661	10516
UN05198	Rsa1.0_00421.1	725	86365	0	701	726	96	+	1	725	11839	12562
UN05199	Rsa1.0_00044.1	784	271658	1.00E-118	219	220	99	+	422	641	57062	57281
UN05200	Rsa1.0_01438.1	598	45882	0	402	408	98	−	189	596	32813	33220
UN05201	Rsa1.0_00870.1	1141	61606	0	851	860	98	+	280	1139	9589	10448
UN05202	Rsa1.0_00516.1	857	78417	1.00E-105	195	195	100	+	588	782	31115	31309
UN05203	Rsa1.0_01675.1	898	41155	0	357	364	98	−	535	898	30271	30634
UN05204	Rsa1.0_00069.1	1548	403561	0	1497	1540	97	+	2	1536	247195	248731
UN05205	Rsa1.0_01602.1	650	42503	0	565	585	96	−	50	633	39978	40562
UN05206	Rsa1.0_00065.1	1197	454616	0	361	361	100	−	837	1197	190404	190764
UN05207	Rsa1.0_00253.1	1263	231292	0	838	849	98	+	36	883	193624	194472
UN05208	Rsa1.0_00076.1	958	146021	1.00E-180	329	332	99	+	625	956	35148	35479
UN05209	Rsa1.0_00342.1	1701	93695	0	817	827	98	+	876	1701	35879	36705
UN05210	Rsa1.0_00347.1	1220	93048	0	440	442	99	+	3	444	90999	91440
UN05211	Rsa1.0_00237.1	691	235974	0	430	438	98	−	255	691	188285	188722
UN05212	Rsa1.0_00387.1	711	495407	1.00E-111	250	265	94	−	351	615	342037	342297
UN05213	Rsa1.0_10607.1	1197	5786	0	382	385	99	+	813	1197	2758	3142
UN05214	Rsa1.0_06774.1	921	10381	0	506	525	96	+	393	917	7069	7593
UN05215	Rsa1.0_00001.1	1292	363028	0	355	358	99	+	937	1292	329335	329691
UN05216	Rsa1.0_04848.1	756	15727	0	679	692	98	+	3	694	5785	6475
UN05217	Rsa1.0_00100.1	1070	249232	0	719	738	97	−	2	739	195789	196523
UN05218	Rsa1.0_00019.1	743	301669	7.00E-85	208	222	93	−	2	219	107363	107584
UN05219	Rsa1.0_00649.1	1509	70263	1.00E-129	253	259	97	+	209	467	24617	24875
UN05220	Rsa1.0_00552.1	1290	206757	0	594	623	95	−	651	1263	67991	68611
UN05221	Rsa1.0_03390.1	738	23270	0	459	473	97	−	265	734	6595	7066
UN05222	Rsa1.0_02807.1	946	27680	1.00E-136	255	258	98	+	389	646	10903	11160
UN05223	Rsa1.0_01104.1	945	285480	1.00E-148	289	294	98	+	651	942	9410	9703
UN05224	Rsa1.0_01296.1	703	49035	0	426	438	97	+	236	671	12562	12999

UN05225	Rsa1.0_00224.1	1289	327530	1.00E-141	270	275	98	+	340	614	208584	208858
UN05226	Rsa1.0_00529.1	416	77487	0	387	396	97	−	19	414	10180	10575
UN05227	Rsa1.0_01407.1	1655	46425	0	900	906	99	−	2	907	19638	20543
UN05228	Rsa1.0_01971.1	1229	36641	0	1203	1230	97	−	3	1229	23421	24649
UN05229	Rsa1.0_00383.1	1443	398431	1.00E-124	242	247	97	+	727	973	2510	2756
UN05230	Rsa1.0_01537.1	1675	187285	1.00E-160	294	295	99	+	234	527	19498	19792
UN05231	Rsa1.0_00615.1	820	72235	1.00E-175	320	323	99	−	351	673	55080	55402
UN05232	Rsa1.0_01351.1	1154	47498	0	1074	1105	97	−	2	1106	2865	3969
UN05233	Rsa1.0_06797.1	2285	10342	0	735	774	94	+	598	1370	4651	5406
UN05234	Rsa1.0_00585.1	950	74164	0	906	933	97	−	1	930	60726	61655
UN05235	Rsa1.0_05267.1	827	14319	0	775	798	97	+	1	798	6606	7402
UN05236	Rsa1.0_03491.1	719	22541	4.00E-86	357	422	84	−	52	473	9114	9535
UN05237	Rsa1.0_03544.1	1071	22182	0	1019	1074	94	+	2	1070	19603	20669
UN05238	Rsa1.0_02909.1	661	26856	0	641	652	98	+	7	658	10343	10994
UN05239	Rsa1.0_00313.1	1101	96745	0	569	573	99	−	529	1101	59603	60174
UN05240	Rsa1.0_07236.1	851	9680	0	457	458	99	+	388	845	2988	3445
UN05241	Rsa1.0_01397.1	883	183276	1.00E-147	264	264	100	−	618	881	164309	164572
UN05242	Rsa1.0_16218.1	955	2240	1.00E-166	297	297	100	−	632	928	78	374
UN05243	Rsa1.0_14974.1	689	2919	0	404	404	100	+	3	406	1485	1888
UN05244	Rsa1.0_00184.1	834	218748	0	537	537	100	−	298	834	2634	3170
UN05245	Rsa1.0_00090.1	1348	273859	0	560	604	92	+	737	1334	138997	139590
UN05246	Rsa1.0_00973.1	1278	57913	0	1140	1167	97	+	113	1278	40860	42025
UN05247	Rsa1.0_00168.1	851	115870	0	394	400	98	−	104	503	111590	111989
UN05248	Rsa1.0_00047.1	1268	168582	0	434	434	100	+	835	1268	4911	5344
UN05249	Rsa1.0_04981.1	753	15250	0	386	399	96	−	3	400	7395	7793
UN05250	Rsa1.0_00149.1	486	305196	1.00E-105	208	212	98	+	278	486	8505	8716
UN05251	Rsa1.0_00802.1	770	63846	0	372	383	97	+	3	382	56821	57203
UN05252	Rsa1.0_02231.1	1317	33661	0	1185	1269	93	−	3	1264	18252	19507
UN05253	Rsa1.0_00141.1	941	121494	2.00E-95	270	304	88	−	647	941	29536	29823
UN05254	Rsa1.0_00390.1	779	88681	1.00E-121	227	229	99	+	252	480	35102	35330
UN05255	Rsa1.0_00128.1	2312	228675	0	1363	1371	99	−	1	1371	159174	160544
UN05256	Rsa1.0_01779.1	803	39448	0	601	693	86	−	98	790	9732	10424
UN05257	Rsa1.0_17997.1	1149	1693	0	886	890	99	+	2	889	804	1693
UN05258	Rsa1.0_09698.1	1077	6203	0	383	399	95	−	435	833	1296	1694
UN05259	Rsa1.0_00589.1	1391	209965	1.00E-139	317	335	94	−	1033	1365	162792	163124
UN05260	Rsa1.0_00006.1	720	396176	0	441	465	94	−	255	719	307636	308096
UN05261	Rsa1.0_05093.1	1262	14876	1.00E-149	298	307	97	−	958	1262	11047	11353
UN05262	Rsa1.0_00792.1	871	64163	9.00E-57	167	185	90	−	314	498	4551	4735
UN05263	Rsa1.0_01043.1	895	55893	0	385	385	100	+	511	895	48633	49017

UN05264	Rsa1.0_23922.1	1152	1017	0	769	770	99	−	2	771	1	770
UN05265	Rsa1.0_01750.1	1433	39816	0	611	627	97	−	1	626	24343	24969
UN05266	Rsa1.0_01586.1	838	42804	1.00E−171	357	373	95	−	16	388	29493	29864
UN05267	Rsa1.0_00821.1	675	62891	0	561	563	99	−	114	675	5065	5627
UN05268	Rsa1.0_01209.1	795	50668	0	772	794	97	−	1	794	24794	25570
UN05269	Rsa1.0_00018.1	694	410899	0	373	385	96	+	311	694	193614	193997
UN05270	Rsa1.0_02991.1	817	26178	0	796	847	93	+	3	816	8733	9573
UN05271	Rsa1.0_00817.1	1425	62961	1.00E−148	284	290	97	−	446	735	39075	39364
UN05272	Rsa1.0_01888.1	1535	37563	1.00E−151	283	287	98	−	236	522	17199	17485
UN05273	Rsa1.0_00675.1	910	145590	0	469	480	97	−	431	910	67441	67920
UN05274	Rsa1.0_02165.1	1041	34423	0	545	604	90	−	375	962	3037	3636
UN05275	Rsa1.0_00239.1	939	104332	0	462	482	95	−	458	939	34699	35180
UN05276	Rsa1.0_02879.1	875	27047	1.00E−114	219	221	99	−	3	222	26493	26713
UN05277	Rsa1.0_00033.1	1060	515811	0	914	937	97	−	125	1060	485085	486009
UN05278	Rsa1.0_04794.1	802	15960	0	383	383	100	+	80	462	6544	6926
UN05279	Rsa1.0_00985.1	657	161950	1.00E−98	220	231	95	+	427	657	39464	39693
UN05280	Rsa1.0_01141.1	969	52530	1.00E−166	318	324	98	−	643	965	39275	39598
UN05281	Rsa1.0_00001.1	821	363028	1.00E−108	200	200	100	+	203	402	318208	318407
UN05282	Rsa1.0_01467.1	994	45161	0	959	994	96	−	1	991	23856	24849
UN05283	Rsa1.0_02873.1	1248	27077	7.00E−43	103	106	97	+	1140	1244	15909	16014
UN05284	Rsa1.0_01382.1	1387	46877	0	513	520	98	−	867	1386	24876	25394
UN05285	Rsa1.0_00107.1	888	431659	0	558	584	95	+	300	876	337025	337604
UN05286	Rsa1.0_22717.1	1016	1101	1.00E−164	299	301	99	+	716	1016	773	1073
UN05287	Rsa1.0_13819.1	971	4264	0	424	434	97	+	2	435	1006	1439
UN05288	Rsa1.0_18379.1	866	1611	0	773	777	99	−	90	866	271	1047
UN05289	Rsa1.0_00146.1	808	149343	0	794	808	98	+	1	808	67589	68396
UN05290	Rsa1.0_00522.1	980	78103	0	949	1000	94	−	3	980	38739	39734
UN05291	Rsa1.0_00284.1	1038	323267	0	463	507	91	+	527	1033	275018	275498
UN05292	Rsa1.0_00067.1	1434	415055	0	516	541	95	−	302	839	252590	253130
UN05293	Rsa1.0_00777.1	751	191865	1.00E−125	256	264	96	+	486	749	182543	182805
UN05294	Rsa1.0_15050.1	1547	2868	1.00E−159	345	359	96	−	1195	1547	454	811
UN05295	Rsa1.0_03256.1	898	24305	1.00E−147	306	321	95	−	3	315	23203	23523
UN05296	Rsa1.0_00010.1	974	294827	1.00E−117	228	231	98	+	744	974	243062	243291
UN05297	Rsa1.0_03028.1	978	25872	0	696	715	97	−	3	713	18683	19391
UN05298	Rsa1.0_00154.1	1796	189461	0	517	546	94	+	5	536	127025	127570
UN05299	Rsa1.0_00544.1	693	76787	1.00E−109	204	205	99	−	5	209	60369	60573
UN05300	Rsa1.0_03490.1	1055	22545	0	483	488	98	−	1	488	20433	20919
UN05301	Rsa1.0_00133.1	1409	123768	0	698	720	96	−	666	1385	13416	14134
UN05302	Rsa1.0_01516.1	1118	44196	0	530	556	95	−	3	558	36762	37315

UN05303	Rsa1.0_01827.1	1644	143321	1.00E-170	383	409	93	+	937	1342	114713	115121
UN05304	Rsa1.0_03451.1	934	22828	1.00E-156	298	304	98	−	1	304	18258	18561
UN05305	Rsa1.0_01094.1	979	167285	0	621	644	96	+	346	977	49222	49865
UN05306	Rsa1.0_00697.1	1027	67913	0	879	962	91	+	10	960	40876	41807
UN05307	Rsa1.0_01063.1	1024	55301	1.00E-153	300	306	98	−	720	1024	54642	54946
UN05308	Rsa1.0_01626.1	1040	41989	1.00E-143	264	266	99	+	775	1040	2329	2594
UN05309	Rsa1.0_00072.1	1355	148743	1.00E-179	353	362	97	+	995	1355	72748	73107
UN05310	Rsa1.0_00301.1	1203	365315	0	1166	1211	96	+	2	1203	354111	355307
UN05311	Rsa1.0_14363.1	2202	3477	0	764	772	98	−	1432	2202	608	1375
UN05312	Rsa1.0_00125.1	876	253043	0	459	464	98	−	268	731	4714	5177
UN05313	Rsa1.0_00526.1	766	169279	0	761	762	99	−	3	764	126150	126911
UN05314	Rsa1.0_02397.1	637	31778	4.00E-21	68	73	93	−	511	583	19073	19145
UN05315	Rsa1.0_00891.1	1323	60724	0	882	915	96	+	267	1177	46240	47142
UN05316	Rsa1.0_01634.1	858	41865	1.00E-102	204	209	97	−	396	604	39398	39606
UN05317	Rsa1.0_01593.1	849	150996	0	404	430	93	−	55	484	5028	5454
UN05318	Rsa1.0_00762.1	793	165246	1.00E-172	322	327	98	−	467	793	51055	51381
UN05319	Rsa1.0_00781.1	979	64480	1.00E-148	267	267	100	+	576	842	31184	31450
UN05320	Rsa1.0_02199.1	1220	33982	0	1158	1176	98	+	4	1175	25722	26897
UN05321	Rsa1.0_00722.1	1635	66840	0	1013	1047	96	−	259	1301	15926	16966
UN05322	Rsa1.0_00303.1	1016	97468	0	574	594	96	−	2	595	13792	14382
UN05323	Rsa1.0_56486.1	1035	405	1.00E-108	199	199	100	+	201	399	63	261
UN05324	Rsa1.0_03916.1	801	20117	0	567	582	97	−	147	728	18468	19049
UN05325	Rsa1.0_05890.1	760	12317	1.00E-111	253	272	93	−	497	753	708	976
UN05326	Rsa1.0_00097.1	1071	138858	0	559	566	98	−	502	1067	92732	93297
UN05327	Rsa1.0_00651.1	2198	247766	1.00E-169	309	311	99	−	1887	2197	92723	93033
UN05328	Rsa1.0_00022.1	834	200461	0	449	472	95	−	364	833	86036	86504
UN05329	Rsa1.0_00387.1	825	495407	1.00E-120	229	232	98	−	473	704	344428	344659
UN05330	Rsa1.0_21508.1	1059	1197	1.00E-112	212	214	99	−	846	1059	936	1149
UN05331	Rsa1.0_00307.1	1245	199003	0	341	341	100	−	905	1245	80051	80391
UN05332	Rsa1.0_26044.1	657	908	0	426	452	94	−	3	450	1	452
UN05333	Rsa1.0_07000.1	886	10049	1.00E-139	275	283	97	+	310	592	5676	5958
UN05334	Rsa1.0_00384.1	1551	89278	0	471	482	97	+	250	731	82326	82807
UN05335	Rsa1.0_00377.1	1118	90038	0	411	412	99	−	707	1118	27976	28387
UN05336	Rsa1.0_03613.1	981	21773	0	527	528	99	−	454	981	19514	20041
UN05337	Rsa1.0_05102.1	1585	14843	0	1144	1209	94	+	2	1203	4089	5277
UN05338	Rsa1.0_00073.1	1047	240924	1.00E-160	306	311	98	+	33	343	187846	188155
UN05339	Rsa1.0_00003.1	1009	340054	0	934	949	98	−	60	1008	266950	267897
UN05340	Rsa1.0_01262.1	970	178815	0	441	451	97	−	523	970	10475	10924
UN05341	Rsa1.0_01783.1	883	39383	0	483	515	93	+	380	881	24517	25025

UN05342	Rsa1.0_21881.1	877	1167	0	337	341	98	-	4	343	13	353
UN05343	Rsa1.0_05506.1	1167	13495	0	903	914	98	+	39	952	1	914
UN05344	Rsa1.0_10669.1	1006	5762	0	357	363	98	-	644	1006	58	420
UN05345	Rsa1.0_45087.1	816	500	0	492	500	98	-	12	511	1	500
UN05346	Rsa1.0_01995.1	690	36347	2.33E-156	292	302	96	+	8	309	25096	25397
UN05347	Rsa1.0_01064.1	1423	55295	1.00E-177	329	332	99	-	391	721	15482	15813
UN05348	Rsa1.0_00146.1	967	149343	0	374	375	99	-	3	376	55443	55817
UN05349	Rsa1.0_00327.1	738	94892	2.00E-66	141	145	97	-	87	231	27389	27533
UN05350	Rsa1.0_02341.1	1453	32387	1.00E-154	295	301	98	-	1153	1453	31192	31492
UN05351	Rsa1.0_07340.1	1023	9530	0	508	524	96	-	326	847	5003	5523
UN05352	Rsa1.0_01199.1	1087	163576	1.00E-173	396	422	93	+	675	1086	20827	21247
UN05353	Rsa1.0_00190.1	1083	318240	0	1022	1083	94	+	3	1083	288624	289705
UN05354	Rsa1.0_00339.1	997	222974	6.00E-83	197	209	94	+	1	209	176118	176325
UN05355	Rsa1.0_07811.1	1037	8496	1.00E-160	286	286	100	+	310	595	4751	5036
UN05356	Rsa1.0_02562.1	1061	30061	0	1039	1059	98	+	3	1061	9155	10206
UN05357	Rsa1.0_04387.1	1022	17625	0	342	346	98	+	671	1016	16703	17048
UN05358	Rsa1.0_19257.1	705	1455	0	379	380	99	-	5	384	108	487
UN05359	Rsa1.0_01417.1	1106	46252	0	406	417	97	+	690	1106	20533	20945
UN05360	Rsa1.0_03107.1	1255	25349	0	594	601	98	-	4	604	17268	17868
UN05361	Rsa1.0_02874.1	870	27070	0	696	696	100	+	175	870	16196	16891
UN05362	Rsa1.0_00111.1	935	390088	0	331	332	99	+	598	929	7100	7431
UN05363	Rsa1.0_00451.1	1430	322533	0	383	385	99	-	1046	1430	283300	283684
UN05364	Rsa1.0_00278.1	1211	231896	0	415	426	97	+	276	701	35432	35856
UN05365	Rsa1.0_03396.1	1052	23242	0	369	378	97	-	657	1034	931	1306
UN05366	Rsa1.0_00470.1	1101	81968	1.00E-117	250	261	95	-	838	1098	30120	30377
UN05367	Rsa1.0_00079.1	765	606987	0	505	514	98	+	20	533	15767	16280
UN05368	Rsa1.0_03278.1	1054	24125	1.00E-132	322	348	92	-	709	1050	1924	2271
UN05369	Rsa1.0_00233.1	1280	104918	7.00E-40	364	456	79	-	17	463	49472	49924
UN05370	Rsa1.0_02878.1	1956	27048	0	454	475	95	+	988	1451	21455	21929
UN05371	Rsa1.0_00608.1	810	172672	1.00E-101	202	207	97	+	319	525	116504	116710
UN05372	Rsa1.0_02074.1	845	35370	3.00E-87	185	188	98	-	635	819	12340	12527
UN05373	Rsa1.0_03725.1	1812	21170	0	1039	1061	97	-	1	1061	15948	17005
UN05374	Rsa1.0_04366.1	994	17731	0	668	724	92	+	105	822	11946	12661
UN05375	Rsa1.0_00169.1	982	274298	0	433	436	99	-	337	772	20667	21102
UN05376	Rsa1.0_07063.1	489	9958	0	487	489	99	+	1	489	5007	5495
UN05377	Rsa1.0_00351.1	1392	413096	0	364	367	99	+	1026	1392	148179	148544
UN05378	Rsa1.0_00010.1	1451	294827	0	416	425	97	-	930	1354	121889	122313
UN05379	Rsa1.0_08961.1	937	6750	1.00E-126	275	288	95	-	585	871	4467	4752
UN05380	Rsa1.0_00002.1	1163	601661	0	414	427	96	+	3	426	97407	97833

UN05381	Rsa1.0_02585.1	964	29865	0	494	509	97	+	1	506	25269	25776
UN05382	Rsa1.0_18581.1	811	1571	0	540	554	97	−	163	715	862	1413
UN05383	Rsa1.0_00079.1	762	606987	0	677	704	96	−	63	760	814	1512
UN05384	Rsa1.0_00116.1	972	365886	0	441	448	98	−	13	460	205570	206017
UN05385	Rsa1.0_19954.1	1224	1360	1.00E−145	277	281	98	−	1	279	541	821
UN05386	Rsa1.0_00342.1	1406	93695	1.00E−171	412	438	94	+	975	1406	57424	57856
UN05387	Rsa1.0_00632.1	732	183777	4.00E−89	210	221	95	+	514	732	97	314
UN05388	Rsa1.0_01366.1	1444	47139	0	1066	1068	99	−	371	1438	33506	34573
UN05389	Rsa1.0_00293.1	1795	339294	0	456	466	97	−	1289	1754	82364	82829
UN05390	Rsa1.0_03401.1	691	23191	6.00E−42	124	136	91	−	553	688	5987	6122
UN05391	Rsa1.0_00098.1	858	349239	0	457	458	99	−	3	460	237652	238108
UN05392	Rsa1.0_05262.1	1268	14334	0	552	580	95	+	689	1268	5964	6531
UN05393	Rsa1.0_08083.1	1108	7936	3.00E−60	196	221	88	−	3	222	7372	7575
UN05394	Rsa1.0_01888.1	1873	37563	1.00E−178	317	317	100	−	2	318	19119	19435
UN05395	Rsa1.0_03416.1	1188	23112	1.00E−172	318	322	98	+	186	507	13423	13744
UN05396	Rsa1.0_00191.1	984	316976	0	942	984	95	−	2	984	172092	173064
UN05397	Rsa1.0_00236.1	1223	104771	1.00E−138	345	373	92	+	856	1220	82285	82651
UN05398	Rsa1.0_02165.1	1025	34423	0	509	527	96	+	121	647	3110	3636
UN05399	Rsa1.0_01070.1	535	55099	1.00E−161	314	323	97	−	3	325	28010	28332
UN05400	Rsa1.0_14298.1	854	3561	4.00E−99	187	188	99	+	493	680	3278	3465
UN05401	Rsa1.0_05978.1	1322	12053	0	1223	1253	97	+	59	1311	477	1729
UN05402	Rsa1.0_01380.1	754	155003	0	582	585	99	+	1	585	8894	9478
UN05403	Rsa1.0_01717.1	830	143162	1.00E−147	273	276	98	+	398	673	15704	15979
UN05404	Rsa1.0_00187.1	1383	393093	1.00E−157	324	338	95	+	1043	1380	211557	211889
UN05405	Rsa1.0_12341.1	941	5250	1.00E−169	320	325	98	+	3	327	113	436
UN05406	Rsa1.0_00031.1	1481	179862	0	896	898	99	+	585	1481	60443	61340
UN05407	Rsa1.0_02039.1	661	35699	1.00E−125	264	274	96	−	8	279	10945	11212
UN05408	Rsa1.0_00166.1	1178	240912	0	756	785	96	+	346	1130	153565	154342
UN05409	Rsa1.0_03321.1	743	23755	0	731	733	99	+	9	741	22453	23184
UN05410	Rsa1.0_00004.1	993	253996	0	480	480	100	+	1	480	196461	196940
UN05411	Rsa1.0_00224.1	865	327530	1.00E−173	314	316	99	−	1	316	210516	210831
UN05412	Rsa1.0_00140.1	435	121541	1.00E−132	284	301	94	+	3	288	56150	56449
UN05413	Rsa1.0_38780.1	787	580	1.00E−141	254	254	100	+	534	787	1	254
UN05414	Rsa1.0_00190.1	761	318240	0	723	738	97	−	1	738	289380	290117
UN05415	Rsa1.0_01904.1	711	37450	0	613	637	96	−	86	710	1915	2548
UN05416	Rsa1.0_00301.1	760	365315	0	345	345	100	+	414	758	346680	347024
UN05417	Rsa1.0_68490.1	856	335	1.00E−177	321	323	99	+	42	364	1	323
UN05418	Rsa1.0_00541.1	736	76863	1.00E−180	323	324	99	−	169	492	34580	34903
UN05419	Rsa1.0_00995.1	1453	57243	0	626	662	94	−	2	662	21109	21751

UN05420	Rsa1.0_01739.1	1065	40068	0	989	1047	94	−	38	1065	8378	9413
UN05421	Rsa1.0_05920.1	1062	12239	0	584	586	99	+	1	586	1252	1837
UN05422	Rsa1.0_00075.1	1126	146680	1.00E−105	349	392	89	+	1	381	141869	142242
UN05423	Rsa1.0_12933.1	1216	5071	0	579	622	93	+	1	619	3333	3941
UN05424	Rsa1.0_00034.1	750	260119	0	406	416	97	+	1	416	154035	154449
UN05425	Rsa1.0_02136.1	801	34707	1.00E−102	198	201	98	−	237	437	2798	2998
UN05426	Rsa1.0_00019.1	897	301669	0	515	520	99	+	1	519	218714	219233
UN05427	Rsa1.0_00302.1	845	97521	0	362	368	98	−	1	368	85022	85388
UN05428	Rsa1.0_04773.1	1040	16037	0	657	659	99	+	1	659	10630	11288
UN05429	Rsa1.0_00275.1	403	210104	1.00E−153	313	324	96	−	3	326	178612	178931
UN05430	Rsa1.0_07813.1	961	8493	0	806	827	97	+	3	827	918	1744
UN05431	Rsa1.0_00333.1	657	94500	0	559	559	100	−	2	560	61826	62384
UN05432	Rsa1.0_00695.1	1020	68019	0	365	366	99	+	655	1020	53951	54316
UN05433	Rsa1.0_00608.1	805	172672	1.00E−165	307	311	98	−	1	311	118055	118365
UN05434	Rsa1.0_03241.1	1101	24423	0	487	489	99	−	1	487	14637	15125
UN05435	Rsa1.0_00139.1	1099	357786	1.00E−176	612	711	86	−	93	802	59566	60264
UN05436	Rsa1.0_15250.1	746	2727	0	450	450	100	−	3	452	1092	1541
UN05437	Rsa1.0_19592.1	734	1410	0	458	487	94	+	30	513	1	480
UN05438	Rsa1.0_03282.1	901	24107	0	552	568	97	−	3	566	10595	11159
UN05439	Rsa1.0_00971.1	838	57973	1.00E−12	39	39	100	−	783	821	43519	43557
UN05440	Rsa1.0_00241.1	678	104000	1.00E−127	276	290	95	+	3	290	65527	65816
UN05441	Rsa1.0_00159.1	721	431686	0	437	441	99	−	1	440	15770	16210
UN05442	Rsa1.0_01876.1	812	37800	0	677	691	97	−	16	706	34625	35314
UN05443	Rsa1.0_05262.1	769	14334	0	551	578	95	−	1	577	5964	6532
UN05444	Rsa1.0_05689.1	1457	12927	1.00E−124	226	226	100	−	1	226	9560	9785
UN05445	Rsa1.0_00201.1	511	232851	1.00E−129	327	355	92	+	3	356	17967	18311
UN05446	Rsa1.0_02020.1	1475	173211	0	1028	1045	98	+	124	1168	137400	138444
UN05447	Rsa1.0_03503.1	718	22445	0	441	458	96	+	2	458	19857	20313
UN05448	Rsa1.0_00646.1	796	70473	4.00E−71	149	153	97	+	4	156	6102	6254
UN05449	Rsa1.0_00708.1	733	67366	0	696	704	98	−	3	706	43756	44459
UN05450	Rsa1.0_01135.1	948	52799	0	762	765	99	−	186	948	24295	25059
UN05451	Rsa1.0_02020.1	1021	173211	1.00E−124	292	312	93	−	708	1019	171655	171962
UN05452	Rsa1.0_00040.1	1320	324196	0	1220	1230	99	−	3	1232	127997	129223
UN05453	Rsa1.0_02589.1	560	300828	1.00E−159	288	289	99	−	6	294	16343	16631
UN05454	Rsa1.0_01724.1	935	40292	1.00E−109	204	205	99	+	51	255	6838	7042
UN05455	Rsa1.0_01061.1	799	55349	1.00E−154	429	475	90	+	7	472	4834	5306
UN05456	Rsa1.0_02142.1	983	34633	0	351	356	98	−	1	356	16828	17183
UN05457	Rsa1.0_02746.1	890	28256	0	688	698	98	−	3	700	3134	3831
UN05458	Rsa1.0_02661.1	1288	28964	0	1283	1288	99	+	1	1288	19277	20564

UN05459	Rsa1.0_07559.1	986	9131	0	981	984	99	+	1	984	1423	2406
UN05460	Rsa1.0_03191.1	456	145762	0	346	348	99	−	1	348	21630	21976
UN05461	Rsa1.0_02811.1	921	27669	1.00E−120	269	282	95	+	1	281	1244	1521
UN05462	Rsa1.0_26967.1	641	871	1.00E−156	357	381	93	−	2	375	308	680
UN05463	Rsa1.0_03927.1	1296	19997	0	549	549	100	+	1	549	16154	16702
UN05464	Rsa1.0_03624.1	815	21706	0	474	486	97	+	4	486	15700	16181
UN05465	Rsa1.0_00269.1	1126	341668	0	571	575	99	−	30	603	18901	19475
UN05466	Rsa1.0_00122.1	1423	265937	1.00E−114	309	337	91	+	681	1015	124131	124459
UN05467	Rsa1.0_03506.1	1095	22416	0	490	491	99	+	2	491	7388	7878
UN05468	Rsa1.0_00740.1	923	215972	0	662	691	95	+	2	682	2956	3643
UN05469	Rsa1.0_00821.1	1036	62891	0	365	365	100	−	671	1035	42502	42866
UN05470	Rsa1.0_02513.1	967	30643	0	926	964	96	+	2	965	10829	11789
UN05471	Rsa1.0_21366.1	1446	1211	0	846	856	98	−	591	1446	356	1211
UN05472	Rsa1.0_00130.1	788	125737	0	443	445	99	−	2	446	42201	42645
UN05473	Rsa1.0_01923.1	875	37233	0	831	877	94	−	1	875	23234	24106
UN05474	Rsa1.0_01261.1	829	49744	0	809	826	97	−	3	826	30840	31660
UN05475	Rsa1.0_00710.1	985	67325	0	691	691	100	+	295	985	5338	6028
UN05476	Rsa1.0_02929.1	838	26673	0	333	334	99	+	505	838	20275	20608
UN05477	Rsa1.0_00203.1	1127	109129	0	947	958	98	+	172	1127	46993	47950
UN05478	Rsa1.0_41744.1	742	538	1.00E−167	334	346	96	+	367	712	193	538
UN05479	Rsa1.0_00030.1	954	250988	0	688	698	98	+	2	698	15958	16655
UN05480	Rsa1.0_08655.1	836	7078	1.00E−129	235	235	100	−	237	471	5600	5834
UN05481	Rsa1.0_00920.1	755	601703	1.00E−114	212	213	99	−	543	755	443622	443834
UN05482	Rsa1.0_00096.1	611	198773	1.00E−180	341	347	98	+	266	611	159088	159434
UN05483	Rsa1.0_00107.1	857	431659	0	479	482	99	−	376	857	135950	136431
UN05484	Rsa1.0_00788.1	1371	64237	0	888	891	99	+	481	1371	22869	23759
UN05485	Rsa1.0_02179.1	1165	34188	0	609	626	97	−	66	691	24331	24956
UN05486	Rsa1.0_04235.1	1295	18373	0	822	866	94	−	434	1295	11442	12303
UN05487	Rsa1.0_00930.1	1219	59224	1.00E−122	270	283	95	−	925	1206	52016	52297
UN05488	Rsa1.0_00323.1	1115	159650	0	1108	1114	99	+	3	1115	148992	150105
UN05489	Rsa1.0_02113.1	847	34890	0	790	823	95	+	4	825	22694	23509
UN05490	Rsa1.0_00085.1	1125	142226	0	1048	1123	93	−	4	1125	121841	122954
UN05491	Rsa1.0_00040.1	1272	324196	0	545	558	97	+	715	1272	114552	115108
UN05492	Rsa1.0_03384.1	744	23291	0	740	743	99	−	2	744	12671	13413
UN05493	Rsa1.0_00017.1	927	752458	0	367	380	96	−	3	379	292600	292977
UN05494	Rsa1.0_03780.1	1072	20899	0	1051	1070	98	+	2	1068	15890	16959
UN05495	Rsa1.0_03081.1	877	25523	1.00E−153	274	274	100	−	1	274	21472	21745
UN05496	Rsa1.0_00184.1	734	218748	4.00E−83	157	157	100	−	185	341	212050	212206
UN05497	Rsa1.0_02433.1	1446	158318	0	393	397	98	−	1036	1432	20016	20411

UN05498	Rsa1.0_07515.1	1072	9221	0	1042	1075	96	+	3	1070	5653	6727
UN05499	Rsa1.0_14881.1	1128	2995	0	468	483	96	+	647	1128	2323	2804
UN05500	Rsa1.0_00890.1	651	323870	0	451	496	90	+	78	555	215367	215859
UN05501	Rsa1.0_00135.1	917	178077	0	456	457	99	−	4	460	82825	83281
UN05502	Rsa1.0_00046.1	842	320315	0	803	841	95	−	2	836	30017	30857
UN05503	Rsa1.0_00742.1	1548	66106	0	435	459	94	−	1092	1548	25739	26188
UN05504	Rsa1.0_00100.1	1402	249232	0	421	430	97	−	971	1400	140938	141367
UN05505	Rsa1.0_08717.1	1549	7008	1.00E−120	286	305	93	+	1250	1549	1261	1564
UN05506	Rsa1.0_12805.1	1140	5121	2.00E−40	116	126	92	+	438	563	4576	4701
UN05507	Rsa1.0_01191.1	1137	51235	0	469	471	99	+	13	483	12138	12608
UN05508	Rsa1.0_01339.1	1261	47813	0	386	388	99	+	874	1261	29690	30077
UN05509	Rsa1.0_02266.1	1335	33195	0	782	796	98	+	4	798	19496	20280
UN05510	Rsa1.0_15580.1	974	2535	0	321	321	100	−	3	323	1727	2047
UN05511	Rsa1.0_29122.1	756	792	0	431	438	98	−	235	672	360	792
UN05512	Rsa1.0_02903.1	2033	26901	0	912	932	97	−	452	1383	12531	13462
UN05513	Rsa1.0_00211.1	848	228189	1.00E−151	283	287	98	−	156	442	213634	213920
UN05514	Rsa1.0_32341.1	1372	704	1.00E−173	309	309	100	+	1064	1372	330	638
UN05515	Rsa1.0_12541.1	758	5198	0	361	370	97	+	1	370	375	744
UN05516	Rsa1.0_05305.1	1141	14207	0	503	527	95	−	3	518	13315	13835
UN05517	Rsa1.0_02355.1	1329	32260	0	612	633	96	−	697	1329	6912	7544
UN05518	Rsa1.0_01389.1	1413	46716	0	368	375	98	−	1038	1412	20717	21090
UN05519	Rsa1.0_01287.1	1517	49195	0	836	836	100	+	1	836	2346	3181
UN05520	Rsa1.0_03369.1	1314	23381	0	1001	1006	99	−	305	1309	10307	11312
UN05521	Rsa1.0_01553.1	755	43379	1.00E−165	294	294	100	+	462	755	26451	26744
UN05522	Rsa1.0_02707.1	676	28520	2.00E−84	213	229	93	−	451	675	172	400
UN05523	Rsa1.0_06775.1	1043	10379	0	457	463	98	+	583	1043	6834	7296
UN05524	Rsa1.0_01514.1	742	44222	0	723	740	97	+	3	740	3978	4717
UN05525	Rsa1.0_08606.1	931	7133	0	439	451	97	−	8	458	6441	6891
UN05526	Rsa1.0_01469.1	878	45139	0	453	455	99	+	422	876	14247	14701
UN05527	Rsa1.0_01452.1	1114	45459	0	465	497	93	+	619	1113	9485	9973
UN05528	Rsa1.0_01085.1	869	54539	0	392	400	98	+	2	401	9663	10062
UN05529	Rsa1.0_00001.1	1268	363028	0	476	476	100	+	569	1044	357923	358398
UN05530	Rsa1.0_51330.1	1229	443	2.00E−77	187	200	93	−	1030	1229	143	342
UN05531	Rsa1.0_00092.1	672	659932	0	660	674	97	+	3	672	353793	354465
UN05532	Rsa1.0_00016.1	881	831256	0	449	449	100	+	1	449	264152	264600
UN05533	Rsa1.0_03693.1	1461	21337	0	738	742	99	+	174	915	563	1303
UN05534	Rsa1.0_00321.1	1132	95671	0	777	789	98	+	353	1132	48387	49175
UN05535	Rsa1.0_02402.1	1236	31710	0	956	961	99	−	276	1236	10375	11335
UN05536	Rsa1.0_02099.1	1141	35030	1.00E−139	264	268	98	+	797	1064	2291	2558

UN05537	Rsa1.0_00105.1	772	134138	0	627	639	98	-	3	631	123747	124385
UN05538	Rsa1.0_06345.1	1486	11205	0	948	951	99	-	276	1226	2128	3078
UN05539	Rsa1.0_02368.1	864	32096	1.00E-119	239	245	97	+	5	248	4841	5085
UN05540	Rsa1.0_05150.1	804	14673	1.00E-177	351	363	96	-	283	645	6013	6375
UN05541	Rsa1.0_00077.1	1851	145005	0	1548	1603	96	+	4	1604	134272	135871
UN05542	Rsa1.0_01608.1	1153	170540	0	361	366	98	-	361	726	35343	35708
UN05543	Rsa1.0_00765.1	945	65029	0	817	827	98	-	3	828	30048	30874
UN05544	Rsa1.0_03216.1	967	24540	1.00E-163	292	292	100	-	676	967	12039	12330
UN05545	Rsa1.0_01636.1	935	41863	1.00E-135	251	252	99	-	684	935	36754	37004
UN05546	Rsa1.0_01114.1	803	53602	0	790	807	97	+	3	803	49683	50489
UN05547	#	#	#	#	#	#	#	#	#	#	#	#
UN05548	Rsa1.0_00533.1	774	77375	0	545	562	96	-	212	773	54830	55391
UN05549	Rsa1.0_06049.1	1093	11851	0	415	417	99	+	677	1093	9709	10125
UN05550	Rsa1.0_11824.1	467	5396	1.00E-102	235	253	92	+	232	466	3655	3907
UN05551	Rsa1.0_08507.1	591	7257	0	501	504	99	+	3	506	4584	5087
UN05552	Rsa1.0_00114.1	815	131330	1.00E-129	298	315	94	-	12	325	86104	86416
UN05553	Rsa1.0_00181.1	1139	114269	0	1090	1136	95	-	3	1135	91480	92614
UN05554	Rsa1.0_01957.1	614	36844	1.00E-121	238	244	97	-	371	614	13022	13265
UN05555	Rsa1.0_08811.1	793	6904	1.00E-123	245	252	97	+	168	419	3280	3531
UN05556	Rsa1.0_02858.1	1006	27246	0	980	1002	97	+	3	1004	14127	15128
UN05557	Rsa1.0_00153.1	1102	192931	0	508	552	92	+	548	1092	26879	27421
UN05558	Rsa1.0_01690.1	751	40939	0	437	438	99	+	313	750	23468	23905
UN05559	Rsa1.0_00132.1	1277	124299	0	327	327	100	-	3	329	104645	104971
UN05560	Rsa1.0_00395.1	1206	214466	1.00E-151	278	280	99	-	12	291	5892	6171
UN05561	Rsa1.0_00299.1	1432	97770	0	1427	1429	99	+	3	1431	41871	43299
UN05562	Rsa1.0_10577.1	597	5796	1.00E-164	380	408	93	+	190	597	621	1027
UN05563	Rsa1.0_00157.1	462	245832	0	406	415	97	+	48	462	239149	239563
UN05564	Rsa1.0_01376.1	926	47004	0	728	738	98	-	1	738	33259	33993
UN05565	Rsa1.0_01489.1	1012	44764	0	484	497	97	-	519	1010	16344	16834
UN05566	Rsa1.0_00714.1	644	67187	1.00E-174	312	313	99	-	1	313	3535	3847
UN05567	Rsa1.0_05199.1	527	14529	1.00E-145	301	313	96	+	2	314	6772	7083
UN05568	Rsa1.0_17607.1	1258	1785	1.00E-173	308	308	100	-	951	1258	1416	1723
UN05569	Rsa1.0_03736.1	1199	21081	1.00E-113	316	348	90	-	850	1189	9918	10265
UN05570	Rsa1.0_01523.1	813	43959	1.00E-168	302	303	99	+	511	813	20541	20843
UN05571	Rsa1.0_00493.1	805	80007	1.00E-133	250	253	98	+	205	457	75604	75856
UN05572	Rsa1.0_02870.1	1016	27102	0	643	644	99	+	3	646	26052	26694
UN05573	Rsa1.0_15516.1	1159	2563	1.00E-178	345	352	98	+	810	1159	1144	1495
UN05574	Rsa1.0_00825.1	783	62855	0	356	356	100	-	223	578	38368	38723
UN05575	Rsa1.0_00043.1	659	264937	2.00E-51	135	144	93	+	413	556	8948	9085

UN05576	Rsa1.0_00770.1	952	64900	0	949	952	99	+	3	952	53371	54322
UN05577	Rsa1.0_01732.1	1029	40194	0	404	404	100	−	1	404	15549	15952
UN05578	Rsa1.0_00016.1	1047	831256	1.00E−162	311	317	98	+	448	764	752718	753032
UN05579	Rsa1.0_14170.1	1158	3709	0	1154	1158	99	+	2	1157	1690	2847
UN05580	Rsa1.0_02377.1	996	31994	0	502	521	96	+	476	996	3419	3939
UN05581	Rsa1.0_00010.1	1507	294827	1.00E−114	221	225	98	−	208	432	219087	219311
UN05582	Rsa1.0_00463.1	751	189177	1.00E−165	327	337	97	−	413	749	169663	169997
UN05583	Rsa1.0_00035.1	1609	176449	0	998	1006	99	+	404	1409	125394	126399
UN05584	Rsa1.0_02107.1	517	34940	1.00E−135	361	395	91	+	134	514	9809	10197
UN05585	Rsa1.0_00850.1	1358	62193	0	694	718	96	−	1	718	25983	26700
UN05586	Rsa1.0_00101.1	988	267193	0	939	946	99	−	3	948	131119	132063
UN05587	Rsa1.0_00939.1	1057	185227	1.00E−130	237	237	100	−	435	671	39141	39377
UN05588	Rsa1.0_02247.1	1246	33427	0	508	529	96	−	240	765	2209	2737
UN05589	Rsa1.0_00005.1	800	353093	1.00E−142	307	321	95	+	482	800	130794	131114
UN05590	Rsa1.0_00805.1	1683	63724	0	634	654	96	−	1025	1678	13362	14012
UN05591	Rsa1.0_00971.1	1209	57973	0	766	783	97	+	2	779	16351	17127
UN05592	Rsa1.0_01687.1	929	40979	0	927	929	99	−	1	929	33077	34005
UN05593	Rsa1.0_02664.1	569	28917	0	509	541	94	+	1	540	7482	8022
UN05594	Rsa1.0_02105.1	1160	156953	0	392	395	99	+	1	395	26596	26990
UN05595	Rsa1.0_02065.1	1021	35467	0	379	382	99	+	236	617	15310	15691
UN05596	Rsa1.0_00102.1	765	135497	1.00E−135	331	361	91	−	3	359	44767	45107
UN05597	Rsa1.0_00037.1	1177	307838	1.00E−169	304	305	99	+	541	845	284445	284749
UN05598	Rsa1.0_04055.1	649	19354	0	629	649	96	−	1	647	11125	11773
UN05599	Rsa1.0_12966.1	1503	5062	0	813	855	95	−	663	1502	780	1628
UN05600	Rsa1.0_00090.1	875	273859	0	653	660	98	−	217	875	38868	39527
UN05601	Rsa1.0_00276.1	984	100160	4.00E−25	111	128	86	−	400	527	33975	34102
UN05602	Rsa1.0_02605.1	881	29637	0	872	879	99	−	4	881	1808	2685
UN05603	Rsa1.0_00904.1	816	180594	1.00E−174	316	317	99	−	26	341	149577	149893
UN05604	Rsa1.0_00414.1	1232	202190	0	467	492	94	−	3	494	201313	201804
UN05605	Rsa1.0_00479.1	1377	81377	0	685	695	98	+	684	1377	27298	27992
UN05606	Rsa1.0_01882.1	603	37697	0	589	601	98	+	1	601	24263	24860
UN05607	Rsa1.0_02651.1	1255	29046	0	502	502	100	+	20	521	25001	25502
UN05608	Rsa1.0_04210.1	717	18442	1.00E−179	362	370	97	−	353	717	3764	4133
UN05609	Rsa1.0_00308.1	870	96915	0	386	387	99	−	484	870	70984	71370
UN05610	Rsa1.0_00087.1	861	761073	1.00E−176	334	341	97	−	515	855	533426	533766
UN05611	Rsa1.0_00656.1	935	69968	0	537	551	97	+	381	931	36888	37434
UN05612	Rsa1.0_00061.1	1105	218685	0	561	566	99	+	118	683	99301	99866
UN05613	Rsa1.0_02213.1	1480	33824	0	880	908	96	−	576	1480	16450	17357
UN05614	Rsa1.0_02730.1	969	28385	0	456	501	91	+	347	823	391	891

UN05615	Rsa1.0_00347.1	1213	93048	0	354	357	99	+	858	1212	92076	92432
UN05616	Rsa1.0_01220.1	1405	161775	0	1037	1046	99	+	4	1048	115795	116839
UN05617	Rsa1.0_02927.1	1047	26698	0	531	556	95	+	1	550	8585	9136
UN05618	Rsa1.0_00525.1	761	78030	0	753	764	98	−	3	761	65524	66287
UN05619	Rsa1.0_12999.1	3539	5050	0	563	563	100	−	2535	3097	1	563
UN05620	Rsa1.0_01656.1	896	41494	0	413	413	100	−	419	831	36323	36735
UN05621	Rsa1.0_00997.1	1210	57190	0	725	726	99	−	485	1210	44267	44992
UN05622	Rsa1.0_02910.1	1579	26843	0	398	400	99	−	649	1047	24197	24596
UN05623	Rsa1.0_01153.1	607	52264	0	601	604	99	+	1	604	7807	8410
UN05624	Rsa1.0_52761.1	827	432	4.00E−28	115	130	88	+	3	132	184	308
UN05625	Rsa1.0_02069.1	1099	35425	0	386	400	96	−	704	1098	748	1146
UN05626	Rsa1.0_02490.1	1672	30885	0	409	418	97	+	1255	1672	28607	29023
UN05627	Rsa1.0_00472.1	1058	398134	1.00E−131	247	250	98	+	433	682	271666	271915
UN05628	Rsa1.0_09404.1	513	6392	0	499	512	97	−	2	513	854	1365
UN05629	Rsa1.0_01075.1	760	156953	1.00E−123	237	241	98	+	473	713	23131	23371
UN05630	Rsa1.0_04293.1	835	18046	1.00E−145	335	357	93	+	3	356	12294	12640
UN05631	Rsa1.0_01249.1	831	188432	0	677	683	99	+	149	831	157690	158372
UN05632	Rsa1.0_03964.1	723	19835	9.00E−72	217	244	88	−	491	723	13100	13337
UN05633	Rsa1.0_03981.1	1356	19724	1.00E−139	335	359	93	−	1003	1356	18184	18533
UN05634	Rsa1.0_00476.1	1693	280966	0	346	347	99	+	1347	1692	156495	156841
UN05635	Rsa1.0_00341.1	1147	249309	0	347	354	98	+	3	353	9179	9532
UN05636	Rsa1.0_02923.1	1364	26736	0	655	697	93	+	631	1316	10816	11508
UN05637	Rsa1.0_67954.1	1060	337	0	327	328	99	+	140	467	10	337
UN05638	Rsa1.0_00028.1	718	293984	0	362	370	97	+	350	718	173266	173635
UN05639	Rsa1.0_34876.1	759	649	3.00E−93	174	174	100	−	194	367	122	295
UN05640	Rsa1.0_01861.1	954	38031	1.00E−170	347	361	96	+	17	377	29580	29937
UN05641	Rsa1.0_04611.1	1254	16647	1.00E−169	307	309	99	−	17	325	14537	14845
UN05642	Rsa1.0_10881.1	1583	5691	0	739	742	99	−	844	1583	4950	5691
UN05643	Rsa1.0_00092.1	1513	659932	0	509	514	99	−	364	877	348844	349357
UN05644	Rsa1.0_00629.1	1062	71381	0	396	405	97	−	3	407	38665	39066
UN05645	Rsa1.0_04297.1	507	18030	1.00E−121	220	220	100	+	1	220	16488	16707
UN05646	Rsa1.0_00283.1	1180	183797	1.00E−144	265	267	99	−	1	267	174880	175146
UN05647	Rsa1.0_01492.1	986	44704	2.33E−156	312	326	95	+	1	320	26463	26786
UN05648	Rsa1.0_01476.1	1235	172928	1.00E−112	276	299	92	+	1	291	137462	137754
UN05649	Rsa1.0_00108.1	1014	478491	0	639	680	93	+	1	677	144384	145050
UN05650	Rsa1.0_00461.1	781	82509	0	431	439	98	−	332	769	19933	20371
UN05651	Rsa1.0_01606.1	718	42432	0	560	572	97	+	18	588	37724	38292
UN05652	Rsa1.0_01971.1	844	36641	0	419	428	97	−	1	423	10966	11391
UN05653	Rsa1.0_01191.1	1154	51235	0	551	555	99	−	1	555	19276	19827

UN05654	Rsa1.0_02229.1	787	33672	0	345	345	100	-	3	347	26818	27162
UN05655	Rsa1.0_01537.1	716	187285	0	519	520	99	+	2	521	165458	165977
UN05656	Rsa1.0_04934.1	641	143962	1.00E-174	367	385	95	+	255	639	142052	142435
UN05657	Rsa1.0_08675.1	474	7054	0	474	474	100	+	1	474	2185	2658
UN05658	Rsa1.0_00383.1	837	398431	1.00E-117	294	318	92	+	2	303	182089	182404
UN05659	Rsa1.0_00294.1	797	144860	0	575	583	98	-	1	583	67251	67833
UN05660	Rsa1.0_03437.1	868	22947	0	380	383	99	+	484	866	18572	18954
UN05661	Rsa1.0_04848.1	1211	15727	0	855	869	98	+	342	1210	6563	7431
UN05662	Rsa1.0_00262.1	734	101906	0	377	380	99	-	2	381	51801	52180
UN05663	Rsa1.0_00136.1	1759	122464	0	502	502	100	-	1258	1759	31374	31875
UN05664	Rsa1.0_01779.1	774	39448	0	392	404	97	-	2	405	25472	25871
UN05665	Rsa1.0_00045.1	704	328694	1.00E-142	269	272	98	-	3	274	232551	232821
UN05666	Rsa1.0_00354.1	983	232005	0	958	983	97	-	1	982	180229	181205
UN05667	Rsa1.0_00546.1	783	76523	0	444	472	94	+	100	571	60591	61062
UN05668	Rsa1.0_00589.1	985	209965	0	535	560	95	-	427	985	9746	10294
UN05669	Rsa1.0_00366.1	1360	90880	1.00E-154	277	277	100	+	1070	1346	7884	8160
UN05670	Rsa1.0_00026.1	782	327835	0	570	593	96	+	5	595	27208	27800
UN05671	Rsa1.0_01892.1	751	37526	0	641	650	98	-	1	650	26443	27091
UN05672	Rsa1.0_04997.1	540	15182	0	525	539	97	+	1	539	3486	4023
UN05673	Rsa1.0_00517.1	1323	142790	0	1220	1287	94	+	1	1280	65140	66425
UN05674	Rsa1.0_01024.1	811	56569	0	761	770	98	-	43	811	32275	33044
UN05675	Rsa1.0_01750.1	814	39816	1.00E-120	252	261	96	-	1	261	22530	22786
UN05676	Rsa1.0_00211.1	1051	228189	1.00E-118	303	332	91	+	739	1050	147827	148158
UN05677	Rsa1.0_24113.1	845	1006	0	481	487	98	+	1	485	507	993
UN05678	Rsa1.0_01248.1	1057	154103	0	425	428	99	-	125	552	11605	12032
UN05679	Rsa1.0_00767.1	746	64980	0	605	611	99	-	1	611	14548	15158
UN05680	Rsa1.0_00279.1	750	100039	0	544	544	100	-	1	544	39261	39804
UN05681	Rsa1.0_01796.1	1140	39154	0	1125	1139	98	-	2	1140	19170	20307
UN05682	Rsa1.0_00430.1	1291	85453	1.00E-178	335	341	98	-	231	571	42843	43183
UN05683	Rsa1.0_00531.1	954	77387	0	945	954	99	+	1	954	61149	62100
UN05684	Rsa1.0_00040.1	949	324196	0	595	605	98	-	1	605	246545	247147
UN05685	Rsa1.0_13693.1	857	4469	0	772	805	95	-	58	857	3195	3997
UN05686	Rsa1.0_02319.1	1408	32580	0	1354	1434	94	-	1	1408	401	1807
UN05687	Rsa1.0_00050.1	1172	237174	0	888	891	99	+	281	1170	186179	187069
UN05688	Rsa1.0_01829.1	1349	166063	0	438	440	99	-	910	1349	128650	129089
UN05689	Rsa1.0_00069.1	670	403561	0	377	388	97	+	269	656	86497	86884
UN05690	Rsa1.0_15510.1	1091	2565	0	724	731	99	+	1	731	1333	2063
UN05691	Rsa1.0_00035.1	994	176449	0	393	396	99	-	599	994	37380	37775
UN05692	Rsa1.0_00281.1	1109	99908	1.00E-147	316	336	94	-	1	322	6793	7128

UN05693	Rsa1.0_03183.1	1241	24755	0	589	590	99	-	652	1241	15263	15852
UN05694	Rsa1.0_00866.1	672	61676	1.00E-123	247	254	97	-	1	251	56474	56727
UN05695	Rsa1.0_02672.1	761	28884	1.00E-106	208	212	98	-	394	605	15855	16066
UN05696	Rsa1.0_01173.1	1229	51727	0	386	402	96	-	39	439	1555	1955
UN05697	Rsa1.0_01580.1	1177	42852	0	400	417	95	-	1	414	31149	31563
UN05698	Rsa1.0_03309.1	1069	23852	0	519	524	99	+	3	526	16961	17479
UN05699	Rsa1.0_14703.1	763	3143	1.00E-104	251	267	94	+	1	264	1017	1282
UN05700	Rsa1.0_00990.1	1342	57445	0	414	416	99	+	579	993	15812	16227
UN05701	Rsa1.0_01340.1	909	47805	0	466	477	97	+	2	478	24238	24712
UN05702	Rsa1.0_01630.1	677	41913	1.00E-133	259	264	98	+	1	264	943	1204
UN05703	Rsa1.0_01280.1	1466	49321	0	1013	1051	96	-	6	1048	36579	37629
UN05704	Rsa1.0_00662.1	1255	192183	0	585	598	97	-	1	598	189833	190426
UN05705	Rsa1.0_03402.1	1032	23188	1.00E-112	296	333	88	+	5	321	11297	11614
UN05706	Rsa1.0_18038.1	995	1684	1.00E-174	316	318	99	-	1	318	735	1052
UN05707	Rsa1.0_00787.1	821	64239	0	814	819	99	-	5	821	32330	33148
UN05708	Rsa1.0_01234.1	570	50183	0	556	574	96	-	1	570	43259	43831
UN05709	Rsa1.0_00673.1	1195	69325	0	395	405	97	-	1	402	50953	51357
UN05710	Rsa1.0_12663.1	588	5160	1.00E-133	314	338	92	+	1	336	935	1260
UN05711	Rsa1.0_01825.1	774	38725	1.00E-164	349	363	96	+	1	358	13989	14350
UN05712	Rsa1.0_02655.1	859	29027	1.00E-176	331	337	98	-	1	337	16785	17121
UN05713	Rsa1.0_00111.1	477	390088	1.00E-111	304	336	90	-	129	459	174813	175148
UN05714	Rsa1.0_06170.1	851	11575	0	394	396	99	-	456	851	6714	7109
UN05715	Rsa1.0_00039.1	631	296927	4.00E-92	188	192	97	-	440	630	18136	18327
UN05716	Rsa1.0_00438.1	1294	84861	1.00E-156	324	337	96	+	110	445	15680	16012
UN05717	Rsa1.0_12454.1	1346	5222	0	1128	1203	93	+	13	1215	1326	2526
UN05718	Rsa1.0_08691.1	880	7034	0	427	431	99	-	3	433	5704	6133
UN05719	Rsa1.0_00651.1	785	247766	1.00E-137	259	263	98	-	415	677	218653	218915
UN05720	Rsa1.0_32832.1	756	693	0	337	341	98	-	1	341	325	665
UN05721	Rsa1.0_00055.1	1374	520193	1.00E-149	380	422	90	+	315	736	395420	395817
UN05722	Rsa1.0_00075.1	1668	146680	0	1316	1318	99	+	1	1318	127832	129149
UN05723	Rsa1.0_00106.1	1629	133916	0	858	873	98	+	1	870	5576	6445
UN05724	Rsa1.0_13332.1	729	4911	1.00E-155	285	286	99	+	1	285	4122	4407
UN05725	Rsa1.0_00009.1	1097	232558	0	337	340	99	-	1	340	142538	142876
UN05726	Rsa1.0_00427.1	496	189678	0	448	482	92	-	2	476	41004	41464
UN05727	Rsa1.0_01526.1	1633	43919	0	574	576	99	+	1058	1631	23866	24441
UN05728	Rsa1.0_00524.1	392	169613	1.00E-136	354	390	90	-	5	391	152026	152398
UN05729	Rsa1.0_07704.1	935	8754	0	663	682	97	+	2	680	3432	4101
UN05730	Rsa1.0_08717.1	1486	7008	1.00E-123	292	310	94	-	3	304	1261	1566
UN05731	Rsa1.0_00001.1	866	363028	0	370	370	100	-	1	370	130696	131065

UN05732	Rsa1.0_02796.1	1336	27805	0	1323	1334	99	−	2	1335	22972	24304
UN05733	Rsa1.0_01044.1	1248	55861	0	1214	1249	97	+	1	1247	16741	17989
UN05734	Rsa1.0_00744.1	951	66084	2.00E−85	179	185	96	+	424	608	58549	58733
UN05735	Rsa1.0_00604.1	1198	146282	0	1180	1187	99	−	3	1189	21925	23111
UN05736	Rsa1.0_02903.1	1304	26901	0	734	750	97	+	471	1220	19384	20133
UN05737	Rsa1.0_00119.1	407	467509	1.00E−103	218	227	96	+	178	404	361786	362012
UN05738	Rsa1.0_00018.1	1086	410899	1.00E−155	322	333	96	−	679	1011	70285	70612
UN05739	Rsa1.0_00701.1	1164	67691	0	389	401	97	−	2	402	23226	23626
UN05740	Rsa1.0_03853.1	641	20447	1.00E−174	374	391	95	−	1	385	10994	11384
UN05741	Rsa1.0_02404.1	850	31702	0	577	580	99	+	1	579	5090	5669
UN05742	Rsa1.0_02374.1	1431	32043	1.00E−106	326	367	88	+	4	365	25644	25991
UN05743	Rsa1.0_00656.1	1270	69968	0	411	413	99	+	237	649	49589	50001
UN05744	Rsa1.0_00395.1	966	214466	1.00E−117	330	367	89	+	4	370	62452	62817
UN05745	Rsa1.0_00555.1	719	75920	0	567	600	94	+	34	632	51205	51801
UN05746	Rsa1.0_00842.1	1331	62493	0	393	410	95	−	250	659	3019	3428
UN05747	Rsa1.0_00988.1	690	57525	1.00E−158	283	283	100	+	1	283	49551	49833
UN05748	Rsa1.0_05689.1	766	12927	1.00E−123	240	245	97	−	29	273	12604	12848
UN05749	Rsa1.0_00838.1	1299	153820	0	1268	1300	97	+	1	1298	59940	61238
UN05750	Rsa1.0_00583.1	1062	74309	1.00E−168	312	316	98	+	496	811	13328	13643
UN05751	Rsa1.0_00525.1	962	78030	0	375	376	99	+	1	376	4852	5227
UN05752	Rsa1.0_04470.1	808	17204	0	700	704	99	−	1	704	14662	15364
UN05753	Rsa1.0_14201.1	400	3670	1.00E−173	378	400	94	+	4	400	573	969
UN05754	Rsa1.0_00071.1	1462	345714	0	1131	1152	98	−	1	1149	75943	77091
UN05755	Rsa1.0_04516.1	1618	17018	0	620	635	97	+	794	1428	2760	3394
UN05756	Rsa1.0_00509.1	1315	78829	0	441	444	99	+	643	1086	57392	57835
UN05757	Rsa1.0_00427.1	978	189678	1.00E−145	272	274	99	−	1	274	171614	171886
UN05758	Rsa1.0_00120.1	884	277288	1.00E−116	213	213	100	+	672	884	93711	93923
UN05759	Rsa1.0_02648.1	847	29073	0	425	441	96	+	1	441	25785	26225
UN05760	Rsa1.0_03360.1	712	23459	0	430	436	98	+	3	438	21550	21984
UN05761	Rsa1.0_00564.1	511	75428	1.00E−143	257	257	100	−	255	511	33555	33811
UN05762	Rsa1.0_00172.1	1184	115476	0	722	728	99	+	1	728	68378	69105
UN05763	Rsa1.0_00220.1	919	192936	1.00E−142	268	272	98	−	2	273	183983	184254
UN05764	Rsa1.0_00387.1	791	495407	0	399	405	98	−	3	407	146424	146826
UN05765	Rsa1.0_32744.1	951	695	9.00E−17	95	110	86	+	489	598	486	594
UN05766	Rsa1.0_00091.1	1197	139699	1.00E−173	384	404	95	+	7	408	15256	15657
UN05767	Rsa1.0_01140.1	556	52586	1.00E−94	229	243	94	+	8	250	6447	6685
UN05768	Rsa1.0_06004.1	1299	11961	1.00E−177	361	376	96	+	856	1231	5996	6371
UN05769	Rsa1.0_05878.1	1095	12344	1.00E−171	373	390	95	+	1	387	8117	8501
UN05770	Rsa1.0_01061.1	874	55349	1.00E−119	220	221	99	−	1	221	48481	48701

UN05771	Rsa1.0_00239.1	671	104332	0	407	409	99	+	4	412	97175	97583
UN05772	Rsa1.0_00417.1	1118	395068	0	927	985	94	+	9	987	386665	387638
UN05773	Rsa1.0_03263.1	406	24268	0	376	389	96	−	17	405	21178	21563
UN05774	Rsa1.0_06056.1	586	137271	1.00E−145	335	359	93	−	199	554	128419	128777
UN05775	Rsa1.0_00438.1	886	84861	0	518	550	94	+	355	886	69255	69798
UN05776	Rsa1.0_00301.1	822	365315	1.00E−102	204	209	97	−	427	635	327848	328056
UN05777	Rsa1.0_00123.1	1022	325650	1.00E−132	257	263	97	−	460	722	216442	216704
UN05778	Rsa1.0_00832.1	1309	62622	0	368	377	97	−	672	1048	42284	42660
UN05779	Rsa1.0_00246.1	1478	103541	0	1193	1250	95	−	1	1241	29514	30762
UN05780	Rsa1.0_00277.1	1046	100132	0	607	615	98	−	41	654	7609	8223
UN05781	Rsa1.0_01006.1	664	163670	1.00E−160	290	291	99	+	243	533	138108	138398
UN05782	Rsa1.0_00602.1	1044	73302	0	565	566	99	−	477	1042	28645	29210
UN05783	Rsa1.0_00201.1	1399	232851	1.00E−148	284	289	98	−	1	286	72009	72297
UN05784	Rsa1.0_03833.1	1061	20575	0	551	559	98	−	5	563	19050	19608
UN05785	Rsa1.0_01060.1	1144	55368	0	1140	1143	99	−	1	1143	25007	26148
UN05786	Rsa1.0_00441.1	1124	84607	0	631	650	97	+	7	656	31044	31687
UN05787	Rsa1.0_01285.1	938	172709	1.00E−142	259	260	99	+	677	936	158476	158735
UN05788	Rsa1.0_01801.1	755	39094	0	524	554	94	−	215	754	292	845
UN05789	Rsa1.0_00019.1	984	301669	1.00E−159	294	297	98	+	2	298	112749	113045
UN05790	Rsa1.0_00502.1	1183	204180	0	369	375	98	+	1	375	9295	9665
UN05791	Rsa1.0_02198.1	1384	33989	0	824	851	96	+	466	1316	6893	7743
UN05792	Rsa1.0_00324.1	1033	218805	1.00E−139	254	255	99	−	2	256	154604	154858
UN05793	Rsa1.0_00093.1	834	214962	0	417	418	99	+	3	420	206576	206993
UN05794	Rsa1.0_03998.1	1071	19651	4.00E−47	128	137	93	+	884	1020	12404	12539
UN05795	Rsa1.0_06133.1	1121	11663	1.00E−169	305	306	99	−	309	614	8879	9184
UN05796	Rsa1.0_00319.1	1469	167331	0	560	589	95	−	3	591	60716	61293
UN05797	Rsa1.0_03883.1	1358	20282	0	1030	1065	96	−	203	1261	9507	10562
UN05798	Rsa1.0_00446.1	846	167829	0	824	856	96	−	3	844	149433	150288
UN05799	Rsa1.0_00394.1	933	148426	1.00E−139	299	313	95	+	1	311	122830	123140
UN05800	Rsa1.0_01896.1	1428	37512	0	1049	1061	98	−	1	1059	30506	31566
UN05801	Rsa1.0_00448.1	1796	84048	0	1754	1796	97	+	1	1795	40467	42259
UN05802	Rsa1.0_02247.1	627	33427	1.00E−143	321	341	94	+	1	330	8468	8807
UN05803	Rsa1.0_04482.1	1014	17175	0	905	956	94	+	3	953	14962	15912
UN05804	Rsa1.0_06911.1	1374	10178	0	732	758	96	−	630	1374	4913	5669
UN05805	Rsa1.0_03289.1	870	24011	1.00E−172	318	322	98	−	1	322	20280	20601
UN05806	#	#	#	#	#	#	#	#	#	#	#	#
UN05807	Rsa1.0_01024.1	900	56569	0	894	900	99	+	1	900	31281	32180
UN05808	Rsa1.0_02841.1	576	27383	0	348	351	99	+	2	352	4965	5314
UN05809	Rsa1.0_00362.1	1341	299497	0	775	793	97	+	79	871	220963	221755

UN05810	Rsa1.0_02473.1	598	31083	0	596	598	99	−	1	598	8040	8637
UN05811	Rsa1.0_00679.1	2011	68971	0	499	518	96	+	1323	1840	51892	52409
UN05812	Rsa1.0_00067.1	1184	415055	0	740	769	96	−	414	1182	247368	248130
UN05813	Rsa1.0_01727.1	1014	40243	1.00E−132	304	322	94	−	1	319	7333	7652
UN05814	Rsa1.0_01471.1	855	45101	1.00E−168	367	387	94	+	1	387	18491	18874
UN05815	Rsa1.0_00570.1	1069	75002	0	580	585	99	+	1	584	34605	35189
UN05816	Rsa1.0_02174.1	849	34316	0	826	848	97	−	2	849	4385	5231
UN05817	Rsa1.0_00117.1	1244	213118	0	815	918	88	−	207	1112	211681	212592
UN05818	Rsa1.0_00816.1	843	403350	1.00E−129	259	266	97	−	17	282	391532	391796
UN05819	Rsa1.0_01635.1	998	41865	1.00E−172	307	307	100	−	692	998	38710	39016
UN05820	Rsa1.0_00589.1	1190	209965	1.00E−154	277	277	100	−	396	672	189295	189571
UN05821	Rsa1.0_02871.1	859	27085	0	464	464	100	−	2	465	6285	6748
UN05822	Rsa1.0_00058.1	668	159921	1.00E−110	212	214	99	−	456	668	83148	83361
UN05823	Rsa1.0_01895.1	939	37512	1.00E−152	357	384	92	+	558	934	10218	10601
UN05824	Rsa1.0_05457.1	784	13687	0	431	438	98	+	3	440	2411	2848
UN05825	Rsa1.0_00881.1	768	61260	0	727	770	94	−	3	760	21610	22372
UN05826	Rsa1.0_05039.1	1015	15056	0	573	573	100	−	443	1015	4260	4832
UN05827	Rsa1.0_00596.1	1816	73578	0	640	646	99	−	221	863	52467	53112
UN05828	Rsa1.0_01175.1	887	51674	0	617	637	96	+	2	638	18537	19173
UN05829	Rsa1.0_01247.1	704	49934	1.00E−171	349	364	95	+	6	369	25027	25390
UN05830	Rsa1.0_15314.1	493	2690	1.00E−111	207	208	99	+	3	210	354	561
UN05831	Rsa1.0_00157.1	588	245832	1.00E−159	291	293	99	+	296	588	51058	51350
UN05832	Rsa1.0_01595.1	766	175383	1.00E−134	283	295	95	+	7	296	38730	39024
UN05833	Rsa1.0_01405.1	953	46445	0	685	717	95	+	244	948	8905	9621
UN05834	Rsa1.0_00921.1	710	59599	0	347	349	99	+	362	710	9703	10051
UN05835	Rsa1.0_00871.1	587	61539	0	445	474	93	+	126	587	4048	4516
UN05836	Rsa1.0_00259.1	818	163204	3.00E−78	167	173	96	+	514	686	149484	149656
UN05837	Rsa1.0_01232.1	871	155185	0	413	419	98	−	453	871	108745	109163
UN05838	Rsa1.0_01414.1	1482	149508	1.00E−169	363	382	95	+	1106	1477	115743	116123
UN05839	Rsa1.0_00999.1	917	57162	0	459	461	99	+	2	462	24939	25399
UN05840	Rsa1.0_00087.1	1079	761073	0	354	360	98	−	720	1079	730624	730983
UN05841	Rsa1.0_01249.1	1939	188432	0	1134	1182	95	+	770	1938	149233	150411
UN05842	Rsa1.0_20900.1	1506	1257	0	532	548	97	+	287	834	5	552
UN05843	Rsa1.0_03565.1	1059	22062	1.00E−166	362	383	94	−	13	395	3775	4155
UN05844	Rsa1.0_13838.1	721	4236	0	489	539	90	+	8	543	1128	1640
UN05845	Rsa1.0_00081.1	1277	143261	0	353	353	100	−	925	1277	108963	109315
UN05846	Rsa1.0_03853.1	1236	20447	0	465	502	92	+	759	1236	10994	11494
UN05847	Rsa1.0_08338.1	534	7511	0	488	520	93	−	3	521	490	989
UN05848	Rsa1.0_03843.1	1088	20496	0	401	427	93	+	6	418	6097	6521

UN05849	Rsa1.0_24098.1	944	1007	0	354	355	99	−	589	943	597	951
UN05850	Rsa1.0_02662.1	732	28956	0	501	514	97	+	5	518	9090	9603
UN05851	Rsa1.0_00427.1	1066	189678	0	356	361	98	+	697	1057	110951	111311
UN05852	Rsa1.0_05604.1	934	13204	0	778	782	99	+	102	883	11682	12463
UN05853	Rsa1.0_00288.1	813	98659	0	492	519	94	+	310	811	89851	90368
UN05854	Rsa1.0_00684.1	972	141701	0	510	547	93	−	5	532	66962	67507
UN05855	Rsa1.0_00998.1	851	57168	1.00E−142	287	297	96	+	378	674	9918	10214
UN05856	Rsa1.0_01368.1	781	47129	0	404	408	99	+	54	461	25973	26380
UN05857	Rsa1.0_00630.1	1050	71281	1.00E−150	272	273	99	−	778	1050	34386	34658
UN05858	Rsa1.0_00124.1	1061	262503	1.00E−116	260	276	94	−	13	288	21650	21919
UN05859	Rsa1.0_06463.1	1192	10968	0	412	419	98	+	774	1192	8511	8928
UN05860	Rsa1.0_00019.1	1594	301669	0	1584	1596	99	+	3	1594	229403	230997
UN05861	Rsa1.0_00419.1	1283	86562	1.00E−137	271	276	98	−	1009	1283	39968	40242
UN05862	Rsa1.0_31661.1	1224	722	0	407	408	99	+	818	1224	1	408
UN05863	Rsa1.0_01943.1	934	37042	0	835	844	98	−	91	934	14217	15060
UN05864	Rsa1.0_00025.1	957	277312	1.00E−173	315	317	99	+	387	703	112270	112586
UN05865	Rsa1.0_00688.1	995	68396	2.00E−76	210	232	90	+	769	995	14822	15046
UN05866	Rsa1.0_02111.1	1337	34913	1.00E−121	237	242	97	+	9	250	833	1074
UN05867	Rsa1.0_03390.1	780	23270	1.00E−174	400	430	93	−	156	585	17528	17957
UN05868	Rsa1.0_00374.1	886	165738	0	390	392	99	+	1	392	128271	128662
UN05869	Rsa1.0_03199.1	690	24640	0	459	466	98	+	227	690	16239	16704
UN05870	Rsa1.0_06289.1	1372	11298	0	511	530	96	+	200	725	4493	5021
UN05871	Rsa1.0_01701.1	532	162121	0	487	493	98	+	4	496	1466	1956
UN05872	Rsa1.0_02120.1	1070	34819	1.00E−81	170	175	97	+	896	1070	9297	9471
UN05873	Rsa1.0_00079.1	2225	606987	0	1161	1204	96	+	1031	2224	56834	58031
UN05874	Rsa1.0_00070.1	712	150321	1.00E−156	289	292	98	−	384	675	59726	60017
UN05875	Rsa1.0_02783.1	1075	27927	0	1036	1075	96	−	2	1075	21274	22327
UN05876	Rsa1.0_13789.1	1442	4332	0	1029	1045	98	−	113	1154	3288	4332
UN05877	Rsa1.0_02508.1	1314	30675	0	545	563	96	+	3	557	20162	20724
UN05878	Rsa1.0_11525.1	1241	5479	0	836	910	91	+	76	985	4434	5343
UN05879	Rsa1.0_00348.1	1200	92818	2.00E−49	110	113	97	+	137	249	10541	10653
UN05880	Rsa1.0_06362.1	1377	11154	0	826	870	94	−	95	951	7607	8460
UN05881	Rsa1.0_00161.1	957	116891	1.00E−149	284	288	98	+	671	957	8114	8401
UN05882	Rsa1.0_00342.1	648	93695	2.00E−87	164	164	100	+	485	648	32258	32421
UN05883	Rsa1.0_04510.1	1118	17033	1.00E−145	306	321	95	+	806	1116	12403	12723
UN05884	Rsa1.0_00612.1	800	72376	0	593	607	97	+	192	797	34827	35433
UN05885	Rsa1.0_00191.1	1293	316976	0	729	818	89	+	27	844	8982	9799
UN05886	Rsa1.0_08768.1	850	6941	1.00E−158	323	334	96	−	453	786	1	326
UN05887	Rsa1.0_01295.1	1162	49040	0	540	561	96	−	100	654	8398	8958

UN05888	Rsa1.0_00417.1	1015	395068	0	477	506	94	+	510	1015	259259	259761
UN05889	Rsa1.0_00017.1	1351	752458	1.00E-160	364	387	94	-	972	1351	661836	662218
UN05890	Rsa1.0_01042.1	1033	293264	0	993	1038	95	-	3	1032	141035	142066
UN05891	Rsa1.0_09029.1	1233	6700	1.00E-114	275	293	93	-	942	1233	176	461
UN05892	Rsa1.0_02358.1	945	32213	1.00E-167	314	319	98	-	426	744	394	712
UN05893	Rsa1.0_01192.1	1429	51150	0	930	953	97	+	476	1428	7058	8003
UN05894	Rsa1.0_02840.1	1049	27386	0	745	779	95	-	3	772	26285	27061
UN05895	Rsa1.0_01159.1	825	52115	1.00E-126	241	245	98	+	313	557	32156	32400
UN05896	Rsa1.0_06072.1	1427	11811	0	516	547	94	-	178	718	11099	11641
UN05897	Rsa1.0_00364.1	1471	90951	1.00E-157	290	293	98	+	683	975	25324	25616
UN05898	Rsa1.0_03298.1	791	23956	0	395	396	99	+	396	791	16319	16714
UN05899	Rsa1.0_00136.1	730	122464	0	397	411	96	+	18	428	83043	83453
UN05900	Rsa1.0_05614.1	1163	13191	5.00E-93	174	174	100	-	23	196	6971	7144
UN05901	Rsa1.0_03982.1	1086	19723	1.00E-131	262	270	97	+	3	272	836	1105
UN05902	Rsa1.0_00092.1	1073	659932	0	643	673	95	-	417	1073	141021	141690
UN05903	Rsa1.0_00525.1	948	78030	1.00E-178	352	363	96	-	586	948	4873	5227
UN05904	Rsa1.0_03828.1	1315	20588	0	1267	1300	97	+	1	1300	14279	15572
UN05905	Rsa1.0_03731.1	894	21136	1.00E-130	242	244	99	+	648	891	1440	1683
UN05906	Rsa1.0_01581.1	506	42833	1.00E-161	346	361	95	-	114	472	27739	28095
UN05907	Rsa1.0_00157.1	1502	245832	0	1118	1170	95	-	343	1490	224410	225579
UN05908	Rsa1.0_01052.1	1192	55548	1.00E-166	318	325	97	-	268	592	45259	45583
UN05909	Rsa1.0_00967.1	673	58049	0	424	440	96	-	3	438	49368	49803
UN05910	Rsa1.0_01537.1	1413	187285	3.00E-14	141	174	81	-	247	420	17912	18085
UN05911	Rsa1.0_01296.1	1052	49035	0	656	677	96	+	377	1052	19219	19890
UN05912	Rsa1.0_00515.1	1053	78417	9.00E-76	145	145	100	+	1	145	57299	57443
UN05913	Rsa1.0_01174.1	720	51696	0	396	406	97	-	3	408	35988	36393
UN05914	Rsa1.0_00466.1	1023	82132	0	717	740	96	-	284	1023	23508	24246
UN05915	Rsa1.0_03612.1	1285	21784	0	1134	1191	95	+	109	1285	9263	10439
UN05916	Rsa1.0_01773.1	909	39546	0	874	910	96	+	1	909	17788	18693
UN05917	Rsa1.0_05842.1	1177	12461	0	1138	1179	96	-	1	1175	10198	11375
UN05918	Rsa1.0_01210.1	1126	50663	0	402	412	97	-	3	410	34618	35029
UN05919	Rsa1.0_09388.1	728	6405	2.00E-94	286	322	88	-	10	313	4675	4991
UN05920	Rsa1.0_01996.1	550	36327	1.00E-116	282	300	94	+	9	304	28087	28381
UN05921	Rsa1.0_08020.1	1023	8075	0	808	823	98	-	203	1022	1041	1863
UN05922	Rsa1.0_00276.1	431	100160	0	426	431	98	+	1	431	3828	4258
UN05923	Rsa1.0_00087.1	959	761073	0	954	960	99	-	1	959	322147	323106
UN05924	Rsa1.0_00632.1	821	183777	0	668	695	96	-	1	694	10503	11197
UN05925	Rsa1.0_01884.1	1102	37659	1.00E-108	282	309	91	+	641	949	25528	25833
UN05926	Rsa1.0_05260.1	1403	14337	0	923	935	98	-	470	1403	3508	4442

UN05927	Rsa1.0_00092.1	1006	659932	0	871	906	96	+	3	902	531020	531925
UN05928	Rsa1.0_03147.1	706	25001	0	699	706	99	+	1	706	13626	14331
UN05929	Rsa1.0_02606.1	1411	29635	0	385	387	99	−	1	387	7554	7940
UN05930	Rsa1.0_00461.1	904	82509	0	591	607	97	−	309	903	48011	48617
UN05931	Rsa1.0_00205.1	855	193401	1.00E−107	328	364	90	−	4	364	77712	78060
UN05932	Rsa1.0_00160.1	1299	346678	0	884	925	95	+	2	920	137671	138591
UN05933	Rsa1.0_00348.1	1482	92818	0	1478	1486	99	−	1	1481	32351	33836
UN05934	Rsa1.0_02544.1	1224	30290	0	339	342	99	+	1	342	28816	29157
UN05935	Rsa1.0_04892.1	839	15573	1.00E−133	253	257	98	+	449	705	10170	10426
UN05936	Rsa1.0_00015.1	1373	561803	0	468	468	100	+	1	468	373914	374381
UN05937	Rsa1.0_00447.1	1231	84051	0	1179	1241	95	−	1	1229	56648	57874
UN05938	Rsa1.0_58400.1	1022	392	0	330	330	100	−	1	330	8	337
UN05939	Rsa1.0_00625.1	1454	71672	0	643	674	95	+	1	655	12121	12794
UN05940	Rsa1.0_01294.1	1486	49090	0	791	808	97	+	365	1171	705	1512
UN05941	Rsa1.0_00083.1	849	442773	0	772	804	96	+	50	849	359544	360342
UN05942	Rsa1.0_02267.1	1213	33194	1.00E−138	265	270	98	−	768	1032	26825	27094
UN05943	Rsa1.0_00920.1	1198	601703	0	1006	1023	98	−	53	1068	296388	297410
UN05944	Rsa1.0_00546.1	881	76523	0	592	632	93	−	248	879	2622	3233
UN05945	Rsa1.0_03487.1	1074	22564	0	1070	1070	100	+	3	1072	3850	4919
UN05946	Rsa1.0_00112.1	1029	132199	0	524	525	99	+	440	964	45141	45665
UN05947	Rsa1.0_00456.1	563	82826	0	545	561	97	−	1	561	19174	19733
UN05948	Rsa1.0_09755.1	1325	6166	0	492	514	95	−	1	510	1225	1733
UN05949	Rsa1.0_02858.1	1046	27246	0	375	380	98	+	666	1044	1688	2064
UN05950	Rsa1.0_01883.1	1551	37661	1.00E−130	307	326	94	−	1221	1539	13162	13482
UN05951	Rsa1.0_06073.1	1345	11807	0	653	677	96	+	663	1339	4362	5024
UN05952	Rsa1.0_00423.1	761	86138	0	408	419	97	+	33	451	38231	38646
UN05953	Rsa1.0_05181.1	1265	14574	0	861	899	95	+	147	1035	234	1132
UN05954	Rsa1.0_00567.1	1179	75260	0	378	399	94	−	767	1148	31710	32108
UN05955	Rsa1.0_01286.1	781	49209	0	749	780	96	+	1	780	6167	6928
UN05956	Rsa1.0_00163.1	964	116847	0	843	869	97	−	3	869	28990	29855
UN05957	Rsa1.0_00472.1	1070	398134	1.00E−166	319	325	98	−	1	325	3801	4124
UN05958	Rsa1.0_01898.1	931	37491	0	465	476	97	+	10	484	1562	2036
UN05959	Rsa1.0_06485.1	1386	10929	0	359	365	98	+	579	943	2190	2554
UN05960	Rsa1.0_00549.1	357	76473	1.00E−47	141	155	90	−	5	159	34751	34897
UN05961	Rsa1.0_02961.1	997	26435	0	881	888	99	−	1	888	15306	16193
UN05962	Rsa1.0_29119.1	1244	792	0	474	488	97	−	601	1088	143	630
UN05963	Rsa1.0_01081.1	1340	499226	0	1115	1180	94	−	177	1338	18453	19620
UN05964	Rsa1.0_01857.1	807	38103	0	617	632	97	+	4	635	25188	25815
UN05965	Rsa1.0_02529.1	881	30437	8.00E−79	168	174	96	−	424	597	22816	22989

UN05966	Rsa1.0_00251.1	1708	303968	3.00E-64	126	126	100	-	143	268	102992	103117
UN05967	Rsa1.0_00286.1	696	98709	1.00E-103	190	190	100	-	303	492	12496	12685
UN05968	Rsa1.0_07396.1	852	9465	0	569	572	99	+	2	573	68	639
UN05969	Rsa1.0_02276.1	1308	33098	0	1123	1184	94	-	1	1177	12207	13388
UN05970	Rsa1.0_00069.1	919	403561	0	665	666	99	+	252	917	22792	23457
UN05971	Rsa1.0_01895.1	767	37512	0	753	771	97	+	1	765	25882	26651
UN05972	Rsa1.0_00316.1	984	96264	0	464	488	95	+	24	504	30625	31108
UN05973	Rsa1.0_02358.1	1631	32213	1.00E-168	349	370	94	+	1281	1631	12217	12586
UN05974	Rsa1.0_01389.1	1267	46716	5.00E-53	128	135	94	+	613	747	16433	16567
UN05975	Rsa1.0_03728.1	1366	21147	1.00E-174	403	433	93	+	1	427	667	1092
UN05976	Rsa1.0_00504.1	683	79334	0	638	650	98	-	7	655	62846	63494
UN05977	Rsa1.0_03520.1	753	22327	1.00E-106	275	299	91	+	455	752	10242	10539
UN05978	Rsa1.0_00190.1	577	318240	2.00E-87	177	180	98	+	43	222	280521	280699
UN05979	Rsa1.0_03588.1	837	21908	1.00E-161	335	350	95	-	489	835	3145	3494
UN05980	Rsa1.0_00179.1	1149	393324	1.00E-123	256	265	96	-	1	265	218140	218403
UN05981	Rsa1.0_03711.1	799	21237	0	464	470	98	-	1	466	20005	20473
UN05982	Rsa1.0_02257.1	837	33318	0	322	322	100	-	346	667	7615	7936
UN05983	Rsa1.0_02855.1	809	27271	0	375	383	97	-	333	715	10359	10741
UN05984	Rsa1.0_00088.1	769	141341	1.00E-124	274	288	95	+	76	359	19012	19299
UN05985	Rsa1.0_01112.1	1482	310601	0	344	348	98	+	1135	1482	176919	177266
UN05986	Rsa1.0_00741.1	1056	66117	0	1021	1079	94	-	1	1054	33376	34444
UN05987	Rsa1.0_56800.1	523	403	0	403	403	100	-	10	412	1	403
UN05988	Rsa1.0_00899.1	1445	60404	0	1437	1441	99	-	5	1444	17545	18984
UN05989	Rsa1.0_00578.1	1227	74581	0	357	357	100	+	871	1227	18938	19294
UN05990	Rsa1.0_02515.1	1299	30616	1.00E-138	249	249	100	+	1049	1297	3055	3303
UN05991	Rsa1.0_02404.1	1077	31702	0	1040	1086	95	-	1	1075	12816	13901
UN05992	Rsa1.0_03567.1	2251	22049	0	401	416	96	-	40	455	21196	21602
UN05993	Rsa1.0_42819.1	866	525	0	451	456	98	+	1	456	70	525
UN05994	Rsa1.0_00301.1	1104	365315	0	1098	1101	99	+	3	1103	85605	86705
UN05995	Rsa1.0_00604.1	730	146282	0	423	436	97	-	2	434	16045	16478
UN05996	Rsa1.0_00944.1	1076	58733	0	379	381	99	-	1	381	54332	54712
UN05997	Rsa1.0_00471.1	1514	81897	0	737	746	98	+	3	744	34504	35249
UN05998	Rsa1.0_00633.1	1514	71250	0	497	519	95	-	1	519	24438	24938
UN05999	Rsa1.0_00743.1	823	177459	0	785	838	93	-	2	823	6117	6942
UN06000	Rsa1.0_18177.1	834	1651	1.00E-126	266	276	96	-	562	834	744	1019
UN06001	Rsa1.0_01090.1	1283	54385	0	558	596	93	-	2	583	45269	45856
UN06002	Rsa1.0_13438.1	806	4828	0	657	678	96	-	110	784	4127	4802
UN06003	Rsa1.0_38359.1	1126	587	0	393	397	98	+	193	589	1	397
UN06004	Rsa1.0_04018.1	914	19551	0	449	454	98	-	423	875	2468	2921

UN06005	Rsa1.0_06008.1	779	11950	1.00E-117	218	219	99	−	538	756	1917	2135
UN06006	Rsa1.0_00098.1	1086	349239	0	636	651	97	−	3	653	344507	345157
UN06007	Rsa1.0_00938.1	1104	193891	0	596	596	100	−	1	596	188142	188737
UN06008	Rsa1.0_01046.1	1211	152989	0	470	471	99	+	476	946	142322	142791
UN06009	Rsa1.0_00767.1	828	64980	1.00E-92	176	177	99	+	1	177	12733	12909
UN06010	Rsa1.0_01402.1	1199	46509	0	950	1033	91	−	2	1034	18403	19435
UN06011	Rsa1.0_01249.1	1380	188432	0	404	415	97	−	3	416	174715	175126
UN06012	Rsa1.0_01904.1	549	37450	0	540	543	99	+	2	543	1918	2460
UN06013	Rsa1.0_07446.1	1198	9384	0	1027	1056	97	−	3	1056	1226	2273
UN06014	Rsa1.0_02003.1	1053	36171	0	719	752	95	−	140	891	19755	20506
UN06015	Rsa1.0_00017.1	1398	752458	0	560	584	95	+	815	1398	416373	416956
UN06016	Rsa1.0_01958.1	808	261128	1.00E-129	304	330	92	−	3	326	1802	2119
UN06017	Rsa1.0_02027.1	900	35869	0	784	784	100	+	1	784	27289	28072
UN06018	Rsa1.0_07304.1	937	9579	0	791	813	97	−	4	815	7455	8264
UN06019	Rsa1.0_03292.1	768	23977	0	743	768	96	−	2	768	13615	14374
UN06020	Rsa1.0_00250.1	705	273771	0	535	535	100	−	170	704	223434	223968
UN06021	Rsa1.0_00065.1	1247	454616	0	1215	1244	97	+	7	1246	414610	415847
UN06022	Rsa1.0_00350.1	1236	92346	0	406	425	95	−	2	421	51776	52197
UN06023	Rsa1.0_00552.1	862	206757	0	398	418	95	+	3	417	195140	195549
UN06024	Rsa1.0_00287.1	1016	98687	1.00E-175	513	574	89	−	398	964	29725	30293
UN06025	Rsa1.0_00689.1	851	68305	1.00E-180	347	356	97	+	395	750	42469	42824
UN06026	Rsa1.0_00154.1	938	189461	1.00E-115	252	262	96	+	3	262	20401	20660
UN06027	Rsa1.0_00067.1	1335	415055	0	474	474	100	+	862	1335	250853	251326
UN06028	Rsa1.0_02860.1	1035	27207	1.00E-169	305	306	99	+	2	307	24990	25295
UN06029	Rsa1.0_00351.1	1599	413096	0	459	473	97	+	412	884	393233	393705
UN06030	Rsa1.0_07861.1	740	8406	0	472	487	96	−	255	740	4280	4758
UN06031	Rsa1.0_02430.1	879	31506	0	559	598	93	+	92	685	12980	13576
UN06032	Rsa1.0_00387.1	942	495407	1.00E-100	204	210	97	−	600	809	337148	337357
UN06033	Rsa1.0_00278.1	1385	231896	1.00E-124	288	303	95	+	3	298	152569	152870
UN06034	Rsa1.0_00584.1	615	74295	0	590	593	99	+	22	613	31159	31751
UN06035	Rsa1.0_20534.1	1508	1295	1.00E-112	215	218	98	+	951	1168	813	1030
UN06036	Rsa1.0_05878.1	1110	12344	1.00E-156	337	350	96	−	759	1108	8159	8501
UN06037	Rsa1.0_01335.1	965	47894	1.00E-133	244	245	99	+	309	553	423	667
UN06038	Rsa1.0_14042.1	933	3881	0	901	910	99	−	25	933	2972	3881
UN06039	Rsa1.0_00044.1	1232	271658	0	752	803	93	+	452	1229	164316	165117
UN06040	Rsa1.0_00414.1	770	202190	1.00E-113	238	247	96	−	180	426	79780	80020
UN06041	Rsa1.0_00911.1	1307	59943	0	548	574	95	+	578	1150	51133	51703
UN06042	Rsa1.0_07831.1	784	8461	0	514	518	99	+	267	784	5029	5546
UN06043	Rsa1.0_00112.1	1351	132199	0	539	539	100	+	773	1311	84794	85332

UN06044	Rsa1.0_14881.1	1178	2995	1.00E-120	420	481	87	-	15	490	2323	2786
UN06045	Rsa1.0_03345.1	1285	23590	1.00E-108	291	326	89	-	5	322	21924	22228
UN06046	Rsa1.0_00073.1	1612	240924	1.00E-161	336	350	96	-	3	349	183586	183929
UN06047	Rsa1.0_19964.1	1724	1359	0	440	447	98	+	194	638	371	817
UN06048	Rsa1.0_00092.1	1297	659932	1.00E-138	303	317	95	-	1	317	626655	626968
UN06049	Rsa1.0_01651.1	1039	41586	0	1005	1034	97	-	6	1039	31227	32257
UN06050	Rsa1.0_01422.1	848	46172	1.00E-107	210	214	98	-	1	214	36174	36387
UN06051	Rsa1.0_01581.1	1046	42833	0	1016	1031	98	+	16	1046	5053	6082
UN06052	Rsa1.0_20335.1	922	1316	0	526	528	99	-	395	922	671	1198
UN06053	Rsa1.0_06542.1	851	10805	0	412	413	99	+	439	851	8536	8948
UN06054	Rsa1.0_00409.1	966	146788	1.00E-115	214	215	99	+	525	739	95483	95697
UN06055	Rsa1.0_01444.1	503	169539	0	496	501	99	+	3	503	12619	13119
UN06056	Rsa1.0_40774.1	1325	550	1.00E-167	356	369	96	-	924	1286	182	550
UN06057	Rsa1.0_00722.1	1008	66840	1.00E-179	327	330	99	-	293	622	50627	50956
UN06058	Rsa1.0_00480.1	709	81325	1.00E-154	276	276	100	-	282	557	53555	53830
UN06059	Rsa1.0_05630.1	799	13129	0	751	763	98	-	37	799	4058	4820
UN06060	Rsa1.0_01021.1	1838	56610	0	1673	1731	96	-	114	1838	38654	40384
UN06061	Rsa1.0_05153.1	860	14663	1.00E-129	249	253	98	+	611	860	2743	2995
UN06062	Rsa1.0_00302.1	587	97521	1.00E-143	276	282	97	-	306	587	82334	82615
UN06063	Rsa1.0_04032.1	1245	19489	0	476	495	96	-	751	1245	6286	6776
UN06064	Rsa1.0_00014.1	1285	348985	0	640	644	99	+	644	1283	164648	165291
UN06065	Rsa1.0_00128.1	911	228675	0	870	924	94	-	1	911	68778	69684
UN06066	Rsa1.0_00149.1	1509	305196	0	389	397	97	-	1114	1509	26603	26998
UN06067	Rsa1.0_00758.1	975	65477	0	342	345	99	-	628	972	33817	34160
UN06068	Rsa1.0_01903.1	2190	37451	0	842	852	98	+	1338	2189	34532	35383
UN06069	Rsa1.0_01192.1	1529	51150	0	396	414	95	+	1061	1473	23563	23976
UN06070	Rsa1.0_00038.1	1472	314568	1.00E-141	258	259	99	+	576	834	166217	166475
UN06071	Rsa1.0_01761.1	1161	39717	4.00E-35	122	137	89	-	905	1041	1436	1572
UN06072	Rsa1.0_08002.1	851	8094	0	701	735	95	-	2	724	4197	4931
UN06073	Rsa1.0_01850.1	1214	162224	0	457	467	97	+	1	467	10270	10736
UN06074	Rsa1.0_01739.1	1547	40068	1.00E-112	210	211	99	+	263	473	9878	10088
UN06075	Rsa1.0_03581.1	1299	21965	1.00E-165	298	299	99	+	3	301	14211	14509
UN06076	Rsa1.0_00817.1	677	62961	1.00E-152	288	293	98	-	385	677	37013	37305
UN06077	Rsa1.0_00152.1	687	257730	1.00E-98	233	249	93	+	32	280	200278	200523
UN06078	Rsa1.0_01029.1	1067	256254	0	368	375	98	+	3	377	241022	241396
UN06079	Rsa1.0_00519.1	1712	197864	0	526	529	99	+	358	885	53017	53545
UN06080	Rsa1.0_01234.1	1474	50183	1.00E-171	308	309	99	-	1166	1474	25998	26306
UN06081	Rsa1.0_00608.1	1446	172672	0	774	786	98	-	627	1404	163174	163958
UN06082	Rsa1.0_00469.1	615	82084	0	613	613	100	+	3	615	56540	57152

UN06083	Rsa1.0_00089.1	1219	406142	0	1032	1063	97	-	159	1219	299835	300895
UN06084	Rsa1.0_00289.1	797	98620	1.00E-150	286	290	98	+	508	797	97044	97332
UN06085	Rsa1.0_00382.1	791	251946	0	731	761	96	-	3	762	63466	64225
UN06086	Rsa1.0_01206.1	1504	50772	0	779	826	94	-	106	902	27195	28020
UN06087	Rsa1.0_02955.1	2188	26484	0	1229	1254	98	+	249	1500	16651	17899
UN06088	Rsa1.0_08762.1	952	6947	1.00E-142	280	288	97	+	1	288	2440	2727
UN06089	Rsa1.0_03671.1	1146	21459	0	387	387	100	-	2	388	9838	10224
UN06090	Rsa1.0_00136.1	626	122464	1.00E-101	247	264	93	+	20	282	110531	110793
UN06091	Rsa1.0_01042.1	1006	293264	0	927	958	96	-	50	1006	137012	137961
UN06092	Rsa1.0_03572.1	1108	146346	0	873	886	98	-	3	888	141058	141943
UN06093	Rsa1.0_00476.1	1522	280966	0	626	634	98	-	3	636	214536	215169
UN06094	Rsa1.0_00100.1	650	249232	4.00E-98	256	279	91	-	375	643	21279	21557
UN06095	Rsa1.0_01990.1	775	36431	0	490	490	100	+	286	775	3552	4041
UN06096	Rsa1.0_01070.1	816	55099	1.00E-110	203	203	100	-	612	814	31368	31570
UN06097	Rsa1.0_03298.1	924	23956	0	466	490	95	+	435	924	20879	21362
UN06098	Rsa1.0_02922.1	711	26753	1.00E-132	274	285	96	+	1	285	8804	9084
UN06099	Rsa1.0_03241.1	1226	24423	1.00E-141	272	277	98	+	3	279	3186	3459
UN06100	Rsa1.0_08933.1	656	6772	1.00E-152	273	273	100	-	382	654	5565	5837
UN06101	Rsa1.0_00165.1	685	116466	0	523	554	94	+	1	548	93786	94339
UN06102	Rsa1.0_01588.1	1117	42724	5.00E-96	196	199	98	-	62	260	33164	33360
UN06103	Rsa1.0_01116.1	1158	142088	0	380	394	96	-	763	1154	119063	119455
UN06104	Rsa1.0_00087.1	1482	761073	0	1069	1095	97	+	1	1093	214392	215486
UN06105	Rsa1.0_00492.1	927	80178	0	774	796	97	-	132	927	63645	64433
UN06106	Rsa1.0_00281.1	1474	99908	0	1001	1113	89	+	350	1448	96671	97757
UN06107	Rsa1.0_00043.1	951	264937	1.00E-176	317	318	99	-	634	951	142234	142551
UN06108	Rsa1.0_00734.1	1785	66508	1.00E-169	336	346	97	+	1440	1785	6513	6857
UN06109	Rsa1.0_01411.1	437	46326	0	431	442	97	+	3	437	29720	30161
UN06110	Rsa1.0_01081.1	821	499226	1.00E-167	304	306	99	+	516	821	473293	473598
UN06111	Rsa1.0_00894.1	1396	269700	0	842	854	98	+	542	1395	124967	125816
UN06112	Rsa1.0_09661.1	883	6226	1.00E-134	243	243	100	-	635	877	761	1003
UN06113	Rsa1.0_00012.1	1164	222148	0	429	429	100	-	736	1164	12400	12828
UN06114	Rsa1.0_00123.1	1149	325650	0	590	613	96	+	3	603	246068	246680
UN06115	Rsa1.0_20598.1	1540	1289	0	486	496	97	-	3	498	1	490
UN06116	Rsa1.0_01535.1	985	43750	0	530	537	98	+	446	981	23633	24169
UN06117	Rsa1.0_05633.1	1128	13118	0	703	712	98	-	418	1128	4525	5236
UN06118	Rsa1.0_01139.1	1095	52601	0	407	410	99	-	3	410	1259	1668
UN06119	Rsa1.0_00071.1	767	345714	1.00E-129	235	235	100	+	458	692	19342	19576
UN06120	Rsa1.0_02362.1	774	32190	0	355	360	98	-	408	765	24786	25145
UN06121	Rsa1.0_02041.1	1238	163634	0	750	796	94	+	216	1011	30110	30889

UN06122	Rsa1.0_07660.1	1424	8891	0	827	833	99	+	594	1424	7634	8466
UN06123	Rsa1.0_01234.1	1255	50183	0	883	889	99	+	1	889	19600	20488
UN06124	Rsa1.0_24918.1	1457	961	0	641	672	95	−	790	1457	290	961
UN06125	Rsa1.0_00026.1	976	327835	2.00E−82	159	160	99	−	117	276	5346	5505
UN06126	Rsa1.0_02919.1	1160	26780	0	377	384	98	+	774	1155	7688	8071
UN06127	Rsa1.0_00067.1	992	415055	0	759	822	92	−	163	953	175393	176214
UN06128	Rsa1.0_00030.1	755	250988	1.00E−131	240	241	99	+	509	749	156217	156457
UN06129	Rsa1.0_00016.1	959	831256	1.00E−83	167	170	98	−	785	954	436547	436716
UN06130	Rsa1.0_01435.1	751	45942	3.00E−78	203	221	91	−	127	347	44559	44779
UN06131	Rsa1.0_01179.1	927	51549	0	807	862	93	+	77	927	17298	18151
UN06132	Rsa1.0_02003.1	697	36171	0	692	695	99	+	1	695	20698	21391
UN06133	Rsa1.0_00162.1	1119	239935	1.00E−114	251	262	95	−	842	1100	23302	23559
UN06134	Rsa1.0_04192.1	1058	18572	0	1051	1057	99	+	1	1056	12192	13248
UN06135	Rsa1.0_14434.1	1161	3394	8.00E−58	184	206	89	−	782	985	2574	2779
UN06136	Rsa1.0_07837.1	830	8449	0	419	433	96	+	1	433	6439	6870
UN06137	Rsa1.0_00277.1	1478	100132	0	1304	1305	99	+	1	1305	16011	17315
UN06138	Rsa1.0_02396.1	744	31783	0	533	561	95	−	183	742	18385	18945
UN06139	Rsa1.0_01502.1	677	44519	0	362	368	98	+	1	368	8033	8397
UN06140	Rsa1.0_63151.1	842	363	0	322	322	100	+	519	840	8	329
UN06141	Rsa1.0_00069.1	957	403561	1.00E−152	288	293	98	+	601	893	179274	179566
UN06142	Rsa1.0_00284.1	785	323267	0	760	801	94	+	3	785	261441	262240
UN06143	Rsa1.0_16332.1	824	2195	0	819	822	99	−	1	822	568	1387
UN06144	Rsa1.0_00206.1	1358	109040	0	632	633	99	−	724	1356	95667	96299
UN06145	Rsa1.0_00048.1	1115	167185	1.00E−154	285	288	98	+	1	288	4871	5158
UN06146	Rsa1.0_10067.1	410	6015	1.00E−139	254	255	99	+	2	256	707	961
UN06147	Rsa1.0_02987.1	1482	26196	0	628	645	97	−	21	665	21354	21998
UN06148	Rsa1.0_01581.1	931	42833	0	396	400	99	−	531	929	32418	32815
UN06149	Rsa1.0_06892.1	1223	10197	0	744	754	98	+	468	1221	1	746
UN06150	Rsa1.0_00050.1	807	237174	0	573	573	100	+	1	573	28502	29074
UN06151	Rsa1.0_00382.1	642	251946	1.00E−171	307	308	99	+	1	308	34541	34848
UN06152	Rsa1.0_05614.1	819	13191	0	804	823	97	−	3	819	2077	2898
UN06153	Rsa1.0_00190.1	896	318240	0	476	501	95	+	3	499	314286	314786
UN06154	Rsa1.0_02097.1	983	35036	0	375	376	99	−	607	982	4257	4632
UN06155	Rsa1.0_01565.1	800	155447	0	795	798	99	+	3	800	146476	147272
UN06156	Rsa1.0_00339.1	1301	222974	0	618	627	98	+	589	1215	74938	75564
UN06157	Rsa1.0_37661.1	1294	598	0	585	598	97	−	455	1050	1	598
UN06158	Rsa1.0_01077.1	905	54772	1.00E−141	299	311	96	+	1	310	10739	11048
UN06159	Rsa1.0_01590.1	1241	42719	0	633	636	99	−	2	637	26643	27278
UN06160	Rsa1.0_00637.1	1326	71038	7.00E−80	357	421	84	+	885	1303	17759	18174

UN06161	Rsa1.0_00277.1	1374	100132	1.00E-174	372	391	95	-	3	392	76194	76584
UN06162	Rsa1.0_00125.1	1244	253043	0	635	646	98	+	131	775	232140	232785
UN06163	Rsa1.0_03843.1	1074	20496	1.00E-102	293	327	89	-	35	361	4472	4790
UN06164	Rsa1.0_00058.1	907	159921	0	902	906	99	+	1	906	81117	82022
UN06165	Rsa1.0_00127.1	864	127446	0	417	423	98	-	442	864	105773	106195
UN06166	Rsa1.0_03290.1	982	24011	1.00E-147	276	280	98	+	703	982	4892	5171
UN06167	Rsa1.0_03167.1	926	24858	0	402	402	100	-	1	402	5682	6083
UN06168	Rsa1.0_13724.1	816	4420	0	776	828	93	+	2	816	992	1814
UN06169	Rsa1.0_01835.1	562	38542	1.00E-169	360	375	96	-	1	372	20857	21228
UN06170	Rsa1.0_00048.1	890	167185	1.00E-149	328	347	94	+	1	347	7215	7559
UN06171	Rsa1.0_00697.1	514	67913	7.00E-75	143	143	100	+	247	389	43648	43790
UN06172	Rsa1.0_02309.1	1215	32744	0	715	719	99	+	2	720	18637	19353
UN06173	Rsa1.0_23904.1	1453	1018	0	802	812	98	-	1	812	53	861
UN06174	Rsa1.0_00802.1	1255	63846	0	1237	1254	98	-	1	1254	24183	25436
UN06175	Rsa1.0_03560.1	1417	22092	0	463	467	99	+	949	1415	11897	12363
UN06176	Rsa1.0_00154.1	1033	189461	1.00E-114	228	232	98	+	804	1033	169282	169512
UN06177	Rsa1.0_00341.1	850	249309	0	464	470	98	+	385	850	178139	178608
UN06178	Rsa1.0_06671.1	1235	10563	1.00E-171	311	313	99	-	3	315	6199	6511
UN06179	Rsa1.0_00616.1	660	195713	0	449	472	95	-	1	467	181777	182245
UN06180	Rsa1.0_01119.1	1340	53459	0	353	355	99	+	1	355	43241	43594
UN06181	Rsa1.0_00531.1	1809	77387	0	684	708	96	+	1105	1805	11646	12353
UN06182	Rsa1.0_07882.1	770	8363	1.00E-178	424	458	92	+	1	457	7179	7618
UN06183	Rsa1.0_02116.1	1097	34853	0	380	380	100	+	1	380	30559	30938
UN06184	Rsa1.0_05367.1	892	14010	0	359	359	100	-	531	889	864	1222
UN06185	Rsa1.0_75173.1	728	305	4.00E-34	75	75	100	-	652	726	231	305
UN06186	Rsa1.0_02599.1	1202	29660	1.00E-152	277	278	99	-	622	899	6935	7212
UN06187	Rsa1.0_02439.1	1425	31379	0	1407	1421	99	-	3	1423	8711	10131
UN06188	Rsa1.0_03172.1	1330	24816	0	452	453	99	+	877	1329	16988	17440
UN06189	Rsa1.0_00159.1	498	431686	0	480	486	98	-	12	497	228703	229188
UN06190	Rsa1.0_01487.1	1302	44784	1.00E-158	340	355	95	-	1	351	2584	2934
UN06191	Rsa1.0_00906.1	1411	178966	0	540	550	98	-	1	547	131974	132523
UN06192	Rsa1.0_02777.1	738	27986	0	391	408	95	-	5	411	18410	18817
UN06193	Rsa1.0_02146.1	1430	34625	1.00E-101	259	283	91	-	1156	1428	10460	10736
UN06194	Rsa1.0_01409.1	1047	151595	1.00E-162	339	354	95	-	382	734	133502	133855
UN06195	Rsa1.0_02269.1	762	33168	1.00E-176	325	328	99	-	1	326	16204	16531
UN06196	Rsa1.0_01539.1	1412	43698	0	669	678	98	-	2	677	4616	5293
UN06197	Rsa1.0_00087.1	789	761073	1.00E-161	321	330	97	-	1	330	190659	190984
UN06198	Rsa1.0_00092.1	996	659932	0	366	366	100	-	1	366	192222	192587
UN06199	Rsa1.0_00087.1	1015	761073	0	387	388	99	-	626	1013	621049	621436

UN06200	Rsa1.0_05870.1	1263	146055	0	593	608	97	-	5	611	6380	6985
UN06201	Rsa1.0_00016.1	1297	831256	0	943	963	97	+	3	960	375561	376517
UN06202	Rsa1.0_00077.1	769	145005	1.00E-169	307	309	99	-	3	311	71685	71993
UN06203	Rsa1.0_11811.1	909	5401	0	889	899	98	+	2	899	3772	4668
UN06204	Rsa1.0_04254.1	969	18258	0	675	698	96	-	275	969	13998	14695
UN06205	Rsa1.0_30865.1	892	742	1.00E-151	272	272	100	-	2	273	1	272
UN06206	Rsa1.0_00601.1	947	177045	0	694	712	97	-	2	709	168330	169041
UN06207	Rsa1.0_02435.1	1062	31454	0	1015	1062	95	-	1	1062	27541	28602
UN06208	Rsa1.0_05842.1	1174	12461	0	1100	1131	97	-	44	1172	10200	11328
UN06209	Rsa1.0_00542.1	1079	76831	0	478	495	96	-	17	511	35582	36076
UN06210	Rsa1.0_04102.1	995	19048	0	364	366	99	+	1	366	18258	18623
UN06211	Rsa1.0_02991.1	1264	26178	0	811	812	99	-	1	812	12893	13704
UN06212	Rsa1.0_01674.1	1005	168300	0	640	660	96	-	21	678	19673	20330
UN06213	Rsa1.0_01137.1	630	52655	1.00E-123	230	232	99	-	256	487	51348	51579
UN06214	Rsa1.0_02319.1	566	32580	1.00E-145	261	261	100	+	1	261	6481	6741
UN06215	Rsa1.0_02142.1	1331	34633	0	352	361	97	+	914	1274	27753	28110
UN06216	Rsa1.0_01857.1	632	38103	0	411	434	94	-	25	448	4693	5124
UN06217	Rsa1.0_01591.1	1129	42718	0	893	896	99	-	1	896	25166	26061
UN06218	Rsa1.0_00577.1	1198	174827	1.00E-114	213	214	99	+	716	929	137715	137928
UN06219	Rsa1.0_14881.1	1093	2995	0	457	466	98	-	1	465	2323	2787
UN06220	Rsa1.0_01646.1	1009	41735	1.00E-101	229	243	94	-	1	243	16887	17129
UN06221	Rsa1.0_00040.1	709	324196	0	425	430	98	+	94	523	51764	52192
UN06222	Rsa1.0_12205.1	598	5289	1.00E-149	302	311	97	+	2	312	331	634
UN06223	Rsa1.0_01386.1	855	46765	0	474	486	97	-	368	853	19943	20427
UN06224	Rsa1.0_00964.1	1351	316648	0	847	853	99	-	500	1349	311189	312041
UN06225	Rsa1.0_02212.1	689	33840	1.00E-144	263	264	99	-	3	266	30764	31027
UN06226	Rsa1.0_01513.1	1044	44234	0	895	904	99	+	141	1044	41225	42128
UN06227	Rsa1.0_00104.1	976	368722	3.00E-78	223	245	91	-	189	431	201374	201618
UN06228	Rsa1.0_00323.1	1069	159650	0	582	588	98	-	1	588	114114	114701
UN06229	Rsa1.0_00897.1	1135	60498	0	560	574	97	-	1	574	38106	38677
UN06230	Rsa1.0_00215.1	1489	201775	0	495	522	94	-	1	519	50928	51434
UN06231	Rsa1.0_01667.1	1198	41315	1.00E-35	105	114	92	-	336	449	1948	2061
UN06232	Rsa1.0_00816.1	1318	403350	1.00E-148	300	310	96	+	20	328	398603	398912
UN06233	Rsa1.0_00045.1	791	328694	0	493	498	98	+	1	498	131720	132215
UN06234	Rsa1.0_06488.1	816	141774	0	607	662	91	-	69	720	5997	6651
UN06235	Rsa1.0_00504.1	1353	79334	0	765	783	97	-	3	781	24076	24857
UN06236	Rsa1.0_01520.1	1002	44087	0	946	958	98	+	5	960	39576	40532
UN06237	Rsa1.0_02394.1	925	31789	3.00E-32	84	88	95	+	290	377	5746	5833
UN06238	Rsa1.0_02565.1	1150	30046	1.00E-132	314	341	92	+	1	337	25157	25485

UN06239	Rsa1.0_00009.1	1031	232558	0	958	996	96	−	24	1016	138126	139110
UN06240	Rsa1.0_00237.1	1347	235974	1.00E−166	296	296	100	−	3	298	192337	192632
UN06241	Rsa1.0_00151.1	1318	119541	0	645	658	98	−	1	658	13346	14003
UN06242	Rsa1.0_04646.1	814	16541	0	399	415	96	+	106	520	443	857
UN06243	Rsa1.0_04123.1	721	18947	0	687	721	95	+	3	721	11838	12545
UN06244	Rsa1.0_00570.1	1395	75002	0	728	728	100	−	3	730	31697	32424
UN06245	Rsa1.0_01898.1	956	37491	0	460	474	97	+	483	956	6437	6910
UN06246	Rsa1.0_04076.1	1404	19209	1.00E−179	321	322	99	+	2	323	8158	8479
UN06247	Rsa1.0_16298.1	1315	2209	0	551	562	98	−	656	1217	473	1034
UN06248	Rsa1.0_03044.1	928	25791	0	468	476	98	−	453	927	5744	6219
UN06249	Rsa1.0_01403.1	783	46453	0	407	407	100	−	1	407	3064	3470
UN06250	Rsa1.0_05867.1	1055	12375	0	1048	1065	98	+	1	1052	10454	11518
UN06251	Rsa1.0_00362.1	521	299497	0	512	519	98	−	3	521	9630	10145
UN06252	Rsa1.0_00201.1	631	232851	0	390	396	98	−	237	631	72600	72994
UN06253	Rsa1.0_00906.1	785	178966	1.00E−163	309	314	98	+	1	312	135566	135879
UN06254	Rsa1.0_02889.1	764	27019	0	599	605	99	−	160	764	8081	8685
UN06255	Rsa1.0_67781.1	1248	338	3.00E−94	176	176	100	−	316	491	160	335
UN06256	Rsa1.0_01441.1	1198	45790	0	339	341	99	−	858	1198	26449	26789
UN06257	Rsa1.0_00632.1	895	183777	1.00E−177	315	315	100	−	4	318	152593	152907
UN06258	Rsa1.0_02779.1	897	27968	0	614	616	99	−	282	897	10303	10918
UN06259	Rsa1.0_16628.1	813	2085	0	752	760	98	−	1	760	1322	2081
UN06260	Rsa1.0_00476.1	999	280966	0	357	358	99	−	641	998	236866	237223
UN06261	Rsa1.0_10516.1	418	5818	1.00E−109	217	221	98	−	110	329	4536	4756
UN06262	Rsa1.0_04528.1	1278	16958	1.00E−168	300	300	100	+	454	753	14192	14491
UN06263	Rsa1.0_00387.1	365	495407	0	346	353	98	−	12	364	389014	389366
UN06264	Rsa1.0_00077.1	1511	145005	0	1462	1498	97	−	2	1497	19276	20768
UN06265	Rsa1.0_05594.1	1177	13229	0	1167	1175	99	−	2	1176	4045	5219
UN06266	Rsa1.0_02306.1	910	32776	0	822	827	99	−	85	910	21208	22034
UN06267	Rsa1.0_01126.1	811	53127	1.00E−178	332	336	98	+	443	777	50776	51111
UN06268	Rsa1.0_00347.1	520	93048	0	517	521	99	−	3	520	66841	67361
UN06269	Rsa1.0_00067.1	1096	415055	0	740	758	97	+	3	748	247373	248130
UN06270	Rsa1.0_00453.1	1323	83031	1.00E−158	284	284	100	+	3	286	79184	79467
UN06271	Rsa1.0_05023.1	582	15105	0	580	580	100	+	3	582	8579	9158
UN06272	Rsa1.0_03435.1	659	22958	1.00E−143	284	293	96	+	274	566	21985	22277
UN06273	Rsa1.0_00378.1	1161	89892	0	419	420	99	+	742	1161	81481	81900
UN06274	Rsa1.0_00748.1	891	65983	1.00E−125	263	276	95	+	1	267	14299	14574
UN06275	Rsa1.0_01276.1	648	49384	0	645	648	99	−	1	648	24952	25599
UN06276	Rsa1.0_05221.1	947	14452	1.00E−77	148	148	100	−	1	148	8530	8677
UN06277	Rsa1.0_02753.1	613	28215	0	580	620	93	+	1	611	8121	8728

UN06278	Rsa1.0_00425.1	1735	147080	0	701	722	97	+	615	1327	106336	107057
UN06279	Rsa1.0_00454.1	1960	216970	0	508	519	97	+	1444	1956	10708	11226
UN06280	Rsa1.0_00154.1	694	189461	0	501	525	95	+	180	694	171027	171546
UN06281	Rsa1.0_00044.1	1371	271658	0	444	452	98	+	54	505	44363	44814
UN06282	Rsa1.0_00479.1	1363	81377	0	1002	1021	98	−	1	1021	80363	81371
UN06283	Rsa1.0_02421.1	1372	31579	0	517	535	96	−	806	1340	8466	9000
UN06284	Rsa1.0_07474.1	1332	9294	0	1219	1224	99	+	111	1331	4914	6137
UN06285	Rsa1.0_02928.1	776	26680	1.00E−180	360	371	97	−	391	761	17090	17458
UN06286	Rsa1.0_00678.1	714	193163	0	377	383	98	+	334	714	164828	165210
UN06287	Rsa1.0_01092.1	815	54348	2.00E−69	137	138	99	+	402	539	51202	51339
UN06288	Rsa1.0_41382.1	1059	543	0	366	369	99	+	209	577	1	369
UN06289	Rsa1.0_00304.1	1176	97429	1.00E−118	232	237	97	−	426	662	25354	25590
UN06290	Rsa1.0_21578.1	1458	1190	1.00E−173	317	319	99	−	1142	1458	526	844
UN06291	Rsa1.0_00440.1	883	84723	1.00E−164	325	335	97	−	548	882	25067	25398
UN06292	Rsa1.0_00549.1	1257	76473	0	804	823	97	−	2	815	42366	43188
UN06293	Rsa1.0_02077.1	794	35328	1.00E−111	219	224	97	+	367	590	19971	20194
UN06294	Rsa1.0_01171.1	1108	51752	1.00E−110	210	211	99	+	3	212	51542	51752
UN06295	Rsa1.0_00742.1	846	66106	0	328	328	100	−	519	846	16522	16849
UN06296	Rsa1.0_01567.1	970	43090	0	965	968	99	−	3	970	25446	26413
UN06297	Rsa1.0_00921.1	1019	59599	0	1014	1021	99	+	3	1019	29788	30808
UN06298	Rsa1.0_00168.1	563	115870	0	427	450	94	−	115	563	90769	91213
UN06299	Rsa1.0_00425.1	1575	147080	0	728	728	100	+	2	729	110296	111023
UN06300	Rsa1.0_00655.1	742	195840	0	354	361	98	−	11	371	168054	168412
UN06301	Rsa1.0_00369.1	499	266462	0	381	381	100	+	119	499	136393	136773
UN06302	Rsa1.0_00007.1	939	239325	1.00E−123	261	272	95	+	585	854	13415	13686
UN06303	Rsa1.0_10792.1	827	5719	1.00E−166	330	340	97	−	487	826	1327	1664
UN06304	Rsa1.0_01940.1	754	37073	1.00E−159	324	334	97	+	420	752	9146	9475
UN06305	Rsa1.0_00522.1	1145	78103	1.00E−101	200	204	98	+	307	510	35226	35429
UN06306	Rsa1.0_00616.1	1134	195713	0	471	472	99	−	663	1134	183059	183530
UN06307	Rsa1.0_05526.1	954	13431	0	697	706	98	+	230	935	5038	5741
UN06308	Rsa1.0_03728.1	1409	21147	0	405	408	99	−	1002	1409	687	1092
UN06309	Rsa1.0_01832.1	806	38581	0	708	715	99	−	91	800	31912	32625
UN06310	Rsa1.0_00497.1	898	79830	0	398	400	99	+	499	898	67322	67720
UN06311	Rsa1.0_05623.1	1813	13141	0	981	1019	96	−	1	1004	10323	11339
UN06312	Rsa1.0_01131.1	1047	52928	1.00E−117	246	255	96	+	228	482	685	938
UN06313	Rsa1.0_00095.1	611	283384	0	609	609	100	+	3	611	23215	23823
UN06314	Rsa1.0_00762.1	1328	165246	0	424	425	99	+	904	1328	25166	25590
UN06315	Rsa1.0_00077.1	1151	145005	0	773	773	100	−	2	774	109765	110537
UN06316	Rsa1.0_01928.1	1289	37192	0	391	395	98	−	896	1289	6981	7375

UN06317	Rsa1.0_00645.1	1556	189716	0	325	325	100	+	703	1027	150795	151119
UN06318	Rsa1.0_01008.1	1022	56924	0	416	433	96	−	519	951	12453	12875
UN06319	Rsa1.0_00104.1	1131	368722	2.00E−89	217	232	93	−	860	1080	334705	334936
UN06320	Rsa1.0_00100.1	1469	249232	0	1057	1078	98	−	347	1424	195789	196862
UN06321	Rsa1.0_02975.1	738	26322	1.00E−145	304	315	96	+	3	317	16518	16827
UN06322	Rsa1.0_01261.1	704	49744	2.00E−79	176	184	95	−	349	532	43082	43261
UN06323	Rsa1.0_00284.1	1572	323267	0	1240	1285	96	−	295	1570	261975	263256
UN06324	Rsa1.0_00377.1	788	90038	0	447	449	99	+	3	451	25239	25686
UN06325	Rsa1.0_03750.1	1550	21027	0	352	354	99	+	1196	1549	5932	6285
UN06326	Rsa1.0_08439.1	1204	7353	0	813	814	99	+	391	1204	6315	7128
UN06327	Rsa1.0_00403.1	1246	87701	0	484	501	96	−	416	916	26353	26847
UN06328	Rsa1.0_04059.1	1037	19323	0	337	342	98	−	256	597	12352	12693
UN06329	Rsa1.0_00115.1	882	131321	0	330	332	99	+	551	882	36126	36457
UN06330	Rsa1.0_00356.1	836	91521	0	473	486	97	+	353	836	5207	5691
UN06331	Rsa1.0_00201.1	774	232851	0	756	767	98	−	8	774	140321	141087
UN06332	Rsa1.0_00479.1	1634	81377	0	1080	1131	95	−	502	1632	62298	63422
UN06333	Rsa1.0_00079.1	879	606987	1.00E−168	334	344	97	−	532	875	464203	464545
UN06334	Rsa1.0_00024.1	862	188018	0	853	862	98	+	1	862	37289	38149
UN06335	Rsa1.0_00034.1	681	260119	3.00E−90	245	273	89	−	115	387	157135	157389
UN06336	Rsa1.0_00016.1	668	831256	0	584	635	91	+	27	660	766975	767589
UN06337	Rsa1.0_01220.1	1441	161775	0	1374	1438	95	+	2	1436	112913	114349
UN06338	Rsa1.0_01606.1	1510	42432	0	662	672	98	−	840	1510	39525	40195
UN06339	Rsa1.0_05338.1	1368	144511	0	424	440	96	−	145	584	6891	7318
UN06340	Rsa1.0_01382.1	1573	46877	0	462	476	97	+	3	466	11323	11798
UN06341	Rsa1.0_19648.1	1442	1403	0	626	631	99	+	3	633	773	1403
UN06342	Rsa1.0_01185.1	1067	149842	0	1048	1066	98	−	2	1066	7338	8401
UN06343	Rsa1.0_00215.1	958	201775	0	383	388	98	+	19	403	25528	25915
UN06344	Rsa1.0_01252.1	1322	49847	0	402	403	99	−	918	1320	35397	35799
UN06345	Rsa1.0_02345.1	537	32337	1.00E−144	269	271	99	+	267	535	17017	17287
UN06346	Rsa1.0_00003.1	1482	340054	0	519	525	98	−	6	529	278263	278787
UN06347	Rsa1.0_01559.1	666	43262	1.00E−172	342	353	96	−	315	664	28796	29148
UN06348	Rsa1.0_00999.1	1412	57162	0	1406	1415	99	+	2	1412	38126	39540
UN06349	Rsa1.0_09504.1	956	6328	0	670	670	100	−	287	956	4335	5004
UN06350	Rsa1.0_00710.1	986	67325	1.00E−105	235	247	95	+	4	245	25381	25627
UN06351	Rsa1.0_00037.1	1251	307838	0	462	465	99	−	788	1251	241190	241654
UN06352	Rsa1.0_01779.1	713	39448	0	691	692	99	−	22	713	16688	17379
UN06353	Rsa1.0_05569.1	954	13311	1.00E−142	265	268	98	−	382	649	5786	6053
UN06354	Rsa1.0_01611.1	939	42269	0	367	370	99	−	571	939	24989	25358
UN06355	Rsa1.0_04166.1	752	18757	0	387	394	98	+	3	394	6382	6771

UN06356	Rsa1.0_02812.1	977	27660	1.00E-25	136	161	84	-	270	430	13956	14116
UN06357	Rsa1.0_03906.1	1489	20161	0	1008	1011	99	-	479	1489	10588	11598
UN06358	Rsa1.0_00216.1	701	197563	0	626	640	97	+	65	701	91238	91877
UN06359	Rsa1.0_00087.1	1022	761073	0	389	405	96	-	278	682	731598	732002
UN06360	Rsa1.0_02180.1	825	34176	0	458	479	95	+	353	825	8790	9268
UN06361	Rsa1.0_00758.1	1074	65477	0	461	464	99	+	611	1074	18698	19161
UN06362	Rsa1.0_04064.1	1248	19297	0	1090	1117	97	+	129	1245	16324	17435
UN06363	Rsa1.0_02808.1	1073	27677	1.00E-176	359	373	96	+	626	998	22072	22437
UN06364	Rsa1.0_04397.1	991	17555	0	951	1021	93	-	2	991	4497	5513
UN06365	Rsa1.0_08380.1	901	7446	1.00E-135	244	244	100	-	658	901	2655	2898
UN06366	Rsa1.0_03241.1	797	24423	1.00E-92	173	173	100	+	3	175	13639	13811
UN06367	Rsa1.0_01276.1	1204	49384	0	552	552	100	+	3	554	23020	23571
UN06368	Rsa1.0_01373.1	764	47052	0	741	758	97	-	2	755	9293	10050
UN06369	Rsa1.0_00803.1	1306	144228	3.00E-91	187	191	97	-	890	1080	2731	2920
UN06370	Rsa1.0_00139.1	1337	357786	0	421	422	99	-	916	1337	24405	24826
UN06371	Rsa1.0_09320.1	836	6457	1.00E-160	307	314	97	+	521	834	1368	1681
UN06372	Rsa1.0_07311.1	1436	9567	0	1419	1433	99	-	3	1435	4643	6075
UN06373	Rsa1.0_00155.1	1374	118465	1.00E-160	286	286	100	+	611	896	104732	105017
UN06374	Rsa1.0_00708.1	888	67366	0	553	554	99	+	86	639	23908	24461
UN06375	Rsa1.0_01471.1	1212	45101	0	485	500	97	-	717	1212	18495	18994
UN06376	Rsa1.0_01883.1	931	37661	2.00E-73	173	185	93	-	481	665	32860	33035
UN06377	Rsa1.0_01727.1	1196	40243	1.00E-167	343	355	96	+	17	369	11162	11516
UN06378	Rsa1.0_03216.1	1321	24540	0	881	918	95	+	1	915	21424	22339
UN06379	Rsa1.0_02874.1	957	27070	4.00E-62	168	181	92	-	766	943	1710	1890
UN06380	Rsa1.0_02114.1	1299	34873	1.00E-113	221	224	98	-	1	224	13073	13295
UN06381	Rsa1.0_00981.1	1010	57700	1.00E-137	257	260	98	-	3	262	15605	15864
UN06382	Rsa1.0_01254.1	1611	49801	0	821	822	99	-	1	821	15174	15995
UN06383	Rsa1.0_01214.1	1314	50599	0	860	915	93	+	24	924	2332	3228
UN06384	Rsa1.0_00207.1	606	248736	0	600	604	99	-	1	604	14991	15594
UN06385	Rsa1.0_03561.1	830	22089	1.00E-119	231	234	98	+	200	432	10198	10431
UN06386	Rsa1.0_01483.1	1716	44826	0	398	405	98	+	1	403	22082	22486
UN06387	Rsa1.0_00007.1	725	239325	0	344	344	100	-	347	690	118158	118501
UN06388	Rsa1.0_01464.1	1066	45193	4.00E-87	211	226	93	-	675	900	16256	16478
UN06389	Rsa1.0_00044.1	1647	271658	0	970	1071	90	+	566	1634	69278	70308
UN06390	Rsa1.0_00123.1	972	325650	0	591	626	94	-	68	678	262756	263381
UN06391	Rsa1.0_00089.1	538	406142	0	500	517	96	-	26	536	80693	81207
UN06392	Rsa1.0_14037.1	1372	3887	0	894	928	96	-	1	928	1	926
UN06393	Rsa1.0_06166.1	1276	11583	0	532	540	98	+	354	888	7173	7711
UN06394	Rsa1.0_01248.1	772	154103	0	752	770	97	-	2	767	7034	7803

UN06395	Rsa1.0_00076.1	648	146021	0	618	671	92	−	2	648	76062	76718
UN06396	Rsa1.0_02457.1	989	31208	0	441	465	94	+	1	456	12457	12914
UN06397	Rsa1.0_00180.1	881	114581	0	373	383	97	+	1	379	89956	90335
UN06398	Rsa1.0_01173.1	847	51727	1.00E−129	360	395	91	−	1	390	27024	27409
UN06399	Rsa1.0_00396.1	606	88242	1.00E−118	311	341	91	−	144	484	65465	65795
UN06400	Rsa1.0_02503.1	577	30709	4.00E−67	136	138	98	−	254	391	26114	26251
UN06401	Rsa1.0_03019.1	1476	25987	0	636	648	98	−	525	1172	575	1222
UN06402	Rsa1.0_02930.1	1362	26668	0	1305	1362	95	−	3	1362	8680	10020
UN06403	Rsa1.0_00273.1	1027	326186	1.00E−104	335	378	88	+	488	855	34780	35151
UN06404	Rsa1.0_08655.1	1159	7078	1.00E−135	287	301	95	+	3	303	4143	4443
UN06405	Rsa1.0_00240.1	982	104175	0	896	940	95	−	1	938	72298	73237
UN06406	Rsa1.0_00019.1	937	301669	0	663	696	95	+	4	682	88347	89039
UN06407	Rsa1.0_00457.1	1766	337301	0	499	569	87	+	102	652	302544	303076
UN06408	Rsa1.0_00033.1	917	515811	1.00E−128	306	326	93	−	1	326	469201	469519
UN06409	Rsa1.0_00571.1	1267	329018	0	772	803	96	−	321	1120	145042	145844
UN06410	Rsa1.0_00536.1	1427	77199	0	375	390	96	+	1042	1427	23555	23944
UN06411	Rsa1.0_03521.1	1578	396204	0	481	488	98	−	450	937	15137	15624
UN06412	Rsa1.0_02099.1	1344	35030	1.00E−147	376	412	91	+	936	1343	2291	2689
UN06413	Rsa1.0_01081.1	798	499226	0	776	802	96	−	2	798	318588	319386
UN06414	Rsa1.0_01262.1	1106	178815	0	610	626	97	−	1	623	11265	11890
UN06415	Rsa1.0_00118.1	572	130637	0	560	566	98	−	1	566	36394	36959
UN06416	Rsa1.0_00739.1	1586	66155	0	508	509	99	−	3	511	6859	7367
UN06417	Rsa1.0_00582.1	1182	74333	0	1126	1162	96	+	23	1169	39429	40587
UN06418	Rsa1.0_01061.1	874	55349	1.00E−155	287	290	98	+	585	874	28685	28974
UN06419	Rsa1.0_03752.1	1195	21022	1.00E−134	246	247	99	+	946	1192	1341	1587
UN06420	Rsa1.0_01382.1	770	46877	0	424	424	100	−	1	424	13890	14313
UN06421	Rsa1.0_01039.1	1334	55946	0	1265	1299	97	+	1	1293	37312	38608
UN06422	Rsa1.0_00114.1	1084	131330	0	706	711	99	+	373	1082	85728	86436
UN06423	Rsa1.0_00136.1	729	122464	1.00E−109	365	417	87	+	3	410	81360	81754
UN06424	Rsa1.0_00658.1	1296	69740	0	770	806	95	+	3	802	11737	12534
UN06425	Rsa1.0_01945.1	792	36989	0	771	793	97	−	1	791	31362	32153
UN06426	Rsa1.0_03992.1	1086	19675	0	1071	1088	98	−	1	1084	14276	15356
UN06427	Rsa1.0_01359.1	851	47271	0	361	368	98	−	1	362	21247	21614
UN06428	Rsa1.0_00341.1	1530	249309	0	440	447	98	−	3	446	243047	243493
UN06429	Rsa1.0_00136.1	753	122464	0	429	444	96	+	7	449	60388	60829
UN06430	Rsa1.0_00208.1	1035	108565	1.00E−163	300	303	99	+	93	395	7689	7991
UN06431	Rsa1.0_00326.1	922	171955	0	574	579	99	−	1	577	16257	16834
UN06432	Rsa1.0_03517.1	1412	22337	0	1207	1226	98	+	2	1222	2988	4213
UN06433	Rsa1.0_00806.1	1129	63674	0	1082	1128	95	−	3	1128	47284	48407

UN06434	Rsa1.0_05709.1	713	12867	0	391	399	97	-	6	403	327	724
UN06435	Rsa1.0_00215.1	1685	201775	0	887	900	98	-	3	902	78157	79055
UN06436	Rsa1.0_00419.1	1106	86562	1.00E-120	329	365	90	-	751	1104	13956	14303
UN06437	Rsa1.0_02209.1	913	33873	1.00E-172	330	337	97	+	1	334	26621	26957
UN06438	Rsa1.0_38359.1	949	587	1.00E-128	238	240	99	-	608	847	1	240
UN06439	Rsa1.0_07377.1	841	9494	1.00E-174	321	325	98	+	461	785	6338	6662
UN06440	Rsa1.0_08080.1	695	137363	1.00E-129	273	283	96	+	414	695	1006	1283
UN06441	Rsa1.0_00018.1	1344	410899	0	832	930	89	-	329	1245	144212	145136
UN06442	Rsa1.0_00002.1	752	601661	0	731	754	96	-	2	752	177388	178141
UN06443	Rsa1.0_00888.1	729	60978	0	534	595	89	-	9	571	50445	51030
UN06444	Rsa1.0_01144.1	856	52431	3.00E-41	90	91	98	-	380	470	17706	17796
UN06445	Rsa1.0_08215.1	1057	7711	0	517	527	98	+	22	545	3655	4181
UN06446	Rsa1.0_01272.1	748	49553	1.00E-159	319	329	96	-	1	329	12117	12444
UN06447	Rsa1.0_00019.1	579	301669	1.00E-130	239	240	99	+	1	240	53770	54009
UN06448	Rsa1.0_00278.1	1159	231896	0	750	770	97	-	327	1092	25555	26321
UN06449	Rsa1.0_01173.1	1454	51727	0	390	392	99	-	1065	1454	25002	25393
UN06450	Rsa1.0_00156.1	837	118383	0	522	540	96	-	85	623	56991	57529
UN06451	Rsa1.0_14725.1	908	3125	0	538	552	97	+	356	906	1471	2022
UN06452	Rsa1.0_02317.1	750	32600	0	737	746	98	+	3	748	12707	13451
UN06453	Rsa1.0_00369.1	1065	266462	0	519	562	92	-	1	553	180846	181403
UN06454	Rsa1.0_01139.1	839	52601	1.00E-108	387	444	87	-	1	423	50365	50805
UN06455	Rsa1.0_00267.1	562	101509	0	415	419	99	-	1	419	32068	32486
UN06456	Rsa1.0_72343.1	1034	317	2.00E-67	134	135	99	-	423	557	29	163
UN06457	Rsa1.0_00451.1	1585	322533	0	527	548	96	-	1018	1565	261558	262097
UN06458	Rsa1.0_17324.1	797	1861	1.00E-147	271	273	99	+	525	797	379	651
UN06459	Rsa1.0_00909.1	1486	59976	0	372	381	97	+	1104	1484	34722	35102
UN06460	Rsa1.0_01949.1	1061	36960	1.00E-172	357	375	95	-	695	1060	16875	17249
UN06461	Rsa1.0_00221.1	136	106972	6.00E-38	96	100	96	-	38	136	106735	106834
UN06462	Rsa1.0_00243.1	1011	103651	1.00E-139	299	313	95	+	61	369	46401	46713
UN06463	Rsa1.0_00332.1	1246	94575	0	447	478	93	-	97	561	59905	60380
UN06464	Rsa1.0_00083.1	1007	442773	0	858	905	94	+	22	926	359544	360429
UN06465	Rsa1.0_02268.1	1320	33172	1.00E-154	318	329	96	-	2	329	16898	17225
UN06466	Rsa1.0_02494.1	786	132923	0	438	450	97	-	3	451	132101	132548
UN06467	Rsa1.0_03005.1	1104	26082	0	1097	1108	99	+	2	1104	12260	13364
UN06468	Rsa1.0_03860.1	705	20407	3.00E-93	238	260	91	+	52	311	14095	14347
UN06469	Rsa1.0_01315.1	840	48361	0	623	645	96	+	2	643	45698	46341
UN06470	Rsa1.0_00487.1	960	80674	0	516	525	98	+	230	752	70783	71306
UN06471	Rsa1.0_00048.1	694	167185	0	453	457	99	-	1	457	37243	37699
UN06472	Rsa1.0_00058.1	891	159921	0	867	876	98	+	15	890	104481	105356

UN06473	Rsa1.0_00037.1	1197	307838	0	663	678	97	-	1	678	192289	192964
UN06474	Rsa1.0_22665.1	716	1104	0	688	728	94	+	2	715	106	833
UN06475	Rsa1.0_01198.1	1056	51029	0	862	903	95	+	17	912	40402	41300
UN06476	Rsa1.0_00002.1	828	601661	0	490	521	94	+	283	793	177153	177673
UN06477	Rsa1.0_00115.1	1430	131321	0	401	418	95	-	1020	1430	49236	49653
UN06478	Rsa1.0_04247.1	1174	18295	0	587	634	92	-	465	1097	5069	5696
UN06479	Rsa1.0_00300.1	293	97747	1.00E-148	283	289	97	-	3	291	67357	67645
UN06480	Rsa1.0_00130.1	755	125737	1.00E-137	311	332	93	+	3	330	10704	11029
UN06481	Rsa1.0_00289.1	946	98620	0	894	909	98	+	39	946	58254	59161
UN06482	Rsa1.0_04229.1	1023	18389	0	966	1020	94	-	3	993	13747	14762
UN06483	Rsa1.0_19257.1	750	1455	1.00E-178	365	379	96	-	4	381	108	484
UN06484	Rsa1.0_10893.1	978	5687	0	523	523	100	+	455	977	236	758
UN06485	Rsa1.0_04210.1	741	18442	1.00E-163	294	295	99	-	439	733	1610	1904
UN06486	Rsa1.0_02211.1	1312	33857	1.00E-155	355	380	93	+	942	1312	32613	32992
UN06487	Rsa1.0_00873.1	982	61459	0	492	502	98	+	480	981	46323	46824
UN06488	Rsa1.0_37263.1	1033	605	0	581	606	95	-	371	973	1	605
UN06489	Rsa1.0_00976.1	1479	57809	0	1000	1016	98	-	197	1212	15917	16932
UN06490	Rsa1.0_00568.1	586	75219	1.00E-153	275	275	100	+	312	586	63277	63551
UN06491	Rsa1.0_00300.1	835	97747	1.00E-164	296	297	99	+	531	827	30484	30780
UN06492	Rsa1.0_01240.1	1221	50075	0	410	418	98	-	807	1221	40593	41009
UN06493	Rsa1.0_00298.1	1233	349131	0	472	477	98	+	2	478	331436	331912
UN06494	Rsa1.0_00679.1	766	68971	3.00E-96	194	199	97	+	358	556	33672	33870
UN06495	Rsa1.0_36989.1	616	610	1.00E-129	240	242	99	+	375	616	1	242
UN06496	Rsa1.0_00001.1	876	363028	1.00E-157	318	329	96	+	287	609	323550	323878
UN06497	Rsa1.0_00470.1	1240	81968	0	340	340	100	+	901	1240	50852	51191
UN06498	Rsa1.0_02161.1	1434	34459	0	1014	1060	95	-	2	1060	21364	22423
UN06499	Rsa1.0_00329.1	1288	222544	1.00E-156	292	296	98	+	993	1288	149718	150013
UN06500	Rsa1.0_00639.1	911	165873	0	556	565	98	+	15	572	107996	108560
UN06501	Rsa1.0_00464.1	1090	82253	0	452	453	99	-	636	1088	37148	37600
UN06502	Rsa1.0_00017.1	779	752458	1.00E-108	221	227	97	+	1	225	26740	26966
UN06503	Rsa1.0_06149.1	1345	11615	0	390	406	96	+	76	475	5349	5754
UN06504	Rsa1.0_00067.1	621	415055	1.00E-145	264	265	99	-	357	621	142400	142664
UN06505	Rsa1.0_02707.1	1075	28520	0	532	549	96	+	220	768	15178	15726
UN06506	Rsa1.0_00136.1	603	122464	9.00E-87	169	171	98	+	47	217	54790	54960
UN06507	Rsa1.0_00374.1	2524	165738	0	916	942	97	+	100	1039	101017	101952
UN06508	Rsa1.0_00218.1	841	201480	0	800	828	96	-	17	841	157964	158790
UN06509	Rsa1.0_00358.1	1047	91293	0	1034	1043	99	+	1	1043	88945	89987
UN06510	Rsa1.0_08929.1	1504	6777	0	451	458	98	-	1047	1504	368	825
UN06511	Rsa1.0_00055.1	1735	520193	1.00E-162	290	290	100	+	1446	1735	70791	71080

UN06512	Rsa1.0_00540.1	1241	76895	0	1225	1238	98	+	2	1239	62828	64065
UN06513	Rsa1.0_00086.1	601	439854	0	355	360	98	+	2	361	362071	362430
UN06514	Rsa1.0_01546.1	1341	43586	0	771	801	96	−	540	1339	33997	34797
UN06515	Rsa1.0_00043.1	1462	264937	0	1411	1461	96	−	1	1461	165208	166648
UN06516	Rsa1.0_04022.1	288	19534	6.00E−74	180	192	93	+	93	282	18148	18339
UN06517	Rsa1.0_01006.1	1340	163670	0	586	608	96	+	735	1340	119738	120336
UN06518	Rsa1.0_03168.1	928	24827	0	652	653	99	+	1	652	4395	5047
UN06519	Rsa1.0_02540.1	874	30344	1.00E−168	310	312	99	−	563	874	18312	18622
UN06520	Rsa1.0_01217.1	1202	50554	0	893	899	99	−	293	1191	22108	23006
UN06521	Rsa1.0_05221.1	979	14452	1.00E−145	277	282	98	−	698	979	9177	9458
UN06522	Rsa1.0_04478.1	845	17183	0	710	712	99	−	134	845	10162	10873
UN06523	Rsa1.0_00207.1	627	248736	0	546	548	99	−	81	627	194289	194836
UN06524	Rsa1.0_00015.1	937	561803	0	691	711	97	+	8	715	447119	447823
UN06525	Rsa1.0_02996.1	2544	26152	0	578	591	97	−	396	983	20697	21285
UN06526	Rsa1.0_11525.1	816	5479	0	566	630	89	+	1	630	4715	5343
UN06527	Rsa1.0_06127.1	1051	11675	0	623	629	99	−	374	1002	2866	3492
UN06528	Rsa1.0_00079.1	779	606987	0	761	780	97	−	1	779	187718	188494
UN06529	Rsa1.0_00789.1	497	64233	0	480	514	93	−	3	497	13047	13560
UN06530	Rsa1.0_08553.1	793	7210	1.00E−114	212	213	99	−	3	215	2666	2878
UN06531	Rsa1.0_00326.1	969	171955	0	389	403	96	−	568	969	66188	66589
UN06532	Rsa1.0_02851.1	1157	27295	0	375	391	95	−	23	413	18959	19346
UN06533	Rsa1.0_00630.1	1044	71281	1.00E−114	254	265	95	−	782	1044	34396	34658
UN06534	Rsa1.0_02310.1	659	32735	0	428	455	94	−	3	452	13010	13454
UN06535	Rsa1.0_00110.1	963	132719	0	637	655	97	−	1	655	115774	116428
UN06536	Rsa1.0_00165.1	535	116466	1.00E−151	292	299	97	−	237	535	4445	4743
UN06537	Rsa1.0_65061.1	792	352	1.00E−160	333	352	94	+	42	378	1	352
UN06538	Rsa1.0_00722.1	1463	66840	1.00E−151	307	315	97	+	851	1162	41419	41733
UN06539	Rsa1.0_01063.1	1417	55301	0	500	535	93	−	886	1414	16258	16779
UN06540	Rsa1.0_02392.1	1017	152517	1.00E−153	297	302	98	−	717	1017	25844	26143
UN06541	Rsa1.0_00132.1	1536	124299	0	498	518	96	+	276	791	99696	100209
UN06542	Rsa1.0_00816.1	1113	403350	1.00E−126	235	237	99	+	1	237	109947	110183
UN06543	Rsa1.0_01801.1	806	39094	0	510	535	95	−	1	529	20432	20966
UN06544	Rsa1.0_01529.1	606	43871	1.00E−147	350	381	91	−	226	606	38315	38678
UN06545	Rsa1.0_00065.1	971	454616	0	621	650	95	+	326	968	254496	255145
UN06546	Rsa1.0_09407.1	1284	6390	2.33E−156	269	271	99	−	2	272	4676	4946
UN06547	Rsa1.0_00964.1	1018	316648	0	396	408	97	−	611	1018	18327	18732
UN06548	Rsa1.0_01325.1	952	48131	5.00E−46	191	223	85	+	537	759	17631	17853
UN06549	Rsa1.0_00087.1	1384	761073	0	455	460	98	+	112	571	439545	440004
UN06550	Rsa1.0_01652.1	854	41579	0	705	708	99	−	5	712	1692	2399

UN06551	Rsa1.0_07604.1	600	9041	0	582	592	98	−	3	594	3098	3689
UN06552	Rsa1.0_00275.1	691	210104	4.00E−83	210	228	92	−	238	465	155730	155951
UN06553	Rsa1.0_00262.1	1356	101906	0	1222	1248	97	+	118	1356	44451	45698
UN06554	Rsa1.0_04552.1	886	16878	0	849	893	95	+	1	886	10057	10935
UN06555	Rsa1.0_03271.1	1165	24187	0	445	469	94	−	697	1163	1265	1716
UN06556	Rsa1.0_00201.1	620	232851	1.00E−177	329	334	98	+	4	337	92986	93319
UN06557	Rsa1.0_01360.1	864	47246	0	348	357	97	+	508	864	37242	37598
UN06558	Rsa1.0_00329.1	1203	222544	0	403	404	99	−	3	406	84859	85262
UN06559	Rsa1.0_00002.1	1475	601661	0	652	668	97	−	397	1064	278342	279009
UN06560	Rsa1.0_01608.1	1195	170540	0	366	366	100	−	385	750	35343	35708
UN06561	Rsa1.0_02069.1	713	35425	1.00E−141	335	360	93	−	293	642	20713	21072
UN06562	Rsa1.0_00231.1	642	105381	0	384	406	94	−	1	406	28173	28569
UN06563	Rsa1.0_05576.1	1471	13287	0	743	748	99	+	617	1364	7059	7805
UN06564	Rsa1.0_00589.1	1333	209965	0	466	482	96	+	852	1333	152952	153431
UN06565	Rsa1.0_00460.1	457	206387	1.00E−130	248	252	98	+	153	404	187529	187780
UN06566	Rsa1.0_05122.1	949	14791	0	573	584	98	+	2	584	4823	5406
UN06567	Rsa1.0_00810.1	443	63289	1.00E−75	177	188	94	−	14	201	19139	19326
UN06568	Rsa1.0_00842.1	737	62493	0	419	442	94	+	7	439	37528	37969
UN06569	Rsa1.0_01137.1	595	52655	1.00E−121	229	232	98	−	221	452	51348	51579
UN06570	Rsa1.0_00354.1	844	232005	0	410	428	95	+	25	452	194557	194983
UN06571	Rsa1.0_00104.1	1522	368722	0	1465	1484	98	+	41	1522	350829	352312
UN06572	Rsa1.0_00251.1	1096	303968	0	1039	1093	95	+	2	1093	10565	11655
UN06573	Rsa1.0_01847.1	777	38346	0	653	667	97	+	1	663	10548	11214
UN06574	Rsa1.0_01338.1	1301	155644	0	1067	1073	99	+	219	1291	123367	124439
UN06575	Rsa1.0_00037.1	1376	307838	0	415	432	96	+	555	982	148174	148601
UN06576	Rsa1.0_00048.1	763	167185	0	343	344	99	−	280	623	115727	116070
UN06577	Rsa1.0_04679.1	1245	16430	0	747	765	97	+	471	1229	1561	2319
UN06578	Rsa1.0_00382.1	1064	251946	0	628	639	98	+	383	1021	19589	20226
UN06579	Rsa1.0_69554.1	765	329	1.00E−162	318	325	97	+	275	598	6	329
UN06580	Rsa1.0_00384.1	903	89278	1.00E−162	322	333	96	−	1	333	62852	63184
UN06581	Rsa1.0_03000.1	843	26125	1.00E−166	408	445	91	+	278	707	7874	8318
UN06582	Rsa1.0_02808.1	1009	27677	0	946	1050	90	+	3	1008	16385	17416
UN06583	Rsa1.0_00262.1	861	101906	1.00E−147	368	401	91	−	13	398	33151	33551
UN06584	Rsa1.0_00813.1	892	63202	0	819	859	95	+	31	888	18471	19313
UN06585	Rsa1.0_02479.1	1152	31038	2.00E−95	216	228	94	−	458	682	15298	15525
UN06586	Rsa1.0_02288.1	1189	32984	0	472	481	98	−	708	1187	29812	30292
UN06587	Rsa1.0_05354.1	811	14073	0	569	599	94	+	68	660	4752	5350
UN06588	Rsa1.0_05161.1	779	14631	1.00E−138	321	345	93	+	13	357	8351	8695
UN06589	Rsa1.0_00673.1	1047	69325	0	421	435	96	+	613	1047	10045	10479

UN06590	Rsa1.0_03517.1	772	22337	1.00E-155	288	290	99	+	2	290	14570	14859
UN06591	Rsa1.0_01274.1	787	49419	1.00E-112	212	214	99	+	247	460	39937	40150
UN06592	Rsa1.0_04051.1	1551	19375	0	443	464	95	+	1090	1549	8731	9188
UN06593	Rsa1.0_05160.1	1465	14637	0	581	618	94	−	211	828	13452	14051
UN06594	Rsa1.0_00100.1	998	249232	3.00E-91	206	217	94	+	69	282	139414	139630
UN06595	Rsa1.0_00679.1	801	68971	1.00E-130	302	324	93	+	1	306	65779	66100
UN06596	Rsa1.0_13662.1	1181	4542	0	729	757	96	+	14	759	3398	4154
UN06597	Rsa1.0_07541.1	1288	9167	1.00E-134	519	611	84	−	140	750	5749	6359
UN06598	Rsa1.0_02496.1	618	30814	0	520	575	90	−	43	616	21060	21622
UN06599	Rsa1.0_00081.1	807	143261	0	638	668	95	−	17	666	136892	137559
UN06600	Rsa1.0_00224.1	902	327530	0	378	391	96	+	1	388	38875	39264
UN06601	Rsa1.0_08309.1	1169	7565	0	582	605	96	−	566	1169	5445	6039
UN06602	Rsa1.0_00916.1	767	59789	0	508	529	96	−	3	524	15473	15998
UN06603	Rsa1.0_03162.1	844	24895	0	801	834	96	+	9	842	20685	21508
UN06604	Rsa1.0_02681.1	688	28711	1.00E-124	294	312	94	−	75	384	20446	20753
UN06605	Rsa1.0_00301.1	863	365315	6.00E-98	215	226	95	+	8	228	165177	165402
UN06606	Rsa1.0_01303.1	930	48853	0	511	533	95	−	14	543	15249	15781
UN06607	Rsa1.0_04019.1	760	19550	0	737	757	97	−	4	760	14445	15199
UN06608	Rsa1.0_00344.1	785	191774	0	401	418	95	−	77	492	108987	109402
UN06609	Rsa1.0_01556.1	495	163858	0	476	515	92	−	1	495	160813	161316
UN06610	Rsa1.0_00690.1	787	140527	0	532	566	93	−	3	565	33220	33781
UN06611	Rsa1.0_00121.1	949	278029	1.00E-139	315	335	94	+	1	335	43403	43735
UN06612	Rsa1.0_08919.1	1332	6788	0	538	538	100	−	781	1318	5898	6435
UN06613	Rsa1.0_28998.1	1192	796	0	346	347	99	−	495	840	435	781
UN06614	Rsa1.0_01049.1	705	55655	1.00E-176	365	380	96	−	327	703	21138	21517
UN06615	Rsa1.0_05702.1	761	12884	1.00E-144	285	293	97	−	1	293	712	1001
UN06616	Rsa1.0_03738.1	1152	21071	1.00E-140	265	269	98	+	570	838	12425	12693
UN06617	Rsa1.0_02626.1	757	29387	0	427	451	94	+	1	444	22846	23293
UN06618	Rsa1.0_00087.1	994	761073	1.00E-138	389	431	90	+	32	448	98823	99250
UN06619	Rsa1.0_03581.1	1743	21965	0	413	420	98	+	3	422	21095	21514
UN06620	Rsa1.0_03981.1	1413	19724	0	1114	1159	96	+	10	1166	14310	15464
UN06621	Rsa1.0_03565.1	1360	22062	0	1102	1143	96	+	81	1223	17456	18590
UN06622	Rsa1.0_00988.1	846	57525	1.00E-123	257	264	97	+	1	263	27411	27672
UN06623	Rsa1.0_00705.1	1245	67505	1.00E-127	250	256	97	−	603	858	29792	30047
UN06624	Rsa1.0_00019.1	604	301669	0	597	600	99	+	3	601	43414	44013
UN06625	Rsa1.0_00825.1	670	62855	0	581	601	96	−	1	599	35580	36171
UN06626	Rsa1.0_01388.1	705	46741	0	658	676	97	−	7	681	7147	7820
UN06627	Rsa1.0_00947.1	859	58664	0	543	581	93	+	4	567	47242	47811
UN06628	Rsa1.0_02312.1	1539	32675	0	526	544	96	−	421	952	21111	21654

UN06629	Rsa1.0_01104.1	1504	285480	0	577	577	100	−	928	1504	283733	284309
UN06630	Rsa1.0_02282.1	896	163951	1.00E−114	216	218	99	+	287	504	149855	150072
UN06631	Rsa1.0_01969.1	1303	153351	1.00E−151	274	275	99	+	835	1109	13008	13282
UN06632	Rsa1.0_00758.1	986	65477	0	513	525	97	+	464	986	38591	39115
UN06633	Rsa1.0_04632.1	1250	16589	0	1218	1254	97	−	6	1250	10093	11343
UN06634	Rsa1.0_02835.1	1402	27491	2.33E−156	300	312	96	+	18	329	21937	22243
UN06635	Rsa1.0_04835.1	820	15766	1.00E−180	360	372	96	−	3	374	4608	4978
UN06636	Rsa1.0_18005.1	915	1691	1.00E−126	245	250	98	−	231	480	1	250
UN06637	Rsa1.0_03624.1	615	21706	0	494	514	96	−	1	509	12386	12899
UN06638	Rsa1.0_00107.1	728	431659	0	523	554	94	−	7	542	337025	337578
UN06639	Rsa1.0_00807.1	1008	63607	1.00E−103	200	203	98	−	688	890	46342	46544
UN06640	Rsa1.0_03029.1	1788	25858	0	535	557	96	−	537	1090	19494	20050
UN06641	Rsa1.0_00521.1	844	78191	0	423	452	93	−	1	450	64098	64531
UN06642	Rsa1.0_00675.1	728	145590	4.00E−52	209	246	84	+	472	705	140335	140580
UN06643	Rsa1.0_00001.1	983	363028	1.00E−110	265	285	92	−	671	952	190868	191142
UN06644	Rsa1.0_01949.1	845	36960	1.00E−137	280	291	96	+	555	845	21094	21384
UN06645	Rsa1.0_00365.1	905	90902	0	565	579	97	+	2	577	73446	74022
UN06646	Rsa1.0_00122.1	767	265937	0	749	760	98	−	1	760	9573	10332
UN06647	Rsa1.0_03569.1	1354	22041	1.00E−164	349	365	95	+	985	1348	14787	15149
UN06648	Rsa1.0_01710.1	865	40586	0	799	824	96	+	2	824	3521	4338
UN06649	Rsa1.0_04600.1	933	16694	1.00E−133	268	275	97	−	590	860	11338	11612
UN06650	Rsa1.0_00409.1	1428	146788	0	930	949	97	+	480	1427	90871	91819
UN06651	Rsa1.0_00019.1	947	301669	0	445	461	96	−	5	460	272068	272528
UN06652	Rsa1.0_02179.1	1487	34188	0	673	692	97	+	416	1107	24265	24956
UN06653	Rsa1.0_02625.1	754	139104	2.00E−91	225	238	94	−	6	242	8676	8909
UN06654	Rsa1.0_00215.1	1063	201775	1.00E−157	306	314	97	+	739	1052	19770	20083
UN06655	Rsa1.0_09067.1	1260	6672	1.00E−109	220	225	97	+	3	227	2354	2577
UN06656	#	#	#	#	#	#	#	#	#	#	#	#
UN06657	Rsa1.0_00187.1	1848	393093	0	649	677	95	−	9	685	208971	209647
UN06658	Rsa1.0_00541.1	907	76863	1.00E−77	208	227	91	−	668	892	9120	9341
UN06659	Rsa1.0_01419.1	985	167402	0	937	974	96	+	1	970	159804	160768
UN06660	Rsa1.0_00938.1	1119	193891	0	421	433	97	+	540	972	50229	50661
UN06661	Rsa1.0_01938.1	850	264018	0	577	609	94	+	2	606	159667	160265
UN06662	Rsa1.0_00982.1	641	57640	0	639	640	99	−	1	640	34050	34689
UN06663	Rsa1.0_14436.1	942	3393	0	537	562	95	+	7	560	2230	2791
UN06664	Rsa1.0_00198.1	860	222963	1.00E−179	358	369	97	+	3	367	17917	18285
UN06665	Rsa1.0_00175.1	1045	166121	0	356	362	98	−	683	1044	37710	38071
UN06666	Rsa1.0_19050.1	946	1488	1.00E−165	321	330	97	−	464	793	976	1305
UN06667	Rsa1.0_01466.1	786	45161	1.00E−160	303	307	98	−	1	307	28487	28792

UN06668	Rsa1.0_00512.1	1319	200277	1.00E-101	199	202	98	+	1116	1317	66192	66390
UN06669	Rsa1.0_00190.1	895	318240	0	395	413	95	+	1	410	211843	212253
UN06670	Rsa1.0_04064.1	695	19297	0	435	451	96	-	37	481	18501	18951
UN06671	Rsa1.0_01435.1	647	45942	0	377	392	96	-	1	388	28028	28419
UN06672	Rsa1.0_02367.1	1042	32106	0	806	839	96	+	1	838	11160	11995
UN06673	Rsa1.0_00330.1	910	94666	0	869	926	93	+	1	908	33158	34077
UN06674	Rsa1.0_01924.1	1384	37226	1.00E-145	271	274	98	-	841	1114	15462	15735
UN06675	Rsa1.0_07232.1	1544	9684	0	376	382	98	+	1164	1544	1213	1593
UN06676	Rsa1.0_00728.1	1055	66704	0	404	415	97	-	1	415	5094	5508
UN06677	Rsa1.0_01896.1	814	37512	0	457	478	95	-	344	813	6000	6477
UN06678	Rsa1.0_13705.1	592	4452	2.00E-93	232	249	93	+	2	247	2560	2803
UN06679	Rsa1.0_00592.1	1065	73788	3.00E-75	159	164	96	+	328	491	10983	11146
UN06680	Rsa1.0_00838.1	564	153820	0	510	524	97	+	1	522	52223	52744
UN06681	Rsa1.0_01707.1	1285	40686	0	641	659	97	+	482	1140	4816	5474
UN06682	Rsa1.0_02073.1	1263	35386	0	951	991	95	-	1	988	20440	21430
UN06683	Rsa1.0_11073.1	785	5625	1.00E-166	335	348	96	+	437	784	4853	5200
UN06684	Rsa1.0_05986.1	1031	12027	0	364	374	97	+	460	833	5450	5823
UN06685	Rsa1.0_00108.1	1201	478491	2.33E-156	372	402	92	-	2	397	463726	464122
UN06686	Rsa1.0_00802.1	786	63846	2.00E-73	147	149	98	+	4	152	14297	14445
UN06687	Rsa1.0_02490.1	821	30885	0	423	425	99	-	2	426	28607	29031
UN06688	Rsa1.0_01949.1	922	36960	1.00E-164	405	435	93	-	1	431	27763	28188
UN06689	Rsa1.0_01997.1	857	158416	0	631	644	97	-	4	644	145646	146287
UN06690	Rsa1.0_02710.1	1299	28492	1.00E-138	302	317	95	+	982	1297	25405	25715
UN06691	Rsa1.0_00171.1	754	115622	1.00E-162	334	347	96	+	410	752	52232	52578
UN06692	Rsa1.0_00622.1	906	71822	0	709	748	94	-	1	746	60939	61677
UN06693	Rsa1.0_01415.1	445	46263	1.00E-111	268	287	93	+	1	276	19591	19876
UN06694	Rsa1.0_01271.1	847	49569	0	470	484	97	-	354	836	27299	27781
UN06695	Rsa1.0_01064.1	896	55295	1.00E-137	291	304	95	+	2	304	43782	44085
UN06696	Rsa1.0_00087.1	1020	761073	0	955	956	99	-	7	962	35915	36870
UN06697	Rsa1.0_00951.1	1212	164588	1.00E-151	289	295	97	-	323	617	116154	116448
UN06698	Rsa1.0_02320.1	1460	32575	6.00E-99	253	276	91	-	1038	1313	22169	22444
UN06699	Rsa1.0_00866.1	1605	61676	0	1045	1049	99	+	559	1605	30351	31399
UN06700	Rsa1.0_01285.1	447	172709	0	438	447	97	-	3	447	156762	157208
UN06701	Rsa1.0_05418.1	499	13816	1.00E-94	242	265	91	-	245	497	1462	1725
UN06702	Rsa1.0_01612.1	849	42255	0	793	804	98	-	3	795	35219	36022
UN06703	Rsa1.0_01313.1	858	48387	0	554	569	97	-	2	570	2100	2666
UN06704	Rsa1.0_00879.1	796	61297	3.00E-93	259	279	92	+	528	796	38364	38642
UN06705	Rsa1.0_00312.1	1414	96765	0	359	363	98	-	1	363	25817	26178
UN06706	Rsa1.0_00576.1	737	178949	4.00E-83	178	185	96	-	253	437	3257	3441

UN06707	Rsa1.0_00620.1	1551	71985	0	487	502	97	−	213	714	55327	55828
UN06708	Rsa1.0_00269.1	789	341668	1.00E-155	293	298	98	+	241	538	18359	18656
UN06709	Rsa1.0_00596.1	782	73578	0	340	343	99	−	441	782	29144	29486
UN06710	Rsa1.0_07073.1	822	9944	0	817	822	99	+	1	822	66	887
UN06711	Rsa1.0_04768.1	1095	16066	0	1057	1094	96	+	2	1095	4357	5447
UN06712	Rsa1.0_14615.1	821	3224	0	799	812	98	−	10	821	2391	3202
UN06713	Rsa1.0_61124.1	935	375	4.00E-87	279	318	87	−	435	745	58	375
UN06714	Rsa1.0_00336.1	633	445051	0	572	595	96	−	40	633	174757	175351
UN06715	Rsa1.0_03995.1	1016	19654	1.00E-153	280	282	99	−	2	283	3701	3982
UN06716	Rsa1.0_00618.1	1240	72195	0	372	373	99	−	864	1236	3305	3677
UN06717	Rsa1.0_00286.1	1136	98709	0	642	691	92	−	350	1040	27069	27739
UN06718	Rsa1.0_01065.1	670	55264	0	500	519	96	−	153	669	30878	31396
UN06719	Rsa1.0_00023.1	1074	194764	1.00E-126	239	242	98	+	833	1074	70561	70802
UN06720	Rsa1.0_00026.1	1448	327835	1.00E-162	368	394	93	+	1041	1434	176398	176786
UN06721	Rsa1.0_00376.1	931	90101	0	880	929	94	+	12	931	13151	14076
UN06722	Rsa1.0_00324.1	1280	218805	0	540	547	98	+	1	544	864	1408
UN06723	Rsa1.0_00620.1	1943	71985	0	632	636	99	−	1	636	13154	13787
UN06724	Rsa1.0_06052.1	1107	11848	1.00E-145	358	390	91	−	614	1003	7486	7875
UN06725	Rsa1.0_01377.1	1096	46967	1.00E-173	352	363	96	+	735	1094	21581	21941
UN06726	Rsa1.0_07780.1	1612	8589	0	605	661	91	−	13	665	748	1386
UN06727	Rsa1.0_05751.1	1300	12733	0	566	568	99	−	733	1300	2681	3248
UN06728	Rsa1.0_00157.1	739	245832	0	721	745	96	−	2	738	216614	217358
UN06729	Rsa1.0_01749.1	1119	39822	1.00E-176	382	405	94	+	2	397	430	833
UN06730	Rsa1.0_35192.1	647	643	1.00E-104	243	256	94	−	6	259	40	293
UN06731	Rsa1.0_01147.1	928	52391	1.00E-110	272	300	90	−	633	928	46084	46362
UN06732	Rsa1.0_01051.1	934	55577	0	400	420	95	+	513	923	3695	4114
UN06733	Rsa1.0_01079.1	1381	54753	1.00E-143	299	312	95	−	1	309	24471	24782
UN06734	Rsa1.0_05051.1	1414	15018	1.00E-163	368	390	94	−	1039	1414	13927	14315
UN06735	Rsa1.0_00743.1	1606	177459	0	1287	1347	95	−	269	1605	6082	7416
UN06736	Rsa1.0_03356.1	686	23487	0	370	381	97	+	307	686	1225	1605
UN06737	Rsa1.0_07636.1	1205	8971	0	674	716	94	−	1	709	3559	4258
UN06738	Rsa1.0_01878.1	824	37768	0	481	486	98	+	340	824	27356	27841
UN06739	Rsa1.0_01674.1	931	168300	0	581	601	96	+	332	930	19673	20265
UN06740	Rsa1.0_00140.1	926	121541	0	698	707	98	+	172	878	83368	84074
UN06741	Rsa1.0_00360.1	764	91190	1.00E-153	296	303	97	−	4	306	78998	79300
UN06742	Rsa1.0_11556.1	1128	5471	1.00E-127	231	231	100	+	8	238	1100	1330
UN06743	Rsa1.0_00306.1	828	146760	0	350	354	98	−	100	453	136637	136990
UN06744	Rsa1.0_00661.1	679	213041	0	515	535	96	+	145	678	66549	67079
UN06745	Rsa1.0_02855.1	882	27271	0	540	573	94	+	319	881	10684	11254

UN06746	Rsa1.0_00241.1	1325	104000	0	428	441	97	+	249	689	11604	12041
UN06747	Rsa1.0_01121.1	885	53407	0	732	774	94	+	121	884	19076	19846
UN06748	Rsa1.0_03309.1	1880	23852	0	481	482	99	+	1	482	6302	6783
UN06749	Rsa1.0_02553.1	1732	30190	0	430	445	96	+	5	447	16579	17023
UN06750	Rsa1.0_00248.1	1447	163568	0	548	563	97	−	335	897	42448	43010
UN06751	Rsa1.0_02170.1	976	34352	0	488	493	98	−	6	497	18484	18976
UN06752	Rsa1.0_00922.1	771	59588	0	601	611	98	+	82	691	39245	39854
UN06753	Rsa1.0_17884.1	1297	1718	1.00E−109	265	286	92	−	886	1171	1433	1718
UN06754	Rsa1.0_00556.1	790	207212	0	403	418	96	−	339	746	147815	148232
UN06755	Rsa1.0_00002.1	1210	601661	3.00E−76	251	286	87	−	324	609	1864	2149
UN06756	Rsa1.0_00480.1	666	81325	0	371	375	98	+	291	665	1280	1653
UN06757	Rsa1.0_02650.1	513	29047	0	508	510	99	−	3	512	25808	26317
UN06758	Rsa1.0_02284.1	1217	33011	0	696	725	96	+	489	1213	13022	13731
UN06759	Rsa1.0_05943.1	1171	12158	0	361	374	96	+	275	648	1366	1739
UN06760	Rsa1.0_08388.1	1686	7436	0	1019	1086	93	+	2	1062	4673	5748
UN06761	Rsa1.0_01474.1	1195	45070	0	404	405	99	−	791	1195	197	601
UN06762	Rsa1.0_13748.1	860	4388	0	471	477	98	+	386	860	2775	3251
UN06763	Rsa1.0_01563.1	1024	170823	0	498	516	96	+	290	801	38791	39306
UN06764	Rsa1.0_01468.1	1039	45146	1.00E−80	162	165	98	+	645	809	13817	13981
UN06765	Rsa1.0_02474.1	1442	31076	0	692	693	99	+	1	692	164	856
UN06766	Rsa1.0_00101.1	1078	267193	0	929	932	99	−	147	1078	18795	19726
UN06767	Rsa1.0_01970.1	1163	36648	0	1124	1174	95	+	3	1163	30243	31413
UN06768	Rsa1.0_52123.1	751	436	1.00E−174	333	341	97	−	1	341	35	375
UN06769	Rsa1.0_00533.1	869	77375	0	824	872	94	−	1	866	49047	49917
UN06770	Rsa1.0_02513.1	977	30643	0	964	990	97	+	3	973	18771	19757
UN06771	Rsa1.0_00006.1	1370	396176	1.00E−170	404	436	92	+	675	1109	312060	312481
UN06772	Rsa1.0_07149.1	826	9824	0	415	426	97	−	408	824	820	1245
UN06773	Rsa1.0_00223.1	1425	106231	0	1059	1095	96	+	331	1421	2967	4060
UN06774	Rsa1.0_00065.1	949	454616	1.00E−131	259	266	97	−	2	267	144340	144605
UN06775	Rsa1.0_00342.1	849	93695	0	724	741	97	+	1	737	62330	63070
UN06776	Rsa1.0_01000.1	950	57140	1.00E−101	188	188	100	−	574	761	3798	3985
UN06777	Rsa1.0_02177.1	910	34262	0	433	441	98	+	389	829	10037	10474
UN06778	Rsa1.0_00078.1	742	175053	1.00E−129	251	255	98	−	3	256	46017	46271
UN06779	Rsa1.0_00134.1	826	390219	1.00E−123	258	269	95	+	211	479	4759	5027
UN06780	Rsa1.0_00046.1	741	320315	0	681	708	96	−	45	737	193705	194412
UN06781	Rsa1.0_07441.1	1036	9388	1.00E−154	285	288	98	+	748	1035	1725	2012
UN06782	Rsa1.0_00035.1	1491	176449	0	508	536	94	−	954	1487	41092	41624
UN06783	Rsa1.0_00058.1	1070	159921	0	482	520	92	+	555	1070	119077	119584
UN06784	Rsa1.0_00167.1	1550	116000	0	1066	1103	96	−	1	1103	33824	34925

UN06785	Rsa1.0_02931.1	987	26654	0	542	562	96	+	431	986	5953	6508
UN06786	Rsa1.0_02236.1	1006	33558	0	531	541	98	+	3	543	11373	11913
UN06787	Rsa1.0_00096.1	1491	198773	1.00E-157	284	285	99	+	1207	1491	173724	174008
UN06788	Rsa1.0_00033.1	760	515811	2.00E-73	206	226	91	-	7	223	35164	35388
UN06789	Rsa1.0_00157.1	1267	245832	0	641	649	98	-	613	1261	146800	147446
UN06790	Rsa1.0_03487.1	1518	22564	1.00E-156	299	304	98	+	1140	1443	1708	2010
UN06791	Rsa1.0_01179.1	743	51549	5.00E-27	81	87	93	-	642	728	42459	42545
UN06792	Rsa1.0_03990.1	557	19676	1.00E-102	227	236	96	-	48	283	8885	9116
UN06793	Rsa1.0_03773.1	914	20937	1.00E-112	377	434	86	+	36	469	7161	7594
UN06794	Rsa1.0_00025.1	826	277312	1.00E-138	267	273	97	+	156	428	115742	116014
UN06795	Rsa1.0_04323.1	1024	17915	5.00E-99	208	216	96	-	3	218	15646	15861
UN06796	Rsa1.0_01198.1	804	51029	0	432	453	95	-	341	793	43871	44312
UN06797	Rsa1.0_00016.1	1027	831256	0	583	583	100	+	1	583	17362	17944
UN06798	Rsa1.0_00237.1	1143	235974	1.00E-147	337	356	94	-	788	1143	17780	18131
UN06799	Rsa1.0_00054.1	1485	162053	0	1076	1107	97	+	320	1423	105351	106456
UN06800	Rsa1.0_07439.1	1097	9392	1.00E-149	268	268	100	+	329	596	4581	4848
UN06801	Rsa1.0_02074.1	1149	35370	0	461	496	92	-	655	1145	12043	12527
UN06802	Rsa1.0_00482.1	845	81162	3.00E-78	179	189	94	-	1	189	6470	6658
UN06803	Rsa1.0_03077.1	952	25558	1.00E-93	212	223	95	-	36	257	8108	8330
UN06804	Rsa1.0_00041.1	799	186197	5.00E-92	295	335	88	-	466	798	114770	115104
UN06805	Rsa1.0_01947.1	659	36977	0	399	409	97	+	1	407	23160	23567
UN06806	Rsa1.0_11621.1	1183	5457	0	824	876	94	+	250	1116	1	865
UN06807	Rsa1.0_00724.1	984	66789	1.00E-83	249	272	91	+	716	982	54634	54897
UN06808	Rsa1.0_00453.1	1345	83031	4.00E-94	194	200	97	-	900	1099	72257	72456
UN06809	Rsa1.0_00339.1	885	222974	0	503	533	94	-	351	883	202302	202834
UN06810	Rsa1.0_18657.1	1059	1558	1.00E-151	286	291	98	-	645	935	559	849
UN06811	Rsa1.0_16460.1	1270	2148	0	1060	1085	97	-	27	1107	1	1081
UN06812	Rsa1.0_00323.1	1345	159650	0	1281	1330	96	+	3	1330	154434	155762
UN06813	Rsa1.0_00977.1	935	198001	0	724	732	98	-	48	779	180527	181258
UN06814	Rsa1.0_00545.1	1119	189649	1.00E-164	328	337	97	+	374	707	35589	35925
UN06815	Rsa1.0_01289.1	236	49181	8.00E-45	101	104	97	+	130	233	6324	6427
UN06816	Rsa1.0_00498.1	1026	79746	0	708	727	97	+	300	1026	10335	11058
UN06817	Rsa1.0_07863.1	1112	8404	0	424	451	94	-	661	1110	961	1395
UN06818	Rsa1.0_00253.1	764	231292	0	404	434	93	+	1	434	16533	16942
UN06819	Rsa1.0_00301.1	645	365315	0	445	481	92	+	183	645	320945	321420
UN06820	Rsa1.0_04034.1	1162	19481	1.00E-137	257	260	98	-	691	950	12812	13071
UN06821	Rsa1.0_13604.1	1094	4657	2.00E-61	127	129	98	+	897	1025	4058	4186
UN06822	Rsa1.0_00645.1	1120	189716	0	1079	1122	96	-	1	1119	29581	30696
UN06823	Rsa1.0_00252.1	1254	102760	1.00E-141	325	347	93	-	8	351	64920	65253

UN06824	Rsa1.0_00524.1	1334	169613	2.00E-95	237	254	93	-	1	252	155579	155827
UN06825	Rsa1.0_01459.1	879	45277	0	835	878	95	+	1	875	35841	36705
UN06826	Rsa1.0_01416.1	768	46262	0	740	775	95	+	1	766	26511	27276
UN06827	Rsa1.0_03302.1	1073	23898	0	370	386	95	+	1	382	11619	11999
UN06828	Rsa1.0_00656.1	1275	69968	0	365	367	99	+	1	367	61879	62245
UN06829	Rsa1.0_03264.1	359	145959	1.00E-53	148	159	93	-	201	359	134684	134840
UN06830	Rsa1.0_01058.1	1161	55430	1.00E-118	236	240	98	-	487	724	28778	29017
UN06831	Rsa1.0_00149.1	1376	305196	1.00E-164	323	333	96	+	20	352	107014	107346
UN06832	Rsa1.0_03242.1	866	24415	0	411	424	96	+	26	448	1652	2074
UN06833	Rsa1.0_00825.1	1565	62855	0	880	947	92	-	590	1517	948	1894
UN06834	Rsa1.0_11435.1	1148	5508	0	585	600	97	-	7	605	4766	5359
UN06835	Rsa1.0_00400.1	1237	87858	0	378	384	98	+	1	384	54696	55078
UN06836	Rsa1.0_00615.1	912	72235	1.00E-129	277	291	95	+	7	297	45703	45993
UN06837	Rsa1.0_00244.1	1386	103590	0	1191	1251	95	+	1	1237	77047	78290
UN06838	Rsa1.0_01729.1	1631	304246	0	1065	1067	99	-	1	1067	283966	285032
UN06839	Rsa1.0_00760.1	1396	65267	0	614	668	91	-	164	818	36639	37296
UN06840	Rsa1.0_00329.1	736	222544	0	732	735	99	-	1	735	20350	21084
UN06841	Rsa1.0_01245.1	1087	149868	0	731	755	96	-	141	895	21270	22024
UN06842	Rsa1.0_02480.1	959	31036	0	401	407	98	-	1	405	4946	5351
UN06843	Rsa1.0_00483.1	1354	81102	1.00E-180	366	380	96	+	7	386	37176	37553
UN06844	Rsa1.0_02756.1	1161	28202	1.00E-106	220	227	96	-	82	306	15023	15249
UN06845	Rsa1.0_06770.1	1252	10389	0	705	728	96	+	5	732	4761	5487
UN06846	Rsa1.0_00770.1	842	64900	1.00E-149	323	340	95	-	501	840	9382	9720
UN06847	Rsa1.0_01559.1	1337	43262	0	687	703	97	-	333	1035	18985	19686
UN06848	Rsa1.0_00593.1	998	143463	0	502	502	100	+	1	502	59459	59960
UN06849	Rsa1.0_06054.1	677	11847	1.00E-157	329	340	96	-	1	340	1351	1685
UN06850	Rsa1.0_02605.1	1327	29637	1.00E-103	207	211	98	-	89	298	8473	8683
UN06851	Rsa1.0_03362.1	1220	23453	0	801	806	99	-	1	806	11300	12105
UN06852	Rsa1.0_02435.1	1342	31454	1.00E-154	346	367	94	-	1	366	17710	18074
UN06853	Rsa1.0_00215.1	789	201775	0	380	386	98	-	399	784	162468	162853
UN06854	Rsa1.0_06218.1	1341	11461	0	1316	1340	98	+	1	1340	5583	6919
UN06855	Rsa1.0_01844.1	744	38421	0	533	535	99	-	1	535	10289	10823
UN06856	Rsa1.0_02608.1	645	29613	0	458	482	95	-	164	645	23961	24438
UN06857	Rsa1.0_00131.1	861	432233	1.00E-128	279	289	96	-	8	293	412408	412695
UN06858	Rsa1.0_05618.1	1081	13165	1.00E-141	332	352	94	+	737	1079	7995	8343
UN06859	Rsa1.0_00084.1	1346	142338	1.00E-172	316	318	99	-	262	578	72082	72399
UN06860	Rsa1.0_00119.1	1361	467509	1.00E-129	268	278	96	-	2	279	390249	390525
UN06861	Rsa1.0_05061.1	1442	14960	0	560	581	96	-	855	1435	12558	13132
UN06862	Rsa1.0_04318.1	574	17947	1.00E-150	272	273	99	+	1	273	7746	8018

UN06863	Rsa1.0_03826.1	939	20614	0	442	446	99	+	38	483	10707	11152
UN06864	Rsa1.0_01129.1	963	52949	0	380	381	99	−	583	963	15840	16220
UN06865	Rsa1.0_00928.1	1918	59293	0	710	730	97	+	606	1334	8481	9207
UN06866	Rsa1.0_00507.1	1424	79074	0	409	416	98	−	525	940	59354	59769
UN06867	Rsa1.0_02784.1	1410	27881	0	800	820	97	+	591	1410	12596	13409
UN06868	Rsa1.0_49161.1	798	461	1.00E−113	248	259	95	+	42	295	6	262
UN06869	Rsa1.0_00721.1	1153	66918	1.00E−170	331	340	97	−	571	910	1020	1359
UN06870	Rsa1.0_00341.1	1228	249309	1.00E−168	384	407	94	+	1	404	125750	126149
UN06871	Rsa1.0_00122.1	1232	265937	1.00E−169	334	342	97	−	1	341	41203	41543
UN06872	Rsa1.0_00165.1	1192	116466	0	514	541	95	+	2	530	93799	94339
UN06873	Rsa1.0_00325.1	915	95371	1.00E−140	278	285	97	−	559	842	73729	74013
UN06874	Rsa1.0_00506.1	1452	79106	0	369	375	98	−	852	1226	19513	19887
UN06875	Rsa1.0_04250.1	1720	18264	0	1152	1157	99	−	1	1157	14687	15843
UN06876	Rsa1.0_06967.1	759	10093	0	479	492	97	+	262	753	7110	7601
UN06877	Rsa1.0_00064.1	1025	240927	2.00E−88	202	214	94	−	12	225	60195	60408
UN06878	Rsa1.0_00975.1	860	57865	0	414	424	97	−	1	424	6741	7161
UN06879	Rsa1.0_00605.1	1570	73199	1.00E−162	313	320	97	−	1251	1570	68945	69261
UN06880	Rsa1.0_00271.1	886	101125	0	455	498	91	+	38	519	26584	27065
UN06881	Rsa1.0_00429.1	1278	85559	1.00E−179	358	369	97	+	314	678	13508	13876
UN06882	Rsa1.0_01461.1	733	45240	0	703	733	95	−	1	733	27346	28074
UN06883	Rsa1.0_00324.1	1004	218805	2.00E−95	187	190	98	−	498	687	188774	188963
UN06884	Rsa1.0_00964.1	1349	316648	0	622	639	97	−	576	1214	158869	159501
UN06885	Rsa1.0_03090.1	963	25464	0	534	548	97	−	3	550	17603	18150
UN06886	Rsa1.0_00055.1	1370	520193	0	672	705	95	+	1	700	385970	386668
UN06887	Rsa1.0_01750.1	1106	39816	1.00E−135	254	257	98	+	66	322	25486	25742
UN06888	Rsa1.0_03927.1	1603	19997	0	452	455	99	+	1	455	10302	10756
UN06889	Rsa1.0_06643.1	1445	10615	0	542	545	99	+	308	852	8723	9267
UN06890	Rsa1.0_00125.1	953	253043	1.00E−119	238	242	98	+	712	953	183436	183675
UN06891	Rsa1.0_01701.1	908	162121	0	355	367	96	−	2	357	145137	145503
UN06892	Rsa1.0_00040.1	1139	324196	1.00E−177	415	451	92	−	286	736	171584	172016
UN06893	Rsa1.0_03081.1	1102	25523	0	485	495	97	+	362	856	19744	20238
UN06894	Rsa1.0_01230.1	1254	50221	0	441	441	100	+	2	442	20322	20762
UN06895	Rsa1.0_00265.1	1229	220635	1.00E−163	363	389	93	+	1	384	188871	189247
UN06896	Rsa1.0_00011.1	638	225044	0	613	637	96	−	1	637	222385	223003
UN06897	Rsa1.0_02128.1	1408	34758	1.00E−155	328	347	94	−	1	347	16324	16657
UN06898	Rsa1.0_01967.1	1012	36672	0	683	710	96	−	314	1012	15881	16589
UN06899	Rsa1.0_01378.1	1228	46959	0	543	550	98	+	1	550	12611	13159
UN06900	Rsa1.0_05509.1	637	13472	1.00E−166	566	641	88	+	23	637	12079	12705
UN06901	Rsa1.0_00055.1	1185	520193	0	952	1015	93	−	204	1185	168945	169959

UN06902	Rsa1.0_00705.1	1049	67505	1.00E-130	242	244	99	-	445	688	25656	25899
UN06903	Rsa1.0_00720.1	525	66931	1.00E-131	251	253	99	+	1	251	18491	18743
UN06904	Rsa1.0_47151.1	1197	479	1.00E-122	245	253	96	+	1	253	106	351
UN06905	Rsa1.0_00039.1	1102	296927	2.00E-82	187	196	95	+	103	297	210893	211088
UN06906	Rsa1.0_00777.1	844	191865	1.00E-169	373	394	94	+	72	462	129755	130145
UN06907	Rsa1.0_00362.1	1881	299497	0	740	754	98	+	602	1355	19860	20612
UN06908	Rsa1.0_01803.1	793	173846	0	415	415	100	-	2	416	143938	144352
UN06909	Rsa1.0_04166.1	869	18757	1.00E-162	347	371	93	-	1	371	8039	8389
UN06910	Rsa1.0_02041.1	1172	163634	0	942	1005	93	-	2	984	30110	31107
UN06911	Rsa1.0_03778.1	541	20908	1.00E-165	307	310	99	-	232	540	8218	8527
UN06912	Rsa1.0_04753.1	838	16124	1.00E-92	182	185	98	+	63	247	3064	3248
UN06913	Rsa1.0_00251.1	1214	303968	0	548	564	97	+	559	1120	142909	143472
UN06914	Rsa1.0_00072.1	1154	148743	1.00E-156	301	308	97	-	845	1152	101731	102038
UN06915	Rsa1.0_01313.1	874	48387	1.00E-141	303	319	94	+	556	874	16151	16469
UN06916	Rsa1.0_00855.1	817	62047	1.00E-134	288	303	95	+	14	312	15749	16051
UN06917	Rsa1.0_02362.1	764	32190	1.00E-74	152	155	98	+	233	387	3474	3628
UN06918	Rsa1.0_00124.1	1081	262503	0	651	654	99	-	3	656	65540	66193
UN06919	Rsa1.0_00632.1	952	183777	0	679	692	98	-	1	692	65729	66418
UN06920	Rsa1.0_00917.1	1357	182012	0	725	731	99	-	254	983	128772	129502
UN06921	Rsa1.0_02219.1	1115	33798	1.00E-177	315	315	100	+	431	745	13598	13912
UN06922	Rsa1.0_08143.1	1529	7836	1.00E-126	300	320	93	+	1203	1521	4649	4964
UN06923	Rsa1.0_08592.1	1211	7150	0	1178	1204	97	-	3	1202	1471	2670
UN06924	Rsa1.0_00015.1	889	561803	0	515	522	98	-	3	524	259145	259663
UN06925	Rsa1.0_07812.1	865	8496	0	585	588	99	-	1	588	2402	2989
UN06926	Rsa1.0_00990.1	1485	57445	0	809	899	89	+	163	1030	18057	18934
UN06927	Rsa1.0_00287.1	706	98687	0	462	493	93	+	203	682	15492	15982
UN06928	Rsa1.0_00009.1	1182	232558	1.00E-111	211	213	99	+	523	735	142063	142275
UN06929	Rsa1.0_00211.1	1115	228189	1.00E-139	260	263	98	-	3	265	14336	14598
UN06930	Rsa1.0_09960.1	1207	6065	0	358	358	100	-	850	1207	755	1112
UN06931	Rsa1.0_04497.1	1346	17103	0	394	402	98	+	3	404	6213	6614
UN06932	Rsa1.0_14168.1	768	3714	0	722	760	95	+	2	749	764	1522
UN06933	Rsa1.0_00886.1	792	167350	0	625	656	95	-	145	786	13047	13702
UN06934	Rsa1.0_00119.1	908	467509	0	459	502	91	-	32	527	386218	386716
UN06935	Rsa1.0_00178.1	1419	114861	0	1016	1067	95	-	354	1413	93342	94397
UN06936	Rsa1.0_01126.1	909	53127	0	523	547	95	+	334	878	7555	8096
UN06937	Rsa1.0_03005.1	761	26082	1.00E-108	215	219	98	+	17	235	7720	7937
UN06938	Rsa1.0_00817.1	1490	62961	0	386	386	100	+	1101	1486	20948	21333
UN06939	Rsa1.0_01193.1	1154	51147	0	774	807	95	-	330	1124	27510	28312
UN06940	Rsa1.0_01540.1	1292	151890	0	1203	1285	93	-	21	1292	142475	143746

UN06941	Rsa1.0_00112.1	699	132199	1.00E-132	289	303	95	+	367	665	118993	119292
UN06942	Rsa1.0_03666.1	1344	21485	0	434	435	99	+	1	434	12813	13247
UN06943	Rsa1.0_00673.1	1049	69325	1.00E-173	379	403	94	-	652	1049	53356	53747
UN06944	Rsa1.0_06165.1	838	11584	0	822	831	98	+	9	838	9166	9996
UN06945	Rsa1.0_00015.1	875	561803	0	855	883	96	+	2	875	349586	350465
UN06946	Rsa1.0_02486.1	1025	30965	1.00E-101	219	231	94	+	18	248	9757	9978
UN06947	Rsa1.0_00052.1	1117	162771	0	1000	1038	96	-	2	1032	81968	83003
UN06948	Rsa1.0_00439.1	761	142776	0	367	379	96	+	27	405	57766	58144
UN06949	Rsa1.0_00128.1	799	228675	0	629	667	94	-	27	690	204000	204660
UN06950	Rsa1.0_03643.1	1959	21607	0	1265	1286	98	-	674	1959	20324	21607
UN06951	Rsa1.0_09607.1	914	6256	0	866	874	99	+	39	911	1	874
UN06952	Rsa1.0_01801.1	768	39094	1.00E-131	238	238	100	-	357	594	21087	21324
UN06953	Rsa1.0_00078.1	976	175053	1.00E-147	273	276	98	-	1	276	32901	33176
UN06954	Rsa1.0_00876.1	726	61328	0	709	725	97	-	2	726	23612	24336
UN06955	Rsa1.0_01209.1	931	50668	1.00E-107	250	269	92	-	20	273	47015	47283
UN06956	Rsa1.0_00035.1	1155	176449	1.00E-175	348	357	97	+	800	1155	156806	157158
UN06957	Rsa1.0_04629.1	731	16595	1.00E-86	173	175	98	+	531	705	8842	9015
UN06958	Rsa1.0_00197.1	1193	109714	0	365	373	97	+	820	1192	13353	13725
UN06959	Rsa1.0_00568.1	1106	75219	0	822	834	98	-	273	1106	58649	59479
UN06960	Rsa1.0_00080.1	814	190437	0	471	480	98	+	1	477	84985	85464
UN06961	Rsa1.0_00127.1	1085	127446	1.00E-145	355	388	91	+	33	411	113030	113405
UN06962	Rsa1.0_05124.1	1528	14790	1.00E-127	234	235	99	-	938	1172	2110	2344
UN06963	Rsa1.0_14327.1	659	3518	0	404	421	95	+	238	658	231	645
UN06964	Rsa1.0_00244.1	1308	103590	0	957	960	99	+	349	1308	333	1292
UN06965	Rsa1.0_01635.1	631	41865	0	459	495	92	-	146	625	7307	7801
UN06966	Rsa1.0_46011.1	544	490	0	402	411	97	+	141	544	1	409
UN06967	Rsa1.0_01185.1	1566	149842	0	1537	1567	98	+	1	1566	1682	3247
UN06968	Rsa1.0_00390.1	622	88681	0	417	422	98	-	171	592	10838	11259
UN06969	Rsa1.0_00642.1	1332	201230	0	436	463	94	-	877	1332	62514	62971
UN06970	Rsa1.0_03267.1	1274	24230	0	705	727	96	+	548	1274	16677	17399
UN06971	Rsa1.0_00350.1	1087	92346	1.00E-137	288	301	95	-	764	1064	53956	54252
UN06972	Rsa1.0_02212.1	1414	33840	1.00E-149	289	295	97	+	101	392	18328	18622
UN06973	Rsa1.0_02662.1	695	28956	1.00E-109	200	200	100	-	496	695	10374	10573
UN06974	Rsa1.0_00206.1	902	109040	0	470	476	98	-	427	902	45612	46086
UN06975	Rsa1.0_00326.1	1202	171955	0	443	454	97	-	3	454	22464	22915
UN06976	Rsa1.0_02566.1	758	30039	0	753	756	99	+	3	758	13221	13976
UN06977	Rsa1.0_00065.1	1093	454616	0	659	745	88	+	394	1091	240790	241531
UN06978	Rsa1.0_01707.1	758	40686	0	395	403	98	+	1	400	11784	12186
UN06979	Rsa1.0_01034.1	854	56046	1.00E-140	268	272	98	+	1	270	24544	24815

UN06980	Rsa1.0_00623.1	1357	207630	1.00E-123	233	236	98	-	172	407	172010	172245
UN06981	Rsa1.0_03785.1	553	20883	0	391	398	98	+	5	400	11311	11708
UN06982	Rsa1.0_00355.1	1178	92038	0	503	504	99	-	50	553	64491	64994
UN06983	Rsa1.0_00731.1	971	66638	2.00E-70	139	140	99	-	831	970	1186	1325
UN06984	Rsa1.0_04910.1	1315	15484	0	740	786	94	+	1	785	6509	7282
UN06985	Rsa1.0_00002.1	862	601661	0	413	413	100	-	448	860	561832	562244
UN06986	Rsa1.0_00455.1	739	224823	2.00E-82	203	217	93	+	1	217	183334	183545
UN06987	Rsa1.0_00518.1	775	78360	1.00E-114	213	214	99	+	560	773	20635	20848
UN06988	Rsa1.0_00022.1	729	200461	2.00E-94	256	287	89	+	3	266	123609	123895
UN06989	Rsa1.0_00017.1	955	752458	0	559	568	98	-	1	567	696586	697153
UN06990	Rsa1.0_08910.1	1236	6798	5.00E-84	171	175	97	+	374	548	1064	1238
UN06991	Rsa1.0_03673.1	1078	21448	1.00E-163	317	325	97	+	756	1077	2710	3034
UN06992	Rsa1.0_00280.1	814	99966	1.00E-180	572	655	87	-	5	643	38104	38752
UN06993	Rsa1.0_02170.1	1107	34352	0	604	639	94	+	2	639	30209	30835
UN06994	Rsa1.0_00005.1	1318	353093	0	402	408	98	+	1	407	50638	51041
UN06995	Rsa1.0_02797.1	757	27800	0	442	458	96	+	3	459	8978	9435
UN06996	Rsa1.0_01781.1	896	39410	0	382	382	100	+	515	896	22750	23131
UN06997	Rsa1.0_00616.1	660	195713	0	601	607	99	-	3	609	144445	145049
UN06998	Rsa1.0_00319.1	864	167331	0	852	863	98	+	3	864	91306	92168
UN06999	Rsa1.0_00010.1	1203	294827	2.00E-21	60	62	96	-	950	1011	207266	207327
UN07000	Rsa1.0_15767.1	1084	2435	0	454	459	98	+	1	459	91	549
UN07001	Rsa1.0_00699.1	1044	184398	0	375	378	99	+	402	779	121768	122145
UN07002	Rsa1.0_00301.1	1335	365315	0	413	414	99	+	923	1335	57406	57819
UN07003	Rsa1.0_01811.1	871	38977	1.00E-145	366	394	92	-	1	387	1187	1577
UN07004	Rsa1.0_01824.1	877	38733	1.00E-122	279	294	94	+	1	294	7738	8023
UN07005	Rsa1.0_00124.1	1785	262503	0	803	837	95	+	53	885	56753	57579
UN07006	Rsa1.0_01779.1	969	39448	0	737	753	97	-	1	750	11033	11785
UN07007	Rsa1.0_02208.1	427	33875	1.00E-105	209	214	97	-	2	215	17960	18173
UN07008	Rsa1.0_02858.1	954	27246	1.00E-128	300	318	94	+	641	951	1699	2016
UN07009	Rsa1.0_00053.1	911	263459	1.00E-137	266	272	97	-	401	672	63729	64000
UN07010	Rsa1.0_01403.1	986	46453	1.00E-154	394	431	91	+	454	876	25846	26261
UN07011	Rsa1.0_02330.1	1234	32452	0	495	498	99	+	736	1233	1503	2000
UN07012	Rsa1.0_02064.1	1015	35476	0	332	332	100	+	240	571	5466	5797
UN07013	Rsa1.0_00017.1	1396	752458	0	734	734	100	+	1	734	391644	392377
UN07014	Rsa1.0_00892.1	924	60703	0	441	454	97	+	471	922	21370	21823
UN07015	Rsa1.0_01422.1	1049	46172	1.00E-162	340	354	96	+	1	353	24781	25133
UN07016	Rsa1.0_00986.1	1038	57571	0	586	589	99	-	2	589	31853	32441
UN07017	Rsa1.0_02646.1	1207	29123	0	553	605	91	-	601	1205	13903	14507
UN07018	Rsa1.0_00273.1	1165	326186	0	1147	1163	98	-	3	1165	144767	145929

UN07019	Rsa1.0_01512.1	810	44234	0	527	559	94	−	3	559	34074	34615
UN07020	Rsa1.0_02816.1	976	27649	0	947	979	96	+	1	976	944	1909
UN07021	Rsa1.0_00194.1	1735	110851	0	1679	1740	96	+	1	1735	60250	61989
UN07022	Rsa1.0_05311.1	786	14181	0	640	661	96	−	1	660	6590	7245
UN07023	Rsa1.0_02345.1	824	32337	1.00E−119	326	360	90	−	1	348	15392	15744
UN07024	Rsa1.0_00066.1	654	152418	1.00E−116	252	265	95	+	4	268	91382	91646
UN07025	Rsa1.0_00187.1	924	393093	0	408	421	96	+	14	433	212110	212530
UN07026	Rsa1.0_00694.1	909	68074	1.00E−122	223	223	100	+	443	665	5480	5702
UN07027	Rsa1.0_12291.1	947	5264	0	455	456	99	+	1	456	4166	4621
UN07028	Rsa1.0_00437.1	961	84879	0	382	397	96	−	87	483	39669	40065
UN07029	Rsa1.0_00013.1	1191	292129	1.00E−169	317	322	98	−	1	322	281575	281896
UN07030	Rsa1.0_00114.1	1160	131330	0	603	634	95	−	1	631	34658	35291
UN07031	Rsa1.0_35417.1	1222	639	0	607	620	97	−	550	1169	20	639
UN07032	Rsa1.0_04458.1	959	17255	0	913	968	94	+	1	955	9436	10395
UN07033	Rsa1.0_00550.1	775	210790	0	360	362	99	+	2	363	188722	189083
UN07034	Rsa1.0_10607.1	1802	5786	0	1050	1116	94	+	206	1312	693	1790
UN07035	Rsa1.0_00248.1	925	163568	1.00E−163	316	324	97	+	2	325	1050	1373
UN07036	Rsa1.0_05077.1	1289	14913	0	604	612	98	+	682	1287	2341	2952
UN07037	Rsa1.0_03997.1	1116	19652	0	394	410	96	−	700	1109	2468	2877
UN07038	Rsa1.0_02171.1	731	167557	1.00E−140	283	292	96	+	1	292	13006	13296
UN07039	Rsa1.0_00522.1	795	78103	1.00E−131	241	242	99	−	2	243	68506	68747
UN07040	Rsa1.0_02384.1	868	31917	0	548	602	91	+	272	867	13168	13751
UN07041	Rsa1.0_01082.1	1305	54640	0	472	479	98	+	825	1303	15720	16198
UN07042	Rsa1.0_18032.1	662	1686	1.00E−108	205	207	99	−	314	520	1291	1497
UN07043	Rsa1.0_02365.1	1008	32118	1.00E−123	234	237	98	+	772	1008	10500	10736
UN07044	Rsa1.0_00849.1	1198	62212	1.00E−170	328	336	97	−	862	1197	21303	21638
UN07045	Rsa1.0_00721.1	1265	66918	0	423	442	95	−	517	958	918	1359
UN07046	Rsa1.0_15757.1	631	2442	0	524	531	98	−	1	529	1463	1993
UN07047	Rsa1.0_00506.1	1449	79106	1.00E−144	303	318	95	−	1	311	6009	6326
UN07048	Rsa1.0_00629.1	1938	71381	0	800	831	96	+	694	1523	45375	46186
UN07049	#	#	#	#	#	#	#	#	#	#	#	#
UN07050	Rsa1.0_01029.1	933	256254	0	886	904	98	+	30	933	230191	231088
UN07051	Rsa1.0_00515.1	549	78417	1.00E−158	304	311	97	−	1	306	55788	56098
UN07052	Rsa1.0_00009.1	1060	232558	0	519	547	94	+	10	546	117345	117890
UN07053	Rsa1.0_01613.1	1226	182542	0	499	503	99	−	1	502	162642	163144
UN07054	Rsa1.0_03391.1	764	23251	0	727	767	94	−	1	762	1652	2408
UN07055	Rsa1.0_00001.1	945	363028	0	762	775	98	+	171	945	238678	239452
UN07056	Rsa1.0_00211.1	585	228189	0	565	568	99	−	18	585	179347	179912
UN07057	Rsa1.0_00522.1	912	78103	1.00E−116	238	246	96	−	626	871	57158	57399

UN07058	Rsa1.0_00516.1	1322	78417	1.00E-155	295	298	98	+	2	299	45139	45433
UN07059	Rsa1.0_05978.1	509	12053	1.00E-103	212	219	96	−	278	496	5919	6137
UN07060	Rsa1.0_00605.1	1141	73199	0	484	490	98	+	1	488	49637	50124
UN07061	Rsa1.0_01504.1	2073	189978	0	463	490	94	−	1582	2071	36465	36941
UN07062	Rsa1.0_00624.1	757	71773	1.00E-121	230	233	98	−	170	402	20764	20996
UN07063	Rsa1.0_01006.1	1148	163670	0	520	530	98	+	27	554	25599	26128
UN07064	Rsa1.0_01251.1	1142	49873	1.00E-133	253	257	98	−	486	742	29907	30163
UN07065	#	#	#	#	#	#	#	#	#	#	#	#
UN07066	Rsa1.0_00163.1	1415	116847	0	558	578	96	+	844	1415	37091	37665
UN07067	Rsa1.0_00201.1	1025	232851	6.00E-86	168	170	98	−	567	736	3232	3401
UN07068	Rsa1.0_00167.1	1204	116000	0	1089	1140	95	+	1	1122	87398	88534
UN07069	Rsa1.0_00029.1	1096	272992	0	1057	1094	96	+	4	1095	230488	231574
UN07070	Rsa1.0_26999.1	751	870	0	332	332	100	−	1	332	154	485
UN07071	Rsa1.0_01284.1	1356	49231	0	389	391	99	+	518	908	45549	45939
UN07072	Rsa1.0_01667.1	1151	41315	1.00E-136	280	288	97	−	867	1151	8241	8526
UN07073	Rsa1.0_00424.1	1442	86005	0	360	365	98	−	343	707	76607	76971
UN07074	Rsa1.0_02110.1	1615	34919	0	760	812	93	+	2	800	23093	23891
UN07075	Rsa1.0_03856.1	498	20429	3.00E-95	215	225	95	−	275	498	15618	15841
UN07076	Rsa1.0_00827.1	758	62790	0	743	757	98	−	2	758	46407	47163
UN07077	Rsa1.0_01218.1	643	50536	0	640	643	99	+	1	641	12503	13145
UN07078	Rsa1.0_00087.1	1088	761073	0	719	749	95	−	5	751	731598	732345
UN07079	Rsa1.0_01477.1	1211	45008	0	1003	1044	96	+	175	1209	42412	43455
UN07080	Rsa1.0_02379.1	1244	150705	0	1224	1244	98	+	3	1244	146234	147476
UN07081	Rsa1.0_00803.1	1249	144228	1.00E-139	278	286	97	+	226	508	12208	12493
UN07082	Rsa1.0_00545.1	1483	189649	0	783	813	96	−	1	804	55735	56545
UN07083	Rsa1.0_00051.1	1706	258225	0	391	398	98	−	1	395	18495	18892
UN07084	Rsa1.0_00532.1	1058	77384	1.00E-131	247	250	98	−	684	933	12854	13103
UN07085	Rsa1.0_02358.1	984	32213	1.00E-168	326	335	97	−	460	794	384	712
UN07086	Rsa1.0_05320.1	1298	14158	0	383	384	99	−	215	598	3179	3562
UN07087	Rsa1.0_06253.1	972	11385	0	426	430	99	−	3	431	7588	8017
UN07088	Rsa1.0_04411.1	1340	142979	1.00E-124	263	274	95	−	11	284	137399	137671
UN07089	Rsa1.0_00326.1	994	171955	1.00E-116	301	331	90	+	649	979	65824	66133
UN07090	Rsa1.0_03193.1	1265	24690	0	487	540	90	−	7	538	21087	21602
UN07091	Rsa1.0_00005.1	1253	353093	1.00E-178	317	317	100	−	162	478	51852	52168
UN07092	Rsa1.0_00254.1	680	102600	1.00E-126	280	297	94	+	9	305	73233	73523
UN07093	Rsa1.0_02236.1	1257	33558	1.00E-179	386	408	94	−	830	1237	13202	13605
UN07094	Rsa1.0_01143.1	689	52456	4.00E-77	165	171	96	+	383	553	46053	46223
UN07095	Rsa1.0_03545.1	917	147543	1.00E-125	273	287	95	−	621	907	147220	147505
UN07096	Rsa1.0_00055.1	747	520193	1.00E-117	249	260	95	−	416	675	211435	211690

UN07097	Rsa1.0_01769.1	901	39568	1.00E-133	244	245	99	-	104	348	33607	33851
UN07098	Rsa1.0_00893.1	1027	60626	0	501	510	98	+	15	522	46398	46907
UN07099	Rsa1.0_00332.1	1232	94575	0	500	521	95	-	716	1230	62006	62524
UN07100	Rsa1.0_00047.1	1505	168582	0	495	498	99	+	2	499	124961	125458
UN07101	Rsa1.0_03057.1	820	25724	2.00E-73	156	161	96	-	534	694	139	299
UN07102	Rsa1.0_00121.1	750	278029	1.00E-154	290	292	99	+	1	291	112289	112579
UN07103	Rsa1.0_00845.1	853	62346	1.00E-128	240	241	99	+	542	781	12049	12289
UN07104	Rsa1.0_00040.1	859	324196	0	526	565	93	-	271	826	36385	36939
UN07105	Rsa1.0_00194.1	1509	110851	0	1240	1297	95	-	184	1471	60327	61623
UN07106	Rsa1.0_00107.1	980	431659	0	513	546	93	-	442	980	165615	166159
UN07107	Rsa1.0_01853.1	1218	147800	1.00E-157	303	310	97	+	498	807	128102	128411
UN07108	Rsa1.0_55790.1	545	410	1.00E-122	273	290	94	-	90	379	1	290
UN07109	Rsa1.0_01382.1	1317	46877	0	406	426	95	+	1	423	11373	11798
UN07110	Rsa1.0_00213.1	1552	108167	0	363	363	100	-	3	365	99017	99379
UN07111	Rsa1.0_03111.1	1074	25327	0	401	419	95	-	658	1074	3066	3481
UN07112	Rsa1.0_00001.1	1442	363028	0	955	980	97	+	469	1442	280589	281568
UN07113	Rsa1.0_00435.1	1269	317579	0	402	404	99	-	394	797	54645	55047
UN07114	Rsa1.0_02726.1	1547	28404	1.00E-133	301	320	94	-	1230	1547	25547	25866
UN07115	Rsa1.0_00976.1	837	57809	1.00E-89	177	180	98	+	516	695	7053	7232
UN07116	Rsa1.0_02179.1	972	34188	1.00E-177	393	417	94	-	543	947	23623	24037
UN07117	Rsa1.0_00114.1	1001	131330	0	702	713	98	-	79	791	95546	96258
UN07118	Rsa1.0_01476.1	1118	172928	1.00E-123	281	297	94	+	819	1111	9187	9481
UN07119	Rsa1.0_00104.1	918	368722	0	915	915	100	-	1	915	155070	155984
UN07120	Rsa1.0_03668.1	412	21483	4.00E-63	135	139	97	+	73	211	19585	19723
UN07121	Rsa1.0_03209.1	1190	24592	0	538	546	98	+	3	547	5163	5708
UN07122	Rsa1.0_00179.1	955	393324	0	751	778	96	-	179	951	241686	242456
UN07123	Rsa1.0_00720.1	957	66931	0	516	517	99	+	439	955	2953	3469
UN07124	Rsa1.0_12814.1	1197	5119	0	553	561	98	+	638	1197	3092	3652
UN07125	Rsa1.0_04111.1	1035	18991	1.00E-106	251	268	93	-	17	283	13843	14110
UN07126	Rsa1.0_01749.1	1103	39822	0	391	400	97	+	704	1103	7472	7869
UN07127	Rsa1.0_01728.1	633	40239	0	484	510	94	-	1	509	21789	22295
UN07128	Rsa1.0_06010.1	1552	11944	0	885	895	98	+	656	1550	7741	8635
UN07129	Rsa1.0_01860.1	1387	38057	2.00E-89	168	168	100	-	414	581	23461	23628
UN07130	Rsa1.0_00324.1	1225	218805	1.00E-167	305	307	99	+	919	1225	182825	183131
UN07131	Rsa1.0_00322.1	1297	212233	1.00E-120	222	223	99	-	838	1060	80171	80393
UN07132	Rsa1.0_06677.1	940	10560	0	911	941	96	-	3	940	2190	3124
UN07133	Rsa1.0_00243.1	748	103651	1.00E-136	334	366	91	+	403	748	23261	23623
UN07134	Rsa1.0_03577.1	862	143993	0	451	454	99	-	409	862	7984	8437
UN07135	Rsa1.0_00162.1	1175	239935	1.00E-126	258	264	97	+	913	1174	25199	25460

UN07136	Rsa1.0_02247.1	963	33427	1.00E-118	267	282	94	+	590	870	28646	28924
UN07137	Rsa1.0_00229.1	1198	105471	0	1078	1094	98	−	1	1094	82850	83941
UN07138	Rsa1.0_00477.1	845	81556	1.00E-125	242	247	97	+	132	378	21683	21929
UN07139	Rsa1.0_00473.1	1069	212204	0	653	672	97	+	233	904	31308	31979
UN07140	Rsa1.0_00044.1	804	271658	0	788	798	98	+	7	804	24744	25540
UN07141	Rsa1.0_00737.1	982	66432	3.00E-97	261	284	91	−	713	982	31720	32003
UN07142	Rsa1.0_00522.1	957	78103	1.00E-110	233	242	96	+	86	326	57158	57399
UN07143	Rsa1.0_01589.1	815	42721	1.00E-150	300	310	96	+	209	518	29072	29381
UN07144	Rsa1.0_03138.1	1115	25092	0	554	558	99	−	556	1113	9101	9658
UN07145	Rsa1.0_00190.1	1268	318240	0	405	406	99	−	863	1268	196708	197113
UN07146	Rsa1.0_02304.1	869	32793	0	404	405	99	−	240	644	15443	15847
UN07147	Rsa1.0_01034.1	667	56046	0	359	367	97	−	60	426	35993	36359
UN07148	Rsa1.0_00218.1	1322	201480	1.00E-108	206	208	99	−	370	577	114870	115077
UN07149	Rsa1.0_00447.1	963	84051	0	379	379	100	−	552	930	56160	56538
UN07150	Rsa1.0_03166.1	1525	24863	0	468	468	100	+	1058	1525	19446	19913
UN07151	Rsa1.0_00423.1	352	86138	1.00E-141	266	270	98	−	83	352	10887	11156
UN07152	Rsa1.0_01837.1	1162	38502	0	1141	1162	98	−	2	1161	27628	28789
UN07153	Rsa1.0_01059.1	1468	55410	0	1228	1281	95	−	195	1468	28279	29538
UN07154	Rsa1.0_00065.1	1786	454616	0	1711	1767	96	−	3	1769	132950	134713
UN07155	Rsa1.0_01245.1	862	149868	0	554	562	98	+	302	862	45970	46531
UN07156	Rsa1.0_00013.1	1262	292129	0	441	444	99	+	818	1261	90182	90625
UN07157	Rsa1.0_06497.1	1061	10890	0	768	782	98	−	2	782	351	1131
UN07158	Rsa1.0_05864.1	1466	12387	0	340	340	100	−	1	340	7543	7882
UN07159	Rsa1.0_04299.1	1157	18027	0	712	713	99	−	1	713	11917	12629
UN07160	Rsa1.0_03475.1	1055	22653	1.00E-158	308	316	97	+	418	733	16055	16370
UN07161	Rsa1.0_03790.1	958	20844	0	412	426	96	−	345	770	8657	9074
UN07162	Rsa1.0_03552.1	817	22151	3.00E-84	168	171	98	+	456	626	14663	14833
UN07163	Rsa1.0_00523.1	1556	178770	0	409	411	99	+	1144	1554	166967	167377
UN07164	Rsa1.0_01282.1	1657	49304	0	1605	1655	96	−	6	1654	30123	31768
UN07165	Rsa1.0_02852.1	691	27287	0	635	668	95	+	3	670	15228	15884
UN07166	Rsa1.0_00378.1	1179	89892	0	415	436	95	−	3	436	81481	81900
UN07167	Rsa1.0_00033.1	1490	515811	0	1169	1179	99	−	315	1490	497657	498835
UN07168	Rsa1.0_00529.1	851	77487	1.00E-105	300	333	90	+	521	851	65173	65489
UN07169	Rsa1.0_01249.1	1038	188432	0	1016	1038	97	+	1	1036	182099	183129
UN07170	Rsa1.0_00750.1	2061	190548	0	654	661	98	+	288	948	16410	17070
UN07171	Rsa1.0_00888.1	852	60978	0	392	398	98	−	229	626	23903	24300
UN07172	Rsa1.0_02295.1	742	149884	1.00E-150	270	270	100	−	473	742	13556	13825
UN07173	Rsa1.0_00012.1	1120	222148	0	448	465	96	−	656	1118	28069	28530
UN07174	Rsa1.0_00754.1	927	65689	0	405	413	98	+	1	407	59411	59822

UN07175	#	#	#	#	#	#	#	#	#	#	#	#
UN07176	Rsa1.0_19503.1	1655	1422	0	870	927	93	−	74	1000	218	1144
UN07177	Rsa1.0_01586.1	1088	42804	1.00E−178	398	420	94	−	672	1088	28079	28496
UN07178	Rsa1.0_00051.1	1240	258225	0	651	667	97	−	574	1240	52532	53198
UN07179	Rsa1.0_03381.1	811	23306	1.00E−142	263	265	99	−	3	267	5379	5643
UN07180	Rsa1.0_04817.1	601	15869	0	595	598	99	+	1	597	12103	12700
UN07181	Rsa1.0_00651.1	1165	247766	0	722	730	98	+	436	1165	98435	99164
UN07182	Rsa1.0_08401.1	1565	7418	0	681	684	99	−	1	683	678	1361
UN07183	Rsa1.0_13478.1	1204	4790	0	1081	1105	97	+	6	1109	2571	3668
UN07184	Rsa1.0_00028.1	769	293984	0	574	576	99	−	195	769	71347	71922
UN07185	Rsa1.0_00471.1	1078	81897	0	870	885	98	+	198	1078	76439	77323
UN07186	Rsa1.0_00389.1	872	88795	0	862	862	100	−	11	872	44597	45458
UN07187	Rsa1.0_03192.1	1797	24695	0	1063	1100	96	+	659	1745	11044	12142
UN07188	Rsa1.0_05553.1	1132	13344	0	1115	1130	98	+	2	1131	10865	11994
UN07189	Rsa1.0_02566.1	763	30039	0	730	760	96	−	3	761	13189	13940
UN07190	Rsa1.0_00092.1	867	659932	1.00E−150	273	274	99	−	129	402	14484	14757
UN07191	Rsa1.0_05676.1	967	12969	0	493	509	96	+	447	954	9434	9937
UN07192	Rsa1.0_01789.1	1185	39250	0	452	463	97	+	717	1178	5483	5943
UN07193	Rsa1.0_01901.1	805	37472	0	484	489	98	−	3	491	26858	27346
UN07194	Rsa1.0_02846.1	814	27338	0	790	815	96	+	1	814	19909	20722
UN07195	Rsa1.0_00021.1	1253	263655	0	621	621	100	+	129	749	10057	10677
UN07196	Rsa1.0_00552.1	598	206757	1.00E−105	271	291	93	−	300	588	72818	73102
UN07197	Rsa1.0_00108.1	1144	478491	0	1001	1049	95	+	3	1051	70429	71470
UN07198	Rsa1.0_02505.1	968	30703	1.00E−135	245	245	100	−	149	393	8840	9084
UN07199	Rsa1.0_02150.1	1784	34580	0	792	797	99	−	990	1784	17993	18789
UN07200	Rsa1.0_00711.1	746	67223	0	520	520	100	+	227	746	15004	15523
UN07201	Rsa1.0_00152.1	1375	257730	0	645	663	97	−	3	665	230931	231593
UN07202	Rsa1.0_00551.1	1404	76227	1.00E−177	430	464	92	−	10	465	20659	21118
UN07203	Rsa1.0_01416.1	896	46262	0	543	556	97	−	7	562	16597	17152
UN07204	Rsa1.0_05401.1	957	13876	0	526	564	93	+	413	957	1410	1973
UN07205	Rsa1.0_00632.1	1457	183777	0	1422	1463	97	+	13	1457	144872	146327
UN07206	Rsa1.0_00344.1	1204	191774	0	1168	1202	97	−	1	1200	127033	128233
UN07207	Rsa1.0_00886.1	998	167350	0	493	500	98	+	7	506	11220	11719
UN07208	Rsa1.0_00414.1	1176	202190	0	967	997	96	−	140	1134	56594	57589
UN07209	Rsa1.0_00976.1	897	57809	0	326	326	100	−	571	896	48272	48597
UN07210	Rsa1.0_01835.1	722	38542	0	656	703	93	+	1	695	12839	13536
UN07211	Rsa1.0_01753.1	1558	39794	1.00E−103	198	200	99	+	440	639	19842	20041
UN07212	Rsa1.0_00278.1	632	231896	0	566	602	94	+	5	603	183530	184118
UN07213	Rsa1.0_00108.1	889	478491	1.00E−132	240	240	100	+	650	889	51602	51841

UN07214	Rsa1.0_00017.1	840	752458	1.00E-150	314	325	96	-	3	320	11197	11519
UN07215	Rsa1.0_05320.1	810	14158	0	809	810	99	+	1	810	8531	9339
UN07216	Rsa1.0_07444.1	585	9386	1.00E-149	276	279	98	+	68	346	7105	7383
UN07217	Rsa1.0_01203.1	1080	50848	0	848	882	96	+	103	974	1079	1960
UN07218	Rsa1.0_00877.1	1323	61305	0	1300	1317	98	+	10	1323	40786	42096
UN07219	Rsa1.0_00392.1	1950	88523	0	685	721	95	-	976	1691	13962	14671
UN07220	Rsa1.0_00731.1	912	66638	0	515	528	97	-	5	531	55615	56140
UN07221	Rsa1.0_00028.1	745	293984	0	653	662	98	-	84	745	86862	87523
UN07222	Rsa1.0_09779.1	1070	6154	1.00E-121	231	233	99	+	5	236	734	966
UN07223	Rsa1.0_02589.1	1491	300828	0	746	841	88	-	476	1316	151178	152018
UN07224	Rsa1.0_20721.1	1550	1276	0	488	501	97	+	355	853	406	906
UN07225	Rsa1.0_02192.1	1145	171291	0	517	538	96	+	571	1104	9538	10075
UN07226	Rsa1.0_00064.1	875	240927	0	869	880	98	-	1	875	232180	233059
UN07227	Rsa1.0_01417.1	1031	46252	0	409	413	99	-	1	412	20533	20944
UN07228	Rsa1.0_05254.1	1378	14362	0	1358	1379	98	-	1	1378	3811	5189
UN07229	Rsa1.0_00497.1	460	79830	1.00E-130	236	236	100	+	1	236	43623	43858
UN07230	Rsa1.0_15414.1	1349	2620	0	663	675	98	+	179	853	1	674
UN07231	Rsa1.0_01570.1	880	43015	1.00E-159	288	289	99	-	377	665	32273	32561
UN07232	Rsa1.0_01352.1	1268	47443	1.00E-109	216	221	97	+	618	838	12792	13012
UN07233	Rsa1.0_02648.1	1059	29073	0	629	647	97	+	422	1058	11052	11698
UN07234	Rsa1.0_01984.1	855	36491	0	850	851	99	+	5	855	31611	32461
UN07235	Rsa1.0_00494.1	676	79967	0	498	521	95	-	2	521	3932	4449
UN07236	Rsa1.0_00029.1	535	272992	0	526	547	96	+	3	535	124469	125015
UN07237	Rsa1.0_00100.1	1583	249232	0	455	470	96	+	1	470	13957	14423
UN07238	Rsa1.0_02706.1	1106	28522	0	470	476	98	-	3	478	14012	14483
UN07239	Rsa1.0_04456.1	1567	17260	0	762	763	99	+	804	1566	15708	16469
UN07240	Rsa1.0_00025.1	641	277312	0	373	377	98	+	265	639	17353	17728
UN07241	Rsa1.0_06355.1	686	11180	0	592	601	98	-	1	598	515	1115
UN07242	Rsa1.0_02245.1	644	33478	0	394	410	96	-	235	642	6316	6725
UN07243	Rsa1.0_05530.1	732	13409	0	721	730	98	+	2	730	11827	12556
UN07244	Rsa1.0_00233.1	847	104918	1.00E-31	83	87	95	-	723	809	1959	2045
UN07245	Rsa1.0_10012.1	1233	6039	0	451	457	98	-	778	1233	341	797
UN07246	Rsa1.0_05984.1	1167	12031	0	454	469	96	-	677	1139	2482	2947
UN07247	Rsa1.0_00939.1	887	185227	0	423	442	95	-	451	887	51026	51466
UN07248	Rsa1.0_04116.1	635	18971	0	405	415	97	+	1	415	18340	18754
UN07249	Rsa1.0_00090.1	1736	273859	1.00E-149	268	268	100	+	456	723	143363	143630
UN07250	Rsa1.0_00551.1	1290	76227	0	530	538	98	-	753	1290	54758	55293
UN07251	Rsa1.0_00022.1	1234	200461	0	1220	1236	98	-	1	1233	141333	142562
UN07252	Rsa1.0_00469.1	797	82084	0	483	510	94	+	292	796	77636	78139

UN07253	Rsa1.0_00837.1	917	62568	0	505	505	100	+	1	505	19284	19788
UN07254	Rsa1.0_02836.1	949	27481	1.00E-150	280	282	99	−	667	948	12895	13175
UN07255	Rsa1.0_25015.1	1133	956	0	467	482	96	+	319	800	475	956
UN07256	Rsa1.0_06200.1	1449	11506	0	920	922	99	+	176	1096	10585	11506
UN07257	Rsa1.0_06990.1	880	10060	0	848	879	96	+	1	868	4363	5235
UN07258	Rsa1.0_02944.1	1181	26553	1.00E-172	310	311	99	+	1	311	9056	9366
UN07259	Rsa1.0_00893.1	1262	60626	0	717	756	94	+	33	783	43045	43797
UN07260	Rsa1.0_00419.1	845	86562	0	427	429	99	+	2	429	39883	40311
UN07261	Rsa1.0_00233.1	1456	104918	0	639	640	99	−	1	640	40246	40885
UN07262	Rsa1.0_17196.1	1023	1893	0	843	868	97	+	158	1023	1	862
UN07263	Rsa1.0_00639.1	1023	165873	0	1011	1011	100	+	13	1023	128422	129432
UN07264	Rsa1.0_01920.1	859	158533	0	575	593	96	−	12	598	135428	136016
UN07265	Rsa1.0_00821.1	1457	62891	1.00E-170	347	361	96	−	1105	1457	55683	56042
UN07266	Rsa1.0_00092.1	1340	659932	0	479	482	99	−	1	480	42020	42501
UN07267	Rsa1.0_00978.1	1290	57739	0	891	891	100	−	400	1290	24460	25350
UN07268	Rsa1.0_00166.1	971	240912	1.00E-175	317	319	99	+	653	971	224857	225175
UN07269	Rsa1.0_00393.1	1013	158674	0	511	511	100	+	63	573	135841	136351
UN07270	Rsa1.0_00108.1	1072	478491	0	976	996	97	−	78	1072	448266	449256
UN07271	Rsa1.0_00004.1	1025	253996	0	1015	1025	99	+	1	1024	158833	159856
UN07272	Rsa1.0_00420.1	1395	86496	1.00E-127	232	232	100	−	11	242	11931	12162
UN07273	Rsa1.0_01494.1	716	44641	0	429	431	99	−	3	432	14397	14827
UN07274	Rsa1.0_08811.1	854	6904	0	437	437	100	+	168	604	3266	3702
UN07275	Rsa1.0_00128.1	658	228675	1.00E-162	298	300	99	+	358	657	139177	139474
UN07276	Rsa1.0_03185.1	755	24739	5.00E-89	179	183	97	−	242	424	9033	9215
UN07277	Rsa1.0_00748.1	1141	65983	0	523	534	97	−	365	898	45626	46159
UN07278	Rsa1.0_01198.1	1039	51029	0	873	897	97	+	1	896	40408	41300
UN07279	Rsa1.0_00387.1	692	495407	0	692	693	99	+	1	692	143940	144632
UN07280	Rsa1.0_07439.1	316	9392	1.00E-81	154	154	100	−	41	194	9239	9392
UN07281	Rsa1.0_00194.1	1156	110851	1.00E-157	292	295	98	−	862	1156	96169	96460
UN07282	Rsa1.0_00075.1	1344	146680	0	515	526	97	−	1	525	19296	19815
UN07283	Rsa1.0_02289.1	1461	32981	0	582	604	96	+	858	1461	17563	18154
UN07284	Rsa1.0_00088.1	1452	141341	0	632	649	97	−	810	1452	97198	97835
UN07285	Rsa1.0_17335.1	903	1857	0	702	713	98	+	109	819	1146	1857
UN07286	Rsa1.0_10108.1	497	5996	1.00E-116	255	266	95	+	216	478	111	374
UN07287	Rsa1.0_00173.1	771	288743	0	395	403	98	+	371	770	203683	204082
UN07288	Rsa1.0_01750.1	932	39816	8.00E-42	107	112	95	+	429	539	18559	18670
UN07289	Rsa1.0_01203.1	1190	50848	1.00E-140	256	257	99	+	1	257	38676	38932
UN07290	Rsa1.0_00110.1	905	132719	0	878	905	97	−	4	905	49653	50556
UN07291	Rsa1.0_01521.1	886	142147	0	448	448	100	+	1	448	137675	138122

UN07292	Rsa1.0_00124.1	761	262503	0	444	446	99	+	1	446	16138	16583
UN07293	Rsa1.0_02929.1	740	26673	1.00E-119	251	261	96	−	474	734	21072	21330
UN07294	Rsa1.0_00807.1	450	63607	5.00E-60	153	162	94	−	292	450	40888	41045
UN07295	Rsa1.0_00359.1	1336	91256	0	570	574	99	+	563	1136	22160	22733
UN07296	Rsa1.0_00784.1	1513	64359	0	921	922	99	−	593	1513	2126	3047
UN07297	Rsa1.0_00027.1	897	283362	0	357	366	97	+	282	645	20864	21229
UN07298	Rsa1.0_02120.1	559	34819	1.00E-175	347	359	96	−	1	359	9113	9471
UN07299	Rsa1.0_00657.1	886	69858	0	859	883	97	+	8	884	17707	18583
UN07300	Rsa1.0_02969.1	1138	26390	0	585	587	99	+	550	1136	6035	6621
UN07301	Rsa1.0_00086.1	981	439854	1.00E-155	284	286	99	+	696	981	284247	284532
UN07302	Rsa1.0_00168.1	687	115870	1.00E-168	299	299	100	+	389	687	38162	38460
UN07303	Rsa1.0_00214.1	631	107508	1.00E-140	300	314	95	−	309	621	36818	37127
UN07304	Rsa1.0_00067.1	1145	415055	0	524	525	99	−	621	1145	34474	34997
UN07305	Rsa1.0_08572.1	597	7184	0	405	416	97	+	180	595	6112	6523
UN07306	Rsa1.0_00385.1	1199	89160	0	409	426	96	−	1	424	12113	12538
UN07307	Rsa1.0_00299.1	1228	97770	0	1185	1228	96	+	1	1228	9212	10432
UN07308	Rsa1.0_00469.1	1097	82084	0	758	765	99	+	1	765	46985	47748
UN07309	Rsa1.0_02916.1	1535	26809	0	372	382	97	+	1	373	6047	6428
UN07310	Rsa1.0_05758.1	1129	12721	0	988	1004	98	+	125	1128	442	1445
UN07311	Rsa1.0_18041.1	843	1683	0	521	522	99	−	322	843	1162	1683
UN07312	Rsa1.0_00111.1	1630	390088	0	723	746	96	−	1	742	178097	178842
UN07313	Rsa1.0_01137.1	1296	52655	0	1291	1297	99	+	1	1296	43969	45262
UN07314	Rsa1.0_02908.1	1308	26859	0	817	826	98	−	482	1307	26034	26859
UN07315	Rsa1.0_00821.1	1148	62891	1.00E-173	318	320	99	+	829	1148	32814	33131
UN07316	Rsa1.0_00286.1	1057	98709	1.00E-177	384	409	93	−	658	1057	54908	55309
UN07317	Rsa1.0_01698.1	800	40830	1.00E-161	288	288	100	−	1	288	30279	30566
UN07318	Rsa1.0_00100.1	1299	249232	1.00E-175	385	412	93	+	284	695	174836	175235
UN07319	Rsa1.0_01855.1	1643	38204	0	1043	1077	96	−	554	1630	26509	27583
UN07320	Rsa1.0_37875.1	1802	595	0	593	595	99	−	758	1352	1	595
UN07321	Rsa1.0_03538.1	1678	22204	0	506	506	100	+	199	704	2685	3190
UN07322	Rsa1.0_00092.1	1933	659932	1.00E-136	272	279	97	+	283	560	630024	630302
UN07323	Rsa1.0_00121.1	1290	278029	1.00E-171	314	317	99	−	974	1290	34412	34728
UN07324	Rsa1.0_01397.1	1378	183276	0	654	671	97	+	412	1082	639	1309
UN07325	Rsa1.0_02076.1	772	153121	0	485	489	99	+	1	489	14561	15048
UN07326	Rsa1.0_05577.1	904	13284	0	878	905	97	+	1	903	11449	12352
UN07327	Rsa1.0_14327.1	1395	3518	0	1065	1068	99	+	239	1306	231	1297
UN07328	Rsa1.0_00073.1	1099	240924	0	656	667	98	−	1	667	96789	97449
UN07329	Rsa1.0_05498.1	1449	13523	7.00E-65	127	127	100	−	590	716	2975	3101
UN07330	Rsa1.0_03900.1	804	133221	0	705	745	94	−	3	746	6943	7663

UN07331	Rsa1.0_04438.1	573	17361	1.00E-143	267	270	98	-	304	573	14944	15213
UN07332	Rsa1.0_02274.1	1360	33109	0	492	494	99	-	334	827	8221	8714
UN07333	Rsa1.0_00006.1	877	396176	0	857	878	97	+	3	877	296014	296891
UN07334	Rsa1.0_16677.1	135	2069	2.00E-68	134	135	99	-	1	135	999	1133
UN07335	Rsa1.0_00059.1	1437	159650	0	795	833	95	-	628	1437	63397	64229
UN07336	Rsa1.0_03610.1	859	21797	1.00E-127	245	249	98	-	38	283	2138	2386
UN07337	Rsa1.0_04080.1	1676	19198	0	519	556	93	+	505	1060	4470	5023
UN07338	Rsa1.0_02642.1	1625	29181	0	1073	1092	98	+	335	1423	13097	14188
UN07339	Rsa1.0_01496.1	994	44595	0	982	994	98	+	2	994	1641	2634
UN07340	Rsa1.0_43703.1	746	514	1.00E-162	293	294	99	-	451	744	221	514
UN07341	Rsa1.0_00392.1	1078	88523	0	911	1003	90	+	18	1003	63833	64811
UN07342	Rsa1.0_01493.1	1183	44670	0	754	786	95	+	217	998	32666	33447
UN07343	Rsa1.0_00848.1	981	152362	0	658	676	97	-	3	677	14944	15619
UN07344	Rsa1.0_09912.1	1195	6092	0	604	614	98	-	1	613	1	614
UN07345	Rsa1.0_33453.1	1436	678	0	466	467	99	+	572	1038	212	678
UN07346	Rsa1.0_00139.1	972	357786	0	877	900	97	+	3	895	263046	263942
UN07347	Rsa1.0_00703.1	810	67597	0	789	805	98	-	6	810	26615	27419
UN07348	Rsa1.0_01400.1	1313	46560	0	815	845	96	-	479	1308	26212	27056
UN07349	Rsa1.0_03434.1	948	22960	0	601	611	98	+	2	612	20817	21423
UN07350	Rsa1.0_01119.1	603	53459	1.00E-162	322	333	96	+	63	395	42022	42348
UN07351	Rsa1.0_00985.1	781	161950	1.00E-134	305	324	94	-	7	330	54597	54915
UN07352	Rsa1.0_00143.1	680	121313	0	642	677	94	-	2	665	31003	31679
UN07353	Rsa1.0_00197.1	1256	109714	0	596	601	99	-	14	614	47701	48301
UN07354	Rsa1.0_00118.1	999	130637	2.00E-89	168	168	100	-	1	168	114153	114320
UN07355	Rsa1.0_00002.1	1308	601661	0	1285	1301	98	+	4	1304	19784	21084
UN07356	Rsa1.0_00831.1	928	62673	0	415	421	98	-	510	928	22690	23110
UN07357	Rsa1.0_01322.1	768	48166	0	403	403	100	-	362	764	28212	28614
UN07358	Rsa1.0_01591.1	853	42718	1.00E-134	345	379	91	-	20	398	24117	24486
UN07359	Rsa1.0_00038.1	874	314568	1.00E-159	319	328	97	+	3	329	46501	46827
UN07360	Rsa1.0_01414.1	653	149508	0	554	575	96	-	79	651	143134	143701
UN07361	Rsa1.0_01481.1	952	184562	0	949	951	99	+	4	952	2409	3359
UN07362	Rsa1.0_01882.1	742	37697	1.00E-155	294	298	98	-	446	742	12510	12807
UN07363	Rsa1.0_07270.1	1100	9634	0	561	582	96	-	8	578	4435	5013
UN07364	Rsa1.0_67954.1	1082	337	1.00E-178	325	328	99	+	225	552	10	337
UN07365	Rsa1.0_00661.1	684	213041	0	415	437	94	-	104	539	66549	66981
UN07366	Rsa1.0_03915.1	1409	20117	0	398	414	96	-	982	1390	19463	19872
UN07367	Rsa1.0_01091.1	873	54383	0	758	758	100	-	116	873	50727	51484
UN07368	Rsa1.0_00605.1	1151	73199	0	1105	1141	96	-	11	1151	33796	34936
UN07369	Rsa1.0_43429.1	1372	517	1.00E-118	225	228	98	+	1126	1353	290	517

UN07370	Rsa1.0_00753.1	1266	65705	0	603	603	100	−	1	603	64071	64673
UN07371	Rsa1.0_01430.1	838	46034	0	615	615	100	−	224	838	40473	41087
UN07372	Rsa1.0_11525.1	1125	5479	0	797	910	87	+	30	939	4434	5343
UN07373	Rsa1.0_00429.1	802	85559	1.00E−177	357	369	96	+	277	641	13508	13876
UN07374	Rsa1.0_02471.1	1182	31104	0	1153	1182	97	−	1	1182	8870	10049
UN07375	Rsa1.0_02142.1	943	34633	0	377	384	98	−	560	943	6294	6677
UN07376	Rsa1.0_01220.1	951	161775	0	947	951	99	−	1	951	113660	114610
UN07377	Rsa1.0_04218.1	413	18423	1.00E−106	215	219	98	+	1	217	13440	13658
UN07378	Rsa1.0_02621.1	921	156120	0	593	614	96	+	3	614	12833	13446
UN07379	Rsa1.0_00350.1	551	92346	0	347	348	99	−	204	551	73658	74005
UN07380	Rsa1.0_02201.1	1271	33981	1.00E−170	333	343	97	+	3	345	21928	22270
UN07381	Rsa1.0_00051.1	869	258225	0	868	868	100	+	2	869	113729	114596
UN07382	Rsa1.0_16652.1	1143	2078	6.00E−71	189	204	92	−	543	745	568	769
UN07383	Rsa1.0_01866.1	880	37961	0	479	492	97	−	389	880	10742	11233
UN07384	Rsa1.0_09755.1	2100	6166	0	585	586	99	−	985	1570	1	586
UN07385	Rsa1.0_05823.1	2071	12521	0	1430	1465	97	−	1	1464	1	1458
UN07386	Rsa1.0_00596.1	595	73578	0	577	591	97	+	5	593	25972	26562
UN07387	Rsa1.0_00635.1	1461	175826	0	1110	1164	95	−	3	1165	106336	107492
UN07388	Rsa1.0_02415.1	1100	31609	0	362	369	98	−	3	369	14	382
UN07389	Rsa1.0_01160.1	2380	52068	0	1058	1108	95	−	1273	2380	24440	25545
UN07390	Rsa1.0_00578.1	521	74581	0	504	522	96	+	3	519	57295	57811
UN07391	Rsa1.0_02207.1	951	33914	0	461	468	98	+	487	951	12982	13449
UN07392	Rsa1.0_07214.1	666	9708	0	423	436	97	+	1	436	2079	2514
UN07393	Rsa1.0_00713.1	983	158017	0	481	491	97	−	493	983	49880	50365
UN07394	Rsa1.0_00911.1	1606	59943	0	1563	1597	97	+	7	1602	40315	41908
UN07395	Rsa1.0_01295.1	655	49040	0	615	647	95	−	3	647	48351	48997
UN07396	Rsa1.0_02670.1	823	28887	0	435	441	98	+	1	438	15754	16192
UN07397	Rsa1.0_01832.1	1110	38581	0	1100	1106	99	−	4	1108	35770	36874
UN07398	Rsa1.0_00681.1	1505	68779	1.00E−121	280	297	94	+	1	296	52436	52730
UN07399	Rsa1.0_00902.1	765	60241	0	754	766	98	−	1	765	16025	16790
UN07400	Rsa1.0_00123.1	1487	325650	0	625	672	93	−	821	1487	27406	28056
UN07401	Rsa1.0_01179.1	820	51549	0	702	707	99	+	114	820	16747	17450
UN07402	Rsa1.0_15831.1	1473	2398	0	578	580	99	−	650	1229	1	580
UN07403	Rsa1.0_00748.1	596	65983	1.00E−120	250	257	97	+	341	596	65464	65716
UN07404	Rsa1.0_02586.1	2148	29856	0	965	971	99	−	1	971	3551	4521
UN07405	Rsa1.0_00131.1	795	432233	0	774	796	97	−	1	795	79118	79913
UN07406	Rsa1.0_00220.1	1012	192936	0	573	587	97	+	427	1012	28752	29333
UN07407	Rsa1.0_01247.1	1508	49934	0	621	622	99	+	513	1134	31079	31700
UN07408	Rsa1.0_00178.1	836	114861	0	813	839	96	+	1	836	90963	91790

UN07409	Rsa1.0_00136.1	949	122464	0	594	602	98	−	1	602	77555	78155
UN07410	Rsa1.0_00105.1	1219	134138	0	614	628	97	−	1	625	107748	108366
UN07411	Rsa1.0_02823.1	1189	27588	0	720	736	97	+	1	736	8563	9298
UN07412	Rsa1.0_00418.1	1108	215377	0	1098	1112	98	−	1	1108	24292	25403
UN07413	Rsa1.0_01481.1	1668	184562	0	1655	1669	99	−	2	1668	166739	168407
UN07414	Rsa1.0_01302.1	738	48907	0	370	370	100	−	140	509	4012	4381
UN07415	Rsa1.0_00583.1	1488	74309	1.00E−152	392	433	90	−	1056	1487	23689	24093
UN07416	Rsa1.0_01735.1	737	40128	0	546	546	100	−	189	734	32412	32957
UN07417	Rsa1.0_02251.1	1752	33404	0	1630	1688	96	−	1	1673	19974	21656
UN07418	Rsa1.0_00087.1	1182	761073	0	511	545	93	+	645	1181	213537	214070
UN07419	Rsa1.0_00384.1	827	89278	0	791	796	99	−	34	827	24218	25013
UN07420	Rsa1.0_00144.1	1073	120708	4.00E−78	155	157	98	+	340	496	66013	66169
UN07421	Rsa1.0_04184.1	1663	18598	5.00E−75	158	160	98	+	3	160	3488	3647
UN07422	Rsa1.0_01207.1	813	141053	0	526	526	100	+	7	532	116977	117502
UN07423	Rsa1.0_00213.1	1319	108167	0	644	659	97	−	661	1319	81596	82254
UN07424	Rsa1.0_00512.1	987	200277	0	987	988	99	+	1	987	32090	33077
UN07425	Rsa1.0_04554.1	1138	16876	0	763	777	98	−	1	774	3567	4343
UN07426	Rsa1.0_03176.1	1290	24801	1.00E−138	287	299	95	+	635	930	14352	14650
UN07427	Rsa1.0_00116.1	1393	365886	0	1356	1390	97	+	1	1389	211992	213375
UN07428	Rsa1.0_02810.1	921	27671	1.00E−160	287	287	100	+	296	582	17019	17305
UN07429	Rsa1.0_00636.1	1428	71113	0	441	454	97	+	905	1358	11206	11659
UN07430	Rsa1.0_01440.1	1291	45807	0	661	661	100	−	1	661	24779	25439
UN07431	Rsa1.0_00104.1	1232	368722	0	843	881	95	+	362	1232	213055	213934
UN07432	Rsa1.0_01016.1	1701	181422	2.33E−156	347	371	93	+	399	768	47315	47677
UN07433	Rsa1.0_00154.1	1391	189461	0	835	854	97	−	2	855	105326	106176
UN07434	Rsa1.0_00018.1	1445	410899	1.00E−111	238	250	95	+	1195	1444	244894	245136
UN07435	Rsa1.0_00583.1	1116	74309	0	422	423	99	+	441	862	19134	19556
UN07436	Rsa1.0_00019.1	1407	301669	0	1388	1405	98	+	1	1403	204380	205784
UN07437	Rsa1.0_02810.1	1192	27671	0	639	639	100	+	3	641	13145	13783
UN07438	Rsa1.0_01361.1	1173	47226	1.00E−132	249	252	98	−	241	492	19655	19906
UN07439	Rsa1.0_00293.1	1667	339294	0	447	448	99	−	1	447	306995	307442
UN07440	Rsa1.0_00978.1	926	57739	1.00E−170	516	586	88	+	25	601	42017	42599
UN07441	Rsa1.0_00015.1	1492	561803	0	1478	1480	99	−	11	1488	478247	479726
UN07442	Rsa1.0_01232.1	558	155185	0	374	384	97	−	4	386	150401	150783
UN07443	Rsa1.0_01467.1	804	45161	0	560	568	98	+	6	572	39833	40400
UN07444	Rsa1.0_00710.1	608	67325	1.00E−143	383	425	90	+	10	434	43826	44250
UN07445	Rsa1.0_00653.1	1183	70029	0	581	601	96	−	585	1172	54979	55579
UN07446	Rsa1.0_02303.1	1496	32818	0	556	558	99	+	11	566	22242	22799
UN07447	Rsa1.0_00182.1	1210	142305	0	1208	1208	100	+	3	1210	35081	36288

UN07448	Rsa1.0_05498.1	771	13523	7.00E-79	218	238	91	−	536	771	3005	3242
UN07449	Rsa1.0_01444.1	1586	169539	0	1240	1273	97	−	321	1586	132361	133633
UN07450	Rsa1.0_05687.1	1379	12932	0	576	583	98	+	798	1376	3372	3954
UN07451	Rsa1.0_06124.1	1423	11681	0	1418	1421	99	+	1	1421	6262	7682
UN07452	Rsa1.0_01170.1	1185	161131	1.00E-176	313	313	100	+	464	776	131509	131821
UN07453	Rsa1.0_00243.1	1304	103651	0	922	922	100	−	383	1304	20395	21316
UN07454	Rsa1.0_00091.1	1107	139699	0	1083	1107	97	+	1	1107	51334	52440
UN07455	Rsa1.0_33295.1	871	682	1.00E-142	257	257	100	−	582	838	112	368
UN07456	Rsa1.0_04236.1	917	18370	0	670	694	96	+	30	723	14257	14950
UN07457	Rsa1.0_00058.1	1538	159921	0	1112	1161	95	−	388	1534	81157	82313
UN07458	Rsa1.0_00175.1	623	166121	0	611	624	97	+	1	623	82963	83580
UN07459	Rsa1.0_13183.1	835	4979	1.00E-154	282	284	99	−	1	284	4420	4703
UN07460	Rsa1.0_02867.1	1095	27121	1.00E-178	317	317	100	−	2	318	17565	17881
UN07461	Rsa1.0_00188.1	661	112020	1.00E-128	300	321	93	−	1	321	72058	72377
UN07462	Rsa1.0_00421.1	1488	86365	4.00E-91	180	183	98	−	531	713	37110	37292
UN07463	Rsa1.0_00051.1	1209	258225	0	400	402	99	−	1	401	104805	105206
UN07464	Rsa1.0_01204.1	1750	50843	0	876	903	97	+	477	1378	28520	29422
UN07465	Rsa1.0_00110.1	890	132719	0	758	758	100	+	1	758	131239	131996
UN07466	Rsa1.0_00305.1	1232	97308	0	761	761	100	+	1	761	92414	93174
UN07467	Rsa1.0_00071.1	939	345714	0	866	875	98	+	65	939	141582	142456
UN07468	Rsa1.0_00397.1	1295	88189	0	709	709	100	−	587	1295	64534	65242
UN07469	Rsa1.0_04794.1	666	15960	0	665	665	100	+	2	666	4544	5208
UN07470	Rsa1.0_00075.1	1116	146680	0	1058	1128	93	+	1	1116	140486	141604
UN07471	Rsa1.0_00011.1	1249	225044	0	1224	1243	98	+	8	1249	40734	41976
UN07472	Rsa1.0_00096.1	1330	198773	1.00E-148	270	271	99	+	3	273	171654	171924
UN07473	Rsa1.0_00275.1	1401	210104	1.00E-169	339	349	97	+	3	350	176395	176741
UN07474	Rsa1.0_02228.1	880	33688	0	793	793	100	−	88	880	13513	14305
UN07475	Rsa1.0_00105.1	1597	134138	0	715	773	92	−	829	1597	62079	62819
UN07476	Rsa1.0_03280.1	1374	145083	0	1334	1384	96	−	1	1374	3	1383
UN07477	Rsa1.0_00071.1	1101	345714	0	1085	1101	98	+	4	1101	147814	148914
UN07478	Rsa1.0_00087.1	1327	761073	0	472	492	95	−	838	1327	119142	119633
UN07479	Rsa1.0_00531.1	1718	77387	0	886	898	98	−	1	898	9144	10037
UN07480	Rsa1.0_00758.1	1811	65477	0	627	628	99	+	1184	1811	2037	2664
UN07481	Rsa1.0_02867.1	1517	27121	0	737	789	93	−	710	1492	9763	10535
UN07482	Rsa1.0_01873.1	1851	37903	0	566	566	100	+	4	569	5295	5860
UN07483	Rsa1.0_00913.1	1312	59914	0	749	796	94	−	520	1312	726	1495
UN07484	Rsa1.0_00046.1	1162	320315	0	1066	1091	97	+	72	1162	23945	25035
UN07485	Rsa1.0_01403.1	1335	46453	0	698	714	97	−	74	787	39504	40217
UN07486	Rsa1.0_00361.1	1292	91181	0	769	779	98	+	176	951	23667	24445

UN07487	Rsa1.0_03387.1	1479	23278	0	593	593	100	+	1	593	336	928
UN07488	Rsa1.0_06489.1	1232	10917	0	888	899	98	+	334	1232	3538	4436
UN07489	#	#	#	#	#	#	#	#	#	#	#	#
UN07490	Rsa1.0_00017.1	1457	752458	0	661	681	97	−	3	683	368688	369367
UN07491	Rsa1.0_15995.1	1189	2328	0	836	841	99	−	349	1189	1171	2011
UN07492	Rsa1.0_00635.1	1609	175826	0	1053	1062	99	+	114	1174	106336	107397
UN07493	Rsa1.0_07618.1	938	9015	2.00E−82	159	160	99	+	1	160	4938	5097
UN07494	Rsa1.0_00222.1	929	172263	1.00E−174	316	318	99	−	3	320	131334	131651
UN07495	Rsa1.0_00565.1	1157	75420	0	1138	1171	97	+	9	1155	47886	49056
UN07496	Rsa1.0_04032.1	1040	19489	0	406	422	96	+	2	423	6359	6776
UN07497	Rsa1.0_00286.1	1189	98709	0	380	383	99	+	807	1189	61172	61554
UN07498	Rsa1.0_01524.1	988	43949	0	356	361	98	−	629	987	43079	43439
UN07499	Rsa1.0_01400.1	930	46560	0	357	363	98	−	191	553	822	1184
UN07500	Rsa1.0_00642.1	1083	201230	0	644	646	99	+	2	647	173316	173961
UN07501	Rsa1.0_03200.1	1111	24634	0	581	581	100	−	529	1109	3607	4187
UN07502	Rsa1.0_00022.1	1236	200461	0	959	962	99	+	275	1236	139496	140457
UN07503	Rsa1.0_00218.1	1344	201480	1.00E−144	263	264	99	+	1	264	109770	110033
UN07504	Rsa1.0_00402.1	1271	385315	0	1214	1256	96	−	3	1252	228250	229499
UN07505	Rsa1.0_00065.1	585	454616	1.00E−178	361	373	96	−	1	367	439453	439824
UN07506	Rsa1.0_01117.1	901	53465	0	380	381	99	+	1	381	10874	11254
UN07507	Rsa1.0_38494.1	753	585	0	581	585	99	+	39	623	1	584
UN07508	Rsa1.0_00001.1	829	363028	0	807	838	96	−	3	829	281745	282569
UN07509	Rsa1.0_00650.1	1741	70235	1.00E−155	314	326	96	+	756	1081	2442	2767
UN07510	Rsa1.0_00061.1	698	218685	1.00E−108	309	341	90	+	16	352	118933	119266
UN07511	Rsa1.0_03420.1	1449	23090	1.00E−151	274	275	99	+	7	281	4797	5071
UN07512	Rsa1.0_45543.1	1579	495	0	494	495	99	+	470	964	1	495
UN07513	Rsa1.0_10284.1	1453	5904	0	457	471	97	+	984	1453	1260	1730
UN07514	Rsa1.0_03075.1	1365	25560	0	493	510	96	−	857	1365	16382	16890
UN07515	Rsa1.0_00033.1	694	515811	0	638	670	95	+	1	661	352589	353258
UN07516	Rsa1.0_01767.1	1020	39615	0	458	466	98	−	365	830	32312	32777
UN07517	Rsa1.0_16049.1	1464	2306	0	1289	1292	99	−	1	1291	695	1986
UN07518	Rsa1.0_02345.1	1491	32337	0	519	551	94	−	854	1400	21144	21688
UN07519	Rsa1.0_00606.1	1564	73150	0	567	569	99	+	286	854	64517	65085
UN07520	Rsa1.0_00017.1	782	752458	0	391	393	99	+	388	780	444997	445389
UN07521	Rsa1.0_01655.1	1309	41508	0	481	481	100	+	829	1309	6342	6822
UN07522	Rsa1.0_04057.1	1750	19333	0	634	649	97	+	395	1043	16812	17460
UN07523	Rsa1.0_00114.1	844	131330	0	840	843	99	+	1	842	88134	88976
UN07524	Rsa1.0_07440.1	1212	9389	0	1180	1214	97	+	3	1211	4099	5312
UN07525	Rsa1.0_00372.1	1710	90418	0	780	815	95	−	1	814	47277	48069

UN07526	Rsa1.0_12214.1	2682	5287	0	1053	1073	98	−	558	1624	4153	5225
UN07527	Rsa1.0_19376.1	1061	1441	2.00E−85	167	169	98	+	445	613	633	801
UN07528	Rsa1.0_07157.1	1064	9805	1.00E−176	313	313	100	−	127	439	4990	5302
UN07529	Rsa1.0_01498.1	2048	44558	0	452	453	99	+	1	453	38005	38457
UN07530	Rsa1.0_00060.1	1554	502678	0	1544	1560	98	+	1	1553	483821	485380
UN07531	Rsa1.0_00128.1	834	228675	1.00E−162	314	322	97	−	392	713	116359	116680
UN07532	Rsa1.0_00740.1	2092	215972	0	860	861	99	+	2	862	29410	30270
UN07533	Rsa1.0_00452.1	1186	83201	1.00E−119	224	226	99	+	662	887	77985	78210
UN07534	Rsa1.0_00322.1	1092	212233	1.00E−142	314	328	95	−	745	1068	92264	92586
UN07535	Rsa1.0_02191.1	1323	34078	0	455	455	100	+	869	1323	23641	24095
UN07536	Rsa1.0_08388.1	1168	7436	0	1059	1114	95	+	1	1093	4633	5745
UN07537	Rsa1.0_06004.1	931	11961	1.00E−93	175	175	100	−	110	284	8503	8677
UN07538	Rsa1.0_02659.1	1431	28971	0	549	560	98	−	867	1426	22635	23193
UN07539	Rsa1.0_00349.1	852	92621	1.00E−157	351	373	94	+	484	850	45076	45447
UN07540	Rsa1.0_00211.1	1409	228189	0	570	603	94	+	718	1311	8747	9346
UN07541	Rsa1.0_00322.1	1220	212233	0	614	635	96	−	583	1211	63652	64286
UN07542	Rsa1.0_04630.1	1131	16595	0	778	785	99	+	2	783	13109	13893
UN07543	Rsa1.0_01855.1	1845	38204	0	486	510	95	+	1314	1820	12699	13208
UN07544	Rsa1.0_01035.1	1031	56019	0	531	540	98	+	490	1029	34973	35511
UN07545	Rsa1.0_03706.1	1742	21255	0	354	361	98	+	627	987	10986	11346
UN07546	Rsa1.0_01785.1	2003	39368	0	641	644	99	−	1	644	21165	21805
UN07547	Rsa1.0_17771.1	1421	1744	0	886	913	97	+	3	915	281	1193
UN07548	Rsa1.0_06341.1	1058	11209	1.00E−180	345	352	98	+	1	351	2347	2698
UN07549	Rsa1.0_04292.1	1044	18050	1.00E−121	233	237	98	+	1	237	7886	8122
UN07550	Rsa1.0_55259.1	1466	413	0	407	413	98	+	30	442	1	413
UN07551	Rsa1.0_16602.1	1350	2094	0	782	808	96	−	74	876	1	800
UN07552	Rsa1.0_00196.1	1198	109960	0	762	786	96	+	420	1196	90095	90880
UN07553	Rsa1.0_02039.1	2447	35699	0	1437	1501	95	+	608	2098	2375	3859
UN07554	Rsa1.0_02374.1	847	32043	0	772	806	95	+	46	847	24237	25038
UN07555	Rsa1.0_00835.1	1065	278058	0	792	807	98	+	254	1053	265120	265926
UN07556	Rsa1.0_00825.1	1071	62855	0	456	494	92	+	1	484	49159	49643
UN07557	Rsa1.0_00817.1	536	62961	0	534	537	99	−	1	536	44954	45490
UN07558	Rsa1.0_01220.1	1188	161775	0	566	568	99	−	1	568	27217	27784
UN07559	Rsa1.0_00228.1	1009	105511	0	419	427	98	−	2	425	77923	78349
UN07560	Rsa1.0_02186.1	1247	34150	1.00E−162	337	349	96	−	899	1246	13886	14231
UN07561	Rsa1.0_06048.1	1162	11854	1.00E−148	270	271	99	−	626	896	2079	2349
UN07562	Rsa1.0_00081.1	1259	143261	0	759	760	99	+	346	1105	118585	119344
UN07563	Rsa1.0_00302.1	1076	97521	0	552	552	100	+	2	553	32203	32754
UN07564	Rsa1.0_00177.1	1717	143394	0	765	804	95	+	362	1165	36345	37135

UN07565	Rsa1.0_00323.1	1150	159650	0	512	526	97	+	3	528	99362	99878
UN07566	Rsa1.0_04664.1	973	16471	1.00E-131	253	258	98	+	10	267	2331	2588
UN07567	Rsa1.0_00012.1	1017	222148	0	357	360	99	+	658	1017	189098	189457
UN07568	Rsa1.0_00413.1	994	86921	2.33E-156	316	330	95	+	1	329	37460	37781
UN07569	Rsa1.0_24496.1	1435	985	0	526	530	99	−	2	531	216	744
UN07570	Rsa1.0_01540.1	2276	151890	0	1294	1371	94	−	86	1431	139025	140386
UN07571	Rsa1.0_00250.1	728	273771	0	686	699	98	+	3	700	249892	250590
UN07572	Rsa1.0_00651.1	1050	247766	1.00E-158	302	307	98	+	744	1050	219195	219500
UN07573	Rsa1.0_00481.1	990	81316	1.00E-77	154	156	98	−	3	158	24855	25010
UN07574	Rsa1.0_03755.1	1643	21000	0	1619	1640	98	−	3	1642	9663	11298
UN07575	Rsa1.0_00296.1	948	97854	0	559	566	98	−	384	948	70192	70757
UN07576	Rsa1.0_00064.1	1233	240927	0	677	698	96	−	535	1232	48554	49243
UN07577	Rsa1.0_00058.1	698	159921	0	425	430	98	−	3	432	135516	135945
UN07578	Rsa1.0_00676.1	971	69166	0	489	504	97	+	470	971	53234	53736
UN07579	Rsa1.0_00069.1	1438	403561	0	473	495	95	−	950	1438	190538	191025
UN07580	Rsa1.0_03379.1	1201	23321	0	596	620	96	−	583	1201	13593	14204
UN07581	Rsa1.0_00548.1	1067	76518	0	473	488	96	−	1	488	35530	36017
UN07582	Rsa1.0_03029.1	1477	25858	0	1064	1083	98	+	1	1080	1296	2378
UN07583	Rsa1.0_00937.1	908	59061	0	350	357	98	−	545	897	6816	7172
UN07584	Rsa1.0_00050.1	1463	237174	0	587	609	96	+	1	609	28676	29282
UN07585	Rsa1.0_00440.1	1621	84723	0	379	385	98	+	120	504	56532	56916
UN07586	Rsa1.0_00181.1	1052	114269	1.00E-128	431	493	87	−	227	719	31685	32174
UN07587	Rsa1.0_02404.1	1188	31702	5.00E-16	123	149	82	+	843	991	5901	6049
UN07588	Rsa1.0_00055.1	1488	520193	0	808	813	99	+	1	813	289692	290503
UN07589	Rsa1.0_09932.1	1373	6085	0	1289	1355	95	+	2	1339	927	2264
UN07590	Rsa1.0_01625.1	866	42019	1.00E-102	319	361	88	+	482	842	34139	34483
UN07591	Rsa1.0_00208.1	1100	108565	0	531	545	97	+	559	1100	12221	12761
UN07592	Rsa1.0_00127.1	1278	127446	1.00E-136	260	263	98	−	1015	1277	48690	48951
UN07593	Rsa1.0_01855.1	1281	38204	0	625	638	97	−	647	1281	30495	31132
UN07594	Rsa1.0_01452.1	1089	45459	1.00E-139	254	255	99	+	834	1088	38660	38914
UN07595	Rsa1.0_02941.1	1264	26583	0	580	592	97	+	673	1264	13703	14294
UN07596	Rsa1.0_00558.1	1447	75819	0	1218	1266	96	+	1	1264	34745	35995
UN07597	Rsa1.0_00426.1	1104	85668	0	396	405	97	−	1	405	59558	59959
UN07598	Rsa1.0_02654.1	1561	29028	0	893	908	98	−	5	912	23477	24384
UN07599	Rsa1.0_00026.1	1125	327835	1.00E-108	216	221	97	+	1	221	183744	183961
UN07600	Rsa1.0_02259.1	1354	33299	0	749	758	98	−	284	1041	25779	26536
UN07601	Rsa1.0_00516.1	1429	78417	0	728	739	98	−	2	740	40369	41107
UN07602	Rsa1.0_00022.1	1237	200461	0	1206	1236	97	−	1	1236	193769	194985
UN07603	Rsa1.0_07630.1	1972	8988	0	417	418	99	+	1555	1972	4066	4483

UN07604	Rsa1.0_00012.1	2446	222148	0	598	600	99	−	789	1388	88471	89069
UN07605	Rsa1.0_01614.1	953	42238	0	411	427	96	−	527	953	33551	33966
UN07606	Rsa1.0_00915.1	1588	194446	0	1270	1330	95	+	2	1331	180468	181796
UN07607	Rsa1.0_05628.1	959	13130	0	513	524	97	+	439	959	11862	12383
UN07608	Rsa1.0_00027.1	1341	283362	0	353	357	98	−	1	357	153443	153798
UN07609	Rsa1.0_00440.1	2093	84723	0	487	522	93	+	1031	1549	24888	25398
UN07610	Rsa1.0_08424.1	941	7375	0	612	625	97	+	1	624	782	1406
UN07611	Rsa1.0_00164.1	1081	116642	0	1061	1078	98	+	3	1080	6177	7251
UN07612	Rsa1.0_00038.1	1394	314568	0	571	580	98	−	814	1392	240906	241485
UN07613	Rsa1.0_25563.1	2195	931	0	779	779	100	−	511	1289	153	931
UN07614	Rsa1.0_07827.1	1410	8466	0	746	754	98	+	10	762	4273	5025
UN07615	Rsa1.0_02514.1	928	30642	0	882	926	95	−	1	925	8822	9737
UN07616	Rsa1.0_00072.1	1186	148743	1.00E−129	344	381	90	+	807	1186	68937	69307
UN07617	Rsa1.0_00049.1	1245	167018	0	370	373	99	−	873	1245	57777	58149
UN07618	Rsa1.0_00627.1	1090	71566	0	697	719	96	+	118	835	60232	60950
UN07619	Rsa1.0_00537.1	807	186980	0	700	734	95	−	49	765	173882	174614
UN07620	Rsa1.0_00017.1	1324	752458	0	974	1021	95	−	297	1302	56037	57054
UN07621	Rsa1.0_00096.1	1397	198773	0	617	617	100	−	781	1397	119398	120014
UN07622	Rsa1.0_03721.1	1551	21185	1.00E−141	258	259	99	−	1260	1518	20888	21146
UN07623	Rsa1.0_00001.1	1391	363028	0	554	558	99	−	833	1390	139974	140531
UN07624	Rsa1.0_06130.1	1300	11667	0	656	660	99	+	124	783	680	1339
UN07625	Rsa1.0_01004.1	1180	56975	0	488	489	99	+	692	1180	3551	4039
UN07626	Rsa1.0_00211.1	563	228189	1.00E−114	228	233	97	−	326	558	134586	134817
UN07627	Rsa1.0_22066.1	884	1152	1.00E−173	338	348	97	−	516	863	805	1152
UN07628	Rsa1.0_00019.1	1073	301669	0	932	949	98	−	135	1072	57759	58707
UN07629	Rsa1.0_00359.1	1297	91256	0	1081	1089	99	+	210	1297	21708	22796
UN07630	Rsa1.0_01480.1	1713	44926	0	432	436	99	−	1278	1713	31947	32382
UN07631	Rsa1.0_00188.1	1171	112020	0	445	460	96	+	529	985	74179	74638
UN07632	Rsa1.0_08437.1	914	7357	0	909	914	99	−	2	914	1867	2780
UN07633	Rsa1.0_00201.1	1095	232851	1.00E−169	316	320	98	+	1	320	212076	212392
UN07634	Rsa1.0_01586.1	1267	42804	0	658	658	100	+	140	797	20465	21122
UN07635	Rsa1.0_04552.1	1133	16878	0	1130	1135	99	+	3	1133	1775	2909
UN07636	Rsa1.0_00668.1	1625	69421	0	394	399	98	+	1227	1625	47385	47782
UN07637	Rsa1.0_04739.1	975	16167	0	476	479	99	+	1	478	12747	13224
UN07638	Rsa1.0_02544.1	1264	30290	0	398	404	98	−	860	1263	5350	5753
UN07639	Rsa1.0_00341.1	804	249309	5.00E−86	177	182	97	+	418	599	121074	121255
UN07640	Rsa1.0_00692.1	1298	68151	0	561	571	98	+	123	693	27013	27583
UN07641	Rsa1.0_24802.1	1002	967	0	641	646	99	+	4	649	325	967
UN07642	Rsa1.0_00127.1	1068	127446	0	522	523	99	−	546	1068	16886	17408

UN07643	Rsa1.0_01189.1	415	51358	0	327	328	99	+	88	415	42574	42901
UN07644	Rsa1.0_02515.1	1467	30616	0	450	452	99	+	1016	1467	9025	9476
UN07645	Rsa1.0_00860.1	1248	61789	0	785	806	97	−	451	1248	30854	31659
UN07646	Rsa1.0_01233.1	1451	50184	0	1419	1451	97	−	3	1449	7711	9159
UN07647	Rsa1.0_00146.1	1047	149343	0	525	547	95	+	501	1047	63621	64163
UN07648	Rsa1.0_00925.1	949	59481	0	782	827	94	−	39	857	3312	4137
UN07649	Rsa1.0_00013.1	1528	292129	0	540	547	98	+	3	549	194728	195272
UN07650	Rsa1.0_00822.1	925	62891	0	739	787	93	+	2	785	4892	5678
UN07651	Rsa1.0_00013.1	1188	292129	1.00E−62	204	229	89	+	709	937	199158	199382
UN07652	Rsa1.0_02165.1	916	34423	0	913	920	99	−	3	915	15019	15938
UN07653	Rsa1.0_01930.1	735	37189	0	727	737	98	−	6	735	1049	1785
UN07654	Rsa1.0_00733.1	1300	66583	0	660	661	99	−	639	1299	53663	54323
UN07655	Rsa1.0_00168.1	1645	115870	1.00E−140	263	266	98	+	1379	1644	51784	52049
UN07656	Rsa1.0_01286.1	764	49209	7.00E−82	161	163	98	−	294	456	11594	11756
UN07657	Rsa1.0_03302.1	659	23898	1.00E−149	354	381	92	−	288	659	11619	11999
UN07658	Rsa1.0_03959.1	884	19866	0	345	349	98	−	5	353	165	512
UN07659	Rsa1.0_05570.1	1120	13301	1.00E−128	243	245	99	+	876	1120	6286	6529
UN07660	Rsa1.0_00123.1	1950	325650	0	1372	1373	99	−	230	1602	94779	96151
UN07661	Rsa1.0_00194.1	958	110851	0	550	553	99	−	1	553	85703	86255
UN07662	Rsa1.0_00172.1	1498	115476	0	870	882	98	−	200	1080	48895	49775
UN07663	Rsa1.0_00581.1	1241	163575	1.00E−108	251	262	95	−	982	1240	28074	28332
UN07664	Rsa1.0_08563.1	945	7193	0	929	941	98	−	4	944	632	1571
UN07665	Rsa1.0_00071.1	1016	345714	0	394	401	98	−	616	1016	5731	6127
UN07666	Rsa1.0_08979.1	919	6739	0	889	919	96	−	2	919	4259	5177
UN07667	Rsa1.0_01011.1	1765	56883	0	839	847	99	+	917	1763	22128	22974
UN07668	Rsa1.0_00221.1	839	106972	0	327	329	99	+	1	329	62498	62826
UN07669	Rsa1.0_01148.1	933	52375	0	514	522	98	−	415	933	27927	28448
UN07670	Rsa1.0_00016.1	661	831256	1.00E−64	210	238	88	−	159	396	685024	685261
UN07671	Rsa1.0_01311.1	1184	48456	0	543	553	98	−	632	1184	46824	47376
UN07672	Rsa1.0_00352.1	1328	92266	0	741	750	98	−	581	1328	13887	14636
UN07673	Rsa1.0_00575.1	896	74791	0	885	901	98	−	2	896	29333	30232
UN07674	Rsa1.0_02499.1	668	30774	0	343	347	98	−	1	347	21561	21907
UN07675	Rsa1.0_01560.1	1073	43256	0	1060	1072	98	−	1	1072	19325	20396
UN07676	Rsa1.0_00714.1	1188	67187	0	1003	1010	99	+	179	1188	41615	42624
UN07677	Rsa1.0_00046.1	1798	320315	0	695	723	96	+	1080	1798	52012	52728
UN07678	Rsa1.0_01384.1	1093	46803	0	364	372	97	−	721	1092	29348	29719
UN07679	Rsa1.0_00128.1	1088	228675	0	578	597	96	+	2	597	188508	189104
UN07680	Rsa1.0_01776.1	1097	39511	0	539	579	93	−	518	1095	26754	27327
UN07681	Rsa1.0_00963.1	804	58176	0	342	348	98	−	18	365	44669	45016

UN07682	Rsa1.0_02160.1	1758	34468	1.00E-177	349	360	96	+	912	1271	20593	20952
UN07683	Rsa1.0_00025.1	1134	277312	0	461	464	99	+	3	466	121941	122403
UN07684	Rsa1.0_00053.1	1119	263459	0	375	391	95	+	729	1119	207739	208128
UN07685	Rsa1.0_03885.1	657	20256	0	435	442	98	−	214	655	10316	10756
UN07686	Rsa1.0_10685.1	1557	5756	0	439	440	99	+	25	464	354	793
UN07687	Rsa1.0_00100.1	984	249232	1.00E-143	359	392	91	+	523	907	8737	9127
UN07688	Rsa1.0_00372.1	1175	90418	0	503	526	95	+	642	1160	20630	21154
UN07689	Rsa1.0_00675.1	1132	145590	0	460	490	93	−	644	1132	45253	45726
UN07690	Rsa1.0_01884.1	697	37659	0	350	354	98	+	1	354	32322	32675
UN07691	Rsa1.0_01171.1	1421	51752	0	449	470	95	+	6	464	35724	36193
UN07692	Rsa1.0_00808.1	1099	63351	0	349	354	98	−	542	895	60275	60627
UN07693	Rsa1.0_00653.1	726	70029	0	716	734	97	−	1	725	41924	42656
UN07694	Rsa1.0_34912.1	1156	648	0	647	648	99	−	54	701	1	648
UN07695	Rsa1.0_04356.1	796	17764	0	707	772	91	−	32	796	10449	11204
UN07696	Rsa1.0_04364.1	1094	17734	0	1051	1091	96	+	3	1093	4195	5278
UN07697	Rsa1.0_01798.1	1146	173909	1.00E-174	313	314	99	−	665	978	162337	162650
UN07698	Rsa1.0_00092.1	1044	659932	0	468	471	99	−	3	472	173928	174396
UN07699	Rsa1.0_02492.1	1140	30859	0	596	610	97	−	534	1140	18258	18867
UN07700	Rsa1.0_02382.1	1323	31969	0	1300	1318	98	−	2	1319	18123	19440
UN07701	Rsa1.0_05554.1	316	13343	1.00E-147	304	316	96	−	2	316	12366	12681
UN07702	Rsa1.0_00810.1	715	63289	0	547	577	94	−	129	699	26680	27247
UN07703	Rsa1.0_14690.1	961	3155	0	390	396	98	−	26	417	137	532
UN07704	Rsa1.0_05931.1	845	12210	0	482	494	97	+	2	491	7419	7912
UN07705	Rsa1.0_22303.1	1496	1132	0	619	634	97	+	749	1382	499	1132
UN07706	Rsa1.0_00050.1	1037	237174	0	775	799	96	−	1	799	165538	166334
UN07707	Rsa1.0_00234.1	728	197060	0	555	601	92	−	135	728	14744	15331
UN07708	Rsa1.0_00146.1	1378	149343	0	1268	1308	96	+	2	1309	138139	139422
UN07709	Rsa1.0_13606.1	684	4648	0	480	482	99	+	202	683	369	850
UN07710	Rsa1.0_00325.1	1268	95371	0	1259	1268	99	−	1	1268	10700	11967
UN07711	Rsa1.0_02995.1	930	26163	1.00E-89	433	514	84	−	201	709	9948	10458
UN07712	Rsa1.0_00139.1	1429	357786	0	731	745	98	+	679	1422	7572	8316
UN07713	Rsa1.0_02903.1	655	26901	1.00E-106	202	204	99	−	452	655	3348	3551
UN07714	Rsa1.0_00113.1	1657	131574	0	410	411	99	−	1240	1650	69445	69855
UN07715	Rsa1.0_01069.1	1516	55113	0	642	644	99	+	250	893	52790	53433
UN07716	Rsa1.0_00178.1	963	114861	0	408	420	97	+	547	963	88533	88950
UN07717	Rsa1.0_03836.1	880	20564	0	864	881	98	+	2	878	16989	17868
UN07718	Rsa1.0_01385.1	1131	46782	0	1112	1128	98	−	2	1129	25913	27040
UN07719	Rsa1.0_00797.1	610	63959	0	380	381	99	+	176	556	17835	18215
UN07720	Rsa1.0_03376.1	1274	23326	0	371	376	98	−	2	377	14936	15311

UN07721	Rsa1.0_04224.1	1220	18402	0	494	521	94	-	696	1204	10838	11357
UN07722	Rsa1.0_00152.1	1435	257730	0	1261	1293	97	+	142	1434	68322	69602
UN07723	Rsa1.0_00056.1	1777	332604	0	493	503	98	+	1275	1777	141016	141512
UN07724	Rsa1.0_08186.1	1041	7770	1.00E-56	125	128	97	-	894	1019	7038	7165
UN07725	Rsa1.0_01817.1	952	38849	1.00E-164	297	298	99	-	1	298	34315	34612
UN07726	Rsa1.0_00175.1	653	166121	1.00E-133	299	317	94	-	3	319	104859	105174
UN07727	Rsa1.0_00706.1	1137	291570	0	458	461	99	+	280	740	14249	14709
UN07728	Rsa1.0_00078.1	1216	175053	0	1156	1223	94	+	16	1215	8988	10201
UN07729	Rsa1.0_02057.1	782	35540	1.00E-105	216	222	97	+	4	224	22336	22557
UN07730	Rsa1.0_00305.1	516	97308	1.00E-169	336	344	97	-	1	342	91981	92322
UN07731	Rsa1.0_00339.1	1082	222974	0	541	548	98	+	3	550	202288	202834
UN07732	Rsa1.0_01997.1	830	158416	0	808	857	94	-	1	830	3758	4612
UN07733	Rsa1.0_04358.1	1288	17759	0	399	399	100	+	890	1288	2339	2737
UN07734	Rsa1.0_02276.1	1641	33098	0	1456	1462	99	-	180	1641	22981	24442
UN07735	Rsa1.0_00626.1	1661	71582	0	1020	1022	99	+	1	1022	51792	52813
UN07736	Rsa1.0_00402.1	1156	385315	0	623	659	94	+	514	1155	233226	233884
UN07737	Rsa1.0_00980.1	1915	57729	0	1064	1066	99	+	1	1064	3677	4742
UN07738	Rsa1.0_01636.1	881	41863	0	570	583	97	-	299	881	24299	24878
UN07739	Rsa1.0_00089.1	977	406142	0	434	455	95	+	524	977	351768	352214
UN07740	Rsa1.0_00048.1	650	167185	1.00E-132	255	260	98	+	391	650	4502	4761
UN07741	Rsa1.0_00049.1	1728	167018	0	1152	1193	96	-	84	1276	45113	46302
UN07742	Rsa1.0_00433.1	948	85381	0	869	889	97	-	46	933	10340	11228
UN07743	Rsa1.0_00476.1	728	280966	1.00E-104	219	228	96	-	267	494	165837	166064
UN07744	Rsa1.0_06010.1	1522	11944	0	666	750	88	+	627	1375	4866	5615
UN07745	Rsa1.0_00359.1	1528	91256	0	491	493	99	+	354	846	3010	3502
UN07746	Rsa1.0_00108.1	1106	478491	1.00E-132	245	247	99	+	860	1106	43807	44053
UN07747	Rsa1.0_00017.1	1862	752458	0	416	416	100	-	1020	1435	627344	627759
UN07748	Rsa1.0_00052.1	503	162771	0	497	501	99	+	3	503	72079	72578
UN07749	Rsa1.0_02685.1	1361	28669	0	1216	1257	96	-	63	1319	6968	8219
UN07750	Rsa1.0_00911.1	744	59943	1.00E-126	250	257	97	-	488	744	11489	11745
UN07751	Rsa1.0_01609.1	1659	42280	0	1327	1331	99	+	3	1333	34645	35975
UN07752	Rsa1.0_08503.1	659	7259	0	645	647	99	+	13	659	749	1395
UN07753	Rsa1.0_01092.1	2800	54348	0	769	828	92	+	1955	2761	43979	44800
UN07754	Rsa1.0_01722.1	675	40320	0	427	429	99	+	246	673	36699	37126
UN07755	Rsa1.0_14798.1	430	3060	1.00E-106	209	212	98	+	2	212	135	346
UN07756	Rsa1.0_04210.1	1385	18442	1.00E-153	292	297	98	-	1085	1381	1611	1904
UN07757	Rsa1.0_02645.1	1457	29158	0	918	918	100	-	540	1457	20181	21098
UN07758	Rsa1.0_00896.1	816	60509	1.00E-174	319	322	99	+	270	591	16503	16824
UN07759	Rsa1.0_00124.1	1109	262503	1.00E-172	309	310	99	+	1	310	91257	91566

UN07760	Rsa1.0_00782.1	1068	64468	0	385	390	98	+	3	392	58826	59215
UN07761	Rsa1.0_00576.1	765	178949	0	714	749	95	+	3	747	61942	62672
UN07762	Rsa1.0_00706.1	1454	291570	1.00E-151	284	288	98	+	1167	1454	163168	163455
UN07763	Rsa1.0_02299.1	1218	32857	0	775	779	99	−	1	779	28565	29343
UN07764	Rsa1.0_15710.1	1463	2468	0	659	667	98	−	258	924	820	1486
UN07765	Rsa1.0_00564.1	1950	75428	0	1441	1478	97	+	39	1516	34347	35821
UN07766	Rsa1.0_08046.1	859	8014	0	465	511	90	+	371	859	472	970
UN07767	Rsa1.0_02714.1	1556	28470	0	442	442	100	−	1	442	1989	2430
UN07768	#	#	#	#	#	#	#	#	#	#	#	#
UN07769	Rsa1.0_00061.1	972	218685	0	344	344	100	−	629	972	166142	166485
UN07770	Rsa1.0_00273.1	1659	326186	0	837	843	99	−	817	1658	274597	275439
UN07771	Rsa1.0_00548.1	962	76518	0	351	352	99	+	281	632	30215	30566
UN07772	Rsa1.0_02541.1	1434	30342	1.00E-177	318	319	99	+	68	386	24690	25008
UN07773	Rsa1.0_00145.1	468	261008	0	390	395	98	+	72	466	77467	77861
UN07774	Rsa1.0_01323.1	1643	48152	0	561	563	99	+	411	973	1420	1982
UN07775	Rsa1.0_00368.1	1536	90669	0	853	884	96	+	610	1480	8923	9804
UN07776	Rsa1.0_00266.1	942	163932	0	935	946	98	−	3	942	128758	129703
UN07777	Rsa1.0_00102.1	1071	135497	0	1060	1070	99	−	3	1070	60039	61108
UN07778	Rsa1.0_00078.1	1357	175053	0	1004	1013	99	+	345	1357	119583	120595
UN07779	Rsa1.0_01961.1	1086	36761	0	1083	1084	99	+	1	1084	27368	28451
UN07780	Rsa1.0_00025.1	750	277312	0	737	749	98	+	2	750	242719	243464
UN07781	Rsa1.0_01104.1	661	285480	6.00E-17	118	141	83	+	118	258	277247	277380
UN07782	Rsa1.0_24258.1	1399	997	0	512	518	98	+	857	1374	374	891
UN07783	Rsa1.0_00026.1	738	327835	0	731	736	99	−	3	738	8175	8910
UN07784	Rsa1.0_00564.1	555	75428	0	541	547	98	−	10	555	9198	9744
UN07785	Rsa1.0_25842.1	703	917	0	456	459	99	−	245	703	460	917
UN07786	Rsa1.0_00457.1	788	337301	4.00E-77	150	151	99	+	3	153	297696	297846
UN07787	Rsa1.0_00019.1	731	301669	0	719	730	98	+	3	731	44469	45197
UN07788	Rsa1.0_00131.1	1354	432233	9.00E-98	185	186	99	−	366	551	416768	416953
UN07789	Rsa1.0_00498.1	1355	79746	0	863	890	96	+	2	891	44460	45349
UN07790	Rsa1.0_07127.1	601	9863	0	432	446	96	−	130	574	8986	9430
UN07791	Rsa1.0_00382.1	2203	251946	1.00E-147	297	305	97	−	1826	2128	113820	114124
UN07792	Rsa1.0_05428.1	1128	13789	1.00E-154	286	289	98	−	828	1116	6198	6486
UN07793	Rsa1.0_03842.1	861	20503	0	832	863	96	−	1	861	8206	9064
UN07794	Rsa1.0_00236.1	1571	104771	0	1103	1162	94	−	127	1284	73950	75085
UN07795	Rsa1.0_00250.1	1456	273771	1.00E-155	279	279	100	+	536	814	241495	241773
UN07796	Rsa1.0_00131.1	995	432233	0	452	452	100	−	1	452	73557	74008
UN07797	Rsa1.0_09746.1	729	6174	0	554	557	99	+	173	729	86	642
UN07798	Rsa1.0_00122.1	1625	265937	0	580	607	95	−	1	596	233492	234097

UN07799	Rsa1.0_00159.1	1157	431686	1.00E-141	295	306	96	+	854	1157	418639	418943
UN07800	Rsa1.0_00793.1	1148	64103	1.00E-178	367	381	96	+	1	380	38945	39324
UN07801	Rsa1.0_01361.1	1312	47226	0	1090	1112	98	+	12	1106	36939	38050
UN07802	Rsa1.0_00381.1	1025	89718	0	394	414	95	+	619	1024	29797	30210
UN07803	Rsa1.0_00319.1	1187	167331	0	947	1079	87	−	6	1073	91408	92486
UN07804	Rsa1.0_00332.1	1293	94575	1.00E-174	332	337	98	−	958	1291	77800	78136
UN07805	Rsa1.0_03353.1	1285	23496	0	540	586	92	−	668	1251	18990	19561
UN07806	Rsa1.0_01431.1	1273	167877	0	346	350	98	+	345	694	23662	24011
UN07807	Rsa1.0_04463.1	1730	17247	0	553	569	97	−	2	569	15847	16407
UN07808	Rsa1.0_00512.1	1509	200277	2.33E-156	276	280	98	+	1230	1509	71604	71883
UN07809	Rsa1.0_03101.1	786	25410	0	709	717	98	−	4	720	390	1105
UN07810	Rsa1.0_03090.1	841	25464	0	432	449	96	+	5	450	21757	22205
UN07811	Rsa1.0_00327.1	1101	94892	0	493	513	96	+	2	508	24938	25448
UN07812	Rsa1.0_00215.1	1222	201775	0	602	609	98	−	612	1220	10591	11197
UN07813	Rsa1.0_01539.1	1344	43698	0	461	461	100	−	135	595	23834	24294
UN07814	Rsa1.0_10233.1	1456	5928	0	484	511	94	−	2	512	2369	2861
UN07815	Rsa1.0_00131.1	903	432233	1.00E-122	249	254	98	+	3	254	6381	6632
UN07816	Rsa1.0_00152.1	1161	257730	0	424	440	96	+	725	1161	38296	38735
UN07817	Rsa1.0_00040.1	988	324196	1.00E-107	201	202	99	+	403	604	320994	321195
UN07818	Rsa1.0_56101.1	929	408	0	361	369	97	−	33	399	40	408
UN07819	Rsa1.0_00321.1	1031	95671	1.00E-111	277	298	92	+	626	923	60426	60718
UN07820	Rsa1.0_00089.1	960	406142	1.00E-175	435	475	91	−	482	934	352489	352958
UN07821	Rsa1.0_01696.1	1169	301850	1.00E-169	399	423	94	−	751	1167	298765	299179
UN07822	Rsa1.0_03084.1	1082	25506	0	595	607	98	+	471	1077	12462	13060
UN07823	Rsa1.0_00123.1	1389	325650	0	389	389	100	−	54	442	4758	5146
UN07824	Rsa1.0_11626.1	1118	5456	0	481	511	94	−	603	1105	3158	3663
UN07825	Rsa1.0_02587.1	862	156090	0	443	448	98	−	38	485	16390	16837
UN07826	Rsa1.0_23327.1	1184	1056	0	519	535	97	+	654	1182	1	533
UN07827	Rsa1.0_00065.1	1037	454616	0	456	472	96	+	132	600	207651	208122
UN07828	Rsa1.0_00207.1	1362	248736	1.00E-108	288	313	92	+	282	590	24166	24475
UN07829	Rsa1.0_28441.1	1606	815	0	782	815	95	−	564	1376	1	815
UN07830	Rsa1.0_07336.1	1803	9540	0	405	406	99	+	544	949	1	406
UN07831	Rsa1.0_01093.1	879	54312	0	551	553	99	+	324	876	26466	27018
UN07832	Rsa1.0_01198.1	1449	51029	1.00E-166	380	406	93	−	1039	1442	803	1196
UN07833	Rsa1.0_00016.1	1198	831256	0	644	662	97	+	543	1198	809000	809661
UN07834	Rsa1.0_00245.1	684	103569	0	663	682	97	+	1	682	97557	98232
UN07835	Rsa1.0_00040.1	1272	324196	0	918	927	99	−	342	1268	93894	94820
UN07836	Rsa1.0_02677.1	924	167801	0	438	444	98	−	3	446	153778	154218
UN07837	Rsa1.0_00146.1	1293	149343	0	657	662	99	−	6	664	86415	87076

UN07838	Rsa1.0_01783.1	1803	39383	0	719	743	96	+	438	1180	33735	34475
UN07839	Rsa1.0_13382.1	1014	4874	3.00E-66	141	145	97	−	257	401	1445	1589
UN07840	Rsa1.0_00057.1	788	160473	1.00E-86	163	163	100	−	565	727	146663	146825
UN07841	Rsa1.0_00898.1	1268	202772	0	536	559	95	−	43	599	182006	182562
UN07842	Rsa1.0_00040.1	774	324196	2.00E-76	146	146	100	−	85	230	264034	264179
UN07843	Rsa1.0_01997.1	1765	158416	0	1694	1787	94	−	3	1765	19326	21081
UN07844	Rsa1.0_00107.1	840	431659	0	595	597	99	+	1	596	69339	69935
UN07845	Rsa1.0_12910.1	1138	5081	0	353	357	98	+	3	356	2347	2703
UN07846	Rsa1.0_04955.1	1408	15337	0	1023	1046	97	+	88	1130	8163	9205
UN07847	Rsa1.0_03576.1	476	21986	0	469	476	98	−	1	476	3383	3855
UN07848	Rsa1.0_00057.1	718	160473	0	709	718	98	+	2	718	24327	25043
UN07849	Rsa1.0_03125.1	1287	147978	0	649	671	96	−	76	743	131472	132131
UN07850	Rsa1.0_01099.1	1238	158481	0	363	363	100	−	380	742	131327	131689
UN07851	Rsa1.0_00014.1	1236	348985	0	364	367	99	+	1	367	343861	344224
UN07852	Rsa1.0_00056.1	838	332604	0	566	582	97	−	253	831	291284	291863
UN07853	Rsa1.0_00642.1	714	201230	0	709	714	99	+	2	714	165129	165842
UN07854	Rsa1.0_00090.1	1394	273859	0	512	530	96	+	868	1394	138994	139519
UN07855	Rsa1.0_00555.1	1945	75920	0	592	611	96	−	874	1482	1049	1652
UN07856	Rsa1.0_01753.1	848	39794	0	766	787	97	−	11	791	23080	23866
UN07857	Rsa1.0_00410.1	1248	86957	0	1246	1251	99	+	1	1248	52544	53794
UN07858	Rsa1.0_04772.1	552	16040	0	500	519	96	+	1	515	13563	14079
UN07859	Rsa1.0_00882.1	848	61249	0	844	846	99	−	3	848	28143	28988
UN07860	Rsa1.0_00525.1	886	78030	0	424	426	99	−	461	886	10815	11240
UN07861	Rsa1.0_00053.1	714	263459	0	686	703	97	−	10	712	78527	79226
UN07862	Rsa1.0_30434.1	1233	754	0	559	577	96	−	1	577	3	579
UN07863	Rsa1.0_00940.1	780	179946	0	775	788	98	+	1	780	24188	24975
UN07864	Rsa1.0_00549.1	1381	76473	0	1023	1059	96	+	326	1381	33161	34211
UN07865	Rsa1.0_00405.1	819	202864	0	642	672	95	−	3	656	85752	86423
UN07866	Rsa1.0_00827.1	771	62790	4.00E-80	174	180	96	−	153	332	42331	42509
UN07867	Rsa1.0_00983.1	1353	57586	0	863	900	95	+	2	901	10494	11393
UN07868	Rsa1.0_04060.1	559	19313	1.00E-156	300	307	97	+	253	559	16471	16777
UN07869	Rsa1.0_02374.1	846	32043	0	826	853	96	−	2	846	4803	5650
UN07870	Rsa1.0_05180.1	1422	14575	0	725	790	91	−	409	1187	4725	5479
UN07871	Rsa1.0_16653.1	1160	2077	0	976	1017	95	−	126	1133	99	1115
UN07872	Rsa1.0_20177.1	1131	1331	0	687	700	98	−	370	1069	130	829
UN07873	Rsa1.0_00140.1	1813	121541	0	999	1004	99	+	3	1006	81953	82956
UN07874	Rsa1.0_05874.1	1756	12351	0	1149	1202	95	+	2	1200	6996	8185
UN07875	Rsa1.0_00401.1	1343	244410	0	400	409	97	+	939	1343	243012	243420
UN07876	Rsa1.0_00015.1	600	561803	0	364	370	98	+	3	372	200435	200804

UN07877	Rsa1.0_00201.1	917	232851	0	387	399	96	+	3	399	22034	22432
UN07878	Rsa1.0_01927.1	1665	37199	0	1109	1155	96	+	118	1271	23470	24616
UN07879	Rsa1.0_02279.1	1343	33069	0	736	755	97	−	332	1086	1	755
UN07880	Rsa1.0_02367.1	1058	32106	0	984	996	98	+	63	1058	7291	8282
UN07881	Rsa1.0_03754.1	1261	21005	0	1114	1215	91	−	14	1218	8962	10176
UN07882	Rsa1.0_02527.1	1886	177225	0	863	897	96	+	3	886	148129	149024
UN07883	Rsa1.0_16318.1	535	2202	0	356	360	98	+	176	535	1839	2196
UN07884	Rsa1.0_00827.1	1562	62790	1.00E−155	348	370	94	−	1193	1559	52062	52427
UN07885	Rsa1.0_00807.1	868	63607	1.00E−135	280	289	96	−	438	725	19934	20221
UN07886	Rsa1.0_02080.1	1236	35297	0	627	652	96	+	17	667	13243	13891
UN07887	Rsa1.0_00676.1	972	69166	1.00E−105	198	199	99	+	1	199	15584	15782
UN07888	Rsa1.0_00373.1	680	90296	1.00E−165	318	324	98	−	357	680	19124	19442
UN07889	Rsa1.0_00486.1	1634	80875	0	548	553	99	+	479	1031	13173	13724
UN07890	Rsa1.0_00588.1	847	73939	0	549	565	97	−	1	560	54483	55041
UN07891	Rsa1.0_00709.1	1553	67329	0	694	730	95	+	810	1527	30393	31121
UN07892	Rsa1.0_00411.1	1570	86944	0	583	605	96	+	969	1566	46561	47165
UN07893	Rsa1.0_00727.1	1096	66724	1.00E−150	291	298	97	+	553	850	24666	24963
UN07894	Rsa1.0_02139.1	1369	34686	0	381	387	98	−	979	1365	7615	8000
UN07895	Rsa1.0_22870.1	1910	1087	0	827	839	98	+	462	1300	1	839
UN07896	Rsa1.0_00675.1	1617	145590	0	516	531	97	+	995	1524	58355	58881
UN07897	Rsa1.0_04739.1	1413	16167	0	372	381	97	+	9	387	6309	6689
UN07898	Rsa1.0_00275.1	1771	210104	0	490	491	99	+	4	494	184311	184801
UN07899	Rsa1.0_01485.1	1043	44797	0	430	430	100	−	614	1043	22104	22533
UN07900	Rsa1.0_00392.1	1714	88523	0	564	594	94	+	1109	1692	65484	66072
UN07901	Rsa1.0_00069.1	1013	403561	0	863	880	98	−	135	1013	206030	206905
UN07902	Rsa1.0_01338.1	1643	155644	0	1224	1230	99	−	1	1230	133631	134860
UN07903	Rsa1.0_00086.1	700	439854	0	690	699	98	+	3	700	129698	130396
UN07904	Rsa1.0_00015.1	1861	561803	0	1352	1515	89	−	186	1700	222002	223516
UN07905	Rsa1.0_00065.1	890	454616	1.00E−101	205	211	97	+	50	260	159767	159977
UN07906	Rsa1.0_00093.1	977	214962	0	666	690	96	−	238	927	173992	174680
UN07907	Rsa1.0_03697.1	971	21304	1.00E−59	142	150	94	−	266	415	5454	5603
UN07908	Rsa1.0_01363.1	617	47195	1.00E−137	270	276	97	+	341	616	8682	8956
UN07909	Rsa1.0_03671.1	1574	21459	0	360	364	98	+	3	363	11189	11552
UN07910	Rsa1.0_00037.1	967	307838	1.00E−137	270	276	97	+	3	278	286938	287212
UN07911	Rsa1.0_08969.1	718	6744	0	701	720	97	−	2	718	3090	3808
UN07912	Rsa1.0_06663.1	1436	10576	1.00E−159	323	335	96	−	1102	1436	10093	10424
UN07913	Rsa1.0_03406.1	1342	23159	1.00E−163	456	510	89	−	634	1128	5534	6031
UN07914	Rsa1.0_00189.1	1260	111830	0	371	372	99	+	1	372	42070	42441
UN07915	Rsa1.0_01354.1	745	47342	0	654	669	97	−	3	671	30879	31547

UN07916	Rsa1.0_00002.1	858	601661	0	837	856	97	+	2	856	416755	417610
UN07917	Rsa1.0_01663.1	1598	168589	0	525	526	99	+	1	526	138820	139345
UN07918	Rsa1.0_00768.1	958	64969	1.00E-133	282	292	96	+	85	375	49455	49743
UN07919	Rsa1.0_00494.1	1004	79967	0	458	462	99	+	543	1004	68676	69137
UN07920	Rsa1.0_00938.1	581	193891	0	570	580	98	+	3	581	175161	175740
UN07921	Rsa1.0_17254.1	973	1878	0	470	474	99	+	258	728	1405	1878
UN07922	Rsa1.0_01308.1	868	48596	0	537	548	97	+	108	655	17350	17896
UN07923	Rsa1.0_00773.1	966	64839	0	567	583	97	+	384	966	6287	6869
UN07924	Rsa1.0_00919.1	505	59675	0	496	505	98	+	1	505	33437	33932
UN07925	Rsa1.0_00180.1	596	114581	1.00E-129	257	262	98	+	331	591	98334	98594
UN07926	Rsa1.0_00061.1	864	218685	0	841	862	97	−	3	862	958	1815
UN07927	Rsa1.0_01875.1	1008	37849	0	798	821	97	−	1	814	18314	19131
UN07928	Rsa1.0_00464.1	1463	82253	0	464	475	97	+	114	587	67303	67777
UN07929	Rsa1.0_30865.1	850	742	9.00E-97	273	300	91	+	552	850	1	288
UN07930	Rsa1.0_02525.1	736	30489	0	427	429	99	+	307	734	14810	15237
UN07931	Rsa1.0_02043.1	1281	163754	1.00E-166	333	343	97	+	940	1280	23393	23734
UN07932	Rsa1.0_00213.1	1703	108167	0	470	485	96	+	1218	1698	86924	87405
UN07933	Rsa1.0_00992.1	806	57419	0	468	487	96	+	320	806	25453	25928
UN07934	Rsa1.0_00085.1	1574	142226	0	1465	1558	94	−	3	1553	121914	123453
UN07935	Rsa1.0_02162.1	1273	34431	0	1023	1025	99	+	3	1025	8263	9287
UN07936	Rsa1.0_01883.1	951	37661	1.00E-157	288	290	99	+	2	291	10628	10917
UN07937	Rsa1.0_00550.1	764	210790	1.00E-116	298	319	93	+	455	764	31765	32080
UN07938	Rsa1.0_01221.1	1236	50421	0	744	770	96	+	188	957	44672	45438
UN07939	Rsa1.0_00002.1	1226	601661	0	918	951	96	+	279	1226	177153	178101
UN07940	Rsa1.0_00582.1	596	74333	0	569	596	95	−	3	588	50361	50955
UN07941	Rsa1.0_00930.1	945	59224	0	387	395	97	−	2	396	52811	53204
UN07942	Rsa1.0_01838.1	1404	38500	0	422	442	95	−	6	443	17537	17978
UN07943	Rsa1.0_00015.1	817	561803	0	474	475	99	+	6	480	257369	257843
UN07944	Rsa1.0_03724.1	1525	21171	0	502	504	99	−	1022	1525	16463	16966
UN07945	Rsa1.0_02456.1	702	31222	1.00E-122	303	327	92	+	378	700	26023	26337
UN07946	Rsa1.0_03974.1	740	19770	0	591	604	97	−	137	740	1460	2061
UN07947	Rsa1.0_01483.1	1241	44826	1.00E-147	271	273	99	−	546	818	22727	22999
UN07948	Rsa1.0_00241.1	1122	104000	1.00E-176	319	321	99	+	801	1121	27774	28094
UN07949	Rsa1.0_00193.1	1081	194198	1.00E-145	315	333	94	+	753	1081	102472	102797
UN07950	Rsa1.0_00978.1	808	57739	0	783	817	95	+	2	807	23545	24354
UN07951	Rsa1.0_00527.1	1047	77733	0	1026	1059	96	−	3	1047	12296	13352
UN07952	Rsa1.0_02711.1	1169	28475	1.00E-153	338	359	94	+	729	1087	20055	20413
UN07953	Rsa1.0_00651.1	844	247766	1.00E-133	326	360	90	−	485	844	92697	93034
UN07954	Rsa1.0_00720.1	871	66931	0	360	368	97	+	5	371	55086	55453

UN07955	Rsa1.0_02387.1	573	31890	1.00E-54	136	145	93	-	3	147	30227	30371
UN07956	Rsa1.0_00562.1	590	75565	0	583	588	99	+	3	590	65791	66378
UN07957	Rsa1.0_03861.1	1239	20402	0	450	459	98	-	723	1181	12424	12882
UN07958	Rsa1.0_01752.1	758	39805	0	612	629	97	+	50	675	23241	23869
UN07959	Rsa1.0_00070.1	1387	150321	1.00E-119	224	226	99	-	943	1168	119907	120132
UN07960	Rsa1.0_13893.1	744	4139	0	434	437	99	-	3	439	2358	2794
UN07961	Rsa1.0_00190.1	1322	318240	0	567	585	96	+	129	713	82	662
UN07962	Rsa1.0_02410.1	788	31659	0	403	419	96	-	236	654	20565	20983
UN07963	Rsa1.0_05547.1	915	13369	0	574	582	98	-	1	581	919	1500
UN07964	Rsa1.0_00581.1	1581	163575	0	874	878	99	+	704	1581	15342	16219
UN07965	Rsa1.0_00664.1	902	69573	0	563	566	99	+	1	566	55264	55829
UN07966	Rsa1.0_00524.1	760	169613	0	474	494	95	-	4	493	141781	142271
UN07967	Rsa1.0_02329.1	891	32474	0	856	869	98	+	1	867	882	1747
UN07968	Rsa1.0_00740.1	876	215972	2.00E-98	230	245	93	-	2	246	32317	32558
UN07969	Rsa1.0_00169.1	1286	274298	1.00E-166	331	341	97	+	1	340	229363	229703
UN07970	Rsa1.0_00476.1	477	280966	1.00E-131	296	312	94	-	39	347	150634	150943
UN07971	Rsa1.0_00847.1	1447	62270	0	491	493	99	-	1	491	50627	51119
UN07972	Rsa1.0_01326.1	1379	48072	0	333	333	100	+	915	1247	34414	34746
UN07973	Rsa1.0_36762.1	1330	613	0	608	613	99	+	533	1145	1	613
UN07974	Rsa1.0_07655.1	520	8913	0	513	515	99	+	4	518	6354	6868
UN07975	Rsa1.0_25166.1	1294	949	0	520	521	99	+	1	521	429	949
UN07976	Rsa1.0_09317.1	1944	6459	0	696	732	95	+	1217	1942	1016	1741
UN07977	Rsa1.0_10540.1	648	5811	0	642	646	99	-	3	648	3651	4296
UN07978	Rsa1.0_02628.1	1110	29369	1.00E-149	277	280	98	+	503	782	7499	7778
UN07979	Rsa1.0_02082.1	868	35228	0	528	558	94	-	149	705	17741	18297
UN07980	Rsa1.0_02900.1	1339	26928	0	728	746	97	-	593	1338	25075	25819
UN07981	Rsa1.0_00222.1	1285	172263	0	597	605	98	-	3	605	4258	4862
UN07982	Rsa1.0_00106.1	1638	133916	0	1080	1103	97	+	2	1103	19192	20291
UN07983	Rsa1.0_04411.1	1009	142979	0	809	817	99	+	1	814	137806	138622
UN07984	Rsa1.0_00308.1	1116	96915	0	446	461	96	+	30	484	13156	13615
UN07985	Rsa1.0_01880.1	1141	37745	0	927	941	98	-	4	943	26258	27198
UN07986	Rsa1.0_00205.1	2138	193401	0	661	682	96	+	1456	2137	55100	55778
UN07987	Rsa1.0_01356.1	522	47287	2.00E-81	160	162	98	-	1	162	3539	3700
UN07988	Rsa1.0_00285.1	970	149638	0	563	590	95	-	1	590	142472	143050
UN07989	Rsa1.0_00099.1	1154	159619	0	586	592	98	-	3	593	136397	136988
UN07990	Rsa1.0_01082.1	1186	54640	0	433	433	100	+	752	1184	15720	16152
UN07991	Rsa1.0_06108.1	809	11730	0	457	462	98	-	2	463	2383	2844
UN07992	Rsa1.0_00197.1	1417	109714	1.00E-159	342	360	95	+	206	559	99351	99709
UN07993	Rsa1.0_00472.1	895	398134	0	464	493	94	-	410	895	314464	314947

UN07994	Rsa1.0_03073.1	1225	25574	0	817	818	99	−	101	917	82	899
UN07995	Rsa1.0_00533.1	1814	77375	0	769	789	97	+	1	789	32030	32815
UN07996	Rsa1.0_02841.1	1379	27383	0	750	764	98	−	138	901	18551	19314
UN07997	Rsa1.0_29367.1	912	785	0	780	786	99	−	125	910	1	785
UN07998	Rsa1.0_01782.1	738	39384	0	681	691	98	−	13	703	33047	33737
UN07999	Rsa1.0_00735.1	911	66503	0	907	910	99	−	1	910	60231	61140
UN08000	Rsa1.0_02609.1	1166	29600	0	406	412	98	−	1	411	21111	21522
UN08001	Rsa1.0_00303.1	2053	97468	0	644	651	98	−	431	1081	26781	27431
UN08002	Rsa1.0_00588.1	600	73939	0	386	393	98	+	1	391	44020	44411
UN08003	Rsa1.0_01015.1	1338	56752	2.33E−156	291	300	97	+	666	965	6238	6537
UN08004	Rsa1.0_03511.1	711	22373	0	442	445	99	−	1	444	806	1249
UN08005	Rsa1.0_01108.1	923	53865	1.00E−141	263	266	98	+	657	922	15154	15419
UN08006	Rsa1.0_01422.1	1239	46172	1.00E−118	222	224	99	+	832	1055	35856	36079
UN08007	Rsa1.0_15660.1	912	2496	0	827	842	98	+	44	883	563	1401
UN08008	Rsa1.0_02030.1	591	179909	0	575	592	97	+	1	590	27041	27632
UN08009	Rsa1.0_01251.1	768	49873	0	496	505	98	+	2	506	27199	27701
UN08010	Rsa1.0_01091.1	1285	54383	0	1123	1124	99	+	160	1283	20814	21937
UN08011	Rsa1.0_07458.1	985	9352	0	693	706	98	−	36	741	8647	9352
UN08012	Rsa1.0_17302.1	1551	1865	0	739	741	99	−	811	1551	1125	1865
UN08013	Rsa1.0_00045.1	630	328694	0	478	492	97	+	112	602	192774	193265
UN08014	Rsa1.0_01117.1	1305	53465	0	531	534	99	+	2	535	45563	46096
UN08015	Rsa1.0_00055.1	781	520193	0	574	577	99	+	204	780	422501	423077
UN08016	Rsa1.0_01740.1	1031	39986	0	367	376	97	+	4	376	22135	22509
UN08017	Rsa1.0_08538.1	1861	7220	0	969	1049	92	−	374	1397	1434	2455
UN08018	Rsa1.0_03940.1	1273	19962	0	433	450	96	−	779	1225	1	444
UN08019	Rsa1.0_03292.1	1253	23977	0	439	440	99	+	812	1251	3716	4155
UN08020	Rsa1.0_01781.1	765	39410	0	551	561	98	+	162	722	6978	7538
UN08021	Rsa1.0_06522.1	1040	10856	1.00E−119	243	251	96	+	19	269	2538	2784
UN08022	Rsa1.0_03566.1	929	22050	0	891	930	95	+	1	928	15859	16780
UN08023	Rsa1.0_11280.1	982	5558	0	374	380	98	+	483	862	4869	5248
UN08024	Rsa1.0_00536.1	1075	77199	0	871	910	95	−	4	908	23937	24846
UN08025	Rsa1.0_02039.1	879	35699	1.00E−176	343	352	97	−	528	879	31463	31812
UN08026	Rsa1.0_00570.1	1529	75002	0	549	560	98	+	496	1055	70246	70805
UN08027	Rsa1.0_14736.1	734	3116	1.00E−168	339	351	96	+	382	732	1970	2319
UN08028	Rsa1.0_00116.1	1164	365886	0	862	872	98	−	3	874	21612	22483
UN08029	Rsa1.0_06230.1	855	11435	0	821	855	96	−	1	855	4439	5280
UN08030	Rsa1.0_00184.1	1144	218748	0	376	393	95	−	2	394	17859	18241
UN08031	Rsa1.0_00862.1	887	61715	0	481	485	99	−	403	887	28518	29002
UN08032	Rsa1.0_00059.1	922	159650	0	477	529	90	+	225	732	134007	134528

UN08033	Rsa1.0_01536.1	1372	43747	0	658	668	98	+	704	1371	16387	17054
UN08034	Rsa1.0_02181.1	428	34172	0	414	429	96	+	3	425	1019	1443
UN08035	Rsa1.0_02829.1	910	27541	2.00E-45	273	331	82	+	194	522	19828	20140
UN08036	Rsa1.0_02927.1	1231	26698	0	1207	1234	97	+	2	1231	23078	24309
UN08037	Rsa1.0_01890.1	953	37542	0	336	340	98	−	584	923	12677	13016
UN08038	Rsa1.0_00221.1	1065	106972	1.00E-156	329	344	95	−	3	340	93919	94262
UN08039	Rsa1.0_00446.1	844	167829	0	833	843	98	−	1	843	149439	150280
UN08040	Rsa1.0_00584.1	1287	74295	0	791	824	95	+	2	825	44010	44833
UN08041	Rsa1.0_05767.1	1329	12701	0	1299	1326	97	−	3	1328	3398	4718
UN08042	Rsa1.0_01102.1	455	53987	0	450	453	99	−	1	453	8658	9110
UN08043	Rsa1.0_00576.1	2144	178949	0	437	439	99	+	166	604	63282	63720
UN08044	Rsa1.0_00497.1	1772	79830	0	777	784	99	+	989	1771	48252	49035
UN08045	Rsa1.0_04014.1	1238	19572	0	484	490	98	−	749	1238	13584	14073
UN08046	Rsa1.0_00373.1	1346	90296	0	683	698	97	−	2	696	42120	42817
UN08047	Rsa1.0_02677.1	989	167801	0	685	719	95	−	277	989	10875	11581
UN08048	Rsa1.0_05577.1	975	13284	0	938	955	98	−	21	975	11449	12403
UN08049	Rsa1.0_00626.1	753	71582	2.33E-156	279	284	98	+	1	280	69542	69825
UN08050	Rsa1.0_00282.1	1835	177799	1.00E-109	201	201	100	+	1304	1504	116639	116839
UN08051	Rsa1.0_01352.1	1091	47443	5.00E-99	219	229	95	+	102	326	9356	9583
UN08052	Rsa1.0_31125.1	1132	735	0	670	677	98	−	52	727	59	735
UN08053	Rsa1.0_03756.1	925	143061	0	647	649	99	+	3	649	10358	11006
UN08054	Rsa1.0_01199.1	1251	163576	0	561	577	97	−	673	1249	126967	127541
UN08055	Rsa1.0_00674.1	1054	193753	1.00E-141	279	287	97	−	467	753	188457	188743
UN08056	Rsa1.0_15443.1	491	2603	0	490	494	99	−	1	490	1965	2458
UN08057	Rsa1.0_04760.1	575	16105	0	353	356	99	+	3	358	10661	11013
UN08058	Rsa1.0_00156.1	992	118383	0	685	704	97	+	1	702	54333	55035
UN08059	Rsa1.0_01979.1	788	36545	0	424	437	97	−	5	438	36007	36441
UN08060	Rsa1.0_01119.1	968	53459	0	434	449	96	−	2	444	19591	20037
UN08061	Rsa1.0_03081.1	851	25523	5.00E-83	169	173	97	+	169	341	13289	13461
UN08062	Rsa1.0_00485.1	1223	80937	0	413	414	99	+	1	413	6623	7036
UN08063	Rsa1.0_02711.1	1495	28475	1.00E-150	320	336	95	−	1158	1492	16349	16672
UN08064	Rsa1.0_00087.1	886	761073	0	511	536	95	+	1	531	312461	312993
UN08065	Rsa1.0_00362.1	1012	299497	0	561	570	98	−	1	570	281999	282568
UN08066	Rsa1.0_01009.1	621	56922	1.00E-151	283	287	98	+	1	287	29237	29523
UN08067	Rsa1.0_00107.1	595	431659	1.00E-137	256	259	98	−	253	511	84232	84490
UN08068	Rsa1.0_00352.1	1441	92266	0	558	573	97	+	868	1439	26252	26824
UN08069	Rsa1.0_00278.1	1036	231896	0	532	537	99	+	2	537	150871	151407
UN08070	Rsa1.0_00066.1	1245	152418	1.00E-151	278	279	99	+	1	278	40911	41189
UN08071	Rsa1.0_00107.1	1093	431659	0	474	504	94	−	12	510	342550	343050

UN08072	Rsa1.0_00071.1	667	345714	1.00E-123	260	269	96	-	371	637	66097	66365
UN08073	Rsa1.0_00080.1	971	190437	0	432	440	98	-	534	971	19235	19674
UN08074	Rsa1.0_00103.1	830	135304	0	805	830	96	-	1	830	30695	31509
UN08075	Rsa1.0_01507.1	903	44361	2.00E-91	184	187	98	-	551	736	24828	25014
UN08076	Rsa1.0_00550.1	1380	210790	1.00E-169	356	373	95	-	14	385	184237	184609
UN08077	Rsa1.0_00143.1	1392	121313	1.00E-167	340	353	96	-	1039	1388	97993	98345
UN08078	Rsa1.0_00940.1	461	179946	0	417	422	98	+	38	459	17311	17732
UN08079	Rsa1.0_00488.1	689	80490	0	368	372	98	+	1	372	77214	77585
UN08080	Rsa1.0_02687.1	900	28648	0	869	908	95	+	3	900	13257	14153
UN08081	Rsa1.0_00703.1	875	67597	4.00E-71	155	161	96	+	500	660	22981	23141
UN08082	Rsa1.0_01194.1	1305	51113	0	675	689	97	-	517	1205	26678	27366
UN08083	Rsa1.0_00382.1	1252	251946	0	345	346	99	-	3	348	168724	169069
UN08084	Rsa1.0_00022.1	1444	200461	0	751	771	97	+	2	772	93920	94690
UN08085	Rsa1.0_00002.1	775	601661	1.00E-134	242	242	100	-	384	625	455292	455533
UN08086	Rsa1.0_00595.1	1869	341648	0	387	387	100	-	1308	1694	12663	13049
UN08087	Rsa1.0_01503.1	1286	44467	0	448	473	94	-	821	1286	35739	36210
UN08088	Rsa1.0_00356.1	1267	91521	0	550	553	99	-	714	1266	25417	25969
UN08089	Rsa1.0_00124.1	1796	262503	0	820	822	99	+	976	1796	64100	64921
UN08090	Rsa1.0_00018.1	915	410899	1.00E-131	244	246	99	-	1	246	125814	126059
UN08091	Rsa1.0_00243.1	990	103651	1.00E-120	253	265	95	-	2	266	12271	12527
UN08092	Rsa1.0_00016.1	1567	831256	0	509	512	99	+	1	512	244882	245392
UN08093	Rsa1.0_00305.1	1589	97308	1.00E-143	312	328	95	-	1262	1587	94377	94696
UN08094	Rsa1.0_00108.1	1045	478491	2.33E-156	312	328	95	-	621	939	7747	8074
UN08095	Rsa1.0_04334.1	1326	17852	0	395	400	98	+	927	1326	10377	10776
UN08096	Rsa1.0_00775.1	1404	64793	0	656	701	93	-	4	685	5368	6068
UN08097	Rsa1.0_09074.1	1371	6670	0	459	474	96	+	614	1084	4110	4577
UN08098	Rsa1.0_00566.1	1481	75325	0	438	470	93	-	415	880	24398	24860
UN08099	Rsa1.0_02703.1	1384	28544	0	1081	1148	94	-	1	1143	9801	10920
UN08100	Rsa1.0_00006.1	1704	396176	0	577	629	91	-	396	1010	223817	224437
UN08101	Rsa1.0_00254.1	1626	102600	0	440	449	97	+	696	1142	20119	20567
UN08102	Rsa1.0_02524.1	1235	30496	0	410	418	98	-	183	599	16107	16524
UN08103	Rsa1.0_03867.1	725	20369	0	402	413	97	-	302	712	15803	16215
UN08104	Rsa1.0_25507.1	796	933	0	380	389	97	-	3	391	454	839
UN08105	Rsa1.0_00454.1	1084	216970	0	704	736	95	-	349	1084	187174	187900
UN08106	Rsa1.0_01330.1	1006	47960	0	350	356	98	+	1	356	43076	43431
UN08107	Rsa1.0_11505.1	1432	5483	0	924	940	98	+	456	1388	1	940
UN08108	Rsa1.0_00160.1	1186	346678	0	387	400	96	+	3	398	145891	146290
UN08109	Rsa1.0_00469.1	473	82084	0	401	420	95	-	1	420	3747	4163
UN08110	Rsa1.0_01986.1	1297	36489	0	834	867	96	-	447	1295	23438	24301

UN08111	Rsa1.0_01750.1	1321	39816	0	409	411	99	+	2	411	22856	23266
UN08112	Rsa1.0_02557.1	1455	30127	0	685	697	98	+	759	1455	10172	10867
UN08113	Rsa1.0_03943.1	598	19931	1.00E-157	323	335	96	−	268	598	8508	8842
UN08114	Rsa1.0_01749.1	1325	39822	0	426	440	96	−	1	440	28271	28706
UN08115	Rsa1.0_00525.1	587	78030	1.00E-130	236	236	100	+	352	587	23409	23644
UN08116	Rsa1.0_01442.1	1429	45776	1.00E-160	286	286	100	+	84	369	20988	21273
UN08117	Rsa1.0_00027.1	738	283362	0	466	472	98	+	4	475	18991	19462
UN08118	Rsa1.0_00421.1	1552	86365	1.00E-140	324	344	94	−	1207	1548	75833	76174
UN08119	Rsa1.0_00165.1	1741	116466	0	1082	1083	99	+	660	1741	47654	48736
UN08120	Rsa1.0_01368.1	1404	47129	0	1377	1398	98	+	17	1402	29056	30453
UN08121	Rsa1.0_03385.1	1654	23280	0	701	714	98	+	888	1601	3669	4381
UN08122	Rsa1.0_01768.1	932	138463	1.00E-176	314	314	100	−	617	930	122648	122961
UN08123	Rsa1.0_03081.1	1446	25523	0	1155	1179	97	−	126	1303	3706	4884
UN08124	Rsa1.0_01772.1	1247	39557	0	1040	1096	94	−	169	1247	160	1238
UN08125	Rsa1.0_01129.1	973	52949	0	927	938	98	+	1	936	13920	14857
UN08126	Rsa1.0_00111.1	1529	390088	0	917	1006	91	−	216	1173	60205	61189
UN08127	Rsa1.0_00076.1	1132	146021	0	1119	1130	99	+	3	1132	258	1387
UN08128	Rsa1.0_00303.1	1371	97468	0	357	359	99	−	2	360	9636	9994
UN08129	Rsa1.0_00011.1	1248	225044	0	646	662	97	+	587	1248	26245	26906
UN08130	Rsa1.0_00013.1	1380	292129	0	400	414	96	−	2	414	281575	281981
UN08131	Rsa1.0_05668.1	867	12994	0	864	867	99	+	1	867	6164	7029
UN08132	Rsa1.0_43462.1	1208	517	3.00E-23	75	81	92	+	990	1070	348	428
UN08133	Rsa1.0_01424.1	1211	46139	0	597	622	95	−	411	1032	43607	44228
UN08134	Rsa1.0_01111.1	1101	53669	1.00E-142	337	360	93	−	746	1101	39889	40244
UN08135	Rsa1.0_00251.1	1101	303968	0	360	372	96	+	160	531	140959	141330
UN08136	Rsa1.0_00980.1	820	57729	0	770	795	96	−	28	820	39709	40492
UN08137	Rsa1.0_02575.1	1018	29944	1.00E-170	357	374	95	+	648	1018	24000	24369
UN08138	Rsa1.0_01932.1	1217	37177	1.00E-110	274	293	93	−	928	1215	10948	11239
UN08139	Rsa1.0_00002.1	1150	601661	1.00E-166	340	352	96	+	800	1150	98981	99331
UN08140	Rsa1.0_05862.1	1520	12390	1.00E-148	270	271	99	−	1250	1520	3824	4094
UN08141	Rsa1.0_00427.1	1106	189678	0	870	916	94	−	194	1106	32890	33793
UN08142	Rsa1.0_00021.1	1045	263655	1.00E-107	255	275	92	+	25	287	14051	14325
UN08143	Rsa1.0_00131.1	1064	432233	0	712	729	97	+	280	1008	205231	205959
UN08144	Rsa1.0_01242.1	1572	50066	0	1377	1397	98	−	3	1393	48281	49677
UN08145	Rsa1.0_00033.1	1192	515811	0	564	591	95	−	4	584	432520	433107
UN08146	Rsa1.0_00064.1	1348	240927	0	529	539	98	+	379	916	132115	132653
UN08147	Rsa1.0_01998.1	1112	36276	0	649	677	95	+	283	959	10886	11561
UN08148	Rsa1.0_00043.1	1412	264937	0	407	424	95	+	766	1189	143187	143609
UN08149	Rsa1.0_00017.1	1210	752458	0	544	599	90	+	621	1206	268430	269007

UN08150	#	#	#	#	#	#	#	#	#		#	#	#
UN08151	Rsa1.0_00065.1		900	454616	0	426	433	98	−	468	900	248976	249408
UN08152	Rsa1.0_00103.1		1251	135304	0	352	352	100	+	338	689	77912	78263
UN08153	Rsa1.0_03242.1		947	24415	0	355	359	98	+	1	359	3770	4128
UN08154	Rsa1.0_01328.1		2200	48021	0	583	613	95	+	1591	2196	23421	24026
UN08155	Rsa1.0_00013.1		745	292129	1.00E−168	335	348	96	+	3	350	279714	280052
UN08156	Rsa1.0_00498.1		1175	79746	0	410	422	97	−	1	419	49512	49931
UN08157	Rsa1.0_00380.1		1460	89748	0	1004	1021	98	−	429	1449	25550	26570
UN08158	Rsa1.0_00030.1		1237	250988	1.00E−140	268	273	98	+	5	277	104240	104512
UN08159	Rsa1.0_00071.1		1591	345714	1.00E−155	319	329	96	−	1268	1591	39307	39635
UN08160	Rsa1.0_00517.1		1182	142790	0	438	443	98	+	739	1181	101484	101925
UN08161	Rsa1.0_01100.1		1210	54113	1.00E−123	277	293	94	−	829	1113	313	603
UN08162	Rsa1.0_00647.1		960	70440	0	923	962	95	−	3	951	44495	45455
UN08163	Rsa1.0_00387.1		1194	495407	0	335	336	99	+	3	338	384402	384737
UN08164	Rsa1.0_03552.1		881	22151	1.00E−107	206	209	98	+	390	598	4290	4498
UN08165	Rsa1.0_14340.1		973	3501	0	449	472	95	−	498	965	300	770
UN08166	Rsa1.0_00192.1		1107	278200	0	445	456	97	+	214	669	260592	261047
UN08167	Rsa1.0_14791.1		651	3064	0	412	414	99	+	232	645	1427	1840
UN08168	Rsa1.0_00055.1		1249	520193	0	417	419	99	−	64	482	349414	349832
UN08169	Rsa1.0_04943.1		1000	15376	0	892	916	97	−	8	920	5795	6708
UN08170	Rsa1.0_01506.1		1338	44370	0	453	454	99	−	1	453	3669	4122
UN08171	Rsa1.0_00616.1		1601	195713	0	518	524	98	−	534	1057	175996	176519
UN08172	Rsa1.0_01017.1		1271	56714	0	456	459	99	−	333	791	33346	33804
UN08173	Rsa1.0_01537.1		1161	187285	0	425	434	97	−	264	697	16266	16699
UN08174	Rsa1.0_00574.1		1152	74797	0	499	508	98	−	2	508	70561	71068
UN08175	Rsa1.0_00909.1		1018	59976	0	426	442	96	+	1	431	38115	38554
UN08176	Rsa1.0_02192.1		1839	171291	0	444	463	95	−	92	548	12268	12724
UN08177	Rsa1.0_00497.1		1256	79830	1.00E−150	270	270	100	+	986	1255	65567	65836
UN08178	Rsa1.0_00970.1		2042	57994	0	1348	1414	95	−	637	2042	22385	23798
UN08179	Rsa1.0_00191.1		1350	316976	0	554	560	98	+	258	817	145135	145694
UN08180	Rsa1.0_01872.1		721	37908	1.00E−103	225	235	95	+	483	717	16040	16268
UN08181	Rsa1.0_01300.1		951	48942	0	638	684	93	+	1	664	46859	47536
UN08182	Rsa1.0_10575.1		1946	5797	1.00E−125	257	264	97	+	842	1103	21	284
UN08183	Rsa1.0_05605.1		724	13202	0	423	424	99	−	1	424	12770	13193
UN08184	Rsa1.0_00167.1		1158	116000	1.00E−155	323	337	95	+	721	1057	17177	17511
UN08185	Rsa1.0_00207.1		736	248736	0	373	384	97	−	2	385	11395	11778
UN08186	Rsa1.0_00233.1		1474	104918	0	631	643	98	+	691	1332	9694	10336
UN08187	Rsa1.0_06009.1		1046	11950	0	833	834	99	−	210	1043	8494	9326
UN08188	Rsa1.0_16140.1		1743	2268	0	974	1008	96	−	744	1742	1274	2268

UN08189	Rsa1.0_01435.1	1422	45942	1.00E-149	282	285	98	+	3	286	15970	16254
UN08190	Rsa1.0_00045.1	1370	328694	0	521	551	94	+	256	803	168766	169302
UN08191	Rsa1.0_07809.1	1906	8503	0	1064	1132	93	+	23	1120	1020	2144
UN08192	Rsa1.0_01727.1	1258	40243	1.00E-141	378	409	92	−	846	1244	11118	11516
UN08193	Rsa1.0_20689.1	2192	1279	0	389	393	98	+	879	1271	15	407
UN08194	Rsa1.0_01522.1	1721	157436	0	973	997	97	+	727	1719	145054	146047
UN08195	Rsa1.0_00722.1	825	66840	0	430	444	96	−	352	795	20493	20936
UN08196	Rsa1.0_00282.1	953	177799	1.00E-170	546	627	87	+	316	942	119453	120079
UN08197	Rsa1.0_00467.1	1316	156542	1.00E-130	268	277	96	−	183	458	127047	127323
UN08198	Rsa1.0_00447.1	1050	84051	1.00E-150	270	270	100	+	781	1050	12107	12376
UN08199	Rsa1.0_01719.1	1335	40388	0	761	789	96	−	543	1331	31932	32716
UN08200	Rsa1.0_02478.1	1897	150183	0	597	642	92	+	596	1223	136567	137203
UN08201	Rsa1.0_00516.1	1000	78417	1.00E-158	283	283	100	+	1	283	6069	6351
UN08202	Rsa1.0_00017.1	1134	752458	0	664	688	96	+	449	1134	617269	617938
UN08203	Rsa1.0_00108.1	1716	478491	0	793	826	96	−	903	1716	284903	285727
UN08204	Rsa1.0_26911.1	914	873	1.00E-113	207	207	100	+	408	614	248	454
UN08205	Rsa1.0_04340.1	1192	17831	0	615	655	93	+	32	681	12631	13260
UN08206	Rsa1.0_00251.1	2207	303968	1.00E-135	254	257	98	+	732	988	87950	88206
UN08207	Rsa1.0_01675.1	1467	41155	1.00E-112	207	207	100	−	391	597	8774	8980
UN08208	Rsa1.0_00219.1	951	106983	0	946	951	99	+	2	951	51023	51973
UN08209	Rsa1.0_51591.1	1355	441	0	441	441	100	+	792	1232	1	441
UN08210	Rsa1.0_00552.1	719	206757	1.00E-151	282	284	99	+	3	285	51528	51811
UN08211	Rsa1.0_01440.1	1653	45807	1.00E-137	248	248	100	+	1406	1653	30644	30891
UN08212	Rsa1.0_00143.1	988	121313	1.00E-143	313	329	95	−	18	343	87223	87551
UN08213	Rsa1.0_00396.1	1759	88242	0	700	706	99	+	584	1288	5961	6666
UN08214	Rsa1.0_14273.1	1421	3588	1.00E-135	289	301	96	−	1113	1413	2208	2506
UN08215	Rsa1.0_01506.1	1494	44370	1.00E-173	317	319	99	−	1178	1494	31429	31747
UN08216	Rsa1.0_00108.1	1640	478491	0	1623	1641	98	+	1	1640	113582	115219
UN08217	Rsa1.0_00320.1	769	95784	0	732	752	97	+	13	764	3267	4015
UN08218	Rsa1.0_00152.1	1358	257730	0	704	739	95	−	2	736	84804	85538
UN08219	Rsa1.0_01419.1	1456	167402	0	1020	1052	96	+	1	1046	159720	160768
UN08220	Rsa1.0_00179.1	875	393324	1.00E-128	241	244	98	−	498	741	49376	49619
UN08221	Rsa1.0_01100.1	2037	54113	0	491	518	94	−	1432	1948	28675	29180
UN08222	Rsa1.0_04649.1	1199	16538	0	409	424	96	+	1	424	10581	11001
UN08223	Rsa1.0_00851.1	1194	62181	1.00E-172	321	326	98	−	558	883	11159	11484
UN08224	Rsa1.0_15352.1	1261	2663	0	656	668	98	−	7	670	1335	2002
UN08225	Rsa1.0_00285.1	1399	149638	0	373	380	98	−	1022	1399	146432	146811
UN08226	Rsa1.0_02477.1	936	31058	0	580	590	98	−	347	935	13066	13654
UN08227	Rsa1.0_00062.1	1105	299291	2.00E-83	759	941	80	−	109	1036	37611	38534

UN08228	Rsa1.0_02043.1	1310	163754	0	332	332	100	+	2	333	140853	141184
UN08229	Rsa1.0_01025.1	1108	56471	1.00E-37	357	433	82	-	112	537	7410	7832
UN08230	Rsa1.0_00537.1	1416	186980	0	1415	1415	100	+	2	1416	164734	166148
UN08231	Rsa1.0_22522.1	1455	1114	0	936	1007	92	+	122	1125	121	1114
UN08232	Rsa1.0_04342.1	934	17828	0	430	447	96	+	16	459	7660	8103
UN08233	Rsa1.0_00663.1	979	164969	1.00E-120	268	284	94	-	699	978	126421	126704
UN08234	Rsa1.0_06471.1	1619	10954	0	1186	1221	97	+	279	1499	776	1984
UN08235	Rsa1.0_00049.1	1702	167018	0	546	579	94	-	1	579	134071	134636
UN08236	Rsa1.0_00425.1	1275	147080	0	1167	1207	96	-	29	1235	95758	96961
UN08237	Rsa1.0_00873.1	1098	61459	0	564	572	98	+	530	1098	46323	46894
UN08238	Rsa1.0_04079.1	522	19202	1.00E-54	146	159	91	-	7	165	15519	15670
UN08239	Rsa1.0_01704.1	1031	40711	0	532	541	98	+	4	543	21920	22459
UN08240	Rsa1.0_03292.1	965	23977	0	939	963	97	-	3	965	11234	12194
UN08241	Rsa1.0_00190.1	1019	318240	0	378	378	100	+	1	378	134844	135221
UN08242	Rsa1.0_00339.1	730	222974	0	374	389	96	-	342	730	52010	52386
UN08243	Rsa1.0_00111.1	884	390088	1.00E-100	228	237	96	-	643	875	77424	77659
UN08244	Rsa1.0_00079.1	966	606987	1.00E-132	292	307	95	-	4	304	41618	41923
UN08245	Rsa1.0_01326.1	765	48072	0	413	419	98	-	342	760	43752	44166
UN08246	Rsa1.0_02157.1	522	149652	0	366	376	97	+	85	457	11588	11963
UN08247	Rsa1.0_04121.1	747	18951	0	477	499	95	-	7	505	13882	14379
UN08248	Rsa1.0_01743.1	1014	39926	1.00E-132	265	272	97	-	1	271	15993	16264
UN08249	Rsa1.0_02316.1	837	32608	0	474	491	96	+	345	834	6330	6817
UN08250	Rsa1.0_00904.1	1239	180594	0	439	448	97	+	788	1234	177571	178018
UN08251	Rsa1.0_00417.1	1024	395068	0	480	487	98	+	535	1017	235258	235744
UN08252	Rsa1.0_00963.1	975	58176	1.00E-134	246	247	99	-	729	975	26299	26545
UN08253	Rsa1.0_02867.1	735	27121	2.00E-78	283	331	85	-	11	327	3466	3780
UN08254	Rsa1.0_00356.1	822	91521	1.00E-154	300	308	97	+	321	628	34979	35286
UN08255	Rsa1.0_00079.1	694	606987	1.00E-122	275	291	94	-	404	694	198839	199128
UN08256	Rsa1.0_00381.1	763	89718	1.00E-165	391	421	92	-	3	420	75167	75586
UN08257	Rsa1.0_00800.1	640	63868	0	402	402	100	-	239	640	19287	19688
UN08258	Rsa1.0_00888.1	595	60978	0	386	386	100	-	202	587	54469	54854
UN08259	Rsa1.0_07379.1	709	9492	2.00E-91	215	227	94	-	1	226	3308	3533
UN08260	Rsa1.0_00737.1	843	66432	1.00E-126	293	319	91	-	525	843	11909	12208
UN08261	Rsa1.0_00273.1	819	326186	1.00E-165	394	420	93	+	13	427	38743	39154
UN08262	Rsa1.0_00252.1	1117	102760	0	398	406	98	-	1	405	44777	45179
UN08263	Rsa1.0_00105.1	757	134138	1.00E-100	324	366	88	+	13	376	10943	11292
UN08264	Rsa1.0_01586.1	1168	42804	1.00E-172	397	425	93	-	746	1168	28079	28496
UN08265	Rsa1.0_00715.1	447	67116	1.00E-69	198	213	92	-	226	434	40371	40581
UN08266	Rsa1.0_00833.1	984	62621	2.00E-92	245	272	90	-	3	255	9801	10072

UN08267	Rsa1.0_02735.1	1221	28344	0	522	553	94	+	7	557	2745	3290
UN08268	Rsa1.0_01754.1	709	39792	0	656	671	97	-	40	707	13161	13827
UN08269	Rsa1.0_02125.1	1283	34775	1.00E-161	292	293	99	+	498	790	1907	2199
UN08270	Rsa1.0_00545.1	1010	189649	1.00E-71	153	157	97	-	760	914	35141	35297
UN08271	Rsa1.0_02183.1	1288	34170	0	1258	1286	97	-	2	1286	20423	21705
UN08272	Rsa1.0_06443.1	970	11008	1.00E-148	279	283	98	-	3	285	6909	7191
UN08273	Rsa1.0_02922.1	945	26753	1.00E-178	374	395	94	+	1	395	12310	12689
UN08274	Rsa1.0_19257.1	902	1455	0	381	383	99	-	3	385	108	490
UN08275	Rsa1.0_02340.1	1109	32388	1.00E-154	276	276	100	+	834	1109	23880	24155
UN08276	Rsa1.0_00615.1	1050	72235	1.00E-173	321	325	98	-	562	886	25400	25724
UN08277	Rsa1.0_00651.1	558	247766	1.00E-107	223	231	96	-	177	407	81983	82210
UN08278	Rsa1.0_02901.1	1262	26909	1.00E-136	261	266	98	+	266	531	20016	20281
UN08279	Rsa1.0_06373.1	928	11138	1.00E-151	280	283	98	-	601	883	751	1033
UN08280	Rsa1.0_01116.1	771	142088	0	680	712	95	-	62	765	129752	130462
UN08281	Rsa1.0_01580.1	1291	42852	0	491	495	99	-	1	494	31149	31642
UN08282	Rsa1.0_00118.1	874	130637	0	455	468	97	+	353	817	90339	90806
UN08283	Rsa1.0_01585.1	925	42806	0	546	546	100	-	1	546	15623	16168
UN08284	Rsa1.0_01031.1	1104	56236	1.00E-135	262	268	97	+	649	916	18106	18373
UN08285	Rsa1.0_06535.1	1397	10827	0	1325	1398	94	+	11	1387	2629	4010
UN08286	Rsa1.0_00018.1	1446	410899	0	336	339	99	+	1108	1446	189085	189421
UN08287	Rsa1.0_03184.1	1222	24750	1.00E-135	290	307	94	+	926	1221	21274	21580
UN08288	Rsa1.0_04961.1	644	15316	1.00E-165	294	294	100	+	350	643	13226	13519
UN08289	Rsa1.0_04988.1	1058	15221	0	352	352	100	-	632	983	499	850
UN08290	Rsa1.0_00016.1	1638	831256	0	546	554	98	-	887	1440	509054	509606
UN08291	Rsa1.0_03388.1	1078	23277	0	389	401	97	-	662	1062	17445	17845
UN08292	Rsa1.0_04524.1	1258	16981	0	507	511	99	+	750	1258	5653	6163
UN08293	Rsa1.0_00543.1	1527	76788	0	350	357	98	+	1173	1527	43302	43658
UN08294	Rsa1.0_00805.1	936	63724	1.00E-165	361	379	95	-	548	925	35498	35874
UN08295	Rsa1.0_38361.1	1729	587	0	569	602	94	-	284	884	1	587
UN08296	Rsa1.0_01630.1	598	41913	0	593	597	99	-	3	598	34843	35439
UN08297	Rsa1.0_00438.1	738	84861	1.00E-174	408	439	92	-	16	445	76921	77354
UN08298	Rsa1.0_02148.1	392	34610	1.00E-35	95	101	94	+	97	197	22799	22899
UN08299	Rsa1.0_03122.1	581	25207	0	576	577	99	+	3	579	12843	13419
UN08300	Rsa1.0_02431.1	679	31504	0	441	456	96	+	1	456	29916	30368
UN08301	Rsa1.0_00339.1	1609	222974	0	805	921	87	-	398	1318	28076	28996
UN08302	Rsa1.0_04282.1	677	18100	0	513	522	98	+	1	520	12088	12609
UN08303	Rsa1.0_00028.1	1212	293984	0	449	477	94	+	728	1204	281443	281904
UN08304	Rsa1.0_00311.1	1800	96847	0	541	552	98	+	1245	1796	35682	36229
UN08305	Rsa1.0_01022.1	1967	56606	1.00E-176	392	415	94	+	332	740	27924	28338

UN08306	Rsa1.0_00651.1	589	247766	1.00E-109	224	231	96	+	152	382	81983	82210
UN08307	Rsa1.0_00124.1	743	262503	1.00E-177	401	424	94	−	305	726	16165	16583
UN08308	Rsa1.0_00197.1	699	109714	1.00E-177	314	314	100	−	83	396	78615	78928
UN08309	Rsa1.0_00025.1	834	277312	1.00E-137	310	328	94	+	1	327	149589	149915
UN08310	Rsa1.0_00636.1	743	71113	1.00E-114	237	245	96	−	459	703	14377	14620
UN08311	Rsa1.0_00317.1	983	218290	2.00E-95	245	266	92	+	53	310	72659	72921
UN08312	Rsa1.0_01188.1	1046	51362	0	328	328	100	−	1	328	31071	31398
UN08313	Rsa1.0_00002.1	861	601661	0	426	429	99	−	413	840	140251	140677
UN08314	Rsa1.0_00131.1	1196	432233	1.00E-167	310	314	98	−	532	845	408531	408844
UN08315	Rsa1.0_00457.1	996	337301	0	379	384	98	−	6	389	128787	129170
UN08316	Rsa1.0_09097.1	696	6650	0	448	448	100	+	249	696	4943	5390
UN08317	Rsa1.0_00480.1	1207	81325	0	497	501	99	+	385	885	4323	4823
UN08318	Rsa1.0_05494.1	1007	13547	0	448	469	95	+	1	461	7633	8101
UN08319	Rsa1.0_25755.1	694	922	0	513	531	96	+	138	660	1	531
UN08320	Rsa1.0_00742.1	847	66106	1.00E-145	311	325	95	−	525	847	16526	16849
UN08321	Rsa1.0_00190.1	1903	318240	0	748	749	99	−	400	1148	105023	105771
UN08322	Rsa1.0_08364.1	811	7472	0	453	454	99	−	1	454	5218	5671
UN08323	Rsa1.0_03114.1	787	25318	0	527	527	100	−	1	527	6565	7091
UN08324	Rsa1.0_02652.1	1472	29038	0	353	355	99	−	1	355	21682	22036
UN08325	Rsa1.0_00150.1	847	154416	0	390	407	95	+	432	837	60307	60710
UN08326	Rsa1.0_04876.1	1321	15631	1.00E-126	242	246	98	+	948	1193	3072	3317
UN08327	Rsa1.0_01561.1	1176	43227	0	907	914	99	−	6	918	20971	21884
UN08328	Rsa1.0_00001.1	1112	363028	0	959	966	99	−	32	996	173735	174700
UN08329	Rsa1.0_00131.1	882	432233	0	569	582	97	−	301	882	259017	259598
UN08330	Rsa1.0_00869.1	1804	61627	0	556	581	95	+	1196	1767	16008	16585
UN08331	Rsa1.0_01382.1	1331	46877	0	357	357	100	−	975	1331	16729	17085
UN08332	Rsa1.0_05293.1	1478	14237	0	414	416	99	−	1065	1478	9512	9927
UN08333	Rsa1.0_08361.1	1747	7477	0	811	812	99	−	937	1747	5189	6000
UN08334	Rsa1.0_06438.1	659	11017	0	460	492	93	+	141	625	1531	2018
UN08335	Rsa1.0_00087.1	1210	761073	1.00E-145	265	266	99	−	271	536	274293	274558
UN08336	Rsa1.0_00079.1	673	606987	1.00E-171	304	304	100	+	1	304	198825	199128
UN08337	Rsa1.0_03694.1	924	21336	0	713	730	97	+	200	924	549	1278
UN08338	Rsa1.0_00235.1	845	154696	0	633	633	100	+	213	845	139087	139719
UN08339	Rsa1.0_00009.1	660	232558	1.00E-124	261	272	95	−	5	276	207990	208258
UN08340	Rsa1.0_01754.1	707	39792	0	685	705	97	+	3	699	13161	13863
UN08341	Rsa1.0_00545.1	1323	189649	0	525	530	99	+	9	538	37254	37783
UN08342	Rsa1.0_00786.1	822	64285	2.00E-97	196	201	97	+	396	596	41596	41796
UN08343	Rsa1.0_04415.1	1105	147889	1.00E-152	309	321	96	−	786	1100	141040	141355
UN08344	Rsa1.0_00234.1	1332	197060	0	400	415	96	+	502	915	144150	144564

UN08345	Rsa1.0_00505.1	1635	79323	0	1564	1576	99	−	57	1632	38536	40111
UN08346	Rsa1.0_00118.1	779	130637	0	513	513	100	−	2	514	95060	95572
UN08347	Rsa1.0_06303.1	1169	11268	0	746	793	94	−	255	1045	4869	5657
UN08348	Rsa1.0_01595.1	670	175383	1.00E−171	331	342	96	+	2	343	41001	41331
UN08349	Rsa1.0_00096.1	1581	198773	0	1576	1579	99	+	4	1580	40964	42542
UN08350	Rsa1.0_01414.1	900	149508	1.00E−170	348	361	96	−	2	359	115743	116102
UN08351	Rsa1.0_02675.1	562	28852	7.00E−75	143	143	100	−	183	325	17929	18071
UN08352	Rsa1.0_00031.1	1353	179862	0	399	410	97	−	3	412	161267	161676
UN08353	Rsa1.0_00079.1	660	606987	1.00E−138	279	288	96	−	373	658	198841	199128
UN08354	Rsa1.0_03956.1	684	19872	1.00E−180	331	335	98	+	317	651	16858	17192
UN08355	Rsa1.0_00340.1	802	305782	0	784	800	98	+	3	802	173358	174157
UN08356	Rsa1.0_00010.1	901	294827	1.00E−114	222	226	98	−	61	286	248372	248597
UN08357	Rsa1.0_01294.1	1107	49090	0	348	348	100	+	755	1102	40470	40817
UN08358	Rsa1.0_00480.1	772	81325	0	484	486	99	+	281	765	18939	19424
UN08359	Rsa1.0_29902.1	973	768	0	423	423	100	+	551	973	291	713
UN08360	Rsa1.0_03959.1	899	19866	0	871	914	95	+	1	899	14869	15769
UN08361	Rsa1.0_00179.1	770	393324	2.00E−72	252	284	88	+	483	752	250660	250934
UN08362	Rsa1.0_01534.1	2179	43764	0	1122	1127	99	+	3	1127	9347	10472
UN08363	Rsa1.0_00055.1	477	520193	1.00E−106	196	196	100	−	282	477	324254	324449
UN08364	Rsa1.0_02675.1	582	28852	6.00E−88	177	181	97	−	3	183	18163	18343
UN08365	Rsa1.0_04275.1	779	18136	1.00E−124	263	274	95	−	503	776	9501	9773
UN08366	Rsa1.0_00068.1	876	151813	1.00E−157	348	368	94	+	1	364	121729	122096
UN08367	Rsa1.0_00651.1	1054	247766	1.00E−65	176	188	93	−	867	1052	90200	90386
UN08368	Rsa1.0_05653.1	1028	13043	0	1004	1028	97	−	1	1028	11373	12394
UN08369	Rsa1.0_18647.1	885	1560	1.00E−170	386	412	93	−	485	885	521	932
UN08370	Rsa1.0_02797.1	732	27800	1.00E−150	300	310	96	+	9	318	652	961
UN08371	Rsa1.0_00528.1	1086	164755	0	540	564	95	−	524	1084	100787	101332
UN08372	Rsa1.0_01196.1	773	51097	1.00E−123	224	224	100	−	2	225	33470	33693
UN08373	Rsa1.0_02593.1	712	29744	1.00E−100	246	262	93	−	450	706	2905	3164
UN08374	Rsa1.0_03175.1	1202	24802	0	558	560	99	+	642	1201	4236	4793
UN08375	Rsa1.0_02673.1	1275	28877	2.00E−86	175	179	97	−	483	661	26953	27131
UN08376	Rsa1.0_01454.1	894	45440	1.00E−116	213	213	100	−	2	214	16053	16265
UN08377	Rsa1.0_05049.1	1024	15025	0	487	503	96	−	1	503	4126	4624
UN08378	Rsa1.0_05260.1	1975	14337	0	904	942	95	+	570	1508	3507	4442
UN08379	Rsa1.0_00327.1	1297	94892	0	1074	1118	96	−	209	1297	69964	71076
UN08380	Rsa1.0_01410.1	1810	46345	0	791	793	99	+	134	926	44531	45323
UN08381	Rsa1.0_06392.1	649	11104	0	466	480	97	−	1	479	10528	11005
UN08382	Rsa1.0_02774.1	665	28038	0	642	679	94	−	3	663	13469	14147
UN08383	Rsa1.0_01876.1	868	37800	0	685	717	95	+	174	868	36310	37026

UN08384	Rsa1.0_02658.1	1332	28978	0	387	405	95	-	888	1291	11639	12043
UN08385	Rsa1.0_00178.1	1469	114861	0	643	661	97	+	364	1021	42445	43105
UN08386	Rsa1.0_02953.1	866	26487	1.00E-156	295	300	98	+	567	866	2339	2638
UN08387	Rsa1.0_02432.1	1340	31499	0	1336	1340	99	+	1	1340	7301	8640
UN08388	Rsa1.0_00030.1	611	250988	1.00E-151	277	279	99	+	333	611	124651	124929
UN08389	Rsa1.0_01506.1	464	44370	2.00E-47	244	287	85	-	178	456	17898	18180
UN08390	Rsa1.0_00059.1	622	159650	1.00E-126	298	318	93	-	310	621	123646	123957
UN08391	Rsa1.0_20105.1	983	1340	0	395	401	98	+	584	981	457	857
UN08392	Rsa1.0_00088.1	775	141341	1.00E-131	253	258	98	-	411	668	19042	19299
UN08393	Rsa1.0_04098.1	750	19078	1.00E-95	304	341	89	+	17	337	11218	11553
UN08394	Rsa1.0_00660.1	808	69688	1.00E-101	233	247	94	-	568	808	52535	52781
UN08395	Rsa1.0_07826.1	1671	8466	0	1530	1543	99	-	131	1671	6924	8466
UN08396	Rsa1.0_05741.1	586	12773	0	364	364	100	+	1	364	3068	3431
UN08397	Rsa1.0_00179.1	1057	393324	1.00E-132	273	283	96	-	693	974	51891	52173
UN08398	Rsa1.0_00104.1	1572	368722	0	482	482	100	-	796	1277	278715	279196
UN08399	Rsa1.0_01857.1	650	38103	1.00E-140	363	399	90	+	178	554	4693	5090
UN08400	Rsa1.0_04518.1	1820	17005	0	517	536	96	+	680	1215	11675	12209
UN08401	Rsa1.0_00247.1	926	103408	1.00E-147	332	352	94	-	3	351	9307	9653
UN08402	Rsa1.0_00041.1	685	186197	1.00E-158	286	287	99	-	32	318	27167	27453
UN08403	Rsa1.0_03860.1	730	20407	0	330	330	100	+	1	330	14018	14347
UN08404	Rsa1.0_00745.1	1459	66067	0	701	726	96	+	207	932	42176	42898
UN08405	Rsa1.0_00001.1	1425	363028	1.00E-103	212	216	98	-	600	813	285546	285761
UN08406	Rsa1.0_00490.1	1731	186713	6.00E-41	87	87	100	+	1495	1581	33066	33152
UN08407	Rsa1.0_00888.1	603	60978	1.00E-172	335	343	97	+	76	417	54512	54854
UN08408	Rsa1.0_19230.1	972	1460	1.00E-154	298	305	97	-	667	971	967	1271
UN08409	Rsa1.0_04352.1	665	17779	0	530	557	95	-	16	572	12118	12667
UN08410	Rsa1.0_03001.1	697	26108	1.00E-103	211	218	96	+	41	253	9490	9707
UN08411	Rsa1.0_01888.1	1779	37563	0	771	772	99	-	1007	1777	14893	15664
UN08412	Rsa1.0_00760.1	924	65267	1.00E-154	277	277	100	+	1	277	51840	52116
UN08413	Rsa1.0_02316.1	813	32608	0	782	805	97	+	6	807	6017	6817
UN08414	Rsa1.0_00097.1	1141	138858	0	353	353	100	+	789	1141	120893	121245
UN08415	Rsa1.0_00978.1	973	57739	1.00E-166	516	588	87	+	49	627	42015	42599
UN08416	Rsa1.0_01460.1	1322	45255	0	617	640	96	+	51	689	34289	34925
UN08417	Rsa1.0_00048.1	1105	167185	1.00E-164	329	340	96	+	772	1105	159151	159489
UN08418	Rsa1.0_00394.1	932	148426	1.00E-132	276	286	96	+	3	286	122856	123140
UN08419	Rsa1.0_00383.1	435	398431	1.00E-174	336	346	97	-	90	435	211813	212149
UN08420	Rsa1.0_02319.1	839	32580	1.00E-145	280	285	98	+	556	839	19826	20110
UN08421	Rsa1.0_36118.1	694	625	1.00E-141	277	283	97	-	413	694	14	296
UN08422	Rsa1.0_00087.1	929	761073	1.00E-125	283	301	94	+	1	301	291671	291957

UN08423	Rsa1.0_00524.1	777	169613	1.00E-137	353	391	90	+	14	385	161710	162096
UN08424	Rsa1.0_03605.1	795	21827	1.00E-150	371	397	93	+	17	408	14371	14765
UN08425	Rsa1.0_01563.1	1132	170823	0	370	379	97	+	3	381	14874	15252
UN08426	Rsa1.0_03491.1	755	22541	0	573	599	95	−	49	640	8983	9580
UN08427	Rsa1.0_07261.1	762	9648	0	761	762	99	+	1	762	2696	3457
UN08428	Rsa1.0_02105.1	960	156953	0	467	487	95	+	3	485	150681	151165
UN08429	Rsa1.0_01635.1	713	41865	0	674	717	94	−	3	713	15907	16610
UN08430	Rsa1.0_00972.1	1190	57937	2.00E-98	196	199	98	+	3	201	45886	46083
UN08431	Rsa1.0_00552.1	1277	206757	2.00E-89	201	212	94	−	212	423	187055	187266
UN08432	Rsa1.0_08717.1	864	7008	0	413	423	97	+	311	733	2884	3306
UN08433	Rsa1.0_02139.1	821	34686	0	371	371	100	+	1	371	5542	5912
UN08434	Rsa1.0_02143.1	690	34632	0	415	416	99	+	275	690	13042	13457
UN08435	Rsa1.0_00344.1	611	191774	1.00E-101	190	191	99	−	213	403	156563	156753
UN08436	Rsa1.0_02662.1	741	28956	8.00E-97	186	188	98	−	301	488	10660	10847
UN08437	Rsa1.0_00115.1	759	131321	0	575	593	96	+	169	759	34041	34619
UN08438	Rsa1.0_01659.1	1857	164577	0	615	630	97	−	154	780	143506	144135
UN08439	Rsa1.0_01687.1	2159	40979	0	1937	1939	99	−	221	2159	1391	3329
UN08440	Rsa1.0_00807.1	1322	63607	0	725	740	97	+	562	1299	61983	62721
UN08441	Rsa1.0_05482.1	939	13595	1.00E-164	329	341	96	−	131	471	12266	12606
UN08442	Rsa1.0_04651.1	1478	16516	0	551	573	96	−	349	921	13816	14388
UN08443	Rsa1.0_00577.1	892	174827	0	458	461	99	−	2	462	126027	126487
UN08444	Rsa1.0_00472.1	1338	398134	0	394	403	97	+	359	761	166818	167220
UN08445	Rsa1.0_00092.1	612	659932	0	606	611	99	−	1	610	650842	651448
UN08446	Rsa1.0_01484.1	1071	44806	0	558	585	95	+	430	1012	40748	41326
UN08447	Rsa1.0_05631.1	802	13127	1.00E-143	327	346	94	−	41	384	4477	4821
UN08448	Rsa1.0_05631.1	800	13127	1.00E-143	323	342	94	+	418	757	4477	4818
UN08449	Rsa1.0_01887.1	1023	37564	0	502	509	98	+	514	1022	26538	27046
UN08450	Rsa1.0_27679.1	468	843	1.00E-171	317	320	99	−	119	437	208	527
UN08451	Rsa1.0_00178.1	655	114861	1.00E-114	276	296	93	−	1	284	12165	12459
UN08452	Rsa1.0_00111.1	648	390088	0	379	381	99	−	1	380	75630	76010
UN08453	Rsa1.0_00571.1	1694	329018	0	587	614	95	+	2	612	13286	13899
UN08454	Rsa1.0_08223.1	760	7700	0	469	492	95	+	263	753	6338	6820
UN08455	Rsa1.0_02064.1	999	35476	1.00E-171	372	391	95	−	542	929	16635	17023
UN08456	Rsa1.0_01536.1	863	43747	0	416	436	95	−	292	727	18915	19349
UN08457	Rsa1.0_00139.1	1077	357786	1.00E-147	264	264	100	−	181	444	265190	265453
UN08458	Rsa1.0_00343.1	1291	93570	0	765	781	97	+	457	1237	90963	91743
UN08459	Rsa1.0_00888.1	997	60978	1.00E-151	300	307	97	−	676	980	13819	14124
UN08460	Rsa1.0_00940.1	1081	179946	0	611	629	97	−	4	631	175916	176544
UN08461	Rsa1.0_01811.1	847	38977	1.00E-159	368	390	94	−	2	385	1187	1575

UN08462	Rsa1.0_00002.1	1520	601661	0	349	351	99	+	1	350	55273	55623
UN08463	Rsa1.0_00230.1	885	105389	1.00E-166	343	356	96	−	456	810	2571	2925
UN08464	Rsa1.0_02434.1	455	31464	0	451	453	99	−	3	455	1745	2197
UN08465	Rsa1.0_02508.1	1233	30675	0	823	831	99	−	1	831	13374	14204
UN08466	Rsa1.0_00026.1	1192	327835	0	1173	1191	98	+	2	1191	2422	3612
UN08467	Rsa1.0_07877.1	1030	8373	2.00E-79	151	151	100	+	429	579	5171	5321
UN08468	Rsa1.0_07344.1	1149	9528	0	396	408	97	−	17	423	523	930
UN08469	Rsa1.0_03236.1	1345	24441	0	1158	1177	98	+	150	1326	5427	6602
UN08470	Rsa1.0_00512.1	1572	200277	0	539	548	98	−	3	550	66192	66734
UN08471	Rsa1.0_05878.1	1065	12344	1.00E-165	336	346	97	−	721	1065	8157	8499
UN08472	Rsa1.0_00248.1	1377	163568	1.00E-177	327	331	98	+	1045	1375	71990	72320
UN08473	Rsa1.0_00126.1	921	127534	1.00E-154	306	316	96	+	497	812	37046	37361
UN08474	Rsa1.0_00017.1	953	752458	1.00E-139	318	339	93	−	618	950	719238	719576
UN08475	Rsa1.0_01145.1	1043	149247	0	502	519	96	−	527	1043	123235	123750
UN08476	Rsa1.0_00638.1	953	71037	1.00E-111	258	275	93	+	341	613	9032	9306
UN08477	Rsa1.0_00065.1	907	454616	0	561	561	100	−	347	907	452719	453279
UN08478	Rsa1.0_09218.1	1196	6550	0	519	541	95	+	2	530	276	816
UN08479	Rsa1.0_00464.1	869	82253	1.00E-162	305	309	98	−	563	869	14642	14950
UN08480	Rsa1.0_02532.1	1219	30403	0	854	859	99	−	321	1178	6583	7440
UN08481	Rsa1.0_09169.1	1026	6589	0	381	400	95	−	5	404	5660	6059
UN08482	Rsa1.0_08358.1	1473	7484	0	452	467	96	+	334	793	6492	6958
UN08483	Rsa1.0_00939.1	1959	185227	0	685	732	93	+	346	1058	37737	38465
UN08484	Rsa1.0_01882.1	1279	37697	0	938	971	96	+	326	1278	23907	24875
UN08485	Rsa1.0_01789.1	1192	39250	0	456	473	96	+	721	1188	19826	20296
UN08486	Rsa1.0_00147.1	1188	249503	0	545	575	94	−	612	1186	155695	156259
UN08487	Rsa1.0_00016.1	1164	831256	1.00E-152	317	328	96	+	25	350	370678	371003
UN08488	Rsa1.0_02139.1	1184	34686	0	494	510	96	+	3	509	24905	25414
UN08489	Rsa1.0_00165.1	1453	116466	1.00E-149	318	332	95	+	9	338	5412	5742
UN08490	Rsa1.0_03097.1	1338	25434	1.00E-139	273	279	97	+	893	1169	20254	20532
UN08491	Rsa1.0_02670.1	1353	28887	0	422	423	99	−	931	1353	3782	4204
UN08492	Rsa1.0_00026.1	1713	327835	0	464	468	99	−	1246	1713	240917	241382
UN08493	Rsa1.0_01975.1	1081	36578	0	399	422	94	−	671	1080	13994	14415
UN08494	Rsa1.0_00821.1	981	62891	0	570	574	99	−	407	979	46782	47355
UN08495	Rsa1.0_02481.1	1361	31035	0	735	768	95	+	2	760	17765	18531
UN08496	Rsa1.0_00938.1	994	193891	0	507	526	96	+	443	966	172122	172647
UN08497	Rsa1.0_00320.1	1115	95784	1.00E-133	379	426	88	+	297	710	37469	37882
UN08498	Rsa1.0_00251.1	537	303968	1.00E-101	276	299	92	−	242	532	120925	121221
UN08499	Rsa1.0_01328.1	1587	48021	2.00E-74	149	151	98	+	916	1066	43897	44047
UN08500	Rsa1.0_02177.1	914	34262	3.00E-75	168	176	95	−	434	609	14545	14720

UN08501	Rsa1.0_00589.1	1473	209965	0	403	415	97	+	1004	1418	152952	153365
UN08502	Rsa1.0_01185.1	1479	149842	0	779	779	100	+	232	1010	12499	13277
UN08503	Rsa1.0_00015.1	1086	561803	1.00E-114	245	257	95	−	376	621	186923	187179
UN08504	Rsa1.0_00104.1	1174	368722	0	425	441	96	−	740	1174	41579	42018
UN08505	Rsa1.0_00144.1	1009	120708	0	449	464	96	−	542	1005	45107	45567
UN08506	Rsa1.0_00552.1	868	206757	0	390	410	95	+	3	400	195140	195549
UN08507	Rsa1.0_00047.1	1192	168582	0	436	455	95	+	734	1188	67922	68376
UN08508	Rsa1.0_02840.1	1006	27386	0	710	736	96	−	4	729	26285	27020
UN08509	Rsa1.0_00430.1	1133	85453	0	860	887	96	−	247	1133	44137	45023
UN08510	Rsa1.0_02474.1	1324	31076	0	423	424	99	+	1	424	19878	20301
UN08511	Rsa1.0_03267.1	1321	24230	0	748	776	96	+	549	1321	16677	17444
UN08512	Rsa1.0_00069.1	1055	403561	1.00E-179	358	371	96	−	441	811	395707	396077
UN08513	Rsa1.0_00064.1	1271	240927	0	859	900	95	+	381	1271	232169	233058
UN08514	Rsa1.0_02163.1	1711	34427	1.00E-167	299	299	100	−	1	299	12765	13063
UN08515	Rsa1.0_04524.1	1142	16981	0	402	417	96	+	698	1105	5653	6069
UN08516	Rsa1.0_01773.1	919	39546	1.00E-108	229	239	95	−	213	451	28417	28655
UN08517	Rsa1.0_00269.1	1635	341668	0	540	541	99	+	1095	1635	190483	191023
UN08518	Rsa1.0_33279.1	1031	682	0	409	420	97	+	1	419	263	682
UN08519	Rsa1.0_00355.1	1061	92038	0	482	488	98	−	1	488	64491	64978
UN08520	Rsa1.0_04466.1	1281	17236	0	1196	1196	100	+	84	1279	7946	9141
UN08521	Rsa1.0_02053.1	785	35554	0	379	380	99	+	406	785	32532	32910
UN08522	Rsa1.0_01946.1	1343	36982	1.00E-176	414	449	92	+	877	1311	33540	33982
UN08523	Rsa1.0_02851.1	1207	27295	2.33E-156	317	332	95	+	1	325	8869	9196
UN08524	Rsa1.0_00080.1	1268	190437	0	502	520	96	+	406	925	84945	85464
UN08525	Rsa1.0_02072.1	992	35401	1.00E-132	278	289	96	+	7	292	5412	5699
UN08526	Rsa1.0_00048.1	1290	167185	1.00E-175	345	356	96	+	3	358	96417	96772
UN08527	Rsa1.0_00646.1	923	70473	6.00E-61	158	168	94	−	659	825	6088	6254
UN08528	Rsa1.0_00295.1	887	152597	6.00E-95	270	298	90	+	569	855	140727	141019
UN08529	Rsa1.0_00523.1	617	178770	0	533	548	97	−	3	550	101654	102201
UN08530	Rsa1.0_11519.1	828	5480	0	375	391	95	−	3	390	4924	5308
UN08531	Rsa1.0_01229.1	1130	50235	0	1115	1130	98	−	1	1129	3374	4502
UN08532	Rsa1.0_00656.1	1225	69968	0	614	615	99	−	8	622	46621	47235
UN08533	Rsa1.0_00025.1	1537	277312	0	789	853	92	+	9	836	251308	252153
UN08534	Rsa1.0_05477.1	924	13613	1.00E-130	284	298	95	−	636	924	4150	4447
UN08535	Rsa1.0_00328.1	2049	224661	0	548	558	98	+	3	560	140650	141205
UN08536	Rsa1.0_01803.1	869	173846	0	399	412	96	+	461	869	143938	144349
UN08537	Rsa1.0_13994.1	1070	3941	0	331	333	99	−	7	339	2090	2422
UN08538	Rsa1.0_00205.1	1239	193401	0	469	482	97	+	85	566	97462	97943
UN08539	Rsa1.0_00418.1	1243	215377	1.00E-142	271	276	98	−	4	279	47449	47724

UN08540	Rsa1.0_03469.1	1043	22681	0	786	805	97	+	199	1000	11058	11858
UN08541	Rsa1.0_01293.1	1861	49139	0	573	575	99	+	1285	1859	23051	23625
UN08542	Rsa1.0_01573.1	1134	42964	1.00E-135	318	341	93	+	780	1119	4044	4369
UN08543	Rsa1.0_00149.1	1040	305196	0	506	506	100	+	535	1040	104054	104559
UN08544	Rsa1.0_00101.1	763	267193	0	344	349	98	−	416	762	216292	216640
UN08545	Rsa1.0_02415.1	1403	31609	0	784	799	98	+	604	1402	27010	27808
UN08546	Rsa1.0_03952.1	908	19891	0	562	567	99	+	171	736	3722	4288
UN08547	Rsa1.0_07515.1	629	9221	0	627	629	99	−	3	629	7004	7632
UN08548	Rsa1.0_01833.1	1117	38555	1.00E-116	213	213	100	+	830	1042	16525	16737
UN08549	Rsa1.0_02058.1	1133	35532	0	535	537	99	−	1	537	13428	13964
UN08550	Rsa1.0_00917.1	964	182012	0	444	467	95	−	498	964	175675	176128
UN08551	Rsa1.0_00086.1	1243	439854	0	583	601	97	+	641	1237	234282	234875
UN08552	Rsa1.0_00725.1	819	66757	0	633	648	97	−	14	660	40226	40872
UN08553	Rsa1.0_02406.1	806	145276	1.00E-169	327	333	98	+	472	803	1739	2070
UN08554	Rsa1.0_02605.1	1107	29637	0	545	578	94	−	3	566	25931	26504
UN08555	Rsa1.0_00954.1	1125	189536	1.00E-174	316	318	99	+	808	1125	155715	156032
UN08556	Rsa1.0_03500.1	1264	22461	0	1251	1260	99	−	5	1264	7828	9086
UN08557	Rsa1.0_00269.1	1290	341668	0	892	899	99	+	368	1266	48310	49208
UN08558	Rsa1.0_00629.1	1454	71381	0	788	812	97	+	292	1102	45375	46186
UN08559	Rsa1.0_00037.1	1279	307838	0	951	954	99	−	325	1278	175872	176825
UN08560	Rsa1.0_04218.1	708	18423	6.00E-82	164	167	98	−	234	400	17583	17749
UN08561	Rsa1.0_02707.1	1282	28520	0	348	350	99	+	433	782	12735	13084
UN08562	Rsa1.0_10478.1	1086	5832	1.00E-154	277	277	100	−	1	277	5140	5416
UN08563	Rsa1.0_01403.1	903	46453	0	449	457	98	+	448	903	3064	3518
UN08564	Rsa1.0_00113.1	914	131574	0	704	723	97	+	1	720	31560	32282
UN08565	Rsa1.0_00416.1	2214	86772	0	508	510	99	+	247	756	74497	75006
UN08566	Rsa1.0_00031.1	728	179862	1.00E-163	391	422	92	+	1	421	73397	73816
UN08567	Rsa1.0_02033.1	1133	35828	0	1087	1130	96	−	2	1124	20373	21501
UN08568	Rsa1.0_00038.1	1389	314568	1.00E-126	285	299	95	+	1065	1359	52376	52671
UN08569	Rsa1.0_00051.1	1068	258225	0	1014	1088	93	+	3	1068	113688	114762
UN08570	Rsa1.0_01434.1	890	45951	0	781	802	97	+	95	889	351	1151
UN08571	Rsa1.0_00039.1	1247	296927	4.00E-63	202	227	88	−	984	1198	223067	223293
UN08572	Rsa1.0_00161.1	1198	116891	0	449	452	99	+	3	454	30418	30869
UN08573	Rsa1.0_02418.1	747	31590	1.00E-169	305	306	99	−	1	306	5189	5494
UN08574	Rsa1.0_00344.1	1647	191774	0	817	847	96	−	780	1614	102407	103253
UN08575	Rsa1.0_00615.1	1018	72235	0	383	384	99	+	633	1016	26377	26760
UN08576	Rsa1.0_04121.1	770	18951	0	436	455	95	−	55	509	13882	14336
UN08577	Rsa1.0_01136.1	577	52724	2.00E-84	229	252	90	+	315	553	14179	14429
UN08578	Rsa1.0_00710.1	562	67325	1.00E-153	317	329	96	−	237	562	65792	66119

UN08579	Rsa1.0_00589.1	1318	209965	1.00E-170	312	315	99	+	984	1298	194796	195110
UN08580	Rsa1.0_00017.1	1229	752458	0	680	716	94	+	491	1204	416018	416733
UN08581	Rsa1.0_00678.1	604	193163	1.00E-153	275	275	100	+	330	604	23909	24183
UN08582	Rsa1.0_00851.1	734	62181	0	443	475	93	−	3	468	8707	9171
UN08583	Rsa1.0_01026.1	2152	171624	0	2144	2154	99	−	1	2152	138812	140965
UN08584	Rsa1.0_02280.1	613	33063	2.00E-53	225	264	85	+	93	356	29423	29677
UN08585	Rsa1.0_00323.1	534	159650	1.00E-134	286	299	95	−	234	530	61652	61947
UN08586	Rsa1.0_00089.1	1095	406142	0	515	515	100	+	581	1095	196391	196905
UN08587	Rsa1.0_03605.1	696	21827	0	407	412	98	−	288	696	17738	18149
UN08588	Rsa1.0_00065.1	741	454616	1.00E-148	293	302	97	+	356	657	231797	232098
UN08589	Rsa1.0_00302.1	1378	97521	1.00E-158	344	363	94	+	3	362	33883	34242
UN08590	Rsa1.0_01322.1	746	48166	1.00E-168	326	335	97	−	35	369	28900	29234
UN08591	Rsa1.0_00071.1	991	345714	0	372	378	98	−	469	846	311954	312331
UN08592	Rsa1.0_00610.1	976	72740	0	491	513	95	+	8	513	35082	35589
UN08593	Rsa1.0_00337.1	813	143174	1.00E-137	260	264	98	+	134	397	1585	1848
UN08594	Rsa1.0_03406.1	846	23159	0	488	499	97	+	3	501	15506	16002
UN08595	Rsa1.0_05708.1	995	12871	0	623	651	95	−	10	660	11144	11794
UN08596	Rsa1.0_28133.1	1259	826	0	417	426	97	−	3	428	1	426
UN08597	Rsa1.0_01335.1	1104	47894	1.00E-157	368	397	92	−	708	1104	18291	18681
UN08598	Rsa1.0_00162.1	529	239935	0	406	407	99	−	1	407	12315	12721
UN08599	Rsa1.0_01088.1	736	54416	2.00E-97	195	199	97	−	3	198	30917	31115
UN08600	Rsa1.0_02606.1	1412	29635	1.00E-175	356	366	97	+	1050	1412	7554	7916
UN08601	Rsa1.0_00433.1	799	85381	5.00E-95	253	273	92	+	28	295	65209	65477
UN08602	Rsa1.0_00136.1	626	122464	1.00E-110	206	207	99	+	2	208	54754	54960
UN08603	Rsa1.0_00077.1	1039	145005	0	799	849	94	+	177	1022	33313	34143
UN08604	Rsa1.0_00306.1	1019	146760	1.00E-164	417	459	90	−	155	595	133202	133651
UN08605	Rsa1.0_00900.1	906	60367	0	507	511	99	+	3	513	19835	20345
UN08606	Rsa1.0_15466.1	775	2589	0	748	774	96	−	9	773	983	1752
UN08607	Rsa1.0_00262.1	688	101906	1.00E-121	248	256	96	−	417	670	46408	46663
UN08608	Rsa1.0_25755.1	671	922	0	507	531	95	+	115	637	1	531
UN08609	Rsa1.0_03462.1	1023	22725	1.00E-130	258	264	97	+	758	1021	15190	15452
UN08610	Rsa1.0_02956.1	1288	26483	0	540	541	99	−	748	1288	3074	3614
UN08611	Rsa1.0_07528.1	645	9195	0	632	644	98	+	3	645	3308	3950
UN08612	Rsa1.0_15363.1	617	2655	0	591	613	96	−	1	608	1692	2304
UN08613	Rsa1.0_00931.1	920	59210	0	916	927	98	−	3	920	44659	45585
UN08614	Rsa1.0_01655.1	874	41508	0	411	429	95	−	448	874	1660	2088
UN08615	Rsa1.0_06732.1	800	10449	0	748	763	98	−	41	800	8829	9591
UN08616	Rsa1.0_16475.1	933	2143	1.00E-103	209	215	97	+	625	839	1084	1298
UN08617	Rsa1.0_01340.1	1120	47805	0	558	605	92	+	520	1120	46060	46664

UN08618	Rsa1.0_00017.1	1565	752458	0	694	751	92	+	750	1497	352440	353175
UN08619	Rsa1.0_00194.1	1216	110851	0	518	519	99	−	1	519	50578	51096
UN08620	Rsa1.0_00066.1	868	152418	0	429	451	95	−	8	450	19638	20088
UN08621	Rsa1.0_09211.1	914	6556	8.00E−76	157	161	97	+	580	740	4357	4517
UN08622	Rsa1.0_00121.1	1240	278029	1.00E−167	411	444	92	−	240	683	44291	44727
UN08623	Rsa1.0_03794.1	662	20825	0	659	660	99	+	3	662	16628	17286
UN08624	Rsa1.0_01373.1	1702	47052	0	481	481	100	+	1	481	3444	3924
UN08625	Rsa1.0_02482.1	1113	31000	0	612	634	96	+	329	947	26970	27603
UN08626	Rsa1.0_01529.1	891	43871	1.00E−145	359	393	91	−	499	891	38298	38675
UN08627	Rsa1.0_02365.1	1174	32118	0	555	596	93	−	568	1157	29274	29863
UN08628	Rsa1.0_01803.1	1104	173846	1.00E−167	335	347	96	+	6	352	169093	169439
UN08629	Rsa1.0_04787.1	520	15987	9.00E−99	246	264	93	−	261	519	3082	3344
UN08630	Rsa1.0_01352.1	784	47443	0	460	464	99	−	4	467	954	1417
UN08631	Rsa1.0_00971.1	1450	57973	0	1448	1451	99	−	1	1450	14055	15505
UN08632	Rsa1.0_02684.1	1474	28676	0	512	552	92	−	447	984	336	882
UN08633	Rsa1.0_05782.1	1028	12646	1.00E−108	211	215	98	+	147	361	8062	8276
UN08634	Rsa1.0_09130.1	708	6623	0	615	645	95	+	20	661	3395	4028
UN08635	Rsa1.0_01058.1	879	55430	0	575	594	96	−	288	879	31998	32588
UN08636	Rsa1.0_00039.1	1134	296927	0	368	374	98	−	119	492	290917	291290
UN08637	Rsa1.0_00460.1	1064	206387	0	540	560	96	+	3	555	17702	18261
UN08638	Rsa1.0_34683.1	1386	653	0	649	653	99	+	3	654	1	653
UN08639	Rsa1.0_00579.1	1240	148308	1.00E−155	279	279	100	−	1	279	71242	71520
UN08640	Rsa1.0_01388.1	1263	46741	0	342	346	98	+	62	407	25410	25755
UN08641	Rsa1.0_00769.1	1000	64911	1.00E−157	297	302	98	+	3	304	12971	13272
UN08642	Rsa1.0_01581.1	675	42833	1.00E−160	298	302	98	+	86	387	7614	7915
UN08643	Rsa1.0_00820.1	945	62911	0	562	564	99	+	382	944	24795	25358
UN08644	Rsa1.0_00448.1	1089	84048	0	1086	1087	99	+	3	1089	19792	20878
UN08645	Rsa1.0_01881.1	683	37721	2.00E−51	113	116	97	−	129	244	23401	23516
UN08646	Rsa1.0_02505.1	649	30703	1.00E−89	180	184	97	−	159	342	29797	29980
UN08647	Rsa1.0_00938.1	931	193891	0	784	801	97	−	2	800	179936	180735
UN08648	Rsa1.0_04051.1	1147	19375	0	486	491	98	+	611	1101	10627	11117
UN08649	Rsa1.0_01519.1	1565	44144	0	612	622	98	−	951	1565	14902	15523
UN08650	Rsa1.0_00980.1	1054	57729	1.00E−142	256	256	100	−	799	1054	26581	26836
UN08651	Rsa1.0_01857.1	1260	38103	1.00E−149	356	383	92	+	361	737	6745	7124
UN08652	Rsa1.0_01578.1	1231	42880	0	780	790	98	−	400	1188	4209	4998
UN08653	Rsa1.0_00213.1	561	108167	1.00E−168	302	303	99	+	259	561	39811	40113
UN08654	Rsa1.0_00064.1	1239	240927	1.00E−132	246	248	99	−	651	898	185539	185786
UN08655	Rsa1.0_00135.1	585	178077	0	350	351	99	−	236	585	2957	3307
UN08656	Rsa1.0_00370.1	1196	90472	1.00E−132	258	263	98	+	85	345	42	304

UN08657	Rsa1.0_00375.1	1113	90207	0	379	387	97	+	120	506	56410	56796
UN08658	Rsa1.0_00201.1	790	232851	0	741	775	95	+	8	779	140320	141089
UN08659	Rsa1.0_03355.1	868	23489	0	563	565	99	−	304	868	14147	14711
UN08660	Rsa1.0_01454.1	1348	45440	1.00E−175	359	373	96	+	985	1348	12955	13326
UN08661	Rsa1.0_03049.1	1267	25771	1.00E−166	296	296	100	−	970	1265	9212	9507
UN08662	Rsa1.0_01493.1	1281	44670	0	781	782	99	+	156	937	32666	33447
UN08663	Rsa1.0_25895.1	567	914	0	370	371	99	−	195	565	161	531
UN08664	Rsa1.0_00018.1	765	410899	1.00E−165	323	329	98	+	437	765	259091	259415
UN08665	Rsa1.0_03476.1	1527	22646	0	1365	1397	97	−	88	1479	12628	14022
UN08666	Rsa1.0_02201.1	607	33981	0	598	606	98	−	3	606	26536	27137
UN08667	Rsa1.0_01359.1	1009	47271	1.00E−122	280	299	93	−	1	299	33556	33849
UN08668	Rsa1.0_03253.1	925	24321	1.00E−103	203	207	98	−	49	255	15147	15353
UN08669	Rsa1.0_01958.1	560	261128	1.00E−117	294	318	92	−	55	368	110198	110502
UN08670	Rsa1.0_00050.1	472	237174	1.00E−125	350	388	90	−	6	383	180825	181210
UN08671	Rsa1.0_00005.1	684	353093	1.00E−101	198	200	99	+	1	200	280457	280655
UN08672	Rsa1.0_00880.1	515	61271	0	453	463	97	+	52	510	40587	41049
UN08673	Rsa1.0_02976.1	621	26315	0	378	382	98	−	240	621	18436	18816
UN08674	Rsa1.0_02983.1	440	26268	1.00E−103	190	190	100	−	87	276	809	998
UN08675	Rsa1.0_00838.1	686	153820	0	395	396	99	−	291	686	149595	149990
UN08676	Rsa1.0_00050.1	1676	237174	0	1034	1042	99	−	635	1676	10213	11254
UN08677	Rsa1.0_04070.1	937	19244	0	497	508	97	+	438	937	2567	3074
UN08678	Rsa1.0_00176.1	687	240984	0	524	560	93	−	3	547	9869	10426
UN08679	Rsa1.0_02037.1	815	35717	0	362	368	98	+	2	368	25463	25830
UN08680	Rsa1.0_04794.1	767	15960	1.00E−131	306	328	93	−	120	447	2694	3014
UN08681	Rsa1.0_00033.1	516	515811	7.00E−78	247	280	88	+	248	516	33139	33414
UN08682	Rsa1.0_02500.1	822	30768	1.00E−152	359	390	92	+	444	816	7770	8159
UN08683	Rsa1.0_02360.1	1047	32203	0	588	630	93	+	1	609	23949	24578
UN08684	Rsa1.0_01492.1	1056	44704	0	329	329	100	−	3	331	35636	35964
UN08685	Rsa1.0_08224.1	503	7697	0	452	472	95	+	3	472	4459	4925
UN08686	Rsa1.0_01136.1	652	52724	0	377	378	99	+	276	652	14179	14556
UN08687	Rsa1.0_02869.1	1042	27109	0	447	470	95	+	28	496	3367	3823
UN08688	Rsa1.0_00301.1	746	365315	1.00E−132	346	382	90	+	368	737	346680	347051
UN08689	Rsa1.0_00237.1	1129	235974	0	396	401	98	−	4	399	99025	99425
UN08690	Rsa1.0_00679.1	977	68971	0	877	921	95	−	3	923	701	1605
UN08691	Rsa1.0_00410.1	771	86957	0	421	421	100	+	19	439	33168	33588
UN08692	Rsa1.0_00867.1	819	61635	1.00E−147	267	268	99	−	3	270	45036	45303
UN08693	Rsa1.0_02848.1	573	27329	1.00E−116	222	225	98	+	204	428	8638	8862
UN08694	Rsa1.0_00066.1	1433	152418	0	568	568	100	−	866	1433	65065	65632
UN08695	Rsa1.0_07606.1	937	9035	1.00E−133	332	362	91	−	584	937	2792	3142

UN08696	Rsa1.0_00306.1	1075	146760	0	546	555	98	−	521	1072	135593	136147
UN08697	Rsa1.0_00743.1	661	177459	4.00E−61	132	136	97	+	259	394	613	748
UN08698	Rsa1.0_00250.1	1306	273771	0	370	371	99	+	935	1305	230593	230963
UN08699	Rsa1.0_00179.1	671	393324	1.00E−166	365	383	95	−	17	392	248844	249223
UN08700	Rsa1.0_18177.1	800	1651	1.00E−165	309	314	98	−	487	800	706	1019
UN08701	Rsa1.0_00598.1	673	73425	0	662	669	98	−	5	673	66879	67541
UN08702	Rsa1.0_00017.1	626	752458	9.00E−87	220	237	92	+	393	624	661547	661779
UN08703	Rsa1.0_11534.1	1190	5477	0	454	467	97	−	452	918	1259	1725
UN08704	Rsa1.0_00639.1	982	165873	0	956	976	97	−	6	981	126875	127849
UN08705	Rsa1.0_00056.1	744	332604	0	722	736	98	+	5	739	1485	2220
UN08706	Rsa1.0_00059.1	1317	159650	0	560	572	97	−	748	1315	121057	121628
UN08707	Rsa1.0_03467.1	1396	22687	0	581	592	98	+	756	1346	13100	13691
UN08708	Rsa1.0_00145.1	1143	261008	0	612	619	98	+	3	616	48222	48840
UN08709	Rsa1.0_00224.1	704	327530	1.00E−95	218	227	96	−	411	634	178415	178637
UN08710	Rsa1.0_00662.1	1012	192183	0	476	485	98	+	392	876	29861	30342
UN08711	Rsa1.0_00111.1	861	390088	0	487	487	100	−	375	861	219082	219568
UN08712	Rsa1.0_02831.1	1427	27533	0	1381	1381	100	+	47	1427	13241	14621
UN08713	Rsa1.0_00644.1	874	136881	1.00E−144	321	339	94	+	545	874	31373	31708
UN08714	Rsa1.0_17354.1	576	1851	2.33E−156	278	282	98	+	157	437	420	701
UN08715	Rsa1.0_00065.1	951	454616	0	681	690	98	−	260	949	58813	59501
UN08716	Rsa1.0_00405.1	1194	202864	0	1146	1172	97	+	22	1193	154165	155336
UN08717	Rsa1.0_00078.1	1289	175053	0	524	531	98	+	3	533	91043	91570
UN08718	Rsa1.0_00407.1	994	191544	0	447	457	97	+	535	990	25043	25499
UN08719	Rsa1.0_00655.1	1356	195840	1.00E−120	329	369	89	+	1009	1356	163505	163873
UN08720	Rsa1.0_02630.1	1274	29349	0	504	515	97	+	1	515	21173	21687
UN08721	Rsa1.0_00179.1	830	393324	1.00E−123	276	289	95	−	65	351	250660	250943
UN08722	Rsa1.0_12975.1	1097	5057	0	486	497	97	−	194	690	1	496
UN08723	Rsa1.0_00472.1	1469	398134	0	1065	1100	96	−	8	1084	174721	175820
UN08724	Rsa1.0_03865.1	837	20373	1.00E−126	230	230	100	−	258	487	15975	16204
UN08725	Rsa1.0_04493.1	868	17137	0	848	868	97	−	6	868	5195	6056
UN08726	Rsa1.0_00323.1	1006	159650	0	527	527	100	−	68	594	78555	79081
UN08727	Rsa1.0_00171.1	788	115622	0	603	609	99	−	180	788	113756	114364
UN08728	Rsa1.0_07638.1	1575	8967	1.00E−168	426	466	91	−	1115	1573	4103	4555
UN08729	Rsa1.0_00130.1	583	125737	1.00E−110	253	264	95	+	326	583	50495	50757
UN08730	Rsa1.0_05082.1	1385	14896	0	1338	1367	97	+	5	1368	9751	11116
UN08731	Rsa1.0_00316.1	731	96264	6.00E−45	108	112	96	+	177	286	57738	57849
UN08732	Rsa1.0_00037.1	1642	307838	0	550	601	91	+	1	578	236111	236705
UN08733	Rsa1.0_02840.1	929	27386	0	432	458	94	+	278	726	26285	26742
UN08734	Rsa1.0_33333.1	684	681	0	578	613	94	−	53	661	76	681

UN08735	Rsa1.0_14327.1	694	3518	1.00E-152	394	428	92	+	254	679	231	645
UN08736	Rsa1.0_08796.1	749	6916	0	446	463	96	+	288	749	4101	4562
UN08737	Rsa1.0_09528.1	919	6309	0	405	405	100	+	515	919	1	405
UN08738	Rsa1.0_02601.1	552	29654	1.00E-60	119	119	100	+	432	550	2926	3044
UN08739	Rsa1.0_05074.1	2119	14925	0	517	527	98	+	1	525	902	1427
UN08740	Rsa1.0_00456.1	1224	82826	0	980	1013	96	−	210	1222	78585	79582
UN08741	Rsa1.0_00124.1	624	262503	0	621	624	99	+	1	624	142943	143565
UN08742	Rsa1.0_04520.1	525	16995	1.00E-156	289	291	99	−	3	291	11580	11870
UN08743	Rsa1.0_00041.1	1067	186197	1.00E-139	279	288	96	+	371	658	33067	33354
UN08744	Rsa1.0_00078.1	1229	175053	0	502	508	98	+	605	1112	41372	41879
UN08745	Rsa1.0_00293.1	939	339294	0	465	471	98	−	83	553	65205	65675
UN08746	Rsa1.0_13353.1	475	4894	7.00E-84	161	162	99	−	230	391	4060	4221
UN08747	Rsa1.0_00001.1	1024	363028	1.00E-123	255	263	96	+	718	978	322377	322638
UN08748	Rsa1.0_00121.1	1235	278029	0	423	437	96	−	233	666	44291	44727
UN08749	Rsa1.0_03028.1	706	25872	0	585	602	97	−	68	668	16430	17031
UN08750	Rsa1.0_00920.1	966	601703	0	920	940	97	+	1	939	13704	14643
UN08751	Rsa1.0_06728.1	916	10455	1.00E-173	342	352	97	−	565	916	9098	9448
UN08752	Rsa1.0_05895.1	1261	12305	0	342	342	100	+	920	1261	4214	4555
UN08753	Rsa1.0_00741.1	1443	66117	0	1055	1233	85	−	212	1438	30392	31621
UN08754	Rsa1.0_05440.1	512	13749	6.00E-66	128	128	100	−	3	130	13262	13389
UN08755	Rsa1.0_00800.1	490	63868	1.00E-136	248	249	99	+	45	293	14037	14285
UN08756	Rsa1.0_00777.1	965	191865	0	424	436	97	−	178	613	128527	128962
UN08757	Rsa1.0_00197.1	699	109714	0	376	382	98	+	224	605	94811	95190
UN08758	Rsa1.0_05819.1	1281	12543	0	1243	1270	97	+	12	1280	2234	3494
UN08759	Rsa1.0_00157.1	566	245832	0	433	456	94	−	62	516	239149	239590
UN08760	Rsa1.0_00198.1	568	222963	0	554	562	98	−	6	567	134425	134980
UN08761	Rsa1.0_00181.1	1028	114269	1.00E-124	248	255	97	−	3	257	103461	103715
UN08762	Rsa1.0_00126.1	1159	127534	0	596	600	99	+	560	1159	17416	18015
UN08763	Rsa1.0_02931.1	951	26654	0	517	539	95	−	10	541	5953	6486
UN08764	Rsa1.0_01172.1	890	51734	1.00E-128	236	237	99	+	1	237	20089	20325
UN08765	Rsa1.0_02825.1	868	27568	0	418	436	95	−	432	866	26073	26506
UN08766	Rsa1.0_00161.1	956	116891	1.00E-151	287	291	98	−	1	291	8114	8403
UN08767	Rsa1.0_00401.1	850	244410	0	403	405	99	−	446	850	243396	243800
UN08768	Rsa1.0_00121.1	774	278029	1.00E-122	232	235	98	−	1	235	86793	87027
UN08769	Rsa1.0_75692.1	805	303	1.00E-122	233	235	99	−	457	690	52	286
UN08770	Rsa1.0_14821.1	765	3044	0	344	345	99	−	1	345	1310	1654
UN08771	Rsa1.0_00447.1	1594	84051	1.00E-140	300	315	95	+	1	312	34487	34801
UN08772	Rsa1.0_01774.1	1563	39528	0	770	772	99	+	1	771	5756	6526
UN08773	Rsa1.0_01650.1	613	41589	0	538	579	92	−	32	607	27296	27871

UN08774	Rsa1.0_03126.1	1479	25192	1.00E-176	314	314	100	+	1166	1479	6646	6959
UN08775	Rsa1.0_02976.1	614	26315	1.00E-98	280	314	89	+	298	596	18887	19200
UN08776	Rsa1.0_01143.1	1006	52456	0	1005	1007	99	+	1	1006	19034	20039
UN08777	Rsa1.0_04325.1	1397	17885	1.00E-136	294	312	94	+	3	302	13731	14042
UN08778	Rsa1.0_15967.1	658	2337	1.00E-141	279	283	98	+	377	658	538	818
UN08779	Rsa1.0_00592.1	559	73788	0	517	563	91	−	6	558	21189	21733
UN08780	Rsa1.0_00518.1	2014	78360	0	837	978	85	+	423	1361	57799	58776
UN08781	Rsa1.0_00335.1	490	94371	0	401	407	98	+	82	488	50290	50696
UN08782	Rsa1.0_00197.1	878	109714	1.00E-139	273	280	97	+	7	286	76243	76522
UN08783	Rsa1.0_00196.1	1727	109960	0	535	537	99	−	1047	1583	59198	59734
UN08784	Rsa1.0_00344.1	526	191774	1.00E-140	255	256	99	−	2	257	136306	136561
UN08785	Rsa1.0_01552.1	650	43396	1.00E-112	262	279	93	+	372	645	12725	13003
UN08786	Rsa1.0_00414.1	476	202190	0	451	477	94	−	4	466	180872	181348
UN08787	Rsa1.0_00792.1	1120	64163	0	555	556	99	−	4	559	27760	28315
UN08788	Rsa1.0_00697.1	982	67913	1.00E-124	307	322	95	−	670	982	39690	40011
UN08789	Rsa1.0_02406.1	822	145276	1.00E-163	420	459	91	+	365	822	14630	15080
UN08790	Rsa1.0_05838.1	1357	12479	0	954	998	95	−	366	1357	8369	9365
UN08791	Rsa1.0_05146.1	1362	14678	1.00E-177	321	323	99	−	1	323	3072	3394
UN08792	Rsa1.0_00184.1	645	218748	1.00E-178	333	338	98	+	3	340	213946	214279
UN08793	Rsa1.0_52876.1	1224	431	0	386	386	100	+	428	813	40	425
UN08794	Rsa1.0_00396.1	1369	88242	0	385	403	95	−	112	514	19721	20114
UN08795	Rsa1.0_03781.1	731	20891	0	463	474	97	−	259	728	5091	5562
UN08796	Rsa1.0_01117.1	1291	53465	0	463	464	99	+	1	464	37870	38333
UN08797	Rsa1.0_17040.1	568	1944	1.00E-178	386	411	93	+	158	565	1207	1605
UN08798	Rsa1.0_00010.1	922	294827	0	454	475	95	−	274	747	247813	248287
UN08799	Rsa1.0_03246.1	777	24379	0	760	783	97	+	2	777	14687	15467
UN08800	Rsa1.0_08562.1	564	7193	3.00E-80	170	175	97	+	45	219	3782	3954
UN08801	Rsa1.0_00855.1	451	62047	0	432	449	96	−	1	449	42814	43250
UN08802	Rsa1.0_00180.1	1161	114581	0	393	406	96	+	756	1161	111408	111810
UN08803	Rsa1.0_00571.1	605	329018	1.00E-142	272	276	98	+	194	468	35755	36030
UN08804	Rsa1.0_05951.1	1048	12128	1.00E-160	310	318	97	−	13	324	5602	5919
UN08805	Rsa1.0_18790.1	1171	1533	0	986	995	99	−	1	994	528	1522
UN08806	Rsa1.0_00233.1	747	104918	0	714	744	95	+	1	739	31462	32201
UN08807	Rsa1.0_04741.1	702	16164	1.00E-106	219	226	96	+	475	698	8098	8323
UN08808	Rsa1.0_01297.1	772	49029	5.00E-92	289	328	88	+	138	465	30755	31082
UN08809	Rsa1.0_00190.1	667	318240	1.00E-149	282	287	98	+	44	330	28703	28989
UN08810	Rsa1.0_01539.1	585	43698	1.00E-123	230	232	99	−	354	585	5486	5717
UN08811	Rsa1.0_00307.1	1013	199003	9.00E-82	326	381	85	−	647	1001	87295	87662
UN08812	Rsa1.0_00890.1	731	323870	0	396	414	95	−	320	728	166555	166964

UN08813	Rsa1.0_04603.1	878	16682	1.00E-160	311	319	97	−	560	878	15427	15745
UN08814	Rsa1.0_03378.1	863	23322	0	369	377	97	−	27	402	21304	21680
UN08815	Rsa1.0_02413.1	1583	31628	0	393	417	94	+	250	666	6450	6853
UN08816	Rsa1.0_31664.1	618	722	1.00E-110	214	218	98	+	61	278	500	717
UN08817	Rsa1.0_02431.1	687	31504	1.00E-156	396	428	92	+	270	682	26607	27030
UN08818	Rsa1.0_73022.1	853	314	1.00E-123	236	240	98	−	227	466	75	314
UN08819	Rsa1.0_12161.1	665	5297	1.00E-98	201	206	97	+	442	645	4345	4550
UN08820	Rsa1.0_02194.1	688	34029	0	378	390	96	+	292	680	33393	33779
UN08821	Rsa1.0_00155.1	850	118465	0	382	390	97	−	1	388	96670	97053
UN08822	Rsa1.0_01319.1	993	48273	0	687	714	96	+	3	715	10899	11594
UN08823	Rsa1.0_00090.1	773	273859	0	499	510	97	−	3	512	148579	149088
UN08824	Rsa1.0_00167.1	600	116000	1.00E-109	229	237	96	−	365	600	90133	90369
UN08825	Rsa1.0_03401.1	1381	23191	3.00E-48	296	361	81	+	128	469	4703	5059
UN08826	Rsa1.0_03062.1	996	25678	0	448	450	99	+	326	775	6369	6818
UN08827	Rsa1.0_06521.1	1485	10856	0	1470	1485	98	−	1	1485	680	2164
UN08828	Rsa1.0_00522.1	532	78103	1.00E-153	435	485	89	−	2	477	60118	60582
UN08829	Rsa1.0_03964.1	717	19835	1.00E-114	210	210	100	+	2	211	13128	13337
UN08830	Rsa1.0_15118.1	798	2814	1.00E-144	281	288	97	+	88	375	934	1221
UN08831	Rsa1.0_00240.1	632	104175	0	524	527	99	−	100	625	52522	53048
UN08832	Rsa1.0_01980.1	612	36544	1.00E-122	334	370	90	+	255	612	32527	32891
UN08833	Rsa1.0_04318.1	1197	17947	1.00E-128	239	241	99	+	2	242	4048	4288
UN08834	Rsa1.0_00670.1	697	69371	3.00E-90	181	185	97	+	275	459	39326	39510
UN08835	Rsa1.0_00345.1	868	93226	1.00E-149	310	323	95	+	4	324	84267	84589
UN08836	Rsa1.0_01881.1	686	37721	3.00E-93	265	288	92	−	325	607	22834	23113
UN08837	Rsa1.0_00060.1	771	502678	0	335	335	100	+	433	767	500151	500485
UN08838	Rsa1.0_00273.1	1312	326186	1.00E-172	344	355	96	−	3	355	4768	5119
UN08839	Rsa1.0_01466.1	687	45161	0	634	681	93	−	7	687	38699	39353
UN08840	Rsa1.0_00632.1	598	183777	1.00E-108	267	291	91	−	318	595	149149	149438
UN08841	Rsa1.0_18647.1	846	1560	1.00E-140	326	345	94	−	499	842	591	932
UN08842	Rsa1.0_15148.1	579	2797	1.00E-150	296	304	97	−	276	579	1946	2247
UN08843	Rsa1.0_00332.1	683	94575	1.00E-92	182	185	98	+	144	328	61370	61554
UN08844	Rsa1.0_02945.1	1340	26548	0	1109	1116	99	+	225	1340	8447	9562
UN08845	Rsa1.0_00055.1	520	520193	1.00E-126	238	241	98	−	280	520	328674	328914
UN08846	#	#	#	#	#	#	#	#	#	#	#	#
UN08847	Rsa1.0_00044.1	1330	271658	1.00E-113	272	294	92	−	1	294	6694	6980
UN08848	Rsa1.0_07850.1	644	8423	1.00E-136	268	274	97	−	1	274	6410	6682
UN08849	Rsa1.0_01965.1	699	36698	0	682	697	97	+	3	699	3467	4155
UN08850	Rsa1.0_01923.1	451	37233	1.00E-121	221	221	100	+	1	221	27800	28020
UN08851	Rsa1.0_00042.1	1284	171507	1.00E-176	390	410	95	+	878	1282	159615	160020

UN08852	Rsa1.0_00163.1	1091	116847	0	402	408	98	−	1	405	62264	62671
UN08853	Rsa1.0_00817.1	886	62961	0	873	876	99	−	1	876	26779	27654
UN08854	Rsa1.0_01832.1	462	38581	1.00E−142	255	255	100	−	4	258	14445	14699
UN08855	Rsa1.0_00201.1	783	232851	0	460	487	94	−	3	486	140594	141080
UN08856	Rsa1.0_26382.1	530	894	1.00E−136	254	257	98	−	274	530	547	803
UN08857	Rsa1.0_02050.1	583	35570	0	570	576	98	−	1	576	30797	31367
UN08858	Rsa1.0_03964.1	701	19835	9.00E−72	196	215	91	+	1	205	13127	13337
UN08859	Rsa1.0_06402.1	827	11080	0	556	565	98	+	259	820	3431	3995
UN08860	Rsa1.0_01352.1	488	47443	4.00E−70	223	247	90	+	249	488	35006	35240
UN08861	Rsa1.0_01769.1	1000	39568	0	422	443	95	+	3	442	32131	32571
UN08862	Rsa1.0_00016.1	1103	831256	0	1093	1114	98	−	3	1101	537699	538810
UN08863	Rsa1.0_00807.1	536	63607	1.00E−139	258	259	99	−	278	536	40788	41045
UN08864	Rsa1.0_02975.1	866	26322	1.00E−129	255	261	97	+	235	493	4821	5081
UN08865	Rsa1.0_01016.1	609	181422	1.00E−149	288	295	97	−	66	360	44377	44671
UN08866	Rsa1.0_04111.1	1547	18991	0	559	579	96	+	135	705	11440	12017
UN08867	Rsa1.0_00196.1	961	109960	1.00E−123	303	323	93	+	633	951	72827	73141
UN08868	Rsa1.0_00077.1	740	145005	1.00E−89	184	188	97	+	452	639	71685	71871
UN08869	Rsa1.0_01026.1	675	171624	0	541	574	94	−	109	675	14047	14614
UN08870	Rsa1.0_02606.1	1005	29635	1.00E−131	264	273	96	+	40	312	5960	6226
UN08871	Rsa1.0_01568.1	679	43077	1.00E−174	336	345	97	−	335	679	21139	21483
UN08872	Rsa1.0_05745.1	781	12750	0	766	783	97	+	3	781	6801	7580
UN08873	Rsa1.0_01941.1	802	37069	5.00E−89	249	276	90	+	546	802	28851	29126
UN08874	Rsa1.0_00457.1	799	337301	3.00E−96	221	231	95	−	567	797	198587	198814
UN08875	Rsa1.0_00524.1	900	169613	1.00E−116	264	279	94	−	586	862	133346	133623
UN08876	Rsa1.0_00327.1	675	94892	6.00E−88	196	205	95	+	449	653	20149	20352
UN08877	Rsa1.0_04511.1	873	17032	0	391	392	99	+	482	873	4585	4975
UN08878	Rsa1.0_00065.1	724	454616	3.00E−99	184	184	100	+	325	508	446607	446790
UN08879	Rsa1.0_00192.1	805	278200	0	455	455	100	−	1	455	33911	34365
UN08880	Rsa1.0_02346.1	770	32337	1.00E−95	181	182	99	−	153	334	7207	7388
UN08881	Rsa1.0_05707.1	706	12872	0	685	713	96	+	1	706	407	1119
UN08882	Rsa1.0_04346.1	789	17813	0	542	552	98	+	1	552	12956	13502
UN08883	Rsa1.0_14881.1	1176	2995	0	454	469	96	+	707	1173	2323	2790
UN08884	Rsa1.0_04591.1	941	16717	1.00E−150	277	278	99	+	665	941	1	278
UN08885	Rsa1.0_00214.1	894	107508	0	397	404	98	+	44	447	75595	75993
UN08886	Rsa1.0_00056.1	935	332604	0	358	370	96	+	577	935	107099	107468
UN08887	Rsa1.0_10540.1	925	5811	0	542	552	98	+	2	553	2219	2769
UN08888	Rsa1.0_16229.1	536	2235	1.00E−109	201	201	100	−	2	202	130	330
UN08889	Rsa1.0_03990.1	320	19676	1.00E−120	234	238	98	+	1	238	8880	9116
UN08890	Rsa1.0_15163.1	758	2786	0	690	701	98	−	58	756	2091	2786

UN08891	Rsa1.0_00446.1	733	167829	0	409	426	96	−	32	456	129961	130381
UN08892	Rsa1.0_00006.1	842	396176	0	762	764	99	+	64	827	186848	187611
UN08893	Rsa1.0_06141.1	650	11644	0	612	654	93	+	1	650	6587	7225
UN08894	Rsa1.0_02157.1	947	149652	1.00E−114	233	238	97	−	473	710	133616	133851
UN08895	Rsa1.0_06071.1	474	11813	1.00E−168	344	355	96	+	120	474	2582	2932
UN08896	Rsa1.0_01081.1	620	499226	0	589	613	96	+	4	615	456338	456948
UN08897	Rsa1.0_00920.1	849	601703	0	456	465	98	+	385	849	439756	440216
UN08898	Rsa1.0_00385.1	578	89160	0	324	325	99	−	1	325	31104	31428
UN08899	Rsa1.0_00323.1	840	159650	0	393	413	95	+	432	840	148155	148558
UN08900	Rsa1.0_00013.1	850	292129	0	651	669	97	−	181	849	191168	191832
UN08901	Rsa1.0_02648.1	572	29073	4.00E−58	240	285	84	+	306	572	28376	28643
UN08902	Rsa1.0_00740.1	743	215972	0	715	744	96	+	1	743	35235	35977
UN08903	Rsa1.0_00875.1	1073	61371	0	729	750	97	−	165	905	1411	2157
UN08904	Rsa1.0_01026.1	705	171624	0	555	588	94	−	107	687	14033	14614
UN08905	Rsa1.0_00002.1	1744	601661	0	1040	1057	98	+	502	1557	289625	290680
UN08906	Rsa1.0_07863.1	583	8404	0	558	588	94	−	1	583	2635	3218
UN08907	Rsa1.0_00254.1	713	102600	1.00E−112	227	233	97	−	98	328	85303	85535
UN08908	Rsa1.0_00016.1	669	831256	0	666	668	99	+	2	669	505433	506098
UN08909	Rsa1.0_00793.1	383	64103	1.00E−113	217	219	99	−	165	383	59208	59425
UN08910	Rsa1.0_06541.1	702	10806	1.00E−169	313	317	98	−	386	702	10056	10372
UN08911	Rsa1.0_07617.1	880	9015	0	852	889	95	+	3	876	2174	3059
UN08912	Rsa1.0_00253.1	768	231292	0	737	769	95	−	2	768	196671	197431
UN08913	Rsa1.0_00985.1	741	161950	0	374	389	96	−	3	388	32823	33211
UN08914	Rsa1.0_00351.1	693	413096	1.00E−177	404	430	93	−	274	692	244400	244827
UN08915	Rsa1.0_09520.1	1336	6318	0	541	560	96	−	20	578	3074	3633
UN08916	Rsa1.0_00678.1	786	193163	1.00E−150	277	278	99	+	510	786	167678	167955
UN08917	Rsa1.0_00502.1	557	204180	0	460	493	93	−	67	557	63890	64372
UN08918	Rsa1.0_03552.1	1312	22151	2.33E−156	341	366	93	−	90	454	12808	13161
UN08919	Rsa1.0_00063.1	1104	156150	0	523	550	95	+	551	1100	142261	142810
UN08920	Rsa1.0_02452.1	850	31280	0	417	432	96	+	420	848	13666	14097
UN08921	Rsa1.0_00253.1	688	231292	0	639	677	94	−	7	681	196759	197423
UN08922	Rsa1.0_03982.1	763	19723	0	551	577	95	−	185	760	17040	17616
UN08923	Rsa1.0_00010.1	1030	294827	0	460	494	93	+	1	488	116518	116996
UN08924	Rsa1.0_02130.1	887	34755	0	434	437	99	+	451	887	14820	15253
UN08925	Rsa1.0_00417.1	830	395068	0	335	338	99	−	493	830	286876	287213
UN08926	Rsa1.0_04504.1	804	17069	1.00E−126	273	292	93	−	4	277	2573	2864
UN08927	Rsa1.0_00019.1	766	301669	1.00E−113	390	455	85	+	4	449	293078	293498
UN08928	Rsa1.0_02662.1	1246	28956	0	1106	1184	93	+	60	1231	8640	9822
UN08929	Rsa1.0_00082.1	731	237584	0	343	349	98	−	32	377	231097	231445

UN08930	Rsa1.0_08397.1	819	7427	1.00E-89	179	182	98	+	264	442	5025	5206
UN08931	Rsa1.0_02757.1	636	28187	0	476	485	98	+	154	636	16196	16680
UN08932	Rsa1.0_02114.1	1076	34873	0	504	507	99	+	570	1076	16049	16555
UN08933	Rsa1.0_01591.1	711	42718	0	588	607	96	+	106	709	14788	15392
UN08934	Rsa1.0_01106.1	947	53916	0	552	565	97	+	383	947	12267	12828
UN08935	Rsa1.0_00522.1	1122	78103	5.00E-99	199	204	97	+	306	509	35226	35429
UN08936	Rsa1.0_00098.1	741	349239	0	416	420	99	−	322	741	220987	221405
UN08937	Rsa1.0_00515.1	725	78417	0	356	357	99	−	369	725	43479	43835
UN08938	Rsa1.0_01482.1	782	44855	0	706	722	97	−	3	721	12165	12886
UN08939	Rsa1.0_00835.1	882	278058	0	666	682	97	−	203	881	261815	262495
UN08940	Rsa1.0_00101.1	907	267193	0	451	452	99	+	456	907	44122	44573
UN08941	Rsa1.0_00017.1	1009	752458	0	459	462	99	+	1	460	466023	466483
UN08942	Rsa1.0_00119.1	1342	467509	1.00E-164	328	338	97	−	1	337	302973	303310
UN08943	Rsa1.0_00002.1	1781	601661	0	1283	1346	95	+	247	1590	289356	290680
UN08944	Rsa1.0_00120.1	832	277288	1.00E-174	348	361	96	−	458	818	120631	120991
UN08945	Rsa1.0_05811.1	661	12572	0	479	522	91	−	3	523	11594	12091
UN08946	Rsa1.0_00082.1	836	237584	0	488	490	99	−	1	490	217985	218474
UN08947	Rsa1.0_01595.1	787	175383	1.00E-101	212	218	97	+	1	218	4059	4273
UN08948	Rsa1.0_00163.1	1323	116847	0	575	587	97	+	738	1321	73974	74557
UN08949	Rsa1.0_05804.1	683	12586	0	634	666	95	+	2	667	9611	10276
UN08950	Rsa1.0_02158.1	700	154841	0	336	341	98	+	331	671	122311	122651
UN08951	Rsa1.0_06604.1	850	10681	0	550	553	99	+	1	552	5922	6474
UN08952	Rsa1.0_07591.1	871	9060	0	817	849	96	−	1	841	1287	2132
UN08953	Rsa1.0_00144.1	758	120708	0	361	366	98	+	393	758	115794	116159
UN08954	Rsa1.0_03722.1	637	21184	1.00E-149	314	329	95	−	34	359	20756	21084
UN08955	Rsa1.0_00662.1	700	192183	1.00E-104	251	268	93	+	7	267	7993	8260
UN08956	Rsa1.0_03669.1	828	147114	0	384	402	95	−	3	403	17855	18248
UN08957	Rsa1.0_01737.1	733	40101	1.00E-106	219	227	96	+	479	705	17336	17562
UN08958	Rsa1.0_00978.1	826	57739	0	659	678	97	−	1	678	26879	27556
UN08959	Rsa1.0_01943.1	1123	37042	0	327	327	100	−	796	1122	4779	5105
UN08960	Rsa1.0_02198.1	759	33989	1.00E-144	291	299	97	+	4	296	13910	14207
UN08961	Rsa1.0_03569.1	607	22041	1.00E-128	232	232	100	+	374	605	10002	10233
UN08962	Rsa1.0_01675.1	1542	41155	1.00E-164	303	306	99	−	279	584	40809	41114
UN08963	Rsa1.0_00059.1	934	159650	1.00E-167	386	415	93	+	518	932	2240	2644
UN08964	Rsa1.0_00239.1	1191	104332	0	496	530	93	−	652	1172	67906	68429
UN08965	Rsa1.0_00807.1	770	63607	0	349	351	99	−	1	351	48916	49266
UN08966	Rsa1.0_01029.1	1324	256254	0	442	444	99	−	1	444	2337	2779
UN08967	Rsa1.0_00331.1	582	176754	4.00E-98	206	214	96	−	192	405	159068	159281
UN08968	Rsa1.0_01230.1	801	50221	0	791	793	99	−	7	799	16959	17750

UN08969	Rsa1.0_00142.1	706	121474	1.00E-132	258	264	97	+	308	571	64267	64530
UN08970	Rsa1.0_01016.1	739	181422	0	484	516	93	+	226	739	171865	172361
UN08971	Rsa1.0_02249.1	909	33412	0	908	909	99	+	1	909	16786	17694
UN08972	Rsa1.0_03152.1	634	24974	1.00E-156	293	296	98	+	1	296	3422	3716
UN08973	Rsa1.0_07921.1	1199	8259	0	452	459	98	−	7	465	2634	3092
UN08974	Rsa1.0_01666.1	840	173972	1.00E-163	340	354	96	+	90	442	6729	7080
UN08975	Rsa1.0_13538.1	1392	4746	1.00E-78	150	150	100	−	222	371	1	150
UN08976	Rsa1.0_00067.1	1322	415055	0	739	749	98	−	1	745	7421	8168
UN08977	Rsa1.0_05097.1	660	14864	1.00E-119	270	288	93	+	1	285	1264	1542
UN08978	Rsa1.0_03435.1	682	22958	1.00E-153	296	303	97	+	220	522	21975	22277
UN08979	Rsa1.0_00048.1	890	167185	1.00E-142	274	280	97	+	480	759	8087	8366
UN08980	Rsa1.0_00148.1	1094	120234	0	657	663	99	+	433	1094	8606	9268
UN08981	Rsa1.0_03652.1	405	21565	0	398	404	98	−	1	403	28	429
UN08982	Rsa1.0_03356.1	1070	23487	1.00E-177	332	337	98	+	2	335	20116	20452
UN08983	Rsa1.0_00107.1	734	431659	0	731	732	99	+	1	732	425791	426522
UN08984	Rsa1.0_05078.1	696	14912	0	386	401	96	−	273	673	7480	7880
UN08985	Rsa1.0_00101.1	973	267193	1.00E-120	266	281	94	+	7	287	131428	131700
UN08986	Rsa1.0_04520.1	471	16995	1.00E-103	207	210	98	+	3	212	10798	11005
UN08987	Rsa1.0_04234.1	854	18378	0	594	603	98	+	1	603	7512	8114
UN08988	Rsa1.0_00711.1	739	67223	0	478	521	91	+	225	739	15004	15517
UN08989	Rsa1.0_00154.1	588	189461	1.00E-131	271	283	95	−	314	588	79500	79782
UN08990	Rsa1.0_00387.1	1118	495407	0	849	888	95	+	198	1085	409522	410403
UN08991	Rsa1.0_08498.1	634	7270	0	423	445	95	−	198	634	5572	6016
UN08992	Rsa1.0_00453.1	554	83031	1.00E-128	260	269	96	+	3	271	534	802
UN08993	Rsa1.0_01149.1	1330	160459	1.00E-111	228	235	97	+	786	1020	131080	131311
UN08994	Rsa1.0_02903.1	569	26901	1.00E-103	266	288	92	+	267	549	17844	18120
UN08995	Rsa1.0_00389.1	922	88795	1.00E-114	210	210	100	+	713	922	68854	69063
UN08996	Rsa1.0_04871.1	862	15662	0	838	852	98	−	3	848	2842	3692
UN08997	Rsa1.0_00416.1	1038	86772	0	488	494	98	+	545	1038	41164	41653
UN08998	Rsa1.0_00642.1	1108	201230	1.00E-172	361	379	95	+	733	1108	161688	162055
UN08999	Rsa1.0_09895.1	716	6101	0	703	722	97	−	3	716	4875	5596
UN09000	Rsa1.0_00192.1	654	278200	0	339	339	100	+	3	341	16376	16714
UN09001	Rsa1.0_05013.1	1370	15131	0	940	963	97	+	415	1370	1	963
UN09002	Rsa1.0_15782.1	991	2423	0	762	764	99	−	1	763	861	1623
UN09003	Rsa1.0_02274.1	2284	33109	0	522	556	93	+	1732	2284	277	814
UN09004	Rsa1.0_00740.1	577	215972	1.00E-148	266	266	100	+	310	575	1704	1969
UN09005	Rsa1.0_00006.1	729	396176	1.00E-121	284	302	94	+	1	295	9150	9447
UN09006	Rsa1.0_01845.1	1058	38415	1.00E-110	214	218	98	+	527	744	36772	36989
UN09007	Rsa1.0_09470.1	986	6345	1.00E-156	304	311	97	−	123	432	2894	3204

UN09008	Rsa1.0_01228.1	458	50238	1.00E-132	281	293	95	-	2	294	733	1021
UN09009	Rsa1.0_00273.1	823	326186	0	819	821	99	-	2	821	296578	297398
UN09010	Rsa1.0_00033.1	545	515811	1.00E-104	285	313	91	-	2	312	33139	33446
UN09011	Rsa1.0_01626.1	1022	41989	0	470	488	96	-	534	1021	12327	12814
UN09012	Rsa1.0_01740.1	971	39986	1.00E-141	280	288	97	+	582	866	27598	27885
UN09013	Rsa1.0_00069.1	1002	403561	1.00E-164	318	326	97	-	1	326	135862	136187
UN09014	Rsa1.0_01926.1	1055	37206	0	597	613	97	-	361	973	13122	13734
UN09015	Rsa1.0_03738.1	856	21071	0	461	486	94	-	29	502	10785	11267
UN09016	Rsa1.0_00628.1	647	214360	1.00E-149	334	350	95	+	7	353	34412	34758
UN09017	Rsa1.0_03107.1	834	25349	1.00E-159	358	378	94	-	324	699	18569	18942
UN09018	Rsa1.0_03958.1	625	19868	1.00E-119	336	373	90	-	6	367	16483	16844
UN09019	Rsa1.0_03477.1	923	22635	1.00E-176	327	331	98	+	1	331	4856	5183
UN09020	Rsa1.0_02269.1	878	33168	0	325	326	99	-	2	327	3021	3346
UN09021	Rsa1.0_01319.1	969	48273	0	639	696	91	-	283	968	10927	11594
UN09022	Rsa1.0_01591.1	714	42718	0	595	615	96	-	32	645	14800	15413
UN09023	Rsa1.0_01002.1	1320	57063	0	1311	1319	99	+	1	1318	17461	18779
UN09024	Rsa1.0_03022.1	1143	25968	0	517	536	96	-	610	1142	1571	2106
UN09025	Rsa1.0_01084.1	967	54605	0	404	405	99	-	561	965	44683	45087
UN09026	Rsa1.0_12520.1	892	5205	1.00E-166	361	384	94	+	194	577	1	374
UN09027	Rsa1.0_01611.1	870	42269	0	866	867	99	+	3	869	3996	4862
UN09028	Rsa1.0_00165.1	1054	116466	1.00E-157	307	314	97	-	3	315	7613	7926
UN09029	Rsa1.0_02482.1	952	31000	0	597	627	95	-	1	624	26970	27572
UN09030	Rsa1.0_14791.1	608	3064	1.00E-152	282	285	98	+	296	580	2779	3063
UN09031	Rsa1.0_00228.1	463	105511	1.00E-141	347	375	92	+	28	397	13172	13545
UN09032	Rsa1.0_00549.1	1341	76473	1.00E-132	251	255	98	-	132	386	9511	9765
UN09033	Rsa1.0_01099.1	894	158481	0	457	462	98	-	1	460	153098	153559
UN09034	Rsa1.0_16218.1	898	2240	1.00E-126	258	265	97	+	630	892	1645	1908
UN09035	Rsa1.0_00369.1	843	266462	1.00E-111	217	221	98	+	335	555	20557	20777
UN09036	Rsa1.0_01390.1	617	46695	1.00E-139	290	301	96	+	104	404	5172	5469
UN09037	Rsa1.0_00197.1	689	109714	1.00E-160	309	315	98	-	91	405	78615	78928
UN09038	Rsa1.0_00571.1	932	329018	0	482	496	97	-	436	931	48784	49277
UN09039	Rsa1.0_00157.1	859	245832	2.00E-94	182	184	98	-	421	604	162373	162556
UN09040	Rsa1.0_00191.1	649	316976	1.00E-178	425	459	92	+	163	604	140127	140583
UN09041	Rsa1.0_03376.1	1290	23326	1.00E-166	436	476	91	-	828	1290	18752	19224
UN09042	Rsa1.0_00896.1	844	60509	0	697	697	100	+	1	697	38747	39443
UN09043	Rsa1.0_06083.1	1384	11780	0	332	334	99	+	2	334	7849	8182
UN09044	Rsa1.0_43457.1	886	517	0	369	373	98	-	165	536	145	517
UN09045	Rsa1.0_00051.1	1047	258225	0	984	1077	91	-	1	1045	113688	114741
UN09046	Rsa1.0_06563.1	577	10765	1.00E-73	165	172	95	+	361	532	863	1032

UN09047	Rsa1.0_00603.1	685	73299	0	417	431	96	−	49	478	32629	33057
UN09048	Rsa1.0_00420.1	638	86496	0	349	353	98	−	286	638	36503	36853
UN09049	Rsa1.0_03722.1	816	21184	1.00E−148	314	329	95	+	474	799	20756	21084
UN09050	Rsa1.0_08933.1	648	6772	1.00E−108	218	223	97	−	6	228	6266	6487
UN09051	Rsa1.0_00009.1	435	232558	1.00E−128	293	310	94	−	126	435	185030	185329
UN09052	Rsa1.0_14302.1	820	3558	0	488	488	100	+	333	820	1424	1911
UN09053	Rsa1.0_01301.1	1389	169072	0	496	498	99	+	890	1387	13992	14489
UN09054	Rsa1.0_00899.1	1050	60404	0	474	496	95	−	429	921	56656	57151
UN09055	Rsa1.0_00438.1	740	84861	1.00E−151	416	458	90	−	5	451	76921	77368
UN09056	Rsa1.0_04937.1	1420	15401	0	625	632	98	+	791	1420	12077	12708
UN09057	Rsa1.0_00690.1	616	140527	1.00E−179	321	322	99	−	293	614	34106	34427
UN09058	Rsa1.0_00585.1	870	74164	0	360	367	98	+	504	870	59736	60102
UN09059	Rsa1.0_00265.1	653	220635	0	384	387	99	+	3	389	24197	24583
UN09060	Rsa1.0_00329.1	783	222544	1.00E−120	293	316	92	−	461	769	178090	178397
UN09061	Rsa1.0_02975.1	592	26322	1.00E−140	274	280	97	+	313	592	23332	23609
UN09062	Rsa1.0_00644.1	860	136881	1.00E−148	309	321	96	+	539	858	31373	31686
UN09063	Rsa1.0_03163.1	986	24894	0	473	474	99	−	513	986	4477	4950
UN09064	Rsa1.0_01701.1	717	162121	0	393	393	100	−	1	393	13837	14229
UN09065	Rsa1.0_01415.1	722	46263	1.00E−130	273	286	95	−	443	721	17573	17858
UN09066	Rsa1.0_01481.1	773	184562	1.00E−162	301	305	98	−	467	771	39106	39410
UN09067	Rsa1.0_03503.1	796	22445	1.00E−163	315	320	98	+	3	320	9930	10249
UN09068	Rsa1.0_00568.1	1396	75219	0	445	445	100	−	948	1392	3698	4142
UN09069	Rsa1.0_01373.1	770	47052	0	410	422	97	+	318	736	21559	21980
UN09070	Rsa1.0_00015.1	1167	561803	0	417	434	96	−	739	1167	362103	362533
UN09071	Rsa1.0_00152.1	1414	257730	0	604	609	99	+	808	1414	118021	118629
UN09072	Rsa1.0_13798.1	807	4320	1.00E−126	242	246	98	+	337	582	3764	4009
UN09073	Rsa1.0_03074.1	970	25568	1.00E−166	335	345	97	+	624	968	21332	21674
UN09074	Rsa1.0_00069.1	955	403561	0	941	953	98	+	1	953	231058	232007
UN09075	Rsa1.0_03821.1	1140	20643	1.00E−110	275	294	93	−	849	1138	12652	12940
UN09076	Rsa1.0_00173.1	812	288743	1.00E−153	274	274	100	+	128	401	41089	41362
UN09077	Rsa1.0_18035.1	1284	1685	0	626	647	96	+	3	646	450	1094
UN09078	Rsa1.0_05271.1	960	14306	0	333	334	99	+	558	890	10323	10656
UN09079	Rsa1.0_13498.1	849	4764	1.00E−175	320	322	99	+	3	322	555	876
UN09080	Rsa1.0_06509.1	937	10873	2.33E−156	263	263	100	+	1	263	7125	7387
UN09081	Rsa1.0_00381.1	867	89718	0	530	547	96	+	323	867	79836	80382
UN09082	Rsa1.0_00369.1	866	266462	0	591	593	99	−	274	866	258502	259094
UN09083	Rsa1.0_01315.1	673	48361	0	324	324	100	−	350	673	11564	11887
UN09084	Rsa1.0_00748.1	773	65983	0	442	464	95	−	25	485	63996	64453
UN09085	Rsa1.0_00221.1	837	106972	1.00E−148	282	287	98	+	550	836	52627	52913

UN09086	Rsa1.0_00736.1	1025	66455	0	857	911	94	+	119	1023	50684	51591
UN09087	Rsa1.0_04123.1	782	18947	0	759	768	98	−	1	768	11871	12637
UN09088	Rsa1.0_00205.1	1134	193401	0	609	617	98	−	520	1133	181776	182392
UN09089	Rsa1.0_02431.1	687	31504	0	655	687	95	−	3	683	12950	13631
UN09090	Rsa1.0_07305.1	1614	9578	0	376	381	98	−	4	381	8667	9045
UN09091	Rsa1.0_00434.1	1495	85171	0	701	701	100	−	129	829	30393	31093
UN09092	Rsa1.0_01632.1	1123	181224	1.00E−123	311	339	91	−	3	335	7459	7791
UN09093	Rsa1.0_02017.1	1344	35999	1.00E−132	269	280	96	+	761	1040	26577	26847
UN09094	Rsa1.0_00850.1	548	62193	0	415	430	96	+	2	430	5918	6346
UN09095	Rsa1.0_04504.1	767	17069	1.00E−154	276	276	100	+	492	767	2573	2848
UN09096	Rsa1.0_00663.1	359	164969	1.00E−143	324	338	95	+	4	337	120751	121082
UN09097	Rsa1.0_00087.1	662	761073	1.00E−128	302	323	93	−	14	323	217289	217610
UN09098	Rsa1.0_02282.1	979	163951	1.00E−129	316	339	93	+	641	979	154467	154802
UN09099	Rsa1.0_03422.1	678	23076	0	443	457	96	+	233	678	9861	10317
UN09100	Rsa1.0_01827.1	696	143321	0	677	704	96	+	10	695	139171	139873
UN09101	Rsa1.0_15780.1	1389	2424	0	475	477	99	+	914	1389	1216	1691
UN09102	Rsa1.0_00803.1	1196	144228	0	489	494	98	+	667	1158	37985	38478
UN09103	Rsa1.0_04218.1	600	18423	1.00E−164	410	449	91	−	155	597	15845	16280
UN09104	Rsa1.0_00038.1	618	314568	1.00E−163	292	292	100	+	327	618	141728	142019
UN09105	Rsa1.0_00677.1	492	69159	1.00E−140	311	333	93	−	173	492	20365	20693
UN09106	Rsa1.0_04689.1	1628	16376	0	969	1006	96	+	623	1628	475	1469
UN09107	Rsa1.0_07724.1	808	8708	0	381	390	97	+	32	421	6618	7007
UN09108	Rsa1.0_02449.1	1047	31292	9.00E−79	189	198	95	+	1	196	13263	13459
UN09109	Rsa1.0_01190.1	646	194501	1.00E−167	356	372	95	−	274	642	157947	158316
UN09110	Rsa1.0_01824.1	838	38733	1.00E−122	228	230	99	+	375	604	8413	8642
UN09111	Rsa1.0_02865.1	762	27133	0	451	484	93	+	290	761	22643	23116
UN09112	Rsa1.0_01778.1	1120	39509	1.00E−74	149	151	98	+	456	606	16810	16960
UN09113	Rsa1.0_00055.1	1124	520193	1.00E−127	259	268	96	−	467	734	207861	208128
UN09114	Rsa1.0_00907.1	1023	60062	0	515	539	95	−	4	534	2211	2749
UN09115	Rsa1.0_08007.1	1399	8087	1.00E−139	259	260	99	+	1138	1397	2203	2461
UN09116	Rsa1.0_00294.1	685	144860	1.00E−130	299	317	94	−	298	605	54782	55096
UN09117	Rsa1.0_00049.1	998	167018	0	642	656	97	−	343	994	137812	138467
UN09118	Rsa1.0_03563.1	1277	22070	0	632	635	99	+	3	637	15461	16095
UN09119	Rsa1.0_00123.1	907	325650	0	463	480	96	−	430	906	109845	110321
UN09120	Rsa1.0_29830.1	1041	770	1.00E−126	289	309	93	+	625	928	114	422
UN09121	Rsa1.0_00132.1	648	124299	1.00E−143	298	310	96	+	338	646	99696	100001
UN09122	Rsa1.0_00207.1	820	248736	0	335	335	100	+	478	812	55294	55628
UN09123	Rsa1.0_00533.1	695	77375	0	602	642	93	+	3	644	49299	49937
UN09124	Rsa1.0_08151.1	949	7830	0	920	951	96	−	3	949	1053	2000

UN09125	Rsa1.0_00903.1	1066	60228	0	565	575	98	+	495	1066	38141	38714
UN09126	Rsa1.0_08221.1	1532	7700	0	1234	1234	100	+	291	1524	5416	6649
UN09127	Rsa1.0_00104.1	520	368722	1.00E-138	313	333	93	−	183	513	225000	225328
UN09128	Rsa1.0_22492.1	956	1117	1.00E-114	213	214	99	+	23	236	685	898
UN09129	Rsa1.0_02099.1	707	35030	1.00E-166	297	297	100	+	10	306	17742	18038
UN09130	Rsa1.0_02064.1	1021	35476	1.00E-138	339	363	93	−	1	351	9518	9879
UN09131	Rsa1.0_01856.1	555	38181	0	399	415	96	+	141	555	12953	13366
UN09132	Rsa1.0_00333.1	1043	94500	0	896	901	99	−	143	1043	44005	44905
UN09133	Rsa1.0_03552.1	878	22151	4.00E-93	202	210	96	+	3	211	13461	13670
UN09134	Rsa1.0_00381.1	1471	89718	1.00E-121	249	258	96	+	3	260	39724	39981
UN09135	Rsa1.0_48558.1	864	466	0	353	355	99	+	52	406	1	355
UN09136	Rsa1.0_00283.1	1134	183797	1.00E-161	351	369	95	+	3	366	10947	11313
UN09137	Rsa1.0_00482.1	2132	81162	0	1366	1371	99	+	1	1371	3813	5180
UN09138	Rsa1.0_00552.1	1100	206757	0	588	593	99	−	506	1098	68022	68611
UN09139	Rsa1.0_01941.1	1114	37069	1.00E-129	240	242	99	−	164	405	32330	32571
UN09140	Rsa1.0_00706.1	1197	291570	1.00E-167	304	306	99	−	3	308	231041	231346
UN09141	Rsa1.0_15024.1	1020	2885	1.00E-141	328	346	94	−	667	1007	2252	2592
UN09142	Rsa1.0_00007.1	1299	239325	1.00E-154	321	333	96	−	965	1297	14225	14555
UN09143	Rsa1.0_00146.1	996	149343	0	918	951	96	−	14	961	67449	68388
UN09144	Rsa1.0_02287.1	1237	32986	1.00E-168	336	348	96	+	278	625	7095	7442
UN09145	Rsa1.0_01696.1	1029	301850	2.00E-98	219	229	95	+	780	1005	32637	32864
UN09146	Rsa1.0_00369.1	1036	266462	1.00E-136	382	424	90	+	1	410	146164	146575
UN09147	Rsa1.0_02139.1	1134	34686	0	534	534	100	−	601	1134	24881	25414
UN09148	Rsa1.0_14744.1	1620	3111	0	489	506	96	−	303	808	724	1229
UN09149	Rsa1.0_03447.1	825	22857	1.00E-141	255	255	100	−	571	825	19460	19714
UN09150	Rsa1.0_07472.1	1222	9306	0	446	447	99	−	1	447	6661	7107
UN09151	Rsa1.0_02213.1	1052	33824	4.00E-53	107	107	100	−	946	1052	33603	33709
UN09152	Rsa1.0_03218.1	1679	24539	6.00E-78	159	161	98	−	3	163	9502	9661
UN09153	Rsa1.0_24098.1	924	1007	1.00E-114	280	305	91	−	597	887	643	947
UN09154	Rsa1.0_01786.1	956	133324	1.00E-126	242	246	98	+	522	767	101482	101727
UN09155	Rsa1.0_00131.1	1123	432233	1.00E-169	393	423	92	+	539	961	34875	35288
UN09156	Rsa1.0_15591.1	985	2530	0	587	619	94	+	383	985	1224	1838
UN09157	Rsa1.0_00089.1	562	406142	0	548	564	97	−	1	560	80693	81252
UN09158	Rsa1.0_02858.1	985	27246	1.00E-113	357	395	90	−	3	380	1699	2086
UN09159	Rsa1.0_00028.1	1063	293984	1.00E-147	268	269	99	+	1	269	263729	263997
UN09160	Rsa1.0_21236.1	1228	1222	0	471	476	98	−	752	1227	605	1080
UN09161	Rsa1.0_00548.1	1069	76518	6.00E-80	214	234	91	−	16	249	62227	62457
UN09162	Rsa1.0_00894.1	1104	269700	1.00E-110	300	326	92	−	718	1039	128038	128357
UN09163	Rsa1.0_00163.1	1061	116847	1.00E-160	368	393	93	+	4	390	25822	26212

UN09164	Rsa1.0_00712.1	1722	67217	0	575	597	96	+	2	597	16542	17138
UN09165	Rsa1.0_14737.1	874	3115	1.00E-154	310	321	96	+	256	576	2055	2375
UN09166	Rsa1.0_04384.1	1301	17629	1.00E-172	333	342	97	+	415	756	3729	4070
UN09167	Rsa1.0_02146.1	880	34625	0	747	771	96	+	112	879	17169	17931
UN09168	Rsa1.0_00561.1	1238	75653	0	844	849	99	−	390	1236	4205	5053
UN09169	Rsa1.0_00757.1	1621	65559	1.00E-108	199	199	100	+	1	199	12480	12678
UN09170	Rsa1.0_00167.1	934	116000	5.00E-80	374	449	83	+	125	573	28365	28795
UN09171	Rsa1.0_01214.1	1037	50599	0	595	623	95	−	3	625	49762	50384
UN09172	Rsa1.0_03940.1	1291	19962	0	931	938	99	+	359	1291	11912	12849
UN09173	Rsa1.0_00353.1	1035	92242	1.00E-104	237	248	95	−	789	1035	55684	55928
UN09174	Rsa1.0_01687.1	1014	40979	0	787	841	93	−	2	829	1551	2390
UN09175	Rsa1.0_00116.1	1052	365886	1.00E-130	252	256	98	+	4	258	239004	239259
UN09176	Rsa1.0_00066.1	1024	152418	1.00E-145	268	269	99	+	755	1022	60921	61189
UN09177	Rsa1.0_03860.1	833	20407	0	479	507	94	+	8	509	15820	16316
UN09178	Rsa1.0_02068.1	1144	35432	1.00E-59	121	122	99	−	559	680	3872	3993
UN09179	Rsa1.0_02048.1	741	35605	0	473	474	99	−	191	664	24818	25291
UN09180	Rsa1.0_03380.1	1208	23320	1.00E-166	319	326	97	−	883	1208	4293	4615
UN09181	Rsa1.0_01666.1	1237	173972	1.00E-115	249	259	96	+	219	477	23919	24175
UN09182	Rsa1.0_00020.1	1361	283099	1.00E-132	291	309	94	+	3	303	90045	90353
UN09183	Rsa1.0_00797.1	1054	63959	0	372	376	98	+	186	561	10750	11125
UN09184	Rsa1.0_21842.1	1268	1169	1.00E-135	244	244	100	−	1	244	480	723
UN09185	Rsa1.0_00131.1	1179	432233	0	392	396	98	−	510	905	194264	194659
UN09186	Rsa1.0_00609.1	1320	72785	0	445	450	98	−	129	578	70202	70651
UN09187	Rsa1.0_39035.1	893	576	1.00E-121	257	268	95	+	595	862	316	576
UN09188	Rsa1.0_07256.1	927	9655	0	471	477	98	+	2	478	4824	5300
UN09189	Rsa1.0_07775.1	860	8594	0	347	349	99	−	1	348	614	962
UN09190	Rsa1.0_03352.1	1487	23506	1.00E-128	276	286	96	+	1201	1485	4054	4337
UN09191	Rsa1.0_02009.1	905	36100	0	421	438	96	−	140	577	35107	35544
UN09192	Rsa1.0_00133.1	1085	123768	1.00E-179	318	318	100	−	1	318	29742	30059
UN09193	Rsa1.0_14298.1	947	3561	1.00E-96	186	188	98	+	618	805	3278	3465
UN09194	Rsa1.0_00226.1	1287	105884	0	602	622	96	−	618	1238	63959	64580
UN09195	Rsa1.0_00645.1	1507	189716	1.00E-176	322	325	99	−	528	852	150795	151119
UN09196	Rsa1.0_02058.1	1295	35532	0	452	463	97	−	4	466	13428	13890
UN09197	Rsa1.0_00497.1	460	79830	1.00E-111	238	248	95	+	1	247	43611	43858
UN09198	Rsa1.0_01130.1	1220	52929	0	371	385	96	−	1	379	44531	44915
UN09199	Rsa1.0_00620.1	1379	71985	0	491	506	97	+	728	1233	55327	55832
UN09200	Rsa1.0_03937.1	1247	19969	1.00E-119	280	298	93	+	5	291	3005	3301
UN09201	Rsa1.0_01282.1	970	49304	1.00E-148	311	321	96	−	654	970	28012	28331
UN09202	Rsa1.0_00020.1	750	283099	0	401	403	99	−	348	750	60863	61265

UN09203	Rsa1.0_01170.1	834	161131	1.00E-171	332	341	97	+	494	834	129391	129727
UN09204	Rsa1.0_01058.1	832	55430	0	804	845	95	−	1	828	34187	35029
UN09205	Rsa1.0_00034.1	1100	260119	1.00E-149	340	358	94	−	59	413	841	1189
UN09206	Rsa1.0_00002.1	951	601661	0	566	597	94	+	254	829	289356	289949
UN09207	Rsa1.0_01713.1	1112	40564	0	1108	1108	100	+	3	1110	26203	27310
UN09208	Rsa1.0_01420.1	2291	46210	0	1083	1086	99	−	1	1086	8650	9735
UN09209	Rsa1.0_36749.1	922	613	0	340	340	100	−	352	691	186	525
UN09210	Rsa1.0_00269.1	1028	341668	0	456	470	97	+	21	486	323926	324393
UN09211	Rsa1.0_00002.1	1940	601661	0	794	824	96	+	821	1626	289098	289921
UN09212	Rsa1.0_01564.1	877	43169	2.33E-156	275	279	98	−	597	875	36245	36523
UN09213	Rsa1.0_11573.1	1576	5468	1.00E-167	326	335	97	−	376	710	5108	5442
UN09214	Rsa1.0_15481.1	1058	2582	0	631	642	98	+	1	631	1050	1691
UN09215	Rsa1.0_05496.1	929	13533	0	626	634	98	+	302	929	453	1086
UN09216	Rsa1.0_00093.1	1407	214962	1.00E-143	354	383	92	−	3	380	81375	81740
UN09217	Rsa1.0_00644.1	1138	136881	0	387	390	99	+	749	1138	52173	52561
UN09218	Rsa1.0_01947.1	687	36977	0	387	392	98	−	296	687	23177	23567
UN09219	Rsa1.0_05624.1	705	13140	1.00E-163	397	433	91	−	268	699	3783	4199
UN09220	Rsa1.0_00894.1	1163	269700	1.00E-101	215	222	96	−	856	1075	128137	128357
UN09221	Rsa1.0_00425.1	942	147080	0	926	943	98	+	1	942	897	1829
UN09222	Rsa1.0_01537.1	691	187285	1.00E-160	364	387	94	+	249	634	839	1220
UN09223	Rsa1.0_00341.1	931	249309	3.00E-75	156	160	97	−	356	515	115284	115443
UN09224	Rsa1.0_00049.1	973	167018	0	651	656	99	+	1	655	137814	138468
UN09225	Rsa1.0_26642.1	949	883	0	477	486	98	−	284	769	294	779
UN09226	Rsa1.0_00728.1	1085	66704	1.00E-171	327	333	98	−	753	1085	2998	3329
UN09227	Rsa1.0_01082.1	504	54640	0	477	497	95	−	8	502	17718	18212
UN09228	Rsa1.0_00204.1	721	109056	1.00E-171	439	478	91	−	1	473	17525	17990
UN09229	Rsa1.0_03560.1	522	22092	1.00E-109	212	216	98	+	27	242	3019	3234
UN09230	Rsa1.0_00502.1	705	204180	0	616	650	94	−	58	705	63729	64372
UN09231	Rsa1.0_00092.1	1059	659932	0	546	578	94	−	416	988	141118	141690
UN09232	Rsa1.0_00009.1	1000	232558	0	413	416	99	−	1	416	53021	53436
UN09233	Rsa1.0_05376.1	885	13977	0	620	631	98	−	1	631	5384	6014
UN09234	Rsa1.0_00104.1	1072	368722	3.00E-94	239	260	91	+	634	893	201374	201633
UN09235	Rsa1.0_04568.1	1418	16805	0	1045	1083	96	+	337	1418	4609	5681
UN09236	Rsa1.0_03686.1	1364	21387	0	357	361	98	−	1005	1364	8981	9340
UN09237	Rsa1.0_52605.1	939	433	0	432	433	99	−	157	589	1	433
UN09238	Rsa1.0_00140.1	474	121541	1.00E-164	295	296	99	+	179	474	579	874
UN09239	Rsa1.0_02813.1	538	27656	0	415	426	97	+	122	538	25679	26104
UN09240	Rsa1.0_01774.1	718	39528	1.00E-135	256	260	98	+	450	709	23650	23909
UN09241	Rsa1.0_01651.1	1036	41586	0	1029	1036	99	+	1	1036	31227	32262

UN09242	Rsa1.0_01176.1	687	51638	1.00E-123	236	240	98	-	448	687	47334	47573
UN09243	Rsa1.0_00083.1	883	442773	0	391	394	99	-	490	883	261482	261875
UN09244	Rsa1.0_07309.1	821	9572	6.00E-98	185	186	99	-	635	820	7736	7921
UN09245	Rsa1.0_02955.1	780	26484	7.00E-82	217	235	92	-	547	778	20351	20581
UN09246	Rsa1.0_01097.1	1651	173349	1.00E-180	404	432	93	-	1233	1648	32232	32663
UN09247	Rsa1.0_02043.1	900	163754	1.00E-114	237	246	96	-	377	622	145291	145536
UN09248	Rsa1.0_00060.1	1416	502678	1.00E-155	350	371	94	-	1046	1415	368884	369249
UN09249	Rsa1.0_00855.1	1236	62047	0	378	392	96	-	3	394	55971	56359
UN09250	Rsa1.0_03168.1	1141	24827	1.00E-123	243	249	97	+	10	258	4696	4944
UN09251	Rsa1.0_01212.1	1222	50622	0	1193	1218	97	+	5	1220	44889	46106
UN09252	Rsa1.0_02622.1	1155	142402	0	683	699	97	+	455	1153	9528	10226
UN09253	Rsa1.0_01545.1	1047	43599	0	558	559	99	-	6	564	32611	33169
UN09254	Rsa1.0_02474.1	1267	31076	0	436	438	99	+	828	1265	21211	21648
UN09255	Rsa1.0_00054.1	1226	162053	1.00E-122	388	436	88	-	792	1218	5405	5820
UN09256	Rsa1.0_18628.1	945	1562	0	910	946	96	-	2	944	367	1307
UN09257	Rsa1.0_04286.1	1022	18084	0	375	376	99	-	647	1022	4017	4392
UN09258	Rsa1.0_00108.1	1223	478491	1.00E-144	371	403	92	-	2	394	463726	464123
UN09259	Rsa1.0_09250.1	1397	6528	0	449	458	98	+	215	672	3462	3916
UN09260	Rsa1.0_00119.1	1337	467509	1.00E-173	333	340	97	+	998	1337	302973	303310
UN09261	Rsa1.0_00198.1	1066	222963	0	897	944	95	+	120	1054	138000	138942
UN09262	Rsa1.0_01683.1	1388	41059	1.00E-166	339	353	96	-	529	881	23260	23612
UN09263	Rsa1.0_13895.1	1363	4135	0	401	401	100	+	1	401	155	555
UN09264	Rsa1.0_00332.1	969	94575	0	507	507	100	+	1	507	62018	62524
UN09265	Rsa1.0_01431.1	910	167877	0	904	907	99	-	3	909	36772	37678
UN09266	Rsa1.0_01696.1	1409	301850	1.00E-171	328	333	98	-	6	338	297579	297908
UN09267	Rsa1.0_03318.1	1096	23780	1.00E-168	334	344	97	-	718	1060	2397	2740
UN09268	Rsa1.0_00356.1	937	91521	0	490	511	95	-	19	523	34979	35485
UN09269	Rsa1.0_00525.1	1322	78030	0	441	452	97	+	2	453	30668	31119
UN09270	Rsa1.0_18957.1	1235	1505	1.00E-158	295	299	98	+	248	546	1203	1501
UN09271	Rsa1.0_00048.1	858	167185	1.00E-137	272	280	97	+	470	749	8087	8366
UN09272	Rsa1.0_00615.1	986	72235	0	620	639	97	+	1	639	54770	55402
UN09273	Rsa1.0_08807.1	872	6906	1.00E-120	229	232	98	-	273	504	4140	4371
UN09274	Rsa1.0_02922.1	907	26753	0	364	364	100	-	544	907	12326	12689
UN09275	Rsa1.0_05804.1	739	12586	0	737	737	100	-	3	739	9607	10343
UN09276	Rsa1.0_04794.1	773	15960	0	461	462	99	+	312	773	2694	3155
UN09277	Rsa1.0_00067.1	1036	415055	1.00E-150	333	352	94	+	641	991	301029	301368
UN09278	Rsa1.0_00140.1	713	121541	1.00E-152	316	329	96	-	383	711	21968	22295
UN09279	Rsa1.0_37242.1	943	605	1.00E-150	317	330	96	+	42	367	10	337
UN09280	Rsa1.0_00642.1	1316	201230	0	969	1017	95	-	1	1013	157947	158961

UN09281	Rsa1.0_14319.1	1104	3529	1.00E-179	491	548	89	+	293	840	2316	2860
UN09282	Rsa1.0_01091.1	1268	54383	0	562	572	98	+	4	575	11319	11890
UN09283	Rsa1.0_01530.1	814	43820	0	514	527	97	−	288	814	5929	6455
UN09284	Rsa1.0_00026.1	411	327835	2.00E-12	61	68	89	+	16	80	305879	305946
UN09285	Rsa1.0_00049.1	1463	167018	0	700	716	97	+	3	718	144469	145184
UN09286	Rsa1.0_01537.1	1001	187285	1.00E-129	333	361	92	−	648	1001	38620	38977
UN09287	Rsa1.0_03728.1	1423	21147	0	454	462	98	+	1	462	631	1092
UN09288	Rsa1.0_08045.1	1451	8014	0	341	343	99	−	1109	1450	4994	5336
UN09289	Rsa1.0_00147.1	1143	249503	0	462	497	92	−	658	1136	155766	156259
UN09290	Rsa1.0_02165.1	1036	34423	0	556	600	92	−	374	960	3037	3636
UN09291	Rsa1.0_00865.1	1293	61686	0	471	471	100	−	2	472	2371	2841
UN09292	Rsa1.0_02043.1	1503	163754	0	450	486	92	+	653	1123	145937	146415
UN09293	Rsa1.0_00096.1	973	198773	1.00E-176	320	322	99	+	4	325	14856	15177
UN09294	Rsa1.0_00715.1	515	67116	1.00E-100	238	250	95	+	14	259	40324	40573
UN09295	Rsa1.0_02433.1	1464	158318	0	413	440	93	+	1	426	19973	20411
UN09296	Rsa1.0_00772.1	1136	64856	0	433	464	93	−	618	1080	47121	47580
UN09297	Rsa1.0_06898.1	1007	10191	4.00E-93	189	194	97	−	1	194	5050	5243
UN09298	Rsa1.0_01519.1	960	44144	0	503	521	96	−	441	960	11634	12151
UN09299	Rsa1.0_00166.1	1193	240912	0	410	422	97	+	521	942	221531	221952
UN09300	Rsa1.0_03971.1	1284	19788	1.00E-151	293	299	97	+	981	1279	3924	4221
UN09301	Rsa1.0_16493.1	1305	2136	1.00E-118	228	232	98	+	1	232	1336	1567
UN09302	Rsa1.0_00017.1	921	752458	6.00E-92	209	220	95	−	7	226	298464	298682
UN09303	Rsa1.0_01144.1	1280	52431	1.00E-155	325	340	95	+	3	339	22926	23265
UN09304	Rsa1.0_00684.1	935	141701	0	463	469	98	+	466	934	9100	9568
UN09305	Rsa1.0_05449.1	1074	13711	0	440	471	93	+	5	473	13194	13646
UN09306	Rsa1.0_00132.1	978	124299	1.00E-172	353	367	96	−	2	367	99700	100066
UN09307	Rsa1.0_00147.1	1218	249503	0	567	609	93	+	1	607	155663	156259
UN09308	Rsa1.0_09273.1	1087	6511	1.00E-107	216	222	97	−	1	222	1960	2181
UN09309	Rsa1.0_01377.1	1070	46967	0	356	363	98	+	708	1070	21582	21943
UN09310	Rsa1.0_00040.1	1881	324196	1.00E-172	334	343	97	+	1539	1881	315220	315562
UN09311	Rsa1.0_00473.1	1293	212204	1.00E-148	278	282	98	+	485	766	48003	48284
UN09312	Rsa1.0_00895.1	1032	60615	1.00E-159	308	313	98	+	722	1032	16603	16915
UN09313	Rsa1.0_00338.1	1098	94109	1.00E-110	261	280	93	−	819	1098	34847	35117
UN09314	Rsa1.0_11428.1	1305	5511	1.00E-167	391	424	92	−	1	402	4231	4654
UN09315	Rsa1.0_00218.1	1092	201480	0	847	857	98	+	235	1090	174782	175637
UN09316	Rsa1.0_02165.1	1067	34423	0	550	600	91	+	114	693	3037	3636
UN09317	Rsa1.0_00760.1	1125	65267	1.00E-139	263	267	98	+	401	667	19685	19951
UN09318	Rsa1.0_02209.1	1078	33873	1.00E-155	340	362	93	−	720	1078	26608	26957
UN09319	Rsa1.0_36506.1	1096	618	1.00E-141	293	306	95	−	786	1091	313	618

UN09320	Rsa1.0_02550.1	1072	291051	0	364	367	99	+	706	1072	6190	6556
UN09321	Rsa1.0_06303.1	1025	11268	0	792	814	97	+	2	815	4844	5657
UN09322	Rsa1.0_00497.1	1223	79830	0	720	741	97	+	487	1223	73170	73910
UN09323	Rsa1.0_11811.1	1369	5401	1.00E-113	224	229	97	+	1140	1364	2702	2930
UN09324	Rsa1.0_08617.1	867	7124	0	837	880	95	−	2	867	284	1161
UN09325	Rsa1.0_00145.1	765	261008	0	428	428	100	−	336	763	8526	8953
UN09326	Rsa1.0_00816.1	1042	403350	0	562	583	96	−	3	582	127627	128209
UN09327	Rsa1.0_02379.1	845	150705	1.00E-141	380	424	89	−	352	753	141733	142156
UN09328	Rsa1.0_01104.1	1192	285480	0	452	456	99	+	736	1191	35015	35470
UN09329	Rsa1.0_09048.1	769	6683	0	667	679	98	+	95	769	5245	5923
UN09330	Rsa1.0_01618.1	625	42118	1.00E-108	266	290	91	+	1	280	17298	17587
UN09331	Rsa1.0_09325.1	956	6454	0	340	344	98	+	613	956	716	1059
UN09332	Rsa1.0_08255.1	801	7652	0	522	524	99	+	1	524	3734	4257
UN09333	Rsa1.0_00387.1	709	495407	1.00E-163	314	320	98	+	1	320	349283	349601
UN09334	Rsa1.0_02658.1	1294	28978	1.00E-161	319	329	96	+	60	388	17755	18083
UN09335	Rsa1.0_01958.1	1345	261128	0	410	414	99	+	1	414	129275	129688
UN09336	Rsa1.0_01397.1	595	183276	8.00E-81	162	165	98	+	73	237	171641	171805
UN09337	Rsa1.0_00558.1	1335	75819	0	739	751	98	+	311	1061	28073	28823
UN09338	Rsa1.0_00364.1	839	90951	0	440	443	99	+	397	839	63260	63702
UN09339	Rsa1.0_00097.1	837	138858	0	375	386	97	+	458	837	42576	42958
UN09340	Rsa1.0_02345.1	743	32337	1.00E-106	262	282	92	+	460	738	15392	15673
UN09341	Rsa1.0_02848.1	786	27329	0	517	530	97	−	254	782	12523	13050
UN09342	Rsa1.0_02548.1	1077	30228	1.00E-153	330	348	94	+	743	1077	10107	10454
UN09343	Rsa1.0_00086.1	1034	439854	0	865	865	100	+	3	867	341743	342607
UN09344	Rsa1.0_02888.1	1639	27020	0	426	430	99	+	2	431	8692	9121
UN09345	Rsa1.0_00636.1	736	71113	1.00E-158	286	287	99	+	2	288	14334	14620
UN09346	Rsa1.0_00192.1	865	278200	0	452	453	99	−	1	453	223102	223554
UN09347	Rsa1.0_00045.1	1305	328694	2.33E-156	275	279	98	−	799	1077	161663	161941
UN09348	Rsa1.0_08619.1	784	7123	1.00E-108	243	255	95	−	538	784	5465	5719
UN09349	Rsa1.0_00025.1	667	277312	0	661	661	100	+	5	665	79953	80613
UN09350	Rsa1.0_00398.1	861	88007	1.00E-141	270	274	98	+	588	860	28102	28375
UN09351	Rsa1.0_01144.1	1626	52431	0	414	420	98	−	2	420	42305	42724
UN09352	Rsa1.0_01806.1	1124	39021	0	567	569	99	−	1	569	24551	25118
UN09353	Rsa1.0_02152.1	884	34558	0	597	603	99	+	3	603	26442	27044
UN09354	Rsa1.0_06911.1	2022	10178	0	599	609	98	−	861	1469	9273	9881
UN09355	Rsa1.0_02392.1	1369	152517	1.00E-163	298	300	99	+	6	305	7771	8070
UN09356	Rsa1.0_29217.1	622	789	1.00E-104	250	268	93	+	353	618	335	602
UN09357	Rsa1.0_08909.1	477	6799	8.00E-96	246	264	93	−	14	270	5387	5646
UN09358	Rsa1.0_01388.1	1016	46741	1.00E-128	321	349	91	+	1	341	16555	16900

UN09359	Rsa1.0_00055.1	871	520193	0	695	719	96	+	152	867	339997	340715
UN09360	Rsa1.0_01574.1	1136	42930	0	672	673	99	+	29	701	17701	18373
UN09361	Rsa1.0_02377.1	902	31994	1.00E-160	361	382	94	+	2	377	17523	17902
UN09362	Rsa1.0_00050.1	1920	237174	0	418	440	95	−	1488	1919	33927	34366
UN09363	Rsa1.0_05973.1	1132	12065	0	1061	1136	93	−	1	1129	3811	4935
UN09364	Rsa1.0_06373.1	942	11138	1.00E-148	298	307	97	−	641	942	727	1033
UN09365	Rsa1.0_02108.1	1336	163029	0	637	643	99	+	693	1335	154209	154848
UN09366	Rsa1.0_00708.1	1496	67366	0	410	426	96	−	285	710	13811	14236
UN09367	Rsa1.0_03818.1	1259	20668	1.00E-121	303	327	92	−	934	1256	6603	6923
UN09368	Rsa1.0_36506.1	704	618	1.00E-152	297	305	97	+	3	307	314	618
UN09369	Rsa1.0_00134.1	1308	390219	0	407	407	100	+	902	1308	381305	381711
UN09370	Rsa1.0_00178.1	1530	114861	0	796	804	99	+	727	1530	67460	68258
UN09371	Rsa1.0_01388.1	973	46741	1.00E-120	274	291	94	+	15	302	16613	16900
UN09372	Rsa1.0_00321.1	1360	95671	0	408	419	97	−	1	419	21420	21835
UN09373	Rsa1.0_00798.1	968	63945	0	504	510	98	+	459	968	43764	44273
UN09374	Rsa1.0_12060.1	958	5328	1.00E-138	253	254	99	−	3	256	97	350
UN09375	Rsa1.0_00868.1	1163	61630	0	445	450	98	+	717	1163	32742	33191
UN09376	Rsa1.0_02138.1	608	34695	1.00E-171	315	317	99	−	3	318	7722	8038
UN09377	Rsa1.0_09709.1	2137	6198	0	661	662	99	+	1	662	4025	4686
UN09378	Rsa1.0_00594.1	1703	73729	0	889	905	98	−	800	1703	4073	4973
UN09379	Rsa1.0_00193.1	1387	194198	0	433	441	98	−	414	854	98615	99055
UN09380	Rsa1.0_00260.1	1226	102021	0	902	940	95	−	4	938	26336	27275
UN09381	Rsa1.0_04281.1	1620	18107	0	1122	1124	99	−	497	1620	15446	16568
UN09382	Rsa1.0_15993.1	646	2329	0	407	419	97	+	220	638	1900	2315
UN09383	Rsa1.0_06408.1	1198	11070	0	345	348	99	−	10	357	1608	1955
UN09384	Rsa1.0_01438.1	601	45882	0	388	401	96	+	3	403	32818	33216
UN09385	Rsa1.0_00541.1	885	76863	1.00E-119	254	265	95	+	1	265	9084	9341
UN09386	Rsa1.0_01156.1	796	52147	1.00E-132	283	299	94	+	180	469	33026	33324
UN09387	Rsa1.0_01458.1	954	175719	0	362	371	97	−	585	952	33186	33554
UN09388	Rsa1.0_04126.1	1171	18939	0	548	561	97	−	2	560	17402	17959
UN09389	Rsa1.0_01097.1	548	173349	8.00E-81	156	157	99	+	189	345	8872	9028
UN09390	Rsa1.0_01876.1	1119	37800	0	351	356	98	−	1	356	9471	9825
UN09391	Rsa1.0_00950.1	694	58547	1.00E-76	179	187	95	+	3	186	54197	54380
UN09392	Rsa1.0_00451.1	917	322533	0	341	343	99	−	573	915	48180	48522
UN09393	Rsa1.0_00033.1	977	515811	7.00E-98	262	289	90	−	94	380	462808	463082
UN09394	Rsa1.0_03607.1	1230	21817	1.00E-129	287	301	95	+	928	1226	3939	4237
UN09395	Rsa1.0_03001.1	769	26108	1.00E-125	276	292	94	−	483	769	9416	9707
UN09396	Rsa1.0_03738.1	749	21071	0	738	744	99	+	3	746	19580	20323
UN09397	Rsa1.0_00519.1	1092	197864	0	416	423	98	−	673	1090	191658	192080

UN09398	Rsa1.0_00655.1	912	195840	1.00E-163	368	390	94	+	525	912	163505	163892
UN09399	Rsa1.0_07935.1	1561	8229	0	1282	1334	96	-	3	1321	6844	8177
UN09400	Rsa1.0_00059.1	637	159650	1.00E-129	301	319	94	-	319	634	123644	123957
UN09401	Rsa1.0_00339.1	1121	222974	7.00E-92	181	184	98	-	907	1090	39246	39429
UN09402	Rsa1.0_02064.1	961	35476	1.00E-102	285	311	91	-	3	306	9520	9825
UN09403	Rsa1.0_20672.1	1102	1281	1.00E-133	242	242	100	-	457	698	1020	1261
UN09404	Rsa1.0_00271.1	807	101125	0	804	809	99	+	1	806	90528	91336
UN09405	Rsa1.0_01546.1	956	43586	0	954	956	99	+	3	956	30240	31195
UN09406	Rsa1.0_00203.1	1108	109129	3.00E-82	183	192	95	+	138	329	21777	21968
UN09407	Rsa1.0_07030.1	940	10008	3.00E-47	158	178	88	-	324	497	5301	5478
UN09408	Rsa1.0_00058.1	1445	159921	1.00E-120	231	235	98	+	759	993	92939	93173
UN09409	Rsa1.0_00094.1	2069	139042	0	772	803	96	+	70	866	90319	91115
UN09410	Rsa1.0_00593.1	526	143463	1.00E-133	240	240	100	-	287	526	60989	61228
UN09411	Rsa1.0_11621.1	625	5457	1.00E-166	308	312	98	-	2	313	601	912
UN09412	Rsa1.0_01023.1	1707	56571	1.00E-117	221	223	99	-	1094	1316	51375	51597
UN09413	Rsa1.0_02176.1	598	34291	0	590	596	98	-	1	596	21001	21596
UN09414	Rsa1.0_01054.1	921	55470	0	916	918	99	-	3	920	23592	24509
UN09415	Rsa1.0_01263.1	494	49703	1.00E-85	164	165	99	+	70	234	39806	39970
UN09416	Rsa1.0_04655.1	1701	16495	0	625	625	100	+	1077	1701	11787	12411
UN09417	Rsa1.0_04027.1	591	19501	1.00E-161	366	387	94	-	1	380	6649	7033
UN09418	Rsa1.0_00301.1	679	365315	0	427	430	99	-	249	676	207737	208166
UN09419	Rsa1.0_00457.1	1485	337301	0	584	588	99	-	896	1483	334144	334731
UN09420	Rsa1.0_01625.1	715	42019	1.00E-116	267	280	95	-	438	715	20455	20732
UN09421	Rsa1.0_00157.1	924	245832	0	407	422	96	+	503	924	160498	160908
UN09422	Rsa1.0_01553.1	630	43379	1.00E-140	253	253	100	+	68	320	14846	15098
UN09423	Rsa1.0_07389.1	850	9479	0	480	510	94	-	339	848	555	1045
UN09424	Rsa1.0_01926.1	1304	37206	0	463	464	99	+	840	1303	17508	17971
UN09425	Rsa1.0_00201.1	1627	232851	0	389	403	96	+	748	1148	4150	4548
UN09426	Rsa1.0_00275.1	1314	210104	1.00E-166	304	305	99	+	8	311	176437	176741
UN09427	Rsa1.0_00341.1	670	249309	1.00E-107	206	209	98	-	189	397	128945	129153
UN09428	Rsa1.0_00035.1	1465	176449	0	1268	1332	95	-	134	1464	51613	52916
UN09429	Rsa1.0_00999.1	1411	57162	1.00E-150	285	290	98	+	2	291	14247	14536
UN09430	Rsa1.0_02074.1	1190	35370	0	527	532	99	+	9	540	11996	12527
UN09431	Rsa1.0_00128.1	855	228675	0	846	853	99	-	3	855	185139	185991
UN09432	Rsa1.0_01635.1	742	41865	0	707	720	98	-	3	722	15877	16592
UN09433	Rsa1.0_01955.1	1255	36850	1.00E-169	454	497	91	+	211	701	13073	13563
UN09434	Rsa1.0_00813.1	1370	63202	1.00E-167	326	335	97	-	1040	1370	10828	11162
UN09435	Rsa1.0_27069.1	430	867	0	367	380	96	+	56	430	31	404
UN09436	Rsa1.0_16574.1	798	2106	0	372	378	98	+	323	700	1729	2106

UN09437	Rsa1.0_04004.1	491	19594	0	466	466	100	-	3	468	15169	15634
UN09438	Rsa1.0_02648.1	745	29073	1.00E-175	368	390	94	+	360	745	12514	12887
UN09439	Rsa1.0_00014.1	719	348985	0	564	571	98	-	149	719	241634	242204
UN09440	Rsa1.0_02113.1	744	34890	0	734	757	96	-	2	744	22769	23525
UN09441	Rsa1.0_04411.1	1300	142979	0	392	414	94	+	260	661	127303	127716
UN09442	Rsa1.0_02768.1	1723	28110	0	387	388	99	+	1332	1719	26304	26691
UN09443	Rsa1.0_00228.1	546	105511	0	477	482	98	-	65	546	13065	13545
UN09444	Rsa1.0_01235.1	605	50180	0	573	603	95	-	3	605	21049	21646
UN09445	Rsa1.0_00761.1	2377	65133	0	894	922	96	-	1454	2365	23616	24535
UN09446	Rsa1.0_01453.1	894	45449	0	803	840	95	+	43	880	28437	29273
UN09447	Rsa1.0_01630.1	916	41913	1.00E-155	281	282	99	-	633	914	923	1204
UN09448	Rsa1.0_00047.1	1075	168582	0	551	552	99	+	1	551	33970	34521
UN09449	Rsa1.0_00145.1	1491	261008	0	507	539	94	+	390	913	83077	83607
UN09450	Rsa1.0_18611.1	1297	1565	0	403	412	97	-	1	412	957	1368
UN09451	Rsa1.0_01896.1	800	37512	0	581	610	95	+	191	800	19518	20127
UN09452	Rsa1.0_00083.1	1069	442773	0	951	954	99	+	5	958	359476	360429
UN09453	Rsa1.0_00275.1	998	210104	1.00E-114	210	210	100	+	79	288	164586	164795
UN09454	Rsa1.0_00971.1	1216	57973	0	895	899	99	-	316	1214	47270	48167
UN09455	Rsa1.0_00760.1	959	65267	6.00E-86	247	277	89	-	677	953	51860	52116
UN09456	Rsa1.0_19592.1	1332	1410	0	482	488	98	-	753	1240	1	488
UN09457	Rsa1.0_00018.1	620	410899	0	500	521	95	+	97	608	140603	141116
UN09458	Rsa1.0_05443.1	720	13737	1.00E-113	221	225	98	+	496	720	4067	4288
UN09459	Rsa1.0_01985.1	1396	36491	0	1269	1282	98	+	115	1396	25011	26292
UN09460	Rsa1.0_00323.1	1068	159650	0	570	577	98	+	3	579	138237	138813
UN09461	Rsa1.0_02123.1	496	149053	0	493	493	100	-	4	496	1171	1663
UN09462	Rsa1.0_02898.1	1465	26958	0	384	387	99	+	100	486	4910	5296
UN09463	Rsa1.0_00245.1	847	103569	0	454	475	95	+	1	474	55038	55503
UN09464	Rsa1.0_01245.1	973	149868	0	641	647	99	+	328	973	45960	46606
UN09465	Rsa1.0_02275.1	1040	33107	0	496	505	98	+	539	1040	30103	30606
UN09466	Rsa1.0_00796.1	1805	63997	0	335	335	100	+	1	335	53537	53871
UN09467	Rsa1.0_01389.1	909	46716	0	382	395	96	+	3	397	9775	10165
UN09468	Rsa1.0_01340.1	1242	47805	0	347	351	98	-	892	1242	12797	13147
UN09469	Rsa1.0_02855.1	890	27271	0	559	572	97	-	2	573	10684	11254
UN09470	Rsa1.0_00002.1	1418	601661	0	330	330	100	+	3	332	114970	115299
UN09471	#	#	#	#	#	#	#	#	#	#	#	#
UN09472	Rsa1.0_00100.1	2112	249232	1.00E-106	216	220	98	+	378	596	115618	115836
UN09473	Rsa1.0_03191.1	979	145762	0	357	359	99	+	621	979	16325	16683
UN09474	Rsa1.0_04110.1	764	18998	1.00E-128	232	232	100	-	1	232	6562	6793
UN09475	Rsa1.0_00335.1	1499	94371	3.00E-73	175	185	94	-	1268	1452	35199	35382

UN09476	Rsa1.0_01196.1	872	51097	1.00E-171	352	370	95	-	501	870	32479	32837
UN09477	Rsa1.0_00272.1	1070	447836	0	593	625	94	+	444	1068	78070	78673
UN09478	Rsa1.0_01295.1	999	49040	0	398	405	98	-	595	999	40213	40616
UN09479	Rsa1.0_00088.1	767	141341	1.00E-114	221	225	98	+	354	578	19396	19620
UN09480	Rsa1.0_02139.1	812	34686	1.00E-132	337	364	92	-	448	805	5551	5912
UN09481	Rsa1.0_00017.1	422	752458	1.00E-127	267	277	96	-	11	287	539206	539479
UN09482	Rsa1.0_00438.1	605	84861	1.00E-156	285	287	99	-	36	322	2602	2888
UN09483	Rsa1.0_01387.1	950	46745	1.00E-145	262	262	100	+	1	262	31059	31320
UN09484	Rsa1.0_04886.1	1113	15597	0	669	674	99	+	439	1112	2209	2882
UN09485	Rsa1.0_00386.1	1847	89134	1.00E-171	350	365	95	+	633	997	42823	43187
UN09486	Rsa1.0_00713.1	1085	158017	0	1078	1083	99	+	1	1083	38893	39974
UN09487	Rsa1.0_02872.1	1105	27080	0	499	519	96	-	568	1080	11232	11749
UN09488	Rsa1.0_00114.1	936	131330	1.00E-140	289	301	96	+	452	752	117617	117917
UN09489	Rsa1.0_08988.1	1488	6732	0	597	649	91	-	856	1486	612	1243
UN09490	Rsa1.0_02018.1	657	35994	0	645	646	99	+	4	649	4756	5401
UN09491	Rsa1.0_00354.1	834	232005	1.00E-114	258	272	94	+	565	833	170578	170849
UN09492	Rsa1.0_01373.1	660	47052	0	580	589	98	+	1	589	31451	32039
UN09493	Rsa1.0_00789.1	1090	64233	1.00E-132	246	248	99	-	3	250	44999	45246
UN09494	Rsa1.0_17016.1	1007	1952	1.00E-171	374	395	94	-	596	987	220	613
UN09495	Rsa1.0_00027.1	970	283362	0	448	479	93	+	3	480	56539	57005
UN09496	Rsa1.0_00485.1	1274	80937	0	518	533	97	-	605	1137	30531	31063
UN09497	Rsa1.0_08165.1	840	7806	1.00E-101	288	317	90	-	2	309	2878	3192
UN09498	Rsa1.0_02152.1	729	34558	0	594	625	95	-	117	729	26446	27067
UN09499	Rsa1.0_21881.1	945	1167	1.00E-143	276	281	98	-	2	280	13	293
UN09500	Rsa1.0_00025.1	695	277312	0	679	683	99	+	14	695	39139	39821
UN09501	Rsa1.0_00035.1	496	176449	1.00E-150	335	353	94	-	1	352	156806	157155
UN09502	Rsa1.0_00276.1	754	100160	0	745	753	98	-	3	754	89077	89829
UN09503	Rsa1.0_00089.1	1039	406142	0	333	335	99	+	3	337	162002	162336
UN09504	Rsa1.0_01199.1	819	163576	1.00E-170	303	303	100	-	517	819	124415	124717
UN09505	Rsa1.0_02253.1	1343	33356	1.00E-131	251	254	98	-	1091	1343	23321	23574
UN09506	Rsa1.0_45348.1	311	497	1.00E-110	240	253	94	-	59	311	237	489
UN09507	Rsa1.0_00354.1	822	232005	1.00E-114	262	279	93	+	546	822	170578	170849
UN09508	Rsa1.0_02923.1	832	26736	1.00E-127	329	356	92	+	56	407	15750	16101
UN09509	Rsa1.0_03408.1	1832	23154	1.00E-167	444	494	89	-	70	543	3835	4328
UN09510	Rsa1.0_02449.1	1076	31292	1.00E-84	194	200	97	-	879	1075	13261	13459
UN09511	Rsa1.0_01774.1	749	39528	1.00E-168	316	321	98	+	3	323	23015	23331
UN09512	Rsa1.0_00009.1	570	232558	0	431	437	98	-	135	570	184896	185329
UN09513	Rsa1.0_00329.1	1347	222544	0	652	690	94	-	677	1347	193045	193732
UN09514	Rsa1.0_01296.1	788	49035	1.00E-161	371	398	93	-	403	788	35557	35953

UN09515	Rsa1.0_00098.1	849	349239	0	399	419	95	+	23	434	228880	229298
UN09516	Rsa1.0_00082.1	915	237584	0	325	325	100	+	591	915	38549	38873
UN09517	Rsa1.0_02349.1	1103	32312	0	644	668	96	+	1	668	14862	15510
UN09518	Rsa1.0_15363.1	454	2655	0	393	405	97	+	53	454	1072	1476
UN09519	Rsa1.0_06898.1	1134	10191	0	332	332	100	+	1	332	3152	3483
UN09520	Rsa1.0_01007.1	778	56933	0	732	765	95	-	20	777	6796	7545
UN09521	Rsa1.0_00028.1	1878	293984	0	358	358	100	+	296	653	20828	21185
UN09522	Rsa1.0_07345.1	925	9526	0	914	922	99	-	3	924	6239	7160
UN09523	Rsa1.0_00015.1	827	561803	0	611	645	94	-	3	641	185641	186284
UN09524	Rsa1.0_05812.1	1047	12571	0	962	995	96	+	3	997	2554	3548
UN09525	Rsa1.0_01022.1	2061	56606	0	484	494	97	+	893	1386	28582	29075
UN09526	Rsa1.0_00200.1	709	162016	0	497	498	99	-	6	502	119257	119754
UN09527	Rsa1.0_00065.1	1490	454616	0	441	454	97	+	1038	1488	213563	214016
UN09528	Rsa1.0_02252.1	1131	33375	0	495	508	97	-	523	1030	27492	27999
UN09529	Rsa1.0_06513.1	1134	10869	1.00E-103	191	191	100	-	868	1058	1	191
UN09530	Rsa1.0_06803.1	1190	10330	1.00E-121	269	278	96	+	22	295	7266	7542
UN09531	Rsa1.0_00419.1	771	86562	1.00E-165	302	303	99	+	470	771	6011	6313
UN09532	Rsa1.0_00410.1	927	86957	1.00E-167	323	330	97	-	599	927	39714	40043
UN09533	Rsa1.0_00007.1	841	239325	1.00E-125	250	256	97	-	580	834	30263	30518
UN09534	Rsa1.0_00221.1	1101	106972	0	531	546	97	+	63	608	37064	37609
UN09535	Rsa1.0_01660.1	1293	41400	1.00E-144	438	492	89	+	804	1293	32286	32760
UN09536	Rsa1.0_01815.1	2439	38897	3.00E-37	81	81	100	-	381	461	23439	23519
UN09537	Rsa1.0_02494.1	1617	132923	0	874	918	95	+	446	1363	4498	5410
UN09538	Rsa1.0_00851.1	1318	62181	1.00E-69	173	182	95	-	1	181	29745	29923
UN09539	Rsa1.0_00153.1	1330	192931	0	1064	1072	99	+	1	1072	13929	15000
UN09540	Rsa1.0_00201.1	1270	232851	0	1181	1237	95	-	1	1229	208685	209911
UN09541	Rsa1.0_01336.1	922	47853	0	738	747	98	-	1	747	39106	39852
UN09542	Rsa1.0_03853.1	812	20447	0	348	348	100	-	11	358	14042	14389
UN09543	Rsa1.0_23843.1	1313	1022	0	693	719	96	+	561	1269	1	717
UN09544	Rsa1.0_07439.1	3141	9392	1.00E-148	268	268	100	+	329	596	4581	4848
UN09545	Rsa1.0_01403.1	782	46453	1.00E-132	273	284	96	-	398	681	32717	33000
UN09546	Rsa1.0_00434.1	826	85171	0	425	434	97	+	394	826	69751	70184
UN09547	Rsa1.0_00900.1	1223	60367	0	551	569	96	-	653	1220	17378	17936
UN09548	Rsa1.0_00727.1	663	66724	1.00E-107	201	202	99	-	180	381	15710	15911
UN09549	Rsa1.0_00351.1	747	413096	0	414	434	95	+	1	428	244397	244827
UN09550	Rsa1.0_00107.1	1007	431659	0	573	577	99	-	1	575	196976	197552
UN09551	Rsa1.0_00123.1	872	325650	1.00E-107	294	326	90	+	415	740	305227	305552
UN09552	Rsa1.0_33464.1	975	678	1.00E-148	278	282	98	-	208	489	154	435
UN09553	Rsa1.0_01448.1	943	45645	5.00E-71	154	159	96	-	22	180	41275	41430

UN09554	Rsa1.0_01007.1	955	56933	0	692	721	95	−	224	943	48054	48774
UN09555	Rsa1.0_06541.1	752	10806	1.00E−110	315	345	91	−	385	714	10030	10372
UN09556	Rsa1.0_36204.1	914	623	1.00E−110	209	211	99	−	418	628	154	364
UN09557	Rsa1.0_00247.1	1068	103408	0	388	402	96	−	3	396	66969	67369
UN09558	Rsa1.0_00017.1	1399	752458	0	1391	1395	99	+	2	1396	285204	286594
UN09559	Rsa1.0_05141.1	489	14710	0	347	348	99	−	143	489	5485	5832
UN09560	Rsa1.0_02105.1	1012	156953	1.00E−177	354	367	96	−	242	608	149700	150066
UN09561	Rsa1.0_02248.1	847	33414	0	361	361	100	+	1	361	26046	26406
UN09562	Rsa1.0_00467.1	1081	156542	0	549	562	97	−	6	564	77638	78199
UN09563	Rsa1.0_02966.1	996	135126	0	515	532	96	−	2	528	17972	18502
UN09564	Rsa1.0_00226.1	1293	105884	1.00E−101	188	188	100	−	1106	1293	79137	79324
UN09565	Rsa1.0_02248.1	832	33414	1.00E−163	342	360	95	+	1	351	26047	26406
UN09566	Rsa1.0_03911.1	1510	20133	0	584	584	100	+	927	1510	16851	17434
UN09567	Rsa1.0_02625.1	1098	139104	0	1050	1051	99	−	43	1093	24665	25715
UN09568	Rsa1.0_00243.1	757	103651	1.00E−175	343	352	97	−	1	352	23261	23606
UN09569	Rsa1.0_00977.1	1301	198001	0	1211	1309	92	+	3	1285	47149	48442
UN09570	Rsa1.0_04649.1	1195	16538	0	418	420	99	−	776	1195	10582	11001
UN09571	#	#	#	#	#	#	#	#	#	#	#	#
UN09572	Rsa1.0_02922.1	925	26753	1.00E−154	357	383	93	+	1	381	12322	12689
UN09573	Rsa1.0_00422.1	1348	86144	0	569	610	93	−	331	926	83575	84184
UN09574	Rsa1.0_02722.1	1094	28417	0	403	422	95	+	1	416	266	687
UN09575	Rsa1.0_01881.1	1383	37721	0	1353	1381	97	−	3	1383	7714	9090
UN09576	Rsa1.0_00152.1	1004	257730	0	568	572	99	−	1	572	256945	257513
UN09577	Rsa1.0_00020.1	1475	283099	0	427	430	99	+	3	432	89927	90353
UN09578	Rsa1.0_00359.1	1016	91256	0	435	470	92	−	309	778	47430	47899
UN09579	Rsa1.0_00313.1	738	96745	0	321	321	100	+	414	734	79688	80008
UN09580	Rsa1.0_00092.1	1307	659932	0	350	358	97	−	956	1306	634946	635303
UN09581	Rsa1.0_02671.1	1177	28887	1.00E−151	322	339	94	+	5	343	9126	9449
UN09582	Rsa1.0_03231.1	1428	24456	0	428	428	100	−	3	430	23696	24123
UN09583	Rsa1.0_07894.1	1437	8347	0	861	879	97	+	1	878	5528	6406
UN09584	Rsa1.0_09284.1	764	6496	1.00E−132	246	248	99	+	517	764	589	836
UN09585	Rsa1.0_02065.1	1101	35467	0	410	413	99	+	691	1101	15761	16173
UN09586	Rsa1.0_00179.1	1078	393324	1.00E−145	357	389	91	−	696	1064	51785	52173
UN09587	Rsa1.0_03241.1	1067	24423	0	484	489	98	+	579	1067	14637	15125
UN09588	Rsa1.0_00307.1	1798	199003	0	831	868	95	+	1	867	170975	171841
UN09589	Rsa1.0_02658.1	1282	28978	0	358	364	98	−	907	1268	17720	18083
UN09590	Rsa1.0_00194.1	1061	110851	1.00E−62	168	182	92	−	880	1061	100172	100351
UN09591	Rsa1.0_24098.1	940	1007	1.00E−172	340	351	96	−	589	939	597	947
UN09592	Rsa1.0_03555.1	1620	22129	0	658	674	97	+	164	837	16383	17056

UN09593	Rsa1.0_00078.1	1762	175053	0	1454	1512	96	−	207	1715	78925	80430
UN09594	Rsa1.0_00473.1	1339	212204	0	774	789	98	−	1	789	76768	77555
UN09595	Rsa1.0_01166.1	1215	51877	0	599	618	96	+	3	620	36965	37579
UN09596	Rsa1.0_00529.1	951	77487	1.00E−151	296	304	97	+	652	951	23092	23395
UN09597	Rsa1.0_00379.1	808	89865	1.00E−150	382	423	90	−	3	416	52174	52575
UN09598	Rsa1.0_00844.1	1289	62403	0	571	580	98	+	711	1289	19070	19646
UN09599	Rsa1.0_03887.1	1130	20247	0	1004	1037	96	−	102	1130	17980	19014
UN09600	Rsa1.0_00097.1	889	138858	0	430	446	96	−	1	441	42576	43021
UN09601	Rsa1.0_08668.1	1529	7059	1.00E−114	212	213	99	+	472	684	5662	5874
UN09602	Rsa1.0_03552.1	1527	22151	0	511	519	98	−	1	519	15663	16181
UN09603	Rsa1.0_00866.1	1520	61676	0	626	662	94	−	1	649	30853	31511
UN09604	Rsa1.0_00349.1	1149	92621	0	1047	1086	96	+	66	1149	691	1773
UN09605	Rsa1.0_01668.1	903	41299	1.00E−108	266	288	92	−	125	412	40402	40689
UN09606	Rsa1.0_01502.1	1112	44519	1.00E−149	330	349	94	+	765	1111	39463	39808
UN09607	Rsa1.0_01077.1	1136	54772	0	583	606	96	−	534	1136	10739	11340
UN09608	Rsa1.0_00642.1	1090	201230	0	615	635	96	−	1	635	180069	180702
UN09609	Rsa1.0_01034.1	660	56046	0	356	367	97	−	60	426	35993	36359
UN09610	Rsa1.0_00501.1	1045	79545	1.00E−173	315	317	99	+	1	317	73172	73488
UN09611	Rsa1.0_01541.1	841	43682	0	413	436	94	−	8	442	1663	2095
UN09612	Rsa1.0_18172.1	1375	1653	0	382	385	99	−	993	1375	719	1103
UN09613	Rsa1.0_03150.1	1460	24980	0	421	421	100	−	1	421	23709	24129
UN09614	Rsa1.0_02362.1	1223	32190	0	468	493	94	−	1	475	23950	24440
UN09615	Rsa1.0_00198.1	1245	222963	0	398	417	95	−	837	1245	37691	38100
UN09616	Rsa1.0_11462.1	1286	5500	0	1094	1153	94	−	160	1284	2183	3334
UN09617	Rsa1.0_00056.1	1409	332604	0	760	762	99	+	646	1407	24496	25257
UN09618	Rsa1.0_01492.1	952	44704	2.00E−91	236	258	91	−	14	265	6008	6265
UN09619	Rsa1.0_00896.1	901	60509	0	688	709	97	−	2	710	29277	29984
UN09620	Rsa1.0_00761.1	951	65133	1.00E−167	335	346	96	+	602	947	62157	62495
UN09621	Rsa1.0_02870.1	1250	27102	1.00E−132	255	259	98	+	1	258	12901	13159
UN09622	Rsa1.0_05106.1	1839	14828	0	649	653	99	−	685	1337	9829	10481
UN09623	Rsa1.0_00642.1	1027	201230	0	411	424	96	+	3	424	181249	181667
UN09624	Rsa1.0_02428.1	1840	31542	0	697	697	100	−	406	1102	28430	29126
UN09625	Rsa1.0_08023.1	976	8071	0	531	562	94	−	422	976	4838	5399
UN09626	Rsa1.0_00710.1	743	67325	1.00E−153	393	430	91	+	320	743	28163	28591
UN09627	Rsa1.0_03401.1	1261	23191	0	410	412	99	−	851	1261	4660	5070
UN09628	Rsa1.0_00092.1	1357	659932	0	468	504	92	+	650	1141	494917	495420
UN09629	Rsa1.0_00008.1	978	358124	0	624	630	99	−	350	978	50872	51499
UN09630	Rsa1.0_00007.1	1265	239325	0	542	572	94	+	696	1265	58424	58990
UN09631	Rsa1.0_00253.1	1871	231292	0	536	587	91	+	811	1381	86630	87195

UN09632	Rsa1.0_02125.1	825	34775	9.00E-94	182	183	99	+	574	755	24927	25109
UN09633	Rsa1.0_00253.1	1286	231292	1.00E-103	192	192	100	+	405	596	90275	90466
UN09634	Rsa1.0_04458.1	1007	17255	0	556	566	98	−	1	566	3967	4532
UN09635	Rsa1.0_02605.1	753	29637	1.00E-128	239	241	99	−	2	242	13844	14084
UN09636	Rsa1.0_00017.1	1140	752458	0	544	548	99	−	593	1139	293263	293810
UN09637	Rsa1.0_03326.1	785	23710	0	537	555	96	+	232	785	17095	17641
UN09638	Rsa1.0_02943.1	1349	153598	0	639	647	98	+	1	646	128867	129513
UN09639	Rsa1.0_00495.1	1083	79934	0	560	589	95	−	2	576	11988	12570
UN09640	Rsa1.0_02295.1	1033	149884	1.00E-127	290	310	93	−	1	303	12460	12765
UN09641	Rsa1.0_00425.1	1356	147080	0	1208	1241	97	+	114	1354	95740	96980
UN09642	Rsa1.0_00646.1	1274	70473	1.00E-163	377	407	92	+	3	406	22444	22838
UN09643	Rsa1.0_00025.1	1518	277312	0	797	810	98	−	1	810	226330	227135
UN09644	Rsa1.0_02505.1	1084	30703	1.00E-145	270	273	98	+	113	385	10093	10365
UN09645	Rsa1.0_05282.1	1615	14270	1.00E-172	334	343	97	+	1	343	4132	4474
UN09646	Rsa1.0_08575.1	1549	7180	0	458	458	100	+	249	706	5922	6379
UN09647	Rsa1.0_00032.1	972	306326	0	940	974	96	−	1	970	144545	145509
UN09648	Rsa1.0_00336.1	1173	445051	1.00E-152	302	309	97	−	1	307	218905	219212
UN09649	Rsa1.0_00925.1	995	59481	0	566	566	100	+	430	995	13819	14384
UN09650	Rsa1.0_03608.1	1357	21804	0	525	556	94	−	813	1356	924	1478
UN09651	Rsa1.0_00522.1	1158	78103	1.00E-105	209	214	97	−	592	805	48991	49204
UN09652	Rsa1.0_03344.1	1031	23593	1.00E-120	292	315	92	+	717	1031	12303	12615
UN09653	Rsa1.0_01588.1	1168	42724	0	462	466	99	−	701	1166	10024	10489
UN09654	Rsa1.0_04474.1	885	17191	0	393	395	99	−	490	884	15117	15511
UN09655	Rsa1.0_01839.1	1170	38482	0	1086	1117	97	−	2	1118	9010	10126
UN09656	Rsa1.0_00214.1	1693	107508	0	782	797	98	+	891	1687	103190	103982
UN09657	Rsa1.0_01151.1	769	52268	1.00E-106	199	200	99	+	63	262	38493	38692
UN09658	Rsa1.0_03955.1	842	19884	0	384	392	97	−	449	840	12308	12697
UN09659	Rsa1.0_14912.1	944	2970	0	342	344	99	+	600	943	1944	2287
UN09660	Rsa1.0_00362.1	719	299497	1.00E-174	343	352	97	+	371	719	228930	229281
UN09661	Rsa1.0_33878.1	795	669	1.00E-128	265	272	97	+	423	693	161	430
UN09662	Rsa1.0_00348.1	1411	92818	0	376	377	99	−	10	386	65410	65786
UN09663	Rsa1.0_01119.1	1279	53459	1.00E-120	233	236	98	+	1035	1269	23849	24084
UN09664	Rsa1.0_00049.1	1727	167018	0	1225	1246	98	+	482	1726	74435	75680
UN09665	Rsa1.0_03506.1	1382	22416	0	527	548	96	−	1	542	8413	8952
UN09666	Rsa1.0_00088.1	783	141341	0	767	780	98	−	4	783	44467	45238
UN09667	Rsa1.0_05792.1	757	12612	1.00E-173	336	344	97	+	414	757	1150	1492
UN09668	Rsa1.0_05415.1	523	13830	0	515	519	99	+	4	522	6191	6709
UN09669	Rsa1.0_04258.1	831	18254	0	425	439	96	−	394	829	2338	2776
UN09670	Rsa1.0_00179.1	1094	393324	1.00E-71	255	294	86	−	259	552	19880	20173

UN09671	Rsa1.0_02658.1	951	28978	0	405	407	99	+	1	406	5440	5846
UN09672	Rsa1.0_04220.1	863	18421	0	510	530	96	+	1	528	9467	9982
UN09673	Rsa1.0_00107.1	2470	431659	0	1498	1523	98	+	640	2160	13765	15287
UN09674	Rsa1.0_02248.1	884	33414	0	355	361	98	+	1	361	26046	26406
UN09675	Rsa1.0_00315.1	1509	96426	0	1286	1287	99	+	155	1441	89766	91052
UN09676	Rsa1.0_02020.1	1105	173211	0	572	575	99	+	1	575	153343	153915
UN09677	Rsa1.0_00108.1	980	478491	1.00E-120	288	301	95	−	3	298	51602	51897
UN09678	Rsa1.0_01170.1	815	161131	0	385	394	97	+	3	395	128366	128759
UN09679	Rsa1.0_00832.1	1819	62622	0	959	1047	91	−	600	1646	34580	35626
UN09680	Rsa1.0_00042.1	1172	171507	0	527	550	95	−	635	1171	55333	55880
UN09681	Rsa1.0_01868.1	984	150904	1.00E-147	288	296	97	+	107	402	138768	139063
UN09682	Rsa1.0_01341.1	2402	47764	0	899	901	99	+	1502	2402	44618	45518
UN09683	Rsa1.0_00002.1	515	601661	4.00E-64	134	137	97	+	15	151	242456	242592
UN09684	Rsa1.0_00223.1	856	106231	0	735	746	98	−	113	856	11528	12273
UN09685	Rsa1.0_02039.1	1190	35699	0	1161	1193	97	+	1	1174	19033	20224
UN09686	Rsa1.0_38361.1	877	587	0	437	437	100	−	370	806	1	437
UN09687	Rsa1.0_00480.1	738	81325	1.00E-159	288	289	99	+	3	291	619	907
UN09688	Rsa1.0_02105.1	1059	156953	0	367	367	100	−	270	636	149700	150066
UN09689	Rsa1.0_00816.1	811	403350	0	364	368	98	−	428	795	267878	268245
UN09690	Rsa1.0_00382.1	1118	251946	0	499	504	99	+	1	503	172844	173347
UN09691	Rsa1.0_00741.1	1195	66117	1.00E-164	351	371	94	−	832	1187	52571	52938
UN09692	Rsa1.0_01006.1	661	163670	1.00E-156	288	291	98	−	174	464	138108	138398
UN09693	Rsa1.0_03433.1	1116	22966	0	631	647	97	−	3	649	11809	12446
UN09694	Rsa1.0_07429.1	1087	9411	0	505	505	100	+	157	661	7	511
UN09695	Rsa1.0_00003.1	2113	340054	0	686	706	97	+	5	710	8264	8966
UN09696	Rsa1.0_01080.1	1177	54752	0	553	557	99	−	610	1166	7919	8475
UN09697	Rsa1.0_00322.1	1740	212233	0	487	491	99	−	1248	1738	4310	4800
UN09698	Rsa1.0_00577.1	1508	174827	0	353	353	100	−	1	353	148897	149249
UN09699	Rsa1.0_00186.1	1550	112669	0	1251	1259	99	+	292	1550	106712	107970
UN09700	Rsa1.0_01036.1	540	55978	1.00E-125	255	262	97	−	281	540	26497	26757
UN09701	Rsa1.0_08848.1	455	6865	1.00E-106	195	195	100	−	1	195	1	195
UN09702	Rsa1.0_01845.1	967	38415	1.00E-114	216	218	99	−	320	537	36772	36989
UN09703	Rsa1.0_00425.1	816	147080	0	395	396	99	+	421	816	17416	17811
UN09704	Rsa1.0_00159.1	971	431686	1.00E-173	317	320	99	−	222	541	268820	269139
UN09705	Rsa1.0_02180.1	933	34176	0	442	460	96	−	1	460	27171	27626
UN09706	Rsa1.0_00031.1	803	179862	1.00E-160	306	313	97	+	1	313	129331	129637
UN09707	Rsa1.0_01023.1	870	56571	0	853	866	98	−	3	868	49493	50352
UN09708	Rsa1.0_01875.1	659	37849	1.00E-179	342	350	97	+	310	659	25024	25373
UN09709	Rsa1.0_00056.1	1134	332604	0	1130	1132	99	−	2	1132	73652	74783

UN09710	Rsa1.0_00121.1	670	278029	1.00E-106	297	325	91	+	342	662	54300	54615
UN09711	Rsa1.0_00451.1	1344	322533	1.00E-171	336	345	97	−	998	1342	283341	283684
UN09712	Rsa1.0_00990.1	1148	57445	0	1143	1149	99	+	1	1148	38652	39798
UN09713	Rsa1.0_00108.1	1663	478491	0	879	879	100	+	783	1661	460044	460922
UN09714	Rsa1.0_04254.1	698	18258	1.00E-113	258	270	95	−	433	698	5139	5406
UN09715	Rsa1.0_16553.1	418	2115	2.00E-80	161	164	98	−	176	339	106	269
UN09716	Rsa1.0_11859.1	1205	5389	0	573	573	100	−	630	1202	4393	4965
UN09717	Rsa1.0_00044.1	810	271658	1.00E-178	316	316	100	−	361	676	13553	13868
UN09718	Rsa1.0_00608.1	1052	172672	0	733	792	92	+	244	1010	3169	3956
UN09719	Rsa1.0_00490.1	849	186713	1.00E-139	313	331	94	−	6	335	153661	153985
UN09720	Rsa1.0_51905.1	707	438	1.00E-112	212	214	99	−	492	705	225	438
UN09721	Rsa1.0_00571.1	756	329018	1.00E-151	301	310	97	+	3	310	158790	159099
UN09722	Rsa1.0_00097.1	858	138858	1.00E-179	382	400	95	+	468	858	54312	54710
UN09723	Rsa1.0_00127.1	1568	127446	1.00E-160	391	424	92	+	1147	1567	21412	21828
UN09724	Rsa1.0_00492.1	1191	80178	1.00E-118	261	278	93	+	7	273	69385	69662
UN09725	Rsa1.0_02215.1	1350	33822	0	391	401	97	+	1	399	8395	8795
UN09726	Rsa1.0_00961.1	997	58185	0	594	609	97	+	135	743	1047	1655
UN09727	Rsa1.0_00066.1	472	152418	9.00E-68	134	135	99	−	123	257	140160	140294
UN09728	Rsa1.0_03795.1	1116	20818	1.00E-103	203	207	98	+	470	676	14751	14957
UN09729	Rsa1.0_00198.1	894	222963	0	595	599	99	−	296	894	176266	176864
UN09730	Rsa1.0_01005.1	822	56967	0	470	483	97	−	1	483	39743	40224
UN09731	Rsa1.0_00394.1	1275	148426	0	478	491	97	+	751	1238	67486	67976
UN09732	Rsa1.0_37428.1	1164	602	0	525	550	95	+	1	545	6	554
UN09733	Rsa1.0_00014.1	1014	348985	1.00E-142	341	362	94	+	659	1014	323900	324259
UN09734	Rsa1.0_02586.1	771	29856	0	686	716	95	+	56	771	15528	16236
UN09735	Rsa1.0_00699.1	855	184398	0	474	477	99	−	358	834	121669	122145
UN09736	Rsa1.0_00425.1	1031	147080	0	622	669	92	+	18	664	125711	126375
UN09737	Rsa1.0_03681.1	1795	21405	0	1280	1307	97	−	424	1728	664	1965
UN09738	Rsa1.0_08947.1	444	6761	0	380	386	98	+	4	389	5535	5920
UN09739	Rsa1.0_00863.1	1025	254535	1.00E-162	290	290	100	−	728	1017	239512	239801
UN09740	Rsa1.0_00525.1	2274	78030	0	1023	1024	99	−	1250	2273	47680	48703
UN09741	Rsa1.0_01046.1	980	152989	1.00E-170	306	307	99	+	489	795	128362	128668
UN09742	Rsa1.0_00168.1	1124	115870	1.00E-179	376	395	95	+	730	1124	31046	31440
UN09743	Rsa1.0_02609.1	1218	29600	0	439	473	92	−	1	458	21111	21581
UN09744	Rsa1.0_00113.1	892	131574	0	383	391	97	−	2	392	72531	72920
UN09745	Rsa1.0_01581.1	674	42833	1.00E-165	300	302	99	+	84	385	7614	7915
UN09746	Rsa1.0_01549.1	1182	43543	0	692	705	98	−	478	1182	341	1042
UN09747	Rsa1.0_00120.1	1206	277288	1.00E-131	269	276	97	+	1	273	92236	92509
UN09748	Rsa1.0_01705.1	1079	40710	0	344	347	99	−	1	347	38579	38924

UN09749	Rsa1.0_02085.1	1282	35198	0	1247	1293	96	+	3	1282	27913	29201
UN09750	Rsa1.0_00389.1	713	88795	1.00E-145	261	261	100	+	2	262	40534	40794
UN09751	Rsa1.0_07066.1	1503	9954	1.00E-161	327	340	96	+	2	341	5833	6161
UN09752	Rsa1.0_00048.1	1479	167185	2.33E-156	267	268	99	−	3	270	154401	154668
UN09753	Rsa1.0_01895.1	1138	37512	1.00E-105	266	288	92	−	3	290	34522	34794
UN09754	Rsa1.0_00065.1	1029	454616	1.00E-112	306	336	91	+	704	1027	438582	438914
UN09755	Rsa1.0_01926.1	1070	37206	0	601	613	98	+	71	683	13122	13734
UN09756	Rsa1.0_00198.1	1182	222963	0	573	576	99	+	607	1182	35760	36335
UN09757	Rsa1.0_01292.1	850	175783	0	626	653	95	−	146	795	144715	145360
UN09758	Rsa1.0_20013.1	961	1352	1.00E-116	213	213	100	−	746	958	294	506
UN09759	Rsa1.0_00031.1	1127	179862	0	637	639	99	+	1	639	43037	43675
UN09760	Rsa1.0_00133.1	1153	123768	1.00E-136	271	279	97	+	468	746	9256	9534
UN09761	Rsa1.0_01930.1	706	37189	0	638	645	98	−	3	643	1141	1785
UN09762	Rsa1.0_52720.1	963	432	0	430	432	99	+	112	543	1	432
UN09763	Rsa1.0_03205.1	1531	24614	0	438	439	99	+	1	439	1618	2056
UN09764	Rsa1.0_00005.1	861	353093	0	833	859	96	+	3	860	288477	289330
UN09765	Rsa1.0_01196.1	1179	51097	1.00E-150	306	317	96	−	1	317	40985	41294
UN09766	Rsa1.0_03599.1	1045	21857	0	585	593	98	−	2	594	2346	2938
UN09767	Rsa1.0_00686.1	841	68511	0	458	468	97	+	35	502	26807	27271
UN09768	Rsa1.0_00008.1	1075	358124	1.00E-111	230	237	97	+	569	804	321744	321980
UN09769	Rsa1.0_02825.1	1430	27568	0	1393	1405	99	−	1	1405	16906	18309
UN09770	Rsa1.0_00239.1	1126	104332	0	455	457	99	−	261	717	97669	98125
UN09771	Rsa1.0_00026.1	986	327835	0	451	472	95	−	484	952	240917	241382
UN09772	Rsa1.0_02753.1	674	28215	0	637	681	93	+	1	672	8063	8731
UN09773	Rsa1.0_03311.1	1231	23850	1.00E-129	253	259	97	+	4	262	17488	17746
UN09774	Rsa1.0_08518.1	1858	7244	0	574	599	95	+	1125	1723	5069	5659
UN09775	Rsa1.0_06936.1	1547	10143	0	866	908	95	+	643	1546	1	906
UN09776	Rsa1.0_04353.1	696	138437	1.00E-107	209	213	98	+	480	692	132600	132812
UN09777	Rsa1.0_00943.1	925	58862	0	569	575	98	−	351	925	6507	7081
UN09778	Rsa1.0_02969.1	1794	26390	1.00E-179	318	318	100	−	1477	1794	10032	10349
UN09779	Rsa1.0_03427.1	1520	22992	0	1190	1206	98	+	2	1204	21787	22992
UN09780	Rsa1.0_04404.1	830	17510	0	680	687	98	+	143	828	9475	10161
UN09781	Rsa1.0_03009.1	764	26065	0	357	357	100	−	402	758	2650	3006
UN09782	Rsa1.0_02314.1	1055	32642	0	528	540	97	−	515	1054	4774	5305
UN09783	Rsa1.0_10577.1	1696	5796	0	492	502	98	+	1195	1696	535	1035
UN09784	Rsa1.0_01819.1	672	38834	2.00E-38	82	82	100	+	1	82	19030	19111
UN09785	Rsa1.0_03869.1	725	20365	0	515	529	97	+	198	725	4022	4550
UN09786	Rsa1.0_00595.1	1253	341648	1.00E-137	257	260	98	−	407	666	142866	143125
UN09787	Rsa1.0_14183.1	2430	132309	0	1165	1166	99	−	305	1470	1395	2560

UN09788	Rsa1.0_06185.1	956	11540	0	410	436	94	+	182	617	3892	4327
UN09789	Rsa1.0_01529.1	1010	43871	0	400	408	98	+	30	437	11420	11821
UN09790	Rsa1.0_01175.1	734	51674	1.00E-154	338	352	96	−	387	734	35951	36301
UN09791	Rsa1.0_00026.1	566	327835	7.00E-78	151	152	99	+	127	278	207342	207493
UN09792	Rsa1.0_01655.1	768	41508	0	330	330	100	−	438	767	6891	7220
UN09793	Rsa1.0_01016.1	833	181422	0	517	524	98	−	1	523	27020	27541
UN09794	Rsa1.0_02774.1	766	28038	0	749	766	97	+	1	764	13384	14147
UN09795	Rsa1.0_00663.1	1084	164969	0	590	591	99	−	492	1082	120771	121360
UN09796	Rsa1.0_00340.1	1515	305782	1.00E-120	248	257	96	+	3	256	188899	189155
UN09797	Rsa1.0_05593.1	1618	13235	0	749	751	99	+	87	836	1140	1890
UN09798	Rsa1.0_00425.1	1136	147080	0	388	389	99	−	3	390	121247	121635
UN09799	Rsa1.0_00017.1	1192	752458	1.00E-108	225	232	96	−	924	1155	1810	2040
UN09800	Rsa1.0_06054.1	774	11847	0	400	400	100	+	351	750	1351	1750
UN09801	Rsa1.0_14535.1	627	3289	0	600	613	97	+	15	627	477	1089
UN09802	Rsa1.0_00336.1	581	445051	1.00E-125	288	309	93	+	1	309	150272	150562
UN09803	Rsa1.0_02404.1	852	31702	0	413	418	98	−	299	716	907	1324
UN09804	Rsa1.0_01402.1	771	46509	0	367	378	97	−	101	478	6199	6576
UN09805	Rsa1.0_01158.1	1436	52121	0	609	615	99	−	268	882	31439	32053
UN09806	Rsa1.0_00870.1	762	61606	1.00E-151	272	272	100	+	3	274	24795	25066
UN09807	Rsa1.0_26775.1	514	878	1.00E-153	278	279	99	+	1	279	600	878
UN09808	Rsa1.0_00632.1	826	183777	0	442	458	96	+	375	824	37301	37758
UN09809	Rsa1.0_00646.1	1153	70473	0	899	928	96	−	63	990	4449	5375
UN09810	Rsa1.0_06382.1	1321	11121	3.00E-45	100	102	98	+	993	1094	10909	11010
UN09811	Rsa1.0_00128.1	1337	228675	0	709	725	97	−	544	1266	154949	155671
UN09812	Rsa1.0_01292.1	673	175783	0	481	482	99	+	4	485	152615	153096
UN09813	Rsa1.0_00153.1	1140	192931	1.00E-122	233	235	99	−	905	1138	168976	169210
UN09814	Rsa1.0_06275.1	711	11342	0	707	709	99	+	3	711	3859	4565
UN09815	Rsa1.0_00952.1	691	58450	0	329	330	99	+	2	331	41887	42216
UN09816	Rsa1.0_00331.1	561	176754	1.00E-162	359	381	94	+	182	561	159068	159442
UN09817	Rsa1.0_01539.1	623	43698	1.00E-135	244	244	100	−	3	246	6459	6702
UN09818	Rsa1.0_00171.1	728	115622	0	362	371	97	+	355	725	52232	52602
UN09819	Rsa1.0_01101.1	1486	54031	0	696	719	96	−	415	1132	14928	15645
UN09820	Rsa1.0_03081.1	750	25523	1.00E-159	356	376	94	+	384	750	22991	23361
UN09821	Rsa1.0_05379.1	1333	13968	0	824	837	98	+	495	1331	8413	9247
UN09822	Rsa1.0_03381.1	812	23306	0	672	675	99	−	136	810	15740	16412
UN09823	Rsa1.0_01179.1	714	51549	0	709	713	99	−	3	713	42457	43169
UN09824	Rsa1.0_02979.1	1449	26298	0	934	946	98	−	504	1449	13517	14462
UN09825	Rsa1.0_00107.1	711	431659	1.00E-144	265	267	99	+	8	274	82089	82355
UN09826	Rsa1.0_02057.1	790	35540	1.00E-120	256	270	94	−	531	789	22288	22557

UN09827	Rsa1.0_00037.1	1289	307838	1.00E-145	286	294	97	+	516	809	305123	305416
UN09828	Rsa1.0_28844.1	902	801	0	647	659	98	+	243	901	1	659
UN09829	Rsa1.0_04223.1	1300	18407	1.00E-115	273	290	94	−	1004	1293	9537	9816
UN09830	Rsa1.0_02001.1	932	36198	0	578	609	94	+	333	932	31940	32544
UN09831	Rsa1.0_00251.1	1360	303968	0	534	588	90	−	799	1360	83651	84232
UN09832	Rsa1.0_03869.1	778	20365	0	558	589	94	−	1	573	4022	4610
UN09833	Rsa1.0_00192.1	1012	278200	0	652	685	95	+	328	1012	25299	25966
UN09834	Rsa1.0_05273.1	1442	14304	1.00E-144	267	268	99	+	1	267	13724	13991
UN09835	Rsa1.0_00589.1	892	209965	1.00E-153	361	384	94	+	2	381	179877	180257
UN09836	Rsa1.0_01168.1	507	153765	3.00E-52	114	117	97	−	34	150	24410	24526
UN09837	Rsa1.0_02607.1	1772	29629	0	366	366	100	−	19	384	9268	9633
UN09838	Rsa1.0_03448.1	660	22844	1.00E-117	298	323	92	+	321	634	1239	1559
UN09839	Rsa1.0_02020.1	757	173211	1.00E-128	252	256	98	+	501	755	21498	21751
UN09840	Rsa1.0_08622.1	842	7122	0	433	435	99	+	408	842	5862	6295
UN09841	Rsa1.0_03694.1	1133	21336	0	828	864	95	+	288	1132	358	1221
UN09842	Rsa1.0_06694.1	1092	10526	0	815	820	99	+	1	819	3918	4737
UN09843	Rsa1.0_00949.1	754	58549	0	743	751	98	+	3	753	54271	55021
UN09844	Rsa1.0_00130.1	1540	125737	0	561	567	98	+	963	1527	117675	118240
UN09845	Rsa1.0_01172.1	1281	51734	1.00E-167	307	310	99	−	3	312	51035	51344
UN09846	Rsa1.0_00668.1	1398	69421	7.00E-77	210	229	91	+	932	1157	54729	54951
UN09847	Rsa1.0_01861.1	956	38031	1.00E-170	345	359	96	+	293	651	32331	32689
UN09848	Rsa1.0_00635.1	743	175826	1.00E-167	311	314	99	+	430	743	15994	16306
UN09849	Rsa1.0_00005.1	1348	353093	0	348	348	100	+	1000	1347	130794	131141
UN09850	Rsa1.0_02180.1	917	34176	0	532	539	98	+	381	917	17424	17962
UN09851	Rsa1.0_00208.1	1012	108565	1.00E-111	240	248	96	−	766	1012	7723	7968
UN09852	Rsa1.0_00913.1	1282	59914	0	426	438	97	+	1	436	19782	20210
UN09853	Rsa1.0_02648.1	721	29073	0	344	347	99	−	1	345	12514	12859
UN09854	Rsa1.0_03401.1	529	23191	0	525	528	99	−	3	529	15968	16493
UN09855	Rsa1.0_00238.1	945	193193	0	534	543	98	+	3	544	192085	192627
UN09856	Rsa1.0_04319.1	1475	17944	0	515	534	96	+	942	1475	16581	17113
UN09857	Rsa1.0_03581.1	2580	21965	0	1456	1457	99	+	452	1908	1	1457
UN09858	Rsa1.0_00910.1	677	59975	0	540	578	93	+	81	647	30391	30964
UN09859	Rsa1.0_00266.1	1297	163932	0	1279	1294	98	+	3	1295	109372	110665
UN09860	Rsa1.0_00144.1	1347	120708	0	1018	1041	97	−	307	1347	47301	48338
UN09861	Rsa1.0_02939.1	1249	26595	0	523	551	94	−	1	551	18298	18839
UN09862	Rsa1.0_00017.1	1192	752458	0	981	1044	93	−	149	1192	71068	72111
UN09863	Rsa1.0_01651.1	1019	41586	1.00E-163	294	295	99	−	9	303	30453	30747
UN09864	Rsa1.0_00326.1	1372	171955	0	401	411	97	+	23	432	66179	66589
UN09865	Rsa1.0_01749.1	948	39822	0	376	391	96	+	25	412	32662	33052

UN09866	Rsa1.0_03089.1	959	25465	0	490	497	98	−	1	497	8730	9225
UN09867	Rsa1.0_01171.1	1075	51752	1.00E−147	354	378	93	−	1	371	50943	51315
UN09868	Rsa1.0_00523.1	1526	178770	0	360	370	97	−	1	369	144517	144886
UN09869	Rsa1.0_00421.1	589	86365	1.00E−61	156	165	94	−	245	408	33753	33916
UN09870	Rsa1.0_04086.1	1702	19166	0	1206	1226	98	−	2	1227	11442	12667
UN09871	Rsa1.0_00055.1	1109	520193	0	760	764	99	+	3	766	64614	65377
UN09872	Rsa1.0_01129.1	1401	52949	0	470	472	99	+	575	1046	15750	16220
UN09873	Rsa1.0_03722.1	906	21184	1.00E−148	314	329	95	−	34	359	20756	21084
UN09874	Rsa1.0_00467.1	845	156542	1.00E−176	386	407	94	−	445	845	80113	80514
UN09875	Rsa1.0_07440.1	1247	9389	0	1165	1207	96	+	3	1208	4133	5339
UN09876	Rsa1.0_00330.1	837	94666	1.00E−171	305	305	100	−	533	837	42444	42748
UN09877	Rsa1.0_05510.1	835	13470	1.00E−124	247	253	97	+	581	833	12012	12262
UN09878	Rsa1.0_05365.1	1144	14040	0	582	582	100	−	1	582	11967	12548
UN09879	Rsa1.0_09775.1	914	6156	0	420	424	99	+	3	423	4670	5093
UN09880	Rsa1.0_01595.1	804	175383	3.00E−87	176	180	97	−	81	260	5166	5345
UN09881	Rsa1.0_02750.1	1027	28239	1.00E−93	188	191	98	−	714	904	22935	23124
UN09882	Rsa1.0_05668.1	1007	12994	0	381	382	99	+	624	1005	12064	12445
UN09883	Rsa1.0_05570.1	997	13301	0	997	997	100	−	1	997	10562	11558
UN09884	Rsa1.0_00031.1	617	179862	1.00E−176	316	317	99	+	1	317	73500	73816
UN09885	Rsa1.0_00017.1	1797	752458	0	384	385	99	−	1413	1797	661834	662218
UN09886	Rsa1.0_01724.1	1365	40292	0	418	419	99	+	947	1365	37306	37724
UN09887	Rsa1.0_01327.1	1199	48033	1.00E−130	267	276	96	+	6	281	14703	14977
UN09888	Rsa1.0_03347.1	761	23588	1.00E−125	255	261	97	+	36	295	20326	20582
UN09889	Rsa1.0_01465.1	1244	45175	0	966	1037	93	+	219	1243	28909	29925
UN09890	Rsa1.0_00112.1	1533	132199	1.00E−160	358	376	95	+	1160	1529	12848	13221
UN09891	Rsa1.0_00546.1	1005	76523	1.00E−117	227	231	98	−	724	954	73168	73398
UN09892	Rsa1.0_04098.1	977	19078	1.00E−132	311	329	94	−	3	328	10291	10614
UN09893	Rsa1.0_00122.1	1473	265937	0	442	471	93	+	32	489	247401	247866
UN09894	Rsa1.0_00078.1	922	175053	0	387	392	98	−	2	390	137160	137551
UN09895	Rsa1.0_10587.1	762	5794	0	443	446	99	+	317	762	234	678
UN09896	Rsa1.0_03395.1	1245	23243	0	402	414	97	+	827	1240	21042	21453
UN09897	Rsa1.0_00656.1	859	69968	0	492	493	99	−	1	493	39109	39601
UN09898	Rsa1.0_00224.1	1026	327530	1.00E−139	340	365	93	+	4	363	228452	228815
UN09899	Rsa1.0_00710.1	559	67325	1.00E−168	324	330	98	+	1	329	65793	66119
UN09900	Rsa1.0_21831.1	899	1169	1.00E−119	230	233	98	+	208	438	793	1025
UN09901	Rsa1.0_04005.1	1573	19586	0	840	843	99	−	731	1573	2113	2955
UN09902	Rsa1.0_00240.1	1108	104175	0	365	370	98	−	4	373	45959	46327
UN09903	Rsa1.0_01976.1	926	36570	1.00E−175	320	322	99	−	605	926	25270	25589
UN09904	Rsa1.0_01997.1	702	158416	3.00E−62	163	177	92	+	4	169	7959	8135

UN09905	Rsa1.0_01536.1	811	43747	1.00E-157	319	330	96	+	410	739	28390	28718
UN09906	Rsa1.0_00678.1	1138	193163	0	1094	1133	96	+	4	1136	155003	156130
UN09907	Rsa1.0_00056.1	843	332604	0	389	391	99	+	451	841	206057	206446
UN09908	Rsa1.0_00328.1	1045	224661	0	575	575	100	+	471	1045	207122	207696
UN09909	Rsa1.0_06382.1	605	11121	1.00E-160	286	286	100	−	1	286	10119	10404
UN09910	Rsa1.0_02794.1	2088	27820	0	1409	1433	98	−	671	2088	25524	26956
UN09911	Rsa1.0_02872.1	742	27080	1.00E-128	236	237	99	−	504	740	25658	25894
UN09912	Rsa1.0_09875.1	802	6109	1.00E-141	272	278	97	−	523	800	5095	5372
UN09913	Rsa1.0_00108.1	1286	478491	0	454	480	94	−	7	472	91768	92247
UN09914	Rsa1.0_02950.1	938	26506	1.00E-136	271	279	97	−	284	562	6575	6853
UN09915	Rsa1.0_01963.1	1001	36719	0	440	467	94	−	296	761	19722	20188
UN09916	Rsa1.0_00707.1	707	190112	1.00E-95	212	223	95	−	34	252	16479	16701
UN09917	Rsa1.0_02750.1	955	28239	1.00E-140	253	253	100	+	2	254	22872	23124
UN09918	Rsa1.0_00055.1	1481	520193	1.00E-129	241	243	99	−	621	863	334867	335109
UN09919	Rsa1.0_20105.1	1084	1340	0	461	487	94	−	1	484	457	931
UN09920	Rsa1.0_21673.1	752	1183	1.00E-43	415	514	80	−	239	737	280	780
UN09921	Rsa1.0_00121.1	669	278029	1.00E-118	299	326	91	+	336	659	54300	54615
UN09922	Rsa1.0_00925.1	1042	59481	0	450	459	98	−	7	464	13819	14277
UN09923	Rsa1.0_00480.1	813	81325	0	376	383	98	−	430	811	3549	3931
UN09924	Rsa1.0_01389.1	1054	46716	0	391	393	99	+	4	396	9773	10165
UN09925	Rsa1.0_00012.1	1034	222148	0	525	535	98	−	500	1034	54569	55103
UN09926	Rsa1.0_00604.1	696	146282	0	455	471	96	+	226	696	16045	16511
UN09927	Rsa1.0_03842.1	848	20503	0	428	447	95	−	1	445	17620	18056
UN09928	Rsa1.0_07536.1	919	9178	0	404	406	99	+	1	406	6488	6893
UN09929	Rsa1.0_01119.1	725	53459	0	479	489	97	−	3	491	1103	1591
UN09930	Rsa1.0_00139.1	585	357786	0	451	453	99	+	1	453	16916	17368
UN09931	Rsa1.0_02362.1	1362	32190	0	392	402	97	−	4	403	5146	5544
UN09932	Rsa1.0_26477.1	581	890	3.00E-80	245	280	87	+	283	558	1	262
UN09933	Rsa1.0_00863.1	1150	254535	0	680	691	98	+	3	693	209202	209892
UN09934	Rsa1.0_01263.1	534	49703	1.00E-159	285	285	100	+	249	533	40157	40441
UN09935	Rsa1.0_02748.1	2067	28242	0	1308	1334	98	−	4	1335	15482	16815
UN09936	Rsa1.0_00397.1	814	88189	6.00E-73	161	168	95	−	645	812	64276	64443
UN09937	Rsa1.0_04510.1	901	17033	1.00E-157	347	367	94	−	5	361	12403	12769
UN09938	Rsa1.0_00294.1	762	144860	1.00E-153	307	318	96	−	66	383	116969	117286
UN09939	Rsa1.0_00227.1	836	105849	0	833	833	100	−	2	834	77950	78782
UN09940	Rsa1.0_00036.1	1039	176186	0	1017	1040	97	+	1	1039	112138	113174
UN09941	Rsa1.0_04961.1	903	15316	0	507	517	98	−	2	517	10674	11190
UN09942	Rsa1.0_00069.1	999	403561	1.00E-147	273	276	98	−	117	392	179274	179549
UN09943	Rsa1.0_02349.1	1021	32312	0	454	466	97	−	1	463	26844	27303

UN09944	Rsa1.0_00019.1	992	301669	0	661	670	98	+	323	992	273565	274234
UN09945	Rsa1.0_00500.1	479	272308	1.00E-124	245	251	97	−	98	348	102407	102654
UN09946	Rsa1.0_00033.1	716	515811	1.00E-143	257	257	100	−	458	714	33602	33858
UN09947	Rsa1.0_00454.1	822	216970	0	805	822	97	+	3	822	158096	158915
UN09948	Rsa1.0_01410.1	650	46345	1.00E-172	310	311	99	+	66	376	14359	14669
UN09949	Rsa1.0_00585.1	1070	74164	0	386	386	100	+	685	1070	59736	60121
UN09950	Rsa1.0_26314.1	886	897	0	412	430	95	+	11	438	109	537
UN09951	Rsa1.0_04641.1	1202	268012	1.00E-178	332	337	98	−	665	1001	260501	260837
UN09952	Rsa1.0_40008.1	990	562	0	344	350	98	+	340	689	1	350
UN09953	Rsa1.0_06500.1	736	10887	0	588	596	98	+	6	601	6670	7265
UN09954	Rsa1.0_02982.1	2431	26270	0	1602	1607	99	+	3	1609	8888	10494
UN09955	Rsa1.0_07440.1	590	9389	1.00E-178	332	336	98	−	3	337	6268	6603
UN09956	Rsa1.0_00589.1	1170	209965	1.00E-155	278	278	100	+	472	749	197377	197654
UN09957	Rsa1.0_18906.1	1178	1514	0	375	381	98	−	771	1151	58	438
UN09958	Rsa1.0_03656.1	784	21552	0	422	430	98	+	3	432	1446	1871
UN09959	Rsa1.0_00294.1	2238	144860	0	560	588	95	+	551	1136	60107	60694
UN09960	Rsa1.0_00437.1	1381	84879	0	840	849	98	+	534	1381	62710	63556
UN09961	Rsa1.0_00642.1	2293	201230	0	683	686	99	−	1609	2293	195794	196479
UN09962	Rsa1.0_00250.1	1320	273771	1.00E-154	362	385	94	+	925	1307	230593	230971
UN09963	Rsa1.0_00322.1	811	212233	1.00E-162	290	290	100	−	233	522	206966	207255
UN09964	Rsa1.0_00143.1	1446	121313	0	1394	1452	96	−	5	1446	32757	34188
UN09965	Rsa1.0_00926.1	926	195072	0	850	865	98	+	63	925	890	1754
UN09966	Rsa1.0_02252.1	797	33375	0	489	522	93	+	106	627	27494	27999
UN09967	Rsa1.0_02584.1	778	29877	0	376	388	96	+	391	778	806	1189
UN09968	Rsa1.0_00380.1	545	89748	1.00E-106	233	242	96	+	304	543	8474	8712
UN09969	Rsa1.0_06155.1	1267	11609	1.00E-126	232	233	99	−	1	233	10248	10480
UN09970	Rsa1.0_01743.1	837	39926	0	420	451	93	+	3	440	6763	7212
UN09971	Rsa1.0_62053.1	539	369	6.00E-63	123	123	100	+	379	501	215	337
UN09972	Rsa1.0_00646.1	925	70473	7.00E-73	257	294	87	−	648	923	5972	6254
UN09973	Rsa1.0_01135.1	843	52799	0	565	588	96	−	258	840	41650	42235
UN09974	Rsa1.0_00727.1	784	66724	0	736	780	94	+	3	769	44953	45714
UN09975	Rsa1.0_01883.1	1352	37661	0	738	755	97	−	511	1265	35516	36270
UN09976	Rsa1.0_02813.1	617	27656	0	490	492	99	+	123	614	25679	26170
UN09977	Rsa1.0_04073.1	771	19238	1.00E-108	214	219	97	−	1	219	13359	13577
UN09978	Rsa1.0_08562.1	538	7193	3.00E-92	181	184	98	+	40	223	3771	3954
UN09979	Rsa1.0_00017.1	1841	752458	0	1076	1145	93	−	543	1687	70609	71753
UN09980	Rsa1.0_18568.1	960	1573	1.00E-138	277	286	96	+	3	288	623	908
UN09981	Rsa1.0_01716.1	776	40483	1.00E-163	332	345	96	+	4	345	17530	17868
UN09982	Rsa1.0_00285.1	566	149638	1.00E-162	293	294	99	+	273	566	71820	72113

UN09983	Rsa1.0_00580.1	1324	74515	0	722	836	86	−	182	1011	54379	55208
UN09984	Rsa1.0_01913.1	769	37363	1.00E−160	315	323	97	+	448	769	27662	27984
UN09985	Rsa1.0_11068.1	1464	5627	0	445	447	99	+	362	808	5174	5620
UN09986	Rsa1.0_00045.1	1252	328694	0	1196	1199	99	+	54	1252	45322	46520
UN09987	Rsa1.0_00470.1	724	81968	1.00E−178	411	439	93	−	288	724	27934	28369
UN09988	Rsa1.0_01384.1	764	46803	1.00E−175	366	378	96	−	1	374	41768	42142
UN09989	Rsa1.0_00502.1	1106	204180	1.00E−153	284	287	98	+	1	287	20714	21000
UN09990	Rsa1.0_02717.1	961	28458	1.00E−131	289	303	95	+	1	303	19174	19465
UN09991	Rsa1.0_00135.1	640	178077	1.00E−133	262	268	97	−	243	508	3040	3307
UN09992	Rsa1.0_05559.1	1425	13329	0	561	563	99	−	2	564	1716	2278
UN09993	Rsa1.0_00287.1	757	98687	1.00E−159	287	288	99	+	1	288	12801	13088
UN09994	Rsa1.0_00686.1	837	68511	0	497	497	100	+	3	499	26775	27271
UN09995	Rsa1.0_00237.1	1169	235974	0	809	809	100	+	1	809	188234	189042
UN09996	Rsa1.0_01086.1	797	54509	0	762	817	93	−	3	795	36460	37269
UN09997	Rsa1.0_19197.1	803	1466	1.00E−124	241	246	97	+	3	248	1104	1349
UN09998	Rsa1.0_00394.1	901	148426	1.00E−119	347	386	89	−	533	900	105856	106241
UN09999	Rsa1.0_16597.1	666	2098	1.00E−162	328	340	96	−	186	518	1416	1755
UN10000	Rsa1.0_00118.1	1421	130637	0	576	598	96	+	3	600	28806	29403
UN10001	Rsa1.0_03944.1	1239	19926	0	463	463	100	−	776	1238	10167	10629
UN10002	Rsa1.0_02411.1	1563	31655	0	819	838	97	+	307	1144	27638	28475
UN10003	Rsa1.0_01544.1	924	185602	0	376	383	98	−	3	385	9690	10069
UN10004	Rsa1.0_00226.1	925	105884	0	574	575	99	−	351	925	97447	98021
UN10005	Rsa1.0_03410.1	804	23147	0	613	667	91	+	155	804	10247	10905
UN10006	Rsa1.0_00550.1	846	210790	0	350	354	98	−	44	397	60958	61311
UN10007	Rsa1.0_04235.1	874	18373	0	854	875	97	−	1	874	16330	17193
UN10008	Rsa1.0_17272.1	966	1874	0	469	475	98	−	1	475	1	475
UN10009	Rsa1.0_02282.1	1043	163951	1.00E−113	436	507	85	−	28	524	150175	150666
UN10010	Rsa1.0_04027.1	514	19501	1.00E−158	289	291	99	+	223	513	6649	6939
UN10011	Rsa1.0_00803.1	1292	144228	0	389	389	100	−	612	1000	52682	53070
UN10012	Rsa1.0_00180.1	1703	114581	1.00E−154	350	372	94	+	493	864	14427	14795
UN10013	Rsa1.0_03487.1	1033	22564	0	495	495	100	+	539	1033	10540	11034
UN10014	Rsa1.0_00092.1	750	659932	0	480	490	97	−	261	750	242810	243295
UN10015	Rsa1.0_00666.1	1863	69505	0	1169	1170	99	+	3	1172	25947	27116
UN10016	Rsa1.0_06100.1	1503	11746	2.00E−92	173	173	100	−	280	452	1076	1248
UN10017	Rsa1.0_03738.1	629	21071	1.00E−165	314	319	98	+	312	629	6129	6447
UN10018	Rsa1.0_01798.1	1215	173909	0	1031	1053	97	−	1	1049	5150	6202
UN10019	Rsa1.0_01077.1	739	54772	0	571	598	95	+	99	690	21078	21673
UN10020	Rsa1.0_01958.1	969	261128	1.00E−153	352	376	93	−	608	968	119362	119737
UN10021	Rsa1.0_01920.1	1577	158533	0	469	492	95	+	1093	1577	128073	128554

UN10022	Rsa1.0_00145.1	1541	261008	3.00E-95	200	206	97	-	39	244	73241	73445
UN10023	Rsa1.0_17015.1	1172	1953	1.00E-112	265	281	94	-	2	278	874	1148
UN10024	Rsa1.0_28434.1	1236	815	0	420	439	95	+	800	1233	48	486
UN10025	Rsa1.0_00005.1	1282	353093	0	580	583	99	-	138	720	138648	139230
UN10026	Rsa1.0_09210.1	1115	6556	1.00E-180	335	340	98	-	99	438	650	989
UN10027	Rsa1.0_05948.1	1369	12142	0	342	347	98	-	415	761	4859	5205
UN10028	Rsa1.0_08598.1	1535	7143	1.00E-161	300	304	98	-	521	824	6199	6502
UN10029	Rsa1.0_01144.1	1216	52431	0	358	363	98	+	852	1214	42305	42667
UN10030	Rsa1.0_05622.1	1519	13145	0	570	583	97	-	1	576	3626	4206
UN10031	Rsa1.0_04323.1	1079	17915	1.00E-108	227	236	96	-	4	239	15646	15881
UN10032	Rsa1.0_00719.1	1178	66952	0	375	381	98	-	1	381	47331	47711
UN10033	Rsa1.0_00303.1	1660	97468	0	828	909	91	+	758	1660	44008	44900
UN10034	Rsa1.0_05228.1	1258	14437	0	398	415	95	-	846	1258	4899	5313
UN10035	Rsa1.0_00016.1	964	831256	1.00E-154	291	295	98	+	1	295	225483	225775
UN10036	Rsa1.0_06421.1	943	11053	1.00E-147	336	357	94	-	587	943	3887	4231
UN10037	Rsa1.0_01507.1	1627	44361	1.00E-137	252	253	99	-	569	821	26446	26698
UN10038	Rsa1.0_01539.1	1384	43698	0	676	693	97	+	704	1384	4616	5308
UN10039	Rsa1.0_00018.1	1929	410899	0	840	939	89	-	778	1707	144212	145134
UN10040	Rsa1.0_00010.1	1016	294827	0	667	718	92	-	301	1015	90136	90830
UN10041	Rsa1.0_00755.1	1454	192559	0	847	847	100	+	3	849	138020	138866
UN10042	Rsa1.0_00732.1	1263	66631	1.00E-111	208	209	99	-	339	547	4101	4309
UN10043	Rsa1.0_00529.1	983	77487	0	330	331	99	-	653	983	21764	22094
UN10044	Rsa1.0_02744.1	1180	28268	8.00E-92	365	430	84	+	751	1180	15581	15983
UN10045	Rsa1.0_01270.1	1509	49574	6.00E-87	179	184	97	-	1	184	18017	18200
UN10046	Rsa1.0_05947.1	1990	12142	0	1259	1328	94	+	675	1990	3191	4512
UN10047	Rsa1.0_01877.1	1046	37789	1.00E-153	278	279	99	+	768	1046	34868	35146
UN10048	Rsa1.0_00173.1	986	288743	1.00E-144	260	260	100	+	3	262	109113	109372
UN10049	Rsa1.0_05329.1	1193	14130	1.00E-133	302	321	94	+	273	592	2124	2429
UN10050	Rsa1.0_03294.1	1043	23971	0	503	508	99	+	460	967	16378	16885
UN10051	Rsa1.0_08516.1	1552	7245	1.00E-161	328	341	96	+	899	1239	891	1231
UN10052	Rsa1.0_03809.1	1173	20731	0	780	831	93	+	347	1172	19080	19907
UN10053	Rsa1.0_00335.1	454	94371	0	375	386	97	-	2	383	50290	50674
UN10054	Rsa1.0_26155.1	928	904	0	632	634	99	+	293	926	1	634
UN10055	Rsa1.0_03292.1	1313	23977	1.00E-161	306	312	98	+	1	312	4625	4936
UN10056	Rsa1.0_04772.1	1603	16040	0	468	501	93	-	1115	1602	13583	14079
UN10057	Rsa1.0_00223.1	1277	106231	0	663	669	99	-	1	669	69796	70464
UN10058	Rsa1.0_01004.1	2183	56975	0	984	992	99	+	2	993	49782	50773
UN10059	Rsa1.0_00270.1	2093	101236	0	1144	1157	98	+	3	1158	39775	40931
UN10060	Rsa1.0_00011.1	1459	225044	0	448	448	100	-	1011	1458	177239	177686

UN10061	Rsa1.0_00111.1	1333	390088	1.00E-161	405	431	93	-	915	1333	32713	33134
UN10062	Rsa1.0_02821.1	1519	27602	0	371	380	97	+	3	380	14874	15251
UN10063	Rsa1.0_00078.1	1040	175053	0	1037	1038	99	+	3	1040	3932	4969
UN10064	Rsa1.0_02914.1	516	26815	1.00E-155	340	358	94	+	3	358	26110	26467
UN10065	Rsa1.0_02928.1	1421	26680	0	788	795	99	+	1	794	15338	16132
UN10066	Rsa1.0_00143.1	960	121313	1.00E-164	309	313	98	+	648	960	87223	87534
UN10067	Rsa1.0_08285.1	1393	7606	0	553	563	98	+	491	1053	2625	3187
UN10068	Rsa1.0_00019.1	973	301669	0	490	498	98	-	478	972	26632	27129
UN10069	Rsa1.0_00059.1	1628	159650	0	1281	1337	95	-	301	1627	38598	39932
UN10070	Rsa1.0_02267.1	550	33194	0	473	494	95	+	65	547	11925	12416
UN10071	Rsa1.0_00466.1	1001	82132	0	739	740	99	-	262	1001	23507	24246
UN10072	Rsa1.0_08259.1	1126	7641	1.00E-140	336	364	92	-	3	351	3089	3451
UN10073	Rsa1.0_00911.1	1081	59943	0	1045	1084	96	-	3	1081	56159	57234
UN10074	Rsa1.0_00171.1	834	115622	1.00E-123	269	279	96	+	48	324	41358	41632
UN10075	Rsa1.0_11285.1	1156	5556	0	385	395	97	+	761	1155	3883	4277
UN10076	Rsa1.0_01033.1	990	56173	0	606	620	97	-	369	988	48472	49090
UN10077	Rsa1.0_03606.1	933	21818	1.00E-165	306	309	99	-	3	309	1339	1647
UN10078	Rsa1.0_16774.1	755	2038	1.00E-137	259	263	98	+	132	394	1396	1658
UN10079	Rsa1.0_00492.1	1278	80178	0	669	679	98	-	3	681	23202	23880
UN10080	Rsa1.0_09091.1	1212	6656	0	363	366	99	-	810	1174	3746	4111
UN10081	Rsa1.0_00269.1	1333	341668	0	542	542	100	+	317	858	189849	190390
UN10082	Rsa1.0_00735.1	1457	66503	0	596	597	99	+	860	1456	4823	5419
UN10083	Rsa1.0_00058.1	1353	159921	1.00E-139	349	376	92	+	965	1339	139467	139830
UN10084	Rsa1.0_00674.1	1549	193753	0	514	517	99	-	11	527	181840	182356
UN10085	Rsa1.0_00040.1	1376	324196	0	522	546	95	+	824	1360	114552	115096
UN10086	Rsa1.0_01731.1	1089	40208	1.00E-127	250	256	97	+	3	253	18260	18515
UN10087	Rsa1.0_34603.1	1247	654	1.00E-126	233	234	99	-	99	332	223	456
UN10088	Rsa1.0_01005.1	982	56967	1.00E-132	258	264	97	-	424	687	19291	19554
UN10089	Rsa1.0_02312.1	1795	32675	0	882	882	100	-	267	1148	20773	21654
UN10090	Rsa1.0_06976.1	1356	10073	0	331	331	100	-	2	332	1316	1646
UN10091	Rsa1.0_00080.1	1070	190437	0	393	397	98	-	1	394	65785	66181
UN10092	Rsa1.0_00011.1	1023	225044	1.00E-93	184	187	98	-	616	802	50686	50872
UN10093	Rsa1.0_00596.1	1024	73578	1.00E-90	173	174	99	-	849	1022	9140	9313
UN10094	Rsa1.0_05890.1	711	12317	1.00E-114	212	213	99	+	1	213	6836	7048
UN10095	Rsa1.0_03479.1	1010	22631	0	785	833	94	+	8	836	21398	22217
UN10096	Rsa1.0_00076.1	601	146021	0	573	595	96	+	14	601	100578	101171
UN10097	Rsa1.0_01469.1	860	45139	1.00E-120	219	219	100	+	1	219	42143	42361
UN10098	Rsa1.0_00838.1	1872	153820	0	412	418	98	+	9	426	54187	54603
UN10099	Rsa1.0_00264.1	850	101674	1.00E-148	285	290	98	+	3	291	68831	69120

UN10100	Rsa1.0_00189.1	1261	111830	1.00E-167	302	303	99	−	958	1260	55224	55526
UN10101	Rsa1.0_00141.1	888	121494	0	461	474	97	+	1	473	82243	82714
UN10102	Rsa1.0_05013.1	905	15131	1.00E-154	298	303	98	−	602	904	1039	1338
UN10103	Rsa1.0_03627.1	964	21658	1.00E-179	400	423	94	+	549	964	8013	8434
UN10104	Rsa1.0_00474.1	825	81731	0	399	417	95	+	15	428	30343	30753
UN10105	Rsa1.0_01081.1	892	499226	1.00E-155	290	294	98	−	599	892	323183	323476
UN10106	Rsa1.0_01656.1	1373	41494	0	438	455	96	+	1	455	35728	36181
UN10107	Rsa1.0_00384.1	1083	89278	0	325	326	99	−	32	357	48409	48734
UN10108	Rsa1.0_01855.1	1885	38204	0	635	642	98	−	2	643	12693	13332
UN10109	Rsa1.0_03790.1	1744	20844	0	426	436	97	−	582	1013	7623	8058
UN10110	Rsa1.0_01459.1	2732	45277	0	2165	2186	99	+	1	2186	35839	38024
UN10111	Rsa1.0_01126.1	960	53127	0	696	716	97	+	3	710	41996	42709
UN10112	Rsa1.0_01272.1	1944	49553	0	1044	1087	96	+	2	1087	17182	18268
UN10113	Rsa1.0_00017.1	954	752458	0	414	434	95	−	525	953	412620	413048
UN10114	Rsa1.0_18957.1	1285	1505	1.00E-160	296	299	98	+	354	652	1203	1501
UN10115	Rsa1.0_00100.1	1604	249232	2.00E-99	185	185	100	+	632	816	220066	220250
UN10116	Rsa1.0_00169.1	1281	274298	0	718	741	96	+	3	743	26954	27690
UN10117	Rsa1.0_03971.1	1294	19788	1.00E-159	291	293	99	+	3	295	1772	2064
UN10118	Rsa1.0_14151.1	1104	3731	0	490	493	99	+	612	1104	981	1472
UN10119	Rsa1.0_01358.1	1478	47278	0	506	506	100	+	548	1053	15623	16128
UN10120	Rsa1.0_08957.1	1280	6754	0	411	429	95	−	854	1280	6326	6754
UN10121	Rsa1.0_07766.1	1247	8612	0	382	383	99	+	49	431	5866	6248
UN10122	Rsa1.0_00068.1	1514	151813	1.00E-111	246	260	94	−	1	251	35329	35586
UN10123	Rsa1.0_00336.1	1217	445051	0	484	488	99	+	1	488	4046	4532
UN10124	Rsa1.0_00045.1	895	328694	1.00E-150	276	278	99	+	1	278	165161	165438
UN10125	Rsa1.0_05410.1	1127	13840	0	507	509	99	−	124	632	5729	6237
UN10126	Rsa1.0_01354.1	692	47342	1.00E-138	265	270	98	−	423	692	9360	9629
UN10127	Rsa1.0_13204.1	1555	4970	0	504	504	100	+	102	605	4329	4832
UN10128	Rsa1.0_01907.1	855	37431	0	513	519	98	+	337	855	17877	18395
UN10129	Rsa1.0_02438.1	1399	31392	0	474	496	95	−	3	497	13585	14074
UN10130	Rsa1.0_00816.1	1315	403350	0	694	719	96	+	3	716	139083	139801
UN10131	Rsa1.0_00272.1	1332	447836	0	371	377	98	−	957	1332	65635	66010
UN10132	Rsa1.0_01943.1	1056	37042	1.00E-164	309	313	98	−	744	1056	4794	5105
UN10133	Rsa1.0_02703.1	1150	28544	0	526	532	98	−	40	569	25964	26495
UN10134	Rsa1.0_28315.1	1144	820	1.00E-119	310	334	92	−	818	1144	446	771
UN10135	Rsa1.0_08102.1	1301	7901	1.00E-130	260	267	97	−	1035	1301	718	981
UN10136	Rsa1.0_02615.1	758	29547	0	753	756	99	−	3	758	24432	25187
UN10137	Rsa1.0_00098.1	969	349239	0	447	467	95	−	419	881	220945	221405
UN10138	Rsa1.0_01156.1	603	52147	1.00E-128	280	296	94	−	316	602	33030	33324

UN10139	Rsa1.0_00063.1	536	156150	1.00E-121	229	232	98	+	116	347	3494	3725
UN10140	Rsa1.0_13982.1	949	3960	0	944	948	99	+	2	949	649	1596
UN10141	Rsa1.0_05367.1	1157	14010	0	475	490	96	+	3	492	2676	3156
UN10142	Rsa1.0_01124.1	2100	53295	0	369	370	99	−	378	747	27263	27632
UN10143	Rsa1.0_02756.1	1743	28202	1.00E-176	334	338	98	+	1	336	20785	21122
UN10144	Rsa1.0_04772.1	1004	16040	1.00E-157	291	293	99	+	6	298	11870	12160
UN10145	Rsa1.0_06319.1	1654	11243	0	471	481	97	−	3	482	9996	10476
UN10146	Rsa1.0_01389.1	1270	46716	1.00E-69	135	135	100	−	494	628	16433	16567
UN10147	Rsa1.0_00189.1	692	111830	1.00E-158	380	405	93	+	1	397	491	890
UN10148	Rsa1.0_00654.1	905	70012	1.00E-166	296	296	100	−	610	905	40143	40438
UN10149	Rsa1.0_00008.1	1198	358124	0	609	625	97	−	574	1198	6476	7100
UN10150	Rsa1.0_00112.1	1002	132199	0	703	750	93	+	253	993	55783	56507
UN10151	Rsa1.0_07657.1	1148	8896	0	536	551	97	+	607	1148	3516	4066
UN10152	Rsa1.0_00838.1	527	153820	0	366	376	97	+	1	376	146488	146863
UN10153	Rsa1.0_13767.1	944	4361	0	552	553	99	−	391	943	566	1118
UN10154	Rsa1.0_03204.1	1138	24622	0	492	492	100	−	238	729	6070	6561
UN10155	Rsa1.0_38829.1	431	579	1.00E-91	212	222	95	−	8	226	32	252
UN10156	Rsa1.0_05696.1	1154	12896	0	520	540	96	−	369	906	351	890
UN10157	Rsa1.0_19770.1	963	1385	0	343	344	99	−	619	962	465	808
UN10158	Rsa1.0_02222.1	532	152798	0	503	556	90	+	1	514	4560	5115
UN10159	Rsa1.0_02273.1	1076	140964	1.00E-178	319	320	99	+	756	1075	4741	5060
UN10160	Rsa1.0_00746.1	924	66044	1.00E-139	309	328	94	+	599	919	22302	22626
UN10161	Rsa1.0_01735.1	1056	40128	0	678	708	95	−	3	710	33988	34675
UN10162	Rsa1.0_02247.1	964	33427	1.00E-150	341	370	92	−	2	371	28646	28995
UN10163	Rsa1.0_01211.1	1274	50646	0	620	665	93	+	612	1274	41797	42450
UN10164	Rsa1.0_00387.1	635	495407	0	411	415	99	−	1	415	475782	476195
UN10165	Rsa1.0_00014.1	633	348985	0	348	352	98	−	188	539	315790	316137
UN10166	Rsa1.0_02777.1	1387	27986	0	596	625	95	+	766	1387	15700	16316
UN10167	Rsa1.0_01700.1	1011	40745	0	420	429	97	−	3	431	31534	31959
UN10168	Rsa1.0_01561.1	721	43227	0	532	552	96	+	156	705	84	633
UN10169	Rsa1.0_04124.1	1294	18945	1.00E-106	359	407	88	+	1	393	6518	6906
UN10170	Rsa1.0_00086.1	952	439854	0	322	322	100	+	2	323	325322	325643
UN10171	Rsa1.0_07339.1	1020	9534	1.00E-179	351	359	97	+	2	354	6432	6790
UN10172	Rsa1.0_00374.1	533	165738	0	526	530	99	+	4	533	160631	161160
UN10173	Rsa1.0_00128.1	807	228675	0	742	763	97	−	45	806	203865	204627
UN10174	Rsa1.0_21673.1	720	1183	0	703	725	96	+	11	720	226	950
UN10175	Rsa1.0_00240.1	1073	104175	0	615	623	98	+	425	1047	14974	15595
UN10176	Rsa1.0_00425.1	524	147080	0	378	388	97	−	2	388	24733	25120
UN10177	Rsa1.0_00537.1	1893	186980	0	1831	1893	96	−	1	1893	164273	166150

UN10178	Rsa1.0_05218.1	874	14457	1.00E-169	321	326	98	+	7	332	9535	9859
UN10179	Rsa1.0_11463.1	1145	5500	1.00E-172	309	310	99	−	836	1145	677	986
UN10180	Rsa1.0_02379.1	867	150705	0	371	383	96	+	392	774	140540	140922
UN10181	Rsa1.0_00675.1	1383	145590	0	1229	1253	98	−	131	1383	67398	68649
UN10182	Rsa1.0_01169.1	1055	51829	0	556	570	97	−	486	1055	36443	37012
UN10183	Rsa1.0_00464.1	731	82253	0	573	579	98	−	3	581	64562	65140
UN10184	Rsa1.0_00071.1	1041	345714	0	361	362	99	−	3	364	317193	317554
UN10185	Rsa1.0_03307.1	797	23872	0	467	480	97	+	320	791	16405	16881
UN10186	Rsa1.0_02506.1	1081	30700	0	495	524	94	−	558	1081	5605	6119
UN10187	Rsa1.0_01937.1	1464	37147	1.00E-132	296	314	94	−	3	316	32247	32557
UN10188	Rsa1.0_06032.1	632	11886	2.00E-78	219	240	91	−	399	632	7622	7859
UN10189	Rsa1.0_00749.1	1295	65933	0	1261	1301	96	+	2	1293	20487	21776
UN10190	Rsa1.0_00550.1	1867	210790	0	460	475	96	−	707	1179	177713	178187
UN10191	Rsa1.0_00108.1	722	478491	0	684	715	95	−	15	722	460205	460913
UN10192	Rsa1.0_00985.1	1382	161950	0	562	581	96	+	6	586	141740	142317
UN10193	Rsa1.0_00930.1	1564	59224	0	389	397	97	+	1	392	38934	39330
UN10194	Rsa1.0_10565.1	1453	5800	0	464	475	97	+	3	476	4451	4919
UN10195	Rsa1.0_07246.1	1884	9665	1.00E-163	315	320	98	+	851	1168	122	441
UN10196	Rsa1.0_01778.1	1123	39509	1.00E-168	306	308	99	−	6	313	23431	23738
UN10197	Rsa1.0_03467.1	1142	22687	0	509	521	97	−	1	521	13100	13620
UN10198	Rsa1.0_00351.1	851	413096	0	375	375	100	+	476	850	242921	243295
UN10199	Rsa1.0_03633.1	927	21644	1.00E-125	230	231	99	−	1	231	18297	18527
UN10200	Rsa1.0_00638.1	670	71037	0	629	640	98	+	2	641	36377	37012
UN10201	Rsa1.0_01788.1	842	39296	0	362	363	99	−	271	633	15792	16154
UN10202	Rsa1.0_02413.1	1288	31628	0	360	371	97	−	459	829	8130	8498
UN10203	Rsa1.0_00078.1	794	175053	0	741	765	96	+	3	762	3934	4695
UN10204	Rsa1.0_00419.1	1399	86562	0	638	647	98	−	752	1396	15419	16064
UN10205	Rsa1.0_01303.1	1196	48853	0	623	626	99	+	571	1196	15251	15876
UN10206	Rsa1.0_00453.1	1016	83031	0	633	643	98	+	375	1016	75551	76193
UN10207	Rsa1.0_06313.1	1545	11252	0	340	341	99	−	441	781	6081	6421
UN10208	Rsa1.0_02901.1	1171	26909	1.00E-109	266	287	92	−	893	1171	13866	14152
UN10209	Rsa1.0_00108.1	1233	478491	0	424	428	99	+	808	1233	463726	464153
UN10210	Rsa1.0_07757.1	944	8633	0	572	589	97	+	1	588	925	1512
UN10211	Rsa1.0_22271.1	1728	1135	0	415	422	98	+	1306	1727	548	966
UN10212	Rsa1.0_00033.1	963	515811	0	576	594	96	−	371	963	485021	485610
UN10213	Rsa1.0_01785.1	913	39368	0	585	585	100	+	2	586	28416	29000
UN10214	Rsa1.0_00119.1	1123	467509	0	477	514	92	+	378	885	386218	386716
UN10215	Rsa1.0_00232.1	1273	104936	1.00E-152	295	301	98	+	907	1206	48471	48771
UN10216	Rsa1.0_06310.1	1589	11262	0	811	963	84	+	386	1345	5160	6122

UN10217	Rsa1.0_00412.1	1015	86935	1.00E-135	252	253	99	+	762	1013	71685	71937
UN10218	Rsa1.0_00037.1	1165	307838	0	779	786	99	+	9	792	293238	294022
UN10219	Rsa1.0_07577.1	1098	9092	0	423	423	100	+	3	425	5723	6145
UN10220	Rsa1.0_00329.1	965	222544	1.00E-166	411	449	91	+	1	439	29883	30321
UN10221	Rsa1.0_00660.1	705	69688	1.00E-80	162	165	98	−	362	526	52782	52946
UN10222	Rsa1.0_00504.1	977	79334	1.00E-119	223	225	99	+	750	974	64280	64504
UN10223	Rsa1.0_00478.1	1487	81419	0	403	403	100	−	1079	1481	60585	60987
UN10224	Rsa1.0_00195.1	1388	251884	0	596	629	94	−	3	628	248803	249431
UN10225	Rsa1.0_04436.1	1216	17364	0	617	637	96	−	1	623	15688	16324
UN10226	Rsa1.0_00340.1	1418	305782	1.00E-132	249	252	98	−	1165	1416	188904	189155
UN10227	Rsa1.0_00041.1	1319	186197	0	447	448	99	−	867	1313	56659	57106
UN10228	Rsa1.0_01789.1	1201	39250	0	459	473	97	−	4	474	19826	20296
UN10229	Rsa1.0_00012.1	769	222148	1.00E-147	318	340	93	+	1	322	49635	49974
UN10230	Rsa1.0_10523.1	1649	5816	0	366	373	98	−	1269	1640	5304	5676
UN10231	Rsa1.0_01543.1	1154	43645	0	1036	1098	94	+	64	1146	24619	25697
UN10232	Rsa1.0_00382.1	910	251946	1.00E-154	277	277	100	−	634	910	112485	112761
UN10233	Rsa1.0_36296.1	1303	621	0	354	358	98	+	550	907	1	358
UN10234	Rsa1.0_01690.1	1448	40939	0	763	798	95	+	112	900	5950	6747
UN10235	Rsa1.0_01081.1	856	499226	0	845	854	98	−	3	854	318528	319381
UN10236	Rsa1.0_05541.1	1084	139959	1.00E-151	301	310	97	−	775	1084	2800	3107
UN10237	Rsa1.0_00483.1	1273	81102	1.00E-155	320	331	96	−	943	1271	56262	56592
UN10238	Rsa1.0_00328.1	1483	224661	0	832	855	97	−	3	855	176338	177191
UN10239	Rsa1.0_02101.1	1038	35017	0	992	1009	98	−	3	1010	15755	16763
UN10240	Rsa1.0_00577.1	1400	174827	0	734	746	98	−	566	1311	29514	30259
UN10241	Rsa1.0_01058.1	1125	55430	1.00E-110	238	247	96	−	1	246	29543	29788
UN10242	Rsa1.0_01776.1	767	39511	0	600	613	97	+	162	767	9082	9693
UN10243	Rsa1.0_00193.1	1081	194198	1.00E-128	283	296	95	+	784	1076	102524	102818
UN10244	Rsa1.0_48139.1	1162	470	0	382	392	97	+	489	880	76	467
UN10245	Rsa1.0_00928.1	1089	59293	1.00E-81	211	230	91	+	805	1034	32258	32481
UN10246	Rsa1.0_00314.1	801	96472	1.00E-105	228	238	95	+	565	801	56253	56490
UN10247	Rsa1.0_01200.1	1214	50895	0	869	914	95	+	305	1212	42501	43412
UN10248	Rsa1.0_00662.1	1589	192183	0	1335	1399	95	−	1	1392	59159	60550
UN10249	Rsa1.0_03001.1	787	26108	1.00E-125	305	327	93	−	468	787	5505	5824
UN10250	Rsa1.0_01591.1	776	42718	1.00E-77	204	224	91	−	560	774	15670	15893
UN10251	Rsa1.0_00737.1	804	66432	1.00E-126	296	316	93	+	491	804	25538	25847
UN10252	Rsa1.0_00075.1	870	146680	1.00E-110	219	222	98	−	649	868	26141	26362
UN10253	Rsa1.0_00529.1	1591	77487	1.00E-162	294	295	99	+	1	295	15484	15778
UN10254	Rsa1.0_00457.1	901	337301	1.00E-128	232	232	100	+	1	232	159587	159818
UN10255	Rsa1.0_06535.1	1336	10827	0	1321	1336	98	−	1	1336	2711	4044

UN10256	Rsa1.0_00727.1	1661	66724	0	359	361	99	−	1301	1661	61914	62274
UN10257	Rsa1.0_02226.1	814	33726	1.00E−61	159	169	94	−	609	775	10227	10395
UN10258	Rsa1.0_01278.1	1039	49370	4.00E−93	180	182	98	+	766	947	20617	20798
UN10259	Rsa1.0_00111.1	862	390088	0	384	394	97	+	265	658	381107	381500
UN10260	Rsa1.0_01846.1	1324	38367	0	550	567	97	+	758	1324	33297	33863
UN10261	Rsa1.0_05384.1	1437	13960	0	778	785	99	+	4	788	1086	1870
UN10262	Rsa1.0_00224.1	1699	327530	0	940	947	99	+	753	1699	192182	193126
UN10263	Rsa1.0_04626.1	1271	16611	0	604	631	95	−	3	631	14781	15406
UN10264	Rsa1.0_00056.1	871	332604	9.00E−60	133	138	96	−	336	473	309803	309940
UN10265	Rsa1.0_00218.1	998	201480	0	441	451	97	−	1	451	153355	153804
UN10266	Rsa1.0_00719.1	2450	66952	0	474	474	100	+	82	555	23347	23820
UN10267	Rsa1.0_00505.1	1071	79323	1.00E−106	217	224	96	+	1	224	4309	4532
UN10268	Rsa1.0_00421.1	1275	86365	0	564	564	100	+	421	984	17163	17726
UN10269	Rsa1.0_01561.1	1925	43227	1.00E−126	233	234	99	+	1692	1925	27114	27347
UN10270	Rsa1.0_00925.1	1770	59481	0	445	451	98	+	247	693	56289	56739
UN10271	Rsa1.0_16574.1	890	2106	0	526	538	97	−	352	888	1573	2106
UN10272	Rsa1.0_00023.1	1559	194764	0	331	332	99	−	1226	1557	182540	182871
UN10273	Rsa1.0_01931.1	810	37187	1.00E−169	385	412	93	+	391	802	32029	32428
UN10274	Rsa1.0_00794.1	833	64063	1.00E−179	361	373	96	−	458	827	39651	40023
UN10275	Rsa1.0_00652.1	946	70089	0	373	385	96	−	2	385	47920	48304
UN10276	Rsa1.0_01440.1	883	45807	0	750	775	96	+	1	774	13700	14471
UN10277	Rsa1.0_01200.1	1123	50895	0	1113	1121	99	−	1	1121	42229	43347
UN10278	Rsa1.0_05884.1	700	12337	0	669	702	95	+	3	697	10020	10716
UN10279	Rsa1.0_00108.1	1322	478491	0	429	454	94	+	868	1320	279966	280404
UN10280	Rsa1.0_01985.1	524	36491	1.00E−143	258	258	100	−	267	524	1761	2018
UN10281	Rsa1.0_00066.1	888	152418	0	518	536	96	+	22	557	20936	21465
UN10282	Rsa1.0_13888.1	1142	4146	0	879	879	100	+	1	879	1792	2670
UN10283	Rsa1.0_00502.1	1345	204180	0	541	546	99	+	1	546	60925	61469
UN10284	Rsa1.0_00991.1	1319	143101	0	634	668	94	−	1	664	137872	138532
UN10285	Rsa1.0_00773.1	957	64839	0	433	460	94	−	515	957	52202	52660
UN10286	Rsa1.0_24540.1	808	982	0	710	732	96	−	47	778	251	982
UN10287	Rsa1.0_00022.1	1375	200461	1.00E−170	316	320	98	−	1036	1355	96365	96684
UN10288	Rsa1.0_04156.1	1411	18799	0	505	505	100	−	905	1409	242	746
UN10289	Rsa1.0_01058.1	1379	55430	1.00E−156	304	310	98	+	1070	1379	44751	45056
UN10290	Rsa1.0_12394.1	1320	5237	1.00E−160	325	337	96	−	1	337	4534	4862
UN10291	Rsa1.0_02358.1	913	32213	0	477	478	99	+	1	478	236	712
UN10292	Rsa1.0_05161.1	1132	14631	1.00E−138	320	345	92	+	16	351	8351	8695
UN10293	Rsa1.0_01668.1	898	41299	1.00E−108	266	288	92	−	122	409	40402	40689
UN10294	Rsa1.0_00967.1	823	58049	0	783	784	99	+	3	786	3374	4156

UN10295	Rsa1.0_11577.1	937	5467	1.00E-114	275	294	93	-	646	937	723	1012
UN10296	Rsa1.0_07808.1	849	8504	0	593	614	96	-	239	849	117	727
UN10297	Rsa1.0_02098.1	1031	35032	1.00E-112	256	270	94	+	142	410	17299	17567
UN10298	Rsa1.0_02020.1	922	173211	0	707	720	98	-	1	720	164608	165327
UN10299	Rsa1.0_03123.1	891	25207	0	561	582	96	-	3	584	22210	22786
UN10300	Rsa1.0_00129.1	891	126831	1.00E-125	289	304	95	+	23	324	113583	113884
UN10301	Rsa1.0_00876.1	1390	61328	1.00E-127	279	295	94	-	1032	1320	49382	49676
UN10302	Rsa1.0_00407.1	1046	191544	0	395	403	98	-	3	402	154691	155093
UN10303	Rsa1.0_00024.1	1304	188018	1.00E-155	400	443	90	-	424	845	84667	85097
UN10304	Rsa1.0_00873.1	1429	61459	1.00E-126	293	313	93	+	2	308	11603	11915
UN10305	Rsa1.0_00515.1	979	78417	0	940	982	95	-	3	979	59809	60784
UN10306	Rsa1.0_00086.1	1185	439854	1.00E-84	195	204	95	+	3	205	429	631
UN10307	Rsa1.0_00329.1	995	222544	1.00E-153	389	426	91	-	523	937	29906	30321
UN10308	Rsa1.0_00041.1	814	186197	1.00E-143	341	369	92	+	3	361	156429	156786
UN10309	Rsa1.0_00369.1	1124	266462	0	437	450	97	-	360	809	169354	169802
UN10310	Rsa1.0_02022.1	1286	35923	1.00E-158	329	343	95	-	1	343	14054	14395
UN10311	Rsa1.0_07835.1	1438	8453	0	364	364	100	-	1	364	6351	6714
UN10312	Rsa1.0_01502.1	1125	44519	1.00E-131	325	354	91	+	775	1125	39463	39804
UN10313	Rsa1.0_00470.1	1566	81968	1.00E-158	283	283	100	+	248	530	48972	49254
UN10314	Rsa1.0_00336.1	1209	445051	1.00E-164	350	368	95	-	723	1080	441	805
UN10315	Rsa1.0_00339.1	1614	222974	0	781	920	84	+	337	1255	28077	28996
UN10316	Rsa1.0_00371.1	1074	90463	0	409	421	97	-	648	1067	73347	73767
UN10317	Rsa1.0_02954.1	1146	26485	0	369	385	95	-	404	788	13577	13961
UN10318	Rsa1.0_03589.1	1383	21893	0	434	452	96	+	3	450	604	1055
UN10319	Rsa1.0_23451.1	1196	1048	1.00E-156	371	402	92	-	1	393	184	572
UN10320	Rsa1.0_00008.1	1015	358124	0	598	635	94	-	382	1015	50872	51499
UN10321	Rsa1.0_01248.1	1077	154103	7.00E-95	211	221	95	-	855	1075	41446	41660
UN10322	Rsa1.0_01338.1	1545	155644	0	465	487	95	+	548	1034	142949	143435
UN10323	Rsa1.0_24295.1	1252	994	0	598	611	97	+	97	707	1	611
UN10324	Rsa1.0_00180.1	956	114581	1.00E-156	310	319	97	+	2	320	34754	35066
UN10325	Rsa1.0_01000.1	1507	57140	0	476	479	99	-	1031	1507	35248	35726
UN10326	Rsa1.0_03347.1	1860	23588	0	968	1006	96	+	2	1003	7901	8903
UN10327	Rsa1.0_54025.1	894	422	0	413	422	97	+	210	630	1	422
UN10328	Rsa1.0_00306.1	786	146760	0	555	629	88	+	189	784	76532	77144
UN10329	Rsa1.0_01920.1	1612	158533	0	482	485	99	+	3	487	125759	126241
UN10330	Rsa1.0_00816.1	1059	403350	1.00E-148	286	290	98	+	124	411	390143	390432
UN10331	Rsa1.0_02884.1	1185	27034	0	645	682	94	-	2	681	12569	13240
UN10332	Rsa1.0_01896.1	940	37512	0	901	938	96	+	2	939	20688	21623
UN10333	Rsa1.0_05909.1	1132	12265	1.00E-157	319	327	97	-	1	324	6137	6460

UN10334	Rsa1.0_00094.1	1383	139042	0	543	563	96	−	3	564	62288	62850
UN10335	Rsa1.0_00383.1	861	398431	1.00E−134	297	316	93	−	558	861	182089	182404
UN10336	Rsa1.0_04882.1	1204	15606	0	341	341	100	−	857	1197	7132	7472
UN10337	Rsa1.0_00152.1	1653	257730	0	1236	1241	99	+	411	1651	201609	202849
UN10338	Rsa1.0_00002.1	922	601661	0	586	593	98	−	31	623	527335	527927
UN10339	Rsa1.0_14918.1	985	2967	1.00E−178	316	316	100	−	2	317	1	316
UN10340	Rsa1.0_00689.1	991	68305	0	621	632	98	−	338	969	12770	13401
UN10341	Rsa1.0_00070.1	1713	150321	0	577	583	98	−	1131	1713	59034	59616
UN10342	Rsa1.0_02322.1	1515	32555	0	619	629	98	+	5	633	4740	5365
UN10343	Rsa1.0_00073.1	867	240924	1.00E−133	250	252	99	+	2	251	97580	97831
UN10344	Rsa1.0_01889.1	600	37562	1.00E−167	313	318	98	+	277	594	23938	24255
UN10345	Rsa1.0_02377.1	973	31994	1.00E−127	303	325	93	−	1	322	10248	10571
UN10346	Rsa1.0_00250.1	714	273771	1.00E−171	313	316	99	+	1	316	16202	16517
UN10347	Rsa1.0_00637.1	1579	71038	0	848	851	99	+	1	851	2355	3204
UN10348	Rsa1.0_04998.1	1110	15180	0	1085	1096	98	−	3	1098	6674	7765
UN10349	Rsa1.0_02583.1	1151	29887	0	435	437	99	−	432	868	24108	24544
UN10350	Rsa1.0_00571.1	967	329018	0	928	980	94	+	4	967	173767	174742
UN10351	Rsa1.0_01534.1	897	43764	0	727	757	96	+	7	755	10709	11464
UN10352	Rsa1.0_02505.1	667	30703	1.00E−149	367	393	93	+	266	658	21664	22048
UN10353	Rsa1.0_00157.1	1152	245832	1.00E−179	408	431	94	+	2	431	64001	64422
UN10354	Rsa1.0_03646.1	902	21598	0	868	905	95	−	3	902	18024	18918
UN10355	Rsa1.0_01632.1	652	181224	0	364	371	98	+	275	645	27928	28294
UN10356	Rsa1.0_05282.1	1696	14270	1.00E−154	276	276	100	+	1419	1694	6690	6965
UN10357	Rsa1.0_01047.1	1223	55705	0	1178	1232	95	+	2	1218	39313	40541
UN10358	Rsa1.0_01097.1	1674	173349	0	420	423	99	+	1	422	32242	32663
UN10359	Rsa1.0_00219.1	1487	106983	1.00E−121	238	242	98	+	80	321	42325	42565
UN10360	Rsa1.0_00065.1	1436	454616	0	610	610	100	+	1	610	26103	26712
UN10361	Rsa1.0_12642.1	1540	5165	0	505	506	99	+	825	1330	18	523
UN10362	Rsa1.0_00635.1	1205	175826	0	592	618	95	+	2	619	19297	19903
UN10363	Rsa1.0_32803.1	1506	693	0	677	705	96	+	554	1258	1	693
UN10364	Rsa1.0_00302.1	908	97521	1.00E−165	362	382	94	−	329	705	79283	79656
UN10365	Rsa1.0_00886.1	919	167350	0	455	491	92	+	7	488	11230	11719
UN10366	Rsa1.0_04446.1	1288	17317	0	712	723	98	−	264	986	2267	2989
UN10367	Rsa1.0_00033.1	1040	515811	1.00E−132	270	279	96	−	762	1036	41403	41679
UN10368	Rsa1.0_09091.1	708	6656	0	500	501	99	−	1	501	2634	3134
UN10369	Rsa1.0_02000.1	1386	36210	1.00E−112	273	295	92	+	1059	1343	4950	5244
UN10370	Rsa1.0_00810.1	990	63289	0	534	553	96	+	441	990	57632	58175
UN10371	Rsa1.0_02901.1	1172	26909	1.00E−158	293	295	99	−	879	1172	13858	14152
UN10372	Rsa1.0_05956.1	928	12111	1.00E−156	282	283	99	−	642	924	3203	3485

UN10373	Rsa1.0_02859.1	1116	162118	1.00E-150	366	396	92	-	5	390	144252	144639
UN10374	Rsa1.0_00244.1	1617	103590	0	1600	1619	98	-	1	1617	21064	22671
UN10375	Rsa1.0_00169.1	1004	274298	0	614	642	95	-	1	641	87856	88495
UN10376	Rsa1.0_00659.1	740	69733	1.00E-127	322	347	92	-	42	383	20560	20902
UN10377	Rsa1.0_00863.1	1240	254535	0	557	564	98	+	3	566	126471	127032
UN10378	Rsa1.0_01527.1	1221	43905	1.00E-164	328	341	96	-	3	343	37951	38282
UN10379	Rsa1.0_01448.1	876	45645	0	460	489	94	+	2	488	37833	38314
UN10380	Rsa1.0_00602.1	1131	73302	0	561	570	98	+	3	565	28641	29210
UN10381	Rsa1.0_00273.1	1226	326186	0	659	663	99	+	1	659	287923	288585
UN10382	Rsa1.0_05100.1	1051	14853	1.00E-117	253	264	95	+	10	268	1926	2189
UN10383	Rsa1.0_01094.1	1702	167285	0	586	586	100	-	1117	1702	162962	163547
UN10384	Rsa1.0_01836.1	1468	38503	0	404	405	99	-	1064	1467	1674	2078
UN10385	Rsa1.0_01559.1	958	43262	2.00E-61	121	121	100	-	34	154	23194	23314
UN10386	Rsa1.0_00051.1	665	258225	0	656	663	98	+	1	663	100708	101370
UN10387	Rsa1.0_15691.1	1315	2479	1.00E-134	252	255	98	-	1061	1315	1418	1672
UN10388	Rsa1.0_01549.1	821	43543	0	778	805	96	-	18	819	31477	32275
UN10389	Rsa1.0_02068.1	1200	35432	0	399	401	99	+	802	1200	8081	8481
UN10390	Rsa1.0_02561.1	1357	30080	0	403	415	97	+	462	876	14681	15090
UN10391	Rsa1.0_00087.1	1074	761073	1.00E-170	416	449	92	+	4	443	620991	621436
UN10392	Rsa1.0_00623.1	959	207630	0	453	458	98	-	503	959	11311	11768
UN10393	Rsa1.0_00329.1	794	222544	1.00E-137	248	248	100	-	3	250	179292	179539
UN10394	Rsa1.0_00361.1	1324	91181	1.00E-147	274	277	98	-	834	1110	12174	12450
UN10395	Rsa1.0_01667.1	1572	41315	0	728	740	98	-	1	740	33670	34407
UN10396	Rsa1.0_00258.1	1235	102381	1.00E-178	331	336	98	+	27	362	45308	45643
UN10397	Rsa1.0_00660.1	757	69688	0	395	399	98	-	362	757	52548	52946
UN10398	Rsa1.0_00277.1	430	100132	1.00E-158	291	294	98	+	112	405	8930	9223
UN10399	Rsa1.0_02753.1	710	28215	0	675	713	94	-	3	708	8050	8744
UN10400	Rsa1.0_04034.1	1162	19481	1.00E-147	264	264	100	+	213	476	12812	13075
UN10401	Rsa1.0_00541.1	912	76863	1.00E-163	316	324	97	-	200	523	34580	34903
UN10402	Rsa1.0_12343.1	895	5249	1.00E-65	190	208	91	+	688	895	2222	2427
UN10403	Rsa1.0_14175.1	1170	3704	0	443	444	99	+	273	715	2887	3330
UN10404	Rsa1.0_05287.1	1883	14264	0	1516	1606	94	+	11	1614	3998	5587
UN10405	Rsa1.0_04806.1	683	15898	0	465	481	96	+	203	683	7176	7647
UN10406	Rsa1.0_00354.1	1464	232005	0	1436	1457	98	+	4	1460	171044	172500
UN10407	Rsa1.0_00415.1	743	86795	1.00E-179	375	394	95	-	1	394	4549	4942
UN10408	Rsa1.0_00722.1	734	66840	0	333	336	99	-	3	338	61473	61808
UN10409	Rsa1.0_00047.1	1246	168582	1.00E-137	298	313	95	-	933	1244	113998	114306
UN10410	Rsa1.0_15127.1	632	2809	0	403	403	100	-	4	406	2023	2425
UN10411	Rsa1.0_00855.1	1229	62047	0	797	812	98	+	414	1225	22692	23503

UN10412	Rsa1.0_00215.1	734	201775	0	384	390	98	+	343	732	24023	24412
UN10413	Rsa1.0_01835.1	707	38542	0	419	419	100	−	288	706	24220	24638
UN10414	Rsa1.0_00833.1	1219	62621	1.00E−119	244	252	96	+	969	1217	7472	7723
UN10415	Rsa1.0_00198.1	1104	222963	0	387	390	99	−	715	1103	178618	179007
UN10416	Rsa1.0_01037.1	1100	55969	0	445	451	98	+	19	469	51914	52364
UN10417	Rsa1.0_03009.1	751	26065	1.00E−180	366	379	96	+	7	382	2628	3006
UN10418	Rsa1.0_00260.1	1968	102021	0	880	921	95	+	732	1652	27623	28531
UN10419	Rsa1.0_00195.1	676	251884	1.00E−156	286	288	99	+	389	676	83583	83870
UN10420	Rsa1.0_02212.1	1008	33840	1.00E−179	319	319	100	+	1	319	27406	27724
UN10421	Rsa1.0_00059.1	1324	159650	1.00E−157	319	331	96	−	995	1322	131088	131418
UN10422	Rsa1.0_00120.1	854	277288	0	405	406	99	−	1	406	121696	122100
UN10423	Rsa1.0_05991.1	500	12013	1.00E−115	238	246	96	−	254	499	1321	1564
UN10424	Rsa1.0_00328.1	1337	224661	0	921	927	99	−	37	963	184283	185209
UN10425	Rsa1.0_00088.1	1293	141341	1.00E−151	389	424	91	−	868	1283	66452	66870
UN10426	Rsa1.0_00163.1	1511	116847	0	439	464	94	+	1033	1493	83512	83968
UN10427	Rsa1.0_00545.1	1278	189649	0	589	596	98	+	3	598	29512	30105
UN10428	Rsa1.0_01872.1	904	37908	0	481	483	99	−	1	483	9035	9517
UN10429	Rsa1.0_07639.1	1432	8966	0	1427	1433	99	−	1	1432	6028	7460
UN10430	Rsa1.0_00902.1	1157	60241	0	665	667	99	−	3	669	16185	16851
UN10431	Rsa1.0_00360.1	1411	91190	1.00E−129	389	434	89	+	299	711	43686	44119
UN10432	Rsa1.0_00040.1	1499	324196	0	961	965	99	−	535	1499	217469	218433
UN10433	Rsa1.0_00413.1	749	86921	0	373	373	100	−	375	747	20449	20821
UN10434	Rsa1.0_01467.1	1828	45161	1.00E−105	267	294	90	+	1554	1828	23046	23339
UN10435	Rsa1.0_00884.1	772	61150	0	765	769	99	−	4	772	56311	57078
UN10436	Rsa1.0_00933.1	1320	59164	1.00E−178	343	350	98	−	1	345	2856	3205
UN10437	Rsa1.0_00328.1	614	224661	0	407	407	100	−	6	412	861	1267
UN10438	Rsa1.0_00949.1	797	58549	0	330	331	99	+	467	797	39561	39891
UN10439	Rsa1.0_03455.1	1712	22804	0	1668	1732	96	+	4	1711	1286	3007
UN10440	Rsa1.0_03413.1	1524	23137	0	1286	1313	97	+	3	1312	17992	19304
UN10441	Rsa1.0_13478.1	922	4790	0	383	385	99	−	1	385	615	999
UN10442	Rsa1.0_00088.1	866	141341	1.00E−133	259	265	97	−	38	302	6655	6919
UN10443	Rsa1.0_00211.1	1375	228189	0	494	516	95	−	315	830	130199	130714
UN10444	Rsa1.0_03401.1	1300	23191	8.00E−52	248	293	84	+	626	917	5986	6277
UN10445	Rsa1.0_00190.1	896	318240	0	383	400	95	−	408	805	314391	314786
UN10446	Rsa1.0_01271.1	1552	49569	0	482	497	96	−	825	1320	22174	22670
UN10447	Rsa1.0_00293.1	1103	339294	0	385	392	98	−	712	1103	4126	4515
UN10448	Rsa1.0_00419.1	1495	86562	1.00E−117	234	238	98	−	1241	1477	15435	15671
UN10449	Rsa1.0_01295.1	881	49040	0	411	425	96	+	458	881	21320	21740
UN10450	Rsa1.0_18906.1	1215	1514	0	374	381	98	−	831	1211	58	438

UN10451	Rsa1.0_03618.1	1016	21744	1.00E-136	247	247	100	-	770	1016	11073	11319
UN10452	Rsa1.0_04022.1	922	19534	2.00E-85	214	235	91	+	703	922	6268	6502
UN10453	Rsa1.0_00797.1	1032	63959	0	565	565	100	-	468	1032	51725	52289
UN10454	Rsa1.0_01537.1	699	187285	1.00E-166	407	444	91	+	257	698	841	1261
UN10455	Rsa1.0_00030.1	1663	250988	0	567	571	99	-	1093	1663	44636	45206
UN10456	Rsa1.0_04395.1	592	17570	1.00E-135	253	256	98	-	306	561	10029	10284
UN10457	Rsa1.0_00066.1	1024	152418	0	568	589	96	-	45	633	112082	112670
UN10458	Rsa1.0_02266.1	1114	33195	1.00E-174	339	346	97	-	759	1104	21919	22262
UN10459	Rsa1.0_00434.1	1260	85171	0	639	658	97	+	617	1259	71294	71949
UN10460	Rsa1.0_00190.1	1636	318240	0	417	417	100	-	1219	1635	211837	212253
UN10461	Rsa1.0_01997.1	1182	158416	1.00E-116	295	315	93	-	8	318	7533	7843
UN10462	Rsa1.0_00294.1	1814	144860	0	841	871	96	-	300	1167	57575	58442
UN10463	Rsa1.0_00090.1	764	273859	0	492	511	96	+	256	760	148579	149086
UN10464	Rsa1.0_02907.1	1222	26861	0	572	575	99	-	616	1190	15223	15794
UN10465	Rsa1.0_03560.1	1586	22092	0	456	464	98	+	1122	1585	379	841
UN10466	Rsa1.0_00215.1	1622	201775	0	922	941	97	+	684	1622	26877	27817
UN10467	Rsa1.0_00589.1	850	209965	0	793	836	94	+	12	847	154838	155658
UN10468	Rsa1.0_00483.1	898	81102	0	381	385	98	-	514	898	46548	46932
UN10469	Rsa1.0_00706.1	797	291570	0	498	510	97	+	2	511	159001	159504
UN10470	Rsa1.0_00145.1	739	261008	0	406	428	94	+	6	424	8528	8953
UN10471	Rsa1.0_01926.1	1358	37206	1.00E-178	380	402	94	+	3	402	15739	16124
UN10472	Rsa1.0_00560.1	1334	75685	7.00E-71	184	197	93	-	1	191	3912	4107
UN10473	Rsa1.0_00451.1	1514	322533	0	1445	1550	93	+	1	1512	249756	251305
UN10474	Rsa1.0_11429.1	506	5511	0	376	389	96	+	127	506	4743	5131
UN10475	Rsa1.0_01951.1	1274	36941	0	766	767	99	-	492	1258	10719	11485
UN10476	Rsa1.0_00826.1	1253	166736	0	525	525	100	+	1	525	130428	130952
UN10477	Rsa1.0_01154.1	907	52249	0	560	570	98	-	1	570	38105	38670
UN10478	Rsa1.0_00808.1	1331	63351	0	799	799	100	+	1	799	39339	40137
UN10479	Rsa1.0_11046.1	1330	5635	0	339	341	99	-	11	351	3092	3432
UN10480	Rsa1.0_02143.1	1026	34632	0	1011	1021	99	+	4	1024	8838	9856
UN10481	Rsa1.0_00734.1	1009	66508	1.00E-56	116	117	99	+	388	504	15185	15301
UN10482	Rsa1.0_00054.1	1177	162053	0	336	336	100	-	841	1176	5485	5820
UN10483	Rsa1.0_01559.1	625	43262	1.00E-105	219	225	97	+	389	611	2268	2491
UN10484	Rsa1.0_01339.1	1726	47813	1.00E-110	307	342	89	+	1336	1661	14806	15137
UN10485	Rsa1.0_01448.1	1130	45645	0	1119	1138	98	-	1	1128	15660	16796
UN10486	Rsa1.0_01569.1	1340	43029	1.00E-167	301	302	99	-	5	306	38883	39184
UN10487	Rsa1.0_02265.1	1665	33233	1.00E-124	239	243	98	+	1196	1438	9083	9325
UN10488	Rsa1.0_00176.1	1525	240984	1.00E-154	297	304	97	+	3	306	184021	184324
UN10489	Rsa1.0_00707.1	943	190112	0	367	369	99	+	1	369	65242	65610

UN10490	Rsa1.0_03581.1	1709	21965	0	401	406	98	+	1269	1674	20231	20636
UN10491	Rsa1.0_01055.1	1445	55466	0	1444	1446	99	−	1	1445	25304	26748
UN10492	Rsa1.0_00941.1	1136	58891	1.00E−162	297	298	99	−	1	298	44924	45220
UN10493	Rsa1.0_00849.1	1043	62212	0	406	425	95	−	617	1037	9683	10099
UN10494	Rsa1.0_00113.1	1603	131574	1.00E−172	319	323	98	−	1281	1603	74503	74825
UN10495	Rsa1.0_00782.1	1618	64468	0	474	494	95	−	553	1046	30043	30536
UN10496	Rsa1.0_04199.1	890	18501	0	860	889	96	+	12	889	8176	9064
UN10497	Rsa1.0_05846.1	700	12434	2.00E−94	179	180	99	+	317	496	7562	7741
UN10498	Rsa1.0_00381.1	1173	89718	8.00E−98	255	276	92	−	904	1173	71999	72270
UN10499	Rsa1.0_04696.1	6086	16339	0	617	694	88	+	3857	4550	11817	12499
UN10500	Rsa1.0_00682.1	1377	68680	0	822	834	98	+	16	849	46123	46956
UN10501	Rsa1.0_09388.1	1093	6405	1.00E−164	367	390	94	+	3	389	2824	3210
UN10502	Rsa1.0_25509.1	1217	933	0	394	405	97	−	1	405	498	902
UN10503	Rsa1.0_09951.1	917	6072	0	539	540	99	−	378	917	2117	2656
UN10504	Rsa1.0_00500.1	836	272308	0	613	618	99	−	4	621	157120	157735
UN10505	Rsa1.0_02928.1	1597	26680	0	363	369	98	−	1220	1582	7240	7608
UN10506	Rsa1.0_16693.1	1042	2064	0	493	494	99	−	549	1042	747	1240
UN10507	Rsa1.0_01737.1	1320	40101	0	463	490	94	+	826	1315	19997	20463
UN10508	Rsa1.0_02358.1	1636	32213	0	922	946	97	−	198	1138	7909	8854
UN10509	Rsa1.0_00697.1	768	67913	0	750	769	97	+	1	767	46052	46817
UN10510	Rsa1.0_05199.1	1524	14529	0	761	807	94	+	1	805	6531	7324
UN10511	Rsa1.0_00162.1	1144	239935	0	618	620	99	+	4	623	112749	113368
UN10512	Rsa1.0_00317.1	1022	218290	0	327	328	99	+	1	328	72594	72921
UN10513	Rsa1.0_00484.1	635	203294	6.00E−91	242	262	92	+	1	262	143450	143708
UN10514	Rsa1.0_06278.1	807	11333	0	387	389	99	+	418	806	1864	2251
UN10515	Rsa1.0_02949.1	1309	26506	0	799	816	97	−	500	1309	12852	13664
UN10516	Rsa1.0_00571.1	1412	329018	0	824	853	96	−	94	942	204578	205423
UN10517	Rsa1.0_00101.1	1019	267193	0	382	396	96	+	623	1017	208637	209032
UN10518	Rsa1.0_01768.1	1205	138463	1.00E−138	282	290	97	−	916	1205	120013	120300
UN10519	Rsa1.0_00451.1	2310	322533	3.00E−99	297	336	88	−	1132	1467	293086	293411
UN10520	Rsa1.0_00002.1	810	601661	0	433	461	93	+	1	461	240858	241305
UN10521	Rsa1.0_03067.1	901	25651	2.00E−94	236	256	92	+	149	404	5352	5607
UN10522	Rsa1.0_00507.1	1229	79074	0	468	476	98	−	754	1229	42740	43214
UN10523	Rsa1.0_00077.1	1252	145005	0	1231	1250	98	−	3	1252	129688	130936
UN10524	Rsa1.0_01426.1	658	46112	0	333	333	100	−	326	658	44168	44500
UN10525	Rsa1.0_02789.1	1578	27850	0	383	385	99	+	239	623	22916	23300
UN10526	Rsa1.0_00706.1	784	291570	0	468	484	96	+	2	485	159033	159504
UN10527	Rsa1.0_05755.1	918	12730	0	529	544	97	−	377	916	9387	9928
UN10528	Rsa1.0_00351.1	789	413096	0	413	413	100	−	377	789	244415	244827

UN10529	Rsa1.0_00344.1	1079	191774	0	528	529	99	+	550	1078	108987	109515
UN10530	Rsa1.0_01005.1	1177	56967	0	627	631	99	+	546	1176	37403	38033
UN10531	Rsa1.0_00021.1	1090	263655	0	358	367	97	−	244	610	238503	238869
UN10532	Rsa1.0_00073.1	1484	240924	0	657	660	99	−	825	1484	118465	119124
UN10533	Rsa1.0_08790.1	882	6918	0	479	517	92	+	367	872	4980	5496
UN10534	Rsa1.0_00273.1	1382	326186	1.00E−143	363	401	90	+	988	1379	4768	5148
UN10535	Rsa1.0_00675.1	1035	145590	0	551	553	99	−	3	555	23696	24248
UN10536	Rsa1.0_02934.1	760	26627	0	472	480	98	−	1	480	25244	25722
UN10537	Rsa1.0_00116.1	940	365886	8.00E−79	150	150	100	−	720	869	353829	353978
UN10538	Rsa1.0_01135.1	1053	52799	0	500	501	99	−	209	709	24574	25074
UN10539	Rsa1.0_00828.1	828	62744	1.00E−165	395	428	92	−	406	828	47929	48337
UN10540	Rsa1.0_00110.1	787	132719	0	672	750	89	+	44	760	116490	117227
UN10541	Rsa1.0_00198.1	860	222963	0	426	427	99	−	4	429	174650	175076
UN10542	Rsa1.0_00041.1	1138	186197	0	786	803	97	+	335	1137	157067	157869
UN10543	Rsa1.0_01406.1	429	46439	0	340	341	99	−	89	429	40283	40623
UN10544	Rsa1.0_02290.1	1426	32971	0	469	470	99	−	955	1424	21744	22213
UN10545	Rsa1.0_47400.1	910	477	0	393	393	100	+	168	560	1	393
UN10546	Rsa1.0_01701.1	541	162121	2.00E−32	96	104	92	+	3	106	9023	9126
UN10547	Rsa1.0_02365.1	1164	32118	0	543	556	97	−	3	557	12194	12741
UN10548	Rsa1.0_00471.1	785	81897	1.00E−178	344	352	97	+	429	779	65986	66337
UN10549	Rsa1.0_00394.1	1267	148426	0	466	491	94	+	775	1265	67486	67964
UN10550	Rsa1.0_73802.1	938	310	1.00E−172	309	310	99	−	99	408	1	310
UN10551	Rsa1.0_00347.1	1415	93048	1.00E−176	340	348	97	−	1071	1415	72503	72850
UN10552	Rsa1.0_03878.1	1027	20305	1.00E−178	316	316	100	−	681	996	11883	12198
UN10553	Rsa1.0_26708.1	1627	881	0	571	602	94	−	1011	1591	4	605
UN10554	Rsa1.0_00485.1	775	80937	2.00E−66	243	279	87	+	435	709	37393	37671
UN10555	Rsa1.0_00394.1	1236	148426	0	399	413	96	+	827	1236	99869	100278
UN10556	Rsa1.0_00805.1	1346	63724	0	855	925	92	−	6	907	57517	58441
UN10557	Rsa1.0_00387.1	1216	495407	0	874	901	97	−	8	907	409522	410422
UN10558	Rsa1.0_00706.1	1146	291570	0	623	657	94	−	493	1145	23308	23961
UN10559	Rsa1.0_00330.1	1263	94666	0	830	843	98	−	1	843	16817	17659
UN10560	Rsa1.0_00529.1	1613	77487	0	538	539	99	+	2	540	69325	69863
UN10561	Rsa1.0_02117.1	1422	34843	1.00E−150	273	274	99	−	238	511	15985	16258
UN10562	Rsa1.0_00028.1	1423	293984	5.00E−87	173	176	98	+	1187	1362	175533	175708
UN10563	Rsa1.0_00697.1	1487	67913	1.00E−115	229	235	97	−	765	999	57671	57905
UN10564	Rsa1.0_13353.1	1356	4894	0	567	569	99	+	245	813	4326	4894
UN10565	Rsa1.0_00407.1	1728	191544	0	1241	1242	99	−	3	1244	166770	168011
UN10566	Rsa1.0_00710.1	2319	67325	0	943	964	97	+	865	1822	11528	12490
UN10567	Rsa1.0_32838.1	1240	693	0	380	380	100	−	506	885	314	693

UN10568	Rsa1.0_01958.1	750	261128	7.00E-88	195	205	95	+	527	731	134810	135009
UN10569	Rsa1.0_02173.1	1235	34318	1.00E-118	225	228	98	+	1	228	15510	15737
UN10570	Rsa1.0_33997.1	1559	666	0	486	488	99	+	3	490	179	666
UN10571	Rsa1.0_04845.1	1462	15747	1.00E-111	220	225	97	−	1055	1279	4169	4393
UN10572	Rsa1.0_00896.1	896	60509	0	650	652	99	−	184	835	38792	39443
UN10573	Rsa1.0_00850.1	526	62193	0	401	406	98	+	1	406	5941	6346
UN10574	Rsa1.0_03062.1	1638	25678	0	465	467	99	−	3	469	8175	8641
UN10575	Rsa1.0_00122.1	590	265937	0	559	575	97	+	10	583	224759	225333
UN10576	Rsa1.0_01837.1	793	38502	0	789	791	99	−	3	793	3739	4529
UN10577	Rsa1.0_00893.1	1047	60626	0	495	506	97	+	38	543	46402	46907
UN10578	Rsa1.0_00064.1	852	240927	0	845	850	99	+	1	850	123684	124533
UN10579	Rsa1.0_00240.1	1200	104175	1.00E-123	274	289	94	−	913	1200	53548	53836
UN10580	Rsa1.0_00050.1	1404	237174	0	875	876	99	−	1	876	124201	125076
UN10581	Rsa1.0_00108.1	1288	478491	1.00E-155	335	350	95	−	114	459	463726	464071
UN10582	Rsa1.0_03140.1	1166	25081	0	387	390	99	+	506	895	12030	12419
UN10583	Rsa1.0_02730.1	1186	28385	0	373	373	100	−	814	1186	2508	2880
UN10584	Rsa1.0_02794.1	1186	27820	1.00E-176	317	318	99	+	869	1186	18902	19219
UN10585	Rsa1.0_00351.1	1104	413096	0	567	568	99	−	2	569	376778	377345
UN10586	Rsa1.0_00001.1	1294	363028	1.00E-177	325	327	99	−	1	327	319592	319916
UN10587	Rsa1.0_00281.1	1120	99908	1.00E-150	276	278	99	−	74	351	6793	7070
UN10588	Rsa1.0_01591.1	1268	42718	1.00E-168	370	393	94	+	863	1249	24117	24506
UN10589	Rsa1.0_00019.1	1291	301669	0	612	621	98	+	67	687	100107	100727
UN10590	Rsa1.0_12292.1	1023	5263	0	743	744	99	+	280	1023	1	744
UN10591	Rsa1.0_01412.1	949	46311	0	511	520	98	−	7	526	2268	2787
UN10592	Rsa1.0_01418.1	1006	46237	1.00E-173	312	313	99	+	694	1006	7186	7498
UN10593	Rsa1.0_02430.1	1013	31506	0	735	740	99	−	274	1013	12837	13576
UN10594	Rsa1.0_00018.1	1043	410899	1.00E-106	268	288	93	+	741	1025	262610	262892
UN10595	Rsa1.0_00063.1	950	156150	1.00E-178	359	373	96	−	1	373	138349	138721
UN10596	Rsa1.0_00756.1	785	65628	0	397	402	98	+	376	776	57538	57939
UN10597	Rsa1.0_03539.1	963	22199	1.00E-138	276	282	97	+	680	961	7072	7351
UN10598	Rsa1.0_00380.1	833	89748	0	663	679	97	−	1	676	83924	84598
UN10599	Rsa1.0_00144.1	1282	120708	1.00E-113	217	220	98	−	464	683	13724	13943
UN10600	Rsa1.0_01853.1	1298	147800	0	489	497	98	+	804	1298	128509	129003
UN10601	Rsa1.0_05321.1	2279	14154	5.00E-73	153	157	97	+	1749	1905	6323	6479
UN10602	Rsa1.0_00706.1	1130	291570	0	642	664	96	+	15	674	23300	23961
UN10603	Rsa1.0_07444.1	1633	9386	0	470	473	99	−	604	1076	918	1390
UN10604	Rsa1.0_15033.1	687	2880	0	542	573	94	−	115	683	128	700
UN10605	Rsa1.0_05945.1	1227	12149	0	366	366	100	+	27	392	11752	12117
UN10606	Rsa1.0_01106.1	941	53916	0	552	560	98	+	383	939	30702	31260

UN10607	Rsa1.0_01089.1	1734	142191	0	696	696	100	+	1030	1725	103236	103931
UN10608	Rsa1.0_02945.1	1529	26548	0	1001	1005	99	−	289	1293	8396	9400
UN10609	Rsa1.0_08916.1	1555	6792	0	1263	1279	98	+	276	1548	5181	6458
UN10610	Rsa1.0_03978.1	1088	19753	0	359	368	97	−	16	380	11983	12350
UN10611	Rsa1.0_07381.1	967	9488	0	590	590	100	+	1	590	3898	4487
UN10612	Rsa1.0_00302.1	1221	97521	1.00E−159	300	305	98	+	915	1219	67021	67325
UN10613	Rsa1.0_18510.1	1041	1586	0	560	602	93	−	444	1040	1001	1586
UN10614	Rsa1.0_04243.1	1329	18321	1.00E−165	295	295	100	+	1	295	2892	3186
UN10615	Rsa1.0_02994.1	674	26168	0	654	658	99	+	12	669	2139	2795
UN10616	Rsa1.0_03114.1	805	25318	0	515	551	93	−	4	552	6565	7106
UN10617	Rsa1.0_00116.1	1502	365886	0	606	621	97	−	887	1501	242226	242843
UN10618	Rsa1.0_02563.1	1339	30050	0	531	535	99	−	4	538	29101	29635
UN10619	Rsa1.0_60174.1	858	381	1.00E−149	268	268	100	−	556	823	1	268
UN10620	Rsa1.0_03860.1	756	20407	0	496	517	95	−	245	755	15804	16316
UN10621	Rsa1.0_02435.1	928	31454	0	353	358	98	−	3	360	20980	21334
UN10622	Rsa1.0_01439.1	1002	45841	0	1000	1002	99	+	1	1002	27658	28659
UN10623	Rsa1.0_00701.1	1100	67691	0	632	635	99	−	466	1100	5281	5915
UN10624	Rsa1.0_01051.1	1175	55577	0	711	733	96	−	184	912	3695	4422
UN10625	Rsa1.0_00793.1	661	64103	1.00E−119	241	249	96	+	413	661	24186	24434
UN10626	Rsa1.0_04518.1	1825	17005	0	517	543	95	−	600	1141	11675	12209
UN10627	Rsa1.0_01931.1	726	37187	1.00E−154	338	358	94	−	7	364	32029	32373
UN10628	Rsa1.0_03843.1	1049	20496	1.00E−157	370	398	92	+	17	408	6124	6521
UN10629	Rsa1.0_01078.1	1900	316910	0	812	843	96	−	1059	1900	280066	280905
UN10630	Rsa1.0_01786.1	1173	133324	0	821	868	94	−	226	1087	98698	99561
UN10631	Rsa1.0_00097.1	1058	138858	0	342	344	99	−	14	357	65577	65920
UN10632	Rsa1.0_00101.1	689	267193	1.00E−122	243	247	98	−	444	689	119126	119371
UN10633	Rsa1.0_01896.1	1819	37512	0	1234	1251	98	+	351	1600	20513	21763
UN10634	Rsa1.0_08846.1	436	6867	4.00E−48	107	110	97	+	99	208	1959	2068
UN10635	Rsa1.0_01522.1	742	157436	0	407	413	98	−	330	741	3059	3471
UN10636	Rsa1.0_00434.1	1017	85171	1.00E−135	278	288	96	−	2	287	9575	9857
UN10637	Rsa1.0_01536.1	448	43747	0	404	405	99	+	4	408	25749	26153
UN10638	Rsa1.0_00900.1	820	60367	3.00E−96	185	187	98	+	537	723	29648	29834
UN10639	Rsa1.0_25306.1	862	942	1.00E−175	388	411	94	−	340	746	329	735
UN10640	Rsa1.0_00595.1	789	341648	0	623	631	98	+	159	789	12663	13287
UN10641	Rsa1.0_00263.1	826	224968	0	432	450	96	+	368	815	151524	151964
UN10642	Rsa1.0_07775.1	430	8594	0	425	430	98	+	1	430	281	709
UN10643	Rsa1.0_02405.1	785	31692	1.00E−156	348	370	94	+	3	369	17028	17383
UN10644	Rsa1.0_03843.1	1126	20496	0	444	488	90	+	5	471	6051	6521
UN10645	Rsa1.0_02212.1	793	33840	0	752	790	95	−	4	793	25149	25932

UN10646	Rsa1.0_00715.1	593	67116	1.00E-172	475	530	89	+	10	538	63627	64136
UN10647	Rsa1.0_00354.1	900	232005	1.00E-147	307	320	95	+	569	887	170578	170897
UN10648	Rsa1.0_32300.1	954	705	5.00E-68	135	136	99	−	601	736	44	179
UN10649	Rsa1.0_01740.1	893	39986	0	360	360	100	−	428	787	26707	27066
UN10650	Rsa1.0_03851.1	1476	20451	0	452	452	100	+	1022	1473	11491	11942
UN10651	Rsa1.0_00223.1	679	106231	3.00E-93	177	178	99	−	502	679	72335	72512
UN10652	Rsa1.0_08259.1	1112	7641	1.00E-154	346	369	93	−	2	358	3089	3455
UN10653	Rsa1.0_00041.1	711	186197	1.00E-141	276	282	97	−	430	711	127152	127432
UN10654	Rsa1.0_01588.1	289	42724	1.00E-145	282	288	97	−	2	289	12918	13204
UN10655	Rsa1.0_03460.1	837	22747	0	425	440	96	−	208	646	12965	13404
UN10656	Rsa1.0_00337.1	629	143174	7.00E-69	148	153	96	−	477	629	128208	128360
UN10657	Rsa1.0_02103.1	725	35006	1.00E-139	263	267	98	+	459	725	24076	24342
UN10658	Rsa1.0_00944.1	925	58733	0	595	627	94	+	302	923	10746	11368
UN10659	Rsa1.0_06488.1	382	141774	1.00E-109	215	219	98	+	166	382	136121	136339
UN10660	Rsa1.0_00247.1	975	103408	0	487	502	97	−	474	975	6006	6507
UN10661	Rsa1.0_00281.1	678	99908	1.00E-168	313	316	99	+	3	318	11309	11623
UN10662	Rsa1.0_01433.1	912	45976	0	362	363	99	+	3	365	13494	13856
UN10663	Rsa1.0_00882.1	1330	61249	1.00E-129	257	262	98	+	1	260	49147	49407
UN10664	Rsa1.0_01326.1	432	48072	1.00E-127	253	260	97	+	173	432	10896	11151
UN10665	Rsa1.0_01046.1	803	152989	0	535	569	94	+	1	568	118630	119184
UN10666	Rsa1.0_05893.1	1379	12306	1.00E-170	392	419	93	−	8	419	8742	9154
UN10667	Rsa1.0_03344.1	1032	23593	0	432	456	94	+	578	1032	12154	12607
UN10668	Rsa1.0_04235.1	1345	18373	0	1247	1396	89	−	1	1345	11381	12739
UN10669	Rsa1.0_00537.1	471	186980	0	451	475	94	−	2	471	176098	176562
UN10670	Rsa1.0_01101.1	1064	54031	3.00E-94	238	256	92	+	60	311	40325	40577
UN10671	Rsa1.0_01211.1	845	50646	1.00E-147	419	466	89	+	387	835	41797	42258
UN10672	Rsa1.0_01549.1	964	43543	0	442	451	98	+	109	559	592	1042
UN10673	Rsa1.0_01453.1	969	45449	0	375	375	100	+	3	377	25076	25450
UN10674	Rsa1.0_00235.1	501	154696	3.00E-77	223	248	89	−	250	486	143801	144045
UN10675	Rsa1.0_00668.1	905	69421	1.00E-110	352	403	87	+	521	901	55635	56035
UN10676	Rsa1.0_07955.1	830	8180	1.00E-174	309	309	100	+	522	830	6422	6730
UN10677	Rsa1.0_00016.1	1137	831256	1.00E-138	289	300	96	−	841	1137	348675	348974
UN10678	Rsa1.0_03956.1	665	19872	1.00E-115	292	317	92	−	47	357	16858	17171
UN10679	Rsa1.0_00213.1	948	108167	0	766	807	94	−	144	948	34732	35527
UN10680	Rsa1.0_01221.1	1360	50421	1.00E-147	318	335	94	+	1030	1360	49364	49694
UN10681	Rsa1.0_02392.1	910	152517	1.00E-127	274	287	95	−	619	905	7785	8070
UN10682	Rsa1.0_01058.1	867	55430	0	864	867	99	−	1	865	34187	35053
UN10683	Rsa1.0_00061.1	1408	218685	0	1252	1285	97	+	125	1408	185100	186383
UN10684	Rsa1.0_02105.1	679	156953	1.00E-162	347	363	95	+	320	679	9327	9686

UN10685	Rsa1.0_02903.1	831	26901	0	432	451	95	−	3	451	14022	14466
UN10686	Rsa1.0_28307.1	1802	820	0	773	841	91	+	706	1540	1	820
UN10687	Rsa1.0_06386.1	609	140583	0	415	422	98	−	186	607	134600	135021
UN10688	Rsa1.0_00152.1	1136	257730	0	836	845	98	−	4	848	81107	81951
UN10689	Rsa1.0_02265.1	717	33233	1.00E−122	238	243	97	+	199	441	9083	9325
UN10690	Rsa1.0_00059.1	762	159650	1.00E−166	302	304	99	−	204	507	45276	45579
UN10691	Rsa1.0_01545.1	910	43599	1.00E−174	322	326	98	−	98	423	32611	32936
UN10692	Rsa1.0_00087.1	1018	761073	1.00E−153	287	291	98	−	407	697	729346	729636
UN10693	Rsa1.0_04874.1	735	15652	1.00E−134	259	262	98	+	3	264	15379	15637
UN10694	Rsa1.0_00394.1	916	148426	1.00E−118	347	385	90	−	543	914	105823	106207
UN10695	Rsa1.0_01058.1	1223	55430	5.00E−96	185	187	98	−	126	312	22337	22523
UN10696	Rsa1.0_00310.1	1288	96852	0	452	474	95	−	815	1288	82816	83289
UN10697	Rsa1.0_04535.1	1348	136600	0	710	729	97	+	618	1346	126575	127303
UN10698	Rsa1.0_42534.1	1289	528	0	525	528	99	+	132	659	1	528
UN10699	Rsa1.0_02372.1	932	32053	1.00E−142	269	272	98	+	662	932	23597	23868
UN10700	Rsa1.0_00022.1	750	200461	0	516	542	95	+	3	540	22593	23133
UN10701	Rsa1.0_04395.1	1604	17570	0	1531	1605	95	−	3	1604	6924	8512
UN10702	Rsa1.0_06323.1	807	11236	0	653	662	98	−	147	807	7477	8138
UN10703	Rsa1.0_17779.1	1136	1742	0	809	829	97	+	67	892	914	1742
UN10704	Rsa1.0_00092.1	940	659932	0	795	836	95	−	115	940	21377	22206
UN10705	Rsa1.0_04279.1	760	18113	1.00E−145	286	293	97	+	7	298	14291	14583
UN10706	Rsa1.0_02320.1	1620	32575	1.00E−143	314	331	94	−	1290	1620	6264	6588
UN10707	Rsa1.0_01287.1	1185	49195	0	488	501	97	−	683	1183	5836	6336
UN10708	Rsa1.0_08609.1	825	7132	0	495	509	97	−	304	809	5515	6023
UN10709	Rsa1.0_02204.1	1114	33930	0	486	505	96	+	2	498	20620	21120
UN10710	Rsa1.0_03843.1	1243	20496	0	769	779	98	+	1	779	3640	4418
UN10711	Rsa1.0_00041.1	1300	186197	1.00E−167	420	458	91	−	604	1060	159530	159986
UN10712	Rsa1.0_03166.1	1226	24863	0	391	405	96	−	800	1200	5364	5765
UN10713	Rsa1.0_01726.1	390	40263	1.00E−125	352	387	90	−	4	385	6807	7178
UN10714	Rsa1.0_01740.1	747	39986	0	709	768	92	+	1	746	11555	12315
UN10715	Rsa1.0_00979.1	756	57733	0	573	599	95	−	156	754	27178	27762
UN10716	Rsa1.0_00196.1	1231	109960	0	780	800	97	+	435	1231	90095	90894
UN10717	Rsa1.0_00110.1	591	132719	0	451	482	93	−	1	482	32257	32727
UN10718	Rsa1.0_00467.1	686	156542	0	630	676	93	−	2	676	94473	95139
UN10719	Rsa1.0_03627.1	776	21658	1.00E−163	381	408	93	+	376	776	8024	8431
UN10720	Rsa1.0_00012.1	1063	222148	0	786	848	92	+	133	965	110286	111109
UN10721	Rsa1.0_03439.1	830	22930	0	756	756	100	+	68	823	14985	15740
UN10722	Rsa1.0_03477.1	1174	22635	0	545	590	92	+	303	875	15370	15958
UN10723	Rsa1.0_01293.1	705	49139	1.00E−166	376	395	95	−	321	705	34533	34924

UN10724	Rsa1.0_04973.1	951	15267	0	774	784	98	+	1	784	12214	12996
UN10725	Rsa1.0_07559.1	906	9131	1.00E-102	248	265	93	−	174	437	1879	2142
UN10726	Rsa1.0_02803.1	942	27715	1.00E-153	343	366	93	−	577	942	2352	2717
UN10727	Rsa1.0_02604.1	786	29638	1.00E-171	327	333	98	−	2	333	28224	28556
UN10728	Rsa1.0_00385.1	704	89160	0	683	701	97	−	5	704	16178	16878
UN10729	Rsa1.0_05653.1	550	13043	0	474	478	99	+	73	550	9994	10471
UN10730	Rsa1.0_00807.1	678	63607	0	374	376	99	−	303	678	54492	54866
UN10731	Rsa1.0_04504.1	994	17069	0	684	714	95	−	290	994	8748	9457
UN10732	Rsa1.0_15167.1	1474	2783	0	598	606	98	+	214	818	896	1501
UN10733	Rsa1.0_00012.1	925	222148	1.00E-156	347	374	92	−	259	632	76837	77192
UN10734	Rsa1.0_02413.1	1728	31628	0	692	716	96	−	463	1177	7791	8498
UN10735	Rsa1.0_01130.1	1191	52929	1.00E-167	350	366	95	+	832	1191	44531	44896
UN10736	Rsa1.0_03969.1	472	19797	1.00E-136	322	347	92	−	126	472	16653	16996
UN10737	Rsa1.0_00661.1	951	213041	1.00E-151	311	323	96	−	9	331	195844	196165
UN10738	Rsa1.0_00176.1	651	240984	0	570	636	89	−	4	620	9780	10392
UN10739	Rsa1.0_01543.1	799	43645	1.00E-143	306	322	95	−	478	799	7865	8186
UN10740	Rsa1.0_00586.1	1135	163802	0	422	453	93	−	6	458	119295	119746
UN10741	Rsa1.0_02190.1	1228	34084	0	843	844	99	−	385	1228	28789	29632
UN10742	Rsa1.0_00539.1	785	76926	1.00E-177	358	372	96	−	414	785	57283	57650
UN10743	Rsa1.0_02414.1	864	31625	0	736	750	98	−	114	863	19100	19848
UN10744	Rsa1.0_00312.1	889	96765	1.00E-175	320	323	99	−	566	888	77984	78306
UN10745	Rsa1.0_02672.1	828	28884	1.00E-115	229	235	97	+	3	237	15539	15773
UN10746	Rsa1.0_00414.1	748	202190	0	395	407	97	+	343	748	22226	22628
UN10747	Rsa1.0_02835.1	862	27491	1.00E-114	271	293	92	−	3	288	21042	21327
UN10748	Rsa1.0_05572.1	489	13291	3.00E-98	201	206	97	−	59	263	11854	12059
UN10749	Rsa1.0_01239.1	1502	50107	1.00E-147	283	289	97	−	1214	1502	20875	21163
UN10750	Rsa1.0_00449.1	1772	83863	0	439	454	96	+	1319	1770	2417	2867
UN10751	Rsa1.0_00048.1	798	167185	0	430	461	93	−	13	470	146592	147043
UN10752	Rsa1.0_00480.1	806	81325	0	599	622	96	+	2	623	7037	7640
UN10753	Rsa1.0_00348.1	788	92818	0	584	617	94	−	93	699	3332	3946
UN10754	Rsa1.0_00216.1	1093	197563	1.00E-120	229	232	98	+	582	813	88910	89141
UN10755	Rsa1.0_01536.1	784	43747	0	565	593	95	−	193	784	18759	19349
UN10756	Rsa1.0_01063.1	974	55301	0	543	548	99	+	1	547	2774	3321
UN10757	Rsa1.0_00802.1	906	63846	0	450	460	97	+	447	906	26567	27020
UN10758	Rsa1.0_01607.1	765	42328	9.00E-32	100	107	93	−	14	120	30704	30808
UN10759	Rsa1.0_17040.1	746	1944	1.00E-162	333	347	95	−	20	363	195	541
UN10760	Rsa1.0_00340.1	786	305782	2.00E-91	180	183	98	−	247	429	195108	195290
UN10761	Rsa1.0_01898.1	614	37491	0	394	404	97	+	160	563	33926	34329
UN10762	Rsa1.0_00987.1	814	57543	0	802	815	98	+	1	813	402	1215

UN10763	Rsa1.0_00344.1	898	191774	0	586	586	100	+	313	898	155554	156139
UN10764	Rsa1.0_00019.1	1546	301669	0	1084	1163	93	+	3	1164	276419	277557
UN10765	Rsa1.0_11912.1	1468	5372	0	841	844	99	+	513	1356	1377	2220
UN10766	Rsa1.0_00105.1	823	134138	0	593	621	95	+	209	823	81104	81721
UN10767	Rsa1.0_00107.1	1169	431659	0	391	402	97	+	3	402	298704	299101
UN10768	Rsa1.0_01544.1	1575	185602	0	724	739	97	−	3	738	33890	34627
UN10769	Rsa1.0_00457.1	1172	337301	0	1102	1173	93	+	2	1164	308152	309307
UN10770	Rsa1.0_00695.1	790	68019	2.00E−26	74	78	94	+	334	411	49728	49805
UN10771	Rsa1.0_26056.1	406	908	1.00E−109	219	225	97	+	2	226	528	752
UN10772	Rsa1.0_00016.1	1000	831256	1.00E−157	307	314	97	−	687	999	447826	448139
UN10773	Rsa1.0_05352.1	1148	14079	0	634	673	94	−	22	689	3381	4040
UN10774	Rsa1.0_00286.1	465	98709	1.00E−116	219	220	99	+	247	465	11606	11825
UN10775	Rsa1.0_01388.1	1017	46741	1.00E−160	330	342	96	+	3	344	16561	16900
UN10776	Rsa1.0_00138.1	823	247040	0	437	451	96	+	372	822	234197	234645
UN10777	Rsa1.0_00703.1	1141	67597	1.00E−169	320	326	98	+	593	918	18006	18331
UN10778	Rsa1.0_13696.1	756	4467	0	579	613	94	+	3	609	3855	4467
UN10779	Rsa1.0_01232.1	1073	155185	1.00E−150	323	340	95	−	4	343	134026	134363
UN10780	Rsa1.0_00477.1	961	81556	1.00E−107	222	230	96	−	732	961	61904	62133
UN10781	Rsa1.0_00437.1	1605	84879	1.00E−117	310	342	90	+	807	1148	23217	23558
UN10782	Rsa1.0_10038.1	707	6029	0	419	424	98	−	284	707	5124	5543
UN10783	Rsa1.0_00082.1	1026	237584	1.00E−107	226	233	96	+	795	1026	38683	38913
UN10784	Rsa1.0_01061.1	1389	55349	0	368	377	97	+	1015	1389	17393	17769
UN10785	Rsa1.0_00336.1	853	445051	0	475	477	99	−	3	479	16575	17051
UN10786	Rsa1.0_00107.1	1037	431659	0	555	557	99	−	480	1036	165603	166159
UN10787	Rsa1.0_01408.1	454	46383	1.00E−177	368	383	96	+	59	439	34615	34997
UN10788	Rsa1.0_00380.1	832	89748	1.00E−135	247	248	99	−	204	451	78451	78698
UN10789	Rsa1.0_00302.1	1477	97521	0	918	936	98	+	544	1477	61024	61959
UN10790	Rsa1.0_04179.1	837	18633	0	666	682	97	+	119	800	15932	16613
UN10791	Rsa1.0_01230.1	795	50221	0	678	695	97	−	36	726	15664	16358
UN10792	Rsa1.0_00582.1	1594	74333	0	386	398	96	+	90	486	15883	16274
UN10793	Rsa1.0_26314.1	885	897	0	414	429	96	−	439	861	109	537
UN10794	Rsa1.0_00063.1	1180	156150	0	619	626	98	+	554	1178	142261	142886
UN10795	Rsa1.0_00342.1	1626	93695	1.00E−174	418	453	92	−	62	492	15020	15471
UN10796	Rsa1.0_09538.1	879	6301	1.00E−121	224	225	99	−	645	869	2206	2430
UN10797	Rsa1.0_50623.1	286	449	5.00E−31	93	101	92	−	162	262	349	449
UN10798	Rsa1.0_01705.1	1007	40710	1.00E−176	709	844	84	−	6	834	19076	19919
UN10799	Rsa1.0_01456.1	747	45372	1.00E−165	313	318	98	+	432	747	9266	9583
UN10800	Rsa1.0_05376.1	779	13977	0	463	483	95	+	273	754	5384	5865
UN10801	Rsa1.0_01328.1	1163	48021	1.00E−132	247	248	99	+	3	249	19715	19962

UN10802	Rsa1.0_00154.1	628	189461	6.00E-82	200	215	93	-	144	358	52420	52634
UN10803	Rsa1.0_00377.1	619	90038	1.00E-155	287	290	98	+	5	294	67689	67978
UN10804	Rsa1.0_27986.1	826	831	0	345	348	99	+	478	825	346	692
UN10805	Rsa1.0_00487.1	1130	80674	0	906	918	98	-	214	1129	70391	71306
UN10806	Rsa1.0_05569.1	1055	13311	0	1030	1053	97	+	2	1050	1990	3041
UN10807	Rsa1.0_03634.1	478	145192	1.00E-113	207	207	100	-	1	207	129362	129568
UN10808	Rsa1.0_01355.1	552	47303	6.00E-97	282	313	90	-	241	552	25895	26192
UN10809	Rsa1.0_00224.1	1417	327530	0	414	421	98	-	1	420	195632	196052
UN10810	Rsa1.0_00106.1	1699	133916	0	1062	1100	96	-	601	1699	19193	20287
UN10811	Rsa1.0_44104.1	1323	510	2.00E-52	170	190	89	+	1067	1256	29	217
UN10812	Rsa1.0_00065.1	924	454616	0	827	861	96	-	2	862	414616	415468
UN10813	Rsa1.0_03175.1	1558	24802	0	979	1004	97	+	3	1003	20687	21683
UN10814	Rsa1.0_01173.1	1017	51727	1.00E-118	225	227	99	+	793	1017	10160	10386
UN10815	Rsa1.0_05467.1	636	13649	0	491	509	96	+	130	636	11334	11842
UN10816	Rsa1.0_03190.1	576	24708	0	380	386	98	+	191	576	19575	19960
UN10817	Rsa1.0_06254.1	699	11384	2.00E-57	138	146	94	-	265	410	1306	1451
UN10818	Rsa1.0_02876.1	517	27062	0	504	516	97	+	2	517	5057	5572
UN10819	Rsa1.0_00001.1	931	363028	4.00E-90	229	249	91	-	665	910	190906	191142
UN10820	Rsa1.0_00364.1	523	90951	0	432	432	100	-	1	432	16823	17254
UN10821	Rsa1.0_00110.1	812	132719	0	511	558	91	-	271	803	35301	35850
UN10822	Rsa1.0_00131.1	711	432233	0	668	710	94	-	4	711	45508	46202
UN10823	Rsa1.0_01340.1	885	47805	1.00E-104	254	272	93	+	8	272	20648	20919
UN10824	Rsa1.0_03122.1	782	25207	4.00E-83	163	165	98	-	283	447	7333	7497
UN10825	Rsa1.0_02170.1	434	34352	0	432	435	99	+	1	434	32948	33382
UN10826	Rsa1.0_00978.1	1435	57739	0	973	1059	91	-	417	1435	17066	18112
UN10827	Rsa1.0_00044.1	1030	271658	2.00E-86	172	175	98	+	855	1029	264749	264923
UN10828	Rsa1.0_05890.1	642	12317	2.00E-72	223	251	88	+	1	235	735	976
UN10829	Rsa1.0_00252.1	942	102760	0	334	335	99	-	3	337	64920	65254
UN10830	Rsa1.0_12954.1	403	5066	3.00E-39	86	87	98	+	197	283	4552	4638
UN10831	Rsa1.0_01085.1	1356	54539	1.00E-115	237	243	97	-	3	243	12212	12454
UN10832	Rsa1.0_01239.1	2744	50107	0	1565	1624	96	-	3	1619	18949	20553
UN10833	Rsa1.0_02068.1	596	35432	0	406	406	100	+	1	406	15206	15611
UN10834	Rsa1.0_01004.1	879	56975	0	802	876	91	+	18	877	17034	17899
UN10835	Rsa1.0_00930.1	812	59224	1.00E-153	284	286	99	-	2	287	49934	50218
UN10836	Rsa1.0_00060.1	1411	502678	1.00E-176	356	368	96	+	1	366	368883	369249
UN10837	Rsa1.0_00481.1	469	81316	0	375	377	99	+	3	379	10479	10855
UN10838	Rsa1.0_00234.1	1137	197060	0	404	415	97	-	374	788	144150	144564
UN10839	Rsa1.0_02372.1	1057	32053	0	512	524	97	-	1	523	24847	25369
UN10840	Rsa1.0_00757.1	881	65559	0	569	585	97	-	1	580	35781	36364

UN10841	Rsa1.0_01422.1	762	46172	8.00E-91	223	234	95	−	3	234	1	231
UN10842	Rsa1.0_00329.1	1433	222544	0	1069	1157	92	−	286	1429	48995	50142
UN10843	Rsa1.0_02011.1	969	36088	0	665	667	99	+	301	967	24827	25493
UN10844	Rsa1.0_01857.1	859	38103	1.00E-138	298	313	95	−	542	852	6812	7124
UN10845	Rsa1.0_26014.1	1110	910	1.00E-160	289	290	99	−	821	1110	236	525
UN10846	Rsa1.0_01514.1	1235	44222	0	890	902	98	−	1	901	22059	22960
UN10847	Rsa1.0_02411.1	645	31655	0	373	375	99	+	1	375	27171	27544
UN10848	Rsa1.0_01578.1	1096	42880	1.00E-151	283	287	98	+	202	488	11676	11962
UN10849	Rsa1.0_01565.1	912	155447	0	887	918	96	+	1	912	146485	147395
UN10850	Rsa1.0_03473.1	814	22665	0	690	692	99	+	12	703	7841	8532
UN10851	Rsa1.0_01534.1	1880	43764	0	892	916	97	−	121	1033	10551	11464
UN10852	Rsa1.0_00254.1	902	102600	0	492	512	96	+	4	515	52348	52845
UN10853	Rsa1.0_03079.1	1595	25545	0	926	932	99	+	2	931	9087	10016
UN10854	Rsa1.0_01149.1	867	160459	1.00E-108	220	227	96	+	258	484	154480	154706
UN10855	Rsa1.0_00947.1	729	58664	0	427	440	97	−	1	438	48225	48664
UN10856	Rsa1.0_00460.1	820	206387	0	495	514	96	+	16	529	27284	27783
UN10857	Rsa1.0_08558.1	994	7198	1.00E-134	359	392	91	+	8	393	2337	2721
UN10858	Rsa1.0_01130.1	1328	52929	1.00E-116	254	265	95	−	1	262	44655	44915
UN10859	Rsa1.0_01061.1	964	55349	1.00E-105	230	237	97	−	1	233	42974	43209
UN10860	Rsa1.0_00082.1	1138	237584	0	1091	1146	95	−	2	1138	105717	106850
UN10861	Rsa1.0_00495.1	668	79934	1.00E-179	328	330	99	−	238	566	14814	15143
UN10862	Rsa1.0_01110.1	805	195852	0	572	603	94	+	204	805	16834	17432
UN10863	Rsa1.0_48630.1	824	466	1.00E-166	339	353	96	−	18	370	1	353
UN10864	Rsa1.0_03646.1	1175	21598	1.00E-127	240	243	98	−	1	243	15617	15859
UN10865	Rsa1.0_01541.1	808	43682	0	500	509	98	−	300	808	4271	4779
UN10866	Rsa1.0_01697.1	1001	40837	1.00E-164	347	362	95	−	35	396	19131	19485
UN10867	Rsa1.0_00466.1	920	82132	0	786	831	94	−	1	818	27152	27979
UN10868	Rsa1.0_00161.1	1097	116891	0	622	643	96	+	456	1097	99195	99825
UN10869	Rsa1.0_04737.1	1425	16173	0	630	656	96	−	128	783	2705	3359
UN10870	Rsa1.0_07446.1	957	9384	0	415	423	98	+	533	955	1803	2225
UN10871	Rsa1.0_02964.1	1207	26407	1.00E-149	289	296	97	−	1	296	14541	14836
UN10872	Rsa1.0_03164.1	540	24887	0	517	540	95	−	1	540	11429	11968
UN10873	Rsa1.0_00157.1	799	245832	1.00E-151	297	304	97	−	11	314	187327	187629
UN10874	Rsa1.0_00869.1	671	61627	1.00E-107	221	229	96	+	142	370	53993	54221
UN10875	Rsa1.0_17551.1	876	1799	0	856	868	98	+	7	870	368	1234
UN10876	Rsa1.0_00898.1	653	202772	1.00E-161	288	288	100	−	15	302	8777	9064
UN10877	Rsa1.0_02505.1	1050	30703	1.00E-153	330	347	95	−	704	1050	10020	10365
UN10878	Rsa1.0_08630.1	1525	7104	0	1480	1519	97	−	11	1524	4794	6312
UN10879	Rsa1.0_06492.1	608	10913	0	498	508	98	+	1	508	6242	6745

UN10880	Rsa1.0_00191.1	915	316976	0	417	421	99	-	1	421	160979	161398
UN10881	Rsa1.0_01061.1	865	55349	0	391	405	96	-	1	403	17654	18057
UN10882	Rsa1.0_53975.1	846	422	2.00E-94	228	243	93	-	2	242	37	278
UN10883	Rsa1.0_11545.1	478	5474	1.00E-122	352	393	89	-	1	384	4900	5282
UN10884	Rsa1.0_00615.1	762	72235	0	728	761	95	+	2	762	60743	61480
UN10885	Rsa1.0_00589.1	1239	209965	1.00E-110	278	304	91	+	1	286	188569	188872
UN10886	Rsa1.0_07334.1	940	9547	1.00E-164	327	336	97	-	1	333	683	1018
UN10887	Rsa1.0_00377.1	597	90038	0	560	589	95	-	9	597	69290	69874
UN10888	Rsa1.0_00040.1	1883	324196	0	662	676	97	+	824	1494	261473	262142
UN10889	Rsa1.0_00203.1	1112	109129	7.00E-86	251	282	89	+	760	1041	89591	89863
UN10890	Rsa1.0_08575.1	1542	7180	0	457	458	99	-	897	1354	5922	6379
UN10891	Rsa1.0_00015.1	1330	561803	0	1134	1171	96	-	162	1330	478490	479655
UN10892	Rsa1.0_00060.1	1102	502678	0	493	520	94	-	13	517	144937	145456
UN10893	Rsa1.0_00083.1	1045	442773	0	857	893	95	-	129	1015	359544	360429
UN10894	Rsa1.0_73033.1	715	314	1.00E-152	309	317	97	-	251	567	1	314
UN10895	Rsa1.0_00701.1	1170	67691	3.00E-94	282	319	88	+	791	1100	23226	23528
UN10896	Rsa1.0_02064.1	1044	35476	0	482	505	95	-	545	1043	16521	17023
UN10897	Rsa1.0_01536.1	1063	43747	0	801	865	92	-	16	860	18915	19773
UN10898	Rsa1.0_01339.1	864	47813	2.00E-57	139	145	95	+	3	144	12190	12334
UN10899	Rsa1.0_00736.1	1185	66455	0	1012	1041	97	+	146	1185	50684	51717
UN10900	Rsa1.0_04041.1	751	19430	8.00E-97	213	224	95	-	1	224	463	686
UN10901	Rsa1.0_01248.1	710	154103	1.00E-106	256	276	92	+	367	639	42217	42486
UN10902	Rsa1.0_01476.1	1185	172928	1.00E-125	285	303	94	-	884	1185	137458	137754
UN10903	Rsa1.0_03761.1	843	20982	1.00E-176	328	333	98	+	509	841	722	1054
UN10904	Rsa1.0_01239.1	763	50107	1.00E-105	212	217	97	+	1	215	17160	17376
UN10905	Rsa1.0_00016.1	926	831256	1.00E-150	319	334	95	+	593	926	426234	426566
UN10906	Rsa1.0_03594.1	780	21868	0	489	503	97	-	278	780	15578	16080
UN10907	Rsa1.0_04520.1	462	16995	1.00E-85	234	256	91	+	191	443	11580	11835
UN10908	Rsa1.0_00792.1	550	64163	2.00E-65	186	202	92	+	348	541	7358	7558
UN10909	Rsa1.0_00019.1	424	301669	1.00E-145	357	390	91	-	56	422	286292	286681
UN10910	Rsa1.0_00165.1	1082	116466	1.00E-142	355	389	91	-	3	376	51074	51454
UN10911	Rsa1.0_00533.1	1224	77375	1.00E-122	266	279	95	-	124	402	66092	66369
UN10912	Rsa1.0_00307.1	1044	199003	1.00E-108	268	287	93	-	10	293	88766	89052
UN10913	Rsa1.0_00345.1	815	93226	0	645	687	93	-	136	806	23826	24499
UN10914	Rsa1.0_00757.1	1304	65559	1.00E-142	265	267	99	-	1038	1304	58108	58372
UN10915	Rsa1.0_01328.1	664	48021	0	535	555	96	+	111	662	17568	18114
UN10916	Rsa1.0_03042.1	942	25803	0	429	430	99	+	513	942	9536	9965
UN10917	Rsa1.0_00285.1	1190	149638	1.00E-124	305	331	92	+	75	396	95084	95414
UN10918	Rsa1.0_04027.1	935	19501	0	689	776	88	+	163	926	6199	6952

UN10919	Rsa1.0_03385.1	979	23280	0	562	571	98	+	3	573	14015	14585
UN10920	Rsa1.0_00329.1	718	222544	4.00E-89	254	280	90	−	450	717	178122	178397
UN10921	Rsa1.0_11872.1	1059	5384	1.00E-126	321	345	93	−	710	1047	606	947
UN10922	Rsa1.0_02987.1	1420	26196	0	584	602	97	+	819	1420	21354	21955
UN10923	Rsa1.0_01294.1	1463	49090	0	1176	1232	95	−	229	1460	284	1512
UN10924	Rsa1.0_02165.1	833	34423	1.00E-166	309	313	98	+	1	313	12551	12863
UN10925	Rsa1.0_03294.1	789	23971	0	492	503	97	+	3	505	21764	22266
UN10926	Rsa1.0_00418.1	1983	215377	0	482	498	96	+	1171	1668	4220	4717
UN10927	Rsa1.0_00324.1	749	218805	0	637	651	97	−	99	749	216761	217409
UN10928	Rsa1.0_00425.1	1094	147080	0	663	668	99	+	1	668	85979	86646
UN10929	Rsa1.0_02608.1	891	29613	0	470	497	94	+	21	512	22131	22620
UN10930	Rsa1.0_05753.1	2229	12732	0	961	966	99	+	489	1451	11407	12372
UN10931	Rsa1.0_01896.1	1398	37512	0	1382	1405	98	+	2	1398	5758	7162
UN10932	Rsa1.0_00040.1	1889	324196	1.00E-108	202	203	99	−	1053	1255	267043	267245
UN10933	Rsa1.0_03143.1	1079	25024	0	608	648	93	+	444	1079	12212	12850
UN10934	Rsa1.0_00410.1	1037	86957	1.00E-123	279	291	95	+	376	663	20313	20600
UN10935	Rsa1.0_07606.1	657	9035	0	629	656	95	−	9	657	1976	2623
UN10936	Rsa1.0_00492.1	1405	80178	0	648	676	95	−	733	1401	26004	26677
UN10937	Rsa1.0_00107.1	819	431659	8.00E-85	235	260	90	−	561	819	254238	254478
UN10938	Rsa1.0_01397.1	862	183276	0	799	831	96	+	7	832	16441	17269
UN10939	Rsa1.0_00404.1	918	87635	0	913	915	99	−	1	915	17495	18409
UN10940	Rsa1.0_00642.1	1405	201230	0	939	1005	93	+	406	1400	157947	158941
UN10941	Rsa1.0_02803.1	724	27715	1.00E-162	333	347	95	+	25	368	18769	19115
UN10942	Rsa1.0_01655.1	896	41508	0	503	503	100	−	394	896	1620	2122
UN10943	Rsa1.0_00394.1	565	148426	0	439	470	93	+	86	549	138987	139445
UN10944	Rsa1.0_00784.1	1022	64359	1.00E-151	370	394	93	−	630	1019	7625	8009
UN10945	Rsa1.0_00060.1	654	502678	1.00E-145	288	297	96	+	3	299	499386	499682
UN10946	Rsa1.0_00218.1	910	201480	1.00E-166	299	300	99	+	611	910	147248	147547
UN10947	Rsa1.0_02104.1	495	34985	1.00E-119	278	292	95	+	192	480	23511	23799
UN10948	Rsa1.0_00767.1	1202	64980	2.00E-92	260	288	90	−	4	289	18547	18819
UN10949	Rsa1.0_66323.1	458	345	1.00E-156	291	295	98	−	3	297	1	295
UN10950	Rsa1.0_00824.1	865	188447	0	583	597	97	+	58	654	154502	155098
UN10951	Rsa1.0_00079.1	1416	606987	0	385	401	96	−	2	402	450064	450464
UN10952	Rsa1.0_00347.1	1600	93048	0	399	419	95	+	1187	1600	12000	12414
UN10953	Rsa1.0_01948.1	711	36966	0	697	707	98	+	2	708	21363	22069
UN10954	Rsa1.0_03781.1	1138	20891	1.00E-176	360	378	95	+	140	517	1213	1578
UN10955	Rsa1.0_02642.1	1002	29181	0	796	818	97	+	179	991	13097	13914
UN10956	Rsa1.0_00025.1	965	277312	0	710	762	93	+	222	964	64911	65667
UN10957	Rsa1.0_00253.1	1170	231292	0	616	645	95	−	222	866	25399	26042

UN10958	Rsa1.0_22094.1	1300	1150	1.00E-180	340	345	98	-	957	1300	725	1069
UN10959	Rsa1.0_00349.1	1451	92621	1.00E-103	191	191	100	-	409	599	41412	41602
UN10960	Rsa1.0_00149.1	1671	305196	0	542	562	96	-	521	1080	200113	200674
UN10961	Rsa1.0_37121.1	1253	607	1.00E-132	338	371	91	+	138	508	237	607
UN10962	Rsa1.0_02785.1	599	27881	1.00E-123	241	246	97	-	353	598	22114	22356
UN10963	Rsa1.0_00639.1	743	165873	0	480	515	93	+	233	742	124129	124625
UN10964	Rsa1.0_05218.1	783	14457	1.00E-113	235	243	96	-	542	783	9617	9859
UN10965	Rsa1.0_00101.1	868	267193	0	821	871	94	+	2	861	192729	193589
UN10966	Rsa1.0_00269.1	848	341668	2.00E-97	202	209	96	+	344	552	322820	323028
UN10967	Rsa1.0_01382.1	963	46877	1.00E-176	369	387	95	-	577	963	16703	17085
UN10968	Rsa1.0_00447.1	924	84051	5.00E-77	162	167	97	-	5	171	26801	26967
UN10969	Rsa1.0_00437.1	937	84879	0	382	396	96	-	26	421	71069	71463
UN10970	Rsa1.0_40049.1	1312	561	2.00E-83	164	166	98	-	951	1116	244	409
UN10971	Rsa1.0_02841.1	1049	27383	1.00E-106	217	220	98	+	833	1049	5500	5719
UN10972	Rsa1.0_00062.1	812	299291	1.00E-108	217	223	97	+	204	426	281388	281610
UN10973	Rsa1.0_00707.1	829	190112	1.00E-138	267	273	97	-	172	444	188378	188650
UN10974	Rsa1.0_01537.1	779	187285	1.00E-125	278	295	94	+	223	517	19498	19792
UN10975	Rsa1.0_00422.1	761	86144	0	451	473	95	+	1	473	32011	32483
UN10976	Rsa1.0_00666.1	1047	69505	0	541	569	95	-	478	1045	41845	42408
UN10977	Rsa1.0_00114.1	736	131330	9.00E-69	142	145	97	+	234	378	117141	117285
UN10978	Rsa1.0_01190.1	665	194501	0	396	414	95	-	246	658	157903	158316
UN10979	Rsa1.0_01013.1	734	56822	1.00E-165	355	370	95	-	369	734	45836	46205
UN10980	Rsa1.0_00014.1	682	348985	7.00E-97	192	196	97	-	11	206	220714	220909
UN10981	Rsa1.0_00914.1	1159	59880	0	1098	1166	94	+	10	1159	12963	14122
UN10982	Rsa1.0_00058.1	804	159921	0	396	410	96	+	307	712	50728	51137
UN10983	Rsa1.0_07314.1	918	9563	0	422	430	98	+	2	431	6290	6719
UN10984	Rsa1.0_00092.1	1001	659932	1.00E-170	428	461	92	+	7	455	504555	505012
UN10985	Rsa1.0_00544.1	1014	76787	1.00E-154	280	281	99	-	418	698	9770	10050
UN10986	Rsa1.0_02767.1	1016	28111	0	979	1033	94	-	2	1016	9776	10806
UN10987	Rsa1.0_01168.1	493	153765	6.00E-75	198	212	93	-	288	493	23484	23689
UN10988	Rsa1.0_00217.1	2017	107079	0	557	578	96	+	14	591	55728	56299
UN10989	Rsa1.0_01382.1	964	46877	1.00E-154	279	280	99	-	685	964	37439	37718
UN10990	Rsa1.0_00224.1	1393	327530	1.00E-180	376	391	96	-	1006	1393	38875	39264
UN10991	Rsa1.0_05827.1	578	12503	1.00E-147	298	309	96	-	2	310	10771	11079
UN10992	Rsa1.0_39537.1	913	569	0	555	569	97	-	346	912	1	569
UN10993	Rsa1.0_00009.1	261	232558	1.00E-62	161	173	93	+	11	183	52203	52373
UN10994	Rsa1.0_06488.1	941	141774	1.00E-167	302	303	99	-	272	574	138123	138425
UN10995	Rsa1.0_01938.1	881	264018	0	584	617	94	-	262	874	159660	160265
UN10996	Rsa1.0_00480.1	576	81325	0	521	523	99	+	54	576	7827	8349

UN10997	Rsa1.0_03081.1	1587	25523	0	805	820	98	−	529	1346	22646	23459
UN10998	Rsa1.0_52681.1	815	432	0	330	332	99	−	80	411	101	432
UN10999	Rsa1.0_07806.1	1165	8506	1.00E−139	278	286	97	−	883	1165	7007	7292
UN11000	Rsa1.0_11948.1	813	5363	1.00E−134	330	359	91	−	475	810	5007	5363
UN11001	Rsa1.0_03842.1	1239	20503	0	599	609	98	+	585	1190	8299	8907
UN11002	Rsa1.0_02128.1	1218	34758	1.00E−127	262	271	96	+	949	1218	16398	16668
UN11003	Rsa1.0_02637.1	1116	29244	1.00E−159	311	319	97	+	799	1114	6155	6473
UN11004	Rsa1.0_01300.1	1373	48942	0	484	498	97	+	882	1373	46684	47181
UN11005	Rsa1.0_02266.1	1578	33195	0	611	631	96	−	620	1250	30338	30965
UN11006	Rsa1.0_09130.1	724	6623	0	626	654	95	−	65	715	3374	4021
UN11007	Rsa1.0_00900.1	819	60367	0	463	479	96	−	341	819	17468	17935
UN11008	Rsa1.0_00330.1	1323	94666	0	651	651	100	+	3	653	26733	27383
UN11009	Rsa1.0_04141.1	983	18864	1.00E−133	250	253	98	−	277	529	16507	16759
UN11010	Rsa1.0_24633.1	1309	976	1.00E−69	161	169	95	+	3	168	742	910
UN11011	Rsa1.0_04873.1	753	15657	1.00E−135	280	289	96	−	13	299	8145	8433
UN11012	Rsa1.0_02803.1	489	27715	1.00E−73	188	203	92	−	290	489	17281	17483
UN11013	Rsa1.0_02939.1	1463	26595	0	769	770	99	−	694	1462	19725	20494
UN11014	Rsa1.0_00855.1	942	62047	0	839	866	96	+	1	862	11433	12295
UN11015	Rsa1.0_01860.1	1064	38057	6.00E−86	165	166	99	−	654	819	23462	23627
UN11016	Rsa1.0_01282.1	1097	49304	0	838	880	95	−	220	1082	30174	31052
UN11017	Rsa1.0_00424.1	428	86005	0	425	440	96	−	1	428	20411	20850
UN11018	Rsa1.0_00486.1	606	80875	0	527	554	95	−	56	605	44160	44700
UN11019	Rsa1.0_15414.1	1104	2620	1.00E−129	332	362	91	−	748	1104	321	674
UN11020	Rsa1.0_05874.1	821	12351	0	728	755	96	−	66	813	9404	10158
UN11021	Rsa1.0_04355.1	1188	146733	0	538	560	96	−	625	1178	3278	3831
UN11022	Rsa1.0_05820.1	1109	12540	1.00E−81	164	167	98	−	681	847	12002	12168
UN11023	Rsa1.0_00609.1	670	72785	1.00E−122	240	246	97	−	15	260	62899	63144
UN11024	Rsa1.0_02756.1	1006	28202	1.00E−101	196	199	98	−	269	467	19920	20118
UN11025	Rsa1.0_00302.1	707	97521	0	686	721	95	−	1	707	61937	62651
UN11026	Rsa1.0_00107.1	1517	431659	0	1190	1224	97	−	294	1512	14065	15287
UN11027	Rsa1.0_23188.1	438	1065	0	347	349	99	−	90	438	228	576
UN11028	Rsa1.0_03723.1	852	21173	0	358	360	99	+	3	362	4087	4446
UN11029	Rsa1.0_01182.1	912	167304	1.00E−179	328	330	99	+	3	332	11267	11595
UN11030	Rsa1.0_00354.1	1117	232005	1.00E−93	210	219	95	+	3	220	166322	166539
UN11031	Rsa1.0_06209.1	1097	11484	1.00E−177	430	471	91	+	33	503	2267	2719
UN11032	Rsa1.0_03898.1	874	20183	1.00E−129	308	327	94	+	549	866	2513	2834
UN11033	Rsa1.0_01960.1	768	36766	1.00E−111	205	205	100	+	116	320	25981	26185
UN11034	Rsa1.0_02113.1	697	34890	0	629	633	99	+	68	697	22890	23522
UN11035	Rsa1.0_01783.1	668	39383	0	646	668	96	−	3	668	19838	20499

UN11036	Rsa1.0_00031.1	924	179862	0	393	403	97	−	5	407	161267	161669
UN11037	Rsa1.0_03865.1	740	20373	1.00E−119	227	230	98	−	188	417	15975	16204
UN11038	Rsa1.0_00108.1	1485	478491	1.00E−160	385	412	93	+	189	597	419082	419489
UN11039	Rsa1.0_00048.1	1316	167185	0	725	745	97	+	2	740	125478	126221
UN11040	Rsa1.0_01285.1	1783	172709	0	478	487	98	+	1280	1766	134715	135200
UN11041	Rsa1.0_00595.1	833	341648	1.00E−168	407	440	92	−	2	424	24913	25348
UN11042	Rsa1.0_01434.1	1092	45951	1.00E−172	352	367	95	+	723	1089	5302	5668
UN11043	Rsa1.0_02734.1	1058	28344	0	1014	1070	94	+	2	1057	1722	2790
UN11044	Rsa1.0_11621.1	602	5457	1.00E−107	278	297	93	+	309	602	601	893
UN11045	Rsa1.0_07645.1	861	8949	2.00E−88	190	198	95	+	638	835	451	648
UN11046	Rsa1.0_00746.1	558	66044	1.00E−168	300	300	100	+	259	558	56840	57139
UN11047	Rsa1.0_00529.1	887	77487	0	623	637	97	−	244	879	53899	54534
UN11048	Rsa1.0_03568.1	901	22048	0	677	688	98	+	214	901	18563	19250
UN11049	Rsa1.0_00887.1	738	61048	1.00E−92	191	197	96	+	190	386	49405	49601
UN11050	Rsa1.0_00608.1	1034	172672	0	605	617	98	+	6	622	112746	113362
UN11051	Rsa1.0_01022.1	589	56606	1.00E−122	252	262	96	−	79	340	37881	38142
UN11052	Rsa1.0_00045.1	1169	328694	1.00E−131	259	266	97	−	294	559	140949	141214
UN11053	Rsa1.0_00539.1	1518	76926	0	555	566	98	+	936	1501	74541	75105
UN11054	Rsa1.0_00092.1	493	659932	0	468	494	94	−	2	492	430883	431376
UN11055	Rsa1.0_00796.1	450	63997	1.00E−124	269	281	95	−	171	450	57203	57482
UN11056	Rsa1.0_02244.1	1444	33494	1.00E−180	373	387	96	+	1026	1408	3842	4228
UN11057	Rsa1.0_00133.1	1040	123768	1.00E−136	297	310	95	−	110	417	11639	11947
UN11058	Rsa1.0_00023.1	815	194764	0	624	640	97	+	177	815	104143	104779
UN11059	Rsa1.0_00097.1	1237	138858	0	355	360	98	−	876	1235	2757	3116
UN11060	Rsa1.0_02051.1	1756	35564	1.00E−100	195	198	98	+	737	934	24062	24259
UN11061	Rsa1.0_00664.1	884	69573	1.00E−165	318	325	97	+	559	880	52733	53057
UN11062	Rsa1.0_01519.1	865	44144	0	501	520	96	−	347	865	11634	12151
UN11063	Rsa1.0_00382.1	1176	251946	1.00E−127	284	297	95	−	878	1174	34559	34848
UN11064	Rsa1.0_00954.1	868	189536	0	733	747	98	−	4	744	8442	9188
UN11065	Rsa1.0_00031.1	1408	179862	4.00E−97	184	185	99	−	1223	1407	86699	86883
UN11066	Rsa1.0_02737.1	856	28325	1.00E−120	277	294	94	+	560	849	8107	8391
UN11067	Rsa1.0_01239.1	798	50107	0	770	796	96	−	3	798	19758	20549
UN11068	Rsa1.0_00964.1	590	316648	0	560	589	95	−	2	580	167634	168213
UN11069	Rsa1.0_00273.1	753	326186	0	731	757	96	−	2	753	274609	275361
UN11070	Rsa1.0_00188.1	1059	112020	0	964	1029	93	+	2	1009	22283	23296
UN11071	Rsa1.0_00097.1	839	138858	0	430	434	99	+	1	434	91322	91755
UN11072	Rsa1.0_01466.1	796	45161	1.00E−157	300	305	98	+	490	794	28487	28790
UN11073	Rsa1.0_04273.1	987	18144	1.00E−103	232	248	93	+	752	987	12147	12394
UN11074	Rsa1.0_00079.1	798	606987	1.00E−108	307	344	89	−	24	364	66614	66942

UN11075	Rsa1.0_00720.1	601	66931	3.00E-71	224	251	89	+	356	599	26183	26426
UN11076	Rsa1.0_00602.1	884	73302	0	858	883	97	-	1	873	30255	31137
UN11077	Rsa1.0_02647.1	1490	29120	0	378	379	99	-	556	934	26972	27350
UN11078	Rsa1.0_02096.1	791	35041	0	564	570	98	+	222	790	14809	15378
UN11079	Rsa1.0_03092.1	1453	25448	0	466	471	98	+	983	1453	4317	4786
UN11080	Rsa1.0_00005.1	914	353093	0	486	490	99	+	5	494	341129	341617
UN11081	Rsa1.0_00497.1	839	79830	1.00E-98	269	290	92	+	550	836	54334	54614
UN11082	Rsa1.0_02756.1	1535	28202	0	673	674	99	+	6	679	16293	16966
UN11083	Rsa1.0_02426.1	796	31564	1.00E-151	289	292	98	+	507	796	12921	13212
UN11084	Rsa1.0_03237.1	1050	24434	0	403	421	95	+	212	629	748	1165
UN11085	Rsa1.0_00430.1	809	85453	0	722	754	95	-	58	809	44272	45025
UN11086	Rsa1.0_06310.1	1151	11262	0	768	913	84	+	1	913	5210	6122
UN11087	Rsa1.0_02280.1	240	33063	1.00E-105	206	210	98	+	31	240	911	1120
UN11088	Rsa1.0_00485.1	752	80937	2.00E-75	203	219	92	+	539	752	37570	37784
UN11089	Rsa1.0_00277.1	524	100132	0	328	329	99	+	197	524	10173	10501
UN11090	Rsa1.0_00191.1	1085	316976	0	533	590	90	+	38	627	9210	9799
UN11091	Rsa1.0_00076.1	1280	146021	1.00E-144	269	272	98	+	767	1038	44210	44481
UN11092	Rsa1.0_01716.1	853	40483	1.00E-141	288	299	96	+	523	821	18921	19219
UN11093	Rsa1.0_04873.1	942	15657	1.00E-116	240	247	97	-	1	243	8145	8391
UN11094	Rsa1.0_00636.1	1045	71113	1.00E-113	207	207	100	+	25	231	694	900
UN11095	Rsa1.0_03820.1	2664	20664	1.00E-137	322	346	93	+	1726	2071	8726	9062
UN11096	Rsa1.0_00027.1	680	283362	5.00E-61	155	165	93	-	520	680	105215	105373
UN11097	Rsa1.0_00311.1	853	96847	0	521	533	97	-	3	535	35682	36210
UN11098	Rsa1.0_33464.1	946	678	1.00E-148	278	282	98	-	196	477	154	435
UN11099	Rsa1.0_00098.1	580	349239	0	489	521	93	-	61	576	234714	235221
UN11100	Rsa1.0_03860.1	628	20407	2.33E-156	272	275	98	+	2	276	14073	14347
UN11101	Rsa1.0_04760.1	862	16105	4.00E-99	229	242	94	+	622	859	7185	7426
UN11102	Rsa1.0_04870.1	758	135142	1.00E-159	312	321	97	+	309	629	638	958
UN11103	Rsa1.0_00502.1	585	204180	0	452	483	93	+	35	516	63883	64361
UN11104	Rsa1.0_05523.1	1039	13438	0	759	807	94	+	3	787	8986	9790
UN11105	Rsa1.0_10176.1	757	5959	1.00E-125	265	275	96	+	1	274	3184	3457
UN11106	Rsa1.0_00184.1	1517	218748	0	882	913	96	+	1	913	58806	59718
UN11107	Rsa1.0_00017.1	564	752458	4.00E-58	222	254	87	-	10	259	40150	40393
UN11108	Rsa1.0_01135.1	801	52799	0	537	546	98	+	2	547	44890	45435
UN11109	Rsa1.0_54418.1	1155	419	3.00E-97	217	229	94	+	68	296	1	229
UN11110	Rsa1.0_00715.1	523	67116	1.00E-176	441	488	90	-	36	523	63649	64119
UN11111	Rsa1.0_00064.1	744	240927	0	491	494	99	-	251	744	112101	112594
UN11112	Rsa1.0_00877.1	1392	61305	0	546	563	96	-	824	1385	6841	7400
UN11113	Rsa1.0_01907.1	585	37431	0	470	488	96	-	3	490	17907	18393

UN11114	Rsa1.0_06392.1	1763	11104	0	1171	1279	91	+	507	1761	2775	4020
UN11115	Rsa1.0_02945.1	939	26548	0	537	540	99	−	165	704	8396	8935
UN11116	Rsa1.0_03233.1	870	24446	0	686	699	98	+	174	870	20170	20868
UN11117	Rsa1.0_00037.1	687	307838	1.00E−117	297	315	94	+	7	317	148299	148601
UN11118	Rsa1.0_00571.1	812	329018	0	733	744	98	+	70	812	165714	166456
UN11119	Rsa1.0_00179.1	822	393324	1.00E−153	344	364	94	+	1	359	334493	334856
UN11120	Rsa1.0_00363.1	572	90968	2.00E−90	195	204	95	−	228	425	53874	54077
UN11121	Rsa1.0_00220.1	949	192936	1.00E−158	330	342	96	+	1	340	143121	143460
UN11122	Rsa1.0_00145.1	872	261008	1.00E−136	266	271	98	+	2	271	50057	50327
UN11123	Rsa1.0_00079.1	858	606987	0	562	564	99	+	1	564	447436	447999
UN11124	Rsa1.0_03833.1	756	20575	1.00E−104	243	259	93	−	22	269	11313	11570
UN11125	Rsa1.0_02584.1	719	29877	1.00E−128	333	365	91	−	1	359	806	1155
UN11126	Rsa1.0_00296.1	1259	97854	0	1187	1259	94	+	3	1258	44704	45960
UN11127	Rsa1.0_01876.1	832	37800	1.00E−128	241	244	98	−	529	772	8719	8962
UN11128	Rsa1.0_00130.1	834	125737	1.00E−156	350	367	95	+	1	366	38241	38602
UN11129	Rsa1.0_00378.1	523	89892	1.00E−126	314	338	92	+	25	358	62119	62453
UN11130	Rsa1.0_00111.1	357	390088	1.00E−161	321	332	96	+	26	357	383857	384188
UN11131	Rsa1.0_00985.1	550	161950	1.00E−164	323	333	96	−	1	333	50364	50696
UN11132	Rsa1.0_00722.1	850	66840	0	384	400	96	+	4	399	58779	59175
UN11133	Rsa1.0_00277.1	998	100132	1.00E−111	314	351	89	+	11	360	83608	83938
UN11134	Rsa1.0_01878.1	783	37768	0	647	652	99	−	1	652	22554	23204
UN11135	Rsa1.0_01679.1	396	146876	1.00E−168	375	401	93	+	3	396	119034	119434
UN11136	Rsa1.0_00522.1	478	78103	1.00E−125	333	368	90	−	108	475	52124	52491
UN11137	Rsa1.0_05282.1	790	14270	8.00E−51	103	103	100	+	535	637	4965	5067
UN11138	Rsa1.0_03193.1	998	24690	1.00E−102	234	244	95	+	1	242	19553	19793
UN11139	Rsa1.0_00833.1	811	62621	1.00E−108	212	216	98	−	428	643	28739	28954
UN11140	Rsa1.0_00615.1	996	72235	0	382	387	98	−	1	387	26377	26761
UN11141	Rsa1.0_02550.1	1236	291051	1.00E−165	334	346	96	+	884	1227	6190	6535
UN11142	Rsa1.0_00360.1	1074	91190	1.00E−128	307	327	93	−	23	345	67270	67594
UN11143	Rsa1.0_00087.1	646	761073	2.00E−91	236	255	92	+	12	265	11767	12015
UN11144	Rsa1.0_19812.1	975	1380	1.00E−142	313	331	94	+	634	964	836	1163
UN11145	Rsa1.0_10606.1	969	5787	1.00E−163	327	336	97	+	1	336	2172	2505
UN11146	Rsa1.0_00960.1	1280	58199	0	383	389	98	−	2	389	30003	30391
UN11147	Rsa1.0_00435.1	580	317579	1.00E−138	344	369	93	+	1	361	70714	71080
UN11148	Rsa1.0_05501.1	309	13513	1.00E−87	206	220	93	−	3	222	6241	6460
UN11149	Rsa1.0_01175.1	875	51674	1.00E−166	360	377	95	−	29	401	979	1351
UN11150	Rsa1.0_00002.1	1185	601661	0	1161	1189	97	−	1	1183	412143	413331
UN11151	Rsa1.0_00387.1	917	495407	0	390	399	97	+	107	501	363148	363546
UN11152	Rsa1.0_01588.1	1510	42724	4.00E−97	184	185	99	−	1326	1510	38439	38623

UN11153	Rsa1.0_02266.1	853	33195	0	485	502	96	+	349	847	30338	30839
UN11154	Rsa1.0_00010.1	1014	294827	2.00E-86	287	324	88	+	7	322	135518	135837
UN11155	Rsa1.0_00913.1	665	59914	1.00E-142	326	353	92	−	332	663	57997	58349
UN11156	Rsa1.0_00157.1	490	245832	1.00E-169	417	455	91	−	44	490	163152	163606
UN11157	Rsa1.0_00040.1	1191	324196	0	648	662	97	−	530	1191	51659	52320
UN11158	Rsa1.0_00108.1	1089	478491	1.00E-161	384	411	93	+	1	398	450924	451332
UN11159	Rsa1.0_00050.1	509	237174	1.00E-148	296	305	97	−	1	303	33432	33736
UN11160	Rsa1.0_04085.1	856	19168	0	465	517	89	+	339	831	755	1268
UN11161	Rsa1.0_00635.1	742	175826	1.00E-86	222	246	90	−	87	314	15994	16239
UN11162	Rsa1.0_24942.1	645	959	1.00E-147	340	362	93	−	1	360	1	358
UN11163	Rsa1.0_29798.1	172	771	4.00E-46	109	114	95	+	1	114	559	672
UN11164	Rsa1.0_07757.1	864	8633	0	512	537	95	+	26	557	867	1403
UN11165	Rsa1.0_01896.1	1383	37512	0	452	466	96	+	832	1297	9812	10277
UN11166	Rsa1.0_00711.1	846	67223	0	427	441	96	+	401	841	30127	30566
UN11167	Rsa1.0_00025.1	716	277312	2.33E-156	299	310	96	−	2	310	113952	114261
UN11168	Rsa1.0_00495.1	749	79934	3.00E-50	120	126	95	+	295	420	16598	16723
UN11169	Rsa1.0_00536.1	849	77199	0	421	437	96	−	4	439	23555	23990
UN11170	Rsa1.0_01384.1	522	46803	1.00E-159	401	432	92	−	8	433	41768	42193
UN11171	Rsa1.0_00385.1	496	89160	1.00E-108	233	242	96	+	255	496	31104	31342
UN11172	Rsa1.0_00040.1	751	324196	0	655	690	94	−	63	751	224194	224883
UN11173	Rsa1.0_00595.1	810	341648	0	517	524	98	+	1	524	23278	23799
UN11174	Rsa1.0_00437.1	814	84879	1.00E-119	259	273	94	−	162	434	23635	23907
UN11175	Rsa1.0_00506.1	913	79106	0	517	531	97	+	383	913	21608	22138
UN11176	Rsa1.0_01156.1	843	52147	0	348	350	99	+	463	812	24757	25106
UN11177	Rsa1.0_03101.1	886	25410	0	878	887	98	−	1	886	19681	20567
UN11178	Rsa1.0_04500.1	1183	17083	1.00E-175	422	455	92	−	50	482	6445	6893
UN11179	Rsa1.0_03299.1	814	23949	0	566	593	95	−	7	590	14331	14919
UN11180	Rsa1.0_00332.1	1323	94575	0	551	600	91	−	7	594	59905	60490
UN11181	Rsa1.0_02157.1	647	149652	2.00E-69	158	166	95	+	1	166	132597	132762
UN11182	Rsa1.0_00208.1	1450	108565	0	1123	1151	97	+	1	1150	100682	101826
UN11183	Rsa1.0_01122.1	463	53369	0	451	462	97	+	1	462	27966	28426
UN11184	Rsa1.0_00023.1	759	194764	0	748	754	99	−	6	759	104960	105713
UN11185	Rsa1.0_00960.1	1442	58199	0	734	788	93	−	585	1365	19404	20167
UN11186	Rsa1.0_02402.1	932	31710	0	721	773	93	+	2	774	23951	24706
UN11187	Rsa1.0_00488.1	1358	80490	0	698	707	98	+	652	1358	6874	7580
UN11188	Rsa1.0_07617.1	1485	9015	0	1303	1352	96	−	5	1344	2699	4049
UN11189	Rsa1.0_01601.1	1285	42519	0	511	512	99	+	31	541	11077	11588
UN11190	Rsa1.0_01796.1	841	39154	1.00E-129	247	251	98	−	482	732	37475	37725
UN11191	Rsa1.0_11948.1	1312	5363	1.00E-117	250	261	95	+	1	261	3922	4180

UN11192	Rsa1.0_01220.1	658	161775	1.00E-131	288	305	94	+	6	296	126160	126462
UN11193	Rsa1.0_02020.1	814	173211	0	773	812	95	-	2	812	148364	149172
UN11194	Rsa1.0_00802.1	813	63846	1.00E-123	289	305	94	-	3	305	17081	17383
UN11195	Rsa1.0_00322.1	1265	212233	1.00E-117	221	223	99	+	187	409	80171	80393
UN11196	Rsa1.0_04230.1	1196	18384	0	457	469	97	-	728	1194	10536	11003
UN11197	Rsa1.0_00056.1	747	332604	1.00E-157	323	337	95	-	1	337	113294	113630
UN11198	Rsa1.0_00018.1	763	410899	1.00E-166	396	424	93	-	3	422	68738	69156
UN11199	Rsa1.0_01146.1	309	163455	2.00E-55	122	126	96	-	90	215	162566	162691
UN11200	Rsa1.0_02803.1	754	27715	1.00E-112	276	297	92	-	1	296	26242	26531
UN11201	Rsa1.0_00580.1	807	74515	1.00E-160	335	353	94	+	4	341	33897	34249
UN11202	Rsa1.0_00335.1	1212	94371	1.00E-179	367	383	95	-	828	1210	42153	42535
UN11203	Rsa1.0_00648.1	877	70351	4.00E-71	177	189	93	-	127	315	19476	19663
UN11204	Rsa1.0_00264.1	1135	101674	0	1105	1137	97	+	2	1131	11991	13127
UN11205	Rsa1.0_00417.1	1168	395068	1.00E-160	336	352	95	+	1	343	225420	225771
UN11206	Rsa1.0_00457.1	1136	337301	1.00E-166	367	389	94	+	283	668	5450	5831
UN11207	Rsa1.0_00339.1	413	222974	2.00E-40	88	89	98	-	305	393	40128	40216
UN11208	Rsa1.0_15801.1	768	2415	0	703	725	96	-	1	724	910	1634
UN11209	Rsa1.0_00453.1	1834	83031	0	466	502	92	+	1050	1532	81190	81689
UN11210	Rsa1.0_00566.1	992	75325	0	366	377	97	+	443	819	20189	20565
UN11211	Rsa1.0_01996.1	796	36327	1.00E-175	321	323	99	+	1	323	22860	23181
UN11212	Rsa1.0_01236.1	678	50156	1.00E-104	199	201	99	+	229	429	44319	44519
UN11213	Rsa1.0_00707.1	842	190112	0	507	515	98	-	1	511	61070	61584
UN11214	Rsa1.0_00573.1	1037	74821	0	990	1037	95	+	9	1036	44746	45782
UN11215	Rsa1.0_00074.1	544	147591	1.00E-137	300	318	94	+	1	318	135599	135905
UN11216	Rsa1.0_07603.1	1103	9043	1.00E-111	348	394	88	+	213	606	2808	3197
UN11217	Rsa1.0_00421.1	780	86365	1.00E-125	290	307	94	+	16	314	82627	82933
UN11218	Rsa1.0_00856.1	975	62037	0	465	491	94	+	1	491	6415	6889
UN11219	Rsa1.0_06373.1	989	11138	1.00E-148	292	299	97	+	26	319	735	1033
UN11220	Rsa1.0_01326.1	1165	48072	0	524	552	94	-	1	545	40139	40681
UN11221	Rsa1.0_00928.1	598	59293	4.00E-95	199	205	97	+	1	205	29534	29737
UN11222	Rsa1.0_00173.1	1204	288743	3.00E-91	266	290	91	+	925	1204	25595	25882
UN11223	Rsa1.0_01493.1	1330	44670	0	505	526	96	+	1	523	13292	13805
UN11224	Rsa1.0_00055.1	1588	520193	0	457	483	94	+	1	479	385970	386446
UN11225	Rsa1.0_01696.1	1088	301850	3.00E-63	139	144	96	-	711	854	291899	292042
UN11226	Rsa1.0_02765.1	1456	28141	0	388	398	97	+	1	392	16308	16705
UN11227	Rsa1.0_00626.1	1252	71582	0	378	380	99	-	872	1251	17748	18127
UN11228	Rsa1.0_01768.1	827	138463	1.00E-117	220	222	99	-	448	669	118797	119018
UN11229	Rsa1.0_00073.1	1526	240924	0	408	424	96	+	1102	1525	226964	227384
UN11230	Rsa1.0_26587.1	1271	885	0	601	647	92	+	1	642	262	885

UN11231	Rsa1.0_00023.1	1167	194764	0	1146	1174	97	+	4	1166	70822	71992
UN11232	Rsa1.0_03568.1	972	22048	0	712	731	97	−	1	731	18651	19379
UN11233	Rsa1.0_55259.1	1584	413	0	405	413	98	+	93	505	1	413
UN11234	Rsa1.0_01593.1	1263	150996	1.00E−155	361	386	93	+	1	385	149467	149850
UN11235	Rsa1.0_13607.1	1238	4645	0	1217	1235	98	−	2	1236	1747	2981
UN11236	Rsa1.0_18598.1	787	1567	0	660	662	99	−	1	662	258	919
UN11237	Rsa1.0_00250.1	1164	273771	0	487	516	94	−	650	1163	253679	254181
UN11238	Rsa1.0_08412.1	792	7404	0	505	537	94	+	10	542	4359	4887
UN11239	Rsa1.0_03623.1	765	21714	0	667	675	98	+	4	678	11647	12321
UN11240	Rsa1.0_01626.1	1146	41989	1.00E−106	196	196	100	−	542	737	22145	22340
UN11241	Rsa1.0_00643.1	814	70658	0	466	466	100	−	1	466	27681	28146
UN11242	Rsa1.0_01682.1	851	41090	0	846	853	99	−	1	851	26026	26877
UN11243	Rsa1.0_00512.1	824	200277	0	555	555	100	−	2	556	55821	56375
UN11244	Rsa1.0_00376.1	825	90101	0	437	458	95	−	31	485	49557	50013
UN11245	Rsa1.0_00048.1	827	167185	1.00E−128	268	280	95	+	429	708	8087	8366
UN11246	Rsa1.0_00699.1	1061	184398	0	368	383	96	−	1	383	145515	145897
UN11247	Rsa1.0_03780.1	1311	20899	2.00E−71	194	206	94	+	69	272	372	574
UN11248	Rsa1.0_00628.1	1029	214360	1.00E−111	207	208	99	−	3	210	48600	48807
UN11249	Rsa1.0_02026.1	907	35896	0	445	450	98	+	1	450	14910	15358
UN11250	Rsa1.0_01271.1	382	49569	0	382	382	100	−	1	382	28301	28682
UN11251	Rsa1.0_04490.1	1172	17144	0	1153	1154	99	−	1	1154	12296	13449
UN11252	Rsa1.0_02623.1	773	29409	0	368	376	97	+	1	376	9606	9981
UN11253	Rsa1.0_00316.1	651	96264	3.00E−40	106	112	94	+	134	243	57738	57849
UN11254	Rsa1.0_00247.1	781	103408	0	468	471	99	+	1	471	6037	6507
UN11255	Rsa1.0_02505.1	830	30703	1.00E−145	348	376	92	−	1	374	17073	17438
UN11256	Rsa1.0_02560.1	1464	30091	0	456	456	100	+	3	458	718	1173
UN11257	Rsa1.0_00248.1	1422	163568	0	605	658	91	+	781	1422	50066	50723
UN11258	Rsa1.0_00490.1	1651	186713	1.00E−117	302	327	92	+	1327	1651	153661	153982
UN11259	Rsa1.0_09108.1	1413	6644	0	907	942	96	−	473	1412	1601	2541
UN11260	Rsa1.0_01062.1	673	55325	1.00E−145	389	429	90	+	1	412	51156	51574
UN11261	Rsa1.0_00336.1	381	445051	0	378	381	99	+	1	380	263448	263828
UN11262	Rsa1.0_00556.1	1173	207212	1.00E−122	356	392	90	+	9	389	33472	33859
UN11263	Rsa1.0_00135.1	647	178077	0	438	447	97	+	201	647	23609	24053
UN11264	Rsa1.0_00081.1	1206	143261	1.00E−107	210	214	98	+	252	465	1376	1589
UN11265	Rsa1.0_01118.1	788	53460	1.00E−117	222	223	99	+	1	223	12659	12880
UN11266	Rsa1.0_00357.1	1387	173671	0	1331	1387	95	+	1	1385	144609	145991
UN11267	Rsa1.0_04051.1	786	19375	0	460	466	98	−	1	465	8731	9194
UN11268	Rsa1.0_00198.1	2219	222963	0	377	383	98	−	1837	2219	17904	18285
UN11269	Rsa1.0_00427.1	1157	189678	1.00E−155	336	351	95	+	2	350	182599	182948

UN11270	Rsa1.0_01177.1	775	51572	0	436	443	98	+	233	675	6753	7195
UN11271	Rsa1.0_02316.1	1173	32608	1.00E-105	221	230	96	−	469	698	17754	17983
UN11272	Rsa1.0_00033.1	972	515811	0	514	549	93	+	7	551	333831	334369
UN11273	Rsa1.0_00145.1	931	261008	0	719	729	98	+	1	729	101336	102061
UN11274	Rsa1.0_00014.1	706	348985	0	693	705	98	−	3	706	174300	175003
UN11275	Rsa1.0_00127.1	741	127446	1.00E-180	345	352	98	−	92	442	106654	107005
UN11276	Rsa1.0_04071.1	789	19243	0	544	554	98	−	1	551	3747	4299
UN11277	Rsa1.0_15608.1	1104	2522	0	945	992	95	+	1	978	154	1144
UN11278	Rsa1.0_00671.1	783	397884	0	548	560	97	−	3	561	176948	177507
UN11279	Rsa1.0_03926.1	1054	20024	1.00E-147	264	264	100	−	789	1052	5352	5615
UN11280	Rsa1.0_00423.1	933	86138	0	658	695	94	−	3	686	48394	49074
UN11281	Rsa1.0_02064.1	1594	35476	0	429	438	97	+	1010	1447	21446	21883
UN11282	Rsa1.0_00627.1	819	71566	3.00E-93	233	251	92	−	1	249	23881	24118
UN11283	Rsa1.0_53997.1	553	422	1.00E-144	313	330	94	−	224	553	95	422
UN11284	Rsa1.0_00210.1	974	108424	0	468	479	97	−	450	924	26237	26714
UN11285	Rsa1.0_00339.1	1379	222974	0	1365	1378	99	+	1	1377	81031	82408
UN11286	Rsa1.0_02248.1	1146	33414	0	536	558	96	−	1	550	3316	3870
UN11287	Rsa1.0_05490.1	1009	13556	1.00E-123	237	240	98	+	515	754	10484	10722
UN11288	Rsa1.0_00707.1	1150	190112	1.00E-168	331	339	97	−	11	349	161352	161687
UN11289	Rsa1.0_22550.1	1400	1111	0	562	583	96	−	1	583	485	1065
UN11290	Rsa1.0_01220.1	475	161775	1.00E-104	214	220	97	−	47	266	117110	117328
UN11291	Rsa1.0_21301.1	918	1217	1.00E-105	275	300	91	−	603	899	79	377
UN11292	Rsa1.0_19077.1	1161	1483	0	430	430	100	+	672	1101	1054	1483
UN11293	Rsa1.0_00103.1	626	135304	0	624	624	100	+	1	624	107507	108130
UN11294	Rsa1.0_01249.1	1610	188432	0	462	488	94	−	555	1030	43045	43532
UN11295	Rsa1.0_27704.1	1348	842	1.00E-148	304	315	96	−	357	670	51	365
UN11296	Rsa1.0_02280.1	1017	33063	0	1011	1012	99	+	1	1012	3266	4277
UN11297	Rsa1.0_02399.1	598	31763	0	556	593	93	−	7	598	13079	13667
UN11298	Rsa1.0_01405.1	1219	46445	0	575	586	98	+	1	586	20659	21238
UN11299	Rsa1.0_01142.1	713	52464	1.00E-111	221	226	97	−	121	346	50939	51161
UN11300	Rsa1.0_02585.1	1211	29865	0	344	346	99	+	759	1103	19802	20147
UN11301	Rsa1.0_00399.1	1238	242951	0	409	423	96	−	1	420	43978	44400
UN11302	Rsa1.0_04934.1	936	143962	0	902	943	95	+	2	936	11089	12028
UN11303	Rsa1.0_04456.1	1168	17260	1.00E-62	361	443	81	−	546	962	8880	9305
UN11304	Rsa1.0_01896.1	991	37512	0	954	991	96	+	3	991	31704	32692
UN11305	Rsa1.0_00130.1	975	125737	0	613	624	98	+	352	975	42198	42821
UN11306	Rsa1.0_00679.1	912	68971	1.00E-162	289	289	100	−	3	291	49245	49533
UN11307	Rsa1.0_00047.1	1360	168582	0	1208	1221	98	+	1	1221	67959	69179
UN11308	Rsa1.0_00046.1	801	320315	0	597	617	96	−	1	605	47138	47754

UN11309	Rsa1.0_02505.1	1315	30703	0	523	549	95	+	4	544	17564	18107
UN11310	Rsa1.0_00059.1	870	159650	0	732	762	96	+	1	760	77216	77963
UN11311	Rsa1.0_00695.1	1076	68019	0	626	627	99	+	2	627	58638	59264
UN11312	Rsa1.0_00042.1	1368	171507	1.00E-174	394	418	94	+	963	1366	159615	160028
UN11313	Rsa1.0_00254.1	1015	102600	0	494	524	94	+	3	514	90254	90773
UN11314	Rsa1.0_02210.1	562	33857	0	559	560	99	+	1	560	1029	1588
UN11315	Rsa1.0_02503.1	542	30709	4.00E-67	136	138	98	-	249	386	26114	26251
UN11316	Rsa1.0_01116.1	697	142088	2.33E-156	376	409	91	-	11	419	128912	129305
UN11317	Rsa1.0_00876.1	840	61328	0	722	760	95	+	1	742	35412	36171
UN11318	Rsa1.0_00009.1	986	232558	1.00E-160	347	366	94	-	120	477	53021	53382
UN11319	Rsa1.0_03709.1	1663	21247	0	974	980	99	-	685	1663	7789	8768
UN11320	Rsa1.0_00165.1	906	116466	1.00E-123	231	233	99	+	1	233	48170	48402
UN11321	Rsa1.0_02795.1	1372	27809	0	760	781	97	+	209	988	8002	8778
UN11322	Rsa1.0_00273.1	701	326186	1.00E-154	345	366	94	+	57	418	113973	114338
UN11323	Rsa1.0_24818.1	424	966	1.00E-163	318	327	97	-	98	424	521	847
UN11324	Rsa1.0_00048.1	1124	167185	0	370	377	98	-	2	376	100204	100579
UN11325	Rsa1.0_00239.1	1092	104332	0	450	450	100	+	1	450	103584	104033
UN11326	Rsa1.0_00186.1	846	112669	0	831	847	98	+	1	846	34682	35525
UN11327	Rsa1.0_03774.1	1097	20932	0	529	577	91	-	513	1079	16175	16736
UN11328	Rsa1.0_00105.1	1628	134138	0	587	623	94	-	3	606	61301	61914
UN11329	Rsa1.0_01536.1	499	43747	0	386	400	96	-	20	419	39543	39942
UN11330	Rsa1.0_00286.1	460	98709	1.00E-108	243	256	94	-	1	247	11606	11861
UN11331	Rsa1.0_00050.1	1461	237174	0	1049	1092	96	+	2	1092	196179	197268
UN11332	Rsa1.0_02555.1	1421	30146	0	1361	1424	95	+	1	1420	2651	4068
UN11333	Rsa1.0_00218.1	1097	201480	0	483	520	92	+	584	1095	180348	180863
UN11334	Rsa1.0_03552.1	1327	22151	1.00E-163	403	438	92	+	899	1327	12808	13231
UN11335	Rsa1.0_01768.1	1410	138463	0	492	523	94	-	1	523	124510	125017
UN11336	Rsa1.0_02224.1	921	33758	0	803	836	96	+	69	903	25744	26576
UN11337	Rsa1.0_01000.1	1323	57140	0	990	1050	94	-	2	1042	52715	53738
UN11338	Rsa1.0_00837.1	1122	62568	0	761	770	98	+	355	1122	4682	5451
UN11339	Rsa1.0_00570.1	1253	75002	0	738	770	95	+	480	1247	72668	73433
UN11340	Rsa1.0_00112.1	1470	132199	0	515	531	96	-	1	531	89572	90100
UN11341	Rsa1.0_00107.1	981	431659	1.00E-117	234	240	97	-	75	314	183555	183791
UN11342	Rsa1.0_00699.1	1235	184398	1.00E-162	328	340	96	-	15	354	11508	11844
UN11343	Rsa1.0_02020.1	732	173211	0	697	728	95	+	1	728	148361	149085
UN11344	Rsa1.0_00041.1	934	186197	1.00E-176	355	368	96	-	227	591	157071	157438
UN11345	Rsa1.0_03357.1	1534	23478	0	483	496	97	+	1043	1532	19472	19966
UN11346	Rsa1.0_00997.1	1270	57190	0	1268	1271	99	+	1	1270	34831	36101
UN11347	Rsa1.0_00394.1	674	148426	1.00E-174	312	313	99	-	362	674	120718	121030

UN11348	Rsa1.0_02671.1	1100	28887	1.00E-126	271	285	95	-	818	1100	9174	9449
UN11349	Rsa1.0_00399.1	1752	242951	0	666	667	99	+	1084	1750	189886	190552
UN11350	Rsa1.0_00247.1	427	103408	1.00E-100	198	202	98	-	226	427	64842	65043
UN11351	Rsa1.0_02858.1	744	27246	1.00E-169	404	435	92	-	321	744	2245	2679
UN11352	Rsa1.0_01996.1	925	36327	6.00E-98	185	186	99	-	256	441	25436	25621
UN11353	Rsa1.0_00955.1	1234	58358	1.00E-135	259	264	98	-	955	1218	8908	9171
UN11354	Rsa1.0_02456.1	767	31222	1.00E-118	247	256	96	-	512	767	23750	24004
UN11355	Rsa1.0_00299.1	1157	97770	0	1090	1120	97	-	40	1157	13734	14844
UN11356	Rsa1.0_05319.1	770	14158	1.00E-98	195	199	97	-	421	619	4536	4734
UN11357	Rsa1.0_00018.1	1305	410899	0	401	406	98	-	358	763	367394	367799
UN11358	Rsa1.0_00035.1	859	176449	5.00E-83	160	161	99	+	242	402	155788	155948
UN11359	Rsa1.0_23763.1	1543	1027	1.00E-162	291	291	100	-	1251	1541	410	700
UN11360	Rsa1.0_00130.1	1036	125737	1.00E-109	210	213	98	+	187	399	34393	34605
UN11361	Rsa1.0_00595.1	1424	341648	0	574	587	97	-	2	588	38530	39116
UN11362	Rsa1.0_05352.1	1328	14079	0	993	1004	98	+	323	1326	1715	2717
UN11363	Rsa1.0_01295.1	1170	49040	0	547	559	97	-	122	677	8398	8956
UN11364	Rsa1.0_01117.1	900	53465	1.00E-151	414	456	90	-	464	900	40147	40598
UN11365	Rsa1.0_00866.1	1180	61676	0	546	570	95	-	614	1180	29469	30037
UN11366	Rsa1.0_00152.1	1326	257730	0	416	432	96	-	3	434	101069	101487
UN11367	Rsa1.0_02020.1	714	173211	1.00E-116	286	303	94	-	413	712	20289	20586
UN11368	Rsa1.0_00923.1	1604	153678	1.00E-115	254	268	94	-	1336	1603	33375	33642
UN11369	Rsa1.0_00472.1	852	398134	2.00E-66	161	169	95	-	5	166	301550	301718
UN11370	Rsa1.0_02072.1	847	35401	1.00E-129	281	295	95	-	527	820	18059	18353
UN11371	Rsa1.0_02474.1	1426	31076	0	801	810	98	+	566	1375	5737	6546
UN11372	Rsa1.0_29181.1	769	790	1.00E-167	310	314	98	+	450	763	44	357
UN11373	Rsa1.0_00464.1	669	82253	0	539	550	98	+	122	669	75573	76121
UN11374	Rsa1.0_00721.1	809	66918	0	359	362	99	-	448	809	13199	13559
UN11375	Rsa1.0_01306.1	1122	48720	1.00E-144	281	287	97	-	834	1119	26057	26343
UN11376	Rsa1.0_01220.1	1062	161775	0	419	445	94	+	640	1062	147689	148133
UN11377	Rsa1.0_01675.1	690	41155	1.00E-155	290	294	98	-	394	687	10316	10609
UN11378	Rsa1.0_04882.1	611	15606	1.00E-144	280	287	97	-	1	287	7553	7839
UN11379	Rsa1.0_00090.1	682	273859	1.00E-109	222	228	97	-	5	232	55632	55858
UN11380	Rsa1.0_02438.1	1427	31392	0	621	654	94	+	496	1145	2419	3072
UN11381	Rsa1.0_00041.1	888	186197	0	875	889	98	+	1	885	30009	30893
UN11382	Rsa1.0_00269.1	1736	341668	0	509	524	97	+	236	759	330610	331133
UN11383	Rsa1.0_02840.1	1103	27386	0	732	774	94	-	3	765	26285	27058
UN11384	Rsa1.0_05911.1	906	12261	1.00E-141	312	332	93	+	588	906	5182	5513
UN11385	Rsa1.0_01688.1	1007	40965	1.00E-158	335	350	95	+	658	1007	36521	36867
UN11386	Rsa1.0_00484.1	823	203294	1.00E-139	261	263	99	-	3	265	43038	43298

UN11387	Rsa1.0_03623.1	708	21714	0	467	478	97	-	3	480	13561	14037
UN11388	Rsa1.0_07811.1	1208	8496	0	418	425	98	+	551	975	6679	7103
UN11389	Rsa1.0_02231.1	529	33661	1.00E-138	249	249	100	-	281	529	33337	33585
UN11390	Rsa1.0_02707.1	561	28520	0	525	554	94	-	11	560	9437	9988
UN11391	Rsa1.0_00746.1	990	66044	1.00E-164	297	298	99	-	693	990	63317	63614
UN11392	Rsa1.0_00017.1	1238	752458	1.00E-152	283	286	98	-	291	576	720938	721223
UN11393	Rsa1.0_03880.1	1462	20293	1.00E-118	226	229	98	+	331	559	18518	18746
UN11394	Rsa1.0_03302.1	1060	23898	0	461	469	98	-	591	1059	5959	6427
UN11395	Rsa1.0_00176.1	1110	240984	0	463	488	94	-	640	1110	151042	151527
UN11396	Rsa1.0_00407.1	921	191544	0	634	663	95	-	259	921	43103	43746
UN11397	Rsa1.0_00483.1	1084	81102	2.00E-52	239	285	83	-	819	1084	32615	32898
UN11398	Rsa1.0_01508.1	664	44334	0	472	474	99	+	191	664	37681	38154
UN11399	Rsa1.0_02267.1	763	33194	1.00E-124	229	230	99	-	331	560	30984	31213
UN11400	Rsa1.0_00618.1	611	72195	1.00E-111	238	249	95	+	26	270	2625	2873
UN11401	Rsa1.0_00224.1	1329	327530	1.00E-102	266	292	91	-	1040	1329	222136	222408
UN11402	Rsa1.0_00501.1	894	79545	1.00E-162	313	321	97	-	477	797	22510	22830
UN11403	Rsa1.0_01696.1	1114	301850	0	717	737	97	-	1	736	273492	274228
UN11404	Rsa1.0_01803.1	713	173846	1.00E-128	274	286	95	+	241	526	34625	34906
UN11405	Rsa1.0_14509.1	1066	3318	1.00E-133	292	308	94	+	98	403	1766	2067
UN11406	Rsa1.0_00123.1	1091	325650	1.00E-179	403	425	94	+	7	427	141835	142252
UN11407	Rsa1.0_00045.1	771	328694	3.00E-62	134	138	97	-	521	658	263471	263608
UN11408	Rsa1.0_00032.1	1391	306326	0	435	436	99	-	954	1389	265453	265888
UN11409	Rsa1.0_02150.1	1372	34580	0	842	862	97	+	89	950	26700	27561
UN11410	Rsa1.0_00467.1	785	156542	1.00E-126	279	296	94	-	484	773	80219	80514
UN11411	Rsa1.0_01328.1	1400	48021	0	431	441	97	+	270	709	16027	16467
UN11412	Rsa1.0_01476.1	1004	172928	0	481	512	93	+	488	995	8955	9464
UN11413	Rsa1.0_02728.1	696	28400	0	399	402	99	-	295	696	17632	18033
UN11414	Rsa1.0_00248.1	1297	163568	0	698	719	97	-	578	1296	50141	50859
UN11415	Rsa1.0_00136.1	1131	122464	1.00E-175	348	358	97	-	3	358	17206	17561
UN11416	Rsa1.0_00006.1	466	396176	0	451	467	96	+	2	466	32240	32706
UN11417	Rsa1.0_00407.1	1937	191544	0	507	507	100	-	1	507	163596	164102
UN11418	Rsa1.0_00589.1	504	209965	1.00E-151	405	451	89	-	80	504	181444	181891
UN11419	Rsa1.0_00068.1	1360	151813	0	979	1037	94	+	1	1022	42912	43944
UN11420	Rsa1.0_00997.1	1206	57190	0	1040	1076	96	+	3	1069	34836	35911
UN11421	Rsa1.0_03536.1	1120	22232	0	697	743	93	+	390	1120	7055	7797
UN11422	Rsa1.0_00473.1	1522	212204	1.00E-165	301	303	99	+	323	625	190581	190883
UN11423	Rsa1.0_00086.1	815	439854	0	780	802	97	+	3	802	434240	435039
UN11424	Rsa1.0_06691.1	759	10532	1.00E-171	329	337	97	+	423	759	6354	6690
UN11425	Rsa1.0_00476.1	1376	280966	0	849	853	99	-	525	1376	7408	8257

UN11426	Rsa1.0_02454.1	2210	31263	0	1427	1485	96	+	50	1524	24099	25580
UN11427	Rsa1.0_07055.1	858	9970	1.00E-153	275	275	100	+	226	500	219	493
UN11428	Rsa1.0_01249.1	726	188432	8.00E-97	186	188	98	−	315	502	151131	151318
UN11429	Rsa1.0_00182.1	1022	142305	1.00E-129	303	326	92	−	430	755	98126	98451
UN11430	Rsa1.0_00195.1	605	251884	1.00E-171	328	335	97	+	3	337	229134	229465
UN11431	Rsa1.0_03790.1	1650	20844	0	410	423	96	−	87	508	8657	9071
UN11432	Rsa1.0_01233.1	898	50184	0	887	913	97	−	3	898	23497	24407
UN11433	Rsa1.0_13999.1	716	3934	1.00E-120	267	284	94	−	99	382	3660	3934
UN11434	Rsa1.0_00662.1	1124	192183	2.00E-89	215	227	94	+	898	1124	53109	53331
UN11435	Rsa1.0_00146.1	929	149343	0	578	582	99	+	2	583	139775	140356
UN11436	Rsa1.0_00286.1	619	98709	1.00E-161	296	298	99	+	322	619	13001	13296
UN11437	Rsa1.0_03686.1	756	21387	1.00E-118	260	277	93	−	497	756	7475	7751
UN11438	Rsa1.0_00013.1	1011	292129	1.00E-136	273	282	96	−	8	289	253557	253838
UN11439	Rsa1.0_23843.1	1116	1022	0	634	655	96	+	459	1112	1	655
UN11440	Rsa1.0_01758.1	762	39747	0	403	415	97	−	342	756	37154	37568
UN11441	Rsa1.0_00109.1	896	132735	1.00E-110	208	210	99	−	424	633	53985	54194
UN11442	Rsa1.0_00010.1	994	294827	1.00E-167	358	379	94	+	316	694	119770	120139
UN11443	Rsa1.0_03424.1	1353	23064	1.00E-166	312	317	98	+	3	319	15398	15710
UN11444	Rsa1.0_09210.1	768	6556	1.00E-166	324	333	97	−	41	373	650	982
UN11445	Rsa1.0_02045.1	1428	35650	1.00E-75	231	254	90	+	1141	1391	22741	22989
UN11446	Rsa1.0_10942.1	1138	5669	0	435	436	99	−	703	1138	968	1403
UN11447	Rsa1.0_01292.1	790	175783	1.00E-131	251	254	98	+	537	790	2296	2548
UN11448	Rsa1.0_05611.1	786	13193	0	555	573	96	−	218	784	2562	3128
UN11449	Rsa1.0_00749.1	1525	65933	0	1480	1545	95	−	3	1525	28027	29566
UN11450	Rsa1.0_00662.1	782	192183	0	769	782	98	+	1	782	54357	55138
UN11451	Rsa1.0_03968.1	707	19804	0	420	425	98	+	3	427	18603	19027
UN11452	Rsa1.0_02384.1	906	31917	0	865	919	94	+	3	906	11014	11920
UN11453	Rsa1.0_02944.1	1057	26553	0	409	425	96	+	609	1025	4612	5036
UN11454	Rsa1.0_00017.1	744	752458	0	455	480	94	+	263	741	222799	223276
UN11455	Rsa1.0_00225.1	1867	105892	1.00E-134	305	322	94	+	1501	1822	20850	21161
UN11456	Rsa1.0_00402.1	1283	385315	0	557	582	95	+	1	567	119064	119645
UN11457	Rsa1.0_07974.1	1298	8147	0	1285	1298	98	−	6	1298	6216	7513
UN11458	Rsa1.0_00923.1	1377	153678	1.00E-109	228	237	96	+	203	439	31496	31732
UN11459	Rsa1.0_00630.1	637	71281	0	560	580	96	+	64	637	18575	19151
UN11460	Rsa1.0_04422.1	842	17423	1.00E-116	270	284	95	−	561	841	10730	11012
UN11461	Rsa1.0_01881.1	803	37721	0	781	809	96	−	3	803	16711	17518
UN11462	Rsa1.0_00385.1	1582	89160	1.00E-168	351	363	96	+	1220	1578	66235	66596
UN11463	Rsa1.0_06047.1	776	11854	0	570	590	96	−	187	773	6802	7390
UN11464	Rsa1.0_34075.1	1390	665	0	334	338	98	−	544	881	9	346

UN11465	Rsa1.0_04286.1	892	18084	2.00E-91	221	237	93	-	1	236	6107	6331
UN11466	Rsa1.0_02846.1	993	27338	0	956	997	95	-	1	993	11314	12305
UN11467	Rsa1.0_00622.1	936	71822	0	859	935	91	-	18	931	25990	26890
UN11468	Rsa1.0_00796.1	759	63997	1.00E-101	236	249	94	-	446	692	58142	58387
UN11469	Rsa1.0_00687.1	775	68494	0	762	771	98	+	3	773	42407	43177
UN11470	Rsa1.0_03466.1	1539	22698	0	1002	1052	95	+	29	1080	17397	18416
UN11471	Rsa1.0_17297.1	1030	1867	4.00E-81	178	186	95	-	607	792	350	535
UN11472	Rsa1.0_01870.1	1400	37919	1.00E-140	297	311	95	+	3	313	14118	14425
UN11473	Rsa1.0_06149.1	778	11615	0	391	403	97	+	376	778	5349	5751
UN11474	Rsa1.0_00570.1	1489	75002	0	1129	1208	93	+	85	1275	20443	21612
UN11475	Rsa1.0_05139.1	1521	14718	1.00E-142	272	277	98	+	661	937	5752	6028
UN11476	Rsa1.0_00380.1	985	89748	0	480	499	96	+	2	497	53812	54310
UN11477	Rsa1.0_01157.1	886	52121	0	392	399	98	-	488	886	28867	29265
UN11478	Rsa1.0_00016.1	1111	831256	0	457	483	94	-	333	814	253037	253513
UN11479	Rsa1.0_04845.1	837	15747	1.00E-108	217	223	97	+	69	291	4171	4393
UN11480	Rsa1.0_01448.1	960	45645	1.00E-173	315	317	99	-	644	960	39297	39613
UN11481	Rsa1.0_00038.1	507	314568	0	372	382	97	+	1	382	283109	283486
UN11482	Rsa1.0_00139.1	950	357786	4.00E-90	202	209	96	-	744	950	290739	290946
UN11483	Rsa1.0_20261.1	806	1323	0	472	495	95	-	292	783	81	575
UN11484	Rsa1.0_00514.1	873	78421	0	852	871	97	-	1	867	65101	65969
UN11485	Rsa1.0_09514.1	1280	6322	0	560	594	94	+	2	595	1150	1742
UN11486	Rsa1.0_05708.1	1419	12871	0	1413	1418	99	+	2	1419	3514	4931
UN11487	Rsa1.0_00505.1	824	79323	0	791	805	98	+	19	823	34066	34870
UN11488	Rsa1.0_00090.1	1270	273859	0	665	668	99	-	1	667	260786	261453
UN11489	Rsa1.0_00603.1	840	73299	1.00E-160	537	620	86	+	4	620	14304	14923
UN11490	Rsa1.0_00145.1	1362	261008	0	385	391	98	-	972	1361	246121	246511
UN11491	Rsa1.0_00470.1	668	81968	1.00E-162	359	383	93	-	291	665	27987	28369
UN11492	Rsa1.0_15660.1	801	2496	0	790	798	98	-	2	797	1451	2246
UN11493	Rsa1.0_00098.1	1057	349239	0	420	426	98	+	630	1054	293541	293966
UN11494	Rsa1.0_03081.1	288	25523	9.00E-76	249	275	90	+	20	285	23144	23409
UN11495	Rsa1.0_05081.1	1467	136103	1.00E-120	263	277	94	-	774	1050	2078	2351
UN11496	Rsa1.0_03460.1	1355	22747	0	662	692	95	-	669	1355	12206	12897
UN11497	Rsa1.0_00192.1	1204	278200	0	1003	1040	96	-	2	1039	225853	226884
UN11498	Rsa1.0_00370.1	884	90472	0	400	420	95	-	356	770	4877	5296
UN11499	Rsa1.0_04002.1	516	19608	5.00E-88	172	173	99	+	124	296	17107	17278
UN11500	Rsa1.0_00730.1	1032	66673	0	884	899	98	+	30	926	52983	53881
UN11501	Rsa1.0_00059.1	1157	159650	0	577	614	93	-	552	1157	136132	136741
UN11502	Rsa1.0_00339.1	1395	222974	0	1085	1110	97	+	10	1119	4026	5135
UN11503	Rsa1.0_05153.1	739	14663	1.00E-105	240	253	94	+	468	714	2744	2994

UN11504	Rsa1.0_00214.1	782	107508	0	509	545	93	−	6	549	20107	20647
UN11505	Rsa1.0_00280.1	1143	99966	0	1135	1144	99	−	2	1143	19098	20241
UN11506	Rsa1.0_00411.1	859	86944	0	804	877	91	+	19	859	61976	62834
UN11507	Rsa1.0_00971.1	938	57973	4.00E−16	45	45	100	−	790	834	43513	43557
UN11508	Rsa1.0_01146.1	733	163455	2.00E−72	192	208	92	−	529	732	20587	20793
UN11509	Rsa1.0_00341.1	2225	249309	0	714	715	99	−	774	1488	22700	23414
UN11510	Rsa1.0_00294.1	728	144860	1.00E−162	316	325	97	−	68	392	116969	117293
UN11511	Rsa1.0_02922.1	954	26753	1.00E−109	226	233	96	−	564	795	12457	12689
UN11512	Rsa1.0_00055.1	853	520193	1.00E−138	267	273	97	+	473	745	310467	310739
UN11513	Rsa1.0_00081.1	1008	143261	1.00E−157	314	321	97	+	691	1008	134326	134645
UN11514	Rsa1.0_01651.1	1064	41586	4.00E−93	177	178	99	−	2	179	39853	40030
UN11515	Rsa1.0_18474.1	1317	1593	1.00E−126	242	246	98	−	2	247	744	989
UN11516	Rsa1.0_00054.1	1117	162053	1.00E−169	307	309	99	−	809	1117	5512	5820
UN11517	Rsa1.0_08183.1	964	7775	0	488	500	97	−	195	694	5000	5498
UN11518	Rsa1.0_03350.1	1185	23522	0	406	420	96	−	769	1185	19888	20307
UN11519	Rsa1.0_03646.1	724	21598	1.00E−178	317	317	100	+	408	724	13555	13871
UN11520	Rsa1.0_04043.1	1413	19416	0	735	790	93	−	3	783	4796	5566
UN11521	Rsa1.0_02105.1	311	156953	1.00E−112	284	303	93	−	15	311	149104	149404
UN11522	Rsa1.0_00934.1	1254	193516	0	1244	1253	99	+	1	1252	145909	147161
UN11523	Rsa1.0_00392.1	1243	88523	0	593	596	99	+	648	1243	65484	66079
UN11524	Rsa1.0_05978.1	531	12053	4.00E−33	94	101	93	−	210	310	2504	2604
UN11525	Rsa1.0_00045.1	898	328694	1.00E−107	214	218	98	+	681	898	313124	313340
UN11526	Rsa1.0_00202.1	1537	185895	1.00E−176	383	403	95	+	2	401	110697	111096
UN11527	Rsa1.0_08486.1	1086	7286	0	654	658	99	−	429	1086	3718	4375
UN11528	Rsa1.0_02203.1	633	164532	1.00E−121	265	280	94	−	31	310	27488	27767
UN11529	Rsa1.0_29345.1	1377	785	0	394	397	99	−	981	1377	36	429
UN11530	Rsa1.0_01832.1	683	38581	1.00E−180	361	374	96	−	7	380	31883	32253
UN11531	Rsa1.0_00054.1	763	162053	0	399	411	97	−	3	413	153867	154275
UN11532	Rsa1.0_00287.1	1164	98687	0	354	362	97	+	808	1164	77890	78251
UN11533	Rsa1.0_02096.1	799	35041	0	771	796	96	+	4	799	3488	4277
UN11534	Rsa1.0_00246.1	994	103541	0	937	1040	90	−	1	994	64497	65527
UN11535	Rsa1.0_04165.1	780	18757	0	377	384	98	−	2	385	613	996
UN11536	Rsa1.0_00407.1	533	191544	0	497	499	99	−	1	499	43246	43744
UN11537	Rsa1.0_00116.1	626	365886	0	424	430	98	−	1	430	139262	139691
UN11538	Rsa1.0_00190.1	1422	318240	0	535	551	97	−	16	566	968	1518
UN11539	Rsa1.0_03726.1	1611	21160	1.00E−115	233	240	97	−	1169	1408	14457	14696
UN11540	Rsa1.0_00921.1	791	59599	0	774	794	97	+	1	791	31024	31816
UN11541	Rsa1.0_01643.1	732	41765	1.00E−167	319	326	97	+	1	326	36731	37056
UN11542	Rsa1.0_00940.1	1367	179946	1.00E−142	295	307	96	+	1064	1367	41158	41464

UN11543	Rsa1.0_05168.1	776	14621	0	652	689	94	−	89	765	8801	9486
UN11544	Rsa1.0_00436.1	2058	85008	0	479	479	100	−	1579	2057	15632	16110
UN11545	Rsa1.0_05081.1	867	136103	1.00E−131	345	384	89	+	486	850	5006	5389
UN11546	Rsa1.0_00960.1	1361	58199	0	437	441	99	+	919	1359	29999	30439
UN11547	Rsa1.0_09781.1	1375	6153	0	762	767	99	+	1	767	4622	5388
UN11548	Rsa1.0_02774.1	1243	28038	1.00E−105	243	255	95	−	992	1243	15516	15770
UN11549	Rsa1.0_00168.1	789	115870	1.00E−156	316	327	96	+	114	440	87952	88277
UN11550	Rsa1.0_00127.1	949	127446	1.00E−157	358	385	92	+	33	407	113030	113405
UN11551	Rsa1.0_09854.1	1502	6118	0	1062	1116	95	+	3	1091	306	1415
UN11552	Rsa1.0_02430.1	962	31506	0	489	495	98	−	392	886	18912	19404
UN11553	Rsa1.0_09314.1	1334	6461	0	772	803	96	+	159	947	3997	4799
UN11554	Rsa1.0_02289.1	1144	32981	0	413	417	99	+	3	419	8949	9365
UN11555	Rsa1.0_00206.1	1310	109040	1.00E−164	294	294	100	+	1	294	33998	34291
UN11556	Rsa1.0_00850.1	1187	62193	0	444	448	99	+	740	1187	49866	50311
UN11557	Rsa1.0_00007.1	1406	239325	0	1393	1411	98	−	1	1406	214053	215462
UN11558	Rsa1.0_02343.1	1371	32374	0	425	441	96	−	932	1371	27735	28172
UN11559	Rsa1.0_01118.1	796	53460	1.00E−145	315	333	94	+	2	334	42466	42788
UN11560	Rsa1.0_00145.1	1295	261008	1.00E−136	250	251	99	−	2	252	42012	42262
UN11561	Rsa1.0_00290.1	866	225096	0	371	377	98	−	3	379	15513	15888
UN11562	Rsa1.0_00087.1	799	761073	1.00E−143	287	294	97	−	498	790	102249	102541
UN11563	Rsa1.0_00046.1	1402	320315	0	644	670	96	+	735	1401	52061	52724
UN11564	Rsa1.0_00128.1	1367	228675	1.00E−150	270	270	100	+	479	748	181348	181617
UN11565	Rsa1.0_00240.1	757	104175	4.00E−80	171	176	97	+	454	629	86117	86291
UN11566	Rsa1.0_00105.1	910	134138	0	598	617	96	+	27	642	106705	107306
UN11567	Rsa1.0_01020.1	1094	56619	1.00E−120	231	235	98	+	209	443	33443	33677
UN11568	Rsa1.0_04831.1	1098	15799	9.00E−76	325	383	84	−	546	927	12431	12805
UN11569	Rsa1.0_03194.1	770	24674	1.00E−147	265	265	100	−	2	266	3262	3526
UN11570	Rsa1.0_00353.1	1232	92242	0	1190	1225	97	−	3	1224	27804	29025
UN11571	Rsa1.0_00368.1	788	90669	1.00E−119	247	256	96	−	1	256	5179	5432
UN11572	Rsa1.0_02326.1	944	32503	1.00E−161	332	348	95	−	34	372	25887	26234
UN11573	Rsa1.0_00494.1	807	79967	0	780	794	98	+	3	796	48939	49724
UN11574	Rsa1.0_00470.1	831	81968	1.00E−138	279	289	96	−	543	831	53615	53903
UN11575	Rsa1.0_00471.1	663	81897	1.00E−180	369	384	96	−	279	658	24754	25135
UN11576	Rsa1.0_09568.1	733	6284	1.00E−123	250	258	96	+	15	272	2860	3114
UN11577	Rsa1.0_00080.1	969	190437	0	680	702	96	−	147	845	104976	105677
UN11578	Rsa1.0_00207.1	1216	248736	3.00E−63	142	148	95	+	461	608	202907	203054
UN11579	Rsa1.0_07826.1	1153	8466	0	985	990	99	+	1	990	7477	8466
UN11580	Rsa1.0_00620.1	766	71985	0	568	617	92	+	154	766	8178	8789
UN11581	Rsa1.0_01151.1	756	52268	1.00E−123	245	252	97	+	25	276	27935	28186

UN11582	Rsa1.0_00014.1	829	348985	1.00E-144	288	297	96	+	2	294	322437	322733
UN11583	Rsa1.0_01976.1	504	36570	5.00E-94	209	218	95	+	273	488	1119	1335
UN11584	Rsa1.0_02672.1	854	28884	0	677	745	90	+	133	846	10847	11584
UN11585	Rsa1.0_00189.1	759	111830	0	457	462	98	+	249	710	110540	111000
UN11586	Rsa1.0_00421.1	684	86365	8.00E-69	249	273	91	−	412	677	82657	82920
UN11587	Rsa1.0_00341.1	818	249309	1.00E-142	324	344	94	+	463	806	190245	190586
UN11588	Rsa1.0_00871.1	1236	61539	0	443	452	98	+	786	1234	4051	4502
UN11589	Rsa1.0_00001.1	953	363028	0	339	342	99	+	610	951	93769	94109
UN11590	Rsa1.0_01883.1	1317	37661	0	804	814	98	−	503	1316	35457	36270
UN11591	#	#	#	#	#	#	#	#	#	#	#	#
UN11592	Rsa1.0_00156.1	853	118383	1.00E-151	334	355	94	+	162	516	41727	42081
UN11593	Rsa1.0_00002.1	1355	601661	0	602	631	95	−	225	848	407155	407779
UN11594	Rsa1.0_07591.1	682	9060	1.00E-137	279	288	96	+	391	677	6177	6464
UN11595	Rsa1.0_05146.1	887	14678	0	843	875	96	−	13	887	3725	4598
UN11596	Rsa1.0_08015.1	676	8078	1.00E-119	218	218	100	−	2	219	2331	2548
UN11597	Rsa1.0_01235.1	844	50180	0	815	840	97	+	5	844	20788	21614
UN11598	Rsa1.0_04888.1	1255	15595	2.00E-31	129	147	87	+	1084	1229	3004	3150
UN11599	Rsa1.0_02725.1	945	28404	0	631	634	99	+	312	945	1939	2572
UN11600	Rsa1.0_01986.1	490	36489	1.00E-173	351	363	96	+	117	478	23647	24007
UN11601	Rsa1.0_06647.1	1093	10604	0	612	638	95	+	463	1093	6307	6942
UN11602	Rsa1.0_00079.1	822	606987	1.00E-178	373	388	96	−	3	390	190876	191255
UN11603	Rsa1.0_03149.1	661	24983	1.00E-111	213	216	98	−	259	474	15485	15700
UN11604	Rsa1.0_03173.1	989	24815	0	951	983	96	+	9	989	18022	19004
UN11605	Rsa1.0_00578.1	476	74581	0	453	469	96	−	6	474	57295	57755
UN11606	Rsa1.0_03706.1	697	21255	0	678	701	96	+	1	697	481	1180
UN11607	Rsa1.0_04483.1	744	17174	1.00E-130	285	298	95	−	449	744	7590	7879
UN11608	Rsa1.0_05984.1	1191	12031	1.00E-138	287	298	96	−	11	302	10469	10766
UN11609	Rsa1.0_00119.1	1221	467509	0	594	610	97	−	611	1220	383281	383890
UN11610	Rsa1.0_01454.1	1238	45440	1.00E-160	364	390	93	+	849	1238	32801	33170
UN11611	Rsa1.0_01679.1	1277	146876	2.00E-95	233	249	93	−	1014	1260	135000	135247
UN11612	Rsa1.0_00005.1	828	353093	1.00E-130	242	244	99	+	239	482	329300	329543
UN11613	Rsa1.0_01294.1	820	49090	0	443	443	100	−	3	445	36747	37189
UN11614	Rsa1.0_00230.1	1171	105389	1.00E-126	238	241	98	+	689	929	87728	87968
UN11615	Rsa1.0_00765.1	892	65029	0	871	891	97	−	2	892	22820	23710
UN11616	Rsa1.0_00457.1	1321	337301	0	861	911	94	−	3	909	317916	318814
UN11617	Rsa1.0_00092.1	1298	659932	1.00E-162	310	314	98	+	985	1298	626655	626966
UN11618	Rsa1.0_00044.1	760	271658	1.00E-148	293	302	97	+	458	759	133405	133706
UN11619	Rsa1.0_00764.1	977	65056	0	521	527	98	+	3	529	39975	40501
UN11620	Rsa1.0_07475.1	953	9292	0	503	520	96	+	312	826	585	1104

UN11621	Rsa1.0_05496.1	1925	13533	0	806	816	98	−	869	1684	453	1265
UN11622	Rsa1.0_07321.1	854	9559	1.00E−176	373	390	95	−	442	824	4937	5326
UN11623	Rsa1.0_07381.1	856	9488	0	526	531	99	−	269	798	7918	8448
UN11624	Rsa1.0_00356.1	982	91521	0	385	404	95	−	201	604	80712	81097
UN11625	Rsa1.0_00216.1	1207	197563	0	545	562	96	+	646	1207	92955	93510
UN11626	Rsa1.0_02694.1	903	28610	0	699	715	97	−	2	716	9378	10092
UN11627	Rsa1.0_00047.1	1249	168582	0	1146	1193	96	−	1	1176	154406	155597
UN11628	Rsa1.0_00419.1	906	86562	3.00E−75	144	144	100	−	361	504	42048	42191
UN11629	Rsa1.0_01960.1	1136	36766	0	396	411	96	−	726	1136	27519	27929
UN11630	Rsa1.0_05056.1	1395	14981	0	373	373	100	−	3	375	9065	9437
UN11631	Rsa1.0_03150.1	1351	24980	0	363	364	99	+	3	366	22091	22454
UN11632	Rsa1.0_01789.1	1098	39250	1.00E−150	281	285	98	−	812	1096	7449	7733
UN11633	Rsa1.0_01957.1	599	36844	1.00E−126	232	233	99	−	87	319	13472	13704
UN11634	Rsa1.0_02173.1	834	34318	0	471	486	96	+	293	777	30693	31178
UN11635	Rsa1.0_08846.1	816	6867	1.00E−58	145	153	94	−	38	185	6654	6806
UN11636	Rsa1.0_33317.1	963	681	1.00E−165	374	399	93	+	3	401	1	398
UN11637	Rsa1.0_02179.1	1086	34188	0	1057	1085	97	+	2	1086	19843	20926
UN11638	Rsa1.0_18402.1	1153	1605	0	737	759	97	−	401	1153	232	990
UN11639	Rsa1.0_04497.1	1194	17103	1.00E−176	313	313	100	+	882	1194	3905	4217
UN11640	Rsa1.0_03125.1	990	147978	0	677	705	96	−	2	704	131175	131879
UN11641	Rsa1.0_00725.1	1787	66757	0	743	825	90	+	20	843	45581	46401
UN11642	Rsa1.0_18069.1	868	1678	1.00E−130	303	319	94	+	549	862	197	504
UN11643	Rsa1.0_00341.1	1653	249309	0	596	600	99	−	1054	1653	207027	207626
UN11644	Rsa1.0_00041.1	1489	186197	1.00E−137	263	268	98	−	253	520	129816	130083
UN11645	Rsa1.0_00185.1	665	248445	0	540	552	97	−	117	665	30579	31130
UN11646	Rsa1.0_01896.1	867	37512	0	627	647	96	+	211	856	19518	20164
UN11647	Rsa1.0_00141.1	772	121494	0	519	537	96	−	6	542	60107	60643
UN11648	Rsa1.0_00487.1	799	80674	9.00E−63	123	123	100	−	1	123	15702	15824
UN11649	Rsa1.0_02174.1	685	34316	0	667	685	97	−	3	683	10485	11169
UN11650	Rsa1.0_00503.1	626	79468	6.00E−88	192	200	96	+	428	625	66553	66752
UN11651	Rsa1.0_01724.1	773	40292	8.00E−97	189	192	98	−	1	192	13121	13312
UN11652	Rsa1.0_00398.1	896	88007	0	411	431	95	+	471	896	63443	63872
UN11653	Rsa1.0_00093.1	1222	214962	0	372	377	98	+	263	639	133459	133835
UN11654	Rsa1.0_02223.1	1191	152421	0	1164	1195	97	+	3	1190	14300	15494
UN11655	Rsa1.0_00800.1	642	63868	0	415	433	95	−	204	636	13864	14285
UN11656	Rsa1.0_01093.1	1628	54312	0	505	508	99	+	1121	1628	47030	47536
UN11657	Rsa1.0_01054.1	815	55470	0	640	642	99	−	171	811	8770	9411
UN11658	Rsa1.0_05305.1	588	14207	0	402	427	94	+	122	535	1015	1438
UN11659	Rsa1.0_00119.1	1304	467509	0	428	439	97	+	31	469	447655	448093

UN11660	Rsa1.0_00485.1	930	80937	0	424	428	99	−	503	930	40721	41148
UN11661	Rsa1.0_00044.1	885	271658	0	858	909	94	+	4	885	260545	261450
UN11662	Rsa1.0_00438.1	881	84861	0	370	380	97	+	501	877	75306	75684
UN11663	Rsa1.0_00068.1	802	151813	0	769	809	95	−	2	801	43681	44485
UN11664	Rsa1.0_01053.1	1150	55483	0	1147	1149	99	−	2	1148	46230	47378
UN11665	Rsa1.0_38108.1	244	591	2.00E−73	185	198	93	+	1	192	31	225
UN11666	Rsa1.0_00395.1	1228	214466	0	358	362	98	−	3	363	5892	6253
UN11667	Rsa1.0_03615.1	1206	21766	0	1004	1061	94	−	1	1057	17207	18246
UN11668	Rsa1.0_02312.1	234	32675	8.00E−85	207	223	92	+	9	231	16058	16280
UN11669	Rsa1.0_02361.1	1445	32190	0	344	346	99	−	2	347	11102	11446
UN11670	Rsa1.0_00066.1	643	152418	0	606	629	96	−	1	620	63377	64003
UN11671	Rsa1.0_02431.1	1367	31504	1.00E−111	262	276	94	−	109	380	2929	3203
UN11672	Rsa1.0_01552.1	778	43396	0	357	365	97	−	414	778	15684	16048
UN11673	Rsa1.0_03005.1	1129	26082	0	539	573	94	+	1	572	3526	4084
UN11674	Rsa1.0_03385.1	756	23280	0	552	582	94	−	2	570	3669	4250
UN11675	Rsa1.0_04236.1	367	18370	1.00E−117	268	284	94	−	17	298	6470	6751
UN11676	Rsa1.0_02633.1	985	29315	0	452	473	95	−	515	983	308	771
UN11677	Rsa1.0_04397.1	1308	17555	0	535	554	96	−	10	561	3221	3767
UN11678	Rsa1.0_02593.1	680	29744	1.00E−83	224	244	91	+	1	236	2922	3164
UN11679	Rsa1.0_00167.1	1615	116000	0	1352	1448	93	+	1	1424	87105	88534
UN11680	Rsa1.0_08919.1	755	6788	0	589	603	97	−	6	608	517	1119
UN11681	Rsa1.0_00087.1	1662	761073	1.00E−134	247	248	99	+	1	248	291710	291957
UN11682	Rsa1.0_00586.1	972	163802	2.00E−76	254	290	87	−	248	537	108878	109167
UN11683	Rsa1.0_18588.1	968	1570	1.00E−106	302	334	90	−	2	331	1176	1508
UN11684	Rsa1.0_00285.1	773	149638	1.00E−174	372	389	95	+	3	388	146424	146811
UN11685	Rsa1.0_05180.1	1019	14575	0	421	436	96	+	144	575	4725	5159
UN11686	Rsa1.0_00522.1	1106	78103	1.00E−101	200	204	98	−	637	840	35226	35429
UN11687	Rsa1.0_01631.1	1650	41882	0	386	386	100	−	1	386	4841	5226
UN11688	Rsa1.0_01219.1	1085	50527	0	835	861	96	−	229	1085	9487	10346
UN11689	Rsa1.0_00114.1	1610	131330	0	577	581	99	+	1	580	7618	8197
UN11690	Rsa1.0_00092.1	680	659932	0	437	457	95	+	1	453	8515	8964
UN11691	Rsa1.0_01537.1	955	187285	0	353	361	97	+	2	360	38620	38980
UN11692	Rsa1.0_01286.1	1170	49209	1.00E−130	297	320	92	−	1	306	3049	3368
UN11693	Rsa1.0_09568.1	845	6284	1.00E−74	220	242	90	−	1	240	5564	5803
UN11694	Rsa1.0_01454.1	841	45440	1.00E−160	295	297	99	+	1	295	14232	14528
UN11695	Rsa1.0_06073.1	1361	11807	0	654	680	96	−	1	678	4362	5029
UN11696	Rsa1.0_03126.1	149	25192	4.00E−67	144	149	96	−	1	149	6718	6866
UN11697	Rsa1.0_00382.1	1180	251946	0	482	489	98	+	693	1180	173937	174423
UN11698	Rsa1.0_02625.1	988	139104	0	907	939	96	+	15	946	24773	25711

UN11699	Rsa1.0_00634.1	512	71222	1.00E-180	380	399	95	-	1	399	57160	57557
UN11700	Rsa1.0_14151.1	603	3731	0	397	409	97	-	1	409	981	1389
UN11701	Rsa1.0_00198.1	965	222963	1.00E-167	358	373	95	+	7	378	135060	135427
UN11702	Rsa1.0_02593.1	1468	29744	1.00E-164	330	342	96	+	331	672	4754	5095
UN11703	Rsa1.0_03631.1	1522	21647	0	811	836	97	-	690	1522	14187	15017
UN11704	Rsa1.0_00351.1	917	413096	0	895	912	98	-	6	917	124066	124977
UN11705	Rsa1.0_18611.1	1329	1565	0	432	447	96	-	1	447	957	1402
UN11706	Rsa1.0_00119.1	988	467509	0	640	669	95	+	1	668	383222	383890
UN11707	Rsa1.0_00029.1	1435	272992	0	1161	1229	94	+	1	1206	257531	258746
UN11708	Rsa1.0_00222.1	1276	172263	0	538	577	93	-	714	1276	39715	40291
UN11709	Rsa1.0_02866.1	1811	27122	0	537	539	99	+	903	1441	9631	10169
UN11710	Rsa1.0_00662.1	900	192183	0	806	838	96	-	68	900	68347	69184
UN11711	Rsa1.0_00329.1	1334	222544	0	682	690	98	+	6	694	193045	193732
UN11712	Rsa1.0_05559.1	693	13329	0	430	444	96	-	61	504	8434	8872
UN11713	Rsa1.0_01482.1	289	44855	1.00E-147	279	284	98	+	6	289	11985	12268
UN11714	Rsa1.0_01419.1	1350	167402	0	627	689	91	+	677	1345	142662	143339
UN11715	Rsa1.0_00045.1	639	328694	0	581	585	99	-	55	639	302570	303154
UN11716	Rsa1.0_26735.1	757	880	1.00E-127	495	583	84	-	175	757	267	849
UN11717	Rsa1.0_00041.1	894	186197	1.00E-100	208	214	97	+	288	501	127713	127925
UN11718	Rsa1.0_00178.1	1103	114861	0	859	902	95	-	1	882	5884	6780
UN11719	Rsa1.0_09471.1	730	6345	1.00E-132	248	251	98	-	383	633	496	746
UN11720	Rsa1.0_00967.1	1072	58049	0	889	911	97	+	160	1070	10028	10937
UN11721	Rsa1.0_06312.1	580	11253	0	433	458	94	-	7	464	6117	6556
UN11722	Rsa1.0_01441.1	952	45790	0	760	768	98	-	1	767	19120	19885
UN11723	Rsa1.0_03388.1	1241	23277	0	417	426	97	+	2	427	17420	17845
UN11724	Rsa1.0_00417.1	722	395068	1.00E-165	336	352	95	+	381	720	14475	14826
UN11725	Rsa1.0_21914.1	151	1163	1.00E-45	135	149	90	-	1	149	326	474
UN11726	Rsa1.0_08811.1	875	6904	0	516	545	94	+	8	548	2993	3531
UN11727	Rsa1.0_07806.1	758	8506	1.00E-175	331	336	98	-	423	758	6161	6495
UN11728	Rsa1.0_01326.1	437	48072	1.00E-104	255	280	91	+	178	435	10896	11175
UN11729	Rsa1.0_00524.1	836	169613	1.00E-111	207	208	99	-	372	579	133897	134104
UN11730	Rsa1.0_16139.1	1009	2268	1.00E-93	209	219	95	-	286	503	684	902
UN11731	Rsa1.0_11716.1	827	5431	1.00E-126	257	262	98	+	568	826	318	579
UN11732	Rsa1.0_02774.1	763	28038	1.00E-154	329	349	94	-	28	376	18910	19246
UN11733	Rsa1.0_00214.1	808	107508	0	388	389	99	-	420	808	69319	69707
UN11734	Rsa1.0_01621.1	1133	42090	0	386	393	98	-	47	439	170	562
UN11735	Rsa1.0_00191.1	612	316976	0	526	574	91	-	39	587	122015	122582
UN11736	Rsa1.0_00151.1	949	119541	0	454	467	97	+	2	467	29920	30385
UN11737	Rsa1.0_00751.1	1405	65759	0	1314	1421	92	+	3	1404	56920	58302

UN11738	Rsa1.0_04426.1	893	17399	0	494	508	97	+	389	893	16809	17316
UN11739	Rsa1.0_09343.1	1159	6439	0	363	365	99	−	793	1157	4332	4696
UN11740	Rsa1.0_00097.1	1166	138858	1.00E−128	249	253	98	+	80	332	116669	116920
UN11741	Rsa1.0_00803.1	988	144228	1.00E−101	213	222	95	−	727	942	2413	2634
UN11742	Rsa1.0_00222.1	1313	172263	0	1171	1221	95	−	103	1313	22625	23839
UN11743	Rsa1.0_01185.1	735	149842	0	724	729	99	−	3	731	142803	143529
UN11744	Rsa1.0_00176.1	810	240984	1.00E−172	339	351	96	+	3	345	183974	184324
UN11745	Rsa1.0_28876.1	924	800	0	682	705	96	+	220	924	79	782
UN11746	Rsa1.0_00025.1	710	277312	0	569	580	98	+	6	584	52186	52765
UN11747	Rsa1.0_03244.1	814	24413	0	437	439	99	+	3	440	10838	11276
UN11748	Rsa1.0_04436.1	1224	17364	0	607	652	93	−	3	640	15688	16328
UN11749	Rsa1.0_00558.1	1881	75819	0	738	751	98	+	774	1524	28073	28823
UN11750	Rsa1.0_00655.1	834	195840	0	421	427	98	−	407	833	35880	36305
UN11751	Rsa1.0_00207.1	1357	248736	0	386	396	97	+	536	929	63444	63839
UN11752	Rsa1.0_00016.1	1145	831256	0	363	370	98	+	773	1141	326365	326734
UN11753	Rsa1.0_01959.1	1357	36798	0	647	684	94	−	621	1304	15377	16060
UN11754	Rsa1.0_11912.1	1164	5372	0	1138	1176	96	−	3	1164	2904	4079
UN11755	Rsa1.0_00006.1	1004	396176	0	462	466	99	+	101	566	210336	210801
UN11756	Rsa1.0_02099.1	733	35030	1.00E−153	327	342	95	−	392	726	17697	18038
UN11757	Rsa1.0_00789.1	1288	64233	4.00E−78	174	181	96	+	887	1061	24697	24877
UN11758	Rsa1.0_02939.1	860	26595	0	622	658	94	−	1	649	15749	16400
UN11759	Rsa1.0_00242.1	762	103956	0	579	596	97	+	2	596	89912	90498
UN11760	Rsa1.0_04369.1	1109	17703	1.00E−99	232	249	93	+	2	250	7015	7254
UN11761	Rsa1.0_42525.1	1252	528	6.00E−74	163	169	96	+	275	441	1	169
UN11762	Rsa1.0_00017.1	1507	752458	0	963	1028	93	−	1	1019	416018	417042
UN11763	Rsa1.0_04873.1	935	15657	1.00E−106	217	224	96	−	9	232	8145	8368
UN11764	Rsa1.0_03410.1	1530	23147	0	1488	1541	96	+	6	1530	20091	21629
UN11765	Rsa1.0_00977.1	961	198001	1.00E−128	246	249	98	+	713	961	29296	29543
UN11766	Rsa1.0_02606.1	991	29635	1.00E−173	314	316	99	+	676	991	13308	13623
UN11767	Rsa1.0_00340.1	654	305782	0	369	369	100	−	286	654	159725	160093
UN11768	Rsa1.0_00720.1	805	66931	1.00E−147	312	324	96	+	3	326	31878	32198
UN11769	Rsa1.0_06619.1	779	10650	0	350	356	98	+	6	361	517	871
UN11770	Rsa1.0_05095.1	947	14869	8.00E−76	170	178	95	+	15	188	14642	14819
UN11771	Rsa1.0_00176.1	971	240984	0	828	860	96	+	4	856	1877	2736
UN11772	Rsa1.0_04542.1	669	16930	1.00E−180	319	319	100	−	1	319	15134	15452
UN11773	Rsa1.0_02410.1	508	31659	0	506	507	99	−	3	508	21387	21893
UN11774	Rsa1.0_00097.1	771	138858	0	400	412	97	+	340	747	116669	117080
UN11775	Rsa1.0_02958.1	779	26443	0	768	788	97	+	2	779	8913	9700
UN11776	Rsa1.0_00123.1	1115	325650	1.00E−160	341	357	95	+	1	355	46100	46450

UN11777	Rsa1.0_00512.1	693	200277	1.00E-112	214	217	98	-	188	404	190936	191152
UN11778	Rsa1.0_00635.1	647	175826	1.00E-134	255	259	98	-	277	535	124757	125015
UN11779	Rsa1.0_06561.1	1159	10772	0	434	448	96	-	2	449	5342	5777
UN11780	Rsa1.0_08849.1	781	6864	1.00E-119	217	217	100	-	4	220	1	217
UN11781	Rsa1.0_00300.1	940	97747	1.00E-166	297	297	100	-	528	824	11210	11506
UN11782	Rsa1.0_06056.1	707	137271	1.00E-107	211	214	98	-	3	215	125553	125766
UN11783	Rsa1.0_00026.1	943	327835	0	425	434	97	-	510	943	178615	179048
UN11784	Rsa1.0_00545.1	1271	189649	0	1000	1045	95	+	193	1233	31865	32902
UN11785	Rsa1.0_00017.1	1611	752458	1.00E-177	367	383	95	+	1229	1611	711970	712351
UN11786	Rsa1.0_01453.1	829	45449	1.00E-130	255	260	98	-	535	793	14857	15116
UN11787	Rsa1.0_00071.1	985	345714	1.00E-119	224	226	99	+	3	228	216763	216988
UN11788	Rsa1.0_03407.1	836	23154	1.00E-141	263	266	98	+	27	292	3652	3917
UN11789	Rsa1.0_01788.1	996	39296	0	982	998	98	+	1	996	19142	20138
UN11790	Rsa1.0_00688.1	793	68396	0	720	739	97	+	19	756	41265	42003
UN11791	Rsa1.0_00269.1	1453	341668	0	514	521	98	-	76	596	316281	316801
UN11792	#	#	#	#	#	#	#	#	#	#	#	#
UN11793	Rsa1.0_00039.1	914	296927	0	548	578	94	-	341	914	11873	12448
UN11794	Rsa1.0_01321.1	976	48248	1.00E-157	376	403	93	+	570	963	8431	8825
UN11795	Rsa1.0_29623.1	783	777	0	537	538	99	+	155	692	240	777
UN11796	Rsa1.0_01883.1	958	37661	2.00E-67	177	192	92	+	543	725	29480	29671
UN11797	Rsa1.0_00947.1	1291	58664	0	457	460	99	-	832	1291	51430	51889
UN11798	Rsa1.0_17015.1	881	1953	1.00E-100	221	235	94	-	4	226	874	1108
UN11799	#	#	#	#	#	#	#	#	#	#	#	#
UN11800	Rsa1.0_00776.1	881	64637	0	768	853	90	+	1	850	6953	7804
UN11801	Rsa1.0_00741.1	980	66117	1.00E-175	344	351	98	+	615	962	50868	51218
UN11802	Rsa1.0_00772.1	806	64856	1.00E-68	142	145	97	+	122	266	6216	6360
UN11803	Rsa1.0_00146.1	605	149343	1.00E-143	258	258	100	-	186	443	57099	57356
UN11804	Rsa1.0_00611.1	1752	72410	0	1364	1419	96	+	3	1414	42889	44294
UN11805	Rsa1.0_00081.1	1769	143261	0	419	443	94	+	1294	1733	129500	129940
UN11806	Rsa1.0_01516.1	1447	44196	4.00E-88	169	170	99	-	1	170	37369	37538
UN11807	Rsa1.0_04835.1	886	15766	1.00E-133	272	281	96	-	160	440	9964	10243
UN11808	Rsa1.0_55259.1	1460	413	1.00E-140	302	317	95	-	1094	1410	1	316
UN11809	Rsa1.0_00037.1	812	307838	1.00E-71	141	142	99	+	281	422	306697	306838
UN11810	Rsa1.0_00890.1	1692	323870	1.00E-156	338	354	95	+	1335	1687	213285	213634
UN11811	Rsa1.0_00913.1	690	59914	1.00E-169	369	390	94	+	228	614	10123	10509
UN11812	Rsa1.0_01028.1	1563	56401	0	900	916	98	+	657	1563	4694	5609
UN11813	Rsa1.0_05561.1	1066	13326	0	471	478	98	+	274	751	3563	4040
UN11814	Rsa1.0_00305.1	683	97308	1.00E-162	310	316	98	-	368	683	87066	87378
UN11815	Rsa1.0_00044.1	1413	271658	6.00E-62	122	122	100	-	624	745	178856	178977

UN11816	Rsa1.0_00413.1	966	86921	0	861	862	99	+	105	966	4445	5306
UN11817	Rsa1.0_01838.1	941	38500	0	828	842	98	+	3	844	7180	8021
UN11818	Rsa1.0_00697.1	845	67913	1.00E-125	236	239	98	+	3	241	54368	54606
UN11819	Rsa1.0_00135.1	1287	178077	0	1250	1290	96	−	3	1285	94525	95810
UN11820	Rsa1.0_01220.1	495	161775	1.00E-131	314	336	93	+	1	336	27368	27698
UN11821	Rsa1.0_00645.1	1315	189716	0	476	480	99	+	836	1315	34408	34887
UN11822	Rsa1.0_00026.1	925	327835	0	439	456	96	−	470	925	240935	241378
UN11823	Rsa1.0_00135.1	820	178077	0	412	414	99	+	3	416	79673	80086
UN11824	Rsa1.0_04773.1	1403	16037	0	1093	1095	99	+	309	1403	9495	10589
UN11825	Rsa1.0_01435.1	1538	45942	0	459	488	94	+	482	965	40444	40931
UN11826	Rsa1.0_00975.1	1263	57865	0	496	509	97	+	753	1261	6741	7247
UN11827	Rsa1.0_07005.1	1416	10044	0	1364	1442	94	+	2	1416	7961	9379
UN11828	Rsa1.0_00135.1	1368	178077	0	521	521	100	+	2	522	102875	103395
UN11829	Rsa1.0_00014.1	628	348985	1.00E-76	155	158	98	−	296	453	220174	220331
UN11830	Rsa1.0_00757.1	676	65559	7.00E-57	116	117	99	−	280	396	60113	60229
UN11831	Rsa1.0_00629.1	685	71381	0	382	385	99	−	3	387	8236	8620
UN11832	Rsa1.0_00610.1	670	72740	0	644	665	96	+	3	667	3651	4315
UN11833	Rsa1.0_00563.1	620	75491	0	615	620	99	−	1	620	22740	23359
UN11834	Rsa1.0_40553.1	369	553	1.00E-142	348	373	93	−	2	369	94	463
UN11835	Rsa1.0_14004.1	896	3929	2.00E-27	94	102	92	−	22	119	869	970
UN11836	Rsa1.0_04419.1	766	17430	0	415	434	95	+	2	435	9162	9586
UN11837	Rsa1.0_01022.1	1152	56606	0	766	810	94	+	11	818	22338	23145
UN11838	Rsa1.0_01957.1	1846	36844	0	1128	1141	98	−	697	1832	23491	24629
UN11839	Rsa1.0_00197.1	838	109714	1.00E-120	219	219	100	+	620	838	93294	93512
UN11840	Rsa1.0_01876.1	1148	37800	0	1137	1147	99	−	2	1148	7315	8458
UN11841	Rsa1.0_00002.1	928	601661	1.00E-150	284	289	98	+	596	884	136152	136440
UN11842	Rsa1.0_07942.1	795	8213	0	380	381	99	−	415	795	3890	4270
UN11843	Rsa1.0_00019.1	1397	301669	1.00E-155	300	306	98	−	1063	1368	176993	177297
UN11844	Rsa1.0_00010.1	1526	294827	0	441	477	92	−	1055	1512	132178	132653
UN11845	Rsa1.0_00518.1	985	78360	0	410	430	95	−	556	985	65054	65481
UN11846	Rsa1.0_04397.1	823	17555	1.00E-144	275	280	98	+	280	559	1800	2079
UN11847	Rsa1.0_02750.1	844	28239	2.00E-91	209	221	94	−	505	722	23211	23431
UN11848	Rsa1.0_01591.1	973	42718	1.00E-133	285	299	95	+	433	728	8053	8351
UN11849	Rsa1.0_00632.1	1459	183777	0	560	571	98	+	889	1459	160327	160897
UN11850	Rsa1.0_00655.1	1280	195840	0	344	344	100	−	1	344	154909	155252
UN11851	Rsa1.0_00351.1	841	413096	1.00E-176	320	322	99	+	520	841	267204	267525
UN11852	Rsa1.0_01100.1	890	54113	0	410	417	98	+	3	419	22249	22663
UN11853	Rsa1.0_00129.1	998	126831	0	339	343	98	+	656	998	88631	88973
UN11854	Rsa1.0_01969.1	1251	153351	0	578	581	99	−	205	785	18539	19119

UN11855	Rsa1.0_01732.1	883	40194	0	528	535	98	+	349	883	35777	36311
UN11856	Rsa1.0_00145.1	1205	261008	0	664	694	95	+	334	1021	252085	252778
UN11857	Rsa1.0_00202.1	918	185895	0	327	328	99	+	2	329	132991	133318
UN11858	Rsa1.0_01077.1	1449	54772	0	1038	1101	94	+	361	1448	48375	49471
UN11859	Rsa1.0_03575.1	1383	22002	0	1376	1382	99	−	2	1383	13685	15065
UN11860	Rsa1.0_04820.1	1564	15857	0	745	784	95	−	798	1563	14846	15622
UN11861	Rsa1.0_00065.1	911	454616	0	582	598	97	+	314	911	254496	255092
UN11862	Rsa1.0_04396.1	1364	17561	1.00E−114	238	246	96	+	1117	1362	2712	2956
UN11863	Rsa1.0_00155.1	1119	118465	0	796	802	99	−	1	801	61433	62234
UN11864	Rsa1.0_04419.1	1502	17430	0	719	766	93	−	41	798	7377	8124
UN11865	Rsa1.0_00826.1	1052	166736	1.00E−121	221	221	100	+	146	366	155658	155878
UN11866	Rsa1.0_04181.1	1299	18615	0	691	720	95	+	255	974	936	1655
UN11867	Rsa1.0_00019.1	694	301669	0	614	659	93	−	41	694	295429	296083
UN11868	Rsa1.0_00131.1	919	432233	0	884	895	98	+	28	919	93710	94604
UN11869	Rsa1.0_00451.1	1035	322533	0	581	599	96	−	442	1033	64916	65513
UN11870	Rsa1.0_02583.1	975	29887	1.00E−130	254	257	98	+	490	746	4643	4897
UN11871	Rsa1.0_00654.1	772	70012	0	763	771	98	+	3	769	9140	9909
UN11872	Rsa1.0_03731.1	1041	21136	0	465	467	99	+	185	651	631	1097
UN11873	Rsa1.0_07499.1	908	9244	0	413	417	99	−	5	421	6510	6926
UN11874	Rsa1.0_06088.1	770	11767	1.00E−132	289	304	95	−	414	716	7185	7488
UN11875	Rsa1.0_01327.1	928	48033	0	510	512	99	+	2	513	22308	22819
UN11876	Rsa1.0_37083.1	374	608	1.00E−141	269	274	98	−	101	374	208	481
UN11877	Rsa1.0_00347.1	718	93048	1.00E−142	300	314	95	+	5	318	8821	9131
UN11878	Rsa1.0_11571.1	1068	5468	1.00E−148	275	278	98	+	5	282	261	538
UN11879	Rsa1.0_02315.1	1041	32618	1.00E−110	217	222	97	+	818	1039	25958	26179
UN11880	Rsa1.0_11924.1	638	5369	1.00E−132	246	248	99	−	391	638	4938	5185
UN11881	Rsa1.0_03692.1	855	21338	1.00E−132	289	304	95	+	555	854	3649	3951
UN11882	Rsa1.0_00193.1	1378	194198	1.00E−148	718	856	83	+	508	1344	13920	14766
UN11883	Rsa1.0_00278.1	1083	231896	0	628	655	95	+	379	1029	56571	57211
UN11884	Rsa1.0_00571.1	1052	329018	0	701	704	99	+	342	1044	143533	144236
UN11885	Rsa1.0_01516.1	746	44196	1.00E−123	264	276	95	−	216	491	36762	37035
UN11886	Rsa1.0_01317.1	761	48287	0	573	589	97	+	9	596	34091	34677
UN11887	Rsa1.0_01005.1	1308	56967	0	705	718	98	−	594	1308	4965	5682
UN11888	Rsa1.0_01925.1	1226	37220	0	482	507	95	+	723	1223	26163	26668
UN11889	Rsa1.0_00022.1	1068	200461	0	651	654	99	+	205	858	23401	24052
UN11890	Rsa1.0_00040.1	1078	324196	1.00E−147	289	297	97	+	392	688	70238	70534
UN11891	Rsa1.0_04296.1	1036	18030	1.00E−107	230	241	95	+	604	844	14729	14969
UN11892	Rsa1.0_04195.1	847	18528	1.00E−126	238	241	98	−	113	353	11857	12097
UN11893	Rsa1.0_00201.1	714	232851	0	671	713	94	−	4	714	140386	141089

UN11894	Rsa1.0_03344.1	819	23593	0	523	566	92	+	1	558	20630	21195
UN11895	Rsa1.0_02061.1	1002	35487	1.00E-120	304	332	91	-	672	1001	23335	23648
UN11896	Rsa1.0_00741.1	1026	66117	0	653	705	92	-	318	1018	32560	33263
UN11897	Rsa1.0_00344.1	804	191774	1.00E-120	240	246	97	+	268	511	179315	179560
UN11898	Rsa1.0_06039.1	1030	11867	1.00E-136	276	284	97	-	749	1030	11470	11750
UN11899	Rsa1.0_15499.1	696	2570	1.00E-134	260	266	97	-	120	385	1132	1397
UN11900	Rsa1.0_00813.1	899	63202	1.00E-125	261	273	95	+	627	899	15290	15554
UN11901	Rsa1.0_00626.1	1332	71582	1.00E-139	522	613	85	+	723	1331	45083	45656
UN11902	Rsa1.0_01477.1	785	45008	0	743	787	94	-	2	782	41030	41809
UN11903	Rsa1.0_01731.1	521	40208	1.00E-66	204	228	89	+	3	230	34880	35105
UN11904	Rsa1.0_00065.1	1402	454616	1.00E-120	220	220	100	+	936	1155	447874	448093
UN11905	Rsa1.0_04043.1	1076	19416	1.00E-147	321	336	95	-	3	336	10618	10946
UN11906	Rsa1.0_00001.1	1105	363028	0	494	531	93	-	2	529	266567	267085
UN11907	Rsa1.0_05599.1	811	143653	0	399	410	97	-	29	438	142328	142737
UN11908	Rsa1.0_02930.1	1192	26668	0	1161	1192	97	+	1	1192	8733	9921
UN11909	Rsa1.0_04458.1	1013	17255	0	984	1015	96	-	4	1013	9436	10442
UN11910	Rsa1.0_01092.1	782	54348	1.00E-117	352	394	89	-	346	712	7601	7993
UN11911	Rsa1.0_04141.1	1041	18864	0	453	456	99	+	3	456	14170	14625
UN11912	Rsa1.0_00468.1	1529	82095	0	542	547	99	-	182	728	12803	13349
UN11913	Rsa1.0_35376.1	891	640	1.00E-170	319	324	98	-	179	502	164	487
UN11914	Rsa1.0_00697.1	900	67913	0	783	839	93	+	3	832	42283	43121
UN11915	Rsa1.0_05367.1	835	14010	0	468	470	99	+	3	472	2687	3156
UN11916	Rsa1.0_03304.1	267	23883	2.00E-92	245	272	90	-	2	267	19525	19784
UN11917	Rsa1.0_00332.1	983	94575	0	475	504	94	+	101	604	54514	55017
UN11918	Rsa1.0_00089.1	1262	406142	0	425	435	97	+	161	595	150722	151156
UN11919	Rsa1.0_01552.1	1044	43396	0	490	498	98	+	213	710	15551	16048
UN11920	Rsa1.0_00269.1	1247	341668	0	913	917	99	+	2	917	37056	37972
UN11921	Rsa1.0_03839.1	1086	20525	4.00E-81	154	154	100	-	2	155	15652	15805
UN11922	Rsa1.0_01343.1	924	47735	0	910	913	99	+	3	915	4082	4994
UN11923	Rsa1.0_04894.1	962	15569	0	945	964	98	+	1	959	3487	4450
UN11924	Rsa1.0_00476.1	1474	280966	0	424	437	97	-	1038	1474	245444	245880
UN11925	Rsa1.0_01164.1	828	51934	0	792	830	95	+	3	828	47385	48204
UN11926	Rsa1.0_03303.1	721	23890	1.00E-148	296	302	98	+	423	721	4111	4412
UN11927	Rsa1.0_06967.1	1411	10093	0	1146	1167	98	-	6	1172	7110	8275
UN11928	Rsa1.0_00040.1	1021	324196	0	549	553	99	+	469	1021	89428	89979
UN11929	Rsa1.0_01836.1	748	38503	1.00E-107	212	217	97	+	280	496	2782	2998
UN11930	Rsa1.0_07840.1	1099	8442	4.00E-53	147	159	92	+	830	987	1451	1609
UN11931	Rsa1.0_00184.1	1249	218748	0	590	617	95	-	642	1249	210843	211456
UN11932	Rsa1.0_01876.1	880	37800	0	688	688	100	+	192	879	36310	36997

UN11933	Rsa1.0_00163.1	942	116847	1.00E-143	277	281	98	-	664	941	10439	10719
UN11934	Rsa1.0_02320.1	724	32575	0	713	725	98	-	4	724	16021	16744
UN11935	Rsa1.0_01600.1	1147	42537	0	375	384	97	-	188	571	38437	38820
UN11936	Rsa1.0_03901.1	604	20170	0	334	337	99	-	268	604	1021	1357
UN11937	Rsa1.0_00275.1	673	210104	1.00E-163	321	331	96	-	3	333	7172	7502
UN11938	Rsa1.0_00051.1	1538	258225	0	1470	1555	94	-	1	1535	119454	120991
UN11939	Rsa1.0_00341.1	1253	249309	0	1240	1250	99	-	4	1253	3216	4465
UN11940	Rsa1.0_10964.1	1155	5663	0	397	406	97	+	645	1050	466	870
UN11941	Rsa1.0_00005.1	1177	353093	0	420	437	96	+	3	430	341181	341617
UN11942	Rsa1.0_05562.1	1119	13323	0	1105	1124	98	-	2	1119	2420	3540
UN11943	Rsa1.0_01236.1	1216	50156	1.00E-107	200	201	99	-	790	990	44319	44519
UN11944	Rsa1.0_00354.1	1494	232005	1.00E-177	408	435	93	+	525	955	191172	191606
UN11945	Rsa1.0_00083.1	1306	442773	0	695	706	98	-	563	1263	359724	360429
UN11946	Rsa1.0_03615.1	1249	21766	0	345	345	100	+	905	1249	6065	6409
UN11947	Rsa1.0_00400.1	1324	87858	1.00E-103	197	199	98	+	1125	1323	61289	61487
UN11948	Rsa1.0_03878.1	1098	20305	1.00E-116	213	213	100	+	555	767	4375	4587
UN11949	Rsa1.0_00326.1	1008	171955	1.00E-179	330	334	98	+	674	1007	65824	66157
UN11950	Rsa1.0_00016.1	1299	831256	1.00E-173	332	340	97	+	555	894	251373	251712
UN11951	Rsa1.0_00038.1	926	314568	1.00E-152	435	494	88	+	275	753	9529	9997
UN11952	Rsa1.0_00121.1	1186	278029	0	693	695	99	-	3	697	241558	242252
UN11953	Rsa1.0_01559.1	1327	43262	0	453	454	99	+	873	1326	4894	5347
UN11954	Rsa1.0_00108.1	1236	478491	0	737	766	96	+	470	1235	108338	109102
UN11955	Rsa1.0_02750.1	1359	28239	8.00E-86	221	237	93	-	1101	1333	25656	25892
UN11956	Rsa1.0_00417.1	765	395068	1.00E-153	389	423	91	+	355	765	228539	228960
UN11957	Rsa1.0_00309.1	1046	96883	5.00E-99	204	208	98	-	840	1046	64164	64370
UN11958	Rsa1.0_00190.1	900	318240	1.00E-154	342	362	94	+	230	582	211050	211411
UN11959	Rsa1.0_24633.1	1241	976	1.00E-99	223	236	94	+	3	238	681	910
UN11960	Rsa1.0_14007.1	770	3925	0	550	551	99	-	2	552	519	1069
UN11961	Rsa1.0_05947.1	1349	12142	0	605	614	98	+	735	1348	3191	3804
UN11962	Rsa1.0_01615.1	1251	42145	3.00E-45	149	166	89	+	443	607	2933	3098
UN11963	Rsa1.0_07021.1	1064	10016	1.00E-151	319	334	95	+	347	677	7093	7426
UN11964	Rsa1.0_00642.1	1096	201230	0	630	630	100	+	467	1096	180069	180698
UN11965	Rsa1.0_01090.1	1231	54385	0	603	631	95	+	595	1220	28191	28818
UN11966	Rsa1.0_00271.1	1043	101125	1.00E-138	289	302	95	-	605	906	36771	37072
UN11967	Rsa1.0_00168.1	831	115870	0	427	437	97	-	396	831	57751	58187
UN11968	Rsa1.0_71997.1	807	318	2.00E-23	81	88	92	-	502	587	26	113
UN11969	Rsa1.0_00187.1	1147	393093	0	1106	1144	96	+	2	1143	245752	246894
UN11970	Rsa1.0_00594.1	172	73729	2.00E-17	55	58	94	-	115	172	30149	30206
UN11971	Rsa1.0_01117.1	899	53465	0	825	841	98	-	66	899	52547	53387

UN11972	Rsa1.0_03365.1	721	23410	0	541	542	99	+	1	542	14847	15388
UN11973	Rsa1.0_06225.1	879	11443	4.00E-31	104	114	91	+	437	550	9455	9567
UN11974	Rsa1.0_00001.1	831	363028	1.00E-163	298	300	99	+	459	758	208174	208473
UN11975	Rsa1.0_00290.1	1135	225096	0	684	706	96	+	1	706	1515	2214
UN11976	Rsa1.0_00803.1	1592	144228	0	1013	1057	95	+	5	1054	135672	136722
UN11977	Rsa1.0_00522.1	1909	78103	0	1078	1100	98	−	1	1082	36235	37334
UN11978	Rsa1.0_00212.1	724	108350	0	708	722	98	+	6	724	66442	67163
UN11979	Rsa1.0_00008.1	825	358124	0	357	357	100	−	128	484	181921	182277
UN11980	Rsa1.0_00079.1	637	606987	0	548	570	96	−	68	637	57329	57898
UN11981	Rsa1.0_00221.1	1236	106972	0	415	426	97	−	1	418	6959	7384
UN11982	Rsa1.0_00005.1	821	353093	0	499	525	95	+	2	524	138046	138562
UN11983	Rsa1.0_00017.1	1045	752458	0	464	464	100	+	1	464	318311	318774
UN11984	Rsa1.0_14319.1	1120	3529	1.00E-169	487	548	88	+	351	898	2316	2860
UN11985	Rsa1.0_00040.1	657	324196	0	510	541	94	−	19	551	114552	115092
UN11986	Rsa1.0_05612.1	1341	13193	0	569	586	97	−	734	1319	2444	3029
UN11987	Rsa1.0_04340.1	305	17831	1.00E-137	291	303	96	−	1	302	505	806
UN11988	Rsa1.0_05360.1	1126	14053	1.00E-149	290	296	97	−	347	641	11840	12135
UN11989	Rsa1.0_01726.1	774	40263	0	442	452	97	+	1	452	172	620
UN11990	Rsa1.0_03995.1	1189	19654	0	348	348	100	+	1	348	8087	8434
UN11991	Rsa1.0_03612.1	1487	21784	0	633	678	93	−	21	693	1189	1862
UN11992	Rsa1.0_01244.1	1704	50061	0	443	451	98	−	1	447	27345	27795
UN11993	Rsa1.0_00284.1	869	323267	1.00E-152	273	273	100	−	3	275	240625	240897
UN11994	Rsa1.0_04110.1	727	18998	1.00E-125	255	264	96	−	462	725	4839	5102
UN11995	Rsa1.0_03309.1	1025	23852	0	1023	1023	100	+	3	1025	18483	19505
UN11996	Rsa1.0_52728.1	766	432	1.00E-172	319	323	98	+	191	513	1	323
UN11997	Rsa1.0_00128.1	1533	228675	0	421	435	96	+	42	473	200446	200880
UN11998	Rsa1.0_00186.1	706	112669	0	403	418	96	−	1	418	47100	47517
UN11999	Rsa1.0_01874.1	668	37853	1.00E-169	333	342	97	+	2	343	23893	24233
UN12000	Rsa1.0_18507.1	955	1586	1.00E-164	323	333	96	+	371	703	552	884
UN12001	Rsa1.0_01103.1	1695	53974	0	468	483	96	−	192	674	17346	17825
UN12002	Rsa1.0_06571.1	895	10750	1.00E-162	322	332	96	−	6	337	1	329
UN12003	Rsa1.0_01236.1	261	50156	1.00E-115	250	262	95	−	1	261	2006	2267
UN12004	Rsa1.0_00371.1	1840	90463	0	1002	1029	97	−	2	1029	51430	52452
UN12005	Rsa1.0_00363.1	580	90968	4.00E-95	197	204	96	−	237	434	53874	54077
UN12006	Rsa1.0_00567.1	1355	75260	0	376	376	100	+	1	376	66917	67292
UN12007	Rsa1.0_01838.1	1556	38500	0	698	701	99	−	43	743	8300	9000
UN12008	Rsa1.0_09081.1	566	6664	1.00E-174	415	446	93	−	1	442	6013	6450
UN12009	Rsa1.0_01989.1	1144	36439	1.00E-175	377	396	95	−	1	396	11214	11593
UN12010	Rsa1.0_00609.1	1203	72785	0	434	448	96	−	112	554	70202	70649

UN12011	Rsa1.0_08265.1	180	7630	1.00E-73	164	172	95	+	5	176	6613	6784
UN12012	Rsa1.0_19598.1	856	1409	0	659	678	97	−	1	678	253	923
UN12013	Rsa1.0_02298.1	1052	32872	0	440	464	94	−	19	481	6363	6819
UN12014	Rsa1.0_00090.1	939	273859	1.00E-100	195	198	98	+	405	602	2039	2236
UN12015	Rsa1.0_00576.1	925	178949	0	749	751	99	−	1	751	25871	26621
UN12016	Rsa1.0_04195.1	1071	18528	1.00E-124	239	243	98	+	736	978	11857	12099
UN12017	Rsa1.0_00038.1	846	314568	1.00E-158	300	303	99	+	542	843	47307	47608
UN12018	Rsa1.0_03777.1	802	20922	0	463	491	94	−	3	479	2336	2823
UN12019	Rsa1.0_01785.1	196	39368	2.00E-66	183	199	91	+	1	196	28462	28660
UN12020	Rsa1.0_00350.1	1114	92346	1.00E-129	244	247	98	+	848	1094	2003	2249
UN12021	Rsa1.0_00155.1	2683	118465	0	2208	2254	97	+	6	2253	60603	62849
UN12022	Rsa1.0_00720.1	749	66931	0	427	436	97	−	1	435	26080	26511
UN12023	Rsa1.0_01900.1	918	37473	0	915	918	99	−	1	918	9796	10712
UN12024	Rsa1.0_01359.1	1327	47271	0	956	1062	90	+	9	1027	32320	33366
UN12025	Rsa1.0_00065.1	435	454616	0	433	433	100	−	1	433	42271	42703
UN12026	Rsa1.0_03846.1	1198	20481	1.00E-173	308	308	100	−	231	538	14102	14409
UN12027	Rsa1.0_08411.1	1464	7404	0	785	791	99	+	675	1464	1676	2466
UN12028	Rsa1.0_00637.1	1436	71038	0	854	888	96	+	2	889	2333	3205
UN12029	Rsa1.0_00190.1	1336	318240	0	536	552	97	+	791	1336	45482	46033
UN12030	Rsa1.0_14273.1	825	3588	1.00E-144	296	306	96	+	1	304	2202	2506
UN12031	Rsa1.0_43896.1	796	512	0	427	435	98	−	362	796	77	511
UN12032	Rsa1.0_02471.1	828	31104	1.00E-178	316	316	100	+	3	318	27908	28223
UN12033	Rsa1.0_00041.1	783	186197	0	468	494	94	+	6	495	151491	151983
UN12034	Rsa1.0_00149.1	879	305196	1.00E-151	287	292	98	+	531	822	179522	179813
UN12035	Rsa1.0_02252.1	1254	33375	0	515	550	93	−	24	563	8074	8622
UN12036	Rsa1.0_00126.1	1352	127534	0	610	622	98	+	728	1348	41116	41737
UN12037	Rsa1.0_03972.1	1120	19787	1.00E-175	355	368	96	−	19	383	13644	14008
UN12038	Rsa1.0_16891.1	1235	1999	2.00E-71	182	193	94	−	1	188	856	1044
UN12039	Rsa1.0_01476.1	884	172928	9.00E-97	272	300	90	+	3	287	137462	137754
UN12040	Rsa1.0_00628.1	1185	214360	0	469	479	97	+	5	479	203522	204000
UN12041	Rsa1.0_00816.1	1184	403350	0	565	585	96	+	129	711	263913	264497
UN12042	Rsa1.0_00837.1	865	62568	0	652	659	98	−	1	659	3086	3737
UN12043	Rsa1.0_02605.1	1733	29637	0	333	335	99	−	3	337	8473	8807
UN12044	Rsa1.0_00616.1	1181	195713	0	474	501	94	+	1	494	183042	183530
UN12045	Rsa1.0_01696.1	775	301850	6.00E-70	141	143	98	+	125	267	291367	291509
UN12046	Rsa1.0_00116.1	1059	365886	1.00E-150	339	362	93	−	278	636	20143	20498
UN12047	Rsa1.0_00773.1	602	64839	1.00E-134	251	254	98	+	1	254	57170	57423
UN12048	Rsa1.0_00094.1	877	139042	3.00E-78	230	254	90	+	42	292	97632	97877
UN12049	Rsa1.0_02273.1	1604	140964	0	1603	1613	99	−	1	1604	136918	138530

UN12050	Rsa1.0_00596.1	1082	73578	1.00E-154	321	335	95	-	746	1080	9140	9467
UN12051	Rsa1.0_00001.1	812	363028	1.00E-132	264	272	97	-	2	273	156998	157269
UN12052	Rsa1.0_00086.1	1111	439854	6.00E-83	192	202	95	+	5	202	431	631
UN12053	Rsa1.0_04434.1	1502	17377	0	1089	1192	91	-	330	1487	7291	8475
UN12054	Rsa1.0_03019.1	1021	25987	0	330	331	99	-	683	1012	2643	2973
UN12055	Rsa1.0_00921.1	1313	59599	3.00E-42	164	187	87	-	133	318	55224	55407
UN12056	Rsa1.0_02282.1	1146	163951	7.00E-86	204	218	93	+	296	513	149855	150072
UN12057	Rsa1.0_02871.1	765	27085	1.00E-113	248	259	95	+	509	765	4928	5186
UN12058	Rsa1.0_01417.1	797	46252	4.00E-40	138	152	90	+	19	168	2265	2414
UN12059	Rsa1.0_00414.1	965	202190	1.00E-117	280	297	94	-	670	964	177489	177782
UN12060	Rsa1.0_00899.1	1003	60404	0	494	496	99	+	130	624	56656	57151
UN12061	Rsa1.0_04123.1	967	18947	1.00E-141	326	350	93	+	632	967	15564	15911
UN12062	Rsa1.0_05353.1	1432	14078	0	512	517	99	-	496	1012	965	1478
UN12063	Rsa1.0_05440.1	592	13749	1.00E-124	235	238	98	-	131	368	12893	13130
UN12064	Rsa1.0_02735.1	1373	28344	1.00E-173	309	309	100	+	3	311	25349	25657
UN12065	Rsa1.0_04991.1	1335	15215	0	798	826	96	+	510	1335	9687	10502
UN12066	Rsa1.0_02883.1	1207	27035	0	719	772	93	-	5	755	3528	4299
UN12067	Rsa1.0_00002.1	719	601661	0	583	591	98	+	129	719	33588	34178
UN12068	Rsa1.0_00449.1	1334	83863	0	696	728	95	-	612	1332	66747	67466
UN12069	Rsa1.0_19998.1	608	1354	0	449	456	98	+	153	608	691	1142
UN12070	Rsa1.0_00577.1	1787	174827	0	658	662	99	-	709	1370	167911	168571
UN12071	Rsa1.0_01067.1	494	55230	1.00E-91	209	215	97	-	278	492	4569	4778
UN12072	Rsa1.0_14220.1	841	3652	3.00E-69	143	146	97	-	197	342	3225	3370
UN12073	Rsa1.0_31728.1	589	720	1.00E-141	306	314	97	-	277	589	413	720
UN12074	Rsa1.0_04937.1	311	15401	1.00E-134	294	308	95	+	1	303	12386	12693
UN12075	Rsa1.0_01090.1	801	54385	0	403	415	97	+	2	416	3212	3626
UN12076	Rsa1.0_00048.1	794	167185	1.00E-163	320	329	97	-	464	784	77345	77673
UN12077	Rsa1.0_00089.1	1044	406142	0	1033	1040	99	+	1	1040	118913	119948
UN12078	Rsa1.0_12087.1	721	5317	0	700	719	97	-	3	721	4366	5084
UN12079	Rsa1.0_00275.1	638	210104	1.00E-105	292	322	90	+	319	628	157269	157585
UN12080	Rsa1.0_03391.1	1306	23251	0	815	820	99	-	487	1306	2329	3148
UN12081	Rsa1.0_00940.1	1549	179946	0	560	564	99	-	986	1549	170747	171310
UN12082	Rsa1.0_02932.1	934	26647	0	535	540	99	-	1	540	14289	14826
UN12083	Rsa1.0_01778.1	876	39509	6.00E-92	193	200	96	+	261	460	8657	8856
UN12084	Rsa1.0_01210.1	1507	50663	2.00E-19	196	243	80	-	754	995	46593	46835
UN12085	Rsa1.0_00509.1	1023	78829	1.00E-131	256	262	97	-	84	345	77983	78244
UN12086	Rsa1.0_12119.1	963	5311	0	330	330	100	-	633	962	4527	4856
UN12087	Rsa1.0_00715.1	950	67116	1.00E-176	317	318	99	-	600	917	23113	23430
UN12088	Rsa1.0_00196.1	933	109960	0	478	483	98	+	451	931	77908	78390

UN12089	Rsa1.0_02180.1	980	34176	1.00E-111	214	217	98	-	513	729	18478	18694
UN12090	Rsa1.0_00211.1	865	228189	0	433	433	100	-	431	863	213042	213474
UN12091	Rsa1.0_00748.1	1588	65983	0	390	396	98	+	674	1069	52034	52429
UN12092	Rsa1.0_01220.1	1070	161775	1.00E-130	260	268	97	+	724	991	7326	7593
UN12093	Rsa1.0_41382.1	1035	543	1.00E-165	356	375	94	+	159	533	1	369
UN12094	Rsa1.0_00204.1	761	109056	1.00E-143	309	323	95	+	436	750	93161	93481
UN12095	Rsa1.0_00012.1	817	222148	1.00E-139	334	362	92	-	453	813	171062	171408
UN12096	Rsa1.0_03369.1	831	23381	1.00E-166	314	317	99	-	517	831	10375	10691
UN12097	Rsa1.0_00421.1	515	86365	1.00E-130	270	275	98	-	242	515	35834	36104
UN12098	Rsa1.0_01649.1	932	41624	1.00E-145	287	293	97	+	640	932	37920	38209
UN12099	Rsa1.0_03631.1	1417	21647	0	577	587	98	+	266	852	14431	15017
UN12100	Rsa1.0_00039.1	508	296927	6.00E-75	194	209	92	+	300	508	20208	20412
UN12101	Rsa1.0_00115.1	1126	131321	0	427	427	100	+	436	862	104800	105226
UN12102	Rsa1.0_06417.1	1007	11056	1.00E-145	264	265	99	-	743	1007	10629	10893
UN12103	Rsa1.0_00361.1	837	91181	1.00E-116	245	253	96	+	363	615	9838	10088
UN12104	Rsa1.0_09709.1	1136	6198	0	419	420	99	-	56	475	1	420
UN12105	Rsa1.0_00070.1	808	150321	0	534	542	98	-	271	808	47733	48274
UN12106	Rsa1.0_00281.1	600	99908	0	591	598	98	+	3	600	47446	48042
UN12107	Rsa1.0_00417.1	1191	395068	0	378	387	97	-	805	1191	296239	296622
UN12108	Rsa1.0_02064.1	712	35476	8.00E-60	118	118	100	-	531	648	8503	8620
UN12109	Rsa1.0_00156.1	767	118383	0	563	580	97	+	3	581	9522	10095
UN12110	Rsa1.0_03632.1	1069	21645	3.00E-94	179	180	99	+	106	285	7183	7362
UN12111	Rsa1.0_00027.1	1078	283362	0	385	385	100	+	692	1076	275355	275739
UN12112	Rsa1.0_00017.1	924	752458	0	434	458	94	-	9	457	354538	354992
UN12113	Rsa1.0_01456.1	1111	45372	0	589	603	97	-	47	649	27459	28061
UN12114	Rsa1.0_03659.1	913	21525	0	881	912	96	+	2	913	11734	12643
UN12115	Rsa1.0_00190.1	850	318240	0	416	441	94	-	418	850	314346	314786
UN12116	Rsa1.0_00107.1	821	431659	3.00E-87	239	266	89	-	555	820	254234	254478
UN12117	Rsa1.0_01691.1	684	40921	1.00E-102	218	226	96	-	3	223	18956	19181
UN12118	Rsa1.0_00047.1	995	168582	0	652	682	95	-	317	994	108374	109049
UN12119	Rsa1.0_00208.1	784	108565	0	769	770	99	-	15	784	20188	20957
UN12120	Rsa1.0_06694.1	864	10526	0	469	475	98	-	375	848	3948	4422
UN12121	Rsa1.0_01719.1	1304	40388	0	1282	1309	97	-	1	1304	9373	10666
UN12122	Rsa1.0_01316.1	1375	48325	0	1370	1374	99	+	2	1375	21370	22743
UN12123	Rsa1.0_00418.1	1262	215377	1.00E-160	287	287	100	-	7	293	47449	47735
UN12124	Rsa1.0_00351.1	837	413096	3.00E-90	280	297	94	-	58	354	133363	133644
UN12125	Rsa1.0_00055.1	526	520193	3.00E-15	83	95	87	+	402	495	180223	180317
UN12126	Rsa1.0_00363.1	1109	90968	0	327	328	99	+	782	1109	20089	20416
UN12127	Rsa1.0_02957.1	1467	26479	0	428	433	98	+	750	1182	26048	26479

UN12128	Rsa1.0_05895.1	830	12305	1.00E-151	278	280	99	+	3	282	2192	2471
UN12129	Rsa1.0_00629.1	1222	71381	0	537	537	100	+	686	1222	58787	59323
UN12130	Rsa1.0_00097.1	723	138858	0	352	352	100	+	372	723	54312	54663
UN12131	Rsa1.0_01803.1	1637	173846	1.00E-128	270	282	95	+	1213	1494	34625	34906
UN12132	Rsa1.0_00074.1	1059	147591	7.00E-95	270	296	91	+	45	335	127846	128141
UN12133	Rsa1.0_01182.1	713	167304	1.00E-117	289	312	92	−	21	332	117900	118207
UN12134	Rsa1.0_69070.1	844	332	7.00E-82	155	155	100	−	194	348	130	284
UN12135	Rsa1.0_02485.1	669	30968	1.00E-132	269	279	96	−	339	617	28448	28726
UN12136	Rsa1.0_03302.1	864	23898	5.00E-52	118	121	97	−	310	430	13234	13353
UN12137	Rsa1.0_04870.1	1374	135142	0	474	487	97	−	735	1220	1045	1531
UN12138	Rsa1.0_00370.1	829	90472	0	458	475	96	−	352	825	17870	18343
UN12139	Rsa1.0_00006.1	1131	396176	0	959	991	96	−	146	1131	385740	386727
UN12140	Rsa1.0_06955.1	848	10111	0	373	380	98	−	468	846	7311	7690
UN12141	Rsa1.0_00061.1	943	218685	0	454	457	99	+	4	460	176225	176681
UN12142	Rsa1.0_00035.1	1044	176449	0	889	924	96	−	3	926	167856	168766
UN12143	Rsa1.0_00727.1	1179	66724	0	591	605	97	+	1	602	5381	5979
UN12144	Rsa1.0_08879.1	546	6831	0	443	449	98	−	97	545	2182	2625
UN12145	Rsa1.0_00273.1	1093	326186	1.00E-107	207	210	98	−	328	537	132133	132342
UN12146	Rsa1.0_01061.1	458	55349	1.00E-137	267	272	98	−	188	458	27285	27556
UN12147	Rsa1.0_00175.1	913	166121	0	375	376	99	+	536	911	67110	67485
UN12148	Rsa1.0_05677.1	600	12968	0	591	599	98	−	2	600	6725	7323
UN12149	Rsa1.0_03740.1	1372	21065	0	364	369	98	+	3	371	15272	15640
UN12150	Rsa1.0_00048.1	787	167185	1.00E-141	254	254	100	+	137	390	117771	118024
UN12151	Rsa1.0_01960.1	901	36766	2.00E-94	224	240	93	+	59	298	7984	8223
UN12152	Rsa1.0_01081.1	845	499226	1.00E-177	337	342	98	+	503	843	473293	473632
UN12153	Rsa1.0_00733.1	1371	66583	0	714	731	97	+	637	1366	47304	48033
UN12154	Rsa1.0_03263.1	836	24268	1.00E-108	223	231	96	−	606	836	3647	3877
UN12155	Rsa1.0_04428.1	596	17399	0	571	605	94	+	1	596	12969	13567
UN12156	Rsa1.0_00189.1	737	111830	1.00E-169	352	367	95	+	3	367	69237	69601
UN12157	Rsa1.0_01557.1	1002	43291	0	531	531	100	+	470	1000	1053	1583
UN12158	Rsa1.0_00721.1	500	66918	0	468	483	96	+	2	480	59368	59844
UN12159	Rsa1.0_00223.1	770	106231	1.00E-174	378	394	95	+	147	540	2967	3355
UN12160	Rsa1.0_68929.1	1265	333	1.00E-121	285	304	93	−	475	776	1	303
UN12161	Rsa1.0_02067.1	824	35451	0	817	821	99	+	4	824	592	1411
UN12162	Rsa1.0_00507.1	722	79074	0	703	712	98	+	11	722	53468	54178
UN12163	Rsa1.0_00017.1	2160	752458	0	783	821	95	+	1353	2160	555179	555989
UN12164	Rsa1.0_01278.1	785	49370	8.00E-91	179	182	98	−	55	236	20617	20798
UN12165	Rsa1.0_00528.1	799	164755	1.00E-174	385	410	93	−	402	799	154833	155234
UN12166	Rsa1.0_00211.1	726	228189	0	672	685	98	−	4	684	6661	7345

UN12167	Rsa1.0_00065.1	1031	454616	1.00E-151	280	283	98	+	3	285	114473	114755
UN12168	Rsa1.0_03263.1	857	24268	0	476	481	98	−	377	857	23594	24074
UN12169	Rsa1.0_00660.1	869	69688	0	862	867	99	−	3	869	39198	40064
UN12170	Rsa1.0_00030.1	825	250988	3.00E-72	142	143	99	+	297	439	174718	174860
UN12171	Rsa1.0_01219.1	957	50527	1.00E-158	319	331	96	+	81	411	39038	39368
UN12172	Rsa1.0_36118.1	1247	625	1.00E-155	290	294	98	+	614	907	3	296
UN12173	Rsa1.0_00629.1	910	71381	0	355	356	99	+	3	358	57277	57631
UN12174	Rsa1.0_05622.1	1437	13145	0	1369	1379	99	+	61	1437	8559	9935
UN12175	Rsa1.0_01168.1	845	153765	1.00E-12	63	71	88	−	316	386	7886	7956
UN12176	Rsa1.0_01954.1	907	145554	1.00E-178	330	333	99	−	575	907	17776	18107
UN12177	Rsa1.0_00485.1	1839	80937	1.00E-145	407	453	89	+	1200	1646	76412	76857
UN12178	Rsa1.0_16218.1	1002	2240	1.00E-169	326	332	98	−	309	636	750	1081
UN12179	Rsa1.0_00483.1	1155	81102	0	547	555	98	+	283	837	2359	2913
UN12180	Rsa1.0_01934.1	816	37162	2.00E-79	160	163	98	−	568	730	4026	4188
UN12181	Rsa1.0_00123.1	823	325650	2.00E-97	202	209	96	+	185	393	127544	127752
UN12182	Rsa1.0_06687.1	1345	10537	3.00E-45	221	267	82	−	752	1002	9902	10168
UN12183	Rsa1.0_05825.1	1617	12509	0	819	840	97	−	333	1171	7872	8701
UN12184	Rsa1.0_00999.1	1357	57162	2.33E-156	307	320	95	+	3	317	14218	14536
UN12185	Rsa1.0_14100.1	1106	3809	0	963	1010	95	−	26	1020	1147	2156
UN12186	Rsa1.0_03994.1	1331	19659	1.00E-173	315	317	99	+	1015	1331	1766	2082
UN12187	Rsa1.0_01766.1	1389	39617	1.00E-126	230	230	100	−	2	231	32199	32428
UN12188	Rsa1.0_02937.1	814	26605	1.00E-140	262	265	98	+	239	503	7574	7838
UN12189	Rsa1.0_01377.1	1039	46967	1.00E-119	250	261	95	−	95	355	1262	1522
UN12190	Rsa1.0_01760.1	606	39730	0	597	612	97	−	3	603	27887	28498
UN12191	Rsa1.0_00640.1	1359	70979	0	1323	1389	95	−	1	1358	57774	59160
UN12192	Rsa1.0_13238.1	704	4960	0	401	416	96	+	297	704	2273	2685
UN12193	Rsa1.0_01094.1	1012	167285	0	580	598	96	+	403	994	49222	49819
UN12194	Rsa1.0_05428.1	841	13789	0	479	495	96	−	2	496	3394	3888
UN12195	Rsa1.0_06571.1	814	10750	1.00E-105	248	265	93	+	550	813	6156	6414
UN12196	Rsa1.0_00679.1	1008	68971	0	997	1005	99	−	4	1008	601	1605
UN12197	Rsa1.0_01302.1	815	48907	0	521	534	97	+	15	547	47056	47586
UN12198	Rsa1.0_01989.1	259	36439	1.00E-102	231	245	94	−	14	258	14985	15229
UN12199	Rsa1.0_01803.1	835	173846	0	409	410	99	+	423	832	31871	32280
UN12200	Rsa1.0_00080.1	985	190437	1.00E-80	192	204	94	+	622	818	65815	66018
UN12201	Rsa1.0_00186.1	758	112669	0	556	567	98	+	183	744	110485	111051
UN12202	Rsa1.0_00121.1	844	278029	0	335	338	99	+	3	338	30090	30427
UN12203	Rsa1.0_00826.1	1134	166736	0	388	392	98	−	318	709	131985	132375
UN12204	Rsa1.0_49724.1	958	456	1.00E-145	268	270	99	−	656	925	1	270
UN12205	Rsa1.0_00211.1	696	228189	1.00E-73	150	153	98	−	476	628	17865	18017

UN12206	Rsa1.0_00630.1	1153	71281	0	692	729	94	+	18	732	20912	21638
UN12207	Rsa1.0_15089.1	799	2840	0	644	677	95	−	19	692	308	981
UN12208	Rsa1.0_01773.1	1136	39546	0	443	454	97	+	683	1136	18314	18763
UN12209	Rsa1.0_00843.1	798	62411	3.00E−87	176	180	97	−	373	552	56484	56663
UN12210	Rsa1.0_02064.1	763	35476	1.00E−120	247	256	96	−	456	711	5542	5797
UN12211	Rsa1.0_18368.1	1149	1614	0	830	859	96	−	291	1149	762	1614
UN12212	Rsa1.0_00990.1	1403	57445	0	421	440	95	+	3	442	19531	19967
UN12213	Rsa1.0_00285.1	845	149638	1.00E−113	229	236	97	−	260	495	148538	148773
UN12214	Rsa1.0_00380.1	808	89748	1.00E−138	252	253	99	−	210	462	78451	78703
UN12215	Rsa1.0_11565.1	1234	5470	1.00E−129	262	270	97	+	807	1075	467	736
UN12216	Rsa1.0_01884.1	787	37659	1.00E−157	323	337	95	−	3	339	16441	16777
UN12217	Rsa1.0_00218.1	1191	201480	1.00E−144	289	299	96	+	453	751	179386	179684
UN12218	Rsa1.0_04322.1	1288	17917	0	487	493	98	+	3	495	7519	8011
UN12219	Rsa1.0_03524.1	1379	22297	0	855	887	96	−	319	1191	14069	14952
UN12220	Rsa1.0_04995.1	806	15199	0	669	672	99	−	135	805	8404	9075
UN12221	Rsa1.0_00110.1	794	132719	6.00E−73	149	152	98	+	505	656	4053	4204
UN12222	Rsa1.0_00670.1	802	69371	0	578	589	98	+	218	802	44010	44596
UN12223	Rsa1.0_00360.1	809	91190	1.00E−170	303	303	100	−	6	308	78998	79300
UN12224	Rsa1.0_00529.1	793	77487	1.00E−177	418	452	92	+	3	448	72563	73001
UN12225	Rsa1.0_00360.1	894	91190	1.00E−138	290	301	96	+	595	894	71408	71707
UN12226	Rsa1.0_05108.1	1080	14820	1.00E−124	226	226	100	+	40	265	13721	13946
UN12227	Rsa1.0_02269.1	1456	33168	1.00E−134	285	300	95	+	3	302	174	460
UN12228	Rsa1.0_00321.1	1000	95671	0	887	902	98	+	2	903	92674	93575
UN12229	Rsa1.0_05652.1	833	13043	0	352	355	99	+	478	832	2619	2972
UN12230	Rsa1.0_00016.1	1198	831256	0	376	377	99	+	233	609	428642	429018
UN12231	Rsa1.0_09002.1	1221	137863	0	751	773	97	−	3	766	4266	5035
UN12232	Rsa1.0_02762.1	1176	28146	1.00E−156	300	306	98	+	836	1138	8443	8748
UN12233	Rsa1.0_00373.1	1105	90296	1.00E−119	317	350	90	+	639	988	71492	71841
UN12234	Rsa1.0_02599.1	1024	29660	0	630	631	99	+	392	1022	4089	4719
UN12235	Rsa1.0_03624.1	770	21706	1.00E−77	160	164	97	+	106	269	2146	2309
UN12236	Rsa1.0_05105.1	1285	14833	0	710	745	95	+	3	741	3014	3758
UN12237	Rsa1.0_00131.1	771	432233	1.00E−129	269	282	95	+	3	284	286431	286703
UN12238	Rsa1.0_15191.1	1113	2770	0	1100	1115	98	−	1	1112	754	1866
UN12239	Rsa1.0_00378.1	815	89892	0	525	529	99	+	3	531	49538	50066
UN12240	Rsa1.0_01468.1	956	45146	0	825	925	89	−	2	925	23938	24862
UN12241	Rsa1.0_02593.1	849	29744	0	352	353	99	+	3	355	12031	12383
UN12242	Rsa1.0_18627.1	1317	1562	0	403	414	97	+	2	415	762	1175
UN12243	Rsa1.0_07091.1	911	9907	1.00E−147	264	264	100	−	636	899	1	264
UN12244	Rsa1.0_00016.1	979	831256	0	349	351	99	+	629	979	521825	522175

UN12245	Rsa1.0_00826.1	877	166736	0	870	874	99	−	3	875	118793	119666
UN12246	Rsa1.0_04027.1	676	19501	0	650	661	98	−	16	672	4294	4954
UN12247	Rsa1.0_00204.1	839	109056	0	781	817	95	+	2	817	104806	105622
UN12248	Rsa1.0_04419.1	999	17430	1.00E−122	309	333	92	+	672	999	9661	9987
UN12249	Rsa1.0_08045.1	1342	8014	1.00E−180	336	340	98	−	1004	1342	4997	5336
UN12250	Rsa1.0_01625.1	939	42019	0	928	937	99	−	3	939	29702	30637
UN12251	Rsa1.0_02383.1	975	31931	1.00E−162	356	378	94	−	601	969	15501	15878
UN12252	Rsa1.0_00021.1	1036	263655	0	360	363	99	−	248	610	238507	238869
UN12253	Rsa1.0_01462.1	570	45231	1.00E−162	311	318	97	+	253	570	18126	18443
UN12254	Rsa1.0_07845.1	436	8434	7.00E−99	219	231	94	−	165	395	6337	6567
UN12255	Rsa1.0_21254.1	557	1221	1.00E−107	247	265	93	−	3	267	1	250
UN12256	Rsa1.0_00328.1	856	224661	1.00E−180	344	350	98	+	507	855	201619	201965
UN12257	Rsa1.0_00744.1	1080	66084	0	453	471	96	−	610	1080	38777	39247
UN12258	Rsa1.0_00341.1	790	249309	0	346	352	98	+	17	367	177596	177947
UN12259	Rsa1.0_03981.1	765	19724	1.00E−110	221	227	97	−	265	491	19277	19503
UN12260	Rsa1.0_00539.1	1673	76926	1.00E−141	291	301	96	+	1374	1673	28381	28678
UN12261	Rsa1.0_05401.1	1369	13876	0	1041	1110	93	+	280	1369	1410	2514
UN12262	Rsa1.0_00025.1	1554	277312	0	617	637	96	−	813	1444	17828	18461
UN12263	Rsa1.0_00248.1	1390	163568	0	575	591	97	−	315	905	42447	43037
UN12264	Rsa1.0_14188.1	1325	3685	1.00E−129	244	246	99	−	1075	1319	3148	3393
UN12265	Rsa1.0_02597.1	857	29694	0	365	370	98	−	169	538	10697	11066
UN12266	Rsa1.0_00322.1	585	212233	4.00E−89	167	167	100	−	22	188	83613	83779
UN12267	Rsa1.0_01958.1	1139	261128	1.00E−169	346	362	95	−	398	759	119970	120323
UN12268	Rsa1.0_01389.1	1050	46716	0	378	393	96	+	6	398	9773	10165
UN12269	Rsa1.0_08334.1	1048	7525	0	825	873	94	+	182	1048	1833	2693
UN12270	Rsa1.0_00017.1	855	752458	0	353	358	98	+	499	855	206408	206765
UN12271	Rsa1.0_04418.1	1169	17434	2.00E−76	255	293	87	+	795	1082	13307	13574
UN12272	Rsa1.0_07600.1	720	9052	2.00E−42	104	109	95	−	327	435	2941	3049
UN12273	Rsa1.0_00091.1	1094	139699	0	768	772	99	−	1	772	85481	86251
UN12274	Rsa1.0_01041.1	1283	55910	0	653	658	99	−	113	770	35356	36012
UN12275	Rsa1.0_03427.1	1294	22992	0	956	977	97	+	2	971	22016	22992
UN12276	Rsa1.0_00293.1	642	339294	1.00E−163	304	307	99	−	336	642	329554	329859
UN12277	Rsa1.0_01338.1	1151	155644	0	586	588	99	−	1	588	134305	134892
UN12278	Rsa1.0_00744.1	1049	66084	1.00E−115	293	317	92	−	737	1047	57794	58101
UN12279	Rsa1.0_13708.1	1009	4447	1.00E−167	299	299	100	−	710	1008	3599	3897
UN12280	Rsa1.0_01298.1	712	48999	0	400	411	97	+	3	412	24192	24598
UN12281	Rsa1.0_01059.1	738	55410	0	707	738	95	−	2	738	28315	29043
UN12282	Rsa1.0_02219.1	763	33798	0	729	758	96	+	7	762	21464	22220
UN12283	Rsa1.0_00110.1	975	132719	0	732	748	97	+	3	748	96935	97674

UN12284	Rsa1.0_01048.1	1035	55684	0	379	379	100	+	655	1033	46755	47133
UN12285	Rsa1.0_00642.1	860	201230	0	600	603	99	−	258	860	21306	21908
UN12286	Rsa1.0_01119.1	961	53459	1.00E−164	305	309	98	−	653	961	12720	13028
UN12287	Rsa1.0_03238.1	748	24432	1.00E−137	259	263	98	−	482	744	12785	13047
UN12288	Rsa1.0_00953.1	1325	145012	0	478	499	95	+	825	1323	12362	12851
UN12289	Rsa1.0_02139.1	1614	34686	0	645	672	95	−	3	671	26074	26736
UN12290	Rsa1.0_03786.1	1071	20879	0	893	933	95	−	2	932	7289	8218
UN12291	Rsa1.0_06061.1	920	11837	0	689	703	98	−	219	918	3206	3902
UN12292	Rsa1.0_04714.1	944	146738	0	407	420	96	+	513	928	139509	139928
UN12293	Rsa1.0_00367.1	544	90750	0	525	559	93	+	1	544	3323	3881
UN12294	Rsa1.0_00990.1	725	57445	0	549	554	99	−	80	633	46214	46765
UN12295	Rsa1.0_01949.1	777	36960	1.00E−67	152	159	95	−	30	188	26254	26412
UN12296	Rsa1.0_05661.1	1089	13011	0	680	681	99	−	3	683	6577	7257
UN12297	Rsa1.0_00672.1	803	69358	1.00E−135	300	316	94	−	378	691	40409	40724
UN12298	Rsa1.0_34075.1	783	665	0	464	470	98	−	3	472	9	478
UN12299	Rsa1.0_00147.1	1232	249503	0	575	585	98	+	1	583	148899	149483
UN12300	Rsa1.0_00028.1	893	293984	0	851	901	94	−	8	893	131879	132778
UN12301	Rsa1.0_00915.1	537	194446	0	535	538	99	+	2	537	44030	44566
UN12302	Rsa1.0_13478.1	884	4790	0	336	336	100	+	3	338	4455	4790
UN12303	Rsa1.0_00118.1	850	130637	0	564	598	94	+	1	598	98363	98945
UN12304	Rsa1.0_02365.1	796	32118	1.00E−132	245	247	99	−	327	573	29954	30200
UN12305	Rsa1.0_00013.1	1055	292129	1.00E−157	313	321	97	+	733	1052	236612	236931
UN12306	Rsa1.0_02179.1	737	34188	0	717	736	97	+	2	737	18828	19561
UN12307	Rsa1.0_00387.1	912	495407	0	334	335	99	−	3	337	342905	343239
UN12308	Rsa1.0_00661.1	1402	213041	1.00E−130	259	266	97	−	842	1104	200599	200864
UN12309	Rsa1.0_00476.1	1043	280966	0	529	587	90	+	457	1037	163758	164336
UN12310	Rsa1.0_00428.1	1184	85599	0	562	590	95	−	22	611	31030	31616
UN12311	Rsa1.0_00791.1	1022	64201	1.00E−157	281	281	100	+	740	1020	55181	55461
UN12312	Rsa1.0_06009.1	995	11950	1.00E−77	157	160	98	+	836	995	3391	3550
UN12313	Rsa1.0_00350.1	1085	92346	2.00E−82	162	164	98	−	619	782	79954	80117
UN12314	Rsa1.0_01219.1	963	50527	0	366	375	97	−	3	377	5476	5850
UN12315	Rsa1.0_00985.1	1352	161950	0	551	552	99	+	1	551	141766	142317
UN12316	Rsa1.0_01632.1	963	181224	1.00E−156	349	375	93	−	36	407	7459	7812
UN12317	Rsa1.0_01121.1	826	53407	1.00E−173	329	336	97	−	2	337	30379	30714
UN12318	Rsa1.0_00203.1	981	109129	0	383	395	96	−	3	397	26020	26413
UN12319	Rsa1.0_01668.1	882	41299	0	533	545	97	+	339	882	30426	30969
UN12320	Rsa1.0_00416.1	1339	86772	0	494	520	95	+	299	812	64686	65205
UN12321	Rsa1.0_23217.1	529	1064	1.00E−149	301	309	97	−	226	529	660	968
UN12322	Rsa1.0_00931.1	828	59210	0	464	484	95	−	127	610	19424	19895

UN12323	Rsa1.0_02202.1	1140	33961	0	484	487	99	+	654	1140	30950	31436
UN12324	Rsa1.0_00069.1	1275	403561	0	809	810	99	+	3	811	234604	235413
UN12325	Rsa1.0_04340.1	851	17831	1.00E-141	360	397	90	+	449	843	16410	16788
UN12326	Rsa1.0_00234.1	1101	197060	0	1085	1101	98	+	1	1101	55622	56722
UN12327	Rsa1.0_00019.1	1555	301669	1.00E-171	509	580	87	−	508	1069	56894	57432
UN12328	Rsa1.0_02197.1	743	33996	1.00E-102	201	205	98	+	287	491	4504	4708
UN12329	Rsa1.0_00784.1	1002	64359	0	489	492	99	+	3	494	36301	36792
UN12330	Rsa1.0_22545.1	934	1111	0	667	690	96	+	254	934	186	875
UN12331	Rsa1.0_00959.1	810	58202	5.00E-86	210	227	92	−	584	810	41528	41746
UN12332	Rsa1.0_01828.1	915	38681	0	901	914	98	+	2	915	6815	7727
UN12333	Rsa1.0_07541.1	776	9167	0	642	660	97	−	105	757	2424	3083
UN12334	Rsa1.0_00098.1	498	349239	0	489	498	98	−	1	498	115012	115509
UN12335	Rsa1.0_00184.1	325	218748	1.00E-145	306	321	95	+	1	321	2851	3171
UN12336	Rsa1.0_01205.1	1279	50795	0	690	698	98	+	580	1277	7529	8226
UN12337	Rsa1.0_01109.1	824	53793	0	576	580	99	+	3	582	50195	50774
UN12338	Rsa1.0_01460.1	1412	45255	0	661	679	97	+	1	676	34248	34925
UN12339	Rsa1.0_04194.1	820	132734	1.00E-149	286	292	97	+	528	819	132027	132318
UN12340	Rsa1.0_01174.1	792	51696	0	519	529	98	−	96	624	12826	13354
UN12341	Rsa1.0_01769.1	1296	39568	1.00E-100	228	242	94	+	903	1144	33607	33848
UN12342	Rsa1.0_00461.1	523	82509	3.00E-86	201	210	95	−	315	523	76296	76503
UN12343	Rsa1.0_01578.1	580	42880	1.00E-132	261	267	97	−	2	267	35273	35539
UN12344	Rsa1.0_01199.1	675	163576	1.00E-148	266	266	100	+	62	327	589	854
UN12345	Rsa1.0_01536.1	900	43747	0	421	429	98	+	2	430	5549	5976
UN12346	Rsa1.0_00417.1	1408	395068	0	441	458	96	+	89	546	296178	296624
UN12347	Rsa1.0_00484.1	730	203294	3.00E-90	187	193	96	−	1	193	63056	63248
UN12348	Rsa1.0_06149.1	620	11615	1.00E-110	237	246	96	−	5	248	6788	7032
UN12349	Rsa1.0_04209.1	664	18442	0	566	584	96	+	1	583	12890	13465
UN12350	Rsa1.0_00122.1	951	265937	0	464	465	99	−	1	465	239713	240177
UN12351	Rsa1.0_01067.1	741	55230	7.00E-85	163	164	99	+	578	741	6328	6491
UN12352	Rsa1.0_00326.1	967	171955	0	381	394	96	+	1	384	21074	21467
UN12353	Rsa1.0_00727.1	653	66724	1.00E-112	278	296	93	+	2	287	14947	15240
UN12354	Rsa1.0_00321.1	768	95671	0	429	452	94	−	3	451	21420	21870
UN12355	Rsa1.0_08790.1	1466	6918	0	507	531	95	−	628	1151	4948	5478
UN12356	Rsa1.0_06133.1	902	11663	0	761	762	99	−	2	763	9464	10225
UN12357	Rsa1.0_00727.1	761	66724	0	484	504	96	−	2	503	6587	7090
UN12358	Rsa1.0_01561.1	604	43227	1.00E-164	305	309	98	−	296	604	36891	37199
UN12359	Rsa1.0_00020.1	886	283099	0	873	884	98	+	3	886	100023	100903
UN12360	Rsa1.0_00384.1	1324	89278	2.33E-156	293	302	97	−	4	303	35218	35519
UN12361	Rsa1.0_00722.1	773	66840	0	754	763	98	−	5	767	23819	24580

UN12362	Rsa1.0_00763.1	808	65121	0	664	696	95	+	1	689	59130	59818
UN12363	Rsa1.0_00248.1	1492	163568	0	675	676	99	−	3	678	2319	2994
UN12364	Rsa1.0_06045.1	912	11858	0	907	917	98	−	1	911	3496	4410
UN12365	Rsa1.0_00733.1	1230	66583	0	715	735	97	−	43	777	47304	48034
UN12366	Rsa1.0_00621.1	809	71840	0	407	412	98	−	1	412	26851	27258
UN12367	Rsa1.0_02341.1	1453	32387	1.00E−114	281	302	93	−	1163	1453	31192	31492
UN12368	Rsa1.0_01036.1	782	55978	1.00E−168	299	299	100	+	482	780	13317	13615
UN12369	Rsa1.0_01847.1	1273	38346	1.00E−121	227	229	99	−	649	877	8990	9218
UN12370	Rsa1.0_00275.1	1365	210104	1.00E−154	325	339	95	−	1	338	178612	178947
UN12371	Rsa1.0_02266.1	1582	33195	1.00E−117	236	243	97	+	1145	1387	13176	13418
UN12372	Rsa1.0_02229.1	1402	33672	0	408	420	97	−	980	1399	12887	13303
UN12373	Rsa1.0_00060.1	1164	502678	1.00E−177	370	385	96	−	6	385	495990	496374
UN12374	Rsa1.0_07541.1	936	9167	0	921	940	97	+	2	936	1610	2549
UN12375	Rsa1.0_00301.1	679	365315	0	661	672	98	+	12	678	320804	321475
UN12376	Rsa1.0_03122.1	1360	25207	0	464	465	99	+	3	467	9395	9859
UN12377	Rsa1.0_01154.1	289	52249	1.00E−138	279	289	96	+	1	289	9044	9332
UN12378	Rsa1.0_00040.1	752	324196	1.00E−137	248	248	100	−	1	248	245385	245632
UN12379	Rsa1.0_19744.1	924	1389	1.00E−108	209	211	99	+	714	924	1179	1388
UN12380	Rsa1.0_15089.1	650	2840	1.00E−159	315	325	96	+	326	650	303	627
UN12381	Rsa1.0_00642.1	965	201230	1.00E−133	271	279	97	+	107	382	162621	162898
UN12382	Rsa1.0_21156.1	1265	1230	0	431	437	98	+	825	1259	1	437
UN12383	Rsa1.0_02064.1	1127	35476	1.00E−151	316	332	95	+	312	634	5466	5797
UN12384	Rsa1.0_00131.1	1096	432233	1.00E−142	262	264	99	−	831	1094	286440	286703
UN12385	Rsa1.0_03191.1	1047	145762	1.00E−151	308	320	96	+	727	1046	135086	135405
UN12386	Rsa1.0_02921.1	878	26779	1.00E−158	331	343	96	−	3	339	22561	22901
UN12387	Rsa1.0_01474.1	1057	45070	1.00E−178	414	441	93	+	214	649	165	601
UN12388	Rsa1.0_00124.1	580	262503	4.00E−89	185	191	96	+	363	553	17080	17270
UN12389	Rsa1.0_00093.1	1225	214962	0	408	408	100	+	815	1222	97155	97562
UN12390	Rsa1.0_06179.1	966	11548	0	496	497	99	+	60	556	8510	9006
UN12391	Rsa1.0_07555.1	745	9139	1.00E−61	269	318	84	+	441	743	4836	5144
UN12392	Rsa1.0_05398.1	483	13885	0	412	412	100	−	2	413	12820	13231
UN12393	Rsa1.0_08192.1	972	7762	0	352	355	99	+	617	970	6351	6705
UN12394	Rsa1.0_01884.1	896	37659	0	478	490	97	+	1	490	14498	14986
UN12395	Rsa1.0_00571.1	1194	329018	0	758	767	98	−	293	1059	145078	145844
UN12396	Rsa1.0_00219.1	982	106983	1.00E−164	303	306	99	+	269	574	29975	30280
UN12397	Rsa1.0_00170.1	1005	179538	0	388	402	96	+	1	398	57760	58161
UN12398	Rsa1.0_00782.1	1336	64468	1.00E−142	317	334	94	+	465	798	19988	20316
UN12399	Rsa1.0_00102.1	1209	135497	0	500	507	98	−	3	509	11483	11987
UN12400	Rsa1.0_02424.1	871	31570	0	484	486	99	−	1	485	6970	7455

UN12401	Rsa1.0_00094.1	291	139042	2.00E-92	277	313	88	+	1	291	41119	41430
UN12402	Rsa1.0_00011.1	1618	225044	0	433	447	96	-	1171	1616	177241	177686
UN12403	Rsa1.0_07540.1	1662	9170	0	841	849	99	-	336	1184	4024	4872
UN12404	Rsa1.0_00228.1	1512	105511	2.00E-86	203	211	96	-	1303	1510	86275	86484
UN12405	Rsa1.0_00502.1	1207	204180	0	544	544	100	+	3	546	159862	160405
UN12406	Rsa1.0_01836.1	1425	38503	0	1151	1154	99	-	272	1425	17887	19040
UN12407	Rsa1.0_00772.1	797	64856	0	365	366	99	+	432	797	51283	51648
UN12408	Rsa1.0_08807.1	981	6906	0	733	746	98	+	1	746	3399	4142
UN12409	Rsa1.0_11769.1	879	5414	0	528	535	98	-	346	879	777	1310
UN12410	Rsa1.0_01111.1	1053	53669	0	354	354	100	-	700	1053	39891	40244
UN12411	Rsa1.0_00279.1	1314	100039	0	355	356	99	-	959	1314	87699	88054
UN12412	Rsa1.0_00449.1	765	83863	1.00E-154	295	301	98	+	465	765	2417	2717
UN12413	Rsa1.0_00011.1	907	225044	0	407	426	95	+	4	426	4124	4549
UN12414	Rsa1.0_03471.1	620	22678	0	397	408	97	+	209	616	14018	14417
UN12415	Rsa1.0_12341.1	886	5250	0	474	496	95	-	79	565	2120	2615
UN12416	Rsa1.0_00018.1	768	410899	0	576	601	95	+	168	768	49999	50599
UN12417	Rsa1.0_01235.1	796	50180	1.00E-155	287	290	98	+	2	291	20002	20291
UN12418	Rsa1.0_01487.1	1284	44784	0	528	571	92	+	300	865	27756	28326
UN12419	Rsa1.0_02941.1	862	26583	0	860	860	100	+	3	862	22061	22920
UN12420	Rsa1.0_00141.1	915	121494	0	515	533	96	-	383	915	94785	95302
UN12421	Rsa1.0_03877.1	919	20318	1.00E-113	213	215	99	-	39	253	9001	9215
UN12422	Rsa1.0_00470.1	1057	81968	0	518	532	97	-	522	1053	78813	79344
UN12423	Rsa1.0_00128.1	731	228675	0	428	431	99	+	3	433	11663	12092
UN12424	Rsa1.0_02372.1	1368	32053	0	1142	1145	99	+	1	1145	8227	9371
UN12425	Rsa1.0_08429.1	968	7370	4.00E-99	196	200	98	+	257	456	568	767
UN12426	Rsa1.0_05881.1	1340	12341	0	441	452	97	-	751	1192	8912	9363
UN12427	Rsa1.0_00138.1	933	247040	0	411	423	97	-	1	415	230305	230727
UN12428	Rsa1.0_00803.1	976	144228	0	609	612	99	+	365	975	38543	39154
UN12429	Rsa1.0_03271.1	1175	24187	0	409	411	99	-	765	1175	1306	1716
UN12430	Rsa1.0_00280.1	799	99966	1.00E-122	252	258	97	-	3	260	65827	66081
UN12431	Rsa1.0_01585.1	780	42806	1.00E-145	276	281	98	+	19	299	14587	14867
UN12432	Rsa1.0_02185.1	263	34150	1.00E-116	255	267	95	-	2	263	27262	27526
UN12433	Rsa1.0_03014.1	859	26018	1.00E-128	301	317	94	-	547	859	7275	7590
UN12434	Rsa1.0_04651.1	1579	16516	1.00E-103	198	200	99	-	3	202	12242	12441
UN12435	Rsa1.0_01097.1	932	173349	1.00E-105	216	222	97	-	1	222	34536	34755
UN12436	Rsa1.0_03159.1	1350	24946	1.00E-180	363	377	96	+	491	867	14985	15361
UN12437	Rsa1.0_01249.1	941	188432	0	341	342	99	+	598	939	40576	40917
UN12438	Rsa1.0_03856.1	608	20429	0	338	342	98	-	268	608	18869	19210
UN12439	Rsa1.0_02858.1	974	27246	1.00E-160	343	353	97	-	3	349	1688	2037

UN12440	Rsa1.0_00522.1	717	78103	4.00E-77	159	163	97	+	342	504	48991	49153
UN12441	Rsa1.0_01716.1	882	40483	0	882	882	100	+	1	882	30051	30932
UN12442	Rsa1.0_02239.1	700	33534	1.00E-153	298	306	97	+	99	404	26202	26507
UN12443	Rsa1.0_58089.1	821	394	0	392	394	99	−	328	721	1	394
UN12444	Rsa1.0_02206.1	1711	33922	0	893	897	99	+	3	898	4082	4978
UN12445	Rsa1.0_00087.1	1213	761073	0	1172	1204	97	+	7	1208	321128	322326
UN12446	Rsa1.0_00204.1	1287	109056	2.33E-156	267	268	99	−	1020	1287	93547	93814
UN12447	Rsa1.0_05911.1	714	12261	1.00E-98	201	207	97	+	159	365	2115	2321
UN12448	Rsa1.0_04573.1	1093	16789	0	375	378	99	+	716	1093	6126	6503
UN12449	Rsa1.0_02359.1	878	32211	0	871	878	99	−	1	878	21824	22698
UN12450	Rsa1.0_03467.1	1029	22687	0	653	683	95	+	356	1029	10718	11394
UN12451	Rsa1.0_00748.1	694	65983	1.00E-101	214	222	96	+	385	606	65464	65682
UN12452	Rsa1.0_00860.1	1604	61789	0	338	338	100	−	437	774	13435	13772
UN12453	Rsa1.0_00664.1	1064	69573	0	451	451	100	−	614	1064	16651	17101
UN12454	Rsa1.0_01165.1	648	51898	0	443	445	99	−	2	446	51358	51802
UN12455	Rsa1.0_03776.1	787	20926	4.00E-80	197	212	92	−	186	397	1375	1586
UN12456	Rsa1.0_09687.1	1306	6212	0	860	867	99	−	3	869	2235	3100
UN12457	Rsa1.0_00778.1	759	196751	0	641	645	99	−	115	759	179980	180624
UN12458	Rsa1.0_03180.1	729	24764	1.00E-102	248	265	93	+	465	726	21745	22005
UN12459	Rsa1.0_00934.1	1088	193516	0	1051	1087	96	+	3	1088	151153	152239
UN12460	Rsa1.0_00484.1	1496	203294	1.00E-108	248	263	94	−	1232	1493	51197	51459
UN12461	Rsa1.0_00480.1	604	81325	0	581	602	96	+	3	604	3549	4146
UN12462	Rsa1.0_02121.1	424	34813	0	350	356	98	−	3	358	22260	22614
UN12463	Rsa1.0_00047.1	928	168582	0	541	549	98	+	2	550	33979	34521
UN12464	Rsa1.0_00804.1	819	63726	0	355	360	98	−	3	359	59947	60306
UN12465	Rsa1.0_00495.1	982	79934	1.00E-173	364	383	95	−	1	377	66097	66474
UN12466	Rsa1.0_00779.1	1532	64568	0	418	419	99	+	1115	1532	31172	31590
UN12467	Rsa1.0_02809.1	579	27674	1.00E-166	311	315	98	−	3	317	4665	4977
UN12468	Rsa1.0_01735.1	705	40128	1.00E-163	327	339	96	−	367	705	338	671
UN12469	Rsa1.0_00569.1	1026	75060	0	558	559	99	−	451	1009	72157	72715
UN12470	Rsa1.0_00178.1	1263	114861	1.00E-117	251	261	96	+	1	260	30323	30580
UN12471	Rsa1.0_00065.1	1354	454616	1.00E-114	264	279	94	+	1074	1352	116989	117262
UN12472	Rsa1.0_00632.1	820	183777	0	464	488	95	+	341	818	14434	14918
UN12473	Rsa1.0_00271.1	729	101125	1.00E-179	365	374	97	−	357	727	36294	36665
UN12474	Rsa1.0_05683.1	827	12944	0	688	690	99	−	3	692	4930	5619
UN12475	Rsa1.0_00476.1	965	280966	0	618	628	98	−	1	628	249081	249708
UN12476	Rsa1.0_00126.1	1089	127534	0	956	978	97	+	113	1089	107493	108470
UN12477	Rsa1.0_04569.1	1017	16798	1.00E-159	284	284	100	+	368	651	7293	7576
UN12478	Rsa1.0_01895.1	1615	37512	0	730	731	99	−	885	1615	10709	11439

UN12479	Rsa1.0_01656.1	913	41494	1.00E-102	193	194	99	-	356	549	12318	12511
UN12480	Rsa1.0_00380.1	807	89748	0	404	419	96	-	389	807	27101	27519
UN12481	Rsa1.0_01850.1	721	162224	1.00E-107	209	213	98	-	226	438	34793	35005
UN12482	Rsa1.0_05123.1	1767	14790	0	1453	1463	99	+	305	1767	13180	14642
UN12483	Rsa1.0_00740.1	776	215972	1.00E-132	240	240	100	+	497	736	212021	212260
UN12484	Rsa1.0_01687.1	879	40979	1.00E-104	195	196	99	-	659	854	13263	13458
UN12485	Rsa1.0_00191.1	663	316976	0	506	553	91	+	141	663	204217	204763
UN12486	Rsa1.0_01360.1	1292	47246	1.00E-160	338	352	96	-	903	1249	24163	24514
UN12487	Rsa1.0_04160.1	1090	18795	0	686	789	86	+	2	786	196	981
UN12488	Rsa1.0_01173.1	1171	51727	0	438	440	99	+	255	694	29674	30113
UN12489	Rsa1.0_00979.1	979	57733	3.00E-60	236	274	86	-	627	898	1113	1386
UN12490	Rsa1.0_32919.1	1140	690	1.00E-176	349	358	97	-	784	1140	334	690
UN12491	Rsa1.0_00347.1	817	93048	1.00E-107	264	283	93	+	422	702	86405	86679
UN12492	Rsa1.0_01996.1	737	36327	1.00E-132	275	287	95	-	117	403	28812	29098
UN12493	Rsa1.0_06403.1	914	11078	0	442	459	96	+	1	446	5766	6224
UN12494	Rsa1.0_06287.1	835	11303	1.00E-171	337	348	96	-	444	791	6397	6744
UN12495	Rsa1.0_01445.1	951	45671	2.00E-88	166	166	100	-	544	709	21373	21538
UN12496	Rsa1.0_22389.1	738	1125	1.00E-109	258	273	94	+	3	272	486	758
UN12497	Rsa1.0_00752.1	1974	65756	0	528	531	99	+	535	1065	49382	49912
UN12498	Rsa1.0_00512.1	1245	200277	3.00E-97	193	197	97	-	1	197	66192	66388
UN12499	Rsa1.0_00285.1	1093	149638	0	445	482	92	-	3	484	87711	88171
UN12500	Rsa1.0_02028.1	1362	35857	1.00E-154	322	335	96	+	3	335	3872	4205
UN12501	Rsa1.0_00698.1	1634	67816	0	937	956	98	+	86	1041	10881	11836
UN12502	Rsa1.0_02228.1	1290	33688	1.00E-151	337	353	95	-	946	1289	10198	10547
UN12503	Rsa1.0_01603.1	659	42468	1.00E-172	339	350	96	+	3	352	36964	37313
UN12504	Rsa1.0_01377.1	828	46967	1.00E-125	236	239	98	+	7	245	22846	23084
UN12505	Rsa1.0_02807.1	669	27680	1.00E-160	298	302	98	+	54	355	7324	7625
UN12506	Rsa1.0_00073.1	734	240924	1.00E-117	240	250	96	+	1	250	91066	91306
UN12507	Rsa1.0_11720.1	1157	5430	1.00E-142	358	390	91	-	763	1150	2325	2696
UN12508	Rsa1.0_02222.1	475	152798	0	455	474	95	+	1	474	4666	5136
UN12509	Rsa1.0_09451.1	767	6359	1.00E-143	263	265	99	-	208	472	6063	6327
UN12510	Rsa1.0_00007.1	712	239325	1.00E-117	255	266	95	+	449	712	41121	41386
UN12511	Rsa1.0_01389.1	1100	46716	0	482	499	96	+	422	913	10246	10744
UN12512	Rsa1.0_00303.1	966	97468	6.00E-52	108	109	99	-	773	881	38623	38731
UN12513	Rsa1.0_00015.1	1128	561803	1.00E-129	257	262	98	-	856	1115	186279	186540
UN12514	Rsa1.0_00366.1	1035	90880	0	885	909	97	+	2	909	80229	81136
UN12515	Rsa1.0_01110.1	1279	195852	0	513	517	99	+	1	517	36509	37025
UN12516	Rsa1.0_01026.1	1246	171624	0	439	444	98	+	3	445	1088	1531
UN12517	Rsa1.0_01505.1	611	44382	1.00E-178	342	350	97	+	262	611	30211	30557

UN12518	Rsa1.0_72328.1	898	317	1.00E-176	314	314	100	−	517	830	4	317
UN12519	Rsa1.0_00083.1	1034	442773	0	413	430	96	−	3	429	252723	253151
UN12520	Rsa1.0_03962.1	1085	19860	0	722	741	97	+	347	1084	7208	7947
UN12521	Rsa1.0_00030.1	854	250988	0	850	863	98	+	1	854	180342	181204
UN12522	Rsa1.0_00967.1	957	58049	0	787	796	98	−	162	957	22951	23746
UN12523	Rsa1.0_13708.1	996	4447	1.00E-110	270	290	93	−	694	980	3612	3897
UN12524	Rsa1.0_03125.1	1139	147978	1.00E-132	251	255	98	−	624	878	138288	138542
UN12525	Rsa1.0_00308.1	700	96915	1.00E-136	324	347	93	+	341	686	81326	81662
UN12526	Rsa1.0_01456.1	964	45372	4.00E-93	198	206	96	+	393	598	42151	42356
UN12527	Rsa1.0_00537.1	998	186980	0	699	713	98	−	287	998	169043	169755
UN12528	Rsa1.0_00134.1	795	390219	1.00E-117	292	319	91	+	483	789	13216	13528
UN12529	Rsa1.0_00558.1	797	75819	0	528	539	97	+	2	540	28285	28823
UN12530	Rsa1.0_04048.1	931	19394	0	839	878	95	+	5	876	13002	13879
UN12531	Rsa1.0_04208.1	758	18444	1.00E-109	234	245	95	+	65	309	12529	12773
UN12532	Rsa1.0_00602.1	868	73302	0	757	784	96	+	82	860	64379	65159
UN12533	Rsa1.0_00759.1	806	65465	1.00E-136	270	278	97	+	3	280	21778	22055
UN12534	Rsa1.0_00002.1	966	601661	0	519	520	99	−	3	522	184017	184536
UN12535	Rsa1.0_00098.1	1380	349239	1.00E-162	355	373	95	−	2	361	237652	238023
UN12536	Rsa1.0_02425.1	841	31567	0	449	451	99	−	390	839	10864	11314
UN12537	Rsa1.0_38008.1	491	593	1.00E-174	321	325	98	−	76	400	1	325
UN12538	Rsa1.0_07058.1	412	9964	1.00E-126	229	229	100	+	3	231	1471	1699
UN12539	Rsa1.0_04511.1	1087	17032	0	405	410	98	−	405	814	8147	8556
UN12540	Rsa1.0_02555.1	745	30146	1.00E-120	242	247	97	+	117	361	18139	18385
UN12541	Rsa1.0_00020.1	786	283099	1.00E-154	313	324	96	+	69	392	90031	90353
UN12542	Rsa1.0_03878.1	629	20305	1.00E-166	315	321	98	+	1	321	2020	2340
UN12543	Rsa1.0_00107.1	891	431659	0	568	598	94	+	311	891	337025	337616
UN12544	Rsa1.0_02810.1	1783	27671	0	990	999	99	−	170	1167	14789	15786
UN12545	Rsa1.0_01414.1	938	149508	0	819	835	98	−	2	836	18058	18892
UN12546	Rsa1.0_00697.1	789	67913	1.00E-103	233	246	94	+	3	243	54368	54606
UN12547	Rsa1.0_01635.1	936	41865	0	680	708	96	−	230	936	17001	17703
UN12548	Rsa1.0_15024.1	955	2885	1.00E-135	273	281	97	−	676	954	2315	2592
UN12549	Rsa1.0_00676.1	1509	69166	0	1199	1206	99	+	3	1208	50311	51516
UN12550	Rsa1.0_00289.1	833	98620	1.00E-65	184	202	91	−	152	353	72035	72233
UN12551	Rsa1.0_00225.1	1041	105892	0	502	521	96	−	315	831	80714	81229
UN12552	Rsa1.0_01439.1	789	45841	0	491	493	99	+	296	788	28055	28547
UN12553	Rsa1.0_04169.1	765	18712	1.00E-131	271	280	96	−	351	629	2082	2358
UN12554	Rsa1.0_00082.1	1000	237584	8.00E-33	76	77	98	−	921	997	1882	1958
UN12555	Rsa1.0_55302.1	823	413	1.00E-109	201	201	100	−	163	363	1	201
UN12556	Rsa1.0_00377.1	928	90038	1.00E-68	139	141	98	+	403	543	9346	9486

UN12557	Rsa1.0_01417.1	1127	46252	0	403	416	96	+	675	1088	20533	20947
UN12558	Rsa1.0_00004.1	1446	253996	0	608	619	98	+	828	1446	29150	29768
UN12559	Rsa1.0_00049.1	1290	167018	0	1218	1294	94	+	1	1288	63754	65043
UN12560	Rsa1.0_00329.1	917	222544	0	405	422	95	−	67	488	194066	194487
UN12561	Rsa1.0_01384.1	1116	46803	0	448	448	100	+	345	792	20167	20614
UN12562	Rsa1.0_00002.1	1084	601661	0	445	464	95	+	623	1084	594694	595157
UN12563	Rsa1.0_00350.1	1209	92346	0	403	425	94	+	787	1209	51776	52197
UN12564	Rsa1.0_10530.1	1063	5813	1.00E−121	247	254	97	−	3	255	4587	4840
UN12565	Rsa1.0_02139.1	1097	34686	0	1044	1101	94	−	1	1092	27658	28740
UN12566	Rsa1.0_04194.1	1066	132734	0	626	654	95	+	413	1066	116698	117350
UN12567	Rsa1.0_00471.1	832	81897	1.00E−123	249	256	97	−	106	361	73515	73769
UN12568	Rsa1.0_00040.1	657	324196	1.00E−171	334	344	97	+	49	392	110483	110826
UN12569	Rsa1.0_00120.1	968	277288	5.00E−34	167	194	86	−	776	968	228729	228919
UN12570	Rsa1.0_02722.1	935	28417	1.00E−89	171	172	99	+	235	406	15844	16015
UN12571	Rsa1.0_07059.1	1081	9962	2.33E−156	302	315	95	−	386	700	3922	4236
UN12572	Rsa1.0_00759.1	1127	65465	0	889	937	94	+	98	1031	8202	9126
UN12573	Rsa1.0_01230.1	1079	50221	0	1052	1083	97	+	1	1079	40487	41569
UN12574	Rsa1.0_01501.1	874	44532	0	426	431	98	−	411	841	32684	33114
UN12575	Rsa1.0_01803.1	870	173846	1.00E−162	358	378	94	+	500	870	137460	137837
UN12576	Rsa1.0_01552.1	934	43396	1.00E−125	317	341	92	+	593	931	24239	24573
UN12577	Rsa1.0_02003.1	983	36171	1.00E−116	518	616	84	−	69	680	19901	20506
UN12578	#	#	#	#	#	#	#	#	#	#	#	#
UN12579	Rsa1.0_03145.1	1118	25020	0	768	786	97	−	118	903	9241	10026
UN12580	Rsa1.0_00130.1	828	125737	0	465	511	90	+	321	814	28245	28750
UN12581	Rsa1.0_01310.1	666	164719	0	516	554	93	−	122	661	6697	7247
UN12582	Rsa1.0_00760.1	945	65267	0	513	531	96	−	53	583	1718	2248
UN12583	Rsa1.0_03235.1	1067	24442	0	585	605	96	+	444	1047	14818	15420
UN12584	Rsa1.0_00425.1	1428	147080	0	1226	1227	99	+	202	1428	95758	96984
UN12585	Rsa1.0_31264.1	798	731	0	386	404	95	−	8	408	105	508
UN12586	Rsa1.0_00502.1	1152	204180	1.00E−157	299	305	98	−	836	1140	9361	9665
UN12587	Rsa1.0_01647.1	737	41645	1.00E−77	160	164	97	−	356	519	31207	31370
UN12588	Rsa1.0_00017.1	810	752458	3.00E−93	211	222	95	+	1	221	26745	26966
UN12589	Rsa1.0_00272.1	670	447836	1.00E−110	205	206	99	−	212	417	58523	58728
UN12590	Rsa1.0_00197.1	868	109714	0	659	684	96	+	3	676	7688	8370
UN12591	Rsa1.0_00191.1	1031	316976	0	1021	1031	99	+	1	1031	208145	209175
UN12592	Rsa1.0_03245.1	1019	24385	0	362	365	99	+	500	864	23943	24307
UN12593	Rsa1.0_07205.1	892	9726	0	408	417	97	−	468	883	259	675
UN12594	Rsa1.0_01092.1	1146	54348	1.00E−166	368	388	94	+	761	1145	53690	54076
UN12595	Rsa1.0_00188.1	965	112020	1.00E−129	271	283	95	+	14	296	9254	9536

UN12596	Rsa1.0_01294.1	605	49090	0	423	437	96	+	169	605	11942	12376
UN12597	Rsa1.0_00013.1	1041	292129	3.00E-69	134	134	100	+	2	135	150538	150671
UN12598	Rsa1.0_01976.1	486	36570	1.00E-128	233	233	100	+	253	485	1117	1349
UN12599	Rsa1.0_14668.1	1053	3173	0	498	507	98	−	547	1053	1837	2343
UN12600	Rsa1.0_04123.1	449	18947	1.00E-149	412	462	89	+	2	448	12312	12762
UN12601	Rsa1.0_02043.1	796	163754	0	720	735	97	+	3	736	156056	156790
UN12602	Rsa1.0_00821.1	782	62891	1.00E-132	310	331	93	−	51	381	43729	44051
UN12603	Rsa1.0_02548.1	902	30228	0	593	594	99	+	307	900	1221	1814
UN12604	Rsa1.0_00773.1	1099	64839	0	377	378	99	−	721	1098	42891	43268
UN12605	Rsa1.0_07030.1	1020	10008	3.00E-35	107	117	91	+	441	557	5301	5417
UN12606	Rsa1.0_03898.1	917	20183	0	412	416	99	+	3	415	6960	7375
UN12607	Rsa1.0_01983.1	1084	36509	0	489	523	93	−	494	1008	21825	22333
UN12608	Rsa1.0_05410.1	780	13840	0	370	376	98	−	308	683	7861	8236
UN12609	Rsa1.0_00190.1	820	318240	1.00E-126	289	302	95	−	504	800	8325	8626
UN12610	Rsa1.0_06648.1	1174	10603	0	447	456	98	+	719	1174	8894	9349
UN12611	Rsa1.0_04434.1	932	17377	0	498	499	99	−	3	500	11412	11910
UN12612	Rsa1.0_02700.1	1021	28584	0	356	366	97	−	658	1021	2911	3276
UN12613	Rsa1.0_00411.1	844	86944	0	448	478	93	+	3	480	41577	42054
UN12614	Rsa1.0_00454.1	1307	216970	1.00E-176	359	374	95	+	461	834	4657	5030
UN12615	Rsa1.0_07636.1	1137	8971	0	902	947	95	−	99	1040	4906	5841
UN12616	Rsa1.0_04462.1	2818	17250	0	899	999	89	−	10	996	4012	4967
UN12617	Rsa1.0_00130.1	1511	125737	0	827	832	99	+	678	1509	93480	94311
UN12618	Rsa1.0_03436.1	1346	22958	0	818	818	100	+	1	818	18408	19225
UN12619	Rsa1.0_00353.1	775	92242	1.00E-58	131	136	96	−	1	136	19022	19157
UN12620	Rsa1.0_01833.1	905	38555	2.00E-82	181	187	96	+	718	904	16525	16708
UN12621	Rsa1.0_17475.1	1112	1820	0	504	508	99	+	293	799	1314	1820
UN12622	Rsa1.0_04005.1	1074	19586	0	573	589	97	+	486	1074	15406	15994
UN12623	Rsa1.0_00016.1	821	831256	4.00E-43	119	128	92	−	134	261	373073	373197
UN12624	Rsa1.0_00087.1	705	761073	0	363	370	98	−	2	371	731598	731967
UN12625	Rsa1.0_00087.1	713	761073	8.00E-66	185	206	89	+	524	713	314989	315194
UN12626	Rsa1.0_02682.1	1310	28711	0	1245	1299	95	−	2	1300	16960	18249
UN12627	Rsa1.0_03860.1	1442	20407	0	1349	1439	93	+	6	1433	12461	13879
UN12628	Rsa1.0_00157.1	949	245832	0	510	536	95	+	1	535	61535	62065
UN12629	Rsa1.0_00782.1	868	64468	9.00E-97	204	212	96	−	352	563	20391	20602
UN12630	Rsa1.0_00115.1	1063	131321	0	945	1001	94	+	66	1063	24263	25246
UN12631	Rsa1.0_06892.1	910	10197	0	423	423	100	+	488	910	5734	6156
UN12632	Rsa1.0_01203.1	1182	50848	1.00E-109	235	245	95	−	939	1182	48526	48770
UN12633	Rsa1.0_01487.1	1277	44784	0	438	465	94	+	817	1276	2584	3042
UN12634	Rsa1.0_02841.1	1326	27383	2.00E-46	102	104	98	+	310	413	3289	3392

UN12635	Rsa1.0_03964.1	986	19835	0	480	481	99 −	312	792	8248	8728
UN12636	Rsa1.0_01121.1	868	53407	0	731	762	95 +	108	868	19076	19836
UN12637	Rsa1.0_03163.1	554	24894	1.00E−157	290	293	98 −	262	554	19033	19325
UN12638	Rsa1.0_02490.1	1207	30885	0	1185	1192	99 −	17	1207	13667	14858
UN12639	Rsa1.0_03776.1	1029	20926	0	940	944	99 +	75	1018	14487	15430
UN12640	Rsa1.0_00612.1	920	72376	0	881	919	95 +	2	920	42047	42962
UN12641	Rsa1.0_00525.1	1382	78030	0	765	781	97 −	2	782	41941	42718
UN12642	Rsa1.0_00555.1	1135	75920	0	498	503	99 +	3	505	33413	33914
UN12643	Rsa1.0_00713.1	589	158017	1.00E−115	232	239	97 −	56	294	31529	31767
UN12644	Rsa1.0_02319.1	774	32580	1.00E−89	171	172	99 −	603	774	28474	28645
UN12645	Rsa1.0_07899.1	953	8335	0	536	536	100 −	1	536	6134	6669
UN12646	Rsa1.0_03783.1	1115	20890	1.00E−156	322	336	95 −	780	1115	17823	18158
UN12647	Rsa1.0_18172.1	839	1653	0	404	408	99 +	1	408	696	1103
UN12648	Rsa1.0_01453.1	995	45449	1.00E−169	359	379	94 −	629	993	25076	25450
UN12649	Rsa1.0_01113.1	1317	53616	1.00E−163	292	292	100 −	1024	1315	2880	3171
UN12650	Rsa1.0_02215.1	168	33822	4.00E−52	110	112	98 +	1	112	8684	8795
UN12651	Rsa1.0_00236.1	696	104771	0	496	514	96 +	5	516	87938	88448
UN12652	Rsa1.0_00010.1	942	294827	0	344	344	100 −	1	344	291011	291354
UN12653	Rsa1.0_01537.1	635	187285	1.00E−149	267	267	100 −	2	268	21663	21929
UN12654	Rsa1.0_01016.1	821	181422	0	344	347	99 −	361	707	54441	54787
UN12655	Rsa1.0_00123.1	829	325650	0	334	338	98 +	411	748	305219	305556
UN12656	Rsa1.0_02239.1	829	33534	1.00E−148	336	359	93 −	2	357	28204	28551
UN12657	Rsa1.0_05569.1	1393	13311	0	511	517	98 +	403	919	2935	3451
UN12658	Rsa1.0_03674.1	834	21442	0	406	407	99 −	1	407	4886	5292
UN12659	Rsa1.0_02447.1	834	31325	0	613	627	97 +	208	834	19325	19951
UN12660	Rsa1.0_00927.1	1140	59312	3.00E−48	155	171	90 +	395	563	37605	37775
UN12661	Rsa1.0_10001.1	1097	6042	0	481	489	98 +	552	1040	376	864
UN12662	Rsa1.0_00472.1	826	398134	0	811	819	99 −	2	819	175008	175826
UN12663	Rsa1.0_00707.1	717	190112	0	710	717	99 +	1	717	13083	13799
UN12664	Rsa1.0_07260.1	808	9650	0	621	638	97 +	175	808	3769	4406
UN12665	Rsa1.0_03467.1	894	22687	0	655	685	95 −	1	682	10718	11395
UN12666	Rsa1.0_00860.1	1319	61789	1.00E−174	329	335	98 +	836	1170	13438	13772
UN12667	Rsa1.0_00886.1	1617	167350	0	1292	1373	94 +	5	1358	41693	43031
UN12668	Rsa1.0_00358.1	647	91293	1.00E−112	295	321	91 −	10	326	34290	34605
UN12669	Rsa1.0_02874.1	924	27070	1.00E−130	242	244	99 −	4	247	3255	3498
UN12670	Rsa1.0_05024.1	1138	15105	0	695	706	98 −	3	708	12297	13002
UN12671	Rsa1.0_00131.1	806	432233	1.00E−142	256	256	100 −	1	256	409494	409749
UN12672	Rsa1.0_01527.1	1898	43905	1.00E−170	335	344	97 −	1452	1795	3571	3908
UN12673	Rsa1.0_00253.1	1337	231292	0	825	831	99 +	503	1333	6842	7672

UN12674	Rsa1.0_08338.1	704	7511	1.00E-134	260	266	97	+	178	443	1645	1910
UN12675	Rsa1.0_01360.1	1155	47246	0	954	962	99	+	1	962	23553	24514
UN12676	Rsa1.0_00039.1	1270	296927	0	1132	1138	99	−	2	1135	43601	44738
UN12677	Rsa1.0_08922.1	824	6788	0	646	649	99	−	11	659	2351	2999
UN12678	Rsa1.0_05854.1	592	12415	2.00E-94	417	498	83	−	121	590	1610	2103
UN12679	Rsa1.0_00277.1	913	100132	0	776	790	98	+	41	830	62070	62858
UN12680	Rsa1.0_17454.1	1287	1825	1.00E-178	338	345	97	+	185	529	397	741
UN12681	Rsa1.0_03753.1	966	21010	0	474	491	96	+	1	487	549	1036
UN12682	Rsa1.0_00168.1	703	115870	0	403	427	94	+	60	477	28692	29117
UN12683	Rsa1.0_02343.1	525	32374	1.00E-118	236	243	97	−	281	523	1010	1252
UN12684	Rsa1.0_01506.1	1015	44370	1.00E-96	300	338	88	+	1	332	34074	34398
UN12685	Rsa1.0_02162.1	961	34431	0	913	913	100	−	1	913	9700	10612
UN12686	Rsa1.0_00308.1	951	96915	0	412	412	100	+	496	907	85014	85425
UN12687	Rsa1.0_00470.1	768	81968	5.00E-95	189	193	97	−	380	572	50362	50554
UN12688	Rsa1.0_00340.1	1617	305782	0	1034	1038	99	+	3	1039	105673	106710
UN12689	Rsa1.0_01414.1	1052	149508	1.00E-159	297	301	98	+	749	1049	18060	18360
UN12690	Rsa1.0_00013.1	515	292129	0	431	450	95	−	1	446	116018	116467
UN12691	Rsa1.0_00773.1	1133	64839	0	375	378	99	+	2	378	42891	43268
UN12692	Rsa1.0_12696.1	637	5151	0	392	398	98	−	4	397	4	401
UN12693	Rsa1.0_00059.1	729	159650	1.00E-167	322	330	97	−	2	331	24598	24927
UN12694	Rsa1.0_07250.1	1370	9663	1.00E-148	274	275	99	+	1097	1370	9079	9353
UN12695	Rsa1.0_02672.1	876	28884	1.00E-133	266	273	97	−	602	874	15502	15773
UN12696	Rsa1.0_00087.1	801	761073	3.00E-75	150	152	98	−	111	262	294730	294881
UN12697	Rsa1.0_02233.1	1178	33658	0	453	457	99	+	720	1176	4344	4800
UN12698	Rsa1.0_00797.1	611	63959	1.00E-139	348	378	92	−	41	406	11646	12022
UN12699	Rsa1.0_02370.1	1113	32071	0	582	587	99	−	264	850	1342	1928
UN12700	Rsa1.0_00759.1	539	65465	1.00E-159	383	414	92	−	1	404	9402	9811
UN12701	Rsa1.0_11046.1	1252	5635	1.00E-99	185	185	100	−	264	448	2762	2946
UN12702	Rsa1.0_00053.1	1484	263459	1.00E-161	395	430	91	−	5	429	84698	85118
UN12703	Rsa1.0_00671.1	859	397884	0	451	452	99	−	1	452	185631	186082
UN12704	Rsa1.0_07638.1	750	8967	7.00E-88	212	227	93	−	83	301	6345	6571
UN12705	Rsa1.0_00467.1	1116	156542	0	532	536	99	−	55	590	77638	78173
UN12706	Rsa1.0_00018.1	1157	410899	0	399	422	94	−	40	457	68738	69159
UN12707	Rsa1.0_00352.1	988	92266	0	533	570	93	+	1	567	24753	25312
UN12708	Rsa1.0_00457.1	888	337301	0	822	825	99	+	2	826	317281	318105
UN12709	Rsa1.0_00169.1	1198	274298	0	703	721	97	+	1	718	26972	27690
UN12710	Rsa1.0_01092.1	815	54348	0	407	416	97	+	365	780	43380	43795
UN12711	Rsa1.0_47960.1	1257	472	1.00E-157	282	282	100	+	575	856	135	416
UN12712	Rsa1.0_01166.1	786	51877	0	485	491	98	−	2	492	8577	9067

UN12713	Rsa1.0_01526.1	1087	43919	0	394	403	97	−		685	1087	1326	1728
UN12714	Rsa1.0_00788.1	912	64237	0	489	498	98	−		1	498	34653	35149
UN12715	Rsa1.0_04655.1	1084	16495	0	619	622	99	−		4	623	11787	12408
UN12716	Rsa1.0_02561.1	1083	30080	1.00E−110	218	222	98	+		1	222	13923	14143
UN12717	Rsa1.0_00087.1	1323	761073	0	505	506	99	−		816	1321	439499	440004
UN12718	Rsa1.0_03895.1	775	20206	1.00E−168	475	527	90	+		1	516	13684	14200
UN12719	Rsa1.0_00214.1	767	107508	0	627	635	98	−		3	636	74183	74817
UN12720	Rsa1.0_01829.1	729	166063	0	698	730	95	−		1	729	161056	161780
UN12721	Rsa1.0_00559.1	1255	190486	0	709	726	97	+		531	1255	182338	183063
UN12722	Rsa1.0_00303.1	376	97468	5.00E−41	101	106	95	+		257	362	39703	39808
UN12723	Rsa1.0_00002.1	586	601661	0	490	501	97	+		1	501	273867	274367
UN12724	Rsa1.0_00280.1	591	99966	1.00E−180	440	475	92	+		1	468	15030	15501
UN12725	Rsa1.0_00060.1	1494	502678	0	636	653	97	+		485	1137	335571	336223
UN12726	Rsa1.0_00152.1	1294	257730	0	493	513	96	−		167	676	84804	85316
UN12727	Rsa1.0_01983.1	820	36509	1.00E−129	277	292	94	−		143	434	31893	32171
UN12728	Rsa1.0_02772.1	1441	28047	0	441	441	100	+		999	1439	4728	5168
UN12729	Rsa1.0_00307.1	727	199003	1.00E−158	331	346	95	−		80	425	70435	70778
UN12730	Rsa1.0_00625.1	631	71672	0	512	544	94	+		4	547	44621	45142
UN12731	Rsa1.0_02269.1	842	33168	0	362	374	96	−		473	842	18650	19023
UN12732	Rsa1.0_01540.1	1214	151890	0	464	471	98	+		3	473	109822	110288
UN12733	Rsa1.0_00002.1	869	601661	0	667	672	99	+		198	869	73556	74224
UN12734	Rsa1.0_01487.1	1330	44784	0	435	463	93	+		846	1303	2584	3046
UN12735	Rsa1.0_00132.1	876	124299	1.00E−168	383	414	92	−		3	411	92484	92882
UN12736	Rsa1.0_00123.1	860	325650	0	744	773	96	+		3	773	93792	94560
UN12737	Rsa1.0_02233.1	148	33658	4.00E−64	144	148	97	+		2	148	22592	22738
UN12738	Rsa1.0_06671.1	1451	10563	0	892	897	99	+		553	1449	3146	4042
UN12739	Rsa1.0_00121.1	880	278029	0	361	365	98	−		476	840	101741	102105
UN12740	Rsa1.0_00224.1	844	327530	0	400	406	98	+		32	437	191268	191673
UN12741	Rsa1.0_00053.1	720	263459	0	351	352	99	+		3	354	142354	142705
UN12742	Rsa1.0_04542.1	1147	16930	0	569	573	99	−		575	1145	14326	14898
UN12743	Rsa1.0_01806.1	794	39021	1.00E−166	309	313	98	+		218	530	11263	11575
UN12744	Rsa1.0_00997.1	1388	57190	0	930	1044	89	+		107	1111	55922	56933
UN12745	Rsa1.0_02001.1	1194	36198	0	562	574	97	−		288	861	18246	18819
UN12746	Rsa1.0_00698.1	931	67816	1.00E−139	281	287	97	+		604	887	65687	65973
UN12747	#	#	#	#	#	#	#	#	#	#	#	#	
UN12748	Rsa1.0_00192.1	896	278200	0	708	761	93	+		137	896	251267	252016
UN12749	Rsa1.0_34603.1	1122	654	1.00E−136	252	254	99	−		68	321	203	456
UN12750	Rsa1.0_00051.1	1040	258225	0	985	1054	93	+		3	1030	113688	114731
UN12751	Rsa1.0_00534.1	675	77361	0	591	596	99	−		81	675	41291	41885

UN12752	Rsa1.0_09314.1	1063	6461	0	522	530	98	+	4	532	5588	6112
UN12753	Rsa1.0_00329.1	717	222544	1.00E-151	322	335	96	+	19	353	69996	70327
UN12754	Rsa1.0_00476.1	1012	280966	0	561	574	97	+	10	581	158349	158922
UN12755	Rsa1.0_05748.1	632	12749	1.00E-129	273	286	95	+	1	286	8246	8531
UN12756	Rsa1.0_04725.1	1066	16221	0	549	554	99	+	513	1066	629	1182
UN12757	Rsa1.0_00744.1	748	66084	1.00E-127	364	408	89	−	3	394	58901	59305
UN12758	Rsa1.0_00366.1	963	90880	0	904	922	98	−	43	963	18961	19882
UN12759	Rsa1.0_02671.1	839	28887	7.00E-76	151	153	98	+	341	493	4160	4312
UN12760	Rsa1.0_00064.1	1077	240927	0	722	737	97	+	343	1077	2395	3122
UN12761	Rsa1.0_03517.1	1267	22337	1.00E-126	258	265	97	−	1	263	17681	17945
UN12762	Rsa1.0_00067.1	978	415055	0	602	672	89	+	331	978	165425	166069
UN12763	Rsa1.0_01751.1	609	39806	1.00E-115	274	290	94	−	318	606	30349	30634
UN12764	Rsa1.0_00173.1	773	288743	7.00E-85	188	196	95	−	1	196	284813	285007
UN12765	Rsa1.0_04981.1	858	15250	0	337	338	99	+	521	858	3452	3789
UN12766	Rsa1.0_00285.1	953	149638	1.00E-74	192	208	92	−	746	953	51931	52134
UN12767	Rsa1.0_08080.1	1084	137363	0	1026	1075	95	+	15	1084	3242	4298
UN12768	Rsa1.0_00687.1	1481	68494	0	842	928	90	−	7	916	23396	24308
UN12769	Rsa1.0_00025.1	804	277312	0	746	747	99	−	5	751	141060	141806
UN12770	Rsa1.0_07056.1	1676	9969	0	377	382	98	−	444	825	3171	3552
UN12771	Rsa1.0_01236.1	810	50156	4.00E-68	196	211	92	−	606	810	40391	40601
UN12772	Rsa1.0_01943.1	786	37042	0	382	398	95	+	386	780	31522	31919
UN12773	Rsa1.0_00828.1	1094	62744	0	695	719	96	+	376	1094	15666	16383
UN12774	Rsa1.0_00002.1	1276	601661	1.00E-157	290	293	98	−	748	1040	56141	56433
UN12775	Rsa1.0_00387.1	886	495407	0	648	657	98	+	226	876	279848	280504
UN12776	Rsa1.0_00183.1	1183	113277	1.00E-164	300	302	99	−	66	367	71078	71379
UN12777	Rsa1.0_03122.1	559	25207	0	545	555	98	−	3	557	14297	14851
UN12778	Rsa1.0_04022.1	1374	19534	0	813	840	96	−	3	839	16373	17212
UN12779	Rsa1.0_00273.1	749	326186	0	619	633	97	−	55	687	239109	239741
UN12780	Rsa1.0_08841.1	1243	6870	1.00E-173	317	320	99	−	924	1243	2715	3034
UN12781	Rsa1.0_00504.1	1087	79334	0	439	454	96	−	632	1085	49866	50319
UN12782	Rsa1.0_00085.1	776	142226	8.00E-91	198	206	96	−	3	208	31612	31816
UN12783	Rsa1.0_08838.1	803	6873	0	416	438	94	+	336	770	2202	2639
UN12784	Rsa1.0_00419.1	1014	86562	1.00E-117	327	364	89	+	2	354	13957	14303
UN12785	Rsa1.0_75419.1	680	304	1.00E-52	126	133	94	−	50	176	172	304
UN12786	Rsa1.0_00789.1	791	64233	1.00E-174	327	333	98	−	2	334	18293	18625
UN12787	Rsa1.0_00255.1	1063	204967	0	394	407	96	+	312	717	202520	202925
UN12788	Rsa1.0_02079.1	1398	35306	0	502	511	98	+	38	548	23541	24051
UN12789	Rsa1.0_00263.1	1712	224968	1.00E-142	326	351	92	−	13	348	143774	144123
UN12790	Rsa1.0_00104.1	815	368722	1.00E-122	343	381	90	+	217	590	68056	68435

UN12791	Rsa1.0_00019.1	759	301669	0	566	592	95	−	174	758	240989	241580
UN12792	Rsa1.0_00055.1	1362	520193	0	414	417	99	+	948	1362	402999	403415
UN12793	Rsa1.0_01151.1	702	52268	1.00E−125	255	262	97	−	443	701	40359	40620
UN12794	Rsa1.0_00245.1	1091	103569	0	462	474	97	+	620	1091	80498	80970
UN12795	Rsa1.0_00673.1	1097	69325	0	474	475	99	−	623	1097	21082	21556
UN12796	Rsa1.0_07321.1	790	9559	1.00E−115	279	304	91	+	395	676	4293	4596
UN12797	Rsa1.0_00252.1	890	102760	1.00E−156	406	443	91	+	463	890	97946	98384
UN12798	Rsa1.0_01825.1	772	38725	1.00E−177	318	319	99	−	2	320	8042	8360
UN12799	Rsa1.0_27953.1	903	833	1.00E−152	300	309	97	−	507	815	4	312
UN12800	Rsa1.0_01318.1	1295	48275	0	931	975	95	+	307	1279	46134	47105
UN12801	Rsa1.0_00048.1	1150	167185	1.00E−101	218	228	95	−	922	1149	4779	5006
UN12802	Rsa1.0_11741.1	807	5423	1.00E−151	351	375	93	−	419	791	4722	5096
UN12803	Rsa1.0_04399.1	1226	17543	0	525	552	95	+	672	1222	9931	10478
UN12804	Rsa1.0_00449.1	1321	83863	0	900	953	94	−	356	1306	69850	70799
UN12805	Rsa1.0_00061.1	751	218685	0	408	425	96	−	334	746	2190	2614
UN12806	#	#	#	#	#	#	#	#	#	#	#	#
UN12807	Rsa1.0_01286.1	1128	49209	1.00E−178	379	402	94	−	743	1127	24662	25063
UN12808	Rsa1.0_01088.1	1322	54416	0	414	422	98	−	2	419	17524	17945
UN12809	Rsa1.0_01477.1	1238	45008	0	876	908	96	+	314	1213	16872	17774
UN12810	Rsa1.0_01482.1	983	44855	7.00E−30	207	247	83	+	733	976	1537	1780
UN12811	Rsa1.0_00017.1	1263	752458	0	426	428	99	−	835	1260	154844	155271
UN12812	Rsa1.0_00935.1	315	59079	1.00E−112	238	246	96	+	70	315	23754	23997
UN12813	Rsa1.0_00178.1	718	114861	0	385	391	98	+	3	392	7147	7537
UN12814	Rsa1.0_03226.1	1105	24494	0	1028	1091	94	+	14	1104	11458	12540
UN12815	Rsa1.0_00026.1	2149	327835	0	405	419	96	+	1	419	168377	168795
UN12816	Rsa1.0_00033.1	861	515811	0	517	544	95	−	318	861	392387	392921
UN12817	Rsa1.0_00068.1	915	151813	0	657	697	94	−	2	695	105616	106300
UN12818	Rsa1.0_00127.1	1117	127446	0	471	497	94	−	626	1117	98617	99109
UN12819	Rsa1.0_00218.1	1308	201480	0	748	766	97	+	545	1308	197280	198045
UN12820	Rsa1.0_00159.1	585	431686	0	551	568	97	+	4	571	402820	403375
UN12821	Rsa1.0_01451.1	868	45459	0	672	706	95	−	6	702	29003	29707
UN12822	Rsa1.0_01279.1	1407	180330	0	580	581	99	−	827	1407	132092	132672
UN12823	Rsa1.0_01097.1	528	173349	9.00E−96	181	182	99	−	347	528	8596	8777
UN12824	Rsa1.0_00002.1	1262	601661	0	641	672	95	−	2	670	454256	454927
UN12825	Rsa1.0_00900.1	688	60367	3.00E−74	145	146	99	−	38	183	28111	28256
UN12826	Rsa1.0_00166.1	693	240912	0	386	388	99	+	307	693	148292	148679
UN12827	Rsa1.0_02013.1	661	36041	0	653	661	98	+	1	661	24618	25278
UN12828	Rsa1.0_00321.1	929	95671	1.00E−104	199	201	99	−	509	709	49435	49635
UN12829	Rsa1.0_00099.1	1528	159619	0	425	427	99	−	1	426	141527	141953

UN12830	Rsa1.0_01563.1	705	170823	0	674	721	93	-	5	705	25362	26066
UN12831	Rsa1.0_00532.1	1099	77384	4.00E-81	178	186	95	-	545	730	35746	35931
UN12832	Rsa1.0_00675.1	713	145590	0	470	484	97	+	4	487	5564	6047
UN12833	Rsa1.0_03752.1	1204	21022	1.00E-105	245	262	93	+	952	1202	1341	1602
UN12834	Rsa1.0_46200.1	793	488	1.00E-110	211	214	98	+	387	600	167	380
UN12835	Rsa1.0_00373.1	848	90296	0	381	384	99	-	466	848	72444	72827
UN12836	Rsa1.0_01942.1	722	37048	0	508	519	97	+	205	722	3600	4118
UN12837	Rsa1.0_00105.1	954	134138	0	460	469	98	-	484	951	122067	122535
UN12838	Rsa1.0_13606.1	1039	4648	0	806	826	97	-	232	1039	3795	4620
UN12839	Rsa1.0_00483.1	961	81102	1.00E-172	388	408	95	-	1	403	51013	51417
UN12840	Rsa1.0_05108.1	1332	14820	1.00E-137	287	299	95	+	6	301	7623	7921
UN12841	Rsa1.0_02387.1	801	31890	0	489	503	97	+	300	801	13695	14197
UN12842	Rsa1.0_00821.1	809	62891	0	466	467	99	+	34	500	46889	47355
UN12843	Rsa1.0_05890.1	622	12317	3.00E-46	137	149	91	-	1	145	1820	1968
UN12844	Rsa1.0_09479.1	1146	6340	0	461	462	99	+	686	1146	1	462
UN12845	Rsa1.0_26922.1	781	873	9.00E-63	126	127	99	+	253	379	178	304
UN12846	Rsa1.0_00744.1	940	66084	1.00E-175	324	327	99	+	1	327	51350	51675
UN12847	Rsa1.0_00949.1	1106	58549	0	886	920	96	+	195	1106	51667	52584
UN12848	Rsa1.0_03327.1	777	23707	0	769	780	98	-	1	777	17824	18603
UN12849	Rsa1.0_00008.1	757	358124	1.00E-86	190	199	95	-	44	242	179685	179883
UN12850	Rsa1.0_02858.1	858	27246	4.00E-99	184	184	100	-	617	800	1	184
UN12851	Rsa1.0_19044.1	789	1490	0	330	331	99	+	449	779	1016	1346
UN12852	Rsa1.0_23774.1	1152	1026	1.00E-145	307	322	95	+	3	312	519	837
UN12853	Rsa1.0_00821.1	1042	62891	1.00E-126	300	321	93	+	20	328	48999	49319
UN12854	Rsa1.0_00323.1	1466	159650	0	446	453	98	+	1013	1465	125184	125636
UN12855	Rsa1.0_00307.1	1488	199003	0	529	548	96	-	942	1487	9419	9963
UN12856	Rsa1.0_00589.1	1345	209965	0	916	930	98	-	417	1345	63245	64174
UN12857	Rsa1.0_00190.1	1405	318240	0	838	840	99	-	566	1405	199849	200688
UN12858	Rsa1.0_01121.1	734	53407	1.00E-171	387	411	94	-	327	732	30379	30789
UN12859	Rsa1.0_01537.1	743	187285	0	496	504	98	+	3	506	14810	15313
UN12860	Rsa1.0_00170.1	757	179538	7.00E-82	155	155	100	-	299	453	28953	29107
UN12861	Rsa1.0_02268.1	1145	33172	0	674	700	96	+	413	1103	29896	30595
UN12862	Rsa1.0_02422.1	784	161122	0	775	784	98	+	3	784	154550	155333
UN12863	Rsa1.0_00008.1	1307	358124	0	830	835	99	-	472	1306	3045	3876
UN12864	Rsa1.0_02494.1	832	132923	3.00E-56	131	136	96	-	698	832	110900	111035
UN12865	Rsa1.0_02081.1	1262	35235	1.00E-164	309	313	98	+	951	1262	16615	16927
UN12866	Rsa1.0_00438.1	1310	84861	1.00E-141	308	319	96	-	965	1278	47498	47816
UN12867	Rsa1.0_05474.1	1182	13620	0	463	474	97	+	711	1181	6888	7361
UN12868	Rsa1.0_00175.1	756	166121	2.00E-75	200	219	91	-	123	341	141878	142090

UN12869	Rsa1.0_00207.1	1544	248736	1.00E-139	299	312	95	+	384	693	24164	24475
UN12870	Rsa1.0_01514.1	1183	44222	1.00E-156	295	300	98	+	12	311	2906	3205
UN12871	Rsa1.0_01474.1	1081	45070	0	984	1001	98	+	81	1081	18278	19277
UN12872	Rsa1.0_33384.1	488	679	0	378	388	97	−	92	479	173	560
UN12873	Rsa1.0_00140.1	772	121541	0	730	756	96	+	5	756	82013	82767
UN12874	Rsa1.0_00020.1	782	283099	0	544	597	91	+	1	594	63641	64224
UN12875	Rsa1.0_01784.1	1049	39371	0	1033	1048	98	+	3	1049	3946	4993
UN12876	Rsa1.0_01564.1	1274	43169	0	1108	1140	97	−	118	1257	19661	20800
UN12877	Rsa1.0_00962.1	1302	184909	0	755	801	94	+	513	1302	19815	20606
UN12878	Rsa1.0_08140.1	823	7845	0	413	430	96	+	394	823	6704	7129
UN12879	Rsa1.0_02119.1	983	34833	0	843	894	94	−	1	872	7164	8055
UN12880	Rsa1.0_04881.1	1198	15608	1.00E-175	353	367	96	+	825	1191	7577	7943
UN12881	Rsa1.0_02655.1	1383	29027	1.00E-104	321	365	87	−	15	376	13789	14132
UN12882	Rsa1.0_29200.1	1262	789	0	695	741	93	+	492	1231	68	788
UN12883	#	#	#	#	#	#	#	#	#	#	#	#
UN12884	Rsa1.0_00372.1	906	90418	0	427	447	95	−	470	905	46191	46637
UN12885	Rsa1.0_01026.1	1175	171624	1.00E-127	265	275	96	−	902	1175	30319	30587
UN12886	Rsa1.0_00108.1	1266	478491	0	512	558	91	−	668	1215	307490	308027
UN12887	Rsa1.0_00519.1	1329	197864	0	447	462	96	+	422	883	192264	192724
UN12888	Rsa1.0_00652.1	906	70089	1.00E-109	229	237	96	−	661	897	22798	23033
UN12889	Rsa1.0_07925.1	750	8245	0	723	756	95	+	2	750	3710	4459
UN12890	Rsa1.0_00016.1	982	831256	1.00E-151	287	292	98	−	691	982	204599	204890
UN12891	Rsa1.0_05505.1	677	13500	0	633	652	97	+	3	652	6227	6878
UN12892	Rsa1.0_00226.1	719	105884	0	445	459	96	−	257	713	42370	42828
UN12893	Rsa1.0_06590.1	1255	10706	0	409	429	95	−	499	927	2006	2428
UN12894	Rsa1.0_00642.1	1490	201230	0	1032	1064	96	+	2	1063	172898	173961
UN12895	Rsa1.0_00196.1	849	109960	0	619	638	97	+	220	849	102104	102740
UN12896	Rsa1.0_01155.1	1097	52182	0	743	766	96	+	4	769	12179	12944
UN12897	Rsa1.0_01585.1	614	42806	0	598	613	97	−	2	614	8561	9173
UN12898	Rsa1.0_14855.1	1348	3011	0	650	674	96	+	259	932	1310	1983
UN12899	Rsa1.0_00890.1	843	323870	1.00E-113	267	284	94	+	560	842	213739	214021
UN12900	Rsa1.0_00218.1	1372	201480	0	997	1085	91	+	285	1364	174782	175837
UN12901	Rsa1.0_00044.1	1150	271658	0	413	425	97	+	718	1140	120064	120488
UN12902	Rsa1.0_04786.1	564	15988	9.00E-90	171	172	99	+	279	450	964	1135
UN12903	Rsa1.0_01667.1	1033	41315	1.00E-155	341	362	94	+	668	1029	2299	2655
UN12904	Rsa1.0_19029.1	775	1493	5.00E-58	169	186	90	−	385	570	55	233
UN12905	Rsa1.0_12866.1	858	5093	0	805	847	95	−	1	824	1621	2461
UN12906	Rsa1.0_00158.1	670	117999	1.00E-168	325	332	97	+	337	668	68825	69155
UN12907	Rsa1.0_05687.1	1304	12932	0	575	589	97	+	718	1304	3372	3956

UN12908	Rsa1.0_02750.1	999	28239	2.00E-89	206	218	94	-	487	701	23214	23431
UN12909	Rsa1.0_00441.1	796	84607	1.00E-156	289	292	98	+	3	294	67630	67921
UN12910	Rsa1.0_06452.1	1207	10994	1.00E-126	276	294	93	-	615	908	2469	2750
UN12911	Rsa1.0_01884.1	469	37659	1.00E-168	317	323	98	+	146	468	23642	23964
UN12912	Rsa1.0_00222.1	1019	172263	0	996	1015	98	+	3	1015	68984	69995
UN12913	Rsa1.0_00284.1	1294	323267	1.00E-103	194	195	99	-	197	391	137125	137319
UN12914	Rsa1.0_00037.1	1874	307838	0	450	451	99	-	938	1388	136526	136976
UN12915	Rsa1.0_00225.1	782	105892	0	434	435	99	-	348	782	47589	48023
UN12916	Rsa1.0_00451.1	603	322533	1.00E-174	309	309	100	-	295	603	26266	26574
UN12917	Rsa1.0_03588.1	671	21908	0	349	352	99	+	3	353	3149	3500
UN12918	Rsa1.0_00215.1	1276	201775	1.00E-161	337	350	96	+	83	428	48897	49244
UN12919	Rsa1.0_00196.1	1177	109960	0	441	449	98	+	731	1177	37671	38114
UN12920	Rsa1.0_03878.1	961	20305	0	517	529	97	+	259	784	1652	2180
UN12921	Rsa1.0_03695.1	1276	21329	0	444	465	95	+	469	927	1	462
UN12922	Rsa1.0_03518.1	1249	22337	0	969	1114	86	-	71	1180	782	1892
UN12923	Rsa1.0_00837.1	1056	62568	0	1006	1060	94	-	2	1056	23094	24150
UN12924	Rsa1.0_27704.1	1233	842	1.00E-177	318	319	99	+	608	926	47	365
UN12925	Rsa1.0_03448.1	1217	22844	0	691	703	98	+	512	1212	3268	3970
UN12926	Rsa1.0_04036.1	298	19473	7.00E-89	277	310	89	+	1	298	2673	2976
UN12927	Rsa1.0_01335.1	1081	47894	0	407	415	98	+	61	475	8128	8542
UN12928	Rsa1.0_00017.1	763	752458	5.00E-18	63	68	92	-	677	744	603147	603214
UN12929	Rsa1.0_00017.1	1349	752458	0	483	489	98	-	3	490	206415	206903
UN12930	Rsa1.0_00088.1	1683	141341	0	332	332	100	+	1	332	126100	126431
UN12931	Rsa1.0_01657.1	1035	41436	0	1012	1043	97	-	2	1034	18371	19411
UN12932	Rsa1.0_06482.1	851	10937	0	579	611	94	+	209	816	8153	8752
UN12933	Rsa1.0_17775.1	876	1743	1.00E-178	492	535	91	-	1	517	1	529
UN12934	Rsa1.0_01078.1	1024	316910	0	443	450	98	+	1	449	263780	264229
UN12935	Rsa1.0_00035.1	1494	176449	0	502	512	98	-	987	1493	41113	41624
UN12936	Rsa1.0_00715.1	254	67116	6.00E-49	120	127	94	+	76	202	41043	41169
UN12937	Rsa1.0_00015.1	533	561803	1.00E-107	267	288	92	-	37	321	323683	323970
UN12938	Rsa1.0_01039.1	1219	55946	1.00E-167	304	306	99	+	912	1217	1887	2192
UN12939	Rsa1.0_34219.1	1388	662	0	551	570	96	+	88	651	93	662
UN12940	Rsa1.0_13940.1	1152	4047	0	952	1001	95	-	1	996	533	1524
UN12941	Rsa1.0_01414.1	572	149508	0	556	568	97	-	3	570	143134	143695
UN12942	Rsa1.0_03594.1	573	21868	0	522	548	95	+	1	543	14605	15150
UN12943	Rsa1.0_01091.1	821	54383	0	421	428	98	+	8	435	3299	3726
UN12944	Rsa1.0_02933.1	747	26641	1.00E-134	330	357	92	-	363	718	14863	15217
UN12945	Rsa1.0_00714.1	348	67187	1.00E-87	192	200	96	+	1	200	22346	22544
UN12946	Rsa1.0_00476.1	687	280966	1.00E-149	381	415	91	+	33	434	31627	32036

UN12947	Rsa1.0_00139.1	1111	357786	0	342	344	99	−	397	740	307381	307724
UN12948	Rsa1.0_00236.1	552	104771	0	388	405	95	−	1	405	3599	4003
UN12949	Rsa1.0_02157.1	924	149652	1.00E−113	252	268	94	+	3	270	12864	13118
UN12950	Rsa1.0_01660.1	1365	41400	0	540	558	96	+	2	558	34367	34923
UN12951	Rsa1.0_09942.1	1454	6077	0	515	521	98	+	404	924	2178	2698
UN12952	Rsa1.0_02688.1	1460	28644	0	581	582	99	−	879	1459	16137	16718
UN12953	Rsa1.0_04658.1	782	16487	0	516	519	99	+	2	520	2415	2933
UN12954	Rsa1.0_01191.1	1300	51235	1.00E−111	216	220	98	+	887	1106	40960	41179
UN12955	Rsa1.0_01117.1	1395	53465	0	852	876	97	−	164	1032	45221	46096
UN12956	Rsa1.0_00114.1	848	131330	1.00E−128	248	252	98	+	595	846	31129	31379
UN12957	Rsa1.0_02952.1	594	26492	0	569	596	95	+	7	592	20510	21105
UN12958	Rsa1.0_00080.1	914	190437	1.00E−126	230	230	100	+	682	911	37681	37910
UN12959	Rsa1.0_00644.1	814	136881	1.00E−74	146	147	99	−	401	547	43911	44057
UN12960	Rsa1.0_01721.1	644	40345	0	644	644	100	−	1	644	24026	24669
UN12961	Rsa1.0_00251.1	1231	303968	1.00E−163	351	368	95	+	1	368	55620	55975
UN12962	Rsa1.0_00347.1	926	93048	1.00E−153	301	310	97	−	366	675	91691	92000
UN12963	Rsa1.0_01481.1	1207	184562	0	596	601	99	+	1	601	180888	181486
UN12964	Rsa1.0_00321.1	710	95671	1.00E−144	285	292	97	+	2	292	47875	48166
UN12965	Rsa1.0_03387.1	989	23278	1.00E−110	378	435	86	−	146	564	1752	2161
UN12966	Rsa1.0_02611.1	1388	29586	0	335	335	100	+	3	337	19139	19473
UN12967	Rsa1.0_00040.1	836	324196	0	586	594	98	+	1	592	36346	36939
UN12968	Rsa1.0_01185.1	1352	149842	1.00E−121	222	222	100	−	388	609	135255	135476
UN12969	Rsa1.0_00552.1	1050	206757	1.00E−162	333	342	97	+	3	340	193044	193385
UN12970	Rsa1.0_00381.1	471	89718	2.00E−65	179	193	92	+	3	194	72068	72256
UN12971	Rsa1.0_07105.1	502	9886	0	417	436	95	+	7	440	1413	1845
UN12972	Rsa1.0_00108.1	1115	478491	1.00E−152	291	297	97	+	776	1072	451984	452280
UN12973	Rsa1.0_18957.1	1293	1505	1.00E−149	293	300	97	+	353	652	1203	1501
UN12974	Rsa1.0_13999.1	1265	3934	0	357	358	99	+	1	358	1800	2157
UN12975	Rsa1.0_02365.1	927	32118	0	918	941	97	−	1	925	13255	14194
UN12976	Rsa1.0_69282.1	1183	331	1.00E−130	252	257	98	+	407	663	71	327
UN12977	Rsa1.0_00139.1	1014	357786	0	957	987	96	−	28	1012	263041	264021
UN12978	Rsa1.0_14538.1	1457	3287	0	385	392	98	+	1	392	2434	2825
UN12979	Rsa1.0_00117.1	1150	213118	0	582	584	99	+	1	584	122790	123373
UN12980	Rsa1.0_02119.1	1422	34833	0	1340	1372	97	+	3	1372	6569	7940
UN12981	Rsa1.0_03321.1	810	23755	0	484	497	97	−	9	503	21986	22480
UN12982	Rsa1.0_00428.1	748	85599	1.00E−164	296	297	99	−	7	303	64925	65221
UN12983	Rsa1.0_03770.1	1472	20952	0	693	715	96	−	1	714	5875	6582
UN12984	Rsa1.0_01571.1	1392	43000	0	416	433	96	+	968	1392	13732	14164
UN12985	Rsa1.0_05184.1	796	14563	1.00E−147	367	394	93	+	3	390	127	518

UN12986	Rsa1.0_01881.1	996	37721	0	794	823	96	+	1	822	7716	8531
UN12987	Rsa1.0_00785.1	1563	64356	0	819	820	99	+	2	821	25927	26746
UN12988	Rsa1.0_01286.1	706	49209	1.00E-157	310	318	97	+	389	706	9368	9684
UN12989	Rsa1.0_02377.1	918	31994	1.00E-167	304	306	99	−	1	306	10248	10553
UN12990	Rsa1.0_00131.1	894	432233	0	406	424	95	+	472	893	96212	96630
UN12991	Rsa1.0_07738.1	680	8686	0	505	535	94	−	1	533	3745	4268
UN12992	Rsa1.0_00925.1	1313	59481	0	705	742	95	−	1	737	21015	21749
UN12993	Rsa1.0_00353.1	767	92242	0	523	542	96	+	1	541	7238	7779
UN12994	Rsa1.0_00435.1	186	317579	7.00E-54	151	165	91	+	1	162	70720	70884
UN12995	Rsa1.0_01310.1	874	164719	0	786	829	94	+	2	825	6052	6865
UN12996	Rsa1.0_00058.1	1307	159921	1.00E-126	306	328	93	−	3	328	139467	139782
UN12997	Rsa1.0_03927.1	525	19997	0	474	491	96	−	1	491	18432	18916
UN12998	Rsa1.0_03318.1	1552	23780	0	410	427	96	+	1119	1545	4296	4722
UN12999	Rsa1.0_00059.1	1330	159650	0	349	357	97	+	1	355	131062	131418
UN13000	Rsa1.0_00389.1	621	88795	5.00E-79	168	174	96	+	151	324	63738	63911
UN13001	Rsa1.0_00816.1	975	403350	4.00E-93	340	396	85	−	606	972	368893	369285
UN13002	Rsa1.0_03134.1	1255	145023	0	412	413	99	−	843	1255	130404	130816
UN13003	Rsa1.0_09270.1	786	6513	0	432	440	98	−	3	442	888	1327
UN13004	Rsa1.0_00437.1	1136	84879	0	428	444	96	+	693	1136	23633	24072
UN13005	Rsa1.0_00018.1	548	410899	1.00E-113	232	238	97	−	14	249	175470	175706
UN13006	Rsa1.0_07021.1	1068	10016	1.00E-133	315	343	91	+	336	666	7093	7426
UN13007	Rsa1.0_00125.1	896	253043	1.00E-157	350	372	94	−	62	433	994	1363
UN13008	Rsa1.0_01136.1	655	52724	2.00E-97	219	229	95	+	3	225	32169	32396
UN13009	Rsa1.0_00175.1	1356	166121	0	674	701	96	+	642	1342	85418	86113
UN13010	Rsa1.0_00105.1	1146	134138	0	385	395	97	+	3	391	126959	127353
UN13011	Rsa1.0_00425.1	1063	147080	0	542	562	96	−	348	909	75128	75689
UN13012	Rsa1.0_00588.1	1252	73939	1.00E-165	381	402	94	+	846	1242	19134	19528
UN13013	Rsa1.0_03543.1	1323	22185	0	494	511	96	−	96	603	3871	4378
UN13014	Rsa1.0_42837.1	730	524	3.00E-93	201	206	97	−	528	730	78	283
UN13015	Rsa1.0_00273.1	780	326186	5.00E-98	289	321	90	−	467	780	307766	308084
UN13016	Rsa1.0_00387.1	627	495407	0	441	457	96	−	172	627	382640	383092
UN13017	Rsa1.0_17794.1	896	1740	1.00E-119	254	265	95	+	632	896	1	264
UN13018	Rsa1.0_00037.1	1267	307838	0	448	467	95	−	803	1267	241190	241654
UN13019	Rsa1.0_00206.1	1387	109040	0	1077	1129	95	−	30	1144	28660	29786
UN13020	Rsa1.0_02921.1	1938	26779	0	1469	1501	97	+	460	1938	23735	25235
UN13021	Rsa1.0_01163.1	1536	51946	1.00E-158	305	310	98	−	1	308	16052	16359
UN13022	Rsa1.0_00069.1	852	403561	0	341	341	100	−	2	342	200655	200995
UN13023	Rsa1.0_08553.1	1748	7210	1.00E-134	253	256	98	−	582	837	1103	1358
UN13024	Rsa1.0_00440.1	851	84723	1.00E-136	278	288	96	+	1	288	25114	25398

UN13025	Rsa1.0_00480.1	1429	81325	0	471	480	98	+	361	837	4323	4802
UN13026	Rsa1.0_01453.1	2020	45449	0	1114	1114	100	+	907	2020	5322	6435
UN13027	Rsa1.0_02493.1	570	30854	0	429	432	99	+	139	570	6059	6490
UN13028	Rsa1.0_02215.1	1364	33822	0	368	371	99	−	3	372	27836	28206
UN13029	Rsa1.0_13994.1	1136	3941	1.00E−176	346	357	96	−	285	641	1610	1966
UN13030	Rsa1.0_03195.1	1648	24673	0	485	506	95	−	29	534	9107	9605
UN13031	Rsa1.0_08557.1	1338	7199	1.00E−126	279	297	93	+	3	299	4756	5042
UN13032	Rsa1.0_03356.1	1024	23487	0	402	413	97	−	4	414	1225	1637
UN13033	Rsa1.0_00427.1	1177	189678	1.00E−142	262	264	99	+	707	970	37925	38188
UN13034	Rsa1.0_04301.1	1431	18022	0	464	474	97	−	864	1337	16876	17344
UN13035	Rsa1.0_00070.1	1028	150321	0	578	588	98	+	1	587	59029	59616
UN13036	Rsa1.0_00008.1	1366	358124	0	810	889	91	+	496	1366	277916	278786
UN13037	Rsa1.0_00518.1	799	78360	1.00E−126	233	234	99	−	3	236	20635	20868
UN13038	Rsa1.0_00863.1	1031	254535	0	387	391	98	−	1	391	213547	213933
UN13039	Rsa1.0_00324.1	1438	218805	0	1389	1446	96	−	2	1432	214977	216417
UN13040	Rsa1.0_14069.1	1184	3842	0	811	835	97	−	351	1180	1982	2810
UN13041	Rsa1.0_00522.1	1148	78103	2.00E−98	207	215	96	+	355	569	48991	49205
UN13042	Rsa1.0_04142.1	740	18861	0	416	434	95	−	8	441	13126	13554
UN13043	Rsa1.0_39426.1	1887	570	0	459	461	99	+	1427	1887	46	506
UN13044	Rsa1.0_00662.1	983	192183	0	671	701	95	−	241	941	182411	183105
UN13045	Rsa1.0_00759.1	964	65465	1.00E−108	268	289	92	−	2	290	63157	63435
UN13046	Rsa1.0_00244.1	949	103590	1.00E−144	292	302	96	+	7	306	29923	30224
UN13047	Rsa1.0_00006.1	1042	396176	0	1039	1041	99	−	3	1042	2835	3875
UN13048	Rsa1.0_10165.1	937	5966	0	413	427	96	−	18	444	365	790
UN13049	Rsa1.0_00484.1	3026	203294	0	1628	1649	98	+	566	2214	140171	141818
UN13050	Rsa1.0_00017.1	1401	752458	0	543	574	94	−	825	1384	743125	743692
UN13051	Rsa1.0_32561.1	1574	699	1.00E−165	311	315	98	+	1	314	385	699
UN13052	Rsa1.0_00229.1	1293	105471	1.00E−176	316	317	99	+	937	1253	43350	43666
UN13053	Rsa1.0_05573.1	1382	13290	0	472	493	95	−	26	509	4157	4646
UN13054	Rsa1.0_00642.1	1454	201230	0	473	477	99	−	977	1452	62495	62971
UN13055	Rsa1.0_05208.1	625	14504	1.00E−153	317	331	95	+	295	625	3908	4234
UN13056	Rsa1.0_03189.1	559	24710	0	421	435	96	−	1	423	9173	9607
UN13057	Rsa1.0_01932.1	1232	37177	1.00E−137	265	270	98	−	966	1232	10970	11239
UN13058	Rsa1.0_00108.1	968	478491	1.00E−126	283	296	95	+	102	390	67372	67663
UN13059	Rsa1.0_00009.1	959	232558	1.00E−135	344	369	93	+	582	937	53021	53382
UN13060	Rsa1.0_06509.1	1049	10873	1.00E−133	254	258	98	−	228	485	6323	6580
UN13061	Rsa1.0_00814.1	1214	63073	0	746	758	98	−	22	778	2802	3557
UN13062	Rsa1.0_02723.1	593	28408	0	342	342	100	−	250	591	2561	2902
UN13063	Rsa1.0_01397.1	1513	183276	0	1131	1146	98	−	3	1148	162124	163267

UN13064	Rsa1.0_00045.1	988	328694	0	429	446	96	−	543	988	131773	132215
UN13065	Rsa1.0_02534.1	997	30396	1.00E−143	261	262	99	−	2	263	5455	5716
UN13066	Rsa1.0_00473.1	1429	212204	1.00E−160	348	367	94	−	1063	1427	47338	47700
UN13067	Rsa1.0_05748.1	916	12749	1.00E−154	313	324	96	+	100	422	6628	6951
UN13068	Rsa1.0_00079.1	2926	606987	0	856	898	95	−	1281	2157	467327	468224
UN13069	Rsa1.0_00264.1	1811	101674	0	408	416	98	−	1097	1512	78413	78828
UN13070	Rsa1.0_02913.1	1070	26816	0	501	501	100	−	570	1070	22091	22591
UN13071	Rsa1.0_00995.1	1754	57243	0	635	643	98	+	1112	1754	21109	21751
UN13072	Rsa1.0_02001.1	848	36198	0	507	515	98	−	2	516	31940	32449
UN13073	Rsa1.0_06273.1	907	11346	0	905	905	100	−	3	907	2931	3835
UN13074	Rsa1.0_08957.1	805	6754	0	515	528	97	+	279	804	5841	6366
UN13075	Rsa1.0_02991.1	705	26178	0	381	391	97	+	306	696	21112	21502
UN13076	Rsa1.0_02229.1	852	33672	0	392	405	96	−	3	407	17849	18253
UN13077	Rsa1.0_02128.1	1352	34758	0	348	348	100	−	3	350	16324	16671
UN13078	Rsa1.0_00287.1	952	98687	0	427	432	98	+	406	837	57224	57655
UN13079	Rsa1.0_04456.1	742	17260	1.00E−157	284	285	99	+	458	742	7706	7990
UN13080	Rsa1.0_00343.1	817	93570	0	530	532	99	+	287	817	84281	84812
UN13081	Rsa1.0_00198.1	1659	222963	0	464	491	94	+	1	485	141259	141746
UN13082	Rsa1.0_01220.1	1484	161775	1.00E−100	196	199	98	−	1030	1228	122655	122853
UN13083	Rsa1.0_00354.1	1134	232005	1.00E−139	270	276	97	−	831	1106	6914	7184
UN13084	Rsa1.0_01295.1	1739	49040	1.00E−156	299	305	98	−	865	1169	23660	23964
UN13085	Rsa1.0_00380.1	849	89748	0	664	692	95	+	160	847	83924	84610
UN13086	Rsa1.0_03740.1	769	21065	0	597	618	96	+	152	767	14480	15091
UN13087	Rsa1.0_00386.1	1515	89134	1.00E−124	248	254	97	−	3	256	67826	68078
UN13088	Rsa1.0_03692.1	570	21338	1.00E−112	244	255	95	−	316	570	6377	6627
UN13089	Rsa1.0_00328.1	1170	224661	1.00E−124	249	256	97	−	915	1167	217156	217411
UN13090	Rsa1.0_00020.1	1495	283099	1.00E−167	374	395	94	+	17	402	60874	61265
UN13091	Rsa1.0_03356.1	2300	23487	0	1345	1383	97	−	538	1912	5698	7080
UN13092	Rsa1.0_00419.1	1272	86562	0	1090	1108	98	−	1	1108	69863	70970
UN13093	Rsa1.0_01166.1	1806	51877	0	632	634	99	−	554	1187	10072	10705
UN13094	Rsa1.0_00316.1	1165	96264	0	590	590	100	−	576	1165	57225	57814
UN13095	Rsa1.0_02872.1	1123	27080	0	538	542	99	−	582	1123	11208	11749
UN13096	Rsa1.0_00734.1	1623	66508	1.00E−164	297	298	99	+	836	1133	59680	59977
UN13097	Rsa1.0_00800.1	649	63868	1.00E−176	365	379	96	+	14	390	19313	19688
UN13098	Rsa1.0_00082.1	1308	237584	1.00E−117	230	235	97	−	497	731	133924	134158
UN13099	Rsa1.0_00507.1	1219	79074	1.00E−157	303	310	97	−	759	1068	42905	43214
UN13100	Rsa1.0_01166.1	1467	51877	0	638	648	98	+	1	647	9132	9779
UN13101	Rsa1.0_01296.1	1621	49035	0	337	339	99	+	5	343	5249	5587
UN13102	Rsa1.0_00451.1	1630	322533	0	476	482	98	−	632	1113	161726	162207

UN13103	Rsa1.0_00026.1	671	327835	1.00E-113	224	228	98	−	442	669	206641	206867
UN13104	Rsa1.0_00556.1	1219	207212	0	422	422	100	−	790	1211	137353	137774
UN13105	Rsa1.0_00296.1	1198	97854	1.00E-161	367	392	93	−	814	1196	51437	51825
UN13106	Rsa1.0_01188.1	1973	51362	0	776	790	98	−	1084	1870	46093	46882
UN13107	Rsa1.0_00413.1	786	86921	1.00E-168	320	326	98	−	5	330	10794	11117
UN13108	Rsa1.0_04014.1	876	19572	0	862	876	98	−	1	876	11437	12306
UN13109	Rsa1.0_00422.1	1089	86144	0	531	541	98	+	1	540	12755	13294
UN13110	Rsa1.0_02477.1	777	31058	0	417	421	99	−	1	418	13987	14407
UN13111	Rsa1.0_00168.1	1347	115870	2.33E-156	303	316	95	−	344	659	56550	56865
UN13112	Rsa1.0_05622.1	1339	13145	0	518	550	94	+	804	1339	3628	4175
UN13113	Rsa1.0_00557.1	758	75865	1.00E-143	276	281	98	+	20	300	16136	16415
UN13114	Rsa1.0_01239.1	1227	50107	0	1199	1217	98	+	7	1223	19332	20546
UN13115	Rsa1.0_00222.1	1314	172263	0	391	394	99	−	921	1314	17043	17436
UN13116	Rsa1.0_02105.1	871	156953	0	476	508	93	−	365	869	150672	151165
UN13117	Rsa1.0_00728.1	842	66704	0	383	396	96	−	1	395	36897	37288
UN13118	Rsa1.0_03279.1	1241	24115	0	620	622	99	−	3	624	7808	8428
UN13119	Rsa1.0_00075.1	2801	146680	0	1704	1801	94	−	1003	2787	140465	142244
UN13120	Rsa1.0_04378.1	819	17651	0	352	354	99	+	331	684	4630	4983
UN13121	Rsa1.0_00019.1	1953	301669	0	543	544	99	−	1408	1950	218690	219233
UN13122	Rsa1.0_02369.1	635	32075	0	483	507	95	+	133	635	5009	5511
UN13123	Rsa1.0_00454.1	895	216970	0	868	893	97	+	5	895	158096	158987
UN13124	Rsa1.0_01185.1	1686	149842	0	425	426	99	−	1260	1685	126522	126947
UN13125	Rsa1.0_01559.1	1263	43262	1.00E-123	333	365	91	+	6	361	20498	20858
UN13126	Rsa1.0_00015.1	1116	561803	1.00E-129	246	249	98	+	3	251	461863	462109
UN13127	Rsa1.0_02756.1	1710	28202	1.00E-175	315	316	99	+	1082	1397	22631	22946
UN13128	Rsa1.0_03299.1	1369	23949	0	476	500	95	−	11	510	14331	14817
UN13129	Rsa1.0_00108.1	1246	478491	0	487	491	99	+	756	1245	383773	384263
UN13130	Rsa1.0_01537.1	973	187285	0	356	358	99	−	617	973	38620	38977
UN13131	Rsa1.0_03054.1	1097	25738	1.00E-160	338	354	95	+	3	344	13554	13907
UN13132	Rsa1.0_01850.1	1547	162224	0	479	484	98	+	867	1350	1286	1768
UN13133	Rsa1.0_00139.1	815	357786	0	810	815	99	−	1	815	141376	142190
UN13134	Rsa1.0_02991.1	773	26178	0	427	438	97	−	3	436	21112	21549
UN13135	Rsa1.0_00045.1	642	328694	3.00E-93	190	194	97	−	449	642	148699	148891
UN13136	Rsa1.0_02946.1	1498	26537	0	928	951	97	+	549	1497	857	1804
UN13137	Rsa1.0_01153.1	1171	52264	0	386	390	98	+	1	390	42506	42893
UN13138	Rsa1.0_04095.1	1172	19095	1.00E-177	344	353	97	+	823	1172	12377	12729
UN13139	Rsa1.0_52681.1	1383	432	0	334	334	100	−	95	428	99	432
UN13140	Rsa1.0_08994.1	929	6724	0	432	447	96	+	477	920	3405	3848
UN13141	Rsa1.0_07617.1	1853	9015	0	1837	1870	98	−	1	1853	2166	4032

UN13142	Rsa1.0_21865.1	853	1168	0	543	548	99	−	154	701	1	548
UN13143	Rsa1.0_00645.1	1621	189716	1.00E−149	272	273	99	+	1349	1621	148716	148988
UN13144	Rsa1.0_00675.1	1020	145590	0	764	764	100	+	1	764	30711	31474
UN13145	Rsa1.0_05510.1	894	13470	1.00E−107	215	221	97	−	121	341	2743	2963
UN13146	Rsa1.0_00198.1	776	222963	1.00E−151	286	291	98	+	136	426	123127	123417
UN13147	Rsa1.0_00482.1	1554	81162	1.00E−164	294	294	100	+	1260	1553	2254	2547
UN13148	Rsa1.0_23933.1	962	1016	1.00E−110	209	211	99	+	752	962	195	405
UN13149	Rsa1.0_00001.1	1069	363028	0	479	483	99	−	3	485	335254	335733
UN13150	Rsa1.0_01249.1	788	188432	1.00E−131	279	293	95	−	506	788	150744	151035
UN13151	Rsa1.0_02901.1	910	26909	1.00E−119	310	337	91	+	575	905	21294	21623
UN13152	Rsa1.0_00398.1	986	88007	0	553	578	95	+	422	986	86395	86970
UN13153	Rsa1.0_02802.1	1433	27736	0	620	656	94	−	719	1355	10025	10678
UN13154	Rsa1.0_00410.1	767	86957	1.00E−119	258	269	95	+	471	737	26432	26699
UN13155	Rsa1.0_02043.1	882	163754	1.00E−147	278	282	98	+	2	280	144930	145211
UN13156	Rsa1.0_03491.1	748	22541	0	585	606	96	+	114	711	8983	9586
UN13157	Rsa1.0_01075.1	1213	156953	0	338	342	98	+	872	1213	23855	24196
UN13158	Rsa1.0_41964.1	835	535	1.00E−124	235	237	99	+	599	835	1	235
UN13159	Rsa1.0_00847.1	996	62270	0	409	434	94	+	568	993	55145	55578
UN13160	Rsa1.0_09273.1	1091	6511	1.00E−147	317	333	95	+	1	323	12	344
UN13161	Rsa1.0_00083.1	1189	442773	0	481	494	97	−	19	509	252723	253215
UN13162	Rsa1.0_04489.1	833	17145	1.00E−150	269	269	100	−	2	270	14197	14465
UN13163	Rsa1.0_03194.1	744	24674	1.00E−142	256	256	100	−	3	258	3262	3517
UN13164	Rsa1.0_00416.1	1020	86772	1.00E−158	295	299	98	−	720	1018	76128	76426
UN13165	Rsa1.0_00030.1	410	250988	1.00E−136	253	254	99	−	157	410	149392	149644
UN13166	Rsa1.0_01220.1	880	161775	1.00E−159	298	301	99	+	4	304	115836	116135
UN13167	Rsa1.0_18356.1	1055	1615	1.00E−165	324	333	97	+	663	995	177	506
UN13168	Rsa1.0_00652.1	857	70089	1.00E−133	277	287	96	+	1	285	59423	59707
UN13169	Rsa1.0_00478.1	785	81419	1.00E−141	258	259	99	+	1	259	28165	28423
UN13170	Rsa1.0_02605.1	1151	29637	0	585	611	95	+	556	1151	25931	26538
UN13171	Rsa1.0_02367.1	421	32106	1.00E−127	242	246	98	−	1	246	27423	27668
UN13172	Rsa1.0_00200.1	961	162016	1.00E−133	297	315	94	−	16	327	96962	97276
UN13173	Rsa1.0_00012.1	1007	222148	1.00E−137	277	286	96	+	1	286	69093	69375
UN13174	Rsa1.0_01554.1	951	43379	0	925	950	97	+	1	950	21188	22136
UN13175	Rsa1.0_02874.1	1236	27070	0	924	948	97	−	202	1146	4410	5357
UN13176	Rsa1.0_01425.1	1283	46136	0	423	427	99	−	857	1283	15494	15919
UN13177	Rsa1.0_02032.1	1129	35830	1.00E−160	292	294	99	−	128	421	5322	5615
UN13178	Rsa1.0_04192.1	1040	18572	0	711	714	99	+	1	714	12150	12863
UN13179	Rsa1.0_04894.1	1002	15569	1.00E−178	372	390	95	−	3	392	11339	11725
UN13180	Rsa1.0_05541.1	1130	139959	1.00E−144	295	307	96	−	824	1130	2801	3107

UN13181	Rsa1.0_07657.1	754	8896	1.00E-165	347	359	96	–	7	357	3516	3874
UN13182	Rsa1.0_09211.1	1634	6556	1.00E-164	296	297	99	–	1134	1430	2282	2578
UN13183	Rsa1.0_00017.1	1536	752458	0	348	352	98	–	3	352	292600	292951
UN13184	Rsa1.0_00132.1	1371	124299	0	1323	1370	96	+	3	1371	52478	53838
UN13185	Rsa1.0_18611.1	1343	1565	0	431	457	94	+	875	1330	957	1409
UN13186	Rsa1.0_01850.1	1115	162224	0	356	361	98	–	5	365	31714	32072
UN13187	Rsa1.0_02053.1	1835	35554	0	431	435	99	+	1400	1834	3919	4353
UN13188	Rsa1.0_00231.1	1425	105381	0	396	396	100	–	1	396	20793	21188
UN13189	Rsa1.0_00344.1	1148	191774	0	550	618	88	–	1	608	108987	109569
UN13190	Rsa1.0_00439.1	1205	142776	0	340	340	100	+	416	755	63119	63458
UN13191	Rsa1.0_01958.1	1047	261128	0	385	398	96	+	1	394	119341	119737
UN13192	Rsa1.0_13229.1	1146	4963	1.00E-175	317	319	99	–	1	319	4414	4732
UN13193	Rsa1.0_05293.1	1439	14237	0	455	486	93	+	59	543	6250	6730
UN13194	Rsa1.0_02435.1	887	31454	1.00E-178	326	329	99	+	3	331	19857	20185
UN13195	Rsa1.0_08265.1	940	7630	0	489	494	98	+	2	494	6565	7058
UN13196	Rsa1.0_03920.1	943	20081	0	619	626	98	–	316	941	13862	14483
UN13197	Rsa1.0_03258.1	808	24302	1.00E-101	221	230	96	–	580	805	11081	11310
UN13198	Rsa1.0_01242.1	981	50066	1.00E-166	320	328	97	+	1	328	16424	16745
UN13199	Rsa1.0_01247.1	1086	49934	0	745	747	99	+	340	1086	45105	45851
UN13200	Rsa1.0_02213.1	1773	33824	0	468	481	97	–	1293	1773	28386	28861
UN13201	Rsa1.0_02825.1	1043	27568	0	510	556	91	–	434	973	25954	26506
UN13202	Rsa1.0_00115.1	2501	131321	0	805	815	98	–	972	1785	48839	49653
UN13203	Rsa1.0_00011.1	1246	225044	0	581	584	99	+	326	909	203328	203911
UN13204	Rsa1.0_00221.1	911	106972	0	345	347	99	+	1	347	51591	51937
UN13205	Rsa1.0_00802.1	1186	63846	1.00E-176	390	414	94	+	7	409	56790	57203
UN13206	Rsa1.0_03524.1	1458	22297	0	682	706	96	–	4	709	14064	14763
UN13207	Rsa1.0_04352.1	662	17779	0	546	549	99	+	114	662	12118	12666
UN13208	Rsa1.0_01964.1	889	36702	0	442	454	97	+	1	454	9330	9776
UN13209	Rsa1.0_00557.1	864	75865	1.00E-147	308	321	95	+	1	318	74489	74807
UN13210	Rsa1.0_00219.1	947	106983	0	425	443	95	+	501	943	67201	67641
UN13211	Rsa1.0_01068.1	912	55208	1.00E-148	282	287	98	–	365	651	46100	46386
UN13212	Rsa1.0_00194.1	1352	110851	0	664	668	99	+	685	1352	54321	54988
UN13213	Rsa1.0_00107.1	855	431659	1.00E-155	284	286	99	+	1	286	180720	181005
UN13214	Rsa1.0_00302.1	1487	97521	0	371	372	99	+	1	372	33875	34246
UN13215	Rsa1.0_02451.1	801	31283	1.00E-170	383	411	93	–	1	407	8148	8548
UN13216	Rsa1.0_00556.1	832	207212	0	815	832	97	–	1	831	14861	15689
UN13217	Rsa1.0_02387.1	913	31890	0	524	553	94	–	1	539	17300	17847
UN13218	Rsa1.0_01206.1	1950	50772	0	1100	1108	99	–	478	1585	33971	35078
UN13219	Rsa1.0_01633.1	1085	41868	0	427	430	99	+	656	1083	3035	3464

UN13220	Rsa1.0_03088.1	539	25476	0	399	410	97	−	8	416	500	909
UN13221	Rsa1.0_00197.1	1107	109714	0	648	672	96	−	439	1107	73165	73828
UN13222	Rsa1.0_00035.1	1632	176449	1.00E−168	323	329	98	−	1304	1632	3179	3506
UN13223	Rsa1.0_26044.1	786	908	0	452	454	99	−	2	455	1	454
UN13224	Rsa1.0_02170.1	1078	34352	0	541	541	100	+	536	1076	28526	29066
UN13225	Rsa1.0_09242.1	851	6534	0	461	464	99	+	1	464	957	1420
UN13226	Rsa1.0_07825.1	1566	8466	0	573	581	98	+	3	583	1349	1929
UN13227	Rsa1.0_00698.1	808	67816	0	596	645	92	−	179	808	21764	22408
UN13228	Rsa1.0_02098.1	1301	35032	0	560	600	93	−	719	1299	751	1349
UN13229	Rsa1.0_11534.1	922	5477	0	488	490	99	−	433	922	1236	1725
UN13230	Rsa1.0_08001.1	2403	8095	0	533	535	99	−	1192	1725	1559	2092
UN13231	Rsa1.0_02074.1	1933	35370	0	513	513	100	−	2	514	17905	18417
UN13232	Rsa1.0_00250.1	2055	273771	0	487	491	99	+	37	525	4510	5000
UN13233	Rsa1.0_11046.1	1307	5635	1.00E−149	314	326	96	+	986	1307	3092	3415
UN13234	Rsa1.0_05624.1	739	13140	1.00E−126	392	447	87	−	284	722	3783	4199
UN13235	Rsa1.0_00250.1	1228	273771	1.00E−100	241	258	93	+	1	257	60326	60583
UN13236	Rsa1.0_01274.1	1005	49419	1.00E−170	332	341	97	+	7	344	30800	31140
UN13237	Rsa1.0_07482.1	1325	9278	0	669	677	98	−	1	677	1002	1677
UN13238	Rsa1.0_00314.1	2114	96472	0	479	494	96	+	750	1243	36322	36815
UN13239	Rsa1.0_01317.1	1916	48287	0	672	697	96	−	21	709	38419	39109
UN13240	Rsa1.0_00728.1	1169	66704	0	360	361	99	+	1	361	15830	16190
UN13241	Rsa1.0_10640.1	1868	5772	0	727	760	95	−	619	1367	1603	2359
UN13242	Rsa1.0_00805.1	1815	63724	0	431	439	98	−	10	447	43181	43619
UN13243	Rsa1.0_00102.1	738	135497	0	549	569	96	−	170	738	40804	41372
UN13244	Rsa1.0_02412.1	1200	31643	0	721	738	97	−	3	740	14291	15025
UN13245	Rsa1.0_00048.1	771	167185	0	400	400	100	+	372	771	13591	13990
UN13246	Rsa1.0_14855.1	1372	3011	0	673	674	99	+	282	955	1310	1983
UN13247	Rsa1.0_01094.1	1620	167285	0	478	518	92	−	1094	1596	163031	163547
UN13248	Rsa1.0_01294.1	1276	49090	1.00E−163	334	346	96	+	933	1274	40470	40815
UN13249	Rsa1.0_00327.1	1053	94892	0	568	568	100	+	435	1002	62360	62927
UN13250	Rsa1.0_21783.1	1000	1173	0	497	519	95	−	3	521	165	679
UN13251	Rsa1.0_00636.1	1851	71113	0	678	702	96	+	14	711	15978	16679
UN13252	Rsa1.0_01739.1	1120	40068	0	1095	1120	97	+	1	1120	13347	14460
UN13253	Rsa1.0_01251.1	1097	49873	0	490	494	99	−	1	494	7549	8042
UN13254	Rsa1.0_07916.1	1194	8288	1.00E−174	392	413	94	−	789	1194	4336	4744
UN13255	Rsa1.0_06571.1	932	10750	1.00E−150	296	305	97	+	626	930	6156	6460
UN13256	Rsa1.0_00055.1	1284	520193	0	921	921	100	−	1	921	163773	164693
UN13257	Rsa1.0_00025.1	903	277312	1.00E−114	213	214	99	−	305	518	157157	157370
UN13258	Rsa1.0_00333.1	1217	94500	0	546	561	97	+	314	874	22948	23506

UN13259	Rsa1.0_01641.1	1736	188213	0	1720	1742	98	−	2	1736	161019	162760
UN13260	Rsa1.0_02420.1	853	31580	0	848	851	99	−	3	853	15881	16729
UN13261	Rsa1.0_00114.1	1037	131330	1.00E−145	261	261	100	+	2	262	127550	127810
UN13262	Rsa1.0_01536.1	878	43747	0	873	876	99	−	1	876	39096	39969
UN13263	Rsa1.0_00740.1	1198	215972	0	565	575	98	−	621	1195	11277	11848
UN13264	Rsa1.0_14637.1	1388	3201	0	1149	1184	97	+	32	1213	2018	3201
UN13265	Rsa1.0_01215.1	1049	166813	0	371	373	99	−	677	1049	9634	10006
UN13266	Rsa1.0_00093.1	648	214962	1.00E−152	276	277	99	+	372	648	48814	49090
UN13267	Rsa1.0_03123.1	1373	25207	0	1367	1370	99	−	3	1372	13492	14861
UN13268	Rsa1.0_01730.1	632	40223	1.00E−105	248	261	95	−	373	632	36577	36828
UN13269	Rsa1.0_17409.1	1132	1837	0	1011	1029	98	+	2	1030	101	1121
UN13270	Rsa1.0_34877.1	1178	649	1.00E−118	217	217	100	+	1	217	361	577
UN13271	Rsa1.0_00358.1	1834	91293	0	454	454	100	+	3	456	32086	32539
UN13272	Rsa1.0_00123.1	850	325650	0	538	540	99	+	311	850	237191	237730
UN13273	Rsa1.0_02543.1	1879	30302	0	375	380	98	−	5	382	3749	4126
UN13274	Rsa1.0_00832.1	854	62622	1.00E−163	334	343	97	+	513	854	41378	41717
UN13275	Rsa1.0_00777.1	963	191865	0	424	436	97	−	168	603	128527	128962
UN13276	Rsa1.0_03898.1	1635	20183	0	1256	1261	99	−	3	1262	5249	6508
UN13277	Rsa1.0_10495.1	1374	5827	0	414	417	99	−	1	417	55	471
UN13278	Rsa1.0_14555.1	1609	3273	1.00E−156	295	300	98	−	1310	1609	576	875
UN13279	Rsa1.0_03009.1	1105	26065	1.00E−130	239	240	99	−	757	996	15478	15717
UN13280	Rsa1.0_05166.1	926	14624	1.00E−128	281	295	95	+	37	328	13068	13361
UN13281	Rsa1.0_00301.1	495	365315	0	493	495	99	−	1	495	283377	283871
UN13282	Rsa1.0_05355.1	757	14069	0	597	613	97	+	145	753	4534	5146
UN13283	Rsa1.0_00019.1	733	301669	1.00E−169	400	430	93	−	293	722	293080	293498
UN13284	Rsa1.0_02085.1	1490	35198	2.00E−83	237	266	89	−	1060	1325	30157	30404
UN13285	Rsa1.0_02253.1	1277	33356	1.00E−101	245	260	94	+	1021	1273	25657	25915
UN13286	Rsa1.0_00870.1	1628	61606	1.00E−131	292	307	95	+	3	308	24761	25066
UN13287	Rsa1.0_00027.1	1894	283362	0	1512	1557	97	−	1	1556	182584	184135
UN13288	Rsa1.0_00959.1	1365	58202	0	762	791	96	+	577	1364	16920	17710
UN13289	Rsa1.0_05908.1	1331	12266	0	1315	1329	98	+	1	1329	10166	11493
UN13290	Rsa1.0_00122.1	1169	265937	0	723	739	97	+	429	1167	82124	82856
UN13291	Rsa1.0_00398.1	1267	88007	0	523	541	96	+	2	538	40985	41523
UN13292	Rsa1.0_00070.1	1013	150321	0	460	465	98	+	549	1013	55838	56300
UN13293	Rsa1.0_04708.1	1191	16286	1.00E−134	246	247	99	+	3	249	15877	16123
UN13294	Rsa1.0_03191.1	1189	145762	0	674	686	98	+	502	1187	134735	135420
UN13295	Rsa1.0_00054.1	1243	162053	0	363	369	98	−	875	1243	92464	92831
UN13296	Rsa1.0_01679.1	1061	146876	0	415	434	95	−	635	1061	122875	123308
UN13297	Rsa1.0_02626.1	918	29387	0	446	448	99	−	471	918	22846	23293

UN13298	Rsa1.0_02207.1	1107	33914	0	470	483	97	−	627	1107	13535	14011
UN13299	Rsa1.0_00037.1	1118	307838	0	701	731	95	−	374	1095	293298	294022
UN13300	Rsa1.0_00198.1	1036	222963	0	369	374	98	−	663	1036	154810	155180
UN13301	Rsa1.0_03044.1	1334	25791	1.00E−144	299	308	97	−	1012	1319	14324	14628
UN13302	Rsa1.0_00732.1	1177	66631	1.00E−172	334	343	97	+	1	343	1781	2123
UN13303	Rsa1.0_02343.1	493	32374	1.00E−125	243	247	98	+	3	249	1007	1252
UN13304	Rsa1.0_00350.1	2245	92346	0	324	324	100	+	47	370	61927	62250
UN13305	Rsa1.0_00468.1	1749	82095	0	591	605	97	−	3	604	36533	37137
UN13306	Rsa1.0_01193.1	1553	51147	0	349	354	98	+	1200	1553	15709	16060
UN13307	Rsa1.0_00065.1	1470	454616	1.00E−153	282	283	99	−	1	283	448185	448466
UN13308	Rsa1.0_00394.1	966	148426	1.00E−166	327	333	98	−	633	964	122810	123140
UN13309	Rsa1.0_08120.1	1520	7876	0	428	447	95	−	1081	1518	6672	7117
UN13310	Rsa1.0_01100.1	1538	54113	0	645	654	98	−	2	655	34687	35337
UN13311	Rsa1.0_00419.1	896	86562	0	886	898	98	+	3	895	55548	56445
UN13312	Rsa1.0_03822.1	1038	20640	1.00E−179	334	339	98	−	698	1036	13557	13895
UN13313	Rsa1.0_00077.1	923	145005	0	558	566	98	−	361	923	38535	39099
UN13314	Rsa1.0_03820.1	1768	20664	1.00E−115	211	211	100	+	42	252	5379	5589
UN13315	Rsa1.0_00191.1	920	316976	0	411	424	96	−	318	735	259268	259691
UN13316	Rsa1.0_02750.1	1548	28239	1.00E−136	267	273	97	+	1	270	25620	25892
UN13317	Rsa1.0_02843.1	1240	27383	0	574	577	99	+	664	1240	23792	24368
UN13318	Rsa1.0_00001.1	1218	363028	1.00E−175	320	323	99	+	894	1216	91340	91662
UN13319	Rsa1.0_00382.1	1465	251946	0	696	716	97	+	264	979	45007	45712
UN13320	Rsa1.0_01679.1	1320	146876	1.00E−81	286	331	86	−	1004	1319	134936	135247
UN13321	Rsa1.0_00585.1	952	74164	0	446	446	100	+	505	950	53696	54141
UN13322	Rsa1.0_00804.1	1256	63726	0	789	797	98	+	1	797	36763	37558
UN13323	Rsa1.0_01140.1	1168	52586	0	698	730	95	−	450	1162	6369	7094
UN13324	Rsa1.0_06004.1	932	11961	1.00E−173	327	333	98	−	596	928	7369	7701
UN13325	Rsa1.0_01019.1	989	56636	1.00E−164	300	302	99	+	669	970	45455	45756
UN13326	Rsa1.0_00017.1	992	752458	0	645	647	99	−	346	992	235957	236603
UN13327	Rsa1.0_01425.1	1527	46136	0	429	438	97	−	1093	1527	10898	11335
UN13328	Rsa1.0_00490.1	1515	186713	0	410	430	95	−	1	428	39915	40340
UN13329	Rsa1.0_01316.1	884	48325	1.00E−119	278	293	94	+	1	290	5498	5788
UN13330	Rsa1.0_00045.1	769	328694	1.00E−156	311	319	97	+	1	316	148573	148891
UN13331	Rsa1.0_04033.1	1455	19481	0	503	507	99	+	259	765	9206	9712
UN13332	Rsa1.0_00104.1	1347	368722	2.00E−92	205	212	96	−	1120	1329	18552	18761
UN13333	Rsa1.0_02536.1	1317	30368	0	1279	1319	96	−	2	1317	19445	20754
UN13334	Rsa1.0_23167.1	1453	1067	0	550	554	99	−	898	1451	345	898
UN13335	Rsa1.0_00045.1	1687	328694	0	555	580	95	−	1070	1642	261133	261705
UN13336	Rsa1.0_03334.1	1009	23659	1.00E−106	247	265	93	+	3	254	18787	19051

UN13337	Rsa1.0_00938.1	1454	193891	0	635	638	99	−	1	637	172122	172759
UN13338	Rsa1.0_00006.1	1389	396176	1.00E−167	308	311	99	+	1079	1389	94003	94313
UN13339	Rsa1.0_00111.1	1943	390088	0	382	394	96	−	1245	1638	381107	381500
UN13340	Rsa1.0_01701.1	959	162121	0	532	538	98	−	3	540	30943	31479
UN13341	Rsa1.0_01981.1	688	36537	0	402	424	94	−	3	425	34741	35156
UN13342	Rsa1.0_00034.1	1278	260119	1.00E−177	316	316	100	−	1	316	6537	6852
UN13343	Rsa1.0_01456.1	884	45372	0	878	882	99	+	3	884	25881	26762
UN13344	Rsa1.0_03387.1	2390	23278	0	671	681	98	+	385	1065	19355	20035
UN13345	Rsa1.0_00035.1	2901	176449	0	870	876	99	+	1368	2243	148096	148971
UN13346	Rsa1.0_00220.1	1042	192936	0	424	432	98	+	374	802	16394	16825
UN13347	Rsa1.0_00890.1	809	323870	1.00E−158	391	427	91	−	80	506	176492	176918
UN13348	Rsa1.0_00418.1	1172	215377	5.00E−99	196	200	98	−	968	1167	40452	40651
UN13349	Rsa1.0_00090.1	1891	273859	1.00E−135	257	261	98	−	1565	1825	184220	184480
UN13350	Rsa1.0_00705.1	1063	67505	0	705	712	99	−	342	1053	9366	10076
UN13351	Rsa1.0_00097.1	1825	138858	0	591	595	99	+	821	1415	21443	22037
UN13352	Rsa1.0_10656.1	921	5768	1.00E−176	569	652	87	+	96	745	3949	4598
UN13353	Rsa1.0_00540.1	1361	76895	1.00E−179	318	318	100	+	1043	1360	33148	33465
UN13354	Rsa1.0_00190.1	995	318240	0	374	384	97	+	1	384	134843	135221
UN13355	Rsa1.0_08255.1	1659	7652	0	407	407	100	−	52	458	2723	3129
UN13356	Rsa1.0_00261.1	1330	151157	0	696	702	99	−	5	706	2607	3308
UN13357	Rsa1.0_16170.1	807	2258	1.00E−105	289	315	91	−	495	799	1231	1542
UN13358	Rsa1.0_02236.1	1322	33558	0	476	476	100	+	845	1320	14207	14682
UN13359	Rsa1.0_00697.1	1561	67913	0	594	602	98	+	376	977	51202	51802
UN13360	Rsa1.0_00453.1	915	83031	0	427	434	98	+	483	914	19304	19734
UN13361	Rsa1.0_00222.1	1695	172263	0	897	897	100	+	797	1693	146662	147558
UN13362	Rsa1.0_00345.1	954	93226	0	354	357	99	+	595	951	85769	86122
UN13363	Rsa1.0_02879.1	1684	27047	0	485	485	100	+	1194	1678	18068	18552
UN13364	Rsa1.0_00232.1	941	104936	1.00E−179	319	319	100	−	2	320	38388	38706
UN13365	Rsa1.0_00249.1	779	153740	1.00E−126	272	286	95	+	1	286	52903	53188
UN13366	Rsa1.0_01387.1	812	46745	1.00E−169	310	313	99	+	499	811	46152	46464
UN13367	Rsa1.0_01376.1	1046	47004	1.00E−153	339	360	94	−	1	357	29440	29789
UN13368	Rsa1.0_00630.1	1148	71281	0	920	956	96	+	209	1146	23790	24743
UN13369	Rsa1.0_01402.1	1109	46509	0	772	788	97	+	1	787	17539	18325
UN13370	Rsa1.0_00198.1	1453	222963	0	362	374	96	−	1081	1453	40461	40834
UN13371	Rsa1.0_04477.1	1002	17183	3.00E−78	200	213	93	−	743	950	5320	5529
UN13372	Rsa1.0_01079.1	1315	54753	0	579	585	98	+	3	587	477	1061
UN13373	Rsa1.0_01116.1	788	142088	0	727	727	100	+	5	731	129729	130455
UN13374	Rsa1.0_00663.1	1127	164969	1.00E−128	236	237	99	−	891	1127	14189	14425
UN13375	Rsa1.0_00009.1	2171	232558	0	956	971	98	−	851	1812	71154	72124

UN13376	Rsa1.0_00991.1	1078	143101	0	455	474	95	+	1	471	133487	133957
UN13377	Rsa1.0_01919.1	939	37272	0	501	514	97	+	1	512	3478	3991
UN13378	Rsa1.0_01838.1	1768	38500	0	482	490	98	+	592	1081	21713	22202
UN13379	Rsa1.0_03567.1	725	22049	0	465	465	100	−	259	723	3399	3863
UN13380	Rsa1.0_03185.1	1404	24739	1.00E−121	260	273	95	+	2	268	7473	7745
UN13381	Rsa1.0_06619.1	1415	10650	1.00E−164	382	406	94	+	426	824	1125	1518
UN13382	Rsa1.0_02288.1	1447	32984	1.00E−173	339	351	96	+	1107	1447	18618	18968
UN13383	Rsa1.0_00340.1	765	305782	0	726	760	95	+	3	762	173360	174118
UN13384	Rsa1.0_00079.1	1238	606987	0	374	382	97	+	801	1181	22253	22631
UN13385	Rsa1.0_10853.1	935	5700	0	350	352	99	+	8	358	1602	1953
UN13386	Rsa1.0_00064.1	946	240927	1.00E−136	268	274	97	+	673	946	170881	171153
UN13387	Rsa1.0_00008.1	1396	358124	0	783	801	97	−	216	1015	67789	68584
UN13388	Rsa1.0_03089.1	940	25465	0	377	382	98	−	1	382	8730	9111
UN13389	Rsa1.0_01777.1	1199	39511	0	381	400	95	+	801	1199	13918	14303
UN13390	Rsa1.0_00058.1	1284	159921	0	380	381	99	−	1	381	116921	117301
UN13391	Rsa1.0_00195.1	1290	251884	0	852	855	99	+	436	1290	85458	86312
UN13392	Rsa1.0_00520.1	1628	78226	0	1532	1540	99	−	88	1627	19279	20818
UN13393	Rsa1.0_02508.1	1042	30675	0	628	655	95	+	406	1041	24669	25323
UN13394	Rsa1.0_00019.1	1488	301669	0	458	463	98	+	1	462	64804	65266
UN13395	Rsa1.0_04911.1	1315	15483	0	422	427	98	+	6	432	7719	8143
UN13396	Rsa1.0_00546.1	818	76523	0	543	549	98	−	270	818	12515	13063
UN13397	Rsa1.0_02929.1	822	26673	1.00E−109	248	262	94	−	1	262	20275	20531
UN13398	Rsa1.0_00773.1	1514	64839	0	439	455	96	+	1061	1514	32802	33252
UN13399	Rsa1.0_01707.1	1281	40686	0	452	460	98	−	821	1280	7606	8065
UN13400	Rsa1.0_00437.1	1321	84879	0	637	648	98	+	603	1250	55056	55702
UN13401	Rsa1.0_00088.1	926	141341	1.00E−142	269	272	98	−	3	274	108001	108271
UN13402	Rsa1.0_00027.1	947	283362	1.00E−151	310	322	96	+	624	945	275355	275674
UN13403	Rsa1.0_01923.1	1035	37233	0	328	329	99	+	695	1023	36457	36785
UN13404	Rsa1.0_01494.1	2546	44641	0	1812	1852	97	+	705	2545	5834	7683
UN13405	Rsa1.0_02518.1	1084	30586	0	335	337	99	+	2	338	27755	28091
UN13406	Rsa1.0_00087.1	784	761073	1.00E−100	188	189	99	−	596	784	278160	278348
UN13407	Rsa1.0_00030.1	976	250988	1.00E−128	242	245	98	−	270	514	156217	156461
UN13408	Rsa1.0_00111.1	874	390088	0	642	644	99	+	2	645	383564	384207
UN13409	Rsa1.0_00651.1	1070	247766	0	738	760	97	−	318	1070	105172	105930
UN13410	Rsa1.0_00092.1	933	659932	7.00E−36	235	281	83	+	594	868	163749	164024
UN13411	Rsa1.0_00417.1	1284	395068	0	389	399	97	+	889	1283	376170	376568
UN13412	Rsa1.0_00451.1	775	322533	0	740	743	99	+	33	773	318149	318891
UN13413	Rsa1.0_01014.1	845	56762	6.00E−95	183	185	98	−	12	196	43013	43197
UN13414	Rsa1.0_01469.1	1599	45139	0	397	398	99	−	1203	1599	17649	18046

UN13415	Rsa1.0_22190.1	565	1143	1.00E-115	232	238	97	-	328	565	908	1143
UN13416	Rsa1.0_00123.1	1303	325650	0	751	766	98	-	538	1301	284045	284809
UN13417	Rsa1.0_06333.1	1162	11217	2.33E-156	319	334	95	-	807	1139	2122	2452
UN13418	Rsa1.0_01140.1	1101	52586	0	676	687	98	+	3	686	6409	7094
UN13419	Rsa1.0_00049.1	1160	167018	0	1128	1162	97	+	2	1160	74527	75688
UN13420	Rsa1.0_01648.1	1155	41635	0	1136	1141	99	+	11	1147	5972	7112
UN13421	Rsa1.0_01217.1	1531	50554	0	1466	1512	96	-	4	1509	46734	48238
UN13422	Rsa1.0_00058.1	1316	159921	0	341	342	99	-	976	1316	46523	46864
UN13423	Rsa1.0_01540.1	1780	151890	1.00E-176	463	507	91	-	1034	1539	127704	128198
UN13424	Rsa1.0_01331.1	1232	47944	0	416	422	98	-	813	1232	29232	29653
UN13425	Rsa1.0_00038.1	1428	314568	0	721	728	99	+	703	1427	158194	158921
UN13426	Rsa1.0_02660.1	1279	28969	1.00E-160	313	320	97	-	3	319	12641	12959
UN13427	Rsa1.0_00323.1	1463	159650	0	1429	1461	97	-	3	1463	134581	136036
UN13428	Rsa1.0_00121.1	874	278029	1.00E-117	230	235	97	-	634	868	59505	59739
UN13429	Rsa1.0_02492.1	746	30859	1.00E-166	353	368	95	+	379	745	12178	12542
UN13430	Rsa1.0_00028.1	2072	293984	1.00E-159	348	361	96	+	336	692	20828	21185
UN13431	Rsa1.0_02272.1	973	33131	0	528	550	96	+	427	972	26860	27409
UN13432	Rsa1.0_00008.1	1334	358124	0	758	773	98	+	378	1147	287628	288397
UN13433	Rsa1.0_00741.1	1368	66117	0	516	538	95	-	341	878	60657	61194
UN13434	Rsa1.0_04064.1	703	19297	0	473	483	97	+	222	703	18501	18981
UN13435	Rsa1.0_09725.1	673	6186	0	369	372	99	-	302	673	944	1315
UN13436	Rsa1.0_01875.1	536	37849	1.00E-158	292	295	98	+	240	534	25879	26173
UN13437	Rsa1.0_00039.1	1510	296927	0	596	608	98	-	901	1508	11841	12448
UN13438	Rsa1.0_06133.1	1167	11663	1.00E-172	306	306	100	+	614	919	8879	9184
UN13439	Rsa1.0_01016.1	1398	181422	0	369	378	97	+	1022	1398	164149	164526
UN13440	Rsa1.0_02392.1	779	152517	0	752	778	96	+	3	779	29654	30422
UN13441	Rsa1.0_01274.1	400	49419	2.00E-83	235	259	90	+	149	400	21348	21599
UN13442	Rsa1.0_04205.1	778	138772	0	435	436	99	-	343	778	133814	134249
UN13443	Rsa1.0_00205.1	857	193401	1.00E-124	241	246	97	+	291	536	98206	98451
UN13444	Rsa1.0_01516.1	1441	44196	1.00E-136	331	354	93	+	940	1291	23523	23873
UN13445	Rsa1.0_01707.1	1158	40686	0	391	401	97	-	338	738	25818	26218
UN13446	Rsa1.0_08927.1	1970	6782	0	799	868	92	+	1121	1967	87	949
UN13447	Rsa1.0_00467.1	1313	156542	0	566	566	100	+	748	1313	77638	78203
UN13448	Rsa1.0_00648.1	912	70351	1.00E-163	301	303	99	+	608	910	24866	25166
UN13449	Rsa1.0_01609.1	1116	42280	0	1107	1111	99	+	3	1113	25585	26693
UN13450	Rsa1.0_13888.1	974	4146	1.00E-154	276	276	100	-	699	974	3871	4146
UN13451	Rsa1.0_00817.1	920	62961	0	493	507	97	-	374	879	53828	54331
UN13452	Rsa1.0_05494.1	1023	13547	0	499	505	98	-	519	1023	7600	8102
UN13453	Rsa1.0_05489.1	1345	13562	0	551	564	97	+	393	956	6625	7188

UN13454	Rsa1.0_00808.1	1160	63351	0	427	431	99	−	675	1105	35075	35505
UN13455	Rsa1.0_00484.1	1252	203294	0	407	420	96	−	770	1189	127050	127468
UN13456	Rsa1.0_02043.1	1991	163754	0	607	631	96	+	348	973	140557	141184
UN13457	Rsa1.0_04116.1	767	18971	0	423	430	98	+	3	432	612	1041
UN13458	Rsa1.0_01595.1	574	175383	1.00E−178	317	317	100	+	2	318	41015	41331
UN13459	Rsa1.0_00240.1	1060	104175	1.00E−173	309	309	100	+	752	1060	69794	70102
UN13460	Rsa1.0_00970.1	1350	57994	0	946	950	99	+	90	1039	18920	19869
UN13461	Rsa1.0_00476.1	1340	280966	1.00E−148	292	298	97	+	3	300	236929	237223
UN13462	Rsa1.0_00354.1	1525	232005	0	476	494	96	+	2	488	197353	197846
UN13463	Rsa1.0_01743.1	1099	39926	1.00E−176	334	340	98	−	1	340	15993	16330
UN13464	Rsa1.0_01950.1	533	36950	0	394	405	97	+	128	532	7846	8247
UN13465	Rsa1.0_16368.1	1539	2175	0	940	951	98	−	586	1535	315	1265
UN13466	Rsa1.0_06615.1	1802	10659	0	724	744	97	−	156	889	9364	10107
UN13467	Rsa1.0_02487.1	1533	30928	0	849	858	98	−	1	857	22194	23048
UN13468	Rsa1.0_01335.1	2043	47894	1.00E−121	235	238	98	+	656	892	430	667
UN13469	Rsa1.0_01078.1	1211	316910	1.00E−164	322	330	97	−	3	331	290400	290729
UN13470	Rsa1.0_14292.1	1429	3569	0	492	494	99	+	16	509	7	500
UN13471	Rsa1.0_08003.1	887	8094	1.00E−139	251	251	100	+	197	447	5286	5536
UN13472	Rsa1.0_03873.1	732	20360	5.00E−98	273	306	89	+	443	731	9247	9541
UN13473	Rsa1.0_01945.1	1446	36989	0	1360	1445	94	+	6	1446	25694	27124
UN13474	Rsa1.0_02378.1	1041	31991	1.00E−178	368	383	96	−	659	1038	12955	13336
UN13475	Rsa1.0_12942.1	980	5068	1.00E−176	326	330	98	−	651	980	1312	1641
UN13476	Rsa1.0_03694.1	908	21336	0	656	661	99	+	220	876	549	1209
UN13477	Rsa1.0_00459.1	955	82580	0	508	509	99	+	1	509	40992	41500
UN13478	Rsa1.0_00827.1	1452	62790	0	398	421	94	−	1044	1452	49150	49567
UN13479	Rsa1.0_10868.1	1533	5695	1.00E−172	348	358	97	−	1166	1522	1661	2015
UN13480	Rsa1.0_01842.1	903	38430	0	387	392	98	−	472	863	2281	2670
UN13481	Rsa1.0_00977.1	860	198001	1.00E−106	205	207	99	+	3	207	197674	197880
UN13482	Rsa1.0_00630.1	913	71281	0	496	514	96	+	1	510	63634	64143
UN13483	Rsa1.0_00093.1	847	214962	0	838	840	99	−	6	845	83901	84740
UN13484	Rsa1.0_00974.1	684	57893	0	534	538	99	−	147	683	38809	39346
UN13485	Rsa1.0_03375.1	612	23332	2.00E−69	134	134	100	−	1	134	7874	8007
UN13486	Rsa1.0_00868.1	746	61630	0	366	367	99	−	249	615	46733	47099
UN13487	Rsa1.0_02074.1	1889	35370	0	666	697	95	+	7	692	33161	33857
UN13488	Rsa1.0_01889.1	634	37562	1.00E−153	293	298	98	−	1	298	23938	24234
UN13489	Rsa1.0_04234.1	642	18378	1.00E−107	237	250	94	+	393	642	8210	8459
UN13490	Rsa1.0_01537.1	965	187285	0	736	748	98	−	217	964	33372	34119
UN13491	Rsa1.0_03216.1	925	24540	8.00E−82	267	302	88	−	623	916	12029	12330
UN13492	Rsa1.0_01855.1	946	38204	0	929	942	98	−	5	945	6567	7508

UN13493	Rsa1.0_08003.1	890	8094	1.00E-132	261	267	97	-	625	890	6154	6420
UN13494	Rsa1.0_01909.1	1085	37417	1.00E-151	274	275	99	-	1	275	13	287
UN13495	Rsa1.0_02052.1	939	35555	0	347	353	98	-	5	357	20165	20517
UN13496	Rsa1.0_01220.1	1770	161775	0	496	518	95	+	1	506	11304	11819
UN13497	Rsa1.0_00105.1	1320	134138	0	462	466	99	+	1	465	126890	127353
UN13498	Rsa1.0_02855.1	1406	27271	0	420	420	100	-	920	1339	10359	10778
UN13499	Rsa1.0_36951.1	1028	610	0	385	386	99	-	3	388	1	386
UN13500	Rsa1.0_04293.1	1400	18046	1.00E-172	338	346	97	-	5	350	11123	11465
UN13501	Rsa1.0_00351.1	855	413096	1.00E-135	289	303	95	-	2	304	269242	269542
UN13502	Rsa1.0_02690.1	1034	28629	1.00E-127	254	259	98	+	775	1033	17941	18197
UN13503	Rsa1.0_04462.1	6364	17250	0	1030	1088	94	+	5036	6121	6961	8026
UN13504	Rsa1.0_02185.1	749	34150	0	725	749	96	-	3	749	27251	27988
UN13505	Rsa1.0_00132.1	1369	124299	0	1298	1346	96	-	3	1342	52496	53834
UN13506	Rsa1.0_01958.1	1135	261128	1.00E-118	261	275	94	+	3	277	111078	111349
UN13507	Rsa1.0_04379.1	1195	17646	1.00E-173	347	356	97	+	1	354	16744	17097
UN13508	Rsa1.0_05049.1	1115	15025	1.00E-163	387	418	92	+	1	416	8807	9209
UN13509	Rsa1.0_02304.1	727	32793	1.00E-108	206	207	99	-	519	724	17374	17580
UN13510	Rsa1.0_03053.1	1412	25745	0	451	452	99	-	1	452	24134	24585
UN13511	Rsa1.0_00341.1	1000	249309	1.00E-153	274	274	100	+	727	1000	6828	7101
UN13512	Rsa1.0_01031.1	1080	56236	0	385	398	96	-	683	1080	17186	17573
UN13513	Rsa1.0_01286.1	1357	49209	1.00E-136	287	298	96	+	1060	1357	39922	40212
UN13514	Rsa1.0_64913.1	993	353	1.00E-163	304	308	98	-	596	903	37	344
UN13515	Rsa1.0_00419.1	1379	86562	0	489	526	92	+	105	629	15539	16064
UN13516	Rsa1.0_00752.1	1530	65756	0	405	420	96	-	813	1230	35504	35920
UN13517	Rsa1.0_00317.1	994	218290	1.00E-104	249	266	93	+	37	294	72658	72921
UN13518	Rsa1.0_01251.1	1229	49873	1.00E-107	197	197	100	+	1	197	7949	8145
UN13519	Rsa1.0_00339.1	1068	222974	0	650	659	98	+	137	795	4477	5135
UN13520	Rsa1.0_01730.1	787	40223	5.00E-92	207	217	95	-	7	221	26029	26242
UN13521	Rsa1.0_00191.1	2532	316976	0	583	584	99	+	942	1525	133763	134346
UN13522	Rsa1.0_07334.1	1452	9547	0	393	397	98	+	1057	1452	683	1079
UN13523	Rsa1.0_00216.1	851	197563	0	376	380	98	-	472	851	187821	188199
UN13524	Rsa1.0_00252.1	1371	102760	0	446	453	98	-	921	1370	93584	94036
UN13525	Rsa1.0_00119.1	1148	467509	1.00E-165	307	310	99	+	1	310	32133	32440
UN13526	Rsa1.0_00891.1	1250	60724	0	587	590	99	+	4	593	54503	55091
UN13527	Rsa1.0_00173.1	1070	288743	0	551	553	99	-	518	1070	29272	29824
UN13528	Rsa1.0_26211.1	664	901	1.00E-111	231	239	96	+	6	242	133	371
UN13529	Rsa1.0_12442.1	1121	5225	1.00E-105	204	207	98	-	317	523	163	369
UN13530	Rsa1.0_06892.1	919	10197	0	354	362	97	+	505	866	9775	10136
UN13531	Rsa1.0_00307.1	1568	199003	0	888	916	96	+	6	921	5352	6260

UN13532	Rsa1.0_03291.1	810	24000	0	377	378	99	+	9	386	18131	18508
UN13533	Rsa1.0_00586.1	1848	163802	0	425	427	99	+	623	1049	153732	154158
UN13534	Rsa1.0_01190.1	1547	194501	0	885	895	98	+	653	1546	10236	11130
UN13535	Rsa1.0_00482.1	1161	81162	0	390	398	97	−	765	1161	60320	60717
UN13536	Rsa1.0_00092.1	1276	659932	0	471	488	96	+	81	568	20395	20882
UN13537	Rsa1.0_17040.1	607	1944	0	384	399	96	+	157	549	1207	1605
UN13538	Rsa1.0_00604.1	773	146282	1.00E−106	240	255	94	−	6	260	113696	113950
UN13539	Rsa1.0_00779.1	925	64568	0	919	923	99	−	3	925	49345	50267
UN13540	Rsa1.0_00131.1	1252	432233	0	582	606	96	+	636	1241	34864	35463
UN13541	Rsa1.0_13999.1	1436	3934	1.00E−167	344	357	96	−	1077	1433	1805	2157
UN13542	Rsa1.0_04338.1	1216	17840	0	334	334	100	+	883	1216	12133	12466
UN13543	Rsa1.0_01177.1	1476	51572	0	354	355	99	+	1	355	47173	47527
UN13544	Rsa1.0_00108.1	1300	478491	1.00E−168	333	342	97	+	964	1300	467659	467996
UN13545	Rsa1.0_03365.1	827	23410	0	819	832	98	−	1	827	12912	13740
UN13546	Rsa1.0_08677.1	1499	7048	1.00E−167	325	334	97	−	1164	1497	2193	2526
UN13547	Rsa1.0_14674.1	1746	3167	1.00E−136	360	393	91	−	1	386	372	760
UN13548	Rsa1.0_01499.1	1239	44555	1.00E−175	332	339	97	+	1	339	14518	14856
UN13549	Rsa1.0_01115.1	1064	183545	0	418	440	95	−	1	434	6514	6952
UN13550	Rsa1.0_18154.1	799	1655	4.00E−80	158	160	98	−	527	686	1333	1492
UN13551	Rsa1.0_01431.1	712	167877	0	404	405	99	+	308	711	12009	12413
UN13552	Rsa1.0_00889.1	1413	60978	0	686	696	98	+	716	1411	55897	56592
UN13553	Rsa1.0_00608.1	1455	172672	0	815	817	99	−	638	1454	163142	163958
UN13554	Rsa1.0_00678.1	486	193163	4.00E−67	133	134	99	−	353	486	24426	24559
UN13555	Rsa1.0_14087.1	819	3821	0	680	702	96	−	107	804	1455	2153
UN13556	Rsa1.0_02345.1	825	32337	0	361	364	99	−	464	825	385	747
UN13557	Rsa1.0_00292.1	714	98141	9.00E−75	158	162	97	−	42	201	21932	22093
UN13558	Rsa1.0_03818.1	1236	20668	1.00E−106	203	205	99	−	726	930	10042	10246
UN13559	Rsa1.0_00979.1	1406	57733	0	333	336	99	−	1069	1404	40278	40613
UN13560	Rsa1.0_00180.1	855	114581	1.00E−135	253	256	98	−	534	789	62118	62373
UN13561	Rsa1.0_04063.1	1207	19300	1.00E−149	283	285	99	+	3	286	12085	12368
UN13562	Rsa1.0_01521.1	1093	142147	1.00E−141	260	262	99	−	360	621	7693	7954
UN13563	Rsa1.0_03334.1	946	23659	1.00E−150	273	274	99	−	1	274	20257	20530
UN13564	Rsa1.0_00933.1	1382	59164	0	341	342	99	+	1039	1379	2856	3197
UN13565	Rsa1.0_00910.1	1491	59975	0	550	563	97	−	931	1491	49138	49700
UN13566	Rsa1.0_00804.1	1363	63726	0	459	465	98	+	1	464	21629	22092
UN13567	Rsa1.0_01440.1	972	45807	0	479	493	97	+	470	954	19323	19815
UN13568	Rsa1.0_02233.1	1615	33658	0	675	707	95	+	225	931	2790	3496
UN13569	Rsa1.0_02605.1	762	29637	1.00E−138	276	285	96	+	3	287	12918	13202
UN13570	Rsa1.0_35458.1	1301	638	1.00E−132	270	276	97	−	1025	1299	365	638

UN13571	Rsa1.0_00055.1	715	520193	0	346	351	98	−	365	715	153888	154237
UN13572	Rsa1.0_01119.1	1272	53459	1.00E−114	216	218	99	+	708	925	23345	23562
UN13573	Rsa1.0_03890.1	993	20223	0	973	995	97	−	1	993	1392	2384
UN13574	Rsa1.0_01172.1	904	51734	1.00E−171	321	326	98	−	282	607	6286	6607
UN13575	Rsa1.0_00222.1	1220	172263	0	789	816	96	+	410	1220	65105	65915
UN13576	Rsa1.0_01684.1	1186	41044	3.00E−29	160	187	85	+	184	363	5126	5311
UN13577	Rsa1.0_02020.1	1600	173211	0	1046	1088	96	−	512	1596	139262	140348
UN13578	Rsa1.0_10836.1	1180	5707	0	892	929	96	−	4	923	4133	5061
UN13579	Rsa1.0_00805.1	3718	63724	0	391	396	98	+	2985	3379	11592	11987
UN13580	Rsa1.0_02581.1	1481	29896	0	736	747	98	−	1	747	11138	11882
UN13581	Rsa1.0_00630.1	1653	71281	0	500	505	99	+	1027	1530	18575	19078
UN13582	Rsa1.0_00568.1	1012	75219	0	771	795	96	−	215	1007	1873	2658
UN13583	Rsa1.0_02295.1	1665	149884	0	761	797	95	+	177	968	118181	118968
UN13584	Rsa1.0_00387.1	1216	495407	0	909	909	100	+	308	1216	409522	410430
UN13585	Rsa1.0_01067.1	1193	55230	1.00E−117	232	235	98	−	959	1193	4545	4777
UN13586	Rsa1.0_00066.1	1235	152418	0	1218	1233	98	+	1	1233	35808	37040
UN13587	Rsa1.0_02146.1	873	34625	0	740	764	96	+	110	872	17172	17931
UN13588	Rsa1.0_18413.1	1020	1603	0	362	367	98	+	251	617	1151	1517
UN13589	Rsa1.0_00052.1	1007	162771	1.00E−120	249	259	96	+	101	359	57659	57917
UN13590	Rsa1.0_02200.1	1773	33981	0	364	364	100	+	1408	1771	10438	10801
UN13591	Rsa1.0_00688.1	1412	68396	0	592	602	98	+	811	1412	37596	38197
UN13592	Rsa1.0_00562.1	1994	75565	0	1417	1440	98	−	1	1436	5411	6850
UN13593	Rsa1.0_04892.1	1492	15573	0	370	381	97	−	1052	1430	6086	6463
UN13594	Rsa1.0_05200.1	556	14522	1.00E−142	256	256	100	−	301	556	7534	7789
UN13595	Rsa1.0_00175.1	1575	166121	1.00E−156	286	288	99	+	1288	1575	28735	29022
UN13596	Rsa1.0_00122.1	1009	265937	0	870	874	99	+	136	1009	239355	240227
UN13597	Rsa1.0_01030.1	1196	56290	1.00E−144	294	304	96	+	893	1196	40732	41029
UN13598	Rsa1.0_10890.1	924	5688	0	844	857	98	−	1	857	1	856
UN13599	Rsa1.0_00728.1	919	66704	1.00E−172	354	366	96	−	553	918	15828	16190
UN13600	Rsa1.0_28780.1	1558	803	0	423	423	100	+	1135	1557	1	423
UN13601	Rsa1.0_00065.1	1001	454616	1.00E−156	324	332	97	+	1	330	243703	244031
UN13602	Rsa1.0_02139.1	876	34686	2.33E−156	346	370	93	−	509	876	5545	5912
UN13603	Rsa1.0_03235.1	1099	24442	0	610	624	97	+	477	1099	14818	15441
UN13604	Rsa1.0_00128.1	1265	228675	0	383	390	98	+	876	1265	135383	135767
UN13605	Rsa1.0_02070.1	1344	158516	1.00E−130	261	268	97	+	2	268	16293	16560
UN13606	Rsa1.0_05569.1	1117	13311	0	731	742	98	−	375	1116	2710	3451
UN13607	Rsa1.0_00002.1	923	601661	0	692	724	95	−	7	726	231774	232495
UN13608	Rsa1.0_01313.1	1259	48387	0	463	472	98	−	789	1259	506	977
UN13609	Rsa1.0_00273.1	1903	326186	0	619	629	98	−	503	1131	137570	138198

UN13610	Rsa1.0_00342.1	1337	93695	0	735	735	100	+	3	737	12430	13164
UN13611	Rsa1.0_01220.1	869	161775	1.00E-131	289	304	95	−	564	867	115836	116135
UN13612	Rsa1.0_00002.1	2100	601661	0	505	509	99	−	1	509	138033	138540
UN13613	Rsa1.0_07877.1	1107	8373	6.00E-77	147	147	100	+	238	384	4515	4661
UN13614	Rsa1.0_18628.1	1618	1562	0	1006	1011	99	−	608	1618	346	1355
UN13615	Rsa1.0_00064.1	1515	240927	0	888	906	98	−	1	906	65164	66069
UN13616	Rsa1.0_05745.1	1597	12750	0	1041	1055	98	+	541	1595	7883	8937
UN13617	Rsa1.0_00252.1	1282	102760	0	360	372	96	+	911	1282	44777	45148
UN13618	Rsa1.0_00090.1	807	273859	1.00E-119	236	242	97	−	3	244	41355	41596
UN13619	Rsa1.0_01209.1	936	50668	0	585	593	98	−	344	936	48588	49180
UN13620	Rsa1.0_09067.1	1057	6672	1.00E-120	234	239	97	−	3	241	4293	4531
UN13621	Rsa1.0_00048.1	1581	167185	0	411	416	98	+	2	417	154805	155219
UN13622	Rsa1.0_00015.1	1141	561803	1.00E-140	322	346	93	+	1	346	190551	190878
UN13623	Rsa1.0_04223.1	935	18407	1.00E-148	348	374	93	+	1	374	9455	9816
UN13624	Rsa1.0_02456.1	803	31222	1.00E-127	277	290	95	+	1	290	23723	24004
UN13625	Rsa1.0_00339.1	891	222974	5.00E-80	158	160	98	+	648	807	63385	63544
UN13626	Rsa1.0_01048.1	1773	55684	0	913	914	99	−	1	914	44113	45026
UN13627	Rsa1.0_01789.1	1030	39250	0	501	522	95	+	283	804	9785	10306
UN13628	Rsa1.0_00363.1	1163	90968	0	432	461	93	−	700	1160	9266	9713
UN13629	Rsa1.0_14319.1	1149	3529	1.00E-172	488	548	89	−	267	814	2316	2860
UN13630	Rsa1.0_00154.1	1702	189461	0	331	333	99	−	1370	1702	41236	41568
UN13631	Rsa1.0_00407.1	1098	191544	0	840	873	96	+	2	869	155496	156357
UN13632	Rsa1.0_01091.1	1240	54383	0	1214	1237	98	+	4	1239	49136	50372
UN13633	Rsa1.0_08072.1	1083	7955	1.00E-151	319	330	96	+	3	331	6001	6326
UN13634	Rsa1.0_00017.1	1136	752458	2.00E-77	148	148	100	−	764	911	603348	603495
UN13635	Rsa1.0_01540.1	1229	151890	1.00E-104	213	220	96	+	781	1000	129408	129627
UN13636	Rsa1.0_00127.1	1371	127446	0	771	808	95	+	492	1298	102847	103654
UN13637	Rsa1.0_01493.1	1377	44670	0	379	381	99	−	1	380	7846	8226
UN13638	Rsa1.0_01485.1	872	44797	0	861	875	98	−	1	872	50	924
UN13639	Rsa1.0_01872.1	676	37908	3.00E-96	217	227	95	−	1	226	16038	16258
UN13640	Rsa1.0_18254.1	1454	1636	1.00E-151	329	347	94	+	1	334	682	1024
UN13641	Rsa1.0_00933.1	1323	59164	0	498	502	99	−	819	1320	52183	52684
UN13642	Rsa1.0_00033.1	1228	515811	0	349	352	99	−	1	350	131617	131968
UN13643	Rsa1.0_00152.1	1461	257730	0	821	848	96	+	618	1459	59089	59936
UN13644	Rsa1.0_05235.1	830	14421	0	405	411	98	−	425	830	10660	11070
UN13645	Rsa1.0_00841.1	1537	183065	0	777	786	98	+	2	787	48464	49248
UN13646	Rsa1.0_01198.1	834	51029	0	827	831	99	+	1	831	25776	26606
UN13647	Rsa1.0_02192.1	1828	171291	0	441	463	95	+	1283	1736	12268	12724
UN13648	Rsa1.0_00064.1	1716	240927	0	413	422	97	+	767	1183	60195	60615

UN13649	Rsa1.0_10168.1	1118	5964	1.00E-144	262	263	99	-	4	266	934	1196
UN13650	Rsa1.0_03826.1	1248	20614	0	805	805	100	-	3	807	16090	16894
UN13651	Rsa1.0_00101.1	1033	267193	1.00E-124	283	299	94	-	735	1032	131413	131700
UN13652	Rsa1.0_07622.1	903	9012	0	560	560	100	-	344	903	4238	4797
UN13653	Rsa1.0_00675.1	1235	145590	0	351	359	97	-	1	359	144665	145019
UN13654	Rsa1.0_06887.1	873	10208	1.00E-159	297	301	98	+	24	324	4091	4391
UN13655	Rsa1.0_00819.1	1502	62918	0	1312	1389	94	+	13	1388	3643	5030
UN13656	Rsa1.0_03570.1	1116	22040	0	444	461	96	+	657	1116	9422	9875
UN13657	Rsa1.0_04097.1	1481	19090	0	660	683	96	-	1	682	6602	7283
UN13658	Rsa1.0_00705.1	1018	67505	1.00E-144	277	283	97	+	105	387	53881	54163
UN13659	Rsa1.0_00625.1	1078	71672	0	602	616	97	-	465	1077	14860	15475
UN13660	Rsa1.0_00627.1	1039	71566	0	360	362	99	-	8	369	63662	64023
UN13661	Rsa1.0_01670.1	1333	41260	1.00E-101	200	204	98	-	1031	1234	25218	25421
UN13662	Rsa1.0_00948.1	589	58620	1.00E-104	199	201	99	+	134	334	50390	50590
UN13663	Rsa1.0_00991.1	1425	143101	0	404	404	100	-	105	508	142698	143101
UN13664	Rsa1.0_00697.1	766	67913	1.00E-174	372	392	94	+	3	394	59555	59944
UN13665	Rsa1.0_00385.1	1178	89160	0	567	569	99	+	3	569	47346	47914
UN13666	Rsa1.0_02916.1	1488	26809	1.00E-177	324	327	99	+	329	655	4488	4814
UN13667	Rsa1.0_02568.1	1234	30015	0	615	615	100	-	618	1232	9866	10480
UN13668	Rsa1.0_07464.1	1053	9328	1.00E-151	352	375	93	+	682	1053	4	377
UN13669	Rsa1.0_00624.1	957	71773	1.00E-126	232	233	99	+	477	709	20764	20996
UN13670	Rsa1.0_42545.1	1182	527	0	341	344	99	-	8	351	1	344
UN13671	Rsa1.0_05950.1	832	12130	1.00E-111	222	228	97	-	297	524	2709	2936
UN13672	Rsa1.0_00197.1	1274	109714	1.00E-168	342	356	96	+	535	890	36533	36888
UN13673	Rsa1.0_04933.1	910	15410	0	631	650	97	-	1	649	6309	6958
UN13674	Rsa1.0_04325.1	1508	17885	0	382	382	100	+	4	385	13661	14042
UN13675	Rsa1.0_04220.1	843	18421	0	516	517	99	-	328	843	9466	9982
UN13676	Rsa1.0_00048.1	772	167185	0	469	489	95	+	2	474	154732	155219
UN13677	Rsa1.0_05570.1	744	13301	0	458	467	98	-	279	744	6801	7266
UN13678	Rsa1.0_00017.1	908	752458	0	456	461	98	-	450	908	412583	413043
UN13679	Rsa1.0_00095.1	717	283384	0	633	645	98	-	2	645	96617	97258
UN13680	Rsa1.0_13767.1	951	4361	0	451	466	96	-	431	896	653	1118
UN13681	Rsa1.0_08280.1	1015	7610	0	974	1007	96	+	1	1006	753	1755
UN13682	Rsa1.0_00324.1	1131	218805	1.00E-133	270	278	97	+	855	1131	189680	189957
UN13683	Rsa1.0_03669.1	886	147114	0	763	791	96	+	102	886	20525	21309
UN13684	Rsa1.0_14348.1	953	3490	1.00E-142	307	320	95	+	637	953	1994	2313
UN13685	Rsa1.0_01773.1	1529	39546	0	666	674	98	-	3	675	4503	5174
UN13686	Rsa1.0_00087.1	673	761073	1.00E-174	318	320	99	-	1	318	217289	217608
UN13687	Rsa1.0_00387.1	2181	495407	0	551	564	97	-	1011	1574	396750	397310

UN13688	Rsa1.0_00273.1	1569	326186	0	855	864	98	+	1	859	237423	238286
UN13689	Rsa1.0_11510.1	1275	5482	0	626	635	98	+	643	1274	4642	5276
UN13690	Rsa1.0_07974.1	656	8147	1.00E-177	326	330	98	−	327	656	6368	6697
UN13691	Rsa1.0_02027.1	1259	35869	1.00E-167	328	334	98	+	1	331	10181	10513
UN13692	Rsa1.0_01540.1	1321	151890	1.00E-152	274	274	100	−	1048	1321	127925	128198
UN13693	Rsa1.0_00381.1	1253	89718	1.00E-160	287	287	100	−	3	289	73706	73992
UN13694	Rsa1.0_14436.1	1640	3393	0	522	540	96	−	1106	1637	2252	2791
UN13695	Rsa1.0_01881.1	892	37721	0	841	871	96	−	24	890	16713	17578
UN13696	Rsa1.0_00440.1	1039	84723	1.00E-150	305	316	96	−	722	1037	25086	25398
UN13697	Rsa1.0_00067.1	1696	415055	1.00E-120	226	228	99	+	1	228	40082	40309
UN13698	Rsa1.0_05217.1	1090	142938	0	417	440	94	−	655	1084	136328	136763
UN13699	Rsa1.0_01836.1	1037	38503	1.00E-162	346	364	95	−	675	1035	2184	2547
UN13700	Rsa1.0_01171.1	1165	51752	0	402	413	97	+	755	1165	50943	51352
UN13701	Rsa1.0_42713.1	972	526	0	413	414	99	+	560	972	1	414
UN13702	Rsa1.0_04310.1	1451	17994	1.00E-157	290	293	98	−	438	730	1739	2031
UN13703	Rsa1.0_00602.1	1759	73302	1.00E-112	219	223	98	+	621	843	39855	40077
UN13704	Rsa1.0_01164.1	2667	51934	0	551	552	99	+	1697	2248	14403	14954
UN13705	Rsa1.0_13438.1	1260	4828	2.33E-156	275	279	98	−	982	1260	943	1221
UN13706	Rsa1.0_00191.1	1455	316976	0	1241	1277	97	+	2	1278	265865	267134
UN13707	Rsa1.0_00456.1	580	82826	0	540	557	96	+	30	578	24528	25082
UN13708	Rsa1.0_00263.1	1534	224968	0	456	460	99	−	1067	1526	6735	7191
UN13709	Rsa1.0_05311.1	1250	14181	0	656	666	98	+	583	1248	6590	7248
UN13710	Rsa1.0_00022.1	846	200461	0	396	402	98	−	5	403	46372	46773
UN13711	Rsa1.0_02802.1	1949	27736	0	1004	1045	96	+	892	1935	8951	9978
UN13712	Rsa1.0_05812.1	1030	12571	0	1028	1030	99	+	1	1028	2560	3589
UN13713	Rsa1.0_00417.1	1052	395068	0	738	739	99	+	313	1051	376215	376953
UN13714	Rsa1.0_00111.1	1328	390088	0	407	421	96	−	1	420	139323	139742
UN13715	Rsa1.0_00001.1	1796	363028	0	1772	1795	98	+	2	1795	280765	282559
UN13716	Rsa1.0_02319.1	1485	32580	0	784	806	97	+	680	1483	2476	3278
UN13717	Rsa1.0_00462.1	1367	82288	0	404	417	96	+	5	419	36788	37202
UN13718	Rsa1.0_00013.1	1830	292129	0	823	826	99	+	1005	1830	155315	156140
UN13719	Rsa1.0_01212.1	1222	50622	0	1180	1221	96	−	1	1221	46722	47939
UN13720	Rsa1.0_02085.1	1286	35198	1.00E-142	280	286	97	+	1000	1285	17654	17935
UN13721	Rsa1.0_06354.1	903	11186	1.00E-178	486	536	90	+	336	870	9549	10064
UN13722	Rsa1.0_11152.1	539	5602	2.00E-87	223	242	92	+	295	536	5016	5249
UN13723	Rsa1.0_01234.1	988	50183	0	513	514	99	+	449	962	8898	9411
UN13724	Rsa1.0_02081.1	1816	35235	0	1120	1127	99	+	688	1814	29049	30175
UN13725	Rsa1.0_02647.1	695	29120	0	672	674	99	+	6	679	25168	25840
UN13726	Rsa1.0_58354.1	769	392	1.00E-180	343	349	98	+	425	768	1	349

UN13727	Rsa1.0_00550.1	597	210790	0	586	598	97	-	3	595	65628	66225
UN13728	Rsa1.0_00265.1	1748	220635	0	614	658	93	-	698	1355	175101	175758
UN13729	Rsa1.0_00416.1	1054	86772	0	432	436	99	-	2	437	81176	81608
UN13730	Rsa1.0_05070.1	1006	14933	0	814	823	98	-	1	823	1434	2256
UN13731	Rsa1.0_02201.1	1306	33981	1.00E-149	272	273	99	+	1034	1306	31757	32029
UN13732	Rsa1.0_01540.1	1294	151890	0	1236	1302	94	+	4	1294	142472	143763
UN13733	Rsa1.0_01468.1	1307	45146	0	440	441	99	-	481	921	34289	34729
UN13734	Rsa1.0_08170.1	935	7799	0	921	937	98	+	1	935	1623	2557
UN13735	Rsa1.0_03776.1	967	20926	0	520	555	93	-	2	549	80	625
UN13736	Rsa1.0_01382.1	1459	46877	1.00E-160	342	358	95	-	1103	1459	37445	37801
UN13737	Rsa1.0_00301.1	956	365315	0	468	500	93	+	460	956	215181	215669
UN13738	Rsa1.0_00220.1	1139	192936	0	414	429	96	+	400	822	16394	16819
UN13739	Rsa1.0_07756.1	619	8634	1.00E-116	221	224	98	-	396	619	6499	6722
UN13740	Rsa1.0_00484.1	931	203294	0	463	480	96	-	1	480	19530	20005
UN13741	Rsa1.0_04667.1	845	16460	0	658	667	98	+	4	669	13322	13983
UN13742	Rsa1.0_11893.1	1659	5379	0	504	507	99	+	1081	1587	3929	4435
UN13743	Rsa1.0_00739.1	1595	66155	0	481	505	95	-	617	1119	51817	52312
UN13744	Rsa1.0_00383.1	681	398431	0	619	630	98	-	41	670	186021	186649
UN13745	Rsa1.0_00017.1	1220	752458	0	531	531	100	+	362	892	313714	314244
UN13746	Rsa1.0_00652.1	1173	70089	0	1155	1173	98	-	1	1172	26210	27382
UN13747	Rsa1.0_00413.1	966	86921	0	771	833	92	-	3	827	48011	48818
UN13748	Rsa1.0_26066.1	1243	908	0	901	921	97	-	13	933	1	908
UN13749	Rsa1.0_03447.1	582	22857	1.00E-147	265	265	100	-	317	581	21380	21644
UN13750	Rsa1.0_00005.1	914	353093	0	898	912	98	+	1	912	199701	200612
UN13751	Rsa1.0_01389.1	1000	46716	0	382	390	97	-	606	995	9776	10165
UN13752	Rsa1.0_00055.1	770	520193	1.00E-143	265	266	99	+	505	769	202154	202419
UN13753	Rsa1.0_05134.1	1137	143181	0	712	739	96	+	9	737	5686	6420
UN13754	Rsa1.0_00040.1	1665	324196	4.00E-91	171	171	100	+	511	681	243684	243854
UN13755	Rsa1.0_03960.1	1463	19864	1.00E-108	277	302	91	-	1	289	10400	10701
UN13756	Rsa1.0_01515.1	3197	170864	0	1285	1349	95	-	299	1631	142791	144127
UN13757	Rsa1.0_02865.1	546	27133	0	430	434	99	+	113	546	1804	2237
UN13758	Rsa1.0_00127.1	1790	127446	0	496	500	99	-	1	500	64912	65411
UN13759	Rsa1.0_00190.1	997	318240	1.00E-150	284	289	98	-	645	933	180338	180626
UN13760	Rsa1.0_01963.1	858	36719	0	424	436	97	-	425	856	31868	32302
UN13761	Rsa1.0_05427.1	1045	13790	0	393	423	92	-	650	1044	12596	13018
UN13762	Rsa1.0_00069.1	1076	403561	0	428	433	98	+	2	433	169601	170031
UN13763	Rsa1.0_00630.1	1346	71281	0	426	426	100	-	202	627	62354	62779
UN13764	Rsa1.0_00082.1	895	237584	1.00E-148	270	271	99	-	107	377	54496	54766
UN13765	Rsa1.0_06604.1	1817	10681	0	394	406	97	-	1057	1462	8849	9252

UN13766	Rsa1.0_01836.1	870	38503	1.00E-148	314	327	96	-	545	870	5091	5416
UN13767	Rsa1.0_00215.1	796	201775	0	379	391	96	-	3	392	24023	24412
UN13768	Rsa1.0_00053.1	1365	263459	0	455	461	98	+	3	461	31999	32458
UN13769	Rsa1.0_00473.1	1368	212204	1.00E-107	251	267	94	-	20	282	73604	73864
UN13770	Rsa1.0_04405.1	1167	17508	0	1120	1170	95	+	3	1166	3076	4226
UN13771	Rsa1.0_39987.1	908	562	0	362	362	100	+	394	755	201	562
UN13772	Rsa1.0_05161.1	1158	14631	0	340	345	98	+	20	364	8351	8695
UN13773	Rsa1.0_02955.1	813	26484	2.00E-73	274	318	86	-	1	297	21585	21894
UN13774	Rsa1.0_03191.1	1812	145762	0	1681	1720	97	+	94	1812	136988	138707
UN13775	Rsa1.0_06438.1	651	11017	0	445	476	93	-	41	511	1531	2002
UN13776	Rsa1.0_01252.1	822	49847	1.00E-112	215	218	98	-	370	587	19015	19232
UN13777	Rsa1.0_02157.1	883	149652	5.00E-80	155	156	99	-	6	161	134731	134886
UN13778	Rsa1.0_01947.1	664	36977	0	384	394	97	+	12	403	23170	23560
UN13779	Rsa1.0_01397.1	1446	183276	0	563	584	96	+	863	1444	11118	11700
UN13780	Rsa1.0_03428.1	1424	22987	0	519	522	99	+	29	548	14396	14917
UN13781	Rsa1.0_01000.1	1236	57140	0	489	495	98	-	747	1236	40270	40763
UN13782	Rsa1.0_00001.1	858	363028	0	591	597	98	+	262	858	210947	211540
UN13783	Rsa1.0_01201.1	1090	50856	1.00E-153	305	314	97	-	5	318	4	316
UN13784	Rsa1.0_00144.1	2231	120708	0	683	695	98	+	401	1095	8173	8867
UN13785	Rsa1.0_05694.1	903	12903	1.00E-118	219	220	99	+	1	220	7117	7336
UN13786	Rsa1.0_00047.1	1276	168582	0	431	451	95	+	834	1276	120050	120500
UN13787	Rsa1.0_03554.1	1284	22137	0	561	583	96	+	703	1284	20026	20596
UN13788	Rsa1.0_00137.1	1137	122457	1.00E-173	309	309	100	+	2	310	81318	81626
UN13789	Rsa1.0_06482.1	904	10937	0	585	611	95	+	256	866	8153	8752
UN13790	Rsa1.0_03900.1	1040	133221	1.00E-151	321	335	95	+	680	1014	3917	4249
UN13791	Rsa1.0_07344.1	1971	9528	0	621	679	91	+	666	1335	525	1195
UN13792	Rsa1.0_02557.1	1334	30127	0	704	775	90	-	2	772	14219	14983
UN13793	Rsa1.0_02500.1	1136	30768	1.00E-155	314	324	96	+	814	1133	24051	24374
UN13794	Rsa1.0_00148.1	947	120234	0	504	552	91	-	393	944	87155	87705
UN13795	Rsa1.0_03253.1	1397	24321	0	579	581	99	-	440	1020	23060	23640
UN13796	Rsa1.0_00707.1	978	190112	1.00E-131	273	281	97	-	695	975	181039	181315
UN13797	Rsa1.0_00231.1	1993	105381	1.00E-161	291	292	99	-	263	554	32328	32619
UN13798	Rsa1.0_00440.1	898	84723	1.00E-169	352	369	95	-	528	896	25036	25398
UN13799	Rsa1.0_01022.1	822	56606	0	396	407	97	-	415	821	36882	37278
UN13800	Rsa1.0_03391.1	1663	23251	0	791	824	95	+	2	812	2328	3148
UN13801	Rsa1.0_00931.1	1072	59210	0	365	377	96	+	701	1072	55356	55732
UN13802	Rsa1.0_01335.1	1402	47894	0	477	478	99	-	542	1019	8065	8542
UN13803	Rsa1.0_00130.1	1442	125737	0	477	489	97	+	956	1438	117675	118163
UN13804	Rsa1.0_00070.1	1700	150321	0	400	400	100	-	885	1284	59705	60104

UN13805	Rsa1.0_03698.1	930	21302	0	376	394	95	+	2	389	6367	6760
UN13806	Rsa1.0_02457.1	1439	31208	1.00E-163	312	318	98	+	1121	1436	12120	12437
UN13807	Rsa1.0_00484.1	1497	203294	0	616	617	99	+	550	1166	63056	63672
UN13808	Rsa1.0_00353.1	1015	92242	1.00E-96	214	223	95	+	1	222	55708	55928
UN13809	Rsa1.0_31415.1	1392	728	2.33E-156	279	284	98	−	1094	1377	1	284
UN13810	Rsa1.0_03264.1	1190	145959	0	521	521	100	−	2	522	134518	135038
UN13811	Rsa1.0_05217.1	1022	142938	0	441	447	98	+	1	447	129767	130211
UN13812	Rsa1.0_07894.1	1424	8347	0	844	878	96	−	550	1423	5534	6406
UN13813	Rsa1.0_00481.1	1189	81316	0	420	445	94	+	71	514	71997	72427
UN13814	Rsa1.0_01995.1	1062	36347	0	502	503	99	+	560	1062	22162	22663
UN13815	Rsa1.0_00364.1	1278	90951	0	561	572	98	−	500	1071	77149	77717
UN13816	Rsa1.0_00529.1	1216	77487	0	394	411	95	−	1	408	75987	76395
UN13817	Rsa1.0_00042.1	960	171507	4.00E-87	219	234	93	+	1	231	88317	88548
UN13818	Rsa1.0_01975.1	1040	36578	0	415	422	98	+	2	423	13994	14415
UN13819	Rsa1.0_04225.1	1631	18400	0	444	445	99	+	741	1185	10237	10681
UN13820	Rsa1.0_00013.1	1250	292129	0	506	511	99	+	6	516	114283	114793
UN13821	Rsa1.0_00090.1	1631	273859	0	1132	1156	97	+	194	1349	231858	232998
UN13822	Rsa1.0_06444.1	1150	11008	0	409	413	99	−	1	412	6961	7373
UN13823	Rsa1.0_00354.1	1369	232005	0	471	497	94	+	9	493	13044	13540
UN13824	Rsa1.0_01605.1	735	42442	0	509	517	98	−	224	733	13424	13940
UN13825	Rsa1.0_00015.1	1358	561803	1.00E-180	420	449	93	−	11	456	550661	551100
UN13826	Rsa1.0_03738.1	1157	21071	1.00E-138	297	311	95	−	3	310	12767	13075
UN13827	Rsa1.0_01899.1	1245	37474	0	336	337	99	−	1	337	35128	35464
UN13828	Rsa1.0_00382.1	979	251946	0	620	627	98	+	351	977	111146	111771
UN13829	Rsa1.0_04625.1	1430	16613	0	603	615	98	−	277	891	14784	15398
UN13830	Rsa1.0_00122.1	1093	265937	0	1079	1090	98	+	3	1091	239090	240178
UN13831	Rsa1.0_01799.1	1112	39127	0	1093	1114	98	+	2	1112	31541	32654
UN13832	Rsa1.0_00169.1	990	274298	1.00E-167	344	357	96	−	2	357	31558	31911
UN13833	Rsa1.0_00618.1	1478	72195	0	392	405	96	−	1076	1478	3276	3677
UN13834	Rsa1.0_00399.1	1714	242951	1.00E-167	314	319	98	+	1	319	51523	51841
UN13835	Rsa1.0_01062.1	1244	55325	1.00E-139	257	259	99	+	986	1244	38724	38982
UN13836	Rsa1.0_10507.1	1836	5822	0	879	903	97	−	424	1313	2792	3694
UN13837	Rsa1.0_03290.1	1270	24011	1.00E-165	336	349	96	−	925	1270	7207	7555
UN13838	Rsa1.0_06784.1	1102	10359	0	506	509	99	+	1	509	39	547
UN13839	Rsa1.0_00010.1	1015	294827	4.00E-81	277	314	88	−	698	1001	135527	135837
UN13840	Rsa1.0_03631.1	1351	21647	0	437	449	97	+	848	1293	2780	3228
UN13841	Rsa1.0_11822.1	668	5397	0	407	407	100	+	261	667	4170	4576
UN13842	Rsa1.0_03556.1	1677	22121	0	700	700	100	−	346	1045	14573	15272
UN13843	Rsa1.0_07645.1	790	8949	1.00E-178	325	328	99	−	3	330	4425	4752

UN13844	Rsa1.0_00124.1	667	262503	1.00E-148	290	296	97	+	1	295	14152	14444
UN13845	Rsa1.0_01530.1	1159	43820	1.00E-118	230	233	98	−	20	251	5090	5322
UN13846	Rsa1.0_00166.1	590	240912	0	507	527	96	−	67	590	131072	131598
UN13847	Rsa1.0_02125.1	863	34775	1.00E-145	262	262	100	+	602	863	24927	25188
UN13848	Rsa1.0_03195.1	849	24673	0	460	473	97	−	1	473	8990	9459
UN13849	Rsa1.0_00237.1	583	235974	0	381	395	96	+	189	583	58852	59246
UN13850	Rsa1.0_00681.1	1786	68779	1.00E-180	408	434	94	+	15	432	42433	42866
UN13851	Rsa1.0_00959.1	1465	58202	0	365	365	100	−	753	1117	1503	1867
UN13852	Rsa1.0_00863.1	883	254535	1.00E-172	371	393	94	−	4	396	213547	213933
UN13853	Rsa1.0_03860.1	1501	20407	0	479	514	93	+	4	495	15804	16316
UN13854	Rsa1.0_43878.1	730	512	2.00E-23	63	65	96	−	177	241	43	107
UN13855	Rsa1.0_00285.1	1233	149638	1.00E-152	301	310	97	−	20	329	56337	56646
UN13856	Rsa1.0_00132.1	1115	124299	0	355	357	99	−	759	1115	108386	108742
UN13857	Rsa1.0_00891.1	880	60724	0	457	465	98	+	26	490	32887	33348
UN13858	Rsa1.0_00085.1	1016	142226	1.00E-152	294	299	98	−	643	940	62524	62819
UN13859	Rsa1.0_21688.1	1159	1181	0	583	599	97	−	560	1158	583	1181
UN13860	Rsa1.0_04088.1	1212	19150	0	498	504	98	−	1	504	18429	18929
UN13861	Rsa1.0_00503.1	909	79468	0	456	456	100	+	454	909	57977	58432
UN13862	Rsa1.0_00180.1	1531	114581	1.00E-160	333	349	95	+	114	456	10716	11058
UN13863	Rsa1.0_04097.1	1480	19090	0	671	697	96	+	789	1478	6602	7298
UN13864	Rsa1.0_00089.1	1610	406142	1.00E-168	337	348	96	+	1251	1597	231223	231570
UN13865	Rsa1.0_42296.1	777	531	1.00E-125	253	259	97	+	461	719	2	258
UN13866	Rsa1.0_00178.1	1061	114861	0	404	409	98	+	1	408	84562	84969
UN13867	Rsa1.0_00694.1	809	68074	8.00E-91	190	196	96	+	3	198	1462	1654
UN13868	Rsa1.0_03416.1	1809	23112	0	345	347	99	+	1453	1797	16130	16476
UN13869	Rsa1.0_48565.1	1333	466	0	349	349	100	−	983	1331	91	439
UN13870	Rsa1.0_01566.1	1326	43134	0	595	606	98	+	658	1263	34060	34665
UN13871	Rsa1.0_00097.1	1162	138858	2.00E-89	177	180	98	+	129	308	119408	119587
UN13872	Rsa1.0_12152.1	561	5300	0	476	477	99	+	3	479	4824	5300
UN13873	Rsa1.0_00340.1	1563	305782	0	401	424	94	−	878	1301	191839	192262
UN13874	Rsa1.0_00786.1	944	64285	1.00E-102	198	201	98	+	362	562	41596	41796
UN13875	Rsa1.0_00134.1	973	390219	0	340	343	99	−	3	344	157266	157607
UN13876	Rsa1.0_00914.1	1010	59880	0	359	366	98	+	1	360	21661	22026
UN13877	Rsa1.0_00104.1	1301	368722	0	767	774	99	+	484	1256	257026	257799
UN13878	Rsa1.0_00145.1	1383	261008	0	1348	1381	97	−	1	1381	44389	45766
UN13879	Rsa1.0_01273.1	1693	49464	0	1152	1184	97	+	466	1644	8684	9866
UN13880	Rsa1.0_00389.1	1052	88795	0	546	554	98	+	497	1050	18741	19294
UN13881	Rsa1.0_00601.1	909	177045	0	392	402	97	−	513	909	26095	26496
UN13882	Rsa1.0_00420.1	1526	86496	2.33E-156	277	279	99	−	2	278	45120	45398

UN13883	Rsa1.0_00693.1	1223	161311	0	1096	1121	97	-	103	1223	41355	42475
UN13884	Rsa1.0_00378.1	1111	89892	1.00E-172	307	307	100	+	3	309	62147	62453
UN13885	Rsa1.0_00451.1	1232	322533	0	430	463	92	+	3	450	300468	300930
UN13886	Rsa1.0_00152.1	1481	257730	0	851	853	99	+	629	1481	59089	59941
UN13887	Rsa1.0_01984.1	1750	36491	0	392	399	98	-	78	476	30118	30516
UN13888	Rsa1.0_00417.1	722	395068	0	350	356	98	+	355	710	14474	14829
UN13889	Rsa1.0_01947.1	721	36977	0	392	405	96	-	293	694	23163	23567
UN13890	Rsa1.0_06740.1	1446	10439	0	527	538	97	-	2	538	8581	9118
UN13891	Rsa1.0_06273.1	921	11346	0	799	850	94	-	68	917	2974	3810
UN13892	Rsa1.0_07471.1	1522	9307	0	910	935	97	+	590	1520	1	935
UN13893	Rsa1.0_11160.1	1077	5600	0	349	353	98	-	3	352	134	486
UN13894	Rsa1.0_00406.1	1117	87398	0	371	372	99	-	3	374	53293	53664
UN13895	Rsa1.0_05570.1	1158	13301	0	558	576	96	-	61	634	12647	13218
UN13896	Rsa1.0_01170.1	1232	161131	1.00E-130	254	260	97	+	24	283	130486	130745
UN13897	Rsa1.0_00015.1	782	561803	1.00E-142	268	272	98	-	509	780	480988	481259
UN13898	Rsa1.0_06603.1	1045	10682	1.00E-84	160	160	100	+	108	267	3481	3640
UN13899	Rsa1.0_00139.1	959	357786	1.00E-130	246	249	98	+	1	249	290698	290946
UN13900	Rsa1.0_13828.1	785	4254	0	498	519	95	+	3	512	1999	2516
UN13901	Rsa1.0_00337.1	730	143174	1.00E-164	300	301	99	+	429	728	2366	2666
UN13902	Rsa1.0_01648.1	804	41635	0	541	551	98	+	99	649	15739	16289
UN13903	Rsa1.0_04273.1	1039	18144	2.33E-156	273	275	99	+	766	1039	12147	12421
UN13904	Rsa1.0_05243.1	728	14399	0	390	394	98	-	333	726	5705	6098
UN13905	Rsa1.0_00026.1	2206	327835	0	1111	1119	99	-	75	1189	176398	177516
UN13906	Rsa1.0_35192.1	742	643	1.00E-122	241	246	97	-	3	247	40	285
UN13907	Rsa1.0_00052.1	892	162771	1.00E-165	309	311	99	+	584	892	58551	58861
UN13908	Rsa1.0_00114.1	1832	131330	1.00E-174	342	351	97	-	588	938	44560	44909
UN13909	Rsa1.0_00222.1	1379	172263	1.00E-123	239	244	97	-	581	824	137688	137931
UN13910	Rsa1.0_01050.1	1232	55651	0	581	595	97	+	1	595	10695	11285
UN13911	Rsa1.0_03633.1	995	21644	1.00E-168	306	308	99	+	686	993	18297	18604
UN13912	Rsa1.0_00096.1	1548	198773	0	372	372	100	+	1175	1546	138020	138391
UN13913	Rsa1.0_03647.1	1602	21597	0	815	840	97	-	2	837	2776	3613
UN13914	Rsa1.0_01154.1	1209	52249	0	556	576	96	+	3	577	9022	9597
UN13915	Rsa1.0_00546.1	551	76523	1.00E-177	354	366	96	-	1	365	11385	11750
UN13916	Rsa1.0_01743.1	881	39926	0	456	466	97	-	417	879	6748	7212
UN13917	Rsa1.0_00912.1	1388	59923	1.00E-173	308	308	100	+	1	308	36975	37282
UN13918	Rsa1.0_00448.1	1919	84048	0	1396	1479	94	-	2	1480	7460	8925
UN13919	Rsa1.0_05319.1	975	14158	1.00E-130	288	305	94	+	18	318	398	702
UN13920	Rsa1.0_00991.1	849	143101	0	463	479	96	+	49	524	133482	133957
UN13921	Rsa1.0_01168.1	535	153765	0	364	373	97	+	160	532	44255	44627

UN13922	Rsa1.0_01799.1	1423	39127	0	590	591	99	-	833	1423	2450	3040
UN13923	Rsa1.0_03382.1	1721	23299	1.00E-180	326	328	99	+	31	358	5210	5537
UN13924	Rsa1.0_01948.1	1257	36966	0	533	538	99	+	72	609	15111	15648
UN13925	Rsa1.0_00397.1	786	88189	1.00E-121	261	273	95	+	514	786	54834	55105
UN13926	Rsa1.0_00657.1	1308	69858	1.00E-148	311	325	95	+	3	327	37501	37818
UN13927	Rsa1.0_00230.1	1011	105389	1.00E-145	300	310	96	+	683	991	5859	6167
UN13928	Rsa1.0_00734.1	1015	66508	1.00E-116	215	216	99	+	65	280	25377	25592
UN13929	Rsa1.0_05818.1	974	12544	0	398	398	100	-	575	972	6711	7108
UN13930	Rsa1.0_02991.1	753	26178	0	438	438	100	-	3	440	21112	21549
UN13931	Rsa1.0_02400.1	1297	31750	0	628	633	99	-	3	631	9576	10208
UN13932	Rsa1.0_14356.1	872	3485	0	467	480	97	-	395	872	3009	3485
UN13933	Rsa1.0_00366.1	593	90880	0	590	593	99	-	1	591	25888	26480
UN13934	Rsa1.0_01923.1	1019	37233	1.00E-158	343	361	95	-	1	358	36457	36816
UN13935	Rsa1.0_01281.1	1079	49313	1.00E-145	264	265	99	-	815	1079	33489	33753
UN13936	Rsa1.0_00120.1	1376	277288	0	463	475	97	+	898	1370	96041	96513
UN13937	Rsa1.0_00599.1	1296	73418	0	438	471	92	+	8	477	52966	53420
UN13938	Rsa1.0_00078.1	1397	175053	0	445	445	100	-	226	670	38368	38812
UN13939	Rsa1.0_01353.1	1431	47351	0	799	805	99	-	3	804	37255	38059
UN13940	Rsa1.0_00296.1	1220	97854	0	971	1038	93	+	180	1217	44816	45851
UN13941	Rsa1.0_00662.1	1074	192183	0	411	433	94	+	647	1071	2200	2630
UN13942	Rsa1.0_02976.1	622	26315	1.00E-100	197	201	98	+	373	573	18887	19087
UN13943	Rsa1.0_02177.1	952	34262	3.00E-75	168	176	95	+	306	481	14545	14720
UN13944	Rsa1.0_00268.1	1505	101470	0	444	450	98	-	1056	1505	43077	43526
UN13945	Rsa1.0_08402.1	1353	7417	1.00E-166	302	304	99	+	447	750	5352	5655
UN13946	Rsa1.0_02206.1	1169	33922	1.00E-170	303	303	100	+	864	1166	19902	20204
UN13947	Rsa1.0_00139.1	2003	357786	0	1125	1173	95	-	827	1993	66423	67583
UN13948	Rsa1.0_10264.1	1101	5913	0	375	382	98	+	722	1101	605	986
UN13949	Rsa1.0_00673.1	2998	69325	1.00E-153	291	296	98	-	2341	2636	47441	47736
UN13950	Rsa1.0_00381.1	953	89718	1.00E-142	257	257	100	+	695	951	62649	62905
UN13951	Rsa1.0_01601.1	1158	42519	1.00E-124	242	246	98	+	2	246	17151	17396
UN13952	Rsa1.0_00797.1	961	63959	0	565	603	93	+	3	598	51699	52289
UN13953	Rsa1.0_04028.1	1075	19493	1.00E-110	209	211	99	-	246	456	3399	3609
UN13954	Rsa1.0_01636.1	830	41863	0	327	328	99	-	503	830	34093	34420
UN13955	Rsa1.0_08293.1	1389	7593	0	553	565	97	-	436	1000	2270	2834
UN13956	Rsa1.0_00091.1	1892	139699	0	482	495	97	-	1399	1892	136921	137413
UN13957	Rsa1.0_00017.1	1311	752458	1.00E-151	336	354	94	+	959	1311	578918	579267
UN13958	Rsa1.0_00880.1	515	61271	0	506	512	98	-	2	513	40535	41046
UN13959	Rsa1.0_00524.1	994	169613	0	449	457	98	+	537	993	129773	130229
UN13960	Rsa1.0_01559.1	702	43262	1.00E-103	262	282	92	-	34	314	2268	2547

UN13961	Rsa1.0_00812.1	613	63233	1.00E-172	309	310	99	+	4	313	17146	17455
UN13962	Rsa1.0_04218.1	840	18423	0	477	484	98	+	359	840	8963	9445
UN13963	Rsa1.0_01512.1	792	44234	0	486	511	95	+	282	792	18084	18592
UN13964	Rsa1.0_00823.1	1188	62889	0	834	835	99	−	1	835	45658	46492
UN13965	Rsa1.0_07208.1	1347	9719	0	548	558	98	−	790	1347	7437	7993
UN13966	Rsa1.0_00505.1	538	79323	0	388	389	99	+	146	534	13080	13468
UN13967	Rsa1.0_00954.1	1078	189536	1.00E-81	161	163	98	+	273	435	37320	37482
UN13968	Rsa1.0_08048.1	1031	137630	0	372	384	96	−	651	1031	135285	135667
UN13969	Rsa1.0_00551.1	1448	76227	0	524	530	98	−	920	1448	54764	55293
UN13970	Rsa1.0_01768.1	1379	138463	0	377	381	98	+	406	786	103736	104116
UN13971	Rsa1.0_00111.1	1333	390088	0	473	481	98	−	230	710	383729	384207
UN13972	Rsa1.0_01516.1	7762	44196	0	2199	2330	94	+	3888	6204	25718	28029
UN13973	Rsa1.0_02670.1	798	28887	1.00E-153	347	372	93	+	444	798	1836	2206
UN13974	Rsa1.0_00029.1	958	272992	0	559	559	100	+	4	562	246766	247324
UN13975	Rsa1.0_08196.1	2403	7753	2.00E-84	205	217	94	+	1615	1827	605	819
UN13976	Rsa1.0_00441.1	1140	84607	2.33E-156	303	316	95	−	1	307	69639	69954
UN13977	Rsa1.0_04311.1	878	17984	0	723	736	98	−	135	870	1418	2152
UN13978	Rsa1.0_00605.1	1190	73199	0	609	620	98	−	3	620	28192	28811
UN13979	Rsa1.0_01806.1	936	39021	1.00E-101	187	187	100	−	74	260	32010	32196
UN13980	Rsa1.0_09347.1	857	6437	0	508	510	99	+	1	510	4886	5395
UN13981	Rsa1.0_30957.1	1582	740	0	479	500	95	+	19	516	1	500
UN13982	Rsa1.0_06553.1	1220	10795	0	525	545	96	+	597	1134	5503	6046
UN13983	Rsa1.0_03800.1	1838	20768	0	528	533	99	+	455	987	18375	18907
UN13984	Rsa1.0_01171.1	965	51752	1.00E-102	237	246	96	+	11	253	14998	15241
UN13985	Rsa1.0_02633.1	936	29315	1.00E-160	391	424	92	−	516	927	361	771
UN13986	Rsa1.0_01846.1	1411	38367	0	744	749	99	−	182	930	35711	36459
UN13987	Rsa1.0_01515.1	2601	170864	0	558	596	93	−	1811	2405	140858	141453
UN13988	Rsa1.0_00466.1	1140	82132	0	404	422	95	+	187	608	63363	63783
UN13989	Rsa1.0_02545.1	1185	30286	0	520	542	95	−	649	1185	13581	14116
UN13990	Rsa1.0_01065.1	719	55264	0	529	542	97	−	180	719	30865	31396
UN13991	Rsa1.0_01302.1	708	48907	0	363	370	98	−	103	472	4012	4381
UN13992	Rsa1.0_05161.1	910	14631	1.00E-136	252	254	99	+	103	356	4	257
UN13993	Rsa1.0_00060.1	1388	502678	1.00E-173	409	431	94	+	969	1388	398264	398694
UN13994	Rsa1.0_00812.1	1033	63233	0	377	382	98	+	2	383	1591	1972
UN13995	Rsa1.0_01482.1	913	44855	0	898	903	99	−	4	906	11984	12886
UN13996	Rsa1.0_00425.1	523	147080	1.00E-178	371	389	95	−	1	389	24733	25121
UN13997	Rsa1.0_00105.1	1409	134138	9.00E-89	173	175	98	−	464	638	73020	73194
UN13998	Rsa1.0_03380.1	1290	23320	0	418	430	97	+	3	432	4190	4615
UN13999	Rsa1.0_02179.1	1223	34188	0	509	518	98	−	185	702	24265	24782

UN14000	Rsa1.0_01668.1	846	41299	2.33E-156	284	291	97	+	511	801	40399	40689
UN14001	Rsa1.0_00387.1	1079	495407	0	508	521	97	−	561	1079	341781	342297
UN14002	Rsa1.0_00180.1	992	114581	1.00E-179	324	326	99	−	2	327	42152	42477
UN14003	Rsa1.0_00240.1	944	104175	0	655	666	98	−	280	937	46807	47471
UN14004	Rsa1.0_00190.1	1489	318240	1.00E-163	335	347	96	−	1143	1489	162006	162344
UN14005	Rsa1.0_00119.1	1116	467509	1.00E-121	239	245	97	+	773	1017	97222	97466
UN14006	Rsa1.0_00467.1	1238	156542	0	1177	1217	96	+	1	1216	98486	99695
UN14007	Rsa1.0_00137.1	1062	122457	1.00E-172	325	332	97	−	2	326	5329	5660
UN14008	Rsa1.0_00095.1	1017	283384	0	642	654	98	+	363	1016	4713	5366
UN14009	Rsa1.0_03241.1	1251	24423	1.00E-171	320	324	98	+	3	324	3136	3459
UN14010	Rsa1.0_00213.1	849	108167	0	362	366	98	+	3	368	14589	14954
UN14011	Rsa1.0_10036.1	890	6030	1.00E-153	426	477	89	−	1	456	496	967
UN14012	Rsa1.0_00206.1	946	109040	1.00E-163	306	310	98	−	639	946	45059	45368
UN14013	Rsa1.0_29537.1	1049	780	0	467	490	95	−	560	1049	199	688
UN14014	Rsa1.0_02628.1	1082	29369	1.00E-132	242	243	99	+	1	243	27702	27944
UN14015	Rsa1.0_00385.1	951	89160	1.00E-162	302	306	98	−	373	678	9032	9337
UN14016	Rsa1.0_00178.1	1736	114861	0	806	830	97	+	911	1734	5884	6709
UN14017	Rsa1.0_01517.1	891	44170	1.00E-136	351	381	92	−	485	851	29482	29855
UN14018	Rsa1.0_02848.1	1039	27329	0	1024	1029	99	+	11	1039	16975	18003
UN14019	Rsa1.0_02204.1	1049	33930	1.00E-153	343	361	95	+	4	362	20564	20915
UN14020	Rsa1.0_00065.1	1579	454616	1.00E-105	204	206	99	−	1283	1487	155644	155849
UN14021	Rsa1.0_00417.1	911	395068	1.00E-122	246	253	97	−	544	793	228530	228782
UN14022	Rsa1.0_00168.1	999	115870	1.00E-175	376	396	94	−	571	963	113976	114364
UN14023	Rsa1.0_05784.1	1027	12643	1.00E-167	405	424	95	+	618	1026	1645	2068
UN14024	Rsa1.0_03100.1	1592	25411	0	721	723	99	+	606	1326	1871	2593
UN14025	Rsa1.0_03361.1	798	23456	0	689	699	98	−	98	796	3196	3893
UN14026	Rsa1.0_02518.1	533	30586	1.00E-51	119	124	95	+	268	391	25939	26062
UN14027	Rsa1.0_01096.1	1385	54266	0	431	454	94	−	227	680	2669	3122
UN14028	Rsa1.0_02565.1	1563	30046	1.00E-149	299	310	96	+	1	302	25176	25485
UN14029	Rsa1.0_00989.1	1095	57494	1.00E-179	328	330	99	+	4	331	10671	11000
UN14030	Rsa1.0_06492.1	1028	10913	0	401	410	97	+	41	447	6336	6745
UN14031	Rsa1.0_00663.1	1061	164969	0	517	520	99	+	542	1061	100742	101261
UN14032	Rsa1.0_02241.1	1022	33520	1.00E-170	334	343	97	+	2	344	10272	10613
UN14033	Rsa1.0_04888.1	1265	15595	0	765	816	93	+	469	1265	1757	2571
UN14034	Rsa1.0_00152.1	752	257730	2.00E-48	152	168	90	+	4	167	116224	116390
UN14035	Rsa1.0_00336.1	1420	445051	0	554	561	98	+	2	562	219222	219780
UN14036	Rsa1.0_01679.1	1314	146876	0	1302	1311	99	−	4	1314	118982	120292
UN14037	Rsa1.0_05004.1	1394	15172	1.00E-117	214	214	100	−	704	917	12015	12228
UN14038	Rsa1.0_00630.1	1385	71281	0	830	833	99	−	1	833	60102	60934

UN14039	Rsa1.0_01576.1	711	42923	0	327	327	100	+	385	711	22319	22645
UN14040	Rsa1.0_04470.1	1179	17204	1.00E-142	311	329	94	+	851	1179	4468	4787
UN14041	Rsa1.0_04519.1	1261	17004	0	570	572	99	+	2	573	3922	4493
UN14042	Rsa1.0_00092.1	725	659932	1.00E-137	270	276	97	+	2	276	43189	43464
UN14043	Rsa1.0_00558.1	607	75819	0	597	608	98	−	1	606	43386	43986
UN14044	Rsa1.0_02676.1	931	28834	0	551	556	99	−	1	556	13630	14185
UN14045	Rsa1.0_21865.1	860	1168	0	537	546	98	−	158	703	3	548
UN14046	Rsa1.0_00341.1	1658	249309	1.00E-142	256	256	100	+	1403	1658	123448	123703
UN14047	Rsa1.0_02667.1	1945	28910	0	428	444	96	−	1	443	15395	15826
UN14048	Rsa1.0_02199.1	1389	33982	1.00E-131	241	242	99	+	1148	1389	13929	14170
UN14049	Rsa1.0_01634.1	1604	41865	1.00E-114	272	289	94	+	1317	1604	38465	38745
UN14050	Rsa1.0_00065.1	1427	454616	0	402	404	99	−	1015	1417	417264	417667
UN14051	Rsa1.0_00449.1	1510	83863	0	768	779	98	+	491	1269	67568	68346
UN14052	Rsa1.0_02072.1	817	35401	1.00E-114	260	274	94	−	541	812	18080	18353
UN14053	Rsa1.0_00225.1	1181	105892	0	503	504	99	+	167	670	43573	44076
UN14054	Rsa1.0_02186.1	818	34150	0	418	424	98	−	395	818	12067	12490
UN14055	Rsa1.0_01090.1	868	54385	0	420	444	94	+	3	446	3190	3626
UN14056	Rsa1.0_00790.1	1418	148693	1.00E-107	288	313	92	−	11	313	21937	22249
UN14057	Rsa1.0_20207.1	1030	1327	1.00E-119	233	237	98	−	1	237	161	396
UN14058	Rsa1.0_04272.1	1390	18144	0	420	421	99	+	966	1386	14816	15236
UN14059	Rsa1.0_01193.1	1537	51147	1.00E-149	281	285	98	−	64	348	15709	15993
UN14060	Rsa1.0_63077.1	1121	364	0	364	364	100	−	738	1101	1	364
UN14061	Rsa1.0_02939.1	1650	26595	0	752	810	92	−	12	797	18298	19101
UN14062	Rsa1.0_00666.1	876	69505	0	457	470	97	−	406	875	34781	35250
UN14063	Rsa1.0_01029.1	1909	256254	0	334	334	100	−	1	334	245893	246226
UN14064	Rsa1.0_00028.1	711	293984	0	691	704	98	−	8	709	64960	65662
UN14065	Rsa1.0_01696.1	957	301850	0	703	727	96	−	232	957	158936	159651
UN14066	Rsa1.0_46563.1	595	485	3.00E-65	134	135	99	+	56	189	1	135
UN14067	Rsa1.0_00368.1	1474	90669	1.00E-180	330	333	99	+	3	335	81664	81996
UN14068	Rsa1.0_00093.1	705	214962	0	694	696	99	+	3	697	135054	135749
UN14069	Rsa1.0_00016.1	1035	831256	1.00E-136	308	324	95	−	580	901	353891	354210
UN14070	Rsa1.0_02853.1	755	27281	1.00E-140	256	257	99	+	14	270	27025	27281
UN14071	Rsa1.0_00407.1	1067	191544	0	674	686	98	+	48	730	43063	43746
UN14072	Rsa1.0_14856.1	1207	3010	1.00E-166	371	396	93	−	344	739	1797	2192
UN14073	Rsa1.0_00060.1	839	502678	0	329	330	99	+	1	330	385886	386215
UN14074	Rsa1.0_00451.1	1304	322533	1.00E-56	122	125	97	−	740	864	294101	294225
UN14075	Rsa1.0_00762.1	1348	165246	0	418	433	96	+	655	1084	47997	48423
UN14076	Rsa1.0_01062.1	1274	55325	1.00E-136	255	257	99	−	3	259	38724	38978
UN14077	Rsa1.0_00558.1	804	75819	0	515	526	97	+	1	525	28298	28823

UN14078	Rsa1.0_00339.1	1362	222974	0	629	648	97	−	469	1116	89885	90532
UN14079	Rsa1.0_15452.1	1082	2597	0	970	1017	95	+	55	1070	1581	2597
UN14080	Rsa1.0_00252.1	1414	102760	0	472	478	98	−	936	1412	93559	94036
UN14081	Rsa1.0_00094.1	1330	139042	1.00E−100	216	226	95	+	302	521	134442	134667
UN14082	Rsa1.0_00822.1	1069	62891	1.00E−172	309	310	99	−	31	340	47514	47823
UN14083	Rsa1.0_03783.1	1234	20890	0	335	336	99	+	4	339	17823	18158
UN14084	Rsa1.0_01374.1	872	47047	1.00E−145	321	338	94	+	25	356	27445	27782
UN14085	Rsa1.0_01536.1	868	43747	0	462	462	100	+	407	868	28390	28851
UN14086	Rsa1.0_01688.1	964	40965	0	336	338	99	+	626	963	36521	36858
UN14087	Rsa1.0_02464.1	1106	31153	0	1067	1105	96	+	3	1106	11857	12961
UN14088	Rsa1.0_02934.1	770	26627	0	767	770	99	−	1	770	24954	25722
UN14089	Rsa1.0_00257.1	1692	102407	0	552	561	98	−	3	562	97010	97570
UN14090	Rsa1.0_01549.1	869	43543	0	797	829	96	−	17	840	31488	32312
UN14091	Rsa1.0_08645.1	1495	7087	0	754	788	95	−	448	1229	6133	6916
UN14092	Rsa1.0_00294.1	1310	144860	0	650	653	99	−	527	1179	138501	139153
UN14093	Rsa1.0_00197.1	1417	109714	1.00E−178	329	333	98	−	3	335	5607	5939
UN14094	Rsa1.0_05893.1	1437	12306	0	401	412	97	+	771	1182	8140	8551
UN14095	Rsa1.0_14124.1	810	3781	1.00E−178	319	320	99	−	1	320	239	558
UN14096	Rsa1.0_01188.1	1000	51362	0	335	338	99	+	663	1000	31071	31408
UN14097	Rsa1.0_00131.1	891	432233	1.00E−148	384	420	91	−	1	412	96212	96609
UN14098	Rsa1.0_01196.1	1420	51097	1.00E−130	246	248	99	+	1164	1410	26796	27043
UN14099	Rsa1.0_04890.1	1220	15591	1.00E−125	275	291	94	−	3	287	11260	11550
UN14100	Rsa1.0_00380.1	735	89748	1.00E−144	346	373	92	+	364	733	88066	88422
UN14101	Rsa1.0_00105.1	1610	134138	0	519	519	100	+	480	998	110004	110522
UN14102	Rsa1.0_00123.1	1864	325650	0	372	380	97	−	1	378	10390	10766
UN14103	Rsa1.0_00720.1	1267	66931	0	740	743	99	−	525	1267	14940	15682
UN14104	Rsa1.0_04027.1	515	19501	1.00E−115	288	308	93	+	209	513	6649	6952
UN14105	Rsa1.0_00949.1	850	58549	1.00E−169	323	329	98	+	522	850	39561	39887
UN14106	Rsa1.0_02082.1	2187	35228	0	341	342	99	−	1846	2187	29648	29989
UN14107	Rsa1.0_07894.1	1531	8347	0	858	877	97	+	5	878	5533	6406
UN14108	Rsa1.0_06807.1	857	10324	0	358	366	97	+	492	857	6307	6672
UN14109	Rsa1.0_01529.1	1163	43871	0	356	364	97	+	3	362	24125	24488
UN14110	Rsa1.0_00477.1	1133	81556	0	359	361	99	+	770	1130	38643	39002
UN14111	Rsa1.0_00221.1	1003	106972	0	478	511	93	+	502	1002	23856	24353
UN14112	Rsa1.0_01000.1	2016	57140	0	466	466	100	+	603	1068	6059	6524
UN14113	Rsa1.0_00216.1	803	197563	0	777	794	97	−	11	803	100207	100999
UN14114	Rsa1.0_02185.1	749	34150	0	736	737	99	−	4	740	27261	27997
UN14115	Rsa1.0_00313.1	638	96745	1.00E−142	259	260	99	−	379	638	24757	25016
UN14116	Rsa1.0_00176.1	1186	240984	0	619	623	99	−	567	1186	210440	211062

UN14117	Rsa1.0_00065.1	1327	454616	0	457	474	96	+	781	1254	235414	235887
UN14118	Rsa1.0_00018.1	1657	410899	0	1621	1656	97	+	1	1656	79100	80752
UN14119	Rsa1.0_01146.1	1020	163455	1.00E-101	202	207	97	−	279	485	24783	24989
UN14120	Rsa1.0_01538.1	1433	43733	1.00E-174	310	310	100	+	1	310	38509	38818
UN14121	Rsa1.0_00133.1	925	123768	0	921	924	99	−	1	924	84817	85740
UN14122	Rsa1.0_00087.1	1442	761073	1.00E-163	340	356	95	−	414	769	433300	433655
UN14123	Rsa1.0_01409.1	1868	151595	0	457	479	95	−	1388	1866	118264	118731
UN14124	Rsa1.0_02503.1	598	30709	1.00E-64	135	138	97	+	196	333	26114	26251
UN14125	Rsa1.0_00115.1	625	131321	0	618	627	98	−	3	625	63408	64031
UN14126	Rsa1.0_00079.1	1816	606987	0	996	1032	96	+	789	1816	38936	39952
UN14127	Rsa1.0_00347.1	933	93048	1.00E-157	298	302	98	+	326	627	68684	68984
UN14128	Rsa1.0_00483.1	940	81102	1.00E-141	327	344	95	+	572	910	51013	51353
UN14129	Rsa1.0_06939.1	791	10137	1.00E-175	318	319	99	+	472	790	4572	4889
UN14130	Rsa1.0_00111.1	709	390088	1.00E-152	404	445	90	+	270	709	75630	76052
UN14131	Rsa1.0_18035.1	1446	1685	0	689	709	97	−	731	1439	386	1094
UN14132	Rsa1.0_02020.1	1115	173211	0	546	552	98	+	564	1115	154385	154936
UN14133	Rsa1.0_00920.1	774	601703	2.00E-94	185	188	98	+	131	318	441092	441279
UN14134	Rsa1.0_03257.1	1115	24305	1.00E-130	250	253	98	+	1	252	13779	14031
UN14135	Rsa1.0_00684.1	1453	141701	0	649	671	96	−	433	1102	9545	10215
UN14136	Rsa1.0_05453.1	878	13696	0	381	386	98	−	494	878	11835	12220
UN14137	Rsa1.0_00012.1	1838	222148	0	388	390	99	−	809	1198	52405	52794
UN14138	Rsa1.0_20028.1	825	1350	0	610	629	96	−	196	823	722	1350
UN14139	Rsa1.0_04670.1	1914	16453	1.00E-179	355	366	96	−	371	735	5440	5805
UN14140	Rsa1.0_00867.1	1524	61635	0	532	547	97	+	20	566	32339	32884
UN14141	Rsa1.0_00200.1	718	162016	1.00E-168	332	340	97	+	380	718	104953	105291
UN14142	Rsa1.0_00037.1	1607	307838	1.00E-127	240	243	98	−	2	244	23059	23301
UN14143	Rsa1.0_06021.1	1433	11911	0	552	579	95	+	853	1431	5321	5899
UN14144	Rsa1.0_03951.1	622	19894	1.00E-134	287	302	95	−	241	537	10099	10400
UN14145	Rsa1.0_01879.1	1039	37748	0	383	393	97	+	645	1037	23051	23438
UN14146	Rsa1.0_00575.1	640	74791	0	404	413	97	+	3	415	62003	62415
UN14147	Rsa1.0_00089.1	1332	406142	1.00E-163	297	299	99	−	1	299	161534	161832
UN14148	Rsa1.0_15449.1	962	2599	0	937	957	97	−	10	962	1246	2194
UN14149	Rsa1.0_00477.1	956	81556	1.00E-129	242	243	99	−	474	715	43576	43818
UN14150	Rsa1.0_06311.1	782	11254	1.00E-134	263	269	97	−	514	780	1289	1557
UN14151	Rsa1.0_00273.1	1013	326186	1.00E-159	290	292	99	+	507	798	137142	137433
UN14152	Rsa1.0_01060.1	907	55368	0	570	596	95	+	323	907	42018	42613
UN14153	Rsa1.0_00096.1	1420	198773	1.00E-109	217	222	97	−	1177	1398	171703	171924
UN14154	Rsa1.0_00777.1	893	191865	1.00E-171	336	345	97	−	2	346	190877	191220
UN14155	Rsa1.0_07217.1	1647	9703	1.00E-147	373	411	90	+	727	1136	4498	4893

UN14156	Rsa1.0_02343.1	483	32374	1.00E-100	201	206	97	-	247	452	1047	1252
UN14157	Rsa1.0_04692.1	1137	16357	0	1046	1076	97	-	1	1057	12091	13163
UN14158	Rsa1.0_03415.1	782	23117	0	761	786	96	+	3	782	17879	18659
UN14159	Rsa1.0_10708.1	1616	5750	0	841	871	96	+	750	1616	1323	2188
UN14160	Rsa1.0_00007.1	941	239325	0	388	394	98	+	547	939	13415	13807
UN14161	Rsa1.0_00016.1	995	831256	1.00E-161	304	308	98	-	683	990	204584	204890
UN14162	Rsa1.0_00048.1	1097	167185	1.00E-144	306	319	95	+	779	1095	159151	159469
UN14163	Rsa1.0_04141.1	1739	18864	1.00E-141	294	306	96	+	1	299	15811	16116
UN14164	Rsa1.0_01942.1	823	37048	0	557	561	99	+	263	823	3757	4317
UN14165	Rsa1.0_00409.1	1128	146788	0	1012	1087	93	-	39	1121	145664	146716
UN14166	Rsa1.0_00630.1	1485	71281	0	592	593	99	+	4	595	59091	59683
UN14167	Rsa1.0_14339.1	783	3501	0	534	534	100	+	1	534	167	700
UN14168	Rsa1.0_00802.1	1592	63846	1.00E-145	281	286	98	+	650	934	16017	16302
UN14169	Rsa1.0_00684.1	1200	141701	0	697	724	96	-	2	725	9100	9822
UN14170	Rsa1.0_00009.1	637	232558	5.00E-79	156	158	98	-	405	562	207505	207662
UN14171	Rsa1.0_00644.1	1132	136881	1.00E-142	392	433	90	-	691	1105	62650	63077
UN14172	Rsa1.0_02079.1	1337	35306	0	524	544	96	+	794	1337	24546	25080
UN14173	Rsa1.0_00684.1	1831	141701	1.00E-136	253	255	99	+	1576	1830	24722	24976
UN14174	Rsa1.0_00009.1	1195	232558	0	460	479	96	+	1	473	29397	29875
UN14175	Rsa1.0_00303.1	1001	97468	1.00E-93	199	207	96	-	285	491	31704	31910
UN14176	Rsa1.0_00978.1	924	57739	0	353	354	99	+	571	924	20422	20775
UN14177	Rsa1.0_00016.1	1052	831256	0	443	443	100	+	2	444	353768	354210
UN14178	Rsa1.0_00032.1	1092	306326	0	602	635	94	-	440	1064	284767	285401
UN14179	Rsa1.0_00221.1	745	106972	0	497	497	100	-	32	528	8699	9195
UN14180	Rsa1.0_00097.1	735	138858	7.00E-14	98	117	83	-	229	345	106303	106419
UN14181	Rsa1.0_00272.1	1225	447836	1.00E-120	260	270	96	-	35	304	219273	219537
UN14182	Rsa1.0_00079.1	883	606987	0	459	478	96	+	405	880	66614	67086
UN14183	Rsa1.0_03463.1	1375	22724	0	528	535	98	-	841	1375	14080	14614
UN14184	Rsa1.0_03956.1	1274	19872	0	530	532	99	-	743	1274	17792	18323
UN14185	Rsa1.0_02214.1	841	161150	1.00E-124	233	234	99	+	1	233	212	445
UN14186	Rsa1.0_01434.1	1344	45951	0	778	796	97	+	550	1338	23019	23814
UN14187	Rsa1.0_00978.1	608	57739	0	565	614	92	+	12	608	12073	12666
UN14188	Rsa1.0_02962.1	992	26419	1.00E-109	263	285	92	-	717	992	11966	12250
UN14189	Rsa1.0_00396.1	816	88242	0	505	509	99	+	306	814	7161	7668
UN14190	Rsa1.0_00250.1	853	273771	0	366	371	98	-	131	500	186945	187315
UN14191	Rsa1.0_02012.1	1588	36084	1.00E-169	339	348	97	-	1	345	18461	18806
UN14192	Rsa1.0_00222.1	1327	172263	1.00E-150	280	282	99	-	1047	1327	138855	139136
UN14193	Rsa1.0_00886.1	1312	167350	0	334	337	99	-	552	888	33611	33947
UN14194	Rsa1.0_00816.1	1020	403350	0	381	382	99	+	1	382	390051	390432

UN14195	Rsa1.0_00183.1	692	113277	0	688	698	98	−	1	692	56094	56791
UN14196	Rsa1.0_00069.1	1666	403561	0	719	719	100	+	510	1228	102812	103530
UN14197	Rsa1.0_00137.1	1677	122457	0	1351	1360	99	+	123	1482	25462	26821
UN14198	Rsa1.0_01938.1	948	264018	0	650	658	98	+	6	662	156709	157365
UN14199	Rsa1.0_00477.1	1570	81556	0	461	464	99	−	1107	1570	23821	24283
UN14200	Rsa1.0_02923.1	850	26736	1.00E−128	327	354	92	+	104	453	15750	16101
UN14201	Rsa1.0_00095.1	748	283384	0	654	669	97	+	77	744	96594	97259
UN14202	Rsa1.0_02325.1	794	160025	0	357	358	99	+	436	793	20740	21097
UN14203	Rsa1.0_01937.1	769	37147	0	504	559	90	−	253	769	33650	34208
UN14204	Rsa1.0_00451.1	1079	322533	1.00E−154	360	381	94	+	705	1079	301659	302035
UN14205	Rsa1.0_00644.1	1457	136881	1.00E−151	277	279	99	+	1178	1456	44565	44843
UN14206	Rsa1.0_00604.1	979	146282	0	473	483	97	−	3	484	111511	111993
UN14207	Rsa1.0_01969.1	1362	153351	1.00E−151	274	275	99	−	242	516	13008	13282
UN14208	Rsa1.0_06653.1	1596	10596	0	681	688	98	+	908	1595	2350	3036
UN14209	Rsa1.0_03185.1	1537	24739	1.00E−136	247	247	100	+	3	249	2968	3214
UN14210	Rsa1.0_00107.1	2182	431659	1.00E−166	300	301	99	+	1882	2182	347678	347978
UN14211	Rsa1.0_04021.1	711	19536	1.00E−160	298	301	99	+	19	317	4146	4446
UN14212	Rsa1.0_02343.1	1252	32374	0	414	416	99	−	369	784	9746	10161
UN14213	Rsa1.0_00055.1	935	520193	0	767	789	97	+	149	935	339997	340776
UN14214	Rsa1.0_03249.1	1260	146254	0	433	438	98	+	2	436	22377	22812
UN14215	Rsa1.0_00012.1	2072	222148	1.00E−173	403	431	93	−	101	520	108799	109225
UN14216	Rsa1.0_00013.1	1631	292129	0	469	477	98	−	3	477	122837	123313
UN14217	Rsa1.0_01610.1	1077	42274	0	810	818	99	−	262	1073	11387	12204
UN14218	Rsa1.0_00926.1	1180	195072	0	555	564	98	−	250	813	176937	177500
UN14219	Rsa1.0_00692.1	910	68151	0	486	530	91	−	4	533	52276	52805
UN14220	Rsa1.0_00380.1	2741	89748	0	1410	1463	96	−	2	1457	56277	57727
UN14221	Rsa1.0_07591.1	715	9060	1.00E−139	308	323	95	+	392	711	6177	6499
UN14222	Rsa1.0_00015.1	682	561803	1.00E−159	422	469	89	+	102	563	483122	483579
UN14223	Rsa1.0_00569.1	747	75060	0	395	400	98	−	346	745	52754	53149
UN14224	Rsa1.0_15250.1	955	2727	0	735	751	97	+	1	750	1977	2727
UN14225	Rsa1.0_00628.1	1627	214360	0	462	464	99	+	919	1382	204637	205100
UN14226	Rsa1.0_01000.1	1393	57140	1.00E−139	276	283	97	+	2	284	16819	17100
UN14227	Rsa1.0_08811.1	1461	6904	1.00E−149	269	269	100	+	112	380	3263	3531
UN14228	Rsa1.0_01458.1	1121	175719	0	413	414	99	−	1	414	34495	34908
UN14229	Rsa1.0_04538.1	1454	16940	1.00E−131	261	267	97	−	1	266	12644	12910
UN14230	Rsa1.0_02165.1	1301	34423	0	515	516	99	+	1	516	4827	5342
UN14231	Rsa1.0_00339.1	1137	222974	1.00E−133	255	258	98	+	880	1137	40928	41184
UN14232	Rsa1.0_00751.1	875	65759	1.00E−129	266	274	97	−	603	874	42533	42806
UN14233	Rsa1.0_13800.1	1945	4315	1.00E−142	256	256	100	−	1688	1943	327	582

UN14234	Rsa1.0_00293.1	1210	339294	0	411	438	93	-	1	437	254540	254976
UN14235	Rsa1.0_01790.1	1344	39248	1.00E-160	313	320	97	-	1029	1344	17207	17526
UN14236	Rsa1.0_00676.1	985	69166	0	357	363	98	-	624	984	6802	7162
UN14237	Rsa1.0_00109.1	870	132735	0	437	442	98	-	2	443	52839	53280
UN14238	Rsa1.0_00642.1	1010	201230	4.00E-99	219	230	95	+	482	711	161379	161605
UN14239	Rsa1.0_00170.1	685	179538	0	363	364	99	-	321	684	90988	91351
UN14240	Rsa1.0_04652.1	837	16515	0	514	534	96	-	10	539	10530	11063
UN14241	Rsa1.0_00813.1	1555	63202	1.00E-140	260	262	99	-	862	1123	47779	48040
UN14242	Rsa1.0_24305.1	1095	994	1.00E-159	292	293	99	+	3	295	55	346
UN14243	Rsa1.0_09317.1	764	6459	0	571	574	99	+	188	761	5300	5873
UN14244	Rsa1.0_00205.1	695	193401	1.00E-86	211	222	95	-	456	676	99221	99438
UN14245	Rsa1.0_00294.1	1177	144860	0	1116	1174	95	+	4	1176	115494	116649
UN14246	Rsa1.0_07385.1	1064	9485	0	360	361	99	-	704	1064	2802	3162
UN14247	Rsa1.0_01838.1	1398	38500	0	429	452	94	-	1	442	17537	17988
UN14248	Rsa1.0_10486.1	1709	5830	0	396	405	97	-	1	403	1667	2071
UN14249	Rsa1.0_01053.1	1081	55483	0	749	763	98	+	324	1081	46655	47416
UN14250	Rsa1.0_03529.1	1423	22277	0	899	1010	89	-	101	1110	15606	16615
UN14251	Rsa1.0_00385.1	900	89160	1.00E-160	315	322	97	+	580	900	45744	46064
UN14252	Rsa1.0_00439.1	815	142776	0	394	395	99	+	419	813	59312	59706
UN14253	Rsa1.0_00730.1	1054	66673	0	1024	1052	97	+	1	1052	7013	8056
UN14254	Rsa1.0_03297.1	587	23969	1.00E-141	317	334	94	-	226	558	14221	14552
UN14255	Rsa1.0_00519.1	1115	197864	0	688	690	99	+	426	1115	192264	192953
UN14256	Rsa1.0_00190.1	1233	318240	0	407	408	99	+	3	410	116660	117067
UN14257	Rsa1.0_00409.1	858	146788	0	540	564	95	-	3	565	80743	81289
UN14258	Rsa1.0_01546.1	1360	43586	0	813	831	97	-	530	1360	33968	34797
UN14259	Rsa1.0_00487.1	1299	80674	0	370	383	96	-	915	1297	37979	38360
UN14260	Rsa1.0_01896.1	940	37512	1.00E-150	278	281	98	-	1	281	23896	24176
UN14261	Rsa1.0_01463.1	2923	45213	0	1113	1113	100	-	34	1146	9762	10874
UN14262	Rsa1.0_00438.1	1264	84861	0	1216	1264	96	-	25	1263	69391	70651
UN14263	Rsa1.0_00067.1	1062	415055	0	442	443	99	+	2	444	46411	46853
UN14264	Rsa1.0_01337.1	1388	47829	0	412	416	99	-	973	1388	40094	40509
UN14265	Rsa1.0_00326.1	1966	171955	0	637	655	97	+	141	795	45447	46101
UN14266	Rsa1.0_00780.1	991	147795	0	546	561	97	-	433	991	42502	43061
UN14267	Rsa1.0_00287.1	1424	98687	0	690	707	97	+	5	708	29686	30391
UN14268	Rsa1.0_01384.1	852	46803	0	449	455	98	+	400	852	41768	42222
UN14269	Rsa1.0_00644.1	1601	136881	0	349	351	99	-	3	353	41427	41777
UN14270	Rsa1.0_01259.1	1334	49754	0	421	427	98	+	907	1332	35262	35688
UN14271	Rsa1.0_05586.1	736	13263	0	446	456	97	-	281	736	9659	10111
UN14272	Rsa1.0_11549.1	1065	5473	0	513	516	99	-	548	1063	4479	4993

UN14273	Rsa1.0_09769.1	1307	6159	0	544	545	99	−	547	1091	3931	4475
UN14274	Rsa1.0_02221.1	637	33796	0	629	636	98	+	3	637	26997	27632
UN14275	Rsa1.0_06865.1	1118	10240	0	629	638	98	−	482	1118	8544	9181
UN14276	Rsa1.0_00098.1	1716	349239	0	1679	1728	97	−	1	1716	306601	308305
UN14277	Rsa1.0_00153.1	1440	192931	0	588	608	96	−	9	615	24233	24837
UN14278	Rsa1.0_23608.1	544	1037	1.00E−137	248	248	100	+	2	249	790	1037
UN14279	Rsa1.0_00868.1	1101	61630	0	649	689	94	−	412	1100	58194	58874
UN14280	Rsa1.0_04286.1	1183	18084	0	448	459	97	−	554	1011	13076	13534
UN14281	Rsa1.0_04588.1	1026	16739	9.00E−85	160	160	100	+	148	307	14592	14751
UN14282	Rsa1.0_03401.1	566	23191	1.00E−155	317	330	96	−	8	337	16158	16487
UN14283	Rsa1.0_00549.1	1183	76473	1.00E−122	289	312	92	−	866	1177	13029	13327
UN14284	Rsa1.0_01207.1	1687	141053	0	406	407	99	+	1	407	131180	131586
UN14285	Rsa1.0_01273.1	2477	49464	1.00E−124	247	251	98	−	2228	2477	18243	18492
UN14286	Rsa1.0_01792.1	1344	39227	0	784	810	96	+	537	1344	15907	16696
UN14287	Rsa1.0_00503.1	1462	79468	0	490	511	95	−	959	1462	39995	40505
UN14288	Rsa1.0_00473.1	1270	212204	1.00E−147	281	284	98	+	989	1270	73604	73886
UN14289	Rsa1.0_02569.1	1292	30002	0	793	811	97	+	483	1290	20247	21050
UN14290	Rsa1.0_02461.1	1113	31166	1.00E−173	327	333	98	−	784	1111	19449	19781
UN14291	Rsa1.0_00244.1	1438	103590	0	1289	1335	96	−	110	1438	76957	78290
UN14292	Rsa1.0_13767.1	981	4361	0	537	578	92	−	421	979	549	1118
UN14293	Rsa1.0_04141.1	999	18864	1.00E−165	311	315	98	+	2	315	15810	16124
UN14294	Rsa1.0_01585.1	1129	42806	0	513	531	96	+	589	1109	15623	16153
UN14295	Rsa1.0_00813.1	846	63202	0	814	861	94	−	3	845	18462	19321
UN14296	Rsa1.0_03643.1	1215	21607	0	634	653	97	+	13	656	20955	21607
UN14297	Rsa1.0_02060.1	1088	35528	0	675	676	99	+	413	1088	19474	20149
UN14298	Rsa1.0_01943.1	1460	37042	0	455	471	96	−	3	472	31522	31987
UN14299	Rsa1.0_00620.1	1668	71985	0	500	506	98	−	329	834	55327	55832
UN14300	Rsa1.0_00516.1	1172	78417	1.00E−143	357	386	92	+	792	1161	20048	20431
UN14301	Rsa1.0_00102.1	1337	135497	1.00E−115	248	262	94	−	1	262	51183	51434
UN14302	Rsa1.0_05326.1	764	14139	0	389	395	98	−	2	396	5288	5682
UN14303	Rsa1.0_30502.1	1296	752	1.00E−144	266	268	99	+	870	1137	483	750
UN14304	Rsa1.0_01740.1	940	39986	1.00E−127	274	287	95	−	72	352	27598	27884
UN14305	Rsa1.0_01121.1	1919	53407	0	1850	1894	97	+	32	1918	6767	8655
UN14306	Rsa1.0_02226.1	884	33726	1.00E−115	275	292	94	−	598	884	10106	10395
UN14307	Rsa1.0_05302.1	810	14212	0	449	452	99	−	359	810	6178	6629
UN14308	Rsa1.0_00224.1	1117	327530	0	462	463	99	+	655	1117	13608	14070
UN14309	Rsa1.0_00011.1	1090	225044	0	580	588	98	+	436	1023	94900	95485
UN14310	Rsa1.0_01412.1	918	46311	0	508	521	97	+	397	917	2268	2786
UN14311	Rsa1.0_16338.1	662	2190	0	345	347	99	+	4	350	392	737

UN14312	Rsa1.0_00097.1	695	138858	2.33E-156	281	287	97	−	51	337	14814	15100
UN14313	Rsa1.0_00108.1	962	478491	0	939	962	97	+	1	962	46801	47761
UN14314	Rsa1.0_00064.1	979	240927	0	655	665	98	+	315	979	132731	133392
UN14315	Rsa1.0_05217.1	1111	142938	0	428	428	100	−	682	1109	136336	136763
UN14316	Rsa1.0_05421.1	1036	13806	1.00E-123	242	248	97	−	311	558	8527	8774
UN14317	Rsa1.0_00078.1	1823	175053	0	1385	1431	96	−	391	1821	66490	67910
UN14318	Rsa1.0_02606.1	1384	29635	0	381	389	97	+	997	1384	7554	7938
UN14319	Rsa1.0_00091.1	1056	139699	0	502	526	95	−	538	1056	80669	81190
UN14320	Rsa1.0_03074.1	1190	25568	1.00E-144	262	263	99	+	659	921	11008	11270
UN14321	Rsa1.0_00008.1	1352	358124	0	422	424	99	+	7	430	14721	15144
UN14322	Rsa1.0_14939.1	1594	2948	0	481	482	99	+	799	1280	559	1040
UN14323	Rsa1.0_05778.1	1216	12656	0	836	848	98	−	2	847	7	854
UN14324	Rsa1.0_04741.1	812	16164	1.00E-156	396	431	91	−	5	419	4356	4783
UN14325	Rsa1.0_00079.1	908	606987	0	434	455	95	+	447	895	66614	67064
UN14326	Rsa1.0_36749.1	866	613	1.00E-173	327	332	98	−	352	683	195	525
UN14327	Rsa1.0_00224.1	737	327530	1.00E-107	246	260	94	+	56	312	178383	178637
UN14328	Rsa1.0_10253.1	1057	5919	0	535	539	99	+	23	561	5381	5919
UN14329	Rsa1.0_00092.1	1609	659932	1.00E-148	442	506	87	−	375	880	443401	443865
UN14330	Rsa1.0_02278.1	1156	33085	0	331	334	99	−	1	334	20639	20972
UN14331	Rsa1.0_20045.1	1122	1348	0	598	641	93	+	225	865	720	1346
UN14332	Rsa1.0_02888.1	2011	27020	0	1011	1019	99	+	993	2011	6751	7767
UN14333	Rsa1.0_00153.1	1115	192931	0	371	372	99	−	744	1115	108610	108981
UN14334	Rsa1.0_03334.1	1036	23659	1.00E-129	285	299	95	+	4	296	18754	19051
UN14335	Rsa1.0_02215.1	739	33822	0	334	335	99	−	4	338	27836	28170
UN14336	Rsa1.0_01825.1	1942	38725	0	1268	1274	99	+	622	1892	27077	28350
UN14337	Rsa1.0_03054.1	1636	25738	0	490	492	99	−	2	493	15390	15881
UN14338	Rsa1.0_03278.1	974	24125	1.00E-107	225	229	98	−	699	923	2043	2271
UN14339	Rsa1.0_13747.1	1035	4388	0	373	373	100	−	615	987	1574	1946
UN14340	Rsa1.0_03185.1	1516	24739	9.00E-86	165	166	99	−	350	515	4793	4958
UN14341	Rsa1.0_03090.1	759	25464	0	752	759	99	+	1	756	14136	14894
UN14342	Rsa1.0_00435.1	1310	317579	0	376	387	97	−	3	389	68865	69251
UN14343	Rsa1.0_00015.1	1905	561803	0	891	900	99	−	835	1728	185641	186540
UN14344	Rsa1.0_00387.1	1810	495407	0	940	981	95	−	828	1805	90908	91884
UN14345	Rsa1.0_01006.1	814	163670	1.00E-100	185	185	100	+	630	814	25073	25257
UN14346	Rsa1.0_02372.1	1515	32053	0	351	353	99	−	367	719	29549	29901
UN14347	Rsa1.0_00879.1	797	61297	1.00E-138	265	269	98	+	1	267	27909	28177
UN14348	Rsa1.0_00510.1	744	78718	0	364	367	99	−	112	478	53847	54213
UN14349	Rsa1.0_00707.1	859	190112	1.00E-124	236	238	99	−	623	859	181078	181315
UN14350	Rsa1.0_01705.1	1629	40710	1.00E-120	231	235	98	−	473	707	12391	12625

UN14351	Rsa1.0_06443.1	1043	11008	1.00E-148	298	308	96	-	737	1041	3689	3996
UN14352	Rsa1.0_00469.1	1045	82084	1.00E-118	219	220	99	-	551	770	57894	58113
UN14353	Rsa1.0_04696.1	2032	16339	0	686	765	89	+	1290	2032	10706	11462
UN14354	Rsa1.0_19120.1	1412	1477	1.00E-143	271	275	98	-	882	1156	326	600
UN14355	Rsa1.0_03668.1	1139	21483	0	634	659	96	-	18	669	2579	3235
UN14356	Rsa1.0_03513.1	1878	22349	0	395	407	97	-	1475	1878	14004	14409
UN14357	Rsa1.0_03352.1	1582	23506	1.00E-117	277	294	94	+	1285	1577	9339	9625
UN14358	Rsa1.0_05168.1	1443	14621	0	677	686	98	-	45	727	8801	9486
UN14359	Rsa1.0_00752.1	902	65756	1.00E-163	335	348	96	+	544	891	14186	14532
UN14360	Rsa1.0_01716.1	1115	40483	0	359	362	99	-	3	363	33499	33860
UN14361	Rsa1.0_00827.1	2393	62790	0	1234	1236	99	-	471	1706	22381	23616
UN14362	Rsa1.0_00533.1	1076	77375	0	562	571	98	+	1	570	1837	2406
UN14363	Rsa1.0_02918.1	977	26797	0	338	339	99	-	632	970	12962	13300
UN14364	Rsa1.0_02500.1	1494	30768	0	1428	1489	95	+	1	1483	26868	28353
UN14365	Rsa1.0_00475.1	615	81678	1.00E-172	325	330	98	+	1	330	26870	27198
UN14366	Rsa1.0_00017.1	838	752458	0	364	371	98	+	471	834	516052	516422
UN14367	Rsa1.0_03001.1	1021	26108	0	516	569	90	-	470	1021	5273	5824
UN14368	Rsa1.0_00018.1	637	410899	0	528	540	97	-	2	541	140603	141134
UN14369	Rsa1.0_00071.1	1751	345714	0	598	602	99	+	1150	1751	129522	130123
UN14370	Rsa1.0_01867.1	1023	37960	1.00E-169	337	349	96	+	235	583	17218	17566
UN14371	Rsa1.0_00133.1	1015	123768	1.00E-154	301	309	97	-	115	423	11639	11947
UN14372	Rsa1.0_00079.1	900	606987	1.00E-122	244	251	97	+	121	371	464295	464545
UN14373	Rsa1.0_00008.1	703	358124	0	332	334	99	+	96	429	194944	195277
UN14374	Rsa1.0_19809.1	815	1380	0	436	436	100	-	379	814	175	610
UN14375	Rsa1.0_06166.1	886	11583	1.00E-160	349	367	95	+	1	356	6734	7094
UN14376	Rsa1.0_00213.1	282	108167	3.00E-63	177	193	91	+	5	196	58561	58745
UN14377	Rsa1.0_00380.1	464	89748	1.00E-126	251	257	97	+	5	261	48776	49031
UN14378	Rsa1.0_00247.1	1590	103408	9.00E-86	189	198	95	-	71	268	12486	12683
UN14379	Rsa1.0_00356.1	1109	91521	0	386	386	100	-	347	732	80712	81097
UN14380	Rsa1.0_62301.1	1072	368	0	359	368	97	+	305	672	1	368
UN14381	Rsa1.0_00705.1	875	67505	0	435	451	96	+	87	536	9626	10076
UN14382	Rsa1.0_00421.1	2263	86365	1.00E-144	292	299	97	+	4	299	70153	70449
UN14383	Rsa1.0_01880.1	1148	37745	0	959	999	95	-	1	995	18288	19285
UN14384	Rsa1.0_00722.1	1252	66840	0	505	514	98	-	3	516	51173	51686
UN14385	Rsa1.0_00336.1	1151	445051	0	1094	1157	94	+	3	1149	308915	310062
UN14386	Rsa1.0_00092.1	1280	659932	1.00E-167	655	764	85	+	336	1079	124559	125307
UN14387	Rsa1.0_01296.1	1072	49035	1.00E-118	243	249	97	+	761	1009	13384	13630
UN14388	Rsa1.0_02430.1	998	31506	0	338	339	99	+	659	997	12388	12726
UN14389	Rsa1.0_18748.1	816	1541	1.00E-176	343	353	97	-	95	447	186	538

UN14390	Rsa1.0_02931.1	1163	26654	0	589	612	96	+	537	1142	5953	6560
UN14391	Rsa1.0_09236.1	1145	6540	1.00E-155	313	324	96	-	406	729	5496	5816
UN14392	Rsa1.0_00506.1	1389	79106	1.00E-148	269	270	99	-	1	270	6009	6278
UN14393	Rsa1.0_00492.1	1319	80178	1.00E-136	385	430	89	+	3	424	69251	69662
UN14394	Rsa1.0_00045.1	821	328694	1.00E-162	316	324	97	-	1	322	139224	139547
UN14395	Rsa1.0_00931.1	1302	59210	0	490	509	96	+	3	506	47749	48246
UN14396	Rsa1.0_01198.1	738	51029	0	651	654	99	-	82	735	25781	26434
UN14397	Rsa1.0_03487.1	985	22564	0	982	984	99	-	2	985	2197	3179
UN14398	Rsa1.0_00738.1	2717	66322	0	460	469	98	+	2245	2712	26036	26504
UN14399	Rsa1.0_00092.1	814	659932	1.00E-179	355	366	96	+	352	716	198161	198526
UN14400	Rsa1.0_00213.1	1220	108167	1.00E-158	293	296	98	-	925	1220	93859	94154
UN14401	Rsa1.0_09490.1	740	6335	0	699	739	94	-	3	737	4796	5510
UN14402	Rsa1.0_01471.1	773	45101	0	593	625	94	+	154	773	13923	14546
UN14403	Rsa1.0_04367.1	546	17728	1.00E-113	220	224	98	+	4	227	13666	13889
UN14404	Rsa1.0_30664.1	1251	748	0	564	566	99	+	119	684	1	566
UN14405	Rsa1.0_08529.1	2042	7233	0	520	552	94	+	824	1371	6697	7233
UN14406	Rsa1.0_00963.1	679	58176	0	667	677	98	+	1	677	10188	10863
UN14407	Rsa1.0_01595.1	1064	175383	1.00E-129	257	262	98	+	3	263	5818	6078
UN14408	Rsa1.0_01287.1	4544	49195	0	743	838	88	+	63	893	18785	19604
UN14409	Rsa1.0_00863.1	1124	254535	1.00E-171	321	324	99	-	3	324	210902	211224
UN14410	Rsa1.0_02601.1	1547	29654	0	523	559	93	+	1	549	3232	3790
UN14411	Rsa1.0_34178.1	635	663	0	406	407	99	-	226	632	7	412
UN14412	Rsa1.0_02916.1	1465	26809	0	419	432	96	-	1	428	5584	6013
UN14413	Rsa1.0_47960.1	1093	472	1.00E-115	226	231	97	+	447	677	135	365
UN14414	Rsa1.0_07055.1	1595	9970	0	458	459	99	+	1137	1594	2701	3159
UN14415	Rsa1.0_03282.1	894	24107	0	558	571	97	-	3	573	10595	11159
UN14416	Rsa1.0_04258.1	784	18254	0	408	416	98	+	3	415	2361	2776
UN14417	Rsa1.0_09003.1	1474	6717	0	344	344	100	-	3	346	2979	3322
UN14418	Rsa1.0_01573.1	1470	42964	1.00E-171	335	345	97	-	1126	1470	2655	2999
UN14419	Rsa1.0_00066.1	976	152418	1.00E-124	254	261	97	-	709	967	53160	53419
UN14420	Rsa1.0_05489.1	1135	13562	0	733	751	97	-	388	1135	12145	12894
UN14421	Rsa1.0_01403.1	759	46453	5.00E-95	216	227	95	-	3	228	26363	26586
UN14422	Rsa1.0_05611.1	817	13193	0	538	554	97	+	1	554	2576	3128
UN14423	Rsa1.0_00896.1	1387	60509	0	608	625	97	+	173	797	16901	17525
UN14424	Rsa1.0_02080.1	1448	35297	0	1324	1360	97	-	38	1396	22495	23851
UN14425	Rsa1.0_02389.1	1335	146428	0	1021	1039	98	+	3	1036	137834	138869
UN14426	Rsa1.0_02362.1	713	32190	0	414	414	100	-	299	712	24732	25145
UN14427	Rsa1.0_00869.1	1647	61627	0	333	333	100	-	848	1180	42825	43157
UN14428	Rsa1.0_00087.1	927	761073	1.00E-170	348	358	97	-	1	353	225356	225712

UN14429	Rsa1.0_00340.1	636	305782	1.00E-153	352	375	93	-	277	636	159725	160093
UN14430	Rsa1.0_00178.1	678	114861	1.00E-130	288	302	95	+	374	674	12165	12462
UN14431	Rsa1.0_10893.1	907	5687	1.00E-170	312	315	99	+	431	745	236	550
UN14432	Rsa1.0_01144.1	1490	52431	0	419	432	96	+	1060	1490	42305	42732
UN14433	Rsa1.0_00163.1	1524	116847	0	446	471	94	+	1059	1523	83512	83980
UN14434	Rsa1.0_00216.1	901	197563	1.00E-89	213	227	93	-	3	229	51876	52090
UN14435	Rsa1.0_00718.1	1051	279335	0	515	515	100	-	2	516	34460	34974
UN14436	Rsa1.0_00576.1	760	178949	1.00E-148	296	302	98	+	41	339	2749	3049
UN14437	Rsa1.0_01033.1	965	56173	0	555	570	97	+	1	568	48521	49090
UN14438	Rsa1.0_03261.1	1005	24281	0	439	441	99	-	2	442	5213	5653
UN14439	Rsa1.0_00595.1	822	341648	1.00E-138	272	278	97	-	1	278	4003	4279
UN14440	Rsa1.0_02365.1	886	32118	0	540	593	91	+	20	587	29274	29863
UN14441	Rsa1.0_00324.1	1261	218805	1.00E-168	326	333	97	+	929	1261	182824	183151
UN14442	Rsa1.0_00527.1	955	77733	0	474	477	99	-	479	955	24551	25027
UN14443	Rsa1.0_00128.1	563	228675	1.00E-103	237	248	95	-	9	252	61319	61564
UN14444	Rsa1.0_00526.1	1266	169279	0	322	322	100	-	945	1266	139659	139980
UN14445	Rsa1.0_00022.1	1465	200461	0	546	556	98	+	497	1052	120556	121111
UN14446	Rsa1.0_00447.1	939	84051	0	381	382	99	+	1	382	56157	56538
UN14447	Rsa1.0_01173.1	1386	51727	0	399	412	96	+	968	1379	1555	1966
UN14448	Rsa1.0_00014.1	1608	348985	0	898	915	98	-	707	1608	267787	268701
UN14449	Rsa1.0_02963.1	1387	26407	1.00E-132	263	271	97	-	889	1159	13077	13347
UN14450	Rsa1.0_03491.1	756	22541	0	560	585	95	-	38	615	8996	9580
UN14451	Rsa1.0_00197.1	695	109714	1.00E-174	313	314	99	-	101	414	78615	78928
UN14452	Rsa1.0_00033.1	1250	515811	1.00E-145	265	266	99	+	274	539	354853	355118
UN14453	Rsa1.0_02692.1	634	28626	0	517	535	96	+	5	539	1031	1561
UN14454	Rsa1.0_01374.1	826	47047	0	524	532	98	+	294	824	32469	32996
UN14455	Rsa1.0_00082.1	1849	237584	1.00E-114	225	230	97	+	727	956	137067	137296
UN14456	Rsa1.0_04652.1	825	16515	0	359	361	99	-	1	361	4833	5193
UN14457	Rsa1.0_00387.1	1221	495407	0	885	924	95	-	1	924	409522	410423
UN14458	Rsa1.0_00851.1	738	62181	0	443	454	97	-	5	451	8707	9160
UN14459	Rsa1.0_00009.1	1267	232558	1.00E-167	298	298	100	-	970	1267	53644	53941
UN14460	Rsa1.0_00663.1	1808	164969	0	523	528	99	-	944	1471	96870	97397
UN14461	Rsa1.0_02678.1	1281	135053	1.00E-179	318	318	100	+	964	1281	112508	112825
UN14462	Rsa1.0_00312.1	912	96765	0	578	579	99	-	1	579	67883	68461
UN14463	Rsa1.0_01469.1	1106	45139	1.00E-117	223	226	98	+	61	286	18180	18405
UN14464	Rsa1.0_01707.1	866	40686	5.00E-86	162	162	100	-	682	843	30412	30573
UN14465	Rsa1.0_00022.1	1421	200461	0	822	838	98	-	584	1419	67747	68575
UN14466	Rsa1.0_05654.1	1512	13042	0	497	503	98	+	694	1196	8195	8697
UN14467	Rsa1.0_00001.1	1531	363028	0	400	400	100	-	1132	1531	284448	284847

UN14468	Rsa1.0_00147.1	1302	249503	0	1268	1304	97	-	2	1301	240704	242005
UN14469	Rsa1.0_00891.1	1208	60724	1.00E-152	274	274	100	-	3	276	57400	57673
UN14470	Rsa1.0_24632.1	826	976	1.00E-149	283	288	98	+	451	738	570	857
UN14471	Rsa1.0_04806.1	709	15898	0	559	586	95	+	130	709	7069	7646
UN14472	Rsa1.0_05784.1	1001	12643	0	411	425	96	+	576	1000	1645	2068
UN14473	Rsa1.0_08115.1	928	7882	0	634	641	98	+	281	921	3894	4534
UN14474	Rsa1.0_00067.1	941	415055	0	591	592	99	+	349	940	82824	83415
UN14475	Rsa1.0_00019.1	1921	301669	1.00E-145	275	278	98	+	42	319	33034	33310
UN14476	Rsa1.0_00028.1	1309	293984	0	902	912	98	-	404	1309	234590	235501
UN14477	Rsa1.0_00592.1	1045	73788	0	369	370	99	-	676	1045	24088	24457
UN14478	Rsa1.0_02560.1	925	30091	0	341	343	99	+	374	716	7721	8063
UN14479	Rsa1.0_00187.1	912	393093	0	403	413	97	+	49	460	212118	212530
UN14480	Rsa1.0_00100.1	781	249232	1.00E-179	406	432	93	+	300	728	13427	13856
UN14481	Rsa1.0_05864.1	849	12387	1.00E-108	257	272	94	+	1	272	9901	10165
UN14482	Rsa1.0_00688.1	1052	68396	1.00E-110	260	278	93	-	1	273	14821	15097
UN14483	Rsa1.0_00253.1	1168	231292	1.00E-110	246	259	94	-	907	1164	124380	124638
UN14484	Rsa1.0_00782.1	1619	64468	0	530	567	93	+	1062	1619	28205	28758
UN14485	Rsa1.0_00582.1	979	74333	0	588	604	97	+	1	604	18904	19507
UN14486	Rsa1.0_00407.1	835	191544	1.00E-160	325	335	97	-	497	829	24358	24688
UN14487	Rsa1.0_03916.1	1346	20117	0	567	572	99	-	777	1345	17771	18342
UN14488	Rsa1.0_00266.1	2855	163932	0	1167	1212	96	-	951	2158	9927	11135
UN14489	Rsa1.0_00049.1	1580	167018	0	625	639	97	-	942	1580	127386	128019
UN14490	Rsa1.0_00252.1	1321	102760	1.00E-142	275	281	97	+	377	657	43752	44032
UN14491	Rsa1.0_00144.1	1008	120708	0	448	451	99	+	3	453	45117	45567
UN14492	Rsa1.0_03981.1	474	19724	1.00E-127	296	315	93	+	98	411	17481	17789
UN14493	Rsa1.0_01717.1	755	143162	1.00E-140	270	276	97	-	140	415	15704	15979
UN14494	Rsa1.0_01303.1	998	48853	0	385	400	96	+	599	998	15291	15689
UN14495	Rsa1.0_01273.1	1402	49464	0	358	362	98	-	21	382	32716	33077
UN14496	Rsa1.0_00110.1	1237	132719	0	549	579	94	+	649	1227	6854	7417
UN14497	Rsa1.0_00764.1	1434	65056	0	1213	1232	98	-	204	1434	51993	53224
UN14498	Rsa1.0_00471.1	1165	81897	0	397	397	100	+	765	1161	4146	4542
UN14499	Rsa1.0_00008.1	494	358124	1.00E-134	283	295	95	+	200	493	30632	30926
UN14500	Rsa1.0_01376.1	976	47004	1.00E-96	226	238	94	+	59	295	22550	22778
UN14501	Rsa1.0_00009.1	1496	232558	1.00E-131	270	278	97	-	1220	1496	40696	40972
UN14502	Rsa1.0_00026.1	829	327835	0	820	827	99	-	5	829	44003	44829
UN14503	Rsa1.0_08154.1	707	7823	1.00E-131	247	250	98	+	3	252	4183	4432
UN14504	Rsa1.0_00120.1	754	277288	0	370	382	96	-	22	403	266327	266707
UN14505	Rsa1.0_00190.1	693	318240	1.00E-141	356	388	91	+	3	379	91417	91801
UN14506	Rsa1.0_01546.1	942	43586	0	889	918	96	+	7	921	30264	31174

UN14507	Rsa1.0_00707.1	866	190112	0	625	634	98	+	17	648	17632	18264
UN14508	Rsa1.0_00019.1	1760	301669	0	1104	1160	95	−	475	1629	280293	281443
UN14509	Rsa1.0_01420.1	2143	46210	0	847	884	95	−	1	868	8853	9736
UN14510	Rsa1.0_15692.1	822	2478	1.00E−147	350	375	93	+	2	369	686	1057
UN14511	Rsa1.0_02357.1	797	32220	0	332	335	99	−	452	786	11445	11779
UN14512	Rsa1.0_00408.1	1713	176752	0	441	463	95	−	272	734	134345	134803
UN14513	Rsa1.0_00509.1	1056	78829	0	363	372	97	−	685	1056	17051	17419
UN14514	Rsa1.0_00110.1	970	132719	1.00E−168	325	331	98	−	637	966	91299	91627
UN14515	Rsa1.0_00284.1	1553	323267	0	624	642	97	+	911	1552	243413	244042
UN14516	Rsa1.0_00426.1	1074	85668	1.00E−101	253	270	93	+	8	274	32315	32577
UN14517	Rsa1.0_03263.1	1404	24268	0	1045	1128	92	+	295	1403	20569	21667
UN14518	Rsa1.0_00879.1	846	61297	1.00E−153	300	307	97	+	541	846	38364	38670
UN14519	Rsa1.0_00804.1	792	63726	1.00E−141	275	282	97	−	364	645	16086	16367
UN14520	Rsa1.0_14032.1	980	3893	1.00E−157	322	334	96	+	647	980	3098	3426
UN14521	Rsa1.0_01244.1	1535	50061	0	417	419	99	−	1	419	30687	31105
UN14522	Rsa1.0_00184.1	1415	218748	0	587	600	97	−	675	1274	80617	81213
UN14523	Rsa1.0_01104.1	1637	285480	1.00E−130	345	383	90	+	1	363	266601	266983
UN14524	Rsa1.0_00337.1	933	143174	0	525	535	98	+	403	931	4654	5188
UN14525	Rsa1.0_03837.1	1900	20559	0	387	388	99	−	1253	1640	20172	20559
UN14526	Rsa1.0_00366.1	599	90880	0	570	597	95	+	3	597	25892	26478
UN14527	Rsa1.0_00140.1	2242	121541	1.00E−158	343	363	94	−	106	468	49081	49443
UN14528	Rsa1.0_06546.1	1248	10801	1.00E−145	274	278	98	−	538	815	4522	4799
UN14529	Rsa1.0_07681.1	1076	8808	1.00E−176	314	314	100	−	58	371	2441	2754
UN14530	Rsa1.0_00298.1	1685	349131	1.00E−111	211	213	99	+	960	1172	177311	177523
UN14531	Rsa1.0_01754.1	714	39792	0	658	673	97	−	43	712	13161	13830
UN14532	Rsa1.0_00805.1	804	63724	0	428	454	94	+	351	804	43181	43621
UN14533	Rsa1.0_01034.1	737	56046	0	362	367	98	+	312	678	35993	36359
UN14534	Rsa1.0_00163.1	814	116847	1.00E−169	302	302	100	+	135	436	66573	66874
UN14535	Rsa1.0_00191.1	826	316976	0	418	426	98	−	2	427	42419	42844
UN14536	Rsa1.0_00316.1	1297	96264	0	499	509	98	−	3	511	85542	86050
UN14537	Rsa1.0_00032.1	684	306326	1.00E−130	266	276	96	+	36	311	154752	155027
UN14538	Rsa1.0_00354.1	835	232005	9.00E−97	256	279	91	−	1	273	170578	170849
UN14539	Rsa1.0_00191.1	1567	316976	0	419	424	98	−	233	655	259269	259692
UN14540	Rsa1.0_00928.1	1156	59293	0	359	359	100	−	796	1154	19222	19580
UN14541	Rsa1.0_01005.1	1409	56967	1.00E−162	302	306	98	−	1089	1394	2548	2853
UN14542	Rsa1.0_00235.1	1517	154696	0	415	415	100	+	1092	1506	101650	102064
UN14543	Rsa1.0_00001.1	405	363028	1.00E−91	171	171	100	+	229	399	314318	314488
UN14544	Rsa1.0_00034.1	870	260119	1.00E−157	320	330	96	−	1	327	67803	68129
UN14545	Rsa1.0_07657.1	1182	8896	0	549	564	97	+	622	1180	3516	4079

UN14546	Rsa1.0_01422.1	1157	46172	0	481	501	96	−	657	1157	10164	10663
UN14547	Rsa1.0_01519.1	989	44144	0	579	594	97	−	400	989	11559	12151
UN14548	Rsa1.0_00425.1	1565	147080	0	1236	1246	99	+	320	1565	2361	3603
UN14549	Rsa1.0_00545.1	1078	189649	1.00E−148	282	287	98	−	792	1078	35014	35300
UN14550	Rsa1.0_02944.1	1322	26553	0	573	576	99	−	418	993	4612	5186
UN14551	Rsa1.0_00145.1	958	261008	0	348	349	99	+	2	349	38513	38861
UN14552	Rsa1.0_07601.1	969	9051	0	857	875	97	−	95	969	1300	2173
UN14553	Rsa1.0_01071.1	1198	166914	0	402	402	100	−	797	1198	14006	14407
UN14554	Rsa1.0_03093.1	1545	25445	0	816	839	97	+	709	1541	14489	15324
UN14555	Rsa1.0_01521.1	1724	142147	0	1102	1117	98	+	3	1119	105710	106826
UN14556	Rsa1.0_01364.1	1490	47172	0	641	661	96	−	3	660	35965	36617
UN14557	Rsa1.0_00050.1	1863	237174	0	350	353	99	+	4	355	34014	34366
UN14558	Rsa1.0_00169.1	935	274298	0	503	513	98	−	423	935	270670	271182
UN14559	Rsa1.0_00326.1	1005	171955	4.00E−96	282	314	89	−	1	313	65824	66115
UN14560	Rsa1.0_00947.1	1000	58664	0	473	499	94	+	484	979	10169	10666
UN14561	Rsa1.0_00300.1	1307	97747	0	545	565	96	+	73	625	81510	82074
UN14562	Rsa1.0_09069.1	1601	6671	0	470	483	97	+	413	895	2813	3295
UN14563	Rsa1.0_00169.1	1239	274298	0	437	460	95	−	2	460	239066	239524
UN14564	Rsa1.0_01462.1	542	45231	2.00E−69	207	228	90	−	3	227	25946	26166
UN14565	Rsa1.0_08961.1	1019	6750	0	407	429	94	−	591	1017	4329	4752
UN14566	Rsa1.0_02306.1	958	32776	0	834	958	87	−	1	947	31298	32254
UN14567	Rsa1.0_01214.1	1270	50599	0	442	473	93	−	1	456	46274	46746
UN14568	Rsa1.0_54426.1	1024	419	1.00E−147	312	324	96	−	3	325	1	322
UN14569	Rsa1.0_00224.1	1604	327530	0	882	897	98	−	711	1604	38370	39264
UN14570	Rsa1.0_01981.1	645	36537	0	396	410	96	−	3	412	34743	35147
UN14571	Rsa1.0_00038.1	969	314568	0	431	435	99	−	535	969	252468	252902
UN14572	Rsa1.0_00420.1	1234	86496	1.00E−151	296	302	98	−	934	1232	67335	67635
UN14573	Rsa1.0_00015.1	737	561803	1.00E−155	419	468	89	+	158	614	483122	483579
UN14574	Rsa1.0_08265.1	878	7630	1.00E−179	419	436	96	−	458	878	6623	7058
UN14575	Rsa1.0_03325.1	2018	23720	0	393	396	99	−	1623	2018	6758	7150
UN14576	Rsa1.0_01666.1	1621	173972	0	435	447	97	+	1174	1620	4179	4625
UN14577	Rsa1.0_08225.1	1246	7695	0	746	770	96	−	481	1246	4138	4907
UN14578	Rsa1.0_02812.1	1120	27660	0	511	534	95	−	6	530	2883	3415
UN14579	Rsa1.0_00012.1	1399	222148	0	1352	1396	96	−	2	1394	102565	103958
UN14580	Rsa1.0_00121.1	816	278029	0	584	585	99	+	2	586	55530	56114
UN14581	Rsa1.0_01717.1	803	143162	1.00E−138	265	270	98	+	1	270	14895	15164
UN14582	Rsa1.0_00179.1	809	393324	1.00E−160	308	315	97	−	495	809	200102	200416
UN14583	Rsa1.0_08102.1	1541	7901	1.00E−114	408	475	85	+	12	465	521	969
UN14584	Rsa1.0_03668.1	1840	21483	0	834	854	97	+	510	1363	13253	14106

UN14585	Rsa1.0_00079.1	866	606987	1.00E-160	334	347	96	-	517	863	464201	464545
UN14586	Rsa1.0_11741.1	1501	5423	0	482	500	96	-	412	908	4597	5096
UN14587	Rsa1.0_02142.1	1268	34633	1.00E-121	224	225	99	+	884	1108	16828	17052
UN14588	Rsa1.0_00646.1	882	70473	1.00E-58	140	148	94	+	731	878	7599	7746
UN14589	Rsa1.0_00321.1	864	95671	0	418	431	96	+	2	424	26911	27341
UN14590	Rsa1.0_00890.1	591	323870	1.00E-131	278	290	95	+	14	303	178708	178996
UN14591	Rsa1.0_00204.1	1262	109056	0	479	494	96	+	769	1262	9815	10308
UN14592	Rsa1.0_00065.1	1063	454616	0	980	991	98	-	73	1063	430845	431834
UN14593	Rsa1.0_03770.1	1138	20952	0	472	475	99	-	9	483	5875	6349
UN14594	Rsa1.0_00390.1	1030	88681	1.00E-176	328	332	98	-	696	1027	14406	14735
UN14595	Rsa1.0_02253.1	1240	33356	1.00E-119	224	226	99	+	1015	1240	25657	25882
UN14596	Rsa1.0_01193.1	920	51147	0	893	912	97	+	5	916	39664	40573
UN14597	Rsa1.0_00526.1	1121	169279	0	498	511	97	+	433	943	148049	148559
UN14598	Rsa1.0_23965.1	1059	1014	0	393	401	98	+	665	1059	374	773
UN14599	Rsa1.0_00102.1	1352	135497	0	1320	1357	97	+	1	1351	39742	41095
UN14600	Rsa1.0_00964.1	1237	316648	0	966	1000	96	+	253	1237	301610	302604
UN14601	Rsa1.0_00015.1	1917	561803	0	863	902	95	+	189	1083	185641	186540
UN14602	Rsa1.0_00263.1	1796	224968	0	380	392	96	+	1407	1796	151524	151911
UN14603	Rsa1.0_00866.1	1335	61676	0	524	544	96	-	798	1335	29494	30037
UN14604	Rsa1.0_00315.1	990	96426	1.00E-138	265	270	98	-	502	771	2999	3268
UN14605	Rsa1.0_02101.1	1476	35017	0	412	412	100	-	403	814	28859	29270
UN14606	Rsa1.0_02210.1	765	33857	0	484	500	96	+	15	512	30230	30727
UN14607	Rsa1.0_00978.1	806	57739	0	454	476	95	+	337	806	14018	14493
UN14608	Rsa1.0_00373.1	1008	90296	1.00E-118	222	224	99	-	303	526	73915	74138
UN14609	Rsa1.0_10516.1	484	5818	1.00E-114	218	221	98	-	119	339	4536	4756
UN14610	Rsa1.0_28434.1	1241	815	0	438	438	100	+	802	1239	48	485
UN14611	Rsa1.0_00002.1	1341	601661	1.00E-161	292	293	99	-	1047	1339	293447	293739
UN14612	Rsa1.0_00558.1	1916	75819	0	771	771	100	-	8	778	56040	56810
UN14613	Rsa1.0_05611.1	993	13193	0	524	538	97	-	456	993	9250	9781
UN14614	Rsa1.0_00398.1	1039	88007	0	507	539	94	-	1	524	86450	86984
UN14615	Rsa1.0_05544.1	849	13381	1.00E-169	307	309	99	-	3	311	8507	8815
UN14616	Rsa1.0_02959.1	1403	26441	0	393	417	94	+	14	421	20181	20597
UN14617	Rsa1.0_00271.1	1460	101125	1.00E-131	239	239	100	+	96	334	95742	95980
UN14618	Rsa1.0_00179.1	1591	393324	0	546	555	98	-	3	556	336310	336860
UN14619	Rsa1.0_02366.1	754	32110	1.00E-149	271	272	99	-	481	752	9096	9367
UN14620	Rsa1.0_00048.1	1145	167185	0	372	378	98	+	378	755	75719	76096
UN14621	Rsa1.0_00727.1	1537	66724	0	686	727	94	+	3	708	44991	45714
UN14622	Rsa1.0_01195.1	1095	147988	0	569	590	96	+	1	586	8316	8901
UN14623	Rsa1.0_00480.1	1387	81325	0	348	349	99	-	1039	1387	41821	42169

UN14624	Rsa1.0_01054.1	2075	55470	0	1212	1215	99	−	3	1216	9825	11039
UN14625	Rsa1.0_14764.1	1724	3084	0	748	753	99	−	972	1724	1882	2634
UN14626	Rsa1.0_00328.1	1318	224661	0	543	559	97	+	489	1047	136232	136790
UN14627	Rsa1.0_03325.1	1814	23720	1.00E−165	318	324	98	−	1489	1811	14655	14978
UN14628	Rsa1.0_00017.1	927	752458	1.00E−179	324	326	99	−	238	563	467038	467363
UN14629	Rsa1.0_02237.1	1136	33544	0	387	395	97	−	344	738	17873	18264
UN14630	Rsa1.0_00302.1	1619	97521	0	631	653	96	−	844	1490	62037	62689
UN14631	Rsa1.0_04989.1	697	15217	0	523	535	97	−	1	534	2322	2853
UN14632	Rsa1.0_04662.1	1069	16477	0	652	660	98	−	2	661	4459	5118
UN14633	Rsa1.0_03713.1	737	21221	1.00E−153	280	282	99	+	6	287	16173	16454
UN14634	Rsa1.0_04332.1	842	17869	0	834	840	99	−	3	842	10897	11736
UN14635	Rsa1.0_00079.1	991	606987	0	338	340	99	+	648	987	41615	41954
UN14636	Rsa1.0_01783.1	1597	39383	0	382	399	95	+	1094	1492	15244	15632
UN14637	Rsa1.0_15775.1	1073	2429	1.00E−116	218	220	99	+	547	766	1	220
UN14638	Rsa1.0_00251.1	791	303968	0	462	498	92	+	1	484	114386	114876
UN14639	Rsa1.0_00111.1	788	390088	1.00E−129	235	235	100	+	554	788	321110	321344
UN14640	Rsa1.0_01214.1	1100	50599	0	680	695	97	+	404	1098	49762	50456
UN14641	Rsa1.0_03609.1	1560	21798	0	484	495	97	−	4	496	8147	8641
UN14642	Rsa1.0_09813.1	1760	6138	0	524	529	99	+	1141	1669	5191	5719
UN14643	Rsa1.0_02373.1	1580	32048	0	689	689	100	−	6	694	30160	30848
UN14644	Rsa1.0_00221.1	2034	106972	0	1349	1394	96	−	87	1474	101680	103060
UN14645	Rsa1.0_07573.1	1181	9101	0	785	796	98	+	28	823	5990	6785
UN14646	Rsa1.0_00063.1	932	156150	1.00E−175	321	323	99	+	610	932	138349	138670
UN14647	Rsa1.0_01410.1	740	46345	0	334	336	99	+	110	445	33684	34019
UN14648	Rsa1.0_00816.1	1265	403350	1.00E−128	336	366	91	+	5	363	109825	110180
UN14649	Rsa1.0_00021.1	1106	263655	1.00E−121	271	290	93	+	829	1106	186306	186595
UN14650	Rsa1.0_02442.1	520	31355	1.00E−132	261	267	97	+	1	267	12539	12804
UN14651	Rsa1.0_00008.1	1696	358124	0	1616	1711	94	+	1	1696	62808	64514
UN14652	Rsa1.0_00048.1	735	167185	1.00E−178	349	357	97	−	116	471	2507	2862
UN14653	Rsa1.0_01070.1	1789	55099	2.00E−71	189	203	93	−	1590	1789	31371	31570
UN14654	Rsa1.0_00571.1	1334	329018	0	739	767	96	−	349	1112	145078	145844
UN14655	Rsa1.0_00502.1	1054	204180	0	456	464	98	+	595	1054	46632	47095
UN14656	Rsa1.0_00888.1	1601	60978	0	606	615	98	−	576	1190	22815	23429
UN14657	Rsa1.0_00071.1	1826	345714	0	875	906	96	−	1	903	105124	106018
UN14658	Rsa1.0_00601.1	869	177045	0	361	361	100	−	509	869	26136	26496
UN14659	Rsa1.0_00657.1	1263	69858	1.00E−168	318	323	98	−	1	323	9457	9777
UN14660	Rsa1.0_00942.1	786	58873	1.00E−152	341	357	95	+	429	784	3175	3527
UN14661	Rsa1.0_00414.1	983	202190	0	485	517	93	−	96	607	66934	67447
UN14662	Rsa1.0_00079.1	1255	606987	0	703	739	95	+	519	1253	4648	5383

UN14663	Rsa1.0_02681.1	729	28711	1.00E-137	263	268	98	+	160	427	6017	6284
UN14664	Rsa1.0_00110.1	934	132719	1.00E-151	308	320	96	+	590	909	92532	92851
UN14665	Rsa1.0_02931.1	933	26654	0	886	927	95	−	6	932	12851	13774
UN14666	Rsa1.0_02700.1	1633	28584	1.00E-133	256	261	98	+	1	261	12453	12713
UN14667	Rsa1.0_00359.1	1274	91256	1.00E-118	267	279	95	+	997	1271	90578	90854
UN14668	Rsa1.0_07344.1	1484	9528	0	868	904	96	−	1	904	5897	6793
UN14669	Rsa1.0_03045.1	871	25787	0	545	569	95	+	1	566	5846	6403
UN14670	Rsa1.0_01020.1	1544	56619	1.00E-162	291	291	100	+	3	293	32289	32579
UN14671	Rsa1.0_00169.1	1449	274298	1.00E-156	341	358	95	−	3	359	31558	31911
UN14672	Rsa1.0_00855.1	1483	62047	0	381	394	96	+	1098	1483	18514	18903
UN14673	Rsa1.0_00027.1	1698	283362	1.00E-163	295	296	99	+	1402	1697	137431	137726
UN14674	Rsa1.0_00221.1	1456	106972	0	525	539	97	−	925	1454	49096	49634
UN14675	Rsa1.0_05643.1	880	13065	0	443	454	97	−	430	879	2340	2793
UN14676	Rsa1.0_01596.1	1435	42595	1.00E-131	239	239	100	+	3	241	37756	37994
UN14677	Rsa1.0_01626.1	1029	41989	1.00E-106	214	220	97	+	1	220	716	935
UN14678	Rsa1.0_00213.1	904	108167	1.00E-170	306	307	99	−	3	309	13319	13625
UN14679	Rsa1.0_00533.1	807	77375	0	401	403	99	−	399	801	52421	52822
UN14680	Rsa1.0_00486.1	1502	80875	0	564	590	95	+	3	587	14780	15369
UN14681	Rsa1.0_00491.1	930	80219	0	436	457	95	−	466	920	15740	16192
UN14682	Rsa1.0_00816.1	1075	403350	0	366	368	99	−	2	369	4316	4683
UN14683	Rsa1.0_01060.1	868	55368	0	554	561	98	+	304	861	42018	42578
UN14684	Rsa1.0_00237.1	961	235974	0	444	454	97	+	76	527	83061	83514
UN14685	Rsa1.0_00382.1	1541	251946	0	440	458	96	−	7	461	107859	108310
UN14686	Rsa1.0_00018.1	1289	410899	0	521	522	99	+	768	1289	115333	115854
UN14687	Rsa1.0_01436.1	1781	45894	0	798	897	88	−	380	1272	35342	36229
UN14688	Rsa1.0_01480.1	1391	44926	0	912	919	99	−	3	921	26422	27340
UN14689	Rsa1.0_00001.1	866	363028	1.00E-166	344	361	95	−	485	836	314899	315259
UN14690	Rsa1.0_01716.1	2399	40483	0	1012	1041	97	−	821	1854	31373	32410
UN14691	Rsa1.0_00018.1	1450	410899	0	620	621	99	+	3	623	61841	62461
UN14692	Rsa1.0_04470.1	1205	17204	0	922	936	98	+	22	953	13580	14515
UN14693	Rsa1.0_01735.1	1344	40128	0	463	470	98	+	98	567	25629	26098
UN14694	Rsa1.0_01008.1	1471	56924	0	398	398	100	+	1	398	3644	4041
UN14695	Rsa1.0_11168.1	1002	5597	1.00E-131	293	310	94	+	693	998	541	848
UN14696	Rsa1.0_00734.1	801	66508	1.00E-142	260	261	99	+	3	263	56019	56279
UN14697	Rsa1.0_03377.1	1911	23322	0	889	892	99	+	714	1605	16518	17409
UN14698	Rsa1.0_00121.1	596	278029	6.00E-88	213	229	93	−	380	594	110609	110836
UN14699	Rsa1.0_00111.1	1621	390088	0	377	394	95	−	917	1310	381107	381500
UN14700	Rsa1.0_01094.1	796	167285	0	367	370	99	−	1	369	12558	12927
UN14701	Rsa1.0_05336.1	927	14117	0	863	892	96	+	37	927	3678	4556

UN14702	Rsa1.0_00344.1	866	191774	1.00E-166	311	316	98	+	466	781	135888	136203
UN14703	Rsa1.0_34955.1	1304	647	0	358	359	99	−	947	1304	33	391
UN14704	Rsa1.0_00716.1	1022	67078	0	437	439	99	−	385	823	80	518
UN14705	Rsa1.0_08779.1	1360	6929	0	1287	1301	98	−	1	1301	2033	3332
UN14706	Rsa1.0_25122.1	1726	950	0	516	516	100	−	394	909	313	828
UN14707	Rsa1.0_03405.1	3678	23166	0	357	357	100	−	2331	2687	18369	18725
UN14708	Rsa1.0_00469.1	1187	82084	1.00E-101	199	202	98	+	986	1187	70000	70199
UN14709	Rsa1.0_16159.1	1294	2262	0	363	367	98	+	497	863	1340	1706
UN14710	Rsa1.0_02910.1	1285	26843	1.00E-167	298	298	100	−	986	1283	20202	20499
UN14711	Rsa1.0_00080.1	1568	190437	0	1405	1406	99	+	1	1406	104272	105677
UN14712	Rsa1.0_00640.1	1721	70979	1.00E-161	313	322	97	+	1	322	42873	43186
UN14713	Rsa1.0_00765.1	898	65029	1.00E-133	241	241	100	+	1	241	25088	25328
UN14714	Rsa1.0_00143.1	716	121313	0	658	676	97	+	41	716	31025	31699
UN14715	Rsa1.0_01632.1	639	181224	1.00E-161	350	371	94	−	4	362	27928	28294
UN14716	Rsa1.0_00964.1	555	316648	0	540	557	96	−	2	555	167658	168214
UN14717	Rsa1.0_04542.1	1463	16930	0	388	389	99	+	1075	1463	13253	13641
UN14718	Rsa1.0_01484.1	947	44806	0	538	539	99	+	407	945	21799	22337
UN14719	Rsa1.0_01608.1	1075	170540	0	360	366	98	−	348	713	35343	35708
UN14720	Rsa1.0_00550.1	1244	210790	1.00E-143	285	294	96	−	394	687	55698	55991
UN14721	Rsa1.0_00001.1	2030	363028	0	1167	1193	97	+	509	1700	93769	94955
UN14722	Rsa1.0_02355.1	1329	32260	0	632	667	94	−	668	1329	6881	7545
UN14723	Rsa1.0_02942.1	1416	26577	1.00E-109	213	217	98	−	936	1152	6404	6620
UN14724	Rsa1.0_00092.1	2072	659932	0	1007	1013	99	+	1060	2072	238480	239492
UN14725	Rsa1.0_00065.1	1212	454616	1.00E-153	275	275	100	+	382	656	39827	40101
UN14726	Rsa1.0_01976.1	692	36570	1.00E-125	233	235	99	+	127	361	29697	29931
UN14727	Rsa1.0_62560.1	820	367	0	340	342	99	−	1	342	1	342
UN14728	Rsa1.0_00203.1	975	109129	0	346	349	99	+	24	372	80679	81027
UN14729	Rsa1.0_00384.1	1395	89278	1.00E-144	294	303	97	+	1095	1394	35218	35520
UN14730	Rsa1.0_03847.1	1150	20479	0	544	553	98	+	479	1030	3845	4397
UN14731	Rsa1.0_01666.1	1207	173972	1.00E-113	220	224	98	−	761	984	23952	24175
UN14732	Rsa1.0_02478.1	2232	150183	0	437	449	97	+	1	445	6671	7117
UN14733	Rsa1.0_00126.1	1369	127534	1.00E-161	309	316	97	+	492	807	37046	37361
UN14734	Rsa1.0_02036.1	1093	35731	0	573	575	99	−	1	575	8134	8707
UN14735	Rsa1.0_00061.1	1127	218685	1.00E-138	285	296	96	−	826	1121	75762	76055
UN14736	Rsa1.0_03156.1	800	24956	0	495	506	97	+	138	639	2777	3278
UN14737	Rsa1.0_53975.1	980	422	1.00E-120	283	303	93	−	1	303	1	302
UN14738	Rsa1.0_00703.1	1000	67597	0	540	595	90	−	12	592	19623	20198
UN14739	Rsa1.0_00647.1	1463	70440	0	1059	1068	99	−	1	1068	42016	43083
UN14740	Rsa1.0_00139.1	987	357786	0	437	437	100	−	550	986	313072	313508

UN14741	Rsa1.0_00229.1	576	105471	1.00E-163	349	367	95	-	1	367	43352	43717
UN14742	Rsa1.0_14263.1	1154	3600	1.00E-164	294	294	100	+	1	294	1223	1516
UN14743	Rsa1.0_02231.1	528	33661	1.00E-116	245	253	96	+	1	251	33333	33585
UN14744	Rsa1.0_03278.1	923	24125	3.00E-72	166	173	95	+	68	238	2101	2271
UN14745	Rsa1.0_00959.1	1378	58202	0	898	911	98	+	1	909	56856	57763
UN14746	Rsa1.0_00537.1	1004	186980	1.00E-135	268	276	97	+	390	665	17079	17354
UN14747	Rsa1.0_02446.1	727	31330	0	426	436	97	+	3	436	16621	17055
UN14748	Rsa1.0_00145.1	719	261008	1.00E-134	287	298	96	-	9	302	83077	83369
UN14749	Rsa1.0_00035.1	1998	176449	0	1814	1859	97	+	142	1997	82921	84770
UN14750	Rsa1.0_02586.1	1227	29856	0	1213	1225	99	-	2	1222	15013	16236
UN14751	Rsa1.0_02859.1	1297	162118	0	470	470	100	-	828	1297	24378	24847
UN14752	Rsa1.0_00104.1	1226	368722	0	1206	1224	98	-	4	1226	71719	72942
UN14753	Rsa1.0_07935.1	1603	8229	0	1310	1349	97	-	3	1330	6844	8189
UN14754	Rsa1.0_01394.1	690	46641	0	371	383	96	-	308	690	21732	22112
UN14755	Rsa1.0_16093.1	639	2288	0	346	351	98	+	3	351	1232	1581
UN14756	Rsa1.0_00970.1	1436	57994	1.00E-78	230	256	89	-	1099	1351	40381	40636
UN14757	Rsa1.0_00699.1	906	184398	1.00E-128	303	326	92	-	585	893	55759	56083
UN14758	Rsa1.0_04203.1	720	18479	1.00E-135	262	268	97	+	226	493	15931	16198
UN14759	Rsa1.0_00376.1	804	90101	1.00E-148	272	274	99	-	2	275	15971	16244
UN14760	Rsa1.0_01724.1	1087	40292	0	509	545	93	-	2	546	19668	20189
UN14761	Rsa1.0_00706.1	1057	291570	0	359	359	100	-	1	359	59656	60014
UN14762	Rsa1.0_00343.1	2626	93570	0	731	752	97	+	301	1052	68624	69375
UN14763	Rsa1.0_01118.1	1121	53460	2.00E-27	76	80	95	+	23	102	26036	26115
UN14764	Rsa1.0_25222.1	1541	946	0	424	437	97	+	273	709	1	437
UN14765	Rsa1.0_00098.1	613	349239	0	398	414	96	-	111	524	234808	235221
UN14766	Rsa1.0_18964.1	1158	1503	1.00E-156	338	355	95	-	583	934	1	355
UN14767	Rsa1.0_03235.1	1993	24442	0	584	592	98	+	1404	1993	21156	21747
UN14768	Rsa1.0_01511.1	828	44235	1.00E-135	245	245	100	+	3	247	27325	27569
UN14769	Rsa1.0_02648.1	730	29073	1.00E-137	342	372	91	+	357	722	12514	12865
UN14770	Rsa1.0_00143.1	1762	121313	0	396	412	96	+	427	838	91078	91486
UN14771	Rsa1.0_01252.1	1407	49847	1.00E-155	279	279	100	-	1129	1407	17823	18101
UN14772	Rsa1.0_01144.1	901	52431	1.00E-169	330	339	97	+	46	381	22927	23265
UN14773	Rsa1.0_00583.1	1948	74309	1.00E-180	398	417	95	-	11	421	2938	3351
UN14774	Rsa1.0_06149.1	692	11615	0	672	690	97	-	2	691	3841	4530
UN14775	Rsa1.0_01220.1	1102	161775	1.00E-155	278	278	100	-	675	952	30176	30453
UN14776	Rsa1.0_04198.1	874	18504	0	398	407	97	+	3	409	11194	11599
UN14777	Rsa1.0_00316.1	1834	96264	0	519	529	98	-	1307	1834	30580	31108
UN14778	Rsa1.0_06179.1	1138	11548	0	492	515	95	+	61	575	8510	9006
UN14779	Rsa1.0_29623.1	1450	777	0	518	535	96	+	53	587	243	777

UN14780	Rsa1.0_00182.1	472	142305	1.00E-102	192	193	99	+	1	193	131693	131885
UN14781	Rsa1.0_00386.1	1415	89134	1.00E-142	259	260	99	+	1154	1413	72114	72373
UN14782	Rsa1.0_00560.1	1518	75685	0	497	500	99	−	4	503	44093	44590
UN14783	Rsa1.0_01536.1	1003	43747	0	453	460	98	+	21	480	21808	22267
UN14784	Rsa1.0_02739.1	1224	28304	5.00E-16	215	269	79	+	275	542	15657	15924
UN14785	Rsa1.0_00335.1	1048	94371	0	1024	1047	97	−	1	1047	19032	20077
UN14786	Rsa1.0_00001.1	1524	363028	1.00E-162	314	319	98	−	1	319	14633	14949
UN14787	Rsa1.0_00138.1	1041	247040	0	604	613	98	−	432	1040	59252	59863
UN14788	Rsa1.0_00637.1	812	71038	0	487	502	97	−	268	767	3282	3783
UN14789	Rsa1.0_02396.1	832	31783	0	327	328	99	+	196	523	8502	8829
UN14790	Rsa1.0_02195.1	707	34024	0	687	690	99	+	18	707	30589	31278
UN14791	Rsa1.0_00071.1	943	345714	1.00E-119	233	237	98	−	707	942	143202	143438
UN14792	Rsa1.0_00838.1	1072	153820	0	668	678	98	−	9	686	49065	49742
UN14793	Rsa1.0_00027.1	1178	283362	1.00E-121	365	412	88	−	31	420	150170	150581
UN14794	Rsa1.0_06160.1	2131	11596	0	696	723	96	+	685	1394	8631	9353
UN14795	Rsa1.0_01167.1	1192	51859	0	699	705	99	+	489	1192	27703	28407
UN14796	Rsa1.0_00035.1	1074	176449	0	491	495	99	−	579	1073	10603	11097
UN14797	Rsa1.0_00031.1	1138	179862	0	876	997	87	+	39	1025	92394	93340
UN14798	Rsa1.0_01138.1	1291	190615	1.00E-169	308	309	99	−	981	1288	10419	10727
UN14799	Rsa1.0_18159.1	1567	1654	0	631	668	94	−	7	652	1	665
UN14800	Rsa1.0_00699.1	925	184398	0	533	547	97	+	380	925	168017	168562
UN14801	Rsa1.0_07569.1	1385	9106	0	380	380	100	+	844	1223	4562	4941
UN14802	Rsa1.0_05774.1	1066	12667	0	764	770	99	−	1	770	3452	4221
UN14803	Rsa1.0_00926.1	1039	195072	0	513	514	99	+	2	515	41560	42072
UN14804	Rsa1.0_07541.1	1468	9167	1.00E-157	783	953	82	−	206	1155	5430	6361
UN14805	Rsa1.0_00336.1	893	445051	1.00E-160	295	298	98	−	9	306	281490	281787
UN14806	Rsa1.0_01214.1	1452	50599	0	960	1032	93	−	434	1451	2222	3228
UN14807	Rsa1.0_05062.1	1360	14960	0	1339	1348	99	+	10	1357	8672	10019
UN14808	Rsa1.0_02575.1	1297	29944	1.00E-124	269	282	95	+	1018	1296	24000	24277
UN14809	Rsa1.0_01442.1	1477	45776	1.00E-156	296	300	98	−	3	301	23065	23364
UN14810	Rsa1.0_05070.1	1586	14933	0	334	334	100	−	1253	1586	2639	2972
UN14811	Rsa1.0_01305.1	1360	48730	0	503	503	100	−	376	878	18860	19362
UN14812	Rsa1.0_04194.1	2199	132734	0	807	857	94	+	353	1206	116698	117546
UN14813	Rsa1.0_00058.1	1314	159921	0	396	407	97	−	642	1048	50728	51134
UN14814	Rsa1.0_05653.1	546	13043	0	463	482	96	+	69	539	9994	10474
UN14815	Rsa1.0_05398.1	1102	13885	0	581	598	97	+	485	1081	3154	3747
UN14816	Rsa1.0_08385.1	852	7439	1.00E-170	312	315	99	+	3	317	3129	3443
UN14817	Rsa1.0_73312.1	391	313	1.00E-122	229	230	99	−	7	236	1	229
UN14818	Rsa1.0_07981.1	805	8132	0	561	575	97	+	1	575	3287	3852

UN14819	Rsa1.0_01957.1	807	36844	1.00E-148	273	275	99	+	522	796	23850	24124
UN14820	Rsa1.0_00660.1	696	69688	1.00E-129	308	331	93	+	376	696	17669	17999
UN14821	Rsa1.0_00422.1	838	86144	0	565	600	94	−	1	597	54119	54705
UN14822	Rsa1.0_02913.1	1263	26816	0	1023	1037	98	−	224	1260	9449	10484
UN14823	Rsa1.0_00343.1	751	93570	0	474	492	96	+	248	739	2493	2980
UN14824	Rsa1.0_04378.1	819	17651	0	362	362	100	−	124	485	4630	4991
UN14825	Rsa1.0_00337.1	1397	143174	0	422	422	100	+	3	424	139838	140259
UN14826	Rsa1.0_00652.1	923	70089	1.00E-163	359	386	93	−	1	363	47920	48305
UN14827	Rsa1.0_12178.1	796	5294	0	414	427	96	−	371	794	3547	3970
UN14828	Rsa1.0_00052.1	906	162771	1.00E-119	238	245	97	+	36	280	57673	57917
UN14829	Rsa1.0_00422.1	877	86144	0	639	639	100	+	239	877	54119	54757
UN14830	Rsa1.0_00890.1	515	323870	1.00E-88	221	234	94	−	284	514	178764	178996
UN14831	Rsa1.0_19203.1	951	1465	1.00E-179	330	334	98	−	1	334	795	1128
UN14832	Rsa1.0_00355.1	817	92038	0	680	689	98	+	1	689	77741	78429
UN14833	Rsa1.0_00881.1	1706	61260	0	604	635	95	−	826	1460	18614	19248
UN14834	Rsa1.0_07142.1	1471	9832	0	1452	1454	99	−	3	1455	4619	6072
UN14835	Rsa1.0_00979.1	1411	57733	1.00E-139	283	291	97	+	1	288	40182	40472
UN14836	Rsa1.0_04518.1	1639	17005	0	360	365	98	+	885	1249	11846	12209
UN14837	Rsa1.0_00111.1	687	390088	0	414	429	96	+	260	687	75630	76057
UN14838	Rsa1.0_00522.1	1170	78103	1.00E-131	306	325	94	−	1	321	36235	36549
UN14839	Rsa1.0_04123.1	1030	18947	1.00E-157	288	290	99	+	384	673	15187	15476
UN14840	Rsa1.0_04085.1	915	19168	1.00E-153	332	351	94	+	1	351	1560	1901
UN14841	Rsa1.0_02411.1	1602	31655	0	816	854	95	−	443	1296	27638	28475
UN14842	Rsa1.0_02375.1	1019	32026	0	738	754	97	−	5	755	28994	29747
UN14843	Rsa1.0_00706.1	1003	291570	0	459	463	99	+	540	1001	41885	42347
UN14844	Rsa1.0_14210.1	1732	3660	0	580	580	100	+	601	1180	654	1233
UN14845	Rsa1.0_00222.1	1284	172263	0	407	415	98	−	34	445	31532	31945
UN14846	Rsa1.0_00111.1	489	390088	0	415	443	93	+	4	446	174815	175245
UN14847	Rsa1.0_00093.1	826	214962	0	530	530	100	−	297	826	11283	11812
UN14848	Rsa1.0_00787.1	1319	64239	0	562	577	97	−	743	1319	32499	33075
UN14849	Rsa1.0_00211.1	1088	228189	0	578	617	93	+	1	616	179317	179912
UN14850	Rsa1.0_00645.1	488	189716	2.00E-99	184	184	100	+	257	440	35226	35409
UN14851	Rsa1.0_00647.1	1088	70440	0	461	461	100	−	628	1088	5071	5531
UN14852	Rsa1.0_00447.1	1062	84051	1.00E-102	218	226	96	−	3	227	26801	27026
UN14853	Rsa1.0_02981.1	707	26285	1.00E-116	219	221	99	+	487	707	5587	5807
UN14854	Rsa1.0_01553.1	1067	43379	0	651	666	97	+	401	1066	2337	3002
UN14855	Rsa1.0_00336.1	1812	445051	0	461	466	98	+	3	468	11085	11550
UN14856	Rsa1.0_01070.1	1730	55099	1.00E-111	204	204	100	−	1526	1729	31367	31570
UN14857	Rsa1.0_06978.1	976	10073	0	628	656	95	−	1	646	1165	1819

UN14858	Rsa1.0_08078.1	1546	7944	0	423	429	98	−	1118	1546	3015	3442
UN14859	Rsa1.0_00200.1	1032	162016	0	323	323	100	+	708	1030	96962	97284
UN14860	Rsa1.0_01016.1	788	181422	0	563	563	100	−	2	564	171865	172427
UN14861	Rsa1.0_00055.1	921	520193	0	740	764	96	+	30	792	289747	290503
UN14862	Rsa1.0_00810.1	805	63289	0	527	560	94	−	2	560	6634	7178
UN14863	Rsa1.0_00546.1	599	76523	1.00E−168	352	366	96	−	1	363	11385	11749
UN14864	Rsa1.0_00033.1	711	515811	0	373	373	100	+	337	709	365844	366216
UN14865	Rsa1.0_02286.1	1203	32993	0	854	872	97	−	334	1202	24132	25003
UN14866	Rsa1.0_35276.1	1672	641	1.00E−121	227	229	99	+	48	276	367	595
UN14867	Rsa1.0_09899.1	1409	6099	0	1181	1201	98	+	216	1409	2789	3989
UN14868	Rsa1.0_00101.1	723	267193	1.00E−137	348	378	92	+	353	720	47241	47613
UN14869	Rsa1.0_00881.1	924	61260	1.00E−159	342	358	95	+	544	891	20053	20410
UN14870	Rsa1.0_07606.1	663	9035	0	638	667	95	−	6	663	1971	2628
UN14871	Rsa1.0_00331.1	1311	176754	0	806	810	99	+	1	810	22158	22967
UN14872	Rsa1.0_09472.1	1342	6344	1.00E−136	322	342	94	−	1004	1342	6005	6344
UN14873	Rsa1.0_01173.1	1189	51727	0	402	409	98	−	2	410	1555	1962
UN14874	Rsa1.0_00168.1	1294	115870	1.00E−170	313	316	99	+	662	977	56550	56865
UN14875	Rsa1.0_00087.1	941	761073	0	695	699	99	+	243	941	182792	183489
UN14876	Rsa1.0_00184.1	1185	218748	0	870	877	99	+	309	1185	43850	44724
UN14877	Rsa1.0_00295.1	807	152597	1.00E−89	202	212	95	+	540	750	140727	140938
UN14878	Rsa1.0_00646.1	937	70473	1.00E−116	267	279	95	−	660	933	5977	6254
UN14879	Rsa1.0_17320.1	829	1861	0	341	347	98	+	16	362	254	600
UN14880	Rsa1.0_01126.1	2877	53127	0	2720	2724	99	−	1	2724	35534	38257
UN14881	Rsa1.0_01272.1	967	49553	0	564	604	93	+	332	932	18652	19240
UN14882	Rsa1.0_05217.1	1016	142938	0	439	443	99	−	141	583	130269	130711
UN14883	Rsa1.0_01100.1	1377	54113	0	836	843	99	+	534	1375	52303	53145
UN14884	Rsa1.0_00059.1	655	159650	1.00E−135	304	322	94	+	4	319	123644	123957
UN14885	Rsa1.0_02723.1	1230	28408	0	644	654	98	−	549	1201	7650	8303
UN14886	Rsa1.0_00112.1	767	132199	0	498	505	98	−	262	766	72844	73348
UN14887	Rsa1.0_00154.1	1141	189461	0	623	632	98	+	508	1139	24514	25144
UN14888	Rsa1.0_00662.1	1496	192183	0	1346	1398	96	−	2	1398	59159	60549
UN14889	Rsa1.0_02043.1	1122	163754	0	858	859	99	+	264	1122	131101	131959
UN14890	Rsa1.0_00385.1	1299	89160	0	559	569	98	+	3	567	47349	47914
UN14891	Rsa1.0_03527.1	1765	22278	0	472	478	98	−	6	483	10601	11075
UN14892	Rsa1.0_01182.1	1328	167304	1.00E−152	332	348	95	−	980	1327	71	409
UN14893	Rsa1.0_00773.1	1523	64839	0	716	752	95	−	59	810	1579	2330
UN14894	Rsa1.0_00001.1	893	363028	0	377	381	98	+	32	412	200236	200616
UN14895	Rsa1.0_00871.1	1091	61539	0	448	464	96	−	621	1083	43277	43732
UN14896	Rsa1.0_02650.1	1063	29047	0	495	511	96	+	1	504	11523	12032

UN14897	Rsa1.0_00077.1	678	145005	0	676	676	100	+	3	678	1851	2526
UN14898	Rsa1.0_00664.1	712	69573	3.00E-96	259	281	92	+	435	712	1722	1994
UN14899	Rsa1.0_14871.1	654	2999	1.00E-95	205	214	95	-	44	257	2735	2948
UN14900	Rsa1.0_06596.1	761	10699	0	357	360	99	-	2	361	5401	5760
UN14901	Rsa1.0_00078.1	1431	175053	0	429	445	96	-	257	701	38368	38812
UN14902	Rsa1.0_01270.1	660	49574	1.00E-130	373	410	90	-	269	657	29161	29567
UN14903	Rsa1.0_03424.1	839	23064	0	604	604	100	+	229	832	79	682
UN14904	Rsa1.0_00589.1	992	209965	0	571	595	95	-	398	992	9704	10294
UN14905	Rsa1.0_17884.1	1831	1718	1.00E-110	267	287	93	+	680	966	1433	1718
UN14906	Rsa1.0_01778.1	1017	39509	7.00E-98	218	230	94	-	788	1017	16288	16512
UN14907	Rsa1.0_00179.1	1058	393324	1.00E-142	279	285	97	+	3	286	188688	188972
UN14908	Rsa1.0_01958.1	1011	261128	4.00E-90	193	201	96	+	127	327	113925	114125
UN14909	Rsa1.0_02901.1	741	26909	0	389	400	97	-	6	404	25992	26389
UN14910	Rsa1.0_11724.1	709	5428	0	421	424	99	-	287	709	5005	5428
UN14911	Rsa1.0_01506.1	1040	44370	0	654	654	100	+	1	654	13549	14202
UN14912	Rsa1.0_01800.1	1377	39109	0	555	564	98	+	293	856	6664	7227
UN14913	Rsa1.0_00026.1	667	327835	0	571	571	100	-	9	579	306356	306926
UN14914	Rsa1.0_00097.1	604	138858	0	520	529	98	+	76	604	110448	110975
UN14915	Rsa1.0_00094.1	1157	139042	0	362	365	99	+	790	1154	5876	6238
UN14916	Rsa1.0_00143.1	715	121313	0	686	709	96	-	6	713	30990	31697
UN14917	Rsa1.0_02083.1	1201	35216	0	866	905	95	-	308	1199	34309	35212
UN14918	Rsa1.0_00302.1	744	97521	0	743	744	99	-	1	744	38111	38854
UN14919	Rsa1.0_02041.1	903	163634	0	890	897	99	+	3	899	157069	157965
UN14920	Rsa1.0_00124.1	1191	262503	0	445	448	99	+	742	1189	29622	30069
UN14921	Rsa1.0_00009.1	429	232558	1.00E-136	291	305	95	-	130	429	185026	185329
UN14922	Rsa1.0_01866.1	693	37961	0	412	419	98	+	273	691	36635	37049
UN14923	Rsa1.0_01276.1	1536	49384	0	557	558	99	-	1	558	18570	19127
UN14924	Rsa1.0_03795.1	1211	20818	1.00E-164	304	306	99	-	2	306	15481	15786
UN14925	Rsa1.0_03149.1	662	24983	1.00E-154	288	292	98	-	364	655	15409	15700
UN14926	Rsa1.0_13824.1	1136	4259	0	483	503	96	-	637	1134	2033	2534
UN14927	Rsa1.0_03662.1	519	21492	3.00E-92	184	188	97	-	69	256	11532	11719
UN14928	Rsa1.0_75718.1	2000	303	1.00E-122	239	244	97	+	913	1156	42	285
UN14929	Rsa1.0_00002.1	1214	601661	0	793	793	100	-	422	1214	182851	183643
UN14930	Rsa1.0_00253.1	1090	231292	1.00E-153	310	318	97	+	1	316	23517	23833
UN14931	Rsa1.0_00178.1	1223	114861	0	559	584	95	-	654	1220	84388	84969
UN14932	Rsa1.0_14432.1	1055	3395	0	1023	1045	97	-	2	1046	1262	2305
UN14933	Rsa1.0_00751.1	1344	65759	0	470	475	98	+	279	753	3228	3702
UN14934	Rsa1.0_12933.1	1585	5071	0	549	550	99	-	1036	1585	3392	3941
UN14935	Rsa1.0_01567.1	1504	43090	0	1486	1510	98	+	3	1502	3586	5095

UN14936	Rsa1.0_00231.1	1277	105381	1.00E-180	323	324	99	-	3	326	32718	33041
UN14937	Rsa1.0_00961.1	1054	58185	0	335	336	99	+	1	336	18029	18364
UN14938	Rsa1.0_30995.1	1108	739	1.00E-117	226	230	98	+	3	232	510	739
UN14939	Rsa1.0_00745.1	1426	66067	0	698	727	96	+	119	844	42176	42902
UN14940	Rsa1.0_06968.1	1429	10090	0	651	676	96	-	755	1429	7748	8421
UN14941	Rsa1.0_00251.1	1057	303968	1.00E-175	329	335	98	+	494	828	141398	141732
UN14942	Rsa1.0_01696.1	1723	301850	1.00E-153	294	300	98	-	1428	1723	153034	153333
UN14943	Rsa1.0_01087.1	1674	54508	0	747	749	99	-	661	1409	18244	18992
UN14944	Rsa1.0_00039.1	1530	296927	0	558	590	94	-	3	574	32084	32673
UN14945	Rsa1.0_00170.1	1527	179538	1.00E-158	358	381	93	-	1154	1527	24375	24753
UN14946	Rsa1.0_07829.1	1259	8462	0	347	349	99	+	911	1259	4627	4974
UN14947	Rsa1.0_00293.1	1627	339294	0	548	604	90	+	75	678	88473	89076
UN14948	Rsa1.0_00027.1	771	283362	1.00E-178	349	356	98	+	1	355	167567	167919
UN14949	Rsa1.0_00860.1	906	61789	0	897	906	99	-	1	906	45040	45945
UN14950	Rsa1.0_00397.1	1246	88189	0	541	564	95	+	1	555	28141	28698
UN14951	Rsa1.0_01351.1	1247	47498	0	468	484	96	-	735	1214	1320	1801
UN14952	Rsa1.0_04070.1	1437	19244	0	426	434	98	-	1004	1435	10639	11071
UN14953	Rsa1.0_02745.1	1174	28263	5.00E-62	249	289	86	-	17	292	10794	11082
UN14954	Rsa1.0_00288.1	1504	98659	0	667	675	98	+	831	1504	80376	81050
UN14955	Rsa1.0_00722.1	1237	66840	1.00E-149	274	276	99	+	962	1237	21060	21335
UN14956	Rsa1.0_00840.1	1316	62514	0	688	694	99	-	4	697	6307	7000
UN14957	Rsa1.0_14703.1	1190	3143	0	555	574	96	+	187	760	1	573
UN14958	Rsa1.0_03876.1	505	20334	0	499	506	98	-	1	504	16808	17313
UN14959	Rsa1.0_05461.1	1447	13674	0	399	406	98	+	1044	1447	9168	9573
UN14960	Rsa1.0_00123.1	935	325650	1.00E-134	327	356	91	+	581	935	128148	128481
UN14961	Rsa1.0_00047.1	1286	168582	0	554	597	92	-	627	1213	122835	123416
UN14962	Rsa1.0_11581.1	1435	5466	0	439	464	94	-	812	1274	557	1010
UN14963	Rsa1.0_15781.1	1390	2424	0	389	391	99	+	949	1339	2034	2424
UN14964	Rsa1.0_00123.1	1019	325650	1.00E-163	339	352	96	+	666	1017	2558	2907
UN14965	Rsa1.0_01921.1	1898	168088	1.00E-111	243	253	96	-	1647	1898	141166	141417
UN14966	Rsa1.0_08355.1	1237	7487	0	424	427	99	-	809	1235	6505	6931
UN14967	Rsa1.0_04478.1	1084	17183	0	1070	1084	98	+	1	1084	9836	10919
UN14968	Rsa1.0_00123.1	1125	325650	1.00E-148	288	295	97	+	1	295	224045	224339
UN14969	Rsa1.0_40698.1	1846	551	0	541	552	98	-	1165	1714	1	551
UN14970	Rsa1.0_03125.1	1315	147978	0	339	341	99	-	288	628	139075	139415
UN14971	Rsa1.0_00471.1	1619	81897	0	987	990	99	+	1	990	27017	28005
UN14972	Rsa1.0_00374.1	1302	165738	0	467	472	98	+	526	997	93436	93907
UN14973	Rsa1.0_06430.1	1398	11034	1.00E-176	327	330	99	+	1070	1398	9389	9718
UN14974	Rsa1.0_00752.1	1023	65756	0	1006	1019	98	-	3	1021	23450	24467

UN14975	Rsa1.0_00286.1	581	98709	2.00E-96	295	329	89	+	259	581	29363	29681
UN14976	Rsa1.0_15608.1	1095	2522	0	957	967	98	-	129	1095	178	1144
UN14977	Rsa1.0_01511.1	1399	44235	0	620	620	100	-	227	846	7364	7983
UN14978	Rsa1.0_00876.1	1131	61328	0	1096	1132	96	+	2	1129	25989	27115
UN14979	Rsa1.0_00047.1	1269	168582	0	1202	1281	93	-	1	1269	149243	150497
UN14980	Rsa1.0_15167.1	897	2783	0	881	896	98	-	1	893	835	1729
UN14981	Rsa1.0_01344.1	655	142535	0	647	649	99	+	3	651	117286	117934
UN14982	Rsa1.0_03524.1	1983	22297	0	680	682	99	-	909	1590	19400	20081
UN14983	Rsa1.0_06175.1	1415	11569	0	423	436	97	+	45	476	4547	4981
UN14984	Rsa1.0_00102.1	755	135497	2.00E-97	184	185	99	+	225	409	88065	88249
UN14985	Rsa1.0_03659.1	978	21525	1.00E-123	234	237	98	-	584	820	13404	13640
UN14986	Rsa1.0_04086.1	565	19166	0	490	533	91	+	10	535	10482	11003
UN14987	Rsa1.0_01545.1	906	43599	0	523	537	97	-	1	530	27015	27548
UN14988	Rsa1.0_00347.1	1315	93048	1.00E-173	366	385	95	+	827	1211	54584	54968
UN14989	Rsa1.0_01610.1	1585	42274	0	869	872	99	-	59	930	18789	19660
UN14990	Rsa1.0_02609.1	1306	29600	0	580	599	96	+	714	1306	24446	25044
UN14991	Rsa1.0_00071.1	1182	345714	0	703	779	90	-	40	805	11652	12412
UN14992	Rsa1.0_01024.1	1303	56569	0	402	430	93	-	874	1303	10984	11395
UN14993	Rsa1.0_00017.1	561	752458	0	542	565	95	+	1	561	363690	364245
UN14994	Rsa1.0_03785.1	1509	20883	0	370	370	100	-	1	370	15659	16028
UN14995	Rsa1.0_00006.1	1028	396176	0	596	606	98	-	1	606	66782	67387
UN14996	Rsa1.0_00071.1	1411	345714	0	990	1006	98	+	387	1388	297779	298784
UN14997	Rsa1.0_00159.1	1193	431686	1.00E-140	356	390	91	-	351	736	333967	334356
UN14998	Rsa1.0_01115.1	825	183545	0	392	398	98	+	1	398	7262	7659
UN14999	Rsa1.0_00473.1	1749	212204	0	398	413	96	+	274	683	190475	190883
UN15000	Rsa1.0_01451.1	716	45459	0	698	715	97	+	3	713	42339	43053
UN15001	Rsa1.0_01699.1	838	40816	0	368	369	99	+	1	368	16162	16530
UN15002	Rsa1.0_01575.1	877	42924	1.00E-155	290	294	98	+	2	295	30820	31113
UN15003	Rsa1.0_07106.1	1090	9882	1.00E-158	286	287	99	+	804	1090	416	702
UN15004	Rsa1.0_00047.1	1310	168582	1.00E-133	247	249	99	-	1062	1310	79354	79602
UN15005	#	#	#	#	#	#	#	#	#	#	#	#
UN15006	Rsa1.0_00953.1	895	145012	0	487	493	98	+	352	844	26538	27030
UN15007	Rsa1.0_00363.1	1677	90968	1.00E-112	239	251	95	-	642	884	30983	31233
UN15008	Rsa1.0_02811.1	801	27669	0	499	513	97	+	289	801	26244	26744
UN15009	Rsa1.0_00013.1	489	292129	1.00E-154	307	316	97	-	5	320	228465	228779
UN15010	Rsa1.0_00739.1	936	66155	1.00E-157	323	339	95	+	3	329	49406	49744
UN15011	Rsa1.0_02725.1	1621	28404	0	1118	1119	99	+	1	1119	23754	24872
UN15012	Rsa1.0_02784.1	1167	27881	1.00E-124	226	226	100	+	25	250	6347	6572
UN15013	Rsa1.0_25122.1	1404	950	0	424	425	99	-	311	735	313	737

UN15014	Rsa1.0_03354.1	931	23491	0	659	670	98	−	10	679	13474	14138
UN15015	Rsa1.0_02975.1	1068	26322	0	577	593	97	+	176	767	23819	24410
UN15016	Rsa1.0_01635.1	824	41865	0	408	415	98	+	1	413	20348	20762
UN15017	Rsa1.0_00702.1	968	67600	0	602	607	99	+	14	620	62881	63487
UN15018	Rsa1.0_00139.1	774	357786	2.00E−76	191	206	92	+	406	611	44950	45155
UN15019	Rsa1.0_01814.1	1267	38917	0	505	509	99	−	4	512	16947	17455
UN15020	Rsa1.0_00353.1	1226	92242	0	1197	1219	98	−	3	1219	27804	29019
UN15021	Rsa1.0_00191.1	542	316976	0	535	542	98	+	2	542	18342	18883
UN15022	Rsa1.0_04561.1	1596	16849	0	1060	1109	95	+	492	1592	4119	5224
UN15023	Rsa1.0_18356.1	978	1615	1.00E−171	339	349	97	−	29	376	177	525
UN15024	Rsa1.0_00554.1	1156	150953	0	864	882	97	−	274	1155	51447	52328
UN15025	Rsa1.0_00793.1	1321	64103	1.00E−96	243	264	92	−	99	362	5308	5571
UN15026	Rsa1.0_20758.1	1147	1272	0	519	523	99	+	625	1147	557	1079
UN15027	Rsa1.0_01949.1	1447	36960	0	417	426	97	+	1022	1447	27763	28188
UN15028	Rsa1.0_04516.1	790	17018	0	613	618	99	+	1	618	16316	16933
UN15029	Rsa1.0_00926.1	849	195072	0	361	364	99	−	170	532	35123	35485
UN15030	Rsa1.0_00703.1	703	67597	1.00E−174	317	318	99	−	1	318	10307	10623
UN15031	Rsa1.0_01002.1	997	57063	0	431	446	96	−	343	788	50140	50582
UN15032	Rsa1.0_00012.1	809	222148	1.00E−165	342	353	96	−	459	808	83199	83548
UN15033	Rsa1.0_04132.1	572	18917	1.00E−117	248	257	96	−	4	260	3215	3468
UN15034	Rsa1.0_00119.1	901	467509	0	468	474	98	−	3	474	8515	8988
UN15035	Rsa1.0_00115.1	1643	131321	0	507	529	95	−	105	625	54113	54641
UN15036	Rsa1.0_00004.1	855	253996	0	475	478	99	−	38	515	198366	198840
UN15037	Rsa1.0_04355.1	881	146733	1.00E−157	323	336	96	+	3	336	129240	129570
UN15038	Rsa1.0_00483.1	1480	81102	0	350	354	98	−	283	636	30497	30850
UN15039	Rsa1.0_05019.1	1417	15114	0	679	680	99	−	1	680	4471	5150
UN15040	Rsa1.0_04199.1	2255	18501	0	2138	2164	98	−	92	2255	8231	10394
UN15041	Rsa1.0_02952.1	672	26492	0	658	672	97	−	1	672	19790	20461
UN15042	Rsa1.0_00019.1	2070	301669	0	545	554	98	−	1	554	298888	299440
UN15043	Rsa1.0_02036.1	542	35731	0	533	537	99	+	3	539	11888	12424
UN15044	Rsa1.0_01296.1	623	49035	0	352	356	98	+	270	623	3720	4075
UN15045	Rsa1.0_06138.1	1148	11651	1.00E−178	320	321	99	−	1	321	8144	8464
UN15046	Rsa1.0_00065.1	1082	454616	0	548	551	99	+	1	551	152180	152730
UN15047	Rsa1.0_00858.1	836	61892	1.00E−108	205	207	99	+	49	255	14979	15185
UN15048	Rsa1.0_13600.1	967	4672	1.00E−170	328	336	97	−	421	756	3970	4305
UN15049	Rsa1.0_00974.1	930	57893	0	483	489	98	+	441	929	5213	5701
UN15050	Rsa1.0_00017.1	1139	752458	0	1084	1170	92	+	2	1139	312127	313293
UN15051	Rsa1.0_01229.1	941	50235	0	937	946	99	+	1	941	2586	3531
UN15052	Rsa1.0_03268.1	552	24197	0	536	550	97	−	3	552	12409	12956

UN15053	Rsa1.0_00964.1	950	316648	0	543	579	93	+	3	568	261724	262289
UN15054	Rsa1.0_00529.1	1402	77487	0	667	730	91	+	2	730	52460	53186
UN15055	Rsa1.0_19438.1	606	1432	3.00E-83	186	195	95	+	3	194	382	576
UN15056	Rsa1.0_00108.1	1528	478491	0	993	1028	96	+	511	1526	305432	306459
UN15057	Rsa1.0_10655.1	1208	5768	1.00E-161	295	296	99	−	911	1206	435	729
UN15058	Rsa1.0_09183.1	1040	6573	4.00E-81	257	284	90	−	762	1040	3968	4243
UN15059	Rsa1.0_18896.1	1396	1515	0	417	427	97	−	1	426	406	831
UN15060	Rsa1.0_07863.1	888	8404	1.00E-126	233	234	99	−	653	886	862	1095
UN15061	Rsa1.0_01442.1	670	45776	0	392	405	96	−	1	405	25486	25890
UN15062	Rsa1.0_00329.1	468	222544	1.00E-135	369	410	90	+	1	388	2552	2960
UN15063	Rsa1.0_03342.1	728	23600	0	389	405	96	+	1	401	17824	18227
UN15064	Rsa1.0_00025.1	1012	277312	0	394	408	96	−	1	397	14017	14423
UN15065	Rsa1.0_04397.1	775	17555	0	600	613	97	−	157	760	4519	5131
UN15066	Rsa1.0_01241.1	1665	50067	0	569	575	98	−	334	908	5667	6241
UN15067	Rsa1.0_00644.1	1067	136881	1.00E-169	404	434	93	−	642	1064	62650	63077
UN15068	Rsa1.0_01301.1	594	169072	0	409	421	97	+	177	594	42572	42989
UN15069	Rsa1.0_00061.1	914	218685	4.00E-93	238	263	90	−	653	914	166243	166485
UN15070	Rsa1.0_00686.1	760	68511	0	723	803	90	+	1	760	32781	33583
UN15071	Rsa1.0_00173.1	978	288743	9.00E-14	86	101	85	+	803	903	112554	112654
UN15072	Rsa1.0_01382.1	831	46877	0	423	440	96	+	359	795	21635	22071
UN15073	Rsa1.0_03944.1	806	19926	0	790	806	98	−	1	806	16353	17154
UN15074	Rsa1.0_00032.1	594	306326	2.00E-47	203	234	86	+	98	330	215913	216125
UN15075	Rsa1.0_00253.1	957	231292	2.00E-67	137	139	98	+	463	601	116001	116139
UN15076	Rsa1.0_02291.1	1074	32959	0	843	849	99	+	7	855	14784	15630
UN15077	Rsa1.0_00127.1	911	127446	0	467	479	97	+	3	479	13924	14395
UN15078	Rsa1.0_01882.1	819	37697	0	429	449	95	−	3	445	36182	36630
UN15079	Rsa1.0_00131.1	851	432233	1.00E-121	340	376	90	−	48	422	8545	8907
UN15080	Rsa1.0_00107.1	788	431659	0	784	791	99	+	1	788	153045	153833
UN15081	Rsa1.0_01625.1	863	42019	0	370	375	98	−	1	374	34139	34513
UN15082	Rsa1.0_09302.1	1108	6470	0	584	606	96	+	3	607	2966	3566
UN15083	Rsa1.0_00768.1	513	64969	1.00E-111	285	310	91	+	1	296	60638	60947
UN15084	Rsa1.0_00733.1	1000	66583	0	645	664	97	−	338	1000	59761	60423
UN15085	Rsa1.0_00577.1	698	174827	1.00E-134	265	272	97	−	101	368	173422	173693
UN15086	Rsa1.0_05050.1	908	15022	1.00E-128	256	264	96	+	639	902	6127	6390
UN15087	Rsa1.0_00369.1	1364	266462	0	370	382	96	+	1	374	143048	143428
UN15088	Rsa1.0_00516.1	778	78417	1.00E-119	241	249	96	−	4	252	37437	37685
UN15089	Rsa1.0_04646.1	753	16541	2.00E-88	166	166	100	+	156	321	9517	9682
UN15090	Rsa1.0_02296.1	2022	32920	0	476	482	98	−	1	479	16072	16553
UN15091	Rsa1.0_00829.1	701	158305	0	461	503	91	+	10	509	47474	47954

UN15092	Rsa1.0_08019.1	725	8075	3.00E-84	159	159	100	-	564	722	2709	2867
UN15093	Rsa1.0_00652.1	1019	70089	0	528	567	93	-	424	989	18097	18649
UN15094	Rsa1.0_00088.1	1040	141341	6.00E-89	245	271	90	-	776	1039	62671	62928
UN15095	Rsa1.0_02185.1	749	34150	0	503	517	97	-	231	745	32514	33030
UN15096	Rsa1.0_01222.1	815	50398	9.00E-63	138	143	96	-	383	525	3523	3665
UN15097	Rsa1.0_00294.1	802	144860	0	529	546	96	+	67	612	57030	57575
UN15098	Rsa1.0_01436.1	857	45894	0	339	342	99	-	1	342	33611	33952
UN15099	Rsa1.0_13590.1	783	4689	0	747	768	97	-	3	767	3665	4431
UN15100	Rsa1.0_00718.1	837	279335	1.00E-168	303	304	99	-	1	304	235035	235338
UN15101	Rsa1.0_02362.1	1493	32190	0	613	630	97	+	1	629	21896	22525
UN15102	Rsa1.0_00482.1	1775	81162	0	766	780	98	-	256	1035	8800	9579
UN15103	Rsa1.0_08234.1	1576	7681	0	383	395	96	+	1	395	7210	7596
UN15104	Rsa1.0_00377.1	950	90038	0	374	387	96	+	7	390	17057	17443
UN15105	Rsa1.0_00104.1	1555	368722	1.00E-167	345	358	96	+	137	492	32684	33041
UN15106	Rsa1.0_00053.1	1070	263459	1.00E-173	362	381	95	+	666	1046	84698	85069
UN15107	Rsa1.0_00632.1	1281	183777	1.00E-176	356	370	96	+	911	1280	157543	157912
UN15108	Rsa1.0_00413.1	1052	86921	0	377	379	99	-	675	1052	37404	37782
UN15109	Rsa1.0_03302.1	976	23898	1.00E-174	350	361	96	-	610	967	6069	6429
UN15110	Rsa1.0_01425.1	1278	46136	0	355	362	98	+	59	420	15558	15919
UN15111	Rsa1.0_01053.1	902	55483	1.00E-165	309	311	99	+	1	311	40466	40774
UN15112	Rsa1.0_00407.1	1305	191544	0	337	338	99	+	968	1305	30155	30492
UN15113	Rsa1.0_01920.1	758	158533	0	738	749	98	-	13	758	4769	5515
UN15114	Rsa1.0_00426.1	1110	85668	0	390	405	96	-	3	407	59557	59955
UN15115	Rsa1.0_18100.1	1211	1667	1.00E-130	344	381	90	-	833	1211	278	641
UN15116	Rsa1.0_03937.1	1523	19969	1.00E-154	295	301	98	-	1223	1523	3001	3301
UN15117	Rsa1.0_00088.1	1180	141341	0	456	462	98	+	106	567	94765	95226
UN15118	Rsa1.0_01969.1	1592	153351	0	440	446	98	-	1	446	16782	17227
UN15119	Rsa1.0_00246.1	1023	103541	0	555	581	95	+	444	1023	78385	78965
UN15120	Rsa1.0_02191.1	800	34078	0	412	418	98	-	53	466	23641	24058
UN15121	Rsa1.0_00186.1	1286	112669	7.00E-83	227	247	91	-	95	336	10420	10666
UN15122	Rsa1.0_00739.1	438	66155	1.00E-145	260	260	100	-	120	379	31439	31698
UN15123	Rsa1.0_01752.1	1359	39805	1.00E-154	298	305	97	+	2	306	19218	19522
UN15124	Rsa1.0_03959.1	810	19866	1.00E-172	324	330	98	-	3	332	165	494
UN15125	Rsa1.0_00427.1	496	189678	1.00E-122	281	297	94	-	2	294	14547	14842
UN15126	Rsa1.0_00684.1	1399	141701	0	540	557	96	+	1	556	54156	54698
UN15127	Rsa1.0_07376.1	970	9496	1.00E-110	202	202	100	+	385	586	7283	7484
UN15128	Rsa1.0_03498.1	779	22480	0	549	550	99	-	230	779	18285	18833
UN15129	Rsa1.0_00708.1	1011	67366	0	451	464	97	-	1	462	25154	25615
UN15130	Rsa1.0_00418.1	1283	215377	0	381	396	96	+	363	758	2935	3330

UN15131	Rsa1.0_03019.1	1365	25987	1.00E-135	257	261	98	−	7	267	21947	22207
UN15132	Rsa1.0_04156.1	1401	18799	1.00E-142	286	296	96	−	1105	1400	15209	15498
UN15133	Rsa1.0_02295.1	1325	149884	1.00E-154	298	305	97	+	528	832	9436	9740
UN15134	Rsa1.0_03068.1	863	25620	0	837	866	96	+	1	862	10839	11697
UN15135	Rsa1.0_00389.1	1361	88795	0	1066	1084	98	−	1	1084	61128	62211
UN15136	Rsa1.0_05293.1	1508	14237	0	490	515	95	+	103	617	6224	6730
UN15137	Rsa1.0_01188.1	1791	51362	0	833	851	97	+	941	1777	33730	34580
UN15138	Rsa1.0_00001.1	940	363028	0	392	397	98	+	1	397	249958	250354
UN15139	Rsa1.0_00100.1	1564	249232	1.00E-162	398	429	92	+	1088	1509	13427	13852
UN15140	Rsa1.0_01509.1	1537	44258	1.00E-178	317	317	100	+	1221	1537	2566	2882
UN15141	Rsa1.0_01554.1	906	43379	0	879	907	96	−	1	905	39958	40863
UN15142	Rsa1.0_01553.1	1200	43379	0	1008	1057	95	−	148	1200	26896	27945
UN15143	Rsa1.0_00045.1	555	328694	1.00E-98	198	203	97	+	6	208	148689	148891
UN15144	Rsa1.0_00163.1	783	116847	1.00E-174	315	317	99	+	467	783	67121	67437
UN15145	Rsa1.0_02911.1	1450	142461	0	603	624	96	−	1	619	23423	24042
UN15146	Rsa1.0_04073.1	1222	19238	1.00E-129	259	267	97	+	959	1220	16886	17152
UN15147	Rsa1.0_00487.1	882	80674	1.00E-178	363	378	96	+	506	880	75851	76228
UN15148	Rsa1.0_10201.1	1743	5946	0	1403	1436	97	+	1	1436	418	1851
UN15149	Rsa1.0_00596.1	698	73578	0	420	425	98	+	5	429	42103	42527
UN15150	Rsa1.0_00277.1	1627	100132	1.00E-132	326	351	92	+	1	349	88382	88725
UN15151	Rsa1.0_00900.1	1028	60367	1.00E-145	306	321	95	−	1	321	29910	30230
UN15152	Rsa1.0_00029.1	758	272992	1.00E-168	367	385	95	−	374	750	10187	10568
UN15153	Rsa1.0_39426.1	713	570	0	384	398	96	−	1	398	48	445
UN15154	Rsa1.0_00469.1	1035	82084	1.00E-164	322	331	97	+	405	732	66858	67188
UN15155	Rsa1.0_01335.1	376	47894	0	344	351	98	+	26	376	29219	29569
UN15156	Rsa1.0_00275.1	773	210104	1.00E-162	326	337	96	+	10	346	176412	176741
UN15157	Rsa1.0_00446.1	736	167829	0	369	382	96	+	355	736	129961	130339
UN15158	Rsa1.0_03966.1	1745	19823	0	392	403	97	−	831	1233	2619	3021
UN15159	Rsa1.0_02991.1	628	26178	2.00E-88	209	219	95	−	1	216	4393	4607
UN15160	Rsa1.0_04835.1	1077	15766	1.00E-132	270	280	96	−	179	458	9964	10243
UN15161	Rsa1.0_00153.1	706	192931	1.00E-127	284	301	94	+	1	295	143120	143418
UN15162	Rsa1.0_04542.1	891	16930	1.00E-147	290	297	97	−	3	299	16072	16362
UN15163	Rsa1.0_03195.1	1354	24673	0	619	620	99	−	1	619	8990	9609
UN15164	Rsa1.0_00549.1	1395	76473	0	328	329	99	−	1067	1395	7914	8242
UN15165	Rsa1.0_01960.1	1862	36766	1.00E-153	287	291	98	−	592	882	9530	9820
UN15166	Rsa1.0_00407.1	1040	191544	0	771	822	93	−	218	1026	155550	156357
UN15167	Rsa1.0_02629.1	1383	29350	0	504	504	100	−	555	1058	5350	5853
UN15168	Rsa1.0_01786.1	1469	133324	0	540	562	96	+	913	1469	113313	113870
UN15169	Rsa1.0_02070.1	864	158516	5.00E-83	160	161	99	−	130	290	141340	141500

UN15170	Rsa1.0_00539.1	1683	76926	1.00E-180	320	320	100	-	1	320	28381	28700
UN15171	Rsa1.0_01651.1	867	41586	0	866	869	99	+	1	867	20839	21706
UN15172	Rsa1.0_01376.1	901	47004	0	369	374	98	-	1	374	41024	41397
UN15173	Rsa1.0_04272.1	766	18144	1.00E-156	315	326	96	+	2	325	9481	9806
UN15174	Rsa1.0_01740.1	892	39986	1.00E-140	279	287	97	-	71	354	27598	27884
UN15175	Rsa1.0_03569.1	600	22041	1.00E-100	201	205	98	-	395	598	9056	9260
UN15176	Rsa1.0_00071.1	1092	345714	0	381	382	99	-	331	712	311950	312331
UN15177	Rsa1.0_15363.1	1716	2655	0	931	975	95	-	744	1713	1688	2655
UN15178	Rsa1.0_03795.1	1178	20818	1.00E-129	267	275	97	-	1	271	15481	15753
UN15179	Rsa1.0_07955.1	932	8180	1.00E-160	290	291	99	+	642	932	6422	6712
UN15180	Rsa1.0_02829.1	872	27541	0	452	469	96	-	408	870	3313	3781
UN15181	Rsa1.0_01611.1	838	42269	1.00E-153	326	345	94	-	67	411	17493	17821
UN15182	Rsa1.0_02367.1	362	32106	1.00E-112	211	213	99	-	1	213	27423	27635
UN15183	Rsa1.0_00049.1	1047	167018	0	640	672	95	-	395	1045	7057	7728
UN15184	Rsa1.0_01065.1	750	55264	0	457	466	98	+	15	478	30932	31396
UN15185	Rsa1.0_01977.1	1213	36559	1.00E-155	278	278	100	-	936	1213	9556	9833
UN15186	Rsa1.0_01512.1	934	44234	1.00E-167	379	405	93	+	528	932	32378	32769
UN15187	Rsa1.0_02163.1	1206	34427	0	356	356	100	+	851	1206	5688	6043
UN15188	Rsa1.0_00813.1	571	63202	1.00E-164	440	487	90	-	1	472	51191	51668
UN15189	Rsa1.0_00773.1	1002	64839	1.00E-141	325	350	92	-	649	997	10104	10440
UN15190	Rsa1.0_12809.1	938	5119	1.00E-155	383	417	91	+	529	936	4445	4848
UN15191	Rsa1.0_01586.1	1157	42804	0	437	452	96	-	708	1157	28046	28496
UN15192	Rsa1.0_02626.1	918	29387	0	396	401	98	-	488	888	22893	23293
UN15193	Rsa1.0_30348.1	1038	756	1.00E-121	227	229	99	+	534	762	364	592
UN15194	Rsa1.0_01435.1	670	45942	0	387	389	99	-	1	389	28028	28416
UN15195	Rsa1.0_00579.1	789	148308	1.00E-102	207	213	97	-	5	217	65939	66151
UN15196	Rsa1.0_00476.1	970	280966	1.00E-171	353	366	96	-	613	967	241651	242016
UN15197	Rsa1.0_08230.1	673	7688	1.00E-144	272	276	98	-	1	276	5351	5626
UN15198	Rsa1.0_00758.1	1053	65477	1.00E-155	281	282	99	+	3	284	48370	48651
UN15199	Rsa1.0_63049.1	938	364	1.00E-149	278	280	99	-	659	938	64	342
UN15200	Rsa1.0_00652.1	941	70089	0	373	387	96	+	557	940	47920	48306
UN15201	Rsa1.0_02113.1	844	34890	0	824	830	99	-	12	841	22694	23522
UN15202	Rsa1.0_16435.1	542	2156	1.00E-102	201	205	98	+	5	209	315	519
UN15203	Rsa1.0_01469.1	1301	45139	1.00E-161	310	316	98	-	632	945	18477	18792
UN15204	Rsa1.0_03261.1	765	24281	1.00E-104	235	246	95	-	24	264	5213	5458
UN15205	Rsa1.0_50581.1	780	449	0	355	363	97	-	218	580	87	449
UN15206	Rsa1.0_00029.1	749	272992	1.00E-163	329	339	97	-	374	710	10231	10568
UN15207	Rsa1.0_00142.1	862	121474	0	438	445	98	-	42	486	62984	63427
UN15208	Rsa1.0_02101.1	970	35017	0	888	922	96	-	58	970	15791	16711

UN15209	Rsa1.0_00396.1	707	88242	1.00E-126	253	261	96	−	391	651	20281	20541
UN15210	Rsa1.0_19203.1	867	1465	1.00E-151	287	292	98	+	576	867	795	1086
UN15211	Rsa1.0_06056.1	806	137271	0	344	344	100	+	463	806	127496	127839
UN15212	Rsa1.0_01327.1	1291	48033	1.00E-145	334	354	94	−	940	1291	14629	14977
UN15213	Rsa1.0_00351.1	667	413096	1.00E-160	364	386	94	+	1	375	244444	244827
UN15214	Rsa1.0_00698.1	825	67816	0	613	646	94	−	186	825	21763	22408
UN15215	Rsa1.0_01827.1	806	143321	1.00E-101	187	187	100	−	355	541	128843	129029
UN15216	Rsa1.0_00179.1	804	393324	1.00E-116	234	240	97	−	29	267	200962	201201
UN15217	Rsa1.0_00385.1	1055	89160	0	403	405	99	−	1	404	45744	46148
UN15218	Rsa1.0_00020.1	1296	283099	1.00E-142	278	285	97	−	1012	1296	90069	90353
UN15219	Rsa1.0_05162.1	1733	14631	1.00E-100	206	211	97	+	1524	1733	4180	4390
UN15220	Rsa1.0_33317.1	882	681	1.00E-153	380	413	92	+	330	733	1	409
UN15221	Rsa1.0_00275.1	1098	210104	0	995	1043	95	−	1	1042	205236	206257
UN15222	Rsa1.0_01370.1	1686	47093	0	578	590	97	+	1096	1683	6095	6683
UN15223	Rsa1.0_00825.1	942	62855	1.00E-166	302	304	99	−	107	410	37215	37518
UN15224	Rsa1.0_00128.1	997	228675	1.00E-145	318	334	95	+	664	997	135383	135710
UN15225	Rsa1.0_03813.1	1458	20708	0	398	403	98	−	1059	1457	4888	5290
UN15226	Rsa1.0_02358.1	932	32213	0	452	462	97	−	479	932	251	712
UN15227	Rsa1.0_00754.1	908	65689	1.00E-166	296	296	100	−	1	296	53130	53425
UN15228	Rsa1.0_17687.1	1026	1765	0	922	961	95	−	37	994	1	956
UN15229	Rsa1.0_26461.1	1271	891	1.00E-90	231	247	93	−	1031	1271	490	730
UN15230	Rsa1.0_06715.1	1185	10485	0	519	547	94	−	1	539	2804	3349
UN15231	Rsa1.0_00190.1	1249	318240	0	606	647	93	−	606	1249	140909	141530
UN15232	Rsa1.0_27652.1	850	844	0	460	500	92	−	351	842	268	767
UN15233	Rsa1.0_00833.1	1204	62621	1.00E-114	223	226	98	−	978	1202	5189	5414
UN15234	Rsa1.0_00059.1	1064	159650	1.00E-104	232	243	95	+	1	240	147724	147965
UN15235	Rsa1.0_00047.1	1193	168582	1.00E-169	355	371	95	+	815	1184	4911	5278
UN15236	Rsa1.0_00168.1	900	115870	0	483	495	97	−	1	495	40022	40514
UN15237	Rsa1.0_00503.1	1077	79468	1.00E-145	338	359	94	+	643	998	69177	69532
UN15238	Rsa1.0_03455.1	1327	22804	1.00E-126	240	242	99	+	1	241	11896	12137
UN15239	Rsa1.0_05918.1	1124	12244	0	1097	1137	96	−	3	1119	8537	9667
UN15240	Rsa1.0_04123.1	851	18947	0	778	830	93	+	21	850	11889	12681
UN15241	Rsa1.0_02660.1	1253	28969	1.00E-115	235	243	96	+	951	1193	12641	12883
UN15242	Rsa1.0_01407.1	1537	46425	0	1465	1537	95	−	1	1537	9376	10911
UN15243	Rsa1.0_49117.1	1180	461	1.00E-141	261	263	99	−	11	273	121	383
UN15244	Rsa1.0_01806.1	1032	39021	1.00E-150	270	270	100	+	762	1031	24779	25048
UN15245	Rsa1.0_00055.1	1587	520193	0	1271	1299	97	+	4	1301	331692	332984
UN15246	Rsa1.0_06563.1	1059	10765	1.00E-154	329	347	94	−	1	347	2187	2527
UN15247	Rsa1.0_02222.1	494	152798	0	472	488	96	−	3	481	4650	5136

UN15248	Rsa1.0_08346.1	891	7497	1.00E-133	244	245	99	−	1	245	3302	3546
UN15249	Rsa1.0_00707.1	686	190112	6.00E-76	179	189	94	−	3	185	182523	182711
UN15250	Rsa1.0_06213.1	876	11478	0	482	519	92	−	371	867	1021	1535
UN15251	Rsa1.0_01454.1	921	45440	1.00E-133	280	291	96	+	1	282	14235	14524
UN15252	Rsa1.0_00577.1	894	174827	0	537	550	97	−	1	550	132266	132812
UN15253	Rsa1.0_00931.1	1049	59210	1.00E-169	349	365	95	+	335	693	54903	55267
UN15254	Rsa1.0_01185.1	656	149842	1.00E-119	260	273	95	−	23	293	11446	11718
UN15255	Rsa1.0_01143.1	1359	52456	0	1286	1367	94	−	2	1356	18964	20302
UN15256	Rsa1.0_02129.1	967	34757	0	388	399	97	−	502	891	5631	6029
UN15257	Rsa1.0_00220.1	1116	192936	0	471	487	96	−	275	752	16394	16880
UN15258	Rsa1.0_00376.1	336	90101	3.00E-60	127	130	97	−	5	134	978	1107
UN15259	Rsa1.0_00550.1	1181	210790	0	447	475	94	−	712	1180	177713	178187
UN15260	Rsa1.0_00002.1	825	601661	1.00E-135	279	288	96	+	117	403	140391	140677
UN15261	Rsa1.0_03005.1	1177	26082	0	523	539	97	+	3	538	23151	23688
UN15262	Rsa1.0_00033.1	821	515811	0	455	455	100	−	3	457	365760	366214
UN15263	Rsa1.0_01459.1	640	45277	2.00E-88	275	313	87	−	345	640	42955	43259
UN15264	Rsa1.0_00894.1	700	269700	7.00E-97	244	265	92	−	13	276	127216	127472
UN15265	Rsa1.0_00317.1	549	218290	3.00E-83	195	205	95	−	345	549	52669	52871
UN15266	Rsa1.0_02198.1	706	33989	1.00E-103	234	247	94	−	460	706	13971	14207
UN15267	Rsa1.0_17040.1	571	1944	0	389	397	97	+	154	550	1207	1602
UN15268	Rsa1.0_07506.1	1137	9233	0	539	555	97	−	583	1137	2689	3242
UN15269	Rsa1.0_03155.1	874	24958	0	443	460	96	−	417	873	9973	10426
UN15270	Rsa1.0_00503.1	763	79468	0	759	763	99	+	1	761	61033	61793
UN15271	Rsa1.0_00044.1	996	271658	1.00E-108	224	230	97	−	1	228	156762	156990
UN15272	Rsa1.0_00730.1	690	66673	1.00E-168	327	335	97	−	357	690	28375	28709
UN15273	Rsa1.0_05510.1	985	13470	1.00E-148	299	310	96	+	197	506	1981	2290
UN15274	Rsa1.0_00087.1	1235	761073	1.00E-145	265	266	99	+	675	940	274293	274558
UN15275	Rsa1.0_00047.1	700	168582	0	410	413	99	−	1	413	43362	43774
UN15276	Rsa1.0_02138.1	495	34695	5.00E-54	134	142	94	−	63	204	7722	7860
UN15277	Rsa1.0_04415.1	965	147889	0	922	933	98	+	3	934	143690	144621
UN15278	Rsa1.0_03656.1	1220	21552	0	749	757	98	+	463	1219	12500	13256
UN15279	Rsa1.0_14744.1	1117	3111	0	352	356	98	−	762	1117	2187	2542
UN15280	Rsa1.0_04281.1	826	18107	0	609	650	93	+	177	826	15672	16321
UN15281	Rsa1.0_06227.1	1103	11442	0	571	572	99	+	502	1073	4251	4822
UN15282	Rsa1.0_00645.1	834	189716	0	808	836	96	−	4	834	24190	25016
UN15283	Rsa1.0_02633.1	932	29315	0	404	422	95	+	3	415	350	771
UN15284	Rsa1.0_03099.1	1326	139424	0	440	443	99	+	1	443	4169	4611
UN15285	Rsa1.0_14032.1	875	3893	1.00E-160	304	310	98	+	564	873	3098	3407
UN15286	Rsa1.0_07565.1	792	9122	0	550	554	99	−	2	553	4744	5297

UN15287	Rsa1.0_02201.1	1405	33981	1.00E-172	445	494	90	+	310	784	30288	30776
UN15288	Rsa1.0_00707.1	1252	190112	0	899	911	98	−	345	1252	155128	156038
UN15289	Rsa1.0_03709.1	958	21247	1.00E-173	330	336	98	−	623	958	18566	18900
UN15290	Rsa1.0_20672.1	866	1281	1.00E-155	281	282	99	+	1	282	246	527
UN15291	Rsa1.0_00655.1	1410	195840	0	422	425	99	−	1	425	17675	18098
UN15292	Rsa1.0_02901.1	1609	26909	0	415	420	98	+	1	420	11160	11577
UN15293	Rsa1.0_04400.1	1319	17529	0	838	879	95	+	63	938	16666	17529
UN15294	Rsa1.0_00826.1	713	166736	0	572	592	96	−	116	705	118806	119397
UN15295	Rsa1.0_00697.1	1032	67913	1.00E-178	356	367	97	+	1	363	39649	40015
UN15296	Rsa1.0_00409.1	857	146788	0	467	478	97	+	387	856	87558	88034
UN15297	Rsa1.0_00418.1	718	215377	0	552	556	99	−	163	718	201867	202422
UN15298	Rsa1.0_00253.1	1240	231292	0	978	983	99	+	1	982	95972	96954
UN15299	Rsa1.0_00569.1	1007	75060	0	568	579	98	+	429	1006	65107	65682
UN15300	Rsa1.0_20024.1	977	1351	8.00E-36	141	162	87	−	2	163	56	217
UN15301	Rsa1.0_05217.1	1116	142938	0	533	553	96	+	564	1116	132903	133451
UN15302	Rsa1.0_07692.1	909	132338	0	721	736	97	−	167	902	6716	7450
UN15303	Rsa1.0_03610.1	979	21797	0	334	335	99	+	335	669	17230	17564
UN15304	Rsa1.0_02223.1	775	152421	1.00E-153	290	295	98	+	352	646	19316	19610
UN15305	Rsa1.0_07444.1	875	9386	1.00E-116	242	249	97	−	628	875	199	446
UN15306	Rsa1.0_00495.1	860	79934	7.00E-70	147	151	97	−	127	277	19840	19990
UN15307	Rsa1.0_01346.1	1022	47618	0	442	453	97	−	2	454	7627	8079
UN15308	Rsa1.0_00092.1	898	659932	0	458	489	93	+	413	896	42020	42499
UN15309	Rsa1.0_00858.1	802	61892	0	401	422	95	−	14	434	33273	33686
UN15310	Rsa1.0_00215.1	954	201775	2.00E-85	216	227	95	+	727	945	2172	2398
UN15311	Rsa1.0_04374.1	701	17670	6.00E-85	160	160	100	+	542	701	8075	8234
UN15312	Rsa1.0_00168.1	1150	115870	0	478	496	96	−	334	826	98954	99439
UN15313	Rsa1.0_01454.1	1068	45440	1.00E-176	378	400	94	+	667	1066	32801	33183
UN15314	Rsa1.0_00253.1	905	231292	1.00E-105	219	226	96	−	3	228	206344	206568
UN15315	Rsa1.0_01001.1	560	57077	1.00E-179	357	370	96	−	191	560	23252	23621
UN15316	Rsa1.0_04172.1	1251	18679	1.00E-150	276	278	99	−	311	588	11435	11712
UN15317	Rsa1.0_00556.1	673	207212	1.00E-121	221	221	100	−	78	298	146894	147114
UN15318	Rsa1.0_07250.1	863	9663	0	692	717	96	+	3	719	5384	6096
UN15319	Rsa1.0_00954.1	2294	189536	1.00E-163	331	342	96	−	7	348	40588	40925
UN15320	Rsa1.0_01540.1	1322	151890	0	1236	1328	93	+	1	1320	142474	143775
UN15321	Rsa1.0_00017.1	1732	752458	1.00E-152	323	336	96	+	80	415	272077	272408
UN15322	Rsa1.0_00003.1	764	340054	1.00E-119	304	332	91	+	403	733	4541	4856
UN15323	Rsa1.0_00087.1	810	761073	1.00E-119	308	335	91	−	485	810	615087	615419
UN15324	Rsa1.0_01105.1	559	53958	1.00E-152	275	276	99	−	138	413	1682	1957
UN15325	Rsa1.0_00883.1	836	61230	0	429	441	97	+	1	438	29866	30306

UN15326	Rsa1.0_09314.1	1403	6461	0	799	810	98	−	408	1216	3997	4803
UN15327	Rsa1.0_00330.1	701	94666	1.00E-129	240	242	99	+	1	242	42507	42748
UN15328	Rsa1.0_00135.1	687	178077	0	491	518	94	−	4	511	8955	9472
UN15329	Rsa1.0_02301.1	880	32835	0	794	837	94	−	44	878	20259	21093
UN15330	Rsa1.0_01995.1	855	36347	0	479	502	95	−	2	494	22162	22663
UN15331	Rsa1.0_00194.1	738	110851	0	604	641	94	−	3	625	54321	54961
UN15332	Rsa1.0_63101.1	638	364	4.00E-89	173	175	98	+	61	235	1	175
UN15333	Rsa1.0_00409.1	603	146788	0	365	365	100	−	11	375	1659	2023
UN15334	Rsa1.0_16093.1	718	2288	0	357	358	99	+	1	357	1223	1580
UN15335	Rsa1.0_03887.1	1293	20247	0	975	982	99	+	311	1292	2579	3560
UN15336	Rsa1.0_00087.1	658	761073	1.00E-109	290	319	90	−	3	304	217289	217606
UN15337	Rsa1.0_00659.1	707	69733	1.00E-137	321	346	92	−	1	346	55176	55500
UN15338	Rsa1.0_11435.1	933	5508	1.00E-147	277	281	98	−	490	770	248	528
UN15339	Rsa1.0_00082.1	953	237584	1.00E-155	339	356	95	−	608	953	53592	53947
UN15340	Rsa1.0_03484.1	714	22618	1.00E-122	229	231	99	+	31	261	4972	5202
UN15341	Rsa1.0_04194.1	1175	132734	7.00E-89	214	227	94	−	950	1175	117319	117544
UN15342	Rsa1.0_01674.1	721	168300	1.00E-132	296	313	94	+	410	721	4084	4393
UN15343	Rsa1.0_11462.1	1135	5500	0	959	1022	93	−	137	1135	2312	3331
UN15344	Rsa1.0_00500.1	811	272308	0	586	599	97	−	1	599	157120	157717
UN15345	Rsa1.0_00198.1	863	222963	0	440	443	99	−	3	445	123517	123959
UN15346	Rsa1.0_08846.1	1491	6867	1.00E-145	324	342	94	+	11	350	3969	4300
UN15347	Rsa1.0_01412.1	1089	46311	0	569	586	97	+	506	1086	11181	11766
UN15348	Rsa1.0_18647.1	893	1560	0	389	389	100	−	504	892	544	932
UN15349	Rsa1.0_02239.1	921	33534	0	379	381	99	+	1	380	9569	9949
UN15350	Rsa1.0_04138.1	794	18898	0	561	562	99	+	1	562	3225	3786
UN15351	Rsa1.0_06978.1	933	10073	0	645	645	100	+	289	933	1165	1809
UN15352	Rsa1.0_00699.1	516	184398	0	507	517	98	−	1	516	127808	128324
UN15353	Rsa1.0_00358.1	1208	91293	0	574	578	99	−	2	579	74541	75118
UN15354	Rsa1.0_01400.1	666	46560	4.00E-95	180	181	99	−	484	664	5934	6114
UN15355	Rsa1.0_14826.1	1144	3040	0	443	456	97	+	689	1142	894	1347
UN15356	Rsa1.0_03870.1	795	20362	0	750	819	91	−	3	794	10660	11461
UN15357	Rsa1.0_02663.1	793	169243	0	629	643	97	−	5	647	7861	8502
UN15358	Rsa1.0_06288.1	2101	11302	0	1736	1773	97	+	1	1763	313	2084
UN15359	Rsa1.0_02629.1	1040	29350	1.00E-156	286	287	99	−	755	1040	4981	5267
UN15360	Rsa1.0_00034.1	790	260119	0	755	813	92	+	2	790	67162	67960
UN15361	Rsa1.0_02647.1	685	29120	0	617	638	96	−	48	684	25184	25819
UN15362	Rsa1.0_01469.1	1594	45139	0	811	837	96	−	759	1594	30986	31819
UN15363	Rsa1.0_01995.1	931	36347	0	478	504	94	+	439	930	22162	22664
UN15364	Rsa1.0_00006.1	799	396176	0	768	799	96	+	1	799	298200	298996

UN15365	Rsa1.0_03326.1	653	23710	0	645	653	98	+	1	653	16896	17548
UN15366	Rsa1.0_00805.1	994	63724	0	964	1008	95	−	2	994	42546	43543
UN15367	Rsa1.0_06802.1	640	10330	0	491	507	96	+	136	639	8965	9468
UN15368	Rsa1.0_01355.1	709	47303	0	621	671	92	−	41	703	39409	40068
UN15369	Rsa1.0_03713.1	671	21221	1.00E−117	220	222	99	+	220	441	16746	16967
UN15370	Rsa1.0_00205.1	547	193401	2.00E−99	218	228	95	−	307	533	130115	130342
UN15371	Rsa1.0_07617.1	866	9015	0	815	835	97	+	2	833	3003	3836
UN15372	Rsa1.0_00253.1	686	231292	0	658	689	95	−	2	686	196757	197431
UN15373	Rsa1.0_00012.1	890	222148	1.00E−131	244	246	99	−	643	888	69119	69364
UN15374	Rsa1.0_01887.1	818	37564	1.00E−137	277	287	96	+	159	445	26172	26458
UN15375	Rsa1.0_19809.1	810	1380	1.00E−138	264	269	98	−	106	374	715	983
UN15376	Rsa1.0_04470.1	1122	17204	1.00E−140	310	328	94	−	3	330	4465	4785
UN15377	Rsa1.0_00299.1	1117	97770	0	407	412	98	−	706	1117	19341	19751
UN15378	Rsa1.0_10940.1	511	5670	3.00E−77	172	177	97	+	238	414	5499	5670
UN15379	Rsa1.0_04542.1	961	16930	1.00E−105	235	246	95	+	719	961	16072	16312
UN15380	Rsa1.0_00358.1	988	91293	7.00E−98	188	190	98	−	177	366	22173	22362
UN15381	Rsa1.0_00001.1	1449	363028	0	1414	1447	97	+	5	1449	34790	36236
UN15382	Rsa1.0_13580.1	1778	4711	0	1059	1077	98	+	3	1077	1390	2464
UN15383	Rsa1.0_00642.1	682	201230	0	675	684	98	−	1	682	62519	63201
UN15384	Rsa1.0_00163.1	799	116847	0	552	581	95	−	230	793	8550	9130
UN15385	Rsa1.0_00127.1	1506	127446	0	472	502	94	+	2	492	62979	63478
UN15386	Rsa1.0_00502.1	1062	204180	0	368	372	98	−	690	1061	11653	12024
UN15387	Rsa1.0_20478.1	1616	1301	0	608	623	97	+	994	1616	5	623
UN15388	Rsa1.0_01349.1	1317	47513	0	357	358	99	−	960	1317	45089	45446
UN15389	Rsa1.0_07974.1	689	8147	1.00E−119	264	279	94	−	412	687	6419	6697
UN15390	Rsa1.0_04140.1	483	18879	1.00E−164	298	300	99	+	183	482	3855	4154
UN15391	Rsa1.0_02966.1	980	135126	0	489	512	95	+	461	971	17972	18475
UN15392	Rsa1.0_00049.1	761	167018	0	376	386	97	−	378	761	7072	7451
UN15393	Rsa1.0_00732.1	838	66631	1.00E−110	304	331	91	+	484	812	1460	1787
UN15394	Rsa1.0_03355.1	861	23489	0	560	572	97	−	290	861	14140	14711
UN15395	Rsa1.0_01773.1	919	39546	1.00E−132	239	239	100	−	213	451	28417	28655
UN15396	Rsa1.0_00301.1	1520	365315	0	370	376	98	+	23	398	249	624
UN15397	Rsa1.0_00037.1	1133	307838	0	518	519	99	−	124	642	182054	182572
UN15398	Rsa1.0_36865.1	1389	612	1.00E−144	295	306	96	+	58	360	140	445
UN15399	Rsa1.0_00050.1	1119	237174	0	366	367	99	+	1	367	103878	104244
UN15400	Rsa1.0_00524.1	911	169613	0	420	430	97	+	450	874	129773	130202
UN15401	Rsa1.0_00777.1	909	191865	0	395	396	99	+	126	521	180530	180925
UN15402	Rsa1.0_00492.1	813	80178	4.00E−99	215	222	96	+	42	260	916	1135
UN15403	Rsa1.0_02550.1	1170	291051	1.00E−169	330	339	97	−	22	360	6190	6525

UN15404	Rsa1.0_12060.1	1085	5328	1.00E-102	235	250	94	+	832	1081	97	346
UN15405	Rsa1.0_02807.1	1289	27680	0	440	455	96	−	783	1236	5010	5464
UN15406	Rsa1.0_00502.1	1209	204180	0	451	468	96	+	15	479	61012	61469
UN15407	Rsa1.0_00648.1	1187	70351	1.00E-134	249	251	99	−	1	251	13289	13539
UN15408	Rsa1.0_00457.1	962	337301	1.00E-108	273	297	91	−	55	341	130569	130856
UN15409	Rsa1.0_00001.1	1051	363028	1.00E-163	378	406	93	−	649	1051	190757	191142
UN15410	Rsa1.0_00009.1	1097	232558	1.00E-110	224	231	96	−	867	1097	32980	33210
UN15411	Rsa1.0_02928.1	755	26680	1.00E-177	358	370	96	−	382	749	7245	7608
UN15412	Rsa1.0_00386.1	858	89134	1.00E-150	301	309	97	+	550	856	44310	44617
UN15413	Rsa1.0_07499.1	943	9244	0	414	421	98	+	513	933	6510	6930
UN15414	Rsa1.0_03218.1	1021	24539	1.00E-167	358	374	95	−	651	1021	3739	4112
UN15415	Rsa1.0_00675.1	1070	145590	0	418	422	99	−	648	1069	143628	144049
UN15416	Rsa1.0_00504.1	1160	79334	1.00E-100	213	222	95	−	265	486	31151	31372
UN15417	Rsa1.0_00059.1	1309	159650	1.00E-170	345	357	96	+	1	353	131062	131418
UN15418	Rsa1.0_02219.1	899	33798	0	887	889	99	−	9	897	21460	22347
UN15419	Rsa1.0_05559.1	1206	13329	0	430	440	97	−	87	526	8434	8872
UN15420	Rsa1.0_00110.1	1040	132719	1.00E-173	377	395	95	−	3	397	4280	4669
UN15421	Rsa1.0_01632.1	537	181224	0	533	535	99	−	1	535	145738	146271
UN15422	Rsa1.0_00526.1	1062	169279	0	495	499	99	−	199	697	148049	148547
UN15423	Rsa1.0_00324.1	1213	218805	0	1181	1227	96	+	3	1212	214955	216176
UN15424	Rsa1.0_03781.1	899	20891	0	703	728	96	+	171	897	11400	12127
UN15425	Rsa1.0_03158.1	1201	24950	0	447	472	94	−	732	1197	8092	8563
UN15426	Rsa1.0_00101.1	1082	267193	0	638	640	99	−	4	643	74600	75239
UN15427	Rsa1.0_00339.1	1031	222974	1.00E-93	220	231	95	−	800	1028	176096	176325
UN15428	Rsa1.0_00909.1	774	59976	0	501	514	97	−	8	521	26084	26597
UN15429	Rsa1.0_00285.1	971	149638	0	368	371	99	+	231	601	86908	87278
UN15430	Rsa1.0_02700.1	1088	28584	0	414	420	98	−	634	1053	2935	3354
UN15431	Rsa1.0_02097.1	757	35036	0	457	461	99	−	1	461	16158	16618
UN15432	Rsa1.0_00308.1	678	96915	1.00E-145	292	302	96	−	1	298	80355	80656
UN15433	Rsa1.0_00087.1	636	761073	0	630	636	99	−	1	634	580712	581347
UN15434	Rsa1.0_08358.1	1092	7484	0	408	424	96	+	2	418	6535	6958
UN15435	Rsa1.0_01104.1	1425	285480	0	844	879	96	−	547	1425	263864	264742
UN15436	Rsa1.0_01883.1	1021	37661	1.00E-156	367	394	93	−	3	390	22302	22692
UN15437	Rsa1.0_30502.1	1160	752	1.00E-158	361	383	94	−	35	416	372	752
UN15438	Rsa1.0_00556.1	726	207212	0	695	725	95	−	1	720	43742	44462
UN15439	Rsa1.0_03325.1	510	23720	1.00E-170	305	306	99	−	205	510	19985	20290
UN15440	Rsa1.0_00917.1	747	182012	0	434	448	96	+	6	449	175681	176128
UN15441	Rsa1.0_01096.1	868	54266	0	436	454	96	−	172	625	2669	3122
UN15442	Rsa1.0_00168.1	824	115870	0	497	512	97	+	316	823	40005	40513

UN15443	Rsa1.0_00195.1	729	251884	2.33E-156	282	288	97	+	445	729	83583	83870
UN15444	Rsa1.0_00482.1	868	81162	1.00E-130	239	240	99	+	268	507	63861	64100
UN15445	Rsa1.0_00121.1	1119	278029	1.00E-150	269	269	100	+	619	887	265346	265614
UN15446	Rsa1.0_00204.1	883	109056	0	510	539	94	−	1	528	9815	10350
UN15447	Rsa1.0_00802.1	778	63846	0	382	383	99	+	69	451	26121	26503
UN15448	Rsa1.0_00087.1	1307	761073	1.00E-123	240	245	97	−	806	1050	293120	293364
UN15449	Rsa1.0_07756.1	588	8634	6.00E-91	210	221	95	+	3	221	6503	6722
UN15450	Rsa1.0_08922.1	1073	6788	0	1041	1066	97	−	8	1073	1939	3002
UN15451	Rsa1.0_00078.1	836	175053	1.00E-171	308	309	99	+	248	556	47230	47538
UN15452	Rsa1.0_08410.1	1559	7406	0	354	358	98	−	2	358	6034	6390
UN15453	Rsa1.0_00762.1	910	165246	0	399	402	99	+	3	404	25816	26217
UN15454	Rsa1.0_05491.1	1074	13554	4.00E-93	180	182	98	+	681	862	9307	9488
UN15455	Rsa1.0_04478.1	735	17183	0	693	727	95	−	1	727	3347	4068
UN15456	Rsa1.0_03574.1	472	22004	4.00E-79	267	302	88	+	1	290	17888	18178
UN15457	Rsa1.0_00579.1	1029	148308	1.00E-133	257	262	98	−	300	561	70548	70809
UN15458	Rsa1.0_00092.1	1122	659932	0	1118	1119	99	−	3	1121	175649	176766
UN15459	Rsa1.0_02178.1	421	156197	2.00E-99	205	212	96	+	3	214	870	1081
UN15460	Rsa1.0_02987.1	1478	26196	0	481	510	94	−	979	1478	24975	25473
UN15461	Rsa1.0_00235.1	913	154696	0	443	463	95	+	27	488	139911	140365
UN15462	Rsa1.0_00040.1	699	324196	0	443	454	97	−	60	510	224430	224883
UN15463	Rsa1.0_02500.1	793	30768	1.00E-159	333	347	95	+	444	787	7770	8115
UN15464	Rsa1.0_00662.1	588	192183	1.00E-167	310	314	98	+	1	314	11320	11633
UN15465	Rsa1.0_00097.1	646	138858	1.00E-168	303	304	99	+	5	308	14049	14352
UN15466	Rsa1.0_37078.1	805	608	0	374	385	97	−	422	805	225	608
UN15467	Rsa1.0_00807.1	711	63607	1.00E-180	361	371	97	−	340	709	11803	12170
UN15468	Rsa1.0_00135.1	763	178077	1.00E-172	330	338	97	+	223	560	8955	9292
UN15469	Rsa1.0_01214.1	918	50599	0	878	922	95	−	2	917	49192	50111
UN15470	Rsa1.0_03517.1	1041	22337	1.00E-130	295	312	94	−	1	312	8366	8674
UN15471	Rsa1.0_11599.1	897	5462	1.00E-133	253	257	98	−	637	893	4673	4929
UN15472	Rsa1.0_08358.1	1096	7484	0	392	409	95	−	694	1095	6550	6958
UN15473	Rsa1.0_01931.1	1086	37187	0	541	573	94	+	390	960	32029	32590
UN15474	Rsa1.0_15178.1	790	2778	0	505	515	98	+	3	516	1791	2305
UN15475	Rsa1.0_00055.1	806	520193	1.00E-128	263	273	96	+	457	729	310467	310739
UN15476	Rsa1.0_00168.1	748	115870	1.00E-123	224	224	100	+	3	226	108565	108788
UN15477	Rsa1.0_00205.1	627	193401	1.00E-143	336	358	93	+	271	627	38432	38777
UN15478	Rsa1.0_00223.1	1393	106231	0	1068	1107	96	−	4	1109	2967	4073
UN15479	Rsa1.0_13708.1	1000	4447	1.00E-113	263	279	94	−	725	999	3623	3897
UN15480	Rsa1.0_01762.1	1150	39682	1.00E-142	378	416	90	−	720	1116	6860	7267
UN15481	Rsa1.0_06898.1	954	10191	3.00E-84	224	244	91	−	676	918	3249	3483

UN15482	Rsa1.0_01090.1	1629	54385	0	625	636	98	+	147	782	44296	44931
UN15483	Rsa1.0_00577.1	1325	174827	0	423	444	95	−	789	1229	149759	150199
UN15484	Rsa1.0_00246.1	1159	103541	1.00E−169	318	323	98	+	838	1157	34114	34436
UN15485	Rsa1.0_00615.1	1030	72235	0	668	690	96	+	1	684	54716	55402
UN15486	Rsa1.0_00184.1	1210	218748	0	864	873	98	+	339	1210	43850	44722
UN15487	Rsa1.0_02910.1	1115	26843	1.00E−164	307	309	99	−	209	516	1620	1927
UN15488	Rsa1.0_44116.1	929	510	0	347	350	99	−	324	673	81	430
UN15489	Rsa1.0_00038.1	1054	314568	0	426	435	97	+	1	435	252468	252902
UN15490	Rsa1.0_01050.1	807	55651	0	783	815	96	−	3	807	12073	12887
UN15491	Rsa1.0_03191.1	1000	145762	1.00E−133	348	382	91	+	633	1000	16325	16695
UN15492	Rsa1.0_00161.1	684	116891	0	660	685	96	+	1	682	32514	33198
UN15493	Rsa1.0_00135.1	1219	178077	0	478	478	100	+	741	1218	19426	19903
UN15494	Rsa1.0_11907.1	919	5374	0	487	492	98	−	1	492	1101	1591
UN15495	Rsa1.0_14913.1	1151	2969	0	461	482	95	+	666	1141	666	1145
UN15496	Rsa1.0_03295.1	1209	23970	1.00E−126	311	338	92	−	206	538	21467	21804
UN15497	Rsa1.0_02022.1	1261	35923	1.00E−151	324	340	95	+	864	1203	14042	14380
UN15498	Rsa1.0_02550.1	912	291051	0	323	323	100	−	1	323	6190	6512
UN15499	Rsa1.0_00060.1	1139	502678	1.00E−168	395	424	93	−	16	437	495990	496403
UN15500	Rsa1.0_00807.1	1631	63607	0	626	632	99	−	1001	1627	16993	17624
UN15501	Rsa1.0_08717.1	856	7008	0	497	534	93	−	33	558	2884	3412
UN15502	Rsa1.0_00777.1	947	191865	0	414	436	94	−	168	603	128527	128962
UN15503	Rsa1.0_00534.1	950	77361	0	529	563	93	+	1	551	8745	9305
UN15504	Rsa1.0_00027.1	838	283362	0	354	354	100	+	1	354	279811	280164
UN15505	Rsa1.0_05204.1	871	14516	0	427	459	93	−	108	551	6647	7105
UN15506	Rsa1.0_03499.1	1268	22462	0	731	775	94	−	497	1268	8399	9161
UN15507	Rsa1.0_00215.1	916	201775	7.00E−73	146	148	98	+	458	605	1921	2068
UN15508	Rsa1.0_02883.1	1116	27035	0	375	386	97	−	320	705	3528	3913
UN15509	Rsa1.0_03263.1	1366	24268	0	1061	1100	96	−	1	1087	20569	21661
UN15510	Rsa1.0_01537.1	462	187285	0	431	460	93	+	1	455	175370	175826
UN15511	Rsa1.0_00254.1	965	102600	1.00E−107	254	271	93	−	698	965	77343	77601
UN15512	Rsa1.0_01209.1	1002	50668	1.00E−176	351	361	97	+	648	1002	40043	40402
UN15513	Rsa1.0_00971.1	1410	57973	1.00E−148	322	338	95	−	4	336	51430	51764
UN15514	Rsa1.0_02019.1	1111	35983	1.00E−133	316	336	94	+	1	335	13309	13635
UN15515	Rsa1.0_01062.1	811	55325	1.00E−100	195	198	98	+	294	491	37369	37566
UN15516	Rsa1.0_01758.1	1116	39747	0	407	416	97	+	339	754	37154	37568
UN15517	Rsa1.0_00140.1	1518	121541	1.00E−165	346	363	95	+	1057	1419	49081	49443
UN15518	Rsa1.0_04034.1	1149	19481	1.00E−132	255	260	98	+	212	471	12812	13071
UN15519	Rsa1.0_02671.1	1185	28887	1.00E−154	322	336	95	−	850	1185	9126	9449
UN15520	Rsa1.0_00710.1	903	67325	1.00E−122	268	279	96	−	35	311	26519	26796

UN15521	Rsa1.0_00026.1	1168	327835	0	1029	1059	97	−	110	1167	2423	3481
UN15522	Rsa1.0_01783.1	521	39383	1.00E−173	314	316	99	+	61	376	19912	20227
UN15523	Rsa1.0_00842.1	1258	62493	1.00E−124	307	326	94	−	9	330	29546	29868
UN15524	Rsa1.0_01581.1	1225	42833	0	575	581	98	+	647	1225	1828	2408
UN15525	Rsa1.0_00119.1	1297	467509	1.00E−104	208	213	97	−	34	246	390249	390461
UN15526	Rsa1.0_00838.1	726	153820	0	414	426	97	+	5	428	52156	52579
UN15527	Rsa1.0_03127.1	1042	25191	0	830	865	95	−	1	847	18507	19371
UN15528	Rsa1.0_03282.1	492	24107	1.00E−109	220	224	98	−	269	491	9551	9773
UN15529	Rsa1.0_01696.1	755	301850	2.00E−75	144	144	100	−	596	739	291899	292042
UN15530	Rsa1.0_01850.1	1071	162224	1.00E−157	344	361	95	+	707	1065	31714	32072
UN15531	Rsa1.0_08558.1	1092	7198	0	436	446	97	+	3	446	2280	2721
UN15532	Rsa1.0_01512.1	1023	44234	0	526	530	99	+	1	529	20885	21414
UN15533	Rsa1.0_00113.1	705	131574	0	364	370	98	−	1	369	73859	74228
UN15534	Rsa1.0_00383.1	1240	398431	0	794	798	99	−	121	918	147000	147797
UN15535	Rsa1.0_00054.1	655	162053	1.00E−70	145	148	97	+	507	654	87400	87547
UN15536	Rsa1.0_00092.1	743	659932	0	652	676	96	+	1	675	126031	126699
UN15537	Rsa1.0_01435.1	1096	45942	0	384	397	96	+	702	1096	41112	41507
UN15538	Rsa1.0_03579.1	1086	21969	1.00E−109	366	403	90	+	704	1086	18958	19359
UN15539	Rsa1.0_00007.1	640	239325	0	530	553	95	+	95	640	58424	58966
UN15540	Rsa1.0_00301.1	731	365315	1.00E−129	337	370	91	+	373	729	346680	347040
UN15541	Rsa1.0_00165.1	666	116466	1.00E−116	225	228	98	−	440	666	26440	26667
UN15542	Rsa1.0_02443.1	930	31353	4.00E−65	146	151	96	−	1	151	20043	20192
UN15543	Rsa1.0_08807.1	947	6906	1.00E−150	295	302	97	−	647	947	3529	3830
UN15544	Rsa1.0_02022.1	1220	35923	1.00E−135	290	305	95	+	916	1220	14054	14358
UN15545	Rsa1.0_00251.1	1388	303968	0	607	620	97	+	2	621	83614	84232
UN15546	Rsa1.0_53223.1	1208	428	1.00E−151	312	322	96	−	3	321	1	321
UN15547	Rsa1.0_00473.1	1310	212204	0	414	423	97	+	881	1300	73422	73844
UN15548	Rsa1.0_02398.1	822	31766	0	814	822	99	+	1	821	4865	5684
UN15549	Rsa1.0_00447.1	1483	84051	0	1459	1480	98	−	3	1482	44335	45814
UN15550	Rsa1.0_01546.1	963	43586	0	527	528	99	−	2	528	34986	35513
UN15551	Rsa1.0_00222.1	1582	172263	0	485	489	99	−	3	491	15395	15882
UN15552	Rsa1.0_26066.1	786	908	0	560	570	98	−	13	582	339	908
UN15553	Rsa1.0_03500.1	827	22461	1.00E−178	316	316	100	−	511	826	10033	10348
UN15554	Rsa1.0_00008.1	626	358124	1.00E−118	234	239	97	−	1	237	67627	67865
UN15555	Rsa1.0_10460.1	1430	5842	0	814	825	98	+	604	1428	4237	5061
UN15556	Rsa1.0_06701.1	1920	10514	0	512	526	97	+	939	1464	707	1232
UN15557	Rsa1.0_05449.1	2007	13711	0	382	382	100	−	1626	2007	13265	13646
UN15558	Rsa1.0_00793.1	586	64103	1.00E−121	248	257	96	+	330	586	24186	24442
UN15559	Rsa1.0_06488.1	1822	141774	0	598	602	99	−	1222	1822	137824	138425

UN15560	Rsa1.0_05819.1	1319	12543	0	382	383	99	-	937	1319	10924	11305
UN15561	Rsa1.0_01618.1	649	42118	2.00E-87	241	259	93	-	380	636	17334	17587
UN15562	Rsa1.0_00111.1	706	390088	1.00E-169	412	449	91	+	262	706	75630	76057
UN15563	Rsa1.0_00034.1	1314	260119	1.00E-138	256	258	99	+	740	997	8763	9020
UN15564	Rsa1.0_00344.1	676	191774	1.00E-98	218	228	95	-	444	668	156236	156461
UN15565	Rsa1.0_20960.1	467	1250	0	437	455	96	-	10	462	521	974
UN15566	Rsa1.0_00848.1	335	152362	1.00E-151	285	290	98	+	1	290	48054	48343
UN15567	Rsa1.0_02020.1	1161	173211	0	584	596	97	-	1	595	154385	154980
UN15568	Rsa1.0_00171.1	636	115622	1.00E-144	322	345	93	-	293	626	50076	50420
UN15569	Rsa1.0_00841.1	803	183065	1.00E-140	253	253	100	+	1	253	1054	1306
UN15570	Rsa1.0_01946.1	977	36982	0	468	471	99	+	506	976	33540	34010
UN15571	Rsa1.0_00056.1	1225	332604	1.00E-152	303	312	97	-	914	1222	81919	82230
UN15572	Rsa1.0_00197.1	879	109714	0	684	702	97	+	178	879	64829	65530
UN15573	Rsa1.0_00636.1	1349	71113	1.00E-180	334	337	99	-	1	337	3145	3480
UN15574	Rsa1.0_02588.1	1307	29840	0	404	404	100	-	904	1307	14286	14689
UN15575	Rsa1.0_00033.1	1139	515811	2.33E-156	263	263	100	+	877	1139	131617	131879
UN15576	Rsa1.0_00630.1	1046	71281	1.00E-176	335	341	98	-	704	1044	59344	59683
UN15577	Rsa1.0_00971.1	1184	57973	1.00E-157	309	317	97	-	1	316	48427	48743
UN15578	Rsa1.0_03258.1	902	24302	1.00E-138	271	277	97	-	625	900	11034	11310
UN15579	Rsa1.0_00066.1	594	152418	1.00E-152	297	304	97	-	290	593	139751	140053
UN15580	Rsa1.0_00546.1	1132	76523	1.00E-127	235	236	99	-	28	263	67867	68102
UN15581	Rsa1.0_03001.1	1202	26108	2.33E-156	389	425	91	-	756	1172	5410	5824
UN15582	Rsa1.0_02955.1	988	26484	1.00E-167	351	363	96	+	625	986	13654	14013
UN15583	Rsa1.0_00759.1	894	65465	1.00E-111	205	205	100	-	42	246	63153	63357
UN15584	Rsa1.0_03940.1	1243	19962	0	842	843	99	-	1	842	12036	12878
UN15585	Rsa1.0_01355.1	837	47303	1.00E-166	311	316	98	+	414	729	23751	24066
UN15586	Rsa1.0_03344.1	1160	23593	0	423	448	94	-	8	455	12154	12601
UN15587	Rsa1.0_01581.1	669	42833	0	425	441	96	+	229	669	28862	29301
UN15588	Rsa1.0_07597.1	850	9055	7.00E-73	146	148	98	-	136	283	487	634
UN15589	Rsa1.0_00473.1	1115	212204	1.00E-111	254	268	94	-	15	281	73604	73864
UN15590	Rsa1.0_00451.1	951	322533	1.00E-176	354	371	95	+	2	372	48167	48522
UN15591	Rsa1.0_01955.1	1183	36850	0	964	993	97	+	190	1180	13035	14015
UN15592	Rsa1.0_05812.1	982	12571	0	907	940	96	-	46	982	2609	3548
UN15593	Rsa1.0_00020.1	1151	283099	1.00E-176	385	406	94	+	731	1130	140893	141292
UN15594	Rsa1.0_03134.1	1174	145023	0	513	516	99	+	260	775	136616	137131
UN15595	Rsa1.0_63936.1	1113	359	1.00E-152	297	305	97	+	669	973	55	359
UN15596	Rsa1.0_02616.1	802	29542	1.00E-138	266	270	98	-	2	270	22169	22438
UN15597	Rsa1.0_01112.1	939	310601	0	520	547	95	+	402	937	31685	32228
UN15598	Rsa1.0_00798.1	751	63945	1.00E-130	266	272	97	+	455	723	43764	44035

UN15599	Rsa1.0_07127.1	758	9863	0	725	756	95	+	1	755	8993	9738
UN15600	Rsa1.0_00333.1	1353	94500	0	532	565	94	+	803	1353	69143	69706
UN15601	Rsa1.0_01997.1	564	158416	3.00E-86	168	170	98	+	395	564	16696	16865
UN15602	Rsa1.0_06124.1	785	11681	1.00E-122	246	254	96	+	2	255	6814	7067
UN15603	Rsa1.0_00630.1	1071	71281	1.00E-168	306	308	99	−	764	1071	59376	59683
UN15604	Rsa1.0_12060.1	802	5328	1.00E-111	249	264	94	−	3	266	97	360
UN15605	Rsa1.0_00132.1	1470	124299	0	599	621	96	+	3	622	90920	91530
UN15606	Rsa1.0_00921.1	1114	59599	0	569	583	97	+	533	1114	2810	3388
UN15607	Rsa1.0_04342.1	760	17828	1.00E-166	320	328	97	−	2	329	422	749
UN15608	Rsa1.0_00133.1	582	123768	1.00E-119	218	218	100	+	365	582	66900	67117
UN15609	Rsa1.0_00032.1	622	306326	1.00E-158	285	286	99	+	3	288	154750	155035
UN15610	Rsa1.0_01249.1	757	188432	0	480	501	95	+	247	743	149428	149921
UN15611	Rsa1.0_08862.1	933	6847	0	351	356	98	+	3	358	4153	4508
UN15612	Rsa1.0_08565.1	865	7189	2.00E-91	224	238	94	+	1	230	313	549
UN15613	Rsa1.0_00913.1	586	59914	1.00E-167	358	376	95	−	9	384	16421	16793
UN15614	Rsa1.0_16367.1	752	2176	0	728	740	98	−	2	738	1412	2145
UN15615	Rsa1.0_00048.1	405	167185	1.00E-168	368	387	95	−	1	384	73806	74191
UN15616	Rsa1.0_03818.1	1121	20668	1.00E-129	237	238	99	−	884	1121	6686	6923
UN15617	Rsa1.0_03940.1	638	19962	0	452	467	96	−	172	638	12248	12712
UN15618	Rsa1.0_00017.1	741	752458	1.00E-147	264	264	100	+	1	264	141391	141654
UN15619	Rsa1.0_01340.1	844	47805	4.00E-65	256	301	85	+	8	298	20630	20919
UN15620	Rsa1.0_00018.1	760	410899	0	395	401	98	−	1	401	68738	69135
UN15621	Rsa1.0_00182.1	1393	142305	0	755	755	100	+	639	1393	94201	94955
UN15622	Rsa1.0_00603.1	481	73299	0	398	416	95	+	66	481	32629	33039
UN15623	Rsa1.0_00119.1	626	467509	0	462	487	94	+	141	626	374988	375470
UN15624	Rsa1.0_12119.1	903	5311	1.00E-130	272	284	95	−	568	851	4573	4856
UN15625	Rsa1.0_02707.1	1045	28520	1.00E-100	226	237	95	+	812	1045	26163	26399
UN15626	Rsa1.0_00111.1	815	390088	0	540	567	95	−	232	795	383652	384207
UN15627	Rsa1.0_00962.1	727	184909	0	341	344	99	+	385	727	13117	13460
UN15628	Rsa1.0_00728.1	684	66704	0	527	548	96	−	141	684	10712	11258
UN15629	Rsa1.0_02647.1	679	29120	0	631	637	99	−	42	678	25183	25819
UN15630	Rsa1.0_01195.1	731	147988	0	703	733	95	−	3	731	15402	16127
UN15631	Rsa1.0_02693.1	197	28617	4.00E-37	103	112	91	−	60	163	26854	26965
UN15632	Rsa1.0_00324.1	692	218805	0	482	485	99	+	2	486	213316	213800
UN15633	Rsa1.0_01015.1	883	56752	1.00E-131	246	249	98	+	9	257	1739	1987
UN15634	Rsa1.0_01209.1	1091	50668	0	652	685	95	+	5	677	48496	49180
UN15635	Rsa1.0_00091.1	1548	139699	0	913	966	94	−	605	1548	137530	138479
UN15636	Rsa1.0_00364.1	771	90951	1.00E-154	353	377	93	+	397	770	63260	63629
UN15637	Rsa1.0_01561.1	723	43227	0	536	560	95	+	156	713	82	638

UN15638	Rsa1.0_13593.1	754	4685	0	724	754	96	+	1	745	45	795
UN15639	Rsa1.0_03271.1	1061	24187	1.00E-158	293	296	98	+	1	296	14691	14986
UN15640	Rsa1.0_06092.1	298	11756	1.00E-144	291	300	97	−	1	298	2803	3100
UN15641	Rsa1.0_00013.1	1114	292129	3.00E-88	172	174	98	−	524	697	72142	72315
UN15642	Rsa1.0_00019.1	771	301669	7.00E-82	164	167	98	+	322	488	113991	114157
UN15643	Rsa1.0_02079.1	1169	35306	0	481	493	97	−	2	494	24546	25038
UN15644	Rsa1.0_00163.1	1390	116847	1.00E-155	321	334	96	−	1	334	83512	83844
UN15645	Rsa1.0_00355.1	706	92038	0	538	581	92	−	123	703	77872	78429
UN15646	Rsa1.0_02872.1	1057	27080	0	461	504	91	+	1	497	11254	11749
UN15647	Rsa1.0_63936.1	1151	359	1.00E-161	301	305	98	+	695	999	55	359
UN15648	Rsa1.0_01651.1	1095	41586	1.00E-153	281	283	99	−	571	853	15937	16219
UN15649	Rsa1.0_04892.1	1300	15573	1.00E-126	278	289	96	+	2	288	6177	6463
UN15650	Rsa1.0_01713.1	994	40564	6.00E-86	171	174	98	+	3	176	30522	30695
UN15651	Rsa1.0_00048.1	1176	167185	0	407	411	99	+	512	922	115660	116070
UN15652	Rsa1.0_02917.1	784	159058	0	662	681	97	+	2	682	144119	144797
UN15653	Rsa1.0_16726.1	906	2053	1.00E-124	290	313	92	−	1	313	1664	1961
UN15654	Rsa1.0_00066.1	987	152418	3.00E-94	182	184	98	+	582	765	41862	42045
UN15655	Rsa1.0_02966.1	750	135126	0	469	482	97	+	269	750	17972	18448
UN15656	Rsa1.0_32803.1	1488	693	0	538	578	93	−	326	888	1	572
UN15657	Rsa1.0_06709.1	1070	10501	0	501	505	99	+	564	1068	267	771
UN15658	Rsa1.0_01478.1	182	44991	4.00E-80	176	183	96	+	1	182	31650	31832
UN15659	Rsa1.0_07193.1	1107	9752	1.00E-122	226	227	99	+	825	1051	4357	4583
UN15660	Rsa1.0_00399.1	1174	242951	0	1130	1179	95	+	3	1168	16373	17538
UN15661	Rsa1.0_05713.1	828	12855	1.00E-132	250	252	99	−	12	262	2417	2668
UN15662	Rsa1.0_00480.1	743	81325	1.00E-166	309	313	98	−	1	313	43625	43937
UN15663	Rsa1.0_00692.1	666	68151	1.00E-148	377	414	91	−	2	415	52276	52689
UN15664	Rsa1.0_00451.1	510	322533	1.00E-140	277	285	97	+	92	376	993	1277
UN15665	Rsa1.0_02040.1	167	35684	1.00E-61	156	166	93	+	1	163	13306	13470
UN15666	Rsa1.0_00482.1	423	81162	0	421	425	99	+	2	423	41471	41895
UN15667	Rsa1.0_01101.1	1354	54031	1.00E-139	263	267	98	−	861	1127	22500	22766
UN15668	Rsa1.0_00265.1	763	220635	1.00E-116	306	336	91	+	386	715	195833	196168
UN15669	Rsa1.0_01659.1	1785	164577	0	605	633	95	−	157	781	143506	144135
UN15670	Rsa1.0_01568.1	715	43077	0	556	566	98	−	87	652	40968	41533
UN15671	Rsa1.0_01588.1	1084	42724	1.00E-162	321	331	96	−	756	1082	28807	29137
UN15672	Rsa1.0_00369.1	402	266462	4.00E-85	189	198	95	+	208	402	199451	199648
UN15673	Rsa1.0_00473.1	1245	212204	1.00E-159	288	289	99	+	955	1243	73607	73895
UN15674	Rsa1.0_01954.1	845	145554	0	538	580	92	−	1	562	13390	13963
UN15675	Rsa1.0_00040.1	538	324196	0	452	491	92	+	2	489	227886	228355
UN15676	Rsa1.0_00122.1	367	265937	1.00E-156	349	359	97	−	1	359	82125	82473

UN15677	Rsa1.0_02963.1	974	26407	1.00E-107	242	254	95	-	721	974	3757	4008
UN15678	Rsa1.0_13843.1	647	4225	0	369	373	98	+	3	373	960	1332
UN15679	Rsa1.0_00347.1	834	93048	0	371	382	97	+	451	832	86405	86776
UN15680	Rsa1.0_00198.1	823	222963	1.00E-104	201	204	98	-	3	206	42326	42529
UN15681	Rsa1.0_02505.1	1057	30703	1.00E-157	375	407	92	-	668	1057	9964	10365
UN15682	Rsa1.0_00644.1	1078	136881	0	449	449	100	+	1	449	62629	63077
UN15683	Rsa1.0_04192.1	1015	18572	0	692	712	97	-	304	1015	12152	12863
UN15684	Rsa1.0_00198.1	435	222963	0	370	372	99	+	1	372	40464	40834
UN15685	Rsa1.0_00143.1	797	121313	0	506	517	97	+	281	797	31129	31645
UN15686	Rsa1.0_02550.1	905	291051	1.00E-178	320	321	99	+	115	435	4853	5173
UN15687	Rsa1.0_00104.1	744	368722	6.00E-73	140	140	100	-	3	142	21169	21308
UN15688	Rsa1.0_00596.1	797	73578	5.00E-98	223	233	95	+	561	791	28854	29084
UN15689	Rsa1.0_00376.1	716	90101	1.00E-171	314	317	99	+	243	559	49173	49489
UN15690	Rsa1.0_01494.1	897	44641	1.00E-152	273	273	100	-	625	897	1605	1877
UN15691	Rsa1.0_07150.1	1115	9817	0	457	464	98	-	653	1115	1301	1764
UN15692	Rsa1.0_00900.1	519	60367	1.00E-128	273	286	95	+	2	284	12161	12446
UN15693	Rsa1.0_00985.1	549	161950	0	524	531	98	+	3	533	133141	133671
UN15694	Rsa1.0_00058.1	1366	159921	1.00E-148	272	274	99	-	1	274	93528	93801
UN15695	Rsa1.0_00029.1	727	272992	1.00E-166	305	308	99	-	85	392	10647	10954
UN15696	Rsa1.0_00497.1	694	79830	1.00E-156	285	287	99	+	1	287	50795	51081
UN15697	Rsa1.0_03422.1	268	23076	1.00E-149	267	267	100	+	2	268	10050	10316
UN15698	Rsa1.0_01245.1	1349	149868	0	546	547	99	-	1	546	37644	38190
UN15699	Rsa1.0_00240.1	1033	104175	1.00E-112	255	271	94	-	753	1022	53574	53836
UN15700	Rsa1.0_00342.1	702	93695	0	451	454	99	-	42	495	15020	15470
UN15701	Rsa1.0_00482.1	1346	81162	0	656	692	94	+	1	687	67345	68021
UN15702	Rsa1.0_00439.1	771	142776	1.00E-167	319	326	97	+	382	707	63131	63456
UN15703	Rsa1.0_01576.1	634	42923	1.00E-105	243	258	94	+	384	634	22319	22572
UN15704	Rsa1.0_00007.1	2091	239325	0	669	672	99	-	322	993	16013	16684
UN15705	Rsa1.0_02586.1	766	29856	0	698	709	98	-	1	709	15528	16236
UN15706	Rsa1.0_00437.1	542	84879	2.00E-87	213	230	92	+	3	225	64125	64354
UN15707	Rsa1.0_00018.1	535	410899	0	515	536	96	+	1	535	79154	79687
UN15708	Rsa1.0_39888.1	855	563	0	490	501	97	-	2	502	1	499
UN15709	Rsa1.0_02080.1	1429	35297	0	1413	1429	98	-	1	1428	19034	20458
UN15710	Rsa1.0_00001.1	777	363028	1.00E-132	240	240	100	-	1	240	91340	91579
UN15711	Rsa1.0_00107.1	361	431659	1.00E-109	244	256	95	-	101	352	180753	181005
UN15712	Rsa1.0_00773.1	1042	64839	0	457	467	97	+	3	469	25178	25644
UN15713	Rsa1.0_00017.1	1039	752458	0	423	442	95	-	587	1027	138929	139369
UN15714	Rsa1.0_03513.1	850	22349	0	490	503	97	-	349	850	20289	20791
UN15715	Rsa1.0_00388.1	797	88890	1.00E-142	299	311	96	+	490	797	56221	56525

UN15716	Rsa1.0_08459.1	998	7315	0	493	524	94	-	476	998	6317	6834
UN15717	Rsa1.0_00381.1	1157	89718	3.00E-97	214	224	95	-	3	224	73705	73928
UN15718	Rsa1.0_00042.1	1073	171507	0	1022	1036	98	-	39	1073	53369	54404
UN15719	Rsa1.0_00112.1	754	132199	1.00E-126	251	258	97	-	42	299	73976	74233
UN15720	Rsa1.0_00777.1	787	191865	0	417	429	97	-	24	452	128527	128955
UN15721	Rsa1.0_03962.1	1325	19860	0	1001	1034	96	-	1	1030	13047	14079
UN15722	Rsa1.0_04744.1	1260	16154	1.00E-169	338	348	97	-	1	345	7259	7606
UN15723	Rsa1.0_02913.1	977	26816	0	420	437	96	+	3	438	22158	22591
UN15724	Rsa1.0_00019.1	703	301669	0	452	468	96	-	1	466	233346	233812
UN15725	Rsa1.0_00105.1	767	134138	1.00E-101	188	188	100	-	374	561	68475	68662
UN15726	Rsa1.0_02641.1	1158	29191	0	331	332	99	+	3	334	5567	5898
UN15727	Rsa1.0_00552.1	897	206757	0	398	421	94	+	3	420	195137	195549
UN15728	Rsa1.0_01484.1	763	44806	0	736	763	96	+	1	763	28469	29218
UN15729	Rsa1.0_00073.1	794	240924	2.00E-88	187	194	96	-	297	490	98132	98325
UN15730	Rsa1.0_00294.1	602	144860	0	387	388	99	+	215	602	28240	28627
UN15731	Rsa1.0_01236.1	1184	50156	0	510	516	98	+	662	1177	12195	12709
UN15732	Rsa1.0_00707.1	860	190112	1.00E-148	267	267	100	-	164	430	155739	156005
UN15733	Rsa1.0_03036.1	1086	25826	0	914	923	99	-	167	1086	20398	21320
UN15734	Rsa1.0_00005.1	1657	353093	0	581	584	99	+	523	1105	138648	139230
UN15735	Rsa1.0_00707.1	801	190112	1.00E-172	346	359	96	-	3	361	156145	156503
UN15736	Rsa1.0_02398.1	610	31766	1.00E-102	257	276	93	+	335	610	5418	5689
UN15737	Rsa1.0_00087.1	745	761073	1.00E-143	283	288	98	-	458	743	191386	191671
UN15738	Rsa1.0_02410.1	1064	31659	0	971	1001	97	-	11	1009	20161	21156
UN15739	Rsa1.0_00501.1	532	79545	0	510	532	95	-	1	532	68684	69215
UN15740	Rsa1.0_00340.1	614	305782	1.00E-143	347	374	92	+	1	359	159726	160093
UN15741	Rsa1.0_00107.1	1529	431659	0	503	531	94	-	572	1097	148415	148945
UN15742	Rsa1.0_01949.1	692	36960	1.00E-103	233	246	94	+	6	250	23210	23455
UN15743	Rsa1.0_04850.1	713	15722	0	613	636	96	-	1	636	7334	7969
UN15744	Rsa1.0_00058.1	724	159921	0	538	545	98	+	3	547	49006	49550
UN15745	Rsa1.0_01403.1	657	46453	2.00E-87	179	184	97	-	297	480	25846	26029
UN15746	Rsa1.0_00152.1	681	257730	1.00E-160	293	294	99	+	389	681	214460	214753
UN15747	Rsa1.0_05611.1	933	13193	0	523	541	96	-	390	930	9253	9785
UN15748	Rsa1.0_00035.1	1630	176449	0	433	445	97	-	60	504	96531	96974
UN15749	Rsa1.0_00163.1	858	116847	0	506	533	94	-	330	858	72621	73151
UN15750	Rsa1.0_00453.1	831	83031	0	490	503	97	-	51	553	68504	69006
UN15751	Rsa1.0_01611.1	775	42269	1.00E-166	320	328	97	-	256	583	25466	25793
UN15752	Rsa1.0_01497.1	1354	44559	1.00E-143	331	352	94	+	1003	1352	42441	42784
UN15753	Rsa1.0_16574.1	627	2106	0	384	390	98	+	58	446	1717	2106
UN15754	Rsa1.0_25951.1	1118	912	0	496	507	97	+	238	742	77	583

UN15755	Rsa1.0_00175.1	815	166121	0	638	667	95	+	149	813	38440	39106
UN15756	Rsa1.0_05120.1	271	14793	1.00E-124	259	270	95	−	2	271	14397	14666
UN15757	Rsa1.0_02859.1	638	162118	1.00E-175	339	348	97	−	291	638	137969	138316
UN15758	Rsa1.0_03581.1	683	21965	0	395	407	97	−	53	454	20231	20636
UN15759	Rsa1.0_00793.1	1016	64103	1.00E-156	279	279	100	+	738	1016	38449	38727
UN15760	Rsa1.0_04124.1	1523	18945	1.00E-133	360	394	91	−	3	383	8363	8751
UN15761	Rsa1.0_00034.1	1782	260119	0	779	779	100	+	446	1224	66394	67172
UN15762	Rsa1.0_00216.1	857	197563	1.00E-119	296	316	93	+	1	314	50380	50690
UN15763	Rsa1.0_03581.1	588	21965	0	389	406	95	−	52	452	20231	20636
UN15764	Rsa1.0_00603.1	691	73299	0	662	693	95	−	3	691	58641	59327
UN15765	Rsa1.0_06852.1	1351	10265	0	538	565	95	+	792	1351	4327	4888
UN15766	Rsa1.0_02878.1	328	27048	1.00E-126	260	269	96	−	1	269	8374	8641
UN15767	Rsa1.0_01716.1	989	40483	1.00E-150	294	300	98	−	112	407	32951	33250
UN15768	Rsa1.0_00845.1	1464	62346	1.00E-166	335	348	96	−	350	697	12349	12696
UN15769	Rsa1.0_00136.1	1219	122464	0	463	480	96	−	689	1164	112511	112990
UN15770	Rsa1.0_01559.1	615	43262	2.00E-81	225	246	91	+	374	615	2268	2510
UN15771	Rsa1.0_00651.1	529	247766	1.00E-107	223	231	96	+	148	378	81983	82210
UN15772	Rsa1.0_00516.1	492	78417	1.00E-137	307	326	94	+	177	491	20048	20372
UN15773	Rsa1.0_02642.1	1565	29181	0	1232	1241	99	−	1	1240	13097	14337
UN15774	Rsa1.0_02173.1	771	34318	1.00E-110	230	239	96	−	394	632	16707	16945
UN15775	Rsa1.0_01015.1	1234	56752	1.00E-175	325	328	99	+	1	327	9980	10307
UN15776	Rsa1.0_03615.1	196	21766	3.00E-81	192	200	96	−	1	196	6198	6396
UN15777	Rsa1.0_00437.1	1095	84879	0	388	404	96	+	603	1006	39662	40065
UN15778	Rsa1.0_01882.1	682	37697	0	517	520	99	−	104	623	5482	6001
UN15779	Rsa1.0_54025.1	862	422	0	419	422	99	+	215	636	1	422
UN15780	Rsa1.0_04223.1	949	18407	0	417	421	99	−	3	421	6198	6618
UN15781	Rsa1.0_00239.1	834	104332	0	438	445	98	+	390	834	95757	96201
UN15782	Rsa1.0_05262.1	936	14334	1.00E-170	363	377	96	+	562	935	11001	11372
UN15783	Rsa1.0_00039.1	678	296927	0	661	676	97	−	3	678	288748	289421
UN15784	Rsa1.0_04524.1	1030	16981	0	413	435	94	+	604	1030	5653	6087
UN15785	Rsa1.0_02584.1	864	29877	0	436	450	96	+	412	861	806	1252
UN15786	Rsa1.0_04768.1	770	16066	0	750	771	97	+	2	770	4694	5463
UN15787	Rsa1.0_00075.1	1473	146680	0	387	394	98	−	1076	1469	29354	29743
UN15788	Rsa1.0_02152.1	654	34558	0	551	588	93	+	4	573	26481	27067
UN15789	Rsa1.0_00078.1	1308	175053	1.00E-138	365	402	90	−	1	393	74758	75152
UN15790	Rsa1.0_04538.1	714	16940	1.00E-116	258	270	95	−	1	267	12644	12910
UN15791	Rsa1.0_00434.1	841	85171	0	367	368	99	−	431	798	35769	36136
UN15792	Rsa1.0_00227.1	890	105849	0	660	660	100	−	231	890	9773	10432
UN15793	Rsa1.0_04192.1	569	18572	0	440	477	92	+	94	569	17147	17622

UN15794	Rsa1.0_00339.1	806	222974	2.00E-82	159	160	99	−	146	305	63385	63544
UN15795	Rsa1.0_00130.1	775	125737	1.00E-161	304	308	98	−	469	775	32083	32390
UN15796	Rsa1.0_00750.1	857	190548	0	637	651	97	−	189	836	184595	185244
UN15797	Rsa1.0_06465.1	1376	10962	0	516	528	97	−	35	562	4224	4749
UN15798	Rsa1.0_00065.1	749	454616	1.00E-121	268	278	96	−	473	749	232830	233100
UN15799	Rsa1.0_16574.1	731	2106	0	383	390	98	−	277	666	1717	2106
UN15800	Rsa1.0_04169.1	625	18712	1.00E-172	315	318	99	+	1	318	3208	3525
UN15801	Rsa1.0_02934.1	654	26627	0	635	657	96	+	1	654	24955	25610
UN15802	Rsa1.0_00153.1	808	192931	0	606	607	99	−	1	606	24233	24839
UN15803	Rsa1.0_00121.1	333	278029	1.00E-120	295	317	93	+	18	332	54300	54614
UN15804	Rsa1.0_00750.1	1189	190548	0	968	988	97	−	188	1172	184257	185239
UN15805	Rsa1.0_00013.1	938	292129	1.00E-107	205	206	99	+	665	870	73022	73226
UN15806	Rsa1.0_01453.1	804	45449	1.00E-113	266	282	94	+	523	800	6149	6430
UN15807	Rsa1.0_00269.1	910	341668	1.00E-117	225	227	99	+	685	910	323318	323544
UN15808	Rsa1.0_00224.1	859	327530	1.00E-92	182	185	98	+	3	187	36681	36865
UN15809	Rsa1.0_58152.1	604	393	9.00E-90	183	188	97	+	14	201	1	188
UN15810	Rsa1.0_07425.1	558	9414	1.00E-135	254	257	98	−	218	474	4720	4976
UN15811	Rsa1.0_00088.1	1172	141341	1.00E-114	237	246	96	−	926	1171	104118	104363
UN15812	Rsa1.0_00348.1	733	92818	0	356	359	99	+	2	360	49675	50033
UN15813	Rsa1.0_02001.1	492	36198	0	482	492	97	−	1	492	31958	32449
UN15814	Rsa1.0_00140.1	479	121541	1.00E-100	188	189	99	+	3	191	114	302
UN15815	Rsa1.0_00480.1	724	81325	0	394	407	96	−	1	404	29204	29607
UN15816	Rsa1.0_00326.1	791	171955	1.00E-148	284	290	97	−	1	290	82742	83031
UN15817	Rsa1.0_02633.1	828	29315	1.00E-161	349	369	94	+	100	456	405	771
UN15818	Rsa1.0_00147.1	1240	249503	0	338	341	99	−	900	1239	217846	218186
UN15819	Rsa1.0_00630.1	1450	71281	0	804	829	96	−	1	829	12763	13582
UN15820	Rsa1.0_01219.1	1049	50527	0	690	716	96	+	332	1045	39455	40167
UN15821	Rsa1.0_03380.1	1163	23320	0	350	356	98	+	3	358	4260	4615
UN15822	Rsa1.0_03718.1	710	21195	3.00E-90	181	185	97	−	479	663	1723	1907
UN15823	Rsa1.0_02676.1	289	28834	1.00E-137	277	287	96	+	3	289	12369	12655
UN15824	Rsa1.0_00130.1	323	125737	3.00E-97	209	216	96	+	110	323	618	833
UN15825	Rsa1.0_03303.1	544	23890	1.00E-130	245	248	98	+	297	544	7060	7307
UN15826	Rsa1.0_05199.1	752	14529	1.00E-158	429	479	89	+	277	752	7773	8230
UN15827	Rsa1.0_00653.1	750	70029	1.00E-122	265	277	95	−	202	472	37747	38020
UN15828	Rsa1.0_00192.1	428	278200	0	410	428	95	−	1	428	13722	14148
UN15829	Rsa1.0_00015.1	1557	561803	0	1061	1067	99	+	492	1557	362642	363708
UN15830	Rsa1.0_00336.1	1046	445051	1.00E-169	317	321	98	−	718	1038	164262	164581
UN15831	Rsa1.0_02963.1	978	26407	1.00E-111	243	254	95	+	727	976	5185	5437
UN15832	Rsa1.0_01656.1	526	41494	1.00E-82	176	182	96	−	19	197	14762	14943

UN15833	Rsa1.0_04518.1	859	17005	1.00E-148	290	298	97	-	1	298	11675	11972
UN15834	Rsa1.0_00246.1	995	103541	1.00E-148	341	359	94	-	627	981	26557	26905
UN15835	Rsa1.0_03142.1	896	25043	0	486	503	96	-	348	846	1430	1932
UN15836	Rsa1.0_00023.1	901	194764	0	600	636	94	-	1	627	118142	118765
UN15837	Rsa1.0_05510.1	1032	13470	1.00E-150	285	290	98	-	475	764	1981	2270
UN15838	Rsa1.0_03799.1	535	20775	9.00E-93	191	197	96	-	164	360	17302	17498
UN15839	Rsa1.0_00790.1	487	148693	1.00E-119	227	230	98	+	218	447	135658	135887
UN15840	Rsa1.0_00226.1	1018	105884	0	383	396	96	+	596	990	32605	32999
UN15841	Rsa1.0_01047.1	1005	55705	1.00E-170	316	320	98	+	686	1005	2010	2329
UN15842	Rsa1.0_16567.1	767	2109	8.00E-94	188	191	98	+	545	734	1316	1506
UN15843	Rsa1.0_00253.1	824	231292	1.00E-127	272	286	95	-	2	285	28152	28428
UN15844	Rsa1.0_00020.1	513	283099	1.00E-164	354	371	95	-	144	513	60898	61265
UN15845	Rsa1.0_01171.1	1068	51752	0	362	362	100	+	706	1067	50943	51304
UN15846	Rsa1.0_02834.1	695	27493	1.00E-174	368	382	96	-	1	380	24286	24665
UN15847	Rsa1.0_01908.1	1475	37427	0	911	957	95	+	276	1232	7707	8663
UN15848	Rsa1.0_01951.1	638	36941	1.00E-174	332	339	97	-	1	339	13558	13893
UN15849	Rsa1.0_00153.1	652	192931	0	556	578	96	-	1	574	157109	157686
UN15850	Rsa1.0_23535.1	631	1042	2.00E-63	130	132	98	+	212	343	98	229
UN15851	Rsa1.0_01068.1	638	55208	1.00E-98	200	205	97	+	40	244	5127	5328
UN15852	Rsa1.0_00163.1	692	116847	0	463	476	97	+	213	688	46561	47036
UN15853	Rsa1.0_02910.1	375	26843	5.00E-35	82	84	97	+	160	243	21401	21484
UN15854	Rsa1.0_00707.1	910	190112	1.00E-125	236	238	99	-	675	910	181078	181315
UN15855	Rsa1.0_00236.1	1293	104771	1.00E-163	372	395	94	-	16	403	82285	82673
UN15856	Rsa1.0_01655.1	686	41508	0	462	480	96	-	2	481	6342	6818
UN15857	Rsa1.0_02663.1	732	169243	1.00E-171	308	309	99	+	359	667	9930	10238
UN15858	Rsa1.0_00477.1	822	81556	0	416	429	96	+	1	425	23857	24283
UN15859	Rsa1.0_00087.1	1254	761073	1.00E-167	377	393	95	-	868	1254	615116	615505
UN15860	Rsa1.0_01245.1	540	149868	0	389	392	99	+	5	394	41022	41413
UN15861	Rsa1.0_00087.1	647	761073	1.00E-134	328	352	93	-	1	343	225356	225700
UN15862	Rsa1.0_00248.1	371	163568	1.00E-143	344	371	92	+	1	371	40147	40513
UN15863	Rsa1.0_02442.1	489	31355	1.00E-122	248	255	97	+	1	254	12550	12804
UN15864	Rsa1.0_11912.1	517	5372	0	491	514	95	+	1	514	1605	2115
UN15865	Rsa1.0_00451.1	661	322533	2.00E-57	196	225	87	+	1	225	293299	293508
UN15866	Rsa1.0_01104.1	1013	285480	1.00E-121	236	241	97	-	1	241	268644	268884
UN15867	Rsa1.0_00388.1	877	88890	0	396	398	99	-	3	400	56221	56618
UN15868	Rsa1.0_02389.1	716	146428	0	697	729	95	+	1	716	138078	138806
UN15869	Rsa1.0_00220.1	782	192936	0	418	426	98	-	193	615	16394	16819
UN15870	Rsa1.0_00018.1	732	410899	1.00E-174	330	337	97	+	87	423	114918	115254
UN15871	Rsa1.0_01937.1	1116	37147	0	423	439	96	-	5	437	21081	21517

UN15872	Rsa1.0_03579.1	1162	21969	0	403	422	95	-	1	421	18958	19378
UN15873	Rsa1.0_02434.1	437	31464	0	423	441	95	-	3	437	1757	2197
UN15874	Rsa1.0_01786.1	1020	133324	0	536	547	97	+	221	767	96281	96827
UN15875	Rsa1.0_00296.1	770	97854	1.00E-148	310	323	95	-	1	322	89589	89911
UN15876	Rsa1.0_00012.1	1121	222148	0	677	677	100	+	443	1119	130812	131488
UN15877	Rsa1.0_01232.1	332	155185	1.00E-117	295	322	91	+	11	332	108755	109076
UN15878	Rsa1.0_00977.1	490	198001	0	413	432	95	+	18	449	50788	51219
UN15879	Rsa1.0_00496.1	870	79834	0	458	461	99	+	197	654	30141	30601
UN15880	Rsa1.0_00651.1	669	247766	0	438	475	92	-	1	472	207681	208143
UN15881	Rsa1.0_00762.1	823	165246	1.00E-163	327	339	96	+	9	347	25879	26217
UN15882	Rsa1.0_31708.1	806	721	1.00E-115	214	215	99	+	259	473	43	257
UN15883	Rsa1.0_01434.1	594	45951	0	575	596	96	+	3	594	7332	7923
UN15884	Rsa1.0_00659.1	260	69733	3.00E-60	128	130	98	+	55	183	20021	20150
UN15885	Rsa1.0_00417.1	1522	395068	0	801	811	98	-	1	811	294903	295713
UN15886	Rsa1.0_00133.1	784	123768	1.00E-174	309	309	100	-	124	432	11639	11947
UN15887	Rsa1.0_00407.1	337	191544	1.00E-157	324	336	96	+	3	337	43105	43438
UN15888	Rsa1.0_08909.1	464	6799	1.00E-85	242	264	91	-	5	257	5387	5646
UN15889	Rsa1.0_03352.1	1520	23506	1.00E-128	279	289	96	-	1	288	4054	4339
UN15890	Rsa1.0_00038.1	851	314568	0	453	460	98	-	1	460	253300	253756
UN15891	Rsa1.0_43698.1	752	514	1.00E-163	298	300	99	+	254	553	215	514
UN15892	Rsa1.0_00897.1	747	60498	1.00E-112	238	249	95	-	349	597	22614	22856
UN15893	Rsa1.0_07536.1	1112	9178	0	419	419	100	-	687	1105	6475	6893
UN15894	Rsa1.0_00005.1	839	353093	0	829	837	99	+	1	837	288482	289318
UN15895	Rsa1.0_03738.1	1174	21071	1.00E-172	306	306	100	-	6	311	12767	13072
UN15896	Rsa1.0_01234.1	701	50183	0	509	509	100	-	3	511	8897	9405
UN15897	Rsa1.0_06571.1	767	10750	3.00E-81	157	158	99	+	38	195	4754	4911
UN15898	Rsa1.0_02839.1	1273	27391	0	365	365	100	-	235	599	18841	19205
UN15899	Rsa1.0_39888.1	936	563	0	491	501	98	+	435	935	1	499
UN15900	Rsa1.0_06201.1	1275	11506	1.00E-167	302	303	99	+	46	348	1	303
UN15901	Rsa1.0_03191.1	1061	145762	0	947	965	98	-	97	1061	137620	138583
UN15902	Rsa1.0_02373.1	270	32048	1.00E-116	260	274	94	-	1	270	30407	30680
UN15903	Rsa1.0_02908.1	253	26859	9.00E-91	214	228	93	-	27	252	9514	9741
UN15904	Rsa1.0_23451.1	1128	1048	0	396	396	100	-	2	397	184	579
UN15905	Rsa1.0_00385.1	634	89160	1.00E-76	167	174	95	+	262	435	50842	51015
UN15906	Rsa1.0_05228.1	864	14437	1.00E-163	356	377	94	+	2	364	4937	5313
UN15907	Rsa1.0_00002.1	513	601661	0	466	483	96	-	1	477	289127	289606
UN15908	Rsa1.0_00130.1	438	125737	1.00E-145	375	409	91	+	1	391	115167	115570
UN15909	Rsa1.0_02910.1	985	26843	1.00E-108	244	258	94	+	3	260	20245	20499
UN15910	Rsa1.0_00602.1	804	73302	1.00E-154	295	301	98	-	3	303	720	1020

UN15911	Rsa1.0_03738.1	1143	21071	1.00E-142	266	269	98	−	306	574	12425	12693
UN15912	Rsa1.0_00288.1	583	98659	1.00E-102	192	193	99	+	391	583	52056	52248
UN15913	Rsa1.0_00632.1	556	183777	2.00E-56	115	116	99	+	374	489	60381	60496
UN15914	Rsa1.0_01865.1	596	37992	0	580	595	97	+	1	594	25937	26528
UN15915	Rsa1.0_00098.1	352	349239	1.00E-149	330	350	94	+	3	352	292708	293055
UN15916	Rsa1.0_00200.1	591	162016	0	495	512	96	+	75	586	119257	119754
UN15917	Rsa1.0_00463.1	1540	189177	1.00E-141	255	255	100	−	928	1182	170242	170496
UN15918	Rsa1.0_03887.1	646	20247	1.00E-131	251	254	98	+	1	254	2246	2498
UN15919	Rsa1.0_00247.1	834	103408	0	378	380	99	−	447	825	65465	65844
UN15920	Rsa1.0_00382.1	882	251946	1.00E-159	285	285	100	+	1	285	245728	246012
UN15921	Rsa1.0_01171.1	776	51752	0	395	395	100	+	382	776	50943	51337
UN15922	Rsa1.0_00121.1	1335	278029	0	396	396	100	+	940	1335	31815	32210
UN15923	Rsa1.0_01578.1	524	42880	1.00E-161	333	348	95	−	2	349	31335	31682
UN15924	Rsa1.0_00013.1	804	292129	1.00E-163	305	308	99	+	497	804	159602	159908
UN15925	Rsa1.0_00222.1	650	172263	0	503	519	96	−	1	519	69472	69990
UN15926	Rsa1.0_03740.1	501	21065	0	462	492	93	+	1	489	14534	15023
UN15927	Rsa1.0_01166.1	458	51877	0	446	459	97	+	1	458	9156	9614
UN15928	Rsa1.0_00015.1	904	561803	0	427	428	99	−	1	428	401953	402379
UN15929	Rsa1.0_00139.1	387	357786	0	380	386	98	−	2	387	128742	129127
UN15930	Rsa1.0_07808.1	766	8504	0	565	574	98	+	82	655	154	727
UN15931	Rsa1.0_00126.1	1257	127534	1.00E-156	308	316	97	+	255	569	37046	37361
UN15932	Rsa1.0_00122.1	1123	265937	1.00E-117	247	254	97	+	3	255	5366	5617
UN15933	Rsa1.0_07396.1	812	9465	0	490	506	96	−	310	812	94	598
UN15934	Rsa1.0_02384.1	1285	31917	0	797	811	98	−	478	1285	5571	6378
UN15935	Rsa1.0_00893.1	640	60626	0	395	396	99	−	245	640	8442	8837
UN15936	Rsa1.0_01649.1	1145	41624	0	562	575	97	−	2	576	18793	19367
UN15937	Rsa1.0_00782.1	697	64468	1.00E-137	301	318	94	−	301	618	60301	60615
UN15938	Rsa1.0_30388.1	416	755	1.00E-118	216	216	100	−	2	217	533	748
UN15939	Rsa1.0_07344.1	744	9528	0	718	743	96	−	2	743	6004	6744
UN15940	Rsa1.0_02398.1	617	31766	1.00E-142	278	284	97	−	1	284	5418	5700
UN15941	Rsa1.0_00660.1	617	69688	1.00E-129	243	246	98	−	6	251	60390	60635
UN15942	Rsa1.0_00821.1	931	62891	1.00E-126	242	246	98	+	2	247	49074	49319
UN15943	Rsa1.0_00071.1	710	345714	1.00E-126	301	324	92	−	29	352	317193	317513
UN15944	Rsa1.0_03552.1	911	22151	0	419	426	98	−	1	425	12808	13231
UN15945	Rsa1.0_01896.1	793	37512	0	573	597	95	−	1	597	19518	20114
UN15946	Rsa1.0_00962.1	576	184909	0	334	334	100	+	243	576	13117	13450
UN15947	Rsa1.0_03347.1	868	23588	0	865	868	99	+	1	868	8101	8968
UN15948	Rsa1.0_01389.1	1024	46716	0	414	424	97	+	3	422	9743	10165
UN15949	Rsa1.0_39335.1	663	572	0	339	340	99	−	1	339	11	350

UN15950	Rsa1.0_00382.1	909	251946	1.00E-138	279	287	97	+	3	288	34566	34848
UN15951	Rsa1.0_00022.1	608	200461	1.00E-151	295	303	97	+	304	606	45805	46107
UN15952	Rsa1.0_10036.1	813	6030	1.00E-117	243	252	96	+	419	667	496	747
UN15953	Rsa1.0_00356.1	743	91521	0	721	743	97	+	1	743	4650	5391
UN15954	Rsa1.0_00071.1	777	345714	0	362	373	97	−	1	372	142456	142821
UN15955	Rsa1.0_00070.1	695	150321	1.00E-128	262	272	96	+	420	691	59705	59976
UN15956	Rsa1.0_01002.1	730	57063	0	724	731	99	−	1	730	20631	21361
UN15957	Rsa1.0_02020.1	686	173211	0	396	405	97	−	1	404	154513	154917
UN15958	Rsa1.0_02040.1	880	35684	1.00E-178	323	325	99	+	554	878	13231	13555
UN15959	Rsa1.0_12866.1	1229	5093	0	1021	1059	96	+	6	1062	1044	2094
UN15960	Rsa1.0_00128.1	530	228675	8.00E-87	219	233	93	−	4	232	61319	61549
UN15961	Rsa1.0_01078.1	524	316910	1.00E-153	309	318	97	+	207	522	290400	290717
UN15962	Rsa1.0_05611.1	813	13193	0	537	555	96	−	261	813	2578	3128
UN15963	Rsa1.0_00168.1	616	115870	1.00E-168	320	327	97	−	4	330	54746	55072
UN15964	Rsa1.0_01182.1	668	167304	5.00E-76	192	209	91	−	5	213	3474	3673
UN15965	Rsa1.0_01281.1	537	49313	1.00E-109	210	213	98	+	285	497	34295	34507
UN15966	Rsa1.0_01412.1	872	46311	0	479	498	96	−	5	501	9158	9650
UN15967	Rsa1.0_02496.1	735	30814	0	392	400	98	−	1	394	28985	29384
UN15968	Rsa1.0_03191.1	382	145762	1.00E-148	301	312	96	−	1	312	16325	16633
UN15969	Rsa1.0_12954.1	1009	5066	1.00E-107	256	272	94	+	17	287	3809	4076
UN15970	Rsa1.0_00630.1	562	71281	1.00E-158	314	319	98	−	203	517	62461	62779
UN15971	Rsa1.0_00171.1	600	115622	0	330	330	100	−	271	600	50091	50420
UN15972	Rsa1.0_29830.1	1085	770	0	409	437	93	+	649	1085	114	534
UN15973	Rsa1.0_00348.1	890	92818	0	444	454	97	−	440	889	49580	50033
UN15974	Rsa1.0_04396.1	1424	17561	0	1251	1287	97	+	50	1336	16277	17561
UN15975	Rsa1.0_00040.1	1314	324196	0	505	506	99	−	3	507	175165	175670
UN15976	Rsa1.0_00601.1	792	177045	0	394	404	97	+	1	400	26093	26496
UN15977	Rsa1.0_00341.1	631	249309	1.00E-73	225	249	90	−	375	620	123991	124239
UN15978	Rsa1.0_03900.1	1024	133221	1.00E-164	319	327	97	+	698	1024	3917	4239
UN15979	Rsa1.0_03465.1	1175	22700	0	508	522	97	−	569	1090	9803	10324
UN15980	Rsa1.0_00601.1	787	177045	0	404	404	100	−	384	787	26093	26496
UN15981	Rsa1.0_00163.1	1252	116847	0	1207	1249	96	−	5	1252	48260	49508
UN15982	Rsa1.0_31664.1	582	722	1.00E-111	204	204	100	+	60	263	514	717
UN15983	Rsa1.0_37263.1	1040	605	0	592	605	97	−	376	980	1	605
UN15984	Rsa1.0_00526.1	1440	169279	0	496	497	99	−	944	1440	128412	128908
UN15985	Rsa1.0_00456.1	638	82826	1.00E-134	263	270	97	+	19	288	65935	66204
UN15986	Rsa1.0_00001.1	1143	363028	0	549	549	100	+	595	1143	266567	267115
UN15987	Rsa1.0_00632.1	699	183777	1.00E-128	265	278	95	−	434	699	55976	56253
UN15988	Rsa1.0_00647.1	1008	70440	1.00E-170	303	303	100	+	706	1008	57755	58057

UN15989	Rsa1.0_00071.1	746	345714	1.00E-132	263	268	98	+	5	271	324470	324736
UN15990	Rsa1.0_13595.1	1543	4681	0	1127	1141	98	+	3	1143	3541	4681
UN15991	Rsa1.0_00182.1	978	142305	1.00E-139	252	252	100	−	320	571	74844	75095
UN15992	Rsa1.0_00075.1	1548	146680	0	457	482	94	+	1068	1548	17393	17857
UN15993	Rsa1.0_30668.1	1243	748	0	423	423	100	−	326	748	1	423
UN15994	Rsa1.0_06136.1	1668	11659	0	388	388	100	−	1001	1388	150	537
UN15995	Rsa1.0_05257.1	1857	14343	0	806	812	99	−	1031	1842	1937	2748
UN15996	Rsa1.0_00825.1	1624	62855	0	421	426	98	+	2	427	53120	53545
UN15997	Rsa1.0_03726.1	898	21160	4.00E-96	213	223	95	−	3	220	17199	17420
UN15998	Rsa1.0_59294.1	1232	386	5.00E-56	135	142	95	−	813	951	141	282
UN15999	Rsa1.0_01536.1	955	43747	0	347	349	99	+	482	830	28390	28738
UN16000	Rsa1.0_00246.1	1217	103541	1.00E-151	318	331	96	−	889	1216	89269	89594
UN16001	Rsa1.0_11549.1	1100	5473	0	529	530	99	−	571	1100	4464	4993
UN16002	Rsa1.0_01439.1	1521	45841	0	638	640	99	−	5	644	1211	1850
UN16003	Rsa1.0_00565.1	1182	75420	0	464	465	99	−	378	842	35259	35723
UN16004	Rsa1.0_03173.1	870	24815	1.00E-124	350	390	89	−	1	389	18617	19006
UN16005	Rsa1.0_01007.1	1029	56933	0	808	808	100	+	1	808	47967	48774
UN16006	Rsa1.0_00407.1	1610	191544	1.00E-174	310	310	100	−	3	312	9694	10003
UN16007	Rsa1.0_02314.1	1285	32642	1.00E-152	285	289	98	−	1	289	1471	1759
UN16008	Rsa1.0_05189.1	1124	14545	0	374	376	99	+	1	376	412	785
UN16009	Rsa1.0_00480.1	1114	81325	0	700	712	98	−	401	1111	3543	4254
UN16010	Rsa1.0_02878.1	1727	27048	0	607	630	96	−	1118	1727	8252	8880
UN16011	Rsa1.0_00156.1	704	118383	0	678	692	97	−	11	702	9517	10202
UN16012	Rsa1.0_03613.1	794	21773	1.00E-147	280	284	98	−	513	794	16649	16932
UN16013	Rsa1.0_00111.1	1551	390088	0	1506	1554	96	+	2	1551	189316	190869
UN16014	Rsa1.0_00247.1	1285	103408	2.00E-83	164	166	98	+	769	934	74405	74570
UN16015	Rsa1.0_03504.1	1117	22443	0	383	384	99	−	3	386	10641	11024
UN16016	Rsa1.0_02222.1	1279	152798	0	390	391	99	−	889	1279	14593	14983
UN16017	Rsa1.0_01015.1	1063	56752	0	612	618	99	+	65	682	8793	9410
UN16018	Rsa1.0_01829.1	1711	166063	0	1674	1728	96	+	3	1709	160068	161794
UN16019	Rsa1.0_00875.1	896	61371	0	856	890	96	+	1	890	6887	7775
UN16020	Rsa1.0_01382.1	1707	46877	0	508	509	99	+	586	1094	27502	28010
UN16021	Rsa1.0_02976.1	625	26315	1.00E-113	220	223	98	+	402	623	18887	19109
UN16022	Rsa1.0_08265.1	909	7630	0	483	488	98	−	422	908	6571	7058
UN16023	Rsa1.0_02663.1	1095	169243	0	366	372	98	−	725	1095	26577	26947
UN16024	Rsa1.0_00001.1	2871	363028	0	607	609	99	+	1	609	26313	26921
UN16025	Rsa1.0_00056.1	712	332604	1.00E-151	289	292	98	+	2	291	90001	90292
UN16026	Rsa1.0_01550.1	709	43443	1.00E-169	321	325	98	+	387	709	14090	14414
UN16027	Rsa1.0_00226.1	1989	105884	0	392	393	99	−	1175	1567	30643	31035

UN16028	Rsa1.0_00638.1	966	71037	1.00E-139	269	275	97	+	307	581	9032	9306
UN16029	Rsa1.0_03009.1	1173	26065	1.00E-114	230	237	97	−	823	1059	15481	15717
UN16030	Rsa1.0_01553.1	638	43379	1.00E-161	328	338	97	+	301	633	15175	15512
UN16031	Rsa1.0_00224.1	1064	327530	1.00E-118	223	225	99	+	1	225	36641	36865
UN16032	Rsa1.0_00959.1	1355	58202	0	958	997	96	+	3	984	56777	57763
UN16033	Rsa1.0_00832.1	1745	62622	0	1243	1252	99	+	1	1252	34375	35626
UN16034	Rsa1.0_02930.1	911	26668	0	886	908	97	+	3	910	12340	13247
UN16035	Rsa1.0_04542.1	952	16930	1.00E-155	319	331	96	+	623	950	16072	16395
UN16036	Rsa1.0_00398.1	1685	88007	0	732	776	94	+	410	1174	70194	70965
UN16037	Rsa1.0_04194.1	1364	132734	1.00E-151	287	292	98	−	801	1092	131500	131791
UN16038	Rsa1.0_01482.1	2060	44855	0	448	452	99	−	1607	2058	22594	23044
UN16039	Rsa1.0_00689.1	935	68305	0	567	581	97	+	130	710	12824	13401
UN16040	Rsa1.0_01047.1	1058	55705	1.00E-133	248	250	99	−	807	1056	27263	27512
UN16041	Rsa1.0_02378.1	1122	31991	0	414	428	96	+	677	1104	13753	14179
UN16042	Rsa1.0_03114.1	2214	25318	0	790	791	99	+	1191	1981	9889	10679
UN16043	Rsa1.0_04383.1	1652	17631	0	502	503	99	+	1	503	9306	9808
UN16044	Rsa1.0_00153.1	897	192931	0	569	575	98	+	324	895	157109	157683
UN16045	Rsa1.0_01249.1	1579	188432	0	1163	1163	100	+	417	1579	149233	150395
UN16046	Rsa1.0_01286.1	773	49209	0	580	580	100	+	2	581	3945	4524
UN16047	Rsa1.0_00134.1	1132	390219	0	554	602	92	−	535	1131	45107	45694
UN16048	Rsa1.0_02329.1	1439	32474	0	784	795	98	+	645	1437	10253	11044
UN16049	Rsa1.0_02400.1	933	31750	1.00E-175	322	324	99	−	610	933	19678	20000
UN16050	Rsa1.0_00734.1	718	66508	1.00E-107	278	305	91	+	216	514	38631	38935
UN16051	Rsa1.0_04544.1	2471	16917	0	751	751	100	+	1033	1783	6890	7640
UN16052	Rsa1.0_05448.1	1631	13712	1.00E-111	210	212	99	+	399	610	11459	11670
UN16053	Rsa1.0_01134.1	1933	52829	0	761	785	96	−	585	1365	23135	23912
UN16054	Rsa1.0_00180.1	1445	114581	0	1092	1119	97	−	331	1445	89957	91072
UN16055	Rsa1.0_08427.1	1121	7371	1.00E-180	320	320	100	−	1	320	1	320
UN16056	Rsa1.0_00900.1	918	60367	0	390	404	96	−	3	399	13294	13696
UN16057	Rsa1.0_13747.1	961	4388	0	405	407	99	+	1	407	1540	1946
UN16058	Rsa1.0_00003.1	1192	340054	0	435	436	99	+	1	436	276970	277405
UN16059	Rsa1.0_08532.1	1186	7228	1.00E-163	322	332	96	−	854	1185	2741	3072
UN16060	Rsa1.0_17922.1	667	1708	0	428	438	97	+	229	666	315	752
UN16061	Rsa1.0_02707.1	1073	28520	1.00E-114	232	237	97	−	7	243	26163	26396
UN16062	Rsa1.0_03278.1	1001	24125	1.00E-159	285	285	100	+	1	285	1987	2271
UN16063	Rsa1.0_01947.1	655	36977	0	614	633	96	+	4	636	24868	25499
UN16064	Rsa1.0_00720.1	896	66931	1.00E-135	244	244	100	+	151	394	35053	35296
UN16065	Rsa1.0_20365.1	1398	1313	0	462	474	97	+	87	555	1	474
UN16066	Rsa1.0_01376.1	1087	47004	3.00E-97	187	189	98	−	428	616	38534	38722

UN16067	Rsa1.0_04169.1	921	18712	1.00E-160	359	381	94	-	543	921	3161	3525
UN16068	Rsa1.0_02913.1	1057	26816	0	508	523	97	-	538	1057	22069	22591
UN16069	Rsa1.0_00979.1	1409	57733	0	372	372	100	+	1	372	30341	30712
UN16070	Rsa1.0_13917.1	834	4095	0	360	366	98	+	460	825	1	366
UN16071	Rsa1.0_01713.1	1272	40564	1.00E-130	332	365	90	-	919	1271	30338	30695
UN16072	Rsa1.0_00500.1	1211	272308	0	756	760	99	+	219	978	143032	143791
UN16073	Rsa1.0_01679.1	1308	146876	1.00E-160	307	313	98	-	998	1308	134935	135247
UN16074	Rsa1.0_00858.1	1735	61892	0	425	432	98	+	1304	1735	33273	33704
UN16075	Rsa1.0_02580.1	805	29909	0	790	799	98	-	6	803	20985	21783
UN16076	Rsa1.0_00029.1	760	272992	0	361	364	99	+	397	760	23298	23661
UN16077	Rsa1.0_02001.1	857	36198	0	495	514	96	+	345	855	31940	32448
UN16078	Rsa1.0_00333.1	1399	94500	1.00E-125	273	284	96	+	1044	1326	50447	50728
UN16079	Rsa1.0_01376.1	962	47004	1.00E-139	301	315	95	+	17	325	22466	22778
UN16080	Rsa1.0_00239.1	1636	104332	0	484	488	99	+	1151	1636	95757	96244
UN16081	Rsa1.0_00602.1	1283	73302	0	714	715	99	-	1	715	29563	30276
UN16082	Rsa1.0_00948.1	510	58620	6.00E-63	208	235	88	-	13	240	50679	50904
UN16083	Rsa1.0_02157.1	705	149652	4.00E-83	200	215	93	-	1	215	134118	134320
UN16084	Rsa1.0_02401.1	419	138425	1.00E-106	251	266	94	+	158	416	14881	15144
UN16085	Rsa1.0_01376.1	1066	47004	1.00E-127	273	285	95	-	778	1062	37678	37957
UN16086	Rsa1.0_06803.1	700	10330	0	616	624	98	+	77	700	4636	5259
UN16087	Rsa1.0_00102.1	1749	135497	0	988	993	99	-	758	1749	39624	40616
UN16088	Rsa1.0_03292.1	1385	23977	0	1283	1330	96	-	57	1385	10858	12173
UN16089	Rsa1.0_00058.1	1464	159921	1.00E-117	230	235	97	+	712	946	92939	93173
UN16090	Rsa1.0_06433.1	1258	11031	1.00E-105	209	213	98	+	1046	1258	8829	9039
UN16091	Rsa1.0_00175.1	1299	166121	1.00E-123	253	261	96	-	28	287	28735	28995
UN16092	Rsa1.0_00913.1	2060	59914	0	425	435	97	-	11	440	16421	16855
UN16093	Rsa1.0_00327.1	1414	94892	0	1192	1205	98	+	3	1206	69881	71076
UN16094	Rsa1.0_02556.1	1765	30133	0	560	562	99	+	92	653	2960	3521
UN16095	Rsa1.0_00471.1	1116	81897	0	395	395	100	-	1	395	4146	4540
UN16096	Rsa1.0_01484.1	1103	44806	0	662	663	99	-	3	665	40748	41410
UN16097	Rsa1.0_00186.1	1330	112669	0	620	628	98	+	2	629	111162	111789
UN16098	Rsa1.0_03303.1	1623	23890	0	405	407	99	+	847	1253	6082	6488
UN16099	Rsa1.0_00123.1	1022	325650	0	400	400	100	+	622	1021	267980	268379
UN16100	Rsa1.0_00592.1	1124	73788	1.00E-142	257	257	100	-	868	1124	10503	10759
UN16101	Rsa1.0_06803.1	1158	10330	1.00E-107	264	279	94	+	15	287	7267	7542
UN16102	Rsa1.0_00104.1	1867	368722	0	348	354	98	-	88	441	35597	35950
UN16103	Rsa1.0_60372.1	930	379	0	376	379	99	-	361	739	1	379
UN16104	Rsa1.0_01786.1	1948	133324	0	1119	1120	99	+	829	1948	97427	98546
UN16105	Rsa1.0_00019.1	1171	301669	0	471	474	99	-	11	484	233346	233819

UN16106	Rsa1.0_34603.1	1128	654	1.00E-113	230	237	97	-	86	322	223	456
UN16107	Rsa1.0_02070.1	1266	158516	1.00E-148	270	271	99	+	1	271	16290	16560
UN16108	Rsa1.0_01413.1	1417	46275	0	853	865	98	-	554	1410	40290	41154
UN16109	Rsa1.0_02825.1	843	27568	0	423	434	97	-	408	841	26073	26506
UN16110	Rsa1.0_03257.1	1191	24305	1.00E-160	293	294	99	-	897	1189	13738	14031
UN16111	Rsa1.0_05501.1	842	13513	6.00E-70	150	155	96	+	516	670	8665	8819
UN16112	Rsa1.0_01947.1	1940	36977	0	986	998	98	-	1	998	11580	12576
UN16113	Rsa1.0_73985.1	885	310	1.00E-144	259	259	100	-	1	259	1	259
UN16114	Rsa1.0_02950.1	1366	26506	6.00E-96	191	194	98	-	1171	1364	17900	18091
UN16115	Rsa1.0_00030.1	1659	250988	0	917	921	99	-	740	1659	141374	142293
UN16116	Rsa1.0_14230.1	1516	3638	1.00E-126	244	248	98	-	3	247	1860	2107
UN16117	Rsa1.0_17016.1	1008	1952	0	408	408	100	-	591	998	206	613
UN16118	Rsa1.0_08293.1	1056	7593	0	638	663	96	-	395	1056	5325	5983
UN16119	Rsa1.0_05221.1	975	14452	1.00E-148	266	266	100	+	3	268	9197	9462
UN16120	Rsa1.0_05510.1	1192	13470	0	341	346	98	-	847	1192	1552	1895
UN16121	Rsa1.0_00513.1	980	78579	1.00E-117	236	242	97	-	5	246	58775	59015
UN16122	Rsa1.0_03752.1	1188	21022	1.00E-109	226	234	96	-	16	249	1341	1574
UN16123	Rsa1.0_00676.1	1503	69166	0	1315	1361	96	+	46	1403	1037	2395
UN16124	Rsa1.0_01635.1	797	41865	1.00E-98	189	191	98	-	146	336	19217	19407
UN16125	Rsa1.0_02975.1	543	26322	1.00E-113	244	253	96	-	2	254	23332	23578
UN16126	Rsa1.0_00203.1	817	109129	9.00E-60	139	146	95	+	478	623	3653	3798
UN16127	Rsa1.0_01669.1	1008	41286	1.00E-166	306	309	99	-	700	1008	5328	5636
UN16128	Rsa1.0_02935.1	1092	26621	1.00E-176	320	322	99	-	155	476	20469	20790
UN16129	Rsa1.0_02149.1	1351	148330	1.00E-125	248	255	97	+	507	755	17448	17702
UN16130	Rsa1.0_01117.1	1779	53465	0	453	481	94	+	1	464	40118	40598
UN16131	Rsa1.0_04355.1	687	146733	0	398	411	96	-	277	687	14261	14671
UN16132	Rsa1.0_02404.1	1150	31702	0	1133	1137	99	+	12	1148	5089	6225
UN16133	Rsa1.0_00707.1	1038	190112	1.00E-145	300	312	96	-	728	1037	155116	155427
UN16134	Rsa1.0_01170.1	893	161131	1.00E-127	251	257	97	+	30	286	130492	130745
UN16135	Rsa1.0_01456.1	785	45372	0	726	750	96	+	3	752	25876	26624
UN16136	Rsa1.0_01946.1	2324	36982	0	716	724	98	-	1601	2323	35168	35891
UN16137	Rsa1.0_02730.1	714	28385	0	584	587	99	+	112	698	13862	14448
UN16138	Rsa1.0_00204.1	1352	109056	1.00E-115	217	219	99	+	960	1178	52555	52773
UN16139	Rsa1.0_00094.1	1681	139042	0	491	515	95	+	1	513	42328	42835
UN16140	Rsa1.0_03738.1	821	21071	1.00E-147	268	269	99	-	314	582	12425	12693
UN16141	Rsa1.0_02205.1	812	33923	0	770	816	94	-	4	811	5195	6007
UN16142	Rsa1.0_04852.1	1823	15712	0	1127	1169	96	+	387	1555	8979	10147
UN16143	Rsa1.0_02254.1	1584	33344	0	469	479	97	+	1106	1583	2379	2854
UN16144	Rsa1.0_00250.1	801	273771	0	536	548	97	+	3	542	39182	39729

UN16145	Rsa1.0_00066.1	1332	152418	0	560	589	95	+	695	1282	112082	112670
UN16146	Rsa1.0_01453.1	872	45449	0	821	849	96	−	5	847	5585	6430
UN16147	Rsa1.0_03482.1	1107	22625	0	364	364	100	+	3	366	4787	5150
UN16148	Rsa1.0_00205.1	1631	193401	1.00E−122	224	224	100	+	983	1206	128919	129142
UN16149	Rsa1.0_05632.1	804	13120	0	482	489	98	+	310	798	9527	10015
UN16150	Rsa1.0_00016.1	892	831256	0	400	407	98	−	456	860	403615	404021
UN16151	Rsa1.0_00804.1	882	63726	0	493	495	99	−	388	882	15873	16367
UN16152	Rsa1.0_03543.1	1113	22185	0	395	398	99	−	714	1111	10470	10867
UN16153	Rsa1.0_01740.1	945	39986	1.00E−133	277	288	96	−	101	385	27598	27885
UN16154	Rsa1.0_03366.1	1339	23401	0	497	506	98	−	1	505	10204	10709
UN16155	Rsa1.0_00714.1	1444	67187	1.00E−161	291	292	99	+	1151	1442	393	684
UN16156	Rsa1.0_00201.1	789	232851	0	412	418	98	+	372	789	30998	31414
UN16157	Rsa1.0_00276.1	1622	100160	0	444	447	99	+	2	447	77970	78416
UN16158	Rsa1.0_01537.1	1121	187285	0	1086	1120	96	+	2	1121	33171	34289
UN16159	Rsa1.0_00135.1	783	178077	0	357	368	97	−	150	517	169208	169575
UN16160	Rsa1.0_00471.1	720	81897	0	430	434	99	−	277	710	24702	25135
UN16161	Rsa1.0_00962.1	1897	184909	0	362	365	99	−	1	364	37364	37726
UN16162	Rsa1.0_00793.1	1696	64103	1.00E−155	278	278	100	−	1415	1692	12995	13272
UN16163	Rsa1.0_01011.1	1992	56883	0	622	629	98	+	546	1174	25569	26195
UN16164	Rsa1.0_00381.1	744	89718	0	375	376	99	+	6	381	65776	66151
UN16165	Rsa1.0_07824.1	996	8467	0	356	358	99	−	638	995	2585	2940
UN16166	Rsa1.0_05660.1	1343	13012	0	599	603	99	−	3	605	4750	5352
UN16167	Rsa1.0_03305.1	1188	147552	0	455	463	98	+	707	1168	141859	142321
UN16168	Rsa1.0_00369.1	988	266462	0	417	427	97	−	563	988	4549	4974
UN16169	Rsa1.0_00265.1	1990	220635	0	621	658	94	−	698	1355	175101	175758
UN16170	Rsa1.0_03759.1	1338	20987	0	1311	1335	98	+	1	1331	15445	16779
UN16171	Rsa1.0_04156.1	1388	18799	0	455	476	95	+	8	479	273	746
UN16172	Rsa1.0_00176.1	1166	240984	0	551	561	98	−	567	1127	210502	211062
UN16173	Rsa1.0_02727.1	2123	28404	0	1672	1715	97	+	1	1708	3361	5070
UN16174	Rsa1.0_00589.1	1028	209965	0	1004	1026	97	−	3	1028	154641	155658
UN16175	Rsa1.0_03326.1	751	23710	0	518	520	99	+	232	751	17095	17614
UN16176	Rsa1.0_02254.1	1231	33344	0	422	422	100	+	27	448	32196	32617
UN16177	Rsa1.0_00107.1	2055	431659	0	675	682	98	−	731	1412	292597	293278
UN16178	Rsa1.0_07250.1	1008	9663	0	967	1006	96	+	3	1008	5296	6293
UN16179	Rsa1.0_01435.1	688	45942	1.00E−166	374	395	94	+	289	678	28028	28421
UN16180	Rsa1.0_00822.1	1374	62891	2.00E−98	198	203	97	+	10	212	29926	30128
UN16181	Rsa1.0_00807.1	1428	63607	0	817	834	97	+	589	1422	61983	62811
UN16182	Rsa1.0_00690.1	821	140527	1.00E−126	265	273	97	+	549	820	31841	32111
UN16183	Rsa1.0_17740.1	604	1752	1.00E−160	308	314	98	−	290	602	1182	1495

UN16184	Rsa1.0_01512.1	1026	44234	0	379	380	99	−	647	1026	31276	31655
UN16185	Rsa1.0_00261.1	824	151157	0	809	825	98	−	1	822	116726	117547
UN16186	Rsa1.0_03477.1	964	22635	0	414	433	95	−	534	964	8439	8869
UN16187	Rsa1.0_01407.1	2405	46425	0	1351	1361	99	+	1	1361	14933	16293
UN16188	Rsa1.0_01786.1	1052	133324	1.00E−145	375	405	92	+	2	403	100602	100988
UN16189	Rsa1.0_02949.1	1423	26506	0	609	632	96	−	575	1206	13033	13664
UN16190	Rsa1.0_04390.1	1025	17598	0	398	405	98	−	619	1023	15931	16335
UN16191	Rsa1.0_03548.1	1019	22164	2.00E−51	157	172	91	−	820	990	1995	2165
UN16192	Rsa1.0_00007.1	1004	239325	1.00E−111	207	208	99	+	442	649	108295	108502
UN16193	Rsa1.0_00748.1	1416	65983	0	479	483	99	+	934	1416	59992	60474
UN16194	Rsa1.0_00923.1	1382	153678	1.00E−129	247	251	98	+	211	461	31482	31732
UN16195	Rsa1.0_01421.1	850	46197	0	677	680	99	−	166	845	18146	18825
UN16196	Rsa1.0_02267.1	2040	33194	0	556	586	94	+	1	573	22181	22764
UN16197	Rsa1.0_00059.1	806	159650	1.00E−119	232	234	99	−	1	233	70204	70436
UN16198	Rsa1.0_00429.1	1815	85559	0	629	635	99	+	535	1169	60450	61084
UN16199	Rsa1.0_03491.1	684	22541	0	588	612	96	+	52	653	8979	9590
UN16200	Rsa1.0_00476.1	1725	280966	1.00E−152	279	281	99	−	4	284	239317	239597
UN16201	Rsa1.0_00175.1	806	166121	0	643	669	96	−	1	667	38440	39108
UN16202	Rsa1.0_00361.1	807	91181	1.00E−152	276	277	99	+	161	437	12174	12450
UN16203	Rsa1.0_00190.1	1219	318240	0	608	623	97	−	603	1219	140909	141530
UN16204	Rsa1.0_01434.1	1194	45951	0	1166	1181	98	+	3	1180	7329	8506
UN16205	Rsa1.0_00094.1	1406	139042	1.00E−108	210	212	99	+	1191	1401	60270	60481
UN16206	Rsa1.0_01344.1	1553	142535	0	441	441	100	+	29	469	109904	110344
UN16207	Rsa1.0_00387.1	1152	495407	1.00E−179	325	326	99	+	827	1152	134727	135051
UN16208	Rsa1.0_09117.1	1258	6637	0	405	420	96	−	846	1258	819	1236
UN16209	Rsa1.0_01581.1	1855	42833	0	524	549	95	−	1276	1817	8519	9067
UN16210	Rsa1.0_00002.1	744	601661	0	709	722	98	−	3	721	273785	274505
UN16211	Rsa1.0_03562.1	755	22084	0	638	642	99	+	31	672	5820	6461
UN16212	Rsa1.0_00770.1	935	64900	1.00E−156	313	324	96	−	610	933	9397	9720
UN16213	Rsa1.0_04128.1	1141	18927	0	517	539	95	−	4	541	12414	12952
UN16214	Rsa1.0_00069.1	845	403561	1.00E−166	332	343	96	−	4	343	151346	151688
UN16215	Rsa1.0_00171.1	743	115622	0	367	371	98	−	3	373	50726	51096
UN16216	Rsa1.0_00060.1	850	502678	0	821	838	97	−	1	838	64710	65547
UN16217	Rsa1.0_03726.1	847	21160	0	421	429	98	+	1	429	14268	14696
UN16218	Rsa1.0_00301.1	1751	365315	1.00E−165	328	336	97	+	1417	1751	189263	189597
UN16219	Rsa1.0_06213.1	872	11478	0	474	509	93	+	1	502	1038	1532
UN16220	Rsa1.0_00152.1	1421	257730	0	448	461	97	+	966	1421	66795	67255
UN16221	Rsa1.0_01866.1	990	37961	1.00E−103	206	211	97	+	208	418	12996	13206
UN16222	Rsa1.0_00789.1	1036	64233	0	404	425	95	+	615	1034	24999	25420

UN16223	Rsa1.0_00526.1	1332	169279	0	586	599	97	−	2	600	110767	111365
UN16224	Rsa1.0_35238.1	624	642	1.00E−101	208	214	97	−	353	564	129	342
UN16225	Rsa1.0_03028.1	1104	25872	1.00E−113	267	282	94	+	88	363	20177	20458
UN16226	Rsa1.0_00571.1	891	329018	1.00E−148	293	302	97	+	1	302	188139	188440
UN16227	Rsa1.0_00127.1	1354	127446	0	784	810	96	−	70	879	102847	103656
UN16228	Rsa1.0_23719.1	1623	1029	0	378	388	97	−	1	388	175	559
UN16229	Rsa1.0_00524.1	1309	169613	0	715	725	98	−	582	1306	151027	151750
UN16230	Rsa1.0_00866.1	731	61676	6.00E−76	152	153	99	−	578	729	55378	55530
UN16231	Rsa1.0_02178.1	896	156197	0	499	503	99	−	61	563	1510	2012
UN16232	Rsa1.0_00281.1	1796	99908	0	781	781	100	−	477	1257	83409	84189
UN16233	Rsa1.0_00577.1	1281	174827	0	344	344	100	+	937	1280	118729	119072
UN16234	Rsa1.0_01553.1	1233	43379	1.00E−150	295	302	97	+	926	1226	5534	5835
UN16235	Rsa1.0_02029.1	1106	35857	0	576	590	97	+	517	1105	13320	13907
UN16236	Rsa1.0_00251.1	1272	303968	1.00E−145	270	273	98	−	1000	1272	287414	287686
UN16237	Rsa1.0_01896.1	1289	37512	0	485	498	97	+	748	1245	9812	10308
UN16238	Rsa1.0_02874.1	931	27070	0	528	532	99	−	397	927	26412	26943
UN16239	Rsa1.0_00751.1	1364	65759	0	492	493	99	−	286	778	46552	47044
UN16240	Rsa1.0_00679.1	934	68971	9.00E−85	166	168	98	+	169	336	65933	66100
UN16241	Rsa1.0_00928.1	2266	59293	0	722	743	97	+	1523	2265	23621	24363
UN16242	Rsa1.0_01416.1	1024	46262	0	998	1009	98	−	14	1022	38989	39997
UN16243	Rsa1.0_00627.1	791	71566	1.00E−103	283	311	90	−	463	764	4453	4756
UN16244	Rsa1.0_20651.1	2014	1283	0	389	392	99	+	1	392	732	1120
UN16245	Rsa1.0_63163.1	891	363	0	362	363	99	+	487	849	1	363
UN16246	Rsa1.0_00001.1	798	363028	1.00E−142	296	306	96	−	3	308	101796	102096
UN16247	Rsa1.0_00087.1	744	761073	1.00E−109	211	213	99	+	533	744	314989	315201
UN16248	Rsa1.0_03925.1	784	20025	0	340	340	100	−	10	349	3816	4155
UN16249	Rsa1.0_00221.1	1009	106972	0	484	509	95	−	8	515	23885	24385
UN16250	Rsa1.0_02706.1	523	28522	0	520	524	99	−	1	523	26835	27358
UN16251	Rsa1.0_00714.1	801	67187	0	404	407	99	+	395	801	46605	47010
UN16252	Rsa1.0_00642.1	1062	201230	1.00E−155	358	379	94	+	688	1061	161688	162063
UN16253	Rsa1.0_00947.1	1159	58664	0	409	418	97	+	586	1003	58154	58571
UN16254	Rsa1.0_10789.1	954	5720	9.00E−14	96	113	84	−	175	287	1056	1167
UN16255	Rsa1.0_03735.1	1254	21087	0	671	673	99	−	581	1253	7918	8590
UN16256	Rsa1.0_02178.1	638	156197	1.00E−137	297	310	95	+	2	310	151574	151879
UN16257	Rsa1.0_02085.1	1340	35198	1.00E−130	275	284	96	+	1057	1339	17654	17935
UN16258	Rsa1.0_04584.1	879	16756	1.00E−118	235	240	97	+	641	879	11165	11404
UN16259	Rsa1.0_00652.1	887	70089	1.00E−165	322	330	97	−	558	887	59380	59707
UN16260	Rsa1.0_00934.1	1096	193516	0	568	582	97	+	509	1090	176062	176637
UN16261	Rsa1.0_32205.1	1049	707	0	435	441	98	+	2	442	269	707

UN16262	Rsa1.0_00119.1	1366	467509	1.00E-111	275	296	92	+	1072	1364	390249	390544
UN16263	Rsa1.0_00244.1	852	103590	2.00E-45	150	168	89	−	102	269	73674	73838
UN16264	Rsa1.0_00013.1	947	292129	3.00E-81	169	174	97	−	432	605	72142	72315
UN16265	Rsa1.0_00485.1	1194	80937	0	392	404	97	−	2	405	16634	17037
UN16266	Rsa1.0_04223.1	1268	18407	0	1224	1268	96	−	1	1267	2341	3607
UN16267	Rsa1.0_01420.1	1403	46210	1.00E-122	229	231	99	+	776	1006	8010	8240
UN16268	Rsa1.0_00287.1	950	98687	0	420	429	97	−	121	549	57224	57652
UN16269	Rsa1.0_07607.1	1110	9034	0	674	680	99	−	1	680	4506	5185
UN16270	Rsa1.0_01031.1	1286	56236	1.00E-72	158	164	96	+	888	1051	6523	6686
UN16271	Rsa1.0_02231.1	902	33661	0	515	525	98	−	378	902	18154	18678
UN16272	Rsa1.0_00017.1	1013	752458	1.00E-130	316	337	93	+	679	1008	11197	11531
UN16273	Rsa1.0_00167.1	1458	116000	0	550	560	98	+	563	1122	84148	84707
UN16274	Rsa1.0_16915.1	1401	1991	1.00E-102	203	206	98	+	79	284	5	209
UN16275	Rsa1.0_00175.1	1264	166121	0	445	449	99	+	1	449	139806	140253
UN16276	Rsa1.0_00913.1	1384	59914	0	440	470	93	−	933	1383	54248	54717
UN16277	Rsa1.0_03485.1	1147	22606	1.00E-120	223	224	99	−	332	555	586	809
UN16278	Rsa1.0_03163.1	1428	24894	0	582	594	97	−	1	593	22793	23386
UN16279	Rsa1.0_01156.1	1521	52147	0	685	701	97	+	19	719	28060	28754
UN16280	Rsa1.0_01352.1	1308	47443	1.00E-102	219	229	95	+	357	585	12214	12442
UN16281	Rsa1.0_00180.1	848	114581	1.00E-172	334	342	97	+	507	848	63449	63789
UN16282	Rsa1.0_01954.1	790	145554	1.00E-163	297	299	99	−	491	789	12997	13295
UN16283	Rsa1.0_05523.1	896	13438	0	707	751	94	+	3	729	9041	9791
UN16284	Rsa1.0_02730.1	1228	28385	0	371	374	99	+	854	1226	3777	4150
UN16285	Rsa1.0_02316.1	911	32608	0	890	910	97	−	3	911	5912	6817
UN16286	Rsa1.0_02122.1	1378	34797	0	405	409	99	−	973	1378	26094	26501
UN16287	Rsa1.0_04883.1	2002	15603	0	792	797	99	−	1204	1999	10313	11109
UN16288	Rsa1.0_04395.1	644	17570	1.00E-173	320	324	98	−	321	644	9961	10284
UN16289	Rsa1.0_04622.1	1222	16618	0	492	496	99	+	1	496	14007	14502
UN16290	Rsa1.0_00163.1	1219	116847	0	756	771	98	+	1	771	7919	8685
UN16291	Rsa1.0_00577.1	1948	174827	1.00E-176	380	397	95	+	1165	1554	148897	149290
UN16292	Rsa1.0_03109.1	1723	25336	0	688	703	97	+	1022	1723	3130	3826
UN16293	Rsa1.0_01719.1	1802	40388	0	868	886	97	−	915	1800	31856	32737
UN16294	Rsa1.0_03209.1	707	24592	0	417	439	94	+	270	707	18885	19318
UN16295	Rsa1.0_00223.1	1305	106231	0	354	358	98	−	1	358	41017	41374
UN16296	Rsa1.0_03569.1	663	22041	1.00E-135	251	253	99	−	411	663	19456	19708
UN16297	Rsa1.0_48139.1	1227	470	0	387	395	97	−	424	818	76	470
UN16298	Rsa1.0_00369.1	839	266462	0	647	667	97	−	176	839	186458	187123
UN16299	Rsa1.0_01115.1	1107	183545	0	450	462	97	+	647	1107	6514	6974
UN16300	Rsa1.0_00354.1	1364	232005	8.00E-89	241	265	90	+	975	1232	168195	168453

UN16301	Rsa1.0_04794.1	827	15960	0	450	459	98	−	13	464	2694	3151
UN16302	Rsa1.0_00132.1	1396	124299	0	342	345	99	+	194	538	91186	91530
UN16303	Rsa1.0_00132.1	853	124299	0	821	851	96	+	3	853	25441	26275
UN16304	Rsa1.0_03184.1	1161	24750	1.00E−139	251	251	100	−	1	251	21274	21524
UN16305	Rsa1.0_16978.1	1567	1964	1.00E−116	263	276	95	−	476	749	1215	1488
UN16306	Rsa1.0_00296.1	883	97854	1.00E−142	269	273	98	+	173	445	21946	22218
UN16307	Rsa1.0_00876.1	1207	61328	0	802	812	98	−	4	815	46191	47002
UN16308	Rsa1.0_00798.1	1352	63945	0	1227	1244	98	−	112	1352	19654	20896
UN16309	Rsa1.0_00017.1	1434	752458	0	678	682	99	−	753	1434	296906	297586
UN16310	Rsa1.0_01015.1	1281	56752	0	466	468	99	−	1	468	11172	11639
UN16311	Rsa1.0_00462.1	598	82288	1.00E−152	286	289	98	−	308	596	35985	36272
UN16312	Rsa1.0_00270.1	822	101236	2.00E−82	162	164	98	+	581	744	1852	2015
UN16313	Rsa1.0_01537.1	1843	187285	2.33E−156	307	319	96	+	18	336	9519	9829
UN16314	Rsa1.0_03278.1	986	24125	1.00E−122	270	283	95	−	706	986	1987	2269
UN16315	Rsa1.0_00416.1	907	86772	0	382	386	98	+	522	907	50850	51235
UN16316	Rsa1.0_00046.1	779	320315	0	748	776	96	−	3	777	22151	22926
UN16317	Rsa1.0_00296.1	1575	97854	0	1252	1262	99	+	1	1261	44613	45874
UN16318	Rsa1.0_26314.1	827	897	0	419	429	97	−	389	817	109	537
UN16319	Rsa1.0_00698.1	1189	67816	4.00E−84	295	336	87	+	837	1168	47244	47571
UN16320	Rsa1.0_04515.1	1282	17021	0	1275	1281	99	+	2	1282	1606	2886
UN16321	Rsa1.0_08484.1	720	7288	0	556	563	98	+	2	564	6567	7129
UN16322	Rsa1.0_01286.1	634	49209	0	559	580	96	−	48	626	3951	4524
UN16323	Rsa1.0_00058.1	699	159921	0	520	537	96	−	166	698	49021	49550
UN16324	Rsa1.0_01313.1	951	48387	1.00E−154	319	333	95	−	44	376	16151	16483
UN16325	Rsa1.0_14746.1	1448	3110	0	442	469	94	−	326	786	1379	1841
UN16326	Rsa1.0_02096.1	1002	35041	0	608	620	98	+	382	1000	14809	15426
UN16327	Rsa1.0_04905.1	962	15507	1.00E−148	364	400	91	+	480	859	5642	6040
UN16328	Rsa1.0_00050.1	968	237174	1.00E−127	279	292	95	−	684	968	133695	133986
UN16329	Rsa1.0_00188.1	728	112020	1.00E−142	290	299	96	+	394	691	47540	47836
UN16330	Rsa1.0_04600.1	964	16694	1.00E−137	285	296	96	+	621	915	12273	12568
UN16331	Rsa1.0_00524.1	811	169613	1.00E−140	336	364	92	+	56	419	161744	162096
UN16332	Rsa1.0_02967.1	777	26395	0	765	774	98	−	4	777	6619	7388
UN16333	Rsa1.0_14009.1	955	3924	0	450	450	100	−	506	955	2437	2886
UN16334	Rsa1.0_76149.1	1076	301	1.00E−166	300	301	99	−	710	1010	1	301
UN16335	Rsa1.0_00686.1	1375	68511	0	485	485	100	−	891	1375	27918	28402
UN16336	Rsa1.0_00007.1	1195	239325	0	368	371	99	−	3	373	108872	109242
UN16337	Rsa1.0_01104.1	1465	285480	0	446	447	99	+	1019	1465	10223	10669
UN16338	Rsa1.0_00273.1	1453	326186	0	606	629	96	+	776	1403	137570	138198
UN16339	Rsa1.0_01564.1	1295	43169	1.00E−168	315	320	98	−	976	1295	36204	36523

UN16340	Rsa1.0_00017.1	687	752458	1.00E-168	316	320	98	-	368	687	398420	398738
UN16341	Rsa1.0_00198.1	1096	222963	0	567	570	99	+	1	570	87389	87958
UN16342	Rsa1.0_00483.1	1283	81102	1.00E-171	379	399	94	-	891	1283	56194	56592
UN16343	Rsa1.0_00833.1	1139	62621	0	352	352	100	+	788	1139	46926	47277
UN16344	Rsa1.0_00078.1	1537	175053	1.00E-155	307	318	96	-	1220	1537	140374	140681
UN16345	Rsa1.0_00090.1	875	273859	0	694	718	96	-	158	874	171766	172481
UN16346	Rsa1.0_01408.1	1102	46383	0	732	749	97	-	3	750	17109	17853
UN16347	Rsa1.0_00338.1	1149	94109	1.00E-166	311	315	98	-	2	313	36386	36700
UN16348	Rsa1.0_00001.1	1766	363028	0	455	461	98	-	1308	1766	303151	303611
UN16349	Rsa1.0_00017.1	661	752458	0	494	511	96	+	153	661	534070	534576
UN16350	Rsa1.0_00605.1	1235	73199	0	396	399	99	+	837	1235	2322	2719
UN16351	Rsa1.0_00855.1	1384	62047	0	799	840	95	-	1	838	22692	23523
UN16352	Rsa1.0_03290.1	1004	24011	1.00E-138	268	274	97	-	1	274	4892	5165
UN16353	Rsa1.0_03668.1	1456	21483	0	507	519	97	-	941	1454	3785	4303
UN16354	Rsa1.0_01306.1	1226	48720	1.00E-154	298	303	98	+	1	303	26045	26343
UN16355	Rsa1.0_00571.1	743	329018	0	432	444	97	+	303	742	159412	159851
UN16356	Rsa1.0_01710.1	479	40586	0	379	390	97	+	1	388	19357	19746
UN16357	Rsa1.0_00042.1	1299	171507	1.00E-180	391	415	94	-	2	403	71838	72249
UN16358	Rsa1.0_01876.1	1173	37800	2.00E-64	153	162	94	-	521	682	10773	10934
UN16359	Rsa1.0_00608.1	1656	172672	0	1309	1323	98	-	1	1323	135636	136956
UN16360	Rsa1.0_00481.1	1185	81316	0	341	342	99	-	842	1183	33786	34127
UN16361	Rsa1.0_05044.1	1609	15041	0	760	762	99	+	1	762	8705	9466
UN16362	Rsa1.0_10681.1	865	5757	0	650	656	99	-	3	655	4487	5142
UN16363	Rsa1.0_04462.1	2768	17250	0	872	908	96	+	1617	2524	3091	3996
UN16364	Rsa1.0_01156.1	2230	52147	0	520	546	95	-	963	1502	25221	25757
UN16365	Rsa1.0_00096.1	2174	198773	0	655	657	99	-	466	1122	31167	31823
UN16366	Rsa1.0_00255.1	2031	204967	0	851	861	98	-	1154	2008	22419	23279
UN16367	Rsa1.0_02356.1	595	141500	0	579	589	98	-	3	590	136345	136932
UN16368	Rsa1.0_01578.1	897	42880	0	680	687	98	-	209	895	11499	12185
UN16369	Rsa1.0_04126.1	1112	18939	0	469	493	95	+	620	1112	17402	17890
UN16370	Rsa1.0_01865.1	1039	37992	1.00E-117	224	226	99	+	456	680	32692	32917
UN16371	Rsa1.0_00007.1	1340	239325	0	627	631	99	+	710	1340	58424	59054
UN16372	Rsa1.0_01540.1	880	151890	0	482	489	98	-	179	667	144792	145280
UN16373	Rsa1.0_01536.1	980	43747	0	612	633	96	-	346	978	18778	19406
UN16374	Rsa1.0_04890.1	924	15591	1.00E-123	237	241	98	+	473	713	10910	11150
UN16375	Rsa1.0_20365.1	1448	1313	0	468	474	98	+	69	541	1	474
UN16376	Rsa1.0_10201.1	880	5946	0	781	798	97	+	43	840	389	1184
UN16377	Rsa1.0_00484.1	1117	203294	0	794	832	95	+	284	1115	56116	56941
UN16378	Rsa1.0_00819.1	2104	62918	0	651	657	99	-	3	659	22482	23138

UN16379	Rsa1.0_08250.1	1183	7658	0	574	602	95	+	3	604	2538	3121
UN16380	Rsa1.0_00667.1	1059	69460	0	483	496	97	+	83	578	24055	24549
UN16381	Rsa1.0_02135.1	618	34713	1.00E-147	267	268	99	−	1	268	17461	17728
UN16382	Rsa1.0_02989.1	819	26187	0	441	449	98	−	124	568	3228	3675
UN16383	Rsa1.0_02173.1	1129	34318	6.00E-74	167	174	95	+	50	222	15564	15737
UN16384	Rsa1.0_04512.1	640	17031	1.00E-135	275	285	96	−	263	547	12659	12943
UN16385	Rsa1.0_00140.1	1200	121541	0	453	460	98	+	712	1171	11145	11603
UN16386	Rsa1.0_08928.1	615	6781	0	590	615	95	+	2	615	493	1099
UN16387	Rsa1.0_01846.1	1344	38367	0	415	421	98	+	5	423	34627	35047
UN16388	Rsa1.0_02700.1	727	28584	0	416	419	99	+	309	727	6214	6632
UN16389	Rsa1.0_09126.1	827	6625	0	440	447	98	−	380	826	5262	5707
UN16390	Rsa1.0_00001.1	1232	363028	0	666	700	95	−	547	1232	26222	26921
UN16391	Rsa1.0_08285.1	1059	7606	0	401	416	96	−	7	420	6676	7088
UN16392	Rsa1.0_00168.1	1427	115870	0	835	835	100	−	593	1427	38894	39728
UN16393	Rsa1.0_02506.1	1215	30700	1.00E-172	350	364	96	−	402	765	6452	6812
UN16394	Rsa1.0_00769.1	874	64911	1.00E-119	227	230	98	+	12	241	40086	40315
UN16395	Rsa1.0_04377.1	782	17658	0	760	781	97	−	3	782	5366	6145
UN16396	Rsa1.0_01513.1	411	44234	0	393	411	95	−	1	411	29195	29605
UN16397	Rsa1.0_02700.1	1178	28584	0	449	470	95	+	30	498	2886	3354
UN16398	Rsa1.0_00821.1	1400	62891	0	458	489	93	−	913	1398	48838	49319
UN16399	Rsa1.0_02017.1	1525	35999	1.00E-124	242	246	98	+	1276	1519	29901	30146
UN16400	Rsa1.0_04238.1	1093	18352	4.00E-93	247	262	94	−	24	273	2712	2973
UN16401	Rsa1.0_02593.1	729	29744	1.00E-116	266	280	95	+	1	278	2886	3164
UN16402	Rsa1.0_00343.1	842	93570	0	513	538	95	+	305	842	2493	3024
UN16403	Rsa1.0_00269.1	910	341668	1.00E-112	324	359	90	+	571	910	45768	46123
UN16404	Rsa1.0_00769.1	965	64911	0	950	961	98	+	1	958	11598	12558
UN16405	Rsa1.0_00380.1	737	89748	1.00E-159	367	393	93	+	347	736	88066	88441
UN16406	Rsa1.0_00719.1	1872	66952	1.00E-159	294	297	98	−	1207	1503	42432	42728
UN16407	Rsa1.0_01836.1	919	38503	1.00E-158	320	331	96	+	1	330	5086	5416
UN16408	Rsa1.0_03294.1	944	23971	0	446	471	94	−	478	944	5093	5558
UN16409	Rsa1.0_02811.1	697	27669	0	651	686	94	+	1	684	21138	21813
UN16410	Rsa1.0_06056.1	796	137271	8.00E-91	176	178	98	+	67	244	126368	126545
UN16411	Rsa1.0_02373.1	1381	32048	0	481	495	97	+	884	1376	30160	30654
UN16412	Rsa1.0_02395.1	772	31785	1.00E-162	358	381	93	+	407	771	8334	8714
UN16413	Rsa1.0_76149.1	1006	301	1.00E-169	301	301	100	+	67	367	1	301
UN16414	Rsa1.0_00375.1	997	90207	0	451	458	98	+	540	997	2405	2861
UN16415	Rsa1.0_00568.1	1464	75219	0	806	836	96	−	630	1464	1834	2658
UN16416	Rsa1.0_18413.1	1065	1603	1.00E-172	352	367	95	+	242	608	1151	1517
UN16417	Rsa1.0_01895.1	814	37512	1.00E-122	223	223	100	+	3	225	9117	9339

UN16418	Rsa1.0_00833.1	1216	62621	1.00E-138	260	262	99	+	1	261	5160	5421
UN16419	Rsa1.0_00301.1	942	365315	0	482	482	100	+	460	941	215181	215662
UN16420	Rsa1.0_07914.1	1203	8294	0	747	755	98	+	449	1203	1896	2650
UN16421	Rsa1.0_05494.1	912	13547	0	378	389	97	+	9	395	7715	8102
UN16422	Rsa1.0_00035.1	1053	176449	1.00E-133	276	285	96	−	53	336	156806	157089
UN16423	Rsa1.0_00657.1	1527	69858	0	433	436	99	−	1092	1527	3121	3556
UN16424	Rsa1.0_00630.1	755	71281	0	423	439	96	+	2	433	63707	64143
UN16425	Rsa1.0_00928.1	1059	59293	2.00E-92	214	228	93	−	1	228	32261	32482
UN16426	Rsa1.0_00006.1	726	396176	1.00E-101	261	282	92	+	1	275	9173	9447
UN16427	Rsa1.0_00170.1	857	179538	0	397	408	97	−	451	857	24343	24750
UN16428	Rsa1.0_04264.1	974	18207	0	430	449	95	−	168	615	5591	6039
UN16429	Rsa1.0_05494.1	888	13547	0	425	450	94	−	439	881	7654	8102
UN16430	Rsa1.0_05499.1	1154	13520	1.00E-104	234	246	95	+	890	1132	12343	12587
UN16431	Rsa1.0_00931.1	1010	59210	1.00E-130	266	276	96	−	141	416	7084	7359
UN16432	Rsa1.0_00041.1	1333	186197	0	411	427	96	+	45	470	56687	57106
UN16433	Rsa1.0_03887.1	1199	20247	0	391	404	96	+	795	1195	4347	4750
UN16434	Rsa1.0_00549.1	1113	76473	0	340	340	100	−	772	1111	12988	13327
UN16435	Rsa1.0_00265.1	749	220635	1.00E-141	288	298	96	+	422	718	197596	197893
UN16436	Rsa1.0_08170.1	564	7799	0	541	566	95	+	1	564	1626	2189
UN16437	Rsa1.0_02041.1	1024	163634	1.00E-152	273	273	100	+	1	273	18760	19032
UN16438	Rsa1.0_08529.1	1018	7233	7.00E-98	198	202	98	−	5	206	6697	6897
UN16439	Rsa1.0_04085.1	959	19168	1.00E-160	364	388	93	−	569	956	1528	1901
UN16440	Rsa1.0_01484.1	898	44806	0	534	544	98	−	33	576	40748	41289
UN16441	Rsa1.0_08558.1	760	7198	0	403	422	95	−	329	736	473	894
UN16442	Rsa1.0_00168.1	1459	115870	0	611	616	99	+	549	1164	88359	88974
UN16443	Rsa1.0_00280.1	813	99966	1.00E-178	333	336	99	−	481	813	12148	12483
UN16444	Rsa1.0_04198.1	1099	18504	0	407	418	97	+	682	1099	15733	16149
UN16445	Rsa1.0_00817.1	598	62961	0	570	594	95	+	5	598	23201	23792
UN16446	Rsa1.0_05095.1	906	14869	1.00E-102	253	277	91	+	644	906	773	1049
UN16447	Rsa1.0_00370.1	662	90472	0	464	484	95	−	35	517	21311	21794
UN16448	Rsa1.0_06488.1	1117	141774	0	619	628	98	−	365	992	4048	4675
UN16449	Rsa1.0_01049.1	1008	55655	1.00E-128	257	264	97	−	744	1007	41045	41306
UN16450	Rsa1.0_00139.1	364	357786	1.00E-171	353	367	96	+	2	364	149438	149804
UN16451	Rsa1.0_00251.1	770	303968	1.00E-156	331	347	95	−	5	349	147121	147463
UN16452	Rsa1.0_00045.1	1435	328694	0	362	363	99	+	8	370	160661	161022
UN16453	Rsa1.0_00100.1	1176	249232	1.00E-157	282	282	100	−	202	483	117508	117789
UN16454	Rsa1.0_04128.1	1195	18927	0	523	563	92	−	13	570	12414	12973
UN16455	Rsa1.0_03185.1	848	24739	3.00E-90	178	181	98	−	216	396	5034	5214
UN16456	Rsa1.0_00887.1	1262	61048	8.00E-95	192	197	97	−	801	997	49405	49601

UN16457	Rsa1.0_00008.1	760	358124	0	371	372	99	+	387	758	73510	73881
UN16458	Rsa1.0_01704.1	1745	40711	0	357	363	98	+	904	1266	5532	5894
UN16459	Rsa1.0_08529.1	1002	7233	2.00E-95	197	203	97	-	798	1000	4941	5139
UN16460	Rsa1.0_00094.1	1380	139042	1.00E-112	221	226	97	-	869	1094	134442	134667
UN16461	Rsa1.0_02217.1	1379	33812	0	1256	1261	99	+	1	1260	30263	31523
UN16462	Rsa1.0_02268.1	1167	33172	0	435	436	99	-	1	436	8776	9211
UN16463	Rsa1.0_01801.1	1427	39094	0	545	566	96	-	579	1138	20432	20997
UN16464	Rsa1.0_01887.1	1001	37564	0	439	457	96	+	510	966	26538	26992
UN16465	Rsa1.0_00566.1	445	75325	1.00E-175	320	323	99	-	2	324	61768	62090
UN16466	Rsa1.0_00234.1	1221	197060	1.00E-175	343	352	97	+	2	350	151100	151449
UN16467	Rsa1.0_08388.1	975	7436	0	548	575	95	+	1	566	2994	3563
UN16468	Rsa1.0_09608.1	992	6256	1.00E-142	304	317	95	+	677	992	172	487
UN16469	Rsa1.0_02486.1	1642	30965	0	757	806	93	-	1	795	11659	12462
UN16470	Rsa1.0_00557.1	2202	75865	1.00E-173	309	309	100	-	3	311	19484	19792
UN16471	Rsa1.0_01762.1	1196	39682	0	403	417	96	-	685	1101	6859	7267
UN16472	Rsa1.0_00280.1	1368	99966	1.00E-144	352	385	91	+	1	366	12102	12483
UN16473	Rsa1.0_01187.1	1181	51377	1.00E-169	307	309	99	+	2	310	30063	30371
UN16474	Rsa1.0_00925.1	1696	59481	0	492	514	95	+	3	516	5571	6084
UN16475	Rsa1.0_01958.1	950	261128	1.00E-153	275	275	100	+	320	594	132443	132717
UN16476	Rsa1.0_05049.1	740	15025	0	490	493	99	-	1	493	4126	4618
UN16477	Rsa1.0_00329.1	1479	222544	1.00E-130	236	236	100	-	3	238	2056	2291
UN16478	Rsa1.0_02347.1	1852	32332	0	1349	1366	98	-	1	1363	29131	30496
UN16479	Rsa1.0_00031.1	920	179862	0	495	505	98	-	72	576	82447	82951
UN16480	Rsa1.0_00343.1	763	93570	0	473	493	95	-	17	507	2493	2979
UN16481	Rsa1.0_00121.1	1104	278029	0	565	596	94	+	3	597	55537	56114
UN16482	Rsa1.0_00642.1	1119	201230	0	385	388	99	+	2	386	160121	160508
UN16483	Rsa1.0_00313.1	725	96745	3.00E-93	287	318	90	+	409	718	79688	80003
UN16484	Rsa1.0_00437.1	1179	84879	0	484	494	97	-	11	502	50510	51002
UN16485	Rsa1.0_01115.1	892	183545	0	504	519	97	+	375	892	174692	175209
UN16486	Rsa1.0_00277.1	1444	100132	0	861	881	97	+	3	881	61968	62846
UN16487	Rsa1.0_02223.1	1066	152421	0	432	452	95	+	618	1066	17952	18403
UN16488	Rsa1.0_02756.1	818	28202	1.00E-179	322	323	99	-	3	325	23023	23345
UN16489	Rsa1.0_00758.1	748	65477	0	374	379	98	-	189	567	3991	4368
UN16490	Rsa1.0_00577.1	840	174827	5.00E-86	181	186	97	+	15	200	116900	117084
UN16491	Rsa1.0_01195.1	414	147988	0	404	415	97	-	2	413	8344	8758
UN16492	Rsa1.0_00112.1	607	132199	0	582	610	95	-	1	607	105199	105804
UN16493	Rsa1.0_00813.1	1378	63202	1.00E-147	290	298	97	-	1043	1340	10869	11162
UN16494	Rsa1.0_00232.1	1134	104936	1.00E-118	241	249	96	-	546	794	47669	47917
UN16495	Rsa1.0_04107.1	1280	19007	0	761	764	99	+	1	764	6776	7539

UN16496	Rsa1.0_00773.1	943	64839	1.00E-179	382	400	95	-	31	429	32802	33197
UN16497	Rsa1.0_00645.1	462	189716	2.00E-99	187	188	99	-	42	229	35226	35413
UN16498	Rsa1.0_01879.1	489	37748	1.00E-170	362	380	95	-	3	382	23051	23425
UN16499	Rsa1.0_02212.1	960	33840	1.00E-90	186	190	97	+	770	958	29047	29236
UN16500	Rsa1.0_00998.1	1898	57168	1.00E-159	294	297	98	+	418	714	9918	10214
UN16501	Rsa1.0_00484.1	971	203294	0	445	451	98	+	3	452	19013	19462
UN16502	Rsa1.0_08947.1	458	6761	0	390	394	98	-	62	455	5535	5928
UN16503	Rsa1.0_14214.1	1194	3656	0	1146	1165	98	-	3	1167	2003	3159
UN16504	Rsa1.0_02304.1	693	32793	6.00E-82	183	191	95	-	504	693	17395	17580
UN16505	Rsa1.0_01410.1	741	46345	1.00E-164	326	336	97	+	110	443	33684	34019
UN16506	Rsa1.0_00483.1	756	81102	0	372	378	98	-	1	378	13909	14286
UN16507	Rsa1.0_00630.1	701	71281	1.00E-108	235	247	95	+	203	449	16575	16821
UN16508	Rsa1.0_00087.1	677	761073	1.00E-86	187	195	95	-	407	601	13132	13326
UN16509	Rsa1.0_01413.1	1292	46275	1.00E-136	256	259	98	-	1034	1292	42171	42429
UN16510	Rsa1.0_01696.1	767	301850	0	551	574	95	-	193	766	159083	159651
UN16511	Rsa1.0_00420.1	715	86496	1.00E-128	250	256	97	-	195	450	43112	43367
UN16512	Rsa1.0_00583.1	767	74309	1.00E-132	255	259	98	-	510	767	68504	68762
UN16513	Rsa1.0_00170.1	767	179538	1.00E-117	236	243	97	-	511	753	142304	142546
UN16514	Rsa1.0_01460.1	747	45255	1.00E-154	321	331	96	+	417	743	3258	3587
UN16515	Rsa1.0_03299.1	1334	23949	0	466	488	95	+	854	1332	14331	14814
UN16516	Rsa1.0_00384.1	1412	89278	1.00E-133	292	304	96	+	1111	1411	35218	35518
UN16517	Rsa1.0_00807.1	975	63607	1.00E-110	203	203	100	+	119	321	46342	46544
UN16518	Rsa1.0_07998.1	921	8105	1.00E-154	303	312	97	+	213	524	4011	4322
UN16519	Rsa1.0_01701.1	525	162121	4.00E-27	90	99	90	+	3	101	9028	9126
UN16520	Rsa1.0_00417.1	1056	395068	0	484	521	92	-	22	542	259263	259778
UN16521	Rsa1.0_01180.1	983	51517	0	357	366	97	-	9	371	17037	17401
UN16522	Rsa1.0_00235.1	839	154696	0	401	418	95	-	12	425	101650	102064
UN16523	Rsa1.0_18141.1	1912	1658	0	458	462	99	-	1451	1912	923	1384
UN16524	Rsa1.0_01280.1	856	49321	0	824	855	96	-	5	856	36779	37629
UN16525	Rsa1.0_47144.1	860	479	1.00E-147	292	301	97	+	313	613	179	479
UN16526	Rsa1.0_00192.1	659	278200	0	404	405	99	+	254	658	22625	23029
UN16527	Rsa1.0_00605.1	1160	73199	0	574	580	98	+	581	1160	28192	28765
UN16528	Rsa1.0_01947.1	634	36977	0	607	623	97	-	6	628	24868	25488
UN16529	Rsa1.0_00533.1	716	77375	3.00E-81	175	182	96	+	139	320	52641	52822
UN16530	Rsa1.0_05239.1	768	14410	0	408	434	94	+	4	422	11690	12121
UN16531	Rsa1.0_02503.1	742	30709	1.00E-98	242	258	93	+	3	259	27268	27522
UN16532	Rsa1.0_04379.1	1055	17646	1.00E-132	324	347	93	+	4	348	16759	17097
UN16533	Rsa1.0_13800.1	1379	4315	1.00E-93	190	195	97	+	174	368	1605	1799
UN16534	Rsa1.0_00601.1	756	177045	0	721	754	95	-	3	756	134705	135454

UN16535	Rsa1.0_00114.1	839	131330	1.00E-144	298	307	97	-	533	837	29607	29912
UN16536	Rsa1.0_00549.1	744	76473	1.00E-137	306	325	94	+	3	327	13012	13327
UN16537	Rsa1.0_00069.1	1326	403561	0	558	570	97	-	757	1326	353470	354037
UN16538	Rsa1.0_04378.1	777	17651	0	348	354	98	+	290	643	4630	4983
UN16539	Rsa1.0_01060.1	1488	55368	1.00E-116	225	229	98	+	388	616	38736	38964
UN16540	Rsa1.0_00483.1	1700	81102	0	558	564	98	+	432	995	13909	14472
UN16541	Rsa1.0_01959.1	1473	36798	0	517	530	97	+	942	1471	23374	23902
UN16542	Rsa1.0_00262.1	634	101906	0	410	424	96	+	214	633	53264	53684
UN16543	Rsa1.0_01185.1	731	149842	1.00E-122	244	249	97	+	3	248	131815	132062
UN16544	Rsa1.0_01376.1	1086	47004	1.00E-104	285	315	90	-	778	1080	37648	37957
UN16545	Rsa1.0_00137.1	1089	122457	1.00E-130	269	277	97	-	813	1089	81352	81626
UN16546	Rsa1.0_13492.1	1044	4775	0	400	405	98	-	638	1042	2425	2829
UN16547	Rsa1.0_00190.1	773	318240	1.00E-111	214	217	98	-	1	217	165006	165222
UN16548	Rsa1.0_00417.1	879	395068	1.00E-141	292	304	96	-	165	468	14475	14775
UN16549	Rsa1.0_00353.1	901	92242	0	545	556	98	-	346	901	59354	59906
UN16550	Rsa1.0_08557.1	460	7199	1.00E-127	252	258	97	+	200	457	6714	6970
UN16551	Rsa1.0_30260.1	1477	759	1.00E-126	251	257	97	-	1221	1477	500	755
UN16552	Rsa1.0_00328.1	1820	224661	0	698	791	88	+	662	1426	218269	219024
UN16553	Rsa1.0_16623.1	496	2086	0	453	458	98	-	40	496	217	671
UN16554	Rsa1.0_01168.1	642	153765	0	636	638	99	-	5	642	43989	44626
UN16555	Rsa1.0_03303.1	791	23890	2.33E-156	318	333	95	+	457	787	4111	4440
UN16556	Rsa1.0_04396.1	1225	17561	0	1133	1154	98	+	3	1153	16408	17561
UN16557	Rsa1.0_02588.1	643	29840	1.00E-104	220	228	96	-	10	236	16005	16232
UN16558	Rsa1.0_00265.1	716	220635	5.00E-92	288	324	88	+	399	716	195833	196155
UN16559	Rsa1.0_00422.1	827	86144	0	432	454	95	+	3	456	12844	13294
UN16560	Rsa1.0_08677.1	642	7048	0	371	380	97	-	27	399	4149	4528
UN16561	Rsa1.0_02438.1	994	31392	0	501	515	97	-	488	994	20171	20681
UN16562	Rsa1.0_00015.1	835	561803	1.00E-132	245	247	99	-	525	771	190632	190878
UN16563	Rsa1.0_00122.1	1423	265937	0	1203	1225	98	+	197	1421	30383	31607
UN16564	Rsa1.0_01143.1	516	52456	1.00E-149	330	347	95	-	1	347	15912	16255
UN16565	Rsa1.0_01376.1	618	47004	1.00E-137	247	247	100	-	372	618	37711	37957
UN16566	Rsa1.0_02865.1	1106	27133	0	411	430	95	-	682	1105	18829	19258
UN16567	Rsa1.0_08811.1	568	6904	1.00E-125	233	235	99	+	326	560	5210	5444
UN16568	Rsa1.0_00171.1	487	115622	3.00E-95	211	221	95	+	250	469	50726	50941
UN16569	Rsa1.0_02269.1	719	33168	0	413	423	97	-	1	423	12865	13287
UN16570	Rsa1.0_00021.1	1461	263655	0	1251	1309	95	-	28	1326	123592	124899
UN16571	Rsa1.0_01536.1	770	43747	1.00E-144	312	327	95	+	421	745	28390	28715
UN16572	Rsa1.0_35376.1	907	640	1.00E-166	318	324	98	-	372	694	164	487
UN16573	Rsa1.0_01335.1	665	47894	0	480	503	95	-	4	496	3912	4412

UN16574	Rsa1.0_00816.1	1111	403350	1.00E-161	316	325	97	-	787	1111	382478	382802
UN16575	Rsa1.0_00014.1	654	348985	2.00E-87	176	180	97	+	472	651	220714	220893
UN16576	Rsa1.0_02430.1	294	31506	5.00E-99	204	211	96	-	64	274	12090	12300
UN16577	Rsa1.0_13916.1	1318	4098	0	544	565	96	-	6	570	531	1093
UN16578	Rsa1.0_08079.1	1191	7942	0	1156	1187	97	-	1	1187	4974	6155
UN16579	Rsa1.0_09269.1	787	6514	1.00E-130	239	240	99	-	450	689	35	274
UN16580	Rsa1.0_01005.1	694	56967	0	612	633	96	-	3	635	37403	38025
UN16581	Rsa1.0_08841.1	729	6870	1.00E-161	347	362	95	+	1	358	2675	3034
UN16582	Rsa1.0_00016.1	772	831256	1.00E-71	144	146	98	-	540	685	204992	205137
UN16583	Rsa1.0_00690.1	215	140527	9.00E-63	187	204	91	+	16	215	31841	32042
UN16584	Rsa1.0_00890.1	651	323870	1.00E-167	325	334	97	+	317	650	162561	162894
UN16585	Rsa1.0_17794.1	1062	1740	0	386	386	100	+	677	1062	1	386
UN16586	Rsa1.0_00120.1	780	277288	2.00E-66	138	141	97	-	282	422	119078	119218
UN16587	Rsa1.0_00482.1	759	81162	1.00E-132	274	285	96	-	5	286	61822	62106
UN16588	Rsa1.0_36744.1	786	614	0	411	416	98	+	179	594	94	509
UN16589	Rsa1.0_00939.1	974	185227	0	923	972	94	-	1	972	152983	153950
UN16590	Rsa1.0_00437.1	894	84879	1.00E-116	258	273	94	-	198	470	23635	23907
UN16591	Rsa1.0_00017.1	886	752458	1.00E-142	282	289	97	-	1	288	154565	154853
UN16592	Rsa1.0_00054.1	750	162053	3.00E-84	162	163	99	-	3	165	7231	7393
UN16593	Rsa1.0_01654.1	891	41532	0	406	407	99	-	485	890	24818	25224
UN16594	Rsa1.0_03698.1	127	21302	1.00E-26	64	65	98	+	63	127	7933	7997
UN16595	Rsa1.0_00682.1	845	68680	0	503	519	96	-	2	519	37871	38387
UN16596	Rsa1.0_00568.1	1116	75219	0	1078	1122	96	-	2	1116	1853	2971
UN16597	Rsa1.0_00913.1	787	59914	7.00E-85	160	160	100	-	231	390	29028	29187
UN16598	Rsa1.0_00913.1	1093	59914	1.00E-143	293	304	96	-	661	964	33903	34203
UN16599	Rsa1.0_00804.1	533	63726	0	408	423	96	-	111	533	21676	22092
UN16600	Rsa1.0_00476.1	1237	280966	0	519	523	99	+	290	812	160084	160606
UN16601	Rsa1.0_00699.1	666	184398	1.00E-128	266	277	96	-	6	278	56459	56735
UN16602	Rsa1.0_23405.1	1103	1052	1.00E-178	340	348	97	+	288	635	682	1029
UN16603	Rsa1.0_00657.1	1384	69858	0	406	417	97	-	16	431	36865	37281
UN16604	Rsa1.0_03569.1	666	22041	1.00E-76	192	207	92	+	3	200	9050	9256
UN16605	Rsa1.0_00816.1	1245	403350	1.00E-109	300	335	89	-	925	1244	109855	110183
UN16606	Rsa1.0_00979.1	852	57733	1.00E-170	335	344	97	+	1	341	30371	30712
UN16607	Rsa1.0_08170.1	783	7799	0	735	755	97	+	28	782	1728	2482
UN16608	Rsa1.0_00153.1	877	192931	0	374	387	96	+	488	874	125252	125636
UN16609	Rsa1.0_00382.1	703	251946	1.00E-175	314	315	99	-	389	703	45398	45712
UN16610	Rsa1.0_00467.1	1012	156542	0	477	512	93	-	6	514	77638	78146
UN16611	Rsa1.0_01344.1	783	142535	1.00E-151	327	343	95	+	1	342	129977	130312
UN16612	Rsa1.0_09695.1	732	6206	3.00E-65	162	171	94	+	564	732	577	747

UN16613	Rsa1.0_00211.1	375	228189	1.00E-140	349	376	92	-	1	375	129494	129865
UN16614	Rsa1.0_08565.1	1443	7189	7.00E-74	178	186	95	-	1259	1442	365	549
UN16615	Rsa1.0_01732.1	1207	40194	0	723	742	97	-	105	846	24772	25513
UN16616	Rsa1.0_04125.1	727	18944	1.00E-178	319	320	99	+	3	322	9445	9764
UN16617	Rsa1.0_07425.1	735	9414	2.00E-82	204	214	95	+	494	702	4352	4563
UN16618	Rsa1.0_00409.1	676	146788	0	468	495	94	+	183	676	80743	81219
UN16619	Rsa1.0_00639.1	719	165873	0	468	494	94	+	229	716	124129	124622
UN16620	Rsa1.0_00244.1	623	103590	8.00E-41	148	168	88	-	87	254	73674	73838
UN16621	Rsa1.0_12391.1	1237	5238	1.00E-126	257	266	96	-	401	666	4081	4346
UN16622	Rsa1.0_03019.1	1208	25987	1.00E-111	271	291	93	-	917	1205	19495	19784
UN16623	Rsa1.0_25475.1	690	934	3.00E-68	135	136	99	-	265	400	28	163
UN16624	Rsa1.0_10890.1	697	5688	0	672	699	96	+	2	697	19	714
UN16625	Rsa1.0_00019.1	906	301669	0	500	523	95	+	7	524	218712	219233
UN16626	Rsa1.0_00269.1	824	341668	1.00E-106	256	271	94	-	1	268	45768	46036
UN16627	Rsa1.0_03369.1	851	23381	0	424	443	95	-	412	851	10354	10790
UN16628	Rsa1.0_00928.1	1424	59293	0	482	500	96	+	849	1348	24314	24813
UN16629	Rsa1.0_01364.1	1458	47172	0	1143	1161	98	-	301	1458	33909	35065
UN16630	Rsa1.0_42543.1	1256	527	1.00E-170	313	316	99	+	941	1256	3	318
UN16631	Rsa1.0_02593.1	971	29744	1.00E-106	226	235	96	+	5	237	28815	29049
UN16632	Rsa1.0_45675.1	812	494	1.00E-98	192	195	98	-	454	648	300	494
UN16633	Rsa1.0_00223.1	880	106231	0	852	880	96	+	2	880	3164	4040
UN16634	Rsa1.0_07829.1	686	8462	1.00E-122	231	234	98	+	232	465	3137	3370
UN16635	Rsa1.0_15591.1	978	2530	0	580	614	94	+	380	977	1224	1833
UN16636	Rsa1.0_00240.1	740	104175	1.00E-106	213	219	97	-	221	439	54590	54808
UN16637	Rsa1.0_05606.1	956	13202	0	480	485	98	-	3	487	11672	12156
UN16638	Rsa1.0_00659.1	661	69733	1.00E-113	271	295	91	+	359	653	55176	55451
UN16639	Rsa1.0_00354.1	1034	232005	1.00E-117	215	215	100	+	818	1032	9448	9662
UN16640	Rsa1.0_04095.1	1177	19095	1.00E-168	321	328	97	-	3	330	12377	12704
UN16641	Rsa1.0_03900.1	900	133221	1.00E-62	208	224	92	-	1	211	3894	4117
UN16642	Rsa1.0_00979.1	1444	57733	1.00E-155	298	302	98	-	1140	1439	40171	40472
UN16643	Rsa1.0_00891.1	696	60724	1.00E-165	345	359	96	+	1	356	32993	33348
UN16644	Rsa1.0_00065.1	1260	454616	1.00E-141	270	275	98	-	576	850	39827	40101
UN16645	Rsa1.0_02500.1	686	30768	1.00E-152	303	313	96	-	1	313	24051	24363
UN16646	Rsa1.0_02670.1	689	28887	7.00E-91	193	200	96	+	423	622	1836	2032
UN16647	Rsa1.0_01316.1	799	48325	1.00E-117	221	222	99	-	3	224	6845	7065
UN16648	Rsa1.0_00002.1	638	601661	1.00E-149	320	337	94	-	3	339	558227	558560
UN16649	Rsa1.0_01245.1	457	149868	0	430	450	95	+	2	451	21666	22114
UN16650	Rsa1.0_01183.1	772	51465	0	752	774	97	-	3	772	41526	42291
UN16651	Rsa1.0_00992.1	541	57419	1.00E-79	151	151	100	-	364	514	4746	4896

UN16652	Rsa1.0_00014.1	875	348985	0	804	833	96	−	45	875	267872	268701
UN16653	Rsa1.0_03005.1	871	26082	0	535	536	99	−	336	871	23153	23688
UN16654	Rsa1.0_02254.1	553	33344	1.00E−159	331	347	95	−	2	347	2377	2713
UN16655	Rsa1.0_04542.1	1014	16930	1.00E−161	295	297	99	+	187	483	14602	14898
UN16656	Rsa1.0_18326.1	760	1620	0	677	700	96	−	61	760	927	1620
UN16657	Rsa1.0_03912.1	1419	135266	0	373	379	98	+	22	400	847	1225
UN16658	Rsa1.0_02429.1	456	31527	3.00E−49	185	211	87	+	11	203	29110	29320
UN16659	Rsa1.0_05076.1	829	14916	0	592	617	95	+	228	829	2709	3325
UN16660	Rsa1.0_03990.1	1116	19676	0	446	456	97	−	1	456	4678	5131
UN16661	Rsa1.0_00876.1	1236	61328	1.00E−176	314	314	100	+	923	1236	51226	51539
UN16662	Rsa1.0_00027.1	742	283362	1.00E−129	278	290	95	−	441	727	279874	280163
UN16663	Rsa1.0_06213.1	760	11478	1.00E−177	380	398	95	−	368	759	1140	1535
UN16664	Rsa1.0_00816.1	783	403350	2.00E−63	139	144	96	+	357	500	110538	110681
UN16665	Rsa1.0_00124.1	530	262503	1.00E−153	305	315	96	+	3	317	162596	162910
UN16666	Rsa1.0_09928.1	946	6087	0	740	745	99	+	199	943	527	1271
UN16667	Rsa1.0_03633.1	548	21644	4.00E−95	198	205	96	+	293	497	17773	17977
UN16668	Rsa1.0_02981.1	676	26285	2.00E−85	170	173	98	−	502	674	4358	4530
UN16669	Rsa1.0_02807.1	1249	27680	0	515	516	99	+	2	517	4945	5460
UN16670	Rsa1.0_01581.1	1408	42833	0	499	519	96	+	1	519	8549	9067
UN16671	Rsa1.0_00635.1	867	175826	2.33E−156	359	387	92	+	4	386	14025	14407
UN16672	Rsa1.0_03982.1	379	19723	1.00E−35	86	89	96	−	217	305	3165	3253
UN16673	Rsa1.0_00152.1	658	257730	1.00E−138	287	297	96	−	1	295	214460	214756
UN16674	Rsa1.0_00190.1	1099	318240	0	536	568	94	+	3	568	140985	141530
UN16675	Rsa1.0_04779.1	841	16008	1.00E−103	200	203	98	−	639	841	9561	9763
UN16676	Rsa1.0_00947.1	1021	58664	0	410	418	98	−	175	592	58154	58571
UN16677	Rsa1.0_01937.1	726	37147	1.00E−141	308	325	94	+	3	320	19950	20274
UN16678	Rsa1.0_00262.1	863	101906	0	455	473	96	+	395	863	53296	53766
UN16679	Rsa1.0_04128.1	757	18927	0	541	585	92	−	1	580	12414	12995
UN16680	Rsa1.0_00382.1	748	251946	1.00E−120	273	293	93	+	4	296	245731	246012
UN16681	Rsa1.0_00035.1	654	176449	0	632	648	97	−	4	651	49852	50498
UN16682	Rsa1.0_00139.1	183	357786	7.00E−54	158	172	91	−	16	182	149611	149781
UN16683	Rsa1.0_00483.1	708	81102	1.00E−140	268	273	98	+	436	708	13909	14181
UN16684	Rsa1.0_00402.1	509	385315	1.00E−158	322	335	96	−	1	335	236413	236747
UN16685	Rsa1.0_00294.1	313	144860	1.00E−129	264	274	96	−	1	274	28240	28513
UN16686	Rsa1.0_00119.1	1914	467509	1.00E−152	337	354	95	+	1560	1910	59938	60291
UN16687	Rsa1.0_08970.1	513	6743	2.33E−156	307	321	95	−	1	318	415	731
UN16688	Rsa1.0_00351.1	672	413096	0	386	395	97	−	6	400	3047	3441
UN16689	Rsa1.0_03148.1	809	24989	0	354	361	98	+	1	361	12153	12513
UN16690	Rsa1.0_04852.1	791	15712	0	758	791	95	−	1	791	9102	9883

UN16691	Rsa1.0_00719.1	876	66952	1.00E-165	303	306	99	+	399	704	46708	47013
UN16692	Rsa1.0_00708.1	749	67366	0	736	748	98	+	1	747	53292	54036
UN16693	Rsa1.0_00827.1	691	62790	1.00E-119	286	305	93	−	3	303	51187	51481
UN16694	Rsa1.0_02178.1	809	156197	0	479	503	95	−	38	540	1510	2012
UN16695	Rsa1.0_00777.1	1113	191865	0	715	752	95	−	5	753	128527	129275
UN16696	Rsa1.0_03100.1	969	25411	0	367	381	96	+	584	964	1871	2250
UN16697	Rsa1.0_00056.1	383	332604	2.00E-80	170	176	96	+	163	338	312898	313073
UN16698	Rsa1.0_00044.1	983	271658	7.00E-98	221	231	95	+	757	983	156762	156990
UN16699	Rsa1.0_02286.1	639	32993	1.00E-157	304	309	98	−	333	639	24695	25003
UN16700	Rsa1.0_00131.1	1271	432233	0	394	396	99	−	519	914	194264	194659
UN16701	Rsa1.0_00197.1	774	109714	1.00E-180	327	328	99	+	3	329	2464	2791
UN16702	Rsa1.0_02103.1	446	35006	0	438	444	98	+	3	446	13486	13929
UN16703	Rsa1.0_00381.1	1228	89718	1.00E-106	274	298	91	−	934	1227	71983	72270
UN16704	Rsa1.0_00116.1	1501	365886	0	599	609	98	−	1	609	83226	83833
UN16705	Rsa1.0_07577.1	944	9092	1.00E-153	278	279	99	−	626	904	3907	4185
UN16706	Rsa1.0_00569.1	728	75060	1.00E-175	373	394	94	+	3	396	64241	64628
UN16707	Rsa1.0_00526.1	795	169279	1.00E-106	208	212	98	−	78	289	141137	141348
UN16708	Rsa1.0_01558.1	896	43287	0	536	537	99	−	360	896	39101	39637
UN16709	Rsa1.0_00101.1	940	267193	1.00E-132	239	239	100	−	700	938	131462	131700
UN16710	Rsa1.0_32735.1	815	695	1.00E-170	303	303	100	+	107	409	1	303
UN16711	Rsa1.0_01846.1	821	38367	0	361	362	99	+	459	820	35711	36072
UN16712	Rsa1.0_00280.1	1333	99966	0	702	707	99	+	626	1332	18404	19110
UN16713	Rsa1.0_05049.1	739	15025	0	497	509	97	+	232	739	4126	4634
UN16714	Rsa1.0_09117.1	625	6637	0	396	399	99	−	226	624	839	1236
UN16715	Rsa1.0_00190.1	732	318240	0	711	728	97	−	3	727	314971	315695
UN16716	Rsa1.0_00168.1	733	115870	0	389	402	96	+	298	696	39841	40242
UN16717	Rsa1.0_00108.1	1128	478491	1.00E-115	311	346	89	+	1	321	134112	134456
UN16718	Rsa1.0_07516.1	693	9218	0	645	664	97	+	7	668	6378	7040
UN16719	Rsa1.0_00926.1	919	195072	0	503	504	99	+	1	504	41569	42072
UN16720	Rsa1.0_00126.1	1528	127534	1.00E-180	326	328	99	+	1201	1528	38254	38581
UN16721	Rsa1.0_02431.1	748	31504	1.00E-166	393	423	92	−	343	745	17503	17922
UN16722	Rsa1.0_00345.1	847	93226	0	322	322	100	+	3	324	84266	84587
UN16723	Rsa1.0_03387.1	717	23278	1.00E-168	333	339	98	+	376	710	19355	19693
UN16724	Rsa1.0_00071.1	714	345714	1.00E-153	301	307	98	+	406	711	142456	142761
UN16725	Rsa1.0_00067.1	1174	415055	0	375	389	96	−	3	390	379553	379940
UN16726	Rsa1.0_13800.1	1054	4315	4.00E-96	179	179	100	+	209	387	2063	2241
UN16727	Rsa1.0_02105.1	885	156953	0	412	422	97	−	460	881	150746	151165
UN16728	Rsa1.0_06939.1	813	10137	1.00E-162	344	359	95	+	3	361	3491	3838
UN16729	Rsa1.0_00185.1	1186	248445	1.00E-121	271	283	95	−	4	278	10314	10593

UN16730	Rsa1.0_00249.1	687	153740	1.00E-134	255	259	98	+	1	259	52930	53188
UN16731	Rsa1.0_00358.1	292	91293	1.00E-134	242	242	100	+	1	242	20908	21149
UN16732	Rsa1.0_02692.1	885	28626	0	402	429	93	+	456	884	21962	22390
UN16733	Rsa1.0_00158.1	562	117999	2.00E-93	262	289	90	−	1	284	100334	100619
UN16734	Rsa1.0_00069.1	1359	403561	1.00E-160	354	374	94	−	1	363	205680	206052
UN16735	Rsa1.0_00015.1	621	561803	1.00E-141	401	454	88	−	123	569	483083	483516
UN16736	Rsa1.0_00333.1	807	94500	0	529	533	99	−	1	533	22949	23481
UN16737	Rsa1.0_00931.1	736	59210	0	362	365	99	+	345	708	54903	55267
UN16738	Rsa1.0_00187.1	686	393093	1.00E-179	363	378	96	−	141	518	47170	47547
UN16739	Rsa1.0_00110.1	749	132719	1.00E-151	304	315	96	−	7	321	92532	92846
UN16740	Rsa1.0_01595.1	464	175383	1.00E-119	220	221	99	+	3	223	41111	41331
UN16741	Rsa1.0_01934.1	639	37162	1.00E-159	323	332	97	+	1	329	1961	2291
UN16742	Rsa1.0_03873.1	420	20360	0	373	386	96	+	5	386	9573	9957
UN16743	Rsa1.0_00147.1	607	249503	0	603	605	99	−	3	607	241358	241962
UN16744	Rsa1.0_00244.1	558	103590	1.00E-117	286	304	94	+	8	309	29926	30224
UN16745	Rsa1.0_01352.1	1722	47443	0	1485	1518	97	−	4	1520	6219	7735
UN16746	Rsa1.0_00949.1	714	58549	1.00E-132	286	299	95	+	417	714	39561	39853
UN16747	Rsa1.0_04779.1	830	16008	7.00E-73	150	152	98	−	333	484	10245	10395
UN16748	Rsa1.0_00044.1	960	271658	1.00E-137	267	272	98	−	1	272	156101	156371
UN16749	Rsa1.0_09126.1	758	6625	0	412	431	95	−	327	756	5284	5707
UN16750	Rsa1.0_03377.1	677	23322	1.00E-110	206	207	99	+	467	673	16048	16254
UN16751	Rsa1.0_01896.1	749	37512	1.00E-175	353	361	97	−	389	742	35284	35644
UN16752	Rsa1.0_00154.1	587	189461	1.00E-133	269	278	96	+	1	274	79505	79782
UN16753	Rsa1.0_02217.1	888	33812	0	862	896	96	+	1	888	30263	31158
UN16754	Rsa1.0_13604.1	823	4657	3.00E-81	207	222	93	+	9	230	2040	2259
UN16755	Rsa1.0_01217.1	912	50554	0	884	913	96	−	1	912	47142	48040
UN16756	Rsa1.0_01803.1	680	173846	0	399	414	96	−	1	411	31867	32278
UN16757	Rsa1.0_01835.1	669	38542	0	391	405	96	−	256	659	24235	24638
UN16758	Rsa1.0_01958.1	1019	261128	3.00E-72	151	155	97	−	109	263	112507	112661
UN16759	Rsa1.0_03570.1	836	22040	1.00E-145	292	300	97	+	58	356	8517	8813
UN16760	Rsa1.0_00759.1	1040	65465	0	791	801	98	−	1	801	55681	56480
UN16761	Rsa1.0_00047.1	1101	168582	1.00E-127	247	252	98	−	1	252	115947	116198
UN16762	Rsa1.0_00044.1	951	271658	1.00E-108	220	227	96	+	723	949	156762	156988
UN16763	Rsa1.0_06468.1	923	10958	0	833	845	98	−	3	845	6517	7359
UN16764	Rsa1.0_00191.1	659	316976	0	494	499	98	−	5	501	140127	140625
UN16765	Rsa1.0_03440.1	732	22928	3.00E-68	141	144	97	+	470	613	12891	13034
UN16766	Rsa1.0_00101.1	660	267193	1.00E-129	300	321	93	−	7	315	47241	47560
UN16767	Rsa1.0_00167.1	618	116000	0	610	618	98	−	1	618	43630	44247
UN16768	Rsa1.0_01296.1	682	49035	1.00E-132	257	263	97	−	9	271	23171	23433

UN16769	Rsa1.0_14354.1	746	3487	0	738	746	98	+	2	746	1273	2017
UN16770	Rsa1.0_00718.1	792	279335	0	410	430	95	+	362	791	34463	34889
UN16771	Rsa1.0_01387.1	730	46745	1.00E-132	266	275	96	+	434	708	46152	46426
UN16772	Rsa1.0_03191.1	1004	145762	1.00E-150	330	348	94	+	626	973	16325	16663
UN16773	Rsa1.0_02458.1	806	31197	1.00E-160	322	332	96	+	474	804	9247	9575
UN16774	Rsa1.0_00039.1	1524	296927	1.00E-87	174	177	98	−	337	513	49432	49608
UN16775	Rsa1.0_04890.1	825	15591	1.00E-109	231	241	95	−	238	478	10910	11150
UN16776	Rsa1.0_00714.1	1333	67187	0	388	398	97	+	2	399	22328	22719
UN16777	Rsa1.0_00805.1	730	63724	1.00E-103	206	211	97	+	492	702	41298	41508
UN16778	Rsa1.0_00954.1	725	189536	2.00E-63	124	124	100	−	590	713	152040	152163
UN16779	Rsa1.0_37263.1	647	605	0	572	589	97	−	1	589	1	589
UN16780	Rsa1.0_17775.1	693	1743	0	571	623	91	+	6	611	1132	1743
UN16781	Rsa1.0_00019.1	1124	301669	0	497	521	95	+	1	514	218714	219233
UN16782	Rsa1.0_00182.1	980	142305	0	916	984	93	−	12	976	35043	36008
UN16783	Rsa1.0_00132.1	1044	124299	1.00E-147	325	342	95	+	3	339	108401	108742
UN16784	Rsa1.0_00611.1	940	72410	0	486	501	97	+	438	938	60559	61059
UN16785	Rsa1.0_00218.1	1007	201480	1.00E-141	332	353	94	+	665	1007	173202	173553
UN16786	Rsa1.0_00213.1	689	108167	1.00E-117	217	218	99	−	468	685	14737	14954
UN16787	Rsa1.0_00848.1	795	152362	5.00E-95	234	249	93	−	1	248	38270	38516
UN16788	Rsa1.0_00394.1	1185	148426	1.00E-172	395	423	93	−	3	415	99869	100286
UN16789	Rsa1.0_17040.1	550	1944	0	379	396	95	−	9	396	1207	1602
UN16790	Rsa1.0_00008.1	475	358124	1.00E-147	276	280	98	−	3	282	30632	30911
UN16791	Rsa1.0_01286.1	568	49209	0	504	520	96	+	2	520	4008	4521
UN16792	Rsa1.0_02917.1	789	159058	0	728	740	98	+	1	740	144062	144799
UN16793	Rsa1.0_01274.1	357	49419	1.00E-72	202	221	91	−	1	214	21348	21566
UN16794	Rsa1.0_00418.1	652	215377	6.00E-88	174	177	98	+	291	467	46155	46331
UN16795	Rsa1.0_00113.1	646	131574	1.00E-165	322	330	97	−	1	330	73859	74187
UN16796	Rsa1.0_03894.1	796	20207	0	728	749	97	+	2	749	2591	3337
UN16797	Rsa1.0_02214.1	866	161150	5.00E-83	178	185	96	+	296	480	695	879
UN16798	Rsa1.0_00883.1	689	61230	0	432	440	98	+	1	440	29870	30306
UN16799	Rsa1.0_00490.1	1546	186713	0	402	428	93	−	1	411	39915	40340
UN16800	Rsa1.0_00549.1	1299	76473	1.00E-129	250	255	98	−	149	403	9511	9765
UN16801	Rsa1.0_02144.1	748	34630	5.00E-89	185	191	96	+	148	338	1304	1494
UN16802	Rsa1.0_10199.1	506	5948	0	460	489	94	+	1	484	5385	5869
UN16803	Rsa1.0_00860.1	965	61789	0	547	556	98	+	408	963	6973	7528
UN16804	Rsa1.0_00041.1	729	186197	9.00E-75	149	151	98	−	420	570	57669	57819
UN16805	Rsa1.0_00327.1	739	94892	1.00E-140	275	280	98	−	460	739	70364	70641
UN16806	Rsa1.0_00401.1	640	244410	0	391	394	99	+	5	398	178052	178445
UN16807	Rsa1.0_06663.1	728	10576	0	642	671	95	+	1	671	6933	7602

UN16808	Rsa1.0_01874.1	777	37853	0	766	776	98	−	3	777	21339	22109
UN16809	Rsa1.0_02105.1	895	156953	1.00E−179	355	367	96	−	126	492	149700	150066
UN16810	Rsa1.0_00185.1	522	248445	1.00E−128	251	257	97	−	3	259	10314	10566
UN16811	Rsa1.0_00686.1	737	68511	3.00E−99	219	230	95	−	10	236	29900	30129
UN16812	Rsa1.0_00605.1	617	73199	0	611	617	99	−	1	617	30938	31554
UN16813	Rsa1.0_15262.1	1318	2722	0	473	481	98	−	9	489	1765	2245
UN16814	Rsa1.0_00071.1	668	345714	1.00E−111	235	243	96	−	423	662	324494	324736
UN16815	Rsa1.0_04584.1	764	16756	0	469	476	98	−	283	757	12391	12866
UN16816	Rsa1.0_00816.1	696	403350	4.00E−83	210	227	92	+	3	223	109964	110183
UN16817	Rsa1.0_02158.1	832	154841	0	526	538	97	+	142	679	131881	132414
UN16818	Rsa1.0_00416.1	956	86772	4.00E−93	242	266	90	−	705	956	76161	76426
UN16819	Rsa1.0_00175.1	738	166121	5.00E−98	207	214	96	−	126	339	141878	142090
UN16820	Rsa1.0_02911.1	754	142461	0	601	622	96	−	1	618	23423	24039
UN16821	Rsa1.0_07692.1	2069	132338	0	543	553	98	+	544	1095	131072	131624
UN16822	Rsa1.0_03326.1	737	23710	0	446	461	96	−	47	506	17095	17547
UN16823	Rsa1.0_00737.1	1214	66432	1.00E−161	332	345	96	+	874	1214	30743	31086
UN16824	Rsa1.0_01874.1	1201	37853	0	500	513	97	+	1	510	23722	24233
UN16825	Rsa1.0_00111.1	1233	390088	1.00E−142	323	342	94	−	2	342	139323	139660
UN16826	Rsa1.0_00087.1	710	761073	1.00E−110	251	263	95	−	448	710	191412	191671
UN16827	Rsa1.0_01611.1	1027	42269	1.00E−164	362	387	93	−	3	383	17493	17863
UN16828	Rsa1.0_00369.1	867	266462	0	414	437	94	−	431	867	4549	4974
UN16829	Rsa1.0_08230.1	1439	7688	0	878	878	100	+	562	1439	5127	6004
UN16830	Rsa1.0_00635.1	854	175826	1.00E−152	313	320	97	+	46	359	14088	14407
UN16831	Rsa1.0_02019.1	1062	35983	1.00E−99	254	273	93	−	798	1062	13360	13627
UN16832	Rsa1.0_00001.1	735	363028	1.00E−112	312	346	90	+	4	348	190816	191142
UN16833	Rsa1.0_00055.1	788	520193	1.00E−155	290	294	98	+	1	294	207483	207776
UN16834	Rsa1.0_05448.1	738	13712	1.00E−116	212	212	100	−	195	406	11459	11670
UN16835	Rsa1.0_02650.1	352	29047	0	338	340	99	−	13	352	11566	11904
UN16836	Rsa1.0_00847.1	912	62270	1.00E−164	393	426	92	−	2	419	55145	55569
UN16837	Rsa1.0_02707.1	1051	28520	7.00E−95	215	225	95	−	8	230	26167	26391
UN16838	Rsa1.0_03120.1	1000	25269	7.00E−95	198	205	96	+	81	285	20575	20779
UN16839	Rsa1.0_03966.1	828	19823	1.00E−171	369	387	95	+	35	418	1948	2332
UN16840	Rsa1.0_00121.1	562	278029	0	540	561	96	+	3	562	55537	56091
UN16841	Rsa1.0_00780.1	774	147795	1.00E−154	295	300	98	−	447	745	42762	43061
UN16842	Rsa1.0_00038.1	1555	314568	0	1423	1450	98	+	2	1451	283204	284648
UN16843	Rsa1.0_02453.1	848	31271	0	359	361	99	−	5	364	14906	15266
UN16844	Rsa1.0_02660.1	742	28969	1.00E−115	235	243	96	−	65	307	12641	12883
UN16845	Rsa1.0_08846.1	648	6867	6.00E−88	248	275	90	+	382	645	3201	3473
UN16846	Rsa1.0_00190.1	986	318240	0	792	811	97	−	1	807	94238	95048

UN16847	Rsa1.0_03668.1	290	21483	1.00E-138	279	289	96	-	1	289	11564	11852
UN16848	Rsa1.0_09017.1	1045	6706	0	353	359	98	-	685	1043	822	1180
UN16849	Rsa1.0_03770.1	706	20952	0	505	522	96	-	1	522	5875	6392
UN16850	Rsa1.0_00116.1	771	365886	0	439	443	99	+	329	771	344422	344864
UN16851	Rsa1.0_00017.1	917	752458	0	618	639	96	-	279	917	616126	616764
UN16852	Rsa1.0_01540.1	943	151890	0	495	507	97	+	208	714	144792	145298
UN16853	Rsa1.0_02723.1	295	28408	1.00E-143	288	297	96	+	3	293	2563	2859
UN16854	Rsa1.0_00827.1	1340	62790	0	691	718	96	-	2	719	22600	23299
UN16855	Rsa1.0_00855.1	1108	62047	0	451	458	98	+	649	1104	55676	56132
UN16856	Rsa1.0_01731.1	1078	40208	0	536	538	99	-	1	537	19840	20377
UN16857	Rsa1.0_00307.1	255	199003	1.00E-132	252	255	98	+	1	255	5805	6058
UN16858	Rsa1.0_01727.1	1423	40243	0	371	371	100	+	3	373	5093	5463
UN16859	Rsa1.0_00552.1	848	206757	0	822	847	97	-	2	848	65984	66826
UN16860	Rsa1.0_06021.1	1278	11911	0	588	590	99	-	689	1278	4387	4976
UN16861	Rsa1.0_00040.1	822	324196	1.00E-150	386	426	90	-	421	820	227177	227600
UN16862	Rsa1.0_00521.1	831	78191	0	372	376	98	+	1	376	71471	71846
UN16863	Rsa1.0_00168.1	1441	115870	0	606	615	98	+	521	1135	88360	88974
UN16864	Rsa1.0_00321.1	1611	95671	0	352	354	99	+	1260	1611	40671	41024
UN16865	Rsa1.0_04518.1	1796	17005	1.00E-170	482	545	88	+	687	1206	11675	12209
UN16866	Rsa1.0_16623.1	1024	2086	0	411	424	96	+	378	801	1196	1619
UN16867	Rsa1.0_00132.1	766	124299	0	431	434	99	-	1	434	46291	46723
UN16868	Rsa1.0_01184.1	1848	51452	0	416	425	97	+	1424	1848	7609	8030
UN16869	Rsa1.0_00767.1	1377	64980	0	385	385	100	-	1	385	18547	18931
UN16870	Rsa1.0_00120.1	1124	277288	1.00E-159	291	293	99	-	2	294	119581	119873
UN16871	Rsa1.0_02144.1	756	34630	1.00E-180	351	361	97	-	3	363	9429	9786
UN16872	Rsa1.0_03873.1	861	20360	1.00E-121	266	282	94	-	121	400	9247	9517
UN16873	Rsa1.0_00329.1	1428	222544	0	458	463	98	-	298	760	41377	41839
UN16874	Rsa1.0_00059.1	2146	159650	0	599	603	99	+	1047	1648	23292	23894
UN16875	Rsa1.0_00052.1	967	162771	1.00E-134	255	259	98	+	61	319	57659	57917
UN16876	Rsa1.0_06373.1	898	11138	1.00E-164	312	317	98	+	1	316	717	1033
UN16877	Rsa1.0_00123.1	719	325650	0	658	665	98	+	9	673	168294	168957
UN16878	Rsa1.0_01881.1	1305	37721	0	774	782	98	-	525	1305	32960	33741
UN16879	Rsa1.0_16623.1	1151	2086	0	454	466	97	-	687	1151	210	671
UN16880	Rsa1.0_00556.1	890	207212	0	351	356	98	+	519	873	5867	6222
UN16881	Rsa1.0_00020.1	1590	283099	0	1579	1584	99	+	1	1582	53213	54796
UN16882	Rsa1.0_05049.1	865	15025	0	485	498	97	+	351	848	4126	4619
UN16883	Rsa1.0_00046.1	1374	320315	1.00E-154	301	309	97	-	1071	1374	72846	73154
UN16884	Rsa1.0_06303.1	967	11268	1.00E-124	241	246	97	-	200	445	9882	10127
UN16885	Rsa1.0_01572.1	811	42995	0	796	810	98	+	2	811	6078	6879

UN16886	Rsa1.0_00373.1	1180	90296	1.00E-132	261	268	97	-	536	803	60869	61136
UN16887	Rsa1.0_00045.1	876	328694	0	868	878	98	-	7	874	302345	303222
UN16888	Rsa1.0_01958.1	1122	261128	1.00E-142	318	337	94	+	785	1115	115205	115537
UN16889	Rsa1.0_00015.1	703	561803	1.00E-147	461	530	86	-	57	568	483124	483640
UN16890	Rsa1.0_01145.1	1517	149247	1.00E-178	380	396	95	+	1	395	123359	123750
UN16891	Rsa1.0_14674.1	945	3167	0	399	408	97	-	1	401	372	779
UN16892	Rsa1.0_00206.1	1310	109040	0	560	612	91	+	32	636	95688	96299
UN16893	Rsa1.0_00272.1	1003	447836	1.00E-135	258	261	98	-	5	265	219273	219532
UN16894	Rsa1.0_04407.1	1326	17504	0	436	444	98	-	868	1311	4181	4623
UN16895	Rsa1.0_00094.1	1458	139042	1.00E-175	319	320	99	-	1	320	99912	100230
UN16896	Rsa1.0_00034.1	2080	260119	4.00E-98	264	291	90	-	151	441	161164	161454
UN16897	Rsa1.0_04291.1	1111	18055	0	473	475	99	-	1	475	8239	8711
UN16898	Rsa1.0_09859.1	482	6115	1.00E-130	324	353	91	-	1	353	5753	6102
UN16899	Rsa1.0_00910.1	1206	59975	1.00E-155	389	426	91	-	590	1015	55926	56351
UN16900	Rsa1.0_00887.1	1419	61048	0	406	411	98	-	9	419	50891	51299
UN16901	Rsa1.0_01814.1	1327	38917	0	473	486	97	-	831	1316	5704	6180
UN16902	Rsa1.0_01512.1	2048	44234	0	591	609	97	-	1443	2045	20810	21414
UN16903	Rsa1.0_07914.1	1257	8294	0	779	801	97	+	454	1250	1883	2683
UN16904	Rsa1.0_18254.1	1443	1636	1.00E-168	337	345	97	-	1100	1442	681	1024
UN16905	Rsa1.0_00384.1	1052	89278	3.00E-94	179	180	99	+	563	742	81079	81258
UN16906	Rsa1.0_03282.1	857	24107	0	539	561	96	+	300	852	10595	11155
UN16907	Rsa1.0_04198.1	816	18504	0	811	817	99	+	1	816	15298	16113
UN16908	Rsa1.0_03195.1	1766	24673	0	625	654	95	-	41	694	8990	9634
UN16909	Rsa1.0_05095.1	956	14869	1.00E-149	299	309	96	+	648	956	773	1077
UN16910	Rsa1.0_02688.1	904	28644	0	494	512	96	-	1	509	26861	27371
UN16911	Rsa1.0_00135.1	1222	178077	0	460	474	97	-	2	473	19429	19899
UN16912	Rsa1.0_02201.1	1279	33981	1.00E-119	267	280	95	-	1	276	31757	32035
UN16913	Rsa1.0_00382.1	912	251946	0	587	613	95	-	4	607	111146	111756
UN16914	Rsa1.0_00734.1	843	66508	0	544	575	94	+	1	574	62091	62665
UN16915	Rsa1.0_01220.1	861	161775	1.00E-140	283	293	96	-	1	293	117108	117400
UN16916	Rsa1.0_03638.1	1792	21634	0	370	376	98	+	1420	1792	11607	11982
UN16917	Rsa1.0_00624.1	1018	71773	1.00E-172	322	327	98	+	692	1018	21070	21396
UN16918	Rsa1.0_00370.1	1020	90472	0	534	542	98	+	1	542	71034	71575
UN16919	Rsa1.0_00211.1	1452	228189	0	1442	1452	99	-	2	1452	211359	212810
UN16920	Rsa1.0_00087.1	1473	761073	0	493	493	100	+	981	1473	568132	568624
UN16921	Rsa1.0_00102.1	1633	135497	0	385	388	99	+	1	388	42149	42536
UN16922	Rsa1.0_03191.1	999	145762	1.00E-118	259	272	95	+	5	275	15383	15654
UN16923	Rsa1.0_01273.1	1387	49464	1.00E-163	305	308	99	-	1074	1380	18185	18492
UN16924	Rsa1.0_01901.1	1094	37472	0	989	1003	98	+	2	1004	25164	26166

UN16925	Rsa1.0_39538.1	1677	569	1.00E-174	332	338	98	+	11	346	90	427
UN16926	Rsa1.0_00457.1	856	337301	0	436	436	100	+	420	855	142257	142692
UN16927	Rsa1.0_08430.1	1132	7367	0	391	408	95	-	1	407	2169	2573
UN16928	Rsa1.0_03120.1	1027	25269	1.00E-155	338	353	95	+	677	1027	22032	22381
UN16929	Rsa1.0_02910.1	975	26843	1.00E-118	216	216	100	-	3	218	2761	2976
UN16930	Rsa1.0_00050.1	1626	237174	0	380	381	99	+	1	380	31140	31520
UN16931	Rsa1.0_02362.1	1148	32190	0	390	402	97	+	749	1148	21332	21730
UN16932	Rsa1.0_04873.1	1516	15657	1.00E-139	269	274	98	+	3	276	12262	12532
UN16933	Rsa1.0_00951.1	658	164588	1.00E-149	279	282	98	+	379	658	114506	114787
UN16934	Rsa1.0_00568.1	1831	75219	0	1020	1050	97	+	543	1584	1622	2658
UN16935	Rsa1.0_05336.1	1680	14117	0	335	335	100	-	1	335	11121	11455
UN16936	Rsa1.0_02247.1	952	33427	2.33E-156	336	361	93	-	1	361	28646	28994
UN16937	Rsa1.0_00913.1	793	59914	0	390	393	99	+	228	620	10123	10512
UN16938	Rsa1.0_01434.1	1260	45951	0	1152	1196	96	-	66	1258	7329	8511
UN16939	Rsa1.0_03504.1	1102	22443	1.00E-166	334	342	97	-	36	376	10641	10977
UN16940	Rsa1.0_00300.1	1349	97747	0	536	566	94	-	703	1256	81512	82074
UN16941	Rsa1.0_01203.1	1147	50848	0	337	340	99	-	2	341	39876	40215
UN16942	Rsa1.0_00999.1	2300	57162	0	486	527	92	+	1759	2263	56248	56765
UN16943	Rsa1.0_00546.1	1497	76523	0	709	716	99	-	111	826	61328	62042
UN16944	Rsa1.0_04873.1	1655	15657	1.00E-152	315	327	96	+	1319	1642	15059	15384
UN16945	Rsa1.0_01462.1	989	45231	0	523	544	96	-	1	532	27589	28132
UN16946	Rsa1.0_00484.1	949	203294	0	537	546	98	-	404	949	49802	50347
UN16947	Rsa1.0_00017.1	1172	752458	0	1154	1171	98	+	2	1172	330369	331538
UN16948	Rsa1.0_00548.1	1091	76518	1.00E-138	259	262	98	+	3	264	60500	60761
UN16949	Rsa1.0_00302.1	1127	97521	1.00E-166	378	406	93	-	1	389	85022	85427
UN16950	Rsa1.0_05628.1	1184	13130	0	426	432	98	-	2	432	1627	2058
UN16951	Rsa1.0_00994.1	2532	165189	0	553	563	98	+	1843	2405	1248	1810
UN16952	Rsa1.0_00580.1	803	74515	1.00E-163	343	356	96	-	451	803	33899	34249
UN16953	Rsa1.0_00861.1	764	61759	1.00E-140	302	317	95	-	2	314	44481	44796
UN16954	Rsa1.0_65165.1	487	352	1.00E-91	204	213	95	+	278	487	1	212
UN16955	Rsa1.0_00084.1	1100	142338	0	694	711	97	-	395	1098	132713	133423
UN16956	Rsa1.0_01280.1	1537	49321	0	1091	1092	99	+	444	1535	36579	37670
UN16957	Rsa1.0_02859.1	554	162118	0	456	457	99	-	1	457	154217	154673
UN16958	Rsa1.0_00333.1	1446	94500	0	536	561	95	+	889	1446	69143	69703
UN16959	Rsa1.0_02431.1	816	31504	0	437	458	95	-	360	812	17474	17922
UN16960	Rsa1.0_02103.1	1001	35006	0	574	598	95	+	14	605	13499	14094
UN16961	Rsa1.0_09270.1	936	6513	0	884	941	93	-	3	936	5294	6201
UN16962	Rsa1.0_00437.1	1048	84879	0	427	452	94	+	593	1043	23633	24067
UN16963	Rsa1.0_09434.1	1294	6371	0	335	336	99	-	498	833	1196	1531

UN16964	Rsa1.0_02455.1	957	31262	0	556	564	98	−	1	563	25408	25971
UN16965	Rsa1.0_06663.1	1506	10576	0	666	673	98	−	834	1506	6927	7599
UN16966	Rsa1.0_00476.1	1328	280966	0	775	803	96	+	279	1077	160084	160884
UN16967	Rsa1.0_11046.1	844	5635	1.00E−155	329	343	95	−	9	347	3092	3432
UN16968	Rsa1.0_01092.1	1190	54348	1.00E−166	364	385	94	+	805	1189	53693	54076
UN16969	Rsa1.0_04400.1	1318	17529	0	846	865	97	+	75	933	16666	17529
UN16970	Rsa1.0_00995.1	1594	57243	0	1588	1593	99	−	2	1594	46530	48122
UN16971	Rsa1.0_00184.1	1331	218748	0	623	635	98	−	697	1331	210822	211456
UN16972	Rsa1.0_00069.1	1544	403561	0	463	464	99	−	1081	1544	394775	395238
UN16973	Rsa1.0_00268.1	1554	101470	0	507	533	95	+	4	518	42996	43526
UN16974	Rsa1.0_01108.1	1588	53865	0	1213	1224	99	−	93	1316	1	1224
UN16975	Rsa1.0_00524.1	1517	169613	0	676	683	98	−	426	1106	129773	130455
UN16976	Rsa1.0_03264.1	1179	145959	1.00E−139	275	283	97	−	496	778	134142	134424
UN16977	Rsa1.0_00108.1	1315	478491	0	381	385	98	−	931	1315	450948	451332
UN16978	Rsa1.0_01029.1	1064	256254	1.00E−155	320	332	96	−	1	329	245893	246223
UN16979	Rsa1.0_00592.1	1187	73788	0	1157	1188	97	+	1	1186	61537	62724
UN16980	Rsa1.0_00232.1	1236	104936	1.00E−167	304	306	99	+	931	1236	48471	48776
UN16981	Rsa1.0_00111.1	1167	390088	0	376	385	97	+	782	1166	373585	373965
UN16982	Rsa1.0_00167.1	1274	116000	0	653	670	97	−	3	672	43588	44257
UN16983	Rsa1.0_00777.1	1049	191865	0	420	424	99	+	4	427	129722	130145
UN16984	Rsa1.0_09656.1	1018	6229	0	940	986	95	+	2	987	5101	6086
UN16985	Rsa1.0_00619.1	1708	71985	0	1526	1556	98	−	153	1708	13958	15500
UN16986	Rsa1.0_00071.1	1150	345714	0	540	551	98	−	600	1146	336436	336985
UN16987	Rsa1.0_00153.1	769	192931	1.00E−116	244	251	97	+	517	765	168694	168941
UN16988	Rsa1.0_15323.1	1242	2685	5.00E−96	188	190	98	+	3	190	1634	1823
UN16989	Rsa1.0_00381.1	1988	89718	1.00E−115	219	220	99	+	1770	1988	37380	37599
UN16990	Rsa1.0_00107.1	2084	431659	0	527	534	98	−	788	1321	299930	300463
UN16991	Rsa1.0_01377.1	1023	46967	1.00E−117	283	307	92	−	3	308	24880	25174
UN16992	Rsa1.0_18948.1	1109	1506	6.00E−80	182	189	96	−	17	203	686	870
UN16993	Rsa1.0_01005.1	1434	56967	0	642	648	99	−	756	1403	5035	5682
UN16994	Rsa1.0_20420.1	872	1306	0	774	800	96	+	1	800	84	861
UN16995	Rsa1.0_01676.1	966	41140	0	942	964	97	−	2	962	32458	33420
UN16996	Rsa1.0_14001.1	908	3933	0	436	446	97	+	3	448	1367	1810
UN16997	Rsa1.0_04121.1	670	18951	0	422	452	93	−	51	502	13877	14328
UN16998	Rsa1.0_01831.1	1777	38628	1.00E−155	293	298	98	+	1173	1470	32851	33148
UN16999	Rsa1.0_01000.1	1085	57140	1.00E−118	238	244	97	+	54	297	37929	38171
UN17000	Rsa1.0_00566.1	524	75325	0	387	399	96	−	1	395	61768	62165
UN17001	Rsa1.0_00006.1	1001	396176	1.00E−179	365	377	96	−	625	1000	371008	371376
UN17002	Rsa1.0_00827.1	1758	62790	0	488	496	98	−	1262	1757	57646	58141

UN17003	Rsa1.0_00646.1	1201	70473	0	903	927	97	−	77	1003	4449	5375
UN17004	Rsa1.0_02319.1	1501	32580	0	773	794	97	+	708	1499	2476	3267
UN17005	Rsa1.0_01675.1	1197	41155	1.00E−134	243	243	100	+	7	249	12330	12572
UN17006	Rsa1.0_15262.1	1212	2722	0	468	486	96	−	9	494	1765	2247
UN17007	Rsa1.0_04872.1	1295	15659	1.00E−104	214	221	96	+	818	1038	14076	14296
UN17008	Rsa1.0_00050.1	1421	237174	1.00E−133	251	254	98	−	3	256	179575	179828
UN17009	Rsa1.0_01668.1	934	41299	1.00E−105	267	291	91	−	96	386	40399	40689
UN17010	Rsa1.0_01867.1	1557	37960	0	485	496	97	+	788	1283	19568	20063
UN17011	Rsa1.0_00151.1	1000	119541	1.00E−123	287	303	94	+	7	303	109633	109935
UN17012	Rsa1.0_01000.1	1631	57140	0	412	413	99	+	17	429	2338	2750
UN17013	Rsa1.0_00033.1	683	515811	1.00E−155	328	343	95	+	1	337	365283	365622
UN17014	Rsa1.0_01286.1	1092	49209	0	438	441	99	−	651	1091	40337	40777
UN17015	Rsa1.0_01425.1	919	46136	1.00E−175	328	332	98	−	21	352	8221	8551
UN17016	Rsa1.0_00524.1	1533	169613	0	671	683	98	−	393	1074	129773	130455
UN17017	Rsa1.0_00476.1	2144	280966	0	551	565	97	−	676	1239	242668	243228
UN17018	Rsa1.0_02807.1	1669	27680	0	425	443	95	−	1	441	12120	12548
UN17019	Rsa1.0_01997.1	1738	158416	0	674	683	98	−	3	684	145620	146299
UN17020	Rsa1.0_03698.1	2715	21302	0	575	580	99	−	2136	2712	9537	10116
UN17021	Rsa1.0_01183.1	1363	51465	0	1158	1191	97	−	3	1191	41109	42291
UN17022	Rsa1.0_00197.1	906	109714	0	606	620	97	−	292	906	87937	88556
UN17023	Rsa1.0_01012.1	571	56836	1.00E−131	274	285	96	+	237	521	40013	40296
UN17024	Rsa1.0_01151.1	665	52268	0	451	451	100	−	214	664	26191	26641
UN17025	Rsa1.0_00108.1	1337	478491	1.00E−179	397	422	94	−	912	1332	450922	451332
UN17026	Rsa1.0_01762.1	719	39682	0	465	492	94	+	144	632	1493	1973
UN17027	Rsa1.0_02089.1	1276	35163	0	331	333	99	+	941	1273	10642	10973
UN17028	Rsa1.0_00119.1	1382	467509	0	1259	1328	94	−	64	1382	346461	347782
UN17029	Rsa1.0_00822.1	1346	62891	1.00E−114	260	275	94	+	1050	1324	32221	32491
UN17030	Rsa1.0_00482.1	1291	81162	1.00E−133	252	254	99	+	1	254	25693	25945
UN17031	Rsa1.0_00611.1	985	72410	0	491	497	98	−	3	499	60559	61055
UN17032	Rsa1.0_00175.1	1108	166121	0	592	607	97	+	502	1108	151998	152597
UN17033	Rsa1.0_01085.1	864	54539	0	433	450	96	+	400	846	15815	16253
UN17034	Rsa1.0_02135.1	1285	34713	0	597	606	98	−	680	1285	15164	15766
UN17035	Rsa1.0_00237.1	897	235974	0	387	396	97	−	1	396	58852	59246
UN17036	Rsa1.0_00017.1	1176	752458	1.00E−144	337	359	93	+	30	382	661863	662218
UN17037	Rsa1.0_00374.1	945	165738	0	425	437	97	−	509	945	95783	96217
UN17038	Rsa1.0_06604.1	1682	10681	0	487	495	98	−	4	497	10140	10634
UN17039	Rsa1.0_01610.1	1018	42274	0	791	814	97	+	2	810	11392	12204
UN17040	Rsa1.0_00128.1	1699	228675	0	1424	1428	99	−	271	1698	207255	208682
UN17041	Rsa1.0_06151.1	1397	11612	0	377	380	99	−	1	379	10276	10655

UN17042	Rsa1.0_00017.1	875	752458	0	322	322	100	−	554	875	141333	141654
UN17043	Rsa1.0_01180.1	1021	51517	1.00E−166	312	317	98	+	2	318	30232	30548
UN17044	Rsa1.0_01701.1	709	162121	0	552	562	98	+	147	705	20038	20599
UN17045	Rsa1.0_00988.1	984	57525	0	336	340	98	+	1	338	27333	27672
UN17046	Rsa1.0_12119.1	2095	5311	2.33E−156	279	284	98	+	1170	1453	4573	4856
UN17047	Rsa1.0_00991.1	1621	143101	0	593	611	97	−	836	1445	28345	28955
UN17048	Rsa1.0_04238.1	1125	18352	1.00E−101	266	292	91	+	848	1125	2706	2996
UN17049	Rsa1.0_01335.1	388	47894	1.00E−172	366	386	94	−	1	386	29200	29585
UN17050	Rsa1.0_00168.1	1684	115870	0	378	386	97	+	16	398	113980	114364
UN17051	Rsa1.0_01707.1	1151	40686	0	394	401	98	−	333	733	25818	26218
UN17052	Rsa1.0_02041.1	991	163634	1.00E−121	240	246	97	−	746	991	18791	19032
UN17053	Rsa1.0_01786.1	981	133324	0	351	356	98	−	623	978	100633	100988
UN17054	Rsa1.0_00348.1	1377	92818	1.00E−111	217	220	98	−	104	322	39668	39887
UN17055	Rsa1.0_01600.1	871	42537	1.00E−159	294	297	98	−	1	297	25097	25393
UN17056	Rsa1.0_01743.1	1098	39926	1.00E−173	321	324	99	+	775	1098	15993	16315
UN17057	Rsa1.0_05051.1	1491	15018	0	456	464	98	+	1	460	13852	14315
UN17058	Rsa1.0_02196.1	1522	34005	0	470	484	97	−	1042	1522	9453	9934
UN17059	Rsa1.0_00457.1	902	337301	1.00E−122	230	231	99	+	3	232	140560	140790
UN17060	Rsa1.0_00132.1	753	124299	0	415	431	96	−	1	422	46291	46718
UN17061	Rsa1.0_00021.1	911	263655	0	473	477	99	−	2	478	78215	78691
UN17062	Rsa1.0_01958.1	1171	261128	1.00E−104	261	282	92	−	3	278	115205	115482
UN17063	Rsa1.0_01262.1	936	178815	0	775	783	98	+	1	783	162983	163765
UN17064	Rsa1.0_00008.1	1261	358124	1.00E−177	333	337	98	−	1	333	39412	39748
UN17065	Rsa1.0_31415.1	1402	728	1.00E−145	271	274	98	−	1123	1396	11	284
UN17066	Rsa1.0_00462.1	1333	82288	0	481	486	98	−	35	518	68395	68880
UN17067	Rsa1.0_01009.1	877	56922	1.00E−156	301	308	97	−	3	310	3612	3919
UN17068	Rsa1.0_00436.1	750	85008	0	532	532	100	+	1	532	368	899
UN17069	Rsa1.0_05030.1	863	15088	0	820	866	94	−	3	863	10854	11698
UN17070	Rsa1.0_00479.1	713	81377	1.00E−144	263	264	99	+	155	418	28662	28925
UN17071	Rsa1.0_00104.1	513	368722	1.00E−169	325	333	97	−	181	513	224996	225328
UN17072	Rsa1.0_00006.1	984	396176	0	957	971	98	−	3	971	279153	280120
UN17073	Rsa1.0_01515.1	837	170864	0	801	834	96	+	4	837	140612	141438
UN17074	Rsa1.0_02494.1	1113	132923	0	1024	1024	100	−	89	1112	6031	7054
UN17075	Rsa1.0_02911.1	952	142461	1.00E−121	224	225	99	+	639	863	129556	129780
UN17076	Rsa1.0_00369.1	1519	266462	1.00E−102	211	217	97	+	3	219	165218	165433
UN17077	Rsa1.0_00071.1	1036	345714	1.00E−158	328	343	95	−	3	345	317193	317535
UN17078	Rsa1.0_03951.1	791	19894	1.00E−155	296	302	98	−	365	666	10099	10400
UN17079	Rsa1.0_00765.1	923	65029	1.00E−144	260	260	100	−	664	923	25069	25328
UN17080	Rsa1.0_03612.1	694	21784	1.00E−138	290	302	96	+	392	692	11617	11918

UN17081	Rsa1.0_11019.1	769	5646	0	370	379	97	+	314	692	463	841
UN17082	Rsa1.0_07597.1	798	9055	0	554	562	98	−	239	798	7494	8053
UN17083	Rsa1.0_00465.1	1278	82138	1.00E−167	308	311	99	−	940	1250	15255	15565
UN17084	Rsa1.0_02433.1	889	158318	1.00E−92	173	173	100	−	277	449	131433	131605
UN17085	Rsa1.0_01335.1	1293	47894	0	459	460	99	−	1	460	3909	4368
UN17086	Rsa1.0_08230.1	834	7688	2.33E−156	263	263	100	−	1	263	5363	5625
UN17087	Rsa1.0_00131.1	943	432233	0	412	418	98	+	526	943	96212	96626
UN17088	Rsa1.0_00651.1	689	247766	0	544	557	97	−	1	555	178480	179032
UN17089	Rsa1.0_01004.1	1348	56975	0	562	583	96	−	748	1328	18969	19547
UN17090	Rsa1.0_00782.1	1690	64468	1.00E−102	240	259	92	−	1448	1690	32971	33228
UN17091	Rsa1.0_04198.1	1456	18504	0	628	628	100	−	829	1456	12882	13509
UN17092	Rsa1.0_00031.1	1403	179862	0	468	468	100	−	1	468	161267	161734
UN17093	Rsa1.0_08609.1	1295	7132	0	525	533	98	+	524	1056	5492	6024
UN17094	Rsa1.0_00136.1	1176	122464	1.00E−121	222	222	100	−	955	1176	68773	68994
UN17095	Rsa1.0_28441.1	1565	815	0	808	815	99	−	559	1373	1	815
UN17096	Rsa1.0_01724.1	985	40292	0	512	536	95	−	1	536	19668	20191
UN17097	Rsa1.0_01866.1	1384	37961	0	573	600	95	−	785	1378	12330	12926
UN17098	Rsa1.0_02883.1	1001	27035	0	376	386	97	−	266	651	3528	3913
UN17099	Rsa1.0_01559.1	721	43262	1.00E−166	343	355	96	−	369	721	28796	29148
UN17100	Rsa1.0_00230.1	1286	105389	0	596	603	98	+	640	1242	32582	33183
UN17101	Rsa1.0_00056.1	695	332604	1.00E−150	287	292	98	−	407	695	90001	90292
UN17102	Rsa1.0_00879.1	734	61297	0	708	720	98	+	14	732	59152	59869
UN17103	Rsa1.0_00082.1	1391	237584	0	762	772	98	−	1	770	228596	229366
UN17104	Rsa1.0_02608.1	1217	29613	0	462	470	98	−	604	1073	24637	25105
UN17105	Rsa1.0_00159.1	1604	431686	0	489	515	94	−	1092	1598	407448	407952
UN17106	Rsa1.0_01185.1	1119	149842	1.00E−148	267	267	100	−	397	663	132558	132824
UN17107	Rsa1.0_01943.1	1106	37042	1.00E−127	232	232	100	+	404	635	30663	30894
UN17108	Rsa1.0_03375.1	1204	23332	0	393	398	98	−	1	398	15136	15532
UN17109	Rsa1.0_05469.1	991	13644	1.00E−149	313	328	95	−	17	344	10994	11321
UN17110	Rsa1.0_01964.1	710	36702	0	554	559	99	−	153	710	20109	20666
UN17111	Rsa1.0_14714.1	1110	3134	8.00E−98	218	229	95	+	1	229	2908	3134
UN17112	Rsa1.0_06468.1	706	10958	0	416	425	97	−	283	704	6358	6782
UN17113	Rsa1.0_00036.1	1066	176186	1.00E−133	245	246	99	+	820	1065	30358	30603
UN17114	Rsa1.0_00065.1	1492	454616	1.00E−160	290	291	99	−	552	842	234141	234431
UN17115	Rsa1.0_00033.1	1225	515811	1.00E−178	328	332	98	+	879	1210	131617	131948
UN17116	Rsa1.0_04564.1	883	16837	0	454	456	99	+	428	883	3115	3570
UN17117	Rsa1.0_18565.1	1748	1573	1.00E−143	315	331	95	−	166	492	1014	1342
UN17118	Rsa1.0_00744.1	370	66084	3.00E−70	138	139	99	−	81	219	16390	16528
UN17119	Rsa1.0_00401.1	982	244410	0	387	395	97	−	585	978	178052	178445

UN17120	Rsa1.0_02252.1	1473	33375	0	539	540	99	−	1	540	8074	8613
UN17121	Rsa1.0_01638.1	1352	41857	1.00E−179	334	339	98	+	609	947	36262	36600
UN17122	Rsa1.0_05766.1	821	12708	0	662	668	99	+	148	815	3550	4217
UN17123	Rsa1.0_23487.1	1258	1045	0	730	762	95	−	411	1172	280	1041
UN17124	Rsa1.0_02404.1	972	31702	0	550	553	99	+	116	668	774	1324
UN17125	Rsa1.0_00418.1	1992	215377	0	490	498	98	−	345	842	4220	4717
UN17126	Rsa1.0_04027.1	782	19501	1.00E−120	225	227	99	+	54	280	3004	3230
UN17127	Rsa1.0_01540.1	1427	151890	0	489	490	99	+	938	1427	146855	147344
UN17128	Rsa1.0_03887.1	1390	20247	0	402	404	99	+	985	1388	4347	4750
UN17129	Rsa1.0_00484.1	1504	203294	0	782	802	97	−	1	802	56116	56910
UN17130	Rsa1.0_01887.1	1035	37564	0	504	531	94	−	1	527	26538	27062
UN17131	Rsa1.0_00323.1	1049	159650	0	401	417	96	−	1	412	32229	32645
UN17132	Rsa1.0_01783.1	995	39383	0	445	465	95	−	541	995	9401	9865
UN17133	Rsa1.0_00101.1	1000	267193	0	499	512	97	+	8	517	211676	212182
UN17134	Rsa1.0_02381.1	1860	31972	1.00E−151	362	389	93	+	4	391	13537	13910
UN17135	Rsa1.0_00254.1	1204	102600	1.00E−178	317	317	100	−	888	1204	74047	74363
UN17136	Rsa1.0_00861.1	1007	61759	1.00E−147	268	269	99	−	326	594	44124	44392
UN17137	Rsa1.0_01047.1	1068	55705	0	325	326	99	−	1	326	2010	2335
UN17138	Rsa1.0_00396.1	780	88242	1.00E−170	318	323	98	−	452	774	20219	20541
UN17139	Rsa1.0_03782.1	1161	20891	1.00E−171	315	317	99	+	846	1161	13909	14225
UN17140	Rsa1.0_00549.1	827	76473	0	360	363	99	+	3	365	26130	26492
UN17141	Rsa1.0_00128.1	1047	228675	0	393	393	100	−	653	1045	217824	218216
UN17142	Rsa1.0_00837.1	1384	62568	0	388	399	97	+	2	397	54460	54856
UN17143	Rsa1.0_00058.1	1407	159921	0	395	404	97	+	331	734	50728	51131
UN17144	Rsa1.0_00962.1	744	184909	0	380	380	100	+	2	381	12660	13039
UN17145	Rsa1.0_01242.1	1718	50066	0	357	359	99	+	94	452	4509	4867
UN17146	Rsa1.0_00351.1	1351	413096	1.00E−157	282	282	100	−	450	731	133363	133644
UN17147	Rsa1.0_26967.1	645	871	1.00E−180	355	367	96	+	279	645	308	668
UN17148	Rsa1.0_01328.1	816	48021	0	754	794	94	−	27	816	46022	46794
UN17149	Rsa1.0_00140.1	2017	121541	0	377	379	99	−	1093	1471	117693	118071
UN17150	Rsa1.0_00204.1	1139	109056	1.00E−165	422	464	90	−	2	456	59835	60283
UN17151	Rsa1.0_01326.1	842	48072	0	588	594	98	−	249	842	41525	42118
UN17152	Rsa1.0_04040.1	1366	19439	0	447	451	99	+	689	1139	9830	10280
UN17153	Rsa1.0_00769.1	865	64911	1.00E−160	338	352	96	+	516	865	41514	41861
UN17154	Rsa1.0_00240.1	1286	104175	0	569	594	95	−	1	591	92182	92773
UN17155	Rsa1.0_00577.1	1344	174827	0	386	388	99	+	954	1341	118729	119116
UN17156	Rsa1.0_07591.1	707	9060	1.00E−173	317	320	99	+	388	707	6177	6496
UN17157	Rsa1.0_09269.1	1237	6514	0	439	441	99	−	1	440	1082	1521
UN17158	Rsa1.0_08583.1	1321	7161	0	383	390	98	+	1	390	940	1329

UN17159	Rsa1.0_00204.1	1384	109056	1.00E-108	214	219	97	-	216	434	52555	52773
UN17160	Rsa1.0_05482.1	601	13595	1.00E-125	257	263	97	-	339	601	13336	13595
UN17161	Rsa1.0_03005.1	1155	26082	0	543	581	93	-	579	1155	23125	23688
UN17162	Rsa1.0_00170.1	894	179538	0	493	503	98	-	44	546	22985	23487
UN17163	Rsa1.0_00121.1	716	278029	1.00E-122	268	280	95	+	1	279	122848	123123
UN17164	Rsa1.0_00475.1	1620	81678	0	827	890	92	-	738	1620	39188	40051
UN17165	Rsa1.0_01249.1	1656	188432	1.00E-168	321	326	98	-	690	1015	176676	176997
UN17166	Rsa1.0_00002.1	1604	601661	0	445	454	98	+	884	1337	247632	248085
UN17167	Rsa1.0_00090.1	1322	273859	0	371	377	98	+	3	379	241913	242289
UN17168	Rsa1.0_06883.1	1808	10210	0	335	339	98	+	117	455	5507	5845
UN17169	Rsa1.0_01086.1	1068	54509	3.00E-66	148	153	96	+	916	1067	29892	30044
UN17170	Rsa1.0_00913.1	1799	59914	0	843	862	97	-	938	1799	24410	25271
UN17171	Rsa1.0_00045.1	1421	328694	1.00E-177	341	349	97	+	3	348	160674	161022
UN17172	Rsa1.0_01374.1	1566	47047	1.00E-171	321	325	98	-	1164	1488	27459	27782
UN17173	Rsa1.0_00189.1	1555	111830	0	1259	1278	98	+	2	1279	70151	71425
UN17174	Rsa1.0_02433.1	1088	158318	0	387	395	97	+	2	393	128385	128779
UN17175	Rsa1.0_42062.1	1390	533	0	441	445	99	-	4	447	11	455
UN17176	Rsa1.0_00002.1	2549	601661	0	952	953	99	+	1596	2547	558227	559179
UN17177	Rsa1.0_00522.1	1232	78103	1.00E-105	209	214	97	-	665	878	48991	49204
UN17178	Rsa1.0_16140.1	1343	2268	0	980	1003	97	+	3	997	1271	2268
UN17179	Rsa1.0_00033.1	844	515811	1.00E-135	264	269	98	+	3	270	130066	130334
UN17180	Rsa1.0_00656.1	837	69968	1.00E-174	391	414	94	+	1	411	45247	45651
UN17181	Rsa1.0_29097.1	1203	793	0	335	336	99	-	868	1203	458	793
UN17182	Rsa1.0_03120.1	1006	25269	1.00E-110	284	309	91	-	30	334	22032	22336
UN17183	Rsa1.0_15977.1	1367	2333	0	401	413	97	+	1	408	1157	1568
UN17184	Rsa1.0_02955.1	963	26484	1.00E-119	332	366	90	-	1	351	13654	14011
UN17185	Rsa1.0_00596.1	1730	73578	1.00E-147	265	265	100	+	1466	1730	24395	24659
UN17186	Rsa1.0_02869.1	977	27109	0	340	343	99	+	43	384	3356	3698
UN17187	Rsa1.0_00247.1	782	103408	0	629	630	99	-	1	630	14550	15179
UN17188	Rsa1.0_00344.1	1030	191774	0	472	480	98	-	1	478	108987	109465
UN17189	Rsa1.0_00678.1	1648	193163	0	659	666	98	+	29	694	161085	161750
UN17190	Rsa1.0_01157.1	830	52121	0	334	335	99	+	1	335	42503	42837
UN17191	Rsa1.0_07998.1	962	8105	1.00E-156	304	312	97	+	221	532	4011	4322
UN17192	Rsa1.0_04194.1	2327	132734	0	845	848	99	+	499	1346	116698	117545
UN17193	Rsa1.0_06468.1	1015	10958	0	993	1022	97	-	3	1015	6342	7359
UN17194	Rsa1.0_00477.1	1518	81556	0	448	451	99	+	1069	1518	17513	17962
UN17195	Rsa1.0_05303.1	2154	14211	0	1257	1263	99	+	3	1265	893	2155
UN17196	Rsa1.0_00605.1	696	73199	0	353	358	98	-	333	690	30515	30872
UN17197	Rsa1.0_01586.1	1288	42804	0	421	428	98	+	2	427	30177	30604

UN17198	Rsa1.0_00674.1	1260	193753	0	507	508	99	−	232	739	4532	5039
UN17199	Rsa1.0_02097.1	954	35036	0	943	953	98	+	2	954	15577	16528
UN17200	Rsa1.0_00601.1	1304	177045	0	1258	1275	98	−	3	1277	134182	135456
UN17201	Rsa1.0_00705.1	950	67505	1.00E−161	320	327	97	+	25	349	17701	18025
UN17202	Rsa1.0_00321.1	1628	95671	0	503	512	98	+	1116	1627	21420	21931
UN17203	Rsa1.0_02267.1	1317	33194	1.00E−109	249	262	95	+	1	258	28519	28778
UN17204	Rsa1.0_01085.1	853	54539	1.00E−178	322	324	99	−	248	571	13646	13969
UN17205	Rsa1.0_00187.1	2047	393093	0	454	454	100	−	1	454	48682	49135
UN17206	Rsa1.0_00033.1	1293	515811	1.00E−176	329	333	98	+	962	1293	356168	356500
UN17207	Rsa1.0_00789.1	1379	64233	0	384	386	99	+	995	1379	53473	53858
UN17208	Rsa1.0_02900.1	1490	26928	0	466	469	99	+	1	469	17172	17640
UN17209	Rsa1.0_04073.1	1339	19238	0	390	396	98	−	3	393	16757	17152
UN17210	Rsa1.0_00056.1	1259	332604	1.00E−101	203	208	97	+	836	1043	313940	314147
UN17211	Rsa1.0_00666.1	1209	69505	0	734	750	97	−	462	1209	41678	42418
UN17212	Rsa1.0_04846.1	1013	15739	0	569	570	99	+	442	1011	13280	13849
UN17213	Rsa1.0_00813.1	926	63202	0	536	548	97	−	1	547	51191	51732
UN17214	Rsa1.0_00097.1	1266	138858	0	600	632	94	−	653	1266	111486	112113
UN17215	Rsa1.0_00010.1	1499	294827	0	428	442	96	+	254	695	121872	122313
UN17216	Rsa1.0_02455.1	740	31262	1.00E−173	370	388	95	+	352	738	25408	25794
UN17217	Rsa1.0_00159.1	1616	431686	0	355	361	98	−	1	361	210507	210861
UN17218	Rsa1.0_00667.1	1051	69460	0	549	594	92	−	477	1051	23959	24549
UN17219	Rsa1.0_03302.1	1216	23898	0	361	362	99	−	849	1210	17711	18072
UN17220	Rsa1.0_03334.1	988	23659	1.00E−130	285	306	93	−	1	306	20257	20543
UN17221	Rsa1.0_01083.1	678	54617	1.00E−132	307	324	94	+	358	677	4951	5274
UN17222	Rsa1.0_07739.1	1855	8684	0	460	466	98	−	400	865	7273	7738
UN17223	Rsa1.0_02404.1	784	31702	0	467	482	96	−	300	776	843	1324
UN17224	Rsa1.0_02501.1	793	30764	0	459	482	95	−	274	749	10592	11072
UN17225	Rsa1.0_09200.1	800	6562	1.00E−143	280	286	97	−	476	761	6029	6313
UN17226	Rsa1.0_00476.1	1006	280966	1.00E−163	343	358	95	+	3	354	241508	241858
UN17227	Rsa1.0_01126.1	1344	53127	0	1203	1241	96	+	105	1344	51583	52822
UN17228	Rsa1.0_00093.1	983	214962	0	622	660	94	−	342	981	11159	11812
UN17229	Rsa1.0_16093.1	774	2288	0	349	355	98	−	65	419	1665	2019
UN17230	Rsa1.0_00476.1	536	280966	1.00E−113	319	357	89	−	2	354	7043	7380
UN17231	Rsa1.0_09695.1	851	6206	1.00E−159	324	336	96	+	512	845	603	934
UN17232	Rsa1.0_02455.1	523	31262	0	501	524	95	−	1	523	25426	25949
UN17233	Rsa1.0_01135.1	344	52799	1.00E−102	205	209	98	−	3	210	25251	25459
UN17234	Rsa1.0_02429.1	894	31527	2.00E−70	199	215	92	+	1	205	29106	29320
UN17235	Rsa1.0_00417.1	855	395068	0	347	355	97	−	5	359	14475	14826
UN17236	Rsa1.0_01000.1	990	57140	1.00E−114	225	230	97	−	761	990	37942	38171

UN17237	Rsa1.0_00381.1	652	89718	0	368	376	97	+	3	378	65776	66151
UN17238	Rsa1.0_00241.1	1431	104000	0	579	592	97	−	1	586	13582	14172
UN17239	Rsa1.0_00900.1	807	60367	1.00E−135	273	281	97	−	9	288	13294	13574
UN17240	Rsa1.0_02904.1	500	26883	8.00E−93	221	237	93	−	1	237	6100	6336
UN17241	Rsa1.0_05049.1	863	15025	0	507	509	99	−	1	509	4126	4634
UN17242	Rsa1.0_00524.1	705	169613	0	462	477	96	−	108	584	151835	152310
UN17243	Rsa1.0_00686.1	715	68511	0	404	426	94	+	10	435	27977	28402
UN17244	Rsa1.0_26967.1	453	871	2.00E−87	205	216	94	−	1	214	308	523
UN17245	Rsa1.0_00002.1	734	601661	0	403	424	95	−	38	461	36398	36814
UN17246	Rsa1.0_01371.1	731	47079	0	471	478	98	−	70	547	25451	25927
UN17247	Rsa1.0_07482.1	751	9278	1.00E−151	278	280	99	−	3	282	2864	3143
UN17248	Rsa1.0_00006.1	865	396176	0	493	499	98	−	367	865	301524	302022
UN17249	Rsa1.0_01146.1	518	163455	3.00E−74	184	198	92	+	1	198	41288	41485
UN17250	Rsa1.0_00749.1	792	65933	0	610	631	96	−	164	792	44807	45433
UN17251	Rsa1.0_00144.1	1316	120708	0	521	528	98	+	1	528	12334	12860
UN17252	Rsa1.0_02144.1	581	34630	1.00E−132	258	264	97	+	318	581	9429	9692
UN17253	Rsa1.0_02246.1	679	33435	1.00E−141	312	330	94	−	1	324	8242	8571
UN17254	Rsa1.0_00050.1	816	237174	1.00E−168	302	303	99	+	1	303	133684	133986
UN17255	Rsa1.0_00017.1	1036	752458	0	762	783	97	−	1	783	21347	22127
UN17256	Rsa1.0_00050.1	889	237174	1.00E−119	274	294	93	−	603	883	133690	133982
UN17257	Rsa1.0_10681.1	517	5757	1.00E−178	327	329	99	+	190	517	4487	4815
UN17258	Rsa1.0_00459.1	652	82580	1.00E−137	257	260	98	−	150	409	57153	57412
UN17259	Rsa1.0_00019.1	562	301669	1.00E−137	388	432	89	+	1	430	293082	293498
UN17260	Rsa1.0_00019.1	404	301669	1.00E−102	259	286	90	+	5	287	112778	113045
UN17261	Rsa1.0_00484.1	1448	203294	0	1008	1027	98	−	424	1448	126950	127973
UN17262	Rsa1.0_04377.1	970	17658	0	862	897	96	+	72	968	5228	6124
UN17263	Rsa1.0_00353.1	716	92242	5.00E−55	116	118	98	−	507	624	56359	56476
UN17264	Rsa1.0_00869.1	722	61627	0	379	380	99	+	1	380	53842	54221
UN17265	Rsa1.0_00123.1	637	325650	0	571	600	95	−	39	637	168360	168957
UN17266	Rsa1.0_03016.1	1450	26000	0	406	411	98	−	1038	1448	1133	1542
UN17267	Rsa1.0_00446.1	900	167829	1.00E−153	274	274	100	+	625	898	121449	121722
UN17268	Rsa1.0_00002.1	243	601661	1.00E−109	233	242	96	−	5	242	36553	36794
UN17269	Rsa1.0_00025.1	1634	277312	0	827	838	98	+	796	1633	271676	272513
UN17270	Rsa1.0_01016.1	780	181422	0	365	368	99	−	4	371	164149	164515
UN17271	Rsa1.0_00008.1	567	358124	1.00E−154	311	320	97	−	4	323	85294	85611
UN17272	Rsa1.0_02029.1	439	35857	1.00E−176	361	375	96	+	15	387	15219	15591
UN17273	Rsa1.0_05900.1	834	12295	1.00E−102	250	266	93	−	1	265	9713	9976
UN17274	Rsa1.0_04905.1	281	15507	1.00E−157	280	280	100	+	2	281	4974	5253
UN17275	Rsa1.0_00863.1	1133	254535	0	609	641	95	+	1	636	251930	252566

UN17276	Rsa1.0_03317.1	765	23782	0	695	742	93	−	3	736	7527	8247
UN17277	Rsa1.0_01335.1	786	47894	0	461	495	93	−	14	496	3909	4394
UN17278	Rsa1.0_05893.1	1590	12306	0	404	412	98	−	412	823	8140	8551
UN17279	Rsa1.0_08388.1	931	7436	0	457	483	94	−	452	931	2955	3434
UN17280	Rsa1.0_01558.1	914	43287	0	533	547	97	−	370	914	39092	39637
UN17281	Rsa1.0_00034.1	1349	260119	0	716	722	99	−	3	724	122108	122829
UN17282	Rsa1.0_02336.1	1047	32397	0	416	425	97	+	1	424	357	781
UN17283	Rsa1.0_01408.1	535	46383	0	511	527	96	−	7	532	34574	35100
UN17284	Rsa1.0_00014.1	660	348985	0	459	460	99	+	5	464	241604	242063
UN17285	Rsa1.0_00077.1	687	145005	1.00E−103	228	240	95	−	340	576	54043	54282
UN17286	Rsa1.0_00636.1	677	71113	2.00E−85	179	185	96	+	218	402	14731	14915
UN17287	Rsa1.0_00583.1	867	74309	1.00E−165	301	302	99	−	530	830	68461	68762
UN17288	Rsa1.0_01060.1	1578	55368	0	356	359	99	−	3	361	40139	40495
UN17289	Rsa1.0_03292.1	909	23977	1.00E−145	273	277	98	−	1	277	7108	7384
UN17290	Rsa1.0_00782.1	785	64468	2.00E−79	169	175	96	−	1	170	36483	36657
UN17291	Rsa1.0_00125.1	597	253043	1.00E−112	277	300	92	+	1	300	190696	190983
UN17292	Rsa1.0_00067.1	824	415055	0	735	743	98	+	83	823	177423	178165
UN17293	Rsa1.0_02989.1	731	26187	1.00E−149	268	268	100	+	248	515	3252	3519
UN17294	Rsa1.0_00682.1	1720	68680	0	564	575	98	−	1145	1719	7486	8060
UN17295	Rsa1.0_00654.1	3285	70012	0	627	633	99	−	2141	2769	41560	42191
UN17296	Rsa1.0_04104.1	603	19043	0	595	606	98	−	1	601	4942	5546
UN17297	Rsa1.0_02089.1	1258	35163	1.00E−166	321	329	97	+	928	1256	10642	10970
UN17298	Rsa1.0_00469.1	892	82084	0	416	432	96	−	463	892	45058	45487
UN17299	Rsa1.0_00744.1	686	66084	1.00E−174	309	309	100	−	378	686	18733	19041
UN17300	Rsa1.0_02593.1	715	29744	1.00E−116	251	261	96	−	452	711	2905	3164
UN17301	Rsa1.0_05491.1	1174	13554	1.00E−112	210	211	99	−	139	349	8427	8637
UN17302	Rsa1.0_02917.1	829	159058	0	816	830	98	+	1	829	144071	144900
UN17303	Rsa1.0_00042.1	1509	171507	0	382	397	96	−	3	393	90435	90831
UN17304	Rsa1.0_09695.1	984	6206	0	419	422	99	+	563	984	577	998
UN17305	Rsa1.0_00141.1	2158	121494	0	753	772	97	+	841	1608	104511	105282
UN17306	Rsa1.0_00705.1	474	67505	0	467	472	98	+	1	471	6457	6928
UN17307	Rsa1.0_00179.1	861	393324	0	515	526	97	−	342	861	45013	45538
UN17308	Rsa1.0_01096.1	1282	54266	1.00E−172	319	322	99	−	959	1280	8874	9194
UN17309	Rsa1.0_05319.1	1065	14158	1.00E−153	314	327	96	+	3	329	380	702
UN17310	Rsa1.0_03043.1	1324	130531	1.00E−118	229	233	98	−	212	444	129647	129879
UN17311	Rsa1.0_01376.1	1082	47004	0	478	481	99	+	602	1082	41027	41507
UN17312	Rsa1.0_03218.1	1724	24539	0	374	374	100	+	1	374	6444	6817
UN17313	Rsa1.0_01803.1	1039	173846	1.00E−171	429	461	93	+	584	1039	137460	137913
UN17314	Rsa1.0_00772.1	1155	64856	0	530	536	98	+	3	538	47045	47580

UN17315	Rsa1.0_05208.1	608	14504	1.00E-156	315	326	96	+	283	608	3908	4230
UN17316	Rsa1.0_00437.1	1369	84879	1.00E-102	210	217	96	−	29	245	15436	15652
UN17317	Rsa1.0_02103.1	865	35006	1.00E-151	303	311	97	−	21	329	24076	24386
UN17318	Rsa1.0_02671.1	1215	28887	1.00E-160	305	311	98	−	7	317	5055	5365
UN17319	Rsa1.0_00601.1	779	177045	1.00E-134	361	394	91	−	391	779	26119	26496
UN17320	Rsa1.0_08133.1	1297	7852	0	495	496	99	+	487	982	1307	1802
UN17321	Rsa1.0_01083.1	710	54617	1.00E-172	334	342	97	−	2	342	4951	5292
UN17322	Rsa1.0_08230.1	986	7688	1.00E-149	306	316	96	−	22	334	5325	5636
UN17323	Rsa1.0_06213.1	523	11478	8.00E-90	215	227	94	+	297	521	10901	11120
UN17324	Rsa1.0_02411.1	1413	31655	0	491	502	97	+	898	1399	5948	6441
UN17325	Rsa1.0_00281.1	1684	99908	0	1674	1685	99	−	1	1684	15586	17270
UN17326	Rsa1.0_00234.1	984	197060	1.00E-107	218	225	96	−	760	984	15854	16073
UN17327	Rsa1.0_00398.1	480	88007	1.00E-156	321	333	96	−	149	480	1596	1925
UN17328	Rsa1.0_00175.1	1692	166121	0	599	634	94	+	1071	1692	38440	39071
UN17329	Rsa1.0_03247.1	1267	24371	0	880	905	97	+	2	905	11640	12544
UN17330	Rsa1.0_00154.1	611	189461	1.00E-167	298	298	100	−	314	611	79485	79782
UN17331	Rsa1.0_04876.1	1132	15631	1.00E-141	279	286	97	−	846	1131	4222	4506
UN17332	Rsa1.0_03223.1	1067	24512	0	406	406	100	−	656	1061	9379	9784
UN17333	Rsa1.0_06258.1	610	11371	0	587	597	98	+	13	609	5683	6278
UN17334	Rsa1.0_00017.1	978	752458	0	389	409	95	−	573	978	138965	139373
UN17335	Rsa1.0_00157.1	1273	245832	0	622	631	98	+	642	1271	163052	163682
UN17336	Rsa1.0_00200.1	1191	162016	0	687	709	96	+	484	1190	108700	109406
UN17337	Rsa1.0_00767.1	814	64980	1.00E-160	326	339	96	−	5	337	18547	18882
UN17338	Rsa1.0_00218.1	1049	201480	0	379	383	98	−	1	382	173202	173584
UN17339	Rsa1.0_00211.1	1492	228189	0	450	468	96	−	6	472	201586	202046
UN17340	Rsa1.0_00033.1	801	515811	0	371	381	97	+	5	383	365245	365622
UN17341	Rsa1.0_00427.1	1045	189678	2.00E-98	201	207	97	−	303	509	10947	11153
UN17342	Rsa1.0_04793.1	684	15963	0	362	362	100	−	96	457	2167	2528
UN17343	Rsa1.0_02574.1	1325	29954	0	658	680	96	+	646	1325	27348	28009
UN17344	Rsa1.0_00034.1	803	260119	1.00E-132	254	258	98	+	549	803	89248	89505
UN17345	Rsa1.0_07236.1	1621	9680	0	408	418	97	+	769	1186	2465	2882
UN17346	Rsa1.0_00224.1	1243	327530	1.00E-101	275	307	89	+	33	325	204842	205139
UN17347	Rsa1.0_00900.1	873	60367	1.00E-159	290	292	99	+	58	349	12155	12446
UN17348	Rsa1.0_05890.1	998	12317	1.00E-150	301	310	97	+	3	312	11601	11904
UN17349	Rsa1.0_11019.1	1130	5646	0	373	383	97	+	357	739	463	845
UN17350	Rsa1.0_11599.1	944	5462	1.00E-159	292	293	99	−	618	910	4638	4929
UN17351	Rsa1.0_00257.1	946	102407	0	489	508	96	+	1	508	70304	70802
UN17352	Rsa1.0_00771.1	2458	64870	0	747	768	97	−	395	1162	47309	48076
UN17353	Rsa1.0_00045.1	1374	328694	1.00E-162	313	318	98	+	2	318	178691	179007

UN17354	Rsa1.0_02728.1	1015	28400	0	327	327	100	−	1	327	26853	27179
UN17355	Rsa1.0_00015.1	699	561803	1.00E−153	418	470	88	+	83	545	483083	483532
UN17356	Rsa1.0_01884.1	1811	37659	0	795	814	97	+	151	962	17507	18320
UN17357	Rsa1.0_01651.1	1299	41586	0	591	612	96	+	1	611	20826	21433
UN17358	Rsa1.0_01581.1	730	42833	0	458	459	99	−	10	468	28862	29320
UN17359	Rsa1.0_00596.1	959	73578	3.00E−97	236	248	95	−	22	265	24395	24640
UN17360	Rsa1.0_01997.1	1407	158416	0	648	662	97	+	746	1406	145642	146298
UN17361	Rsa1.0_00071.1	921	345714	0	436	460	94	+	477	921	142456	142908
UN17362	Rsa1.0_63369.1	967	362	1.00E−175	317	319	99	+	477	795	44	362
UN17363	Rsa1.0_09583.1	1061	6270	0	385	385	100	−	129	513	5886	6270
UN17364	Rsa1.0_06586.1	1243	10712	0	563	564	99	+	1	564	8594	9157
UN17365	Rsa1.0_00170.1	797	179538	1.00E−100	231	241	95	−	531	767	142306	142546
UN17366	Rsa1.0_01505.1	2008	44382	1.00E−180	458	499	91	+	1519	2008	38967	39454
UN17367	Rsa1.0_14772.1	1069	3077	0	1067	1070	99	+	1	1069	1242	2311
UN17368	Rsa1.0_00340.1	748	305782	0	419	428	97	−	90	511	186295	186722
UN17369	Rsa1.0_03521.1	1306	396204	1.00E−154	323	333	96	+	977	1306	139674	140005
UN17370	Rsa1.0_00429.1	1351	85559	0	722	723	99	−	1	723	61177	61899
UN17371	Rsa1.0_00135.1	939	178077	0	359	368	97	−	251	618	169208	169575
UN17372	Rsa1.0_01100.1	726	54113	0	425	445	95	−	289	726	33553	33997
UN17373	Rsa1.0_00247.1	1629	103408	0	951	1044	91	−	621	1629	13063	14090
UN17374	Rsa1.0_01335.1	826	47894	0	473	505	93	−	9	504	3909	4402
UN17375	Rsa1.0_00192.1	2259	278200	0	772	793	97	+	432	1222	41407	42196
UN17376	Rsa1.0_00148.1	1192	120234	0	932	935	99	−	1	935	63550	64484
UN17377	Rsa1.0_00284.1	1079	323267	0	726	750	96	+	330	1079	316783	317532
UN17378	Rsa1.0_03951.1	665	19894	1.00E−156	297	302	98	−	275	574	10099	10400
UN17379	Rsa1.0_04138.1	1011	18898	0	778	788	98	−	220	1007	3167	3953
UN17380	Rsa1.0_01761.1	1426	39717	0	890	924	96	+	410	1333	25093	26009
UN17381	Rsa1.0_04374.1	870	17670	0	446	453	98	+	422	870	7853	8305
UN17382	Rsa1.0_03077.1	1078	25558	1.00E−170	317	321	98	+	758	1078	8100	8417
UN17383	Rsa1.0_03818.1	835	20668	1.00E−145	292	301	97	+	5	299	9553	9853
UN17384	Rsa1.0_00092.1	1141	659932	1.00E−172	328	335	97	−	709	1043	13028	13362
UN17385	Rsa1.0_01344.1	981	142535	0	438	464	94	+	529	979	130981	131441
UN17386	Rsa1.0_04794.1	874	15960	0	384	393	97	−	9	397	7004	7395
UN17387	Rsa1.0_00397.1	874	88189	0	532	536	99	+	10	545	7409	7944
UN17388	Rsa1.0_06968.1	1513	10090	0	704	741	95	+	1	740	7686	8421
UN17389	Rsa1.0_00040.1	1922	324196	0	332	333	99	−	1	333	315220	315552
UN17390	Rsa1.0_00171.1	1471	115622	1.00E−140	276	282	97	+	12	293	90858	91138
UN17391	Rsa1.0_00336.1	1930	445051	0	482	484	99	+	876	1359	134298	134781
UN17392	Rsa1.0_03149.1	1753	24983	0	1392	1436	96	+	306	1734	13791	15224

UN17393	Rsa1.0_03634.1	1973	145192	0	370	384	96	-	1593	1972	133526	133901
UN17394	Rsa1.0_03887.1	1253	20247	0	379	394	96	+	861	1251	4347	4740
UN17395	Rsa1.0_00301.1	1228	365315	0	608	617	98	+	612	1228	304665	305281
UN17396	Rsa1.0_00476.1	803	280966	1.00E-144	272	275	98	+	530	803	242668	242942
UN17397	Rsa1.0_00179.1	905	393324	0	423	437	96	-	456	892	196266	196702
UN17398	Rsa1.0_03491.1	678	22541	1.00E-115	406	471	86	+	105	575	9065	9535
UN17399	Rsa1.0_01773.1	933	39546	0	392	397	98	-	535	931	27322	27718
UN17400	Rsa1.0_01513.1	1020	44234	0	534	561	95	+	6	556	28412	28969
UN17401	Rsa1.0_00923.1	1768	153678	1.00E-155	294	299	98	+	301	599	53741	54039
UN17402	Rsa1.0_01963.1	884	36719	0	641	651	98	+	5	655	5467	6117
UN17403	Rsa1.0_00101.1	992	267193	1.00E-152	273	273	100	+	7	279	131428	131700
UN17404	Rsa1.0_03163.1	1304	24894	0	487	502	97	+	801	1301	22793	23294
UN17405	Rsa1.0_03713.1	845	21221	0	433	447	96	-	401	845	10458	10904
UN17406	Rsa1.0_00123.1	642	325650	0	570	597	95	-	45	641	168363	168957
UN17407	Rsa1.0_17886.1	1717	1718	0	758	804	94	+	914	1715	1	794
UN17408	Rsa1.0_02215.1	1361	33822	1.00E-160	323	334	96	-	505	836	27167	27500
UN17409	Rsa1.0_00418.1	1069	215377	1.00E-107	257	270	95	+	807	1069	45045	45312
UN17410	Rsa1.0_00805.1	1447	63724	0	435	437	99	-	1011	1447	6952	7388
UN17411	Rsa1.0_00019.1	752	301669	1.00E-175	400	428	93	+	12	437	293080	293498
UN17412	Rsa1.0_00113.1	1252	131574	0	742	779	95	+	484	1252	52836	53613
UN17413	Rsa1.0_00270.1	528	101236	0	514	531	96	+	3	528	69420	69947
UN17414	Rsa1.0_01397.1	1319	183276	1.00E-167	332	341	97	-	3	343	175579	175916
UN17415	Rsa1.0_02228.1	1317	33688	0	384	384	100	+	1	384	10171	10554
UN17416	Rsa1.0_02029.1	1400	35857	0	579	581	99	+	819	1398	16287	16867
UN17417	Rsa1.0_15018.1	825	2889	1.00E-145	283	290	97	+	331	620	2384	2673
UN17418	Rsa1.0_02092.1	1077	35135	0	801	823	97	-	1	819	27152	27974
UN17419	Rsa1.0_01384.1	604	46803	1.00E-109	222	228	97	-	4	230	33810	34037
UN17420	Rsa1.0_00875.1	855	61371	0	809	853	94	-	3	855	6927	7779
UN17421	Rsa1.0_00848.1	955	152362	0	801	807	99	+	1	807	48987	49792
UN17422	Rsa1.0_04612.1	1236	16647	1.00E-164	305	309	98	+	667	975	14693	15001
UN17423	Rsa1.0_02683.1	1479	28679	1.00E-180	383	399	95	+	1076	1471	16865	17261
UN17424	Rsa1.0_03741.1	994	143933	0	591	605	97	+	391	994	4881	5484
UN17425	Rsa1.0_01737.1	731	40101	1.00E-141	254	254	100	+	476	729	17336	17589
UN17426	Rsa1.0_01310.1	1066	164719	1.00E-150	286	290	98	-	1	290	13319	13607
UN17427	Rsa1.0_00477.1	1342	81556	1.00E-139	300	312	96	+	1	310	10538	10848
UN17428	Rsa1.0_04630.1	743	16595	1.00E-141	385	423	91	+	1	402	14481	14895
UN17429	Rsa1.0_01326.1	755	48072	1.00E-158	391	421	92	-	335	754	43752	44166
UN17430	Rsa1.0_04511.1	1233	17032	1.00E-130	237	237	100	-	664	900	8958	9194
UN17431	Rsa1.0_01884.1	996	37659	0	717	738	97	-	1	738	17512	18249

UN17432	Rsa1.0_00856.1	1327	62037	2.00E-83	216	232	93	+	1015	1243	16952	17176
UN17433	Rsa1.0_12809.1	995	5119	0	457	458	99	−	1	457	4445	4902
UN17434	Rsa1.0_04238.1	1166	18352	1.00E-172	326	331	98	+	837	1166	2706	3036
UN17435	Rsa1.0_00583.1	843	74309	1.00E-141	327	346	94	−	502	841	68420	68762
UN17436	Rsa1.0_00231.1	1387	105381	1.00E-171	345	357	96	+	1	356	29946	30302
UN17437	Rsa1.0_02688.1	807	28644	0	513	519	98	+	292	807	26861	27379
UN17438	Rsa1.0_00017.1	1888	752458	1.00E-165	301	303	99	−	568	870	686521	686823
UN17439	Rsa1.0_00049.1	604	167018	7.00E-66	151	158	95	−	3	157	80313	80470
UN17440	Rsa1.0_00188.1	1339	112020	0	479	518	92	+	372	889	24708	25224
UN17441	Rsa1.0_00281.1	889	99908	0	414	439	94	−	461	889	24104	24539
UN17442	Rsa1.0_02400.1	635	31750	0	598	632	94	−	3	633	12369	12988
UN17443	Rsa1.0_07536.1	1113	9178	0	401	424	94	+	1	416	6471	6893
UN17444	Rsa1.0_08388.1	971	7436	0	558	576	96	−	404	971	2994	3563
UN17445	Rsa1.0_00197.1	863	109714	0	615	625	98	+	1	625	87932	88556
UN17446	Rsa1.0_02895.1	816	26979	1.00E-128	253	259	97	−	1	257	11384	11642
UN17447	Rsa1.0_03853.1	841	20447	1.00E-173	339	348	97	+	3	350	13326	13672
UN17448	Rsa1.0_00178.1	2004	114861	0	1118	1137	98	+	867	2002	26941	28077
UN17449	Rsa1.0_00679.1	800	68971	7.00E-76	151	153	98	−	564	716	65948	66100
UN17450	Rsa1.0_00476.1	1178	280966	1.00E-142	342	372	91	−	60	429	7043	7397
UN17451	Rsa1.0_01783.1	870	39383	1.00E-129	269	279	96	+	1	278	12978	13256
UN17452	Rsa1.0_06306.1	1087	11266	0	533	535	99	−	19	553	4950	5484
UN17453	Rsa1.0_00472.1	420	398134	1.00E-122	223	223	100	−	1	223	163465	163687
UN17454	Rsa1.0_02365.1	1009	32118	0	431	442	97	−	1	440	12194	12628
UN17455	Rsa1.0_00126.1	786	127534	1.00E-163	310	316	98	+	345	660	37046	37361
UN17456	Rsa1.0_02398.1	618	31766	1.00E-115	260	273	95	−	9	281	5418	5686
UN17457	Rsa1.0_00326.1	1075	171955	1.00E-127	263	271	97	−	807	1075	28314	28583
UN17458	Rsa1.0_01431.1	854	167877	1.00E-169	311	313	99	+	539	850	157684	157996
UN17459	Rsa1.0_02281.1	962	292576	1.00E-129	237	238	99	−	476	713	263640	263877
UN17460	Rsa1.0_04107.1	1158	19007	0	378	388	97	−	775	1158	6895	7282
UN17461	Rsa1.0_00065.1	1452	454616	0	602	634	94	+	1	628	26082	26712
UN17462	Rsa1.0_00190.1	1581	318240	0	590	595	99	−	821	1415	14943	15537
UN17463	Rsa1.0_00546.1	702	76523	0	505	506	99	−	1	506	11381	11886
UN17464	Rsa1.0_00463.1	888	189177	1.00E-152	292	297	98	+	589	884	181422	181718
UN17465	Rsa1.0_00557.1	759	75865	0	510	523	97	+	205	725	69508	70030
UN17466	Rsa1.0_01180.1	1172	51517	0	423	447	94	−	731	1172	32258	32704
UN17467	Rsa1.0_00291.1	977	172129	0	451	456	98	+	1	453	64277	64732
UN17468	Rsa1.0_00017.1	1694	752458	0	328	328	100	−	1329	1656	272081	272408
UN17469	Rsa1.0_01005.1	666	56967	0	608	640	95	−	1	637	37403	38029
UN17470	Rsa1.0_00002.1	835	601661	0	497	513	96	−	260	768	28847	29359

UN17471	Rsa1.0_03521.1	776	396204	0	494	515	95	+	266	776	269243	269756
UN17472	Rsa1.0_03356.1	936	23487	1.00E-155	323	338	95	+	477	814	20820	21157
UN17473	Rsa1.0_02246.1	2405	33435	0	1417	1472	96	+	944	2405	8242	9705
UN17474	Rsa1.0_01047.1	1207	55705	0	375	383	97	+	753	1135	2010	2392
UN17475	Rsa1.0_01218.1	745	50536	0	719	743	96	+	3	743	20143	20874
UN17476	Rsa1.0_00277.1	1831	100132	0	573	579	98	−	1	579	50424	50999
UN17477	Rsa1.0_00960.1	787	58199	1.00E-141	343	368	93	−	3	364	14176	14542
UN17478	Rsa1.0_00593.1	1048	143463	1.00E-145	329	352	93	−	662	1012	56991	57332
UN17479	Rsa1.0_02750.1	2114	28239	1.00E-107	242	253	95	−	1707	1956	25641	25892
UN17480	Rsa1.0_00019.1	1119	301669	0	423	436	97	+	685	1119	233346	233777
UN17481	Rsa1.0_08867.1	1334	6843	0	1184	1196	98	+	137	1332	1	1196
UN17482	Rsa1.0_00426.1	840	85668	2.00E-82	254	285	89	+	25	308	32312	32577
UN17483	Rsa1.0_00039.1	1873	296927	1.00E-149	281	284	98	+	1	284	47162	47443
UN17484	Rsa1.0_01397.1	904	183276	2.00E-57	120	122	98	+	253	374	173954	174075
UN17485	Rsa1.0_16832.1	1577	2019	1.00E-148	290	298	97	+	1280	1577	122	419
UN17486	Rsa1.0_00097.1	750	138858	0	359	366	98	+	1	366	26228	26593
UN17487	Rsa1.0_00060.1	1078	502678	0	426	436	97	+	644	1078	398264	398697
UN17488	Rsa1.0_04371.1	1028	17700	1.00E-140	300	315	95	−	68	382	2476	2787
UN17489	Rsa1.0_00526.1	918	169279	0	585	600	97	+	1	596	126177	126774
UN17490	Rsa1.0_00948.1	760	58620	1.00E-173	318	320	99	−	442	760	49928	50247
UN17491	Rsa1.0_00002.1	1052	601661	0	518	527	98	−	127	653	28644	29170
UN17492	Rsa1.0_00121.1	949	278029	9.00E-91	199	206	96	−	745	948	104309	104514
UN17493	Rsa1.0_00280.1	2022	99966	0	821	926	88	+	719	1644	28824	29749
UN17494	Rsa1.0_05863.1	818	12388	0	358	361	99	−	458	818	7089	7449
UN17495	Rsa1.0_03581.1	873	21965	0	664	690	96	−	3	692	1557	2229
UN17496	Rsa1.0_00067.1	972	415055	1.00E-126	268	281	95	+	211	491	226623	226903
UN17497	Rsa1.0_00821.1	1499	62891	0	351	352	99	−	1146	1496	48968	49319
UN17498	Rsa1.0_01543.1	1181	43645	0	567	619	91	−	397	1009	24990	25584
UN17499	Rsa1.0_00269.1	924	341668	0	353	359	98	+	566	924	45769	46123
UN17500	Rsa1.0_00037.1	1109	307838	0	717	736	97	+	1	736	293287	294022
UN17501	Rsa1.0_04140.1	1335	18879	1.00E-106	211	216	97	+	329	544	7286	7501
UN17502	Rsa1.0_03956.1	685	19872	0	348	348	100	−	332	679	16185	16532
UN17503	Rsa1.0_00013.1	970	292129	1.00E-177	315	315	100	−	656	970	204483	204797
UN17504	Rsa1.0_12642.1	1552	5165	0	491	506	97	−	215	720	18	523
UN17505	Rsa1.0_00173.1	904	288743	0	459	463	99	−	443	904	83946	84408
UN17506	Rsa1.0_01228.1	644	50238	6.00E-91	217	233	93	−	412	644	21404	21625
UN17507	Rsa1.0_05321.1	879	14154	1.00E-129	237	238	99	+	237	474	399	636
UN17508	Rsa1.0_01895.1	964	37512	0	385	386	99	−	2	387	10218	10603
UN17509	Rsa1.0_01076.1	1396	54835	0	542	542	100	+	534	1075	44925	45466

UN17510	Rsa1.0_02158.1	1518	154841	0	534	536	99	+	182	717	131881	132416
UN17511	Rsa1.0_02964.1	788	26407	3.00E-90	211	225	93	+	190	414	19004	19228
UN17512	Rsa1.0_00958.1	1891	58243	0	1247	1263	98	−	346	1608	2102	3364
UN17513	Rsa1.0_00092.1	1297	659932	1.00E-177	330	335	98	+	246	580	13028	13362
UN17514	Rsa1.0_00042.1	1414	171507	0	901	915	98	+	506	1414	161589	162503
UN17515	Rsa1.0_13498.1	1193	4764	0	417	435	95	−	1	434	1593	2024
UN17516	Rsa1.0_01026.1	1210	171624	1.00E-114	308	339	90	−	1	330	31953	32291
UN17517	Rsa1.0_05653.1	604	13043	0	572	604	94	−	1	601	10806	11405
UN17518	Rsa1.0_19257.1	712	1455	1.00E-159	366	392	93	+	319	710	108	487
UN17519	Rsa1.0_03605.1	759	21827	0	427	429	99	+	331	759	20342	20768
UN17520	Rsa1.0_00799.1	1501	63918	0	1364	1391	98	+	75	1455	38059	39447
UN17521	Rsa1.0_00055.1	709	520193	1.00E-124	253	260	97	+	450	709	202154	202409
UN17522	Rsa1.0_00251.1	799	303968	1.00E-173	359	373	96	−	1	372	147121	147488
UN17523	Rsa1.0_06336.1	910	11215	0	648	659	98	+	246	904	6954	7611
UN17524	Rsa1.0_00353.1	974	92242	3.00E-97	216	224	96	−	751	973	55708	55928
UN17525	Rsa1.0_01939.1	956	37124	0	364	375	97	−	1	375	33165	33533
UN17526	Rsa1.0_01419.1	1045	167402	0	813	833	97	−	215	1045	6718	7548
UN17527	Rsa1.0_02000.1	1300	36210	1.00E-155	282	283	99	−	1	283	4950	5232
UN17528	Rsa1.0_04358.1	1713	17759	0	1685	1720	97	−	3	1713	10290	12001
UN17529	Rsa1.0_00070.1	1987	150321	0	539	557	96	+	961	1513	115433	115989
UN17530	Rsa1.0_01069.1	814	55113	0	419	430	97	−	386	814	17637	18064
UN17531	Rsa1.0_07516.1	772	9218	0	758	763	99	+	1	762	6344	7105
UN17532	Rsa1.0_05124.1	1177	14790	1.00E-129	235	235	100	+	420	654	2110	2344
UN17533	Rsa1.0_02859.1	356	162118	1.00E-156	339	358	94	−	1	356	154282	154639
UN17534	Rsa1.0_06303.1	982	11268	1.00E-117	238	246	96	+	529	774	9882	10127
UN17535	Rsa1.0_02253.1	1351	33356	0	438	459	95	−	5	457	11611	12065
UN17536	Rsa1.0_14855.1	1198	3011	0	772	787	98	+	1	785	1172	1958
UN17537	Rsa1.0_01414.1	901	149508	0	349	352	99	−	9	360	115743	116094
UN17538	Rsa1.0_00008.1	821	358124	0	359	365	98	+	180	544	85289	85653
UN17539	Rsa1.0_00646.1	1183	70473	0	906	933	97	−	80	1009	4443	5375
UN17540	Rsa1.0_00163.1	1513	116847	0	1512	1513	99	+	1	1513	48101	49613
UN17541	Rsa1.0_03482.1	1004	22625	1.00E-178	361	374	96	+	2	372	4778	5150
UN17542	Rsa1.0_00067.1	1126	415055	1.00E-133	271	281	96	−	367	647	226623	226903
UN17543	Rsa1.0_01482.1	1072	44855	0	504	540	93	+	3	525	15512	16044
UN17544	Rsa1.0_01884.1	670	37659	0	408	415	98	−	72	486	17511	17925
UN17545	Rsa1.0_02490.1	1222	30885	0	342	348	98	−	439	786	5982	6329
UN17546	Rsa1.0_00293.1	1259	339294	0	674	676	99	+	54	729	177088	177763
UN17547	Rsa1.0_06123.1	901	11681	1.00E-102	189	189	100	+	3	191	11241	11429
UN17548	Rsa1.0_00153.1	772	192931	1.00E-108	229	236	97	+	538	772	168694	168928

UN17549	Rsa1.0_00472.1	545	398134	1.00E-150	269	269	100	-	208	476	162878	163146
UN17550	Rsa1.0_04194.1	1627	132734	0	636	683	93	-	953	1625	116870	117544
UN17551	Rsa1.0_00077.1	791	145005	0	502	506	99	+	286	791	82456	82959
UN17552	Rsa1.0_07250.1	920	9663	0	907	919	98	+	1	918	5357	6273
UN17553	Rsa1.0_00713.1	1481	158017	0	513	515	99	+	56	570	19379	19893
UN17554	Rsa1.0_03831.1	1036	20579	2.00E-98	267	292	91	-	760	1035	18129	18420
UN17555	Rsa1.0_02085.1	710	35198	1.00E-116	252	263	95	+	4	266	15843	16097
UN17556	Rsa1.0_00289.1	1367	98620	1.00E-123	254	264	96	+	48	311	12905	13168
UN17557	Rsa1.0_09661.1	834	6226	1.00E-141	264	267	98	+	568	834	1	267
UN17558	Rsa1.0_00651.1	948	247766	0	357	365	97	+	552	915	231957	232321
UN17559	Rsa1.0_00108.1	1116	478491	0	348	349	99	-	738	1085	134108	134456
UN17560	Rsa1.0_00067.1	1009	415055	0	341	343	99	+	667	1009	227217	227559
UN17561	Rsa1.0_03439.1	846	22930	0	767	767	100	+	80	846	14985	15751
UN17562	Rsa1.0_01362.1	992	47217	1.00E-124	252	259	97	-	695	953	28906	29163
UN17563	Rsa1.0_00251.1	775	303968	1.00E-149	297	306	97	+	423	725	147121	147426
UN17564	Rsa1.0_00089.1	1618	406142	0	984	1007	97	-	613	1618	322151	323152
UN17565	Rsa1.0_05311.1	814	14181	1.00E-163	330	340	97	-	4	342	3429	3767
UN17566	Rsa1.0_01039.1	1470	55946	0	395	399	98	+	3	401	37258	37653
UN17567	Rsa1.0_02688.1	780	28644	0	516	534	96	+	247	780	26861	27390
UN17568	Rsa1.0_07706.1	764	8752	0	592	600	98	-	165	764	8153	8752
UN17569	Rsa1.0_29951.1	1835	767	0	644	666	96	+	1175	1835	1	666
UN17570	Rsa1.0_01512.1	845	44234	1.00E-141	296	310	95	+	536	845	9997	10306
UN17571	Rsa1.0_00131.1	844	432233	1.00E-137	297	310	95	+	540	844	96212	96517
UN17572	Rsa1.0_00789.1	826	64233	1.00E-105	200	202	99	+	428	629	52566	52767
UN17573	Rsa1.0_00019.1	723	301669	2.00E-72	160	167	95	-	308	474	113991	114157
UN17574	Rsa1.0_03077.1	932	25558	1.00E-74	185	195	94	-	736	929	6795	6987
UN17575	Rsa1.0_01674.1	855	168300	0	507	523	96	+	335	855	19673	20190
UN17576	Rsa1.0_00786.1	1584	64285	0	389	391	99	+	1	390	52320	52710
UN17577	Rsa1.0_03965.1	1237	19828	0	518	534	97	-	699	1232	1289	1810
UN17578	Rsa1.0_00069.1	851	403561	0	489	511	95	+	5	515	353538	354037
UN17579	Rsa1.0_00331.1	1358	176754	0	868	887	97	-	474	1358	22206	23092
UN17580	Rsa1.0_03951.1	593	19894	1.00E-134	287	302	95	-	256	557	10099	10400
UN17581	Rsa1.0_02467.1	670	31141	1.00E-113	273	291	93	+	382	670	3808	4091
UN17582	Rsa1.0_00002.1	291	601661	4.00E-56	146	156	93	+	3	158	245979	246129
UN17583	Rsa1.0_00407.1	786	191544	0	718	756	94	-	35	784	155490	156225
UN17584	Rsa1.0_09422.1	775	6381	0	619	640	96	+	131	769	1654	2293
UN17585	Rsa1.0_00316.1	1272	96264	0	496	509	97	-	4	512	85542	86050
UN17586	Rsa1.0_01176.1	591	51638	0	395	405	97	-	1	402	28134	28538
UN17587	Rsa1.0_00094.1	782	139042	1.00E-138	312	334	93	-	3	323	99912	100245

UN17588	Rsa1.0_00870.1	975	61606	0	377	380	99	+	2	381	27510	27889
UN17589	Rsa1.0_03625.1	1689	21692	0	731	742	98	+	948	1689	14698	15436
UN17590	Rsa1.0_02267.1	893	33194	0	479	483	99	−	411	893	22282	22764
UN17591	Rsa1.0_01505.1	556	44382	1.00E−171	305	305	100	+	252	556	38967	39271
UN17592	Rsa1.0_01482.1	1047	44855	0	426	440	96	+	608	1047	16982	17414
UN17593	Rsa1.0_00524.1	960	169613	0	483	497	97	+	465	960	129773	130269
UN17594	Rsa1.0_00910.1	671	59975	0	585	586	99	−	1	586	30391	30976
UN17595	Rsa1.0_01977.1	1169	36559	1.00E−111	254	269	94	−	901	1169	9566	9833
UN17596	Rsa1.0_03254.1	999	24311	1.00E−159	292	293	99	+	1	293	2867	3158
UN17597	Rsa1.0_00008.1	839	358124	0	435	456	95	−	222	674	53991	54446
UN17598	Rsa1.0_00104.1	785	368722	0	390	393	99	+	394	785	265144	265536
UN17599	Rsa1.0_00068.1	682	151813	0	427	438	97	+	248	680	73640	74076
UN17600	Rsa1.0_05523.1	864	13438	0	709	713	99	−	152	864	9079	9791
UN17601	Rsa1.0_00752.1	603	65756	1.00E−129	322	345	93	+	3	342	12935	13277
UN17602	Rsa1.0_00108.1	1049	478491	1.00E−126	317	350	90	+	31	359	134108	134456
UN17603	Rsa1.0_02246.1	1305	33435	0	590	599	98	+	708	1305	7690	8288
UN17604	Rsa1.0_00427.1	1346	189678	1.00E−161	330	342	96	−	1009	1345	11818	12159
UN17605	Rsa1.0_09599.1	1231	6260	0	720	725	99	+	1	725	4431	5152
UN17606	Rsa1.0_00179.1	886	393324	1.00E−92	224	237	94	+	1	235	214015	214250
UN17607	Rsa1.0_00240.1	836	104175	0	515	538	95	−	5	541	92181	92715
UN17608	Rsa1.0_01068.1	1425	55208	0	480	493	97	+	934	1425	9670	10153
UN17609	Rsa1.0_01751.1	606	39806	1.00E−120	274	290	94	−	314	603	30348	30634
UN17610	Rsa1.0_00363.1	598	90968	1.00E−103	224	235	95	+	3	237	28268	28502
UN17611	Rsa1.0_00071.1	569	345714	0	459	479	95	+	1	478	336508	336985
UN17612	Rsa1.0_02750.1	841	28239	1.00E−101	253	270	93	+	1	266	25624	25892
UN17613	Rsa1.0_03028.1	779	25872	4.00E−71	143	145	98	−	142	286	21997	22141
UN17614	Rsa1.0_00923.1	863	153678	0	684	722	94	−	4	699	45727	46448
UN17615	Rsa1.0_01078.1	961	316910	0	941	971	96	+	1	961	136904	137871
UN17616	Rsa1.0_00646.1	601	70473	0	412	425	96	+	177	601	4452	4876
UN17617	Rsa1.0_02601.1	864	29654	0	386	403	95	+	462	863	26835	27237
UN17618	Rsa1.0_00300.1	1307	97747	0	633	636	99	+	292	927	31675	32310
UN17619	Rsa1.0_00451.1	2381	322533	0	1187	1193	99	−	1189	2381	9181	10373
UN17620	Rsa1.0_01377.1	834	46967	0	605	646	93	+	1	643	10023	10647
UN17621	Rsa1.0_03141.1	991	25061	0	399	415	96	+	3	413	15103	15517
UN17622	Rsa1.0_02158.1	765	154841	0	520	536	97	+	169	704	131881	132416
UN17623	Rsa1.0_15420.1	902	2614	1.00E−169	331	341	97	−	408	748	1557	1897
UN17624	Rsa1.0_00162.1	506	239935	1.00E−176	362	377	96	−	1	377	12315	12685
UN17625	Rsa1.0_00833.1	864	62621	1.00E−153	358	383	93	−	1	382	46926	47297
UN17626	Rsa1.0_00143.1	772	121313	0	704	718	98	+	3	715	30988	31705

UN17627	Rsa1.0_02396.1	1250	31783	1.00E-149	319	335	95	−	1	335	27105	27431
UN17628	Rsa1.0_01403.1	762	46453	5.00E-92	181	184	98	+	217	400	25846	26029
UN17629	Rsa1.0_02399.1	1652	31763	1.00E-141	287	295	97	−	1355	1643	14145	14438
UN17630	Rsa1.0_00816.1	1196	403350	0	563	573	98	+	5	577	132495	133066
UN17631	Rsa1.0_10348.1	1187	5880	1.00E-131	254	258	98	−	930	1187	478	734
UN17632	Rsa1.0_01146.1	1250	163455	0	878	890	98	−	360	1248	152929	153818
UN17633	Rsa1.0_04377.1	1046	17658	0	931	944	98	+	104	1046	5220	6160
UN17634	Rsa1.0_55790.1	539	410	1.00E-143	282	290	97	+	162	451	1	290
UN17635	Rsa1.0_02362.1	711	32190	2.33E-156	379	410	92	−	296	696	24739	25145
UN17636	Rsa1.0_04729.1	1000	16201	1.00E-133	248	249	99	+	3	251	11808	12055
UN17637	Rsa1.0_00323.1	1028	159650	0	441	442	99	−	587	1028	11013	11454
UN17638	Rsa1.0_02719.1	990	28428	0	706	730	96	+	262	990	9837	10554
UN17639	Rsa1.0_00228.1	1264	105511	0	1213	1228	98	+	3	1229	97601	98827
UN17640	Rsa1.0_00331.1	1492	176754	0	976	1013	96	−	482	1487	22081	23092
UN17641	Rsa1.0_00385.1	1331	89160	0	465	466	99	+	867	1331	15758	16223
UN17642	Rsa1.0_03069.1	1352	25615	0	549	564	97	−	398	960	18568	19131
UN17643	Rsa1.0_00744.1	644	66084	1.00E-124	258	268	96	+	5	269	18774	19041
UN17644	Rsa1.0_00295.1	1394	152597	0	457	458	99	−	1	458	87629	88086
UN17645	Rsa1.0_00119.1	650	467509	0	494	510	96	−	1	510	374988	375497
UN17646	Rsa1.0_00407.1	697	191544	0	437	451	96	−	44	493	182904	183344
UN17647	Rsa1.0_54025.1	957	422	0	411	422	97	+	244	662	1	422
UN17648	Rsa1.0_00449.1	1210	83863	0	391	395	98	−	806	1200	54568	54962
UN17649	Rsa1.0_15420.1	1763	2614	0	549	567	96	+	823	1389	1334	1900
UN17650	Rsa1.0_01180.1	1203	51517	0	460	465	98	−	741	1202	32240	32704
UN17651	Rsa1.0_01084.1	1523	54605	0	438	443	98	+	1079	1521	18255	18694
UN17652	Rsa1.0_00733.1	1130	66583	0	652	662	98	−	53	714	59548	60209
UN17653	Rsa1.0_00651.1	951	247766	0	364	365	99	−	29	393	231957	232321
UN17654	Rsa1.0_09200.1	941	6562	0	399	402	99	−	540	941	5913	6313
UN17655	Rsa1.0_00128.1	806	228675	3.00E-50	111	114	97	−	693	806	218103	218216
UN17656	Rsa1.0_01513.1	1028	44234	0	548	561	97	−	464	1024	28412	28969
UN17657	Rsa1.0_01696.1	1872	301850	0	844	861	98	+	925	1785	279164	280024
UN17658	Rsa1.0_00739.1	950	66155	1.00E-150	328	342	95	−	607	948	51979	52316
UN17659	Rsa1.0_01696.1	1145	301850	6.00E-77	200	213	93	+	934	1144	293478	293686
UN17660	Rsa1.0_00603.1	861	73299	0	703	718	97	−	7	724	42400	43117
UN17661	Rsa1.0_03077.1	1024	25558	1.00E-117	271	286	94	+	1	285	6704	6987
UN17662	Rsa1.0_03120.1	972	25269	2.33E-156	298	309	96	−	1	309	22032	22337
UN17663	Rsa1.0_01536.1	909	43747	0	620	622	99	+	1	622	18728	19349
UN17664	Rsa1.0_01389.1	1534	46716	0	383	399	95	+	182	575	27804	28202
UN17665	Rsa1.0_00391.1	975	183853	0	391	400	97	+	3	396	72481	72880

UN17666	Rsa1.0_01076.1	1058	54835	1.00E-179	355	363	97	-	11	370	39978	40340
UN17667	Rsa1.0_02405.1	832	31692	0	362	362	100	-	469	830	17022	17383
UN17668	Rsa1.0_03101.1	734	25410	0	561	568	98	+	3	570	17206	17773
UN17669	Rsa1.0_00237.1	884	235974	1.00E-159	284	284	100	-	599	882	4688	4971
UN17670	Rsa1.0_07506.1	775	9233	0	533	535	99	+	6	539	2669	3203
UN17671	Rsa1.0_00316.1	1252	96264	0	537	540	99	-	706	1245	83816	84355
UN17672	Rsa1.0_00472.1	671	398134	0	397	402	98	+	3	404	162745	163146
UN17673	Rsa1.0_02097.1	1395	35036	1.00E-174	310	310	100	-	1	310	19172	19481
UN17674	Rsa1.0_03831.1	1155	20579	1.00E-160	292	294	99	+	3	296	18127	18420
UN17675	Rsa1.0_00640.1	1225	70979	0	1200	1225	97	+	1	1225	16846	18067
UN17676	Rsa1.0_02144.1	1787	34630	0	678	690	98	-	78	766	2872	3558
UN17677	Rsa1.0_06648.1	911	10603	0	372	385	96	-	527	911	4432	4814
UN17678	Rsa1.0_00855.1	1355	62047	0	791	839	94	+	524	1355	22692	23522
UN17679	Rsa1.0_00622.1	822	71822	0	802	819	97	+	6	822	23241	24055
UN17680	Rsa1.0_01414.1	892	149508	1.00E-160	340	353	96	+	544	890	115743	116095
UN17681	Rsa1.0_00360.1	1289	91190	0	515	532	96	-	183	712	49756	50287
UN17682	Rsa1.0_00113.1	793	131574	1.00E-168	299	299	100	+	3	301	59749	60047
UN17683	Rsa1.0_02000.1	909	36210	1.00E-116	281	302	93	-	3	295	4950	5251
UN17684	Rsa1.0_00239.1	808	104332	1.00E-142	281	289	97	-	329	617	28438	28726
UN17685	Rsa1.0_04779.1	947	16008	1.00E-110	279	298	93	+	10	305	9472	9765
UN17686	Rsa1.0_05847.1	1465	12433	0	524	535	97	+	931	1464	8210	8743
UN17687	Rsa1.0_00139.1	897	357786	0	885	894	98	-	2	894	148941	149834
UN17688	Rsa1.0_00480.1	934	81325	0	461	468	98	+	1	468	30781	31248
UN17689	Rsa1.0_00729.1	712	66690	0	697	713	97	-	1	712	56546	57252
UN17690	Rsa1.0_00078.1	1354	175053	0	544	550	98	-	804	1353	130154	130703
UN17691	Rsa1.0_00092.1	990	659932	0	362	363	99	+	628	990	234653	235015
UN17692	Rsa1.0_01553.1	1065	43379	0	652	665	98	-	3	665	2337	3001
UN17693	Rsa1.0_00730.1	806	66673	0	413	423	97	+	2	422	28287	28709
UN17694	Rsa1.0_00717.1	904	67051	0	449	463	96	-	3	463	8485	8945
UN17695	Rsa1.0_03303.1	1129	23890	0	645	663	97	+	1	661	9589	10250
UN17696	Rsa1.0_00525.1	1328	78030	1.00E-151	274	275	99	-	875	1149	25800	26074
UN17697	Rsa1.0_00348.1	892	92818	0	424	437	97	+	2	436	49598	50033
UN17698	Rsa1.0_03834.1	607	20572	1.00E-166	370	391	94	-	228	606	17625	18013
UN17699	Rsa1.0_00753.1	1232	65705	0	368	373	98	-	3	374	1957	2329
UN17700	Rsa1.0_01743.1	692	39926	1.00E-177	328	331	99	+	362	692	12660	12989
UN17701	Rsa1.0_00635.1	925	175826	0	403	424	95	-	503	921	13997	14407
UN17702	Rsa1.0_00382.1	2220	251946	0	622	674	92	-	900	1561	34180	34848
UN17703	Rsa1.0_00239.1	809	104332	1.00E-152	285	289	98	+	196	484	28438	28726
UN17704	Rsa1.0_49554.1	1231	458	0	424	425	99	-	807	1231	26	450

UN17705	Rsa1.0_04200.1	934	18490	0	392	402	97	+	1	401	768	1169
UN17706	Rsa1.0_01126.1	914	53127	1.00E-162	308	313	98	-	604	914	14941	15253
UN17707	Rsa1.0_02685.1	784	28669	0	386	389	99	-	393	781	27822	28210
UN17708	Rsa1.0_02431.1	923	31504	0	485	502	96	-	396	895	17432	17922
UN17709	Rsa1.0_00121.1	1875	278029	0	547	547	100	-	1329	1875	9593	10139
UN17710	Rsa1.0_00485.1	1673	80937	0	860	883	97	-	8	890	71868	72738
UN17711	Rsa1.0_01293.1	1304	49139	0	604	623	96	+	1	611	34534	35155
UN17712	Rsa1.0_02185.1	1266	34150	0	422	436	96	+	36	469	20599	21034
UN17713	Rsa1.0_02429.1	1004	31527	1.00E-154	319	332	96	+	676	1004	12869	13197
UN17714	Rsa1.0_03543.1	1720	22185	0	430	437	98	-	1269	1705	1769	2205
UN17715	Rsa1.0_01209.1	1053	50668	1.00E-173	351	364	96	-	10	366	40043	40406
UN17716	Rsa1.0_00342.1	1359	93695	4.00E-97	268	298	89	-	1	297	1568	1848
UN17717	Rsa1.0_00024.1	1453	188018	1.00E-123	253	259	97	-	4	258	108783	109040
UN17718	Rsa1.0_05043.1	800	15044	1.00E-180	355	364	97	+	438	798	332	691
UN17719	Rsa1.0_03069.1	1354	25615	0	559	564	99	+	414	977	18568	19131
UN17720	Rsa1.0_02964.1	1519	26407	1.00E-154	326	339	96	-	2	340	20559	20893
UN17721	Rsa1.0_03623.1	841	21714	0	528	535	98	-	308	841	3269	3803
UN17722	Rsa1.0_01004.1	720	56975	0	352	355	99	-	364	718	16989	17342
UN17723	Rsa1.0_00277.1	1411	100132	0	768	778	98	-	558	1335	62070	62846
UN17724	Rsa1.0_00141.1	792	121494	1.00E-169	367	388	94	+	409	792	20677	21051
UN17725	Rsa1.0_08259.1	819	7641	1.00E-171	350	360	97	-	8	367	3089	3443
UN17726	Rsa1.0_00792.1	2073	64163	0	655	665	98	-	1281	1945	3157	3821
UN17727	Rsa1.0_04518.1	1829	17005	0	525	536	97	-	610	1145	11675	12209
UN17728	Rsa1.0_00744.1	669	66084	1.00E-131	274	284	96	+	4	284	18759	19041
UN17729	Rsa1.0_04238.1	1100	18352	1.00E-108	254	268	94	-	1	266	2706	2972
UN17730	Rsa1.0_03247.1	719	24371	0	708	719	98	+	1	719	11700	12418
UN17731	Rsa1.0_24929.1	718	960	1.00E-141	281	290	96	+	54	343	159	448
UN17732	Rsa1.0_00224.1	1286	327530	1.00E-180	332	336	98	+	29	364	204804	205139
UN17733	Rsa1.0_00175.1	1325	166121	0	685	690	99	+	621	1309	140783	141471
UN17734	Rsa1.0_01707.1	416	40686	0	384	388	98	+	1	387	25316	25703
UN17735	Rsa1.0_02606.1	980	29635	0	382	387	98	+	252	637	14421	14807
UN17736	Rsa1.0_01776.1	1236	39511	0	361	371	97	+	42	410	35159	35529
UN17737	Rsa1.0_08717.1	929	7008	0	603	606	99	-	2	607	2884	3489
UN17738	Rsa1.0_04104.1	575	19043	0	548	574	95	-	8	574	4942	5515
UN17739	Rsa1.0_03634.1	755	145192	3.00E-90	175	177	98	-	321	497	136040	136216
UN17740	Rsa1.0_09406.1	1153	6391	1.00E-145	288	293	98	+	700	990	6100	6391
UN17741	Rsa1.0_00125.1	493	253043	5.00E-94	187	191	97	+	1	191	190793	190983
UN17742	Rsa1.0_16978.1	1138	1964	1.00E-141	305	319	95	-	1	319	1215	1527
UN17743	Rsa1.0_01049.1	770	55655	1.00E-116	235	241	97	+	7	246	33850	34090

UN17744	Rsa1.0_00017.1	717	752458	2.33E-156	274	278	98	-	314	591	139458	139735
UN17745	Rsa1.0_05894.1	1346	12306	0	625	626	99	+	2	627	6858	7483
UN17746	Rsa1.0_01753.1	1478	39794	0	396	418	94	-	1	411	16947	17364
UN17747	Rsa1.0_00804.1	765	63726	1.00E-162	299	302	99	+	214	515	52476	52777
UN17748	Rsa1.0_01096.1	885	54266	1.00E-117	220	222	99	-	364	585	9756	9977
UN17749	Rsa1.0_01737.1	230	40101	8.00E-54	140	150	93	+	1	150	16476	16623
UN17750	Rsa1.0_03513.1	1305	22349	0	500	506	98	-	364	869	20286	20791
UN17751	Rsa1.0_16723.1	1388	2053	1.00E-147	309	321	96	-	1068	1388	1043	1356
UN17752	Rsa1.0_00025.1	780	277312	0	490	495	98	+	257	751	271079	271573
UN17753	Rsa1.0_00771.1	647	64870	0	460	464	99	-	184	647	47613	48076
UN17754	Rsa1.0_00077.1	541	145005	1.00E-108	199	199	100	-	343	541	54084	54282
UN17755	Rsa1.0_04512.1	703	17031	0	390	403	96	-	301	703	12545	12943
UN17756	Rsa1.0_04140.1	747	18879	1.00E-104	210	216	97	+	292	507	7286	7501
UN17757	Rsa1.0_02788.1	1067	27852	1.00E-136	320	344	93	+	736	1066	5593	5935
UN17758	Rsa1.0_02395.1	725	31785	0	359	361	99	-	1	361	8334	8692
UN17759	Rsa1.0_00459.1	616	82580	1.00E-132	258	264	97	+	270	533	57149	57412
UN17760	Rsa1.0_01026.1	807	171624	1.00E-176	316	317	99	-	3	319	31953	32269
UN17761	Rsa1.0_00132.1	1094	124299	1.00E-150	334	352	94	-	743	1090	108391	108742
UN17762	Rsa1.0_00040.1	892	324196	1.00E-169	321	326	98	+	567	892	308714	309038
UN17763	Rsa1.0_00782.1	1469	64468	1.00E-101	188	188	100	+	72	259	33382	33569
UN17764	Rsa1.0_00017.1	740	752458	2.00E-91	194	201	96	-	204	401	274013	274213
UN17765	Rsa1.0_00224.1	872	327530	1.00E-142	263	265	99	+	5	269	204875	205139
UN17766	Rsa1.0_00417.1	740	395068	1.00E-101	194	196	98	+	317	512	258984	259179
UN17767	Rsa1.0_00123.1	861	325650	0	356	359	99	-	1	359	227015	227373
UN17768	Rsa1.0_00725.1	1726	66757	0	474	489	96	-	54	542	10669	11155
UN17769	Rsa1.0_00116.1	724	365886	0	584	584	100	+	141	724	154724	155307
UN17770	Rsa1.0_02601.1	879	29654	0	397	403	98	-	2	404	26835	27237
UN17771	Rsa1.0_01213.1	978	50616	0	465	474	98	+	226	699	10242	10712
UN17772	Rsa1.0_00501.1	1763	79545	0	466	485	96	-	1	482	20922	21401
UN17773	Rsa1.0_09695.1	803	6206	1.00E-131	352	388	90	+	411	795	577	948
UN17774	Rsa1.0_00034.1	983	260119	0	553	572	96	-	1	572	122108	122679
UN17775	Rsa1.0_00240.1	864	104175	1.00E-113	216	219	98	-	264	482	54590	54808
UN17776	Rsa1.0_03741.1	622	143933	0	550	566	97	-	1	564	4881	5440
UN17777	Rsa1.0_01146.1	729	163455	1.00E-109	247	257	96	-	1	255	25092	25346
UN17778	Rsa1.0_00006.1	751	396176	1.00E-127	285	302	94	-	452	751	9146	9447
UN17779	Rsa1.0_02431.1	443	31504	0	401	418	95	+	13	429	17508	17922
UN17780	Rsa1.0_03713.1	766	21221	1.00E-176	360	373	96	-	396	766	10532	10904
UN17781	Rsa1.0_00230.1	786	105389	1.00E-122	315	334	94	-	460	784	2593	2925
UN17782	Rsa1.0_11366.1	722	5532	1.00E-119	249	259	96	+	1	259	2668	2923

UN17783	Rsa1.0_19324.1	1674	1447	1.00E-130	267	276	96	+	963	1237	1029	1304
UN17784	Rsa1.0_01389.1	795	46716	1.00E-127	299	316	94	−	5	315	30335	30647
UN17785	Rsa1.0_14772.1	383	3077	0	373	383	97	+	1	383	1557	1939
UN17786	Rsa1.0_14856.1	1314	3010	0	426	429	99	−	3	431	2281	2709
UN17787	Rsa1.0_03292.1	701	23977	1.00E-139	279	287	97	−	2	288	7104	7389
UN17788	Rsa1.0_00895.1	1249	60615	0	474	483	98	+	452	934	39139	39621
UN17789	Rsa1.0_00224.1	1141	327530	1.00E-147	264	264	100	+	2	265	204876	205139
UN17790	Rsa1.0_08293.1	791	7593	0	371	378	98	+	414	791	2270	2647
UN17791	Rsa1.0_02728.1	450	28400	1.00E-163	328	338	97	−	1	336	26853	27189
UN17792	Rsa1.0_00192.1	792	278200	0	397	415	95	+	12	426	41784	42196
UN17793	Rsa1.0_02501.1	505	30764	0	486	506	96	+	1	505	10542	11045
UN17794	Rsa1.0_00013.1	849	292129	1.00E-105	207	211	98	+	1	211	204587	204797
UN17795	Rsa1.0_00355.1	696	92038	0	531	576	92	−	129	695	77872	78429
UN17796	Rsa1.0_02964.1	728	26407	5.00E-95	213	225	94	+	198	422	19004	19228
UN17797	Rsa1.0_05893.1	765	12306	1.00E-174	343	350	98	−	419	765	8202	8551
UN17798	Rsa1.0_06968.1	633	10090	0	598	630	94	+	9	633	7775	8402
UN17799	Rsa1.0_00220.1	959	192936	1.00E-147	348	371	93	+	3	362	18977	19347
UN17800	Rsa1.0_03356.1	474	23487	1.00E-121	248	257	96	+	1	257	20196	20452
UN17801	Rsa1.0_00415.1	675	86795	1.00E-135	304	321	94	−	15	335	6755	7069
UN17802	Rsa1.0_00281.1	560	99908	0	439	462	95	+	11	465	24079	24539
UN17803	Rsa1.0_09583.1	937	6270	0	372	385	96	−	140	519	5886	6270
UN17804	Rsa1.0_00003.1	1382	340054	0	615	629	97	−	750	1378	257546	258174
UN17805	Rsa1.0_00190.1	461	318240	0	385	400	96	+	1	400	314387	314786
UN17806	Rsa1.0_01843.1	758	265131	0	479	481	99	−	13	493	25363	25843
UN17807	Rsa1.0_00127.1	866	127446	0	480	481	99	−	386	866	62999	63478
UN17808	Rsa1.0_00605.1	1022	73199	0	573	580	98	−	23	602	30975	31554
UN17809	Rsa1.0_02312.1	1064	32675	0	1060	1060	100	+	2	1061	15843	16902
UN17810	Rsa1.0_32336.1	746	704	2.00E-76	197	213	92	+	1	201	451	663
UN17811	Rsa1.0_01768.1	1307	138463	1.00E-141	308	321	95	−	987	1307	119986	120300
UN17812	Rsa1.0_03028.1	979	25872	1.00E-118	271	286	94	+	26	307	20174	20458
UN17813	Rsa1.0_00067.1	430	415055	1.00E-157	303	308	98	+	121	428	351902	352206
UN17814	Rsa1.0_00397.1	396	88189	0	380	395	96	+	2	396	7409	7802
UN17815	Rsa1.0_02054.1	970	35548	1.00E-142	271	276	98	+	203	478	27079	27354
UN17816	Rsa1.0_01761.1	761	39717	0	421	425	99	+	334	758	25093	25516
UN17817	Rsa1.0_00960.1	683	58199	1.00E-107	245	257	95	−	6	260	14176	14431
UN17818	Rsa1.0_00123.1	678	325650	1.00E-179	364	376	96	+	1	374	59913	60285
UN17819	Rsa1.0_01618.1	693	42118	0	485	505	96	−	2	500	1	505
UN17820	Rsa1.0_00300.1	782	97747	0	544	574	94	+	73	640	81510	82074
UN17821	Rsa1.0_01068.1	696	55208	1.00E-106	246	267	92	+	2	268	5080	5328

UN17822	Rsa1.0_09406.1	781	6391	1.00E-113	222	227	97	+	165	391	5073	5299
UN17823	Rsa1.0_00475.1	510	81678	1.00E-115	266	282	94	+	1	280	26917	27198
UN17824	Rsa1.0_27096.1	862	866	0	411	421	97	−	1	420	335	755
UN17825	Rsa1.0_02258.1	378	33307	1.00E-121	242	249	97	+	1	249	23474	23722
UN17826	Rsa1.0_00284.1	749	323267	0	582	600	97	−	3	599	316787	317386
UN17827	Rsa1.0_02398.1	298	31766	1.00E-103	233	245	95	−	3	247	5418	5659
UN17828	Rsa1.0_03356.1	615	23487	1.00E-172	330	338	97	−	123	460	20820	21157
UN17829	Rsa1.0_00439.1	1074	142776	0	571	585	97	+	490	1073	52240	52824
UN17830	Rsa1.0_03160.1	452	24916	2.00E-80	169	172	98	+	280	450	18697	18867
UN17831	Rsa1.0_10348.1	733	5880	1.00E-128	257	264	97	+	1	264	478	740
UN17832	Rsa1.0_00014.1	336	348985	1.00E-164	325	336	96	+	1	336	241629	241964
UN17833	Rsa1.0_00805.1	860	63724	1.00E-138	256	258	99	+	327	584	8627	8884
UN17834	Rsa1.0_00753.1	673	65705	1.00E-144	282	290	97	−	384	673	588	870
UN17835	Rsa1.0_38832.1	1608	579	1.00E-121	224	225	99	+	74	298	1	225
UN17836	Rsa1.0_06306.1	609	11266	0	521	535	97	−	17	551	4950	5484
UN17837	Rsa1.0_02281.1	804	292576	1.00E-116	222	224	99	−	459	681	263654	263877
UN17838	Rsa1.0_02608.1	1063	29613	0	430	465	92	−	603	1063	24658	25105
UN17839	Rsa1.0_11366.1	877	5532	1.00E-116	224	228	98	−	106	333	3977	4204
UN17840	Rsa1.0_10348.1	476	5880	2.33E-156	263	263	100	+	1	263	478	740
UN17841	Rsa1.0_00821.1	701	62891	1.00E-115	240	250	96	+	3	252	49070	49319
UN17842	Rsa1.0_01803.1	651	173846	1.00E-76	175	186	94	+	158	334	136428	136613
UN17843	Rsa1.0_02381.1	803	31972	1.00E-178	364	380	95	+	1	380	13541	13909
UN17844	Rsa1.0_00141.1	557	121494	1.00E-149	291	299	97	+	259	557	103874	104172
UN17845	Rsa1.0_30763.1	491	745	0	401	402	99	−	2	403	135	535
UN17846	Rsa1.0_00105.1	279	134138	1.00E-93	203	210	96	+	7	215	71490	71698
UN17847	Rsa1.0_00144.1	569	120708	0	505	528	95	+	20	546	12334	12860
UN17848	Rsa1.0_07536.1	1090	9178	0	399	423	94	+	5	417	6471	6893
UN17849	Rsa1.0_01047.1	862	55705	4.00E-96	200	207	96	+	208	414	1138	1344
UN17850	Rsa1.0_00848.1	855	152362	0	699	722	96	+	1	722	49072	49792
UN17851	Rsa1.0_01176.1	577	51638	0	389	404	96	+	174	575	28134	28534
UN17852	Rsa1.0_00630.1	1453	71281	0	520	520	100	−	229	748	62260	62779
UN17853	Rsa1.0_00270.1	454	101236	0	454	454	100	+	1	454	69450	69903
UN17854	Rsa1.0_01094.1	1145	167285	0	414	418	99	+	730	1145	12558	12975
UN17855	Rsa1.0_03873.1	1190	20360	0	701	715	98	−	35	746	9247	9957
UN17856	Rsa1.0_00289.1	832	98620	5.00E-86	180	186	96	+	417	602	14777	14962
UN17857	Rsa1.0_00111.1	741	390088	0	727	748	97	+	1	741	373996	374743
UN17858	Rsa1.0_02779.1	681	27968	0	664	683	97	+	1	681	3437	4119
UN17859	Rsa1.0_19861.1	822	1373	1.00E-154	277	277	100	−	11	287	448	724
UN17860	Rsa1.0_00055.1	704	520193	3.00E-65	148	155	95	+	512	666	180177	180331

UN17861	Rsa1.0_04371.1	839	17700	1.00E-151	349	374	93	−	1	374	2476	2841
UN17862	Rsa1.0_01707.1	1158	40686	0	398	401	99	+	426	826	25818	26218
UN17863	Rsa1.0_03741.1	987	143933	0	552	566	97	−	3	568	4881	5443
UN17864	Rsa1.0_02054.1	953	35548	1.00E-142	272	277	98	−	482	758	27078	27354
UN17865	Rsa1.0_00055.1	1158	520193	0	368	368	100	+	791	1158	106239	106606
UN17866	Rsa1.0_01325.1	1086	48131	0	631	652	96	+	429	1080	17437	18088
UN17867	Rsa1.0_01094.1	810	167285	1.00E-80	159	161	98	+	457	617	11257	11417
UN17868	Rsa1.0_00339.1	766	222974	0	391	394	99	+	3	395	210669	211062
UN17869	Rsa1.0_00689.1	982	68305	0	360	365	98	−	617	981	23514	23878
UN17870	Rsa1.0_02601.1	875	29654	0	400	416	96	−	1	416	26835	27249
UN17871	Rsa1.0_00041.1	691	186197	1.00E-156	324	338	95	+	357	691	157071	157408
UN17872	Rsa1.0_00729.1	1130	66690	0	616	627	98	−	1	627	56634	57260
UN17873	Rsa1.0_01811.1	554	38977	0	405	420	96	+	135	554	26394	26808
UN17874	Rsa1.0_07193.1	1044	9752	1.00E-166	302	304	99	+	741	1044	4357	4660
UN17875	Rsa1.0_01084.1	922	54605	1.00E-144	263	264	99	+	655	918	17676	17939
UN17876	Rsa1.0_00253.1	846	231292	0	448	455	98	+	2	456	204509	204960
UN17877	Rsa1.0_00903.1	1555	60228	0	413	415	99	−	1	415	9118	9532
UN17878	Rsa1.0_00033.1	705	515811	0	393	394	99	−	312	705	366451	366843
UN17879	Rsa1.0_02239.1	938	33534	1.00E-139	302	316	95	−	625	938	9639	9949
UN17880	Rsa1.0_00116.1	755	365886	5.00E-95	225	236	95	+	520	753	315711	315943
UN17881	Rsa1.0_00988.1	846	57525	1.00E-139	277	283	97	−	564	845	27392	27672
UN17882	Rsa1.0_04994.1	1007	15199	0	392	397	98	+	612	1007	5955	6351
UN17883	Rsa1.0_00652.1	922	70089	1.00E-107	229	239	95	−	671	909	22798	23033
UN17884	Rsa1.0_03831.1	942	20579	1.00E-109	271	291	93	+	3	279	18131	18420
UN17885	Rsa1.0_04462.1	422	17250	1.00E-144	323	343	94	−	1	343	13370	13711
UN17886	Rsa1.0_04194.1	777	132734	0	578	589	98	−	190	777	123400	123988
UN17887	Rsa1.0_03148.1	834	24989	0	380	399	95	+	368	766	21013	21408
UN17888	Rsa1.0_00141.1	765	121494	1.00E-140	318	339	93	+	432	765	20677	21003
UN17889	Rsa1.0_00253.1	604	231292	0	584	594	98	+	11	604	97765	98358
UN17890	Rsa1.0_00122.1	453	265937	1.00E-179	425	461	92	+	3	451	224882	225339
UN17891	Rsa1.0_03296.1	744	23969	0	389	394	98	+	3	396	14345	14738
UN17892	Rsa1.0_00501.1	300	79545	1.00E-131	283	297	95	+	4	299	58691	58987
UN17893	Rsa1.0_00437.1	629	84879	1.00E-103	227	240	94	−	397	629	64119	64358
UN17894	Rsa1.0_00884.1	901	61150	0	874	899	97	−	3	901	48091	48983
UN17895	Rsa1.0_00162.1	525	239935	0	384	398	96	+	128	525	12315	12703
UN17896	Rsa1.0_01311.1	756	48456	3.00E-96	222	233	95	+	65	295	20313	20542
UN17897	Rsa1.0_00501.1	1019	79545	0	557	573	97	−	448	1019	58698	59270
UN17898	Rsa1.0_02622.1	848	142402	1.00E-165	331	341	97	+	3	342	6671	7008
UN17899	Rsa1.0_13702.1	867	4456	1.00E-132	259	265	97	+	1	265	1756	2017

UN17900	Rsa1.0_01146.1	778	163455	0	740	779	94	+	1	771	152988	153766
UN17901	Rsa1.0_16906.1	721	1993	1.00E-134	258	263	98	−	1	263	4	266
UN17902	Rsa1.0_00622.1	817	71822	0	802	823	97	−	1	817	23235	24055
UN17903	Rsa1.0_01651.1	360	41586	1.00E-176	350	361	96	+	1	360	20839	21199
UN17904	Rsa1.0_01509.1	848	44258	0	503	516	97	+	331	845	16019	16530
UN17905	Rsa1.0_00083.1	846	442773	0	496	516	96	−	332	846	323904	324417
UN17906	Rsa1.0_02072.1	801	35401	1.00E-132	264	272	97	−	528	799	18082	18353
UN17907	Rsa1.0_03783.1	1107	20890	1.00E-144	295	303	97	−	794	1096	17848	18147
UN17908	Rsa1.0_01299.1	529	162028	1.00E-106	202	204	99	+	326	529	159762	159965
UN17909	Rsa1.0_00123.1	958	325650	0	651	677	96	+	277	953	272132	272808
UN17910	Rsa1.0_01232.1	705	155185	0	487	497	97	−	210	705	108724	109218
UN17911	Rsa1.0_10547.1	863	5807	1.00E-151	346	373	92	−	502	863	104	476
UN17912	Rsa1.0_00190.1	611	318240	4.00E-58	115	115	100	−	289	403	90400	90514
UN17913	Rsa1.0_00150.1	827	154416	1.00E-117	218	219	99	−	448	666	59697	59915
UN17914	Rsa1.0_03191.1	946	145762	1.00E-123	270	284	95	−	437	720	133541	133818
UN17915	Rsa1.0_01119.1	757	53459	0	475	489	97	+	269	757	1103	1589
UN17916	Rsa1.0_01874.1	784	37853	1.00E-116	255	268	95	−	422	688	24318	24585
UN17917	Rsa1.0_00240.1	1010	104175	1.00E-157	318	331	96	−	3	326	54892	55222
UN17918	Rsa1.0_00358.1	721	91293	7.00E-88	234	251	93	+	3	248	35095	35343
UN17919	Rsa1.0_04415.1	674	147889	1.00E-103	271	295	91	−	373	664	141075	141355
UN17920	Rsa1.0_14400.1	678	3430	0	617	669	92	−	1	660	1	662
UN17921	Rsa1.0_00220.1	874	192936	1.00E-141	274	278	98	+	2	278	143184	143460
UN17922	Rsa1.0_01926.1	831	37206	1.00E-154	276	276	100	+	556	831	6451	6726
UN17923	Rsa1.0_04218.1	750	18423	1.00E-151	340	358	94	−	1	358	12805	13157
UN17924	Rsa1.0_00501.1	1051	79545	0	595	599	99	−	451	1049	58672	59270
UN17925	Rsa1.0_24632.1	747	976	1.00E-142	280	288	97	+	389	676	570	857
UN17926	Rsa1.0_00635.1	930	175826	1.00E-175	380	399	95	+	3	397	14013	14407
UN17927	Rsa1.0_02625.1	726	139104	0	558	628	88	+	1	602	138419	139030
UN17928	Rsa1.0_01843.1	263	265131	1.00E-125	254	263	96	−	1	263	25510	25772
UN17929	Rsa1.0_00497.1	1149	79830	0	502	525	95	−	3	525	41564	42079
UN17930	Rsa1.0_02362.1	766	32190	1.00E-128	338	365	92	+	1	356	24783	25145
UN17931	Rsa1.0_00992.1	1762	57419	1.00E-138	253	254	99	−	1509	1762	1930	2183
UN17932	Rsa1.0_01016.1	747	181422	0	400	408	98	+	341	747	18736	19143
UN17933	Rsa1.0_04371.1	847	17700	2.33E-156	348	374	93	−	5	378	2476	2841
UN17934	Rsa1.0_16723.1	804	2053	0	362	367	98	+	1	366	991	1356
UN17935	Rsa1.0_00139.1	858	357786	0	827	852	97	−	4	854	148943	149794
UN17936	Rsa1.0_00657.1	772	69858	0	396	410	96	−	336	744	4196	4605
UN17937	Rsa1.0_00733.1	548	66583	1.00E-106	226	238	94	−	2	239	59548	59774
UN17938	Rsa1.0_04200.1	927	18490	0	395	402	98	+	1	402	768	1169

UN17939	Rsa1.0_48656.1	1042	465	1.00E-111	216	220	98	+	245	464	152	371
UN17940	Rsa1.0_00729.1	927	66690	0	607	622	97	−	4	625	56634	57253
UN17941	Rsa1.0_00719.1	650	66952	0	427	440	97	−	2	441	25642	26081
UN17942	Rsa1.0_01801.1	833	39094	0	554	566	97	−	21	579	20432	20997
UN17943	Rsa1.0_19861.1	805	1373	1.00E-120	258	271	95	+	535	805	448	712
UN17944	Rsa1.0_02399.1	798	31763	0	384	397	96	+	402	798	12071	12467
UN17945	Rsa1.0_07679.1	727	8811	0	348	348	100	+	29	376	1	348
UN17946	Rsa1.0_00067.1	813	415055	0	777	798	97	−	3	796	614	1411
UN17947	Rsa1.0_03783.1	1192	20890	1.00E-147	314	325	96	−	869	1189	17823	18147
UN17948	Rsa1.0_04846.1	969	15739	0	503	525	95	+	442	965	13280	13801
UN17949	Rsa1.0_19050.1	749	1488	1.00E-114	213	214	99	+	350	563	257	470
UN17950	Rsa1.0_01482.1	1111	44855	0	442	446	99	+	666	1111	16982	17427
UN17951	Rsa1.0_00055.1	744	520193	1.00E-116	237	245	96	+	3	247	103853	104097
UN17952	Rsa1.0_00132.1	725	124299	0	687	710	96	+	14	723	25491	26200
UN17953	Rsa1.0_55790.1	397	410	1.00E-160	289	290	99	+	25	314	1	290
UN17954	Rsa1.0_04386.1	1397	17627	0	511	521	98	+	881	1397	15113	15633
UN17955	Rsa1.0_00114.1	677	131330	1.00E-165	424	464	91	−	7	468	117617	118072
UN17956	Rsa1.0_00250.1	775	273771	0	368	372	98	−	103	473	186945	187315
UN17957	Rsa1.0_00010.1	1109	294827	1.00E-170	411	447	91	+	2	448	81197	81627
UN17958	Rsa1.0_01707.1	978	40686	0	391	401	97	−	178	578	25818	26218
UN17959	Rsa1.0_01377.1	509	46967	0	470	509	92	+	1	508	10117	10601
UN17960	Rsa1.0_00324.1	772	218805	1.00E-100	205	210	97	+	18	226	188072	188281
UN17961	Rsa1.0_00270.1	1028	101236	1.00E-129	298	315	94	−	718	1028	57803	58114
UN17962	Rsa1.0_01115.1	820	183545	0	439	459	95	+	371	820	174692	175150
UN17963	Rsa1.0_00128.1	761	228675	1.00E-102	198	201	98	−	558	758	216628	216828
UN17964	Rsa1.0_01549.1	819	43543	0	755	781	96	−	37	817	41915	42691
UN17965	Rsa1.0_03723.1	744	21173	3.00E-13	55	60	91	+	137	196	16991	17050
UN17966	Rsa1.0_00644.1	1641	136881	1.00E-179	359	377	95	−	3	379	41427	41785
UN17967	Rsa1.0_03574.1	464	22004	2.00E-77	264	299	88	+	3	289	17891	18178
UN17968	Rsa1.0_01934.1	761	37162	0	424	438	96	−	318	752	1854	2291
UN17969	Rsa1.0_00128.1	1102	228675	1.00E-161	307	312	98	−	789	1099	216103	216414
UN17970	Rsa1.0_00825.1	834	62855	0	829	833	99	+	2	834	48140	48972
UN17971	Rsa1.0_00407.1	595	191544	0	380	382	99	+	214	595	182904	183285
UN17972	Rsa1.0_23101.1	816	1071	0	379	383	98	−	427	809	1	383
UN17973	Rsa1.0_03016.1	916	26000	1.00E-167	368	384	95	−	9	388	3315	3694
UN17974	Rsa1.0_15840.1	773	2394	0	403	403	100	+	371	773	288	690
UN17975	Rsa1.0_00363.1	619	90968	1.00E-101	231	243	95	+	21	261	28260	28502
UN17976	Rsa1.0_00630.1	1109	71281	0	512	520	98	−	226	745	62260	62779
UN17977	Rsa1.0_00104.1	1475	368722	1.00E-174	368	383	96	+	1098	1475	197643	198022

UN17978	Rsa1.0_03303.1	542	23890	0	523	547	95	+	1	541	9575	10118
UN17979	Rsa1.0_00228.1	832	105511	0	797	825	96	+	3	826	97604	98424
UN17980	Rsa1.0_01126.1	819	53127	1.00E-137	264	268	98	+	2	268	14986	15253
UN17981	Rsa1.0_01874.1	834	37853	1.00E-113	254	268	94	-	421	687	24318	24585
UN17982	Rsa1.0_00953.1	805	145012	1.00E-169	360	379	94	+	3	376	11206	11581
UN17983	Rsa1.0_00194.1	597	110851	1.00E-108	319	360	88	+	253	590	56294	56652
UN17984	Rsa1.0_00083.1	818	442773	0	799	822	97	+	1	817	401735	402551
UN17985	Rsa1.0_03953.1	775	19889	0	693	728	95	+	1	709	6295	7021
UN17986	Rsa1.0_01730.1	577	40223	0	506	545	92	-	17	548	33434	33974
UN17987	Rsa1.0_02685.1	757	28669	0	361	364	99	-	392	755	27849	28210
UN17988	Rsa1.0_01783.1	788	39383	1.00E-159	294	297	98	+	1	297	12960	13256
UN17989	Rsa1.0_00730.1	448	66673	6.00E-81	162	165	98	+	1	165	28545	28709
UN17990	Rsa1.0_02470.1	772	31108	1.00E-55	123	127	96	+	319	445	24543	24669
UN17991	Rsa1.0_00739.1	1364	66155	0	506	516	98	-	598	1112	51804	52318
UN17992	Rsa1.0_07704.1	765	8754	0	517	544	95	-	227	765	3569	4101
UN17993	Rsa1.0_02092.1	747	35135	0	746	747	99	-	1	747	27231	27976
UN17994	Rsa1.0_00410.1	753	86957	5.00E-55	110	110	100	-	375	484	18062	18171
UN17995	Rsa1.0_49554.1	1177	458	0	384	397	96	-	779	1174	59	450
UN17996	Rsa1.0_00863.1	1173	254535	0	636	636	100	-	537	1172	251931	252566
UN17997	Rsa1.0_00123.1	899	325650	1.00E-159	327	339	96	+	450	786	305219	305556
UN17998	Rsa1.0_19861.1	514	1373	1.00E-119	265	281	94	-	1	281	448	722
UN17999	Rsa1.0_00037.1	1134	307838	0	599	618	96	+	1	618	16677	17290
UN18000	Rsa1.0_00490.1	1074	186713	0	621	623	99	-	450	1072	52225	52847
UN18001	Rsa1.0_00262.1	288	101906	9.00E-79	164	169	97	-	52	220	69618	69786
UN18002	Rsa1.0_00407.1	1039	191544	0	836	838	99	-	192	1028	155520	156357
UN18003	Rsa1.0_05199.1	1060	14529	0	537	584	91	+	9	592	7797	8369
UN18004	Rsa1.0_02693.1	782	28617	0	416	434	95	+	1	433	24856	25279
UN18005	Rsa1.0_01081.1	599	499226	0	592	594	99	+	6	599	456332	456925
UN18006	Rsa1.0_05199.1	891	14529	0	779	837	93	-	55	891	8003	8835
UN18007	Rsa1.0_00713.1	1165	158017	0	419	422	99	+	436	857	19367	19788
UN18008	Rsa1.0_04462.1	560	17250	0	417	435	95	+	94	528	13640	14074
UN18009	Rsa1.0_00449.1	809	83863	1.00E-173	344	356	96	-	3	358	56110	56465
UN18010	Rsa1.0_02312.1	761	32675	0	731	749	97	+	13	761	15856	16603
UN18011	Rsa1.0_08916.1	371	6792	1.00E-149	332	354	93	-	18	371	5995	6342
UN18012	Rsa1.0_00744.1	969	66084	0	423	443	95	-	2	442	54853	55295
UN18013	Rsa1.0_04793.1	659	15963	0	355	362	98	+	205	566	2167	2528
UN18014	Rsa1.0_00501.1	905	79545	0	449	466	96	-	11	476	20921	21386
UN18015	Rsa1.0_01206.1	880	50772	1.00E-154	326	341	95	+	1	341	38827	39166
UN18016	Rsa1.0_00360.1	797	91190	2.00E-94	210	220	95	-	1	220	49756	49974

UN18017	Rsa1.0_00127.1	543	127446	1.00E-133	240	240	100	+	234	473	35697	35936
UN18018	Rsa1.0_00917.1	687	182012	0	419	438	95	−	255	686	175692	176128
UN18019	Rsa1.0_00924.1	441	59524	3.00E-58	118	119	99	−	125	243	4369	4487
UN18020	Rsa1.0_01061.1	538	55349	0	532	540	98	−	1	538	29539	30075
UN18021	Rsa1.0_00038.1	835	314568	6.00E-67	186	200	93	+	3	198	254440	254637
UN18022	Rsa1.0_03158.1	726	24950	0	456	474	96	+	6	479	8090	8563
UN18023	Rsa1.0_00471.1	639	81897	1.00E-138	265	269	98	−	370	637	2892	3160
UN18024	Rsa1.0_05287.1	870	14264	0	445	467	95	+	279	745	5123	5587
UN18025	Rsa1.0_07877.1	733	8373	1.00E-154	309	320	96	+	420	733	5639	5958
UN18026	Rsa1.0_00876.1	711	61328	1.00E-134	283	297	95	+	67	357	49380	49676
UN18027	Rsa1.0_01126.1	837	53127	1.00E-125	262	271	96	−	570	837	14975	15245
UN18028	Rsa1.0_05332.1	866	14123	1.00E-129	271	283	95	+	186	468	8338	8620
UN18029	Rsa1.0_08133.1	733	7852	0	438	451	97	−	283	733	1352	1802
UN18030	Rsa1.0_16834.1	976	2019	0	514	526	97	−	442	966	606	1131
UN18031	Rsa1.0_00078.1	857	175053	0	380	390	97	−	23	412	131869	132258
UN18032	Rsa1.0_02362.1	695	32190	1.00E-119	361	400	90	−	298	684	24747	25145
UN18033	Rsa1.0_01233.1	1247	50184	0	433	445	97	−	1	445	42742	43186
UN18034	Rsa1.0_00651.1	1945	247766	0	711	711	100	+	1235	1945	80258	80968
UN18035	Rsa1.0_02710.1	1552	28492	0	662	662	100	−	1	662	20922	21583
UN18036	Rsa1.0_03751.1	833	21025	0	564	571	98	−	263	833	16682	17252
UN18037	Rsa1.0_04111.1	1556	18991	0	794	794	100	+	1	794	11224	12017
UN18038	Rsa1.0_00363.1	1397	90968	0	534	548	97	−	750	1297	67294	67840
UN18039	Rsa1.0_06495.1	624	10899	1.00E-140	290	304	95	+	4	296	5240	5540
UN18040	Rsa1.0_01945.1	1388	36989	0	409	426	96	−	1	421	6140	6557
UN18041	Rsa1.0_00304.1	964	97429	0	540	540	100	+	425	964	15850	16389
UN18042	Rsa1.0_02101.1	1169	35017	0	390	394	98	+	778	1168	14474	14867
UN18043	Rsa1.0_00198.1	1578	222963	0	617	627	98	−	627	1253	152016	152642
UN18044	Rsa1.0_00513.1	739	78579	1.00E-132	257	260	98	+	1	259	57408	57666
UN18045	Rsa1.0_00603.1	803	73299	0	798	800	99	+	2	801	58541	59340
UN18046	Rsa1.0_00547.1	1124	76521	0	471	476	98	−	647	1122	54262	54737
UN18047	Rsa1.0_01668.1	958	41299	1.00E-111	267	288	92	+	553	840	40402	40689
UN18048	Rsa1.0_00097.1	751	138858	0	445	448	99	+	306	751	59426	59873
UN18049	Rsa1.0_00144.1	811	120708	0	371	384	96	−	52	435	68637	69018
UN18050	Rsa1.0_00092.1	790	659932	1.00E-161	316	320	98	+	2	317	627090	627409
UN18051	Rsa1.0_01250.1	1210	49874	0	327	329	99	+	1	329	31238	31566
UN18052	Rsa1.0_01204.1	1403	50843	0	877	902	97	−	369	1270	28520	29421
UN18053	Rsa1.0_02040.1	886	35684	1.00E-157	316	326	96	+	560	885	13231	13555
UN18054	Rsa1.0_00153.1	994	192931	0	533	534	99	+	1	534	124372	124905
UN18055	Rsa1.0_00324.1	894	218805	0	893	893	100	+	1	893	214998	215890

UN18056	Rsa1.0_00434.1	1152	85171	0	370	373	99	+	782	1152	9575	9947
UN18057	Rsa1.0_02467.1	512	31141	0	487	527	92	+	3	509	15460	15978
UN18058	Rsa1.0_03898.1	880	20183	1.00E-111	225	231	97	+	1	231	12147	12375
UN18059	Rsa1.0_01766.1	2159	39617	0	399	424	94	−	1	403	26519	26942
UN18060	Rsa1.0_02926.1	1044	26716	0	803	805	99	+	1	804	2187	2991
UN18061	Rsa1.0_01016.1	1051	181422	0	500	508	98	+	402	907	18736	19243
UN18062	Rsa1.0_00009.1	1292	232558	0	411	420	97	+	191	606	8865	9284
UN18063	Rsa1.0_00107.1	1630	431659	0	720	720	100	+	424	1143	148415	149134
UN18064	Rsa1.0_01860.1	1616	38057	1.00E-131	285	302	94	+	3	293	21475	21772
UN18065	Rsa1.0_01678.1	1278	162174	0	417	422	98	+	857	1278	161267	161687
UN18066	Rsa1.0_00131.1	1268	432233	1.00E-169	342	356	96	+	913	1268	11596	11940
UN18067	Rsa1.0_00792.1	585	64163	1.00E-109	237	246	96	+	342	582	7358	7603
UN18068	Rsa1.0_04200.1	1432	18490	0	358	360	99	−	1	360	10181	10540
UN18069	Rsa1.0_00065.1	1556	454616	0	500	530	94	−	1032	1555	26185	26712
UN18070	Rsa1.0_00001.1	789	363028	3.00E-96	202	209	96	−	9	214	252923	253131
UN18071	Rsa1.0_01937.1	1071	37147	0	387	400	96	+	676	1071	21081	21477
UN18072	Rsa1.0_01881.1	923	37721	0	523	542	96	+	12	553	13978	14513
UN18073	Rsa1.0_01185.1	830	149842	2.00E-82	246	274	89	−	435	708	126678	126947
UN18074	Rsa1.0_00190.1	1927	318240	0	405	405	100	+	30	434	17920	18324
UN18075	Rsa1.0_12638.1	1599	5167	0	558	562	99	+	797	1358	4606	5167
UN18076	Rsa1.0_02067.1	885	35451	0	425	440	96	−	375	814	2132	2571
UN18077	Rsa1.0_00715.1	438	67116	5.00E-66	195	211	92	−	219	423	40371	40581
UN18078	Rsa1.0_00305.1	1659	97308	0	1612	1663	96	−	10	1655	15753	17399
UN18079	Rsa1.0_00106.1	1647	133916	0	651	674	96	+	36	709	91915	92585
UN18080	Rsa1.0_00030.1	611	250988	8.00E-81	232	262	88	+	9	249	123871	124132
UN18081	Rsa1.0_03241.1	1017	24423	0	775	787	98	−	233	1017	11435	12221
UN18082	Rsa1.0_14001.1	905	3933	0	397	403	98	+	39	441	1408	1810
UN18083	Rsa1.0_00724.1	867	66789	0	862	868	99	−	3	867	60470	61337
UN18084	Rsa1.0_00848.1	792	152362	1.00E-149	268	268	100	+	3	270	139681	139948
UN18085	Rsa1.0_01504.1	1316	189978	0	732	747	97	−	277	1023	25772	26517
UN18086	Rsa1.0_02623.1	855	29409	0	482	489	98	−	2	490	21515	22003
UN18087	Rsa1.0_05546.1	1523	13379	0	528	538	98	+	988	1523	5177	5710
UN18088	Rsa1.0_00663.1	897	164969	1.00E-171	330	337	97	+	3	339	126367	126702
UN18089	Rsa1.0_01101.1	1532	54031	1.00E-148	267	267	100	+	352	618	22500	22766
UN18090	Rsa1.0_01517.1	1461	44170	0	412	424	97	+	1036	1458	23723	24143
UN18091	Rsa1.0_00189.1	871	111830	1.00E-151	285	288	98	−	585	871	26274	26561
UN18092	Rsa1.0_00515.1	1293	78417	1.00E-107	211	214	98	−	3	216	50318	50530
UN18093	Rsa1.0_00740.1	1836	215972	0	1201	1209	99	+	625	1833	34795	36003
UN18094	Rsa1.0_01214.1	839	50599	0	432	442	97	+	396	835	35740	36180

UN18095	Rsa1.0_03143.1	744	25024	1.00E-138	289	302	95	+	166	467	11910	12211
UN18096	Rsa1.0_00307.1	1451	199003	0	375	381	98	+	1071	1451	70435	70815
UN18097	Rsa1.0_02782.1	1001	27929	1.00E-176	325	329	98	+	3	331	13477	13805
UN18098	Rsa1.0_01423.1	1968	46170	0	434	437	99	+	1533	1968	28713	29149
UN18099	Rsa1.0_02586.1	1709	29856	1.00E-145	277	282	98	−	339	620	8030	8311
UN18100	Rsa1.0_00706.1	1464	291570	0	543	559	97	+	219	777	181310	181868
UN18101	Rsa1.0_06102.1	1374	11740	1.00E-167	318	324	98	−	3	323	6539	6862
UN18102	Rsa1.0_00001.1	819	363028	2.00E-85	194	203	95	+	14	212	99180	99382
UN18103	Rsa1.0_01214.1	1226	50599	0	385	390	98	+	837	1226	46274	46660
UN18104	Rsa1.0_00218.1	1115	201480	5.00E-68	135	136	99	+	1	136	35114	35249
UN18105	Rsa1.0_00913.1	2232	59914	0	880	880	100	−	1352	2231	616	1495
UN18106	Rsa1.0_16335.1	1062	2193	1.00E-150	275	277	99	−	786	1062	933	1209
UN18107	Rsa1.0_00810.1	784	63289	0	537	544	98	+	239	782	6634	7176
UN18108	Rsa1.0_01182.1	1045	167304	0	355	360	98	−	688	1044	165185	165544
UN18109	Rsa1.0_02928.1	864	26680	0	474	479	98	−	381	858	25575	26053
UN18110	Rsa1.0_00194.1	729	110851	1.00E-132	249	252	98	+	3	254	83885	84136
UN18111	Rsa1.0_01999.1	1196	36237	0	1184	1194	99	+	3	1194	16309	17501
UN18112	Rsa1.0_00699.1	902	184398	0	878	899	97	−	4	902	146307	147204
UN18113	Rsa1.0_00061.1	1491	218685	0	1331	1372	97	+	126	1491	185101	186471
UN18114	Rsa1.0_02166.1	1204	34414	0	346	346	100	−	416	761	20683	21028
UN18115	Rsa1.0_00296.1	844	97854	1.00E-131	264	273	96	+	149	421	21946	22218
UN18116	Rsa1.0_05338.1	1283	144511	0	385	391	98	−	894	1283	9087	9476
UN18117	Rsa1.0_38645.1	687	582	0	354	354	100	−	20	373	1	354
UN18118	Rsa1.0_00037.1	2222	307838	0	1107	1133	97	+	1	1133	213823	214949
UN18119	Rsa1.0_00273.1	1665	326186	1.00E-137	267	273	97	−	513	785	152695	152967
UN18120	Rsa1.0_01997.1	1389	158416	1.00E-179	353	362	97	−	3	363	9828	10187
UN18121	Rsa1.0_04303.1	1439	18007	0	394	408	96	−	3	404	11136	11537
UN18122	Rsa1.0_01641.1	1140	188213	0	389	393	98	−	748	1140	174759	175151
UN18123	Rsa1.0_00105.1	945	134138	4.00E-99	196	200	98	+	170	369	67944	68143
UN18124	Rsa1.0_07482.1	945	9278	0	355	361	98	−	88	448	2864	3224
UN18125	Rsa1.0_00090.1	1207	273859	0	1194	1204	99	−	2	1205	161589	162792
UN18126	Rsa1.0_00331.1	591	176754	1.00E-104	219	228	96	−	186	413	159068	159295
UN18127	Rsa1.0_00173.1	733	288743	1.00E-148	288	294	97	+	166	458	23958	24251
UN18128	Rsa1.0_02848.1	611	27329	0	410	411	99	+	201	611	8638	9048
UN18129	Rsa1.0_02505.1	751	30703	1.00E-171	305	305	100	+	3	307	29167	29471
UN18130	Rsa1.0_00018.1	1318	410899	0	420	441	95	+	871	1307	68738	69175
UN18131	Rsa1.0_00207.1	1196	248736	0	596	609	97	−	1	609	64174	64779
UN18132	Rsa1.0_00376.1	928	90101	1.00E-138	253	254	99	+	602	855	7299	7552
UN18133	Rsa1.0_00838.1	836	153820	0	609	632	96	−	197	820	15935	16558

UN18134	Rsa1.0_00247.1	1097	103408	1.00E-159	388	421	92	-	3	415	66969	67376
UN18135	Rsa1.0_04108.1	707	19001	0	695	705	98	+	1	704	7385	8088
UN18136	Rsa1.0_02020.1	1072	173211	1.00E-135	260	264	98	+	1	264	162244	162506
UN18137	Rsa1.0_15257.1	1233	2724	0	1133	1233	91	-	2	1233	258	1490
UN18138	Rsa1.0_01164.1	619	51934	2.00E-90	271	297	91	-	2	294	43900	44189
UN18139	Rsa1.0_34719.1	1752	652	1.00E-145	273	277	98	+	868	1144	355	631
UN18140	Rsa1.0_02846.1	1528	27338	0	427	434	98	-	1	434	18619	19052
UN18141	Rsa1.0_02655.1	2698	29027	1.00E-170	317	320	99	+	942	1261	13789	14107
UN18142	Rsa1.0_00213.1	748	108167	1.00E-153	306	315	97	+	1	315	14641	14954
UN18143	Rsa1.0_03962.1	1187	19860	0	413	419	98	+	127	545	14221	14639
UN18144	Rsa1.0_00363.1	605	90968	1.00E-125	231	232	99	-	1	232	55162	55393
UN18145	Rsa1.0_00727.1	1023	66724	1.00E-156	295	300	98	-	601	900	30138	30437
UN18146	Rsa1.0_00066.1	1267	152418	7.00E-80	176	184	95	+	1082	1265	143576	143759
UN18147	Rsa1.0_05769.1	1828	12694	1.00E-143	273	278	98	-	2	279	6632	6909
UN18148	Rsa1.0_00228.1	697	105511	1.00E-168	307	308	99	-	390	697	50155	50461
UN18149	Rsa1.0_00628.1	815	214360	1.00E-125	237	240	98	-	204	443	66081	66320
UN18150	Rsa1.0_11080.1	1152	5623	0	379	380	99	+	395	774	1789	2168
UN18151	Rsa1.0_00287.1	969	98687	0	599	679	88	-	213	891	53406	54084
UN18152	Rsa1.0_06738.1	1712	10441	0	940	943	99	+	769	1710	4253	5195
UN18153	Rsa1.0_00329.1	1345	222544	0	669	689	97	-	657	1344	193046	193732
UN18154	Rsa1.0_03565.1	990	22062	1.00E-163	295	296	99	-	1	296	16874	17169
UN18155	Rsa1.0_08707.1	1031	7018	0	735	737	99	+	293	1029	1	737
UN18156	Rsa1.0_00275.1	1663	210104	0	352	360	97	+	81	439	149312	149671
UN18157	Rsa1.0_00772.1	1278	64856	0	493	501	98	-	417	917	28581	29081
UN18158	Rsa1.0_00857.1	1869	61904	0	724	743	97	-	824	1563	14129	14871
UN18159	Rsa1.0_01042.1	995	293264	0	575	592	97	+	403	993	141476	142066
UN18160	Rsa1.0_04489.1	748	17145	1.00E-106	225	233	96	+	521	748	14197	14429
UN18161	Rsa1.0_00886.1	714	167350	1.00E-149	274	276	99	-	2	277	8901	9176
UN18162	Rsa1.0_14220.1	1463	3652	1.00E-105	195	195	100	-	74	268	3458	3652
UN18163	Rsa1.0_05467.1	1238	13649	0	557	559	99	-	673	1231	6289	6847
UN18164	Rsa1.0_00136.1	1332	122464	0	665	679	97	+	5	682	56038	56713
UN18165	Rsa1.0_00208.1	989	108565	1.00E-126	395	446	88	+	1	434	28175	28605
UN18166	Rsa1.0_02494.1	1176	132923	1.00E-165	316	323	97	-	801	1123	107655	107977
UN18167	Rsa1.0_00427.1	1406	189678	0	413	421	98	-	986	1406	53848	54266
UN18168	Rsa1.0_00016.1	1420	831256	0	416	418	99	-	769	1186	253037	253454
UN18169	Rsa1.0_00107.1	1229	431659	0	429	434	98	+	1	433	14103	14536
UN18170	Rsa1.0_14436.1	1672	3393	0	494	515	95	+	536	1050	2867	3381
UN18171	Rsa1.0_01511.1	840	44235	0	664	668	99	+	2	669	19782	20449
UN18172	Rsa1.0_00533.1	987	77375	0	362	364	99	-	623	986	43209	43572

UN18173	Rsa1.0_04281.1	1603	18107	0	849	898	94	+	187	1084	15671	16568
UN18174	Rsa1.0_38435.1	1670	586	0	583	586	99	−	153	738	1	586
UN18175	Rsa1.0_00087.1	994	761073	1.00E−147	368	400	92	+	47	442	98854	99250
UN18176	Rsa1.0_00175.1	1921	166121	0	948	969	97	−	954	1921	84197	85165
UN18177	Rsa1.0_00253.1	1767	231292	0	587	617	95	+	755	1359	86583	87195
UN18178	Rsa1.0_02490.1	1241	30885	1.00E−180	341	348	97	+	440	787	5982	6329
UN18179	Rsa1.0_00326.1	1455	171955	1.00E−133	242	242	100	−	691	932	41797	42038
UN18180	Rsa1.0_00562.1	1334	75565	0	1167	1210	96	+	4	1201	23842	25047
UN18181	Rsa1.0_00163.1	1730	116847	1.00E−112	296	325	91	−	754	1061	64188	64512
UN18182	Rsa1.0_46357.1	885	487	1.00E−136	262	267	98	+	613	879	221	487
UN18183	Rsa1.0_00230.1	849	105389	0	394	394	100	+	1	394	2532	2925
UN18184	Rsa1.0_00092.1	1024	659932	2.00E−86	172	175	98	+	691	865	24958	25132
UN18185	Rsa1.0_06771.1	717	10388	0	699	709	98	−	5	712	2586	3293
UN18186	Rsa1.0_00235.1	813	154696	0	563	590	95	−	21	609	139087	139668
UN18187	Rsa1.0_00190.1	1133	318240	0	408	417	97	+	419	835	8210	8626
UN18188	Rsa1.0_04545.1	702	146799	0	335	337	99	−	366	702	7535	7869
UN18189	Rsa1.0_03673.1	996	21448	0	509	516	98	+	3	518	4077	4589
UN18190	Rsa1.0_00079.1	632	606987	1.00E−179	330	334	98	+	280	613	536507	536840
UN18191	Rsa1.0_00010.1	1387	294827	2.33E−156	283	288	98	+	1100	1386	291011	291298
UN18192	Rsa1.0_06840.1	1270	10286	1.00E−162	308	314	98	+	490	803	9049	9362
UN18193	Rsa1.0_00971.1	647	57973	0	621	622	99	−	3	623	19834	20455
UN18194	Rsa1.0_00105.1	799	134138	1.00E−168	384	411	93	+	398	798	91785	92188
UN18195	Rsa1.0_00278.1	935	231896	0	490	501	97	+	432	932	49869	50369
UN18196	Rsa1.0_04811.1	2200	15885	0	417	420	99	−	1469	1888	12070	12489
UN18197	Rsa1.0_02794.1	967	27820	1.00E−157	281	281	100	−	685	965	26676	26956
UN18198	Rsa1.0_04369.1	1673	17703	1.00E−168	338	349	96	−	1326	1673	1786	2134
UN18199	Rsa1.0_05714.1	1415	12850	1.00E−124	230	231	99	+	98	328	8830	9060
UN18200	Rsa1.0_00104.1	619	368722	0	614	619	99	+	1	619	76335	76952
UN18201	Rsa1.0_00088.1	1248	141341	0	693	703	98	−	547	1248	96279	96981
UN18202	Rsa1.0_00253.1	744	231292	1.00E−179	399	423	94	−	309	722	79564	79984
UN18203	Rsa1.0_00472.1	1883	398134	1.00E−163	322	332	96	−	16	347	309961	310292
UN18204	Rsa1.0_00067.1	1878	415055	0	923	944	97	+	3	946	181422	182365
UN18205	Rsa1.0_00662.1	1325	192183	1.00E−172	319	323	98	+	3	325	51398	51720
UN18206	Rsa1.0_00182.1	1489	142305	0	699	702	99	−	247	948	132407	133108
UN18207	Rsa1.0_00037.1	1904	307838	0	868	906	95	−	1021	1904	33020	33925
UN18208	Rsa1.0_00010.1	1430	294827	0	453	458	98	+	202	659	87840	88297
UN18209	Rsa1.0_02214.1	1286	161150	0	377	391	96	−	894	1283	13132	13521
UN18210	Rsa1.0_00719.1	1699	66952	1.00E−145	288	297	96	+	368	664	42432	42728
UN18211	Rsa1.0_02570.1	753	30000	0	565	573	98	−	166	738	1046	1615

UN18212	Rsa1.0_00087.1	1503	761073	1.00E-165	341	356	95	+	706	1061	433300	433655
UN18213	Rsa1.0_01996.1	885	36327	0	369	379	97	+	3	373	3452	3830
UN18214	Rsa1.0_01908.1	780	37427	0	518	521	99	+	260	780	16804	17324
UN18215	Rsa1.0_00069.1	813	403561	0	443	465	95	+	352	812	162099	162559
UN18216	Rsa1.0_01454.1	952	45440	1.00E-162	296	297	99	−	657	952	14232	14528
UN18217	Rsa1.0_01850.1	810	162224	2.00E-67	134	135	99	+	116	250	32969	33103
UN18218	Rsa1.0_07756.1	1481	8634	1.00E-177	327	331	98	+	1151	1481	5774	6104
UN18219	Rsa1.0_00805.1	1517	63724	0	783	829	94	−	29	851	57517	58345
UN18220	Rsa1.0_02786.1	800	27880	0	491	512	95	−	290	800	18684	19185
UN18221	Rsa1.0_01569.1	959	43029	0	675	676	99	+	1	676	22759	23434
UN18222	Rsa1.0_01376.1	984	47004	0	418	447	93	−	522	965	40139	40582
UN18223	Rsa1.0_00459.1	1350	82580	0	351	355	98	−	2	356	20821	21174
UN18224	Rsa1.0_01970.1	1259	36648	0	939	983	95	−	5	985	2521	3495
UN18225	Rsa1.0_00013.1	1552	292129	0	499	519	96	+	51	569	225224	225739
UN18226	Rsa1.0_00522.1	960	78103	2.00E-98	207	215	96	−	392	606	48991	49205
UN18227	Rsa1.0_09091.1	1153	6656	0	401	404	99	+	4	407	3708	4111
UN18228	Rsa1.0_00668.1	2559	69421	0	1132	1152	98	−	297	1448	23289	24437
UN18229	Rsa1.0_02454.1	2428	31263	0	1580	1584	99	+	96	1679	24099	25681
UN18230	Rsa1.0_00402.1	1951	385315	0	737	755	97	+	824	1578	88949	89697
UN18231	Rsa1.0_00179.1	1138	393324	0	412	414	99	−	724	1137	211659	212072
UN18232	Rsa1.0_01060.1	1383	55368	1.00E-111	223	229	97	−	894	1122	38736	38964
UN18233	Rsa1.0_00724.1	929	66789	0	605	619	97	−	317	927	4427	5045
UN18234	Rsa1.0_01139.1	1665	52601	0	431	441	97	−	1186	1626	48424	48864
UN18235	Rsa1.0_07344.1	1567	9528	0	848	858	98	−	1	858	5897	6754
UN18236	Rsa1.0_00414.1	862	202190	0	492	515	95	+	358	861	36763	37276
UN18237	Rsa1.0_01382.1	1258	46877	0	387	404	95	−	12	411	13890	14293
UN18238	Rsa1.0_04458.1	1211	17255	1.00E-179	319	319	100	+	668	986	7725	8043
UN18239	Rsa1.0_00606.1	1184	73150	1.00E-123	237	240	98	+	945	1183	32638	32877
UN18240	Rsa1.0_00500.1	970	272308	0	798	803	99	−	1	802	143320	144121
UN18241	Rsa1.0_03655.1	1839	21557	0	349	356	98	−	798	1153	8466	8821
UN18242	Rsa1.0_00440.1	1430	84723	1.00E-151	275	276	99	−	1	276	68557	68832
UN18243	Rsa1.0_01352.1	1779	47443	0	1388	1415	98	+	1	1413	7065	8479
UN18244	Rsa1.0_04189.1	1052	18584	1.00E-178	320	321	99	−	3	323	4224	4544
UN18245	Rsa1.0_01659.1	1090	164577	0	404	409	98	+	1	408	148556	148963
UN18246	Rsa1.0_01546.1	890	43586	0	857	887	96	−	7	888	30231	31115
UN18247	Rsa1.0_00078.1	1450	175053	1.00E-103	203	207	98	+	648	854	43779	43985
UN18248	Rsa1.0_32222.1	1201	707	0	364	365	99	−	1	365	1	364
UN18249	Rsa1.0_03718.1	1612	21195	0	473	503	94	−	1109	1611	450	935
UN18250	Rsa1.0_04985.1	1061	15232	0	1041	1055	98	−	7	1061	4170	5219

UN18251	Rsa1.0_00709.1	522	67329	0	497	520	95	−	1	520	13757	14268
UN18252	Rsa1.0_00493.1	1421	80007	1.00E−136	249	250	99	+	143	392	47779	48028
UN18253	Rsa1.0_00333.1	820	94500	1.00E−151	306	315	97	−	4	318	34823	35135
UN18254	Rsa1.0_01705.1	922	40710	1.00E−130	267	273	97	+	1	272	10482	10752
UN18255	Rsa1.0_00990.1	1428	57445	0	533	551	96	+	787	1333	42846	43396
UN18256	Rsa1.0_48565.1	1283	466	1.00E−119	306	338	90	+	5	320	108	439
UN18257	Rsa1.0_02802.1	1269	27736	0	631	657	96	+	1	648	10030	10678
UN18258	Rsa1.0_02916.1	1171	26809	0	341	343	99	−	10	352	1634	1976
UN18259	Rsa1.0_03874.1	979	20360	1.00E−165	310	314	98	+	49	360	381	694
UN18260	Rsa1.0_00734.1	2349	66508	0	718	732	98	+	1015	1745	27246	27974
UN18261	Rsa1.0_01788.1	1083	39296	1.00E−173	355	374	94	+	1	358	23988	24361
UN18262	Rsa1.0_01424.1	1246	46139	0	1222	1253	97	+	1	1246	30927	32176
UN18263	Rsa1.0_00038.1	1279	314568	1.00E−163	309	315	98	+	914	1228	194906	195220
UN18264	Rsa1.0_02791.1	1498	27832	0	892	924	96	−	26	935	20235	21158
UN18265	Rsa1.0_00176.1	777	240984	0	531	550	96	−	11	557	232410	232959
UN18266	Rsa1.0_00682.1	933	68680	0	542	546	99	+	388	933	2986	3531
UN18267	Rsa1.0_00848.1	1062	152362	5.00E−71	146	149	97	−	676	824	36953	37101
UN18268	Rsa1.0_13143.1	1539	4993	1.00E−144	334	354	94	+	1	353	702	1050
UN18269	Rsa1.0_01491.1	1415	44718	0	414	427	96	+	9	431	42154	42578
UN18270	Rsa1.0_02696.1	1233	28600	1.00E−164	310	314	98	−	920	1233	8254	8566
UN18271	Rsa1.0_00001.1	1239	363028	1.00E−172	318	321	99	+	922	1239	319592	319912
UN18272	Rsa1.0_00978.1	1936	57739	1.00E−165	310	312	99	+	1371	1680	35725	36036
UN18273	Rsa1.0_01104.1	1330	285480	0	388	390	99	−	915	1304	121482	121871
UN18274	Rsa1.0_00094.1	1576	139042	0	503	520	96	−	1033	1551	28273	28789
UN18275	Rsa1.0_05360.1	852	14053	1.00E−133	279	287	97	+	556	836	11840	12126
UN18276	Rsa1.0_00565.1	934	75420	0	534	537	99	−	3	538	21245	21781
UN18277	Rsa1.0_00032.1	1443	306326	0	1060	1072	98	−	330	1401	192599	193670
UN18278	Rsa1.0_00040.1	1895	324196	2.33E−156	269	271	99	+	88	358	312608	312878
UN18279	Rsa1.0_03702.1	825	21292	1.00E−128	233	233	100	+	338	570	7814	8046
UN18280	Rsa1.0_00224.1	1284	327530	0	946	959	98	+	327	1284	85779	86736
UN18281	Rsa1.0_00173.1	682	288743	0	668	678	98	−	4	678	261503	262180
UN18282	Rsa1.0_00822.1	1099	62891	4.00E−84	259	292	88	+	818	1093	32221	32509
UN18283	Rsa1.0_00141.1	993	121494	0	387	395	97	−	157	551	105801	106195
UN18284	Rsa1.0_01270.1	1217	49574	1.00E−152	390	427	91	−	796	1217	29160	29567
UN18285	Rsa1.0_05827.1	969	12503	0	721	739	97	−	89	827	6485	7221
UN18286	Rsa1.0_00171.1	1254	115622	0	400	402	99	+	3	403	33584	33984
UN18287	Rsa1.0_01949.1	1036	36960	0	402	418	96	+	604	1021	27763	28176
UN18288	Rsa1.0_03738.1	816	21071	1.00E−135	263	269	97	−	312	580	12425	12693
UN18289	Rsa1.0_00111.1	1006	390088	0	382	394	96	+	331	724	381107	381500

UN18290	Rsa1.0_00785.1	1599	64356	0	432	438	98	−	5	440	33751	34184
UN18291	Rsa1.0_03697.1	1532	21304	0	431	437	98	−	1096	1532	16445	16881
UN18292	Rsa1.0_01806.1	1542	39021	0	771	833	92	+	2	811	34071	34889
UN18293	Rsa1.0_01067.1	659	55230	1.00E−82	162	164	98	−	1	164	6328	6491
UN18294	Rsa1.0_00457.1	913	337301	1.00E−123	278	292	95	+	604	889	130569	130858
UN18295	Rsa1.0_01668.1	1069	41299	0	468	477	98	−	597	1069	33622	34096
UN18296	Rsa1.0_00365.1	1670	90902	0	985	1011	97	−	1	1010	59535	60545
UN18297	Rsa1.0_05505.1	1877	13500	0	464	487	95	−	1392	1875	7318	7801
UN18298	Rsa1.0_01146.1	682	163455	1.00E−161	328	339	96	+	341	679	162766	163101
UN18299	Rsa1.0_12642.1	1536	5165	0	497	504	98	+	834	1337	23	523
UN18300	Rsa1.0_03160.1	873	24916	0	866	871	99	+	3	873	2929	3799
UN18301	Rsa1.0_00645.1	2251	189716	0	1192	1204	99	−	4	1206	23763	24966
UN18302	Rsa1.0_00200.1	1495	162016	0	816	871	93	+	581	1445	85676	86546
UN18303	Rsa1.0_04811.1	1275	15885	0	402	404	99	−	872	1275	1248	1651
UN18304	Rsa1.0_00010.1	1148	294827	0	422	435	97	+	708	1142	244411	244838
UN18305	Rsa1.0_00476.1	1408	280966	0	491	493	99	−	771	1263	44981	45473
UN18306	Rsa1.0_00351.1	1057	413096	0	403	419	96	+	621	1038	3047	3457
UN18307	Rsa1.0_03448.1	921	22844	1.00E−169	313	317	98	−	340	656	5438	5754
UN18308	Rsa1.0_00071.1	687	345714	1.00E−101	268	291	92	+	397	687	22598	22875
UN18309	Rsa1.0_00271.1	773	101125	1.00E−160	311	319	97	+	218	536	60726	61044
UN18310	Rsa1.0_00143.1	736	121313	0	723	736	98	−	1	736	32757	33491
UN18311	Rsa1.0_00214.1	1394	107508	1.00E−179	339	345	98	+	1051	1392	42267	42611
UN18312	Rsa1.0_01435.1	912	45942	1.00E−126	255	263	96	+	3	265	24576	24835
UN18313	Rsa1.0_00037.1	2518	307838	0	964	994	96	−	562	1550	180789	181781
UN18314	Rsa1.0_00611.1	827	72410	0	436	455	95	−	370	823	15018	15467
UN18315	Rsa1.0_00323.1	1216	159650	1.00E−132	257	263	97	+	322	584	43088	43350
UN18316	Rsa1.0_01375.1	863	47038	9.00E−60	124	126	98	−	395	520	13143	13268
UN18317	Rsa1.0_00632.1	1330	183777	0	344	347	99	+	986	1330	867	1212
UN18318	Rsa1.0_00185.1	1046	248445	1.00E−159	288	289	99	+	756	1044	4378	4666
UN18319	Rsa1.0_00642.1	1412	201230	0	599	648	92	+	781	1408	158305	158949
UN18320	Rsa1.0_00187.1	961	393093	0	959	961	99	−	1	961	355923	356883
UN18321	Rsa1.0_00335.1	872	94371	1.00E−126	263	270	97	−	603	871	33581	33848
UN18322	Rsa1.0_00082.1	1253	237584	0	1211	1268	95	−	2	1247	46737	48000
UN18323	Rsa1.0_02860.1	856	27207	1.00E−163	308	313	98	+	161	473	16123	16432
UN18324	Rsa1.0_00003.1	1371	340054	0	592	593	99	+	779	1371	263741	264333
UN18325	Rsa1.0_05977.1	1081	12056	0	369	371	99	−	589	959	8430	8799
UN18326	Rsa1.0_03384.1	898	23291	0	831	848	97	+	7	851	10618	11465
UN18327	Rsa1.0_04936.1	846	15403	1.00E−104	199	200	99	+	590	788	8752	8951
UN18328	Rsa1.0_00920.1	1511	601703	0	554	567	97	+	945	1511	35940	36496

UN18329	Rsa1.0_02105.1	1215	156953	3.00E-91	174	175	99	+	744	918	8913	9087
UN18330	Rsa1.0_00081.1	1736	143261	1.00E-158	286	287	99	-	1450	1736	140556	140842
UN18331	Rsa1.0_06341.1	1541	11209	0	373	383	97	+	1	380	2316	2698
UN18332	Rsa1.0_01635.1	683	41865	0	645	645	100	+	35	679	15909	16553
UN18333	Rsa1.0_02281.1	1391	292576	0	349	354	98	+	3	356	1968	2321
UN18334	Rsa1.0_00043.1	835	264937	1.00E-105	218	225	96	+	49	273	243772	243993
UN18335	Rsa1.0_00990.1	1318	57445	0	1250	1322	94	-	3	1302	55447	56753
UN18336	Rsa1.0_00344.1	1683	191774	0	1103	1130	97	+	552	1681	129614	130743
UN18337	Rsa1.0_00071.1	1062	345714	0	370	382	96	+	369	750	311950	312331
UN18338	Rsa1.0_00481.1	1131	81316	1.00E-138	501	585	85	+	282	866	29295	29879
UN18339	Rsa1.0_00476.1	1474	280966	0	406	416	97	+	1	416	245467	245880
UN18340	Rsa1.0_04561.1	1660	16849	1.00E-117	250	262	95	+	30	286	5399	5660
UN18341	Rsa1.0_07919.1	1388	8261	1.00E-167	349	365	95	+	232	596	250	611
UN18342	Rsa1.0_74475.1	1433	308	1.00E-158	283	283	100	+	1	283	26	308
UN18343	Rsa1.0_02766.1	1629	28128	0	586	592	98	-	571	1159	22540	23131
UN18344	Rsa1.0_00247.1	1458	103408	0	544	576	94	-	3	574	30209	30767
UN18345	Rsa1.0_00081.1	1033	143261	1.00E-151	307	317	96	-	1	313	134327	134643
UN18346	Rsa1.0_11907.1	913	5374	1.00E-179	430	455	94	-	1	445	1101	1551
UN18347	Rsa1.0_36803.1	1539	613	1.00E-179	322	323	99	+	1	323	291	613
UN18348	Rsa1.0_01092.1	1177	54348	0	1149	1175	97	+	3	1175	47736	48909
UN18349	Rsa1.0_01591.1	520	42718	1.00E-104	296	326	90	+	10	329	8914	9233
UN18350	Rsa1.0_03099.1	1459	139424	0	509	543	93	-	917	1457	4079	4611
UN18351	Rsa1.0_00763.1	1194	65121	0	356	359	99	+	1	359	38841	39199
UN18352	Rsa1.0_00961.1	1736	58185	0	472	482	97	-	393	874	14537	15018
UN18353	Rsa1.0_00266.1	987	163932	1.00E-123	236	240	98	-	272	511	7133	7372
UN18354	Rsa1.0_04369.1	1407	17703	2.33E-156	332	351	94	+	3	352	1792	2134
UN18355	Rsa1.0_00714.1	1110	67187	0	470	489	96	-	87	566	10569	11054
UN18356	Rsa1.0_00154.1	1880	189461	1.00E-147	283	288	98	+	1	285	157488	157775
UN18357	Rsa1.0_00046.1	1546	320315	0	1503	1542	97	+	7	1546	192831	194366
UN18358	Rsa1.0_03353.1	1799	23496	0	624	640	97	+	1163	1799	18009	18647
UN18359	Rsa1.0_00307.1	1067	199003	0	878	915	95	+	14	926	5353	6260
UN18360	Rsa1.0_00783.1	1830	64459	0	905	927	97	+	904	1827	20058	20984
UN18361	Rsa1.0_01536.1	761	43747	0	436	444	98	+	283	718	39543	39986
UN18362	Rsa1.0_20222.1	1987	1326	0	587	597	98	-	1145	1741	1	597
UN18363	Rsa1.0_00194.1	938	110851	0	413	432	95	+	183	614	7999	8430
UN18364	Rsa1.0_00621.1	1707	71840	1.00E-144	347	369	94	+	3	365	48836	49191
UN18365	Rsa1.0_00609.1	1320	72785	0	729	756	96	+	544	1296	17899	18637
UN18366	Rsa1.0_52653.1	1239	433	1.00E-166	305	308	99	-	1	308	1	308
UN18367	Rsa1.0_01305.1	1504	48730	0	476	477	99	-	1028	1504	15327	15803

UN18368	Rsa1.0_00386.1	1211	89134	1.00E-143	295	305	96	−	3	307	29431	29732
UN18369	Rsa1.0_02803.1	960	27715	0	447	454	98	+	505	958	2231	2684
UN18370	Rsa1.0_00086.1	1168	439854	0	892	899	99	−	3	900	318961	319859
UN18371	Rsa1.0_00448.1	924	84048	0	903	922	97	+	3	924	32812	33732
UN18372	Rsa1.0_00162.1	1266	239935	0	731	744	98	−	523	1265	38543	39286
UN18373	Rsa1.0_00087.1	640	761073	1.00E-86	172	175	98	−	204	378	278974	279148
UN18374	Rsa1.0_00089.1	1059	406142	1.00E-130	261	267	97	+	795	1059	342938	343203
UN18375	Rsa1.0_00047.1	856	168582	1.00E-107	229	239	95	+	63	298	114068	114306
UN18376	Rsa1.0_00067.1	1203	415055	0	408	414	98	−	789	1202	147119	147531
UN18377	Rsa1.0_00153.1	761	192931	1.00E-95	222	232	95	+	531	759	168694	168922
UN18378	Rsa1.0_02634.1	1427	29299	0	1074	1082	99	−	2	1081	16743	17824
UN18379	Rsa1.0_00033.1	1141	515811	1.00E-130	268	275	97	+	20	294	467569	467839
UN18380	Rsa1.0_10503.1	1208	5824	1.00E-141	277	283	97	−	860	1141	1980	2262
UN18381	Rsa1.0_03451.1	998	22828	0	497	503	98	−	494	995	17165	17667
UN18382	Rsa1.0_27081.1	1404	866	1.00E-149	367	401	91	+	1018	1404	437	833
UN18383	Rsa1.0_00104.1	1062	368722	1.00E-142	259	260	99	+	7	266	65010	65269
UN18384	Rsa1.0_00280.1	1319	99966	0	630	665	94	−	661	1319	6002	6666
UN18385	Rsa1.0_00082.1	1057	237584	0	413	431	95	−	633	1051	53517	53947
UN18386	Rsa1.0_06839.1	1078	10286	0	531	542	97	−	537	1075	1902	2443
UN18387	Rsa1.0_02558.1	611	30112	0	601	625	96	+	3	611	7161	7785
UN18388	Rsa1.0_11362.1	991	5532	1.00E-147	295	305	96	+	3	307	4613	4917
UN18389	Rsa1.0_03319.1	1195	23767	0	554	574	96	−	574	1144	4081	4649
UN18390	Rsa1.0_00160.1	1260	346678	0	1196	1225	97	+	1	1225	144590	145814
UN18391	Rsa1.0_02410.1	1279	31659	0	1269	1277	99	−	2	1278	19948	21224
UN18392	Rsa1.0_00345.1	948	93226	0	447	467	95	−	388	854	74468	74934
UN18393	Rsa1.0_01015.1	1841	56752	0	457	466	98	+	1376	1841	7528	7990
UN18394	Rsa1.0_00676.1	1120	69166	0	486	519	93	−	99	603	54623	55138
UN18395	Rsa1.0_00739.1	1098	66155	1.00E-114	246	257	95	−	448	704	38886	39141
UN18396	Rsa1.0_00397.1	768	88189	1.00E-106	263	284	92	+	483	764	54834	55109
UN18397	Rsa1.0_00421.1	767	86365	1.00E-174	316	318	99	−	450	767	14715	15032
UN18398	Rsa1.0_44085.1	1286	510	1.00E-161	325	337	96	−	311	647	70	406
UN18399	Rsa1.0_00667.1	1861	69460	0	403	409	98	+	1	406	22479	22887
UN18400	Rsa1.0_36357.1	615	620	1.00E-101	187	187	100	+	429	615	1	187
UN18401	Rsa1.0_00127.1	1326	127446	1.00E-142	263	265	99	+	830	1094	16292	16556
UN18402	Rsa1.0_07528.1	679	9195	0	641	668	95	+	5	671	3310	3975
UN18403	Rsa1.0_00128.1	1601	228675	0	460	467	98	+	1138	1599	202406	202871
UN18404	Rsa1.0_01767.1	1122	39615	1.00E-104	192	192	100	−	1	192	15425	15616
UN18405	Rsa1.0_00467.1	961	156542	1.00E-120	249	259	96	−	593	851	91014	91272
UN18406	Rsa1.0_02505.1	1179	30703	0	417	418	99	−	755	1172	9948	10365

UN18407	Rsa1.0_00009.1	1238	232558	0	434	445	97	−	763	1207	22905	23349
UN18408	Rsa1.0_00200.1	1031	162016	0	345	346	99	−	1	346	115230	115575
UN18409	Rsa1.0_00038.1	1058	314568	0	429	439	97	−	36	474	253300	253735
UN18410	Rsa1.0_00286.1	844	98709	0	763	814	93	+	54	844	4958	5770
UN18411	Rsa1.0_02079.1	941	35306	0	404	410	98	−	242	651	18111	18517
UN18412	Rsa1.0_02871.1	791	27085	1.00E−145	262	262	100	+	530	791	18241	18502
UN18413	Rsa1.0_08023.1	937	8071	0	492	521	94	−	422	937	4879	5399
UN18414	Rsa1.0_01116.1	775	142088	0	672	695	96	−	89	775	129744	130438
UN18415	Rsa1.0_01144.1	854	52431	0	407	425	95	−	2	424	42305	42724
UN18416	Rsa1.0_03191.1	1079	145762	0	740	747	99	−	332	1078	20691	21437
UN18417	Rsa1.0_00418.1	680	215377	1.00E−89	221	237	93	−	9	241	42754	42989
UN18418	Rsa1.0_02808.1	1714	27677	0	553	604	91	−	493	1092	17030	17614
UN18419	Rsa1.0_01146.1	1253	163455	0	419	423	99	+	1	422	16387	16809
UN18420	Rsa1.0_00031.1	1089	179862	0	420	420	100	−	670	1089	165989	166408
UN18421	Rsa1.0_00348.1	1587	92818	0	1512	1544	97	−	3	1544	16030	17570
UN18422	Rsa1.0_00045.1	628	328694	1.00E−115	226	230	98	−	5	233	232551	232780
UN18423	Rsa1.0_00214.1	1179	107508	0	401	411	97	−	1	407	67033	67442
UN18424	Rsa1.0_00991.1	991	143101	0	533	539	98	−	1	537	34643	35181
UN18425	Rsa1.0_00937.1	1501	59061	1.00E−133	266	274	97	+	14	287	26612	26885
UN18426	Rsa1.0_01648.1	941	41635	0	718	723	99	+	222	941	10802	11524
UN18427	Rsa1.0_01997.1	1094	158416	1.00E−124	258	267	96	+	300	565	152201	152467
UN18428	Rsa1.0_01896.1	939	37512	0	910	938	97	+	1	937	31721	32658
UN18429	Rsa1.0_01511.1	1152	44235	1.00E−175	311	311	100	−	1	311	30182	30492
UN18430	Rsa1.0_01559.1	2031	43262	0	1181	1189	99	+	840	2026	33099	34287
UN18431	Rsa1.0_05355.1	745	14069	0	580	609	95	−	1	600	4534	5139
UN18432	Rsa1.0_01323.1	1144	48152	1.00E−151	298	306	97	+	1	304	26865	27170
UN18433	Rsa1.0_02074.1	1115	35370	0	462	496	93	+	5	493	12043	12527
UN18434	Rsa1.0_02026.1	1200	35896	0	580	589	98	−	1	583	22657	23242
UN18435	Rsa1.0_01270.1	1796	49574	0	779	779	100	−	435	1213	29988	30766
UN18436	Rsa1.0_01172.1	1020	51734	0	418	424	98	−	10	433	27421	27844
UN18437	Rsa1.0_00107.1	1167	431659	1.00E−166	338	352	96	−	806	1157	314611	314962
UN18438	Rsa1.0_03799.1	1437	20775	0	426	439	97	+	793	1231	18208	18646
UN18439	Rsa1.0_01634.1	1025	41865	1.00E−147	332	349	95	+	2	345	38759	39103
UN18440	Rsa1.0_00863.1	1513	254535	0	562	574	97	+	2	575	126460	127032
UN18441	Rsa1.0_01506.1	1140	44370	1.00E−141	312	327	95	−	815	1140	34074	34398
UN18442	Rsa1.0_01169.1	818	51829	0	813	817	99	+	1	816	5715	6531
UN18443	Rsa1.0_00065.1	1441	454616	1.00E−142	276	280	98	−	1	279	116989	117267
UN18444	Rsa1.0_01931.1	766	37187	1.00E−144	368	400	92	−	1	398	32029	32413
UN18445	Rsa1.0_00336.1	1214	445051	1.00E−126	245	250	98	+	462	711	164694	164943

UN18446	Rsa1.0_03547.1	1358	22167	0	625	635	98	−	1	634	1874	2508
UN18447	Rsa1.0_03037.1	1735	25825	0	612	630	97	−	521	1145	11252	11881
UN18448	Rsa1.0_01696.1	1207	301850	1.00E−175	384	402	95	−	801	1197	298784	299179
UN18449	Rsa1.0_05864.1	930	12387	1.00E−123	299	322	92	−	608	928	9853	10165
UN18450	Rsa1.0_02023.1	1423	35922	0	1343	1423	94	−	6	1422	32669	34090
UN18451	Rsa1.0_02835.1	1122	27491	0	517	537	96	−	591	1120	9213	9749
UN18452	Rsa1.0_15018.1	801	2889	1.00E−148	284	290	97	−	222	511	2384	2673
UN18453	Rsa1.0_02476.1	1515	31063	8.00E−80	491	606	81	+	98	677	23812	24412
UN18454	Rsa1.0_05110.1	1208	14814	1.00E−163	308	312	98	+	245	555	5291	5602
UN18455	Rsa1.0_00038.1	994	314568	1.00E−172	309	310	99	+	535	844	249454	249763
UN18456	Rsa1.0_02246.1	972	33435	1.00E−167	356	375	94	−	597	971	4753	5127
UN18457	Rsa1.0_00312.1	1033	96765	1.00E−145	320	345	92	+	1	324	88011	88355
UN18458	Rsa1.0_01170.1	1679	161131	1.00E−163	316	324	97	+	868	1191	10604	10927
UN18459	Rsa1.0_03305.1	1016	147552	0	404	405	99	+	3	407	12490	12894
UN18460	Rsa1.0_00023.1	1583	194764	0	791	800	98	−	686	1484	104143	104941
UN18461	Rsa1.0_01331.1	1712	47944	0	1698	1715	99	−	1	1712	17681	19395
UN18462	Rsa1.0_01173.1	1251	51727	0	561	569	98	−	1	569	32928	33493
UN18463	Rsa1.0_00294.1	2316	144860	0	479	499	95	+	3	500	59118	59616
UN18464	Rsa1.0_14096.1	914	3814	1.00E−112	255	266	95	+	80	342	2615	2879
UN18465	Rsa1.0_00026.1	821	327835	0	808	818	98	+	3	820	41922	42737
UN18466	Rsa1.0_01024.1	1032	56569	0	1025	1030	99	−	2	1031	33919	34948
UN18467	Rsa1.0_01512.1	1650	44234	0	495	516	95	+	635	1150	17477	17992
UN18468	Rsa1.0_01660.1	1888	41400	0	485	521	93	+	1365	1876	30402	30919
UN18469	Rsa1.0_03554.1	1549	22137	0	1544	1547	99	+	1	1547	10349	11895
UN18470	Rsa1.0_00336.1	849	445051	0	739	777	95	−	34	810	146903	147660
UN18471	Rsa1.0_19807.1	933	1380	1.00E−145	325	343	94	−	595	933	365	700
UN18472	Rsa1.0_01884.1	1474	37659	0	565	583	96	−	122	704	20785	21367
UN18473	Rsa1.0_07122.1	941	9866	0	345	352	98	−	590	938	6612	6963
UN18474	Rsa1.0_05338.1	1363	144511	0	414	436	94	−	195	627	6891	7314
UN18475	Rsa1.0_00301.1	1293	365315	1.00E−115	217	219	99	+	22	240	87944	88162
UN18476	Rsa1.0_03668.1	1542	21483	0	508	528	96	−	942	1469	20227	20754
UN18477	Rsa1.0_00086.1	752	439854	0	375	379	98	+	3	381	240929	241307
UN18478	Rsa1.0_00359.1	1406	91256	0	446	446	100	+	961	1406	84690	85135
UN18479	Rsa1.0_00087.1	1259	761073	0	403	405	99	+	854	1258	352672	353076
UN18480	Rsa1.0_04518.1	915	17005	1.00E−159	314	320	98	+	598	915	11675	11992
UN18481	Rsa1.0_00399.1	1095	242951	0	748	763	98	+	2	764	72869	73631
UN18482	Rsa1.0_03851.1	1192	20451	0	377	391	96	+	740	1129	11491	11880
UN18483	Rsa1.0_04428.1	1896	17399	0	1146	1167	98	+	416	1582	6851	8014
UN18484	Rsa1.0_01559.1	1143	43262	1.00E−127	304	327	92	+	3	329	3014	3329

UN18485	Rsa1.0_03985.1	1185	19704	0	949	975	97	+	3	977	14374	15342
UN18486	Rsa1.0_00347.1	783	93048	0	443	454	97	−	330	782	70009	70462
UN18487	Rsa1.0_00045.1	1174	328694	1.00E−152	304	312	97	−	1	310	16514	16823
UN18488	Rsa1.0_00800.1	950	63868	0	370	381	97	+	570	950	21908	22279
UN18489	Rsa1.0_00550.1	1708	210790	0	421	423	99	+	1284	1706	60958	61380
UN18490	Rsa1.0_01434.1	1460	45951	0	446	470	94	−	1	462	39461	39930
UN18491	Rsa1.0_00687.1	1366	68494	1.00E−133	270	279	96	−	1088	1366	1925	2200
UN18492	Rsa1.0_03555.1	841	22129	0	673	677	99	+	160	835	16383	17059
UN18493	Rsa1.0_00050.1	1106	237174	1.00E−106	324	368	88	−	745	1104	103893	104244
UN18494	Rsa1.0_00018.1	923	410899	1.00E−156	308	316	97	−	18	328	128266	128580
UN18495	Rsa1.0_03201.1	993	157656	0	897	996	90	−	12	993	20750	21737
UN18496	Rsa1.0_00420.1	768	86496	0	566	568	99	−	160	727	41171	41738
UN18497	Rsa1.0_02836.1	1093	27481	1.00E−125	234	236	99	−	510	745	13279	13514
UN18498	Rsa1.0_01068.1	1045	55208	1.00E−125	244	249	97	−	7	251	10091	10339
UN18499	Rsa1.0_01976.1	987	36570	1.00E−133	349	379	92	−	606	979	25218	25588
UN18500	Rsa1.0_28177.1	928	825	1.00E−164	317	324	97	+	607	928	459	782
UN18501	Rsa1.0_00067.1	1343	415055	1.00E−108	214	219	97	−	368	586	41964	42182
UN18502	Rsa1.0_09929.1	569	6087	0	566	567	99	+	1	567	173	739
UN18503	Rsa1.0_00646.1	931	70473	0	678	691	98	−	238	927	1728	2417
UN18504	Rsa1.0_00121.1	2002	278029	1.00E−140	259	261	99	+	1742	2002	80339	80599
UN18505	Rsa1.0_01803.1	943	173846	0	404	426	94	−	3	421	137460	137885
UN18506	Rsa1.0_00028.1	975	293984	0	334	336	99	−	639	974	253781	254116
UN18507	Rsa1.0_00806.1	1343	63674	1.00E−154	313	322	97	−	3	323	29072	29389
UN18508	Rsa1.0_00544.1	683	76787	1.00E−144	277	283	97	+	377	659	445	721
UN18509	Rsa1.0_01411.1	925	46326	1.00E−170	309	311	99	+	11	321	36020	36330
UN18510	Rsa1.0_01593.1	1351	150996	0	421	421	100	−	902	1322	149430	149850
UN18511	Rsa1.0_01412.1	1631	46311	0	548	549	99	−	502	1050	11181	11729
UN18512	Rsa1.0_00653.1	1199	70029	0	396	403	98	+	1	400	26626	27027
UN18513	Rsa1.0_00442.1	1198	84596	0	593	600	98	−	2	600	13141	13740
UN18514	Rsa1.0_02079.1	1170	35306	0	418	419	99	+	751	1169	7765	8183
UN18515	Rsa1.0_00786.1	1017	64285	0	364	366	99	−	3	368	50723	51087
UN18516	Rsa1.0_36951.1	1079	610	0	414	440	94	−	3	422	1	440
UN18517	Rsa1.0_15111.1	1481	2818	1.00E−151	272	272	100	−	1208	1479	1444	1715
UN18518	Rsa1.0_02969.1	808	26390	0	667	697	95	+	3	692	23180	23875
UN18519	Rsa1.0_08085.1	1317	7934	0	721	739	97	−	562	1300	5075	5812
UN18520	Rsa1.0_01211.1	812	50646	0	803	816	98	−	1	812	15179	15989
UN18521	Rsa1.0_00153.1	1189	192931	1.00E−96	282	314	89	−	4	307	170695	171004
UN18522	Rsa1.0_00938.1	1314	193891	0	688	703	97	+	612	1314	50748	51450
UN18523	Rsa1.0_03376.1	1226	23326	0	461	482	95	+	13	488	18750	19225

UN18524	Rsa1.0_00139.1	1352	357786	0	736	750	98	−	601	1350	120058	120807
UN18525	Rsa1.0_05324.1	1026	14145	0	405	411	98	+	195	605	11922	12332
UN18526	Rsa1.0_00193.1	1685	194198	0	439	441	99	−	418	858	98615	99055
UN18527	Rsa1.0_00182.1	1350	142305	0	698	717	97	−	2	718	94201	94913
UN18528	Rsa1.0_00087.1	651	761073	1.00E−145	279	285	97	+	327	611	532516	532800
UN18529	Rsa1.0_02984.1	1654	26263	1.00E−171	350	357	98	−	242	592	5683	6039
UN18530	Rsa1.0_00838.1	1108	153820	1.00E−108	230	239	96	−	1	238	35295	35533
UN18531	Rsa1.0_03158.1	2092	24950	0	533	542	98	+	7	546	15094	15633
UN18532	Rsa1.0_00098.1	926	349239	0	400	400	100	+	1	400	228899	229298
UN18533	Rsa1.0_00119.1	1436	467509	0	1262	1302	96	+	1	1293	346494	347789
UN18534	Rsa1.0_00654.1	969	70012	0	435	439	99	+	1	438	32608	33046
UN18535	Rsa1.0_01301.1	1134	169072	0	493	508	97	−	620	1124	41711	42218
UN18536	Rsa1.0_01124.1	1667	53295	0	1202	1231	97	+	1	1228	50492	51721
UN18537	Rsa1.0_14084.1	1128	3822	0	463	468	98	+	454	921	281	748
UN18538	Rsa1.0_00141.1	1142	121494	1.00E−178	325	328	99	+	1	328	110498	110825
UN18539	Rsa1.0_00042.1	990	171507	0	485	510	95	+	2	509	143914	144412
UN18540	Rsa1.0_02786.1	904	27880	0	480	482	99	+	3	483	18704	19185
UN18541	Rsa1.0_00540.1	1261	76895	1.00E−136	249	250	99	−	590	839	35535	35784
UN18542	Rsa1.0_00466.1	986	82132	0	867	897	96	−	1	889	27152	28045
UN18543	Rsa1.0_01196.1	2048	51097	0	515	526	97	−	1030	1554	26796	27317
UN18544	Rsa1.0_03664.1	840	21487	0	539	550	98	−	1	549	10815	11364
UN18545	Rsa1.0_00545.1	1331	189649	0	526	538	97	−	789	1326	37246	37783
UN18546	Rsa1.0_03640.1	1476	21624	1.00E−176	368	385	95	+	2	385	17081	17454
UN18547	Rsa1.0_09407.1	1273	6390	0	537	551	97	−	45	593	1529	2077
UN18548	Rsa1.0_01747.1	1135	39881	0	1097	1112	98	+	1	1111	15949	17059
UN18549	Rsa1.0_00001.1	1492	363028	0	392	406	96	+	1	402	249949	250354
UN18550	Rsa1.0_00053.1	913	263459	1.00E−107	229	240	95	−	1	234	50661	50900
UN18551	Rsa1.0_00676.1	1464	69166	0	1001	1011	99	−	453	1462	61418	62428
UN18552	Rsa1.0_00240.1	1050	104175	1.00E−170	307	308	99	+	742	1049	20254	20561
UN18553	Rsa1.0_01296.1	1023	49035	1.00E−111	205	205	100	+	561	765	13100	13304
UN18554	Rsa1.0_00013.1	779	292129	4.00E−83	172	176	97	−	1	176	206086	206259
UN18555	Rsa1.0_00065.1	1827	454616	0	356	357	99	−	1096	1452	224536	224892
UN18556	Rsa1.0_18047.1	1462	1682	5.00E−78	149	149	100	+	165	313	1431	1579
UN18557	Rsa1.0_00235.1	967	154696	0	408	410	99	+	4	413	146397	146806
UN18558	Rsa1.0_10100.1	1569	5998	0	517	518	99	+	1052	1569	877	1394
UN18559	Rsa1.0_00044.1	1269	271658	0	355	358	99	+	911	1268	133405	133762
UN18560	Rsa1.0_00055.1	1648	520193	0	615	615	100	+	1	615	401231	401845
UN18561	Rsa1.0_00608.1	1574	172672	0	1365	1372	99	+	202	1573	19560	20931
UN18562	Rsa1.0_05172.1	2093	14608	0	1806	1831	98	+	106	1931	9667	11495

UN18563	Rsa1.0_00846.1	1633	62342	0	1157	1170	98	−	460	1629	31233	32398
UN18564	Rsa1.0_03001.1	1099	26108	0	454	454	100	−	646	1099	5371	5824
UN18565	Rsa1.0_00196.1	1444	109960	0	675	678	99	−	767	1444	53072	53749
UN18566	Rsa1.0_07881.1	1389	8369	1.00E−149	313	325	96	+	78	399	2930	3251
UN18567	Rsa1.0_00608.1	802	172672	0	527	546	96	+	239	780	3169	3708
UN18568	Rsa1.0_02129.1	525	34757	0	394	407	96	+	119	525	29381	29785
UN18569	Rsa1.0_01091.1	1475	54383	0	456	472	96	−	1	472	5374	5845
UN18570	Rsa1.0_02223.1	877	152421	0	875	878	99	+	1	877	25626	26503
UN18571	Rsa1.0_03165.1	994	24866	0	722	741	97	+	260	994	21465	22200
UN18572	Rsa1.0_02005.1	809	36137	1.00E−104	229	241	95	+	3	238	6393	6633
UN18573	Rsa1.0_00467.1	833	156542	0	798	843	94	+	1	832	92934	93769
UN18574	Rsa1.0_05389.1	1763	13943	0	991	1052	94	+	3	1037	7219	8249
UN18575	Rsa1.0_00667.1	1044	69460	0	537	560	95	−	477	1036	23995	24549
UN18576	Rsa1.0_00088.1	1276	141341	0	776	789	98	−	7	795	42241	43029
UN18577	Rsa1.0_00087.1	1742	761073	0	1723	1751	98	+	2	1742	275772	277513
UN18578	Rsa1.0_00599.1	1249	73418	0	1206	1219	98	+	2	1220	39408	40625
UN18579	Rsa1.0_00102.1	1093	135497	2.00E−95	204	212	96	+	843	1051	51183	51394
UN18580	Rsa1.0_00066.1	1621	152418	0	694	709	97	+	30	737	17596	18304
UN18581	Rsa1.0_03341.1	940	23604	0	543	581	93	+	360	939	2950	3527
UN18582	Rsa1.0_00487.1	730	80674	7.00E−85	166	168	98	+	276	443	8246	8413
UN18583	Rsa1.0_07193.1	1338	9752	0	633	638	99	−	5	642	3	639
UN18584	Rsa1.0_04768.1	1379	16066	0	1370	1384	98	+	1	1379	4080	5462
UN18585	Rsa1.0_00065.1	1168	454616	0	688	716	96	−	459	1168	150657	151371
UN18586	Rsa1.0_01940.1	1651	37073	0	482	482	100	+	500	981	8086	8567
UN18587	Rsa1.0_03506.1	1330	22416	0	478	498	95	+	834	1330	8413	8907
UN18588	Rsa1.0_02092.1	573	35135	0	376	377	99	−	197	573	30332	30708
UN18589	Rsa1.0_07959.1	778	8173	1.00E−125	236	239	98	+	2	240	5603	5841
UN18590	Rsa1.0_00251.1	2196	303968	0	353	355	99	−	3	357	39415	39769
UN18591	Rsa1.0_04934.1	1611	143962	0	1487	1531	97	+	16	1543	11179	12707
UN18592	Rsa1.0_07314.1	1713	9563	0	630	632	99	+	580	1211	5776	6407
UN18593	Rsa1.0_07251.1	1359	9662	0	442	449	98	−	1	449	8993	9436
UN18594	Rsa1.0_02439.1	1033	31379	0	965	981	98	−	1	979	22953	23933
UN18595	Rsa1.0_02420.1	1287	31580	0	737	749	98	+	9	751	13687	14435
UN18596	Rsa1.0_04925.1	963	15424	1.00E−160	397	429	92	−	525	943	9282	9704
UN18597	Rsa1.0_07636.1	1182	8971	0	691	691	100	−	3	693	3556	4246
UN18598	Rsa1.0_00059.1	981	159650	0	405	423	95	+	1	416	130999	131418
UN18599	Rsa1.0_00127.1	1196	127446	0	432	434	99	−	763	1196	46527	46960
UN18600	Rsa1.0_02434.1	1698	31464	0	755	765	98	−	2	765	27271	28032
UN18601	Rsa1.0_00838.1	1007	153820	1.00E−171	330	336	98	+	3	336	38110	38444

UN18602	Rsa1.0_00971.1	1036	57973	0	385	385	100	+	3	387	47270	47654
UN18603	Rsa1.0_18022.1	567	1687	0	357	361	98	−	207	567	419	779
UN18604	Rsa1.0_06728.1	968	10455	1.00E−161	366	387	94	−	579	961	9063	9448
UN18605	Rsa1.0_04488.1	1461	17155	0	657	672	97	+	3	674	13391	14062
UN18606	Rsa1.0_02248.1	1545	33414	0	463	470	98	−	566	1033	12478	12947
UN18607	Rsa1.0_01489.1	1271	44764	1.00E−174	335	343	97	+	92	430	127	469
UN18608	Rsa1.0_00113.1	498	131574	0	490	498	98	−	1	498	60382	60879
UN18609	Rsa1.0_01412.1	916	46311	0	519	519	100	−	2	520	2268	2786
UN18610	Rsa1.0_01533.1	1040	43765	0	385	390	98	−	104	493	9229	9618
UN18611	Rsa1.0_04772.1	1600	16040	1.00E−127	245	247	99	+	1	245	5454	5700
UN18612	Rsa1.0_01779.1	1710	39448	0	709	802	88	+	899	1699	9724	10521
UN18613	Rsa1.0_00017.1	876	752458	1.00E−99	200	204	98	−	4	207	608166	608367
UN18614	Rsa1.0_00098.1	1048	349239	0	449	485	92	+	6	475	235871	236346
UN18615	Rsa1.0_00383.1	827	398431	3.00E−84	255	283	90	+	5	269	182122	182404
UN18616	Rsa1.0_00201.1	1410	232851	0	412	419	98	−	3	421	4812	5228
UN18617	Rsa1.0_00715.1	1320	67116	0	473	473	100	+	1	473	26725	27197
UN18618	Rsa1.0_00048.1	919	167185	1.00E−123	233	236	98	−	2	237	33623	33858
UN18619	Rsa1.0_00367.1	1629	90750	0	646	648	99	−	983	1629	78116	78763
UN18620	Rsa1.0_08837.1	1484	6874	0	351	354	99	+	1	353	2905	3258
UN18621	Rsa1.0_00180.1	1113	114581	0	398	406	98	−	1	406	111408	111810
UN18622	Rsa1.0_00837.1	1470	62568	1.00E−143	336	358	93	+	32	383	50670	51024
UN18623	Rsa1.0_06102.1	1302	11740	0	550	563	97	−	431	993	3255	3817
UN18624	Rsa1.0_00412.1	1492	86935	0	712	714	99	−	762	1475	14941	15654
UN18625	Rsa1.0_01326.1	753	48072	0	412	417	98	+	1	416	43750	44166
UN18626	Rsa1.0_09203.1	724	6559	0	719	727	98	+	1	724	650	1376
UN18627	Rsa1.0_02269.1	1677	33168	0	621	640	97	+	416	1055	23759	24398
UN18628	Rsa1.0_11276.1	1193	5560	0	356	361	98	+	118	478	4	364
UN18629	Rsa1.0_01845.1	1151	38415	8.00E−98	224	234	95	−	1	232	8035	8267
UN18630	Rsa1.0_00546.1	581	76523	0	462	482	95	+	111	581	58516	58997
UN18631	Rsa1.0_03211.1	1670	24591	0	608	617	98	−	2	617	9059	9675
UN18632	Rsa1.0_20083.1	835	1343	0	390	397	98	+	1	397	152	547
UN18633	Rsa1.0_00234.1	1258	197060	0	462	465	99	+	792	1256	152696	153160
UN18634	Rsa1.0_00972.1	1500	57937	0	579	584	99	+	229	811	52610	53193
UN18635	Rsa1.0_03894.1	871	20207	0	335	335	100	+	1	335	18461	18795
UN18636	Rsa1.0_00797.1	708	63959	1.00E−119	236	241	97	−	2	241	41753	41993
UN18637	Rsa1.0_00001.1	1991	363028	0	498	512	97	−	1	509	203498	204009
UN18638	Rsa1.0_00002.1	1245	601661	1.00E−168	328	336	97	−	910	1244	13141	13476
UN18639	Rsa1.0_49161.1	1337	461	1.00E−106	241	253	95	−	1063	1313	14	262
UN18640	Rsa1.0_05554.1	637	13343	0	597	627	95	−	10	635	12429	13049

UN18641	Rsa1.0_00628.1	611	214360	1.00E-119	228	231	98	−	3	233	53812	54039
UN18642	Rsa1.0_00092.1	1266	659932	0	948	959	98	−	311	1266	530973	531931
UN18643	Rsa1.0_00546.1	837	76523	0	816	827	98	+	8	834	24901	25724
UN18644	Rsa1.0_00706.1	1133	291570	1.00E-145	262	262	100	−	586	847	1	262
UN18645	Rsa1.0_01396.1	1894	151624	1.00E-177	337	342	98	+	3	341	136190	136530
UN18646	Rsa1.0_00017.1	2192	752458	0	1557	1576	98	+	617	2192	360274	361838
UN18647	Rsa1.0_02383.1	1967	31931	0	922	922	100	−	229	1150	16329	17250
UN18648	Rsa1.0_00668.1	879	69421	1.00E-143	303	314	96	+	545	856	14760	15072
UN18649	Rsa1.0_01938.1	857	264018	0	600	621	96	−	240	856	159646	160265
UN18650	Rsa1.0_00741.1	1049	66117	0	365	365	100	+	685	1049	50868	51232
UN18651	Rsa1.0_00589.1	1283	209965	1.00E-140	271	277	97	−	422	698	189295	189571
UN18652	Rsa1.0_00497.1	920	79830	0	429	433	99	+	488	920	73170	73602
UN18653	Rsa1.0_01090.1	779	54385	1.00E-175	396	424	93	−	354	777	3212	3626
UN18654	Rsa1.0_02625.1	920	139104	0	390	398	97	−	38	432	13985	14381
UN18655	Rsa1.0_00374.1	1319	165738	0	1314	1317	99	+	3	1319	163177	164493
UN18656	Rsa1.0_00220.1	891	192936	0	335	336	99	−	556	891	143125	143460
UN18657	Rsa1.0_00194.1	782	110851	0	383	384	99	+	398	781	42338	42721
UN18658	Rsa1.0_01468.1	710	45146	0	377	387	97	+	1	387	33809	34195
UN18659	#	#	#	#	#	#	#	#	#	#	#	#
UN18660	Rsa1.0_00128.1	1769	228675	0	419	423	99	−	1342	1764	100520	100941
UN18661	Rsa1.0_01363.1	2091	47195	0	775	786	98	−	97	882	1763	2548
UN18662	Rsa1.0_02107.1	556	34940	0	412	419	98	+	139	556	11867	12285
UN18663	Rsa1.0_00653.1	1223	70029	0	628	646	97	−	587	1223	54934	55579
UN18664	Rsa1.0_00265.1	1114	220635	0	990	1000	99	−	115	1114	207966	208965
UN18665	Rsa1.0_00222.1	891	172263	1.00E-170	307	308	99	+	113	420	28919	29226
UN18666	Rsa1.0_01785.1	1095	39368	0	496	499	99	+	589	1086	27142	27640
UN18667	Rsa1.0_00550.1	1068	210790	1.00E-118	249	259	96	+	3	259	58216	58474
UN18668	Rsa1.0_01363.1	1305	47195	0	492	495	99	−	17	511	674	1168
UN18669	Rsa1.0_00853.1	1468	62101	0	1463	1467	99	−	1	1466	35741	37205
UN18670	Rsa1.0_00714.1	1017	67187	0	368	368	100	+	648	1015	14249	14616
UN18671	Rsa1.0_00954.1	804	189536	0	794	803	98	−	2	804	31811	32612
UN18672	Rsa1.0_00044.1	868	271658	0	336	336	100	−	461	796	264749	265084
UN18673	Rsa1.0_00740.1	1735	215972	0	1042	1046	99	−	1	1046	198503	199548
UN18674	Rsa1.0_02910.1	1455	26843	0	445	452	98	−	1004	1455	10823	11273
UN18675	Rsa1.0_09707.1	824	6199	1.00E-135	355	389	91	+	436	824	5205	5577
UN18676	Rsa1.0_01661.1	1183	41400	0	773	783	98	−	319	1101	32175	32957
UN18677	Rsa1.0_01827.1	845	143321	9.00E-94	184	187	98	−	345	531	128843	129029
UN18678	Rsa1.0_00895.1	1281	60615	0	477	483	98	+	429	911	39139	39621
UN18679	Rsa1.0_02198.1	1066	33989	1.00E-166	306	309	99	−	635	943	23969	24277

UN18680	Rsa1.0_02726.1	826	28404	0	664	670	99	−	144	813	8406	9075
UN18681	Rsa1.0_00746.1	1185	66044	1.00E−167	307	310	99	+	206	515	21064	21373
UN18682	Rsa1.0_00399.1	1990	242951	0	1946	1994	97	+	1	1990	238572	240562
UN18683	Rsa1.0_04194.1	786	132734	0	570	604	94	+	2	601	123389	123988
UN18684	Rsa1.0_00040.1	1401	324196	0	668	711	93	+	693	1400	15517	16201
UN18685	Rsa1.0_06825.1	1974	10297	0	788	791	99	−	398	1188	7176	7966
UN18686	Rsa1.0_00031.1	1059	179862	1.00E−142	271	276	98	+	146	421	172803	173078
UN18687	Rsa1.0_01105.1	694	53958	1.00E−155	278	278	100	−	411	688	7703	7980
UN18688	Rsa1.0_00417.1	1432	395068	0	587	592	99	−	4	592	364722	365313
UN18689	Rsa1.0_00870.1	1219	61606	0	593	600	98	+	592	1187	27218	27817
UN18690	Rsa1.0_07122.1	849	9866	0	399	403	99	−	448	849	5080	5482
UN18691	Rsa1.0_00842.1	1204	62493	0	476	484	98	−	719	1202	14572	15054
UN18692	Rsa1.0_00119.1	1561	467509	1.00E−157	316	325	97	−	164	487	62711	63034
UN18693	Rsa1.0_00284.1	1468	323267	1.00E−136	258	262	98	+	1	262	237097	237358
UN18694	Rsa1.0_00104.1	1550	368722	0	975	1034	94	−	2	1015	68555	69588
UN18695	Rsa1.0_07645.1	1073	8949	0	609	622	97	+	1	622	2495	3116
UN18696	Rsa1.0_06597.1	894	10697	0	426	430	99	−	67	496	4936	5365
UN18697	Rsa1.0_00354.1	1012	232005	0	978	990	98	+	22	1011	76109	77095
UN18698	Rsa1.0_28295.1	2518	821	0	360	361	99	−	446	806	5	365
UN18699	Rsa1.0_36296.1	1156	621	0	351	358	98	+	618	974	1	358
UN18700	Rsa1.0_00070.1	853	150321	1.00E−174	310	310	100	−	3	312	32070	32379
UN18701	Rsa1.0_00602.1	819	73302	0	804	817	98	−	1	817	35784	36596
UN18702	Rsa1.0_00201.1	1310	232851	0	662	684	96	+	481	1164	211	894
UN18703	Rsa1.0_13994.1	919	3941	1.00E−178	347	357	97	−	227	583	1610	1966
UN18704	Rsa1.0_00129.1	1436	126831	0	758	770	98	−	668	1433	34355	35124
UN18705	Rsa1.0_03172.1	1772	24816	0	1686	1705	98	−	1	1701	2171	3875
UN18706	Rsa1.0_01196.1	892	51097	1.00E−176	370	390	94	+	1	387	32459	32837
UN18707	Rsa1.0_00023.1	945	194764	0	385	387	99	−	370	756	96929	97315
UN18708	Rsa1.0_00426.1	1381	85668	1.00E−124	297	318	93	+	14	328	30271	30583
UN18709	Rsa1.0_03155.1	1495	24958	0	1385	1394	99	+	1	1394	20481	21874
UN18710	Rsa1.0_00642.1	1400	201230	0	510	516	98	+	883	1398	30064	30578
UN18711	Rsa1.0_00116.1	1451	365886	1.00E−115	289	315	91	−	45	359	67695	67993
UN18712	Rsa1.0_00648.1	1108	70351	0	398	407	97	−	1	407	31295	31701
UN18713	Rsa1.0_30970.1	1626	740	0	326	327	99	−	1011	1337	259	585
UN18714	Rsa1.0_01476.1	842	172928	1.00E−164	320	329	97	+	9	337	134299	134627
UN18715	Rsa1.0_04235.1	1649	18373	0	378	385	98	−	1016	1399	1875	2259
UN18716	Rsa1.0_04641.1	800	268012	1.00E−180	327	328	99	+	472	798	132021	132348
UN18717	Rsa1.0_05784.1	1027	12643	0	398	414	96	−	2	414	1645	2057
UN18718	Rsa1.0_02452.1	950	31280	0	545	545	100	+	406	950	13663	14207

UN18719	Rsa1.0_00483.1	1227	81102	0	553	555	99	+	440	994	2359	2913
UN18720	Rsa1.0_00105.1	896	134138	0	644	684	94	−	226	889	1925	2604
UN18721	Rsa1.0_13941.1	1639	4043	0	482	491	98	+	1093	1583	3554	4043
UN18722	Rsa1.0_00307.1	1223	199003	0	383	402	95	−	1	399	70435	70829
UN18723	Rsa1.0_00077.1	2318	145005	0	522	524	99	−	1795	2318	40534	41057
UN18724	Rsa1.0_00017.1	1984	752458	1.00E−140	396	435	91	+	1556	1984	718544	718965
UN18725	Rsa1.0_01812.1	1474	38956	0	1465	1472	99	−	1	1472	23997	25468
UN18726	Rsa1.0_00321.1	434	95671	1.00E−135	254	256	99	+	178	433	56950	57204
UN18727	Rsa1.0_00006.1	1200	396176	0	1190	1199	99	+	1	1199	266273	267471
UN18728	Rsa1.0_00414.1	1381	202190	0	1316	1401	93	+	1	1381	14739	16134
UN18729	Rsa1.0_00387.1	1045	495407	1.00E−167	319	326	97	−	689	1014	1271	1596
UN18730	Rsa1.0_02393.1	1448	31795	0	1442	1447	99	+	1	1447	14243	15689
UN18731	Rsa1.0_00053.1	1905	263459	0	1180	1193	98	−	713	1905	229615	230807
UN18732	Rsa1.0_03353.1	1400	23496	0	637	640	99	−	1	638	18009	18648
UN18733	Rsa1.0_07056.1	1071	9969	1.00E−173	393	424	92	−	651	1062	1789	2206
UN18734	Rsa1.0_14752.1	687	3100	1.00E−113	225	231	97	+	456	686	33	263
UN18735	Rsa1.0_01583.1	899	156297	8.00E−85	172	176	97	−	109	284	130377	130552
UN18736	Rsa1.0_02135.1	1829	34713	0	705	716	98	−	1036	1750	7000	7715
UN18737	Rsa1.0_06938.1	905	10140	2.00E−60	178	195	91	−	682	874	9785	9979
UN18738	Rsa1.0_10918.1	1504	5679	0	900	929	96	−	580	1504	2063	2989
UN18739	Rsa1.0_00433.1	1880	85381	0	573	587	97	−	3	586	79697	80279
UN18740	Rsa1.0_02103.1	1746	35006	0	488	500	97	−	137	636	34046	34545
UN18741	Rsa1.0_00318.1	1130	96094	0	356	359	99	+	771	1128	76649	77007
UN18742	Rsa1.0_00078.1	1169	175053	0	405	428	94	−	388	812	101013	101438
UN18743	Rsa1.0_06590.1	1246	10706	0	686	709	96	−	244	951	2006	2708
UN18744	Rsa1.0_00371.1	1236	90463	0	1221	1235	98	+	2	1236	19905	21138
UN18745	Rsa1.0_00480.1	1172	81325	0	441	456	96	+	385	840	4323	4778
UN18746	Rsa1.0_01236.1	825	50156	1.00E−132	239	239	100	−	587	825	40363	40601
UN18747	Rsa1.0_00662.1	1249	192183	1.00E−101	246	264	93	−	883	1145	4988	5251
UN18748	Rsa1.0_01876.1	1282	37800	1.00E−103	199	200	99	−	1083	1282	9795	9993
UN18749	Rsa1.0_10764.1	1101	5726	1.00E−147	325	350	92	−	735	1064	2496	2845
UN18750	Rsa1.0_00545.1	1837	189649	0	489	497	98	−	1340	1835	44747	45243
UN18751	Rsa1.0_00900.1	1010	60367	1.00E−141	294	306	96	+	703	1007	29910	30215
UN18752	Rsa1.0_00818.1	1575	62939	0	783	809	96	+	768	1575	36240	37036
UN18753	Rsa1.0_00805.1	1067	63724	0	363	371	97	−	212	582	15725	16095
UN18754	Rsa1.0_02682.1	1333	28711	0	1003	1045	95	−	210	1251	16904	17947
UN18755	Rsa1.0_08551.1	2110	7210	0	1965	1977	99	−	3	1970	3	1979
UN18756	Rsa1.0_04600.1	1132	16694	0	482	498	96	+	633	1130	1433	1930
UN18757	Rsa1.0_01041.1	682	55910	1.00E−123	232	233	99	−	3	234	9812	10044

UN18758	Rsa1.0_03786.1	1218	20879	0	664	716	92	−	108	821	13224	13939
UN18759	Rsa1.0_00144.1	779	120708	0	386	388	99	−	392	779	79316	79702
UN18760	Rsa1.0_00067.1	1214	415055	0	494	506	97	−	9	514	237446	237951
UN18761	Rsa1.0_15455.1	1349	2595	0	604	618	97	+	1	615	1697	2313
UN18762	Rsa1.0_00329.1	1318	222544	1.00E−164	617	725	85	−	197	903	139552	140273
UN18763	Rsa1.0_00107.1	1382	431659	0	811	853	95	+	17	862	86606	87435
UN18764	Rsa1.0_00490.1	1174	186713	0	569	573	99	−	1	572	31223	31795
UN18765	Rsa1.0_03141.1	1187	25061	1.00E−177	338	345	97	−	841	1185	4256	4597
UN18766	Rsa1.0_00006.1	1267	396176	0	348	352	98	+	1	351	100733	101082
UN18767	Rsa1.0_00916.1	950	59789	1.00E−130	243	245	99	+	158	402	13276	13520
UN18768	Rsa1.0_00262.1	967	101906	0	415	424	97	−	1	423	66278	66700
UN18769	Rsa1.0_00732.1	1291	66631	1.00E−157	321	334	96	+	12	345	1795	2123
UN18770	Rsa1.0_01376.1	838	47004	0	392	400	98	−	1	400	41027	41426
UN18771	Rsa1.0_00021.1	1799	263655	1.00E−166	312	317	98	−	559	875	15808	16124
UN18772	Rsa1.0_00149.1	1162	305196	1.00E−65	216	244	88	+	741	984	12986	13228
UN18773	Rsa1.0_00194.1	826	110851	0	520	527	98	+	1	527	7904	8430
UN18774	Rsa1.0_12259.1	1442	5273	1.00E−162	334	348	95	+	164	511	487	831
UN18775	Rsa1.0_00247.1	909	103408	0	347	347	100	+	561	907	9307	9653
UN18776	Rsa1.0_00017.1	1562	752458	0	877	879	99	−	651	1529	615886	616764
UN18777	Rsa1.0_08544.1	1105	7215	0	354	358	98	−	4	361	5274	5630
UN18778	Rsa1.0_02110.1	1022	34919	1.00E−132	239	239	100	−	784	1022	23653	23891
UN18779	Rsa1.0_00058.1	1324	159921	1.00E−167	355	372	95	+	511	877	118433	118804
UN18780	Rsa1.0_00002.1	1461	601661	0	575	600	95	+	864	1457	33588	34173
UN18781	Rsa1.0_00079.1	1428	606987	0	520	525	99	+	2	526	81236	81760
UN18782	Rsa1.0_00361.1	1544	91181	0	1198	1242	96	+	317	1543	47136	48374
UN18783	Rsa1.0_00002.1	830	601661	0	510	515	99	−	316	830	240792	241305
UN18784	Rsa1.0_01004.1	813	56975	0	430	461	93	−	365	806	16883	17342
UN18785	Rsa1.0_03828.1	1141	20588	0	1112	1143	97	+	3	1141	10638	11780
UN18786	Rsa1.0_06101.1	1268	11745	1.00E−103	197	199	98	−	160	358	1332	1530
UN18787	Rsa1.0_00980.1	2450	57729	0	1910	1943	98	−	508	2446	7089	9026
UN18788	Rsa1.0_01876.1	782	37800	0	568	590	96	+	194	782	20518	21107
UN18789	Rsa1.0_02579.1	1069	29923	0	486	489	99	+	583	1069	28379	28867
UN18790	Rsa1.0_00155.1	1675	118465	0	975	1009	96	−	2	1010	61433	62438
UN18791	Rsa1.0_00017.1	798	752458	1.00E−103	203	207	98	−	100	306	644525	644731
UN18792	Rsa1.0_00460.1	1069	206387	1.00E−152	279	281	99	+	309	589	35604	35884
UN18793	Rsa1.0_02839.1	1387	27391	0	355	365	97	−	236	600	18841	19205
UN18794	Rsa1.0_00946.1	862	58666	1.00E−142	281	289	97	−	557	845	48137	48425
UN18795	Rsa1.0_01118.1	2599	53460	0	446	467	95	+	353	819	11739	12205
UN18796	Rsa1.0_00710.1	1003	67325	0	353	356	99	+	646	1001	26519	26874

UN18797	Rsa1.0_00875.1	1851	61371	0	834	856	97	+	998	1850	37621	38474
UN18798	Rsa1.0_00434.1	802	85171	0	560	566	98	−	237	802	1504	2069
UN18799	Rsa1.0_03434.1	631	22960	1.00E−140	339	364	93	−	276	631	12113	12476
UN18800	Rsa1.0_19324.1	1560	1447	9.00E−86	165	166	99	−	502	667	1029	1194
UN18801	Rsa1.0_05423.1	1045	13800	1.00E−114	248	263	94	−	10	272	9118	9368
UN18802	Rsa1.0_00379.1	1226	89865	0	527	529	99	+	454	982	11782	12310
UN18803	Rsa1.0_00070.1	713	150321	0	376	376	100	+	1	376	146028	146403
UN18804	Rsa1.0_00716.1	1196	67078	0	1027	1076	95	+	122	1194	1	1058
UN18805	Rsa1.0_00226.1	922	105884	0	499	512	97	+	56	567	97510	98021
UN18806	Rsa1.0_01134.1	1002	52829	0	992	1003	98	+	1	1001	1608	2610
UN18807	Rsa1.0_03541.1	1375	22194	0	1359	1378	98	+	3	1375	9567	10939
UN18808	Rsa1.0_00977.1	553	198001	0	463	492	94	−	3	486	50788	51279
UN18809	Rsa1.0_01236.1	859	50156	1.00E−104	199	201	99	+	220	420	44319	44519
UN18810	Rsa1.0_00690.1	815	140527	1.00E−74	207	224	92	−	52	271	31841	32061
UN18811	Rsa1.0_00001.1	1182	363028	0	478	480	99	−	703	1182	349184	349663
UN18812	Rsa1.0_05546.1	1119	13379	1.00E−135	282	292	96	+	381	670	7603	7894
UN18813	Rsa1.0_00686.1	1464	68511	1.00E−154	285	287	99	+	1176	1462	59729	60013
UN18814	Rsa1.0_00429.1	1605	85559	0	735	750	98	−	69	818	23587	24336
UN18815	Rsa1.0_06489.1	1005	10917	0	482	482	100	−	29	510	4840	5321
UN18816	Rsa1.0_00628.1	657	214360	1.00E−163	326	335	97	+	1	333	34425	34758
UN18817	Rsa1.0_03016.1	1065	26000	1.00E−148	272	274	99	−	17	290	24452	24725
UN18818	Rsa1.0_01675.1	744	41155	1.00E−153	287	290	98	+	1	289	10320	10609
UN18819	Rsa1.0_00543.1	1186	76788	0	452	476	94	+	2	468	17171	17643
UN18820	Rsa1.0_05631.1	762	13127	1.00E−139	310	326	95	−	38	359	4477	4802
UN18821	Rsa1.0_03916.1	1103	20117	0	463	465	99	+	1	465	13460	13924
UN18822	Rsa1.0_00120.1	1358	277288	0	763	776	98	−	393	1164	272480	273251
UN18823	Rsa1.0_00741.1	1529	66117	1.00E−162	309	314	98	+	13	325	46297	46610
UN18824	Rsa1.0_01453.1	1055	45449	1.00E−132	333	369	90	−	702	1046	25082	25450
UN18825	Rsa1.0_00010.1	968	294827	0	381	383	99	+	33	415	269513	269894
UN18826	Rsa1.0_00108.1	1279	478491	1.00E−96	264	286	92	+	99	371	67382	67663
UN18827	Rsa1.0_03005.1	679	26082	0	654	685	95	+	3	679	19677	20352
UN18828	Rsa1.0_00586.1	897	163802	1.00E−116	244	253	96	+	161	412	154481	154733
UN18829	Rsa1.0_08526.1	1298	7236	0	376	382	98	+	916	1297	1300	1681
UN18830	Rsa1.0_00363.1	770	90968	0	753	770	97	−	1	770	14384	15153
UN18831	Rsa1.0_00190.1	873	318240	1.00E−175	353	367	96	−	177	543	4626	4992
UN18832	Rsa1.0_00494.1	866	79967	0	856	865	98	+	3	866	48939	49803
UN18833	Rsa1.0_00026.1	1032	327835	1.00E−127	294	312	94	−	731	1030	191591	191902
UN18834	Rsa1.0_04387.1	1187	17625	0	478	498	95	−	1	488	7587	8082
UN18835	Rsa1.0_01469.1	1340	45139	1.00E−166	311	316	98	+	355	670	18477	18792

UN18836	Rsa1.0_15186.1	828	2772	4.00E-74	154	158	97	-	74	231	1966	2123
UN18837	Rsa1.0_00094.1	836	139042	1.00E-157	330	346	95	+	13	358	4652	4992
UN18838	Rsa1.0_00821.1	1069	62891	0	454	468	97	+	3	469	46889	47355
UN18839	Rsa1.0_00521.1	1390	78191	0	448	449	99	-	1	449	64098	64546
UN18840	Rsa1.0_00662.1	843	192183	1.00E-155	300	306	98	-	1	305	190197	190502
UN18841	Rsa1.0_01851.1	603	38275	1.00E-161	321	328	97	-	278	603	28477	28803
UN18842	Rsa1.0_03356.1	3393	23487	0	1318	1321	99	+	133	1453	3614	4934
UN18843	Rsa1.0_00228.1	1379	105511	0	484	503	96	+	873	1375	82494	82991
UN18844	Rsa1.0_01101.1	1267	54031	1.00E-112	206	206	100	+	1060	1265	24383	24588
UN18845	Rsa1.0_03228.1	898	24471	0	406	419	96	+	475	892	22305	22722
UN18846	Rsa1.0_01494.1	2041	44641	0	753	783	96	-	1250	2027	11207	11988
UN18847	Rsa1.0_00422.1	626	86144	0	477	488	97	+	141	626	16094	16580
UN18848	Rsa1.0_00413.1	1465	86921	0	612	615	99	-	351	965	31378	31992
UN18849	Rsa1.0_74600.1	825	307	1.00E-164	299	301	99	-	73	373	7	307
UN18850	Rsa1.0_03133.1	1330	25135	0	669	679	98	+	408	1086	11553	12231
UN18851	Rsa1.0_04073.1	1637	19238	1.00E-132	243	244	99	-	1085	1328	11043	11286
UN18852	Rsa1.0_04095.1	1108	19095	0	353	353	100	-	3	355	12377	12729
UN18853	Rsa1.0_00234.1	1952	197060	0	1551	1603	96	-	131	1725	13733	15331
UN18854	Rsa1.0_06247.1	1806	11395	0	778	841	92	+	784	1623	7116	7931
UN18855	Rsa1.0_14744.1	1284	3111	0	485	506	95	-	340	845	724	1229
UN18856	Rsa1.0_00173.1	957	288743	1.00E-116	260	268	97	-	675	936	26009	26275
UN18857	Rsa1.0_01762.1	1967	39682	7.00E-81	175	182	96	-	1576	1757	7088	7269
UN18858	Rsa1.0_00603.1	684	73299	0	465	475	97	+	209	683	32629	33097
UN18859	Rsa1.0_00517.1	830	142790	1.00E-144	285	291	97	+	540	829	69988	70276
UN18860	Rsa1.0_05943.1	1056	12158	0	588	603	97	-	8	610	6907	7509
UN18861	Rsa1.0_00457.1	899	337301	0	556	573	97	-	1	573	335355	335927
UN18862	Rsa1.0_02188.1	1725	34104	0	498	518	96	-	1212	1723	2416	2933
UN18863	Rsa1.0_00086.1	1310	439854	1.00E-112	215	218	98	-	5	222	373324	373541
UN18864	Rsa1.0_00009.1	984	232558	0	976	984	99	+	2	983	197731	198714
UN18865	Rsa1.0_00056.1	1215	332604	0	1012	1083	93	+	1	1078	84534	85600
UN18866	Rsa1.0_00095.1	1279	283384	1.00E-170	352	367	95	-	1	360	16815	17181
UN18867	Rsa1.0_14668.1	1679	3173	0	1188	1199	99	-	481	1679	1153	2343
UN18868	Rsa1.0_21467.1	1454	1200	0	639	672	95	+	588	1256	1	663
UN18869	Rsa1.0_00025.1	841	277312	0	327	327	100	+	1	327	149589	149915
UN18870	Rsa1.0_00972.1	1171	57937	0	464	494	93	+	674	1167	53820	54309
UN18871	Rsa1.0_00308.1	703	96915	1.00E-76	266	302	88	-	1	289	80355	80656
UN18872	Rsa1.0_21660.1	1698	1184	0	477	503	94	+	309	805	506	1000
UN18873	Rsa1.0_00107.1	1546	431659	0	510	531	96	-	590	1114	148415	148945
UN18874	Rsa1.0_01969.1	512	153351	1.00E-125	298	318	93	-	1	314	22253	22565

UN18875	Rsa1.0_01232.1	1317	155185	1.00E-115	220	223	98	−	272	494	131817	132039
UN18876	Rsa1.0_01455.1	1603	45440	0	647	679	95	+	931	1603	24534	25195
UN18877	Rsa1.0_00775.1	1960	64793	0	363	367	98	−	1595	1960	9000	9366
UN18878	Rsa1.0_02176.1	1520	34291	0	628	644	97	+	2	644	14455	15098
UN18879	Rsa1.0_00418.1	1266	215377	1.00E-156	298	304	98	−	1	304	47449	47752
UN18880	Rsa1.0_00620.1	1082	71985	0	906	913	99	+	168	1080	8178	9090
UN18881	Rsa1.0_01029.1	1091	256254	1.00E-153	336	354	94	+	741	1091	245893	246242
UN18882	Rsa1.0_00199.1	1453	249604	0	387	388	99	+	311	698	60822	61209
UN18883	Rsa1.0_00981.1	1836	57700	0	518	522	99	−	1315	1836	48205	48726
UN18884	Rsa1.0_02954.1	1296	26485	0	1282	1295	98	−	3	1294	16411	17704
UN18885	Rsa1.0_01198.1	828	51029	0	820	821	99	−	7	827	20993	21813
UN18886	Rsa1.0_01247.1	2017	49934	0	1160	1185	97	−	834	2017	7059	8223
UN18887	Rsa1.0_12119.1	959	5311	1.00E-138	264	269	98	+	64	332	4588	4856
UN18888	Rsa1.0_00183.1	1719	113277	0	764	788	96	+	3	790	62438	63223
UN18889	Rsa1.0_00237.1	565	235974	1.00E-172	358	375	95	+	191	565	58852	59226
UN18890	Rsa1.0_14939.1	1182	2948	0	478	482	99	−	303	783	559	1040
UN18891	Rsa1.0_02454.1	804	31263	0	556	571	97	+	227	797	27132	27702
UN18892	Rsa1.0_08062.1	1078	7978	0	489	520	94	−	26	531	6822	7336
UN18893	Rsa1.0_00357.1	1626	173671	1.00E-168	322	328	98	+	746	1073	8462	8788
UN18894	Rsa1.0_00425.1	624	147080	1.00E-134	314	337	93	+	2	337	132376	132701
UN18895	Rsa1.0_03199.1	1150	24640	0	541	551	98	+	599	1149	16239	16789
UN18896	Rsa1.0_00114.1	557	131330	0	532	553	96	−	10	557	65804	66351
UN18897	Rsa1.0_03671.1	1083	21459	1.00E-142	358	384	93	−	1	374	9842	10224
UN18898	Rsa1.0_02103.1	1416	35006	0	1193	1217	98	+	10	1222	29031	30243
UN18899	Rsa1.0_00002.1	1073	601661	1.00E-176	368	387	95	+	3	389	264137	264511
UN18900	Rsa1.0_05878.1	1305	12344	0	1258	1300	96	+	8	1305	7156	8451
UN18901	Rsa1.0_01760.1	1040	39730	0	351	354	99	−	488	841	1443	1796
UN18902	Rsa1.0_00048.1	1292	167185	0	630	642	98	+	643	1284	63567	64203
UN18903	Rsa1.0_00615.1	1811	72235	0	898	902	99	+	3	902	21727	22628
UN18904	Rsa1.0_00038.1	1107	314568	1.00E-145	262	262	100	−	844	1105	8948	9209
UN18905	Rsa1.0_00773.1	1165	64839	1.00E-96	201	208	96	−	422	629	58210	58417
UN18906	Rsa1.0_02807.1	1247	27680	0	464	500	92	−	736	1217	4965	5464
UN18907	Rsa1.0_16685.1	1687	2065	0	1130	1159	97	−	2	1160	670	1826
UN18908	Rsa1.0_00512.1	1358	200277	1.00E-170	315	319	98	+	1035	1353	66192	66510
UN18909	Rsa1.0_58003.1	899	394	3.00E-84	191	199	95	−	678	875	117	314
UN18910	Rsa1.0_00495.1	787	79934	2.00E-76	149	150	99	−	107	256	19840	19989
UN18911	Rsa1.0_02717.1	1439	28458	0	624	648	96	+	804	1439	26715	27360
UN18912	Rsa1.0_11116.1	1734	5612	0	627	638	98	+	410	1047	4936	5573
UN18913	Rsa1.0_00637.1	1116	71038	0	451	459	98	−	615	1073	46365	46823

UN18914	Rsa1.0_02528.1	1165	30452	0	406	411	98	+		754	1164	21646	22056
UN18915	Rsa1.0_03499.1	1679	22462	0	711	744	95	−		887	1628	8424	9161
UN18916	Rsa1.0_02486.1	885	30965	0	551	584	94	−		302	879	11945	12522
UN18917	Rsa1.0_00114.1	906	131330	0	453	456	99	+		449	904	117617	118072
UN18918	Rsa1.0_01496.1	623	44595	0	615	622	98	+		1	622	15261	15878
UN18919	Rsa1.0_00354.1	1305	232005	0	548	560	97	−		251	810	230428	230987
UN18920	Rsa1.0_10159.1	1120	5971	0	410	414	99	+		707	1120	298	711
UN18921	Rsa1.0_03259.1	1852	24290	0	540	568	95	+		20	579	10286	10841
UN18922	Rsa1.0_23805.1	516	1024	4.00E−64	125	125	100	−		11	135	635	759
UN18923	Rsa1.0_02545.1	1359	30286	0	459	467	98	+		727	1193	1356	1822
UN18924	Rsa1.0_00215.1	822	201775	0	409	413	99	+		3	415	162441	162853
UN18925	Rsa1.0_00472.1	1773	398134	0	1099	1117	98	−		2	1118	329133	330246
UN18926	Rsa1.0_00783.1	1189	64459	0	1171	1194	98	−		3	1189	19848	21041
UN18927	Rsa1.0_01969.1	508	153351	1.00E−170	309	310	99	+		199	508	22253	22561
UN18928	Rsa1.0_01148.1	1101	52375	0	533	542	98	+		561	1101	33458	33998
UN18929	Rsa1.0_00706.1	1218	291570	0	667	686	97	+		498	1183	47607	48289
UN18930	Rsa1.0_00913.1	1316	59914	1.00E−155	383	416	92	−		2	414	16421	16825
UN18931	Rsa1.0_01182.1	1414	167304	1.00E−141	307	326	94	−		1082	1407	11280	11595
UN18932	Rsa1.0_00498.1	974	79746	0	448	451	99	−		522	972	65535	65985
UN18933	Rsa1.0_00044.1	1400	271658	0	834	858	97	+		1	854	68372	69227
UN18934	Rsa1.0_00447.1	1489	84051	0	369	371	99	−		3	373	14586	14956
UN18935	Rsa1.0_00457.1	2676	337301	0	1987	2111	94	+		575	2676	316699	318784
UN18936	Rsa1.0_00312.1	1021	96765	0	485	486	99	−		528	1013	57361	57846
UN18937	Rsa1.0_00154.1	772	189461	0	367	375	97	+		151	522	104819	105193
UN18938	Rsa1.0_00005.1	1506	353093	0	357	357	100	−		100	456	118786	119142
UN18939	Rsa1.0_07217.1	1964	9703	1.00E−173	387	415	93	+		181	594	4498	4897
UN18940	Rsa1.0_01546.1	881	43586	0	820	847	96	+		11	853	30259	31099
UN18941	Rsa1.0_00254.1	1070	102600	5.00E−96	254	277	91	+		3	261	77326	77601
UN18942	Rsa1.0_03801.1	1340	20762	0	356	357	99	−		982	1338	249	605
UN18943	Rsa1.0_00481.1	927	81316	0	590	603	97	−		35	637	40269	40871
UN18944	Rsa1.0_00215.1	2583	201775	0	736	798	92	+		179	966	168643	169409
UN18945	Rsa1.0_01180.1	1172	51517	0	482	492	97	−		1	492	15698	16187
UN18946	Rsa1.0_02451.1	1490	31283	1.00E−117	301	325	92	−		8	330	9158	9479
UN18947	Rsa1.0_15160.1	1567	2789	0	560	585	95	+		912	1492	1	585
UN18948	Rsa1.0_03562.1	1125	22084	0	1098	1123	97	+		1	1123	16092	17214
UN18949	Rsa1.0_03183.1	827	24755	0	360	361	99	+		296	656	24205	24565
UN18950	Rsa1.0_00981.1	905	57700	1.00E−153	275	275	100	+		82	356	13052	13326
UN18951	Rsa1.0_01075.1	1326	156953	0	1307	1325	98	−		2	1326	126690	128009
UN18952	#	#	#	#	#	#	#	#	#	#	#	#	

UN18953	Rsa1.0_00284.1	1132	323267	1.00E-130	406	461	88	−	21	464	275018	275460
UN18954	Rsa1.0_00710.1	806	67325	1.00E-168	435	479	90	−	1	473	28170	28648
UN18955	Rsa1.0_01028.1	919	56401	0	644	657	98	+	1	656	7710	8365
UN18956	Rsa1.0_05336.1	1498	14117	0	1000	1075	93	−	3	1062	5128	6181
UN18957	Rsa1.0_01397.1	839	183276	0	744	775	96	+	38	800	16441	17215
UN18958	Rsa1.0_03562.1	1160	22084	0	1102	1143	96	−	21	1160	16086	17225
UN18959	Rsa1.0_00590.1	1267	73835	0	480	488	98	−	780	1265	23232	23718
UN18960	Rsa1.0_03237.1	1594	24434	1.00E-166	356	372	95	−	1227	1594	4566	4937
UN18961	Rsa1.0_04711.1	1188	16282	0	822	833	98	+	356	1188	11996	12828
UN18962	Rsa1.0_00218.1	1022	201480	1.00E-131	244	246	99	+	223	468	172495	172740
UN18963	Rsa1.0_03227.1	887	24478	0	863	886	97	+	3	887	4854	5737
UN18964	Rsa1.0_00040.1	1261	324196	0	646	656	98	+	606	1261	33258	33911
UN18965	Rsa1.0_00028.1	1604	293984	0	439	452	97	+	1160	1604	276975	277426
UN18966	Rsa1.0_00111.1	1810	390088	0	498	523	95	+	1286	1804	334412	334932
UN18967	Rsa1.0_00213.1	1694	108167	0	1053	1099	95	+	2	1100	9922	11001
UN18968	Rsa1.0_00206.1	916	109040	1.00E-137	257	260	98	−	655	914	45109	45368
UN18969	Rsa1.0_00103.1	1259	135304	4.00E-44	186	216	86	−	86	301	31552	31766
UN18970	Rsa1.0_00028.1	2943	293984	0	672	684	98	−	658	1337	54913	55596
UN18971	Rsa1.0_07603.1	1288	9043	1.00E-120	354	397	89	−	655	1051	2808	3200
UN18972	Rsa1.0_00453.1	1706	83031	0	682	684	99	+	1022	1705	16425	17107
UN18973	Rsa1.0_02428.1	1384	31542	0	478	497	96	−	180	667	28430	28923
UN18974	Rsa1.0_02411.1	1267	31655	0	604	612	98	−	651	1261	16712	17323
UN18975	Rsa1.0_00437.1	1870	84879	0	400	424	94	+	1447	1870	23633	24042
UN18976	Rsa1.0_00153.1	897	192931	0	555	580	95	+	317	892	157109	157682
UN18977	Rsa1.0_01370.1	1050	47093	1.00E-177	315	315	100	+	736	1050	9921	10235
UN18978	Rsa1.0_00301.1	1193	365315	0	528	551	95	+	602	1152	304665	305208
UN18979	Rsa1.0_00827.1	1170	62790	0	780	805	96	−	366	1170	17354	18140
UN18980	Rsa1.0_02882.1	1031	27039	0	584	587	99	−	446	1031	1457	2042
UN18981	Rsa1.0_01156.1	2095	52147	0	639	667	95	+	1438	2094	32888	33553
UN18982	Rsa1.0_03205.1	1191	24614	0	359	362	99	−	3	364	4230	4591
UN18983	Rsa1.0_00063.1	1904	156150	0	945	1016	93	−	833	1840	62977	63986
UN18984	Rsa1.0_09689.1	1436	6210	0	411	424	96	−	963	1374	1784	2207
UN18985	Rsa1.0_04801.1	1252	15918	0	344	347	99	+	6	352	6890	7236
UN18986	Rsa1.0_00763.1	1293	65121	1.00E-145	268	269	99	−	1013	1280	59137	59405
UN18987	Rsa1.0_01405.1	1490	46445	0	415	431	96	−	1061	1489	37002	37432
UN18988	Rsa1.0_01141.1	2410	52530	0	376	390	96	+	36	422	3569	3955
UN18989	Rsa1.0_16203.1	932	2246	0	859	885	97	−	3	886	995	1875
UN18990	Rsa1.0_00278.1	1322	231896	0	935	963	97	+	344	1306	82162	83121
UN18991	Rsa1.0_05076.1	491	14916	1.00E-119	217	217	100	−	1	217	7475	7691

UN18992	Rsa1.0_00026.1	1217	327835	1.00E-158	298	302	98	+	683	981	24412	24713
UN18993	Rsa1.0_00206.1	1177	109040	0	507	518	97	−	2	519	68752	69269
UN18994	Rsa1.0_01555.1	759	166456	0	385	386	99	+	7	392	144938	145323
UN18995	Rsa1.0_00538.1	1514	77149	0	473	475	99	−	1040	1514	54717	55191
UN18996	Rsa1.0_09160.1	1057	6599	1.00E-172	312	314	99	−	249	562	1109	1422
UN18997	Rsa1.0_07124.1	1439	9865	0	516	527	97	−	913	1439	7506	8031
UN18998	Rsa1.0_02402.1	1260	31710	0	650	654	99	+	1	654	5332	5984
UN18999	Rsa1.0_02446.1	1816	31330	0	1372	1416	96	−	290	1701	3866	5281
UN19000	Rsa1.0_05319.1	999	14158	0	324	324	100	+	3	326	379	702
UN19001	Rsa1.0_00020.1	1561	283099	0	1040	1069	97	+	6	1073	94582	95650
UN19002	Rsa1.0_03627.1	1757	21658	1.00E-147	312	325	96	−	1433	1757	661	983
UN19003	Rsa1.0_00015.1	1131	561803	1.00E-166	297	297	100	+	835	1131	529332	529628
UN19004	Rsa1.0_00053.1	1557	263459	0	818	857	95	−	706	1556	242869	243720
UN19005	Rsa1.0_05792.1	1731	12612	0	550	570	96	−	1	568	8012	8581
UN19006	Rsa1.0_01354.1	658	47342	3.00E-74	169	174	97	−	352	523	9457	9629
UN19007	Rsa1.0_04576.1	1475	16783	1.00E-127	234	235	99	−	1241	1475	393	627
UN19008	Rsa1.0_00548.1	957	76518	2.00E-21	244	301	81	−	285	580	62952	63251
UN19009	Rsa1.0_00655.1	1365	195840	0	362	362	100	+	1004	1365	163505	163866
UN19010	Rsa1.0_03555.1	1542	22129	0	653	674	96	+	160	833	16383	17056
UN19011	Rsa1.0_00857.1	1427	61904	0	620	695	89	−	617	1308	12884	13578
UN19012	Rsa1.0_00010.1	1218	294827	0	956	990	96	−	3	984	102962	103944
UN19013	Rsa1.0_00460.1	1100	206387	0	1089	1100	99	−	1	1099	196168	197267
UN19014	Rsa1.0_00389.1	1900	88795	0	1361	1443	94	+	1	1424	16759	18177
UN19015	Rsa1.0_15068.1	1550	2854	0	1056	1086	97	−	473	1548	999	2084
UN19016	Rsa1.0_00037.1	1460	307838	1.00E-168	304	305	99	+	750	1054	284445	284749
UN19017	Rsa1.0_00639.1	1963	165873	0	1076	1093	98	+	2	1090	134022	135114
UN19018	Rsa1.0_03589.1	1421	21893	0	428	436	98	−	986	1419	624	1055
UN19019	Rsa1.0_00302.1	1326	97521	0	362	371	97	+	3	373	64950	65312
UN19020	Rsa1.0_01113.1	1321	53616	1.00E-127	258	267	96	+	859	1125	4376	4642
UN19021	Rsa1.0_03261.1	1602	24281	0	1596	1598	99	−	2	1597	20779	22376
UN19022	Rsa1.0_01448.1	834	45645	0	446	446	100	−	388	833	37869	38314
UN19023	Rsa1.0_72585.1	490	316	1.00E-107	200	201	99	+	289	489	1	201
UN19024	Rsa1.0_03311.1	1405	23850	0	442	447	98	+	500	946	1218	1664
UN19025	Rsa1.0_02663.1	1141	169243	1.00E-139	255	256	99	−	133	388	168988	169243
UN19026	Rsa1.0_00826.1	1630	166736	0	1517	1522	99	−	109	1630	14148	15665
UN19027	Rsa1.0_00107.1	1495	431659	0	1004	1032	97	−	2	1033	326713	327744
UN19028	Rsa1.0_11964.1	1004	5359	9.00E-85	311	361	86	−	655	1001	108	468
UN19029	Rsa1.0_03503.1	1769	22445	0	922	984	93	−	794	1769	19337	20315
UN19030	Rsa1.0_02769.1	1255	28107	0	369	377	97	+	370	746	10869	11239

UN19031	Rsa1.0_00374.1	1057	165738	1.00E-127	254	259	98	-	85	343	5796	6051
UN19032	Rsa1.0_03646.1	1484	21598	1.00E-159	306	314	97	+	1	314	7610	7915
UN19033	Rsa1.0_00033.1	1079	515811	0	462	468	98	+	1	467	431431	431898
UN19034	Rsa1.0_00092.1	1703	659932	1.00E-124	257	267	96	+	1105	1371	189112	189378
UN19035	Rsa1.0_00615.1	1440	72235	0	1314	1352	97	+	89	1439	60843	62177
UN19036	Rsa1.0_00050.1	679	237174	0	610	672	90	-	11	677	84436	85092
UN19037	Rsa1.0_00377.1	1113	90038	0	422	435	97	-	1	435	9787	10211
UN19038	Rsa1.0_02670.1	1366	28887	1.00E-180	413	437	94	+	1	431	3772	4204
UN19039	Rsa1.0_00045.1	1186	328694	0	509	509	100	+	1	509	184209	184717
UN19040	Rsa1.0_00844.1	1633	62403	0	560	570	98	-	1	570	41735	42304
UN19041	Rsa1.0_01016.1	945	181422	1.00E-123	227	228	99	-	211	438	3575	3802
UN19042	Rsa1.0_00893.1	1056	60626	0	680	706	96	+	1	703	43093	43797
UN19043	Rsa1.0_13471.1	1635	4802	0	497	526	94	-	2	526	3829	4346
UN19044	Rsa1.0_00332.1	746	94575	0	741	743	99	-	3	744	68951	69693
UN19045	Rsa1.0_00039.1	2141	296927	0	671	687	97	+	4	690	67268	67953
UN19046	Rsa1.0_49549.1	504	458	3.00E-86	165	166	99	+	339	504	75	240
UN19047	Rsa1.0_06272.1	3254	11351	5.00E-86	163	163	100	+	2949	3111	1	163
UN19048	Rsa1.0_06645.1	1420	10607	0	963	977	98	-	1	977	8052	9028
UN19049	Rsa1.0_00079.1	912	606987	1.00E-117	260	274	94	-	18	285	41618	41891
UN19050	Rsa1.0_00732.1	1180	66631	0	359	359	100	+	3	361	140	498
UN19051	Rsa1.0_00773.1	1386	64839	0	859	930	92	+	476	1385	25726	26634
UN19052	Rsa1.0_00406.1	1065	87398	0	370	373	99	-	5	377	53293	53665
UN19053	Rsa1.0_01295.1	810	49040	1.00E-142	257	257	100	-	554	810	15483	15739
UN19054	Rsa1.0_03688.1	1661	143676	1.00E-137	314	333	94	-	1324	1650	3787	4119
UN19055	Rsa1.0_04692.1	1195	16357	0	1066	1073	99	+	127	1195	12091	13163
UN19056	Rsa1.0_00046.1	1066	320315	0	475	492	96	-	3	493	31869	32359
UN19057	Rsa1.0_00043.1	1454	264937	0	455	467	97	-	998	1454	259731	260197
UN19058	Rsa1.0_01494.1	1202	44641	0	887	888	99	-	315	1202	11101	11988
UN19059	Rsa1.0_02663.1	1616	169243	0	710	710	100	+	907	1616	7793	8502
UN19060	Rsa1.0_00273.1	717	326186	1.00E-161	347	366	94	-	283	645	113973	114338
UN19061	Rsa1.0_01005.1	921	56967	0	482	482	100	+	440	921	39743	40224
UN19062	Rsa1.0_00939.1	1424	185227	0	484	537	90	-	237	773	171556	172059
UN19063	Rsa1.0_02490.1	1542	30885	1.00E-143	262	263	99	+	226	488	26954	27216
UN19064	Rsa1.0_00117.1	1107	213118	0	380	380	100	-	728	1107	202418	202797
UN19065	Rsa1.0_02536.1	1273	30368	1.00E-171	370	388	95	-	880	1265	21481	21861
UN19066	Rsa1.0_00753.1	1486	65705	0	834	852	97	+	2	853	57301	58152
UN19067	Rsa1.0_07521.1	1537	9206	0	521	526	99	-	3	528	7907	8429
UN19068	Rsa1.0_00833.1	1069	62621	0	1043	1069	97	+	1	1069	37633	38699
UN19069	Rsa1.0_00041.1	2190	186197	1.00E-111	324	359	90	+	1758	2106	148191	148548

UN19070	Rsa1.0_00816.1	1266	403350	0	668	680	98	−	587	1265	137456	138135
UN19071	Rsa1.0_00089.1	1560	406142	0	810	829	97	+	716	1542	374035	374863
UN19072	Rsa1.0_02615.1	714	29547	1.00E−171	305	305	100	−	407	711	15642	15946
UN19073	Rsa1.0_00191.1	834	316976	0	438	442	99	−	393	834	197443	197882
UN19074	Rsa1.0_03825.1	1373	20623	0	332	333	99	+	1041	1373	6154	6486
UN19075	Rsa1.0_00034.1	1154	260119	0	416	421	98	−	2	419	841	1261
UN19076	Rsa1.0_01773.1	1565	39546	0	1500	1540	97	+	28	1564	17250	18789
UN19077	Rsa1.0_01307.1	710	48685	0	704	707	99	−	4	710	11808	12514
UN19078	Rsa1.0_03280.1	1578	145083	0	608	620	98	+	2	621	125643	126262
UN19079	Rsa1.0_00124.1	672	262503	1.00E−108	273	292	93	−	382	671	14159	14444
UN19080	Rsa1.0_02797.1	1647	27800	0	537	563	95	−	1086	1642	13743	14301
UN19081	Rsa1.0_00198.1	616	222963	1.00E−162	334	346	96	+	1	346	37757	38100
UN19082	Rsa1.0_02541.1	1446	30342	0	481	489	98	−	3	491	11560	12046
UN19083	Rsa1.0_02841.1	1422	27383	1.00E−137	380	414	91	−	676	1079	78	485
UN19084	Rsa1.0_01513.1	1966	44234	0	1869	1909	97	−	1	1900	41226	43133
UN19085	Rsa1.0_02922.1	1462	26753	0	368	373	98	−	1	373	11365	11734
UN19086	Rsa1.0_02113.1	856	34890	0	852	854	99	−	3	855	14224	15077
UN19087	Rsa1.0_00005.1	1333	353093	1.00E−150	311	322	96	−	3	322	130794	131115
UN19088	Rsa1.0_01772.1	1306	39557	1.00E−118	217	217	100	−	3	219	8198	8414
UN19089	Rsa1.0_00006.1	1485	396176	0	454	460	98	+	2	457	107049	107508
UN19090	Rsa1.0_02735.1	1551	28344	1.00E−107	248	261	95	−	87	345	28008	28266
UN19091	Rsa1.0_09270.1	1283	6513	0	509	520	97	+	469	988	1	520
UN19092	Rsa1.0_00178.1	720	114861	1.00E−151	328	343	95	−	7	347	12165	12501
UN19093	Rsa1.0_00467.1	1316	156542	0	388	388	100	+	106	493	134097	134484
UN19094	Rsa1.0_09067.1	1315	6672	1.00E−114	248	257	96	+	3	258	2323	2577
UN19095	Rsa1.0_19308.1	1176	1449	0	933	939	99	+	1	936	511	1449
UN19096	Rsa1.0_00817.1	871	62961	0	520	537	96	−	335	871	53797	54331
UN19097	Rsa1.0_04256.1	857	18257	1.00E−147	267	268	99	−	590	857	13574	13841
UN19098	Rsa1.0_03117.1	1095	25295	3.00E−23	103	116	88	+	117	232	17004	17116
UN19099	Rsa1.0_05293.1	1343	14237	0	392	406	96	−	800	1205	6325	6730
UN19100	Rsa1.0_01168.1	738	153765	1.00E−175	337	344	97	+	398	738	41780	42121
UN19101	Rsa1.0_00035.1	1366	176449	0	1347	1368	98	−	2	1366	78863	80230
UN19102	Rsa1.0_01760.1	923	39730	0	468	468	100	+	456	923	1864	2331
UN19103	Rsa1.0_01298.1	1307	48999	1.00E−160	290	291	99	+	13	303	6681	6971
UN19104	Rsa1.0_03131.1	887	25142	0	859	859	100	−	27	885	3582	4440
UN19105	Rsa1.0_00910.1	801	59975	0	501	501	100	−	3	503	58026	58526
UN19106	Rsa1.0_08342.1	1300	7502	0	344	346	99	+	955	1300	5075	5420
UN19107	Rsa1.0_01606.1	862	42432	0	794	828	95	−	46	862	38803	39630
UN19108	Rsa1.0_00009.1	2331	232558	0	1467	1509	97	+	2	1509	26775	28283

UN19109	Rsa1.0_00039.1	1559	296927	0	1552	1560	99	+	2	1559	146905	148464
UN19110	Rsa1.0_04894.1	1257	15569	0	785	786	99	+	1	786	7358	8142
UN19111	Rsa1.0_02377.1	1449	31994	0	631	648	97	+	358	1005	4733	5380
UN19112	Rsa1.0_04651.1	1468	16516	0	561	581	96	−	335	914	13808	14388
UN19113	Rsa1.0_07811.1	1071	8496	0	588	588	100	+	482	1069	1308	1895
UN19114	Rsa1.0_00728.1	917	66704	1.00E−136	268	274	97	+	2	275	33849	34120
UN19115	Rsa1.0_03469.1	718	22681	0	685	715	95	+	1	712	11103	11814
UN19116	Rsa1.0_00122.1	981	265937	2.00E−98	205	211	97	+	772	981	7192	7402
UN19117	Rsa1.0_03111.1	859	25327	0	375	377	99	−	284	660	4629	5005
UN19118	Rsa1.0_04073.1	1803	19238	1.00E−131	248	251	98	−	1	251	13359	13609
UN19119	Rsa1.0_00203.1	1112	109129	1.00E−114	212	213	99	+	467	679	22484	22696
UN19120	Rsa1.0_00790.1	844	148693	0	831	841	98	−	4	844	43541	44381
UN19121	Rsa1.0_04371.1	647	17700	0	626	649	96	+	2	646	14353	14997
UN19122	Rsa1.0_00073.1	901	240924	6.00E−98	191	194	98	+	357	550	98132	98325
UN19123	Rsa1.0_04181.1	1340	18615	0	714	723	98	+	281	1003	936	1658
UN19124	Rsa1.0_00003.1	1267	340054	0	405	418	96	−	850	1265	276988	277405
UN19125	Rsa1.0_00683.1	1539	68638	1.00E−155	296	302	98	−	1237	1538	8503	8804
UN19126	Rsa1.0_00010.1	976	294827	0	419	421	99	−	558	976	81207	81627
UN19127	Rsa1.0_00336.1	899	445051	0	629	631	99	−	267	897	194552	195182
UN19128	Rsa1.0_06819.1	1282	10302	0	1229	1271	96	+	10	1280	1017	2284
UN19129	Rsa1.0_00896.1	1973	60509	0	662	664	99	−	643	1306	20944	21607
UN19130	Rsa1.0_00727.1	957	66724	0	938	950	98	−	16	956	36814	37763
UN19131	Rsa1.0_06715.1	1030	10485	0	396	410	96	+	19	426	3413	3822
UN19132	Rsa1.0_06603.1	1406	10682	0	393	400	98	−	1	399	7727	8126
UN19133	Rsa1.0_00556.1	1403	207212	0	573	617	92	−	4	608	18791	19403
UN19134	Rsa1.0_00024.1	1064	188018	1.00E−138	280	289	96	+	1	288	75329	75612
UN19135	Rsa1.0_00179.1	1123	393324	1.00E−151	403	439	91	−	693	1123	280262	280692
UN19136	Rsa1.0_00211.1	911	228189	0	357	358	99	−	93	450	145393	145750
UN19137	Rsa1.0_00015.1	1568	561803	0	1305	1331	98	+	239	1568	252554	253883
UN19138	Rsa1.0_02645.1	1289	29158	1.00E−178	335	341	98	+	949	1289	3773	4113
UN19139	Rsa1.0_01043.1	1192	55893	0	549	572	95	+	622	1192	33225	33789
UN19140	Rsa1.0_00748.1	832	65983	0	457	483	94	−	28	504	63996	64475
UN19141	Rsa1.0_00093.1	1550	214962	0	549	574	95	−	975	1540	128325	128896
UN19142	Rsa1.0_00195.1	587	251884	1.00E−178	330	333	99	+	3	335	229134	229465
UN19143	Rsa1.0_00002.1	866	601661	0	864	865	99	−	3	866	113211	114075
UN19144	Rsa1.0_00562.1	1930	75565	0	1264	1285	98	+	546	1824	8259	9537
UN19145	Rsa1.0_02033.1	1452	35828	0	710	736	96	−	7	741	31310	32043
UN19146	Rsa1.0_00439.1	818	142776	0	414	423	97	+	3	422	57722	58144
UN19147	Rsa1.0_00759.1	1030	65465	1.00E−179	339	346	97	−	665	1010	61787	62132

UN19148	Rsa1.0_00454.1	1289	216970	1.00E-121	221	221	100	+	1069	1289	146032	146252
UN19149	Rsa1.0_00083.1	1373	442773	0	1213	1215	99	+	3	1217	432305	433519
UN19150	Rsa1.0_39138.1	1232	575	0	337	339	99	−	2	340	1	338
UN19151	Rsa1.0_00212.1	984	108350	0	704	705	99	+	280	984	14132	14836
UN19152	Rsa1.0_00372.1	1519	90418	0	486	486	100	+	1	486	14572	15057
UN19153	Rsa1.0_00751.1	1272	65759	0	419	430	97	−	640	1069	3274	3702
UN19154	Rsa1.0_01606.1	1194	42432	1.00E-166	378	402	94	+	1	389	16041	16440
UN19155	Rsa1.0_02552.1	1939	30191	0	463	466	99	+	1474	1939	21612	22077
UN19156	Rsa1.0_00035.1	1003	176449	0	660	695	94	+	1	676	51397	52091
UN19157	Rsa1.0_00063.1	608	156150	1.00E-125	231	232	99	−	258	489	3494	3725
UN19158	Rsa1.0_00016.1	852	831256	0	839	855	98	+	3	850	684570	685421
UN19159	Rsa1.0_01387.1	981	46745	6.00E-95	262	289	90	+	1	275	31034	31320
UN19160	Rsa1.0_15067.1	1610	2854	0	613	615	99	+	2	616	646	1260
UN19161	Rsa1.0_03288.1	1001	24016	0	386	394	97	−	2	393	6221	6611
UN19162	Rsa1.0_01730.1	977	40223	0	373	381	97	+	154	534	22751	23131
UN19163	Rsa1.0_00366.1	715	90880	0	360	368	97	+	1	368	9189	9556
UN19164	Rsa1.0_00066.1	638	152418	0	563	582	96	−	3	579	63404	63985
UN19165	Rsa1.0_00055.1	1504	520193	1.00E-120	282	298	94	−	1	295	27909	28204
UN19166	Rsa1.0_22514.1	1234	1114	0	587	619	94	+	614	1232	212	830
UN19167	Rsa1.0_02040.1	1207	35684	1.00E-126	285	302	94	+	57	347	13775	14076
UN19168	Rsa1.0_00495.1	945	79934	1.00E-135	248	249	99	+	1	249	16085	16333
UN19169	Rsa1.0_00188.1	576	112020	0	504	555	90	−	11	561	4438	4977
UN19170	Rsa1.0_01191.1	1142	51235	0	778	779	99	+	1	779	48414	49192
UN19171	Rsa1.0_00047.1	1305	168582	0	1221	1249	97	−	57	1304	134145	135393
UN19172	Rsa1.0_00136.1	1449	122464	0	551	565	97	−	1	565	84856	85417
UN19173	Rsa1.0_33830.1	1009	670	0	408	411	99	+	133	543	1	411
UN19174	Rsa1.0_00179.1	1115	393324	0	750	759	98	+	357	1115	320006	320764
UN19175	Rsa1.0_09755.1	705	6166	0	480	504	95	−	1	500	1225	1723
UN19176	Rsa1.0_07895.1	1679	8344	0	1514	1666	90	−	1	1656	5934	7587
UN19177	Rsa1.0_07783.1	1043	8578	0	898	900	99	+	142	1041	7277	8176
UN19178	Rsa1.0_01220.1	764	161775	0	368	370	99	+	396	764	138711	139080
UN19179	Rsa1.0_02505.1	1173	30703	1.00E-166	391	418	93	−	741	1154	9950	10365
UN19180	Rsa1.0_00727.1	871	66724	0	800	831	96	−	4	823	13535	14361
UN19181	Rsa1.0_00011.1	824	225044	0	402	412	97	−	3	414	207085	207496
UN19182	Rsa1.0_16460.1	1867	2148	0	1044	1118	93	+	583	1674	669	1773
UN19183	Rsa1.0_00651.1	552	247766	1.00E-125	228	228	100	+	178	405	81983	82210
UN19184	Rsa1.0_15387.1	1306	2642	0	390	402	97	−	906	1306	2008	2407
UN19185	Rsa1.0_00033.1	1283	515811	0	903	946	95	+	6	939	350359	351298
UN19186	Rsa1.0_28951.1	1674	798	1.00E-151	309	320	96	+	3	322	78	396

UN19187	Rsa1.0_00195.1	1878	251884	0	726	729	99	+	1	729	75462	76189
UN19188	Rsa1.0_00001.1	1779	363028	0	722	756	95	−	1025	1777	287292	288046
UN19189	Rsa1.0_00111.1	889	390088	0	395	404	97	+	493	889	85380	85783
UN19190	Rsa1.0_02493.1	879	30854	1.00E−180	332	336	98	−	3	338	3991	4326
UN19191	Rsa1.0_02409.1	1777	31663	1.00E−161	445	492	90	+	63	553	22841	23323
UN19192	Rsa1.0_01387.1	750	46745	1.00E−162	305	309	98	−	442	750	2575	2881
UN19193	Rsa1.0_00289.1	1018	98620	1.00E−145	268	270	99	−	443	712	26980	27249
UN19194	Rsa1.0_02421.1	1319	31579	0	543	545	99	+	3	547	8459	9003
UN19195	Rsa1.0_04203.1	1314	18479	0	1285	1313	97	+	1	1311	10105	11417
UN19196	Rsa1.0_07621.1	1284	9014	0	495	503	98	−	782	1284	7421	7922
UN19197	Rsa1.0_16086.1	931	2289	1.00E−109	221	228	96	+	682	903	1078	1305
UN19198	Rsa1.0_03878.1	1137	20305	0	732	752	97	+	264	1014	1652	2399
UN19199	Rsa1.0_01245.1	1712	149868	0	865	866	99	+	846	1711	21270	22134
UN19200	Rsa1.0_02298.1	1068	32872	0	489	492	99	+	575	1066	6363	6854
UN19201	Rsa1.0_00841.1	1070	183065	1.00E−138	265	269	98	−	804	1070	1038	1306
UN19202	Rsa1.0_23598.1	1697	1038	1.00E−173	315	317	99	+	3	319	579	895
UN19203	Rsa1.0_00192.1	1962	278200	1.00E−169	312	314	99	−	1648	1960	90832	91145
UN19204	Rsa1.0_00041.1	1345	186197	0	696	711	97	+	635	1345	84481	85191
UN19205	Rsa1.0_05005.1	1448	15169	0	379	381	99	−	1	381	14659	15039
UN19206	Rsa1.0_02280.1	557	33063	0	549	554	99	−	1	554	29316	29869
UN19207	Rsa1.0_00087.1	1934	761073	0	489	496	98	−	1445	1933	624624	625119
UN19208	Rsa1.0_20417.1	1711	1307	1.00E−127	249	252	98	+	669	918	362	613
UN19209	Rsa1.0_01853.1	1289	147800	1.00E−126	232	233	99	+	401	633	8320	8552
UN19210	Rsa1.0_06915.1	729	10174	0	440	444	99	−	1	444	3810	4253
UN19211	Rsa1.0_17507.1	934	1814	0	427	430	99	+	1	430	283	712
UN19212	Rsa1.0_07619.1	1428	9015	0	348	354	98	−	982	1335	3415	3766
UN19213	Rsa1.0_06313.1	1627	11252	0	1038	1044	99	+	2	1044	1213	2256
UN19214	Rsa1.0_26642.1	902	883	0	490	490	100	+	160	649	290	779
UN19215	Rsa1.0_00226.1	1449	105884	0	635	641	99	+	566	1203	42188	42828
UN19216	Rsa1.0_00031.1	1364	179862	0	484	486	99	+	165	650	76038	76523
UN19217	Rsa1.0_06839.1	1130	10286	3.00E−97	267	298	89	+	3	283	6343	6640
UN19218	Rsa1.0_10890.1	1549	5688	0	759	767	98	−	466	1232	3525	4291
UN19219	Rsa1.0_02126.1	2251	34765	0	375	376	99	−	1535	1910	4849	5224
UN19220	Rsa1.0_00518.1	1074	78360	1.00E−120	223	224	99	+	1	224	66713	66936
UN19221	Rsa1.0_00001.1	1250	363028	0	369	375	98	+	875	1249	88739	89112
UN19222	Rsa1.0_13630.1	2004	4607	0	864	875	98	+	1	874	1878	2752
UN19223	Rsa1.0_01202.1	1905	158825	0	674	691	97	+	1218	1903	45033	45719
UN19224	Rsa1.0_00817.1	1065	62961	0	618	627	98	+	437	1058	53165	53791
UN19225	Rsa1.0_04979.1	976	15257	1.00E−114	210	210	100	+	374	583	8714	8923

UN19226	Rsa1.0_01355.1	782	47303	0	708	745	95	+	3	741	39399	40140
UN19227	Rsa1.0_00087.1	1067	761073	1.00E-101	199	203	98	+	628	830	292656	292858
UN19228	Rsa1.0_01185.1	1117	149842	1.00E-122	256	267	95	+	447	713	132558	132824
UN19229	Rsa1.0_00123.1	1104	325650	0	459	477	96	−	5	474	47946	48416
UN19230	Rsa1.0_01234.1	620	50183	0	468	478	97	−	1	473	43366	43843
UN19231	Rsa1.0_01825.1	893	38725	2.00E-85	185	193	95	+	168	360	24330	24522
UN19232	Rsa1.0_02461.1	1760	31166	0	1196	1209	98	−	552	1760	4244	5452
UN19233	Rsa1.0_08043.1	1457	8019	0	486	486	100	+	972	1457	3725	4210
UN19234	Rsa1.0_01130.1	1253	52929	1.00E-175	374	393	95	+	867	1253	44531	44915
UN19235	Rsa1.0_00670.1	1715	69371	0	479	479	100	+	523	1001	31040	31518
UN19236	Rsa1.0_00762.1	1432	165246	0	912	969	94	+	481	1432	32881	33840
UN19237	Rsa1.0_02443.1	1037	31353	1.00E-135	244	244	100	−	793	1036	18128	18371
UN19238	Rsa1.0_00573.1	610	74821	1.00E-131	241	242	99	+	1	242	29923	30164
UN19239	Rsa1.0_00044.1	1241	271658	0	333	334	99	+	908	1241	139508	139841
UN19240	Rsa1.0_00915.1	917	194446	9.00E-94	211	223	94	+	148	370	178495	178717
UN19241	Rsa1.0_00009.1	1656	232558	0	994	1025	96	−	629	1653	130857	131878
UN19242	Rsa1.0_37455.1	1497	602	0	406	416	97	+	93	508	1	416
UN19243	Rsa1.0_00917.1	1453	182012	0	660	666	99	−	218	883	7047	7711
UN19244	Rsa1.0_02022.1	752	35923	1.00E-103	210	216	97	+	3	214	4698	4913
UN19245	Rsa1.0_00398.1	1373	88007	0	645	645	100	+	280	924	62692	63336
UN19246	Rsa1.0_00007.1	1803	239325	0	816	863	94	+	946	1803	29997	30851
UN19247	Rsa1.0_00101.1	616	267193	1.00E-125	274	290	94	−	328	616	4429	4708
UN19248	Rsa1.0_00167.1	1436	116000	0	404	406	99	+	4	407	12093	12498
UN19249	Rsa1.0_08756.1	2495	6955	8.00E-69	234	268	87	+	281	537	6119	6385
UN19250	Rsa1.0_00723.1	808	66806	0	724	743	97	+	48	790	58018	58756
UN19251	Rsa1.0_00572.1	1593	74969	0	1281	1293	99	−	301	1593	60862	62154
UN19252	Rsa1.0_15644.1	1219	2505	0	598	619	96	+	599	1215	577	1195
UN19253	Rsa1.0_07058.1	1093	9964	0	832	855	97	−	70	920	3048	3901
UN19254	Rsa1.0_00869.1	666	61627	1.00E-129	344	372	92	+	18	376	53852	54221
UN19255	Rsa1.0_10900.1	740	5684	0	722	733	98	+	1	733	466	1198
UN19256	Rsa1.0_00009.1	1702	232558	0	731	743	98	+	1	742	202456	203198
UN19257	Rsa1.0_20028.1	1468	1350	0	768	781	98	−	51	830	406	1186
UN19258	Rsa1.0_00367.1	971	90750	0	967	969	99	−	1	969	20755	21722
UN19259	Rsa1.0_00131.1	1092	432233	0	350	350	100	+	1	350	398586	398935
UN19260	Rsa1.0_01018.1	799	56685	1.00E-102	192	193	99	+	606	798	54766	54958
UN19261	Rsa1.0_00008.1	1751	358124	0	830	860	96	−	1	857	67627	68485
UN19262	Rsa1.0_00712.1	1747	67217	0	1083	1083	100	−	215	1297	40531	41613
UN19263	Rsa1.0_01233.1	891	50184	0	444	472	94	−	283	751	23507	23969
UN19264	Rsa1.0_04455.1	1280	17266	0	433	439	98	−	3	440	12968	13406

UN19265	Rsa1.0_01117.1	2884	53465	0	1325	1338	99	+	765	2099	52128	53465
UN19266	Rsa1.0_08532.1	738	7228	0	716	737	97	+	1	734	5235	5968
UN19267	Rsa1.0_01198.1	824	51029	0	805	831	96	−	3	824	25781	26606
UN19268	Rsa1.0_00145.1	1648	261008	0	604	628	96	−	604	1231	101443	102061
UN19269	Rsa1.0_00064.1	1163	240927	0	482	489	98	−	3	490	220651	221139
UN19270	Rsa1.0_00002.1	815	601661	2.33E−156	282	287	98	−	403	688	140391	140677
UN19271	Rsa1.0_47151.1	1233	479	0	479	479	100	+	70	548	1	479
UN19272	Rsa1.0_00158.1	1223	117999	0	448	448	100	−	1	448	14475	14922
UN19273	Rsa1.0_00556.1	1287	207212	0	510	527	96	+	3	528	193419	193935
UN19274	Rsa1.0_00836.1	1007	185968	0	641	676	94	+	21	687	161658	162333
UN19275	Rsa1.0_20661.1	1535	1282	0	988	1005	98	+	530	1534	1	999
UN19276	Rsa1.0_01283.1	1007	49257	0	461	464	99	+	526	989	7067	7530
UN19277	Rsa1.0_14140.1	1716	3748	0	549	594	92	−	513	1102	1121	1705
UN19278	Rsa1.0_02079.1	806	35306	0	567	574	98	+	206	779	24546	25119
UN19279	Rsa1.0_07786.1	771	8569	1.00E−103	190	190	100	+	578	767	173	362
UN19280	Rsa1.0_00463.1	872	189177	1.00E−135	265	272	97	+	601	872	115301	115572
UN19281	Rsa1.0_00959.1	1519	58202	0	360	365	98	+	415	779	1503	1867
UN19282	Rsa1.0_01168.1	1327	153765	0	1097	1160	94	+	172	1322	20887	22031
UN19283	Rsa1.0_00883.1	1286	61230	1.00E−127	343	376	91	−	5	374	54391	54764
UN19284	Rsa1.0_02798.1	1392	27792	0	446	450	99	+	3	452	5375	5823
UN19285	Rsa1.0_03356.1	1275	23487	0	647	654	98	+	622	1274	11582	12235
UN19286	Rsa1.0_00257.1	1258	102407	0	515	525	98	−	16	540	17702	18225
UN19287	Rsa1.0_01768.1	752	138463	0	369	376	98	−	1	375	110146	110520
UN19288	Rsa1.0_03420.1	1191	23090	1.00E−134	253	255	99	−	2	256	668	921
UN19289	Rsa1.0_00026.1	1830	327835	1.00E−168	330	340	97	−	3	342	186323	186662
UN19290	Rsa1.0_33000.1	1347	689	1.00E−165	301	303	99	+	12	314	281	583
UN19291	Rsa1.0_01806.1	476	39021	0	474	474	100	−	1	474	25710	26183
UN19292	Rsa1.0_03355.1	1767	23489	0	838	845	99	−	3	847	2596	3440
UN19293	Rsa1.0_25135.1	1596	950	0	883	969	91	+	66	1034	1	950
UN19294	Rsa1.0_00526.1	791	169279	0	482	499	96	−	198	696	148049	148547
UN19295	Rsa1.0_01632.1	1749	181224	1.00E−116	232	238	97	−	3	240	154142	154379
UN19296	Rsa1.0_09098.1	1588	6647	0	501	524	95	+	1	524	6125	6647
UN19297	Rsa1.0_02605.1	1138	29637	0	571	606	94	−	3	590	25931	26536
UN19298	Rsa1.0_01073.1	841	55030	0	827	840	98	+	2	841	34942	35781
UN19299	Rsa1.0_15681.1	1592	2484	1.00E−111	213	216	98	+	1097	1312	348	563
UN19300	Rsa1.0_02248.1	1095	33414	0	1057	1100	96	+	1	1093	31699	32788
UN19301	Rsa1.0_05870.1	1269	146055	0	477	507	94	−	3	502	142951	143450
UN19302	Rsa1.0_02589.1	990	300828	0	373	376	99	−	17	392	155784	156159
UN19303	Rsa1.0_00675.1	1113	145590	1.00E−179	434	468	92	−	651	1113	45266	45726

UN19304	Rsa1.0_04027.1		981	19501	3.00E-75	147	148	99	+		136	283	14002	14149
UN19305	Rsa1.0_03085.1		1555	25500	0	738	768	96	+		808	1555	18211	18978
UN19306	Rsa1.0_00168.1		1532	115870	1.00E-110	209	211	99	+		1071	1281	35634	35844
UN19307	Rsa1.0_01070.1		614	55099	0	590	607	97	+		13	614	29901	30507
UN19308	Rsa1.0_00179.1		786	393324	0	777	788	98	−		2	784	73880	74665
UN19309	Rsa1.0_00016.1		1016	831256	1.00E-124	253	258	98	+		763	1016	246882	247139
UN19310	Rsa1.0_00663.1		836	164969	0	700	721	97	+		119	836	125061	125775
UN19311	Rsa1.0_02670.1		826	28887	1.00E-179	363	375	96	+		452	825	1836	2209
UN19312	Rsa1.0_00874.1		1905	179723	0	938	943	99	−		2	944	44911	45853
UN19313	Rsa1.0_01402.1		1715	46509	0	785	844	93	−		2	845	18403	19246
UN19314	Rsa1.0_01566.1		1236	43134	0	381	382	99	−		855	1236	30363	30744
UN19315	Rsa1.0_00351.1		872	413096	1.00E-152	364	389	93	+		490	872	267204	267584
UN19316	Rsa1.0_02486.1		1100	30965	1.00E-120	245	251	97	+		3	251	9729	9978
UN19317	Rsa1.0_01157.1		808	52121	1.00E-122	307	335	91	−		487	808	42504	42837
UN19318	Rsa1.0_00236.1		1249	104771	1.00E-129	346	379	91	−		8	375	82285	82651
UN19319	Rsa1.0_01720.1		1049	40358	1.00E-141	286	295	96	+		3	296	31601	31895
UN19320	Rsa1.0_00705.1		1032	67505	0	578	598	96	+		62	659	9479	10076
UN19321	Rsa1.0_00227.1		2070	105849	0	601	608	98	+		812	1419	30551	31158
UN19322	Rsa1.0_04405.1		1555	17508	0	405	409	99	+		1	409	9041	9449
UN19323	Rsa1.0_01352.1		1413	47443	1.00E-126	229	229	100	+		348	576	12214	12442
UN19324	Rsa1.0_08285.1		1381	7606	0	551	563	97	−		337	897	2625	3187
UN19325	Rsa1.0_00871.1		1559	61539	0	1526	1557	98	−		3	1558	40032	41582
UN19326	Rsa1.0_06092.1		1955	11756	0	493	506	97	−		3	506	2635	3140
UN19327	Rsa1.0_03615.1		1023	21766	0	339	340	99	−		2	341	6065	6404
UN19328	Rsa1.0_00947.1		1711	58664	0	530	565	93	−		1157	1709	47261	47811
UN19329	Rsa1.0_00196.1		1156	109960	0	563	581	96	+		587	1156	102144	102722
UN19330	Rsa1.0_02914.1		1288	26815	0	360	366	98	−		15	378	25036	25401
UN19331	Rsa1.0_08429.1		895	7370	1.00E-128	235	236	99	+		70	305	5517	5752
UN19332	Rsa1.0_00130.1		1702	125737	1.00E-177	316	316	100	+		1	316	99030	99345
UN19333	Rsa1.0_00801.1		845	63861	0	462	466	99	+		1	466	37111	37575
UN19334	#	#	#	#	#	#	#		#	#	#	#	#	
UN19335	Rsa1.0_00758.1		1021	65477	1.00E-167	355	373	95	+		1	373	50034	50404
UN19336	Rsa1.0_01965.1		775	36698	0	348	352	98	−		1	352	24177	24526
UN19337	Rsa1.0_00363.1		579	90968	1.00E-82	191	203	94	−		238	434	53874	54076
UN19338	Rsa1.0_00152.1		871	257730	0	735	781	94	−		91	869	210117	210889
UN19339	Rsa1.0_06868.1		1353	10234	0	558	572	97	+		782	1351	3835	4406
UN19340	Rsa1.0_06311.1		457	11254	5.00E-94	219	233	93	−		91	323	2605	2834
UN19341	Rsa1.0_00730.1		789	66673	1.00E-163	374	398	93	+		2	384	28312	28709
UN19342	Rsa1.0_03292.1		1386	23977	0	439	457	96	−		1	448	20331	20782

UN19343	Rsa1.0_00928.1	1117	59293	1.00E-118	229	233	98	-	770	1002	19347	19579
UN19344	Rsa1.0_00199.1	890	249604	0	530	539	98	+	360	890	164308	164846
UN19345	Rsa1.0_00086.1	1217	439854	0	717	723	99	-	494	1216	290243	290964
UN19346	Rsa1.0_02399.1	1600	31763	0	710	751	94	+	851	1600	12962	13708
UN19347	Rsa1.0_02009.1	905	36100	0	418	438	95	-	143	580	35107	35544
UN19348	Rsa1.0_01296.1	915	49035	0	422	422	100	-	494	915	35532	35953
UN19349	Rsa1.0_00053.1	1540	263459	0	969	986	98	-	550	1535	210955	211935
UN19350	Rsa1.0_02716.1	1960	28465	1.00E-149	329	349	94	+	5	353	17871	18219
UN19351	Rsa1.0_04190.1	1747	18581	1.00E-120	227	228	99	-	1	227	1	228
UN19352	Rsa1.0_00502.1	779	204180	0	567	591	95	-	191	779	181060	181649
UN19353	Rsa1.0_01272.1	867	49553	1.00E-176	314	314	100	-	553	866	36802	37115
UN19354	Rsa1.0_01465.1	1131	45175	0	629	631	99	+	470	1099	3759	4389
UN19355	Rsa1.0_02550.1	785	291051	1.00E-168	311	315	98	-	163	477	275718	276032
UN19356	Rsa1.0_00887.1	1346	61048	0	363	369	98	-	979	1346	29110	29478
UN19357	Rsa1.0_01519.1	1069	44144	0	586	608	96	+	466	1069	39374	39964
UN19358	Rsa1.0_00019.1	766	301669	1.00E-175	401	428	93	-	324	751	293082	293498
UN19359	Rsa1.0_05066.1	905	14943	0	428	439	97	+	475	905	419	855
UN19360	Rsa1.0_00285.1	1153	149638	0	849	866	98	-	288	1153	74970	75834
UN19361	Rsa1.0_00413.1	2099	86921	0	1158	1192	97	+	319	1510	2979	4170
UN19362	Rsa1.0_00985.1	1308	161950	0	492	506	97	+	5	510	141815	142317
UN19363	Rsa1.0_01236.1	1121	50156	0	665	694	95	+	440	1121	35121	35814
UN19364	Rsa1.0_01348.1	1876	47569	0	851	886	96	-	921	1799	16734	17618
UN19365	Rsa1.0_00278.1	1359	231896	0	516	528	97	+	1	526	162428	162951
UN19366	Rsa1.0_02981.1	1137	26285	0	476	481	98	+	2	482	23059	23539
UN19367	Rsa1.0_00722.1	1240	66840	0	474	493	96	+	749	1240	51177	51669
UN19368	Rsa1.0_02305.1	652	32783	0	648	650	99	-	3	652	22360	23009
UN19369	Rsa1.0_00968.1	1427	58020	0	425	436	97	-	242	677	49013	49448
UN19370	Rsa1.0_00380.1	898	89748	4.00E-99	231	248	93	+	444	682	78451	78698
UN19371	Rsa1.0_00222.1	1177	172263	0	783	815	96	-	2	807	65105	65914
UN19372	Rsa1.0_00037.1	1912	307838	0	365	369	98	-	919	1287	289028	289395
UN19373	Rsa1.0_09490.1	823	6335	0	815	822	99	+	2	822	4727	5548
UN19374	Rsa1.0_08988.1	1246	6732	1.00E-161	426	476	89	+	1	458	782	1243
UN19375	Rsa1.0_08661.1	940	7068	0	923	923	100	-	2	924	4632	5554
UN19376	Rsa1.0_00517.1	1068	142790	0	397	410	96	+	1	401	135260	135669
UN19377	Rsa1.0_00823.1	1263	62889	0	358	358	100	-	873	1230	36289	36646
UN19378	Rsa1.0_00159.1	910	431686	0	658	681	96	+	4	684	173431	174103
UN19379	Rsa1.0_01206.1	2992	50772	0	839	846	99	+	2147	2991	13869	14714
UN19380	Rsa1.0_00752.1	2567	65756	2.00E-91	281	317	88	+	2037	2349	49569	49885
UN19381	Rsa1.0_00193.1	987	194198	0	826	865	95	-	125	986	23793	24642

UN19382	Rsa1.0_02716.1	881	28465	2.00E-67	149	155	96	+	575	729	1935	2089
UN19383	Rsa1.0_62786.1	1169	365	0	344	349	98	−	3	351	1	349
UN19384	Rsa1.0_09527.1	1099	6311	1.00E-134	295	311	94	−	527	837	5870	6179
UN19385	Rsa1.0_02504.1	780	30706	1.00E-134	243	243	100	−	538	780	6846	7088
UN19386	Rsa1.0_00114.1	580	131330	0	576	578	99	+	3	580	53520	54097
UN19387	Rsa1.0_00440.1	905	84723	1.00E-166	324	332	97	−	572	903	25068	25398
UN19388	Rsa1.0_13229.1	1826	4963	1.00E-165	317	324	97	+	655	978	3129	3452
UN19389	Rsa1.0_01221.1	1018	50421	0	365	365	100	+	654	1018	49364	49728
UN19390	Rsa1.0_01994.1	1184	36374	0	721	734	98	+	450	1182	10029	10762
UN19391	Rsa1.0_03065.1	686	25656	0	677	684	98	−	3	686	16139	16821
UN19392	Rsa1.0_00754.1	904	65689	1.00E-106	209	213	98	+	3	211	51674	51886
UN19393	Rsa1.0_03837.1	1799	20559	0	379	386	98	+	1369	1754	20172	20556
UN19394	Rsa1.0_00365.1	832	90902	0	376	384	97	−	450	832	58706	59089
UN19395	Rsa1.0_03355.1	956	23489	0	598	611	97	−	346	956	19179	19789
UN19396	Rsa1.0_02236.1	1396	33558	1.00E-136	300	319	94	−	4	313	19567	19885
UN19397	Rsa1.0_00050.1	859	237174	0	587	626	93	−	3	610	7595	8214
UN19398	Rsa1.0_03111.1	1054	25327	0	461	464	99	+	1	464	3018	3481
UN19399	Rsa1.0_00668.1	1025	69421	0	1001	1025	97	+	1	1025	64358	65378
UN19400	Rsa1.0_00154.1	1194	189461	0	965	1009	95	−	151	1159	125784	126792
UN19401	Rsa1.0_00224.1	539	327530	1.00E-97	257	280	91	−	125	402	221387	221660
UN19402	Rsa1.0_00690.1	1472	140527	0	1262	1280	98	+	2	1279	11466	12743
UN19403	Rsa1.0_31349.1	2130	729	0	561	578	97	−	118	688	152	729
UN19404	Rsa1.0_00801.1	1477	63861	0	1233	1337	92	−	8	1313	61300	62604
UN19405	Rsa1.0_51850.1	816	438	2.00E-97	234	248	94	+	5	248	22	269
UN19406	Rsa1.0_01035.1	1075	56019	0	1025	1074	95	+	1	1073	43847	44908
UN19407	Rsa1.0_00130.1	1419	125737	0	1415	1417	99	+	1	1417	29471	30885
UN19408	Rsa1.0_02424.1	572	31570	1.00E-162	303	306	99	−	267	572	6573	6877
UN19409	Rsa1.0_00656.1	876	69968	1.00E-147	285	292	97	−	39	330	46621	46912
UN19410	Rsa1.0_03834.1	1420	20572	0	1373	1418	96	+	3	1420	11804	13211
UN19411	Rsa1.0_00967.1	1764	58049	0	945	948	99	−	3	950	7511	8458
UN19412	Rsa1.0_03241.1	1181	24423	0	1000	1059	94	−	125	1180	5601	6646
UN19413	Rsa1.0_11710.1	1180	5433	0	647	651	99	−	299	949	1873	2523
UN19414	Rsa1.0_00339.1	845	222974	5.00E-80	158	160	98	−	85	244	63385	63544
UN19415	Rsa1.0_05995.1	1566	11997	0	361	367	98	+	1202	1566	8577	8943
UN19416	Rsa1.0_05620.1	557	13148	0	543	555	97	−	3	557	8128	8680
UN19417	Rsa1.0_02156.1	1235	34517	0	383	385	99	−	474	858	20207	20591
UN19418	Rsa1.0_01453.1	1757	45449	0	885	966	91	+	2	929	9979	10921
UN19419	Rsa1.0_01105.1	1481	53958	0	600	615	97	−	554	1168	27951	28565
UN19420	Rsa1.0_00559.1	1512	190486	0	1019	1136	89	−	38	1173	178452	179587

UN19421	Rsa1.0_01012.1	1285	56836	0	624	624	100	+	660	1283	7489	8112
UN19422	Rsa1.0_01371.1	1681	47079	0	1205	1316	91	−	125	1440	42895	44210
UN19423	Rsa1.0_00008.1	1510	358124	0	1086	1134	95	+	320	1450	28198	29322
UN19424	Rsa1.0_16880.1	732	2004	0	528	528	100	+	1	528	468	995
UN19425	Rsa1.0_00284.1	1822	323267	0	459	487	94	−	1	464	134274	134760
UN19426	Rsa1.0_01770.1	1208	39563	0	1168	1210	96	+	1	1207	31815	33002
UN19427	Rsa1.0_03271.1	1441	24187	0	1396	1446	96	+	1	1441	12134	13579
UN19428	Rsa1.0_01170.1	573	161131	0	360	362	99	−	3	364	117909	118270
UN19429	Rsa1.0_00649.1	1494	70263	0	442	445	99	+	1050	1494	62855	63299
UN19430	Rsa1.0_18218.1	2080	1643	0	873	913	95	−	408	1320	1	912
UN19431	Rsa1.0_01602.1	2187	42503	0	560	565	99	−	446	1010	11562	12125
UN19432	Rsa1.0_01500.1	1546	141844	0	621	630	98	−	1	628	10187	10815
UN19433	Rsa1.0_00165.1	1580	116466	0	684	719	95	+	405	1113	27250	27960
UN19434	Rsa1.0_01450.1	471	144251	1.00E−91	180	183	98	+	1	183	133312	133494
UN19435	Rsa1.0_01368.1	2120	47129	0	566	579	97	+	1277	1848	29056	29634
UN19436	Rsa1.0_04617.1	967	16625	0	904	989	91	−	7	967	15283	16262
UN19437	Rsa1.0_01184.1	1268	51452	1.00E−175	375	394	95	+	882	1268	42359	42746
UN19438	Rsa1.0_05442.1	1303	13741	0	407	433	93	+	872	1303	1778	2196
UN19439	Rsa1.0_00841.1	1264	183065	0	595	598	99	−	2	598	49678	50275
UN19440	Rsa1.0_00475.1	1583	81678	0	867	869	99	−	714	1582	39183	40051
UN19441	Rsa1.0_01709.1	1239	40587	1.00E−179	337	343	98	+	758	1100	10369	10711
UN19442	Rsa1.0_01071.1	1169	166914	0	366	372	98	+	1	372	14036	14407
UN19443	Rsa1.0_00482.1	1445	81162	0	475	485	97	−	1	485	66121	66598
UN19444	Rsa1.0_00920.1	675	601703	1.00E−132	245	247	99	+	427	673	448348	448594
UN19445	Rsa1.0_00057.1	1319	160473	1.00E−170	312	315	99	−	208	522	71413	71727
UN19446	Rsa1.0_08861.1	1744	6847	0	1719	1744	98	−	1	1744	2308	4050
UN19447	Rsa1.0_00071.1	1132	345714	0	488	495	98	−	67	560	207419	207913
UN19448	Rsa1.0_03290.1	2124	24011	1.00E−157	329	341	96	+	1785	2124	18991	19328
UN19449	Rsa1.0_03663.1	1562	21488	0	749	760	98	+	792	1551	6262	7020
UN19450	Rsa1.0_00065.1	1484	454616	1.00E−163	291	291	100	+	1	291	8919	9209
UN19451	Rsa1.0_00008.1	1285	358124	0	740	749	98	−	531	1274	283032	283780
UN19452	Rsa1.0_00543.1	867	76788	0	563	569	98	−	299	867	15007	15572
UN19453	Rsa1.0_00339.1	1099	222974	0	372	389	95	−	711	1098	180014	180389
UN19454	Rsa1.0_01728.1	1345	40239	0	1298	1335	97	−	15	1345	3956	5287
UN19455	Rsa1.0_01042.1	798	293264	0	527	527	100	+	1	527	153984	154510
UN19456	Rsa1.0_49744.1	1617	456	1.00E−140	313	329	95	+	39	363	1	325
UN19457	Rsa1.0_08305.1	1151	7571	1.00E−126	286	301	95	−	1	295	5615	5912
UN19458	Rsa1.0_04574.1	1421	16786	0	515	526	97	−	402	921	5374	5899
UN19459	Rsa1.0_00453.1	1481	83031	0	1466	1479	99	−	1	1479	77210	78685

UN19460	Rsa1.0_00236.1	1869	104771	0	576	583	98	−	1287	1867	61616	62198
UN19461	Rsa1.0_00497.1	1455	79830	0	990	990	100	+	466	1455	70653	71642
UN19462	Rsa1.0_04934.1	991	143962	0	964	987	97	−	7	991	11783	12768
UN19463	Rsa1.0_04740.1	2135	16165	0	773	777	99	−	1343	2119	468	1243
UN19464	Rsa1.0_00616.1	2016	195713	0	471	471	100	−	1544	2014	147120	147590
UN19465	Rsa1.0_00016.1	1354	831256	0	561	562	99	−	1	561	504338	504899
UN19466	Rsa1.0_00394.1	1523	148426	0	1497	1524	98	−	3	1523	102166	103686
UN19467	Rsa1.0_01295.1	1192	49040	0	1159	1194	97	+	1	1187	46793	47986
UN19468	Rsa1.0_07654.1	837	8917	0	408	416	98	−	1	408	3857	4272
UN19469	Rsa1.0_14764.1	1971	3084	0	1109	1156	95	+	814	1961	1483	2634
UN19470	Rsa1.0_00116.1	1264	365886	0	340	340	100	+	924	1263	67695	68034
UN19471	Rsa1.0_00329.1	1543	222544	0	671	691	97	−	146	830	20262	20952
UN19472	Rsa1.0_15087.1	2002	2842	0	638	644	99	+	173	816	2062	2705
UN19473	Rsa1.0_03476.1	1416	22646	0	401	411	97	+	132	541	10297	10707
UN19474	Rsa1.0_00707.1	1372	190112	0	722	726	99	+	644	1369	149667	150392
UN19475	Rsa1.0_00684.1	1353	141701	0	497	531	93	−	828	1353	54180	54698
UN19476	Rsa1.0_01094.1	1450	167285	0	629	648	97	+	3	648	1140	1782
UN19477	Rsa1.0_00128.1	1692	228675	0	503	505	99	−	3	507	97144	97648
UN19478	Rsa1.0_03029.1	1409	25858	0	868	906	95	+	512	1408	20341	21241
UN19479	Rsa1.0_00657.1	2189	69858	1.00E−109	201	201	100	−	1989	2189	58010	58210
UN19480	Rsa1.0_00027.1	1265	283362	0	514	519	99	+	402	920	51034	51552
UN19481	Rsa1.0_00047.1	1729	168582	0	570	605	94	+	99	703	29923	30513
UN19482	Rsa1.0_00904.1	995	180594	0	968	985	98	+	11	993	16478	17459
UN19483	Rsa1.0_06127.1	1564	11675	0	656	685	95	+	1	682	6082	6758
UN19484	Rsa1.0_05882.1	1915	12340	0	437	441	99	+	1	440	4283	4723
UN19485	Rsa1.0_02695.1	915	28605	0	650	660	98	−	255	914	7026	7685
UN19486	Rsa1.0_01331.1	3147	47944	0	1844	1931	95	−	2	1898	17217	19146
UN19487	Rsa1.0_02643.1	1153	29176	0	714	714	100	+	56	769	27641	28354
UN19488	Rsa1.0_00056.1	1644	332604	0	375	378	99	+	1269	1644	94692	95069
UN19489	Rsa1.0_08430.1	2431	7367	0	444	479	92	+	1243	1710	5345	5821
UN19490	Rsa1.0_01263.1	1477	49703	0	973	1017	95	−	3	1005	1553	2569
UN19491	Rsa1.0_03978.1	1014	19753	1.00E−138	291	303	96	+	712	1011	11983	12284
UN19492	Rsa1.0_00434.1	1395	85171	0	358	363	98	+	727	1089	34846	35208
UN19493	Rsa1.0_01844.1	1412	38421	1.00E−163	340	356	95	−	612	967	15108	15463
UN19494	Rsa1.0_00262.1	1717	101906	0	483	496	97	−	1223	1716	94662	95156
UN19495	Rsa1.0_00216.1	594	197563	0	470	474	99	−	3	476	186705	187178
UN19496	Rsa1.0_01056.1	1207	55449	0	402	417	96	+	3	419	14440	14849
UN19497	Rsa1.0_15814.1	1299	2409	1.00E−155	279	279	100	+	302	580	740	1018
UN19498	Rsa1.0_00435.1	572	317579	0	352	353	99	−	220	572	70728	71080

UN19499	Rsa1.0_00686.1	1367	68511	1.00E-140	279	286	97	−	1	285	42629	42914
UN19500	Rsa1.0_00506.1	1401	79106	0	1015	1038	97	−	1	1038	64312	65342
UN19501	Rsa1.0_00363.1	1352	90968	0	397	407	97	+	944	1349	22565	22971
UN19502	Rsa1.0_00245.1	1508	103569	0	1069	1073	99	+	1	1073	61676	62748
UN19503	Rsa1.0_00144.1	765	120708	1.00E-152	349	374	93	−	391	763	79342	79702
UN19504	Rsa1.0_05723.1	1048	12817	0	646	678	95	−	7	678	5257	5932
UN19505	Rsa1.0_00017.1	1566	752458	1.00E-171	312	314	99	+	1253	1566	54639	54952
UN19506	Rsa1.0_00340.1	512	305782	1.00E-153	291	296	98	+	2	294	109342	109637
UN19507	Rsa1.0_00240.1	1276	104175	0	555	574	96	+	702	1274	92182	92747
UN19508	Rsa1.0_03956.1	1334	19872	0	819	841	97	−	493	1332	13126	13956
UN19509	Rsa1.0_15623.1	1209	2515	0	759	766	99	−	1	766	14	778
UN19510	Rsa1.0_14460.1	1139	3366	0	483	523	92	−	3	508	942	1460
UN19511	Rsa1.0_14737.1	1497	3115	0	611	631	96	−	646	1261	1328	1958
UN19512	Rsa1.0_01756.1	1441	39779	0	568	570	99	−	874	1441	26923	27492
UN19513	Rsa1.0_00532.1	949	77384	0	924	943	97	+	7	947	30126	31068
UN19514	Rsa1.0_00791.1	1061	64201	0	859	868	98	+	14	880	56706	57573
UN19515	Rsa1.0_00038.1	1153	314568	2.00E-27	67	68	98	+	54	121	27750	27817
UN19516	Rsa1.0_36275.1	1020	622	0	475	482	98	+	155	635	1	481
UN19517	Rsa1.0_00429.1	1091	85559	1.00E-178	322	324	99	+	2	325	25745	26068
UN19518	Rsa1.0_00292.1	924	98141	0	343	343	100	+	1	343	63912	64254
UN19519	Rsa1.0_02894.1	2157	26997	0	535	552	96	−	1606	2157	12015	12557
UN19520	Rsa1.0_08079.1	1600	7942	0	1568	1603	97	+	1	1599	4554	6154
UN19521	Rsa1.0_01319.1	857	48273	1.00E-162	289	289	100	+	79	367	40946	41234
UN19522	Rsa1.0_01245.1	1143	149868	0	604	604	100	+	539	1142	42586	43189
UN19523	Rsa1.0_03063.1	1308	25673	0	564	584	96	−	1	580	9182	9765
UN19524	Rsa1.0_05273.1	1379	14304	1.00E-117	233	239	97	−	1139	1377	13753	13991
UN19525	Rsa1.0_11912.1	1454	5372	0	818	837	97	+	617	1453	2521	3351
UN19526	Rsa1.0_08645.1	987	7087	0	530	531	99	−	457	987	60	590
UN19527	Rsa1.0_03926.1	1012	20024	1.00E-123	228	229	99	+	34	262	5387	5615
UN19528	Rsa1.0_01310.1	900	164719	0	440	444	99	−	459	899	159252	159695
UN19529	Rsa1.0_03294.1	1652	23971	1.00E-136	258	261	98	+	1392	1652	4753	5011
UN19530	Rsa1.0_00716.1	684	67078	0	535	549	97	−	137	684	37365	37913
UN19531	Rsa1.0_01274.1	950	49419	1.00E-114	271	290	93	+	649	937	37166	37455
UN19532	Rsa1.0_05755.1	966	12730	0	531	545	97	+	10	550	9386	9928
UN19533	Rsa1.0_11741.1	1412	5423	0	464	500	92	−	414	913	4597	5096
UN19534	Rsa1.0_00532.1	1404	77384	1.00E-136	247	247	100	−	3	249	15300	15546
UN19535	Rsa1.0_19147.1	874	1473	0	553	557	99	+	1	557	888	1441
UN19536	Rsa1.0_03978.1	1115	19753	0	370	370	100	+	718	1087	11983	12352
UN19537	Rsa1.0_00402.1	899	385315	0	372	374	99	−	1	374	86468	86841

UN19538	Rsa1.0_00038.1	889	314568	1.00E-120	305	330	92	+	571	889	16997	17323
UN19539	Rsa1.0_04835.1	993	15766	0	434	442	98	+	6	447	9140	9578
UN19540	Rsa1.0_11005.1	886	5650	1.00E-151	301	310	97	+	580	886	766	1075
UN19541	Rsa1.0_13978.1	1130	3965	0	674	678	99	−	146	823	1	678
UN19542	Rsa1.0_02226.1	908	33726	1.00E-126	287	305	94	−	610	903	10091	10395
UN19543	Rsa1.0_00922.1	871	59588	0	705	718	98	−	4	716	39245	39961
UN19544	Rsa1.0_35031.1	1354	646	0	594	596	99	−	706	1301	1	596
UN19545	Rsa1.0_09024.1	833	6703	0	628	633	99	+	28	660	6072	6703
UN19546	Rsa1.0_00021.1	1038	263655	0	361	367	98	−	246	612	238503	238869
UN19547	Rsa1.0_12442.1	1035	5225	1.00E-144	260	260	100	+	776	1035	461	720
UN19548	Rsa1.0_00211.1	1373	228189	0	499	516	96	−	265	778	130199	130714
UN19549	Rsa1.0_21670.1	1082	1183	1.00E-124	236	239	98	−	844	1082	71	309
UN19550	Rsa1.0_03169.1	775	24826	0	471	477	98	−	304	775	2578	3054
UN19551	Rsa1.0_00315.1	1033	96426	0	597	601	99	−	433	1033	46896	47493
UN19552	Rsa1.0_02377.1	1202	31994	0	647	656	98	−	11	666	6670	7325
UN19553	Rsa1.0_01238.1	944	50120	0	904	915	98	−	30	944	26916	27828
UN19554	Rsa1.0_02090.1	1427	35161	1.00E-133	242	242	100	−	157	398	20970	21211
UN19555	Rsa1.0_38361.1	899	587	0	430	437	98	−	384	820	1	437
UN19556	Rsa1.0_07331.1	710	9548	0	704	708	99	−	3	710	3176	3883
UN19557	Rsa1.0_02772.1	1143	28047	1.00E-163	348	368	94	−	776	1143	17257	17615
UN19558	Rsa1.0_00604.1	863	146282	1.00E-151	314	327	96	+	536	861	113696	114022
UN19559	Rsa1.0_00241.1	590	104000	1.00E-137	273	279	97	−	2	279	65104	65381
UN19560	Rsa1.0_01876.1	910	37800	0	690	699	98	+	212	910	36310	37006
UN19561	Rsa1.0_00093.1	1831	214962	0	741	743	99	−	1088	1830	22719	23461
UN19562	Rsa1.0_01660.1	913	41400	0	434	450	96	+	476	912	27246	27695
UN19563	Rsa1.0_34219.1	1389	662	0	644	653	98	−	739	1389	10	662
UN19564	Rsa1.0_33878.1	830	669	0	338	340	99	−	71	410	161	500
UN19565	Rsa1.0_00383.1	675	398431	0	621	658	94	−	16	664	186026	186678
UN19566	Rsa1.0_00387.1	814	495407	0	344	345	99	+	1	345	339943	340287
UN19567	Rsa1.0_21904.1	982	1164	1.00E-171	311	313	99	+	4	316	840	1152
UN19568	Rsa1.0_00281.1	638	99908	1.00E-125	234	235	99	+	376	609	12443	12677
UN19569	Rsa1.0_03995.1	1130	19654	1.00E-138	305	317	96	−	654	964	2310	2626
UN19570	Rsa1.0_00347.1	858	93048	0	410	417	98	+	444	858	86405	86821
UN19571	Rsa1.0_04064.1	893	19297	0	413	427	96	+	286	709	18501	18925
UN19572	Rsa1.0_01491.1	1265	44718	0	452	458	98	+	414	871	19029	19486
UN19573	Rsa1.0_01171.1	1138	51752	0	377	394	95	+	755	1134	50943	51336
UN19574	Rsa1.0_01525.1	1243	43925	0	440	441	99	−	1	441	32428	32867
UN19575	Rsa1.0_00080.1	909	190437	1.00E-133	265	273	97	−	185	457	34258	34530
UN19576	Rsa1.0_02944.1	868	26553	1.00E-165	306	310	98	+	28	337	24455	24764

UN19577	Rsa1.0_00361.1	1261	91181	1.00E-154	277	277	100	−	851	1127	12174	12450
UN19578	Rsa1.0_08558.1	1150	7198	0	483	526	91	−	633	1149	2202	2721
UN19579	Rsa1.0_01113.1	1983	53616	0	861	873	98	+	1110	1982	48955	49825
UN19580	Rsa1.0_05231.1	1816	14424	1.00E-113	227	233	97	+	1399	1631	7214	7446
UN19581	Rsa1.0_00612.1	1760	72376	1.00E-152	273	273	100	−	848	1120	63354	63626
UN19582	Rsa1.0_00295.1	1408	152597	0	615	615	100	+	274	888	33905	34519
UN19583	Rsa1.0_00061.1	964	218685	0	584	585	99	+	1	585	2031	2614
UN19584	Rsa1.0_02826.1	2330	27560	0	484	484	100	−	256	739	25714	26197
UN19585	Rsa1.0_01604.1	918	42461	1.00E-175	343	352	97	+	3	353	1092	1443
UN19586	Rsa1.0_00382.1	914	251946	1.00E-129	244	247	98	−	284	530	113183	113429
UN19587	Rsa1.0_04852.1	2884	15712	0	923	977	94	+	414	1390	8979	9942
UN19588	Rsa1.0_00325.1	1016	95371	1.00E-153	284	287	98	+	174	460	73729	74015
UN19589	Rsa1.0_00205.1	1514	193401	0	593	599	98	−	114	712	175714	176311
UN19590	Rsa1.0_05324.1	1147	14145	0	444	449	98	−	1	449	13120	13568
UN19591	Rsa1.0_00006.1	683	396176	1.00E-106	294	321	91	−	368	677	275841	276159
UN19592	Rsa1.0_01434.1	1163	45951	0	889	905	98	−	1	905	23021	23925
UN19593	Rsa1.0_04969.1	935	15283	1.00E-105	209	214	97	+	288	501	14289	14502
UN19594	Rsa1.0_00525.1	714	78030	0	393	393	100	−	321	713	23993	24385
UN19595	Rsa1.0_08459.1	1077	7315	0	594	595	99	+	2	596	6240	6834
UN19596	Rsa1.0_04437.1	787	17362	0	780	785	99	−	3	785	1600	2382
UN19597	Rsa1.0_02398.1	1366	31766	0	1099	1188	92	−	72	1250	16806	17987
UN19598	Rsa1.0_00589.1	1636	209965	0	1014	1051	96	−	402	1448	9243	10291
UN19599	Rsa1.0_04381.1	1015	17639	0	531	577	92	−	445	1011	12731	13281
UN19600	Rsa1.0_00065.1	1934	454616	0	1028	1040	98	+	887	1924	126502	127541
UN19601	Rsa1.0_03260.1	1212	24284	0	544	555	98	−	658	1212	16797	17349
UN19602	Rsa1.0_02276.1	898	33098	1.00E-129	271	283	95	+	3	280	28453	28735
UN19603	Rsa1.0_05539.1	1591	13389	0	563	566	99	+	1024	1589	8363	8928
UN19604	Rsa1.0_00494.1	936	79967	0	927	935	99	+	1	934	49884	50817
UN19605	Rsa1.0_12391.1	1247	5238	0	382	397	96	−	3	398	4768	5164
UN19606	Rsa1.0_03592.1	1686	21878	0	789	806	97	+	883	1686	4363	5168
UN19607	Rsa1.0_03110.1	1356	25335	0	463	466	99	+	890	1355	8179	8644
UN19608	Rsa1.0_00615.1	1549	72235	0	356	358	99	−	202	559	26723	27079
UN19609	Rsa1.0_00067.1	567	415055	0	368	379	97	−	55	428	209269	209647
UN19610	Rsa1.0_00302.1	713	97521	0	346	350	98	+	1	346	79307	79656
UN19611	Rsa1.0_01390.1	747	46695	0	435	435	100	−	313	747	18984	19418
UN19612	Rsa1.0_03352.1	1039	23506	0	1029	1048	98	+	1	1039	14261	15308
UN19613	Rsa1.0_01590.1	857	42719	0	778	791	98	−	67	857	9983	10773
UN19614	Rsa1.0_03090.1	1610	25464	0	997	1035	96	−	389	1419	14474	15505
UN19615	Rsa1.0_02754.1	591	28213	0	426	429	99	−	164	591	8589	9017

UN19616	Rsa1.0_05501.1	1110	13513	1.00E-77	169	176	96	-	202	377	9065	9240
UN19617	Rsa1.0_04242.1	674	18340	1.00E-141	261	263	99	-	1	263	6626	6888
UN19618	Rsa1.0_05122.1	967	14791	1.00E-159	284	284	100	+	1	284	9010	9293
UN19619	Rsa1.0_01466.1	1249	45161	1.00E-120	289	313	92	+	935	1247	14125	14430
UN19620	Rsa1.0_00546.1	681	76523	0	396	402	98	+	200	598	69605	70006
UN19621	Rsa1.0_00475.1	746	81678	0	736	755	97	+	1	746	38102	38856
UN19622	Rsa1.0_00273.1	969	326186	0	358	359	99	-	1	359	125342	125700
UN19623	Rsa1.0_01293.1	506	49139	1.00E-144	290	299	96	-	3	294	28083	28381
UN19624	Rsa1.0_00630.1	1718	71281	0	627	634	98	+	1086	1718	5334	5967
UN19625	Rsa1.0_00121.1	687	278029	1.00E-131	300	317	94	+	373	687	54300	54614
UN19626	Rsa1.0_16574.1	754	2106	0	386	390	98	+	76	465	1717	2106
UN19627	Rsa1.0_01123.1	713	53359	0	420	423	99	+	1	422	14635	15057
UN19628	Rsa1.0_00353.1	1003	92242	0	551	562	98	+	2	563	61983	62538
UN19629	Rsa1.0_00512.1	764	200277	0	560	578	96	+	187	764	72466	73043
UN19630	Rsa1.0_00021.1	644	263655	1.00E-179	363	376	96	-	267	640	80113	80486
UN19631	Rsa1.0_48937.1	1394	463	0	439	444	98	-	918	1361	1	441
UN19632	Rsa1.0_00224.1	1292	327530	0	561	566	99	+	716	1281	4810	5375
UN19633	Rsa1.0_00567.1	1443	75260	1.00E-115	280	303	92	+	47	339	66990	67292
UN19634	Rsa1.0_05436.1	1240	13758	0	844	854	98	+	332	1185	9953	10797
UN19635	Rsa1.0_00362.1	1155	299497	0	691	703	98	+	401	1103	238849	239549
UN19636	Rsa1.0_00782.1	1946	64468	1.00E-174	473	521	90	+	476	983	19171	19678
UN19637	Rsa1.0_00077.1	1006	145005	1.00E-149	271	272	99	-	732	1003	66829	67100
UN19638	Rsa1.0_00086.1	1221	439854	0	379	387	97	+	755	1140	299867	300253
UN19639	Rsa1.0_01633.1	1229	41868	0	739	755	97	+	14	765	18059	18813
UN19640	Rsa1.0_01459.1	662	45277	1.00E-100	287	321	89	-	346	657	42950	43259
UN19641	Rsa1.0_00123.1	965	325650	1.00E-136	257	259	99	+	706	964	143131	143388
UN19642	Rsa1.0_00022.1	576	200461	1.00E-104	192	192	100	+	385	576	191050	191241
UN19643	Rsa1.0_39888.1	925	563	0	467	482	96	+	427	905	1	482
UN19644	Rsa1.0_03010.1	776	26054	1.00E-123	245	252	97	-	525	776	20626	20877
UN19645	Rsa1.0_01377.1	1025	46967	1.00E-130	299	321	93	+	704	1023	24880	25188
UN19646	Rsa1.0_01175.1	748	51674	0	365	365	100	-	375	739	35937	36301
UN19647	Rsa1.0_05792.1	762	12612	1.00E-143	266	269	98	+	162	430	642	910
UN19648	Rsa1.0_00211.1	1212	228189	1.00E-130	242	244	99	-	414	657	16536	16779
UN19649	Rsa1.0_02295.1	684	149884	0	675	692	97	+	1	680	1496	2187
UN19650	Rsa1.0_00037.1	1363	307838	0	984	1026	95	+	1	1022	175802	176825
UN19651	Rsa1.0_00458.1	572	82717	0	568	568	100	-	3	570	74870	75437
UN19652	Rsa1.0_00678.1	646	193163	6.00E-17	97	114	85	-	428	541	23509	23622
UN19653	Rsa1.0_01936.1	595	37151	9.00E-90	220	236	93	+	315	546	18530	18763
UN19654	Rsa1.0_05482.1	914	13595	1.00E-150	279	281	99	-	634	914	4273	4552

UN19655	Rsa1.0_00848.1	1075	152362	1.00E-141	282	291	96	+	785	1075	35655	35945
UN19656	Rsa1.0_00596.1	752	73578	1.00E-164	353	373	94	+	123	495	42155	42527
UN19657	Rsa1.0_00179.1	736	393324	1.00E-150	287	292	98	−	22	310	199770	200061
UN19658	Rsa1.0_00414.1	946	202190	0	628	632	99	+	315	946	36763	37394
UN19659	Rsa1.0_00152.1	939	257730	1.00E-128	299	320	93	−	620	937	96159	96463
UN19660	Rsa1.0_01882.1	852	37697	0	817	858	95	−	3	851	10955	11799
UN19661	Rsa1.0_02829.1	961	27541	0	499	539	92	−	440	952	3243	3781
UN19662	Rsa1.0_00782.1	688	64468	1.00E-132	249	252	98	−	435	686	17720	17971
UN19663	Rsa1.0_00134.1	610	390219	0	374	377	99	+	1	377	21793	22169
UN19664	Rsa1.0_01786.1	692	133324	1.00E-119	232	237	97	−	23	259	106421	106657
UN19665	Rsa1.0_02450.1	934	31288	0	831	842	98	−	1	839	16916	17755
UN19666	Rsa1.0_00030.1	882	250988	0	464	468	99	+	417	882	87453	87920
UN19667	Rsa1.0_00695.1	1688	68019	0	667	673	99	−	1017	1686	58664	59336
UN19668	Rsa1.0_43909.1	645	512	0	396	398	99	+	3	400	77	473
UN19669	Rsa1.0_02813.1	1095	27656	2.00E-86	222	245	90	+	691	935	25680	25909
UN19670	Rsa1.0_04600.1	1634	16694	0	618	619	99	+	650	1268	12273	12891
UN19671	Rsa1.0_00194.1	1036	110851	0	393	397	98	−	641	1036	72527	72923
UN19672	Rsa1.0_00657.1	1303	69858	1.00E-154	319	329	96	+	977	1303	9457	9784
UN19673	Rsa1.0_02486.1	1051	30965	1.00E-150	284	289	98	+	762	1050	11659	11947
UN19674	Rsa1.0_00294.1	2294	144860	0	587	588	99	+	563	1150	60107	60694
UN19675	Rsa1.0_00318.1	628	96094	1.00E-108	202	203	99	+	208	410	43395	43597
UN19676	Rsa1.0_00204.1	1054	109056	1.00E-173	309	309	100	+	598	906	48140	48448
UN19677	Rsa1.0_00453.1	868	83031	0	498	503	99	−	53	555	68504	69006
UN19678	Rsa1.0_00023.1	1407	194764	0	683	685	99	+	602	1286	180083	180767
UN19679	Rsa1.0_00911.1	1056	59943	0	499	504	99	−	552	1050	44346	44849
UN19680	Rsa1.0_00712.1	1138	67217	0	435	436	99	+	2	437	66455	66889
UN19681	Rsa1.0_00179.1	1647	393324	0	359	361	99	+	3	363	51814	52173
UN19682	Rsa1.0_01721.1	1599	40345	0	691	695	99	−	905	1599	23089	23783
UN19683	Rsa1.0_00185.1	1145	248445	0	1012	1077	93	−	1	1073	204336	205411
UN19684	Rsa1.0_03216.1	1360	24540	0	1281	1289	99	+	1	1288	23252	24540
UN19685	Rsa1.0_02079.1	1388	35306	0	504	511	98	+	103	613	23541	24051
UN19686	Rsa1.0_00101.1	954	267193	0	811	874	92	+	65	932	103685	104554
UN19687	Rsa1.0_00354.1	1376	232005	0	407	408	99	−	1	407	168195	168602
UN19688	Rsa1.0_00447.1	937	84051	1.00E-130	271	281	96	−	625	905	14956	15235
UN19689	Rsa1.0_00634.1	1816	71222	0	387	392	98	+	1421	1812	57160	57550
UN19690	Rsa1.0_00897.1	1088	60498	1.00E-118	318	348	91	−	747	1088	21846	22191
UN19691	Rsa1.0_05150.1	966	14673	0	367	369	99	+	1	369	3240	3608
UN19692	Rsa1.0_05130.1	1474	14758	0	1123	1126	99	+	1	1125	11444	12569
UN19693	Rsa1.0_01068.1	1732	55208	0	399	410	97	+	4	407	28140	28548

UN19694	Rsa1.0_00719.1	1227	66952	0	417	435	95	+	791	1225	47331	47760
UN19695	Rsa1.0_04030.1	823	19489	2.00E-66	158	167	94	+	659	822	304	470
UN19696	Rsa1.0_02389.1	1252	146428	0	474	476	99	−	4	479	694	1169
UN19697	Rsa1.0_06443.1	942	11008	1.00E-112	239	248	96	−	615	859	5835	6081
UN19698	Rsa1.0_04961.1	686	15316	1.00E-108	269	294	91	−	2	276	13226	13518
UN19699	Rsa1.0_00002.1	1179	601661	0	1110	1118	99	−	5	1121	289127	290241
UN19700	Rsa1.0_00468.1	1450	82095	0	1344	1385	97	−	65	1447	66625	68007
UN19701	Rsa1.0_01701.1	1309	162121	0	1069	1069	100	−	1	1069	144523	145591
UN19702	Rsa1.0_02151.1	896	34575	0	350	350	100	−	547	896	4597	4946
UN19703	Rsa1.0_00014.1	1781	348985	1.00E-133	400	452	88	+	672	1116	252326	252776
UN19704	Rsa1.0_01901.1	1324	37472	0	567	593	95	+	693	1284	20156	20727
UN19705	Rsa1.0_00238.1	773	193193	0	527	547	96	−	1	543	190030	190576
UN19706	Rsa1.0_00403.1	1187	87701	0	740	740	100	+	1	740	43676	44415
UN19707	Rsa1.0_00819.1	949	62918	0	360	367	98	−	582	948	5151	5516
UN19708	Rsa1.0_01887.1	988	37564	1.00E-174	370	390	94	+	3	392	23860	24249
UN19709	Rsa1.0_01484.1	1028	44806	1.00E-170	318	320	99	+	1	319	14353	14671
UN19710	Rsa1.0_00179.1	1185	393324	0	739	747	98	+	2	747	277903	278648
UN19711	Rsa1.0_04252.1	1456	18262	1.00E-148	275	278	98	−	574	851	5104	5381
UN19712	Rsa1.0_03122.1	1372	25207	1.00E-180	326	328	99	−	758	1085	6320	6647
UN19713	Rsa1.0_00258.1	1113	102381	1.00E-161	304	309	98	−	157	465	46518	46826
UN19714	Rsa1.0_00059.1	1344	159650	0	412	423	97	−	921	1342	84772	85194
UN19715	Rsa1.0_00065.1	1231	454616	0	445	447	99	−	3	449	191624	192070
UN19716	Rsa1.0_01522.1	1252	157436	0	923	936	98	+	1	936	3042	3976
UN19717	Rsa1.0_01536.1	658	43747	0	639	658	97	−	1	656	25562	26207
UN19718	Rsa1.0_00087.1	1288	761073	0	489	491	99	−	798	1288	300483	300973
UN19719	Rsa1.0_10932.1	1023	5673	0	493	500	98	+	522	1021	2552	3051
UN19720	Rsa1.0_12394.1	1406	5237	0	378	393	96	+	977	1369	4534	4918
UN19721	Rsa1.0_02210.1	1646	33857	0	1037	1050	98	+	597	1646	5810	6859
UN19722	Rsa1.0_01240.1	1515	50075	0	674	683	98	−	836	1515	40540	41221
UN19723	Rsa1.0_01178.1	2267	51571	0	655	674	97	+	837	1509	19574	20234
UN19724	Rsa1.0_01795.1	1378	39171	0	628	640	98	+	508	1146	18095	18733
UN19725	Rsa1.0_00056.1	872	332604	0	790	836	94	+	44	872	7983	8808
UN19726	Rsa1.0_03175.1	1206	24802	0	524	540	97	+	667	1205	4236	4772
UN19727	Rsa1.0_03835.1	1400	20567	0	605	621	97	+	768	1382	8140	8760
UN19728	Rsa1.0_01198.1	680	51029	2.00E-97	312	353	88	−	292	629	7545	7885
UN19729	Rsa1.0_02333.1	624	32431	1.00E-105	358	405	88	+	234	624	29979	30370
UN19730	Rsa1.0_00977.1	589	198001	0	587	587	100	−	1	587	50728	51314
UN19731	Rsa1.0_06166.1	1789	11583	0	789	825	95	+	968	1788	5941	6749
UN19732	Rsa1.0_00002.1	983	601661	1.00E-136	261	265	98	+	717	981	255585	255847

UN19733	Rsa1.0_00015.1	881	561803	0	497	499	99	−	385	881	193292	193790
UN19734	Rsa1.0_01349.1	1213	47513	0	758	759	99	+	454	1212	34812	35570
UN19735	Rsa1.0_02725.1	762	28404	1.00E−86	182	186	97	+	2	185	6572	6756
UN19736	Rsa1.0_01363.1	1717	47195	1.00E−177	337	344	97	+	3	346	20177	20520
UN19737	Rsa1.0_01178.1	1040	51571	7.00E−98	260	286	90	+	443	728	25592	25877
UN19738	Rsa1.0_00062.1	1319	299291	0	396	407	97	−	806	1211	263778	264184
UN19739	Rsa1.0_00105.1	1981	134138	0	665	674	98	−	2	670	123747	124420
UN19740	Rsa1.0_16059.1	890	2302	1.00E−136	246	246	100	+	645	890	345	590
UN19741	Rsa1.0_05679.1	1759	12958	1.00E−174	311	311	100	−	1448	1758	499	809
UN19742	Rsa1.0_00055.1	863	520193	0	661	666	99	+	198	863	179688	180352
UN19743	Rsa1.0_00634.1	1392	71222	0	369	379	97	+	1015	1392	3677	4051
UN19744	Rsa1.0_00547.1	754	76521	0	374	376	99	+	3	378	74160	74535
UN19745	Rsa1.0_07634.1	913	8979	0	430	438	98	−	3	440	5114	5548
[truncated: 518,452 more chars]
