# Supplementary material for: Draft Sequences of the Radish (Raphanus sativus L.) Genome
Source: DNA Res. 2014 May 16;21(5):481–90. doi: 10.1093/dnares/dsu014 (PMC4195494; doi:10.1093/dnares/dsu014)
Supplement: Supplementary Data [file supp_dsu014_dsu014supp.pdf]

Supplementary Data

Supplementary data listed below are available at www.dnaresearch.oxfordjournals.org.

Table S1 Statistics of the assembled scaffolds only by short-read sequences

Table S2 Summary of BAC clones

Table S3 Linkages of both ends of scaffolds

Table S4 Genes of 80,521 predicted by Augustus.

Table S5 Gene prediction by Augustus 2.7 with *A. thaliana* training set

Table S6 Numbers of predicted tRNAs

Table S7 Blast search of RSA_r1.0 with RadishBase

Table S8 Comparison of repetitive sequences in three Brassicaceae species

Table S9 Classification of predicted gene into Clusters of Orthologous Groups of proteins (KOG)

Table S10 Numbers of SNPs between sequences of RSA_r1.0 and reads of Sayatori

Table S11 List of SNP DNA markers

Table S12 List of RGB PCR-RFLP markers

Table S13 List of RGC PCR-RFLP markers.

Table S14 Integrated linkage map

Table S15 A summary of genetic map

Table S16 Assignment of scaffolds to integrated linkage map

Table S17 Number of SNPs between different lines of radish

Table S18 Number of common amplicons containing SNPs between different lines of radish

Fig. S1 Distribution of the number of distinct kmers in each multiplicity (kmer = 17).

Fig. S2 A linkage map of *R. sativus*.

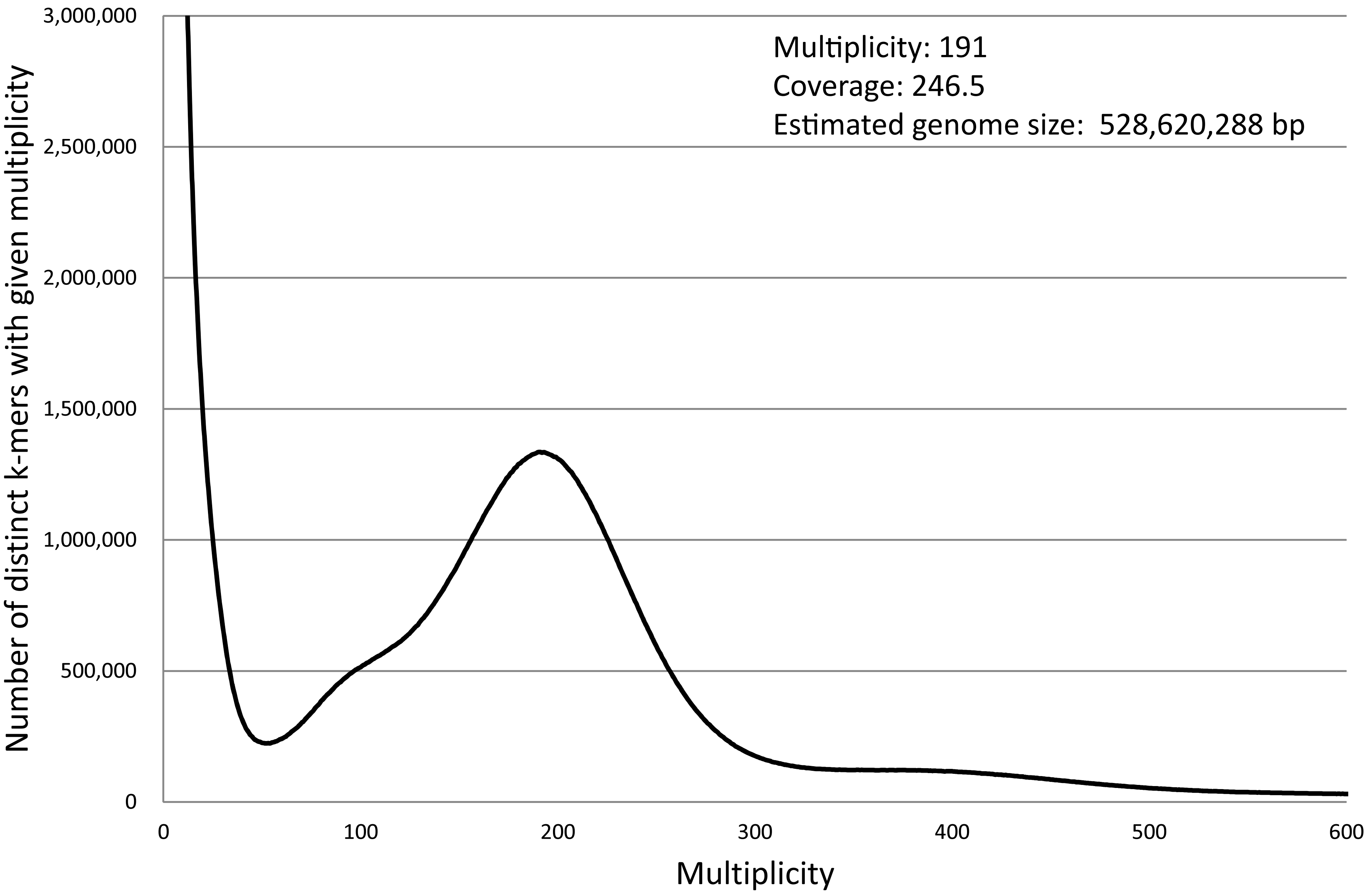

Fig. S1 Distribution of the number of distinct kmers in each multiplicity (kmer = 17).

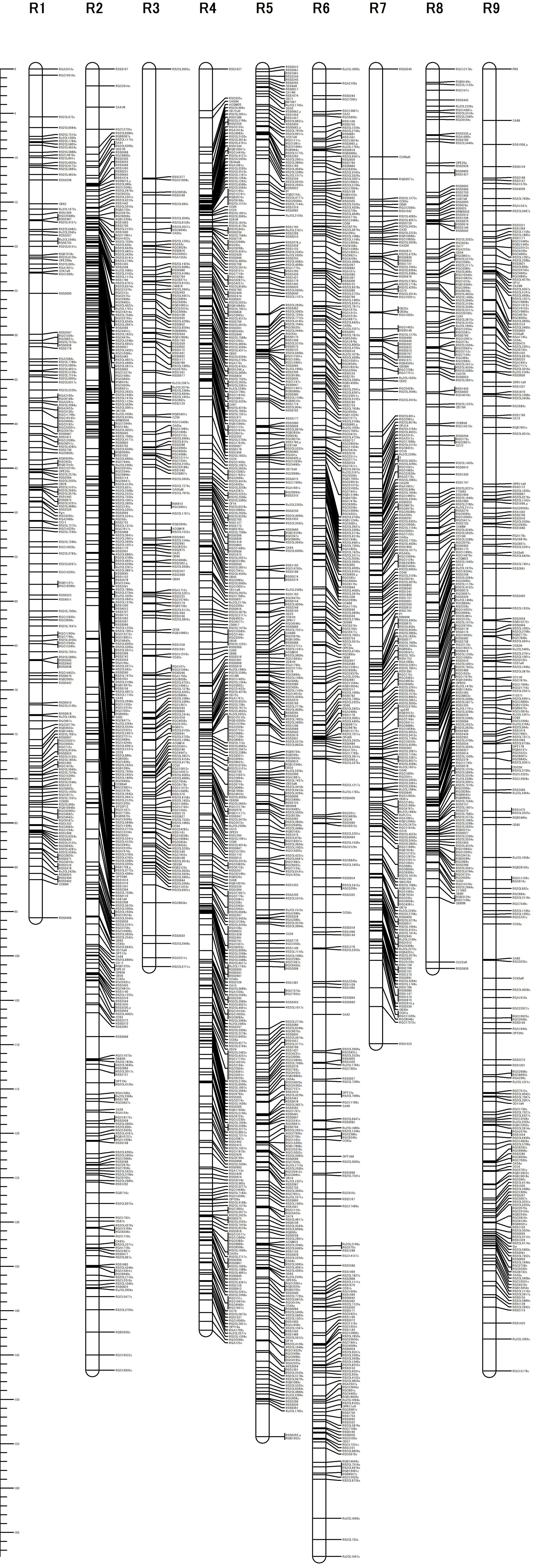

Fig. S2 A linkage map of R. sativus.
Nine linkage groups are labelled as R1–R9 according to Li et al. (2011). Scale of genetic distance (in cM) are shown on the left side of the figure. Marker names are shown on the right side of each linkage group.
