# Supplementary material for: Draft Sequences of the Radish (Raphanus sativus L.) Genome
Source: DNA Res. 2014 May 16;21(5):481–90. doi: 10.1093/dnares/dsu014 (PMC4195494; doi:10.1093/dnares/dsu014)
Supplement: Supplementary Data [file supp_dsu014_dsu014supp_tables8.pdf]

Table S8 Comparison of repetitive sequences in three Brassicaceae species

					R. sativus (RSA_r1.0)		*B. rapa* (v 1.2)		*A. thaliana* (TAIR10)	
					Length occupied (bp)	Percentage of sequence	Length occupied (bp)	Percentage of sequence	Length occupied (bp)	Percentage of sequence
Known repeats	Interspread repeats	Class I	SINEs:	Total	830,222	0.2	657,234	0.2	126,746	0.1
			LINEs:	Total	6,002,395	1.5	5,368,571	1.9	1,359,015	1.4
		Class I		Total	16,418,718	4.1	12,490,461	4.4	8,165,644	8.7
			LTR elements	Copia	8,186,444	2.0	7,173,353	2.5	1,826,674	1.9
				Gypsy	7,679,430	1.9	4,854,533	1.7	6,243,686	6.6
		Class II	DNA elements		8,044,795	2.0	7,309,301	2.6	5,392,948	5.7
			Unclassified		1,820	0.0	2,360	0.0	0	0.0
		Helitrons			1,058,771	0.3	671,961	0.2	2,205,279	2.3
		Low complexity			15,206,887	3.8	12,241,121	4.3	1,434,843	1.5
		Unknown			15,488	0.0	13,217	0.0	5,676	0.0
		Subtotal			48,164,975	12.0	39,231,687	13.8	18,791,499	20.0
Unique repeats		Unknown			59,040,800	14.7	54,198,683	19.1	4,832,479	5.1
		Low complexity			525	0.0	803	0.0	43	0.0
		Subtotal			59,041,325	14.7	54,199,486	19.1	4,832,522	5.1
		Total			107,206,300	26.6	93,431,173	32.9	23,624,021	25.1

Table S9 Classification of predicted gene into eukaryotic Clusters of Orthologous Groups of proteins (KOG)

KOG ID	Name	*R. sativus* (RSA_r1.0)		*B. rapa* (v 1.2)		*B. oleracea* (unigene)		*R. raphanistrum* (unigene)		*A. thaliana* (TAIR10)		KOG
		Number of KOGs	%	Number of KOGs	%	Number of KOGs	%	Number of KOGs	%	Number of KOGs	%	
J	Information storage and processing	1,343	2.64	1,140	2.82	1,056	3.57	1,045	5.27	977	2.72	J: Translation, ribosomal structure and biogenesis
A		950	1.87	815	2.02	799	2.70	396	2.00	798	2.22	A: RNA processing and modification
K		1,896	3.73	1,645	4.08	1,082	3.65	697	3.51	1,391	3.88	K: Transcription
L		855	1.68	463	1.15	450	1.52	192	0.97	462	1.29	L: Replication, recombination and repair
B		345	0.68	286	0.71	257	0.87	155	0.78	294	0.82	B: Chromatin structure and dynamics
D	Cellular processes and signaling	1,075	2.11	698	1.73	509	1.72	261	1.32	547	1.52	D: Cell cycle control, cell division, chromosome partitioning
Y		108	0.21	81	0.20	88	0.30	41	0.21	63	0.18	Y: Nuclear structure
V		227	0.45	190	0.47	135	0.46	93	0.47	177	0.49	V: Defense mechanisms
T		2,645	5.20	2,197	5.44	1,778	6.00	950	4.79	1,864	5.19	T: Signal transduction mechanisms
M		338	0.66	296	0.73	259	0.87	161	0.81	242	0.67	M: Cell wall/membrane/envelope biogenesis
Z		649	1.28	474	1.17	402	1.36	274	1.38	407	1.13	Z: Cytoskeleton
W		83	0.16	83	0.21	54	0.18	20	0.10	59	0.16	W: Extracellular structures
U		1,364	2.68	1,174	2.91	1,059	3.58	761	3.84	970	2.70	U: Intracellular trafficking, secretion, and vesicular transport
O		2,974	5.85	2,601	6.44	2,173	7.34	1,594	8.03	2,300	6.41	O: Posttranslational modification, protein turnover, chaperones
C	Metabolism	978	1.92	842	2.09	810	2.73	665	3.35	720	2.01	C: Energy production and conversion
G		1,482	2.91	1,235	3.06	1,117	3.77	669	3.37	1,076	3.00	G: Carbohydrate transport and metabolism
E		1,097	2.16	885	2.19	873	2.95	531	2.68	787	2.19	E: Amino acid transport and metabolism
F		272	0.53	224	0.56	206	0.70	126	0.64	208	0.58	F: Nucleotide transport and metabolism
H		223	0.44	180	0.45	198	0.67	130	0.66	197	0.55	H: Coenzyme transport and metabolism
I		987	1.94	880	2.18	764	2.58	492	2.48	781	2.18	I: Lipid transport and metabolism
P		839	1.65	704	1.74	509	1.72	349	1.76	592	1.65	P: Inorganic ion transport and metabolism
Q		1,098	2.16	974	2.41	593	2.00	368	1.85	775	2.16	Q: Secondary metabolites biosynthesis, transport and catabolism
R	Poorly characterized	6,667	13.11	4,327	10.72	3,309	11.17	1,985	10.00	3,730	10.39	R: General function prediction only
S		1,304	2.56	1,127	2.79	949	3.20	637	3.21	1,090	3.04	S: Function unknown
–		21,040	41.38	16,830	41.70	10,184	34.38	7,247	36.52	15,380	42.85	No hits

Table S10 Numbers of SNPs between sequences of RSA_r1.0 and reads of Sayatori

Total size of RSA_r1.0 (bp)	402,330,269
Total size of regions mapped by reads of Sayatori with the read depth of 3 to 18 times (bp)	151,012,313
Number of SNPs	1,137,732
Total size of genic region of RSA_r1.0 (bp)	121,807,005
Total size of regions mapped by reads of Sayatori with the read depth of 3 to 18 times (bp)	77,327,761
Number of SNPs	500,113
Total size of non-genic region of RSA_r1.0 (bp)	280,523,264
Total size of regions mapped by reads of Sayatori with the read depth of 3 to 18 times (bp)	73,684,552
Number of SNPs	637,619

Table S11 List of SNP DNA markers

Marker name	Primer sequence (5'-3')		Probe sequence	Hybridization condition		Probe sequence	Hybridization condition	
	Forward	Reverse	**Sayatori**	Temperature	SSC	**Aokubi**	Temperature	SSC
RGA01256	CGAGTTCTTCTTGGTGAGCA	ACCTGACATCATGAAGCGAAT	AAGTCAGAATCTCACAC	45	0.5	AAGTCAGAGTCTCACAC	45	0.5
RGA00888	GGAGGATGAAGCAAGCAGAG	GGATGCAGATTCCGATCAGT	TACGAGTGGGCATATAG	45	0.5	TACGAGTGTGCATATAG	45	0.5
RGA02693	CGATTAGCAGCTCTCATGGAT	TTGTGGTGAGTGCTCGAAAA	TGACCTATGTCAATGTG	35	1	TGACCTATCTCAATGTG	35	0.1
RGA01388	ATGGGAGCGTCAATTTCAAC	AGAGCCTAACGCTGGTCTTG	ACTATACGATCTCCATC	35	0.5	ACTATACGTTCTCCATC	35	0.1
RGA07052	CGACACCGAAAAGGAAAAAC	TGGTATGGTGCCATGTCATT	CATGATATTTATACAGG	35	1	CATGATATATATACAGG	35	1
RGA00413	CCAGGGAAGTGGAAATCTCA	AGGACCAATTGACACGGCTA	ATCGGAGATTTTGGTGT	45	0.5	ATCGGAGAGTTTGGTGT	45	0.5
RGA01339	TCCCCACACCACATCTTACA	AATGATCGGCCTTTGATTTG	TTTACTTCGGTTTTGAG	45	1	TTTACTTCCGTTTTGTG	45	1
RGA01095	CGGAAAGCCAACCATCTCTA	CGCAATGCTCAATCCTAACA	AGGATCTTTTTCTCCAA	45	1	AGGATCTTCTTCTCCAA	45	1
RGA00073	CTTCTCTGCTTTGCCTCCAC	ATGCTCTTGTGAGCCGTCTT	TTTCTAAACAATCTAAT	35	1	CTTCTAAATAATCTAAA	35	1
RGA00793	CGCATTGCTCTCATACTCCA	TCGCTCACACACTTCTCACC	TTGAAAATGGAAACATG	35	0.5	TTGAAAATACAAACATG	35	0.5
RGA00105	CACCCCTGCTTTCACTAAGG	GCAGATCCATTCCTTGAGGT	CACCACATGAAGTTAAA	45	1	CACCACATCAAGTAAAA	45	1
RGA06967	TGGACTCCCGGATGATAAAC	TCGCAATCTCAACAGTCGTC	ACTGGCCAGAGACTAAT	45	0.1	ACTGGCCATAGCCTAAT	45	0.1
RGA02490	GTCCCTTCCCTTCCAGATTC	CACACACAGCTTCCATGCTT	ATCTTGATACCAAACTC	45	0.1	ATCTTGATGCCAAACTC	45	0.1
RGA00645	TTTGCTGTCGTCACGTTTTC	ACTGAACGGCTGCTCCTAAA	CATTTAATACTGATAAC	35	0.5	CATTTAATGCTGATAAC	35	0.5
RGA07150	TCACCACATCCGCTATGAGA	AGGTTCTTCCCCGTTCACTT	TGCAAGATCGAAGTAGC	45	0.1	TGCAAGATGGAAGTAGC	45	0.1
RGA03718	TTGAGGCAGTCAAGATGTCG	TTGAAACAAGTTGCAGCACA	ACCTGAGATAGCTTTGT	45	1	ACCTGAGACAGCTTTCT	45	1
RGA00159	GGCCAAATTTAACCTCCACA	AAAATCTGAACTGTCCACAAGC	TGTCTGTTATTATAAAC	35	1	TGTCTGTTCTTATAAAC	35	1
RGA00979	ACAATCCATGCGTGTGATGA	GGCTGGAGAAAATAGCCAGA	AAATGTCGTAATTTAAT	40	0.5	AAATGTCGCAATTTAAC	40	0.5
RGA00250	TGGCATGTTTTAGGGATGGT	AAGGATGCATGTTGGTCGAT	TATATGTGAACAAATGT	35	0.5	TATATGTGTACAAATGT	35	0.5
RGA01191	TAGGCAGTGGCTTCATTGTG	GGTTCGTGTGTGTGGGTACA	ATCAAGGTCACAGAAGC	45	1	ATCAAGGTTACAGAAGC	45	1
RGA00046	TCGCTGGAAACAAACTTGAA	AGATAACCACGACCGCAAAC	CACTTTAGCCCTAGACA	45	0.5	CACTTTAGTCCTAGACA	45	0.5
RGA13794	GAAAACTCTGCGATGGGAAA	CGGCCCTAGAGGTATAAAGG	TAGCCAAATTTCGCAAT	45	0.5	TAGCCAAACTTCGCAAT	45	0.5
RGA00945	CGAAACTGGAAGTTGTCGTG	CGGGGAAATCCAAATGTTTA	AAAACATAAGATCCCAG	45	1	AAAACATAGGATCCCAG	45	1
RGA03572	CGAATAAACGGGCATGAGTT	CCGGGTTTGAGACCTTGTTA	TTTACATGCTTTACCCA	45	1	TTTACATGATTTACCCA	45	1
RGA00454	CGCAAGTGCAAGCCTACTAC	GTGGTGCAGGACAAATCTCA	CCATCGTCCATGCGTTC	45	0.1	CCATCGTCGATGCGTTC	45	0.1
RGA01070	TGCTGGCATAGCTAAACTCC	CTTGTCAACGTAGGGCCAAT	TAATAGTAGGTAAGCCC	45	1	TAATAGTAAGTAAGCCC	45	1
RGA00089	CCGGAAGAACTCCACTGAAA	TGGTAGGTGCAGTTCCGACT	CTCATGTCCAGAAGTGA	45	0.1	CTCATGTCGAGAAGTGA	45	0.1
RGA05944	CAGTGGGATCAAGTGCATCA	GAAACGGCTGATCAAAGACC	TCATAAGCTTCTGTGTA	40	0.5	TCATAAGCGTCTGTGTA	45	0.5
RGA01214	TGAACAGCAGCTCAAAGACG	CAGTCGTGGGTGAGTGAGTG	CTTCATTCTGGATTTTA	35	0.5	CTTCATTATAGATTTTA	35	0.5
RGA00018	GAGGTGGAGACTCCTGGTGA	CGTGCAACGGACATTACATC	AACACTGTAATCAATAA	35	0.5	AACACTGTTATCAATAA	35	0.5
RGA00016	TTAGATTCGGTTTGCCTTGG	ACACTGGACGTGGAAAGAGG	AGGTGTATAAAACCCTA	45	0.5	TGGTGTATGAAACCCTA	45	0.5
RGA00035	CTCATCAAACAACTAATGCACCA	TCAAAACAGAACAACCAACAAAA	ACATTAACTAAGTAACT	40	1	ACATTAACCAAGTAACT	40	1

RGA03229	AAACCGGACCTAACCAAACA	GGGAGGTCAGAGAGAAGCAA	GGTTTACTCTATGGTTT	40	1	GGTTTACTTTATGGTTT	40	1
RGA00237	TCAAAAGCCACGTTTTCATTT	GCTAACCCGCCATTATCAAA	CCAATCAAGAATGTCCA	45	1	CCAATCAACAATGTCCA	45	1
RGA00019	TTGCAGCGTTTCTGTTATGC	CACATTGTTTGAGGGTGCAG	GAAGGAATGATACAAGG	35	0.5	GAAGGAATAATACAAGG	35	0.5
RGA00043	AGCAATGCTTCGAGTCCTTT	ATGCGAGTCCGAGAAGCTAA	ATGTGAAGGGAAGTTCT	45	1	ATGTGAAGAGAAGTTCT	45	1
RGA01945	TCCAACATATACGCCGTTCA	CGATTTCTCCACGTGGTCTT	GGCACTAAACTAAAAGT	45	1	GGCACTAACCTAAAAGT	45	1
RGA00842	CGCACGCAGTGATAAGAAAG	AAAGAAACTCGCGAGGAACA	GTTATAACTCATACTGT	45	1	GTTATAACGCATACTGT	45	1
RGA01070	CAATCACTCCGACTCCCACT	TGATGACTGCTGGAAACAGG	TCCCTGTTCTTCTTCCT	45	1	TCCCTGTTTTTCTTCCT	45	1
RGA01025	CATGGCGAGGAATTGGTACT	ATCATTGAGAACCGGGTCAA	GCTTAACTTCAATGTAT	35	0.5	GCTTAACTACAATGTAT	35	0.5
RGA00092	AACAAGGGGATGTGATTTGA	TAGGCACGGCTTTCACTCTT	CTAAAGTATGAGTGGAC	45	1	CTAAAGTACGAGTGGAC	45	1
RGA02573	TTTCTGCGCCTAACACAATG	AGCAGAAAGCCTGTGGAGAA	TCCAACAACAGAAAATC	45	1	TCCAACAAAAGAAAATC	45	1
RGA01233	GCCATCTCCAAAGCAAAATA	AATTCTTGTCTGCCAAATCG	TCAAAATTTTCAGTCTC	40	0.5	TCAAAATTATCAGTCTC	40	0.5
RGA02492	CGGACTCAATGCCTCTTCTT	AACGACGCTTCTTCTTCATCA	GGTTAAACTTAAACCCA	45	0.5	GGTTAAACCTAAACCCA	45	0.5
RGA10835	CCAAACGAGCAAAAGAAAGC	GGAAAGACCGCAAGTGATGT	AGTTTAACATCACGTGT	45	1	AGTTTAACGTCACGTGT	45	0.5
RGA01378	TTCCCACTTAATTCGCGTTG	TTACTGGAAATCTTTCGACTGC	GATGTCTTTTGAAAATT	35	0.5	GATGTCTTCTGAAAATT	35	0.5
RGA00304	TGGAACAACAAAGCATGAGC	CGTTGGTGAACTTTGCTTCA	ATCGTCTACGTTCATTC	45	0.5	ATCGTCTATGTTCATTC	45	0.5
RGA02393	AATTCCTGCCTCAGAGCAAA	CTCCAGAAAGCGTGAACACA	TCATTGTTAAGTAGTAC	35	0.2	TCATTGTTCAGTAGTAC	35	0.2
RGA02777	CCGGGAGAGACAGTCATGTT	TGGACTACCTGCACAATGGA	TTTTGACAAGCCAAAGT	45	0.5	TTTTGACAGGCCAAAGT	45	0.5
RGA00141	AACCGCATGTGAAGTTTTCC	ATCATTCAACGGTTGGGGTA	TGAGGAATATGTAGAGA	40	0.5	TGAGGAATGTGTAGAGA	40	0.5
RGA01783	AGAAAGCCACCTGCGTCTAA	CACGTGGTTTATGCTTGTGG	GATTCATATGACACCAA	45	1	GATTCATACGACACCAA	45	1
RGA02221	TTGTCCACGTGTCAGTCGTT	CCTTCCACCAGTTCCACCTA	GCTAGTTAGTATCATAT	35	0.5	GCTAGTTAATATCATAT	35	0.5
RGA00051	AGTCGTTCCAAGGGGAGAGT	CAAAGCACAAAAACCCGATT	CTCTTTTTGACTGTCTT	35	0.5	CTCTTTTTTACTGTCTT	35	0.5
RGA00236	ACGTGGACATGTTTGGATCA	ATGGTGAAAGGCCAAGACTG	CACCTTCAAGAGATCAT	45	1	CACCTTCACGAGATCAT	45	1
RGA00150	CGCCCTTAGAACAGCTTCAT	TGTTCATGGGATGAGTTCCA	GCCAATGTGAATTCTAA	45	1	GCCAATGTAAATTCTAA	45	1
RGA00721	CGAAAACGAACAGCATTTTT	TTTCACTGTCGTCGCATTTA	CGGTGCAAGAGTAAATA	45	0.5	CGGTGCAATAGTAAATA	35	0.5
RGA01154	TGGGCAAATTTTTGAATCAT	CCCATTCAACATACGACCAT	AATCTAGAATAGAGAAG	45	0.5	AATCTAGAGTAGAGAAG	45	0.5
RGA00011	ATGTTGGGTCCTTCAACAAA	AAGCATCGAAATGCAGAATC	AGTATTATACTATTATT	35	0.5	AGTATTATGCTATTATT	35	0.5
RGA00323	CAGAGTGGGAAGCTTTGGAG	GCACCCAGTCGTATGAACCT	ACATAAGGCTCTCCCTA	35	0.5	ACATAAGGTTCTCCCTA	35	0.5
RGA00606	AATCCATAATCGCAGATCCA	ATCTTGGATTGAGCAGCAAG	TTGATATAGATCATAGT	45	0.5	TTGATATAAATCATAGT	45	0.5
RGA01128	TTGCTCTTCGTTGTCTGTGG	TCCTCGGGAGTCTGAGTGAT	TCTCGTCGACGAACTCA	45	0.2	TCTCGTCGGCGAACTCA	35	1
RGA00397	TCGATTGAACCACTGACCAA	GAACCAGGCAACTTTCAGGA	ACTAAACTTTTTTATTT	45	1	ACTAAACTATTTTATTT	45	1
RGA01510	TCGAACTGAAAAGGGCTCAC	AGCCCTCACAATTTTTGGAA	CTTTTCATGGATCTTGC	35	0.5	CTTTTCATAGATCTTGC	35	0.5
RGA03795	TCAAAGAAGAGAGCGGGTGT	TTGCAGCCCTAGCCTTCTAA	CTCATATCCAATCTGTG	35	0.5	CTCATATCGAATCTGTG	35	0.1
RGA00832	AGCTCTCTGGATCACGCCTA	ATCTGCATATTGCCCTGGAG	ATAAAGTCAGCTCTTTT	35	0.5	ATAAAGTCGGCTCTTTT	35	0.5
RS2CL1103s	GTGGTGCAGGACAAATCTCAAA	AGATTGCTCACAACGGAAGGAT	AGTCAAGGGTCGCCCTA	50	0.1	AGTCAAGGCTCGCCCTA	50	0.1
RS2CL1107s	TGGTCCAGCTCCAGAAAGTGTA	TTGCTCGCTCTCGAATTGTAAC	ACCACATGAAAATATCC	35	0.5	ACCACATGGAAATATCC	35	0.5

RS2CL1124s	ACCTAAACCAAACCCTCCGATT	GGTCTGCGTACCGAAAAAGATT	TCCAAACGCGTCGAGAC	40	0.5	TCCAAACGTGTCGAGAC	40	0.5
Rs2CL1221s	ATGCAAATCGCTACAGCTTCTG	TGCTCACTGGCGAAAAGAGTAA	ATGAATAACTTTTACGG	35	0.5	ATGAATAAATTTTACGG	35	0.5
Rs2CL1337s	CCACGAAGCTCTTCTTCACCTT	CCTTTGCCATCAGAGAACAACT	TTCTTGTTCTACTGAAA	35	0.5	TTCTTGTTGTACTGAAA	30	0.5
Rs2CL1347s	TGACAAGCACATGTACCACGAC	GCGCAGAGTAGGTGTTTTGACA	GCTAGCCGAGAAAGGAA	50	0.5	GCTAGCCGTGAAAGGAA	50	0.5
RS2CL1353s	AAAGAACCGATGGTGATTGGAC	ATCCCATCTGCACCTAGCTGTT	GGAGTGAAACATCTAGT	40	0.5	GGAGTGAAGCATCTAGT	40	0.5
Rs2CL1468s	TGAAGAAGGGAAACCTCTTTCG	CGACATAAAAGCATCCAAGTGC	TTATTCACCTGCGTTCT	45	0.5	TTATTCACATGCGTTCT	45	0.5
Rs2CL1478s	GAAGCTGCAGCAAACAACCTAA	ACTTGGAGCACGTGATTTCTGA	CTAAAGCTGTTGACCTG	45	0.5	CTAAAGCTCTTGACCTG	40	0.5
Rs2CL1480s	GGCACGCTTTTCTACAGTGGAT	AACAGAATCATCAACCGGAGTC	TACTTGTTCGTTTTCTT	40	0.5	TACTTGTTTGTTTTCTT	40	0.5
RS2CL1483s	GGGATGATCGATTACGACAACA	CCTCAGGGGAAATGAAAGGATA	GTGGAGGAAAAGAAGGT	40	0.5	GTGGAGGAGAAGAAGGT	40	0.5
Rs2CL1500s	AGGAACTCCTTTTGCAGACGAC	AAGATGCGTACCTTGTGCATGT	TGCATTTTCTTAACGAT	45	0.5	TGCATTTTGTTAGCGAT	40	0.5
RS2CL1509s	ACGGATGCAAGCTTGAAGAACT	GAGCCAAGGTTCTCAAACCACT	TGGAACAGTGTTGGCTG	45	0.5	TGGAAGAGCGTCGGCTG	45	0.5
Rs2CL1513s	CTCGTTGTCTTCACCACCAACT	TCTTCAACTAGAACGCCACTCG	GATTGTTCATATAGAGA	35	0.5	GATTGTTCGTATAGAGA	35	0.5
Rs2CL1517s	TCGGAATACCCTTTCTGGAGAC	GCTTAACTACTGACCGCAGCAA	TGATCTCACTGTATATA	35	0.5	TGATCTCATTGTATATA	35	0.5
Rs2CL1549s	TCGAACCATCTTGGTGTGAACT	GCTTAAAGAGGTGTGGCATGTG	TAACTCATGATTTGATA	40	0.5	TAACTCATAATTTGACA	40	0.5
Rs2CL1620s	GCCAATTACGGACCTTCTCTGT	TACTCTTGGGTTCCATCGCTTT	TGGAGTCAGTTCCTTAT	45	0.5	TGGAGTCACTTATAATG	40	0.5
Rs2CL1633s	GCCGCAAAGCTTATCATCAA	CGATACTCAGCAAGTGCCCATA	AGAAACACTTTCCCATT	50	0.5	AGAAACACCTTCCCATT	50	0.5
Rs2CL1639s	TGTTGCACAACGCAATGACTAC	CAGTGTGAAGCAAAAGGGAATG	GTGTTAACCATATGCGA	40	0.5	GTGTTAACAATATGTGA	35	0.5
Rs2CL1647s	CCAAACAGAAAACAACCAGACG	CGGGAAAAGTTATGGCTAAACC	ATAATGGTCAACTTTCT	40	0.5	ATAATGGTTAACTTTCT	35	0.5
Rs2CL1650s	ACCTTTCGAGACAAGCCTCATC	ACCCCCAAGGAGAAAGAGAAAG	TCTCGCTGGACAGATAA	50	0.5	TCTCGCTGAACAGATAA	40	0.5
Rs2CL1651s	TCTTGTCATGTGCGGCTATCTT	GATACCGGAAACCATTTCTTCG	ATTAGGTACGTATAAGA	30	0.5	ATTAGGTAAGTATAAGA	30	0.5
Rs2CL1671s	ATGTCCAGAGACGCCGATAGTT	CCTCAGGCAACCAATTCAGATT	GTTTTTGTGTTAAAGAG	35	0.5	GTTTTTGTTGTTAAAGA	35	0.5
Rs2CL1672s	GGGTCACGTGAAAGTTTGGATT	CACGGATCTGCTCAACTTTCTT	GAAGCCATCTCGAGGCA	50	0.5	GAAGCCATGTCGAGGCA	50	0.5
Rs2CL1678s	ATCCTCACATTCATGTCGAACG	TACTCTGACAACTTCCCGCAGA	ATCTTGGTTATTAAATT	35	0.5	ATCTTGGTGATCAGCGT	35	0.5
RS2CL1685s	GAGATGGAGACTGTGCAAATGG	GGGTGGCTCAAAAGGATTAACA	CTGCAAAAAAATTCACA	40	0.5	CTGCAAAATTTTCACAC	35	0.5
Rs2CL1694s	GATCTGTTCGCCATGGAAGATA	GCATCCGATCAAAAAGTCTCTG	TACGATCTTCTTCATCC	40	0.5	TATGATCTACTTCATCC	35	0.5
Rs2CL1695s	TCGATCACGTCATCATCTCCTT	CGTAACCTCTTCAAACCCCAAT	ACTCGATTCGTGTTTAT	40	0.5	ACTCGATTTGTGTTTAT	40	0.5
Rs2CL1713s	CAGATTGTTTGCTTCCTGCAAC	CATTTGCTCAAGATGCTCCAAC	TCGTAACCCGAAGCTGC	45	0.5	TCGTAACCTGAAGCTGC	40	0.5
Rs2CL1741s	AGGAAAACTTGGCATGGGAAC	CCACGAGGTTACAAAGCCCTTA	GTCACACTGAATGGAAT	40	0.5	GTCACACTCAATGGAAT	40	0.5
Rs2CL1743s	CATGCCATTCTTCCATCATAGC	TTCCAGTACAAGCTGGCCATTA	AAGCTTAACTGGCCAAT	45	0.5	AAGCTTAATTGGCCAAT	40	0.5
Rs2CL1745s	AGCGAGCCATTGCTTCTATTTC	CGACTCCGAGATTCAACACAAC	TTTTACTCCATGAATAG	40	0.5	TTTCAGTCTATGAATAG	40	0.5
Rs2CL1765s	AGCTTGAACCTTTCTTGCCGTA	GGAGGCTCGTTCTATCTTCCAA	CTCACATCGTCCGGGTC	50	0.5	CTCACATCATCCGGGTC	50	0.5
Rs2CL1769s	GCTGACATGGGTAATGCAGATG	TAATGAAACGCTTGCTGGTCTG	GAGCTTTCCCTTTAGCT	50	0.5	GAGCTTTCTCTTTAGCT	45	0.5
RS2CL1784s	CTGCTTCGCAATTCCCTTATCT	GACGGAGATGACAGTTCCAAAA	AGTTAAAGTTGATGGAG	45	0.5	AGTTAAAGCTGATCGAG	45	0.5
Rs2CL1785s	TTTCTGGCAACCGTAAGGCTAT	AGTTTCCTGTACACACCGACCA	GAGGATGTGGCTTACCC	50	0.5	GAGGATGTCGCTTACCC	50	0.5
Rs2CL1791s	AAGACGAAGTCTGCATTTGTGC	AGTCGCAGTAACACGTTTTTGG	CTGTTTGGGTTAACATA	40	0.5	CTGTTTGGCTTAACATA	40	0.5

Rs2CL1793s	GGATCGTTACAACGCATCTCAA	AGCGTAAACCAGAGCTCTCACA	CTCTCTCTTTCTGTTTG	35	0.5	CTTTCTTTCTCTGTTTG	35	0.5
Rs2CL1800s	ACATGTGAGACGGCAATACAGC	GGCCAAAGCCAAGTGATCATA	CTAAATAAGTTCGAGTG	35	0.5	CTAAATAAATGCGAGTG	35	0.5
Rs2CL1806s	AAGGAGCGTGTTGAGGAGAAAG	ACAAGAACCACCCCTAAGAGCA	GATTCTCTGTGTATTTT	35	0.5	GATTCTCTCTGTATTTT	35	0.5
Rs2CL1826s	ACCACCACCTCTAAAAGCAAGG	CTCACAGGCAAAGAGATCGAAA	TGTTTTCCGGCGTAGAT	50	0.5	TGTTTTCCAGCGTAGAT	50	0.5
Rs2CL1833s	AGTGCAGGTTGTGTATGGGAAG	TGCAGGAAGCTTACTCCTTCAA	TCTTCCATTTGATAACC	35	0.5	TCTTCCACCAGATAACC	35	0.5
Rs2CL1849s	TTGATATCGAACCAACCGACAC	AAACACAACCACCACCCCTAAG	TACTTGTTGGAATTTTA	35	0.5	ACTTGTTTGGATTTTTA	35	0.5
RS2CL1855s	TCTCCTCCTCGCCTACAAAATC	CCCAAACGTGTAAGAGACACCA	GATCTGCTCTGGTCTGA	45	0.5	GATCTGCTTTGGTCTGA	45	0.5
Rs2CL1884s	AACCGTTCTGCTGAAGTTTTCC	GACAAGCCGGTTGAAGCTAATC	ACGGTTGCCCGCATAGA	50	0.5	ACGGTTGCTCGCATAGA	50	0.5
Rs2CL1936s	AACGAGTTCCATGACAAGACCA	TATAGCAACATGACGGACACCA	CATATCTTCACCATCGT	30	0.5	CATATCTTAACCATCGT	30	0.5
Rs2CL1958s	ACTTCAACGCCGTTTCTTCTTC	CGTTTGTTCTTGACCTTGGAGA	GTGTTTAATCAACAAGA	35	0.5	TTAATCAAAGAACCAAG	35	0.5
Rs2CL1966s	AACACGAGCTGTGGACATGTTT	GAAGGAAGCTCTCGGTGATGAT	CATGTTCCCGAAGCTTC	45	0.5	CATGTTCCTGAAGCTTC	45	0.5
Rs2CL1970s	GACGTTGGAGGCAAGATCTCTA	GCTGTCCAGCTAGCCAAAATCT	ATCCCCTTTGCATCAAG	45	0.5	ATCCCCTTCGCATCAAG	45	0.5
Rs2CL1980s	TCGTTCGTGTTTCTTGTGTCCT	TTGAGATCTCTCGTCCAGTTCG	GGTCAACAATACATGGC	40	0.5	GGTCAACAGTACATGGC	40	0.5
Rs2CL1984s	CGAAGATCACAGAGGTTGATCG	TGAGTCCGCTTCTTCTTCAATG	CTGAGATTGAAAAAGGT	45	0.5	CTGAGATTTGAAAGGGT	45	0.5
Rs2CL2028s	TTAGCACTTTCTTCCCCCAAGA	TAACCCAAGAAGCACGACTTTG	GAGCTGAAATTTTTATC	35	0.5	GAGCTGAAGTTTTTATC	35	0.5
Rs2CL2045s	TGGGACCAAAGTGTCGAATATG	TTAAGCAGCAACGTCTTTGTCC	GAGACAATACCGCCGCC	50	0.5	GAGACAATGCCGCCGCC	55	0.5
Rs2CL2052s	GTACCCACCCTTCACAATGGTT	TGGTTGGAGTCCTCCTTATGGT	AGATGTTACCTATCTAT	30	0.5	AGATATTATCTATCTAT	30	0.5
Rs2CL2077s	GCAGACAAAACTGGGATTGTTC	GTTCGCCTACACTCCCAAAAAC	GGAAATGATATTGAATG	35	0.5	GGAAATGAAATTGAATG	30	0.5
Rs2CL2106s	CTGAAGTGCTTTTGTTCGGTTG	CTGGTTAGATGGAAGGCATGTG	TCAATCTTATAGACAAC	40	0.5	TCAATCTTGTAGACAAC	45	0.5
Rs2CL2111s	GTGGCAAAGCTTCAGTATTTGG	CTGCTGAACGAGCATTCAAGAT	ATGTAAGACATACTTCC	40	0.5	GTAAGAGACGTACTTCC	40	0.5
Rs2CL2150s	GGTTCACTTCTCCCTCTCCAAA	ACAGCATCCCTTTTATCCATCC	AACCCATCACAACTAGG	45	0.5	AACCCATCCGAACTAGG	45	0.5
Rs2CL2167s	CTTTGTCTCCAATGGCGTTC	GTTAACAAAGCCGCCGATCTT	TTCGTTGAAATTTTGGA	40	0.5	TTCGTTGATGTTTTGGA	40	0.5
Rs2CL2178s	GGGGTGCGTTATACGAAGTTGT	TCCAGAGCATGATGTTGTTGTC	CCGCGCGAGGAGAGATG	50	0.5	CGCCGCGACAAGAGATG	50	0.5
Rs2CL2196s	CATGGGTGGAATGGAAGAGTTT	TCTGGAACGAACAGCAACTTCT	ATAACGTATTGAAATGG	35	0.5	ATAAGGTAGTGAAATGG	40	0.5
Rs2CL2197s	AAGTCACTTGAGAGCAGCAACG	ACTACAAAAGCCTCACGTGCTC	CAGGAAGTGGACGAGGA	40	0.5	CAGGAAGTTGATGAGGA	40	0.5
Rs2CL2199s	GTGCAGAAGATTTGGAGAAGGA	ACTGCATCATCAACTCCAGGAA	AGGTTAATAATGTCTAT	35	0.5	GGTTAATTATTGTCTAT	35	0.5
RS2CL2201s	AGGAGGAAACGAAGAAGCATTG	GGAAGAGTGAACATTCCACGAA	AAGGAAGCGAACAAGTC	35	0.5	AAGGAAGCTAACAAGTC	35	0.5
Rs2CL2203s	TAAAGGGCACAGTCAGCATCAT	TCTCCCGGAGTTCAAACATTTC	CCCCCGGCTAAGTTCGC	50	0.5	CCCCCGGCCAAGTTCGC	50	0.5
Rs2CL2226s	ACCCAACAGAGGAGGAACTTTG	TGGGAGGAATGTTACAACAACG	TGAGATCACGCCGTCCT	45	0.5	TGAGATCAAGCCTCCTT	45	0.5
Rs2CL2236s	ACCATTACCATGTTCGTCATGC	TGATTCCCTTCCCTGGATCTAA	GATTTTTGTCTGTAATT	35	0.5	GATTTTTGCCTGTAATT	35	0.5
Rs2CL2240s	CTGCAGATCCATCTCCACAAAT	ATGGAATCTTCACCAGGAGGAA	TCTCTTTACGTTTTCAA	35	0.5	TCTCTTTATGTTTTCAA	35	0.5
Rs2CL2263s	GGAAATTCAACCTGAAGCTGGT	CGCATGTCTTCCATTTCTTCAC	CTACGCACGCATATGAT	45	0.5	CTACGCAGACATATGAT	45	0.5
Rs2CL2264s	CATGGGTGAGAAAGGTCTGATG	GAAAACAACCGGGAACATCACT	CATCTTTGGACAAGATC	40	0.5	CATCTTTGAACAAGATC	35	0.5
Rs2CL2267s	GGGAATTTGCACTCCAGAAAGT	AGATGCTCAATGGTGTCGATGA	TAAAATCAATCTATTCT	30	0.5	TAGAATCAGTCTATTCT	35	0.5
Rs2CL2268s	TTGAGGCCTGCTGAGTACAAGA	GAAACGTGAAATGTGGACGAGA	TACGGCTCATTGACCAA	45	0.5	TTCTGCTCTTTGACCAA	40	0.5

Rs2CL2272s	AATCTGAAACCCCAGAACAGGA	ACTTGAATGCGTCTGCAACAAC	AAACCAGCCAAGAAGAA	45	0.5	AAACCAGCGAAGAAGAA	45	0.5
Rs2CL2284s	CCCTCGATTCCCAAACTACTTG	TCAGTGAGAGGCTGCTTGTCTT	CAATAGTACAACTTGCT	35	0.5	CAATAGTAGAACTTGCT	35	0.5
Rs2CL2299s	AATAAAGTAATGGGGCGTGTGG	AATCACACCAGGAAGGCTAAGG	GTTTTAACTAGAGATTT	35	0.5	GTTTTAACGAGAGATTT	35	0.5
Rs2CL2329s	CACGACCATCCACTCTTCAATC	GATTTTAGCTACACGCCCAAGG	TCCTCAACTGGTACAGC	45	0.5	TCCTCAACAGGTACAGC	45	0.5
Rs2CL2337s	CGTTCGCCAGACGTAATGTATC	TCTGTGACTCATTTCCGCTTTC	CAACCGGGACAAGAATC	35	0.5	CAACCGGGCCAAGAATC	50	0.5
Rs2CL2338s	CTTCTTGTTCCCAAACCTCCAC	GGATCCACCTTTAAATCCCACA	ATATAATAATGGACATT	30	0.5	ATATAATAGTGGACATT	35	0.5
Rs2CL2348s	TCCATGCGTGGAAGACTTTG	CAATCGTCGGATCAGCAAATAG	AGCTATGTTTTTCTATA	35	0.5	AGCTATATATTTCTATA	35	0.5
Rs2CL2367s	GCGATGGCTTCCATTACTTCTT	ACACAGCCAACCAACAAACAAC	TTAACTGTAGCTCTCTA	40	0.5	TAAGTGTGAGCTCTCTA	35	0.5
Rs2CL2368s	CCCTAGCAAAGTTTTGGAGCAT	ACGTTGGTACAAATGAGCCTGA	TCATTGATGGCAACGCA	45	0.5	TCATTGATCGCAACGCA	45	0.5
Rs2CL2374s	AAGAGAACCTTGCCACCAGTTC	TCTTCTCCTCCATTTTCCCTCA	AGAAATCACAGCCATTC	45	0.5	AAAAATCAAAGCCGTTC	45	0.5
Rs2CL2383s	ATGGAAGTGTTGCGGGACTTAT	ATTCGTGTAGCCTGCTTCCTTC	TAGTATTGGTTAGGATC	35	0.5	TAGTATGGTTTAGGATC	35	0.5
RS2CL2387s	AGAACTTGTAACGGGGCAAAGA	TCTCCCATACACACACACTCCA	CTTCAGGGTGTAATAAG	50	0.5	CTTCAGTGTATAATAAG	50	0.5
Rs2CL2399s	CGTGAGTTTATCCATGCCAAAG	TGGACTCTTTAAACGACCACGA	ATTTTGAAGGAGAAGCC	40	0.5	ATTTTGAAAGAGAAGCC	45	0.5
Rs2CL2428s	ACACTTTGGTGAAGCCTCTTCC	CAGCGTCCATTCAAATCTCAAC	AAAGCTCGTCGTGTTAG	45	0.5	AAAGCTCGCCGTGTGAG	45	0.5
Rs2CL2460s	GCCTGCTCATGGAAAAGAAAGA	GAAGAAGAGGCCTCAATTGCTC	CATCCAGCCCATTCTAT	45	0.5	CATCAATCACATTCTAT	35	0.5
Rs2CL2464s	AACAGATGTCCGATCCCAGAGT	AGATTTTAGCTCCCTCCCCATC	CCTTTTTATGAGTGTTT	35	0.5	GCCTTTTACGAGTCTTT	30	0.1
Rs2CL2475s	AGGGTAAGATTGTCCCTGAGCA	GTTTCGGATCCGTTGTTGTTCT	AAGTTCTGATTTTAAGC	35	0.5	AAGTTCTGGTTTTAAGC	35	0.5
Rs2CL2508s	GAAATGTTTGGCTGAGATGGTG	CTTTCAAAGCTCCGTCTTCCAT	AAAGAGAGATTTTTGCT	40	0.5	AGAGAGAGATTTTTGCT	40	0.5
Rs2CL2530s	GTGTTCCAATGTCGTGATGGAT	CTGCTTGTCTGTCTCAACTTCG	TGGTGATGAAGGTAAGT	35	0.5	TGGTGATGCAGGTAAGT	40	0.5
Rs2CL2589s	TCGGAAACCGTTTGGTACAGAT	CTCACGAGCTTACGGTTTTGTG	AGAGTGGTATTTTAGAG	40	0.5	GAGCTGGTGTTTTAGAG	30	0.5
Rs2CL2604s	TGTCACCCCTTCCACTATAAGC	AATGAAGTCGGCGACAATATCC	AAACTCACTAGGGTAAA	40	0.5	AAACTCACAAGGGTAAA	40	0.5
RS2CL2631s	ATCCCACCATGAGTGAGGCTAT	TTCTTTGAGCTCATCCCACATC	ACTTTCCCGTTGATGTG	45	0.5	ACTTTCTAATTGATGTG	45	0.5
RS2CL2655s	GTCCGAGAAACCGATCACAGTA	GTTAGGCTGCTGCTCTATTTGC	TCGAGAATGACTTGATC	40	0.5	TCGAGAATCACTTGATC	40	0.5
RS2CL2656s	GATCGATGGAAGGGATCGTTAC	GCACGTGATTCAGCATCTTTCT	GTTAAACCTTTTGTTCC	40	0.5	GTTAAAACCTCTGTTCC	40	0.5
RS2CL2659s	CAAACAGCAACAACAGCTCCTC	ATTATCGGTGTGCTTCGATGG	GAAGCGGGTGTATACGG	50	0.5	GAAGCAGGCGGATACGG	50	0.5
RS2CL2669s	ACAACGTGTGGAACTCTCATCC	TCTGCAGCAGTTGAGACCATAC	TAGAATTCGATCTTTTT	35	0.5	TAGAATTCAACTTTTTT	35	0.5
RS2CL2676s	CCATCTTCACCTCCACTTGAGA	CTGTAAATGCCGTCTCCATGTC	TTTCCAGCACGATGATC	50	0.5	TTCCCAGCTCGATGATC	50	0.5
RS2CL2679s	AATCCACAAGCTCGAAAAGGAG	ATGTGTGTTGGCTTCTCAGCAT	CAAGAGATTCCCCGAAC	45	0.5	CAAGAGATCCCCCGAAC	50	0.5
RS2CL2685s	ACCAGCTCTCACATCAAGAGGA	CGTGTGGATTCACTTGACGAAT	TACAAAGAATAGTTCCA	40	0.5	TACAAAGAGTAGTTCCA	35	0.5
RS2CL2687s	GAGCCAAGCATTGGAGATAAGC	TAACAGCACCGTGAGTGTTCCT	CAAATCAACAGTGAGAG	35	0.5	CAAATCAATAGTGATAG	35	0.5
RS2CL2691s	CTGGCCTATCCTGCTTTGCTAT	AGCTGCATAGATCTGTCCGTCA	CTCAACTTATCCTCATT	35	0.5	CTCAACTTTTCCCCATT	35	0.5
RS2CL2717s	ATCGGTTTGGCAGTAGCTTCA	GCCTTCAAACTACGCAGCTCTT	ATGGAAGCTGATTTGAT	40	0.5	ATGGAAGCGGATTTGAT	45	0.5
RS2CL2718s	AGGGATCTCCGATCTCAATGAA	GCTGCTGCTGTCATCCAAATTA	GCCTGTGAAGCATATAC	40	0.5	GCCTGTAACGAATATAC	40	0.5
RS2CL2738s	TACCACAAATGGCTGCAACTCT	CGTCTGATCCTGCAAGAATGTT	TTTTTCTTCAAGAGTCT	35	0.5	TTTCTCTTAAGGAATCT	0	0
RS2CL2740s	GTTGTGAAACTTCCGCGTTC	CGCCATAAACAGTTGAAGGTGA	GATGCTGTCGGTGAAAT	50	0.5	GATGCTGTGGGTGAAAT	50	0.5

RS2CL2788s	GAAATCAGCCGTTTCAGTAACC	CTCTCTCCTGCTCCAAAGGAAT	AGTCTCAAGTGTTGCAG	45	0.5	AGTCTCAAATGTTGCAG	45	0.5
RS2CL3112s	TCATGGCCTTAAGCTTCTCCTC	GTGAGGCTGTTCGTTGACATCT	CTCCACGCTTCAAACTG	45	0.5	CTCCACGCCTCAAACTG	45	0.5
RS2CL3543s	TTAAGGGTCTTCCTTCCATGCT	GAGGTCAAGATGGAGCAAGGTT	ATCAGCAGTTGCGGTTT	50	0.5	ATCAGCAAATGCGGTTT	50	0.5
RS2CL3605s	GAAGATGGTGTATGCCAGCTCA	ACAGCTTATCCCAAGCCAGAGT	GCCTGATTCCTTATCAT	45	0.5	GCCTGATTTCTTATCAT	45	0.5
RS2CL3610s	ATGGGGATGAATCGAACTCAGA	CTGCAAAGGGTGGCTTGATT	AAGCCCAATGGGGGTAA	50	0.1	AAGCCCAAAGGGGGTAA	50	0.1
RS2CL3614s	ACCGGTATGTACATGCAACAGC	TCCATTCCCCGTAATAATCACC	ATACATATATGTTATCG	35	0.5	ATACATATGTGATATCG	35	0.5
RS2CL3771s	ACGCTAAGAAGTTGGGAGGAGA	GAGCATTCTCTTGTTCGGTCAT	ACACAACATATCCACCA	40	0.5	ACACAACACATCCACCA	40	0.5
RS2CL3783s	TCTTGACCATCTTTCTCCACCA	TGGAAGAAACAACAGAGGAGGA	GCACTCCTCTGTCCAGA	50	0.1	GCACTCCTTTGCCCAGA	50	0.1
RS2CL3894s	AGAGGTTACGAACGGAAGGAAA	ATCCCCATGCCTATTCTCTTCA	TTGACTTATTTAAAACC	35	1	TTGACTTACTTAAAACC	35	1
RS2CL4118s	TGTCTTCCATGTGAATCCAACC	TGAATCTCTCACGCAGGTTTTC	CACGAAGGCTTCAAAAT	45	0.5	CACGAAGGTTTCAAAAT	45	0.5
RS2CL4559s	AGATAGTGCAGCGATCACCAGA	TGCAACAGAGACATTGGTTACG	GGGTGGTGCTGATGATG	50	0.5	GGGTGATGATGATGATG	50	0.5
RS2CL4635s	GTTCACGGAAACCTCAAATCCT	CATTCCGAATTTATCCCTGTCC	GGTGAGAAGAATGGGAA	40	0.5	GGTGAGAAAAAATGGGAA	40	0.5
RS2CL4689s	ACTGCCACAGCTGTTGAAAGAA	TACGCTTGGGGCTTATAAGAGG	TGAAGCGAAGCCTAGAG	45	0.5	TGAAGCTACGCCTAGAG	45	0.5
RS2CL4701s	AAAGGTGCGTTTACGGTAGCAG	ACTCCTGTGAGGCCAAAACAA	GCTGAAACCTTGTTTTT	40	0.5	CTGAAACGTTTGTTTTT	40	0.5
RS2CL4777s	GGGTACTTCCACCACCACATTT	TCACCAGCTCCGATATCTTCAG	GAGACATGTACCAAGAC	40	0.5	GAGACATGCACCAAGAC	40	0.5
RS2CL4906s	CGTCTGAAACCAAAGACACTGG	GGACTGGTTACAGTTTCGTGGA	ACAATATGAGCTGATTC	40	0.5	ACAATATTTGCTGATTC	40	0.5
RS2CL5024s	TAACATAAACCCCCAAGGCGTA	TGAAGAAGAGTGGCGATGTCTC	TTCCGTCACTGAAATCG	45	0.5	TTCCGTCGTTGAAATCG	45	0.5
RS2CL5083s	ACGGAGTTTGAGGAACAGAAGG	TCCTTCCGAGAATGCCTAACTC	GCAAGGAATTTGCGTAT	50	0.5	GCAAGGAACTTGCGTAT	50	0.5
RS2CL5121s	AAGTTCCCTTCGGTTTTTAGCC	AACCGTCCTCATCGTTAAGCTC	CAGCAGCGGCTTGAAGA	50	0.5	CAGCAGCGGCTTGAAGA	50	0.5
RS2CL5128s	GACTTACTTTGGAGGCGAAACG	ATGATGACTCCGAACAGAGCAC	TGGGCTGACAGGTGTCT	50	0.1	TGGGCTGAAAGGTGTCT	50	0.1
RS2CL5137s	TTCATTGCATGTGAGGAGGAGT	GAATGGAAAAAGCAGTGAGGAG	GCTGAAGCTACAAGTTC	40	0.5	GCTGAAGCAACAAGTTC	40	0.5
RS2CL5411s	GGGCAGAACTGGTGTTCTGTAA	CAACAAACACAAGGTTGGAAGC	ACCAGAAGAAGCAGCAG	50	0.5	ACCAGAAGCAGCAGCAG	50	0.5
RS2CL5524s	AGCAAGCAAACCTCCCAAACTA	GAAGCCTTCCCACAAAACAGAG	TCGAGGTTACTCCTCCT	50	0.5	TCGAGGTTACCTCTCTT	50	0.5
RS2CL5831s	TCCAAACCCATCAAGACAAGTG	AACGTACCCGAAACTGTGCTTT	TTTGCTCGAGCCAAGCT	50	0.5	TTCGCTCGGGCCAAGCT	50	0.5
RS2CL6133s	CGAACTGAATCAGCATCAAAGG	GGAGCCCTTTTACCTCATCAAA	CGGACACGTGCTAGACG	45	0.5	CGGGCACGGGCTAGACG	45	0.5
RS2CL6161s	GCTTGACAAGGTTACATGCTGA	AAGAGCAAGTTAGCGGTGTTCA	CACGAGGCGTTTGTATG	50	0.5	CACGAGGCTTTTGTATG	50	0.5
RS2CL6191s	TAGGATTGGCTGGTCAACAAGA	ACCATGGTTGGTTTGTCAACTG	GTTTCAACTAACCAACC	40	0.5	GTTTCAACCAACCAACC	40	0.5
RS2CL6239s	TTTGTCCAGATGGATGAAGTGG	GTGCGCAAAAGAATTGGTGAC	TTCACTAAAAAGTCGCC	40	0.5	TTCACTAAGAAGTCGCC	40	0.5
RS2CL6266s	ATAATCTGCGACGAGAAGCTGA	GGAGAAACGATGCATCAAAGAG	TTCAAATTATCTTTGTT	35	0.5	TTCAAATTTTCTTTGTT	35	0.5
RS2CL6287s	TGTGGTTCAGATCGGTCTGTTT	CCTCCAGTGACATTGGTTCAAG	CGATGTTGAGAGGTTTA	40	0.5	CGATGTTGCGAGGTTTA	40	0.5
RS2CL6294s	TTGCAAACGCTCTTCTCTATCG	ACCCTCGTGCTAGTCACATGAA	AGAATATTCTGACATCT	35	0.5	AGAATCTTCCGACACCT	35	0.5
RS2CL6354s	CAGAAGCCTTTCTTCCCACCTA	TTACAATGCCTTAACCGCTGTG	CTTCTTGATTTGAAAAC	45	0.5	CTTCTTGACTTGAAAAC	45	0.5
RS2CL6683s	GAAGAAAGTCGAAATGCGTGTG	GATTCCACGCAAACTCTCAATG	GGAGACTGAGGAGGGTG	45	0.5	GGAGACTGGGGAGAGTG	45	0.5
RS2CL6931s	AGCATTCCCAACAGGAGGATTA	GAATTTCCCGACTCAATGTCAG	TTCACATGCCACAAAAA	45	0.5	TTCACATGGCACAAAAA	45	0.5
RS2CL7090s	AAAAGGGCAGGCTTGAGAACTT	ACACACAAAAGCAGGCCAGATA	TTTGTATTTTACTATTA	45	0.5	TTTGTGTTATACTATTA	45	0.5

RS2CL7544s	CGGGTTTGATGGTTCACCTAAT	ACTGCAAAAAGACAGCACGAAG	TTTTATGAGATAGTGTT	35	0.5	TTTTATGACATAGTTAT	35	0.5
RS2CL8063s	CTTCTCCGGATCTTCCTTTTCA	CATGGGGCTTATTCACCAATAC	TGGTTGTGGGATGACAG	45	0.5	TGGTTGTGAGATGACAG	45	0.5
RS2CL8102s	AGGTCTCCCTCGCCTTAAAAAC	GATGCAAGAAGTGAGCAGGAAA	GGGAGCTTCGCGAGGAA	50	0.5	GGGAGCTTTGCGAGGAA	50	0.5
RS2CL8104s	TACGAGACGAAATGGGATCTGA	CTGGGTCCATAAACGCAGAGAT	CTAGCCATAGGGCTCGT	50	0.1	CTAGCCATCGGGCTCGT	50	0.1
RS2CL8169s	ATTGTGGAGGTGATGATCCAGA	CAAGGCCAAGCCATTCTTTAAC	GAGCTCGGTAATCGGGT	35	0.5	GAGCTCGGCAATCAGGT	35	0.5
RS2CL8214s	GCCAGGATATCAAATCGGCTTA	TCCTACGTAACACGAACCAAGC	CGATCTGTCTACGATCG	40	0.5	CGATCTGTATACGATCG	40	0.5
RS2CL8380s	CTGAACTGGCATCTTCACTTGG	GGCTTATCACCTTGCTTGATTG	CGTGAAAATTTGGTGTA	40	0.5	CGTGAAAAGTTGGTGTA	40	0.5
RS2CL8395s	ACGAACTCAGGACAGAGCAACA	AAACACTAAACCGCCTGAAACC	TCCAACCGCTAAACTCT	45	0.5	TCCAACCGTTAAACTCT	45	0.5
RS2CL8405s	CACTTTCGAGGTGGAGATGATG	TATCTGCATTCTCCGGTAGTCC	GATGGACATGACTCTGC	35	0.5	GATGCACAGGACTCTGC	45	0.5
RS2CL8540s	TGGTGGTACATCTCTGGCTACA[illegible]	[illegible]GTAAGCAACGCCATGAACTGA[illegible]	GCTGACGTCGGCGTAGC	55	0.5	GCTGACGTTGGCGTAGC	55	0.5
RS2CL8726s	GAAAGAGCCGTTGAAGAACCTG	TGTTGCTGTGTGTTTTCCTCCT	CTGTGTAAACTGTCTTC	40	0.5	CTGTGTAACTGTCTTCT	40	0.5
RS2CL8976s	TTAGAGGACGGATGTACCACGA	ACATAACGCAGTCTCTGCTCCA	GGGGTTATTATATATGT	35	1	GGGGTTATATTATATAT	35	1
RS2CL966s	CCGACTTCTTCATCGACACATC	TCACCAAGACTATGGGCGTATG	CCAGCCGCGGAAACAAG	50	0.5	CTAGCCGCTGTAACAAG	50	0.5
RS2CL996s	GTTTTATGCCCTTTGCTCCAC	TTGGTGGGATTCTCACTTCCT	CGCAGCGCTTTGTTTAT	55	0.5	CGCAGCGCCTTGGTTAT	55	0.5

Table S12 List of RGB PCR-RFLP markers

Marker loci	Restriction Enzyme	forwerd primer sequence (5'→3')	reverse primer sequence (5'→3')
RGB10057c	XbaI	GACTGATCCGAACTTGTTAGACATT	ACAGTACCCACAAGATCAAGACTTC
RGB10350c	PstI	CGAGCTGTGAAATGATAGAGGTATT	CAGCTTCTTACGTTTCAGATCATTT
RGB10737c	HindIII	GGAAAACTCTAACATGAGTCCAAGA	GATGATAACGATGACGATTGTTGTA
RGB10851c	HindIII	AGCTTTAATTAGGTTCCCTCTTCCT	GACAAAGACAAGGCATCTATGTTCT
RGB10862c	EcoRI	GTATATGATGCTCAAAAGCCACATT	AATCTAATTCCCGAGAGACAAAGTC
RGB1089c	HindIII	ATGTACTCGAGGAAAATGCTGTAAG	CGGCTGCACATATTATAAATTAAGG
RGB10934c	EcoRI	ACTCCCCTGATATTTTCTTCTTCAC	CTCGAGGTAATGACTCTTAGCAGTG
RGB10948c	XbaI	GGATTTATATCCGCTTAGTCCATTT	CATGTACTACAAGATCAACGTCTGC
RGB11029c	EcoRI	GAATTGGTGAGTCTCTGAGATTTGT	CCCTACTTTTACATCACTGAACACG
RGB11177c	PstI	TAATACCTTAGGAGATCCACCATCA	AGTCTAGACAAATGCTTGGGAGAAG
RGB11249c	EcoRI	GGTTCTAGTTCAGGTTTGTGTTACG	ACTGTGGCTAGAAAAGAAACAGCTA
RGB1137c	XhoI	GCTTCGTCAAAGTTAGGAATTTACA	ACAAGTCAAAGTACCATCCAAGATT
RGB11843c	XbaI	GGAGAAAAGAAAAATAACCGAACTG	TTAGTTCCACCTTGTATTCGTTGTT
RGB12060c	SalI	AAGACTCTGTCTTTGACCTCAGCTA	ACTGGTTATCGTTCATTCTCACATT
RGB12172c	XbaI	AACAGGTTCTTGAAGAGAAGAATCA	ATAAACCACTCAGACTCCAGATAGC
RGB12188c	HindIII	CAGAAAAAGAATGTCACGAATCATC	AATCTTGCTCTTTATCGGTCCATAC
RGB12209c	PstI	AACTCCGAAGCAGTACTCTCTAACA	ACCGAGGTCTAATAGCTTTGTTTTT
RGB12346c	SacI	AAAAGGATAAGAGGGCAGAAGATAA	ATGCGCATCTGTAAGTAGTCTAAGC
RGB1253c	EcoRI	TTTGGAGTTAGCTTGAGATGTTTTC	AGCCTTCTCGGATATGCTTACTACT
RGB12822c	EcoRI	TGATCAGTTCAGAGCTTAGTGTTGA	GTTTCTGATCACGTTTGACCTACTC
RGB12858c	EcoRI	GAGAGAGTGGGAATCACATTGTAAA	CTTATTGATTACCACCGCGTATAAC
RGB13054c	SacI	TCACGGCAAACTAACCTAATACCTA	CGAGTCGTAGTTCTCGACTTCTATC
RGB1306c	XbaI	GAGATCGTTAACCAAATCATCTTCC	CAGTTTCCTAGGTTTAACTTCCCTA
RGB13975c	EcoRI	GTTTTTGTCGAATAAGTCGAAGATG	AGTCCAATCATGTAAGGCAAACTAA
RGB1399c	HindIII	TCACGGGTCTTTTTGTTCTTATTAC	TCATTTAAAGACCTCCTCCTAAACA
RGB14019c	EcoRI	AAGGTAGAAGACGATGTGATTCTTG	ATGTTGACATCAAGAGGACACTACC
RGB14399c	HindIII	CGTAAAGTCTGATTCCCTACAGCTC	AATTGGTGTTGATATGTGTCACTTG
RGB1443c	EcoRI	CTAGTTTGGAGTTTGCGTCAGAGAG	TTATTGGCTATTTCGTTTAGCTTTG
RGB14459c	PstI	CCCAAAATTAGCATAGAGATAGCTG	GGAGAAGGTGAAGTTTTTGGATAAT
RGB14649c	EcoRI	GCAGAGAAAACTATAGTGGTGTGGT	GGGCTTGGATAGATTGGAATATAAA
RGB15203c	EcoRI	CATGATGGTTCAGGTCATATGTTTA	CATCACCATCTATAGCATCAATGTC
RGB15272c	XbaI	CTAAGCCAACTAAATGCTTTTCAAG	CAGATTTGCTAAATTCTCCTGCTAC
RGB15964c	EcoRI	GTCAAATTTGGGTTGAATAGAAGTG	CGTTTTTATGACTGTTGTGTGTGAG
RGB16366c	HindIII	TAAAGGTTATTAGGATTTGCGAGGA	CTCGATTACTAGACGAACTCGAAGA
RGB16666c	HindIII	CTACTCAACAATCTCTTGGCTCAGT	AGGAGAAGATGAGGGTAGTGAAGAT
RGB16843c	EcoRI	TACTTCTGAACAGACAAACAATCCA	CAAAACTCAAACTACGAAAAGAGGA
RGB17458c	HindIII	TATCTAAAGGAACGATAGCGACAAC	GATCCTAAACACAGGGACTAAAACC
RGB17888c	EcoRI	CAATCATGTGTTCGAAGAAGTTCAA	CAGCCAACAAAAACTAAACATCTCT
RGB17905c	HindIII	ATCTATTCGGTATTTTGCAGTTCAG	GTTGATAAATGACGAGGAAAGACAT
RGB18830c	XbaI	ATCGATATCAATAGGGAAACGAAAC	ATTAGATTTGTGATAGCGTGTGTGA
RGB19108c	EcoRI	TAATCTAAGGGGCGAGTTTTAGTCT	TCCCTACAGTTTATTTCCCTAAACC
RGB19113c	EcoRI	CTTACCGTGTGGTTAGATTTGTACC	CATCTTACCAAACCATTAGCTGTTC
RGB19481c	XhoI	TCTTATAATATTGCAAGCCAAGACC	AGGAGAAGAAGAAGAAAGGAAACAG
RGB1952c	EcoRI	CGGTATCAATATCTCATTCATGCTT	GTCTGAAAGAAGGCAAATCTTTGAT
RGB19618c	PstI	GTATCAAAGAAAGAAGGAGGAGGAG	TCCAGCTATAGCGAACAGATAGTTT
RGB2012c	PstI	GAATCATCAAGACTCGTATCCATCT	TTCTTCTTTCAAAGTACGACCCACT
RGB2029c	HindIII	GTGCAATTGATGTTGTCTATTTGGT	AGAGTCTTGTTTTGGATCACCACTT
RGB2123c	EcoRI	GTGTTTTCGGAAGGTTATGTTATTG	AACAGTGCTAAGGAGATTGAACTTG
RGB21440c	SacI	CCCACCTCTAAAAATCTAACCTTTC	AACTTTCGATGTGTAGTACCTCGTC

RGB21550c	EcoRI	TAATTAACCGTTGTAAAGGAACACG	GATCTCGCGAATGATTAACTAGAAG
RGB2163c	HindIII	CGACCTATTTCTACCTTCTTCTTCC	TGAAATCGAAGTCATAGACAGCATA
RGB24589c	BamH I	CCAGAAAGGTTGTCACAAGTAGAAT	CCTGACCAGTAATTGAAGAAACTGT
RGB25198c	HindIII	GATCTCCGCTTTTTACTTTACTTCC	ATGTTGACCTAATTGTGGAACTGAT
RGB2543c	PstI	TCAGCAACTAGTCTCTTCTCCTGAT	GGTGTGGGTATGTGTTAATTTTGAT
RGB25459c	HindIII	AGAGATTTTGTATACCCCGAAATGT	TATGTTCTGATCACATGTGCTTCTT
RGB2659c	HindIII	TCTGTTATGGGAATTCTTGTTGTTC	ATCTTGAGACAAAGGTTAGATGTCG
RGB2673c	EcoRI	AGTAAAACTCATTACGTGGAAACCA	GGGTCTCATAACTGTGTGTCTGTTT
RGB2724c	EcoRI	AGTATAAGCAGGAGAAGGTGAGGAT	CAGAGTCTGAATCAGTAGCATTCCT
RGB2773c	HindIII	TACGGGTAAGGCTGTATTTAGGTTT	ACGGTACTGTATAACTTGACGAAGC
RGB28103c	HindIII	GGTTGTGAGTATAAGTTCACGCTCT	TCTTGAGATACTTTTGCATCATCAC
RGB2849c	EcoRI	CCTGTTCATAAATCAGGAAGAGACT	CTACAGCTTTGTCGGTTACATACGA
RGB28864c	XbaI	TGGAGAGGAGTATAAATGGGTACAA	CTGTTGGGACTTGGTTTAACTTCTA
RGB2900c	SacI	CTCTATGCATTGGCGTTATTTTAGT	CTGAGTCTGTATATGTCTCCGGATT
RGB299c	XbaI	CTTTGGATTCTTCTTCTTCTTCTCC	GCTGTTGTTGAGGTAGAGGTACTGT
RGB3040c	PstI	TCTGTTTCTGAGACTCCATGAACTT	TCATTCACAATCCCTAGTTTCTCTC
RGB3124c	EcoRI	TTTACTACAAAACACTGCGTCACAT	CCACAGTTTATATCCTTTTTGCTTG
RGB3215c	EcoRI	TCCAAAGTAAGACCAAAAGAGACAC	TATGTAGATGGGATATGGTTCTGCT
RGB3288c	HindIII	CTGATAACCAGATCACAGGAGAGAT	ATGTCTATGAAGGAGAGTGATTTGG
RGB3655c	EcoRI	GAATCGGCCTATTAGTTGTGAATAA	TGCGTAAAGTCAGAAAGCTGTAACT
RGB3739c	EcoRI	CAGAAATTCATCGATATTGCTCTCT	GTTACAGCTGGATCTTAGGAGGATT
RGB3765c	HindIII	CCAGATCCATTAGATGTTTCGACTA	TCTCCAACTTTTGTGTAGTTCGATT
RGB37c	EcoRI	TTGATGGTTTAAAGTGGAAGTCCTA	AATGTGAGGGTTGAACTTGTTAATG
RGB3806c	HindIII	AACGAGAACTAATTCAATGATCTGC	GTCTCAGAAGAGTGGAACCATAAAA
RGB4147c	EcoRI	CAGGATAATCATCATTTCCAGTTTC	AGACCAGCTCAGCTATAATCAGTTC
RGB4153c	PstI	CTACAATGCTTAGGCAAGAAGAGAG	AAGCAGAGTGAGTGAGACCTCTAAA
RGB419c	SacI	GATTTTTCGCTTACTCTCCAGCTAC	TGCTTGTAATGACTTAGCCTTCTCT
RGB4201c	BamH I	TGTTGTTCAAGTACGTAAAACGATG	TAAAACTCACACTCCAGAAGGATTC
RGB4595c	XbaI	AGAGAAGAATCAAGAAGGAGCTTTT	TTGGTCTCATTTTCTCCTGTTTTAG
RGB4736c	EcoRI	GATCATGTAGACAATCAAAACACCA	GTCTCTGCTCAATTTTTAAGGTCAA
RGB4922c	EcoRI	GTATTACTATCAGGGGAGGCAAGTT	GTGTAGAGCATTGCTGCTCTCTTAT
RGB500c	XbaI	GATGCCTGTCCATTTACTCACTATC	CTGAAGATGGAGATCACTTCAAAAC
RGB520c	EcoRI	CTCTCCTTGGAAGTAAGTGTAACGA	GTGTCTTCTAACTGTCGATCTCACG
RGB5240c	EcoRI	TAATACCTTGAAGACGCGAATAAGA	ATTTAGGTTTTGGATGGCGTATTAG
RGB5484c	HindIII	ATTCATAAACTCCAAGTCGCTGCTA	AAATATCTCCTACGTACACGCACAA
RGB5491c	EcoRI	CACCTTCATCCTTTCATAACAAAAC	CGGAAGAGATGAGTCAGAACTTAAA
RGB5549c	SacI	AGTTTCTCTGCTGGGTAATAGAACA	TTTGTGGATATGTTTTGTAGGGACT
RGB5639c	SacI	GTTCATAAGCCTAAGTGTGACTGGT	GTTTCGAACCCCTTTATCAATCTAT
RGB5705c	EcoRI	CTCCGTTTAAGTGTTCACCAGTAGT	GAAACTTAAAACCAAGCTCTGTTGC
RGB5729c	EcoRI	ATATGACCACACACGTTTTAGATGA	CAATACCTCTCTGTCTCTCTCTCCA
RGB6149c	HindIII	TGTACCGTTCCTACAGATAAAGGAG	CTGTCTGCAAAGATACAAAGAAACA
RGB645c	EcoRI	ATGGGAGAGAGAGAGTGGTACTTTT	GGGAGTAAAGTTACAGAACGAAGGT
RGB6475c	HindIII	ACTTCGAATTTGCAGTCTCTTTTCT	AATAAGTCAAGTAGCCGATTGGTTC
RGB6680c	EcoRI	TCAACAAGTGGAATAATGAAGCTCT	ACAGTCTTCATCAGGTTAGCATTTG
RGB7078c	EcoRI	CTTTTGTCGTAGAGGGAAGGTTATT	ATAAACTTTCGTCACGCTGAATTAG
RGB716c	HindIII	TCCTTTGGAATTGACTTGACAATAG	ATTACCAGAGAGGAAATCCGTAGAC
RGB7310c	EcoRI	AGCCTATCAAGCCCAACTAGTATCT	GGATCGGATTCCTACTCTGTTTAAT
RGB7653c	EcoRI	CTAAGTATAAGCAAGCGACATTGGT	ACGTAGATGGTGTAAAAACAAGCTC
RGB7818c	EcoRI	TACCTTGAATGGAGTTAGCTTTGAC	TCGAAACTTGTAGGAAAGAGAGAGA
RGB7858c	HindIII	ATGTAGGGCTGTTATCATGCTATTC	GATATGCTTTGCATTCATCTTAGGT
RGB7901c	BamH I	AGAGAAGAAGATGACACCAGTCAAG	AAGAAACCCTAGTCGATTTCTGGAG

RGB8057c	HindIII	AACAGATCGCCTACATAATCAGAAG	AAGAGAGAAGAGGTAGATCGCTAGG
RGB8387c	HindIII	AGAAGTGTATAGGGCAAAACATGAG	ACGCATAGAAGAATGATTTAGGTTG
RGB8501c	XhoI	TTAGGGCTAAAAGTATCAACAATCG	GAGATTTTGGTGAAAGGGTTTTTAG
RGB8510c	EcoRI	CAGTAACAAGCAAACACCTAATCCT	CCCCTTTGATGACTTAATTCTCTCT
RGB8542c	HindIII	TTCCTTCAACGTAATGTTCATATCC	GAGGGTTTCCACAGACTATGATGTA
RGB8990c	XhoI	AATAAACCCCCTCCTCTTATCACTA	GTTCATAGATTCGCTAAAGCAAAAG
RGB8994c	HindIII	AACTTAAATGCAAAAAGACCTCACC	CTTTCGATCAGTTCAAGAACAAGAC
RGB90c	HindIII	AAAGAGTGAGAGAATGGAACACTCA	TCCTTTCCCTGAAAGAGTAAAACTT
RGB921c	HindIII	ACGCATATACGCACCAATCTATATC	ACTCACGTAGAGAACACAGGAGAAC
RGB9500c	EcoRI	GTAAAAACGTCATTAAACGCAGACA	AAGAAAAAGAACGTTAACACACGAC
RGB964c	HindIII	ACTGTAGACAGTCGTTGAAGTCGTC	CTTCTTGGCCCGTCTATTATTATTT
RGB966c	PstI	CAGTTGGTACTGTTTAAGACGAACA	ACGTCAACAGAAAAATCAGAGAAAC
RGB9670c	HindIII	GGTCCCATATCTTCTCCTTGTAACT	CTGTCAAGAAAGAAGGCTTGAGTAA
RGB9734c	EcoRI	CCGGACATGTTGAAGAATTAATAGA	TCAAAGTGACGAAGAAAAAGTTGAC
RGB9877c	HindIII	TTCGACATCTGCGACTCAAG	AGGCATCGCTGAATACCAAA
RGB9971c	HindIII	CAATGTCGTATCTTACTTTCGCTTT	AAACTAGGTTAGAGGAGTCGAGGAA

Table S13 List of RGC PCR-RFLP markers.

Marker loci	Restriction Enzyme	forwerd primer sequence（5'→3'）	reverse primer sequence（5'→3'）
RGC10000c	EcoRI	AGAGAGAGAAAGATGATGAATGGTG	ATCAACACAATTGTCATGTGTTAGG
RGC10051c	HindIII	TTAGAAGAATACAGCTGGCAAAAAC	CTAAAGAGGTTAGCAGAAACCATCC
RGC10056c	EcoRI	GGCTCGTATTAAGAAGATTATGCAA	GGTGTAGGACACAGTCAATATAGGG
RGC10060c	EcoRI	CTCCTCATCTTATCTCCTTCCTCTG	ATTACGCAATTACGTGCTTAGAGAC
RGC10094c	HindIII	GTATACTTGTATCCCAAATCCACCA	CTAAACCGAAAGAGACACCACTAAA
RGC10110c	EcoRI	GGAATCATGTATGCCTTCTTCTCTA	CACCTAAAAGCTAACTCATGAATGG
RGC10301c	EcoRI	GTTTGTAACGTTTGTTGAGAATGGT	AAATTGATGGATAGGAGTCGTAGGT
RGC10310c	EcoRI	TAAGTTTGCTGAATAGTGGAACACA	AAGAAAATGAGAACTGTCACCTGAA
RGC10321c	HindIII	TAGACAGATTGATGGTCAACTCAAA	GATACGCGACTTTTGTCTGAAGTAT
RGC10385c	HindIII	CCAACTGGAGATGCTAAATTATGAG	GTGGCAGGCTACATCAGATTTTACT
RGC10400c	HindIII	CCAGTTGATCCAAGTGTAGTTATCC	TCCAACCTATATATGCTTTGCAAGT
RGC1041c	HindIII	ATTGCTCATCACACAAGATGACTTA	GCTTTGTTGCCATACTTTGTCTACT
RGC10518c	EcoRI	GAAAGAAGTGTCAAGGGTAGAATCA	GGACACTAGCAGCGTATGAGATATT
RGC1052c	HindIII	GCATTTTTGTCTCTGTTAGATGAGC	CACTCGTTAAGTTTTCTTGAGCTTC
RGC10531c	HindIII	ATGGAGTGTGGAGGATAGATGATAA	TCAAAACACGTTGAGACTTGAGATA
RGC10544c	HindIII	AGGAGAATACCGTCCTAAAGAGAAA	TAGTCAGATACTGTTCGTTGGGAGT
RGC10583c	EcoRI	TACTTGAGGAAAATTGCTGGTAAAG	CTAAGCTGATATCACCATCTTGCTT
RGC1058c	HindIII	CGTGTAAGTTTGATTCTTTCTACCG	CTGTAAATCTTAGGCTGCACATCTT
RGC10645c	EcoRI	CCATTATACAACTCACATCATTCCA	AAGTAAGGGAATCCAAGAAGAGAAA
RGC10740c	HindIII	ATTAACCTAGTTTGCCTCTCTTTGC	TTAAGAGAAAGAGTTGATTGCCGTA
RGC107c	EcoRI	AATGAATAAGCATGGAAGGCTAATC	ATATCAAGCCGTTCTTGAAAATCTG
RGC10800c	HindIII	AACCATTGTCTCACCTAATAGACCA	ACAACACTGCTAGCTGCAATAATAA
RGC10825c	EcoRI	AGACAATGTTATTCCGTCAGAGAAG	TGTGAGGGAGTAATTTCTTTGCTAC
RGC10838c	EcoRI	AGTTCAGTCCTATTTGACAGACCAG	GATTGTGAGATTTTGGAAGGTTCTC
RGC10842c	EcoRI	TTATTGTCAGGAAGAGGAGAGTTTG	GTTTTTACCATGGATGCTTGATTAG
RGC10902c	SacI	TGGACGTGGTTGTAGTTGTAGTAGA	CAGTCTGAATCTATCATCGTTGTTG
RGC10939c	HindIII	TAGCTTGAACTTGGTTGATATGGTT	TCAAAAAGATTGCTAGGAGCTAAGA
RGC10969c	HindIII	TTTGTTTTTGGGGAATTAGTCTGGT	TAAGCAGTAGACCATCTCCTTTCAG
RGC11016c	EcoRI	ATAGAACTCACGCGTCTTCATAAAC	CAAGACATACACAAGGAGTCTGATG
RGC11044c	EcoRI	GTCATGGAATGTAGAGTCACCATCT	AACCTGCATTATTGTTGTCTCCTAA
RGC1110c	HindIII	TCCTCCAGACAAAAAGAACTGTATC	TATCATCACACGGTTAAGATTTGTG
RGC1111c	EcoRI	TATTACTCTGTTCAGAATGGGAAGC	CGAATCTCTGAAGTATCAATCAGGT
RGC1115c	HindIII	GAAGTCACCTGTGGAAGTAAAAGAA	TCCAAAAATGATAGAAGGGTAAAGG
RGC11199c	HindIII	AGACTCAGTTACAGCAGGTGAAAAC	ACAGTCAAACAAAATAGGGTCACAT
RGC1119c	HindIII	TAGCAACTGCAATACCAACTATCTG	TTTCAAAGGGTAAAAGTCTCTCTCA
RGC1124c	EcoRI	ATCATTTCAGTTTGAGGACAAACAC	CTGAAACCAGCTATCCAAAAGTTTA
RGC11312c	EcoRI	GCACTGCACTATCAGTTTCTCTTTT	AAGTTGAAGCGTATTGTAACTCAGC
RGC11328c	EcoRI	CAAAGCTGACAAGAAGAGAATCAAT	GAACTTTGCTTCCTGTTGAGAATAA
RGC11372c	HindIII	TGTTAACCTGTTTGGAGAGATAAGC	TTGCAAGATACCCTTTGATAGTTGT
RGC11405c	HindIII	TGATATTATCCCAGGATTCCTTCTT	CAGTAACTGGAAAATGGATCTTTTG
RGC11410c	HindIII	CTTGGATGTAACCTATCCTTGCTAA	CGTTGCTAGTGTTCTAATTGGAGTT
RGC11438c	EcoRI	AAGATGAGAAAGAAGAGAGCAATGA	GATTGAGCAGGTGAGACAAATAAAG
RGC11447c	PstI	AGCATGTACTAAAAGGAGTGACCTG	TTGAGCATGTCTATGATCTGAAGAG
RGC1147c	EcoRI	ACTATCATCCCCCAAGTTTTGTATT	ATTGGAGTTGAGTACGAACCAGTAG
RGC11501c	HindIII	AGCACAAGTGGTATTAAGCAAAAAG	GCGGCATACTCTTACTAATCTTCAA
RGC11504c	HindIII	TGATTTTCTTCCATGCTTCCTATTC	TGATTTAGCAATTCGGACTGTCATA
RGC11533c	EcoRI	TTTAGGGCATTATGGTAATCTGATG	ACTAAACCGAATGGAACAGAGTTTT
RGC11618c	HindIII	TCAGCAGATCTTCTTCATTCTCTTT	CAGAGTCTCTAAGGTGAGACAGGAG
RGC1165c	EcoRI	TGGTTACTTGGTTTGTTCCTATGTT	GTCACGGTTACCTTCTTTAGCTGTA

RGC11660c	HindIII	CACTGAAGACAACAATTTTGTAACG	AAGAAGAAGACGAAATGCTGAGTCT
RGC11673c	HindIII	ATTTTATGTCTATCTTCGGTGGTCA	CATAGAATCAGCAAACTTCATCCTT
RGC1167c	EcoRI	ACTCTCTTTTTCATGATTGTCACCT	CGGGTATTAACAAACTTAGATTCTG
RGC11743c	EcoRI	ATGTGACACTCATCAAGTCATCATC	TCGAACTCCCATTTTACATAGTCAT
RGC11789c	EcoRI	GTTTAAAGTGTTAAATGACGCGACT	ATAAAAGCACAGAGCATGTCGTTAT
RGC11798c	HindIII	TGTGATACAAAATCTTCCTCCTAGC	AGAGATCCTTGACTGACATTCCTCT
RGC11816c	EcoRI	CATGGTGAGTGATACTGAGAGGAAT	TGAAGAAAACTCTATTTGCCACATC
RGC11873c	PstI	TATCTTGGTGTCTCAGAGAGTGATG	CATAGGGTACGTAAAAGACCAAGAA
RGC11890c	EcoRI	AGATCACAGTGTACAACAACAATGC	TATTGATAAAAGTAACCCCGACTCC
RGC11896c	XbaI	TTCTTTCTTTCCAGATGACGTAGAT	TCCTTCTCCTCTAACTCTTTCACTC
RGC11904c	SacI	GACGCTCTCCCATAAGTGTATAGAA	ACAGAACCTCAAGAAGACCCTAGAT
RGC11938c	HindIII	GACCAAGAACTTGAGTCTACTGAGC	TCCAACGCATTATAAGTAACCTTGT
RGC1195c	HindIII	TCTCTTGAATGATCTCACCGAGTAT	GTGTTTCTTAAGGTTGAGAATGTGG
RGC11985c	EcoRI	TTATACTTAAGGTTATCCGCACACG	CAAGTGCAAGCTGTGTTAATAGTGA
RGC12020c	SacI	GTGTACAACGACATTTTCTGCATAG	CAGAGATGTTCTTTCTCTCCAAAAG
RGC12097c	HindIII	GTCTCTTTATCATTTCTCGCTCCTC	CGCTAAATCTGCTTCTACTTTGTTC
RGC12111c	HindIII	TACAGAGGAAACAAGAGGAAACAAG	GTGTAGAAACAAGACAAGAGGTTCG
RGC12174c	EcoRI	TCATCACAAAAGTCATCATCAGAGT	GCTGGATCAACTTGCTTAGTAGAAG
RGC12178c	EcoRI	GCTTGTTCTGTTCTTTCTCTGAATC	AAGAAAACAGAGTGCCATTGACTAC
RGC12216c	EcoRI	ACAAGACGCCAAAGAGTTTAACATA	ACCTAGTCATGGCCTTTTAGTTACC
RGC12230c	HindIII	CTTCAAGACCGTTGAACTTAAAGAA	AGAGAAAGTAAGATGAGTGGCAGTG
RGC12272c	XbaI	AGTGAACACGAGTCGTAAAACAAGT	ATAGAGAGAGAGGGAGAGGATACCG
RGC12310c	EcoRI	ATGTGTGGTGTTTGTGTAGGTTTTA	TTCCAAGAACGATGACTGTAGTGTA
RGC12323c	SacI	TCGCTCTAGAGAAAGGTAAACAAAA	CCCTCTTCAGTAAACAGAAAATCAA
RGC1234c	EcoRI	AACTGAACCAACTTTACAGAACCAG	AACCTATGATGTCAAATTCGTATGC
RGC12354c	HindIII	AGGAGGGTGAGTTTTTCTTTTCTAA	AGTCCCGGCATTAGTTTATATTCTC
RGC12381c	HindIII	GTATCAGAAGTCTTCCCCCAAATAG	ATTTCTACAGGAACGAGTCTTCTCA
RGC12389c	EcoRI	GTTTAATTGAAAACTAACCGACGTG	TCACTAACTCAAGTCAGACGAATCA
RGC12399c	EcoRI	CCGTCTGTTGGTCTAGTACTGTTTT	TTTAGTATTTAGGGGCCAATGTTGT
RGC1244c	XbaI	TCCTTGAAGAAGCTTAGTGTTCTGT	TTGCAATGACTTTTGAGATAGACTG
RGC12486c	HindIII	TCTACAAGTTTAAGCTCGGTTTCAG	GTTCGATTGCAGAGACTAATTTGAT
RGC12560c	HindIII	AGTTCAGCGAGTATAATGAGTCTGC	TCACTTGTCCTTGTATCTGGAGTTT
RGC12573c	EcoRI	AGTGAGGAAACAAAGACGTGTTAAA	GCTTGTTTGGAATCGAAATTAGTCA
RGC12577c	HindIII	TCCGGAGGAAGAGTATTAGAAGATT	ACATCTACACCCAAATTGTTCAGAT
RGC12603c	HindIII	TAATGCTAAGCCTCGAATGATTATG	GCTTGATCTCTCGACTAACTCTTTG
RGC12639c	HindIII	TTTCGTTGGATATGCTTGTTAGTTC	ACTGCTGCTTTTATCTCATTGTTTC
RGC12711c	HindIII	GTTCCGGTTTACAGATTACCCTATC	AAGGATCCATCCAATTCAGTCATTA
RGC12721c	EcoRI	GGATTCAAATCAATACAGCCACTAA	GTTTGATGTTTGGGAATGTAGAATG
RGC12735c	EcoRI	GAAACGTAAACATCTGAATCGAACC	CGATGCTGCTCTCGTATCTACTACT
RGC12742c	EcoRI	AACTAACCCAAATGAAATGCTCAAC	TAGGAGACTTTGAGTACACATGCAA
RGC12804c	EcoRI	AAAAGCAAATGACTTTAGGGAGAGT	AAGGTCTTTCACTCTCTCTCGTTTT
RGC12828c	EcoRI	GTTTTGGATCTTTCTAGTCACAACA	GGATCTTCCAATATTGTCTACAAGG
RGC12869c	BamH I	GTTGTACCAATGTGGATGTTTTAAG	TTCATGCATGTCATGTACAATTGAG
RGC12875c	EcoRI	CGTAATTCCAGTATTGAAATCCAAC	ATAGAACTGAAAGAGCGAGTTGCTA
RGC1288c	XbaI	CACCAGTTTGTCTATTTTAGGCATC	ATTGTTGGAAAGATAAAGCCTCTTC
RGC12943c	HindIII	CGAGTAGTTTCCTCTACCACGATAA	CATAGATGGAAACCCTCTTTCTTCT
RGC12949c	SacI	CCACATCCTCCTGAATTAGTCAATA	CGTGTATAAACCAAGAAATGGGTAG
RGC13028c	SalI	CTGTAGAAGGAGGTTCAGAGACTTG	CTTAATTGTCTGCAGGTAATGGTCT
RGC1312c	HindIII	CACGTTCGAGATGAATATATGATGA	CAAGTTCGTCCTTACACTTGTTGAT
RGC13134c	EcoRI	TCAACTTGACATGTATTTGATGCTC	GCTATTGGTACAACCTTTCAACAAC
RGC13159c	EcoRI	GTTGTTATTGGAGTTGTTTTTACGG	TGGTCAAGAGATCAGAGAATGATTT

RGC1319c	EcoRI	GAAAAGGTGCATGTATTGTGAAACT	CAAAAAGTTGTTTTGACCTCGTACA
RGC13248c	EcoRI	AGGGAATATGGACTAACGAAGTTGA	GTCTTCTTCTCTTCATCCCAAATCT
RGC13289c	EcoRI	ATCAATGGAGGTCACGTCATATACT	CCACGGTTAACTTGATCTTATCCTA
RGC13305c	SacI	TAACTTATTTGGTGGAGGCAAGATA	CTGAACAACCTTAATGAATCTTTCG
RGC1333c	SalI	TGTGATATGATCATGAGTAGCTGGT	GGGACTGATTAACTCTTTCCTTTCT
RGC13382c	EcoRI	CTACAAAATAACGTCACCAGGTTTC	ATATGGTCTCATCACGAAGTTGTTC
RGC13395c	EcoRI	CTTCCTTCCTCCTCATCTCTATGAT	TCACCTCATGATATCAAAGTCTCAA
RGC13408c	XhoI	GTAAGTTGCAGGATGATGTTCTCTT	ATAAAACCGGTTACCCACTACTTTC
RGC13420c	EcoRI	ACTGTAAGATCGGGGTAAAGGTTTT	GCTATAGCTCATTTTGACACCCTAA
RGC13599c	PstI	CTTCTAGCGTACCGATTTGAATAGA	ATATAGGGGATTTGATTTCTGGATG
RGC1367c	HindIII	CAAAATTTCTGGTAGACAAACAAGG	GAAACTATTGCTGTGTTCTTGTCCT
RGC13684c	SacI	GTGTATGAAAGAGGATGCAAATCAT	AATGATGGGAGAAAATCTAAAGGAG
RGC13764c	HindIII	GTACTATAGCTGGTCCTGCTTCTTG	ATCAGTACAAAGAACTGTCGGAGAC
RGC13794c	XbaI	ATCATGTTAATCAGAGGGACTCTTG	GACCGAACCCTATTTTGAATTTATG
RGC13816c	EcoRI	CGTGTGGTAGCTTTCTAGTATGGTT	TCGCACTTTGAATTTTCTGTCTACT
RGC13819c	EcoRI	AGCCTTATGAGACCTTCATCTATTG	AACGATGACTTTGCTTACATCACTT
RGC1381c	HindIII	GCTCTATTCTGGTAAGTGAAGCAAG	GGTACAAGCTTAGCCAAAACAATAC
RGC13867c	PstI	TTAGGTGTGACGTTGATTGAAGATA	TACTTCTTAGGGATAGCAACACACC
RGC1397c	EcoRI	TGCAGACTGGTTTATAGGTTTTGTT	CTTATCTTCCGCCTTACTCATTTTT
RGC139c	HindIII	GAAACGGTGGTGTGTAATAGAAAAG	TTCCAATAACAGAACACAGGAATCT
RGC14010c	EcoRI	TTCTTTTCTTCTACCGTTCACAAAC	GCTTGTCGTTGTCTACATTGGTTTA
RGC1402c	EcoRI	AATATGAGCGGAAAATCTAAGCAAC	AAACTGCTCAAAAGGTTTTCTAGGT
RGC14157c	HindIII	GTGGTGGCTTCATTTCACATTACTT	GTTGAGAATGAAAGATACCTGGAGA
RGC14226c	XbaI	CTATTTATTCCACTTGCTGTTTGCT	TGATGAGGAATGAACTAGAACATGA
RGC14244c	EcoRI	CTTAACTGATGGCTTAGGAAAATCA	GTTAATATCAGACTGGACGTTGGTC
RGC14293c	EcoRI	ATACTTCGTTGATGGTTCTTGACAT	TAGTTTCTATTCGGGTCGGGTAATA
RGC14352c	HindIII	AAGAAGTATCTATGCAAAACCGAAG	CGTAACCGAATAAGAGAGAGATGAA
RGC14381c	HindIII	ACTACCAAGTGCAACTACAATGGAC	TGCTACACGAGAGATCCTAGTTTGT
RGC14398c	EcoRI	AAGAGGAACATTTTGCATTAGGTCT	GCATTATTTTCGGTGGACATCTTAT
RGC14416c	HindIII	GGAGTCAGATTATCATCACAACACA	CATAAGCTCCGATTACGATAATTCA
RGC14440c	SalI	ATATGAACATCAGAACATGTTGCAC	GACATCTAAACAAATGAACGAGGAC
RGC14452c	BamH I	GGTACCAGAAATATCTCGGTTCTCT	AAATAGTTTTGCAGTGGAAAGTACG
RGC14479c	EcoRI	ATATCATTCACAAGCGACCATAGAC	AGTTCTTTGAAATCGGGTCATTTCT
RGC1448c	PstI	CTCATATGTGGATCCTTTCAACATT	ACATTAGCTTTATAAGTCGGGAGGT
RGC1455c	EcoRI	GCTCTCGTAGAATATGAAGATTGGA	AATTTTCTATCTTTGGCCTGCTAGT
RGC14573c	HindIII	ATGCTTATGGAAAGATATGGCTTCT	AAGCATCTTTCTACATTCATCTTGC
RGC14676c	HindIII	AGGCTGATAAGCTGACTACAACTGA	ACTGTGATAATAGGTGGACAACACC
RGC147c	EcoRI	TAACTCCTATTCTTCCACTTTGTCG	AGAGACGAAAGTAACTTCTGAAGCA
RGC148c	HindIII	GGCCTCAACCTACTGTAATCTTCTT	AGAGACAATGAGTGCTTCAAAGTTC
RGC14901c	HindIII	GAGATGACTAGCCAAACGATTTAGA	CTGCTTTTAGATCGTCGCATACAAT
RGC14934c	EcoRI	GGAAGTTCACTTGTTCCACATAAAA	CATATGTACACAAACTCGAAGCAAA
RGC15052c	EcoRI	GACAAAGAGAAAAGAAACGGTAAGG	CATTAAACCCACTGTCGATAATACG
RGC15061c	EcoRI	AGGTTAGTAGGTCTCGGAGTCACTT	AGCTTGTGAGAGAGAAGTGAAAGAA
RGC1509c	HindIII	GTGTTAAATGAAGAATGTTGCCAAG	ATAGTTGTTAGATCCCAGCTGCTCT
RGC15178c	EcoRI	ATGTAAGCGACTAACCGAACACTAC	TGATGAGACTTCTGACATACCTTGA
RGC151c	XbaI	CTCCATAGAAAATCGCAAGTAACAT	GAATCTCTGTACATCACAATGATCG
RGC15219c	HindIII	GGGGTGTACTGTTAGGTTTCTCTCT	GGAGATTGTCCATAATTTCTTGATG
RGC15280c	EcoRI	ATTTGTTCAGGCAAATGTAGAGACA	CAGTAAGACTTTTCTTCAGGGTTCA
RGC15296c	SacI	TGGTTAAGGTATCTCTCCTCCTTCT	TATTGATGAACTTCTTGAAGGGAAC
RGC15300c	BamH I	ATAGTAGCTTTGAGCTGACAAAGGA	GAAAACAGCTGATTCAAACTAAAGG
RGC15346c	PstI	TATTGAACGCACCAACTTATGTCTA	ATGAAAGTCTCTAATTGGGTTTTCC

RGC1537c	HindIII	TCTTGTGTAGAGGTTGGAGAAGAAC	AAAGGATCAAACAGGTAAAGGAGAT
RGC15408c	EcoRI	AGTTTCTATGAGGAACTTTCAGTGC	TGGAATCTTTTTGGTTACAGTTAGG
RGC15411c	EcoRI	GACGTGAGAACCAAATTCTAAAAGA	GCATCTGTCACTGAGTTAGGGTATT
RGC1543c	HindIII	GGCCTGAAGAATCAAAATTACAGTT	GGACTTGGAGAAAAATCGAGTTAGA
RGC15477c	HindIII	TAAACAAGGAGAGTGGAGTGAGAAC	ATATGTATATTGTTTGGGAGGAGCA
RGC15483c	XbaI	ATACGTGGATAGTTTTCTTGTACGC	CCCTCAGTTTATAGCATCTCAATGT
RGC1566c	EcoRI	TCAGAGTTTTCCATTCCAGAGTAAG	ACCACTCATGTCAAAGTTGTTCAGT
RGC15683c	EcoRI	TACAGCTAACCTCAAACATTTCCAT	GATAGAGATGACACAAAGGAGCAAT
RGC15702c	HindIII	TGTTCTTCTGTCACCTTAACAATCA	CTCAATGACATCAACTCATCAAAAG
RGC15741c	HindIII	CCTACATTTTTGTTGTTTTGGCTTG	GTCTACTACTGGTTGAGACCCAAGA
RGC15769c	PstI	TATACATACCTCGTCCCTCTCAAAG	TAAAGAAAAACCCAAGAGTTACACG
RGC15812c	EcoRI	GCACAAATCCACTAATTTTTGAACC	TTAGAGTTGACCGGAAGAGTACAAG
RGC15844c	XhoI	GTAGTCGAGTTTAGTTTGCACCTTC	TATGTACTTTTATGCGTTTCCCTTC
RGC15890c	EcoRI	AGGGCATATCGATTATGAGTTTCTT	CGGAGGTAGAGTTGAGAAGCATATT
RGC15930c	EcoRI	CGACTATGGTAGTACACATCAGCAC	GAAACCTCTGCAACTGTCAGTAAAT
RGC15937c	EcoRI	GTGTTATGGTCAAACTCAATTGTCA	CAAGCCAATTTTATTCATAGTCTGC
RGC15951c	XbaI	GTTTCTTGGTAACAAGGATGTATGG	TTCTCTGCAAGTGTTATCCTTCTCT
RGC15962c	HindIII	AAGATAGTGGCTTAGTGCATCAAAA	GTATTCTGCTACGAGGTTTGTGTCT
RGC1596c	HindIII	ATGAAGTAAGTGATGACGAGGATCA	CCACGACTACGACAACAACTCTCTA
RGC16041c	EcoRI	AGTTGTGCAGCTGAGAGAGTAAGAT	GTACAAGAAGAGTGTGGTTTGGTCT
RGC1609c	EcoRI	GAAGGAGGGAGAGATAGATGACTTC	CTCTTGAGAGGGTTGTTTTTGATAA
RGC16154c	EcoRI	AGTGTGTGATCTTCAAAACCTTACC	GTTTGTGGTCTTTGGATAACTCAAT
RGC16204c	EcoRI	ACAACTGCTCACATTTCACATACAT	GTACAACACGCTCTTATCAAGCTAC
RGC16214c	HindIII	ACGATTTGACAACCGACAATATTAC	ACGACACTTATTCAGAAACAAAGGA
RGC16310c	SalI	GCACATGCCCTAATGATATTTAATG	ACACATACAACTACACCTTGACTGG
RGC16329c	EcoRI	CTTTGAGCCAACTAAAGTGAAATGT	CAAGCCTTTCTTTGTAGTGTCTTGT
RGC16357c	PstI	CATACCTTCTCCTCCTATTTCCTTC	ATCATAGCAGCCTATAAACTTGCAC
RGC16522c	SacI	TTCCTTCCGTCTTCTCTCTATATTC	TAAGCATCAATTCAATCTCTGTGAG
RGC1653c	EcoRI	AGTCAAACAAAGGTCAGAGAGCTTA	CTCTCTCTCTCCCTCTATTGTTCCT
RGC1662c	HindIII	TAACAGGCCAAAAGATTCACTACCT	GTTAAACTCAGCCACTCCAATTATG
RGC16642c	XbaI	CCAATGTGGCAGTCTTAAGTAGAAT	CCTGCTTGTATTCTCAATTTCATGT
RGC16913c	EcoRI	TAGTGTTTGGTTCGTTTGGTTTAGT	TCTCTTTTCTTGGATTTCTGCATAG
RGC16935c	BamH I	TCTTCTTCTTCTTCTTCGTCTGTGT	GATCTTGATCAGTTTGGGTTGTATT
RGC1718c	EcoRI	TGCTAACGTGGTCTTGTGATATAGA	GAGCTGAGATCGTTACAAACTGAAT
RGC17231c	EcoRI	ACAGAACTTTGTGAATATGCTGGTT	TATGGGTTTAAAAGCTTAGCAGATG
RGC1736c	EcoRI	GAGGATTAGTGGTCTTAAACTGCAA	TGGAGGTTGTGGTCTTTGTTACTTA
RGC17372c	HindIII	GCCACGTGACTGATTATAGTTTCTG	CTTTTGTACTGCCTTTAGTGATCGT
RGC17385c	XhoI	AGCCCCTTCTCTACATTCATAGAGT	AAGCTATAAGGGCGTAGTTCTTCAT
RGC173c	XbaI	GATCATCAACCATAGTCCAGGATAG	TATTTGCATCTGGAAACCTAGAGAC
RGC17489c	HindIII	AGGGTGGAACAAGATTCATAACATA	AATTAACAGTGTGGTCTCTGGCTAC
RGC175c	EcoRI	ATGTTTTGCTATTGAAGAACAGGAG	GTCTGGCAAGTAGAGTCAGTTTGTT
RGC17618c	HindIII	GATTTGATGACACCTCAAAGTATCC	ATTCAGCAGAGGTACTGTGTCAGTT
RGC17631c	HindIII	AGACTCTGGATACTTGGTTTTCTCC	TGGATGTTACTCCAAAGATTCTCAT
RGC17676c	EcoRI	ACATTTGTATATTGGCTTGACCTGT	CATGCTTCTTATGTTGTAGCCTTGT
RGC17695c	HindIII	TTAGCCTTCTTTTGCAGTCTCTCTA	GAGTGATCGGAAGCTAAAAACATTA
RGC17698c	HindIII	ACTCAATTAGACGGCTCCTAAAATC	GAGATTTATGGAGGCTTGAAACAAT
RGC17699c	HindIII	TATCACTAAGCTGAAACGGATTCTC	AGCAATGAAGTAACGGTCAAGATAG
RGC17710c	EcoRI	TCGTTGCTTAAAAGACCCAATAAGT	ACTCTGGTGGTTATGTGATTCATCT
RGC17769c	HindIII	GTGCTATTACCATTTGCTTTACGAT	TACGGTAGGCAATATCCAATAAAGA
RGC17861c	SacI	ATGTTTGTCTTCAAAAGACTTACCG	TAGTCTCAAACTTATGTGCGACTTG
RGC1792c	EcoRI	TACGCAGTCTTGAACTAAGGACTCT	CAAATGGCTTATTGTAGGTGAACTC

RGC1797c	EcoRI	GTTCCAGATTGTCTCATGGAAATAG	GTTGACTGCTCATCCTACTCAGTCT
RGC17999c	HindIII	AAGCACCATGAGAGTTGTTCTACTT	CAAACCTAGATGCTAAGATCCAAGA
RGC1800c	EcoRI	AGTCTCAAAGCTGTGTACAATCCTC	GTTGAAACTTTTGGTGTTTGTTCTC
RGC18142c	EcoRI	ACTGCATCTTCCTGATTGATTAGAT	CGTTTATGAAAGCGTAAGACTTGTT
RGC18172c	EcoRI	TAGCTTACCAGAGGAGTGTAAATGC	AATCAGAGAAACCACAAACAAAGAG
RGC18238c	EcoRI	CGAAGCTCGGTAATTTCATAGATAA	GTTATAGGTTGGAGATGCCCTAATG
RGC18316c	HindIII	AGCCATTGTAAGTAACAGAGACCAG	GTCTCTTTCTACGTTGTCGTTTTGT
RGC18337c	EcoRI	AGGGTTTTCAAGTTAACAACACAAG	CTCTCTCTCTCTCCTTTTCCTTGAT
RGC1833c	HindIII	CTGGCCTTCAAATGTAAATCCTAAA	GTCTTAAGATTTTGGGGGTTGTAAA
RGC1836c	HindIII	TGATTTTGAGACTCCTATCTCCATC	ATCAGCGTCTTTGTCTATCACTCTT
RGC1838c	EcoRI	GAACATCTTAATTTGCGTTGATACG	GGTGACAGGAAACCAGTCATATTCT
RGC18537c	EcoRI	CCTTCCTAAACTCTGCAATAAAACA	TTCTATTTTTCTCCTCTCTCCGAAT
RGC18584c	EcoRI	CAGGGAAAAATATCAGTTCATGTGT	AAAGGTATGCTGGTTCTTAAATTCC
RGC18589c	HindIII	TAGTTAGTTGCTTTTGATTGCAGGT	GGTAAGGAGTTTCCATCTGAAAGTT
RGC18651c	EcoRI	GGTTTTCTAGAAGCTCTCTTTGCTC	CTTTCTCCCTCTCGATTTATCATTT
RGC1871c	EcoRI	CACGAGCTGATGTTAATGACTTATG	AAAGTGCTTTCTTCAAGGACTCTTC
RGC1879c	EcoRI	ATACAGTTTCAAGGAATGTCTTCCA	AGCTGACTTATCGACATTTCAAAAC
RGC1883c	PstI	CGGATATGTGATAGAAGTCTTGTCC	GGAAATGAAAAACAAACACAGTACC
RGC18965c	SacI	CTGAGACCTAGCAAAGAACTCAAAC	AACGTCTAATCTCAACGAAGAGCTA
RGC19084c	EcoRI	TCTTCTCTGTTTCTAACGGCTCTAA	CGACCATAAGCATAGACTCTTTTGT
RGC19152c	EcoRI	AGTCTACCTTTGAAAATCATTGCAG	CATGTGAAACTAATCAAGCTCAACA
RGC19350c	HindIII	AATGTATGTAGGCCACACAGGTTAT	GGCAACCTCAATATTGTTTTACAGT
RGC19516c	EcoRI	TAGTGCATGAATGTCTGCTTAGTGT	GCGATTGTTCTAACAAAATCATGTG
RGC19563c	EcoRI	GTAGATTTCTAACGCATGCAAATTC	ATCTCGATGATCAAAGATTCAACAG
RGC19763c	EcoRI	ATAAATCATAATGGGACTGGAGACA	GTTTTGAAACTCGGTGTGTTATGTT
RGC1979c	HindIII	TATGAATGTGTGTGTTTTCAGGACA	TTACCTACCGTGTTTAGCAAGAAAG
RGC1982c	EcoRI	TTTATCCGCTCAATCTTTATCTGTG	CCAACCGACTCCAACTCTTATAAAT
RGC19850c	HindIII	CTGCAATCGTTGTTCTGATTGTACT	TGATGCTGTTCCTTCCTTAGACTTA
RGC19895c	HindIII	TCGTACAAAAGAGACTGGAGAGAAC	CTGGCGACTTAGGAAAAACTATGTA
RGC19925c	HindIII	CAAATAACTAATCTTCTGGGGGTTC	AGGGAGTTGGACAGAGTAGGTAGTT
RGC1995c	XhoI	GCAGATACATAGAGAAGGGAGTCTG	AGGAATTGGATATTTCTGGGTTTAG
RGC19961c	EcoRI	CTACTCTTCATTTTTGGAGTTCCTG	ACTCACAATGGATAGTTGGTATGGT
RGC1996c	EcoRI	AGCCAATGAGTTGCATAAGATAAAG	GTCGAACTAAACGTTAAAAAGCTGA
RGC1999c	HindIII	AGTGTTTCAAAGTATCCATGTCAGA	CGGATAGATACCCTAATCCAAGAAA
RGC20165c	EcoRI	TTATCACTTTGGGTGGTATATCGAA	GGCTAGCTTCATACAGAAATCCATA
RGC20183c	XhoI	CAGCTACTGGCTTCTTCTCATTTTA	TACAACTCAGTCAGATCCTTCCTCT
RGC201c	EcoRI	AGAAGATTTCTGTCTTCATGTACGG	GCCCTCTGTTTCTTGAAATTGTTAC
RGC20400c	PstI	TACTATACAACAATGAACGGAAGCA	CTGGTTATTCCTCAAGAACCTACAA
RGC20412c	XbaI	CGAGAACCAACAGTTAGAGGATAAA	AGTAAACATATAAATGGCGTGTGGT
RGC20489c	HindIII	ATGTGAAACCTAGTGATGACCAAAT	ACTTCATAACGTCTTGGAACTTGAC
RGC20497c	XhoI	TACAATGTATACGACCAACCAGCTA	TGTAACAGCATACCCTCTGATACAA
RGC2049c	SacI	GGAGAGTTATTGAGCATGAGGATAA	ATGATGTGACGTACAAAGAAGATGA
RGC20584c	HindIII	ACCGAGTTTTATCCTGTTTCCTTAC	GTCGACACGTTACGTTGATTAAGAG
RGC20593c	EcoRI	TATTGCATTCTCCGTTTCAGTTTAC	AACTTCACTTCTCAGGTTTTTGTTG
RGC206c	HindIII	CCCTTTTACAACGATATTCATTCTG	TGTTTGGTGAACTGACCAATATGTA
RGC2070c	EcoRI	ATCTGACCTGTATCCTGAGAGATTG	AGTTATGAAAGCGCACATTTTACTC
RGC2076c	EcoRI	GGCTTCTTTGGTTCGAATATCTTAT	CCTTGTACCCTAAATCACACTCAAC
RGC20779c	HindIII	TCAGAATAATGAACACTCCTGAACA	AACAGTCGAGAACCATCATATCAAT
RGC21003c	HindIII	GTCACACAAGCAAACAAAAAGTATG	ACGTGTTACAGTGTGAGGAAGGTAT
RGC21070c	EcoRI	TATTTCACATAACCCTGACAACGAC	ACAAGAAGCTGTGGTCTACAAGAAT
RGC21094c	HindIII	TATATGATCAGTATGCACTCGTTCG	TGGTTCTAATGGACAAGACATACCT

RGC2115c	EcoRI	AAACAATAATACCAATGAGGGCTTC	AAACTTGTCTTCGCGTTGTTAATAG
RGC2126c	HindIII	GATGAACCTTGTTTGAGAAATGAGA	ACTCTCACAAGTTGAAAGGAATCAC
RGC212c	HindIII	ACTATCCCATCACCACCACTAAGTA	GTTTTCAACTTTACCCCAAGTCTTC
RGC21373c	EcoRI	CTTCCAATTAAACAATCCATCACTC	AGAGTTAGCTTTTAGCAATGTGGTG
RGC21423c	EcoRI	GTACCTCCAAAAATTTAGCGATTGT	GTTCCGACGTGGATAGTAGTAATTG
RGC21459c	HindIII	CATGCACGTAGACATTGTAGAGAGA	CAGAGCAAATAAAAGCTTCTCACAT
RGC2145c	EcoRI	GTTACTTCAGCAACTTGATCTGCTT	GGATCAATAACACAGAAAGCAAAAA
RGC2146c	EcoRI	CTGAGAGATGGTCATTAGTGAATCC	TTAGAAACGCCATTAGAGAACAAAC
RGC21483c	HindIII	TGATATTATTCTTGTGGTGGGAAGT	TGAGGGAAAGTGTAAATAGGACAAG
RGC2153c	HindIII	AGACATTTGCTTATGGACTTGTGAC	AAGGGTTTGTACACACAATTACGTT
RGC2155c	HindIII	AACCAAACCAAGTGTTATATGAGGA	CCACAGTTTTCTTTTGGGATAATTG
RGC21614c	EcoRI	TTGGAGAAAGAGATGTTGAATTAGG	GTTTGGTTTGGTTTGTTTCTTCTGT
RGC21683c	EcoRI	AATTAGCGATGTATTTGTCAGAGGA	GACTTCACTCATCAGCTAGTGGTCT
RGC21684c	HindIII	CTCCAGCATATGGTTAGTTTCAGTT	GCTTCAGTTTCTTTTTCCTAAGGTC
RGC2170c	HindIII	CGGATTCTGATTTTATGTGGTTAAG	GAGTTTTCAAGACGAACAAAAGTGT
RGC21710c	HindIII	CCTCACGAGTCTCATAAATGAAGTT	TTTTATTCGGTGAGTTTGCTCTAAG
RGC21716c	HindIII	TTAGTAATGCAAGAGAGGAGTTTCG	TGCTCAACAGGTGACTACAGTAAAA
RGC21796c	PstI	TTACTGTGCTGTTGAACCTCTATGA	ACAGTAAACCAGCTTATGTGTCCTC
RGC21838c	EcoRI	GATACAGATAAAACATGGACGCAGT	GTTTGTCTCGATCTCTCTTTATCCA
RGC2185c	HindIII	CAGACTGCAAAGGAGTTAGAGTTTC	CCATGTCAATAATCTCCACATACAA
RGC2192c	XbaI	TGCTAGAGGAAGAGAGATCAGACAT	TCTGAGAGTTACTGGCAAACTTCTT
RGC2194c	EcoRI	AAGATTGTTGTCTTGTGAGTGATGA	CAAAGTAGTAAGCTTCTCCAACTGC
RGC21966c	HindIII	GGCAGATTGTAACGCTAAGGTAGTA	GCTTCTCTTCCATGAGATTATTCAA
RGC22105c	HindIII	TCTTTTCCATTGACCTAGTTTTCAG	CGAAAATCATGACTTATTCTCCACA
RGC2219c	EcoRI	TGAATCCAACAGATATCATGAACAC	CTAGTGCATAGTGGGACTCGTACTT
RGC22331c	HindIII	GGTAAACTTTCGATTTCACAGAAGA	TAGCTTAAATCTCTGCCACGTAAAC
RGC22415c	EcoRI	TTGTGTCTCTTGTTGCTGTCTGTAT	ATGCATAATTACAGCAAGGTCTCAT
RGC22512c	HindIII	CTGTTGGTTACAAATCCACCTTTAC	CTCATTCTGGTAAAAGAAAACCTGA
RGC22627c	HindIII	TGACCGATAATCAGGTAAGTTCATT	TCAGCTTCTCTATCTCAAAGCTCAT
RGC22628c	EcoRI	GTGTCACCATGAAGAAAAGAAAACT	GAGCTGCTGGTTATGTTATAAGGAA
RGC22795c	SacI	GATTCCTTGTCAACAACCTCATTAC	ATCATCACCAGAAACTCTATCAAGC
RGC2286c	EcoRI	CTGTTTTAATGCTTCAACGTCAGTT	TTTATAGAGCGGACAAACTCAACTC
RGC22968c	PstI	GATGGAGAGAGAGGGAGAGATAAAG	AAGCTGGTGATTGCATCGTTTAATA
RGC23031c	HindIII	GTATCACACCGTGGTGTTTATCATC	GCGAGGTCTCGTTCTTCTATTTACT
RGC23150c	HindIII	GCTGAGAAAGCACAAATTTTACAGT	CTAGACCTTCTTTCTCCTGAGAGGT
RGC23188c	BamH I	ATAACGAGGTCCATATGTTTTCAGA	ATTCCTGGGAAAAACTAATTAGACG
RGC23207c	HindIII	TCGGGTAAATGATTCGAGATTAGTA	TAATGCATGTACGTAAGAAGGGAAG
RGC2328c	HindIII	ACAGCATCTTTGGTTCAGTTATCTC	TACGATATAGGTATCTGGCGCTTTA
RGC2343c	HindIII	TTATCTAGCAGCGGACAAATATGAG	CACTGATATACGAATTGCACGAATA
RGC2344c	EcoRI	ACGTAGACAAGACATAACCCACAGT	GAGAGGTAGATCGACTGATATTGGA
RGC2356c	SalI	AACATTATCAATGGTAGGTCCTTCA	AGCTCTTTCTTTGCCTTCTCTAATC
RGC235c	HindIII	TCCACGATTGGAGATAGTACTTGTT	CCACGTTCACGGTATATTGACTTAT
RGC23650c	HindIII	CGTTTTTGTTCATGGATCAGATAGT	ACACACACACATCTCAAAATCATTC
RGC2369c	HindIII	CAAAGGTTACGTTCAGAACTTGTTT	AGCTTACCAACATGAAGTTACAAGG
RGC23821c	XbaI	ACTATTCTTGTTTTTGCCACTATCG	TGAGTCTCTCAAATGGAAACTCTTC
RGC2383c	EcoRI	GAATAAAAGGCTTGGGACATAACTT	ACACGTAGTGGAATGTTTACAAGGT
RGC2386c	BamH I/SacI	ATTTCCATTTTCCTCAACAGATCCT	GGAATCGATCAGTCGTATAATCTTC
RGC23911c	EcoRI	GATGGATATTGACTACATGGATGGT	ACATCAAGACAAAGACATGACAAGA
RGC24023c	HindIII	CCTCTCTCTCTATGTGCGTCTTTAG	CTCTCCTCTTCTTCTCTTTCCAGAT
RGC24282c	EcoRI	ACTTCCCAAACTAAGGACAAAGATT	CTGTTTCCGTCTATGAGATTGTTCT
RGC2431c	HindIII	TCTTACTACAAGAGCTTCGACCAAC	ATGTCGTCTCTTATGATAACGCTTC

RGC24370c	PstI	CACTAGCAGATCCTGAAGATCGTAT	GCTACTCCTCATAGCGACAAAAATA
RGC2467c	HindIII	CTAACGTTGCTATACTCCCAGCTAA	CAACACCATTGTTTTTATCTTCTCC
RGC2472c	PstI	GACAAACGTCTACAAGTTGAAGGAT	TGATTTAGGTACTTGTTGGGTTGTT
RGC24745c	BamH I	AACGACGACATATAATAGGTGAAGC	ACTTATACCATCCTTCCTCTGGTCT
RGC24823c	EcoRI	GTTGAGCCATGCAATACATTATACA	AACCAGTCTCCCTATTTTCCTTCTC
RGC2498c	EcoRI	TAGGTGCAGCTTCTTACTTTCATCT	ACCATGAATAGAAAACAACATCAGG
RGC2511c	EcoRI	ATGGAGATACGGCTCTACAGTTCAC	TACTTCATGTTTGTGTCATTTTTGC
RGC25318c	EcoRI	CTTGCATGACTCTATGAACATTCTG	CACACATGTGGGAATGAAACTATTA
RGC25363c	XbaI	TGGGTATTATAGATTTACGGTCGAA	GTTGATTCTTGAAGGTCTAGTCGAG
RGC2546c	XhoI	ACACCAACTCCTTCATATTTGACAT	CTCTGTGTGTGTTTTTGATTCTTTC
RGC2574c	EcoRI	GTTTTTGTTTGAACCTATTCAGTCG	TTTCTGATCTGAATCTTCTCTCTCG
RGC2575c	EcoRI	AAAATCTTATGCAACTAACGTAGCC	CTGTTATAGATGCGATGAGACGAGT
RGC258c	EcoRI	AGGTTATTGGTCCAAACGATATGTA	GACATTGATCAAAACACCAGATGTA
RGC2630c	XbaI	CTTTTACTCTTGTGCTTGCTCTTCT	AAAGGAACCAGTATGCCAGTATGTA
RGC26589c	HindIII	ATCTCTGTTGTGTTTCGTATGTTCA	TAGCAATCCCTATTGAAGCAGATAC
RGC265c	EcoRI	ACCTCACTCGACAATGACAATTTAT	ATCAATGGTACGACTCTAAACCAAA
RGC2666c	EcoRI	CATGAGAACTTTTCACGTATGATTG	TGTGAAAGCTCTTTTAATCCATTGT
RGC26764c	HindIII	ATGGTTCTGACGAATAACGAGTTTA	CCGAGGAAGCTGTTCTTATACATCT
RGC2676c	EcoRI	TATTTATTTTGCATTAAGTGCAGTG	TCCAATATGTCTTTTGACACTAGAA
RGC2683c	EcoRI	ACAGCTTATAAGTGGCAGTCTGTTC	CTGCGATGGTTAAGTTTAAAGAAGA
RGC2693c	HindIII	AACGAACAACCTAAACTAGCAAATG	TATGTTGAAGATTGAGACTGCTGTG
RGC2698c	HindIII	ATCTCTTGTTCTTCGAAGTGTTCAG	ATGATGGCTATGTATCTCCAACTGT
RGC2699c	HindIII	CACGAAGTCTGGTTATGCAATTTAT	ATTGGTCTCTTATTAGCAGGGTTTC
RGC2709c	EcoRI	CCACTACTGTTGGCTACTTCTGATT	CATGTTACCACCTACGTTCTTAACC
RGC2715c	HindIII	GGTTTCAGTACGAGACATGAAAGTG	TCAACTGGAGACTACTCTGCTTTCT
RGC27211c	EcoRI	AGTTTGTCTCCAATAAGACCTGTCA	AGCATCCTCACCATTATTCTCTTCT
RGC2728c	EcoRI	AAAGAAGTAGCTTCTGGTCTGCATT	GATGATCTAAAAGAAAGGCATGTGA
RGC27467c	PstI	AACATAGCAAACTAGAATCCGTAAG	ATTTCATATTTACGCTGTGGTTAGG
RGC2749c	EcoRI	CTTTCTGCTTCTTAATGTTCTCCAA	TTGTAGGAAAGTTGAGTCCTAACCA
RGC274c	EcoRI	CCCTTTCAAATTGTTGTTGAGTAAC	TCCAGATGGATTAGGTTTTGATGTA
RGC2758c	EcoRI	TATTTTGTTCACATCGATTTTGTTG	ATATGGTGTTTTCACATGTTTTACG
RGC2779c	EcoRI	AGCAGGATTCGTAACTTGACAATAG	ATCCGTCCCTTTTATTTTATCACTC
RGC27939c	PstI	CTGCAGGATCAACATGTAATGTAAC	GTTATTCAGGATTCTTGAGCACATT
RGC28054c	HindIII	AGCCTCAAATACATTTTTCACTCAG	GCTTCAATATCTTGTCCTTCAGTTC
RGC2807c	HindIII	GAAGCTACAATCACCGATGTTACTC	ATGTATAAGGACAGCCAATGTGTTT
RGC2833c	PstI	TTTGGTCTGTTGTAGGTTACCAAGT	TCTTCTTCATTGACCAGGAAGTATC
RGC2885c	XhoI	TTGGGAATTGGTGGTAGTTAGTTTA	ATCAATCAACACTCAACCTAATGTG
RGC2894c	EcoRI	AAGACAGAGACAAAAGAAGACGAGA	TGTAACTGGAGAGGCTGTTATTACC
RGC2914c	EcoRI	GGCACTTTCACCGTCTCTAAATAAT	TACCACACACACATCCTTGTTTTAC
RGC2932c	HindIII	GCATTTTCTAGACGAAGTTGATGTT	GCACCCAAATCAAGGTATATGTATG
RGC2981c	HindIII	TAATAATCTGCCTCTTGGTCATCTC	CAGTGGCAGGTTCTATTGTAGTTCT
RGC2987c	HindIII	CTATTAGTTTCCTGGATTTCCCTTT	GTCTCAAGATCTCCCCTAATGTTGT
RGC29914c	HindIII	CTACACAAACCTCTGAACTTGTGGT	GATTTATATCCAGCTGAAACCATGA
RGC2994c	EcoRI	TCTGTTAGGCACCCTGATATTGTAT	CATTCATCTTGAACTTTCCTCAGTT
RGC2998c	EcoRI	AGAAAAACTAGGAAAAGGAGTGCTG	TAGAGAAAAAGCAGCAAAAATGTGG
RGC3002c	SacI	TACGTTTCTCTCTCGGTATAAGCAT	ATGAACTTTGACTTGATCTTTCCAG
RGC3008c	EcoRI	TTTGGTAGATGAGGTCAGAATTAGC	AGTTCATAGTAAAACAGGCCATGAA
RGC3016c	EcoRI	TAATGTCTAACGCAGATAGGGAGTC	TTGAAGAATCTCTTCTCATCCATGT
RGC3045c	PstI	CTAAAACCCTTAAATCGATTCGTGT	AACTGAGATTGAGAGACAGCAAAGT
RGC3063c	EcoRI	AGTTACATAGATACACGTCCCCAAA	AAGCAGAGATATGAACAGTTCTTCG
RGC3069c	EcoRI	GCAGTGTTGAGTACAATTTGCTTTA	AATACAGCCTGGTAATTTCAGTGAG

RGC3081c	HindIII	AATATTAAAGCCCTTTGCGAGTGTT	ATATCTAGAGAACCAAAACCGAACC
RGC3130c	HindIII	CTACGCTTTGAACATTACCAGTTTC	CAGAGATTGGAGGTGGAGATAGATA
RGC3132c	BamH I	TCCTTTTACAGTTTTGTACCTTTGC	TTCTAAGCATGATAGAGAGCCAAAT
RGC3153c	PstI	AATGCTCCATGATATCTTTACCTGA	AGTTAAGGTACAGCTCTCGGATTCT
RGC32005c	XhoI	TGCTAATGACCAAAGGACATACATA	AAGGTGATATCAATGGAAGGTATGA
RGC32186c	HindIII	TAATGACAGGTAGGTCCATTTCAAG	CCTAACCTCATGTAGAAATCCTGGT
RGC3235c	EcoRI	CAAGAAGATTCTCCAAGTCTTCAAC	CCAGATACCTTCTTTCACTACCTCA
RGC32871c	PstI	ACCCAGAGCACATATGAAGTAGAAT	CGTATGGTTTGCTACTACAGGAGTT
RGC3295c	SalI	CAAACTTGAGTTTCCTTTTGTCTGT	ATCTTGACCTGTTTCGTTACTTTTG
RGC3356c	EcoRI	GAGACCCTTTCAGGAAACATAAGAT	TCCTTGACTCCTCAGAGAATAGAGA
RGC3380c	HindIII	GAGTAGATTCACAATGCTTTCAGGT	CCATTAGTCAACATCAGAAACATCA
RGC3382c	PstI	CTCTCTGCTCGTTATATCACTGGTC	ATCATGTTCTCATCAGAGGTTCCTT
RGC3412c	HindIII	ACTGGCAAGATCAATGTTTGATAAC	TTGATGAGAGTGGAACAATTGACTA
RGC3413c	HindIII	GTTTTCAATCTTCTCCAAACAACTG	AAGGAGAAAGTGAGATTGAGAGGTT
RGC3431c	HindIII	AGTTCCATTCAACCTATTACATCCA	TCCTTTGGAGATCTTGACTTTATTG
RGC347c	SalI	CAAGTGGAGATGATATCTCAGTGAC	TTCTTTGTTTACACACTTGTTGACC
RGC3490c	EcoRI	CCCACAAACAAAAGAGAAGACTTTA	TGGACAAGAGATAGTGGGTCTAAAA
RGC355c	HindIII	ACATTCAACGCTATCTTGACCTAAG	GTAACGAGTGTGCATCTATTTGTTG
RGC3577c	XbaI	ACTTAGACCCTCAGTTGTCGTTTTA	TCGAAAAGTAATCCTCTGAAACATC
RGC3637c	HindIII	CGACTCCAGATGCATAATAAAGACT	ACACGATGAGATGATGAAGAAAGAT
RGC3643c	HindIII	TTAGTGTACCAATCAACAACACACC	TACAGGGAAATGATTACGAAAGAGA
RGC364c	XbaI	TATTATACCACAACCTGCTCGATTT	AAGCTAATGTCCATTACAGGAAAGA
RGC3691c	HindIII	GCATTCTTGAACCAGAGACTTGTAG	GTGCTTGTTCTTTAGCTTTTTGAAG
RGC3696c	HindIII	ACATAAGCAAAGCAGAAGGAAATCT	TAGGGTTCTAGACATTAGGGGAGAG
RGC3758c	HindIII	GATGTGCTGGAGATAGTACTGCTTG	AATCTGTACAAGGTGAGAAAAACCA
RGC3773c	EcoRI	ATATAAACTCGATGAAACCGTACCC	GAAGAGTGTGCTCAGAGAGGTTTTC
RGC3820c	SacI	GTGTCAGCTACTGAGTTTGTGTGTT	CCTCATCTGCTGAGCTACTTCTTAC
RGC3886c	SacI	AACCAGAGTGTAATTCTCAAACCAG	TAACAACTCCAACAACTATCAGCAC
RGC3942c	HindIII	AGACAAAAACCATCCCTCTAACAAT	TATAACTCAAATTCTAACCGGACCA
RGC395c	EcoRI	AAGACAGAGATTTAGGAAGGGAAGA	AGACAAGACCTCTTTTGTCCTTTTT
RGC3985c	EcoRI	AGGTTCAAGATGCATACAAGAGTTC	ATGGTTCTATTCCACCAAATATTCC
RGC3995c	PstI	GTAGGTAGGAAACCAGTGATGAAGA	GGTGGTTGTTCCTAAGACTAATTGA
RGC4004c	HindIII	GTATGTCCTATGCTCAACAAACCAT	GGAGCTGATGACTTACAACATTCTC
RGC4050c	SacI	TTAATGCAGGAGCTATTACAAATGC	GAATCAAGATAAAGAGTGGAACCAA
RGC4056c	HindIII	TTATGTTAGCTGGAACCATTCCTAA	CTACTATCGTTTTCCACATTTGTCC
RGC4058c	EcoRI	ACTTGCTCTGAGTTAACAACATTCC	ACGATTTCCTCCTCTAGTTTCTGTT
RGC4062c	PstI	TGTGTTCTTATTACCGCTAAGGTTC	TGGACTTTGAAAGAACGAGTAATTC
RGC4083c	EcoRI	GAAGAAAACGTCTCTGTGTTTTGAT	GATAATGCAAATGGTACGCTAAATC
RGC4098c	HindIII	AAGTGAACATACACTGTGATGGATG	ATCTGATTAGCCAACTGTGATTGAT
RGC4102c	SalI	CACGTCATACCTTGCTGTTTACATA	CCTGTTTTATTGAGCATTCTAGCTG
RGC4124c	HindIII	CAGAACTGGTCCTAGGTTTGTTAAA	GTACACGACCGTACAATATACCACA
RGC4166c	EcoRI	TGAAGTAACATTTATCCATGTGGTG	TAAAGTGAATGACTGCAACTTCTCC
RGC4195c	EcoRI	AACTTAACAAAATACCTTGCGGTCT	TCAACGAGTAATTTGGATTCTGTCT
RGC4199c	PstI	GTTGGAATGATAAATATGCACTTGG	GTTTGTTAAAAGGGGTAAGATGTGA
RGC4219c	XbaI	CAAGTTTCTCTTGGAACTTCTTGAG	TGTAGCTCCCAAAATTTTACTTGTC
RGC4231c	HindIII	TTTACCACTCCTACACTCTCCATGT	CAAGGGTTAGTGACAGCCATATTTA
RGC4257c	EcoRI	TCACAAATATTCGGGATCTAAGTGT	AGTAGTGGTAGTGGGGATAGGTTGA
RGC4350c	HindIII	CAGATCGCTAAACCCTATTCTTCTT	GATATACACAAAGGAAAACGCAAAC
RGC4352c	EcoRI	TATTCTATGTGTATTTGCCGGTGAG	GTTATTAAAAATGCAGTTGGGGAAC
RGC4381c	EcoRI	TATTCGTTTTGAAAGTCCAAGTAGG	GCTTGATTATTATCCAGAGGAAGGT
RGC4388c	EcoRI	ATAAGACATGAATGATCGAACCAAC	ACTATTTGGGAGAACTACGCTTTTC

RGC4391c	EcoRI	GTTACTTGAACATAAAAGGGTGTGC	CTGGCTTTAAGGCTTTTAAGATTGT
RGC439c	PstI	GTGGATATAATTGTCTGAAGCAACC	GCTGTGAATGTGATACATGAGAGAC
RGC43c	EcoRI	TGCATATAGCACACATGTAACACAC	ATAGGTCACTGAAAATTGAAGATCG
RGC4403c	HindIII	CAGAAGATCAGCCATTTTGAGAATA	AGATCCAATCAAAGCTATGTGGTAA
RGC4411c	HindIII	GGCCTTACCTTTTCGATATCTGATT	TTGATCTCTCCACTATGATCGTACA
RGC4447c	EcoRI	ATGGGTTTGATCGATCTGTTATCAT	CAAATCATGCTCTAAGCTTCTTCTC
RGC4451c	EcoRI	TACTTAGCTTGTGAGAATCGTGATG	CGGTCGAGCTAAGACTAAAGATAGA
RGC4476c	EcoRI	TTACTGTCTGTTTCCTCTGGATGTT	TCGTAAGGTAAACAAAACAGCCTTG
RGC4480c	BamH I	GGTGTCTCGTCATATCTTTTGAACT	CAATTGAGATGTTTGACAGAAAGTG
RGC4495c	EcoRI	TAGTTGCTTGAAAGCGATTAGAGAT	AGTTGAGATCATTATCCGATGTAGC
RGC4505c	XbaI	CTATATCAGAGTCATGGTGGTGTTG	AGTAAGATACCAAAACTGGCATTTG
RGC4518c	EcoRI	CTGCCAGAAATAGTTGAGCAGTAAT	CTGAAAAGGATCTTCGAATGAGTTA
RGC453c	EcoRI	TGGCTTAAGTGATGAGTGCTTAAAT	CGTCAAAATATGGGTGAAAAAGTCT
RGC4546c	XhoI	TGATATCCTCTCTCAGCTCTCTGAT	GTGAACACCGATCATTACATCACTA
RGC4566c	EcoRI	TGGCAGATGATATGAAAGAGAAAGT	ACTAACCCGAGTTGCTAGCTTTATT
RGC45c	HindIII	GGTATTGGTCCCATTGATTGTAAAG	CATGCATCAATAGTCCTAAGCATTT
RGC4610c	EcoRI	CAACCAATAATTTCGTCGTTGATAC	ACCGGTTTATTTAACTTCCAGTTTC
RGC4639c	EcoRI	TAGAAAGGACGATTACAAGGCTATG	GTGCATGAATCCTCAATCTTATGTT
RGC4640c	PstI	GTCTTGTGTGTATGAGTTGAACGAC	GGAATCAACTTGTTAAGAGCATTGT
RGC4642c	EcoRI	CATAGCGTCCTATCAGCTACTCATT	TACTAACGGTGACGGTAACAGAAAT
RGC4643c	PstI	AGCCCTTTACACTATGTTGACACTC	TACCTGGTGAACATCTCCGATATAA
RGC4665c	EcoRI	TATTTGGTCAGATGATGGGTAAAGT	ATAATGAATCCCTTGCACATACAGT
RGC4679c	HindIII	TCAAATAATGTAACGCTTCTTCTCC	GAGCACAAGATCGATAGAGCTAAAG
RGC4684c	XhoI	ACACACACAATCTTTCTTCTTCTCC	TGACTATATTGATTGCTGCTGCTAC
RGC4695c	PstI	CTCCATTCTTCATCACCATATAACC	GATGTAATGAAGAGGAGAAGACCAA
RGC4722c	EcoRI	AGGCATAATAACTTGGAGGAAATGT	TACACATGCAGCTACAACCTAGAGA
RGC4797c	EcoRI	CAACAGTTTCCAGTTAATCAAGAGC	CGTATTGGCTTCTAATCAAATCATC
RGC4812c	EcoRI	TAACAACCCACATTAAATTGTCTCC	TCACTAACTAGAAAAAGGTCACTCG
RGC484c	EcoRI	GACCACCTAACCACTTAATTCCTTT	GACCATACCGATGATGTTTCTTTAC
RGC4853c	EcoRI	GAGAACGCTAGACAGTATGACTCAA	TATCTTCTCCTCTATTCGCACTGTT
RGC4895c	EcoRI	TGTTTCGTGTTCTTCTTCTTACACA	GAGAAAGCAACTTCCTTGAAAAGAT
RGC4940c	EcoRI	GGAGGTTCTTTGTTCTAACTTGTGA	GAAAAACAAGAGCTTTGGAGAGAGA
RGC4948c	EcoRI	CATGTGCCTACAATGATTTACCTTT	CAATAGTTTGGCCATCAAATCACTA
RGC4977c	EcoRI	CCGTTCCTGTAGTGATATTTTGTTC	AATAGACGTGTGTATTCCGAGTTCC
RGC5007c	XhoI	TTGTCTTCTTACTTGCTGAGTTCCT	GACTGAATCATTGTTAATCGGAATC
RGC5019c	EcoRI	TCCACCATATTATTGGAAACAGTCT	TATTTGTATAGCTGCTTGCTTCGAC
RGC5031c	HindIII	ATTTGAGCGAGTCTTATGTGACAGT	AGTTTGTCTGAGGCTAGTACCAATG
RGC5105c	HindIII	AATATGCTAGCTTGTTGCAGAGACT	CTCCTTCAACTTCAAGAAATACAGC
RGC5183c	EcoRI	TTATTTGATACGGGGTTTAGATCCT	ATGCCACATTTTCATGTAGCTTGTA
RGC5191c	HindIII	GTGACCAAAGGTTATCTCAGAATTG	CCAAACTAAACAAGAAGATCCACAC
RGC5211c	HindIII	GTGAAAGCATACCAAAGAAGAGTGT	ACAAGCATACACCTAGTCATGTTCA
RGC5236c	HindIII	GTAGCAAATTACCATCAGACAAACC	AGCTTGTACCATTTTATTGTGTGCT
RGC5241c	HindIII	TTCTCCTATTTCTCTTTTGGTTTCC	TGAAGAAACTGGTTACATGTCAATG
RGC5299c	EcoRI	AACGGAAACTGTCTTTCTTTCTTCT	GAAGGTACTTGAGGCGTTTTATTTT
RGC5306c	HindIII	ACCTTCGTCTTGTTTGTGTTAGAAG	CTTTTATCCTCTGTTTCTTGGTGAA
RGC5308c	EcoRI	GGAATGTGGAAGTGGTATATTTCAA	GTCTGATAACTCGAATGACCTCAAC
RGC5414c	HindIII	TTACTGCTGTATGTCATCCCCTAAT	GCACCTTTGTACACCAAAATAAAAG
RGC5417c	EcoRI	TGTATGCTTCCGATGATACTAACCT	TAATGGTGAGAGAGGTGGAGATAAG
RGC5424c	PstI	GGAGTCGATATGAGTCACTATGAAA	CATAGACATGTTTGGGTTGTTGTTA
RGC5428c	EcoRI	ACATAAACGAAGTTTCATCCACTTG	TTTGGACTCCCAACATAAGCTATTA
RGC5431c	EcoRI	GTGAGTCAGTGAGTGAATCTGTCAT	CACAAAAGGTATTGTTTCCAACTTC

RGC5476c	SalI	AACCTCACTACTTCATCACTGGAAG	AACAAAAGAAGGTAATCCACAGAGA
RGC5481c	SacI	ACGTCTCTCAGGTCTGTTATCAAAT	ATACCCTCATCCTCTGAATCAAAAT
RGC54c	HindIII	GAAGCAAACCAGACAGAACCTATTA	GTGGTAATGGTTTTTAAGGTCAATG
RGC5509c	EcoRI	CCAATAATTAACGAATGCTTCTCTG	TATTTGGGCTCACTTATGGAGTAGA
RGC5522c	HindIII	GAAGGTGCTTCCTACAGTTTTGTTA	TCCGTCTTCATCTGTAAATATTCTG
RGC5530c	HindIII	GATGAATCTCAAGGTCATCTGCTAT	TGGAGTATCCTATTGATGGTAGTGG
RGC5561c	HindIII	TGCAGACACTCAATGAACTACTGAT	CTTTCAGTGCTATACTCGTTTTCGT
RGC5574c	XbaI	AACTTTTACGACCTCAAAACTTCCT	AGTTAAACCCAGTTTATTGCACCTT
RGC5581c	SacI	ACGTCTCTCAGGTCTGTTATCAAAT	ATACCCTCATCCTCTGAATCAAAAT
RGC5600c	HindIII	AGGCAATTTGAACGATATCCTCTAT	CTGGTTTCTTTGAAGCAAGTCTAAA
RGC560c	SacI	ATCTCTTACCTACTTGGGTCTTCGT	AGAGAGATCCAGAGAGACTAAATGC
RGC5664c	EcoRI	TTTCCGGTTTAGGTACTTTTTAAGG	CCGAACGCAGAGTAGTAGTAGAAGA
RGC5676c	EcoRI	GGAGTCGGATTATTTACTTCTGGTT	ACGATAATCAGTTTTGAGCACAATC
RGC5691c	EcoRI	ACTTGACACAGATGACATTTGGATA	AGTCAAGTCCTACTTCAGCAAAGAA
RGC5695c	HindIII	CCAAAGGAAATTCTATTGATGAGAG	GTCAGAGTAGGTCTCTTCCGTTGTA
RGC5701c	HindIII	TGATGCATAAGTAACCAGCAAGATA	AAGTAAGAAAGCTCCCACTTTTCAT
RGC570c	EcoRI	ACTCTATCACGGACATTGTTTGATT	CCTACAAAACATTCTCGCAGATTTA
RGC5712c	EcoRI	AAGCCTCTAGGTCTATGTTCAGAGC	ATGAAACAAAATGAGTACTGGCTTG
RGC5762c	SalI	GCAAAACCAAGAACTTACAAATCTC	GGAGATCATGTCTAAGAAGCTGTTC
RGC5771c	EcoRI	GCCTGTGATCAGCTTTTTATTTTCT	ATGTTTTCTCTGTAGCAATGTCGTC
RGC5774c	HindIII	AGTAGAACCCCAAAAAGACAAAAAC	GAAACCCAAGATTGTGTAACCATAA
RGC5793c	HindIII	TAGGGTAGAAAGAAAGCATTGTCAC	CCTGAGATAGATTCCCACAACTAGA
RGC5810c	XhoI	GTCTCTCCAAATTCTCTACCAAAAA	CAGATCAACGATGTAGGTAACCTCT
RGC5894c	EcoRI	CCAAAGCATCAATAGATCAGAAAAG	TATCGTGACAAACTTGAGATGCTTA
RGC5898c	EcoRI	ACGAATATGATTGGGTTAGTCTCAA	CATATGTTTGGTTATCTTCGGATGT
RGC591c	EcoRI	AGTGAGACACTGGTCTTTGATTTCT	TGTAGACCACATACGAGTCTCCATA
RGC5939c	HindIII	CTTGGCTTCACCACTTACACTATCT	TACCTGAGGTTCTTTGTCATTTGTT
RGC5945c	BamH I	TTCAGAACATTGAAGAAGAGATGGT	CCCAACCTGAAGCTCTCTATAAAAT
RGC5954c	PstI	AGACCACAACGTTAACATTCAAGAT	AACAACAACTCTTTCTTCATTCAGC
RGC5970c	HindIII	ATATTTCTCTTCCGCTTTTTGTCTC	TGCAAGGTACAACTTATCCCTAAAA
RGC5978c	PstI	GAGGTTTGTGTAGAGAAAGCTGAAG	ACTTCACCTGATAACGCAAGAGTAT
RGC6011c	XbaI	TGTTTGGAGCTATTATATCGGTGAT	AGCTTTGTGAGAATCTTATTGGATG
RGC6032c	HindIII	TCTCTCCAGTTCCTTATCTTGAATG	AGTTACCCCATAGAAGCAAGCTAGT
RGC6034c	EcoRI	AGACGGAGAATTAATATTGGTGTCA	ACGAGCAAACATGGCTATAACTTTT
RGC6043c	XbaI	GTTTCTCTCAAGCATTTCATCGAAC	AGAGTTATCACATTTTGGTCGAGAT
RGC6058c	EcoRI	AGCTATGATGCGATTATTAAGCAGA	CGCTAAACTATTTTCACTGAACCAG
RGC607c	HindIII	GGAGAGTATGATTCCCTTCCTTTCT	AAGAAGCGTGAGAATATGAGTTTTG
RGC6104c	EcoRI	ATTTTCTTGTTTCTTACCCTCTTGG	TAATGCAACTTCCAATTACCACTTC
RGC6156c	EcoRI	ACAATCAAAAGCAAGACCTAGCAAT	ATAGTGGCGATATTGATCTTGGATG
RGC6170c	EcoRI	GCAGATTTCTACAAACAAGGTGTCT	TTGAGTTTTTCGTAGACTTTGGCTA
RGC6178c	EcoRI	CGAAACAGTTGAAGAGCAAGTTAAT	GTTGACTAAATAAGCCACACGACTT
RGC6186c	EcoRI	GCTTCTGTATTCATTTCTGGTATGG	CTAGCTTCAACAGGTAGCTCAAGTC
RGC6220c	HindIII	ATCGAGATAATGGCAATCAAGAAAG	CAAGGCACACTTAGTCAAAAGGTAT
RGC6279c	SacI	AAAGCAAAGTAGACGTATTCCACAC	TTACCTAACAATGAACGACTTAGCC
RGC6292c	EcoRI	TAAAATCAAATTCGAGTTGGGAGAG	ATGAGGTCTCGAAAGTTTATTGACA
RGC629c	EcoRI	AGCTTCATGAATAACAAGTTTGGAG	TAGGTGTATCACATGTTGTTTCCAG
RGC6336c	HindIII	GTAGGTCGGAATCTATAAGCCAAGT	ATGAGCTTGTGTATTTCTTGAGTCC
RGC6353c	XbaI	TATCATCCTTTCATTATTCCTGCTC	ATCAATATGCCAAATGAGTACACTG
RGC6359c	HindIII	ACATCCCAATATTCACCCTCTTTAT	TAATCCTGATTCTGTCGGTAAATGT
RGC6399c	XbaI	TTGTCATCTAACGGAAGTCATAACA	GTATGGTGGTAAATCTGACAGAACC
RGC6433c	HindIII	CCTTTGGAAACTAAACCGTGTACTA	ATGGAGTACGTTCTTGATGTCTCTC

RGC6462c	PstI	GAATAGCTGTCAGACTTTGGACTGT	AGCTAGAACCATTCTCTGAAACTGA
RGC6470c	EcoRI	GGGTTAAAAGGTTGCTGCTATTTAT	AATACTTCAAAGGGCTATTGCTTCT
RGC6477c	HindIII	CCATACTGTTTTGCAGTTCCTTAAA	AATTTCACACTAGTCACACCCTGTT
RGC6482c	HindIII	AGAAGCAAGCAAGAGAAATAGATGA	GCCTTTTATGTTCCTCAATTAGGTT
RGC6519c	HindIII	AATCTCCTCGTCTCAGTATTTAGGG	GATGTCGTCATAAAATTTTGGAGTG
RGC6520c	EcoRI	GAGTTGTCTGGTAGTTCACCTGTCT	TTGATAGTTTTCGGAAAGTCATAGC
RGC6540c	HindIII	TCATAGGCTAAATTGAGGACAAAAG	TGACTCTATAGCTTCCCATACCTTG
RGC6547c	EcoRI	GTGGATGAAGTTGATGGATAAGTTT	ATCGTCTTCAAAGTCCTTAACACTG
RGC6603c	EcoRI	TAAAACAAGGGATAACGCACTCTAC	GGTTGGACCAATTATGCTTAAAAAC
RGC662c	EcoRI	TGCACTCCTCATAGGTCAAAATAGT	TCGATGGAGACTTCACTTCTAACTC
RGC6716c	EcoRI	CAAATGATTTTAGGGCTAGTTCAGA	ATCTCTGTAACAACCTCAACAGGAA
RGC6719c	EcoRI	CTTTCTATAGAGCGGGTTTAACCTT	GTTTCCAGTACACAACTACCGTTTT
RGC6745c	EcoRI	GTAAAGTAGCAGGCTTTTCTTCCAC	TACAGGTCTTGAAATATTCGTTGGT
RGC6748c	HindIII	GGATATAGGATTGGTTAAGCAAGGT	GTGCAACCTAAGTACACAACAACAG
RGC6749c	EcoRI	TATGAGTGGCAAATATGTTTCATCC	AAGGGATGTTATAAAAGGCAATAGG
RGC674c	HindIII	AGAGCAGAACAAAGAGCTGAAAGTA	AACATGACGCTCTGTTTCTATCAAT
RGC680c	XbaI	TCTCAAAGTCTTCCCTGTTTCATAG	CTGTTAAAGTAACCAAAGACGGTGT
RGC6821c	EcoRI	CTCCTGCATTATAAGGAAAACAGAA	AGTACCTTTTGAAATCCATCCACTT
RGC683c	EcoRI	ACAGAGGCAATGTAATCATGAAATC	TGAGCACCTATTTTGTCAAACACTT
RGC684c	EcoRI	ATGTATCCTTGTCCTTATGTCCGTA	ATTCCTCCTCCTTTCCCTAGTAAAT
RGC6858c	HindIII	AAATGATTTTGCAGCCTATGTAGAG	AGTTGAAAGTCTTGAGCTCTTTGTG
RGC6864c	HindIII	AAAGAATGTGCTATTGACCTTTTGG	TGCTATCGGTAAAGTAGACAACTCC
RGC6866c	EcoRI	ACCAGGATAGTTCTTGTGAGTTTCA	AATGGGTGTGAAGTTGACCTAGTTA
RGC6878c	HindIII	CTAATTCACAAACGTGTCAAACAAG	CTAAGCCGACTACTCAGCCATAATA
RGC6888c	HindIII	CTAATCAACAAAGCTAAGGATGGTG	ATGTACCTGAGATAGGATGATTGGA
RGC6897c	HindIII	ATTATTCCTCCTCGATCTTCTATGG	AACTCTCTGAGGATGATGATTTTTG
RGC6940c	EcoRI	TGAGCAGATTCATTTAAACTGGTTG	CTACTCTCCCTGCTCTGGAATTTAG
RGC6949c	SacI	GAAGAGTAGGAACGGGAGATAAGTC	CTGCAATTTAGAAGCTAAAGCTAGG
RGC6966c	EcoRI	ATATTGATCTCGTGTAAGTCGCAAA	AGAAAAGCGAGCTATTTCAGGAATC
RGC6994c	PstI	CCATAGTGCAGTCCATCAAATATAA	ATCATGTTTATGTTGAGGAAGTTGG
RGC7029c	EcoRI	TTATATACCTGCATGAGAACCCAGT	GAGTATCAACTGGTTGGTTGTTTTC
RGC7030c	EcoRI	ATTAACCGGAGGAACAAACTTAAAC	GTTGCTAATACTGTTGTTGCTGTTG
RGC7035c	EcoRI	TAGCTACGTAATGATGCTTTTACCC	TGAAGTTTTCGTTACACTTACCACA
RGC7054c	XhoI	ATTCCTTCTACCTTAGGAAACCTCA	GGAACTTGTTGTTGCTTAGATCAAT
RGC7108c	EcoRI	ATAGGCAGACAGGAAACATAATTCA	GTTGTGAACTCATAAGAAGCAACCT
RGC7124c	EcoRI	CATGTGACCCGTAATACTTTGAGAA	TTGCTATTTAGCTGGAGAAATGAAG
RGC7132c	EcoRI	AAGAATCTGGTGAGTTAATGCTGTC	ATAAAAGGTACGAAACACGATACGA
RGC7139c	EcoRI	GTTGTTGGACCGTACCTATACAAAA	GAAGGAGAAGCAGATATTGTGGTTA
RGC7156c	BamH I	ATCTCATGCTGTGTTGTGTCACTAT	CCGAGTTTACTAGACCGGTTATTTT
RGC7157c	EcoRI	AGTTGCTGATTAAGTGTTCGTCAAT	GATTAGAATCTTCAAACCCATGTTG
RGC715c	EcoRI	CCTATTTGGGTTCTACAATCATTTG	AAAACCCTGCTCCAATAGAATTTAC
RGC7168c	EcoRI	ATCTAATGACCAAAAAGGTTTAACA	CATGTTTTTAAACCTCTCACCATCT
RGC7232c	HindIII	CCAAAGGGTGACCAAACTATAAAAA	CGGAAACGAGAAGATAATAGATGAA
RGC7238c	HindIII	TGGACAAGAAGGTAACCCTACATAA	TCCGAACCAACTGTAGAATGTAATC
RGC7277c	EcoRI	CCACAGATTAGTGTAGGTTCAGGTC	AGAGCAAATATAACAGCAACCATGA
RGC7282c	HindIII	AAGGATATAAAAGAAAGGACCACA	CATTCTCAGTTTCATCTTCTTCCAT
RGC7305c	PstI	CGGTAACATTTAATCCCTGTTCTAA	ACTAAAGATCACATGGTGAAGTGGT
RGC7341c	HindIII	CATCCTAGAAACAAGGTTCAACAGT	AGCAAAAGGTTAGATTATCGAGTGA
RGC7374c	EcoRI	CTAAGTCACAGTTTCAGTTCCCAAC	GGTCAGGAGTAAAGGAGAAGAGAGA
RGC7411c	EcoRI	CTTGTATTTCCATTCTCAGCTTCAC	CGCAACTACTAAGGCTAACGTTTTT
RGC7441c	EcoRI	CTTAATTGCCACATCCCATATTTAC	AAATAATATGGCCTTCTAAGCCAAG

RGC7444c	HindIII	GGAGGCTGAAGTACACAGAAAAATA	ATAGGGATCTCCAAACACATAGACA
RGC7461c	EcoRI	AGCAGCTTTAGATTCAACCCTACTT	AGTGGTGTCTCTAGTCAATCCAAAG
RGC7478c	XhoI	AACGACAATTAGTGTTTTAGGCAAC	ATCGGACACATGAGAGTTTTATGAT
RGC7515c	HindIII	TCCTTGGTAAGTACATGGTCTGTTT	GATGTGGTAAGAGAAAGCAACATCT
RGC7548c	EcoRI	TTCCAGCTTCAAGTTTTGTCTGATA	GTTGGAGATCATTTATGTTTTGGTC
RGC7607c	EcoRI	AAAGATGAGCAGACCTGACAGTATC	CTTCTCTCTTCACCACACACTCTCT
RGC7617c	EcoRI	GTAATGGACCAAACACAATACGAGT	GATGACTATGTTCTGGTCTTGAAGG
RGC7717c	HindIII	CTTCTCAGAGCTGTTCAAGGTAAAG	TTGAAGACACCAATGATAACAGAGA
RGC7718c	HindIII	TCTTTGAGAGTGAGACATCACTACG	ATCTGGCAGAATACAGGATACAAAG
RGC7750c	PstI	GCTGAAAACCTGTACAAGGTAGGTA	CCTCCTACTAAACCACTACCAAACA
RGC7757c	HindIII	AAAGGGAGAAGACACTCTCTGAAAT	GTTTTGAGTGAGAAGAGAGAGGTTG
RGC7796c	HindIII	GTTGGAGGGTAAACACAAAGTTCTC	TTTGAAGCTTTTCTGCAGTCTTACT
RGC7805c	SacI	TTGTGGAGAAGAATAGTGACATTGA	TAAATGGATCATCTAGCCTCATACC
RGC7838c	EcoRI	GGAAAGTCTGTCATCAGTTCTTGTT	GTGTTGAAGCTAAGGACATTGATCT
RGC7851c	HindIII	GTTCGACTTTGGAAGTTTGTATTTG	AGTTATCGAGTGGGAAGAAACTTTG
RGC7876c	HindIII	TGCAATTATTTTCCAGGAACTAGAG	AATTGGAAAGGCTGTAGTAAGTTCC
RGC7922c	HindIII	AAGGAGCTTTTGGTTCTTTAGGTTA	AACATCCAACATTGTCTCCATAAAC
RGC793c	HindIII	ATCCACATAACAAGGAGGTCTACAA	GATTTCTCAAGCTCATCTGTGTTTT
RGC7955c	HindIII	TAAGTTATTTGTCCCTTCTGATTCC	TGTTTTCAGCTTTTGTGATGTATTC
RGC7958c	EcoRI	GATACGAAGTTGCATAAACGAACAT	TTGTAAACATTTATACCGCGTATGG
RGC7982c	PstI	CATTTCTTTGAACAGATCTTCCATC	GTTAAACCCTTGTCTAGTGTCGTGT
RGC801c	HindIII	CTGGTTCATGTATCCAGGCTTATAG	TCTCTAATTTGCGAAACTCTTATGG
RGC8038c	HindIII	GTTTCTCTCTAACAGTGCAGTTTCC	GAACCGTATCTAATCTTCAAGTCCA
RGC8082c	HindIII	GTTTTAACAATTCTCCCATCAACAC	AGAAGAAAAGAAGGCCGTTTAAGTA
RGC8128c	EcoRI	AGGATGTCTTGATCCAGTCAGATAC	GCTTGTTAGTAGTGGAACGGATTTA
RGC8188c	XhoI	GGTATATCAGCTATTTTGTGCCATT	TAGTTTTATTGCTCTGGTTTTCACC
RGC820c	HindIII	ACCGGTAAGTACCCATTTAACTTTC	AGACAGAGGAGAGACTGTTCTCAAA
RGC8225c	EcoRI	AAAACCAACTCTGATTTTGGCATAC	TCAACCGTACCTAAATTCATCTCTC
RGC8228c	HindIII	GTTACTTGTCAGCTGGTGATAATCC	AGTGTAACTGCCTGTTTTATGTGCT
RGC8280c	SacI	GGTTTCTTGACAGCCAAGTTATTAG	AGTTCAATGGGAAAAGGAAACTATC
RGC8307c	EcoRI	GATTTTGTGGTTTAATGGTACTGGA	GCAGTCTAGCACCATATTCTCAGTT
RGC8308c	EcoRI	TCGTAACTACTCCCACAACTCCTAA	AGCTTGCAATCAGTCAATCAATATG
RGC8374c	HindIII	TAGTTTTCCTCAAATTCACAGAAGG	GTTTAGATTACTGATGCTGTGATGG
RGC8396c	HindIII	CGTGAACAGAGAATACTAAGCACCT	TTTTGATTCATCTCGTTCTAGTTCC
RGC8422c	EcoRI	CATCTTTGAATCCAGTTCTCACTTT	CTTCAAAAGTGTCCCAAACATTTAC
RGC8507c	HindIII	GGTTTTATATAAGGGAAAGGGGAAA	CCGTACTTTGAAAAACACTCAATTC
RGC8508c	SacI	GTATGACCAATCGTAAACCGTAAAT	CAAACTTTCAATTACACTCCAAACC
RGC8546c	HindIII	TGGTTGTACAGATGGTAGAATACGC	CAGTCTTCTCTGTCATCTTCTCCTC
RGC8553c	XbaI	CTTTGATAGGTATAAGCCAGGATCA	GGACTAAAGGTGATAGCCATAGGAT
RGC8563c	EcoRI	AATGCCTCATGATGTATAGGGTAAG	TGAAACGGTATAGAAGGAGAATGAC
RGC8606c	SacI	ATAGCTATAGCAACAAGCCCGTCTA	CAGGTCCATAATTAGACAAAACGAC
RGC8634c	EcoRI	AGATAGTTGCTCCTTATGAGCCTTT	CTAAGAGTAGGAATCCCCGGTTAGT
RGC8651c	EcoRI	CGGTTAAATGCTGTTATATTTCTGG	GATCTCATGTCATTCCAGACCAAGT
RGC8742c	HindIII	ACAGTGACATACATGACCTGCTAGA	GCTCTATGCCACGTTAGAAAAATAA
RGC8830c	EcoRI	ATGACCTGATATAAAAGCTGTCCAA	GCTTCTCTTGAAGAAGTTAATCTCG
RGC8839c	HindIII	AACGAATCAAATCACAATAGCTCTC	AGGATGAATTCTAGGGTTCTTGTTC
RGC8845c	HindIII	CTGAGAGAAATATGGTTTCTTGGAA	AACTCTTCAACCATCAGAGCATAGT
RGC8853c	HindIII	ATTGCCAATAGTGATCTCTTCGTTA	TTCAGAACTAAAGACTGTGCAGGAT
RGC8873c	EcoRI	TGCTATCTTGGTCTCTTGAAGAAGT	ACTACATAAGGTTCGTTGGTGATGT
RGC8922c	XbaI	AGAAAGGAGCCGATCATATATCTTT	ACAATCAGTAAAAGTTTGCAGCTCT
RGC893c	XbaI	TGAGTGATTCTCCTGTTCTTCACTA	TGGGTTTCGAATCTTATCTCATATC

RGC897c	EcoRI	CGAAACATATGAAGGATTAAGACGA	ACACAGAACTGGTACAATTGGAAAT
RGC8995c	EcoRI	CATATATGTGCATTGGGAAAATCTC	CATATCCCTTGTATGATCTGCAAAA
RGC9028c	EcoRI	ATAAGAATCCCCATAGAAAGACCAG	AGTTGTCTTCCCTTTTAATTCTTCG
RGC9047c	HindIII	GAAGTTTAGTGTTTGGTTTCAGTGG	CACCTAACATATTCAAGCACACAAG
RGC9063c	EcoRI	TGAAGGAGGGAGAGAGAGTGTATAA	CAAAAAGCATGTGTAGCTTCCTAGT
RGC9070c	EcoRI	AATGACTGCAAGATATGGTTATTCG	TTGGTTTAGAAGCATATAGTGGGTA
RGC908c	EcoRI	CAGCTGGTAAGTCATATGTGTTTTG	CTGTCATCCTTGGAAAATATACACC
RGC9094c	HindIII	CTCATTGAAACAAAACCTGAAAGAC	TATGGCTTCGAGTCTTTGAATTAGA
RGC9137c	HindIII	GCACAAGACTAGAACACAAGATGGT	TTATAACAACGTGGAGGAGGTGTAT
RGC916c	XbaI	TACTACAAGAATGGTCTGTGACGAA	CAACATTTGTTTCTGTGACATTAGC
RGC9187c	PstI	AAACTCTCACACTGAAAACACCAAT	GTGACCTCTATGGATTTGTGAAACT
RGC9198c	EcoRI	AAAGCTGAAGTTAAGAACAGAGCAA	TTCTTCCTATCTTCGGTTAAGGAAA
RGC9240c	EcoRI	GTCTAAATGAAGGAGAGATGGTGAA	TAATTCACAGTCAAAGTGGGTTTCT
RGC9253c	HindIII	ATAGGATATGGCATGCTAATAACGA	AATGCCAGACTCTTTCTACATTCAC
RGC9298c	HindIII	TCGGGTAGATCGTAGAAGTAGATCA	TCCAATATACCCTCCCAGTTATCTT
RGC9306c	SacI	GAGAATCTCCTTTGTTATCATCGAA	CAACCCACTATACACAGTGAAGCTA
RGC9315c	HindIII	TATGAAGGAAAACATAACCATGACC	ACAAACCAAAGCAAGTAGGTCAATA
RGC9337c	EcoRI	CTTATCCTTGGTTCTTTCTGGACAT	TTTATACTTCCGGTTCACTTTCTCC
RGC9339c	XbaI	TCATGAGAGGCTTAGGGATTATAGA	CTAAACAAGGGACTGAACTGACCTA
RGC9371c	EcoRI	ACAGCAGTGATATTGGTTTTACCAT	GTGATGATTTTATGAAGTGGACACA
RGC9387c	EcoRI	GACAGCAAATAAAAAGTCTTCGTGT	AGGTGAAGAATTGGTGTTAATGAGA
RGC9390c	HindIII	AAGTAGCCAGAGTCTCTTCTTCGAT	ACTCCAAATTTCGTTTCTCTTTACC
RGC9409c	EcoRI	AACGTGGAGATGAAATACAATGAGT	GTGTTTTGTTCTGTGTCTCTGTCTG
RGC9453c	EcoRI	GCGGTAGAAGTACCTACAGTGATGT	TTCTCATTTCACGATTACTCACTCA
RGC948c	EcoRI	GGAGCTATTAAATGATTTGGGTTTT	CTGAGAAAGAGATTGTCCATGAATC
RGC9493c	HindIII	TAAGAGAATATGCATGGCGATTAAG	GATCCTTCATCCATTTTGTTTCTCA
RGC9543c	EcoRI	GCATGGTCTGATATTTCTACTGGTT	TTTATTGGTCAGAGAAGGTTGTTGT
RGC9577c	EcoRI	AGCACTAATCTCTTGCATCTCAACT	TTTATCCTCTGGTGAACAAAGTTTC
RGC9615c	PstI	GAGTTTTCAGATTTGAGCGTAAGAC	GAGCTTACAAGTTCCAGTGATTGAT
RGC9621c	EcoRI	ACTGATTATTTGAACTGGTTGTGGT	CAAGTAAGTTCTAATCTCGCCTGAG
RGC9697c	EcoRI	AACCAGTGTAACCTTTGGTGATAAA	GAGCCATCCTATAAAATTCATGTTC
RGC9723c	EcoRI	ACGAATTAAACCCTAAAATCTGGTC	AGAAGCTCCTTTGATTCCTTTATGT
RGC9763c	HindIII	GTCCCTTATAACCTCTGGTCTCAAT	ATACCAGAGTGGCACAAACAATAAT
RGC9765c	HindIII	ATGCAGATGAAAAGTGAAATAGAGG	GCGTGTTCAGATATGTCAAAAATTC
RGC9800c	XhoI	CGCAAATCCGACGAGTATTATTTAT	TTCTTGCAATCTAAGGGATTCATAG
RGC9843c	SacI	TCCTCAACTACATTCGATTACAACA	GTGGTGGATACAGCTCAAACTATCT
RGC9866c	EcoRI	TGTCTTTATGATAGGGGGAAATGTA	ATGTGTAAAAGATGTAGCCCTTGAG
RGC9878c	XbaI	GCTGAAAAAGATCTTACAGAAGCAC	TAGATCCCAACACCGTTCTATGTAT
RGC989c	EcoRI	CGAGATAATAAACATTCCGTTCTTG	GGATCATTTTATAAGTCAACCGATG
RGC9973c	EcoRI	ATAGAAAACTCGACAGAGACCTTCC	GAGAGAAGTTGAGGATGACTTGAGA
RGC9986c	EcoRI	TCCTGCTTTAACCATAGATGTTTGT	GAGAGAGTTTGCAAGACATACCATT
RGC9998c	EcoRI	GTGCAAATGGTCTCTTGTTTCTACT	TGGTGTTAAAACAGTGGAGGTACAA

Table S14 Integrated linkage map

Linkage group	Marker loci	Position (cM)	Homologous gene in *B.rapa*		Synteny region (SR)
			Gene ID	Gene position (Mb)	
R1	RGA2415s	0	Bra011173	A01-3.498	
R1	RGC19516c	0.68			
R1	RS2CL672s	5.4	Bra015547	A10-0.658	1
R1	RS2CL6084s	6.61	Bra015472	A10-1.097	1
R1	RS2CL7513s	7.64	Bra033215	A10-3.502	1
R1	Rs2CL1500s	7.86	Bra032628	A09-38.681	1
R1	RS2CL1784s	8.15	Bra033287	A10-3.142	1
R1	RS2CL5805s	8.3	Bra033283	A10-3.151	1
R1	RS2CL4624s	8.57	Bra033337	A10-2.806	1
R1	RS2CL5888s	9.37	Bra015297	A10-2.137	1
R1	RGC14676c	9.78	Bra015431	A10-1.333	1
R1	RS2CL841s	9.99	Bra015367	A10-1.778	1
R1	RS2CL6024s	10.66	Bra015515	A10-0.83	1
R1	RS2CL6172s	10.88	Bra015548	A10-0.654	1
R1	RS2CL5895s	11.05	Bra015499	A10-0.906	1
R1	RS2CL4654s	11.89	Bra022662	A02-7.161	
R1	RSS3298	12.45	Bra018674	A06-2.693	
R1	CB62	15.15			
R1	Rs2CL1672s	15.78	Bra020022	A06-3.246	
R1	RSS1309	16.39	Bra033367	A10-2.69	
R1	RGC20489c	16.99			
R1	RSS2487	17.02	Bra033408	A10-2.469	
R1	RS2CL4157s	17.05	Bra031701	A09-36.925	
R1	RS2CL6482s	18.07	Bra019938	A06-3.6	
R1	Rs2CL2045s	18.34	Bra019956	A06-3.531	
R1	RSS2037	18.71	Bra030629	A08-20.247	
R1	Rs2CL2348s	19.35	Bra016757	A08-19.054	
R1	RSS0755	19.41	Bra015469	A10-1.116	
R1	RS2CL6151s	19.97	Bra019875	A06-3.933	2
R1	RGB7078c	20.98			2
R1	RS2CL6126s	21.21	Bra019720	A06-4.802	2
R1	OPE20Ha	21.39			2
R1	RS2CL895s	21.97	Bra019792	A06-4.398	2
R1	RGA1821s	22.51			2
R1	CC67aH	22.94			2
R1	RGC1996c	22.96			2
R1	RSS3090	25.32	Bra019853	A06-4.02	2
R1	RSS2047	29.92	Bra026838	A09-35.687	2
R1	RGC1509c	30.59			2
R1	RGC6821c	30.59			2
R1	RS2CL7575s	30.89	Bra026222	A06-5.209	2
R1	RSS0938	30.91	Bra026186	A06-5.395	2

R1	RSS3778	31.31	Bra026167	A06-5.488	2
R1	RGA2384s	32.66	Bra026010	A06-6.237	2
R1	RS2CL7786s	32.99	Bra026099	A06-5.857	2
R1	RS2CL5804s	33.35	Bra026110	A06-5.769	2
R1	RS2CL4651s	34	Bra025982	A06-6.351	2
R1	RS2CL5128s	34.14	Bra025994	A06-6.302	2
R1	RS2CL7214s	34.53	Bra026085	A06-5.908	2
R1	RS2CL6905s	34.74	Bra026062	A06-5.99	2
R1	RS2CL6317s	35.31	Bra026046	A06-6.058	2
R1	RS2CL5226s	36.21	Bra025939	A06-6.576	2
R1	RGA2190s	37.05			2
R1	RGC6748c	37.13	Bra026031	A06-6.137	2
R1	RGA2261s	37.39	Bra025920	A06-6.693	2
R1	RS2CL2084s	37.59	Bra025919	A06-6.704	2
R1	RGA2423s	38.18			2
R1	RGC3130c	38.24			2
R1	RGC11798c	38.54	Bra026127	A06-5.652	2
R1	RGC18142c	38.54			2
R1	RGC9409c	38.54			2
R1	RGC2192c	39.98	Bra025690	A06-7.026	2
R1	RGC23031c	39.98			2
R1	RGC5476c	39.98	Bra025937	A06-6.583	2
R1	RSS0987	40.32	Bra025719	A06-7.172	2
R1	RSS1811	41.12	Bra025804	A06-7.799	2
R1	RGC13289c	41.33			2
R1	RGC6011c	41.33	Bra025769	A06-7.5	2
R1	RS2CL4282s	41.98	Bra025798	A06-7.776	2
R1	RSS2736	42.7	Bra017911	A06-8.51	
R1	RGC6858c	43.24	Bra024709	A09-24.618	3
R1	RGB9500c	44.09	Bra024635	A09-25.121	3
R1	RGC453c	44.55			3
R1	RGB7310c	44.55			3
R1	RGC12216c	45.06			3
R1	OPE20b	45.24			3
R1	RS2CL2578s	45.38	Bra025873	A06-8.335	3
R1	RSS2094	45.53	Bra031364	A09-26.126	3
R1	RS2CL2020s	46.15	Bra031370	A09-26.077	3
R1	CB28	46.91			3
R1	RS2CL5101s	47.07	Bra016401	A08-17.511	3
R1	RS2CL2643s	47.2	Bra024542	A09-25.732	3
R1	RS2CL3579s	47.2	Bra024559	A09-25.636	3
R1	RSS1945	47.4	Bra024542	A09-25.732	3
R1	RSS3875	47.5	Bra024537	A09-25.754	3
R1	RS2CL5231s	47.52	Bra031387	A09-25.973	3
R1	RS2CL4686s	48.3	Bra024547	A09-25.699	3
R1	RSS2326	48.67	Bra040067	A08-12.415	3

R1	Pgm	48.77			3
R1	RGC3295c	49.44	Bra024687	A09-24.715	3
R1	RGA2466s	50.02			3
R1	CC11	50.93			3
R1	RS2CL7272s	50.99	Bra031377	A09-26.029	3
R1	RGC14226c	51.66			3
R1	RS2CL7240s	52.01	Bra024659	A09-24.975	3
R1	RS2CL7289s	53.29	Bra024686	A09-24.743	3
R1	RGC13028c	53.92	Bra024722	A09-24.558	3
R1	RS2CL3783s	54.59	Bra024724	A09-24.546	3
R1	RS2CL8267s	55.77	Bra024665	A09-24.939	3
R1	RGC14293c	56.66			3
R1	RGB1137c	58.06	Bra032807	A09-23.829	3
R1	RGC18589c	58.06	Bra032847	A09-23.599	3
R1	RSS0325	59.29	Bra024735	A09-24.451	3
R1	RSS4011	59.79	Bra023199	A09-20.875	3
R1	RS2CL7006s	61.13	Bra032859	A09-23.52	3
R1	RGC15930c	61.77	Bra026040	A06-6.09	3
R1	RGC3696c	62.07			3
R1	RS2CL7647s	62.83	Bra032873	A09-23.409	3
R1	RGC11904c	63.88	Bra032856	A09-23.545	3
R1	RGC7796c	63.88	Bra032883	A09-23.312	3
R1	RS2CL4233s	64.51			3
R1	RGC12943c	64.75			3
R1	RGC15280c	64.75			3
R1	RS2CL7501s	65.71	Bra032368	A09-22.176	3
R1	RS2CL5860s	66.58	Bra010776	A08-15.605	3
R1	RSS2045	66.67	Bra032310	A09-22.702	3
R1	RSS2843	66.67	Bra032316	A09-22.665	3
R1	RSS3638	66.67	Bra010829	A08-15.894	3
R1	RGC14452c	68.11			3
R1	RSS0679	68.37	Bra032396	A09-21.823	3
R1	RGB2900c	68.82	Bra026838	A09-35.687	3
R1	RSS0442	69.17	Bra032364	A09-22.198	3
R1	RSS0913	71.47	Bra033662	A06-24.692	3
R1	RS2CL2130s	71.65	Bra023176	A09-21.038	3
R1	RSS3818	72.83			
R1	Rs2CL1826s	72.89	Bra008122	A02-12.257	
R1	RGC897c	73.47			
R1	RGC20779c	74.35			
R1	RGB3655c	75.57			
R1	RGB1443c	76.34	Bra028651	A02-1.311	
R1	RS2CL7691s	76.63	Bra023306	A09-19.748	3
R1	RSS0997	77.39	Bra036948	Scaffold000123-0.259	3
R1	RGC23207c	77.65			3
R1	RGC4403c	77.65			3

R1	RGC715c	77.65			3
R1	RS2CL8104s	78.05	Bra028013	A09-19.437	3
R1	RSS0923	78.13	Bra040009	Scaffold000185-0.133	3
R1	RS2CL5132s	78.61	Bra028004	A09-19.545	3
R1	RS2CL1854s	78.75	Bra028012	A09-19.447	3
R1	RSS1995	79.15			3
R1	RGC1879c	79.18			3
R1	RS2CL5940s	79.53	Bra026107	A06-5.79	3
R1	RS2CL7378s	79.58	Bra028046	A09-19.094	3
R1	RGC15296c	79.98	Bra036965	Scaffold000123-0.509	3
R1	RSS2552	80.3	Bra028057	A09-18.975	3
R1	RS2CL5546s	80.93	Bra018735	A06-2.268	
R1	RSS1912	81	Bra015610	A10-0.175	
R1	RGC6603c	81.24			
R1	RS2CL1420s	81.55	Bra037299	A09-5.071	
R1	RGC1871c	81.98	Bra040749	Scaffold000249-0.015	
R1	RS2CL7231s	82.45	Bra040753	Scaffold000249-0.037	
R1	CC60H	82.7			
R1	RS2CL996s	82.92	Bra040955	Scaffold000337-0.006	
R1	RGB10948c	83.21			
R1	RGC32005c	83.73	Bra018866	A06-1.539	4
R1	RGC4062c	83.73			4
R1	RGC4642c	83.73	Bra018906	A06-1.34	4
R1	RGC9187c	84.21	Bra015613	A10-0.125	4
R1	RSS1252	84.4	Bra006164	A03-2.312	4
R1	RGC3063c	85.17	Bra018885	A06-1.445	4
R1	RGC1234c	85.32	Bra018872	A06-1.513	4
R1	RSS1998	85.8	Bra037273	A09-5.231	4
R1	RS2CL6354s	85.86	Bra018932	A06-1.19	4
R1	RSS0430	86	Bra018860	A06-1.585	4
R1	RS2CL2122s	86.08	Bra030419	A05-11.191	4
R1	RGC6043c	86.3	Bra018979	A06-0.918	4
R1	RS2CL4281s	86.63	Bra019002	A06-0.813	4
R1	RS2CL5593s	87.28	Bra019013	A06-0.771	4
R1	RGC3820c	87.88	Bra037970	A06-0.361	4
R1	RSS0471	87.9	Bra018964	A06-1.005	4
R1	RGC3016c	88	Bra037994	A06-0.24	4
R1	RSS2516	88.4	Bra022927	A03-7.813	
R1	RSS3378	88.4	Bra041018	Scaffold000383-0.003	
R1	Rs2CL2428s	89	Bra028015	A09-19.419	
R1	RSS0925	89.9	Bra037969	A06-0.365	
R1	RSS2494	90.7	Bra040781	Scaffold000257-0.009	
R1	RSS3402	90.7	Bra040781	Scaffold000257-0.009	
R1	CC89H	91.4			
R1	RSS0496	95.7	Bra037966	A06-0.376	

R2	RSS3197	0	Bra011256	A01-3.057	
R2	RGC2914c	1.88			
R2	CA41N	4.3			
R2	RGC12735c	6.75	Bra011588	A01-1.531	
R2	RS2CL8380s	8.75	Bra030055	A07-9.162	
R2	RGB8387c	9.56	Bra034641	A08-11.537	
R2	RS2CL1172s	10.39	Bra037884	A09-15.742	
R2	CA41	10.9			
R2	RS2CL6266s	10.92	Bra011519	A01-1.846	5
R2	CD57H	11.1			5
R2	RSS0368	12	Bra011744	A01-0.776	5
R2	RGC9800c	12.25			5
R2	RSS2503	12.9	Bra013783	A01-7.747	5
R2	RSS3053	12.9	Bra011830	A01-0.331	5
R2	RSS3449	12.9	Bra011776	A01-0.608	5
R2	RSS1595	13	Bra011839	A01-0.279	5
R2	RSS0251	13.1	Bra011808	A01-0.421	5
R2	RSS0845	13.1	Bra011833	A01-0.316	5
R2	RSS1374	13.1	Bra011813	A01-0.406	5
R2	RSS0073_a	13.2	Bra010632	A08-14.92	5
R2	RS2CL986s	13.61	Bra011843	A01-0.264	5
R2	RGC15346c	13.81	Bra011784	A01-0.553	5
R2	RS2CL2676s	14.27	Bra033012	A02-22.161	5
R2	RGC9253c	14.68	Bra011410	A01-2.288	5
R2	RS2CL2351s	15.12	Bra011545	A01-1.74	5
R2	RS2CL6213s	15.6	Bra011537	A01-1.782	5
R2	RSS1442	15.7	Bra011709	A01-0.945	5
R2	RS2CL5010s	16.14	Bra011805	A01-0.431	5
R2	RGB5729c	16.47			5
R2	RGC5970c	16.47	Bra011887	A01-0.073	5
R2	RGC8308c	16.47			5
R2	RGB12209c	16.67	Bra011669	A01-1.151	5
R2	RSS1863	16.8	Bra011620	A01-1.375	5
R2	RSS2730	16.8	Bra011643	A01-1.292	5
R2	RS2CL2152s	16.82	Bra014487	A04-0.989	5
R2	RSS1640	16.9	Bra011645	A01-1.285	5
R2	RGC17861c	17.15			5
R2	RGA1922s	17.24	Bra033555	A06-25.188	5
R2	RS2CL7559s	17.37	Bra011851	A01-0.233	5
R2	RS2CL826s	17.52	Bra011824	A01-0.354	5
R2	RS2CL6429s	17.75	Bra011783	A01-0.563	5
R2	RS2CL4831s	17.76	Bra011816	A01-0.395	5
R2	RS2CL3429s	17.76	Bra011812	A01-0.407	5
R2	RS2CL6141s	17.92	Bra011779	A01-0.593	5
R2	RS2CL2740s	18.06	Bra011751	A01-0.732	5
R2	RSS1435	18.1	Bra011540	A01-1.767	5

R2	RSS2508	18.2	Bra011545	A01-1.74	5
R2	Rs2CL1685s	18.75	Bra011643	A01-1.292	5
R2	RS2CL2162s	19			5
R2	RS2CL1115s	19.23	Bra011434	A01-2.182	5
R2	RSS1796	19.4	Bra011459	A01-2.087	5
R2	RS2CL6767s	19.46	Bra011683	A01-1.067	5
R2	RS2CL8214s	19.55	Bra011656	A01-1.234	5
R2	RGC21070c	19.59			5
R2	RGC212c	19.59			5
R2	RGC2575c	19.59	Bra011630	A01-1.344	5
R2	RGC2666c	19.59			5
R2	RGC6462c	19.59	Bra037291	A09-5.116	5
R2	RS2CL4022s	19.85	Bra011605	A01-1.439	5
R2	Rs2CL1791s	19.86			5
R2	RGC16214c	19.95			5
R2	RS2CL7540s	20.16	Bra011573	A01-1.626	5
R2	RGC22795c	20.7	Bra011424	A01-2.237	5
R2	RS2CL6543s	20.72	Bra011417	A01-2.257	5
R2	RS2CL3997s	20.74	Bra011544	A01-1.745	5
R2	RSS3566	20.9	Bra011250	A01-3.083	5
R2	RGC4451c	21.13	Bra011536	A01-1.787	5
R2	RS2CL1822s	21.21	Bra011319	A01-2.744	5
R2	RGC4476c	21.51	Bra011178	A01-3.465	5
R2	RS2CL5248s	21.66	Bra011353	A01-2.597	5
R2	RS2CL6952s	21.76	Bra011350	A01-2.616	5
R2	RSS0957	21.9	Bra011236	A01-3.156	5
R2	RS2CL5432s	22.08	Bra011349	A01-2.621	5
R2	RGC10385c	22.23	Bra011221	A01-3.246	5
R2	RSS2483	22.5			5
R2	RS2CL4657s	22.51	Bra016914	A04-17.737	5
R2	RS2CL4960s	22.85	Bra011222	A01-3.242	5
R2	RS2CL3611s	23.07	Bra011228	A01-3.196	5
R2	RSS3805	23.1			5
R2	RGC12573c	23.27			5
R2	RGC1566c	23.27			5
R2	RGC3643c	23.27			5
R2	RGB419c	23.27	Bra011154	A01-3.63	5
R2	RGC4350c	23.27			5
R2	RGB921c	23.27	Bra011387	A01-2.402	5
R2	RS2CL2632s	23.29	Bra011250	A01-3.083	5
R2	RS2CL2446s	23.61	Bra011168	A01-3.542	5
R2	RS2CL1476s	23.8	Bra011172	A01-3.518	5
R2	RS2CL1124s	23.94	Bra024095	A03-27.718	5
R2	RS2CL5639s	24.16	Bra011179	A01-3.463	5
R2	RS2CL3061s	24.43	Bra011170	A01-3.531	5
R2	CB72H	24.5			5

R2	Rs2CL1639s	24.64	Bra011118	A01-3.814	5
R2	RS2CL8169s	24.94	Bra011240	A01-3.13	5
R2	BRMS036	25.2			5
R2	RGC12949c	25.36	Bra011065	A01-4.127	5
R2	RGC148c	25.36	Bra011072	A01-4.087	5
R2	RS2CL3052s	25.46	Bra011056	A01-4.183	5
R2	RSS0445	25.8	Bra010339	A08-13.182	5
R2	RS2CL7090s	25.88	Bra034810	A05-22.249	5
R2	RS2CL4177s	26.05	Bra011069	A01-4.109	5
R2	RSS0802	26.3	Bra011042	A01-4.276	5
R2	RSS3702	26.5	Bra011034	A01-4.327	5
R2	RS2CL6599s	26.65	Bra011037	A01-4.315	5
R2	RGB3040c	26.97	Bra026274	A01-10.045	6
R2	RSS1032	27			6
R2	RS2CL6683s	27.19	Bra026262	A01-10.118	
R2	RGC7444c	27.36	Bra011038	A01-4.293	6
R2	Rs2CL2399s	27.68	Bra026304	A01-9.912	6
R2	RGC20165c	27.88	Bra011026	A01-4.355	6
R2	RGC2344c	27.88	Bra026386	A01-9.404	6
R2	RGC9866c	27.88			6
R2	RSS2114	27.9	Bra026335	A01-9.74	6
R2	RGC9047c	28			6
R2	RS2CL4120s	28	Bra026301	A01-9.926	6
R2	RS2CL831s	28.34	Bra026294	A01-9.968	6
R2	RS2CL2568s	28.61	Bra019085	A03-26.439	6
R2	RS2CL2222s	28.81	Bra026340	A01-9.724	6
R2	RS2CL7550s	29.06	Bra026349	A01-9.657	6
R2	RS2CL1001s	29.17	Bra026350	A01-9.655	6
R2	RS2CL2803s	29.61	Bra026426	A01-9.161	6
R2	RGC2145c	30.12	Bra013948	A01-8.657	6
R2	RS2CL3202s	30.38	Bra013924	A01-8.479	6
R2	RSS3094	30.5	Bra013945	A01-8.648	6
R2	RS2CL3241s	30.7	Bra026450	A01-9.041	6
R2	RGC43c	31.12			6
R2	RSS2752	31.2	Bra013912	A01-8.42	6
R2	RS2CL1313s	31.21	Bra026376	A01-9.44	6
R2	RGC7617c	31.35			6
R2	RSS2898	31.4	Bra013918	A01-8.448	6
R2	RS2CL3641s	31.7	Bra013900	A01-8.374	6
R2	RS2CL2961s	32.19	Bra013888	A01-8.258	6
R2	RS2CL3459s	32.49	Bra019192	A03-25.814	6
R2	RS2CL1051s	32.92	Bra013907	A01-8.405	6
R2	RSS1170	33.1	Bra013836	A01-8.021	6
R2	RSS2001	33.5	Bra013832	A01-8.003	6
R2	RS2CL6882s	33.69	Bra013939	A01-8.565	6
R2	RS2CL4789s	35.07	Bra013786	A01-7.756	6

R2	RS2CL4931s	35.07	Bra011180	A01-3.456	6
R2	RS2CL6206s	35.07	Bra013790	A01-7.77	6
R2	Rs2CL2267s	35.07	Bra013797	A01-7.796	6
R2	RS2CL3897s	35.7	Bra013690	A01-7.161	6
R2	RSS0645	36	Bra011765	A01-0.672	6
R2	RSS1152	36	Bra013706	A01-7.29	6
R2	RSS2678	36			6
R2	RGC1244c	36.02	Bra013636	A01-6.785	6
R2	RSS2341	36.1	Bra019300	A03-25.12	6
R2	RS2CL7884s	36.19	Bra013734	A01-7.5	6
R2	RS2CL3734s	36.37	Bra013652	A01-6.886	6
R2	Rs2CL1620s	36.37	Bra013642	A01-6.816	6
R2	RS2CL5841s	36.66	Bra013708	A01-7.315	6
R2	RS2CL1379s	37.03	Bra013631	A01-6.755	6
R2	RSS1500	37.3	Bra013598	A01-6.562	6
R2	RSS4012	37.3	Bra013606	A01-6.615	6
R2	RSS3707	37.6	Bra013549	A01-6.278	6
R2	RSS0715	37.7	Bra013539	A01-6.204	6
R2	RSS1692	37.7	Bra013576	A01-6.434	6
R2	RSS2367	38.7	Bra013453	A01-5.769	6
R2	RSS3086	38.7	Bra013456	A01-5.801	6
R2	RS2CL1253s	39.05	Bra013617	A01-6.652	6
R2	RS2CL7301s	39.34	Bra013602	A01-6.599	6
R2	RGC12272c	39.68			6
R2	RGC18651c	39.68			6
R2	RGC4643c	39.68	Bra013541	A01-6.21	6
R2	RS2CL8395s	39.86	Bra013556	A01-6.34	6
R2	RS2CL3283s	40.08	Bra013468	A01-5.847	6
R2	RS2CL5011s	40.59	Bra013456	A01-5.801	6
R2	RSS2368	40.62	Bra039543	A01-11.525	6
R2	RSS3742	40.62	Bra012716	A03-22.602	6
R2	RSS3354	40.72	Bra013269	A01-4.645	6
R2	RGA106s	40.91			6
R2	RS2CL5071s	41.08	Bra013373	A01-5.273	6
R2	RS2CL942s	41.4	Bra013440	A01-5.683	6
R2	RSS0590	41.62	Bra036882	A01-12.234	6
R2	RS2CL7475s	42.8	Bra013303	A01-4.876	6
R2	RSS1031	42.81	Bra023563	A02-2.913	6
R2	RS2CL2108s	43.74	Bra040093	A01-10.535	6
R2	RGB13975c	44.12	Bra013470	A01-5.853	6
R2	RSS0156	44.14	Bra019095	A03-26.375	6
R2	RS2CL4957s	44.62	Bra040182	A01-4.539	6
R2	RS2CL1710s	45.89	Bra036878	A01-12.263	6
R2	CA43	46.15			6
R2	RS2CL2938s	46.32	Bra038877	A01-15.461	6
R2	RGC11533c	46.32	Bra013287	A01-4.752	6

R2	RGC15219c	46.32			6
R2	RGC7035c	46.75			6
R2	RGB966c	46.75	Bra038448	A01-10.949	6
R2	S582	46.95			6
R2	RGC6477c	47.53	Bra013313	A01-4.932	6
R2	Rs2CL2264s	48.06	Bra039721	A08-6.427	6
R2	RS2CL5524s	48.33	Bra036878	A01-12.263	6
R2	RGC22331c	49.21	Bra013717	A01-7.372	6
R2	RS2CL2407s	49.8	Bra006362	A03-3.184	6
R2	RGC27211c	50.63	Bra036900	A01-12.05	6
R2	RGC4684c	51.43			6
R2	RGC14244c	52.4	Bra013443	A01-5.695	6
R2	RS2CL9061s	53.2	Bra023433	A02-2.328	7
R2	RS2CL5137s	53.59	Bra023435	A02-2.334	7
R2	CC29	54.54			7
R2	RS2CL868s	54.62	Bra023413	A02-2.244	7
R2	RGB500c	55.34	Bra023518	A02-2.708	7
R2	RGC1836c	55.7	Bra038172	Scaffold000140-0.346	7
R2	RS2CL2432s	56.46	Bra028585	A02-1.601	7
R2	RGB1399c	57.19			7
R2	RS2CL7359s	57.82	Bra028570	A02-1.664	7
R2	RS2CL5452s	58.64			7
R2	RS2CL1869s	58.94	Bra028665	A02-1.256	7
R2	RGC9493c	59.59			7
R2	CC98bH	60.48			7
R2	RGC23821c	60.48			7
R2	RGC1979c	63.35	Bra028578	A02-1.634	7
R2	RS2CL2645s	64.21	Bra028825	A02-0.483	7
R2	RS2CL4947s	64.26	Bra028860	A02-0.333	7
R2	RS2CL2123s	65.65	Bra028906	A02-0.106	7
R2	RGC1333c	66.38			
R2	STG2F12	67.38			8
R2	RGC1167c	67.88			8
R2	RGC7750c	67.88	Bra022709	A02-6.852	8
R2	RGB9670c	67.88	Bra022717	A02-6.816	8
R2	RGC13599c	69.68	Bra040900	Scaffold000302-0.016	8
R2	RS2CL5848s	70.53	Bra022683	A02-7.027	8
R2	RGC11438c	71.44	Bra035582	A02-6.396	8
R2	RS2CL2713s	72.33	Bra035587	A02-6.385	8
R2	RGC13134c	73.11	Bra035619	A02-6.212	8
R2	RGC820c	73.71	Bra020286	A02-5.001	8
R2	RS2CL5541s	74.31	Bra035650	A02-5.918	8
R2	RS2CL4123s	74.58	Bra035637	A02-6.045	8
R2	RGC5945c	74.85	Bra020377	A02-5.436	8
R2	RGC22105c	75.04			8
R2	RGA1974s	75.6			8

R2	RS2CL5783s	75.94	Bra020433	A02-5.708	8
R2	RS2CL4762s	76.11	Bra002737	A10-7.995	8
R2	RS2CL5589s	76.67	Bra020411	A02-5.56	8
R2	RS2CL3263s	77.93	Bra020332	A02-5.227	8
R2	RSS1758_a	78.06	Bra002241	A10-10.779	8
R2	RS2CL3772s	78.07	Bra020341	A02-5.287	8
R2	RS2CL4384s	79.33	Bra020311	A02-5.13	8
R2	OPT04H	80.88			8
R2	RSS1614	81.58	Bra006692	A03-4.655	8
R2	RSS1076	82.28	Bra020239	A02-4.768	8
R2	RSS1341	82.68	Bra020237	A02-4.734	8
R2	RSS2394	82.78	Bra020239	A02-4.768	8
R2	RS2CL7538s	83.28	Bra020232	A02-4.681	8
R2	RSS0119	83.98	Bra020184	A02-4.417	8
R2	CA87aH	85.68			8
R2	RSS2308	86.78	Bra002280	A10-10.59	8
R2	RS2CL3410s	89.36	Bra040902	Scaffold000302-0.028	8
R2	RS2CL2004s	89.93	Bra020155	A02-4.272	8
R2	RS2CL1934s	90.29	Bra020136	A02-4.174	8
R2	RGC10518c	90.29	Bra020037	A02-3.715	8
R2	RS2CL5545s	90.94	Bra020190	A02-4.444	8
R2	RSS3936	91.67	Bra029968	A01-14.826	8
R2	RS2CL3191s	91.85	Bra020082	A02-3.938	8
R2	RGC3758c	92.25	Bra020079	A02-3.919	8
R2	RGC10400c	92.64			8
R2	RS2CL3650s	93.11	Bra023697	A02-3.554	8
R2	RS2CL2542s	93.48	Bra002216	A10-10.894	8
R2	CB93	93.64			8
R2	CC45b	95.5			8
R2	RS2CL6642s	95.63	Bra023641	A02-3.307	8
R2	CD72aH	96.43			
R2	OPT12b	97.63			
R2	CA68	98.73			
R2	RS2CL6884s	98.86	Bra029972	A01-14.882	
R2	CO-2	99.2			
R2	RGB1253c	99.55			
R2	OPE10	99.57			
R2	OPK06	99.87			
R2	CB56	99.97			
R2	CC42a	100.37			
R2	RGC2431c	100.78	Bra008529	A02-15.229	9
R2	RSS2455	101.41	Bra033767	A01-13.892	9
R2	RGC4812c	101.88			9
R2	RSS1140	101.97	Bra037606	A01-12.628	9
R2	RS2CL1225s	102.08	Bra008517	A02-15.127	9
R2	RSS2316	102.43	Bra018212	A06-10.808	9

R2	RSS2594	102.73			9
R2	RSS1635	102.83			9
R2	RSS3530_a	102.83			9
R2	RSS3934	102.83	Bra008520	A02-15.163	9
R2	RS2CL4062s	103.51	Bra018469	A05-8.384	9
R2	CC62	104.33			9
R2	RSS2572	105.33	Bra033122	A02-16.365	9
R2	RSS0373	105.53	Bra029435	A09-17.241	9
R2	RSS2081	106.43	Bra033134	A02-16.482	9
R2	RSS0968	109.13	Bra033173	A02-16.895	9
R2	RGC11673c	111.59			9
R2	CB82H	111.74			9
R2	RS2CL7828s	111.76			9
R2	RS2CL3640s	112.16	Bra033156	A02-16.716	9
R2	RSS2889	112.19	Bra022549	A02-8.048	9
R2	RS2CL3017s	112.82	Bra033117	A02-16.288	9
R2	RSS2721	112.83			9
R2	OPT16b	114.33			9
R2	RS2CL4126s	114.34	Bra033197	A02-17.244	9
R2	RGA1566s	115.56			9
R2	Rs2CL2368s	116.12	Bra040656	Scaffold000230-0.014	9
R2	RSS1798	116.12	Bra040624	A02-8.929	10
R2	RGC3637c	116.43			10
R2	CA39	117.35			10
R2	RGA154s	117.59			10
R2	RGC18172c	118.6	Bra038305	A02-10.366	10
R2	RSS2958	118.61	Bra034018	A02-9.172	10
R2	RS2CL3903s	118.85	Bra039748	A02-8.709	10
R2	RS2CL836s	119.25	Bra039740	A02-8.663	10
R2	RGC13420c	120			10
R2	RS2CL4187s	120.34	Bra033998	A02-9.34	10
R2	RGB10737c	120.78	Bra034033	A02-9.046	10
R2	RGC11938c	120.78	Bra034012	A02-9.208	10
R2	RSS3138	120.86	Bra022542	A02-8.131	10
R2	RS2CL6395s	122.3	Bra033938	A02-9.772	10
R2	RS2CL3405s	122.76	Bra038348	A02-10.088	10
R2	RGC13684c	123.32			10
R2	RSS3268	123.53	Bra038316	A02-10.323	10
R2	RGC2676c	123.61			10
R2	RGC7958c	124.04			10
R2	RS2CL5422s	124.16	Bra038321	A02-10.294	10
R2	RS2CL3768s	124.53	Bra007881	A02-10.552	10
R2	RGC9765c	124.71	Bra007865	A02-10.393	10
R2	RS2CL2980s	125.34	Bra007872	A02-10.454	10
R2	RSS1262	125.5	Bra038308	A02-10.357	10
R2	RGB716c	126.74	Bra032574	A09-38.447	10

R2	RS2CL6075s	127.94	Bra010286	A08-12.873	10
R2	RGC1792c	129.51			10
R2	CD87b	129.91			10
R2	RS2CL4370s	130.56	Bra008072	A02-12.007	10
R2	RGC15769c	130.68	Bra020218	A02-4.587	10
R2	RGC6336c	130.71			10
R2	RGC1718c	131.61	Bra008241	A02-13.025	10
R2	CA95b	132.47			10
R2	RS2CL2371s	132.51	Bra008202	A02-12.773	10
R2	RGA1718s	132.9			10
R2	RGC2467c	133.04	Bra008184	A02-12.643	10
R2	RSS0477	133.16	Bra008172	A02-12.578	10
R2	RS2CL867s	133.81	Bra008152	A02-12.453	10
R2	RSS1885	134.86	Bra008246	A02-13.064	10
R2	RS2CL4248s	134.95	Bra003672	A07-17.663	10
R2	RGC15951c	135.1			10
R2	RGC6949c	135.1			10
R2	RS2CL2716s	135.95	Bra008353	A02-13.978	10
R2	RGC12310c	136.55	Bra035131	A07-25.592	10
R2	RS2CL1046s	137.05	Bra008407	A02-14.411	10
R2	RSS0822	137.36	Bra008097	A02-12.123	10
R2	Rs2CL2604s	137.46	Bra008476	A02-14.882	10
R2	RGC15477c	138.39			10
R2	RS2CL3703s	139.86	Bra008411	A02-14.449	10
R2	RGB5639c	142.35			10
R2	RGC16522c	144.97	Bra033987	A02-9.415	10
R2	RGC16935c	146.66			
R3	RS2CL3605s	0	Bra022587	A02-7.703	
R3	RSS1377	12.27	Bra005691	A03-0.143	11
R3	RGC21838c	12.38			11
R3	RGC6058c	13.93			11
R3	RSS3168	14.17	Bra005984	A03-1.499	11
R3	RS2CL683s	15.22	Bra009536	A10-15.947	11
R3	RS2CL3040s	16.78	Bra005719	A03-0.272	11
R3	RS2CL6139s	17.34	Bra005661	A03-0.034	11
R3	RS2CL3557s	18.05	Bra005998	A03-1.543	11
R3	RGC4640c	18.08			11
R3	CA05b	18.37			11
R3	RS2CL1245s	19.46	Bra005901	A03-1.117	11
R3	RGA93s	19.93			11
R3	RGC9028c	19.95			11
R3	RSS0816	20.27	Bra005929	A03-1.273	11
R3	RGC3942c	20.67			11
R3	RGA1553s	21.24			11
R3	RS2CL1424s	22.26	Bra006082	A03-1.905	11

R3	RS2CL6686s	22.71	Bra006120	A03-2.1	11
R3	RSS0486	23.17	Bra006103	A03-2.014	11
R3	RS2CL4186s	23.23	Bra006190	A03-2.432	11
R3	RSS2764	23.37	Bra006107	A03-2.046	11
R3	RGC9371c	23.92	Bra006129	A03-2.134	11
R3	RS2CL6155s	23.97	Bra006117	A03-2.088	11
R3	Z46819	24.27			11
R3	RS2CL2845s	24.88	Bra006237	A03-2.646	11
R3	RSS2403	25.17	Bra006215	A03-2.555	11
R3	RS2CL4457s	25.37	Bra006235	A03-2.637	11
R3	RGC5894c	25.43			11
R3	RGC5939c	25.43	Bra006227	A03-2.61	11
R3	RGB10851c	25.97	Bra006334	A03-3.059	11
R3	RGC22415c	25.97	Bra006241	A03-2.659	11
R3	RGC3069c	25.97	Bra006453	A03-3.559	11
R3	RSS2108	26.17	Bra006352	A03-3.142	11
R3	RSS2935	26.97	Bra006415	A03-3.412	11
R3	RS2CL4299s	27.74			11
R3	RSS0832	27.87	Bra020363	A02-5.384	11
R3	RS2CL6594s	28.31	Bra006454	A03-3.563	11
R3	RGB8990c	29.16	Bra006158	A03-2.282	11
R3	RSS2634	29.67	Bra006505	A03-3.825	11
R3	RGB12858c	29.83			11
R3	RSS1743	30.37	Bra020043	A02-3.757	11
R3	RSS2359	30.67	Bra006571	A03-4.097	11
R3	RSS3002	30.67	Bra006537	A03-3.959	11
R3	RSS3172	30.67	Bra006553	A03-4.014	11
R3	RSS1931	30.77	Bra006524	A03-3.906	11
R3	RSS0692	31.37	Bra006603	A03-4.225	11
R3	RSS1117	31.37	Bra006603	A03-4.225	11
R3	RSS0789	32.17	Bra020169	A02-4.355	11
R3	RS2CL1360s	32.23	Bra006618	A03-4.297	11
R3	RGC5762c	33	Bra006641	A03-4.421	11
R3	RSS1261	33.96	Bra006629	A03-4.354	11
R3	RSS0977	34.16	Bra006626	A03-4.334	11
R3	Rs2CL2367s	35.4	Bra011523	A01-1.834	11
R3	Rs2CL2374s	36.41	Bra006727	A03-4.81	11
R3	RS2CL2369s	36.41	Bra020353	A02-5.337	11
R3	RS2CL6944s	36.6	Bra006753	A03-4.912	11
R3	RS2CL1855s	36.78	Bra006731	A03-4.831	11
R3	RGC662c	36.95			11
R3	RSS2129	37.7	Bra006819	A03-5.188	11
R3	RGB5491c	39.04			11
R3	CZ09	39.21			11
R3	RGC15408c	39.83			11
R3	CA05a	40.92			11

R3	RGC11985c	41.21			11
R3	RGC1838c	41.21			11
R3	RGC2115c	41.21			11
R3	RS2CL3966s	41.97	Bra028924	A03-5.44	11
R3	RS2CL812s	42.64	Bra028957	A03-5.597	11
R3	RSS3288	42.74	Bra028922	A03-5.433	11
R3	RGC2693c	42.9	Bra029095	A03-6.336	11
R3	RGC4050c	42.9	Bra028971	A03-5.656	11
R3	RGC4388c	43.29			11
R3	RGC12111c	43.34			11
R3	RGC7232c	43.5			11
R3	RS2CL2195s	43.62	Bra029000	A03-5.808	11
R3	RGC23188c	44.01			11
R3	RSS1540	44.56			11
R3	RGC6897c	44.92	Bra028982	A03-5.707	11
R3	RS2CL5856s	46.17	Bra029091	A03-6.32	11
R3	RS2CL7274s	47.01	Bra029107	A03-6.403	11
R3	CA05aN	47.4			11
R3	RS2CL7810s	47.79	Bra028301	A10-4.801	11
R3	H36913	49.21			11
R3	RGC3691c	49.24	Bra022751	A03-6.825	11
R3	RS2CL1767s	50.07	Bra022816	A03-7.233	11
R3	RGB2849c	51.25	Bra029121	A03-6.464	11
R3	ACGM18	51.95			11
R3	RS2CL1552s	52.03	Bra022751	A03-6.825	11
R3	RSS2841	52.85	Bra022808	A03-7.176	11
R3	RS2CL1544s	53.12	Bra022815	A03-7.23	11
R3	RSS2981	53.56	Bra022870	A03-7.491	11
R3	RGC8225c	53.73			11
R3	RSS2675	53.96	Bra005564	A05-6.25	11
R3	CA25	54.26			11
R3	CB96	54.76			11
R3	RGC4948c	55.47			11
R3	RSS2362_a	55.57	Bra022931	A03-7.83	11
R3	RS2CL5646s	56.07	Bra022826	A03-7.294	11
R3	RSS2207	56.58	Bra022993	A03-8.148	11
R3	RSS2468	56.98	Bra022958	A03-7.951	11
R3	CB94	57.48			11
R3	RGA1712s	58.99	Bra023072	A03-8.539	11
R3	RS2CL5207s	59.06	Bra022981	A03-8.083	11
R3	RSS2225	59.3			11
R3	RGC10740c	59.51			11
R3	RSS2174	60.19	Bra023091	A03-8.657	11
R3	RGB3739c	60.33			11
R3	RS2CL3113s	61.11	Bra023084	A03-8.601	11
R3	RSS1494	61.19	Bra000011	A03-8.843	11

R3	RSS3166	61.59	Bra000064	A03-9.149	11
R3	RS2CL3855s	61.86	Bra000015	A03-8.872	11
R3	CF06	63.27			11
R3	RGB10862c	63.47			11
R3	RSS1258	64.87	Bra000102	A03-9.269	11
R3	RSS1041	65.37	Bra000102	A03-9.269	11
R3	RGC11016c	66.07			11
R3	CC42b	66.24			11
R3	RGC107c	68.06			11
R3	RGC21094c	68.06			11
R3	RSS0596	68.25			11
R3	RGA1793s	68.4			11
R3	RGC8038c	68.78	Bra000071	A03-9.168	11
R3	RS2CL4752s	69.57	Bra016951	A04-17.526	11
R3	RGC19961c	70.03	Bra000218	A03-9.967	11
R3	RS2CL7278s	70.4	Bra000211	A03-9.913	11
R3	RS2CL4292s	70.81	Bra000190	A03-9.82	11
R3	RGC6864c	71.16	Bra000222	A03-10.001	11
R3	RS2CL1086s	72.51	Bra000300	A03-10.438	11
R3	RS2CL5384s	72.63	Bra000311	A03-10.508	11
R3	RGC1367c	73.14	Bra000302	A03-10.45	11
R3	RSS2543	73.19	Bra000248	A03-10.152	11
R3	RSS0860	73.29	Bra000256	A03-10.215	11
R3	RS2CL2414s	73.89	Bra000339	A03-10.644	11
R3	RSS1018	74.26	Bra000283	A03-10.351	11
R3	RGC4056c	74.38	Bra037728	A04-18.143	11
R3	RGC5105c	74.79			11
R3	RGB37c	75.59			11
R3	RS2CL6133s	75.61	Bra000365	A03-10.767	11
R3	RSS0441	75.73	Bra022754	A03-6.853	11
R3	RS2CL5720s	76.79	Bra000393	A03-10.918	11
R3	RS2CL1398s	77.71	Bra000428	A03-11.078	11
R3	ACGM14	78.13			11
R3	RGC5561c	78.52			11
R3	RSS3186	78.72	Bra004950	A05-2.616	11
R3	RGC4447c	79.22			11
R3	RS2CL2857s	79.26	Bra000508	A03-11.519	11
R3	RS2CL4154s	79.62	Bra000571	A03-11.855	11
R3	RS2CL4178s	79.71	Bra000566	A03-11.831	11
R3	CC55	79.89			11
R3	RGC15812c	79.91			11
R3	RGC16357c	80.36			11
R3	RS2CL4585s	80.4	Bra000524	A03-11.595	11
R3	RS2CL4689s	81.12	Bra000616	A03-12.095	11
R3	RGC7054c	81.5	Bra000657	A03-12.346	11
R3	RGC6178c	81.75			11

R3	RGC11372c	81.97			11
R3	RGC13408c	81.97			11
R3	RSS1570	82.01	Bra000483	A03-11.354	11
R3	RS2CL4158s	82.28	Bra000625	A03-12.143	11
R3	RS2CL7432s	82.98	Bra000675	A03-12.421	11
R3	RGC11660c	83.31	Bra000786	A03-13.119	11
R3	RGC12742c	83.31			11
R3	RGC1543c	83.31			11
R3	RSS3173	83.56	Bra000651	A03-12.324	11
R3	RSS3827	84.06	Bra035769	A05-17.795	11
R3	RS2CL7533s	84.39	Bra000701	A03-12.565	11
R3	RS2CL1880s	84.55	Bra000713	A03-12.642	11
R3	CC34	84.56			11
R3	RGC24282c	85.01	Bra000673	A03-12.412	11
R3	RSS1161	85.63	Bra000646	A03-12.27	11
R3	RGC18238c	86.01			11
R3	RGC19084c	86.01	Bra000971	A03-14.205	11
R3	RGC5954c	86.01	Bra000852	A03-13.525	11
R3	RGC8553c	86.09	Bra000835	A03-13.432	11
R3	RS2CL3230s	86.71	Bra033138	A02-16.503	11
R3	RSS1549	87.26	Bra000787	A03-13.126	11
R3	RGC7922c	87.46	Bra000932	A03-14.018	11
R3	RSS0198	87.66	Bra000825	A03-13.392	11
R3	RS2CL3016s	88.22	Bra000842	A03-13.463	11
R3	CB53H	88.66			11
R3	RGB2123c	89.04	Bra022768	A03-6.948	11
R3	RS2CL3633s	89.42	Bra000940	A03-14.064	11
R3	RS2CL2659s	89.47	Bra000947	A03-14.109	11
R3	RS2CL2865s	89.84	Bra000884	A03-13.717	11
R3	RSS3644	90.06	Bra000895	A03-13.792	11
R3	RS2CL2009s	90.52	Bra000867	A03-13.605	11
R3	RGC11410c	90.76	Bra000868	A03-13.618	11
R3	RS2CL6931s	92.01	Bra000950	A03-14.121	11
R3	RGC8634c	93.98			
R3	RSS3933	97.66			
R3	RS2CL5949s	98.6	Bra037722	A04-18.176	
R3	RGC5211c	100.2			
R3	RS2CL6711s	101.22	Bra000388	A03-10.892	
R4	RSS1627	0	Bra023968	A03-28.583	
R4	RGC355c	4.7			
R4	CA83H	5.5			
R4	ACGM20	8.8			
R4	RS2CL966s	9.32	Bra033596	A06-25.01	
R4	CD72cH	9.6			
R4	RS2CL2691s	10.56	Bra010695	A08-15.25	

R4	RSS1586	11.7	Bra011552	A01-1.712	
R4	RS2CL5798s	11.98	Bra019211	A03-25.712	12
R4	RSS2559	12	Bra017742	A03-30.194	12
R4	RGC2155c	12.54	Bra024154	A03-27.312	12
R4	RGA1914s	12.79			12
R4	RGC4083c	13.03			12
R4	RS2CL2103s	13.47	Bra017761	A03-30.349	12
R4	RS2CL3614s	14.17	Bra011378	A01-2.455	12
R4	RS2CL4701s	14.25	Bra024062	A03-27.94	12
R4	RSS1544	14.4	Bra017782	A03-30.493	12
R4	RGB10057c	14.55	Bra017840	A03-30.918	12
R4	RGC14416c	14.55			12
R4	RS2CL4127s	14.75	Bra017687	A03-29.818	12
R4	RS2CL5450s	14.9	Bra037006	A03-29.039	12
R4	CD34aH	15			12
R4	RGA1981s	15.06			12
R4	RS2CL3293s	15.26	Bra037007	A03-29.037	12
R4	RS2CL5513s	15.43	Bra024057	A03-27.973	12
R4	RS2CL1187s	15.53	Bra009857	A06-18.337	12
R4	RS2CL1264s	15.6	Bra024012	A03-28.265	12
R4	RS2CL1262s	15.72	Bra018929	A06-1.213	12
R4	RS2CL6586s	15.76	Bra024020	A03-28.226	12
R4	RS2CL5563s	15.87	Bra024058	A03-27.968	12
R4	RGC1195c	16.09			12
R4	RGC15741c	16.09			12
R4	RGB2673c	16.09	Bra017646	A03-29.563	12
R4	RGC9198c	16.09			12
R4	RS2CL1215s	16.1	Bra024071	A03-27.885	12
R4	CC00	16.2			12
R4	CC23	16.4			12
R4	RS2CL1831s	16.48	Bra024153	A03-27.325	12
R4	RS2CL3995s	16.98	Bra024183	A03-27.1	12
R4	S281N	17.2			12
R4	RS2CL1816s	17.34	Bra019196	A03-25.783	12
R4	RS2CL4170s	17.7	Bra024230	A03-26.799	12
R4	RS2CL842s	17.86	Bra019045	A03-26.608	12
R4	RSS0234	17.9	Bra019039	A03-26.636	12
R4	RS2CL7584s	17.95	Bra019144	A03-26.114	12
R4	RSS3716	18.1	Bra024209	A03-26.907	12
R4	RGC22968c	18.34			12
R4	RGC45c	18.34			12
R4	RGC9298c	18.34	Bra019069	A03-26.503	12
R4	RS2CL4241s	18.38	Bra019054	A03-26.578	12
R4	RSS2439	18.9	Bra019210	A03-25.718	12
R4	RSS3649	19.4	Bra019250	A03-25.478	12
R4	RS2CL4850s	19.57	Bra019267	A03-25.395	12

R4	RSS2708	20.24	Bra038769	A03-24.199	12
R4	RS2CL5615s	20.76	Bra019316	A03-25.051	12
R4	RGC9137c	21.04	Bra033606	A06-24.971	12
R4	RGC7718c	21.77	Bra038784	A03-24.083	12
R4	RGC1288c	21.99			12
R4	RGC32871c	21.99	Bra019372	A03-24.637	12
R4	RGC4257c	21.99			12
R4	RS2CL8540s	22.23	Bra021503	A03-24.5	12
R4	RSS2783	22.26	Bra012523	A03-23.847	12
R4	RSS2409	22.76	Bra012558	A03-23.578	12
R4	RSS2474	22.76	Bra012558	A03-23.578	12
R4	RSS0031	23.16	Bra012581	A03-23.458	12
R4	RS2CL5025s	23.45	Bra038761	A03-24.251	12
R4	RGB16843c	23.8			12
R4	RS2CL7051s	23.81	Bra038792	A03-24.053	12
R4	RSS0858	23.99	Bra012600	A03-23.315	12
R4	RGC3995c	24.04			12
R4	RS2CL4212s	24.6	Bra012563	A03-23.557	12
R4	RSS3799	24.91	Bra012624	A03-23.141	12
R4	RS2CL3610s	25.64	Bra012661	A03-22.899	12
R4	RSS3591	25.75	Bra038503	A01-10.628	12
R4	RGB5549c	25.85	Bra012621	A03-23.195	12
R4	RSS2846	26.7	Bra012600	A03-23.315	12
R4	RS2CL7588s	26.76	Bra012619	A03-23.204	12
R4	RGC1455c	27.17			12
R4	RS2CL4804s	27.78	Bra012663	A03-22.895	12
R4	RS2CL3599s	28.31	Bra012684	A03-22.775	12
R4	RS2CL4311s	28.72	Bra012671	A03-22.859	12
R4	CB92	28.89			12
R4	RS2CL6294s	28.95	Bra012680	A03-22.8	12
R4	RS2CL2726s	29.25	Bra012722	A03-22.587	12
R4	RGA173s	29.73	Bra012755	A03-22.432	12
R4	RS2CL4162s	30.03	Bra012752	A03-22.454	12
R4	RSS1249_a	30.36	Bra038895	A01-15.621	12
R4	RS2CL3606s	30.37	Bra012807	A03-22.151	12
R4	RSS3924	30.9	Bra012857	A03-21.921	12
R4	RS2CL7525s	30.95	Bra012848	A03-21.952	12
R4	RS2CL2655s	31.06	Bra012867	A03-21.885	12
R4	RS2CL2361s	31.12	Bra012861	A03-21.912	12
R4	Rs2CL2299s	31.5	Bra012885	A03-21.764	12
R4	RGC21796c	31.84			12
R4	RGC11816c	32.14			12
R4	RGC5691c	32.14	Bra012781	A03-22.324	12
R4	RS2CL3326s	32.59	Bra012978	A03-21.259	12
R4	CA87	32.86			12
R4	RS2CL4218s	32.9	Bra012951	A03-21.388	12

R4	RS2CL7603s	33.33	Bra012993	A03-21.186	12
R4	RS2CL7351s	33.61	Bra012989	A03-21.212	12
R4	RSS1971	33.82	Bra012946	A03-21.407	12
R4	RS2CL6125s	34.15	Bra009722	A06-17.463	
R4	RGB15964c	34.42			
R4	RGC1115c	34.88	Bra012996	A03-21.174	
R4	RGC21614c	34.88			
R4	RGC9615c	34.88			
R4	RSS2202	35.03	Bra009703	A06-17.266	13
R4	RS2CL5759s	35.06	Bra009698	A06-17.235	13
R4	RGC17618c	35.16	Bra013017	A03-20.999	13
R4	RSS0067	35.99	Bra009671	A06-17.087	13
R4	RSS0178	36.89	Bra009758	A06-17.672	13
R4	RSS1636	37.29	Bra009799	A06-17.962	13
R4	RS2CL1832s	39.19	Bra009858	A06-18.342	13
R4	CA24	41.29			13
R4	RGC21459c	42.16	Bra009833	A06-18.202	13
R4	RS2CL1097s	42.88	Bra009895	A06-18.572	13
R4	RS2CL5633s	43.92	Bra009965	A06-18.971	13
R4	RGC13382c	44.44	Bra010028	A06-19.332	13
R4	RGC17631c	44.44	Bra037432	A06-20.045	13
R4	RGC2699c	44.44	Bra038947	A06-18.744	13
R4	RS2CL4419s	45.05	Bra037428	A06-20.022	13
R4	RGC4505c	45.62			13
R4	RS2CL3258s	46.01	Bra037467	A06-20.233	13
R4	RGA2287s	46.24			13
R4	RS2CL5222s	46.57	Bra037461	A06-20.21	13
R4	RS2CL2500s	46.6	Bra037509	A06-20.404	13
R4	RS2CL3360s	46.73	Bra037480	A06-20.282	13
R4	CF16	47			13
R4	RS2CL6318s	47.2	Bra037511	A06-20.413	13
R4	RGC18965c	47.36	Bra025307	A06-21.194	13
R4	RS2CL2926s	47.48	Bra037506	A06-20.386	13
R4	RS2CL6239s	47.77	Bra037492	A06-20.341	13
R4	RSS2705	47.83	Bra036216	A09-1.911	13
R4	RSS1127	48.13	Bra025383	A06-20.787	13
R4	RSS3772	48.43			13
R4	RGB3765c	48.58	Bra025314	A06-21.167	13
R4	RS2CL7398s	49.31	Bra037490	A06-20.331	13
R4	RSS0396	49.97	Bra025248	A06-21.51	13
R4	RSS0839	49.97	Bra032981	A02-21.97	13
R4	RSS1418	50.35			13
R4	RSS3863	50.35	Bra025260	A06-21.46	13
R4	RGC21483c	50.8	Bra030338	A04-9.388	13
R4	RSS0936	50.82	Bra025233	A06-21.561	13
R4	RSS1675	51.12			13

R4	RS2CL2021s	52.06	Bra025265	A06-21.422	13
R4	RGB25198c	52.36	Bra037518	A06-20.438	13
R4	RSS0033	53.1	Bra025181	A06-21.843	13
R4	RS2CL5851s	53.33	Bra025284	A06-21.324	13
R4	RGC7341c	53.7	Bra025283	A06-21.331	13
R4	RS2CL4350s	53.79	Bra025239	A06-21.541	13
R4	CB05	53.92			13
R4	RGC2885c	54.63			13
R4	RS2CL6990s	54.9	Bra025201	A06-21.737	13
R4	RGC10051c	55.53	Bra025179	A06-21.85	13
R4	CD11aH	56.72			13
R4	RS2CL2925s	56.83	Bra025140	A06-22.193	13
R4	RGC17385c	57.02			13
R4	RSS3637	57.69	Bra037460	A06-20.208	13
R4	Rs2CL2178s	58.46	Bra024808	A06-22.493	13
R4	RGC2356c	59.5	Bra030204	A04-10.397	13
R4	RSS3616	59.85	Bra033693	A06-24.531	13
R4	RS2CL5879s	60.87	Bra024887	A06-23.042	13
R4	RS2CL5717s	61.19	Bra024854	A06-22.787	13
R4	RGB4922c	61.51	Bra022133	A02-19.284	13
R4	RGC9577c	62.06			13
R4	Z30817	62.64			13
R4	RS2CL7473s	62.89	Bra024927	A06-23.216	13
R4	RGC10583c	63.35	Bra024908	A06-23.149	13
R4	RGC2146c	63.35	Bra024868	A06-22.918	13
R4	RGC3356c	63.35	Bra024849	A06-22.746	13
R4	CB39	64.56			13
R4	RS2CL1232s	64.61	Bra024975	A06-23.428	13
R4	RSS0369	65.4	Bra025078	A06-23.966	13
R4	CA83	66			13
R4	CB86	66.6			13
R4	RSS3516	68.1	Bra033680	A06-24.603	13
R4	RSS1061	69.2	Bra028126	A04-6.316	13
R4	RSS0008	69.5			13
R4	RSS3919	69.6			13
R4	Rs2CL1980s	70.35	Bra040776	Scaffold000256-0.021	13
R4	RS2CL5596s	71.35	Bra002165	A10-11.22	13
R4	CC19N	71.79			13
R4	RGC11405c	72.03	Bra025106	A06-24.124	13
R4	RS2CL1344s	72.44	Bra039506	Scaffold000167-0.011	13
R4	RGC3002c	72.8	Bra025067	A06-23.91	13
R4	RGC21423c	73.12			13
R4	RS2CL4378s	73.31	Bra033712	A06-24.419	13
R4	CD72b	73.38			13
R4	RGA1767s	73.47			13
R4	RS2CL6532s	73.68	Bra027532	A09-11.586	13

R4	RS2CL738s	74.44	Bra033654	A06-24.728	13
R4	RS2CL7671s	74.49	Bra033667	A06-24.668	13
R4	RS2CL4635s	74.62	Bra033656	A06-24.722	13
R4	Rs2CL2052s	74.78	Bra033749	A06-24.209	13
R4	RGC10110c	75.04			13
R4	RGB10350c	75.04	Bra035359	Scaffold000104-0.158	13
R4	RGC13794c	75.04			13
R4	RGC2328c	75.04	Bra033663	A06-24.689	13
R4	RGC2715c	75.04	Bra033700	A06-24.484	13
R4	RGC3886c	75.04			
R4	RGC7139c	75.04	Bra035365	Scaffold000104-0.211	
R4	RGC8082c	75.04			
R4	RS2CL713s	75.22	Bra034466	Scaffold000096-0.054	
R4	RS2CL6965s	75.46	Bra017881	A03-31.278	
R4	RS2CL2947s	75.76	Bra035354	Scaffold000104-0.114	
R4	RS2CL2388s	76.25	Bra038153	Scaffold000140-0.14	
R4	RGC9337c	76.67	Bra025617	A04-7.83	14
R4	RGC3045c	77.19	Bra028125	A04-6.306	14
R4	RGC8922c	77.19			14
R4	RS2CL6195s	77.6	Bra028124	A04-6.291	14
R4	RS2CL5121s	78.06	Bra028241	A04-7.357	14
R4	RSS3739	78.06	Bra025454	A04-9.016	14
R4	RGC15937c	78.59			14
R4	RGC2894c	78.59	Bra025507	A04-8.641	14
R4	RGC364c	78.59			14
R4	RGC6719c	78.59			14
R4	RGC6966c	78.59	Bra004818	A05-1.944	14
R4	RGB7858c	78.59			14
R4	RGC4610c	78.64			14
R4	RS2CL4033s	79.02	Bra025654	A04-7.59	14
R4	CD63H	79.42			14
R4	RS2CL5842s	79.52	Bra025636	A04-7.731	14
R4	RGC12174c	79.74			14
R4	RSS2079	80.1	Bra025446	A04-9.052	14
R4	RGB11177c	80.11	Bra025599	A04-7.937	14
R4	RSS0347	80.49	Bra030308	A04-9.579	14
R4	RS2CL3470s	80.8	Bra025599	A04-7.937	14
R4	RGC2472c	81.04	Bra028221	A04-7.146	14
R4	Rs2CL2508s	81.4	Bra025600	A04-7.912	14
R4	CC15	81.96			14
R4	OPE01	82.76			14
R4	RSS1284	83.86			14
R4	RS2CL1961s	84.09	Bra025446	A04-9.052	14
R4	CC89	84.65			14
R4	RS2CL4014s	85.38	Bra030249	A04-9.995	14
R4	RSS3847	85.63	Bra039195	A09-33.1	14

R4	RGC15702c	85.89	Bra021660	A04-13.79	14
R4	RSS1710	86.62			14
R4	RSS3515	87.12	Bra039237	A09-32.854	14
R4	RS2CL3313s	88.51	Bra032174	A04-10.705	14
R4	RGC24823c	88.69			14
R4	RGC6716c	88.69	Bra021783	A04-14.543	14
R4	RSS3297	89.19	Bra032055	A04-11.576	14
R4	RGC32186c	89.65	Bra032035	A04-11.719	14
R4	RGB520c	90.52	Bra035700	A04-12.801	14
R4	CA98	90.86			14
R4	RGB14019c	91.07	Bra021831	A04-14.868	14
R4	RSS3234	91.65	Bra034298	A04-12.102	14
R4	RSS1064	91.85	Bra034298	A04-12.102	14
R4	RGC27467c	92.02			14
R4	RGC2779c	92.02			14
R4	RGC5481c	92.02	Bra034373	A04-12.569	14
R4	RGC206c	92.28			14
R4	RGC2343c	92.28	Bra021691	A04-13.976	14
R4	RGC2932c	92.28	Bra021779	A04-14.519	14
R4	RS2CL6948s	92.3	Bra021743	A04-14.314	14
R4	RSS2397	92.42	Bra034308	A04-12.186	14
R4	RS2CL4453s	93.08	Bra034290	A04-12.06	14
R4	RGC13764c	93.44	Bra021941	A04-15.403	14
R4	RS2CL3226s	94.1	Bra011936	A07-13.399	14
R4	RGC139c	94.27			14
R4	RSS0625	94.3			14
R4	RSS1628	94.3	Bra034346	A04-12.412	14
R4	RSS2491	94.3	Bra034371	A04-12.555	14
R4	RSS3741	94.3	Bra034368	A04-12.551	14
R4	RGC1537c	94.36	Bra021855	A04-14.978	14
R4	RGC6353c	94.36			14
R4	RS2CL6090s	94.57	Bra018371	A05-7.817	14
R4	RS2CL2598s	94.76	Bra003592	A07-17.242	14
R4	RS2CL6646s	94.86	Bra034325	A04-12.27	14
R4	RS2CL5488s	94.98	Bra034309	A04-12.191	14
R4	RS2CL8917s	95.07	Bra039995	A04-13.367	14
R4	Rs2CL1743s	95.16	Bra021685	A04-13.948	14
R4	RSS0393	95.22	Bra022772	A03-6.981	14
R4	RSS0341	95.82	Bra005031	A05-3.073	14
R4	RSS2667	95.82	Bra021715	A04-14.165	14
R4	CC43b	96.52			14
R4	RSS3109	97.62	Bra021723	A04-14.217	14
R4	CA15	99.42			14
R4	RS2CL3689s	100.43	Bra021688	A04-13.966	14
R4	RGA1556s	100.61	Bra021821	A04-14.809	14
R4	RS2CL2599s	100.81	Bra034343	A04-12.404	14

R4	RSS2245	101.28	Bra021654	A04-13.771	14
R4	RS2CL2069s	101.29	Bra021837	A04-14.898	14
R4	RS2CL6037s	101.56	Bra022937	A03-7.867	14
R4	RS2CL4491s	102.02	Bra039965	A04-13.208	14
R4	RGC16154c	102.46	Bra021870	A04-15.056	14
R4	RGC19350c	102.46			14
R4	RGC9063c	102.46			14
R4	RS2CL3466s	102.69	Bra021850	A04-14.966	14
R4	Rs2CL2383s	103.26	Bra021896	A04-15.191	14
R4	RSS3247	103.38	Bra021634	A04-13.669	14
R4	RS2CL3308s	103.73	Bra021912	A04-15.265	14
R4	RS2CL2278s	104.72	Bra017321	A04-15.49	14
R4	RS2CL8405s	105.31	Bra017292	A04-15.617	14
R4	CC56a	105.59			14
R4	RS2CL6277s	106.41	Bra017241	A04-15.908	14
R4	RS2CL3794s	106.71	Bra017215	A04-16.103	14
R4	CD24	107.49			14
R4	RS2CL2462s	107.89	Bra017113	A04-16.659	14
R4	RS2CL4251s	107.91	Bra017107	A04-16.684	14
R4	RGC17710c	108.16	Bra017130	A04-16.562	14
R4	RGC16310c	108.21	Bra017139	A04-16.472	14
R4	RGC2194c	108.21	Bra017307	A04-15.538	14
R4	RGC2833c	108.21			14
R4	RGC4391c	108.21	Bra017245	A04-15.874	14
R4	RGC5031c	108.21			14
R4	RGC6220c	108.21			14
R4	RS2CL2730s	108.25	Bra017135	A04-16.526	14
R4	RS2CL6009s	108.46	Bra017109	A04-16.676	14
R4	RS2CL1897s	108.78	Bra017126	A04-16.581	14
R4	RS2CL5094s	109.2	Bra017008	A04-17.272	14
R4	RGC9763c	109.49	Bra017297	A04-15.601	14
R4	RSS3305	109.57	Bra016897	A04-17.815	14
R4	RGC2574c	109.94			14
R4	RS2CL1636s	110.31	Bra016989	A04-17.346	14
R4	RSS3500	111.08	Bra017018	A04-17.213	14
R4	RGB17458c	111.59			14
R4	RS2CL5146s	112.69	Bra016946	A04-17.566	14
R4	RGC9723c	113.08			14
R4	RGC12230c	113.41	Bra016976	A04-17.406	14
R4	RS2CL1956s	114.11	Bra016860	A04-17.969	14
R4	RS2CL5598s	114.15			14
R4	RS2CL2277s	114.21	Bra016862	A04-17.966	14
R4	RS2CL8851s	114.3			14
R4	RS2CL7217s	115.77	Bra037664	A04-18.379	14
R4	RGC2987c	116.69	Bra004887	A05-2.305	14
R4	RSS1902	116.96	Bra016981	A04-17.384	14

R4	RSS2415	117.66	Bra017005	A04-17.282	14
R4	RS2CL1437s	118.37	Bra039291	A04-18.852	14
R4	RGC11873c	118.75	Bra037647	A04-18.448	14
R4	RSS2929	119.67	Bra016935	A04-17.638	14
R4	RGC6749c	120.83			14
R4	RSS3668	120.97	Bra016891	A04-17.839	14
R4	RS2CL2439s	121.57	Bra011819	A01-0.386	14
R4	RSS0993	122.28	Bra037715	A04-18.206	14
R4	RGA1715s	123.77			14
R4	RSS1828	124.02	Bra040312	A04-18.654	14
R4	RSS3914	124.62	Bra039338	A04-18.676	14
R4	RGC4219c	125			
R4	RS2CL983s	125.63	Bra032770	A04-5.073	15
R4	RS2CL3277s	126.29	Bra007025	A09-28.395	15
R4	RGC14440c	126.65			15
R4	RS2CL7183s	126.95	Bra014828	A04-3.435	15
R4	RGC14398c	127.38			15
R4	OPT09a	127.47			15
R4	RS2CL4168s	127.88	Bra019639	A04-4.163	15
R4	RS2CL1373s	128.17	Bra040688	A04-4.004	15
R4	RGC7805c	128.8			15
R4	RS2CL5077s	128.82	Bra022419	A05-18.536	15
R4	RS2CL2423s	129.15	Bra014841	A04-3.55	15
R4	RSS0375	129.25			15
R4	RS2CL2532s	129.29	Bra014819	A04-3.379	15
R4	RS2CL7410s	129.43			15
R4	RS2CL6216s	129.9	Bra014719	A04-2.505	15
R4	RSS2818	130.28	Bra016728	A08-18.916	15
R4	RGC12577c	130.65			15
R4	RGC12869c	130.65			15
R4	RGC2383c	130.65			15
R4	RGC6866c	130.65			15
R4	RGC8508c	130.65			15
R4	RS2CL1846s	130.8	Bra014808	A04-3.305	15
R4	CA35b	130.81			15
R4	RS2CL2717s	131.12	Bra014721	A04-2.513	15
R4	RSS3394	131.17	Bra033462	A04-4.678	15
R4	RSS3897	131.17	Bra033420	A04-4.387	15
R4	RS2CL1003s	131.21	Bra014681	A04-2.202	15
R4	RS2CL1386s	131.67	Bra014741	A04-2.736	15
R4	RS2CL4891s	131.97	Bra015981	A07-23.242	15
R4	RSS0649	132.1	Bra039808	A04-3.76	15
R4	RSS0371	132.6	Bra003163	A07-14.916	15
R4	RS2CL4267s	133.5	Bra014601	A04-1.697	15
R4	RSS2126	134.1	Bra007274	A09-29.825	15
R4	RSS0912	134.5	Bra014427	A04-0.589	15

R4	RS2CL2291s	134.57	Bra014598	A04-1.682	15
R4	RS2CL2308s	135.38	Bra021448	A04-19.23	15
R4	RGC151c	135.73	Bra014543	A04-1.327	15
R4	RGC16913c	135.73			15
R4	RGC4940c	135.73	Bra014385	A04-0.272	15
R4	RGC7607c	135.73	Bra040433	A04-0.091	15
R4	CC79	135.87			15
R4	RS2CL5873s	136.57	Bra040435	A04-0.085	15
R4	RSS1537	136.72	Bra040440	A04-0.047	15
R4	RGC10060c	137.23	Bra014508	A04-1.093	15
R4	RS2CL2821s	137.64	Bra014410	A04-0.381	15
R4	OPT16a	138.02			15
R4	RGA1700s	138.08			15
R4	Rs2CL2077s	138.8	Bra040448	A04-0.016	15
R4	RS2CL1358s	140.21	Bra014406	A04-0.363	15
R4	RGC2546c	141.22	Bra014619	A04-1.809	15
R4	RGA125s	142.16			
R5	RSS3015	0	Bra001221	A03-15.42	16
R5	RSS3061	0.3	Bra000989	A03-14.308	16
R5	RSS1661	0.8	Bra040292	A01-25.62	16
R5	RSS3244	0.8	Bra001137	A03-14.944	16
R5	RSS3245	0.8	Bra001159	A03-15.037	16
R5	RSS0292	1.1	Bra039144	A05-25.141	16
R5	CD34bH	1.4			16
R5	RSS0017	1.6	Bra029787	A05-23.003	16
R5	CA74H	2			16
R5	RSS1674	2.1	Bra001406	A03-16.249	16
R5	CD71	2.5			16
R5	N97067	2.8			16
R5	Rs2CL1745s	2.81	Bra001526	A03-16.881	16
R5	CD20	3.1			16
R5	RSS0662_a	4	Bra039342	Scaffold000164-0.012	16
R5	RSS1054	4.2	Bra030394	A05-10.94	16
R5	RSS1457	4.6	Bra001664	A03-17.768	16
R5	RS2CL8651s	4.64	Bra001194	A03-15.26	16
R5	RSS3450	5	Bra001530	A03-16.899	16
R5	RSS0662_b	5.5	Bra039342	Scaffold000164-0.012	16
R5	RS2CL7919s	5.86	Bra001153	A03-15.021	16
R5	RS2CL6011s	6.39	Bra001186	A03-15.218	16
R5	CC37bH	6.5			16
R5	RGC1111c	7.06	Bra001418	A03-16.306	16
R5	RGC1381c	7.06	Bra034869	A05-22.536	16
R5	RGC21684c	7.06	Bra001229	A03-15.453	16
R5	RGB964c	7.06			16
R5	RS2CL5773s	7.17	Bra001040	A03-14.519	16

R5	RSS2263	7.2	Bra001813	A03-18.628	16
R5	RS2CL2901s	7.31	Bra001065	A03-14.597	16
R5	RS2CL3894s	7.44	Bra001057	A03-14.574	16
R5	RSS1183	7.9	Bra001809	A03-18.615	16
R5	RS2CL4044s	7.95	Bra001057	A03-14.574	16
R5	RS2CL5289s	8.81	Bra001411	A03-16.268	16
R5	Rs2CL1517s	9.19	Bra001418	A03-16.306	16
R5	RSS2534	9.7	Bra001849	A03-18.877	16
R5	RS2CL6910s	10.05	Bra001421	A03-16.318	16
R5	RS2CL2032s	11.89	Bra001505	A03-16.739	16
R5	RSS0922	11.9	Bra028345	A01-18.227	16
R5	Pf	12.7			16
R5	RGB2724c	12.95			16
R5	RS2CL4777s	13.68	Bra001661	A03-17.755	16
R5	RGC20584c	13.86			16
R5	RS2CL7144s	14.51	Bra001600	A03-17.321	16
R5	RSS1319	15	Bra013224	A03-19.798	16
R5	RSS3682	15.7	Bra013241	A03-19.717	16
R5	Rs2CL2150s	16.48	Bra001817	A03-18.65	16
R5	RSS1795	17.9	Bra013215	A03-19.85	16
R5	Rs2CL2167s	18.29	Bra001952	A03-19.59	16
R5	RSS2289	18.6	Bra019594	A06-13.589	17
R5	RSS0310	18.8	Bra019594	A06-13.589	17
R5	RSS2078_a	19.7	Bra019616	A06-13.744	17
R5	RSS2459	20.3	Bra010084	A06-14.267	17
R5	RSS1155	20.5	Bra010053	A06-13.89	17
R5	RS2CL2345s	20.91	Bra013218	A03-19.828	17
R5	Rs2CL1650s	21.12	Bra013219	A03-19.825	17
R5	RSS0255	21.5	Bra010123	A06-14.547	17
R5	RS2CL4383s	21.86	Bra029925	A01-14.392	17
R5	RS2CL4098s	22.38	Bra019582	A06-13.458	17
R5	RGA1775s	22.64			17
R5	RSS1293	22.7	Bra024256	A06-15.187	17
R5	RSS2428	22.7	Bra024255	A06-15.184	17
R5	RSS1421	23	Bra024270	A06-15.269	17
R5	RSS2179	23.4	Bra024286	A06-15.357	17
R5	RSS1445	23.6	Bra024295	A06-15.388	17
R5	RSS0020	24.1	Bra024331	A06-15.627	17
R5	RS2CL6459s	24.65	Bra038615	A06-14.804	17
R5	RSS0640	25.1	Bra038639	A06-14.926	17
R5	RS2CL1107s	25.48	Bra038614	A06-14.8	17
R5	RS2CL2656s	27.64	Bra024274	A06-15.279	17
R5	RSS3462	27.7	Bra013229	A03-19.775	17
R5	RS2CL5063s	28.95	Bra024326	A06-15.606	17
R5	RS2CL7234s	29.1	Bra024320	A06-15.544	17
R5	RS2CL7712s	29.4	Bra024325	A06-15.596	17

R5	RS2CL4145s	29.82	Bra024416	A06-16.264	17
R5	RS2CL7340s	30.09			17
R5	RGC6520c	30.12			17
R5	RS2CL3444s	30.63	Bra024389	A06-16.052	17
R5	RGC54c	31.11	Bra024345	A06-15.701	17
R5	RSS0173	31.6	Bra040870	Scaffold000289-0.024	17
R5	RSS1336	31.6	Bra002086	A07-1.97	17
R5	RS2CL5210s	31.68	Bra013051	A03-20.791	17
R5	RSS0575	31.7	Bra040870	Scaffold000289-0.024	17
R5	RSS0125	31.8	Bra009653	A06-17.017	17
R5	RS2CL6408s	31.85	Bra009666	A06-17.065	17
R5	RGC11501c	32.07			17
R5	RGC1596c	32.07	Bra024337	A06-15.656	17
R5	RGC15962c	32.07	Bra024442	A06-16.402	17
R5	RSS2665	32.2	Bra002021	A07-2.493	17
R5	Rs2CL1966s	32.35	Bra024511	A06-16.942	17
R5	RSS2209	32.6	Bra013048	A03-20.805	18
R5	RGC8188c	33.13			18
R5	RSS3635	33.8	Bra024325	A06-15.596	18
R5	RGC1147c	34.2			18
R5	RSS0647	34.8	Bra013161	A03-20.149	18
R5	RGC11447c	35.27	Bra013141	A03-20.25	18
R5	RGB3806c	35.27			18
R5	RSS0380	36	Bra013186	A03-19.999	18
R5	RS2CL1106s	36.01	Bra018068	A06-9.706	
R5	RGB8510c	36.65			
R5	RSS1774	37.1			
R5	RS2CL804s	37.28	Bra014361	A08-1.409	
R5	RSS0192	37.5	Bra031790	A09-36.497	
R5	RSS3177	39.4	Bra030365	A05-10.72	19
R5	RSS2660	40.2	Bra030366	A05-10.722	19
R5	RSS0609	40.3	Bra030366	A05-10.722	19
R5	RS2CL8976s	40.42	Bra040815	Scaffold000267-0.035	19
R5	RGB2659c	40.99			19
R5	R30624b	41.1			19
R5	RGC8374c	41.67	Bra030433	A05-11.272	19
R5	RSS1763_a	42.5	Bra014663	A04-2.103	19
R5	CC67bH	42.8			19
R5	RS2CL5232s	42.8	Bra030429	A05-11.246	19
R5	RSS0485	43.5	Bra039648	Scaffold000172-0.212	19
R5	RGA91s	43.58	Bra014251	A08-2.196	19
R5	RSS0430_a	43.8			19
R5	RGC12639c	44.01	Bra032247	A05-12.493	19
R5	RGC4480c	44.3			19
R5	CD72bH	45.1			19
R5	RGC9986c	45.6			19

R5	RSS3072	46.2	Bra040663	A05-13.239	19
R5	RGC17999c	46.55	Bra007713	A09-32.022	20
R5	RGA1661s	47.18	Bra038401	A09-15.313	20
R5	RGC9094c	47.89	Bra007614	A09-31.562	20
R5	RSS2310	47.9	Bra007762	A09-32.293	20
R5	Rs2CL2263s	49.11	Bra032210	A05-13.009	20
R5	RSS3250	49.8	Bra007669	A09-31.852	20
R5	RS2CL4099s	50.48	Bra007758	A09-32.259	20
R5	RSS1955	50.6	Bra003487	A07-16.727	20
R5	RS2CL3592s	51.19	Bra007736	A09-32.146	20
R5	RSS3666	52.1	Bra019756	A06-4.607	20
R5	RGB19108c	52.6			20
R5	RGC347c	52.6			20
R5	RGC8606c	52.6	Bra007563	A09-31.322	20
R5	RS2CL3943s	53.18	Bra007677	A09-31.882	20
R5	CA64	54.1			20
R5	RS2CL6006s	54.22	Bra007609	A09-31.525	20
R5	RSS1195	56.1	Bra007514	A09-31.097	20
R5	RGC19763c	56.37	Bra014532	A04-1.266	20
R5	RSS2188	56.5	Bra007494	A09-31.008	20
R5	RSS0574	57.4	Bra007432	A09-30.691	20
R5	RSS2974	57.4	Bra007423	A09-30.652	20
R5	Rs2CL2589s	58.74	Bra007067	A09-28.649	20
R5	RSS1763	59.2	Bra014663	A04-2.103	20
R5	RGC6470c	59.95			20
R5	RSS0154	60.3	Bra003314	A07-15.839	20
R5	RS2CL4004s	60.33	Bra007154	A09-29.113	20
R5	RSS2249	61.2	Bra007276	A09-29.832	20
R5	RSS0193	61.3	Bra007276	A09-29.832	20
R5	CD23	61.8			20
R5	CC61H	62.8			20
R5	OPR17	63.3			20
R5	RGC4546c	63.81	Bra007323	A09-30.1	20
R5	RSS0866	64.18	Bra007221	A09-29.572	20
R5	RS2CL7551s	64.45	Bra007273	A09-29.823	20
R5	CA66H	64.85			20
R5	RGC5978c	64.88	Bra007177	A09-29.276	20
R5	RGB1306c	65.46			20
R5	RSS0068	65.49	Bra007218	A09-29.562	20
R5	RGC12711c	65.74			20
R5	RS2CL1241s	65.81	Bra007173	A09-29.242	20
R5	ACGM16	65.98			20
R5	RS2CL3809s	66.03	Bra003204	A07-15.142	20
R5	RGC16642c	66.43			20
R5	CD81H	66.83			20
R5	RGA1790s	67.11			20

R5	RGC21710c	67.56			20
R5	CD78	67.87			20
R5	RGC5664c	67.98			20
R5	RS2CL1583s	68.25	Bra007027	A09-28.413	20
R5	RGC948c	68.41	Bra019446	A06-11.868	20
R5	RSS2089	69.01	Bra007053	A09-28.574	20
R5	RGC439c	69.14			20
R5	RS2CL1103s	69.16			20
R5	RGC14010c	69.74			20
R5	RS2CL4593s	70.03	Bra006989	A09-28.219	20
R5	RSS2970	70.12			20
R5	RSS0769	70.32	Bra007002	A09-28.278	20
R5	RS2CL2778s	70.36	Bra019447	A06-11.882	20
R5	RS2CL4036s	70.75	Bra006965	A09-28.077	20
R5	RSS0295	71.17	Bra019445	A06-11.858	20
R5	RSS3324	71.17	Bra006938	A09-27.861	20
R5	RS2CL7602s	71.33	Bra019491	A06-12.561	
R5	RS2CL3400s	71.45	Bra031740	A09-36.737	21
R5	RSS1246	71.94	Bra019447	A06-11.882	21
R5	RSS3089	73.14	Bra018096	A06-9.921	21
R5	RSS3043	73.74	Bra031705	A09-36.905	21
R5	RSS2679	73.84	Bra031722	A09-36.808	21
R5	RSS2831	74.04	Bra031747	A09-36.704	21
R5	RS2CL5573s	75.09	Bra026884	A09-35.862	21
R5	RS2CL8653s	75.77	Bra031965	A05-24.585	21
R5	RGB9734c	77.27			21
R5	RGB299c	77.57	Bra031693	A09-36.971	21
R5	RGC9453c	77.74	Bra031684	A09-37.013	21
R5	RSS0810	77.98	Bra026779	A09-35.405	21
R5	RGB12346c	78.96			21
R5	CD32	79			21
R5	Rs2CL2329s	79.26	Bra031632	A09-37.241	21
R5	RSS2090	79.62	Bra015511	A10-0.848	21
R5	RGA1887s	79.93	Bra031575	A09-37.512	21
R5	RS2CL7437s	80.03	Bra020251	A02-4.844	21
R5	CB25aN	80.23			21
R5	RS2CL3418s	80.53	Bra031558	A09-37.593	21
R5	RS2CL6413s	80.74	Bra031573	A09-37.529	21
R5	RS2CL4294s	81.65	Bra032424	A09-37.782	21
R5	RSS0606	81.69	Bra022183	A05-19.945	21
R5	RGC5898c	81.86			21
R5	RSS3723	81.9	Bra015308	A10-2.088	21
R5	N95848	82.6			21
R5	RGC6482c	82.83	Bra032468	A09-37.964	21
R5	RS2CL5033s	82.88	Bra030520	A08-20.715	21
R5	RGC8128c	83.47			21

R5	RSS2992	83.74	Bra032591	A09-38.511	21
R5	RS2CL3856s	83.87	Bra032547	A09-38.322	21
R5	RGC20400c	83.92	Bra032588	A09-38.501	21
R5	RS2CL3486s	84.57	Bra032640	A09-38.737	21
R5	RGB2163c	85.28	Bra033236	A10-3.395	21
R5	RSS1973	86.18	Bra032653	A09-38.794	21
R5	RS2CL876s	86.59	Bra032650	A09-38.782	21
R5	RGC18337c	86.85	Bra033217	A10-3.498	21
R5	RS2CL3834s	86.87	Bra032659	A09-38.812	21
R5	RS2CL1002s	87.15	Bra032668	A09-38.865	21
R5	RGC13305c	87.84			
R5	RS2CL4231s	88.98	Bra034432	A05-14.025	22
R5	RS2CL8487s	89.15	Bra034448	A05-14.235	22
R5	RGC1982c	89.53			22
R5	RGC6032c	89.53	Bra034446	A05-14.224	22
R5	RGC7124c	89.53	Bra010195	A05-14.605	22
R5	RS2CL6191s	89.91	Bra010203	A05-14.53	22
R5	RGA1924s	90.52	Bra010195	A05-14.605	22
R5	RSS1355	92.03	Bra012223	A07-11.44	22
R5	RSS2290	93.13	Bra010152	A05-15.216	22
R5	RS2CL5313s	93.45	Bra033870	A05-15.621	22
R5	Rs2CL1513s	94.83	Bra030114	A07-8.682	
R5	RGA2486s	95.37			
R5	RSS3328	95.74	Bra027315	A05-21.006	
R5	RGA2386s	95.75			
R5	RS2CL3218s	96.23	Bra015149	A07-5.281	23
R5	RS2CL3664s	96.44	Bra015135	A07-5.419	23
R5	CC59	97.52			23
R5	RSS2770	98.32	Bra022856	A03-7.422	23
R5	RGC1058c	98.62	Bra027325	A05-21.041	23
R5	RSS1146	99.14	Bra015086	A07-5.806	23
R5	RS2CL7110s	99.54	Bra015104	A07-5.684	23
R5	RS2CL1096s	99.98	Bra006672	A03-4.558	23
R5	RGC2286c	100.36	Bra015091	A07-5.774	23
R5	RGC1397c	100.58			23
R5	RGC6433c	101.17	Bra023433	A02-2.328	23
R5	RSS2008	101.24	Bra015061	A07-6.071	23
R5	RSS1381	103.04			
R5	RGC7374c	104.07	Bra037544	A01-20.958	
R5	RGC7955c	104.07			
R5	RSS3453	105.19	Bra031294	A05-16.971	24
R5	RS2CL1017s	105.83	Bra014972	A07-6.896	24
R5	RS2CL2718s	108.19	Bra031283	A05-17.067	24
R5	RSS2080	108.93	Bra039947	A05-16.503	24
R5	RS2CL6248s	109.54	Bra031279	A05-17.101	24
R5	RGC9070c	109.89			24

R5	RSS0835	110.06	Bra031313	A05-16.796	24
R5	RS2CL4474s	110.66	Bra041034	Scaffold000402-0.001	24
R5	RSS1657	111.19	Bra031269	A05-17.177	24
R5	RS2CL3771s	111.23	Bra033915	A05-16.146	24
R5	RSS0799	111.5	Bra001804	A03-18.595	24
R5	RSS1437	112.1	Bra035786	A05-17.918	24
R5	RGC4231c	112.77	Bra035745	A05-17.609	24
R5	RGC3008c	113.12			24
R5	RGC15844c	113.61			24
R5	RS2CL7568s	113.78	Bra035799	A05-17.983	24
R5	RSS2076	113.98			24
R5	RGC793c	114.27	Bra031273	A05-17.147	24
R5	RGC4102c	114.8	Bra035779	A05-17.852	24
R5	RGB28864c	115.22	Bra022381	A05-18.776	24
R5	CA04a	115.62			24
R5	RGC24023c	115.72	Bra022445	A05-18.351	24
R5	RGC25363c	115.72	Bra031305	A05-16.866	24
R5	RGC7157c	115.72	Bra031331	A05-16.655	24
R5	RSS1610	116.43	Bra022436	A05-18.423	24
R5	RS2CL4228s	116.53	Bra022416	A05-18.563	24
R5	RSS2461	117.75	Bra022338	A05-19.028	24
R5	RSS3878	117.75	Bra022358	A05-18.912	24
R5	RS2CL3657s	117.86	Bra022326	A05-19.106	24
R5	RSS3562	117.95	Bra022336	A05-19.041	24
R5	RGC1797c	118.51	Bra027293	A05-20.881	24
R5	RSS0491	118.75	Bra022255	A05-19.52	24
R5	RSS0607	118.85	Bra022246	A05-19.586	24
R5	RGC5241c	119.04	Bra022212	A05-19.758	24
R5	RGC9697c	119.69			24
R5	RSS0794	120.35	Bra022321	A05-19.139	24
R5	RS2CL2631s	120.38	Bra022204	A05-19.815	24
R5	RGC27939c	120.85	Bra022188	A05-19.925	24
R5	RGC2709c	120.96			24
R5	RGC1165c	120.98	Bra027247	A05-20.657	24
R5	RGC15300c	120.98			24
R5	RGB17888c	120.98	Bra027250	A05-20.667	24
R5	RGC25318c	120.98	Bra021117	A01-23.03	24
R5	RGC15052c	121.1			24
R5	RS2CL3363s	121.34	Bra022228	A05-19.658	24
R5	RSS0599	121.85	Bra027174	A05-20.257	24
R5	RGC7029c	122.02	Bra027173	A05-20.252	24
R5	Rs2CL1713s	122.09	Bra027168	A05-20.197	24
R5	RS2CL2036s	122.64	Bra022160	A05-20.133	24
R5	RGC20412c	123.21			24
R5	RGC3985c	123.21	Bra027369	A05-21.286	24
R5	CB14	123.25			24

R5	Rs2CL1337s	123.75	Bra022223	A05-19.674	24
R5	RSS2087	124.05			24
R5	RSS2733	124.15			24
R5	RS2CL3095s	125.44	Bra027325	A05-21.041	24
R5	RGC7851c	125.81			24
R5	Rs2CL1671s	126.08	Bra027294	A05-20.89	24
R5	RSS3895	126.45	Bra027355	A05-21.214	24
R5	RS2CL1405s	126.99	Bra027357	A05-21.221	24
R5	RSS1697	127.25	Bra027313	A05-20.998	24
R5	RGC1110c	128.21			24
R5	RGC5424c	128.21			24
R5	CA74	128.25			24
R5	RS2CL4817s	128.42	Bra027410	A05-21.492	24
R5	RSS0139	129.25	Bra021517	A01-23.998	24
R5	RS2CL3543s	129.83	Bra027432	A05-21.614	24
R5	RS2CL6094s	130.35	Bra027437	A05-21.631	24
R5	RGB90c	130.75	Bra027409	A05-21.484	24
R5	RSS0258	131.05	Bra027421	A05-21.543	24
R5	RS2CL2801s	131.77	Bra034804	A05-22.228	24
R5	ACGM13	132.35			24
R5	RS2CL2046s	132.49	Bra034812	A05-22.255	24
R5	RS2CL6493s	132.63	Bra034805	A05-22.232	24
R5	RSS1153	132.95	Bra034716	A05-21.843	24
R5	RSS3828	133.15	Bra034748	A05-21.989	24
R5	RS2CL5254s	133.67	Bra034844	A05-22.408	24
R5	CA04b	133.85			24
R5	RS2CL5065s	133.9	Bra034849	A05-22.44	24
R5	RS2CL4091s	134.28	Bra034863	A05-22.511	24
R5	RS2CL4365s	134.82	Bra029859	A05-22.696	24
R5	CD63	135.05			24
R5	Rs2CL2530s	135.15	Bra034863	A05-22.511	24
R5	OPE03b	135.25			24
R5	RGC15061c	136.17			24
R5	RGB2029c	136.17			24
R5	RGB5705c	136.17			24
R5	RSS2040	136.35	Bra034845	A05-22.423	24
R5	RS2CL7735s	136.88	Bra029743	A05-23.175	24
R5	RS2CL6813s	137.44	Bra029805	A05-22.946	24
R5	RGC4124c	137.99			24
R5	CC45a	138.45			24
R5	RSS0084	139.15	Bra029867	A05-22.663	24
R5	RS2CL5459s	139.19	Bra029703	A05-23.321	24
R5	RS2CL5893s	139.4	Bra029671	A05-23.446	24
R5	RS2CL1331s	139.71	Bra029670	A05-23.45	24
R5	RSS1903	140.05	Bra029765	A05-23.083	24
R5	RGA1658s	140.08	Bra029667	A05-23.463	24

R5	RS2CL1047s	140.55	Bra029675	A05-23.436	24
R5	RSS1242	140.85	Bra029704	A05-23.319	24
R5	RSS1489	140.85	Bra001347	A03-15.967	24
R5	RS2CL5612s	141.78	Bra029596	A05-23.813	24
R5	CF19	142.05			24
R5	RS2CL4128s	142.13	Bra029602	A05-23.771	24
R5	RS2CL1648s	143.3	Bra020801	A05-24.172	24
R5	RGC16329c	143.35	Bra039440	A05-24.316	24
R5	RGC2498c	143.35			24
R5	RGC4098c	143.35	Bra020774	A05-24.07	24
R5	RGC4195c	143.35	Bra039413	A05-24.227	24
R5	RGA2325s	144.54	Bra007069	A09-28.657	24
R5	RSS3694	144.65	Bra020752	A05-23.971	24
R5	RSS1361	145.65	Bra029603	A05-23.768	24
R5	RS2CL5529s	145.69	Bra001113	A03-14.837	24
R5	RS2CL5178s	146.41	Bra040406	Scaffold000203-0.056	24
R5	RS2CL6670s	146.79	Bra031946	A05-24.507	24
R5	RGB1089c	147.24	Bra040210	Scaffold000191-0.124	24
R5	RS2CL5532s	147.65	Bra031968	A05-24.595	24
R5	RS2CL6564s	148.26	Bra039164	A05-25.052	24
R5	RS2CL4868s	148.73	Bra040594	A05-24.937	24
R5	Rs2CL2284s	149.12	Bra039118	A05-25.237	24
R5	RGC908c	149.41	Bra039123	A05-25.216	24
R5	RSS2296	149.85	Bra040410	Scaffold000203-0.07	24
R5	RSS3026	149.95	Bra040193	Scaffold000191-0.05	24
R5	RSS0361	150.15	Bra039464	A05-24.436	24
R5	Rs2CL1785s	150.52	Bra039182	A05-24.96	24
R5	RSS0292_a	154.15	Bra039144	A05-25.141	24
R5	RGB1952c	154.21	Bra036450	A01-26.253	
R6	Rs2CL1800s	0	Bra034122	A01-24.89	25
R6	RGA2109s	1.63			25
R6	RSS0284	3.02			25
R6	RGC7282c	3.44	Bra021485	A01-26.675	25
R6	RGC13867c	4.8			25
R6	CA22	5.12			25
R6	RGC5600c	5.27	Bra036502	A07-0.036	25
R6	RSS1106	6.52	Bra036497	A07-0.064	25
R6	RSS0750	6.52	Bra039176	A05-24.986	25
R6	RS2CL1259s	7.06	Bra040701	Scaffold000237-0.018	25
R6	RS2CL2749s	7.84	Bra021411	A01-26.478	25
R6	RSS0881	8.02	Bra036503	A07-0.035	25
R6	RSS1501	8.02	Bra021420	A01-26.508	25
R6	RGC13819c	8.2	Bra034092	A01-25.04	25
R6	RSS3965_a	8.82	Bra040135	A01-25.995	25
R6	Rs2CL1793s	9	Bra021373	A01-26.372	25

R6	RSS0818	9.12	Bra040298	A01-25.598	25
R6	RGB6680c	9.17			25
R6	RS2CL6567s	9.92	Bra040531	Scaffold000215-0.038	25
R6	RSS2859	10.32	Bra029757	A05-23.106	25
R6	RSS2760	10.32	Bra034078	A01-25.096	25
R6	RSS0864	10.32			25
R6	RGC6359c	10.59	Bra034092	A01-25.04	25
R6	RS2CL4163s	10.95	Bra040285	A01-25.638	25
R6	RS2CL3329s	11.58	Bra040376	A01-25.483	25
R6	RS2CL5007s	12.62	Bra040019	A01-25.439	25
R6	RGC10902c	12.91	Bra034046	A01-25.244	25
R6	RS2CL5739s	13.18	Bra034093	A01-25.038	25
R6	RGC12804c	13.49	Bra034046	A01-25.244	25
R6	RSS2138	13.72	Bra038724	A01-24.295	25
R6	RGB4153c	13.85	Bra034081	A01-25.088	25
R6	RSS0662	13.92	Bra039342	Scaffold000164-0.012	25
R6	RSS2505	14.32	Bra038738	A01-24.244	25
R6	RS2CL5533s	14.67	Bra002039	A07-2.348	25
R6	RS2CL4358s	14.98	Bra034153	A01-24.73	25
R6	RGC2728c	15.34			25
R6	RS2CL4648s	16.17	Bra039394	Scaffold000164-0.26	25
R6	RGC5774c	16.49			25
R6	RGC12486c	16.98	Bra039379	Scaffold000164-0.195	25
R6	CC68	17.43			25
R6	RGC6279c	17.74	Bra001368	A03-16.051	25
R6	RS2CL3679s	17.81	Bra021593	A01-23.485	25
R6	RGC395c	18.06			25
R6	RS2CL1088s	18.31	Bra021110	A01-23.106	25
R6	RS2CL1149s	18.56	Bra021125	A01-22.993	25
R6	RGC10056c	18.77	Bra038724	A01-24.295	25
R6	RGC6186c	19.41	Bra021144	A01-22.921	25
R6	RGC12389c	20.04			25
R6	RGC16041c	20.04			25
R6	RGC9621c	20.04			25
R6	RGC4166c	20.04			25
R6	RS2CL3998s	20.73	Bra021222	A01-22.436	25
R6	RSS2497	21.32	Bra037588	A01-21.309	25
R6	RSS0895	21.82	Bra038260	A01-20.416	25
R6	RGA107s	22.47			25
R6	RSS2680	22.71	Bra023895	A01-20.047	25
R6	RSS1367	22.81	Bra023821	A01-19.357	25
R6	RSS3306	22.81	Bra023902	A01-20.078	25
R6	RSS3125	23.01	Bra023895	A01-20.047	25
R6	RS2CL8378s	23.28	Bra038236	A01-20.559	25
R6	RSS3489	23.61			25
R6	RS2CL5725s	24.27	Bra021300	A01-21.858	25

R6	RSS0768	24.6	Bra023830	A01-19.421	25
R6	RS2CL1483s	25.01	Bra023854	A01-19.616	25
R6	RS2CL6241s	26.23	Bra023862	A01-19.69	25
R6	RS2CL7817s	26.73	Bra023854	A01-19.616	25
R6	RGA1973s	26.81			25
R6	RGC1312c	27	Bra038237	A01-20.554	25
R6	RGC2219c	27			25
R6	RGC4977c	27.32			25
R6	RS2CL6432s	27.84	Bra023817	A01-19.34	25
R6	CA09a	27.86			25
R6	Rs2CL2337s	28.18	Bra023732	A01-18.6	25
R6	RSS3301	28.35	Bra023830	A01-19.421	25
R6	RS2CL7813s	28.43	Bra028324	A01-18.404	25
R6	RGB5240c	28.79	Bra021246	A01-22.251	25
R6	RGA1878s	29.4			25
R6	RS2CL8063s	29.63	Bra031448	A01-17.187	25
R6	RS2CL4703s	30.21	Bra028361	A01-18.041	25
R6	RSS0518	30.33	Bra031401	A01-17.617	25
R6	Rs2CL1678s	30.94	Bra031502	A01-16.784	25
R6	RS2CL4308s	31.06	Bra031523	A01-16.588	25
R6	RGC10301c	31.56	Bra023813	A01-19.334	25
R6	RGC6540c	31.56			25
R6	RGC18316c	31.56	Bra023813	A01-19.334	25
R6	RGA2264s	32.04	Bra031501	A01-16.789	25
R6	RSS0124	32.73	Bra035464	A01-15.801	25
R6	RSS2701	32.93	Bra035427	A01-16.23	25
R6	RS2CL2683s	33.48			
R6	RGB14399c	33.64			
R6	CB22	34.22			
R6	RS2CL2041s	34.53	Bra021322	A05-9.524	
R6	RS2CL6287s	34.63	Bra038153	Scaffold000140-0.14	
R6	RGC23911c	34.9	Bra028330	A01-18.312	
R6	RS2CL3132s	35.26	Bra038170	Scaffold000140-0.322	
R6	RSS3180	35.49	Bra038161	Scaffold000140-0.214	
R6	RSS2724	35.99	Bra037182	A09-4.805	
R6	RS2CL7658s	36.11	Bra031520	A01-16.604	
R6	RGC9543c	36.42			
R6	RGB11029c	37.42			
R6	RGC7277c	38.15			
R6	RS2CL2168s	38.51	Bra031898	A02-26.314	26
R6	RSS0895_a	39.42	Bra038260	A01-20.416	26
R6	Rs2CL1936s	39.42	Bra029245	A02-25.812	26
R6	RGC17695c	40.16			26
R6	RGC3412c	40.71	Bra029376	A02-25.011	26
R6	RS2CL4724s	41.51	Bra029272	A02-25.659	26
R6	RSS0727	41.58	Bra029287	A02-25.575	26

R6	RGC28054c	41.79			26
R6	RGC11504c	41.79			26
R6	RGC5414c	41.79	Bra031805	A02-26.91	26
R6	RSS2078	42.57	Bra019616	A06-13.744	26
R6	RGC2511c	42.69	Bra020724	A02-22.953	26
R6	RGC5771c	42.99			26
R6	RSS0254	43.26	Bra009697	A06-17.229	26
R6	RGC7411c	43.37	Bra003495	A07-16.758	26
R6	RS2CL5813s	43.75	Bra020472	A02-24.624	26
R6	Rs2CL2197s	43.82	Bra029371	A02-25.035	26
R6	RGC5509c	44.09			26
R6	RGB17905c	44.09			26
R6	RGC29914c	44.09	Bra020494	A02-24.476	26
R6	RGC21373c	44.09	Bra020557	A02-24.04	26
R6	RGC21003c	44.09			26
R6	RGC7441c	44.09			26
R6	RGB12188c	44.09			26
R6	RGB4736c	44.09	Bra020737	A02-22.861	26
R6	RGC7478c	44.09			26
R6	RGC6399c	44.09			26
R6	RGC5236c	44.09	Bra009983	A06-19.082	26
R6	RGC10969c	44.09	Bra020587	A02-23.875	26
R6	RGC4352c	44.13			26
R6	RS2CL3740s	44.73	Bra020529	A02-24.205	26
R6	RGB11249c	45.18	Bra033016	A02-22.194	26
R6	RGC3081c	45.61			26
R6	RS2CL3937s	46.07	Bra020621	A02-23.608	26
R6	RS2CL3306s	46.23	Bra020651	A02-23.395	26
R6	RS2CL3718s	46.39	Bra020589	A02-23.868	26
R6	RS2CL6218s	46.68	Bra020628	A02-23.563	26
R6	RGC1448c	47.23			26
R6	RS2CL6481s	47.72	Bra020724	A02-22.953	26
R6	RS2CL7263s	48.1	Bra033092	A02-22.63	26
R6	RGA1805s	48.13			26
R6	RS2CL7425s	48.43	Bra033097	A02-22.642	26
R6	RS2CL2511s	49.5	Bra033089	A02-22.617	26
R6	Rs2CL2028s	50.62	Bra033035	A02-22.304	26
R6	RS2CL5945s	51.52	Bra032932	A02-21.657	26
R6	RSS2865	51.81	Bra033005	A02-22.131	26
R6	RS2CL4000s	52.73	Bra036345	A02-21.346	26
R6	RS2CL6161s	53.18	Bra036350	A02-21.265	26
R6	RSS0839_a	53.55	Bra032981	A02-21.97	26
R6	RGC265c	53.78			26
R6	RGC4004c	53.78	Bra022053	A02-18.586	26
R6	RGC6170c	53.78			26
R6	RSS3844	54.55	Bra032986	A02-22.007	26

R6	RS2CL4583s	54.66	Bra026577	A02-20.356	26
R6	RS2CL3419s	55.3	Bra026579	A02-20.329	26
R6	RSS1338	55.35	Bra032984	A02-21.995	26
R6	RGC2998c	56.11	Bra003495	A07-16.758	26
R6	CC46H	58.44			26
R6	RGA1719s	58.61			26
R6	RSS0049	59.34	Bra026647	A02-19.64	26
R6	RS2CL3836s	60.14	Bra014330	A08-1.583	26
R6	RSS3534	60.64	Bra024918	A06-23.181	26
R6	RS2CL2788s	60.89	Bra022146	A02-19.342	26
R6	CD99H	61.04			26
R6	RGC5574c	61.82			26
R6	RGC5793c	62.06			26
R6	RGC3577c	62.06			26
R6	RS2CL7662s	62.39	Bra021992	A02-17.93	26
R6	RSS3754	62.54	Bra022067	A02-18.677	26
R6	RS2CL3015s	63.23	Bra021979	A02-17.778	26
R6	CD72a	63.43			
R6	OPE03a	64.63			
R6	RS2CL4740s	64.92	Bra035988	Scaffold000111-0.299	
R6	RGC893c	65.03			
R6	CB89	65.83			
R6	RSS2657	67.03	Bra039620	A07-1.514	
R6	RSS3589	67.43	Bra011059	A01-4.171	
R6	RGC12381c	67.76			
R6	RGC8396c	68.5			
R6	RGC24370c	68.79			
R6	Rs2CL2226s	69.23	Bra041043	Scaffold000410-0.01	
R6	RSS2881	69.33	Bra040674	Scaffold000232-0.015	
R6	RGC11044c	70.26			
R6	RGB10934c	71.09			
R6	RSS2217	71.42	Bra038850	A07-0.854	27
R6	RS2CL1066s	72.37	Bra038817	A07-1.141	27
R6	RSS0782	72.42	Bra017741	A03-30.19	27
R6	RS2CL7383s	72.89	Bra015209	A07-3.706	27
R6	RS2CL2325s	73.45	Bra039913	A07-1.671	27
R6	CD66	73.92			27
R6	RS2CL2832s	73.95	Bra002080	A07-2.008	27
R6	RGA2488s	74.57			27
R6	RSS1178	74.62	Bra031017	A09-34.672	27
R6	RS2CL3082s	74.94	Bra036406	A07-4.57	27
R6	RS2CL4493s	75.18	Bra002022	A07-2.489	27
R6	RGB9877c	75.31			27
R6	RGC9878c	75.31			27
R6	RGB12172c	75.31	Bra015233	A07-3.426	27
R6	RS2CL7011s	75.39	Bra036409	A07-4.638	27

R6	OPJ09	75.52			27
R6	RS2CL2923s	75.63	Bra036405	A07-4.565	27
R6	RSS0489	75.81	Bra031031	A09-34.604	
R6	RS2CL3164s	75.96	Bra040613	Scaffold000223-0.025	
R6	RGA1701s	76.25			
R6	RGC17769c	76.93			
R6	RS2CL3911s	77.46	Bra012317	A07-10.795	
R6	RSS2949_a	77.71	Bra001995	A07-2.775	
R6	RS2CL5376s	77.83	Bra002663	A10-8.412	
R6	RS2CL1217s	80.65	Bra012369	A07-10.374	
R6	Rs2CL1765s	81.47	Bra038815	A07-1.162	
R6	RSS0406	82.2	Bra026736	A09-35.203	28
R6	RGC201c	84.1			28
R6	RGC4639c	84.33			28
R6	CA51H	84.49			28
R6	RSS2960	84.99	Bra006933	A09-27.822	28
R6	RGC9315c	85.11	Bra026869	A09-35.804	28
R6	RS2CL2201s	86.23	Bra026705	A09-35.058	28
R6	RSS0494	86.61	Bra006917	A09-27.656	29
R6	RS2CL1536s	87.18	Bra026708	A09-35.074	29
R6	RGC2126c	87.73			29
R6	RGC8845c	89.19	Bra036817	A09-27.146	29
R6	RS2CL2405s	89.66	Bra036092	A09-26.735	29
R6	RSS0654	91.15	Bra036813	A09-27.165	29
R6	RS2CL2415s	92.15	Bra017991	A06-9.027	
R6	RGC5299c	92.23			
R6	RSS2335	93.11	Bra021109	A01-23.112	
R6	CC56b	95.11			
R6	RSS0318	96.81	Bra018086	A06-9.841	30
R6	RSS1066	97.31	Bra018099	A06-9.951	30
R6	RSS0144	97.61	Bra018141	A06-10.28	30
R6	RSS1276	99.11	Bra018168	A06-10.479	30
R6	RS2CL2353s	99.2	Bra018190	A06-10.639	30
R6	RGA2249s	102.93			30
R6	RSS1104	103.07	Bra019442	A06-11.788	30
R6	RSS1095	103.47	Bra034972	Scaffold000100-0.446	
R6	RSS3864	104.67	Bra007782	A09-32.371	31
R6	RSS0660	105.17	Bra010099	A06-14.397	31
R6	CA92	106.57			31
R6	RS2CL2669s	110.66	Bra034985	Scaffold000100-0.661	31
R6	RGC3431c	110.9	Bra018128	A06-10.182	31
R6	RS2CL2529s	110.93	Bra019425	A06-11.556	31
R6	RSS3405	112.02	Bra038518	A09-33.476	31
R6	RSS1020	112.02	Bra038518	A09-33.476	31
R6	Rs2CL1769s	112.07	Bra007788	A09-32.393	31
R6	RGC7305c	112.65			31

R6	RSS3937	113.85	Bra031205	A09-33.77	31
R6	RS2CL7280s	114.14	Bra007846	A09-32.746	31
R6	OPT12a	115.58			
R6	RS2CL7089s	115.59	Bra039216	A09-32.964	31
R6	RGC11789c	116.51			31
R6	CA96	116.89			
R6	RS2CL6447s	118.47	Bra031143	A09-34.063	31
R6	RSS3082	118.61	Bra031137	A09-34.075	31
R6	Rs2CL1480s	119.54	Bra031139	A09-34.069	31
R6	RS2CL1246s	119.59	Bra031127	A09-34.116	31
R6	RGC2386c	120.35	Bra034672	A08-11.369	31
R6	RGC9240c	120.35			
R6	CC65a	120.73			
R6	OPT16H	122.63			
R6	RS2CL3093s	123.21	Bra012077	A07-12.394	
R6	RSS2968	124.52			
R6	RS2CL7541s	124.65	Bra034318	A04-12.239	
R6	RGC916c	126.72	Bra018273	A05-7.08	32
R6	RSS2167	127.44	Bra018640	A06-2.865	32
R6	RGC17489c	128.24	Bra005056	A05-3.202	32
R6	Rs2CL2106s	132.7	Bra018353	A05-7.715	32
R6	RGC570c	132.71			32
R6	RSS1288	133.22	Bra018368	A05-7.797	32
R6	RGC14157c	133.77	Bra005645	A05-6.713	32
R6	RSS2586	134.89	Bra018329	A05-7.5	32
R6	RSS1469	135.6	Bra005611	A05-6.527	32
R6	RS2CL7837s	136.74	Bra005609	A05-6.514	32
R6	RSS2868	137.34	Bra005543	A05-6.132	32
R6	RS2CL1111s	137.74	Bra005613	A05-6.532	32
R6	RSS1670	138.04	Bra018239	A05-6.883	32
R6	CC61b	138.14			32
R6	RGC1609c	138.62	Bra005613	A05-6.532	32
R6	RSS1084	138.84	Bra005522	A05-5.997	32
R6	RSS0942	138.84	Bra005505	A05-5.861	32
R6	RSS2362	139.24	Bra022931	A03-7.83	32
R6	RS2CL7123s	139.45	Bra005542	A05-6.124	32
R6	RSS3503	139.64	Bra022981	A03-8.083	32
R6	RSS0171	140.04	Bra005408	A05-5.352	32
R6	RGC5431c	140.86			32
R6	RSS1190	141.24	Bra005306	A05-4.692	32
R6	RSS3372	142.54	Bra023061	A03-8.469	32
R6	RGC1119c	142.83	Bra005269	A05-4.425	32
R6	RGC1052c	143.71	Bra005105	A05-3.444	32
R6	RSS1193	143.84	Bra005191	A05-3.908	32
R6	RGC15890c	143.93			32
R6	RS2CL1853s	144.22	Bra005092	A05-3.388	32

R6	RGC23650c	144.24	Bra005286	A05-4.556	32
R6	RGC7461c	144.69			32
R6	RGC4566c	145.16			32
R6	RSS0454	145.24	Bra005060	A05-3.229	32
R6	RS2CL6557s	145.65	Bra005207	A05-4.011	32
R6	RS2CL2395s	145.71	Bra005236	A05-4.201	32
R6	RS2CL3438s	146.12	Bra005168	A05-3.806	32
R6	RS2CL1346s	146.24	Bra005141	A05-3.685	32
R6	RS2CL8252s	146.39	Bra006768	A03-4.978	32
R6	RSS3153	146.44	Bra004993	A05-2.856	32
R6	RS2CL6331s	146.81	Bra005143	A05-3.704	32
R6	RS2CL934s	147.02	Bra005159	A05-3.761	32
R6	RS2CL4132s	147.49	Bra005102	A05-3.433	32
R6	RS2CL4854s	148.37	Bra005054	A05-3.197	32
R6	RGA2337s	149.59			32
R6	RGC10645c	150.02			32
R6	RGC801c	150.02	Bra005006	A05-2.932	32
R6	RGC4495c	150.16	Bra004987	A05-2.835	32
R6	RGB18830c	150.21			32
R6	Rs2CL1694s	150.39	Bra004998	A05-2.893	32
R6	RS2CL8102s	150.96	Bra004587	A05-0.907	32
R6	OPR11cH	151.04			32
R6	RGC9387c	151.43			32
R6	RSS2756	151.84	Bra004795	A05-1.863	32
R6	RSS1754	151.84	Bra004786	A05-1.822	32
R6	RSS3095	151.84			32
R6	RSS2532	151.84	Bra000321	A03-10.559	32
R6	RS2CL5874s	152.34	Bra004654	A05-1.253	32
R6	RGC7168c	152.49			32
R6	RSS0166	153.24	Bra004935	A05-2.562	32
R6	RSS0050	153.34	Bra004930	A05-2.542	32
R6	RGC5183c	153.72			32
R6	CD57	153.84			32
R6	RGC17231c	154.38	Bra004925	A05-2.52	32
R6	RSS1221	154.54	Bra004546	A05-0.743	32
R6	RS2CL6859s	154.77	Bra004727	A05-1.536	32
R6	RGC5676c	155.53			32
R6	RGB14649c	157.06			32
R6	RS2CL7316s	157.61	Bra004461	A05-0.257	32
R6	RS2CL6976s	157.63	Bra004481	A05-0.36	32
R6	RGB19481c	158.25	Bra004415	A05-0.022	32
R6	RGB8057c	158.25			32
R6	RGC12020c	158.47	Bra021391	A04-19.116	32
R6	RS2CL8726s	159.04	Bra004531	A05-0.67	32
R6	Rs2CL1849s	163.38	Bra033420	A04-4.387	32
R6	RS2CL755s	165.82	Bra004497	A05-0.452	32

R6	Rs2CL1647s	167.73	Bra004926	A05-2.522	32
R7	RSS3540	0	Bra028508	A07-14.473	33
R7	CC98aH	9.9			33
R7	RGB9971c	12.43			33
R7	RS2CL1375s	14.69	Bra028434	A07-13.973	33
R7	CC65b	14.9			33
R7	CB85	15.3			33
R7	RGC12560c	15.52			33
R7	RSS1042	16	Bra028547	A07-14.692	33
R7	RS2CL4083s	16.56	Bra028511	A07-14.487	33
R7	RS2CL4057s	17.21	Bra025495	A04-8.709	33
R7	RSS3729	17.8			33
R7	RS2CL5433s	18.08	Bra028506	A07-14.454	33
R7	CA30	18.3			33
R7	RS2CL2223s	18.54	Bra028519	A07-14.527	33
R7	RGB16666c	19.03	Bra007084	A09-28.734	33
R7	RGC19850c	19.22	Bra003138	A07-14.826	33
R7	RS2CL5628s	19.36	Bra003139	A07-14.828	33
R7	CA32H	19.7			33
R7	RSS2871	20.8	Bra007019	A09-28.372	33
R7	RS2CL4729s	21.14	Bra003220	A07-15.246	33
R7	RSS0830	21.2	Bra003187	A07-15.018	33
R7	RSS0953	21.2	Bra003158	A07-14.897	33
R7	RSS1721	21.2	Bra003181	A07-15	33
R7	RS2CL1045s	21.43	Bra014743	A04-2.747	33
R7	RS2CL6364s	21.73	Bra003239	A07-15.367	33
R7	RS2CL4386s	22.6	Bra003289	A07-15.689	33
R7	RSS0476	23.1	Bra003300	A07-15.754	33
R7	RS2CL5785s	24.13	Bra003319	A07-15.86	33
R7	RS2CL1776s	24.81	Bra003435	A07-16.45	33
R7	RS2CL4295s	24.88	Bra003397	A07-16.291	33
R7	CC75	24.93			33
R7	RS2CL3314s	25.05	Bra003402	A07-16.312	33
R7	RGC10321c	25.45			33
R7	CB25b	27.39			33
R7	CB30a	27.39			33
R7	RGC1833c	27.47			33
R7	RGC1402c	29.25			33
R7	RSS0148	29.26	Bra003466	A07-16.62	33
R7	RS2CL1270s	30.34	Bra003527	A07-16.917	33
R7	RSS0466	30.48	Bra003560	A07-17.072	33
R7	RSS0449	30.58	Bra003564	A07-17.088	33
R7	RSS3262	30.68	Bra003541	A07-16.979	33
R7	RSS3922	30.68	Bra003542	A07-16.981	33
R7	RSS0313	31.28	Bra007679	A09-31.883	33

R7	RSS0797	31.28	Bra003502	A07-16.78	33
R7	RSS3963	31.28	Bra003498	A07-16.77	33
R7	RSS1371	31.98	Bra003502	A07-16.78	33
R7	RGB4595c	32.21	Bra003556	A07-17.047	33
R7	CB38H	32.74			33
R7	RGC7238c	33.65	Bra003645	A07-17.498	33
R7	RGC7757c	33.65	Bra003687	A07-17.759	33
R7	Rs2CL1633s	34.88	Bra003630	A07-17.422	33
R7	CD02	35.09			33
R7	RGC5428c	36.07			33
R7	RS2CL2646s	36.27	Bra003647	A07-17.503	33
R7	RS2CL3416s	37.33	Bra003692	A07-17.788	33
R7	RS2CL891s	40.94	Bra003779	A07-18.304	33
R7	RGC2981c	41.38			33
R7	RS2CL6078s	42.78	Bra003804	A07-18.435	33
R7	OPJ14	43.31			33
R7	RS2CL2164s	43.56	Bra003821	A07-18.495	33
R7	RS2CL4632s	43.73	Bra003818	A07-18.482	33
R7	RGA2312s	44.31	Bra003861	A07-18.696	33
R7	RGA2511s	44.91	Bra021154	A01-22.851	33
R7	RGC17698c	45.51			33
R7	RS2CL2115s	46.17	Bra003864	A07-18.718	33
R7	RGC8422c	47.25			33
R7	CC18	47.59			33
R7	Rs2CL2268s	48.3	Bra003991	A07-19.431	33
R7	S	48.56			33
R7	RS2CL5832s	48.58	Bra003981	A07-19.396	33
R7	RS2CL4290s	49.03	Bra004036	A07-19.728	33
R7	RGC1041c	49.35	Bra004020	A07-19.64	33
R7	RGC1883c	49.35			33
R7	RGC22628c	49.35	Bra004016	A07-19.613	33
R7	RGC3773c	49.35			33
R7	RS2CL2685s	49.47	Bra003928	A07-19.084	33
R7	CA52H	49.99			33
R7	RGA1801s	50.05			33
R7	RS2CL848s	50.34	Bra004067	A07-19.927	33
R7	RS2CL7649s	50.38	Bra004064	A07-19.903	33
R7	RGC14901c	50.95			33
R7	RS2CL2344s	51.05	Bra004105	A07-20.151	33
R7	RSS0553	51.43	Bra004125	A07-20.253	33
R7	RGC8873c	51.81	Bra004128	A07-20.265	33
R7	RGC9973c	51.81	Bra004100	A07-20.127	33
R7	RGC26589c	52.28	Bra004085	A07-20.031	33
R7	RGA200s	52.69	Bra006651	A03-4.478	33
R7	RSS0291	52.99	Bra004146	A07-20.348	33
R7	RS2CL6332s	53.21	Bra004127	A07-20.26	33

R7	RGC10939c	53.74			33
R7	RS2CL1154s	54.33	Bra004143	A07-20.341	33
R7	RGC5695c	54.52			33
R7	RGC7132c	54.73	Bra004135	A07-20.309	33
R7	CA95a	55.04			33
R7	RS2CL7750s	55.11	Bra004174	A07-20.491	33
R7	RS2CL4436s	55.83	Bra004175	A07-20.495	33
R7	RSS1809	56.46			33
R7	RS2CL1077s	56.55	Bra004214	A07-20.748	33
R7	RGA63s	57.03	Bra004185	A07-20.568	33
R7	RGC10094c	57.07			33
R7	RGC1124c	57.07			33
R7	RGB24589c	57.07			33
R7	RGB25459c	57.07	Bra004214	A07-20.748	33
R7	RS2CL6603s	58.03	Bra004236	A07-20.861	33
R7	CC46	58.38			33
R7	RS2CL2158s	58.67	Bra004238	A07-20.87	33
R7	RGC5019c	58.84			33
R7	RS2CL3876s	58.98	Bra004263	A07-20.984	33
R7	RSS0893	59.49	Bra040803	Scaffold000262-0.012	
R7	RSS0043	59.69	Bra015711	A07-24.511	34
R7	RSS1241	59.89	Bra015733	A07-24.392	34
R7	RSS1896	60.29	Bra015739	A07-24.364	34
R7	RSS1737	60.69	Bra015779	A07-24.177	34
R7	RSS3091	61.49	Bra015784	A07-24.152	34
R7	RSS2593	61.59	Bra015784	A07-24.152	34
R7	RSS3615	62.19	Bra003748	A07-18.118	34
R7	RSS1781	62.99	Bra015878	A07-23.671	34
R7	Ph	65.09			34
R7	R89998	65.59			34
R7	RS2CL4343s	66.48	Bra008243	A02-13.043	34
R7	RSS0675	66.51	Bra015797	A07-24.097	34
R7	RS2CL859s	66.54	Bra015799	A07-24.092	34
R7	RS2CL6039s	66.88	Bra015829	A07-23.943	34
R7	RS2CL3860s	67.05	Bra015835	A07-23.901	34
R7	Rs2CL1833s	67.15	Bra015826	A07-23.958	34
R7	RSS0577	67.36	Bra015846	A07-23.829	34
R7	RS2CL7600s	67.42	Bra015832	A07-23.914	34
R7	RS2CL7455s	67.69	Bra002474	A10-9.385	35
R7	RGB645c	67.77	Bra015750	A07-24.317	35
R7	RGC6547c	67.77			35
R7	RS2CL762s	68.17	Bra015897	A07-23.604	35
R7	RSS1752	68.17	Bra002290	A10-10.527	35
R7	RGA1809s	68.47			35
R7	RS2CL5281s	68.86	Bra002440	A10-9.627	35
R7	RS2CL7601s	69.33	Bra002421	A10-9.759	35

R7	RSS1395	70.08	Bra002468	A10-9.413	35
R7	RGC175c	70.15	Bra015894	A07-23.614	35
R7	RGC3382c	70.15	Bra003807	A07-18.443	35
R7	RGC4853c	70.15	Bra002412	A10-9.817	35
R7	RGC6940c	70.15			35
R7	RS2CL5291s	70.76	Bra002382	A10-9.991	35
R7	RS2CL896s	71.48	Bra002352	A10-10.197	35
R7	RS2CL1576s	71.8	Bra002347	A10-10.219	35
R7	RS2CL8862s	72.13	Bra002314	A10-10.429	35
R7	RS2CL2861s	72.54	Bra002333	A10-10.313	35
R7	RS2CL3030s	73.07	Bra002332	A10-10.327	35
R7	RS2CL6700s	73.6	Bra009434	A10-15.435	36
R7	nga249	73.64			36
R7	RGB2012c	74.45	Bra002347	A10-10.219	36
R7	RGC21683c	74.45	Bra009189	A10-14.897	36
R7	RGC2749c	74.45	Bra009151	A10-15.045	36
R7	RGC4411c	74.45			36
R7	RGC6888c	74.45	Bra002325	A10-10.368	36
R7	RGC18584c	74.93	Bra002293	A10-10.512	36
R7	RS2CL6652s	75.18	Bra009412	A10-15.339	36
R7	RS2CL3415s	75.68	Bra009116	A10-15.178	36
R7	RS2CL7185s	75.95	Bra009127	A10-15.131	36
R7	RGC10310c	76.37			36
R7	RS2CL4596s	76.64	Bra009152	A10-15.041	36
R7	RSS3362	76.72	Bra035210	A09-17.026	36
R7	RS2CL2687s	77.19	Bra009244	A10-14.691	36
R7	RS2CL7134s	77.57	Bra009248	A10-14.655	36
R7	RS2CL2387s	77.69	Bra009240	A10-14.7	36
R7	RS2CL6027s	77.69	Bra009243	A10-14.693	36
R7	RS2CL4568s	77.82	Bra009227	A10-14.753	36
R7	Rs2CL1651s	78.15	Bra009265	A10-14.596	36
R7	CA66	78.65			36
R7	RGC1800c	78.93	Bra009366	A10-14.12	36
R7	RGC591c	79.41	Bra030964	A08-1.162	36
R7	RS2CL5950s	79.93	Bra009311	A10-14.365	36
R7	RS2CL3381s	80.6	Bra009351	A10-14.178	36
R7	RS2CL3384s	81.07	Bra009366	A10-14.12	36
R7	Rs2CL1984s	81.23	Bra009373	A10-14.092	36
R7	RSS3640	81.25	Bra009393	A10-14.029	36
R7	RGC12603c	82.14	Bra009064	A10-13.902	36
R7	CD87a	82.62			36
R7	RGC2185c	82.75			36
R7	RGC21966c	82.75	Bra009058	A10-13.88	36
R7	RGC4797c	82.75	Bra009044	A10-13.808	36
R7	Rs2CL1884s	82.82	Bra009063	A10-13.898	36
R7	RsFLC1s	83.04			36

R7	RGA1861s	83.25	Bra009006	A10-13.637	36
R7	RGB12060c	83.54			36
R7	Rs2CL2475s	83.84	Bra006061	A03-1.813	36
R7	RGC11618c	84.2	Bra009240	A10-14.7	36
R7	CC19	84.63			36
R7	RS2CL4523s	85.11	Bra008889	A10-13.11	36
R7	RS2CL6940s	85.26	Bra008859	A10-12.969	36
R7	RS2CL3345s	85.37	Bra023394	A02-2.137	36
R7	RS2CL5802s	85.95	Bra008821	A10-12.789	36
R7	RGC11896c	86.53			36
R7	RGB7818c	86.53	Bra008957	A10-13.422	36
R7	RGC12721c	87.71			36
R7	RGC12875c	87.71			36
R7	RGC15411c	87.71			36
R7	RGC1662c	87.71	Bra008774	A10-12.6	36
R7	RGC680c	87.71	Bra008750	A10-12.5	36
R7	RGC8830c	87.71	Bra008841	A10-12.867	36
R7	RGC9306c	87.71	Bra008820	A10-12.785	36
R7	RS2CL1610s	87.74	Bra008750	A10-12.5	36
R7	RSS1194	88.57	Bra008701	A10-12.263	36
R7	SWG7B3	89.07			36
R7	RS2CL7086s	89.22	Bra006242	A03-2.661	36
R7	RGB19113c	89.63	Bra015781	A07-24.169	36
R7	RGC1995c	90.67	Bra002154	A10-11.27	36
R7	RGB7653c	90.67	Bra008624	A10-11.934	36
R7	RGC235c	90.7			36
R7	RGC4058c	90.7	Bra008609	A10-11.858	36
R7	RGC4381c	90.7	Bra008599	A10-11.796	36
R7	CB74	90.8			36
R7	Rs2CL2240s	91.06	Bra008564	A10-11.643	36
R7	RS2CL2738s	91.23	Bra002133	A10-11.386	36
R7	RSS0403	92.25	Bra008600	A10-11.804	36
R7	RS2CL5276s	92.65	Bra008581	A10-11.718	36
R7	RSS0011	92.65	Bra008592	A10-11.763	36
R7	RS2CL1994s	93.06	Bra006490	A03-3.764	36
R7	RS2CL4131s	93.24	Bra002198	A10-11.015	36
R7	RS2CL7414s	93.6	Bra006500	A03-3.804	36
R7	RSS2945	93.75	Bra002141	A10-11.359	36
R7	Rs2CL2199s	93.75	Bra020059	A02-3.832	36
R7	RSS1012	93.85	Bra002179	A10-11.13	36
R7	RGC5308c	93.93	Bra009447	A10-15.491	36
R7	Rs2CL2272s	94.18	Bra002255	A10-10.706	36
R7	RGB4201c	94.61	Bra009571	A10-16.072	36
R7	RS2CL2679s	94.78	Bra005806	A03-0.73	36
R7	RSS2692	96.05	Bra002223	A10-10.855	36
R7	RGC3153c	96.24	Bra002170	A10-11.174	36

R7	RSS1758	96.45	Bra002241	A10-10.779	36
R7	RSS2706	96.55	Bra020038	A02-3.721	36
R7	RSS2731	96.55	Bra018298	A05-7.282	36
R7	RSS1273	96.75	Bra002237	A10-10.799	36
R7	RSS3991	97.05	Bra009474	A10-15.652	37
R7	RS2CL3584s	97.21	Bra009607	A10-16.233	37
R7	RS2CL1749s	98.06			37
R7	RSS1786	98.25	Bra009629	A10-16.317	37
R7	RSS0080	98.35	Bra028854	A02-0.352	37
R7	RSS1147	98.35	Bra009568	A10-16.06	37
R7	RSS1574	98.35	Bra009554	A10-16.019	37
R7	RSS3973	98.35	Bra009586	A10-16.136	37
R7	RSS2914_a	98.75	Bra009533	A10-15.929	37
R7	RSS0239	99.05	Bra009491	A10-15.709	37
R7	CB30b	99.65			
R7	CC61a	100.45			
R7	RGC11328c	100.46			
R7	RGC8546c	103.63	Bra013962	A08-4.951	
R7	RGC17372c	105.7	Bra013977	A08-4.906	
R7	RSS1523	109.85	Bra008918	A10-13.208	
R8	RGC12178c	0	Bra014304	A08-1.819	38
R8	RGB6149c	1.5			38
R8	RS2CL1123s	1.72	Bra014320	A08-1.669	38
R8	RGC147c	2.44			38
R8	RSS3445	3.54	Bra014278	A08-2.019	38
R8	Rs2CL2236s	4.21	Bra018920	A06-1.267	38
R8	RGC14381c	4.67			38
R8	RS2CL5518s	4.94	Bra030877	A08-0.464	38
R8	RS2CL2580s	5.35	Bra030905	A08-0.688	38
R8	RGC6104c	5.67			
R8	RSS2533_a	7.42	Bra025124	A06-22.26	39
R8	RGA1690s	7.53			39
R8	RSS0760	7.88	Bra025167	A06-21.924	39
R8	RS2CL5440s	8.21	Bra014243	A08-2.248	39
R8	OPE20a	10.61			39
R8	RSS2626	10.91	Bra024817	A06-22.54	39
R8	RSS0805	11.71	Bra024828	A06-22.593	39
R8	RSS1427	11.71	Bra024832	A06-22.616	39
R8	RSS0092	13.91	Bra024863	A06-22.87	39
R8	RSS2684	14.31			39
R8	RSS2703	14.51	Bra014192	A08-2.682	39
R8	RSS1899	15.21	Bra024910	A06-23.154	39
R8	CA87bH	15.31			39
R8	RSS3003	15.81	Bra024899	A06-23.108	39
R8	R30624a	16.01			

R8	R30025	16.31			
R8	BRMS033	16.51			
R8	RSS2613	17.01	Bra030889	A08-0.573	40
R8	RSS1208	17.11	Bra030893	A08-0.604	40
R8	RSS3870	17.21	Bra024967	A06-23.384	40
R8	RSS0974	17.41	Bra014278	A08-2.019	40
R8	RSS2196	17.41	Bra014292	A08-1.885	40
R8	RSS0042	17.51	Bra014295	A08-1.865	40
R8	RS2CL3262s	19.21	Bra014097	A08-3.551	40
R8	RGC629c	20.26	Bra035538	A08-8.363	40
R8	CA77	20.76			40
R8	RGA2253s	21.22			40
R8	CD04	21.41			40
R8	RGC8651c	21.79			40
R8	RS2CL5213s	21.81	Bra014080	A08-3.739	40
R8	RGC2369c	22.71	Bra036751	A08-7.355	40
R8	RS2CL2117s	23.07	Bra034897	A08-5.551	40
R8	RGC3380c	23.17	Bra034880	A08-5.762	40
R8	RS2CL809s	23.49	Bra034873	A08-5.881	40
R8	RGC10544c	23.95			40
R8	RGC10825c	23.95			40
R8	RGC1319c	23.95			40
R8	RGB16366c	23.95			40
R8	RGC2994c	23.95	Bra014016	A08-4.555	40
R8	RGC8228c	23.95			40
R8	RS2CL4855s	24.03	Bra036771	A08-7.139	40
R8	RS2CL1406s	24.21	Bra036763	A08-7.233	40
R8	RS2CL3940s	24.73			40
R8	RS2CL2104s	24.88	Bra012397	A07-10.224	40
R8	RS2CL3538s	25.82	Bra038939	A08-8.444	40
R8	RS2CL5211s	26.82	Bra038952	A08-8.541	40
R8	RGC20183c	27.25			40
R8	CA91	28.52			40
R8	RS2CL3875s	28.66	Bra038439	A08-8.787	40
R8	RS2CL3155s	29.12	Bra038418	A08-8.978	40
R8	Rs2CL1806s	29.56	Bra008122	A02-12.257	40
R8	RGC12354c	29.8			40
R8	RGC5581c	29.8			40
R8	RSS0790	29.82	Bra039880	A08-6.121	40
R8	RGC12097c	30.29	Bra038949	A08-8.525	40
R8	RGC274c	31.31			40
R8	RS2CL5594s	31.39	Bra038093	A08-6.624	40
R8	RGC20497c	31.5			40
R8	RGC5712c	31.5			40
R8	RGC7156c	31.5			40
R8	RGC484c	32			40

R8	RGC2683c	32.63			40
R8	RS2CL5411s	32.94	Bra021506	A01-24.07	40
R8	RS2CL4559s	33.61	Bra021852	A04-14.973	40
R8	RS2CL5083s	33.61	Bra021067	A08-9.273	40
R8	CA57H	33.7			40
R8	RSS0053	34.1	Bra040572	Scaffold000219-0.025	40
R8	RS2CL5831s	34.39	Bra019179	A03-25.875	40
R8	RSS1433	36.43	Bra040111	A01-10.373	40
R8	RSS4002	36.43	Bra026237	A01-10.267	40
R8	RGC4518c	36.63			40
R8	RS2CL1252s	37.79	Bra013262	A01-4.604	40
R8	CB75H	38.06			40
R8	CC88bH	39.96			40
R8	RGC14573c	40.23	Bra020935	A08-10.307	40
R8	RSS0294	41.44	Bra020933	A08-10.32	40
R8	RGC173c	41.86	Bra034664	A08-11.419	40
R8	RGC2807c	41.86			40
R8	CC72	42.41			40
R8	RS2CL1403s	44.41	Bra010681	A08-15.172	40
R8	RSS0615	44.92	Bra010222	A08-12.496	40
R8	RSS1330	45.72	Bra034675	A08-11.343	40
R8	RSS1747	46.62			40
R8	RS2CL6221s	47.94	Bra034595	A08-11.822	40
R8	T75662	48.28			40
R8	RSS1844	48.88	Bra024567	A09-25.573	40
R8	RS2CL1646s	49.21	Bra040090	A08-12.246	40
R8	RSS1680	49.37	Bra034616	A08-11.727	40
R8	RS2CL2780s	50.36	Bra040079	A08-12.296	40
R8	Rs2CL2111s	50.69	Bra040068	A08-12.411	40
R8	RSS3270	51.11	Bra010246	A08-12.624	40
R8	RGC9390c	51.14			40
R8	RGC10000c	51.69	Bra010327	A08-13.113	40
R8	RGC5417c	51.69	Bra023955	A03-28.649	40
R8	RSS2735	51.76	Bra040087	A08-12.256	40
R8	CA88N	52.46			40
R8	RS2CL4240s	52.61	Bra010229	A08-12.525	40
R8	RS2CL5626s	53.34	Bra010245	A08-12.621	40
R8	CC15H	53.49			40
R8	RS2CL5288s	54.18	Bra010248	A08-12.628	40
R8	RGC2076c	55.59	Bra010546	A08-14.305	40
R8	RSS0305	56.55	Bra026273	A01-10.047	40
R8	RSS1175	56.55			40
R8	RGC11890c	56.96			40
R8	RGC14479c	58.07			40
R8	ACGM23	58.99			40
R8	RS2CL1940s	59.21	Bra011642	A01-1.296	40

R8	CC13	60.13			40
R8	Rs2CL1970s	60.24	Bra010818	A08-15.843	40
R8	RGC16204c	60.99	Bra010628	A08-14.9	40
R8	RSS2248	61.37	Bra010548	A08-14.319	40
R8	RS2CL3284s	61.75	Bra010554	A08-14.371	40
R8	RGC3490c	62.11			40
R8	RS2CL1179s	62.29	Bra010574	A08-14.491	40
R8	RS2CL4152s	62.99	Bra010600	A08-14.671	40
R8	RSS0073	63	Bra010632	A08-14.92	40
R8	RGC8307c	63.9			40
R8	RSS3326	64.45			40
R8	Rs2CL1468s	64.49	Bra010982	A08-16.622	40
R8	RGC8839c	65.26			40
R8	RGC258c	65.62	Bra010759	A08-15.506	40
R8	RGC14352c	65.76	Bra010775	A08-15.579	40
R8	RGC6994c	65.76	Bra010745	A08-15.448	40
R8	RS2CL881s	66.05	Bra010719	A08-15.331	40
R8	RGC2153c	66.26			40
R8	RS2CL1475s	66.86	Bra010706	A08-15.287	40
R8	RGC26764c	67.25	Bra010867	A08-16.068	40
R8	RSS3178	67.32	Bra010811	A08-15.793	40
R8	RSS3174	67.72	Bra010829	A08-15.894	40
R8	RGC19563c	68.79			40
R8	RSS0382	69.5	Bra010851	A08-16.007	40
R8	RSS3738	69.5	Bra010848	A08-16	40
R8	RGC2170c	69.95			40
R8	RGC18537c	70.29			40
R8	RSS0919	71.15	Bra010979	A08-16.608	40
R8	RS2CL1250s	71.23	Bra010899	A08-16.232	40
R8	RGC15683c	71.86			40
R8	RSS2610	72.12	Bra010936	A08-16.428	40
R8	RS2CL5401s	72.43	Bra010950	A08-16.464	40
R8	RS2CL7655s	72.61	Bra010940	A08-16.437	40
R8	RSS2489	72.61	Bra010956	A08-16.49	40
R8	RGC1653c	73.01	Bra016271	A08-16.862	40
R8	RGC17676c	73.01			40
R8	RGB14459c	73.15	Bra010976	A08-16.58	40
R8	OPR20	73.34			40
R8	Rs2CL1478s	73.69	Bra010996	A08-16.718	40
R8	RGB11843c	73.9	Bra016376	A08-17.353	40
R8	RSS1585	74.03	Bra011009	A08-16.794	40
R8	RGC8853c	74.38			40
R8	RS2CL3498s	74.55	Bra016293	A08-16.962	40
R8	RS2CL5877s	75.73	Bra016372	A08-17.334	40
R8	RSS1295	75.84	Bra011002	A08-16.754	40
R8	Rs2CL1741s	76.98	Bra016436	A08-17.688	40

R8	RS2CL3268s	77.62	Bra016401	A08-17.511	40
R8	RSS0229	78.36	Bra025871	A06-8.324	40
R8	RS2CL5368s	78.58	Bra016448	A08-17.752	40
R8	RSS0066	78.94	Bra037224	A09-5.526	40
R8	RS2CL2922s	79.04	Bra016490	A08-17.924	40
R8	RS2CL3626s	79.45	Bra016457	A08-17.794	40
R8	RSS2460	79.51	Bra016574	A08-18.347	40
R8	RSS2434	79.81	Bra025940	A06-6.575	40
R8	RSS2901	80.01	Bra016586	A08-18.393	40
R8	RS2CL4706s	80.14	Bra016502	A08-18.009	40
R8	RSS3034	80.67	Bra016604	A08-18.483	40
R8	RS2CL5634s	80.88	Bra016521	A08-18.09	40
R8	RSS0327	80.95	Bra016607	A08-18.494	40
R8	RSS3614	81.25	Bra016593	A08-18.427	40
R8	RS2CL1509s	82.09	Bra016749	A08-19.031	40
R8	RSS2378	82.3	Bra016653	A08-18.661	40
R8	RGC11743c	82.42			40
R8	RSS3078	82.59	Bra026205	A06-5.288	40
R8	RS2CL1319s	82.66	Bra016653	A08-18.661	40
R8	RS2CL5849s	82.67	Bra016644	A08-18.632	40
R8	RS2CL5318s	82.87	Bra015597	A10-0.275	40
R8	Rs2CL2338s	82.96	Bra016664	A08-18.706	40
R8	RS2CL3001s	83.11	Bra016739	A08-18.987	40
R8	RS2CL7379s	83.72			40
R8	RGC5306c	83.97	Bra016640	A08-18.615	40
R8	RGC8280c	83.97			40
R8	RGB8542c	83.97	Bra016836	A08-19.432	40
R8	RGB8994c	83.97	Bra016609	A08-18.501	40
R8	RS2CL7544s	84.27	Bra030780	A08-19.622	40
R8	RSS2157	84.62	Bra016755	A08-19.047	40
R8	RS2CL1601s	84.74	Bra016848	A08-19.501	40
R8	RSS3273	84.81	Bra016750	A08-19.034	40
R8	RS2CL7685s	85.42	Bra031639	A09-37.196	40
R8	RS2CL2573s	85.44	Bra030756	A08-19.707	40
R8	RS2CL1983s	85.58	Bra030799	A08-19.559	40
R8	RS2CL5298s	85.75	Bra030718	A08-19.899	40
R8	RS2CL5635s	85.91	Bra016570	A08-18.33	40
R8	RGB3215c	86.15	Bra018437	A05-8.218	40
R8	RSS0027	86.26	Bra030729	A08-19.845	40
R8	RS2CL2296s	86.45	Bra030790	A08-19.587	40
R8	RS2CL3554s	86.87	Bra018629	A06-2.91	40
R8	RSS1843	87.06	Bra018414	A05-8.108	40
R8	RS2CL4310s	87.42	Bra030705	A08-19.959	40
R8	RGC10842c	87.68	Bra030801	A08-19.552	40
R8	RGC13395c	87.68	Bra030709	A08-19.934	40
R8	RGC3413c	87.68	Bra030742	A08-19.775	40

R8	RGB3288c	87.87			40
R8	RGC989c	87.87	Bra030784	A08-19.608	40
R8	RSS2286	88.06	Bra030584	A08-20.441	40
R8	RS2CL3341s	88.21	Bra030599	A08-20.389	40
R8	RS2CL6784s	88.37	Bra015302	A10-2.107	40
R8	RGC4722c	88.59	Bra030560	A08-20.545	40
R8	RGC9339c	88.59	Bra030517	A08-20.722	40
R8	CC50	89.36			40
R8	RGC13159c	89.51	Bra018418	A05-8.121	40
R8	RS2CL2944s	90.15	Bra016834	A08-19.429	40
R8	Z17993	90.36			40
R8	CB80	91.26			40
R8	RGB3124c	92.24	Bra030183	A07-8.107	40
R8	RGC7108c	92.24	Bra011858	A01-0.21	40
R8	CB80N	93.36			40
R8	CA22bH	100.66			40
R8	RSS3836	101.36	Bra016534	A08-18.138	40
R9	PK9	0			
R9	CA88	5.8			
R9	RSS1006_a	8.5	Bra019484	A06-12.444	
R9	RSS0129	11	Bra035194	A07-25.819	41
R9	RSS2168	12.3	Bra035159	A07-25.695	41
R9	RSS2147	12.5	Bra012094	A07-12.281	41
R9	RGA1576s	12.9			41
R9	RSS4036	13.5	Bra035052	A07-25.255	41
R9	RS2CL7699s	14.59	Bra035095	A07-25.426	41
R9	RGA1547s	15.36			41
R9	RS2CL3487s	16.04	Bra035048	A07-25.232	41
R9	RSS2321	17.62			41
R9	RSS1369	18.26	Bra004367	A07-21.549	42
R9	RS2CL1156s	18.9	Bra004320	A07-21.257	42
R9	RS2CL1885s	19.14	Bra035407	A01-16.476	42
R9	RSS1177	19.52	Bra004402	A07-21.747	42
R9	RGC15483c	20.02			42
R9	RGB21440c	20.02	Bra015640	A07-24.885	42
R9	RGC22512c	20.02	Bra026703	A09-35.045	42
R9	RGC4199c	20.02			42
R9	RGC4665c	20.02	Bra003612	A07-17.345	42
R9	RS2CL1281s	20.2	Bra004397	A07-21.72	42
R9	RS2CL2288s	21.02	Bra006371	A03-3.222	42
R9	RSS3821	21.87	Bra016190	A07-22.261	42
R9	RS2CL808s	21.94	Bra016264	A07-21.845	42
R9	RGC24745c	22.32			42
R9	RGC4695c	22.32	Bra004294	A07-21.098	42
R9	RGC4895c	22.32	Bra004301	A07-21.133	42

R9	RS2CL4579s	22.68	Bra016263	A07-21.848	42
R9	CF12	22.82			42
R9	CA24N	22.92			42
R9	RS2CL4567s	23.1	Bra016255	A07-21.911	42
R9	RS2CL5197s	23.69	Bra016216	A07-22.115	42
R9	RS2CL4906s	23.91	Bra016216	A07-22.115	42
R9	RS2CL1327s	24.22	Bra016195	A07-22.219	42
R9	RGC10838c	24.23			42
R9	RGC11312c	24.23	Bra016213	A07-22.127	42
R9	RGC19152c	24.23			42
R9	RGC19895c	24.23	Bra016114	A07-22.622	42
R9	RGC4679c	24.23	Bra016237	A07-21.983	42
R9	RGC8563c	24.23			42
R9	RS2CL3830s	24.61	Bra016187	A07-22.277	42
R9	RSS1513	26.01	Bra016092	A07-22.746	42
R9	RS2CL3460s	26.12			42
R9	RSS3835	27.27	Bra016093	A07-22.742	42
R9	CC56aN	27.57			42
R9	RGA1745s	27.66			42
R9	CA34c	28.07			42
R9	RSS0475	29.07	Bra015994	A07-23.188	42
R9	RS2CL2301s	29.21	Bra015953	A07-23.362	42
R9	RGC6745c	29.99	Bra002566	A10-8.912	43
R9	RSS1532	30.9	Bra020332	A02-5.227	43
R9	RS2CL8479s	31.66	Bra002509	A10-9.218	43
R9	RSS0225	32.18	Bra002518	A10-9.139	43
R9	RGA1941s	32.47	Bra002561	A10-8.934	43
R9	RGC7717c	32.9	Bra002596	A10-8.722	43
R9	RS2CL3316s	33.32	Bra002616	A10-8.607	43
R9	Rs2CL1549s	34.13	Bra002568	A10-8.897	43
R9	RSS3830	34.16	Bra002528	A10-9.112	43
R9	OPR11bH	35.26			43
R9	RSS1337	35.76	Bra002582	A10-8.828	43
R9	RSS1870	36.56	Bra002622	A10-8.579	43
R9	RS2CL1098s	36.65	Bra002684	A10-8.319	43
R9	RS2CL3458s	36.88	Bra002673	A10-8.368	43
R9	RGC683c	37.96			43
R9	RSS1790	39.11	Bra002749	A10-7.939	43
R9	CA73	39.31			43
R9	RGB7901c	40.43	Bra002861	A10-7.141	43
R9	RS2CL4013s	41.08	Bra002841	A10-7.264	43
R9	OPR11aH	46.84			43
R9	RSS2112	47.14	Bra002924	A10-6.781	43
R9	RS2CL1950s	47.68	Bra002904	A10-6.922	43
R9	RSS0897	48.06			43
R9	RS2CL6276s	48.47	Bra002897	A10-6.955	43

R9	RS2CL1162s	48.59	Bra002928	A10-6.752	43
R9	RSS1696_a	48.78	Bra038099	A05-10.647	43
R9	RGC20593c	49.53			43
R9	RSS1592	50.09	Bra003055	A10-5.7	43
R9	RSS0706	50.49	Bra003069	A10-5.533	43
R9	RSS2916	50.69	Bra003086	A10-5.364	43
R9	RS2CL5504s	51.19	Bra002995	A10-6.254	43
R9	RGC2630c	51.3			43
R9	RSS1660	51.61			43
R9	RGA178s	52.61			43
R9	RGC6878c	53.04			43
R9	RGC607c	53.57	Bra003090	A10-5.334	43
R9	RS2CL5261s	53.82			43
R9	CA53aH	54.57			43
R9	RS2CL6424s	54.76	Bra003079	A10-5.439	43
R9	RS2CL7401s	55.84	Bra028269	A10-4.473	43
R9	RSS2661	56.33	Bra028279	A10-4.591	43
R9	RSS3495	59.43	Bra028862	A02-0.325	43
R9	RS2CL1353s	60.82	Bra028288	A10-4.673	43
R9	RSS2934	62.05	Bra040885	Scaffold000295-0.022	
R9	RGB15272c	62.2			
R9	RSS0404	63.19	Bra040885	Scaffold000295-0.022	
R9	RS2CL1092s	63.52	Bra037892	A09-15.831	44
R9	RS2CL3798s	63.86	Bra039774	A01-8.912	44
R9	RGB2773c	63.88			44
R9	RGC7838c	63.88	Bra037920	A09-16.149	44
R9	RS2CL4941s	63.97	Bra037951	A10-3.593	44
R9	CC62H	65.01			44
R9	Rs2CL2460s	65.4	Bra035259	A09-16.53	44
R9	RS2CL3791s	66.13	Bra035247	A09-16.619	44
R9	RS2CL1061s	66.44	Bra025359	A06-20.931	44
R9	RGC3132c	66.69	Bra035237	A09-16.753	44
R9	CC37aH	66.77			44
R9	RS2CL1946s	67.44	Bra035213	A09-17.001	44
R9	RS2CL3879s	67.92	Bra014871	A07-7.949	
R9	CC11H	68.69			
R9	RGC7876c	68.84			
R9	RGC17699c	69.55	Bra036257	A09-1.009	
R9	RGC21716c	69.55			
R9	RS2CL2047s	70.11	Bra029574	A09-18.405	
R9	F15571	70.94			
R9	RS2CL6991s	71.22	Bra026881	A09-35.842	
R9	RGC10800c	71.67			
R9	RGB21550c	71.67			
R9	RGB6475c	71.67			
R9	RGC13816c	71.99			

R9	Rs2CL1347s	72.64	Bra036285	A09-0.838	45
R9	CC83	73.17			45
R9	RGC12399c	73.22			45
R9	Rs2CL2196s	73.8	Bra000879	A03-13.686	45
R9	RS2CL964s	74.73	Bra000933	A03-14.02	45
R9	RSS1474	75.03	Bra037338	A09-0.146	45
R9	RGC7982c	75.19	Bra037404	A09-0.45	45
R9	RS2CL7317s	75.75	Bra037366	A09-0.292	45
R9	RSS1063	76.03	Bra039831	Scaffold000178-0.122	45
R9	RS2CL6758s	76.06	Bra037389	A09-0.388	45
R9	OPT17H	77.07			45
R9	RGB4147c	77.1	Bra037211	A09-5.008	45
R9	RGC5522c	77.1	Bra037306	A09-0.007	45
R9	RGC8507c	77.1			45
R9	RGC9843c	77.67	Bra036148	A09-2.235	45
R9	RS2CL3567s	77.68	Bra037326	A09-0.085	45
R9	CD32H	78.87			45
R9	RS2CL3739s	78.89	Bra039830	Scaffold000178-0.119	45
R9	RGC12323c	79.54	Bra024511	A06-16.942	45
R9	RGC12828c	80.03	Bra037282	A09-5.171	45
R9	RSS2466	81.29	Bra037403	A09-0.444	45
R9	Rs2CL2464s	81.6	Bra037306	A09-0.007	45
R9	RSS1475	83.65	Bra034212	A09-1.31	45
R9	RS2CL3325s	83.73	Bra037270	A09-5.245	46
R9	RGB5484c	84.38			46
R9	CD85	85.22			46
R9	Rs2CL1958s	88.92	Bra036700	A09-5.965	46
R9	RGB28103c	89.98	Bra027759	A09-6.851	46
R9	RGC11199c	91.4	Bra036646	A09-6.31	46
R9	RGC674c	91.4			46
R9	RS2CL832s	92.4	Bra027661	A09-7.483	46
R9	RGC684c	93.01			46
R9	RS2CL2118s	93.38	Bra027664	A09-7.472	46
R9	RGC7548c	94.07	Bra027745	A09-6.926	46
R9	RS2CL1196s	94.92	Bra027741	A09-6.946	46
R9	RS2CL1395s	95.28	Bra027717	A09-7.107	46
R9	RGC5191c	95.61			46
R9	CC43a	96.44			46
R9	CA80	100.44			46
R9	RGC3235c	100.54			46
R9	CC43aN	102.49			46
R9	RS2CL4058s	103.77	Bra027021	A09-8.112	46
R9	RGA1616s	104.73	Bra029601	A05-23.773	
R9	RGC22627c	105.86			
R9	RGC19925c	107.01			
R9	RGC2049c	107.01			

R9	RSS0140	107.54	Bra026489	A09-3.228	47
R9	RGA1944s	108.16			47
R9	OPT09b	108.7			47
R9	RSS2575	111.7	Bra036604	A09-2.612	47
R9	RSS1301	112.3	Bra036607	A09-2.596	47
R9	RGC2698c	113.43	Bra036604	A09-2.612	47
R9	RGC8995c	113.43			47
R9	RGA236s	113.58			47
R9	Rs2CL1221s	114.17	Bra036570	A09-2.772	47
R9	RGC7515c	115.38			47
R9	RS2CL4042s	115.45	Bra036111	A09-2.419	47
R9	RS2CL7387s	115.76	Bra036137	A09-2.317	47
R9	RS2CL2007s	115.93	Bra036125	A09-2.365	47
R9	CD11bH	116.39			47
R9	RGC1736c	117.63			47
R9	RS2CL7327s	117.9	Bra036223	A09-1.878	47
R9	RS2CL6327s	118.34	Bra036177	A09-2.077	47
R9	RGC13248c	118.48	Bra039071	A09-1.616	47
R9	Rs2CL2203s	118.74	Bra036221	A09-1.888	47
R9	RGB15203c	118.97	Bra017346	A09-14.707	47
R9	RS2CL2614s	119.09	Bra039046	A09-1.769	47
R9	RGA2478s	119.76			
R9	RSS1954	119.83	Bra017336	A09-14.756	
R9	RS2CL6430s	120.43	Bra036154	A09-2.19	
R9	RGC14934c	121.15	Bra026462	A09-3.092	
R9	RS2CL5708s	121.78	Bra017380	A09-14.316	
R9	RGC6292c	123.13			
R9	RGC9998c	123.13			
R9	RSS2285	123.16	Bra025140	A06-22.193	
R9	RGC6034c	123.28			
R9	RGC7030c	123.28			
R9	CA34a	123.73			
R9	CC16	124.23			
R9	RGC5701c	124.31	Bra035945	A09-3.404	
R9	RGB12822c	125.73	Bra038389	A09-15.488	48
R9	RGB19618c	125.77	Bra017539	A09-12.935	48
R9	RGC560c	126.31	Bra017563	A09-12.615	48
R9	RS2CL4118s	127.34	Bra027569	A09-11.817	48
R9	RSS1035	127.52	Bra017511	A09-13.238	48
R9	RS2CL5486s	127.93	Bra017581	A09-12.492	48
R9	RGC1999c	128.49	Bra017567	A09-12.561	48
R9	RSS3287	128.67	Bra017502	A09-13.31	48
R9	RGC5007c	128.91			48
R9	RS2CL3231s	129.25	Bra027460	A09-10.943	48
R9	RS2CL5203s	129.51	Bra027497	A09-11.289	48
R9	RGC2070c	130	Bra027450	A09-10.872	48

R9	RGC23150c	130			48
R9	RGB2543c	130			48
R9	RGC5810c	130	Bra027462	A09-10.955	48
R9	RGC6156c	130			48
R9	RGB8501c	130	Bra027570	A09-11.826	48
R9	RSS1133	130.12	Bra017577	A09-12.514	48
R9	RS2CL5024s	130.79	Bra027882	A09-10	48
R9	RSS0093	130.8	Bra027536	A09-11.614	48
R9	RS2CL3112s	131.1	Bra027877	A09-9.941	48
R9	RSS1329	131.1	Bra027544	A09-11.661	48
R9	RS2CL4174s	131.25	Bra027877	A09-9.941	48
R9	Gpi	131.48			48
R9	RS2CL5963s	131.63	Bra027880	A09-9.978	48
R9	RSS3041	131.77	Bra027509	A09-11.376	48
R9	RS2CL7452s	131.96	Bra027836	A09-9.538	48
R9	RSS0603	132.16	Bra027506	A09-11.362	48
R9	RS2CL1949s	132.17	Bra027125	A09-9.094	48
R9	RGC2758c	132.35			48
R9	RGC5530c	132.35	Bra027908	A09-10.177	48
R9	RGC8742c	132.56			48
R9	CA35a	132.74			48
R9	RS2CL5908s	133.03	Bra027821	A09-9.45	48
R9	RGC10531c	133.65	Bra027869	A09-9.853	48
R9	RGC6519c	134.07			48
R9	RGB13054c	134.36	Bra035838	A09-3.97	48
R9	RS2CL1116s	134.82	Bra027932	A09-10.377	48
R9	RS2CL3917s	135.53	Bra027860	A09-9.763	48
R9	RSS0155	136.73	Bra035877	A09-3.76	49
R9	RS2CL5880s	137.21	Bra038912	Scaffold000155-0.01	49
R9	RSS1128	138.23	Bra037843	A09-4.459	49
R9	RS2CL2842s	138.53	Bra037157	A09-4.715	49
R9	RSS2113	139.63			49
R9	RSS1425	141.43	Bra035875	A09-3.79	49
R9	Rs2CL1695s	143.22	Bra027462	A09-10.955	
R9	RGC15178c	146.77	Bra017388	A09-14.269	

Table S15 A summary of genetic map

LG	Number of markers	Length of linkage (cM)	Corresopondence<br>Shirasawa et al (2011)
R1	179	95.7	LG9
R2	349	146.7	LG5
R3	194	101.2	LG8
R4	392	142.2	LG4
R5	379	154.2	LG3
R6	339	167.7	LG2
R7	248	109.9	LG1
R8	226	101.4	LG7
R9	247	146.8	LG6
Total	2,553	1165.8	

Table S16 Assignment of scaffolds to integrated linkage map													
Linkage group	Marker loci(new)	ᐳosition (cM	strand	Q size	Q start	Q end	T name	T size	T start	T end	match ratio	Q ratio	ratio
R1	RGA2415s	0											
R1	RGC19516c	0.68	+	486	0	486	Rsa1.0_00059.1	159650	55585	56071	100.0	100.0	100.0
R1	RS2CL672s	5.4	+	1249	2	1249	Rsa1.0_00821.1	62891	49069	50975	97.8	99.8	152.9
R1	RS2CL6084s	6.61	−	1168	2	1168	Rsa1.0_00991.1	143101	17501	20481	76.5	99.8	255.6
R1	RS2CL7513s	7.64	−	775	0	775	Rsa1.0_00305.1	97308	15777	16540	96.7	100.0	98.5
R1	Rs2CL1500s	7.86	+	838	2	837	Rsa1.0_00949.1	58549	38318	39872	91.5	99.6	186.1
R1	RS2CL1784s	8.15	+	1061	0	1060	Rsa1.0_02722.1	28417	265	1578	99.6	99.9	123.9
R1	RS2CL5805s	8.3	+	1015	1	1015	Rsa1.0_01185.1	149842	2203	3243	75.7	99.9	102.6
R1	RS2CL4624s	8.57	+	919	0	914	Rsa1.0_00457.1	337301	176889	177758	92.7	99.5	95.1
R1	RS2CL5888s	9.37	+	1106	2	1106		101906	65557	66658	96.4	99.8	99.7
R1	RGC14676c	9.78	+	490	0	490	Rsa1.0_00085.1	142226	96079	96569	100.0	100.0	100.0
R1	RS2CL841s	9.99	+	689	1	689	Rsa1.0_00190.1	318240	190857	191807	90.3	99.9	138.1
R1	RS2CL6024s	10.66	−	541	1	402	Rsa1.0_00112.1	132199	84900	85301	56.2	74.1	100.0
R1	RS2CL6172s	10.88	+	909	0	909	Rsa1.0_00821.1	62891	46260	48030	98.8	100.0	194.7
R1	RS2CL5895s	11.05	−	905	1	905	Rsa1.0_00773.1	64839	52201	53278	90.9	99.9	119.1
R1	RS2CL4654s	11.89	+	1177	3	1177	Rsa1.0_00821.1	62891	54266	55665	89.0	99.8	119.2
R1	RSS3298	12.45	−	861	0	861	Rsa1.0_00832.1	62622	53953	55393	92.5	100.0	167.3
R1	CB62	15.15											
R1	Rs2CL1672s	15.78	−	1382	3	1382	Rsa1.0_00197.1	109714	23785	25730	97.9	99.8	141.0
R1	RSS1309	16.39	+	740	0	740	Rsa1.0_00336.1	445051	217080	218597	99.6	100.0	205.0
R1	RGC20489c	16.99	+	489	0	489	Rsa1.0_00018.1	410899	406915	407404	100.0	100.0	100.0
R1	RSS2487	17.02	−	845	15	845	Rsa1.0_00556.1	207212	31920	32947	78.8	98.2	123.7
R1	RS2CL4157s	17.05	+	1301	1	1301	Rsa1.0_01328.1	48021	41797	45013	94.8	99.9	247.4
R1	RS2CL6482s	18.07	+	1043	0	1042	Rsa1.0_00605.1	73199	26010	27269	97.0	99.9	120.8
R1	Rs2CL2045s	18.34	−	879	2	873	Rsa1.0_01537.1	187285	175377	176660	98.5	99.1	147.3
R1	RSS2037	18.71	+	909	0	909		222544	195313	196222	96.9	100.0	100.0
R1	Rs2CL2348s	19.35	−	1337	6	1337	Rsa1.0_00018.1	410899	404244	406641	85.3	99.6	180.1
R1	RSS0755	19.41	+	680	0	680	Rsa1.0_00991.1	143101	33841	34744	99.9	100.0	132.8
R1	RS2CL6151s	19.97	+	1073	1	1073	Rsa1.0_01166.1	51877	36964	38196	92.9	99.9	114.9
R1	RGC7078c	20.98	−	490	0	490	Rsa1.0_00615.1	72235	38718	39208	100.0	100.0	100.0
R1	RS2CL6126s	21.21	−	905	1	905	Rsa1.0_00018.1	410899	57045	58076	99.2	99.9	114.1
R1	OPE20Ha	21.39											
R1	RS2CL895s	21.97											
R1	RGA1821s	22.51											

R1	CC67aH	22.94											
R1	RGC1996c	22.96	−	458	0	458	Rsa1.0_00419.1	86562	43130	43588	100.0	100.0	100.0
R1	RSS3090	25.32	−	482	0	482	Rsa1.0_01749.1	39822	33735	34376	94.0	100.0	133.0
R1	RSS2047	29.92	+	861	0	861	Rsa1.0_00453.1	83031	64322	65512	70.0	100.0	138.2
R1	RGC1509c	30.59	−	426	0	426	Rsa1.0_00018.1	410899	259731	260154	98.4	100.0	99.3
R1	RGC6821c	30.59	−	412	0	412	Rsa1.0_00018.1	410899	11311	11723	100.0	100.0	100.0
R1	RS2CL7575s	30.89	+	871	1	871	Rsa1.0_00453.1	83031	18117	19715	50.6	99.9	183.7
R1	RSS0938	30.91	+	839	0	839	Rsa1.0_00911.1	59943	14883	16152	91.4	100.0	151.3
R1	RSS3778	31.31	−	419	0	419	Rsa1.0_05026.1	15095	10013	10436	89.0	100.0	101.0
R1	RGA2384s	32.66											
R1	RS2CL7786s	32.99	+	956	3	956	Rsa1.0_00065.1	454616	254014	255149	95.3	99.7	119.1
R1	RS2CL5804s	33.35	−	985	2	984	Rsa1.0_00965.1	58139	49431	50413	89.3	99.7	100.0
R1	RS2CL4651s	34	−	758	1	726	Rsa1.0_01724.1	40292	384	1486	92.6	95.7	152.0
R1	RS2CL5128s	34.14	−	838	29	838	Rsa1.0_02928.1	26680	25587	26484	79.0	96.5	110.9
R1	RS2CL7214s	34.53	+	1017	0	1017	Rsa1.0_00065.1	454616	207434	208714	99.8	100.0	125.9
R1	RS2CL6905s	34.74	−	1061	1	1061	Rsa1.0_00065.1	454616	132936	133989	95.9	99.9	99.3
R1	RS2CL6317s	35.31	−	947	39	947	Rsa1.0_00065.1	454616	58810	59890	90.8	95.9	118.9
R1	RS2CL5226s	36.21	−	1230	2	1228		77375	54316	55700	96.8	99.7	112.9
R1	RGA2190s	37.05											
R1	RGC6748c	37.13	+	440	0	440	Rsa1.0_00734.1	66508	10958	11398	100.0	100.0	100.0
R1	RGA2261s	37.39											
R1	RS2CL2084s	37.59	−	740	4	736	Rsa1.0_04142.1	18861	12654	13558	97.2	98.9	123.5
R1	RGA2423s	38.18											
R1	RGC3130c	38.24	+	461	0	461	Rsa1.0_00911.1	59943	16867	17328	100.0	100.0	100.0
R1	RGC11798c	38.54	−	440	0	440	Rsa1.0_00065.1	454616	412135	412575	100.0	100.0	100.0
R1	RGC18142c	38.54	−	515	1	515	Rsa1.0_00380.1	89748	66872	67386	99.8	99.8	100.0
R1	RGC9409c	38.54	−	429	0	429	Rsa1.0_00065.1	454616	250496	250925	98.8	100.0	100.0
R1	RGC2192c	39.98	+	475	0	475	Rsa1.0_00015.1	561803	446513	446988	100.0	100.0	100.0
R1	RGC23031c	39.98	+	452	0	452	Rsa1.0_00015.1	561803	284971	285423	100.0	100.0	100.0
R1	RGC5476c	39.98	−	495	0	495		77375	62331	62826	84.7	100.0	100.0
R1	RSS0987	40.32	+	894	68	894	Rsa1.0_00015.1	561803	352322	353148	81.9	92.4	100.0
R1	RSS1811	41.12	−	451	1	451	Rsa1.0_00805.1	63724	43178	43615	93.8	99.8	97.1
R1	RGC13289c	41.33	+	472	0	472	Rsa1.0_00067.1	415055	65195	65667	100.0	100.0	100.0
R1	RGC6011c	41.33	−	422	0	422	Rsa1.0_00876.1	61328	8099	8521	100.0	100.0	100.0
R1	RS2CL4282s	41.98	+	693	5	693	Rsa1.0_00805.1	63724	13361	14114	96.3	99.3	109.5
R1	RSS2736	42.7	+	812	0	812	Rsa1.0_00067.1	415055	247438	248459	96.1	100.0	125.7

R1	RGC6858c	43.24	+	449	0	449	Rsa1.0_00204.1	109056	58645	59094	45.0	100.0	100.0
R1	RGB9500c	44.09	+	408	0	408	Rsa1.0_00600.1	73395	69576	70364	99.8	100.0	193.1
R1	RGC453c	44.55	+	406	53	406	Rsa1.0_00059.1	159650	14824	15177	81.5	87.0	100.0
R1	RGB7310c	44.55	−	452	0	452	Rsa1.0_02253.1	33356	11895	12357	95.4	100.0	102.2
R1	RGC12216c	45.06	+	402	0	402	Rsa1.0_00346.1	93130	57437	57839	100.0	100.0	100.0
R1	OPE20b	45.24											
R1	RS2CL2578s	45.38	−	1175	2	1174	Rsa1.0_00067.1	415055	377758	379940	95.9	99.7	186.2
R1	RSS2094	45.53	+	811	0	811	Rsa1.0_01344.1	142535	109884	110835	84.3	100.0	117.3
R1	RS2CL2020s	46.15	−	1326	1	1324	Rsa1.0_03366.1	23401	8628	10694	99.2	99.8	156.2
R1	CB28	46.91											
R1	RS2CL5101s	47.07	+	984	0	984	Rsa1.0_00067.1	415055	6328	7816	98.1	100.0	151.2
R1	RS2CL2643s	47.2	+	832	2	832	Rsa1.0_00059.1	159650	121124	122276	86.7	99.8	138.8
R1	RS2CL3579s	47.2	−	1628	1	1627	Rsa1.0_00059.1	159650	38597	40319	96.4	99.9	105.9
R1	RSS1945	47.4	+	794	0	794	Rsa1.0_00059.1	159650	121139	122234	92.6	100.0	137.9
R1	RSS3875	47.5	−	866	0	866	Rsa1.0_00059.1	159650	133924	135118	98.9	100.0	137.9
R1	RS2CL5231s	47.52	+	952	3	952	Rsa1.0_02210.1	33857	2694	5231	90.8	99.7	267.3
R1	RS2CL4686s	48.3	−	1219	1	1206	Rsa1.0_00059.1	159650	88691	90890	97.8	98.9	182.5
R1	RSS2326	48.67	−	826	0	826		49419	30932	32156	99.3	100.0	148.2
R1	Pgm	48.77											
R1	RGC3295c	49.44	+	426	0	426	Rsa1.0_01600.1	42537	4897	5323	100.0	100.0	100.0
R1	RGA2466s	50.02											
R1	CC11	50.93											
R1	RS2CL7272s	50.99	−	718	0	717	Rsa1.0_02210.1	33857	30253	31059	96.8	99.9	112.4
R1	RGC14226c	51.66	−	562	0	562	Rsa1.0_00204.1	109056	77526	78087	99.8	100.0	99.8
R1	RS2CL7240s	52.01	−	685	0	682	Rsa1.0_02387.1	31890	16724	17783	86.0	99.6	155.3
R1	RS2CL7289s	53.29	+	754	1	754	Rsa1.0_07613.1	9021	2349	3509	75.7	99.9	154.1
R1	RGC13028c	53.92	−	519	0	519	Rsa1.0_00204.1	109056	14089	14608	100.0	100.0	100.0
R1	RS2CL3783s	54.59	+	860	0	859	Rsa1.0_01091.1	54383	48071	49970	84.0	99.9	221.1
R1	RS2CL8267s	55.77	+	887	0	887	Rsa1.0_02939.1	26595	10284	11478	96.8	100.0	134.6
R1	RGC14293c	56.66	−	499	0	499	Rsa1.0_00050.1	237174	87408	87907	100.0	100.0	100.0
R1	RGB1137c	58.06	−	581	0	581	Rsa1.0_02910.1	26843	25508	26089	100.0	100.0	100.0
R1	RGC18589c	58.06	+	429	0	429	Rsa1.0_00021.1	263655	7664	8093	100.0	100.0	100.0
R1	RSS0325	59.29	−	826	0	593	Rsa1.0_04734.1	16184	4508	5101	52.5	71.8	100.0
R1	RSS4011	59.79	+	909	1	909	Rsa1.0_02110.1	34919	22282	23191	90.5	99.9	100.1
R1	RS2CL7006s	61.13	+	626	4	625	Rsa1.0_00021.1	263655	80112	80991	96.8	99.2	141.6
R1	RGC15930c	61.77	−	549	0	549	Rsa1.0_00065.1	454616	26253	26802	100.0	100.0	100.0

R1	RGC3696c	62.07												
R1	RS2CL7647s	62.83	+	693	0	693	Rsa1.0_00021.1	263655	179001	179866	97.4	100.0	124.8
R1	RGC11904c	63.88	+	430	0	430	Rsa1.0_00021.1	263655	66560	66990	100.0	100.0	100.0
R1	RGC7796c	63.88	−	467	0	245	Rsa1.0_00021.1	263655	257072	257317	52.5	52.5	100.0
R1	RS2CL4233s	64.51	−	1026	35	1025	Rsa1.0_03193.1	24690	8281	9272	88.2	96.5	100.1
R1	RGC12943c	64.75	+	437	0	437	Rsa1.0_03633.1	21644	5961	6398	100.0	100.0	100.0
R1	RGC15280c	64.75	−	408	0	408	Rsa1.0_01190.1	194501	157316	157724	100.0	100.0	100.0
R1	RS2CL7501s	65.71	+	908	3	902	Rsa1.0_02054.1	35548	23043	24021	93.3	99.0	108.8
R1	RS2CL5860s	66.58	−	460	99	460	Rsa1.0_00519.1	197864	65704	66065	77.6	78.5	100.0
R1	RSS2045	66.67	−	744	0	744		606987	447549	448386	96.2	100.0	112.5
R1	RSS2843	66.67	−	861	10	861		606987	468762	469853	95.4	98.8	128.2
R1	RSS3638	66.67	+	691	1	691		396176	186772	187491	95.5	99.9	104.2
R1	RGC14452c	68.11											
R1	RSS0679	68.37	+	747	9	747	Rsa1.0_01190.1	194501	9120	10343	98.8	98.8	165.7
R1	RGB2900c	68.82	+	429	0	429	Rsa1.0_00453.1	83031	64685	65114	100.0	100.0	100.0
R1	RSS0442	69.17	−	819	0	819	Rsa1.0_16049.1	2306	1040	1859	99.8	100.0	100.0
R1	RSS0913	71.47	−	710	0	708		88681	34712	35733	99.7	99.7	144.2
R1	RS2CL2130s	71.65	−	883	3	883	Rsa1.0_00459.1	82580	41014	42357	99.6	99.7	152.6
R1	RSS3818	72.83	−	894	0	884	Rsa1.0_02209.1	33873	10295	11180	61.6	98.9	100.1
R1	Rs2CL1826s	72.89	+	708	0	708	Rsa1.0_00459.1	82580	56624	57741	71.6	100.0	157.8
R1	RGC897c	73.47	−	512	0	512	Rsa1.0_00462.1	82288	41837	42349	100.0	100.0	100.0
R1	RGC20779c	74.35	+	456	0	456	Rsa1.0_01499.1	44555	42687	43143	70.4	100.0	100.0
R1	RGB3655c	75.57	−	420	0	420	Rsa1.0_12212.1	5287	3558	3978	46.7	100.0	100.0
R1	RGB1443c	76.34	+	407	0	407		69858	62749	63227	88.7	100.0	117.4
R1	RS2CL7691s	76.63	−	624	1	624	Rsa1.0_00943.1	58862	6633	8828	97.3	99.8	352.3
R1	RSS0997	77.39	−	872	0	872		329018	206985	208282	97.3	100.0	148.7
R1	RGC23207c	77.65	−	594	0	296	Rsa1.0_01017.1	56714	38900	39196	42.3	49.8	100.0
R1	RGC4403c	77.65	+	421	0	421	Rsa1.0_00808.1	63351	39275	39696	100.0	100.0	100.0
R1	RGC715c	77.65	+	468	0	468	Rsa1.0_01346.1	47618	13473	13941	100.0	100.0	100.0
R1	RS2CL8104s	78.05	+	1114	1	1114	Rsa1.0_00462.1	82288	1235	2411	98.7	99.9	105.7
R1	RSS0923	78.13	+	844	0	843	Rsa1.0_00155.1	118465	51111	52940	98.7	99.9	217.0
R1	RS2CL5132s	78.61	+	835	1	835	Rsa1.0_01999.1	36237	14503	15788	96.1	99.9	154.1
R1	RS2CL1854s	78.75	+	651	1	644	Rsa1.0_01673.1	41197	7446	8102	96.8	98.8	102.0
R1	RSS1995	79.15	−	539	3	403	Rsa1.0_01215.1	166813	160027	160482	72.9	74.2	113.8
R1	RGC1879c	79.18											
R1	RS2CL5940s	79.53	+	740	0	655	Rsa1.0_01780.1	39411	2824	3469	83.5	88.5	98.5

R1	RS2CL7378s	79.58	−	664	1	664	Rsa1.0_00106.1	133916	95839	96586	98.5	99.9	112.7
R1	RGC15296c	79.98	−	472	0	472	Rsa1.0_08003.1	8094	6502	6974	100.0	100.0	100.0
R1	RSS2552	80.3	+	863	0	863	Rsa1.0_00106.1	133916	5575	6438	99.1	100.0	100.0
R1	RS2CL5546s	80.93											
R1	RSS1912	81	−	870	53	870	Rsa1.0_00904.1	180594	17758	18557	86.0	93.9	97.8
R1	RGC6603c	81.24	−	568	0	568	Rsa1.0_05366.1	14036	10068	10636	37.0	100.0	100.0
R1	RS2CL1420s	81.55											
R1	RGC1871c	81.98	−	454	0	454	Rsa1.0_03062.1	25678	6994	7448	86.3	100.0	100.0
R1	RS2CL7231s	82.45	−	740	2	739	Rsa1.0_04862.1	15688	3297	4240	98.2	99.6	128.0
R1	CC60H	82.7											
R1	RS2CL996s	82.92	−	948	0	945	Rsa1.0_07998.1	8105	3133	4977	96.8	99.7	195.1
R1	RGB10948c	83.21	−	460	0	460	Rsa1.0_00778.1	196751	185987	186447	100.0	100.0	100.0
R1	RGC32005c	83.73	+	435	0	435	Rsa1.0_00731.1	66638	13018	13453	100.0	100.0	100.0
R1	RGC4062c	83.73	+	561	0	561	Rsa1.0_00769.1	64911	45346	45907	57.0	100.0	100.0
R1	RGC4642c	83.73	+	557	0	557	Rsa1.0_04111.1	18991	13676	14235	94.6	100.0	100.4
R1	RGC9187c	84.21	+	452	0	452	Rsa1.0_00904.1	180594	145545	145997	94.7	100.0	100.0
R1	RSS1252	84.4	−	635	0	635		78829	75487	76222	81.9	100.0	115.8
R1	RGC3063c	85.17	−	502	46	502	Rsa1.0_02067.1	35451	29061	29517	90.8	90.8	100.0
R1	RGC1234c	85.32	+	442	0	442	Rsa1.0_01009.1	56922	39925	40367	100.0	100.0	100.0
R1	RSS1998	85.8	+	866	8	866	Rsa1.0_05663.1	13005	8450	9309	99.0	99.1	100.1
R1	RS2CL6354s	85.86	−	1069	1	1069	Rsa1.0_00859.1	61795	59674	60986	98.9	99.9	122.9
R1	RSS0430	86	+	808	0	808		12173	2326	3128	97.3	100.0	99.3
R1	RS2CL2122s	86.08	+	845	0	845	Rsa1.0_07808.1	8504	118	1517	97.6	100.0	165.6
R1	RGC6043c	86.3	+	410	0	410	Rsa1.0_00890.1	323870	313428	313838	100.0	100.0	100.0
R1	RS2CL4281s	86.63	−	934	18	810	Rsa1.0_00135.1	178077	168916	170096	82.3	84.8	149.0
R1	RS2CL5593s	87.28	+	1059	2	1059	Rsa1.0_01416.1	46262	45010	46072	97.0	99.8	100.5
R1	RGC3820c	87.88	−	518	0	518	Rsa1.0_01436.1	45894	10102	10620	100.0	100.0	100.0
R1	RSS0471	87.9	−	576	10	568	Rsa1.0_00890.1	323870	200883	201448	75.5	96.9	101.3
R1	RGC3016c	88	−	493	0	493	Rsa1.0_01556.1	163858	161343	161836	99.6	100.0	100.0
R1	RSS2516	88.4											
R1	RSS3378	88.4	−	816	1	816	Rsa1.0_00518.1	78360	14556	15422	94.2	99.9	106.3
R1	Rs2CL2428s	89	+	512	2	512	Rsa1.0_00462.1	82288	36045	36741	97.5	99.6	136.5
R1	RSS0925	89.9	+	839	0	839	Rsa1.0_01436.1	45894	13429	15109	99.9	100.0	200.2
R1	RSS2494	90.7	−	863	0	863	Rsa1.0_01641.1	188213	178281	179864	100.0	100.0	183.4
R1	RSS3402	90.7	+	691	44	687	Rsa1.0_01641.1	188213	177955	179008	93.1	93.1	163.8
R1	CC89H	91.4											

R1	RSS0496	95.7	−	723	0	723	Rsa1.0_01436.1	45894	36125	37418	100.0	100.0	178.8
R2	RSS3197	0	−	852	0	852	Rsa1.0_00017.1	752458	3298	4151	97.5	100.0	100.1
R2	RGC2914c	1.88	−	476	0	476	Rsa1.0_03625.1	21692	1176	1652	99.6	100.0	100.0
R2	CA41N	4.3											
R2	RGC12735c	6.75	+	470	0	470	Rsa1.0_00094.1	139042	24718	25188	100.0	100.0	100.0
R2	RS2CL8380s	8.75	−	1031	0	1031	Rsa1.0_04097.1	19090	16083	18186	98.7	100.0	204.0
R2	RGB8387c	9.56	−	520	0	520		167185	148584	149104	100.0	100.0	100.0
R2	RS2CL1172s	10.39											
R2	CA41	10.9											
R2	RS2CL6266s	10.92	+	454	1	454		167185	13099	13949	90.3	99.8	187.6
R2	CD57H	11.1											
R2	RSS0368	12											
R2	RGC9800c	12.25	−	462	0	462	Rsa1.0_00529.1	77487	29394	29856	92.4	100.0	100.0
R2	RSS2503	12.9											
R2	RSS3053	12.9	−	828	0	788		301669	219589	220653	95.1	95.2	135.0
R2	RSS3449	12.9	−	837	0	837	Rsa1.0_01053.1	55483	13784	14708	100.0	100.0	110.4
R2	RSS1595	13	+	583	0	579	Rsa1.0_03169.1	24826	605	1531	99.3	99.3	159.9
R2	RSS0251	13.1	+	833	0	833	Rsa1.0_06073.1	11807	3582	4602	96.4	100.0	122.5
R2	RSS0845	13.1	+	849	0	849	Rsa1.0_01670.1	41260	14398	15247	80.0	100.0	100.0
R2	RSS1374	13.1	−	674	0	674		301669	293105	294367	39.0	100.0	187.2
R2	RSS0073_a	13.2	+	684	0	680		365886	323535	324434	95.2	99.4	132.2
R2	RS2CL986s	13.61	−	1679	4	1679	Rsa1.0_03169.1	24826	15793	18358	99.0	99.8	153.1
R2	RGC15346c	13.81	−	548	0	548	Rsa1.0_00461.1	82509	32324	32872	100.0	100.0	100.0
R2	RS2CL2676s	14.27	+	1274	0	1272		152362	39167	41218	96.2	99.8	161.2
R2	RGC9253c	14.68	+	409	0	409	Rsa1.0_00017.1	752458	711870	712279	100.0	100.0	100.0
R2	RS2CL2351s	15.12	−	925	0	924		167185	125427	126354	96.7	99.9	100.3
R2	RS2CL6213s	15.6	−	793	0	764		167185	77363	78539	93.3	96.3	153.9
R2	RSS1442	15.7	+	819	0	819	Rsa1.0_00037.1	307838	186275	187094	99.5	100.0	100.0
R2	RS2CL5010s	16.14	−	717	0	717	Rsa1.0_00426.1	85668	72903	73812	93.0	100.0	126.8
R2	RGB5729c	16.47	+	404	0	404	Rsa1.0_00426.1	85668	12394	12798	93.8	100.0	100.0
R2	RGC5970c	16.47	−	568	0	568		301669	55455	56021	98.6	100.0	99.7
R2	RGC8308c	16.47	−	484	0	484		301669	228492	228976	100.0	100.0	100.0
R2	RGB12209c	16.67	−	447	0	447	Rsa1.0_00037.1	307838	21814	22261	100.0	100.0	100.0
R2	RSS1863	16.8	−	836	0	836	Rsa1.0_03964.1	19835	2326	3450	95.5	100.0	134.5
R2	RSS2730	16.8											

R2	RS2CL2152s	16.82	−	1042	2	1042	Rsa1.0_00693.1	161311	60094	61424	90.2	99.8	127.9
R2	RSS1640	16.9	−	824	0	824	Rsa1.0_01634.1	41865	38910	40639	88.4	100.0	209.8
R2	RGC17861c	17.15	+	542	0	542	Rsa1.0_01010.1	56906	8931	9473	60.3	100.0	100.0
R2	RGA1922s	17.24											
R2	RS2CL7559s	17.37	−	1390	0	1390		301669	191693	193217	57.2	100.0	109.6
R2	RS2CL826s	17.52	−	1077	6	1077		301669	238083	239810	95.8	99.4	161.3
R2	RS2CL6429s	17.75	−	780	0	780	Rsa1.0_00461.1	82509	19937	20815	99.0	100.0	112.6
R2	RS2CL4831s	17.76	−	1083	1	1078		301669	282414	283492	97.5	99.5	100.1
R2	RS2CL3429s	17.76	+	500	4	499		301669	294783	295429	94.0	99.0	130.5
R2	RS2CL6141s	17.92	+	947	112	945	Rsa1.0_04478.1	17183	2594	3587	84.8	88.0	119.2
R2	RS2CL2740s	18.06	+	1225	2	1225	Rsa1.0_01010.1	56906	23346	25927	96.2	99.8	211.0
R2	RSS1435	18.1	+	840	0	840		167185	96460	97706	75.7	100.0	148.3
R2	RSS2508	18.2	+	718	2	718		167185	125681	126378	93.5	99.7	97.4
R2	Rs2CL1685s	18.75	−	1012	0	1012	Rsa1.0_01634.1	41865	33091	34550	96.5	100.0	144.2
R2	RS2CL2162s	19	+	801	1	801	Rsa1.0_00487.1	80674	70327	71133	98.9	99.9	100.8
R2	RS2CL1115s	19.23	+	900	2	900	Rsa1.0_00127.1	127446	112975	115148	94.0	99.8	242.0
R2	RSS1796	19.4	+	493	4	493	Rsa1.0_00127.1	127446	24931	25592	88.0	99.2	135.2
R2	RS2CL6767s	19.46	−	656	0	656	Rsa1.0_00487.1	80674	17359	18280	99.4	100.0	140.4
R2	RS2CL8214s	19.55	−	833	2	832	Rsa1.0_00611.1	72410	15017	15859	95.6	99.6	101.5
R2	RGC21070c	19.59	+	518	0	518	Rsa1.0_00611.1	72410	60010	60528	100.0	100.0	100.0
R2	RGC212c	19.59	−	503	0	503	Rsa1.0_00094.1	139042	79373	79876	100.0	100.0	100.0
R2	RGC2575c	19.59	+	407	0	407	Rsa1.0_02931.1	26654	14437	14844	100.0	100.0	100.0
R2	RGC2666c	19.59											
R2	RGC6462c	19.59	−	410	0	410	Rsa1.0_00464.1	82253	11150	11560	100.0	100.0	100.0
R2	RS2CL4022s	19.85	+	981	2	897	Rsa1.0_00094.1	139042	89983	91232	57.5	91.2	139.6
R2	Rs2CL1791s	19.86	+	458	1	457	Rsa1.0_00762.1	165246	64387	64970	87.3	99.6	127.9
R2	RGC16214c	19.95	−	504	196	475	Rsa1.0_00083.1	442773	309461	309740	55.4	55.4	100.0
R2	RS2CL7540s	20.16	+	746	0	746	Rsa1.0_00464.1	82253	47661	49076	58.6	100.0	189.7
R2	RGC22795c	20.7	+	557	0	557	Rsa1.0_00668.1	69421	61597	62154	100.0	100.0	100.0
R2	RS2CL6543s	20.72	+	1273	0	1273	Rsa1.0_00017.1	752458	743111	745235	96.9	100.0	166.9
R2	RS2CL3997s	20.74	−	878	0	876		167185	119899	121160	96.9	99.8	144.0
R2	RSS3566	20.9	−	866	3	866	Rsa1.0_02358.1	32213	8176	9440	85.0	99.7	146.5
R2	RGC4451c	21.13	−	497	0	497		167185	75879	76376	100.0	100.0	100.0
R2	RS2CL1822s	21.21	+	1755	1	1755	Rsa1.0_03209.1	24592	16799	19317	98.8	99.9	143.6
R2	RGC4476c	21.51	+	453	0	453	Rsa1.0_00729.1	66690	59266	59719	100.0	100.0	100.0
R2	RS2CL5248s	21.66	−	820	0	815	Rsa1.0_00017.1	752458	416174	416989	93.8	99.4	100.0

R2	RS2CL6952s	21.76	−	730	2	717	Rsa1.0_00017.1	752458	398380	399827	97.0	98.0	202.4
R2	RSS0957	21.9	−	871	2	871	Rsa1.0_00356.1	91521	4827	5696	99.8	99.8	100.0
R2	RS2CL5432s	22.08	−	1033	2	1033	Rsa1.0_00017.1	752458	391648	394148	97.7	99.8	242.5
R2	RGC10385c	22.23	−	402	0	402	Rsa1.0_00277.1	100132	34002	34404	100.0	100.0	100.0
R2	RSS2483	22.5	−	862	0	862	Rsa1.0_00277.1	100132	39422	40284	100.0	100.0	100.0
R2	RS2CL4657s	22.51	−	948	0	948	Rsa1.0_02840.1	27386	23809	25381	91.8	100.0	165.8
R2	RS2CL4960s	22.85	−	657	0	657	Rsa1.0_00277.1	100132	35699	36435	98.6	100.0	112.0
R2	RS2CL3611s	23.07	+	763	1	763	Rsa1.0_00277.1	100132	61993	62792	98.4	99.9	104.9
R2	RSS3805	23.1	−	842	1	842	Rsa1.0_00729.1	66690	27221	28062	96.6	99.9	100.0
R2	RGC12573c	23.27	+	471	0	471	Rsa1.0_01015.1	56752	18181	18652	100.0	100.0	100.0
R2	RGC1566c	23.27	−	463	0	463	Rsa1.0_00721.1	66918	20575	21038	100.0	100.0	100.0
R2	RGC3643c	23.27	+	458	0	458	Rsa1.0_02358.1	32213	4211	4669	98.3	100.0	100.0
R2	RGB419c	23.27	+	464	0	464	Rsa1.0_00897.1	60498	19835	20366	100.0	100.0	114.4
R2	RGC4350c	23.27	+	446	0	446	Rsa1.0_00017.1	752458	69766	70212	100.0	100.0	100.0
R2	RGB921c	23.27	+	402	0	402	Rsa1.0_00017.1	752458	607929	608331	100.0	100.0	100.0
R2	RS2CL2632s	23.29											
R2	RS2CL2446s	23.61	+	1250	2	1250	Rsa1.0_02703.1	28544	4006	10261	89.2	99.8	501.2
R2	RS2CL1476s	23.8	−	1317	0	1316	Rsa1.0_00721.1	66918	58130	59802	60.0	99.9	127.1
R2	RS2CL1124s	23.94	+	2428	0	2427	Rsa1.0_01141.1	52530	3533	7345	92.8	100.0	157.1
R2	RS2CL5639s	24.16	+	1064	3	1059	Rsa1.0_00729.1	66690	55928	57170	70.7	99.3	117.6
R2	RS2CL3061s	24.43	+	1150	2	1111	Rsa1.0_02703.1	28544	24847	26495	95.4	96.4	148.6
R2	CB72H	24.5											
R2	Rs2CL1639s	24.64	+	779	0	779	Rsa1.0_00744.1	66084	52412	53621	98.5	100.0	155.2
R2	RS2CL8169s	24.94	−	941	4	941	Rsa1.0_00744.1	66084	3356	5405	95.9	99.6	218.7
R2	BRMS036	25.2											
R2	RGC12949c	25.36	+	580	0	580	Rsa1.0_02295.1	149884	132359	132939	100.0	100.0	100.0
R2	RGC148c	25.36	+	501	0	501	Rsa1.0_02295.1	149884	22238	22739	100.0	100.0	100.0
R2	RS2CL3052s	25.46	−	728	3	728	Rsa1.0_00171.1	115622	51138	52602	97.7	99.6	201.9
R2	RSS0445	25.8	+	681	0	681	Rsa1.0_04450.1	17297	11689	12594	100.0	100.0	132.9
R2	RS2CL7090s	25.88	+	748	2	748	Rsa1.0_00909.1	59976	3985	5053	89.7	99.7	143.2
R2	RS2CL4177s	26.05	+	767	17	767	Rsa1.0_02295.1	149884	117484	118774	95.2	97.8	172.0
R2	RSS0802	26.3	−	718	1	716	Rsa1.0_03304.1	23883	20725	21785	99.6	99.6	148.3
R2	RSS3702	26.5	−	856	0	856	Rsa1.0_00476.1	280966	7920	9372	99.8	100.0	169.6
R2	RS2CL6599s	26.65	+	1097	2	1097	Rsa1.0_03784.1	20887	12821	13958	91.6	99.8	103.8
R2	RGB3040c	26.97	−	502	0	502	Rsa1.0_02400.1	31750	14321	14833	98.2	100.0	102.0
R2	RSS1032	27	+	547	0	547	Rsa1.0_00476.1	280966	147114	147663	100.0	100.0	100.4

R2	RS2CL6683s	27.19	+	694	1	694	Rsa1.0_00283.1	183797	65958	66735	99.7	99.9	112.1
R2	RGC7444c	27.36	−	409	9	409	Rsa1.0_03304.1	23883	9427	9827	97.8	97.8	100.0
R2	Rs2CL2399s	27.68											
R2	RGC20165c	27.88	−	587	0	587	Rsa1.0_00476.1	280966	35824	36411	99.8	100.0	100.0
R2	RGC2344c	27.88	−	416	0	416	Rsa1.0_00121.1	278029	91060	91476	100.0	100.0	100.0
R2	RGC9866c	27.88	−	515	0	515	Rsa1.0_00630.1	71281	22897	23412	100.0	100.0	100.0
R2	RSS2114	27.9	−	860	0	860	Rsa1.0_00630.1	71281	4928	5965	100.0	100.0	120.6
R2	RGC9047c	28	+	471	345	471	Rsa1.0_00630.1	71281	54590	54716	26.8	26.8	100.0
R2	RS2CL4120s	28	+	824	1	794	Rsa1.0_01460.1	45255	32135	33019	90.4	96.2	111.5
R2	RS2CL831s	28.34	−	766	9	765	Rsa1.0_01460.1	45255	2071	3605	64.1	98.7	202.9
R2	RS2CL2568s	28.61	−	1243	2	1243	Rsa1.0_00121.1	278029	43470	45226	93.4	99.8	141.5
R2	RS2CL2222s	28.81	−	989	0	959	Rsa1.0_00630.1	71281	21089	22524	94.4	97.0	149.6
R2	RS2CL7550s	29.06	−	1049	2	1049	Rsa1.0_00630.1	71281	61634	63089	96.7	99.8	139.0
R2	RS2CL1001s	29.17	−	756	0	752	Rsa1.0_00630.1	71281	63710	64978	98.4	99.5	168.6
R2	RS2CL2803s	29.61	+	1017	5	1003	Rsa1.0_00838.1	153820	52227	53407	49.9	98.1	118.2
R2	RGC2145c	30.12	+	421	0	421	Rsa1.0_00102.1	135497	41306	41727	100.0	100.0	100.0
R2	RS2CL3202s	30.38	+	838	0	836	Rsa1.0_00198.1	222963	84022	85419	99.8	99.8	167.1
R2	RSS3094	30.5	−	844	0	844	Rsa1.0_00102.1	135497	28034	29083	99.1	100.0	124.3
R2	RS2CL3241s	30.7	+	1124	2	1124	Rsa1.0_03290.1	24011	22635	23808	34.2	99.8	104.6
R2	RGC43c	31.12	−	449	0	449	Rsa1.0_00001.1	363028	53225	53674	100.0	100.0	100.0
R2	RSS2752	31.2	+	777	0	777	Rsa1.0_00198.1	222963	128619	130033	95.1	100.0	182.0
R2	RS2CL1313s	31.21	+	719	0	717	Rsa1.0_00121.1	278029	122847	124309	90.4	99.7	203.9
R2	RGC7617c	31.35	+	414	0	414	Rsa1.0_00001.1	363028	311136	311550	100.0	100.0	100.0
R2	RSS2898	31.4	+	836	2	836	Rsa1.0_00198.1	222963	105082	106319	97.6	99.8	148.3
R2	RS2CL3641s	31.7	−	894	4	894	Rsa1.0_00198.1	222963	176265	177333	77.7	99.6	120.0
R2	RS2CL2961s	32.19	+	1187	1	1187	Rsa1.0_01701.1	162121	144250	145583	99.5	99.9	112.4
R2	RS2CL3459s	32.49	−	707	0	707	Rsa1.0_01701.1	162121	19813	20603	97.3	100.0	111.7
R2	RS2CL1051s	32.92	+	1356	2	1356	Rsa1.0_00198.1	222963	141543	143411	97.2	99.9	138.0
R2	RSS1170	33.1	+	811	4	811	Rsa1.0_00001.1	363028	279762	280931	97.7	99.5	144.9
R2	RSS2001	33.5	−	866	0	866	Rsa1.0_00001.1	363028	265429	267044	94.8	100.0	186.5
R2	RS2CL6882s	33.69	−	1125	1	1125	Rsa1.0_00198.1	222963	13519	14634	91.4	99.9	99.2
R2	RS2CL4789s	35.07											
R2	RS2CL4931s	35.07	+	1085	3	1085	Rsa1.0_00729.1	66690	48844	50228	73.7	99.7	127.9
R2	RS2CL6206s	35.07	+	874	2	874	Rsa1.0_00001.1	363028	73735	74608	97.4	99.8	100.1
R2	Rs2CL2267s	35.07	+	878	11	878	Rsa1.0_00001.1	363028	92859	94113	93.4	98.8	144.6
R2	RS2CL3897s	35.7	−	1258	2	1258	Rsa1.0_02257.1	33318	18907	22221	94.1	99.8	263.9

R2	RSS0645	36											
R2	RSS1152	36	−	843	0	843	Rsa1.0_00356.1	91521	90671	91514	100.0	100.0	100.0
R2	RSS2678	36	+	791	0	773	Rsa1.0_00356.1	91521	80148	81230	97.6	97.7	140.0
R2	RGC1244c	36.02	+	492	0	492	Rsa1.0_00706.1	291570	186654	187146	100.0	100.0	100.0
R2	RSS2341	36.1	−	433	8	433	Rsa1.0_00441.1	84607	19085	19513	97.5	98.2	100.7
R2	RS2CL7884s	36.19	+	613	2	611	Rsa1.0_00697.1	67913	59554	61392	96.6	99.4	301.8
R2	RS2CL3734s	36.37	+	648	3	648	Rsa1.0_00473.1	212204	76081	76866	94.1	99.5	121.7
R2	Rs2CL1620s	36.37	−	1198	2	1198	Rsa1.0_00706.1	291570	229284	231346	98.3	99.8	172.4
R2	RS2CL5841s	36.66	−	1115	3	1115	Rsa1.0_03138.1	25092	9098	10683	98.7	99.7	142.5
R2	RS2CL1379s	37.03	+	745	2	744	Rsa1.0_00706.1	291570	159073	160169	97.6	99.6	147.7
R2	RSS1500	37.3	−	834	0	834	Rsa1.0_00706.1	291570	7623	8460	85.6	100.0	100.4
R2	RSS4012	37.3	−	835	0	835	Rsa1.0_00706.1	291570	32116	33245	97.1	100.0	135.2
R2	RSS3707	37.6	−	877	0	877	Rsa1.0_00115.1	131321	110663	111541	100.0	100.0	100.1
R2	RSS0715	37.7	+	745	4	745	Rsa1.0_00108.1	478491	458858	459708	99.5	99.5	114.7
R2	RSS1692	37.7	−	880	0	880	Rsa1.0_02329.1	32474	9694	10996	98.8	100.0	148.0
R2	RSS2367	38.7	+	855	0	855	Rsa1.0_00108.1	478491	9476	11627	94.2	100.0	251.6
R2	RSS3086	38.7	+	881	1	881	Rsa1.0_00108.1	478491	36389	38860	99.1	99.9	280.8
R2	RS2CL1253s	39.05	−	1032	0	1030	Rsa1.0_00706.1	291570	58067	60014	99.4	99.8	189.0
R2	RS2CL7301s	39.34	−	816	0	816	Rsa1.0_00706.1	291570	23305	24200	96.9	100.0	109.7
R2	RGC12272c	39.68	−	480	0	480	Rsa1.0_00108.1	478491	407111	407591	100.0	100.0	100.0
R2	RGC18651c	39.68	+	421	0	421	Rsa1.0_00473.1	212204	200373	200794	100.0	100.0	100.0
R2	RGC4643c	39.68	+	399	0	399	Rsa1.0_00108.1	478491	466397	466796	100.0	100.0	100.0
R2	RS2CL8395s	39.86	−	837	1	837	Rsa1.0_00115.1	131321	73166	74397	98.7	99.9	147.3
R2	RS2CL3283s	40.08	+	1158	0	1156	Rsa1.0_00108.1	478491	90484	92227	96.7	99.8	150.8
R2	RS2CL5011s	40.59	+	833	1	832	Rsa1.0_00108.1	478491	39781	41051	95.2	99.8	152.8
R2	RSS2368	40.62	+	653	0	653	Rsa1.0_00033.1	515811	149172	150441	100.0	100.0	194.3
R2	RSS3742	40.62	−	478	3	478	Rsa1.0_02976.1	26315	18529	19043	89.5	99.4	108.2
R2	RSS3354	40.72	+	439	9	318		316976	204963	205281	66.3	70.4	102.9
R2	RGA106s	40.91											
R2	RS2CL5071s	41.08	+	1018	3	1018	Rsa1.0_00107.1	431659	280742	281857	98.8	99.7	109.9
R2	RS2CL942s	41.4	−	638	3	637		167018	79406	80502	95.6	99.4	172.9
R2	RSS0590	41.62	+	466	0	466	Rsa1.0_00033.1	515811	474675	475135	98.7	100.0	98.7
R2	RS2CL7475s	42.8	−	816	1	816		316976	41738	42844	98.2	99.9	135.7
R2	RSS1031	42.81	+	694	0	694	Rsa1.0_01170.1	161131	130481	131738	99.6	100.0	181.1
R2	RS2CL2108s	43.74	+	1479	1	1479	Rsa1.0_00235.1	154696	99382	102037	99.9	99.9	179.6
R2	RGB13975c	44.12	+	532	0	532	Rsa1.0_00108.1	478491	95215	95747	100.0	100.0	100.0

R2	RSS0156	44.14											
R2	RS2CL4957s	44.62	+	1119	3	1119	Rsa1.0_03593.1	21869	1676	3014	93.1	99.7	119.9
R2	RS2CL1710s	45.89	+	894	0	892	Rsa1.0_00033.1	515811	485073	486356	87.7	99.8	143.8
R2	CA43	46.15											
R2	RS2CL2938s	46.32	−	1006	5	997	Rsa1.0_00240.1	104175	14152	15595	96.5	98.6	145.5
R2	RGC11533c	46.75	−	413	0	413		316976	122851	123264	100.0	100.0	100.0
R2	RGC15219c	46.75	+	511	0	511	Rsa1.0_00326.1	171955	26116	26627	100.0	100.0	100.0
R2	RGC7035c	46.75	−	450	0	450	Rsa1.0_03593.1	21869	12766	13216	54.0	100.0	100.0
R2	RGB966c	46.75	−	450	0	450	Rsa1.0_00077.1	145005	122150	122600	100.0	100.0	100.0
R2	S582	46.95											
R2	RGC6477c	47.53	+	433	0	433	Rsa1.0_00107.1	431659	9970	10403	99.8	100.0	100.0
R2	Rs2CL2264s	48.06	+	774	0	774	Rsa1.0_00033.1	515811	481294	482563	98.8	100.0	164.0
R2	RS2CL5524s	48.33	−	1060	2	1060	Rsa1.0_00033.1	515811	485084	486330	88.2	99.8	117.8
R2	RGC22331c	49.21	−	482	0	482	Rsa1.0_01937.1	37147	19723	20223	93.2	100.0	103.7
R2	RS2CL2407s	49.8	+	1040	11	1040	Rsa1.0_03688.1	143676	3786	5499	43.1	98.9	166.5
R2	RGC27211c	50.63	+	405	0	405	Rsa1.0_00033.1	515811	389278	389683	100.0	100.0	100.0
R2	RGC4684c	51.43	−	412	0	412	Rsa1.0_00775.1	64793	12236	12648	94.4	100.0	100.0
R2	RGC14244c	52.4	+	508	0	508		167018	67562	68070	98.2	100.0	100.0
R2	RS2CL9061s	53.2	+	1078	0	1078		21706	11078	12631	98.6	100.0	144.1
R2	RS2CL5137s	53.59	−	790	6	785		21706	15728	16631	97.6	98.6	115.9
R2	CC29	54.54											
R2	RS2CL868s	54.62	−	621	2	621	Rsa1.0_01786.1	133324	101113	102300	98.6	99.7	191.8
R2	RGB500c	55.34	−	450	0	450	Rsa1.0_00301.1	365315	180769	181219	100.0	100.0	100.0
R2	RGC1836c	55.7	+	462	0	462		120234	118164	118626	100.0	100.0	100.0
R2	RS2CL2432s	56.46	+	1262	9	1250	Rsa1.0_00893.1	60626	43002	44863	94.4	98.3	150.0
R2	RGB1399c	57.19	+	451	0	451	Rsa1.0_01786.1	133324	105722	106197	98.2	100.0	105.3
R2	RS2CL7359s	57.82	−	930	1	839	Rsa1.0_00082.1	237584	19675	20877	88.4	90.1	143.4
R2	RS2CL5452s	58.64	−	390	2	390		69858	9751	10140	98.7	99.5	100.3
R2	RS2CL1869s	58.94	+	810	0	810		69858	7722	8874	72.2	100.0	142.2
R2	RGC9493c	59.59											
R2	CC98bH	60.48											
R2	RGC23821c	62.48	+	440	0	440	Rsa1.0_02208.1	33875	9188	9628	100.0	100.0	100.0
R2	RGC1979c	63.35	−	416	0	416	Rsa1.0_00893.1	60626	8440	8856	100.0	100.0	100.0
R2	RS2CL2645s	64.21	−	1044	1	1044	Rsa1.0_02122.1	34797	22599	23872	89.0	99.9	122.1
R2	RS2CL4947s	64.26	−	753	0	753	Rsa1.0_00303.1	97468	9423	10260	96.2	100.0	111.2
R2	RS2CL2123s	65.65	+	816	2	809	Rsa1.0_01513.1	44234	28411	29591	72.2	98.9	146.2

R2	RGC1333c	66.38	+	403	0	403	Rsa1.0_04578.1	16774	4097	4500	100.0	100.0	100.0
R2	STG2F12	67.38											
R2	RGC1167c	67.88	+	400	0	400	Rsa1.0_00164.1	116642	77199	77599	80.5	100.0	100.0
R2	RGC7750c	67.88	−	450	0	450	Rsa1.0_00226.1	105884	81235	81685	100.0	100.0	100.0
R2	RGB9670c	67.88	+	425	0	425	Rsa1.0_00226.1	105884	42013	42438	100.0	100.0	100.0
R2	RGC13599c	69.68	−	514	0	503	Rsa1.0_01611.1	42269	13865	14368	97.9	97.9	100.0
R2	RS2CL5848s	70.53	−	781	21	780	Rsa1.0_00428.1	85599	30765	31616	91.7	97.2	112.1
R2	RGC11438c	71.44	−	403	88	403	Rsa1.0_00026.1	327835	55259	55574	78.2	78.2	100.0
R2	RS2CL2713s	72.33	+	795	3	795	Rsa1.0_00026.1	327835	45483	46269	96.0	99.6	99.2
R2	RGC13134c	73.11											
R2	RGC820c	73.71	+	447	0	447		300828	290937	291384	100.0	100.0	100.0
R2	RS2CL5541s	74.31	+	1160	2	1160		188018	83976	85777	85.6	99.8	155.5
R2	RS2CL4123s	74.58	−	704	2	704	Rsa1.0_02786.1	27880	18736	20242	92.9	99.7	214.5
R2	RGC5945c	74.85											
R2	RGC22105c	75.04											
R2	RGA1974s	75.6											
R2	RS2CL5783s	75.94											
R2	RS2CL4762s	76.11	−	1148	1	1148		188018	4637	7189	93.3	99.9	222.5
R2	RS2CL5589s	76.67	−	723	0	723	Rsa1.0_00877.1	61305	14387	15621	98.8	100.0	170.7
R2	RS2CL3263s	77.93	+	861	4	861		300828	15426	16566	98.6	99.5	133.0
R2	RSS1758_a	78.06	−	785	0	761		68971	65835	67193	60.0	96.9	178.5
R2	RS2CL3772s	78.07	−	782	2	782	Rsa1.0_02114.1	34873	3164	4483	97.6	99.7	169.1
R2	RS2CL4384s	79.33	−	854	21	854	Rsa1.0_11462.1	5500	2311	3152	92.5	97.5	101.0
R2	OPT04H	80.88											
R2	RSS1614	81.58	+	595	0	595	Rsa1.0_03605.1	21827	11207	11805	100.0	100.0	100.5
R2	RSS1076	82.28	−	878	0	878	Rsa1.0_03736.1	21081	11746	12868	91.5	100.0	127.8
R2	RSS1341	82.68	−	751	0	751	Rsa1.0_01086.1	54509	36477	37222	96.4	100.0	99.2
R2	RSS2394	82.78	−	440	0	440	Rsa1.0_03736.1	21081	12428	12868	83.2	100.0	100.0
R2	RS2CL7538s	83.28	−	1023	6	1022	Rsa1.0_01194.1	51113	26555	28294	95.3	99.3	171.2
R2	RSS0119	83.98	+	851	0	851	Rsa1.0_00673.1	69325	46905	48060	96.5	100.0	135.7
R2	CA87aH	85.68											
R2	RSS2308	86.78	−	776	0	776		325650	246462	247408	79.1	100.0	121.9
R2	RS2CL3410s	89.36	−	714	1	714	Rsa1.0_01611.1	42269	4116	4822	92.6	99.9	99.0
R2	RS2CL2004s	89.93	−	684	0	684	Rsa1.0_00355.1	92038	77883	78949	45.5	100.0	155.9
R2	RS2CL1934s	90.29	−	1174	0	1174	Rsa1.0_04238.1	18352	425	3036	97.2	100.0	222.4
R2	RGC10518c	90.47	+	547	0	547		66155	5725	6272	99.6	100.0	100.0

R2	RS2CL5545s	90.94	+	1123	1	1123	Rsa1.0_02430.1	31506	25170	26522	97.9	99.9	120.5
R2	RSS3936	91.67	+	676	0	676	Rsa1.0_02954.1	26485	16509	17185	98.1	100.0	100.0
R2	RS2CL3191s	91.85	+	1051	4	1051	Rsa1.0_01750.1	39816	28173	29221	93.5	99.6	100.1
R2	RGC3758c	92.25	−	497	0	497	Rsa1.0_01060.1	55368	37197	37694	95.4	100.0	100.0
R2	RGC10400c	92.64	+	410	0	410	Rsa1.0_00062.1	299291	261730	262140	100.0	100.0	100.0
R2	RS2CL3650s	93.11	−	1187	2	1180	Rsa1.0_00550.1	210790	177712	179518	96.3	99.2	153.3
R2	RS2CL2542s	93.48	−	1046	10	1044	Rsa1.0_01876.1	37800	6988	9746	97.1	98.9	266.7
R2	CB93	93.64											
R2	CC45b	95.5											
R2	RS2CL6642s	95.63	+	717	0	717	Rsa1.0_01021.1	56610	38695	39412	99.4	100.0	100.0
R2	CD72aH	96.43											
R2	OPT12b	97.63											
R2	CA68	98.73											
R2	RS2CL6884s	98.86	−	861	1	776	Rsa1.0_06645.1	10607	7569	8468	89.4	90.0	116.0
R2	CO-2	99.2											
R2	RGB1253c	99.55											
R2	OPE10	99.57											
R2	OPK06	99.87											
R2	CB56	99.97											
R2	CC42a	100.37											
R2	RGC2431c	100.78	+	442	0	442	Rsa1.0_00633.1	71250	4898	5340	100.0	100.0	100.0
R2	RSS2455	101.41	−	880	0	880	Rsa1.0_01587.1	42789	29604	31315	99.1	100.0	194.4
R2	RGC4812c	101.88	−	475	0	475	Rsa1.0_00230.1	105389	26549	27024	100.0	100.0	100.0
R2	RSS1140	101.97	+	792	0	792	Rsa1.0_01984.1	36491	31605	32396	97.7	100.0	99.9
R2	RS2CL1225s	102.08	+	853	0	853	Rsa1.0_00677.1	69159	45110	45967	99.7	100.0	100.5
R2	RSS2316	102.43	−	865	0	865	Rsa1.0_03280.1	145083	1610	2866	99.3	100.0	145.2
R2	RSS2594	102.73	+	909	0	909	Rsa1.0_05782.1	12646	7692	9403	99.2	100.0	188.2
R2	RSS1635	102.83											
R2	RSS3530_a	102.83	+	496	2	468	Rsa1.0_02302.1	32823	14157	14764	81.7	94.0	130.3
R2	RSS3934	102.83	−	870	0	870	Rsa1.0_05498.1	13523	2593	4394	99.5	100.0	207.0
R2	RS2CL4062s	103.51	−	688	0	687	Rsa1.0_01407.1	46425	16859	17632	97.1	99.9	112.5
R2	CC62	104.33											
R2	RSS2572	105.33	−	848	0	848	Rsa1.0_01103.1	53974	16628	17986	97.8	100.0	160.1
R2	RSS0373	105.53	+	640	10	568	Rsa1.0_03568.1	22048	18038	18585	83.8	87.2	98.0
R2	RSS2081	106.43	−	884	0	884	Rsa1.0_01099.1	158481	44486	45249	76.1	100.0	86.3
R2	RSS0968	109.13	+	832	0	832	Rsa1.0_00855.1	62047	15779	17725	94.8	100.0	233.9

R2	RGC11673c	111.59	+	555	208	466	Rsa1.0_00633.1	71250	46185	46443	32.1	46.5	100.0
R2	CB82H	111.74											
R2	RS2CL7828s	111.76	+	1066	8	1066	Rsa1.0_00147.1	249503	129148	131231	96.6	99.3	196.9
R2	RS2CL3640s	112.16	+	1095	7	1093	Rsa1.0_00147.1	249503	155765	157314	96.4	99.2	142.6
R2	RSS2889	112.19	−	849	0	849	Rsa1.0_03232.1	24450	8717	10144	100.0	100.0	168.1
R2	RS2CL3017s	112.82	+	1395	2	1395	Rsa1.0_02290.1	32971	21743	23967	99.1	99.9	159.7
R2	RSS2721	112.83	+	835	30	834	Rsa1.0_00877.1	61305	40806	41610	77.4	96.3	100.0
R2	OPT16b	114.33											
R2	RS2CL4126s	114.34	+	1195	6	1195	Rsa1.0_02735.1	28344	2744	4215	94.1	99.5	123.7
R2	RGA1566s	115.56											
R2	Rs2CL2368s	116.12	−	1182	0	1181	Rsa1.0_05573.1	13290	2917	4696	98.0	99.9	150.6
R2	RSS1798	116.12	−	908	0	842	Rsa1.0_03087.1	25478	21512	22388	79.6	92.7	104.0
R2	RGC3637c	116.43	−	406	75	406	Rsa1.0_01095.1	54276	24217	24548	48.5	81.5	100.0
R2	CA39	117.35											
R2	RGA154s	117.59											
R2	RGC18172c	118.6	+	477	0	477	Rsa1.0_06373.1	11138	6368	6845	100.0	100.0	100.0
R2	RSS2958	118.61	−	827	1	827	Rsa1.0_01064.1	55295	51314	52510	96.9	99.9	144.8
R2	RS2CL3903s	118.85	−	1114	2	1114	Rsa1.0_05878.1	12344	8156	9924	96.1	99.8	159.0
R2	RS2CL836s	119.25	+	758	0	753	Rsa1.0_00594.1	73729	29256	30313	96.3	99.3	140.4
R2	RGC13420c	120											
R2	RS2CL4187s	120.34	+	1684	0	1682	Rsa1.0_05593.1	13235	442	3208	98.5	99.9	164.5
R2	RGB10737c	120.78	+	600	0	600	Rsa1.0_01047.1	55705	39513	40113	100.0	100.0	100.0
R2	RGC11938c	120.78	+	538	0	538	Rsa1.0_01576.1	42923	26736	27274	100.0	100.0	100.0
R2	RSS3138	120.86	−	835	2	835	Rsa1.0_00375.1	90207	65073	66803	99.6	99.8	207.7
R2	RS2CL6395s	122.3	−	782	0	782	Rsa1.0_00004.1	253996	197687	198804	99.1	100.0	142.8
R2	RS2CL3405s	122.76	−	1180	0	1180	Rsa1.0_01030.1	56290	38433	41014	98.1	100.0	218.7
R2	RGC13684c	123.32	−	514	0	514	Rsa1.0_02108.1	163029	155330	155844	100.0	100.0	100.0
R2	RSS3268	123.53	−	804	0	804	Rsa1.0_00829.1	158305	29183	29986	98.4	100.0	99.9
R2	RGC2676c	123.61	−	401	0	401	Rsa1.0_01471.1	45101	26170	26571	90.0	100.0	100.0
R2	RGC7958c	124.04	+	496	0	496	Rsa1.0_01826.1	38700	22280	22776	69.6	100.0	100.0
R2	RS2CL5422s	124.16	+	639	0	638	Rsa1.0_00829.1	158305	101486	102629	96.1	99.8	179.2
R2	RS2CL3768s	124.53	+	1207	2	1207	Rsa1.0_02108.1	163029	152693	154723	99.6	99.8	168.5
R2	RGC9765c	124.71	−	544	0	544	Rsa1.0_03267.1	24230	18534	19078	100.0	100.0	100.0
R2	RS2CL2980s	125.34	+	1140	2	1128	Rsa1.0_02178.1	156197	148496	151019	95.3	98.8	224.1
R2	RSS1262	125.5	+	444	0	442	Rsa1.0_03781.1	20891	11682	12145	96.2	99.6	104.8
R2	RGB716c	126.74	−	406	0	406	Rsa1.0_04034.1	19481	18803	19209	100.0	100.0	100.0

R2	RS2CL6075s	127.94	+	904	342	904	Rsa1.0_00257.1	102407	97009	97572	61.2	62.2	100.2
R2	RGC1792c	129.51	+	523	0	523	Rsa1.0_01855.1	38204	18598	19121	100.0	100.0	100.0
R2	CD87b	129.91											
R2	RS2CL4370s	130.56											
R2	RGC15769c	130.68	+	493	0	493	Rsa1.0_01351.1	47498	19413	19906	100.0	100.0	100.0
R2	RGC6336c	130.71	+	456	0	420	Rsa1.0_00063.1	156150	27311	27731	82.5	92.1	100.0
R2	RGC1718c	131.61	−	439	0	439	Rsa1.0_03513.1	22349	14138	14577	100.0	100.0	100.0
R2	CA95b	132.47											
R2	RS2CL2371s	132.51	+	1103	2	1103	Rsa1.0_03570.1	22040	8485	9875	97.6	99.8	126.3
R2	RGA1718s	132.9											
R2	RGC2467c	133.04	+	427	0	427	Rsa1.0_02159.1	34471	30172	30599	100.0	100.0	100.0
R2	RSS0477	133.16	−	748	0	748	Rsa1.0_09160.1	6599	576	1741	91.4	100.0	155.8
R2	RS2CL867s	133.81	+	1225	28	1120		368722	278550	280555	78.5	89.1	183.6
R2	RSS1885	134.86	−	858	1	858	Rsa1.0_04337.1	17845	10013	11235	99.9	99.9	142.6
R2	RS2CL4248s	134.95	+	653	2	653	Rsa1.0_00365.1	90902	80844	82092	99.1	99.7	191.7
R2	RGC15951c	135.1	−	448	0	448	Rsa1.0_00343.1	93570	87740	88188	100.0	100.0	100.0
R2	RGC6949c	135.1	−	524	0	524	Rsa1.0_00343.1	93570	41236	41760	100.0	100.0	100.0
R2	RS2CL2716s	135.95	−	974	2	957	Rsa1.0_00343.1	93570	88602	90103	92.4	98.1	157.2
R2	RGC12310c	136.55	−	404	0	404	Rsa1.0_01004.1	56975	39815	40219	100.0	100.0	100.0
R2	RS2CL1046s	137.05	+	726	3	718	Rsa1.0_05401.1	13876	542	1735	37.9	98.5	166.9
R2	RSS0822	137.36	−	797	0	797	Rsa1.0_00687.1	68494	2707	4333	99.9	100.0	204.0
R2	Rs2CL2604s	137.46											
R2	RGC15477c	138.39	−	462	0	462	Rsa1.0_01637.1	41862	40568	41030	100.0	100.0	100.0
R2	RS2CL3703s	139.86	−	1189	2	1189	Rsa1.0_00969.1	58014	21460	23158	93.1	99.8	143.1
R2	RGB5639c	142.35	−	448	0	448	Rsa1.0_00230.1	105389	73130	73578	52.5	100.0	100.0
R2	RGC16522c	144.97	−	425	0	425	Rsa1.0_04193.1	18571	10056	10481	100.0	100.0	100.0
R2	RGC16935c	146.66											
R3	RS2CL3605s	0	−	765	1	765	Rsa1.0_00173.1	288743	84007	85294	76.6	99.9	168.5
R3	RSS1377	12.27	+	869	0	869	Rsa1.0_03448.1	22844	12215	13084	96.8	100.0	100.0
R3	RGC21838c	12.38	+	481	0	481	Rsa1.0_00192.1	278200	772	1253	100.0	100.0	100.0
R3	RGC6058c	13.93	+	547	0	547	Rsa1.0_02663.1	169243	11068	11615	100.0	100.0	100.0
R3	RSS3168	14.17											
R3	RS2CL683s	15.22	+	916	0	896		99966	12147	13568	87.6	97.8	158.6
R3	RS2CL3040s	16.78	+	842	0	841	Rsa1.0_01300.1	48942	33422	34835	97.5	99.9	168.0
R3	RS2CL6139s	17.34	−	1303	5	1302	Rsa1.0_00727.1	66724	50809	52418	92.3	99.5	124.1

R3	RS2CL3557s	18.05											
R3	RGC4640c	18.08	−	419	0	419	Rsa1.0_00192.1	278200	248517	248936	93.3	100.0	100.0
R3	CA05b	18.37											
R3	RS2CL1245s	19.46	−	1226	0	1225	Rsa1.0_00192.1	278200	224880	228241	98.9	99.9	274.4
R3	RGA93s	19.93											
R3	RGC9028c	19.95	−	464	0	464	Rsa1.0_01220.1	161775	17840	18303	99.4	100.0	99.8
R3	RSS0816	20.27	−	828	0	828	Rsa1.0_00192.1	278200	98708	99992	97.6	100.0	155.1
R3	RGC3942c	20.67	−	481	0	481	Rsa1.0_00661.1	213041	57093	57574	100.0	100.0	100.0
R3	RGA1553s	21.24											
R3	RS2CL1424s	22.26	+	1185	0	1184	Rsa1.0_02884.1	27034	11429	13240	81.9	99.9	153.0
R3	RS2CL6686s	22.71	−	726	5	710	Rsa1.0_02356.1	141500	4993	5689	89.9	97.1	98.7
R3	RSS0486	23.17	+	574	0	574	Rsa1.0_02879.1	27047	17843	18502	100.0	100.0	114.8
R3	RS2CL4186s	23.23	−	947	2	946	Rsa1.0_01310.1	164719	137111	139211	97.9	99.7	222.5
R3	RSS2764	23.37	+	794	0	794	Rsa1.0_02750.1	28239	25623	26916	96.4	100.0	162.9
R3	RGC9371c	23.92	−	417	0	417	Rsa1.0_02808.1	27677	23689	24106	99.3	100.0	100.0
R3	RS2CL6155s	23.97	−	879	37	879	Rsa1.0_16063.1	2301	780	2301	91.9	95.8	180.6
R3	Z46819	24.27											
R3	RS2CL2845s	24.88	−	1308	17	1308	Rsa1.0_02903.1	26901	18651	20543	97.0	98.7	146.6
R3	RSS2403	25.17	−	762	10	762	Rsa1.0_01310.1	164719	9836	10873	92.3	98.7	137.9
R3	RS2CL4457s	25.37	+	885	4	882	Rsa1.0_07000.1	10049	4934	6599	92.4	99.2	189.6
R3	RGC22415c	25.97	−	550	0	550	Rsa1.0_02903.1	26901	7329	7879	100.0	100.0	100.0
R3	RGC3069c	25.97	+	406	0	406	Rsa1.0_06803.1	10330	2860	3266	100.0	100.0	100.0
R3	RGC5894c	25.43	−	492	0	492	Rsa1.0_03249.1	146254	4048	4540	100.0	100.0	100.0
R3	RGC5939c	25.43	−	415	0	415	Rsa1.0_03302.1	23898	13929	14344	100.0	100.0	100.0
R3	RGB10851c	25.97	+	497	0	497	Rsa1.0_03090.1	25464	14283	14822	68.2	100.0	108.5
R3	RSS2108	26.17	+	872	0	872	Rsa1.0_07021.1	10016	6320	8332	99.0	100.0	230.7
R3	RSS2935	26.97	−	807	0	807	Rsa1.0_06677.1	10560	5704	6511	83.4	100.0	100.0
R3	RS2CL4299s	27.74	+	1305	12	1305	Rsa1.0_04852.1	15712	8257	9860	90.0	99.1	124.0
R3	RSS0832	27.87	−	779	23	779	Rsa1.0_04662.1	16477	144	899	93.6	97.1	99.9
R3	RS2CL6594s	28.31	+	629	15	629	Rsa1.0_06803.1	10330	4639	5258	73.9	97.6	100.8
R3	RGB8990c	29.16	−	455	0	455		78829	48930	49385	100.0	100.0	100.0
R3	RSS2634	29.67											
R3	RGB12858c	29.83	+	462	0	397	Rsa1.0_31666.1	722	325	722	85.7	85.9	100.0
R3	RSS1743	30.37	−	862	0	862		66155	52006	53429	71.9	100.0	165.1
R3	RSS2359	30.67	−	428	10	428	Rsa1.0_04550.1	16893	828	1754	97.7	97.7	221.5
R3	RSS3002	30.67	+	864	59	864	Rsa1.0_02268.1	33172	10444	11407	93.2	93.2	119.6

R3	RSS3172	30.67	−	821	1	821	Rsa1.0_05332.1	14123	7891	9155	81.4	99.9	154.2
R3	RSS1931	30.77	−	416	0	416	Rsa1.0_40889.1	549	40	458	99.3	100.0	100.5
R3	RSS0692	31.37	+	805	1	805	Rsa1.0_00728.1	66704	38874	40228	68.0	99.9	168.4
R3	RSS1117	31.37	+	822	0	822		294827	118102	119664	70.9	100.0	190.0
R3	RSS0789	32.17	+	713	0	713	Rsa1.0_01731.1	40208	18230	19318	100.0	100.0	152.6
R3	RS2CL1360s	32.23	−	871	2	871	Rsa1.0_00883.1	61230	29865	31418	96.2	99.8	178.7
R3	RGC5762c	33	+	408	0	408	Rsa1.0_00412.1	86935	47608	48016	100.0	100.0	100.0
R3	RSS1261	33.96	−	836	0	836	Rsa1.0_08153.1	7828	5634	7003	96.4	100.0	163.8
R3	RSS0977	34.16											
R3	Rs2CL2367s	35.4	+	955	1	954		167185	32132	33858	93.9	99.8	181.1
R3	Rs2CL2374s	36.41	+	704	9	702	Rsa1.0_02099.1	35030	17741	18715	98.3	98.4	140.6
R3	RS2CL2369s	36.41	−	1018	0	951	Rsa1.0_03261.1	24281	10622	15566	77.3	93.4	519.9
R3	RS2CL6944s	36.6	+	929	2	929	Rsa1.0_04399.1	17543	11125	12581	97.0	99.8	157.1
R3	RS2CL1855s	36.78	−	1347	2	1347	Rsa1.0_03069.1	25615	17941	19872	49.3	99.9	143.6
R3	RGC662c	36.95	+	446	0	446	Rsa1.0_02099.1	35030	5768	6214	98.9	100.0	100.0
R3	RSS2129	37.7	−	672	0	672	Rsa1.0_00322.1	212233	65166	65839	100.0	100.0	100.2
R3	RGB5491c	39.04	−	464	0	464	Rsa1.0_00090.1	273859	17347	18015	66.4	100.0	144.0
R3	CZ09	39.21											
R3	RGC15408c	39.83	−	424	0	424	Rsa1.0_00090.1	273859	173120	173544	100.0	100.0	100.0
R3	CA05a	40.92											
R3	RGC11985c	41.21	+	420	0	420	Rsa1.0_00090.1	273859	81593	82013	99.8	100.0	100.0
R3	RGC1838c	41.21	+	491	0	491	Rsa1.0_04463.1	17247	9666	10157	100.0	100.0	100.0
R3	RGC2115c	41.21	−	398	0	398	Rsa1.0_00090.1	273859	50908	51306	100.0	100.0	100.0
R3	RS2CL3966s	41.97	+	1126	2	747	Rsa1.0_01896.1	37512	4581	5561	17.0	66.2	131.5
R3	RS2CL812s	42.64	−	787	47	786	Rsa1.0_01896.1	37512	35257	36969	93.0	93.9	231.7
R3	RSS3288	42.74	+	735	0	623	Rsa1.0_02870.1	27102	11643	12612	77.4	84.8	155.5
R3	RGC2693c	42.9	+	423	0	423	Rsa1.0_00695.1	68019	60931	61354	100.0	100.0	100.0
R3	RGC4050c	42.9	−	509	118	509	Rsa1.0_00233.1	104918	88132	88523	76.8	76.8	100.0
R3	RGC4388c	43.29	−	451	221	451	Rsa1.0_00828.1	62744	45935	46165	51.0	51.0	100.0
R3	RGC12111c	43.34	+	563	0	563		78829	22382	22945	100.0	100.0	100.0
R3	RGC7232c	43.5	+	493	0	493	Rsa1.0_00207.1	248736	84044	84537	100.0	100.0	100.0
R3	RS2CL2195s	43.62	+	467	2	467	Rsa1.0_00207.1	248736	196514	196979	99.6	99.6	100.0
R3	RGC23188c	44.01											
R3	RSS1540	44.56	+	794	315	794	Rsa1.0_03261.1	24281	21086	21565	60.3	60.3	100.0
R3	RGC6897c	44.92	+	441	0	441	Rsa1.0_00905.1	60110	31918	32359	100.0	100.0	100.0
R3	RS2CL5856s	46.17	+	1120	1	1120	Rsa1.0_00695.1	68019	48162	50570	97.3	99.9	215.2

R3	RS2CL7274s	47.01	−	926	78	926	Rsa1.0_01570.1	43015	7257	8105	35.2	91.6	100.0
R3	CA05aN	47.4											
R3	RS2CL7810s	47.79	+	787	2	787	Rsa1.0_00173.1	288743	60856	65214	80.6	99.8	555.2
R3	H36913	49.21											
R3	RGC3691c	49.24	+	429	0	429	Rsa1.0_01884.1	37659	21212	21641	99.8	100.0	100.0
R3	RS2CL1767s	50.07	−	835	2	835	Rsa1.0_01938.1	264018	156771	158134	96.8	99.8	163.6
R3	RGB2849c	51.25	+	409	0	409	Rsa1.0_00173.1	288743	82248	82657	100.0	100.0	100.0
R3	ACGM18	51.95											
R3	RS2CL1552s	52.03	−	1458	1	1458	Rsa1.0_01884.1	37659	17346	18837	93.8	99.9	102.3
R3	RSS2841	52.85	−	842	6	842	Rsa1.0_04762.1	16101	4361	5197	96.7	99.3	100.0
R3	RS2CL1544s	53.12	−	891	2	887	Rsa1.0_01938.1	264018	159637	160700	94.6	99.3	120.1
R3	RSS2981	53.56	−	926	0	926	Rsa1.0_08551.1	7210	781	1716	95.8	100.0	101.0
R3	RGC8225c	53.73	+	400	124	400	Rsa1.0_03748.1	21035	17688	17964	69.0	69.0	100.0
R3	RSS2675	53.96	−	613	84	613	Rsa1.0_10495.1	5827	5158	5811	86.1	86.3	123.4
R3	CA25	54.26											
R3	CB96	54.76											
R3	RGC4948c	55.47	−	406	0	406	Rsa1.0_01356.1	47287	15215	15621	53.7	100.0	100.0
R3	RSS2362_a	55.57	−	530	5	515	Rsa1.0_03218.1	24539	22154	23021	93.0	96.2	170.0
R3	RS2CL5646s	56.07	−	896	161	896	Rsa1.0_01356.1	47287	8247	8982	78.1	82.0	100.0
R3	RSS2207	56.58											
R3	RSS2468	56.98	+	844	0	844	Rsa1.0_02398.1	31766	584	1660	98.5	100.0	127.5
R3	CB94	57.48											
R3	RGA1712s	58.99											
R3	RS2CL5207s	59.06	−	606	50	606		155185	137578	138293	87.5	91.8	128.6
R3	RSS2225	59.3	−	841	0	332	Rsa1.0_63300.1	362	0	332	39.5	39.5	100.0
R3	RGC10740c	59.51	−	402	0	402	Rsa1.0_00732.1	66631	30568	30970	100.0	100.0	100.0
R3	RSS2174	60.19	+	643	9	643	Rsa1.0_09388.1	6405	3530	4952	98.6	98.6	224.3
R3	RGB3739c	60.33	−	506	90	506	Rsa1.0_00158.1	117999	26141	26557	80.6	82.2	100.0
R3	RS2CL3113s	61.11	−	1170	0	1169	Rsa1.0_00619.1	71985	14030	15858	68.7	99.9	156.4
R3	RSS1494	61.19	−	851	0	851	Rsa1.0_00456.1	82826	78621	79473	100.0	100.0	100.1
R3	RSS3166	61.59	+	689	17	689	Rsa1.0_00539.1	76926	56537	57301	93.5	97.5	113.7
R3	RS2CL3855s	61.86	−	1355	1	1355	Rsa1.0_06218.1	11461	5565	6919	39.0	99.9	100.0
R3	CF06	63.27											
R3	RGB10862c	63.47	+	402	0	402	Rsa1.0_00207.1	248736	4742	5144	100.0	100.0	100.0
R3	RSS1258	64.87	−	203	0	203	Rsa1.0_01771.1	172709	12737	12951	87.2	100.0	105.4
R3	RSS1041	65.37	−	670	0	670	Rsa1.0_01771.1	172709	8960	12980	93.9	100.0	600.0

R3	RGC11016c	66.07	+	468	0	468	Rsa1.0_03723.1	21173	6464	6932	100.0	100.0	100.0
R3	CC42b	66.24											
R3	RGC107c	68.06	+	460	51	460	Rsa1.0_04411.1	142979	10112	10521	37.2	88.9	100.0
R3	RGC21094c	68.06	+	416	0	416	Rsa1.0_01977.1	36559	33816	34232	100.0	100.0	100.0
R3	RSS0596	68.25											
R3	RGA1793s	68.4											
R3	RGC8038c	68.78	−	502	0	502	Rsa1.0_00539.1	76926	74214	74716	100.0	100.0	100.0
R3	RS2CL4752s	69.57	−	1059	2	1025	Rsa1.0_02937.1	26605	22563	24225	95.9	96.6	162.5
R3	RGC19961c	70.03	−	523	0	523	Rsa1.0_00029.1	272992	146933	147456	100.0	100.0	100.0
R3	RS2CL7278s	70.4	+	727	3	724	Rsa1.0_00029.1	272992	246780	247983	98.2	99.2	166.9
R3	RS2CL4292s	70.81	−	736	0	736	Rsa1.0_00743.1	177459	6116	6855	95.0	100.0	100.4
R3	RGC6864c	71.16	+	537	0	537	Rsa1.0_00029.1	272992	107808	108345	100.0	100.0	100.0
R3	RS2CL1086s	72.51	−	1239	1	1239	Rsa1.0_09069.1	6671	2073	4429	98.1	99.9	190.3
R3	RS2CL5384s	72.63	+	1085	2	1081	Rsa1.0_01325.1	48131	16462	18088	91.2	99.5	150.7
R3	RGC1367c	73.14	+	460	0	460	Rsa1.0_03552.1	22151	14759	15219	100.0	100.0	100.0
R3	RSS2543	73.19	+	874	0	636	Rsa1.0_01029.1	256254	130119	131157	72.7	72.8	163.2
R3	RSS0860	73.29	−	755	10	755	Rsa1.0_03407.1	23154	9585	10331	74.2	98.7	100.1
R3	RS2CL2414s	73.89	−	938	8	938	Rsa1.0_00646.1	70473	1742	3631	89.6	99.2	203.1
R3	RSS1018	74.26	+	665	0	665	Rsa1.0_02246.1	33435	22769	23778	100.0	100.0	151.7
R3	RGC4056c	74.38	+	487	0	487	Rsa1.0_02412.1	31643	19981	20468	99.2	100.0	100.0
R3	RGC5105c	74.79	−	491	0	491	Rsa1.0_02022.1	35923	14225	14716	100.0	100.0	100.0
R3	RGB37c	75.59	+	507	0	483	Rsa1.0_01373.1	47052	31981	32464	95.3	95.3	100.0
R3	RS2CL6133s	75.61	+	709	3	708	Rsa1.0_00500.1	272308	143296	144004	97.7	99.4	100.4
R3	RSS0441	75.73	+	697	0	697	Rsa1.0_01220.1	161775	112671	113371	97.6	100.0	100.4
R3	RS2CL5720s	76.79											
R3	RS2CL1398s	77.71	−	747	63	747	Rsa1.0_01195.1	147988	15401	16135	88.0	91.6	107.3
R3	ACGM14	78.13											
R3	RGC5561c	78.52	−	518	0	518	Rsa1.0_00430.1	85453	27282	27800	100.0	100.0	100.0
R3	RSS3186	78.72	+	893	0	893	Rsa1.0_00710.1	67325	254	1817	51.0	100.0	175.0
R3	RGC4447c	79.22	−	453	0	453	Rsa1.0_01364.1	47172	41888	42341	62.7	100.0	100.0
R3	RS2CL2857s	79.26	−	788	0	787	Rsa1.0_06345.1	11205	925	2655	99.5	99.9	219.8
R3	RS2CL4154s	79.62	−	1073	2	1073	Rsa1.0_00934.1	193516	175206	176640	98.4	99.8	133.9
R3	RS2CL4178s	79.71	−	1050	4	1050	Rsa1.0_01612.1	42255	34975	36022	99.2	99.6	100.1
R3	CC55	79.89											
R3	RGC15812c	79.91	−	438	0	438	Rsa1.0_00452.1	83201	54975	55413	100.0	100.0	100.0
R3	RGC16357c	80.36	+	451	0	224	Rsa1.0_02119.1	34833	18772	18996	49.7	49.7	100.0

R3	RS2CL4585s	80.4	+	1016	2	1015	Rsa1.0_03442.1	22927	21657	22675	99.4	99.7	100.5
R3	RS2CL4689s	81.12	+	1017	2	1017	Rsa1.0_02119.1	34833	5614	8012	96.3	99.8	236.3
R3	RGC7054c	81.5	+	496	69	496	Rsa1.0_03404.1	23177	9160	9587	86.1	86.1	100.0
R3	RGC6178c	81.75	+	480	5	480	Rsa1.0_00607.1	73146	43552	44027	67.9	99.0	100.0
R3	RGC11372c	81.97	−	503	0	503	Rsa1.0_00263.1	224968	177144	177647	100.0	100.0	100.0
R3	RGC13408c	81.97	−	447	0	343	Rsa1.0_03734.1	21103	16781	17124	38.5	76.7	100.0
R3	RSS1570	82.01	+	260	0	168	Rsa1.0_01754.1	39792	13218	13389	63.9	64.6	101.8
R3	RS2CL4158s	82.28	+	1170	3	1170	Rsa1.0_00158.1	117999	12952	14870	99.2	99.7	164.4
R3	RS2CL7432s	82.98	−	1021	1	1021	Rsa1.0_02407.1	31684	9268	10562	97.4	99.9	126.9
R3	RGC11660c	83.31	+	431	0	431	Rsa1.0_00316.1	96264	82673	83104	100.0	100.0	100.0
R3	RGC12742c	83.31											
R3	RGC1543c	83.31	+	432	0	432	Rsa1.0_00511.1	78616	35860	36292	100.0	100.0	100.0
R3	RSS3173	83.56	+	502	1	502	Rsa1.0_04049.1	19385	17902	18404	95.4	99.8	100.2
R3	RSS3827	84.06	−	376	0	376	Rsa1.0_01552.1	43396	35214	35555	89.6	100.0	90.7
R3	RS2CL7533s	84.39	−	1193	2	1192	Rsa1.0_00851.1	62181	20311	21501	93.9	99.8	100.0
R3	RS2CL1880s	84.55	−	906	0	906	Rsa1.0_00607.1	73146	21253	22235	100.0	100.0	108.4
R3	CC34	84.56											
R3	RGC24282c	85.01	−	446	0	446	Rsa1.0_02407.1	31684	27458	27904	100.0	100.0	100.0
R3	RSS1161	85.63	−	689	0	689	Rsa1.0_00263.1	224968	178599	179812	99.7	100.0	176.1
R3	RGC18238c	86.01	+	456	0	435	Rsa1.0_02283.1	33024	23646	24081	95.4	95.4	100.0
R3	RGC19084c	86.01	+	554	0	554	Rsa1.0_03528.1	22278	19759	20313	93.3	100.0	100.0
R3	RGC5954c	86.01	+	491	0	491	Rsa1.0_02399.1	31763	20318	20809	100.0	100.0	100.0
R3	RGC8553c	86.09	+	595	0	595	Rsa1.0_00467.1	156542	103948	104543	100.0	100.0	100.0
R3	RS2CL3230s	86.71	−	867	2	867	Rsa1.0_00511.1	78616	34987	36128	99.1	99.8	131.9
R3	RSS1549	87.26	+	843	0	843	Rsa1.0_00316.1	96264	57233	58469	93.4	100.0	146.6
R3	RGC7922c	87.46	+	485	0	485	Rsa1.0_01857.1	38103	7320	7805	100.0	100.0	100.0
R3	RSS0198	87.66	+	819	53	819	Rsa1.0_00467.1	156542	142692	143766	93.5	93.5	140.2
R3	RS2CL3016s	88.22	−	1505	2	1490	Rsa1.0_04434.1	17377	7290	8978	87.5	98.9	113.4
R3	CB53H	88.66											
R3	RGB2123c	89.04	−	435	0	435		358124	146014	146449	100.0	100.0	100.0
R3	RS2CL3633s	89.42	−	1067	0	1067	Rsa1.0_00187.1	393093	206988	208775	99.2	100.0	167.5
R3	RS2CL2659s	89.47	−	654	0	654	Rsa1.0_07936.1	8228	3330	4444	97.4	100.0	170.3
R3	RS2CL2865s	89.84	+	675	2	675	Rsa1.0_00603.1	73299	32351	33090	97.9	99.7	109.8
R3	RSS3644	90.06	−	826	0	826	Rsa1.0_01558.1	43287	39126	40847	60.1	100.0	208.4
R3	RS2CL2009s	90.52	−	798	0	798	Rsa1.0_05239.1	14410	11681	13080	92.5	100.0	175.3
R3	RGC11410c	90.76	−	560	0	560	Rsa1.0_01008.1	56924	28901	29461	100.0	100.0	100.0

R3	RS2CL6931s	92.01	−	1382	2	1378	Rsa1.0_02215.1	33822	8381	10064	74.6	99.6	122.3
R3	RGC8634c	93.98	+	533	0	533	Rsa1.0_00103.1	135304	68095	68628	74.1	100.0	100.0
R3	RSS3933	97.66	−	782	240	782	Rsa1.0_00452.1	83201	57372	57914	69.3	69.3	100.0
R3	RS2CL5949s	98.6	+	975	2	975	Rsa1.0_17397.1	1840	412	1383	87.8	99.8	99.8
R3	RGC5211c	100.2											
R3	RS2CL6711s	101.22	−	1067	0	1067	Rsa1.0_00275.1	210104	14871	15941	99.1	100.0	100.3
R4	RSS1627	0	+	645	1	645	Rsa1.0_00092.1	659932	182526	183740	99.8	99.8	188.5
R4	RGC355c	4.7	+	693	0	693	Rsa1.0_00340.1	305782	115174	115867	86.4	100.0	100.0
R4	CA83H	5.5											
R4	ACGM20	8.8											
R4	RS2CL966s	9.32	+	1121	2	1080	Rsa1.0_00211.1	228189	179312	180707	93.1	96.2	129.4
R4	CD72cH	9.6											
R4	RS2CL2691s	10.56	−	1072	4	1071	Rsa1.0_00211.1	228189	144636	146110	94.8	99.5	138.1
R4	RSS1586	11.7	+	857	0	857		167185	162187	163122	99.8	100.0	109.1
R4	RS2CL5798s	11.98	+	975	1	975	Rsa1.0_06209.1	11484	7932	9555	89.1	99.9	166.6
R4	RSS2559	12	+	820	0	820		341668	282404	283224	99.9	100.0	100.0
R4	RGC2155c	12.54	−	507	0	507	Rsa1.0_00011.1	225044	28429	28936	100.0	100.0	100.0
R4	RGA1914s	12.79											
R4	RGC4083c	13.03	−	471	0	471	Rsa1.0_00237.1	235974	168752	169223	80.9	100.0	100.0
R4	RS2CL2103s	13.47	−	1278	3	1278	Rsa1.0_00327.1	94892	22326	23851	75.1	99.8	119.6
R4	RS2CL3614s	14.17	−	743	0	743	Rsa1.0_00237.1	235974	188233	189221	99.9	100.0	133.0
R4	RS2CL4701s	14.25											
R4	RSS1544	14.4	+	848	0	848		341668	47623	48829	84.7	100.0	142.2
R4	RGB10057c	14.55	+	438	0	438	Rsa1.0_00712.1	67217	20748	21148	91.3	100.0	91.3
R4	RGC14416c	14.55	+	406	89	406	Rsa1.0_00621.1	71840	41096	41413	78.1	78.1	100.0
R4	RS2CL4127s	14.75											
R4	RS2CL5450s	14.9	+	712	117	711	Rsa1.0_00237.1	235974	40735	41422	81.5	83.4	115.7
R4	CD34aH	15											
R4	RGA1981s	15.06											
R4	RS2CL3293s	15.26	−	1285	1	1285	Rsa1.0_00237.1	235974	41503	43369	97.5	99.9	145.3
R4	RS2CL5513s	15.43	−	721	0	717	Rsa1.0_05923.1	12230	1985	3092	96.5	99.5	154.4
R4	RS2CL1187s	15.53	−	776	3	760	Rsa1.0_00870.1	61606	24774	26176	91.6	97.6	185.2
R4	RS2CL1264s	15.6	−	1125	2	1124	Rsa1.0_01448.1	45645	20602	21798	96.8	99.7	106.6
R4	RS2CL1262s	15.72											
R4	RS2CL6586s	15.76	−	931	1	930	Rsa1.0_02220.1	176031	156517	157541	85.6	99.8	110.2

R4	RS2CL5563s	15.87	−	1020	2	1020	Rsa1.0_05923.1	12230	4126	5529	98.2	99.8	137.8
R4	RGC1195c	16.09	+	468	0	468	Rsa1.0_00327.1	94892	33329	33797	100.0	100.0	100.0
R4	RGC15741c	16.09	−	492	0	492	Rsa1.0_00011.1	225044	7220	7712	100.0	100.0	100.0
R4	RGB2673c	16.09	+	428	0	428	Rsa1.0_00678.1	193163	178772	179238	99.5	100.0	108.9
R4	RGC9198c	16.09	+	420	0	420	Rsa1.0_05923.1	12230	1332	1752	100.0	100.0	100.0
R4	RS2CL1215s	16.1	−	759	0	757	Rsa1.0_00092.1	659932	627110	628500	95.7	99.7	183.6
R4	CC00	16.2											
R4	CC23	16.4											
R4	RS2CL1831s	16.48	−	1215	0	1210	Rsa1.0_00011.1	225044	25532	26906	98.1	99.6	113.6
R4	RS2CL3995s	16.98	−	806	2	797	Rsa1.0_00011.1	225044	205926	207482	90.9	98.6	195.7
R4	S281N	17.2											
R4	RS2CL1816s	17.34	−	1345	7	1335	Rsa1.0_00807.1	63607	60823	62810	95.5	98.7	149.6
R4	RS2CL4170s	17.7	+	954	0	953	Rsa1.0_00593.1	143463	64256	65200	95.9	99.9	99.1
R4	RS2CL842s	17.86	−	1320	1	1318	Rsa1.0_00341.1	249309	189276	190754	95.6	99.8	112.2
R4	RSS0234	17.9	−	783	0	783	Rsa1.0_00341.1	249309	213508	214786	99.9	100.0	163.2
R4	RS2CL7584s	17.95	−	974	1	974		267193	253264	254575	96.1	99.9	134.7
R4	RSS3716	18.1	−	472	0	472	Rsa1.0_00113.1	131574	53009	53481	100.0	100.0	100.0
R4	RGC22968c	18.34	−	510	0	510	Rsa1.0_00011.1	225044	104695	105205	100.0	100.0	100.0
R4	RGC45c	18.34	−	422	0	422	Rsa1.0_00113.1	131574	122034	122456	100.0	100.0	100.0
R4	RGC9298c	18.34	−	538	0	538	Rsa1.0_00341.1	249309	127361	127899	100.0	100.0	100.0
R4	RS2CL4241s	18.38	−	775	2	775	Rsa1.0_02349.1	32312	25979	27271	98.6	99.7	167.1
R4	RSS2439	18.9	+	805	0	805	Rsa1.0_06209.1	11484	2288	3661	99.9	100.0	170.6
R4	RSS3649	19.4	−	522	0	522	Rsa1.0_00340.1	305782	31221	31743	95.0	100.0	100.0
R4	RS2CL4850s	19.57	−	809	2	808	Rsa1.0_00340.1	305782	109390	110191	96.7	99.6	99.4
R4	RSS2708	20.24	−	630	0	630	Rsa1.0_00708.1	67366	53303	53933	98.3	100.0	100.0
R4	RS2CL5615s	20.76	−	644	0	642	Rsa1.0_00340.1	305782	289426	290068	71.3	99.7	100.0
R4	RGC9137c	21.04	−	431	0	431	Rsa1.0_00211.1	228189	142464	142895	84.9	100.0	100.0
R4	RGC7718c	21.77	+	502	0	502	Rsa1.0_00286.1	98709	20583	21085	99.8	100.0	100.0
R4	RGC1288c	21.99	−	403	0	400	Rsa1.0_00119.1	467509	305013	305413	58.1	99.3	100.0
R4	RGC32871c	21.99	−	403	0	403	Rsa1.0_00200.1	162016	148614	149017	100.0	100.0	100.0
R4	RGC4257c	21.99	−	494	249	494	Rsa1.0_00119.1	467509	243610	243855	49.6	49.6	100.0
R4	RS2CL8540s	22.23	−	601	2	601		174827	2548	3148	96.7	99.7	100.2
R4	RSS2783	22.26	+	536	0	523	Rsa1.0_00003.1	340054	326332	326947	97.6	97.6	117.6
R4	RSS2409	22.76	+	769	0	758	Rsa1.0_00119.1	467509	61932	63034	95.5	98.6	145.4
R4	RSS2474	22.76											
R4	RSS0031	23.16											

R4	RS2CL5025s	23.45	+	1330	1	1329	Rsa1.0_00708.1	67366	12730	14579	97.1	99.9	139.2
R4	RGB16843c	23.8	−	410	0	410	Rsa1.0_00003.1	340054	256661	257071	100.0	100.0	100.0
R4	RS2CL7051s	23.81	+	712	2	712	Rsa1.0_08460.1	7315	4224	5487	99.7	99.7	177.9
R4	RSS0858	23.99	+	599	15	599		316976	33937	34806	96.8	97.5	148.8
R4	RGC3995c	24.04											
R4	RS2CL4212s	24.6	+	1314	2	1314	Rsa1.0_00119.1	467509	90515	92289	96.8	99.9	135.2
R4	RSS3799	24.91	+	771	0	771	Rsa1.0_00042.1	171507	2942	3713	99.9	100.0	100.0
R4	RS2CL3610s	25.64	−	1099	2	1098	Rsa1.0_01949.1	36960	20439	22086	96.5	99.7	150.3
R4	RSS3591	25.75	+	722	7	722	Rsa1.0_01777.1	39511	21604	22624	97.9	99.0	142.7
R4	RGB5549c	25.85	+	442	0	442	Rsa1.0_00119.1	467509	392627	393095	99.3	100.0	105.9
R4	RSS2846	26.7											
R4	RS2CL7588s	26.76	−	1119	2	1119	Rsa1.0_00119.1	467509	385647	387377	92.5	99.8	154.9
R4	RGC1455c	27.17	+	494	0	494	Rsa1.0_00042.1	171507	169928	170422	100.0	100.0	100.0
R4	RS2CL4804s	27.78	+	1197	2	1197	Rsa1.0_01949.1	36960	10403	11867	98.8	99.8	122.5
R4	RS2CL3599s	28.31	+	1037	2	1037	Rsa1.0_03818.1	20668	16600	18540	98.6	99.8	187.4
R4	RS2CL4311s	28.72	+	1205	1	1205	Rsa1.0_00524.1	169613	151107	152386	84.6	99.9	106.2
R4	CB92	28.89											
R4	RS2CL6294s	28.95	+	997	1	989	Rsa1.0_00524.1	169613	97545	99764	92.2	99.1	224.6
R4	RS2CL2726s	29.25	−	692	20	691	Rsa1.0_01355.1	47303	39403	40080	87.7	97.0	100.9
R4	RGA173s	29.73											
R4	RS2CL4162s	30.03	+	984	0	984	Rsa1.0_01191.1	51235	16630	19656	99.0	100.0	307.5
R4	RSS1249_a	30.36	+	474	0	406	Rsa1.0_02860.1	27207	15218	16432	85.4	85.7	299.0
R4	RS2CL3606s	30.37	+	1206	2	1203	Rsa1.0_00347.1	93048	90998	92429	94.4	99.6	119.2
R4	RSS3924	30.9	+	891	1	891	Rsa1.0_00369.1	266462	203222	204992	88.8	99.9	198.9
R4	RS2CL7525s	30.95	+	641	2	631	Rsa1.0_04066.1	19262	7130	8580	83.8	98.1	230.5
R4	RS2CL2655s	31.06	+	1428	2	1428	Rsa1.0_00369.1	266462	165219	168399	97.8	99.9	223.0
R4	RS2CL2361s	31.12	+	759	0	759	Rsa1.0_00369.1	266462	192289	193480	96.8	100.0	156.9
R4	Rs2CL2299s	31.5	−	986	2	986	Rsa1.0_00369.1	266462	4548	5879	97.3	99.8	135.3
R4	RGC21796c	31.84	+	425	0	425	Rsa1.0_04027.1	19501	9765	10190	100.0	100.0	100.0
R4	RGC11816c	32.14	+	437	0	437	Rsa1.0_01191.1	51235	29970	30407	100.0	100.0	100.0
R4	RGC5691c	32.14	−	411	0	411	Rsa1.0_16327.1	2198	712	1123	100.0	100.0	100.0
R4	RS2CL3326s	32.59	+	1317	2	1312	Rsa1.0_02228.1	33688	15710	17716	97.7	99.5	153.1
R4	CA87	32.86											
R4	RS2CL4218s	32.9	+	1024	0	1024	Rsa1.0_06797.1	10342	3819	5057	93.9	100.0	120.9
R4	RS2CL7603s	33.33	+	712	1	712	Rsa1.0_00576.1	178949	62962	63934	92.1	99.9	136.7
R4	RS2CL7351s	33.61	−	914	4	914	Rsa1.0_00576.1	178949	39367	40658	99.0	99.6	141.9

R4	RSS1971	33.82	−	866	4	866	Rsa1.0_00383.1	398431	2241	3430	93.4	99.5	137.9
R4	RS2CL6125s	34.15	+	888	2	888	Rsa1.0_00146.1	149343	135719	136611	95.6	99.8	100.7
R4	RGB15964c	34.42	−	550	136	550	Rsa1.0_00576.1	178949	54823	55237	75.3	75.3	100.0
R4	RGC1115c	34.88	+	421	0	421	Rsa1.0_06842.1	10285	9128	9548	98.1	100.0	99.8
R4	RGC21614c	34.88	+	402	0	381	Rsa1.0_00383.1	398431	165886	166267	94.8	94.8	100.0
R4	RGC9615c	34.88	−	407	0	407	Rsa1.0_04066.1	19262	1224	1631	100.0	100.0	100.0
R4	RSS2202	35.03	+	826	0	826	Rsa1.0_00146.1	149343	43374	45392	91.8	100.0	244.3
R4	RS2CL5759s	35.06	−	835	2	835		42964	15015	16178	96.2	99.8	139.6
R4	RGC17618c	35.16	−	400	0	400	Rsa1.0_00302.1	97521	17760	18160	100.0	100.0	100.0
R4	RSS0067	35.99	+	751	10	751	Rsa1.0_00302.1	97521	86296	87537	91.1	98.7	167.5
R4	RSS0178	36.89	+	804	1	804	Rsa1.0_00837.1	62568	4114	5140	99.9	99.9	127.8
R4	RSS1636	37.29	+	498	1	497	Rsa1.0_04159.1	18795	12148	12636	94.8	99.6	98.4
R4	RS2CL1832s	39.19	−	1073	0	1073	Rsa1.0_00870.1	61606	27393	28837	97.8	100.0	134.6
R4	CA24	41.29											
R4	RGC21459c	42.16	−	542	0	542	Rsa1.0_00182.1	142305	63791	64333	100.0	100.0	100.0
R4	RS2CL1097s	42.88	+	822	2	820	Rsa1.0_00044.1	271658	137918	139348	97.0	99.5	174.8
R4	RS2CL5633s	43.92	−	980	1	980	Rsa1.0_00251.1	303968	58128	60009	96.8	99.9	192.1
R4	RGC13382c	44.44	−	435	0	435	Rsa1.0_00362.1	299497	18932	19367	100.0	100.0	100.0
R4	RGC17631c	44.44	−	527	0	527	Rsa1.0_00818.1	62939	5383	5910	100.0	100.0	100.0
R4	RGC2699c	44.44	+	458	0	458	Rsa1.0_01566.1	43134	32982	33440	100.0	100.0	100.0
R4	RS2CL4419s	45.05	−	1212	1	1212	Rsa1.0_03132.1	25142	7930	9489	98.0	99.9	128.7
R4	RGC4505c	45.62	+	487	0	487	Rsa1.0_00044.1	271658	111698	112185	71.7	100.0	100.0
R4	RS2CL3258s	46.01	+	956	0	954	Rsa1.0_02777.1	27986	6280	8154	98.0	99.8	196.4
R4	RGA2287s	46.24											
R4	RS2CL5222s	46.57											
R4	RS2CL2500s	46.6	−	744	31	418	Rsa1.0_05821.1	12531	4015	4502	52.0	52.0	125.8
R4	RS2CL3360s	46.73	+	1016	11	1016	Rsa1.0_00243.1	103651	42167	43169	94.7	98.9	99.7
R4	CF16	47											
R4	RS2CL6318s	47.2	−	1050	1	1050	Rsa1.0_05821.1	12531	8087	9762	98.2	99.9	159.7
R4	RGC18965c	47.36	+	442	72	442	Rsa1.0_01509.1	44258	22521	22891	83.7	83.7	100.0
R4	RS2CL2926s	47.48	−	557	2	557	Rsa1.0_00923.1	153678	23046	24295	98.6	99.6	225.1
R4	RS2CL6239s	47.77	−	659	27	659	Rsa1.0_00243.1	103651	92356	93103	85.4	95.9	118.2
R4	RSS2705	47.83	−	856	0	856		75219	2039	3002	100.0	100.0	112.5
R4	RSS1127	48.13	−	831	0	831		495407	401649	402846	96.9	100.0	144.0
R4	RSS3772	48.43	+	550	3	550	Rsa1.0_04789.1	15981	10007	10559	90.7	99.5	100.9
R4	RGB3765c	48.58	+	412	0	412	Rsa1.0_01110.1	195852	179047	179459	100.0	100.0	100.0

R4	RS2CL7398s	49.31	+	1227	1	1227	Rsa1.0_00243.1	103651	85901	87118	86.2	99.9	99.3
R4	RSS0396	49.97	+	745	0	745		97747	81041	82435	96.6	100.0	187.1
R4	RSS0839	49.97	+	806	0	806		97747	81636	82882	96.5	100.0	154.6
R4	RSS1418	50.35	+	577	11	577		97747	53036	53679	98.1	98.1	113.6
R4	RSS3863	50.35	+	775	0	775	Rsa1.0_02798.1	27792	5380	6688	96.3	100.0	168.8
R4	RGC21483c	50.8	−	407	0	308	Rsa1.0_04200.1	18490	10290	10598	75.7	75.7	100.0
R4	RSS0936	50.82	−	786	0	786	Rsa1.0_01696.1	301850	28625	29953	95.0	100.0	169.0
R4	RSS1675	51.12	−	849	77	838		97747	4014	4775	56.4	89.6	100.0
R4	RS2CL2021s	52.06	−	915	2	915	Rsa1.0_02247.1	33427	27283	28994	95.4	99.8	187.4
R4	RGB25198c	52.36	+	454	0	454	Rsa1.0_00294.1	144860	24720	25174	100.0	100.0	100.0
R4	RSS0033	53.1	+	853	1	853	Rsa1.0_03843.1	20496	9905	11432	96.0	99.9	179.2
R4	RS2CL5851s	53.33	+	620	1	620	Rsa1.0_01110.1	195852	28210	28833	99.4	99.8	100.7
R4	RGC7341c	53.7	+	537	0	537	Rsa1.0_01110.1	195852	19273	19810	100.0	100.0	100.0
R4	RS2CL4350s	53.79	−	684	0	684		97747	22867	23714	99.4	100.0	123.8
R4	CB05	53.92											
R4	RGC2885c	54.63	+	402	0	402	Rsa1.0_00886.1	167350	9294	9696	100.0	100.0	100.0
R4	RS2CL6990s	54.9	−	741	2	735	Rsa1.0_00985.1	161950	32296	33211	91.9	98.9	124.8
R4	RGC10051c	55.53	+	454	0	454	Rsa1.0_03843.1	20496	3239	3693	100.0	100.0	100.0
R4	CD11aH	56.72											
R4	RS2CL2925s	56.83	−	1372	0	1369	Rsa1.0_01459.1	45277	35840	37209	75.4	99.8	100.0
R4	RGC17385c	57.02	−	447	0	447	Rsa1.0_07638.1	8967	8112	8559	100.0	100.0	100.0
R4	RSS3637	57.69	+	793	4	793	Rsa1.0_14327.1	3518	597	1449	99.4	99.5	108.0
R4	Rs2CL2178s	58.46	−	816	3	816		66432	11911	13472	48.2	99.6	192.0
R4	RGC2356c	59.5	+	485	0	485	Rsa1.0_00126.1	127534	37800	38285	100.0	100.0	100.0
R4	RSS3616	59.85	−	847	0	847	Rsa1.0_00057.1	160473	70666	72057	99.1	100.0	164.2
R4	RS2CL5879s	60.87	−	1137	1	1137	Rsa1.0_00238.1	193193	99096	100517	99.6	99.9	125.1
R4	RS2CL5717s	61.19	+	1148	0	1147		80937	30206	31713	97.0	99.9	131.4
R4	RGB4922c	61.51	−	511	0	511	Rsa1.0_04846.1	15739	5259	5770	100.0	100.0	100.0
R4	RGC9577c	62.06	+	507	0	507		101125	44990	45497	100.0	100.0	100.0
R4	Z30817	62.64											
R4	RS2CL7473s	62.89	+	782	8	764		101125	60423	61232	87.6	96.7	107.0
R4	RGC10583c	63.35	−	552	0	552		106231	66280	66832	100.0	100.0	100.0
R4	RGC2146c	63.35	+	403	0	403	Rsa1.0_13824.1	4259	2721	3124	100.0	100.0	100.0
R4	RGC3356c	63.35	−	561	0	561	Rsa1.0_06215.1	11478	5208	5769	99.6	100.0	100.0
R4	CB39	64.56											
R4	RS2CL1232s	64.61	+	887	1	885	Rsa1.0_00884.1	61150	48101	48981	82.4	99.7	99.6

R4	RSS0369	65.4	−	815	0	815	Rsa1.0_00526.1	169279	165179	165994	100.0	100.0	100.0
R4	CA83	66											
R4	CB86	66.6											
R4	RSS3516	68.1	+	856	0	856	Rsa1.0_00584.1	74295	9334	10186	97.4	100.0	99.5
R4	RSS1061	69.2	−	881	0	881	Rsa1.0_00520.1	78226	2471	3355	98.1	100.0	100.3
R4	RSS0008	69.5	+	732	45	732	Rsa1.0_01189.1	51358	8605	9325	85.7	93.9	104.8
R4	RSS3919	69.6	−	789	0	789	Rsa1.0_04167.1	18757	17259	18389	99.9	100.0	143.2
R4	Rs2CL1980s	70.35	+	1237	2	1236	Rsa1.0_01298.1	48999	6687	8921	99.4	99.8	181.0
R4	RS2CL5596s	71.35											
R4	CC19N	71.79											
R4	RGC11405c	72.03	−	497	0	497	Rsa1.0_01646.1	41735	31905	32402	98.6	100.0	100.0
R4	RS2CL1344s	72.44											
R4	RGC3002c	72.8	−	407	0	407	Rsa1.0_01096.1	54266	5593	6000	100.0	100.0	100.0
R4	RGC21423c	73.12	−	442	0	442		251946	36857	37299	93.2	100.0	100.0
R4	RS2CL4378s	73.31	+	850	2	850	Rsa1.0_02462.1	31158	2654	3675	99.3	99.8	120.4
R4	CD72b	73.38											
R4	RGA1767s	73.47											
R4	RS2CL6532s	73.68	−	826	1	825	Rsa1.0_02301.1	32835	28179	29148	99.5	99.8	117.6
R4	RS2CL738s	74.44	−	1506	0	1506	Rsa1.0_05431.1	13780	5275	8243	99.8	100.0	197.1
R4	RS2CL7671s	74.49	−	784	1	784	Rsa1.0_02615.1	29547	15678	16914	98.9	99.9	157.9
R4	RS2CL4635s	74.62	+	660	0	660	Rsa1.0_00274.1	210119	66474	67561	99.6	100.0	164.7
R4	Rs2CL2052s	74.78	−	1456	2	1456	Rsa1.0_00592.1	73788	20106	21723	99.6	99.9	111.2
R4	RGC10110c	75.04											
R4	RGB10350c	75.04	+	488	0	488	Rsa1.0_00906.1	178966	137182	137670	100.0	100.0	100.0
R4	RGC13794c	75.04	−	448	0	413	Rsa1.0_02574.1	29954	12820	13233	55.4	92.2	100.0
R4	RGC2328c	75.04	−	403	0	403		88681	14804	15207	100.0	100.0	100.0
R4	RGC2715c	75.04	−	445	0	445	Rsa1.0_00057.1	160473	146989	147434	99.1	100.0	100.0
R4	RGC3886c	75.04	+	402	182	402	Rsa1.0_01598.1	42553	15173	15393	54.7	54.7	100.0
R4	RGC7139c	75.04	+	448	0	448	Rsa1.0_02857.1	27254	13638	14086	100.0	100.0	100.0
R4	RGC8082c	75.04	−	472	0	472	Rsa1.0_00852.1	62179	53321	53793	100.0	100.0	100.0
R4	RS2CL713s	75.22	+	937	0	688	Rsa1.0_03681.1	21405	665	1350	72.8	73.4	99.6
R4	RS2CL6965s	75.46	−	786	1	786	Rsa1.0_01205.1	50795	8543	9329	94.5	99.9	100.1
R4	RS2CL2947s	75.76	+	1114	0	1114	Rsa1.0_01071.1	166914	14041	16450	99.7	100.0	216.3
R4	RS2CL2388s	76.25	−	1434	0	1434	Rsa1.0_00852.1	62179	27332	28823	92.1	100.0	104.0
R4	RGC9337c	76.67	+	460	0	460	Rsa1.0_01989.1	36439	22682	23142	100.0	100.0	100.0
R4	RGC3045c	77.19	−	469	0	469	Rsa1.0_00520.1	78226	15274	15743	100.0	100.0	100.0

R4	RGC8922c	77.19	−	458	0	458	Rsa1.0_00210.1	108424	21919	22377	100.0	100.0	100.0
R4	RS2CL6195s	77.6	−	757	0	756	Rsa1.0_00520.1	78226	19413	20171	98.9	99.9	100.3
R4	RS2CL5121s	78.06	−	939	1	939	Rsa1.0_01287.1	49195	2347	3349	99.6	99.9	106.8
R4	RSS3739	78.06	−	844	0	844	Rsa1.0_00350.1	92346	47201	48635	97.6	100.0	169.9
R4	RGC15937c	78.59	−	576	0	576	Rsa1.0_19177.1	1469	473	1049	100.0	100.0	100.0
R4	RGC2894c	78.59	−	548	0	548	Rsa1.0_00260.1	102021	69750	70298	100.0	100.0	100.0
R4	RGC364c	78.59	−	430	0	430	Rsa1.0_00350.1	92346	22423	22853	55.6	100.0	100.0
R4	RGC6719c	78.59	−	431	0	431	Rsa1.0_02746.1	28256	20796	21227	100.0	100.0	100.0
R4	RGC6966c	78.59	−	400	0	400	Rsa1.0_01361.1	47226	30750	31150	100.0	100.0	100.0
R4	RGB7858c	78.59	+	420	0	420	Rsa1.0_01083.1	54617	18966	19386	100.0	100.0	100.0
R4	RGC4610c	78.64	+	425	0	425	Rsa1.0_01778.1	39509	10035	10460	93.9	100.0	100.0
R4	RS2CL4033s	79.02	+	815	13	815	Rsa1.0_01302.1	48907	47055	47869	94.1	98.4	101.5
R4	CD63H	79.42											
R4	RS2CL5842s	79.52	+	1117	6	1117	Rsa1.0_00802.1	63846	56789	58273	88.5	99.5	133.6
R4	RGC12174c	79.74	+	473	0	473	Rsa1.0_03048.1	25777	10535	11008	100.0	100.0	100.0
R4	RSS2079	80.1	−	873	0	873	Rsa1.0_00350.1	92346	79679	81232	98.2	100.0	177.9
R4	RGB11177c	80.11	+	453	0	453	Rsa1.0_00516.1	78417	39967	40420	100.0	100.0	100.0
R4	RSS0347	80.49	−	797	0	797	Rsa1.0_00427.1	189678	33179	34321	100.0	100.0	143.3
R4	RS2CL3470s	80.8	+	1428	0	1427	Rsa1.0_00516.1	78417	39526	41107	97.9	99.9	110.8
R4	RGC2472c	81.04	−	568	0	568	Rsa1.0_00290.1	225096	158409	158977	100.0	100.0	100.0
R4	Rs2CL2508s	81.4	+	435	0	429	Rsa1.0_00516.1	78417	19865	20386	95.9	98.6	121.5
R4	CC15	81.96											
R4	OPE01	82.76											
R4	RSS1284	83.86	+	484	0	484	Rsa1.0_00126.1	127534	94230	94714	75.6	100.0	100.0
R4	RS2CL1961s	84.09	+	1100	1	1095	Rsa1.0_00350.1	92346	79571	81287	92.6	99.5	156.9
R4	CC89	84.65											
R4	RS2CL4014s	85.38	+	962	4	959	Rsa1.0_00401.1	244410	178051	179385	96.8	99.3	139.7
R4	RSS3847	85.63	−	860	0	783	Rsa1.0_07855.1	8417	106	1233	90.6	91.1	143.9
R4	RGC15702c	85.89	+	583	0	583	Rsa1.0_02125.1	34775	17676	18259	93.5	100.0	100.0
R4	RSS1710	86.62	+	558	0	558	Rsa1.0_00229.1	105471	39470	40028	100.0	100.0	100.0
R4	RSS3515	87.12	−	821	0	821	Rsa1.0_04646.1	16541	1433	2723	99.6	100.0	157.1
R4	RS2CL3313s	88.51	+	1267	5	1267	Rsa1.0_04266.1	18195	4593	6034	91.6	99.6	114.2
R4	RGC24823c	88.69	+	461	0	461	Rsa1.0_02518.1	30586	16267	16728	100.0	100.0	100.0
R4	RGC6716c	88.69	−	422	0	422	Rsa1.0_00140.1	121541	90036	90458	100.0	100.0	100.0
R4	RSS3297	89.19	−	509	0	509	Rsa1.0_00122.1	265937	224826	225335	99.6	100.0	100.0
R4	RGC32186c	89.65	+	402	0	402	Rsa1.0_02367.1	32106	17906	18307	90.3	100.0	99.8

R4	RGB520c	90.52	−	496	0	496	Rsa1.0_00076.1	146021	107386	107882	100.0	100.0	100.0
R4	CA98	90.86											
R4	RGB14019c	91.07	+	431	0	431	Rsa1.0_01339.1	47813	6038	6500	99.8	100.0	107.2
R4	RSS3234	91.65	−	785	0	785	Rsa1.0_00013.1	292129	155049	156057	61.9	100.0	128.4
R4	RSS1064	91.85	+	829	0	715	Rsa1.0_00013.1	292129	154649	155587	86.3	86.3	131.2
R4	RGC27467c	92.02	−	436	0	436	Rsa1.0_01990.1	36431	24325	24761	100.0	100.0	100.0
R4	RGC2779c	92.02	+	407	0	407	Rsa1.0_00013.1	292129	134615	135022	100.0	100.0	100.0
R4	RGC5481c	92.02	+	457	0	457	Rsa1.0_00477.1	81556	21597	22054	92.8	100.0	100.0
R4	RGC206c	92.28	+	425	0	425	Rsa1.0_03007.1	26070	17568	17993	100.0	100.0	100.0
R4	RGC2343c	92.28	+	421	0	421	Rsa1.0_01144.1	52431	21023	21444	100.0	100.0	100.0
R4	RGC2932c	92.28	−	545	0	545	Rsa1.0_01027.1	56402	7812	8357	100.0	100.0	100.0
R4	RS2CL6948s	92.3	+	606	1	591	Rsa1.0_00581.1	163575	26286	28062	88.3	97.4	301.0
R4	RSS2397	92.42	−	208	0	208	Rsa1.0_00013.1	292129	120586	120794	100.0	100.0	100.0
R4	RS2CL4453s	93.08	+	786	0	784	Rsa1.0_00013.1	292129	191217	192404	98.6	99.8	151.4
R4	RGC13764c	93.44	+	421	0	421	Rsa1.0_00752.1	65756	11770	12191	100.0	100.0	100.0
R4	RS2CL3226s	94.1	−	752	0	752	Rsa1.0_08151.1	7830	2088	2845	98.7	100.0	100.7
R4	RGC139c	94.27	+	432	0	432	Rsa1.0_01936.1	37151	23667	24099	100.0	100.0	100.0
R4	RSS0625	94.3	+	251	0	251	Rsa1.0_04617.1	16625	15282	15546	95.6	100.0	105.2
R4	RSS1628	94.3	−	865	0	865	Rsa1.0_04617.1	16625	15384	16269	93.9	100.0	102.3
R4	RSS2491	94.3	+	674	0	674	Rsa1.0_00477.1	81556	13737	14544	93.6	100.0	119.7
R4	RSS3741	94.3	−	516	2	516	Rsa1.0_00477.1	81556	10595	11407	92.1	99.6	158.0
R4	RGC1537c	94.36	−	421	0	421		390088	77935	78356	100.0	100.0	100.0
R4	RGC6353c	94.36	−	502	149	502		188018	2603	2956	70.3	70.3	100.0
R4	RS2CL6090s	94.57	+	678	22	678	Rsa1.0_16231.1	131890	0	764	91.9	96.8	116.5
R4	RS2CL2598s	94.76	+	783	2	783	Rsa1.0_06619.1	10650	527	1475	94.6	99.7	121.4
R4	RS2CL6646s	94.86	−	1049	1	1047	Rsa1.0_00013.1	292129	15338	16888	96.4	99.7	148.2
R4	RS2CL5488s	94.98	+	1194	2	1194	Rsa1.0_00013.1	292129	114305	116481	99.3	99.8	182.6
R4	RS2CL8917s	95.07	−	733	6	733	Rsa1.0_00370.1	90472	43128	43876	96.2	99.2	102.9
R4	Rs2CL1743s	95.16	−	1612	0	1609	Rsa1.0_01144.1	52431	39379	42732	66.8	99.8	208.4
R4	RSS0393	95.22	+	708	0	708	Rsa1.0_03436.1	22958	18416	19124	94.1	100.0	100.0
R4	RSS0341	95.82	+	828	0	828		40243	5200	6833	98.7	100.0	197.2
R4	RSS2667	95.82	+	824	45	824	Rsa1.0_00385.1	89160	81269	82755	86.0	94.5	190.8
R4	CC43b	96.52											
R4	RSS3109	97.62	+	863	0	860	Rsa1.0_00385.1	89160	47404	48348	98.5	99.7	109.8
R4	CA15	99.42											
R4	RS2CL3689s	100.43	+	802	8	792	Rsa1.0_01144.1	52431	22887	24462	93.3	97.8	200.9

R4	RGA1556s	100.61											
R4	RS2CL2599s	100.81	−	1222	2	1190	Rsa1.0_02907.1	26861	15222	16610	82.3	97.2	116.8
R4	RSS2245	101.28	−	847	0	847	Rsa1.0_00407.1	191544	155701	156635	100.0	100.0	110.3
R4	RS2CL2069s	101.29	+	953	4	952		390088	5959	7451	75.5	99.5	157.4
R4	RS2CL6037s	101.56	+	1013	0	1013		390088	55219	56497	90.4	100.0	126.2
R4	RS2CL4491s	102.02	−	1121	0	1120	Rsa1.0_00159.1	431686	407496	408969	97.2	99.9	131.5
R4	RGC16154c	102.46	+	466	0	466		390088	177718	178184	100.0	100.0	100.0
R4	RGC19350c	102.46	−	421	0	421		390088	141848	142269	100.0	100.0	100.0
R4	RGC9063c	102.46	−	478	0	478	Rsa1.0_03504.1	22443	8297	8775	100.0	100.0	100.0
R4	RS2CL3466s	102.69	+	1590	2	1586		390088	59689	61436	95.8	99.6	110.3
R4	Rs2CL2383s	103.26	+	623	0	622		390088	320345	321331	99.8	99.8	158.5
R4	RSS3247	103.38	+	755	3	755	Rsa1.0_01254.1	49801	13792	14998	99.6	99.6	160.4
R4	RS2CL3308s	103.73	+	863	2	863		390088	380683	381776	98.6	99.8	127.0
R4	RS2CL2278s	104.72	−	1064	2	1064	Rsa1.0_07208.1	9719	7728	9009	93.8	99.8	120.6
R4	RS2CL8405s	105.31	−	887	2	887	Rsa1.0_02803.1	27715	17271	18452	98.1	99.8	133.5
R4	CC56a	105.59											
R4	RS2CL6277s	106.41	+	619	1	619	Rsa1.0_00498.1	79746	48401	49900	97.6	99.8	242.6
R4	RS2CL3794s	106.71	−	991	5	958	Rsa1.0_00788.1	64237	58539	59946	83.4	96.2	147.6
R4	CD24	107.49											
R4	RS2CL2462s	107.89	−	623	3	623	Rsa1.0_15127.1	2809	1503	2425	98.9	99.5	148.7
R4	RS2CL4251s	107.91	−	879	0	879	Rsa1.0_01061.1	55349	21837	22983	98.1	100.0	130.4
R4	RGC17710c	108.16	−	522	0	522	Rsa1.0_01154.1	52249	49615	50136	88.9	100.0	99.8
R4	RGC16310c	108.21	+	412	0	412	Rsa1.0_00328.1	224661	185668	186080	100.0	100.0	100.0
R4	RGC2194c	108.21	+	451	0	451	Rsa1.0_01413.1	46275	6310	6761	100.0	100.0	100.0
R4	RGC2833c	108.21	−	477	0	477	Rsa1.0_00788.1	64237	49407	49884	87.4	100.0	100.0
R4	RGC4391c	108.21	+	493	0	493	Rsa1.0_00498.1	79746	25957	26450	100.0	100.0	100.0
R4	RGC5031c	108.21	−	509	0	509	Rsa1.0_07208.1	9719	5945	6454	100.0	100.0	100.0
R4	RGC6220c	108.21	+	450	0	450	Rsa1.0_03437.1	22947	16801	17251	100.0	100.0	100.0
R4	RS2CL2730s	108.25	+	1237	3	1235	Rsa1.0_00328.1	224661	135239	137083	95.7	99.6	149.7
R4	RS2CL6009s	108.46	+	1389	2	1389	Rsa1.0_01061.1	55349	15445	17769	96.4	99.9	167.6
R4	RS2CL1897s	108.78	+	734	0	694	Rsa1.0_01154.1	52249	37203	38568	71.8	94.6	196.7
R4	RS2CL5094s	109.2	+	1151	2	1151	Rsa1.0_01118.1	53460	1850	3244	89.8	99.8	121.3
R4	RGC9763c	109.49	−	467	0	467	Rsa1.0_04203.1	18479	5357	5824	100.0	100.0	100.0
R4	RSS3305	109.57	+	826	0	826	Rsa1.0_02432.1	31499	17554	18575	99.8	100.0	123.6
R4	RGC2574c	109.94	+	430	0	430	Rsa1.0_01930.1	37189	18509	18939	85.1	100.0	100.0
R4	RS2CL1636s	110.31	−	749	2	749	Rsa1.0_01200.1	50895	30781	31941	92.3	99.7	155.3

R4	RSS3500	111.08	+	850	3	850	Rsa1.0_02402.1	31710	28973	30627	97.3	99.7	195.3
R4	RGB17458c	111.59	−	466	0	466	Rsa1.0_02152.1	34558	27402	27868	58.2	100.0	100.0
R4	RS2CL5146s	112.69											
R4	RGC9723c	113.08	+	445	0	445	Rsa1.0_00962.1	184909	39642	40087	100.0	100.0	100.0
R4	RGC12230c	113.41	−	461	0	461	Rsa1.0_03420.1	23090	7451	7912	99.8	100.0	100.0
R4	RS2CL1956s	114.11	+	963	9	961	Rsa1.0_00647.1	70440	44494	45455	88.7	98.9	101.0
R4	RS2CL5598s	114.15	−	1211	5	934	Rsa1.0_04811.1	15885	13002	14514	76.0	76.7	162.8
R4	RS2CL2277s	114.21	−	714	1	705	Rsa1.0_00647.1	70440	41485	42356	96.1	98.6	123.7
R4	RS2CL8851s	114.3	+	935	0	935	Rsa1.0_04811.1	15885	10981	12797	99.1	100.0	194.2
R4	RS2CL7217s	115.77	−	772	1	772	Rsa1.0_00910.1	59975	57347	58498	99.7	99.9	149.3
R4	RGC2987c	116.69	−	430	0	430	Rsa1.0_05398.1	13885	12986	13416	99.8	100.0	100.0
R4	RSS1902	116.96	−	632	0	632	Rsa1.0_06032.1	11886	7572	8622	99.5	100.0	166.1
R4	RSS2415	117.66	+	845	0	845	Rsa1.0_01118.1	53460	10217	11858	98.1	100.0	194.2
R4	RS2CL1437s	118.37											
R4	RGC11873c	118.75	−	438	0	404	Rsa1.0_01305.1	48730	18621	19025	92.2	92.2	100.0
R4	RSS2929	119.67	−	828	0	828	Rsa1.0_07974.1	8147	6681	7510	97.0	100.0	100.1
R4	RGC6749c	120.83	−	452	0	452	Rsa1.0_00916.1	59789	4373	4825	100.0	100.0	100.0
R4	RSS3668	120.97	−	850	3	850	Rsa1.0_23904.1	1018	58	915	91.4	99.7	101.2
R4	RS2CL2439s	121.57	+	935	1	935		301669	271373	272522	97.1	99.9	123.0
R4	RSS0993	122.28	+	846	0	821	Rsa1.0_08530.1	7232	19	1772	92.9	97.0	213.5
R4	RGA1715s	123.77											
R4	RSS1828	124.02	+	837	0	837	Rsa1.0_00358.1	91293	65917	66755	100.0	100.0	100.1
R4	RSS3914	124.62	+	840	0	840	Rsa1.0_00358.1	91293	88944	89784	99.5	100.0	100.0
R4	RGC4219c	125	−	517	0	517	Rsa1.0_01384.1	46803	21031	21548	100.0	100.0	100.0
R4	RS2CL983s	125.63	+	782	1	770	Rsa1.0_00095.1	283384	26010	27356	96.3	98.3	175.0
R4	RS2CL3277s	126.29	+	1381	3	1381		152418	93035	95375	21.9	99.8	169.8
R4	RGC14440c	126.65	+	504	0	504	Rsa1.0_00422.1	86144	17537	18041	100.0	100.0	100.0
R4	RS2CL7183s	126.95											
R4	RGC14398c	127.38	−	528	0	279	Rsa1.0_01676.1	41140	38459	38738	52.8	52.8	100.0
R4	OPT09a	127.47											
R4	RS2CL4168s	127.88	+	866	75	866	Rsa1.0_04568.1	16805	4889	5680	64.3	91.3	100.0
R4	RS2CL1373s	128.17	+	959	2	959	Rsa1.0_00143.1	121313	85782	87534	98.8	99.8	183.1
R4	RGC7805c	128.8	−	444	0	424	Rsa1.0_00313.1	96745	17880	18304	65.3	95.5	100.0
R4	RS2CL5077s	128.82	+	1056	1	1056	Rsa1.0_00836.1	185968	168536	170215	91.3	99.9	159.2
R4	RS2CL2423s	129.15	+	1369	1	1367	Rsa1.0_04033.1	19481	8910	11083	97.7	99.8	159.1
R4	RSS0375	129.25											

R4	RS2CL2532s	129.29	−	817	0	817	Rsa1.0_00836.1	185968	182124	183567	95.5	100.0	176.6
R4	RS2CL7410s	129.43	−	646	3	642	Rsa1.0_01514.1	44222	4068	4695	96.4	98.9	98.1
R4	RS2CL6216s	129.9	−	970	6	970	Rsa1.0_00836.1	185968	17828	19202	93.8	99.4	142.5
R4	RSS2818	130.28	−	842	0	842		48033	42387	43231	100.0	100.0	100.2
R4	RGC12577c	130.65											
R4	RGC12869c	130.65											
R4	RGC2383c	130.65	+	555	0	555	Rsa1.0_00313.1	96745	79841	80396	95.0	100.0	100.0
R4	RGC6866c	130.65	−	447	0	447	Rsa1.0_02160.1	34468	3507	3954	100.0	100.0	100.0
R4	RGC8508c	130.65	+	525	0	392	Rsa1.0_01366.1	47139	28112	28504	51.8	74.7	100.0
R4	RS2CL1846s	130.8	−	623	2	622	Rsa1.0_01065.1	55264	30933	32367	74.5	99.5	231.3
R4	CA35b	130.81											
R4	RS2CL2717s	131.12	−	918	2	831	Rsa1.0_00847.1	62270	54052	55578	87.5	90.3	184.1
R4	RSS3394	131.17	−	845	155	845	Rsa1.0_00364.1	90951	5906	6723	79.8	81.7	118.4
R4	RSS3897	131.17	+	537	0	537	Rsa1.0_01384.1	46803	32825	34016	49.5	100.0	221.8
R4	RS2CL1003s	131.21	−	892	5	892	Rsa1.0_04785.1	15993	7242	8743	95.6	99.4	169.2
R4	RS2CL1386s	131.67	−	1165	2	1164	Rsa1.0_00227.1	105849	102220	104784	94.4	99.7	220.7
R4	RS2CL4891s	131.97	−	1341	2	1341	Rsa1.0_02459.1	31185	18210	20256	92.1	99.9	152.8
R4	RSS0649	132.1	−	651	1	651	Rsa1.0_03028.1	25872	20810	22684	95.2	99.9	288.3
R4	RSS0371	132.6	−	635	0	635	Rsa1.0_03194.1	24674	2759	3496	93.5	100.0	116.1
R4	RS2CL4267s	133.5	+	775	3	775	Rsa1.0_01157.1	52121	44784	45562	78.1	99.6	100.8
R4	RSS2126	134.1	+	830	0	830	Rsa1.0_00422.1	86144	34812	35643	99.3	100.0	100.1
R4	RSS0912	134.5	+	285	13	271	Rsa1.0_01555.1	166456	17908	18260	88.1	90.5	136.4
R4	RS2CL2291s	134.57	+	1047	3	1047	Rsa1.0_04235.1	18373	11397	12739	97.5	99.7	128.5
R4	RS2CL2308s	135.38	+	721	1	719	Rsa1.0_09709.1	6198	2932	3656	99.0	99.6	100.8
R4	RGC151c	135.73	+	420	0	420	Rsa1.0_00645.1	189716	11058	11478	100.0	100.0	100.0
R4	RGC16913c	135.73	−	472	0	472	Rsa1.0_00554.1	150953	121061	121533	100.0	100.0	100.0
R4	RGC4940c	135.73	+	522	0	522	Rsa1.0_00031.1	179862	85292	85814	93.7	100.0	100.0
R4	RGC7607c	135.73	+	437	0	437	Rsa1.0_00031.1	179862	161066	161559	100.0	100.0	112.8
R4	CC79	135.87											
R4	RS2CL5873s	136.57	+	1011	1	1010	Rsa1.0_00031.1	179862	166009	167292	99.5	99.8	127.2
R4	RSS1537	136.72	−	812	0	812	Rsa1.0_01489.1	44764	885	1995	100.0	100.0	136.7
R4	RGC10060c	137.23	+	447	0	447	Rsa1.0_03733.1	21108	1634	2081	100.0	100.0	100.0
R4	RS2CL2821s	137.64	−	1169	0	1167	Rsa1.0_00270.1	101236	40699	41940	79.2	99.8	106.3
R4	OPT16a	138.02											
R4	RGA1700s	138.08											
R4	Rs2CL2077s	138.8	−	661	3	653	Rsa1.0_04073.1	19238	15161	16112	94.7	98.3	146.3

R4	RS2CL1358s	140.21	−	696	3	694	Rsa1.0_00270.1	101236	23111	24645	93.1	99.3	222.0
R4	RGC2546c	141.22	+	486	0	486	Rsa1.0_00424.1	86005	77194	77680	100.0	100.0	100.0
R4	RGA125s	142.16											
R5	RSS3015	0	+	861	176	861	Rsa1.0_04026.1	19508	6450	7135	51.9	79.6	100.0
R5	RSS3061	0.3											
R5	RSS1661	0.8	−	853	0	853	Rsa1.0_07499.1	9244	5637	6863	91.3	100.0	143.7
R5	RSS3244	0.8	+	776	0	776	Rsa1.0_00055.1	520193	162514	164222	94.3	100.0	220.1
R5	RSS3245	0.8	−	439	0	397		283362	269838	270532	80.9	90.4	174.8
R5	RSS0292	1.1	−	662	0	662	Rsa1.0_03325.1	23720	16433	17382	90.6	100.0	143.4
R5	CD34bH	1.4											
R5	RSS0017	1.6	+	728	9	728	Rsa1.0_10522.1	5816	4428	5414	94.8	98.8	137.1
R5	CA74H	2											
R5	RSS1674	2.1	+	695	1	695	Rsa1.0_00179.1	393324	249543	250936	65.3	99.9	200.7
R5	CD71	2.5											
R5	N97067	2.8											
R5	Rs2CL1745s	2.81	+	729	2	729	Rsa1.0_04288.1	18077	12915	14097	99.7	99.7	162.6
R5	CD20	3.1											
R5	RSS0662_a	4	−	838	0	838		59481	8137	9280	99.4	100.0	136.4
R5	RSS1054	4.2											
R5	RSS1457	4.6	−	838	0	838	Rsa1.0_04396.1	17561	4330	5316	98.1	100.0	117.7
R5	RS2CL8651s	4.64	+	597	173	597	Rsa1.0_00703.1	67597	26997	27421	69.9	71.0	100.0
R5	RSS3450	5											
R5	RSS0662_b	5.5											
R5	RS2CL7919s	5.86	+	481	1	476	Rsa1.0_02017.1	35999	32830	33315	97.3	98.8	102.1
R5	RS2CL6011s	6.39	+	1261	48	1261	Rsa1.0_00055.1	520193	401657	403415	90.3	96.2	144.9
R5	CC37bH	6.5											
R5	RGC1111c	7.06	+	474	0	474	Rsa1.0_00179.1	393324	205601	206075	100.0	100.0	100.0
R5	RGC1381c	7.06	−	429	0	429	Rsa1.0_00179.1	393324	365750	366179	79.0	100.0	100.0
R5	RGC21684c	7.06	−	520	0	520	Rsa1.0_00542.1	76831	19505	20025	100.0	100.0	100.0
R5	RGB964c	7.06	+	535	0	535	Rsa1.0_00542.1	76831	70503	71038	99.6	100.0	100.0
R5	RS2CL5773s	7.17	+	989	1	989	Rsa1.0_06489.1	10917	3347	4436	88.0	99.9	110.2
R5	RSS2263	7.2	+	777	49	777	Rsa1.0_00977.1	198001	30168	30928	86.2	93.7	104.4
R5	RS2CL2901s	7.31	+	1689	14	1685	Rsa1.0_04364.1	17734	4249	6573	70.3	98.9	139.1
R5	RS2CL3894s	7.44											
R5	RSS1183	7.9	+	494	0	494	Rsa1.0_00977.1	198001	50820	51314	100.0	100.0	100.0

R5	RS2CL4044s	7.95											
R5	RS2CL5289s	8.81	+	945	2	944	Rsa1.0_00179.1	393324	230333	232864	67.3	99.7	268.7
R5	Rs2CL1517s	9.19	+	855	2	855	Rsa1.0_00179.1	393324	203929	205837	90.1	99.8	223.7
R5	RSS2534	9.7											
R5	RS2CL6910s	10.05	−	764	4	764	Rsa1.0_00179.1	393324	196239	197641	97.1	99.5	184.5
R5	RS2CL2032s	11.89	−	806	3	806	Rsa1.0_01206.1	50772	39547	41243	98.1	99.6	211.2
R5	RSS0922	11.9											
R5	Pf	12.7											
R5	RGB2724c	12.95	+	492	0	492	Rsa1.0_00642.1	201230	164830	165322	100.0	100.0	100.0
R5	RS2CL4777s	13.68											
R5	RGC20584c	13.86	−	420	0	420	Rsa1.0_02628.1	29369	27144	27564	100.0	100.0	100.0
R5	RS2CL7144s	14.51	+	608	6	608	Rsa1.0_01603.1	42468	8935	9536	97.5	99.0	99.8
R5	RSS1319	15	−	724	7	723	Rsa1.0_13800.1	4315	2319	3386	92.1	98.9	149.0
R5	RSS3682	15.7											
R5	Rs2CL2150s	16.48	−	510	2	510	Rsa1.0_05572.1	13291	11314	12104	94.1	99.6	155.5
R5	RSS1795	17.9	−	562	0	562	Rsa1.0_03782.1	20891	13576	14225	97.5	100.0	115.5
R5	Rs2CL2167s	18.29	−	800	1	800	Rsa1.0_06136.1	11659	1334	2544	97.8	99.9	151.4
R5	RSS2289	18.6	+	886	0	886	Rsa1.0_00130.1	125737	115183	117108	92.1	100.0	217.3
R5	RSS0310	18.8	−	798	1	798	Rsa1.0_00130.1	125737	116623	118143	91.0	99.9	190.7
R5	RSS2078_a	19.7	+	821	20	810		240912	223707	225014	90.3	96.2	165.4
R5	RSS2459	20.3	−	719	0	719	Rsa1.0_00913.1	59914	54249	55221	90.0	100.0	135.2
R5	RSS1155	20.5	+	810	0	810	Rsa1.0_09141.1	6612	672	2389	100.0	100.0	212.0
R5	RS2CL2345s	20.91											
R5	Rs2CL1650s	21.12											
R5	RSS0255	21.5	+	767	0	767	Rsa1.0_03005.1	26082	7789	8938	99.2	100.0	149.8
R5	RS2CL4383s	21.86	+	709	0	708	Rsa1.0_03759.1	20987	15444	16156	97.9	99.9	100.6
R5	RS2CL4098s	22.38	+	1275	1	1275	Rsa1.0_00130.1	125737	52565	55020	98.4	99.9	192.7
R5	RGA1775s	22.64											
R5	RSS1293	22.7	−	459	1	459	Rsa1.0_00632.1	183777	638	1205	99.6	99.8	123.8
R5	RSS2428	22.7	+	384	6	384	Rsa1.0_08203.1	7742	5070	5547	95.8	98.4	126.2
R5	RSS1421	23	+	616	0	616	Rsa1.0_00632.1	183777	55991	60779	94.0	100.0	777.3
R5	RSS2179	23.4	+	833	0	833	Rsa1.0_00632.1	183777	145494	146327	100.0	100.0	100.0
R5	RSS1445	23.6	−	815	0	815	Rsa1.0_00632.1	183777	176179	176994	95.7	100.0	100.0
R5	RSS0020	24.1	+	798	0	798	Rsa1.0_00098.1	349239	232345	233312	95.1	100.0	121.2
R5	RS2CL6459s	24.65	+	916	2	916	Rsa1.0_04544.1	16917	12702	13615	97.9	99.8	99.9
R5	RSS0640	25.1	+	395	13	395	Rsa1.0_02592.1	29751	6020	6404	92.9	96.7	100.5

R5	RS2CL1107s	25.48	+	1371	0	1369	Rsa1.0_04544.1	16917	5919	8075	40.3	99.9	157.5
R5	RS2CL2656s	27.64											
R5	RSS3462	27.7	−	909	0	909	Rsa1.0_11534.1	5477	1235	2228	86.4	100.0	109.2
R5	RS2CL5063s	28.95	+	526	0	521	Rsa1.0_00098.1	349239	253666	254320	93.5	99.1	125.5
R5	RS2CL7234s	29.1	+	1024	2	1023	Rsa1.0_00098.1	349239	292727	293964	95.4	99.7	121.2
R5	RS2CL7712s	29.4	−	847	6	847	Rsa1.0_00098.1	349239	255475	256370	72.9	99.3	106.4
R5	RS2CL4145s	29.82	−	1299	2	1299	Rsa1.0_03581.1	21965	14208	16201	98.1	99.9	153.7
R5	RS2CL7340s	30.09	+	852	1	852	Rsa1.0_00098.1	349239	294602	295487	95.4	99.9	104.0
R5	RGC6520c	30.12	+	448	0	448	Rsa1.0_00632.1	183777	152797	153245	100.0	100.0	100.0
R5	RS2CL3444s	30.63	−	993	2	992	Rsa1.0_05410.1	13840	5178	6808	95.1	99.7	164.7
R5	RGC54c	31.11	+	474	0	474	Rsa1.0_01707.1	40686	30892	31366	100.0	100.0	100.0
R5	RSS0173	31.6	+	847	0	847	Rsa1.0_00626.1	71582	51775	52623	96.6	100.0	100.1
R5	RSS1336	31.6	−	862	3	862	Rsa1.0_01142.1	52464	49764	51296	94.6	99.7	178.4
R5	RS2CL5210s	31.68	−	926	2	926	Rsa1.0_00391.1	183853	72478	74289	90.0	99.8	196.0
R5	RSS0575	31.7	+	876	0	876	Rsa1.0_00626.1	71582	51841	52718	96.7	100.0	100.1
R5	RSS0125	31.8	+	866	0	866	Rsa1.0_02196.1	34005	6419	7660	63.4	100.0	143.3
R5	RS2CL6408s	31.85	−	890	4	890	Rsa1.0_04773.1	16037	10745	13723	88.3	99.6	336.1
R5	RGC11501c	32.07	−	483	0	483	Rsa1.0_05663.1	13005	11577	12060	100.0	100.0	100.0
R5	RGC1596c	32.07	−	548	0	548	Rsa1.0_00098.1	349239	214483	215031	100.0	100.0	100.0
R5	RGC15962c	32.07	−	401	0	401	Rsa1.0_02955.1	26484	2140	2541	92.0	100.0	100.0
R5	RSS2665	32.2	+	850	0	850		92266	13886	15051	100.0	100.0	137.1
R5	Rs2CL1966s	32.35	+	1029	0	1029		329018	14080	15374	99.0	100.0	125.8
R5	RSS2209	32.6	−	855	27	855	Rsa1.0_00391.1	183853	64038	65077	94.9	96.8	125.5
R5	RGC8188c	33.13	+	422	0	422		329018	139869	140291	76.1	100.0	100.0
R5	RSS3635	33.8	+	827	0	667	Rsa1.0_00098.1	349239	255468	256141	74.0	80.7	100.9
R5	RGC1147c	34.2	−	409	0	409	Rsa1.0_03282.1	24107	20044	20453	94.9	100.0	100.0
R5	RSS0647	34.8	−	710	10	710	Rsa1.0_01005.1	56967	5855	7479	97.8	98.6	232.0
R5	RGC11447c	35.27	−	499	0	499	Rsa1.0_00713.1	158017	46109	46608	100.0	100.0	100.0
R5	RGB3806c	35.27											
R5	RSS0380	36											
R5	RS2CL1106s	36.01											
R5	RGB8510c	36.65	−	426	0	419	Rsa1.0_01656.1	41494	10044	10463	98.4	98.4	100.0
R5	RSS1774	37.1	−	382	0	268	Rsa1.0_09168.1	6590	5392	5660	70.2	70.2	100.0
R5	RS2CL804s	37.28	−	379	0	379	Rsa1.0_03870.1	20362	718	1097	94.5	100.0	100.0
R5	RSS0192	37.5	+	659	0	659	Rsa1.0_00081.1	143261	140583	141614	96.5	100.0	156.5
R5	RSS3177	39.4											

R5	RSS2660	40.2	+	862	0	862	Rsa1.0_03016.1	26000	1173	2723	98.8	100.0	179.8
R5	RSS0609	40.3	−	683	0	683	Rsa1.0_03016.1	26000	2222	3601	99.4	100.0	201.9
R5	RS2CL8976s	40.42	−	698	1	698	Rsa1.0_10460.1	5842	3567	4298	84.1	99.9	104.9
R5	RGB2659c	40.99	+	506	0	506	Rsa1.0_02071.1	35409	5053	5559	100.0	100.0	100.0
R5	R30624b	41.1											
R5	RGC8374c	41.67	+	400	0	400	Rsa1.0_07592.1	9059	8023	8423	83.8	100.0	100.0
R5	RSS1763_a	42.5	+	573	0	573	Rsa1.0_04989.1	15217	1951	2779	99.8	100.0	144.5
R5	CC67bH	42.8											
R5	RS2CL5232s	42.8	−	897	12	897	Rsa1.0_02343.1	32374	8809	10166	98.6	98.7	153.3
R5	RSS0485	43.5											
R5	RGA91s	43.58											
R5	RSS0430_a	43.8	+	808	0	808		12173	2326	3128	97.3	100.0	99.3
R5	RGC12639c	44.01	−	471	0	471	Rsa1.0_04298.1	147466	6676	7147	100.0	100.0	100.0
R5	RGC4480c	44.3											
R5	CD72bH	45.1											
R5	RGC9986c	45.6	+	457	0	457	Rsa1.0_00332.1	94575	53643	54100	100.0	100.0	100.0
R5	RSS3072	46.2	+	874	0	874	Rsa1.0_00947.1	58664	47318	48610	93.3	100.0	147.8
R5	RGC17999c	46.55	−	476	0	476	Rsa1.0_00332.1	94575	39392	39868	100.0	100.0	100.0
R5	RGA1661s	47.18											
R5	RGC9094c	47.89	−	428	0	428	Rsa1.0_01018.1	56685	21633	22061	100.0	100.0	100.0
R5	RSS2310	47.9	−	788	0	788	Rsa1.0_02314.1	32642	30106	31494	100.0	100.0	176.1
R5	Rs2CL2263s	49.11	+	833	0	831	Rsa1.0_00759.1	65465	10764	12531	96.8	99.8	212.6
R5	RSS3250	49.8	+	808	0	808	Rsa1.0_02867.1	27121	9734	10535	93.1	100.0	99.1
R5	RS2CL4099s	50.48	+	808	5	806	Rsa1.0_01286.1	49209	40520	41770	95.8	99.1	156.1
R5	RSS1955	50.6	+	416	0	407	Rsa1.0_01960.1	36766	7656	8444	97.6	97.8	193.6
R5	RS2CL3592s	51.19											
R5	RSS3666	52.1	+	462	0	462	Rsa1.0_15163.1	2786	2148	2607	98.3	100.0	99.4
R5	RGB19108c	52.6											
R5	RGC347c	52.6	−	535	0	535	Rsa1.0_01249.1	188432	38816	39351	69.9	100.0	100.0
R5	RGC8606c	52.6	−	581	0	581	Rsa1.0_01249.1	188432	174779	175360	86.6	100.0	100.0
R5	RS2CL3943s	53.18	+	505	2	505	Rsa1.0_05653.1	13043	9441	10445	99.0	99.6	199.6
R5	CA64	54.1											
R5	RS2CL6006s	54.22	+	1052	53	1052	Rsa1.0_01018.1	56685	10	2258	94.5	95.0	225.0
R5	RSS1195	56.1	−	503	0	480	Rsa1.0_07909.1	8315	0	480	95.2	95.4	100.0
R5	RGC19763c	56.37	−	459	0	459	Rsa1.0_03861.1	20402	17871	18330	100.0	100.0	100.0
R5	RSS2188	56.5	+	590	0	590	Rsa1.0_04778.1	16010	8904	9510	92.0	100.0	102.7

R5	RSS0574	57.4											
R5	RSS2974	57.4	+	841	0	841	Rsa1.0_14144.1	3745	859	2001	97.5	100.0	135.8
R5	Rs2CL2589s	58.74	+	950	0	949	Rsa1.0_01783.1	39383	9454	11696	78.5	99.9	236.3
R5	RSS1763	59.2											
R5	RGC6470c	59.95	−	518	0	518	Rsa1.0_01783.1	39383	9147	9665	100.0	100.0	100.0
R5	RSS0154	60.3	−	838	0	838	Rsa1.0_05707.1	12872	1347	2403	100.0	100.0	126.0
R5	RS2CL4004s	60.33	−	1136	7	1124	Rsa1.0_01034.1	56046	18566	20469	95.3	98.3	170.4
R5	RSS2249	61.2	−	853	95	853	Rsa1.0_00100.1	249232	193365	194128	86.6	88.9	100.7
R5	RSS0193	61.3	−	529	0	529	Rsa1.0_00100.1	249232	193393	193920	97.0	100.0	99.6
R5	CD23	61.8											
R5	CC61H	62.8											
R5	OPR17	63.3											
R5	RGC4546c	63.81	−	433	0	433	Rsa1.0_03462.1	22725	20272	20705	100.0	100.0	100.0
R5	RSS0866	64.18	−	656	0	553	Rsa1.0_07814.1	8487	259	1368	84.3	84.3	200.5
R5	RS2CL7551s	64.45	−	840	0	840	Rsa1.0_00100.1	249232	191214	192266	99.8	100.0	125.2
R5	CA66H	64.85											
R5	RGC5978c	64.88	+	579	0	579	Rsa1.0_01803.1	173846	13565	14144	100.0	100.0	100.0
R5	RGB1306c	65.46	−	411	0	411	Rsa1.0_00324.1	218805	190019	190671	99.8	100.0	158.6
R5	RSS0068	65.49	−	841	0	841	Rsa1.0_01453.1	45449	10838	12831	100.0	100.0	237.0
R5	RGC12711c	65.74	+	430	62	430	Rsa1.0_01034.1	56046	44291	44659	85.6	85.6	100.0
R5	RS2CL1241s	65.81	−	876	0	875	Rsa1.0_02040.1	35684	11985	13555	95.9	99.9	179.4
R5	ACGM16	65.98											
R5	RS2CL3809s	66.03	+	641	2	641	Rsa1.0_00447.1	84051	12844	13659	99.5	99.7	127.5
R5	RGC16642c	66.43	−	556	0	556	Rsa1.0_05766.1	12708	5905	6461	88.3	100.0	100.0
R5	CD81H	66.83											
R5	RGA1790s	67.11											
R5	RGC21710c	67.56	+	504	0	504		152418	148637	149141	100.0	100.0	100.0
R5	CD78	67.87											
R5	RGC5664c	67.98	+	437	0	437	Rsa1.0_01134.1	52829	23448	23885	100.0	100.0	100.0
R5	RS2CL1583s	68.25	+	1343	3	1340		152418	110674	112729	95.5	99.6	153.7
R5	RGC948c	68.41	+	405	0	405		247766	217646	218051	100.0	100.0	100.0
R5	RSS2089	69.01	+	455	8	455	Rsa1.0_01540.1	151890	111937	112388	91.7	98.2	100.9
R5	RGC439c	69.14	+	467	0	467	Rsa1.0_03614.1	21773	10422	10889	100.0	100.0	100.0
R5	RS2CL1103s	69.16	+	1292	2	1292	Rsa1.0_00454.1	216970	212660	215026	69.1	99.9	183.4
R5	RGC14010c	69.74	−	527	0	527	Rsa1.0_00454.1	216970	158630	159157	98.5	100.0	100.0
R5	RS2CL4593s	70.03	−	1004	1	1004	Rsa1.0_01134.1	52829	18262	19953	82.6	99.9	168.6

R5	RSS2970	70.12	+	804	6	804		152418	1718	2487	87.6	99.3	96.4
R5	RSS0769	70.32	−	729	129	729		152418	11460	12415	78.6	82.3	159.2
R5	RS2CL2778s	70.36	−	882	3	870		247766	206972	208112	90.4	98.3	131.5
R5	RS2CL4036s	70.75											
R5	RSS0295	71.17	−	739	9	739		247766	221495	222357	96.2	98.8	118.1
R5	RSS3324	71.17	+	860	0	860	Rsa1.0_02935.1	26621	6487	8342	86.4	100.0	215.7
R5	RS2CL7602s	71.33	−	797	0	797	Rsa1.0_03477.1	22635	4900	5890	95.4	100.0	124.2
R5	RS2CL3400s	71.45	+	862	196	861	Rsa1.0_00745.1	66067	52614	53451	60.0	77.2	125.9
R5	RSS1246	71.94	−	428	0	428		247766	207482	208098	99.8	100.0	143.9
R5	RSS3089	73.14	+	855	0	760		146428	127303	128155	83.0	88.9	112.1
R5	RSS3043	73.74											
R5	RSS2679	73.84	+	751	6	751	Rsa1.0_01878.1	37768	26765	27841	97.9	99.2	144.4
R5	RSS2831	74.04	+	876	0	876	Rsa1.0_00454.1	216970	155239	156567	91.3	100.0	151.6
R5	RS2CL5573s	75.09											
R5	RS2CL8653s	75.77	−	890	1	890	Rsa1.0_00833.1	62621	8599	10085	96.5	99.9	167.2
R5	RGC9734c	77.27	−	418	31	418	Rsa1.0_02411.1	31655	31157	31544	92.6	92.6	100.0
R5	RGB299c	77.57	−	414	61	414	Rsa1.0_03751.1	21025	19419	19794	81.2	85.3	106.2
R5	RGC9453c	77.74	+	433	0	433	Rsa1.0_00470.1	81968	43387	43820	100.0	100.0	100.0
R5	RSS0810	77.98											
R5	RGB12346c	78.96	−	494	0	494	Rsa1.0_00627.1	71566	57620	58114	100.0	100.0	100.0
R5	CD32	79											
R5	Rs2CL2329s	79.26	−	906	1	906	Rsa1.0_01539.1	43698	4189	5258	98.1	99.9	118.1
R5	RSS2090	79.62	+	740	3	740	Rsa1.0_00863.1	254535	112235	113046	95.3	99.6	110.0
R5	RGA1887s	79.93											
R5	RS2CL7437s	80.03	−	719	569	718	Rsa1.0_01230.1	50221	5850	5990	18.4	20.7	94.0
R5	CB25aN	80.23											
R5	RS2CL3418s	80.53	+	1010	0	1010	Rsa1.0_00689.1	68305	41868	44067	99.4	100.0	217.7
R5	RS2CL6413s	80.74	−	992	5	970	Rsa1.0_00689.1	68305	12769	13825	57.9	97.3	109.4
R5	RS2CL4294s	81.65	+	949	2	941	Rsa1.0_03123.1	25207	21615	22836	45.5	99.0	130.0
R5	RSS0606	81.69	+	842	0	842	Rsa1.0_03188.1	24722	9735	10947	99.9	100.0	143.9
R5	RGC5898c	81.86	−	538	0	368	Rsa1.0_00165.1	116466	10610	10978	67.3	68.4	100.0
R5	RSS3723	81.9	+	818	0	818		101906	25269	26089	68.6	100.0	100.2
R5	N95848	82.6											
R5	RGC6482c	82.83	−	426	0	426	Rsa1.0_00946.1	58666	54057	54483	100.0	100.0	100.0
R5	RS2CL5033s	82.88											
R5	RGC8128c	83.47	+	412	0	412	Rsa1.0_00653.1	70029	10577	10989	52.9	100.0	100.0

R5	RSS2992	83.74	−	859	4	859	Rsa1.0_00798.1	63945	21398	22325	83.1	99.5	108.4
R5	RS2CL3856s	83.87	−	1045	2	1044	Rsa1.0_00653.1	70029	35915	36953	97.0	99.7	99.6
R5	RGC20400c	83.92	−	453	0	453	Rsa1.0_00798.1	63945	40218	40671	90.5	100.0	100.0
R5	RS2CL3486s	84.57	−	1186	0	1185	Rsa1.0_00069.1	403561	102672	104056	99.2	99.9	116.8
R5	RGB2163c	85.28	+	404	0	265	Rsa1.0_00069.1	403561	162865	163130	65.6	65.6	100.0
R5	RSS1973	86.18	+	776	0	776	Rsa1.0_00069.1	403561	187771	188634	100.0	100.0	111.2
R5	RS2CL876s	86.59	+	809	2	808	Rsa1.0_00069.1	403561	169706	171073	88.1	99.6	169.6
R5	RGC18337c	86.85	−	439	0	439	Rsa1.0_01313.1	48387	13659	14098	100.0	100.0	100.0
R5	RS2CL3834s	86.87	−	1013	5	1013	Rsa1.0_00069.1	403561	206029	207119	97.3	99.5	108.1
R5	RS2CL1002s	87.15	+	801	0	801	Rsa1.0_01313.1	48387	12951	14262	96.6	100.0	163.7
R5	RGC13305c	87.84	−	504	0	504	Rsa1.0_00232.1	104936	55033	55537	100.0	100.0	100.0
R5	RS2CL4231s	88.98	+	1211	0	1207	Rsa1.0_04911.1	15483	7753	9474	97.7	99.7	142.6
R5	RS2CL8487s	89.15	−	1245	5	1238	Rsa1.0_00960.1	58199	14657	16736	94.9	99.0	168.6
R5	RGC1982c	89.53	−	579	0	579	Rsa1.0_01873.1	37903	14697	15276	49.9	100.0	100.0
R5	RGC6032c	89.53	+	466	0	466	Rsa1.0_00960.1	58199	9376	9842	100.0	100.0	100.0
R5	RGC7124c	89.53	+	423	0	423	Rsa1.0_00043.1	264937	236764	237187	100.0	100.0	100.0
R5	RS2CL6191s	89.91	+	1232	0	1232	Rsa1.0_01518.1	141402	131239	132545	99.9	100.0	106.0
R5	RGA1924s	90.52											
R5	RSS1355	92.03	+	427	0	427	Rsa1.0_13918.1	4094	423	1061	100.0	100.0	149.4
R5	RSS2290	93.13	−	560	0	560	Rsa1.0_02028.1	35857	34549	35240	91.4	100.0	123.4
R5	RS2CL5313s	93.45	−	1379	2	1376	Rsa1.0_03524.1	22297	13551	15759	94.6	99.6	160.7
R5	Rs2CL1513s	94.83	−	905	12	905	Rsa1.0_14009.1	3924	2436	3850	96.4	98.7	158.3
R5	RGA2486s	95.37											
R5	RSS3328	95.74	+	857	0	857	Rsa1.0_00725.1	66757	25518	26874	98.3	100.0	158.2
R5	RGA2386s	95.75											
R5	RS2CL3218s	96.23	+	811	2	808	Rsa1.0_03380.1	23320	4233	5277	97.2	99.4	129.5
R5	RS2CL3664s	96.44	+	805	2	805	Rsa1.0_00641.1	208241	145717	146749	97.1	99.8	128.5
R5	CC59	97.52											
R5	RSS2770	98.32											
R5	RGC1058c	98.62	+	424	0	424	Rsa1.0_01965.1	36698	25119	25543	100.0	100.0	100.0
R5	RSS1146	99.14	+	863	0	863	Rsa1.0_02435.1	31454	16180	17600	96.1	100.0	164.5
R5	RS2CL7110s	99.54	+	815	1	815	Rsa1.0_05569.1	13311	2709	3600	71.2	99.9	109.5
R5	RS2CL1096s	99.98											
R5	RGC2286c	100.36	+	419	0	419	Rsa1.0_01428.1	46071	24303	24722	100.0	100.0	100.0
R5	RGC1397c	100.58	−	442	0	442	Rsa1.0_00150.1	154416	20467	20909	100.0	100.0	100.0
R5	RGC6433c	101.17	−	439	0	439		21706	11046	11485	100.0	100.0	100.0

R5	RSS2008	101.24	+	807	0	807	Rsa1.0_00819.1	62918	20432	21752	98.8	100.0	163.6
R5	RSS1381	103.04	−	877	0	342	Rsa1.0_00468.1	82095	18113	18455	18.4	39.0	100.0
R5	RGC7374c	104.07	−	482	0	482	Rsa1.0_09919.1	6090	4523	5005	100.0	100.0	100.0
R5	RGC7955c	104.07	−	401	0	401		194198	130086	130487	100.0	100.0	100.0
R5	RSS3453	105.19	−	761	1	761	Rsa1.0_00186.1	112669	111695	112549	96.9	99.9	112.4
R5	RS2CL1017s	105.83	−	1129	1	1129	Rsa1.0_02710.1	28492	20036	21555	92.8	99.9	134.7
R5	RS2CL2718s	108.19	+	720	0	720	Rsa1.0_02374.1	32043	25677	26901	99.3	100.0	170.0
R5	RSS2080	108.93	−	481	0	481	Rsa1.0_01026.1	171624	140036	140518	87.1	100.0	100.2
R5	RS2CL6248s	109.54	+	1197	3	1197	Rsa1.0_00629.1	71381	44546	45859	96.4	99.8	110.0
R5	RGC9070c	109.89	+	421	0	421	Rsa1.0_00506.1	79106	48473	48894	85.0	100.0	100.0
R5	RSS0835	110.06	+	795	0	795	Rsa1.0_00186.1	112669	7686	9134	96.2	100.0	182.1
R5	RS2CL4474s	110.66	−	1087	2	1087	Rsa1.0_00685.1	68515	26875	28063	99.5	99.8	109.5
R5	RSS1657	111.19	−	840	0	722	Rsa1.0_04317.1	17967	2169	2891	57.1	86.0	100.0
R5	RS2CL3771s	111.23	+	1020	2	1020	Rsa1.0_02508.1	30675	23820	25284	69.0	99.8	143.8
R5	RSS0799	111.5	+	593	6	593	Rsa1.0_00767.1	64980	34719	35588	89.2	99.0	148.0
R5	RSS1437	112.1	−	811	0	806	Rsa1.0_04542.1	16930	14605	16284	94.3	99.4	208.3
R5	RGC4231c	112.77	−	442	0	442	Rsa1.0_01679.1	146876	139593	140035	100.0	100.0	100.0
R5	RGC3008c	113.12	+	542	0	542	Rsa1.0_04491.1	17140	4485	5027	100.0	100.0	100.0
R5	RGC15844c	113.61	−	478	0	478	Rsa1.0_03647.1	21597	10656	11134	100.0	100.0	100.0
R5	RS2CL7568s	113.78	−	1336	0	1336	Rsa1.0_00990.1	57445	15065	16652	95.1	100.0	118.8
R5	RSS2076	113.98	−	841	0	841	Rsa1.0_00871.1	61539	48609	49450	100.0	100.0	100.0
R5	RGC793c	114.27	+	487	107	437	Rsa1.0_00514.1	78421	57413	57743	67.8	67.8	100.0
R5	RGC4102c	114.8	+	402	0	402	Rsa1.0_02315.1	32618	10836	11238	100.0	100.0	100.0
R5	RGB28864c	115.22	+	508	0	508	Rsa1.0_00217.1	107079	6955	7463	100.0	100.0	100.0
R5	CA04a	115.62											
R5	RGC24023c	115.72	−	477	0	477	Rsa1.0_02599.1	29660	9078	9555	100.0	100.0	100.0
R5	RGC25363c	115.72	+	415	0	415	Rsa1.0_00186.1	112669	46437	46852	100.0	100.0	100.0
R5	RGC7157c	115.72	+	462	0	462	Rsa1.0_00685.1	68515	7836	8298	100.0	100.0	100.0
R5	RSS1610	116.43	−	606	1	606		194198	185130	185738	91.6	99.8	100.5
R5	RS2CL4228s	116.53	−	810	0	797	Rsa1.0_08412.1	7404	4360	5456	95.6	98.4	137.5
R5	RSS2461	117.75	−	869	1	869	Rsa1.0_00279.1	100039	83466	85220	95.2	99.9	202.1
R5	RSS3878	117.75	+	791	0	791	Rsa1.0_00546.1	76523	45699	46491	100.0	100.0	100.1
R5	RS2CL3657s	117.86	−	803	34	799	Rsa1.0_00279.1	100039	5703	7209	90.9	95.3	196.9
R5	RSS3562	117.95	−	466	0	466	Rsa1.0_00279.1	100039	43989	44525	88.2	100.0	115.0
R5	RGC1797c	118.51	+	402	0	402	Rsa1.0_00377.1	90038	82355	82757	100.0	100.0	100.0
R5	RSS0491	118.75	−	699	0	699	Rsa1.0_05293.1	14237	7455	8771	99.9	100.0	188.3

R5	RSS0607	118.85	−	815	0	815	Rsa1.0_00484.1	203294	136731	137836	98.9	100.0	135.6
R5	RGC5241c	119.04	−	416	0	416	Rsa1.0_00151.1	119541	37377	37793	100.0	100.0	100.0
R5	RGC9697c	119.69	−	456	135	284	Rsa1.0_00618.1	72195	36815	36964	32.7	32.7	100.0
R5	RSS0794	120.35	+	691	0	691	Rsa1.0_07440.1	9389	4109	4800	100.0	100.0	100.0
R5	RS2CL2631s	120.38	−	1034	1	1034	Rsa1.0_10201.1	5946	417	1448	97.3	99.9	99.8
R5	RGC27939c	120.85	+	453	0	453	Rsa1.0_06002.1	11976	7094	7547	100.0	100.0	100.0
R5	RGC2709c	120.96	−	425	0	425	Rsa1.0_00484.1	203294	161141	161566	100.0	100.0	100.0
R5	RGC1165c	120.98	−	458	0	458	Rsa1.0_00978.1	57739	21904	22362	100.0	100.0	100.0
R5	RGC15300c	120.98											
R5	RGB17888c	120.98	−	477	0	477	Rsa1.0_00978.1	57739	13357	13834	100.0	100.0	100.0
R5	RGC25318c	120.98	−	537	0	537	Rsa1.0_01386.1	46765	20563	21100	100.0	100.0	100.0
R5	RGC15052c	121.1	+	575	0	575	Rsa1.0_00151.1	119541	108743	109318	100.0	100.0	100.0
R5	RS2CL3363s	121.34	−	1415	0	1415	Rsa1.0_03834.1	20572	11806	13211	96.5	100.0	99.3
R5	RSS0599	121.85	−	796	0	796	Rsa1.0_01234.1	50183	31582	33075	97.4	100.0	187.6
R5	RGC7029c	122.02	−	456	0	456	Rsa1.0_01234.1	50183	35720	36176	100.0	100.0	100.0
R5	Rs2CL1713s	122.09	−	1209	0	1207	Rsa1.0_00628.1	214360	182830	184389	97.4	99.8	129.2
R5	RS2CL2036s	122.64	+	1482	1	1482	Rsa1.0_00628.1	214360	201779	203526	76.8	99.9	118.0
R5	RGC20412c	123.21	+	414	0	414	Rsa1.0_00032.1	306326	261698	262112	100.0	100.0	100.0
R5	RGC3985c	123.21	−	589	0	589	Rsa1.0_00032.1	306326	295635	296224	99.8	100.0	100.0
R5	CB14	123.25											
R5	Rs2CL1337s	123.75	−	757	1	757	Rsa1.0_00151.1	119541	109865	111654	98.3	99.9	236.6
R5	RSS2087	124.05	−	870	0	870	Rsa1.0_00978.1	57739	24459	25330	96.7	100.0	100.1
R5	RSS2733	124.15	+	460	0	460	Rsa1.0_00978.1	57739	25289	25980	100.0	100.0	150.2
R5	RS2CL3095s	125.44	+	784	2	783	Rsa1.0_01965.1	36698	22561	24540	96.1	99.6	253.4
R5	RGC7851c	125.81	−	464	0	464	Rsa1.0_00817.1	62961	43133	43597	100.0	100.0	100.0
R5	Rs2CL1671s	126.08	−	1115	1	1115	Rsa1.0_01712.1	154210	1953	3293	99.2	99.9	120.3
R5	RSS3895	126.45	+	897	4	813	Rsa1.0_02411.1	31655	27331	28233	87.7	90.2	111.5
R5	RS2CL1405s	126.99	−	734	0	634	Rsa1.0_02411.1	31655	17404	18255	86.0	86.4	134.2
R5	RSS1697	127.25	−	766	1	766	Rsa1.0_00725.1	66757	26327	27163	96.1	99.9	109.3
R5	RGC1110c	128.21	−	511	0	511	Rsa1.0_01440.1	45807	10572	11083	81.6	100.0	100.0
R5	RGC5424c	128.21	+	537	0	537	Rsa1.0_00080.1	190437	13918	14455	100.0	100.0	100.0
R5	CA74	128.25											
R5	RS2CL4817s	128.42	+	786	2	776	Rsa1.0_00602.1	73302	35792	36565	97.1	98.5	99.9
R5	RSS0139	129.25	−	656	441	656	Rsa1.0_00032.1	306326	192394	192609	32.8	32.8	100.0
R5	RS2CL3543s	129.83	−	1023	0	958	Rsa1.0_00789.1	64233	33702	34945	68.8	93.7	129.8
R5	RS2CL6094s	130.35	−	953	1	953	Rsa1.0_00789.1	64233	49233	50813	88.0	99.9	166.0

R5	RGB90c	130.75	−	403	0	403	Rsa1.0_00602.1	73302	43958	44361	100.0	100.0	100.0
R5	RSS0258	131.05	−	863	0	863	Rsa1.0_00602.1	73302	1678	2643	99.2	100.0	111.8
R5	RS2CL2801s	131.77	−	649	0	649	Rsa1.0_04486.1	17161	8412	9061	99.5	100.0	100.0
R5	ACGM13	132.35											
R5	RS2CL2046s	132.49	−	825	2	825	Rsa1.0_01440.1	45807	13754	14806	91.5	99.8	127.8
R5	RS2CL6493s	132.63	+	1096	1	1096	Rsa1.0_04486.1	17161	12613	14123	96.4	99.9	137.9
R5	RSS1153	132.95	+	818	0	818	Rsa1.0_03990.1	19676	3566	4888	98.4	100.0	161.6
R5	RSS3828	133.15	+	736	0	736	Rsa1.0_00817.1	62961	26769	27504	99.6	100.0	99.9
R5	RS2CL5254s	133.67	+	822	107	822	Rsa1.0_01308.1	48596	17349	18138	64.5	87.0	110.4
R5	CA04b	133.85											
R5	RS2CL5065s	133.9	+	1125	3	1124	Rsa1.0_00784.1	64359	26029	28124	94.7	99.6	186.9
R5	RS2CL4091s	134.28	−	834	3	832	Rsa1.0_05595.1	13228	4395	5624	90.2	99.4	148.3
R5	RS2CL4365s	134.82	−	1297	2	1297	Rsa1.0_01591.1	42718	22514	24555	99.2	99.9	157.6
R5	CD63	135.05											
R5	Rs2CL2530s	135.15	+	661	0	661	Rsa1.0_05595.1	13228	3233	4172	94.9	100.0	142.1
R5	OPE03b	135.25											
R5	RGC15061c	136.17	−	593	0	593	Rsa1.0_00339.1	222974	20595	21188	100.0	100.0	100.0
R5	RGB2029c	136.17	−	534	189	534	Rsa1.0_00339.1	222974	75448	75746	55.8	64.6	86.4
R5	RGB5705c	136.17	+	467	0	467	Rsa1.0_00339.1	222974	175734	176201	100.0	100.0	100.0
R5	RSS2040	136.35	−	822	0	822	Rsa1.0_00784.1	64359	2795	3712	94.7	100.0	111.6
R5	RS2CL7735s	136.88	+	1318	2	1318	Rsa1.0_00409.1	146788	123783	125151	95.8	99.9	104.0
R5	RS2CL6813s	137.44	−	880	0	879	Rsa1.0_00339.1	222974	180013	181129	96.3	99.9	127.0
R5	RGC4124c	137.99	+	444	0	444	Rsa1.0_00032.1	306326	185276	185720	100.0	100.0	100.0
R5	CC45a	138.45											
R5	RSS0084	139.15	+	521	1	521	Rsa1.0_14855.1	3011	636	1588	95.4	99.8	183.1
R5	RS2CL5459s	139.19	−	1180	3	1180	Rsa1.0_00213.1	108167	36367	38863	96.7	99.8	212.1
R5	RS2CL5893s	139.4	+	554	9	554	Rsa1.0_00105.1	134138	81169	81720	93.5	98.4	101.1
R5	RS2CL1331s	139.71	+	1038	17	1037	Rsa1.0_00105.1	134138	71490	73331	95.0	98.3	180.5
R5	RSS1903	140.05	+	407	0	336	Rsa1.0_00363.1	90968	53657	54108	82.6	82.6	134.2
R5	RGA1658s	140.08											
R5	RS2CL1047s	140.55	+	799	0	798	Rsa1.0_00105.1	134138	90483	92188	92.0	99.9	213.7
R5	RSS1242	140.85	−	862	2	862	Rsa1.0_00213.1	108167	35048	35921	97.3	99.8	101.5
R5	RSS1489	140.85	+	652	0	652	Rsa1.0_01168.1	153765	40994	42117	59.1	100.0	172.2
R5	RS2CL5612s	141.78	−	1148	0	1148	Rsa1.0_00793.1	64103	38944	40562	94.9	100.0	140.9
R5	CF19	142.05											
R5	RS2CL4128s	142.13	+	744	2	744	Rsa1.0_02392.1	152517	29661	30400	98.1	99.7	99.6

R5	RS2CL1648s	143.3	−	1158	0	1158	Rsa1.0_00420.1	86496	40528	42407	91.2	100.0	162.3
R5	RGC16329c	143.35	−	410	0	410	Rsa1.0_00087.1	761073	56207	56617	100.0	100.0	100.0
R5	RGC2498c	143.35	+	463	0	463	Rsa1.0_00087.1	761073	200708	201171	100.0	100.0	100.0
R5	RGC4098c	143.35	+	402	0	402	Rsa1.0_01737.1	40101	6243	6645	100.0	100.0	100.0
R5	RGC4195c	143.35	−	519	0	519	Rsa1.0_01811.1	38977	31311	31830	100.0	100.0	100.0
R5	RGA2325s	144.54											
R5	RSS3694	144.65	+	872	0	872	Rsa1.0_02180.1	34176	26552	27617	97.1	100.0	122.1
R5	RSS1361	145.65	−	880	45	880	Rsa1.0_02392.1	152517	25981	27504	94.9	94.9	182.4
R5	RS2CL5529s	145.69											
R5	RS2CL5178s	146.41	−	887	2	887	Rsa1.0_00087.1	761073	312460	313447	94.4	99.8	111.5
R5	RS2CL6670s	146.79	+	944	1	939	Rsa1.0_00087.1	761073	621070	622821	96.4	99.4	186.7
R5	RGB1089c	147.24	+	503	29	503	Rsa1.0_00087.1	761073	585789	586263	58.1	94.2	100.0
R5	RS2CL5532s	147.65	−	1079	5	1079	Rsa1.0_00087.1	761073	730623	732298	92.8	99.5	156.0
R5	RS2CL6564s	148.26	+	900	2	900	Rsa1.0_00078.1	175053	136069	137566	83.6	99.8	166.7
R5	RS2CL4868s	148.73	−	1140	0	1140	Rsa1.0_00078.1	175053	37459	38791	98.8	100.0	116.8
R5	Rs2CL2284s	149.12	−	956	2	955	Rsa1.0_01068.1	55208	45677	46845	95.7	99.7	122.6
R5	RGC908c	149.41	+	529	0	529	Rsa1.0_04405.1	17508	7344	7873	100.0	100.0	100.0
R5	RSS2296	149.85	+	842	0	842	Rsa1.0_00087.1	761073	295735	296583	95.0	100.0	100.7
R5	RSS3026	149.95	−	846	66	787	Rsa1.0_00087.1	761073	184673	185388	52.8	85.2	99.2
R5	RSS0361	150.15	+	654	0	654	Rsa1.0_00087.1	761073	186488	187738	95.6	100.0	191.1
R5	Rs2CL1785s	150.52											
R5	RSS0292_a	154.15											
R5	RGB1952c	154.21	−	468	0	468	Rsa1.0_01285.1	172709	31880	32348	100.0	100.0	100.0
R6	Rs2CL1800s	0	+	658	0	658	Rsa1.0_00951.1	164588	113344	114787	99.5	100.0	219.3
R6	RGA2109s	1.63											
R6	RSS0284	3.02											
R6	RGC7282c	3.44	−	420	0	420	Rsa1.0_00609.1	72785	57279	57698	99.8	100.0	99.8
R6	RGC13867c	4.8	+	560	0	560	Rsa1.0_01233.1	50184	30754	31314	100.0	100.0	100.0
R6	CA22	5.12											
R6	RGC5600c	5.27	+	448	0	448	Rsa1.0_01806.1	39021	13688	14136	90.2	100.0	100.0
R6	RSS0750	6.52	+	214	0	214		358124	29394	29611	99.5	100.0	101.4
R6	RSS1106	6.52	−	533	3	513	Rsa1.0_01806.1	39021	24550	25044	90.6	95.7	96.9
R6	RS2CL1259s	7.06	+	736	0	736	Rsa1.0_01117.1	53465	38990	39893	99.9	100.0	122.7
R6	RS2CL2749s	7.84	−	1488	0	1487		358124	86342	89007	79.7	99.9	179.2
R6	RSS0881	8.02	−	509	0	509	Rsa1.0_01806.1	39021	12196	12993	89.8	100.0	156.6

R6	RSS1501	8.02	+	330	0	330		358124	66030	66362	100.0	100.0	100.6
R6	RGC13819c	8.2	−	404	0	404	Rsa1.0_00030.1	250988	50016	50420	100.0	100.0	100.0
R6	RSS3965_a	8.82	+	544	0	544	Rsa1.0_01602.1	42503	35794	36336	99.3	100.0	99.6
R6	Rs2CL1793s	9	−	775	0	774		358124	201197	203343	98.7	99.9	277.3
R6	RSS0818	9.12	−	839	0	839	Rsa1.0_00030.1	250988	184441	185348	99.6	100.0	108.1
R6	RGB6680c	9.17											
R6	RS2CL6567s	9.92	+	1331	2	1331	Rsa1.0_01553.1	43379	33373	35255	96.7	99.9	141.6
R6	RSS0864	10.32	+	740	0	740	Rsa1.0_00131.1	432233	33938	34927	100.0	100.0	133.7
R6	RSS2760	10.32	+	870	0	839	Rsa1.0_00131.1	432233	267849	269037	92.9	96.4	141.6
R6	RSS2859	10.32	−	858	0	735	Rsa1.0_01097.1	173349	33474	34744	75.3	85.7	172.8
R6	RGC6359c	10.59	+	465	0	465	Rsa1.0_00030.1	250988	188044	188509	100.0	100.0	100.0
R6	RS2CL4163s	10.95	+	777	0	777	Rsa1.0_00030.1	250988	141373	142152	99.2	100.0	100.3
R6	RS2CL3329s	11.58	−	869	3	867	Rsa1.0_00196.1	109960	18592	19738	60.3	99.4	132.6
R6	RS2CL5007s	12.62	−	725	0	725	Rsa1.0_00196.1	109960	51496	52397	71.0	100.0	124.3
R6	RGC10902c	12.91	−	472	0	472	Rsa1.0_00131.1	432233	164405	164877	100.0	100.0	100.0
R6	RS2CL5739s	13.18	−	776	0	773	Rsa1.0_00937.1	59061	22551	23330	98.7	99.6	100.8
R6	RGC12804c	13.49	−	441	0	441	Rsa1.0_00131.1	432233	91877	92318	100.0	100.0	100.0
R6	RSS2138	13.72	+	856	0	856	Rsa1.0_01126.1	53127	12828	14670	96.9	100.0	215.2
R6	RGB4153c	13.85	−	530	0	530	Rsa1.0_00131.1	432233	275922	276452	76.6	100.0	100.0
R6	RSS0662	13.92	−	838	0	838		59481	8137	9280	99.4	100.0	136.4
R6	RSS2505	14.32	+	862	0	862		59481	56093	57283	96.5	100.0	138.1
R6	RS2CL5533s	14.67	−	1035	85	1030	Rsa1.0_00025.1	277312	240456	241286	51.6	91.3	87.8
R6	RS2CL4358s	14.98	+	1086	9	1086	Rsa1.0_00110.1	132719	93130	95045	90.5	99.2	177.8
R6	RGC2728c	15.34	+	402	0	402	Rsa1.0_03502.1	22446	14050	14452	100.0	100.0	100.0
R6	RS2CL4648s	16.17	+	1106	2	1104	Rsa1.0_02706.1	28522	11821	14483	98.3	99.6	241.6
R6	RGC5774c	16.49	+	553	0	553	Rsa1.0_00110.1	132719	65184	65737	100.0	100.0	100.0
R6	RGC12486c	16.98	−	511	0	511		174827	163535	164046	100.0	100.0	100.0
R6	CC68	17.43											
R6	RGC6279c	17.74	+	428	0	428	Rsa1.0_00951.1	164588	37254	37682	100.0	100.0	100.0
R6	RS2CL3679s	17.81	+	794	2	793	Rsa1.0_02584.1	29877	247	1201	97.7	99.6	120.6
R6	RGC395c	18.06	+	478	0	478	Rsa1.0_04626.1	16611	9407	9885	100.0	100.0	100.0
R6	RS2CL1088s	18.31	−	999	2	999	Rsa1.0_00749.1	65933	20460	21782	96.6	99.8	132.6
R6	RS2CL1149s	18.56	−	824	2	824	Rsa1.0_00203.1	109129	2797	4234	99.4	99.8	174.8
R6	RGC10056c	18.77	+	476	0	476	Rsa1.0_01126.1	53127	13404	13880	100.0	100.0	100.0
R6	RGC6186c	19.41	−	444	0	444	Rsa1.0_00203.1	109129	80012	80456	100.0	100.0	100.0
R6	RGC12389c	20.04	+	529	0	529	Rsa1.0_00239.1	104332	23516	24045	94.7	100.0	100.0

R6	RGC16041c	20.04	−	492	0	492	Rsa1.0_00604.1	146282	11378	11870	100.0	100.0	100.0
R6	RGC4166c	20.04	+	404	0	404	Rsa1.0_00239.1	104332	46210	46614	100.0	100.0	100.0
R6	RGC9621c	20.04	+	522	0	522	Rsa1.0_00604.1	146282	124978	125500	100.0	100.0	100.0
R6	RS2CL3998s	20.73	−	861	2	860		88189	7406	8747	96.8	99.7	156.3
R6	RSS2497	21.32	−	774	53	774	Rsa1.0_00195.1	251884	215691	216379	83.0	93.2	95.4
R6	RSS0895	21.82											
R6	RGA107s	22.47											
R6	RSS2680	22.71	−	854	0	854	Rsa1.0_00005.1	353093	144539	146349	100.0	100.0	211.9
R6	RSS1367	22.81	+	821	26	821	Rsa1.0_00181.1	114269	77841	78636	96.8	96.8	100.0
R6	RSS3306	22.81	−	879	0	878	Rsa1.0_00005.1	353093	122794	124442	87.5	99.9	187.7
R6	RSS3125	23.01	−	882	0	882	Rsa1.0_00005.1	353093	144540	146379	100.0	100.0	208.5
R6	RS2CL8378s	23.28	+	1186	0	1186	Rsa1.0_01740.1	39986	12689	14758	99.7	100.0	174.5
R6	RSS3489	23.61	+	749	0	749	Rsa1.0_00181.1	114269	42149	42898	92.5	100.0	100.0
R6	RS2CL5725s	24.27	+	1048	12	1046	Rsa1.0_06819.1	10302	1253	2284	95.3	98.7	99.7
R6	RSS0768	24.6	+	754	0	754	Rsa1.0_00812.1	63233	34146	34902	89.1	100.0	100.3
R6	RS2CL1483s	25.01	−	1042	2	1042	Rsa1.0_00701.1	67691	5310	6453	67.4	99.8	109.9
R6	RS2CL6241s	26.23	−	1161	2	1148	Rsa1.0_00701.1	67691	40933	42842	96.3	98.7	166.6
R6	RS2CL7817s	26.73	−	546	2	530	Rsa1.0_00701.1	67691	5923	6451	70.3	96.7	100.0
R6	RGA1973s	26.81											
R6	RGC1312c	27	−	470	0	470	Rsa1.0_01740.1	39986	16935	17405	100.0	100.0	100.0
R6	RGC2219c	27	−	405	0	405	Rsa1.0_00005.1	353093	333479	333884	93.6	100.0	100.0
R6	RGC4977c	27.32	+	545	0	545	Rsa1.0_00888.1	60978	5678	6223	76.9	100.0	100.0
R6	RS2CL6432s	27.84	+	777	9	777	Rsa1.0_00181.1	114269	65747	66676	96.1	98.8	121.0
R6	CA09a	27.86											
R6	Rs2CL2337s	28.18	−	778	0	778	Rsa1.0_03726.1	21160	14301	15470	92.4	100.0	150.3
R6	RSS3301	28.35	+	798	0	798	Rsa1.0_00812.1	63233	34616	35406	94.7	100.0	99.0
R6	RS2CL7813s	28.43	−	687	1	687	Rsa1.0_00169.1	274298	270923	272129	96.7	99.9	175.8
R6	RGB5240c	28.79	−	535	0	535	Rsa1.0_02851.1	27295	5690	6225	100.0	100.0	100.0
R6	RGA1878s	29.4											
R6	RS2CL8063s	29.63	−	1044	2	1044	Rsa1.0_00035.1	176449	167699	168766	96.3	99.8	102.4
R6	RS2CL4703s	30.21	+	864	2	860	Rsa1.0_08909.1	6799	4802	5646	92.7	99.3	98.4
R6	RSS0518	30.33	−	601	0	601	Rsa1.0_02536.1	30368	28715	29317	100.0	100.0	100.2
R6	Rs2CL1678s	30.94	−	1259	1	1259	Rsa1.0_02393.1	31795	14420	15689	99.5	99.9	100.9
R6	RS2CL4308s	31.06	−	1290	0	1283	Rsa1.0_01217.1	50554	46718	48000	64.9	99.5	99.9
R6	RGC10301c	31.56	+	505	0	505	Rsa1.0_00181.1	114269	50227	50732	100.0	100.0	100.0
R6	RGC18316c	31.56	+	445	0	445	Rsa1.0_00181.1	114269	92550	92995	100.0	100.0	100.0

R6	RGC6540c	31.56	+	475	0	326	Rsa1.0_01125.1	53193	33484	33810	27.8	68.6	100.0
R6	RGA2264s	32.04											
R6	RSS0124	32.73	+	837	0	837	Rsa1.0_00023.1	194764	83325	84532	88.3	100.0	144.2
R6	RSS2701	32.93	+	817	0	817	Rsa1.0_00835.1	278058	163058	164061	98.4	100.0	122.8
R6	RS2CL2683s	33.48											
R6	RGB14399c	33.64	+	453	0	453	Rsa1.0_00117.1	213118	48906	49441	83.9	100.0	118.1
R6	CB22	34.22											
R6	RS2CL2041s	34.53	−	631	0	507		120234	63977	64493	78.3	80.4	101.8
R6	RS2CL6287s	34.63	+	1196	2	1075	Rsa1.0_00023.1	194764	179568	180767	88.8	89.7	111.7
R6	RGC23911c	34.9	−	525	0	525	Rsa1.0_00169.1	274298	242778	243303	100.0	100.0	100.0
R6	RS2CL3132s	35.26	−	906	2	885	Rsa1.0_00900.1	60367	12162	13696	95.4	97.5	173.7
R6	RSS3180	35.5	−	348	0	343	Rsa1.0_00900.1	60367	40013	40392	87.9	98.6	110.5
R6	RSS2724	36	−	784	0	784	Rsa1.0_01196.1	51097	25847	27051	100.0	100.0	153.6
R6	RS2CL7658s	36.11	+	1284	8	1284	Rsa1.0_01217.1	50554	13008	14464	97.5	99.4	114.1
R6	RGC9543c	36.42	−	480	0	480	Rsa1.0_00035.1	176449	139024	139504	100.0	100.0	100.0
R6	RGB11029c	37.42	+	474	0	474	Rsa1.0_00073.1	240924	141057	141531	100.0	100.0	100.0
R6	RGC7277c	38.15	+	546	0	546	Rsa1.0_02477.1	31058	6990	7536	85.7	100.0	100.0
R6	RS2CL2168s	38.51	+	1227	1	1227	Rsa1.0_00022.1	200461	45194	46761	98.1	99.9	127.8
R6	Rs2CL1936s	39.42	−	1057	0	1055	Rsa1.0_01613.1	182542	164106	165855	99.3	99.8	165.8
R6	RSS0895_a	39.42	−	656	0	656	Rsa1.0_00655.1	195840	136556	137707	96.2	100.0	175.5
R6	RGC17695c	40.16	−	586	0	586	Rsa1.0_00073.1	240924	231144	231730	89.8	100.0	100.0
R6	RGC3412c	40.71	+	542	0	317	Rsa1.0_00208.1	108565	40651	40968	58.5	58.5	100.0
R6	RS2CL4724s	41.51	+	900	2	898	Rsa1.0_10199.1	5948	4333	5841	98.6	99.6	168.3
R6	RSS0727	41.58	+	742	0	742	Rsa1.0_00411.1	86944	44447	46038	91.2	100.0	214.4
R6	RGC11504c	41.79	−	550	0	550	Rsa1.0_00889.1	60978	3638	4188	50.7	100.0	100.0
R6	RGC28054c	41.79											
R6	RGC5414c	41.79	−	433	0	433	Rsa1.0_01196.1	51097	29384	29817	99.3	100.0	100.0
R6	RSS2078	42.57	+	821	20	810		240912	223707	225014	90.3	96.2	165.4
R6	RGC2511c	42.69	−	419	0	419	Rsa1.0_07573.1	9101	5681	6100	91.7	100.0	100.0
R6	RGC5771c	42.99	+	461	0	461	Rsa1.0_00501.1	79545	61953	62414	100.0	100.0	100.0
R6	RSS0254	43.26	−	836	14	836		42964	13293	14615	98.3	98.3	160.8
R6	RGC7411c	43.37	+	483	0	483	Rsa1.0_00293.1	339294	255439	255922	79.9	100.0	100.0
R6	RS2CL5813s	43.75	−	996	2	996	Rsa1.0_00585.1	74164	60680	61666	95.9	99.8	99.2
R6	Rs2CL2197s	43.82	−	1043	2	949	Rsa1.0_00208.1	108565	7688	9480	86.4	90.8	189.2
R6	RGC10969c	44.09	−	502	0	502	Rsa1.0_00421.1	86365	77105	77607	100.0	100.0	100.0
R6	RGB12188c	44.09	−	446	0	446	Rsa1.0_02188.1	34104	10003	10449	100.0	100.0	100.0

R6	RGC21003c	44.09	−	438	0	438	Rsa1.0_00028.1	293984	233974	234412	100.0	100.0	100.0
R6	RGC21373c	44.09	+	503	0	503	Rsa1.0_02169.1	34367	20036	20539	100.0	100.0	100.0
R6	RGC29914c	44.09	−	473	0	473	Rsa1.0_00367.1	90750	90173	90646	100.0	100.0	100.0
R6	RGC4352c	44.09	+	401	0	401	Rsa1.0_01819.1	38834	22832	23233	100.0	100.0	100.0
R6	RGB4736c	44.09	+	527	0	527	Rsa1.0_00968.1	58020	51858	52385	100.0	100.0	100.0
R6	RGC5236c	44.09	−	448	0	448	Rsa1.0_00730.1	66673	23187	23635	99.1	100.0	100.0
R6	RGC5509c	44.09	+	472	0	472	Rsa1.0_00028.1	293984	262096	262568	94.1	100.0	100.0
R6	RGC6399c	44.09	−	591	0	591	Rsa1.0_00282.1	177799	133145	133736	100.0	100.0	100.0
R6	RGC7441c	44.09	−	497	0	497	Rsa1.0_00028.1	293984	127210	127707	100.0	100.0	100.0
R6	RGC7478c	44.09	−	405	0	405	Rsa1.0_00912.1	59923	42487	42892	100.0	100.0	100.0
R6	RGB17905c	44.13	−	420	0	420	Rsa1.0_00293.1	339294	233990	234410	100.0	100.0	100.0
R6	RS2CL3740s	44.73	−	939	0	939	Rsa1.0_00293.1	339294	64751	66350	98.5	100.0	170.3
R6	RGB11249c	45.18	−	401	0	401		152362	51456	51857	100.0	100.0	100.0
R6	RGC3081c	45.61	+	563	0	173	Rsa1.0_02538.1	30364	19470	19643	30.7	30.7	100.0
R6	RS2CL3937s	46.07	+	1499	2	1499	Rsa1.0_00028.1	293984	274461	277426	97.9	99.9	198.1
R6	RS2CL3306s	46.23	+	893	0	886	Rsa1.0_00028.1	293984	131878	132778	85.2	99.2	101.6
R6	RS2CL3718s	46.39	+	770	0	770	Rsa1.0_00421.1	86365	80322	81191	99.5	100.0	112.9
R6	RS2CL6218s	46.68	+	964	3	964	Rsa1.0_00028.1	293984	220625	221587	87.1	99.7	100.1
R6	RGC1448c	47.23	+	446	0	446	Rsa1.0_00540.1	76895	57024	57470	100.0	100.0	100.0
R6	RS2CL6481s	47.72	+	1067	0	1067	Rsa1.0_07573.1	9101	6007	7159	95.3	100.0	108.0
R6	RS2CL7263s	48.1	−	1063	0	1063	Rsa1.0_00564.1	75428	73601	74777	96.8	100.0	110.6
R6	RGA1805s	48.13											
R6	RS2CL7425s	48.43	−	848	150	848	Rsa1.0_00564.1	75428	60615	61342	79.6	82.3	104.2
R6	RS2CL2511s	49.5	−	663	2	657	Rsa1.0_01996.1	36327	28097	29381	96.4	98.8	196.0
R6	Rs2CL2028s	50.62	+	1328	2	1328	Rsa1.0_00540.1	76895	30884	33427	93.6	99.9	191.8
R6	RS2CL5945s	51.52	−	641	1	623	Rsa1.0_00501.1	79545	21616	22321	93.5	97.0	113.3
R6	RSS2865	51.81	+	852	0	852		152362	10790	12308	99.8	100.0	178.2
R6	RS2CL4000s	52.73	−	836	3	128	Rsa1.0_06738.1	10441	5069	5194	15.0	15.0	100.0
R6	RS2CL6161s	53.18	+	822	0	822	Rsa1.0_00562.1	75565	657	1858	99.0	100.0	146.1
R6	RSS0839_a	53.55	+	806	0	806		97747	81636	82882	96.5	100.0	154.6
R6	RGC265c	53.78	−	495	0	495	Rsa1.0_00250.1	273771	182714	183209	100.0	100.0	100.0
R6	RGC4004c	53.78	+	477	0	477	Rsa1.0_01147.1	52391	42255	42732	100.0	100.0	100.0
R6	RGC6170c	53.78											
R6	RSS3844	54.55	−	786	0	653	Rsa1.0_00252.1	102760	64039	65198	80.9	83.1	177.5
R6	RS2CL4583s	54.66	+	653	2	653	Rsa1.0_00068.1	151813	72412	74048	98.8	99.7	251.3
R6	RS2CL3419s	55.3	−	1211	13	1210	Rsa1.0_00068.1	151813	97150	99692	96.9	98.8	212.4

R6	RSS1338	55.35	−	708	0	708	Rsa1.0_00748.1	65983	63633	64407	93.5	100.0	109.3
R6	RGC2998c	56.11	−	457	0	457	Rsa1.0_00293.1	339294	336263	336720	100.0	100.0	100.0
R6	CC46H	58.44											
R6	RGA1719s	58.61											
R6	RSS0049	59.34	−	833	0	829	Rsa1.0_00250.1	273771	5094	6645	98.7	99.5	187.1
R6	RS2CL3836s	60.14	+	903	0	902	Rsa1.0_00250.1	273771	255187	256431	97.9	99.9	137.9
R6	RSS3534	60.64	+	894	0	894		101125	12025	12995	93.2	100.0	108.5
R6	RS2CL2788s	60.89	+	649	8	645	Rsa1.0_00152.1	257730	203801	204727	93.5	98.2	145.4
R6	CD99H	61.04											
R6	RGC5574c	61.82	−	542	0	542	Rsa1.0_00259.1	163204	101488	102030	81.6	100.0	100.0
R6	RGC3577c	62.06	−	463	0	328	Rsa1.0_01551.1	43411	39072	39400	41.3	70.8	100.0
R6	RGC5793c	62.06											
R6	RS2CL7662s	62.39	+	731	0	731	Rsa1.0_01869.1	37928	6693	7424	98.2	100.0	100.0
R6	RSS3754	62.54	+	629	0	629	Rsa1.0_03626.1	160993	3901	4534	81.9	100.0	100.6
R6	RS2CL3015s	63.23	+	1405	1	1404	Rsa1.0_02080.1	35297	19033	20433	98.7	99.9	99.8
R6	CD72a	63.43											
R6	OPE03a	64.63											
R6	RS2CL4740s	64.92	+	814	7	814	Rsa1.0_05205.1	14513	13144	13953	94.2	99.1	100.3
R6	RGC893c	65.04	+	446	5	446	Rsa1.0_00199.1	249604	27368	27809	98.9	98.9	100.0
R6	CB89	65.83											
R6	RSS2657	67.03	+	342	0	342	Rsa1.0_04456.1	17260	14531	15055	97.4	100.0	153.2
R6	RSS3589	67.43	−	895	0	895	Rsa1.0_01716.1	40483	32213	33725	100.0	100.0	168.9
R6	RGC12381c	67.76	+	428	0	428	Rsa1.0_01716.1	40483	29280	29708	100.0	100.0	100.0
R6	RGC8396c	68.5	−	444	0	444	Rsa1.0_01070.1	55099	16043	16487	100.0	100.0	100.0
R6	RGC24370c	68.79	+	493	0	493	Rsa1.0_00846.1	62342	57321	57814	100.0	100.0	100.0
R6	Rs2CL2226s	69.24	−	1145	15	1145	Rsa1.0_02671.1	28887	9147	10961	97.6	98.7	160.5
R6	RSS2881	69.33	−	858	0	858	Rsa1.0_01163.1	51946	15058	16317	88.6	100.0	146.7
R6	RGC11044c	70.26											
R6	RGB10934c	71.09											
R6	RSS2217	71.42	−	874	0	874	Rsa1.0_00450.1	139397	124138	125054	99.0	100.0	104.8
R6	RS2CL1066s	72.37	+	1017	2	1016	Rsa1.0_03978.1	19753	10156	12287	99.0	99.7	210.2
R6	RSS0782	72.42	+	670	0	670		341668	286189	286864	98.2	100.0	100.8
R6	RS2CL7383s	72.89	+	758	2	758	Rsa1.0_05581.1	13272	3853	4843	98.8	99.7	131.0
R6	RS2CL2325s	73.46	+	744	0	744	Rsa1.0_00439.1	142776	57721	59666	96.6	100.0	261.4
R6	CD66	73.92											
R6	RS2CL2832s	73.95	+	1110	3	1109	Rsa1.0_00492.1	80178	65020	66126	95.1	99.6	100.0

R6	RGA2488s	74.57											
R6	RSS1178	74.62	+	730	0	730	Rsa1.0_04210.1	18442	1610	2657	99.5	100.0	143.4
R6	RS2CL3082s	74.94	−	1422	2	1422	Rsa1.0_03589.1	21893	621	2777	97.1	99.9	151.8
R6	RS2CL4493s	75.18	+	848	2	848		92266	22086	22941	76.3	99.8	101.1
R6	RGB12172c	75.31	−	458	0	458	Rsa1.0_00785.1	64356	35834	36312	98.3	100.0	104.4
R6	RGB9877c	75.31	+	562	0	234	Rsa1.0_04487.1	17160	11950	12184	41.6	41.6	100.0
R6	RGC9878c	75.31	+	427	0	427	Rsa1.0_00298.1	349131	19752	20179	100.0	100.0	100.0
R6	RS2CL7011s	75.39	−	1042	2	1038	Rsa1.0_01521.1	142147	6875	8387	95.0	99.4	146.0
R6	OPJ09	75.51											
R6	RS2CL2923s	75.63	−	1375	6	1375	Rsa1.0_03755.1	21000	9825	11194	80.4	99.6	100.0
R6	RSS0489	75.81	−	774	0	774	Rsa1.0_02622.1	142402	13169	14040	97.6	100.0	112.5
R6	RS2CL3164s	75.96	−	799	1	796	Rsa1.0_03045.1	25787	5906	7910	83.6	99.5	252.1
R6	RGA1701s	76.25											
R6	RGC17769c	76.93	+	421	0	421	Rsa1.0_01427.1	46083	33630	34051	100.0	100.0	100.0
R6	RS2CL3911s	77.46	+	737	0	737	Rsa1.0_00258.1	102381	41383	42511	97.7	100.0	153.1
R6	RSS2949_a	77.71	−	650	0	650	Rsa1.0_07122.1	9866	5114	6056	57.9	100.0	144.9
R6	RS2CL5376s	77.83	−	868	1	605	Rsa1.0_00878.1	330303	113221	113976	68.2	69.6	125.0
R6	RS2CL1217s	80.65	+	855	2	855	Rsa1.0_02941.1	26583	4943	6672	98.6	99.8	202.7
R6	Rs2CL1765s	81.47	−	960	0	959	Rsa1.0_01624.1	42064	4061	5361	98.7	99.9	135.6
R6	RSS0406	82.2											
R6	RGC201c	84.1	−	537	0	537	Rsa1.0_01335.1	47894	11996	12533	100.0	100.0	100.0
R6	RGC4639c	84.49	+	517	0	517	Rsa1.0_02086.1	35198	5908	6425	89.8	100.0	100.0
R6	CA51H	84.49											
R6	RSS2960	84.99											
R6	RGC9315c	85.11	+	487	21	487	Rsa1.0_03486.1	22573	20831	21297	95.7	95.7	100.0
R6	RS2CL2201s	86.23											
R6	RSS0494	86.61	+	738	2	722	Rsa1.0_17112.1	1920	645	1365	81.0	97.6	100.0
R6	RS2CL1536s	87.18	+	1097	197	1097	Rsa1.0_03279.1	24115	7289	8415	81.8	82.0	125.1
R6	RGC2126c	87.73	−	417	0	417	Rsa1.0_09270.1	6513	4845	5262	100.0	100.0	100.0
R6	RGC8845c	89.19	−	511	0	511	Rsa1.0_04819.1	140505	11597	12108	100.0	100.0	100.0
R6	RS2CL2405s	89.66	−	1497	1	1497	Rsa1.0_01439.1	45841	141	1829	97.9	99.9	112.8
R6	RSS0654	91.15	+	842	0	842	Rsa1.0_06604.1	10681	8422	9481	99.1	100.0	125.8
R6	RS2CL2415s	92.15	+	804	2	804	Rsa1.0_09566.1	6285	817	1626	97.8	99.8	100.9
R6	RGC5299c	92.23	−	476	0	476	Rsa1.0_03099.1	139424	6473	6949	93.9	100.0	100.0
R6	RSS2335	93.11	−	863	0	766	Rsa1.0_00749.1	65933	28271	29037	87.6	88.8	100.0
R6	CC56b	95.11											

R6	RSS0318	96.81	+	851	0	851	Rsa1.0_03263.1	24268	20145	21184	100.0	100.0	122.1
R6	RSS1066	97.31	+	879	0	879		146428	130565	131525	75.3	100.0	109.2
R6	RSS0144	97.61	+	836	0	836	Rsa1.0_00861.1	61759	21693	22999	76.2	100.0	156.2
R6	RSS1276	99.11	+	589	0	589	Rsa1.0_16822.1	2022	233	833	90.7	100.0	101.9
R6	RS2CL2353s	99.2											
R6	RGA2249s	102.93											
R6	RSS1104	103.07	−	820	0	793	Rsa1.0_28309.1	820	0	794	89.5	96.7	100.1
R6	RSS1095	103.47	+	844	0	844	Rsa1.0_04293.1	18046	12236	13624	100.0	100.0	164.5
R6	RSS3864	104.67	−	892	3	892	Rsa1.0_02911.1	142461	23036	24062	98.4	99.7	115.4
R6	RSS0660	105.17											
R6	CA92	106.57											
R6	RS2CL2669s	110.66											
R6	RGC3431c	110.9											
R6	RS2CL2529s	110.93	−	889	2	886	Rsa1.0_12809.1	5119	3401	4848	92.8	99.4	163.7
R6	RSS3405	112.02	−	684	0	684	Rsa1.0_07521.1	9206	6492	7391	94.7	100.0	131.4
R6	RSS1020	112.02	−	615	10	614	Rsa1.0_07521.1	9206	7688	8418	93.3	98.2	120.9
R6	Rs2CL1769s	112.07	+	774	0	774	Rsa1.0_03223.1	24512	9371	10716	99.7	100.0	173.8
R6	RGC7305c	112.65											
R6	RSS3937	113.85	+	736	0	736	Rsa1.0_05482.1	13595	4343	5505	99.2	100.0	157.9
R6	RS2CL7280s	114.14	+	1062	0	1062	Rsa1.0_05590.1	13242	3699	4761	99.9	100.0	100.0
R6	OPT12a	115.58											
R6	RS2CL7089s	115.59	+	685	2	685	Rsa1.0_03216.1	24540	23261	23945	99.0	99.7	100.2
R6	RGC11789c	116.51	−	438	0	438	Rsa1.0_03762.1	20976	6037	6475	100.0	100.0	100.0
R6	CA96	116.89											
R6	RS2CL6447s	118.47											
R6	RSS3082	118.61	−	868	0	864	Rsa1.0_12370.1	5243	392	1407	99.2	99.5	117.5
R6	Rs2CL1480s	119.54	+	632	58	631	Rsa1.0_11621.1	5457	0	912	89.7	90.7	159.2
R6	RS2CL1246s	119.59	+	983	2	983	Rsa1.0_05947.1	12142	2347	3800	79.3	99.8	148.1
R6	RGC2386c	120.35											
R6	RGC9240c	120.35	−	529	0	529	Rsa1.0_00418.1	215377	60462	60991	90.6	100.0	100.0
R6	CC65a	120.73											
R6	OPT16H	122.63											
R6	RS2CL3093s	123.21	−	1056	0	1056	Rsa1.0_03264.1	145959	133495	135003	99.2	100.0	142.8
R6	RSS2968	124.52											
R6	RS2CL7541s	124.65	−	1237	1	1237	Rsa1.0_01000.1	57140	40267	42348	99.5	99.9	168.4
R6	RGC916c	126.72	−	476	0	476	Rsa1.0_02007.1	36130	30993	31469	100.0	100.0	100.0

R6	RSS2167	127.44	−	805	0	805	Rsa1.0_00612.1	72376	36601	37848	94.4	100.0	154.9
R6	RGC17489c	128.24	−	425	0	425	Rsa1.0_03225.1	24500	18038	18463	100.0	100.0	100.0
R6	Rs2CL2106s	132.7	+	783	1	781	Rsa1.0_00348.1	92818	49682	51071	97.3	99.6	178.1
R6	RGC570c	132.71	−	497	0	464	Rsa1.0_03612.1	21784	7367	7831	93.4	93.4	100.0
R6	RSS1288	133.22	−	841	0	841	Rsa1.0_00348.1	92818	5503	6343	97.7	100.0	99.9
R6	RGC14157c	133.77	+	443	0	443	Rsa1.0_02711.1	28475	24847	25290	100.0	100.0	100.0
R6	RSS2586	134.89											
R6	RSS1469	135.6	+	580	0	580	Rsa1.0_08080.1	137363	3714	4294	100.0	100.0	100.0
R6	RS2CL7837s	136.74	+	1119	2	1113	Rsa1.0_07984.1	8130	4564	6329	98.6	99.3	158.9
R6	RSS2868	137.34	+	851	177	851	Rsa1.0_05995.1	11997	2711	4104	78.7	79.2	206.7
R6	RS2CL1111s	137.74	−	1378	0	1376	Rsa1.0_01581.1	42833	391	2408	97.8	99.9	146.6
R6	RSS1670	138.04	−	881	0	713	Rsa1.0_17275.1	1874	0	714	78.1	80.9	100.1
R6	CC61b	138.14											
R6	RGC1609c	138.62	−	453	0	453	Rsa1.0_01581.1	42833	1788	2241	100.0	100.0	100.0
R6	RSS0942	138.84	+	870	0	870	Rsa1.0_03300.1	23937	10198	11405	99.1	100.0	138.7
R6	RSS1084	138.84	−	743	0	743	Rsa1.0_01458.1	175719	37330	38853	100.0	100.0	205.0
R6	RSS2362	139.24	−	530	5	515	Rsa1.0_03218.1	24539	22154	23021	93.0	96.2	170.0
R6	RS2CL7123s	139.45	+	710	7	708	Rsa1.0_02834.1	27493	23473	24685	96.1	98.7	172.9
R6	RSS3503	139.64	+	446	3	446		155185	137602	138127	96.2	99.3	118.5
R6	RSS0171	140.04	−	701	118	699	Rsa1.0_11073.1	5625	5044	5625	81.5	82.9	100.0
R6	RGC5431c	140.86	−	522	0	522	Rsa1.0_00639.1	165873	163086	163608	100.0	100.0	100.0
R6	RSS1190	141.24	−	823	0	823	Rsa1.0_00672.1	69358	39959	41514	100.0	100.0	188.9
R6	RSS3372	142.54	+	862	16	862	Rsa1.0_01481.1	184562	2487	3334	87.5	98.1	100.1
R6	RGC1119c	142.83	+	411	0	411	Rsa1.0_00333.1	94500	26446	26857	100.0	100.0	100.0
R6	RGC1052c	143.71	−	492	0	492	Rsa1.0_03545.1	147543	16297	16789	100.0	100.0	100.0
R6	RSS1193	143.84	+	902	0	902	Rsa1.0_03506.1	22416	7445	8559	96.1	100.0	123.5
R6	RGC15890c	143.93	+	468	0	468	Rsa1.0_04022.1	19534	18955	19423	100.0	100.0	100.0
R6	RS2CL1853s	144.22	+	855	2	855	Rsa1.0_00741.1	66117	63648	64653	99.3	99.8	117.8
R6	RGC23650c	144.24	+	412	0	412	Rsa1.0_00333.1	94500	68365	68777	99.3	100.0	100.0
R6	RGC7461c	144.69	−	453	0	453	Rsa1.0_00222.1	172263	67123	67576	100.0	100.0	100.0
R6	RGC4566c	145.16	+	548	0	548	Rsa1.0_00707.1	190112	182788	183336	77.4	100.0	100.0
R6	RSS0454	145.24	+	898	3	898	Rsa1.0_00472.1	398134	314463	316366	95.6	99.7	212.6
R6	RS2CL6557s	145.65	−	1007	0	1007	Rsa1.0_00865.1	61686	45497	46665	96.9	100.0	116.0
R6	RS2CL2395s	145.71	−	433	0	433	Rsa1.0_00707.1	190112	52291	52722	98.4	100.0	99.5
R6	RS2CL3438s	146.12	+	1004	0	325	Rsa1.0_02086.1	35198	27598	27923	29.8	32.4	100.0
R6	RS2CL1346s	146.24	−	868	0	868	Rsa1.0_03355.1	23489	14146	15084	99.4	100.0	108.1

R6	RS2CL8252s	146.39											
R6	RSS3153	146.44	+	442	0	442	Rsa1.0_11907.1	5374	1121	1559	95.0	100.0	99.1
R6	RS2CL6331s	146.81	+	693	1	693	Rsa1.0_03355.1	23489	867	1711	97.4	99.9	122.0
R6	RS2CL934s	147.02	+	1283	82	1279	Rsa1.0_00589.1	209965	188613	190419	82.5	93.3	150.9
R6	RS2CL4132s	147.49	−	1294	0	1292	Rsa1.0_03545.1	147543	7330	8799	98.9	99.9	113.7
R6	RS2CL4854s	148.37	+	1070	1	1065	Rsa1.0_03225.1	24500	13609	15068	94.3	99.4	137.1
R6	RGA2337s	149.59											
R6	RGC10645c	150.02	+	409	0	409	Rsa1.0_00222.1	172263	25497	25906	100.0	100.0	100.0
R6	RGC801c	150.02	−	593	0	593	Rsa1.0_00354.1	232005	211654	212247	100.0	100.0	100.0
R6	RGC4495c	150.16	−	470	0	470	Rsa1.0_02221.1	33796	14834	15304	73.4	100.0	100.0
R6	RGB18830c	150.21	−	535	0	199	Rsa1.0_01743.1	39926	29782	29981	37.2	37.2	100.0
R6	Rs2CL1694s	150.39	+	723	2	723	Rsa1.0_00354.1	232005	168936	170776	97.9	99.7	255.2
R6	RS2CL8102s	150.96	+	748	2	746	Rsa1.0_00222.1	172263	39658	41294	94.4	99.5	219.9
R6	OPR11cH	151.04											
R6	RGC9387c	151.43	+	420	0	420		40243	22893	23313	100.0	100.0	100.0
R6	RSS2756	151.84	−	864	0	864	Rsa1.0_00284.1	323267	132143	133526	94.9	100.0	160.1
R6	RSS1754	151.84	+	422	0	422	Rsa1.0_06010.1	11944	332	758	93.4	100.0	101.0
R6	RSS3095	151.84	−	287	0	287	Rsa1.0_10379.1	5866	42	329	100.0	100.0	100.0
R6	RSS2532	151.84											
R6	RS2CL5874s	152.34	−	1037	2	1036	Rsa1.0_01408.1	46383	16660	17853	90.7	99.7	115.4
R6	RGC7168c	152.49	+	401	0	401	Rsa1.0_00183.1	113277	40294	40695	38.2	100.0	100.0
R6	RSS0166	153.24											
R6	RSS0050	153.34	−	778	0	363	Rsa1.0_02273.1	140964	28473	28836	46.5	46.7	100.0
R6	RGC5183c	153.72											
R6	CD57	153.84											
R6	RGC17231c	154.38	−	482	0	482	Rsa1.0_07379.1	9492	3675	4157	88.4	100.0	100.0
R6	RSS1221	154.54	−	479	75	479	Rsa1.0_00201.1	232851	72717	73234	70.4	84.3	128.0
R6	RS2CL6859s	154.77	−	631	0	623	Rsa1.0_08183.1	7775	4885	5650	96.4	98.7	122.8
R6	RGC5676c	155.53	−	466	0	466	Rsa1.0_00201.1	232851	31486	31952	100.0	100.0	100.0
R6	RGB14649c	157.06	+	500	0	500	Rsa1.0_00201.1	232851	103002	103502	100.0	100.0	100.0
R6	RS2CL7316s	157.61	−	1157	0	1157	Rsa1.0_00064.1	240927	219809	221135	98.3	100.0	114.6
R6	RS2CL6976s	157.63	−	965	1	957	Rsa1.0_00064.1	240927	119509	120739	84.4	99.1	128.7
R6	RGB19481c	158.25	+	538	0	538	Rsa1.0_01184.1	51452	37623	38161	100.0	100.0	100.0
R6	RGB8057c	158.25	+	441	0	441	Rsa1.0_00221.1	106972	64914	65355	100.0	100.0	100.0
R6	RGC12020c	158.47	+	487	0	487	Rsa1.0_00064.1	240927	168782	169269	100.0	100.0	100.0
R6	RS2CL8726s	159.04	+	608	1	608	Rsa1.0_00201.1	232851	151369	152082	99.2	99.8	117.5

R6	Rs2CL1849s	163.38	+	700	1	670	Rsa1.0_00479.1	81377	28329	29268	67.7	95.6	140.4
R6	RS2CL755s	165.82	+	856	1	856	Rsa1.0_00064.1	240927	58831	60254	96.0	99.9	166.4
R6	Rs2CL1647s	167.73	+	712	2	709	Rsa1.0_07379.1	9492	1907	3533	94.5	99.3	230.0
R7	RSS3540	0	−	725	0	725	Rsa1.0_07133.1	9856	7071	7795	98.5	100.0	99.9
R7	CC98aH	9.9											
R7	RGB9971c	12.43											
R7	RS2CL1375s	14.69	+	964	2	963	Rsa1.0_01688.1	40965	35335	36858	98.7	99.7	158.5
R7	CC65b	14.9											
R7	CB85	15.3											
R7	RGC12560c	15.52	+	406	0	406	Rsa1.0_00830.1	62685	57378	57783	99.8	100.0	99.8
R7	RSS1042	16	+	566	1	566	Rsa1.0_03259.1	24290	675	1396	99.8	99.8	127.6
R7	RS2CL4083s	16.56	−	1306	1	1306	Rsa1.0_03391.1	23251	2328	4051	99.4	99.9	132.0
R7	RS2CL4057s	17.21											
R7	RSS3729	17.8	+	862	0	862	Rsa1.0_10300.1	5899	2349	3211	63.2	100.0	100.0
R7	RS2CL5433s	18.08	+	1218	1	1218	Rsa1.0_01717.1	143162	124543	125760	98.9	99.9	100.0
R7	CA30	18.3											
R7	RS2CL2223s	18.54											
R7	RGB16666c	19.03	−	406	0	406	Rsa1.0_00840.1	62514	28518	28924	99.0	100.0	100.0
R7	RGC19850c	19.22	−	570	0	570	Rsa1.0_06715.1	10485	3199	3767	96.8	100.0	99.7
R7	RS2CL5628s	19.36	+	1096	1	1096	Rsa1.0_06715.1	10485	1996	3358	99.2	99.9	124.4
R7	CA32H	19.7											
R7	RSS2871	20.8	+	786	0	786		152418	65105	65964	99.9	100.0	109.3
R7	RS2CL4729s	21.14	−	960	14	960	Rsa1.0_02979.1	26298	13516	14462	86.4	98.5	100.0
R7	RSS0830	21.2	−	805	0	805	Rsa1.0_02428.1	31542	27702	28878	100.0	100.0	146.1
R7	RSS0953	21.2											
R7	RSS1721	21.2	−	209	0	209	Rsa1.0_02428.1	31542	20733	21096	86.6	100.0	173.7
R7	RS2CL1045s	21.43											
R7	RS2CL6364s	21.73	+	1032	1	1032	Rsa1.0_06227.1	11442	3354	7673	98.3	99.9	418.9
R7	RS2CL4386s	22.6											
R7	RSS0476	23.1	+	623	10	623	Rsa1.0_08919.1	6788	5892	6585	98.4	98.4	113.1
R7	RS2CL5785s	24.13											
R7	RS2CL1776s	24.81	+	1143	0	1143	Rsa1.0_01958.1	261128	113575	115457	97.1	100.0	164.7
R7	RS2CL4295s	24.88	+	854	1	854	Rsa1.0_14157.1	3726	1052	2442	99.0	99.9	163.0
R7	CC75	24.93											
R7	RS2CL3314s	25.05											

R7	RGC10321c	25.45												
R7	CB25b	27.39												
R7	CB30a	27.39												
R7	RGC1833c	27.47	+	490	149	342	Rsa1.0_01470.1	45108	9499	9692	39.4	39.4	100.0
R7	RGC1402c	29.25												
R7	RSS0148	29.26	+	815	2	815	Rsa1.0_03134.1	145023	1312	2920	99.6	99.8	197.8
R7	RS2CL1270s	30.34	−	1356	0	1355	Rsa1.0_02043.1	163754	156604	158049	99.7	99.9	106.6
R7	RSS0466	30.48	+	693	1	693	Rsa1.0_04618.1	16623	960	1652	99.9	99.9	100.0
R7	RSS0449	30.58	−	595	10	595	Rsa1.0_01207.1	141053	132968	133681	92.1	98.3	121.9
R7	RSS3262	30.68	−	844	0	844	Rsa1.0_09314.1	6461	4334	5258	98.6	100.0	109.5
R7	RSS3922	30.68											
R7	RSS0313	31.28	+	732	0	732	Rsa1.0_14739.1	3113	2345	3081	96.0	100.0	100.6
R7	RSS0797	31.28	−	684	46	684	Rsa1.0_07100.1	9896	4980	5621	89.9	93.3	100.5
R7	RSS3963	31.28	+	451	48	451	Rsa1.0_15363.1	2655	1071	1476	84.9	89.4	100.5
R7	RSS1371	31.98	−	861	3	861	Rsa1.0_07100.1	9896	4759	5621	96.8	99.7	100.5
R7	RGB4595c	32.21	−	432	0	432	Rsa1.0_03750.1	21027	4672	5622	99.1	100.0	219.9
R7	CB38H	32.74											
R7	RGC7238c	33.65	+	482	0	482	Rsa1.0_01412.1	46311	1967	2449	100.0	100.0	100.0
R7	RGC7757c	33.65	+	409	0	409	Rsa1.0_01525.1	43925	31153	31562	100.0	100.0	100.0
R7	Rs2CL1633s	34.88											
R7	CD02	35.09											
R7	RGC5428c	36.07	+	434	0	224	Rsa1.0_02053.1	35554	12883	13107	51.6	51.6	100.0
R7	RS2CL2646s	36.27	−	774	57	772	Rsa1.0_01412.1	46311	8237	9540	74.3	92.4	182.2
R7	RS2CL3416s	37.33	−	833	0	832	Rsa1.0_03566.1	22050	13792	14770	99.6	99.9	117.6
R7	RS2CL891s	40.94	+	838	4	814	Rsa1.0_01963.1	36719	5466	6375	94.9	96.7	112.2
R7	RGC2981c	41.38	−	400	0	400	Rsa1.0_00573.1	74821	44037	44437	100.0	100.0	100.0
R7	RS2CL6078s	42.78	+	1004	0	1004	Rsa1.0_00573.1	74821	40770	41788	93.2	100.0	101.4
R7	OPJ14	43.31											
R7	RS2CL2164s	43.56	−	753	0	750	Rsa1.0_15250.1	2727	602	1543	99.3	99.6	125.5
R7	RS2CL4632s	43.73	−	1227	2	1194	Rsa1.0_08867.1	6843	0	1196	36.3	97.2	100.3
R7	RGA2312s	44.31											
R7	RGA2511s	44.91											
R7	RGC17698c	45.51	+	469	0	469	Rsa1.0_00060.1	502678	488830	489299	94.0	100.0	100.0
R7	RS2CL2115s	46.17	+	762	25	762	Rsa1.0_02003.1	36171	8859	20354	63.0	96.7	1559.7
R7	RGC8422c	47.25	+	417	0	417	Rsa1.0_01476.1	172928	130776	131193	100.0	100.0	100.0
R7	CC18	47.59											

R7	Rs2CL2268s	48.3	−	616	2	614	Rsa1.0_00291.1	172129	17348	18460	97.9	99.4	181.7
R7	S	48.56											
R7	RS2CL5832s	48.58	−	860	0	860	Rsa1.0_01246.1	49968	34280	35478	96.4	100.0	139.3
R7	RS2CL4290s	49.03	−	1055	2	1051	Rsa1.0_00666.1	69505	41831	43364	98.6	99.4	146.1
R7	RGC1041c	49.35	+	495	0	495	Rsa1.0_00486.1	80875	48596	49091	100.0	100.0	100.0
R7	RGC1883c	49.35	+	459	0	459	Rsa1.0_00486.1	80875	11635	12094	100.0	100.0	100.0
R7	RGC22628c	49.35	−	430	0	430	Rsa1.0_04913.1	15464	13826	14256	100.0	100.0	100.0
R7	RGC3773c	49.35	−	423	0	423	Rsa1.0_00291.1	172129	92000	92423	100.0	100.0	100.0
R7	RS2CL2685s	49.47	+	1518	0	1513	Rsa1.0_00060.1	502678	493350	496374	96.4	99.7	199.9
R7	CA52H	49.99											
R7	RGA1801s	50.05											
R7	RS2CL848s	50.34	+	762	1	762	Rsa1.0_00089.1	406142	228381	230027	97.8	99.9	216.3
R7	RS2CL7649s	50.38	−	800	0	754	Rsa1.0_00089.1	406142	276504	277341	93.5	94.3	111.0
R7	RGC14901c	50.95	−	509	0	509	Rsa1.0_00089.1	406142	188966	189475	91.6	100.0	100.0
R7	RS2CL2344s	51.05	−	1186	1	1186	Rsa1.0_01228.1	50238	1666	3113	73.4	99.9	122.1
R7	RSS0553	51.43	−	827	0	827	Rsa1.0_01882.1	37697	13439	14774	97.7	100.0	161.4
R7	RGC8873c	51.81	+	431	0	431	Rsa1.0_01882.1	37697	1130	1561	100.0	100.0	100.0
R7	RGC9973c	51.81	+	519	0	519	Rsa1.0_01228.1	50238	8482	9001	100.0	100.0	100.0
R7	RGC26589c	52.28	−	562	0	562	Rsa1.0_00089.1	406142	100019	100581	100.0	100.0	100.0
R7	RGA200s	52.69											
R7	RSS0291	52.99	−	640	9	640	Rsa1.0_00175.1	166121	65077	71614	98.0	98.6	1036.0
R7	RS2CL6332s	53.21	−	1248	2	1248	Rsa1.0_01882.1	37697	4755	6001	93.0	99.8	100.0
R7	RGC10939c	53.74	+	425	0	425	Rsa1.0_00502.1	204180	73218	73643	100.0	100.0	100.0
R7	RS2CL1154s	54.33	−	1035	1	1033	Rsa1.0_00175.1	166121	56235	63296	88.8	99.7	684.2
R7	RGC5695c	54.52	−	418	0	418	Rsa1.0_00175.1	166121	131751	132169	84.5	100.0	100.0
R7	RGC7132c	54.73	−	416	0	416	Rsa1.0_00175.1	166121	32954	33370	100.0	100.0	100.0
R7	CA95a	55.04											
R7	RS2CL7750s	55.11	−	691	0	553	Rsa1.0_01586.1	42804	4637	5172	57.7	80.0	96.8
R7	RS2CL4436s	55.83	+	1146	18	1146	Rsa1.0_01586.1	42804	20464	21683	94.8	98.4	108.1
R7	RSS1809	56.46	+	909	98	909	Rsa1.0_02104.1	34985	25036	25808	39.6	89.2	95.2
R7	RS2CL1077s	56.55	+	1380	2	1371	Rsa1.0_02097.1	35036	17204	19468	97.3	99.2	165.4
R7	RGA63s	57.03											
R7	RGC10094c	57.07	+	508	0	508	Rsa1.0_01321.1	48248	12575	13083	100.0	100.0	100.0
R7	RGC1124c	57.07	+	458	0	458	Rsa1.0_01410.1	46345	21463	21921	100.0	100.0	100.0
R7	RGB24589c	57.07											
R7	RGB25459c	57.07	−	418	0	418	Rsa1.0_02097.1	35036	18446	18864	100.0	100.0	100.0

R7	RS2CL6603s	58.03	+	790	9	790		606987	82145	83390	97.5	98.9	159.4
R7	CC46	58.38											
R7	RS2CL2158s	58.67	−	1200	0	1198		606987	70233	71809	93.9	99.8	131.6
R7	RGC5019c	58.84	−	414	0	414	Rsa1.0_02104.1	34985	20368	20782	100.0	100.0	100.0
R7	RS2CL3876s	58.98	−	870	1	870	Rsa1.0_02355.1	32260	7129	8615	96.6	99.9	171.0
R7	RSS0893	59.49	−	632	0	632	Rsa1.0_01238.1	50120	47449	48693	100.0	100.0	196.8
R7	RSS0043	59.69	−	703	0	703	Rsa1.0_01321.1	48248	5991	6695	97.0	100.0	100.1
R7	RSS1241	59.89	+	400	0	389	Rsa1.0_00253.1	231292	132058	132447	97.3	97.3	100.0
R7	RSS1896	60.29	−	823	0	823	Rsa1.0_00253.1	231292	97440	98339	100.0	100.0	109.2
R7	RSS1737	60.69	+	791	0	791	Rsa1.0_06213.1	11478	1043	1939	96.1	100.0	113.3
R7	RSS3091	61.49	−	835	2	835	Rsa1.0_04383.1	17631	10917	12511	99.6	99.8	191.4
R7	RSS2593	61.59	−	845	0	845	Rsa1.0_04383.1	17631	11236	12499	100.0	100.0	149.5
R7	RSS3615	62.19	+	769	0	769	Rsa1.0_03260.1	24284	16796	17830	99.4	100.0	134.5
R7	RSS1781	62.99	+	787	0	787	Rsa1.0_01214.1	50599	35213	36182	97.6	100.0	123.1
R7	Ph	65.09											
R7	R89998	65.59											
R7	RS2CL4343s	66.48	+	733	0	733	Rsa1.0_06213.1	11478	7236	8206	97.1	100.0	132.3
R7	RSS0675	66.51	−	644	26	644	Rsa1.0_02020.1	173211	168269	169361	92.4	96.0	176.7
R7	RS2CL859s	66.54	+	938	0	938	Rsa1.0_02144.1	34630	1055	2703	95.6	100.0	175.7
R7	RS2CL6039s	66.88	−	1107	1	1101	Rsa1.0_00014.1	348985	204532	205632	95.4	99.4	100.0
R7	RS2CL3860s	67.05	+	1054	0	1054	Rsa1.0_00014.1	348985	163873	165235	99.8	100.0	129.2
R7	Rs2CL1833s	67.15	−	675	2	675	Rsa1.0_00014.1	348985	219912	220928	96.4	99.7	151.0
R7	RSS0577	67.36	+	817	0	817	Rsa1.0_00014.1	348985	26937	28106	99.5	100.0	143.1
R7	RS2CL7600s	67.42	+	577	2	577	Rsa1.0_00014.1	348985	174430	175005	60.7	99.7	100.0
R7	RS2CL7455s	67.69	−	820	0	820		368722	68759	69593	95.5	100.0	101.7
R7	RGB645c	67.77	−	409	178	409	Rsa1.0_07698.1	8763	2502	2733	56.5	56.5	100.0
R7	RGC6547c	67.77	−	439	0	439	Rsa1.0_02020.1	173211	4046	4485	100.0	100.0	100.0
R7	RS2CL762s	68.17	−	778	2	749	Rsa1.0_00502.1	204180	181088	181956	92.8	96.0	116.2
R7	RSS1752	68.17	+	542	0	542		325650	304666	305381	99.6	100.0	131.9
R7	RGA1809s	68.47											
R7	RS2CL5281s	68.86	+	1129	2	1129	Rsa1.0_00306.1	146760	54411	56154	97.4	99.8	154.7
R7	RS2CL7601s	69.33	−	1118	0	1118	Rsa1.0_01898.1	37491	18808	20247	99.2	100.0	128.7
R7	RSS1395	70.08	+	450	0	450		368722	33228	33866	87.6	100.0	141.8
R7	RGC175c	70.15	+	483	0	483	Rsa1.0_00502.1	204180	169492	169975	100.0	100.0	100.0
R7	RGC3382c	70.15	−	449	0	449		368722	215660	216109	100.0	100.0	100.0
R7	RGC4853c	70.15	−	404	0	404		294827	256808	257212	100.0	100.0	100.0

R7	RGC6940c	70.15	−	500	0	500	Rsa1.0_14410.1	3420	1223	1723	87.4	100.0	100.0
R7	RS2CL5291s	70.76	+	1201	0	1200		294827	116377	117712	92.8	99.9	111.3
R7	RS2CL896s	71.48	+	1344	2	1342	Rsa1.0_00247.1	103408	72149	75182	99.6	99.7	226.3
R7	RS2CL1576s	71.8	−	1182	1	1182	Rsa1.0_00247.1	103408	94048	95561	99.7	99.9	128.1
R7	RS2CL8862s	72.13	+	562	1	562	Rsa1.0_01549.1	43543	41739	42303	94.1	99.8	100.5
R7	RS2CL2861s	72.54	−	1061	1	1061	Rsa1.0_00483.1	81102	27454	29750	96.7	99.9	216.6
R7	RS2CL3030s	73.07											
R7	RS2CL6700s	73.6	−	918	2	918		325650	175948	176864	99.7	99.8	100.0
R7	nga249	73.64											
R7	RGB2012c	74.45	+	547	0	547	Rsa1.0_00247.1	103408	98357	98904	100.0	100.0	100.0
R7	RGC21683c	74.45	−	400	0	400	Rsa1.0_00816.1	403350	379804	380204	100.0	100.0	100.0
R7	RGC2749c	74.45	−	413	0	413	Rsa1.0_01933.1	37172	13243	13656	100.0	100.0	100.0
R7	RGC4411c	74.45	−	475	0	475	Rsa1.0_01182.1	167304	118700	119173	99.0	100.0	99.6
R7	RGC6888c	74.45	−	458	84	458	Rsa1.0_03698.1	21302	12840	13214	81.7	81.7	100.0
R7	RGC18584c	74.93	+	516	0	516		325650	320016	320532	100.0	100.0	100.0
R7	RS2CL6652s	75.18	−	892	2	891		325650	109844	111119	96.3	99.7	143.4
R7	RS2CL3415s	75.68	−	838	2	838	Rsa1.0_01023.1	56571	49490	50322	98.6	99.8	99.5
R7	RS2CL7185s	75.95	−	937	1	937	Rsa1.0_00570.1	75002	2447	3610	96.6	99.9	124.3
R7	RGC10310c	76.37	+	413	0	413	Rsa1.0_02664.1	28917	4762	5175	100.0	100.0	100.0
R7	RS2CL4596s	76.64	−	1240	1	1235	Rsa1.0_01933.1	37172	5060	6466	96.2	99.5	113.9
R7	RSS3362	76.72	+	865	0	865		368722	272711	273581	98.8	100.0	100.6
R7	RS2CL2687s	77.19	+	1238	4	1235	Rsa1.0_00816.1	403350	109824	111849	93.3	99.4	164.5
R7	RS2CL7134s	77.57	−	809	0	809	Rsa1.0_01078.1	316910	132829	133638	99.4	100.0	100.0
R7	RS2CL2387s	77.69	+	1338	0	1338	Rsa1.0_00816.1	403350	119747	121173	86.3	100.0	106.6
R7	RS2CL6027s	77.69	+	963	1	960	Rsa1.0_00816.1	403350	113311	114334	92.7	99.6	106.7
R7	RS2CL4568s	77.82	−	842	2	838	Rsa1.0_02570.1	30000	5723	6556	97.5	99.3	99.6
R7	Rs2CL1651s	78.15	+	771	0	771	Rsa1.0_00480.1	81325	52340	53356	85.0	100.0	131.8
R7	CA66	78.65											
R7	RGC1800c	78.93	−	402	0	402	Rsa1.0_00722.1	66840	19073	19475	100.0	100.0	100.0
R7	RGC591c	79.41	+	529	0	529	Rsa1.0_00287.1	98687	57086	57615	93.8	100.0	100.0
R7	RS2CL5950s	79.93	−	1170	2	1170	Rsa1.0_00676.1	69166	53701	55584	94.6	99.8	161.2
R7	RS2CL3381s	80.6	−	1677	0	1677	Rsa1.0_02269.1	33168	23244	25388	96.7	100.0	127.9
R7	RS2CL3384s	81.07	+	1003	0	1003	Rsa1.0_00722.1	66840	20201	21375	99.2	100.0	117.1
R7	Rs2CL1984s	81.23	−	873	0	873	Rsa1.0_01719.1	40388	27266	28921	99.7	100.0	189.6
R7	RSS3640	81.25	−	284	0	284	Rsa1.0_00168.1	115870	41695	41979	100.0	100.0	100.0
R7	RGC12603c	82.14	−	401	0	401	Rsa1.0_01411.1	46326	16717	17118	100.0	100.0	100.0

R7	CD87a	82.62												
R7	RGC2185c	82.75	+	404	0	404	Rsa1.0_00016.1	831256	692591	692995	100.0	100.0	100.0
R7	RGC21966c	82.75											
R7	RGC4797c	82.75											
R7	Rs2CL1884s	82.82	+	1030	0	1030	Rsa1.0_01411.1	46326	22910	24368	99.3	100.0	141.6
R7	RsFLC1s	83.04											
R7	RGA1861s	83.25											
R7	RGB12060c	83.54	+	419	0	419	Rsa1.0_00016.1	831256	438800	439219	86.9	100.0	100.0
R7	Rs2CL2475s	83.84	+	898	0	895	Rsa1.0_00907.1	60062	1045	1936	91.3	99.7	99.6
R7	RGC11618c	84.2	+	410	0	410	Rsa1.0_00816.1	403350	118151	118561	98.8	100.0	100.0
R7	CC19	84.63											
R7	RS2CL4523s	85.11	+	1247	0	1247	Rsa1.0_00016.1	831256	58860	60779	89.7	100.0	153.9
R7	RS2CL6940s	85.26	−	462	1	462	Rsa1.0_00224.1	327530	26158	27943	96.8	99.8	387.2
R7	RS2CL3345s	85.37	+	1227	2	1227	Rsa1.0_00224.1	327530	3043	5325	99.4	99.8	186.3
R7	RS2CL5802s	85.95	+	976	3	976	Rsa1.0_00218.1	201480	152478	153800	97.4	99.7	135.9
R7	RGC11896c	86.53											
R7	RGB7818c	86.53											
R7	RGC12721c	87.71											
R7	RGC12875c	87.71											
R7	RGC15411c	87.71											
R7	RGC1662c	87.71											
R7	RGC680c	87.71											
R7	RGC8830c	87.71											
R7	RGC9306c	87.71											
R7	RS2CL1610s	87.74	+	1160	2	1160	Rsa1.0_00088.1	141341	94446	96175	96.6	99.8	149.3
R7	RSS1194	88.57	+	862	0	862	Rsa1.0_00040.1	324196	39285	40382	100.0	100.0	127.3
R7	SWG7B3	89.07											
R7	RS2CL7086s	89.22	+	718	1	711	Rsa1.0_00218.1	201480	22455	23290	95.7	98.9	117.6
R7	RGB19113c	89.63											
R7	RGC1995c	90.67											
R7	RGB7653c	90.67											
R7	RGC235c	90.7											
R7	RGC4058c	90.7											
R7	RGC4381c	90.7											
R7	CB74	90.8											
R7	Rs2CL2240s	91.06	−	585	0	585	Rsa1.0_00047.1	168582	4450	5349	98.1	100.0	153.7

R7	RS2CL2738s	91.23	+	696	2	692	Rsa1.0_04353.1	138437	131186	132812	97.8	99.1	235.7
R7	RSS0403	92.25	−	714	0	701	Rsa1.0_00047.1	168582	149805	150497	93.0	98.2	98.7
R7	RS2CL5276s	92.65	+	1293	1	1293	Rsa1.0_00047.1	168582	66688	68502	64.9	99.9	140.4
R7	RSS0011	92.65											
R7	RS2CL1994s	93.06	+	1355	0	1355	Rsa1.0_00075.1	146680	15883	17836	96.2	100.0	144.1
R7	RS2CL4131s	93.24	−	844	0	842	Rsa1.0_00075.1	146680	26140	27221	99.4	99.8	128.4
R7	RS2CL7414s	93.6	+	1236	0	1236	Rsa1.0_04248.1	18287	3484	5852	99.1	100.0	191.6
R7	RSS2945	93.75	−	867	34	867	Rsa1.0_00373.1	90296	65873	66900	96.1	96.1	123.3
R7	Rs2CL2199s	93.75	−	1206	0	1036	Rsa1.0_03560.1	22092	6182	7868	82.4	85.9	162.7
R7	RSS1012	93.85	+	854	0	854	Rsa1.0_00075.1	146680	125344	127106	99.9	100.0	206.3
R7	RGC5308c	93.93											
R7	Rs2CL2272s	94.18	+	1031	1	1029	Rsa1.0_01877.1	37789	33433	35144	99.0	99.7	166.4
R7	RGB4201c	94.61											
R7	RS2CL2679s	94.78											
R7	RSS2692	96.05	−	889	0	889	Rsa1.0_03945.1	19924	15513	17381	100.0	100.0	210.1
R7	RGC3153c	96.24											
R7	RSS1758	96.45	−	785	0	761		68971	65835	67193	60.0	96.9	178.5
R7	RSS2706	96.55	+	514	0	513		66155	18272	18963	98.6	99.8	134.7
R7	RSS2731	96.55	−	819	0	819	Rsa1.0_04971.1	15273	6715	8640	99.9	100.0	235.0
R7	RSS1273	96.75	−	898	10	862		68971	51542	52851	67.8	94.9	153.6
R7	RSS3991	97.05	−	858	0	858	Rsa1.0_03997.1	19652	11069	11838	80.5	100.0	89.6
R7	RS2CL3584s	97.21	−	786	2	774	Rsa1.0_00931.1	59210	19149	20497	95.3	98.2	174.6
R7	RS2CL1749s	98.06	+	938	2	938	Rsa1.0_03162.1	24895	18782	20173	99.0	99.8	148.6
R7	RSS1786	98.25	+	771	0	771	Rsa1.0_00129.1	126831	67022	67815	56.2	100.0	102.9
R7	RSS0080	98.35	+	721	0	622	Rsa1.0_00178.1	114861	4417	5397	82.7	86.3	157.6
R7	RSS1147	98.35	+	891	0	891	Rsa1.0_00178.1	114861	66259	67652	98.8	100.0	156.3
R7	RSS1574	98.35	+	854	0	854	Rsa1.0_00178.1	114861	4482	5893	83.0	100.0	165.2
R7	RSS3973	98.35	−	811	0	811	Rsa1.0_03292.1	23977	19412	20774	98.8	100.0	167.9
R7	RSS2914_a	98.75	−	858	0	854		99966	785	2202	84.4	99.5	165.9
R7	RSS0239	99.05	+	857	17	857	Rsa1.0_01059.1	55410	28292	29120	93.0	98.0	98.6
R7	CB30b	99.65											
R7	CC61a	100.45											
R7	RGC11328c	100.46											
R7	RGC8546c	103.63											
R7	RGC17372c	105.7											
R7	RSS1523	109.85	−	840	3	840	Rsa1.0_00224.1	327530	242166	243003	99.6	99.6	100.0

R8	RGC12178c	0	+	430	0	430	Rsa1.0_00998.1	57168	13823	14253	100.0	100.0	100.0
R8	RGB6149c	1.5	−	526	0	526	Rsa1.0_00471.1	81897	58976	59502	100.0	100.0	100.0
R8	RS2CL1123s	1.72	−	965	4	965	Rsa1.0_00125.1	253043	181930	183672	96.3	99.6	181.3
R8	RGC147c	2.44	−	586	0	586	Rsa1.0_03689.1	21354	16497	17083	100.0	100.0	100.0
R8	RSS3445	3.54	+	769	1	769	Rsa1.0_03894.1	20207	2551	3318	89.1	99.9	99.9
R8	Rs2CL2236s	4.21	+	823	2	822	Rsa1.0_02944.1	26553	3608	4884	91.1	99.6	155.6
R8	RGC14381c	4.67	+	486	0	486	Rsa1.0_06817.1	10307	8118	8604	68.9	100.0	100.0
R8	RS2CL5518s	4.94	−	928	2	928	Rsa1.0_01444.1	169539	4693	5619	99.5	99.8	100.0
R8	RS2CL2580s	5.35	+	1458	1	1458	Rsa1.0_03820.1	20664	10041	11510	94.7	99.9	100.8
R8	RGC6104c	5.67	+	511	0	511	Rsa1.0_00658.1	69740	38849	39360	100.0	100.0	100.0
R8	RSS2533_a	7.42	−	581	0	577	Rsa1.0_01016.1	181422	44039	44904	91.6	99.3	149.9
R8	RGA1690s	7.53											
R8	RSS0760	7.88	−	725	0	725	Rsa1.0_00950.1	58547	39573	40298	99.9	100.0	100.0
R8	RS2CL5440s	8.21	+	848	0	848	Rsa1.0_03042.1	25803	8642	9965	99.9	100.0	156.0
R8	OPE20a	10.61											
R8	RSS2626	10.91	+	707	0	659		66432	61611	62621	81.2	93.2	153.3
R8	RSS0805	11.71	−	645	0	645	Rsa1.0_02783.1	27927	8353	9224	97.7	100.0	135.0
R8	RSS1427	11.71	+	450	0	450	Rsa1.0_02813.1	27656	25359	26104	99.1	100.0	165.6
R8	RSS0092	13.91	+	718	12	718		80937	3409	4411	95.1	98.3	141.9
R8	RSS2684	14.31	−	860	0	860	Rsa1.0_00724.1	66789	8711	13143	84.1	100.0	515.4
R8	RSS2703	14.51	+	839	0	685	Rsa1.0_03884.1	20274	18022	19712	81.6	81.6	246.7
R8	RSS1899	15.21	+	748	0	748		106231	70581	71442	93.6	100.0	115.1
R8	CA87bH	15.31											
R8	RSS3003	15.81	+	857	0	857	Rsa1.0_02922.1	26753	1412	2352	100.0	100.0	109.7
R8	R30624a	16.01											
R8	R30025	16.31											
R8	BRMS033	16.51											
R8	RSS2613	17.01	−	929	0	928	Rsa1.0_04483.1	17174	9881	11393	78.3	99.9	162.9
R8	RSS1208	17.11	+	854	0	180	Rsa1.0_01444.1	169539	132379	132560	21.0	21.1	100.6
R8	RSS3870	17.21	+	360	0	360		251946	134173	134533	82.2	100.0	100.0
R8	RSS0974	17.41	−	864	0	864	Rsa1.0_03894.1	20207	2592	4549	91.0	100.0	226.5
R8	RSS2196	17.41	−	571	0	571	Rsa1.0_02944.1	26553	10430	11223	100.0	100.0	138.9
R8	RSS0042	17.51	−	747	0	747	Rsa1.0_04493.1	17137	15711	16459	87.4	100.0	100.1
R8	RS2CL3262s	19.21	+	1177	2	1172	Rsa1.0_01616.1	42137	23432	24968	97.8	99.4	131.3
R8	RGC629c	20.26	−	403	0	403	Rsa1.0_00312.1	96765	27747	28150	100.0	100.0	100.0

R8	CA77	20.76												
R8	RGA2253s	21.22												
R8	CD04	21.41												
R8	RGC8651c	21.79												
R8	RS2CL5213s	21.81	+	1107	2	1106	Rsa1.0_05024.1	15105	11367	13003	98.0	99.7	148.2
R8	RGC2369c	22.71	+	459	0	459	Rsa1.0_02514.1	30642	5769	6228	100.0	100.0	100.0
R8	RS2CL2117s	23.07	+	1236	2	1236	Rsa1.0_00975.1	57865	5589	7040	97.7	99.8	117.6
R8	RGC3380c	23.17	−	481	0	481	Rsa1.0_02121.1	34813	19694	20175	100.0	100.0	100.0
R8	RS2CL809s	23.49	+	1500	2	1500	Rsa1.0_01420.1	46210	6554	8942	97.6	99.9	159.4
R8	RGC10544c	23.95	+	420	0	420	Rsa1.0_02904.1	26883	25506	25926	100.0	100.0	100.0
R8	RGC10825c	23.95	+	445	0	445	Rsa1.0_00399.1	242951	2592	3037	100.0	100.0	100.0
R8	RGC1319c	23.95	−	568	0	568	Rsa1.0_02897.1	26967	3793	4361	49.7	100.0	100.0
R8	RGB16366c	23.95											
R8	RGC2994c	23.95	−	477	0	477	Rsa1.0_02583.1	29887	6282	6759	100.0	100.0	100.0
R8	RGC8228c	23.95	−	485	0	485	Rsa1.0_05333.1	14122	10937	11422	36.3	100.0	100.0
R8	RS2CL4855s	24.03	+	1238	1	1238	Rsa1.0_00572.1	74969	61170	62482	99.0	99.9	106.1
R8	RS2CL1406s	24.21	+	808	9	806	Rsa1.0_02458.1	31197	8204	9566	97.9	98.6	170.9
R8	RS2CL3940s	24.73	+	710	7	562	Rsa1.0_01129.1	52949	8750	9370	73.1	78.2	111.7
R8	RS2CL2104s	24.88	+	737	2	691	Rsa1.0_03326.1	23710	16531	17547	91.2	93.5	147.5
R8	RS2CL3538s	25.82	−	1033	0	1033	Rsa1.0_00312.1	96765	88010	89867	98.6	100.0	179.8
R8	RS2CL5211s	26.82	+	613	2	613	Rsa1.0_05871.1	12356	4420	5353	96.7	99.7	152.7
R8	RGC20183c	27.25	+	479	107	422	Rsa1.0_07507.1	9231	2974	3289	65.8	65.8	100.0
R8	CA91	28.52											
R8	RS2CL3875s	28.66	−	582	2	582	Rsa1.0_02893.1	26999	24275	25484	94.5	99.7	208.5
R8	RS2CL3155s	29.12	−	852	37	844	Rsa1.0_00142.1	121474	62548	63430	91.0	94.7	109.3
R8	Rs2CL1806s	29.56											
R8	RGC12354c	29.8	+	452	0	452	Rsa1.0_03121.1	25230	23859	24311	100.0	100.0	100.0
R8	RGC5581c	29.8	+	507	0	479	Rsa1.0_00794.1	64063	12363	12842	94.5	94.5	100.0
R8	RSS0790	29.82	+	771	0	771	Rsa1.0_01683.1	41059	22062	23545	99.1	100.0	192.4
R8	RGC12097c	30.29	+	515	0	515	Rsa1.0_00399.1	242951	75864	76379	100.0	100.0	100.0
R8	RGC274c	31.31	+	505	0	357	Rsa1.0_02203.1	164532	1149	1506	33.1	70.7	100.0
R8	RS2CL5594s	31.39	−	1027	4	1024	Rsa1.0_02883.1	27035	2227	4340	97.0	99.3	207.2
R8	RGC20497c	31.5	−	412	0	412	Rsa1.0_03243.1	24414	11321	11733	100.0	100.0	100.0
R8	RGC5712c	31.5	−	477	0	460	Rsa1.0_00648.1	70351	36816	37276	82.8	96.4	100.0
R8	RGC7156c	31.5											
R8	RGC484c	32	−	516	0	516	Rsa1.0_03842.1	20503	1709	2225	100.0	100.0	100.0

R8	RGC2683c	32.63	−	470	0	470	Rsa1.0_00142.1	121474	33486	33956	100.0	100.0	100.0
R8	RS2CL5411s	32.94	−	995	4	980	Rsa1.0_01702.1	40722	17654	18640	95.8	98.1	101.0
R8	RS2CL4559s	33.61	+	898	1	898		390088	70420	71980	96.4	99.9	173.9
R8	RS2CL5083s	33.61	+	829	1	829	Rsa1.0_00288.1	98659	79985	81050	98.2	99.9	128.6
R8	CA57H	33.7											
R8	RSS0053	34.1	−	544	0	544	Rsa1.0_02802.1	27736	10820	11525	96.5	100.0	129.6
R8	RS2CL5831s	34.39	+	809	8	795		267193	103761	104554	90.1	97.3	100.8
R8	RSS1433	36.43	−	848	0	848	Rsa1.0_00288.1	98659	88951	90352	96.0	100.0	165.2
R8	RSS4002	36.43	+	600	0	600	Rsa1.0_00246.1	103541	42070	42670	100.0	100.0	100.0
R8	RGC4518c	36.63	−	464	0	464	Rsa1.0_02556.1	30133	5066	5530	68.1	100.0	100.0
R8	RS2CL1252s	37.79	−	780	17	780	Rsa1.0_08317.1	7554	1841	2605	96.0	97.8	100.1
R8	CB75H	38.06											
R8	CC88bH	39.96											
R8	RGC14573c	40.23	−	454	0	454	Rsa1.0_02503.1	30709	27639	28093	100.0	100.0	100.0
R8	RSS0294	41.44	−	693	10	693		167018	31475	32158	94.2	98.6	100.0
R8	RGC173c	41.86	−	432	0	432	Rsa1.0_00997.1	57190	37779	38211	100.0	100.0	100.0
R8	RGC2807c	41.86	−	421	0	399	Rsa1.0_00523.1	178770	36457	36856	42.8	94.8	100.0
R8	CC72	42.41											
R8	RS2CL1403s	44.41	+	1224	2	1224	Rsa1.0_03607.1	21817	1505	4226	99.4	99.8	222.7
R8	RSS0615	44.92	+	728	13	718	Rsa1.0_01487.1	44784	313	1390	83.0	96.8	152.8
R8	RSS1330	45.72											
R8	RSS1747	46.62	+	838	0	838	Rsa1.0_01041.1	55910	37195	38034	99.6	100.0	100.1
R8	RS2CL6221s	47.94	+	1281	1	1281	Rsa1.0_01722.1	40320	32574	33855	99.3	99.9	100.1
R8	T75662	48.28											
R8	RSS1844	48.88	+	820	0	820	Rsa1.0_02688.1	28644	16173	17249	100.0	100.0	131.2
R8	RS2CL1646s	49.21	−	941	3	939	Rsa1.0_01112.1	310601	31111	32228	89.3	99.5	119.3
R8	RSS1680	49.37	−	795	1	795	Rsa1.0_02092.1	35135	27158	27953	99.9	99.9	100.1
R8	RS2CL2780s	50.36	+	871	2	810	Rsa1.0_01660.1	41400	31295	32703	86.7	92.8	174.3
R8	Rs2CL2111s	50.69	+	955	2	953		49419	35521	37478	98.2	99.6	205.8
R8	RSS3270	51.11	+	827	0	827	Rsa1.0_01377.1	46967	13163	14171	96.9	100.0	121.9
R8	RGC9390c	51.14	−	426	0	426		283362	66423	66849	100.0	100.0	100.0
R8	RGC10000c	51.69	+	417	13	417	Rsa1.0_00410.1	86957	25583	25987	96.9	96.9	100.0
R8	RGC5417c	51.69											
R8	RSS2735	51.76	+	756	0	756	Rsa1.0_01112.1	310601	48350	49184	99.3	100.0	110.3
R8	CA88N	52.46											
R8	RS2CL4240s	52.61	−	1333	1	1297	Rsa1.0_01633.1	41868	6820	8814	95.5	97.2	153.9

R8	RS2CL5626s	53.34	+	935	0	935	Rsa1.0_01377.1	46967	19801	21943	97.5	100.0	229.1
R8	CC15H	53.49											
R8	RS2CL5288s	54.18	+	1302	1	1302	Rsa1.0_01377.1	46967	8613	10006	89.3	99.9	107.1
R8	RGC2076c	55.59	−	485	0	485	Rsa1.0_00007.1	239325	224811	225296	100.0	100.0	100.0
R8	RSS0305	56.55	+	261	0	261	Rsa1.0_00188.1	112020	4679	4941	100.0	100.0	100.4
R8	RSS1175	56.55	+	337	0	337	Rsa1.0_06288.1	11302	312	649	100.0	100.0	100.0
R8	RGC11890c	56.96	−	465	0	465	Rsa1.0_21829.1	1170	94	559	86.7	100.0	100.0
R8	RGC14479c	58.07	+	424	0	424	Rsa1.0_00007.1	239325	98262	98686	100.0	100.0	100.0
R8	ACGM23	58.99											
R8	RS2CL1940s	59.21											
R8	CC13	60.13											
R8	Rs2CL1970s	60.24	−	657	0	657		396176	93367	94313	98.2	100.0	144.0
R8	RGC16204c	60.99	−	548	0	548		365886	334329	334877	100.0	100.0	100.0
R8	RSS2248	61.37	−	820	0	686	Rsa1.0_00007.1	239325	214773	215462	77.9	83.7	100.4
R8	RS2CL3284s	61.75	+	608	0	593	Rsa1.0_00007.1	239325	118003	118885	84.7	97.5	148.7
R8	RGC3490c	62.11	−	596	0	596	Rsa1.0_02161.1	34459	13112	13708	100.0	100.0	100.0
R8	RS2CL1179s	62.29	+	1450	0	1449	Rsa1.0_00007.1	239325	14077	16181	93.9	99.9	145.2
R8	RS2CL4152s	62.99	−	1044	20	1041	Rsa1.0_00403.1	87701	43775	45321	96.3	97.8	151.4
R8	RSS0073	63	+	684	0	680		365886	323535	324434	95.2	99.4	132.2
R8	RGC8307c	63.9	−	495	111	495		365886	191819	192203	77.6	77.6	100.0
R8	RSS3326	64.45	+	272	0	272	Rsa1.0_01828.1	38681	33028	33302	94.9	100.0	100.7
R8	Rs2CL1468s	64.49	−	794	0	794	Rsa1.0_01923.1	37233	35386	36734	89.7	100.0	169.8
R8	RGC8839c	65.26	−	516	0	516		365886	140708	141224	100.0	100.0	100.0
R8	RGC258c	65.62	+	418	0	418	Rsa1.0_00128.1	228675	66405	66823	100.0	100.0	100.0
R8	RGC14352c	65.76	+	467	0	467	Rsa1.0_04198.1	18504	13658	14125	99.8	100.0	100.0
R8	RGC6994c	65.76	+	414	0	414	Rsa1.0_00128.1	228675	118269	118683	100.0	100.0	100.0
R8	RS2CL881s	66.05	−	831	3	831	Rsa1.0_00944.1	58733	41317	42542	82.2	99.6	148.0
R8	RGC2153c	66.26	+	405	0	405		396176	292160	292565	79.3	100.0	100.0
R8	RS2CL1475s	66.86	−	621	0	621	Rsa1.0_08027.1	8058	6814	7435	92.1	100.0	100.0
R8	RGC26764c	67.25	−	437	0	437		396176	376963	377400	100.0	100.0	100.0
R8	RSS3178	67.32	+	572	0	572		396176	18606	19179	96.5	100.0	100.2
R8	RSS3174	67.72	+	870	20	870		396176	186748	187737	94.1	97.7	116.4
R8	RGC19563c	68.79	+	423	0	423	Rsa1.0_00215.1	201775	93860	94283	100.0	100.0	100.0
R8	RSS0382	69.5	−	644	0	644		396176	304667	306127	100.0	100.0	226.7
R8	RSS3738	69.5	+	744	0	744		396176	296029	296774	68.6	100.0	100.1
R8	RGC2170c	69.95	−	566	0	566	Rsa1.0_00504.1	79334	16654	17220	100.0	100.0	100.0

R8	RGC18537c	70.29	+	478	0	478	Rsa1.0_02198.1	33989	15073	15551	100.0	100.0	100.0
R8	RSS0919	71.15	+	736	0	736	Rsa1.0_00114.1	131330	124837	126422	99.7	100.0	215.4
R8	RS2CL1250s	71.23	+	1168	2	1168	Rsa1.0_00914.1	59880	12954	14120	45.8	99.8	100.0
R8	RGC15683c	71.86	−	595	46	595	Rsa1.0_00437.1	84879	62178	62727	77.5	92.3	100.0
R8	RSS2610	72.12	+	374	0	374	Rsa1.0_00215.1	201775	3123	3594	100.0	100.0	125.9
R8	RS2CL5401s	72.43	−	1293	1	1293	Rsa1.0_01976.1	36570	7806	10214	98.1	99.9	186.4
R8	RS2CL7655s	72.61	−	824	1	824	Rsa1.0_01976.1	36570	33617	34533	99.9	99.9	111.3
R8	RSS2489	72.61	−	814	10	814	Rsa1.0_00114.1	131330	8757	11009	98.8	98.8	280.1
R8	RGC1653c	73.01	−	533	0	533	Rsa1.0_01143.1	52456	42346	42879	72.6	100.0	100.0
R8	RGC17676c	73.01	−	429	0	429	Rsa1.0_02449.1	31292	8268	8697	99.1	100.0	100.0
R8	RGB14459c	73.15	−	448	0	448	Rsa1.0_00114.1	131330	100984	101432	100.0	100.0	100.0
R8	OPR20	73.34											
R8	Rs2CL1478s	73.69	−	1018	1	1018	Rsa1.0_04243.1	18321	2896	4619	98.4	99.9	169.4
R8	RGB11843c	73.9	+	456	0	456	Rsa1.0_00133.1	123768	52751	53207	93.4	100.0	100.0
R8	RSS1585	74.03	+	845	1	845	Rsa1.0_01422.1	46172	16387	17717	67.8	99.9	157.6
R8	RGC8853c	74.38	+	408	0	408	Rsa1.0_01422.1	46172	27604	28012	100.0	100.0	100.0
R8	RS2CL3498s	74.55	−	651	1	648	Rsa1.0_02179.1	34188	23622	24419	97.1	99.4	123.2
R8	RS2CL5877s	75.73	+	799	1	799	Rsa1.0_00133.1	123768	66297	67096	97.9	99.9	100.1
R8	RSS1295	75.84	−	437	0	238	Rsa1.0_18041.1	1683	1441	1679	54.5	54.5	100.0
R8	Rs2CL1741s	76.98	−	746	3	745	Rsa1.0_04741.1	16164	7118	8332	99.1	99.5	163.6
R8	RS2CL3268s	77.62	+	935	3	840	Rsa1.0_01668.1	41299	18574	19690	82.5	89.5	133.3
R8	RSS0229	78.36	+	777	0	777	Rsa1.0_04741.1	16164	5332	6388	94.9	100.0	135.9
R8	RS2CL5368s	78.58	−	1046	1	1046	Rsa1.0_00034.1	260119	121393	122572	98.0	99.9	112.8
R8	RSS0066	78.94	+	685	9	685	Rsa1.0_05641.1	13077	7245	7902	94.3	98.7	97.2
R8	RS2CL2922s	79.04	+	1283	1	1283	Rsa1.0_00549.1	76473	33491	34901	96.3	99.9	110.0
R8	RS2CL3626s	79.45	+	805	0	805	Rsa1.0_00034.1	260119	77363	78246	71.4	100.0	109.7
R8	RSS2460	79.51	−	927	49	926	Rsa1.0_01561.1	43227	38328	39984	90.4	94.6	188.8
R8	RSS2434	79.81	−	734	0	734		77375	52445	53875	91.7	100.0	194.8
R8	RSS2901	80.01	+	850	0	850	Rsa1.0_00153.1	192931	4421	5271	75.4	100.0	100.0
R8	RS2CL4706s	80.14	+	777	1	776	Rsa1.0_04128.1	18927	11475	12891	99.7	99.7	182.7
R8	RSS3034	80.67	+	542	0	542	Rsa1.0_00153.1	192931	86721	87264	99.8	100.0	100.2
R8	RS2CL5634s	80.88	+	870	0	869	Rsa1.0_01789.1	39250	12709	14191	96.4	99.9	170.5
R8	RSS0327	80.95	−	804	0	804	Rsa1.0_00153.1	192931	110170	111937	98.3	100.0	219.8
R8	RSS3614	81.25	+	858	14	858	Rsa1.0_00153.1	192931	37475	38357	92.1	98.4	104.5
R8	RS2CL1509s	82.09	+	1888	5	1887	Rsa1.0_00020.1	283099	54912	57213	93.1	99.7	122.3
R8	RSS2378	82.3	+	898	0	898	Rsa1.0_00163.1	116847	44448	45740	100.0	100.0	143.9

R8	RGC11743c	82.42	−	498	0	498	Rsa1.0_00153.1	192931	18859	19357	100.0	100.0	100.0
R8	RSS3078	82.59	+	875	0	875	Rsa1.0_02453.1	31271	12105	14237	99.9	100.0	243.7
R8	RS2CL1319s	82.66											
R8	RS2CL5849s	82.67	−	348	156	348	Rsa1.0_00163.1	116847	75920	76111	50.3	55.2	99.5
R8	RS2CL5318s	82.87	+	1240	1	1240		48033	25520	26759	99.8	99.9	100.0
R8	Rs2CL2338s	82.96	+	946	1	942	Rsa1.0_00163.1	116847	10438	11897	97.2	99.5	155.1
R8	RS2CL3001s	83.11	−	853	0	853	Rsa1.0_00020.1	283099	14473	15326	78.7	100.0	100.0
R8	RS2CL7379s	83.72	−	716	2	491	Rsa1.0_00020.1	283099	101648	102448	66.5	68.3	163.6
R8	RGC5306c	83.97	+	462	0	462	Rsa1.0_00163.1	116847	98693	99155	100.0	100.0	100.0
R8	RGC8280c	83.97	+	431	0	431	Rsa1.0_00497.1	79830	16297	16728	100.0	100.0	100.0
R8	RGB8542c	83.97	+	525	0	525	Rsa1.0_01920.1	158533	29914	30451	97.3	100.0	102.3
R8	RGB8994c	83.97	−	451	0	451	Rsa1.0_00153.1	192931	113499	113950	100.0	100.0	100.0
R8	RS2CL7544s	84.27	−	885	1	885	Rsa1.0_06021.1	11911	4386	5466	97.4	99.9	122.2
R8	RSS2157	84.62	−	651	19	651	Rsa1.0_00020.1	283099	75564	77039	97.1	97.1	233.4
R8	RS2CL1601s	84.74	−	1098	2	1098	Rsa1.0_01758.1	39747	36639	38270	96.6	99.8	148.8
R8	RSS3273	84.81	+	888	0	888	Rsa1.0_00020.1	283099	58219	59281	83.5	100.0	119.6
R8	RS2CL7685s	85.42	−	729	0	729	Rsa1.0_00157.1	245832	76427	77641	95.3	100.0	166.5
R8	RS2CL2573s	85.44	−	1108	3	1087	Rsa1.0_00273.1	326186	3138	5148	92.2	97.8	185.4
R8	RS2CL1983s	85.58	−	1262	3	1262	Rsa1.0_00273.1	326186	137299	139314	98.0	99.8	160.1
R8	RS2CL5298s	85.75	+	1163	3	1163	Rsa1.0_00157.1	245832	182201	184322	87.9	99.7	182.8
R8	RS2CL5635s	85.91	+	705	0	705	Rsa1.0_01561.1	43227	20408	21392	96.5	100.0	139.6
R8	RGB3215c	86.15	−	428	0	428	Rsa1.0_00273.1	326186	305626	306054	100.0	100.0	100.0
R8	RSS0027	86.26	−	661	0	661	Rsa1.0_01326.1	48072	44499	45168	94.0	100.0	101.2
R8	RS2CL2296s	86.45											
R8	RS2CL3554s	86.87	+	588	0	588	Rsa1.0_00157.1	245832	160095	160881	97.1	100.0	133.7
R8	RSS1843	87.06	+	843	0	843	Rsa1.0_02544.1	30290	464	1883	100.0	100.0	168.3
R8	RS2CL4310s	87.42	−	707	0	707	Rsa1.0_00157.1	245832	236064	236848	76.8	100.0	110.9
R8	RGC10842c	87.68	+	408	0	408	Rsa1.0_00273.1	326186	146279	146687	100.0	100.0	100.0
R8	RGC13395c	87.68	−	499	0	499	Rsa1.0_00157.1	245832	215954	216453	100.0	100.0	100.0
R8	RGC3413c	87.68	−	428	0	428	Rsa1.0_00157.1	245832	61029	61457	100.0	100.0	100.0
R8	RGB3288c	87.87											
R8	RGC989c	87.87	+	464	0	464	Rsa1.0_01435.1	45942	16462	16926	100.0	100.0	100.0
R8	RSS2286	88.06											
R8	RS2CL3341s	88.21	−	955	2	955		222544	29888	31613	98.9	99.8	181.0
R8	RS2CL6784s	88.37	+	792	2	792	Rsa1.0_00097.1	138858	49719	51303	99.8	99.8	200.5
R8	RGC4722c	88.59	−	537	0	537	Rsa1.0_00097.1	138858	137097	137634	100.0	100.0	100.0

R8	RGC9339c	88.59	−	530	0	530	Rsa1.0_00161.1	116891	105405	105935	100.0	100.0	100.0
R8	CC50	89.36											
R8	RGC13159c	89.51	+	415	0	415	Rsa1.0_01920.1	158533	132946	133361	100.0	100.0	100.0
R8	RS2CL2944s	90.15	−	1216	2	1216	Rsa1.0_01920.1	158533	22466	23718	86.3	99.8	103.1
R8	Z17993	90.36											
R8	CB80	91.26											
R8	RGB3124c	92.24	+	418	0	418		396176	2267	2685	100.0	100.0	100.0
R8	RGC7108c	92.24	+	400	0	400	Rsa1.0_02143.1	34632	17843	18243	100.0	100.0	100.0
R8	CB80N	93.36											
R8	CA22bH	100.66											
R8	RSS3836	101.36	+	845	19	775	Rsa1.0_00145.1	261008	232062	232914	85.7	89.5	112.7
R9	PK9	0											
R9	CA88	5.8											
R9	RSS1006_a	8.5	+	831	0	831		247766	78313	79707	97.5	100.0	167.8
R9	RSS0129	11	+	678	0	662	Rsa1.0_02529.1	30437	20009	20981	97.5	97.6	146.8
R9	RSS2168	12.3	−	434	0	434	Rsa1.0_00525.1	78030	9014	9450	100.0	100.0	100.5
R9	RSS2147	12.5	+	476	0	394	Rsa1.0_01538.1	43733	28494	28886	80.0	82.8	99.5
R9	RGA1576s	12.9											
R9	RSS4036	13.5	−	150	0	150	Rsa1.0_01388.1	46741	12576	12726	98.7	100.0	100.0
R9	RS2CL7699s	14.59	−	854	0	854	Rsa1.0_00688.1	68396	37209	38191	93.9	100.0	115.0
R9	RGA1547s	15.36											
R9	RS2CL3487s	16.04	−	1045	0	1045	Rsa1.0_02680.1	28721	17984	19028	99.3	100.0	99.9
R9	RSS2321	17.62	+	846	0	846	Rsa1.0_00264.1	101674	39504	40350	74.4	100.0	100.0
R9	RSS1369	18.26	−	870	0	852	Rsa1.0_00012.1	222148	170982	172306	96.0	97.9	155.4
R9	RS2CL1156s	18.9	−	1171	0	1170	Rsa1.0_01947.1	36977	10478	12576	41.2	99.9	179.3
R9	RS2CL1885s	19.14	+	952	0	951	Rsa1.0_05411.1	13838	7560	8511	97.9	99.9	100.0
R9	RSS1177	19.52	+	493	0	493	Rsa1.0_00170.1	179538	83207	83802	73.6	100.0	120.7
R9	RGC15483c	20.02	−	468	0	468	Rsa1.0_01273.1	49464	38274	38742	100.0	100.0	100.0
R9	RGB21440c	20.02	−	531	0	531	Rsa1.0_00920.1	601703	12282	12813	96.1	100.0	100.0
R9	RGC22512c	20.02	+	567	0	567	Rsa1.0_00857.1	61904	30303	30870	100.0	100.0	100.0
R9	RGC4199c	20.02	−	400	0	400	Rsa1.0_03310.1	23851	23022	23422	100.0	100.0	100.0
R9	RGC4665c	20.02	+	497	0	497	Rsa1.0_01980.1	36544	32515	33012	99.4	100.0	100.0
R9	RS2CL1281s	20.2	+	1311	1	1309	Rsa1.0_03454.1	149870	144058	145866	99.5	99.8	138.2
R9	RS2CL2288s	21.02	+	1245	2	1245	Rsa1.0_02800.1	27760	12372	13978	81.7	99.8	129.2
R9	RSS3821	21.87	−	840	2	840	Rsa1.0_00699.1	184398	40640	41463	91.0	99.8	98.2

R9	RS2CL808s	21.94	+	787	0	787	Rsa1.0_02139.1	34686	5575	6993	43.7	100.0	180.2
R9	RGC24745c	22.32											
R9	RGC4695c	22.32	+	429	0	429	Rsa1.0_00920.1	601703	445861	446290	100.0	100.0	100.0
R9	RGC4895c	22.32	+	429	0	429	Rsa1.0_05564.1	13319	2478	2907	100.0	100.0	100.0
R9	RS2CL4579s	22.68	−	931	10	927	Rsa1.0_02139.1	34686	7614	8746	96.4	98.5	123.5
R9	CF12	22.82											
R9	CA24N	22.92											
R9	RS2CL4567s	23.1	+	906	2	906	Rsa1.0_01967.1	36672	15957	17134	99.5	99.8	130.2
R9	RS2CL5197s	23.69	+	795	2	795	Rsa1.0_01081.1	499226	321155	322640	96.5	99.8	187.3
R9	RS2CL4906s	23.91	+	1065	0	1065	Rsa1.0_01081.1	499226	321268	323133	97.0	100.0	175.1
R9	RS2CL1327s	24.22	+	818	2	813	Rsa1.0_01081.1	499226	471924	473615	98.2	99.1	208.5
R9	RGC10838c	24.23	−	476	0	476	Rsa1.0_02924.1	26736	21134	21610	100.0	100.0	100.0
R9	RGC11312c	24.23	+	494	0	494	Rsa1.0_01081.1	499226	332161	332655	100.0	100.0	100.0
R9	RGC19152c	24.23	−	450	0	450	Rsa1.0_00170.1	179538	137910	138360	81.3	100.0	100.0
R9	RGC19895c	24.23	+	541	0	541	Rsa1.0_03231.1	24456	3545	4086	98.9	100.0	100.0
R9	RGC4679c	24.23	−	426	0	426	Rsa1.0_01204.1	50843	8194	8620	100.0	100.0	100.0
R9	RGC8563c	24.23	+	405	0	405	Rsa1.0_01221.1	50421	32974	33379	100.0	100.0	100.0
R9	RS2CL3830s	24.61	−	876	2	876	Rsa1.0_00699.1	184398	55771	56738	97.8	99.8	110.6
R9	RSS1513	26.01	+	787	0	787	Rsa1.0_00139.1	357786	6687	7680	94.0	100.0	126.2
R9	RS2CL3460s	26.12	−	1418	2	1418	Rsa1.0_03895.1	20206	13691	15565	97.7	99.9	132.3
R9	RSS3835	27.27	−	773	0	773	Rsa1.0_00139.1	357786	1678	2531	100.0	100.0	110.4
R9	CC56aN	27.57											
R9	RGA1745s	27.66											
R9	CA34c	28.07											
R9	RSS0475	29.07	+	691	0	691	Rsa1.0_02683.1	28679	14328	15747	99.4	100.0	205.4
R9	RS2CL2301s	29.21	−	754	2	754	Rsa1.0_02244.1	33494	10212	11146	99.6	99.7	124.2
R9	RGC6745c	29.99	−	453	0	453	Rsa1.0_01945.1	36989	3868	4321	99.8	100.0	100.0
R9	RSS1532	30.9	+	598	0	598		300828	15711	16591	99.8	100.0	147.2
R9	RS2CL8479s	31.66	−	727	1	727	Rsa1.0_00099.1	159619	33431	34157	87.4	99.9	100.0
R9	RSS0225	32.18	+	646	0	646	Rsa1.0_02431.1	31504	547	1825	99.9	100.0	197.8
R9	RGA1941s	32.47											
R9	RGC7717c	32.9	+	528	0	528	Rsa1.0_03483.1	22621	18412	18940	81.4	100.0	100.0
R9	RS2CL3316s	33.32	+	1093	1	1093	Rsa1.0_06049.1	11851	8555	10125	99.3	99.9	143.8
R9	Rs2CL1549s	34.13	+	672	0	672	Rsa1.0_01919.1	37272	3536	4644	98.2	100.0	164.9
R9	RSS3830	34.16	−	596	0	596	Rsa1.0_03900.1	133221	6921	7488	92.1	100.0	95.1
R9	OPR11bH	35.26											

R9	RSS1337	35.76	−	718	8	718	Rsa1.0_00610.1	72740	44429	45158	95.8	98.9	102.7
R9	RSS1870	36.56											
R9	RS2CL1098s	36.65	+	959	2	959	Rsa1.0_13994.1	3941	838	2341	97.3	99.8	157.1
R9	RS2CL3458s	36.88	−	785	1	626	Rsa1.0_21931.1	1162	73	698	77.7	79.6	100.0
R9	RGC683c	37.96	+	449	0	449	Rsa1.0_04234.1	18378	712	1161	67.5	100.0	100.0
R9	RSS1790	39.11	−	316	0	316	Rsa1.0_06508.1	10874	3035	3429	90.5	100.0	124.7
R9	CA73	39.31											
R9	RGB7901c	40.43											
R9	RS2CL4013s	41.08											
R9	OPR11aH	46.84											
R9	RSS2112	47.14	−	677	16	673	Rsa1.0_00254.1	102600	73227	74045	93.4	97.1	124.5
R9	RS2CL1950s	47.68											
R9	RSS0897	48.06											
R9	RS2CL6276s	48.47	+	1308	44	1308	Rsa1.0_02276.1	33098	11898	13388	76.4	96.6	117.9
R9	RS2CL1162s	48.59	+	598	2	595	Rsa1.0_00254.1	102600	83341	84508	96.2	99.2	196.8
R9	RSS1696_a	48.78											
R9	RGC20593c	49.53	−	541	0	541	Rsa1.0_03359.1	23466	8512	9053	78.6	100.0	100.0
R9	RSS1592	50.09											
R9	RSS0706	50.49	−	780	0	780	Rsa1.0_00561.1	75653	4647	5687	95.0	100.0	133.3
R9	RSS2916	50.69	−	837	0	833	Rsa1.0_03639.1	21629	2457	3420	93.8	99.5	115.6
R9	RS2CL5504s	51.19	+	1093	4	1093	Rsa1.0_03972.1	19787	11929	13999	97.8	99.6	190.1
R9	RGC2630c	51.3	+	499	0	499	Rsa1.0_04873.1	15657	2775	3274	100.0	100.0	100.0
R9	RSS1660	51.61	−	911	0	911	Rsa1.0_08001.1	8095	611	1503	69.6	100.0	97.9
R9	RGA178s	52.61											
R9	RGC6878c	53.04											
R9	RGC607c	53.57	+	425	0	425	Rsa1.0_00231.1	105381	15704	16129	100.0	100.0	100.0
R9	RS2CL5261s	53.82	+	670	2	670	Rsa1.0_03361.1	23456	3213	3881	69.7	99.7	100.0
R9	CA53aH	54.57											
R9	RS2CL6424s	54.76	−	1234	0	1233	Rsa1.0_02927.1	26698	23077	24309	98.4	99.9	99.9
R9	RS2CL7401s	55.84	−	1103	2	1103	Rsa1.0_03572.1	146346	140674	141943	94.6	99.8	115.3
R9	RSS2661	56.33											
R9	RSS3495	59.43											
R9	RS2CL1353s	60.82	−	1261	0	1261	Rsa1.0_08609.1	7132	4843	6433	98.3	100.0	126.1
R9	RSS2934	62.05	+	818	0	816	Rsa1.0_00606.1	73150	29752	30846	99.8	99.8	134.1
R9	RGB15272c	62.2	−	532	307	532	Rsa1.0_06409.1	11067	4147	4372	36.5	42.3	100.0
R9	RSS0404	63.19	+	820	0	820	Rsa1.0_00606.1	73150	29776	31133	100.0	100.0	165.5

R9	RS2CL1092s	63.52	+	1141	2	1141	Rsa1.0_05815.1	12563	8444	10050	99.7	99.8	141.0
R9	RS2CL3798s	63.86	+	748	1	744	Rsa1.0_01812.1	38956	24043	24787	91.8	99.3	100.1
R9	RGB2773c	63.88	+	426	0	426	Rsa1.0_04299.1	18027	17498	17924	84.5	100.0	100.0
R9	RGC7838c	63.88	+	585	0	585	Rsa1.0_10699.1	5752	3639	4224	100.0	100.0	100.0
R9	RS2CL4941s	63.97	−	1177	0	1177	Rsa1.0_02330.1	32452	415	1944	98.8	100.0	129.9
R9	CC62H	65.01											
R9	Rs2CL2460s	65.4	+	654	0	653	Rsa1.0_06052.1	11848	6622	7513	98.2	99.9	136.5
R9	RS2CL3791s	66.13	−	617	2	615	Rsa1.0_00994.1	165189	136079	136901	97.6	99.4	134.1
R9	RS2CL1061s	66.44	−	1093	0	1092		495407	250482	251576	67.5	99.9	100.2
R9	RGC3132c	66.69											
R9	CC37aH	66.77											
R9	RS2CL1946s	67.44	−	776	1	776	Rsa1.0_06535.1	10827	3273	4045	96.9	99.9	99.6
R9	RS2CL3879s	67.92	+	1326	1	1326	Rsa1.0_04565.1	16837	2922	4732	92.7	99.9	136.6
R9	CC11H	68.69											
R9	RGC7876c	68.84	−	475	0	475	Rsa1.0_00132.1	124299	30474	30949	100.0	100.0	100.0
R9	RGC17699c	69.55	−	526	0	526	Rsa1.0_00132.1	124299	114729	115254	98.9	100.0	99.8
R9	RGC21716c	69.55	−	494	0	494	Rsa1.0_02695.1	28605	27902	28396	85.0	100.0	100.0
R9	RS2CL2047s	70.11	−	822	2	796	Rsa1.0_06663.1	10576	7668	8993	88.7	96.6	166.9
R9	F15571	70.94											
R9	RS2CL6991s	71.22	+	1172	2	1172	Rsa1.0_10113.1	5992	1703	3027	98.3	99.8	113.2
R9	RGC10800c	71.67	+	464	0	464	Rsa1.0_00061.1	218685	65016	65480	100.0	100.0	100.0
R9	RGB21550c	71.67	+	456	0	456	Rsa1.0_02128.1	34758	20966	21422	100.0	100.0	100.0
R9	RGB6475c	71.67	−	520	0	520	Rsa1.0_00435.1	317579	62135	62655	100.0	100.0	100.0
R9	RGC13816c	71.99	−	509	0	509	Rsa1.0_00435.1	317579	26116	26625	91.6	100.0	100.0
R9	Rs2CL1347s	72.64	+	848	2	848	Rsa1.0_00276.1	100160	94494	96125	97.9	99.8	192.8
R9	CC83	73.17											
R9	RGC12399c	73.22	−	528	0	528	Rsa1.0_01620.1	42091	4893	5421	83.5	100.0	100.0
R9	Rs2CL2196s	73.8	+	881	2	881	Rsa1.0_04511.1	17032	3480	5212	90.6	99.8	197.0
R9	RS2CL964s	74.73	−	585	2	585	Rsa1.0_00435.1	317579	70702	71725	98.3	99.7	175.5
R9	RSS1474	75.03	−	788	0	788	Rsa1.0_00378.1	89892	72451	73334	100.0	100.0	112.1
R9	RGC7982c	75.19	+	462	0	462		194198	30114	30576	100.0	100.0	100.0
R9	RS2CL7317s	75.75	+	1355	1	1355	Rsa1.0_11496.1	5485	1373	2739	32.8	99.9	100.9
R9	RSS1063	76.03	+	866	1	866	Rsa1.0_03546.1	22172	12865	14073	95.6	99.9	139.7
R9	RS2CL6758s	76.06	−	680	2	680	Rsa1.0_03191.1	145762	134763	135439	95.3	99.7	99.7
R9	OPT17H	77.07											
R9	RGB4147c	77.1	−	470	0	470	Rsa1.0_00954.1	189536	8277	8747	93.2	100.0	100.0

R9	RGC5522c	77.1	−	418	0	418	Rsa1.0_01001.1	57077	23727	24145	94.5	100.0	100.0
R9	RGC8507c	77.1	−	480	0	479	Rsa1.0_02185.1	34150	13107	13586	99.8	99.8	100.0
R9	RGC9843c	77.67	−	430	0	430	Rsa1.0_00045.1	328694	314657	315087	100.0	100.0	100.0
R9	RS2CL3567s	77.68	−	931	9	931	Rsa1.0_03354.1	23491	13058	14138	79.4	99.0	117.1
R9	CD32H	78.87											
R9	RS2CL3739s	78.89	+	546	2	545	Rsa1.0_05811.1	12572	11186	11991	91.4	99.5	148.3
R9	RGC12323c	79.54	−	537	0	537	Rsa1.0_02381.1	31972	23653	24190	100.0	100.0	100.0
R9	RGC12828c	80.03	+	434	0	434	Rsa1.0_01504.1	189978	20633	21067	85.0	100.0	100.0
R9	RSS2466	81.29	−	799	1	799		194198	23791	24586	94.5	99.9	99.6
R9	Rs2CL2464s	81.6	+	594	1	593	Rsa1.0_01001.1	57077	23257	24104	92.4	99.7	143.1
R9	RSS1475	83.65	−	849	33	849	Rsa1.0_00417.1	395068	386834	387638	90.7	96.1	98.5
R9	RS2CL3325s	83.73											
R9	RGB5484c	84.38	+	401	0	401	Rsa1.0_00841.1	183065	153070	153471	50.4	100.0	100.0
R9	CD85	85.22											
R9	Rs2CL1958s	88.92											
R9	RGB28103c	89.98	−	502	0	502	Rsa1.0_01879.1	37748	10808	11310	100.0	100.0	100.0
R9	RGC11199c	91.4	+	434	0	434	Rsa1.0_08038.1	8034	4353	4787	100.0	100.0	100.0
R9	RGC674c	91.4	+	406	99	406	Rsa1.0_01006.1	163670	40270	40577	75.6	75.6	100.0
R9	RS2CL832s	92.4	+	1647	4	1625	Rsa1.0_01467.1	45161	13042	15829	94.8	98.4	171.9
R9	RGC684c	93.01	+	428	0	428		88189	8023	8451	93.5	100.0	100.0
R9	RS2CL2118s	93.38	−	754	2	681	Rsa1.0_03344.1	23593	15206	15887	89.8	90.1	100.3
R9	RGC7548c	94.07	−	435	0	435	Rsa1.0_02189.1	34098	28239	28674	99.8	100.0	100.0
R9	RS2CL1196s	94.92	−	753	48	753	Rsa1.0_05884.1	12337	478	1183	85.7	93.6	100.0
R9	RS2CL1395s	95.28	+	791	2	786	Rsa1.0_00557.1	75865	69196	70122	88.1	99.1	118.1
R9	RGC5191c	95.61	−	481	0	481	Rsa1.0_01090.1	54385	34077	34558	84.4	100.0	100.0
R9	CC43a	96.44											
R9	CA80	100.44											
R9	RGC3235c	100.54											
R9	CC43aN	102.49											
R9	RS2CL4058s	103.77	+	1066	1	1065	Rsa1.0_02032.1	35830	2986	4138	98.1	99.8	108.3
R9	RGA1616s	104.73											
R9	RGC22627c	105.86	−	472	0	453	Rsa1.0_00056.1	332604	14690	15143	96.0	96.0	100.0
R9	RGC19925c	107.01	+	406	0	406	Rsa1.0_01881.1	37721	25907	26313	100.0	100.0	100.0
R9	RGC2049c	107.01	+	579	83	298	Rsa1.0_00753.1	65705	6533	6748	37.1	37.1	100.0
R9	RSS0140	107.54	−	579	83	579	Rsa1.0_06726.1	10460	9881	10381	77.0	85.7	100.8
R9	RGA1944s	108.16											

R9	OPT09b	108.7												
R9	RSS2575	111.7	+	874	0	874	Rsa1.0_00942.1	58873	48955	49830	100.0	100.0	100.1
R9	RSS1301	112.3	−	710	0	710	Rsa1.0_00942.1	58873	24148	24858	97.5	100.0	100.0
R9	RGC2698c	113.43	+	455	0	455	Rsa1.0_00942.1	58873	48716	49171	100.0	100.0	100.0
R9	RGC8995c	113.43	+	401	0	401	Rsa1.0_00942.1	58873	9690	10090	55.6	100.0	99.8
R9	RGA236s	113.58											
R9	Rs2CL1221s	114.17	−	935	2	935	Rsa1.0_02820.1	27629	22434	24062	98.3	99.8	174.5
R9	RGC7515c	115.38	−	435	0	435	Rsa1.0_00045.1	328694	196928	197363	68.5	100.0	100.0
R9	RS2CL4042s	115.45	−	687	1	687	Rsa1.0_00045.1	328694	131772	132624	79.3	99.9	124.2
R9	RS2CL7387s	115.76	+	813	0	813	Rsa1.0_00045.1	328694	231371	232758	94.2	100.0	170.6
R9	RS2CL2007s	115.93	+	954	1	842	Rsa1.0_00045.1	328694	175470	176392	85.9	88.2	109.6
R9	CD11bH	116.39											
R9	RGC1736c	117.63	+	540	0	540	Rsa1.0_00560.1	75685	52946	53486	100.0	100.0	100.0
R9	RS2CL7327s	117.9	−	1057	60	1056		75219	26633	27890	74.2	94.2	126.2
R9	RS2CL6327s	118.34	−	925	1	922	Rsa1.0_00323.1	159650	113373	115513	98.2	99.6	232.4
R9	RGC13248c	118.48	+	405	9	405	Rsa1.0_04138.1	18898	8896	9292	97.8	97.8	100.0
R9	Rs2CL2203s	118.74	−	811	2	811		75219	16111	17983	99.6	99.8	231.4
R9	RGB15203c	118.97	−	437	0	437	Rsa1.0_00038.1	314568	243571	244008	100.0	100.0	100.0
R9	RS2CL2614s	119.09	−	1045	0	1044	Rsa1.0_02312.1	32675	15842	16886	99.8	99.9	100.0
R9	RGA2478s	119.76											
R9	RSS1954	119.83	+	837	0	837	Rsa1.0_00038.1	314568	264516	266426	98.1	100.0	228.2
R9	RS2CL6430s	120.43	+	1009	0	1009	Rsa1.0_02130.1	34755	20489	21902	85.6	100.0	140.0
R9	RGC14934c	121.15	−	442	0	442	Rsa1.0_00753.1	65705	42738	43180	100.0	100.0	100.0
R9	RS2CL5708s	121.78	+	626	4	626	Rsa1.0_01119.1	53459	35842	36562	98.2	99.4	115.8
R9	RGC6292c	123.13	+	531	0	531	Rsa1.0_00414.1	202190	47223	47754	64.0	100.0	100.0
R9	RGC9998c	123.13	−	529	0	529	Rsa1.0_04103.1	19048	3572	4101	83.2	100.0	100.0
R9	RSS2285	123.16	−	858	0	858	Rsa1.0_00038.1	314568	146073	146959	84.9	100.0	103.3
R9	RGC6034c	123.28	+	405	0	405	Rsa1.0_00118.1	130637	129809	130214	100.0	100.0	100.0
R9	RGC7030c	123.28	+	521	0	484	Rsa1.0_00118.1	130637	50086	50570	92.9	92.9	100.0
R9	CA34a	123.73											
R9	CC16	124.23											
R9	RGC5701c	124.31	−	411	0	411	Rsa1.0_00056.1	332604	111788	112199	100.0	100.0	100.0
R9	RGB12822c	125.73	+	569	0	569	Rsa1.0_04095.1	19095	10902	11421	91.2	100.0	91.2
R9	RGB19618c	125.77	+	429	0	429	Rsa1.0_00154.1	189461	49401	49830	100.0	100.0	100.0
R9	RGC560c	126.31	−	472	0	472	Rsa1.0_00154.1	189461	106274	106746	100.0	100.0	100.0
R9	RS2CL4118s	127.34	+	746	0	746	Rsa1.0_00662.1	192183	1271	2379	97.2	100.0	148.5

R9	RSS1035	127.52	+	574	0	574	Rsa1.0_00414.1	202190	45335	46091	100.0	100.0	131.7
R9	RS2CL5486s	127.93	+	585	0	585	Rsa1.0_00154.1	189461	181967	182547	95.6	100.0	99.2
R9	RGC1999c	128.49	−	400	0	400	Rsa1.0_00154.1	189461	151787	152187	100.0	100.0	100.0
R9	RSS3287	128.67	+	778	0	778	Rsa1.0_01376.1	47004	37625	39039	98.6	100.0	181.8
R9	RGC5007c	128.91	−	453	0	453	Rsa1.0_00351.1	413096	245705	246158	100.0	100.0	100.0
R9	RS2CL3231s	129.25	+	691	4	691	Rsa1.0_01236.1	50156	11646	12709	97.3	99.4	154.7
R9	RS2CL5203s	129.51	+	1264	2	1230	Rsa1.0_01199.1	163576	126966	128986	53.6	97.2	164.5
R9	RGC2070c	130	+	425	0	425	Rsa1.0_00351.1	413096	385602	386027	100.0	100.0	100.0
R9	RGC23150c	130	−	499	0	499	Rsa1.0_00002.1	601661	43509	44008	95.8	100.0	100.0
R9	RGB2543c	130	+	476	0	476	Rsa1.0_00662.1	192183	33434	33910	100.0	100.0	100.0
R9	RGC5810c	130	+	445	0	445	Rsa1.0_01236.1	50156	1173	1618	100.0	100.0	100.0
R9	RGC6156c	130	+	494	0	494	Rsa1.0_01799.1	39127	8241	8735	89.7	100.0	100.0
R9	RGB8501c	130	−	548	0	548	Rsa1.0_01639.1	41850	4814	5362	100.0	100.0	100.0
R9	RSS1133	130.12	+	592	0	592	Rsa1.0_00154.1	189461	172747	173818	99.8	100.0	180.9
R9	RS2CL5024s	130.79	−	899	5	899	Rsa1.0_00002.1	601661	419967	421720	96.4	99.4	196.1
R9	RSS0093	130.8	−	278	0	278	Rsa1.0_01799.1	39127	4082	4360	100.0	100.0	100.0
R9	RS2CL3112s	131.1	+	899	0	898	Rsa1.0_00002.1	601661	453710	454610	99.7	99.9	100.2
R9	RSS1329	131.1	+	852	2	198	Rsa1.0_00475.1	81678	39192	39389	23.0	23.0	100.5
R9	RS2CL4174s	131.25	−	729	0	729	Rsa1.0_00002.1	601661	454953	456192	96.8	100.0	170.0
R9	Gpi	131.48											
R9	RS2CL5963s	131.63	−	1382	2	1381	Rsa1.0_00002.1	601661	436514	437893	45.2	99.8	100.0
R9	RSS3041	131.77	+	862	0	862	Rsa1.0_00662.1	192183	181768	182802	100.0	100.0	120.0
R9	RS2CL7452s	131.96	+	662	7	661	Rsa1.0_00053.1	263459	211034	211688	97.4	98.8	100.0
R9	RSS0603	132.16	+	769	0	769	Rsa1.0_00351.1	413096	1787	3249	100.0	100.0	190.1
R9	RS2CL1949s	132.17	+	1368	0	1366	Rsa1.0_03185.1	24739	7505	9914	98.0	99.9	176.4
R9	RGC2758c	132.35	+	405	0	405	Rsa1.0_00002.1	601661	480951	481356	100.0	100.0	100.0
R9	RGC5530c	132.35	+	505	0	505	Rsa1.0_01242.1	50066	22601	23106	100.0	100.0	100.0
R9	RGC8742c	132.56	+	472	0	472	Rsa1.0_00002.1	601661	551663	552135	100.0	100.0	100.0
R9	CA35a	132.74											
R9	RS2CL5908s	133.03	+	877	1	877	Rsa1.0_00053.1	263459	144852	145830	99.8	99.9	111.6
R9	RGC10531c	133.65	−	586	0	586	Rsa1.0_00002.1	601661	506667	507253	100.0	100.0	100.0
R9	RGC6519c	134.07	+	402	0	402	Rsa1.0_00071.1	345714	36175	36577	100.0	100.0	100.0
R9	RGB13054c	134.36	−	465	0	465	Rsa1.0_04057.1	19333	11930	12395	100.0	100.0	100.0
R9	RS2CL1116s	134.82	+	1035	7	1033	Rsa1.0_00002.1	601661	264134	265939	94.9	99.1	175.9
R9	RS2CL3917s	135.53	+	1176	1	1176	Rsa1.0_00002.1	601661	573673	575147	98.3	99.9	125.5
R9	RSS0155	136.73	+	869	0	869	Rsa1.0_00039.1	296927	67740	68681	94.8	100.0	108.3

R9	RS2CL5880s	137.21	+	1144	2	1139	Rsa1.0_00002.1	601661	243969	245223	97.1	99.4	110.3
R9	RSS1128	138.23	+	748	1	748	Rsa1.0_00071.1	345714	334641	335384	95.7	99.9	99.5
R9	RS2CL2842s	138.53	+	744	0	744	Rsa1.0_00176.1	240984	188320	189155	97.6	100.0	112.2
R9	RSS2113	139.63	+	891	268	891	Rsa1.0_01073.1	55030	11306	11917	64.8	69.9	98.1
R9	RSS1425	141.43	−	843	29	842	Rsa1.0_00039.1	296927	48748	50478	95.5	96.4	212.8
R9	Rs2CL1695s	143.22	+	717	2	717	Rsa1.0_01236.1	50156	1258	2273	96.8	99.7	142.0
R9	RGC15178c	146.77	−	479	0	479	Rsa1.0_01119.1	53459	8047	8526	91.0	100.0	100.0

Table S17 Number of SNPs between different lines of radish

	Taibyosobutori	Yumehomare	Sakurajima	AZ26H	N1-3	Nishimachiriso	Aokubi	Sayatori
Taibyosobutori	–	2,387 (3.49)	3,289 (3.68)	3,028 (3.64)	2,494 (3.36)	2,659 (3.52)	2,066 (2.85)	5,569 (6.04)
Yumehomare		–	3,441 (3.76)	3,034 (3.64)	2,730 (3.50)	2,842 (3.64)	2,681 (3.31)	5,681 (5.97)
Sakurajima			–	3,386 (3.59)	3,542 (3.78)	3,459 (3.65)	3,568 (3.55)	5,457 (5.65)
AZ26H				–	3,129 (3.66)	3,003 (3.44)	3,144 (3.44)	5,321 (5.78)
N1-3					–	2,763 (3.43)	2,882 (3.41)	5,642 (5.98)
Nishimachiriso						–	2,938 (3.39)	5,436 (5.81)
Aokubi							–	7,873 (6.95)

Values in parenthes represent the number of SNPs per a common amplicon.

Table S18 Number of common amplicons containing SNPs between different lines of Radish

	Taibyosobutori	Yumehomare	Sakurajima	AZ26H	N1-3	Nishimachiriso	Aokubi	Sayatori
Number of fragments	1,484	1,506	1,487	1,494	1,496	1,498	2,880	1,722
Taibyosobutori	–	684	894	832	743	755	724	922
Yumehomare		–	915	834	780	780	809	952
Sakurajima			–	943	938	947	1,004	965
AZ26H				–	854	872	913	921
N1-3					–	806	845	943
Nishimachiriso						–	866	936
Aokubi							–	1,133
